# Genome-wide siRNA screening reveals several host receptors for the binding of human gut commensal *Bifidobacterium bifidum*

**DOI:** 10.1038/s41522-022-00312-0

**Published:** 2022-06-29

**Authors:** Veera Kainulainen, Carina von Schantz-Fant, Ruusu-Maria Kovanen, Swapnil Potdar, Karoliina Laamanen, Jani Saarela, Reetta Satokari

**Affiliations:** 1 Human Microbiome Research Program, Faculty of Medicine, University of Helsinki, Helsinki, Finland; 2 Institute for Molecular Medicine Finland (FIMM), University of Helsinki, Helsinki, Finland

**Keywords:** Cellular microbiology, Applied microbiology

## Abstract

*Bifidobacterium* spp. are abundant gut commensals, especially in breast-fed infants. Bifidobacteria are associated with many health-promoting effects including maintenance of epithelial barrier and integrity as well as immunomodulation. However, the protective mechanisms of bifidobacteria on intestinal epithelium at molecular level are poorly understood. In this study, we developed a high-throughput in vitro screening assay to explore binding receptors of intestinal epithelial cells for *Bifidobacterium bifidum. S*hort interfering RNAs (siRNA) were used to silence expression of each gene in the Caco-2 cell line one by one. The screen yielded four cell surface proteins, SERPINB3, LGICZ1, PKD1 and PAQR6, which were identified as potential receptors as the siRNA knock-down of their expression decreased adhesion of *B. bifidum* to the cell line repeatedly during the three rounds of siRNA screening. Furthermore, blocking of these host cell proteins by specific antibodies decreased the binding of *B. bifidum* significantly to Caco-2 and HT29 cell lines. All these molecules are located on the surface of epithelial cells and three out of four, SERPINB3, PKD1 and PAQR6, are involved in the regulation of cellular processes related to proliferation, differentiation and apoptosis as well as inflammation and immunity. Our results provide leads to the first steps in the mechanistic cascade of *B. bifidum*-host interactions leading to regulatory effects in the epithelium and may partly explain how this commensal bacterium is able to promote intestinal homeostasis.

## Introduction

The gastrointestinal tract of human and animals is colonized by a dense microbiota, which regulates numerous facets of intestinal biology including immunity, epithelial barrier function and colonization resistance^1,2^. Mucosa-associated microbiota is considered to play a key role in intestinal homeostasis due to its intimate contact with the host^3,4^. Bifidobacteria are an integral part of the human intestinal microbiota constituting even up to 90% of all bacteria in breast-fed infants and in average 4% in adults^5^. Bifidobacteria form temporarily stable populations in healthy adults^6,7^ and colonize also the intestinal mucosa^8^. *Bifidobacterium bifidum* is one of the most dominant *Bifidobacterium* species found in humans^9^.

*Bifidobacterium* spp. are associated with many health-promoting effects at local and systemic levels such as prevention of pathogen colonization, maintenance of epithelial barrier and integrity, and influencing the immune system through changes in innate and/or adaptive immune responses^8,10,11^. Concerning *B. bifidum* in particular, strains belonging to the species has been reported to reduce apoptosis in the intestinal epithelium both in vivo in rat model of necrotizing enterocolitis and in vitro in enterocyte cultures^12^. Furthermore, *B. bifidum* strains have been shown to suppress intestinal inflammation in a mouse colitis model^13^ and enforce epithelial integrity in vitro^14^. The mechanisms of interaction of bifidobacteria with the host have been studied extensively during the past years and a variety of secreted or surface-associated molecules that act as mediators in the bifidobacteria-host interaction have been uncovered^10^. Still, the current knowledge on bifidobacterial-host interactions is superficial and particularly the host side molecular mechanisms remain poorly studied. The ability of bifidobacteria to adhere to the intestinal mucosa is considered as an important trait, which facilitates intimate interaction with the host. Bifidobacteria in general and specifically strains of *B. bifidum* adhere strongly to intestinal epithelial cell lines Caco-2 and HT-29 and to intestinal mucus^15,16^. *B. bifidum* has been found to carry two functional pili gene clusters encoding for extracellular string-like appendices or pili (also called fimbriae), which mediate the binding of bacteria to host cells and tissues^10,17^. The expression of *B. bifidum* pili is upregulated upon colonization of mice^17^, but the pilin proteins are also expressed during growth in standard laboratory medium^18^, which may explain the high adherence of the bacterium also under in vitro conditions^15,16^. In addition to pili, *B. bifidum* harbors other adhesins including specific cytosolic proteins, which perform a moonlighting binding function when localized on the cell surface^19^. While the different adhesins of *B. bifidum* have been actively investigated, the identification of binding receptors in the host epithelium have received little attention. As receptor-ligand binding is one mechanism by which cells sense and respond to external stimuli, the identification of binding sites could provide intriguing insights into the mechanisms by which this commensal bacterium affects the host.

RNA interference (RNAi) is a widely used technique to knockdown gene expression in eukaryotic cells and to study loss-of-function phenotypes (reviewed in 20). In the endogenous pathway of RNAi, non-coding, double-stranded RNAs are processed into short, RNA duplexes or microRNAs by a conserved cellular machinery. Subsequently, one strand of the miRNA associates with the RNA-induced silencing complex (RISC) to guide the cleaving of homologous messenger RNA (mRNA)^20^. RNAi technology utilizes this endogenous RNAi pathway to control the activity of genes through post-transcriptional downregulation, by transfecting cells with exogenous dsRNA. The introduction of large-scale synthetic libraries of short interfering RNAs (siRNA) has enabled the screening of gene functions on a genome-wide scale^21^. siRNA molecules enter the RNAi pathway by joining the RISC, which then degrades the associated mRNA thereby allowing the linkage of a specific gene with a phenotype. Large scale siRNA screening has already been used to decipher the invasion mechanisms and host pathways that support or limit the infection of intracellular parasitic bacteria such as *Coxiella burnetii* and *Listeria monocytogenes*^22–24^. However, the use of genome-wide screening to study host-bacteria interactions is still scarce and limited to pathogens.

In this study, we developed an assay to use genome-wide siRNA screening to explore binding receptors of intestinal epithelial cells for adherent human commensal bacteria. The screening method allowed us to reveal several binding receptors for the gut commensal *B. bifidum* and provided molecular level insights into the interactions between the bacterium and its host. The developed assay can be adapted to a wide range of bacteria. To our knowledge, this is the first study to report the use of genome-wide siRNA screening to survey host receptors for the binding of commensal bacterium to survey the molecular mechanisms possibly underlying its reported health-promoting effects.

## Results

**Fig. 1:**
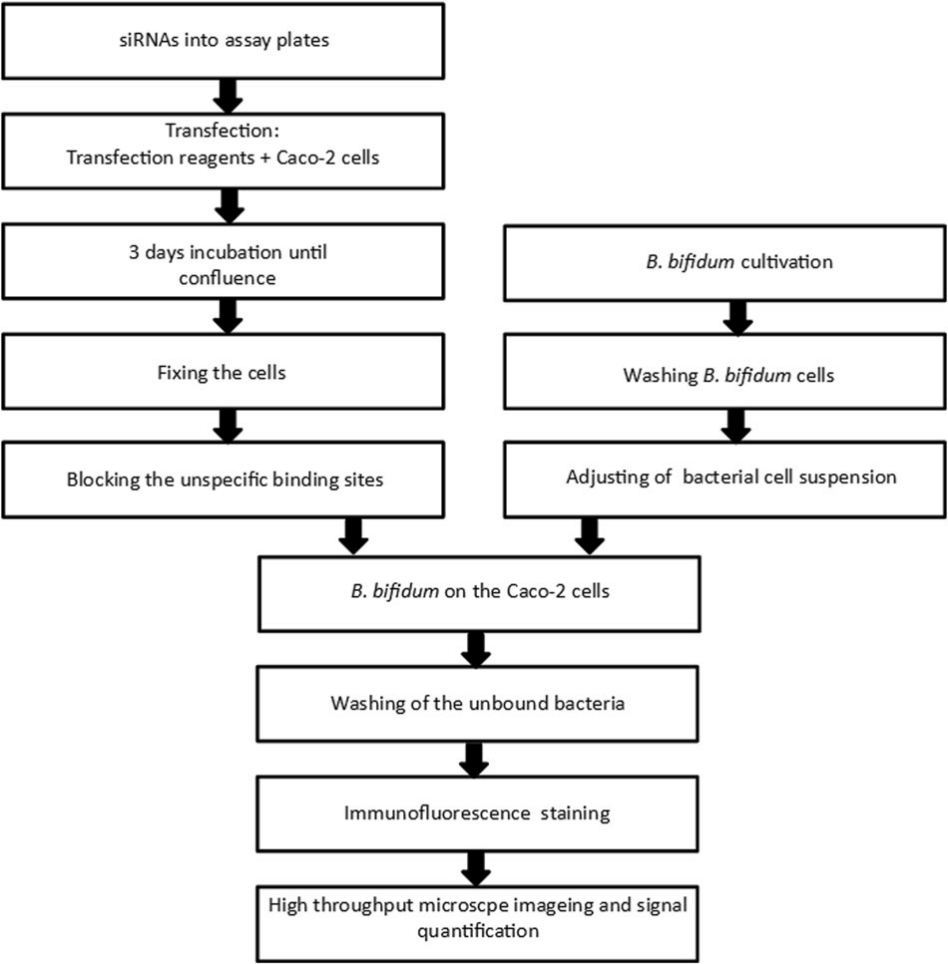
Workflow of the screening assays. All three screenings were performed using the same protocol.

We developed a genome-wide screening assay to reveal receptors used by commensal *B. bifidum* on the surface of intestinal epithelial cells for adhesion. The workflow of the genome-wide screen is shown in Fig. 1. The Caco-2 cells were transfected using three siRNAs for each gene expressed in intestinal epithelial cells. The cells were grown on plate for three days, which is sufficient to reach confluence and confluence of the cell layer was verified in the subsequent steps when high-throughput microscopy was performed (see below and Fig. 3B). After the cell layer had reached confluence, the cells were fixed and the unspecific binding sites were blocked and after cultivation, *B. bifidum* cells were washed and the cell concentration was adjusted. Bacterial cells were allowed to adhere to Caco-2 cells and unbound bacteria were washed away. The adhered bacteria and nuclei of Caco-2 cells were stained using immunofluorescence/fluorescence staining and detected using high-throughput microscope imaging.

**Fig. 2:**
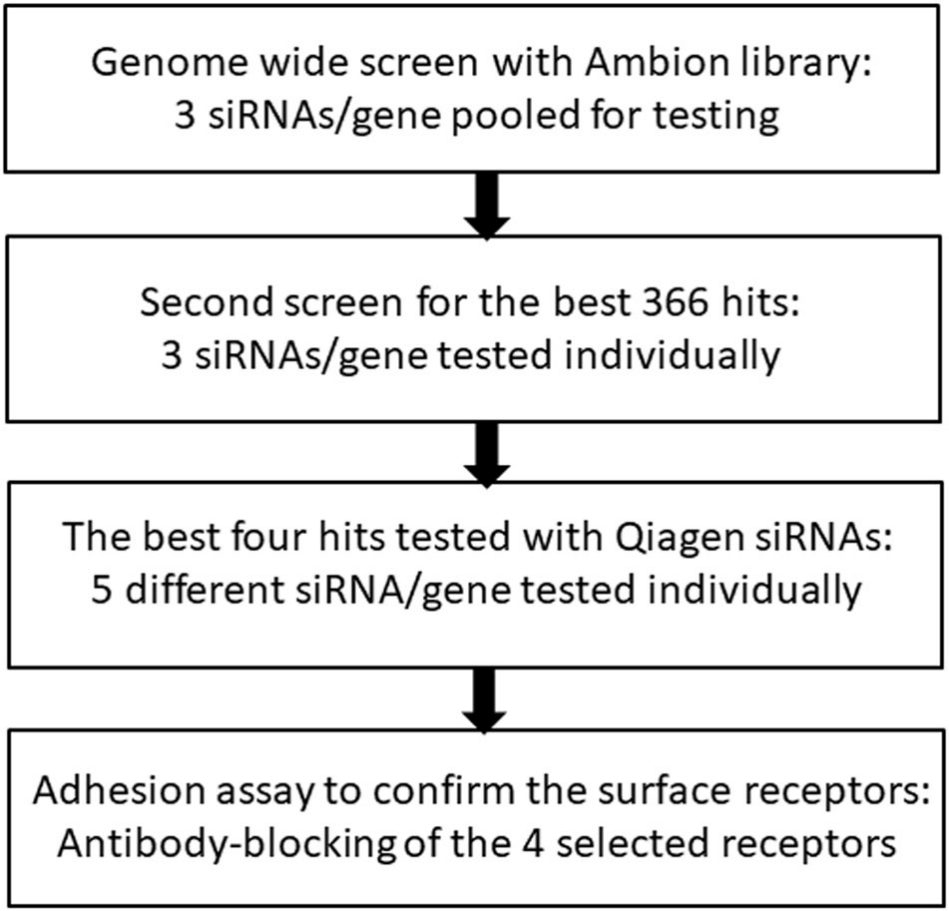
Workflow of a high-throughput siRNA silencing assay. The assay was used to screen proteins that might serve as host surface receptors for bacterial binding and uncovered four receptors for the commensal *B. bifidum.*

The screening procedure consisted of three individual screens (Fig. 2). In the first screening round, whole-genome screening was performed and three siRNAs per gene were pooled and used to knock down individually the expression of each gene to reveal putative receptors which are involved in adhesion of *B. bifidum* to epithelial cells. The hits which resulted in more than 30% decrease in adhesion were selected for the second screen, where the three individual siRNAs per gene were tested separately. The best hits from the second screen we further tested with 5 different siRNAs per gene, using a custom designed siRNA library purchased from another manufacturer. Finally, the most promising receptor molecules were validated in an adhesion assay in which specific antibodies were used to block the surface proteins.

### Genome-wide siRNA screen

**Fig. 3:**
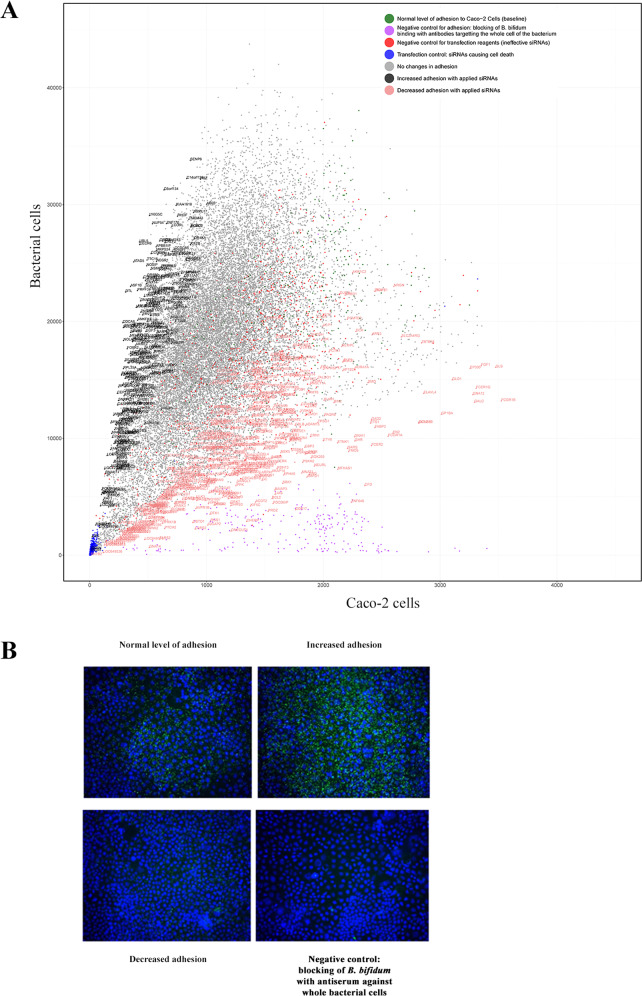
Genome-wide siRNA screen reveals the silencing effect of different siRNAs in Caco-2 cell line to the adhesion level of *B. bifidum.* **A** Results of the genome-wide siRNA screen. The samples in which siRNAs didn´t affect the bacterial adhesion are indicated as grey and normal baseline adhesion without any siRNAs is indicated as green. Increased and decreased adhesion with specific siRNAs are indicated with light purple and pink, respectively. Dark purple dots indicate the control samples in which the bacterial surface proteins were blocked with the antiserum produced against whole *B. bifidum* cells (no gene name on the dots). The samples with ineffective siRNAs (to test the effect of transfection reagents) are indicated with red dots and the samples with death siRNAs (transfection efficiency controls) are indicated as bright blue dots. **B** Microscopic images of representative wells with *B. bifidum* adhered to siRNA-transfected Caco-2 cells and controls. The nuclei of Caco-2 cells are DAPI-stained and the *B. bifidum* cells are immunostained using the antiserum produced against whole bacterial cells and a secondary antibody with Alexa448 label and these cells appear blue and green, respectively.

We used the Ambion Silencer Select v4.0 siRNA library to perform a knock-down screen of gene expression in Caco-2 cell line, to explore binding receptors for *B. bifidum* DSM20456. The arrayed genome-wide siRNA library contains 64,755 siRNA duplexes covering 21,585 genes with three duplexes per gene. The three siRNA sequences per gene, were pooled into the same well for the transfection of Caco-2 cells. Positive and negative controls for transfection and bacterial adhesion were added on every 384-well plate. As each gene was targeted individually, the screen included in total 57 plates with 384 wells and 21 822 samples (wells). Subsequently, transfected Caco-2 cells were grown, *B. bifidum* was allowed to adhere to the enterocytes and the level of adhesion was calculated by comparing the ratio of ALEXA 488 -stained bacteria and DAPI-labelled nuclei of enterocytes (Figs. 1 and 3). The samples were analyzed by using high-throughput microscope imaging and signal quantification. The control samples gave expected results; transfection reagents did not affect the growth of the cell line and bacteria adhered to enterocytes transfected with ineffective siRNAs similarly as compared to the untreated samples. Also as expected, antiserum against whole *B. bifidum* cells blocked the bacterial adhesion indicating that adhesion under the assay conditions is specific and dependent on bacterial cell-surface molecules. In the siRNA transfected samples, *B. bifidum* adhesion was decreased for more than 70% in 16 samples, 50–70% in 36 samples and 25–50% in 449 samples as compared to the normal level of adhesion to enterocytes without siRNA transfection (Fig. 3A., Supplementary Fig. 2, Supplementary Table 1). The genes, whose targeting by siRNA decreased the bacterial adhesion by more than 30 %, in total 376 genes (Supplementary Table 2), were considered as positive hits. Interestingly, in a substantial number of samples, the adhesion increased as compared to the baseline (Fig. 3, Supplementary Fig. 2). The targeted genes included for example transcriptional regulators and thus, the expression of binding receptors could have been affected in these samples too. However, we didn´t study these genes in detail, but chose to continue the survey only with the siRNA hits that decreased adhesion, which we considered as potential candidates for the direct identification of host binding receptors.

### Validation siRNA screens

In order to confirm the results of the genome-wide screen, we next targeted all the genes whose siRNA knock-down decreased the bacterial adhesion with at least 30 %. In the second screen, we targeted 366 genes by using the three different siRNAs per gene separately. The adhesion was decreased in case of 78 out of 366 genes when siRNAs were tested separately. The criterium for a positive result was that 2/3 of the separated siRNAs for a specific gene showed a positive effect in decreased bacterial adhesion. A second validation screen was carried out, using a custom designed siRNA library from (Qiagen), containing five individual siRNAs per gene for each of the 78 “hit” genes from the previous screens. A different siRNA supplier was used in this screen to exclude the possible supplier effect and to confirm the decreased adhesion seen with previous screens.

**Fig. 4:**
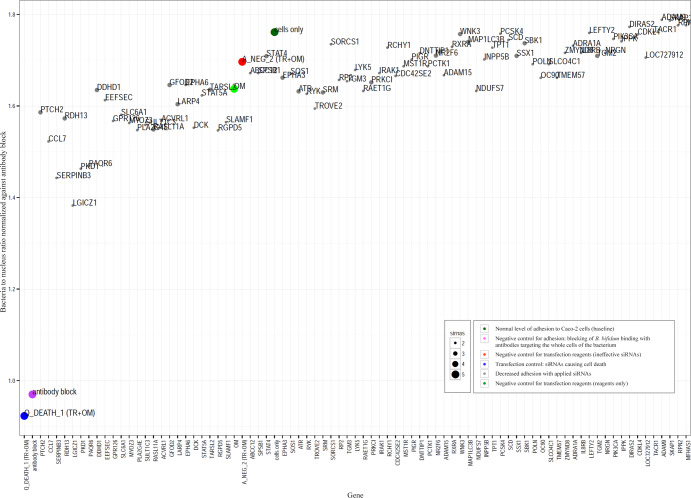
Results of the validation siRNA screen. The normal baseline adhesion without any siRNAs is indicated as dark green. Decreased adhesion with specific siRNAs is indicated with grey. Dark purple dots indicate the control samples in which the bacterial surface proteins were blocked with the antiserum produced against whole *B. bifidum* cells. The samples with ineffective siRNAs (to test the effect of transfection reagents) are indicated with red and light green dots and the samples with death siRNAs (transfection efficiency controls) are indicated as bright blue dots. Y axis presents bacteria to nucleus ratio normalized against antibody block control and gene abbreviations are given on X axis.

In the last round of siRNA screening, the silencing of SERPINB3, LGICZ1, PKD1 and PAQR6 expression in the Caco-2 cells resulted in decreased adhesion of *B. bifidum* to the cell line most as compared to baseline adhesion (Fig. 4), and two out the five used siRNAs resulted in decreased adhesion. For the other 74 genes, siRNA silencing didn´t result in decreased adhesion or resulted in more modest decrease as compared to the aforementioned four genes. We decided to continue to further experiments with the four genes, whose knock down resulted in clear and distinctive decrease in adhesion (Fig. 4). Three out of these four genes encode for extracellular or plasma membrane proteins; LGICZ1 for Ligand-Gated Ion-Channel receptor L2, PKD1 for Polycystic Kidney Disease -associated Protein and PAQR6 for Progestin and AdipoQ receptor Family member IV. SERPINB3 codes for serine protease inhibitor SERPIN B3, which is mainly found in the cytosol.

### Validation of the binding receptors by using antibody blocking

**Fig. 5:**
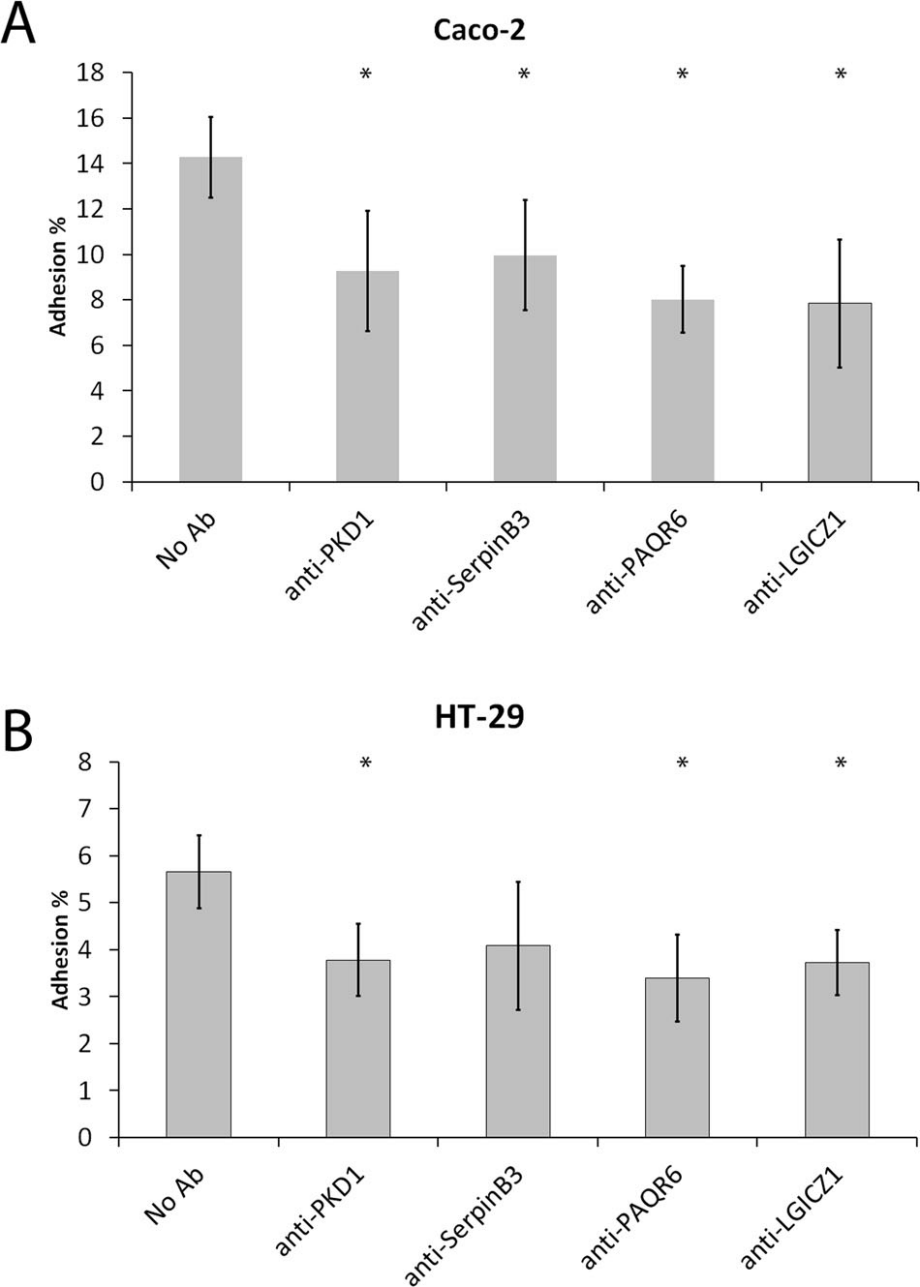
Results of the adhesion blocking assay. Adhesion of *B. bifidum* to Caco-2 (**A**) and HT-29 (**B**) cell lines and the effect of antibody-blocking of the four specific receptors on adhesion level. The results of 5 technical replicates (parallel wells) from the representative experiments are expressed as means ± standard deviations. Significant reductions (*P* < 0.05) in the adhesion in the inhibition assays as compared to the basic adhesion assay are indicated with asterisks. A pairwise Student’s t-test was used to determine the significant difference (*P* < 0.05).

In order to further validate SERPINB3, LGICZ1, PKD1 and PAQR6 as binding receptors for *B. bifidum* we used specific antibodies against these proteins to block the adhesion of bacteria to them. In addition to the Caco-2 cell line, which was used in the screening assay, we also used another enterocyte cell line, HT-29 in the validation. When antibodies against SERPINB3, LGICZ1, PKD1 and PAQR6 were used to block the receptors, the adhesion of *B. bifidum* to Caco-2 cells decreased significantly in all cases (*p* < 0.05, Fig. 5A). Repeating the experiment, using the HT-29 cell line instead, confirmed the results for LGICZ1, PKD1 and PAQR6, as their blocking significantly reduced *B. bifidum* adhesion (*p* < 0.05). The antibody against the SERPINB3 did also decrease the adhesion to HT-29, but the change wasn´t statistically significant. Thus, the binding receptors identified in the siRNA screen were validated also at protein level.

Next, we performed the adhesion assay with antibody blocking of the selected four receptors by using additional four other *Bifidobacterium* species/strains as well as with *Lacticaseibacillus rhamnosus* GG, which is a probiotic strain studied extensively in the past, to study whether the receptors identified for *B. bifidum* DSM20456 serve as binding receptors also for other adherent strains. Our results showed that antibody blocking of the selected four proteins decreased the adhesion of the other species and strains too in a strain-dependent manner i.e. each protein seemed to act as a receptor for at least one of the tested additional strains (Supplementary Fig. 1). The results reinforce the previous observations that adhesion properties (and molecules) are strain specific and consequently, the host receptors to which bacteria bind differ from strain to strain.

## Discussion

In this study we developed a high-throughput siRNA silencing assay to screen proteins that might serve as host surface receptors for bacterial binding and uncovered four receptors for the commensal *B. bifidum*. Bifidobacteria are members of normal gut microbiota and they are regarded as health-promoting bacteria and for that they are widely used as probiotics^10^. However, their interactions with the human host are mostly uncovered at a molecular level. Our study showed that *B. bifidum* binds to several surface proteins on human enterocytes, providing the first insights into the direct bacterium-host communication mechanisms.

Gene silencing through siRNAs has enabled genome-wide functional screens in cultured cells and in vivo in model organisms. Whole genome siRNA screening has previously been used to identify new genes, or gene networks, that are involved in a wide variety of biological processes. These networks are involved in for example signal transduction, cell viability, cell or organelle morphology, organelle or protein localization and/or function, drug resistance, and responses of host cells to pathogens^25–27^.

In this study we first performed a genome-wide screen with 21,822 samples in total. Those genes whose inhibition by siRNA decreased the bacterial adhesion by more than 30 %, were defined as positive hits. The selection criteria produced 366 genes to be tested separately with each siRNA. In the study of Moser et al.^28^ a genome-wide siRNA screen was used to identify host factors necessary for growth of the parasite *Toxoplasma gondii*. Their preliminary screen identified 34 genes whose inhibition by siRNA lowered parasite replication more than 50%. In another study, in which a genome-wide siRNA screen was used to identify the retromer as a cellular entry factor for human papillomavirus, 216 genes were selected for a validation screen based on the preliminary screen^29^. In general, genome-wide siRNA screening seems to yield reasonable numbers of genes for further validation and can be considered as a viable approach in surveying host-microbe interaction mechanisms.

In our last round of siRNA screening, four proteins, SERPINB3, LGICZ1, PKD1 and PAQR6, were identified as potential receptors, as their siRNA targeting significantly decreased adhesion of *B. bifidum* to a cell line as compared to baseline adhesion. All these receptor candidates have been found to be located on the surface of the host cells according to the GeneCards database (www.genecards.org). Validation of the four proteins as binding receptors was obtained by blocking of them with specific antibodies in adhesion experiments, which showed significantly decreased adhesion of *B. bifidum* to Caco2 and HT-29 intestinal cell lines for all four, or three out of the four proteins, respectively. Based on these results, *B. bifidum* has several specific binding receptors on the surface of intestinal epithelial cells. Our study provides the first insights into the direct communication of *B. bifidum* with the host epithelial cells and although further studies on the downstream signaling pathways after binding to the receptors were beyond the reach of this study, the obtained results provide intriguing prospects on microbe-host interactions when considering the known biological functions of the revealed receptors.

SERPINB3 belongs to the family of serine peptidase inhibitors that inhibit their target protease by forming an irreversible covalent complex with it^30^. These endogenous protease inhibitors have broad biological functions. SERPINB3 expression has been reported to be elevated in inflammatory diseases and cancer, but its expression and function is still largely unclear in normal epithelial tissues^30^. According to the Human Protein Atlas (proteinatlas.org), SERPINB3 is mainly localized to the cytosol, but also to the plasma membrane. In general, extracellular serpins have been described to regulate the proteolytic cascades linked to blood clotting, inflammatory and immune responses and tissue remodeling^31^. SERPINB3 is thought to modulate epidermal cell homeostasis and modulate immune responses in skin^30^, and its functions could be similar in the intestinal epithelium. Putatively, the binding of *B. bifidum* to SERPINB3 could lead to the modulation of regulatory functions of the protein.

The polycystic kidney disease 1 (PKD1) gene encodes the membrane bound protein polycystin 1, which is a C-type lectin and has multiple cell recognition domains, and thus the binding of *B. bifidum* to this molecule seems plausible. Polycystin-1 forms a complex with polycystin-2 (PKD1-PKD2 complex) to form a transient receptor potential channel, where PKD1 is proposed to be a sensor of chemical and mechanical force stimuli, and PKD2 a calcium ion channel^32^. Mutations of the PKD1 gene are involved in polycystic kidney disease, but the role of polycystins as mechanosensor molecules still needs to be clarified. The current knowledge suggests that PKD1 stimulation could affect proliferation, differentiation and apoptosis of cells^33^, and it is intriguing to speculate that *B. bifidum* could affect these processes by binding to the receptor.

PAQR6 belongs the family of the class II progestin and adipoQ receptor. This family contains nonclassical progesterone signaling molecules also called membrane progestin. Progesterone and it´s receptors are well-studied in the nervous system, because of their role in regulating gonadotropin-releasing hormone (GnRH) release. In addition to the classical progestin receptor, progesterone exerts effects through multiple non-classical receptors^34^. Porcine PAQR6 is showed to be even more expressed in small intestinal than in other tissues^35^. Zhou et al.^36^ reported that progesterone treatment increased intestinal trans-epithelial electrical resistance (TER) in primary human colon tissues and Caco-2 cells in vitro, through upregulating tight junction protein occludin expression. It has been shown that bifidobacteria could increase intestinal barrier function in vitro and in murine inflammation models^37^, and the possibility that binding of *B. bifidum* to progesterone receptors affect the mucosal permeability warrants further studies.

LGICZ1 is a zinc-activated ligand-gated ion channel that defines a new subgroup of the cysteine-loop superfamily of ligand-gated ion channels. Very little is known about LGICZ1 and the function of the protein is still unknown^38^, but it shares partial homology with serotonin receptors 5HT3A and 5HT3B^39^. Whether LGICZ1 could be involved in serotonergic signalling is unknown, but it might be an interesting study target. In this line, gut microbiota is known to be able to modulate the GI serotonergic system^40^, and *Bifidobacterium dentium* is capable of regulating key components of the serotonergic system in multiple host tissues^41^.

To our knowledge, this is the first study to search for specific surface receptor molecules on the human intestinal epithelial cells for gut commensal species by using genome-wide gene silencing screening. Four binding receptors, PKD1, SERPINB3, PAQR6 and LGICZ1, were identified and antibodies produced against them reduced adhesion of *B. bifidum* to the epithelial cell lines. Also, the blocking of these proteins reduced the adhesion of other *Bifidobacterium* species and *L. rhamnosus* in a strain-dependent manner showing that also other species and strains can bind to these proteins and share receptors, although none of the other tested strains seemed to use all of the four receptors identified for *B. bifidum*. Furthermore, antibody-blocking of the receptors didn´t block the adhesion completely indicating that there are also other receptor molecules.

Bacterial pili are known to bind effectively to the carbohydrate structures of glycoproteins, which are the main components of secreted intestinal mucus and together with glycolipids from the glycocalyx i.e. array of glycosylated biomolecules expressed on the membrane of epithelial cells^42^. Bifidobacteria, including the investigated *B. bifidum* strain, adhere effectively to mucus^15^ and their adhesion to the mucus-deficient enterocyte cell lines Caco-2 and HT-29 is also likely to be mediated to a large extent by their interaction with the host´s glycosylated biomolecules. Involvement of pili in bifidobacterial adhesion has been for *B. bifidum* and *Bifidobacterium breve*^17,43^. For example, the *B. bifidum* PRL2010 genome harbors three pilus clusters^17^. However, bifidobacteria use also moonlighting proteins as adhesin-like factors^44^. In this regard, surface-exposed glycolytic enzymes, including transaldolase from *B. bifidum* and enolase from *Bifidobacterium animalis*, bind to mucin and plasminogen, respectively^45,46^. Other surface-exposed moonlighting proteins, including the chaperone DnaK from *B. animalis* and the elongation factor Tu from *Bifidobacterium longum* showed high affinity for human plasminogen in vitro and have been proposed as mediators of intestinal attachment^45,47^. Also, the probiotic strain *Lacticaseibacillus rhamnosus* GG, which has been isolated from the human intestine, is known to use both pili and moonlighting proteins to adhere the intestinal epithelium^19,48^ illustrating the multifaceted adhesion mechanisms of intestinal bacteria.

In our genome-wide siRNA knock-down of host proteins to identify binding sites for *B. bifidum* the best four hits were structural proteins, but our screen revealed decreased adhesion also when glycosylating enzymes glycosyltransferase 1 domain (GLT1D1, 34%) or glucosidase, alpha; acid (Pompe disease, glycogen storage disease type II) (9,5%) were knocked down (Fig. 3, Supplementary Fig. 2, Supplementary Table 1). However, the knock down of only GLT1D1 decreased the adhesion of *B. bifidum* by more than 30% and was included in the second siRNA screen, where it´s knock down decreased the adhesion in average by 24% (Supplementary Tables 1 and 2) and thus, wasn´t included in the third siRNA screen. The results indicate, however, that enterocyte glycosylation activity may also affect bacterial binding, but we didn´t investigate this further as we continued with the siRNA hits that yielded more than 30 % decrease in the bacterial adhesion. Finally, antibody blocking of the four proteins, SERPINB3, LGICZ1, PKD1 and PAQR6, decreased the adhesion of *B. bifidum* to the enterocyte cell lines by approximately 30–45% and 28–40% in Caco-2 and HT-29 cells, respectively. It is comprehensible that the knock-down of individual proteins can decrease the adhesion only to a minor part, as binding to other proteins and glycocalyx still remains unaffected.

Three out of the four proteins that were found to be receptors for *B. bifidum* binding, PKD1, SERPINB3, PAQR6, are involved in the regulation of cellular processes related to proliferation, differentiation, and apoptosis as well as inflammation and immunity. On the other hand, in vitro and in vivo animal models have shown that *B. bifidum* is able to reduce epithelial apoptosis in necrotizing enterocolitis^12^, reinforce epithelial barrier function^14^, induce the secretion on anti-inflammatory cytokine IL-10 from enterocytes^49^, inhibit inflammatory responses in enteric glial cells^50^ and ameliorate intestinal inflammation^51^. Our study may provide leads to the first steps in the mechanistic cascade of *B. bifidum*-host interactions leading to regulatory effects in the epithelium, and the results on binding receptors for the species may partly explain how this commensal bacterium is able to promote intestinal homeostasis.

We acknowledge that the developed assay has some limitations. Firstly, the cell lines used in the assay are derived from cancer cells and therefore, their use as a model for healthy epithelium has limitations and the translation of results to reflect microbe-host interactions in vivo in a physiological state remains speculative. Furthermore, in the siRNA knock-down experiments, the cell lines were grown only for three days and thus, full differentiation of the Caco-2 cells wasn´t reached. Another limitation is that immunofluorescence staining, which was found to be the most practical and reliable method for bacterial quantification, necessitates prior production of antiserum/antibodies against the strain to be investigated. This may limit the application of the technique to a large number of strains even if targeted siRNA libraries would be used to facilitate the performance of the screening assay with a large number of bacterial strains. The growing of cell lines as spots on plastic dish to enable even more high density siRNA experiments has been described previously^52^. However, despite of several attempts we couldn´t grow Caco-2 cell line as regular spots and the use of plate format in the assay resulted in a laborious protocol. Furthermore, regarding the adhesion of bacteria to intestinal epithelium, glycocalyx of the host cells is expected to play a major role. In the gene-by-gene knock-down, we observed rather modest decrease in bacterial adhesion when glycosylation enzymes were knocked down individually (see above), which indicates that the overall glycocalyx may not be affected drastically by an individual enzyme. Simultaneous knock-down of several glycosylation enzymes could be considered in the future studies. However, in many respects these cell lines provide an excellent in vitro model for the investigation of host-microbe interactions and the possibility to discover unexpected protein level interactions could be considered as the main strength of genome-wide siRNA knock down screening.

## Material and methods

### Bacterial strains, colonic epithelial cell lines and culture conditions

*Bifidobacterium bifidum* type strain (DSM20456) was cultivated in De Man, Rogosa, and Sharpe broth (MRS; Difco) supplemented with 500 μg ml−1 L-cysteine (Sigma-Aldrich) and were incubated at 37 °C in an anaerobic chamber. The human intestinal cell lines Caco-2 and HT-29 were obtained from DSMZ and were grown at 37 °C in a 95% air–5% CO2 atmosphere. Caco-2 cells were grown in RPMI 1640 medium (Sigma-Aldrich) supplemented with 2 mM L-glutamine (Lonza), 20% heat-inactivated (30 min at 56 °C) fetal calf serum (Integro B.V), 100 U ml−1 penicillin-streptomycin (Lonza), and 1% (vol/vol) nonessential amino acids (Lonza), and HT-29 cells were grown in McCoy 5 A medium (Lonza) supplemented with 10% fetal calf serum and 100 U ml−1 penicillin-streptomycin.

### Transfection of siRNAs to Caco-2 cells

siRNAs used in this study were obtained from Ambion (Silencer Select Human Druggable Genome V4 siRNA library) and QIAGEN (custom designed library) and three or five different siRNA sequences were used for each gene. In the primary whole genome screen, Ambion whole-genome siRNA library was used and all three siRNA sequences for each gene were pooled to the same well. In the secondary screen all three siRNAs were tested separately in different wells. A “scrambled” siRNA that doesn’t target any gene in the human genome (Allstars negative siRNA, Qiagen) was used as a negative control and cell death siRNAs (Allstars positive siRNA, Qiagen) were used as positive control for transfection. A few wells with only cells were used to compare this phenotype to the one from the negative control. The final volume on the assay plates was 25 μL/well (including transfection reagent, siRNA and cell suspension). The final concentration of siRNAs was 10 nM. The stock concentration for control siRNAs was 10 μM, whereas the stock concentration for library siRNAs was 2.5 μM. 25 nL of each control siRNA and 100 nL of library siRNAs were transferred to the assay plates into at least 16–32 wells per plate. Then the transfection reagent was dispensed into the assay plate prior to adding 20 μL of Caco-2 cells (25,000 cells/mL) on the plate. The assay plates were placed into a humidified 37 °C cell incubator with 5% CO_2_ for incubation for 96 h.

### Adhesion of B. bifidum to siRNA transfected Caco-2 cells

The adhesion assay was carried out with an established protocol^15,53,54^ with minor modifications. In essence, bacteria were collected from growth medium by centrifugation, and washed with RPMI 1640 without supplements. The optical density (A600 = 0.25) was adjusted to the same culture medium. Caco-2 cells were blocked using 1% bovine serum albumin (BSA) and 0,005% Tween20 in phosphate buffered saline (PBS) to prevent the unspecific binding of bacteria to the plastic walls of the wells. Blocking was done at 37 °C under an oxic atmosphere in an incubator supplemented with 5% CO_2_ for one hour. Then bacterial suspension (30 μl) was added to the wells and incubated in a CO_2_ incubator at 37 °C for 1 h. Bacterial suspension with the addition of serum produced against whole *B. bifidum* cells was used as a control to block all adhesins of the bacterium. Polyclonal rabbit antiserum against *B. bifidum* was produced at the Laboratory Animal Centre, University of Helsinki and immunization was carried out following standard procedures and by using^15^ 100 μl of Freund’s complete adjuvant (first injection) or Freund’s incomplete adjuvant (three booster injections) together with 100 μl volume of the cell suspension (10^9^ ml^−1^ in PBS) to inoculate the rabbit once every 3 weeks, and the animal was sacrificed and blood collected 10 days after the third booster injection. After incubation, nonadherent bacteria were removed by washing the wells three times with PBS. After washing, the cells were fixed using 4% paraformaldehyde (PFA) overnight at + 4 C.

### Immunofluorescence staining and high-throughput microscope imaging

The cells were washed three times with PBS to remove PFA, prior to adding 10 ul per well of 1:1000 PBS-diluted primary antibody produced against whole *B. bifidum* cells. The antibody was allowed to bind to the bacteria for one hour at room temperature prior to washing the cells three times with PBS to remove unbound antibody. Next, the cells were stained for one hour at room temperature with the ALEXA 488 -labeled goat anti-rabbit IgG (Invitrogen) and 4′,6-diamidino-2-phenylindole (DAPI) in PBS (each at 1 μg ml^−1^). Unbound stains were removed by 3 washes with PBS.

ScanR (Olympus), a modular epifluorescence microscope designed for fully automated high-content image acquisition was utilized for imaging the plates. Several different fields of view per well were analyzed using ScanR Analysis software 1.3.0.3.2.3 (Olympus). An automated background correction and thresholding was applied to each channel. The cell nuclei stained with Dapi (wavelength 358 nm/361 nm) were defined and counted as main objects and ALEXA 488 -stained (wavelength 495 nm/519 nm) adherent bacteria were defined and counted as subobjects. ALEXA 488-positive bacteria were analyzed for their total size, number and intensity. The average and standard deviation, for the number of objects as well as for the mean intensity of the objects, were calculated for each sample. The number of adherent bacteria per each cell was calculated as the ratio of the total amount of Alexa 488-positive bacteria divided by the total amount of Dapi positive nuclei for each well.

### Bacterial adhesion to enterocytes with antibody-blocking of specific receptors

Caco-2 and HT-29 cells were cultivated on 96-well tissue culture plate (30,000 cells/well; Nunc) for 4 days and washed once with culture medium before the adhesion assay. *B. bifidum* cells were metabolically radiolabeled by cultivating bacteria with 10 μl/ml [5′-3H] thymidine (17.0 Ci/mmol; Perkin Elmer). The adhesion assay was performed with established protocols^15,54^ and as detailed above. Briefly, after cultivation, bacteria were collected by centrifugation and washed with RPMI without supplements. The optical density was adjusted (OD600 nm = 0.25) to the same culture medium was used for washing. Bacteria (100 μl) were incubated with the IECs in a CO_2_ incubator at 37 °C for one hour, and the non-adherent bacteria were removed by washing the wells three times with PBS. To assess the effect of blocking specific receptors to the bacterial adhesion, antibodies against PKD1 (Pierce/Thermo Scientific), SerpinB3 (Abcam), PAQR6 (Pierce/Thermo Scientific) and LGIZCI (Santa Cruz Biotechnology) were added in 1:100 to enterocytes at the same time with the bacterial suspensions. Bacteria bound to Caco-2 cells were lysed with 1% SDS–0.1 M NaOH by incubating at 37 °C for 12–14 h. The radioactivity of the suspension was measured by liquid scintillation. Five parallel wells (i.e., technical replicates) were used in each experiment. The percent of bacterial adhesion was determined by calculating the ratio between the radioactivity of the adherent bacteria and that of the added bacteria.

### Bioinformatics and data analysis

Data generated in the primary screen with the whole genome Ambion library plates, read by ScanR microscope was combined with the annotations of library plates. As a part of quality control check, distribution and performance of the controls was analysed per plate with the help of the well signals from wells marked with positive, negative controls and internal controls. For each of the plate, the ratio of the bacterial_green_objects (ALEXA 488) to nuclei blue objects (DAPI) was calculated resulting in bac_nuc_ratio. To take care of plate wise differences, Percent to Negative Control normalization was used^55^ in which the bac_nuc_ratio values were normalized to the median value of wells marked with the scrambled siRNA controls (negative control). The resulting ratios were used to filter and select interesting hits for the secondary screen.

In the secondary screen along with the negative siRNA controls, the bac_nuc_ratio data was normalized to the median value of the wells, where bacterial cells were treated with antiserum to block their adhesion (Antibody block).

The data was processed with the help of R programming language and the plots were generated using GGPlot2 R package.

## Supplementary information

Supplementary figures and tables

## Data Availability

The R scripts used for the data collection and analysis are available on Github repository at https://github.com/potdarswapnil/siRNA.
