# Supplementary material for: Genome-wide siRNA screening reveals several host receptors for the binding of human gut commensal Bifidobacterium bifidum
Source: NPJ Biofilms Microbiomes. 2022 Jun 29;8:50. doi: 10.1038/s41522-022-00312-0 (PMC9243078; doi:10.1038/s41522-022-00312-0)
Supplement: Supplementary file 1 — Supplementary figures and tables [file 41522_2022_312_MOESM1_ESM.pdf]

Supplementary figure 1.

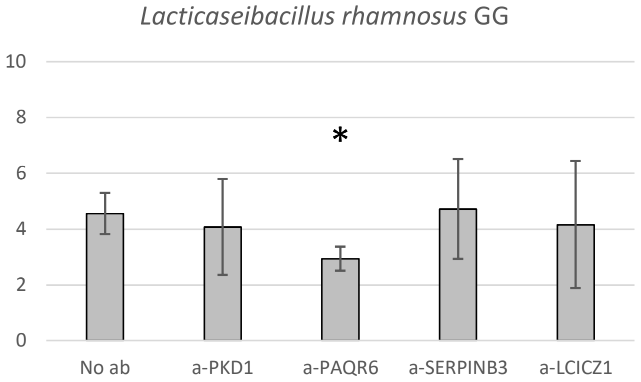


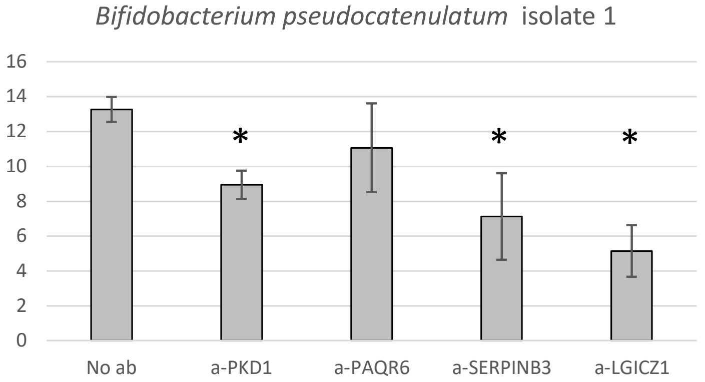


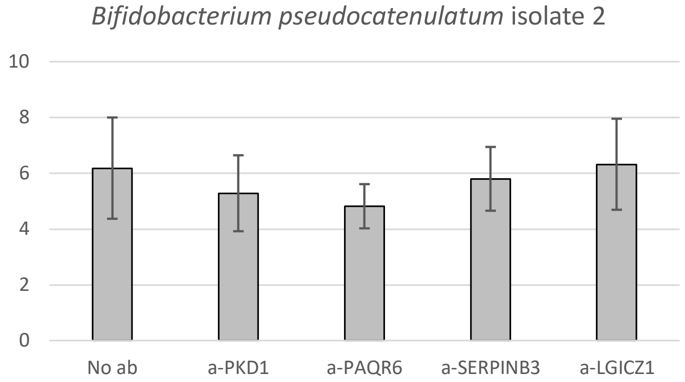


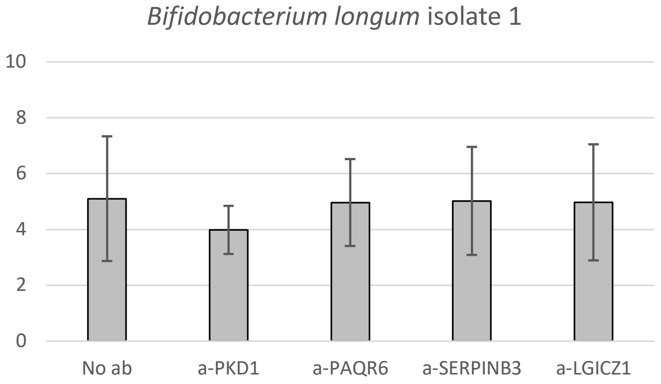


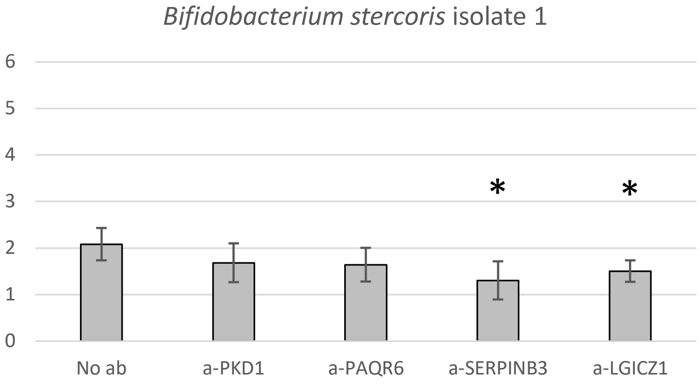


Caco-2 cells were grown as described in the M&M section of the main article. *Lacticaseibacillus rhamnosus* GG was obtained from the DSMZ Culture collection,Germany and the *Bifidobacterium* strains are recent isolates from two healthy adults and they were tentatively identified by 16S rRNA gene sequencing (Jouhten et al 2020 Front Microbiol 11:1663, Hiippala et al 2020 Nutrients 12, 935). All strains were grown and adhesion experiments done similarly to *B. bifidum* (see M&M). The results of 5 technical replicates (parallel wells) from the representative experiments are expressed as means ± standard deviations. Significant reductions ($P < 0.05$) in the adhesion in the antibody blocking assays (a-PKD1, a-PAQR6, a-SERPIN3 and a-LGICZ1) as compared to the basic adhesion assay (No ab) are indicated with asterisks. A pairwise Student’s t test was used to determine the significant difference ($P < 0.05$).

**Supplementary Figure 1**. **The effect of antibody blocking of specific protein on the adhesion of different bacterial strains to the Caco-2 cell line.** Main article by Kainulainen et al. “Genome-wide siRNA screening reveals several host receptors for the binding of human gut commensal *Bifidobacterium bifidum*”

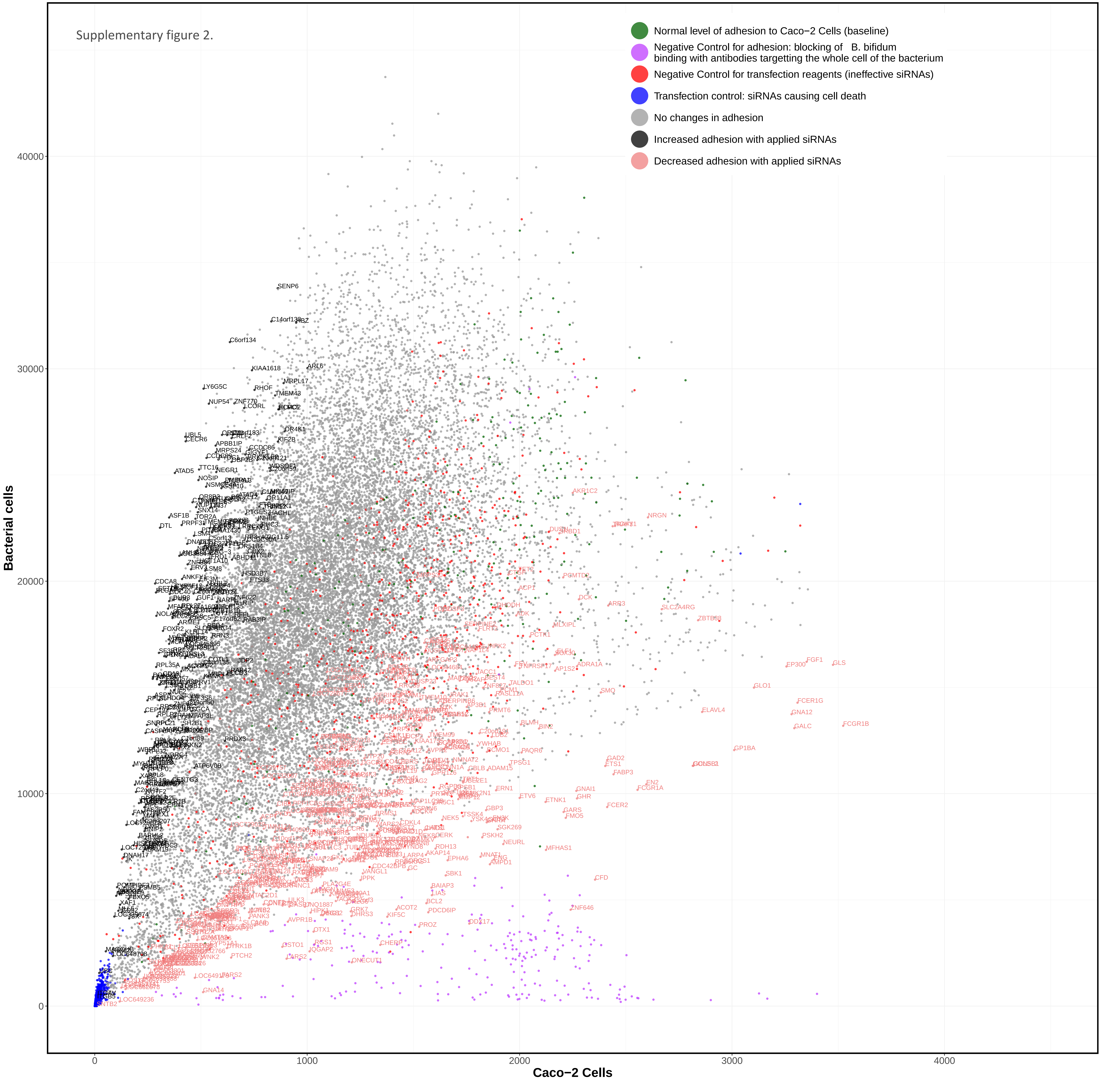


Supplementary figure 2.

# Supplementary table 1.

| | src well r | DWell | Dapi_Objects | Dapi objects intensity mea | baku_green_objects | baku green intensity | bac_nuc_ratio | bac_nuc_ratio_neg_ratio | Plate_Id | content | Plate.Nam | Plate.Nam | Plate.Nam | Plate.ID 1 | Plate.ID 2 | Plate.ID 3 | RefSeq.Acc | RefSeq.Ac | RefSeq.Ac | Gene.Sym | Gene.Sym | Gene.Sym | Full.Gene.Name | Full.Gene. | Full.Gene. | Gene.ID 1 | Gene.ID 2 | Gene.ID 3 | siRNA.ID | siRNA.ID | siRNA.ID_3 |
|---|---|---|---|---|---|---|---|---|---|---|---|---|---|---|---|---|---|---|---|---|---|---|---|---|---|---|---|---|---|---|---|
| 10529 | G24 | G1 | 2096 | 954 | 7516 | 142,1 | 3,6 | 0,407 | H5A034 C | cells | Hm Drgbl | Hm Drgbl | Hm Drgbl | CPE002EB | CPE002EO | CPE002FR | | | | | | | | | | | | | | | |
| 2920 | J23 | J24 | 2071 | 752,6 | 10358 | 475,9 | 5 | 0,588 | H5A034 C | cells | Hm Drgbl | Hm Drgbl | Hm Drgbl | CPE002DP | CPE002EF | CPE002F5 | | | | | | | | | | | | | | | |
| 21737 | J24 | J1 | 520 | 910,4 | 2950 | 414,8 | 5,7 | 0,6 | H5A034 C | cells | Hm Gen E | Hm Gen E | Hm Gen E | CPE002JU | CPE002O9 | CPE002KP | | | | | | | | | | | | | | | |
| 10601 | J24 | J1 | 2141 | 959,3 | 13206 | 159,3 | 6,2 | 0,701 | H5A034 C | cells | Hm Drgbl | Hm Drgbl | Hm Drgbl | CPE002EB | CPE002EO | CPE002FR | | | | | | | | | | | | | | | |
| 7912 | J23 | J24 | 2021 | 643,2 | 12601 | 368,7 | 6,2 | 0,947 | H5A034 C | cells | Hm Drgbl | Hm Drgbl | Hm Drgbl | CPE002E4 | CPE002EV | CPE002FG | | | | | | | | | | | | | | | |
| 617 | J24 | J1 | 3251 | 1989 | 21404 | 365,2 | 6,6 | 0,964 | H5A034 C | cells | Hm Drgbl | Hm Drgbl | Hm Drgbl | CPE002E0 | CPE002EZ | CPE002FK | | | | | | | | | | | | | | | |
| 7913 | J24 | J1 | 1681 | 665,3 | 11067 | 409,4 | 6,6 | 1,008 | H5A034 C | cells | Hm Drgbl | Hm Drgbl | Hm Drgbl | CPE002E4 | CPE002EV | CPE002FG | | | | | | | | | | | | | | | |
| 16360 | J23 | J24 | 1991 | 1023 | 13913 | 189 | 7 | 0,642 | H5A034 C | cells | Hm Gen E | Hm Gen E | Hm Gen E | CPE002JG | CPE002NV | CPE002KB | | | | | | | | | | | | | | | |
| 11752 | J23 | J24 | 2289 | 982,4 | 16282 | 108,2 | 7,1 | 0,634 | H5A034 C | cells | Hm Gen E | Hm Gen E | Hm Gen E | CPE002JA | CPE002NJ | CPE002JZ | | | | | | | | | | | | | | | |
| 1768 | J23 | J24 | 2681 | 877,9 | 19165 | 372,6 | 7,1 | 0,743 | H5A034 C | cells | Hm Drgbl | Hm Drgbl | Hm Drgbl | CPE002FW | CPE002G0 | CPE002G4 | | | | | | | | | | | | | | | |
| 10528 | G23 | G24 | 2137 | 986,8 | 15259 | 177,4 | 7,1 | 0,802 | H5A034 C | cells | Hm Drgbl | Hm Drgbl | Hm Drgbl | CPE002EB | CPE002EO | CPE002FR | | | | | | | | | | | | | | | |
| 2921 | J24 | J1 | 1853 | 703,8 | 13409 | 558,6 | 7,2 | 0,847 | H5A034 C | cells | Hm Drgbl | Hm Drgbl | Hm Drgbl | CPE002DP | CPE002EF | CPE002F5 | | | | | | | | | | | | | | | |
| 4000 | G23 | G24 | 2789 | 835 | 20488 | 388,7 | 7,3 | 0,649 | H5A034 C | cells | Hm Drgbl | Hm Drgbl | Hm Drgbl | CPE002DS | CPE002EI | CPE002F8 | | | | | | | | | | | | | | | |
| 1696 | G23 | G24 | 2914 | 907,9 | 21382 | 439 | 7,3 | 0,764 | H5A034 C | cells | Hm Drgbl | Hm Drgbl | Hm Drgbl | CPE002FW | CPE002G0 | CPE002G4 | | | | | | | | | | | | | | | |
| 2848 | G23 | G24 | 2260 | 753,6 | 16556 | 595,5 | 7,3 | 0,859 | H5A034 C | cells | Hm Drgbl | Hm Drgbl | Hm Drgbl | CPE002DP | CPE002EF | CPE002F5 | | | | | | | | | | | | | | | |
| 545 | G24 | G1 | 2501 | 1964 | 18135 | 331,4 | 7,3 | 1,066 | H5A034 C | cells | Hm Drgbl | Hm Drgbl | Hm Drgbl | CPE002E0 | CPE002EZ | CPE002FK | | | | | | | | | | | | | | | |
| 616 | J23 | J24 | 2699 | 1942 | 19703 | 360,5 | 7,3 | 1,066 | H5A034 C | cells | Hm Drgbl | Hm Drgbl | Hm Drgbl | CPE002E0 | CPE002EZ | CPE002FK | | | | | | | | | | | | | | | |
| 161 | G24 | G1 | 656 | 1765 | 4937 | 644,8 | 7,5 | 0,638 | H5A034 C | cells | Hm Drgbl | Hm Drgbl | Hm Drgbl | CPE002DT | CPE002EJ | CPE002F9 | | | | | | | | | | | | | | | |
| 20129 | G24 | G1 | 1374 | 1174 | 10451 | 108,5 | 7,6 | 0,927 | H5A034 C | cells | Hm Gen E | Hm Gen E | Hm Gen E | CPE002JQ | CPE002O5 | CPE002KL | | | | | | | | | | | | | | | |
| 20201 | J24 | J1 | 901 | 1106 | 6937 | 131,4 | 7,7 | 0,939 | H5A034 C | cells | Hm Gen E | Hm Gen E | Hm Gen E | CPE002JQ | CPE002O5 | CPE002KL | | | | | | | | | | | | | | | |
| 7840 | G23 | G24 | 1772 | 613,4 | 13693 | 396,5 | 7,7 | 1,176 | H5A034 C | cells | Hm Drgbl | Hm Drgbl | Hm Drgbl | CPE002E4 | CPE002EV | CPE002FG | | | | | | | | | | | | | | | |
| 14369 | G24 | G1 | 2275 | 1027 | 18227 | 105,3 | 8 | 0,569 | H5A034 C | cells | Hm Gen E | Hm Gen E | Hm Gen E | CPE002J7 | CPE002NC | CPE002K6 | | | | | | | | | | | | | | | |
| 544 | G23 | G24 | 2667 | 1959 | 21379 | 392,6 | 8 | 1,168 | H5A034 C | cells | Hm Drgbl | Hm Drgbl | Hm Drgbl | CPE002E0 | CPE002EZ | CPE002FK | | | | | | | | | | | | | | | |
| 2080 | G23 | G24 | 2905 | 847,5 | 23760 | 469,9 | 8,2 | 0,713 | H5A034 C | cells | Hm Drgbl | Hm Drgbl | Hm Drgbl | CPE002FX | CPE002G1 | CPE002G5 | | | | | | | | | | | | | | | |
| 4072 | J23 | J24 | 2359 | 793,9 | 19245 | 381 | 8,2 | 0,729 | H5A034 C | cells | Hm Drgbl | Hm Drgbl | Hm Drgbl | CPE002DS | CPE002EI | CPE002F8 | | | | | | | | | | | | | | | |
| 13601 | G24 | G1 | 2062 | 1208 | 16819 | 136,9 | 8,2 | 0,77 | H5A034 C | cells | Hm Gen E | Hm Gen E | Hm Gen E | CPE002JC | CPE002NC | CPE002K4 | | | | | | | | | | | | | | | |
| 4840 | J23 | J24 | 1490 | 922,2 | 12336 | 436,6 | 8,3 | 0,912 | H5A034 C | cells | Hm Drgbl | Hm Drgbl | Hm Drgbl | CPE002DV | CPE002EL | CPE002FP | | | | | | | | | | | | | | | |
| 1312 | G23 | G24 | 2753 | 840,1 | 23092 | 490,7 | 8,4 | 0,771 | H5A034 C | cells | Hm Drgbl | Hm Drgbl | Hm Drgbl | CPE002FV | CPE002FZ | CPE002G3 | | | | | | | | | | | | | | | |
| 1769 | J24 | J1 | 1850 | 803,4 | 15570 | 383,6 | 8,4 | 0,88 | H5A034 C | cells | Hm Drgbl | Hm Drgbl | Hm Drgbl | CPE002FW | CPE002G0 | CPE002G4 | | | | | | | | | | | | | | | |
| 4841 | J24 | J1 | 1929 | 972,6 | 16245 | 430,7 | 8,4 | 0,923 | H5A034 C | cells | Hm Drgbl | Hm Drgbl | Hm Drgbl | CPE002DV | CPE002EL | CPE002FP | | | | | | | | | | | | | | | |
| 3616 | G23 | G24 | 2864 | 1716 | 24532 | 454,5 | 8,6 | 0,827 | H5A034 C | cells | Hm Drgbl | Hm Drgbl | Hm Drgbl | CPE002DR | CPE002EH | CPE002F7 | | | | | | | | | | | | | | | |
| 20200 | J23 | J24 | 1514 | 1207 | 13021 | 157,3 | 8,6 | 1,049 | H5A034 C | cells | Hm Gen E | Hm Gen E | Hm Gen E | CPE002JQ | CPE002O5 | CPE002KL | | | | | | | | | | | | | | | |
| 4384 | G23 | G24 | 2755 | 860,8 | 23879 | 449,3 | 8,7 | 0,734 | H5A034 C | cells | Hm Drgbl | Hm Drgbl | Hm Drgbl | CPE002DU | CPE002EK | CPE002FQ | | | | | | | | | | | | | | | |
| 21736 | J23 | J24 | 408 | 945,1 | 3569 | 635,6 | 8,7 | 0,916 | H5A034 C | cells | Hm Gen E | Hm Gen E | Hm Gen E | CPE002JU | CPE002O9 | CPE002KP | | | | | | | | | | | | | | | |
| 2464 | G23 | G24 | 2790 | 873,1 | 24592 | 435,7 | 8,8 | 0,693 | H5A034 C | cells | Hm Drgbl | Hm Drgbl | Hm Drgbl | CPE002DC | CPE002EE | CPE002F4 | | | | | | | | | | | | | | | |
| 1384 | J23 | J24 | 2313 | 798,9 | 20268 | 441,7 | 8,8 | 0,807 | H5A034 C | cells | Hm Drgbl | Hm Drgbl | Hm Drgbl | CPE002FV | CPE002FZ | CPE002G3 | | | | | | | | | | | | | | | |
| 232 | J23 | J24 | 695 | 1689 | 6218 | 723,4 | 8,9 | 0,757 | H5A034 C | cells | Hm Drgbl | Hm Drgbl | Hm Drgbl | CPE002DT | CPE002EJ | CPE002F9 | | | | | | | | | | | | | | | |
| 10600 | J23 | J24 | 2016 | 946,1 | 17985 | 193,6 | 8,9 | 1,006 | H5A034 C | cells | Hm Drgbl | Hm Drgbl | Hm Drgbl | CPE002EB | CPE002EO | CPE002FR | | | | | | | | | | | | | | | |
| 3688 | J23 | J24 | 2496 | 1573 | 22431 | 427,8 | 9 | 0,865 | H5A034 C | cells | Hm Drgbl | Hm Drgbl | Hm Drgbl | CPE002DR | CPE002EH | CPE002F7 | | | | | | | | | | | | | | | |
| 10216 | J23 | J24 | 2128 | 965,8 | 19406 | 184,2 | 9,1 | 0,875 | H5A034 C | cells | Hm Drgbl | Hm Drgbl | Hm Drgbl | CPE002EA | CPE002EP | CPE002FA | | | | | | | | | | | | | | | |
| 4768 | G23 | G24 | 1862 | 929,4 | 16987 | 452,1 | 9,1 | 1 | H5A034 C | cells | Hm Drgbl | Hm Drgbl | Hm Drgbl | CPE002DV | CPE002EL | CPE002FP | | | | | | | | | | | | | | | |
| 3689 | J24 | J1 | 1945 | 1437 | 17903 | 334,3 | 9,2 | 0,885 | H5A034 C | cells | Hm Drgbl | Hm Drgbl | Hm Drgbl | CPE002DR | CPE002EH | CPE002F7 | | | | | | | | | | | | | | | |
| 12449 | G24 | G1 | 2328 | 1244 | 21740 | 133,5 | 9,3 | 0,699 | H5A034 C | cells | Hm Gen E | Hm Gen E | Hm Gen E | CPE002J5 | CPE002NK | CPE002K1 | | | | | | | | | | | | | | | |
| 2152 | J23 | J24 | 2074 | 823,1 | 19208 | 445,6 | 9,3 | 0,809 | H5A034 C | cells | Hm Drgbl | Hm Drgbl | Hm Drgbl | CPE002FX | CPE002G1 | CPE002G5 | | | | | | | | | | | | | | | |
| 2849 | G24 | G1 | 1444 | 696,6 | 13443 | 632,7 | 9,3 | 1,094 | H5A034 C | cells | Hm Drgbl | Hm Drgbl | Hm Drgbl | CPE002DP | CPE002EF | CPE002F5 | | | | | | | | | | | | | | | |
| 2081 | G24 | G1 | 1595 | 781,6 | 14945 | 433,1 | 9,4 | 0,817 | H5A034 C | cells | Hm Drgbl | Hm Drgbl | Hm Drgbl | CPE002FX | CPE002G1 | CPE002G5 | | | | | | | | | | | | | | | |
| 21665 | G24 | G1 | 222 | 896 | 2077 | 516,6 | 9,4 | 0,989 | H5A034 C | cells | Hm Gen E | Hm Gen E | Hm Gen E | CPE002JU | CPE002O9 | CPE002KP | | | | | | | | | | | | | | | |
| 4457 | J24 | J1 | 2197 | 773,3 | 20767 | 421,9 | 9,5 | 0,802 | H5A034 C | cells | Hm Drgbl | Hm Drgbl | Hm Drgbl | CPE002DU | CPE002EK | CPE002FQ | | | | | | | | | | | | | | | |
| 15592 | J23 | J24 | 1910 | 939 | 18141 | 121,2 | 9,5 | 0,864 | H5A034 C | cells | Hm Gen E | Hm Gen E | Hm Gen E | CPE002JE | CPE002NT | CPE002K9 | | | | | | | | | | | | | | | |
| 8224 | G23 | G24 | 2210 | 728 | 20924 | 415,4 | 9,5 | 0,913 | H5A034 C | cells | Hm Drgbl | Hm Drgbl | Hm Drgbl | CPE002E5 | CPE002EU | CPE002FF | | | | | | | | | | | | | | | |
| 2536 | J23 | J24 | 2552 | 805 | 24872 | 420,5 | 9,7 | 0,764 | H5A034 C | cells | Hm Drgbl | Hm Drgbl | Hm Drgbl | CPE002DC | CPE002EE | CPE002F4 | | | | | | | | | | | | | | | |
| 1000 | J23 | J24 | 1939 | 767,1 | 18824 | 417,6 | 9,7 | 0,919 | H5A034 C | cells | Hm Drgbl | Hm Drgbl | Hm Drgbl | CPE002FU | CPE002FY | CPE002G2 | | | | | | | | | | | | | | | |
| 8297 | J24 | J1 | 1740 | 767,2 | 16791 | 308 | 9,7 | 0,933 | H5A034 C | cells | Hm Drgbl | Hm Drgbl | Hm Drgbl | CPE002E5 | CPE002EU | CPE002FF | | | | | | | | | | | | | | | |
| 14440 | J23 | J24 | 2262 | 1043 | 22175 | 125,4 | 9,8 | 0,698 | H5A034 C | cells | Hm Gen E | Hm Gen E | Hm Gen E | CPE002J7 | CPE002NC | CPE002K6 | | | | | | | | | | | | | | | |
| 233 | J24 | J1 | 781 | 1727 | 7748 | 591,5 | 9,9 | 0,843 | H5A034 C | cells | Hm Drgbl | Hm Drgbl | Hm Drgbl | CPE002DT | CPE002EJ | CPE002F9 | | | | | | | | | | | | | | | |
| 1313 | G24 | G1 | 1706 | 777,1 | 16862 | 417 | 9,9 | 0,908 | H5A034 C | cells | Hm Drgbl | Hm Drgbl | Hm Drgbl | CPE002FV | CPE002FZ | CPE002G3 | | | | | | | | | | | | | | | |
| 5536 | G23 | G24 | 2361 | 828,4 | 23706 | 584,3 | 10 | 0,952 | H5A034 C | cells | Hm Drgbl | Hm Drgbl | Hm Drgbl | CPE002DX | CPE002F2 | CPE002FN | | | | | | | | | | | | | | | |
| 20128 | G23 | G24 | 1101 | 1146 | 11046 | 183,9 | 10 | 1,22 | H5A034 C | cells | Hm Gen E | Hm Gen E | Hm Gen E | CPE002JQ | CPE002O5 | CPE002KL | | | | | | | | | | | | | | | |
| 1385 | J24 | J1 | 2065 | 802,2 | 20886 | 458,9 | 10,1 | 0,927 | H5A034 C | cells | Hm Drgbl | Hm Drgbl | Hm Drgbl | CPE002FV | CPE002FZ | CPE002G3 | | | | | | | | | | | | | | | |
| 8296 | J23 | J24 | 1867 | 710,9 | 18911 | 403,8 | 10,1 | 0,971 | H5A034 C | cells | Hm Drgbl | Hm Drgbl | Hm Drgbl | CPE002E5 | CPE002EU | CPE002FF | | | | | | | | | | | | | | | |
| 5993 | J24 | J1 | 1563 | 816,2 | 15940 | 435,8 | 10,2 | 0,932 | H5A034 C | cells | Hm Drgbl | Hm Drgbl | Hm Drgbl | CPE002DY | CPE002F1 | CPE002FM | | | | | | | | | | | | | | | |
| 3304 | J23 | J24 | 1988 | 1494 | 20404 | 400,1 | 10,3 | 0,638 | H5A034 C | cells | Hm Drgbl | Hm Drgbl | Hm Drgbl | CPE002DC | CPE002EG | CPE002F6 | | | | | | | | | | | | | | | |
| 4001 | G24 | G1 | 1513 | 724,2 | 15590 | 403,1 | 10,3 | 0,916 | H5A034 C | cells | Hm Drgbl | Hm Drgbl | Hm Drgbl | CPE002DS | CPE002EI | CPE002F8 | | | | | | | | | | | | | | | |
| 2153 | J24 | J1 | 2224 | 779,9 | 23170 | 467,5 | 10,4 | 0,904 | H5A034 C | cells | Hm Drgbl | Hm Drgbl | Hm Drgbl | CPE002FX | CPE002G1 | CPE002G5 | | | | | | | | | | | | | | | |
| 11753 | J24 | J1 | 1944 | 878 | 20149 | 118,7 | 10,4 | 0,929 | H5A034 C | cells | Hm Gen E | Hm Gen E | Hm Gen E | CPE002JA | CPE002NJ | CPE002JZ | | | | | | | | | | | | | | | |
| 10144 | G23 | G24 | 2308 | 1067 | 24113 | 171,2 | 10,4 | 1 | H5A034 C | cells | Hm Drgbl | Hm Drgbl | Hm Drgbl | CPE002EA | CPE002EP | CPE002FA | | | | | | | | | | | | | | | |
| 12520 | J23 | J24 | 2338 | 1201 | 24873 | 183,6 | 10,6 | 0,797 | H5A034 C | cells | Hm Gen E | Hm Gen E | Hm Gen E | CPE002J5 | CPE002NK | CPE002K1 | | | | | | | | | | | | | | | |
| 8992 | G23 | G24 | 2781 | 2307 | 29459 | 192,3 | 10,6 | 0,851 | H5A034 C | cells | Hm Drgbl | Hm Drgbl | Hm Drgbl | CPE002E7 | CPE002ES | CPE002FD | | | | | | | | | | | | | | | |
| 1001 | J24 | J1 | 1810 | 748 | 19125 | 428,2 | 10,6 | 1,005 | H5A034 C | cells | Hm Drgbl | Hm Drgbl | Hm Drgbl | CPE002FU | CPE002FY | CPE002G2 | | | | | | | | | | | | | | | |
| 13673 | J24 | J1 | 2069 | 1178 | 22091 | 150,4 | 10,7 | 1,005 | H5A034 C | cells | Hm Gen E | Hm Gen E | Hm Gen E | CPE002JC | CPE002NC | CPE002K4 | | | | | | | | | | | | | | | |
| 10145 | G24 | G1 | 1999 | 941,1 | 21487 | 133,6 | 10,7 | 1,029 | H5A034 C | cells | Hm Drgbl | Hm Drgbl | Hm Drgbl | CPE002EA | CPE002EP | CPE002FA | | | | | | | | | | | | | | | |
| 20968 | J23 | J24 | 1541 | 1128 | 16572 | 335,8 | 10,8 | 0,766 | H5A034 C | cells | Hm Gen E | Hm Gen E | Hm Gen E | CPE002JS | CPE002O7 | CPE002KN | | | | | | | | | | | | | | | |
| 14368 | G23 | G24 | 2033 | 930,7 | 22052 | 141,1 | 10,8 | 0,769 | H5A034 C | cells | Hm Gen E | Hm Gen E | Hm Gen E | CPE002J7 | CPE002NC | CPE002K6 | | | | | | | | | | | | | | | |
| 9377 | G24 | G1 | 2312 | 1247 | 25544 | 134,1 | 11 | 0,764 | H5A034 C | cells | Hm Drgbl | Hm Drgbl | Hm Drgbl | CPE002E8 | CPE002ER | CPE002FC | | | | | | | | | | | | | | | |
| 2537 | J24 | J1 | 2182 | 781,8 | 23926 | 481,3 | 11 | 0,866 | H5A034 C | cells | Hm Drgbl | Hm Drgbl | Hm Drgbl | CPE002DC | CPE002EE | CPE002F4 | | | | | | | | | | | | | | | |
| 17897 | J24 | J1 | 1190 | 1019 | 13113 | 212,9 | 11 | 0,87 | H5A034 C | cells | Hm Gen E | Hm Gen E | Hm Gen E | CPE002JK | CPE002NZ | CPE002KF | | | | | | | | | | | | | | | |
| 13600 | G23 | G24 | 2057 | 1151 | 22613 | 161,2 | 11 | 1,033 | H5A034 C | cells | Hm Gen E | Hm Gen E | Hm Gen E | CPE002JC | CPE002NC | CPE002K4 | | | | | | | | | | | | | | | |
| 3617 | G24 | G1 | 1722 | 1470 | 18862 | 405,2 | 11 | 1,058 | H5A034 C | cells | Hm Drgbl | Hm Drgbl | Hm Drgbl | CPE002DR | CPE002EH | CPE002F7 | | | | | | | | | | | | | | | |
| 7528 | J23 | J24 | 1879 | 673,7 | 20837 | 526,3 | 11,1 | 0,847 | H5A034 C | cells | Hm Drgbl | Hm Drgbl | Hm Drgbl | CPE002E3 | CPE002EW | CPE002FH | | | | | | | | | | | | | | | |
| 4456 | J23 | J24 | 1778 | 749 | 19692 | 481,8 | 11,1 | 0,937 | H5A034 C | cells | Hm Drgbl | Hm Drgbl | Hm Drgbl | CPE002DU | CPE002EK | CPE002FQ | | | | | | | | | | | | | | | |
| 11681 | G24 | G1 | 2162 | 990,8 | 23916 | 150,4 | 11,1 | 0,991 | H5A034 C | cells | Hm Gen E | Hm Gen E | Hm Gen E | CPE002JA | CPE002NJ | CPE002JZ | | | | | | | | | | | | | | | |
| 10913 | G24 | G1 | 2214 | 973,1 | 24864 | 470,8 | 11,2 | 0,848 | H5A034 C | cells | Hm Drgbl | Hm Drgbl | Hm Drgbl | CPE002EC | CPE002EN | CPE002FS | | | | | | | | | | | | | | | |
| 4073 | J24 | J1 | 1854 | 731,7 | 20688 | 397,5 | 11,2 | 0,996 | H5A034 C | cells | Hm Drgbl | Hm Drgbl | Hm Drgbl | CPE002DS | CPE002EI | CPE002F8 | | | | | | | | | | | | | | | |
| 3232 | G23 | G24 | 1908 | 1444 | 21530 | 426,5 | 11,3 | 0,7 | H5A034 C | cells | Hm Drgbl | Hm Drgbl | Hm Drgbl | CPE002DC | CPE002EG | CPE002F6 | | | | | | | | | | | | | | | |
| 15208 | J23 | J24 | 2005 | 953,9 | 22642 | 121,6 | 11,3 | 0,79 | H5A034 C | cells | Hm Gen E | Hm Gen E | Hm Gen E | CPE002J8 | CPE002NS | CPE002K8 | | | | | | | | | | | | | | | |
| 7529 | J24 | J1 | 899 | 684,4 | 10199 | 266,3 | 11,3 | 0,863 | H5A034 C | cells | Hm Drgbl | Hm Drgbl | Hm Drgbl | CPE002E3 | CPE002EW | CPE002FH | | | | | | | | | | | | | | | |
| 2465 | G24 | G1 | 1252 | 706,3 | 14179 | 459,2 | 11,3 | 0,89 | H5A034 C | cells | Hm Drgbl | Hm Drgbl | Hm Drgbl | CPE002DC | CPE002EE | CPE002F4 | | | | | | | | | | | | | | | |
| 16288 | G23 | G24 | 1679 | 1066 | 18895 | 292,6 | 11,3 | 1,037 | H5A034 C | cells | Hm Gen E | Hm Gen E | Hm Gen E | CPE002JG | CPE002NV | CPE002KB | | | | | | | | | | | | | | | |
| 7841 | G24 | G1 | 395 | 602,7 | 4463 | 416,9 | 11,3 | 1,725 | H5A034 C | cells | Hm Drgbl | Hm Drgbl | Hm Drgbl | CPE002E4 | CPE002EV | CPE002FG | | | | | | | | | | | | | | | |
| 15137 | G24 | G1 | 2176 | 979 | 24856 | 121,8 | 11,4 | 0,797 | H5A034 C | cells | Hm Gen E | Hm Gen E | Hm Gen E | CPE002J8 | CPE002NS | CPE002K8 | | | | | | | | | | | | | | | |
| 928 | G23 | G24 | 2075 | 756,8 | 23663 | 420,8 | 11,4 | 1,081 | H5A034 C | cells | Hm Drgbl | Hm Drgbl | Hm Drgbl | CPE002FU | CPE002FY | CPE002G2 | | | | | | | | | | | | | | | |
| 9376 | G23 | G24 | 2101 | 1151 | 24253 | 151 | 11,5 | 0,799 | H5A034 C | cells | Hm Drgbl | Hm Drgbl | Hm Drgbl | CPE002E8 | CPE002ER | CPE002FC | | | | | | | | | | | | | | | |
| 8608 | G23 | G24 | 2194 | 1309 | 25281 | 178,6 | 11,5 | 0,813 | H5A034 C | cells | Hm Drgbl | Hm Drgbl | Hm Drgbl | CPE002E6 | CPE002ET | CPE002FE | | | | | | | | | | | | | | | |
| 11297 | G24 | G1 | 2224 | 990,6 | 25561 | 138,4 | 11,5 | 0,833 | H5A034 C | cells | Hm Drgbl | Hm Drgbl | Hm Drgbl | CPE002ED | CPE002F3 | CPE002FT | | | | | | | | | | | | | | | |
| 12448 | G23 | G24 | 1993 | 1126 | 22985 | 194,3 | 11,5 | 0,865 | H5A034 C | cells | Hm Gen E | Hm Gen E | Hm Gen E | CPE002J5 | CPE002NK | CPE002K1 | | | | | | | | | | | | | | | |
| 8993 | G24 | G1 | 1877 | 1849 | 21832 | 124,8 | 11,6 | 0,932 | H5A034 C | cells | Hm Drgbl | Hm Drgbl | Hm Drgbl | CPE002E7 | CPE002ES | CPE002FD | | | | | | | | | | | | | | | |
| 6688 | G23 | G24 | 2088 | 706,8 | 24126 | 502,9 | 11,6 | 1,018 | H5A034 C | cells | Hm Drgbl | Hm Drgbl | Hm Drgbl | CPE002E1 | CPE002EY | CPE002FJ | | | | | | | | | | | | | | | |
| 4769 | G24 | G1 | 274 | 708,7 | 3184 | 404 | 11,6 | 1,275 | H5A034 C | cells | Hm Drgbl | Hm Drgbl | Hm Drgbl | CPE002DV | CPE002EL | CPE002FP | | | | | | | | | | | | | | | |
| 15904 | G23 | G24 | 2183 | 997,6 | 25550 | 156,5 | 11,7 | 0,652 | H5A034 C | cells | Hm Gen E | Hm Gen E | Hm Gen E | CPE002J9 | CPE002NU | CPE002KA | | | | | | | | | | | | | | | |
| 10217 | J24 | J1 | 1909 | 888,5 | 22423 | 138,2 | 11,7 | 1,125 | H5A034 C | cells | Hm Drgbl | Hm Drgbl | Hm Drgbl | CPE002EA | CPE002EP | CPE002FA | | | | | | | | | | | | | | | |
| 10912 | G23 | G24 | 2004 | 943,1 | 23741 | 566,9 | 11,8 | 0,894 | H5A034 C | cells | Hm Drgbl | Hm Drgbl | Hm Drgbl | CPE002EC | CPE002EN | CPE002FS | | | | | | | | | | | | | | | |
| 15520 | G23 | G24 | 1736 | 932,7 | 20458 | 147,9 | 11,8 | 1,073 | H5A034 C | cells | Hm Gen E | Hm Gen E | Hm Gen E | CPE002JE | CPE002NT | CPE002K9 | | | | | | | | | | | | | | | |
| 5608 | J23 | J24 | 1803 | 875 | 21193 | 596,8 | 11,8 | 1,124 | H5A034 C | cells | Hm Drgbl | Hm Drgbl | Hm Drgbl | CPE002DX | CPE002F2 | CPE002FN | | | | | | | | | | | | | | | |
| 12137 | J24 | J1 | 2120 | 894,5 | 25226 | 98,56 | 11,9 | 0,763 | H5A034 C | cells | Hm Gen E | Hm Gen E | Hm Gen E | CPE002J4 | CPE002NL | CPE002K0 | | | | | | | | | | | | | | | |
| 9448 | J23 | J24 | 1709 | 1229 | 20279 | 175,1 | 11,9 | 0,826 | H5A034 C | cells | Hm Drgbl | Hm Drgbl | Hm Drgbl | CPE002E8 | CPE002ER | CPE002FC | | | | | | | | | | | | | | | |
| 12521 | J24 | J1 | 1990 | 1164 | 23778 | 139,8 | 11,9 | 0,895 | H5A034 C | cells | Hm Gen E | Hm Gen E | Hm Gen E | CPE002J5 | CPE002NK | CPE002K1 | | | | | | | | | | | | | | | |
| 5225 | J24 | J1 | 1535 | 719,4 | 18325 | 318,2 | 11,9 | 0,933 | H5A034 C | cells | Hm Drgbl | Hm Drgbl | Hm Drgbl | CPE002DV | CPE002EM | CPE002FO | | | | | | | | | | | | | | | |
| 5920 | G23 | G24 | 2563 | 967,3 | 30515 | 553,4 | 11,9 | 1,087 | H5A034 C | cells | Hm Drgbl | Hm Drgbl | Hm Drgbl | CPE002DY | CPE002F1 | CPE002FM | | | | | | | | | | | | | | | |
| 5609 | J24 | J1 | 1934 | 789,4 | 23086 | 576,4 | 11,9 | 1,133 | H5A034 C | cells | Hm Drgbl | Hm Drgbl | Hm Drgbl | CPE002DX | CPE002F2 | CPE002FN | | | | | | | | | | | | | | | |
| 10985 | J24 | J1 | 2113 | 1016 | 25436 | 448,6 | 12 | 0,909 | H5A034 C | cells | Hm Drgbl | Hm Drgbl | Hm Drgbl | CPE002EC | CPE002EN | CPE002FS | | | | | | | | | | | | | | | |
| 9832 | J23 | J24 | 2307 | 1246 | 27952 | 193,7 | 12,1 | 0,846 | H5A034 C | cells | Hm Drgbl | Hm Drgbl | Hm Drgbl | CPE002E9 | CPE002EQ | CPE002FB | | | | | | | | | | | | | | | |
| 17056 | G23 | G24 | 2150 | 1081 | 26070 | 244,4 | 12,1 | 0,934 | H5A034 C | cells | Hm Gen E | Hm Gen E | Hm Gen E | CPE002JI | CPE002NZ | CPE002KD | | | | | | | | | | | | | | | |
| 16361 | J24 | J1 | 1211 | 1025 | 14627 | 278,4 | 12,1 | 1,11 | H5A034 C | cells | Hm Gen E | Hm Gen E | Hm Gen E | CPE002JG | CPE002NV | CPE002KB | | | | | | | | | | | | | | | |
| 16744 | J23 | J24 | 1663 | 1044 | 20391 | 217,9 | 12,3 | 0,972 | H5A034 C | cells | Hm Gen E | Hm Gen E | Hm Gen E | CPE002JH | CPE002NV | CPE002KC | | | | | | | | | | | | | | | |
| 20896 | G23 | G24 | 1665 | 1067 | 20581 | 268 | 12,4 | 0,879 | H5A034 C | cells | Hm Gen E | Hm Gen E | Hm Gen E | CPE002JS | CPE002O7 | CPE002KN | | | | | | | | | | | | | | | |
| 160 | G23 | G24 | 740 | 1759 | 9189 | 809,1 | 12,4 | 1,055 | H5A034 C | cells | Hm Drgbl | Hm Drgbl | Hm Drgbl | CPE002DT | CPE002EJ | CPE002F9 | | | | | | | | | | | | | | | |
| 15521 | G24 | G1 | 1923 | 982,4 | 23917 | 157,3 | 12,4 | 1,127 | H5A034 C | cells | Hm Gen E | Hm Gen E | Hm Gen E | CPE002JE | CPE002NT | CPE002K9 | | | | | | | | | | | | | | | |
| 1697 | G24 | G1 | 1281 | 772,1 | 15907 | 436,5 | 12,4 | 1,298 | H5A034 C | cells | Hm Drgbl | Hm Drgbl | Hm Drgbl | CPE002FW | CPE002G0 | CPE002G4 | | | | | | | | | | | | | | | |
| 6376 | J23 | J24 | 1543 | 633,3 | 19314 | 452,1 | 12,5 | 0,825 | H5A034 C | cells | Hm Drgbl | Hm Drgbl | Hm Drgbl | CPE002DZ | CPE002F0 | CPE002FL | | | | | | | | | | | | | | | |
| 9760 | G23 | G24 | 2360 | 1227 | 29561 | 159,9 | 12,5 | 0,874 | H5A034 C | cells | Hm Drgbl | Hm Drgbl | Hm Drgbl | CPE002E9 | CPE002EQ | CPE002FB | | | | | | | | | | | | | | | |
| 5152 | G23 | G24 | 2097 | 696,5 | 26142 | 554,7 | 12,5 | 0,98 | H5A034 C | cells | Hm Drgbl | Hm Drgbl | Hm Drgbl | CPE002DV | CPE002EM | CPE002FO | | | | | | | | | | | | | | | |
| 5224 | J23 | J24 | 1971 | 689,6 | 24602 | 531,8 | 12,5 | 0,98 | H5A034 C | cells | Hm Drgbl | Hm Drgbl | Hm Drgbl | CPE002DV | CPE002EM | CPE002FO | | | | | | | | | | | | | | | |
| 15136 | G23 | G24 | 2193 | 974,6 | 27786 | 139,4 | 12,7 | 0,888 | H5A034 C | cells | Hm Gen E | Hm Gen E | Hm Gen E | CPE002J8 | CPE002NS | CPE002K8 | | | | | | | | | | | | | | | |
| 7144 | J23 | J24 | 1690 | 636,7 | 21381 | 501,4 | 12,7 | 0,988 | H5A034 C | cells | Hm Drgbl | Hm Drgbl | Hm Drgbl | CPE002E2 | CPE002EX | CPE002FI | | | | | | | | | | | | | | | |
| 11680 | G23 | G24 | 2096 | 959 | 26723 | 172,6 | 12,7 | 1,134 | H5A034 C | cells | Hm Gen E | Hm Gen E | Hm Gen E | CPE002JA | CPE002NJ | CPE002JZ | | | | | | | | | | | | | | | |
| 14824 | J23 | J24 | 2175 | 1008 | 27854 | 137,9 | 12,8 | 0,729 | H5A034 C | cells | Hm Gen E | Hm Gen E | Hm Gen E | CPE002JD | CPE002NR | CPE002K7 | | | | | | | | | | | | | | | |
| 12833 | G24 | G1 | 2147 | 1230 | 27665 | 130,3 | 12,9 | 0,875 | H5A034 C | cells | Hm Gen E | Hm Gen E | Hm Gen E | CPE002J6 | CPE002NM | CPE002K2 | | | | | | | | | | | | | | | |
| 17128 | J23 | J24 | 2213 | 1113 | 28440 | 275,3 | 12,9 | 0,996 | H5A034 C | cells | Hm Gen E | Hm Gen E | Hm Gen E | CPE002JI | CPE002NZ | CPE002KD | | | | | | | | | | | | | | | |
| 9065 | J24 | J1 | 2139 | 1967 | 27888 | 168 | 13 | 1,044 | H5A034 C | cells | Hm Drgbl | Hm Drgbl | Hm Drgbl | CPE002E7 | CPE002ES | CPE002FD | | | | | | | | | | | | | | | |
| 5992 | J23 | J24 | 1730 | 869,5 | 22409 | 670,3 | 13 | 1,187 | H5A034 C | cells | Hm Drgbl | Hm Drgbl | Hm Drgbl | CPE002DY | CPE002F1 | CPE002FM | | | | | | | | | | | | | | | |
| 16672 | G23 | G24 | 1566 | 1039 | 20475 | 323,4 | 13,1 | 1,036 | H5A034 C | cells | Hm Gen E | Hm Gen E | Hm Gen E | CPE002JH | CPE002NV | CPE002KC | | | | | | | | | | | | | | | |
| 11296 | G23 | G24 | 2103 | 988,9 | 27692 | 178,1 | 13,2 | 0,957 | H5A034 C | cells | Hm Drgbl | Hm Drgbl | Hm Drgbl | CPE002ED | CPE002F3 | CPE002FT | | | | | | | | | | | | | | | |
| 10984 | J23 | J24 | 2072 | 932,1 | 27448 | 815 | 13,2 | 1 | H5A034 C | cells | Hm Drgbl | Hm Drgbl | Hm Drgbl | CPE002EC | CPE002EN | CPE002FS | | | | | | | | | | | | | | | |
| 8225 | G24 | G1 | 676 | 630,2 | 8902 | 472,2 | 13,2 | 1,269 | H5A034 C | cells | Hm Drgbl | Hm Drgbl | Hm Drgbl | CPE002E5 | CPE002EU | CPE002FF | | | | | | | | | | | | | | | |
| 21664 | G23 | G24 | 291 | 889,4 | 3849 | 732,3 | 13,2 | 1,389 | H5A034 C | cells | Hm Gen E | Hm Gen E | Hm Gen E | CPE002JU | CPE002O9 | CPE002KP | | | | | | | | | | | | | | | |
| 8680 | J23 | J24 | 2061 | 1190 | 27702 | 231,8 | 13,4 | 0,947 | H5A034 C | cells | Hm Drgbl | Hm Drgbl | Hm Drgbl | CPE002E6 | CPE002ET | CPE002FE | | | | | | | | | | | | | | | |
| 20897 | G24 | G1 | 1712 | 1061 | 22981 | 315,6 | 13,4 | 0,95 | H5A034 C | cells | Hm Gen E | Hm Gen E | Hm Gen E | CPE002JS | CPE002O7 | CPE002KN | | | | | | | | | | | | | | | |
| 14441 | J24 | J1 | 1907 | 886,8 | 25506 | 117,4 | 13,4 | 0,954 | H5A034 C | cells | Hm Gen E | Hm Gen E | Hm Gen E | CPE002J7 | CPE002NC | CPE002K6 | | | | | | | | | | | | | | | |
| 17057 | G24 | G1 | 1490 | 1019 | 19987 | 136,9 | 13,4 | 1,035 | H5A034 C | cells | Hm Gen E | Hm Gen E | Hm Gen E | CPE002JI | CPE002NZ | CPE002KD | | | | | | | | | | | | | | | |
| 17129 | J24 | J1 | 1473 | 1011 | 19699 | 219,1 | 13,4 | 1,035 | H5A034 C | cells | Hm Gen E | Hm Gen E | Hm Gen E | CPE002JI | CPE002NZ | CPE002KD | | | | | | | | | | | | | | | |
| 16745 | J24 | J1 | 953 | 1027 | 12781 | 284,3 | 13,4 | 1,059 | H5A034 C | cells | Hm Gen E | Hm Gen E | Hm Gen E | CPE002JH | CPE002NV | CPE002KC | | | | | | | | | | | | | | | |
| 16289 | G24 | G1 | 714 | 999,4 | 9534 | 227,3 | 13,4 | 1,229 | H5A034 C | cells | Hm Gen E | Hm Gen E | Hm Gen E | CPE002JG | CPE002NV | CPE002KB | | | | | | | | | | | | | | | |
| 15905 | G24 | G1 | 2007 | 945,2 | 27234 | 102,1 | 13,6 | 0,758 | H5A034 C | cells | Hm Gen E | Hm Gen E | Hm Gen E | CPE002J9 | CPE002NU | CPE002KA | | | | | | | | | | | | | | | |
| 20513 | G24 | G1 | 811 | 809,9 | 11026 | 149,8 | 13,6 | 0,907 | H5A034 C | cells | Hm Gen E | Hm Gen E | Hm Gen E | CPE002JR | CPE002O6 | CPE002KM | | | | | | | | | | | | | | | |
| 21353 | J24 | J1 | 1224 | 954 | 16702 | 271,4 | 13,6 | 0,965 | H5A034 C | cells | Hm Gen E | Hm Gen E | Hm Gen E | CPE002JT | CPE002O8 | CPE002KO | | | | | | | | | | | | | | | |
| 7456 | G23 | G24 | 1695 | 681,5 | 23121 | 374,3 | 13,6 | 1,038 | H5A034 C | cells | Hm Drgbl | Hm Drgbl | Hm Drgbl | CPE002E3 | CPE002EW | CPE002FH | | | | | | | | | | | | | | | |
| 15593 | J24 | J1 | 1878 | 917,6 | 25499 | 149,9 | 13,6 | 1,236 | H5A034 C | cells | Hm Gen E | Hm Gen E | Hm Gen E | CPE002JE | CPE002NT | CPE002K9 | | | | | | | | | | | | | | | |
| 18976 | G23 | G24 | 1771 | 1085 | 24232 | 277,7 | 13,7 | 0,955 | H5A034 C | cells | Hm Gen E | Hm Gen E | Hm Gen E | CPE002JN | CPE002O2 | CPE002KI | | | | | | | | | | | | | | | |
| 13672 | J23 | J24 | 1677 | 1179 | 23058 | 220,4 | 13,7 | 1,286 | H5A034 C | cells | Hm Gen E | Hm Gen E | Hm Gen E | CPE002JC | CPE002NC | CPE002K4 | | | | | | | | | | | | | | | |
| 18208 | G23 | G24 | 1972 | 1088 | 27161 | 266,2 | 13,8 | 0,887 | H5A034 C | cells | Hm Gen E | Hm Gen E | Hm Gen E | CPE002JL | CPE002O0 | CPE002KG | | | | | | | | | | | | | | | |
| 11369 | J24 | J1 | 1934 | 903,8 | 26739 | 175,4 | 13,8 | 1 | H5A034 C | cells | Hm Drgbl | Hm Drgbl | Hm Drgbl | CPE002ED | CPE002F3 | CPE002FT | | | | | | | | | | | | | | | |
| 6760 | J23 | J24 | 1624 | 652,5 | 22409 | 546,2 | 13,8 | 1,211 | H5A034 C | cells | Hm Drgbl | Hm Drgbl | Hm Drgbl | CPE002E1 | CPE002EY | CPE002FJ | | | | | | | | | | | | | | | |
| 9449 | J24 | J1 | 2040 | 1165 | 28277 | 147,1 | 13,9 | 0,965 | H5A034 C | cells | Hm Drgbl | Hm Drgbl | Hm Drgbl | CPE002E8 | CPE002ER | CPE002FC | | | | | | | | | | | | | | | |
| 5153 | G24 | G1 | 525 | 612,9 | 7283 | 480 | 13,9 | 1,09 | H5A034 C | cells | Hm Drgbl | Hm Drgbl | Hm Drgbl | CPE002DV | CPE002EM | CPE002FO | | | | | | | | | | | | | | | |
| 929 | G24 | G1 | 1860 | 834,9 | 25846 | 341,7 | 13,9 | 1,318 | H5A034 C | cells | Hm Drgbl | Hm Drgbl | Hm Drgbl | CPE002FU | CPE002FY | CPE002G2 | | | | | | | | | | | | | | | |
| 12904 | J23 | J24 | 2053 | 1146 | 28821 | 188,6 | 14 | 0,949 | H5A034 C | cells | Hm Gen E | Hm Gen E | Hm Gen E | CPE002J6 | CPE002NM | CPE002K2 | | | | | | | | | | | | | | | |
| 21280 | G23 | G24 | 1889 | 1063 | 26402 | 395,2 | 14 | 0,993 | H5A034 C | cells | Hm Gen E | Hm Gen E | Hm Gen E | CPE002JT | CPE002O8 | CPE002KO | | | | | | | | | | | | | | | |
| 13985 | G24 | G1 | 2197 | 1222 | 30930 | 135,5 | 14,1 | 0,808 | H5A034 C | cells | Hm Gen E | Hm Gen E | Hm Gen E | CPE002JF | CPE002NP | CPE002K5 | | | | | | | | | | | | | | | |
| 20512 | G23 | G24 | 1409 | 870,5 | 19869 | 248,5 | 14,1 | 0,94 | H5A034 C | cells | Hm Gen E | Hm Gen E | Hm Gen E | CPE002JR | CPE002O6 | CPE002KM | | | | | | | | | | | | | | | |
| 17824 | G23 | G24 | 1711 | 1113 | 24190 | 300,9 | 14,1 | 1,115 | H5A034 C | cells | Hm Gen E | Hm Gen E | Hm Gen E | CPE002JK | CPE002NZ | CPE002KF | | | | | | | | | | | | | | | |
| 9064 | J23 | J24 | 2179 | 2102 | 30803 | 237,5 | 14,1 | 1,133 | H5A034 C | cells | Hm Drgbl | Hm Drgbl | Hm Drgbl | CPE002E7 | CPE002ES | CPE002FD | | | | | | | | | | | | | | | |
| 6304 | G23 | G24 | 1963 | 655,6 | 27780 | 516,3 | 14,2 | 0,937 | H5A034 C | cells | Hm Drgbl | Hm Drgbl | Hm Drgbl | CPE002DZ | CPE002F0 | CPE002FL | | | | | | | | | | | | | | | |
| 11368 | J23 | J24 | 2170 | 978,2 | 30803 | 202,3 | 14,2 | 1,029 | H5A034 C | cells | Hm Drgbl | Hm Drgbl | Hm Drgbl | CPE002ED | CPE002F3 | CPE002FT | | | | | | | | | | | | | | | |
| 19744 | G23 | G24 | 1688 | 373,5 | 24196 | 378,3 | 14,3 | 0,932 | H5A034 C | cells | Hm Gen E | Hm Gen E | Hm Gen E | CPE002JP | CPE002O4 | CPE002KK | | | | | | | | | | | | | | | |
| 20585 | J24 | J1 | 680 | 810,3 | 9737 | 168,5 | 14,3 | 0,953 | H5A034 C | cells | Hm Gen E | Hm Gen E | Hm Gen E | CPE002JR | CPE002O6 | CPE002KM | | | | | | | | | | | | | | | |
| 12905 | J24 | J1 | 2236 | 1238 | 32076 | 171,9 | 14,3 | 0,969 | H5A034 C | cells | Hm Gen E | Hm Gen E | Hm Gen E | CPE002J6 | CPE002NM | CPE002K2 | | | | | | | | | | | | | | | |
| 19048 | J23 | J24 | 1648 | 1032 | 23493 | 268,4 | 14,3 | 0,997 | H5A034 C | cells | Hm Gen E | Hm Gen E | Hm Gen E | CPE002JN | CPE002O2 | CPE002KI | | | | | | | | | | | | | | | |
| 17896 | J23 | J24 | 1384 | 1113 | 19821 | 317,7 | 14,3 | 1,13 | H5A034 C | cells | Hm Gen E | Hm Gen E | Hm Gen E | CPE002JK | CPE002NZ | CPE002KF | | | | | | | | | | | | | | | |
| 14057 | J24 | J1 | 2001 | 1191 | 28861 | 146 | 14,4 | 0,825 | H5A034 C | cells | Hm Gen E | Hm Gen E | Hm Gen E | CPE002JF | CPE002NP | CPE002K5 | | | | | | | | | | | | | | | |
| 22504 | J23 | J24 | 1005 | 1284 | 14619 | 348,8 | 14,5 | 0,747 | H5A034 C | cells | Hm Gen E | Hm Gen E | Hm Gen E | CPE002JW | CPE002OB | CPE002KR | | | | | | | | | | | | | | | |
| 17440 | G23 | G24 | 1989 | 1132 | 28926 | 217,5 | 14,5 | 0,99 | H5A034 C | cells | Hm Gen E | Hm Gen E | Hm Gen E | CPE002JJ | CPE002NY | CPE002KE | | | | | | | | | | | | | | | |
| 17512 | J23 | J24 | 1449 | 1043 | 20977 | 245,5 | 14,5 | 0,99 | H5A034 C | cells | Hm Gen E | Hm Gen E | Hm Gen E | CPE002JJ | CPE002NY | CPE002KE | | | | | | | | | | | | | | | |
| 14056 | J23 | J24 | 1820 | 1152 | 26495 | 182,9 | 14,6 | 0,837 | H5A034 C | cells | Hm Gen E | Hm Gen E | Hm Gen E | CPE002JF | CPE002NP | CPE002K5 | | | | | | | | | | | | | | | |
| 12832 | G23 | G24 | 1830 | 1127 | 26801 | 193,9 | 14,6 | 0,99 | H5A034 C | cells | Hm Gen E | Hm Gen E | Hm Gen E | CPE002J6 | CPE002NM | CPE002K2 | | | | | | | | | | | | | | | |
| 17441 | G24 | G1 | 1431 | 965,9 | 20871 | 210,7 | 14,6 | 0,997 | H5A034 C | cells | Hm Gen E | Hm Gen E | Hm Gen E | CPE002JJ | CPE002NY | CPE002KE | | | | | | | | | | | | | | | |
| 7145 | J24 | J1 | 1236 | 623,6 | 18029 | 547,6 | 14,6 | 1,136 | H5A034 C | cells | Hm Drgbl | Hm Drgbl | Hm Drgbl | CPE002E2 | CPE002EX | CPE002FI | | | | | | | | | | | | | | | |
| 15976 | J23 | J24 | 1866 | 950,4 | 27502 | 141,4 | 14,7 | 0,819 | H5A034 C | cells | Hm Gen E | Hm Gen E | Hm Gen E | CPE002J9 | CPE002NU | CPE002KA | | | | | | | | | | | | | | | |
| 7072 | G23 | G24 | 1581 | 640,3 | 23177 | 530,9 | 14,7 | 1,144 | H5A034 C | cells | Hm Drgbl | Hm Drgbl | Hm Drgbl | CPE002E2 | CPE002EX | CPE002FI | | | | | | | | | | | | | | | |
| 13288 | J23 | J24 | 2084 | 1220 | 30754 | 174 | 14,8 | 0,742 | H5A034 C | cells | Hm Gen E | Hm Gen E | Hm Gen E | CPE002JB | CPE002NN | CPE002K3 | | | | | | | | | | | | | | | |
| 8681 | J24 | J1 | 1872 | 1170 | 27719 | 160 | 14,8 | 1,046 | H5A034 C | cells | Hm Drgbl | Hm Drgbl | Hm Drgbl | CPE002E6 | CPE002ET | CPE002FE | | | | | | | | | | | | | | | |
| 12136 | J23 | J24 | 2114 | 910 | 31596 | 150,8 | 14,9 | 0,955 | H5A034 C | cells | Hm Gen E | Hm Gen E | Hm Gen E | CPE002J4 | CPE002NL | CPE002K0 | | | | | | | | | | | | | | | |
| 14825 | J24 | J1 | 2183 | 994,3 | 32674 | 132,4 | 15 | 0,855 | H5A034 C | cells | Hm Gen E | Hm Gen E | Hm Gen E | CPE002JD | CPE002NR | CPE002K7 | | | | | | | | | | | | | | | |
| 21281 | G24 | G1 | 1171 | 979,9 | 17532 | 300,6 | 15 | 1,064 | H5A034 C | cells | Hm Gen E | Hm Gen E | Hm Gen E | CPE002JT | CPE002O8 | CPE002KO | | | | | | | | | | | | | | | |
| 4385 | G24 | G1 | 1195 | 721,4 | 17892 | 464,5 | 15 | 1,266 | H5A034 C | cells | Hm Drgbl | Hm Drgbl | Hm Drgbl | CPE002DU | CPE002EK | CPE002FQ | | | | | | | | | | | | | | | |
| 17825 | G24 | G1 | 751 | 972,1 | 11395 | 186,6 | 15,2 | 1,202 | H5A034 C | cells | Hm Gen E | Hm Gen E | Hm Gen E | CPE002JK | CPE002NZ | CPE002KF | | | | | | | | | | | | | | | |
| 19816 | J23 | J24 | 1340 | 357,1 | 20462 | 391,5 | 15,3 | 0,997 | H5A034 C | cells | Hm Gen E | Hm Gen E | Hm Gen E | CPE002JP | CPE002O4 | CPE002KK | | | | | | | | | | | | | | | |
| 18592 | G23 | G24 | 1660 | 1075 | 25452 | 266 | 15,3 | 1,037 | H5A034 C | cells | Hm Gen E | Hm Gen E | Hm Gen E | CPE002JM | CPE002O1 | CPE002KH | | | | | | | | | | | | | | | |
| 20969 | J24 | J1 | 1500 | 1028 | 22977 | 312,8 | 15,3 | 1,085 | H5A034 C | cells | Hm Gen E | Hm Gen E | Hm Gen E | CPE002JS | CPE002O7 | CPE002KN | | | | | | | | | | | | | | | |
| 5921 | G24 | G1 | 852 | 798,3 | 13077 | 552,7 | 15,3 | 1,397 | H5A034 C | cells | Hm Drgbl | Hm Drgbl | Hm Drgbl | CPE002DY | CPE002F1 | CPE002FM | | | | | | | | | | | | | | | |
| 19049 | J24 | J1 | 1289 | 1037 | 19797 | 290,4 | 15,4 | 1,073 | H5A034 C | cells | Hm Gen E | Hm Gen E | Hm Gen E | CPE002JN | CPE002O2 | CPE002KI | | | | | | | | | | | | | | | |
| 9833 | J24 | J1 | 1966 | 1160 | 30293 | 138,6 | 15,4 | 1,077 | H5A034 C | cells | Hm Drgbl | Hm Drgbl | Hm Drgbl | CPE002E9 | CPE002EQ | CPE002FB | | | | | | | | | | | | | | | |
| 15209 | J24 | J1 | 2158 | 947,6 | 33314 | 148,6 | 15,4 | 1,077 | H5A034 C | cells | Hm Gen E | Hm Gen E | Hm Gen E | CPE002J8 | CPE002NS | CPE002K8 | | | | | | | | | | | | | | | |
| 21352 | J23 | J24 | 1171 | 1011 | 18047 | 399,1 | 15,4 | 1,092 | H5A034 C | cells | Hm Gen E | Hm Gen E | Hm Gen E | CPE002JT | CPE002O8 | CPE002KO | | | | | | | | | | | | | | | |
| 9761 | G24 | G1 | 1781 | 1121 | 27591 | 140,8 | 15,5 | 1,084 | H5A034 C | cells | Hm Drgbl | Hm Drgbl | Hm Drgbl | CPE002E9 | CPE002EQ | CPE002FB | | | | | | | | | | | | | | | |
| 13984 | G23 | G24 | 2007 | 1254 | 31381 | 166 | 15,6 | 0,894 | H5A034 C | cells | Hm Gen E | Hm Gen E | Hm Gen E | CPE002JF | CPE002NP | CPE002K5 | | | | | | | | | | | | | | | |
| 18280 | J23 | J24 | 1215 | 1156 | 18909 | 300,2 | 15,6 | 1,003 | H5A034 C | cells | Hm Gen E | Hm Gen E | Hm Gen E | CPE002JL | CPE002O0 | CPE002KG | | | | | | | | | | | | | | | |
| 16673 | G24 | G1 | 1371 | 1042 | 21497 | 289,5 | 15,7 | 1,241 | H5A034 C | cells | Hm Gen E | Hm Gen E | Hm Gen E | CPE002JH | CPE002NV | CPE002KC | | | | | | | | | | | | | | | |
| 22432 | G23 | G24 | 1356 | 1390 | 21476 | 335,5 | 15,8 | 0,814 | H5A034 C | cells | Hm Gen E | Hm Gen E | Hm Gen E | CPE002JW | CPE002OB | CPE002KR | | | | | | | | | | | | | | | |
| 14753 | G24 | G1 | 2251 | 1011 | 35467 | 150,7 | 15,8 | 0,9 | H5A034 C | cells | Hm Gen E | Hm Gen E | Hm Gen E | CPE002JD | CPE002NR | CPE002K7 | | | | | | | | | | | | | | | |
| 12065 | G24 | G1 | 1929 | 950,4 | 30431 | 93,61 | 15,8 | 1,013 | H5A034 C | cells | Hm Gen E | Hm Gen E | Hm Gen E | CPE002J4 | CPE002NL | CPE002K0 | | | | | | | | | | | | | | | |
| 6377 | J24 | J1 | 1452 | 691 | 22883 | 367 | 15,8 | 1,043 | H5A034 C | cells | Hm Drgbl | Hm Drgbl | Hm Drgbl | CPE002DZ | CPE002F0 | CPE002FL | | | | | | | | | | | | | | | |
| 22049 | G24 | G1 | 1547 | 1335 | 24669 | 212,2 | 15,9 | 0,755 | H5A034 C | cells | Hm Gen E | Hm Gen E | Hm Gen E | CPE002JV | CPE002OA | CPE002KQ | | | | | | | | | | | | | | | |
| 15977 | J24 | J1 | 1857 | 890,8 | 29525 | 125,8 | 15,9 | 0,886 | H5A034 C | cells | Hm Gen E | Hm Gen E | Hm Gen E | CPE002J9 | CPE002NU | CPE002KA | | | | | | | | | | | | | | | |
| 3233 | G24 | G1 | 810 | 1285 | 13040 | 485,5 | 16,1 | 0,997 | H5A034 C | cells | Hm Drgbl | Hm Drgbl | Hm Drgbl | CPE002DC | CPE002EG | CPE002F6 | | | | | | | | | | | | | | | |
| 18664 | J23 | J24 | 1508 | 1060 | 24266 | 343,1 | 16,1 | 1,092 | H5A034 C | cells | Hm Gen E | Hm Gen E | Hm Gen E | CPE002JM | CPE002O1 | CPE002KH | | | | | | | | | | | | | | | |
| 14752 | G23 | G24 | 2054 | 980,4 | 33324 | 170,2 | 16,2 | 0,923 | H5A034 C | cells | Hm Gen E | Hm Gen E | Hm Gen E | CPE002JD | CPE002NR | CPE002K7 | | | | | | | | | | | | | | | |
| 19433 | J24 | J1 | 985 | 1331 | 15994 | 300,8 | 16,2 | 1,149 | H5A034 C | cells | Hm Gen E | Hm Gen E | Hm Gen E | CPE002JO | CPE002O3 | CPE002KJ | | | | | | | | | | | | | | | |
| 20584 | J23 | J24 | 905 | 843,5 | 14731 | 243 | 16,3 | 1,087 | H5A034 C | cells | Hm Gen E | Hm Gen E | Hm Gen E | CPE002JR | CPE002O6 | CPE002KM | | | | | | | | | | | | | | | |
| 8609 | G24 | G1 | 2005 | 1288 | 32676 | 156,5 | 16,3 | 1,152 | H5A034 C | cells | Hm Drgbl | Hm Drgbl | Hm Drgbl | CPE002E6 | CPE002ET | CPE002FE | | | | | | | | | | | | | | | |
| 13217 | G24 | G1 | 2304 | 1240 | 38051 | 171,6 | 16,5 | 0,827 | H5A034 C | cells | Hm Gen E | Hm Gen E | Hm Gen E | CPE002JB | CPE002NN | CPE002K3 | | | | | | | | | | | | | | | |
| 18209 | G24 | G1 | 1098 | 1056 | 18160 | 266,8 | 16,5 | 1,061 | H5A034 C | cells | Hm Gen E | Hm Gen E | Hm Gen E | CPE002JL | CPE002O0 | CPE002KG | | | | | | | | | | | | | | | |
| 17513 | J24 | J1 | 1223 | 1036 | 20268 | 286,8 | 16,6 | 1,133 | H5A034 C | cells | Hm Gen E | Hm Gen E | Hm Gen E | CPE002JJ | CPE002NY | CPE002KE | | | | | | | | | | | | | | | |
| 19361 | G24 | G1 | 865 | 1219 | 14410 | 282,4 | 16,7 | 1,184 | H5A034 C | cells | Hm Gen E | Hm Gen E | Hm Gen E | CPE002JO | CPE002O3 | CPE002KJ | | | | | | | | | | | | | | | |
| 5537 | G24 | G1 | 825 | 672,3 | 13898 | 628,4 | 16,8 | 1,6 | H5A034 C | cells | Hm Drgbl | Hm Drgbl | Hm Drgbl | CPE002DX | CPE002F2 | CPE002FN | | | | | | | | | | | | | | | |
| 12064 | G23 | G24 | 1722 | 856,8 | 29282 | 143,6 | 17 | 1,09 | H5A034 C | cells | Hm Gen E | Hm Gen E | Hm Gen E | CPE002J4 | CPE002NL | CPE002K0 | | | | | | | | | | | | | | | |
| 19360 | G23 | G24 | 1609 | 1235 | 27512 | 390,3 | 17,1 | 1,213 | H5A034 C | cells | Hm Gen E | Hm Gen E | Hm Gen E | CPE002JO | CPE002O3 | CPE002KJ | | | | | | | | | | | | | | | |
| 19432 | J23 | J24 | 1291 | 1277 | 22254 | 389,4 | 17,2 | 1,22 | H5A034 C | cells | Hm Gen E | Hm Gen E | Hm Gen E | CPE002JO | CPE002O3 | CPE002KJ | | | | | | | | | | | | | | | |
| 18593 | G24 | G1 | 1025 | 1077 | 17740 | 259,9 | 17,3 | 1,173 | H5A034 C | cells | Hm Gen E | Hm Gen E | Hm Gen E | CPE002JM | CPE002O1 | CPE002KH | | | | | | | | | | | | | | | |
| 23657 | J24 | J1 | 1150 | 1577 | 19974 | 245,2 | 17,4 | 0,923 | H5A034 C | cells | Hm Gen E | Hm Gen E | Hm Gen E | CPE002KV | CPE002OE | CPE002KU | | | | | | | | | | | | | | | |
| 19745 | G24 | G1 | 1374 | 349,2 | 23941 | 355,5 | 17,4 | 1,134 | H5A034 C | cells | Hm Gen E | Hm Gen E | Hm Gen E | CPE002JP | CPE002O4 | CPE002KK | | | | | | | | | | | | | | | |
| 18665 | J24 | J1 | 1131 | 1015 | 19664 | 314,5 | 17,4 | 1,18 | H5A034 C | cells | Hm Gen E | Hm Gen E | Hm Gen E | CPE002JM | CPE002O1 | CPE002KH | | | | | | | | | | | | | | | |
| 22817 | G24 | G1 | 1362 | 1695 | 23839 | 220,1 | 17,5 | 0,822 | H5A034 C | cells | Hm Gen E | Hm Gen E | Hm Gen E | CPE002JX | CPE002OC | CPE002KS | | | | | | | | | | | | | | | |
| 23585 | G24 | G1 | 1328 | 1538 | 23651 | 228,9 | 17,8 | 0,944 | H5A034 C | cells | Hm Gen E | Hm Gen E | Hm Gen E | CPE002KV | CPE002OE | CPE002KU | | | | | | | | | | | | | | | |
| 7457 | G24 | G1 | 740 | 596,4 | 13188 | 378,1 | 17,8 | 1,359 | H5A034 C | cells | Hm Drgbl | Hm Drgbl | Hm Drgbl | CPE002E3 | CPE002EW | CPE002FH | | | | | | | | | | | | | | | |
| 13289 | J24 | J1 | 1788 | 1101 | 32091 | 117,8 | 17,9 | 0,897 | H5A034 C | cells | Hm Gen E | Hm Gen E | Hm Gen E | CPE002JB | CPE002NN | CPE002K3 | | | | | | | | | | | | | | | |

| | | | | | | | | | | | | | | | | | |
|---|---|---|---|---|---|---|---|---|---|---|---|---|---|---|---|---|---|
| 18977 | G24 | G1 | 1411 | 1037 | 25505 | 310,5 | 18,1 | 1,261 | H5A034 | C | cells | Hm Gen E | Hm Gen E | Hm Gen E | CPE002JN | CPE002O2 | CPE002KI |
| 13216 | G23 | G24 | 2001 | 1154 | 36504 | 167,3 | 18,2 | 0,912 | H5A034 | C | cells | Hm Gen E | Hm Gen E | Hm Gen E | CPE002JB | CPE002NN | CPE002K3 |
| 23584 | G23 | G24 | 979 | 1499 | 18506 | 306,2 | 18,9 | 1,003 | H5A034 | C | cells | Hm Gen E | Hm Gen E | Hm Gen E | CPE002KV | CPE002OE | CPE002KU |
| 23201 | G24 | G1 | 1145 | 1120 | 22162 | 270 | 19,4 | 0,838 | H5A034 | C | cells | Hm Gen E | Hm Gen E | Hm Gen E | CPE002JY | CPE002OD | CPE002KT |
| 22816 | G23 | G24 | 1179 | 1680 | 22873 | 340,8 | 19,4 | 0,911 | H5A034 | C | cells | Hm Gen E | Hm Gen E | Hm Gen E | CPE002JX | CPE002OC | CPE002KS |
| 22505 | J24 | J1 | 1041 | 1342 | 20319 | 435,7 | 19,5 | 1,005 | H5A034 | C | cells | Hm Gen E | Hm Gen E | Hm Gen E | CPE002JW | CPE002OB | CPE002KR |
| 3305 | J24 | J1 | 1317 | 1373 | 25738 | 465,3 | 19,5 | 1,207 | H5A034 | C | cells | Hm Drgbl | Hm Drgbl | Hm Drgbl | CPE002DC | CPE002EG | CPE002F6 |
| 19817 | J24 | J1 | 958 | 351 | 18717 | 365,1 | 19,5 | 1,27 | H5A034 | C | cells | Hm Gen E | Hm Gen E | Hm Gen E | CPE002JP | CPE002O4 | CPE002KK |
| 23656 | J23 | J24 | 1035 | 1506 | 20844 | 333,7 | 20,1 | 1,066 | H5A034 | C | cells | Hm Gen E | Hm Gen E | Hm Gen E | CPE002KV | CPE002OE | CPE002KU |
| 22888 | J23 | J24 | 1078 | 1627 | 21913 | 359,3 | 20,3 | 0,953 | H5A034 | C | cells | Hm Gen E | Hm Gen E | Hm Gen E | CPE002JX | CPE002OC | CPE002KS |
| 22889 | J24 | J1 | 804 | 1718 | 16309 | 175,6 | 20,3 | 0,953 | H5A034 | C | cells | Hm Gen E | Hm Gen E | Hm Gen E | CPE002JX | CPE002OC | CPE002KS |
| 22120 | J23 | J24 | 910 | 1346 | 18619 | 357 | 20,5 | 0,974 | H5A034 | C | cells | Hm Gen E | Hm Gen E | Hm Gen E | CPE002JV | CPE002OA | CPE002KQ |
| 22433 | G24 | G1 | 745 | 1328 | 15264 | 361,1 | 20,5 | 1,057 | H5A034 | C | cells | Hm Gen E | Hm Gen E | Hm Gen E | CPE002JW | CPE002OB | CPE002KR |
| 22048 | G23 | G24 | 974 | 1361 | 20256 | 344,3 | 20,8 | 0,988 | H5A034 | C | cells | Hm Gen E | Hm Gen E | Hm Gen E | CPE002JV | CPE002OA | CPE002KQ |
| 22121 | J24 | J1 | 894 | 1303 | 18588 | 245,7 | 20,8 | 0,988 | H5A034 | C | cells | Hm Gen E | Hm Gen E | Hm Gen E | CPE002JV | CPE002OA | CPE002KQ |
| 6761 | J24 | J1 | 998 | 614,5 | 20765 | 568,1 | 20,8 | 1,825 | H5A034 | C | cells | Hm Drgbl | Hm Drgbl | Hm Drgbl | CPE002E1 | CPE002EY | CPE002FJ |
| 23272 | J23 | J24 | 921 | 1083 | 19735 | 247,8 | 21,4 | 0,924 | H5A034 | C | cells | Hm Gen E | Hm Gen E | Hm Gen E | CPE002JY | CPE002OD | CPE002KT |
| 23200 | G23 | G24 | 900 | 1066 | 19506 | 285,2 | 21,7 | 0,937 | H5A034 | C | cells | Hm Gen E | Hm Gen E | Hm Gen E | CPE002JY | CPE002OD | CPE002KT |
| 6305 | G24 | G1 | 686 | 530,6 | 14945 | 490,5 | 21,8 | 1,439 | H5A034 | C | cells | Hm Drgbl | Hm Drgbl | Hm Drgbl | CPE002DZ | CPE002F0 | CPE002FL |
| 23273 | J24 | J1 | 948 | 1097 | 21005 | 243,5 | 22,2 | 0,959 | H5A034 | C | cells | Hm Gen E | Hm Gen E | Hm Gen E | CPE002JY | CPE002OD | CPE002KT |
| 18281 | J24 | J1 | 809 | 945,6 | 18427 | 335 | 22,8 | 1,466 | H5A034 | C | cells | Hm Gen E | Hm Gen E | Hm Gen E | CPE002JL | CPE002O0 | CPE002KG |
| 6689 | G24 | G1 | 377 | 582,8 | 9405 | 497,1 | 24,9 | 2,184 | H5A034 | C | cells | Hm Drgbl | Hm Drgbl | Hm Drgbl | CPE002E1 | CPE002EY | CPE002FJ |
| 7073 | G24 | G1 | 355 | 509,6 | 9581 | 474,3 | 27 | 2,101 | H5A034 | C | cells | Hm Drgbl | Hm Drgbl | Hm Drgbl | CPE002E2 | CPE002EX | CPE002FI |
| 3256 | H23 | H24 | 2104 | 1459 | 295 | 231,5 | 0,1 | 0,006 | H5A034 | C | internal1 | Hm Drgbl | Hm Drgbl | Hm Drgbl | CPE002DC | CPE002EG | CPE002F6 |
| 2512 | I23 | I24 | 2499 | 811,5 | 300 | 241,4 | 0,1 | 0,008 | H5A034 | C | internal1 | Hm Drgbl | Hm Drgbl | Hm Drgbl | CPE002DC | CPE002EE | CPE002F4 |
| 7505 | I24 | I1 | 488 | 501,3 | 71 | 223,8 | 0,1 | 0,008 | H5A034 | C | internal1 | Hm Drgbl | Hm Drgbl | Hm Drgbl | CPE002E3 | CPE002EW | CPE002FH |
| 1337 | H24 | H1 | 2559 | 826,4 | 367 | 277,2 | 0,1 | 0,009 | H5A034 | C | internal1 | Hm Drgbl | Hm Drgbl | Hm Drgbl | CPE002FV | CPE002FZ | CPE002G3 |
| 4024 | H23 | H24 | 2192 | 797,5 | 277 | 239,3 | 0,1 | 0,009 | H5A034 | C | internal1 | Hm Drgbl | Hm Drgbl | Hm Drgbl | CPE002DS | CPE002EI | CPE002F8 |
| 4048 | I23 | I24 | 2369 | 827,8 | 344 | 283,8 | 0,1 | 0,009 | H5A034 | C | internal1 | Hm Drgbl | Hm Drgbl | Hm Drgbl | CPE002DS | CPE002EI | CPE002F8 |
| 1720 | H23 | H24 | 2117 | 812,6 | 254 | 237,8 | 0,1 | 0,01 | H5A034 | C | internal1 | Hm Drgbl | Hm Drgbl | Hm Drgbl | CPE002FW | CPE002G0 | CPE002G4 |
| 2872 | H23 | H24 | 2471 | 805,6 | 332 | 420,1 | 0,1 | 0,012 | H5A034 | C | internal1 | Hm Drgbl | Hm Drgbl | Hm Drgbl | CPE002DP | CPE002EF | CPE002F5 |
| 2873 | H24 | H1 | 2630 | 757,3 | 303 | 494,4 | 0,1 | 0,012 | H5A034 | C | internal1 | Hm Drgbl | Hm Drgbl | Hm Drgbl | CPE002DP | CPE002EF | CPE002F5 |
| 2896 | I23 | I24 | 2476 | 772,3 | 197 | 418,7 | 0,1 | 0,012 | H5A034 | C | internal1 | Hm Drgbl | Hm Drgbl | Hm Drgbl | CPE002DP | CPE002EF | CPE002F5 |
| 569 | H24 | H1 | 3166 | 2041 | 349 | 216 | 0,1 | 0,015 | H5A034 | C | internal1 | Hm Drgbl | Hm Drgbl | Hm Drgbl | CPE002E0 | CPE002EZ | CPE002FK |
| 593 | I24 | I1 | 2900 | 1961 | 282 | 261,9 | 0,1 | 0,015 | H5A034 | C | internal1 | Hm Drgbl | Hm Drgbl | Hm Drgbl | CPE002E0 | CPE002EZ | CPE002FK |
| 2488 | H23 | H24 | 2554 | 788,6 | 384 | 280,6 | 0,2 | 0,016 | H5A034 | C | internal1 | Hm Drgbl | Hm Drgbl | Hm Drgbl | CPE002DC | CPE002EE | CPE002F4 |
| 2104 | H23 | H24 | 1616 | 817,3 | 267 | 249,7 | 0,2 | 0,017 | H5A034 | C | internal1 | Hm Drgbl | Hm Drgbl | Hm Drgbl | CPE002FX | CPE002G1 | CPE002G5 |
| 2105 | H24 | H1 | 2317 | 767 | 400 | 269,7 | 0,2 | 0,017 | H5A034 | C | internal1 | Hm Drgbl | Hm Drgbl | Hm Drgbl | CPE002FX | CPE002G1 | CPE002G5 |
| 2128 | I23 | I24 | 2340 | 811,7 | 399 | 246 | 0,2 | 0,017 | H5A034 | C | internal1 | Hm Drgbl | Hm Drgbl | Hm Drgbl | CPE002FX | CPE002G1 | CPE002G5 |
| 4409 | H24 | H1 | 1950 | 797,3 | 442 | 246,7 | 0,2 | 0,017 | H5A034 | C | internal1 | Hm Drgbl | Hm Drgbl | Hm Drgbl | CPE002DL | CPE002EK | CPE002FQ |
| 4432 | I23 | I24 | 2034 | 765,7 | 382 | 268,3 | 0,2 | 0,017 | H5A034 | C | internal1 | Hm Drgbl | Hm Drgbl | Hm Drgbl | CPE002DL | CPE002EK | CPE002FQ |
| 1336 | H23 | H24 | 1991 | 832,6 | 311 | 275,3 | 0,2 | 0,018 | H5A034 | C | internal1 | Hm Drgbl | Hm Drgbl | Hm Drgbl | CPE002FV | CPE002FZ | CPE002G3 |
| 1360 | I23 | I24 | 2468 | 812,9 | 536 | 288,1 | 0,2 | 0,018 | H5A034 | C | internal1 | Hm Drgbl | Hm Drgbl | Hm Drgbl | CPE002FV | CPE002FZ | CPE002G3 |
| 4025 | H24 | H1 | 1787 | 773,3 | 305 | 253,8 | 0,2 | 0,018 | H5A034 | C | internal1 | Hm Drgbl | Hm Drgbl | Hm Drgbl | CPE002DS | CPE002EI | CPE002F8 |
| 952 | H23 | H24 | 2167 | 794,5 | 508 | 290,3 | 0,2 | 0,019 | H5A034 | C | internal1 | Hm Drgbl | Hm Drgbl | Hm Drgbl | CPE002FU | CPE002FY | CPE002G2 |
| 953 | H24 | H1 | 1779 | 797,9 | 402 | 240,7 | 0,2 | 0,019 | H5A034 | C | internal1 | Hm Drgbl | Hm Drgbl | Hm Drgbl | CPE002FU | CPE002FY | CPE002G2 |
| 976 | I23 | I24 | 2229 | 804,7 | 541 | 292 | 0,2 | 0,019 | H5A034 | C | internal1 | Hm Drgbl | Hm Drgbl | Hm Drgbl | CPE002FU | CPE002FY | CPE002G2 |
| 3640 | H23 | H24 | 2459 | 1638 | 378 | 275,7 | 0,2 | 0,019 | H5A034 | C | internal1 | Hm Drgbl | Hm Drgbl | Hm Drgbl | CPE002DR | CPE002EH | CPE002F7 |
| 3641 | H24 | H1 | 1999 | 1499 | 335 | 317,8 | 0,2 | 0,019 | H5A034 | C | internal1 | Hm Drgbl | Hm Drgbl | Hm Drgbl | CPE002DR | CPE002EH | CPE002F7 |
| 3664 | I23 | I24 | 2528 | 1640 | 456 | 286,2 | 0,2 | 0,019 | H5A034 | C | internal1 | Hm Drgbl | Hm Drgbl | Hm Drgbl | CPE002DR | CPE002EH | CPE002F7 |
| 568 | H23 | H24 | 3127 | 2006 | 594 | 297,8 | 0,2 | 0,029 | H5A034 | C | internal1 | Hm Drgbl | Hm Drgbl | Hm Drgbl | CPE002E0 | CPE002EZ | CPE002FK |
| 592 | I23 | I24 | 3400 | 2074 | 574 | 325,5 | 0,2 | 0,029 | H5A034 | C | internal1 | Hm Drgbl | Hm Drgbl | Hm Drgbl | CPE002E0 | CPE002EZ | CPE002FK |
| 3257 | H24 | H1 | 1815 | 1447 | 546 | 256,6 | 0,3 | 0,019 | H5A034 | C | internal1 | Hm Drgbl | Hm Drgbl | Hm Drgbl | CPE002DC | CPE002EG | CPE002F6 |
| 3280 | I23 | I24 | 1946 | 1496 | 508 | 299,3 | 0,3 | 0,019 | H5A034 | C | internal1 | Hm Drgbl | Hm Drgbl | Hm Drgbl | CPE002DC | CPE002EG | CPE002F6 |
| 2489 | H24 | H1 | 1985 | 770,9 | 536 | 241,6 | 0,3 | 0,024 | H5A034 | C | internal1 | Hm Drgbl | Hm Drgbl | Hm Drgbl | CPE002DC | CPE002EE | CPE002F4 |
| 4408 | H23 | H24 | 2044 | 762,2 | 534 | 238,1 | 0,3 | 0,025 | H5A034 | C | internal1 | Hm Drgbl | Hm Drgbl | Hm Drgbl | CPE002DL | CPE002EK | CPE002FQ |
| 4433 | I24 | I1 | 1131 | 700 | 364 | 263,3 | 0,3 | 0,025 | H5A034 | C | internal1 | Hm Drgbl | Hm Drgbl | Hm Drgbl | CPE002DL | CPE002EK | CPE002FQ |
| 977 | I24 | I1 | 2012 | 795,8 | 565 | 234,8 | 0,3 | 0,028 | H5A034 | C | internal1 | Hm Drgbl | Hm Drgbl | Hm Drgbl | CPE002FU | CPE002FY | CPE002G2 |
| 1721 | H24 | H1 | 1664 | 839,3 | 556 | 260,8 | 0,3 | 0,031 | H5A034 | C | internal1 | Hm Drgbl | Hm Drgbl | Hm Drgbl | CPE002FW | CPE002G0 | CPE002G4 |
| 1744 | I23 | I24 | 2472 | 907,5 | 779 | 295 | 0,3 | 0,031 | H5A034 | C | internal1 | Hm Drgbl | Hm Drgbl | Hm Drgbl | CPE002FW | CPE002G0 | CPE002G4 |
| 4816 | I23 | I24 | 1841 | 920,7 | 635 | 254 | 0,3 | 0,033 | H5A034 | C | internal1 | Hm Drgbl | Hm Drgbl | Hm Drgbl | CPE002DV | CPE002EL | CPE002FP |
| 10577 | I24 | I1 | 2334 | 953 | 751 | 166,1 | 0,3 | 0,034 | H5A034 | C | internal1 | Hm Drgbl | Hm Drgbl | Hm Drgbl | CPE002EB | CPE002EO | CPE002FR |
| 2513 | I24 | I1 | 1115 | 686,8 | 463 | 280,4 | 0,4 | 0,031 | H5A034 | C | internal1 | Hm Drgbl | Hm Drgbl | Hm Drgbl | CPE002DC | CPE002EE | CPE002F4 |
| 2129 | I24 | I1 | 1159 | 664,7 | 515 | 314,8 | 0,4 | 0,035 | H5A034 | C | internal1 | Hm Drgbl | Hm Drgbl | Hm Drgbl | CPE002FX | CPE002G1 | CPE002G5 |
| 5560 | H23 | H24 | 2494 | 772,9 | 915 | 276,5 | 0,4 | 0,038 | H5A034 | C | internal1 | Hm Drgbl | Hm Drgbl | Hm Drgbl | CPE002DX | CPE002F2 | CPE002FN |
| 8248 | H23 | H24 | 1972 | 689,5 | 867 | 294,2 | 0,4 | 0,038 | H5A034 | C | internal1 | Hm Drgbl | Hm Drgbl | Hm Drgbl | CPE002E5 | CPE002EU | CPE002FF |
| 8249 | H24 | H1 | 1615 | 660,3 | 567 | 257,9 | 0,4 | 0,038 | H5A034 | C | internal1 | Hm Drgbl | Hm Drgbl | Hm Drgbl | CPE002E5 | CPE002EU | CPE002FF |
| 10553 | H24 | H1 | 2212 | 930,6 | 960 | 212 | 0,4 | 0,045 | H5A034 | C | internal1 | Hm Drgbl | Hm Drgbl | Hm Drgbl | CPE002EB | CPE002EO | CPE002FR |
| 7864 | H23 | H24 | 2427 | 741,2 | 997 | 184,3 | 0,4 | 0,061 | H5A034 | C | internal1 | Hm Drgbl | Hm Drgbl | Hm Drgbl | CPE002E4 | CPE002EV | CPE002FG |
| 7865 | H24 | H1 | 1437 | 611 | 607 | 261,7 | 0,4 | 0,061 | H5A034 | C | internal1 | Hm Drgbl | Hm Drgbl | Hm Drgbl | CPE002E4 | CPE002EV | CPE002FG |
| 7888 | I23 | I24 | 2302 | 695,4 | 811 | 216,2 | 0,4 | 0,061 | H5A034 | C | internal1 | Hm Drgbl | Hm Drgbl | Hm Drgbl | CPE002E4 | CPE002EV | CPE002FG |
| 3281 | I24 | I1 | 802 | 1196 | 422 | 286,1 | 0,5 | 0,031 | H5A034 | C | internal1 | Hm Drgbl | Hm Drgbl | Hm Drgbl | CPE002DC | CPE002EG | CPE002F6 |
| 5200 | I23 | I24 | 1869 | 674 | 998 | 386 | 0,5 | 0,039 | H5A034 | C | internal1 | Hm Drgbl | Hm Drgbl | Hm Drgbl | CPE002DV | CPE002EM | CPE002FO |
| 7120 | I23 | I24 | 1715 | 643,5 | 895 | 293,1 | 0,5 | 0,039 | H5A034 | C | internal1 | Hm Drgbl | Hm Drgbl | Hm Drgbl | CPE002E2 | CPE002EX | CPE002FI |
| 208 | I23 | I24 | 686 | 1592 | 373 | 201,2 | 0,5 | 0,043 | H5A034 | C | internal1 | Hm Drgbl | Hm Drgbl | Hm Drgbl | CPE002DT | CPE002EJ | CPE002F9 |
| 209 | I24 | I1 | 603 | 1615 | 288 | 179,5 | 0,5 | 0,043 | H5A034 | C | internal1 | Hm Drgbl | Hm Drgbl | Hm Drgbl | CPE002DT | CPE002EJ | CPE002F9 |
| 4049 | I24 | I1 | 933 | 656,7 | 455 | 266,2 | 0,5 | 0,044 | H5A034 | C | internal1 | Hm Drgbl | Hm Drgbl | Hm Drgbl | CPE002DS | CPE002EI | CPE002F8 |
| 6713 | H24 | H1 | 1154 | 604,3 | 607 | 349,3 | 0,5 | 0,044 | H5A034 | C | internal1 | Hm Drgbl | Hm Drgbl | Hm Drgbl | CPE002E1 | CPE002EY | CPE002FJ |
| 5944 | H23 | H24 | 2326 | 932,9 | 1070 | 328,7 | 0,5 | 0,046 | H5A034 | C | internal1 | Hm Drgbl | Hm Drgbl | Hm Drgbl | CPE002DY | CPE002F1 | CPE002FM |
| 3665 | I24 | I1 | 785 | 1301 | 422 | 211,9 | 0,5 | 0,048 | H5A034 | C | internal1 | Hm Drgbl | Hm Drgbl | Hm Drgbl | CPE002DR | CPE002EH | CPE002F7 |
| 5584 | I23 | I24 | 2176 | 791,3 | 1194 | 346,4 | 0,5 | 0,048 | H5A034 | C | internal1 | Hm Drgbl | Hm Drgbl | Hm Drgbl | CPE002DX | CPE002F2 | CPE002FN |
| 8272 | I23 | I24 | 2026 | 734,5 | 981 | 238,4 | 0,5 | 0,048 | H5A034 | C | internal1 | Hm Drgbl | Hm Drgbl | Hm Drgbl | CPE002E5 | CPE002EU | CPE002FF |
| 4792 | H23 | H24 | 2096 | 889,6 | 975 | 245,3 | 0,5 | 0,055 | H5A034 | C | internal1 | Hm Drgbl | Hm Drgbl | Hm Drgbl | CPE002DV | CPE002EL | CPE002FP |
| 4793 | H24 | H1 | 2072 | 1001 | 1058 | 277,3 | 0,5 | 0,055 | H5A034 | C | internal1 | Hm Drgbl | Hm Drgbl | Hm Drgbl | CPE002DV | CPE002EL | CPE002FP |
| 7480 | H23 | H24 | 2010 | 713,3 | 1244 | 301,1 | 0,6 | 0,046 | H5A034 | C | internal1 | Hm Drgbl | Hm Drgbl | Hm Drgbl | CPE002E3 | CPE002EW | CPE002FH |
| 7481 | H24 | H1 | 1225 | 572,4 | 677 | 384,6 | 0,6 | 0,046 | H5A034 | C | internal1 | Hm Drgbl | Hm Drgbl | Hm Drgbl | CPE002E3 | CPE002EW | CPE002FH |
| 7504 | I23 | I24 | 1534 | 701,7 | 945 | 218 | 0,6 | 0,046 | H5A034 | C | internal1 | Hm Drgbl | Hm Drgbl | Hm Drgbl | CPE002E3 | CPE002EW | CPE002FH |
| 7096 | H23 | H24 | 1350 | 579,6 | 864 | 345,2 | 0,6 | 0,047 | H5A034 | C | internal1 | Hm Drgbl | Hm Drgbl | Hm Drgbl | CPE002E2 | CPE002EX | CPE002FI |
| 184 | H23 | H24 | 585 | 1606 | 366 | 270,9 | 0,6 | 0,051 | H5A034 | C | internal1 | Hm Drgbl | Hm Drgbl | Hm Drgbl | CPE002DT | CPE002EJ | CPE002F9 |
| 1745 | I24 | I1 | 1183 | 698,2 | 685 | 236,6 | 0,6 | 0,063 | H5A034 | C | internal1 | Hm Drgbl | Hm Drgbl | Hm Drgbl | CPE002FW | CPE002G0 | CPE002G4 |
| 5176 | H23 | H24 | 1849 | 699,5 | 1350 | 308,5 | 0,7 | 0,055 | H5A034 | C | internal1 | Hm Drgbl | Hm Drgbl | Hm Drgbl | CPE002DV | CPE002EM | CPE002FO |
| 5561 | H24 | H1 | 1613 | 745,3 | 1130 | 375,3 | 0,7 | 0,067 | H5A034 | C | internal1 | Hm Drgbl | Hm Drgbl | Hm Drgbl | CPE002DX | CPE002F2 | CPE002FN |
| 4817 | I24 | I1 | 572 | 740,7 | 378 | 304,6 | 0,7 | 0,077 | H5A034 | C | internal1 | Hm Drgbl | Hm Drgbl | Hm Drgbl | CPE002DV | CPE002EL | CPE002FP |
| 2897 | I24 | I1 | 585 | 637,7 | 392 | 510,9 | 0,7 | 0,082 | H5A034 | C | internal1 | Hm Drgbl | Hm Drgbl | Hm Drgbl | CPE002DP | CPE002EF | CPE002F5 |
| 7889 | I24 | I1 | 648 | 552,7 | 465 | 247,8 | 0,7 | 0,107 | H5A034 | C | internal1 | Hm Drgbl | Hm Drgbl | Hm Drgbl | CPE002E4 | CPE002EV | CPE002FG |
| 6328 | H23 | H24 | 1681 | 614,9 | 1288 | 338,9 | 0,8 | 0,053 | H5A034 | C | internal1 | Hm Drgbl | Hm Drgbl | Hm Drgbl | CPE002DZ | CPE002F0 | CPE002FL |
| 6329 | H24 | H1 | 1327 | 644,5 | 1107 | 350,7 | 0,8 | 0,053 | H5A034 | C | internal1 | Hm Drgbl | Hm Drgbl | Hm Drgbl | CPE002DZ | CPE002F0 | CPE002FL |
| 6353 | I24 | I1 | 479 | 519 | 370 | 404 | 0,8 | 0,053 | H5A034 | C | internal1 | Hm Drgbl | Hm Drgbl | Hm Drgbl | CPE002DZ | CPE002F0 | CPE002FL |
| 10937 | H24 | H1 | 2090 | 934,2 | 1660 | 515,5 | 0,8 | 0,061 | H5A034 | C | internal1 | Hm Drgbl | Hm Drgbl | Hm Drgbl | CPE002EC | CPE002EN | CPE002FS |
| 7097 | H24 | H1 | 1405 | 647,7 | 1079 | 325 | 0,8 | 0,062 | H5A034 | C | internal1 | Hm Drgbl | Hm Drgbl | Hm Drgbl | CPE002E2 | CPE002EX | CPE002FI |
| 11729 | I24 | I1 | 2283 | 963,1 | 1774 | 183,6 | 0,8 | 0,071 | H5A034 | C | internal1 | Hm Gen E | Hm Gen E | Hm Gen E | CPE002JA | CPE002NJ | CPE002JZ |
| 5945 | H24 | H1 | 1797 | 878,4 | 1498 | 290,1 | 0,8 | 0,073 | H5A034 | C | internal1 | Hm Drgbl | Hm Drgbl | Hm Drgbl | CPE002DY | CPE002F1 | CPE002FM |
| 15544 | H23 | H24 | 1973 | 934,3 | 1608 | 159,7 | 0,8 | 0,073 | H5A034 | C | internal1 | Hm Gen E | Hm Gen E | Hm Gen E | CPE002JE | CPE002NT | CPE002K9 |
| 15568 | I23 | I24 | 2264 | 970,7 | 1853 | 143,8 | 0,8 | 0,073 | H5A034 | C | internal1 | Hm Gen E | Hm Gen E | Hm Gen E | CPE002JE | CPE002NT | CPE002K9 |
| 10552 | H23 | H24 | 2023 | 922,4 | 1592 | 218,6 | 0,8 | 0,09 | H5A034 | C | internal1 | Hm Drgbl | Hm Drgbl | Hm Drgbl | CPE002EB | CPE002EO | CPE002FR |
| 7121 | I24 | I1 | 605 | 492,1 | 541 | 381,3 | 0,9 | 0,07 | H5A034 | C | internal1 | Hm Drgbl | Hm Drgbl | Hm Drgbl | CPE002E2 | CPE002EX | CPE002FI |
| 185 | H24 | H1 | 590 | 1668 | 510 | 160,8 | 0,9 | 0,077 | H5A034 | C | internal1 | Hm Drgbl | Hm Drgbl | Hm Drgbl | CPE002DT | CPE002EJ | CPE002F9 |
| 15569 | I24 | I1 | 2325 | 981,7 | 2123 | 180 | 0,9 | 0,082 | H5A034 | C | internal1 | Hm Gen E | Hm Gen E | Hm Gen E | CPE002JE | CPE002NT | CPE002K9 |
| 1361 | I24 | I1 | 1227 | 772,1 | 1077 | 230 | 0,9 | 0,083 | H5A034 | C | internal1 | Hm Drgbl | Hm Drgbl | Hm Drgbl | CPE002FV | CPE002FZ | CPE002G3 |
| 13625 | H24 | H1 | 2011 | 1177 | 1883 | 235,2 | 0,9 | 0,085 | H5A034 | C | internal1 | Hm Gen E | Hm Gen E | Hm Gen E | CPE002JC | CPE002NC | CPE002K4 |
| 6352 | I23 | I24 | 1469 | 640 | 1420 | 268,6 | 1 | 0,066 | H5A034 | C | internal1 | Hm Drgbl | Hm Drgbl | Hm Drgbl | CPE002DZ | CPE002F0 | CPE002FL |
| 12881 | I24 | I1 | 2329 | 1262 | 2397 | 181,9 | 1 | 0,068 | H5A034 | C | internal1 | Hm Gen E | Hm Gen E | Hm Gen E | CPE002J6 | CPE002NN | CPE002K2 |
| 10936 | H23 | H24 | 2256 | 972,8 | 2252 | 605,8 | 1 | 0,076 | H5A034 | C | internal1 | Hm Drgbl | Hm Drgbl | Hm Drgbl | CPE002EC | CPE002EN | CPE002FS |
| 16721 | I24 | I1 | 1763 | 1004 | 1763 | 246,4 | 1 | 0,079 | H5A034 | C | internal1 | Hm Gen E | Hm Gen E | Hm Gen E | CPE002JH | CPE002NV | CPE002KC |
| 9016 | H23 | H24 | 2503 | 2277 | 2396 | 222,4 | 1 | 0,08 | H5A034 | C | internal1 | Hm Drgbl | Hm Drgbl | Hm Drgbl | CPE002E7 | CPE002ES | CPE002FD |
| 6712 | H23 | H24 | 1959 | 732,4 | 1962 | 276,5 | 1 | 0,088 | H5A034 | C | internal1 | Hm Drgbl | Hm Drgbl | Hm Drgbl | CPE002E1 | CPE002EY | CPE002FJ |
| 10193 | I24 | I1 | 2164 | 979,8 | 2213 | 168,2 | 1 | 0,096 | H5A034 | C | internal1 | Hm Drgbl | Hm Drgbl | Hm Drgbl | CPE002EA | CPE002EP | CPE002FA |
| 12880 | I23 | I24 | 2057 | 1167 | 2312 | 192,1 | 1,1 | 0,075 | H5A034 | C | internal1 | Hm Gen E | Hm Gen E | Hm Gen E | CPE002J6 | CPE002NN | CPE002K2 |
| 14393 | H24 | H1 | 2166 | 933,8 | 2320 | 171 | 1,1 | 0,078 | H5A034 | C | internal1 | Hm Gen E | Hm Gen E | Hm Gen E | CPE002J7 | CPE002NC | CPE002K6 |
| 5177 | H24 | H1 | 1321 | 735,1 | 1419 | 347,4 | 1,1 | 0,086 | H5A034 | C | internal1 | Hm Drgbl | Hm Drgbl | Hm Drgbl | CPE002DV | CPE002EM | CPE002FO |
| 6736 | I23 | I24 | 1631 | 677,4 | 1806 | 434,1 | 1,1 | 0,096 | H5A034 | C | internal1 | Hm Drgbl | Hm Drgbl | Hm Drgbl | CPE002E1 | CPE002EY | CPE002FJ |
| 11728 | I23 | I24 | 2013 | 944,1 | 2127 | 194,7 | 1,1 | 0,098 | H5A034 | C | internal1 | Hm Gen E | Hm Gen E | Hm Gen E | CPE002JA | CPE002NJ | CPE002JZ |
| 5968 | I23 | I24 | 2027 | 1009 | 2245 | 256,9 | 1,1 | 0,1 | H5A034 | C | internal1 | Hm Drgbl | Hm Drgbl | Hm Drgbl | CPE002DY | CPE002F1 | CPE002FM |
| 16312 | H23 | H24 | 1968 | 1098 | 2229 | 258,8 | 1,1 | 0,101 | H5A034 | C | internal1 | Hm Gen E | Hm Gen E | Hm Gen E | CPE002JG | CPE002NV | CPE002KB |
| 8273 | I24 | I1 | 885 | 641,7 | 952 | 191,5 | 1,1 | 0,106 | H5A034 | C | internal1 | Hm Drgbl | Hm Drgbl | Hm Drgbl | CPE002E5 | CPE002EU | CPE002FF |
| 10192 | I23 | I24 | 1981 | 938,5 | 2194 | 189,7 | 1,1 | 0,106 | H5A034 | C | internal1 | Hm Drgbl | Hm Drgbl | Hm Drgbl | CPE002EA | CPE002EP | CPE002FA |
| 12113 | I24 | I1 | 2179 | 930,7 | 2658 | 98,92 | 1,2 | 0,077 | H5A034 | C | internal1 | Hm Gen E | Hm Gen E | Hm Gen E | CPE002J4 | CPE002NL | CPE002K0 |
| 9425 | I24 | I1 | 2336 | 1214 | 2900 | 195,7 | 1,2 | 0,083 | H5A034 | C | internal1 | Hm Drgbl | Hm Drgbl | Hm Drgbl | CPE002E8 | CPE002ER | CPE002FC |
| 9784 | H23 | H24 | 2384 | 1259 | 2869 | 212,5 | 1,2 | 0,084 | H5A034 | C | internal1 | Hm Drgbl | Hm Drgbl | Hm Drgbl | CPE002E9 | CPE002EQ | CPE002FB |
| 9785 | H24 | H1 | 2001 | 1116 | 2471 | 192,9 | 1,2 | 0,084 | H5A034 | C | internal1 | Hm Drgbl | Hm Drgbl | Hm Drgbl | CPE002E9 | CPE002EQ | CPE002FB |
| 9808 | I23 | I24 | 2287 | 1200 | 2822 | 178,8 | 1,2 | 0,084 | H5A034 | C | internal1 | Hm Drgbl | Hm Drgbl | Hm Drgbl | CPE002E9 | CPE002EQ | CPE002FB |
| 8633 | H24 | H1 | 2076 | 1196 | 2411 | 160,3 | 1,2 | 0,085 | H5A034 | C | internal1 | Hm Drgbl | Hm Drgbl | Hm Drgbl | CPE002E6 | CPE002ET | CPE002FE |
| 11320 | H23 | H24 | 2122 | 933,8 | 2620 | 216,1 | 1,2 | 0,087 | H5A034 | C | internal1 | Hm Drgbl | Hm Drgbl | Hm Drgbl | CPE002ED | CPE002F3 | CPE002FT |
| 11321 | H24 | H1 | 1837 | 878,7 | 2165 | 219,7 | 1,2 | 0,087 | H5A034 | C | internal1 | Hm Drgbl | Hm Drgbl | Hm Drgbl | CPE002ED | CPE002F3 | CPE002FT |
| 11345 | I24 | I1 | 2020 | 938 | 2325 | 190,6 | 1,2 | 0,087 | H5A034 | C | internal1 | Hm Drgbl | Hm Drgbl | Hm Drgbl | CPE002ED | CPE002F3 | CPE002FT |
| 12497 | I24 | I1 | 2034 | 1190 | 2516 | 206,3 | 1,2 | 0,09 | H5A034 | C | internal1 | Hm Gen E | Hm Gen E | Hm Gen E | CPE002J5 | CPE002NK | CPE002K1 |
| 5201 | I24 | I1 | 381 | 592,6 | 443 | 422,2 | 1,2 | 0,094 | H5A034 | C | internal1 | Hm Drgbl | Hm Drgbl | Hm Drgbl | CPE002DV | CPE002EM | CPE002FO |
| 16313 | H24 | H1 | 1030 | 1077 | 1272 | 187,6 | 1,2 | 0,11 | H5A034 | C | internal1 | Hm Gen E | Hm Gen E | Hm Gen E | CPE002JG | CPE002NV | CPE002KB |
| 10168 | H23 | H24 | 1998 | 923,6 | 2367 | 178,9 | 1,2 | 0,115 | H5A034 | C | internal1 | Hm Drgbl | Hm Drgbl | Hm Drgbl | CPE002EA | CPE002EP | CPE002FA |
| 10576 | I23 | I24 | 1937 | 885,5 | 2252 | 212,6 | 1,2 | 0,136 | H5A034 | C | internal1 | Hm Drgbl | Hm Drgbl | Hm Drgbl | CPE002EB | CPE002EO | CPE002FR |
| 12472 | H23 | H24 | 2351 | 1222 | 3107 | 243,8 | 1,3 | 0,098 | H5A034 | C | internal1 | Hm Gen E | Hm Gen E | Hm Gen E | CPE002J5 | CPE002NK | CPE002K1 |
| 12473 | H24 | H1 | 2128 | 1209 | 2763 | 206,8 | 1,3 | 0,098 | H5A034 | C | internal1 | Hm Gen E | Hm Gen E | Hm Gen E | CPE002J5 | CPE002NK | CPE002K1 |
| 16696 | H23 | H24 | 1985 | 1037 | 2673 | 275,2 | 1,3 | 0,103 | H5A034 | C | internal1 | Hm Gen E | Hm Gen E | Hm Gen E | CPE002JH | CPE002NV | CPE002KC |
| 9017 | H24 | H1 | 2204 | 1906 | 2857 | 219,1 | 1,3 | 0,104 | H5A034 | C | internal1 | Hm Drgbl | Hm Drgbl | Hm Drgbl | CPE002E7 | CPE002ES | CPE002FD |
| 13649 | I24 | I1 | 2067 | 1184 | 2641 | 210,7 | 1,3 | 0,122 | H5A034 | C | internal1 | Hm Gen E | Hm Gen E | Hm Gen E | CPE002JC | CPE002NC | CPE002K4 |
| 14033 | I24 | I1 | 2330 | 1273 | 3242 | 174,3 | 1,4 | 0,08 | H5A034 | C | internal1 | Hm Gen E | Hm Gen E | Hm Gen E | CPE002JF | CPE002NP | CPE002K5 |
| 12857 | H24 | H1 | 1790 | 1083 | 2585 | 204,1 | 1,4 | 0,095 | H5A034 | C | internal1 | Hm Gen E | Hm Gen E | Hm Gen E | CPE002J6 | CPE002NN | CPE002K2 |
| 9041 | I24 | I1 | 1933 | 1910 | 2728 | 203,3 | 1,4 | 0,112 | H5A034 | C | internal1 | Hm Drgbl | Hm Drgbl | Hm Drgbl | CPE002E7 | CPE002ES | CPE002FD |
| 16336 | I23 | I24 | 1967 | 1011 | 2668 | 308,3 | 1,4 | 0,128 | H5A034 | C | internal1 | Hm Gen E | Hm Gen E | Hm Gen E | CPE002JG | CPE002NV | CPE002KB |
| 13624 | H23 | H24 | 2247 | 1218 | 3098 | 237,5 | 1,4 | 0,131 | H5A034 | C | internal1 | Hm Gen E | Hm Gen E | Hm Gen E | CPE002JC | CPE002NC | CPE002K4 |
| 10169 | H24 | H1 | 1891 | 889,2 | 2635 | 176,8 | 1,4 | 0,135 | H5A034 | C | internal1 | Hm Drgbl | Hm Drgbl | Hm Drgbl | CPE002EA | CPE002EP | CPE002FA |
| 14008 | H23 | H24 | 2185 | 1200 | 3179 | 206,5 | 1,5 | 0,086 | H5A034 | C | internal1 | Hm Gen E | Hm Gen E | Hm Gen E | CPE002JF | CPE002NP | CPE002K5 |
| 14009 | H24 | H1 | 1967 | 1211 | 2856 | 178,1 | 1,5 | 0,086 | H5A034 | C | internal1 | Hm Gen E | Hm Gen E | Hm Gen E | CPE002JF | CPE002NP | CPE002K5 |
| 12856 | H23 | H24 | 2056 | 1170 | 3095 | 222,5 | 1,5 | 0,102 | H5A034 | C | internal1 | Hm Gen E | Hm Gen E | Hm Gen E | CPE002J6 | CPE002NN | CPE002K2 |
| 9401 | H24 | H1 | 2049 | 1174 | 3127 | 188,1 | 1,5 | 0,104 | H5A034 | C | internal1 | Hm Drgbl | Hm Drgbl | Hm Drgbl | CPE002E8 | CPE002ER | CPE002FC |
| 8657 | I24 | I1 | 2272 | 1306 | 3508 | 168,9 | 1,5 | 0,106 | H5A034 | C | internal1 | Hm Drgbl | Hm Drgbl | Hm Drgbl | CPE002E6 | CPE002ET | CPE002FE |
| 19409 | I24 | I1 | 1344 | 1239 | 2057 | 324,3 | 1,5 | 0,106 | H5A034 | C | internal1 | Hm Gen E | Hm Gen E | Hm Gen E | CPE002JO | CPE002O3 | CPE002KJ |
| 14417 | I24 | I1 | 1865 | 943,7 | 2848 | 167,7 | 1,5 | 0,107 | H5A034 | C | internal1 | Hm Gen E | Hm Gen E | Hm Gen E | CPE002J7 | CPE002NC | CPE002K6 |
| 11344 | I23 | I24 | 2288 | 981,5 | 3488 | 215,8 | 1,5 | 0,109 | H5A034 | C | internal1 | Hm Drgbl | Hm Drgbl | Hm Drgbl | CPE002ED | CPE002F3 | CPE002FT |
| 12496 | I23 | I24 | 2008 | 1167 | 3059 | 227,9 | 1,5 | 0,113 | H5A034 | C | internal1 | Hm Gen E | Hm Gen E | Hm Gen E | CPE002J5 | CPE002NK | CPE002K1 |
| 10961 | I24 | I1 | 2202 | 1036 | 3344 | 606,4 | 1,5 | 0,114 | H5A034 | C | internal1 | Hm Drgbl | Hm Drgbl | Hm Drgbl | CPE002EC | CPE002EN | CPE002FS |
| 9040 | I23 | I24 | 2077 | 1975 | 3160 | 226,5 | 1,5 | 0,12 | H5A034 | C | internal1 | Hm Drgbl | Hm Drgbl | Hm Drgbl | CPE002E7 | CPE002ES | CPE002FD |
| 6737 | I24 | I1 | 372 | 609,2 | 559 | 215,5 | 1,5 | 0,132 | H5A034 | C | internal1 | Hm Drgbl | Hm Drgbl | Hm Drgbl | CPE002E1 | CPE002EY | CPE002FJ |
| 11704 | H23 | H24 | 1927 | 947,1 | 2919 | 208,3 | 1,5 | 0,134 | H5A034 | C | internal1 | Hm Gen E | Hm Gen E | Hm Gen E | CPE002JA | CPE002NJ | CPE002JZ |
| 21688 | H23 | H24 | 578 | 1163 | 888 | 406,6 | 1,5 | 0,158 | H5A034 | C | internal1 | Hm Gen E | Hm Gen E | Hm Gen E | CPE002JU | CPE002O9 | CPE002KP |
| 21689 | H24 | H1 | 405 | 876,3 | 601 | 369,5 | 1,5 | 0,158 | H5A034 | C | internal1 | Hm Gen E | Hm Gen E | Hm Gen E | CPE002JU | CPE002O9 | CPE002KP |
| 20153 | H24 | H1 | 1475 | 1121 | 2205 | 72,77 | 1,5 | 0,183 | H5A034 | C | internal1 | Hm Gen E | Hm Gen E | Hm Gen E | CPE002JQ | CPE002O5 | CPE002KL |
| 15953 | I24 | I1 | 2179 | 975,8 | 3450 | 126,6 | 1,6 | 0,089 | H5A034 | C | internal1 | Hm Gen E | Hm Gen E | Hm Gen E | CPE002J9 | CPE002NL | CPE002KA |
| 12089 | H24 | H1 | 2053 | 888,1 | 3369 | 134,5 | 1,6 | 0,103 | H5A034 | C | internal1 | Hm Gen E | Hm Gen E | Hm Gen E | CPE002J4 | CPE002NL | CPE002K0 |
| 9809 | I24 | I1 | 1960 | 1071 | 3103 | 202,7 | 1,6 | 0,112 | H5A034 | C | internal1 | Hm Drgbl | Hm Drgbl | Hm Drgbl | CPE002E9 | CPE002EQ | CPE002FB |
| 8632 | H23 | H24 | 2274 | 1238 | 3693 | 202,7 | 1,6 | 0,113 | H5A034 | C | internal1 | Hm Drgbl | Hm Drgbl | Hm Drgbl | CPE002E6 | CPE002ET | CPE002FE |
| 21305 | H24 | H1 | 1332 | 941,6 | 2075 | 227,4 | 1,6 | 0,113 | H5A034 | C | internal1 | Hm Gen E | Hm Gen E | Hm Gen E | CPE002JT | CPE002O8 | CPE002KO |
| 14416 | I23 | I24 | 2176 | 969,7 | 3445 | 198,3 | 1,6 | 0,114 | H5A034 | C | internal1 | Hm Gen E | Hm Gen E | Hm Gen E | CPE002J7 | CPE002NC | CPE002K6 |
| 10960 | I23 | I24 | 2075 | 949,4 | 3247 | 720,9 | 1,6 | 0,121 | H5A034 | C | internal1 | Hm Drgbl | Hm Drgbl | Hm Drgbl | CPE002EC | CPE002EN | CPE002FS |
| 5969 | I24 | I1 | 316 | 788,8 | 502 | 255 | 1,6 | 0,146 | H5A034 | C | internal1 | Hm Drgbl | Hm Drgbl | Hm Drgbl | CPE002DY | CPE002F1 | CPE002FM |
| 15928 | H23 | H24 | 2041 | 972,9 | 3433 | 151,1 | 1,7 | 0,095 | H5A034 | C | internal1 | Hm Gen E | Hm Gen E | Hm Gen E | CPE002J9 | CPE002NL | CPE002KA |
| 14032 | I23 | I24 | 2182 | 1256 | 3755 | 179,7 | 1,7 | 0,097 | H5A034 | C | internal1 | Hm Gen E | Hm Gen E | Hm Gen E | CPE002JF | CPE002NP | CPE002K5 |
| 9400 | H23 | H24 | 2000 | 1208 | 3490 | 194,4 | 1,7 | 0,118 | H5A034 | C | internal1 | Hm Drgbl | Hm Drgbl | Hm Drgbl | CPE002E8 | CPE002ER | CPE002FC |
| 14392 | H23 | H24 | 1974 | 944,8 | 3373 | 169,1 | 1,7 | 0,121 | H5A034 | C | internal1 | Hm Gen E | Hm Gen E | Hm Gen E | CPE002J7 | CPE002NC | CPE002K6 |
| 17080 | H23 | H24 | 2118 | 1049 | 3539 | 144 | 1,7 | 0,131 | H5A034 | C | internal1 | Hm Gen E | Hm Gen E | Hm Gen E | CPE002JI | CPE002NZ | CPE002KD |
| 17081 | H24 | H1 | 1255 | 962,4 | 2162 | 106,7 | 1,7 | 0,131 | H5A034 | C | internal1 | Hm Gen E | Hm Gen E | Hm Gen E | CPE002JI | CPE002NZ | CPE002KD |
| 16697 | H24 | H1 | 1197 | 1005 | 2089 | 217,4 | 1,7 | 0,134 | H5A034 | C | internal1 | Hm Gen E | Hm Gen E | Hm Gen E | CPE002JH | CPE002NV | CPE002KC |
| 16720 | I23 | I24 | 1544 | 1039 | 2574 | 255,8 | 1,7 | 0,134 | H5A034 | C | internal1 | Hm Gen E | Hm Gen E | Hm Gen E | CPE002JH | CPE002NV | CPE002KC |
| 11705 | H24 | H1 | 1945 | 952,9 | 3292 | 166 | 1,7 | 0,152 | H5A034 | C | internal1 | Hm Gen E | Hm Gen E | Hm Gen E | CPE002JA | CPE002NJ | CPE002JZ |
| 16337 | I24 | I1 | 1117 | 997,6 | 1913 | 242,2 | 1,7 | 0,156 | H5A034 | C | internal1 | Hm Gen E | Hm Gen E | Hm Gen E | CPE002JG | CPE002NV | CPE002KB |
| 20945 | I24 | I1 | 1403 | 1013 | 2457 | 206,5 | 1,8 | 0,128 | H5A034 | C | internal1 | Hm Gen E | Hm Gen E | Hm Gen E | CPE002JS | CPE002O7 | CPE002KN |
| 21304 | H23 | H24 | 1638 | 1014 | 2908 | 310,5 | 1,8 | 0,128 | H5A034 | C | internal1 | Hm Gen E | Hm Gen E | Hm Gen E | CPE002JT | CPE002O8 | CPE002KO |
| 17849 | H24 | H1 | 1385 | 1041 | 2522 | 170,4 | 1,8 | 0,142 | H5A034 | C | internal1 | Hm Gen E | Hm Gen E | Hm Gen E | CPE002JK | CPE002NZ | CPE002KF |
| 15545 | H24 | H1 | 1954 | 911,1 | 3440 | 207,7 | 1,8 | 0,164 | H5A034 | C | internal1 | Hm Gen E | Hm Gen E | Hm Gen E | CPE002JE | CPE002NT | CPE002K9 |
| 14777 | H24 | H1 | 2135 | 967,8 | 4033 | 129,9 | 1,9 | 0,108 | H5A034 | C | internal1 | Hm Gen E | Hm Gen E | Hm Gen E | CPE002JD | CPE002NR | CPE002K7 |
| 19793 | I24 | I1 | 1265 | 342,5 | 2363 | 307,8 | 1,9 | 0,124 | H5A034 | C | internal1 | Hm Gen E | Hm Gen E | Hm Gen E | CPE002JP | CPE002O4 | CPE002KK |
| 17489 | I24 | I1 | 1328 | 1028 | 2513 | 172,8 | 1,9 | 0,13 | H5A034 | C | internal1 | Hm Gen E | Hm Gen E | Hm Gen E | CPE002JJ | CPE002NY | CPE002KE |
| 20920 | H23 | H24 | 1731 | 1049 | 3310 | 244,5 | 1,9 | 0,135 | H5A034 | C | internal1 | Hm Gen E | Hm Gen E | Hm Gen E | CPE002JS | CPE002O7 | CPE002KN |
| 17105 | I24 | I1 | 1448 | 1007 | 2765 | 128,2 | 1,9 | 0,147 | H5A034 | C | internal1 | Hm Gen E | Hm Gen E | Hm Gen E | CPE002JI | CPE002NZ | CPE002KD |
| 17873 | I24 | I1 | 1478 | 1005 | 2871 | 185,6 | 1,9 | 0,15 | H5A034 | C | internal1 | Hm Gen E | Hm Gen E | Hm Gen E | CPE002JK | CPE002NZ | CPE002KF |
| 13241 | H24 | H1 | 2150 | 1211 | 4387 | 142 | 2 | 0,1 | H5A034 | C | internal1 | Hm Gen E | Hm Gen E | Hm Gen E | CPE002JB | CPE002NN | CPE002K3 |
| 12112 | I23 | I24 | 2141 | 912,7 | 4313 | 149,1 | 2 | 0,128 | H5A034 | C | internal1 | Hm Gen E | Hm Gen E | Hm Gen E | CPE002J4 | CPE002NL | CPE002K0 |
| 19384 | H23 | H24 | 1423 | 1298 | 2897 | 431,6 | 2 | 0,142 | H5A034 | C | internal1 | Hm Gen E | Hm Gen E | Hm Gen E | CPE002JO | CPE002O3 | CPE002KJ |
| 21329 | I24 | I1 | 1309 | 901,6 | 2571 | 204 | 2 | 0,142 | H5A034 | C | internal1 | Hm Gen E | Hm Gen E | Hm Gen E | CPE002JT | CPE002O8 | CPE002KO |
| 17848 | H23 | H24 | 1802 | 1077 | 3596 | 261 | 2 | 0,158 | H5A034 | C | internal1 | Hm Gen E | Hm Gen E | Hm Gen E | CPE002JK | CPE002NZ | CPE002KF |
| 13648 | I23 | I24 | 1661 | 1134 | 3386 | 224,9 | 2 | 0,188 | H5A034 | C | internal1 | Hm Gen E | Hm Gen E | Hm Gen E | CPE002JC | CPE002NC | CPE002K4 |
| 5585 | I24 | I1 | 655 | 719,3 | 1314 | 242,9 | 2 | 0,19 | H5A034 | C | internal1 | Hm Drgbl | Hm Drgbl | Hm Drgbl | CPE002DX | CPE002F2 | CPE002FN |
| 22457 | H24 | H1 | 1463 | 1349 | 3080 | 282,7 | 2,1 | 0,108 | H5A034 | C | internal1 | Hm Gen E | Hm Gen E | Hm Gen E | CPE002JW | CPE002OB | CPE002KR |
| 22481 | I24 | I1 | 1218 | 1319 | 2606 | 280,2 | 2,1 | 0,108 | H5A034 | C | internal1 | Hm Gen E | Hm Gen E | Hm Gen E | CPE002JW | CPE002OB | CPE002KR |
| 15952 | I23 | I24 | 2017 | 962,8 | 4224 | 157,6 | 2,1 | 0,117 | H5A034 | C | internal1 | Hm Gen E | Hm Gen E | Hm Gen E | CPE002J9 | CPE002NL | CPE002KA |
| 14801 | I24 | I1 | 2249 | 1042 | 4723 | 119,7 | 2,1 | 0,12 | H5A034 | C | internal1 | Hm Gen E | Hm Gen E | Hm Gen E | CPE002JD | CPE002NR | CPE002K7 |
| 18616 | H23 | H24 | 1952 | 1058 | 4036 | 237,4 | 2,1 | 0,142 | H5A034 | C | internal1 | Hm Gen E | Hm Gen E | Hm Gen E | CPE002JM | CPE002O1 | CPE002KH |
| 9424 | I23 | I24 | 1870 | 1176 | 3896 | 178 | 2,1 | 0,146 | H5A034 | C | internal1 | Hm Drgbl | Hm Drgbl | Hm Drgbl | CPE002E8 | CPE002ER | CPE002FC |
| 19000 | H23 | H24 | 1815 | 1042 | 3893 | 226,3 | 2,1 | 0,146 | H5A034 | C | internal1 | Hm Gen E | Hm Gen E | Hm Gen E | CPE002JN | CPE002O2 | CPE002KI |
| 20537 | H24 | H1 | 1016 | 829,7 | 2196 | 161,4 | 2,2 | 0,147 | H5A034 | C | internal1 | Hm Gen E | Hm Gen E | Hm Gen E | CPE002JR | CPE002O6 | CPE002KM |
| 17464 | H23 | H24 | 1576 | 1090 | 3412 | 169,9 | 2,2 | 0,15 | H5A034 | C | internal1 | Hm Gen E | Hm Gen E | Hm Gen E | CPE002JJ | CPE002NY | CPE002KE |
| 19408 | I23 | I24 | 1444 | 1279 | 3112 | 424,6 | 2,2 | 0,156 | H5A034 | C | internal1 | Hm Gen E | Hm Gen E | Hm Gen E | CPE002JO | CPE002O3 | CPE002KJ |
| 21712 | I23 | I24 | 386 | 893,9 | 861 | 376,2 | 2,2 | 0,232 | H5A034 | C | internal1 | Hm Gen E | Hm Gen E | Hm Gen E | CPE002JU | CPE002O9 | CPE002KP |
| 13265 | I24 | I1 | 2140 | 1215 | 4968 | 136 | 2,3 | 0,115 | H5A034 | C | internal1 | Hm Gen E | Hm Gen E | Hm Gen E | CPE002JB | CPE002NN | CPE002K3 |
| 12088 | H23 | H24 | 1814 | 860,6 | 4128 | 126,3 | 2,3 | 0,147 | H5A034 | C | internal1 | Hm Gen E | Hm Gen E | Hm Gen E | CPE002J4 | CPE002NL | CPE002K0 |
| 18640 | I23 | I24 | 1762 | 959,6 | 4009 | 242,9 | 2,3 | 0,156 | H5A034 | C | internal1 | Hm Gen E | Hm Gen E | Hm Gen E | CPE002JM | CPE002O1 | CPE002KH |
| 17465 | H24 | H1 | 1163 | 912,5 | 2729 | 134 | 2,3 | 0,157 | H5A034 | C | internal1 | Hm Gen E | Hm Gen E | Hm Gen E | CPE002JJ | CPE002NY | CPE002KE |
| 8656 | I23 | I24 | 2283 | 1241 | 5217 | 208 | 2,3 | 0,163 | H5A034 | C | internal1 | Hm Drgbl | Hm Drgbl | Hm Drgbl | CPE002E6 | CPE002ET | CPE002FE |
| 19385 | H24 | H1 | 965 | 1173 | 2188 | 255,9 | 2,3 | 0,163 | H5A034 | C | internal1 | Hm Gen E | Hm Gen E | Hm Gen E | CPE002JO | CPE002O3 | CPE002KJ |
| 20921 | H24 | H1 | 1187 | 945,9 | 2754 | 183,2 | 2,3 | 0,163 | H5A034 | C | internal1 | Hm Gen E | Hm Gen E | Hm Gen E | CPE002JS | CPE002O7 | CPE002KN |
| 21713 | I24 | I1 | 288 | 946,8 | 666 | 397,6 | 2,3 | 0,242 | H5A034 | C | internal1 | Hm Gen E | Hm Gen E | Hm Gen E | CPE002JU | CPE002O9 | CPE002KP |
| 20176 | I23 | I24 | 1210 | 1157 | 2819 | 106,2 | 2,3 | 0,28 | H5A034 | C | internal1 | Hm Gen E | Hm Gen E | Hm Gen E | CPE002JQ | CPE002O5 | CPE002KL |
| 20177 | I24 | I1 | 538 | 1113 | 1224 | 105,6 | 2,3 | 0,28 | H5A034 | C | internal1 | Hm Gen E | Hm Gen E | Hm Gen E | CPE002JQ | CPE002O5 | CPE002KL |
| 15929 | H24 | H1 | 1820 | 955,1 | 4294 | 153 | 2,4 | 0,134 | H5A034 | C | internal1 | Hm Gen E | Hm Gen E | Hm Gen E | CPE002J9 | CPE002NL | CPE002KA |
| 18232 | H23 | H24 | 1967 | 1073 | 4707 | 249 | 2,4 | 0,154 | H5A034 | C | internal1 | Hm Gen E | Hm Gen E | Hm Gen E | CPE002JL | CPE002O0 | CPE002KG |
| 19768 | H23 | H24 | 1775 | 412,2 | 4308 | 378,5 | 2,4 | 0,156 | H5A034 | C | internal1 | Hm Gen E | Hm Gen E | Hm Gen E | CPE002JP | CPE002O4 | CPE002KK |

| | | | | | | | | | | | | | | | | | |
|---|---|---|---|---|---|---|---|---|---|---|---|---|---|---|---|---|---|
| 18641 | I24 | I1 | 932 | 1027 | 2273 | 210,5 | 2,4 | 0,163 | H5A034 | C | internal1 | Hm Gen E | Hm Gen E | Hm Gen E | CPE002JM | CPE002O1 | CPE002KH |
| 17488 | I23 | I24 | 1440 | 1022 | 3498 | 261,4 | 2,4 | 0,164 | H5A034 | C | internal1 | Hm Gen E | Hm Gen E | Hm Gen E | CPE002JJ | CPE002NY | CPE002KE |
| 19024 | I23 | I24 | 1385 | 999 | 3272 | 190,5 | 2,4 | 0,167 | H5A034 | C | internal1 | Hm Gen E | Hm Gen E | Hm Gen E | CPE002JN | CPE002O2 | CPE002KI |
| 20944 | I23 | I24 | 1399 | 964,7 | 3371 | 217,9 | 2,4 | 0,17 | H5A034 | C | internal1 | Hm Gen E | Hm Gen E | Hm Gen E | CPE002JS | CPE002O7 | CPE002KN |
| 20152 | H23 | H24 | 1331 | 1122 | 3186 | 99,56 | 2,4 | 0,293 | H5A034 | C | internal1 | Hm Gen E | Hm Gen E | Hm Gen E | CPE002JQ | CPE002O5 | CPE002KL |
| 19792 | I23 | I24 | 1338 | 330,4 | 3325 | 338,9 | 2,5 | 0,163 | H5A034 | C | internal1 | Hm Gen E | Hm Gen E | Hm Gen E | CPE002JP | CPE002O4 | CPE002KK |
| 21328 | I23 | I24 | 1508 | 956,9 | 3822 | 361,1 | 2,5 | 0,177 | H5A034 | C | internal1 | Hm Gen E | Hm Gen E | Hm Gen E | CPE002JT | CPE002O8 | CPE002KO |
| 17104 | I23 | I24 | 2118 | 1069 | 5240 | 211 | 2,5 | 0,193 | H5A034 | C | internal1 | Hm Gen E | Hm Gen E | Hm Gen E | CPE002JI | CPE002NZ | CPE002KD |
| 19769 | H24 | H1 | 918 | 331,9 | 2428 | 261,8 | 2,6 | 0,169 | H5A034 | C | internal1 | Hm Gen E | Hm Gen E | Hm Gen E | CPE002JP | CPE002O4 | CPE002KK |
| 18617 | H24 | H1 | 1269 | 1009 | 3258 | 192,3 | 2,6 | 0,176 | H5A034 | C | internal1 | Hm Gen E | Hm Gen E | Hm Gen E | CPE002JM | CPE002O1 | CPE002KH |
| 22480 | I23 | I24 | 1216 | 1341 | 3308 | 285,7 | 2,7 | 0,139 | H5A034 | C | internal1 | Hm Gen E | Hm Gen E | Hm Gen E | CPE002JW | CPE002OB | CPE002KR |
| 23633 | I24 | I1 | 1051 | 1501 | 2834 | 200,1 | 2,7 | 0,143 | H5A034 | C | internal1 | Hm Gen E | Hm Gen E | Hm Gen E | CPE002KV | CPE002OE | CPE002KU |
| 19001 | H24 | H1 | 1249 | 1020 | 3417 | 187,5 | 2,7 | 0,188 | H5A034 | C | internal1 | Hm Gen E | Hm Gen E | Hm Gen E | CPE002JN | CPE002O2 | CPE002KI |
| 13264 | I23 | I24 | 2227 | 1237 | 6137 | 181,3 | 2,8 | 0,14 | H5A034 | C | internal1 | Hm Gen E | Hm Gen E | Hm Gen E | CPE002JB | CPE002NN | CPE002K3 |
| 20561 | I24 | I1 | 1032 | 817 | 2886 | 120,9 | 2,8 | 0,187 | H5A034 | C | internal1 | Hm Gen E | Hm Gen E | Hm Gen E | CPE002JR | CPE002O6 | CPE002KM |
| 19025 | I24 | I1 | 1386 | 1038 | 3849 | 176,7 | 2,8 | 0,195 | H5A034 | C | internal1 | Hm Gen E | Hm Gen E | Hm Gen E | CPE002JN | CPE002O2 | CPE002KI |
| 23632 | I23 | I24 | 1176 | 1529 | 3412 | 226 | 2,9 | 0,154 | H5A034 | C | internal1 | Hm Gen E | Hm Gen E | Hm Gen E | CPE002KV | CPE002OE | CPE002KU |
| 14776 | H23 | H24 | 1916 | 944,2 | 5572 | 146,3 | 2,9 | 0,165 | H5A034 | C | internal1 | Hm Gen E | Hm Gen E | Hm Gen E | CPE002JD | CPE002NR | CPE002K7 |
| 18257 | I24 | I1 | 1351 | 1063 | 3915 | 199,4 | 2,9 | 0,186 | H5A034 | C | internal1 | Hm Gen E | Hm Gen E | Hm Gen E | CPE002JL | CPE002OO | CPE002KG |
| 20536 | H23 | H24 | 1066 | 823,8 | 3061 | 131 | 2,9 | 0,193 | H5A034 | C | internal1 | Hm Gen E | Hm Gen E | Hm Gen E | CPE002JR | CPE002O6 | CPE002KM |
| 17872 | I23 | I24 | 1177 | 1066 | 3588 | 245 | 3 | 0,237 | H5A034 | C | internal1 | Hm Gen E | Hm Gen E | Hm Gen E | CPE002JK | CPE002NZ | CPE002KF |
| 22097 | I24 | I1 | 1203 | 1349 | 3844 | 203,8 | 3,2 | 0,152 | H5A034 | C | internal1 | Hm Gen E | Hm Gen E | Hm Gen E | CPE002JV | CPE002OA | CPE002KQ |
| 14800 | I23 | I24 | 1787 | 944,4 | 5667 | 154,8 | 3,2 | 0,182 | H5A034 | C | internal1 | Hm Gen E | Hm Gen E | Hm Gen E | CPE002JD | CPE002NR | CPE002K7 |
| 23224 | H23 | H24 | 1636 | 1133 | 5459 | 165,6 | 3,3 | 0,143 | H5A034 | C | internal1 | Hm Gen E | Hm Gen E | Hm Gen E | CPE002JY | CPE002OD | CPE002KT |
| 23609 | H24 | H1 | 901 | 1446 | 2932 | 189,5 | 3,3 | 0,175 | H5A034 | C | internal1 | Hm Gen E | Hm Gen E | Hm Gen E | CPE002KV | CPE002OE | CPE002KU |
| 22864 | I23 | I24 | 1166 | 1679 | 4012 | 260,4 | 3,4 | 0,16 | H5A034 | C | internal1 | Hm Gen E | Hm Gen E | Hm Gen E | CPE002JX | CPE002OC | CPE002KS |
| 22072 | H23 | H24 | 1589 | 1339 | 5438 | 239,6 | 3,4 | 0,162 | H5A034 | C | internal1 | Hm Gen E | Hm Gen E | Hm Gen E | CPE002JV | CPE002OA | CPE002KQ |
| 13240 | H23 | H24 | 1791 | 1121 | 6027 | 167,8 | 3,4 | 0,17 | H5A034 | C | internal1 | Hm Gen E | Hm Gen E | Hm Gen E | CPE002JB | CPE002NN | CPE002K3 |
| 18233 | H24 | H1 | 1004 | 1011 | 3531 | 205,8 | 3,5 | 0,225 | H5A034 | C | internal1 | Hm Gen E | Hm Gen E | Hm Gen E | CPE002JL | CPE002OO | CPE002KG |
| 18256 | I23 | I24 | 1445 | 1032 | 5114 | 225,4 | 3,5 | 0,225 | H5A034 | C | internal1 | Hm Gen E | Hm Gen E | Hm Gen E | CPE002JL | CPE002OO | CPE002KG |
| 22073 | H24 | H1 | 1087 | 1286 | 4072 | 168,7 | 3,7 | 0,176 | H5A034 | C | internal1 | Hm Gen E | Hm Gen E | Hm Gen E | CPE002JV | CPE002OA | CPE002KQ |
| 22456 | H23 | H24 | 1036 | 1381 | 4007 | 296,6 | 3,9 | 0,201 | H5A034 | C | internal1 | Hm Gen E | Hm Gen E | Hm Gen E | CPE002JW | CPE002OB | CPE002KR |
| 22096 | I23 | I24 | 1215 | 1347 | 4900 | 211,3 | 4 | 0,19 | H5A034 | C | internal1 | Hm Gen E | Hm Gen E | Hm Gen E | CPE002JV | CPE002OA | CPE002KQ |
| 23608 | H23 | H24 | 828 | 1519 | 3516 | 200,9 | 4,2 | 0,223 | H5A034 | C | internal1 | Hm Gen E | Hm Gen E | Hm Gen E | CPE002KV | CPE002OE | CPE002KU |
| 20560 | I23 | I24 | 838 | 818,7 | 3536 | 145,6 | 4,2 | 0,28 | H5A034 | C | internal1 | Hm Gen E | Hm Gen E | Hm Gen E | CPE002JR | CPE002O6 | CPE002KM |
| 22865 | I24 | I1 | 895 | 1597 | 3814 | 166,6 | 4,3 | 0,202 | H5A034 | C | internal1 | Hm Gen E | Hm Gen E | Hm Gen E | CPE002JX | CPE002OC | CPE002KS |
| 22841 | H24 | H1 | 948 | 1531 | 4316 | 169,6 | 4,6 | 0,216 | H5A034 | C | internal1 | Hm Gen E | Hm Gen E | Hm Gen E | CPE002JX | CPE002OC | CPE002KS |
| 23248 | I23 | I24 | 1172 | 1044 | 5615 | 131,7 | 4,8 | 0,207 | H5A034 | C | internal1 | Hm Gen E | Hm Gen E | Hm Gen E | CPE002JY | CPE002OD | CPE002KT |
| 23225 | H24 | H1 | 1139 | 1056 | 5664 | 143,6 | 5 | 0,216 | H5A034 | C | internal1 | Hm Gen E | Hm Gen E | Hm Gen E | CPE002JY | CPE002OD | CPE002KT |
| 22840 | H23 | H24 | 1061 | 1566 | 5563 | 193,2 | 5,2 | 0,244 | H5A034 | C | internal1 | Hm Gen E | Hm Gen E | Hm Gen E | CPE002JX | CPE002OC | CPE002KS |
| 23249 | I24 | I1 | 1043 | 1076 | 7141 | 94,37 | 6,8 | 0,294 | H5A034 | C | internal1 | Hm Gen E | Hm Gen E | Hm Gen E | CPE002JY | CPE002OD | CPE002KT |
| 15161 | H24 | H1 | 1902 | 891,1 | 15604 | 103,8 | 8,2 | 0,573 | H5A034 | C | internal1 | Hm Gen E | Hm Gen E | Hm Gen E | CPE002J8 | CPE002NS | CPE002K8 |
| 15185 | I24 | I1 | 2261 | 989,2 | 29604 | 153,5 | 13,1 | 0,916 | H5A034 | C | internal1 | Hm Gen E | Hm Gen E | Hm Gen E | CPE002J8 | CPE002NS | CPE002K8 |
| 15184 | I23 | I24 | 1956 | 941,5 | 27468 | 218,9 | 14 | 0,979 | H5A034 | C | internal1 | Hm Gen E | Hm Gen E | Hm Gen E | CPE002J8 | CPE002NS | CPE002K8 |
| 15160 | H23 | H24 | 2047 | 942,3 | 29048 | 169,4 | 14,2 | 0,993 | H5A034 | C | internal1 | Hm Gen E | Hm Gen E | Hm Gen E | CPE002J8 | CPE002NS | CPE002K8 |
| 8441 | P24 | P1 | 1391 | 597,1 | 2564 | 168 | 1,8 | 0,173 | H5A034 | C | neg1 | Hm Drgbl | Hm Drgbl | Hm Drgbl | CPE002E5 | CPE002EU | CPE002FF |
| 5704 | N23 | N24 | 1487 | 819,8 | 3481 | 155,5 | 2,3 | 0,219 | H5A034 | C | neg1 | Hm Drgbl | Hm Drgbl | Hm Drgbl | CPE002DX | CPE002F2 | CPE002FN |
| 6065 | M24 | M1 | 542 | 774,9 | 2684 | 207,6 | 5 | 0,457 | H5A034 | C | neg1 | Hm Drgbl | Hm Drgbl | Hm Drgbl | CPE002DY | CPE002F1 | CPE002FM |
| 21760 | K23 | K24 | 629 | 1146 | 3212 | 417,6 | 5,1 | 0,537 | H5A034 | C | neg1 | Hm Gen E | Hm Gen E | Hm Gen E | CPE002JU | CPE002O9 | CPE002KP |
| 10721 | O24 | O1 | 1294 | 838,1 | 6544 | 183,6 | 5,1 | 0,576 | H5A034 | C | neg1 | Hm Drgbl | Hm Drgbl | Hm Drgbl | CPE002EB | CPE002EO | CPE002FR |
| 10697 | N24 | N1 | 1631 | 896,8 | 8513 | 189,5 | 5,2 | 0,588 | H5A034 | C | neg1 | Hm Drgbl | Hm Drgbl | Hm Drgbl | CPE002EB | CPE002EO | CPE002FR |
| 20321 | O24 | O1 | 1460 | 1092 | 7526 | 83,8 | 5,2 | 0,634 | H5A034 | C | neg1 | Hm Gen E | Hm Gen E | Hm Gen E | CPE002JQ | CPE002O5 | CPE002KL |
| 2968 | L23 | L24 | 1728 | 710 | 9096 | 474,2 | 5,3 | 0,624 | H5A034 | C | neg1 | Hm Drgbl | Hm Drgbl | Hm Drgbl | CPE002DP | CPE002EF | CPE002F5 |
| 7960 | L23 | L24 | 2229 | 640,4 | 11988 | 391,4 | 5,4 | 0,824 | H5A034 | C | neg1 | Hm Drgbl | Hm Drgbl | Hm Drgbl | CPE002E4 | CPE002EV | CPE002FG |
| 10649 | L24 | L1 | 1172 | 897,8 | 6929 | 186,1 | 5,9 | 0,667 | H5A034 | C | neg1 | Hm Drgbl | Hm Drgbl | Hm Drgbl | CPE002EB | CPE002EO | CPE002FR |
| 2944 | K23 | K24 | 1680 | 691,6 | 9870 | 555,5 | 5,9 | 0,694 | H5A034 | C | neg1 | Hm Drgbl | Hm Drgbl | Hm Drgbl | CPE002DP | CPE002EF | CPE002F5 |
| 20297 | N24 | N1 | 1237 | 1098 | 7392 | 94,01 | 6 | 0,732 | H5A034 | C | neg1 | Hm Gen E | Hm Gen E | Hm Gen E | CPE002JQ | CPE002O5 | CPE002KL |
| 641 | K24 | K1 | 2491 | 1985 | 15048 | 348,5 | 6 | 0,876 | H5A034 | C | neg1 | Hm Drgbl | Hm Drgbl | Hm Drgbl | CPE002E0 | CPE002EZ | CPE002FK |
| 737 | O24 | O1 | 2640 | 2006 | 15861 | 333,4 | 6 | 0,876 | H5A034 | C | neg1 | Hm Drgbl | Hm Drgbl | Hm Drgbl | CPE002E0 | CPE002EZ | CPE002FK |
| 8009 | N24 | N1 | 1802 | 666,9 | 10973 | 328,9 | 6,1 | 0,931 | H5A034 | C | neg1 | Hm Drgbl | Hm Drgbl | Hm Drgbl | CPE002E4 | CPE002EV | CPE002FG |
| 8057 | P24 | P1 | 1329 | 615,3 | 8120 | 415,6 | 6,1 | 0,931 | H5A034 | C | neg1 | Hm Drgbl | Hm Drgbl | Hm Drgbl | CPE002E4 | CPE002EV | CPE002FG |
| 689 | M24 | M1 | 2940 | 2115 | 18134 | 365,3 | 6,2 | 0,905 | H5A034 | C | neg1 | Hm Drgbl | Hm Drgbl | Hm Drgbl | CPE002E0 | CPE002EZ | CPE002FK |
| 7937 | K24 | K1 | 610 | 619,1 | 3784 | 374,7 | 6,2 | 0,947 | H5A034 | C | neg1 | Hm Drgbl | Hm Drgbl | Hm Drgbl | CPE002E4 | CPE002EV | CPE002FG |
| 1121 | O24 | O1 | 1657 | 794,6 | 10467 | 321 | 6,3 | 0,597 | H5A034 | C | neg1 | Hm Drgbl | Hm Drgbl | Hm Drgbl | CPE002FU | CPE002FY | CPE002G2 |
| 10312 | N23 | N24 | 1888 | 996,6 | 12223 | 104,7 | 6,5 | 0,625 | H5A034 | C | neg1 | Hm Drgbl | Hm Drgbl | Hm Drgbl | CPE002EA | CPE002EP | CPE002FA |
| 7936 | K23 | K24 | 1858 | 640,5 | 12010 | 392,6 | 6,5 | 0,992 | H5A034 | C | neg1 | Hm Drgbl | Hm Drgbl | Hm Drgbl | CPE002E4 | CPE002EV | CPE002FG |
| 8008 | N23 | N24 | 1596 | 653,7 | 10295 | 446,3 | 6,5 | 0,992 | H5A034 | C | neg1 | Hm Drgbl | Hm Drgbl | Hm Drgbl | CPE002E4 | CPE002EV | CPE002FG |
| 1864 | N23 | N24 | 2111 | 898 | 14002 | 345,6 | 6,6 | 0,691 | H5A034 | C | neg1 | Hm Drgbl | Hm Drgbl | Hm Drgbl | CPE002FW | CPE002G0 | CPE002G4 |
| 665 | L24 | L1 | 2252 | 2012 | 14802 | 346,9 | 6,6 | 0,964 | H5A034 | C | neg1 | Hm Drgbl | Hm Drgbl | Hm Drgbl | CPE002E0 | CPE002EZ | CPE002FK |
| 8033 | O24 | O1 | 323 | 611,2 | 2124 | 283,8 | 6,6 | 1,008 | H5A034 | C | neg1 | Hm Drgbl | Hm Drgbl | Hm Drgbl | CPE002E4 | CPE002EV | CPE002FG |
| 2992 | M23 | M24 | 1780 | 744,7 | 11927 | 532,3 | 6,7 | 0,788 | H5A034 | C | neg1 | Hm Drgbl | Hm Drgbl | Hm Drgbl | CPE002DP | CPE002EF | CPE002F5 |
| 20273 | M24 | M1 | 808 | 1239 | 5440 | 108 | 6,7 | 0,817 | H5A034 | C | neg1 | Hm Gen E | Hm Gen E | Hm Gen E | CPE002JQ | CPE002O5 | CPE002KL |
| 7961 | L24 | L1 | 1444 | 662,5 | 9732 | 367,3 | 6,7 | 1,023 | H5A034 | C | neg1 | Hm Drgbl | Hm Drgbl | Hm Drgbl | CPE002E4 | CPE002EV | CPE002FG |
| 7984 | M23 | M24 | 1670 | 604,9 | 11125 | 428,9 | 6,7 | 1,023 | H5A034 | C | neg1 | Hm Drgbl | Hm Drgbl | Hm Drgbl | CPE002E4 | CPE002EV | CPE002FG |
| 8032 | O23 | O24 | 1915 | 623,4 | 12853 | 459 | 6,7 | 1,023 | H5A034 | C | neg1 | Hm Drgbl | Hm Drgbl | Hm Drgbl | CPE002E4 | CPE002EV | CPE002FG |
| 4960 | O23 | O24 | 2302 | 889,4 | 15583 | 264,7 | 6,8 | 0,747 | H5A034 | C | neg1 | Hm Drgbl | Hm Drgbl | Hm Drgbl | CPE002DV | CPE002EL | CPE002FP |
| 640 | K23 | K24 | 3170 | 1971 | 21435 | 386,6 | 6,8 | 0,993 | H5A034 | C | neg1 | Hm Drgbl | Hm Drgbl | Hm Drgbl | CPE002E0 | CPE002EZ | CPE002FK |
| 713 | N24 | N1 | 3321 | 2117 | 22618 | 398,5 | 6,8 | 0,993 | H5A034 | C | neg1 | Hm Drgbl | Hm Drgbl | Hm Drgbl | CPE002E0 | CPE002EZ | CPE002FK |
| 15713 | O24 | O1 | 1851 | 896,4 | 12718 | 104,3 | 6,9 | 0,627 | H5A034 | C | neg1 | Hm Gen E | Hm Gen E | Hm Gen E | CPE002JE | CPE002NT | CPE002K9 |
| 20249 | L24 | L1 | 985 | 1149 | 6796 | 106,7 | 6,9 | 0,841 | H5A034 | C | neg1 | Hm Gen E | Hm Gen E | Hm Gen E | CPE002JQ | CPE002O5 | CPE002KL |
| 664 | L23 | L24 | 2547 | 2077 | 17589 | 358,8 | 6,9 | 1,007 | H5A034 | C | neg1 | Hm Drgbl | Hm Drgbl | Hm Drgbl | CPE002E0 | CPE002EZ | CPE002FK |
| 21784 | L23 | L24 | 323 | 1135 | 2266 | 671,8 | 7 | 0,737 | H5A034 | C | neg1 | Hm Gen E | Hm Gen E | Hm Gen E | CPE002JU | CPE002O9 | CPE002KP |
| 4864 | K23 | K24 | 2270 | 899,4 | 15869 | 381,6 | 7 | 0,769 | H5A034 | C | neg1 | Hm Drgbl | Hm Drgbl | Hm Drgbl | CPE002DV | CPE002EL | CPE002FP |
| 688 | M23 | M24 | 2956 | 2014 | 20955 | 412,1 | 7,1 | 1,036 | H5A034 | C | neg1 | Hm Drgbl | Hm Drgbl | Hm Drgbl | CPE002E0 | CPE002EZ | CPE002FK |
| 4985 | P24 | P1 | 1775 | 883,1 | 12839 | 486,5 | 7,2 | 0,791 | H5A034 | C | neg1 | Hm Drgbl | Hm Drgbl | Hm Drgbl | CPE002DV | CPE002EL | CPE002FP |
| 712 | N23 | N24 | 2775 | 1949 | 20053 | 412,6 | 7,2 | 1,051 | H5A034 | C | neg1 | Hm Drgbl | Hm Drgbl | Hm Drgbl | CPE002E0 | CPE002EZ | CPE002FK |
| 8056 | P23 | P24 | 1129 | 609,5 | 8087 | 486,3 | 7,2 | 1,099 | H5A034 | C | neg1 | Hm Drgbl | Hm Drgbl | Hm Drgbl | CPE002E4 | CPE002EV | CPE002FG |
| 13721 | L24 | L1 | 1039 | 1078 | 7679 | 164 | 7,4 | 0,695 | H5A034 | C | neg1 | Hm Gen E | Hm Gen E | Hm Gen E | CPE002JC | CPE002NC | CPE002K4 |
| 1816 | L23 | L24 | 1941 | 923,6 | 14376 | 324 | 7,4 | 0,775 | H5A034 | C | neg1 | Hm Drgbl | Hm Drgbl | Hm Drgbl | CPE002FW | CPE002G0 | CPE002G4 |
| 16456 | N23 | N24 | 1875 | 1078 | 14089 | 292,9 | 7,5 | 0,688 | H5A034 | C | neg1 | Hm Gen E | Hm Gen E | Hm Gen E | CPE002JG | CPE002NV | CPE002KB |
| 13769 | N24 | N1 | 1562 | 1089 | 11694 | 161 | 7,5 | 0,704 | H5A034 | C | neg1 | Hm Gen E | Hm Gen E | Hm Gen E | CPE002JC | CPE002NC | CPE002K4 |
| 760 | P23 | P24 | 3199 | 2002 | 23935 | 435,7 | 7,5 | 1,095 | H5A034 | C | neg1 | Hm Drgbl | Hm Drgbl | Hm Drgbl | CPE002E0 | CPE002EZ | CPE002FK |
| 10240 | K23 | K24 | 2126 | 971,6 | 16094 | 137,9 | 7,6 | 0,731 | H5A034 | C | neg1 | Hm Drgbl | Hm Drgbl | Hm Drgbl | CPE002EA | CPE002EP | CPE002FA |
| 13744 | M23 | M24 | 1433 | 1157 | 11208 | 134,1 | 7,8 | 0,732 | H5A034 | C | neg1 | Hm Gen E | Hm Gen E | Hm Gen E | CPE002JC | CPE002NC | CPE002K4 |
| 3016 | N23 | N24 | 1866 | 754 | 14634 | 626 | 7,8 | 0,918 | H5A034 | C | neg1 | Hm Drgbl | Hm Drgbl | Hm Drgbl | CPE002DP | CPE002EF | CPE002F5 |
| 20296 | N23 | N24 | 1148 | 1199 | 8907 | 142,9 | 7,8 | 0,951 | H5A034 | C | neg1 | Hm Gen E | Hm Gen E | Hm Gen E | CPE002JQ | CPE002O5 | CPE002KL |
| 7193 | L24 | L1 | 1753 | 706,5 | 13901 | 192,1 | 7,9 | 0,615 | H5A034 | C | neg1 | Hm Drgbl | Hm Drgbl | Hm Drgbl | CPE002E2 | CPE002EX | CPE002FI |
| 21832 | N23 | N24 | 399 | 946,4 | 3164 | 459,3 | 7,9 | 0,832 | H5A034 | C | neg1 | Hm Gen E | Hm Gen E | Hm Gen E | CPE002JU | CPE002O9 | CPE002KP |
| 4888 | L23 | L24 | 2131 | 873 | 16815 | 473,4 | 7,9 | 0,868 | H5A034 | C | neg1 | Hm Drgbl | Hm Drgbl | Hm Drgbl | CPE002DV | CPE002EL | CPE002FP |
| 2993 | M24 | M1 | 907 | 678,8 | 7182 | 521,3 | 7,9 | 0,929 | H5A034 | C | neg1 | Hm Drgbl | Hm Drgbl | Hm Drgbl | CPE002DP | CPE002EF | CPE002F5 |
| 11777 | K24 | K1 | 1030 | 968,5 | 8275 | 120,5 | 8 | 0,714 | H5A034 | C | neg1 | Hm Gen E | Hm Gen E | Hm Gen E | CPE002JA | CPE002NJ | CPE002JZ |
| 10313 | N24 | N1 | 1665 | 837 | 13365 | 113,8 | 8 | 0,769 | H5A034 | C | neg1 | Hm Drgbl | Hm Drgbl | Hm Drgbl | CPE002EA | CPE002EP | CPE002FA |
| 10625 | K24 | K1 | 1035 | 857,8 | 8235 | 175,6 | 8 | 0,904 | H5A034 | C | neg1 | Hm Drgbl | Hm Drgbl | Hm Drgbl | CPE002EB | CPE002EO | CPE002FR |
| 761 | P24 | P1 | 2831 | 1961 | 22548 | 425 | 8 | 1,168 | H5A034 | C | neg1 | Hm Drgbl | Hm Drgbl | Hm Drgbl | CPE002E0 | CPE002EZ | CPE002FK |
| 15617 | K24 | K1 | 1378 | 908,4 | 11347 | 122,5 | 8,2 | 0,745 | H5A034 | C | neg1 | Hm Gen E | Hm Gen E | Hm Gen E | CPE002JE | CPE002NT | CPE002K9 |
| 21833 | N24 | N1 | 310 | 994,7 | 2556 | 446,1 | 8,2 | 0,863 | H5A034 | C | neg1 | Hm Gen E | Hm Gen E | Hm Gen E | CPE002JU | CPE002O9 | CPE002KP |
| 20320 | O23 | O24 | 1376 | 1170 | 11329 | 166 | 8,2 | 1 | H5A034 | C | neg1 | Hm Gen E | Hm Gen E | Hm Gen E | CPE002JQ | CPE002O5 | CPE002KL |
| 20345 | P24 | P1 | 524 | 1343 | 4291 | 74,65 | 8,2 | 1 | H5A034 | C | neg1 | Hm Gen E | Hm Gen E | Hm Gen E | CPE002JQ | CPE002O5 | CPE002KL |
| 353 | O24 | O1 | 139 | 1328 | 1148 | 487 | 8,3 | 0,706 | H5A034 | C | neg1 | Hm Drgbl | Hm Drgbl | Hm Drgbl | CPE002DT | CPE002EJ | CPE002F9 |
| 16384 | K23 | K24 | 1839 | 1126 | 15224 | 326,6 | 8,3 | 0,761 | H5A034 | C | neg1 | Hm Gen E | Hm Gen E | Hm Gen E | CPE002JG | CPE002NV | CPE002KB |
| 1840 | M23 | M24 | 2065 | 846,3 | 17168 | 425,1 | 8,3 | 0,869 | H5A034 | C | neg1 | Hm Drgbl | Hm Drgbl | Hm Drgbl | CPE002FW | CPE002G0 | CPE002G4 |
| 20224 | K23 | K24 | 1653 | 1248 | 13741 | 150,9 | 8,3 | 1,012 | H5A034 | C | neg1 | Hm Gen E | Hm Gen E | Hm Gen E | CPE002JQ | CPE002O5 | CPE002KL |
| 20225 | K24 | K1 | 621 | 1186 | 5185 | 150,3 | 8,3 | 1,012 | H5A034 | C | neg1 | Hm Gen E | Hm Gen E | Hm Gen E | CPE002JQ | CPE002O5 | CPE002KL |
| 7985 | M24 | M1 | 550 | 616,1 | 4574 | 354,6 | 8,3 | 1,267 | H5A034 | C | neg1 | Hm Drgbl | Hm Drgbl | Hm Drgbl | CPE002E4 | CPE002EV | CPE002FG |
| 1096 | N23 | N24 | 1899 | 753,5 | 15961 | 399,5 | 8,4 | 0,796 | H5A034 | C | neg1 | Hm Drgbl | Hm Drgbl | Hm Drgbl | CPE002FU | CPE002FY | CPE002G2 |
| 1913 | P24 | P1 | 1953 | 805,9 | 16487 | 475,8 | 8,4 | 0,88 | H5A034 | C | neg1 | Hm Drgbl | Hm Drgbl | Hm Drgbl | CPE002FW | CPE002G0 | CPE002G4 |
| 3017 | N24 | N1 | 1350 | 670,7 | 11319 | 476,1 | 8,4 | 0,988 | H5A034 | C | neg1 | Hm Drgbl | Hm Drgbl | Hm Drgbl | CPE002DP | CPE002EF | CPE002F5 |
| 2584 | L23 | L24 | 2191 | 839,7 | 18537 | 358,3 | 8,5 | 0,669 | H5A034 | C | neg1 | Hm Drgbl | Hm Drgbl | Hm Drgbl | CPE002DC | CPE002EE | CPE002F4 |
| 16432 | M23 | M24 | 2093 | 1069 | 17709 | 351,9 | 8,5 | 0,78 | H5A034 | C | neg1 | Hm Gen E | Hm Gen E | Hm Gen E | CPE002JG | CPE002NV | CPE002KB |
| 10648 | L23 | L24 | 1532 | 888,1 | 13096 | 202,7 | 8,5 | 0,96 | H5A034 | C | neg1 | Hm Drgbl | Hm Drgbl | Hm Drgbl | CPE002EB | CPE002EO | CPE002FR |
| 1865 | N24 | N1 | 2016 | 808,9 | 17429 | 404,5 | 8,6 | 0,901 | H5A034 | C | neg1 | Hm Drgbl | Hm Drgbl | Hm Drgbl | CPE002FW | CPE002G0 | CPE002G4 |
| 21761 | K24 | K1 | 143 | 959,7 | 1236 | 503,7 | 8,6 | 0,905 | H5A034 | C | neg1 | Hm Gen E | Hm Gen E | Hm Gen E | CPE002JU | CPE002O9 | CPE002KP |
| 2945 | K24 | K1 | 906 | 700,5 | 7754 | 563 | 8,6 | 1,012 | H5A034 | C | neg1 | Hm Drgbl | Hm Drgbl | Hm Drgbl | CPE002DP | CPE002EF | CPE002F5 |
| 4912 | M23 | M24 | 1992 | 912,9 | 17418 | 414,5 | 8,7 | 0,956 | H5A034 | C | neg1 | Hm Drgbl | Hm Drgbl | Hm Drgbl | CPE002DV | CPE002EL | CPE002FP |
| 20248 | L23 | L24 | 1185 | 1203 | 10316 | 177,8 | 8,7 | 1,061 | H5A034 | C | neg1 | Hm Gen E | Hm Gen E | Hm Gen E | CPE002JQ | CPE002O5 | CPE002KL |
| 1529 | P24 | P1 | 1701 | 756,3 | 14948 | 470,8 | 8,8 | 0,807 | H5A034 | C | neg1 | Hm Drgbl | Hm Drgbl | Hm Drgbl | CPE002FV | CPE002FZ | CPE002G3 |
| 16481 | O24 | O1 | 1528 | 1054 | 13462 | 318,9 | 8,8 | 0,807 | H5A034 | C | neg1 | Hm Gen E | Hm Gen E | Hm Gen E | CPE002JG | CPE002NV | CPE002KB |
| 10337 | O24 | O1 | 1769 | 892,8 | 15639 | 134,9 | 8,8 | 0,846 | H5A034 | C | neg1 | Hm Drgbl | Hm Drgbl | Hm Drgbl | CPE002EA | CPE002EP | CPE002FA |
| 21809 | M24 | M1 | 253 | 1047 | 2223 | 492,4 | 8,8 | 0,926 | H5A034 | C | neg1 | Hm Gen E | Hm Gen E | Hm Gen E | CPE002JU | CPE002O9 | CPE002KP |
| 4936 | N23 | N24 | 1803 | 863 | 15885 | 462,2 | 8,8 | 0,967 | H5A034 | C | neg1 | Hm Drgbl | Hm Drgbl | Hm Drgbl | CPE002DV | CPE002EL | CPE002FP |
| 10624 | K23 | K24 | 1478 | 904,5 | 13032 | 187,5 | 8,8 | 0,994 | H5A034 | C | neg1 | Hm Drgbl | Hm Drgbl | Hm Drgbl | CPE002EB | CPE002EO | CPE002FR |
| 20272 | M23 | M24 | 1233 | 1188 | 10903 | 180 | 8,8 | 1,073 | H5A034 | C | neg1 | Hm Gen E | Hm Gen E | Hm Gen E | CPE002JQ | CPE002O5 | CPE002KL |
| 736 | O23 | O24 | 2755 | 2075 | 24187 | 441,4 | 8,8 | 1,285 | H5A034 | C | neg1 | Hm Drgbl | Hm Drgbl | Hm Drgbl | CPE002E0 | CPE002EZ | CPE002FK |
| 5297 | M24 | M1 | 680 | 667,6 | 6043 | 232,9 | 8,9 | 0,698 | H5A034 | C | neg1 | Hm Drgbl | Hm Drgbl | Hm Drgbl | CPE002DV | CPE002EM | CPE002FO |
| 17969 | M24 | M1 | 788 | 1108 | 7013 | 103 | 8,9 | 0,704 | H5A034 | C | neg1 | Hm Gen E | Hm Gen E | Hm Gen E | CPE002JK | CPE002NZ | CPE002KF |
| 8417 | O24 | O1 | 378 | 545,2 | 3352 | 471,6 | 8,9 | 0,856 | H5A034 | C | neg1 | Hm Drgbl | Hm Drgbl | Hm Drgbl | CPE002E5 | CPE002EU | CPE002FF |
| 10696 | N23 | N24 | 1846 | 938,8 | 16427 | 177,1 | 8,9 | 1,006 | H5A034 | C | neg1 | Hm Drgbl | Hm Drgbl | Hm Drgbl | CPE002EB | CPE002EO | CPE002FR |
| 11465 | N24 | N1 | 1408 | 843,6 | 12692 | 130,2 | 9 | 0,652 | H5A034 | C | neg1 | Hm Drgbl | Hm Drgbl | Hm Drgbl | CPE002ED | CPE002F3 | CPE002FT |
| 12641 | O24 | O1 | 1781 | 1081 | 15994 | 138,8 | 9 | 0,677 | H5A034 | C | neg1 | Hm Gen E | Hm Gen E | Hm Gen E | CPE002J5 | CPE002NK | CPE002K1 |
| 3065 | P24 | P1 | 1638 | 700,7 | 14756 | 598,6 | 9 | 1,059 | H5A034 | C | neg1 | Hm Drgbl | Hm Drgbl | Hm Drgbl | CPE002DP | CPE002EF | CPE002F5 |
| 1792 | K23 | K24 | 1863 | 758,4 | 17011 | 357,1 | 9,1 | 0,953 | H5A034 | C | neg1 | Hm Drgbl | Hm Drgbl | Hm Drgbl | CPE002FW | CPE002G0 | CPE002G4 |
| 10673 | M24 | M1 | 1488 | 881,8 | 13541 | 182,7 | 9,1 | 1,028 | H5A034 | C | neg1 | Hm Drgbl | Hm Drgbl | Hm Drgbl | CPE002EB | CPE002EO | CPE002FR |
| 257 | K24 | K1 | 482 | 1649 | 4424 | 722,7 | 9,2 | 0,783 | H5A034 | C | neg1 | Hm Drgbl | Hm Drgbl | Hm Drgbl | CPE002DT | CPE002EJ | CPE002F9 |
| 6041 | L24 | L1 | 1415 | 788,9 | 13074 | 291,9 | 9,2 | 0,84 | H5A034 | C | neg1 | Hm Drgbl | Hm Drgbl | Hm Drgbl | CPE002DY | CPE002F1 | CPE002FM |
| 9161 | N24 | N1 | 1811 | 1760 | 16928 | 138,9 | 9,3 | 0,747 | H5A034 | C | neg1 | Hm Drgbl | Hm Drgbl | Hm Drgbl | CPE002E7 | CPE002ES | CPE002FD |
| 377 | P24 | P1 | 899 | 1851 | 8331 | 672,8 | 9,3 | 0,791 | H5A034 | C | neg1 | Hm Drgbl | Hm Drgbl | Hm Drgbl | CPE002DT | CPE002EJ | CPE002F9 |
| 2248 | N23 | N24 | 1646 | 824,3 | 15281 | 421,8 | 9,3 | 0,809 | H5A034 | C | neg1 | Hm Drgbl | Hm Drgbl | Hm Drgbl | CPE002FX | CPE002G1 | CPE002G5 |
| 16408 | L23 | L24 | 2045 | 1145 | 19025 | 311,7 | 9,3 | 0,853 | H5A034 | C | neg1 | Hm Gen E | Hm Gen E | Hm Gen E | CPE002JG | CPE002NV | CPE002KB |
| 13720 | L23 | L24 | 1176 | 1079 | 10921 | 159 | 9,3 | 0,873 | H5A034 | C | neg1 | Hm Gen E | Hm Gen E | Hm Gen E | CPE002JC | CPE002NC | CPE002K4 |
| 5753 | P24 | P1 | 1221 | 849,7 | 11319 | 387 | 9,3 | 0,886 | H5A034 | C | neg1 | Hm Drgbl | Hm Drgbl | Hm Drgbl | CPE002DX | CPE002F2 | CPE002FN |
| 2632 | N23 | N24 | 1815 | 803,4 | 17099 | 346,2 | 9,4 | 0,74 | H5A034 | C | neg1 | Hm Drgbl | Hm Drgbl | Hm Drgbl | CPE002DC | CPE002EE | CPE002F4 |
| 6880 | O23 | O24 | 1430 | 597,4 | 13387 | 448,9 | 9,4 | 0,825 | H5A034 | C | neg1 | Hm Drgbl | Hm Drgbl | Hm Drgbl | CPE002E1 | CPE002EY | CPE002FJ |
| 11897 | P24 | P1 | 1595 | 854,6 | 14970 | 139,4 | 9,4 | 0,839 | H5A034 | C | neg1 | Hm Gen E | Hm Gen E | Hm Gen E | CPE002JA | CPE002NJ | CPE002JZ |
| 1480 | N23 | N24 | 1915 | 803,9 | 17921 | 462,1 | 9,4 | 0,862 | H5A034 | C | neg1 | Hm Drgbl | Hm Drgbl | Hm Drgbl | CPE002FV | CPE002FZ | CPE002G3 |
| 3736 | L23 | L24 | 1792 | 1481 | 16797 | 397,1 | 9,4 | 0,904 | H5A034 | C | neg1 | Hm Drgbl | Hm Drgbl | Hm Drgbl | CPE002DR | CPE002EH | CPE002F7 |
| 8344 | L23 | L24 | 2136 | 709 | 20036 | 479,7 | 9,4 | 0,904 | H5A034 | C | neg1 | Hm Drgbl | Hm Drgbl | Hm Drgbl | CPE002E5 | CPE002EU | CPE002FF |
| 4889 | L24 | L1 | 1819 | 825,6 | 17025 | 460,8 | 9,4 | 1,033 | H5A034 | C | neg1 | Hm Drgbl | Hm Drgbl | Hm Drgbl | CPE002DV | CPE002EL | CPE002FP |
| 4937 | N24 | N1 | 1798 | 782 | 16898 | 450,2 | 9,4 | 1,033 | H5A034 | C | neg1 | Hm Drgbl | Hm Drgbl | Hm Drgbl | CPE002DV | CPE002EL | CPE002FP |
| 15737 | P24 | P1 | 1653 | 890,9 | 15777 | 136,8 | 9,5 | 0,864 | H5A034 | C | neg1 | Hm Gen E | Hm Gen E | Hm Gen E | CPE002JE | CPE002NT | CPE002K9 |
| 2969 | L24 | L1 | 1402 | 745,2 | 13277 | 707,5 | 9,5 | 1,118 | H5A034 | C | neg1 | Hm Drgbl | Hm Drgbl | Hm Drgbl | CPE002DP | CPE002EF | CPE002F5 |
| 4120 | L23 | L24 | 1464 | 754,2 | 14057 | 374,1 | 9,6 | 0,853 | H5A034 | C | neg1 | Hm Drgbl | Hm Drgbl | Hm Drgbl | CPE002DS | CPE002EI | CPE002F8 |
| 15616 | K23 | K24 | 1319 | 860,9 | 12683 | 144,8 | 9,6 | 0,873 | H5A034 | C | neg1 | Hm Gen E | Hm Gen E | Hm Gen E | CPE002JE | CPE002NT | CPE002K9 |
| 13793 | O24 | O1 | 1612 | 1120 | 15423 | 158,7 | 9,6 | 0,901 | H5A034 | C | neg1 | Hm Gen E | Hm Gen E | Hm Gen E | CPE002JC | CPE002NC | CPE002K4 |
| 1145 | P24 | P1 | 1553 | 742,1 | 14976 | 468 | 9,6 | 0,91 | H5A034 | C | neg1 | Hm Drgbl | Hm Drgbl | Hm Drgbl | CPE002FU | CPE002FY | CPE002G2 |
| 8320 | K23 | K24 | 1919 | 692,2 | 18473 | 449,5 | 9,6 | 0,923 | H5A034 | C | neg1 | Hm Drgbl | Hm Drgbl | Hm Drgbl | CPE002E5 | CPE002EU | CPE002FF |
| 15305 | N24 | N1 | 1455 | 822,1 | 14163 | 115,6 | 9,7 | 0,678 | H5A034 | C | neg1 | Hm Gen E | Hm Gen E | Hm Gen E | CPE002J8 | CPE002NS | CPE002K8 |
| 2176 | K23 | K24 | 2098 | 803,7 | 20452 | 469,7 | 9,7 | 0,843 | H5A034 | C | neg1 | Hm Drgbl | Hm Drgbl | Hm Drgbl | CPE002FX | CPE002G1 | CPE002G5 |
| 4144 | M23 | M24 | 1706 | 764,2 | 16513 | 406,6 | 9,7 | 0,862 | H5A034 | C | neg1 | Hm Drgbl | Hm Drgbl | Hm Drgbl | CPE002DS | CPE002EI | CPE002F8 |
| 5656 | L23 | L24 | 2209 | 792,8 | 21318 | 549,4 | 9,7 | 0,924 | H5A034 | C | neg1 | Hm Drgbl | Hm Drgbl | Hm Drgbl | CPE002DX | CPE002F2 | CPE002FN |
| 10745 | P24 | P1 | 514 | 858,4 | 5002 | 188,6 | 9,7 | 1,096 | H5A034 | C | neg1 | Hm Drgbl | Hm Drgbl | Hm Drgbl | CPE002EB | CPE002EO | CPE002FR |
| 20681 | N24 | N1 | 1128 | 757 | 11008 | 113,9 | 9,8 | 0,653 | H5A034 | C | neg1 | Hm Gen E | Hm Gen E | Hm Gen E | CPE002JR | CPE002O6 | CPE002KM |
| 14561 | O24 | O1 | 1631 | 909,9 | 15923 | 118 | 9,8 | 0,698 | H5A034 | C | neg1 | Hm Gen E | Hm Gen E | Hm Gen E | CPE002J7 | CPE002NC | CPE002K6 |
| 1456 | M23 | M24 | 1864 | 856,8 | 18270 | 489,7 | 9,8 | 0,899 | H5A034 | C | neg1 | Hm Drgbl | Hm Drgbl | Hm Drgbl | CPE002FV | CPE002FZ | CPE002G3 |
| 5752 | P23 | P24 | 1910 | 784,7 | 18712 | 623,8 | 9,8 | 0,933 | H5A034 | C | neg1 | Hm Drgbl | Hm Drgbl | Hm Drgbl | CPE002DX | CPE002F2 | CPE002FN |
| 3833 | P24 | P1 | 1250 | 1487 | 12219 | 407,6 | 9,8 | 0,942 | H5A034 | C | neg1 | Hm Drgbl | Hm Drgbl | Hm Drgbl | CPE002DR | CPE002EH | CPE002F7 |
| 7288 | P23 | P24 | 1432 | 624,3 | 14219 | 404,1 | 9,9 | 0,77 | H5A034 | C | neg1 | Hm Drgbl | Hm Drgbl | Hm Drgbl | CPE002E2 | CPE002EX | CPE002FI |
| 5369 | P24 | P1 | 1136 | 624,2 | 11273 | 537,7 | 9,9 | 0,776 | H5A034 | C | neg1 | Hm Drgbl | Hm Drgbl | Hm Drgbl | CPE002DV | CPE002EM | CPE002FO |
| 17993 | N24 | N1 | 1564 | 1121 | 15545 | 169,8 | 9,9 | 0,783 | H5A034 | C | neg1 | Hm Gen E | Hm Gen E | Hm Gen E | CPE002JK | CPE002NZ | CPE002KF |
| 2224 | M23 | M24 | 1840 | 742,8 | 18301 | 442,8 | 9,9 | 0,861 | H5A034 | C | neg1 | Hm Drgbl | Hm Drgbl | Hm Drgbl | CPE002FX | CPE002G1 | CPE002G5 |
| 11417 | L24 | L1 | 1293 | 852 | 12932 | 135,3 | 10 | 0,725 | H5A034 | C | neg1 | Hm Drgbl | Hm Drgbl | Hm Drgbl | CPE002ED | CPE002F3 | CPE002FT |
| 12592 | M23 | M24 | 1874 | 1157 | 18767 | 143,7 | 10 | 0,752 | H5A034 | C | neg1 | Hm Gen E | Hm Gen E | Hm Gen E | CPE002J5 | CPE002NK | CPE002K1 |
| 11776 | K23 | K24 | 1404 | 880,4 | 14001 | 134,3 | 10 | 0,893 | H5A034 | C | neg1 | Hm Gen E | Hm Gen E | Hm Gen E | CPE002JA | CPE002NJ | CPE002JZ |
| 1097 | N24 | N1 | 1479 | 803 | 14788 | 422 | 10 | 0,948 | H5A034 | C | neg1 | Hm Drgbl | Hm Drgbl | Hm Drgbl | CPE002FU | CPE002FY | CPE002G2 |
| 5728 | O23 | O24 | 1885 | 810,9 | 18764 | 530,9 | 10 | 0,952 | H5A034 | C | neg1 | Hm Drgbl | Hm Drgbl | Hm Drgbl | CPE002DX | CPE002F2 | CPE002FN |
| 3760 | M23 | M24 | 1922 | 1560 | 19207 | 392,6 | 10 | 0,962 | H5A034 | C | neg1 | Hm Drgbl | Hm Drgbl | Hm Drgbl | CPE002DR | CPE002EH | CPE002F7 |
| 8393 | N24 | N1 | 1510 | 670,3 | 15173 | 422,7 | 10 | 0,962 | H5A034 | C | neg1 | Hm Drgbl | Hm Drgbl | Hm Drgbl | CPE002E5 | CPE002EU | CPE002FF |
| 1889 | O24 | O1 | 1281 | 782,2 | 12823 | 488,4 | 10 | 1,047 | H5A034 | C | neg1 | Hm Drgbl | Hm Drgbl | Hm Drgbl | CPE002FW | CPE002G0 | CPE002G4 |
| 6784 | K23 | K24 | 1785 | 654,7 | 17946 | 465 | 10,1 | 0,886 | H5A034 | C | neg1 | Hm Drgbl | Hm Drgbl | Hm Drgbl | CPE002E1 | CPE002EY | CPE002FJ |
| 1408 | K23 | K24 | 2178 | 788,1 | 21974 | 440 | 10,1 | 0,927 | H5A034 | C | neg1 | Hm Drgbl | Hm Drgbl | Hm Drgbl | CPE002FV | CPE002FZ | CPE002G3 |
| 16480 | O23 | O24 | 1696 | 1022 | 17194 | 393,7 | 10,1 | 0,927 | H5A034 | C | neg1 | Hm Gen E | Hm Gen E | Hm Gen E | CPE002JG | CPE002NV | CPE002KB |
| 1025 | K24 | K1 | 1387 | 771,8 | 13986 | 396,2 | 10,1 | 0,957 | H5A034 | C | neg1 | Hm Drgbl | Hm Drgbl | Hm Drgbl | CPE002FU | CPE002FY | CPE002G2 |
| 1048 | L23 | L24 | 1650 | 838,3 | 16673 | 416,5 | 10,1 | 0,957 | H5A034 | C | neg1 | Hm Drgbl | Hm Drgbl | Hm Drgbl | CPE002FU | CPE002FY | CPE002G2 |
| 3712 | K23 | K24 | 2146 | 1670 | 21618 | 493,6 | 10,1 | 0,971 | H5A034 | C | neg1 | Hm Drgbl | Hm Drgbl | Hm Drgbl | CPE002DR | CPE002EH | CPE002F7 |
| 8345 | L24 | L1 | 1753 | 649,9 | 17699 | 421,9 | 10,1 | 0,971 | H5A034 | C | neg1 | Hm Drgbl | Hm Drgbl | Hm Drgbl | CPE002E5 | CPE002EU | CPE002FF |
| 11513 | P24 | P1 | 1187 | 847 | 12070 | 139 | 10,2 | 0,739 | H5A034 | C | neg1 | Hm Drgbl | Hm Drgbl | Hm Drgbl | CPE002ED | CPE002F3 | CPE002FT |
| 2560 | K23 | K24 | 2059 | 795,8 | 20900 | 368,4 | 10,2 | 0,803 | H5A034 | C | neg1 | Hm Drgbl | Hm Drgbl | Hm Drgbl | CPE002DC | CPE002EE | CPE002F4 |
| 16864 | O23 | O24 | 1639 | 990,7 | 16763 | 337,7 | 10,2 | 0,806 | H5A034 | C | neg1 | Hm Gen E | Hm Gen E | Hm Gen E | CPE002JH | CPE002NV | CPE002KC |
| 9185 | O24 | O1 | 1978 | 1849 | 20149 | 160,4 | 10,2 | 0,819 | H5A034 | C | neg1 | Hm Drgbl | Hm Drgbl | Hm Drgbl | CPE002E7 | CPE002ES | CPE002FD |
| 4600 | P23 | P24 | 1612 | 751 | 16434 | 485,4 | 10,2 | 0,861 | H5A034 | C | neg1 | Hm Drgbl | Hm Drgbl | Hm Drgbl | CPE002DU | CPE002EK | CPE002FQ |
| 256 | K23 | K24 | 709 | 1691 | 7264 | 770,3 | 10,2 | 0,868 | H5A034 | C | neg1 | Hm Drgbl | Hm Drgbl | Hm Drgbl | CPE002DT | CPE002EJ | CPE002F9 |
| 4192 | O23 | O24 | 1793 | 807,6 | 18369 | 495,5 | 10,2 | 0,907 | H5A034 | C | neg1 | Hm Drgbl | Hm Drgbl | Hm Drgbl | CPE002DS | CPE002EI | CPE002F8 |
| 6137 | P24 | P1 | 1408 | 806,9 | 14423 | 523,8 | 10,2 | 0,932 | H5A034 | C | neg1 | Hm Drgbl | Hm Drgbl | Hm Drgbl | CPE002DY | CPE002F1 | CPE002FM |
| 3784 | N23 | N24 | 1704 | 1456 | 17319 | 382,1 | 10,2 | 0,981 | H5A034 | C | neg1 | Hm Drgbl | Hm Drgbl | Hm Drgbl | CPE002DR | CPE002EH | CPE002F7 |
| 10265 | L24 | L1 | 1567 | 849,3 | 16060 | 133,3 | 10,2 | 0,981 | H5A034 | C | neg1 | Hm Drgbl | Hm Drgbl | Hm Drgbl | CPE002EA | CPE002EP | CPE002FA |
| 21785 | L24 | L1 | 300 | 961,3 | 3065 | 557,3 | 10,2 | 1,074 | H5A034 | C | neg1 | Hm Gen E | Hm Gen E | Hm Gen E | CPE002JU | CPE002O9 | CPE002KP |
| 9496 | L23 | L24 | 1555 | 1217 | 16028 | 120,2 | 10,3 | 0,715 | H5A034 | C | neg1 | Hm Drgbl | Hm Drgbl | Hm Drgbl | CPE002E8 | CPE002ER | CPE002FC |
| 21064 | N23 | N24 | 1519 | 1106 | 15613 | 177,7 | 10,3 | 0,73 | H5A034 | C | neg1 | Hm Gen E | Hm Gen E | Hm Gen E | CPE002JS | CPE002O7 | CPE002KN |
| 12616 | N23 | N24 | 1907 | 1136 | 19556 | 154,9 | 10,3 | 0,774 | H5A034 | C | neg1 | Hm Gen E | Hm Gen E | Hm Gen E | CPE002J5 | CPE002NK | CPE002K1 |
| 5273 | L24 | L1 | 1706 | 673,4 | 17614 | 295 | 10,3 | 0,808 | H5A034 | C | neg1 | Hm Drgbl | Hm Drgbl | Hm Drgbl | CPE002DV | CPE002EM | CPE002FO |
| 18041 | P24 | P1 | 645 | 1081 | 6633 | 227 | 10,3 | 0,814 | H5A034 | C | neg1 | Hm Gen E | Hm Gen E | Hm Gen E | CPE002JK | CPE002NZ | CPE002KF |
| 2297 | P24 | P1 | 1558 | 711,1 | 15975 | 471 | 10,3 | 0,896 | H5A034 | C | neg1 | Hm Drgbl | Hm Drgbl | Hm Drgbl | CPE002FX | CPE002G1 | CPE002G5 |
| 6881 | O24 | O1 | 321 | 632,8 | 3311 | 336,7 | 10,3 | 0,904 | H5A034 | C | neg1 | Hm Drgbl | Hm Drgbl | Hm Drgbl | CPE002E1 | CPE002EY | CPE002FJ |
| 15664 | M23 | M24 | 1636 | 906 | 16867 | 152,7 | 10,3 | 0,936 | H5A034 | C | neg1 | Hm Gen E | Hm Gen E | Hm Gen E | CPE002JE | CPE002NT | CPE002K9 |
| 3832 | P23 | P24 | 2003 | 1568 | 20586 | 459 | 10,3 | 0,99 | H5A034 | C | neg1 | Hm Drgbl | Hm Drgbl | Hm Drgbl | CPE002DR | CPE002EH | CPE002F7 |
| 10289 | M24 | M1 | 2109 | 936,3 | 21758 | 133,5 | 10,3 | 0,99 | H5A034 | C | neg1 | Hm Drgbl | Hm Drgbl | Hm Drgbl | CPE002EA | CPE002EP | CPE002FA |
| 21880 | P23 | P24 | 297 | 1264 | 3072 | 358,3 | 10,3 | 1,084 | H5A034 | C | neg1 | Hm Gen E | Hm Gen E | Hm Gen E | CPE002JU | CPE002O9 | CPE002KP |
| 4984 | P23 | P24 | 1315 | 875,3 | 13542 | 347,2 | 10,3 | 1,132 | H5A034 | C | neg1 | Hm Drgbl | Hm Drgbl | Hm Drgbl | CPE002DV | CPE002EL | CPE002FP |
| 6497 | O24 | O1 | 419 | 486,4 | 4375 | 434 | 10,4 | 0,686 | H5A034 | C | neg1 | Hm Drgbl | Hm Drgbl | Hm Drgbl | CPE002DZ | CPE002F0 | CPE002FL |
| 7673 | P24 | P1 | 716 | 642,5 | 7478 | 417,6 | 10,4 | 0,794 | H5A034 | C | neg1 | Hm Drgbl | Hm Drgbl | Hm Drgbl | CPE002E3 | CPE002EW | CPE002FH |
| 17224 | N23 | N24 | 2137 | 1061 | 22321 | 235,7 | 10,4 | 0,803 | H5A034 | C | neg1 | Hm Gen E | Hm Gen E | Hm Gen E | CPE002JI | CPE002NZ | CPE002KD |
| 11800 | L23 | L24 | 1468 | 894,3 | 15296 | 131,8 | 10,4 | 0,929 | H5A034 | C | neg1 | Hm Gen E | Hm Gen E | Hm Gen E | CPE002JA | CPE002NJ | CPE002JZ |
| 11849 | N24 | N1 | 1328 | 876,5 | 13748 | 123,2 | 10,4 | 0,929 | H5A034 | C | neg1 | Hm Gen E | Hm Gen E | Hm Gen E | CPE002JA | CPE002NJ | CPE002JZ |
| 1481 | N24 | N1 | 1164 | 741,1 | 12113 | 442,2 | 10,4 | 0,954 | H5A034 | C | neg1 | Hm Drgbl | Hm Drgbl | Hm Drgbl | CPE002FV | CPE002FZ | CPE002G3 |
| 13745 | M24 | M1 | 1408 | 1095 | 14590 | 161,1 | 10,4 | 0,977 | H5A034 | C | neg1 | Hm Gen E | Hm Gen E | Hm Gen E | CPE002JC | CPE002NC | CPE002K4 |
| 5729 | O24 | O1 | 636 | 763,5 | 6603 | 604,1 | 10,4 | 0,99 | H5A034 | C | neg1 | Hm Drgbl | Hm Drgbl | Hm Drgbl | CPE002DX | CPE002F2 | CPE002FN |
| 3040 | O23 | O24 | 1469 | 685,4 | 15256 | 609,1 | 10,4 | 1,224 | H5A034 | C | neg1 | Hm Drgbl | Hm Drgbl | Hm Drgbl | CPE002DP | CPE002EF | CPE002F5 |

| | | | | | | | | | | | | | | | | | |
|---|---|---|---|---|---|---|---|---|---|---|---|---|---|---|---|---|---|
| 3064 | P23 | P24 | 1633 | 714,5 | 16994 | 691,8 | 10,4 | 1,224 | H5A034 | C | neg1 | Hm Drgbl | Hm Drgbl | Hm Drgbl | CPE002DP | CPE002EF | CPE002F5 |
| 20344 | P23 | P24 | 915 | 1312 | 9549 | 157,7 | 10,4 | 1,268 | H5A034 | C | neg1 | Hm Gen E | Hm Gen E | Hm Gen E | CPE002JQ | CPE002O5 | CPE002KL |
| 19505 | M24 | M1 | 1303 | 1416 | 13662 | 248,2 | 10,5 | 0,745 | H5A034 | C | neg1 | Hm Gen E | Hm Gen E | Hm Gen E | CPE002JO | CPE002O3 | CPE002KJ |
| 11032 | L23 | L24 | 1698 | 909,6 | 17822 | 516,9 | 10,5 | 0,795 | H5A034 | C | neg1 | Hm Drgbl | Hm Drgbl | Hm Drgbl | CPE002EC | CPE002EN | CPE002FS |
| 18017 | O24 | O1 | 1283 | 994,7 | 13425 | 298,1 | 10,5 | 0,83 | H5A034 | C | neg1 | Hm Gen E | Hm Gen E | Hm Gen E | CPE002JK | CPE002NZ | CPE002KF |
| 3808 | O23 | O24 | 1803 | 1569 | 18909 | 475 | 10,5 | 1,01 | H5A034 | C | neg1 | Hm Drgbl | Hm Drgbl | Hm Drgbl | CPE002DR | CPE002EH | CPE002F7 |
| 10241 | K24 | K1 | 1636 | 920,4 | 17156 | 138 | 10,5 | 1,01 | H5A034 | C | neg1 | Hm Drgbl | Hm Drgbl | Hm Drgbl | CPE002EA | CPE002EP | CPE002FA |
| 1817 | L24 | L1 | 1832 | 799,1 | 19322 | 409,7 | 10,5 | 1,099 | H5A034 | C | neg1 | Hm Drgbl | Hm Drgbl | Hm Drgbl | CPE002FW | CPE002G0 | CPE002G4 |
| 1912 | P23 | P24 | 1481 | 786,2 | 15604 | 496,6 | 10,5 | 1,099 | H5A034 | C | neg1 | Hm Drgbl | Hm Drgbl | Hm Drgbl | CPE002FW | CPE002G0 | CPE002G4 |
| 9569 | O24 | O1 | 1870 | 1147 | 19877 | 107,4 | 10,6 | 0,736 | H5A034 | C | neg1 | Hm Drgbl | Hm Drgbl | Hm Drgbl | CPE002E8 | CPE002ER | CPE002FC |
| 16840 | N23 | N24 | 1653 | 1023 | 17472 | 261 | 10,6 | 0,838 | H5A034 | C | neg1 | Hm Gen E | Hm Gen E | Hm Gen E | CPE002JH | CPE002NV | CPE002KC |
| 329 | N24 | N1 | 572 | 1615 | 6080 | 695,2 | 10,6 | 0,902 | H5A034 | C | neg1 | Hm Drgbl | Hm Drgbl | Hm Drgbl | CPE002DT | CPE002EJ | CPE002F9 |
| 6017 | K24 | K1 | 749 | 800,7 | 7969 | 292,8 | 10,6 | 0,968 | H5A034 | C | neg1 | Hm Drgbl | Hm Drgbl | Hm Drgbl | CPE002DY | CPE002F1 | CPE002FM |
| 6064 | M23 | M24 | 1836 | 818 | 19527 | 583,1 | 10,6 | 0,968 | H5A034 | C | neg1 | Hm Drgbl | Hm Drgbl | Hm Drgbl | CPE002DY | CPE002F1 | CPE002FM |
| 5705 | N24 | N1 | 1644 | 687,3 | 17447 | 554 | 10,6 | 1,01 | H5A034 | C | neg1 | Hm Drgbl | Hm Drgbl | Hm Drgbl | CPE002DX | CPE002F2 | CPE002FN |
| 3809 | O24 | O1 | 912 | 1362 | 9647 | 411 | 10,6 | 1,019 | H5A034 | C | neg1 | Hm Drgbl | Hm Drgbl | Hm Drgbl | CPE002DR | CPE002EH | CPE002F7 |
| 1888 | O23 | O24 | 1979 | 811,2 | 20929 | 430,8 | 10,6 | 1,11 | H5A034 | C | neg1 | Hm Drgbl | Hm Drgbl | Hm Drgbl | CPE002FW | CPE002G0 | CPE002G4 |
| 9544 | N23 | N24 | 1910 | 1148 | 20347 | 128,2 | 10,7 | 0,743 | H5A034 | C | neg1 | Hm Drgbl | Hm Drgbl | Hm Drgbl | CPE002E8 | CPE002ER | CPE002FC |
| 6904 | P23 | P24 | 1246 | 655,3 | 13350 | 514,4 | 10,7 | 0,939 | H5A034 | C | neg1 | Hm Drgbl | Hm Drgbl | Hm Drgbl | CPE002E1 | CPE002EY | CPE002FJ |
| 4168 | N23 | N24 | 1629 | 706,8 | 17452 | 443,5 | 10,7 | 0,951 | H5A034 | C | neg1 | Hm Drgbl | Hm Drgbl | Hm Drgbl | CPE002DS | CPE002EI | CPE002F8 |
| 8368 | M23 | M24 | 1952 | 702,6 | 20822 | 460,5 | 10,7 | 1,029 | H5A034 | C | neg1 | Hm Drgbl | Hm Drgbl | Hm Drgbl | CPE002E5 | CPE002EU | CPE002FF |
| 8416 | O23 | O24 | 1768 | 641,2 | 18904 | 490,3 | 10,7 | 1,029 | H5A034 | C | neg1 | Hm Drgbl | Hm Drgbl | Hm Drgbl | CPE002E5 | CPE002EU | CPE002FF |
| 8440 | P23 | P24 | 1558 | 667,2 | 16738 | 515,9 | 10,7 | 1,029 | H5A034 | C | neg1 | Hm Drgbl | Hm Drgbl | Hm Drgbl | CPE002E5 | CPE002EU | CPE002FF |
| 21808 | M23 | M24 | 309 | 952,1 | 3293 | 721,3 | 10,7 | 1,126 | H5A034 | C | neg1 | Hm Gen E | Hm Gen E | Hm Gen E | CPE002JU | CPE002O9 | CPE002KP |
| 3400 | N23 | N24 | 1830 | 1432 | 19811 | 476,1 | 10,8 | 0,669 | H5A034 | C | neg1 | Hm Drgbl | Hm Drgbl | Hm Drgbl | CPE002DC | CPE002EG | CPE002F6 |
| 8825 | P24 | P1 | 1711 | 1104 | 18467 | 144,2 | 10,8 | 0,763 | H5A034 | C | neg1 | Hm Drgbl | Hm Drgbl | Hm Drgbl | CPE002E6 | CPE002ET | CPE002FE |
| 17225 | N24 | N1 | 1785 | 1010 | 19208 | 153 | 10,8 | 0,834 | H5A034 | C | neg1 | Hm Gen E | Hm Gen E | Hm Gen E | CPE002JI | CPE002NZ | CPE002KD |
| 7289 | P24 | P1 | 1107 | 604,5 | 11974 | 444,5 | 10,8 | 0,84 | H5A034 | C | neg1 | Hm Drgbl | Hm Drgbl | Hm Drgbl | CPE002E2 | CPE002EX | CPE002FI |
| 305 | M24 | M1 | 616 | 1677 | 6668 | 675,7 | 10,8 | 0,919 | H5A034 | C | neg1 | Hm Drgbl | Hm Drgbl | Hm Drgbl | CPE002DT | CPE002EJ | CPE002F9 |
| 6856 | N23 | N24 | 1417 | 606,5 | 15256 | 480,9 | 10,8 | 0,947 | H5A034 | C | neg1 | Hm Drgbl | Hm Drgbl | Hm Drgbl | CPE002E1 | CPE002EY | CPE002FJ |
| 10264 | L23 | L24 | 1837 | 931,1 | 19888 | 140,1 | 10,8 | 1,038 | H5A034 | C | neg1 | Hm Drgbl | Hm Drgbl | Hm Drgbl | CPE002EA | CPE002EP | CPE002FA |
| 14969 | P24 | P1 | 1520 | 867,9 | 16557 | 79,51 | 10,9 | 0,621 | H5A034 | C | neg1 | Hm Gen E | Hm Gen E | Hm Gen E | CPE002JD | CPE002NR | CPE002K7 |
| 15640 | L23 | L24 | 1438 | 894,3 | 15669 | 162,7 | 10,9 | 0,991 | H5A034 | C | neg1 | Hm Gen E | Hm Gen E | Hm Gen E | CPE002JE | CPE002NT | CPE002K9 |
| 6088 | N23 | N24 | 2019 | 831,5 | 22027 | 683,1 | 10,9 | 0,995 | H5A034 | C | neg1 | Hm Drgbl | Hm Drgbl | Hm Drgbl | CPE002DY | CPE002F1 | CPE002FM |
| 1432 | L23 | L24 | 1984 | 760,7 | 21721 | 472,8 | 10,9 | 1 | H5A034 | C | neg1 | Hm Drgbl | Hm Drgbl | Hm Drgbl | CPE002FV | CPE002FZ | CPE002G3 |
| 1505 | O24 | O1 | 1009 | 672,5 | 10969 | 425,6 | 10,9 | 1 | H5A034 | C | neg1 | Hm Drgbl | Hm Drgbl | Hm Drgbl | CPE002FV | CPE002FZ | CPE002G3 |
| 13697 | K24 | K1 | 1246 | 1079 | 13583 | 141,9 | 10,9 | 1,023 | H5A034 | C | neg1 | Hm Gen E | Hm Gen E | Hm Gen E | CPE002JC | CPE002NC | CPE002K4 |
| 5680 | M23 | M24 | 2025 | 830,4 | 22089 | 520,5 | 10,9 | 1,038 | H5A034 | C | neg1 | Hm Drgbl | Hm Drgbl | Hm Drgbl | CPE002DX | CPE002F2 | CPE002FN |
| 21857 | O24 | O1 | 221 | 881,3 | 2407 | 691,8 | 10,9 | 1,147 | H5A034 | C | neg1 | Hm Gen E | Hm Gen E | Hm Gen E | CPE002JU | CPE002O9 | CPE002KP |
| 3041 | O24 | O1 | 1095 | 722,1 | 11913 | 537,8 | 10,9 | 1,282 | H5A034 | C | neg1 | Hm Drgbl | Hm Drgbl | Hm Drgbl | CPE002DP | CPE002EF | CPE002F5 |
| 2200 | L23 | L24 | 1870 | 757,2 | 20654 | 428,4 | 11 | 0,957 | H5A034 | C | neg1 | Hm Drgbl | Hm Drgbl | Hm Drgbl | CPE002FX | CPE002G1 | CPE002G5 |
| 11825 | M24 | M1 | 1868 | 923,4 | 20507 | 133,9 | 11 | 0,982 | H5A034 | C | neg1 | Hm Gen E | Hm Gen E | Hm Gen E | CPE002JA | CPE002NJ | CPE002JZ |
| 6089 | N24 | N1 | 1401 | 738,2 | 15393 | 419,9 | 11 | 1,005 | H5A034 | C | neg1 | Hm Drgbl | Hm Drgbl | Hm Drgbl | CPE002DY | CPE002F1 | CPE002FM |
| 6112 | O23 | O24 | 2291 | 838,7 | 25263 | 649 | 11 | 1,005 | H5A034 | C | neg1 | Hm Drgbl | Hm Drgbl | Hm Drgbl | CPE002DY | CPE002F1 | CPE002FM |
| 1024 | K23 | K24 | 1126 | 781,8 | 12339 | 407,2 | 11 | 1,043 | H5A034 | C | neg1 | Hm Drgbl | Hm Drgbl | Hm Drgbl | CPE002FU | CPE002FY | CPE002G2 |
| 21881 | P24 | P1 | 253 | 1251 | 2791 | 466,2 | 11 | 1,158 | H5A034 | C | neg1 | Hm Gen E | Hm Gen E | Hm Gen E | CPE002JU | CPE002O9 | CPE002KP |
| 4577 | O24 | O1 | 1004 | 734,3 | 11193 | 450,6 | 11,1 | 0,937 | H5A034 | C | neg1 | Hm Drgbl | Hm Drgbl | Hm Drgbl | CPE002DU | CPE002EK | CPE002FQ |
| 4121 | L24 | L1 | 1444 | 709,3 | 16089 | 401,3 | 11,1 | 0,987 | H5A034 | C | neg1 | Hm Drgbl | Hm Drgbl | Hm Drgbl | CPE002DS | CPE002EI | CPE002F8 |
| 4217 | P24 | P1 | 1224 | 676 | 13610 | 386,2 | 11,1 | 0,987 | H5A034 | C | neg1 | Hm Drgbl | Hm Drgbl | Hm Drgbl | CPE002DS | CPE002EI | CPE002F8 |
| 15665 | M24 | M1 | 1783 | 868,3 | 19765 | 127 | 11,1 | 1,009 | H5A034 | C | neg1 | Hm Gen E | Hm Gen E | Hm Gen E | CPE002JE | CPE002NT | CPE002K9 |
| 2249 | N24 | N1 | 1719 | 738,3 | 19233 | 527,7 | 11,2 | 0,974 | H5A034 | C | neg1 | Hm Drgbl | Hm Drgbl | Hm Drgbl | CPE002FX | CPE002G1 | CPE002G5 |
| 6832 | M23 | M24 | 1858 | 685,5 | 20826 | 461,8 | 11,2 | 0,982 | H5A034 | C | neg1 | Hm Drgbl | Hm Drgbl | Hm Drgbl | CPE002E1 | CPE002EY | CPE002FJ |
| 3376 | M23 | M24 | 1770 | 1521 | 19929 | 449,4 | 11,3 | 0,7 | H5A034 | C | neg1 | Hm Drgbl | Hm Drgbl | Hm Drgbl | CPE002DC | CPE002EG | CPE002F6 |
| 5248 | K23 | K24 | 2283 | 748,3 | 25756 | 548,6 | 11,3 | 0,886 | H5A034 | C | neg1 | Hm Drgbl | Hm Drgbl | Hm Drgbl | CPE002DV | CPE002EM | CPE002FO |
| 16816 | M23 | M24 | 1787 | 1008 | 20162 | 299,9 | 11,3 | 0,893 | H5A034 | C | neg1 | Hm Gen E | Hm Gen E | Hm Gen E | CPE002JH | CPE002NV | CPE002KC |
| 16841 | N24 | N1 | 1408 | 1000 | 15891 | 262,6 | 11,3 | 0,893 | H5A034 | C | neg1 | Hm Gen E | Hm Gen E | Hm Gen E | CPE002JH | CPE002NV | CPE002KC |
| 17992 | N23 | N24 | 1679 | 1126 | 18920 | 300,7 | 11,3 | 0,893 | H5A034 | C | neg1 | Hm Gen E | Hm Gen E | Hm Gen E | CPE002JK | CPE002NZ | CPE002KF |
| 9113 | L24 | L1 | 1880 | 1795 | 21262 | 141,4 | 11,3 | 0,908 | H5A034 | C | neg1 | Hm Drgbl | Hm Drgbl | Hm Drgbl | CPE002E7 | CPE002ES | CPE002FD |
| 4552 | N23 | N24 | 1372 | 735,8 | 15489 | 431,6 | 11,3 | 0,954 | H5A034 | C | neg1 | Hm Drgbl | Hm Drgbl | Hm Drgbl | CPE002DU | CPE002EK | CPE002FQ |
| 1433 | L24 | L1 | 1784 | 743,3 | 20217 | 415,9 | 11,3 | 1,037 | H5A034 | C | neg1 | Hm Drgbl | Hm Drgbl | Hm Drgbl | CPE002FV | CPE002FZ | CPE002G3 |
| 1049 | L24 | L1 | 1566 | 754,3 | 17662 | 427,1 | 11,3 | 1,071 | H5A034 | C | neg1 | Hm Drgbl | Hm Drgbl | Hm Drgbl | CPE002FU | CPE002FY | CPE002G2 |
| 9088 | K23 | K24 | 2541 | 2274 | 28991 | 162,8 | 11,4 | 0,916 | H5A034 | C | neg1 | Hm Drgbl | Hm Drgbl | Hm Drgbl | CPE002E7 | CPE002ES | CPE002FD |
| 9137 | M24 | M1 | 2031 | 1822 | 23134 | 141,9 | 11,4 | 0,916 | H5A034 | C | neg1 | Hm Drgbl | Hm Drgbl | Hm Drgbl | CPE002E7 | CPE002ES | CPE002FD |
| 4601 | P24 | P1 | 1435 | 753,2 | 16370 | 482,5 | 11,4 | 0,962 | H5A034 | C | neg1 | Hm Drgbl | Hm Drgbl | Hm Drgbl | CPE002DU | CPE002EK | CPE002FQ |
| 4216 | P23 | P24 | 1650 | 774,1 | 18830 | 497,8 | 11,4 | 1,013 | H5A034 | C | neg1 | Hm Drgbl | Hm Drgbl | Hm Drgbl | CPE002DS | CPE002EI | CPE002F8 |
| 11873 | O24 | O1 | 1447 | 899,3 | 16545 | 186,4 | 11,4 | 1,018 | H5A034 | C | neg1 | Hm Gen E | Hm Gen E | Hm Gen E | CPE002JA | CPE002NJ | CPE002JZ |
| 1528 | P23 | P24 | 1981 | 801,3 | 22630 | 540 | 11,4 | 1,046 | H5A034 | C | neg1 | Hm Drgbl | Hm Drgbl | Hm Drgbl | CPE002FV | CPE002FZ | CPE002G3 |
| 1073 | M24 | M1 | 1464 | 763,7 | 16619 | 436,4 | 11,4 | 1,081 | H5A034 | C | neg1 | Hm Drgbl | Hm Drgbl | Hm Drgbl | CPE002FU | CPE002FY | CPE002G2 |
| 12569 | L24 | L1 | 1253 | 1085 | 14361 | 150,9 | 11,5 | 0,865 | H5A034 | C | neg1 | Hm Gen E | Hm Gen E | Hm Gen E | CPE002J5 | CPE002NK | CPE002K1 |
| 11056 | M23 | M24 | 1604 | 893,3 | 18366 | 568 | 11,5 | 0,871 | H5A034 | C | neg1 | Hm Drgbl | Hm Drgbl | Hm Drgbl | CPE002EC | CPE002EN | CPE002FS |
| 11105 | O24 | O1 | 1514 | 895,9 | 17346 | 639,1 | 11,5 | 0,871 | H5A034 | C | neg1 | Hm Drgbl | Hm Drgbl | Hm Drgbl | CPE002EC | CPE002EN | CPE002FS |
| 16793 | L24 | L1 | 986 | 1042 | 11301 | 255,3 | 11,5 | 0,909 | H5A034 | C | neg1 | Hm Gen E | Hm Gen E | Hm Gen E | CPE002JH | CPE002NV | CPE002KC |
| 4505 | L24 | L1 | 1505 | 696,4 | 17354 | 407,7 | 11,5 | 0,97 | H5A034 | C | neg1 | Hm Drgbl | Hm Drgbl | Hm Drgbl | CPE002DU | CPE002EK | CPE002FQ |
| 19553 | O24 | O1 | 1012 | 1223 | 11771 | 318,6 | 11,6 | 0,823 | H5A034 | C | neg1 | Hm Gen E | Hm Gen E | Hm Gen E | CPE002JO | CPE002O3 | CPE002KJ |
| 7577 | L24 | L1 | 1127 | 594,2 | 13034 | 263,4 | 11,6 | 0,885 | H5A034 | C | neg1 | Hm Drgbl | Hm Drgbl | Hm Drgbl | CPE002E3 | CPE002EW | CPE002FH |
| 5272 | L23 | L24 | 1970 | 771,6 | 22946 | 439,6 | 11,6 | 0,91 | H5A034 | C | neg1 | Hm Drgbl | Hm Drgbl | Hm Drgbl | CPE002DV | CPE002EM | CPE002FO |
| 4504 | L23 | L24 | 1312 | 801,4 | 15177 | 432,9 | 11,6 | 0,979 | H5A034 | C | neg1 | Hm Drgbl | Hm Drgbl | Hm Drgbl | CPE002DU | CPE002EK | CPE002FQ |
| 6857 | N24 | N1 | 1357 | 677,6 | 15685 | 244,4 | 11,6 | 1,018 | H5A034 | C | neg1 | Hm Drgbl | Hm Drgbl | Hm Drgbl | CPE002E1 | CPE002EY | CPE002FJ |
| 6040 | L23 | L24 | 1987 | 887,4 | 23041 | 626,5 | 11,6 | 1,059 | H5A034 | C | neg1 | Hm Drgbl | Hm Drgbl | Hm Drgbl | CPE002DY | CPE002F1 | CPE002FM |
| 10672 | M23 | M24 | 1435 | 877,1 | 16639 | 223,9 | 11,6 | 1,311 | H5A034 | C | neg1 | Hm Drgbl | Hm Drgbl | Hm Drgbl | CPE002EB | CPE002EO | CPE002FR |
| 19193 | P24 | P1 | 966 | 1146 | 11320 | 236,3 | 11,7 | 0,815 | H5A034 | C | neg1 | Hm Gen E | Hm Gen E | Hm Gen E | CPE002JN | CPE002O2 | CPE002KI |
| 7264 | O23 | O24 | 1790 | 669 | 20951 | 506 | 11,7 | 0,911 | H5A034 | C | neg1 | Hm Drgbl | Hm Drgbl | Hm Drgbl | CPE002E2 | CPE002EX | CPE002FI |
| 6905 | P24 | P1 | 1039 | 634,2 | 12190 | 363,3 | 11,7 | 1,026 | H5A034 | C | neg1 | Hm Drgbl | Hm Drgbl | Hm Drgbl | CPE002E1 | CPE002EY | CPE002FJ |
| 16457 | N24 | N1 | 1400 | 997,1 | 16438 | 230,4 | 11,7 | 1,073 | H5A034 | C | neg1 | Hm Gen E | Hm Gen E | Hm Gen E | CPE002JG | CPE002NV | CPE002KB |
| 18761 | N24 | N1 | 1605 | 1076 | 18863 | 243,8 | 11,8 | 0,8 | H5A034 | C | neg1 | Hm Gen E | Hm Gen E | Hm Gen E | CPE002JM | CPE002O1 | CPE002KH |
| 17633 | O24 | O1 | 1327 | 981,1 | 15627 | 249,7 | 11,8 | 0,805 | H5A034 | C | neg1 | Hm Gen E | Hm Gen E | Hm Gen E | CPE002JJ | CPE002NY | CPE002KE |
| 21425 | M24 | M1 | 1165 | 936,8 | 13744 | 237 | 11,8 | 0,837 | H5A034 | C | neg1 | Hm Gen E | Hm Gen E | Hm Gen E | CPE002JT | CPE002O8 | CPE002KO |
| 21449 | N24 | N1 | 1136 | 927,2 | 13458 | 194,9 | 11,8 | 0,837 | H5A034 | C | neg1 | Hm Gen E | Hm Gen E | Hm Gen E | CPE002JT | CPE002O8 | CPE002KO |
| 12617 | N24 | N1 | 1542 | 1059 | 18242 | 144,3 | 11,8 | 0,887 | H5A034 | C | neg1 | Hm Gen E | Hm Gen E | Hm Gen E | CPE002J5 | CPE002NK | CPE002K1 |
| 7649 | O24 | O1 | 577 | 548,3 | 6809 | 516,7 | 11,8 | 0,901 | H5A034 | C | neg1 | Hm Drgbl | Hm Drgbl | Hm Drgbl | CPE002E3 | CPE002EW | CPE002FH |
| 17200 | M23 | M24 | 2210 | 1098 | 26040 | 272,8 | 11,8 | 0,911 | H5A034 | C | neg1 | Hm Gen E | Hm Gen E | Hm Gen E | CPE002JI | CPE002NZ | CPE002KD |
| 17249 | O24 | O1 | 1654 | 1011 | 19448 | 204,8 | 11,8 | 0,911 | H5A034 | C | neg1 | Hm Gen E | Hm Gen E | Hm Gen E | CPE002JI | CPE002NZ | CPE002KD |
| 2273 | O24 | O1 | 920 | 665,1 | 10854 | 462,9 | 11,8 | 1,026 | H5A034 | C | neg1 | Hm Drgbl | Hm Drgbl | Hm Drgbl | CPE002FX | CPE002G1 | CPE002G5 |
| 15689 | N24 | N1 | 1644 | 888,9 | 19347 | 134 | 11,8 | 1,073 | H5A034 | C | neg1 | Hm Gen E | Hm Gen E | Hm Gen E | CPE002JE | CPE002NT | CPE002K9 |
| 6016 | K23 | K24 | 1772 | 875,3 | 20881 | 414,6 | 11,8 | 1,078 | H5A034 | C | neg1 | Hm Drgbl | Hm Drgbl | Hm Drgbl | CPE002DY | CPE002F1 | CPE002FM |
| 1457 | M24 | M1 | 1047 | 712,7 | 12387 | 421 | 11,8 | 1,083 | H5A034 | C | neg1 | Hm Drgbl | Hm Drgbl | Hm Drgbl | CPE002FV | CPE002FZ | CPE002G3 |
| 3761 | M24 | M1 | 819 | 1341 | 9636 | 364,7 | 11,8 | 1,135 | H5A034 | C | neg1 | Hm Drgbl | Hm Drgbl | Hm Drgbl | CPE002DR | CPE002EH | CPE002F7 |
| 10361 | P24 | P1 | 1488 | 813,5 | 17598 | 148,8 | 11,8 | 1,135 | H5A034 | C | neg1 | Hm Drgbl | Hm Drgbl | Hm Drgbl | CPE002EA | CPE002EP | CPE002FA |
| 19529 | N24 | N1 | 1129 | 1150 | 13382 | 214,8 | 11,9 | 0,844 | H5A034 | C | neg1 | Hm Gen E | Hm Gen E | Hm Gen E | CPE002JO | CPE002O3 | CPE002KJ |
| 21376 | K23 | K24 | 1850 | 1068 | 22022 | 397 | 11,9 | 0,844 | H5A034 | C | neg1 | Hm Gen E | Hm Gen E | Hm Gen E | CPE002JT | CPE002O8 | CPE002KO |
| 21473 | O24 | O1 | 1462 | 964,6 | 17412 | 259,2 | 11,9 | 0,844 | H5A034 | C | neg1 | Hm Gen E | Hm Gen E | Hm Gen E | CPE002JT | CPE002O8 | CPE002KO |
| 17201 | M24 | M1 | 1450 | 1060 | 17290 | 162 | 11,9 | 0,919 | H5A034 | C | neg1 | Hm Gen E | Hm Gen E | Hm Gen E | CPE002JI | CPE002NZ | CPE002KD |
| 6808 | L23 | L24 | 1680 | 636,6 | 19986 | 486,2 | 11,9 | 1,044 | H5A034 | C | neg1 | Hm Drgbl | Hm Drgbl | Hm Drgbl | CPE002E1 | CPE002EY | CPE002FJ |
| 10720 | O23 | O24 | 1665 | 859,6 | 19798 | 256,2 | 11,9 | 1,345 | H5A034 | C | neg1 | Hm Drgbl | Hm Drgbl | Hm Drgbl | CPE002EB | CPE002EO | CPE002FR |
| 20608 | K23 | K24 | 1351 | 848,9 | 16165 | 220,9 | 12 | 0,8 | H5A034 | C | neg1 | Hm Gen E | Hm Gen E | Hm Gen E | CPE002JR | CPE002O6 | CPE002KM |
| 9976 | P23 | P24 | 1947 | 1125 | 23295 | 226,1 | 12 | 0,839 | H5A034 | C | neg1 | Hm Drgbl | Hm Drgbl | Hm Drgbl | CPE002E9 | CPE002EQ | CPE002FB |
| 5657 | L24 | L1 | 1999 | 836,4 | 23972 | 589,9 | 12 | 1,143 | H5A034 | C | neg1 | Hm Drgbl | Hm Drgbl | Hm Drgbl | CPE002DX | CPE002F2 | CPE002FN |
| 10288 | M23 | M24 | 1752 | 905,9 | 21105 | 174,7 | 12 | 1,154 | H5A034 | C | neg1 | Hm Drgbl | Hm Drgbl | Hm Drgbl | CPE002EA | CPE002EP | CPE002FA |
| 9184 | O23 | O24 | 1928 | 2005 | 23312 | 240,6 | 12,1 | 0,972 | H5A034 | C | neg1 | Hm Drgbl | Hm Drgbl | Hm Drgbl | CPE002E7 | CPE002ES | CPE002FD |
| 4553 | N24 | N1 | 1218 | 760,5 | 14702 | 452,3 | 12,1 | 1,021 | H5A034 | C | neg1 | Hm Drgbl | Hm Drgbl | Hm Drgbl | CPE002DU | CPE002EK | CPE002FQ |
| 5632 | K23 | K24 | 1767 | 760,6 | 21426 | 533,7 | 12,1 | 1,152 | H5A034 | C | neg1 | Hm Drgbl | Hm Drgbl | Hm Drgbl | CPE002DX | CPE002F2 | CPE002FN |
| 22625 | O24 | O1 | 1044 | 1341 | 12762 | 471,4 | 12,2 | 0,629 | H5A034 | C | neg1 | Hm Gen E | Hm Gen E | Hm Gen E | CPE002JW | CPE002OB | CPE002KR |
| 16097 | O24 | O1 | 1890 | 923,1 | 22966 | 107,8 | 12,2 | 0,68 | H5A034 | C | neg1 | Hm Gen E | Hm Gen E | Hm Gen E | CPE002J9 | CPE002NU | CPE002KA |
| 9545 | N24 | N1 | 1977 | 1138 | 24168 | 121,1 | 12,2 | 0,847 | H5A034 | C | neg1 | Hm Drgbl | Hm Drgbl | Hm Drgbl | CPE002E8 | CPE002ER | CPE002FC |
| 9880 | L23 | L24 | 1979 | 1214 | 24201 | 165 | 12,2 | 0,853 | H5A034 | C | neg1 | Hm Drgbl | Hm Drgbl | Hm Drgbl | CPE002E9 | CPE002EQ | CPE002FB |
| 4576 | O23 | O24 | 1247 | 706,7 | 15181 | 509 | 12,2 | 1,03 | H5A034 | C | neg1 | Hm Drgbl | Hm Drgbl | Hm Drgbl | CPE002DU | CPE002EK | CPE002FQ |
| 19937 | O24 | O1 | 1027 | 359,3 | 12674 | 412,9 | 12,3 | 0,801 | H5A034 | C | neg1 | Hm Gen E | Hm Gen E | Hm Gen E | CPE002JP | CPE002O4 | CPE002KK |
| 9904 | M23 | M24 | 2363 | 1283 | 29135 | 196 | 12,3 | 0,86 | H5A034 | C | neg1 | Hm Drgbl | Hm Drgbl | Hm Drgbl | CPE002E9 | CPE002EQ | CPE002FB |
| 15256 | L23 | L24 | 1795 | 885 | 22120 | 158,4 | 12,3 | 0,86 | H5A034 | C | neg1 | Hm Gen E | Hm Gen E | Hm Gen E | CPE002J8 | CPE002NS | CPE002K8 |
| 14465 | K24 | K1 | 1645 | 920,1 | 20278 | 125,3 | 12,3 | 0,875 | H5A034 | C | neg1 | Hm Gen E | Hm Gen E | Hm Gen E | CPE002J7 | CPE002NC | CPE002K6 |
| 14585 | P24 | P1 | 1359 | 827,3 | 16649 | 148,9 | 12,3 | 0,875 | H5A034 | C | neg1 | Hm Gen E | Hm Gen E | Hm Gen E | CPE002J7 | CPE002NC | CPE002K6 |
| 7168 | K23 | K24 | 1771 | 665,2 | 21797 | 504 | 12,3 | 0,957 | H5A034 | C | neg1 | Hm Drgbl | Hm Drgbl | Hm Drgbl | CPE002E2 | CPE002EX | CPE002FI |
| 16865 | O24 | O1 | 1272 | 969,8 | 15586 | 372 | 12,3 | 0,972 | H5A034 | C | neg1 | Hm Gen E | Hm Gen E | Hm Gen E | CPE002JH | CPE002NV | CPE002KC |
| 2296 | P23 | P24 | 1437 | 774,2 | 17672 | 479,3 | 12,3 | 1,07 | H5A034 | C | neg1 | Hm Drgbl | Hm Drgbl | Hm Drgbl | CPE002FX | CPE002G1 | CPE002G5 |
| 4096 | K23 | K24 | 1662 | 750,8 | 20432 | 468,1 | 12,3 | 1,093 | H5A034 | C | neg1 | Hm Drgbl | Hm Drgbl | Hm Drgbl | CPE002DS | CPE002EI | CPE002F8 |
| 16385 | K24 | K1 | 413 | 968,9 | 5083 | 287,7 | 12,3 | 1,128 | H5A034 | C | neg1 | Hm Gen E | Hm Gen E | Hm Gen E | CPE002JG | CPE002NV | CPE002KB |
| 1072 | M23 | M24 | 1265 | 769,4 | 15519 | 475,2 | 12,3 | 1,166 | H5A034 | C | neg1 | Hm Drgbl | Hm Drgbl | Hm Drgbl | CPE002FU | CPE002FY | CPE002G2 |
| 1144 | P23 | P24 | 1648 | 801,8 | 20269 | 548,2 | 12,3 | 1,166 | H5A034 | C | neg1 | Hm Drgbl | Hm Drgbl | Hm Drgbl | CPE002FU | CPE002FY | CPE002G2 |
| 8392 | N23 | N24 | 1598 | 647,8 | 19576 | 559,5 | 12,3 | 1,183 | H5A034 | C | neg1 | Hm Drgbl | Hm Drgbl | Hm Drgbl | CPE002E5 | CPE002EU | CPE002FF |
| 6400 | K23 | K24 | 2323 | 685,7 | 28704 | 479,8 | 12,4 | 0,818 | H5A034 | C | neg1 | Hm Drgbl | Hm Drgbl | Hm Drgbl | CPE002DZ | CPE002F0 | CPE002FL |
| 20705 | O24 | O1 | 868 | 789,6 | 10755 | 205,3 | 12,4 | 0,827 | H5A034 | C | neg1 | Hm Gen E | Hm Gen E | Hm Gen E | CPE002JR | CPE002O6 | CPE002KM |
| 4913 | M24 | M1 | 542 | 748,9 | 6726 | 468 | 12,4 | 1,363 | H5A034 | C | neg1 | Hm Drgbl | Hm Drgbl | Hm Drgbl | CPE002DV | CPE002EL | CPE002FP |
| 18784 | O23 | O24 | 1999 | 1071 | 24957 | 402,3 | 12,5 | 0,847 | H5A034 | C | neg1 | Hm Gen E | Hm Gen E | Hm Gen E | CPE002JM | CPE002O1 | CPE002KH |
| 17560 | L23 | L24 | 1719 | 1045 | 21429 | 266 | 12,5 | 0,853 | H5A034 | C | neg1 | Hm Gen E | Hm Gen E | Hm Gen E | CPE002JJ | CPE002NY | CPE002KE |
| 17608 | N23 | N24 | 1672 | 1003 | 20889 | 197 | 12,5 | 0,853 | H5A034 | C | neg1 | Hm Gen E | Hm Gen E | Hm Gen E | CPE002JJ | CPE002NY | CPE002KE |
| 17632 | O23 | O24 | 1824 | 1076 | 22833 | 292 | 12,5 | 0,853 | H5A034 | C | neg1 | Hm Gen E | Hm Gen E | Hm Gen E | CPE002JJ | CPE002NY | CPE002KE |
| 19096 | L23 | L24 | 1679 | 1040 | 20949 | 219,8 | 12,5 | 0,871 | H5A034 | C | neg1 | Hm Gen E | Hm Gen E | Hm Gen E | CPE002JN | CPE002O2 | CPE002KI |
| 8801 | O24 | O1 | 1905 | 1206 | 23878 | 136,9 | 12,5 | 0,883 | H5A034 | C | neg1 | Hm Drgbl | Hm Drgbl | Hm Drgbl | CPE002E6 | CPE002ET | CPE002FE |
| 19577 | P24 | P1 | 743 | 1341 | 9300 | 292,4 | 12,5 | 0,887 | H5A034 | C | neg1 | Hm Gen E | Hm Gen E | Hm Gen E | CPE002JO | CPE002O3 | CPE002KJ |
| 11489 | O24 | O1 | 1523 | 813 | 19064 | 151,3 | 12,5 | 0,906 | H5A034 | C | neg1 | Hm Drgbl | Hm Drgbl | Hm Drgbl | CPE002ED | CPE002F3 | CPE002FT |
| 7576 | L23 | L24 | 1873 | 640 | 23458 | 540,2 | 12,5 | 0,954 | H5A034 | C | neg1 | Hm Drgbl | Hm Drgbl | Hm Drgbl | CPE002E3 | CPE002EW | CPE002FH |
| 17248 | O23 | O24 | 1883 | 1020 | 23584 | 337,8 | 12,5 | 0,965 | H5A034 | C | neg1 | Hm Gen E | Hm Gen E | Hm Gen E | CPE002JI | CPE002NZ | CPE002KD |
| 17944 | L23 | L24 | 1633 | 1087 | 20486 | 319,7 | 12,5 | 0,988 | H5A034 | C | neg1 | Hm Gen E | Hm Gen E | Hm Gen E | CPE002JK | CPE002NZ | CPE002KF |
| 2201 | L24 | L1 | 1477 | 707,1 | 18515 | 441,4 | 12,5 | 1,087 | H5A034 | C | neg1 | Hm Drgbl | Hm Drgbl | Hm Drgbl | CPE002FX | CPE002G1 | CPE002G5 |
| 1409 | K24 | K1 | 1558 | 778,7 | 19409 | 473,5 | 12,5 | 1,147 | H5A034 | C | neg1 | Hm Drgbl | Hm Drgbl | Hm Drgbl | CPE002FV | CPE002FZ | CPE002G3 |
| 3328 | K23 | K24 | 1580 | 1408 | 19972 | 470,4 | 12,6 | 0,78 | H5A034 | C | neg1 | Hm Drgbl | Hm Drgbl | Hm Drgbl | CPE002DC | CPE002EG | CPE002F6 |
| 6472 | N23 | N24 | 1624 | 624,9 | 20477 | 373,7 | 12,6 | 0,832 | H5A034 | C | neg1 | Hm Drgbl | Hm Drgbl | Hm Drgbl | CPE002DZ | CPE002F0 | CPE002FL |
| 14464 | K23 | K24 | 1183 | 843,9 | 14922 | 139,3 | 12,6 | 0,897 | H5A034 | C | neg1 | Hm Gen E | Hm Gen E | Hm Gen E | CPE002J7 | CPE002NC | CPE002K6 |
| 5368 | P23 | P24 | 1621 | 627 | 20348 | 623,6 | 12,6 | 0,988 | H5A034 | C | neg1 | Hm Drgbl | Hm Drgbl | Hm Drgbl | CPE002DV | CPE002EM | CPE002FO |
| 2609 | M24 | M1 | 1127 | 747,8 | 14175 | 469,9 | 12,6 | 0,992 | H5A034 | C | neg1 | Hm Drgbl | Hm Drgbl | Hm Drgbl | CPE002DC | CPE002EE | CPE002F4 |
| 2680 | P23 | P24 | 1727 | 762 | 21792 | 522,5 | 12,6 | 0,992 | H5A034 | C | neg1 | Hm Drgbl | Hm Drgbl | Hm Drgbl | CPE002DC | CPE002EE | CPE002F4 |
| 2272 | O23 | O24 | 1525 | 778,2 | 19156 | 540,6 | 12,6 | 1,096 | H5A034 | C | neg1 | Hm Drgbl | Hm Drgbl | Hm Drgbl | CPE002FX | CPE002G1 | CPE002G5 |
| 16409 | L24 | L1 | 845 | 1035 | 10685 | 322 | 12,6 | 1,156 | H5A034 | C | neg1 | Hm Gen E | Hm Gen E | Hm Gen E | CPE002JG | CPE002NV | CPE002KB |
| 3785 | N24 | N1 | 1411 | 1372 | 17713 | 423,5 | 12,6 | 1,212 | H5A034 | C | neg1 | Hm Drgbl | Hm Drgbl | Hm Drgbl | CPE002DR | CPE002EH | CPE002F7 |
| 12977 | M24 | M1 | 1767 | 1100 | 22478 | 126,5 | 12,7 | 0,861 | H5A034 | C | neg1 | Hm Gen E | Hm Gen E | Hm Gen E | CPE002J6 | CPE002NN | CPE002K2 |
| 13049 | P24 | P1 | 1285 | 1108 | 16377 | 120,7 | 12,7 | 0,861 | H5A034 | C | neg1 | Hm Gen E | Hm Gen E | Hm Gen E | CPE002J6 | CPE002NN | CPE002K2 |
| 18760 | N23 | N24 | 1836 | 1087 | 23262 | 276,1 | 12,7 | 0,861 | H5A034 | C | neg1 | Hm Gen E | Hm Gen E | Hm Gen E | CPE002JM | CPE002O1 | CPE002KH |
| 8776 | N23 | N24 | 1816 | 1160 | 22990 | 150,3 | 12,7 | 0,898 | H5A034 | C | neg1 | Hm Drgbl | Hm Drgbl | Hm Drgbl | CPE002E6 | CPE002ET | CPE002FE |
| 21089 | O24 | O1 | 1387 | 1000 | 17579 | 294,4 | 12,7 | 0,901 | H5A034 | C | neg1 | Hm Gen E | Hm Gen E | Hm Gen E | CPE002JS | CPE002O7 | CPE002KN |
| 7241 | N24 | N1 | 1258 | 662,7 | 16008 | 459,1 | 12,7 | 0,988 | H5A034 | C | neg1 | Hm Drgbl | Hm Drgbl | Hm Drgbl | CPE002E2 | CPE002EX | CPE002FI |
| 2585 | L24 | L1 | 1415 | 769,2 | 17913 | 416,6 | 12,7 | 1 | H5A034 | C | neg1 | Hm Drgbl | Hm Drgbl | Hm Drgbl | CPE002DC | CPE002EE | CPE002F4 |
| 2608 | M23 | M24 | 1526 | 792,6 | 19309 | 369,5 | 12,7 | 1 | H5A034 | C | neg1 | Hm Drgbl | Hm Drgbl | Hm Drgbl | CPE002DC | CPE002EE | CPE002F4 |
| 4529 | M24 | M1 | 1054 | 773,7 | 13403 | 435,5 | 12,7 | 1,072 | H5A034 | C | neg1 | Hm Drgbl | Hm Drgbl | Hm Drgbl | CPE002DU | CPE002EK | CPE002FQ |
| 281 | L24 | L1 | 411 | 1413 | 5225 | 563,4 | 12,7 | 1,081 | H5A034 | C | neg1 | Hm Drgbl | Hm Drgbl | Hm Drgbl | CPE002DT | CPE002EJ | CPE002F9 |
| 4145 | M24 | M1 | 822 | 662,5 | 10408 | 373,2 | 12,7 | 1,129 | H5A034 | C | neg1 | Hm Drgbl | Hm Drgbl | Hm Drgbl | CPE002DS | CPE002EI | CPE002F8 |
| 4193 | O24 | O1 | 729 | 677,9 | 9265 | 408 | 12,7 | 1,129 | H5A034 | C | neg1 | Hm Drgbl | Hm Drgbl | Hm Drgbl | CPE002DS | CPE002EI | CPE002F8 |
| 6136 | P23 | P24 | 1309 | 769,5 | 16686 | 565,8 | 12,7 | 1,16 | H5A034 | C | neg1 | Hm Drgbl | Hm Drgbl | Hm Drgbl | CPE002DY | CPE002F1 | CPE002FM |
| 13817 | P24 | P1 | 1402 | 1021 | 17812 | 155,9 | 12,7 | 1,192 | H5A034 | C | neg1 | Hm Gen E | Hm Gen E | Hm Gen E | CPE002JC | CPE002NC | CPE002K4 |
| 18401 | O24 | O1 | 1280 | 1010 | 16411 | 409,4 | 12,8 | 0,823 | H5A034 | C | neg1 | Hm Gen E | Hm Gen E | Hm Gen E | CPE002JL | CPE002O0 | CPE002KG |
| 7672 | P23 | P24 | 1372 | 561,6 | 17568 | 577,4 | 12,8 | 0,977 | H5A034 | C | neg1 | Hm Drgbl | Hm Drgbl | Hm Drgbl | CPE002E3 | CPE002EW | CPE002FH |
| 17968 | M23 | M24 | 1515 | 1156 | 19442 | 273,5 | 12,8 | 1,012 | H5A034 | C | neg1 | Hm Gen E | Hm Gen E | Hm Gen E | CPE002JK | CPE002NZ | CPE002KF |
| 9209 | P24 | P1 | 1440 | 1506 | 18456 | 139,7 | 12,8 | 1,028 | H5A034 | C | neg1 | Hm Drgbl | Hm Drgbl | Hm Drgbl | CPE002E7 | CPE002ES | CPE002FD |
| 7552 | K23 | K24 | 1717 | 636,8 | 22150 | 555 | 12,9 | 0,985 | H5A034 | C | neg1 | Hm Drgbl | Hm Drgbl | Hm Drgbl | CPE002E3 | CPE002EW | CPE002FH |
| 5344 | O23 | O24 | 1813 | 689,3 | 23341 | 633,9 | 12,9 | 1,012 | H5A034 | C | neg1 | Hm Drgbl | Hm Drgbl | Hm Drgbl | CPE002DV | CPE002EM | CPE002FO |
| 280 | L23 | L24 | 505 | 1517 | 6530 | 784,9 | 12,9 | 1,098 | H5A034 | C | neg1 | Hm Drgbl | Hm Drgbl | Hm Drgbl | CPE002DT | CPE002EJ | CPE002F9 |
| 11824 | M23 | M24 | 1422 | 883,8 | 18387 | 154,3 | 12,9 | 1,152 | H5A034 | C | neg1 | Hm Gen E | Hm Gen E | Hm Gen E | CPE002JA | CPE002NJ | CPE002JZ |
| 1504 | O23 | O24 | 2014 | 837,7 | 25912 | 564,2 | 12,9 | 1,183 | H5A034 | C | neg1 | Hm Drgbl | Hm Drgbl | Hm Drgbl | CPE002FV | CPE002FZ | CPE002G3 |
| 19864 | L23 | L24 | 1302 | 352 | 16912 | 237,8 | 13 | 0,847 | H5A034 | C | neg1 | Hm Gen E | Hm Gen E | Hm Gen E | CPE002JP | CPE002O4 | CPE002KK |
| 17584 | M23 | M24 | 1698 | 1056 | 22106 | 281,9 | 13 | 0,887 | H5A034 | C | neg1 | Hm Gen E | Hm Gen E | Hm Gen E | CPE002JJ | CPE002NY | CPE002KE |
| 8777 | N24 | N1 | 1569 | 1131 | 20395 | 110,5 | 13 | 0,919 | H5A034 | C | neg1 | Hm Drgbl | Hm Drgbl | Hm Drgbl | CPE002E6 | CPE002ET | CPE002FE |
| 21088 | O23 | O24 | 1299 | 1068 | 16837 | 382,6 | 13 | 0,922 | H5A034 | C | neg1 | Hm Gen E | Hm Gen E | Hm Gen E | CPE002JS | CPE002O7 | CPE002KN |
| 14513 | M24 | M1 | 1409 | 824,8 | 18365 | 137,4 | 13 | 0,925 | H5A034 | C | neg1 | Hm Gen E | Hm Gen E | Hm Gen E | CPE002J7 | CPE002NC | CPE002K6 |
| 12593 | M24 | M1 | 1584 | 1141 | 20625 | 151 | 13 | 0,977 | H5A034 | C | neg1 | Hm Gen E | Hm Gen E | Hm Gen E | CPE002J5 | CPE002NK | CPE002K1 |
| 7216 | M23 | M24 | 1389 | 573,3 | 18036 | 420,2 | 13 | 1,012 | H5A034 | C | neg1 | Hm Drgbl | Hm Drgbl | Hm Drgbl | CPE002E2 | CPE002EX | CPE002FI |
| 16792 | L23 | L24 | 1614 | 1080 | 21022 | 303,3 | 13 | 1,028 | H5A034 | C | neg1 | Hm Gen E | Hm Gen E | Hm Gen E | CPE002JH | CPE002NV | CPE002KC |
| 11848 | N23 | N24 | 1677 | 933,3 | 21751 | 150,5 | 13 | 1,161 | H5A034 | C | neg1 | Hm Gen E | Hm Gen E | Hm Gen E | CPE002JA | CPE002NJ | CPE002JZ |
| 20704 | O23 | O24 | 860 | 789,3 | 11283 | 239,1 | 13,1 | 0,873 | H5A034 | C | neg1 | Hm Gen E | Hm Gen E | Hm Gen E | CPE002JR | CPE002O6 | CPE002KM |
| 11008 | K23 | K24 | 1402 | 876,9 | 18419 | 599,7 | 13,1 | 0,992 | H5A034 | C | neg1 | Hm Drgbl | Hm Drgbl | Hm Drgbl | CPE002EC | CPE002EN | CPE002FS |
| 11080 | N23 | N24 | 1675 | 872,1 | 21949 | 544,2 | 13,1 | 0,992 | H5A034 | C | neg1 | Hm Drgbl | Hm Drgbl | Hm Drgbl | CPE002EC | CPE002EN | CPE002FS |
| 11129 | P24 | P1 | 1237 | 884,6 | 16228 | 544 | 13,1 | 0,992 | H5A034 | C | neg1 | Hm Drgbl | Hm Drgbl | Hm Drgbl | CPE002EC | CPE002EN | CPE002FS |
| 16768 | K23 | K24 | 1598 | 1057 | 20993 | 380,5 | 13,1 | 1,036 | H5A034 | C | neg1 | Hm Gen E | Hm Gen E | Hm Gen E | CPE002JH | CPE002NV | CPE002KC |
| 16769 | K24 | K1 | 998 | 1040 | 13080 | 259 | 13,1 | 1,036 | H5A034 | C | neg1 | Hm Gen E | Hm Gen E | Hm Gen E | CPE002JH | CPE002NV | CPE002KC |
| 4169 | N24 | N1 | 1274 | 772,4 | 16703 | 416,4 | 13,1 | 1,164 | H5A034 | C | neg1 | Hm Drgbl | Hm Drgbl | Hm Drgbl | CPE002DS | CPE002EI | CPE002F8 |
| 11801 | L24 | L1 | 1211 | 875,8 | 15823 | 130 | 13,1 | 1,17 | H5A034 | C | neg1 | Hm Gen E | Hm Gen E | Hm Gen E | CPE002JA | CPE002NJ | CPE002JZ |
| 4961 | O24 | O1 | 635 | 776 | 8320 | 498,1 | 13,1 | 1,44 | H5A034 | C | neg1 | Hm Drgbl | Hm Drgbl | Hm Drgbl | CPE002DV | CPE002EL | CPE002FP |
| 6424 | L23 | L24 | 1755 | 655,6 | 23243 | 517,4 | 13,2 | 0,871 | H5A034 | C | neg1 | Hm Drgbl | Hm Drgbl | Hm Drgbl | CPE002DZ | CPE002F0 | CPE002FL |
| 9928 | N23 | N24 | 2303 | 1249 | 30445 | 222,3 | 13,2 | 0,923 | H5A034 | C | neg1 | Hm Drgbl | Hm Drgbl | Hm Drgbl | CPE002E9 | CPE002EQ | CPE002FB |
| 5321 | N24 | N1 | 1643 | 688,5 | 21659 | 353,6 | 13,2 | 1,035 | H5A034 | C | neg1 | Hm Drgbl | Hm Drgbl | Hm Drgbl | CPE002DV | CPE002EM | CPE002FO |
| 4528 | M23 | M24 | 1165 | 743,4 | 15333 | 419,6 | 13,2 | 1,114 | H5A034 | C | neg1 | Hm Drgbl | Hm Drgbl | Hm Drgbl | CPE002DU | CPE002EK | CPE002FQ |
| 1120 | O23 | O24 | 1379 | 804,1 | 18184 | 560 | 13,2 | 1,251 | H5A034 | C | neg1 | Hm Drgbl | Hm Drgbl | Hm Drgbl | CPE002FU | CPE002FY | CPE002G2 |
| 9856 | K23 | K24 | 2178 | 1242 | 28889 | 198,8 | 13,3 | 0,93 | H5A034 | C | neg1 | Hm Drgbl | Hm Drgbl | Hm Drgbl | CPE002E9 | CPE002EQ | CPE002FB |
| 8753 | M24 | M1 | 2018 | 1208 | 26884 | 154,5 | 13,3 | 0,94 | H5A034 | C | neg1 | Hm Drgbl | Hm Drgbl | Hm Drgbl | CPE002E6 | CPE002ET | CPE002FE |
| 19456 | K23 | K24 | 1449 | 1228 | 19303 | 310,2 | 13,3 | 0,943 | H5A034 | C | neg1 | Hm Gen E | Hm Gen E | Hm Gen E | CPE002JO | CPE002O3 | CPE002KJ |
| 19528 | N23 | N24 | 1249 | 1193 | 16610 | 265,7 | 13,3 | 0,943 | H5A034 | C | neg1 | Hm Gen E | Hm Gen E | Hm Gen E | CPE002JO | CPE002O3 | CPE002KJ |
| 11081 | N24 | N1 | 1555 | 873,3 | 20734 | 482,7 | 13,3 | 1,008 | H5A034 | C | neg1 | Hm Drgbl | Hm Drgbl | Hm Drgbl | CPE002EC | CPE002EN | CPE002FS |
| 7600 | M23 | M24 | 1507 | 627,6 | 20053 | 526,4 | 13,3 | 1,015 | H5A034 | C | neg1 | Hm Drgbl | Hm Drgbl | Hm Drgbl | CPE002E3 | CPE002EW | CPE002FH |
| 3737 | L24 | L1 | 1254 | 1488 | 16654 | 380,9 | 13,3 | 1,279 | H5A034 | C | neg1 | Hm Drgbl | Hm Drgbl | Hm Drgbl | CPE002DR | CPE002EH | CPE002F7 |
| 10744 | P23 | P24 | 1488 | 877,8 | 19744 | 239,9 | 13,3 | 1,503 | H5A034 | C | neg1 | Hm Drgbl | Hm Drgbl | Hm Drgbl | CPE002EB | CPE002EO | CPE002FR |
| 18376 | N23 | N24 | 1637 | 1098 | 21876 | 294,2 | 13,4 | 0,862 | H5A034 | C | neg1 | Hm Gen E | Hm Gen E | Hm Gen E | CPE002JL | CPE002O0 | CPE002KG |
| 9521 | M24 | M1 | 1717 | 1182 | 22961 | 116,2 | 13,4 | 0,931 | H5A034 | C | neg1 | Hm Drgbl | Hm Drgbl | Hm Drgbl | CPE002E8 | CPE002ER | CPE002FC |
| 9977 | P24 | P1 | 1371 | 1107 | 18394 | 163,3 | 13,4 | 0,937 | H5A034 | C | neg1 | Hm Drgbl | Hm Drgbl | Hm Drgbl | CPE002E9 | CPE002EQ | CPE002FB |
| 15233 | K24 | K1 | 1578 | 890,8 | 21108 | 147,5 | 13,4 | 0,937 | H5A034 | C | neg1 | Hm Gen E | Hm Gen E | Hm Gen E | CPE002J8 | CPE002NS | CPE002K8 |
| 11441 | M24 | M1 | 1617 | 869 | 21681 | 141,8 | 13,4 | 0,971 | H5A034 | C | neg1 | Hm Drgbl | Hm Drgbl | Hm Drgbl | CPE002ED | CPE002F3 | CPE002FT |
| 7648 | O23 | O24 | 1485 | 547,4 | 19877 | 490,3 | 13,4 | 1,023 | H5A034 | C | neg1 | Hm Drgbl | Hm Drgbl | Hm Drgbl | CPE002E3 | CPE002EW | CPE002FH |
| 17153 | K24 | K1 | 1199 | 982,8 | 16042 | 160,5 | 13,4 | 1,035 | H5A034 | C | neg1 | Hm Gen E | Hm Gen E | Hm Gen E | CPE002JI | CPE002NZ | CPE002KD |
| 9160 | N23 | N24 | 2255 | 2117 | 30235 | 249,9 | 13,4 | 1,076 | H5A034 | C | neg1 | Hm Drgbl | Hm Drgbl | Hm Drgbl | CPE002E7 | CPE002ES | CPE002FD |
| 11896 | P23 | P24 | 1521 | 922,5 | 20394 | 207,6 | 13,4 | 1,196 | H5A034 | C | neg1 | Hm Gen E | Hm Gen E | Hm Gen E | CPE002JA | CPE002NJ | CPE002JZ |
| 14177 | O24 | O1 | 1483 | 1051 | 20057 | 142,1 | 13,5 | 0,774 | H5A034 | C | neg1 | Hm Gen E | Hm Gen E | Hm Gen E | CPE002JF | CPE002NP | CPE002K5 |
| 12185 | L24 | L1 | 1485 | 844,8 | 20041 | 90,78 | 13,5 | 0,865 | H5A034 | C | neg1 | Hm Gen E | Hm Gen E | Hm Gen E | CPE002J4 | CPE002NL | CPE002K0 |
| 20633 | L24 | L1 | 930 | 773,8 | 12582 | 171,1 | 13,5 | 0,9 | H5A034 | C | neg1 | Hm Gen E | Hm Gen E | Hm Gen E | CPE002JR | CPE002O6 | CPE002KM |
| 18785 | O24 | O1 | 1226 | 965 | 16530 | 280,2 | 13,5 | 0,915 | H5A034 | C | neg1 | Hm Gen E | Hm Gen E | Hm Gen E | CPE002JM | CPE002O1 | CPE002KH |
| 15281 | M24 | M1 | 1763 | 841 | 23760 | 131,6 | 13,5 | 0,944 | H5A034 | C | neg1 | Hm Gen E | Hm Gen E | Hm Gen E | CPE002J8 | CPE002NS | CPE002K8 |
| 17152 | K23 | K24 | 1738 | 1067 | 23405 | 289,9 | 13,5 | 1,042 | H5A034 | C | neg1 | Hm Gen E | Hm Gen E | Hm Gen E | CPE002JI | CPE002NZ | CPE002KD |
| 4480 | K23 | K24 | 1205 | 785,3 | 16253 | 517,6 | 13,5 | 1,139 | H5A034 | C | neg1 | Hm Drgbl | Hm Drgbl | Hm Drgbl | CPE002DU | CPE002EK | CPE002FQ |
| 15688 | N23 | N24 | 1559 | 909,7 | 21062 | 154,2 | 13,5 | 1,227 | H5A034 | C | neg1 | Hm Gen E | Hm Gen E | Hm Gen E | CPE002JE | CPE002NT | CPE002K9 |
| 3352 | L23 | L24 | 1503 | 1438 | 20480 | 445,7 | 13,6 | 0,842 | H5A034 | C | neg1 | Hm Drgbl | Hm Drgbl | Hm Drgbl | CPE002DC | CPE002EG | CPE002F6 |

| | | | | | | | | | | | | | | | | | |
|---|---|---|---|---|---|---|---|---|---|---|---|---|---|---|---|---|---|
| 17536 | K23 | K24 | 1801 | 1122 | 24459 | 257,5 | 13,6 | 0,928 | HSA034 | C | neg1 | Hm Gen E | Hm Gen E | Hm Gen E | CPE002JJ | CPE002NY | CPE002KE |
| 15232 | K23 | K24 | 1700 | 911,3 | 23054 | 153,9 | 13,6 | 0,951 | HSA034 | C | neg1 | Hm Gen E | Hm Gen E | Hm Gen E | CPE002J8 | CPE002NS | CPE002K8 |
| 21040 | M23 | M24 | 1284 | 1138 | 17476 | 340,5 | 13,6 | 0,965 | HSA034 | C | neg1 | Hm Gen E | Hm Gen E | Hm Gen E | CPE002JS | CPE002O7 | CPE002KN |
| 21448 | N23 | N24 | 1257 | 1163 | 17122 | 315,6 | 13,6 | 0,965 | HSA034 | C | neg1 | Hm Gen E | Hm Gen E | Hm Gen E | CPE002JT | CPE002O8 | CPE002KO |
| 11393 | K24 | K1 | 1499 | 860,8 | 20388 | 149,5 | 13,6 | 0,986 | HSA034 | C | neg1 | Hm Drgbl | Hm Drgbl | Hm Drgbl | CPE002ED | CPE002F3 | CPE002FT |
| 12568 | L23 | L24 | 1471 | 1144 | 20015 | 180 | 13,6 | 1,023 | HSA034 | C | neg1 | Hm Gen E | Hm Gen E | Hm Gen E | CPE002J5 | CPE002NK | CPE002K1 |
| 16817 | M24 | M1 | 1409 | 978,3 | 19189 | 242,1 | 13,6 | 1,075 | HSA034 | C | neg1 | Hm Gen E | Hm Gen E | Hm Gen E | CPE002JH | CPE002NV | CPE002KC |
| 328 | N23 | N24 | 547 | 1558 | 7429 | 787,6 | 13,6 | 1,157 | HSA034 | C | neg1 | Hm Drgbl | Hm Drgbl | Hm Drgbl | CPE002DT | CPE002EJ | CPE002F9 |
| 21856 | O23 | O24 | 206 | 872,7 | 2798 | 732,2 | 13,6 | 1,432 | HSA034 | C | neg1 | Hm Gen E | Hm Gen E | Hm Gen E | CPE002JU | CPE002O9 | CPE002KP |
| 18736 | M23 | M24 | 1349 | 1060 | 18452 | 233 | 13,7 | 0,929 | HSA034 | C | neg1 | Hm Gen E | Hm Gen E | Hm Gen E | CPE002JM | CPE002O1 | CPE002KH |
| 2681 | P24 | P1 | 1332 | 729,5 | 18275 | 493,4 | 13,7 | 1,079 | HSA034 | C | neg1 | Hm Drgbl | Hm Drgbl | Hm Drgbl | CPE002DC | CPE002EE | CPE002F4 |
| 9136 | M23 | M24 | 2089 | 2142 | 28650 | 205,2 | 13,7 | 1,1 | HSA034 | C | neg1 | Hm Drgbl | Hm Drgbl | Hm Drgbl | CPE002E7 | CPE002ES | CPE002FD |
| 12160 | K23 | K24 | 1736 | 844,6 | 23913 | 128,3 | 13,8 | 0,885 | HSA034 | C | neg1 | Hm Gen E | Hm Gen E | Hm Gen E | CPE002J4 | CPE002NL | CPE002K0 |
| 6521 | P24 | P1 | 1320 | 599,2 | 18257 | 495,4 | 13,8 | 0,911 | HSA034 | C | neg1 | Hm Drgbl | Hm Drgbl | Hm Drgbl | CPE002DZ | CPE002F0 | CPE002FL |
| 12976 | M23 | M24 | 1667 | 1038 | 22946 | 144,5 | 13,8 | 0,936 | HSA034 | C | neg1 | Hm Gen E | Hm Gen E | Hm Gen E | CPE002J6 | CPE002NN | CPE002K2 |
| 19120 | M23 | M24 | 1213 | 1043 | 16704 | 185,5 | 13,8 | 0,962 | HSA034 | C | neg1 | Hm Gen E | Hm Gen E | Hm Gen E | CPE002JN | CPE002O2 | CPE002KI |
| 21016 | L23 | L24 | 1163 | 1067 | 16082 | 292,8 | 13,8 | 0,979 | HSA034 | C | neg1 | Hm Gen E | Hm Gen E | Hm Gen E | CPE002JS | CPE002O7 | CPE002KN |
| 5320 | N23 | N24 | 1613 | 660,5 | 22265 | 637,4 | 13,8 | 1,082 | HSA034 | C | neg1 | Hm Drgbl | Hm Drgbl | Hm Drgbl | CPE002DV | CPE002EM | CPE002FO |
| 1841 | M24 | M1 | 972 | 730,4 | 13386 | 456,1 | 13,8 | 1,445 | HSA034 | C | neg1 | Hm Drgbl | Hm Drgbl | Hm Drgbl | CPE002FW | CPE002G0 | CPE002G4 |
| 18377 | N24 | N1 | 1278 | 1014 | 17701 | 284,4 | 13,9 | 0,894 | HSA034 | C | neg1 | Hm Gen E | Hm Gen E | Hm Gen E | CPE002JL | CPE002O0 | CPE002KG |
| 8729 | L24 | L1 | 1780 | 1192 | 24826 | 105,8 | 13,9 | 0,982 | HSA034 | C | neg1 | Hm Drgbl | Hm Drgbl | Hm Drgbl | CPE002E6 | CPE002ET | CPE002FE |
| 20992 | K23 | K24 | 1550 | 1062 | 21499 | 309,5 | 13,9 | 0,986 | HSA034 | C | neg1 | Hm Gen E | Hm Gen E | Hm Gen E | CPE002JS | CPE002O7 | CPE002KN |
| 14489 | L24 | L1 | 1822 | 915,8 | 25315 | 147,5 | 13,9 | 0,989 | HSA034 | C | neg1 | Hm Gen E | Hm Gen E | Hm Gen E | CPE002J7 | CPE002NC | CPE002K6 |
| 5345 | O24 | O1 | 557 | 617,5 | 7747 | 571,3 | 13,9 | 1,09 | HSA034 | C | neg1 | Hm Drgbl | Hm Drgbl | Hm Drgbl | CPE002DV | CPE002EM | CPE002FO |
| 304 | M23 | M24 | 406 | 1582 | 5641 | 691,1 | 13,9 | 1,183 | HSA034 | C | neg1 | Hm Drgbl | Hm Drgbl | Hm Drgbl | CPE002DT | CPE002EJ | CPE002F9 |
| 2225 | M24 | M1 | 811 | 682,2 | 11285 | 440,2 | 13,9 | 1,209 | HSA034 | C | neg1 | Hm Drgbl | Hm Drgbl | Hm Drgbl | CPE002FX | CPE002G1 | CPE002G5 |
| 12233 | N24 | N1 | 1475 | 820,9 | 20631 | 94,31 | 14 | 0,897 | HSA034 | C | neg1 | Hm Gen E | Hm Gen E | Hm Gen E | CPE002J4 | CPE002NL | CPE002K0 |
| 19889 | M24 | M1 | 1193 | 380,9 | 16724 | 351,4 | 14 | 0,912 | HSA034 | C | neg1 | Hm Gen E | Hm Gen E | Hm Gen E | CPE002JP | CPE002O4 | CPE002KK |
| 19144 | N23 | N24 | 1638 | 1065 | 22888 | 257 | 14 | 0,976 | HSA034 | C | neg1 | Hm Gen E | Hm Gen E | Hm Gen E | CPE002JN | CPE002O2 | CPE002KI |
| 19145 | N24 | N1 | 1262 | 969,9 | 17657 | 215,5 | 14 | 0,976 | HSA034 | C | neg1 | Hm Gen E | Hm Gen E | Hm Gen E | CPE002JN | CPE002O2 | CPE002KI |
| 15353 | P24 | P1 | 1087 | 784,7 | 15182 | 151,7 | 14 | 0,979 | HSA034 | C | neg1 | Hm Gen E | Hm Gen E | Hm Gen E | CPE002J8 | CPE002NS | CPE002K8 |
| 21472 | O23 | O24 | 1331 | 1088 | 18678 | 524,9 | 14 | 0,993 | HSA034 | C | neg1 | Hm Gen E | Hm Gen E | Hm Gen E | CPE002JT | CPE002O8 | CPE002KO |
| 11416 | L23 | L24 | 1388 | 858 | 19478 | 155,8 | 14 | 1,014 | HSA034 | C | neg1 | Hm Drgbl | Hm Drgbl | Hm Drgbl | CPE002ED | CPE002F3 | CPE002FT |
| 17177 | L24 | L1 | 1208 | 1051 | 16864 | 190,5 | 14 | 1,081 | HSA034 | C | neg1 | Hm Gen E | Hm Gen E | Hm Gen E | CPE002JI | CPE002NZ | CPE002KD |
| 5296 | M23 | M24 | 1610 | 649,1 | 22497 | 622,7 | 14 | 1,098 | HSA034 | C | neg1 | Hm Drgbl | Hm Drgbl | Hm Drgbl | CPE002DV | CPE002EM | CPE002FO |
| 2657 | O24 | O1 | 704 | 704,5 | 9865 | 455 | 14 | 1,102 | HSA034 | C | neg1 | Hm Drgbl | Hm Drgbl | Hm Drgbl | CPE002DC | CPE002EE | CPE002F4 |
| 4097 | K24 | K1 | 837 | 762 | 11735 | 440,8 | 14 | 1,244 | HSA034 | C | neg1 | Hm Drgbl | Hm Drgbl | Hm Drgbl | CPE002DS | CPE002EI | CPE002F8 |
| 13696 | K23 | K24 | 1199 | 1141 | 16822 | 156,6 | 14 | 1,315 | HSA034 | C | neg1 | Hm Gen E | Hm Gen E | Hm Gen E | CPE002JC | CPE002NC | CPE002K4 |
| 19912 | N23 | N24 | 1720 | 354,6 | 24284 | 344,3 | 14,1 | 0,919 | HSA034 | C | neg1 | Hm Gen E | Hm Gen E | Hm Gen E | CPE002JP | CPE002O4 | CPE002KK |
| 18737 | M24 | M1 | 1353 | 1039 | 19086 | 228,1 | 14,1 | 0,956 | HSA034 | C | neg1 | Hm Gen E | Hm Gen E | Hm Gen E | CPE002JM | CPE002O1 | CPE002KH |
| 9593 | P24 | P1 | 1293 | 1032 | 18205 | 177,6 | 14,1 | 0,979 | HSA034 | C | neg1 | Hm Drgbl | Hm Drgbl | Hm Drgbl | CPE002E8 | CPE002ER | CPE002FC |
| 19072 | K23 | K24 | 1711 | 1049 | 24097 | 298 | 14,1 | 0,983 | HSA034 | C | neg1 | Hm Gen E | Hm Gen E | Hm Gen E | CPE002JN | CPE002O2 | CPE002KI |
| 11009 | K24 | K1 | 1641 | 910,6 | 23059 | 593,2 | 14,1 | 1,068 | HSA034 | C | neg1 | Hm Drgbl | Hm Drgbl | Hm Drgbl | CPE002EC | CPE002EN | CPE002FS |
| 17176 | L23 | L24 | 1897 | 1097 | 26841 | 255,7 | 14,1 | 1,089 | HSA034 | C | neg1 | Hm Gen E | Hm Gen E | Hm Gen E | CPE002JI | CPE002NZ | CPE002KD |
| 7192 | L23 | L24 | 1461 | 632,4 | 20637 | 423,3 | 14,1 | 1,097 | HSA034 | C | neg1 | Hm Drgbl | Hm Drgbl | Hm Drgbl | CPE002E2 | CPE002EX | CPE002FI |
| 2633 | N24 | N1 | 1303 | 764 | 18413 | 478,9 | 14,1 | 1,11 | HSA034 | C | neg1 | Hm Drgbl | Hm Drgbl | Hm Drgbl | CPE002DC | CPE002EE | CPE002F4 |
| 13768 | N23 | N24 | 1468 | 1105 | 20681 | 214,2 | 14,1 | 1,324 | HSA034 | C | neg1 | Hm Gen E | Hm Gen E | Hm Gen E | CPE002JC | CPE002NC | CPE002K4 |
| 21400 | L23 | L24 | 1463 | 989,8 | 20753 | 389,1 | 14,2 | 1,007 | HSA034 | C | neg1 | Hm Gen E | Hm Gen E | Hm Gen E | CPE002JT | CPE002O8 | CPE002KO |
| 14537 | N24 | N1 | 1439 | 933,2 | 20405 | 120,6 | 14,2 | 1,011 | HSA034 | C | neg1 | Hm Gen E | Hm Gen E | Hm Gen E | CPE002J7 | CPE002NC | CPE002K6 |
| 11488 | O23 | O24 | 1342 | 843,6 | 19100 | 229,1 | 14,2 | 1,029 | HSA034 | C | neg1 | Hm Drgbl | Hm Drgbl | Hm Drgbl | CPE002ED | CPE002F3 | CPE002FT |
| 15641 | L24 | L1 | 1304 | 893,1 | 18470 | 154,7 | 14,2 | 1,291 | HSA034 | C | neg1 | Hm Gen E | Hm Gen E | Hm Gen E | CPE002JE | CPE002NT | CPE002K9 |
| 13816 | P23 | P24 | 1319 | 1056 | 18706 | 236,8 | 14,2 | 1,333 | HSA034 | C | neg1 | Hm Gen E | Hm Gen E | Hm Gen E | CPE002JC | CPE002NC | CPE002K4 |
| 13409 | O24 | O1 | 1671 | 1146 | 23836 | 104,4 | 14,3 | 0,717 | HSA034 | C | neg1 | Hm Gen E | Hm Gen E | Hm Gen E | CPE002JB | CPE002NN | CPE002K3 |
| 12953 | L24 | L1 | 1203 | 1035 | 17251 | 124,6 | 14,3 | 0,969 | HSA034 | C | neg1 | Hm Gen E | Hm Gen E | Hm Gen E | CPE002J6 | CPE002NN | CPE002K2 |
| 13001 | N24 | N1 | 1236 | 1062 | 17670 | 143,4 | 14,3 | 0,969 | HSA034 | C | neg1 | Hm Gen E | Hm Gen E | Hm Gen E | CPE002J6 | CPE002NN | CPE002K2 |
| 21041 | M24 | M1 | 1388 | 1053 | 19901 | 286,4 | 14,3 | 1,014 | HSA034 | C | neg1 | Hm Gen E | Hm Gen E | Hm Gen E | CPE002JS | CPE002O7 | CPE002KN |
| 12665 | P24 | P1 | 847 | 1032 | 12084 | 154,7 | 14,3 | 1,075 | HSA034 | C | neg1 | Hm Gen E | Hm Gen E | Hm Gen E | CPE002J5 | CPE002NK | CPE002K1 |
| 9112 | L23 | L24 | 2185 | 2349 | 31205 | 197,1 | 14,3 | 1,149 | HSA034 | C | neg1 | Hm Drgbl | Hm Drgbl | Hm Drgbl | CPE002E7 | CPE002ES | CPE002FD |
| 8369 | M24 | M1 | 755 | 622 | 10791 | 533,7 | 14,3 | 1,375 | HSA034 | C | neg1 | Hm Drgbl | Hm Drgbl | Hm Drgbl | CPE002E5 | CPE002EU | CPE002FF |
| 23777 | O24 | O1 | 765 | 1525 | 10979 | 241,9 | 14,4 | 0,764 | HSA034 | C | neg1 | Hm Gen E | Hm Gen E | Hm Gen E | CPE002KV | CPE002OE | CPE002KU |
| 18352 | M23 | M24 | 1346 | 1048 | 19334 | 379,6 | 14,4 | 0,926 | HSA034 | C | neg1 | Hm Gen E | Hm Gen E | Hm Gen E | CPE002JL | CPE002O0 | CPE002KG |
| 8728 | L23 | L24 | 1830 | 1209 | 26355 | 138,4 | 14,4 | 1,018 | HSA034 | C | neg1 | Hm Drgbl | Hm Drgbl | Hm Drgbl | CPE002E6 | CPE002ET | CPE002FE |
| 4481 | K24 | K1 | 610 | 761,1 | 8767 | 455,6 | 14,4 | 1,215 | HSA034 | C | neg1 | Hm Drgbl | Hm Drgbl | Hm Drgbl | CPE002DU | CPE002EK | CPE002FO |
| 14945 | O24 | O1 | 1453 | 858,5 | 21046 | 96,59 | 14,5 | 0,826 | HSA034 | C | neg1 | Hm Gen E | Hm Gen E | Hm Gen E | CPE002JD | CPE002NR | CPE002K7 |
| 8824 | P23 | P24 | 1577 | 1076 | 22928 | 156,7 | 14,5 | 1,025 | HSA034 | C | neg1 | Hm Drgbl | Hm Drgbl | Hm Drgbl | CPE002E6 | CPE002ET | CPE002FE |
| 11057 | M24 | M1 | 1566 | 929,5 | 22736 | 484,9 | 14,5 | 1,098 | HSA034 | C | neg1 | Hm Drgbl | Hm Drgbl | Hm Drgbl | CPE002EC | CPE002EN | CPE002FS |
| 7624 | N23 | N24 | 1444 | 581,7 | 20903 | 523,1 | 14,5 | 1,107 | HSA034 | C | neg1 | Hm Drgbl | Hm Drgbl | Hm Drgbl | CPE002E3 | CPE002EW | CPE002FH |
| 17920 | K23 | K24 | 1219 | 1041 | 17643 | 328,3 | 14,5 | 1,146 | HSA034 | C | neg1 | Hm Gen E | Hm Gen E | Hm Gen E | CPE002JK | CPE002NZ | CPE002KF |
| 9208 | P23 | P24 | 1604 | 1776 | 23316 | 183,6 | 14,5 | 1,165 | HSA034 | C | neg1 | Hm Drgbl | Hm Drgbl | Hm Drgbl | CPE002E7 | CPE002ES | CPE002FD |
| 12257 | O24 | O1 | 1555 | 828,8 | 22659 | 139,6 | 14,6 | 0,936 | HSA034 | C | neg1 | Hm Gen E | Hm Gen E | Hm Gen E | CPE002J4 | CPE002NL | CPE002K0 |
| 19961 | P24 | P1 | 766 | 381,3 | 11208 | 307 | 14,6 | 0,951 | HSA034 | C | neg1 | Hm Gen E | Hm Gen E | Hm Gen E | CPE002JP | CPE002O4 | CPE002KK |
| 13000 | N23 | N24 | 1621 | 1079 | 23654 | 226,3 | 14,6 | 0,99 | HSA034 | C | neg1 | Hm Gen E | Hm Gen E | Hm Gen E | CPE002J6 | CPE002NN | CPE002K2 |
| 19168 | O23 | O24 | 1484 | 1059 | 21650 | 344,2 | 14,6 | 1,017 | HSA034 | C | neg1 | Hm Gen E | Hm Gen E | Hm Gen E | CPE002JN | CPE002O2 | CPE002KI |
| 15257 | L24 | L1 | 1771 | 871,9 | 25800 | 132,7 | 14,6 | 1,021 | HSA034 | C | neg1 | Hm Gen E | Hm Gen E | Hm Gen E | CPE002J8 | CPE002NS | CPE002K8 |
| 21017 | L24 | L1 | 1002 | 1023 | 14639 | 278,2 | 14,6 | 1,035 | HSA034 | C | neg1 | Hm Gen E | Hm Gen E | Hm Gen E | CPE002JS | CPE002O7 | CPE002KN |
| 18328 | L23 | L24 | 1558 | 1099 | 22927 | 242,9 | 14,7 | 0,945 | HSA034 | C | neg1 | Hm Gen E | Hm Gen E | Hm Gen E | CPE002JL | CPE002O0 | CPE002KG |
| 19840 | K23 | K24 | 1534 | 361,1 | 22576 | 279,2 | 14,7 | 0,958 | HSA034 | C | neg1 | Hm Gen E | Hm Gen E | Hm Gen E | CPE002JP | CPE002O4 | CPE002KK |
| 20632 | L23 | L24 | 1093 | 829,7 | 16118 | 227,9 | 14,7 | 0,98 | HSA034 | C | neg1 | Hm Gen E | Hm Gen E | Hm Gen E | CPE002JR | CPE002O6 | CPE002KM |
| 9520 | M23 | M24 | 1645 | 1137 | 24176 | 175,5 | 14,7 | 1,021 | HSA034 | C | neg1 | Hm Drgbl | Hm Drgbl | Hm Drgbl | CPE002E8 | CPE002ER | CPE002FC |
| 2561 | K24 | K1 | 1009 | 746,6 | 14858 | 432,8 | 14,7 | 1,157 | HSA034 | C | neg1 | Hm Drgbl | Hm Drgbl | Hm Drgbl | CPE002DC | CPE002EE | CPE002F4 |
| 16433 | M24 | M1 | 1206 | 1088 | 17692 | 293,5 | 14,7 | 1,349 | HSA034 | C | neg1 | Hm Gen E | Hm Gen E | Hm Gen E | CPE002JG | CPE002NV | CPE002KB |
| 14081 | K24 | K1 | 1726 | 1149 | 25493 | 130,3 | 14,8 | 0,848 | HSA034 | C | neg1 | Hm Gen E | Hm Gen E | Hm Gen E | CPE002JF | CPE002NP | CPE002K5 |
| 14152 | N23 | N24 | 1571 | 1119 | 23231 | 157,7 | 14,8 | 0,848 | HSA034 | C | neg1 | Hm Gen E | Hm Gen E | Hm Gen E | CPE002JF | CPE002NP | CPE002K5 |
| 12281 | P24 | P1 | 1127 | 790,9 | 16697 | 132 | 14,8 | 0,949 | HSA034 | C | neg1 | Hm Gen E | Hm Gen E | Hm Gen E | CPE002J4 | CPE002NL | CPE002K0 |
| 18400 | O23 | O24 | 1524 | 1063 | 22616 | 457,8 | 14,8 | 0,952 | HSA034 | C | neg1 | Hm Gen E | Hm Gen E | Hm Gen E | CPE002JL | CPE002O0 | CPE002KG |
| 21424 | M23 | M24 | 1352 | 1055 | 20072 | 398,2 | 14,8 | 1,05 | HSA034 | C | neg1 | Hm Gen E | Hm Gen E | Hm Gen E | CPE002JT | CPE002O8 | CPE002KO |
| 12545 | K24 | K1 | 1557 | 1089 | 23047 | 138,3 | 14,8 | 1,113 | HSA034 | C | neg1 | Hm Gen E | Hm Gen E | Hm Gen E | CPE002J5 | CPE002NK | CPE002K1 |
| 13433 | P24 | P1 | 1264 | 1079 | 18880 | 110,6 | 14,9 | 0,747 | HSA034 | C | neg1 | Hm Gen E | Hm Gen E | Hm Gen E | CPE002JB | CPE002NN | CPE002K3 |
| 6448 | M23 | M24 | 1338 | 551,1 | 19955 | 499,6 | 14,9 | 0,983 | HSA034 | C | neg1 | Hm Drgbl | Hm Drgbl | Hm Drgbl | CPE002DZ | CPE002F0 | CPE002FL |
| 13024 | O23 | O24 | 1629 | 1117 | 24255 | 251,7 | 14,9 | 1,01 | HSA034 | C | neg1 | Hm Gen E | Hm Gen E | Hm Gen E | CPE002J6 | CPE002NN | CPE002K2 |
| 19576 | P23 | P24 | 1342 | 1403 | 19981 | 473,1 | 14,9 | 1,057 | HSA034 | C | neg1 | Hm Gen E | Hm Gen E | Hm Gen E | CPE002JO | CPE002O3 | CPE002KJ |
| 16505 | P24 | P1 | 643 | 1138 | 9559 | 293,3 | 14,9 | 1,367 | HSA034 | C | neg1 | Hm Gen E | Hm Gen E | Hm Gen E | CPE002JG | CPE002NV | CPE002KB |
| 10360 | P23 | P24 | 1521 | 855,1 | 22619 | 188,8 | 14,9 | 1,433 | HSA034 | C | neg1 | Hm Drgbl | Hm Drgbl | Hm Drgbl | CPE002EA | CPE002EP | CPE002FA |
| 22241 | O24 | O1 | 976 | 1321 | 14661 | 319,1 | 15 | 0,713 | HSA034 | C | neg1 | Hm Gen E | Hm Gen E | Hm Gen E | CPE002JV | CPE002OA | CPE002KQ |
| 8752 | M23 | M24 | 1914 | 1189 | 28619 | 162,9 | 15 | 1,06 | HSA034 | C | neg1 | Hm Drgbl | Hm Drgbl | Hm Drgbl | CPE002E6 | CPE002ET | CPE002FE |
| 2656 | O23 | O24 | 1917 | 773,5 | 28972 | 557,7 | 15,1 | 1,189 | HSA034 | C | neg1 | Hm Drgbl | Hm Drgbl | Hm Drgbl | CPE002DC | CPE002EE | CPE002F4 |
| 12929 | K24 | K1 | 1656 | 1133 | 25122 | 144,4 | 15,2 | 1,031 | HSA034 | C | neg1 | Hm Gen E | Hm Gen E | Hm Gen E | CPE002J6 | CPE002NN | CPE002K2 |
| 9929 | N24 | N1 | 1737 | 1113 | 26340 | 138,4 | 15,2 | 1,063 | HSA034 | C | neg1 | Hm Drgbl | Hm Drgbl | Hm Drgbl | CPE002E9 | CPE002EQ | CPE002FB |
| 21401 | L24 | L1 | 943 | 913,5 | 14368 | 239,8 | 15,2 | 1,078 | HSA034 | C | neg1 | Hm Gen E | Hm Gen E | Hm Gen E | CPE002JT | CPE002O8 | CPE002KO |
| 23681 | K24 | K1 | 1067 | 1669 | 16347 | 222,9 | 15,3 | 0,812 | HSA034 | C | neg1 | Hm Gen E | Hm Gen E | Hm Gen E | CPE002KV | CPE002OE | CPE002KU |
| 23753 | N24 | N1 | 1056 | 1601 | 16206 | 202,3 | 15,3 | 0,812 | HSA034 | C | neg1 | Hm Gen E | Hm Gen E | Hm Gen E | CPE002KV | CPE002OE | CPE002KU |
| 14080 | K23 | K24 | 1805 | 1174 | 27596 | 166,1 | 15,3 | 0,877 | HSA034 | C | neg1 | Hm Gen E | Hm Gen E | Hm Gen E | CPE002JF | CPE002NP | CPE002K5 |
| 14104 | L23 | L24 | 1551 | 1084 | 23667 | 130,1 | 15,3 | 0,877 | HSA034 | C | neg1 | Hm Gen E | Hm Gen E | Hm Gen E | CPE002JF | CPE002NP | CPE002K5 |
| 20680 | N23 | N24 | 754 | 848,6 | 11505 | 230 | 15,3 | 1,02 | HSA034 | C | neg1 | Hm Gen E | Hm Gen E | Hm Gen E | CPE002JR | CPE002O6 | CPE002KM |
| 19169 | O24 | O1 | 1077 | 1034 | 16497 | 317,2 | 15,3 | 1,066 | HSA034 | C | neg1 | Hm Gen E | Hm Gen E | Hm Gen E | CPE002JN | CPE002O2 | CPE002KI |
| 19481 | L24 | L1 | 1049 | 1215 | 16029 | 274,9 | 15,3 | 1,085 | HSA034 | C | neg1 | Hm Gen E | Hm Gen E | Hm Gen E | CPE002JO | CPE002O3 | CPE002KJ |
| 7240 | N23 | N24 | 1192 | 555,3 | 18191 | 547,1 | 15,3 | 1,191 | HSA034 | C | neg1 | Hm Drgbl | Hm Drgbl | Hm Drgbl | CPE002E2 | CPE002EX | CPE002FI |
| 9089 | K24 | K1 | 1577 | 1740 | 24122 | 169,7 | 15,3 | 1,229 | HSA034 | C | neg1 | Hm Drgbl | Hm Drgbl | Hm Drgbl | CPE002E7 | CPE002ES | CPE002FD |
| 3713 | K24 | K1 | 573 | 1372 | 8780 | 412,4 | 15,3 | 1,471 | HSA034 | C | neg1 | Hm Drgbl | Hm Drgbl | Hm Drgbl | CPE002DR | CPE002EH | CPE002F7 |
| 10336 | O23 | O24 | 1640 | 866,6 | 25054 | 192,6 | 15,3 | 1,471 | HSA034 | C | neg1 | Hm Drgbl | Hm Drgbl | Hm Drgbl | CPE002EA | CPE002EP | CPE002FA |
| 1793 | K24 | K1 | 935 | 741,4 | 14310 | 415,1 | 15,3 | 1,602 | HSA034 | C | neg1 | Hm Drgbl | Hm Drgbl | Hm Drgbl | CPE002FW | CPE002G0 | CPE002G4 |
| 22600 | N23 | N24 | 957 | 1399 | 14764 | 301 | 15,4 | 0,794 | HSA034 | C | neg1 | Hm Gen E | Hm Gen E | Hm Gen E | CPE002JW | CPE002OB | CPE002KR |
| 23729 | M24 | M1 | 1131 | 1538 | 17444 | 191,9 | 15,4 | 0,817 | HSA034 | C | neg1 | Hm Gen E | Hm Gen E | Hm Gen E | CPE002KV | CPE002OE | CPE002KU |
| 12184 | L23 | L24 | 1536 | 889,5 | 23590 | 136,9 | 15,4 | 0,987 | HSA034 | C | neg1 | Hm Gen E | Hm Gen E | Hm Gen E | CPE002J4 | CPE002NL | CPE002K0 |
| 6473 | N24 | N1 | 1581 | 586,5 | 24356 | 516,1 | 15,4 | 1,017 | HSA034 | C | neg1 | Hm Drgbl | Hm Drgbl | Hm Drgbl | CPE002DZ | CPE002F0 | CPE002FL |
| 6496 | O23 | O24 | 1463 | 562,2 | 22462 | 554,1 | 15,4 | 1,017 | HSA034 | C | neg1 | Hm Drgbl | Hm Drgbl | Hm Drgbl | CPE002DZ | CPE002F0 | CPE002FL |
| 18713 | L24 | L1 | 908 | 1059 | 14001 | 198 | 15,4 | 1,044 | HSA034 | C | neg1 | Hm Gen E | Hm Gen E | Hm Gen E | CPE002JM | CPE002O1 | CPE002KH |
| 9497 | L24 | L1 | 1472 | 1188 | 22667 | 157,8 | 15,4 | 1,069 | HSA034 | C | neg1 | Hm Drgbl | Hm Drgbl | Hm Drgbl | CPE002E8 | CPE002ER | CPE002FC |
| 15329 | O24 | O1 | 1410 | 839,6 | 21752 | 156,4 | 15,4 | 1,077 | HSA034 | C | neg1 | Hm Gen E | Hm Gen E | Hm Gen E | CPE002J8 | CPE002NS | CPE002K8 |
| 19480 | L23 | L24 | 1425 | 1234 | 21975 | 346,8 | 15,4 | 1,092 | HSA034 | C | neg1 | Hm Gen E | Hm Gen E | Hm Gen E | CPE002JO | CPE002O3 | CPE002KJ |
| 20993 | K24 | K1 | 1109 | 1015 | 17048 | 325,1 | 15,4 | 1,092 | HSA034 | C | neg1 | Hm Gen E | Hm Gen E | Hm Gen E | CPE002JS | CPE002O7 | CPE002KN |
| 14512 | M23 | M24 | 1706 | 862,9 | 26206 | 204,2 | 15,4 | 1,096 | HSA034 | C | neg1 | Hm Gen E | Hm Gen E | Hm Gen E | CPE002J7 | CPE002NC | CPE002K6 |
| 12664 | P23 | P24 | 1405 | 1030 | 21664 | 214,5 | 15,4 | 1,158 | HSA034 | C | neg1 | Hm Gen E | Hm Gen E | Hm Gen E | CPE002J5 | CPE002NK | CPE002K1 |
| 7553 | K24 | K1 | 477 | 600,9 | 7333 | 288,9 | 15,4 | 1,176 | HSA034 | C | neg1 | Hm Drgbl | Hm Drgbl | Hm Drgbl | CPE002E3 | CPE002EW | CPE002FH |
| 376 | P23 | P24 | 689 | 1779 | 10580 | 824,5 | 15,4 | 1,311 | HSA034 | C | neg1 | Hm Drgbl | Hm Drgbl | Hm Drgbl | CPE002DT | CPE002EJ | CPE002F9 |
| 3448 | P23 | P24 | 1191 | 1395 | 18494 | 585,4 | 15,5 | 0,96 | HSA034 | C | neg1 | Hm Drgbl | Hm Drgbl | Hm Drgbl | CPE002DC | CPE002EG | CPE002F6 |
| 20657 | M24 | M1 | 735 | 792,2 | 11423 | 177 | 15,5 | 1,033 | HSA034 | C | neg1 | Hm Gen E | Hm Gen E | Hm Gen E | CPE002JR | CPE002O6 | CPE002KM |
| 12928 | K23 | K24 | 1800 | 1129 | 27824 | 163,4 | 15,5 | 1,051 | HSA034 | C | neg1 | Hm Gen E | Hm Gen E | Hm Gen E | CPE002J6 | CPE002NN | CPE002K2 |
| 18712 | L23 | L24 | 1453 | 1067 | 22546 | 338,3 | 15,5 | 1,051 | HSA034 | C | neg1 | Hm Gen E | Hm Gen E | Hm Gen E | CPE002JM | CPE002O1 | CPE002KH |
| 9952 | O23 | O24 | 2057 | 1262 | 31911 | 242,8 | 15,5 | 1,084 | HSA034 | C | neg1 | Hm Drgbl | Hm Drgbl | Hm Drgbl | CPE002E9 | CPE002EQ | CPE002FB |
| 15304 | N23 | N24 | 1650 | 858 | 25619 | 171 | 15,5 | 1,084 | HSA034 | C | neg1 | Hm Gen E | Hm Gen E | Hm Gen E | CPE002J8 | CPE002NS | CPE002K8 |
| 21065 | N24 | N1 | 879 | 980,8 | 13634 | 260 | 15,5 | 1,099 | HSA034 | C | neg1 | Hm Gen E | Hm Gen E | Hm Gen E | CPE002JS | CPE002O7 | CPE002KN |
| 7265 | O24 | O1 | 372 | 592,4 | 5757 | 490,2 | 15,5 | 1,206 | HSA034 | C | neg1 | Hm Drgbl | Hm Drgbl | Hm Drgbl | CPE002E2 | CPE002EX | CPE002FI |
| 17945 | L24 | L1 | 907 | 1030 | 14046 | 247,3 | 15,5 | 1,225 | HSA034 | C | neg1 | Hm Gen E | Hm Gen E | Hm Gen E | CPE002JK | CPE002NZ | CPE002KF |
| 18016 | O23 | O24 | 1451 | 1016 | 22442 | 359,4 | 15,5 | 1,225 | HSA034 | C | neg1 | Hm Gen E | Hm Gen E | Hm Gen E | CPE002JK | CPE002NZ | CPE002KF |
| 23009 | O24 | O1 | 937 | 1599 | 14618 | 286,6 | 15,6 | 0,732 | HSA034 | C | neg1 | Hm Gen E | Hm Gen E | Hm Gen E | CPE002JX | CPE002OC | CPE002KS |
| 20609 | K24 | K1 | 523 | 803,5 | 8155 | 175,1 | 15,6 | 1,04 | HSA034 | C | neg1 | Hm Gen E | Hm Gen E | Hm Gen E | CPE002JR | CPE002O6 | CPE002KM |
| 12952 | L23 | L24 | 1604 | 1117 | 25168 | 188,6 | 15,7 | 1,064 | HSA034 | C | neg1 | Hm Gen E | Hm Gen E | Hm Gen E | CPE002J6 | CPE002NN | CPE002K2 |
| 17657 | P24 | P1 | 772 | 1058 | 12121 | 180,7 | 15,7 | 1,072 | HSA034 | C | neg1 | Hm Gen E | Hm Gen E | Hm Gen E | CPE002JJ | CPE002NY | CPE002KE |
| 9473 | K24 | K1 | 1363 | 1074 | 21443 | 161,3 | 15,7 | 1,09 | HSA034 | C | neg1 | Hm Drgbl | Hm Drgbl | Hm Drgbl | CPE002E8 | CPE002ER | CPE002FC |
| 19121 | M24 | M1 | 1559 | 1174 | 24513 | 302,2 | 15,7 | 1,094 | HSA034 | C | neg1 | Hm Gen E | Hm Gen E | Hm Gen E | CPE002JN | CPE002O2 | CPE002KI |
| 9857 | K24 | K1 | 955 | 1081 | 15015 | 113,4 | 15,7 | 1,098 | HSA034 | C | neg1 | Hm Drgbl | Hm Drgbl | Hm Drgbl | CPE002E9 | CPE002EQ | CPE002FB |
| 9881 | L24 | L1 | 1643 | 1159 | 25748 | 137 | 15,7 | 1,098 | HSA034 | C | neg1 | Hm Drgbl | Hm Drgbl | Hm Drgbl | CPE002E9 | CPE002EQ | CPE002FB |
| 5681 | M24 | M1 | 676 | 644,1 | 10595 | 534,8 | 15,7 | 1,495 | HSA034 | C | neg1 | Hm Drgbl | Hm Drgbl | Hm Drgbl | CPE002DX | CPE002F2 | CPE002FN |
| 23801 | P24 | P1 | 700 | 1800 | 11045 | 190,2 | 15,8 | 0,838 | HSA034 | C | neg1 | Hm Gen E | Hm Gen E | Hm Gen E | CPE002KV | CPE002OE | CPE002KU |
| 12161 | K24 | K1 | 1746 | 910,3 | 27567 | 119,7 | 15,8 | 1,013 | HSA034 | C | neg1 | Hm Gen E | Hm Gen E | Hm Gen E | CPE002J4 | CPE002NL | CPE002K0 |
| 12209 | M24 | M1 | 1854 | 853 | 29303 | 121,8 | 15,8 | 1,013 | HSA034 | C | neg1 | Hm Gen E | Hm Gen E | Hm Gen E | CPE002J4 | CPE002NL | CPE002K0 |
| 13025 | O24 | O1 | 1615 | 1126 | 25540 | 174 | 15,8 | 1,071 | HSA034 | C | neg1 | Hm Gen E | Hm Gen E | Hm Gen E | CPE002J6 | CPE002NN | CPE002K2 |
| 18688 | K23 | K24 | 1414 | 1075 | 22273 | 272,1 | 15,8 | 1,071 | HSA034 | C | neg1 | Hm Gen E | Hm Gen E | Hm Gen E | CPE002JM | CPE002O1 | CPE002KH |
| 16888 | P23 | P24 | 959 | 1105 | 15114 | 427,4 | 15,8 | 1,249 | HSA034 | C | neg1 | Hm Gen E | Hm Gen E | Hm Gen E | CPE002JH | CPE002NV | CPE002KC |
| 15736 | P23 | P24 | 1315 | 892,7 | 20721 | 225,6 | 15,8 | 1,436 | HSA034 | C | neg1 | Hm Gen E | Hm Gen E | Hm Gen E | CPE002JE | CPE002NT | CPE002K9 |
| 16121 | P24 | P1 | 1086 | 778,1 | 17318 | 134,8 | 15,9 | 0,886 | HSA034 | C | neg1 | Hm Gen E | Hm Gen E | Hm Gen E | CPE002J9 | CPE002NL | CPE002KA |
| 9953 | O24 | O1 | 1255 | 1012 | 19970 | 153 | 15,9 | 1,112 | HSA034 | C | neg1 | Hm Drgbl | Hm Drgbl | Hm Drgbl | CPE002E9 | CPE002EQ | CPE002FB |
| 11392 | K23 | K24 | 1413 | 863,2 | 22422 | 182,3 | 15,9 | 1,152 | HSA034 | C | neg1 | Hm Drgbl | Hm Drgbl | Hm Drgbl | CPE002ED | CPE002F3 | CPE002FT |
| 12232 | N23 | N24 | 1475 | 866,9 | 23582 | 166 | 16 | 1,026 | HSA034 | C | neg1 | Hm Gen E | Hm Gen E | Hm Gen E | CPE002J4 | CPE002NL | CPE002K0 |
| 19841 | K24 | K1 | 949 | 365,4 | 15139 | 246 | 16 | 1,042 | HSA034 | C | neg1 | Hm Gen E | Hm Gen E | Hm Gen E | CPE002JP | CPE002O4 | CPE002KK |
| 8800 | O23 | O24 | 1457 | 1082 | 23321 | 230,9 | 16 | 1,131 | HSA034 | C | neg1 | Hm Drgbl | Hm Drgbl | Hm Drgbl | CPE002E6 | CPE002ET | CPE002FE |
| 11440 | M23 | M24 | 1479 | 864 | 23694 | 214,2 | 16 | 1,159 | HSA034 | C | neg1 | Hm Drgbl | Hm Drgbl | Hm Drgbl | CPE002ED | CPE002F3 | CPE002FT |
| 11464 | N23 | N24 | 1402 | 940,3 | 22372 | 220,4 | 16 | 1,159 | HSA034 | C | neg1 | Hm Drgbl | Hm Drgbl | Hm Drgbl | CPE002ED | CPE002F3 | CPE002FT |
| 22601 | N24 | N1 | 871 | 1312 | 13988 | 331,3 | 16,1 | 0,83 | HSA034 | C | neg1 | Hm Gen E | Hm Gen E | Hm Gen E | CPE002JW | CPE002OB | CPE002KR |
| 3377 | M24 | M1 | 820 | 1339 | 13187 | 426,6 | 16,1 | 0,997 | HSA034 | C | neg1 | Hm Drgbl | Hm Drgbl | Hm Drgbl | CPE002DC | CPE002EG | CPE002F6 |
| 19504 | M23 | M24 | 1365 | 1211 | 22033 | 311,8 | 16,1 | 1,142 | HSA034 | C | neg1 | Hm Gen E | Hm Gen E | Hm Gen E | CPE002JO | CPE002O3 | CPE002KJ |
| 14536 | N23 | N24 | 1498 | 925,2 | 24079 | 174,7 | 16,1 | 1,146 | HSA034 | C | neg1 | Hm Gen E | Hm Gen E | Hm Gen E | CPE002J7 | CPE002NC | CPE002K6 |
| 17273 | P24 | P1 | 493 | 1132 | 7954 | 154,4 | 16,1 | 1,243 | HSA034 | C | neg1 | Hm Gen E | Hm Gen E | Hm Gen E | CPE002JI | CPE002NZ | CPE002KD |
| 3353 | L24 | L1 | 1159 | 1329 | 18779 | 408 | 16,2 | 1,003 | HSA034 | C | neg1 | Hm Drgbl | Hm Drgbl | Hm Drgbl | CPE002DC | CPE002EG | CPE002F6 |
| 12280 | P23 | P24 | 1314 | 836,3 | 21327 | 162,8 | 16,2 | 1,038 | HSA034 | C | neg1 | Hm Gen E | Hm Gen E | Hm Gen E | CPE002J4 | CPE002NL | CPE002K0 |
| 15352 | P23 | P24 | 1364 | 828,9 | 22058 | 191,9 | 16,2 | 1,133 | HSA034 | C | neg1 | Hm Gen E | Hm Gen E | Hm Gen E | CPE002J8 | CPE002NS | CPE002K8 |
| 19457 | K24 | K1 | 998 | 1301 | 16148 | 292,4 | 16,2 | 1,149 | HSA034 | C | neg1 | Hm Gen E | Hm Gen E | Hm Gen E | CPE002JO | CPE002O3 | CPE002KJ |
| 11033 | L24 | L1 | 1157 | 902,5 | 18768 | 546,2 | 16,2 | 1,227 | HSA034 | C | neg1 | Hm Drgbl | Hm Drgbl | Hm Drgbl | CPE002EC | CPE002EN | CPE002FS |
| 6809 | L24 | L1 | 1252 | 623,9 | 20259 | 412,4 | 16,2 | 1,421 | HSA034 | C | neg1 | Hm Drgbl | Hm Drgbl | Hm Drgbl | CPE002E1 | CPE002EY | CPE002FJ |
| 13792 | O23 | O24 | 1535 | 1052 | 24864 | 237,6 | 16,2 | 1,521 | HSA034 | C | neg1 | Hm Gen E | Hm Gen E | Hm Gen E | CPE002JC | CPE002NC | CPE002K4 |
| 16073 | N24 | N1 | 1464 | 838,5 | 23800 | 90,16 | 16,3 | 0,908 | HSA034 | C | neg1 | Hm Gen E | Hm Gen E | Hm Gen E | CPE002J9 | CPE002NL | CPE002KA |
| 18305 | K24 | K1 | 827 | 1105 | 13490 | 320,5 | 16,3 | 1,048 | HSA034 | C | neg1 | Hm Gen E | Hm Gen E | Hm Gen E | CPE002JL | CPE002O0 | CPE002KG |
| 6520 | P23 | P24 | 1165 | 549 | 18997 | 610 | 16,3 | 1,076 | HSA034 | C | neg1 | Hm Drgbl | Hm Drgbl | Hm Drgbl | CPE002DZ | CPE002F0 | CPE002FL |
| 17921 | K24 | K1 | 722 | 1019 | 11750 | 236,8 | 16,3 | 1,289 | HSA034 | C | neg1 | Hm Gen E | Hm Gen E | Hm Gen E | CPE002JK | CPE002NZ | CPE002KF |
| 6113 | O24 | O1 | 619 | 725,7 | 10118 | 506 | 16,3 | 1,489 | HSA034 | C | neg1 | Hm Drgbl | Hm Drgbl | Hm Drgbl | CPE002DY | CPE002F1 | CPE002FM |
| 16001 | K24 | K1 | 1606 | 882,3 | 26270 | 139,5 | 16,4 | 0,914 | HSA034 | C | neg1 | Hm Gen E | Hm Gen E | Hm Gen E | CPE002J9 | CPE002NL | CPE002KA |
| 16049 | M24 | M1 | 1508 | 889,7 | 24770 | 109,7 | 16,4 | 0,914 | HSA034 | C | neg1 | Hm Gen E | Hm Gen E | Hm Gen E | CPE002J9 | CPE002NL | CPE002KA |
| 14849 | K24 | K1 | 1806 | 937,6 | 29620 | 113,6 | 16,4 | 0,934 | HSA034 | C | neg1 | Hm Gen E | Hm Gen E | Hm Gen E | CPE002JD | CPE002NR | CPE002K7 |
| 6425 | L24 | L1 | 1526 | 599,1 | 25018 | 513,4 | 16,4 | 1,083 | HSA034 | C | neg1 | Hm Drgbl | Hm Drgbl | Hm Drgbl | CPE002DZ | CPE002F0 | CPE002FL |
| 19552 | O23 | O24 | 1271 | 1213 | 20854 | 512,1 | 16,4 | 1,163 | HSA034 | C | neg1 | Hm Gen E | Hm Gen E | Hm Gen E | CPE002JO | CPE002O3 | CPE002KJ |
| 352 | O23 | O24 | 471 | 1525 | 7704 | 747 | 16,4 | 1,396 | HSA034 | C | neg1 | Hm Drgbl | Hm Drgbl | Hm Drgbl | CPE002DT | CPE002EJ | CPE002F9 |
| 15712 | O23 | O24 | 1394 | 924,9 | 22847 | 179,4 | 16,4 | 1,491 | HSA034 | C | neg1 | Hm Gen E | Hm Gen E | Hm Gen E | CPE002JE | CPE002NT | CPE002K9 |
| 5633 | K24 | K1 | 675 | 743,8 | 11155 | 437,1 | 16,5 | 1,571 | HSA034 | C | neg1 | Hm Drgbl | Hm Drgbl | Hm Drgbl | CPE002DX | CPE002F2 | CPE002FN |
| 14848 | K23 | K24 | 1719 | 936,7 | 28470 | 150,8 | 16,6 | 0,946 | HSA034 | C | neg1 | Hm Gen E | Hm Gen E | Hm Gen E | CPE002JD | CPE002NR | CPE002K7 |
| 17561 | L24 | L1 | 998 | 1083 | 16519 | 208,7 | 16,6 | 1,133 | HSA034 | C | neg1 | Hm Gen E | Hm Gen E | Hm Gen E | CPE002JJ | CPE002NY | CPE002KE |
| 3424 | O23 | O24 | 1340 | 1396 | 22320 | 556,9 | 16,7 | 1,034 | HSA034 | C | neg1 | Hm Drgbl | Hm Drgbl | Hm Drgbl | CPE002DC | CPE002EG | CPE002F6 |
| 12208 | M23 | M24 | 1559 | 856,6 | 26031 | 148,6 | 16,7 | 1,071 | HSA034 | C | neg1 | Hm Gen E | Hm Gen E | Hm Gen E | CPE002J4 | CPE002NL | CPE002K0 |
| 17609 | N24 | N1 | 1064 | 1016 | 17792 | 197,1 | 16,7 | 1,14 | HSA034 | C | neg1 | Hm Gen E | Hm Gen E | Hm Gen E | CPE002JJ | CPE002NY | CPE002KE |
| 19936 | O23 | O24 | 1313 | 337,5 | 22040 | 492 | 16,8 | 1,094 | HSA034 | C | neg1 | Hm Gen E | Hm Gen E | Hm Gen E | CPE002JP | CPE002O4 | CPE002KK |
| 14896 | M23 | M24 | 1523 | 923,5 | 25769 | 155,9 | 16,9 | 0,963 | HSA034 | C | neg1 | Hm Gen E | Hm Gen E | Hm Gen E | CPE002JD | CPE002NR | CPE002K7 |
| 21113 | P24 | P1 | 742 | 1135 | 12525 | 286,4 | 16,9 | 1,199 | HSA034 | C | neg1 | Hm Gen E | Hm Gen E | Hm Gen E | CPE002JS | CPE002O7 | CPE002KN |
| 21377 | K24 | K1 | 1040 | 980,2 | 17524 | 267,1 | 16,9 | 1,199 | HSA034 | C | neg1 | Hm Gen E | Hm Gen E | Hm Gen E | CPE002JT | CPE002O8 | CPE002KO |
| 20656 | M23 | M24 | 685 | 849,2 | 11661 | 226,1 | 17 | 1,133 | HSA034 | C | neg1 | Hm Gen E | Hm Gen E | Hm Gen E | CPE002JR | CPE002O6 | CPE002KM |
| 9568 | O23 | O24 | 1575 | 1141 | 26787 | 186 | 17 | 1,181 | HSA034 | C | neg1 | Hm Drgbl | Hm Drgbl | Hm Drgbl | CPE002E8 | CPE002ER | CPE002FC |
| 14560 | O23 | O24 | 1330 | 843,3 | 22653 | 217,3 | 17 | 1,21 | HSA034 | C | neg1 | Hm Gen E | Hm Gen E | Hm Gen E | CPE002J7 | CPE002NC | CPE002K6 |
| 19913 | N24 | N1 | 912 | 331,4 | 15625 | 242,5 | 17,1 | 1,114 | HSA034 | C | neg1 | Hm Gen E | Hm Gen E | Hm Gen E | CPE002JP | CPE002O4 | CPE002KK |
| 9592 | P23 | P24 | 1468 | 1101 | 25105 | 203,5 | 17,1 | 1,188 | HSA034 | C | neg1 | Hm Drgbl | Hm Drgbl | Hm Drgbl | CPE002E8 | CPE002ER | CPE002FC |
| 12544 | K23 | K24 | 1329 | 1105 | 22773 | 183,9 | 17,1 | 1,286 | HSA034 | C | neg1 | Hm Gen E | Hm Gen E | Hm Gen E | CPE002J5 | CPE002NK | CPE002K1 |
| 14201 | P24 | P1 | 1461 | 1068 | 25121 | 149,9 | 17,2 | 0,986 | HSA034 | C | neg1 | Hm Gen E | Hm Gen E | Hm Gen E | CPE002JF | CPE002NP | CPE002K5 |
| 9905 | M24 | M1 | 1432 | 1140 | 24693 | 138,2 | 17,2 | 1,203 | HSA034 | C | neg1 | Hm Drgbl | Hm Drgbl | Hm Drgbl | CPE002E9 | CPE002EQ | CPE002FB |
| 11512 | P23 | P24 | 1577 | 836,7 | 27063 | 273,1 | 17,2 | 1,246 | HSA034 | C | neg1 | Hm Drgbl | Hm Drgbl | Hm Drgbl | CPE002ED | CPE002F3 | CPE002FT |
| 11872 | O23 | O24 | 1315 | 887,6 | 22647 | 225,7 | 17,2 | 1,536 | HSA034 | C | neg1 | Hm Gen E | Hm Gen E | Hm Gen E | CPE002JA | CPE002NJ | CPE002JZ |
| 22576 | M23 | M24 | 922 | 1359 | 15936 | 404 | 17,3 | 0,892 | HSA034 | C | neg1 | Hm Gen E | Hm Gen E | Hm Gen E | CPE002JW | CPE002OB | CPE002KR |
| 12640 | O23 | O24 | 1214 | 1092 | 21025 | 245,2 | 17,3 | 1,301 | HSA034 | C | neg1 | Hm Gen E | Hm Gen E | Hm Gen E | CPE002J5 | CPE002NK | CPE002K1 |
| 13336 | L23 | L24 | 1233 | 1204 | 21467 | 139,5 | 17,4 | 0,872 | HSA034 | C | neg1 | Hm Gen E | Hm Gen E | Hm Gen E | CPE002JB | CPE002NN | CPE002K3 |
| 22577 | M24 | M1 | 873 | 1335 | 15191 | 358,6 | 17,4 | 0,897 | HSA034 | C | neg1 | Hm Gen E | Hm Gen E | Hm Gen E | CPE002JW | CPE002OB | CPE002KR |
| 18304 | K23 | K24 | 1339 | 1061 | 23243 | 308 | 17,4 | 1,119 | HSA034 | C | neg1 | Hm Gen E | Hm Gen E | Hm Gen E | CPE002JL | CPE002O0 | CPE002KG |
| 16504 | P23 | P24 | 690 | 1179 | 11998 | 434,4 | 17,4 | 1,596 | HSA034 | C | neg1 | Hm Gen E | Hm Gen E | Hm Gen E | CPE002JG | CPE002NV | CPE002KB |
| 14872 | L23 | L24 | 1277 | 927,4 | 22291 | 129,6 | 17,5 | 0,997 | HSA034 | C | neg1 | Hm Gen E | Hm Gen E | Hm Gen E | CPE002JD | CPE002NR | CPE002K7 |
| 14920 | N23 | N24 | 1357 | 866,4 | 23855 | 151,2 | 17,6 | 1,003 | HSA034 | C | neg1 | Hm Gen E | Hm Gen E | Hm Gen E | CPE002JD | CPE002NR | CPE002K7 |
| 8705 | K24 | K1 | 1856 | 1199 | 32606 | 150,1 | 17,6 | 1,244 | HSA034 | C | neg1 | Hm Drgbl | Hm Drgbl | Hm Drgbl | CPE002E6 | CPE002ET | CPE002FE |
| 14921 | N24 | N1 | 1501 | 880,2 | 26600 | 118,9 | 17,7 | 1,009 | HSA034 | C | neg1 | Hm Gen E | Hm Gen E | Hm Gen E | CPE002JD | CPE002NR | CPE002K7 |
| 14200 | P23 | P24 | 1421 | 1066 | 25146 | 237 | 17,7 | 1,014 | HSA034 | C | neg1 | Hm Gen E | Hm Gen E | Hm Gen E | CPE002JF | CPE002NP | CPE002K5 |
| 21497 | P24 | P1 | 413 | 1105 | 7304 | 264,1 | 17,7 | 1,255 | HSA034 | C | neg1 | Hm Gen E | Hm Gen E | Hm Gen E | CPE002JT | CPE002O8 | CPE002KO |
| 11128 | P23 | P24 | 1279 | 876,8 | 22590 | 858,3 | 17,7 | 1,341 | HSA034 | C | neg1 | Hm Drgbl | Hm Drgbl | Hm Drgbl | CPE002EC | CPE002EN | CPE002FS |
| 2177 | K24 | K1 | 887 | 688,2 | 15689 | 506,5 | 17,7 | 1,539 | HSA034 | C | neg1 | Hm Drgbl | Hm Drgbl | Hm Drgbl | CPE002FX | CPE002G1 | CPE002G5 |
| 22961 | M24 | M1 | 910 | 1636 | 16183 | 230,8 | 17,8 | 0,836 | HSA034 | C | neg1 | Hm Gen E | Hm Gen E | Hm Gen E | CPE002JX | CPE002OC | CPE002KS |
| 16048 | M23 | M24 | 1442 | 918,7 | 25615 | 198 | 17,8 | 0,992 | HSA034 | C | neg1 | Hm Gen E | Hm Gen E | Hm Gen E | CPE002J9 | CPE002NL | CPE002KA |
| 14129 | M24 | M1 | 1738 | 1144 | 30868 | 152 | 17,8 | 1,02 | HSA034 | C | neg1 | Hm Gen E | Hm Gen E | Hm Gen E | CPE002JF | CPE002NP | CPE002K5 |
| 14176 | O23 | O24 | 1479 | 1052 | 26352 | 217,1 | 17,8 | 1,02 | HSA034 | C | neg1 | Hm Gen E | Hm Gen E | Hm Gen E | CPE002JF | CPE002NP | CPE002K5 |
| 18353 | M24 | M1 | 956 | 1111 | 16974 | 270,3 | 17,8 | 1,145 | HSA034 | C | neg1 | Hm Gen E | Hm Gen E | Hm Gen E | CPE002JL | CPE002O0 | CPE002KG |
| 13048 | P23 | P24 | 1347 | 1032 | 23920 | 254,4 | 17,8 | 1,207 | HSA034 | C | neg1 | Hm Gen E | Hm Gen E | Hm Gen E | CPE002J6 | CPE002NN | CPE002K2 |
| 9472 | K23 | K24 | 1235 | 1197 | 21944 | 192,9 | 17,8 | 1,236 | HSA034 | C | neg1 | Hm Drgbl | Hm Drgbl | Hm Drgbl | CPE002E8 | CPE002ER | CPE002FC |
| 14488 | L23 | L24 | 1007 | 859,6 | 17906 | 145,9 | 17,8 | 1,267 | HSA034 | C | neg1 | Hm Gen E | Hm Gen E | Hm Gen E | CPE002J7 | CPE002NC | CPE002K6 |
| 7217 | M24 | M1 | 364 | 519,5 | 6485 | 377,5 | 17,8 | 1,385 | HSA034 | C | neg1 | Hm Drgbl | Hm Drgbl | Hm Drgbl | CPE002E2 | CPE002EX | CPE002FI |

| | | | | | | | | | | | | | | | | |
|---|---|---|---|---|---|---|---|---|---|---|---|---|---|---|---|---|
| 14105 | L24 | L1 | 1329 | 1093 | 23741 | 153,9 | 17,9 | 1,026 | H5A034 C | neg1 | Hm Gen E | Hm Gen E | Hm Gen E | CPE002JF | CPE002NP | CPE002K5 |
| 3425 | O24 | O1 | 485 | 1329 | 8664 | 551,3 | 17,9 | 1,108 | H5A034 C | neg1 | Hm Drgbl | Hm Drgbl | Hm Drgbl | CPE002DC | CPE002EG | CPE002F6 |
| 18329 | L24 | L1 | 861 | 1045 | 15401 | 305,5 | 17,9 | 1,151 | H5A034 C | neg1 | Hm Gen E | Hm Gen E | Hm Gen E | CPE002JL | CPE002O0 | CPE002KG |
| 6449 | M24 | M1 | 512 | 440,4 | 9153 | 459,8 | 17,9 | 1,182 | H5A034 C | neg1 | Hm Drgbl | Hm Drgbl | Hm Drgbl | CPE002DZ | CPE002F0 | CPE002FL |
| 17272 | P23 | P24 | 961 | 1265 | 17173 | 257 | 17,9 | 1,382 | H5A034 C | neg1 | Hm Gen E | Hm Gen E | Hm Gen E | CPE002JI | CPE002NZ | CPE002KD |
| 12256 | O23 | O24 | 1300 | 834,2 | 23435 | 207,6 | 18 | 1,154 | H5A034 C | neg1 | Hm Gen E | Hm Gen E | Hm Gen E | CPE002J4 | CPE002NL | CPE002K0 |
| 8321 | K24 | K1 | 407 | 634 | 7335 | 339,3 | 18 | 1,731 | H5A034 C | neg1 | Hm Drgbl | Hm Drgbl | Hm Drgbl | CPE002E5 | CPE002EU | CPE002FF |
| 16025 | L24 | L1 | 1539 | 883 | 27790 | 123,1 | 18,1 | 1,008 | H5A034 C | neg1 | Hm Gen E | Hm Gen E | Hm Gen E | CPE002J9 | CPE002NL | CPE002KA |
| 14584 | P23 | P24 | 1229 | 851 | 22212 | 222,7 | 18,1 | 1,288 | H5A034 C | neg1 | Hm Gen E | Hm Gen E | Hm Gen E | CPE002J7 | CPE002NC | CPE002K6 |
| 23728 | M23 | M24 | 1062 | 1519 | 19304 | 330,3 | 18,2 | 0,966 | H5A034 C | neg1 | Hm Gen E | Hm Gen E | Hm Gen E | CPE002KV | CPE002OE | CPE002KU |
| 14944 | O23 | O24 | 1640 | 890,9 | 29872 | 180,8 | 18,2 | 1,037 | H5A034 C | neg1 | Hm Gen E | Hm Gen E | Hm Gen E | CPE002JD | CPE002NR | CPE002K7 |
| 15280 | M23 | M24 | 1626 | 883 | 29564 | 192,7 | 18,2 | 1,273 | H5A034 C | neg1 | Hm Gen E | Hm Gen E | Hm Gen E | CPE002J8 | CPE002NS | CPE002K8 |
| 11104 | O23 | O24 | 1463 | 859,7 | 26642 | 849,7 | 18,2 | 1,379 | H5A034 C | neg1 | Hm Drgbl | Hm Drgbl | Hm Drgbl | CPE002EC | CPE002EN | CPE002FS |
| 14897 | M24 | M1 | 1604 | 925,1 | 29318 | 99,15 | 18,3 | 1,043 | H5A034 C | neg1 | Hm Gen E | Hm Gen E | Hm Gen E | CPE002JD | CPE002NR | CPE002K7 |
| 14153 | N24 | N1 | 1277 | 1078 | 23370 | 156,5 | 18,3 | 1,049 | H5A034 C | neg1 | Hm Gen E | Hm Gen E | Hm Gen E | CPE002JF | CPE002NP | CPE002K5 |
| 13361 | M24 | M1 | 2010 | 1172 | 37039 | 118,2 | 18,4 | 0,922 | H5A034 C | neg1 | Hm Gen E | Hm Gen E | Hm Gen E | CPE002JB | CPE002NN | CPE002K3 |
| 17585 | M24 | M1 | 1247 | 1040 | 22979 | 276,6 | 18,4 | 1,256 | H5A034 C | neg1 | Hm Gen E | Hm Gen E | Hm Gen E | CPE002JJ | CPE002NY | CPE002KE |
| 7601 | M24 | M1 | 228 | 553 | 4193 | 386,6 | 18,4 | 1,405 | H5A034 C | neg1 | Hm Drgbl | Hm Drgbl | Hm Drgbl | CPE002E3 | CPE002EW | CPE002FH |
| 4865 | K24 | K1 | 211 | 744,2 | 3878 | 471,1 | 18,4 | 2,022 | H5A034 C | neg1 | Hm Drgbl | Hm Drgbl | Hm Drgbl | CPE002DV | CPE002EL | CPE002FP |
| 15328 | O23 | O24 | 1361 | 797 | 25179 | 240 | 18,5 | 1,294 | H5A034 C | neg1 | Hm Gen E | Hm Gen E | Hm Gen E | CPE002J8 | CPE002NS | CPE002K8 |
| 22960 | M23 | M24 | 836 | 1805 | 15650 | 379,5 | 18,7 | 0,878 | H5A034 C | neg1 | Hm Gen E | Hm Gen E | Hm Gen E | CPE002JX | CPE002OC | CPE002KS |
| 23368 | N23 | N24 | 1042 | 1098 | 19575 | 162,3 | 18,8 | 0,812 | H5A034 C | neg1 | Hm Gen E | Hm Gen E | Hm Gen E | CPE002JY | CPE002OD | CPE002KT |
| 13360 | M23 | M24 | 1628 | 1168 | 30609 | 158,8 | 18,8 | 0,942 | H5A034 C | neg1 | Hm Gen E | Hm Gen E | Hm Gen E | CPE002JB | CPE002NN | CPE002K3 |
| 16000 | K23 | K24 | 1168 | 901,1 | 22009 | 152,4 | 18,8 | 1,047 | H5A034 C | neg1 | Hm Gen E | Hm Gen E | Hm Gen E | CPE002J9 | CPE002NL | CPE002KA |
| 16024 | L23 | L24 | 1084 | 863,6 | 20471 | 148,8 | 18,9 | 1,053 | H5A034 C | neg1 | Hm Gen E | Hm Gen E | Hm Gen E | CPE002J9 | CPE002NL | CPE002KA |
| 22217 | N24 | N1 | 1059 | 1382 | 20082 | 241,8 | 19 | 0,903 | H5A034 C | neg1 | Hm Gen E | Hm Gen E | Hm Gen E | CPE002JV | CPE002OA | CPE002KQ |
| 14968 | P23 | P24 | 1497 | 872,7 | 28457 | 159,8 | 19 | 1,083 | H5A034 C | neg1 | Hm Gen E | Hm Gen E | Hm Gen E | CPE002JD | CPE002NR | CPE002K7 |
| 18689 | K24 | K1 | 1027 | 989,9 | 19510 | 265,7 | 19 | 1,288 | H5A034 C | neg1 | Hm Gen E | Hm Gen E | Hm Gen E | CPE002JM | CPE002O1 | CPE002KH |
| 22553 | L24 | L1 | 681 | 1306 | 13036 | 433,8 | 19,1 | 0,985 | H5A034 C | neg1 | Hm Gen E | Hm Gen E | Hm Gen E | CPE002JW | CPE002OB | CPE002KR |
| 14873 | L24 | L1 | 1189 | 878,3 | 22712 | 109,3 | 19,1 | 1,088 | H5A034 C | neg1 | Hm Gen E | Hm Gen E | Hm Gen E | CPE002JD | CPE002NR | CPE002K7 |
| 18425 | P24 | P1 | 479 | 1109 | 9139 | 309,6 | 19,1 | 1,228 | H5A034 C | neg1 | Hm Gen E | Hm Gen E | Hm Gen E | CPE002JL | CPE002O0 | CPE002KG |
| 18809 | P24 | P1 | 768 | 1068 | 14671 | 297,8 | 19,1 | 1,295 | H5A034 C | neg1 | Hm Gen E | Hm Gen E | Hm Gen E | CPE002JM | CPE002O1 | CPE002KH |
| 8704 | K23 | K24 | 1471 | 1168 | 28033 | 165,3 | 19,1 | 1,35 | H5A034 C | neg1 | Hm Drgbl | Hm Drgbl | Hm Drgbl | CPE002E6 | CPE002ET | CPE002FE |
| 7169 | K24 | K1 | 441 | 571 | 8431 | 369,3 | 19,1 | 1,486 | H5A034 C | neg1 | Hm Drgbl | Hm Drgbl | Hm Drgbl | CPE002E2 | CPE002EX | CPE002FI |
| 22240 | O23 | O24 | 819 | 1406 | 15713 | 482,9 | 19,2 | 0,912 | H5A034 C | neg1 | Hm Gen E | Hm Gen E | Hm Gen E | CPE002JV | CPE002OA | CPE002KQ |
| 16072 | N23 | N24 | 1629 | 900,9 | 31247 | 204,6 | 19,2 | 1,07 | H5A034 C | neg1 | Hm Gen E | Hm Gen E | Hm Gen E | CPE002J9 | CPE002NL | CPE002KA |
| 13384 | N23 | N24 | 1619 | 1198 | 31218 | 93,93 | 19,3 | 0,967 | H5A034 C | neg1 | Hm Gen E | Hm Gen E | Hm Gen E | CPE002JB | CPE002NN | CPE002K3 |
| 14128 | M23 | M24 | 1083 | 1108 | 20962 | 162,7 | 19,4 | 1,112 | H5A034 C | neg1 | Hm Gen E | Hm Gen E | Hm Gen E | CPE002JF | CPE002NP | CPE002K5 |
| 23752 | N23 | N24 | 733 | 1570 | 14304 | 269,9 | 19,5 | 1,034 | H5A034 C | neg1 | Hm Gen E | Hm Gen E | Hm Gen E | CPE002KV | CPE002OE | CPE002KU |
| 19888 | M23 | M24 | 1212 | 341,2 | 23655 | 326,5 | 19,5 | 1,27 | H5A034 C | neg1 | Hm Gen E | Hm Gen E | Hm Gen E | CPE002JP | CPE002O4 | CPE002KK |
| 23705 | L24 | L1 | 807 | 1496 | 15783 | 229,9 | 19,6 | 1,04 | H5A034 C | neg1 | Hm Gen E | Hm Gen E | Hm Gen E | CPE002KV | CPE002OE | CPE002KU |
| 3449 | P24 | P1 | 902 | 1303 | 17672 | 512,2 | 19,6 | 1,214 | H5A034 C | neg1 | Hm Drgbl | Hm Drgbl | Hm Drgbl | CPE002DC | CPE002EG | CPE002F6 |
| 22193 | M24 | M1 | 721 | 1355 | 14234 | 242,1 | 19,7 | 0,936 | H5A034 C | neg1 | Hm Gen E | Hm Gen E | Hm Gen E | CPE002JV | CPE002OA | CPE002KQ |
| 22529 | K24 | K1 | 621 | 1364 | 12215 | 402,3 | 19,7 | 1,015 | H5A034 C | neg1 | Hm Gen E | Hm Gen E | Hm Gen E | CPE002JW | CPE002OB | CPE002KR |
| 22985 | N24 | N1 | 1039 | 1646 | 20604 | 219,1 | 19,8 | 0,93 | H5A034 C | neg1 | Hm Gen E | Hm Gen E | Hm Gen E | CPE002JX | CPE002OC | CPE002KS |
| 21496 | P23 | P24 | 736 | 1073 | 14627 | 448,8 | 19,9 | 1,411 | H5A034 C | neg1 | Hm Gen E | Hm Gen E | Hm Gen E | CPE002JT | CPE002O8 | CPE002KO |
| 22624 | O23 | O24 | 908 | 1354 | 18130 | 574,5 | 20 | 1,031 | H5A034 C | neg1 | Hm Gen E | Hm Gen E | Hm Gen E | CPE002JW | CPE002OB | CPE002KR |
| 23800 | P23 | P24 | 756 | 1742 | 15134 | 436 | 20 | 1,061 | H5A034 C | neg1 | Hm Gen E | Hm Gen E | Hm Gen E | CPE002KV | CPE002OE | CPE002KU |
| 5249 | K24 | K1 | 442 | 557,2 | 8838 | 641,2 | 20 | 1,569 | H5A034 C | neg1 | Hm Drgbl | Hm Drgbl | Hm Drgbl | CPE002DV | CPE002EM | CPE002FO |
| 22913 | K24 | K1 | 849 | 1683 | 17051 | 240,4 | 20,1 | 0,944 | H5A034 C | neg1 | Hm Gen E | Hm Gen E | Hm Gen E | CPE002JX | CPE002OC | CPE002KS |
| 3401 | N24 | N1 | 1066 | 1305 | 21473 | 410,3 | 20,1 | 1,245 | H5A034 C | neg1 | Hm Drgbl | Hm Drgbl | Hm Drgbl | CPE002DC | CPE002EG | CPE002F6 |
| 23344 | M23 | M24 | 1014 | 1127 | 20448 | 342,5 | 20,2 | 0,873 | H5A034 C | neg1 | Hm Gen E | Hm Gen E | Hm Gen E | CPE002JY | CPE002OD | CPE002KT |
| 18040 | P23 | P24 | 695 | 1159 | 14155 | 242,7 | 20,4 | 1,613 | H5A034 C | neg1 | Hm Gen E | Hm Gen E | Hm Gen E | CPE002JK | CPE002NZ | CPE002KF |
| 19097 | L24 | L1 | 762 | 1085 | 15587 | 160,8 | 20,5 | 1,429 | H5A034 C | neg1 | Hm Gen E | Hm Gen E | Hm Gen E | CPE002JN | CPE002O2 | CPE002KI |
| 23369 | N24 | N1 | 878 | 1105 | 18126 | 232,5 | 20,6 | 0,89 | H5A034 C | neg1 | Hm Gen E | Hm Gen E | Hm Gen E | CPE002JY | CPE002OD | CPE002KT |
| 13385 | N24 | N1 | 1494 | 1113 | 30810 | 106,5 | 20,6 | 1,033 | H5A034 C | neg1 | Hm Gen E | Hm Gen E | Hm Gen E | CPE002JB | CPE002NN | CPE002K3 |
| 19865 | L24 | L1 | 848 | 334,8 | 17506 | 358,9 | 20,6 | 1,342 | H5A034 C | neg1 | Hm Gen E | Hm Gen E | Hm Gen E | CPE002JP | CPE002O4 | CPE002KK |
| 17537 | K24 | K1 | 955 | 1017 | 19660 | 247,6 | 20,6 | 1,406 | H5A034 C | neg1 | Hm Gen E | Hm Gen E | Hm Gen E | CPE002JJ | CPE002NY | CPE002KE |
| 7625 | N24 | N1 | 1123 | 586,8 | 23105 | 582,7 | 20,6 | 1,573 | H5A034 C | neg1 | Hm Drgbl | Hm Drgbl | Hm Drgbl | CPE002E3 | CPE002EW | CPE002FH |
| 22216 | N23 | N24 | 738 | 1338 | 15303 | 374,3 | 20,7 | 0,983 | H5A034 C | neg1 | Hm Gen E | Hm Gen E | Hm Gen E | CPE002JV | CPE002OA | CPE002KQ |
| 6401 | K24 | K1 | 634 | 537 | 13145 | 511,4 | 20,7 | 1,366 | H5A034 C | neg1 | Hm Drgbl | Hm Drgbl | Hm Drgbl | CPE002DZ | CPE002F0 | CPE002FL |
| 19073 | K24 | K1 | 738 | 1085 | 15272 | 282,9 | 20,7 | 1,443 | H5A034 C | neg1 | Hm Gen E | Hm Gen E | Hm Gen E | CPE002JN | CPE002O2 | CPE002KI |
| 13337 | L24 | L1 | 922 | 1052 | 19194 | 105,3 | 20,8 | 1,043 | H5A034 C | neg1 | Hm Gen E | Hm Gen E | Hm Gen E | CPE002JB | CPE002NN | CPE002K3 |
| 23321 | L24 | L1 | 893 | 1076 | 18707 | 227,9 | 20,9 | 0,903 | H5A034 C | neg1 | Hm Gen E | Hm Gen E | Hm Gen E | CPE002JY | CPE002OD | CPE002KT |
| 22912 | K23 | K24 | 793 | 1678 | 16592 | 374,9 | 20,9 | 0,981 | H5A034 C | neg1 | Hm Gen E | Hm Gen E | Hm Gen E | CPE002JX | CPE002OC | CPE002KS |
| 22168 | L23 | L24 | 998 | 1357 | 20818 | 426,5 | 20,9 | 0,993 | H5A034 C | neg1 | Hm Gen E | Hm Gen E | Hm Gen E | CPE002JV | CPE002OA | CPE002KQ |
| 3329 | K24 | K1 | 816 | 1306 | 17131 | 528,8 | 21 | 1,3 | H5A034 C | neg1 | Hm Drgbl | Hm Drgbl | Hm Drgbl | CPE002DC | CPE002EG | CPE002F6 |
| 16889 | P24 | P1 | 585 | 1052 | 12273 | 310,5 | 21 | 1,66 | H5A034 C | neg1 | Hm Gen E | Hm Gen E | Hm Gen E | CPE002JH | CPE002NW | CPE002KC |
| 6833 | M24 | M1 | 476 | 583,1 | 9991 | 463,1 | 21 | 1,842 | H5A034 C | neg1 | Hm Drgbl | Hm Drgbl | Hm Drgbl | CPE002E1 | CPE002EY | CPE002FJ |
| 16120 | P23 | P24 | 1223 | 894,5 | 25834 | 255,1 | 21,1 | 1,175 | H5A034 C | neg1 | Hm Gen E | Hm Gen E | Hm Gen E | CPE002J9 | CPE002NL | CPE002KA |
| 22144 | K23 | K24 | 718 | 1355 | 15234 | 402,5 | 21,2 | 1,007 | H5A034 C | neg1 | Hm Gen E | Hm Gen E | Hm Gen E | CPE002JV | CPE002OA | CPE002KQ |
| 23704 | L23 | L24 | 819 | 1633 | 17333 | 333,5 | 21,2 | 1,125 | H5A034 C | neg1 | Hm Gen E | Hm Gen E | Hm Gen E | CPE002KV | CPE002OE | CPE002KU |
| 13408 | O23 | O24 | 1090 | 1141 | 23498 | 177,8 | 21,6 | 1,083 | H5A034 C | neg1 | Hm Gen E | Hm Gen E | Hm Gen E | CPE002JB | CPE002NN | CPE002K3 |
| 22984 | N23 | N24 | 639 | 1558 | 13868 | 309,4 | 21,7 | 1,019 | H5A034 C | neg1 | Hm Gen E | Hm Gen E | Hm Gen E | CPE002JX | CPE002OC | CPE002KS |
| 22169 | L24 | L1 | 704 | 1411 | 15397 | 282 | 21,9 | 1,04 | H5A034 C | neg1 | Hm Gen E | Hm Gen E | Hm Gen E | CPE002JV | CPE002OA | CPE002KQ |
| 23776 | O23 | O24 | 863 | 1516 | 18983 | 415,2 | 22 | 1,167 | H5A034 C | neg1 | Hm Gen E | Hm Gen E | Hm Gen E | CPE002KV | CPE002OE | CPE002KU |
| 16096 | O23 | O24 | 1122 | 846,9 | 24939 | 219,1 | 22,2 | 1,237 | H5A034 C | neg1 | Hm Gen E | Hm Gen E | Hm Gen E | CPE002J9 | CPE002NL | CPE002KA |
| 19960 | P23 | P24 | 831 | 434,2 | 18442 | 323,4 | 22,2 | 1,446 | H5A034 C | neg1 | Hm Gen E | Hm Gen E | Hm Gen E | CPE002JP | CPE002O4 | CPE002KK |
| 23320 | L23 | L24 | 921 | 1073 | 20897 | 294,2 | 22,7 | 0,981 | H5A034 C | neg1 | Hm Gen E | Hm Gen E | Hm Gen E | CPE002JY | CPE002OD | CPE002KT |
| 23393 | O24 | O1 | 712 | 1072 | 16267 | 328,6 | 22,8 | 0,985 | H5A034 C | neg1 | Hm Gen E | Hm Gen E | Hm Gen E | CPE002JY | CPE002OD | CPE002KT |
| 13312 | K23 | K24 | 742 | 1156 | 16915 | 127,1 | 22,8 | 1,143 | H5A034 C | neg1 | Hm Gen E | Hm Gen E | Hm Gen E | CPE002JB | CPE002NN | CPE002K3 |
| 22192 | M23 | M24 | 590 | 1419 | 13537 | 454,4 | 22,9 | 1,088 | H5A034 C | neg1 | Hm Gen E | Hm Gen E | Hm Gen E | CPE002JV | CPE002OA | CPE002KQ |
| 13313 | K24 | K1 | 861 | 1104 | 19700 | 101,1 | 22,9 | 1,148 | H5A034 C | neg1 | Hm Gen E | Hm Gen E | Hm Gen E | CPE002JB | CPE002NN | CPE002K3 |
| 22937 | L24 | L1 | 640 | 1612 | 14920 | 285,6 | 23,3 | 1,094 | H5A034 C | neg1 | Hm Gen E | Hm Gen E | Hm Gen E | CPE002JX | CPE002OC | CPE002KS |
| 23008 | O23 | O24 | 621 | 1614 | 14523 | 411,9 | 23,4 | 1,099 | H5A034 C | neg1 | Hm Gen E | Hm Gen E | Hm Gen E | CPE002JX | CPE002OC | CPE002KS |
| 23296 | K23 | K24 | 804 | 1133 | 18919 | 277,3 | 23,5 | 1,015 | H5A034 C | neg1 | Hm Gen E | Hm Gen E | Hm Gen E | CPE002JY | CPE002OD | CPE002KT |
| 22145 | K24 | K1 | 538 | 1366 | 12791 | 277,1 | 23,8 | 1,131 | H5A034 C | neg1 | Hm Gen E | Hm Gen E | Hm Gen E | CPE002JV | CPE002OA | CPE002KQ |
| 13432 | P23 | P24 | 1072 | 1113 | 25839 | 192,8 | 24,1 | 1,208 | H5A034 C | neg1 | Hm Gen E | Hm Gen E | Hm Gen E | CPE002JB | CPE002NN | CPE002K3 |
| 23297 | K24 | K1 | 846 | 1121 | 20454 | 286,1 | 24,2 | 1,045 | H5A034 C | neg1 | Hm Gen E | Hm Gen E | Hm Gen E | CPE002JY | CPE002OD | CPE002KT |
| 23345 | M24 | M1 | 613 | 1100 | 14877 | 256,3 | 24,3 | 1,05 | H5A034 C | neg1 | Hm Gen E | Hm Gen E | Hm Gen E | CPE002JY | CPE002OD | CPE002KT |
| 23392 | O23 | O24 | 918 | 1069 | 22431 | 389,2 | 24,4 | 1,054 | H5A034 C | neg1 | Hm Gen E | Hm Gen E | Hm Gen E | CPE002JY | CPE002OD | CPE002KT |
| 23680 | K23 | K24 | 676 | 1562 | 16540 | 348,2 | 24,5 | 1,3 | H5A034 C | neg1 | Hm Gen E | Hm Gen E | Hm Gen E | CPE002KV | CPE002OE | CPE002KU |
| 18808 | P23 | P24 | 643 | 1062 | 15774 | 367,6 | 24,5 | 1,661 | H5A034 C | neg1 | Hm Gen E | Hm Gen E | Hm Gen E | CPE002JM | CPE002O1 | CPE002KH |
| 23033 | P24 | P1 | 347 | 1893 | 8519 | 266 | 24,6 | 1,155 | H5A034 C | neg1 | Hm Gen E | Hm Gen E | Hm Gen E | CPE002JX | CPE002OC | CPE002KS |
| 21112 | P23 | P24 | 446 | 1251 | 11025 | 294,3 | 24,7 | 1,752 | H5A034 C | neg1 | Hm Gen E | Hm Gen E | Hm Gen E | CPE002JS | CPE002O7 | CPE002KN |
| 6785 | K24 | K1 | 365 | 592,7 | 9009 | 310,4 | 24,7 | 2,167 | H5A034 C | neg1 | Hm Drgbl | Hm Drgbl | Hm Drgbl | CPE002E1 | CPE002EY | CPE002FJ |
| 22528 | K23 | K24 | 534 | 1363 | 13413 | 480,1 | 25,1 | 1,294 | H5A034 C | neg1 | Hm Gen E | Hm Gen E | Hm Gen E | CPE002JW | CPE002OB | CPE002KR |
| 17656 | P23 | P24 | 503 | 1108 | 13077 | 196,1 | 26 | 1,775 | H5A034 C | neg1 | Hm Gen E | Hm Gen E | Hm Gen E | CPE002JJ | CPE002NY | CPE002KE |
| 22936 | L23 | L24 | 597 | 1661 | 15577 | 405 | 26,1 | 1,225 | H5A034 C | neg1 | Hm Gen E | Hm Gen E | Hm Gen E | CPE002JX | CPE002OC | CPE002KS |
| 22649 | P24 | P1 | 243 | 1286 | 6432 | 343,7 | 26,5 | 1,366 | H5A034 C | neg1 | Hm Gen E | Hm Gen E | Hm Gen E | CPE002JW | CPE002OB | CPE002KR |
| 22552 | L23 | L24 | 461 | 1400 | 12630 | 497 | 27,4 | 1,412 | H5A034 C | neg1 | Hm Gen E | Hm Gen E | Hm Gen E | CPE002JW | CPE002OB | CPE002KR |
| 18424 | P23 | P24 | 692 | 1134 | 19905 | 365,1 | 28,8 | 1,852 | H5A034 C | neg1 | Hm Gen E | Hm Gen E | Hm Gen E | CPE002JL | CPE002O0 | CPE002KG |
| 20729 | P24 | P1 | 114 | 773,6 | 3305 | 134,1 | 29 | 1,933 | H5A034 C | neg1 | Hm Gen E | Hm Gen E | Hm Gen E | CPE002JR | CPE002O6 | CPE002KM |
| 19192 | P23 | P24 | 398 | 1232 | 12124 | 272 | 30,5 | 2,125 | H5A034 C | neg1 | Hm Gen E | Hm Gen E | Hm Gen E | CPE002JN | CPE002O2 | CPE002KI |
| 20728 | P23 | P24 | 87 | 867,1 | 3150 | 177,3 | 36,2 | 2,413 | H5A034 C | neg1 | Hm Gen E | Hm Gen E | Hm Gen E | CPE002JR | CPE002O6 | CPE002KM |
| 22265 | P24 | P1 | 196 | 1317 | 7534 | 271,1 | 38,4 | 1,824 | H5A034 C | neg1 | Hm Gen E | Hm Gen E | Hm Gen E | CPE002JV | CPE002OA | CPE002KQ |
| 22648 | P23 | P24 | 169 | 1403 | 6504 | 491,3 | 38,5 | 1,985 | H5A034 C | neg1 | Hm Gen E | Hm Gen E | Hm Gen E | CPE002JW | CPE002OB | CPE002KR |
| 23417 | P24 | P1 | 246 | 1000 | 10320 | 266,3 | 42 | 1,814 | H5A034 C | neg1 | Hm Gen E | Hm Gen E | Hm Gen E | CPE002JY | CPE002OD | CPE002KT |
| 23032 | P23 | P24 | 275 | 1915 | 12899 | 249,8 | 46,9 | 2,202 | H5A034 C | neg1 | Hm Gen E | Hm Gen E | Hm Gen E | CPE002JX | CPE002OC | CPE002KS |
| 23416 | P23 | P24 | 130 | 1036 | 6945 | 204,8 | 53,4 | 2,307 | H5A034 C | neg1 | Hm Gen E | Hm Gen E | Hm Gen E | CPE002JY | CPE002OD | CPE002KT |
| 22264 | P23 | P24 | 56 | 1573 | 3383 | 276,7 | 60,4 | 2,869 | H5A034 C | neg1 | Hm Gen E | Hm Gen E | Hm Gen E | CPE002JV | CPE002OA | CPE002KQ |
| 64 | C23 | C24 | 2 | 933,5 | 0 | 0 | 0 | 0 | H5A034 C | pos1 | Hm Drgbl | Hm Drgbl | Hm Drgbl | CPE002DT | CPE002EJ | CPE002F9 |
| 89 | D24 | D1 | 1 | 739,1 | 0 | 0 | 0 | 0 | H5A034 C | pos1 | Hm Drgbl | Hm Drgbl | Hm Drgbl | CPE002DT | CPE002EJ | CPE002F9 |
| 905 | F24 | F1 | 1 | 430,1 | 0 | 0 | 0 | 0 | H5A034 C | pos1 | Hm Drgbl | Hm Drgbl | Hm Drgbl | CPE002FU | CPE002FY | CPE002G2 |
| 1265 | E24 | E1 | 1 | 542,5 | 0 | 0 | 0 | 0 | H5A034 C | pos1 | Hm Drgbl | Hm Drgbl | Hm Drgbl | CPE002FV | CPE002FZ | CPE002G3 |
| 1288 | F23 | F24 | 1 | 477 | 0 | 0 | 0 | 0 | H5A034 C | pos1 | Hm Drgbl | Hm Drgbl | Hm Drgbl | CPE002FV | CPE002FZ | CPE002G3 |
| 1600 | C23 | C24 | 9 | 550,1 | 0 | 0 | 0 | 0 | H5A034 C | pos1 | Hm Drgbl | Hm Drgbl | Hm Drgbl | CPE002FW | CPE002G0 | CPE002G4 |
| 2416 | E23 | E24 | 3 | 415,8 | 0 | 0 | 0 | 0 | H5A034 C | pos1 | Hm Drgbl | Hm Drgbl | Hm Drgbl | CPE002DC | CPE002EE | CPE002F4 |
| 3977 | F24 | F1 | 2 | 508,7 | 0 | 0 | 0 | 0 | H5A034 C | pos1 | Hm Drgbl | Hm Drgbl | Hm Drgbl | CPE002DS | CPE002EI | CPE002F8 |
| 4288 | C23 | C24 | 2 | 440,5 | 0 | 0 | 0 | 0 | H5A034 C | pos1 | Hm Drgbl | Hm Drgbl | Hm Drgbl | CPE002DL | CPE002EK | CPE002FQ |
| 4337 | E24 | E1 | 4 | 477,1 | 0 | 0 | 0 | 0 | H5A034 C | pos1 | Hm Drgbl | Hm Drgbl | Hm Drgbl | CPE002DL | CPE002EK | CPE002FQ |
| 5057 | C24 | C1 | 1 | 371,1 | 0 | 0 | 0 | 0 | H5A034 C | pos1 | Hm Drgbl | Hm Drgbl | Hm Drgbl | CPE002DV | CPE002EM | CPE002FO |
| 5441 | C24 | C1 | 10 | 434,6 | 0 | 0 | 0 | 0 | H5A034 C | pos1 | Hm Drgbl | Hm Drgbl | Hm Drgbl | CPE002DX | CPE002F2 | CPE002FN |
| 5513 | F24 | F1 | 3 | 480,2 | 0 | 0 | 0 | 0 | H5A034 C | pos1 | Hm Drgbl | Hm Drgbl | Hm Drgbl | CPE002DX | CPE002F2 | CPE002FN |
| 9737 | F24 | F1 | 3 | 638,6 | 0 | 0 | 0 | 0 | H5A034 C | pos1 | Hm Drgbl | Hm Drgbl | Hm Drgbl | CPE002E9 | CPE002EQ | CPE002FB |
| 10049 | C24 | C1 | 6 | 632,5 | 0 | 0 | 0 | 0 | H5A034 C | pos1 | Hm Drgbl | Hm Drgbl | Hm Drgbl | CPE002EA | CPE002EP | CPE002FA |
| 10817 | C24 | C1 | 1 | 523,6 | 0 | 0 | 0 | 0 | H5A034 C | pos1 | Hm Drgbl | Hm Drgbl | Hm Drgbl | CPE002EC | CPE002EN | CPE002FS |
| 11176 | B23 | B24 | 1 | 635,6 | 0 | 0 | 0 | 0 | H5A034 C | pos1 | Hm Drgbl | Hm Drgbl | Hm Drgbl | CPE002ED | CPE002F3 | CPE002FT |
| 11608 | D23 | D24 | 1 | 641,6 | 0 | 0 | 0 | 0 | H5A034 C | pos1 | Hm Gen E | Hm Gen E | Hm Gen E | CPE002JA | CPE002NJ | CPE002JZ |
| 12304 | A23 | A24 | 2 | 514,7 | 0 | 0 | 0 | 0 | H5A034 C | pos1 | Hm Gen E | Hm Gen E | Hm Gen E | CPE002J5 | CPE002NK | CPE002K1 |
| 14272 | C23 | C24 | 1 | 566,7 | 0 | 0 | 0 | 0 | H5A034 C | pos1 | Hm Gen E | Hm Gen E | Hm Gen E | CPE002J7 | CPE002NC | CPE002K6 |
| 18905 | D24 | D1 | 1 | 434,9 | 0 | 0 | 0 | 0 | H5A034 C | pos1 | Hm Gen E | Hm Gen E | Hm Gen E | CPE002JN | CPE002O2 | CPE002KI |
| 20776 | B23 | B24 | 1 | 669,4 | 0 | 0 | 0 | 0 | H5A034 C | pos1 | Hm Gen E | Hm Gen E | Hm Gen E | CPE002JS | CPE002O7 | CPE002KN |
| 7696 | A23 | A24 | 4 | 1848 | 1 | 194,1 | 0,2 | 0,031 | H5A034 C | pos1 | Hm Drgbl | Hm Drgbl | Hm Drgbl | CPE002E4 | CPE002EV | CPE002FG |
| 1553 | A24 | A1 | 3 | 758,6 | 1 | 207,5 | 0,3 | 0,031 | H5A034 C | pos1 | Hm Drgbl | Hm Drgbl | Hm Drgbl | CPE002FW | CPE002G0 | CPE002G4 |
| 20417 | C24 | C1 | 2 | 292,2 | 1 | 35,5 | 0,5 | 0,033 | H5A034 C | pos1 | Hm Gen E | Hm Gen E | Hm Gen E | CPE002JR | CPE002O6 | CPE002KM |
| 17393 | E24 | E1 | 2 | 743,1 | 1 | 70,54 | 0,5 | 0,034 | H5A034 C | pos1 | Hm Gen E | Hm Gen E | Hm Gen E | CPE002JJ | CPE002NY | CPE002KE |
| 16985 | D24 | D1 | 3 | 588,8 | 2 | 17,45 | 0,7 | 0,054 | H5A034 C | pos1 | Hm Gen E | Hm Gen E | Hm Gen E | CPE002JI | CPE002NZ | CPE002KD |
| 17008 | E23 | E24 | 1 | 580,4 | 1 | 60,5 | 1 | 0,077 | H5A034 C | pos1 | Hm Gen E | Hm Gen E | Hm Gen E | CPE002JI | CPE002NZ | CPE002KD |
| 3593 | F24 | F1 | 1 | 1020 | 1 | 133,3 | 1 | 0,096 | H5A034 C | pos1 | Hm Drgbl | Hm Drgbl | Hm Drgbl | CPE002DR | CPE002EH | CPE002F7 |
| 1577 | B24 | B1 | 3 | 993,9 | 5 | 346,4 | 1,7 | 0,178 | H5A034 C | pos1 | Hm Drgbl | Hm Drgbl | Hm Drgbl | CPE002FW | CPE002G0 | CPE002G4 |
| 2728 | B23 | B24 | 1 | 593,5 | 2 | 262 | 2 | 0,235 | H5A034 C | pos1 | Hm Drgbl | Hm Drgbl | Hm Drgbl | CPE002DP | CPE002EF | CPE002F5 |
| 1960 | B23 | B24 | 5 | 675,6 | 12 | 476,1 | 2,4 | 0,209 | H5A034 C | pos1 | Hm Drgbl | Hm Drgbl | Hm Drgbl | CPE002FX | CPE002G1 | CPE002G5 |
| 3088 | A23 | A24 | 3 | 1031 | 8 | 382,7 | 2,7 | 0,167 | H5A034 C | pos1 | Hm Drgbl | Hm Drgbl | Hm Drgbl | CPE002DC | CPE002EG | CPE002F6 |
| 3569 | E24 | E1 | 23 | 1239 | 66 | 240,2 | 2,9 | 0,279 | H5A034 C | pos1 | Hm Drgbl | Hm Drgbl | Hm Drgbl | CPE002DR | CPE002EH | CPE002F7 |
| 3113 | B24 | B1 | 2 | 1889 | 6 | 337,5 | 3 | 0,186 | H5A034 C | pos1 | Hm Drgbl | Hm Drgbl | Hm Drgbl | CPE002DC | CPE002EG | CPE002F6 |
| 2729 | B24 | B1 | 11 | 628,7 | 33 | 886,8 | 3 | 0,353 | H5A034 C | pos1 | Hm Drgbl | Hm Drgbl | Hm Drgbl | CPE002DP | CPE002EF | CPE002F5 |
| 1168 | A23 | A24 | 5 | 746,6 | 16 | 183,9 | 3,2 | 0,294 | H5A034 C | pos1 | Hm Drgbl | Hm Drgbl | Hm Drgbl | CPE002FV | CPE002FZ | CPE002G3 |
| 5897 | F24 | F1 | 6 | 666,6 | 20 | 359,6 | 3,3 | 0,301 | H5A034 C | pos1 | Hm Drgbl | Hm Drgbl | Hm Drgbl | CPE002DY | CPE002F1 | CPE002FM |
| 17 | A24 | A1 | 9 | 904,9 | 31 | 198,1 | 3,4 | 0,289 | H5A034 C | pos1 | Hm Drgbl | Hm Drgbl | Hm Drgbl | CPE002DT | CPE002EJ | CPE002F9 |
| 497 | E24 | E1 | 110 | 2006 | 390 | 249,3 | 3,5 | 0,511 | H5A034 C | pos1 | Hm Drgbl | Hm Drgbl | Hm Drgbl | CPE002E0 | CPE002EZ | CPE002FK |
| 7745 | C24 | C1 | 27 | 510 | 109 | 286 | 4 | 0,611 | H5A034 C | pos1 | Hm Drgbl | Hm Drgbl | Hm Drgbl | CPE002E4 | CPE002EV | CPE002FG |
| 4361 | F24 | F1 | 10 | 866 | 41 | 341,6 | 4,1 | 0,346 | H5A034 C | pos1 | Hm Drgbl | Hm Drgbl | Hm Drgbl | CPE002DL | CPE002EK | CPE002FQ |
| 2344 | B23 | B24 | 10 | 623,1 | 43 | 316 | 4,3 | 0,339 | H5A034 C | pos1 | Hm Drgbl | Hm Drgbl | Hm Drgbl | CPE002DC | CPE002EE | CPE002F4 |
| 21617 | E24 | E1 | 29 | 1121 | 129 | 468,8 | 4,4 | 0,463 | H5A034 C | pos1 | Hm Gen E | Hm Gen E | Hm Gen E | CPE002JU | CPE002O9 | CPE002KP |
| 1169 | A24 | A1 | 9 | 576,2 | 43 | 321 | 4,8 | 0,44 | H5A034 C | pos1 | Hm Drgbl | Hm Drgbl | Hm Drgbl | CPE002FV | CPE002FZ | CPE002G3 |
| 14705 | E24 | E1 | 3 | 764,8 | 15 | 96,03 | 5 | 0,285 | H5A034 C | pos1 | Hm Gen E | Hm Gen E | Hm Gen E | CPE002JD | CPE002NR | CPE002K7 |
| 2321 | A24 | A1 | 132 | 782,5 | 660 | 464,3 | 5 | 0,394 | H5A034 C | pos1 | Hm Drgbl | Hm Drgbl | Hm Drgbl | CPE002DC | CPE002EE | CPE002F4 |
| 1984 | C23 | C24 | 2 | 620,4 | 10 | 161,4 | 5 | 0,435 | H5A034 C | pos1 | Hm Drgbl | Hm Drgbl | Hm Drgbl | CPE002FX | CPE002G1 | CPE002G5 |
| 784 | A23 | A24 | 2 | 564 | 10 | 290,2 | 5 | 0,474 | H5A034 C | pos1 | Hm Drgbl | Hm Drgbl | Hm Drgbl | CPE002FU | CPE002FY | CPE002G2 |
| 2752 | C23 | C24 | 1 | 644,2 | 5 | 1169 | 5 | 0,588 | H5A034 C | pos1 | Hm Drgbl | Hm Drgbl | Hm Drgbl | CPE002DP | CPE002EF | CPE002F5 |
| 7793 | E24 | E1 | 1 | 663,9 | 5 | 249,5 | 5 | 0,763 | H5A034 C | pos1 | Hm Drgbl | Hm Drgbl | Hm Drgbl | CPE002E4 | CPE002EV | CPE002FG |
| 18472 | B23 | B24 | 15 | 743,2 | 76 | 302,3 | 5,1 | 0,346 | H5A034 C | pos1 | Hm Gen E | Hm Gen E | Hm Gen E | CPE002JM | CPE002O1 | CPE002KH |
| 3904 | C23 | C24 | 59 | 589 | 304 | 320,4 | 5,2 | 0,462 | H5A034 C | pos1 | Hm Drgbl | Hm Drgbl | Hm Drgbl | CPE002DS | CPE002EI | CPE002F8 |
| 16961 | C24 | C1 | 9 | 874,6 | 49 | 200,6 | 5,4 | 0,417 | H5A034 C | pos1 | Hm Gen E | Hm Gen E | Hm Gen E | CPE002JI | CPE002NZ | CPE002KD |
| 16265 | F24 | F1 | 53 | 924,9 | 285 | 399,6 | 5,4 | 0,495 | H5A034 C | pos1 | Hm Gen E | Hm Gen E | Hm Gen E | CPE002JG | CPE002NV | CPE002KB |
| 7816 | F23 | F24 | 94 | 548,9 | 558 | 452,9 | 5,9 | 0,901 | H5A034 C | pos1 | Hm Drgbl | Hm Drgbl | Hm Drgbl | CPE002E4 | CPE002EV | CPE002FG |
| 3185 | E24 | E1 | 9 | 1552 | 54 | 503,2 | 6 | 0,372 | H5A034 C | pos1 | Hm Drgbl | Hm Drgbl | Hm Drgbl | CPE002DC | CPE002EG | CPE002F6 |
| 7025 | E24 | E1 | 3 | 722,3 | 18 | 393,2 | 6 | 0,467 | H5A034 C | pos1 | Hm Drgbl | Hm Drgbl | Hm Drgbl | CPE002E2 | CPE002EX | CPE002FI |
| 1217 | C24 | C1 | 1 | 534 | 6 | 775,2 | 6 | 0,55 | H5A034 C | pos1 | Hm Drgbl | Hm Drgbl | Hm Drgbl | CPE002FV | CPE002FZ | CPE002G3 |
| 7792 | E23 | E24 | 51 | 569,9 | 306 | 445,8 | 6 | 0,916 | H5A034 C | pos1 | Hm Drgbl | Hm Drgbl | Hm Drgbl | CPE002E4 | CPE002EV | CPE002FG |
| 1241 | D24 | D1 | 12 | 651,6 | 74 | 431,2 | 6,2 | 0,569 | H5A034 C | pos1 | Hm Drgbl | Hm Drgbl | Hm Drgbl | CPE002FV | CPE002FZ | CPE002G3 |
| 2776 | D23 | D24 | 5 | 1045 | 32 | 734,7 | 6,4 | 0,753 | H5A034 C | pos1 | Hm Drgbl | Hm Drgbl | Hm Drgbl | CPE002DP | CPE002EF | CPE002F5 |
| 3136 | C23 | C24 | 32 | 1292 | 214 | 381,8 | 6,7 | 0,415 | H5A034 C | pos1 | Hm Drgbl | Hm Drgbl | Hm Drgbl | CPE002DC | CPE002EG | CPE002F6 |
| 5105 | E24 | E1 | 4 | 666,5 | 27 | 670,1 | 6,8 | 0,533 | H5A034 C | pos1 | Hm Drgbl | Hm Drgbl | Hm Drgbl | CPE002DV | CPE002EM | CPE002FO |
| 20081 | E24 | E1 | 19 | 1080 | 132 | 262,3 | 6,9 | 0,841 | H5A034 C | pos1 | Hm Gen E | Hm Gen E | Hm Gen E | CPE002JQ | CPE002O5 | CPE002KL |
| 785 | A24 | A1 | 3 | 782 | 21 | 430,4 | 7 | 0,664 | H5A034 C | pos1 | Hm Drgbl | Hm Drgbl | Hm Drgbl | CPE002FU | CPE002FY | CPE002G2 |
| 521 | F24 | F1 | 3040 | 2065 | 21303 | 385,4 | 7 | 1,022 | H5A034 C | pos1 | Hm Drgbl | Hm Drgbl | Hm Drgbl | CPE002E0 | CPE002EZ | CPE002FK |
| 520 | F23 | F24 | 3321 | 2111 | 23636 | 375,5 | 7,1 | 1,036 | H5A034 C | pos1 | Hm Drgbl | Hm Drgbl | Hm Drgbl | CPE002E0 | CPE002EZ | CPE002FK |
| 449 | C24 | C1 | 15 | 1786 | 108 | 401,9 | 7,2 | 1,051 | H5A034 C | pos1 | Hm Drgbl | Hm Drgbl | Hm Drgbl | CPE002E0 | CPE002EZ | CPE002FK |
| 425 | B24 | B1 | 87 | 1957 | 635 | 573,6 | 7,3 | 1,066 | H5A034 C | pos1 | Hm Drgbl | Hm Drgbl | Hm Drgbl | CPE002E0 | CPE002EZ | CPE002FK |
| 4697 | D24 | D1 | 11 | 698,7 | 81 | 427,5 | 7,4 | 0,813 | H5A034 C | pos1 | Hm Drgbl | Hm Drgbl | Hm Drgbl | CPE002DV | CPE002EL | CPE002FP |
| 6209 | C24 | C1 | 6 | 672,2 | 45 | 457,6 | 7,5 | 0,495 | H5A034 C | pos1 | Hm Drgbl | Hm Drgbl | Hm Drgbl | CPE002DZ | CPE002F0 | CPE002FL |
| 3473 | A24 | A1 | 2 | 1357 | 15 | 374,2 | 7,5 | 0,721 | H5A034 C | pos1 | Hm Drgbl | Hm Drgbl | Hm Drgbl | CPE002DR | CPE002EH | CPE002F7 |
| 5129 | F24 | F1 | 15 | 862,7 | 116 | 473,4 | 7,7 | 0,604 | H5A034 C | pos1 | Hm Drgbl | Hm Drgbl | Hm Drgbl | CPE002DV | CPE002EM | CPE002FO |
| 10433 | C24 | C1 | 12 | 959,8 | 96 | 221,9 | 8 | 0,904 | H5A034 C | pos1 | Hm Drgbl | Hm Drgbl | Hm Drgbl | CPE002EB | CPE002EO | CPE002FR |
| 2705 | A24 | A1 | 2 | 984,2 | 16 | 722,8 | 8 | 0,941 | H5A034 C | pos1 | Hm Drgbl | Hm Drgbl | Hm Drgbl | CPE002DP | CPE002EF | CPE002F5 |
| 5080 | D23 | D24 | 16 | 777,1 | 129 | 652,1 | 8,1 | 0,635 | H5A034 C | pos1 | Hm Drgbl | Hm Drgbl | Hm Drgbl | CPE002DV | CPE002EM | CPE002FO |
| 472 | D23 | D24 | 156 | 2041 | 1275 | 681,4 | 8,2 | 1,197 | H5A034 C | pos1 | Hm Drgbl | Hm Drgbl | Hm Drgbl | CPE002E0 | CPE002EZ | CPE002FK |
| 4313 | D24 | D1 | 3 | 1241 | 25 | 601,4 | 8,3 | 0,7 | H5A034 C | pos1 | Hm Drgbl | Hm Drgbl | Hm Drgbl | CPE002DL | CPE002EK | CPE002FQ |
| 4312 | D23 | D24 | 5 | 894,5 | 42 | 319,3 | 8,4 | 0,709 | H5A034 C | pos1 | Hm Drgbl | Hm Drgbl | Hm Drgbl | CPE002DL | CPE002EK | CPE002FQ |
| 1552 | A23 | A24 | 10 | 1376 | 84 | 612,3 | 8,4 | 0,88 | H5A034 C | pos1 | Hm Drgbl | Hm Drgbl | Hm Drgbl | CPE002FW | CPE002G0 | CPE002G4 |
| 3976 | F23 | F24 | 26 | 773,4 | 221 | 508,4 | 8,5 | 0,756 | H5A034 C | pos1 | Hm Drgbl | Hm Drgbl | Hm Drgbl | CPE002DS | CPE002EI | CPE002F8 |
| 1264 | E23 | E24 | 2 | 453,7 | 17 | 472,1 | 8,5 | 0,78 | H5A034 C | pos1 | Hm Drgbl | Hm Drgbl | Hm Drgbl | CPE002FV | CPE002FZ | CPE002G3 |
| 20752 | A23 | A24 | 18 | 837,7 | 155 | 418,2 | 8,6 | 0,61 | H5A034 C | pos1 | Hm Gen E | Hm Gen E | Hm Gen E | CPE002JS | CPE002O7 | CPE002KN |
| 15856 | E23 | E24 | 3 | 623,9 | 27 | 95,26 | 9 | 0,501 | H5A034 C | pos1 | Hm Gen E | Hm Gen E | Hm Gen E | CPE002J9 | CPE002NL | CPE002KA |
| 2032 | E23 | E24 | 43 | 614 | 385 | 323,4 | 9 | 0,783 | H5A034 C | pos1 | Hm Drgbl | Hm Drgbl | Hm Drgbl | CPE002FX | CPE002G1 | CPE002G5 |
| 4745 | F24 | F1 | 13 | 987,6 | 117 | 603,4 | 9 | 0,989 | H5A034 C | pos1 | Hm Drgbl | Hm Drgbl | Hm Drgbl | CPE002DV | CPE002EL | CPE002FP |
| 18449 | A24 | A1 | 19 | 851,8 | 175 | 283,6 | 9,2 | 0,624 | H5A034 C | pos1 | Hm Gen E | Hm Gen E | Hm Gen E | CPE002JM | CPE002O1 | CPE002KH |
| 880 | E23 | E24 | 8 | 645 | 74 | 366,4 | 9,2 | 0,872 | H5A034 C | pos1 | Hm Drgbl | Hm Drgbl | Hm Drgbl | CPE002FU | CPE002FY | CPE002G2 |
| 2441 | F24 | F1 | 2 | 1673 | 19 | 437,4 | 9,5 | 0,748 | H5A034 C | pos1 | Hm Drgbl | Hm Drgbl | Hm Drgbl | CPE002DC | CPE002EE | CPE002F4 |
| 112 | E23 | E24 | 2 | 1593 | 19 | 579,6 | 9,5 | 0,809 | H5A034 C | pos1 | Hm Drgbl | Hm Drgbl | Hm Drgbl | CPE002DT | CPE002EJ | CPE002F9 |
| 4240 | A23 | A24 | 5 | 1086 | 48 | 610,1 | 9,6 | 0,81 | H5A034 C | pos1 | Hm Drgbl | Hm Drgbl | Hm Drgbl | CPE002DL | CPE002EK | CPE002FQ |
| 17801 | F24 | F1 | 33 | 909,5 | 319 | 233,8 | 9,7 | 0,767 | H5A034 C | pos1 | Hm Gen E | Hm Gen E | Hm Gen E | CPE002JK | CPE002NZ | CPE002KF |
| 7697 | A24 | A1 | 40 | 739,3 | 389 | 581,8 | 9,7 | 1,481 | H5A034 C | pos1 | Hm Drgbl | Hm Drgbl | Hm Drgbl | CPE002E4 | CPE002EV | CPE002FG |
| 3496 | B23 | B24 | 15 | 1055 | 147 | 333,5 | 9,8 | 0,942 | H5A034 C | pos1 | Hm Drgbl | Hm Drgbl | Hm Drgbl | CPE002DR | CPE002EH | CPE002F7 |
| 881 | E24 | E1 | 5 | 956,5 | 50 | 407,9 | 10 | 0,948 | H5A034 C | pos1 | Hm Drgbl | Hm Drgbl | Hm Drgbl | CPE002FU | CPE002FY | CPE002G2 |
| 5800 | B23 | B24 | 57 | 989,8 | 576 | 592,4 | 10,1 | 0,922 | H5A034 C | pos1 | Hm Drgbl | Hm Drgbl | Hm Drgbl | CPE002DY | CPE002F1 | CPE002FM |
| 5849 | D24 | D1 | 12 | 944,4 | 121 | 513,9 | 10,1 | 0,922 | H5A034 C | pos1 | Hm Drgbl | Hm Drgbl | Hm Drgbl | CPE002DY | CPE002F1 | CPE002FM |
| 11969 | C24 | C1 | 4 | 741 | 41 | 99,49 | 10,2 | 0,654 | H5A034 C | pos1 | Hm Gen E | Hm Gen E | Hm Gen E | CPE002J4 | CPE002NL | CPE002K0 |
| 809 | B24 | B1 | 13 | 706,1 | 133 | 406,5 | 10,2 | 0,967 | H5A034 C | pos1 | Hm Drgbl | Hm Drgbl | Hm Drgbl | CPE002FU | CPE002FY | CPE002G2 |
| 4241 | A24 | A1 | 17 | 729,1 | 175 | 570,1 | 10,3 | 0,869 | H5A034 C | pos1 | Hm Drgbl | Hm Drgbl | Hm Drgbl | CPE002DL | CPE002EK | CPE002FQ |
| 3520 | C23 | C24 | 34 | 1710 | 349 | 508,6 | 10,3 | 0,99 | H5A034 C | pos1 | Hm Drgbl | Hm Drgbl | Hm Drgbl | CPE002DR | CPE002EH | CPE002F7 |
| 20057 | D24 | D1 | 28 | 1001 | 293 | 205,4 | 10,5 | 1,28 | H5A034 C | pos1 | Hm Gen E | Hm Gen E | Hm Gen E | CPE002JQ | CPE002O5 | CPE002KL |
| 10792 | B23 | B24 | 5 | 810 | 53 | 1593 | 10,6 | 0,803 | H5A034 C | pos1 | Hm Drgbl | Hm Drgbl | Hm Drgbl | CPE002EC | CPE002EN | CPE002FS |
| 7433 | F24 | F1 | 9 | 677 | 95 | 498,6 | 10,6 | 0,809 | H5A034 C | pos1 | Hm Drgbl | Hm Drgbl | Hm Drgbl | CPE002E3 | CPE002EW | CPE002FH |
| 5009 | A24 | A1 | 12 | 549 | 127 | 597,9 | 10,6 | 0,831 | H5A034 C | pos1 | Hm Drgbl | Hm Drgbl | Hm Drgbl | CPE002DV | CPE002EM | CPE002FO |
| 6640 | E23 | E24 | 20 | 677,5 | 212 | 534,3 | 10,6 | 0,93 | H5A034 C | pos1 | Hm Drgbl | Hm Drgbl | Hm Drgbl | CPE002E1 | CPE002EY | CPE002FJ |
| 136 | F23 | F24 | 687 | 1662 | 7352 | 728,6 | 10,7 | 0,911 | H5A034 C | pos1 | Hm Drgbl | Hm Drgbl | Hm Drgbl | CPE002DT | CPE002EJ | CPE002F9 |
| 5465 | D24 | D1 | 15 | 871,1 | 161 | 607,2 | 10,7 | 1,019 | H5A034 C | pos1 | Hm Drgbl | Hm Drgbl | Hm Drgbl | CPE002DX | CPE002F2 | CPE002FN |
| 4648 | B23 | B24 | 18 | 1365 | 192 | 330,8 | 10,7 | 1,176 | H5A034 C | pos1 | Hm Drgbl | Hm Drgbl | Hm Drgbl | CPE002DV | CPE002EL | CPE002FP |
| 10384 | A23 | A24 | 3 | 1280 | 32 | 312,5 | 10,7 | 1,209 | H5A034 C | pos1 | Hm Drgbl | Hm Drgbl | Hm Drgbl | CPE002EB | CPE002EO | CPE002FR |
| 2345 | B24 | B1 | 2 | 1154 | 22 | 544,1 | 11 | 0,866 | H5A034 C | pos1 | Hm Drgbl | Hm Drgbl | Hm Drgbl | CPE002DC | CPE002EE | CPE002F4 |
| 2033 | E24 | E1 | 4 | 1032 | 44 | 567,1 | 11 | 0,957 | H5A034 C | pos1 | Hm Drgbl | Hm Drgbl | Hm Drgbl | CPE002FX | CPE002G1 | CPE002G5 |
| 137 | F24 | F1 | 726 | 1648 | 8053 | 668 | 11,1 | 0,945 | H5A034 C | pos1 | Hm Drgbl | Hm Drgbl | Hm Drgbl | CPE002DT | CPE002EJ | CPE002F9 |
| 2824 | F23 | F24 | 11 | 715,3 | 122 | 659,5 | 11,1 | 1,306 | H5A034 C | pos1 | Hm Drgbl | Hm Drgbl | Hm Drgbl | CPE002DP | CPE002EF | CPE002F5 |

| | | | | | | | | | | | | | | | | | |
|---|---|---|---|---|---|---|---|---|---|---|---|---|---|---|---|---|---|
| 496 | E23 | E24 | 96 | 1639 | 1066 | 590,2 | 11,1 | 1,62 | H5A034 | C | pos1 | Hm Drgbl | Hm Drgbl | Hm Drgbl | CPE002E0 | CPE002EZ | CPE002FK |
| 21160 | B23 | B24 | 10 | 870 | 112 | 310,6 | 11,2 | 0,794 | H5A034 | C | pos1 | Hm Gen E | Hm Gen E | Hm Gen E | CPE002JT | CPE002O8 | CPE002KO |
| 8129 | C24 | C1 | 6 | 700,6 | 68 | 591,5 | 11,3 | 1,087 | H5A034 | C | pos1 | Hm Drgbl | Hm Drgbl | Hm Drgbl | CPE002E5 | CPE002EU | CPE002FF |
| 448 | C23 | C24 | 15 | 1779 | 172 | 561,7 | 11,5 | 1,679 | H5A034 | C | pos1 | Hm Drgbl | Hm Drgbl | Hm Drgbl | CPE002E0 | CPE002EZ | CPE002FK |
| 3905 | C24 | C1 | 5 | 600,1 | 59 | 314,7 | 11,8 | 1,049 | H5A034 | C | pos1 | Hm Drgbl | Hm Drgbl | Hm Drgbl | CPE002DS | CPE002EI | CPE002F8 |
| 473 | D24 | D1 | 26 | 2029 | 308 | 619 | 11,8 | 1,723 | H5A034 | C | pos1 | Hm Drgbl | Hm Drgbl | Hm Drgbl | CPE002E0 | CPE002EZ | CPE002FK |
| 7817 | F24 | F1 | 19 | 571,2 | 227 | 301,5 | 11,9 | 1,817 | H5A034 | C | pos1 | Hm Drgbl | Hm Drgbl | Hm Drgbl | CPE002E4 | CPE002EV | CPE002FG |
| 16 | A23 | A24 | 1 | 1972 | 12 | 533,1 | 12 | 1,021 | H5A034 | C | pos1 | Hm Drgbl | Hm Drgbl | Hm Drgbl | CPE002DT | CPE002EJ | CPE002F9 |
| 1937 | A24 | A1 | 2 | 699,3 | 24 | 702,1 | 12 | 1,043 | H5A034 | C | pos1 | Hm Drgbl | Hm Drgbl | Hm Drgbl | CPE002FX | CPE002G1 | CPE002G5 |
| 5776 | A23 | A24 | 50 | 1101 | 600 | 539,6 | 12 | 1,096 | H5A034 | C | pos1 | Hm Drgbl | Hm Drgbl | Hm Drgbl | CPE002DY | CPE002F1 | CPE002FM |
| 21544 | B23 | B24 | 2 | 1361 | 24 | 715,2 | 12 | 1,263 | H5A034 | C | pos1 | Hm Gen E | Hm Gen E | Hm Gen E | CPE002JU | CPE002O9 | CPE002KP |
| 400 | A23 | A24 | 43 | 2025 | 522 | 579,3 | 12,1 | 1,766 | H5A034 | C | pos1 | Hm Drgbl | Hm Drgbl | Hm Drgbl | CPE002E0 | CPE002EZ | CPE002FK |
| 7720 | B23 | B24 | 18 | 643,6 | 217 | 446,9 | 12,1 | 1,847 | H5A034 | C | pos1 | Hm Drgbl | Hm Drgbl | Hm Drgbl | CPE002E4 | CPE002EV | CPE002FG |
| 8201 | F24 | F1 | 30 | 683 | 366 | 785,9 | 12,2 | 1,173 | H5A034 | C | pos1 | Hm Drgbl | Hm Drgbl | Hm Drgbl | CPE002E5 | CPE002EU | CPE002FF |
| 4696 | D23 | D24 | 38 | 1014 | 467 | 945,5 | 12,3 | 1,352 | H5A034 | C | pos1 | Hm Drgbl | Hm Drgbl | Hm Drgbl | CPE002DV | CPE002EL | CPE002FP |
| 857 | D24 | D1 | 44 | 723 | 545 | 373,8 | 12,4 | 1,175 | H5A034 | C | pos1 | Hm Drgbl | Hm Drgbl | Hm Drgbl | CPE002FU | CPE002FY | CPE002G2 |
| 7768 | D23 | D24 | 23 | 727,4 | 285 | 499,1 | 12,4 | 1,893 | H5A034 | C | pos1 | Hm Drgbl | Hm Drgbl | Hm Drgbl | CPE002E4 | CPE002EV | CPE002FG |
| 3953 | E24 | E1 | 2 | 1329 | 25 | 434,8 | 12,5 | 1,111 | H5A034 | C | pos1 | Hm Drgbl | Hm Drgbl | Hm Drgbl | CPE002DS | CPE002EI | CPE002F8 |
| 20033 | C24 | C1 | 6 | 1100 | 75 | 288,5 | 12,5 | 1,524 | H5A034 | C | pos1 | Hm Gen E | Hm Gen E | Hm Gen E | CPE002JQ | CPE002O5 | CPE002KL |
| 20056 | D23 | D24 | 68 | 1258 | 865 | 162,6 | 12,7 | 1,549 | H5A034 | C | pos1 | Hm Gen E | Hm Gen E | Hm Gen E | CPE002JQ | CPE002O5 | CPE002KL |
| 7432 | F23 | F24 | 8 | 714,7 | 103 | 502 | 12,9 | 0,985 | H5A034 | C | pos1 | Hm Drgbl | Hm Drgbl | Hm Drgbl | CPE002E3 | CPE002EW | CPE002FH |
| 4744 | F23 | F24 | 19 | 918,5 | 246 | 804 | 12,9 | 1,418 | H5A034 | C | pos1 | Hm Drgbl | Hm Drgbl | Hm Drgbl | CPE002DV | CPE002EL | CPE002FP |
| 9736 | F23 | F24 | 17 | 1133 | 224 | 241,9 | 13,2 | 0,923 | H5A034 | C | pos1 | Hm Drgbl | Hm Drgbl | Hm Drgbl | CPE002E9 | CPE002EQ | CPE002FB |
| 19984 | A23 | A24 | 54 | 1284 | 713 | 281 | 13,2 | 1,61 | H5A034 | C | pos1 | Hm Gen E | Hm Gen E | Hm Gen E | CPE002JQ | CPE002O5 | CPE002KL |
| 4625 | A24 | A1 | 10 | 937,6 | 133 | 681,5 | 13,3 | 1,462 | H5A034 | C | pos1 | Hm Drgbl | Hm Drgbl | Hm Drgbl | CPE002DV | CPE002EL | CPE002FP |
| 20008 | B23 | B24 | 60 | 1384 | 798 | 175 | 13,3 | 1,622 | H5A034 | C | pos1 | Hm Gen E | Hm Gen E | Hm Gen E | CPE002JQ | CPE002O5 | CPE002KL |
| 20801 | C24 | C1 | 36 | 1231 | 486 | 465,6 | 13,5 | 0,957 | H5A034 | C | pos1 | Hm Gen E | Hm Gen E | Hm Gen E | CPE002JS | CPE002O7 | CPE002KN |
| 5464 | D23 | D24 | 43 | 914,1 | 585 | 782,4 | 13,6 | 1,295 | H5A034 | C | pos1 | Hm Drgbl | Hm Drgbl | Hm Drgbl | CPE002DX | CPE002F2 | CPE002FN |
| 3856 | A23 | A24 | 20 | 772,9 | 273 | 277,4 | 13,7 | 1,218 | H5A034 | C | pos1 | Hm Drgbl | Hm Drgbl | Hm Drgbl | CPE002DS | CPE002EI | CPE002F8 |
| 3184 | E23 | E24 | 12 | 1269 | 166 | 306,7 | 13,8 | 0,854 | H5A034 | C | pos1 | Hm Drgbl | Hm Drgbl | Hm Drgbl | CPE002DC | CPE002EG | CPE002F6 |
| 16625 | E24 | E1 | 20 | 1069 | 276 | 485,5 | 13,8 | 1,091 | H5A034 | C | pos1 | Hm Gen E | Hm Gen E | Hm Gen E | CPE002JH | CPE002NV | CPE002KC |
| 2417 | E24 | E1 | 1 | 764,9 | 14 | 544,4 | 14 | 1,102 | H5A034 | C | pos1 | Hm Drgbl | Hm Drgbl | Hm Drgbl | CPE002DC | CPE002EE | CPE002F4 |
| 2009 | D24 | D1 | 1 | 423,6 | 14 | 575,4 | 14 | 1,217 | H5A034 | C | pos1 | Hm Drgbl | Hm Drgbl | Hm Drgbl | CPE002FX | CPE002G1 | CPE002G5 |
| 20777 | B24 | B1 | 60 | 1047 | 852 | 403,6 | 14,2 | 1,007 | H5A034 | C | pos1 | Hm Gen E | Hm Gen E | Hm Gen E | CPE002JS | CPE002O7 | CPE002KN |
| 5777 | A24 | A1 | 27 | 990 | 383 | 843,9 | 14,2 | 1,297 | H5A034 | C | pos1 | Hm Drgbl | Hm Drgbl | Hm Drgbl | CPE002DY | CPE002F1 | CPE002FM |
| 1240 | D23 | D24 | 5 | 987,9 | 71 | 400,7 | 14,2 | 1,303 | H5A034 | C | pos1 | Hm Drgbl | Hm Drgbl | Hm Drgbl | CPE002FV | CPE002FZ | CPE002G3 |
| 22408 | F23 | F24 | 16 | 1143 | 232 | 424,4 | 14,5 | 0,747 | H5A034 | C | pos1 | Hm Gen E | Hm Gen E | Hm Gen E | CPE002JW | CPE002OB | CPE002KR |
| 20416 | C23 | C24 | 28 | 750,1 | 406 | 259,1 | 14,5 | 0,967 | H5A034 | C | pos1 | Hm Gen E | Hm Gen E | Hm Gen E | CPE002JR | CPE002O6 | CPE002KM |
| 12329 | B24 | B1 | 24 | 1229 | 347 | 181,3 | 14,5 | 1,09 | H5A034 | C | pos1 | Hm Gen E | Hm Gen E | Hm Gen E | CPE002J5 | CPE002NK | CPE002K1 |
| 1216 | C23 | C24 | 13 | 976,4 | 188 | 918,4 | 14,5 | 1,33 | H5A034 | C | pos1 | Hm Drgbl | Hm Drgbl | Hm Drgbl | CPE002FV | CPE002FZ | CPE002G3 |
| 2753 | C24 | C1 | 7 | 883,9 | 102 | 984,7 | 14,6 | 1,718 | H5A034 | C | pos1 | Hm Drgbl | Hm Drgbl | Hm Drgbl | CPE002DP | CPE002EF | CPE002F5 |
| 18113 | C24 | C1 | 17 | 1152 | 253 | 263,2 | 14,9 | 0,958 | H5A034 | C | pos1 | Hm Gen E | Hm Gen E | Hm Gen E | CPE002JL | CPE002O0 | CPE002KG |
| 6208 | C23 | C24 | 74 | 561,3 | 1110 | 464,9 | 15 | 0,99 | H5A034 | C | pos1 | Hm Drgbl | Hm Drgbl | Hm Drgbl | CPE002DZ | CPE002F0 | CPE002FL |
| 17729 | C24 | C1 | 4 | 1340 | 60 | 289,8 | 15 | 1,186 | H5A034 | C | pos1 | Hm Gen E | Hm Gen E | Hm Gen E | CPE002JK | CPE002NZ | CPE002KF |
| 16192 | C23 | C24 | 27 | 1043 | 404 | 386,2 | 15 | 1,376 | H5A034 | C | pos1 | Hm Gen E | Hm Gen E | Hm Gen E | CPE002JG | CPE002NV | CPE002KB |
| 2800 | E23 | E24 | 1 | 1159 | 15 | 666,1 | 15 | 1,765 | H5A034 | C | pos1 | Hm Drgbl | Hm Drgbl | Hm Drgbl | CPE002DP | CPE002EF | CPE002F5 |
| 20825 | D24 | D1 | 41 | 935,5 | 619 | 270,8 | 15,1 | 1,071 | H5A034 | C | pos1 | Hm Gen E | Hm Gen E | Hm Gen E | CPE002JS | CPE002O7 | CPE002KN |
| 5128 | F23 | F24 | 39 | 731,2 | 589 | 761,9 | 15,1 | 1,184 | H5A034 | C | pos1 | Hm Drgbl | Hm Drgbl | Hm Drgbl | CPE002DV | CPE002EM | CPE002FO |
| 21592 | D23 | D24 | 7 | 1117 | 106 | 728,8 | 15,1 | 1,589 | H5A034 | C | pos1 | Hm Gen E | Hm Gen E | Hm Gen E | CPE002JU | CPE002O9 | CPE002KP |
| 19336 | F23 | F24 | 49 | 1430 | 746 | 520,6 | 15,2 | 1,078 | H5A034 | C | pos1 | Hm Gen E | Hm Gen E | Hm Gen E | CPE002JO | CPE002O3 | CPE002KJ |
| 5081 | D24 | D1 | 11 | 934,3 | 167 | 419,9 | 15,2 | 1,192 | H5A034 | C | pos1 | Hm Drgbl | Hm Drgbl | Hm Drgbl | CPE002DV | CPE002EM | CPE002FO |
| 5440 | C23 | C24 | 55 | 852,7 | 842 | 631,3 | 15,3 | 1,457 | H5A034 | C | pos1 | Hm Drgbl | Hm Drgbl | Hm Drgbl | CPE002DX | CPE002F2 | CPE002FN |
| 9712 | E23 | E24 | 5 | 1106 | 77 | 330,6 | 15,4 | 1,077 | H5A034 | C | pos1 | Hm Drgbl | Hm Drgbl | Hm Drgbl | CPE002E9 | CPE002EQ | CPE002FB |
| 8177 | E24 | E1 | 9 | 708,2 | 139 | 938,3 | 15,4 | 1,481 | H5A034 | C | pos1 | Hm Drgbl | Hm Drgbl | Hm Drgbl | CPE002E5 | CPE002EU | CPE002FF |
| 4649 | B24 | B1 | 16 | 890,4 | 246 | 678,2 | 15,4 | 1,692 | H5A034 | C | pos1 | Hm Drgbl | Hm Drgbl | Hm Drgbl | CPE002DV | CPE002EL | CPE002FP |
| 10432 | C23 | C24 | 4 | 1459 | 62 | 232,4 | 15,5 | 1,751 | H5A034 | C | pos1 | Hm Drgbl | Hm Drgbl | Hm Drgbl | CPE002EB | CPE002EO | CPE002FR |
| 7744 | C23 | C24 | 22 | 702,2 | 341 | 481,7 | 15,5 | 2,366 | H5A034 | C | pos1 | Hm Drgbl | Hm Drgbl | Hm Drgbl | CPE002E4 | CPE002EV | CPE002FG |
| 2057 | F24 | F1 | 5 | 763 | 78 | 528,3 | 15,6 | 1,357 | H5A034 | C | pos1 | Hm Drgbl | Hm Drgbl | Hm Drgbl | CPE002FX | CPE002G1 | CPE002G5 |
| 3929 | D24 | D1 | 5 | 913,3 | 78 | 516,3 | 15,6 | 1,387 | H5A034 | C | pos1 | Hm Drgbl | Hm Drgbl | Hm Drgbl | CPE002DS | CPE002EI | CPE002F8 |
| 5848 | D23 | D24 | 21 | 1290 | 332 | 677 | 15,8 | 1,443 | H5A034 | C | pos1 | Hm Drgbl | Hm Drgbl | Hm Drgbl | CPE002DY | CPE002F1 | CPE002FM |
| 5392 | A23 | A24 | 35 | 869,1 | 552 | 659,7 | 15,8 | 1,505 | H5A034 | C | pos1 | Hm Drgbl | Hm Drgbl | Hm Drgbl | CPE002DX | CPE002F2 | CPE002FN |
| 8080 | A23 | A24 | 34 | 865,7 | 540 | 299,4 | 15,9 | 1,529 | H5A034 | C | pos1 | Hm Drgbl | Hm Drgbl | Hm Drgbl | CPE002E5 | CPE002EU | CPE002FF |
| 11248 | E23 | E24 | 3 | 637,8 | 48 | 310,6 | 16 | 1,159 | H5A034 | C | pos1 | Hm Drgbl | Hm Drgbl | Hm Drgbl | CPE002ED | CPE002F3 | CPE002FT |
| 16144 | A23 | A24 | 10 | 1233 | 161 | 434,8 | 16,1 | 1,477 | H5A034 | C | pos1 | Hm Gen E | Hm Gen E | Hm Gen E | CPE002JG | CPE002NV | CPE002KB |
| 16193 | C24 | C1 | 27 | 1008 | 434 | 492 | 16,1 | 1,477 | H5A034 | C | pos1 | Hm Gen E | Hm Gen E | Hm Gen E | CPE002JG | CPE002NV | CPE002KB |
| 2368 | C23 | C24 | 23 | 632,6 | 372 | 470,4 | 16,2 | 1,276 | H5A034 | C | pos1 | Hm Drgbl | Hm Drgbl | Hm Drgbl | CPE002DC | CPE002EE | CPE002F4 |
| 4289 | C24 | C1 | 6 | 996,8 | 97 | 597 | 16,2 | 1,367 | H5A034 | C | pos1 | Hm Drgbl | Hm Drgbl | Hm Drgbl | CPE002DU | CPE002EK | CPE002FQ |
| 11657 | F24 | F1 | 14 | 1217 | 227 | 146 | 16,2 | 1,446 | H5A034 | C | pos1 | Hm Gen E | Hm Gen E | Hm Gen E | CPE002JA | CPE002NJ | CPE002JZ |
| 8153 | D24 | D1 | 28 | 653,6 | 454 | 771,9 | 16,2 | 1,558 | H5A034 | C | pos1 | Hm Drgbl | Hm Drgbl | Hm Drgbl | CPE002E5 | CPE002EU | CPE002FF |
| 8152 | D23 | D24 | 34 | 817,2 | 559 | 645,5 | 16,4 | 1,577 | H5A034 | C | pos1 | Hm Drgbl | Hm Drgbl | Hm Drgbl | CPE002E5 | CPE002EU | CPE002FF |
| 17369 | D24 | D1 | 31 | 1371 | 511 | 366,4 | 16,5 | 1,126 | H5A034 | C | pos1 | Hm Gen E | Hm Gen E | Hm Gen E | CPE002JJ | CPE002NY | CPE002KE |
| 19264 | C23 | C24 | 13 | 1640 | 214 | 446,8 | 16,5 | 1,17 | H5A034 | C | pos1 | Hm Gen E | Hm Gen E | Hm Gen E | CPE002JO | CPE002O3 | CPE002KJ |
| 16913 | A24 | A1 | 145 | 1179 | 2409 | 258 | 16,6 | 1,282 | H5A034 | C | pos1 | Hm Gen E | Hm Gen E | Hm Gen E | CPE002JI | CPE002NZ | CPE002KD |
| 23513 | D24 | D1 | 54 | 1824 | 901 | 358,8 | 16,7 | 0,886 | H5A034 | C | pos1 | Hm Gen E | Hm Gen E | Hm Gen E | CPE002KV | CPE002OE | CPE002KU |
| 19241 | B24 | B1 | 7 | 1065 | 117 | 357,5 | 16,7 | 1,184 | H5A034 | C | pos1 | Hm Gen E | Hm Gen E | Hm Gen E | CPE002JO | CPE002O3 | CPE002KJ |
| 5416 | B23 | B24 | 20 | 1072 | 335 | 652,1 | 16,8 | 1,6 | H5A034 | C | pos1 | Hm Drgbl | Hm Drgbl | Hm Drgbl | CPE002DX | CPE002F2 | CPE002FN |
| 20080 | E23 | E24 | 20 | 1241 | 335 | 311,8 | 16,8 | 2,049 | H5A034 | C | pos1 | Hm Gen E | Hm Gen E | Hm Gen E | CPE002JQ | CPE002O5 | CPE002KL |
| 401 | A24 | A1 | 12 | 1998 | 202 | 826,4 | 16,8 | 2,453 | H5A034 | C | pos1 | Hm Drgbl | Hm Drgbl | Hm Drgbl | CPE002E0 | CPE002EZ | CPE002FK |
| 7049 | F24 | F1 | 1 | 1214 | 17 | 704,5 | 17 | 1,323 | H5A034 | C | pos1 | Hm Drgbl | Hm Drgbl | Hm Drgbl | CPE002E2 | CPE002EX | CPE002FI |
| 424 | B23 | B24 | 22 | 1714 | 375 | 487,1 | 17 | 2,482 | H5A034 | C | pos1 | Hm Drgbl | Hm Drgbl | Hm Drgbl | CPE002E0 | CPE002EZ | CPE002FK |
| 6665 | F24 | F1 | 18 | 681,6 | 312 | 525,6 | 17,3 | 1,518 | H5A034 | C | pos1 | Hm Drgbl | Hm Drgbl | Hm Drgbl | CPE002E1 | CPE002EY | CPE002FJ |
| 21640 | F23 | F24 | 3 | 1183 | 52 | 559 | 17,3 | 1,821 | H5A034 | C | pos1 | Hm Gen E | Hm Gen E | Hm Gen E | CPE002JU | CPE002O9 | CPE002KP |
| 18953 | F24 | F1 | 62 | 1130 | 1083 | 302 | 17,5 | 1,22 | H5A034 | C | pos1 | Hm Gen E | Hm Gen E | Hm Gen E | CPE002JN | CPE002O2 | CPE002KI |
| 5032 | B23 | B24 | 46 | 855,3 | 807 | 762 | 17,5 | 1,373 | H5A034 | C | pos1 | Hm Drgbl | Hm Drgbl | Hm Drgbl | CPE002DV | CPE002EM | CPE002FO |
| 2369 | C24 | C1 | 4 | 994,4 | 70 | 599,8 | 17,5 | 1,378 | H5A034 | C | pos1 | Hm Drgbl | Hm Drgbl | Hm Drgbl | CPE002DC | CPE002EE | CPE002F4 |
| 6544 | A23 | A24 | 41 | 698,2 | 716 | 587,3 | 17,5 | 1,535 | H5A034 | C | pos1 | Hm Drgbl | Hm Drgbl | Hm Drgbl | CPE002E1 | CPE002EY | CPE002FJ |
| 6976 | C23 | C24 | 30 | 715,8 | 527 | 664,8 | 17,6 | 1,37 | H5A034 | C | pos1 | Hm Drgbl | Hm Drgbl | Hm Drgbl | CPE002E2 | CPE002EX | CPE002FI |
| 5896 | F23 | F24 | 63 | 880,4 | 1108 | 664,7 | 17,6 | 1,607 | H5A034 | C | pos1 | Hm Drgbl | Hm Drgbl | Hm Drgbl | CPE002DY | CPE002F1 | CPE002FM |
| 13577 | F24 | F1 | 6 | 1219 | 106 | 128,5 | 17,7 | 1,662 | H5A034 | C | pos1 | Hm Gen E | Hm Gen E | Hm Gen E | CPE002JC | CPE002NC | CPE002K4 |
| 7337 | B24 | B1 | 44 | 603 | 794 | 542,8 | 18 | 1,374 | H5A034 | C | pos1 | Hm Drgbl | Hm Drgbl | Hm Drgbl | CPE002E3 | CPE002EW | CPE002FH |
| 16649 | F24 | F1 | 12 | 1278 | 219 | 382,3 | 18,2 | 1,439 | H5A034 | C | pos1 | Hm Gen E | Hm Gen E | Hm Gen E | CPE002JH | CPE002NV | CPE002KC |
| 19217 | A24 | A1 | 13 | 1178 | 238 | 557,8 | 18,3 | 1,298 | H5A034 | C | pos1 | Hm Gen E | Hm Gen E | Hm Gen E | CPE002JO | CPE002O3 | CPE002KJ |
| 15857 | E24 | E1 | 20 | 1182 | 367 | 116 | 18,4 | 1,025 | H5A034 | C | pos1 | Hm Gen E | Hm Gen E | Hm Gen E | CPE002J9 | CPE002NU | CPE002KA |
| 20105 | F24 | F1 | 7 | 1344 | 129 | 131,9 | 18,4 | 2,244 | H5A034 | C | pos1 | Hm Gen E | Hm Gen E | Hm Gen E | CPE002JQ | CPE002O5 | CPE002KL |
| 3857 | A24 | A1 | 2 | 784,5 | 37 | 235,7 | 18,5 | 1,644 | H5A034 | C | pos1 | Hm Drgbl | Hm Drgbl | Hm Drgbl | CPE002DS | CPE002EI | CPE002F8 |
| 19697 | E24 | E1 | 7 | 392,9 | 130 | 364 | 18,6 | 1,212 | H5A034 | C | pos1 | Hm Gen E | Hm Gen E | Hm Gen E | CPE002JP | CPE002O4 | CPE002KK |
| 5512 | F23 | F24 | 60 | 676,2 | 1121 | 731,5 | 18,7 | 1,781 | H5A034 | C | pos1 | Hm Drgbl | Hm Drgbl | Hm Drgbl | CPE002DX | CPE002F2 | CPE002FN |
| 8104 | B23 | B24 | 75 | 792,4 | 1403 | 727,4 | 18,7 | 1,798 | H5A034 | C | pos1 | Hm Drgbl | Hm Drgbl | Hm Drgbl | CPE002E5 | CPE002EU | CPE002FF |
| 21209 | D24 | D1 | 16 | 1134 | 301 | 306,3 | 18,8 | 1,333 | H5A034 | C | pos1 | Hm Gen E | Hm Gen E | Hm Gen E | CPE002JT | CPE002O8 | CPE002KO |
| 21520 | A23 | A24 | 5 | 1455 | 94 | 1019 | 18,8 | 1,979 | H5A034 | C | pos1 | Hm Gen E | Hm Gen E | Hm Gen E | CPE002JU | CPE002O9 | CPE002KP |
| 16577 | C24 | C1 | 6 | 1017 | 114 | 505,1 | 19 | 1,502 | H5A034 | C | pos1 | Hm Gen E | Hm Gen E | Hm Gen E | CPE002JH | CPE002NV | CPE002KC |
| 16241 | E24 | E1 | 2 | 1915 | 38 | 271,6 | 19 | 1,743 | H5A034 | C | pos1 | Hm Gen E | Hm Gen E | Hm Gen E | CPE002JG | CPE002NV | CPE002KB |
| 833 | C24 | C1 | 1 | 848,6 | 19 | 624,2 | 19 | 1,801 | H5A034 | C | pos1 | Hm Drgbl | Hm Drgbl | Hm Drgbl | CPE002FU | CPE002FY | CPE002G2 |
| 17416 | F23 | F24 | 45 | 1105 | 860 | 300,5 | 19,1 | 1,304 | H5A034 | C | pos1 | Hm Gen E | Hm Gen E | Hm Gen E | CPE002JJ | CPE002NY | CPE002KE |
| 16168 | B23 | B24 | 19 | 986,3 | 363 | 410,9 | 19,1 | 1,752 | H5A034 | C | pos1 | Hm Gen E | Hm Gen E | Hm Gen E | CPE002JG | CPE002NV | CPE002KB |
| 8105 | B24 | B1 | 59 | 715,5 | 1138 | 761,6 | 19,3 | 1,856 | H5A034 | C | pos1 | Hm Drgbl | Hm Drgbl | Hm Drgbl | CPE002E5 | CPE002EU | CPE002FF |
| 4672 | C23 | C24 | 44 | 1091 | 851 | 728,6 | 19,3 | 2,121 | H5A034 | C | pos1 | Hm Drgbl | Hm Drgbl | Hm Drgbl | CPE002DV | CPE002EL | CPE002FP |
| 16216 | D23 | D24 | 14 | 1048 | 272 | 361,7 | 19,4 | 1,78 | H5A034 | C | pos1 | Hm Gen E | Hm Gen E | Hm Gen E | CPE002JG | CPE002NV | CPE002KB |
| 16264 | F23 | F24 | 19 | 1196 | 369 | 335,1 | 19,4 | 1,78 | H5A034 | C | pos1 | Hm Gen E | Hm Gen E | Hm Gen E | CPE002JG | CPE002NV | CPE002KB |
| 5417 | B24 | B1 | 19 | 859,4 | 371 | 695,4 | 19,5 | 1,857 | H5A034 | C | pos1 | Hm Drgbl | Hm Drgbl | Hm Drgbl | CPE002DX | CPE002F2 | CPE002FN |
| 5488 | E23 | E24 | 32 | 968 | 631 | 641,7 | 19,7 | 1,876 | H5A034 | C | pos1 | Hm Drgbl | Hm Drgbl | Hm Drgbl | CPE002DX | CPE002F2 | CPE002FN |
| 11633 | E24 | E1 | 14 | 980,8 | 277 | 240,9 | 19,8 | 1,768 | H5A034 | C | pos1 | Hm Gen E | Hm Gen E | Hm Gen E | CPE002JA | CPE002NJ | CPE002JZ |
| 23489 | C24 | C1 | 51 | 2114 | 1014 | 464,9 | 19,9 | 1,056 | H5A034 | C | pos1 | Hm Gen E | Hm Gen E | Hm Gen E | CPE002KV | CPE002OE | CPE002KU |
| 5033 | B24 | B1 | 23 | 792,6 | 464 | 620,6 | 20,2 | 1,584 | H5A034 | C | pos1 | Hm Drgbl | Hm Drgbl | Hm Drgbl | CPE002DV | CPE002EM | CPE002FO |
| 11273 | F24 | F1 | 8 | 1083 | 163 | 219,5 | 20,4 | 1,478 | H5A034 | C | pos1 | Hm Drgbl | Hm Drgbl | Hm Drgbl | CPE002ED | CPE002F3 | CPE002FT |
| 7769 | D24 | D1 | 30 | 597 | 611 | 470,3 | 20,4 | 3,115 | H5A034 | C | pos1 | Hm Drgbl | Hm Drgbl | Hm Drgbl | CPE002E4 | CPE002EV | CPE002FG |
| 3161 | D24 | D1 | 2 | 1969 | 41 | 1199 | 20,5 | 1,269 | H5A034 | C | pos1 | Hm Drgbl | Hm Drgbl | Hm Drgbl | CPE002DC | CPE002EG | CPE002F6 |
| 16217 | D24 | D1 | 10 | 1070 | 205 | 419,2 | 20,5 | 1,881 | H5A034 | C | pos1 | Hm Gen E | Hm Gen E | Hm Gen E | CPE002JG | CPE002NV | CPE002KB |
| 22768 | E23 | E24 | 19 | 1735 | 394 | 244,1 | 20,7 | 0,972 | H5A034 | C | pos1 | Hm Gen E | Hm Gen E | Hm Gen E | CPE002JX | CPE002OC | CPE002KS |
| 2393 | D24 | D1 | 3 | 1122 | 62 | 739,2 | 20,7 | 1,63 | H5A034 | C | pos1 | Hm Drgbl | Hm Drgbl | Hm Drgbl | CPE002DC | CPE002EE | CPE002F4 |
| 7721 | B24 | B1 | 43 | 648,7 | 889 | 494,5 | 20,7 | 3,16 | H5A034 | C | pos1 | Hm Drgbl | Hm Drgbl | Hm Drgbl | CPE002E4 | CPE002EV | CPE002FG |
| 22001 | E24 | E1 | 40 | 1663 | 834 | 225,8 | 20,9 | 0,993 | H5A034 | C | pos1 | Hm Gen E | Hm Gen E | Hm Gen E | CPE002JV | CPE002OA | CPE002KQ |
| 6161 | A24 | A1 | 10 | 613,1 | 209 | 634,8 | 20,9 | 1,38 | H5A034 | C | pos1 | Hm Drgbl | Hm Drgbl | Hm Drgbl | CPE002DZ | CPE002F0 | CPE002FL |
| 13505 | C24 | C1 | 11 | 869,3 | 230 | 199 | 20,9 | 1,962 | H5A034 | C | pos1 | Hm Gen E | Hm Gen E | Hm Gen E | CPE002JC | CPE002NC | CPE002K4 |
| 18833 | A24 | A1 | 47 | 1020 | 985 | 483,4 | 21 | 1,463 | H5A034 | C | pos1 | Hm Gen E | Hm Gen E | Hm Gen E | CPE002JN | CPE002O2 | CPE002KI |
| 3881 | B24 | B1 | 4 | 1047 | 85 | 677,1 | 21,2 | 1,884 | H5A034 | C | pos1 | Hm Drgbl | Hm Drgbl | Hm Drgbl | CPE002DS | CPE002EI | CPE002F8 |
| 22337 | C24 | C1 | 28 | 1600 | 598 | 511,8 | 21,4 | 1,103 | H5A034 | C | pos1 | Hm Gen E | Hm Gen E | Hm Gen E | CPE002JW | CPE002OB | CPE002KR |
| 23512 | D23 | D24 | 24 | 1967 | 516 | 381,5 | 21,5 | 1,141 | H5A034 | C | pos1 | Hm Gen E | Hm Gen E | Hm Gen E | CPE002KV | CPE002OE | CPE002KU |
| 4624 | A23 | A24 | 45 | 1131 | 969 | 854,1 | 21,5 | 2,363 | H5A034 | C | pos1 | Hm Drgbl | Hm Drgbl | Hm Drgbl | CPE002DV | CPE002EL | CPE002FP |
| 17345 | C24 | C1 | 5 | 1077 | 108 | 330 | 21,6 | 1,474 | H5A034 | C | pos1 | Hm Gen E | Hm Gen E | Hm Gen E | CPE002JJ | CPE002NY | CPE002KE |
| 15401 | B24 | B1 | 31 | 1077 | 674 | 247,9 | 21,7 | 1,973 | H5A034 | C | pos1 | Hm Gen E | Hm Gen E | Hm Gen E | CPE002JE | CPE002NT | CPE002K9 |
| 1672 | F23 | F24 | 7 | 925,1 | 152 | 711,6 | 21,7 | 2,272 | H5A034 | C | pos1 | Hm Drgbl | Hm Drgbl | Hm Drgbl | CPE002FW | CPE002G0 | CPE002G4 |
| 16960 | C23 | C24 | 17 | 1247 | 373 | 326,6 | 21,9 | 1,691 | H5A034 | C | pos1 | Hm Gen E | Hm Gen E | Hm Gen E | CPE002JI | CPE002NZ | CPE002KD |
| 20465 | E24 | E1 | 10 | 735,1 | 220 | 177,1 | 22 | 1,467 | H5A034 | C | pos1 | Hm Gen E | Hm Gen E | Hm Gen E | CPE002JR | CPE002O6 | CPE002KM |
| 19289 | D24 | D1 | 9 | 1662 | 198 | 436,5 | 22 | 1,56 | H5A034 | C | pos1 | Hm Gen E | Hm Gen E | Hm Gen E | CPE002JO | CPE002O3 | CPE002KJ |
| 12400 | E23 | E24 | 3 | 1505 | 66 | 310,7 | 22 | 1,654 | H5A034 | C | pos1 | Hm Gen E | Hm Gen E | Hm Gen E | CPE002J5 | CPE002NK | CPE002K1 |
| 21233 | E24 | E1 | 9 | 1044 | 201 | 266,7 | 22,3 | 1,582 | H5A034 | C | pos1 | Hm Gen E | Hm Gen E | Hm Gen E | CPE002JT | CPE002O8 | CPE002KO |
| 5801 | B24 | B1 | 9 | 1026 | 201 | 799,3 | 22,3 | 2,037 | H5A034 | C | pos1 | Hm Drgbl | Hm Drgbl | Hm Drgbl | CPE002DY | CPE002F1 | CPE002FM |
| 23561 | F24 | F1 | 28 | 1837 | 628 | 269,4 | 22,4 | 1,188 | H5A034 | C | pos1 | Hm Gen E | Hm Gen E | Hm Gen E | CPE002KV | CPE002OE | CPE002KU |
| 17344 | C23 | C24 | 58 | 1135 | 1302 | 442,8 | 22,4 | 1,529 | H5A034 | C | pos1 | Hm Gen E | Hm Gen E | Hm Gen E | CPE002JJ | CPE002NY | CPE002KE |
| 23537 | E24 | E1 | 11 | 2023 | 249 | 438,7 | 22,6 | 1,199 | H5A034 | C | pos1 | Hm Gen E | Hm Gen E | Hm Gen E | CPE002KV | CPE002OE | CPE002KU |
| 21184 | C23 | C24 | 34 | 998,7 | 767 | 431,2 | 22,6 | 1,603 | H5A034 | C | pos1 | Hm Gen E | Hm Gen E | Hm Gen E | CPE002JT | CPE002O8 | CPE002KO |
| 5008 | A23 | A24 | 61 | 688,7 | 1387 | 782 | 22,7 | 1,78 | H5A034 | C | pos1 | Hm Drgbl | Hm Drgbl | Hm Drgbl | CPE002DV | CPE002EM | CPE002FO |
| 7024 | E23 | E24 | 19 | 713,5 | 434 | 583,6 | 22,8 | 1,774 | H5A034 | C | pos1 | Hm Drgbl | Hm Drgbl | Hm Drgbl | CPE002E2 | CPE002EX | CPE002FI |
| 17033 | F24 | F1 | 13 | 1154 | 298 | 251,7 | 22,9 | 1,768 | H5A034 | C | pos1 | Hm Gen E | Hm Gen E | Hm Gen E | CPE002JI | CPE002NZ | CPE002KD |
| 8969 | F24 | F1 | 9 | 3083 | 206 | 216,2 | 22,9 | 1,839 | H5A034 | C | pos1 | Hm Drgbl | Hm Drgbl | Hm Drgbl | CPE002E7 | CPE002ES | CPE002FD |
| 14321 | E24 | E1 | 4 | 910,8 | 92 | 181 | 23 | 1,637 | H5A034 | C | pos1 | Hm Gen E | Hm Gen E | Hm Gen E | CPE002J7 | CPE002NC | CPE002K6 |
| 17752 | D23 | D24 | 44 | 1332 | 1017 | 370,6 | 23,1 | 1,826 | H5A034 | C | pos1 | Hm Gen E | Hm Gen E | Hm Gen E | CPE002JK | CPE002NZ | CPE002KF |
| 16528 | A23 | A24 | 59 | 1030 | 1370 | 500,6 | 23,2 | 1,834 | H5A034 | C | pos1 | Hm Gen E | Hm Gen E | Hm Gen E | CPE002JH | CPE002NV | CPE002KC |
| 7000 | D23 | D24 | 51 | 688 | 1188 | 615,6 | 23,3 | 1,813 | H5A034 | C | pos1 | Hm Drgbl | Hm Drgbl | Hm Drgbl | CPE002E2 | CPE002EX | CPE002FI |
| 17753 | D24 | D1 | 22 | 773,5 | 512 | 315,8 | 23,3 | 1,842 | H5A034 | C | pos1 | Hm Gen E | Hm Gen E | Hm Gen E | CPE002JK | CPE002NZ | CPE002KF |
| 904 | F23 | F24 | 7 | 1098 | 163 | 614,8 | 23,3 | 2,209 | H5A034 | C | pos1 | Hm Drgbl | Hm Drgbl | Hm Drgbl | CPE002FU | CPE002FY | CPE002G2 |
| 8200 | F23 | F24 | 25 | 818,7 | 586 | 673 | 23,4 | 2,25 | H5A034 | C | pos1 | Hm Drgbl | Hm Drgbl | Hm Drgbl | CPE002E5 | CPE002EU | CPE002FF |
| 10385 | A24 | A1 | 15 | 1592 | 351 | 231,8 | 23,4 | 2,644 | H5A034 | C | pos1 | Hm Drgbl | Hm Drgbl | Hm Drgbl | CPE002EB | CPE002EO | CPE002FR |
| 11225 | D24 | D1 | 17 | 1043 | 400 | 152,9 | 23,5 | 1,703 | H5A034 | C | pos1 | Hm Drgbl | Hm Drgbl | Hm Drgbl | CPE002ED | CPE002F3 | CPE002FT |
| 16529 | A24 | A1 | 2 | 1213 | 47 | 417,3 | 23,5 | 1,858 | H5A034 | C | pos1 | Hm Gen E | Hm Gen E | Hm Gen E | CPE002JH | CPE002NV | CPE002KC |
| 20441 | D24 | D1 | 20 | 887 | 472 | 281,4 | 23,6 | 1,573 | H5A034 | C | pos1 | Hm Gen E | Hm Gen E | Hm Gen E | CPE002JR | CPE002O6 | CPE002KM |
| 11609 | D24 | D1 | 3 | 1191 | 71 | 149,6 | 23,7 | 2,116 | H5A034 | C | pos1 | Hm Gen E | Hm Gen E | Hm Gen E | CPE002JA | CPE002NJ | CPE002JZ |
| 15448 | D23 | D24 | 11 | 1035 | 261 | 164,6 | 23,7 | 2,155 | H5A034 | C | pos1 | Hm Gen E | Hm Gen E | Hm Gen E | CPE002JE | CPE002NT | CPE002K9 |
| 13456 | A23 | A24 | 20 | 1330 | 475 | 204,8 | 23,8 | 2,235 | H5A034 | C | pos1 | Hm Gen E | Hm Gen E | Hm Gen E | CPE002JC | CPE002NC | CPE002K4 |
| 5393 | A24 | A1 | 16 | 934,2 | 382 | 757,7 | 23,9 | 2,276 | H5A034 | C | pos1 | Hm Drgbl | Hm Drgbl | Hm Drgbl | CPE002DX | CPE002F2 | CPE002FN |
| 8081 | A24 | A1 | 11 | 840,1 | 263 | 795,2 | 23,9 | 2,298 | H5A034 | C | pos1 | Hm Drgbl | Hm Drgbl | Hm Drgbl | CPE002E5 | CPE002EU | CPE002FF |
| 7312 | A23 | A24 | 24 | 681,9 | 575 | 669,8 | 24 | 1,832 | H5A034 | C | pos1 | Hm Drgbl | Hm Drgbl | Hm Drgbl | CPE002E3 | CPE002EW | CPE002FH |
| 7408 | E23 | E24 | 20 | 773,9 | 480 | 659,9 | 24 | 1,832 | H5A034 | C | pos1 | Hm Drgbl | Hm Drgbl | Hm Drgbl | CPE002E3 | CPE002EW | CPE002FH |
| 7385 | D24 | D1 | 5 | 969,2 | 121 | 644,3 | 24,2 | 1,847 | H5A034 | C | pos1 | Hm Drgbl | Hm Drgbl | Hm Drgbl | CPE002E3 | CPE002EW | CPE002FH |
| 8176 | E23 | E24 | 62 | 726,7 | 1501 | 991,2 | 24,2 | 2,327 | H5A034 | C | pos1 | Hm Drgbl | Hm Drgbl | Hm Drgbl | CPE002E5 | CPE002EU | CPE002FF |
| 6568 | B23 | B24 | 26 | 911,1 | 632 | 553,5 | 24,3 | 2,132 | H5A034 | C | pos1 | Hm Drgbl | Hm Drgbl | Hm Drgbl | CPE002E1 | CPE002EY | CPE002FJ |
| 16240 | E23 | E24 | 4 | 1439 | 98 | 372,5 | 24,5 | 2,248 | H5A034 | C | pos1 | Hm Gen E | Hm Gen E | Hm Gen E | CPE002JG | CPE002NV | CPE002KB |
| 22361 | D24 | D1 | 25 | 1634 | 615 | 520,8 | 24,6 | 1,268 | H5A034 | C | pos1 | Hm Gen E | Hm Gen E | Hm Gen E | CPE002JW | CPE002OB | CPE002KR |
| 23441 | A24 | A1 | 69 | 1861 | 1697 | 408,3 | 24,6 | 1,305 | H5A034 | C | pos1 | Hm Gen E | Hm Gen E | Hm Gen E | CPE002KV | CPE002OE | CPE002KU |
| 20489 | F24 | F1 | 9 | 622,7 | 222 | 331,7 | 24,7 | 1,647 | H5A034 | C | pos1 | Hm Gen E | Hm Gen E | Hm Gen E | CPE002JR | CPE002O6 | CPE002KM |
| 20872 | F23 | F24 | 66 | 1119 | 1633 | 298,4 | 24,7 | 1,752 | H5A034 | C | pos1 | Hm Gen E | Hm Gen E | Hm Gen E | CPE002JS | CPE002O7 | CPE002KN |
| 17009 | E24 | E1 | 6 | 1120 | 148 | 470,7 | 24,7 | 1,907 | H5A034 | C | pos1 | Hm Gen E | Hm Gen E | Hm Gen E | CPE002JI | CPE002NZ | CPE002KD |
| 22721 | C24 | C1 | 10 | 2265 | 248 | 597,5 | 24,8 | 1,164 | H5A034 | C | pos1 | Hm Gen E | Hm Gen E | Hm Gen E | CPE002JX | CPE002OC | CPE002KS |
| 16553 | B24 | B1 | 23 | 1093 | 571 | 572,8 | 24,8 | 1,96 | H5A034 | C | pos1 | Hm Gen E | Hm Gen E | Hm Gen E | CPE002JH | CPE002NV | CPE002KC |
| 21232 | E23 | E24 | 41 | 1333 | 1022 | 601,5 | 24,9 | 1,766 | H5A034 | C | pos1 | Hm Gen E | Hm Gen E | Hm Gen E | CPE002JT | CPE002O8 | CPE002KO |
| 10072 | D23 | D24 | 10 | 959,8 | 249 | 201,6 | 24,9 | 2,394 | H5A034 | C | pos1 | Hm Drgbl | Hm Drgbl | Hm Drgbl | CPE002EA | CPE002EP | CPE002FA |
| 18089 | B24 | B1 | 31 | 1230 | 774 | 435,2 | 25 | 1,608 | H5A034 | C | pos1 | Hm Gen E | Hm Gen E | Hm Gen E | CPE002JL | CPE002O0 | CPE002KG |
| 19673 | D24 | D1 | 16 | 435,5 | 402 | 469,1 | 25,1 | 1,635 | H5A034 | C | pos1 | Hm Gen E | Hm Gen E | Hm Gen E | CPE002JP | CPE002O4 | CPE002KK |
| 7001 | D24 | D1 | 9 | 640,4 | 226 | 603,6 | 25,1 | 1,953 | H5A034 | C | pos1 | Hm Drgbl | Hm Drgbl | Hm Drgbl | CPE002E2 | CPE002EX | CPE002FI |
| 2320 | A23 | A24 | 7 | 1024 | 176 | 591,5 | 25,1 | 1,976 | H5A034 | C | pos1 | Hm Drgbl | Hm Drgbl | Hm Drgbl | CPE002DC | CPE002EE | CPE002F4 |
| 11584 | C23 | C24 | 11 | 877 | 276 | 201,2 | 25,1 | 2,241 | H5A034 | C | pos1 | Hm Gen E | Hm Gen E | Hm Gen E | CPE002JA | CPE002NJ | CPE002JZ |
| 808 | B23 | B24 | 17 | 962 | 426 | 809,1 | 25,1 | 2,379 | H5A034 | C | pos1 | Hm Drgbl | Hm Drgbl | Hm Drgbl | CPE002FU | CPE002FY | CPE002G2 |
| 17417 | F24 | F1 | 24 | 892,8 | 606 | 318,1 | 25,2 | 1,72 | H5A034 | C | pos1 | Hm Gen E | Hm Gen E | Hm Gen E | CPE002JJ | CPE002NY | CPE002KE |
| 17296 | A23 | A24 | 25 | 1040 | 636 | 360,2 | 25,4 | 1,734 | H5A034 | C | pos1 | Hm Gen E | Hm Gen E | Hm Gen E | CPE002JJ | CPE002NY | CPE002KE |
| 1576 | B23 | B24 | 12 | 1013 | 307 | 728,1 | 25,6 | 2,681 | H5A034 | C | pos1 | Hm Drgbl | Hm Drgbl | Hm Drgbl | CPE002FW | CPE002G0 | CPE002G4 |
| 6664 | F23 | F24 | 19 | 665,5 | 489 | 531 | 25,7 | 2,254 | H5A034 | C | pos1 | Hm Drgbl | Hm Drgbl | Hm Drgbl | CPE002E1 | CPE002EY | CPE002FJ |
| 22696 | B23 | B24 | 17 | 1774 | 438 | 340,7 | 25,8 | 1,211 | H5A034 | C | pos1 | Hm Gen E | Hm Gen E | Hm Gen E | CPE002JX | CPE002OC | CPE002KS |
| 13552 | E23 | E24 | 37 | 1054 | 954 | 225,2 | 25,8 | 2,423 | H5A034 | C | pos1 | Hm Gen E | Hm Gen E | Hm Gen E | CPE002JC | CPE002NC | CPE002K4 |
| 4720 | E23 | E24 | 37 | 1065 | 957 | 988,8 | 25,9 | 2,846 | H5A034 | C | pos1 | Hm Drgbl | Hm Drgbl | Hm Drgbl | CPE002DV | CPE002EL | CPE002FP |
| 16937 | B24 | B1 | 24 | 1189 | 623 | 426,4 | 26 | 2,008 | H5A034 | C | pos1 | Hm Gen E | Hm Gen E | Hm Gen E | CPE002JI | CPE002NZ | CPE002KD |
| 6616 | D23 | D24 | 8 | 818,7 | 209 | 604 | 26,1 | 2,289 | H5A034 | C | pos1 | Hm Drgbl | Hm Drgbl | Hm Drgbl | CPE002E1 | CPE002EY | CPE002FJ |
| 6977 | C24 | C1 | 5 | 493,9 | 131 | 592,6 | 26,2 | 2,039 | H5A034 | C | pos1 | Hm Drgbl | Hm Drgbl | Hm Drgbl | CPE002E2 | CPE002EX | CPE002FI |
| 6281 | F24 | F1 | 13 | 665,3 | 342 | 471,7 | 26,3 | 1,736 | H5A034 | C | pos1 | Hm Drgbl | Hm Drgbl | Hm Drgbl | CPE002DZ | CPE002F0 | CPE002FL |
| 15449 | D24 | D1 | 9 | 1227 | 238 | 239,6 | 26,4 | 2,4 | H5A034 | C | pos1 | Hm Gen E | Hm Gen E | Hm Gen E | CPE002JE | CPE002NT | CPE002K9 |
| 11177 | B24 | B1 | 6 | 1136 | 159 | 246,1 | 26,5 | 1,92 | H5A034 | C | pos1 | Hm Drgbl | Hm Drgbl | Hm Drgbl | CPE002ED | CPE002F3 | CPE002FT |
| 5872 | E23 | E24 | 21 | 979,1 | 557 | 789,8 | 26,5 | 2,42 | H5A034 | C | pos1 | Hm Drgbl | Hm Drgbl | Hm Drgbl | CPE002DY | CPE002F1 | CPE002FM |
| 12352 | C23 | C24 | 20 | 1197 | 531 | 231,8 | 26,6 | 2 | H5A034 | C | pos1 | Hm Gen E | Hm Gen E | Hm Gen E | CPE002J5 | CPE002NK | CPE002K1 |
| 21977 | D24 | D1 | 12 | 1516 | 320 | 253,3 | 26,7 | 1,268 | H5A034 | C | pos1 | Hm Gen E | Hm Gen E | Hm Gen E | CPE002JV | CPE002OA | CPE002KQ |
| 11920 | A23 | A24 | 15 | 926,8 | 401 | 178,5 | 26,7 | 1,712 | H5A034 | C | pos1 | Hm Gen E | Hm Gen E | Hm Gen E | CPE002J4 | CPE002NL | CPE002K0 |
| 18904 | D23 | D24 | 13 | 1265 | 347 | 418,6 | 26,7 | 1,861 | H5A034 | C | pos1 | Hm Gen E | Hm Gen E | Hm Gen E | CPE002JN | CPE002O2 | CPE002KI |
| 17680 | A23 | A24 | 20 | 1354 | 535 | 334,7 | 26,8 | 2,119 | H5A034 | C | pos1 | Hm Gen E | Hm Gen E | Hm Gen E | CPE002JK | CPE002NZ | CPE002KF |
| 17776 | E23 | E24 | 16 | 1235 | 429 | 372,2 | 26,8 | 2,119 | H5A034 | C | pos1 | Hm Gen E | Hm Gen E | Hm Gen E | CPE002JK | CPE002NZ | CPE002KF |
| 22360 | D23 | D24 | 132 | 1472 | 3560 | 556,8 | 27 | 1,392 | H5A034 | C | pos1 | Hm Gen E | Hm Gen E | Hm Gen E | CPE002JW | CPE002OB | CPE002KR |
| 23464 | B23 | B24 | 22 | 1893 | 593 | 401,8 | 27 | 1,432 | H5A034 | C | pos1 | Hm Gen E | Hm Gen E | Hm Gen E | CPE002KV | CPE002OE | CPE002KU |
| 19288 | D23 | D24 | 30 | 1362 | 809 | 522,5 | 27 | 1,915 | H5A034 | C | pos1 | Hm Gen E | Hm Gen E | Hm Gen E | CPE002JO | CPE002O3 | CPE002KJ |
| 21256 | F23 | F24 | 27 | 1039 | 729 | 519,2 | 27 | 1,915 | H5A034 | C | pos1 | Hm Gen E | Hm Gen E | Hm Gen E | CPE002JT | CPE002O8 | CPE002KO |
| 5104 | E23 | E24 | 65 | 673,4 | 1752 | 663 | 27 | 2,118 | H5A034 | C | pos1 | Hm Drgbl | Hm Drgbl | Hm Drgbl | CPE002DV | CPE002EM | CPE002FO |
| 20464 | E23 | E24 | 68 | 940,3 | 1848 | 342,3 | 27,2 | 1,813 | H5A034 | C | pos1 | Hm Gen E | Hm Gen E | Hm Gen E | CPE002JR | CPE002O6 | CPE002KM |
| 20873 | F24 | F1 | 10 | 733,5 | 272 | 272,1 | 27,2 | 1,929 | H5A034 | C | pos1 | Hm Gen E | Hm Gen E | Hm Gen E | CPE002JS | CPE002O7 | CPE002KN |
| 12328 | B23 | B24 | 11 | 1315 | 299 | 236,2 | 27,2 | 2,045 | H5A034 | C | pos1 | Hm Gen E | Hm Gen E | Hm Gen E | CPE002J5 | CPE002NK | CPE002K1 |
| 6928 | A23 | A24 | 25 | 715,3 | 684 | 748 | 27,4 | 2,132 | H5A034 | C | pos1 | Hm Drgbl | Hm Drgbl | Hm Drgbl | CPE002E2 | CPE002EX | CPE002FI |
| 18928 | E23 | E24 | 32 | 1104 | 879 | 458,6 | 27,5 | 1,916 | H5A034 | C | pos1 | Hm Gen E | Hm Gen E | Hm Gen E | CPE002JN | CPE002O2 | CPE002KI |
| 1625 | D24 | D1 | 27 | 642,1 | 743 | 502,1 | 27,5 | 2,88 | H5A034 | C | pos1 | Hm Drgbl | Hm Drgbl | Hm Drgbl | CPE002FW | CPE002G0 | CPE002G4 |
| 5489 | E24 | E1 | 5 | 728,6 | 138 | 515,8 | 27,6 | 2,629 | H5A034 | C | pos1 | Hm Drgbl | Hm Drgbl | Hm Drgbl | CPE002DX | CPE002F2 | CPE002FN |
| 21161 | B24 | B1 | 23 | 1070 | 639 | 355,3 | 27,8 | 1,972 | H5A034 | C | pos1 | Hm Gen E | Hm Gen E | Hm Gen E | CPE002JT | CPE002O8 | CPE002KO |
| 21952 | C23 | C24 | 18 | 1613 | 502 | 229,1 | 27,9 | 1,325 | H5A034 | C | pos1 | Hm Gen E | Hm Gen E | Hm Gen E | CPE002JV | CPE002OA | CPE002KQ |
| 23177 | F24 | F1 | 22 | 1211 | 617 | 166 | 28 | 1,21 | H5A034 | C | pos1 | Hm Gen E | Hm Gen E | Hm Gen E | CPE002JY | CPE002OD | CPE002KT |
| 11201 | C24 | C1 | 11 | 1201 | 308 | 183,5 | 28 | 2,029 | H5A034 | C | pos1 | Hm Drgbl | Hm Drgbl | Hm Drgbl | CPE002ED | CPE002F3 | CPE002FT |
| 2056 | F23 | F24 | 6 | 1083 | 169 | 562,9 | 28,2 | 2,452 | H5A034 | C | pos1 | Hm Drgbl | Hm Drgbl | Hm Drgbl | CPE002FX | CPE002G1 | CPE002G5 |
| 15377 | A24 | A1 | 6 | 925 | 169 | 275,8 | 28,2 | 2,564 | H5A034 | C | pos1 | Hm Gen E | Hm Gen E | Hm Gen E | CPE002JE | CPE002NT | CPE002K9 |
| 6160 | A23 | A24 | 11 | 540,4 | 311 | 517,5 | 28,3 | 1,868 | H5A034 | C | pos1 | Hm Drgbl | Hm Drgbl | Hm Drgbl | CPE002DZ | CPE002F0 | CPE002FL |
| 6569 | B24 | B1 | 9 | 643,2 | 255 | 487 | 28,3 | 2,482 | H5A034 | C | pos1 | Hm Drgbl | Hm Drgbl | Hm Drgbl | CPE002E1 | CPE002EY | CPE002FJ |
| 15473 | E24 | E1 | 18 | 987,3 | 509 | 218,6 | 28,3 | 2,573 | H5A034 | C | pos1 | Hm Gen E | Hm Gen E | Hm Gen E | CPE002JE | CPE002NT | CPE002K9 |
| 12040 | F23 | F24 | 25 | 949 | 711 | 222,6 | 28,4 | 1,821 | H5A034 | C | pos1 | Hm Gen E | Hm Gen E | Hm Gen E | CPE002J4 | CPE002NL | CPE002K0 |
| 18088 | B23 | B24 | 33 | 1204 | 937 | 351,2 | 28,4 | 1,826 | H5A034 | C | pos1 | Hm Gen E | Hm Gen E | Hm Gen E | CPE002JL | CPE002O0 | CPE002KG |

| | | | | | | | | | | | | | | | | | |
|---|---|---|---|---|---|---|---|---|---|---|---|---|---|---|---|---|---|
| 6184 | B23 | B24 | 40 | 733,1 | 1136 | 694,6 | 28,4 | 1,875 | H5A034 | C | pos1 | Hm Drgbl | Hm Drgbl | Hm Drgbl | CPE002DZ | CPE002F0 | CPE002FL |
| 7360 | C23 | C24 | 31 | 661,1 | 880 | 595,6 | 28,4 | 2,168 | H5A034 | C | pos1 | Hm Drgbl | Hm Drgbl | Hm Drgbl | CPE002E3 | CPE002EW | CPE002FH |
| 16169 | B24 | B1 | 8 | 714 | 227 | 334,1 | 28,4 | 2,606 | H5A034 | C | pos1 | Hm Gen E | Hm Gen E | Hm Gen E | CPE002JG | CPE002NV | CPE002KB |
| 8128 | C23 | C24 | 69 | 758,6 | 1982 | 839,5 | 28,7 | 2,76 | H5A034 | C | pos1 | Hm Drgbl | Hm Drgbl | Hm Drgbl | CPE002E5 | CPE002EU | CPE002FF |
| 15832 | D23 | D24 | 16 | 1008 | 460 | 148,1 | 28,8 | 1,604 | H5A034 | C | pos1 | Hm Gen E | Hm Gen E | Hm Gen E | CPE002J9 | CPE002NU | CPE002KA |
| 12424 | F23 | F24 | 20 | 1262 | 575 | 216,8 | 28,8 | 2,165 | H5A034 | C | pos1 | Hm Gen E | Hm Gen E | Hm Gen E | CPE002J5 | CPE002NK | CPE002K1 |
| 11560 | B23 | B24 | 15 | 1125 | 432 | 229,4 | 28,8 | 2,571 | H5A034 | C | pos1 | Hm Gen E | Hm Gen E | Hm Gen E | CPE002JA | CPE002NJ | CPE002JZ |
| 17321 | B24 | B1 | 18 | 1096 | 521 | 424,1 | 28,9 | 1,973 | H5A034 | C | pos1 | Hm Gen E | Hm Gen E | Hm Gen E | CPE002JJ | CPE002NY | CPE002KE |
| 23129 | D24 | D1 | 37 | 1133 | 1072 | 299,1 | 29 | 1,253 | H5A034 | C | pos1 | Hm Gen E | Hm Gen E | Hm Gen E | CPE002JY | CPE002OD | CPE002KT |
| 11585 | C24 | C1 | 3 | 1198 | 87 | 113,6 | 29 | 2,589 | H5A034 | C | pos1 | Hm Gen E | Hm Gen E | Hm Gen E | CPE002JA | CPE002NJ | CPE002JZ |
| 19672 | D23 | D24 | 13 | 338,8 | 378 | 476,3 | 29,1 | 1,896 | H5A034 | C | pos1 | Hm Gen E | Hm Gen E | Hm Gen E | CPE002JP | CPE002O4 | CPE002KK |
| 13912 | D23 | D24 | 5 | 1370 | 146 | 139,7 | 29,2 | 1,673 | H5A034 | C | pos1 | Hm Gen E | Hm Gen E | Hm Gen E | CPE002JF | CPE002NP | CPE002K5 |
| 20369 | A24 | A1 | 5 | 1485 | 147 | 262,7 | 29,4 | 1,96 | H5A034 | C | pos1 | Hm Gen E | Hm Gen E | Hm Gen E | CPE002JR | CPE002O6 | CPE002KM |
| 15809 | C24 | C1 | 4 | 1097 | 118 | 149,1 | 29,5 | 1,643 | H5A034 | C | pos1 | Hm Gen E | Hm Gen E | Hm Gen E | CPE002J9 | CPE002NU | CPE002KA |
| 18881 | C24 | C1 | 8 | 1059 | 236 | 361,8 | 29,5 | 2,056 | H5A034 | C | pos1 | Hm Gen E | Hm Gen E | Hm Gen E | CPE002JN | CPE002O2 | CPE002KI |
| 7384 | D23 | D24 | 36 | 750,8 | 1062 | 647,3 | 29,5 | 2,252 | H5A034 | C | pos1 | Hm Drgbl | Hm Drgbl | Hm Drgbl | CPE002E3 | CPE002EW | CPE002FH |
| 22720 | C23 | C24 | 16 | 1854 | 474 | 339,1 | 29,6 | 1,39 | H5A034 | C | pos1 | Hm Gen E | Hm Gen E | Hm Gen E | CPE002JX | CPE002OC | CPE002KS |
| 22384 | E23 | E24 | 23 | 1805 | 684 | 539,7 | 29,7 | 1,531 | H5A034 | C | pos1 | Hm Gen E | Hm Gen E | Hm Gen E | CPE002JW | CPE002OB | CPE002KR |
| 6185 | B24 | B1 | 38 | 608,1 | 1130 | 672 | 29,7 | 1,96 | H5A034 | C | pos1 | Hm Drgbl | Hm Drgbl | Hm Drgbl | CPE002DZ | CPE002F0 | CPE002FL |
| 8849 | A24 | A1 | 3 | 2803 | 89 | 170,8 | 29,7 | 2,386 | H5A034 | C | pos1 | Hm Drgbl | Hm Drgbl | Hm Drgbl | CPE002E7 | CPE002ES | CPE002FD |
| 20009 | B24 | B1 | 18 | 1157 | 535 | 343,2 | 29,7 | 3,622 | H5A034 | C | pos1 | Hm Gen E | Hm Gen E | Hm Gen E | CPE002JQ | CPE002O5 | CPE002KL |
| 6256 | E23 | E24 | 15 | 539,8 | 452 | 795 | 30,1 | 1,987 | H5A034 | C | pos1 | Hm Drgbl | Hm Drgbl | Hm Drgbl | CPE002DZ | CPE002F0 | CPE002FL |
| 13865 | B24 | B1 | 17 | 1374 | 514 | 232,5 | 30,2 | 1,731 | H5A034 | C | pos1 | Hm Gen E | Hm Gen E | Hm Gen E | CPE002JF | CPE002NP | CPE002K5 |
| 19648 | C23 | C24 | 36 | 382,7 | 1092 | 545,3 | 30,3 | 1,974 | H5A034 | C | pos1 | Hm Gen E | Hm Gen E | Hm Gen E | CPE002JP | CPE002O4 | CPE002KK |
| 6232 | D23 | D24 | 21 | 668,5 | 637 | 581,5 | 30,3 | 2 | H5A034 | C | pos1 | Hm Drgbl | Hm Drgbl | Hm Drgbl | CPE002DZ | CPE002F0 | CPE002FL |
| 6592 | C23 | C24 | 36 | 642,8 | 1093 | 576,9 | 30,4 | 2,667 | H5A034 | C | pos1 | Hm Drgbl | Hm Drgbl | Hm Drgbl | CPE002E1 | CPE002EY | CPE002FJ |
| 8896 | C23 | C24 | 11 | 2545 | 336 | 205,5 | 30,5 | 2,45 | H5A034 | C | pos1 | Hm Drgbl | Hm Drgbl | Hm Drgbl | CPE002E7 | CPE002ES | CPE002FD |
| 18185 | F24 | F1 | 14 | 1262 | 428 | 316,1 | 30,6 | 1,968 | H5A034 | C | pos1 | Hm Gen E | Hm Gen E | Hm Gen E | CPE002JL | CPE002OO | CPE002KG |
| 1936 | A23 | A24 | 18 | 875,7 | 551 | 697,1 | 30,6 | 2,661 | H5A034 | C | pos1 | Hm Drgbl | Hm Drgbl | Hm Drgbl | CPE002FX | CPE002G1 | CPE002G5 |
| 17032 | F23 | F24 | 80 | 1151 | 2455 | 438,2 | 30,7 | 2,371 | H5A034 | C | pos1 | Hm Gen E | Hm Gen E | Hm Gen E | CPE002JI | CPE002NZ | CPE002KD |
| 18569 | F24 | F1 | 32 | 1186 | 988 | 306,3 | 30,9 | 2,095 | H5A034 | C | pos1 | Hm Gen E | Hm Gen E | Hm Gen E | CPE002JM | CPE002O1 | CPE002KH |
| 10408 | B23 | B24 | 8 | 1104 | 248 | 249,6 | 31 | 3,503 | H5A034 | C | pos1 | Hm Drgbl | Hm Drgbl | Hm Drgbl | CPE002EB | CPE002EO | CPE002FR |
| 16624 | E23 | E24 | 16 | 1240 | 497 | 568,1 | 31,1 | 2,458 | H5A034 | C | pos1 | Hm Gen E | Hm Gen E | Hm Gen E | CPE002JH | CPE002NV | CPE002KC |
| 11224 | D23 | D24 | 32 | 917,5 | 997 | 167,9 | 31,2 | 2,261 | H5A034 | C | pos1 | Hm Drgbl | Hm Drgbl | Hm Drgbl | CPE002ED | CPE002F3 | CPE002FT |
| 23465 | B24 | B1 | 61 | 1594 | 1912 | 454,5 | 31,3 | 1,66 | H5A034 | C | pos1 | Hm Gen E | Hm Gen E | Hm Gen E | CPE002KV | CPE002OE | CPE002KU |
| 10793 | B24 | B1 | 21 | 1083 | 658 | 694,9 | 31,3 | 2,371 | H5A034 | C | pos1 | Hm Drgbl | Hm Drgbl | Hm Drgbl | CPE002EC | CPE002EN | CPE002FS |
| 10121 | F24 | F1 | 11 | 1199 | 344 | 240,2 | 31,3 | 3,01 | H5A034 | C | pos1 | Hm Drgbl | Hm Drgbl | Hm Drgbl | CPE002EA | CPE002EP | CPE002FA |
| 11992 | D23 | D24 | 4 | 1033 | 126 | 143,4 | 31,5 | 2,019 | H5A034 | C | pos1 | Hm Gen E | Hm Gen E | Hm Gen E | CPE002J4 | CPE002NL | CPE002K0 |
| 18160 | E23 | E24 | 24 | 1170 | 755 | 407,2 | 31,5 | 2,026 | H5A034 | C | pos1 | Hm Gen E | Hm Gen E | Hm Gen E | CPE002JL | CPE002OO | CPE002KG |
| 15064 | D23 | D24 | 4 | 797,4 | 127 | 175,1 | 31,8 | 2,224 | H5A034 | C | pos1 | Hm Gen E | Hm Gen E | Hm Gen E | CPE002J8 | CPE002NS | CPE002K8 |
| 19337 | F24 | F1 | 26 | 1381 | 828 | 367,2 | 31,8 | 2,255 | H5A034 | C | pos1 | Hm Gen E | Hm Gen E | Hm Gen E | CPE002JO | CPE002O3 | CPE002KJ |
| 23152 | E23 | E24 | 13 | 1322 | 415 | 234,3 | 31,9 | 1,378 | H5A034 | C | pos1 | Hm Gen E | Hm Gen E | Hm Gen E | CPE002JY | CPE002OD | CPE002KT |
| 4265 | B24 | B1 | 10 | 842,5 | 319 | 550,7 | 31,9 | 2,692 | H5A034 | C | pos1 | Hm Drgbl | Hm Drgbl | Hm Drgbl | CPE002DU | CPE002EK | CPE002FQ |
| 19720 | F23 | F24 | 19 | 383,1 | 610 | 472 | 32,1 | 2,091 | H5A034 | C | pos1 | Hm Gen E | Hm Gen E | Hm Gen E | CPE002JP | CPE002O4 | CPE002KK |
| 20368 | A23 | A24 | 32 | 990 | 1026 | 391,3 | 32,1 | 2,14 | H5A034 | C | pos1 | Hm Gen E | Hm Gen E | Hm Gen E | CPE002JR | CPE002O6 | CPE002KM |
| 20753 | A24 | A1 | 13 | 1232 | 417 | 431 | 32,1 | 2,277 | H5A034 | C | pos1 | Hm Gen E | Hm Gen E | Hm Gen E | CPE002JS | CPE002O7 | CPE002KN |
| 20800 | C23 | C24 | 18 | 1322 | 577 | 516,2 | 32,1 | 2,277 | H5A034 | C | pos1 | Hm Gen E | Hm Gen E | Hm Gen E | CPE002JS | CPE002O7 | CPE002KN |
| 15400 | B23 | B24 | 8 | 1064 | 257 | 151,9 | 32,1 | 2,918 | H5A034 | C | pos1 | Hm Gen E | Hm Gen E | Hm Gen E | CPE002JE | CPE002NT | CPE002K9 |
| 17800 | F23 | F24 | 33 | 995,7 | 1063 | 465,5 | 32,2 | 2,545 | H5A034 | C | pos1 | Hm Gen E | Hm Gen E | Hm Gen E | CPE002JK | CPE002NZ | CPE002KF |
| 23128 | D23 | D24 | 34 | 1364 | 1098 | 341,3 | 32,3 | 1,395 | H5A034 | C | pos1 | Hm Gen E | Hm Gen E | Hm Gen E | CPE002JY | CPE002OD | CPE002KT |
| 12305 | A24 | A1 | 7 | 993,8 | 226 | 337,4 | 32,3 | 2,429 | H5A034 | C | pos1 | Hm Gen E | Hm Gen E | Hm Gen E | CPE002J5 | CPE002NK | CPE002K1 |
| 8488 | B23 | B24 | 6 | 1224 | 195 | 224,3 | 32,5 | 2,297 | H5A034 | C | pos1 | Hm Drgbl | Hm Drgbl | Hm Drgbl | CPE002E6 | CPE002ET | CPE002FE |
| 19601 | A24 | A1 | 16 | 418,3 | 522 | 552,4 | 32,6 | 2,124 | H5A034 | C | pos1 | Hm Gen E | Hm Gen E | Hm Gen E | CPE002JP | CPE002O4 | CPE002KK |
| 18496 | C23 | C24 | 35 | 1224 | 1140 | 436,4 | 32,6 | 2,21 | H5A034 | C | pos1 | Hm Gen E | Hm Gen E | Hm Gen E | CPE002JM | CPE002O1 | CPE002KH |
| 7336 | B23 | B24 | 21 | 917,6 | 685 | 616,5 | 32,6 | 2,489 | H5A034 | C | pos1 | Hm Drgbl | Hm Drgbl | Hm Drgbl | CPE002E3 | CPE002EW | CPE002FH |
| 6257 | E24 | E1 | 6 | 595,2 | 197 | 542,7 | 32,8 | 2,165 | H5A034 | C | pos1 | Hm Drgbl | Hm Drgbl | Hm Drgbl | CPE002DZ | CPE002F0 | CPE002FL |
| 6617 | D24 | D1 | 9 | 734,7 | 295 | 556,2 | 32,8 | 2,877 | H5A034 | C | pos1 | Hm Drgbl | Hm Drgbl | Hm Drgbl | CPE002E1 | CPE002EY | CPE002FJ |
| 16936 | B23 | B24 | 29 | 1294 | 954 | 294,5 | 32,9 | 2,541 | H5A034 | C | pos1 | Hm Gen E | Hm Gen E | Hm Gen E | CPE002JI | CPE002NZ | CPE002KD |
| 16984 | D23 | D24 | 50 | 1300 | 1644 | 536 | 32,9 | 2,541 | H5A034 | C | pos1 | Hm Gen E | Hm Gen E | Hm Gen E | CPE002JI | CPE002NZ | CPE002KD |
| 12017 | E24 | E1 | 8 | 1266 | 264 | 122,5 | 33 | 2,115 | H5A034 | C | pos1 | Hm Gen E | Hm Gen E | Hm Gen E | CPE002J4 | CPE002NL | CPE002K0 |
| 16648 | F23 | F24 | 24 | 987,5 | 792 | 440,4 | 33 | 2,609 | H5A034 | C | pos1 | Hm Gen E | Hm Gen E | Hm Gen E | CPE002JH | CPE002NV | CPE002KC |
| 15376 | A23 | A24 | 3 | 1243 | 99 | 246,9 | 33 | 3 | H5A034 | C | pos1 | Hm Gen E | Hm Gen E | Hm Gen E | CPE002JE | CPE002NT | CPE002K9 |
| 23080 | B23 | B24 | 27 | 1385 | 898 | 310,4 | 33,3 | 1,438 | H5A034 | C | pos1 | Hm Gen E | Hm Gen E | Hm Gen E | CPE002JY | CPE002OD | CPE002KT |
| 21953 | C24 | C1 | 41 | 1534 | 1366 | 489,3 | 33,3 | 1,582 | H5A034 | C | pos1 | Hm Gen E | Hm Gen E | Hm Gen E | CPE002JV | CPE002OA | CPE002KQ |
| 22312 | B23 | B24 | 29 | 1536 | 967 | 772,5 | 33,3 | 1,716 | H5A034 | C | pos1 | Hm Gen E | Hm Gen E | Hm Gen E | CPE002JW | CPE002OB | CPE002KR |
| 6233 | D24 | D1 | 21 | 526,8 | 699 | 717,8 | 33,3 | 2,198 | H5A034 | C | pos1 | Hm Drgbl | Hm Drgbl | Hm Drgbl | CPE002DZ | CPE002F0 | CPE002FL |
| 16912 | A23 | A24 | 24 | 1329 | 801 | 306,6 | 33,4 | 2,579 | H5A034 | C | pos1 | Hm Gen E | Hm Gen E | Hm Gen E | CPE002JI | CPE002NZ | CPE002KD |
| 19600 | A23 | A24 | 47 | 401,2 | 1575 | 595 | 33,5 | 2,182 | H5A034 | C | pos1 | Hm Gen E | Hm Gen E | Hm Gen E | CPE002JP | CPE002O4 | CPE002KK |
| 19649 | C24 | C1 | 65 | 362 | 2180 | 712,9 | 33,5 | 2,182 | H5A034 | C | pos1 | Hm Gen E | Hm Gen E | Hm Gen E | CPE002JP | CPE002O4 | CPE002KK |
| 22769 | E24 | E1 | 23 | 2040 | 772 | 375,2 | 33,6 | 1,577 | H5A034 | C | pos1 | Hm Gen E | Hm Gen E | Hm Gen E | CPE002JX | CPE002OC | CPE002KS |
| 19624 | B23 | B24 | 8 | 474,5 | 269 | 546,8 | 33,6 | 2,189 | H5A034 | C | pos1 | Hm Gen E | Hm Gen E | Hm Gen E | CPE002JP | CPE002O4 | CPE002KK |
| 17728 | C23 | C24 | 10 | 1149 | 336 | 263,5 | 33,6 | 2,656 | H5A034 | C | pos1 | Hm Gen E | Hm Gen E | Hm Gen E | CPE002JK | CPE002NZ | CPE002KF |
| 10073 | D24 | D1 | 5 | 1450 | 168 | 259,6 | 33,6 | 3,231 | H5A034 | C | pos1 | Hm Drgbl | Hm Drgbl | Hm Drgbl | CPE002EA | CPE002EP | CPE002FA |
| 18448 | A23 | A24 | 39 | 1159 | 1326 | 449,2 | 34 | 2,305 | H5A034 | C | pos1 | Hm Gen E | Hm Gen E | Hm Gen E | CPE002JM | CPE002O1 | CPE002KH |
| 21137 | A24 | A1 | 19 | 1256 | 647 | 443,2 | 34,1 | 2,418 | H5A034 | C | pos1 | Hm Gen E | Hm Gen E | Hm Gen E | CPE002JT | CPE002O8 | CPE002KO |
| 22409 | F24 | F1 | 13 | 1584 | 444 | 480,4 | 34,2 | 1,763 | H5A034 | C | pos1 | Hm Gen E | Hm Gen E | Hm Gen E | CPE002JW | CPE002OB | CPE002KR |
| 20849 | E24 | E1 | 18 | 1180 | 616 | 299,2 | 34,2 | 2,426 | H5A034 | C | pos1 | Hm Gen E | Hm Gen E | Hm Gen E | CPE002JS | CPE002O7 | CPE002KN |
| 7313 | A24 | A1 | 6 | 589 | 206 | 614,3 | 34,3 | 2,618 | H5A034 | C | pos1 | Hm Drgbl | Hm Drgbl | Hm Drgbl | CPE002E3 | CPE002EW | CPE002FH |
| 18136 | D23 | D24 | 20 | 1189 | 687 | 414 | 34,4 | 2,212 | H5A034 | C | pos1 | Hm Gen E | Hm Gen E | Hm Gen E | CPE002JL | CPE002OO | CPE002KG |
| 22697 | B24 | B1 | 16 | 2069 | 552 | 526,1 | 34,5 | 1,62 | H5A034 | C | pos1 | Hm Gen E | Hm Gen E | Hm Gen E | CPE002JX | CPE002OC | CPE002KS |
| 6280 | F23 | F24 | 21 | 723,5 | 725 | 724,4 | 34,5 | 2,277 | H5A034 | C | pos1 | Hm Drgbl | Hm Drgbl | Hm Drgbl | CPE002DZ | CPE002F0 | CPE002FL |
| 8489 | B24 | B1 | 6 | 1258 | 208 | 205,8 | 34,7 | 2,452 | H5A034 | C | pos1 | Hm Drgbl | Hm Drgbl | Hm Drgbl | CPE002E6 | CPE002ET | CPE002FE |
| 22025 | F24 | F1 | 14 | 1624 | 487 | 263,2 | 34,8 | 1,653 | H5A034 | C | pos1 | Hm Gen E | Hm Gen E | Hm Gen E | CPE002JV | CPE002OA | CPE002KQ |
| 22744 | D23 | D24 | 15 | 1871 | 526 | 443,3 | 35,1 | 1,648 | H5A034 | C | pos1 | Hm Gen E | Hm Gen E | Hm Gen E | CPE002JX | CPE002OC | CPE002KS |
| 13144 | D23 | D24 | 4 | 1186 | 141 | 123,9 | 35,2 | 1,764 | H5A034 | C | pos1 | Hm Gen E | Hm Gen E | Hm Gen E | CPE002JB | CPE002NN | CPE002K3 |
| 14344 | F23 | F24 | 8 | 1012 | 282 | 167 | 35,2 | 2,505 | H5A034 | C | pos1 | Hm Gen E | Hm Gen E | Hm Gen E | CPE002J7 | CPE002NC | CPE002K6 |
| 19312 | E23 | E24 | 71 | 1191 | 2504 | 623 | 35,3 | 2,504 | H5A034 | C | pos1 | Hm Gen E | Hm Gen E | Hm Gen E | CPE002JO | CPE002O3 | CPE002KJ |
| 14249 | B24 | B1 | 13 | 1146 | 459 | 283,5 | 35,3 | 2,512 | H5A034 | C | pos1 | Hm Gen E | Hm Gen E | Hm Gen E | CPE002J7 | CPE002NC | CPE002K6 |
| 10120 | F23 | F24 | 12 | 960,5 | 425 | 215,4 | 35,4 | 3,404 | H5A034 | C | pos1 | Hm Drgbl | Hm Drgbl | Hm Drgbl | CPE002EA | CPE002EP | CPE002FA |
| 19985 | A24 | A1 | 7 | 1316 | 248 | 269,2 | 35,4 | 4,317 | H5A034 | C | pos1 | Hm Gen E | Hm Gen E | Hm Gen E | CPE002JQ | CPE002O5 | CPE002KL |
| 18929 | E24 | E1 | 24 | 1121 | 860 | 429 | 35,8 | 2,495 | H5A034 | C | pos1 | Hm Gen E | Hm Gen E | Hm Gen E | CPE002JN | CPE002O2 | CPE002KI |
| 23536 | E23 | E24 | 37 | 1833 | 1329 | 352,1 | 35,9 | 1,905 | H5A034 | C | pos1 | Hm Gen E | Hm Gen E | Hm Gen E | CPE002KV | CPE002OE | CPE002KU |
| 15472 | E23 | E24 | 22 | 913,7 | 793 | 155,9 | 36 | 3,273 | H5A034 | C | pos1 | Hm Gen E | Hm Gen E | Hm Gen E | CPE002JE | CPE002NT | CPE002K9 |
| 15112 | F23 | F24 | 14 | 829,5 | 505 | 203,9 | 36,1 | 2,524 | H5A034 | C | pos1 | Hm Gen E | Hm Gen E | Hm Gen E | CPE002J8 | CPE002NS | CPE002K8 |
| 21185 | C24 | C1 | 8 | 1148 | 290 | 505,3 | 36,2 | 2,567 | H5A034 | C | pos1 | Hm Gen E | Hm Gen E | Hm Gen E | CPE002JT | CPE002O8 | CPE002KO |
| 12376 | D23 | D24 | 14 | 1623 | 507 | 213,3 | 36,2 | 2,722 | H5A034 | C | pos1 | Hm Gen E | Hm Gen E | Hm Gen E | CPE002J5 | CPE002NK | CPE002K1 |
| 20848 | E23 | E24 | 27 | 1103 | 986 | 456,9 | 36,5 | 2,589 | H5A034 | C | pos1 | Hm Gen E | Hm Gen E | Hm Gen E | CPE002JS | CPE002O7 | CPE002KN |
| 19696 | E23 | E24 | 33 | 432,3 | 1207 | 782,5 | 36,6 | 2,384 | H5A034 | C | pos1 | Hm Gen E | Hm Gen E | Hm Gen E | CPE002JP | CPE002O4 | CPE002KK |
| 22745 | D24 | D1 | 32 | 2118 | 1175 | 427,7 | 36,7 | 1,723 | H5A034 | C | pos1 | Hm Gen E | Hm Gen E | Hm Gen E | CPE002JX | CPE002OC | CPE002KS |
| 6641 | E24 | E1 | 6 | 713,9 | 221 | 554,7 | 36,8 | 3,228 | H5A034 | C | pos1 | Hm Drgbl | Hm Drgbl | Hm Drgbl | CPE002E1 | CPE002EY | CPE002FJ |
| 23153 | E24 | E1 | 9 | 1030 | 332 | 168,4 | 36,9 | 1,594 | H5A034 | C | pos1 | Hm Gen E | Hm Gen E | Hm Gen E | CPE002JY | CPE002OD | CPE002KT |
| 12737 | C24 | C1 | 13 | 1384 | 481 | 191,5 | 37 | 2,508 | H5A034 | C | pos1 | Hm Gen E | Hm Gen E | Hm Gen E | CPE002J6 | CPE002NN | CPE002K2 |
| 18544 | E23 | E24 | 43 | 1311 | 1593 | 428,8 | 37 | 2,508 | H5A034 | C | pos1 | Hm Gen E | Hm Gen E | Hm Gen E | CPE002JM | CPE002O1 | CPE002KH |
| 13528 | D23 | D24 | 12 | 1131 | 444 | 208,1 | 37 | 3,474 | H5A034 | C | pos1 | Hm Gen E | Hm Gen E | Hm Gen E | CPE002JC | CPE002NC | CPE002K4 |
| 18520 | D23 | D24 | 14 | 1233 | 524 | 342 | 37,4 | 2,536 | H5A034 | C | pos1 | Hm Gen E | Hm Gen E | Hm Gen E | CPE002JM | CPE002O1 | CPE002KH |
| 18952 | F23 | F24 | 27 | 1125 | 1011 | 415 | 37,4 | 2,606 | H5A034 | C | pos1 | Hm Gen E | Hm Gen E | Hm Gen E | CPE002JN | CPE002O2 | CPE002KI |
| 9304 | D23 | D24 | 4 | 1390 | 150 | 199,8 | 37,5 | 2,604 | H5A034 | C | pos1 | Hm Drgbl | Hm Drgbl | Hm Drgbl | CPE002E8 | CPE002ER | CPE002FC |
| 11536 | A23 | A24 | 18 | 911,4 | 680 | 197,4 | 37,8 | 3,375 | H5A034 | C | pos1 | Hm Gen E | Hm Gen E | Hm Gen E | CPE002JA | CPE002NJ | CPE002JZ |
| 10768 | A23 | A24 | 6 | 1068 | 228 | 944,9 | 38 | 2,879 | H5A034 | C | pos1 | Hm Drgbl | Hm Drgbl | Hm Drgbl | CPE002EC | CPE002EN | CPE002FS |
| 16552 | B23 | B24 | 8 | 1399 | 305 | 495,9 | 38,1 | 3,012 | H5A034 | C | pos1 | Hm Gen E | Hm Gen E | Hm Gen E | CPE002JH | CPE002NV | CPE002KC |
| 20392 | B23 | B24 | 17 | 939,4 | 649 | 519,7 | 38,2 | 2,547 | H5A034 | C | pos1 | Hm Gen E | Hm Gen E | Hm Gen E | CPE002JR | CPE002O6 | CPE002KM |
| 12760 | D23 | D24 | 15 | 1443 | 573 | 203,2 | 38,2 | 2,59 | H5A034 | C | pos1 | Hm Gen E | Hm Gen E | Hm Gen E | CPE002J6 | CPE002NN | CPE002K2 |
| 18857 | B24 | B1 | 12 | 1186 | 458 | 423,9 | 38,2 | 2,662 | H5A034 | C | pos1 | Hm Gen E | Hm Gen E | Hm Gen E | CPE002JN | CPE002O2 | CPE002KI |
| 21208 | D23 | D24 | 19 | 1161 | 725 | 420,5 | 38,2 | 2,709 | H5A034 | C | pos1 | Hm Gen E | Hm Gen E | Hm Gen E | CPE002JT | CPE002O8 | CPE002KO |
| 22336 | C23 | C24 | 17 | 1627 | 653 | 507,5 | 38,4 | 1,979 | H5A034 | C | pos1 | Hm Gen E | Hm Gen E | Hm Gen E | CPE002JW | CPE002OB | CPE002KR |
| 9305 | D24 | D1 | 8 | 1119 | 307 | 227,6 | 38,4 | 2,667 | H5A034 | C | pos1 | Hm Drgbl | Hm Drgbl | Hm Drgbl | CPE002E8 | CPE002ER | CPE002FC |
| 8873 | B24 | B1 | 18 | 2418 | 693 | 251,7 | 38,5 | 3,092 | H5A034 | C | pos1 | Hm Drgbl | Hm Drgbl | Hm Drgbl | CPE002E7 | CPE002ES | CPE002FD |
| 18856 | B23 | B24 | 37 | 1298 | 1433 | 515,5 | 38,7 | 2,697 | H5A034 | C | pos1 | Hm Gen E | Hm Gen E | Hm Gen E | CPE002JN | CPE002O2 | CPE002KI |
| 17297 | A24 | A1 | 19 | 1191 | 737 | 347 | 38,8 | 2,648 | H5A034 | C | pos1 | Hm Gen E | Hm Gen E | Hm Gen E | CPE002JJ | CPE002NY | CPE002KE |
| 18473 | B24 | B1 | 15 | 1191 | 584 | 593,7 | 38,9 | 2,637 | H5A034 | C | pos1 | Hm Gen E | Hm Gen E | Hm Gen E | CPE002JM | CPE002O1 | CPE002KH |
| 22793 | F24 | F1 | 13 | 2229 | 507 | 437,3 | 39 | 1,831 | H5A034 | C | pos1 | Hm Gen E | Hm Gen E | Hm Gen E | CPE002JX | CPE002OC | CPE002KS |
| 11153 | A24 | A1 | 6 | 1151 | 234 | 245,4 | 39 | 2,826 | H5A034 | C | pos1 | Hm Drgbl | Hm Drgbl | Hm Drgbl | CPE002ED | CPE002F3 | CPE002FT |
| 10889 | F24 | F1 | 3 | 1077 | 117 | 496,4 | 39 | 2,955 | H5A034 | C | pos1 | Hm Drgbl | Hm Drgbl | Hm Drgbl | CPE002EC | CPE002EN | CPE002FS |
| 8897 | C24 | C1 | 1 | 3835 | 39 | 183,9 | 39 | 3,133 | H5A034 | C | pos1 | Hm Drgbl | Hm Drgbl | Hm Drgbl | CPE002E7 | CPE002ES | CPE002FD |
| 13504 | C23 | C24 | 5 | 1053 | 196 | 182,8 | 39,2 | 3,681 | H5A034 | C | pos1 | Hm Gen E | Hm Gen E | Hm Gen E | CPE002JC | CPE002NC | CPE002K4 |
| 19313 | E24 | E1 | 7 | 1124 | 275 | 480,2 | 39,3 | 2,787 | H5A034 | C | pos1 | Hm Gen E | Hm Gen E | Hm Gen E | CPE002JO | CPE002O3 | CPE002KJ |
| 21257 | F24 | F1 | 10 | 1291 | 398 | 341,9 | 39,8 | 2,823 | H5A034 | C | pos1 | Hm Gen E | Hm Gen E | Hm Gen E | CPE002JT | CPE002O8 | CPE002KO |
| 15041 | C24 | C1 | 11 | 933,3 | 439 | 199 | 39,9 | 2,79 | H5A034 | C | pos1 | Hm Gen E | Hm Gen E | Hm Gen E | CPE002J8 | CPE002NS | CPE002K8 |
| 15497 | F24 | F1 | 7 | 1043 | 281 | 198,2 | 40,1 | 3,645 | H5A034 | C | pos1 | Hm Gen E | Hm Gen E | Hm Gen E | CPE002JE | CPE002NT | CPE002K9 |
| 10048 | C23 | C24 | 5 | 1114 | 201 | 236,8 | 40,2 | 3,865 | H5A034 | C | pos1 | Hm Drgbl | Hm Drgbl | Hm Drgbl | CPE002EA | CPE002EP | CPE002FA |
| 17681 | A24 | A1 | 2 | 1131 | 81 | 333,8 | 40,5 | 3,202 | H5A034 | C | pos1 | Hm Gen E | Hm Gen E | Hm Gen E | CPE002JK | CPE002NZ | CPE002KF |
| 8561 | E24 | E1 | 5 | 1349 | 203 | 164,8 | 40,6 | 2,869 | H5A034 | C | pos1 | Hm Drgbl | Hm Drgbl | Hm Drgbl | CPE002E6 | CPE002ET | CPE002FE |
| 22385 | E24 | E1 | 30 | 1430 | 1220 | 547,7 | 40,7 | 2,098 | H5A034 | C | pos1 | Hm Gen E | Hm Gen E | Hm Gen E | CPE002JW | CPE002OB | CPE002KR |
| 6952 | B23 | B24 | 23 | 703,4 | 937 | 613 | 40,7 | 3,167 | H5A034 | C | pos1 | Hm Drgbl | Hm Drgbl | Hm Drgbl | CPE002E2 | CPE002EX | CPE002FI |
| 22792 | F23 | F24 | 19 | 1974 | 776 | 593,8 | 40,8 | 1,915 | H5A034 | C | pos1 | Hm Gen E | Hm Gen E | Hm Gen E | CPE002JX | CPE002OC | CPE002KS |
| 23440 | A23 | A24 | 15 | 2291 | 612 | 449,7 | 40,8 | 2,164 | H5A034 | C | pos1 | Hm Gen E | Hm Gen E | Hm Gen E | CPE002KV | CPE002OE | CPE002KU |
| 13961 | F24 | F1 | 10 | 1497 | 408 | 172 | 40,8 | 2,338 | H5A034 | C | pos1 | Hm Gen E | Hm Gen E | Hm Gen E | CPE002JF | CPE002NP | CPE002K5 |
| 12736 | C23 | C24 | 8 | 1247 | 326 | 139,4 | 40,8 | 2,766 | H5A034 | C | pos1 | Hm Gen E | Hm Gen E | Hm Gen E | CPE002J6 | CPE002NN | CPE002K2 |
| 9329 | E24 | E1 | 4 | 1087 | 163 | 237,9 | 40,8 | 2,833 | H5A034 | C | pos1 | Hm Drgbl | Hm Drgbl | Hm Drgbl | CPE002E8 | CPE002ER | CPE002FC |
| 18568 | F23 | F24 | 18 | 1188 | 737 | 344,7 | 40,9 | 2,773 | H5A034 | C | pos1 | Hm Gen E | Hm Gen E | Hm Gen E | CPE002JM | CPE002O1 | CPE002KH |
| 11200 | C23 | C24 | 3 | 1034 | 123 | 174,3 | 41 | 2,971 | H5A034 | C | pos1 | Hm Drgbl | Hm Drgbl | Hm Drgbl | CPE002ED | CPE002F3 | CPE002FT |
| 21928 | B23 | B24 | 20 | 1950 | 823 | 409,1 | 41,1 | 1,952 | H5A034 | C | pos1 | Hm Gen E | Hm Gen E | Hm Gen E | CPE002JV | CPE002OA | CPE002KQ |
| 8536 | D23 | D24 | 4 | 1153 | 166 | 164,6 | 41,5 | 2,933 | H5A034 | C | pos1 | Hm Drgbl | Hm Drgbl | Hm Drgbl | CPE002E6 | CPE002ET | CPE002FE |
| 21136 | A23 | A24 | 28 | 1448 | 1163 | 606,4 | 41,5 | 2,943 | H5A034 | C | pos1 | Hm Gen E | Hm Gen E | Hm Gen E | CPE002JT | CPE002O8 | CPE002KO |
| 23056 | A23 | A24 | 46 | 1407 | 1916 | 342,7 | 41,7 | 1,801 | H5A034 | C | pos1 | Hm Gen E | Hm Gen E | Hm Gen E | CPE002JY | CPE002OD | CPE002KT |
| 23560 | F23 | F24 | 24 | 1867 | 1000 | 529,8 | 41,7 | 2,212 | H5A034 | C | pos1 | Hm Gen E | Hm Gen E | Hm Gen E | CPE002KV | CPE002OE | CPE002KU |
| 8945 | E24 | E1 | 10 | 2384 | 417 | 214,2 | 41,7 | 3,349 | H5A034 | C | pos1 | Hm Drgbl | Hm Drgbl | Hm Drgbl | CPE002E7 | CPE002ES | CPE002FD |
| 10457 | D24 | D1 | 3 | 1028 | 125 | 241,8 | 41,7 | 4,712 | H5A034 | C | pos1 | Hm Drgbl | Hm Drgbl | Hm Drgbl | CPE002EB | CPE002EO | CPE002FR |
| 8944 | E23 | E24 | 4 | 2324 | 167 | 190,9 | 41,8 | 3,357 | H5A034 | C | pos1 | Hm Drgbl | Hm Drgbl | Hm Drgbl | CPE002E7 | CPE002ES | CPE002FD |
| 15424 | C23 | C24 | 7 | 836,2 | 293 | 262,1 | 41,9 | 3,809 | H5A034 | C | pos1 | Hm Gen E | Hm Gen E | Hm Gen E | CPE002JE | CPE002NT | CPE002K9 |
| 23104 | C23 | C24 | 35 | 1141 | 1474 | 275,9 | 42,1 | 1,819 | H5A034 | C | pos1 | Hm Gen E | Hm Gen E | Hm Gen E | CPE002JY | CPE002OD | CPE002KT |
| 12688 | A23 | A24 | 12 | 1287 | 505 | 193,9 | 42,1 | 2,854 | H5A034 | C | pos1 | Hm Gen E | Hm Gen E | Hm Gen E | CPE002J6 | CPE002NN | CPE002K2 |
| 7048 | F23 | F24 | 22 | 532,6 | 931 | 399 | 42,3 | 3,292 | H5A034 | C | pos1 | Hm Drgbl | Hm Drgbl | Hm Drgbl | CPE002E2 | CPE002EX | CPE002FI |
| 8584 | F23 | F24 | 5 | 1768 | 212 | 204,8 | 42,4 | 2,996 | H5A034 | C | pos1 | Hm Drgbl | Hm Drgbl | Hm Drgbl | CPE002E6 | CPE002ET | CPE002FE |
| 11945 | B24 | B1 | 6 | 1174 | 255 | 134,8 | 42,5 | 2,724 | H5A034 | C | pos1 | Hm Gen E | Hm Gen E | Hm Gen E | CPE002J4 | CPE002NL | CPE002K0 |
| 8921 | D24 | D1 | 8 | 2075 | 340 | 200,2 | 42,5 | 3,414 | H5A034 | C | pos1 | Hm Drgbl | Hm Drgbl | Hm Drgbl | CPE002E7 | CPE002ES | CPE002FD |
| 22313 | B24 | B1 | 33 | 1541 | 1424 | 685,4 | 43,2 | 2,227 | H5A034 | C | pos1 | Hm Gen E | Hm Gen E | Hm Gen E | CPE002JW | CPE002OB | CPE002KR |
| 14657 | C24 | C1 | 10 | 867,8 | 432 | 159,5 | 43,2 | 2,462 | H5A034 | C | pos1 | Hm Gen E | Hm Gen E | Hm Gen E | CPE002JD | CPE002NR | CPE002K7 |
| 12808 | F23 | F24 | 10 | 1158 | 433 | 264,6 | 43,3 | 2,936 | H5A034 | C | pos1 | Hm Gen E | Hm Gen E | Hm Gen E | CPE002J6 | CPE002NN | CPE002K2 |
| 22288 | A23 | A24 | 8 | 1556 | 347 | 595,8 | 43,4 | 2,237 | H5A034 | C | pos1 | Hm Gen E | Hm Gen E | Hm Gen E | CPE002JW | CPE002OB | CPE002KR |
| 20824 | D23 | D24 | 11 | 1134 | 477 | 444,7 | 43,4 | 3,078 | H5A034 | C | pos1 | Hm Gen E | Hm Gen E | Hm Gen E | CPE002JS | CPE002O7 | CPE002KN |
| 18137 | D24 | D1 | 5 | 1023 | 218 | 591,5 | 43,6 | 2,804 | H5A034 | C | pos1 | Hm Gen E | Hm Gen E | Hm Gen E | CPE002JL | CPE002OO | CPE002KG |
| 17320 | B23 | B24 | 19 | 1195 | 833 | 330,3 | 43,8 | 2,99 | H5A034 | C | pos1 | Hm Gen E | Hm Gen E | Hm Gen E | CPE002JJ | CPE002NY | CPE002KE |
| 11632 | E23 | E24 | 5 | 1116 | 219 | 266,9 | 43,8 | 3,911 | H5A034 | C | pos1 | Hm Gen E | Hm Gen E | Hm Gen E | CPE002JA | CPE002NJ | CPE002JZ |
| 10480 | E23 | E24 | 6 | 1074 | 264 | 252,4 | 44 | 4,972 | H5A034 | C | pos1 | Hm Drgbl | Hm Drgbl | Hm Drgbl | CPE002EB | CPE002EO | CPE002FR |
| 14225 | A24 | A1 | 7 | 1208 | 309 | 169,8 | 44,1 | 3,139 | H5A034 | C | pos1 | Hm Gen E | Hm Gen E | Hm Gen E | CPE002J7 | CPE002NC | CPE002K6 |
| 11561 | B24 | B1 | 23 | 946,6 | 1017 | 257,4 | 44,2 | 3,946 | H5A034 | C | pos1 | Hm Gen E | Hm Gen E | Hm Gen E | CPE002JA | CPE002NJ | CPE002JZ |
| 18545 | E24 | E1 | 16 | 931,4 | 710 | 403,1 | 44,4 | 3,01 | H5A034 | C | pos1 | Hm Gen E | Hm Gen E | Hm Gen E | CPE002JM | CPE002O1 | CPE002KH |
| 9664 | C23 | C24 | 5 | 1626 | 222 | 160,7 | 44,4 | 3,105 | H5A034 | C | pos1 | Hm Drgbl | Hm Drgbl | Hm Drgbl | CPE002E9 | CPE002EQ | CPE002FB |
| 14993 | A24 | A1 | 9 | 1096 | 401 | 195,7 | 44,6 | 3,119 | H5A034 | C | pos1 | Hm Gen E | Hm Gen E | Hm Gen E | CPE002J8 | CPE002NS | CPE002K8 |
| 11152 | A23 | A24 | 6 | 1133 | 268 | 207,4 | 44,7 | 3,239 | H5A034 | C | pos1 | Hm Drgbl | Hm Drgbl | Hm Drgbl | CPE002ED | CPE002F3 | CPE002FT |
| 18832 | A23 | A24 | 26 | 1027 | 1166 | 545,6 | 44,8 | 3,122 | H5A034 | C | pos1 | Hm Gen E | Hm Gen E | Hm Gen E | CPE002JN | CPE002O2 | CPE002KI |
| 15016 | B23 | B24 | 17 | 1097 | 762 | 202,5 | 44,8 | 3,133 | H5A034 | C | pos1 | Hm Gen E | Hm Gen E | Hm Gen E | CPE002J8 | CPE002NS | CPE002K8 |
| 22673 | A24 | A1 | 3 | 2594 | 135 | 422,3 | 45 | 2,113 | H5A034 | C | pos1 | Hm Gen E | Hm Gen E | Hm Gen E | CPE002JX | CPE002OC | CPE002KS |
| 18065 | A24 | A1 | 8 | 959,8 | 360 | 444,3 | 45 | 2,894 | H5A034 | C | pos1 | Hm Gen E | Hm Gen E | Hm Gen E | CPE002JL | CPE002OO | CPE002KG |
| 12353 | C24 | C1 | 5 | 1344 | 225 | 236,1 | 45 | 3,383 | H5A034 | C | pos1 | Hm Gen E | Hm Gen E | Hm Gen E | CPE002J5 | CPE002NK | CPE002K1 |
| 15065 | D24 | D1 | 11 | 994,6 | 496 | 204,4 | 45,1 | 3,154 | H5A034 | C | pos1 | Hm Gen E | Hm Gen E | Hm Gen E | CPE002J8 | CPE002NS | CPE002K8 |
| 12784 | E23 | E24 | 14 | 1048 | 633 | 212,6 | 45,2 | 3,064 | H5A034 | C | pos1 | Hm Gen E | Hm Gen E | Hm Gen E | CPE002J6 | CPE002NN | CPE002K2 |
| 10840 | D23 | D24 | 5 | 1245 | 226 | 905,6 | 45,2 | 3,424 | H5A034 | C | pos1 | Hm Drgbl | Hm Drgbl | Hm Drgbl | CPE002EC | CPE002EN | CPE002FS |
| 8513 | C24 | C1 | 4 | 1314 | 182 | 254,9 | 45,5 | 3,216 | H5A034 | C | pos1 | Hm Drgbl | Hm Drgbl | Hm Drgbl | CPE002E6 | CPE002ET | CPE002FE |
| 19721 | F24 | F1 | 33 | 346,3 | 1510 | 495,7 | 45,8 | 2,984 | H5A034 | C | pos1 | Hm Gen E | Hm Gen E | Hm Gen E | CPE002JP | CPE002O4 | CPE002KK |
| 15113 | F24 | F1 | 8 | 627,3 | 366 | 162,5 | 45,8 | 3,203 | H5A034 | C | pos1 | Hm Gen E | Hm Gen E | Hm Gen E | CPE002J8 | CPE002NS | CPE002K8 |
| 13936 | E23 | E24 | 7 | 1457 | 321 | 183,6 | 45,9 | 2,63 | H5A034 | C | pos1 | Hm Gen E | Hm Gen E | Hm Gen E | CPE002JF | CPE002NP | CPE002K5 |
| 20488 | F23 | F24 | 15 | 856,1 | 689 | 463,5 | 45,9 | 3,06 | H5A034 | C | pos1 | Hm Gen E | Hm Gen E | Hm Gen E | CPE002JR | CPE002O6 | CPE002KM |
| 15088 | E23 | E24 | 16 | 1046 | 738 | 237,4 | 46,1 | 3,224 | H5A034 | C | pos1 | Hm Gen E | Hm Gen E | Hm Gen E | CPE002J8 | CPE002NS | CPE002K8 |
| 21929 | B24 | B1 | 20 | 1409 | 925 | 418,3 | 46,2 | 2,195 | H5A034 | C | pos1 | Hm Gen E | Hm Gen E | Hm Gen E | CPE002JV | CPE002OA | CPE002KQ |
| 21905 | A24 | A1 | 19 | 1219 | 879 | 230,8 | 46,3 | 2,2 | H5A034 | C | pos1 | Hm Gen E | Hm Gen E | Hm Gen E | CPE002JV | CPE002OA | CPE002KQ |
| 14680 | D23 | D24 | 5 | 975 | 232 | 150,1 | 46,4 | 2,644 | H5A034 | C | pos1 | Hm Gen E | Hm Gen E | Hm Gen E | CPE002JD | CPE002NR | CPE002K7 |
| 17704 | B23 | B24 | 8 | 1464 | 372 | 442,2 | 46,5 | 3,676 | H5A034 | C | pos1 | Hm Gen E | Hm Gen E | Hm Gen E | CPE002JK | CPE002NZ | CPE002KF |
| 18112 | C23 | C24 | 33 | 1164 | 1545 | 516,6 | 46,8 | 3,01 | H5A034 | C | pos1 | Hm Gen E | Hm Gen E | Hm Gen E | CPE002JL | CPE002OO | CPE002KG |
| 10481 | E24 | E1 | 5 | 1221 | 234 | 231,7 | 46,8 | 5,288 | H5A034 | C | pos1 | Hm Drgbl | Hm Drgbl | Hm Drgbl | CPE002EB | CPE002EO | CPE002FR |
| 22289 | A24 | A1 | 12 | 1458 | 563 | 459,6 | 46,9 | 2,418 | H5A034 | C | pos1 | Hm Gen E | Hm Gen E | Hm Gen E | CPE002JW | CPE002OB | CPE002KR |
| 13457 | A24 | A1 | 1 | 645,5 | 47 | 237,7 | 47 | 4,413 | H5A034 | C | pos1 | Hm Gen E | Hm Gen E | Hm Gen E | CPE002JC | CPE002NC | CPE002K4 |
| 19625 | B24 | B1 | 6 | 427,9 | 283 | 518,7 | 47,2 | 3,075 | H5A034 | C | pos1 | Hm Gen E | Hm Gen E | Hm Gen E | CPE002JP | CPE002O4 | CPE002KK |
| 9256 | B23 | B24 | 37 | 1010 | 1751 | 236,4 | 47,3 | 3,285 | H5A034 | C | pos1 | Hm Drgbl | Hm Drgbl | Hm Drgbl | CPE002E8 | CPE002ER | CPE002FC |
| 23105 | C24 | C1 | 16 | 1086 | 759 | 362,9 | 47,4 | 2,048 | H5A034 | C | pos1 | Hm Gen E | Hm Gen E | Hm Gen E | CPE002JY | CPE002OD | CPE002KT |
| 10456 | D23 | D24 | 6 | 1152 | 285 | 260,4 | 47,5 | 5,367 | H5A034 | C | pos1 | Hm Drgbl | Hm Drgbl | Hm Drgbl | CPE002EB | CPE002EO | CPE002FR |
| 18880 | C23 | C24 | 19 | 1039 | 905 | 373,3 | 47,6 | 3,317 | H5A034 | C | pos1 | Hm Gen E | Hm Gen E | Hm Gen E | CPE002JN | CPE002O2 | CPE002KI |
| 13913 | D24 | D1 | 11 | 1340 | 526 | 192,1 | 47,8 | 2,739 | H5A034 | C | pos1 | Hm Gen E | Hm Gen E | Hm Gen E | CPE002JF | CPE002NP | CPE002K5 |
| 19240 | B23 | B24 | 37 | 1243 | 1773 | 559,4 | 47,9 | 3,397 | H5A034 | C | pos1 | Hm Gen E | Hm Gen E | Hm Gen E | CPE002JO | CPE002O3 | CPE002KJ |
| 13553 | E24 | E1 | 6 | 1112 | 288 | 170,2 | 48 | 4,507 | H5A034 | C | pos1 | Hm Gen E | Hm Gen E | Hm Gen E | CPE002JC | CPE002NC | CPE002K4 |
| 12712 | B23 | B24 | 18 | 1296 | 867 | 155,8 | 48,2 | 3,268 | H5A034 | C | pos1 | Hm Gen E | Hm Gen E | Hm Gen E | CPE002J6 | CPE002NN | CPE002K2 |
| 6953 | B24 | B1 | 20 | 680,2 | 971 | 814,5 | 48,5 | 3,774 | H5A034 | C | pos1 | Hm Drgbl | Hm Drgbl | Hm Drgbl | CPE002E2 | CPE002EX | CPE002FI |
| 14632 | B23 | B24 | 9 | 1245 | 438 | 165,3 | 48,7 | 2,775 | H5A034 | C | pos1 | Hm Gen E | Hm Gen E | Hm Gen E | CPE002JD | CPE002NR | CPE002K7 |
| 14296 | D23 | D24 | 18 | 907,7 | 884 | 243,1 | 49,1 | 3,495 | H5A034 | C | pos1 | Hm Gen E | Hm Gen E | Hm Gen E | CPE002J7 | CPE002NC | CPE002K6 |
| 11272 | F23 | F24 | 8 | 942 | 394 | 240,2 | 49,2 | 3,565 | H5A034 | C | pos1 | Hm Drgbl | Hm Drgbl | Hm Drgbl | CPE002ED | CPE002F3 | CPE002FT |
| 6929 | A24 | A1 | 2 | 423,7 | 99 | 560 | 49,5 | 3,852 | H5A034 | C | pos1 | Hm Drgbl | Hm Drgbl | Hm Drgbl | CPE002E2 | CPE002EX | CPE002FI |
| 13937 | E24 | E1 | 6 | 1299 | 302 | 179,1 | 50,3 | 2,883 | H5A034 | C | pos1 | Hm Gen E | Hm Gen E | Hm Gen E | CPE002JF | CPE002NP | CPE002K5 |
| 18184 | F23 | F24 | 24 | 1008 | 1208 | 414,1 | 50,3 | 3,235 | H5A034 | C | pos1 | Hm Gen E | Hm Gen E | Hm Gen E | CPE002JL | CPE002OO | CPE002KG |
| 22672 | A23 | A24 | 12 | 1978 | 607 | 418,3 | 50,6 | 2,376 | H5A034 | C | pos1 | Hm Gen E | Hm Gen E | Hm Gen E | CPE002JX | CPE002OC | CPE002KS |
| 18064 | A23 | A24 | 17 | 1420 | 861 | 387,2 | 50,6 | 3,254 | H5A034 | C | pos1 | Hm Gen E | Hm Gen E | Hm Gen E | CPE002JL | CPE002OO | CPE002KG |
| 8872 | B23 | B24 | 10 | 1696 | 508 | 254 | 50,8 | 4,08 | H5A034 | C | pos1 | Hm Drgbl | Hm Drgbl | Hm Drgbl | CPE002E7 | CPE002ES | CPE002FD |
| 20393 | B24 | B1 | 19 | 775,8 | 974 | 453,4 | 51,3 | 3,42 | H5A034 | C | pos1 | Hm Gen E | Hm Gen E | Hm Gen E | CPE002JR | CPE002O6 | CPE002KM |
| 14656 | C23 | C24 | 8 | 1098 | 412 | 142,6 | 51,5 | 2,934 | H5A034 | C | pos1 | Hm Gen E | Hm Gen E | Hm Gen E | CPE002JD | CPE002NR | CPE002K7 |
| 14992 | A23 | A24 | 18 | 724,9 | 931 | 161,5 | 51,7 | 3,615 | H5A034 | C | pos1 | Hm Gen E | Hm Gen E | Hm Gen E | CPE002J8 | CPE002NS | CPE002K8 |
| 13888 | C23 | C24 | 6 | 1711 | 311 | 218,1 | 51,8 | 2,968 | H5A034 | C | pos1 | Hm Gen E | Hm Gen E | Hm Gen E | CPE002JF | CPE002NP | CPE002K5 |
| 6545 | A24 | A1 | 4 | 660,5 | 207 | 668,4 | 51,8 | 4,544 | H5A034 | C | pos1 | Hm Drgbl | Hm Drgbl | Hm Drgbl | CPE002E1 | CPE002EY | CPE002FJ |
| 9617 | A24 | A1 | 2 | 875,9 | 105 | 275,2 | 52,5 | 3,671 | H5A034 | C | pos1 | Hm Drgbl | Hm Drgbl | Hm Drgbl | CPE002E9 | CPE002EQ | CPE002FB |
| 13072 | A23 | A24 | 19 | 1370 | 1006 | 171,1 | 52,9 | 2,652 | H5A034 | C | pos1 | Hm Gen E | Hm Gen E | Hm Gen E | CPE002JB | CPE002NN | CPE002K3 |
| 3472 | A23 | A24 | 27 | 1366 | 1444 | 750,6 | 53,5 | 5,144 | H5A034 | C | pos1 | Hm Drgbl | Hm Drgbl | Hm Drgbl | CPE002DR | CPE002EH | CPE002F7 |
| 22000 | E23 | E24 | 12 | 1763 | 643 | 409,9 | 53,6 | 2,546 | H5A034 | C | pos1 | Hm Gen E | Hm Gen E | Hm Gen E | CPE002JV | CPE002OA | CPE002KQ |
| 18521 | D24 | D1 | 10 | 825,9 | 536 | 435 | 53,6 | 3,634 | H5A034 | C | pos1 | Hm Gen E | Hm Gen E | Hm Gen E | CPE002JM | CPE002O1 | CPE002KH |
| 11993 | D24 | D1 | 3 | 821,7 | 161 | 162,2 | 53,7 | 3,442 | H5A034 | C | pos1 | Hm Gen E | Hm Gen E | Hm Gen E | CPE002J4 | CPE002NL | CPE002K0 |
| 9328 | E23 | E24 | 11 | 1037 | 593 | 184,1 | 53,9 | 3,743 | H5A034 | C | pos1 | Hm Drgbl | Hm Drgbl | Hm Drgbl | CPE002E8 | CPE002ER | CPE002FC |
| 10865 | E24 | E1 | 2 | 1067 | 109 | 1199 | 54,5 | 4,129 | H5A034 | C | pos1 | Hm Drgbl | Hm Drgbl | Hm Drgbl | CPE002EC | CPE002EN | CPE002FS |
| 21904 | A23 | A24 | 26 | 1680 | 1420 | 502,9 | 54,6 | 2,594 | H5A034 | C | pos1 | Hm Gen E | Hm Gen E | Hm Gen E | CPE002JV | CPE002OA | CPE002KQ |

| | | | | | | | | | | | | | | | | | | | | | | | | | | | | | | | |
|---|---|---|---|---|---|---|---|---|---|---|---|---|---|---|---|---|---|---|---|---|---|---|---|---|---|---|---|---|---|---|---|
| 8585 | F24 | F1 | 4 | 1492 | 222 | 187,3 | 55,5 | 3,922 | HSA034 C | pos1 | Hm Drgbl | Hm Drgbl | Hm Drgbl | CPE002E6 | CPE002ET | CPE002FE | | | | | | | | | | | | | | | |
| 21976 | D23 | D24 | 30 | 1639 | 1674 | 541,2 | 55,8 | 2,651 | HSA034 C | pos1 | Hm Gen E | Hm Gen E | Hm Gen E | CPE002JV | CPE002OA | CPE002KQ | | | | | | | | | | | | | | | |
| 13121 | C24 | C1 | 14 | 1299 | 782 | 151 | 55,9 | 2,802 | HSA034 C | pos1 | Hm Gen E | Hm Gen E | Hm Gen E | CPE002JB | CPE002NN | CPE002K3 | | | | | | | | | | | | | | | |
| 9640 | B23 | B24 | 17 | 1361 | 954 | 276,8 | 56,1 | 3,923 | HSA034 C | pos1 | Hm Drgbl | Hm Drgbl | Hm Drgbl | CPE002E9 | CPE002EQ | CPE002FB | | | | | | | | | | | | | | | |
| 15761 | A24 | A1 | 13 | 829,4 | 730 | 211,3 | 56,2 | 3,131 | HSA034 C | pos1 | Hm Gen E | Hm Gen E | Hm Gen E | CPE002J9 | CPE002NL | CPE002KA | | | | | | | | | | | | | | | |
| 8848 | A23 | A24 | 6 | 3166 | 337 | 166,1 | 56,2 | 4,514 | HSA034 C | pos1 | Hm Drgbl | Hm Drgbl | Hm Drgbl | CPE002E7 | CPE002ES | CPE002FD | | | | | | | | | | | | | | | |
| 13889 | C24 | C1 | 5 | 1635 | 283 | 150,1 | 56,6 | 3,244 | HSA034 C | pos1 | Hm Gen E | Hm Gen E | Hm Gen E | CPE002JF | CPE002NP | CPE002K5 | | | | | | | | | | | | | | | |
| 14224 | A23 | A24 | 10 | 1150 | 568 | 218,4 | 56,8 | 4,043 | HSA034 C | pos1 | Hm Gen E | Hm Gen E | Hm Gen E | CPE002J7 | CPE002NC | CPE002K6 | | | | | | | | | | | | | | | |
| 23488 | C23 | C24 | 8 | 1523 | 457 | 452,8 | 57,1 | 3,029 | HSA034 C | pos1 | Hm Gen E | Hm Gen E | Hm Gen E | CPE002KV | CPE002OE | CPE002KU | | | | | | | | | | | | | | | |
| 15785 | B24 | B1 | 16 | 869,1 | 922 | 193,7 | 57,6 | 3,209 | HSA034 C | pos1 | Hm Gen E | Hm Gen E | Hm Gen E | CPE002J9 | CPE002NL | CPE002KA | | | | | | | | | | | | | | | |
| 10024 | B23 | B24 | 1 | 938,8 | 58 | 224,4 | 58 | 5,577 | HSA034 C | pos1 | Hm Drgbl | Hm Drgbl | Hm Drgbl | CPE002EA | CPE002EP | CPE002FA | | | | | | | | | | | | | | | |
| 9713 | E24 | E1 | 7 | 1436 | 407 | 224,7 | 58,1 | 4,063 | HSA034 C | pos1 | Hm Drgbl | Hm Drgbl | Hm Drgbl | CPE002E9 | CPE002EQ | CPE002FB | | | | | | | | | | | | | | | |
| 9232 | A23 | A24 | 9 | 1472 | 524 | 170,3 | 58,2 | 4,042 | HSA034 C | pos1 | Hm Drgbl | Hm Drgbl | Hm Drgbl | CPE002E8 | CPE002ER | CPE002FC | | | | | | | | | | | | | | | |
| 22024 | F23 | F24 | 13 | 1235 | 767 | 498,7 | 59 | 2,803 | HSA034 C | pos1 | Hm Gen E | Hm Gen E | Hm Gen E | CPE002JV | CPE002OA | CPE002KQ | | | | | | | | | | | | | | | |
| 13841 | A24 | A1 | 10 | 1301 | 590 | 234,6 | 59 | 3,381 | HSA034 C | pos1 | Hm Gen E | Hm Gen E | Hm Gen E | CPE002JF | CPE002NP | CPE002K5 | | | | | | | | | | | | | | | |
| 13960 | F23 | F24 | 7 | 1355 | 417 | 174,7 | 59,6 | 3,415 | HSA034 C | pos1 | Hm Gen E | Hm Gen E | Hm Gen E | CPE002JF | CPE002NP | CPE002K5 | | | | | | | | | | | | | | | |
| 10841 | D24 | D1 | 5 | 1040 | 298 | 813,7 | 59,6 | 4,515 | HSA034 C | pos1 | Hm Drgbl | Hm Drgbl | Hm Drgbl | CPE002EC | CPE002EN | CPE002FS | | | | | | | | | | | | | | | |
| 12761 | D24 | D1 | 7 | 1171 | 422 | 200,7 | 60,3 | 4,088 | HSA034 C | pos1 | Hm Gen E | Hm Gen E | Hm Gen E | CPE002J6 | CPE002NN | CPE002K2 | | | | | | | | | | | | | | | |
| 23081 | B24 | B1 | 10 | 1451 | 607 | 376,9 | 60,7 | 2,622 | HSA034 C | pos1 | Hm Gen E | Hm Gen E | Hm Gen E | CPE002JY | CPE002OD | CPE002KT | | | | | | | | | | | | | | | |
| 8920 | D23 | D24 | 12 | 2959 | 733 | 248,6 | 61,1 | 4,908 | HSA034 C | pos1 | Hm Drgbl | Hm Drgbl | Hm Drgbl | CPE002E7 | CPE002ES | CPE002FD | | | | | | | | | | | | | | | |
| 23176 | F23 | F24 | 21 | 1248 | 1287 | 398,8 | 61,3 | 2,648 | HSA034 C | pos1 | Hm Gen E | Hm Gen E | Hm Gen E | CPE002JY | CPE002OD | CPE002KT | | | | | | | | | | | | | | | |
| 11968 | C23 | C24 | 7 | 1177 | 430 | 138 | 61,4 | 3,936 | HSA034 C | pos1 | Hm Gen E | Hm Gen E | Hm Gen E | CPE002J4 | CPE002NL | CPE002K0 | | | | | | | | | | | | | | | |
| 13481 | B24 | B1 | 9 | 1330 | 556 | 225,4 | 61,8 | 5,803 | HSA034 C | pos1 | Hm Gen E | Hm Gen E | Hm Gen E | CPE002JC | CPE002NC | CPE002K4 | | | | | | | | | | | | | | | |
| 9281 | C24 | C1 | 8 | 1473 | 495 | 225,7 | 61,9 | 4,299 | HSA034 C | pos1 | Hm Drgbl | Hm Drgbl | Hm Drgbl | CPE002E8 | CPE002ER | CPE002FC | | | | | | | | | | | | | | | |
| 9280 | C23 | C24 | 3 | 1378 | 186 | 179,1 | 62 | 4,306 | HSA034 C | pos1 | Hm Drgbl | Hm Drgbl | Hm Drgbl | CPE002E8 | CPE002ER | CPE002FC | | | | | | | | | | | | | | | |
| 8537 | D24 | D1 | 5 | 1401 | 310 | 233,2 | 62 | 4,382 | HSA034 C | pos1 | Hm Drgbl | Hm Drgbl | Hm Drgbl | CPE002E6 | CPE002ET | CPE002FE | | | | | | | | | | | | | | | |
| 13864 | B23 | B24 | 11 | 1251 | 687 | 186,4 | 62,5 | 3,582 | HSA034 C | pos1 | Hm Gen E | Hm Gen E | Hm Gen E | CPE002JF | CPE002NP | CPE002K5 | | | | | | | | | | | | | | | |
| 14248 | B23 | B24 | 7 | 1371 | 439 | 247,1 | 62,7 | 4,463 | HSA034 C | pos1 | Hm Gen E | Hm Gen E | Hm Gen E | CPE002J7 | CPE002NC | CPE002K6 | | | | | | | | | | | | | | | |
| 14728 | F23 | F24 | 7 | 1044 | 442 | 184,4 | 63,1 | 3,595 | HSA034 C | pos1 | Hm Gen E | Hm Gen E | Hm Gen E | CPE002JD | CPE002NR | CPE002K7 | | | | | | | | | | | | | | | |
| 10864 | E23 | E24 | 4 | 919,3 | 253 | 568,2 | 63,2 | 4,788 | HSA034 C | pos1 | Hm Drgbl | Hm Drgbl | Hm Drgbl | CPE002EC | CPE002EN | CPE002FS | | | | | | | | | | | | | | | |
| 13073 | A24 | A1 | 9 | 1523 | 576 | 225,6 | 64 | 3,208 | HSA034 C | pos1 | Hm Gen E | Hm Gen E | Hm Gen E | CPE002JB | CPE002NN | CPE002K3 | | | | | | | | | | | | | | | |
| 15880 | F23 | F24 | 5 | 1217 | 320 | 211,1 | 64 | 3,565 | HSA034 C | pos1 | Hm Gen E | Hm Gen E | Hm Gen E | CPE002J9 | CPE002NL | CPE002KA | | | | | | | | | | | | | | | |
| 9665 | C24 | C1 | 3 | 1165 | 194 | 235,8 | 64,7 | 4,524 | HSA034 C | pos1 | Hm Drgbl | Hm Drgbl | Hm Drgbl | CPE002E9 | CPE002EQ | CPE002FB | | | | | | | | | | | | | | | |
| 12689 | A24 | A1 | 5 | 1469 | 324 | 267,2 | 64,8 | 4,393 | HSA034 C | pos1 | Hm Gen E | Hm Gen E | Hm Gen E | CPE002J6 | CPE002NN | CPE002K2 | | | | | | | | | | | | | | | |
| 9616 | A23 | A24 | 6 | 1434 | 389 | 253,5 | 64,8 | 4,531 | HSA034 C | pos1 | Hm Drgbl | Hm Drgbl | Hm Drgbl | CPE002E9 | CPE002EQ | CPE002FB | | | | | | | | | | | | | | | |
| 12377 | D24 | D1 | 3 | 1323 | 195 | 220,6 | 65 | 4,887 | HSA034 C | pos1 | Hm Gen E | Hm Gen E | Hm Gen E | CPE002J5 | CPE002NK | CPE002K1 | | | | | | | | | | | | | | | |
| 13168 | E23 | E24 | 14 | 1451 | 915 | 185,1 | 65,4 | 3,278 | HSA034 C | pos1 | Hm Gen E | Hm Gen E | Hm Gen E | CPE002JB | CPE002NN | CPE002K3 | | | | | | | | | | | | | | | |
| 9257 | B24 | B1 | 4 | 1016 | 262 | 235,7 | 65,5 | 4,549 | HSA034 C | pos1 | Hm Drgbl | Hm Drgbl | Hm Drgbl | CPE002E8 | CPE002ER | CPE002FC | | | | | | | | | | | | | | | |
| 10000 | A23 | A24 | 13 | 1342 | 852 | 207,1 | 65,5 | 6,298 | HSA034 C | pos1 | Hm Drgbl | Hm Drgbl | Hm Drgbl | CPE002EA | CPE002EP | CPE002FA | | | | | | | | | | | | | | | |
| 10769 | A24 | A1 | 7 | 1425 | 459 | 1132 | 65,6 | 4,97 | HSA034 C | pos1 | Hm Drgbl | Hm Drgbl | Hm Drgbl | CPE002EC | CPE002EN | CPE002FS | | | | | | | | | | | | | | | |
| 14320 | E23 | E24 | 7 | 1065 | 465 | 197,3 | 66,4 | 4,726 | HSA034 C | pos1 | Hm Gen E | Hm Gen E | Hm Gen E | CPE002J7 | CPE002NC | CPE002K6 | | | | | | | | | | | | | | | |
| 15833 | D24 | D1 | 26 | 813,5 | 1730 | 82,06 | 66,5 | 3,705 | HSA034 C | pos1 | Hm Gen E | Hm Gen E | Hm Gen E | CPE002J9 | CPE002NL | CPE002KA | | | | | | | | | | | | | | | |
| 14729 | F24 | F1 | 4 | 855,8 | 267 | 129,8 | 66,8 | 3,806 | HSA034 C | pos1 | Hm Gen E | Hm Gen E | Hm Gen E | CPE002JD | CPE002NR | CPE002K7 | | | | | | | | | | | | | | | |
| 10001 | A24 | A1 | 7 | 1341 | 470 | 287,1 | 67,1 | 6,452 | HSA034 C | pos1 | Hm Drgbl | Hm Drgbl | Hm Drgbl | CPE002EA | CPE002EP | CPE002FA | | | | | | | | | | | | | | | |
| 14345 | F24 | F1 | 4 | 1235 | 270 | 169,1 | 67,5 | 4,804 | HSA034 C | pos1 | Hm Gen E | Hm Gen E | Hm Gen E | CPE002J7 | CPE002NC | CPE002K6 | | | | | | | | | | | | | | | |
| 10816 | C23 | C24 | 2 | 819,5 | 136 | 763,5 | 68 | 5,152 | HSA034 C | pos1 | Hm Drgbl | Hm Drgbl | Hm Drgbl | CPE002EC | CPE002EN | CPE002FS | | | | | | | | | | | | | | | |
| 15808 | C23 | C24 | 10 | 1124 | 682 | 153,5 | 68,2 | 3,799 | HSA034 C | pos1 | Hm Gen E | Hm Gen E | Hm Gen E | CPE002J9 | CPE002NL | CPE002KA | | | | | | | | | | | | | | | |
| 14681 | D24 | D1 | 7 | 1073 | 481 | 200,9 | 68,7 | 3,915 | HSA034 C | pos1 | Hm Gen E | Hm Gen E | Hm Gen E | CPE002JD | CPE002NR | CPE002K7 | | | | | | | | | | | | | | | |
| 14633 | B24 | B1 | 15 | 1120 | 1034 | 165,4 | 68,9 | 3,926 | HSA034 C | pos1 | Hm Gen E | Hm Gen E | Hm Gen E | CPE002JD | CPE002NR | CPE002K7 | | | | | | | | | | | | | | | |
| 9233 | A24 | A1 | 7 | 1441 | 482 | 251,5 | 68,9 | 4,785 | HSA034 C | pos1 | Hm Drgbl | Hm Drgbl | Hm Drgbl | CPE002E8 | CPE002ER | CPE002FC | | | | | | | | | | | | | | | |
| 10097 | E24 | E1 | 2 | 1048 | 138 | 345,5 | 69 | 6,635 | HSA034 C | pos1 | Hm Drgbl | Hm Drgbl | Hm Drgbl | CPE002EA | CPE002EP | CPE002FA | | | | | | | | | | | | | | | |
| 13193 | F24 | F1 | 6 | 1273 | 417 | 121,5 | 69,5 | 3,484 | HSA034 C | pos1 | Hm Gen E | Hm Gen E | Hm Gen E | CPE002JB | CPE002NN | CPE002K3 | | | | | | | | | | | | | | | |
| 13576 | F23 | F24 | 8 | 1145 | 557 | 248,5 | 69,6 | 6,535 | HSA034 C | pos1 | Hm Gen E | Hm Gen E | Hm Gen E | CPE002JC | CPE002NC | CPE002K4 | | | | | | | | | | | | | | | |
| 8968 | F23 | F24 | 12 | 2185 | 849 | 288 | 70,8 | 5,687 | HSA034 C | pos1 | Hm Drgbl | Hm Drgbl | Hm Drgbl | CPE002E7 | CPE002ES | CPE002FD | | | | | | | | | | | | | | | |
| 19265 | C24 | C1 | 4 | 1704 | 284 | 733,7 | 71 | 5,035 | HSA034 C | pos1 | Hm Gen E | Hm Gen E | Hm Gen E | CPE002JO | CPE002O3 | CPE002KJ | | | | | | | | | | | | | | | |
| 23057 | A24 | A1 | 20 | 1328 | 1422 | 367,1 | 71,1 | 3,071 | HSA034 C | pos1 | Hm Gen E | Hm Gen E | Hm Gen E | CPE002JY | CPE002OD | CPE002KT | | | | | | | | | | | | | | | |
| 15425 | C24 | C1 | 4 | 1185 | 288 | 294,7 | 72 | 6,545 | HSA034 C | pos1 | Hm Gen E | Hm Gen E | Hm Gen E | CPE002JE | CPE002NT | CPE002K9 | | | | | | | | | | | | | | | |
| 18497 | C24 | C1 | 3 | 1694 | 218 | 562,1 | 72,7 | 4,929 | HSA034 C | pos1 | Hm Gen E | Hm Gen E | Hm Gen E | CPE002JM | CPE002O1 | CPE002KH | | | | | | | | | | | | | | | |
| 13840 | A23 | A24 | 9 | 1499 | 658 | 162,5 | 73,1 | 4,189 | HSA034 C | pos1 | Hm Gen E | Hm Gen E | Hm Gen E | CPE002JF | CPE002NP | CPE002K5 | | | | | | | | | | | | | | | |
| 14608 | A23 | A24 | 7 | 1046 | 514 | 171,2 | 73,4 | 4,182 | HSA034 C | pos1 | Hm Gen E | Hm Gen E | Hm Gen E | CPE002JD | CPE002NR | CPE002K7 | | | | | | | | | | | | | | | |
| 9641 | B24 | B1 | 5 | 1471 | 369 | 230,3 | 73,8 | 5,161 | HSA034 C | pos1 | Hm Drgbl | Hm Drgbl | Hm Drgbl | CPE002E9 | CPE002EQ | CPE002FB | | | | | | | | | | | | | | | |
| 12016 | E23 | E24 | 6 | 1093 | 449 | 236,3 | 74,8 | 4,795 | HSA034 C | pos1 | Hm Gen E | Hm Gen E | Hm Gen E | CPE002J4 | CPE002NL | CPE002K0 | | | | | | | | | | | | | | | |
| 9352 | F23 | F24 | 4 | 1463 | 301 | 270,1 | 75,2 | 5,222 | HSA034 C | pos1 | Hm Drgbl | Hm Drgbl | Hm Drgbl | CPE002E8 | CPE002ER | CPE002FC | | | | | | | | | | | | | | | |
| 15881 | F24 | F1 | 28 | 767,5 | 2116 | 150,8 | 75,6 | 4,212 | HSA034 C | pos1 | Hm Gen E | Hm Gen E | Hm Gen E | CPE002J9 | CPE002NL | CPE002KA | | | | | | | | | | | | | | | |
| 11249 | E24 | E1 | 1 | 1397 | 77 | 297,5 | 77 | 5,58 | HSA034 C | pos1 | Hm Drgbl | Hm Drgbl | Hm Drgbl | CPE002ED | CPE002F3 | CPE002FT | | | | | | | | | | | | | | | |
| 15760 | A23 | A24 | 4 | 910,5 | 311 | 170,6 | 77,8 | 4,334 | HSA034 C | pos1 | Hm Gen E | Hm Gen E | Hm Gen E | CPE002J9 | CPE002NL | CPE002KA | | | | | | | | | | | | | | | |
| 12713 | B24 | B1 | 4 | 1223 | 315 | 171,1 | 78,8 | 5,342 | HSA034 C | pos1 | Hm Gen E | Hm Gen E | Hm Gen E | CPE002J6 | CPE002NN | CPE002K2 | | | | | | | | | | | | | | | |
| 19216 | A23 | A24 | 8 | 1499 | 633 | 712,9 | 79,1 | 5,61 | HSA034 C | pos1 | Hm Gen E | Hm Gen E | Hm Gen E | CPE002JO | CPE002O3 | CPE002KJ | | | | | | | | | | | | | | | |
| 13097 | B24 | B1 | 4 | 1345 | 321 | 199 | 80,2 | 4,02 | HSA034 C | pos1 | Hm Gen E | Hm Gen E | Hm Gen E | CPE002JB | CPE002NN | CPE002K3 | | | | | | | | | | | | | | | |
| 9689 | D24 | D1 | 3 | 898,4 | 247 | 236,8 | 82,3 | 5,755 | HSA034 C | pos1 | Hm Drgbl | Hm Drgbl | Hm Drgbl | CPE002E9 | CPE002EQ | CPE002FB | | | | | | | | | | | | | | | |
| 10025 | B24 | B1 | 2 | 1218 | 171 | 197,6 | 85,5 | 8,221 | HSA034 C | pos1 | Hm Drgbl | Hm Drgbl | Hm Drgbl | CPE002EA | CPE002EP | CPE002FA | | | | | | | | | | | | | | | |
| 13192 | F23 | F24 | 7 | 1464 | 606 | 166,1 | 86,6 | 4,341 | HSA034 C | pos1 | Hm Gen E | Hm Gen E | Hm Gen E | CPE002JB | CPE002NN | CPE002K3 | | | | | | | | | | | | | | | |
| 18161 | E24 | E1 | 14 | 1231 | 1218 | 480,9 | 87 | 5,595 | HSA034 C | pos1 | Hm Gen E | Hm Gen E | Hm Gen E | CPE002JL | CPE002O0 | CPE002KG | | | | | | | | | | | | | | | |
| 14297 | D24 | D1 | 6 | 1253 | 529 | 232,6 | 88,2 | 6,278 | HSA034 C | pos1 | Hm Gen E | Hm Gen E | Hm Gen E | CPE002J7 | CPE002NC | CPE002K6 | | | | | | | | | | | | | | | |
| 15784 | B23 | B24 | 4 | 1347 | 358 | 231,3 | 89,5 | 4,986 | HSA034 C | pos1 | Hm Gen E | Hm Gen E | Hm Gen E | CPE002J9 | CPE002NL | CPE002KA | | | | | | | | | | | | | | | |
| 13096 | B23 | B24 | 5 | 1466 | 462 | 142,9 | 92,4 | 4,632 | HSA034 C | pos1 | Hm Gen E | Hm Gen E | Hm Gen E | CPE002JB | CPE002NN | CPE002K3 | | | | | | | | | | | | | | | |
| 13169 | E24 | E1 | 3 | 920,5 | 282 | 137,9 | 94 | 4,712 | HSA034 C | pos1 | Hm Gen E | Hm Gen E | Hm Gen E | CPE002JB | CPE002NN | CPE002K3 | | | | | | | | | | | | | | | |
| 1961 | B24 | B1 | 2 | 628,6 | 194 | 815 | 97 | 8,435 | HSA034 C | pos1 | Hm Drgbl | Hm Drgbl | Hm Drgbl | CPE002FX | CPE002G1 | CPE002G5 | | | | | | | | | | | | | | | |
| 13120 | C23 | C24 | 7 | 1483 | 737 | 167,6 | 105,3 | 5,278 | HSA034 C | pos1 | Hm Gen E | Hm Gen E | Hm Gen E | CPE002JB | CPE002NN | CPE002K3 | | | | | | | | | | | | | | | |
| 12785 | E24 | E1 | 11 | 1273 | 1218 | 204,5 | 110,7 | 7,505 | HSA034 C | pos1 | Hm Gen E | Hm Gen E | Hm Gen E | CPE002J6 | CPE002NN | CPE002K2 | | | | | | | | | | | | | | | |
| 10096 | E23 | E24 | 8 | 1105 | 900 | 383,7 | 112,5 | 10,817 | HSA034 C | pos1 | Hm Drgbl | Hm Drgbl | Hm Drgbl | CPE002EA | CPE002EP | CPE002FA | | | | | | | | | | | | | | | |
| 15017 | B24 | B1 | 1 | 855,6 | 116 | 290,2 | 116 | 8,112 | HSA034 C | pos1 | Hm Gen E | Hm Gen E | Hm Gen E | CPE002J8 | CPE002NS | CPE002K8 | | | | | | | | | | | | | | | |
| 8465 | A24 | A1 | 1 | 586,3 | 126 | 185,2 | 126 | 8,905 | HSA034 C | pos1 | Hm Drgbl | Hm Drgbl | Hm Drgbl | CPE002E6 | CPE002ET | CPE002FE | | | | | | | | | | | | | | | |
| 8560 | E23 | E24 | 4 | 1182 | 513 | 292,9 | 128,2 | 9,06 | HSA034 C | pos1 | Hm Drgbl | Hm Drgbl | Hm Drgbl | CPE002E6 | CPE002ET | CPE002FE | | | | | | | | | | | | | | | |
| 40 | B23 | B24 | 0 | 0 | 0 | 0 | | | HSA034 C | pos1 | Hm Drgbl | Hm Drgbl | Hm Drgbl | CPE002DT | CPE002EJ | CPE002F9 | | | | | | | | | | | | | | | |
| 41 | B24 | B1 | 0 | 0 | 0 | 0 | | | HSA034 C | pos1 | Hm Drgbl | Hm Drgbl | Hm Drgbl | CPE002DT | CPE002EJ | CPE002F9 | | | | | | | | | | | | | | | |
| 65 | C24 | C1 | 0 | 0 | 0 | 0 | | | HSA034 C | pos1 | Hm Drgbl | Hm Drgbl | Hm Drgbl | CPE002DT | CPE002EJ | CPE002F9 | | | | | | | | | | | | | | | |
| 88 | D23 | D24 | 0 | 0 | 0 | 0 | | | HSA034 C | pos1 | Hm Drgbl | Hm Drgbl | Hm Drgbl | CPE002DT | CPE002EJ | CPE002F9 | | | | | | | | | | | | | | | |
| 113 | E24 | E1 | 0 | 0 | 0 | 0 | | | HSA034 C | pos1 | Hm Drgbl | Hm Drgbl | Hm Drgbl | CPE002DT | CPE002EJ | CPE002F9 | | | | | | | | | | | | | | | |
| 832 | C23 | C24 | 0 | 0 | 0 | 0 | | | HSA034 C | pos1 | Hm Drgbl | Hm Drgbl | Hm Drgbl | CPE002FU | CPE002FY | CPE002G2 | | | | | | | | | | | | | | | |
| 856 | D23 | D24 | 0 | 0 | 0 | 0 | | | HSA034 C | pos1 | Hm Drgbl | Hm Drgbl | Hm Drgbl | CPE002FU | CPE002FY | CPE002G2 | | | | | | | | | | | | | | | |
| 1192 | B23 | B24 | 0 | 0 | 0 | 0 | | | HSA034 C | pos1 | Hm Drgbl | Hm Drgbl | Hm Drgbl | CPE002FV | CPE002FZ | CPE002G3 | | | | | | | | | | | | | | | |
| 1193 | B24 | B1 | 0 | 0 | 0 | 0 | | | HSA034 C | pos1 | Hm Drgbl | Hm Drgbl | Hm Drgbl | CPE002FV | CPE002FZ | CPE002G3 | | | | | | | | | | | | | | | |
| 1289 | F24 | F1 | 0 | 0 | 0 | 0 | | | HSA034 C | pos1 | Hm Drgbl | Hm Drgbl | Hm Drgbl | CPE002FV | CPE002FZ | CPE002G3 | | | | | | | | | | | | | | | |
| 1601 | C24 | C1 | 0 | 0 | 0 | 0 | | | HSA034 C | pos1 | Hm Drgbl | Hm Drgbl | Hm Drgbl | CPE002FW | CPE002G0 | CPE002G4 | | | | | | | | | | | | | | | |
| 1624 | D23 | D24 | 0 | 0 | 0 | 0 | | | HSA034 C | pos1 | Hm Drgbl | Hm Drgbl | Hm Drgbl | CPE002FW | CPE002G0 | CPE002G4 | | | | | | | | | | | | | | | |
| 1648 | E23 | E24 | 0 | 0 | 0 | 0 | | | HSA034 C | pos1 | Hm Drgbl | Hm Drgbl | Hm Drgbl | CPE002FW | CPE002G0 | CPE002G4 | | | | | | | | | | | | | | | |
| 1649 | E24 | E1 | 0 | 0 | 0 | 0 | | | HSA034 C | pos1 | Hm Drgbl | Hm Drgbl | Hm Drgbl | CPE002FW | CPE002G0 | CPE002G4 | | | | | | | | | | | | | | | |
| 1673 | F24 | F1 | 0 | 0 | 0 | 0 | | | HSA034 C | pos1 | Hm Drgbl | Hm Drgbl | Hm Drgbl | CPE002FW | CPE002G0 | CPE002G4 | | | | | | | | | | | | | | | |
| 1985 | C24 | C1 | 0 | 0 | 0 | 0 | | | HSA034 C | pos1 | Hm Drgbl | Hm Drgbl | Hm Drgbl | CPE002FX | CPE002G1 | CPE002G5 | | | | | | | | | | | | | | | |
| 2008 | D23 | D24 | 0 | 0 | 0 | 0 | | | HSA034 C | pos1 | Hm Drgbl | Hm Drgbl | Hm Drgbl | CPE002FX | CPE002G1 | CPE002G5 | | | | | | | | | | | | | | | |
| 2392 | D23 | D24 | 0 | 0 | 0 | 0 | | | HSA034 C | pos1 | Hm Drgbl | Hm Drgbl | Hm Drgbl | CPE002DC | CPE002EE | CPE002F4 | | | | | | | | | | | | | | | |
| 2440 | F23 | F24 | 0 | 0 | 0 | 0 | | | HSA034 C | pos1 | Hm Drgbl | Hm Drgbl | Hm Drgbl | CPE002DC | CPE002EE | CPE002F4 | | | | | | | | | | | | | | | |
| 2704 | A23 | A24 | 0 | 0 | 0 | 0 | | | HSA034 C | pos1 | Hm Drgbl | Hm Drgbl | Hm Drgbl | CPE002DP | CPE002EF | CPE002F5 | | | | | | | | | | | | | | | |
| 2777 | D24 | D1 | 0 | 0 | 0 | 0 | | | HSA034 C | pos1 | Hm Drgbl | Hm Drgbl | Hm Drgbl | CPE002DP | CPE002EF | CPE002F5 | | | | | | | | | | | | | | | |
| 2801 | E24 | E1 | 0 | 0 | 0 | 0 | | | HSA034 C | pos1 | Hm Drgbl | Hm Drgbl | Hm Drgbl | CPE002DP | CPE002EF | CPE002F5 | | | | | | | | | | | | | | | |
| 2825 | F24 | F1 | 0 | 0 | 0 | 0 | | | HSA034 C | pos1 | Hm Drgbl | Hm Drgbl | Hm Drgbl | CPE002DP | CPE002EF | CPE002F5 | | | | | | | | | | | | | | | |
| 3089 | A24 | A1 | 0 | 0 | 0 | 0 | | | HSA034 C | pos1 | Hm Drgbl | Hm Drgbl | Hm Drgbl | CPE002DC | CPE002EG | CPE002F6 | | | | | | | | | | | | | | | |
| 3112 | B23 | B24 | 0 | 0 | 0 | 0 | | | HSA034 C | pos1 | Hm Drgbl | Hm Drgbl | Hm Drgbl | CPE002DC | CPE002EG | CPE002F6 | | | | | | | | | | | | | | | |
| 3137 | C24 | C1 | 0 | 0 | 0 | 0 | | | HSA034 C | pos1 | Hm Drgbl | Hm Drgbl | Hm Drgbl | CPE002DC | CPE002EG | CPE002F6 | | | | | | | | | | | | | | | |
| 3160 | D23 | D24 | 0 | 0 | 0 | 0 | | | HSA034 C | pos1 | Hm Drgbl | Hm Drgbl | Hm Drgbl | CPE002DC | CPE002EG | CPE002F6 | | | | | | | | | | | | | | | |
| 3208 | F23 | F24 | 0 | 0 | 0 | 0 | | | HSA034 C | pos1 | Hm Drgbl | Hm Drgbl | Hm Drgbl | CPE002DC | CPE002EG | CPE002F6 | | | | | | | | | | | | | | | |
| 3209 | F24 | F1 | 0 | 0 | 0 | 0 | | | HSA034 C | pos1 | Hm Drgbl | Hm Drgbl | Hm Drgbl | CPE002DC | CPE002EG | CPE002F6 | | | | | | | | | | | | | | | |
| 3497 | B24 | B1 | 0 | 0 | 0 | 0 | | | HSA034 C | pos1 | Hm Drgbl | Hm Drgbl | Hm Drgbl | CPE002DR | CPE002EH | CPE002F7 | | | | | | | | | | | | | | | |
| 3521 | C24 | C1 | 0 | 0 | 0 | 0 | | | HSA034 C | pos1 | Hm Drgbl | Hm Drgbl | Hm Drgbl | CPE002DR | CPE002EH | CPE002F7 | | | | | | | | | | | | | | | |
| 3544 | D23 | D24 | 0 | 0 | 0 | 0 | | | HSA034 C | pos1 | Hm Drgbl | Hm Drgbl | Hm Drgbl | CPE002DR | CPE002EH | CPE002F7 | | | | | | | | | | | | | | | |
| 3545 | D24 | D1 | 0 | 0 | 0 | 0 | | | HSA034 C | pos1 | Hm Drgbl | Hm Drgbl | Hm Drgbl | CPE002DR | CPE002EH | CPE002F7 | | | | | | | | | | | | | | | |
| 3568 | E23 | E24 | 0 | 0 | 0 | 0 | | | HSA034 C | pos1 | Hm Drgbl | Hm Drgbl | Hm Drgbl | CPE002DR | CPE002EH | CPE002F7 | | | | | | | | | | | | | | | |
| 3592 | F23 | F24 | 0 | 0 | 0 | 0 | | | HSA034 C | pos1 | Hm Drgbl | Hm Drgbl | Hm Drgbl | CPE002DR | CPE002EH | CPE002F7 | | | | | | | | | | | | | | | |
| 3880 | B23 | B24 | 0 | 0 | 0 | 0 | | | HSA034 C | pos1 | Hm Drgbl | Hm Drgbl | Hm Drgbl | CPE002DS | CPE002EI | CPE002F8 | | | | | | | | | | | | | | | |
| 3928 | D23 | D24 | 0 | 0 | 0 | 0 | | | HSA034 C | pos1 | Hm Drgbl | Hm Drgbl | Hm Drgbl | CPE002DS | CPE002EI | CPE002F8 | | | | | | | | | | | | | | | |
| 3952 | E23 | E24 | 0 | 0 | 0 | 0 | | | HSA034 C | pos1 | Hm Drgbl | Hm Drgbl | Hm Drgbl | CPE002DS | CPE002EI | CPE002F8 | | | | | | | | | | | | | | | |
| 4264 | B23 | B24 | 0 | 0 | 0 | 0 | | | HSA034 C | pos1 | Hm Drgbl | Hm Drgbl | Hm Drgbl | CPE002DU | CPE002EK | CPE002FQ | | | | | | | | | | | | | | | |
| 4336 | E23 | E24 | 0 | 0 | 0 | 0 | | | HSA034 C | pos1 | Hm Drgbl | Hm Drgbl | Hm Drgbl | CPE002DU | CPE002EK | CPE002FQ | | | | | | | | | | | | | | | |
| 4360 | F23 | F24 | 0 | 0 | 0 | 0 | | | HSA034 C | pos1 | Hm Drgbl | Hm Drgbl | Hm Drgbl | CPE002DU | CPE002EK | CPE002FQ | | | | | | | | | | | | | | | |
| 4673 | C24 | C1 | 0 | 0 | 0 | 0 | | | HSA034 C | pos1 | Hm Drgbl | Hm Drgbl | Hm Drgbl | CPE002DV | CPE002EL | CPE002FP | | | | | | | | | | | | | | | |
| 4721 | E24 | E1 | 0 | 0 | 0 | 0 | | | HSA034 C | pos1 | Hm Drgbl | Hm Drgbl | Hm Drgbl | CPE002DV | CPE002EL | CPE002FP | | | | | | | | | | | | | | | |
| 5056 | C23 | C24 | 0 | 0 | 0 | 0 | | | HSA034 C | pos1 | Hm Drgbl | Hm Drgbl | Hm Drgbl | CPE002DV | CPE002EM | CPE002FO | | | | | | | | | | | | | | | |
| 5824 | C23 | C24 | 0 | 0 | 0 | 0 | | | HSA034 C | pos1 | Hm Drgbl | Hm Drgbl | Hm Drgbl | CPE002DY | CPE002F1 | CPE002FM | | | | | | | | | | | | | | | |
| 5825 | C24 | C1 | 0 | 0 | 0 | 0 | | | HSA034 C | pos1 | Hm Drgbl | Hm Drgbl | Hm Drgbl | CPE002DY | CPE002F1 | CPE002FM | | | | | | | | | | | | | | | |
| 5873 | E24 | E1 | 0 | 0 | 0 | 0 | | | HSA034 C | pos1 | Hm Drgbl | Hm Drgbl | Hm Drgbl | CPE002DY | CPE002F1 | CPE002FM | | | | | | | | | | | | | | | |
| 6593 | C24 | C1 | 0 | 0 | 0 | 0 | | | HSA034 C | pos1 | Hm Drgbl | Hm Drgbl | Hm Drgbl | CPE002E1 | CPE002EY | CPE002FJ | | | | | | | | | | | | | | | |
| 7361 | C24 | C1 | 0 | 0 | 0 | 0 | | | HSA034 C | pos1 | Hm Drgbl | Hm Drgbl | Hm Drgbl | CPE002E3 | CPE002EW | CPE002FH | | | | | | | | | | | | | | | |
| 7409 | E24 | E1 | 0 | 0 | 0 | 0 | | | HSA034 C | pos1 | Hm Drgbl | Hm Drgbl | Hm Drgbl | CPE002E3 | CPE002EW | CPE002FH | | | | | | | | | | | | | | | |
| 8464 | A23 | A24 | 0 | 0 | 0 | 0 | | | HSA034 C | pos1 | Hm Drgbl | Hm Drgbl | Hm Drgbl | CPE002E6 | CPE002ET | CPE002FE | | | | | | | | | | | | | | | |
| 8512 | C23 | C24 | 0 | 0 | 0 | 0 | | | HSA034 C | pos1 | Hm Drgbl | Hm Drgbl | Hm Drgbl | CPE002E6 | CPE002ET | CPE002FE | | | | | | | | | | | | | | | |
| 9353 | F24 | F1 | 0 | 0 | 0 | 0 | | | HSA034 C | pos1 | Hm Drgbl | Hm Drgbl | Hm Drgbl | CPE002E8 | CPE002ER | CPE002FC | | | | | | | | | | | | | | | |
| 9688 | D23 | D24 | 0 | 0 | 0 | 0 | | | HSA034 C | pos1 | Hm Drgbl | Hm Drgbl | Hm Drgbl | CPE002E9 | CPE002EQ | CPE002FB | | | | | | | | | | | | | | | |
| 10409 | B24 | B1 | 0 | 0 | 0 | 0 | | | HSA034 C | pos1 | Hm Drgbl | Hm Drgbl | Hm Drgbl | CPE002EB | CPE002EO | CPE002FR | | | | | | | | | | | | | | | |
| 10504 | F23 | F24 | 0 | 0 | 0 | 0 | | | HSA034 C | pos1 | Hm Drgbl | Hm Drgbl | Hm Drgbl | CPE002EB | CPE002EO | CPE002FR | | | | | | | | | | | | | | | |
| 10505 | F24 | F1 | 0 | 0 | 0 | 0 | | | HSA034 C | pos1 | Hm Drgbl | Hm Drgbl | Hm Drgbl | CPE002EB | CPE002EO | CPE002FR | | | | | | | | | | | | | | | |
| 10888 | F23 | F24 | 0 | 0 | 0 | 0 | | | HSA034 C | pos1 | Hm Drgbl | Hm Drgbl | Hm Drgbl | CPE002EC | CPE002EN | CPE002FS | | | | | | | | | | | | | | | |
| 11537 | A24 | A1 | 0 | 0 | 0 | 0 | | | HSA034 C | pos1 | Hm Gen E | Hm Gen E | Hm Gen E | CPE002JA | CPE002NJ | CPE002JZ | | | | | | | | | | | | | | | |
| 11656 | F23 | F24 | 0 | 0 | 0 | 0 | | | HSA034 C | pos1 | Hm Gen E | Hm Gen E | Hm Gen E | CPE002JA | CPE002NJ | CPE002JZ | | | | | | | | | | | | | | | |
| 11921 | A24 | A1 | 0 | 0 | 0 | 0 | | | HSA034 C | pos1 | Hm Gen E | Hm Gen E | Hm Gen E | CPE002J4 | CPE002NL | CPE002K0 | | | | | | | | | | | | | | | |
| 11944 | B23 | B24 | 0 | 0 | 0 | 0 | | | HSA034 C | pos1 | Hm Gen E | Hm Gen E | Hm Gen E | CPE002J4 | CPE002NL | CPE002K0 | | | | | | | | | | | | | | | |
| 12041 | F24 | F1 | 0 | 0 | 0 | 0 | | | HSA034 C | pos1 | Hm Gen E | Hm Gen E | Hm Gen E | CPE002J4 | CPE002NL | CPE002K0 | | | | | | | | | | | | | | | |
| 12401 | E24 | E1 | 0 | 0 | 0 | 0 | | | HSA034 C | pos1 | Hm Gen E | Hm Gen E | Hm Gen E | CPE002J5 | CPE002NK | CPE002K1 | | | | | | | | | | | | | | | |
| 12425 | F24 | F1 | 0 | 0 | 0 | 0 | | | HSA034 C | pos1 | Hm Gen E | Hm Gen E | Hm Gen E | CPE002J5 | CPE002NK | CPE002K1 | | | | | | | | | | | | | | | |
| 12809 | F24 | F1 | 0 | 0 | 0 | 0 | | | HSA034 C | pos1 | Hm Gen E | Hm Gen E | Hm Gen E | CPE002J6 | CPE002NN | CPE002K2 | | | | | | | | | | | | | | | |
| 13145 | D24 | D1 | 0 | 0 | 0 | 0 | | | HSA034 C | pos1 | Hm Gen E | Hm Gen E | Hm Gen E | CPE002JB | CPE002NN | CPE002K3 | | | | | | | | | | | | | | | |
| 13480 | B23 | B24 | 0 | 0 | 0 | 0 | | | HSA034 C | pos1 | Hm Gen E | Hm Gen E | Hm Gen E | CPE002JC | CPE002NC | CPE002K4 | | | | | | | | | | | | | | | |
| 13529 | D24 | D1 | 0 | 0 | 0 | 0 | | | HSA034 C | pos1 | Hm Gen E | Hm Gen E | Hm Gen E | CPE002JC | CPE002NC | CPE002K4 | | | | | | | | | | | | | | | |
| 14273 | C24 | C1 | 0 | 0 | 0 | 0 | | | HSA034 C | pos1 | Hm Gen E | Hm Gen E | Hm Gen E | CPE002J7 | CPE002NC | CPE002K6 | | | | | | | | | | | | | | | |
| 14609 | A24 | A1 | 0 | 0 | 0 | 0 | | | HSA034 C | pos1 | Hm Gen E | Hm Gen E | Hm Gen E | CPE002JD | CPE002NR | CPE002K7 | | | | | | | | | | | | | | | |
| 14704 | E23 | E24 | 0 | 0 | 0 | 0 | | | HSA034 C | pos1 | Hm Gen E | Hm Gen E | Hm Gen E | CPE002JD | CPE002NR | CPE002K7 | | | | | | | | | | | | | | | |
| 15040 | C23 | C24 | 0 | 0 | 0 | 0 | | | HSA034 C | pos1 | Hm Gen E | Hm Gen E | Hm Gen E | CPE002J8 | CPE002NS | CPE002K8 | | | | | | | | | | | | | | | |
| 15089 | E24 | E1 | 0 | 0 | 0 | 0 | | | HSA034 C | pos1 | Hm Gen E | Hm Gen E | Hm Gen E | CPE002J8 | CPE002NS | CPE002K8 | | | | | | | | | | | | | | | |
| 15496 | F23 | F24 | 0 | 0 | 0 | 0 | | | HSA034 C | pos1 | Hm Gen E | Hm Gen E | Hm Gen E | CPE002JE | CPE002NT | CPE002K9 | | | | | | | | | | | | | | | |
| 16145 | A24 | A1 | 0 | 0 | 0 | 0 | | | HSA034 C | pos1 | Hm Gen E | Hm Gen E | Hm Gen E | CPE002JG | CPE002NV | CPE002KB | | | | | | | | | | | | | | | |
| 16576 | C23 | C24 | 0 | 0 | 0 | 0 | | | HSA034 C | pos1 | Hm Gen E | Hm Gen E | Hm Gen E | CPE002JH | CPE002NV | CPE002KC | | | | | | | | | | | | | | | |
| 16600 | D23 | D24 | 0 | 0 | 0 | 0 | | | HSA034 C | pos1 | Hm Gen E | Hm Gen E | Hm Gen E | CPE002JH | CPE002NV | CPE002KC | | | | | | | | | | | | | | | |
| 16601 | D24 | D1 | 0 | 0 | 0 | 0 | | | HSA034 C | pos1 | Hm Gen E | Hm Gen E | Hm Gen E | CPE002JH | CPE002NV | CPE002KC | | | | | | | | | | | | | | | |
| 17368 | D23 | D24 | 0 | 0 | 0 | 0 | | | HSA034 C | pos1 | Hm Gen E | Hm Gen E | Hm Gen E | CPE002JJ | CPE002NY | CPE002KE | | | | | | | | | | | | | | | |
| 17392 | E23 | E24 | 0 | 0 | 0 | 0 | | | HSA034 C | pos1 | Hm Gen E | Hm Gen E | Hm Gen E | CPE002JJ | CPE002NY | CPE002KE | | | | | | | | | | | | | | | |
| 17705 | B24 | B1 | 0 | 0 | 0 | 0 | | | HSA034 C | pos1 | Hm Gen E | Hm Gen E | Hm Gen E | CPE002JK | CPE002NZ | CPE002KF | | | | | | | | | | | | | | | |
| 17777 | E24 | E1 | 0 | 0 | 0 | 0 | | | HSA034 C | pos1 | Hm Gen E | Hm Gen E | Hm Gen E | CPE002JK | CPE002NZ | CPE002KF | | | | | | | | | | | | | | | |
| 20032 | C23 | C24 | 0 | 0 | 0 | 0 | | | HSA034 C | pos1 | Hm Gen E | Hm Gen E | Hm Gen E | CPE002JQ | CPE002O5 | CPE002KL | | | | | | | | | | | | | | | |
| 20104 | F23 | F24 | 0 | 0 | 0 | 0 | | | HSA034 C | pos1 | Hm Gen E | Hm Gen E | Hm Gen E | CPE002JQ | CPE002O5 | CPE002KL | | | | | | | | | | | | | | | |
| 20440 | D23 | D24 | 0 | 0 | 0 | 0 | | | HSA034 C | pos1 | Hm Gen E | Hm Gen E | Hm Gen E | CPE002JR | CPE002O6 | CPE002KM | | | | | | | | | | | | | | | |
| 21521 | A24 | A1 | 0 | 0 | 0 | 0 | | | HSA034 C | pos1 | Hm Gen E | Hm Gen E | Hm Gen E | CPE002JU | CPE002O9 | CPE002KP | | | | | | | | | | | | | | | |
| 21545 | B24 | B1 | 0 | 0 | 0 | 0 | | | HSA034 C | pos1 | Hm Gen E | Hm Gen E | Hm Gen E | CPE002JU | CPE002O9 | CPE002KP | | | | | | | | | | | | | | | |
| 21568 | C23 | C24 | 0 | 0 | 0 | 0 | | | HSA034 C | pos1 | Hm Gen E | Hm Gen E | Hm Gen E | CPE002JU | CPE002O9 | CPE002KP | | | | | | | | | | | | | | | |
| 21569 | C24 | C1 | 0 | 0 | 0 | 0 | | | HSA034 C | pos1 | Hm Gen E | Hm Gen E | Hm Gen E | CPE002JU | CPE002O9 | CPE002KP | | | | | | | | | | | | | | | |
| 21593 | D24 | D1 | 0 | 0 | 0 | 0 | | | HSA034 C | pos1 | Hm Gen E | Hm Gen E | Hm Gen E | CPE002JU | CPE002O9 | CPE002KP | | | | | | | | | | | | | | | |
| 21616 | E23 | E24 | 0 | 0 | 0 | 0 | | | HSA034 C | pos1 | Hm Gen E | Hm Gen E | Hm Gen E | CPE002JU | CPE002O9 | CPE002KP | | | | | | | | | | | | | | | |
| 21641 | F24 | F1 | 0 | 0 | 0 | 0 | | | HSA034 C | pos1 | Hm Gen E | Hm Gen E | Hm Gen E | CPE002JU | CPE002O9 | CPE002KP | | | | | | | | | | | | | | | |
| 311 | M8 | M9 | 9 | 695 | 0 | 0 | 0 | 0 | HSA034 C | sample | Hm Drgbl | Hm Drgbl | Hm Drgbl | CPE002DT | CPE002EJ | CPE002F9 | NM 00675 | NM 0067 | NM 0067 | SNTB2 | SNTB2 | SNTB2 | syntrophin, beta | syntrophi | syntrophi | 6645 | 6645 | 6645 | s13263 | s13261 | s13262 |
| 7356 | C2 | C3 | 510 | 685,4 | 654 | 201,1 | 1,3 | 0,099 | HSA034 C | sample | Hm Drgbl | Hm Drgbl | Hm Drgbl | CPE002E3 | CPE002EW | CPE002FH | NM 00429 | NM 0042 | NM 0042 | GNA14 | GNA14 | GNA14 | guanine nucleotic | guanine n | guanine n | 9630 | 9630 | 9630 | s18511 | s18510 | s18509 |
| 6570 | B3 | B4 | 1210 | 605,5 | 2058 | 160,2 | 1,7 | 0,149 | HSA034 C | sample | Hm Drgbl | Hm Drgbl | Hm Drgbl | CPE002E1 | CPE002EY | CPE002FJ | NM 00449 | NM 0044 | NM 0044 | ONECUT1 | ONECUT1 | ONECUT1 | one cut domain, f | one cut d | one cut d | 3175 | 3175 | 3175 | s6704 | s6703 | s6702 |
| 21620 | E5 | E6 | 117 | 1247 | 214 | 210,6 | 1,8 | 0,189 | HSA034 C | sample | Hm Gen E | Hm Gen E | Hm Gen E | CPE002JU | CPE002O9 | CPE002KP | XM 00112 | XM 0011 | XM 0011 | LOC64923 | LOC64923 | LOC64923 | similar to G antig | similar to | similar to | 649236 | 649236 | 649236 | s56940 | s56941 | s56942 |
| 1208 | C16 | C17 | 2240 | 779,9 | 4534 | 162,9 | 2 | 0,183 | HSA034 C | sample | Hm Drgbl | Hm Drgbl | Hm Drgbl | CPE002FV | CPE002FZ | CPE002G3 | NM 01469 | NM 0146 | NM 0146 | ZNF646 | ZNF646 | ZNF646 | zinc finger protein | zinc finger | zinc finger | 9726 | 9726 | 9726 | s18749 | s18750 | s18751 |
| 8003 | N19 | N20 | 1344 | 657,9 | 2879 | 177,7 | 2,1 | 0,321 | HSA034 C | sample | Hm Drgbl | Hm Drgbl | Hm Drgbl | CPE002E4 | CPE002EV | CPE002FG | NM 00638 | NM 0063 | NM 0063 | CHERP | CHERP | CHERP | calcium homeosta | calcium h | calcium h | 10523 | 10523 | 10523 | s20626 | s20627 | s20628 |
| 7999 | N15 | N16 | 1763 | 709,1 | 3881 | 182,9 | 2,2 | 0,336 | HSA034 C | sample | Hm Drgbl | Hm Drgbl | Hm Drgbl | CPE002E4 | CPE002EV | CPE002FG | NM 03088 | NM 0308 | NM 0308 | DDX17 | DDX17 | DDX17 | DEAD (Asp-Glu-Al | DEAD (As | DEAD (As | 10521 | 10521 | 10521 | s20622 | s20623 | s20624 |
| 7880 | I16 | I17 | 598 | 592,9 | 1374 | 146,1 | 2,3 | 0,351 | HSA034 C | sample | Hm Drgbl | Hm Drgbl | Hm Drgbl | CPE002E4 | CPE002EV | CPE002FG | NM 00656 | NM 0065 | NM 0065 | FARS2 | FARS2 | FARS2 | phenylalanyl-tRN | phenylala | phenylala | 10667 | 10667 | 10667 | s20977 | s20976 | s20975 |
| 4802 | I10 | I11 | 1527 | 963,3 | 3735 | 151 | 2,4 | 0,264 | HSA034 C | sample | Hm Drgbl | Hm Drgbl | Hm Drgbl | CPE002DV | CPE002EL | CPE002FP | NM 00389 | NM 0038 | NM 0038 | PROZ | PROZ | PROZ | protein Z, vitamin | protein Z, | protein Z, | 8858 | 8858 | 8858 | s16915 | s16916 | s225047 |
| 8098 | B18 | B19 | 903 | 693 | 2229 | 141 | 2,5 | 0,24 | HSA034 C | sample | Hm Drgbl | Hm Drgbl | Hm Drgbl | CPE002E5 | CPE002EU | CPE002FF | NM 01534 | NM 0153 | NM 0153 | LARS2 | LARS2 | LARS2 | leucyl-tRNA synth | leucyl-tRN | leucyl-tRN | 23395 | 23395 | 23395 | s23727 | s23728 | s23729 |
| 4905 | M17 | M18 | 2355 | 1010 | 5951 | 149,8 | 2,5 | 0,275 | HSA034 C | sample | Hm Drgbl | Hm Drgbl | Hm Drgbl | CPE002DV | CPE002EL | CPE002FP | NM 00192 | NM 0019 | NM 0019 | CFD | CFD | CFD | complement fact | compleme | compleme | 1675 | 1675 | 1675 | s223404 | s4054 | s4055 |
| 8028 | O2 | O3 | 1011 | 696,2 | 2574 | 161,5 | 2,5 | 0,382 | HSA034 C | sample | Hm Drgbl | Hm Drgbl | Hm Drgbl | CPE002E4 | CPE002EV | CPE002FG | NM 00663 | NM 0066 | NM 0066 | IQGAP2 | IQGAP2 | IQGAP2 | IQ motif containi | IQ motif c | IQ motif c | 10788 | 10788 | 10788 | s21195 | s21197 | s21196 |
| 7466 | H10 | H11 | 1570 | 589,7 | 4413 | 169,1 | 2,8 | 0,214 | HSA034 C | sample | Hm Drgbl | Hm Drgbl | Hm Drgbl | CPE002E3 | CPE002EW | CPE002FH | NM 01337 | NM 0133 | NM 0133 | PDCD6IP | PDCD6IP | PDCD6IP | programmed cell | programm | programm | 10015 | 10015 | 10015 | s19466 | s19467 | s19465 |
| 3933 | D6 | D7 | 1035 | 679,8 | 2911 | 168,1 | 2,8 | 0,249 | HSA034 C | sample | Hm Drgbl | Hm Drgbl | Hm Drgbl | CPE002DS | CPE002EI | CPE002F8 | NM 00292 | NM 0029 | NM 0029 | RGS1 | RGS1 | RGS1 | regulator of G-pro | regulator | regulator | 5996 | 5996 | 5996 | s11986 | s11987 | s11988 |
| 21561 | C17 | C18 | 471 | 1000 | 1331 | 493,7 | 2,8 | 0,295 | HSA034 C | sample | Hm Gen E | Hm Gen E | Hm Gen E | CPE002JU | CPE002O9 | CPE002KP | XM 93824 | XM 9382 | XM 9382 | LOC64917 | LOC64917 | LOC64917 | similar to leucine | similar to | similar to | 649179 | 649179 | 649179 | s56926 | s56927 | s56928 |
| 6702 | H14 | H15 | 1377 | 554,4 | 4203 | 135,1 | 3,1 | 0,272 | HSA034 C | sample | Hm Drgbl | Hm Drgbl | Hm Drgbl | CPE002E1 | CPE002EY | CPE002FJ | NM 00452 | NM 0045 | NM 0045 | KIF5C | KIF5C | KIF5C | kinesin family me | kinesin fa | kinesin fa | 3800 | 3800 | 3800 | s7842 | s7840 | s7841 |
| 5805 | B6 | B7 | 1561 | 886,5 | 4841 | 188,6 | 3,1 | 0,283 | HSA034 C | sample | Hm Drgbl | Hm Drgbl | Hm Drgbl | CPE002DY | CPE002F1 | CPE002FM | NM 00063 | NM 0006 | NM 0006 | BCL2 | BCL2 | BCL2 | B-cell CLL/lympho | B-cell CLL/ | B-cell CLL/ | 596 | 596 | 596 | s1916 | s1915 | s224526 |
| 7484 | H5 | H6 | 883 | 542,1 | 2788 | 174,6 | 3,2 | 0,244 | HSA034 C | sample | Hm Drgbl | Hm Drgbl | Hm Drgbl | CPE002E3 | CPE002EW | CPE002FH | NM 00483 | NM 0048 | NM 0048 | GSTO1 | GSTO1 | GSTO1 | glutathione S-trar | glutathior | glutathior | 9446 | 9446 | 9446 | s18090 | s18091 | s18089 |
| 7898 | J10 | J11 | 1420 | 632,2 | 4544 | 171,7 | 3,2 | 0,489 | HSA034 C | sample | Hm Drgbl | Hm Drgbl | Hm Drgbl | CPE002E4 | CPE002EV | CPE002FG | NM 00682 | NM 0068 | NM 0068 | ACOT2 | ACOT2 | ACOT2 | acyl-CoA thioeste | acyl-CoA t | acyl-CoA t | 10965 | 10965 | 10965 | s195077 | s195079 | s195078 |
| 8000 | N16 | N17 | 1587 | 675,3 | 5219 | 177,5 | 3,3 | 0,504 | HSA034 C | sample | Hm Drgbl | Hm Drgbl | Hm Drgbl | CPE002E4 | CPE002EV | CPE002FG | NM 00685 | NM 0068 | NM 0068 | LIAS | LIAS | LIAS | lipoic acid synthe | lipoic acid | lipoic acid | 11019 | 11019 | 11019 | s21702 | s21703 | s223178 |
| 7510 | I7 | I8 | 1030 | 582,1 | 3503 | 195,4 | 3,4 | 0,26 | HSA034 C | sample | Hm Drgbl | Hm Drgbl | Hm Drgbl | CPE002E3 | CPE002EW | CPE002FH | NM 01456 | NM 0145 | NM 0145 | OTX1 | OTX1 | OTX1 | orthodenticle hor | orthodent | orthodent | 5013 | 5013 | 5013 | s9928 | s9929 | s9930 |
| 12468 | H2 | H3 | 1585 | 1183 | 5575 | 181,2 | 3,5 | 0,263 | HSA034 C | sample | Hm Gen E | Hm Gen E | Hm Gen E | CPE002J5 | CPE002NK | CPE002K1 | NM 00393 | NM 0039 | NM 0039 | BAIAP3 | BAIAP3 | BAIAP3 | BAI1-associated p | BAI1-asso | BAI1-asso | 8938 | 8938 | 8938 | s17091 | s17092 | s17093 |
| 5600 | J16 | J17 | 1208 | 771,1 | 4235 | 157,3 | 3,5 | 0,333 | HSA034 C | sample | Hm Drgbl | Hm Drgbl | Hm Drgbl | CPE002DX | CPE002F2 | CPE002FN | NM 00475 | NM 0047 | NM 0047 | DHRS3 | DHRS3 | DHRS3 | dehydrogenase/r | dehydrog | dehydrog | 9249 | 9249 | 9249 | s17688 | s17689 | s17690 |
| 2894 | I21 | I22 | 2124 | 685,8 | 7350 | 404,7 | 3,5 | 0,412 | HSA034 C | sample | Hm Drgbl | Hm Drgbl | Hm Drgbl | CPE002DP | CPE002EF | CPE002F5 | NM 00422 | NM 0042 | NM 0042 | MFHAS1 | MFHAS1 | MFHAS1 | malignant fibrous | malignant | malignant | 9258 | 9258 | 9258 | s17706 | s17707 | s17708 |
| 5898 | F3 | F4 | 1860 | 755,9 | 6674 | 163,2 | 3,6 | 0,329 | HSA034 C | sample | Hm Drgbl | Hm Drgbl | Hm Drgbl | CPE002DY | CPE002F1 | CPE002FM | NM 00003 | NM 0000 | NM 0000 | AMPD1 | AMPD1 | AMPD1 | adenosine mono | adenosine | adenosine | 270 | 270 | 270 | s1329 | s1330 | s1328 |
| 5457 | D17 | D18 | 642 | 674 | 2281 | 185,2 | 3,6 | 0,343 | HSA034 C | sample | Hm Drgbl | Hm Drgbl | Hm Drgbl | CPE002DX | CPE002F2 | CPE002FN | NM 00373 | NM 0037 | NM 0037 | PTCH2 | PTCH2 | PTCH2 | patched homolog | patched h | patched h | 8643 | 8643 | 8643 | s16445 | s16446 | s16444 |
| 2773 | D20 | D21 | 1205 | 638,2 | 4457 | 276,1 | 3,7 | 0,435 | HSA034 C | sample | Hm Drgbl | Hm Drgbl | Hm Drgbl | CPE002DP | CPE002EF | CPE002F5 | NM 13920 | NM 1392 | NM 1392 | GRK7 | GRK7 | GRK7 | G protein-couple | G protein | G protein | 131890 | 131890 | 131890 | s43646 | s43647 | s43648 |
| 2954 | L10 | L11 | 1652 | 768,4 | 6149 | 373,9 | 3,7 | 0,435 | HSA034 C | sample | Hm Drgbl | Hm Drgbl | Hm Drgbl | CPE002DP | CPE002EF | CPE002F5 | XM 93756 | XM 9375 | XM 9375 | SBK1 | SBK1 | SBK1 | SH3-binding dom | SH3-bindi | SH3-bindi | 388228 | 388228 | 388228 | s52072 | s52073 | s52074 |
| 672 | L9 | L10 | 3523 | 2000 | 13193 | 258,4 | 3,7 | 0,54 | HSA034 C | sample | Hm Drgbl | Hm Drgbl | Hm Drgbl | CPE002E0 | CPE002EZ | CPE002FK | NM 00100 | NM 0010 | NM 0010 | FCGR1B | FCGR1B | FCGR1B | Fc fragment of IgG | Fc fragme | Fc fragme | 2210 | 2210 | 2210 | s5071 | s194405 | s194406 |
| 696 | M9 | M10 | 1878 | 1745 | 6896 | 237,4 | 3,7 | 0,54 | HSA034 C | sample | Hm Drgbl | Hm Drgbl | Hm Drgbl | CPE002E0 | CPE002EZ | CPE002FK | NM 00011 | NM 0001 | NM 0001 | ENG | ENG | ENG | endoglin (Osler-R | endoglin ( | endoglin ( | 2022 | 2022 | 2022 | s4678 | s4677 | s4679 |
| 7229 | N13 | N14 | 1816 | 652,2 | 7028 | 147,1 | 3,9 | 0,304 | HSA034 C | sample | Hm Drgbl | Hm Drgbl | Hm Drgbl | CPE002E2 | CPE002EX | CPE002FI | NM 00243 | NM 0024 | NM 0024 | MNAT1 | MNAT1 | MNAT1 | menage a trois h | menage a | menage a | 4331 | 4331 | 4331 | s8899 | s224052 | s8898 |
| 668 | L5 | L6 | 2412 | 1763 | 9368 | 263,7 | 3,9 | 0,569 | HSA034 C | sample | Hm Drgbl | Hm Drgbl | Hm Drgbl | CPE002E0 | CPE002EZ | CPE002FK | NM 00200 | NM 0020 | NM 0020 | FCER2 | FCER2 | FCER2 | Fc fragment of IgE | Fc fragme | Fc fragme | 2208 | 2208 | 2208 | s5065 | s5066 | s5067 |
| 12443 | G19 | G20 | 1221 | 982,5 | 4897 | 175,2 | 4 | 0,301 | HSA034 C | sample | Hm Gen E | Hm Gen E | Hm Gen E | CPE002J5 | CPE002NK | CPE002K1 | NM 00320 | NM 0032 | NM 0032 | C2orf3 | C2orf3 | C2orf3 | chromosome 2 op | chromoso | chromoso | 6936 | 6936 | 6936 | s13886 | s13887 | s13888 |
| 5783 | A8 | A9 | 1074 | 935,4 | 4275 | 177,7 | 4 | 0,365 | HSA034 C | sample | Hm Drgbl | Hm Drgbl | Hm Drgbl | CPE002DY | CPE002F1 | CPE002FM | NM 00004 | NM 0000 | NM 0000 | ARG1 | ARG1 | ARG1 | arginase, liver | arginase, | arginase, | 383 | 383 | 383 | s1568 | s1569 | s1570 |
| 5456 | D16 | D17 | 1923 | 739,1 | 7623 | 183,7 | 4 | 0,381 | HSA034 C | sample | Hm Drgbl | Hm Drgbl | Hm Drgbl | CPE002DX | CPE002F2 | CPE002FN | NM 00421 | NM 0042 | NM 0042 | NEURL | NEURL | NEURL | neuralized homol | neuralized | neuralized | 9148 | 9148 | 9148 | s17488 | s17486 | s17487 |
| 10548 | H2 | H3 | 1065 | 914,5 | 4283 | 199,3 | 4 | 0,452 | HSA034 C | sample | Hm Drgbl | Hm Drgbl | Hm Drgbl | CPE002EB | CPE002EO | CPE002FR | NM 20691 | NM 2069 | NM 2069 | DEGS2 | DEGS2 | DEGS2 | degenerative sper | degenerat | degenerat | 123099 | 123099 | 123099 | s42619 | s42618 | s42617 |
| 501 | E6 | E7 | 2217 | 1949 | 8860 | 276,9 | 4 | 0,584 | HSA034 C | sample | Hm Drgbl | Hm Drgbl | Hm Drgbl | CPE002E0 | CPE002EZ | CPE002FK | NM 00146 | NM 0014 | NM 0014 | FMO5 | FMO5 | FMO5 | flavin containing | flavin con | flavin con | 2330 | 2330 | 2330 | s5311 | s5309 | s223582 |
| 645 | K6 | K7 | 3293 | 1947 | 13082 | 248,8 | 4 | 0,584 | HSA034 C | sample | Hm Drgbl | Hm Drgbl | Hm Drgbl | CPE002E0 | CPE002EZ | CPE002FK | NM 00015 | NM 0001 | NM 0001 | GALC | GALC | GALC | galactosylceramic | galactosyl | galactosyl | 2581 | 2581 | 2581 | s5530 | s5531 | s5532 |
| 650 | L10 | L11 | 3010 | 1906 | 12049 | 301 | 4 | 0,584 | HSA034 C | sample | Hm Drgbl | Hm Drgbl | Hm Drgbl | CPE002E0 | CPE002EZ | CPE002FK | NM 00017 | NM 0001 | NM 0001 | GP1BA | GP1BA | GP1BA | glycoprotein Ib (p | glycoprot | glycoprot | 2811 | 2811 | 2811 | s5964 | s5963 | s5965 |
| 667 | L4 | L5 | 2815 | 1798 | 11309 | 252,8 | 4 | 0,584 | HSA034 C | sample | Hm Drgbl | Hm Drgbl | Hm Drgbl | CPE002E0 | CPE002EZ | CPE002FK | NM 00448 | NM 0044 | NM 0044 | GOLGB1 | GOLGB1 | GOLGB1 | golgi autoantigen | golgi auto | golgi auto | 2804 | 2804 | 2804 | s5953 | s5952 | s5951 |
| 670 | L7 | L8 | 2555 | 1809 | 10178 | 232,5 | 4 | 0,584 | HSA034 C | sample | Hm Drgbl | Hm Drgbl | Hm Drgbl | CPE002E0 | CPE002EZ | CPE002FK | NM 00056 | NM 0005 | NM 0005 | FCGR1A | FCGR1A | FCGR1A | Fc fragment of IgG | Fc fragme | Fc fragme | 2209 | 2209 | 2209 | s5070 | s5068 | s5069 |
| 692 | M5 | M6 | 2596 | 1797 | 10434 | 276,4 | 4 | 0,584 | HSA034 C | sample | Hm Drgbl | Hm Drgbl | Hm Drgbl | CPE002E0 | CPE002EZ | CPE002FK | NM 00142 | NM 0014 | NM 0014 | EN2 | EN2 | EN2 | engrailed homeol | engrailed | engrailed | 2020 | 2020 | 2020 | s4674 | s4675 | s4676 |
| 728 | O16 | O17 | 2818 | 1902 | 11311 | 266,2 | 4 | 0,584 | HSA034 C | sample | Hm Drgbl | Hm Drgbl | Hm Drgbl | CPE002E0 | CPE002EZ | CPE002FK | NM 02107 | NM 0210 | NM 0210 | GCN5L2 | GCN5L2 | GCN5L2 | GCN5 general cor | GCN5 gen | GCN5 gen | 2648 | 2648 | 2648 | s5657 | s5658 | s5659 |
| 2919 | J22 | J23 | 1661 | 646,3 | 6862 | 409,7 | 4,1 | 0,482 | HSA034 C | sample | Hm Drgbl | Hm Drgbl | Hm Drgbl | CPE002DP | CPE002EF | CPE002F5 | NM 17365 | NM 1736 | NM 1736 | EPHA6 | EPHA6 | EPHA6 | EPH receptor A6 | EPH recep | EPH recep | 285220 | 285220 | 285220 | s49853 | s49854 | s49855 |
| 20132 | G5 | G6 | 1186 | 1231 | 4823 | 98,36 | 4,1 | 0,5 | HSA034 C | sample | Hm Gen E | Hm Gen E | Hm Gen E | CPE002JQ | CPE002O5 | CPE002KL | NM 00101 | NM 0010 | NM 0010 | OR2G6 | OR2G6 | OR2G6 | olfactory recepto | olfactory | olfactory | 391211 | 391211 | 391211 | s52962 | s52963 | s52964 |
| 695 | M8 | M9 | 2207 | 1768 | 9136 | 302,2 | 4,1 | 0,599 | HSA034 C | sample | Hm Drgbl | Hm Drgbl | Hm Drgbl | CPE002E0 | CPE002EZ | CPE002FK | NM 00204 | NM 0020 | NM 0020 | GARS | GARS | GARS | glycyl-tRNA synth | glycyl-tRN | glycyl-tRN | 2617 | 2617 | 2617 | s5578 | s5579 | s5580 |
| 517 | F20 | F21 | 3280 | 1843 | 13739 | 226,5 | 4,2 | 0,613 | HSA034 C | sample | Hm Drgbl | Hm Drgbl | Hm Drgbl | CPE002E0 | CPE002EZ | CPE002FK | NM 00703 | NM 0070 | NM 0070 | GNA12 | GNA12 | GNA12 | guanine nucleotic | guanine n | guanine n | 2768 | 2768 | 2768 | s5865 | s5866 | s5867 |
| 2916 | J2 | J3 | 1016 | 645,7 | 4401 | 426,6 | 4,3 | 0,506 | HSA034 C | sample | Hm Drgbl | Hm Drgbl | Hm Drgbl | CPE002DP | CPE002EF | CPE002F5 | NM 19826 | NM 1982 | NM 1982 | HIPK1 | HIPK1 | HIPK1 | homeodomain in | homeodo | homeodo | 204851 | 204851 | 204851 | s47547 | s47548 | s47549 |
| 477 | D6 | D7 | 2270 | 1912 | 9773 | 262,9 | 4,3 | 0,628 | HSA034 C | sample | Hm Drgbl | Hm Drgbl | Hm Drgbl | CPE002E0 | CPE002EZ | CPE002FK | NM 00016 | NM 0001 | NM 0001 | GHR | GHR | GHR | growth hormone | growth hc | growth hc | 2690 | 2690 | 2690 | s5738 | s5739 | s5740 |

| | | | | | | | | |
|---|---|---|---|---|---|---|---|---|
| 666 | L3 | L4 | 3308 | 1913 | 14281 | 265 | 4,3 | 0,628 |
| 741 | O6 | O7 | 1475 | 1866 | 6409 | 340,5 | 4,3 | 0,628 |
| 863 | D8 | D9 | 1190 | 675,5 | 5217 | 179,1 | 4,4 | 0,417 |
| 3513 | C17 | C18 | 909 | 1245 | 3971 | 165,2 | 4,4 | 0,423 |
| 2731 | B4 | B5 | 1824 | 701,7 | 7943 | 581,9 | 4,4 | 0,518 |
| 2902 | I7 | I8 | 622 | 728,2 | 2728 | 457,8 | 4,4 | 0,518 |
| 2938 | K18 | K19 | 500 | 687,6 | 2216 | 457,7 | 4,4 | 0,518 |
| 2948 | K5 | K6 | 1135 | 658,1 | 4945 | 511,6 | 4,4 | 0,518 |
| 2999 | M8 | M9 | 1896 | 672 | 8322 | 379,3 | 4,4 | 0,518 |
| 11412 | L2 | L3 | 1756 | 931 | 7950 | 127 | 4,5 | 0,326 |
| 2807 | E8 | E9 | 2122 | 712,1 | 9608 | 445,3 | 4,5 | 0,529 |
| 2907 | J11 | J12 | 1144 | 630,1 | 5118 | 440,4 | 4,5 | 0,529 |
| 564 | H2 | H3 | 2267 | 1754 | 10129 | 280,9 | 4,5 | 0,657 |
| 570 | H3 | H4 | 2442 | 1824 | 10910 | 230,5 | 4,5 | 0,657 |
| 10424 | C16 | C17 | 1456 | 926,3 | 6750 | 202,3 | 4,6 | 0,52 |
| 10698 | N3 | N4 | 1604 | 829,6 | 7412 | 175,6 | 4,6 | 0,52 |
| 2879 | H8 | H9 | 1559 | 675,4 | 7117 | 531,7 | 4,6 | 0,541 |
| 523 | F4 | F5 | 3475 | 1883 | 16063 | 258,8 | 4,6 | 0,672 |
| 5250 | K3 | K4 | 989 | 617,3 | 4678 | 233,8 | 4,7 | 0,369 |
| 2796 | E2 | E3 | 1108 | 668,1 | 5204 | 491,2 | 4,7 | 0,553 |
| 2890 | I18 | I19 | 1873 | 656 | 8762 | 431,6 | 4,7 | 0,553 |
| 2891 | I19 | I20 | 1413 | 638,9 | 6698 | 458,4 | 4,7 | 0,553 |
| 2895 | I22 | I23 | 1251 | 698,4 | 5929 | 442,6 | 4,7 | 0,553 |
| 2962 | L18 | L19 | 1139 | 576,4 | 5326 | 370 | 4,7 | 0,553 |
| 457 | D1 | D2 | 2403 | 1900 | 11302 | 270,5 | 4,7 | 0,686 |
| 479 | D8 | D9 | 1845 | 1979 | 8648 | 294 | 4,7 | 0,686 |
| 671 | L8 | L9 | 1852 | 1817 | 8691 | 289,2 | 4,7 | 0,686 |
| 10729 | P1 | P2 | 1454 | 832,9 | 7005 | 168,2 | 4,8 | 0,542 |
| 516 | F2 | F3 | 3103 | 1847 | 14991 | 244,6 | 4,8 | 0,701 |
| 642 | K3 | K4 | 2859 | 1840 | 13845 | 246,8 | 4,8 | 0,701 |
| 643 | K4 | K5 | 2412 | 1834 | 11579 | 263,9 | 4,8 | 0,701 |
| 703 | N15 | N16 | 3351 | 1935 | 16207 | 267,1 | 4,8 | 0,701 |
| 16273 | G1 | G2 | 1262 | 1101 | 6246 | 143,8 | 4,9 | 0,45 |
| 2989 | M20 | M21 | 1773 | 686,4 | 8708 | 448,9 | 4,9 | 0,576 |
| 480 | D9 | D10 | 2000 | 1728 | 9798 | 279,3 | 4,9 | 0,715 |
| 683 | M19 | M20 | 3256 | 1867 | 15985 | 265,7 | 4,9 | 0,715 |
| 7989 | M6 | M7 | 1075 | 640,4 | 5232 | 188,9 | 4,9 | 0,748 |
| 2939 | K19 | K20 | 1307 | 719,8 | 6496 | 533,5 | 5 | 0,588 |
| 2940 | K2 | K3 | 1603 | 622,1 | 7951 | 482,2 | 5 | 0,588 |
| 20292 | N2 | N3 | 753 | 1119 | 3791 | 87,21 | 5 | 0,61 |
| 732 | O2 | O3 | 1842 | 2040 | 9222 | 323,1 | 5 | 0,73 |
| 7801 | F1 | F2 | 1622 | 577,7 | 8053 | 388,9 | 5 | 0,763 |
| 4818 | I3 | I4 | 916 | 1034 | 4682 | 439,5 | 5,1 | 0,56 |
| 2958 | L14 | L15 | 1378 | 595,2 | 7053 | 378,2 | 5,1 | 0,6 |
| 588 | I2 | I3 | 2757 | 1908 | 14068 | 255,7 | 5,1 | 0,745 |
| 597 | I6 | I7 | 2843 | 1800 | 14632 | 246,7 | 5,1 | 0,745 |
| 636 | K2 | K3 | 2809 | 1988 | 14396 | 300,6 | 5,1 | 0,745 |
| 711 | N22 | N23 | 844 | 2083 | 4300 | 299,3 | 5,1 | 0,745 |
| 7709 | B13 | B14 | 1556 | 656,1 | 7969 | 241,1 | 5,1 | 0,779 |
| 7925 | K13 | K14 | 528 | 638,5 | 2669 | 399,8 | 5,1 | 0,779 |
| 12539 | K19 | K20 | 1461 | 1023 | 7573 | 173,6 | 5,2 | 0,391 |
| 6821 | M13 | M14 | 1040 | 676,9 | 5397 | 136,3 | 5,2 | 0,456 |
| 2914 | J18 | J19 | 1733 | 660,4 | 8934 | 417,8 | 5,2 | 0,612 |
| 2915 | J19 | J20 | 1422 | 635,8 | 7408 | 368,4 | 5,2 | 0,612 |
| 2917 | J20 | J21 | 1523 | 656,2 | 7932 | 405,4 | 5,2 | 0,612 |
| 2943 | K22 | K23 | 1493 | 623,7 | 7737 | 425,8 | 5,2 | 0,612 |
| 20274 | M3 | M4 | 1021 | 1164 | 5319 | 79,07 | 5,2 | 0,634 |
| 490 | E18 | E19 | 1207 | 1624 | 6238 | 322,3 | 5,2 | 0,759 |
| 628 | K12 | K13 | 2827 | 1838 | 14827 | 361,4 | 5,2 | 0,759 |
| 649 | L1 | L2 | 3140 | 1943 | 16440 | 282 | 5,2 | 0,759 |
| 7717 | B20 | B21 | 1380 | 632,9 | 7155 | 392,4 | 5,2 | 0,794 |
| 7917 | J6 | J7 | 1134 | 597,8 | 5859 | 241,8 | 5,2 | 0,794 |
| 9780 | H2 | H3 | 1552 | 1065 | 8288 | 133,3 | 5,3 | 0,371 |
| 21584 | D16 | D17 | 361 | 906,7 | 1920 | 453,2 | 5,3 | 0,558 |
| 21804 | M2 | M3 | 225 | 982 | 1194 | 440,3 | 5,3 | 0,558 |
| 10538 | H10 | H11 | 1321 | 826,5 | 7017 | 187,9 | 5,3 | 0,599 |
| 10542 | H14 | H15 | 1073 | 824,4 | 5651 | 173,6 | 5,3 | 0,599 |
| 2718 | B14 | B15 | 1435 | 630,3 | 7623 | 461,4 | 5,3 | 0,624 |
| 2898 | I3 | I4 | 1562 | 631,4 | 8308 | 446,8 | 5,3 | 0,624 |
| 3015 | N22 | N23 | 541 | 749,2 | 2868 | 641,1 | 5,3 | 0,624 |
| 510 | F14 | F15 | 2822 | 1895 | 14866 | 222,9 | 5,3 | 0,774 |
| 655 | L15 | L16 | 2480 | 1779 | 13261 | 277,2 | 5,3 | 0,774 |
| 693 | M6 | M7 | 2220 | 1864 | 11813 | 264 | 5,3 | 0,774 |
| 694 | M7 | M8 | 2698 | 1870 | 14356 | 291,7 | 5,3 | 0,774 |
| 698 | N10 | N11 | 2645 | 1732 | 14016 | 329,1 | 5,3 | 0,774 |
| 7966 | L7 | L8 | 1113 | 616,2 | 5902 | 340,3 | 5,3 | 0,809 |
| 7976 | M16 | M17 | 1185 | 645,1 | 6270 | 272,4 | 5,3 | 0,809 |
| 21601 | E1 | E2 | 267 | 822,1 | 1451 | 474,9 | 5,4 | 0,568 |
| 21763 | K4 | K5 | 241 | 879,6 | 1308 | 573,8 | 5,4 | 0,568 |
| 2768 | D16 | D17 | 884 | 726,4 | 4732 | 488,4 | 5,4 | 0,635 |
| 2957 | L13 | L14 | 911 | 736,7 | 4907 | 443,2 | 5,4 | 0,635 |
| 2967 | L22 | L23 | 1889 | 661,9 | 10156 | 409,9 | 5,4 | 0,635 |
| 2971 | L4 | L5 | 1636 | 631,1 | 8764 | 450,8 | 5,4 | 0,635 |
| 474 | D3 | D4 | 3050 | 1872 | 16513 | 263,9 | 5,4 | 0,788 |
| 529 | G1 | G2 | 2718 | 2030 | 14712 | 296,6 | 5,4 | 0,788 |
| 594 | I3 | I4 | 2449 | 1900 | 13339 | 330,5 | 5,4 | 0,788 |
| 618 | J3 | J4 | 3322 | 1884 | 17937 | 285,2 | 5,4 | 0,788 |
| 632 | K16 | K17 | 2489 | 1727 | 13363 | 250,4 | 5,4 | 0,788 |
| 634 | K18 | K19 | 1740 | 1749 | 9439 | 297,4 | 5,4 | 0,788 |
| 635 | K19 | K20 | 2552 | 1963 | 13734 | 254 | 5,4 | 0,788 |
| 644 | K5 | K6 | 1994 | 1879 | 10781 | 245 | 5,4 | 0,788 |
| 669 | L6 | L7 | 2378 | 1757 | 12850 | 259,6 | 5,4 | 0,788 |
| 7743 | C22 | C23 | 2061 | 587,2 | 11092 | 352,3 | 5,4 | 0,824 |
| 7909 | J20 | J21 | 1303 | 600,3 | 7035 | 238 | 5,4 | 0,824 |
| 7919 | J8 | J9 | 1953 | 629 | 10616 | 238,4 | 5,4 | 0,824 |
| 265 | L1 | L2 | 697 | 1403 | 3844 | 494,2 | 5,5 | 0,468 |
| 13585 | G1 | G2 | 1307 | 1011 | 7247 | 154,8 | 5,5 | 0,516 |
| 10272 | L9 | L10 | 1424 | 819,7 | 7791 | 118,6 | 5,5 | 0,529 |
| 21560 | C16 | C17 | 145 | 1067 | 801 | 612,1 | 5,5 | 0,579 |
| 21750 | K14 | K15 | 453 | 1124 | 2492 | 538,1 | 5,5 | 0,579 |
| 512 | F16 | F17 | 1763 | 1566 | 9611 | 205 | 5,5 | 0,803 |
| 591 | I22 | I23 | 2122 | 1705 | 11607 | 294,7 | 5,5 | 0,803 |
| 646 | K7 | K8 | 2292 | 1794 | 12709 | 240 | 5,5 | 0,803 |
| 705 | N17 | N18 | 2556 | 1769 | 13951 | 283,7 | 5,5 | 0,803 |
| 714 | N3 | N4 | 2547 | 1827 | 14087 | 360,6 | 5,5 | 0,803 |
| 9108 | L2 | L3 | 1378 | 1715 | 7751 | 164,2 | 5,6 | 0,45 |
| 21828 | N2 | N3 | 340 | 885 | 1908 | 333,9 | 5,6 | 0,589 |
| 10650 | L3 | L4 | 1327 | 805,1 | 7383 | 192 | 5,6 | 0,633 |
| 2744 | C16 | C17 | 1361 | 639,8 | 7603 | 509,6 | 5,6 | 0,659 |
| 2961 | L17 | L18 | 1230 | 589,5 | 6906 | 367,3 | 5,6 | 0,659 |
| 450 | C3 | C4 | 2154 | 1753 | 11998 | 252,5 | 5,6 | 0,818 |
| 563 | H19 | H20 | 2600 | 1930 | 14683 | 264,9 | 5,6 | 0,818 |
| 682 | M18 | M19 | 2969 | 2033 | 16734 | 268,9 | 5,6 | 0,818 |
| 719 | N8 | N9 | 2507 | 1725 | 13977 | 284,1 | 5,6 | 0,818 |
| 7895 | I8 | I9 | 1862 | 664 | 10368 | 334,4 | 5,6 | 0,855 |
| 7963 | L4 | L5 | 1273 | 596,1 | 7169 | 345,8 | 5,6 | 0,855 |
| 6342 | I14 | I15 | 1239 | 627,8 | 7020 | 163,3 | 5,7 | 0,376 |
| 1244 | D5 | D6 | 1420 | 751,2 | 8114 | 200,1 | 5,7 | 0,523 |
| 21756 | K2 | K3 | 192 | 903,4 | 1097 | 538,2 | 5,7 | 0,6 |
| 21758 | K21 | K22 | 231 | 911,4 | 1321 | 571,1 | 5,7 | 0,6 |
| 4787 | H19 | H20 | 1711 | 898,1 | 9737 | 404,6 | 5,7 | 0,626 |
| 2802 | E3 | E4 | 629 | 621,6 | 3557 | 460 | 5,7 | 0,671 |
| 2855 | G8 | G9 | 1261 | 626,6 | 7247 | 438 | 5,7 | 0,671 |
| 2936 | K16 | K17 | 1719 | 626,3 | 9839 | 416 | 5,7 | 0,671 |
| 2988 | M2 | M3 | 727 | 635,5 | 4141 | 470,9 | 5,7 | 0,671 |
| 2996 | M5 | M6 | 1199 | 740,2 | 6811 | 496,8 | 5,7 | 0,671 |
| 20048 | D16 | D17 | 1271 | 1297 | 7203 | 136,2 | 5,7 | 0,695 |
| 500 | E5 | E6 | 2240 | 1804 | 12790 | 269,7 | 5,7 | 0,832 |
| 527 | F8 | F9 | 1422 | 1918 | 8149 | 310,7 | 5,7 | 0,832 |
| 648 | K9 | K10 | 3185 | 1919 | 18195 | 319,2 | 5,7 | 0,832 |
| 674 | M10 | M11 | 2608 | 1795 | 14747 | 335,9 | 5,7 | 0,832 |
| 697 | N1 | N2 | 2864 | 1914 | 16280 | 382,8 | 5,7 | 0,832 |
| 734 | O21 | O22 | 2777 | 1975 | 15702 | 375,6 | 5,7 | 0,832 |
| 6826 | M18 | M19 | 1715 | 696,6 | 9926 | 154 | 5,8 | 0,509 |
| 4879 | L15 | L16 | 1953 | 830 | 11367 | 481,2 | 5,8 | 0,637 |
| 10489 | F1 | F2 | 1406 | 835,6 | 8150 | 164 | 5,8 | 0,655 |
| 10537 | H1 | H2 | 817 | 801,2 | 4748 | 213,7 | 5,8 | 0,655 |
| 2805 | E6 | E7 | 1723 | 639,2 | 9932 | 416,1 | 5,8 | 0,682 |
| 2886 | I14 | I15 | 1302 | 630,1 | 7615 | 367,3 | 5,8 | 0,682 |
| 2892 | I2 | I3 | 1217 | 655,8 | 7040 | 496,5 | 5,8 | 0,682 |
| 2927 | J8 | J9 | 1302 | 642,5 | 7607 | 439,4 | 5,8 | 0,682 |
| 2942 | K21 | K22 | 1162 | 724,4 | 6774 | 427,8 | 5,8 | 0,682 |
| 515 | F19 | F20 | 3107 | 1933 | 17866 | 239,9 | 5,8 | 0,847 |
| 546 | G3 | G4 | 2106 | 1772 | 12136 | 251,2 | 5,8 | 0,847 |
| 573 | H6 | H7 | 2621 | 1802 | 15307 | 277,2 | 5,8 | 0,847 |
| 576 | H9 | H10 | 3010 | 1885 | 17453 | 271,7 | 5,8 | 0,847 |
| 609 | J17 | J18 | 3285 | 1837 | 18976 | 266,6 | 5,8 | 0,847 |
| 717 | N6 | N7 | 2337 | 1708 | 13647 | 354,5 | 5,8 | 0,847 |
| 733 | O20 | O21 | 3357 | 1856 | 19615 | 333,1 | 5,8 | 0,847 |
| 7706 | B10 | B11 | 1368 | 553,4 | 7915 | 404,4 | 5,8 | 0,885 |
| 7956 | L2 | L3 | 1151 | 549,7 | 6705 | 334,1 | 5,8 | 0,885 |
| 8039 | O8 | O9 | 2017 | 598,6 | 11621 | 388,8 | 5,8 | 0,885 |
| 10290 | M3 | M4 | 2009 | 860,1 | 11936 | 118,1 | 5,9 | 0,567 |
| 2785 | E1 | E2 | 367 | 731,4 | 2177 | 479,5 | 5,9 | 0,694 |
| 2973 | L6 | L7 | 1437 | 615,8 | 8547 | 424,7 | 5,9 | 0,694 |
| 403 | A4 | A5 | 2356 | 1935 | 13892 | 412,7 | 5,9 | 0,861 |
| 420 | B2 | B3 | 2841 | 2001 | 16750 | 303,1 | 5,9 | 0,861 |
| 437 | C13 | C14 | 2587 | 1832 | 15250 | 240,5 | 5,9 | 0,861 |
| 444 | C2 | C3 | 2972 | 1963 | 17543 | 371,5 | 5,9 | 0,861 |
| 584 | I16 | I17 | 1743 | 1721 | 10256 | 264,8 | 5,9 | 0,861 |
| 604 | J12 | J13 | 2169 | 1725 | 12873 | 327,9 | 5,9 | 0,861 |
| 631 | K15 | K16 | 2893 | 1822 | 17109 | 271,7 | 5,9 | 0,861 |
| 659 | L19 | L20 | 3318 | 1958 | 19665 | 284,8 | 5,9 | 0,861 |
| 661 | L20 | L21 | 2921 | 1858 | 17209 | 273,7 | 5,9 | 0,861 |
| 687 | M22 | M23 | 2885 | 1909 | 16889 | 354,5 | 5,9 | 0,861 |
| 766 | P7 | P8 | 3067 | 1950 | 18049 | 383,4 | 5,9 | 0,861 |
| 7714 | B18 | B19 | 1001 | 687,7 | 5950 | 377,8 | 5,9 | 0,901 |
| 7767 | D22 | D23 | 1543 | 563,7 | 9088 | 379,4 | 5,9 | 0,901 |
| 7941 | K6 | K7 | 1246 | 571,8 | 7376 | 361,2 | 5,9 | 0,901 |
| 6153 | A17 | A18 | 795 | 625,4 | 4785 | 174,7 | 6 | 0,396 |
| 241 | K1 | K2 | 282 | 1423 | 1702 | 485,3 | 6 | 0,511 |
| 10329 | O17 | O18 | 1697 | 884,5 | 10199 | 113,9 | 6 | 0,577 |
| 21770 | L10 | L11 | 260 | 985,4 | 1567 | 699,8 | 6 | 0,632 |
| 4767 | G22 | G23 | 1849 | 851,3 | 11106 | 469,7 | 6 | 0,659 |
| 10712 | O16 | O17 | 1395 | 845,6 | 8307 | 175,9 | 6 | 0,678 |
| 2882 | I10 | I11 | 1258 | 660,2 | 7518 | 340,9 | 6 | 0,706 |
| 2910 | J14 | J15 | 1300 | 600,6 | 7756 | 444,8 | 6 | 0,706 |
| 2966 | L21 | L22 | 738 | 605,9 | 4426 | 388,8 | 6 | 0,706 |
| 20257 | M1 | M2 | 1594 | 1201 | 9497 | 76,85 | 6 | 0,732 |
| 481 | E1 | E2 | 1969 | 1678 | 11818 | 243,5 | 6 | 0,876 |
| 596 | I5 | I6 | 3155 | 1809 | 18857 | 310,1 | 6 | 0,876 |
| 619 | J4 | J5 | 2443 | 1718 | 14707 | 287,7 | 6 | 0,876 |
| 622 | J7 | J8 | 2853 | 1894 | 17112 | 336 | 6 | 0,876 |
| 652 | L12 | L13 | 1995 | 1638 | 11926 | 248,5 | 6 | 0,876 |
| 762 | P3 | P4 | 2941 | 1850 | 17664 | 398,9 | 6 | 0,876 |
| 7885 | I20 | I21 | 906 | 638,8 | 5470 | 248 | 6 | 0,916 |
| 7887 | I22 | I23 | 1688 | 599,1 | 10121 | 307,4 | 6 | 0,916 |
| 7914 | J3 | J4 | 1181 | 589,6 | 7106 | 357,7 | 6 | 0,916 |
| 8007 | N22 | N23 | 1569 | 580,8 | 9343 | 362,1 | 6 | 0,916 |
| 12577 | M1 | M2 | 1498 | 1091 | 9208 | 141 | 6,1 | 0,459 |
| 5048 | C16 | C17 | 1732 | 642,4 | 10587 | 504,4 | 6,1 | 0,478 |
| 5242 | K18 | K19 | 1734 | 676,8 | 10524 | 175,8 | 6,1 | 0,478 |
| 4387 | G4 | G5 | 726 | 810,5 | 4414 | 468,5 | 6,1 | 0,515 |
| 4812 | I2 | I3 | 1044 | 860,8 | 6336 | 504 | 6,1 | 0,67 |
| 4893 | L6 | L7 | 1336 | 884 | 8164 | 446,9 | 6,1 | 0,67 |
| 2812 | F12 | F13 | 282 | 781,7 | 1728 | 496,3 | 6,1 | 0,718 |
| 2816 | F16 | F17 | 992 | 668,2 | 6027 | 465,9 | 6,1 | 0,718 |
| 2819 | F19 | F20 | 1004 | 607,7 | 6144 | 462 | 6,1 | 0,718 |
| 2868 | H2 | H3 | 1640 | 749,4 | 9972 | 503,9 | 6,1 | 0,718 |
| 2984 | M16 | M17 | 1489 | 657,2 | 9065 | 472,7 | 6,1 | 0,718 |
| 20261 | M13 | M14 | 1443 | 1027 | 8804 | 95,38 | 6,1 | 0,744 |
| 456 | C9 | C10 | 2472 | 1890 | 15084 | 316,9 | 6,1 | 0,891 |
| 492 | E2 | E3 | 2659 | 1837 | 16242 | 247,3 | 6,1 | 0,891 |
| 538 | G18 | G19 | 2718 | 2001 | 16607 | 287,2 | 6,1 | 0,891 |
| 638 | K21 | K22 | 2804 | 1839 | 17173 | 283,5 | 6,1 | 0,891 |
| 739 | O4 | O5 | 2109 | 1903 | 12848 | 383 | 6,1 | 0,891 |
| 753 | P17 | P18 | 3099 | 1903 | 18920 | 379,1 | 6,1 | 0,891 |
| 7719 | B22 | B23 | 1666 | 646,7 | 10211 | 351,3 | 6,1 | 0,931 |
| 7778 | E10 | E11 | 1500 | 637,4 | 9128 | 445,3 | 6,1 | 0,931 |
| 7903 | J15 | J16 | 1400 | 650,3 | 8511 | 357,4 | 6,1 | 0,931 |
| 7993 | N1 | N2 | 1034 | 683,2 | 6277 | 296,7 | 6,1 | 0,931 |
| 1483 | N4 | N5 | 507 | 781,1 | 3143 | 403,9 | 6,2 | 0,569 |
| 3464 | A16 | A17 | 2382 | 1404 | 14760 | 269,9 | 6,2 | 0,596 |
| 1879 | O15 | O16 | 2091 | 758 | 13064 | 351,9 | 6,2 | 0,649 |
| 21730 | J18 | J19 | 584 | 984,3 | 3625 | 325,1 | 6,2 | 0,653 |
| 4986 | P3 | P4 | 1584 | 802,3 | 9900 | 431,6 | 6,2 | 0,681 |
| 2748 | C2 | C3 | 1331 | 747,4 | 8210 | 479,4 | 6,2 | 0,729 |
| 2904 | I9 | I10 | 1012 | 627,1 | 6319 | 385,1 | 6,2 | 0,729 |
| 2906 | J10 | J11 | 1113 | 654,2 | 6908 | 346,9 | 6,2 | 0,729 |
| 2909 | J13 | J14 | 935 | 605 | 5837 | 398,7 | 6,2 | 0,729 |
| 2912 | J16 | J17 | 1015 | 646,6 | 6322 | 328,3 | 6,2 | 0,729 |
| 2963 | L19 | L20 | 950 | 554,2 | 5856 | 363,8 | 6,2 | 0,729 |
| 20220 | K2 | K3 | 1306 | 1087 | 8051 | 99,34 | 6,2 | 0,756 |
| 20226 | K3 | K4 | 740 | 1101 | 4595 | 118,4 | 6,2 | 0,756 |
| 20287 | N15 | N16 | 1148 | 1114 | 7162 | 104,6 | 6,2 | 0,756 |
| 20298 | N3 | N4 | 354 | 1228 | 2197 | 86,02 | 6,2 | 0,756 |

| | | | | | | | | | |
|---|---|---|---|---|---|---|---|---|---|
| 483 | E11 | E12 | 3317 | 1938 | 20426 | 292,2 | 6,2 | 0,905 | EFNA2 |
| 559 | H15 | H16 | 2771 | 1977 | 17150 | 318,3 | 6,2 | 0,905 | ACSL3 |
| 589 | I20 | I21 | 2569 | 1810 | 15878 | 371,6 | 6,2 | 0,905 | GAA |
| 639 | K22 | K23 | 2332 | 1689 | 14367 | 309,7 | 6,2 | 0,905 | GAMT |
| 729 | O17 | O18 | 2372 | 1825 | 14604 | 339,8 | 6,2 | 0,905 | ERCC1 |
| 731 | O19 | O20 | 3234 | 1959 | 19964 | 321,5 | 6,2 | 0,905 | ERCC2 |
| 742 | O7 | O8 | 2748 | 1856 | 16993 | 417,4 | 6,2 | 0,905 | EPO |
| 7699 | A4 | A5 | 1724 | 632,5 | 10752 | 405 | 6,2 | 0,947 | ARPC1A |
| 7724 | B5 | B6 | 1128 | 574,8 | 6947 | 409,3 | 6,2 | 0,947 | HMG20B |
| 7808 | F16 | F17 | 1770 | 541,3 | 10969 | 387,9 | 6,2 | 0,947 | DBF4 |
| 8029 | O20 | O21 | 1721 | 545,5 | 10631 | 330 | 6,2 | 0,947 | SRCAP |
| 8030 | O21 | O22 | 1893 | 624,3 | 11779 | 410,9 | 6,2 | 0,947 | ABCA9 |
| 4319 | D8 | D9 | 374 | 681,2 | 2353 | 180,5 | 6,3 | 0,532 | TRPV4 |
| 121 | F1 | F2 | 556 | 1544 | 3525 | 391,4 | 6,3 | 0,536 | SFTPD |
| 6132 | P2 | P3 | 1759 | 807,2 | 11104 | 229,8 | 6,3 | 0,575 | CBLB |
| 3525 | C6 | C7 | 981 | 1270 | 6221 | 251,6 | 6,3 | 0,606 | TACR1 |
| 10328 | O16 | O17 | 1623 | 891,4 | 10242 | 112,9 | 6,3 | 0,606 | RGPD5 |
| 4753 | G1 | G2 | 327 | 755,9 | 2064 | 487,2 | 6,3 | 0,692 | C1R |
| 10681 | N1 | N2 | 1180 | 813,1 | 7381 | 199,4 | 6,3 | 0,712 | TUBA3E |
| 2804 | E5 | E6 | 1408 | 668,6 | 8821 | 480,8 | 6,3 | 0,741 | MAP2K1IP |
| 2883 | I11 | I12 | 855 | 651,6 | 5410 | 427,9 | 6,3 | 0,741 | MAP3K13 |
| 2951 | K8 | K9 | 1348 | 662,9 | 8454 | 409 | 6,3 | 0,741 | ALS2CR7 |
| 20024 | C16 | C17 | 1314 | 1198 | 8295 | 135,2 | 6,3 | 0,768 | C4orf40 |
| 20106 | F3 | F4 | 1251 | 1139 | 7936 | 110,2 | 6,3 | 0,768 | C11orf38 |
| 20300 | N5 | N6 | 1077 | 1108 | 6784 | 96,36 | 6,3 | 0,768 | C1orf141 |
| 466 | D18 | D19 | 2615 | 1754 | 16562 | 246,9 | 6,3 | 0,92 | GCLM |
| 499 | E4 | E5 | 3213 | 1926 | 20100 | 294,2 | 6,3 | 0,92 | FMO4 |
| 522 | F3 | F4 | 2655 | 1792 | 16640 | 261 | 6,3 | 0,92 | EXTL3 |
| 571 | H4 | H5 | 2534 | 1825 | 16038 | 289 | 6,3 | 0,92 | GNAI2 |
| 575 | H8 | H9 | 2795 | 1788 | 17594 | 250,1 | 6,3 | 0,92 | GNAL |
| 611 | J19 | J20 | 2783 | 1849 | 17579 | 279,4 | 6,3 | 0,92 | FCAR |
| 612 | J2 | J3 | 2919 | 1856 | 18293 | 277 | 6,3 | 0,92 | GNB2 |
| 624 | J9 | J10 | 2614 | 1783 | 16338 | 256,7 | 6,3 | 0,92 | FAT2 |
| 647 | K8 | K9 | 2419 | 1751 | 15294 | 307,4 | 6,3 | 0,92 | GALE |
| 662 | L21 | L22 | 2800 | 1788 | 17718 | 293,3 | 6,3 | 0,92 | FDPS |
| 764 | P5 | P6 | 2754 | 2021 | 17271 | 385,4 | 6,3 | 0,92 | FH |
| 7693 | A20 | A21 | 1832 | 600,2 | 11603 | 380 | 6,3 | 0,962 | AKAP3 |
| 7886 | I21 | I22 | 1536 | 693,9 | 9646 | 325,7 | 6,3 | 0,962 | ABCC4 |
| 7965 | L6 | L7 | 1161 | 569 | 7260 | 316,2 | 6,3 | 0,962 | GCN1L1 |
| 7979 | M19 | M20 | 1777 | 615,7 | 11166 | 387 | 6,3 | 0,962 | CCNU |
| 5228 | J5 | J6 | 1207 | 669,3 | 7738 | 186,1 | 6,4 | 0,502 | TFEB |
| 2079 | G22 | G23 | 2840 | 749,6 | 18161 | 364 | 6,4 | 0,557 | ZBTB38 |
| 2244 | N2 | N3 | 1021 | 711,1 | 6503 | 398,7 | 6,4 | 0,557 | |
| 5964 | I2 | I3 | 865 | 727 | 5579 | 457,5 | 6,4 | 0,584 | SERPINC1 |
| 10314 | N3 | N4 | 1486 | 811,1 | 9553 | 121,8 | 6,4 | 0,615 | MAP1LC3E |
| 21635 | F19 | F20 | 482 | 1092 | 3105 | 527,8 | 6,4 | 0,674 | LOC65138 |
| 21739 | J4 | J5 | 326 | 1070 | 2095 | 685,7 | 6,4 | 0,674 | GPR89A |
| 21745 | K1 | K2 | 324 | 998,6 | 2075 | 552,6 | 6,4 | 0,674 | LOC64993 |
| 21757 | K20 | K21 | 359 | 945,7 | 2294 | 628,3 | 6,4 | 0,674 | LOC65278 |
| 10541 | H13 | H14 | 1329 | 781,5 | 8469 | 166,7 | 6,4 | 0,723 | MARS2 |
| 2769 | D17 | D18 | 1496 | 683,6 | 9517 | 369,4 | 6,4 | 0,753 | NLK |
| 2876 | H5 | H6 | 1074 | 592,3 | 6824 | 491,6 | 6,4 | 0,753 | AAK1 |
| 2925 | J6 | J7 | 1514 | 660,3 | 9750 | 565,6 | 6,4 | 0,753 | IPMK |
| 2965 | L20 | L21 | 751 | 702 | 4769 | 450,2 | 6,4 | 0,753 | PKMYT1 |
| 2991 | M22 | M23 | 1410 | 680,1 | 8976 | 431,4 | 6,4 | 0,753 | HKDC1 |
| 431 | B8 | B9 | 3003 | 1981 | 19087 | 314,9 | 6,4 | 0,934 | GFAP |
| 433 | C1 | C2 | 2167 | 1732 | 13774 | 307,2 | 6,4 | 0,934 | ECGF1 |
| 446 | C21 | C22 | 2328 | 1607 | 14936 | 289,4 | 6,4 | 0,934 | EEF1A2 |
| 468 | D2 | D3 | 2371 | 1800 | 15152 | 284,2 | 6,4 | 0,934 | GH1 |
| 494 | E21 | E22 | 1998 | 1771 | 12874 | 230,2 | 6,4 | 0,934 | MEGF9 |
| 583 | I15 | I16 | 2258 | 1735 | 14475 | 271 | 6,4 | 0,934 | EIF5A |
| 590 | I21 | I22 | 2686 | 1786 | 17155 | 317,7 | 6,4 | 0,934 | ELAVL1 |
| 660 | L2 | L3 | 2707 | 1839 | 17430 | 284,3 | 6,4 | 0,934 | GNS |
| 748 | P12 | P13 | 1973 | 1896 | 12569 | 430,7 | 6,4 | 0,934 | GSN |
| 7695 | A22 | A23 | 2234 | 670,4 | 14287 | 367,3 | 6,4 | 0,977 | SLC34A2 |
| 7746 | C3 | C4 | 950 | 633 | 6067 | 256,9 | 6,4 | 0,977 | CHST4 |
| 8034 | O3 | O4 | 808 | 625,3 | 5163 | 223,4 | 6,4 | 0,977 | RRAGB |
| 6955 | B4 | B5 | 1231 | 643,5 | 7942 | 166 | 6,5 | 0,506 | NDUFA5 |
| 9139 | M4 | M5 | 1846 | 2004 | 11968 | 115,2 | 6,5 | 0,522 | BCMO1 |
| 214 | I7 | I8 | 428 | 1589 | 2762 | 374,4 | 6,5 | 0,553 | SERPINB3 |
| 997 | J20 | J21 | 1185 | 823,5 | 7735 | 350,7 | 6,5 | 0,616 | TAF11 |
| 21752 | K16 | K17 | 330 | 1026 | 2158 | 568,3 | 6,5 | 0,684 | LOC65276 |
| 10554 | H3 | H4 | 977 | 866,5 | 6383 | 191,8 | 6,5 | 0,734 | ZNF276 |
| 2789 | E13 | E14 | 663 | 672,6 | 4287 | 486,9 | 6,5 | 0,765 | DYRK4 |
| 2985 | M17 | M18 | 705 | 685,1 | 4605 | 428,8 | 6,5 | 0,765 | MRC2 |
| 20089 | F1 | F2 | 1084 | 1245 | 7016 | 126,2 | 6,5 | 0,793 | FLJ46266 |
| 20112 | F9 | F10 | 1282 | 1206 | 8282 | 124,2 | 6,5 | 0,793 | LOC40002 |
| 20148 | H2 | H3 | 789 | 1265 | 5114 | 99,52 | 6,5 | 0,793 | OR5K3 |
| 432 | B9 | B10 | 2582 | 1850 | 16750 | 277,2 | 6,5 | 0,949 | ERF |
| 505 | F1 | F2 | 2786 | 1966 | 18240 | 295,6 | 6,5 | 0,949 | EXTL2 |
| 539 | G19 | G20 | 2069 | 1861 | 13456 | 359,8 | 6,5 | 0,949 | EIF4A1 |
| 561 | H17 | H18 | 1544 | 1750 | 10097 | 285,2 | 6,5 | 0,949 | ACSL4 |
| 615 | J22 | J23 | 1991 | 1900 | 12859 | 331,1 | 6,5 | 0,949 | GNRH2 |
| 633 | K17 | K18 | 2038 | 1766 | 13274 | 280 | 6,5 | 0,949 | ELN |
| 637 | K20 | K21 | 2021 | 1712 | 13192 | 290,8 | 6,5 | 0,949 | GALT |
| 691 | M4 | M5 | 1859 | 1829 | 12023 | 309,5 | 6,5 | 0,949 | GAPDH |
| 720 | N9 | N10 | 3007 | 2014 | 19565 | 298,1 | 6,5 | 0,949 | FGA |
| 744 | O9 | O10 | 2203 | 1894 | 14235 | 307,5 | 6,5 | 0,949 | EPOR |
| 7733 | C13 | C14 | 1198 | 621,1 | 7785 | 422,7 | 6,5 | 0,992 | SORBS3 |
| 7753 | D1 | D2 | 2160 | 579,6 | 14148 | 389,4 | 6,5 | 0,992 | CEPT1 |
| 7899 | J11 | J12 | 1354 | 664,2 | 8737 | 277,1 | 6,5 | 0,992 | ZNF238 |
| 49 | C1 | C2 | 296 | 1747 | 1939 | 620,2 | 6,6 | 0,562 | NR3C1 |
| 3661 | I20 | I21 | 845 | 1343 | 5615 | 272,5 | 6,6 | 0,635 | NMUR1 |
| 21715 | I4 | I5 | 370 | 1121 | 2455 | 753,8 | 6,6 | 0,695 | LOC65269 |
| 21793 | M1 | M2 | 391 | 970,5 | 2574 | 486,7 | 6,6 | 0,695 | LOC65013 |
| 4715 | E19 | E20 | 2006 | 807,9 | 13254 | 349,5 | 6,6 | 0,725 | BLMH |
| 10608 | J9 | J10 | 1155 | 855 | 7629 | 204,2 | 6,6 | 0,746 | MGC2187 |
| 10705 | O1 | O2 | 1745 | 807,9 | 11434 | 166,9 | 6,6 | 0,746 | ATP6V1E2 |
| 2745 | C17 | C18 | 786 | 759,6 | 5192 | 532,5 | 6,6 | 0,776 | MKNK1 |
| 2795 | E19 | E20 | 1286 | 667,4 | 8521 | 463,1 | 6,6 | 0,776 | PIM2 |
| 2823 | F22 | F23 | 1645 | 659 | 10882 | 516 | 6,6 | 0,776 | PDIK1L |
| 2861 | H13 | H14 | 1736 | 704,7 | 11423 | 552,4 | 6,6 | 0,776 | STK38L |
| 2964 | L2 | L3 | 586 | 617,4 | 3847 | 573,6 | 6,6 | 0,776 | NRBP2 |
| 20154 | H3 | H4 | 544 | 1156 | 3614 | 91,97 | 6,6 | 0,805 | FLJ22447 |
| 20305 | O1 | O2 | 1282 | 1079 | 8451 | 97,34 | 6,6 | 0,805 | ELFN1 |
| 409 | B1 | B2 | 2425 | 2088 | 16085 | 341,7 | 6,6 | 0,964 | ERCC3 |
| 422 | B21 | B22 | 2100 | 1854 | 13845 | 353,5 | 6,6 | 0,964 | ETFDH |
| 623 | J8 | J9 | 2596 | 1835 | 17258 | 310,2 | 6,6 | 0,964 | GNG4 |
| 653 | L13 | L14 | 2932 | 1861 | 19428 | 322,6 | 6,6 | 0,964 | FCGR2B |
| 663 | L22 | L23 | 2610 | 1842 | 17318 | 304,7 | 6,6 | 0,964 | GPS1 |
| 708 | N2 | N3 | 2269 | 1840 | 15070 | 320,2 | 6,6 | 0,964 | GPS2 |
| 735 | O22 | O23 | 2212 | 1796 | 14563 | 410,2 | 6,6 | 0,964 | MSTN |
| 767 | P8 | P9 | 2123 | 1855 | 13967 | 419,5 | 6,6 | 0,964 | PDIA3 |
| 7931 | K19 | K20 | 1557 | 613,6 | 10316 | 383,6 | 6,6 | 1,008 | SDCCAG1 |
| 7997 | N13 | N14 | 1782 | 631,6 | 11674 | 432,5 | 6,6 | 1,008 | FBLN5 |
| 9150 | N14 | N15 | 1404 | 1643 | 9404 | 177,3 | 6,7 | 0,538 | DIRAS2 |
| 120 | E9 | E10 | 930 | 1638 | 6198 | 330,1 | 6,7 | 0,57 | RXRB |
| 235 | J4 | J5 | 576 | 1528 | 3836 | 469,8 | 6,7 | 0,57 | SSX1 |
| 2094 | H14 | H15 | 331 | 785,4 | 2211 | 376,3 | 6,7 | 0,583 | ZNF772 |
| 21765 | K6 | K7 | 142 | 968,9 | 946 | 546,7 | 6,7 | 0,705 | LOC65274 |
| 4670 | C21 | C22 | 2410 | 1043 | 16109 | 258,5 | 6,7 | 0,736 | ACR |
| 4903 | M15 | M16 | 1339 | 873,2 | 8979 | 223,9 | 6,7 | 0,736 | ACE |
| 10539 | H11 | H12 | 1261 | 832,6 | 8480 | 206,7 | 6,7 | 0,757 | UBE2Q2 |
| 10620 | K2 | K3 | 1248 | 856,5 | 8394 | 184,2 | 6,7 | 0,757 | SLC22A12 |
| 10633 | L1 | L2 | 1371 | 944,2 | 9254 | 185 | 6,7 | 0,757 | ARHGAP1 |
| 2717 | B13 | B14 | 1277 | 644,1 | 8516 | 462,8 | 6,7 | 0,788 | ERN2 |
| 2727 | B22 | B23 | 1823 | 738,6 | 12162 | 505 | 6,7 | 0,788 | SLAMF6 |
| 2787 | E11 | E12 | 1988 | 720,9 | 13376 | 433,2 | 6,7 | 0,788 | RIOK3 |
| 2806 | E7 | E8 | 1102 | 620 | 7415 | 457,2 | 6,7 | 0,788 | TNK1 |
| 2911 | J15 | J16 | 1258 | 679,3 | 8488 | 397,9 | 6,7 | 0,788 | CCRK |
| 2946 | K3 | K4 | 791 | 617,4 | 5296 | 465,5 | 6,7 | 0,788 | STK17A |
| 3013 | N20 | N21 | 1286 | 614,9 | 8608 | 497,4 | 6,7 | 0,788 | CRY1 |
| 20049 | D17 | D18 | 1002 | 1315 | 6757 | 90,42 | 6,7 | 0,817 | LOC39993 |
| 20108 | F5 | F6 | 827 | 1202 | 5506 | 142,3 | 6,7 | 0,817 | LOC39993 |
| 20268 | M2 | M3 | 964 | 1153 | 6464 | 96,73 | 6,7 | 0,817 | C9orf105 |
| 20281 | N1 | N2 | 1487 | 1095 | 9917 | 83,25 | 6,7 | 0,817 | LOC40075 |
| 20283 | N11 | N12 | 1282 | 1138 | 8616 | 114,8 | 6,7 | 0,817 | C1orf220 |
| 20291 | N19 | N20 | 1911 | 1057 | 12841 | 110,2 | 6,7 | 0,817 | DEFB32 |
| 462 | D14 | D15 | 1370 | 1773 | 9198 | 345,1 | 6,7 | 0,978 | GLB1 |
| 524 | F5 | F6 | 1724 | 1816 | 11570 | 289,4 | 6,7 | 0,978 | EYA1 |
| 540 | G2 | G3 | 2187 | 1696 | 14544 | 306,8 | 6,7 | 0,978 | FOS |
| 595 | I4 | I5 | 2864 | 1828 | 19253 | 310,4 | 6,7 | 0,978 | FUT5 |
| 601 | J1 | J2 | 1937 | 1821 | 12940 | 360,8 | 6,7 | 0,978 | FBLN1 |
| 651 | L11 | L12 | 2893 | 1916 | 19493 | 351,1 | 6,7 | 0,978 | FCGR2A |
| 7879 | I15 | I16 | 591 | 562,2 | 3954 | 309,3 | 6,7 | 1,023 | SPRY1 |
| 7964 | L5 | L6 | 1111 | 577,6 | 7471 | 339 | 6,7 | 1,023 | SEC23B |
| 7977 | M17 | M18 | 1515 | 663,5 | 10102 | 356,8 | 6,7 | 1,023 | APBB3 |
| 7983 | M22 | M23 | 1488 | 548,7 | 9947 | 341,7 | 6,7 | 1,023 | SLC17A3 |
| 8054 | P21 | P22 | 1387 | 680,2 | 9225 | 425,2 | 6,7 | 1,023 | PRDX4 |
| 5210 | J10 | J11 | 1802 | 734 | 12260 | 184,6 | 6,8 | 0,533 | YWHAB |
| 1323 | H11 | H12 | 1009 | 784,6 | 6837 | 468,7 | 6,8 | 0,624 | SNAP23 |
| 1434 | L3 | L4 | 1868 | 814,5 | 12670 | 438,1 | 6,8 | 0,624 | LDB2 |
| 13770 | N3 | N4 | 1321 | 1055 | 8983 | 176,6 | 6,8 | 0,638 | BRMS1 |
| 8066 | A10 | A11 | 1686 | 736,1 | 11502 | 240,6 | 6,8 | 0,654 | NMNAT2 |
| 21783 | L22 | L23 | 281 | 1076 | 1906 | 681,8 | 6,8 | 0,716 | LOC65388 |
| 10567 | I15 | I16 | 1408 | 770,6 | 9615 | 165,4 | 6,8 | 0,768 | SERPINB12 |
| 2733 | B6 | B7 | 1262 | 685,5 | 8572 | 540,8 | 6,8 | 0,8 | TPD52L3 |
| 2918 | J21 | J22 | 636 | 675,2 | 4310 | 377,5 | 6,8 | 0,8 | TSSK2 |
| 3018 | N3 | N4 | 1196 | 619,7 | 8106 | 430,6 | 6,8 | 0,8 | RPS6KC1 |
| 20113 | G1 | G2 | 1099 | 1225 | 7469 | 107,7 | 6,8 | 0,829 | OR2M2 |
| 20178 | I3 | I4 | 909 | 1015 | 6163 | 91,67 | 6,8 | 0,829 | LOC39152 |
| 20295 | N22 | N23 | 1650 | 1110 | 11145 | 129 | 6,8 | 0,829 | LOC43996 |
| 20352 | P9 | P10 | 588 | 1479 | 3981 | 77,94 | 6,8 | 0,829 | C22orf16 |
| 427 | B4 | B5 | 2509 | 1954 | 17002 | 339 | 6,8 | 0,993 | GDI2 |
| 435 | C11 | C12 | 2026 | 1716 | 13716 | 262,5 | 6,8 | 0,993 | EDN1 |
| 476 | D5 | D6 | 2136 | 1940 | 14467 | 283,3 | 6,8 | 0,993 | ETV2 |
| 504 | E9 | E10 | 2726 | 1772 | 18449 | 248,8 | 6,8 | 0,993 | LGTN |
| 548 | G5 | G6 | 1682 | 1735 | 11371 | 306,7 | 6,8 | 0,993 | EGR3 |
| 562 | H18 | H19 | 2145 | 1655 | 14490 | 264,7 | 6,8 | 0,993 | GNAT2 |
| 574 | H7 | H8 | 2898 | 1844 | 19788 | 323 | 6,8 | 0,993 | FABP6 |
| 606 | J14 | J15 | 2735 | 1737 | 18465 | 306,2 | 6,8 | 0,993 | GNG11 |
| 621 | J6 | J7 | 2668 | 1882 | 18136 | 317,6 | 6,8 | 0,993 | GNG3 |
| 679 | M15 | M16 | 2445 | 1657 | 16669 | 275,9 | 6,8 | 0,993 | ENO3 |
| 684 | M2 | M3 | 2014 | 1736 | 13784 | 387,4 | 6,8 | 0,993 | GANC |
| 752 | P16 | P17 | 2198 | 1814 | 14837 | 400 | 6,8 | 0,993 | GSR |
| 7759 | D15 | D16 | 1933 | 575,8 | 13190 | 370,3 | 6,8 | 1,038 | PIAS3 |
| 7777 | E1 | E2 | 595 | 585,9 | 4017 | 367,3 | 6,8 | 1,038 | TXNDC9 |
| 7907 | J19 | J20 | 1626 | 659,9 | 11130 | 256,6 | 6,8 | 1,038 | SLC25A17 |
| 7959 | L22 | L23 | 1774 | 538,3 | 12040 | 361,4 | 6,8 | 1,038 | SLC27A2 |
| 7978 | M18 | M19 | 1908 | 635,7 | 12911 | 410 | 6,8 | 1,038 | FUSIP1 |
| 8050 | P18 | P19 | 2037 | 638,5 | 13939 | 407,1 | 6,8 | 1,038 | SMA4 |
| 4245 | A6 | A7 | 1616 | 699,6 | 11148 | 255,8 | 6,9 | 0,582 | SCNN1A |
| 240 | J9 | J10 | 577 | 1476 | 4001 | 456,5 | 6,9 | 0,587 | SLAMF1 |
| 6682 | G18 | G19 | 936 | 591 | 6496 | 146,7 | 6,9 | 0,605 | IL10RA |
| 5987 | J19 | J20 | 834 | 989 | 5740 | 195,9 | 6,9 | 0,63 | APEX1 |
| 3536 | D16 | D17 | 1588 | 1426 | 10893 | 286,2 | 6,9 | 0,663 | GPR126 |
| 8120 | C16 | C17 | 1368 | 611 | 9390 | 458,8 | 6,9 | 0,663 | MCF2L2 |
| 8264 | I16 | I17 | 1125 | 651,3 | 7778 | 164,9 | 6,9 | 0,663 | RHOBTB2 |
| 21524 | A5 | A6 | 125 | 942,1 | 865 | 632,9 | 6,9 | 0,726 | LOC64872 |
| 21759 | K22 | K23 | 322 | 1092 | 2212 | 754,7 | 6,9 | 0,726 | LOC65278 |
| 21790 | L7 | L8 | 395 | 1090 | 2739 | 469,1 | 6,9 | 0,726 | LOC65195 |
| 2798 | E21 | E22 | 1777 | 713,4 | 12341 | 502,6 | 6,9 | 0,812 | CIT |
| 2878 | H7 | H8 | 1398 | 660,4 | 9617 | 458 | 6,9 | 0,812 | LMTK2 |
| 2926 | J7 | J8 | 1135 | 715,2 | 7780 | 472,7 | 6,9 | 0,812 | SNF1LK2 |
| 2947 | K4 | K5 | 894 | 591,5 | 6139 | 373,5 | 6,9 | 0,812 | IQCH |
| 3007 | N15 | N16 | 1116 | 600,4 | 7706 | 417,7 | 6,9 | 0,812 | RPS6KA6 |
| 20250 | L3 | L4 | 824 | 1118 | 5660 | 107,1 | 6,9 | 0,841 | FLJ35767 |
| 402 | A3 | A4 | 2995 | 2043 | 20534 | 407,1 | 6,9 | 1,007 | DTNB |
| 547 | G4 | G5 | 2374 | 1728 | 16289 | 293,5 | 6,9 | 1,007 | FOSB |
| 553 | H1 | H2 | 2467 | 1771 | 16936 | 298,2 | 6,9 | 1,007 | FABP2 |
| 567 | H22 | H23 | 2945 | 1949 | 20311 | 312,7 | 6,9 | 1,007 | GNB1 |
| 586 | I18 | I19 | 2744 | 1849 | 18911 | 313,7 | 6,9 | 1,007 | XRCC6 |
| 598 | I7 | I8 | 2021 | 1902 | 14031 | 308,5 | 6,9 | 1,007 | EIF4EBP2 |
| 630 | K14 | K15 | 960 | 1976 | 6635 | 316,3 | 6,9 | 1,007 | GALNT1 |
| 678 | M14 | M15 | 1732 | 1705 | 11970 | 335,4 | 6,9 | 1,007 | GATM |
| 716 | N5 | N6 | 2958 | 1919 | 20332 | 330,7 | 6,9 | 1,007 | FECH |
| 749 | P13 | P14 | 2667 | 1861 | 18472 | 433,4 | 6,9 | 1,007 | FKBP3 |
| 7682 | A10 | A11 | 1662 | 547,4 | 11473 | 427,7 | 6,9 | 1,053 | RPP30 |
| 7702 | A7 | A8 | 1487 | 659,3 | 10258 | 363,2 | 6,9 | 1,053 | EBI3 |
| 7930 | K18 | K19 | 1721 | 555,2 | 11799 | 346,1 | 6,9 | 1,053 | POLQ |
| 7944 | K9 | K10 | 1683 | 581,5 | 11605 | 320 | 6,9 | 1,053 | RAMP3 |
| 7958 | L21 | L22 | 1716 | 635,8 | 11876 | 340,2 | 6,9 | 1,053 | SEMA6C |
| 7982 | M21 | M22 | 1890 | 643 | 13032 | 374,9 | 6,9 | 1,053 | TCIRG1 |
| 8045 | P13 | P14 | 1905 | 565 | 13080 | 353 | 6,9 | 1,053 | TXNL2 |
| 8055 | P22 | P23 | 1281 | 701 | 8809 | 464,1 | 6,9 | 1,053 | MID2 |
| 3467 | A19 | A20 | 2270 | 1475 | 15996 | 331 | 7 | 0,673 | ADRA1A |
| 3666 | I3 | I4 | 1187 | 1366 | 8256 | 321,1 | 7 | 0,673 | CCR6 |
| 10170 | H3 | H4 | 1344 | 842,8 | 9345 | 139,5 | 7 | 0,673 | DDHD1 |
| 1805 | L13 | L14 | 505 | 933,3 | 3550 | 407,7 | 7 | 0,733 | VPS11 |
| 1862 | N21 | N22 | 2669 | 843,3 | 18681 | 402,5 | 7 | 0,733 | SLC2A4RG |
| 21704 | I16 | I17 | 442 | 962 | 3089 | 489,5 | 7 | 0,737 | LOC65272 |
| 21810 | M3 | M4 | 196 | 963,7 | 1375 | 331,2 | 7 | 0,737 | LOC65015 |
| 21831 | N22 | N23 | 337 | 933,5 | 2357 | 561,5 | 7 | 0,737 | OCM |
| 21840 | N9 | N10 | 374 | 965,4 | 2601 | 510,7 | 7 | 0,737 | LOC65213 |
| 4820 | I5 | I6 | 1130 | 917,8 | 7894 | 505,3 | 7 | 0,769 | CASP8 |
| 2755 | C4 | C5 | 1128 | 717,6 | 7895 | 552,5 | 7 | 0,824 | STYK1 |

| | | | | | | | | | | | | | | |
|---|---|---|---|---|---|---|---|---|---|---|---|---|---|---|
| 2759 | C8 | C9 | 1136 | 660,8 | 7907 | 507 | 7 | 0,824 | H5A034 C | sample | Hm Drgbl | ALS2CR2 | amyotrophic later | 55437 |
| 2845 | G20 | G21 | 1230 | 612,5 | 8624 | 454,2 | 7 | 0,824 | H5A034 C | sample | Hm Drgbl | SCYL1 | SCY1-like 1 (S. cer | 57410 |
| 2885 | I13 | I14 | 866 | 599,7 | 6096 | 442,9 | 7 | 0,824 | H5A034 C | sample | Hm Drgbl | DCLK1 | doublecortin-like | 9201 |
| 2995 | M4 | M5 | 1003 | 681,4 | 6984 | 509,1 | 7 | 0,824 | H5A034 C | sample | Hm Drgbl | FN3KRP | fructosamine-3-ki | 79672 |
| 2997 | M6 | M7 | 1246 | 626,3 | 8784 | 461,8 | 7 | 0,824 | H5A034 C | sample | Hm Drgbl | LRRK1 | leucine-rich repea | 79705 |
| 486 | E14 | E15 | 2992 | 1858 | 20998 | 268,6 | 7 | 1,022 | H5A034 C | sample | Hm Drgbl | FNTA | farnesyltransfera | 2339 |
| 509 | F13 | F14 | 2730 | 1878 | 19194 | 256,1 | 7 | 1,022 | H5A034 C | sample | Hm Drgbl | EZH2 | enhancer of zeste | 2146 |
| 585 | I17 | I18 | 2573 | 1848 | 17965 | 306,9 | 7 | 1,022 | H5A034 C | sample | Hm Drgbl | SERPINB1 | serpin peptidase | 1992 |
| 658 | L18 | L19 | 2370 | 1788 | 16659 | 238,3 | 7 | 1,022 | H5A034 C | sample | Hm Drgbl | GPI | glucose phosphat | 2821 |
| 673 | M1 | M2 | 2065 | 1660 | 14363 | 308,2 | 7 | 1,022 | H5A034 C | sample | Hm Drgbl | EMX2 | empty spiracles h | 2018 |
| 699 | N11 | N12 | 2486 | 1700 | 17317 | 332,2 | 7 | 1,022 | H5A034 C | sample | Hm Drgbl | FGB | fibrinogen beta cl | 2244 |
| 7687 | A15 | A16 | 1008 | 675,9 | 7106 | 287,3 | 7 | 1,069 | H5A034 C | sample | Hm Drgbl | RASA4 | RAS p21 protein a | 10156 |
| 7737 | C17 | C18 | 1023 | 562,5 | 7121 | 331,9 | 7 | 1,069 | H5A034 C | sample | Hm Drgbl | RBM6 | RNA binding moti | 10180 |
| 7809 | F17 | F18 | 1517 | 551,2 | 10644 | 367,9 | 7 | 1,069 | H5A034 C | sample | Hm Drgbl | TMEM147 | transmembrane c | 10430 |
| 7874 | I10 | I11 | 1518 | 563,1 | 10592 | 330,3 | 7 | 1,069 | H5A034 C | sample | Hm Drgbl | CUGBP1 | CUG triplet repea | 10658 |
| 7935 | K22 | K23 | 1406 | 546,3 | 9857 | 383,7 | 7 | 1,069 | H5A034 C | sample | Hm Drgbl | NUDC | nuclear distributi | 10726 |
| 7948 | L12 | L13 | 1212 | 614 | 8438 | 363,5 | 7 | 1,069 | H5A034 C | sample | Hm Drgbl | SDS | serine dehydrata | 10993 |
| 7950 | L14 | L15 | 1660 | 590,9 | 11630 | 352,4 | 7 | 1,069 | H5A034 C | sample | Hm Drgbl | ILVBL | ilvB (bacterial ace | 10994 |
| 7981 | M20 | M21 | 1760 | 616,9 | 12322 | 371,2 | 7 | 1,069 | H5A034 C | sample | Hm Drgbl | POP4 | processing of pre | 10775 |
| 7998 | N14 | N15 | 1006 | 625,4 | 7016 | 321,2 | 7 | 1,069 | H5A034 C | sample | Hm Drgbl | TMED1 | transmembrane e | 11018 |
| 8043 | P11 | P12 | 1345 | 534,3 | 9408 | 383,8 | 7 | 1,069 | H5A034 C | sample | Hm Drgbl | RNASEH2A | ribonuclease H2. | 10535 |
| 8063 | P8 | P9 | 1422 | 727,2 | 9927 | 465,9 | 7 | 1,069 | H5A034 C | sample | Hm Drgbl | LILRA3 | leukocyte immun | 11026 |
| 14778 | H3 | H4 | 1226 | 817,4 | 8654 | 96,28 | 7,1 | 0,405 | H5A034 C | sample | Hm Gen E | TMEM57 | transmembrane p | 55219 |
| 11299 | G4 | G5 | 1033 | 793,7 | 7372 | 153,1 | 7,1 | 0,514 | H5A034 C | sample | Hm Drgbl | SULT1C3 | sulfotransferase f | 442038 |
| 17964 | M2 | M3 | 1811 | 1028 | 12843 | 101,9 | 7,1 | 0,561 | H5A034 C | sample | Hm Gen E | C20orf191 | chromosome 20 c | 149934 |
| 9046 | I7 | I8 | 1565 | 1899 | 11156 | 109,3 | 7,1 | 0,57 | H5A034 C | sample | Hm Drgbl | ASCC1 | activating signal c | 51008 |
| 2226 | M3 | M4 | 726 | 651,2 | 5121 | 333,3 | 7,1 | 0,617 | H5A034 C | sample | Hm Drgbl | MTAC2D1 | membrane target | 123036 |
| 3877 | B20 | B21 | 1241 | 751,8 | 8786 | 328,6 | 7,1 | 0,631 | H5A034 C | sample | Hm Drgbl | RFNG | RFNG O-fucosylxe | 5986 |
| 3999 | G22 | G23 | 1485 | 731,8 | 10504 | 407 | 7,1 | 0,631 | H5A034 C | sample | Hm Drgbl | RAG2 | recombination ac | 5897 |
| 21697 | I1 | I2 | 253 | 1141 | 1789 | 572,7 | 7,1 | 0,747 | H5A034 C | sample | Hm Gen E | LOC64975 | similar to Ribulos | 649755 |
| 21788 | L5 | L6 | 481 | 1018 | 3393 | 540,7 | 7,1 | 0,747 | H5A034 C | sample | Hm Gen E | LOC65192 | similar to ataxia t | 651921 |
| 10487 | E8 | E9 | 1575 | 844,1 | 11160 | 153,1 | 7,1 | 0,802 | H5A034 C | sample | Hm Drgbl | OSBPL8 | oxysterol binding | 114882 |
| 2751 | C22 | C23 | 1547 | 684,8 | 11024 | 569,8 | 7,1 | 0,835 | H5A034 C | sample | Hm Drgbl | BRSK1 | BR serine/threoni | 84446 |
| 2913 | J17 | J18 | 1201 | 614,5 | 8530 | 416,4 | 7,1 | 0,835 | H5A034 C | sample | Hm Drgbl | DAPK2 | death-associated | 23604 |
| 2934 | K14 | K15 | 879 | 691,7 | 6261 | 496,6 | 7,1 | 0,835 | H5A034 C | sample | Hm Drgbl | WNK4 | WNK lysine defici | 65266 |
| 2937 | K17 | K18 | 1921 | 674,7 | 13638 | 488,5 | 7,1 | 0,835 | H5A034 C | sample | Hm Drgbl | ROCK2 | Rho-associated, c | 9475 |
| 20172 | I2 | I3 | 1047 | 1327 | 7439 | 115,1 | 7,1 | 0,866 | H5A034 C | sample | Hm Gen E | LOC40129 | hypothetical prot | 401296 |
| 20175 | I22 | I23 | 1119 | 1191 | 7938 | 108,8 | 7,1 | 0,866 | H5A034 C | sample | Hm Gen E | LOC40141 | hypothetical LOC4 | 401410 |
| 20196 | J2 | J3 | 683 | 1152 | 4854 | 117,6 | 7,1 | 0,866 | H5A034 C | sample | Hm Gen E | C10orf85 | chromosome 10 c | 404216 |
| 20199 | J22 | J23 | 1846 | 1188 | 13105 | 131,8 | 7,1 | 0,866 | H5A034 C | sample | Hm Gen E | HERV-FRD | HERV-FRD proviru | 405754 |
| 20312 | O16 | O17 | 1249 | 1093 | 8929 | 117,2 | 7,1 | 0,866 | H5A034 C | sample | Hm Gen E | LOC40189 | similar to hypoth | 401898 |
| 495 | E22 | E23 | 2995 | 1840 | 21358 | 308,4 | 7,1 | 1,036 | H5A034 C | sample | Hm Drgbl | FOLR3 | folate receptor 3 | 2352 |
| 530 | G10 | G11 | 2760 | 1932 | 19510 | 305,8 | 7,1 | 1,036 | H5A034 C | sample | Hm Drgbl | FSHR | follicle stimulatin | 2492 |
| 565 | H20 | H21 | 2438 | 1751 | 17252 | 267,6 | 7,1 | 1,036 | H5A034 C | sample | Hm Drgbl | GNAZ | guanine nucleotic | 2781 |
| 577 | I1 | I2 | 2211 | 1890 | 15650 | 329,6 | 7,1 | 1,036 | H5A034 C | sample | Hm Drgbl | EIF4B | eukaryotic transla | 1975 |
| 603 | J11 | J12 | 2498 | 1900 | 17717 | 284,3 | 7,1 | 1,036 | H5A034 C | sample | Hm Drgbl | FBLN2 | fibulin 2 | 2199 |
| 654 | L14 | L15 | 2366 | 1703 | 16890 | 268,2 | 7,1 | 1,036 | H5A034 C | sample | Hm Drgbl | GPD1 | glycerol-3-phosph | 2819 |
| 675 | M11 | M12 | 3030 | 2036 | 21600 | 303,1 | 7,1 | 1,036 | H5A034 C | sample | Hm Drgbl | ENO1 | enolase 1, (alpha) | 2023 |
| 685 | M20 | M21 | 2527 | 1797 | 17889 | 304,9 | 7,1 | 1,036 | H5A034 C | sample | Hm Drgbl | GBP1 | guanylate binding | 2633 |
| 700 | N12 | N13 | 2699 | 1780 | 19037 | 374,4 | 7,1 | 1,036 | H5A034 C | sample | Hm Drgbl | GPX4 | glutathione perox | 2879 |
| 755 | P19 | P20 | 2855 | 1909 | 20201 | 424,5 | 7,1 | 1,036 | H5A034 C | sample | Hm Drgbl | FOXG1 | forkhead box G1 | 2290 |
| 758 | P21 | P22 | 3099 | 1945 | 22139 | 402,7 | 7,1 | 1,036 | H5A034 C | sample | Hm Drgbl | FOXF1 | forkhead box F1 | 2294 |
| 7689 | A17 | A18 | 917 | 698,1 | 6499 | 438 | 7,1 | 1,084 | H5A034 C | sample | Hm Drgbl | AASS | aminoadipate-ser | 10157 |
| 7811 | F19 | F20 | 1235 | 569,6 | 8757 | 401,7 | 7,1 | 1,084 | H5A034 C | sample | Hm Drgbl | TIMM23 | translocase of inr | 10431 |
| 7862 | H21 | H22 | 1213 | 623,3 | 8615 | 364 | 7,1 | 1,084 | H5A034 C | sample | Hm Drgbl | PECI | peroxisomal D3,D | 10455 |
| 7910 | J21 | J22 | 1259 | 622,4 | 8913 | 265 | 7,1 | 1,084 | H5A034 C | sample | Hm Drgbl | SLC9A6 | solute carrier fam | 10479 |
| 7938 | K3 | K4 | 429 | 548,6 | 3052 | 307,8 | 7,1 | 1,084 | H5A034 C | sample | Hm Drgbl | IRX5 | iroquois homeob | 10265 |
| 8025 | O17 | O18 | 1027 | 530,6 | 7268 | 355,3 | 7,1 | 1,084 | H5A034 C | sample | Hm Drgbl | ABCA7 | ATP-binding casse | 10347 |
| 9151 | N15 | N16 | 1591 | 1582 | 11475 | 125,8 | 7,2 | 0,578 | H5A034 C | sample | Hm Drgbl | REV1 | REV1 homolog (S. | 51455 |
| 57 | C17 | C18 | 515 | 1424 | 3720 | 464 | 7,2 | 0,613 | H5A034 C | sample | Hm Drgbl | RARA | retinoic acid rece | 5914 |
| 6740 | I5 | I6 | 780 | 669,5 | 5606 | 150,7 | 7,2 | 0,632 | H5A034 C | sample | Hm Drgbl | HBQ1 | hemoglobin, thet | 3049 |
| 4069 | J20 | J21 | 1374 | 588,2 | 9946 | 237,4 | 7,2 | 0,64 | H5A034 C | sample | Hm Drgbl | RPN2 | ribophorin II | 6185 |
| 6108 | O2 | O3 | 1542 | 826,3 | 11091 | 243,9 | 7,2 | 0,658 | H5A034 C | sample | Hm Drgbl | BAAT | bile acid Coenzyn | 570 |
| 3740 | L5 | L6 | 1114 | 1489 | 8068 | 394,5 | 7,2 | 0,692 | H5A034 C | sample | Hm Drgbl | IL8RB | interleukin 8 rece | 3579 |
| 21702 | I14 | I15 | 90 | 1152 | 648 | 607,6 | 7,2 | 0,758 | H5A034 C | sample | Hm Gen E | LOC65272 | similar to LIM hor | 652721 |
| 21714 | I3 | I4 | 366 | 922,7 | 2630 | 600,9 | 7,2 | 0,758 | H5A034 C | sample | Hm Gen E | LOC64977 | hypothetical prot | 649771 |
| 21776 | L16 | L17 | 283 | 877,7 | 2051 | 469,6 | 7,2 | 0,758 | H5A034 C | sample | Hm Gen E | ARP11 | actin-related Arp | 653857 |
| 2959 | L15 | L16 | 1424 | 657 | 10225 | 430,6 | 7,2 | 0,847 | H5A034 C | sample | Hm Drgbl | DAK | dihydroxyacetone | 26007 |
| 2990 | M21 | M22 | 1600 | 740,3 | 11454 | 531,5 | 7,2 | 0,847 | H5A034 C | sample | Hm Drgbl | OXSR1 | oxidative-stress r | 9943 |
| 20173 | I20 | I21 | 709 | 1307 | 5107 | 144,4 | 7,2 | 0,878 | H5A034 C | sample | Hm Gen E | hCG 1651 | hypothetical gene | 401399 |
| 20233 | L1 | L2 | 540 | 1170 | 3895 | 132,2 | 7,2 | 0,878 | H5A034 C | sample | Hm Gen E | FLJ36644 | hypothetical gene | 400617 |
| 20244 | L2 | L3 | 748 | 1133 | 5365 | 119 | 7,2 | 0,878 | H5A034 C | sample | Hm Gen E | FAM19A1 | family with seque | 407738 |
| 20303 | N8 | N9 | 1267 | 1113 | 9156 | 139,1 | 7,2 | 0,878 | H5A034 C | sample | Hm Gen E | RGS21 | regulator of G-pro | 431704 |
| 404 | A5 | A6 | 2712 | 1905 | 19595 | 437 | 7,2 | 1,051 | H5A034 C | sample | Hm Drgbl | HBEGF | heparin-binding E | 1839 |
| 451 | C4 | C5 | 2363 | 1963 | 17043 | 267,8 | 7,2 | 1,051 | H5A034 C | sample | Hm Drgbl | FOXO1 | forkhead box O1 | 2308 |
| 452 | C5 | C6 | 2972 | 2066 | 21504 | 326,1 | 7,2 | 1,051 | H5A034 C | sample | Hm Drgbl | ECHS1 | enoyl Coenzyme A | 1892 |
| 587 | I19 | I20 | 2550 | 1901 | 18299 | 281,3 | 7,2 | 1,051 | H5A034 C | sample | Hm Drgbl | ELAVL2 | ELAV (embryonic | 1993 |
| 614 | J21 | J22 | 2830 | 1841 | 20513 | 303,6 | 7,2 | 1,051 | H5A034 C | sample | Hm Drgbl | FCER1A | Fc fragment of IgE | 2205 |
| 676 | M12 | M13 | 2303 | 1801 | 16628 | 363,3 | 7,2 | 1,051 | H5A034 C | sample | Hm Drgbl | GAS6 | growth arrest-spe | 2621 |
| 727 | O15 | O16 | 2703 | 1814 | 19418 | 360,5 | 7,2 | 1,051 | H5A034 C | sample | Hm Drgbl | EPS15 | epidermal growth | 2060 |
| 7774 | D7 | D8 | 1181 | 633,7 | 8504 | 410,5 | 7,2 | 1,099 | H5A034 C | sample | Hm Drgbl | DLC1 | deleted in liver ca | 10395 |
| 7815 | F22 | F23 | 1291 | 544,7 | 9278 | 412,9 | 7,2 | 1,099 | H5A034 C | sample | Hm Drgbl | APOBEC2 | apolipoprotein B | 10930 |
| 7823 | F8 | F9 | 1017 | 549 | 7323 | 306,3 | 7,2 | 1,099 | H5A034 C | sample | Hm Drgbl | COPS8 | COP9 constitutive | 10920 |
| 7954 | L18 | L19 | 1134 | 507,3 | 8169 | 302,3 | 7,2 | 1,099 | H5A034 C | sample | Hm Drgbl | SLC27A4 | solute carrier fam | 10999 |
| 7990 | M7 | M8 | 1258 | 614 | 9118 | 316,9 | 7,2 | 1,099 | H5A034 C | sample | Hm Drgbl | LILRB2 | leukocyte immun | 10288 |
| 8015 | N8 | N9 | 1074 | 571,6 | 7704 | 354,4 | 7,2 | 1,099 | H5A034 C | sample | Hm Drgbl | KDELR2 | KDEL (Lys-Asp-Glu | 11014 |
| 11263 | F15 | F16 | 1645 | 817,5 | 12085 | 128,4 | 7,3 | 0,529 | H5A034 C | sample | Hm Drgbl | ACSM2B | acyl-CoA syntheta | 348158 |
| 2343 | B22 | B23 | 1680 | 795,4 | 12330 | 394,3 | 7,3 | 0,575 | H5A034 C | sample | Hm Drgbl | PKN2 | protein kinase N2 | 5586 |
| 2533 | J20 | J21 | 931 | 705,4 | 6756 | 258,5 | 7,3 | 0,575 | H5A034 C | sample | Hm Drgbl | RP2 | retinitis pigmentc | 6102 |
| 13628 | H5 | H6 | 1562 | 1129 | 11340 | 154,6 | 7,3 | 0,685 | H5A034 C | sample | Hm Gen E | CXorf27 | chromosome X op | 25763 |
| 5535 | G22 | G23 | 2167 | 674,1 | 15785 | 526,7 | 7,3 | 0,695 | H5A034 C | sample | Hm Drgbl | AP1S2 | adaptor-related c | 8905 |
| 3675 | J11 | J12 | 466 | 1628 | 3386 | 322,6 | 7,3 | 0,702 | H5A034 C | sample | Hm Drgbl | HTR2A | 5-hydroxytryptam | 3356 |
| 3756 | M2 | M3 | 814 | 1164 | 5915 | 286,9 | 7,3 | 0,702 | H5A034 C | sample | Hm Drgbl | HRH3 | histamine recepto | 11255 |
| 21547 | B4 | B5 | 204 | 997,6 | 1487 | 652,3 | 7,3 | 0,768 | H5A034 C | sample | Hm Gen E | ZDHHC11E | zinc finger, DHHC- | 653082 |
| 21636 | F2 | F3 | 448 | 1014 | 3253 | 501,8 | 7,3 | 0,768 | H5A034 C | sample | Hm Gen E | LOC65325 | similar to RAB, m | 653256 |
| 21782 | L21 | L22 | 525 | 1015 | 3816 | 644,8 | 7,3 | 0,768 | H5A034 C | sample | Hm Gen E | LOC65204 | similar to hect (h | 652045 |
| 4988 | P5 | P6 | 2363 | 819 | 17165 | 485,1 | 7,3 | 0,802 | H5A034 C | sample | Hm Drgbl | PSEN2 | presenilin 2 (Alzh | 5664 |
| 10425 | C17 | C18 | 1177 | 895,5 | 8612 | 180,6 | 7,3 | 0,825 | H5A034 C | sample | Hm Drgbl | TIGD5 | tigger transposab | 84948 |
| 10611 | K11 | K12 | 1113 | 835,6 | 8144 | 184,1 | 7,3 | 0,825 | H5A034 C | sample | Hm Drgbl | TRIM15 | tripartite motif-cc | 89870 |
| 2799 | E22 | E23 | 2090 | 753,9 | 15313 | 463,6 | 7,3 | 0,859 | H5A034 C | sample | Hm Drgbl | PBK | PDZ binding kinas | 55872 |
| 2803 | E4 | E5 | 574 | 632,6 | 4164 | 500,8 | 7,3 | 0,859 | H5A034 C | sample | Hm Drgbl | TTBK1 | tau tubulin kinase | 84630 |
| 2888 | I16 | I17 | 1038 | 627,1 | 7590 | 368,7 | 7,3 | 0,859 | H5A034 C | sample | Hm Drgbl | SNX16 | sorting nexin 16 | 64089 |
| 2893 | I20 | I21 | 1204 | 682,3 | 8840 | 451,8 | 7,3 | 0,859 | H5A034 C | sample | Hm Drgbl | MPP5 | membrane protei | 64398 |
| 2930 | K10 | K11 | 385 | 671,3 | 2829 | 502,3 | 7,3 | 0,859 | H5A034 C | sample | Hm Drgbl | WNK1 | WNK lysine defici | 65125 |
| 2976 | L9 | L10 | 1542 | 620,3 | 11196 | 527,8 | 7,3 | 0,859 | H5A034 C | sample | Hm Drgbl | RIPK5 | receptor interacti | 25778 |
| 2982 | M14 | M15 | 1166 | 595 | 8508 | 457,3 | 7,3 | 0,859 | H5A034 C | sample | Hm Drgbl | DCAKD | dephospho-CoA k | 79877 |
| 3014 | N21 | N22 | 1672 | 662,1 | 12282 | 476,8 | 7,3 | 0,859 | H5A034 C | sample | Hm Drgbl | HIPK2 | homeodomain int | 28996 |
| 3034 | O18 | O19 | 2511 | 890,1 | 18361 | 657,5 | 7,3 | 0,859 | H5A034 C | sample | Hm Drgbl | RPS6KL1 | ribosomal protein | 83694 |
| 20156 | H5 | H6 | 1281 | 1081 | 9334 | 129,4 | 7,3 | 0,89 | H5A034 C | sample | Hm Gen E | C14orf181 | chromosome 14 c | 400223 |
| 20166 | I14 | I15 | 1570 | 1036 | 11527 | 108,5 | 7,3 | 0,89 | H5A034 C | sample | Hm Gen E | LOC40138 | hypothetical prot | 401387 |
| 20169 | I17 | I18 | 1323 | 1029 | 9633 | 106,9 | 7,3 | 0,89 | H5A034 C | sample | Hm Gen E | LOC39176 | similar to hCG198 | 391764 |
| 20218 | K18 | K19 | 1573 | 1098 | 11480 | 121,7 | 7,3 | 0,89 | H5A034 C | sample | Hm Gen E | PTPLAD2 | protein tyrosine p | 401494 |
| 20239 | L15 | L16 | 979 | 1033 | 7180 | 113,9 | 7,3 | 0,89 | H5A034 C | sample | Hm Gen E | LOC40068 | similar to zinc fin | 400682 |
| 20271 | M22 | M23 | 1659 | 1085 | 12193 | 127,7 | 7,3 | 0,89 | H5A034 C | sample | Hm Gen E | RP11-114H | hypothetical LOC4 | 401589 |
| 525 | F6 | F7 | 2788 | 1937 | 20413 | 247,8 | 7,3 | 1,066 | H5A034 C | sample | Hm Drgbl | GLRX | glutaredoxin (thic | 2745 |
| 718 | N7 | N8 | 2351 | 1767 | 17218 | 267 | 7,3 | 1,066 | H5A034 C | sample | Hm Drgbl | FEN1 | flap structure-spe | 2237 |
| 743 | O8 | O9 | 2256 | 1925 | 16382 | 370,2 | 7,3 | 1,066 | H5A034 C | sample | Hm Drgbl | GCDH | glutaryl-Coenzym | 2639 |
| 763 | P4 | P5 | 2904 | 1962 | 21183 | 442,2 | 7,3 | 1,066 | H5A034 C | sample | Hm Drgbl | GRINA | glutamate recept | 2907 |
| 7694 | A21 | A22 | 1637 | 617,5 | 11960 | 382,2 | 7,3 | 1,115 | H5A034 C | sample | Hm Drgbl | FARP1 | FERM, RhoGEF (A | 10160 |
| 7739 | C19 | C20 | 1761 | 638,3 | 12891 | 361,9 | 7,3 | 1,115 | H5A034 C | sample | Hm Drgbl | RBM5 | RNA binding moti | 10181 |
| 7875 | I11 | I12 | 775 | 575,2 | 5665 | 304 | 7,3 | 1,115 | H5A034 C | sample | Hm Drgbl | SRRM1 | serine/arginine re | 10250 |
| 7918 | J7 | J8 | 1159 | 592,9 | 8435 | 358,2 | 7,3 | 1,115 | H5A034 C | sample | Hm Drgbl | COG5 | component of oli | 10466 |
| 7952 | L16 | L17 | 1220 | 563 | 8938 | 357,4 | 7,3 | 1,115 | H5A034 C | sample | Hm Drgbl | SLC27A5 | solute carrier fam | 10998 |
| 20432 | D16 | D17 | 1029 | 762,1 | 7613 | 81,59 | 7,4 | 0,493 | H5A034 C | sample | Hm Gen E | LOC64199 | similar to capicua | 641994 |
| 7531 | J4 | J5 | 1548 | 659,7 | 11456 | 219,4 | 7,4 | 0,565 | H5A034 C | sample | Hm Drgbl | TOM1 | target of myb1 (cl | 10043 |
| 315 | N11 | N12 | 553 | 1532 | 4115 | 707,9 | 7,4 | 0,63 | H5A034 C | sample | Hm Drgbl | SLC6A1 | solute carrier fam | 6529 |
| 6575 | B8 | B9 | 1351 | 663,9 | 9994 | 465,9 | 7,4 | 0,649 | H5A034 C | sample | Hm Drgbl | ITGAD | integrin, alpha D | 3681 |
| 13578 | F3 | F4 | 1432 | 1083 | 10643 | 157,1 | 7,4 | 0,695 | H5A034 C | sample | Hm Gen E | C9orf4 | chromosome 9 or | 23732 |
| 3470 | A21 | A22 | 1643 | 1443 | 12107 | 235,5 | 7,4 | 0,712 | H5A034 C | sample | Hm Drgbl | ADRA2A | adrenergic, alpha | 150 |
| 8401 | O1 | O2 | 564 | 550,8 | 4166 | 208,4 | 7,4 | 0,712 | H5A034 C | sample | Hm Drgbl | XPOT | exportin, tRNA (n | 11260 |
| 1819 | L4 | L5 | 1384 | 766,8 | 10254 | 376 | 7,4 | 0,775 | H5A034 C | sample | Hm Drgbl | GTF2IRD2 | GTF2I repeat dom | 84163 |
| 1836 | M2 | M3 | 858 | 752,5 | 6323 | 356,9 | 7,4 | 0,775 | H5A034 C | sample | Hm Drgbl | IKZF5 | IKAROS family zin | 64376 |
| 21539 | B19 | B20 | 200 | 1400 | 1486 | 533 | 7,4 | 0,779 | H5A034 C | sample | Hm Gen E | LOC65083 | similar to mitoger | 650832 |
| 4742 | F21 | F22 | 1859 | 942,3 | 13776 | 491,1 | 7,4 | 0,813 | H5A034 C | sample | Hm Drgbl | MIPEP | mitochondrial int | 4285 |
| 4815 | I22 | I23 | 1886 | 828,6 | 13955 | 320,2 | 7,4 | 0,813 | H5A034 C | sample | Hm Drgbl | USP2 | ubiquitin specific | 9099 |
| 4906 | M18 | M19 | 1937 | 965,1 | 14253 | 228,1 | 7,4 | 0,813 | H5A034 C | sample | Hm Drgbl | USP15 | ubiquitin specific | 9958 |
| 10657 | M1 | M2 | 1453 | 958,6 | 10809 | 199,8 | 7,4 | 0,836 | H5A034 C | sample | Hm Drgbl | RHOT2 | ras homolog gene | 89941 |
| 2767 | D15 | D16 | 448 | 598,4 | 3337 | 484,8 | 7,4 | 0,871 | H5A034 C | sample | Hm Drgbl | MPP6 | membrane protei | 51678 |
| 2793 | E17 | E18 | 1276 | 658,4 | 9417 | 467 | 7,4 | 0,871 | H5A034 C | sample | Hm Drgbl | KSR1 | kinase suppresso | 8844 |
| 2863 | H15 | H16 | 904 | 652,8 | 6727 | 541,2 | 7,4 | 0,871 | H5A034 C | sample | Hm Drgbl | MAST3 | microtubule asso | 23031 |
| 2908 | J12 | J13 | 1445 | 661,1 | 10669 | 489,3 | 7,4 | 0,871 | H5A034 C | sample | Hm Drgbl | MAGI3 | membrane associ | 260425 |
| 2941 | K20 | K21 | 1548 | 676,4 | 11430 | 465,1 | 7,4 | 0,871 | H5A034 C | sample | Hm Drgbl | STK33 | serine/threonine | 65975 |
| 2972 | L5 | L6 | 1248 | 644,4 | 9175 | 454,8 | 7,4 | 0,871 | H5A034 C | sample | Hm Drgbl | SGK3 | serum/glucocorti | 23678 |
| 2977 | M1 | M2 | 830 | 662,1 | 6181 | 484,2 | 7,4 | 0,871 | H5A034 C | sample | Hm Drgbl | AATK | apoptosis-associa | 9625 |
| 20241 | L17 | L18 | 1405 | 1206 | 10349 | 135,1 | 7,4 | 0,902 | H5A034 C | sample | Hm Gen E | FLJ26850 | FLJ26850 protein | 400710 |
| 20286 | N14 | N15 | 904 | 1100 | 6666 | 101,7 | 7,4 | 0,902 | H5A034 C | sample | Hm Gen E | C8orf15 | chromosome 8 op | 439940 |
| 20289 | N17 | N18 | 1373 | 1117 | 10142 | 104,9 | 7,4 | 0,902 | H5A034 C | sample | Hm Gen E | FLJ43505 | FLJ43505 protein | 400823 |
| 20293 | N20 | N21 | 1212 | 1206 | 8936 | 128,7 | 7,4 | 0,902 | H5A034 C | sample | Hm Gen E | LOC43995 | hypothetical gene | 439950 |
| 20319 | O22 | O23 | 1216 | 1132 | 9004 | 168,2 | 7,4 | 0,902 | H5A034 C | sample | Hm Gen E | PRAMEF3 | PRAME family me | 401940 |
| 396 | A2 | A3 | 2713 | 2081 | 19944 | 351,7 | 7,4 | 1,08 | H5A034 C | sample | Hm Drgbl | FOXF2 | forkhead box F2 | 2295 |
| 428 | B5 | B6 | 2369 | 1918 | 17414 | 366,1 | 7,4 | 1,08 | H5A034 C | sample | Hm Drgbl | ERCC5 | excision repair cr | 2073 |
| 464 | D16 | D17 | 1095 | 1699 | 8138 | 311,2 | 7,4 | 1,08 | H5A034 C | sample | Hm Drgbl | GCLC | glutamate-cystein | 2729 |
| 470 | D21 | D22 | 2188 | 1800 | 16202 | 251,7 | 7,4 | 1,08 | H5A034 C | sample | Hm Drgbl | EXTL1 | exostoses (multip | 2134 |
| 484 | E12 | E13 | 2508 | 1861 | 18595 | 265 | 7,4 | 1,08 | H5A034 C | sample | Hm Drgbl | FN1 | fibronectin 1 | 2335 |
| 599 | I8 | I9 | 2117 | 1848 | 15637 | 338,1 | 7,4 | 1,08 | H5A034 C | sample | Hm Drgbl | FUT7 | fucosyltransferase | 2529 |
| 625 | K1 | K2 | 1954 | 1876 | 14418 | 324,1 | 7,4 | 1,08 | H5A034 C | sample | Hm Drgbl | ELAVL3 | ELAV (embryonic | 1995 |
| 626 | K10 | K11 | 1822 | 1789 | 13573 | 278,8 | 7,4 | 1,08 | H5A034 C | sample | Hm Drgbl | B4GALNT1 | beta-1,4-N-acetyl | 2583 |
| 759 | P22 | P23 | 2718 | 1929 | 20104 | 407,1 | 7,4 | 1,08 | H5A034 C | sample | Hm Drgbl | GSTA3 | glutathione S-trar | 2940 |
| 7796 | E5 | E6 | 1463 | 563,1 | 10800 | 399,8 | 7,4 | 1,13 | H5A034 C | sample | Hm Drgbl | PRMT3 | protein arginine r | 10196 |
| 7908 | J2 | J3 | 1108 | 590,6 | 8183 | 249,8 | 7,4 | 1,13 | H5A034 C | sample | Hm Drgbl | TOMM34 | translocase of ou | 10953 |
| 7943 | K8 | K9 | 1635 | 552 | 12068 | 345,6 | 7,4 | 1,13 | H5A034 C | sample | Hm Drgbl | EBP | emopamil binding | 10682 |
| 7995 | N11 | N12 | 1519 | 623,3 | 11212 | 373,8 | 7,4 | 1,13 | H5A034 C | sample | Hm Drgbl | MYBBP1A | MYB binding prot | 10514 |
| 8001 | N17 | N18 | 1327 | 633,6 | 9839 | 393,9 | 7,4 | 1,13 | H5A034 C | sample | Hm Drgbl | DEAF1 | deformed epiderr | 10522 |
| 8004 | N2 | N3 | 1372 | 583,2 | 10153 | 400,7 | 7,4 | 1,13 | H5A034 C | sample | Hm Drgbl | KIF2C | kinesin family me | 11004 |
| 8017 | O1 | O2 | 806 | 580,1 | 5945 | 398,6 | 7,4 | 1,13 | H5A034 C | sample | Hm Drgbl | B3GALT5 | UDP-Gal:betaGlcN | 10317 |
| 8026 | O18 | O19 | 1994 | 567,3 | 14755 | 418,5 | 7,4 | 1,13 | H5A034 C | sample | Hm Drgbl | PDE10A | phosphodiesteras | 10846 |
| 3217 | G1 | G2 | 556 | 1305 | 4151 | 448,9 | 7,5 | 0,464 | H5A034 C | sample | Hm Drgbl | DUSP6 | dual specificity ph | 1848 |
| 9474 | K3 | K4 | 1856 | 1201 | 13836 | 121,6 | 7,5 | 0,521 | H5A034 C | sample | Hm Drgbl | PRMT6 | protein arginine r | 55170 |
| 11324 | H5 | H6 | 1256 | 832,6 | 9380 | 135,8 | 7,5 | 0,543 | H5A034 C | sample | Hm Drgbl | LGICZ1 | ligand-gated ion c | 353174 |
| 7513 | J1 | J2 | 1005 | 661,3 | 7582 | 305,7 | 7,5 | 0,573 | H5A034 C | sample | Hm Drgbl | PCYT1B | phosphate cytidy | 9468 |
| 7657 | P1 | P2 | 990 | 632,5 | 7409 | 294,6 | 7,5 | 0,573 | H5A034 C | sample | Hm Drgbl | BCAR1 | breast cancer ant | 9564 |
| 180 | H2 | H3 | 572 | 1497 | 4282 | 479 | 7,5 | 0,638 | H5A034 C | sample | Hm Drgbl | SRM | spermidine synth | 6723 |
| 231 | J22 | J23 | 545 | 1494 | 4086 | 501,6 | 7,5 | 0,638 | H5A034 C | sample | Hm Drgbl | STAT4 | signal transducer | 6775 |
| 6003 | K11 | K12 | 1139 | 902,8 | 8562 | 182,2 | 7,5 | 0,685 | H5A034 C | sample | Hm Drgbl | ADM | adrenomedullin | 133 |
| 13627 | H4 | H5 | 1404 | 1057 | 10476 | 164,5 | 7,5 | 0,704 | H5A034 C | sample | Hm Gen E | FBXO3 | F-box protein 3 | 26273 |
| 5639 | K8 | K9 | 1658 | 703,5 | 12358 | 214,6 | 7,5 | 0,714 | H5A034 C | sample | Hm Drgbl | AP3D1 | adaptor-related c | 8943 |
| 3725 | L13 | L14 | 1091 | 1490 | 8180 | 341,3 | 7,5 | 0,721 | H5A034 C | sample | Hm Drgbl | MC3R | melanocortin 3 re | 4159 |
| 1746 | I3 | I4 | 1294 | 729,9 | 9757 | 417,8 | 7,5 | 0,785 | H5A034 C | sample | Hm Drgbl | TFCP2L1 | transcription fact | 29842 |
| 21740 | J5 | J6 | 470 | 1083 | 3532 | 690,2 | 7,5 | 0,789 | H5A034 C | sample | Hm Gen E | LOC65174 | hypothetical prot | 651741 |
| 21806 | M21 | M22 | 548 | 1045 | 4107 | 616,6 | 7,5 | 0,789 | H5A034 C | sample | Hm Gen E | LOC65043 | similar to radical | 650433 |
| 21815 | M8 | M9 | 202 | 1105 | 1514 | 641,6 | 7,5 | 0,789 | H5A034 C | sample | Hm Gen E | LOC65279 | similar to Mast/st | 652799 |
| 4887 | L22 | L23 | 1322 | 1062 | 9971 | 406,7 | 7,5 | 0,824 | H5A034 C | sample | Hm Drgbl | PARL | presenilin associa | 55486 |
| 10560 | H9 | H10 | 1129 | 863,5 | 8461 | 189,4 | 7,5 | 0,847 | H5A034 C | sample | Hm Drgbl | HNRPLL | heterogeneous nu | 92906 |
| 10654 | L7 | L8 | 1341 | 837,2 | 10043 | 173,9 | 7,5 | 0,847 | H5A034 C | sample | Hm Drgbl | TSGA14 | testis specific, 14 | 95681 |
| 2746 | C18 | C19 | 815 | 642,5 | 6082 | 481,9 | 7,5 | 0,882 | H5A034 C | sample | Hm Drgbl | TSSK6 | testis-specific ser | 83983 |
| 2837 | G13 | G14 | 939 | 643,5 | 7079 | 522,6 | 7,5 | 0,882 | H5A034 C | sample | Hm Drgbl | CDKL2 | cyclin-dependent | 8999 |
| 2843 | G19 | G20 | 1665 | 660,2 | 12540 | 500,5 | 7,5 | 0,882 | H5A034 C | sample | Hm Drgbl | PAPSS2 | 3-phosphoadeno: | 9060 |
| 2871 | H22 | H23 | 1244 | 636,1 | 9328 | 430,9 | 7,5 | 0,882 | H5A034 C | sample | Hm Drgbl | NRK | Nik related kinase | 203447 |
| 2929 | K1 | K2 | 402 | 700 | 3002 | 558,9 | 7,5 | 0,882 | H5A034 C | sample | Hm Drgbl | STK17B | serine/threonine | 9262 |
| 2987 | M19 | M20 | 1515 | 639,9 | 11341 | 571,4 | 7,5 | 0,882 | H5A034 C | sample | Hm Drgbl | XYLB | xylulokinase hom | 9942 |
| 20045 | D13 | D14 | 985 | 1171 | 7353 | 131,4 | 7,5 | 0,915 | H5A034 C | sample | Hm Gen E | LOC39988 | hypothetical gene | 399888 |
| 20087 | E8 | E9 | 685 | 1306 | 5146 | 173,8 | 7,5 | 0,915 | H5A034 C | sample | Hm Gen E | RGS7BP | regulator of G-pr | 401190 |
| 20098 | F18 | F19 | 1256 | 1045 | 9463 | 127,5 | 7,5 | 0,915 | H5A034 C | sample | Hm Gen E | OR2T27 | olfactory receptor | 403239 |
| 20197 | J20 | J21 | 1412 | 1177 | 10573 | 123,1 | 7,5 | 0,915 | H5A034 C | sample | Hm Gen E | DUOXA2 | dual oxidase mat | 405753 |
| 20255 | L8 | L9 | 1058 | 1107 | 7948 | 137,9 | 7,5 | 0,915 | H5A034 C | sample | Hm Gen E | HCG3 | HCG3 gene | 414061 |
| 20276 | M5 | M6 | 1211 | 1227 | 9131 | 153,1 | 7,5 | 0,915 | H5A034 C | sample | Hm Gen E | LOC39239 | similar to hemice | 392395 |
| 526 | F7 | F8 | 2158 | 1672 | 16127 | 271 | 7,5 | 1,095 | H5A034 C | sample | Hm Drgbl | EYA2 | eyes absent hom | 2139 |
| 534 | G14 | G15 | 2418 | 1839 | 18249 | 295 | 7,5 | 1,095 | H5A034 C | sample | Hm Drgbl | FUCA2 | fucosidase, alpha | 2519 |
| 537 | G17 | G18 | 1230 | 1628 | 9282 | 320,6 | 7,5 | 1,095 | H5A034 C | sample | Hm Drgbl | EIF2S3 | eukaryotic transla | 1968 |
| 610 | J18 | J19 | 2467 | 1766 | 18497 | 312,6 | 7,5 | 1,095 | H5A034 C | sample | Hm Drgbl | GNGT2 | guanine nucleotic | 2793 |
| 629 | K13 | K14 | 1902 | 1757 | 14243 | 319,7 | 7,5 | 1,095 | H5A034 C | sample | Hm Drgbl | ELK3 | ELK3, ETS-domain | 2004 |
| 7684 | A12 | A13 | 1457 | 656,4 | 10988 | 428,7 | 7,5 | 1,145 | H5A034 C | sample | Hm Drgbl | RPP38 | ribonuclease P/M | 10557 |
| 7727 | B8 | B9 | 1216 | 600,5 | 9124 | 390,3 | 7,5 | 1,145 | H5A034 C | sample | Hm Drgbl | CYP46A1 | cytochrome P450, | 10858 |
| 7748 | C5 | C6 | 1381 | 616,3 | 10307 | 420,3 | 7,5 | 1,145 | H5A034 C | sample | Hm Drgbl | SLC25A13 | solute carrier fam | 10165 |
| 7881 | I17 | I18 | 1151 | 552,9 | 8638 | 250 | 7,5 | 1,145 | H5A034 C | sample | Hm Drgbl | SPRY2 | sprouty homolog | 10253 |
| 7920 | J9 | J10 | 831 | 630,5 | 6268 | 283 | 7,5 | 1,145 | H5A034 C | sample | Hm Drgbl | TIMM44 | translocase of inr | 10469 |
| 7933 | K20 | K21 | 1834 | 633,7 | 13791 | 365,9 | 7,5 | 1,145 | H5A034 C | sample | Hm Drgbl | SLC12A7 | solute carrier fam | 10723 |
| 7975 | M15 | M16 | 1558 | 621,8 | 11676 | 376,7 | 7,5 | 1,145 | H5A034 C | sample | Hm Drgbl | MARCH6 | membrane-associ | 10299 |
| 8027 | O19 | O20 | 1442 | 627,1 | 10785 | 400,4 | 7,5 | 1,145 | H5A034 C | sample | Hm Drgbl | ABCA10 | ATP-binding casse | 10349 |
| 11312 | H16 | H17 | 1341 | 775,2 | 10142 | 116,6 | 7,6 | 0,551 | H5A034 C | sample | Hm Drgbl | | | |
| 9117 | L6 | L7 | 885 | 1799 | 6761 | 127,1 | 7,6 | 0,61 | H5A034 C | sample | Hm Drgbl | CRLS1 | cardiolipin syntha | 54675 |
| 118 | E7 | E8 | 885 | 1661 | 6730 | 321,9 | 7,6 | 0,647 | H5A034 C | sample | Hm Drgbl | RXRA | retinoid X recepto | 6256 |
| 127 | F15 | F16 | 554 | 1777 | 4191 | 303,9 | 7,6 | 0,647 | H5A034 C | sample | Hm Drgbl | THBS4 | thrombospondin | 7060 |
| 243 | K11 | K12 | 546 | 1583 | 4166 | 599,2 | 7,6 | 0,647 | H5A034 C | sample | Hm Drgbl | TGM2 | transglutaminase | 7052 |
| 245 | K13 | K14 | 623 | 1527 | 4728 | 429,9 | 7,6 | 0,647 | H5A034 C | sample | Hm Drgbl | TGM3 | transglutaminase | 7053 |
| 1128 | O9 | O10 | 2176 | 810,3 | 16619 | 434 | 7,6 | 0,72 | H5A034 C | sample | Hm Drgbl | ELF1 | E74-like factor 1 ( | 1997 |
| 3462 | A14 | A15 | 1617 | 1263 | 12302 | 255,2 | 7,6 | 0,731 | H5A034 C | sample | Hm Drgbl | SCTR | secretin receptor | 6344 |
| 3515 | C19 | C20 | 1563 | 1424 | 11954 | 362,5 | 7,6 | 0,731 | H5A034 C | sample | Hm Drgbl | AVPR2 | arginine vasopres | 554 |
| 8078 | A21 | A22 | 2166 | 701 | 16527 | 360,7 | 7,6 | 0,731 | H5A034 C | sample | Hm Drgbl | SOX30 | SRY (sex determir | 11063 |
| 21711 | I22 | I23 | 301 | 965,9 | 2293 | 601,3 | 7,6 | 0,8 | H5A034 C | sample | Hm Gen E | LOC65272 | similar to Nitric o | 652728 |
| 21798 | M14 | M15 | 209 | 922,1 | 1589 | 468,6 | 7,6 | 0,8 | H5A034 C | sample | Hm Gen E | LOC65282 | hypothetical prot | 652821 |
| 21837 | N6 | N7 | 269 | 1035 | 2037 | 555,4 | 7,6 | 0,8 | H5A034 C | sample | Hm Gen E | LOC65388 | similar to AF4/FM | 653886 |
| 4771 | G4 | G5 | 1090 | 863,7 | 8234 | 345 | 7,6 | 0,835 | H5A034 C | sample | Hm Drgbl | MMP23B | matrix metallope | 8510 |
| 4786 | H18 | H19 | 1315 | 874,6 | 9990 | 340,6 | 7,6 | 0,835 | H5A034 C | sample | Hm Drgbl | DPP7 | dipeptidyl-peptid | 29952 |
| 4827 | J11 | J12 | 1453 | 813,1 | 11034 | 380,7 | 7,6 | 0,835 | H5A034 C | sample | Hm Drgbl | MST1 | macrophage stim | 4485 |
| 4940 | N5 | N6 | 1531 | 819,7 | 11639 | 456 | 7,6 | 0,835 | H5A034 C | sample | Hm Drgbl | LGMN | legumain | 5641 |

| | | | | | | | | | | | | | | | | | | | | | | | | | | | | | | | |
|---|---|---|---|---|---|---|---|---|---|---|---|---|---|---|---|---|---|---|---|---|---|---|---|---|---|---|---|---|---|---|---|
| 10578 | I3 | I4 | 1441 | 904,8 | 10969 | 199,4 | 7,6 | 0,859 | HSA034 C | sample | Hm Drebl | Hm Drebl | Hm Drebl | CPE002EB | CPE002EO | CPE002FR | NM 02495 | NM 0249 | NM 0249 | GFM1 | GFM1 | GFM1 | G elongation fact | G elongat | G elongat | 85476 | 85476 | 85476 | s40060 | s40061 | s40062 |
| 10586 | J10 | J11 | 1237 | 850,5 | 9398 | 166,5 | 7,6 | 0,859 | HSA034 C | sample | Hm Drebl | Hm Drebl | Hm Drebl | CPE002EB | CPE002EO | CPE002FR | NM 15320 | NM 1532 | NM 1532 | KIF19 | KIF19 | KIF19 | kinesin family me | kinesin fa | kinesin fa | 124602 | 124602 | 124602 | s42772 | s42770 | s42771 |
| 10736 | P16 | P17 | 1168 | 894,2 | 8865 | 183,8 | 7,6 | 0,859 | HSA034 C | sample | Hm Drebl | Hm Drebl | Hm Drebl | CPE002EB | CPE002EO | CPE002FR | NM 13880 | NM 1388 | NM 1388 | TRIM43 | TRIM43 | TRIM43 | tripartite motif-co | tripartite | tripartite | 129868 | 129868 | 129868 | s43448 | s43446 | s43447 |
| 2709 | A6 | A7 | 1875 | 684,3 | 14306 | 539,5 | 7,6 | 0,894 | HSA034 C | sample | Hm Drebl | Hm Drebl | Hm Drebl | CPE002DP | CPE002EF | CPE002F5 | NM 01650 | NM 0165 | NM 0165 | CDKL3 | CDKL3 | CDKL3 | cyclin-dependent | cyclin-dep | cyclin-dep | 51265 | 51265 | 51265 | s27797 | s27798 | s27799 |
| 2794 | E18 | E19 | 2018 | 711,9 | 15253 | 520,4 | 7,6 | 0,894 | HSA034 C | sample | Hm Drebl | Hm Drebl | Hm Drebl | CPE002DP | CPE002EF | CPE002F5 | NM 01798 | NM 0179 | NM 0179 | SCYL2 | SCYL2 | SCYL2 | SCY1-like 2 (S. cer | SCY1-like | SCY1-like | 55681 | 55681 | 55681 | s31239 | s31240 | s31241 |
| 2869 | H20 | H21 | 638 | 596,1 | 4818 | 520,1 | 7,6 | 0,894 | HSA034 C | sample | Hm Drebl | Hm Drebl | Hm Drebl | CPE002DP | CPE002EF | CPE002F5 | NM 17492 | NM 1749 | NM 1749 | ADCK5 | ADCK5 | ADCK5 | aarF domain cont | aarF doma | aarF doma | 203054 | 203054 | 203054 | s47461 | s47462 | s195847 |
| 2900 | I5 | I6 | 825 | 659,6 | 6310 | 560,4 | 7,6 | 0,894 | HSA034 C | sample | Hm Drebl | Hm Drebl | Hm Drebl | CPE002DP | CPE002EF | CPE002F5 | NM 00471 | NM 0047 | NM 0047 | HGS | HGS | HGS | hepatocyte growt | hepatocyt | hepatocyt | 9146 | 9146 | 9146 | s17480 | s17481 | s17482 |
| 2922 | J3 | J4 | 1620 | 671,1 | 12348 | 483,8 | 7,6 | 0,894 | HSA034 C | sample | Hm Drebl | Hm Drebl | Hm Drebl | CPE002DP | CPE002EF | CPE002F5 | NM 01510 | NM 0151 | NM 0151 | PLXND1 | PLXND1 | PLXND1 | plexin D1 | plexin D1 | plexin D1 | 23129 | 23129 | 23129 | s23094 | s23095 | s23096 |
| 20083 | E4 | E5 | 929 | 1112 | 7022 | 146,9 | 7,6 | 0,927 | HSA034 C | sample | Hm Gen E | Hm Gen E | Hm Gen E | CPE002JQ | CPE002O5 | CPE002KL | NM 00100 | NM 0010 | NM 0010 | FLJ33360 | FLJ33360 | FLJ33360 | FLJ33360 protein | FLJ33360 p | FLJ33360 p | 401172 | 401172 | 401172 | s53497 | s53498 | s53499 |
| 20245 | L20 | L21 | 1137 | 1128 | 8623 | 134,4 | 7,6 | 0,927 | HSA034 C | sample | Hm Gen E | Hm Gen E | Hm Gen E | CPE002JQ | CPE002O5 | CPE002KL | NM 00101 | NM 0010 | NM 0010 | C9orf106 | C9orf106 | C9orf106 | chromosome 9 or | chromoso | chromoso | 414318 | 414318 | 414318 | s53929 | s53930 | s53931 |
| 426 | B3 | B4 | 2579 | 1878 | 19668 | 337,1 | 7,6 | 1,109 | HSA034 C | sample | Hm Drebl | Hm Drebl | Hm Drebl | CPE002E0 | CPE002EZ | CPE002FK | NM 00523 | NM 0052 | NM 0052 | ERCC4 | ERCC4 | ERCC4 | excision repair cro | excision re | excision re | 2072 | 2072 | 2072 | s4799 | s4801 | s4800 |
| 465 | D17 | D18 | 2473 | 1712 | 18879 | 277,1 | 7,6 | 1,109 | HSA034 C | sample | Hm Drebl | Hm Drebl | Hm Drebl | CPE002E0 | CPE002EZ | CPE002FK | NM 00012 | NM 0001 | NM 0001 | EXT1 | EXT1 | EXT1 | exostoses (multip | exostoses | exostoses | 2131 | 2131 | 2131 | s4891 | s4889 | s4890 |
| 487 | E15 | E16 | 2645 | 1869 | 20220 | 269,9 | 7,6 | 1,109 | HSA034 C | sample | Hm Drebl | Hm Drebl | Hm Drebl | CPE002E0 | CPE002EZ | CPE002FK | NM 00196 | NM 0019 | NM 0019 | EGF | EGF | EGF | epidermal growth | epidermal | epidermal | 1950 | 1950 | 1950 | s4519 | s4520 | s4521 |
| 558 | H14 | H15 | 2357 | 1816 | 17929 | 331,3 | 7,6 | 1,109 | HSA034 C | sample | Hm Drebl | Hm Drebl | Hm Drebl | CPE002E0 | CPE002EZ | CPE002FK | NM 08042 | NM 0804 | NM 0804 | GNAS | GNAS | GNAS | GNAS complex lo | GNAS com | GNAS com | 2778 | 2778 | 2778 | s5889 | s5891 | s5890 |
| 572 | H5 | H6 | 2112 | 1790 | 16056 | 351,4 | 7,6 | 1,109 | HSA034 C | sample | Hm Drebl | Hm Drebl | Hm Drebl | CPE002E0 | CPE002EZ | CPE002FK | NM 00144 | NM 0014 | NM 0014 | FABP5 | FABP5 | FABP5 | fatty acid binding | fatty acid | fatty acid | 2171 | 2171 | 2171 | s4978 | s4976 | s4977 |
| 600 | I9 | I10 | 1896 | 1673 | 14499 | 299,9 | 7,6 | 1,109 | HSA034 C | sample | Hm Drebl | Hm Drebl | Hm Drebl | CPE002E0 | CPE002EZ | CPE002FK | NM 00495 | NM 0049 | NM 0049 | EIF4G1 | EIF4G1 | EIF4G1 | eukaryotic transla | eukaryoti | eukaryoti | 1981 | 1981 | 1981 | s4585 | s4586 | s4587 |
| 620 | J5 | J6 | 1867 | 1850 | 14213 | 240,3 | 7,6 | 1,109 | HSA034 C | sample | Hm Drebl | Hm Drebl | Hm Drebl | CPE002E0 | CPE002EZ | CPE002FK | NM 00410 | NM 0041 | NM 0041 | FASN | FASN | FASN | fatty acid synthas | fatty acid | fatty acid | 2194 | 2194 | 2194 | s5031 | s5032 | s5030 |
| 706 | N18 | N19 | 2538 | 1844 | 19410 | 343,7 | 7,6 | 1,109 | HSA034 C | sample | Hm Drebl | Hm Drebl | Hm Drebl | CPE002E0 | CPE002EZ | CPE002FK | NM 00103 | NM 0010 | NM 0010 | GRB7 | GRB7 | GRB7 | growth factor rec | growth fa | growth fa | 2886 | 2886 | 2886 | s6122 | s6123 | s6124 |
| 7766 | D21 | D22 | 1119 | 574,4 | 8450 | 416,6 | 7,6 | 1,16 | HSA034 C | sample | Hm Drebl | Hm Drebl | Hm Drebl | CPE002E4 | CPE002EV | CPE002FG | NM 00610 | NM 0061 | NM 0061 | RAPGEF3 | RAPGEF3 | RAPGEF3 | Rap guanine nucl | Rap guani | Rap guani | 10411 | 10411 | 10411 | s20360 | s20361 | s20362 |
| 7803 | F11 | F12 | 965 | 680,4 | 7330 | 482,9 | 7,6 | 1,16 | HSA034 C | sample | Hm Drebl | Hm Drebl | Hm Drebl | CPE002E4 | CPE002EV | CPE002FG | NM 00632 | NM 0063 | NM 0063 | ARIH2 | ARIH2 | ARIH2 | ariadne homolog | ariadne h | ariadne h | 10425 | 10425 | 10425 | s20390 | s20391 | s20392 |
| 7902 | J14 | J15 | 1888 | 614,5 | 14276 | 344,9 | 7,6 | 1,16 | HSA034 C | sample | Hm Drebl | Hm Drebl | Hm Drebl | CPE002E4 | CPE002EV | CPE002FG | NM 00682 | NM 0068 | NM 0068 | YWHAQ | YWHAQ | YWHAQ | tyrosine 3-monoo | tyrosine 3 | tyrosine 3 | 10971 | 10971 | 10971 | s21598 | s21597 | s21599 |
| 7911 | J22 | J23 | 1426 | 581,9 | 10787 | 318,7 | 7,6 | 1,16 | HSA034 C | sample | Hm Drebl | Hm Drebl | Hm Drebl | CPE002E4 | CPE002EV | CPE002FG | NM 00683 | NM 0068 | NM 0068 | CLP1 | CLP1 | CLP1 | CLP1, cleavage an | CLP1, clea | CLP1, clea | 10978 | 10978 | 10978 | s21610 | s21609 | s21611 |
| 7922 | K10 | K11 | 1197 | 591,7 | 9135 | 356,9 | 7,6 | 1,16 | HSA034 C | sample | Hm Drebl | Hm Drebl | Hm Drebl | CPE002E4 | CPE002EV | CPE002FG | NM 00658 | NM 0065 | NM 0065 | FUT9 | FUT9 | FUT9 | fucosyltransferas | fucosyltra | fucosyltra | 10690 | 10690 | 10690 | s21018 | s21017 | s21019 |
| 8012 | N5 | N6 | 1424 | 584 | 10767 | 335,8 | 7,6 | 1,16 | HSA034 C | sample | Hm Drebl | Hm Drebl | Hm Drebl | CPE002E4 | CPE002EV | CPE002FG | NM 00637 | NM 0063 | NM 0063 | SEMA4D | SEMA4D | SEMA4D | sema domain, im | sema dom | sema dom | 10507 | 10507 | 10507 | s20593 | s20592 | s20594 |
| 8018 | O10 | O11 | 1522 | 591,4 | 11492 | 372,5 | 7,6 | 1,16 | HSA034 C | sample | Hm Drebl | Hm Drebl | Hm Drebl | CPE002E4 | CPE002EV | CPE002FG | NM 00664 | NM 0066 | NM 0066 | NOXA1 | NOXA1 | NOXA1 | NADPH oxidase a | NADPH ox | NADPH ox | 10811 | 10811 | 10811 | s21249 | s195068 | s195069 |
| 8035 | O4 | O5 | 1249 | 631,7 | 9490 | 355,1 | 7,6 | 1,16 | HSA034 C | sample | Hm Drebl | Hm Drebl | Hm Drebl | CPE002E4 | CPE002EV | CPE002FG | NM 00104 | NM 0010 | NM 0010 | MTHFD2 | MTHFD2 | MTHFD2 | methylenetetrahy | methylene | methylene | 10797 | 10797 | 10797 | s21210 | s21212 | s21211 |
| 8049 | P17 | P18 | 1574 | 666,1 | 11921 | 410,9 | 7,6 | 1,16 | HSA034 C | sample | Hm Drebl | Hm Drebl | Hm Drebl | CPE002E4 | CPE002EV | CPE002FG | NM 00640 | NM 0064 | NM 0064 | PROCR | PROCR | PROCR | protein C recepto | protein C | protein C | 10544 | 10544 | 10544 | s20680 | s20681 | s20682 |
| 8052 | P2 | P3 | 1616 | 589,6 | 12336 | 462,1 | 7,6 | 1,16 | HSA034 C | sample | Hm Drebl | Hm Drebl | Hm Drebl | CPE002E4 | CPE002EV | CPE002FG | NM 19913 | NM 1991 | NM 1991 | VAX1 | VAX1 | VAX1 | ventral anterior h | ventral an | ventral an | 11023 | 11023 | 11023 | s21715 | s21714 | s223182 |
| 8062 | P7 | P8 | 1288 | 581,2 | 9834 | 397,7 | 7,6 | 1,16 | HSA034 C | sample | Hm Drebl | Hm Drebl | Hm Drebl | CPE002E4 | CPE002EV | CPE002FG | NM 00639 | NM 0063 | NM 0063 | NEBL | NEBL | NEBL | nebulette | nebulette | nebulette | 10529 | 10529 | 10529 | s20646 | s20645 | s223127 |
| 11449 | N1 | N2 | 1887 | 864 | 14619 | 119,3 | 7,7 | 0,558 | HSA034 C | sample | Hm Drebl | Hm Drebl | Hm Drebl | CPE002ED | CPE002F3 | CPE002FT | NM 20682 | NM 2068 | NM 2068 | RASL11A | RASL11A | RASL11A | RAS-like, family 1 | RAS-like, f | RAS-like, f | 387496 | 387496 | 387496 | s51865 | s51866 | s51867 |
| 2460 | G2 | G3 | 762 | 793,1 | 5875 | 513,9 | 7,7 | 0,606 | HSA034 C | sample | Hm Drebl | Hm Drebl | Hm Drebl | CPE002DC | CPE002EE | CPE002F4 | NM 00244 | NM 0024 | NM 0024 | MST1R | MST1R | MST1R | macrophage stim | macropha | macropha | 4486 | 4486 | 4486 | s8996 | s8997 | s8998 |
| 139 | F4 | F5 | 750 | 1602 | 5786 | 392 | 7,7 | 0,655 | HSA034 C | sample | Hm Drebl | Hm Drebl | Hm Drebl | CPE002DT | CPE002EJ | CPE002F9 | NM 00101 | NM 0010 | NM 0010 | SPRR2G | SPRR2G | SPRR2G | small proline-rich | small prol | small prol | 6706 | 6706 | 6706 | s13394 | s13395 | s13396 |
| 141 | F6 | F7 | 573 | 1461 | 4393 | 324,7 | 7,7 | 0,655 | HSA034 C | sample | Hm Drebl | Hm Drebl | Hm Drebl | CPE002DT | CPE002EJ | CPE002F9 | NM 00541 | NM 0054 | NM 0054 | SPRR3 | SPRR3 | SPRR3 | small proline-rich | small prol | small prol | 6707 | 6707 | 6707 | s13398 | s13399 | s13397 |
| 288 | L9 | L10 | 521 | 1412 | 3999 | 437,6 | 7,7 | 0,655 | HSA034 C | sample | Hm Drebl | Hm Drebl | Hm Drebl | CPE002DT | CPE002EJ | CPE002F9 | NM 00693 | NM 0069 | NM 0069 | SLC2A3 | SLC2A3 | SLC2A3 | solute carrier fam | solute car | solute car | 6515 | 6515 | 6515 | s12931 | s12932 | s12933 |
| 292 | M12 | M13 | 655 | 1575 | 5014 | 454,8 | 7,7 | 0,655 | HSA034 C | sample | Hm Drebl | Hm Drebl | Hm Drebl | CPE002DT | CPE002EJ | CPE002F9 | NM 00045 | NM 0004 | NM 0004 | SOD1 | SOD1 | SOD1 | superoxide dismu | superoxid | superoxid | 6647 | 6647 | 6647 | s451 | s453 | s452 |
| 3956 | E5 | E6 | 1953 | 732,8 | 15124 | 301 | 7,7 | 0,684 | HSA034 C | sample | Hm Drebl | Hm Drebl | Hm Drebl | CPE002DS | CPE002EI | CPE002F8 | NM 00675 | NM 0067 | NM 0067 | TALDO1 | TALDO1 | TALDO1 | transaldolase 1 | transaldo | transaldo | 6888 | 6888 | 6888 | s13776 | s224706 | s13775 |
| 6091 | N4 | N5 | 1210 | 828,9 | 9338 | 212,5 | 7,7 | 0,703 | HSA034 C | sample | Hm Drebl | Hm Drebl | Hm Drebl | CPE002DY | CPE002F1 | CPE002FN | NM 00613 | NM 0061 | NM 0061 | CAPZA2 | CAPZA2 | CAPZA2 | capping protein (a | capping p | capping p | 830 | 830 | 830 | s2395 | s2396 | s2397 |
| 8227 | G4 | G5 | 936 | 541,4 | 7175 | 167 | 7,7 | 0,74 | HSA034 C | sample | Hm Drebl | Hm Drebl | Hm Drebl | CPE002E5 | CPE002EU | CPE002FF | NM 00104 | NM 0010 | NM 0010 | MAPK8IP3 | MAPK8IP3 | MAPK8IP3 | mitogen-activated | mitogen-a | mitogen-a | 23162 | 23162 | 23162 | s23167 | s23166 | s23168 |
| 10297 | N1 | N2 | 1473 | 799,5 | 11277 | 127,9 | 7,7 | 0,74 | HSA034 C | sample | Hm Drebl | Hm Drebl | Hm Drebl | CPE002EA | CPE002EP | CPE002FA | NM 03092 | NM 0309 | NM 0309 | ACSBG2 | ACSBG2 | ACSBG2 | acyl-CoA syntheta | acyl-CoA s | acyl-CoA s | 81616 | 81616 | 81616 | s37711 | s37710 | s37712 |
| 1855 | N15 | N16 | 875 | 812,7 | 6724 | 420,3 | 7,7 | 0,806 | HSA034 C | sample | Hm Drebl | Hm Drebl | Hm Drebl | CPE002FW | CPE002G0 | CPE002G4 | NM 01961 | NM 0196 | NM 0196 | PARD3 | PARD3 | PARD3 | par-3 partitioning | par-3 part | par-3 part | 56288 | 56288 | 56288 | s32126 | s32127 | s32128 |
| 21512 | A16 | A17 | 235 | 1341 | 1817 | 750,4 | 7,7 | 0,811 | HSA034 C | sample | Hm Gen E | Hm Gen E | Hm Gen E | CPE002JU | CPE002O9 | CPE002KP | XM 94196 | XM 9419 | XM 9419 | LOC65249 | LOC65249 | LOC65249 | similar to CG4785 | similar to | similar to | 652499 | 652499 | 652499 | s57374 | s57375 | s197896 |
| 21708 | I2 | I3 | 213 | 1019 | 1644 | 494,1 | 7,7 | 0,811 | HSA034 C | sample | Hm Gen E | Hm Gen E | Hm Gen E | CPE002JU | CPE002O9 | CPE002KP | XM 94230 | XM 9423 | XM 9423 | LOC65269 | LOC65269 | LOC65269 | similar to Olfacto | similar to | similar to | 652692 | 652692 | 652692 | s57440 | s57441 | s57442 |
| 21762 | K3 | K4 | 270 | 902,9 | 2076 | 490,2 | 7,7 | 0,811 | HSA034 C | sample | Hm Gen E | Hm Gen E | Hm Gen E | CPE002JU | CPE002O9 | CPE002KP | XM 93901 | XM 9390 | XM 9390 | LOC64993 | LOC64993 | LOC64993 | hypothetical prot | hypotheti | hypotheti | 649935 | 649935 | 649935 | s57026 | s197695 | s197696 |
| 21786 | L3 | L4 | 234 | 1062 | 1805 | 583,3 | 7,7 | 0,811 | HSA034 C | sample | Hm Gen E | Hm Gen E | Hm Gen E | CPE002JU | CPE002O9 | CPE002KP | XM 94112 | XM 9411 | XM 9411 | LOC65186 | LOC65186 | LOC65186 | similar to Histone | similar to | similar to | 651868 | 651868 | 651868 | s57265 | s57266 | s57267 |
| 21817 | N1 | N2 | 326 | 971,5 | 2498 | 391,3 | 7,7 | 0,811 | HSA034 C | sample | Hm Gen E | Hm Gen E | Hm Gen E | CPE002JU | CPE002O9 | CPE002KP | XM 94136 | XM 9413 | XM 9413 | LOC65204 | LOC65204 | LOC65204 | similar to ribosom | similar to | similar to | 652046 | 652046 | 652046 | s57298 | s57299 | s197843 |
| 4639 | B15 | B16 | 1392 | 886,3 | 10708 | 414,7 | 7,7 | 0,846 | HSA034 C | sample | Hm Drebl | Hm Drebl | Hm Drebl | CPE002DV | CPE002EL | CPE002FP | NM 00614 | NM 0061 | NM 0061 | GZMA | GZMA | GZMA | granzyme A (gran | granzyme | granzyme | 3001 | 3001 | 3001 | s6384 | s6385 | s6386 |
| 10566 | I14 | I15 | 1195 | 915,3 | 9258 | 187,6 | 7,7 | 0,87 | HSA034 C | sample | Hm Drebl | Hm Drebl | Hm Drebl | CPE002EB | CPE002EO | CPE002FR | NM 13845 | NM 1384 | NM 1384 | RAB3C | RAB3C | RAB3C | RAB3C, member R | RAB3C, m | RAB3C, m | 115827 | 115827 | 115827 | s41886 | s41885 | s41884 |
| 10668 | M2 | M3 | 1384 | 837,5 | 10654 | 172,6 | 7,7 | 0,87 | HSA034 C | sample | Hm Drebl | Hm Drebl | Hm Drebl | CPE002EB | CPE002EO | CPE002FR | XM 00113 | XM 0011 | XM 0011 | TOP1MT | TOP1MT | TOP1MT | topoisomerase (D | topoisom | topoisom | 116447 | 116447 | 116447 | s42019 | s42018 | s42017 |
| 2712 | A9 | A10 | 698 | 663,4 | 5342 | 449,6 | 7,7 | 0,906 | HSA034 C | sample | Hm Drebl | Hm Drebl | Hm Drebl | CPE002DP | CPE002EF | CPE002F5 | NM 00107 | NM 0010 | NM 0010 | ZAP70 | ZAP70 | ZAP70 | zeta-chain (TCR) a | zeta-chair | zeta-chair | 7535 | 7535 | 7535 | s14973 | s14974 | s14975 |
| 2714 | B10 | B11 | 1638 | 711,5 | 12613 | 456,6 | 7,7 | 0,906 | HSA034 C | sample | Hm Drebl | Hm Drebl | Hm Drebl | CPE002DP | CPE002EF | CPE002F5 | NM 03311 | NM 0331 | NM 0331 | NEK9 | NEK9 | NEK9 | NIMA (never in m | NIMA (ne | NIMA (ne | 91754 | 91754 | 91754 | s40771 | s40772 | s40773 |
| 2720 | B16 | B17 | 1385 | 715,5 | 10672 | 604,1 | 7,7 | 0,906 | HSA034 C | sample | Hm Drebl | Hm Drebl | Hm Drebl | CPE002DP | CPE002EF | CPE002F5 | NM 14504 | NM 1450 | NM 1450 | PRKCDBP | PRKCDBP | PRKCDBP | protein kinase C, | protein ki | protein ki | 112464 | 112464 | 112464 | s41325 | s195582 | s195583 |
| 2813 | F13 | F14 | 1313 | 688,5 | 10171 | 466,7 | 7,7 | 0,906 | HSA034 C | sample | Hm Drebl | Hm Drebl | Hm Drebl | CPE002DP | CPE002EF | CPE002F5 | NM 01771 | NM 0177 | NM 0177 | SNRK | SNRK | SNRK | SNF related kinas | SNF relate | SNF relate | 54861 | 54861 | 54861 | s29614 | s224323 | s29616 |
| 2903 | I8 | I9 | 931 | 724 | 7209 | 477,5 | 7,7 | 0,906 | HSA034 C | sample | Hm Drebl | Hm Drebl | Hm Drebl | CPE002DP | CPE002EF | CPE002F5 | NM 02122 | NM 0212 | NM 0212 | LY6G5B | LY6G5B | LY6G5B | lymphocyte antig | lymphocy | lymphocy | 58496 | 58496 | 58496 | s33861 | s33862 | s33863 |
| 2923 | J4 | J5 | 974 | 692,3 | 7547 | 476 | 7,7 | 0,906 | HSA034 C | sample | Hm Drebl | Hm Drebl | Hm Drebl | CPE002DP | CPE002EF | CPE002F5 | NM 13902 | NM 1390 | NM 1390 | MAPK15 | MAPK15 | MAPK15 | mitogen-activated | mitogen-a | mitogen-a | 225689 | 225689 | 225689 | s48270 | s48271 | s48272 |
| 2956 | L12 | L13 | 1058 | 595,5 | 8097 | 461,4 | 7,7 | 0,906 | HSA034 C | sample | Hm Drebl | Hm Drebl | Hm Drebl | CPE002DP | CPE002EF | CPE002F5 | NM 00100 | NM 0010 | NM 0010 | MAP3K15 | MAP3K15 | MAP3K15 | mitogen-activated | mitogen-a | mitogen-a | 389840 | 389840 | 389840 | s52568 | s52569 | s52570 |
| 2983 | M15 | M16 | 675 | 658,5 | 5165 | 474,9 | 7,7 | 0,906 | HSA034 C | sample | Hm Drebl | Hm Drebl | Hm Drebl | CPE002DP | CPE002EF | CPE002F5 | NM 01229 | NM 0122 | NM 0122 | TLK1 | TLK1 | TLK1 | tousled-like kinas | tousled-lil | tousled-lil | 9874 | 9874 | 9874 | s19122 | s19123 | s19124 |
| 2994 | M3 | M4 | 659 | 656,5 | 5101 | 421 | 7,7 | 0,906 | HSA034 C | sample | Hm Drebl | Hm Drebl | Hm Drebl | CPE002DP | CPE002EF | CPE002F5 | NM 01400 | NM 0140 | NM 0140 | IKBKE | IKBKE | IKBKE | inhibitor of kappa | inhibitor c | inhibitor c | 9641 | 9641 | 9641 | s18536 | s18537 | s18538 |
| 3000 | M9 | M10 | 1259 | 605,5 | 9749 | 435,6 | 7,7 | 0,906 | HSA034 C | sample | Hm Drebl | Hm Drebl | Hm Drebl | CPE002DP | CPE002EF | CPE002F5 | NM 15327 | NM 1532 | NM 1532 | IHPK1 | IHPK1 | IHPK1 | inositol hexaphos | inositol h | inositol h | 9807 | 9807 | 9807 | s18957 | s18958 | s18959 |
| 3022 | N7 | N8 | 1457 | 672,3 | 11154 | 528,8 | 7,7 | 0,906 | HSA034 C | sample | Hm Drebl | Hm Drebl | Hm Drebl | CPE002DP | CPE002EF | CPE002F5 | NM 00104 | NM 0010 | NM 0010 | TPK1 | TPK1 | TPK1 | thiamin pyrophos | thiamin p | thiamin p | 27010 | 27010 | 27010 | s25692 | s25693 | s25694 |
| 20025 | C17 | C18 | 840 | 1175 | 6456 | 149,8 | 7,7 | 0,939 | HSA034 C | sample | Hm Gen E | Hm Gen E | Hm Gen E | CPE002JQ | CPE002O5 | CPE002KL | NM 00108 | NM 0010 | NM 0010 | PRAMEF12 | PRAMEF1 | PRAMEF1 | PRAME family me | PRAME fa | PRAME fa | 390999 | 390999 | 390999 | s52905 | s52906 | s52907 |
| 20117 | G13 | G14 | 1679 | 1115 | 12846 | 139,8 | 7,7 | 0,939 | HSA034 C | sample | Hm Gen E | Hm Gen E | Hm Gen E | CPE002JQ | CPE002O5 | CPE002KL | XM 37291 | XM 3729 | XM 3729 | LOC39134 | LOC39134 | LOC39134 | similar to CG3047 | similar to | similar to | 391347 | 391347 | 391347 | s196379 | s196380 | s196381 |
| 20139 | H11 | H12 | 946 | 1101 | 7241 | 116 | 7,7 | 0,939 | HSA034 C | sample | Hm Gen E | Hm Gen E | Hm Gen E | CPE002JQ | CPE002O5 | CPE002KL | XM 37514 | XM 3751 | XM 3751 | LOC40029 | LOC40029 | LOC40029 | similar to Ribosom | similar to | similar to | 400299 | 400299 | 400299 | s53267 | s53268 | s53269 |
| 20216 | K16 | K17 | 1008 | 1086 | 7786 | 117,2 | 7,7 | 0,939 | HSA034 C | sample | Hm Gen E | Hm Gen E | Hm Gen E | CPE002JQ | CPE002O5 | CPE002KL | XM 37962 | XM 3796 | XM 3796 | FLJ41200 | FLJ41200 | FLJ41200 | hypothetical gene | hypotheti | hypotheti | 401492 | 401492 | 401492 | s53615 | s53616 | s53617 |
| 20301 | N6 | N7 | 1264 | 1081 | 9758 | 113 | 7,7 | 0,939 | HSA034 C | sample | Hm Gen E | Hm Gen E | Hm Gen E | CPE002JQ | CPE002O5 | CPE002KL | NM 00100 | NM 0010 | NM 0010 | MGC7085 | MGC7085 | MGC7085 | similar to RIKEN c | similar to | similar to | 414919 | 414919 | 414919 | s53944 | s53945 | s196499 |
| 398 | A21 | A22 | 3268 | 1934 | 25038 | 461,3 | 7,7 | 1,124 | HSA034 C | sample | Hm Drebl | Hm Drebl | Hm Drebl | CPE002E0 | CPE002EZ | CPE002FK | NM 19825 | NM 1982 | NM 1982 | E2F6 | E2F6 | E2F6 | E2F transcription | E2F transc | E2F transc | 1876 | 1876 | 1876 | s4420 | s4421 | s223456 |
| 478 | D7 | D8 | 1572 | 1773 | 12042 | 360,1 | 7,7 | 1,124 | HSA034 C | sample | Hm Drebl | Hm Drebl | Hm Drebl | CPE002E0 | CPE002EZ | CPE002FK | NM 00524 | NM 0052 | NM 0052 | ETV3 | ETV3 | ETV3 | ets variant gene 3 | ets varian | ets varian | 2117 | 2117 | 2117 | s223505 | s4857 | s4858 |
| 513 | F17 | F18 | 2017 | 1689 | 15566 | 239,9 | 7,7 | 1,124 | HSA034 C | sample | Hm Drebl | Hm Drebl | Hm Drebl | CPE002E0 | CPE002EZ | CPE002FK | NM 00144 | NM 0014 | NM 0014 | FAAH | FAAH | FAAH | fatty acid amide h | fatty acid | fatty acid | 2166 | 2166 | 2166 | s4961 | s4962 | s4963 |
| 555 | H11 | H12 | 2302 | 1771 | 17795 | 268,5 | 7,7 | 1,124 | HSA034 C | sample | Hm Drebl | Hm Drebl | Hm Drebl | CPE002E0 | CPE002EZ | CPE002FK | NM 00013 | NM 0001 | NM 0001 | FANCA | FANCA | FANCA | Fanconi anemia, c | Fanconi a | Fanconi a | 2175 | 2175 | 2175 | s164 | s162 | s163 |
| 715 | N4 | N5 | 2762 | 1945 | 21279 | 358,6 | 7,7 | 1,124 | HSA034 C | sample | Hm Drebl | Hm Drebl | Hm Drebl | CPE002E0 | CPE002EZ | CPE002FK | NM 00530 | NM 0053 | NM 0053 | GPT | GPT | GPT | glutamic-pyruvate | glutamic-p | glutamic-p | 2875 | 2875 | 2875 | s6103 | s6101 | s6102 |
| 721 | O1 | O2 | 2355 | 1830 | 18041 | 391,3 | 7,7 | 1,124 | HSA034 C | sample | Hm Drebl | Hm Drebl | Hm Drebl | CPE002E0 | CPE002EZ | CPE002FK | NM 00011 | NM 0001 | NM 0001 | EPB42 | EPB42 | EPB42 | erythrocyte mem | erythrocyt | erythrocyt | 2038 | 2038 | 2038 | s4711 | s4712 | s4710 |
| 7688 | A16 | A17 | 981 | 667,3 | 7522 | 280,9 | 7,7 | 1,176 | HSA034 C | sample | Hm Drebl | Hm Drebl | Hm Drebl | CPE002E4 | CPE002EV | CPE002FG | NM 00641 | NM 0064 | NM 0064 | SLC35A1 | SLC35A1 | SLC35A1 | solute carrier fam | solute car | solute car | 10559 | 10559 | 10559 | s20713 | s20714 | s20715 |
| 7741 | C20 | C21 | 1491 | 543,6 | 11520 | 357,8 | 7,7 | 1,176 | HSA034 C | sample | Hm Drebl | Hm Drebl | Hm Drebl | CPE002E4 | CPE002EV | CPE002FG | NM 00644 | NM 0064 | NM 0064 | SLCO1B1 | SLCO1B1 | SLCO1B1 | solute carrier org | solute car | solute car | 10599 | 10599 | 10599 | s20806 | s20805 | s20807 |
| 7773 | D6 | D7 | 998 | 576,6 | 7642 | 447,6 | 7,7 | 1,176 | HSA034 C | sample | Hm Drebl | Hm Drebl | Hm Drebl | CPE002E4 | CPE002EV | CPE002FG | NM 00678 | NM 0067 | NM 0067 | MALT1 | MALT1 | MALT1 | mucosa associate | mucosa a | mucosa a | 10892 | 10892 | 10892 | s21397 | s21398 | s21396 |
| 7790 | E21 | E22 | 1305 | 586 | 10085 | 434 | 7,7 | 1,176 | HSA034 C | sample | Hm Drebl | Hm Drebl | Hm Drebl | CPE002E4 | CPE002EV | CPE002FG | NM 00580 | NM 0058 | NM 0058 | DDX39 | DDX39 | DDX39 | DEAD (Asp-Glu-Al | DEAD (Asp | DEAD (Asp | 10212 | 10212 | 10212 | s19916 | s19917 | s19918 |
| 7905 | J17 | J18 | 1272 | 527,7 | 9795 | 283,2 | 7,7 | 1,176 | HSA034 C | sample | Hm Drebl | Hm Drebl | Hm Drebl | CPE002E4 | CPE002EV | CPE002FG | NM 18267 | NM 1826 | NM 1826 | UBE2E3 | UBE2E3 | UBE2E3 | ubiquitin-conjuga | ubiquitin- | ubiquitin- | 10477 | 10477 | 10477 | s20517 | s20519 | s20518 |
| 7942 | K7 | K8 | 1390 | 549,3 | 10670 | 339,9 | 7,7 | 1,176 | HSA034 C | sample | Hm Drebl | Hm Drebl | Hm Drebl | CPE002E4 | CPE002EV | CPE002FG | NM 00585 | NM 0058 | NM 0058 | RAMP1 | RAMP1 | RAMP1 | receptor (G prote | receptor ( | receptor ( | 10267 | 10267 | 10267 | s20060 | s20061 | s195024 |
| 7953 | L17 | L18 | 1278 | 567,4 | 9858 | 324,4 | 7,7 | 1,176 | HSA034 C | sample | Hm Drebl | Hm Drebl | Hm Drebl | CPE002E4 | CPE002EV | CPE002FG | NM 00637 | NM 0063 | NM 0063 | UNC13B | UNC13B | UNC13B | unc-13 homolog B | unc-13 ho | unc-13 ho | 10497 | 10497 | 10497 | s20576 | s20575 | s20574 |
| 7967 | L8 | L9 | 907 | 501,5 | 7008 | 316,4 | 7,7 | 1,176 | HSA034 C | sample | Hm Drebl | Hm Drebl | Hm Drebl | CPE002E4 | CPE002EV | CPE002FG | NM 00684 | NM 0068 | NM 0010 | LILRB5 | LILRB5 | LILRB5 | leukocyte immun | leukocyte | leukocyte | 10990 | 10990 | 10990 | s21638 | s21637 | s223174 |
| 7980 | M2 | M3 | 780 | 553,2 | 6032 | 319 | 7,7 | 1,176 | HSA034 C | sample | Hm Drebl | Hm Drebl | Hm Drebl | CPE002E4 | CPE002EV | CPE002FG | NM 00660 | NM 0066 | NM 0066 | PHTF1 | PHTF1 | PHTF1 | putative homeod | putative h | putative h | 10745 | 10745 | 10745 | s21115 | s21113 | s21114 |
| 8016 | N9 | N10 | 1505 | 588,5 | 11662 | 308,4 | 7,7 | 1,176 | HSA034 C | sample | Hm Drebl | Hm Drebl | Hm Drebl | CPE002E4 | CPE002EV | CPE002FG | NM 00638 | NM 0063 | NM 0063 | APPBP2 | APPBP2 | APPBP2 | amyloid beta pre | amyloid b | amyloid b | 10513 | 10513 | 10513 | s20603 | s20601 | s20602 |
| 12954 | L3 | L4 | 1401 | 995,2 | 10981 | 137,8 | 7,8 | 0,529 | HSA034 C | sample | Hm Gen E | Hm Gen E | Hm Gen E | CPE002J6 | CPE002NM | CPE002K2 | NM 01476 | NM 0147 | NM 0147 | MRPL19 | MRPL19 | MRPL19 | mitochondrial rib | mitochon | mitochon | 9801 | 9801 | 9801 | s18942 | s18943 | s18944 |
| 15138 | G3 | G4 | 1021 | 771,4 | 8002 | 164,3 | 7,8 | 0,545 | HSA034 C | sample | Hm Gen E | Hm Gen E | Hm Gen E | CPE002J8 | CPE002NS | CPE002K8 | NM 03127 | NM 0312 | NM 0312 | RNF17 | RNF17 | RNF17 | ring finger protei | ring finger | ring finger | 56163 | 56163 | 56163 | s32033 | s32034 | s32035 |
| 10842 | D3 | D4 | 1225 | 801,1 | 9508 | 367 | 7,8 | 0,591 | HSA034 C | sample | Hm Drebl | Hm Drebl | Hm Drebl | CPE002EC | CPE002EN | CPE002FS | NM 13349 | NM 1334 | NM 1334 | SLC30A7 | SLC30A7 | SLC30A7 | solute carrier fam | solute car | solute car | 148867 | 148867 | 148867 | s45225 | s45226 | s45224 |
| 2506 | I18 | I19 | 1908 | 695,8 | 14796 | 286,4 | 7,8 | 0,614 | HSA034 C | sample | Hm Drebl | Hm Drebl | Hm Drebl | CPE002DC | CPE002EE | CPE002F4 | NM 00619 | NM 0061 | NM 0061 | PCM1 | PCM1 | PCM1 | pericentriolar ma | pericentri | pericentri | 5108 | 5108 | 5108 | s10127 | s10128 | s10129 |
| 192 | H9 | H10 | 553 | 1449 | 4336 | 520,5 | 7,8 | 0,664 | HSA034 C | sample | Hm Drebl | Hm Drebl | Hm Drebl | CPE002DT | CPE002EJ | CPE002F9 | NM 00303 | NM 1732 | NM 1732 | ST6GAL1 | ST6GAL1 | ST6GAL1 | ST6 beta-galactos | ST6 beta-g | ST6 beta-g | 6480 | 6480 | 6480 | s224629 | s12842 | s12843 |
| 207 | I22 | I23 | 539 | 1617 | 4224 | 447,1 | 7,8 | 0,664 | HSA034 C | sample | Hm Drebl | Hm Drebl | Hm Drebl | CPE002DT | CPE002EJ | CPE002F9 | NM 00308 | NM 0030 | NM 0030 | SNCG | SNCG | SNCG | synuclein, gamma | synuclein, | synuclein, | 6623 | 6623 | 6623 | s194805 | s194806 | s194807 |
| 238 | J7 | J8 | 254 | 1569 | 1983 | 372,3 | 7,8 | 0,664 | HSA034 C | sample | Hm Drebl | Hm Drebl | Hm Drebl | CPE002DT | CPE002EJ | CPE002F9 | NM 00693 | NM 0069 | NM 0069 | SKP1A | SKP1A | SKP1A | S-phase kinase-as | S-phase ki | S-phase ki | 6500 | 6500 | 6500 | s12889 | s12890 | s12891 |
| 276 | L2 | L3 | 645 | 1585 | 5050 | 428,5 | 7,8 | 0,664 | HSA034 C | sample | Hm Drebl | Hm Drebl | Hm Drebl | CPE002DT | CPE002EJ | CPE002F9 | NM 00315 | NM 0031 | NM 0031 | STAT5A | STAT5A | STAT5A | signal transducer | signal trar | signal trar | 6776 | 6776 | 6776 | s13535 | s13534 | s13536 |
| 5766 | A14 | A15 | 1144 | 903,7 | 8929 | 184,7 | 7,8 | 0,712 | HSA034 C | sample | Hm Drebl | Hm Drebl | Hm Drebl | CPE002DY | CPE002F1 | CPE002FN | NM 00404 | NM 0040 | NM 0040 | RHOB | RHOB | RHOB | ras homolog gene | ras homol | ras homol | 388 | 388 | 388 | s1574 | s1575 | s1576 |
| 5823 | C22 | C23 | 2417 | 837,5 | 18854 | 518 | 7,8 | 0,712 | HSA034 C | sample | Hm Drebl | Hm Drebl | Hm Drebl | CPE002DY | CPE002F1 | CPE002FN | NM 00431 | NM 0043 | NM 0043 | ARR3 | ARR3 | ARR3 | arrestin 3, retinal | arrestin 3 | arrestin 3 | 407 | 407 | 407 | s224021 | s1621 | s1619 |
| 841 | D1 | D2 | 1419 | 840,2 | 11031 | 304 | 7,8 | 0,739 | HSA034 C | sample | Hm Drebl | Hm Drebl | Hm Drebl | CPE002FU | CPE002FY | CPE002G2 | NM 00205 | NM 0020 | NM 0020 | GATA3 | GATA3 | GATA3 | GATA binding pro | GATA bin | GATA bin | 2625 | 2625 | 2625 | s5599 | s5600 | s5601 |
| 5721 | O17 | O18 | 1468 | 655,4 | 11435 | 477,1 | 7,8 | 0,743 | HSA034 C | sample | Hm Drebl | Hm Drebl | Hm Drebl | CPE002DX | CPE002F2 | CPE002FN | NM 00369 | NM 0036 | NM 0036 | PRKRA | PRKRA | PRKRA | protein kinase, in | protein ki | protein ki | 8575 | 8575 | 8575 | s16336 | s16334 | s16335 |
| 8144 | D16 | D17 | 1431 | 632,1 | 11189 | 452,7 | 7,8 | 0,75 | HSA034 C | sample | Hm Drebl | Hm Drebl | Hm Drebl | CPE002E5 | CPE002EU | CPE002FF | NM 01221 | NM 0122 | NM 0122 | MLYCD | MLYCD | MLYCD | malonyl-CoA dec | malonyl-C | malonyl-C | 23417 | 23417 | 23417 | s23787 | s23788 | s23789 |
| 21548 | B5 | B6 | 296 | 980,7 | 2315 | 712,2 | 7,8 | 0,821 | HSA034 C | sample | Hm Gen E | Hm Gen E | Hm Gen E | CPE002JU | CPE002O9 | CPE002KP | XM 93980 | XM 9398 | XM 9398 | LOC65072 | LOC65072 | LOC65072 | similar to double | similar to | similar to | 650724 | 650724 | 650724 | s197747 | s197748 | s197749 |
| 21666 | G3 | G4 | 255 | 1056 | 1983 | 315,5 | 7,8 | 0,821 | HSA034 C | sample | Hm Gen E | Hm Gen E | Hm Gen E | CPE002JU | CPE002O9 | CPE002KP | NM 00103 | NM 0010 | NM 0010 | FLJ35776 | FLJ35776 | FLJ35776 | hypothetical prot | hypotheti | hypotheti | 649446 | 649446 | 649446 | s56973 | s56974 | s227319 |
| 21835 | N4 | N5 | 287 | 960,8 | 2243 | 483,8 | 7,8 | 0,821 | HSA034 C | sample | Hm Gen E | Hm Gen E | Hm Gen E | CPE002JU | CPE002O9 | CPE002KP | XM 93624 | XM 9362 | XM 9362 | LOC65388 | LOC65388 | LOC65388 | similar to hypoth | similar to | similar to | 653885 | 653885 | 653885 | s198100 | s198101 | s198102 |
| 21854 | O21 | O22 | 507 | 899,6 | 3943 | 632,7 | 7,8 | 0,821 | HSA034 C | sample | Hm Gen E | Hm Gen E | Hm Gen E | CPE002JU | CPE002O9 | CPE002KP | XM 93977 | XM 9397 | XM 9397 | LOC65068 | LOC65068 | LOC65068 | similar to nuclear | similar to | similar to | 650686 | 650686 | 650686 | s57110 | s57111 | s57112 |
| 4687 | D15 | D16 | 2247 | 930,9 | 17422 | 328,3 | 7,8 | 0,857 | HSA034 C | sample | Hm Drebl | Hm Drebl | Hm Drebl | CPE002DV | CPE002EL | CPE002FP | NM 00020 | NM 0002 | NM 0002 | CFI | CFI | CFI | complement facto | compleme | compleme | 3426 | 3426 | 3426 | s7133 | s7134 | s7135 |
| 4783 | H15 | H16 | 1940 | 885,5 | 15039 | 367 | 7,8 | 0,857 | HSA034 C | sample | Hm Drebl | Hm Drebl | Hm Drebl | CPE002DV | CPE002EL | CPE002FP | NM 00242 | NM 0024 | NM 0024 | MMP10 | MMP10 | MMP10 | matrix metallope | matrix me | matrix me | 4319 | 4319 | 4319 | s8865 | s8866 | s8867 |
| 4796 | H5 | H6 | 1825 | 789,9 | 14190 | 310,6 | 7,8 | 0,857 | HSA034 C | sample | Hm Drebl | Hm Drebl | Hm Drebl | CPE002DV | CPE002EL | CPE002FP | NM 00453 | NM 0045 | NM 0045 | MMP2 | MMP2 | MMP2 | matrix metallope | matrix me | matrix me | 4313 | 4313 | 4313 | s8850 | s8851 | s8852 |
| 4896 | L9 | L10 | 2053 | 802,5 | 16054 | 490,9 | 7,8 | 0,857 | HSA034 C | sample | Hm Drebl | Hm Drebl | Hm Drebl | CPE002DV | CPE002EL | CPE002FP | NM 00100 | NM 0010 | NM 0010 | DDI1 | DDI1 | DDI1 | DDI1, DNA-damag | DDI1, DNA | DDI1, DNA | 414301 | 414301 | 414301 | s53923 | s53924 | s53925 |
| 4967 | O8 | O9 | 1748 | 881,6 | 13671 | 554,2 | 7,8 | 0,857 | HSA034 C | sample | Hm Drebl | Hm Drebl | Hm Drebl | CPE002DV | CPE002EL | CPE002FP | NM 00574 | NM 0057 | NM 0057 | ELA3A | ELA3A | ELA3A | elastase 3A, panc | elastase 3 | elastase 3 | 10136 | 10136 | 10136 | s19727 | s19728 | s19729 |
| 10589 | J13 | J14 | 1520 | 917,2 | 11808 | 172,3 | 7,8 | 0,881 | HSA034 C | sample | Hm Drebl | Hm Drebl | Hm Drebl | CPE002EB | CPE002EO | CPE002FR | NM 02289 | NM 0228 | NM 0228 | ACTR8 | ACTR8 | ACTR8 | ARP8 actin-relate | ARP8 acti | ARP8 acti | 93973 | 93973 | 93973 | s41201 | s41202 | s41203 |
| 2732 | B5 | B6 | 1621 | 697,7 | 12571 | 503,7 | 7,8 | 0,918 | HSA034 C | sample | Hm Drebl | Hm Drebl | Hm Drebl | CPE002DP | CPE002EF | CPE002F5 | NM 00717 | NM 0071 | NM 0071 | TESK2 | TESK2 | TESK2 | testis-specific kina | testis-spe | testis-spe | 10420 | 10420 | 10420 | s20378 | s20379 | s20380 |
| 2738 | C10 | C11 | 1262 | 665,4 | 9868 | 478,8 | 7,8 | 0,918 | HSA034 C | sample | Hm Drebl | Hm Drebl | Hm Drebl | CPE002DP | CPE002EF | CPE002F5 | NM 03148 | NM 0314 | NM 0314 | RIOK1 | RIOK1 | RIOK1 | RIO kinase 1 (yeas | RIO kinase | RIO kinase | 83732 | 83732 | 83732 | s38154 | s38155 | s38156 |
| 2830 | F7 | F8 | 698 | 719,4 | 5475 | 531,4 | 7,8 | 0,918 | HSA034 C | sample | Hm Drebl | Hm Drebl | Hm Drebl | CPE002DP | CPE002EF | CPE002F5 | NM 00100 | NM 0010 | NM 0010 | FGFRL1 | FGFRL1 | FGFRL1 | fibroblast growth | fibroblast | fibroblast | 53834 | 53834 | 53834 | s28780 | s28781 | s28782 |
| 20269 | M20 | M21 | 1547 | 1142 | 12035 | 135,2 | 7,8 | 0,951 | HSA034 C | sample | Hm Gen E | Hm Gen E | Hm Gen E | CPE002JQ | CPE002O5 | CPE002KL | XM 37969 | XM 3796 | XM 3796 | LOC40157 | LOC40157 | LOC40157 | hypothetical prot | hypotheti | hypotheti | 401577 | 401577 | 401577 | s53657 | s53658 | s226570 |
| 20270 | M21 | M22 | 1361 | 1043 | 10670 | 139,5 | 7,8 | 0,951 | HSA034 C | sample | Hm Gen E | Hm Gen E | Hm Gen E | CPE002JQ | CPE002O5 | CPE002KL | XM 37338 | XM 3733 | XM 3733 | LOC39256 | LOC39256 | LOC39256 | similar to neurofi | similar to | similar to | 392563 | 392563 | 392563 | s53102 | s53103 | s196413 |
| 20299 | N4 | N5 | 708 | 1112 | 5539 | 107,9 | 7,8 | 0,951 | HSA034 C | sample | Hm Gen E | Hm Gen E | Hm Gen E | CPE002JQ | CPE002O5 | CPE002KL | NM 00100 | NM 0010 | NM 0010 | FAM116B | FAM116B | FAM116B | family with seque | family wit | family wit | 414918 | 414918 | 414918 | s53941 | s53942 | s53943 |
| 578 | I10 | I11 | 2114 | 1872 | 16450 | 319,8 | 7,8 | 1,139 | HSA034 C | sample | Hm Drebl | Hm Drebl | Hm Drebl | CPE002E0 | CPE002EZ | CPE002FK | NM 17815 | NM 1781 | NM 1781 | FUT8 | FUT8 | FUT8 | fucosyltransferas | fucosyltra | fucosyltra | 2530 | 2530 | 2530 | s5423 | s5424 | s5425 |
| 582 | I14 | I15 | 2088 | 1781 | 16254 | 338,3 | 7,8 | 1,139 | HSA034 C | sample | Hm Drebl | Hm Drebl | Hm Drebl | CPE002E0 | CPE002EZ | CPE002FK | NM 00104 | NM 0010 | NM 0010 | G6PD | G6PD | G6PD | glucose-6-phosph | glucose-6- | glucose-6- | 2539 | 2539 | 2539 | s5448 | s5447 | s5446 |
| 681 | M17 | M18 | 1919 | 1740 | 14940 | 325,7 | 7,8 | 1,139 | HSA034 C | sample | Hm Drebl | Hm Drebl | Hm Drebl | CPE002E0 | CPE002EZ | CPE002FK | NM 00107 | NM 0010 | NM 0010 | SLC29A1 | SLC29A1 | SLC29A1 | solute carrier fam | solute car | solute car | 2030 | 2030 | 2030 | s4693 | s4694 | s4692 |
| 702 | N14 | N15 | 2424 | 1696 | 18925 | 346,5 | 7,8 | 1,139 | HSA034 C | sample | Hm Drebl | Hm Drebl | Hm Drebl | CPE002E0 | CPE002EZ | CPE002FK | NM 00150 | NM 0015 | NM 0015 | GPX5 | GPX5 | GPX5 | glutathione pero | glutathior | glutathior | 2880 | 2880 | 2880 | s6114 | s6115 | s6113 |
| 7701 | A6 | A7 | 1396 | 652 | 10890 | 345,5 | 7,8 | 1,191 | HSA034 C | sample | Hm Drebl | Hm Drebl | Hm Drebl | CPE002E4 | CPE002EV | CPE002FG | NM 00641 | NM 0064 | NM 0064 | AGPAT1 | AGPAT1 | AGPAT1 | 1-acylglycerol-3-p | 1-acylglyc | 1-acylglyc | 10554 | 10554 | 10554 | s7 | s9 | s8 |
| 7716 | B2 | B3 | 1388 | 591,1 | 10875 | 430,1 | 7,8 | 1,191 | HSA034 C | sample | Hm Drebl | Hm Drebl | Hm Drebl | CPE002E4 | CPE002EV | CPE002FG | NM 00666 | NM 0066 | NM 0066 | HPSE | HPSE | HPSE | heparanase | heparana | heparana | 10855 | 10855 | 10855 | s21304 | s21305 | s21306 |
| 7749 | C6 | C7 | 1180 | 560,7 | 9200 | 365,3 | 7,8 | 1,191 | HSA034 C | sample | Hm Drebl | Hm Drebl | Hm Drebl | CPE002E4 | CPE002EV | CPE002FG | NM 00642 | NM 0064 | NM 0064 | SIVA1 | SIVA1 | SIVA1 | SIVA1, apoptosis- | SIVA1, apo | SIVA1, apo | 10572 | 10572 | 10572 | s20746 | s20747 | s20748 |
| 7783 | E15 | E16 | 882 | 559,3 | 6865 | 375,8 | 7,8 | 1,191 | HSA034 C | sample | Hm Drebl | Hm Drebl | Hm Drebl | CPE002E4 | CPE002EV | CPE002FG | NM 17687 | NM 1768 | NM 1768 | INADL | INADL | INADL | InaD-like (Drosop | InaD-like ( | InaD-like ( | 10207 | 10207 | 10207 | s19903 | s19901 | s19902 |
| 7802 | F10 | F11 | 1493 | 567 | 11718 | 434,8 | 7,8 | 1,191 | HSA034 C | sample | Hm Drebl | Hm Drebl | Hm Drebl | CPE002E4 | CPE002EV | CPE002FG | NM 00671 | NM 0067 | NM 0067 | RNPS1 | RNPS1 | RNPS1 | RNA binding prot | RNA bindi | RNA bindi | 10921 | 10921 | 10921 | s21476 | s21474 | s21475 |
| 7810 | F18 | F19 | 1623 | 580,4 | 12692 | 394,6 | 7,8 | 1,191 | HSA034 C | sample | Hm Drebl | Hm Drebl | Hm Drebl | CPE002E4 | CPE002EV | CPE002FG | NM 00678 | NM 0067 | NM 0067 | RALBP1 | RALBP1 | RALBP1 | ralA binding prote | ralA bindi | ralA bindi | 10928 | 10928 | 10928 | s21493 | s21494 | s21492 |
| 7822 | F7 | F8 | 1405 | 551,5 | 10933 | 348,7 | 7,8 | 1,191 | HSA034 C | sample | Hm Drebl | Hm Drebl | Hm Drebl | CPE002E4 | CPE002EV | CPE002FG | NM 00631 | NM 0063 | NM 0063 | CDIPT | CDIPT | CDIPT | CDP-diacylglycero | CDP-diacy | CDP-diacy | 10423 | 10423 | 10423 | s20387 | s20388 | s20389 |
| 8031 | O22 | O23 | 1566 | 534,4 | 12219 | 390,3 | 7,8 | 1,191 | HSA034 C | sample | Hm Drebl | Hm Drebl | Hm Drebl | CPE002E4 | CPE002EV | CPE002FG | NM 00666 | NM 0066 | NM 0066 | CCL27 | CCL27 | CCL27 | chemokine (C-C m | chemokin | chemokin | 10850 | 10850 | 10850 | s21301 | s21303 | s21302 |
| 4329 | E17 | E18 | 1253 | 663,8 | 9850 | 330 | 7,9 | 0,667 | HSA034 C | sample | Hm Drebl | Hm Drebl | Hm Drebl | CPE002DU | CPE002EK | CPE002FQ | NM 02303 | NM 0230 | NM 0230 | CACNA1A | CACNA1A | CACNA1A | calcium channel, | calcium ch | calcium ch | 773 | 773 | 773 | s2277 | s2278 | s2279 |
| 183 | H22 | H23 | 431 | 1730 | 3405 | 622,6 | 7,9 | 0,672 | HSA034 C | sample | Hm Drebl | Hm Drebl | Hm Drebl | CPE002DT | CPE002EJ | CPE002F9 | NM 00314 | NM 0031 | NM 0031 | SSRP1 | SSRP1 | SSRP1 | structure specific | structure | structure | 6749 | 6749 | 6749 | s13489 | s13490 | s13491 |
| 213 | I6 | I7 | 860 | 1614 | 6785 | 418,8 | 7,9 | 0,672 | HSA034 C | sample | Hm Drebl | Hm Drebl | Hm Drebl | CPE002DT | CPE002EJ | CPE002F9 | NM 00307 | NM 0030 | NM 0030 | SMARCE1 | SMARCE1 | SMARCE1 | SWI/SNF related, | SWI/SNF r | SWI/SNF r | 6605 | 6605 | 6605 | s13160 | s13161 | s13162 |
| 3943 | E15 | E16 | 518 | 778,8 | 4071 | 320,5 | 7,9 | 0,702 | HSA034 C | sample | Hm Drebl | Hm Drebl | Hm Drebl | CPE002DS | CPE002EI | CPE002F8 | NM 00054 | NM 0005 | NM 0005 | TAP2 | TAP2 | TAP2 | transporter 2, ATI | transporte | transporte | 6891 | 6891 | 6891 | s13783 | s13782 | s13781 |
| 15697 | O1 | O2 | 1442 | 818,5 | 11419 | 125,2 | 7,9 | 0,718 | HSA034 C | sample | Hm Gen E | Hm Gen E | Hm Gen E | CPE002JE | CPE002NT | CPE002K9 | NM 02245 | NM 0224 | NM 0224 | FBRS | FBRS | FBRS | fibrosin | fibrosin | fibrosin | 64319 | 64319 | 64319 | s34620 | s34621 | s34622 |
| 5433 | C17 | C18 | 1215 | 626,7 | 9608 | 569,8 | 7,9 | 0,752 | HSA034 C | sample | Hm Drebl | Hm Drebl | Hm Drebl | CPE002DX | CPE002F2 | CPE002FN | NM 00356 | NM 0035 | NM 0035 | PLA2G10 | PLA2G10 | PLA2G10 | phospholipase A2 | phosphol | phosphol | 8399 | 8399 | 8399 | s15944 | s15942 | s224923 |
| 3709 | K20 | K21 | 1154 | 1429 | 9081 | 334,5 | 7,9 | 0,76 | HSA034 C | sample | Hm Drebl | Hm Drebl | Hm Drebl | CPE002DR | CPE002EH | CPE002F7 | NM 00722 | NM 0072 | NM 0072 | GPR45 | GPR45 | GPR45 | G protein-couple | G protein | G protein | 11250 | 11250 | 11250 | s22208 | s22209 | s22210 |
| 8372 | M5 | M6 | 1603 | 652,6 | 12660 | 393,4 | 7,9 | 0,76 | HSA034 C | sample | Hm Drebl | Hm Drebl | Hm Drebl | CPE002E5 | CPE002EU | CPE002FF | NM 00721 | NM 0072 | NM 0072 | PRAF2 | PRAF2 | PRAF2 | PRA1 domain fam | PRA1 dom | PRA1 dom | 11230 | 11230 | 11230 | s22164 | s22165 | s22163 |
| 1824 | L9 | L10 | 1763 | 747,8 | 13948 | 344 | 7,9 | 0,827 | HSA034 C | sample | Hm Drebl | Hm Drebl | Hm Drebl | CPE002FW | CPE002G0 | CPE002G4 | NM 01840 | NM 0184 | NM 0184 | DCP1A | DCP1A | DCP1A | DCP1 decapping e | DCP1 deca | DCP1 deca | 55802 | 55802 | 55802 | s31547 | s31548 | s31549 |
| 21749 | K13 | K14 | 316 | 1098 | 2507 | 654 | 7,9 | 0,832 | HSA034 C | sample | Hm Gen E | Hm Gen E | Hm Gen E | CPE002JU | CPE002O9 | CPE002KP | XM 93909 | XM 9390 | XM 9390 | LOC65001 | LOC65001 | LOC65001 | similar to Nucleol | similar to | similar to | 650010 | 650010 | 650010 | s57031 | s57032 | s57033 |
| 21766 | K7 | K8 | 173 | 956,2 | 1361 | 634,2 | 7,9 | 0,832 | HSA034 C | sample | Hm Gen E | Hm Gen E | Hm Gen E | CPE002JU | CPE002O9 | CPE002KP | XM 93906 | XM 9390 | XM 9390 | LOC64997 | LOC64997 | LOC64997 | similar to stereoc | similar to | similar to | 649975 | 649975 | 649975 | s57029 | s197699 | s227335 |
| 21818 | N10 | N11 | 433 | 984,5 | 3416 | 393,3 | 7,9 | 0,832 | HSA034 C | sample | Hm Gen E | Hm Gen E | Hm Gen E | CPE002JU | CPE002O9 | CPE002KP | XM 94304 | XM 9430 | XM 9430 | LOC65390 | LOC65390 | LOC65390 | similar to hypoth | similar to | similar to | 653904 | 653904 | 653904 | s57804 | s57805 | s57806 |
| 4611 | A11 | A12 | 1694 | 898,8 | 13375 | 475 | 7,9 | 0,868 | HSA034 C | sample | Hm Drebl | Hm Drebl | Hm Drebl | CPE002DV | CPE002EL | CPE002FP | NM 00174 | NM 0017 | NM 0017 | CAPN2 | CAPN2 | CAPN2 | calpain 2, (m/II) la | calpain 2, | calpain 2, | 824 | 824 | 824 | s319 | s320 | s321 |
| 4763 | G19 | G20 | 1357 | 816,2 | 10691 | 364 | 7,9 | 0,868 | HSA034 C | sample | Hm Drebl | Hm Drebl | Hm Drebl | CPE002DV | CPE002EL | CPE002FP | NM 00122 | NM 0333 | NM 0333 | CASP4 | CASP4 | CASP4 | caspase 4, apopto | caspase 4 | caspase 4 | 837 | 837 | 837 | s224915 | s2414 | s2415 |
| 4795 | H4 | H5 | 1605 | 813,2 | 12633 | 417,5 | 7,9 | 0,868 | HSA034 C | sample | Hm Drebl | Hm Drebl | Hm Drebl | CPE002DV | CPE002EL | CPE002FP | NM 01567 | NM 0156 | NM 0156 | SENP3 | SENP3 | SENP3 | SUMO1/sentrin/S | SUMO1/se | SUMO1/se | 26168 | 26168 | 26168 | s25208 | s25209 | s25210 |
| 10644 | L2 | L3 | 1112 | 859,3 | 8787 | 201 | 7,9 | 0,893 | HSA034 C | sample | Hm Drebl | Hm Drebl | Hm Drebl | CPE002EB | CPE002EO | CPE002FR | NM 13345 | NM 1334 | NM 1334 | RAVER1 | RAVER1 | RAVER1 | ribonucleoprotei | ribonucle | ribonucle | 125950 | 125950 | 125950 | s42905 | s42907 | s42906 |
| 2821 | F20 | F21 | 1403 | 654,8 | 11026 | 494,2 | 7,9 | 0,929 | HSA034 C | sample | Hm Drebl | Hm Drebl | Hm Drebl | CPE002DP | CPE002EF | CPE002F5 | NM 14468 | NM 1446 | NM 1446 | HIPK4 | HIPK4 | HIPK4 | homeodomain in | homeodo | homeodo | 147746 | 147746 | 147746 | s45052 | s45053 | s45054 |
| 2829 | F6 | F7 | 1704 | 638,6 | 13431 | 513,6 | 7,9 | 0,929 | HSA034 C | sample | Hm Drebl | Hm Drebl | Hm Drebl | CPE002DP | CPE002EF | CPE002F5 | NM 15380 | NM 1538 | NM 1538 | TAF1L | TAF1L | TAF1L | TAF1-like RNA po | TAF1-like | TAF1-like | 138474 | 138474 | 138474 | s44115 | s44116 | s44117 |
| 2865 | H17 | H18 | 1640 | 606,6 | 13014 | 459,8 | 7,9 | 0,929 | HSA034 C | sample | Hm Drebl | Hm Drebl | Hm Drebl | CPE002DP | CPE002EF | CPE002F5 | NM 01502 | NM 0150 | NM 0150 | TNIK | TNIK | TNIK | TRAF2 and NCK in | TRAF2 anc | TRAF2 anc | 23043 | 23043 | 23043 | s22905 | s22906 | s22907 |
| 2874 | H3 | H4 | 1142 | 724 | 9000 | 485,5 | 7,9 | 0,929 | HSA034 C | sample | Hm Drebl | Hm Drebl | Hm Drebl | CPE002DP | CPE002EF | CPE002F5 | NM 00728 | NM 0072 | NM 0072 | TWF2 | TWF2 | TWF2 | twinfilin, actin-bi | twinfilin, a | twinfilin, a | 11344 | 11344 | 11344 | s22382 | s22383 | s22384 |
| 2975 | L8 | L9 | 722 | 580,9 | 5686 | 373,6 | 7,9 | 0,929 | HSA034 C | sample | Hm Drebl | Hm Drebl | Hm Drebl | CPE002DP | CPE002EF | CPE002F5 | NM 17813 | NM 1781 | NM 1781 | TXNDC6 | TXNDC6 | TXNDC6 | thioredoxin doma | thioredox | thioredox | 347736 | 347736 | 347736 | s51301 | s51302 | s51303 |
| 20074 | E18 | E19 | 1095 | 1199 | 8635 | 143,7 | 7,9 | 0,963 | HSA034 C | sample | Hm Gen E | Hm Gen E | Hm Gen E | CPE002JQ | CPE002O5 | CPE002KL | NM 20749 | NM 2074 | NM 2074 | DKFZp686 | DKFZp686 | DKFZp686 | hypothetical prot | hypotheti | hypotheti | 401232 | 401232 | 401232 | s53518 | s53519 | s53520 |
| 20111 | F8 | F9 | 962 | 1057 | 7588 | 131,3 | 7,9 | 0,963 | HSA034 C | sample | Hm Gen E | Hm Gen E | Hm Gen E | CPE002JQ | CPE002O5 | CPE002KL | XM 38000 | XM 3800 | XM 3800 | LOC40266 | LOC40266 | LOC40266 | hCG1651476 | hCG16514 | hCG16514 | 402665 | 402665 | 402665 | s53783 | s53784 | s53785 |
| 20192 | J16 | J17 | 971 | 1160 | 7667 | 158,4 | 7,9 | 0,963 | HSA034 C | sample | Hm Gen E | Hm Gen E | Hm Gen E | CPE002JQ | CPE002O5 | CPE002KL | NM 20711 | NM 2071 | NM 2071 | GTF2H5 | GTF2H5 | GTF2H5 | general transcript | general tr | general tr | 404672 | 404672 | 404672 | s53874 | s53875 | s53876 |
| 20215 | K15 | K16 | 1085 | 1116 | 8580 | 132,5 | 7,9 | 0,963 | HSA034 C | sample | Hm Gen E | Hm Gen E | Hm Gen E | CPE002JQ | CPE002O5 | CPE002KL | XM 37327 | XM 3732 | XM 3732 | LOC39228 | LOC39228 | LOC39228 | similar to microtu | similar to | similar to | 392288 | 392288 | 392288 | s53053 | s53054 | s226504 |
| 20235 | L11 | L12 | 795 | 1097 | 6266 | 145,3 | 7,9 | 0,963 | HSA034 C | sample | Hm Gen E | Hm Gen E | Hm Gen E | CPE002JQ | CPE002O5 | CPE002KL | XM 00112 | XM 0011 | XM 0011 | FLJ37638 | FLJ37638 | FLJ37638 | hypothetical gene | hypotheti | hypotheti | 400660 | 400660 | 400660 | s53334 | s53335 | s226543 |
| 20259 | M11 | M12 | 1259 | 1145 | 9905 | 126,3 | 7,9 | 0,963 | HSA034 C | sample | Hm Gen E | Hm Gen E | Hm Gen E | CPE002JQ | CPE002O5 | CPE002KL | NM 00108 | NM 0010 | NM 0010 | GLOD5 | GLOD5 | GLOD5 | glyoxalase domai | glyoxalase | glyoxalase | 392465 | 392465 | 392465 | s53087 | s53088 | s53089 |
| 20294 | N21 | N22 | 1211 | 1126 | 9538 | 124,4 | 7,9 | 0,963 | HSA034 C | sample | Hm Gen E | Hm Gen E | Hm Gen E | CPE002JQ | CPE002O5 | CPE002KL | XM 93095 | XM 9309 | XM 9309 | LOC40083 | LOC40083 | LOC40083 | hypothetical LOC | hypotheti | hypotheti | 400831 | 400831 | 400831 | s53387 | s53388 | s53389 |
| 475 | D4 | D5 | 2513 | 1891 | 19927 | 353,3 | 7,9 | 1,153 | HSA034 C | sample | Hm Drebl | Hm Drebl | Hm Drebl | CPE002E0 | CPE002EZ | CPE002FK | NM 00205 | NM 0225 | NM 0225 | GH2 | GH2 | GH2 | growth hormone | growth hc | growth hc | 2689 | 2689 | 2689 | s223712 | s5737 | s5735 |
| 507 | F11 | F12 | 1541 | 1619 | 12135 | 267,2 | 7,9 | 1,153 | HSA034 C | sample | Hm Drebl | Hm Drebl | Hm Drebl | CPE002E0 | CPE002EZ | CPE002FK | NM 00199 | NM 0019 | NM 0019 | EZH1 | EZH1 | EZH1 | enhancer of zeste | enhancer | enhancer | 2145 | 2145 | 2145 | s4914 | s4915 | s4913 |
| 543 | G22 | G23 | 2067 | 1761 | 16250 | 317,3 | 7,9 | 1,153 | HSA034 C | sample | Hm Drebl | Hm Drebl | Hm Drebl | CPE002E0 | CPE002EZ | CPE002FK | NM 00014 | NM 0001 | NM 0001 | FUT3 | FUT3 | FUT3 | fucosyltransferas | fucosyltra | fucosyltra | 2525 | 2525 | 2525 | s5410 | s5411 | s5412 |
| 607 | J15 | J16 | 2349 | 1793 | 18627 | 294,8 | 7,9 | 1,153 | HSA034 C | sample | Hm Drebl | Hm Drebl | Hm Drebl | CPE002E0 | CPE002EZ | CPE002FK | NM 00199 | NM 0019 | NM 0019 | FBN2 | FBN2 | FBN2 | fibrillin 2 (congen | fibrillin 2 | fibrillin 2 | 2201 | 2201 | 2201 | s5047 | s5048 | s5049 |
| 657 | L17 | L18 | 1907 | 1625 | 15108 | 260,3 | 7,9 | 1,153 | HSA034 C | sample | Hm Drebl | Hm Drebl | Hm Drebl | CPE002E0 | CPE002EZ | CPE002FK | NM 00410 | NM 0041 | NM 0041 | FCGRT | FCGRT | FCGRT | Fc fragment of IgG | Fc fragme | Fc fragme | 2217 | 2217 | 2217 | s5082 | s5084 | s5083 |
| 686 | M21 | M22 | 2160 | 1733 | 16980 | 346,7 | 7,9 | 1,153 | HSA034 C | sample | Hm Drebl | Hm Drebl | Hm Drebl | CPE002E0 | CPE002EZ | CPE002FK | NM 00143 | NM 0014 | NM 0014 | EPAS1 | EPAS1 | EPAS1 | endothelial PAS d | endotheli | endotheli | 2034 | 2034 | 2034 | s4699 | s4698 | s4700 |
| 707 | N19 | N20 | 2900 | 1922 | 23002 | 337 | 7,9 | 1,153 | HSA034 C | sample | Hm Drebl | Hm Drebl | Hm Drebl | CPE002E0 | CPE002EZ | CPE002FK | NM 00446 | NM 0044 | NM 0044 | FGF10 | FGF10 | FGF10 | fibroblast growth | fibroblast | fibroblast | 2255 | 2255 | 2255 | s5151 | s5149 | s5150 |
| 726 | O14 | O15 | 2648 | 1868 | 20998 | 350,4 | 7,9 | 1,153 | HSA034 C | sample | Hm Drebl | Hm Drebl | Hm Drebl | CPE002E0 | CPE002EZ | CPE002FK | NM 00525 | NM 0052 | NM 0052 | GCHFR | GCHFR | GCHFR | GTP cyclohydrola | GTP cyclol | GTP cyclol | 2644 | 2644 | 2644 | s5647 | s223697 | s223698 |
| 745 | P1 | P2 | 2123 | 1708 | 16743 | 412,9 | 7,9 | 1,153 | HSA034 C | sample | Hm Drebl | Hm Drebl | Hm Drebl | CPE002E0 | CPE002EZ | CPE002FK | NM 02187 | NM 0218 | NM 0218 | FGG | FGG | FGG | fibrinogen gamma | fibrinoger | fibrinoger | 2266 | 2266 | 2266 | s5181 | s5179 | s5180 |
| 7730 | C10 | C11 | 1539 | 574,1 | 12206 | 442,7 | 7,9 | 1,206 | HSA034 C | sample | Hm Drebl | Hm Drebl | Hm Drebl | CPE002E4 | CPE002EV | CPE002FG | NM 00644 | NM 0064 | NM 0064 | TXNRD2 | TXNRD2 | TXNRD2 | thioredoxin redu | thioredox | thioredox | 10587 | 10587 | 10587 | s20781 | s20782 | s223135 |
| 7734 | C14 | C15 | 656 | 607,7 | 5191 | 431 | 7,9 | 1,206 | HSA034 C | sample | Hm Drebl | Hm Drebl | Hm Drebl | CPE002E4 | CPE002EV | CPE002FG | NM 00104 | NM 0010 | NM 0010 | SMC2 | SMC2 | SMC2 | structural mainte | structural | structural | 10592 | 10592 | 10592 | s20793 | s20794 | s20795 |
| 7838 | G21 | G22 | 1617 | 568,1 | 12779 | 416 | 7,9 | 1,206 | HSA034 C | sample | Hm Drebl | Hm Drebl | Hm Drebl | CPE002E4 | CPE002EV | CPE002FG | NM 15381 | NM 1538 | NM 1538 | RASGRP2 | RASGRP2 | RASGRP2 | RAS guanyl releas | RAS guany | RAS guany | 10235 | 10235 | 10235 | s19978 | s19977 | s19976 |
| 7876 | I12 | I13 | 996 | 543,5 | 7902 | 336 | 7,9 | 1,206 | HSA034 C | sample | Hm Drebl | Hm Drebl | Hm Drebl | CPE002E4 | CPE002EV | CPE002FG | NM 00656 | NM 0065 | NM 0065 | CUGBP2 | CUGBP2 | CUGBP2 | CUG triplet repea | CUG triple | CUG triple | 10659 | 10659 | 10659 | s20957 | s20959 | s20958 |
| 7934 | K21 | K22 | 1117 | 581,9 | 8776 | 352,3 | 7,9 | 1,206 | HSA034 C | sample | Hm Drebl | Hm Drebl | Hm Drebl | CPE002E4 | CPE002EV | CPE002FG | NM 00587 | NM 0058 | NM 0058 | SAP18 | SAP18 | SAP18 | Sin3A-associated | Sin3A-ass | Sin3A-ass | 10284 | 10284 | 10284 | s20098 | s20099 | s20100 |
| 7949 | L13 | L14 | 924 | 597,4 | 7326 | 376,9 | 7,9 | 1,206 | HSA034 C | sample | Hm Drebl | Hm Drebl | Hm Drebl | CPE002E4 | CPE002EV | CPE002FG | NM 00637 | NM 0063 | NM 0063 | VAT1 | VAT1 | VAT1 | vesicle amine trar | vesicle am | vesicle am | 10493 | 10493 | 10493 | s20567 | s20566 | s20565 |
| 7970 | M10 | M11 | 1067 | 592,7 | 8478 | 334,7 | 7,9 | 1,206 | HSA034 C | sample | Hm Drebl | Hm Drebl | Hm Drebl | CPE002E4 | CPE002EV | CPE002FG | NM 20246 | NM 2024 | NM 2024 | GIPC1 | GIPC1 | GIPC1 | GIPC PDZ domain | GIPC PDZ | GIPC PDZ | 10755 | 10755 | 10755 | s21130 | s21129 | s21131 |
| 8046 | P14 | P15 | 1686 | 623 | 13354 | 412,6 | 7,9 | 1,206 | HSA034 C | sample | Hm Drebl | Hm Drebl | Hm Drebl | CPE002E4 | CPE002EV | CPE002FG | NM 00686 | NM 0068 | NM 0068 | RAB31 | RAB31 | RAB31 | RAB31, member R | RAB31, m | RAB31, m | 11031 | 11031 | 11031 | s21731 | s21733 | s21732 |
| 19530 | N3 | N4 | 1169 | 1146 | 9318 | 190,1 | 8 | 0,567 | HSA034 C | sample | Hm Gen E | Hm Gen E | Hm Gen E | CPE002JO | CPE002O3 | CPE002KJ | XM 29202 | XM 2920 | XM 2920 | LOC34134 | LOC34134 | LOC34134 | hypothetical LOC | hypotheti | hypotheti | 341346 | 341346 | 341346 | s50826 | s50827 | s50828 |
| 2586 | L3 | L4 | 1012 | 791,7 | 8109 | 300,8 | 8 | 0,63 | HSA034 C | sample | Hm Drebl | Hm Drebl | Hm Drebl | CPE002DC | CPE002EE | CPE002F4 | NM 18264 | NM 1826 | NM 1826 | EPHA3 | EPHA3 | EPHA3 | EPH receptor A3 | EPH recep | EPH recep | 2042 | 2042 | 2042 | s4722 | s4723 | s4724 |
| 2602 | M18 | M19 | 706 | 748,2 | 5680 | 355,1 | 8 | 0,63 | HSA034 C | sample | Hm Drebl | Hm Drebl | Hm Drebl | CPE002DC | CPE002EE | CPE002F4 | NM 00621 | NM 0062 | NM 0062 | PIK3CA | PIK3CA | PIK3CA | phosphoinositide | phosphoi | phosphoi | 5290 | 5290 | 5290 | s10520 | s10521 | s10522 |
| 16818 | M3 | M4 | 783 | 1024 | 6251 | 147,1 | 8 | 0,632 | HSA034 C | sample | Hm Gen E | Hm Gen E | Hm Gen E | CPE002JH | CPE002NW | CPE002KC | NM 03292 | NM 0329 | NM 0329 | TMEM128 | TMEM128 | TMEM128 | transmembrane p | transmem | transmem | 85013 | 85013 | 85013 | s39833 | s39834 | s228409 |
| 5790 | B14 | B15 | 2000 | 858,5 | 15910 | 398 | 8 | 0,731 | HSA034 C | sample | Hm Drebl | Hm Drebl | Hm Drebl | CPE002DY | CPE002F1 | CPE002FN | NM 00119 | NM 0011 | NM 0011 | TNFRSF17 | TNFRSF17 | TNFRSF17 | tumor necrosis fa | tumor nec | tumor nec | 608 | 608 | 608 | s1938 | s1939 | s1940 |
| 1294 | F7 | F8 | 1751 | 784,1 | 14079 | 405,9 | 8 | 0,734 | HSA034 C | sample | Hm Drebl | Hm Drebl | Hm Drebl | CPE002FV | CPE002FZ | CPE002G3 | NM 00366 | NM 0036 | NM 0036 | AP3B1 | AP3B1 | AP3B1 | adaptor-related p | adaptor-r | adaptor-r | 8546 | 8546 | 8546 | s16266 | s16267 | s16268 |
| 10345 | P1 | P2 | 696 | 710,7 | 5538 | 132,5 | 8 | 0,769 | HSA034 C | sample | Hm Drebl | Hm Drebl | Hm Drebl | CPE002EA | CPE002EP | CPE002FA | NM 03096 | NM 0309 | NM 0309 | SPRY4 | SPRY4 | SPRY4 | sprouty homolog | sprouty h | sprouty h | 81848 | 81848 | 81848 | s37826 | s37825 | s37824 |
| 1880 | O16 | O17 | 2462 | 784,8 | 19785 | 385,1 | 8 | 0,838 | HSA034 C | sample | Hm Drebl | Hm Drebl | Hm Drebl | CPE002FW | CPE002G0 | CPE002G4 | NM 02432 | NM 0243 | NM 0243 | EFHD2 | EFHD2 | EFHD2 | EF-hand domain f | EF-hand d | EF-hand d | 79180 | 79180 | 79180 | s35692 | s224815 | s224814 |
| 21649 | G1 | G2 | 299 | 835,2 | 2381 | 415,6 | 8 | 0,842 | HSA034 C | sample | Hm Gen E | Hm Gen E | Hm Gen E | CPE002JU | CPE002O9 | CPE002KP | XM 93850 | XM 9385 | XM 9385 | LOC64942 | LOC64942 | LOC64942 | similar to double | similar to | similar to | 649425 | 649425 | 649425 | s56966 | s197660 | s197661 |
| 4886 | L21 | L22 | 2286 | 814,4 | 18337 | 407,5 | 8 | 0,879 | HSA034 C | sample | Hm Drebl | Hm Drebl | Hm Drebl | CPE002DV | CPE002EL | CPE002FP | NM 00030 | NM 0003 | NM 0003 | CTSA | CTSA | CTSA | cathepsin A | cathepsin | cathepsin | 5476 | 5476 | 5476 | s10904 | s10905 | s224315 |
| 10507 | F4 | F5 | 1126 | 873,1 | 9031 | 180,9 | 8 | 0,904 | HSA034 C | sample | Hm Drebl | Hm Drebl | Hm Drebl | CPE002EB | CPE002EO | CPE002FR | NM 15232 | NM 1523 | NM 1523 | ADSSL1 | ADSSL1 | ADSSL1 | adenylosuccinate | adenylosu | adenylosu | 122622 | 122622 | 122622 | s42551 | s42552 | s42553 |
| 2719 | B15 | B16 | 1394 | 685,4 | 11108 | 557,3 | 8 | 0,941 | HSA034 C | sample | Hm Drebl | Hm Drebl | Hm Drebl | CPE002DP | CPE002EF | CPE002F5 | NM 17221 | NM 0065 | NM 1722 | CAMKK2 | CAMKK2 | CAMKK2 | calcium/calmodul | calcium/ca | calcium/ca | 10645 | 10645 | 10645 | s20925 | s223143 | s20927 |
| 2836 | G12 | G13 | 1316 | 638 | 10479 | 449,2 | 8 | 0,941 | HSA034 C | sample | Hm Drebl | Hm Drebl | Hm Drebl | CPE002DP | CPE002EF | CPE002F5 | NM 02034 | NM 0203 | NM 0203 | PAK7 | PAK7 | PAK7 | p21(CDKN1A)-acti | p21(CDKN | p21(CDKN | 57144 | 57144 | 57144 | s32772 | s32773 | s32774 |
| 20072 | E16 | E17 | 990 | 1000 | 7953 | 106,1 | 8 | 0,976 | HSA034 C | sample | Hm Gen E | Hm Gen E | Hm Gen E | CPE002JQ | CPE002O5 | CPE002KL | XM 37937 | XM 3793 | XM 3793 | LOC40122 | LOC40122 | LOC40122 | hypothetical gene | hypotheti | hypotheti | 401220 | 401220 | 401220 | s53515 | s53516 | s53517 |
| 20110 | F7 | F8 | 1060 | 1068 | 8447 | 128,8 | 8 | 0,976 | HSA034 C | sample | Hm Gen E | Hm Gen E | Hm Gen E | CPE002JQ | CPE002O5 | CPE002KL | XM 94462 | XM 9446 | XM 9446 | hCG 2032 | hCG 2032 | hCG 2032 | hCG2032978 | hCG2032 | hCG2032 | 399978 | 399978 | 399978 | s53222 | s53223 | s53224 |
| 20118 | G14 | G15 | 1688 | 1106 | 13566 | 125,9 | 8 | 0,976 | HSA034 C | sample | Hm Gen E | Hm Gen E | Hm Gen E | CPE002JQ | CPE002O5 | CPE002KL | NM 00100 | NM 0010 | NM 0010 | FLJ42177 | FLJ42177 | FLJ42177 | FLJ42177 protein | FLJ42177 p | FLJ42177 p | 401271 | 401271 | 401271 | s53547 | s53548 | s53549 |
| 20161 | I1 | I2 | 983 | 1083 | 7881 | 106,4 | 8 | 0,976 | HSA034 C | sample | Hm Gen E | Hm Gen E | Hm Gen E | CPE002JQ | CPE002O5 | CPE002KL | XM 00112 | XM 0011 | XM 0011 | LOC39150 | LOC39150 | LOC39150 | similar to Phosph | similar to | similar to | 391508 | 391508 | 391508 | s52991 | s52992 | s52993 |
| 20167 | I15 | I16 | 1139 | 1018 | 9125 | 110,9 | 8 | 0,976 | HSA034 C | sample | Hm Gen E | Hm Gen E | Hm Gen E | CPE002JQ | CPE002O5 | CPE002KL | XM 37305 | XM 3730 | XM 3730 | LOC39174 | LOC39174 | LOC39174 | similar to hCG198 | similar to | similar to | 391742 | 391742 | 391742 | s53015 | s53016 | s53017 |
| 20188 | J12 | J13 | 1203 | 1193 | 9648 | 158,7 | 8 | 0,976 | HSA034 C | sample | Hm Gen E | Hm Gen E | Hm Gen E | CPE002JQ | CPE002O5 | CPE002KL | NM 20700 | NM 2070 | NM 2070 | FAM45A | FAM45A | FAM45A | family with seque | family wit | family wit | 404636 | 404636 | 404636 | s53868 | s53869 | s53870 |
| 20246 | L21 | L22 | 1452 | 1108 | 11634 | 141,5 | 8 | 0,976 | HSA034 C | sample | Hm Gen E | Hm Gen E | Hm Gen E | CPE002JQ | CPE002O5 | CPE002KL | NM 00101 | NM 0010 | NM 0010 | C1orf130 | C1orf130 | C1orf130 | chromosome 1 op | chromoso | chromoso | 400746 | 400746 | 400746 | s53357 | s53358 | s226546 |
| 536 | G16 | G17 | 2287 | 1614 | 18184 | 272,1 | 8 | 1,168 | HSA034 C | sample | Hm Drebl | Hm Drebl | Hm Drebl | CPE002E0 | CPE002EZ | CPE002FK | NM 00496 | NM 0049 | NM 0049 | FUS | FUS | FUS | fusion (involved i | fusion (inv | fusion (inv | 2521 | 2521 | 2521 | s5401 | s5402 | s5403 |
| 550 | G7 | G8 | 2327 | 1826 | 18663 | 291,7 | 8 | 1,168 | HSA034 C | sample | Hm Drebl | Hm Drebl | Hm Drebl | CPE002E0 | CPE002EZ | CPE002FK | NM 00196 | NM 0019 | NM 0019 | EGR4 | EGR4 | EGR4 | early growth respo | early grow | early grow | 1961 | 1961 | 1961 | s4546 | s4547 | s4548 |
| 551 | G8 | G9 | 2260 | 1828 | 18128 | 288,7 | 8 | 1,168 | HSA034 C | sample | Hm Drebl | Hm Drebl | Hm Drebl | CPE002E0 | CPE002EZ | CPE002FK | NM 00101 | NM 0010 | NM 0010 | FPGS | FPGS | FPGS | folylpolyglutamat | folylpolyg | folylpolyg | 2356 | 2356 | 2356 | s5348 | s5349 | s5350 |
| 560 | H16 | H17 | 2454 | 1785 | 19539 | 313,9 | 8 | 1,168 | HSA034 C | sample | Hm Drebl | Hm Drebl | Hm Drebl | CPE002E0 | CPE002EZ | CPE002FK | NM 00017 | NM 0001 | NM 0001 | GNAT1 | GNAT1 | GNAT1 | guanine nucleotic | guanine n | guanine n | 2779 | 2779 | 2779 | s5893 | s5894 | s5892 |
| 765 | P6 | P7 | 2321 | 1737 | 18562 | 422,6 | 8 | 1,168 | HSA034 C | sample | Hm Drebl | Hm Drebl | Hm Drebl | CPE002E0 | CPE002EZ | CPE002FK | NM 00449 | NM 0044 | NM 0044 | GRLF1 | GRLF1 | GRLF1 | glucocorticoid rec | glucocorti | glucocorti | 2909 | 2909 | 2909 | s6190 | s6188 | s6189 |
| 7708 | B12 | B13 | 1760 | 610,8 | 14061 | 409,7 | 8 | 1,221 | HSA034 C | sample | Hm Drebl | Hm Drebl | Hm Drebl | CPE002E4 | CPE002EV | CPE002FG | NM 02204 | NM 0220 | NM 0220 | SLC26A1 | SLC26A1 | SLC26A1 | solute carrier fam | solute car | solute car | 10861 | 10861 | 10861 | s21320 | s21319 | s223158 |
| 7722 | B3 | B4 | 1435 | 591,3 | 11416 | 393,8 | 8 | 1,221 | HSA034 C | sample | Hm Drebl | Hm Drebl | Hm Drebl | CPE002E4 | CPE002EV | CPE002FG | NM 20126 | NM 2012 | NM 2012 | WARS2 | WARS2 | WARS2 | tryptophanyl tRN | tryptopha | tryptopha | 10352 | 10352 | 10352 | s20253 | s20252 | s20251 |
| 7729 | C1 | C2 | 560 | 504,8 | 4474 | 344,8 | 8 | 1,221 | HSA034 C | sample | Hm Drebl | Hm Drebl | Hm Drebl | CPE002E4 | CPE002EV | CPE002FG | NM 00576 | NM 0057 | NM 0057 | MBOAT5 | MBOAT5 | MBOAT5 | membrane bound | membran | membran | 10162 | 10162 | 10162 | s19799 | s19801 | s19800 |
| 7762 | D18 | D19 | 1424 | 572 | 11455 | 330,3 | 8 | 1,221 | HSA034 C | sample | Hm Drebl | Hm Drebl | Hm Drebl | CPE002E4 | CPE002EV | CPE002FG | NM 00670 | NM 0067 | NM 0067 | PNPLA6 | PNPLA6 | PNPLA6 | patatin-like phos | patatin-lik | patatin-lik | 10908 | 10908 | 10908 | s21442 | s21441 | s21443 |
| 7789 | E20 | E21 | 1318 | 626,3 | 10534 | 370,5 | 8 | 1,221 | HSA034 C | sample | Hm Drebl | Hm Drebl | Hm Drebl | CPE002E4 | CPE002EV | CPE002FG | NM 00646 | NM 0064 | NM 0064 | POLR3G | POLR3G | POLR3G | polymerase (RNA | polymeras | polymeras | 10622 | 10622 | 10622 | s20865 | s20864 | s20863 |
| 7890 | I3 | I4 | 761 | 551,5 | 6105 | 304 | 8 | 1,221 | HSA034 C | sample | Hm Drebl | Hm Drebl | Hm Drebl | CPE002E4 | CPE002EV | CPE002FG | NM 00582 | NM 0058 | NM 0058 | AP3S2 | AP3S2 | AP3S2 | adaptor-related p | adaptor-r | adaptor-r | 10239 | 10239 | 10239 | s19988 | s19989 | s19990 |
| 7987 | M4 | M5 | 579 | 555,2 | 4620 | 247,7 | 8 | 1,221 | HSA034 C | sample | Hm Drebl | Hm Drebl | Hm Drebl | CPE002E4 | CPE002EV | CPE002FG | NM 00661 | NM 0066 | NM 0066 | KLRA1 | KLRA1 | KLRA1 | killer cell lectin-lik | killer cell l | killer cell l | 10748 | 10748 | 10748 | s21122 | s21121 | s21120 |
| 8037 | O6 | O7 | 1364 | 583,5 | 10853 | 390,3 | 8 | 1,221 | HSA034 C | sample | Hm Drebl | Hm Drebl | Hm Drebl | CPE002E4 | CPE002EV | CPE002FG | NM 00664 | NM 0066 | NM 0066 | SEPT9 | SEPT9 | SEPT9 | septin 9 | septin 9 | septin 9 | 10801 | 10801 | 10801 | s21222 | s21223 | s21224 |
| 8044 | P12 | P13 | 1281 | 701,1 | 10186 | 499,2 | 8 | 1,221 | HSA034 C | sample | Hm Drebl | Hm Drebl | Hm Drebl | CPE002E4 | CPE002EV | CPE002FG | NM 00686 | NM 0068 | NM 0068 | RBPMS | RBPMS | RBPMS | RNA binding prot | RNA bindi | RNA bindi | 11030 | 11030 | 11030 | s21730 | s21728 | s21729 |
| 8059 | P4 | P5 | 1651 | 561,1 | 13261 | 428,8 | 8 | 1,221 | HSA034 C | sample | Hm Drebl | Hm Drebl | Hm Drebl | CPE002E4 | CPE002EV | CPE002FG | NM 00686 | NM 0068 | NM 0068 | LILRA1 | LILRA1 | LILRA1 | leukocyte immun | leukocyte | leukocyte | 11024 | 11024 | 11024 | s21718 | s21717 | s21716 |
| 11310 | H14 | H15 | 1607 | 853,1 | 12979 | 122,5 | 8,1 | 0,587 | HSA034 C | sample | Hm Drebl | Hm Drebl | Hm Drebl | CPE002ED | CPE002F3 | CPE002FT | | | | | | | | | | | | | | | |
| 12344 | C16 | C17 | 1376 | 1002 | 11129 | 151,3 | 8,1 | 0,609 | HSA034 C | sample | Hm Gen E | Hm Gen E | Hm Gen E | CPE002J5 | CPE002NK | CPE002K1 | NM 00353 | NM 0035 | NM 0035 | HIST1H3I | HIST1H3I | HIST1H3I | histone cluster 1, | histone cl | histone cl | 8354 | 8354 | 8354 | s15876 | s194910 | s194911 |
| 17994 | N3 | N4 | 1573 | 994,1 | 12673 | 110,6 | 8,1 | 0,64 | HSA034 C | sample | Hm Gen E | Hm Gen E | Hm Gen E | CPE002JK | CPE002NZ | CPE002KF | NM 14527 | NM 1452 | NM 1452 | TMEM99 | TMEM99 | TMEM99 | transmembrane p | transmem | transmem | 147184 | 147184 | 147184 | s44977 | s44978 | s44979 |
| 9163 | N4 | N5 | 1978 | 1798 | 16017 | 111,4 | 8,1 | 0,651 | HSA034 C | sample | Hm Drebl | Hm Drebl | Hm Drebl | CPE002E7 | CPE002ES | CPE002FD | NM 01752 | NM 0175 | NM 0175 | FEV | FEV | FEV | FEV (ETS oncogen | FEV (ETS c | FEV (ETS c | 54738 | 54738 | 54738 | s29365 | s29366 | s29367 |
| 206 | I21 | I22 | 799 | 1580 | 6480 | 488,1 | 8,1 | 0,689 | HSA034 C | sample | Hm Drebl | Hm Drebl | Hm Drebl | CPE002DT | CPE002EJ | CPE002F9 | NM 00324 | NM 0032 | NM 0032 | LEFTY2 | LEFTY2 | LEFTY2 | left-right determi | left-right c | left-right c | 7044 | 7044 | 7044 | s14065 | s14066 | s14067 |
| 210 | I3 | I4 | 574 | 1439 | 4667 | 412,8 | 8,1 | 0,689 | HSA034 C | sample | Hm Drebl | Hm Drebl | Hm Drebl | CPE002DT | CPE002EJ | CPE002F9 | NM 05295 | NM 0529 | NM 0529 | DNTTIP1 | DNTTIP1 | DNTTIP1 | deoxynucleotidyl | deoxynuc | deoxynuc | 116092 | 116092 | 116092 | s41922 | s41923 | s223242 |
| 216 | I9 | I10 | 463 | 1683 | 3762 | 446,4 | 8,1 | 0,689 | HSA034 C | sample | Hm Drebl | Hm Drebl | Hm Drebl | CPE002DT | CPE002EJ | CPE002F9 | NM 00506 | NM 0050 | NM 0050 | SCD | SCD | SCD | stearoyl-CoA desa | stearoyl-C | stearoyl-C | 6319 | 6319 | 6319 | s12503 | s12504 | s12505 |
| 266 | L10 | L11 | 630 | 1368 | 5088 | 496,3 | 8,1 | 0,689 | HSA034 C | sample | Hm Drebl | Hm Drebl | Hm Drebl | CPE002DT | CPE002EJ | CPE002F9 | NM 00315 | NM 0031 | NM 0031 | STC1 | STC1 | STC1 | stanniocalcin 1 | stannioca | stannioca | 6781 | 6781 | 6781 | s13549 | s13550 | s13551 |
| 284 | L5 | L6 | 614 | 1487 | 5000 | 389,1 | 8,1 | 0,689 | HSA034 C | sample | Hm Drebl | Hm Drebl | Hm Drebl | CPE002DT | CPE002EJ | CPE002F9 | NM 00651 | NM 0065 | NM 0065 | SLC2A1 | SLC2A1 | SLC2A1 | solute carrier fam | solute car | solute car | 6513 | 6513 | 6513 | s224640 | s12926 | s12927 |
| 295 | M15 | M16 | 328 | 1517 | 2672 | 386,7 | 8,1 | 0,689 | HSA034 C | sample | Hm Drebl | Hm Drebl | Hm Drebl | CPE002DT | CPE002EJ | CPE002F9 | NM 00627 | NM 0062 | NM 0062 | CCL7 | CCL7 | CCL7 | chemokine (C-C m | chemokin | chemokin | 6354 | 6354 | 6354 | s12578 | s12579 | s224578 |
| 348 | O2 | O3 | 137 | 1174 | 1115 | 557,5 | 8,1 | 0,689 | HSA034 C | sample | Hm Drebl | Hm Drebl | Hm Drebl | CPE002DT | CPE002EJ | CPE002F9 | NM 00563 | NM 0056 | NM 0056 | SOS1 | SOS1 | SOS1 | son of sevenless | son of sev | son of sev | 6654 | 6654 | 6654 | s13285 | s13286 | s13287 |
| 1358 | I21 | I22 | 1472 | 749,5 | 11858 | 225,1 | 8,1 | 0,743 | HSA034 C | sample | Hm Drebl | Hm Drebl | Hm Drebl | CPE002FV | CPE002FZ | CPE002G3 | NM 00343 | NM 0034 | NM 0034 | ZNF134 | ZNF134 | ZNF134 | zinc finger protei | zinc finger | zinc finger | 7693 | 7693 | 7693 | s15168 | s15169 | s15170 |
| 13602 | G3 | G4 | 1200 | 1060 | 9770 | 162,7 | 8,1 | 0,761 | HSA034 C | sample | Hm Gen E | Hm Gen E | Hm Gen E | CPE002JC | CPE002NC | CPE002K4 | NM 01531 | NM 0153 | NM 0153 | OBSL1 | OBSL1 | OBSL1 | obscurin-like 1 | obscurin-l | obscurin-l | 23363 | 23363 | 23363 | s23656 | s23657 | s23658 |
| 13705 | L1 | L2 | 1194 | 1080 | 9688 | 164,2 | 8,1 | 0,761 | HSA034 C | sample | Hm Gen E | Hm Gen E | Hm Gen E | CPE002JC | CPE002NC | CPE002K4 | NM 01215 | NM 0121 | NM 0121 | FBXL2 | FBXL2 | FBXL2 | F-box and leucine | F-box and | F-box and | 25827 | 25827 | 25827 | s24563 | s24564 | s24565 |
| 1092 | N2 | N3 | 1500 | 734,5 | 12100 | 466 | 8,1 | 0,768 | HSA034 C | sample | Hm Drebl | Hm Drebl | Hm Drebl | CPE002FU | CPE002FY | CPE002G2 | NM 00565 | NM 0056 | NM 0056 | KLF10 | KLF10 | KLF10 | Kruppel-like facto | Kruppel-li | Kruppel-li | 7071 | 7071 | 7071 | s14128 | s14129 | s14130 |
| 3479 | A8 | A9 | 1273 | 1327 | 10271 | 321,9 | 8,1 | 0,779 | HSA034 C | sample | Hm Drebl | Hm Drebl | Hm Drebl | CPE002DR | CPE002EH | CPE002F7 | NM 00504 | NM 0050 | NM 0050 | PTHR2 | PTHR2 | PTHR2 | parathyroid horm | parathyro | parathyro | 5746 | 5746 | 5746 | s11481 | s11482 | s11483 |
| 3543 | D22 | D23 | 1883 | 1444 | 15299 | 344 | 8,1 | 0,779 | HSA034 C | sample | Hm Drebl | Hm Drebl | Hm Drebl | CPE002DR | CPE002EH | CPE002F7 | NM 02162 | NM 0216 | NM 0216 | HRH4 | HRH4 | HRH4 | histamine recepto | histamine | histamine | 59340 | 59340 | 59340 | s33998 | s33999 | s34000 |
| 1542 | A14 | A15 | 1851 | 891,9 | 14966 | 449,5 | 8,1 | 0,848 | HSA034 C | sample | Hm Drebl | Hm Drebl | Hm Drebl | CPE002FW | CPE002G0 | CPE002G4 | NM 02067 | NM 0206 | NM 0206 | S100A14 | S100A14 | S100A14 | S100 calcium binc | S100 calci | S100 calci | 57402 | 57402 | 57402 | s32989 | s32990 | s32991 |
| 1854 | N14 | N15 | 1631 | 806,9 | 13173 | 423,6 | 8,1 | 0,848 | HSA034 C | sample | Hm Drebl | Hm Drebl | Hm Drebl | CPE002FW | CPE002G0 | CPE002G4 | NM 03257 | NM 0325 | NM 0325 | GLIS2 | GLIS2 | GLIS2 | GLIS family zinc fi | GLIS famil | GLIS famil | 84662 | 84662 | 84662 | s39272 | s39273 | s39274 |
| 21619 | E4 | E5 | 273 | 1024 | 2220 | 536,2 | 8,1 | 0,853 | HSA034 C | sample | Hm Gen E | Hm Gen E | Hm Gen E | CPE002JU | CPE002O9 | CPE002KP | XM 94213 | XM 9421 | XM 9421 | LOC65260 | LOC65260 | LOC65260 | similar to Polyade | similar to | similar to | 652607 | 652607 | 652607 | s57408 | s57409 | s57410 |
| 21622 | E7 | E8 | 175 | 1074 | 1410 | 567,1 | 8,1 | 0,853 | HSA034 C | sample | Hm Gen E | Hm Gen E | Hm Gen E | CPE002JU | CPE002O9 | CPE002KP | XM 93830 | XM 9383 | XM 9383 | LOC64923 | LOC64923 | LOC64923 | similar to hCG164 | similar to | similar to | 649238 | 649238 | 649238 | s56943 | s197644 | s227316 |
| 21755 | K19 | K20 | 410 | 976,9 | 3334 | 615,2 | 8,1 | 0,853 | HSA034 C | sample | Hm Gen E | Hm Gen E | Hm Gen E | CPE002JU | CPE002O9 | CPE002KP | XM 93915 | XM 9391 | XM 9391 | LOC65007 | LOC65007 | LOC65007 | similar to Genera | similar to | similar to | 650070 | 650070 | 650070 | s57039 | s197712 | s197713 |
| 21769 | L1 | L2 | 321 | 1031 | 2601 | 570,3 | 8,1 | 0,853 | HSA034 C | sample | Hm Gen E | Hm Gen E | Hm Gen E | CPE002JU | CPE002O9 | CPE002KP | XM 94110 | XM 9411 | XM 9411 | LOC65184 | LOC65184 | LOC65184 | similar to HLA cla | similar to | similar to | 651845 | 651845 | 651845 | s57261 | s57262 | s57263 |
| 21787 | L4 | L5 | 140 | 840,2 | 1134 | 612,6 | 8,1 | 0,853 | HSA034 C | sample | Hm Gen E | Hm Gen E | Hm Gen E | CPE002JU | CPE002O9 | CPE002KP | XM 92957 | XM 9295 | XM 9295 | LOC65376 | LOC65376 | LOC65376 | similar to Group | similar to | similar to | 653768 | 653768 | 653768 | s57754 | s57755 | s57756 |
| 4623 | A22 | A23 | 2297 | 873,6 | 18687 | 481 | 8,1 | 0,89 | HSA034 C | sample | Hm Drebl | Hm Drebl | Hm Drebl | CPE002DV | CPE002EL | CPE002FP | NM 00318 | NM 0031 | NM 0031 | ADAM17 | ADAM17 | ADAM17 | ADAM metallope | ADAM me | ADAM me | 6868 | 6868 | 6868 | s13718 | s13719 | s13720 |

| | | | | | | | | | | | | | | | | | | | | | | | | | | | | | | | |
|---|---|---|---|---|---|---|---|---|---|---|---|---|---|---|---|---|---|---|---|---|---|---|---|---|---|---|---|---|---|---|---|
| 4946 | O10 | O11 | 1832 | 882,2 | 14924 | 463,8 | 8,1 | 0,89 | H5A034 C | sample | Hm Drgbl | Hm Drgbl | Hm Drgbl | CPE002DV | CPE002EL | CPE002FP | NM 00585 | NM 0058 | NM 0058 | ZMPSTE24 | ZMPSTE24 | ZMPSTE24 | zinc metallopepti | zinc metal | zinc metal | 10269 | 10269 | 10269 | s20065 | s20066 | s20067 |
| 10571 | I19 | I20 | 1055 | 848,6 | 8522 | 132,5 | 8,1 | 0,915 | H5A034 C | sample | Hm Drgbl | Hm Drgbl | Hm Drgbl | CPE002EB | CPE002EO | CPE002FR | NM 03313 | NM 0331 | NM 0331 | WNT3A | WNT3A | WNT3A | wingless-type MM | wingless-t | wingless-t | 89780 | 89780 | 89780 | s195525 | s195523 | s195524 |
| 2698 | A18 | A19 | 2014 | 726,9 | 16246 | 582,8 | 8,1 | 0,953 | H5A034 C | sample | Hm Drgbl | Hm Drgbl | Hm Drgbl | CPE002DP | CPE002EF | CPE002F5 | NM 01821 | NM 0182 | NM 0182 | PANK4 | PANK4 | PANK4 | pantothenate kin | pantother | pantother | 55229 | 55229 | 55229 | s30500 | s30501 | s224353 |
| 2743 | C15 | C16 | 1698 | 721,3 | 13683 | 482,3 | 8,1 | 0,953 | H5A034 C | sample | Hm Drgbl | Hm Drgbl | Hm Drgbl | CPE002DP | CPE002EF | CPE002F5 | NM 13047 | NM 1304 | NM 0036 | MADD | MADD | MADD | MAP-kinase activa | MAP-kina | MAP-kina | 8567 | 8567 | 8567 | s16313 | s16314 | s225002 |
| 2851 | G4 | G5 | 1249 | 663,6 | 10122 | 580,4 | 8,1 | 0,953 | H5A034 C | sample | Hm Drgbl | Hm Drgbl | Hm Drgbl | CPE002DP | CPE002EF | CPE002F5 | NM 02012 | NM 0201 | NM 0201 | SPHK2 | SPHK2 | SPHK2 | sphingosine kinas | sphingosi | sphingosi | 56848 | 56848 | 56848 | s32283 | s32284 | s32285 |
| 2960 | L16 | L17 | 823 | 643,2 | 6679 | 452,6 | 8,1 | 0,953 | H5A034 C | sample | Hm Drgbl | Hm Drgbl | Hm Drgbl | CPE002DP | CPE002EF | CPE002F5 | NM 00101 | NM 0010 | NM 0010 | EIF2AK4 | EIF2AK4 | EIF2AK4 | eukaryotic transla | eukaryoti | eukaryoti | 440275 | 440275 | 440275 | s54067 | s54068 | s54069 |
| 2978 | M10 | M11 | 1156 | 690,4 | 9416 | 442,9 | 8,1 | 0,953 | H5A034 C | sample | Hm Drgbl | Hm Drgbl | Hm Drgbl | CPE002DP | CPE002EF | CPE002F5 | NM 02477 | NM 0247 | NM 0247 | PIP5K2C | PIP5K2C | PIP5K2C | phosphatidylinos | phosphat | phosphat | 79837 | 79837 | 79837 | s36447 | s36448 | s36449 |
| 20084 | E5 | E6 | 1085 | 1135 | 8774 | 141 | 8,1 | 0,988 | H5A034 C | sample | Hm Gen E | Hm Gen E | Hm Gen E | CPE002JQ | CPE002O5 | CPE002KL | NM 00100 | NM 0010 | NM 0010 | OR10K1 | OR10K1 | OR10K1 | olfactory recepto | olfactory r | olfactory r | 391109 | 391109 | 391109 | s52922 | s52923 | s226488 |
| 20165 | I13 | I14 | 1324 | 1163 | 10706 | 143 | 8,1 | 0,988 | H5A034 C | sample | Hm Gen E | Hm Gen E | Hm Gen E | CPE002JQ | CPE002O5 | CPE002KL | XM 37304 | XM 3730 | XM 3730 | LOC39172 | LOC39172 | LOC39172 | similar to myosin | similar to | similar to | 391722 | 391722 | 391722 | s53009 | s53010 | s53011 |
| 20275 | M4 | M5 | 535 | 1091 | 4325 | 150,6 | 8,1 | 0,988 | H5A034 C | sample | Hm Gen E | Hm Gen E | Hm Gen E | CPE002JQ | CPE002O5 | CPE002KL | NM 00100 | NM 0010 | NM 0010 | FLJ45537 | FLJ45537 | FLJ45537 | FLJ45537 protein | FLJ45537 r | FLJ45537 r | 401535 | 401535 | 401535 | s53630 | s53631 | s53632 |
| 20290 | N18 | N19 | 1371 | 1026 | 11141 | 110,1 | 8,1 | 0,988 | H5A034 C | sample | Hm Gen E | Hm Gen E | Hm Gen E | CPE002JQ | CPE002O5 | CPE002KL | XM 00112 | XM 0011 | XM 0011 | LOC43994 | LOC43994 | LOC43994 | hypothetical gene | hypotheti | hypotheti | 439949 | 439949 | 439949 | s53973 | s53974 | s53975 |
| 440 | C16 | C17 | 1923 | 1615 | 15643 | 304,5 | 8,1 | 1,182 | H5A034 C | sample | Hm Drgbl | Hm Drgbl | Hm Drgbl | CPE002E0 | CPE002EZ | CPE002FK | NM 00145 | NM 0014 | NM 0014 | FLNC | FLNC | FLNC | filamin C, gamma | filamin C, | filamin C, | 2318 | 2318 | 2318 | s5281 | s5282 | s5283 |
| 461 | D13 | D14 | 2549 | 1752 | 20648 | 297,4 | 8,1 | 1,182 | H5A034 C | sample | Hm Drgbl | Hm Drgbl | Hm Drgbl | CPE002E0 | CPE002EZ | CPE002FK | NM 00198 | NM 0019 | NM 0019 | EVX1 | EVX1 | EVX1 | even-skipped hor | even-skip | even-skip | 2128 | 2128 | 2128 | s4884 | s4885 | s194403 |
| 751 | P15 | P16 | 2453 | 1915 | 19947 | 438,2 | 8,1 | 1,182 | H5A034 C | sample | Hm Drgbl | Hm Drgbl | Hm Drgbl | CPE002E0 | CPE002EZ | CPE002FK | NM 00201 | NM 0020 | NM 0020 | FKBP4 | FKBP4 | FKBP4 | FK506 binding pro | FK506 bin | FK506 bin | 2288 | 2288 | 2288 | s50 | s49 | s48 |
| 754 | P18 | P19 | 2481 | 1799 | 20212 | 423,2 | 8,1 | 1,182 | H5A034 C | sample | Hm Drgbl | Hm Drgbl | Hm Drgbl | CPE002E0 | CPE002EZ | CPE002FK | NM 00017 | NM 0001 | NM 0001 | GSS | GSS | GSS | glutathione synth | glutathior | glutathior | 2937 | 2937 | 2937 | s6251 | s6252 | s6253 |
| 7728 | B9 | B10 | 1501 | 566,4 | 12087 | 400,9 | 8,1 | 1,237 | H5A034 C | sample | Hm Drgbl | Hm Drgbl | Hm Drgbl | CPE002E4 | CPE002EV | CPE002FG | NM 00608 | NM 0060 | NM 0060 | ISGF3G | ISGF3G | ISGF3G | interferon-stimul | interferor | interferon | 10379 | 10379 | 10379 | s20291 | s20290 | s20292 |
| 7752 | C9 | C10 | 997 | 581,8 | 8031 | 413,2 | 8,1 | 1,237 | H5A034 C | sample | Hm Drgbl | Hm Drgbl | Hm Drgbl | CPE002E4 | CPE002EV | CPE002FG | NM 00577 | NM 0057 | NM 0057 | DHRS9 | DHRS9 | DHRS9 | dehydrogenase/r | dehydroge | dehydroge | 10170 | 10170 | 10170 | s19818 | s19819 | s19817 |
| 7814 | F21 | F22 | 1420 | 603,2 | 11559 | 407,5 | 8,1 | 1,237 | H5A034 C | sample | Hm Drgbl | Hm Drgbl | Hm Drgbl | CPE002E4 | CPE002EV | CPE002FG | NM 00632 | NM 0063 | NM 0063 | RBM14 | RBM14 | RBM14 | RNA binding moti | RNA bindi | RNA bindi | 10432 | 10432 | 10432 | s20406 | s20407 | s20408 |
| 7906 | J18 | J19 | 1446 | 554,1 | 11666 | 305,4 | 8,1 | 1,237 | H5A034 C | sample | Hm Drgbl | Hm Drgbl | Hm Drgbl | CPE002E4 | CPE002EV | CPE002FG | NM 02209 | NM 0220 | NM 0068 | ASCC3 | ASCC3 | ASCC3 | activating signal c | activating | activating | 10973 | 10973 | 10973 | s21604 | s21605 | s223172 |
| 7939 | K4 | K5 | 829 | 569,3 | 6733 | 359,1 | 8,1 | 1,237 | H5A034 C | sample | Hm Drgbl | Hm Drgbl | Hm Drgbl | CPE002E4 | CPE002EV | CPE002FG | NM 00657 | NM 0065 | NM 0065 | B3GNT2 | B3GNT2 | B3GNT2 | UDP-GlcNAc:beta | UDP-GlcN | UDP-GlcN | 10678 | 10678 | 10678 | s21004 | s21002 | s21003 |
| 7947 | L11 | L12 | 1710 | 597,7 | 13775 | 375,2 | 8,1 | 1,237 | H5A034 C | sample | Hm Drgbl | Hm Drgbl | Hm Drgbl | CPE002E4 | CPE002EV | CPE002FG | NM 00637 | NM 0063 | NM 0063 | SYNCRIP | SYNCRIP | SYNCRIP | synaptotagmin bi | synaptota | synaptota | 10492 | 10492 | 10492 | s20564 | s20563 | s20562 |
| 7991 | M8 | M9 | 1317 | 616,4 | 10604 | 290,5 | 8,1 | 1,237 | H5A034 C | sample | Hm Drgbl | Hm Drgbl | Hm Drgbl | CPE002E4 | CPE002EV | CPE002FG | NM 00661 | NM 0066 | NM 0066 | CHL1 | CHL1 | CHL1 | cell adhesion mol | cell adhes | cell adhes | 10752 | 10752 | 10752 | s21123 | s21124 | s21125 |
| 11318 | H21 | H22 | 736 | 768,1 | 6039 | 163,1 | 8,2 | 0,594 | H5A034 C | sample | Hm Drgbl | Hm Drgbl | Hm Drgbl | CPE002ED | CPE002F3 | CPE002FT | NM 0244 | NM 0244 | NM 0244 | NDUFS7 | NDUFS7 | NDUFS7 | NADH dehydroger | NADH deh | NADH deh | 374291 | 374291 | 374291 | s223959 | s51524 | s196147 |
| 5166 | H14 | H15 | 1056 | 614,2 | 8668 | 482,7 | 8,2 | 0,643 | H5A034 C | sample | Hm Drgbl | Hm Drgbl | Hm Drgbl | CPE002DV | CPE002EM | CPE002FO | NM 00037 | NM 0003 | NM 0003 | XDH | XDH | XDH | xanthine dehydro | xanthine ( | xanthine ( | 7498 | 7498 | 7498 | s14916 | s14917 | s14918 |
| 2338 | B18 | B19 | 1104 | 796,3 | 9107 | 444,1 | 8,2 | 0,646 | H5A034 C | sample | Hm Drgbl | Hm Drgbl | Hm Drgbl | CPE002DC | CPE002EE | CPE002F4 | NM 00274 | NM 0027 | NM 0027 | PRKCI | PRKCI | PRKCI | protein kinase C, | protein ki | protein ki | 5584 | 5584 | 5584 | s11110 | s11111 | s11112 |
| 16593 | D17 | D18 | 1507 | 861,6 | 12382 | 194,9 | 8,2 | 0,648 | H5A034 C | sample | Hm Gen E | Hm Gen E | Hm Gen E | CPE002JH | CPE002NV | CPE002KC | NM 00102 | NM 0010 | NM 0010 | KIAA1755 | KIAA1755 | KIAA1755 | KIAA1755 | KIAA1755 | KIAA1755 | 85449 | 85449 | 85449 | s40012 | s40013 | s40014 |
| 17988 | N2 | N3 | 1828 | 1056 | 14983 | 107 | 8,2 | 0,648 | H5A034 C | sample | Hm Gen E | Hm Gen E | Hm Gen E | CPE002JK | CPE002NZ | CPE002KF | NM 17883 | NM 1788 | NM 1788 | ZNF827 | ZNF827 | ZNF827 | zinc finger protei | zinc finger | zinc finger | 152485 | 152485 | 152485 | s45694 | s45695 | s45696 |
| 20 | A5 | A6 | 455 | 1674 | 3720 | 673 | 8,2 | 0,698 | H5A034 C | sample | Hm Drgbl | Hm Drgbl | Hm Drgbl | CPE002DT | CPE002EJ | CPE002F9 | NM 00523 | NM 0052 | NM 0052 | NR2F6 | NR2F6 | NR2F6 | nuclear receptor s | nuclear re | nuclear re | 2063 | 2063 | 2063 | s4776 | s4777 | s194399 |
| 167 | G8 | G9 | 584 | 1469 | 4810 | 644,6 | 8,2 | 0,698 | H5A034 C | sample | Hm Drgbl | Hm Drgbl | Hm Drgbl | CPE002DT | CPE002EJ | CPE002F9 | NM 00306 | NM 0030 | NM 0030 | SNAI2 | SNAI2 | SNAI2 | snail homolog 2 ( | snail hom | snail hom | 6591 | 6591 | 6591 | s13127 | s13128 | s224653 |
| 176 | H16 | H17 | 726 | 1534 | 5987 | 385,4 | 8,2 | 0,698 | H5A034 C | sample | Hm Drgbl | Hm Drgbl | Hm Drgbl | CPE002DT | CPE002EJ | CPE002F9 | NM 00104 | NM 0010 | NM 0010 | TROVE2 | TROVE2 | TROVE2 | TROVE domain fa | TROVE do | TROVE do | 6738 | 6738 | 6738 | s13465 | s13466 | s13467 |
| 239 | J8 | J9 | 651 | 1523 | 5319 | 558,5 | 8,2 | 0,698 | H5A034 C | sample | Hm Drgbl | Hm Drgbl | Hm Drgbl | CPE002DT | CPE002EJ | CPE002F9 | NM 02101 | NM 0210 | NM 0210 | SSX5 | SSX5 | SSX5 | synovial sarcoma, | synovial s | synovial s | 6758 | 6758 | 6758 | s13509 | s194824 | s194823 |
| 286 | L7 | L8 | 601 | 1445 | 4934 | 399,8 | 8,2 | 0,698 | H5A034 C | sample | Hm Drgbl | Hm Drgbl | Hm Drgbl | CPE002DT | CPE002EJ | CPE002F9 | NM 00034 | NM 0003 | NM 0003 | SLC2A2 | SLC2A2 | SLC2A2 | solute carrier fam | solute car | solute car | 6514 | 6514 | 6514 | s12928 | s12929 | s12930 |
| 2082 | G3 | G4 | 852 | 752,7 | 7005 | 443,2 | 8,2 | 0,713 | H5A034 C | sample | Hm Drgbl | Hm Drgbl | Hm Drgbl | CPE002FX | CPE002G1 | CPE002G5 | NM 03319 | NM 0331 | NM 0331 | HSPB9 | HSPB9 | HSPB9 | heat shock protei | heat shoc | heat shoc | 94086 | 94086 | 94086 | s41251 | s41252 | s225127 |
| 3946 | E18 | E19 | 1663 | 665,9 | 13649 | 287,1 | 8,2 | 0,729 | H5A034 C | sample | Hm Drgbl | Hm Drgbl | Hm Drgbl | CPE002DS | CPE002EI | CPE002F8 | NM 00458 | NM 0045 | NM 0045 | RAB5C | RAB5C | RAB5C | RAB5C, member F | RAB5C, m | RAB5C, m | 5878 | 5878 | 5878 | s11709 | s224512 | s11708 |
| 4135 | M15 | M16 | 885 | 619 | 7283 | 331,9 | 8,2 | 0,729 | H5A034 C | sample | Hm Drgbl | Hm Drgbl | Hm Drgbl | CPE002DS | CPE002EI | CPE002F8 | NM 00044 | NM 0004 | NM 0004 | PON1 | PON1 | PON1 | paraoxonase 1 | paraoxona | paraoxona | 5444 | 5444 | 5444 | s10829 | s10830 | s10831 |
| 5808 | B9 | B10 | 883 | 951 | 7280 | 628,8 | 8,2 | 0,749 | H5A034 C | sample | Hm Drgbl | Hm Drgbl | Hm Drgbl | CPE002DY | CPE002F1 | CPE002FM | NM 00069 | NM 0006 | NM 0006 | ALDH9A1 | ALDH9A1 | ALDH9A1 | aldehyde dehydro | aldehyde | aldehyde | 223 | 223 | 223 | s540 | s541 | s542 |
| 1359 | I22 | I23 | 1784 | 801,4 | 14589 | 412,2 | 8,2 | 0,752 | H5A034 C | sample | Hm Drgbl | Hm Drgbl | Hm Drgbl | CPE002FV | CPE002FZ | CPE002G3 | NM 00586 | NM 0058 | NM 0058 | BET1 | BET1 | BET1 | BET1 homolog (S. | BET1 hom | BET1 hom | 10282 | 10282 | 10282 | s20092 | s20093 | s20094 |
| 1477 | N20 | N21 | 1953 | 715,8 | 15958 | 425,6 | 8,2 | 0,752 | H5A034 C | sample | Hm Drgbl | Hm Drgbl | Hm Drgbl | CPE002FV | CPE002FZ | CPE002G3 | NM 01519 | NM 0151 | NM 0151 | DNAJC9 | DNAJC9 | DNAJC9 | DnaJ (Hsp40) hom | DnaJ (Hsp | DnaJ (Hsp | 23234 | 23234 | 23234 | s23352 | s23353 | s23354 |
| 13584 | F9 | F10 | 1493 | 999,2 | 12243 | 157,9 | 8,2 | 0,77 | H5A034 C | sample | Hm Gen E | Hm Gen E | Hm Gen E | CPE002JC | CPE002NC | CPE002K4 | NM 02204 | NM 0220 | NM 0220 | SDF2L1 | SDF2L1 | SDF2L1 | stromal cell-deriv | stromal ce | stromal ce | 23753 | 23753 | 23753 | s24355 | s24357 | s225981 |
| 13729 | M1 | M2 | 1635 | 1088 | 13398 | 153,5 | 8,2 | 0,77 | H5A034 C | sample | Hm Gen E | Hm Gen E | Hm Gen E | CPE002JC | CPE002NC | CPE002K4 | NM 01534 | NM 0153 | NM 0153 | DAAM2 | DAAM2 | DAAM2 | dishevelled assoc | dishevelle | dishevelle | 23500 | 23500 | 23500 | s23940 | s23941 | s23942 |
| 788 | A5 | A6 | 1167 | 724,8 | 9609 | 447 | 8,2 | 0,777 | H5A034 C | sample | Hm Drgbl | Hm Drgbl | Hm Drgbl | CPE002FU | CPE002FY | CPE002G2 | NM 00434 | NM 0043 | NM 0043 | CALR | CALR | CALR | calreticulin | calreticuli | calreticuli | 811 | 811 | 811 | s114 | s115 | s116 |
| 3503 | B8 | B9 | 1673 | 1299 | 13668 | 360 | 8,2 | 0,788 | H5A034 C | sample | Hm Drgbl | Hm Drgbl | Hm Drgbl | CPE002DR | CPE002EH | CPE002F7 | NM 02037 | NM 0203 | NM 0203 | GPR84 | GPR84 | GPR84 | G protein-couplec | G protein- | G protein- | 53831 | 53831 | 53831 | s28771 | s28772 | s28773 |
| 3702 | K14 | K15 | 1148 | 1262 | 9443 | 264,5 | 8,2 | 0,788 | H5A034 C | sample | Hm Drgbl | Hm Drgbl | Hm Drgbl | CPE002DR | CPE002EH | CPE002F7 | NM 00679 | NM 0067 | NM 0067 | GPR75 | GPR75 | GPR75 | G protein-couplec | G protein- | G protein- | 10936 | 10936 | 10936 | s21510 | s21511 | s21512 |
| 8336 | L16 | L17 | 793 | 699,2 | 6512 | 185,7 | 8,2 | 0,788 | H5A034 C | sample | Hm Drgbl | Hm Drgbl | Hm Drgbl | CPE002E5 | CPE002EU | CPE002FF | NM 01209 | NM 0120 | NM 0120 | ADAT1 | ADAT1 | ADAT1 | adenosine deami | adenosine | adenosine | 23536 | 23536 | 23536 | s24033 | s24034 | s223609 |
| 1719 | H22 | H23 | 1904 | 729,1 | 15609 | 351,2 | 8,2 | 0,859 | H5A034 C | sample | Hm Drgbl | Hm Drgbl | Hm Drgbl | CPE002FW | CPE002G0 | CPE002G4 | NM 03130 | NM 0313 | NM 0313 | MXD3 | MXD3 | MXD3 | MAX dimerizatior | MAX dime | MAX dime | 83463 | 83463 | 83463 | s37946 | s37947 | s195436 |
| 1856 | N16 | N17 | 2203 | 795,3 | 18069 | 377,1 | 8,2 | 0,859 | H5A034 C | sample | Hm Drgbl | Hm Drgbl | Hm Drgbl | CPE002FW | CPE002G0 | CPE002G4 | NM 03258 | NM 0325 | NM 0325 | ZNF347 | ZNF347 | ZNF347 | zinc finger protei | zinc finger | zinc finger | 84671 | 84671 | 84671 | s39293 | s39294 | s39295 |
| 21585 | D17 | D18 | 322 | 1017 | 2643 | 487,4 | 8,2 | 0,863 | H5A034 C | sample | Hm Gen E | Hm Gen E | Hm Gen E | CPE002JU | CPE002O9 | CPE002KP | XM 94014 | XM 9401 | XM 9401 | LOC65103 | LOC65103 | LOC65103 | hypothetical LOC | hypotheti | hypotheti | 651033 | 651033 | 651033 | s57160 | s57161 | s57162 |
| 21609 | E17 | E18 | 329 | 1081 | 2704 | 568,9 | 8,2 | 0,863 | H5A034 C | sample | Hm Gen E | Hm Gen E | Hm Gen E | CPE002JU | CPE002O9 | CPE002KP | XM 00113 | XM 0011 | XM 0011 | LOC64929 | LOC64929 | LOC64929 | hypothetical prot | hypotheti | hypotheti | 649294 | 649294 | 649294 | s56955 | s197650 | s197651 |
| 21841 | O1 | O2 | 229 | 927 | 1870 | 667,2 | 8,2 | 0,863 | H5A034 C | sample | Hm Gen E | Hm Gen E | Hm Gen E | CPE002JU | CPE002O9 | CPE002KP | XM 93955 | XM 9395 | XM 9395 | LOC65046 | LOC65046 | LOC65046 | similar to phosph | similar to | similar to | 650468 | 650468 | 650468 | s57072 | s57073 | s197733 |
| 4671 | C22 | C23 | 1588 | 860,1 | 12950 | 251,6 | 8,2 | 0,901 | H5A034 C | sample | Hm Drgbl | Hm Drgbl | Hm Drgbl | CPE002DV | CPE002EL | CPE002FP | NM 00336 | NM 0033 | NM 0033 | UQCRC2 | UQCRC2 | UQCRC2 | ubiquinol-cytochr | ubiquinol | ubiquinol | 7385 | 7385 | 7385 | s14702 | s14703 | s14704 |
| 4738 | F18 | F19 | 1658 | 823,3 | 13517 | 394,5 | 8,2 | 0,901 | H5A034 C | sample | Hm Drgbl | Hm Drgbl | Hm Drgbl | CPE002DV | CPE002EL | CPE002FP | NM 13899 | NM 1389 | NM 1389 | BACE2 | BACE2 | BACE2 | beta-site APP-clea | beta-site | beta-site | 25825 | 25825 | 25825 | s24560 | s24561 | s24562 |
| 4836 | J2 | J3 | 1331 | 750,2 | 10915 | 446,2 | 8,2 | 0,901 | H5A034 C | sample | Hm Drgbl | Hm Drgbl | Hm Drgbl | CPE002DV | CPE002EL | CPE002FP | NM 14589 | NM 1458 | NM 0195 | KLK12 | KLK12 | KLK12 | kallikrein-related | kallikrein- | kallikrein- | 43849 | 43849 | 43849 | s26975 | s26976 | s195195 |
| 4942 | N7 | N8 | 939 | 888,5 | 7668 | 564,8 | 8,2 | 0,901 | H5A034 C | sample | Hm Drgbl | Hm Drgbl | Hm Drgbl | CPE002DV | CPE002EL | CPE002FP | NM 00276 | NM 0027 | NM 0027 | PRSS1 | PRSS1 | PRSS1 | protease, serine, | protease, | protease, | 5644 | 5644 | 5644 | s11253 | s11254 | s11255 |
| 10455 | D22 | D23 | 1207 | 902,2 | 9863 | 167,9 | 8,2 | 0,927 | H5A034 C | sample | Hm Drgbl | Hm Drgbl | Hm Drgbl | CPE002EB | CPE002EO | CPE002FR | NM 21359 | NM 2135 | NM 2135 | FOXN4 | FOXN4 | FOXN4 | forkhead box N4 | forkhead | forkhead | 121643 | 121643 | 121643 | s42499 | s42498 | s42497 |
| 10458 | D3 | D4 | 1327 | 847,3 | 10912 | 183,3 | 8,2 | 0,927 | H5A034 C | sample | Hm Drgbl | Hm Drgbl | Hm Drgbl | CPE002EB | CPE002EO | CPE002FR | NM 13869 | NM 1386 | NM 1386 | LOC91431 | LOC91431 | LOC91431 | prematurely term | prematur | prematur | 91431 | 91431 | 91431 | s40655 | s40654 | s40656 |
| 10546 | H18 | H19 | 1194 | 778,6 | 9746 | 149,5 | 8,2 | 0,927 | H5A034 C | sample | Hm Drgbl | Hm Drgbl | Hm Drgbl | CPE002EB | CPE002EO | CPE002FR | NM 15234 | NM 1523 | NM 1523 | PAQR4 | PAQR4 | PAQR4 | progestin and adi | progestin | progestin | 124222 | 124222 | 124222 | s42715 | s42716 | s42714 |
| 10555 | H4 | H5 | 883 | 895,4 | 7234 | 201 | 8,2 | 0,927 | H5A034 C | sample | Hm Drgbl | Hm Drgbl | Hm Drgbl | CPE002EB | CPE002EO | CPE002FR | NM 13879 | NM 1387 | NM 1387 | LEO1 | LEO1 | LEO1 | Leo1, Paf1/RNA p | Leo1, Paf1 | Leo1, Paf1 | 123169 | 123169 | 123169 | s42625 | s42624 | s42623 |
| 2774 | D21 | D22 | 1106 | 617,4 | 9034 | 548,7 | 8,2 | 0,965 | H5A034 C | sample | Hm Drgbl | Hm Drgbl | Hm Drgbl | CPE002DP | CPE002EF | CPE002F5 | NM 01650 | NM 0165 | NM 0165 | CRKRS | CRKRS | CRKRS | Cdc2-related kina | Cdc2-relat | Cdc2-relat | 51755 | 51755 | 51755 | s28621 | s28622 | s28623 |
| 2833 | G1 | G2 | 932 | 605 | 7622 | 603,9 | 8,2 | 0,965 | H5A034 C | sample | Hm Drgbl | Hm Drgbl | Hm Drgbl | CPE002DP | CPE002EF | CPE002F5 | NM 00657 | NM 0065 | NM 0065 | MAP4K5 | MAP4K5 | MAP4K5 | mitogen-activatec | mitogen-a | mitogen-a | 11183 | 11183 | 11183 | s22077 | s22078 | s22079 |
| 20100 | F2 | F3 | 1265 | 1215 | 10398 | 116,4 | 8,2 | 1 | H5A034 C | sample | Hm Gen E | Hm Gen E | Hm Gen E | CPE002JQ | CPE002O5 | CPE002KL | XM 37988 | XM 3798 | XM 3798 | hCG 2023 | hCG 2023 | hCG 2023 | hCG2023776 | hCG20237 | hCG20237 | 402562 | 402562 | 402562 | s53777 | s53778 | s53779 |
| 20137 | H1 | H2 | 1250 | 1123 | 10212 | 107,1 | 8,2 | 1 | H5A034 C | sample | Hm Gen E | Hm Gen E | Hm Gen E | CPE002JQ | CPE002O5 | CPE002KL | NM 20744 | NM 2074 | NM 2074 | C13orf35 | C13orf35 | C13orf35 | chromosome 13 c | chromoso | chromoso | 400165 | 400165 | 400165 | s53249 | s53250 | s53251 |
| 20213 | K13 | K14 | 817 | 1064 | 6659 | 168,6 | 8,2 | 1 | H5A034 C | sample | Hm Gen E | Hm Gen E | Hm Gen E | CPE002JQ | CPE002O5 | CPE002KL | XM 37326 | XM 3732 | XM 3732 | LOC39227 | LOC39227 | LOC39227 | similar to Sphingo | similar to | similar to | 392275 | 392275 | 392275 | s196408 | s196409 | s196410 |
| 20334 | P14 | P15 | 970 | 1452 | 7951 | 126,8 | 8,2 | 1 | H5A034 C | sample | Hm Gen E | Hm Gen E | Hm Gen E | CPE002JQ | CPE002O5 | CPE002KL | NM 00100 | NM 0010 | NM 0010 | KRTAP5-1 | KRTAP5-1 | KRTAP5-1 | keratin associatec | keratin as | keratin as | 440051 | 440051 | 440051 | s53998 | s196511 | s196512 |
| 503 | E8 | E9 | 1642 | 1821 | 13543 | 272,4 | 8,2 | 1,197 | H5A034 C | sample | Hm Drgbl | Hm Drgbl | Hm Drgbl | CPE002E0 | CPE002EZ | CPE002FK | NM 00202 | NM 0020 | NM 0020 | FMOD | FMOD | FMOD | fibromodulin | fibromodu | fibromodu | 2331 | 2331 | 2331 | s5312 | s223584 | s5314 |
| 757 | P20 | P21 | 2879 | 1919 | 23499 | 441,9 | 8,2 | 1,197 | H5A034 C | sample | Hm Drgbl | Hm Drgbl | Hm Drgbl | CPE002E0 | CPE002EZ | CPE002FK | NM 14574 | NM 1457 | NM 1457 | GSTA1 | GSTA1 | GSTA1 | glutathione S-tran | glutathior | glutathior | 2938 | 2938 | 2938 | s6256 | s6255 | s6254 |
| 7713 | B17 | B18 | 1421 | 569,1 | 11713 | 397,7 | 8,2 | 1,252 | H5A034 C | sample | Hm Drgbl | Hm Drgbl | Hm Drgbl | CPE002E4 | CPE002EV | CPE002FG | NM 00699 | NM 0069 | NM 0069 | BTN3A3 | BTN3A3 | BTN3A3 | butyrophilin, sub | butyrophi | butyrophi | 10384 | 10384 | 10384 | s20306 | s20305 | s20304 |
| 7818 | F3 | F4 | 1215 | 537,4 | 9973 | 335,2 | 8,2 | 1,252 | H5A034 C | sample | Hm Drgbl | Hm Drgbl | Hm Drgbl | CPE002E4 | CPE002EV | CPE002FG | NM 00610 | NM 0061 | NM 0061 | PRMT5 | PRMT5 | PRMT5 | protein arginine r | protein ar | protein ar | 10419 | 10419 | 10419 | s20377 | s20375 | s20376 |
| 7849 | H1 | H2 | 719 | 688 | 5909 | 406,9 | 8,2 | 1,252 | H5A034 C | sample | Hm Drgbl | Hm Drgbl | Hm Drgbl | CPE002E4 | CPE002EV | CPE002FG | NM 00633 | NM 0063 | NM 0063 | LYPLA1 | LYPLA1 | LYPLA1 | lysophospholipas | lysophosp | lysophosp | 10434 | 10434 | 10434 | s20409 | s20410 | s20411 |
| 7855 | H15 | H16 | 977 | 558,8 | 8032 | 392,2 | 8,2 | 1,252 | H5A034 C | sample | Hm Drgbl | Hm Drgbl | Hm Drgbl | CPE002E4 | CPE002EV | CPE002FG | NM 00611 | NM 0061 | NM 0061 | PPIE | PPIE | PPIE | peptidylprolyl iso | peptidylp | peptidylp | 10450 | 10450 | 10450 | s20445 | s20446 | s20447 |
| 7946 | L10 | L11 | 1201 | 557,3 | 9897 | 363,2 | 8,2 | 1,252 | H5A034 C | sample | Hm Drgbl | Hm Drgbl | Hm Drgbl | CPE002E4 | CPE002EV | CPE002FG | NM 00684 | NM 0068 | NM 0068 | SLC38A3 | SLC38A3 | SLC38A3 | solute carrier fam | solute car | solute car | 10991 | 10991 | 10991 | s21639 | s21640 | s21641 |
| 7986 | M3 | M4 | 553 | 609,4 | 4509 | 348,5 | 8,2 | 1,252 | H5A034 C | sample | Hm Drgbl | Hm Drgbl | Hm Drgbl | CPE002E4 | CPE002EV | CPE002FG | NM 00587 | NM 0058 | NM 0058 | BCAS2 | BCAS2 | BCAS2 | breast carcinoma | breast car | breast car | 10286 | 10286 | 10286 | s20105 | s20104 | s20106 |
| 8038 | O7 | O8 | 1493 | 586,6 | 12296 | 418,6 | 8,2 | 1,252 | H5A034 C | sample | Hm Drgbl | Hm Drgbl | Hm Drgbl | CPE002E4 | CPE002EV | CPE002FG | NM 01425 | NM 0142 | NM 0142 | B3GNT3 | B3GNT3 | B3GNT3 | UDP-GlcNAc:beta | UDP-GlcN | UDP-GlcN | 10331 | 10331 | 10331 | s20209 | s20211 | s20210 |
| 8048 | P16 | P17 | 1422 | 562,1 | 11666 | 440,1 | 8,2 | 1,252 | H5A034 C | sample | Hm Drgbl | Hm Drgbl | Hm Drgbl | CPE002E4 | CPE002EV | CPE002FG | NM 00686 | NM 0068 | NM 0068 | CENTA1 | CENTA1 | CENTA1 | centaurin, alpha 1 | centaurin, | centaurin, | 11033 | 11033 | 11033 | s21736 | s21735 | s21734 |
| 19506 | M3 | M4 | 1035 | 1152 | 8567 | 179,5 | 8,3 | 0,589 | H5A034 C | sample | Hm Gen E | Hm Gen E | Hm Gen E | CPE002JO | CPE002O3 | CPE002KJ | NM 00108 | NM 0010 | NM 0010 | C17orf67 | C17orf67 | C17orf67 | chromosome 17 c | chromoso | chromoso | 339210 | 339210 | 339210 | s50477 | s50478 | s50479 |
| 11322 | H3 | H4 | 756 | 884,8 | 6262 | 143,9 | 8,3 | 0,601 | H5A034 C | sample | Hm Drgbl | Hm Drgbl | Hm Drgbl | CPE002ED | CPE002F3 | CPE002FT | NM 00100 | NM 0010 | NM 0010 | RAET1G | RAET1G | RAET1G | retinoic acid early | retinoic a | retinoic a | 353091 | 353091 | 353091 | s51418 | s51419 | s51417 |
| 5167 | H15 | H16 | 1341 | 560,7 | 11144 | 330,8 | 8,3 | 0,651 | H5A034 C | sample | Hm Drgbl | Hm Drgbl | Hm Drgbl | CPE002DV | CPE002EM | CPE002FO | NM 17074 | NM 1707 | NM 1707 | ALDH5A1 | ALDH5A1 | ALDH5A1 | aldehyde dehydro | aldehyde | aldehyde | 7915 | 7915 | 7915 | s15462 | s15461 | s15460 |
| 2601 | M17 | M18 | 1182 | 811,2 | 9772 | 412,4 | 8,3 | 0,654 | H5A034 C | sample | Hm Drgbl | Hm Drgbl | Hm Drgbl | CPE002DC | CPE002EE | CPE002F4 | NM 00107 | NM 0010 | NM 0010 | ACVRL1 | ACVRL1 | ACVRL1 | activin A receptor | activin A r | activin A r | 94 | 94 | 94 | s986 | s987 | s988 |
| 17970 | M3 | M4 | 1154 | 971,6 | 9612 | 104,6 | 8,3 | 0,656 | H5A034 C | sample | Hm Gen E | Hm Gen E | Hm Gen E | CPE002JK | CPE002NZ | CPE002KF | NM 14466 | NM 1446 | NM 1446 | GLT1D1 | GLT1D1 | GLT1D1 | glycosyltransferas | glycosyltra | glycosyltra | 144423 | 144423 | 144423 | s44654 | s44655 | s44656 |
| 9020 | H5 | H6 | 1639 | 1790 | 13637 | 134,4 | 8,3 | 0,667 | H5A034 C | sample | Hm Drgbl | Hm Drgbl | Hm Drgbl | CPE002E7 | CPE002ES | CPE002FD | NM 01658 | NM 0165 | NM 0165 | PCDH12 | PCDH12 | PCDH12 | protocadherin 12 | protocadh | protocadh | 51294 | 51294 | 51294 | s27869 | s27870 | s27871 |
| 9138 | M3 | M4 | 2158 | 1856 | 17859 | 122,6 | 8,3 | 0,667 | H5A034 C | sample | Hm Drgbl | Hm Drgbl | Hm Drgbl | CPE002E7 | CPE002ES | CPE002FD | NM 03295 | NM 0329 | NM 0329 | MLXIPL | MLXIPL | MLXIPL | MLX interacting p | MLX inter | MLX inter | 51085 | 51085 | 51085 | s27386 | s27387 | s27388 |
| 217 | J1 | J2 | 502 | 1448 | 4166 | 586 | 8,3 | 0,706 | H5A034 C | sample | Hm Drgbl | Hm Drgbl | Hm Drgbl | CPE002DT | CPE002EJ | CPE002F9 | NM 00506 | NM 0050 | NM 0050 | SIM2 | SIM2 | SIM2 | single-minded ho | single-mir | single-mir | 6493 | 6493 | 6493 | s12870 | s12869 | s12868 |
| 3975 | F22 | F23 | 1751 | 688,4 | 14591 | 341,5 | 8,3 | 0,738 | H5A034 C | sample | Hm Drgbl | Hm Drgbl | Hm Drgbl | CPE002DS | CPE002EI | CPE002F8 | NM 00293 | NM 0029 | NM 0029 | RNASE2 | RNASE2 | RNASE2 | ribonuclease, RNa | ribonuclea | ribonuclea | 6036 | 6036 | 6036 | s12052 | s12053 | s12054 |
| 5775 | A22 | A23 | 2244 | 894,2 | 18522 | 519,4 | 8,3 | 0,758 | H5A034 C | sample | Hm Drgbl | Hm Drgbl | Hm Drgbl | CPE002DY | CPE002F1 | CPE002FM | NM 00430 | NM 0043 | NM 0043 | ARHGAP1 | ARHGAP1 | ARHGAP1 | Rho GTPase activ | Rho GTPa | Rho GTPa | 392 | 392 | 392 | s1583 | s1585 | s1584 |
| 1209 | C17 | C18 | 2189 | 763,2 | 18125 | 373,8 | 8,3 | 0,761 | H5A034 C | sample | Hm Drgbl | Hm Drgbl | Hm Drgbl | CPE002FV | CPE002FZ | CPE002G3 | NM 14468 | NM 1446 | NM 1446 | ZNF18 | ZNF18 | ZNF18 | zinc finger protei | zinc finger | zinc finger | 7566 | 7566 | 7566 | s15039 | s15040 | s15041 |
| 13754 | N10 | N11 | 1220 | 964 | 10120 | 152,2 | 8,3 | 0,779 | H5A034 C | sample | Hm Gen E | Hm Gen E | Hm Gen E | CPE002JC | CPE002NC | CPE002K4 | NM 03266 | NM 0326 | NM 0326 | BSCL2 | BSCL2 | BSCL2 | Bernardinelli-Seip | Bernardin | Bernardin | 26580 | 26580 | 26580 | s25557 | s25559 | s226083 |
| 855 | D22 | D23 | 1279 | 781,8 | 10667 | 322,5 | 8,3 | 0,787 | H5A034 C | sample | Hm Drgbl | Hm Drgbl | Hm Drgbl | CPE002FU | CPE002FY | CPE002G2 | NM 00296 | NM 0029 | NM 0029 | S100A9 | S100A9 | S100A9 | S100 calcium binc | S100 calci | S100 calci | 6280 | 6280 | 6280 | s12425 | s12426 | s224563 |
| 5652 | L2 | L3 | 1743 | 773,4 | 14416 | 273,5 | 8,3 | 0,79 | H5A034 C | sample | Hm Drgbl | Hm Drgbl | Hm Drgbl | CPE002DX | CPE002F2 | CPE002FN | NM 00422 | NM 0042 | NM 0042 | PSCD3 | PSCD3 | PSCD3 | pleckstrin homolc | pleckstrin | pleckstrin | 9265 | 9265 | 9265 | s17715 | s17716 | s17717 |
| 3565 | E20 | E21 | 2289 | 1537 | 18897 | 397,8 | 8,3 | 0,798 | H5A034 C | sample | Hm Drgbl | Hm Drgbl | Hm Drgbl | CPE002DR | CPE002EH | CPE002F7 | XM 00113 | XM 0011 | XM 0011 | GPR109B | GPR109B | GPR109B | G protein-couplec | G protein- | G protein- | 8843 | 8843 | 8843 | s16882 | s16883 | s16884 |
| 3614 | G21 | G22 | 2689 | 1636 | 22398 | 356,7 | 8,3 | 0,798 | H5A034 C | sample | Hm Drgbl | Hm Drgbl | Hm Drgbl | CPE002DR | CPE002EH | CPE002F7 | NM 00550 | NM 0055 | NM 0055 | CCR4 | CCR4 | CCR4 | chemokine (C-C m | chemokin | chemokin | 1233 | 1233 | 1233 | s3206 | s3207 | s3208 |
| 3716 | K5 | K6 | 958 | 1411 | 7951 | 317 | 8,3 | 0,798 | H5A034 C | sample | Hm Drgbl | Hm Drgbl | Hm Drgbl | CPE002DR | CPE002EH | CPE002F7 | NM 00079 | NM 0007 | NM 0007 | DRD3 | DRD3 | DRD3 | dopamine recepto | dopamine | dopamine | 1814 | 1814 | 1814 | s4287 | s4288 | s223439 |
| 3727 | L15 | L16 | 893 | 1555 | 7449 | 381,4 | 8,3 | 0,798 | H5A034 C | sample | Hm Drgbl | Hm Drgbl | Hm Drgbl | CPE002DR | CPE002EH | CPE002F7 | NM 00591 | NM 0059 | NM 0059 | MC4R | MC4R | MC4R | melanocortin 4 re | melanoco | melanoco | 4160 | 4160 | 4160 | s8565 | s8566 | s8567 |
| 8269 | I20 | I21 | 1698 | 736,7 | 14146 | 233,5 | 8,3 | 0,798 | H5A034 C | sample | Hm Drgbl | Hm Drgbl | Hm Drgbl | CPE002E5 | CPE002EU | CPE002FF | NM 18273 | NM 1827 | NM 1827 | PLCB1 | PLCB1 | PLCB1 | phospholipase C, | phospholi | phospholi | 23236 | 23236 | 23236 | s23358 | s23360 | s23359 |
| 8271 | I22 | I23 | 1278 | 691,7 | 10549 | 421,1 | 8,3 | 0,798 | H5A034 C | sample | Hm Drgbl | Hm Drgbl | Hm Drgbl | CPE002E5 | CPE002EU | CPE002FF | NM 01520 | NM 0152 | NM 0152 | PDS5A | PDS5A | PDS5A | PDS5, regulator o | PDS5, regu | PDS5, regu | 23244 | 23244 | 23244 | s23378 | s23379 | s23380 |
| 8285 | J13 | J14 | 1876 | 690,8 | 15538 | 346,8 | 8,3 | 0,798 | H5A034 C | sample | Hm Drgbl | Hm Drgbl | Hm Drgbl | CPE002E5 | CPE002EU | CPE002FF | NM 00734 | NM 0073 | NM 0073 | ATF6 | ATF6 | ATF6 | activating transcr | activating | activating | 22926 | 22926 | 22926 | s223545 | s22688 | s22690 |
| 1863 | N22 | N23 | 2166 | 799,9 | 17962 | 383,7 | 8,3 | 0,869 | H5A034 C | sample | Hm Drgbl | Hm Drgbl | Hm Drgbl | CPE002FW | CPE002G0 | CPE002G4 | NM 03267 | NM 0326 | NM 0326 | ZNF577 | ZNF577 | ZNF577 | zinc finger protei | zinc finger | zinc finger | 84765 | 84765 | 84765 | s39411 | s39412 | s39413 |
| 21764 | K5 | K6 | 102 | 1159 | 843 | 726,3 | 8,3 | 0,874 | H5A034 C | sample | Hm Gen E | Hm Gen E | Hm Gen E | CPE002JU | CPE002O9 | CPE002KP | XM 93904 | XM 9390 | XM 9390 | LOC64996 | LOC64996 | LOC64996 | similar to stereoc | similar to | similar to | 649965 | 649965 | 649965 | s57027 | s57028 | s197697 |
| 21779 | L19 | L20 | 458 | 1079 | 3809 | 728 | 8,3 | 0,874 | H5A034 C | sample | Hm Gen E | Hm Gen E | Hm Gen E | CPE002JU | CPE002O9 | CPE002KP | XM 94135 | XM 9413 | XM 9413 | LOC65204 | LOC65204 | LOC65204 | similar to 3-phosp | similar to | similar to | 652041 | 652041 | 652041 | s57294 | s197841 | s197842 |
| 21838 | N7 | N8 | 446 | 992,7 | 3717 | 680,8 | 8,3 | 0,874 | H5A034 C | sample | Hm Gen E | Hm Gen E | Hm Gen E | CPE002JU | CPE002O9 | CPE002KP | XM 00113 | XM 0011 | XM 0011 | LOC65209 | LOC65209 | LOC65209 | hypothetical prot | hypotheti | hypotheti | 652095 | 652095 | 652095 | s57304 | s57305 | s227383 |
| 4621 | A20 | A21 | 2016 | 1068 | 16640 | 376,5 | 8,3 | 0,912 | H5A034 C | sample | Hm Drgbl | Hm Drgbl | Hm Drgbl | CPE002DV | CPE002EL | CPE002FP | NM 02197 | NM 0219 | NM 0219 | ST14 | ST14 | ST14 | suppression of tu | suppressi | suppressi | 6768 | 6768 | 6768 | s13519 | s13520 | s224696 |
| 4633 | B1 | B2 | 2039 | 802 | 16938 | 497,7 | 8,3 | 0,912 | H5A034 C | sample | Hm Drgbl | Hm Drgbl | Hm Drgbl | CPE002DV | CPE002EL | CPE002FP | NM 00446 | NM 0044 | NM 0044 | FAP | FAP | FAP | fibroblast activati | fibroblast | fibroblast | 2191 | 2191 | 2191 | s5021 | s5022 | s5023 |
| 4748 | F5 | F6 | 2132 | 823,3 | 17662 | 430 | 8,3 | 0,912 | H5A034 C | sample | Hm Drgbl | Hm Drgbl | Hm Drgbl | CPE002DV | CPE002EL | CPE002FP | NM 00557 | NM 0055 | NM 0055 | LNPEP | LNPEP | LNPEP | leucyl/cystinyl am | leucyl/cys | leucyl/cys | 4012 | 4012 | 4012 | s8245 | s8246 | s8247 |
| 4872 | K9 | K10 | 2120 | 930,6 | 17509 | 357,7 | 8,3 | 0,912 | H5A034 C | sample | Hm Drgbl | Hm Drgbl | Hm Drgbl | CPE002DV | CPE002EL | CPE002FP | NM 19832 | NM 1983 | NM 0010 | CPM | CPM | CPM | carboxypeptidase | carboxype | carboxype | 1368 | 1368 | 1368 | s3450 | s3451 | s223321 |
| 10517 | G13 | G14 | 1091 | 871,6 | 9007 | 190,5 | 8,3 | 0,938 | H5A034 C | sample | Hm Drgbl | Hm Drgbl | Hm Drgbl | CPE002EB | CPE002EO | CPE002FR | NM 03311 | NM 0331 | NM 0331 | ZCRB1 | ZCRB1 | ZCRB1 | zinc finger CCHC-t | zinc finger | zinc finger | 85437 | 85437 | 85437 | s39987 | s39985 | s39986 |
| 10587 | J11 | J12 | 772 | 853,6 | 6420 | 205,4 | 8,3 | 0,938 | H5A034 C | sample | Hm Drgbl | Hm Drgbl | Hm Drgbl | CPE002EB | CPE002EO | CPE002FR | NM 03351 | NM 0335 | NM 0335 | ARHGAP1 | ARHGAP1 | ARHGAP1 | Rho GTPase activ | Rho GTPa | Rho GTPa | 93663 | 93663 | 93663 | s41193 | s41194 | s41192 |
| 10669 | M20 | M21 | 1513 | 907,7 | 12506 | 176,3 | 8,3 | 0,938 | H5A034 C | sample | Hm Drgbl | Hm Drgbl | Hm Drgbl | CPE002EB | CPE002EO | CPE002FR | NM 01477 | NM 0147 | NM 0147 | CENTG1 | CENTG1 | CENTG1 | centaurin, gamma | centaurin, | centaurin, | 116986 | 116986 | 116986 | s42089 | s42091 | s42090 |
| 2693 | A13 | A14 | 848 | 724,5 | 7042 | 591,8 | 8,3 | 0,976 | H5A034 C | sample | Hm Drgbl | Hm Drgbl | Hm Drgbl | CPE002DP | CPE002EF | CPE002F5 | NM 00463 | NM 0046 | NM 0046 | MAPKAPK | MAPKAPK | MAPKAPK | mitogen-activatec | mitogen-a | mitogen-a | 7867 | 7867 | 7867 | s15427 | s15428 | s15429 |
| 2725 | B20 | B21 | 1464 | 689,9 | 12209 | 500 | 8,3 | 0,976 | H5A034 C | sample | Hm Drgbl | Hm Drgbl | Hm Drgbl | CPE002DP | CPE002EF | CPE002F5 | NM 05290 | NM 0529 | NM 0529 | STK11IP | STK11IP | STK11IP | serine/threonine | serine/thr | serine/thr | 114790 | 114790 | 114790 | s41609 | s41610 | s41611 |
| 2790 | E14 | E15 | 1305 | 636,3 | 10783 | 434,8 | 8,3 | 0,976 | H5A034 C | sample | Hm Drgbl | Hm Drgbl | Hm Drgbl | CPE002DP | CPE002EF | CPE002F5 | NM 01756 | NM 0175 | NM 0175 | NAGK | NAGK | NAGK | N-acetylglucosam | N-acetylgl | N-acetylgl | 55577 | 55577 | 55577 | s31007 | s31008 | s224374 |
| 2808 | E9 | E10 | 1244 | 612,5 | 10353 | 448,7 | 8,3 | 0,976 | H5A034 C | sample | Hm Drgbl | Hm Drgbl | Hm Drgbl | CPE002DP | CPE002EF | CPE002F5 | NM 00380 | NM 0038 | NM 0038 | RIPK1 | RIPK1 | RIPK1 | receptor (TNFRSF) | receptor ( | receptor ( | 8737 | 8737 | 8737 | s16651 | s16652 | s16653 |
| 2831 | F8 | F9 | 1365 | 631,8 | 11269 | 446 | 8,3 | 0,976 | H5A034 C | sample | Hm Drgbl | Hm Drgbl | Hm Drgbl | CPE002DP | CPE002EF | CPE002F5 | NM 00101 | NM 0010 | NM 0010 | DGKK | DGKK | DGKK | diacylglycerol kin | diacylglyc | diacylglyc | 139189 | 139189 | 139189 | s44175 | s44176 | s44177 |
| 2884 | I12 | I13 | 501 | 614 | 4177 | 468,7 | 8,3 | 0,976 | H5A034 C | sample | Hm Drgbl | Hm Drgbl | Hm Drgbl | CPE002DP | CPE002EF | CPE002F5 | NM 02173 | NM 0217 | NM 0217 | TSKS | TSKS | TSKS | testis-specific kin | testis-spe | testis-spe | 60385 | 60385 | 60385 | s34052 | s34053 | s34054 |
| 2931 | K11 | K12 | 928 | 637,1 | 7718 | 495,6 | 8,3 | 0,976 | H5A034 C | sample | Hm Drgbl | Hm Drgbl | Hm Drgbl | CPE002DP | CPE002EF | CPE002F5 | NM 01240 | NM 0124 | NM 0124 | PICK1 | PICK1 | PICK1 | protein interactin | protein in | protein in | 9463 | 9463 | 9463 | s18128 | s18129 | s18130 |
| 2932 | K12 | K13 | 1305 | 638,2 | 10780 | 454,2 | 8,3 | 0,976 | H5A034 C | sample | Hm Drgbl | Hm Drgbl | Hm Drgbl | CPE002DP | CPE002EF | CPE002F5 | NM 02301 | NM 0230 | NM 0230 | NADK | NADK | NADK | NAD kinase | NAD kinas | NAD kinas | 65220 | 65220 | 65220 | s35239 | s224642 | s35241 |
| 2952 | K9 | K10 | 951 | 662,6 | 7911 | 448,5 | 8,3 | 0,976 | H5A034 C | sample | Hm Drgbl | Hm Drgbl | Hm Drgbl | CPE002DP | CPE002EF | CPE002F5 | NM 00483 | NM 0048 | NM 0048 | EIF2AK3 | EIF2AK3 | EIF2AK3 | eukaryotic transla | eukaryoti | eukaryoti | 9451 | 9451 | 9451 | s18101 | s18102 | s18103 |
| 3024 | N9 | N10 | 1007 | 683,4 | 8342 | 559,6 | 8,3 | 0,976 | H5A034 C | sample | Hm Drgbl | Hm Drgbl | Hm Drgbl | CPE002DP | CPE002EF | CPE002F5 | NM 01441 | NM 0144 | NM 0144 | EIF2AK1 | EIF2AK1 | EIF2AK1 | eukaryotic transla | eukaryoti | eukaryoti | 27102 | 27102 | 27102 | s25822 | s25823 | s25824 |
| 19997 | B13 | B14 | 1047 | 1104 | 8638 | 158 | 8,3 | 1,012 | H5A034 C | sample | Hm Gen E | Hm Gen E | Hm Gen E | CPE002JQ | CPE002O5 | CPE002KL | NM 20742 | NM 2074 | NM 2074 | FLJ45983 | FLJ45983 | FLJ45983 | FLJ45983 protein | FLJ45983 r | FLJ45983 r | 399717 | 399717 | 399717 | s53165 | s53166 | s226516 |
| 20010 | B3 | B4 | 1668 | 1153 | 13779 | 154,2 | 8,3 | 1,012 | H5A034 C | sample | Hm Gen E | Hm Gen E | Hm Gen E | CPE002JQ | CPE002O5 | CPE002KL | NM 20330 | NM 2033 | NM 2033 | MGC3477 | MGC3477 | MGC3477 | ribosomal protein | ribosomal | ribosomal | 399670 | 399670 | 399670 | s53147 | s53148 | s53149 |
| 20064 | D9 | D10 | 1357 | 1187 | 11215 | 154,4 | 8,3 | 1,012 | H5A034 C | sample | Hm Gen E | Hm Gen E | Hm Gen E | CPE002JQ | CPE002O5 | CPE002KL | XM 94164 | XM 9416 | XM 9416 | LOC39988 | LOC39988 | LOC39988 | similar to heterog | similar to | similar to | 399881 | 399881 | 399881 | s53188 | s53189 | s53190 |
| 20079 | E22 | E23 | 1139 | 1097 | 9482 | 132,2 | 8,3 | 1,012 | H5A034 C | sample | Hm Gen E | Hm Gen E | Hm Gen E | CPE002JQ | CPE002O5 | CPE002KL | XM 37646 | XM 3764 | XM 3764 | FLJ23152 | FLJ23152 | FLJ23152 | hypothetical gene | hypotheti | hypotheti | 401236 | 401236 | 401236 | s53525 | s53526 | s226555 |
| 20086 | E7 | E8 | 1336 | 1088 | 11070 | 149,4 | 8,3 | 1,012 | H5A034 C | sample | Hm Gen E | Hm Gen E | Hm Gen E | CPE002JQ | CPE002O5 | CPE002KL | NM 00100 | NM 0010 | NM 0010 | OR6Y1 | OR6Y1 | OR6Y1 | olfactory recepto | olfactory r | olfactory r | 391112 | 391112 | 391112 | s52925 | s52926 | s52927 |
| 20103 | F22 | F23 | 1233 | 1126 | 10188 | 118,2 | 8,3 | 1,012 | H5A034 C | sample | Hm Gen E | Hm Gen E | Hm Gen E | CPE002JQ | CPE002O5 | CPE002KL | NM 00100 | NM 0010 | NM 0010 | OR4C45 | OR4C45 | OR4C45 | olfactory recepto | olfactory r | olfactory r | 403257 | 403257 | 403257 | s53807 | s53808 | s53809 |
| 20107 | F4 | F5 | 982 | 1090 | 8126 | 141,5 | 8,3 | 1,012 | H5A034 C | sample | Hm Gen E | Hm Gen E | Hm Gen E | CPE002JQ | CPE002O5 | CPE002KL | NM 00100 | NM 0010 | NM 0010 | LOC40257 | LOC40257 | LOC40257 | hypothetical LOC | hypotheti | hypotheti | 402573 | 402573 | 402573 | s53780 | s53781 | s196478 |
| 20168 | I16 | I17 | 924 | 1071 | 7668 | 149,7 | 8,3 | 1,012 | H5A034 C | sample | Hm Gen E | Hm Gen E | Hm Gen E | CPE002JQ | CPE002O5 | CPE002KL | NM 20750 | NM 2075 | NM 2075 | FLJ42280 | FLJ42280 | FLJ42280 | FLJ42280 protein | FLJ42280 r | FLJ42280 r | 401388 | 401388 | 401388 | s53579 | s53581 | s226562 |
| 20181 | I6 | I7 | 548 | 1355 | 4530 | 127 | 8,3 | 1,012 | H5A034 C | sample | Hm Gen E | Hm Gen E | Hm Gen E | CPE002JQ | CPE002O5 | CPE002KL | XM 37947 | XM 3794 | XM 3794 | LOC40131 | LOC40131 | LOC40131 | hypothetical LOC | hypotheti | hypotheti | 401317 | 401317 | 401317 | s53567 | s53568 | s196446 |
| 20186 | J10 | J11 | 1048 | 1093 | 8650 | 148 | 8,3 | 1,012 | H5A034 C | sample | Hm Gen E | Hm Gen E | Hm Gen E | CPE002JQ | CPE002O5 | CPE002KL | NM 20699 | NM 2069 | NM 2069 | SCGB1D4 | SCGB1D4 | SCGB1D4 | secretoglobin, far | secretoglc | secretoglc | 404552 | 404552 | 404552 | s53865 | s53866 | s226580 |
| 20230 | K7 | K8 | 497 | 1231 | 4111 | 166,2 | 8,3 | 1,012 | H5A034 C | sample | Hm Gen E | Hm Gen E | Hm Gen E | CPE002JQ | CPE002O5 | CPE002KL | XM 00113 | XM 0011 | XM 0011 | LOC39224 | LOC39224 | LOC39224 | hypothetical gene | hypotheti | hypotheti | 392242 | 392242 | 392242 | s53045 | s53046 | s226501 |
| 20237 | L13 | L14 | 1081 | 1093 | 9022 | 152,6 | 8,3 | 1,012 | H5A034 C | sample | Hm Gen E | Hm Gen E | Hm Gen E | CPE002JQ | CPE002O5 | CPE002KL | NM 00101 | NM 0010 | NM 0010 | VMAC | VMAC | VMAC | vimentin-type IF-a | vimentin- | vimentin- | 400673 | 400673 | 400673 | s53338 | s53339 | s226545 |
| 20252 | L5 | L6 | 1088 | 1087 | 9077 | 135,1 | 8,3 | 1,012 | H5A034 C | sample | Hm Gen E | Hm Gen E | Hm Gen E | CPE002JQ | CPE002O5 | CPE002KL | XM 00112 | XM 0011 | XM 0011 | FLJ38028 | FLJ38028 | FLJ38028 | hypothetical gene | hypotheti | hypotheti | 400643 | 400643 | 400643 | s53325 | s53326 | s226540 |
| 20256 | L9 | L10 | 677 | 1231 | 5595 | 150,1 | 8,3 | 1,012 | H5A034 C | sample | Hm Gen E | Hm Gen E | Hm Gen E | CPE002JQ | CPE002O5 | CPE002KL | XM 94016 | XM 9401 | XM 9271 | LOC40065 | LOC40065 | LOC40065 | similar to 40S ribo | similar to | similar to | 400652 | 400652 | 400652 | s53331 | s53332 | s226542 |
| 20340 | P2 | P3 | 955 | 1254 | 7928 | 128,2 | 8,3 | 1,012 | H5A034 C | sample | Hm Gen E | Hm Gen E | Hm Gen E | CPE002JQ | CPE002O5 | CPE002KL | NM 00101 | NM 0010 | NM 0010 | LOC43998 | LOC43998 | LOC43998 | hypothetical gene | hypotheti | hypotheti | 439985 | 439985 | 439985 | s53980 | s53981 | s53982 |
| 467 | D19 | D20 | 2119 | 1554 | 17535 | 309,8 | 8,3 | 1,212 | H5A034 C | sample | Hm Drgbl | Hm Drgbl | Hm Drgbl | CPE002E0 | CPE002EZ | CPE002FK | NM 20712 | NM 2071 | NM 2071 | EXT2 | EXT2 | EXT2 | exostoses (multip | exostoses | exostoses | 2132 | 2132 | 2132 | s4892 | s4893 | s4894 |
| 518 | F21 | F22 | 2291 | 1760 | 18942 | 289,4 | 8,3 | 1,212 | H5A034 C | sample | Hm Drgbl | Hm Drgbl | Hm Drgbl | CPE002E0 | CPE002EZ | CPE002FK | NM 00144 | NM 0014 | NM 0014 | FABP1 | FABP1 | FABP1 | fatty acid binding | fatty acid | fatty acid | 2168 | 2168 | 2168 | s4969 | s4967 | s4968 |
| 531 | G11 | G12 | 1478 | 1823 | 12262 | 347 | 8,3 | 1,212 | H5A034 C | sample | Hm Drgbl | Hm Drgbl | Hm Drgbl | CPE002E0 | CPE002EZ | CPE002FK | NM 00141 | NM 0014 | NM 0014 | EIF1AX | EIF1AX | EIF1AX | eukaryotic transla | eukaryoti | eukaryoti | 1964 | 1964 | 1964 | s4552 | s4554 | s4553 |
| 627 | K11 | K12 | 1774 | 1842 | 14775 | 320,2 | 8,3 | 1,212 | H5A034 C | sample | Hm Drgbl | Hm Drgbl | Hm Drgbl | CPE002E0 | CPE002EZ | CPE002FK | NM 00522 | NM 0052 | NM 0052 | ELK1 | ELK1 | ELK1 | ELK1, member of | ELK1, men | ELK1, men | 2002 | 2002 | 2002 | s4632 | s4633 | s4634 |
| 756 | P2 | P3 | 3045 | 1793 | 25271 | 445,1 | 8,3 | 1,212 | H5A034 C | sample | Hm Drgbl | Hm Drgbl | Hm Drgbl | CPE002E0 | CPE002EZ | CPE002FK | NM 00208 | NM 0020 | NM 0020 | GRN | GRN | GRN | granulin | granulin | granulin | 2896 | 2896 | 2896 | s6150 | s6151 | s6149 |
| 7683 | A11 | A12 | 1602 | 587,7 | 13225 | 416,5 | 8,3 | 1,267 | H5A034 C | sample | Hm Drgbl | Hm Drgbl | Hm Drgbl | CPE002E4 | CPE002EV | CPE002FG | NM 00575 | NM 0057 | NM 0057 | ABI2 | ABI2 | ABI2 | abl interactor 2 | abl intera | abl intera | 10152 | 10152 | 10152 | s19771 | s19770 | s19769 |
| 7763 | D19 | D20 | 1115 | 550,9 | 9279 | 365,7 | 8,3 | 1,267 | H5A034 C | sample | Hm Drgbl | Hm Drgbl | Hm Drgbl | CPE002E4 | CPE002EV | CPE002FG | NM 00610 | NM 0061 | NM 0061 | WFDC2 | WFDC2 | WFDC2 | WAP four-disulfid | WAP four- | WAP four- | 10406 | 10406 | 10406 | s20356 | s195032 | s195031 |
| 7795 | E4 | E5 | 893 | 563 | 7427 | 354,6 | 8,3 | 1,267 | H5A034 C | sample | Hm Drgbl | Hm Drgbl | Hm Drgbl | CPE002E4 | CPE002EV | CPE002FG | NM 00645 | NM 0064 | NM 0064 | MXD4 | MXD4 | MXD4 | MAX dimerizatior | MAX dime | MAX dime | 10608 | 10608 | 10608 | s20828 | s20827 | s20826 |
| 7820 | F5 | F6 | 1098 | 521,3 | 9145 | 362 | 8,3 | 1,267 | H5A034 C | sample | Hm Drgbl | Hm Drgbl | Hm Drgbl | CPE002E4 | CPE002EV | CPE002FG | NM 00611 | NM 0061 | NM 0061 | CD2BP2 | CD2BP2 | CD2BP2 | CD2 (cytoplasmic | CD2 (cyto | CD2 (cyto | 10421 | 10421 | 10421 | s20382 | s20383 | s20381 |
| 7915 | J4 | J5 | 1539 | 655,2 | 12700 | 434,1 | 8,3 | 1,267 | H5A034 C | sample | Hm Drgbl | Hm Drgbl | Hm Drgbl | CPE002E4 | CPE002EV | CPE002FG | NM 00681 | NM 0068 | NM 0068 | PDIA5 | PDIA5 | PDIA5 | protein disulfide | protein di | protein di | 10954 | 10954 | 10954 | s21559 | s21560 | s21558 |
| 7940 | K5 | K6 | 1161 | 563,1 | 9605 | 360,6 | 8,3 | 1,267 | H5A034 C | sample | Hm Drgbl | Hm Drgbl | Hm Drgbl | CPE002E4 | CPE002EV | CPE002FG | NM 00585 | NM 0058 | NM 0058 | RAMP2 | RAMP2 | RAMP2 | receptor (G prote | receptor ( | receptor ( | 10266 | 10266 | 10266 | s20057 | s223100 | s223099 |
| 7945 | L1 | L2 | 1487 | 591,6 | 12401 | 376,6 | 8,3 | 1,267 | H5A034 C | sample | Hm Drgbl | Hm Drgbl | Hm Drgbl | CPE002E4 | CPE002EV | CPE002FG | NM 00636 | NM 0063 | NM 0063 | HOXB13 | HOXB13 | HOXB13 | homeobox B13 | homeobo | homeobo | 10481 | 10481 | 10481 | s20529 | s20530 | s20531 |
| 7955 | L19 | L20 | 1058 | 556,8 | 8831 | 347,2 | 8,3 | 1,267 | H5A034 C | sample | Hm Drgbl | Hm Drgbl | Hm Drgbl | CPE002E4 | CPE002EV | CPE002FG | NM 19914 | NM 1991 | NM 1991 | CARM1 | CARM1 | CARM1 | coactivator-assoc | coactivatc | coactivatc | 10498 | 10498 | 10498 | s20577 | s20578 | s20579 |
| 7968 | L9 | L10 | 1110 | 537,4 | 9208 | 322,6 | 8,3 | 1,267 | H5A034 C | sample | Hm Drgbl | Hm Drgbl | Hm Drgbl | CPE002E4 | CPE002EV | CPE002FG | NM 00636 | NM 0063 | NM 0063 | CREB3 | CREB3 | CREB3 | cAMP responsive | cAMP resp | cAMP resp | 10488 | 10488 | 10488 | s20552 | s20551 | s223123 |
| 8005 | N20 | N21 | 955 | 609,1 | 7917 | 376,6 | 8,3 | 1,267 | H5A034 C | sample | Hm Drgbl | Hm Drgbl | Hm Drgbl | CPE002E4 | CPE002EV | CPE002FG | NM 00686 | NM 0068 | NM 0068 | RAB35 | RAB35 | RAB35 | RAB35, member F | RAB35, m | RAB35, m | 11021 | 11021 | 11021 | s21709 | s21707 | s21708 |
| 8019 | O11 | O12 | 839 | 645,8 | 6949 | 449,6 | 8,3 | 1,267 | H5A034 C | sample | Hm Drgbl | Hm Drgbl | Hm Drgbl | CPE002E4 | CPE002EV | CPE002FG | NM 00607 | NM 0060 | NM 0060 | CCL26 | CCL26 | CCL26 | chemokine (C-C m | chemokin | chemokin | 10344 | 10344 | 10344 | s20230 | s20231 | s20232 |
| 8040 | O9 | O10 | 1371 | 610,4 | 11356 | 445 | 8,3 | 1,267 | H5A034 C | sample | Hm Drgbl | Hm Drgbl | Hm Drgbl | CPE002E4 | CPE002EV | CPE002FG | NM 00606 | NM 0060 | NM 0060 | TLR6 | TLR6 | TLR6 | toll-like receptor | toll-like re | toll-like re | 10333 | 10333 | 10333 | s20215 | s20216 | s20217 |
| 3135 | C22 | C23 | 1195 | 1534 | 10082 | 469,2 | 8,4 | 0,52 | H5A034 C | sample | Hm Drgbl | Hm Drgbl | Hm Drgbl | CPE002DC | CPE002EG | CPE002F6 | NM 01767 | NM 0176 | NM 0176 | MTMR8 | MTMR8 | MTMR8 | myotubularin rela | myotubul | myotubul | 55613 | 55613 | 55613 | s31077 | s31078 | s31079 |
| 12369 | D17 | D18 | 822 | 961 | 6927 | 131,5 | 8,4 | 0,632 | H5A034 C | sample | Hm Gen E | Hm Gen E | Hm Gen E | CPE002J5 | CPE002NK | CPE002K1 | NM 00337 | NM 0033 | NM 0033 | VGF | VGF | VGF | VGF nerve growth | VGF nerve | VGF nerve | 7425 | 7425 | 7425 | s14786 | s14787 | s14788 |
| 2414 | E21 | E22 | 2279 | 738,9 | 19146 | 362,9 | 8,4 | 0,661 | H5A034 C | sample | Hm Drgbl | Hm Drgbl | Hm Drgbl | CPE002DC | CPE002EE | CPE002F4 | NM 00078 | NM 0007 | NM 0007 | DCK | DCK | DCK | deoxycytidine kin | deoxycyti | deoxycyti | 1633 | 1633 | 1633 | s186 | s187 | s188 |
| 4452 | J2 | J3 | 1836 | 680,1 | 15372 | 374,5 | 8,4 | 0,709 | H5A034 C | sample | Hm Drgbl | Hm Drgbl | Hm Drgbl | CPE002DU | CPE002EK | CPE002FQ | NM 15327 | NM 1532 | NM 1532 | BEST4 | BEST4 | BEST4 | bestrophin 4 | bestrophi | bestrophi | 266675 | 266675 | 266675 | s49003 | s49004 | s49005 |
| 345 | O17 | O18 | 631 | 1384 | 5288 | 619,2 | 8,4 | 0,715 | H5A034 C | sample | Hm Drgbl | Hm Drgbl | Hm Drgbl | CPE002DT | CPE002EJ | CPE002F9 | NM 00299 | NM 0029 | NM 0029 | CCL24 | CCL24 | CCL24 | chemokine (C-C m | chemokin | chemokin | 6369 | 6369 | 6369 | s12613 | s194796 | s224581 |
| 6555 | B11 | B12 | 1526 | 668,6 | 12803 | 195,9 | 8,4 | 0,737 | H5A034 C | sample | Hm Drgbl | Hm Drgbl | Hm Drgbl | CPE002E1 | CPE002EY | CPE002FJ | NM 03124 | NM 0312 | NM 0312 | HNRPA2B1 | HNRPA2B1 | HNRPA2B1 | heterogeneous nu | heteroger | heteroger | 3181 | 3181 | 3181 | s6713 | s6715 | s6714 |
| 3997 | G20 | G21 | 2321 | 700,1 | 19413 | 297,4 | 8,4 | 0,747 | H5A034 C | sample | Hm Drgbl | Hm Drgbl | Hm Drgbl | CPE002DS | CPE002EI | CPE002F8 | NM 00044 | NM 0004 | NM 0004 | RAG1 | RAG1 | RAG1 | recombination ac | recombina | recombina | 5896 | 5896 | 5896 | s11752 | s11753 | s11754 |
| 4139 | M19 | M20 | 1479 | 686,9 | 12452 | 455,6 | 8,4 | 0,747 | H5A034 C | sample | Hm Drgbl | Hm Drgbl | Hm Drgbl | CPE002DS | CPE002EI | CPE002F8 | NM 00094 | NM 0009 | NM 0009 | PON3 | PON3 | PON3 | paraoxonase 3 | paraoxona | paraoxona | 5446 | 5446 | 5446 | s10837 | s10835 | s10836 |
| 15660 | M2 | M3 | 1513 | 838,4 | 12739 | 119,3 | 8,4 | 0,764 | H5A034 C | sample | Hm Gen E | Hm Gen E | Hm Gen E | CPE002JE | CPE002NT | CPE002K9 | NM 02407 | NM 0240 | NM 0240 | TMEM38A | TMEM38A | TMEM38A | transmembrane p | transmem | transmem | 79041 | 79041 | 79041 | s35471 | s35472 | s35473 |
| 6142 | P7 | P8 | 1777 | 884,1 | 15001 | 413,7 | 8,4 | 0,767 | H5A034 C | sample | Hm Drgbl | Hm Drgbl | Hm Drgbl | CPE002DY | CPE002F1 | CPE002FM | NM 02098 | NM 0209 | NM 0209 | AQP9 | AQP9 | AQP9 | aquaporin 9 | aquaporir | aquaporir | 366 | 366 | 366 | s1535 | s1536 | s223949 |
| 996 | J2 | J3 | 1330 | 714,3 | 11199 | 416,9 | 8,4 | 0,796 | H5A034 C | sample | Hm Drgbl | Hm Drgbl | Hm Drgbl | CPE002FU | CPE002FY | CPE002G2 | NM 00563 | NM 0056 | NM 0056 | SYBL1 | SYBL1 | SYBL1 | synaptobrevin-lik | synaptobr | synaptobr | 6845 | 6845 | 6845 | s13670 | s13671 | s13672 |
| 3755 | M19 | M20 | 2821 | 1603 | 23832 | 361,9 | 8,4 | 0,808 | H5A034 C | sample | Hm Drgbl | Hm Drgbl | Hm Drgbl | CPE002DR | CPE002EH | CPE002F7 | NM 00203 | NM 0020 | NM 0020 | DARC | DARC | DARC | Duffy blood grou | Duffy bloc | Duffy bloc | 2532 | 2532 | 2532 | s5429 | s5430 | s194424 |
| 8083 | A4 | A5 | 1118 | 635,1 | 9434 | 498,9 | 8,4 | 0,808 | H5A034 C | sample | Hm Drgbl | Hm Drgbl | Hm Drgbl | CPE002E5 | CPE002EU | CPE002FF | NM 01503 | NM 0150 | NM 0150 | ZHX3 | ZHX3 | ZHX3 | zinc fingers and h | zinc finger | zinc finger | 23051 | 23051 | 23051 | s22920 | s22921 | s22922 |
| 8126 | C21 | C22 | 1797 | 678,1 | 15056 | 313 | 8,4 | 0,808 | H5A034 C | sample | Hm Drgbl | Hm Drgbl | Hm Drgbl | CPE002E5 | CPE002EU | CPE002FF | NM 00704 | NM 0070 | NM 0070 | KRR1 | KRR1 | KRR1 | KRR1, small subur | KRR1, sma | KRR1, sma | 11103 | 11103 | 11103 | s21894 | s21893 | s21892 |
| 8367 | M22 | M23 | 2171 | 668,6 | 18140 | 451,4 | 8,4 | 0,808 | H5A034 C | sample | Hm Drgbl | Hm Drgbl | Hm Drgbl | CPE002E5 | CPE002EU | CPE002FF | NM 01527 | NM 0152 | NM 0152 | TRIM2 | TRIM2 | TRIM2 | tripartite motif-cc | tripartite | tripartite | 23321 | 23321 | 23321 | s23555 | s23556 | s23554 |
| 8415 | O22 | O23 | 1627 | 583,1 | 13721 | 425,7 | 8,4 | 0,808 | H5A034 C | sample | Hm Drgbl | Hm Drgbl | Hm Drgbl | CPE002E5 | CPE002EU | CPE002FF | XM 37125 | XM 3712 | XM 3712 | USP24 | USP24 | USP24 | ubiquitin specific | ubiquitin | ubiquitin | 23358 | 23358 | 23358 | s23641 | s23642 | s23643 |
| 21805 | M20 | M21 | 409 | 884,4 | 3439 | 433,7 | 8,4 | 0,884 | H5A034 C | sample | Hm Gen E | Hm Gen E | Hm Gen E | CPE002JU | CPE002O9 | CPE002KP | XM 94251 | XM 9425 | XM 9425 | LOC65283 | LOC65283 | LOC65283 | similar to protein | similar to | similar to | 652832 | 652832 | 652832 | s57516 | s57517 | s227419 |
| 21839 | N8 | N9 | 321 | 977,3 | 2712 | 580,6 | 8,4 | 0,884 | H5A034 C | sample | Hm Gen E | Hm Gen E | Hm Gen E | CPE002JU | CPE002O9 | CPE002KP | XM 93625 | XM 9362 | XM 9362 | LOC65388 | LOC65388 | LOC65388 | similar to U5 snRI | similar to | similar to | 653889 | 653889 | 653889 | s57801 | s57802 | s227447 |
| 21865 | P1 | P2 | 205 | 1175 | 1718 | 439,2 | 8,4 | 0,884 | H5A034 C | sample | Hm Gen E | Hm Gen E | Hm Gen E | CPE002JU | CPE002O9 | CPE002KP | XM 94158 | XM 9415 | XM 9415 | LOC65220 | LOC65220 | LOC65220 | hypothetical prot | hypotheti | hypotheti | 652203 | 652203 | 652203 | s57325 | s197858 | s197859 |
| 4636 | B12 | B13 | 2371 | 844,3 | 19838 | 505,6 | 8,4 | 0,923 | H5A034 C | sample | Hm Drgbl | Hm Drgbl | Hm Drgbl | CPE002DV | CPE002EL | CPE002FP | NM 00667 | NM 0066 | NM 0066 | USP19 | USP19 | USP19 | ubiquitin specific | ubiquitin | ubiquitin | 10869 | 10869 | 10869 | s21339 | s21340 | s21341 |
| 4758 | G14 | G15 | 1005 | 842,2 | 8438 | 415,5 | 8,4 | 0,923 | H5A034 C | sample | Hm Drgbl | Hm Drgbl | Hm Drgbl | CPE002DV | CPE002EL | CPE002FP | NM 00381 | NM 0038 | NM 0038 | ADAM23 | ADAM23 | ADAM23 | ADAM metallope | ADAM me | ADAM me | 8745 | 8745 | 8745 | s16669 | s16670 | s16671 |
| 2690 | A10 | A11 | 812 | 611,6 | 6840 | 434,8 | 8,4 | 0,988 | H5A034 C | sample | Hm Drgbl | Hm Drgbl | Hm Drgbl | CPE002DP | CPE002EF | CPE002F5 | NM 01628 | NM 0162 | NM 0162 | TAOK3 | TAOK3 | TAOK3 | TAO kinase 3 | TAO kinas | TAO kinas | 51347 | 51347 | 51347 | s27994 | s27995 | s27996 |
| 2750 | C21 | C22 | 1341 | 694,5 | 11316 | 561 | 8,4 | 0,988 | H5A034 C | sample | Hm Drgbl | Hm Drgbl | Hm Drgbl | CPE002DP | CPE002EF | CPE002F5 | NM 00100 | NM 0010 | NM 0010 | STK16 | STK16 | STK16 | serine/threonine | serine/thr | serine/thr | 8576 | 8576 | 8576 | s16337 | s16338 | s16339 |
| 2760 | C9 | C10 | 1805 | 703,8 | 15161 | 429,8 | 8,4 | 0,988 | H5A034 C | sample | Hm Drgbl | Hm Drgbl | Hm Drgbl | CPE002DP | CPE002EF | CPE002F5 | NM 00365 | NM 0036 | NM 0036 | CAMK1 | CAMK1 | CAMK1 | calcium/calmodul | calcium/ca | calcium/ca | 8536 | 8536 | 8536 | s16236 | s16237 | s16238 |
| 2770 | D18 | D19 | 1054 | 629,5 | 8887 | 435,3 | 8,4 | 0,988 | H5A034 C | sample | Hm Drgbl | Hm Drgbl | Hm Drgbl | CPE002DP | CPE002EF | CPE002F5 | NM 14525 | NM 1452 | NM 1452 | ACVR1C | ACVR1C | ACVR1C | activin A receptor | activin A r | activin A r | 130399 | 130399 | 130399 | s43497 | s43498 | s43499 |
| 2881 | I1 | I2 | 1038 | 658,2 | 8696 | 668 | 8,4 | 0,988 | H5A034 C | sample | Hm Drgbl | Hm Drgbl | Hm Drgbl | CPE002DP | CPE002EF | CPE002F5 | NM 00467 | NM 0046 | NM 0046 | MAP3K6 | MAP3K6 | MAP3K6 | mitogen-activatec | mitogen-a | mitogen-a | 9064 | 9064 | 9064 | s17288 | s17289 | s17290 |
| 3003 | N11 | N12 | 1221 | 633,6 | 10309 | 492,6 | 8,4 | 0,988 | H5A034 C | sample | Hm Drgbl | Hm Drgbl | Hm Drgbl | CPE002DP | CPE002EF | CPE002F5 | NM 01569 | NM 0156 | NM 0156 | STK36 | STK36 | STK36 | serine/threonine | serine/thr | serine/thr | 27148 | 27148 | 27148 | s25906 | s25907 | s25908 |
| 3062 | P21 | P22 | 2344 | 778,4 | 19645 | 630,6 | 8,4 | 0,988 | H5A034 C | sample | Hm Drgbl | Hm Drgbl | Hm Drgbl | CPE002DP | CPE002EF | CPE002F5 | NM 01612 | NM 0161 | NM 0161 | IRAK4 | IRAK4 | IRAK4 | interleukin-1 rece | interleuki | interleuki | 51135 | 51135 | 51135 | s27527 | s27528 | s27529 |
| 20068 | E12 | E13 | 746 | 1248 | 6290 | 186,3 | 8,4 | 1,024 | H5A034 C | sample | Hm Gen E | Hm Gen E | Hm Gen E | CPE002JQ | CPE002O5 | CPE002KL | XM 93278 | XM 9327 | XM 9327 | FLJ44606 | FLJ44606 | FLJ44606 | hypothetical gene | hypotheti | hypotheti | 401207 | 401207 | 401207 | s53509 | s53510 | s53511 |
| 20101 | F20 | F21 | 1033 | 1219 | 8687 | 180,8 | 8,4 | 1,024 | H5A034 C | sample | Hm Gen E | Hm Gen E | Hm Gen E | CPE002JQ | CPE002O5 | CPE002KL | NM 00100 | NM 0010 | NM 0010 | OR4A47 | OR4A47 | OR4A47 | olfactory recepto | olfactory r | olfactory r | 403253 | 403253 | 403253 | s53804 | s53805 | s53806 |
| 20123 | G19 | G20 | 1410 | 1191 | 11914 | 170 | 8,4 | 1,024 | H5A034 C | sample | Hm Gen E | Hm Gen E | Hm Gen E | CPE002JQ | CPE002O5 | CPE002KL | XM 37292 | XM 3729 | XM 3729 | LOC39135 | LOC39135 | LOC39135 | similar to Epitheli | similar to | similar to | 391359 | 391359 | 391359 | s52983 | s52984 | s52985 |
| 20138 | H10 | H11 | 1245 | 1149 | 10408 | 143,7 | 8,4 | 1,024 | H5A034 C | sample | Hm Gen E | Hm Gen E | Hm Gen E | CPE002JQ | CPE002O5 | CPE002KL | NM 20345 | NM 2034 | NM 2034 | PPAPDC2 | PPAPDC2 | PPAPDC2 | phosphatidic acid | phosphat | phosphat | 403313 | 403313 | 403313 | s53828 | s53829 | s53830 |
| 20214 | K14 | K15 | 638 | 1167 | 5386 | 150,7 | 8,4 | 1,024 | H5A034 C | sample | Hm Gen E | Hm Gen E | Hm Gen E | CPE002JQ | CPE002O5 | CPE002KL | XM 94003 | XM 9400 | XM 9400 | FLJ45872 | FLJ45872 | FLJ45872 | FLJ45872 protein | FLJ45872 r | FLJ45872 r | 401478 | 401478 | 401478 | s53612 | s53613 | s53614 |
| 20285 | N13 | N14 | 938 | 1078 | 7865 | 126,4 | 8,4 | 1,024 | H5A034 C | sample | Hm Gen E | Hm Gen E | Hm Gen E | CPE002JQ | CPE002O5 | CPE002KL | NM 00101 | NM 0010 | NM 0010 | C1orf140 | C1orf140 | C1orf140 | chromosome 1 or | chromoso | chromoso | 400804 | 400804 | 400804 | s53375 | s53376 | s53377 |
| 20348 | P5 | P6 | 893 | 1375 | 7480 | 129,1 | 8,4 | 1,024 | H5A034 C | sample | Hm Gen E | Hm Gen E | Hm Gen E | CPE002JQ | CPE002O5 | CPE002KL | NM 20747 | NM 2074 | NM 2074 | FLJ41733 | FLJ41733 | FLJ41733 | FLJ41733 protein | FLJ41733 p | FLJ41733 p | 400870 | 400870 | 400870 | s53399 | s53400 | s53401 |
| 498 | E3 | E4 | 2817 | 1990 | 23596 | 314,7 | 8,4 | 1,226 | H5A034 C | sample | Hm Drgbl | Hm Drgbl | Hm Drgbl | CPE002E0 | CPE002EZ | CPE002FK | NM 00196 | NM 0323 | NM 0323 | EEF1D | EEF1D | EEF1D | eukaryotic transla | eukaryoti | eukaryoti | 1936 | 1936 | 1936 | s223468 | s4487 | s4485 |
| 613 | J20 | J21 | 2323 | 1820 | 19487 | 351,2 | 8,4 | 1,226 | H5A034 C | sample | Hm Drgbl | Hm Drgbl | Hm Drgbl | CPE002E0 | CPE002EZ | CPE002FK | NM 00527 | NM 0052 | NM 0052 | GNL1 | GNL1 | GNL1 | guanine nucleotic | guanine n | guanine n | 2794 | 2794 | 2794 | s5928 | s5927 | s5926 |
| 710 | N21 | N22 | 2177 | 1612 | 18303 | 336,4 | 8,4 | 1,226 | H5A034 C | sample | Hm Drgbl | Hm Drgbl | Hm Drgbl | CPE002E0 | CPE002EZ | CPE002FK | NM 00411 | NM 0336 | NM 0336 | FGF13 | FGF13 | FGF13 | fibroblast growth | fibroblast | fibroblast | 2258 | 2258 | 2258 | s223532 | s5160 | s5159 |
| 724 | O12 | O13 | 2849 | 1878 | 23958 | 380,8 | 8,4 | 1,226 | H5A034 C | sample | Hm Drgbl | Hm Drgbl | Hm Drgbl | CPE002E0 | CPE002EZ | CPE002FK | NM 00102 | NM 0010 | NM 0010 | GCH1 | GCH1 | GCH1 | GTP cyclohydrola | GTP cyclol | GTP cyclol | 2643 | 2643 | 2643 | s5645 | s5643 | s5644 |
| 7685 | A13 | A14 | 995 | 620,3 | 8322 | 460 | 8,4 | 1,282 | H5A034 C | sample | Hm Drgbl | Hm Drgbl | Hm Drgbl | CPE002E4 | CPE002EV | CPE002FG | NM 00576 | NM 0057 | NM 0057 | TRIM28 | TRIM28 | TRIM28 | tripartite motif-cc | tripartite | tripartite | 10155 | 10155 | 10155 | s19779 | s19780 | s19778 |
| 7718 | B21 | B22 | 1092 | 629,6 | 9142 | 387,4 | 8,4 | 1,282 | H5A034 C | sample | Hm Drgbl | Hm Drgbl | Hm Drgbl | CPE002E4 | CPE002EV | CPE002FG | NM 00608 | NM 0060 | NM 0060 | SCML2 | SCML2 | SCML2 | sex comb on midl | sex comb | sex comb | 10389 | 10389 | 10389 | s20313 | s20314 | s20315 |
| 7761 | D17 | D18 | 918 | 535,1 | 7707 | 350,4 | 8,4 | 1,282 | H5A034 C | sample | Hm Drgbl | Hm Drgbl | Hm Drgbl | CPE002E4 | CPE002EV | CPE002FG | NM 00610 | NM 0061 | NM 0061 | ST3GAL6 | ST3GAL6 | ST3GAL6 | ST3 beta-galactos | ST3 beta-g | ST3 beta-g | 10402 | 10402 | 10402 | s20347 | s20348 | s20349 |
| 7824 | F9 | F10 | 994 | 507,6 | 8337 | 354,6 | 8,4 | 1,282 | H5A034 C | sample | Hm Drgbl | Hm Drgbl | Hm Drgbl | CPE002E4 | CPE002EV | CPE002FG | NM 00632 | NM 0063 | NM 0063 | PGRMC2 | PGRMC2 | PGRMC2 | progesterone rec | progester | progester | 10424 | 10424 | 10424 | s704 | s706 | s705 |
| 7848 | G9 | G10 | 1281 | 573,5 | 10794 | 358,7 | 8,4 | 1,282 | H5A034 C | sample | Hm Drgbl | Hm Drgbl | Hm Drgbl | CPE002E4 | CPE002EV | CPE002FG | NM 00581 | NM 0058 | NM 0058 | KLRG1 | KLRG1 | KLRG1 | killer cell lectin-lik | killer cell l | killer cell l | 10219 | 10219 | 10219 | s19934 | s19935 | s19936 |
| 7957 | L20 | L21 | 982 | 534,7 | 8269 | 331,3 | 8,4 | 1,282 | H5A034 C | sample | Hm Drgbl | Hm Drgbl | Hm Drgbl | CPE002E4 | CPE002EV | CPE002FG | NM 02433 | NM 0243 | NM 0243 | SLC27A3 | SLC27A3 | SLC27A3 | solute carrier fam | solute car | solute car | 11000 | 11000 | 11000 | s21657 | s21658 | s21659 |
| 8011 | N4 | N5 | 1177 | 613,1 | 9885 | 325,3 | 8,4 | 1,282 | H5A034 C | sample | Hm Drgbl | Hm Drgbl | Hm Drgbl | CPE002E4 | CPE002EV | CPE002FG | NM 00108 | NM 0010 | NM 0010 | LILRB4 | LILRB4 | LILRB4 | leukocyte immuno | leukocyte | leukocyte | 11006 | 11006 | 11006 | s21669 | s21670 | s21671 |
| 8014 | N7 | N8 | 1117 | 562,1 | 9327 | 349,5 | 8,4 | 1,282 | H5A034 C | sample | Hm Drgbl | Hm Drgbl | Hm Drgbl | CPE002E4 | CPE002EV | CPE002FG | NM 02021 | NM 0202 | NM 0202 | SEMA4B | SEMA4B | SEMA4B | sema domain, im | sema dom | sema dom | 10509 | 10509 | 10509 | s20597 | s20596 | s20595 |
| 8061 | P6 | P7 | 1449 | 545 | 12111 | 434,7 | 8,4 | 1,282 | H5A034 C | sample | Hm Drgbl | Hm Drgbl | Hm Drgbl | CPE002E4 | CPE002EV | CPE002FG | NM 00108 | NM 0010 | NM 0010 | LILRB3 | LILRB3 | LILRB3 | leukocyte immuno | leukocyte | leukocyte | 11025 | 11025 | 11025 | s21719 | s21721 | s21720 |
| 2509 | I20 | I21 | 2049 | 702,2 | 17391 | 313,5 | 8,5 | 0,669 | H5A034 C | sample | Hm Drgbl | Hm Drgbl | Hm Drgbl | CPE002DC | CPE002EE | CPE002F4 | NM 03301 | NM 0330 | NM 0330 | PCTK1 | PCTK1 | PCTK1 | PCTAIRE protein k | PCTAIRE p | PCTAIRE p | 5127 | 5127 | 5127 | s10156 | s10157 | s10158 |
| 16568 | C16 | C17 | 1388 | 979,6 | 11847 | 236,4 | 8,5 | 0,672 | H5A034 C | sample | Hm Gen E | Hm Gen E | Hm Gen E | CPE002JH | CPE002NV | CPE002KC | NM 13835 | NM 1383 | NM 1383 | SCRN2 | SCRN2 | SCRN2 | secernin 2 | secernin 2 | secernin 2 | 90507 | 90507 | 90507 | s40406 | s195552 | s195553 |
| 4444 | J12 | J13 | 773 | 739,2 | 6551 | 397,5 | 8,5 | 0,717 | H5A034 C | sample | Hm Drgbl | Hm Drgbl | Hm Drgbl | CPE002DU | CPE002EK | CPE002FQ | NM 19850 | NM 1985 | NM 1985 | ATP13A5 | ATP13A5 | ATP13A5 | ATPase type 13A5 | ATPase ty | ATPase ty | 344905 | 344905 | 344905 | s51113 | s51114 | s51115 |
| 194 | I10 | I11 | 488 | 1378 | 4163 | 563,7 | 8,5 | 0,723 | H5A034 C | sample | Hm Drgbl | Hm Drgbl | Hm Drgbl | CPE002DT | CPE002EJ | CPE002F9 | NM 00308 | NM 0030 | NM 0030 | SMPD2 | SMPD2 | SMPD2 | sphingomyelin ph | sphingom | sphingom | 6610 | 6610 | 6610 | s13172 | s13170 | s13171 |
| 1976 | C16 | C17 | 1722 | 668 | 14593 | 258 | 8,5 | 0,739 | H5A034 C | sample | Hm Drgbl | Hm Drgbl | Hm Drgbl | CPE002FX | CPE002G1 | CPE002G5 | NM 13347 | NM 1334 | NM 1334 | ZNF431 | ZNF431 | ZNF431 | zinc finger protei | zinc finger | zinc finger | 170959 | 170959 | 170959 | s46878 | s46879 | s46880 |
| 3878 | B21 | B22 | 2029 | 756,1 | 17168 | 365,3 | 8,5 | 0,756 | H5A034 C | sample | Hm Drgbl | Hm Drgbl | Hm Drgbl | CPE002DS | CPE002EI | CPE002F8 | NM 00682 | NM 0068 | NM 0068 | PKIA | PKIA | PKIA | protein kinase (cA | protein ki | protein ki | 5569 | 5569 | 5569 | s11076 | s11075 | s11074 |
| 4040 | I16 | I17 | 918 | 694,6 | 7758 | 241,9 | 8,5 | 0,756 | H5A034 C | sample | Hm Drgbl | Hm Drgbl | Hm Drgbl | CPE002DS | CPE002EI | CPE002F8 | NM 00288 | NM 0028 | NM 0028 | RAP1A | RAP1A | RAP1A | RAP1A, member o | RAP1A, m | RAP1A, m | 5906 | 5906 | 5906 | s11779 | s11780 | s11781 |
| 13732 | M12 | M13 | 1011 | 1056 | 8554 | 131,2 | 8,5 | 0,798 | H5A034 C | sample | Hm Gen E | Hm Gen E | Hm Gen E | CPE002JC | CPE002NC | CPE002K4 | NM 01560 | NM 0156 | NM 0156 | C1orf144 | C1orf144 | C1orf144 | chromosome 1 op | chromoso | chromoso | 26099 | 26099 | 26099 | s25097 | s25098 | s25099 |
| 861 | D6 | D7 | 1592 | 790,4 | 13489 | 331,2 | 8,5 | 0,806 | H5A034 C | sample | Hm Drgbl | Hm Drgbl | Hm Drgbl | CPE002FU | CPE002FY | CPE002G2 | NM 00691 | NM 0069 | NM 0069 | RNF5 | RNF5 | RNF5 | ring finger protei | ring finger | ring finger | 6048 | 6048 | 6048 | s12076 | s12077 | s12078 |
| 1040 | L16 | L17 | 2084 | 747,2 | 17763 | 369,9 | 8,5 | 0,806 | H5A034 C | sample | Hm Drgbl | Hm Drgbl | Hm Drgbl | CPE002FU | CPE002FY | CPE002G2 | NM 00104 | NM 0010 | NM 0010 | TFAP2A | TFAP2A | TFAP2A | transcription fact | transcript | transcript | 7020 | 7020 | 7020 | s14003 | s14004 | s14005 |
| 1058 | M10 | M11 | 1393 | 785,7 | 11790 | 402,9 | 8,5 | 0,806 | H5A034 C | sample | Hm Drgbl | Hm Drgbl | Hm Drgbl | CPE002FU | CPE002FY | CPE002G2 | NM 00269 | NM 0026 | NM 0026 | POU2F1 | POU2F1 | POU2F1 | POU domain, clas | POU dom | POU dom | 5451 | 5451 | 5451 | s10847 | s10848 | s10849 |
| 5559 | H22 | H23 | 1659 | 667 | 14145 | 543,6 | 8,5 | 0,81 | H5A034 C | sample | Hm Drgbl | Hm Drgbl | Hm Drgbl | CPE002DX | CPE002F2 | CPE002FN | NM 00421 | NM 0042 | NM 0042 | RAB11B | RAB11B | RAB11B | RAB11B, member | RAB11B, r | RAB11B, r | 9230 | 9230 | 9230 | s225102 | s17647 | s17649 |
| 5695 | N15 | N16 | 1600 | 759,6 | 13593 | 287,1 | 8,5 | 0,81 | H5A034 C | sample | Hm Drgbl | Hm Drgbl | Hm Drgbl | CPE002DX | CPE002F2 | CPE002FN | NM 00383 | NM 0038 | NM 0038 | SIGLEC5 | SIGLEC5 | SIGLEC5 | sialic acid binding | sialic acid | sialic acid | 8778 | 8778 | 8778 | s16723 | s16724 | s16725 |
| 3466 | A18 | A19 | 1956 | 1431 | 16652 | 310,3 | 8,5 | 0,817 | H5A034 C | sample | Hm Drgbl | Hm Drgbl | Hm Drgbl | CPE002DR | CPE002EH | CPE002F7 | NM 00104 | NM 0010 | NM 0010 | SSTR1 | SSTR1 | SSTR1 | somatostatin rece | somatosta | somatosta | 6751 | 6751 | 6751 | s13495 | s13496 | s13497 |
| 3560 | E16 | E17 | 2015 | 1430 | 17028 | 308,1 | 8,5 | 0,817 | H5A034 C | sample | Hm Drgbl | Hm Drgbl | Hm Drgbl | CPE002DR | CPE002EH | CPE002F7 | NM 00366 | NM 0036 | NM 0036 | LGR5 | LGR5 | LGR5 | leucine-rich repea | leucine-ric | leucine-ric | 8549 | 8549 | 8549 | s16275 | s16276 | s16277 |
| 3571 | E4 | E5 | 1115 | 1350 | 9523 | 294,5 | 8,5 | 0,817 | H5A034 C | sample | Hm Drgbl | Hm Drgbl | Hm Drgbl | CPE002DR | CPE002EH | CPE002F7 | NM 00350 | NM 0035 | NM 0035 | FZD1 | FZD1 | FZD1 | frizzled homolog | frizzled hc | frizzled hc | 8321 | 8321 | 8321 | s15835 | s15836 | s15837 |
| 3591 | F22 | F23 | 2071 | 1522 | 17656 | 385,9 | 8,5 | 0,817 | H5A034 C | sample | Hm Drgbl | Hm Drgbl | Hm Drgbl | CPE002DR | CPE002EH | CPE002F7 | NM 03078 | NM 0307 | NM 0307 | GPR63 | GPR63 | GPR63 | G protein-couplec | G protein- | G protein- | 81491 | 81491 | 81491 | s37565 | s37566 | s224878 |
| 3722 | L10 | L11 | 1174 | 1211 | 9987 | 288,8 | 8,5 | 0,817 | H5A034 C | sample | Hm Drgbl | Hm Drgbl | Hm Drgbl | CPE002DR | CPE002EH | CPE002F7 | NM 17505 | NM 1750 | NM 1750 | TAAR9 | TAAR9 | TAAR9 | trace amine assoc | trace amir | trace amir | 134860 | 134860 | 134860 | s43899 | s43900 | s223304 |
| 21780 | L2 | L3 | 323 | 953,6 | 2747 | 543,1 | 8,5 | 0,895 | H5A034 C | sample | Hm Gen E | Hm Gen E | Hm Gen E | CPE002JU | CPE002O9 | CPE002KP | XM 93050 | XM 9305 | XM 9305 | LOC65374 | LOC65374 | LOC65374 | similar to Dipepti | similar to | similar to | 653748 | 653748 | 653748 | s57752 | s57753 | s227443 |
| 21823 | N15 | N16 | 188 | 905,4 | 1604 | 553,8 | 8,5 | 0,895 | H5A034 C | sample | Hm Gen E | Hm Gen E | Hm Gen E | CPE002JU | CPE002O9 | CPE002KP | XM 94150 | XM 9415 | XM 9415 | LOC65216 | LOC65216 | LOC65216 | similar to phosph | similar to | similar to | 652164 | 652164 | 652164 | s57312 | s57314 | s227384 |
| 21834 | N3 | N4 | 489 | 1022 | 4149 | 414,6 | 8,5 | 0,895 | H5A034 C | sample | Hm Gen E | Hm Gen E | Hm Gen E | CPE002JU | CPE002O9 | CPE002KP | XM 94516 | XM 9451 | XM 9451 | LOC65204 | LOC65204 | LOC65204 | hypothetical prot | hypotheti | hypotheti | 652048 | 652048 | 652048 | s57300 | s57301 | s227381 |
| 4764 | G2 | G3 | 1016 | 782,5 | 8657 | 497,7 | 8,5 | 0,934 | H5A034 C | sample | Hm Drgbl | Hm Drgbl | Hm Drgbl | CPE002DV | CPE002EL | CPE002FP | NM 00361 | NM 0036 | NM 0036 | PRSS12 | PRSS12 | PRSS12 | protease, serine, | protease, | protease, | 8492 | 8492 | 8492 | s16124 | s16125 | s16126 |
| 4881 | L17 | L18 | 2622 | 899,3 | 22364 | 363,7 | 8,5 | 0,934 | H5A034 C | sample | Hm Drgbl | Hm Drgbl | Hm Drgbl | CPE002DV | CPE002EL | CPE002FP | NM 00103 | NM 0010 | NM 0010 | KLK3 | KLK3 | KLK3 | kallikrein-related | kallikrein- | kallikrein- | 354 | 354 | 354 | s200412 | s200413 | s200414 |
| 4882 | L18 | L19 | 2530 | 908,2 | 21624 | 394,6 | 8,5 | 0,934 | H5A034 C | sample | Hm Drgbl | Hm Drgbl | Hm Drgbl | CPE002DV | CPE002EL | CPE002FP | NM 01821 | NM 0182 | NM 0182 | USP40 | USP40 | USP40 | ubiquitin specific | ubiquitin | ubiquitin | 55230 | 55230 | 55230 | s30503 | s30504 | s30505 |
| 10436 | C5 | C6 | 1313 | 916,1 | 11197 | 169,1 | 8,5 | 0,96 | H5A034 C | sample | Hm Drgbl | Hm Drgbl | Hm Drgbl | CPE002EB | CPE002EO | CPE002FR | NM 03284 | NM 0328 | NM 0328 | SPRYD3 | SPRYD3 | SPRYD3 | SPRY domain con | SPRY dom | SPRY dom | 84926 | 84926 | 84926 | s39678 | s39677 | s39676 |
| 10523 | G19 | G20 | 1088 | 864,9 | 9231 | 171,5 | 8,5 | 0,96 | H5A034 C | sample | Hm Drgbl | Hm Drgbl | Hm Drgbl | CPE002EB | CPE002EO | CPE002FR | NM 13899 | NM 1389 | NM 1389 | CNTNAP4 | CNTNAP4 | CNTNAP4 | contactin associat | contactin | contactin | 85445 | 85445 | 85445 | s40011 | s40009 | s40010 |
| 10572 | I2 | I3 | 1523 | 895,4 | 12994 | 176,9 | 8,5 | 0,96 | H5A034 C | sample | Hm Drgbl | Hm Drgbl | Hm Drgbl | CPE002EB | CPE002EO | CPE002FR | NM 05294 | NM 0529 | NM 0529 | SNX26 | SNX26 | SNX26 | sorting nexin 26 | sorting ne | sorting ne | 115703 | 115703 | 115703 | s41853 | s41851 | s41852 |
| 10679 | M8 | M9 | 1298 | 828,7 | 10977 | 144,9 | 8,5 | 0,96 | H5A034 C | sample | Hm Drgbl | Hm Drgbl | Hm Drgbl | CPE002EB | CPE002EO | CPE002FR | NM 05297 | NM 0529 | NM 0529 | LRG1 | LRG1 | LRG1 | leucine-rich alpha | leucine-ric | leucine-ric | 116844 | 116844 | 116844 | s42069 | s42068 | s42070 |
| 2810 | F10 | F11 | 1025 | 635 | 8681 | 422,9 | 8,5 | 1 | H5A034 C | sample | Hm Drgbl | Hm Drgbl | Hm Drgbl | CPE002DP | CPE002EF | CPE002F5 | NM 00103 | NM 0010 | NM 0010 | PNCK | PNCK | PNCK | pregnancy upregu | pregnancy | pregnancy | 139728 | 139728 | 139728 | s44235 | s44236 | s44237 |
| 3001 | N1 | N2 | 922 | 569,5 | 7839 | 481,6 | 8,5 | 1 | H5A034 C | sample | Hm Drgbl | Hm Drgbl | Hm Drgbl | CPE002DP | CPE002EF | CPE002F5 | NM 01437 | NM 0143 | NM 0143 | SRPK3 | SRPK3 | SRPK3 | SFRS protein kina | SFRS prot | SFRS prot | 26576 | 26576 | 26576 | s25545 | s25546 | s25547 |
| 3009 | N17 | N18 | 751 | 659,7 | 6416 | 475,5 | 8,5 | 1 | H5A034 C | sample | Hm Drgbl | Hm Drgbl | Hm Drgbl | CPE002DP | CPE002EF | CPE002F5 | NM 01323 | NM 0132 | NM 0132 | STK39 | STK39 | STK39 | serine threonine k | serine thr | serine thr | 27347 | 27347 | 27347 | s26207 | s26208 | s26209 |

| | | | | | | | |
|---|---|---|---|---|---|---|---|
| 3012 | N2 | N3 | 1161 | 670,7 | 9813 | 473,3 | 8,5 |
| 19996 | B12 | B13 | 833 | 1116 | 7122 | 170,1 | 8,5 |
| 20044 | D12 | D13 | 1214 | 1204 | 10293 | 167,4 | 8,5 |
| 20076 | E2 | E3 | 876 | 1138 | 7452 | 171,7 | 8,5 |
| 20090 | F10 | F11 | 782 | 1221 | 6649 | 148,7 | 8,5 |
| 20115 | G11 | G12 | 1128 | 1045 | 9589 | 138,5 | 8,5 |
| 20191 | J15 | J16 | 689 | 1096 | 5844 | 149,3 | 8,5 |
| 20238 | L14 | L15 | 597 | 1089 | 5056 | 140,8 | 8,5 |
| 488 | E16 | E17 | 2718 | 1914 | 23175 | 283,6 | 8,5 |
| 519 | F22 | F23 | 2576 | 1749 | 22011 | 310 | 8,5 |
| 533 | G13 | G14 | 1453 | 1723 | 12394 | 356 | 8,5 |
| 542 | G21 | G22 | 2614 | 1906 | 22110 | 388,8 | 8,5 |
| 554 | H10 | H11 | 2812 | 1886 | 23809 | 280,1 | 8,5 |
| 656 | L16 | L17 | 2315 | 1796 | 19612 | 310,8 | 8,5 |
| 7692 | A2 | A3 | 1094 | 575,5 | 9353 | 431,8 | 8,5 |
| 7800 | E9 | E10 | 824 | 599 | 6998 | 338,1 | 8,5 |
| 7806 | F14 | F15 | 1236 | 556,6 | 10529 | 431,6 | 8,5 |
| 7813 | F20 | F21 | 1365 | 599,5 | 11539 | 404,3 | 8,5 |
| 11326 | H7 | H8 | 1328 | 814,3 | 11418 | 122,7 | 8,6 |
| 11466 | N3 | N4 | 1394 | 816,7 | 11936 | 135,2 | 8,6 |
| 5340 | O2 | O3 | 1465 | 600,2 | 12603 | 229,6 | 8,6 |
| 2570 | L10 | L11 | 1625 | 729,7 | 13977 | 304,1 | 8,6 |
| 4548 | N2 | N3 | 1064 | 793,8 | 9158 | 436,9 | 8,6 |
| 169 | H1 | H2 | 657 | 1647 | 5621 | 516 | 8,6 |
| 186 | H3 | H4 | 513 | 1330 | 4417 | 458,9 | 8,6 |
| 199 | I15 | I16 | 631 | 1441 | 5417 | 465,5 | 8,6 |
| 260 | K5 | K6 | 668 | 1561 | 5717 | 534,3 | 8,6 |
| 2281 | P1 | P2 | 675 | 675,2 | 5791 | 443,2 | 8,6 |
| 6865 | O1 | O2 | 328 | 650,4 | 2823 | 353,5 | 8,6 |
| 4088 | K16 | K17 | 2146 | 681 | 18524 | 282,7 | 8,6 |
| 4195 | O4 | O5 | 891 | 704,3 | 7706 | 443,1 | 8,6 |
| 5817 | C17 | C18 | 1455 | 768 | 12491 | 456,1 | 8,6 |
| 3465 | A17 | A18 | 2011 | 1385 | 17335 | 329,1 | 8,6 |
| 8075 | A19 | A20 | 1589 | 705,5 | 13637 | 407,8 | 8,6 |
| 1730 | I10 | I11 | 575 | 781,1 | 4921 | 413,3 | 8,6 |
| 1825 | M1 | M2 | 662 | 702,6 | 5675 | 386,2 | 8,6 |
| 1834 | M18 | M19 | 1531 | 764,6 | 13126 | 448,4 | 8,6 |
| 21707 | I19 | I20 | 438 | 1106 | 3753 | 515,3 | 8,6 |
| 21767 | K8 | K9 | 132 | 884,8 | 1134 | 651,4 | 8,6 |
| 21792 | L9 | L10 | 295 | 906 | 2534 | 788,5 | 8,6 |
| 21796 | M12 | M13 | 405 | 941,2 | 3483 | 546,9 | 8,6 |
| 21829 | N20 | N21 | 563 | 1002 | 4830 | 537,3 | 8,6 |
| 4800 | H9 | H10 | 1454 | 816,7 | 12474 | 325,6 | 8,6 |
| 4868 | K5 | K6 | 1306 | 804,1 | 11214 | 470,3 | 8,6 |
| 4909 | M20 | M21 | 2304 | 841,7 | 19844 | 399,5 | 8,6 |
| 4921 | N1 | N2 | 1725 | 778,4 | 14873 | 450,2 | 8,6 |
| 4944 | N9 | N10 | 1514 | 839,5 | 12962 | 415,2 | 8,6 |
| 2740 | C12 | C13 | 731 | 611,5 | 6253 | 387,1 | 8,6 |
| 2797 | E20 | E21 | 1410 | 623,4 | 12113 | 487,5 | 8,6 |
| 2820 | F2 | F3 | 1136 | 666,8 | 9749 | 593,6 | 8,6 |
| 2838 | G14 | G15 | 1009 | 631,3 | 8671 | 480,6 | 8,6 |
| 2852 | G5 | G6 | 1616 | 677,6 | 13843 | 513,9 | 8,6 |
| 3020 | N5 | N6 | 1153 | 595,4 | 9871 | 484,2 | 8,6 |
| 19986 | A3 | A4 | 952 | 1188 | 8166 | 110,4 | 8,6 |
| 20140 | H12 | H13 | 1114 | 1162 | 9604 | 165,7 | 8,6 |
| 20209 | K1 | K2 | 850 | 1122 | 7345 | 121,3 | 8,6 |
| 20217 | K17 | K18 | 558 | 1220 | 4776 | 134,4 | 8,6 |
| 20288 | N16 | N17 | 1190 | 1074 | 10262 | 106,4 | 8,6 |
| 407 | A8 | A9 | 880 | 1756 | 7569 | 405 | 8,6 |
| 438 | C14 | C15 | 1951 | 1717 | 16735 | 257,6 | 8,6 |
| 7703 | A8 | A9 | 1408 | 626,2 | 12093 | 434,6 | 8,6 |
| 7725 | B6 | B7 | 1165 | 546,5 | 10073 | 411,3 | 8,6 |
| 7760 | D16 | D17 | 632 | 585,3 | 5460 | 293,2 | 8,6 |
| 7791 | E22 | E23 | 1155 | 548,5 | 9895 | 404,3 | 8,6 |
| 7798 | E7 | E8 | 1072 | 550,1 | 9166 | 389,2 | 8,6 |
| 7812 | F2 | F3 | 1459 | 562,8 | 12548 | 360,8 | 8,6 |
| 7819 | F4 | F5 | 1148 | 540,8 | 9906 | 342 | 8,6 |
| 7832 | G16 | G17 | 331 | 641,4 | 2843 | 382,7 | 8,6 |
| 7837 | G20 | G21 | 1332 | 607,8 | 11504 | 406,7 | 8,6 |
| 7994 | N10 | N11 | 1035 | 646,5 | 8934 | 415 | 8,6 |
| 8002 | N18 | N19 | 1122 | 551,2 | 9658 | 359,8 | 8,6 |
| 3301 | J20 | J21 | 1642 | 1351 | 14269 | 294,5 | 8,7 |
| 12987 | N11 | N12 | 1587 | 1038 | 13821 | 106,4 | 8,7 |
| 19478 | L21 | L22 | 860 | 1313 | 7457 | 199,3 | 8,7 |
| 11258 | F10 | F11 | 1966 | 937,1 | 17084 | 124,6 | 8,7 |
| 11306 | H10 | H11 | 1510 | 834,7 | 13158 | 127,3 | 8,7 |
| 11316 | H2 | H3 | 1851 | 905,2 | 16188 | 120,3 | 8,7 |
| 12433 | G1 | G2 | 1797 | 1044 | 15637 | 137,4 | 8,7 |
| 12457 | H1 | H2 | 1044 | 1064 | 9095 | 167,2 | 8,7 |
| 7221 | M6 | M7 | 853 | 656,9 | 7455 | 204,1 | 8,7 |
| 2462 | G21 | G22 | 1674 | 713,5 | 14571 | 380,5 | 8,7 |
| 2564 | K5 | K6 | 1754 | 761 | 15264 | 399,4 | 8,7 |
| 4450 | J18 | J19 | 1055 | 633,9 | 9134 | 325,1 | 8,7 |
| 179 | H19 | H20 | 710 | 1508 | 6191 | 388,1 | 8,7 |
| 273 | L17 | L18 | 328 | 1220 | 2854 | 465 | 8,7 |
| 283 | L4 | L5 | 759 | 1509 | 6572 | 372,8 | 8,7 |
| 307 | M4 | M5 | 716 | 1670 | 6252 | 426,5 | 8,7 |
| 6595 | C4 | C5 | 1386 | 648,2 | 11992 | 416,6 | 8,7 |
| 3911 | C8 | C9 | 1914 | 746 | 16639 | 343,8 | 8,7 |
| 4042 | I18 | I19 | 1606 | 770,3 | 13933 | 334,8 | 8,7 |
| 11765 | K13 | K14 | 1597 | 955,7 | 13952 | 129,9 | 8,7 |
| 5868 | E2 | E3 | 1629 | 924,9 | 14126 | 605,3 | 8,7 |
| 1332 | H2 | H3 | 644 | 718,2 | 5582 | 432,6 | 8,7 |
| 1449 | M17 | M18 | 2606 | 775,7 | 22648 | 392,5 | 8,7 |
| 16454 | N21 | N22 | 2046 | 1038 | 17884 | 276,8 | 8,7 |
| 13609 | H1 | H2 | 1425 | 1064 | 12422 | 164,5 | 8,7 |
| 886 | E7 | E8 | 965 | 823,8 | 8356 | 464,9 | 8,7 |
| 969 | I17 | I18 | 1637 | 807,8 | 14207 | 295,9 | 8,7 |
| 5738 | P10 | P11 | 1292 | 733,2 | 11299 | 634,4 | 8,7 |
| 3510 | C14 | C15 | 1708 | 1258 | 14897 | 271,9 | 8,7 |
| 3680 | J16 | J17 | 1147 | 1262 | 9984 | 254,6 | 8,7 |
| 3706 | K18 | K19 | 1100 | 1354 | 9527 | 302,1 | 8,7 |
| 21598 | D7 | D8 | 534 | 1113 | 4665 | 621,6 | 8,7 |
| 21794 | M10 | M11 | 408 | 879,5 | 3544 | 629,4 | 8,7 |
| 21814 | M7 | M8 | 167 | 1001 | 1453 | 666,3 | 8,7 |
| 4957 | O20 | O21 | 2333 | 851 | 20183 | 315,6 | 8,7 |
| 10530 | G3 | G4 | 1178 | 814,4 | 10285 | 180,6 | 8,7 |
| 10574 | I21 | I22 | 931 | 812,9 | 8099 | 171,6 | 8,7 |
| 10626 | K3 | K4 | 878 | 860,1 | 7604 | 203,9 | 8,7 |
| 2756 | C5 | C6 | 1682 | 718 | 14647 | 390,4 | 8,7 |
| 2791 | E15 | E16 | 1092 | 638,8 | 9506 | 528,1 | 8,7 |
| 2981 | M13 | M14 | 646 | 701,7 | 5625 | 480,4 | 8,7 |
| 20055 | D22 | D23 | 1044 | 1063 | 9101 | 77,01 | 8,7 |
| 20065 | E1 | E2 | 408 | 1406 | 3540 | 147,1 | 8,7 |
| 20088 | E9 | E10 | 689 | 1137 | 5985 | 159,3 | 8,7 |
| 20097 | F17 | F18 | 1273 | 1119 | 11136 | 147,9 | 8,7 |
| 20127 | G22 | G23 | 1692 | 1055 | 14790 | 138,1 | 8,7 |
| 20170 | I18 | I19 | 783 | 1257 | 6849 | 175,3 | 8,7 |
| 20227 | K4 | K5 | 573 | 1137 | 5003 | 155,6 | 8,7 |
| 20240 | L16 | L17 | 1062 | 1070 | 9233 | 114,4 | 8,7 |
| 20242 | L18 | L19 | 1370 | 1089 | 11981 | 133,2 | 8,7 |
| 20258 | M10 | M11 | 1274 | 1097 | 11098 | 136,1 | 8,7 |
| 20260 | M12 | M13 | 1057 | 1093 | 9213 | 135,2 | 8,7 |
| 20267 | M19 | M20 | 1236 | 1189 | 10701 | 153,9 | 8,7 |
| 20310 | O14 | O15 | 1041 | 1121 | 9036 | 180,5 | 8,7 |
| 405 | A6 | A7 | 1410 | 1904 | 12298 | 482,4 | 8,7 |
| 439 | C15 | C16 | 1515 | 1804 | 13223 | 257,1 | 8,7 |
| 485 | E13 | E14 | 2091 | 1727 | 18215 | 281 | 8,7 |
| 579 | I11 | I12 | 2322 | 1719 | 20088 | 371,7 | 8,7 |
| 725 | O13 | O14 | 2228 | 1730 | 19411 | 372,6 | 8,7 |
| 730 | O18 | O19 | 1964 | 1741 | 17102 | 314,9 | 8,7 |
| 7765 | D20 | D21 | 1119 | 630,7 | 9789 | 363,1 | 8,7 |
| 7782 | E14 | E15 | 611 | 535,6 | 5305 | 353,2 | 8,7 |
| 7807 | F15 | F16 | 1364 | 540,2 | 11904 | 331,5 | 8,7 |
| 7830 | G14 | G15 | 432 | 555,5 | 3778 | 348 | 8,7 |
| 7835 | G19 | G20 | 1440 | 580,8 | 12508 | 355,2 | 8,7 |
| 7878 | I14 | I15 | 548 | 557,4 | 4775 | 374,6 | 8,7 |
| 7894 | I7 | I8 | 1146 | 541,1 | 9966 | 319,3 | 8,7 |
| 8058 | P3 | P4 | 1572 | 581,6 | 13714 | 424,4 | 8,7 |
| 11327 | H8 | H9 | 1566 | 819,7 | 13808 | 119,4 | 8,8 |
| 10938 | H3 | H4 | 1011 | 786,6 | 8930 | 447,8 | 8,8 |
| 2461 | G20 | G21 | 2604 | 712,8 | 23004 | 327,5 | 8,8 |
| 2505 | I17 | I18 | 1616 | 713,3 | 14275 | 293 | 8,8 |
| 4391 | G8 | G9 | 516 | 847,4 | 4563 | 387,3 | 8,8 |
| 282 | L3 | L4 | 581 | 1423 | 5124 | 477,5 | 8,8 |
| 337 | O1 | O2 | 286 | 1210 | 2523 | 597,2 | 8,8 |
| 2078 | G21 | G22 | 2475 | 715,6 | 21740 | 437,5 | 8,8 |
| 3897 | C17 | C18 | 1508 | 694,6 | 13305 | 377,8 | 8,8 |
| 3996 | G2 | G3 | 1504 | 703 | 13194 | 398,9 | 8,8 |
| 4068 | J2 | J3 | 1236 | 698,2 | 10834 | 327,5 | 8,8 |
| 5963 | I19 | I20 | 1270 | 838,6 | 11178 | 526,6 | 8,8 |
| 6049 | M1 | M2 | 605 | 786,4 | 5324 | 251,4 | 8,8 |
| 6090 | N3 | N4 | 1551 | 736,1 | 13579 | 240,3 | 8,8 |
| 1386 | J3 | J4 | 2146 | 808,3 | 18968 | 383,2 | 8,8 |
| 13737 | M17 | M18 | 1781 | 1071 | 15655 | 135,5 | 8,8 |
| 889 | F1 | F2 | 1363 | 805 | 12015 | 285,1 | 8,8 |
| 3463 | A15 | A16 | 1771 | 1430 | 15545 | 334,5 | 8,8 |
| 3499 | B4 | B5 | 1666 | 1343 | 14654 | 362,5 | 8,8 |
| 3659 | I19 | I20 | 1547 | 1327 | 13549 | 322,3 | 8,8 |
| 3742 | L7 | L8 | 1835 | 1423 | 16232 | 328,2 | 8,8 |
| 1700 | G5 | G6 | 2263 | 816,5 | 20017 | 389,2 | 8,8 |
| 1859 | N19 | N20 | 1562 | 715,4 | 13681 | 399,9 | 8,8 |
| 21830 | N21 | N22 | 474 | 1056 | 4161 | 509,3 | 8,8 |
| 4691 | D19 | D20 | 1535 | 826,3 | 13437 | 439,7 | 8,8 |
| 4976 | P16 | P17 | 1881 | 829,1 | 16566 | 509,6 | 8,8 |
| 10388 | A5 | A6 | 1087 | 936,2 | 9526 | 213,2 | 8,8 |
| 10540 | H12 | H13 | 687 | 998,5 | 6043 | 209,3 | 8,8 |
| 10713 | O17 | O18 | 1215 | 869,7 | 10749 | 175 | 8,8 |
| 2864 | H16 | H17 | 1122 | 645,2 | 9834 | 428,9 | 8,8 |
| 2950 | K7 | K8 | 880 | 617,9 | 7770 | 488,5 | 8,8 |
| 2974 | L7 | L8 | 809 | 636,9 | 7082 | 434 | 8,8 |
| 2979 | M11 | M12 | 981 | 629,3 | 8589 | 469,4 | 8,8 |
| 2986 | M18 | M19 | 1077 | 647,1 | 9430 | 395,6 | 8,8 |
| 3031 | O15 | O16 | 1409 | 668,1 | 12454 | 564,1 | 8,8 |
| 3070 | P7 | P8 | 1423 | 601,2 | 12505 | 507,6 | 8,8 |
| 20157 | H6 | H7 | 1346 | 1065 | 11862 | 136,4 | 8,8 |
| 20190 | J14 | J15 | 624 | 1190 | 5463 | 150,4 | 8,8 |
| 20313 | O17 | O18 | 708 | 1011 | 6259 | 99,47 | 8,8 |
| 434 | C10 | C11 | 2789 | 1918 | 24595 | 330,8 | 8,8 |
| 508 | F12 | F13 | 2020 | 1666 | 17834 | 313,1 | 8,8 |
| 532 | G12 | G13 | 1835 | 1714 | 16206 | 330,8 | 8,8 |
| 7704 | A9 | A10 | 1499 | 608,5 | 13189 | 453 | 8,8 |
| 7742 | C21 | C22 | 1342 | 592 | 11815 | 408,6 | 8,8 |
| 7764 | D2 | D3 | 1597 | 559,7 | 14087 | 376,2 | 8,8 |
| 7821 | F6 | F7 | 1148 | 585,8 | 10086 | 379,5 | 8,8 |
| 7854 | H14 | H15 | 1505 | 551,9 | 13187 | 396,1 | 8,8 |
| 7897 | J1 | J2 | 992 | 551,8 | 8761 | 395,7 | 8,8 |
| 7924 | K12 | K13 | 799 | 547,3 | 7029 | 360,8 | 8,8 |
| 6175 | B15 | B16 | 1503 | 615 | 13405 | 198,8 | 8,9 |
| 20408 | C16 | C17 | 1209 | 814,6 | 10775 | 173,5 | 8,9 |
| 2498 | I10 | I11 | 1718 | 681,4 | 15320 | 308,5 | 8,9 |
| 2604 | M2 | M3 | 999 | 661,7 | 8932 | 345,2 | 8,9 |
| 9214 | P7 | P8 | 1555 | 1695 | 13835 | 136 | 8,9 |
| 4335 | E22 | E23 | 1609 | 719,7 | 14325 | 365,7 | 8,9 |
| 1 | A1 | A2 | 526 | 1598 | 4691 | 786 | 8,9 |
| 43 | B4 | B5 | 519 | 1455 | 4641 | 566,5 | 8,9 |
| 190 | H7 | H8 | 512 | 1362 | 4553 | 656,6 | 8,9 |
| 230 | J21 | J22 | 634 | 1346 | 5624 | 411,9 | 8,9 |
| 2191 | L15 | L16 | 760 | 838 | 6791 | 455 | 8,9 |
| 11760 | J9 | J10 | 1109 | 767,1 | 9912 | 109,2 | 8,9 |
| 11865 | O17 | O18 | 1089 | 936,5 | 9728 | 144,8 | 8,9 |
| 1262 | E21 | E22 | 2067 | 774,2 | 18499 | 405,9 | 8,9 |
| 13626 | H3 | H4 | 1029 | 1080 | 9184 | 183,2 | 8,9 |
| 13632 | H9 | H10 | 1276 | 996,5 | 11362 | 153,9 | 8,9 |
| 5700 | N2 | N3 | 1479 | 671,8 | 13222 | 428,9 | 8,9 |
| 3508 | C12 | C13 | 1103 | 1214 | 9826 | 214,4 | 8,9 |
| 3562 | E18 | E19 | 1403 | 1303 | 12468 | 274 | 8,9 |
| 8294 | J21 | J22 | 1804 | 649,2 | 16017 | 432,8 | 8,9 |
| 10321 | O1 | O2 | 1001 | 875,2 | 8909 | 149,7 | 8,9 |
| 21709 | I20 | I21 | 249 | 931,1 | 2215 | 752,6 | 8,9 |
| 21716 | I5 | I6 | 167 | 1147 | 1484 | 736,3 | 8,9 |
| 21774 | L14 | L15 | 238 | 966,1 | 2126 | 703,1 | 8,9 |
| 21847 | O15 | O16 | 270 | 1002 | 2405 | 865,8 | 8,9 |
| 4752 | F9 | F10 | 1557 | 788,9 | 13867 | 499 | 8,9 |
| 4878 | L14 | L15 | 2716 | 869,6 | 24044 | 361,1 | 8,9 |
| 4969 | P1 | P2 | 1898 | 780,9 | 16896 | 505,7 | 8,9 |
| 10380 | A2 | A3 | 1750 | 907,1 | 15635 | 196,6 | 8,9 |
| 10459 | D4 | D5 | 1490 | 862,4 | 13308 | 165 | 8,9 |
| 10556 | H5 | H6 | 1084 | 890,4 | 9690 | 200,3 | 8,9 |
| 2786 | E10 | E11 | 1557 | 664,8 | 13896 | 516,2 | 8,9 |
| 2811 | F11 | F12 | 792 | 748,9 | 7074 | 518,7 | 8,9 |
| 2847 | G22 | G23 | 1415 | 683,9 | 12526 | 566,9 | 8,9 |
| 20078 | E21 | E22 | 1701 | 1202 | 15071 | 134,1 | 8,9 |
| 20247 | L22 | L23 | 1156 | 1204 | 10328 | 163,2 | 8,9 |
| 20337 | P17 | P18 | 614 | 1276 | 5479 | 117,6 | 8,9 |
| 528 | F9 | F10 | 1747 | 1792 | 15549 | 305,6 | 8,9 |

| | | | | | | | | | | | |
|---|---|---|---|---|---|---|---|---|---|---|---|
| 566 | H21 | H22 | 2210 | 1752 | 19710 | 314,5 | 8,9 | 1,299 | H5A034 C | sample | Hm Drgbl |
| 581 | I13 | I14 | 1193 | 1656 | 10583 | 426,2 | 8,9 | 1,299 | H5A034 C | sample | Hm Drgbl |
| 7797 | E6 | E7 | 1009 | 579,8 | 8952 | 410,1 | 8,9 | 1,359 | H5A034 C | sample | Hm Drgbl |
| 7867 | H4 | H5 | 1095 | 560,7 | 9765 | 397,4 | 8,9 | 1,359 | H5A034 C | sample | Hm Drgbl |
| 8013 | N6 | N7 | 1378 | 595 | 12207 | 375 | 8,9 | 1,359 | H5A034 C | sample | Hm Drgbl |
| 19345 | G1 | G2 | 1052 | 1252 | 9422 | 205,3 | 9 | 0,638 | H5A034 C | sample | Hm Gen E |
| 11307 | H11 | H12 | 728 | 936,3 | 6521 | 141,6 | 9 | 0,652 | H5A034 C | sample | Hm Drgbl |
| 11388 | K2 | K3 | 1465 | 844,5 | 13136 | 143,9 | 9 | 0,652 | H5A034 C | sample | Hm Drgbl |
| 11421 | L6 | L7 | 1516 | 832,4 | 13692 | 121,1 | 9 | 0,652 | H5A034 C | sample | Hm Drgbl |
| 11460 | N2 | N3 | 1764 | 851,3 | 15954 | 116,2 | 9 | 0,652 | H5A034 C | sample | Hm Drgbl |
| 2507 | I19 | I20 | 1207 | 741 | 10812 | 403,7 | 9 | 0,709 | H5A034 C | sample | Hm Drgbl |
| 114 | E3 | E4 | 246 | 1414 | 2202 | 597,3 | 9 | 0,766 | H5A034 C | sample | Hm Drgbl |
| 2050 | F18 | F19 | 1664 | 711,8 | 15058 | 352,9 | 9 | 0,783 | H5A034 C | sample | Hm Drgbl |
| 2247 | N22 | N23 | 2240 | 780,2 | 20266 | 452,1 | 9 | 0,783 | H5A034 C | sample | Hm Drgbl |
| 1160 | A16 | A17 | 2371 | 808,5 | 21385 | 469,5 | 9 | 0,826 | H5A034 C | sample | Hm Drgbl |
| 1237 | D20 | D21 | 1722 | 753,2 | 15529 | 238,4 | 9 | 0,826 | H5A034 C | sample | Hm Drgbl |
| 13716 | L2 | L3 | 1162 | 1005 | 10440 | 163 | 9 | 0,845 | H5A034 C | sample | Hm Gen E |
| 5439 | C22 | C23 | 2287 | 757,2 | 20595 | 557,6 | 9 | 0,857 | H5A034 C | sample | Hm Drgbl |
| 5660 | L5 | L6 | 1150 | 782,8 | 10402 | 226,6 | 9 | 0,857 | H5A034 C | sample | Hm Drgbl |
| 3682 | J18 | J19 | 1604 | 1467 | 14446 | 289,9 | 9 | 0,865 | H5A034 C | sample | Hm Drgbl |
| 3730 | L18 | L19 | 1139 | 1332 | 10226 | 345 | 9 | 0,865 | H5A034 C | sample | Hm Drgbl |
| 8402 | O10 | O11 | 2061 | 586,9 | 18483 | 491,5 | 9 | 0,865 | H5A034 C | sample | Hm Drgbl |
| 1844 | M5 | M6 | 1570 | 677,3 | 14055 | 331,1 | 9 | 0,942 | H5A034 C | sample | Hm Drgbl |
| 21541 | B20 | B21 | 281 | 1009 | 2533 | 344,7 | 9 | 0,947 | H5A034 C | sample | Hm Gen E |
| 21799 | M15 | M16 | 320 | 927,8 | 2869 | 594,2 | 9 | 0,947 | H5A034 C | sample | Hm Gen E |
| 4695 | D22 | D23 | 1834 | 884,9 | 16531 | 482,2 | 9 | 0,989 | H5A034 C | sample | Hm Drgbl |
| 4927 | N15 | N16 | 1632 | 779,9 | 14731 | 425,9 | 9 | 0,989 | H5A034 C | sample | Hm Drgbl |
| 10652 | L5 | L6 | 1222 | 825,8 | 11029 | 179,4 | 9 | 1,017 | H5A034 C | sample | Hm Drgbl |
| 2696 | A16 | A17 | 1663 | 661,7 | 14939 | 663,2 | 9 | 1,059 | H5A034 C | sample | Hm Drgbl |
| 2828 | F5 | F6 | 780 | 614,9 | 7009 | 534,7 | 9 | 1,059 | H5A034 C | sample | Hm Drgbl |
| 2840 | G16 | G17 | 970 | 622,5 | 8767 | 445,8 | 9 | 1,059 | H5A034 C | sample | Hm Drgbl |
| 2842 | G18 | G19 | 1178 | 660,1 | 10552 | 414,4 | 9 | 1,059 | H5A034 C | sample | Hm Drgbl |
| 3046 | O7 | O8 | 1664 | 696,6 | 14916 | 505,2 | 9 | 1,059 | H5A034 C | sample | Hm Drgbl |
| 20073 | E17 | E18 | 1203 | 1193 | 10802 | 152,4 | 9 | 1,098 | H5A034 C | sample | Hm Gen E |
| 20124 | G2 | G3 | 1003 | 1115 | 9042 | 107,8 | 9 | 1,098 | H5A034 C | sample | Hm Gen E |
| 20131 | G4 | G5 | 1021 | 1159 | 9162 | 159,9 | 9 | 1,098 | H5A034 C | sample | Hm Gen E |
| 20189 | J13 | J14 | 928 | 1072 | 8318 | 135,4 | 9 | 1,098 | H5A034 C | sample | Hm Gen E |
| 414 | B14 | B15 | 1558 | 1913 | 14049 | 355,6 | 9 | 1,314 | H5A034 C | sample | Hm Drgbl |
| 430 | B7 | B8 | 2013 | 1828 | 18182 | 322,1 | 9 | 1,314 | H5A034 C | sample | Hm Drgbl |
| 455 | C8 | C9 | 1581 | 1633 | 14254 | 303 | 9 | 1,314 | H5A034 C | sample | Hm Drgbl |
| 458 | D10 | D11 | 2304 | 1786 | 20684 | 317,2 | 9 | 1,314 | H5A034 C | sample | Hm Drgbl |
| 511 | F15 | F16 | 1582 | 1618 | 14168 | 276,4 | 9 | 1,314 | H5A034 C | sample | Hm Drgbl |
| 535 | G15 | G16 | 1947 | 1779 | 17549 | 317,5 | 9 | 1,314 | H5A034 C | sample | Hm Drgbl |
| 602 | J10 | J11 | 1806 | 1672 | 16328 | 339,3 | 9 | 1,314 | H5A034 C | sample | Hm Drgbl |
| 608 | J16 | J17 | 1889 | 1715 | 16966 | 299,1 | 9 | 1,314 | H5A034 C | sample | Hm Drgbl |
| 7779 | E11 | E12 | 1124 | 569,2 | 10170 | 424,9 | 9 | 1,374 | H5A034 C | sample | Hm Drgbl |
| 7787 | E19 | E20 | 1234 | 543,4 | 11136 | 390,2 | 9 | 1,374 | H5A034 C | sample | Hm Drgbl |
| 7900 | J12 | J13 | 1430 | 561,9 | 12818 | 357,7 | 9 | 1,374 | H5A034 C | sample | Hm Drgbl |
| 7928 | K16 | K17 | 829 | 530 | 7479 | 349,8 | 9 | 1,374 | H5A034 C | sample | Hm Drgbl |
| 9296 | D16 | D17 | 1365 | 1075 | 12483 | 107,3 | 9,1 | 0,632 | H5A034 C | sample | Hm Drgbl |
| 9930 | N3 | N4 | 1351 | 1115 | 12339 | 128,3 | 9,1 | 0,636 | H5A034 C | sample | Hm Drgbl |
| 12649 | P1 | P2 | 1255 | 972,5 | 11373 | 153,3 | 9,1 | 0,684 | H5A034 C | sample | Hm Gen E |
| 2459 | G19 | G20 | 1849 | 702,4 | 16851 | 295,5 | 9,1 | 0,717 | H5A034 C | sample | Hm Drgbl |
| 142 | F7 | F8 | 518 | 1344 | 4710 | 399,7 | 9,1 | 0,774 | H5A034 C | sample | Hm Drgbl |
| 193 | I1 | I2 | 423 | 1370 | 3834 | 685,9 | 9,1 | 0,774 | H5A034 C | sample | Hm Drgbl |
| 268 | L12 | L13 | 550 | 1361 | 5027 | 383,7 | 9,1 | 0,774 | H5A034 C | sample | Hm Drgbl |
| 318 | N14 | N15 | 547 | 1381 | 4983 | 545,7 | 9,1 | 0,774 | H5A034 C | sample | Hm Drgbl |
| 369 | P17 | P18 | 616 | 1504 | 5631 | 571 | 9,1 | 0,774 | H5A034 C | sample | Hm Drgbl |
| 4044 | I2 | I3 | 673 | 693,2 | 6121 | 388,6 | 9,1 | 0,809 | H5A034 C | sample | Hm Drgbl |
| 5840 | D16 | D17 | 1864 | 786,6 | 16950 | 497,3 | 9,1 | 0,831 | H5A034 C | sample | Hm Drgbl |
| 6062 | M21 | M22 | 2513 | 847,7 | 22894 | 462 | 9,1 | 0,831 | H5A034 C | sample | Hm Drgbl |
| 6066 | M3 | M4 | 1301 | 769,5 | 11902 | 263,4 | 9,1 | 0,831 | H5A034 C | sample | Hm Drgbl |
| 16185 | C17 | C18 | 1334 | 993,2 | 12122 | 327,1 | 9,1 | 0,835 | H5A034 C | sample | Hm Gen E |
| 5679 | M22 | M23 | 2439 | 780,5 | 22224 | 570,4 | 9,1 | 0,867 | H5A034 C | sample | Hm Drgbl |
| 3763 | M4 | M5 | 752 | 1252 | 6845 | 335 | 9,1 | 0,875 | H5A034 C | sample | Hm Drgbl |
| 3807 | O22 | O23 | 1758 | 1498 | 16075 | 384,1 | 9,1 | 0,875 | H5A034 C | sample | Hm Drgbl |
| 8189 | F13 | F14 | 1106 | 605,9 | 10041 | 474 | 9,1 | 0,875 | H5A034 C | sample | Hm Drgbl |
| 10142 | G21 | G22 | 1497 | 877,3 | 13650 | 125,2 | 9,1 | 0,875 | H5A034 C | sample | Hm Drgbl |
| 10153 | H1 | H2 | 1239 | 845,1 | 11288 | 144,3 | 9,1 | 0,875 | H5A034 C | sample | Hm Drgbl |
| 1759 | J15 | J16 | 1282 | 729,6 | 11706 | 305 | 9,1 | 0,953 | H5A034 C | sample | Hm Drgbl |
| 1848 | M9 | M10 | 1734 | 769,3 | 15705 | 361 | 9,1 | 0,953 | H5A034 C | sample | Hm Drgbl |
| 21621 | E6 | E7 | 256 | 1011 | 2322 | 606 | 9,1 | 0,958 | H5A034 C | sample | Hm Gen E |
| 21637 | F20 | F21 | 500 | 1001 | 4560 | 487,8 | 9,1 | 0,958 | H5A034 C | sample | Hm Gen E |
| 21753 | K17 | K18 | 236 | 1013 | 2155 | 509 | 9,1 | 0,958 | H5A034 C | sample | Hm Gen E |
| 21848 | O16 | O17 | 332 | 861,1 | 3036 | 630,3 | 9,1 | 0,958 | H5A034 C | sample | Hm Gen E |
| 4647 | B22 | B23 | 1745 | 958,8 | 15876 | 402,3 | 9,1 | 1 | H5A034 C | sample | Hm Drgbl |
| 4729 | F1 | F2 | 2004 | 805,2 | 18292 | 468,9 | 9,1 | 1 | H5A034 C | sample | Hm Drgbl |
| 4779 | H11 | H12 | 1325 | 794,4 | 12013 | 422,9 | 9,1 | 1 | H5A034 C | sample | Hm Drgbl |
| 4923 | N11 | N12 | 2011 | 904,8 | 18295 | 375,1 | 9,1 | 1 | H5A034 C | sample | Hm Drgbl |
| 10704 | N9 | N10 | 1307 | 796,2 | 11833 | 189,5 | 9,1 | 1,028 | H5A034 C | sample | Hm Drgbl |
| 2766 | D14 | D15 | 1132 | 657 | 10298 | 465,3 | 9,1 | 1,071 | H5A034 C | sample | Hm Drgbl |
| 2772 | D2 | D3 | 857 | 633 | 7787 | 467,3 | 9,1 | 1,071 | H5A034 C | sample | Hm Drgbl |
| 2780 | D5 | D6 | 1733 | 737,6 | 15853 | 550,7 | 9,1 | 1,071 | H5A034 C | sample | Hm Drgbl |
| 3050 | P10 | P11 | 1966 | 661,9 | 17918 | 522,4 | 9,1 | 1,071 | H5A034 C | sample | Hm Drgbl |
| 19971 | A11 | A12 | 665 | 1154 | 6054 | 167,2 | 9,1 | 1,11 | H5A034 C | sample | Hm Gen E |
| 20069 | E13 | E14 | 788 | 1131 | 7204 | 170,3 | 9,1 | 1,11 | H5A034 C | sample | Hm Gen E |
| 20070 | E14 | E15 | 819 | 1141 | 7427 | 168,4 | 9,1 | 1,11 | H5A034 C | sample | Hm Gen E |
| 20075 | E19 | E20 | 1144 | 1075 | 10422 | 130,2 | 9,1 | 1,11 | H5A034 C | sample | Hm Gen E |
| 20266 | M18 | M19 | 1047 | 1064 | 9520 | 137,1 | 9,1 | 1,11 | H5A034 C | sample | Hm Gen E |
| 20282 | N10 | N11 | 980 | 1115 | 8930 | 164 | 9,1 | 1,11 | H5A034 C | sample | Hm Gen E |
| 7747 | C4 | C5 | 332 | 625,5 | 3019 | 426,8 | 9,1 | 1,389 | H5A034 C | sample | Hm Drgbl |
| 7776 | D9 | D10 | 1460 | 606,2 | 13293 | 354,8 | 9,1 | 1,389 | H5A034 C | sample | Hm Drgbl |
| 7786 | E18 | E19 | 926 | 498,7 | 8466 | 338,4 | 9,1 | 1,389 | H5A034 C | sample | Hm Drgbl |
| 7826 | G10 | G11 | 1584 | 598,4 | 14423 | 370,5 | 9,1 | 1,389 | H5A034 C | sample | Hm Drgbl |
| 7839 | G22 | G23 | 1272 | 539,4 | 11570 | 374,5 | 9,1 | 1,389 | H5A034 C | sample | Hm Drgbl |
| 7904 | J16 | J17 | 1014 | 554,2 | 9251 | 296,5 | 9,1 | 1,389 | H5A034 C | sample | Hm Drgbl |
| 14161 | O1 | O2 | 1269 | 1093 | 11662 | 119,9 | 9,2 | 0,527 | H5A034 C | sample | Hm Gen E |
| 9553 | O1 | O2 | 2205 | 1171 | 20179 | 110,1 | 9,2 | 0,639 | H5A034 C | sample | Hm Drgbl |
| 8785 | O1 | O2 | 2449 | 1208 | 22599 | 130,1 | 9,2 | 0,65 | H5A034 C | sample | Hm Drgbl |
| 14466 | K3 | K4 | 1472 | 815,5 | 13475 | 128 | 9,2 | 0,655 | H5A034 C | sample | Hm Gen E |
| 12462 | H14 | H15 | 1101 | 926 | 10092 | 138,8 | 9,2 | 0,692 | H5A034 C | sample | Hm Gen E |
| 7500 | I2 | I3 | 390 | 572,4 | 3607 | 425,1 | 9,2 | 0,702 | H5A034 C | sample | Hm Drgbl |
| 2439 | F22 | F23 | 1664 | 713,8 | 15348 | 338,8 | 9,2 | 0,724 | H5A034 C | sample | Hm Drgbl |
| 2468 | G5 | G6 | 1402 | 674,2 | 12939 | 374,5 | 9,2 | 0,724 | H5A034 C | sample | Hm Drgbl |
| 2523 | J11 | J12 | 1127 | 760,5 | 10322 | 423,7 | 9,2 | 0,724 | H5A034 C | sample | Hm Drgbl |
| 4247 | A8 | A9 | 1488 | 708,2 | 13746 | 246,3 | 9,2 | 0,776 | H5A034 C | sample | Hm Drgbl |
| 212 | I5 | I6 | 441 | 1379 | 4045 | 452,5 | 9,2 | 0,783 | H5A034 C | sample | Hm Drgbl |
| 343 | O15 | O16 | 311 | 1620 | 2861 | 521,2 | 9,2 | 0,783 | H5A034 C | sample | Hm Drgbl |
| 6598 | C7 | C8 | 903 | 625,3 | 8331 | 435 | 9,2 | 0,807 | H5A034 C | sample | Hm Drgbl |
| 4059 | J11 | J12 | 1181 | 667,6 | 10846 | 306,8 | 9,2 | 0,818 | H5A034 C | sample | Hm Drgbl |
| 4111 | L15 | L16 | 67 | 760,2 | 615 | 352 | 9,2 | 0,818 | H5A034 C | sample | Hm Drgbl |
| 4143 | M22 | M23 | 1770 | 705,5 | 16290 | 332,1 | 9,2 | 0,818 | H5A034 C | sample | Hm Drgbl |
| 1503 | O22 | O23 | 2670 | 850,7 | 24432 | 493,4 | 9,2 | 0,844 | H5A034 C | sample | Hm Drgbl |
| 1534 | P7 | P8 | 1582 | 727,9 | 14546 | 471,1 | 9,2 | 0,844 | H5A034 C | sample | Hm Drgbl |
| 3676 | J12 | J13 | 882 | 1318 | 8093 | 334,2 | 9,2 | 0,885 | H5A034 C | sample | Hm Drgbl |
| 8079 | A22 | A23 | 2064 | 632,8 | 18890 | 428,2 | 9,2 | 0,885 | H5A034 C | sample | Hm Drgbl |
| 8146 | D18 | D19 | 1560 | 591,7 | 14424 | 443,9 | 9,2 | 0,885 | H5A034 C | sample | Hm Drgbl |
| 10172 | H5 | H6 | 1301 | 827 | 11994 | 130,8 | 9,2 | 0,885 | H5A034 C | sample | Hm Drgbl |
| 1813 | L20 | L21 | 1658 | 854,9 | 15175 | 376,8 | 9,2 | 0,963 | H5A034 C | sample | Hm Drgbl |
| 1873 | O1 | O2 | 914 | 715,8 | 8366 | 457,4 | 9,2 | 0,963 | H5A034 C | sample | Hm Drgbl |
| 21638 | F21 | F22 | 486 | 1033 | 4470 | 453,2 | 9,2 | 0,968 | H5A034 C | sample | Hm Gen E |
| 21754 | K18 | K19 | 177 | 841,8 | 1630 | 614,2 | 9,2 | 0,968 | H5A034 C | sample | Hm Gen E |
| 4743 | F22 | F23 | 1793 | 904 | 16501 | 344,8 | 9,2 | 1,011 | H5A034 C | sample | Hm Drgbl |
| 4766 | G21 | G22 | 1842 | 880,6 | 16895 | 440,9 | 9,2 | 1,011 | H5A034 C | sample | Hm Drgbl |
| 4826 | J10 | J11 | 2090 | 811,3 | 19222 | 395,6 | 9,2 | 1,011 | H5A034 C | sample | Hm Drgbl |
| 4830 | J14 | J15 | 2054 | 823 | 18964 | 346,9 | 9,2 | 1,011 | H5A034 C | sample | Hm Drgbl |
| 4883 | L19 | L20 | 1383 | 812 | 12658 | 423,4 | 9,2 | 1,011 | H5A034 C | sample | Hm Drgbl |
| 4884 | L2 | L3 | 1278 | 734,2 | 11815 | 456 | 9,2 | 1,011 | H5A034 C | sample | Hm Drgbl |
| 4907 | M19 | M20 | 2151 | 877,2 | 19885 | 469,3 | 9,2 | 1,011 | H5A034 C | sample | Hm Drgbl |
| 4945 | O1 | O2 | 425 | 823,1 | 3915 | 418,6 | 9,2 | 1,011 | H5A034 C | sample | Hm Drgbl |
| 4970 | P10 | P11 | 1920 | 773,7 | 17612 | 491,8 | 9,2 | 1,011 | H5A034 C | sample | Hm Drgbl |
| 10386 | A3 | A4 | 1828 | 978,5 | 16881 | 182,7 | 9,2 | 1,04 | H5A034 C | sample | Hm Drgbl |
| 10545 | H17 | H18 | 1120 | 765,5 | 10251 | 180,6 | 9,2 | 1,04 | H5A034 C | sample | Hm Drgbl |
| 2901 | I6 | I7 | 1256 | 639,9 | 11571 | 467,8 | 9,2 | 1,082 | H5A034 C | sample | Hm Drgbl |
| 2933 | K13 | K14 | 818 | 635,1 | 7562 | 379,8 | 9,2 | 1,082 | H5A034 C | sample | Hm Drgbl |
| 3006 | N14 | N15 | 1480 | 601,9 | 13636 | 424,9 | 9,2 | 1,082 | H5A034 C | sample | Hm Drgbl |
| 19973 | A13 | A14 | 791 | 1153 | 7283 | 187,5 | 9,2 | 1,122 | H5A034 C | sample | Hm Gen E |
| 20063 | D8 | D9 | 1145 | 1171 | 10487 | 157,5 | 9,2 | 1,122 | H5A034 C | sample | Hm Gen E |
| 20151 | H22 | H23 | 1160 | 1179 | 10614 | 172,8 | 9,2 | 1,122 | H5A034 C | sample | Hm Gen E |
| 20179 | I4 | I5 | 917 | 1072 | 8470 | 149,1 | 9,2 | 1,122 | H5A034 C | sample | Hm Gen E |
| 20222 | K21 | K22 | 1292 | 1082 | 11827 | 139,7 | 9,2 | 1,122 | H5A034 C | sample | Hm Gen E |
| 20232 | K9 | K10 | 864 | 1147 | 7931 | 148,1 | 9,2 | 1,122 | H5A034 C | sample | Hm Gen E |
| 20251 | L4 | L5 | 995 | 1084 | 9183 | 187,8 | 9,2 | 1,122 | H5A034 C | sample | Hm Gen E |
| 20265 | M17 | M18 | 1004 | 1046 | 9258 | 134,7 | 9,2 | 1,122 | H5A034 C | sample | Hm Gen E |
| 20302 | N7 | N8 | 794 | 1135 | 7343 | 180,5 | 9,2 | 1,122 | H5A034 C | sample | Hm Gen E |
| 20336 | P16 | P17 | 281 | 1686 | 2580 | 154,8 | 9,2 | 1,122 | H5A034 C | sample | Hm Gen E |
| 20349 | P6 | P7 | 1090 | 1260 | 10023 | 145,6 | 9,2 | 1,122 | H5A034 C | sample | Hm Gen E |
| 410 | B10 | B11 | 2010 | 1602 | 18559 | 296,6 | 9,2 | 1,343 | H5A034 C | sample | Hm Drgbl |
| 419 | B19 | B20 | 1564 | 1935 | 14328 | 243,5 | 9,2 | 1,343 | H5A034 C | sample | Hm Drgbl |
| 445 | C20 | C21 | 2797 | 2078 | 25613 | 406,9 | 9,2 | 1,343 | H5A034 C | sample | Hm Drgbl |
| 690 | M3 | M4 | 1701 | 1796 | 15697 | 376,4 | 9,2 | 1,343 | H5A034 C | sample | Hm Drgbl |
| 709 | N20 | N21 | 2186 | 1767 | 20164 | 357,6 | 9,2 | 1,343 | H5A034 C | sample | Hm Drgbl |
| 7691 | A19 | A20 | 1043 | 566,9 | 9626 | 415,4 | 9,2 | 1,405 | H5A034 C | sample | Hm Drgbl |
| 7705 | B1 | B2 | 1081 | 528,9 | 9913 | 429,8 | 9,2 | 1,405 | H5A034 C | sample | Hm Drgbl |
| 7758 | D14 | D15 | 1035 | 588,9 | 9482 | 395,3 | 9,2 | 1,405 | H5A034 C | sample | Hm Drgbl |
| 7827 | G11 | G12 | 1684 | 598,2 | 15556 | 362,1 | 9,2 | 1,405 | H5A034 C | sample | Hm Drgbl |
| 8051 | P19 | P20 | 1515 | 564,6 | 13886 | 444,6 | 9,2 | 1,405 | H5A034 C | sample | Hm Drgbl |
| 7537 | K1 | K2 | 409 | 601 | 3809 | 286,4 | 9,3 | 0,71 | H5A034 C | sample | Hm Drgbl |
| 5055 | C22 | C23 | 2438 | 697,2 | 22586 | 591,8 | 9,3 | 0,729 | H5A034 C | sample | Hm Drgbl |
| 5196 | I2 | I3 | 741 | 559,7 | 6928 | 343,2 | 9,3 | 0,729 | H5A034 C | sample | Hm Drgbl |
| 2422 | E7 | E8 | 1361 | 766 | 12639 | 416,1 | 9,3 | 0,732 | H5A034 C | sample | Hm Drgbl |
| 2603 | M19 | M20 | 1984 | 749,9 | 18384 | 324,3 | 9,3 | 0,732 | H5A034 C | sample | Hm Drgbl |
| 17780 | E5 | E6 | 838 | 1010 | 7797 | 262,5 | 9,3 | 0,735 | H5A034 C | sample | Hm Gen E |
| 4334 | E21 | E22 | 1175 | 821 | 10958 | 377,7 | 9,3 | 0,785 | H5A034 C | sample | Hm Drgbl |
| 202 | I18 | I19 | 700 | 1705 | 6537 | 433,2 | 9,3 | 0,791 | H5A034 C | sample | Hm Drgbl |
| 258 | K3 | K4 | 473 | 1501 | 4419 | 406,4 | 9,3 | 0,791 | H5A034 C | sample | Hm Drgbl |
| 264 | K9 | K10 | 323 | 1163 | 3013 | 518,2 | 9,3 | 0,791 | H5A034 C | sample | Hm Drgbl |
| 334 | N7 | N8 | 652 | 1739 | 6050 | 505,2 | 9,3 | 0,791 | H5A034 C | sample | Hm Drgbl |
| 6691 | G4 | G5 | 668 | 630,1 | 6211 | 367,7 | 9,3 | 0,816 | H5A034 C | sample | Hm Drgbl |
| 11577 | C17 | C18 | 1618 | 828,3 | 15018 | 131,5 | 9,3 | 0,83 | H5A034 C | sample | Hm Gen E |
| 11782 | K7 | K8 | 891 | 789 | 8245 | 143,4 | 9,3 | 0,83 | H5A034 C | sample | Hm Gen E |
| 5764 | A12 | A13 | 978 | 824 | 9132 | 418,7 | 9,3 | 0,849 | H5A034 C | sample | Hm Drgbl |
| 5773 | A20 | A21 | 2004 | 845,5 | 18672 | 531,8 | 9,3 | 0,849 | H5A034 C | sample | Hm Drgbl |
| 1444 | M12 | M13 | 1228 | 737,9 | 11386 | 474,7 | 9,3 | 0,853 | H5A034 C | sample | Hm Drgbl |
| 1501 | O20 | O21 | 2140 | 746,2 | 19950 | 426 | 9,3 | 0,853 | H5A034 C | sample | Hm Drgbl |
| 13556 | E5 | E6 | 1044 | 991,8 | 9683 | 168,2 | 9,3 | 0,873 | H5A034 C | sample | Hm Gen E |
| 781 | A20 | A21 | 1162 | 857,7 | 10834 | 533,8 | 9,3 | 0,882 | H5A034 C | sample | Hm Drgbl |
| 5658 | L3 | L4 | 671 | 706,2 | 6273 | 551 | 9,3 | 0,886 | H5A034 C | sample | Hm Drgbl |
| 3572 | E5 | E6 | 1214 | 1309 | 11315 | 285,8 | 9,3 | 0,894 | H5A034 C | sample | Hm Drgbl |
| 3584 | F16 | F17 | 1204 | 1293 | 11204 | 359,4 | 9,3 | 0,894 | H5A034 C | sample | Hm Drgbl |
| 3668 | I5 | I6 | 1389 | 1290 | 12850 | 349,5 | 9,3 | 0,894 | H5A034 C | sample | Hm Drgbl |
| 3720 | K9 | K10 | 973 | 1313 | 9036 | 341,2 | 9,3 | 0,894 | H5A034 C | sample | Hm Drgbl |
| 8391 | N22 | N23 | 1759 | 621 | 16435 | 493,4 | 9,3 | 0,894 | H5A034 C | sample | Hm Drgbl |
| 10308 | N2 | N3 | 1048 | 814,6 | 9745 | 149 | 9,3 | 0,894 | H5A034 C | sample | Hm Drgbl |
| 21703 | I15 | I16 | 386 | 912,1 | 3583 | 689,8 | 9,3 | 0,979 | H5A034 C | sample | Hm Gen E |
| 21746 | K10 | K11 | 351 | 932,9 | 3267 | 660,8 | 9,3 | 0,979 | H5A034 C | sample | Hm Gen E |
| 21775 | L15 | L16 | 303 | 846,5 | 2821 | 677,8 | 9,3 | 0,979 | H5A034 C | sample | Hm Gen E |
| 21777 | L17 | L18 | 397 | 978,2 | 3689 | 449,8 | 9,3 | 0,979 | H5A034 C | sample | Hm Gen E |
| 21781 | L20 | L21 | 119 | 1048 | 1107 | 587,7 | 9,3 | 0,979 | H5A034 C | sample | Hm Gen E |
| 4737 | F17 | F18 | 2005 | 810,4 | 18564 | 402,5 | 9,3 | 1,022 | H5A034 C | sample | Hm Drgbl |
| 4784 | H16 | H17 | 1946 | 763,2 | 18180 | 283,5 | 9,3 | 1,022 | H5A034 C | sample | Hm Drgbl |
| 4834 | J18 | J19 | 1746 | 866,6 | 16277 | 351,2 | 9,3 | 1,022 | H5A034 C | sample | Hm Drgbl |
| 4862 | K21 | K22 | 2241 | 865 | 20751 | 472,7 | 9,3 | 1,022 | H5A034 C | sample | Hm Drgbl |
| 4863 | K22 | K23 | 1994 | 814,4 | 18548 | 463,9 | 9,3 | 1,022 | H5A034 C | sample | Hm Drgbl |
| 4941 | N6 | N7 | 1566 | 828,3 | 14519 | 402,8 | 9,3 | 1,022 | H5A034 C | sample | Hm Drgbl |
| 10463 | D8 | D9 | 889 | 786,1 | 8253 | 170,7 | 9,3 | 1,051 | H5A034 C | sample | Hm Drgbl |
| 2782 | D7 | D8 | 1331 | 717,8 | 12329 | 652,1 | 9,3 | 1,094 | H5A034 C | sample | Hm Drgbl |
| 2857 | H1 | H2 | 1430 | 650,2 | 13284 | 615,7 | 9,3 | 1,094 | H5A034 C | sample | Hm Drgbl |
| 2877 | H6 | H7 | 1713 | 693,3 | 15929 | 592,2 | 9,3 | 1,094 | H5A034 C | sample | Hm Drgbl |
| 2899 | I4 | I5 | 639 | 614,4 | 5911 | 449,8 | 9,3 | 1,094 | H5A034 C | sample | Hm Drgbl |
| 2905 | J1 | J2 | 910 | 655,6 | 8481 | 457,8 | 9,3 | 1,094 | H5A034 C | sample | Hm Drgbl |
| 2928 | J9 | J10 | 1346 | 626,5 | 12518 | 374,8 | 9,3 | 1,094 | H5A034 C | sample | Hm Drgbl |
| 2949 | K6 | K7 | 707 | 573,1 | 6610 | 430,8 | 9,3 | 1,094 | H5A034 C | sample | Hm Drgbl |
| 19995 | B11 | B12 | 1360 | 1186 | 12636 | 147,1 | 9,3 | 1,134 | H5A034 C | sample | Hm Gen E |
| 20004 | B2 | B3 | 1101 | 1246 | 10230 | 187,5 | 9,3 | 1,134 | H5A034 C | sample | Hm Gen E |
| 20020 | C12 | C13 | 1121 | 1110 | 10477 | 123,7 | 9,3 | 1,134 | H5A034 C | sample | Hm Gen E |
| 20054 | D21 | D22 | 1365 | 1218 | 12706 | 151,5 | 9,3 | 1,134 | H5A034 C | sample | Hm Gen E |
| 20102 | F21 | F22 | 1064 | 1113 | 9895 | 165,9 | 9,3 | 1,134 | H5A034 C | sample | Hm Gen E |
| 20180 | I5 | I6 | 742 | 1116 | 6875 | 149,2 | 9,3 | 1,134 | H5A034 C | sample | Hm Gen E |
| 20204 | J5 | J6 | 229 | 1314 | 2141 | 184,7 | 9,3 | 1,134 | H5A034 C | sample | Hm Gen E |
| 20254 | L7 | L8 | 888 | 1079 | 8262 | 145,6 | 9,3 | 1,134 | H5A034 C | sample | Hm Gen E |
| 395 | A19 | A20 | 2337 | 1888 | 21845 | 442 | 9,3 | 1,358 | H5A034 C | sample | Hm Drgbl |
| 406 | A7 | A8 | 2690 | 1917 | 25120 | 465,9 | 9,3 | 1,358 | H5A034 C | sample | Hm Drgbl |
| 421 | B20 | B21 | 2245 | 1741 | 20913 | 387,4 | 9,3 | 1,358 | H5A034 C | sample | Hm Drgbl |
| 506 | F10 | F11 | 1824 | 1699 | 17049 | 307,4 | 9,3 | 1,358 | H5A034 C | sample | Hm Drgbl |
| 680 | M16 | M17 | 1169 | 1853 | 10898 | 455,4 | 9,3 | 1,358 | H5A034 C | sample | Hm Drgbl |
| 722 | O10 | O11 | 1924 | 1680 | 17865 | 351,7 | 9,3 | 1,358 | H5A034 C | sample | Hm Drgbl |
| 7681 | A1 | A2 | 530 | 599 | 4920 | 465,7 | 9,3 | 1,42 | H5A034 C | sample | Hm Drgbl |
| 7698 | A3 | A4 | 1218 | 601,7 | 11316 | 475,9 | 9,3 | 1,42 | H5A034 C | sample | Hm Drgbl |
| 7732 | C12 | C13 | 1337 | 588,6 | 12394 | 429,2 | 9,3 | 1,42 | H5A034 C | sample | Hm Drgbl |
| 7750 | C7 | C8 | 1170 | 524,8 | 10823 | 341,2 | 9,3 | 1,42 | H5A034 C | sample | Hm Drgbl |
| 7788 | E2 | E3 | 445 | 592,7 | 4121 | 406,3 | 9,3 | 1,42 | H5A034 C | sample | Hm Drgbl |
| 7804 | F12 | F13 | 1358 | 555,4 | 12596 | 443 | 9,3 | 1,42 | H5A034 C | sample | Hm Drgbl |
| 7861 | H20 | H21 | 1428 | 594,5 | 13346 | 350,8 | 9,3 | 1,42 | H5A034 C | sample | Hm Drgbl |
| 8024 | O16 | O17 | 854 | 617,4 | 7908 | 449,2 | 9,3 | 1,42 | H5A034 C | sample | Hm Drgbl |
| 20682 | N3 | N4 | 977 | 781,7 | 9152 | 95,56 | 9,4 | 0,627 | H5A034 C | sample | Hm Gen E |

| | | | | | | | | | | | | | | | |
|---|---|---|---|---|---|---|---|---|---|---|---|---|---|---|---|
| 16916 | A5 | A6 | 1535 | 1161 | 14438 | 264,9 | 9,4 | 0,726 | H5A034 C | sample | Hm Gen E | Hm Gen E | Hm Gen E | CPE002JI | CPE002NZ |
| 6961 | C1 | C2 | 452 | 553,7 | 4260 | 451,6 | 9,4 | 0,732 | H5A034 C | sample | Hm Drgbl | Hm Drgbl | Hm Drgbl | CPE002E2 | CPE002EX |
| 2444 | F5 | F6 | 1218 | 662,3 | 11479 | 371,8 | 9,4 | 0,74 | H5A034 C | sample | Hm Drgbl | Hm Drgbl | Hm Drgbl | CPE002DC | CPE002EE |
| 2478 | H14 | H15 | 1485 | 643,5 | 13977 | 341,6 | 9,4 | 0,74 | H5A034 C | sample | Hm Drgbl | Hm Drgbl | Hm Drgbl | CPE002DC | CPE002EE |
| 2514 | I3 | I4 | 1043 | 663,6 | 9814 | 377,1 | 9,4 | 0,74 | H5A034 C | sample | Hm Drgbl | Hm Drgbl | Hm Drgbl | CPE002DC | CPE002EE |
| 4340 | E5 | E6 | 1451 | 677,8 | 13630 | 347,3 | 9,4 | 0,793 | H5A034 C | sample | Hm Drgbl | Hm Drgbl | Hm Drgbl | CPE002DU | CPE002EK |
| 4537 | N1 | N2 | 1231 | 755,3 | 11521 | 420,8 | 9,4 | 0,793 | H5A034 C | sample | Hm Drgbl | Hm Drgbl | Hm Drgbl | CPE002DU | CPE002EK |
| 144 | F9 | F10 | 504 | 1453 | 4759 | 405,7 | 9,4 | 0,8 | H5A034 C | sample | Hm Drgbl | Hm Drgbl | Hm Drgbl | CPE002DT | CPE002EJ |
| 165 | G6 | G7 | 89 | 1550 | 841 | 640,8 | 9,4 | 0,8 | H5A034 C | sample | Hm Drgbl | Hm Drgbl | Hm Drgbl | CPE002DT | CPE002EJ |
| 336 | N9 | N10 | 590 | 1555 | 5573 | 528,3 | 9,4 | 0,8 | H5A034 C | sample | Hm Drgbl | Hm Drgbl | Hm Drgbl | CPE002DT | CPE002EJ |
| 359 | O8 | O9 | 730 | 1749 | 6896 | 714,4 | 9,4 | 0,8 | H5A034 C | sample | Hm Drgbl | Hm Drgbl | Hm Drgbl | CPE002DT | CPE002EJ |
| 6677 | G13 | G14 | 409 | 533,1 | 3827 | 162,3 | 9,4 | 0,825 | H5A034 C | sample | Hm Drgbl | Hm Drgbl | Hm Drgbl | CPE002E1 | CPE002EY |
| 6852 | N2 | N3 | 782 | 509,9 | 7319 | 457,6 | 9,4 | 0,825 | H5A034 C | sample | Hm Drgbl | Hm Drgbl | Hm Drgbl | CPE002E1 | CPE002EY |
| 4080 | J9 | J10 | 1452 | 735,1 | 13593 | 307,9 | 9,4 | 0,836 | H5A034 C | sample | Hm Drgbl | Hm Drgbl | Hm Drgbl | CPE002DS | CPE002EI |
| 4161 | N17 | N18 | 1531 | 709,4 | 14446 | 317,6 | 9,4 | 0,836 | H5A034 C | sample | Hm Drgbl | Hm Drgbl | Hm Drgbl | CPE002DS | CPE002EI |
| 4164 | N2 | N3 | 491 | 777,4 | 4637 | 457 | 9,4 | 0,836 | H5A034 C | sample | Hm Drgbl | Hm Drgbl | Hm Drgbl | CPE002DS | CPE002EI |
| 5784 | A9 | A10 | 1712 | 973,4 | 16082 | 315,5 | 9,4 | 0,858 | H5A034 C | sample | Hm Drgbl | Hm Drgbl | Hm Drgbl | CPE002DY | CPE002F1 |
| 1233 | D17 | D18 | 1386 | 724,5 | 13010 | 280 | 9,4 | 0,862 | H5A034 C | sample | Hm Drgbl | Hm Drgbl | Hm Drgbl | CPE002FV | CPE002FZ |
| 1311 | G22 | G23 | 2357 | 785,5 | 22163 | 459,3 | 9,4 | 0,862 | H5A034 C | sample | Hm Drgbl | Hm Drgbl | Hm Drgbl | CPE002FV | CPE002FZ |
| 1428 | L2 | L3 | 1439 | 731,3 | 13482 | 392 | 9,4 | 0,862 | H5A034 C | sample | Hm Drgbl | Hm Drgbl | Hm Drgbl | CPE002FV | CPE002FZ |
| 13698 | K3 | K4 | 1131 | 1068 | 10583 | 164,5 | 9,4 | 0,883 | H5A034 C | sample | Hm Gen E | Hm Gen E | Hm Gen E | CPE002JC | CPE002NC |
| 13753 | N1 | N2 | 1414 | 967,9 | 13349 | 153,5 | 9,4 | 0,883 | H5A034 C | sample | Hm Gen E | Hm Gen E | Hm Gen E | CPE002JC | CPE002NC |
| 900 | F2 | F3 | 1633 | 876,7 | 15421 | 283 | 9,4 | 0,891 | H5A034 C | sample | Hm Drgbl | Hm Drgbl | Hm Drgbl | CPE002FU | CPE002FY |
| 903 | F22 | F23 | 1454 | 713,4 | 13738 | 415,3 | 9,4 | 0,891 | H5A034 C | sample | Hm Drgbl | Hm Drgbl | Hm Drgbl | CPE002FU | CPE002FY |
| 1082 | N10 | N11 | 1194 | 728,7 | 11214 | 432,2 | 9,4 | 0,891 | H5A034 C | sample | Hm Drgbl | Hm Drgbl | Hm Drgbl | CPE002FU | CPE002FY |
| 5604 | J2 | J3 | 1668 | 658,9 | 15626 | 567,4 | 9,4 | 0,895 | H5A034 C | sample | Hm Drgbl | Hm Drgbl | Hm Drgbl | CPE002DX | CPE002F2 |
| 5649 | L17 | L18 | 1951 | 748,5 | 18402 | 375 | 9,4 | 0,895 | H5A034 C | sample | Hm Drgbl | Hm Drgbl | Hm Drgbl | CPE002DX | CPE002F2 |
| 3585 | F17 | F18 | 1126 | 1298 | 10597 | 343,2 | 9,4 | 0,904 | H5A034 C | sample | Hm Drgbl | Hm Drgbl | Hm Drgbl | CPE002DR | CPE002EH |
| 3596 | F5 | F6 | 1146 | 1365 | 10800 | 337,7 | 9,4 | 0,904 | H5A034 C | sample | Hm Drgbl | Hm Drgbl | Hm Drgbl | CPE002DR | CPE002EH |
| 3606 | G14 | G15 | 1314 | 1247 | 12364 | 300,6 | 9,4 | 0,904 | H5A034 C | sample | Hm Drgbl | Hm Drgbl | Hm Drgbl | CPE002DR | CPE002EH |
| 3684 | J2 | J3 | 836 | 1442 | 7883 | 394,4 | 9,4 | 0,904 | H5A034 C | sample | Hm Drgbl | Hm Drgbl | Hm Drgbl | CPE002DR | CPE002EH |
| 3701 | K13 | K14 | 1116 | 1135 | 10452 | 241,5 | 9,4 | 0,904 | H5A034 C | sample | Hm Drgbl | Hm Drgbl | Hm Drgbl | CPE002DR | CPE002EH |
| 3793 | O1 | O2 | 942 | 1294 | 8883 | 419,5 | 9,4 | 0,904 | H5A034 C | sample | Hm Drgbl | Hm Drgbl | Hm Drgbl | CPE002DR | CPE002EH |
| 8121 | C17 | C18 | 1252 | 562,1 | 11783 | 464,5 | 9,4 | 0,904 | H5A034 C | sample | Hm Drgbl | Hm Drgbl | Hm Drgbl | CPE002E5 | CPE002EU |
| 8191 | F15 | F16 | 1170 | 602,4 | 11045 | 501,9 | 9,4 | 0,904 | H5A034 C | sample | Hm Drgbl | Hm Drgbl | Hm Drgbl | CPE002E5 | CPE002EU |
| 8414 | O21 | O22 | 2481 | 679,8 | 23392 | 460,4 | 9,4 | 0,904 | H5A034 C | sample | Hm Drgbl | Hm Drgbl | Hm Drgbl | CPE002E5 | CPE002EU |
| 1839 | M22 | M23 | 1969 | 766,6 | 18507 | 352,4 | 9,4 | 0,984 | H5A034 C | sample | Hm Drgbl | Hm Drgbl | Hm Drgbl | CPE002FW | CPE002G0 |
| 1910 | P21 | P22 | 2177 | 865 | 20445 | 344,3 | 9,4 | 0,984 | H5A034 C | sample | Hm Drgbl | Hm Drgbl | Hm Drgbl | CPE002FW | CPE002G0 |
| 1914 | P3 | P4 | 2269 | 750,9 | 21406 | 414,6 | 9,4 | 0,984 | H5A034 C | sample | Hm Drgbl | Hm Drgbl | Hm Drgbl | CPE002FW | CPE002G0 |
| 21623 | E8 | E9 | 317 | 1113 | 2985 | 675,8 | 9,4 | 0,989 | H5A034 C | sample | Hm Gen E | Hm Gen E | Hm Gen E | CPE002JU | CPE002O9 |
| 4626 | A3 | A4 | 2030 | 841,9 | 19020 | 558,7 | 9,4 | 1,033 | H5A034 C | sample | Hm Drgbl | Hm Drgbl | Hm Drgbl | CPE002DV | CPE002EL |
| 4828 | J12 | J13 | 2281 | 824,3 | 21394 | 377,5 | 9,4 | 1,033 | H5A034 C | sample | Hm Drgbl | Hm Drgbl | Hm Drgbl | CPE002DV | CPE002EL |
| 4831 | J15 | J16 | 1416 | 862,4 | 13263 | 267 | 9,4 | 1,033 | H5A034 C | sample | Hm Drgbl | Hm Drgbl | Hm Drgbl | CPE002DV | CPE002EL |
| 4839 | J22 | J23 | 1785 | 821,1 | 16862 | 429,4 | 9,4 | 1,033 | H5A034 C | sample | Hm Drgbl | Hm Drgbl | Hm Drgbl | CPE002DV | CPE002EL |
| 4871 | K8 | K9 | 1201 | 920,7 | 11307 | 263 | 9,4 | 1,033 | H5A034 C | sample | Hm Drgbl | Hm Drgbl | Hm Drgbl | CPE002DV | CPE002EL |
| 10406 | B21 | B22 | 1716 | 948,1 | 16054 | 192,3 | 9,4 | 1,062 | H5A034 C | sample | Hm Drgbl | Hm Drgbl | Hm Drgbl | CPE002EB | CPE002EO |
| 10435 | C4 | C5 | 1464 | 905,6 | 13789 | 186,2 | 9,4 | 1,062 | H5A034 C | sample | Hm Drgbl | Hm Drgbl | Hm Drgbl | CPE002EB | CPE002EO |
| 10565 | I13 | I14 | 1070 | 860,7 | 10074 | 171,5 | 9,4 | 1,062 | H5A034 C | sample | Hm Drgbl | Hm Drgbl | Hm Drgbl | CPE002EB | CPE002EO |
| 10609 | K1 | K2 | 1115 | 832,9 | 10494 | 175,6 | 9,4 | 1,062 | H5A034 C | sample | Hm Drgbl | Hm Drgbl | Hm Drgbl | CPE002EB | CPE002EO |
| 10656 | L9 | L10 | 1085 | 812,8 | 10222 | 187,1 | 9,4 | 1,062 | H5A034 C | sample | Hm Drgbl | Hm Drgbl | Hm Drgbl | CPE002EB | CPE002EO |
| 2730 | B3 | B4 | 411 | 835,4 | 3864 | 778,5 | 9,4 | 1,106 | H5A034 C | sample | Hm Drgbl | Hm Drgbl | Hm Drgbl | CPE002DP | CPE002EF |
| 3039 | O22 | O23 | 881 | 672,9 | 8278 | 623,8 | 9,4 | 1,106 | H5A034 C | sample | Hm Drgbl | Hm Drgbl | Hm Drgbl | CPE002DP | CPE002EF |
| 20019 | C11 | C12 | 1393 | 1105 | 13162 | 158,6 | 9,4 | 1,146 | H5A034 C | sample | Hm Gen E | Hm Gen E | Hm Gen E | CPE002JQ | CPE002O5 |
| 20023 | C15 | C16 | 1314 | 1105 | 12300 | 159,8 | 9,4 | 1,146 | H5A034 C | sample | Hm Gen E | Hm Gen E | Hm Gen E | CPE002JQ | CPE002O5 |
| 20096 | F16 | F17 | 903 | 1100 | 8459 | 134,1 | 9,4 | 1,146 | H5A034 C | sample | Hm Gen E | Hm Gen E | Hm Gen E | CPE002JQ | CPE002O5 |
| 20121 | G17 | G18 | 847 | 1246 | 7997 | 165,2 | 9,4 | 1,146 | H5A034 C | sample | Hm Gen E | Hm Gen E | Hm Gen E | CPE002JQ | CPE002O5 |
| 20306 | O10 | O11 | 1406 | 1095 | 13235 | 156,7 | 9,4 | 1,146 | H5A034 C | sample | Hm Gen E | Hm Gen E | Hm Gen E | CPE002JQ | CPE002O5 |
| 20324 | O5 | O6 | 494 | 1079 | 4623 | 159,7 | 9,4 | 1,146 | H5A034 C | sample | Hm Gen E | Hm Gen E | Hm Gen E | CPE002JQ | CPE002O5 |
| 20325 | O6 | O7 | 367 | 1184 | 3463 | 176,3 | 9,4 | 1,146 | H5A034 C | sample | Hm Gen E | Hm Gen E | Hm Gen E | CPE002JQ | CPE002O5 |
| 412 | B12 | B13 | 2650 | 1856 | 24866 | 372,2 | 9,4 | 1,372 | H5A034 C | sample | Hm Drgbl | Hm Drgbl | Hm Drgbl | CPE002E0 | CPE002EZ |
| 447 | C22 | C23 | 1802 | 1615 | 16907 | 297,3 | 9,4 | 1,372 | H5A034 C | sample | Hm Drgbl | Hm Drgbl | Hm Drgbl | CPE002E0 | CPE002EZ |
| 502 | E7 | E8 | 1504 | 1771 | 14154 | 378,5 | 9,4 | 1,372 | H5A034 C | sample | Hm Drgbl | Hm Drgbl | Hm Drgbl | CPE002E0 | CPE002EZ |
| 514 | F18 | F19 | 1894 | 1723 | 17728 | 283,3 | 9,4 | 1,372 | H5A034 C | sample | Hm Drgbl | Hm Drgbl | Hm Drgbl | CPE002E0 | CPE002EZ |
| 677 | M13 | M14 | 2319 | 1782 | 21824 | 383 | 9,4 | 1,372 | H5A034 C | sample | Hm Drgbl | Hm Drgbl | Hm Drgbl | CPE002E0 | CPE002EZ |
| 7723 | B4 | B5 | 859 | 533,9 | 8082 | 423,2 | 9,4 | 1,435 | H5A034 C | sample | Hm Drgbl | Hm Drgbl | Hm Drgbl | CPE002E4 | CPE002EV |
| 7962 | L3 | L4 | 199 | 570,1 | 1861 | 353 | 9,4 | 1,435 | H5A034 C | sample | Hm Drgbl | Hm Drgbl | Hm Drgbl | CPE002E4 | CPE002EV |
| 8041 | P1 | P2 | 1079 | 506,5 | 10149 | 371,5 | 9,4 | 1,435 | H5A034 C | sample | Hm Drgbl | Hm Drgbl | Hm Drgbl | CPE002E4 | CPE002EV |
| 20491 | F4 | F5 | 968 | 759,4 | 9148 | 126,1 | 9,5 | 0,633 | H5A034 C | sample | Hm Gen E | Hm Gen E | Hm Gen E | CPE002JR | CPE002O6 |
| 20602 | K18 | K19 | 1446 | 759,4 | 13729 | 160,4 | 9,5 | 0,633 | H5A034 C | sample | Hm Gen E | Hm Gen E | Hm Gen E | CPE002JR | CPE002O6 |
| 20684 | N5 | N6 | 1073 | 721 | 10234 | 125,8 | 9,5 | 0,633 | H5A034 C | sample | Hm Gen E | Hm Gen E | Hm Gen E | CPE002JR | CPE002O6 |
| 12612 | N2 | N3 | 1458 | 1044 | 13822 | 141,9 | 9,5 | 0,714 | H5A034 C | sample | Hm Gen E | Hm Gen E | Hm Gen E | CPE002J5 | CPE002NK |
| 5252 | K5 | K6 | 1433 | 591,3 | 13644 | 524,2 | 9,5 | 0,745 | H5A034 C | sample | Hm Drgbl | Hm Drgbl | Hm Drgbl | CPE002DV | CPE002EM |
| 2411 | E19 | E20 | 830 | 802,5 | 7880 | 461,7 | 9,5 | 0,748 | H5A034 C | sample | Hm Drgbl | Hm Drgbl | Hm Drgbl | CPE002DC | CPE002EE |
| 2454 | G14 | G15 | 784 | 686,8 | 7450 | 354,7 | 9,5 | 0,748 | H5A034 C | sample | Hm Drgbl | Hm Drgbl | Hm Drgbl | CPE002DC | CPE002EE |
| 2613 | M6 | M7 | 1773 | 726,4 | 16904 | 335,3 | 9,5 | 0,748 | H5A034 C | sample | Hm Drgbl | Hm Drgbl | Hm Drgbl | CPE002DC | CPE002EE |
| 84 | D2 | D3 | 557 | 1426 | 5294 | 483,7 | 9,5 | 0,809 | H5A034 C | sample | Hm Drgbl | Hm Drgbl | Hm Drgbl | CPE002DT | CPE002EJ |
| 156 | G2 | G3 | 329 | 1388 | 3121 | 541,8 | 9,5 | 0,809 | H5A034 C | sample | Hm Drgbl | Hm Drgbl | Hm Drgbl | CPE002DT | CPE002EJ |
| 237 | J6 | J7 | 391 | 1309 | 3698 | 373,6 | 9,5 | 0,809 | H5A034 C | sample | Hm Drgbl | Hm Drgbl | Hm Drgbl | CPE002DT | CPE002EJ |
| 2083 | G4 | G5 | 2289 | 858,1 | 21706 | 448,3 | 9,5 | 0,826 | H5A034 C | sample | Hm Drgbl | Hm Drgbl | Hm Drgbl | CPE002FX | CPE002G1 |
| 3971 | F19 | F20 | 1211 | 744,4 | 11558 | 444 | 9,5 | 0,844 | H5A034 C | sample | Hm Drgbl | Hm Drgbl | Hm Drgbl | CPE002DS | CPE002EI |
| 4045 | I20 | I21 | 1779 | 707,2 | 16928 | 286,3 | 9,5 | 0,844 | H5A034 C | sample | Hm Drgbl | Hm Drgbl | Hm Drgbl | CPE002DS | CPE002EI |
| 11881 | P1 | P2 | 1112 | 854,5 | 10537 | 154,2 | 9,5 | 0,848 | H5A034 C | sample | Hm Gen E | Hm Gen E | Hm Gen E | CPE002JA | CPE002NJ |
| 5895 | F22 | F23 | 2199 | 807,3 | 20808 | 591 | 9,5 | 0,868 | H5A034 C | sample | Hm Drgbl | Hm Drgbl | Hm Drgbl | CPE002DY | CPE002F1 |
| 6020 | K5 | K6 | 1352 | 719,4 | 12893 | 489,2 | 9,5 | 0,868 | H5A034 C | sample | Hm Drgbl | Hm Drgbl | Hm Drgbl | CPE002DY | CPE002F1 |
| 6073 | N1 | N2 | 1878 | 811,8 | 17909 | 504,3 | 9,5 | 0,868 | H5A034 C | sample | Hm Drgbl | Hm Drgbl | Hm Drgbl | CPE002DY | CPE002F1 |
| 1335 | H22 | H23 | 1358 | 759,8 | 12869 | 415,4 | 9,5 | 0,872 | H5A034 C | sample | Hm Drgbl | Hm Drgbl | Hm Drgbl | CPE002FV | CPE002FZ |
| 1401 | K17 | K18 | 1123 | 769,2 | 10626 | 411,4 | 9,5 | 0,872 | H5A034 C | sample | Hm Drgbl | Hm Drgbl | Hm Drgbl | CPE002FV | CPE002FZ |
| 1497 | O17 | O18 | 2153 | 797,5 | 20430 | 446,1 | 9,5 | 0,872 | H5A034 C | sample | Hm Drgbl | Hm Drgbl | Hm Drgbl | CPE002FV | CPE002FZ |
| 16478 | O21 | O22 | 1518 | 1063 | 14389 | 311,4 | 9,5 | 0,872 | H5A034 C | sample | Hm Gen E | Hm Gen E | Hm Gen E | CPE002JG | CPE002NV |
| 13764 | N2 | N3 | 806 | 921,4 | 7693 | 177,2 | 9,5 | 0,892 | H5A034 C | sample | Hm Gen E | Hm Gen E | Hm Gen E | CPE002JC | CPE002NC |
| 840 | C9 | C10 | 1175 | 805,7 | 11139 | 389,8 | 9,5 | 0,9 | H5A034 C | sample | Hm Drgbl | Hm Drgbl | Hm Drgbl | CPE002FU | CPE002FY |
| 1064 | M16 | M17 | 2029 | 807,9 | 19253 | 387,8 | 9,5 | 0,9 | H5A034 C | sample | Hm Drgbl | Hm Drgbl | Hm Drgbl | CPE002FU | CPE002FY |
| 3490 | B18 | B19 | 1636 | 1358 | 15465 | 342,4 | 9,5 | 0,913 | H5A034 C | sample | Hm Drgbl | Hm Drgbl | Hm Drgbl | CPE002DR | CPE002EH |
| 3501 | B6 | B7 | 1547 | 1360 | 14756 | 356,7 | 9,5 | 0,913 | H5A034 C | sample | Hm Drgbl | Hm Drgbl | Hm Drgbl | CPE002DR | CPE002EH |
| 3539 | D19 | D20 | 1932 | 1446 | 18384 | 362 | 9,5 | 0,913 | H5A034 C | sample | Hm Drgbl | Hm Drgbl | Hm Drgbl | CPE002DR | CPE002EH |
| 3698 | K10 | K11 | 1189 | 1411 | 11321 | 329,5 | 9,5 | 0,913 | H5A034 C | sample | Hm Drgbl | Hm Drgbl | Hm Drgbl | CPE002DR | CPE002EH |
| 1808 | L16 | L17 | 1504 | 697,7 | 14299 | 285,9 | 9,5 | 0,995 | H5A034 C | sample | Hm Drgbl | Hm Drgbl | Hm Drgbl | CPE002FW | CPE002G0 |
| 1811 | L19 | L20 | 1820 | 704,8 | 17268 | 326,4 | 9,5 | 0,995 | H5A034 C | sample | Hm Drgbl | Hm Drgbl | Hm Drgbl | CPE002FW | CPE002G0 |
| 1814 | L21 | L22 | 1164 | 840,7 | 11047 | 311,2 | 9,5 | 0,995 | H5A034 C | sample | Hm Drgbl | Hm Drgbl | Hm Drgbl | CPE002FW | CPE002G0 |
| 1866 | N3 | N4 | 1628 | 665,5 | 15456 | 363,3 | 9,5 | 0,995 | H5A034 C | sample | Hm Drgbl | Hm Drgbl | Hm Drgbl | CPE002FW | CPE002G0 |
| 21608 | E16 | E17 | 455 | 953,3 | 4330 | 473,4 | 9,5 | 1 | H5A034 C | sample | Hm Gen E | Hm Gen E | Hm Gen E | CPE002JU | CPE002O9 |
| 21625 | F1 | F2 | 264 | 1113 | 2511 | 581 | 9,5 | 1 | H5A034 C | sample | Hm Gen E | Hm Gen E | Hm Gen E | CPE002JU | CPE002O9 |
| 21771 | L11 | L12 | 443 | 984,9 | 4219 | 729,1 | 9,5 | 1 | H5A034 C | sample | Hm Gen E | Hm Gen E | Hm Gen E | CPE002JU | CPE002O9 |
| 21822 | N14 | N15 | 261 | 951,4 | 2467 | 591,4 | 9,5 | 1 | H5A034 C | sample | Hm Gen E | Hm Gen E | Hm Gen E | CPE002JU | CPE002O9 |
| 4789 | H20 | H21 | 1792 | 799,6 | 16953 | 327,1 | 9,5 | 1,044 | H5A034 C | sample | Hm Drgbl | Hm Drgbl | Hm Drgbl | CPE002DV | CPE002EL |
| 4822 | I7 | I8 | 1341 | 862,2 | 12797 | 400,4 | 9,5 | 1,044 | H5A034 C | sample | Hm Drgbl | Hm Drgbl | Hm Drgbl | CPE002DV | CPE002EL |
| 4891 | L4 | L5 | 1469 | 795,6 | 13955 | 488,8 | 9,5 | 1,044 | H5A034 C | sample | Hm Drgbl | Hm Drgbl | Hm Drgbl | CPE002DV | CPE002EL |
| 4919 | M8 | M9 | 1875 | 785 | 17743 | 464,7 | 9,5 | 1,044 | H5A034 C | sample | Hm Drgbl | Hm Drgbl | Hm Drgbl | CPE002DV | CPE002EL |
| 4938 | N3 | N4 | 1205 | 870,2 | 11449 | 538,7 | 9,5 | 1,044 | H5A034 C | sample | Hm Drgbl | Hm Drgbl | Hm Drgbl | CPE002DV | CPE002EL |
| 4954 | O18 | O19 | 1945 | 827,2 | 18489 | 491,4 | 9,5 | 1,044 | H5A034 C | sample | Hm Drgbl | Hm Drgbl | Hm Drgbl | CPE002DV | CPE002EL |
| 10508 | F5 | F6 | 1160 | 872,6 | 11076 | 188 | 9,5 | 1,073 | H5A034 C | sample | Hm Drgbl | Hm Drgbl | Hm Drgbl | CPE002EB | CPE002EO |
| 10658 | M10 | M11 | 1158 | 777,1 | 10962 | 149,8 | 9,5 | 1,073 | H5A034 C | sample | Hm Drgbl | Hm Drgbl | Hm Drgbl | CPE002EB | CPE002EO |
| 3008 | N16 | N17 | 877 | 655 | 8288 | 432,4 | 9,5 | 1,118 | H5A034 C | sample | Hm Drgbl | Hm Drgbl | Hm Drgbl | CPE002DP | CPE002EF |
| 3047 | O8 | O9 | 1874 | 782,1 | 17873 | 494,6 | 9,5 | 1,118 | H5A034 C | sample | Hm Drgbl | Hm Drgbl | Hm Drgbl | CPE002DP | CPE002EF |
| 19972 | A12 | A13 | 598 | 1126 | 5667 | 166,1 | 9,5 | 1,159 | H5A034 C | sample | Hm Gen E | Hm Gen E | Hm Gen E | CPE002JQ | CPE002O5 |
| 20005 | B20 | B21 | 1298 | 1225 | 12324 | 161,9 | 9,5 | 1,159 | H5A034 C | sample | Hm Gen E | Hm Gen E | Hm Gen E | CPE002JQ | CPE002O5 |
| 20130 | G3 | G4 | 728 | 1110 | 6910 | 122,8 | 9,5 | 1,159 | H5A034 C | sample | Hm Gen E | Hm Gen E | Hm Gen E | CPE002JQ | CPE002O5 |
| 20228 | K5 | K6 | 898 | 1103 | 8564 | 150,1 | 9,5 | 1,159 | H5A034 C | sample | Hm Gen E | Hm Gen E | Hm Gen E | CPE002JQ | CPE002O5 |
| 20284 | N12 | N13 | 1010 | 1021 | 9619 | 121,7 | 9,5 | 1,159 | H5A034 C | sample | Hm Gen E | Hm Gen E | Hm Gen E | CPE002JQ | CPE002O5 |
| 20311 | O15 | O16 | 1498 | 1177 | 14230 | 169,9 | 9,5 | 1,159 | H5A034 C | sample | Hm Gen E | Hm Gen E | Hm Gen E | CPE002JQ | CPE002O5 |
| 20351 | P8 | P9 | 933 | 1210 | 8898 | 136,4 | 9,5 | 1,159 | H5A034 C | sample | Hm Gen E | Hm Gen E | Hm Gen E | CPE002JQ | CPE002O5 |
| 463 | D15 | D16 | 1901 | 1660 | 18098 | 312,9 | 9,5 | 1,387 | H5A034 C | sample | Hm Drgbl | Hm Drgbl | Hm Drgbl | CPE002E0 | CPE002EZ |
| 7781 | E13 | E14 | 1003 | 544,5 | 9572 | 364,1 | 9,5 | 1,45 | H5A034 C | sample | Hm Drgbl | Hm Drgbl | Hm Drgbl | CPE002E4 | CPE002EV |
| 7921 | K1 | K2 | 175 | 696,9 | 1664 | 424,5 | 9,5 | 1,45 | H5A034 C | sample | Hm Drgbl | Hm Drgbl | Hm Drgbl | CPE002E4 | CPE002EV |
| 3275 | I19 | I20 | 1744 | 1337 | 16661 | 308,2 | 9,6 | 0,594 | H5A034 C | sample | Hm Drgbl | Hm Drgbl | Hm Drgbl | CPE002DC | CPE002EG |
| 12657 | P17 | P18 | 1138 | 1021 | 10902 | 154,4 | 9,6 | 0,722 | H5A034 C | sample | Hm Gen E | Hm Gen E | Hm Gen E | CPE002J5 | CPE002NK |
| 5053 | C20 | C21 | 2341 | 637,9 | 22567 | 413,8 | 9,6 | 0,753 | H5A034 C | sample | Hm Drgbl | Hm Drgbl | Hm Drgbl | CPE002DV | CPE002EM |
| 2425 | F1 | F2 | 1244 | 671,3 | 11996 | 402,9 | 9,6 | 0,756 | H5A034 C | sample | Hm Drgbl | Hm Drgbl | Hm Drgbl | CPE002DC | CPE002EE |
| 2479 | H15 | H16 | 2071 | 654,4 | 19903 | 327,8 | 9,6 | 0,756 | H5A034 C | sample | Hm Drgbl | Hm Drgbl | Hm Drgbl | CPE002DC | CPE002EE |
| 2522 | J10 | J11 | 1883 | 687,7 | 18094 | 287,6 | 9,6 | 0,756 | H5A034 C | sample | Hm Drgbl | Hm Drgbl | Hm Drgbl | CPE002DC | CPE002EE |
| 2679 | P22 | P23 | 1692 | 811,2 | 16232 | 553,7 | 9,6 | 0,756 | H5A034 C | sample | Hm Drgbl | Hm Drgbl | Hm Drgbl | CPE002DC | CPE002EE |
| 16836 | N2 | N3 | 1753 | 997,8 | 16904 | 197,1 | 9,6 | 0,759 | H5A034 C | sample | Hm Gen E | Hm Gen E | Hm Gen E | CPE002JH | CPE002NV |
| 16857 | O17 | O18 | 1262 | 963 | 12054 | 233,9 | 9,6 | 0,759 | H5A034 C | sample | Hm Gen E | Hm Gen E | Hm Gen E | CPE002JH | CPE002NV |
| 4324 | E12 | E13 | 1251 | 720 | 12003 | 400,2 | 9,6 | 0,81 | H5A034 C | sample | Hm Drgbl | Hm Drgbl | Hm Drgbl | CPE002DU | CPE002EK |
| 4330 | E18 | E19 | 1966 | 649 | 18790 | 336,5 | 9,6 | 0,81 | H5A034 C | sample | Hm Drgbl | Hm Drgbl | Hm Drgbl | CPE002DU | CPE002EK |
| 4430 | I21 | I22 | 2138 | 730,8 | 20577 | 347,1 | 9,6 | 0,81 | H5A034 C | sample | Hm Drgbl | Hm Drgbl | Hm Drgbl | CPE002DU | CPE002EK |
| 123 | F11 | F12 | 624 | 1529 | 5994 | 521,1 | 9,6 | 0,817 | H5A034 C | sample | Hm Drgbl | Hm Drgbl | Hm Drgbl | CPE002DT | CPE002EJ |
| 146 | G10 | G11 | 766 | 1647 | 7357 | 310,9 | 9,6 | 0,817 | H5A034 C | sample | Hm Drgbl | Hm Drgbl | Hm Drgbl | CPE002DT | CPE002EJ |
| 272 | L16 | L17 | 631 | 1426 | 6087 | 414,6 | 9,6 | 0,817 | H5A034 C | sample | Hm Drgbl | Hm Drgbl | Hm Drgbl | CPE002DT | CPE002EJ |
| 275 | L19 | L20 | 522 | 1438 | 5012 | 415 | 9,6 | 0,817 | H5A034 C | sample | Hm Drgbl | Hm Drgbl | Hm Drgbl | CPE002DT | CPE002EJ |
| 313 | N1 | N2 | 442 | 1432 | 4249 | 609,5 | 9,6 | 0,817 | H5A034 C | sample | Hm Drgbl | Hm Drgbl | Hm Drgbl | CPE002DT | CPE002EJ |
| 3896 | C16 | C17 | 1802 | 751,9 | 17363 | 377,8 | 9,6 | 0,853 | H5A034 C | sample | Hm Drgbl | Hm Drgbl | Hm Drgbl | CPE002DS | CPE002EI |
| 4054 | I7 | I8 | 1576 | 672,1 | 15063 | 291,4 | 9,6 | 0,853 | H5A034 C | sample | Hm Drgbl | Hm Drgbl | Hm Drgbl | CPE002DS | CPE002EI |
| 4128 | L9 | L10 | 73 | 904,7 | 701 | 400,9 | 9,6 | 0,853 | H5A034 C | sample | Hm Drgbl | Hm Drgbl | Hm Drgbl | CPE002DS | CPE002EI |
| 11693 | H13 | H14 | 860 | 974,7 | 8254 | 125,8 | 9,6 | 0,857 | H5A034 C | sample | Hm Gen E | Hm Gen E | Hm Gen E | CPE002JA | CPE002NJ |
| 5841 | D17 | D18 | 1165 | 824,2 | 11196 | 524,2 | 9,6 | 0,877 | H5A034 C | sample | Hm Drgbl | Hm Drgbl | Hm Drgbl | CPE002DY | CPE002F1 |
| 6026 | L10 | L11 | 1549 | 813,4 | 14902 | 478,9 | 9,6 | 0,877 | H5A034 C | sample | Hm Drgbl | Hm Drgbl | Hm Drgbl | CPE002DY | CPE002F1 |
| 1474 | N18 | N19 | 1607 | 763,2 | 15482 | 447,1 | 9,6 | 0,881 | H5A034 C | sample | Hm Drgbl | Hm Drgbl | Hm Drgbl | CPE002FV | CPE002FZ |
| 16167 | B22 | B23 | 2069 | 1040 | 19768 | 311,1 | 9,6 | 0,881 | H5A034 C | sample | Hm Gen E | Hm Gen E | Hm Gen E | CPE002JG | CPE002NV |
| 16214 | D21 | D22 | 1753 | 1053 | 16885 | 250,8 | 9,6 | 0,881 | H5A034 C | sample | Hm Gen E | Hm Gen E | Hm Gen E | CPE002JG | CPE002NV |
| 1020 | K2 | K3 | 1279 | 767,9 | 12294 | 382,5 | 9,6 | 0,91 | H5A034 C | sample | Hm Drgbl | Hm Drgbl | Hm Drgbl | CPE002FU | CPE002FY |
| 5558 | H21 | H22 | 1410 | 739,6 | 13518 | 259,7 | 9,6 | 0,914 | H5A034 C | sample | Hm Drgbl | Hm Drgbl | Hm Drgbl | CPE002DX | CPE002F2 |
| 3484 | B12 | B13 | 1283 | 1363 | 12304 | 280,2 | 9,6 | 0,923 | H5A034 C | sample | Hm Drgbl | Hm Drgbl | Hm Drgbl | CPE002DR | CPE002EH |
| 3662 | I21 | I22 | 1242 | 1381 | 11870 | 270,4 | 9,6 | 0,923 | H5A034 C | sample | Hm Drgbl | Hm Drgbl | Hm Drgbl | CPE002DR | CPE002EH |
| 3759 | M22 | M23 | 1434 | 1431 | 13826 | 427,5 | 9,6 | 0,923 | H5A034 C | sample | Hm Drgbl | Hm Drgbl | Hm Drgbl | CPE002DR | CPE002EH |
| 1647 | E22 | E23 | 2499 | 841,4 | 23868 | 429,9 | 9,6 | 1,005 | H5A034 C | sample | Hm Drgbl | Hm Drgbl | Hm Drgbl | CPE002FW | CPE002G0 |
| 1717 | H20 | H21 | 2009 | 808,9 | 19190 | 357,2 | 9,6 | 1,005 | H5A034 C | sample | Hm Drgbl | Hm Drgbl | Hm Drgbl | CPE002FW | CPE002G0 |
| 21732 | J2 | J3 | 399 | 880,3 | 3818 | 613 | 9,6 | 1,011 | H5A034 C | sample | Hm Gen E | Hm Gen E | Hm Gen E | CPE002JU | CPE002O9 |
| 21735 | J22 | J23 | 488 | 1098 | 4688 | 647,1 | 9,6 | 1,011 | H5A034 C | sample | Hm Gen E | Hm Gen E | Hm Gen E | CPE002JU | CPE002O9 |
| 21772 | L12 | L13 | 340 | 945,6 | 3262 | 630,9 | 9,6 | 1,011 | H5A034 C | sample | Hm Gen E | Hm Gen E | Hm Gen E | CPE002JU | CPE002O9 |
| 21812 | M5 | M6 | 94 | 1007 | 898 | 682 | 9,6 | 1,011 | H5A034 C | sample | Hm Gen E | Hm Gen E | Hm Gen E | CPE002JU | CPE002O9 |
| 21836 | N5 | N6 | 251 | 876,5 | 2412 | 576,3 | 9,6 | 1,011 | H5A034 C | sample | Hm Gen E | Hm Gen E | Hm Gen E | CPE002JU | CPE002O9 |
| 4911 | M22 | M23 | 1262 | 856,8 | 12080 | 387,4 | 9,6 | 1,055 | H5A034 C | sample | Hm Drgbl | Hm Drgbl | Hm Drgbl | CPE002DV | CPE002EL |
| 10476 | E2 | E3 | 1530 | 895,6 | 14761 | 204,3 | 9,6 | 1,085 | H5A034 C | sample | Hm Drgbl | Hm Drgbl | Hm Drgbl | CPE002EB | CPE002EO |
| 2710 | A7 | A8 | 866 | 847,5 | 8356 | 641 | 9,6 | 1,129 | H5A034 C | sample | Hm Drgbl | Hm Drgbl | Hm Drgbl | CPE002DP | CPE002EF |
| 2726 | B21 | B22 | 1378 | 739,5 | 13279 | 711,6 | 9,6 | 1,129 | H5A034 C | sample | Hm Drgbl | Hm Drgbl | Hm Drgbl | CPE002DP | CPE002EF |
| 2734 | B7 | B8 | 1128 | 716,5 | 10883 | 549,3 | 9,6 | 1,129 | H5A034 C | sample | Hm Drgbl | Hm Drgbl | Hm Drgbl | CPE002DP | CPE002EF |
| 2742 | C14 | C15 | 1592 | 757 | 15228 | 616,7 | 9,6 | 1,129 | H5A034 C | sample | Hm Drgbl | Hm Drgbl | Hm Drgbl | CPE002DP | CPE002EF |
| 2841 | G17 | G18 | 1716 | 633,8 | 16502 | 523,2 | 9,6 | 1,129 | H5A034 C | sample | Hm Drgbl | Hm Drgbl | Hm Drgbl | CPE002DP | CPE002EF |
| 2846 | G21 | G22 | 1661 | 706,4 | 15946 | 537,7 | 9,6 | 1,129 | H5A034 C | sample | Hm Drgbl | Hm Drgbl | Hm Drgbl | CPE002DP | CPE002EF |
| 2858 | H10 | H11 | 1276 | 648,4 | 12284 | 477,3 | 9,6 | 1,129 | H5A034 C | sample | Hm Drgbl | Hm Drgbl | Hm Drgbl | CPE002DP | CPE002EF |
| 2998 | M7 | M8 | 1021 | 649,3 | 9793 | 489 | 9,6 | 1,129 | H5A034 C | sample | Hm Drgbl | Hm Drgbl | Hm Drgbl | CPE002DP | CPE002EF |
| 3057 | P17 | P18 | 1714 | 717,9 | 16502 | 566,6 | 9,6 | 1,129 | H5A034 C | sample | Hm Drgbl | Hm Drgbl | Hm Drgbl | CPE002DP | CPE002EF |
| 19993 | B1 | B2 | 1220 | 1155 | 11701 | 208 | 9,6 | 1,171 | H5A034 C | sample | Hm Gen E | Hm Gen E | Hm Gen E | CPE002JQ | CPE002O5 |
| 20091 | F11 | F12 | 778 | 1152 | 7501 | 162,2 | 9,6 | 1,171 | H5A034 C | sample | Hm Gen E | Hm Gen E | Hm Gen E | CPE002JQ | CPE002O5 |
| 20329 | P1 | P2 | 415 | 1375 | 3998 | 115 | 9,6 | 1,171 | H5A034 C | sample | Hm Gen E | Hm Gen E | Hm Gen E | CPE002JQ | CPE002O5 |
| 20342 | P21 | P22 | 1115 | 1313 | 10741 | 179,9 | 9,6 | 1,171 | H5A034 C | sample | Hm Gen E | Hm Gen E | Hm Gen E | CPE002JQ | CPE002O5 |
| 541 | G20 | G21 | 2423 | 1826 | 23286 | 290,6 | 9,6 | 1,401 | H5A034 C | sample | Hm Drgbl | Hm Drgbl | Hm Drgbl | CPE002E0 | CPE002EZ |
| 7731 | C11 | C12 | 1304 | 567,3 | 12465 | 389,1 | 9,6 | 1,466 | H5A034 C | sample | Hm Drgbl | Hm Drgbl | Hm Drgbl | CPE002E4 | CPE002EV |
| 7738 | C18 | C19 | 849 | 598,2 | 8188 | 432,5 | 9,6 | 1,466 | H5A034 C | sample | Hm Drgbl | Hm Drgbl | Hm Drgbl | CPE002E4 | CPE002EV |
| 7868 | H5 | H6 | 991 | 613,8 | 9556 | 361,2 | 9,6 | 1,466 | H5A034 C | sample | Hm Drgbl | Hm Drgbl | Hm Drgbl | CPE002E4 | CPE002EV |
| 7932 | K2 | K3 | 439 | 512,1 | 4209 | 326,8 | 9,6 | 1,466 | H5A034 C | sample | Hm Drgbl | Hm Drgbl | Hm Drgbl | CPE002E4 | CPE002EV |
| 8053 | P20 | P21 | 1110 | 555,2 | 10651 | 384,3 | 9,6 | 1,466 | H5A034 C | sample | Hm Drgbl | Hm Drgbl | Hm Drgbl | CPE002E4 | CPE002EV |
| 20641 | M1 | M2 | 848 | 747,5 | 8201 | 125,7 | 9,7 | 0,647 | H5A034 C | sample | Hm Gen E | Hm Gen E | Hm Gen E | CPE002JR | CPE002O6 |
| 21282 | G3 | G4 | 700 | 1005 | 6793 | 142,8 | 9,7 | 0,688 | H5A034 C | sample | Hm Gen E | Hm Gen E | Hm Gen E | CPE002JT | CPE002O8 |
| 5268 | L2 | L3 | 1541 | 595,4 | 14898 | 339 | 9,7 | 0,761 | H5A034 C | sample | Hm Drgbl | Hm Drgbl | Hm Drgbl | CPE002DV | CPE002EM |
| 2559 | K22 | K23 | 1974 | 717,4 | 19064 | 393 | 9,7 | 0,764 | H5A034 C | sample | Hm Drgbl | Hm Drgbl | Hm Drgbl | CPE002DC | CPE002EE |
| 4366 | F7 | F8 | 1017 | 696 | 9874 | 388,9 | 9,7 | 0,819 | H5A034 C | sample | Hm Drgbl | Hm Drgbl | Hm Drgbl | CPE002DU | CPE002EK |
| 4497 | L17 | L18 | 1396 | 810,4 | 13589 | 393,1 | 9,7 | 0,819 | H5A034 C | sample | Hm Drgbl | Hm Drgbl | Hm Drgbl | CPE002DU | CPE002EK |
| 119 | E8 | E9 | 1060 | 1846 | 10312 | 398,3 | 9,7 | 0,826 | H5A034 C | sample | Hm Drgbl | Hm Drgbl | Hm Drgbl | CPE002DT | CPE002EJ |
| 131 | F19 | F20 | 740 | 1641 | 7163 | 416,4 | 9,7 | 0,826 | H5A034 C | sample | Hm Drgbl | Hm Drgbl | Hm Drgbl | CPE002DT | CPE002EJ |
| 222 | J14 | J15 | 512 | 1304 | 4970 | 486,2 | 9,7 | 0,826 | H5A034 C | sample | Hm Drgbl | Hm Drgbl | Hm Drgbl | CPE002DT | CPE002EJ |
| 289 | M1 | M2 | 558 | 1454 | 5409 | 624,2 | 9,7 | 0,826 | H5A034 C | sample | Hm Drgbl | Hm Drgbl | Hm Drgbl | CPE002DT | CPE002EJ |
| 355 | O4 | O5 | 958 | 1969 | 9294 | 580,5 | 9,7 | 0,826 | H5A034 C | sample | Hm Drgbl | Hm Drgbl | Hm Drgbl | CPE002DT | CPE002EJ |
| 2302 | P7 | P8 | 1493 | 698,4 | 14537 | 503,4 | 9,7 | 0,843 | H5A034 C | sample | Hm Drgbl | Hm Drgbl | Hm Drgbl | CPE002FX | CPE002G1 |
| 6855 | N22 | N23 | 1453 | 580,8 | 14136 | 459,1 | 9,7 | 0,851 | H5A034 C | sample | Hm Drgbl | Hm Drgbl | Hm Drgbl | CPE002E1 | CPE002EY |
| 4009 | H1 | H2 | 1090 | 772,3 | 10573 | 476,1 | 9,7 | 0,862 | H5A034 C | sample | Hm Drgbl | Hm Drgbl | Hm Drgbl | CPE002DS | CPE002EI |
| 4160 | N16 | N17 | 1262 | 698,9 | 12281 | 379,3 | 9,7 | 0,862 | H5A034 C | sample | Hm Drgbl | Hm Drgbl | Hm Drgbl | CPE002DS | CPE002EI |
| 4178 | O10 | O11 | 1109 | 681,7 | 10776 | 440,1 | 9,7 | 0,862 | H5A034 C | sample | Hm Drgbl | Hm Drgbl | Hm Drgbl | CPE002DS | CPE002EI |
| 4189 | O20 | O21 | 1413 | 769,2 | 13693 | 469,1 | 9,7 | 0,862 | H5A034 C | sample | Hm Drgbl | Hm Drgbl | Hm Drgbl | CPE002DS | CPE002EI |
| 4222 | P7 | P8 | 757 | 739,6 | 7373 | 447,5 | 9,7 | 0,862 | H5A034 C | sample | Hm Drgbl | Hm Drgbl | Hm Drgbl | CPE002DS | CPE002EI |
| 5829 | C6 | C7 | 1644 | 896,3 | 16006 | 648,3 | 9,7 | 0,886 | H5A034 C | sample | Hm Drgbl | Hm Drgbl | Hm Drgbl | CPE002DY | CPE002F1 |
| 6059 | M19 | M20 | 2477 | 928,5 | 23926 | 527 | 9,7 | 0,886 | H5A034 C | sample | Hm Drgbl | Hm Drgbl | Hm Drgbl | CPE002DY | CPE002F1 |
| 1185 | B17 | B18 | 1728 | 905,8 | 16824 | 468 | 9,7 | 0,89 | H5A034 C | sample | Hm Drgbl | Hm Drgbl | Hm Drgbl | CPE002FV | CPE002FZ |
| 1190 | B21 | B22 | 2006 | 788 | 19384 | 448,3 | 9,7 | 0,89 | H5A034 C | sample | Hm Drgbl | Hm Drgbl | Hm Drgbl | CPE002FV | CPE002FZ |
| 1306 | G18 | G19 | 2379 | 808,5 | 23095 | 382,9 | 9,7 | 0,89 | H5A034 C | sample | Hm Drgbl | Hm Drgbl | Hm Drgbl | CPE002FV | CPE002FZ |
| 16407 | L22 | L23 | 1651 | 899,8 | 16063 | 331,3 | 9,7 | 0,89 | H5A034 C | sample | Hm Gen E | Hm Gen E | Hm Gen E | CPE002JG | CPE002NV |
| 13637 | I13 | I14 | 1319 | 931,6 | 12730 | 152,9 | 9,7 | 0,911 | H5A034 C | sample | Hm Gen E | Hm Gen E | Hm Gen E | CPE002JC | CPE002NC |
| 5557 | H20 | H21 | 2268 | 692,1 | 22088 | 441,6 | 9,7 | 0,924 | H5A034 C | sample | Hm Drgbl | Hm Drgbl | Hm Drgbl | CPE002DX | CPE002F2 |
| 5673 | M17 | M18 | 2145 | 743 | 20894 | 490,2 | 9,7 | 0,924 | H5A034 C | sample | Hm Drgbl | Hm Drgbl | Hm Drgbl | CPE002DX | CPE002F2 |
| 3595 | F4 | F5 | 1283 | 1304 | 12451 | 366,4 | 9,7 | 0,933 | H5A034 C | sample | Hm Drgbl | Hm Drgbl | Hm Drgbl | CPE002DR | CPE002EH |
| 3732 | L2 | L3 | 1114 | 1297 | 10813 | 375 | 9,7 | 0,933 | H5A034 C | sample | Hm Drgbl | Hm Drgbl | Hm Drgbl | CPE002DR | CPE002EH |
| 8107 | B4 | B5 | 1635 | 655,2 | 15801 | 478,8 | 9,7 | 0,933 | H5A034 C | sample | Hm Drgbl | Hm Drgbl | Hm Drgbl | CPE002E5 | CPE002EU |
| 8109 | B6 | B7 | 1729 | 663,6 | 16814 | 487,2 | 9,7 | 0,933 | H5A034 C | sample | Hm Drgbl | Hm Drgbl | Hm Drgbl | CPE002E5 | CPE002EU |
| 8174 | E21 | E22 | 2249 | 718,8 | 21857 | 408,5 | 9,7 | 0,933 | H5A034 C | sample | Hm Drgbl | Hm Drgbl | Hm Drgbl | CPE002E5 | CPE002EU |
| 8175 | E22 | E23 | 2065 | 655,2 | 20098 | 436,9 | 9,7 | 0,933 | H5A034 C | sample | Hm Drgbl | Hm Drgbl | Hm Drgbl | CPE002E5 | CPE002EU |
| 10268 | L5 | L6 | 1142 | 773,5 | 11129 | 135,2 | 9,7 | 0,933 | H5A034 C | sample | Hm Drgbl | Hm Drgbl | Hm Drgbl | CPE002EA | CPE002EP |
| 1842 | M3 | M4 | 851 | 700,9 | 8238 | 389,8 | 9,7 | 1,016 | H5A034 C | sample | Hm Drgbl | Hm Drgbl | Hm Drgbl | CPE002FW | CPE002G0 |
| 1860 | N2 | N3 | 1748 | 750,1 | 16936 | 398,5 | 9,7 | 1,016 | H5A034 C | sample | Hm Drgbl | Hm Drgbl | Hm Drgbl | CPE002FW | CPE002G0 |
| 21720 | I9 | I10 | 268 | 1040 | 2598 | 781,6 | 9,7 | 1,021 | H5A034 C | sample | Hm Gen E | Hm Gen E | Hm Gen E | CPE002JU | CPE002O9 |
| 4790 | H21 | H22 | 1749 | 789,8 | 16982 | 430,3 | 9,7 | 1,066 | H5A034 C | sample | Hm Drgbl | Hm Drgbl | Hm Drgbl | CPE002DV | CPE002EL |
| 4899 | M11 | M12 | 1367 | 779,4 | 13202 | 525,5 | 9,7 | 1,066 | H5A034 C | sample | Hm Drgbl | Hm Drgbl | Hm Drgbl | CPE002DV | CPE002EL |
| 4916 | M5 | M6 | 1714 | 835,3 | 16548 | 460 | 9,7 | 1,066 | H5A034 C | sample | Hm Drgbl | Hm Drgbl | Hm Drgbl | CPE002DV | CPE002EL |
| 2771 | D19 | D20 | 1535 | 659,4 | 14840 | 517,2 | 9,7 | 1,141 | H5A034 C | sample | Hm Drgbl | Hm Drgbl | Hm Drgbl | CPE002DP | CPE002EF |
| 2955 | L11 | L12 | 1102 | 702,4 | 10671 | 537 | 9,7 | 1,141 | H5A034 C | sample | Hm Drgbl | Hm Drgbl | Hm Drgbl | CPE002DP | CPE002EF |
| 2970 | L3 | L4 | 1197 | 610,7 | 11665 | 580,4 | 9,7 | 1,141 | H5A034 C | sample | Hm Drgbl | Hm Drgbl | Hm Drgbl | CPE002DP | CPE002EF |

| | | | | | | | | | | | | | | | | | | | | | | | | | | | | | | | |
|---|---|---|---|---|---|---|---|---|---|---|---|---|---|---|---|---|---|---|---|---|---|---|---|---|---|---|---|---|---|---|---|
| 19980 | A2 | A3 | 521 | 1291 | 5065 | 270,3 | 9,7 | 1,183 | H5A034 C | sample | Hm Gen E | Hm Gen E | Hm Gen E | CPE002JQ | CPE002O5 | CPE002KL | XM 93455 | XM 93455 | XM 93455 | LOC400960 | LOC40096 | LOC40096 | hypothetical gene | hypotheti | hypotheti | 400960 | 400960 | 400960 | s53425 | s53426 | s53427 |
| 20211 | K11 | K12 | 958 | 1095 | 9254 | 158,3 | 9,7 | 1,183 | H5A034 C | sample | Hm Gen E | Hm Gen E | Hm Gen E | CPE002JQ | CPE002O5 | CPE002KL | XM 93825 | XM 93825 | XM 37326 | LOC392262 | LOC39226 | LOC39226 | similar to 40S ribo | similar to | similar to | 392262 | 392262 | 392262 | s53050 | s53051 | s226503 |
| 20212 | K12 | K13 | 924 | 994,9 | 8982 | 147,4 | 9,7 | 1,183 | H5A034 C | sample | Hm Gen E | Hm Gen E | Hm Gen E | CPE002JQ | CPE002O5 | CPE002KL | NM 20750 | NM 20750 | NM 20750 | SAMD12 | SAMD12 | SAMD12 | sterile alpha moti | sterile alp | sterile alp | 401474 | 401474 | 401474 | s53609 | s53610 | s53611 |
| 20219 | K19 | K20 | 1393 | 1132 | 13481 | 156,7 | 9,7 | 1,183 | H5A034 C | sample | Hm Gen E | Hm Gen E | Hm Gen E | CPE002JQ | CPE002O5 | CPE002KL | NM 00100 | NM 00100 | NM 00100 | OR13J1 | OR13J1 | OR13J1 | olfactory receptor | olfactory | olfactory | 392309 | 392309 | 392309 | s53059 | s196411 | s196412 |
| 20243 | L19 | L20 | 956 | 1079 | 9274 | 126,9 | 9,7 | 1,183 | H5A034 C | sample | Hm Gen E | Hm Gen E | Hm Gen E | CPE002JQ | CPE002O5 | CPE002KL | XM 93927 | XM 93927 | XM 93927 | SH2D5 | SH2D5 | SH2D5 | SH2 domain conta | SH2 doma | SH2 doma | 400745 | 400745 | 400745 | s53354 | s53355 | s53356 |
| 20277 | M6 | M7 | 804 | 1164 | 7812 | 162,7 | 9,7 | 1,183 | H5A034 C | sample | Hm Gen E | Hm Gen E | Hm Gen E | CPE002JQ | CPE002O5 | CPE002KL | NM 00101 | NM 0010 | NM 0010 | CENPP | CENPP | CENPP | centromere prote | centrome | centrome | 401541 | 401541 | 401541 | s53633 | s53634 | s53635 |
| 20304 | N9 | N10 | 797 | 1079 | 7739 | 142,4 | 9,7 | 1,183 | H5A034 C | sample | Hm Gen E | Hm Gen E | Hm Gen E | CPE002JQ | CPE002O5 | CPE002KL | XM 00113 | XM 00113 | XM 00113 | LOC400796 | LOC40079 | LOC40079 | hypothetical LOC4 | hypotheti | hypotheti | 400796 | 400796 | 400796 | s53369 | s53370 | s53371 |
| 20316 | O2 | O3 | 1024 | 1080 | 9949 | 133,9 | 9,7 | 1,183 | H5A034 C | sample | Hm Gen E | Hm Gen E | Hm Gen E | CPE002JQ | CPE002O5 | CPE002KL | NM 00101 | NM 0010 | NM 0010 | C10orf132 | C10orf132 | C10orf132 | chromosome 10 o | chromoso | chromoso | 401647 | 401647 | 401647 | s53666 | s53667 | s53668 |
| 397 | A20 | A21 | 2334 | 1931 | 22675 | 427,7 | 9,7 | 1,416 | H5A034 C | sample | Hm Drgbl | Hm Drgbl | Hm Drgbl | CPE002E0 | CPE002EZ | CPE002FK | NM 20200 | NM 2020 | NM 2020 | FOXM1 | FOXM1 | FOXM1 | forkhead box M1 | forkhead | forkhead | 2305 | 2305 | 2305 | s5248 | s5249 | s5250 |
| 557 | H13 | H14 | 2273 | 1797 | 21975 | 309,2 | 9,7 | 1,416 | H5A034 C | sample | Hm Drgbl | Hm Drgbl | Hm Drgbl | CPE002E0 | CPE002EZ | CPE002FK | NM 00199 | NM 0019 | NM 0019 | ACSL1 | ACSL1 | ACSL1 | acyl-CoA syntheta | acyl-CoA s | acyl-CoA s | 2180 | 2180 | 2180 | s4994 | s4995 | s4996 |
| 7712 | B16 | B17 | 779 | 537,3 | 7591 | 420,6 | 9,7 | 1,481 | H5A034 C | sample | Hm Drgbl | Hm Drgbl | Hm Drgbl | CPE002E4 | CPE002EV | CPE002FG | NM 00668 | NM 0066 | NM 0066 | ME3 | ME3 | ME3 | malic enzyme 3, N | malic enzy | malic enzy | 10873 | 10873 | 10873 | s21346 | s21347 | s21348 |
| 7884 | I2 | I3 | 559 | 603,2 | 5414 | 351 | 9,7 | 1,481 | H5A034 C | sample | Hm Drgbl | Hm Drgbl | Hm Drgbl | CPE002E4 | CPE002EV | CPE002FG | NM 02110 | NM 0211 | NM 0211 | SPINT2 | SPINT2 | SPINT2 | serine peptidase | serine pep | serine pep | 10653 | 10653 | 10653 | s20939 | s20940 | s20941 |
| 7929 | K17 | K18 | 350 | 573,4 | 3397 | 386,7 | 9,7 | 1,481 | H5A034 C | sample | Hm Drgbl | Hm Drgbl | Hm Drgbl | CPE002E4 | CPE002EV | CPE002FG | NM 00586 | NM 0058 | NM 0058 | OPRS1 | OPRS1 | OPRS1 | opioid receptor, s | opioid rec | opioid rec | 10280 | 10280 | 10280 | s20088 | s20087 | s20086 |
| 8023 | O15 | O16 | 669 | 594,1 | 6509 | 481,4 | 9,7 | 1,481 | H5A034 C | sample | Hm Drgbl | Hm Drgbl | Hm Drgbl | CPE002E4 | CPE002EV | CPE002FG | NM 00607 | NM 0060 | NM 0060 | TRIM22 | TRIM22 | TRIM22 | tripartite motif-co | tripartite | tripartite | 10346 | 10346 | 10346 | s20236 | s20238 | s20237 |
| 3083 | A19 | A20 | 1996 | 1353 | 19595 | 315,7 | 9,8 | 0,607 | H5A034 C | sample | Hm Drgbl | Hm Drgbl | Hm Drgbl | CPE002DC | CPE002EG | CPE002F6 | NM 00430 | NM 0043 | NM 0010 | ACP1 | ACP1 | ACP1 | acid phosphatase | acid phos | acid phos | 52 | 52 | 52 | s927 | s928 | s224267 |
| 6201 | C17 | C18 | 1153 | 626,6 | 11251 | 241,9 | 9,8 | 0,647 | H5A034 C | sample | Hm Drgbl | Hm Drgbl | Hm Drgbl | CPE002DZ | CPE002F0 | CPE002FL | NM 00613 | NM 0061 | NM 0061 | CD7 | CD7 | CD7 | CD7 molecule | CD7 mole | CD7 mole | 924 | 924 | 924 | s2590 | s194330 | s194329 |
| 20628 | L2 | L3 | 1014 | 794 | 9953 | 130,6 | 9,8 | 0,653 | H5A034 C | sample | Hm Gen E | Hm Gen E | Hm Gen E | CPE002JR | CPE002O6 | CPE002KN | XM 93080 | XM 9308 | XM 9308 | LOC642426 | LOC64242 | LOC64242 | hypothetical LOC | hypotheti | hypotheti | 642426 | 642426 | 642426 | s55014 | s55015 | s55016 |
| 8610 | G3 | G4 | 1806 | 1233 | 17678 | 90,49 | 9,8 | 0,693 | H5A034 C | sample | Hm Drgbl | Hm Drgbl | Hm Drgbl | CPE002E6 | CPE002ET | CPE002FE | NM 01323 | NM 0132 | NM 0132 | FLRT2 | FLRT2 | FLRT2 | fibronectin leucin | fibronecti | fibronecti | 23768 | 23768 | 23768 | s24379 | s24380 | s24381 |
| 11418 | L3 | L4 | 1018 | 801,5 | 10002 | 142,6 | 9,8 | 0,71 | H5A034 C | sample | Hm Drgbl | Hm Drgbl | Hm Drgbl | CPE002ED | CPE002F3 | CPE002FT | NM 19868 | NM 1986 | NM 1986 | RAB15 | RAB15 | RAB15 | RAB15, member R | RAB15, m | RAB15, m | 376267 | 376267 | 376267 | s51762 | s51763 | s51761 |
| 17233 | O1 | O2 | 1685 | 978,5 | 16585 | 135,3 | 9,8 | 0,757 | H5A034 C | sample | Hm Gen E | Hm Gen E | Hm Gen E | CPE002JI | CPE002NZ | CPE002KD | NM 02507 | NM 1825 | NM 0250 | TOE1 | C13orf30 | TOE1 | target of EGR1, m | chromoso | target of E | 114034 | 144809 | 114034 | s41523 | s44694 | s41525 |
| 2665 | P1 | P2 | 810 | 743,7 | 7968 | 444,4 | 9,8 | 0,772 | H5A034 C | sample | Hm Drgbl | Hm Drgbl | Hm Drgbl | CPE002DC | CPE002EE | CPE002F4 | NM 00202 | NM 0020 | NM 0020 | FLT4 | FLT4 | FLT4 | fms-related tyrosi | fms-relate | fms-relate | 2324 | 2324 | 2324 | s5294 | s5295 | s5296 |
| 16839 | N22 | N23 | 1749 | 945,7 | 17062 | 275,8 | 9,8 | 0,775 | H5A034 C | sample | Hm Gen E | Hm Gen E | Hm Gen E | CPE002JH | CPE002NV | CPE002KC | NM 00103 | NM 0010 | NM 0010 | DMKN | DMKN | DMKN | dermokine | dermokin | dermokin | 93099 | 93099 | 93099 | s41081 | s228483 | s228484 |
| 4476 | K2 | K3 | 1265 | 756,4 | 12398 | 373,8 | 9,8 | 0,827 | H5A034 C | sample | Hm Drgbl | Hm Drgbl | Hm Drgbl | CPE002DL | CPE002EK | CPE002FQ | NM 01437 | NM 0143 | NM 0143 | KCNV1 | KCNV1 | KCNV1 | potassium chann | potassium | potassium | 27012 | 27012 | 27012 | s25695 | s25696 | s25697 |
| 50 | C10 | C11 | 937 | 1677 | 9221 | 504,2 | 9,8 | 0,834 | H5A034 C | sample | Hm Drgbl | Hm Drgbl | Hm Drgbl | CPE002DT | CPE002EJ | CPE002F9 | NM 00305 | NM 0030 | NM 0030 | SLC16A1 | SLC16A1 | SLC16A1 | solute carrier fam | solute car | solute car | 6566 | 6566 | 6566 | s580 | s579 | s578 |
| 147 | G11 | G12 | 921 | 1816 | 8980 | 476 | 9,8 | 0,834 | H5A034 C | sample | Hm Drgbl | Hm Drgbl | Hm Drgbl | CPE002DT | CPE002EJ | CPE002F9 | NM 02200 | NM 0220 | NM 0220 | NR1I2 | NR1I2 | NR1I2 | nuclear receptor s | nuclear re | nuclear re | 8856 | 8856 | 8856 | s16909 | s16910 | s16911 |
| 170 | H10 | H11 | 659 | 1521 | 6479 | 611,4 | 9,8 | 0,834 | H5A034 C | sample | Hm Drgbl | Hm Drgbl | Hm Drgbl | CPE002DT | CPE002EJ | CPE002F9 | NM 00313 | NM 0031 | NM 0031 | SRPR | SRPR | SRPR | signal recognition | signal rec | signal rec | 6734 | 6734 | 6734 | s13458 | s224687 | s13457 |
| 309 | M6 | M7 | 617 | 1546 | 6056 | 450,4 | 9,8 | 0,834 | H5A034 C | sample | Hm Drgbl | Hm Drgbl | Hm Drgbl | CPE002DT | CPE002EJ | CPE002F9 | NM 02102 | NM 0210 | NM 0210 | SNTB1 | SNTB1 | SNTB1 | syntrophin, beta | syntrophi | syntrophi | 6641 | 6641 | 6641 | s13254 | s224663 | s224664 |
| 2194 | L18 | L19 | 1063 | 664,3 | 10421 | 375,2 | 9,8 | 0,852 | H5A034 C | sample | Hm Drgbl | Hm Drgbl | Hm Drgbl | CPE002FX | CPE002G1 | CPE002G5 | | | | | | | | | | | | | | | |
| 6531 | A11 | A12 | 1323 | 741,2 | 12941 | 244,9 | 9,8 | 0,86 | H5A034 C | sample | Hm Drgbl | Hm Drgbl | Hm Drgbl | CPE002E1 | CPE002EY | CPE002FJ | NM 00085 | NM 0008 | NM 0008 | GSTM5 | GSTM5 | GSTM5 | glutathione S-tran | glutathior | glutathior | 2949 | 2949 | 2949 | s6276 | s223819 | s6274 |
| 3931 | D4 | D5 | 1406 | 739 | 13765 | 255,5 | 9,8 | 0,871 | H5A034 C | sample | Hm Drgbl | Hm Drgbl | Hm Drgbl | CPE002DS | CPE002EI | CPE002F8 | NM 00044 | NM 0004 | NM 0004 | RFX5 | RFX5 | RFX5 | regulatory factor | regulatory | regulatory | 5993 | 5993 | 5993 | s11977 | s11978 | s11979 |
| 4078 | J7 | J8 | 1230 | 637,8 | 12013 | 308,7 | 9,8 | 0,871 | H5A034 C | sample | Hm Drgbl | Hm Drgbl | Hm Drgbl | CPE002DS | CPE002EI | CPE002F8 | NM 20703 | NM 2070 | NM 2070 | TCF12 | TCF12 | TCF12 | transcription fact | transcript | transcript | 6938 | 6938 | 6938 | s13891 | s13889 | s13890 |
| 4133 | M13 | M14 | 1008 | 669,5 | 9868 | 277,9 | 9,8 | 0,871 | H5A034 C | sample | Hm Drgbl | Hm Drgbl | Hm Drgbl | CPE002DS | CPE002EI | CPE002F8 | NM 00093 | NM 0009 | NM 0009 | POMC | POMC | POMC | proopiomelanocc | proopiom | proopiom | 5443 | 5443 | 5443 | s10828 | s194704 | s194703 |
| 4141 | M20 | M21 | 1561 | 739,4 | 15365 | 260,2 | 9,8 | 0,871 | H5A034 C | sample | Hm Drgbl | Hm Drgbl | Hm Drgbl | CPE002DS | CPE002EI | CPE002F8 | NM 00289 | NM 0028 | NM 0028 | RBP1 | RBP1 | RBP1 | retinol binding pr | retinol bir | retinol bir | 5947 | 5947 | 5947 | s11872 | s224524 | s11871 |
| 11798 | L21 | L22 | 499 | 884,8 | 4866 | 149,7 | 9,8 | 0,875 | H5A034 C | sample | Hm Gen E | Hm Gen E | Hm Gen E | CPE002JA | CPE002NJ | CPE002JZ | NM 00158 | NM 0015 | NM 0015 | TEX28 | TEX28 | TEX28 | testis expressed 2 | testis exp | testis exp | 1527 | 1527 | 1527 | s3777 | s3778 | s3779 |
| 11813 | M13 | M14 | 1166 | 752,5 | 11370 | 124,2 | 9,8 | 0,875 | H5A034 C | sample | Hm Gen E | Hm Gen E | Hm Gen E | CPE002JA | CPE002NJ | CPE002JZ | NM 00073 | NM 0007 | NM 0007 | CD3E | CD3E | CD3E | CD3e molecule, e | CD3e mol | CD3e mol | 916 | 916 | 916 | s2569 | s2570 | s2571 |
| 5989 | J20 | J21 | 1785 | 905,3 | 17441 | 239,4 | 9,8 | 0,895 | H5A034 C | sample | Hm Drgbl | Hm Drgbl | Hm Drgbl | CPE002DY | CPE002F1 | CPE002FM | NM 00121 | NM 0012 | NM 0012 | CA6 | CA6 | CA6 | carbonic anhydra | carbonic a | carbonic a | 765 | 765 | 765 | s2261 | s2259 | s2260 |
| 1523 | P19 | P20 | 976 | 757,6 | 9589 | 508,3 | 9,8 | 0,899 | H5A034 C | sample | Hm Drgbl | Hm Drgbl | Hm Drgbl | CPE002FV | CPE002FZ | CPE002G3 | NM 00100 | NM 0048 | NM 0048 | GOSR1 | GOSR1 | GOSR1 | golgi SNAP recept | golgi SNAI | golgi SNAI | 9527 | 9527 | 9527 | s225155 | s18279 | s18280 |
| 16479 | O22 | O23 | 1492 | 1043 | 14580 | 367,1 | 9,8 | 0,899 | H5A034 C | sample | Hm Gen E | Hm Gen E | Hm Gen E | CPE002JG | CPE002NV | CPE002KB | NM 03241 | NM 0324 | NM 0324 | HOOK3 | HOOK3 | HOOK3 | hook homolog 3 ( | hook hom | hook hom | 84376 | 84376 | 84376 | s38972 | s38973 | s228364 |
| 13497 | C17 | C18 | 1434 | 1056 | 14026 | 150,2 | 9,8 | 0,92 | H5A034 C | sample | Hm Gen E | Hm Gen E | Hm Gen E | CPE002JC | CPE002NC | CPE002K4 | XM 92931 | XM 92931 | XM 92931 | TTC28 | TTC28 | TTC28 | tetratricopeptide | tetratrico | tetratrico | 23331 | 23331 | 23331 | s23578 | s23579 | s23580 |
| 13530 | D3 | D4 | 1667 | 1140 | 16332 | 159,4 | 9,8 | 0,92 | H5A034 C | sample | Hm Gen E | Hm Gen E | Hm Gen E | CPE002JC | CPE002NC | CPE002K4 | NM 15283 | NM 1528 | NM 1528 | FAM89B | FAM89B | FAM89B | family with seque | family wit | family wit | 23625 | 23625 | 23625 | s24226 | s24227 | s24228 |
| 13617 | H17 | H18 | 1702 | 1192 | 16752 | 154,6 | 9,8 | 0,92 | H5A034 C | sample | Hm Gen E | Hm Gen E | Hm Gen E | CPE002JC | CPE002NC | CPE002K4 | NM 01210 | NM 0121 | NM 0121 | TMEM59L | TMEM59L | TMEM59L | transmembrane p | transmem | transmem | 25789 | 25789 | 25789 | s24480 | s24481 | s195113 |
| 970 | I18 | I19 | 1619 | 705,8 | 15791 | 286,9 | 9,8 | 0,929 | H5A034 C | sample | Hm Drgbl | Hm Drgbl | Hm Drgbl | CPE002FU | CPE002FY | CPE002G2 | NM 00043 | NM 0004 | NM 0004 | PAFAH2 | PAFAH2 | PAFAH2 | platelet-activatin | platelet-a | platelet-a | 5051 | 5051 | 5051 | s10004 | s10005 | s224163 |
| 1028 | K5 | K6 | 720 | 871,6 | 7060 | 441,8 | 9,8 | 0,929 | H5A034 C | sample | Hm Drgbl | Hm Drgbl | Hm Drgbl | CPE002FU | CPE002FY | CPE002G2 | NM 05716 | NM 0571 | NM 0571 | COL6A3 | COL6A3 | COL6A3 | collagen, type VI, | collagen, | collagen, | 1293 | 1293 | 1293 | s3317 | s3318 | s3319 |
| 1057 | M1 | M2 | 1758 | 765,2 | 17219 | 454,4 | 9,8 | 0,929 | H5A034 C | sample | Hm Drgbl | Hm Drgbl | Hm Drgbl | CPE002FU | CPE002FY | CPE002G2 | NM 00049 | NM 0004 | NM 0004 | COL17A1 | COL17A1 | COL17A1 | collagen, type XVI | collagen, | collagen, | 1308 | 1308 | 1308 | s3359 | s3360 | s3361 |
| 3461 | A13 | A14 | 1521 | 1330 | 14841 | 279,2 | 9,8 | 0,942 | H5A034 C | sample | Hm Drgbl | Hm Drgbl | Hm Drgbl | CPE002DR | CPE002EH | CPE002F7 | NM 00067 | NM 0006 | NM 0006 | ADORA2B | ADORA2B | ADORA2B | adenosine A2b re | adenosine | adenosine | 136 | 136 | 136 | s1091 | s1092 | s1093 |
| 3528 | C9 | C10 | 1477 | 1377 | 14529 | 305,9 | 9,8 | 0,942 | H5A034 C | sample | Hm Drgbl | Hm Drgbl | Hm Drgbl | CPE002DR | CPE002EH | CPE002F7 | NM 03185 | NM 0318 | NM 0318 | AGTR1 | AGTR1 | AGTR1 | angiotensin II rec | angiotens | angiotens | 185 | 185 | 185 | s1180 | s1181 | s1182 |
| 3643 | H4 | H5 | 1392 | 1414 | 13699 | 397,8 | 9,8 | 0,942 | H5A034 C | sample | Hm Drgbl | Hm Drgbl | Hm Drgbl | CPE002DR | CPE002EH | CPE002F7 | NM 05327 | NM 0532 | NM 0532 | TAAR8 | TAAR8 | TAAR8 | trace amine assoc | trace amir | trace amir | 83551 | 83551 | 83551 | s38007 | s38008 | s38009 |
| 3703 | K15 | K16 | 2021 | 1627 | 19750 | 379,1 | 9,8 | 0,942 | H5A034 C | sample | Hm Drgbl | Hm Drgbl | Hm Drgbl | CPE002DR | CPE002EH | CPE002F7 | NM 00140 | NM 0014 | NM 0014 | EDG2 | EDG2 | EDG2 | endothelial differ | endotheli | endotheli | 1902 | 1902 | 1902 | s4450 | s4451 | s4452 |
| 8082 | A3 | A4 | 1176 | 658,6 | 11549 | 575,5 | 9,8 | 0,942 | H5A034 C | sample | Hm Drgbl | Hm Drgbl | Hm Drgbl | CPE002E5 | CPE002EU | CPE002FF | NM 00700 | NM 0070 | NM 0070 | SLC35D2 | SLC35D2 | SLC35D2 | solute carrier fam | solute car | solute car | 11046 | 11046 | 11046 | s21764 | s223185 | s21766 |
| 8111 | B8 | B9 | 1611 | 699,2 | 15859 | 491,7 | 9,8 | 0,942 | H5A034 C | sample | Hm Drgbl | Hm Drgbl | Hm Drgbl | CPE002E5 | CPE002EU | CPE002FF | NM 01531 | NM 0153 | NM 0153 | ARHGEF18 | ARHGEF18 | ARHGEF18 | rho/rac guanine n | rho/rac gu | rho/rac gu | 23370 | 23370 | 23370 | s23674 | s23675 | s23676 |
| 8127 | C22 | C23 | 1606 | 676 | 15762 | 470,7 | 9,8 | 0,942 | H5A034 C | sample | Hm Drgbl | Hm Drgbl | Hm Drgbl | CPE002E5 | CPE002EU | CPE002FF | NM 01509 | NM 0150 | NM 0150 | HIC2 | HIC2 | HIC2 | hypermethylated | hypermet | hypermet | 23119 | 23119 | 23119 | s23077 | s23078 | s23076 |
| 8133 | C6 | C7 | 871 | 647,9 | 8547 | 520,5 | 9,8 | 0,942 | H5A034 C | sample | Hm Drgbl | Hm Drgbl | Hm Drgbl | CPE002E5 | CPE002EU | CPE002FF | NM 17198 | NM 1719 | NM 1719 | TRIM35 | TRIM35 | TRIM35 | tripartite motif-co | tripartite | tripartite | 23087 | 23087 | 23087 | s23002 | s23001 | s23003 |
| 10242 | K3 | K4 | 2155 | 913,4 | 21029 | 131,5 | 9,8 | 0,942 | H5A034 C | sample | Hm Drgbl | Hm Drgbl | Hm Drgbl | CPE002EA | CPE002EP | CPE002FA | NM 02483 | NM 0248 | NM 0248 | THNSL1 | THNSL1 | THNSL1 | threonine syntha | threonine | threonine | 79896 | 79896 | 79896 | s36598 | s36597 | s36596 |
| 21606 | E14 | E15 | 479 | 1057 | 4679 | 620,3 | 9,8 | 1,032 | H5A034 C | sample | Hm Gen E | Hm Gen E | Hm Gen E | CPE002JU | CPE002O9 | CPE002KP | XM 94215 | XM 9421 | XM 94215 | LOC652618 | LOC65261 | LOC65261 | similar to CG4845 | similar to | similar to | 652618 | 652618 | 652618 | s57416 | s57417 | s57418 |
| 21700 | I12 | I13 | 265 | 944 | 2591 | 651,8 | 9,8 | 1,032 | H5A034 C | sample | Hm Gen E | Hm Gen E | Hm Gen E | CPE002JU | CPE002O9 | CPE002KP | XM 94232 | XM 94232 | XM 94232 | LOC652710 | LOC65271 | LOC65271 | similar to Voltage | similar to | similar to | 652710 | 652710 | 652710 | s57449 | s197959 | s197960 |
| 21768 | K9 | K10 | 132 | 1091 | 1294 | 549,6 | 9,8 | 1,032 | H5A034 C | sample | Hm Gen E | Hm Gen E | Hm Gen E | CPE002JU | CPE002O9 | CPE002KP | XM 93906 | XM 93906 | XM 93906 | LOC649984 | LOC64998 | LOC64998 | similar to Glycode | similar to | similar to | 649984 | 649984 | 649984 | s57030 | s197702 | s197703 |
| 4634 | B10 | B11 | 1838 | 774,5 | 17950 | 487,9 | 9,8 | 1,077 | H5A034 C | sample | Hm Drgbl | Hm Drgbl | Hm Drgbl | CPE002DV | CPE002EL | CPE002FP | NM 00100 | NM 0010 | NM 0010 | USP20 | USP20 | USP20 | ubiquitin specific | ubiquitin | ubiquitin | 10868 | 10868 | 10868 | s21336 | s21337 | s21338 |
| 4664 | C16 | C17 | 1207 | 786,3 | 11849 | 431,5 | 9,8 | 1,077 | H5A034 C | sample | Hm Drgbl | Hm Drgbl | Hm Drgbl | CPE002DV | CPE002EL | CPE002FP | NM 00600 | NM 0060 | NM 0060 | UCHL3 | UCHL3 | UCHL3 | ubiquitin carboxy | ubiquitin | ubiquitin | 7347 | 7347 | 7347 | s14619 | s14620 | s14621 |
| 4689 | D17 | D18 | 1523 | 810,1 | 14885 | 416,8 | 9,8 | 1,077 | H5A034 C | sample | Hm Drgbl | Hm Drgbl | Hm Drgbl | CPE002DV | CPE002EL | CPE002FP | NM 00218 | NM 0021 | NM 0021 | IHH | IHH | IHH | Indian hedgehog | Indian he | Indian he | 3549 | 3549 | 3549 | s7257 | s7258 | s7259 |
| 4701 | D6 | D7 | 1412 | 776,1 | 13786 | 521,1 | 9,8 | 1,077 | H5A034 C | sample | Hm Drgbl | Hm Drgbl | Hm Drgbl | CPE002DV | CPE002EL | CPE002FP | NM 13902 | NM 1390 | NM 1390 | ADAMTS1 | ADAMTS1 | ADAMTS1 | ADAM metallopep | ADAM me | ADAM me | 11093 | 11093 | 11093 | s21868 | s21869 | s21870 |
| 4739 | F19 | F20 | 1386 | 721,4 | 13646 | 437,7 | 9,8 | 1,077 | H5A034 C | sample | Hm Drgbl | Hm Drgbl | Hm Drgbl | CPE002DV | CPE002EL | CPE002FP | NM 17752 | NM 1775 | NM 1775 | MEST | MEST | MEST | mesoderm specifi | mesodern | mesodern | 4232 | 4232 | 4232 | s8697 | s8698 | s8699 |
| 4772 | G5 | G6 | 1439 | 809,3 | 14135 | 389,5 | 9,8 | 1,077 | H5A034 C | sample | Hm Drgbl | Hm Drgbl | Hm Drgbl | CPE002DV | CPE002EL | CPE002FP | NM 00006 | NM 0000 | NM 0000 | C2 | C2 | C2 | complement com | compleme | compleme | 717 | 717 | 717 | s2158 | s2159 | s2160 |
| 4781 | H13 | H14 | 1652 | 822,6 | 16254 | 406,7 | 9,8 | 1,077 | H5A034 C | sample | Hm Drgbl | Hm Drgbl | Hm Drgbl | CPE002DV | CPE002EL | CPE002FP | NM 00499 | NM 0049 | NM 0049 | MMP9 | MMP9 | MMP9 | matrix metallope | matrix me | matrix me | 4318 | 4318 | 4318 | s8862 | s8863 | s8864 |
| 10491 | F11 | F12 | 1065 | 909,9 | 10438 | 191,8 | 9,8 | 1,107 | H5A034 C | sample | Hm Drgbl | Hm Drgbl | Hm Drgbl | CPE002EB | CPE002EO | CPE002FR | NM 00108 | NM 0010 | NM 0010 | DUSP27 | DUSP27 | DUSP27 | dual specificity ph | dual speci | dual speci | 92235 | 92235 | 92235 | s40885 | s40886 | s40887 |
| 10619 | K19 | K20 | 1024 | 808,9 | 10001 | 171,5 | 9,8 | 1,107 | H5A034 C | sample | Hm Drgbl | Hm Drgbl | Hm Drgbl | CPE002EB | CPE002EO | CPE002FR | NM 13046 | NM 1304 | NM 1304 | UBE3B | UBE3B | UBE3B | ubiquitin protein | ubiquitin | ubiquitin | 89910 | 89910 | 89910 | s40201 | s40202 | s40200 |
| 10632 | K9 | K10 | 772 | 810,4 | 7593 | 187,9 | 9,8 | 1,107 | H5A034 C | sample | Hm Drgbl | Hm Drgbl | Hm Drgbl | CPE002EB | CPE002EO | CPE002FR | NM 03312 | NM 0331 | NM 0331 | PLCZ1 | PLCZ1 | PLCZ1 | phospholipase C, | phospholi | phospholi | 89869 | 89869 | 89869 | s40168 | s40169 | s40170 |
| 2694 | A14 | A15 | 1470 | 684,6 | 14401 | 552,8 | 9,8 | 1,153 | H5A034 C | sample | Hm Drgbl | Hm Drgbl | Hm Drgbl | CPE002DP | CPE002EF | CPE002F5 | NM 01790 | NM 0179 | NM 0179 | AURKAIP1 | AURKAIP1 | AURKAIP1 | aurora kinase A in | aurora kir | aurora kir | 54998 | 54998 | 54998 | s29953 | s29954 | s224333 |
| 2701 | A20 | A21 | 1418 | 714,3 | 13880 | 565,4 | 9,8 | 1,153 | H5A034 C | sample | Hm Drgbl | Hm Drgbl | Hm Drgbl | CPE002DP | CPE002EF | CPE002F5 | NM 01832 | NM 0183 | NM 0183 | PI4K2B | PI4K2B | PI4K2B | phosphatidylinos | phosphat | phosphat | 55300 | 55300 | 55300 | s30681 | s30682 | s30683 |
| 2741 | C13 | C14 | 942 | 614,9 | 9225 | 413,2 | 9,8 | 1,153 | H5A034 C | sample | Hm Drgbl | Hm Drgbl | Hm Drgbl | CPE002DP | CPE002EF | CPE002F5 | NM 00368 | NM 0036 | NM 0036 | PDXK | PDXK | PDXK | pyridoxal (pyrido: | pyridoxal | pyridoxal | 8566 | 8566 | 8566 | s16310 | s16311 | s16312 |
| 2826 | F3 | F4 | 967 | 636,1 | 9437 | 516 | 9,8 | 1,153 | H5A034 C | sample | Hm Drgbl | Hm Drgbl | Hm Drgbl | CPE002DP | CPE002EF | CPE002F5 | NM 13364 | NM 1336 | NM 1336 | ZAK | ZAK | ZAK | sterile alpha moti | sterile alp | sterile alp | 51776 | 51776 | 51776 | s28651 | s28652 | s28653 |
| 20067 | E11 | E12 | 632 | 1113 | 6216 | 183,3 | 9,8 | 1,195 | H5A034 C | sample | Hm Gen E | Hm Gen E | Hm Gen E | CPE002JQ | CPE002O5 | CPE002KL | NM 00101 | NM 0010 | NM 0010 | VSIG8 | VSIG8 | VSIG8 | V-set and immun | V-set and | V-set and | 391123 | 391123 | 391123 | s52931 | s52933 | s226489 |
| 20077 | E20 | E21 | 1249 | 1167 | 12265 | 162,4 | 9,8 | 1,195 | H5A034 C | sample | Hm Gen E | Hm Gen E | Hm Gen E | CPE002JQ | CPE002O5 | CPE002KL | NM 00101 | NM 0010 | NM 0010 | LOC401233 | LOC40123 | LOC40123 | similar to HIV TAT | similar to | similar to | 401233 | 401233 | 401233 | s53521 | s53522 | s53523 |
| 20095 | F15 | F16 | 687 | 1063 | 6731 | 178,4 | 9,8 | 1,195 | H5A034 C | sample | Hm Gen E | Hm Gen E | Hm Gen E | CPE002JQ | CPE002O5 | CPE002KL | NM 20743 | NM 2074 | NM 2074 | FLJ40142 | FLJ40142 | FLJ40142 | FLJ40142 protein | FLJ40142 | FLJ40142 | 400073 | 400073 | 400073 | s53238 | s53239 | s226525 |
| 20171 | I19 | I20 | 728 | 1111 | 7108 | 160,5 | 9,8 | 1,195 | H5A034 C | sample | Hm Gen E | Hm Gen E | Hm Gen E | CPE002JQ | CPE002O5 | CPE002KL | XM 37307 | XM 37307 | XM 37307 | LOC391767 | LOC39176 | LOC39176 | similar to hCG198 | similar to | similar to | 391767 | 391767 | 391767 | s53025 | s226498 | s226499 |
| 20309 | O13 | O14 | 1335 | 1107 | 13116 | 159,6 | 9,8 | 1,195 | H5A034 C | sample | Hm Gen E | Hm Gen E | Hm Gen E | CPE002JQ | CPE002O5 | CPE002KL | NM 00101 | NM 0010 | NM 0010 | C6orf205 | C6orf205 | C6orf205 | chromosome 6 or | chromoso | chromoso | 394263 | 394263 | 394263 | s53121 | s53122 | s53123 |
| 20314 | O18 | O19 | 1121 | 1186 | 11022 | 189,2 | 9,8 | 1,195 | H5A034 C | sample | Hm Gen E | Hm Gen E | Hm Gen E | CPE002JQ | CPE002O5 | CPE002KL | XM 37752 | XM 37752 | XM 37752 | RPL23AP2 | RPL23AP2 | RPL23AP2 | ribosomal protein | ribosomal | ribosomal | 401904 | 401904 | 401904 | s53695 | s53696 | s53697 |
| 482 | E10 | E11 | 1934 | 1760 | 18903 | 302,3 | 9,8 | 1,431 | H5A034 C | sample | Hm Drgbl | Hm Drgbl | Hm Drgbl | CPE002E0 | CPE002EZ | CPE002FK | NM 00202 | NM 0020 | NM 0020 | FMR1 | FMR1 | FMR1 | fragile X mental r | fragile X m | fragile X m | 2332 | 2332 | 2332 | s5315 | s5316 | s5317 |
| 493 | E20 | E21 | 1990 | 1761 | 19441 | 281,5 | 9,8 | 1,431 | H5A034 C | sample | Hm Drgbl | Hm Drgbl | Hm Drgbl | CPE002E0 | CPE002EZ | CPE002FK | NM 00080 | NM 0008 | NM 0008 | FOLR2 | FOLR2 | FOLR2 | folate receptor 2 | folate rec | folate rec | 2350 | 2350 | 2350 | s5333 | s223605 | s5335 |
| 580 | I12 | I13 | 1939 | 1610 | 18932 | 355,7 | 9,8 | 1,431 | H5A034 C | sample | Hm Drgbl | Hm Drgbl | Hm Drgbl | CPE002E0 | CPE002EZ | CPE002FK | NM 00203 | NM 0020 | NM 0020 | FVT1 | FVT1 | FVT1 | follicular lymphor | follicular l | follicular l | 2531 | 2531 | 2531 | s5428 | s5426 | s5427 |
| 605 | J13 | J14 | 1993 | 1767 | 19494 | 339,9 | 9,8 | 1,431 | H5A034 C | sample | Hm Drgbl | Hm Drgbl | Hm Drgbl | CPE002E0 | CPE002EZ | CPE002FK | NM 00013 | NM 0001 | NM 0001 | FBN1 | FBN1 | FBN1 | fibrillin 1 | fibrillin 1 | fibrillin 1 | 2200 | 2200 | 2200 | s5044 | s5045 | s5046 |
| 7715 | B19 | B20 | 979 | 579 | 9634 | 433,7 | 9,8 | 1,496 | H5A034 C | sample | Hm Drgbl | Hm Drgbl | Hm Drgbl | CPE002E4 | CPE002EV | CPE002FG | NM 00699 | NM 0069 | NM 0069 | BTN2A2 | BTN2A2 | BTN2A2 | butyrophilin, sub | butyrophi | butyrophi | 10385 | 10385 | 10385 | s20308 | s20307 | s20309 |
| 7726 | B7 | B8 | 935 | 589,1 | 9168 | 419,4 | 9,8 | 1,496 | H5A034 C | sample | Hm Drgbl | Hm Drgbl | Hm Drgbl | CPE002E4 | CPE002EV | CPE002FG | NM 00608 | NM 0060 | NM 0060 | TUBA1B | TUBA1B | TUBA1B | tubulin, alpha 1b | tubulin, a | tubulin, a | 10376 | 10376 | 10376 | s20287 | s20288 | s20289 |
| 7775 | D8 | D9 | 550 | 532,3 | 5384 | 423,5 | 9,8 | 1,496 | H5A034 C | sample | Hm Drgbl | Hm Drgbl | Hm Drgbl | CPE002E4 | CPE002EV | CPE002FG | NM 00108 | NM 0010 | NM 0010 | CPSF4 | CPSF4 | CPSF4 | cleavage and poly | cleavage a | cleavage a | 10898 | 10898 | 10898 | s21413 | s21412 | s21411 |
| 7794 | E3 | E4 | 867 | 562 | 8528 | 413,4 | 9,8 | 1,496 | H5A034 C | sample | Hm Drgbl | Hm Drgbl | Hm Drgbl | CPE002E4 | CPE002EV | CPE002FG | NM 00578 | NM 0057 | NM 0057 | ALG3 | ALG3 | ALG3 | asparagine-linked | asparagin | asparagin | 10195 | 10195 | 10195 | s19865 | s19866 | s19867 |
| 7834 | G18 | G19 | 1143 | 597,7 | 11211 | 373,6 | 9,8 | 1,496 | H5A034 C | sample | Hm Drgbl | Hm Drgbl | Hm Drgbl | CPE002E4 | CPE002EV | CPE002FG | NM 00654 | NM 0065 | NM 0065 | IGF2BP1 | IGF2BP1 | IGF2BP1 | insulin-like growt | insulin-lik | insulin-lik | 10642 | 10642 | 10642 | s20916 | s20917 | s20918 |
| 7896 | I9 | I10 | 796 | 481,6 | 7832 | 284,3 | 9,8 | 1,496 | H5A034 C | sample | Hm Drgbl | Hm Drgbl | Hm Drgbl | CPE002E4 | CPE002EV | CPE002FG | NM 00583 | NM 0058 | NM 0058 | GLYAT | GLYAT | GLYAT | glycine-N-acyltrar | glycine-N- | glycine-N- | 10249 | 10249 | 10249 | s175 | s223098 | s176 |
| 7901 | J13 | J14 | 933 | 562,5 | 9190 | 414,1 | 9,8 | 1,496 | H5A034 C | sample | Hm Drgbl | Hm Drgbl | Hm Drgbl | CPE002E4 | CPE002EV | CPE002FG | NM 00635 | NM 0063 | NM 0063 | HMGN4 | HMGN4 | HMGN4 | high mobility grou | high mobi | high mobi | 10473 | 10473 | 10473 | s20507 | s20506 | s20505 |
| 7926 | K14 | K15 | 353 | 565 | 3447 | 316,1 | 9,8 | 1,496 | H5A034 C | sample | Hm Drgbl | Hm Drgbl | Hm Drgbl | CPE002E4 | CPE002EV | CPE002FG | NM 00659 | NM 0065 | NM 0065 | POLD3 | POLD3 | POLD3 | polymerase (DNA | polymeras | polymeras | 10714 | 10714 | 10714 | s21046 | s21045 | s21044 |
| 3319 | K15 | K16 | 1152 | 1259 | 11400 | 330,6 | 9,9 | 0,613 | H5A034 C | sample | Hm Drgbl | Hm Drgbl | Hm Drgbl | CPE002DC | CPE002EG | CPE002F6 | NM 15222 | NM 1522 | NM 1522 | PPEF1 | PPEF1 | PPEF1 | protein phosphat | protein pl | protein pl | 5475 | 5475 | 5475 | s10901 | s10902 | s10903 |
| 6224 | D16 | D17 | 1893 | 613,2 | 18785 | 389,1 | 9,9 | 0,653 | H5A034 C | sample | Hm Drgbl | Hm Drgbl | Hm Drgbl | CPE002DZ | CPE002F0 | CPE002FL | NM 00136 | NM 0013 | NM 0013 | DHODH | DHODH | DHODH | dihydroorotate d | dihydroor | dihydroor | 1723 | 1723 | 1723 | s4085 | s4084 | s4083 |
| 11471 | N8 | N9 | 1862 | 907,4 | 18522 | 126,3 | 9,9 | 0,717 | H5A034 C | sample | Hm Drgbl | Hm Drgbl | Hm Drgbl | CPE002ED | CPE002F3 | CPE002FT | | | | | | | | | | | | | | | |
| 5083 | D4 | D5 | 938 | 620,6 | 9324 | 628 | 9,9 | 0,776 | H5A034 C | sample | Hm Drgbl | Hm Drgbl | Hm Drgbl | CPE002DV | CPE002EM | CPE002FO | NM 00337 | NM 0033 | NM 0033 | VAV2 | VAV2 | VAV2 | vav 2 oncogene | vav 2 onc | vav 2 onc | 7410 | 7410 | 7410 | s14755 | s14754 | s14753 |
| 2318 | A21 | A22 | 1192 | 879,9 | 11850 | 548,6 | 9,9 | 0,78 | H5A034 C | sample | Hm Drgbl | Hm Drgbl | Hm Drgbl | CPE002DC | CPE002EE | CPE002F4 | NM 00520 | NM 0052 | NM 0052 | MAP3K8 | MAP3K8 | MAP3K8 | mitogen-activatec | mitogen-a | mitogen-a | 1326 | 1326 | 1326 | s3383 | s3384 | s3385 |
| 2341 | B20 | B21 | 2171 | 771,3 | 21595 | 396,2 | 9,9 | 0,78 | H5A034 C | sample | Hm Drgbl | Hm Drgbl | Hm Drgbl | CPE002DC | CPE002EE | CPE002F4 | NM 21356 | NM 2135 | NM 2135 | PKN1 | PKN1 | PKN1 | protein kinase N1 | protein ki | protein ki | 5585 | 5585 | 5585 | s11113 | s11114 | s11115 |
| 2528 | J16 | J17 | 1333 | 713 | 13194 | 258,7 | 9,9 | 0,78 | H5A034 C | sample | Hm Drgbl | Hm Drgbl | Hm Drgbl | CPE002DC | CPE002EE | CPE002F4 | NM 00540 | NM 0054 | NM 0054 | ROCK1 | ROCK1 | ROCK1 | Rho-associated, c | Rho-assoc | Rho-assoc | 6093 | 6093 | 6093 | s12097 | s224548 | s12099 |
| 2531 | J19 | J20 | 1333 | 739,2 | 13238 | 317,6 | 9,9 | 0,78 | H5A034 C | sample | Hm Drgbl | Hm Drgbl | Hm Drgbl | CPE002DC | CPE002EE | CPE002F4 | NM 00443 | NM 0044 | NM 0044 | EPHA2 | EPHA2 | EPHA2 | EPH receptor A2 | EPH recep | EPH recep | 1969 | 1969 | 1969 | s4564 | s4565 | s4566 |
| 2553 | K17 | K18 | 979 | 787 | 9662 | 351,4 | 9,9 | 0,78 | H5A034 C | sample | Hm Drgbl | Hm Drgbl | Hm Drgbl | CPE002DC | CPE002EE | CPE002F4 | NM 00265 | NM 0026 | NM 0026 | PIK4CA | PIK4CA | PIK4CA | phosphatidylinos | phosphat | phosphat | 5297 | 5297 | 5297 | s709 | s224265 | s711 |
| 17785 | F1 | F2 | 1669 | 1087 | 16494 | 235,8 | 9,9 | 0,783 | H5A034 C | sample | Hm Gen E | Hm Gen E | Hm Gen E | CPE002JK | CPE002NZ | CPE002KF | NM 13847 | NM 1384 | NM 1384 | CDAN1 | CDAN1 | CDAN1 | congenital dysery | congenita | congenita | 146059 | 146059 | 146059 | s44815 | s44816 | s44817 |
| 9027 | I11 | I12 | 1132 | 1549 | 11234 | 129,6 | 9,9 | 0,795 | H5A034 C | sample | Hm Drgbl | Hm Drgbl | Hm Drgbl | CPE002E7 | CPE002ES | CPE002FD | NM 01604 | NM 0160 | NM 0160 | EXOSC3 | EXOSC3 | EXOSC3 | exosome compon | exosome | exosome | 51010 | 51010 | 51010 | s27229 | s27230 | s27231 |
| 188 | H5 | H6 | 598 | 1425 | 5925 | 479,8 | 9,9 | 0,843 | H5A034 C | sample | Hm Drgbl | Hm Drgbl | Hm Drgbl | CPE002DT | CPE002EJ | CPE002F9 | NM 00303 | NM 0030 | NM 0030 | SIAH1 | SIAH1 | SIAH1 | seven in absentia | seven in a | seven in a | 6477 | 6477 | 6477 | s12835 | s12836 | s12837 |
| 270 | L14 | L15 | 697 | 1566 | 6900 | 521,9 | 9,9 | 0,843 | H5A034 C | sample | Hm Drgbl | Hm Drgbl | Hm Drgbl | CPE002DT | CPE002EJ | CPE002F9 | NM 02272 | NM 0227 | NM 0227 | ELOVL4 | ELOVL4 | ELOVL4 | elongation of ver | elongatio | elongatio | 6785 | 6785 | 6785 | s13558 | s224700 | s13559 |
| 306 | M3 | M4 | 505 | 1353 | 4994 | 620 | 9,9 | 0,843 | H5A034 C | sample | Hm Drgbl | Hm Drgbl | Hm Drgbl | CPE002DT | CPE002EJ | CPE002F9 | NM 00100 | NM 0010 | NM 0010 | SCP2 | SCP2 | SCP2 | sterol carrier prot | sterol car | sterol car | 6342 | 6342 | 6342 | s200457 | s200459 | s200458 |
| 354 | O3 | O4 | 1032 | 1914 | 10250 | 529,8 | 9,9 | 0,843 | H5A034 C | sample | Hm Drgbl | Hm Drgbl | Hm Drgbl | CPE002DT | CPE002EJ | CPE002F9 | NM 00298 | NM 0029 | NM 0029 | CCL17 | CCL17 | CCL17 | chemokine (C-C m | chemokin | chemokin | 6361 | 6361 | 6361 | s194793 | s194794 | s224580 |
| 2180 | K5 | K6 | 1878 | 696,4 | 18547 | 382,9 | 9,9 | 0,861 | H5A034 C | sample | Hm Drgbl | Hm Drgbl | Hm Drgbl | CPE002FX | CPE002G1 | CPE002G5 | NM 05328 | NM 0532 | NM 0532 | DACH2 | DACH2 | DACH2 | dachshund homo | dachshun | dachshun | 117154 | 117154 | 117154 | s42110 | s42111 | s42112 |
| 6554 | B10 | B11 | 1093 | 713,9 | 10874 | 380,4 | 9,9 | 0,868 | H5A034 C | sample | Hm Drgbl | Hm Drgbl | Hm Drgbl | CPE002E1 | CPE002EY | CPE002FJ | NM 00220 | NM 0022 | NM 0022 | ITGAE | ITGAE | ITGAE | integrin, alpha E ( | integrin, a | integrin, a | 3682 | 3682 | 3682 | s7561 | s223951 | s7560 |
| 3860 | A5 | A6 | 1859 | 707,5 | 18485 | 302,3 | 9,9 | 0,88 | H5A034 C | sample | Hm Drgbl | Hm Drgbl | Hm Drgbl | CPE002DS | CPE002EI | CPE002F8 | NM 20736 | NM 2073 | NM 2073 | GPR148 | GPR148 | GPR148 | G protein-couplec | G protein | G protein | 344561 | 344561 | 344561 | s51068 | s51069 | s223906 |
| 3947 | E19 | E20 | 1625 | 785,4 | 16168 | 386,2 | 9,9 | 0,88 | H5A034 C | sample | Hm Drgbl | Hm Drgbl | Hm Drgbl | CPE002DS | CPE002EI | CPE002F8 | NM 18294 | NM 1829 | NM 1829 | PLOD2 | PLOD2 | PLOD2 | procollagen-lysine | procollage | procollage | 5352 | 5352 | 5352 | s10672 | s10673 | s10674 |
| 4066 | J18 | J19 | 1316 | 696,2 | 13089 | 321,7 | 9,9 | 0,88 | H5A034 C | sample | Hm Drgbl | Hm Drgbl | Hm Drgbl | CPE002DS | CPE002EI | CPE002F8 | NM 00295 | NM 0029 | NM 0029 | RPN1 | RPN1 | RPN1 | ribophorin I | ribophori | ribophori | 6184 | 6184 | 6184 | s12246 | s12247 | s12248 |
| 4114 | L18 | L19 | 1179 | 699,1 | 11620 | 375,6 | 9,9 | 0,88 | H5A034 C | sample | Hm Drgbl | Hm Drgbl | Hm Drgbl | CPE002DS | CPE002EI | CPE002F8 | NM 00295 | NM 0029 | NM 0029 | CLIP1 | CLIP1 | CLIP1 | CAP-GLY domain | CAP-GLY c | CAP-GLY c | 6249 | 6249 | 6249 | s12374 | s12373 | s12372 |
| 4140 | M2 | M3 | 629 | 568,3 | 6221 | 368,4 | 9,9 | 0,88 | H5A034 C | sample | Hm Drgbl | Hm Drgbl | Hm Drgbl | CPE002DS | CPE002EI | CPE002F8 | NM 00690 | NM 0069 | NM 0069 | RASGRF2 | RASGRF2 | RASGRF2 | Ras protein-speci | Ras prote | Ras prote | 5924 | 5924 | 5924 | s11828 | s11829 | s11830 |
| 6140 | P5 | P6 | 2023 | 855,4 | 20090 | 355 | 9,9 | 0,904 | H5A034 C | sample | Hm Drgbl | Hm Drgbl | Hm Drgbl | CPE002DY | CPE002F1 | CPE002FM | NM 00117 | NM 0011 | NM 0011 | AQP7 | AQP7 | AQP7 | aquaporin 7 | aquaporir | aquaporir | 364 | 364 | 364 | s1532 | s1533 | s1534 |
| 1165 | A20 | A21 | 2065 | 811,5 | 20471 | 465,3 | 9,9 | 0,908 | H5A034 C | sample | Hm Drgbl | Hm Drgbl | Hm Drgbl | CPE002FV | CPE002FZ | CPE002G3 | NM 00101 | NM 0010 | NM 0010 | GOSR2 | GOSR2 | GOSR2 | golgi SNAP recept | golgi SNAI | golgi SNAI | 9570 | 9570 | 9570 | s18383 | s18384 | s18385 |
| 1222 | C7 | C8 | 789 | 780,8 | 7822 | 509,7 | 9,9 | 0,908 | H5A034 C | sample | Hm Drgbl | Hm Drgbl | Hm Drgbl | CPE002FV | CPE002FZ | CPE002G3 | NM 00695 | NM 0069 | NM 0069 | ZNF12 | ZNF12 | ZNF12 | zinc finger protein | zinc finger | zinc finger | 7559 | 7559 | 7559 | s15024 | s15025 | s15026 |
| 16393 | L1 | L2 | 996 | 1070 | 9866 | 342 | 9,9 | 0,908 | H5A034 C | sample | Hm Gen E | Hm Gen E | Hm Gen E | CPE002JG | CPE002NV | CPE002KB | NM 03213 | NM 0321 | NM 0321 | C12orf25 | C12orf25 | C12orf25 | chromosome 12 o | chromoso | chromoso | 84070 | 84070 | 84070 | s38450 | s38451 | s38452 |
| 13554 | E3 | E4 | 1191 | 1105 | 11741 | 162,5 | 9,9 | 0,93 | H5A034 C | sample | Hm Gen E | Hm Gen E | Hm Gen E | CPE002JC | CPE002NC | CPE002K4 | NM 01528 | NM 0152 | NM 0152 | VPS39 | VPS39 | VPS39 | vacuolar protein s | vacuolar p | vacuolar p | 23339 | 23339 | 23339 | s23599 | s23600 | s23601 |
| 13814 | P21 | P22 | 1357 | 1071 | 13392 | 121,5 | 9,9 | 0,93 | H5A034 C | sample | Hm Gen E | Hm Gen E | Hm Gen E | CPE002JC | CPE002NC | CPE002K4 | NM 01544 | NM 0154 | NM 0154 | CNOT10 | CNOT10 | CNOT10 | CCR4-NOT transcr | CCR4-NOT | CCR4-NOT | 25904 | 25904 | 25904 | s24718 | s24719 | s24720 |
| 838 | C7 | C8 | 1066 | 787,4 | 10600 | 503 | 9,9 | 0,938 | H5A034 C | sample | Hm Drgbl | Hm Drgbl | Hm Drgbl | CPE002FU | CPE002FY | CPE002G2 | NM 00163 | NM 0016 | NM 0016 | APCS | APCS | APCS | amyloid P compo | amyloid P | amyloid P | 325 | 325 | 325 | s1436 | s1437 | s1438 |
| 972 | I2 | I3 | 1078 | 725,5 | 10631 | 401,3 | 9,9 | 0,938 | H5A034 C | sample | Hm Drgbl | Hm Drgbl | Hm Drgbl | CPE002FU | CPE002FY | CPE002G2 | XM 00113 | NM 0025 | XM 00113 | NPTX1 | NPTX1 | NPTX1 | neuronal pentrax | neuronal | neuronal | 4884 | 4884 | 4884 | s9693 | s224133 | s9695 |
| 1093 | N20 | N21 | 1591 | 692,8 | 15829 | 413,9 | 9,9 | 0,938 | H5A034 C | sample | Hm Drgbl | Hm Drgbl | Hm Drgbl | CPE002FU | CPE002FY | CPE002G2 | NM 00331 | NM 0033 | NM 0033 | DNAJC7 | DNAJC7 | DNAJC7 | DnaJ (Hsp40) hom | DnaJ (Hsp | DnaJ (Hsp | 7266 | 7266 | 7266 | s14472 | s14473 | s14474 |
| 5551 | H15 | H16 | 2065 | 638,8 | 20388 | 473 | 9,9 | 0,943 | H5A034 C | sample | Hm Drgbl | Hm Drgbl | Hm Drgbl | CPE002DX | CPE002F2 | CPE002FN | NM 00677 | NM 0067 | NM 0067 | MARCO | MARCO | MARCO | macrophage rece | macropha | macropha | 8685 | 8685 | 8685 | s16549 | s16550 | s16551 |
| 5689 | N1 | N2 | 1498 | 779,5 | 14761 | 522 | 9,9 | 0,943 | H5A034 C | sample | Hm Drgbl | Hm Drgbl | Hm Drgbl | CPE002DX | CPE002F2 | CPE002FN | NM 00381 | NM 0038 | NM 0038 | TNFSF10 | TNFSF10 | TNFSF10 | tumor necrosis fa | tumor nec | tumor nec | 8743 | 8743 | 8743 | s16663 | s16665 | s16664 |
| 5725 | O20 | O21 | 1851 | 652,9 | 18398 | 365,1 | 9,9 | 0,943 | H5A034 C | sample | Hm Drgbl | Hm Drgbl | Hm Drgbl | CPE002DX | CPE002F2 | CPE002FN | NM 00467 | NM 0046 | NM 0046 | DIRAS3 | DIRAS3 | DIRAS3 | DIRAS family, GTF | DIRAS fan | DIRAS fan | 9077 | 9077 | 9077 | s17319 | s17320 | s17318 |
| 5746 | P18 | P19 | 1907 | 650,5 | 18825 | 618,1 | 9,9 | 0,943 | H5A034 C | sample | Hm Drgbl | Hm Drgbl | Hm Drgbl | CPE002DX | CPE002F2 | CPE002FN | NM 00603 | NM 0060 | NM 0060 | CPNE6 | CPNE6 | CPNE6 | copine VI (neuror | copine VI | copine VI | 9362 | 9362 | 9362 | s17906 | s17905 | s17904 |
| 3575 | E8 | E9 | 1090 | 1221 | 10786 | 293,6 | 9,9 | 0,952 | H5A034 C | sample | Hm Drgbl | Hm Drgbl | Hm Drgbl | CPE002DR | CPE002EH | CPE002F7 | NM 00350 | NM 0035 | NM 0035 | FZD7 | FZD7 | FZD7 | frizzled homolog | frizzled hc | frizzled hc | 8324 | 8324 | 8324 | s15841 | s15842 | s15843 |
| 3660 | I2 | I3 | 1104 | 1455 | 10960 | 364,1 | 9,9 | 0,952 | H5A034 C | sample | Hm Drgbl | Hm Drgbl | Hm Drgbl | CPE002DR | CPE002EH | CPE002F7 | NM 00423 | NM 0042 | NM 0042 | EDG5 | EDG5 | EDG5 | endothelial differ | endotheli | endotheli | 9294 | 9294 | 9294 | s17766 | s17767 | s17768 |
| 3678 | J14 | J15 | 983 | 1133 | 9696 | 235,3 | 9,9 | 0,952 | H5A034 C | sample | Hm Drgbl | Hm Drgbl | Hm Drgbl | CPE002DR | CPE002EH | CPE002F7 | NM 05403 | NM 0540 | NM 0540 | MRGPRX2 | MRGPRX2 | MRGPRX2 | MAS-related GPR, | MAS-relat | MAS-relat | 117194 | 117194 | 117194 | s42133 | s42134 | s223248 |
| 3733 | L20 | L21 | 1276 | 1398 | 12578 | 341,3 | 9,9 | 0,952 | H5A034 C | sample | Hm Drgbl | Hm Drgbl | Hm Drgbl | CPE002DR | CPE002EH | CPE002F7 | NM 15252 | NM 1525 | NM 1525 | GPR155 | GPR155 | GPR155 | G protein-couplec | G protein | G protein | 151556 | 151556 | 151556 | s45587 | s45588 | s45589 |
| 3753 | M17 | M18 | 1901 | 1310 | 18735 | 330,7 | 9,9 | 0,952 | H5A034 C | sample | Hm Drgbl | Hm Drgbl | Hm Drgbl | CPE002DR | CPE002EH | CPE002F7 | NM 00203 | NM 0020 | NM 0020 | FPRL2 | FPRL2 | FPRL2 | formyl peptide re | formyl pe | formyl pe | 2359 | 2359 | 2359 | s5357 | s5358 | s5359 |
| 8108 | B5 | B6 | 1579 | 724,1 | 15703 | 389 | 9,9 | 0,952 | H5A034 C | sample | Hm Drgbl | Hm Drgbl | Hm Drgbl | CPE002E5 | CPE002EU | CPE002FF | NM 01427 | NM 0142 | NM 0142 | RBPJL | RBPJL | RBPJL | recombination sig | recombin | recombin | 11317 | 11317 | 11317 | s22310 | s22311 | s22312 |
| 8314 | K18 | K19 | 1196 | 649,2 | 11790 | 434 | 9,9 | 0,952 | H5A034 C | sample | Hm Drgbl | Hm Drgbl | Hm Drgbl | CPE002E5 | CPE002EU | CPE002FF | NM 01523 | NM 0152 | NM 0152 | CSTF2T | CSTF2T | CSTF2T | cleavage stimulat | cleavage s | cleavage s | 23283 | 23283 | 23283 | s23470 | s23471 | s23472 |
| 8431 | P15 | P16 | 2135 | 622,2 | 21151 | 454,1 | 9,9 | 0,952 | H5A034 C | sample | Hm Drgbl | Hm Drgbl | Hm Drgbl | CPE002E5 | CPE002EU | CPE002FF | NM 01502 | NM 0150 | NM 0150 | WDTC1 | WDTC1 | WDTC1 | WD and tetratricc | WD and t | WD and t | 23038 | 23038 | 23038 | s22892 | s22891 | s22890 |
| 10294 | M7 | M8 | 1044 | 774,4 | 10298 | 128,8 | 9,9 | 0,952 | H5A034 C | sample | Hm Drgbl | Hm Drgbl | Hm Drgbl | CPE002EA | CPE002EP | CPE002FA | NM 02490 | NM 0249 | NM 0249 | SCD5 | SCD5 | SCD5 | stearoyl-CoA desa | stearoyl-C | stearoyl-C | 79966 | 79966 | 79966 | s36734 | s36735 | s36733 |
| 1543 | A15 | A16 | 2221 | 821,5 | 22045 | 409,7 | 9,9 | 1,037 | H5A034 C | sample | Hm Drgbl | Hm Drgbl | Hm Drgbl | CPE002FW | CPE002G0 | CPE002G4 | XM 00113 | XM 00113 | XM 00113 | DNAJB5 | DNAJB5 | DNAJB5 | DnaJ (Hsp40) hom | DnaJ (Hsp | DnaJ (Hsp | 25822 | 25822 | 25822 | s24554 | s24555 | s24556 |
| 1657 | F1 | F2 | 1702 | 733,2 | 16808 | 433,1 | 9,9 | 1,037 | H5A034 C | sample | Hm Drgbl | Hm Drgbl | Hm Drgbl | CPE002FW | CPE002G0 | CPE002G4 | NM 00100 | NM 0010 | NM 0010 | SUHW4 | SUHW4 | SUHW4 | suppressor of hai | suppressc | suppressc | 54816 | 54816 | 54816 | s29504 | s29505 | s29506 |
| 1693 | G20 | G21 | 1979 | 771,9 | 19591 | 368,8 | 9,9 | 1,037 | H5A034 C | sample | Hm Drgbl | Hm Drgbl | Hm Drgbl | CPE002FW | CPE002G0 | CPE002G4 | NM 02122 | NM 0212 | NM 0212 | ZNF462 | ZNF462 | ZNF462 | zinc finger protein | zinc finger | zinc finger | 58499 | 58499 | 58499 | s33870 | s33871 | s33872 |
| 1887 | O22 | O23 | 2463 | 847,1 | 24476 | 443,6 | 9,9 | 1,037 | H5A034 C | sample | Hm Drgbl | Hm Drgbl | Hm Drgbl | CPE002FW | CPE002G0 | CPE002G4 | NM 02461 | NM 0246 | NM 0246 | PLEKHF2 | PLEKHF2 | PLEKHF2 | pleckstrin homolo | pleckstrin | pleckstrin | 79666 | 79666 | 79666 | s36049 | s36050 | s36051 |
| 21610 | E18 | E19 | 351 | 980,3 | 3460 | 518,7 | 9,9 | 1,042 | H5A034 C | sample | Hm Gen E | Hm Gen E | Hm Gen E | CPE002JU | CPE002O9 | CPE002KP | XM 94217 | XM 94217 | XM 94217 | LOC652627 | LOC65262 | LOC65262 | similar to dead er | similar to | similar to | 652627 | 652627 | 652627 | s197926 | s197927 | s197928 |
| 21811 | M4 | M5 | 168 | 1135 | 1657 | 614,3 | 9,9 | 1,042 | H5A034 C | sample | Hm Gen E | Hm Gen E | Hm Gen E | CPE002JU | CPE002O9 | CPE002KP | XM 94245 | XM 94245 | XM 94245 | LOC652793 | LOC65279 | LOC65279 | similar to PDZ do | similar to | similar to | 652793 | 652793 | 652793 | s57489 | s57490 | s227416 |
| 4628 | A5 | A6 | 1262 | 922,6 | 12543 | 364,8 | 9,9 | 1,088 | H5A034 C | sample | Hm Drgbl | Hm Drgbl | Hm Drgbl | CPE002DV | CPE002EL | CPE002FP | NM 00190 | NM 0019 | NM 0019 | CTSD | CTSD | CTSD | cathepsin D | cathepsin | cathepsin | 1509 | 1509 | 1509 | s135 | s136 | s137 |
| 4714 | E18 | E19 | 1732 | 889 | 17206 | 454,1 | 9,9 | 1,088 | H5A034 C | sample | Hm Drgbl | Hm Drgbl | Hm Drgbl | CPE002DV | CPE002EL | CPE002FP | NM 00103 | NM 0010 | NM 0010 | USP9X | USP9X | USP9X | ubiquitin specific | ubiquitin | ubiquitin | 8239 | 8239 | 8239 | s15742 | s15743 | s15744 |
| 4733 | F13 | F14 | 1887 | 862,9 | 18672 | 444,2 | 9,9 | 1,088 | H5A034 C | sample | Hm Drgbl | Hm Drgbl | Hm Drgbl | CPE002DV | CPE002EL | CPE002FP | NM 00239 | NM 0023 | NM 0023 | ADAM11 | ADAM11 | ADAM11 | ADAM metallopep | ADAM me | ADAM me | 4185 | 4185 | 4185 | s8610 | s8611 | s8612 |
| 4770 | G3 | G4 | 951 | 744,6 | 9396 | 449,4 | 9,9 | 1,088 | H5A034 C | sample | Hm Drgbl | Hm Drgbl | Hm Drgbl | CPE002DV | CPE002EL | CPE002FP | NM 20144 | NM 2014 | NM 2014 | C1S | C1S | C1S | complement com | compleme | compleme | 716 | 716 | 716 | s2155 | s2156 | s2157 |
| 4880 | L16 | L17 | 1816 | 835,3 | 18011 | 396,9 | 9,9 | 1,088 | H5A034 C | sample | Hm Drgbl | Hm Drgbl | Hm Drgbl | CPE002DV | CPE002EL | CPE002FP | NM 01794 | NM 0179 | NM 0179 | USP47 | USP47 | USP47 | ubiquitin specific | ubiquitin | ubiquitin | 55031 | 55031 | 55031 | s30028 | s30029 | s30030 |
| 4895 | L8 | L9 | 1453 | 726 | 14315 | 428,9 | 9,9 | 1,088 | H5A034 C | sample | Hm Drgbl | Hm Drgbl | Hm Drgbl | CPE002DV | CPE002EL | CPE002FP | NM 01767 | NM 0176 | NM 0176 | GIN1 | GIN1 | GIN1 | gypsy retrotransp | gypsy retr | gypsy retr | 54826 | 54826 | 54826 | s29525 | s29526 | s29527 |
| 10692 | N2 | N3 | 979 | 811 | 9731 | 187 | 9,9 | 1,119 | H5A034 C | sample | Hm Drgbl | Hm Drgbl | Hm Drgbl | CPE002EB | CPE002EO | CPE002FR | NM 08060 | NM 0806 | NM 0806 | B3GALT6 | B3GALT6 | B3GALT6 | UDP-Gal:betaGal | UDP-Gal:b | UDP-Gal:b | 126792 | 126792 | 126792 | s43087 | s43089 | s43088 |
| 2832 | F9 | F10 | 1036 | 609,2 | 10227 | 455,6 | 9,9 | 1,165 | H5A034 C | sample | Hm Drgbl | Hm Drgbl | Hm Drgbl | CPE002DP | CPE002EF | CPE002F5 | NM 02204 | NM 0220 | NM 0220 | CSNK1G1 | CSNK1G1 | CSNK1G1 | casein kinase 1, g | casein kin | casein kin | 53944 | 53944 | 53944 | s28821 | s28822 | s28823 |
| 3033 | O17 | O18 | 1468 | 718 | 14581 | 674,3 | 9,9 | 1,165 | H5A034 C | sample | Hm Drgbl | Hm Drgbl | Hm Drgbl | CPE002DP | CPE002EF | CPE002F5 | NM 02519 | NM 0251 | NM 0251 | TRIB1 | TRIB1 | TRIB1 | tribbles homolog | tribbles h | tribbles h | 10221 | 10221 | 10221 | s19940 | s19941 | s19942 |
| 19970 | A10 | A11 | 717 | 1178 | 7131 | 185,6 | 9,9 | 1,207 | H5A034 C | sample | Hm Gen E | Hm Gen E | Hm Gen E | CPE002JQ | CPE002O5 | CPE002KL | XM 00112 | XM 00112 | XM 00112 | LOC401021 | LOC40102 | LOC40102 | hypothetical gene | hypotheti | hypotheti | 401021 | 401021 | 401021 | s53440 | s53441 | s53442 |
| 19988 | A5 | A6 | 986 | 1211 | 9718 | 212 | 9,9 | 1,207 | H5A034 C | sample | Hm Gen E | Hm Gen E | Hm Gen E | CPE002JQ | CPE002O5 | CPE002KL | NM 00108 | NM 0010 | NM 0010 | LOC390748 | LOC39074 | LOC39074 | similar to poly(A) | similar to | similar to | 390748 | 390748 | 390748 | s52857 | s196339 | s226480 |
| 20011 | B4 | B5 | 1132 | 1138 | 11230 | 240,4 | 9,9 | 1,207 | H5A034 C | sample | Hm Gen E | Hm Gen E | Hm Gen E | CPE002JQ | CPE002O5 | CPE002KL | NM 00100 | NM 0010 | NM 0010 | OR2T2 | OR2T2 | OR2T2 | olfactory receptor | olfactory | olfactory | 401992 | 401992 | 401992 | s53712 | s53713 | s53714 |
| 20193 | J17 | J18 | 803 | 1083 | 7940 | 153,3 | 9,9 | 1,207 | H5A034 C | sample | Hm Gen E | Hm Gen E | Hm Gen E | CPE002JQ | CPE002O5 | CPE002KL | NM 00100 | NM 0010 | NM 0010 | FLJ45831 | FLJ45831 | FLJ45831 | FLJ45831 protein | FLJ45831 | FLJ45831 | 400576 | 400576 | 400576 | s53306 | s53307 | s226534 |
| 20202 | J3 | J4 | 755 | 1146 | 7498 | 149,2 | 9,9 | 1,207 | H5A034 C | sample | Hm Gen E | Hm Gen E | Hm Gen E | CPE002JQ | CPE002O5 | CPE002KL | NM 00101 | NM 0010 | NM 0010 | RUNDC2B | RUNDC2B | RUNDC2B | RUN domain cont | RUN dom | RUN dom | 400509 | 400509 | 400509 | s53288 | s53289 | s53290 |
| 411 | B11 | B12 | 1868 | 1659 | 18424 | 345 | 9,9 | 1,445 | H5A034 C | sample | Hm Drgbl | Hm Drgbl | Hm Drgbl | CPE002E0 | CPE002EZ | CPE002FK | NM 18291 | NM 1829 | NM 1829 | ERG | ERG | ERG | v-ets erythroblast | v-ets eryt | v-ets eryt | 2078 | 2078 | 2078 | s4812 | s4813 | s4811 |
| 489 | E17 | E18 | 1763 | 1650 | 17511 | 282 | 9,9 | 1,445 | H5A034 C | sample | Hm Drgbl | Hm Drgbl | Hm Drgbl | CPE002E0 | CPE002EZ | CPE002FK | NM 00140 | NM 0014 | NM 0014 | MEGF6 | MEGF6 | MEGF6 | multiple EGF-like | multiple E | multiple E | 1953 | 1953 | 1953 | s4528 | s4529 | s4530 |
| 738 | O3 | O4 | 1795 | 1865 | 17770 | 381,2 | 9,9 | 1,445 | H5A034 C | sample | Hm Drgbl | Hm Drgbl | Hm Drgbl | CPE002E0 | CPE002EZ | CPE002FK | NM 00012 | NM 0001 | NM 0001 | EPHX1 | EPHX1 | EPHX1 | epoxide hydrolas | epoxide h | epoxide h | 2052 | 2052 | 2052 | s4749 | s4750 | s4751 |
| 750 | P14 | P15 | 1680 | 1677 | 16582 | 452,5 | 9,9 | 1,445 | H5A034 C | sample | Hm Drgbl | Hm Drgbl | Hm Drgbl | CPE002E0 | CPE002EZ | CPE002FK | NM 00209 | NM 0020 | NM 0020 | GSPT1 | GSPT1 | GSPT1 | G1 to S phase tra | G1 to S ph | G1 to S ph | 2935 | 2935 | 2935 | s6247 | s6246 | s6245 |
| 7707 | B11 | B12 | 1072 | 556,8 | 10585 | 421,9 | 9,9 | 1,511 | H5A034 C | sample | Hm Drgbl | Hm Drgbl | Hm Drgbl | CPE002E4 | CPE002EV | CPE002FG | NM 00608 | NM 0060 | NM 0060 | TUBB3 | TUBB3 | TUBB3 | tubulin, beta 3 | tubulin, b | tubulin, b | 10381 | 10381 | 10381 | s20296 | s20297 | s195028 |
| 7756 | D12 | D13 | 1325 | 512,1 | 13115 | 362,4 | 9,9 | 1,511 | H5A034 C | sample | Hm Drgbl | Hm Drgbl | Hm Drgbl | CPE002E4 | CPE002EV | CPE002FG | NM 13919 | NM 1391 | NM 1391 | BRD8 | BRD8 | BRD8 | bromodomain co | bromodor | bromodor | 10902 | 10902 | 10902 | s21425 | s21424 | s21423 |
| 7836 | G2 | G3 | 729 | 554,1 | 7200 | 384,4 | 9,9 | 1,511 | H5A034 C | sample | Hm Drgbl | Hm Drgbl | Hm Drgbl | CPE002E4 | CPE002EV | CPE002FG | XM 00112 | XM 00112 | XM 00112 | TRIM16 | TRIM16 | TRIM16 | tripartite motif-co | tripartite | tripartite | 10626 | 10626 | 10626 | s20872 | s20873 | s20874 |
| 7845 | G6 | G7 | 1401 | 620,6 | 13865 | 471,2 | 9,9 | 1,511 | H5A034 C | sample | Hm Drgbl | Hm Drgbl | Hm Drgbl | CPE002E4 | CPE002EV | CPE002FG | NM 00647 | NM 0064 | NM 0064 | POSTN | POSTN | POSTN | periostin, osteobl | periostin, | periostin, | 10631 | 10631 | 10631 | s20887 | s20888 | s20889 |
| 7863 | H22 | H23 | 1279 | 562 | 12664 | 348,5 | 9,9 | 1,511 | H5A034 C | sample | Hm Drgbl | Hm Drgbl | Hm Drgbl | CPE002E4 | CPE002EV | CPE002FG | NM 00680 | NM 0068 | NM 0068 | CBX1 | CBX1 | CBX1 | chromobox homo | chromobc | chromobc | 10951 | 10951 | 10951 | s21549 | s21550 | s21551 |
| 7891 | I4 | I5 | 746 | 541,2 | 7368 | 356,7 | 9,9 | 1,511 | H5A034 C | sample | Hm Drgbl | Hm Drgbl | Hm Drgbl | CPE002E4 | CPE002EV | CPE002FG | NM 18187 | NM 1818 | NM 1818 | DMRT2 | DMRT2 | DMRT2 | doublesex and m | doublesex | doublesex | 10655 | 10655 | 10655 | s20945 | s20946 | s20947 |
| 7892 | I5 | I6 | 1172 | 543,9 | 11574 | 436,1 | 9,9 | 1,511 | H5A034 C | sample | Hm Drgbl | Hm Drgbl | Hm Drgbl | CPE002E4 | CPE002EV | CPE002FG | NM 00583 | NM 0058 | NM 0058 | SLC17A2 | SLC17A2 | SLC17A2 | solute carrier fam | solute car | solute car | 10246 | 10246 | 10246 | s20009 | s223097 | s20011 |
| 3327 | K22 | K23 | 1345 | 1239 | 13481 | 395 | 10 | 0,619 | H5A034 C | sample | Hm Drgbl | Hm Drgbl | Hm Drgbl | CPE002DC | CPE002EG | CPE002F6 | NM 15254 | NM 1525 | NM 1525 | PPM1K | PPM1K | PPM1K | protein phosphat | protein pl | protein pl | 152926 | 152926 | 152926 | s45745 | s45746 | s45747 |
| 20679 | N22 | N23 | 906 | 781,7 | 9091 | 182,3 | 10 | 0,667 | H5A034 C | sample | Hm Gen E | Hm Gen E | Hm Gen E | CPE002JR | CPE002O6 | CPE002KN | XM 92610 | XM 92610 | XM 92610 | LOC642636 | LOC64263 | LOC64263 | similar to Double | similar to | similar to | 642636 | 642636 | 642636 | s55074 | s55075 | s55076 |
| 9481 | L1 | L2 | 1658 | 1022 | 16655 | 99,53 | 10 | 0,694 | H5A034 C | sample | Hm Drgbl | Hm Drgbl | Hm Drgbl | CPE002E8 | CPE002ER | CPE002FC | NM 01778 | NM 0177 | NM 0177 | CHD7 | CHD7 | CHD7 | chromodomain h | chromodc | chromodc | 55636 | 55636 | 55636 | s31140 | s31141 | s31142 |
| 20866 | F18 | F19 | 1678 | 909,5 | 16810 | 213,8 | 10 | 0,709 | H5A034 C | sample | Hm Gen E | Hm Gen E | Hm Gen E | CPE002JS | CPE002O7 | CPE002KN | XM 92802 | XM 92802 | XM 92802 | LOC644935 | LOC64493 | LOC64493 | similar to E74-like | similar to | similar to | 644935 | 644935 | 644935 | s55852 | s55853 | s55854 |
| 12552 | K9 | K10 | 512 | 987,8 | 5135 | 111,8 | 10 | 0,752 | H5A034 C | sample | Hm Gen E | Hm Gen E | Hm Gen E | CPE002J5 | CPE002NK | CPE002K1 | NM 00325 | NM 0032 | NM 0032 | THRSP | THRSP | THRSP | thyroid hormone | thyroid hc | thyroid hc | 7069 | 7069 | 7069 | s14122 | s14123 | s14124 |
| 12656 | P16 | P17 | 1181 | 1069 | 11827 | 138,4 | 10 | 0,752 | H5A034 C | sample | Hm Gen E | Hm Gen E | Hm Gen E | CPE002J5 | CPE002NK | CPE002K1 | NM 00509 | NM 0050 | NM 0050 | ZMYM4 | ZMYM4 | ZMYM4 | zinc finger, MYM- | zinc finger | zinc finger | 9202 | 9202 | 9202 | s17587 | s17588 | s17589 |
| 17228 | N5 | N6 | 1946 | 1046 | 19547 | 131 | 10 | 0,772 | H5A034 C | sample | Hm Gen E | Hm Gen E | Hm Gen E | CPE002JI | CPE002NZ | CPE002KD | NM 05295 | NM 1456 | NM 0529 | PANX3 | SCGB1C1 | PANX3 | pannexin 3 | secretoglc | pannexin | 116337 | 147199 | 116337 | s41991 | s44981 | s225509 |
| 2543 | J8 | J9 | 1255 | 615,4 | 12494 | 284,6 | 10 | 0,787 | H5A034 C | sample | Hm Drgbl | Hm Drgbl | Hm Drgbl | CPE002DC | CPE002EE | CPE002F4 | NM 00288 | NM 0028 | NM 0028 | RAF1 | RAF1 | RAF1 | v-raf-1 murine leu | v-raf-1 mu | v-raf-1 mu | 5894 | 5894 | 5894 | s11749 | s11750 | s11751 |
| 16550 | B21 | B22 | 2050 | 1113 | 20580 | 310,6 | 10 | 0,791 | H5A034 C | sample | Hm Gen E | Hm Gen E | Hm Gen E | CPE002JH | CPE002NV | CPE002KC | NM 03236 | NM 0323 | NM 0323 | DNAJC14 | DNAJC14 | DNAJC14 | DnaJ (Hsp40) hom | DnaJ (Hsp | DnaJ (Hsp | 85406 | 85406 | 85406 | s39958 | s39959 | s39960 |
| 9176 | O16 | O17 | 2094 | 1835 | 20934 | 129,7 | 10 | 0,803 | H5A034 C | sample | Hm Drgbl | Hm Drgbl | Hm Drgbl | CPE002E7 | CPE002ES | CPE002FD | NM 14477 | NM 1447 | NM 1447 | RBM11 | RBM11 | RBM11 | RNA binding moti | RNA bindi | RNA bindi | 54033 | 54033 | 54033 | s28838 | s28836 | s28837 |
| 4367 | F8 | F9 | 802 | 809,6 | 8006 | 438,8 | 10 | 0,844 | H5A034 C | sample | Hm Drgbl | Hm Drgbl | Hm Drgbl | CPE002DL | CPE002EK | CPE002FQ | NM 17236 | NM 1723 | NM 1723 | CACNA2D4 | CACNA2D | CACNA2D | calcium channel, | calcium ch | calcium ch | 93589 | 93589 | 93589 | s41162 | s41163 | s41164 |
| 187 | H4 | H5 | 637 | 1395 | 6378 | 480,1 | 10 | 0,851 | H5A034 C | sample | Hm Drgbl | Hm Drgbl | Hm Drgbl | CPE002DT | CPE002EJ | CPE002F9 | NM 00313 | NM 0031 | NM 0031 | SRP14 | SRP14 | SRP14 | signal recognition | signal rec | signal rec | 6727 | 6727 | 6727 | s212 | s224681 | s210 |
| 344 | O16 | O17 | 494 | 1574 | 4956 | 651,4 | 10 | 0,851 | H5A034 C | sample | Hm Drgbl | Hm Drgbl | Hm Drgbl | CPE002DT | CPE002EJ | CPE002F9 | NM 00034 | NM 0003 | NM 0003 | SOX9 | SOX9 | SOX9 | SRY (sex determir | SRY (sex d | SRY (sex d | 6662 | 6662 | 6662 | s13306 | s13307 | s13308 |
| 1933 | A20 | A21 | 1687 | 761,9 | 16921 | 445,5 | 10 | 0,87 | H5A034 C | sample | Hm Drgbl | Hm Drgbl | Hm Drgbl | CPE002FX | CPE002G1 | CPE002G5 | NM 15262 | NM 1526 | NM 1526 | ZNF366 | ZNF366 | ZNF366 | zinc finger protein | zinc finger | zinc finger | 167465 | 167465 | 167465 | s46668 | s46669 | s46670 |
| 1959 | B22 | B23 | 2001 | 745,6 | 20093 | 421,1 | 10 | 0,87 | H5A034 C | sample | Hm Drgbl | Hm Drgbl | Hm Drgbl | CPE002FX | CPE002G1 | CPE002G5 | NM 02088 | NM 0208 | NM 0208 | ZNF530 | ZNF530 | ZNF530 | zinc finger protein | zinc finger | zinc finger | 348327 | 348327 | 348327 | s51343 | s51344 | s51345 |
| 2075 | G19 | G20 | 1914 | 705,9 | 19153 | 415 | 10 | 0,87 | H5A034 C | sample | Hm Drgbl | Hm Drgbl | Hm Drgbl | CPE002FX | CPE002G1 | CPE002G5 | NM 03346 | NM 0334 | NM 0334 | ZNF257 | ZNF257 | ZNF257 | zinc finger protein | zinc finger | zinc finger | 113835 | 113835 | 113835 | s41516 | s41517 | s41518 |
| 2089 | H1 | H2 | 1582 | 743,4 | 15870 | 476,6 | 10 | 0,87 | H5A034 C | sample | Hm Drgbl | Hm Drgbl | Hm Drgbl | CPE002FX | CPE002G1 | CPE002G5 | NM 13346 | NM 1334 | NM 1334 | ZNF418 | ZNF418 | ZNF418 | zinc finger protein | zinc finger | zinc finger | 147686 | 147686 | 147686 | s45027 | s45028 | s45029 |
| 2216 | M16 | M17 | 2133 | 766,5 | 21306 | 388,9 | 10 | 0,87 | H5A034 C | sample | Hm Drgbl | Hm Drgbl | Hm Drgbl | CPE002FX | CPE002G1 | CPE002G5 | NM 17368 | NM 1736 | NM 1736 | ZNF775 | ZNF775 | ZNF775 | zinc finger protein | zinc finger | zinc finger | 285971 | 285971 | 285971 | s50064 | s50065 | s50066 |
| 6608 | D16 | D17 | 1725 | 595,8 | 17169 | 370,4 | 10 | 0,877 | H5A034 C | sample | Hm Drgbl | Hm Drgbl | Hm Drgbl | CPE002E1 | CPE002EY | CPE002FJ | NM 00221 | NM 0022 | NM 0022 | ITIH1 | ITIH1 | ITIH1 | inter-alpha (globu | inter-alph | inter-alph | 3697 | 3697 | 3697 | s7602 | s7603 | s7601 |
| 3848 | A16 | A17 | 2139 | 791,7 | 21324 | 338,8 | 10 | 0,889 | H5A034 C | sample | Hm Drgbl | Hm Drgbl | Hm Drgbl | CPE002DS | CPE002EI | CPE002F8 | NM 00690 | NM 0069 | NM 0069 | PYCR1 | PYCR1 | PYCR1 | pyrroline-5-carbo | pyrroline- | pyrroline- | 5831 | 5831 | 5831 | s63 | s200450 | s194735 |
| 3960 | E9 | E10 | 566 | 652,9 | 5639 | 325,5 | 10 | 0,889 | H5A034 C | sample | Hm Drgbl | Hm Drgbl | Hm Drgbl | CPE002DS | CPE002EI | CPE002F8 | NM 00093 | NM 0009 | NM 0009 | SERPINF2 | SERPINF2 | SERPINF2 | serpin peptidase | serpin pe | serpin pe | 5345 | 5345 | 5345 | s10660 | s10658 | s10659 |
| 4043 | I19 | I20 | 1692 | 656,5 | 16893 | 299,7 | 10 | 0,889 | H5A034 C | sample | Hm Drgbl | Hm Drgbl | Hm Drgbl | CPE002DS | CPE002EI | CPE002F8 | NM 00269 | NM 0026 | NM 0026 | POLB | POLB | POLB | polymerase (DNA | polymeras | polymeras | 5423 | 5423 | 5423 | s10776 | s10777 | s10775 |
| 4093 | K20 | K21 | 1825 | 691,5 | 18219 | 290,4 | 10 | 0,889 | H5A034 C | sample | Hm Drgbl | Hm Drgbl | Hm Drgbl | CPE002DS | CPE002EI | CPE002F8 | NM 00650 | NM 0065 | NM 0065 | RASA2 | RASA2 | RASA2 | RAS p21 protein a | RAS p21 p | RAS p21 p | 5922 | 5922 | 5922 | s11822 | s11823 | s11824 |
| 15614 | K21 | K22 | 1951 | 849,6 | 19494 | 133,1 | 10 | 0,909 | H5A034 C | sample | Hm Gen E | Hm Gen E | Hm Gen E | CPE002JE | CPE002NT | CPE002K9 | NM 02234 | NM 0223 | NM 0223 | C17orf75 | C17orf75 | C17orf75 | chromosome 17 o | chromoso | chromoso | 64149 | 64149 | 64149 | s34510 | s34511 | s34512 |
| 5822 | C21 | C22 | 1692 | 945,5 | 16971 | 562,5 | 10 | 0,913 | H5A034 C | sample | Hm Drgbl | Hm Drgbl | Hm Drgbl | CPE002DY | CPE002F1 | CPE002FM | NM 00160 | NM 0016 | NM 0016 | ACADSB | ACADSB | ACADSB | acyl-Coenzyme A | acyl-Coen | acyl-Coen | 36 | 36 | 36 | s898 | s897 | s899 |
| 6109 | O20 | O21 | 2760 | 911,3 | 27483 | 527,3 | 10 | 0,913 | H5A034 C | sample | Hm Drgbl | Hm Drgbl | Hm Drgbl | CPE002DY | CPE002F1 | CPE002FM | NM 00005 | NM 0000 | NM 0000 | BCHE | BCHE | BCHE | butyrylcholineste | butyrylch | butyrylch | 590 | 590 | 590 | s1906 | s1907 | s1908 |
| 1220 | C5 | C6 | 1442 | 732,7 | 14377 | 257 | 10 | 0,917 | H5A034 C | sample | Hm Drgbl | Hm Drgbl | Hm Drgbl | CPE002FV | CPE002FZ | CPE002G3 | NM 01539 | NM 0153 | NM 0153 | ZNF10 | ZNF10 | ZNF10 | zinc finger protein | zinc finger | zinc finger | 7556 | 7556 | 7556 | s15021 | s15022 | s15023 |
| 1429 | L20 | L21 | 1558 | 705,7 | 15574 | 420,6 | 10 | 0,917 | H5A034 C | sample | Hm Drgbl | Hm Drgbl | Hm Drgbl | CPE002FV | CPE002FZ | CPE002G3 | NM 01507 | NM 0150 | NM 0150 | SARM1 | SARM1 | SARM1 | sterile alpha and | sterile alp | sterile alp | 23098 | 23098 | 23098 | s23031 | s23032 | s23033 |
| 1459 | M4 | M5 | 1574 | 781,5 | 15742 | 388,6 | 10 | 0,917 | H5A034 C | sample | Hm Drgbl | Hm Drgbl | Hm Drgbl | CPE002FV | CPE002FZ | CPE002G3 | NM 01244 | NM 0124 | NM 0124 | SPON2 | SPON2 | SPON2 | spondin 2, extrac | spondin 2 | spondin 2 | 10417 | 10417 | 10417 | s20369 | s20370 | s20371 |
| 16153 | B1 | B2 | 849 | 987,4 | 8456 | 404,1 | 10 | 0,917 | H5A034 C | sample | Hm Gen E | Hm Gen E | Hm Gen E | CPE002JG | CPE002NV | CPE002KB | NM 03147 | NM 0314 | NM 0314 | CRISPLD2 | CRISPLD2 | CRISPLD2 | cysteine-rich secr | cysteine-r | cysteine-r | 83716 | 83716 | 83716 | s38142 | s38143 | s228311 |
| 16184 | C16 | C17 | 1462 | 947,1 | 14619 | 297,6 | 10 | 0,917 | H5A034 C | sample | Hm Gen E | Hm Gen E | Hm Gen E | CPE002JG | CPE002NV | CPE002KB | NM 03227 | NM 0322 | NM 0322 | TMEM126 | TMEM126 | TMEM126 | transmembrane p | transmem | transmem | 84233 | 84233 | 84233 | s38694 | s38695 | s38696 |
| 782 | A21 | A22 | 1825 | 942,1 | 18162 | 544,5 | 10 | 0,948 | H5A034 C | sample | Hm Drgbl | Hm Drgbl | Hm Drgbl | CPE002FU | CPE002FY | CPE002G2 | NM 00163 | NM 0016 | NM 0016 | AMPH | AMPH | AMPH | amphiphysin | amphiphy | amphiphy | 273 | 273 | 273 | s1337 | s1338 | s1339 |
| 5687 | M8 | M9 | 1882 | 649,2 | 18885 | 470,1 | 10 | 0,952 | H5A034 C | sample | Hm Drgbl | Hm Drgbl | Hm Drgbl | CPE002DX | CPE002F2 | CPE002FN | NM 00396 | NM 0039 | NM 0039 | NAT8 | NAT8 | NAT8 | N-acetyltransfera | N-acetyltr | N-acetyltr | 9027 | 9027 | 9027 | s666 | s667 | s665 |
| 3511 | C15 | C16 | 988 | 1285 | 9835 | 312,9 | 10 | 0,962 | H5A034 C | sample | Hm Drgbl | Hm Drgbl | Hm Drgbl | CPE002DR | CPE002EH | CPE002F7 | NM 00070 | NM 0007 | NM 0007 | AVPR1A | AVPR1A | AVPR1A | arginine vasopres | arginine v | arginine v | 552 | 552 | 552 | s1836 | s1837 | s1838 |
| 3526 | C7 | C8 | 935 | 1309 | 9378 | 241,9 | 10 | 0,962 | H5A034 C | sample | Hm Drgbl | Hm Drgbl | Hm Drgbl | CPE002DR | CPE002EH | CPE002F7 | NM 00002 | NM 0000 | NM 0000 | ADRB3 | ADRB3 | ADRB3 | adrenergic, beta-3 | adrenergi | adrenergi | 155 | 155 | 155 | s1124 | s1125 | s1126 |
| 3553 | E1 | E2 | 563 | 1338 | 5651 | 365,9 | 10 | 0,962 | H5A034 C | sample | Hm Drgbl | Hm Drgbl | Hm Drgbl | CPE002DR | CPE002EH | CPE002F7 | NM 00170 | NM 0017 | NM 0017 | BAI2 | BAI2 | BAI2 | brain-specific ang | brain-spe | brain-spe | 576 | 576 | 576 | s1873 | s1874 | s1875 |
| 3580 | F12 | F13 | 323 | 1618 | 3228 | 390,8 | 10 | 0,962 | H5A034 C | sample | Hm Drgbl | Hm Drgbl | Hm Drgbl | CPE002DR | CPE002EH | CPE002F7 | NM 02257 | NM 0225 | NM 0225 | GPR135 | GPR135 | GPR135 | G protein-couplec | G protein | G protein | 64582 | 64582 | 64582 | s34786 | s34787 | s34788 |
| 3608 | G16 | G17 | 1800 | 1336 | 17989 | 307,7 | 10 | 0,962 | H5A034 C | sample | Hm Drgbl | Hm Drgbl | Hm Drgbl | CPE002DR | CPE002EH | CPE002F7 | NM 00103 | NM 0010 | NM 0010 | TAAR2 | TAAR2 | TAAR2 | trace amine assoc | trace amir | trace amir | 9287 | 9287 | 9287 | s17754 | s17755 | s17756 |
| 3613 | G20 | G21 | 2409 | 1536 | 24204 | 377,2 | 10 | 0,962 | H5A034 C | sample | Hm Drgbl | Hm Drgbl | Hm Drgbl | CPE002DR | CPE002EH | CPE002F7 | NM 00568 | NM 0056 | NM 0056 | GPR55 | GPR55 | GPR55 | G protein-couplec | G protein | G protein | 9290 | 9290 | 9290 | s17760 | s17761 | s17762 |
| 3639 | H22 | H23 | 1510 | 1319 | 15171 | 396,3 | 10 | 0,962 | H5A034 C | sample | Hm Drgbl | Hm Drgbl | Hm Drgbl | CPE002DR | CPE002EH | CPE002F7 | NM 03278 | NM 0327 | NM 0327 | GPR128 | GPR128 | GPR128 | G protein-couplec | G protein | G protein | 84873 | 84873 | 84873 | s39550 | s39551 | s39552 |
| 3757 | M20 | M21 | 1731 | 1391 | 17382 | 337,6 | 10 | 0,962 | H5A034 C | sample | Hm Drgbl | Hm Drgbl | Hm Drgbl | CPE002DR | CPE002EH | CPE002F7 | NM 03277 | NM 0327 | NM 0327 | GPR124 | GPR124 | GPR124 | G protein-couplec | G protein | G protein | 25960 | 25960 | 25960 | s24826 | s24827 | s24828 |
| 8433 | P17 | P18 | 2029 | 610 | 20376 | 459,1 | 10 | 0,962 | H5A034 C | sample | Hm Drgbl | Hm Drgbl | Hm Drgbl | CPE002E5 | CPE002EU | CPE002FF | NM 01502 | NM 0150 | NM 0150 | XPO7 | XPO7 | XPO7 | exportin 7 | exportin 7 | exportin 7 | 23039 | 23039 | 23039 | s22894 | s22893 | s223558 |
| 10129 | G1 | G2 | 1811 | 855,5 | 18083 | 136,7 | 10 | 0,962 | H5A034 C | sample | Hm Drgbl | Hm Drgbl | Hm Drgbl | CPE002EA | CPE002EP | CPE002FA | NM 02471 | NM 0247 | NM 0247 | ELMO3 | ELMO3 | ELMO3 | engulfment and c | engulfmer | engulfmer | 79767 | 79767 | 79767 | s36285 | s36287 | s36286 |
| 1558 | A7 | A8 | 1519 | 811,3 | 15192 | 491,2 | 10 | 1,047 | H5A034 C | sample | Hm Drgbl | Hm Drgbl | Hm Drgbl | CPE002FW | CPE002G0 | CPE002G4 | NM 17742 | NM 1774 | NM 1774 | STX12 | STX12 | STX12 | syntaxin 12 | syntaxin 1 | syntaxin 1 | 23673 | 23673 | 23673 | s24307 | s24308 | s24309 |
| 1599 | C22 | C23 | 1807 | 777,3 | 17988 | 405,4 | 10 | 1,047 | H5A034 C | sample | Hm Drgbl | Hm Drgbl | Hm Drgbl | CPE002FW | CPE002G0 | CPE002G4 | XM 04755 | XM 04755 | XM 04755 | ZNF492 | ZNF492 | ZNF492 | zinc finger protein | zinc finger | zinc finger | 57615 | 57615 | 57615 | s33445 | s33446 | s33447 |
| 1660 | F12 | F13 | 1570 | 837,7 | 15763 | 424,6 | 10 | 1,047 | H5A034 C | sample | Hm Drgbl | Hm Drgbl | Hm Drgbl | CPE002FW | CPE002G0 | CPE002G4 | NM 02520 | NM 0252 | NM 0252 | EFHD1 | EFHD1 | EFHD1 | EF-hand domain f | EF-hand d | EF-hand d | 80303 | 80303 | 80303 | s37168 | s37169 | s37170 |
| 1694 | G21 | G22 | 1992 | 796,3 | 19958 | 380,8 | 10 | 1,047 | H5A034 C | sample | Hm Drgbl | Hm Drgbl | Hm Drgbl | CPE002FW | CPE002G0 | CPE002G4 | NM 01437 | NM 0143 | NM 0143 | REPIN1 | REPIN1 | REPIN1 | replication initiat | replicatio | replicatio | 29803 | 29803 | 29803 | s26605 | s26606 | s26607 |
| 1718 | H21 | H22 | 2067 | 780,5 | 20756 | 322,6 | 10 | 1,047 | H5A034 C | sample | Hm Drgbl | Hm Drgbl | Hm Drgbl | CPE002FW | CPE002G0 | CPE002G4 | NM 00108 | NM 0010 | NM 0010 | HSZFP36 | HSZFP36 | HSZFP36 | ZFP-36 for a zinc f | ZFP-36 for | ZFP-36 for | 55552 | 55552 | 55552 | s30965 | s30966 | s30967 |
| 1767 | J22 | J23 | 1714 | 773 | 17141 | 388,8 | 10 | 1,047 | H5A034 C | sample | Hm Drgbl | Hm Drgbl | Hm Drgbl | CPE002FW | CPE002G0 | CPE002G4 | NM 20126 | NM 0166 | NM 2012 | ZNF644 | ZNF644 | ZNF644 | zinc finger protein | zinc finger | zinc finger | 84146 | 84146 | 84146 | s38566 | s224934 | s38568 |
| 1800 | K9 | K10 | 1276 | 660,9 | 12819 | 300,2 | 10 | 1,047 | H5A034 C | sample | Hm Drgbl | Hm Drgbl | Hm Drgbl | CPE002FW | CPE002G0 | CPE002G4 | NM 01459 | NM 0145 | NM 0145 | ZNF354C | ZNF354C | ZNF354C | zinc finger protein | zinc finger | zinc finger | 30832 | 30832 | 30832 | s26938 | s26939 | s26940 |
| 21687 | H22 | H23 | 522 | 1161 | 5220 | 678,7 | 10 | 1,053 | H5A034 C | sample | Hm Gen E | Hm Gen E | Hm Gen E | CPE002JU | CPE002O9 | CPE002KP | XM 93460 | XM 9346 | NM 0011 | SFTPA1 | SFTPA1 | SFTPA1 | surfactant, pulmc | surfactant | surfactant | 653509 | 653509 | 653509 | s57690 | s57692 | s227435 |
| 21717 | I6 | I7 | 144 | 903,7 | 1440 | 726,7 | 10 | 1,053 | H5A034 C | sample | Hm Gen E | Hm Gen E | Hm Gen E | CPE002JU | CPE002O9 | CPE002KP | XM 94230 | XM 9423 | XM 9423 | LOC652698 | LOC65269 | LOC65269 | similar to Tripart | similar to | similar to | 652698 | 652698 | 652698 | s57444 | s57446 | s227410 |
| 4665 | C17 | C18 | 631 | 784,9 | 6334 | 445,3 | 10 | 1,099 | H5A034 C | sample | Hm Drgbl | Hm Drgbl | Hm Drgbl | CPE002DV | CPE002EL | CPE002FP | NM 00115 | NM 0011 | NM 0011 | ANPEP | ANPEP | ANPEP | alanyl (membrane | alanyl (me | alanyl (me | 290 | 290 | 290 | s683 | s684 | s685 |
| 4756 | G12 | G13 | 1833 | 895,5 | 18268 | 363,1 | 10 | 1,099 | H5A034 C | sample | Hm Drgbl | Hm Drgbl | Hm Drgbl | CPE002DV | CPE002EL | CPE002FP | NM 00380 | NM 0038 | NM 0038 | CRADD | CRADD | CRADD | CASP2 and RIPK1 | CASP2 and | CASP2 and | 8738 | 8738 | 8738 | s16654 | s225028 | s16656 |
| 4814 | I21 | I22 | 1550 | 820,1 | 15515 | 397,2 | 10 | 1,099 | H5A034 C | sample | Hm Drgbl | Hm Drgbl | Hm Drgbl | CPE002DV | CPE002EL | CPE002FP | NM 00187 | NM 0018 | NM 0018 | CPA3 | CPA3 | CPA3 | carboxypeptidase | carboxype | carboxype | 1359 | 1359 | 1359 | s3426 | s3427 | s3428 |
| 4829 | J13 | J14 | 1893 | 760,6 | 18979 | 368 | 10 | 1,099 | H5A034 C | sample | Hm Drgbl | Hm Drgbl | Hm Drgbl | CPE002DV | CPE002EL | CPE002FP | NM 00252 | NM 0025 | NM 0025 | NRD1 | NRD1 | NRD1 | nardilysin (N-argi | nardilysin | nardilysin | 4898 | 4898 | 4898 | s9717 | s9718 | s9719 |
| 4832 | J16 | J17 | 1686 | 795,2 | 16817 | 285 | 10 | 1,099 | H5A034 C | sample | Hm Drgbl | Hm Drgbl | Hm Drgbl | CPE002DV | CPE002EL | CPE002FP | NM 01654 | NM 0165 | NM 0165 | C1RL | C1RL | C1RL | complement com | compleme | compleme | 51279 | 51279 | 51279 | s27830 | s27831 | s27832 |
| 4855 | K15 | K16 | 784 | 847,4 | 7870 | 252,7 | 10 | 1,099 | H5A034 C | sample | Hm Drgbl | Hm Drgbl | Hm Drgbl | CPE002DV | CPE002EL | CPE002FP | NM 00190 | NM 0019 | NM 0019 | CTRL | CTRL | CTRL | chymotrypsin-like | chymotryp | chymotryp | 1506 | 1506 | 1506 | s3735 | s3736 | s3737 |
| 4870 | K7 | K8 | 1608 | 812,9 | 16156 | 404,7 | 10 | 1,099 | H5A034 C | sample | Hm Drgbl | Hm Drgbl | Hm Drgbl | CPE002DV | CPE002EL | CPE002FP | NM 00187 | NM 0018 | NM 0018 | CPE | CPE | CPE | carboxypeptidase | carboxype | carboxype | 1363 | 1363 | 1363 | s3438 | s3439 | s3440 |
| 4877 | L13 | L14 | 2281 | 879,9 | 22781 | 320,2 | 10 | 1,099 | H5A034 C | sample | Hm Drgbl | Hm Drgbl | Hm Drgbl | CPE002DV | CPE002EL | CPE002FP | NM 00280 | NM 0028 | NM 0028 | PSMB10 | PSMB10 | PSMB10 | proteasome (pros | proteasor | proteasor | 5699 | 5699 | 5699 | s194721 | s194722 | s194723 |
| 4925 | N13 | N14 | 2418 | 859,3 | 24278 | 426 | 10 | 1,099 | H5A034 C | sample | Hm Drgbl | Hm Drgbl | Hm Drgbl | CPE002DV | CPE002EL | CPE002FP | NM 13927 | NM 1392 | NM 1392 | KLK7 | KLK7 | KLK7 | kallikrein-related | kallikrein- | kallikrein- | 5650 | 5650 | 5650 | s11267 | s11268 | s11269 |
| 4932 | N2 | N3 | 1246 | 824,7 | 12464 | 424 | 10 | 1,099 | H5A034 C | sample | Hm Drgbl | Hm Drgbl | Hm Drgbl | CPE002DV | CPE002EL | CPE002FP | NM 01750 | NM 0175 | NM 0175 | KLK15 | KLK15 | KLK15 | kallikrein-related | kallikrein- | kallikrein- | 55554 | 55554 | 55554 | s30971 | s30972 | s30973 |
| 10383 | A22 | A23 | 1153 | 920,7 | 11515 | 215,1 | 10 | 1,13 | H5A034 C | sample | Hm Drgbl | Hm Drgbl | Hm Drgbl | CPE002EB | CPE002EO | CPE002FR | NM 00100 | NM 0010 | NM 0010 | SLC25A25 | SLC25A25 | SLC25A25 | solute carrier fam | solute car | solute car | 114789 | 114789 | 114789 | s41606 | s41607 | s41608 |

| | | | | | | | | |
|---|---|---|---|---|---|---|---|---|
| 10387 | A4 | A5 | 981 | 952,9 | 9791 | 204,2 | 10 | 1,13 |
| 10389 | A6 | A7 | 1078 | 887,6 | 10757 | 228,5 | 10 | 1,13 |
| 10415 | B8 | B9 | 1706 | 978 | 17035 | 220,3 | 10 | 1,13 |
| 10448 | D16 | D17 | 839 | 886 | 8394 | 203,2 | 10 | 1,13 |
| 2692 | A12 | A13 | 864 | 660,7 | 8609 | 487,2 | 10 | 1,176 |
| 2814 | F14 | F15 | 1518 | 641,2 | 15246 | 486,5 | 10 | 1,176 |
| 3023 | N8 | N9 | 511 | 665,2 | 5135 | 483,5 | 10 | 1,176 |
| 20052 | D2 | D3 | 1187 | 1080 | 11886 | 158,2 | 10 | 1,22 |
| 20061 | D6 | D7 | 999 | 1230 | 10024 | 219,7 | 10 | 1,22 |
| 20120 | G16 | G17 | 1176 | 1035 | 11817 | 112,2 | 10 | 1,22 |
| 20145 | H17 | H18 | 1088 | 1082 | 10837 | 130,8 | 10 | 1,22 |
| 20350 | P7 | P8 | 949 | 1353 | 9521 | 132,2 | 10 | 1,22 |
| 469 | D20 | D21 | 2278 | 1859 | 22691 | 297,6 | 10 | 1,46 |
| 3207 | F22 | F23 | 1434 | 1327 | 14540 | 409,8 | 10,1 | 0,625 |
| 3378 | M3 | M4 | 933 | 1319 | 9455 | 397,1 | 10,1 | 0,625 |
| 20687 | N8 | N9 | 982 | 740,3 | 9880 | 151,4 | 10,1 | 0,673 |
| 9402 | H3 | H4 | 708 | 1218 | 7129 | 149,3 | 10,1 | 0,701 |
| 21080 | O16 | O17 | 1569 | 1022 | 15853 | 231,9 | 10,1 | 0,716 |
| 11113 | P1 | P2 | 1519 | 854,9 | 15417 | 547,8 | 10,1 | 0,765 |
| 17241 | O17 | O18 | 1573 | 926,5 | 15838 | 197,9 | 10,1 | 0,78 |
| 5035 | B4 | B5 | 988 | 646 | 9996 | 594,9 | 10,1 | 0,792 |
| 2510 | I21 | I22 | 2361 | 819 | 23733 | 358 | 10,1 | 0,795 |
| 16675 | G4 | G5 | 1616 | 986,9 | 16281 | 223,2 | 10,1 | 0,798 |
| 9200 | P16 | P17 | 1142 | 1550 | 11480 | 136,8 | 10,1 | 0,811 |
| 4227 | A11 | A12 | 1279 | 685 | 12857 | 363,3 | 10,1 | 0,852 |
| 4458 | J3 | J4 | 1684 | 737,4 | 17049 | 394,6 | 10,1 | 0,852 |
| 4520 | M16 | M17 | 1497 | 770,8 | 15077 | 356 | 10,1 | 0,852 |
| 4522 | M18 | M19 | 2094 | 787,6 | 21208 | 404 | 10,1 | 0,852 |
| 4532 | M5 | M6 | 527 | 652,1 | 5307 | 376,1 | 10,1 | 0,852 |
| 218 | J10 | J11 | 480 | 1518 | 4868 | 372 | 10,1 | 0,86 |
| 2219 | M19 | M20 | 2000 | 729,6 | 20166 | 423,2 | 10,1 | 0,878 |
| 4067 | J19 | J20 | 1320 | 656,8 | 13323 | 279,2 | 10,1 | 0,898 |
| 15612 | K2 | K3 | 1063 | 816 | 10772 | 143,2 | 10,1 | 0,918 |
| 5798 | B21 | B22 | 1754 | 805,2 | 17797 | 592,6 | 10,1 | 0,922 |
| 5984 | J16 | J17 | 2118 | 852,8 | 21434 | 474,7 | 10,1 | 0,922 |
| 5994 | J3 | J4 | 1608 | 823,3 | 16217 | 440,1 | 10,1 | 0,922 |
| 16297 | H1 | H2 | 1278 | 981,7 | 12907 | 236,8 | 10,1 | 0,927 |
| 16402 | L18 | L19 | 684 | 1143 | 6900 | 366 | 10,1 | 0,927 |
| 16473 | O17 | O18 | 1286 | 1018 | 13050 | 234,8 | 10,1 | 0,927 |
| 13588 | G12 | G13 | 1116 | 990,7 | 11308 | 149,6 | 10,1 | 0,948 |
| 5564 | H5 | H6 | 1536 | 693,2 | 15590 | 456,4 | 10,1 | 0,962 |
| 3478 | A7 | A8 | 1361 | 1386 | 13785 | 365,4 | 10,1 | 0,971 |
| 3493 | B20 | B21 | 1789 | 1399 | 18142 | 334,9 | 10,1 | 0,971 |
| 3517 | C20 | C21 | 1694 | 1448 | 17145 | 370,5 | 10,1 | 0,971 |
| 3573 | E6 | E7 | 1475 | 1306 | 14887 | 340,6 | 10,1 | 0,971 |
| 3612 | G2 | G3 | 456 | 1406 | 4589 | 433,2 | 10,1 | 0,971 |
| 3721 | L1 | L2 | 1642 | 1446 | 16661 | 373,8 | 10,1 | 0,971 |
| 8073 | A17 | A18 | 1505 | 662,1 | 15128 | 497,6 | 10,1 | 0,971 |
| 8173 | E20 | E21 | 931 | 676 | 9359 | 433,5 | 10,1 | 0,971 |
| 8413 | O20 | O21 | 2241 | 711,7 | 22625 | 438,5 | 10,1 | 0,971 |
| 8446 | P7 | P8 | 1519 | 529,9 | 15321 | 472,7 | 10,1 | 0,971 |
| 10043 | C19 | C20 | 1676 | 954,6 | 16998 | 109,3 | 10,1 | 0,971 |
| 1691 | G19 | G20 | 1489 | 664,4 | 15013 | 298 | 10,1 | 1,058 |
| 1713 | H17 | H18 | 2135 | 787,3 | 21563 | 318,9 | 10,1 | 1,058 |
| 1791 | K22 | K23 | 2120 | 781,1 | 21463 | 368,9 | 10,1 | 1,058 |
| 1802 | L10 | L11 | 1688 | 749,4 | 17113 | 336,5 | 10,1 | 1,058 |
| 1850 | N10 | N11 | 1125 | 725,1 | 11377 | 386,5 | 10,1 | 1,058 |
| 1851 | N11 | N12 | 1811 | 788 | 18356 | 373,9 | 10,1 | 1,058 |
| 21644 | F5 | F6 | 330 | 952,8 | 3325 | 538,6 | 10,1 | 1,063 |
| 21751 | K15 | K16 | 380 | 989,1 | 3837 | 723,1 | 10,1 | 1,063 |
| 4892 | L5 | L6 | 1313 | 790,4 | 13299 | 458 | 10,1 | 1,11 |
| 10680 | M9 | M10 | 1345 | 827,1 | 13545 | 167,3 | 10,1 | 1,141 |
| 10699 | N4 | N5 | 1207 | 859,6 | 12250 | 204,3 | 10,1 | 1,141 |
| 2707 | A4 | A5 | 761 | 667,4 | 7691 | 542,2 | 10,1 | 1,188 |
| 2711 | A8 | A9 | 1507 | 642,5 | 15170 | 523,1 | 10,1 | 1,188 |
| 2736 | B9 | B10 | 1125 | 677 | 11401 | 672,5 | 10,1 | 1,188 |
| 2783 | D8 | D9 | 1220 | 745,4 | 12371 | 589,1 | 10,1 | 1,188 |
| 2788 | E12 | E13 | 767 | 680,8 | 7774 | 533 | 10,1 | 1,188 |
| 2809 | F1 | F2 | 982 | 704,7 | 9934 | 534,4 | 10,1 | 1,188 |
| 3010 | N18 | N19 | 1384 | 606,7 | 14040 | 534,2 | 10,1 | 1,188 |
| 3021 | N6 | N7 | 1146 | 621,6 | 11532 | 511,2 | 10,1 | 1,188 |
| 3061 | P20 | P21 | 1383 | 603,4 | 13913 | 526,2 | 10,1 | 1,188 |
| 19983 | A22 | A23 | 1411 | 1148 | 14235 | 174,8 | 10,1 | 1,232 |
| 20122 | G18 | G19 | 1126 | 1121 | 11341 | 144,2 | 10,1 | 1,232 |
| 20162 | I10 | I11 | 621 | 1131 | 6290 | 186,9 | 10,1 | 1,232 |
| 20185 | J1 | J2 | 722 | 1054 | 7290 | 119 | 10,1 | 1,232 |
| 20231 | K8 | K9 | 596 | 1138 | 6001 | 155,2 | 10,1 | 1,232 |
| 20278 | M7 | M8 | 931 | 1044 | 9379 | 161,2 | 10,1 | 1,232 |
| 20333 | P13 | P14 | 1159 | 1319 | 11655 | 164,9 | 10,1 | 1,232 |
| 20335 | P15 | P16 | 589 | 1292 | 5927 | 167,8 | 10,1 | 1,232 |
| 740 | O5 | O6 | 2283 | 1898 | 23029 | 376,1 | 10,1 | 1,474 |
| 7686 | A14 | A15 | 878 | 632,3 | 8831 | 483,3 | 10,1 | 1,542 |
| 7710 | B14 | B15 | 1030 | 553,9 | 10406 | 434 | 10,1 | 1,542 |
| 7951 | L15 | L16 | 785 | 569,2 | 7940 | 336 | 10,1 | 1,542 |
| 7974 | M14 | M15 | 993 | 568,2 | 10043 | 380 | 10,1 | 1,542 |
| 7988 | M5 | M6 | 1072 | 565,1 | 10835 | 365,2 | 10,1 | 1,542 |
| 8006 | N21 | N22 | 1019 | 596,9 | 10270 | 417,7 | 10,1 | 1,542 |
| 3128 | C16 | C17 | 1491 | 1266 | 15191 | 281,9 | 10,2 | 0,632 |
| 3191 | E8 | E9 | 385 | 1234 | 3939 | 391 | 10,2 | 0,632 |
| 3307 | J4 | J5 | 265 | 1369 | 2712 | 354,2 | 10,2 | 0,632 |
| 9426 | I3 | I4 | 2187 | 1213 | 22254 | 139,6 | 10,2 | 0,708 |
| 7490 | I10 | I11 | 1522 | 607,8 | 15556 | 346,1 | 10,2 | 0,779 |
| 17031 | F22 | F23 | 2203 | 1146 | 22525 | 195,1 | 10,2 | 0,788 |
| 6968 | C16 | C17 | 634 | 578,4 | 6460 | 240,9 | 10,2 | 0,794 |
| 7261 | O20 | O21 | 1635 | 647,8 | 16656 | 457 | 10,2 | 0,794 |
| 5006 | A21 | A22 | 2443 | 738,9 | 24925 | 570,8 | 10,2 | 0,8 |
| 5299 | M4 | M5 | 1499 | 641,2 | 15220 | 278,7 | 10,2 | 0,8 |
| 2327 | A8 | A9 | 1686 | 686,9 | 17202 | 481 | 10,2 | 0,803 |
| 2410 | E18 | E19 | 1833 | 715,2 | 18737 | 353,7 | 10,2 | 0,803 |
| 2580 | L2 | L3 | 709 | 762 | 7200 | 447 | 10,2 | 0,803 |
| 16642 | F18 | F19 | 1525 | 1004 | 15535 | 259,2 | 10,2 | 0,806 |
| 16717 | I20 | I21 | 913 | 1113 | 9292 | 183,6 | 10,2 | 0,806 |
| 17720 | C16 | C17 | 1010 | 1078 | 10275 | 335,7 | 10,2 | 0,806 |
| 4487 | K8 | K9 | 1551 | 754,5 | 15811 | 422,8 | 10,2 | 0,861 |
| 228 | J2 | J3 | 413 | 1601 | 4227 | 409,3 | 10,2 | 0,868 |
| 1923 | A11 | A12 | 1756 | 745,8 | 17841 | 486,4 | 10,2 | 0,887 |
| 2165 | K13 | K14 | 1687 | 658,5 | 17255 | 409,9 | 10,2 | 0,887 |
| 2175 | K22 | K23 | 2096 | 816,2 | 21462 | 418,4 | 10,2 | 0,887 |
| 4086 | K14 | K15 | 1317 | 652,8 | 13388 | 255,4 | 10,2 | 0,907 |
| 15600 | J9 | J10 | 1023 | 785,1 | 10411 | 139,1 | 10,2 | 0,927 |
| 5789 | B13 | B14 | 1039 | 984,2 | 10604 | 463,4 | 10,2 | 0,932 |
| 5816 | C16 | C17 | 820 | 763,1 | 8397 | 475,2 | 10,2 | 0,932 |
| 5936 | H16 | H17 | 542 | 696 | 5528 | 247,4 | 10,2 | 0,932 |
| 6063 | M22 | M23 | 2212 | 807,1 | 22495 | 582 | 10,2 | 0,932 |
| 1326 | H14 | H15 | 1523 | 710,6 | 15474 | 315,7 | 10,2 | 0,936 |
| 1431 | L22 | L23 | 1722 | 763,5 | 17494 | 480,3 | 10,2 | 0,936 |
| 1476 | N2 | N3 | 1611 | 822,8 | 16456 | 380,3 | 10,2 | 0,936 |
| 16263 | F22 | F23 | 1386 | 1108 | 14186 | 392,4 | 10,2 | 0,936 |
| 16410 | L3 | L4 | 1256 | 1056 | 12780 | 234,3 | 10,2 | 0,936 |
| 13521 | D17 | D18 | 1414 | 1063 | 14486 | 144,4 | 10,2 | 0,958 |
| 13668 | J2 | J3 | 1596 | 1069 | 16319 | 142,9 | 10,2 | 0,958 |
| 830 | C21 | C22 | 1624 | 767,9 | 16620 | 367,3 | 10,2 | 0,967 |
| 858 | D3 | D4 | 1948 | 793,6 | 19797 | 336,1 | 10,2 | 0,967 |
| 1021 | K20 | K21 | 1035 | 812,6 | 10545 | 390,8 | 10,2 | 0,967 |
| 5620 | K12 | K13 | 658 | 753 | 6685 | 646,3 | 10,2 | 0,971 |
| 5674 | M18 | M19 | 2201 | 785,5 | 22361 | 530,1 | 10,2 | 0,971 |
| 5709 | N6 | N7 | 2065 | 654,6 | 20999 | 550,6 | 10,2 | 0,971 |
| 5723 | O19 | O20 | 1886 | 771,8 | 19166 | 504,4 | 10,2 | 0,971 |
| 3600 | F9 | F10 | 809 | 1431 | 8276 | 392,4 | 10,2 | 0,981 |
| 3609 | G17 | G18 | 1371 | 1470 | 13998 | 398,1 | 10,2 | 0,981 |
| 3697 | K1 | K2 | 1232 | 1514 | 12577 | 434,8 | 10,2 | 0,981 |
| 3758 | M21 | M22 | 1651 | 1301 | 16828 | 400,3 | 10,2 | 0,981 |
| 8292 | J2 | J3 | 850 | 578,7 | 8688 | 510,1 | 10,2 | 0,981 |
| 1608 | C9 | C10 | 1382 | 826,9 | 14037 | 470,8 | 10,2 | 1,068 |
| 1748 | I5 | I6 | 1221 | 680,1 | 12404 | 368,6 | 10,2 | 1,068 |
| 1794 | K3 | K4 | 1025 | 656,5 | 10495 | 382,6 | 10,2 | 1,068 |
| 1837 | M20 | M21 | 1987 | 749,1 | 20227 | 340,3 | 10,2 | 1,068 |
| 1901 | P13 | P14 | 2020 | 719,5 | 20511 | 382,1 | 10,2 | 1,068 |
| 21570 | C3 | C4 | 52 | 1371 | 531 | 795,1 | 10,2 | 1,074 |
| 21669 | G6 | G7 | 231 | 967,4 | 2364 | 574,2 | 10,2 | 1,074 |
| 21726 | J14 | J15 | 406 | 951,6 | 4137 | 758,9 | 10,2 | 1,074 |
| 21734 | J21 | J22 | 320 | 936,9 | 3280 | 501,8 | 10,2 | 1,074 |
| 21807 | M22 | M23 | 271 | 1086 | 2774 | 706,9 | 10,2 | 1,074 |
| 21859 | O4 | O5 | 156 | 953,2 | 1595 | 867 | 10,2 | 1,074 |
| 4643 | B19 | B20 | 1653 | 850,1 | 16918 | 467,6 | 10,2 | 1,121 |
| 4688 | D16 | D17 | 1541 | 856,2 | 15705 | 383,3 | 10,2 | 1,121 |
| 4791 | H22 | H23 | 1811 | 805 | 18382 | 400,2 | 10,2 | 1,121 |
| 4813 | I20 | I21 | 1743 | 785,6 | 17808 | 332,7 | 10,2 | 1,121 |
| 4825 | J1 | J2 | 1188 | 807,5 | 12092 | 397 | 10,2 | 1,121 |
| 4853 | K13 | K14 | 887 | 725,7 | 9034 | 467,8 | 10,2 | 1,121 |
| 4953 | O17 | O18 | 1700 | 852,8 | 17275 | 316,1 | 10,2 | 1,121 |
| 10655 | L8 | L9 | 1211 | 802,4 | 12309 | 172,1 | 10,2 | 1,153 |
| 3045 | O6 | O7 | 1071 | 671 | 10941 | 507,4 | 10,2 | 1,2 |
| 20041 | D1 | D2 | 1212 | 1123 | 12410 | 187 | 10,2 | 1,244 |
| 20150 | H21 | H22 | 1267 | 1155 | 12902 | 193,7 | 10,2 | 1,244 |
| 20236 | L12 | L13 | 883 | 1120 | 9043 | 149,6 | 10,2 | 1,244 |
| 7690 | A18 | A19 | 652 | 599,8 | 6682 | 478,9 | 10,2 | 1,557 |
| 7755 | D11 | D12 | 1035 | 504,8 | 10573 | 388,3 | 10,2 | 1,557 |
| 7860 | H2 | H3 | 1056 | 539,5 | 10757 | 368,7 | 10,2 | 1,557 |
| 8047 | P15 | P16 | 1070 | 608,5 | 10936 | 440,5 | 10,2 | 1,557 |
| 3303 | J22 | J23 | 1741 | 1375 | 17888 | 366,2 | 10,3 | 0,638 |
| 17420 | F5 | F6 | 1416 | 968,5 | 14643 | 203,2 | 10,3 | 0,703 |
| 9487 | L15 | L16 | 1388 | 1134 | 14265 | 117,3 | 10,3 | 0,715 |
| 9788 | H5 | H6 | 1634 | 1081 | 16862 | 103,3 | 10,3 | 0,72 |
| 14508 | M2 | M3 | 1993 | 863 | 20467 | 108,4 | 10,3 | 0,733 |
| 11423 | L8 | L9 | 1775 | 887,8 | 18299 | 123,4 | 10,3 | 0,746 |
| 7196 | L5 | L6 | 1059 | 665,9 | 10933 | 207,6 | 10,3 | 0,802 |
| 2591 | L8 | L9 | 909 | 670,2 | 9395 | 312,4 | 10,3 | 0,811 |
| 2605 | M20 | M21 | 1866 | 758,1 | 19174 | 333,4 | 10,3 | 0,811 |
| 16698 | H3 | H4 | 1177 | 980,8 | 12146 | 175 | 10,3 | 0,814 |
| 4356 | F2 | F3 | 1342 | 683,8 | 13791 | 469,9 | 10,3 | 0,869 |
| 4561 | O1 | O2 | 1547 | 676,6 | 15979 | 472,1 | 10,3 | 0,869 |
| 19 | A4 | A5 | 648 | 1593 | 6696 | 832,9 | 10,3 | 0,877 |
| 69 | C6 | C7 | 863 | 1747 | 8930 | 295,1 | 10,3 | 0,877 |
| 140 | F5 | F6 | 352 | 1374 | 3628 | 338,2 | 10,3 | 0,877 |
| 251 | K19 | K20 | 313 | 1505 | 3227 | 368,4 | 10,3 | 0,877 |
| 335 | N8 | N9 | 654 | 1551 | 6751 | 540,4 | 10,3 | 0,877 |
| 349 | O20 | O21 | 769 | 1739 | 7906 | 558,3 | 10,3 | 0,877 |
| 1927 | A15 | A16 | 2145 | 776,9 | 22083 | 479,8 | 10,3 | 0,896 |
| 1970 | C10 | C11 | 892 | 680,7 | 9185 | 487,5 | 10,3 | 0,896 |
| 2018 | E10 | E11 | 1119 | 621,4 | 11544 | 397,2 | 10,3 | 0,896 |
| 2195 | L19 | L20 | 536 | 705,9 | 5513 | 458,2 | 10,3 | 0,896 |
| 2222 | M21 | M22 | 1386 | 661,6 | 14250 | 421,2 | 10,3 | 0,896 |
| 15430 | C7 | C8 | 643 | 855,4 | 6640 | 128,2 | 10,3 | 0,936 |
| 15644 | L5 | L6 | 970 | 926,2 | 9955 | 162,6 | 10,3 | 0,936 |
| 6048 | L9 | L10 | 1275 | 827,1 | 13100 | 521,8 | 10,3 | 0,941 |
| 1485 | N6 | N7 | 1367 | 677,5 | 14129 | 391,4 | 10,3 | 0,945 |
| 16423 | M15 | M16 | 1257 | 883,7 | 12958 | 183,7 | 10,3 | 0,945 |
| 13787 | O19 | O20 | 997 | 1107 | 10244 | 136,5 | 10,3 | 0,967 |
| 860 | D5 | D6 | 1485 | 745,2 | 15273 | 535,2 | 10,3 | 0,976 |
| 1107 | O11 | O12 | 1443 | 760,6 | 14885 | 556,1 | 10,3 | 0,976 |
| 1149 | P6 | P7 | 2134 | 744,8 | 22044 | 431,1 | 10,3 | 0,976 |
| 5415 | B22 | B23 | 2141 | 720,7 | 22009 | 524,2 | 10,3 | 0,981 |
| 5593 | J1 | J2 | 1638 | 705,6 | 16841 | 564,5 | 10,3 | 0,981 |
| 3457 | A1 | A2 | 722 | 1372 | 7469 | 396,8 | 10,3 | 0,99 |
| 3486 | B14 | B15 | 1720 | 1351 | 17797 | 308,8 | 10,3 | 0,99 |
| 3512 | C16 | C17 | 1029 | 1572 | 10586 | 304,7 | 10,3 | 0,99 |
| 3738 | L3 | L4 | 1065 | 1201 | 10994 | 370,3 | 10,3 | 0,99 |
| 8171 | E19 | E20 | 1437 | 591,8 | 14738 | 392,6 | 10,3 | 0,99 |
| 10286 | M21 | M22 | 1633 | 934,2 | 16893 | 127,5 | 10,3 | 0,99 |
| 1646 | E21 | E22 | 1659 | 738,3 | 17066 | 407,5 | 10,3 | 1,079 |
| 1674 | F3 | F4 | 2244 | 797,6 | 23215 | 475,7 | 10,3 | 1,079 |
| 1695 | G22 | G23 | 1748 | 757,9 | 17960 | 357,1 | 10,3 | 1,079 |
| 1742 | I21 | I22 | 1031 | 697,9 | 10665 | 305,1 | 10,3 | 1,079 |
| 1751 | I8 | I9 | 1580 | 787,9 | 16301 | 387,6 | 10,3 | 1,079 |
| 21535 | B15 | B16 | 310 | 1036 | 3192 | 574,4 | 10,3 | 1,084 |
| 21543 | B22 | B23 | 211 | 872,9 | 2165 | 602,3 | 10,3 | 1,084 |
| 21667 | G4 | G5 | 221 | 1028 | 2286 | 408,9 | 10,3 | 1,084 |
| 21846 | O14 | O15 | 465 | 1017 | 4778 | 747,9 | 10,3 | 1,084 |
| 21877 | P20 | P21 | 395 | 1300 | 4060 | 516,4 | 10,3 | 1,084 |
| 4674 | C3 | C4 | 760 | 851,6 | 7838 | 544,3 | 10,3 | 1,132 |
| 4700 | D5 | D6 | 1932 | 833 | 19916 | 486,1 | 10,3 | 1,132 |
| 4717 | E20 | E21 | 1919 | 792,5 | 19786 | 436,8 | 10,3 | 1,132 |
| 4780 | H12 | H13 | 1154 | 894,3 | 11875 | 408,7 | 10,3 | 1,132 |
| 4851 | K11 | K12 | 1129 | 782,3 | 11611 | 448,9 | 10,3 | 1,132 |
| 10585 | J1 | J2 | 1110 | 943,6 | 11446 | 169,7 | 10,3 | 1,164 |
| 10695 | N22 | N23 | 1352 | 845,2 | 13982 | 210,8 | 10,3 | 1,164 |
| 2697 | A17 | A18 | 1722 | 692,7 | 17744 | 626,6 | 10,3 | 1,212 |
| 2854 | G7 | G8 | 1213 | 645,2 | 12499 | 662 | 10,3 | 1,212 |
| 3005 | N13 | N14 | 1357 | 579,8 | 14009 | 432,2 | 10,3 | 1,212 |
| 20051 | D19 | D20 | 928 | 1082 | 9589 | 159,6 | 10,3 | 1,256 |
| 20071 | E15 | E16 | 902 | 1102 | 9299 | 156,5 | 10,3 | 1,256 |
| 20114 | G10 | G11 | 772 | 1142 | 7934 | 159,6 | 10,3 | 1,256 |
| 20119 | G15 | G16 | 1289 | 1110 | 13247 | 155,6 | 10,3 | 1,256 |
| 20208 | J9 | J10 | 531 | 1127 | 5456 | 167 | 10,3 | 1,256 |
| 385 | A1 | A2 | 1625 | 1925 | 16669 | 410,6 | 10,3 | 1,504 |

| | | | | | | | | | |
|---|---|---|---|---|---|---|---|---|---|
| 723 | O11 | O12 | 1768 | 1748 | 18192 | 361,5 | 10,3 | 1,504 | EPRS |
| 7735 | C15 | C16 | 588 | 533,6 | 6042 | 358,5 | 10,3 | 1,573 | RBM7 |
| 7870 | H7 | H8 | 1351 | 602,5 | 13954 | 359 | 10,3 | 1,573 | C1D |
| 7893 | I6 | I7 | 931 | 563,4 | 9559 | 392,8 | 10,3 | 1,573 | KHDRBS3 |
| 8042 | P10 | P11 | 1110 | 489,5 | 11452 | 425,7 | 10,3 | 1,573 | LILRA2 |
| 3379 | M4 | M5 | 1153 | 1174 | 12007 | 338 | 10,4 | 0,644 | CDC14C |
| 6207 | C22 | C23 | 1947 | 595,5 | 20313 | 361,6 | 10,4 | 0,686 | CTGF |
| 20665 | N1 | N2 | 989 | 746,3 | 10327 | 104,1 | 10,4 | 0,693 | LOC44204 |
| 20693 | O13 | O14 | 1306 | 754,4 | 13551 | 193,9 | 10,4 | 0,693 | LOC44105 |
| 9656 | C16 | C17 | 2141 | 1139 | 22355 | 143 | 10,4 | 0,727 | DUS1L |
| 8778 | N3 | N4 | 1566 | 1102 | 16210 | 106,6 | 10,4 | 0,735 | ARFGAP3 |
| 7524 | J2 | J3 | 1016 | 668,3 | 10572 | 313,2 | 10,4 | 0,794 | TOMM1 |
| 2317 | A20 | A21 | 2231 | 778,8 | 23102 | 403,6 | 10,4 | 0,819 | HK3 |
| 2361 | C17 | C18 | 1416 | 682,1 | 14715 | 355,5 | 10,4 | 0,819 | CSNK2A2 |
| 2458 | G18 | G19 | 1787 | 758,9 | 18515 | 342,7 | 10,4 | 0,819 | NME4 |
| 2520 | I9 | I10 | 1854 | 707,7 | 19306 | 297,4 | 10,4 | 0,819 | AK1 |
| 2550 | K14 | K15 | 2089 | 773,3 | 21759 | 348,3 | 10,4 | 0,819 | PDK4 |
| 2583 | L22 | L23 | 1880 | 723 | 19640 | 364,4 | 10,4 | 0,819 | SRPK1 |
| 16742 | J21 | J22 | 1700 | 978,1 | 17740 | 200,9 | 10,4 | 0,822 | KBTBD6 |
| 16814 | M21 | M22 | 1746 | 978 | 18236 | 203 | 10,4 | 0,822 | KRTAP9-5 |
| 16838 | N21 | N22 | 1082 | 1007 | 11232 | 301,3 | 10,4 | 0,822 | FRMD7 |
| 17961 | M17 | M18 | 1123 | 968,4 | 11641 | 179,5 | 10,4 | 0,822 | C12orf60 |
| 4304 | D16 | D17 | 1491 | 738,2 | 15565 | 320,5 | 10,4 | 0,878 | KCNH6 |
| 197 | I13 | I14 | 715 | 1443 | 7405 | 436 | 10,4 | 0,885 | SCML1 |
| 236 | J5 | J6 | 417 | 1414 | 4324 | 377,5 | 10,4 | 0,885 | SKIV2L |
| 360 | O9 | O10 | 333 | 1477 | 3472 | 693,4 | 10,4 | 0,885 | CCL20 |
| 3910 | C7 | C8 | 1125 | 721,5 | 11716 | 353,5 | 10,4 | 0,924 | FFAR1 |
| 3921 | D17 | D18 | 1039 | 698,6 | 10841 | 432,3 | 10,4 | 0,924 | PRL |
| 4034 | I10 | I11 | 1258 | 600 | 13127 | 347,1 | 10,4 | 0,924 | RANBP1 |
| 11730 | I3 | I4 | 1506 | 794,7 | 15679 | 170,3 | 10,4 | 0,929 | C4A |
| 15457 | E1 | E2 | 1566 | 807,5 | 16274 | 116,3 | 10,4 | 0,929 | C12orf44 |
| 5877 | E6 | E7 | 1674 | 807 | 17488 | 576,8 | 10,4 | 0,95 | ARSA |
| 5890 | F18 | F19 | 2256 | 856,7 | 23436 | 547,1 | 10,4 | 0,95 | FOXL2 |
| 5977 | J1 | J2 | 1193 | 829 | 12410 | 351,6 | 10,4 | 0,95 | ANXA6 |
| 6022 | K7 | K8 | 1533 | 711,9 | 15957 | 492,3 | 10,4 | 0,95 | ADH6 |
| 1189 | B20 | B21 | 2132 | 778,2 | 22078 | 438,7 | 10,4 | 0,954 | ZMYND11 |
| 1232 | D16 | D17 | 1561 | 784,1 | 16210 | 354,5 | 10,4 | 0,954 | GJB6 |
| 1259 | E19 | E20 | 2300 | 810,4 | 23887 | 454,8 | 10,4 | 0,954 | ZNF35 |
| 16406 | L21 | L22 | 1250 | 1001 | 13850 | 272,3 | 10,4 | 0,954 | CCDC55 |
| 16469 | O13 | O14 | 1829 | 1013 | 19029 | 475,9 | 10,4 | 0,954 | C12orf32 |
| 805 | B20 | B21 | 1978 | 771,9 | 20621 | 451,6 | 10,4 | 0,986 | RFX4 |
| 854 | D21 | D22 | 1630 | 865,6 | 16892 | 400,3 | 10,4 | 0,986 | GJB1 |
| 1022 | K21 | K22 | 1518 | 769 | 15716 | 309,6 | 10,4 | 0,986 | COL16A1 |
| 1129 | P1 | P2 | 1612 | 672,3 | 16728 | 441,8 | 10,4 | 0,986 | AFF3 |
| 5432 | C16 | C17 | 1466 | 530,3 | 15184 | 460,7 | 10,4 | 0,99 | ALKBH1 |
| 5442 | C3 | C4 | 1458 | 653,3 | 15210 | 545,9 | 10,4 | 0,99 | ACOX3 |
| 5500 | F12 | F13 | 1858 | 707,3 | 19327 | 620,2 | 10,4 | 0,99 | ZW10 |
| 5695 | L7 | L8 | 2104 | 749,2 | 21904 | 518,1 | 10,4 | 0,99 | ABCC3 |
| 3460 | A12 | A13 | 610 | 1359 | 6367 | 385,9 | 10,4 | 1 | RHO |
| 3690 | J3 | J4 | 851 | 1415 | 8891 | 350,1 | 10,4 | 1 | HTR1B |
| 8145 | D17 | D18 | 1342 | 604,2 | 13925 | 438,6 | 10,4 | 1 | MRAS |
| 8364 | M2 | M3 | 789 | 578,2 | 8184 | 358,7 | 10,4 | 1 | SMG6 |
| 10266 | L3 | L4 | 1216 | 798,5 | 12610 | 138,3 | 10,4 | 1 | TXNDC5 |
| 1642 | E18 | E19 | 1790 | 787,3 | 18626 | 388,8 | 10,4 | 1,089 | ZBTB26 |
| 1782 | K14 | K15 | 1644 | 702,1 | 17171 | 332,4 | 10,4 | 1,089 | DNAJC1 |
| 1795 | K4 | K5 | 1517 | 730,9 | 15817 | 410 | 10,4 | 1,089 | TSPYL2 |
| 1820 | L5 | L6 | 952 | 615,9 | 9865 | 353,9 | 10,4 | 1,089 | ZNF83 |
| 1826 | M10 | M11 | 459 | 889,7 | 4790 | 513,2 | 10,4 | 1,089 | ZNF574 |
| 1881 | O17 | O18 | 1688 | 728,8 | 17591 | 400,2 | 10,4 | 1,089 | ZNF588 |
| 1903 | P15 | P16 | 2080 | 779,3 | 21714 | 367,9 | 10,4 | 1,089 | GJA12 |
| 21517 | A20 | A21 | 107 | 903,7 | 1112 | 625,1 | 10,4 | 1,095 | LOC65251 |
| 21529 | B1 | B2 | 162 | 1143 | 1688 | 860,5 | 10,4 | 1,095 | LOC650689 |
| 21615 | E22 | E23 | 307 | 1130 | 3179 | 591,1 | 10,4 | 1,095 | LOC652631 |
| 21643 | F4 | F5 | 201 | 1147 | 2089 | 687 | 10,4 | 1,095 | LOC653303 |
| 21797 | M13 | M14 | 389 | 912 | 4043 | 587,7 | 10,4 | 1,095 | LOC650262 |
| 21803 | M19 | M20 | 312 | 929,2 | 3244 | 323,4 | 10,4 | 1,095 | LOC650428 |
| 4751 | F8 | F9 | 1772 | 844,8 | 18462 | 425,4 | 10,4 | 1,143 | CAPN7 |
| 4803 | I11 | I12 | 1709 | 884 | 17763 | 454,5 | 10,4 | 1,143 | CTSC |
| 4859 | K19 | K20 | 1764 | 749,2 | 18383 | 416,6 | 10,4 | 1,143 | CTSE |
| 4904 | M16 | M17 | 1517 | 820,7 | 15802 | 320,5 | 10,4 | 1,143 | USP52 |
| 10437 | C6 | C7 | 1534 | 824,6 | 16021 | 203,1 | 10,4 | 1,175 | SLITRK1 |
| 10531 | G4 | G5 | 904 | 959,1 | 9357 | 170,8 | 10,4 | 1,175 | MARCH3 |
| 10569 | I17 | I18 | 1430 | 770,7 | 14935 | 172 | 10,4 | 1,175 | SERPINB1 |
| 2775 | D22 | D23 | 587 | 822,3 | 6129 | 378,1 | 10,4 | 1,224 | GLYCTK |
| 2817 | F17 | F18 | 1436 | 658,6 | 14919 | 515,5 | 10,4 | 1,224 | UCKL1 |
| 2860 | H12 | H13 | 1499 | 672,7 | 15587 | 524,2 | 10,4 | 1,224 | DCLK2 |
| 2935 | K15 | K16 | 501 | 724,4 | 5257 | 453,1 | 10,4 | 1,224 | SH3BP5 |
| 3019 | N4 | N5 | 1252 | 602 | 13071 | 519,1 | 10,4 | 1,224 | ALDH1A3 |
| 3036 | O2 | O3 | 1428 | 666,1 | 14799 | 561 | 10,4 | 1,224 | ALPK1 |
| 20026 | C18 | C19 | 1299 | 1118 | 13562 | 212,9 | 10,4 | 1,224 | AMTN |
| 20109 | F6 | F7 | 871 | 1082 | 9015 | 157,5 | 10,4 | 1,224 | LOC40263 |
| 20143 | H15 | H16 | 944 | 1169 | 9843 | 163,8 | 10,4 | 1,268 | C15orf54 |
| 20318 | O21 | O22 | 1016 | 1085 | 10540 | 162,9 | 10,4 | 1,268 | FAM102A |
| 20327 | O8 | O9 | 725 | 1126 | 7550 | 195 | 10,4 | 1,268 | LOC40167 |
| 390 | A14 | A15 | 2501 | 1988 | 26076 | 463,3 | 10,4 | 1,518 | FOXJ1 |
| 418 | B18 | B19 | 2036 | 1837 | 21192 | 345,3 | 10,4 | 1,518 | GFRA3 |
| 471 | D22 | D23 | 2104 | 1855 | 21894 | 349,7 | 10,4 | 1,518 | GLG1 |
| 491 | E19 | E20 | 1807 | 1773 | 18847 | 274,5 | 10,4 | 1,518 | MEGF8 |
| 704 | N16 | N17 | 1942 | 1878 | 20122 | 312,5 | 10,4 | 1,518 | GPX7 |
| 7770 | D3 | D4 | 1228 | 548,9 | 12793 | 383,8 | 10,4 | 1,588 | NOD1 |
| 8036 | O5 | O6 | 745 | 526,1 | 7748 | 421,2 | 10,4 | 1,588 | AKR1A1 |
| 3282 | I3 | I4 | 1156 | 1129 | 12184 | 386,5 | 10,5 | 0,5 | INPP5B |
| 12948 | L2 | L3 | 796 | 1139 | 8352 | 158,7 | 10,5 | 0,712 | TIMM17A |
| 18872 | C16 | C17 | 1076 | 922 | 11272 | 222,5 | 10,5 | 0,735 | TPRX1 |
| 12588 | M2 | M3 | 1053 | 1068 | 11011 | 153 | 10,5 | 0,789 | THOC5 |
| 7417 | F1 | F2 | 1250 | 576,3 | 13088 | 460,7 | 10,5 | 0,789 | WDR57 |
| 7214 | M21 | M22 | 1480 | 564,9 | 15632 | 462 | 10,5 | 0,815 | TACSTD2 |
| 5151 | G22 | G23 | 1915 | 699,7 | 20026 | 423,9 | 10,5 | 0,824 | TUBB2A |
| 2312 | A16 | A17 | 1961 | 722,9 | 20708 | 429,6 | 10,5 | 0,824 | HK1 |
| 2486 | H21 | H22 | 1659 | 667 | 17451 | 347,1 | 10,5 | 0,824 | DGKA |
| 2629 | N20 | N21 | 1205 | 746,1 | 12663 | 427,7 | 10,5 | 0,827 | TAF1 |
| 17981 | N13 | N14 | 1949 | 1070 | 20402 | 154,3 | 10,5 | 0,83 | hCG1643 |
| 17991 | N22 | N23 | 1834 | 1143 | 19330 | 200,8 | 10,5 | 0,83 | C4orf26 |
| 9132 | M2 | M3 | 2173 | 1872 | 22910 | 128,8 | 10,5 | 0,835 | MBD3 |
| 4229 | A13 | A14 | 1312 | 709,1 | 13769 | 375,4 | 10,5 | 0,855 | ATP2B1 |
| 4503 | L22 | L23 | 891 | 738 | 9320 | 402,5 | 10,5 | 0,886 | ICAM5 |
| 223 | J15 | J16 | 530 | 1515 | 5366 | [illegible] | 10,5 | 0,886 | SLC1A3 |
| 261 | L1 | L2 | 281 | 1415 | 2944 | 549 | 10,5 | 0,886 | SLC2A4 |
| 297 | M17 | M18 | 472 | 1586 | 4958 | 450,6 | 10,5 | 0,894 | CCL8 |
| 6573 | B6 | B7 | 890 | 659 | 9321 | 508,7 | 10,5 | 0,894 | ITGA9 |
| 3903 | C22 | C23 | 1564 | 701,2 | 16399 | 445,4 | 10,5 | 0,933 | RAB5A |
| 4003 | G4 | G5 | 1182 | 715,8 | 12380 | 445,4 | 10,5 | 0,933 | RAD9A |
| 4033 | I1 | I2 | 896 | 627,1 | 9377 | 432,6 | 10,5 | 0,933 | PRRX1 |
| 11665 | G1 | G2 | 1588 | 861 | 16632 | 127,7 | 10,5 | 0,938 | BMP3 |
| 5855 | D8 | D9 | 1953 | 803,2 | 20461 | 260,8 | 10,5 | 0,956 | BHMT |
| 5970 | I3 | I4 | 1129 | 752,8 | 11871 | 453,5 | 10,5 | 0,956 | ADCY3 |
| 1245 | D6 | D7 | 1840 | 812,9 | 19395 | 410,4 | 10,5 | 0,963 | ZNF266 |
| 16208 | D16 | D17 | 1221 | 952,9 | 12868 | 282,6 | 10,5 | 0,963 | GHDC |
| 16239 | E22 | E23 | 1627 | 993,2 | 17102 | 313,5 | 10,5 | 0,963 | PSMG3 |
| 16380 | K2 | K3 | 1074 | 1032 | 11290 | 303,9 | 10,5 | 0,963 | C17orf37 |
| 16455 | N22 | N23 | 1540 | 981,2 | 16131 | 309,9 | 10,5 | 0,963 | C10orf53 |
| 13507 | C4 | C5 | 1621 | 1112 | 16943 | 148,4 | 10,5 | 0,986 | NIPSNAP3 |
| 13596 | G2 | G3 | 1330 | 1060 | 13927 | 152,4 | 10,5 | 0,986 | C20orf4 |
| 13650 | I3 | I4 | 1329 | 1020 | 14006 | 170,6 | 10,5 | 0,986 | MED13L |
| 1099 | N4 | N5 | 1239 | 751,7 | 12969 | 426,7 | 10,5 | 0,995 | TJP1 |
| 5487 | E22 | E23 | 2032 | 697,4 | 21283 | 359,1 | 10,5 | 1 | APPBP1 |
| 5552 | H16 | H17 | 2073 | 573,2 | 21814 | 472,6 | 10,5 | 1 | LRAT |
| 5586 | I3 | I4 | 926 | 723 | 9768 | 565,9 | 10,5 | 1 | OFD1 |
| 5677 | M20 | M21 | 2240 | 673,2 | 23464 | 502,7 | 10,5 | 1 | BTAF1 |
| 8706 | N3 | N4 | 1784 | 635,3 | 18766 | 528,4 | 10,5 | 1 | CDS2 |
| 3563 | E19 | E20 | 1500 | 1436 | 15721 | 385,5 | 10,5 | 1,01 | CALCR |
| 3570 | E3 | E4 | 580 | 1369 | 6116 | 360,8 | 10,5 | 1,01 | BAI3 |
| 3586 | F18 | F19 | 1200 | 1336 | 12642 | 356,5 | 10,5 | 1,01 | GPR172A |
| 3801 | O17 | O18 | 1190 | 1218 | 12546 | 403,2 | 10,5 | 1,01 | GNRHR |
| 8199 | F22 | F23 | 1873 | 667,5 | 19605 | 518,3 | 10,5 | 1,01 | ANGPTL2 |
| 10263 | L22 | L23 | 1907 | 915,2 | 19975 | 148 | 10,5 | 1,01 | PIGO |
| 1668 | F2 | F3 | 993 | 794,9 | 10377 | 484,9 | 10,5 | 1,099 | ZNF614 |
| 1787 | K19 | K20 | 1154 | 712,2 | 12099 | 387,8 | 10,5 | 1,099 | PHF20L1 |
| 1909 | P20 | P21 | 2084 | 785,8 | 21802 | 407,1 | 10,5 | 1,099 | ZNRF1 |
| 21526 | A7 | A8 | 139 | 898,5 | 1454 | 574 | 10,5 | 1,105 | LOC64874 |
| 21701 | I13 | I14 | 282 | 910,6 | 2973 | 674,3 | 10,5 | 1,105 | HLA-A29.1 |
| 4718 | E21 | E22 | 1594 | 831,9 | 16717 | 459,4 | 10,5 | 1,154 | BMP1 |
| 4755 | G11 | G12 | 1324 | 867,7 | 13937 | 542,7 | 10,5 | 1,154 | CAPNS1 |
| 4811 | I19 | I20 | 2005 | 783,4 | 21097 | 411,3 | 10,5 | 1,154 | CPA2 |
| 4847 | J8 | J9 | 1167 | 903,3 | 12224 | 450,4 | 10,5 | 1,154 | LAP3 |
| 4858 | K18 | K19 | 1964 | 792,9 | 20663 | 380,7 | 10,5 | 1,154 | ADAMTS4 |
| 4962 | O3 | O4 | 1101 | 776,1 | 11527 | 524,2 | 10,5 | 1,154 | ELA2 |
| 10404 | B2 | B3 | 1095 | 899,4 | 11463 | 199,5 | 10,5 | 1,185 | ANTXR2 |
| 10496 | F16 | F17 | 1171 | 849,1 | 12334 | 168,8 | 10,5 | 1,185 | ACOT4 |
| 2713 | B1 | B2 | 739 | 715,2 | 7766 | 427,4 | 10,5 | 1,235 | BCKDK |
| 2758 | C7 | C8 | 1158 | 644,1 | 12172 | 558,8 | 10,5 | 1,235 | DGKD |
| 2875 | H4 | H5 | 1155 | 627,1 | 12132 | 490,9 | 10,5 | 1,235 | NEK10 |
| 3049 | P1 | P2 | 839 | 643,7 | 8799 | 437,6 | 10,5 | 1,235 | PACSIN3 |
| 3069 | P5 | P6 | 1656 | 675,1 | 17400 | 610,9 | 10,5 | 1,235 | ASAH1 |
| 19999 | B15 | B16 | 969 | 1213 | 10204 | 179,1 | 10,5 | 1,275 | C10orf114 |
| 20116 | G12 | G13 | 1160 | 1075 | 12195 | 148,5 | 10,5 | 1,275 | KLHL31 |
| 20125 | G20 | G21 | 1014 | 1014 | 11964 | 167,8 | 10,5 | 1,275 | LOC40128 |
| 20263 | M15 | M16 | 734 | 1093 | 7681 | 185,6 | 10,5 | 1,275 | ARL13A |
| 416 | B16 | B17 | 2124 | 1762 | 22306 | 326,7 | 10,5 | 1,533 | GFRA2 |
| 747 | P11 | P12 | 2377 | 1900 | 24849 | 411,7 | 10,5 | 1,533 | FKBP2 |
| 7700 | A5 | A6 | 878 | 538,1 | 9187 | 408,3 | 10,5 | 1,603 | SFRS14 |
| 7843 | G4 | G5 | 745 | 602,7 | 7833 | 293 | 10,5 | 1,603 | MRCL3 |
| 7851 | H11 | H12 | 1337 | 600,6 | 13777 | 407,1 | 10,5 | 1,603 | LRRN2 |
| 7969 | M1 | M2 | 228 | 626,8 | 2391 | 360,5 | 10,5 | 1,603 | SMNDC1 |
| 3351 | L22 | L23 | 1337 | 1173 | 14231 | 377,4 | 10,6 | 0,5 | PIGR |
| 3392 | N16 | N17 | 849 | 1238 | 8957 | 366,2 | 10,6 | 0,656 | PKD1 |
| 20658 | M3 | M4 | 587 | 1170 | 6222 | 98,61 | 10,6 | 0,693 | LOC440917 |
| 17569 | M1 | M2 | 1415 | 979,2 | 14997 | 173,9 | 10,6 | 0,724 | LRRC39 |
| 9361 | G1 | G2 | 1746 | 1146 | 18528 | 96,64 | 10,6 | 0,735 | RNF31 |
| 11308 | H12 | H13 | 1722 | 862,5 | 18171 | 116,7 | 10,6 | 0,794 | TMSB4X |
| 12598 | M7 | M8 | 1169 | 948,7 | 12446 | 133,9 | 10,6 | 0,797 | MIB2 |
| 16953 | C17 | C18 | 1879 | 986,6 | 19770 | 195,3 | 10,6 | 0,812 | TTYH2 |
| 5010 | A3 | A4 | 1275 | 669,1 | 13511 | 257,2 | 10,6 | 0,829 | ABHD4 |
| 5101 | E20 | E21 | 1729 | 586,4 | 18399 | 489,9 | 10,6 | 0,829 | TSHB |
| 2342 | B21 | B22 | 1858 | 730,6 | 19576 | 361,3 | 10,6 | 0,835 | CAMK2B |
| 2490 | H3 | H4 | 1150 | 739,7 | 12056 | 394,4 | 10,6 | 0,835 | KIT |
| 2664 | O9 | O10 | 1701 | 662,7 | 17889 | 356,1 | 10,6 | 0,835 | ALK |
| 2682 | P3 | P4 | 1250 | 765,4 | 13250 | 509,5 | 10,6 | 0,835 | FRK |
| 2687 | P8 | P9 | 1216 | 803,6 | 12892 | 523,5 | 10,6 | 0,835 | TGFBR1 |
| 4428 | I2 | I3 | 1060 | 620 | 11188 | 423,7 | 10,6 | 0,891 | SLC34A1 |
| 56 | C16 | C17 | 360 | 1563 | 3808 | 341,3 | 10,6 | 0,898 | ST3GAL1 |
| 171 | H11 | H12 | 445 | 1440 | 4737 | 436,8 | 10,6 | 0,898 | ST3GAL5 |
| 219 | J11 | J12 | 1302 | 1352 | 13706 | 431 | 10,6 | 0,898 | SLC25A10 |
| 2007 | D22 | D23 | 1733 | 724,9 | 18283 | 482,3 | 10,6 | 0,924 | ZNF773 |
| 2084 | G5 | G6 | 2094 | 694,4 | 22285 | 438,2 | 10,6 | 0,924 | SYTL3 |
| 2103 | H22 | H23 | 1568 | 647 | 16597 | 442,4 | 10,6 | 0,924 | SPINK6 |
| 2149 | J20 | J21 | 581 | 644 | 6067 | 349,4 | 10,6 | 0,924 | GLI4 |
| 3853 | A20 | A21 | 1715 | 689,2 | 18102 | 311,5 | 10,6 | 0,939 | PYGB |
| 3879 | B22 | B23 | 1357 | 701,4 | 14357 | 359,3 | 10,6 | 0,939 | TRIM27 |
| 3970 | F18 | F19 | 1156 | 700,2 | 12128 | 382,6 | 10,6 | 0,939 | RLN2 |
| 4188 | O2 | O3 | 724 | 693,9 | 7648 | 419,9 | 10,6 | 0,939 | RBP3 |
| 11799 | L22 | L23 | 1633 | 847,9 | 17385 | 153,8 | 10,6 | 0,944 | HTN1 |
| 5762 | A10 | A11 | 1905 | 901,3 | 20160 | 549,6 | 10,6 | 0,953 | ARG2 |
| 16290 | G3 | G4 | 1062 | 1059 | 11282 | 202,5 | 10,6 | 0,968 | RNF146 |
| 16472 | O16 | O17 | 1041 | 1049 | 10368 | 292,4 | 10,6 | 0,972 | ELOF1 |
| 13561 | F1 | F2 | 1030 | 1043 | 10908 | 171,4 | 10,6 | 0,993 | C9orf5 |
| 13681 | K1 | K2 | 1165 | 955,1 | 12353 | 193,5 | 10,6 | 0,995 | TMED3 |
| 783 | A22 | A23 | 2105 | 833,1 | 22352 | 508,7 | 10,6 | 1,005 | SMAD9 |
| 5462 | D21 | D22 | 1510 | 756,1 | 15975 | 459,9 | 10,6 | 1,007 | NCOA1 |
| 5486 | E21 | E22 | 2312 | 714 | 24570 | 579,8 | 10,6 | 1,007 | DOC2B |
| 5579 | I19 | I20 | 1580 | 718,3 | 16849 | 455,6 | 10,6 | 1,01 | PPFIA1 |
| 5683 | M3 | M4 | 1245 | 683,3 | 13288 | 446,6 | 10,6 | 1,01 | CYP4F2 |
| 5686 | M7 | M8 | 1702 | 786,3 | 18131 | 477,9 | 10,6 | 1,01 | CSDA |
| 3519 | C22 | C23 | 1482 | 1480 | 15772 | 440,2 | 10,6 | 1,019 | FZD3 |
| 3628 | H12 | H13 | 1526 | 1409 | 16240 | 362,2 | 10,6 | 1,019 | GPR123 |
| 3679 | J15 | J16 | 1384 | 1409 | 14750 | 294,9 | 10,6 | 1,019 | HTR2C |
| 3718 | K7 | K8 | 1300 | 1390 | 13847 | 372,3 | 10,6 | 1,019 | DRD4 |
| 3754 | M18 | M19 | 1414 | 1247 | 15007 | 293,3 | 10,6 | 1,019 | NTSR2 |
| 8077 | A20 | A21 | 1718 | 734,9 | 18294 | 381 | 10,6 | 1,019 | HECW1 |
| 8233 | H1 | H2 | 1221 | 673,3 | 12607 | 403,9 | 10,6 | 1,019 | ENPP4 |
| 8317 | K20 | K21 | 2008 | 659,9 | 21220 | 423,3 | 10,6 | 1,019 | AGTPBP1 |
| 8386 | N18 | N19 | 1458 | 616,8 | 15467 | 541,3 | 10,6 | 1,019 | CHST5 |
| 1617 | D17 | D18 | 1532 | 745,7 | 16102 | 452,3 | 10,6 | 1,101 | MBTD1 |
| 1818 | L3 | L4 | 1761 | 753,7 | 18614 | 363,3 | 10,6 | 1,101 | ZNF701 |
| 1846 | M7 | M8 | 1199 | 720,9 | 12711 | 455,7 | 10,6 | 1,101 | WBP5 |
| 1857 | N17 | N18 | 1595 | 791,3 | 16952 | 400,9 | 10,6 | 1,101 | CABP5 |
| 1871 | N8 | N9 | 2047 | 783,6 | 21784 | 385,8 | 10,6 | 1,101 | ZNF587 |
| 1902 | P14 | P15 | 2015 | 749,4 | 21266 | 385,6 | 10,6 | 1,101 | ZNF594 |
| 21551 | B8 | B9 | 210 | 868 | 2220 | 646,3 | 10,6 | 1,107 | LOC652606 |
| 21577 | D1 | D2 | 228 | 980,1 | 2412 | 696,1 | 10,6 | 1,107 | LOC650628 |
| 21600 | D9 | D10 | 401 | 1073 | 4239 | 661,4 | 10,6 | 1,107 | LOC642796 |
| 21748 | K12 | K13 | 404 | 1011 | 4212 | 412,2 | 10,6 | 1,107 | HPX-2 |
| 4638 | B14 | B15 | 1306 | 972,7 | 13895 | 412 | 10,6 | 1,165 | MMP24 |
| 4646 | B21 | B22 | 1763 | 833,9 | 18574 | 472,6 | 10,6 | 1,165 | GZMM |
| 4840 | J2 | J3 | 1536 | 876,2 | 16232 | 366,5 | 10,6 | 1,165 | DHH |
| 4848 | J9 | J10 | 1605 | 792,3 | 17071 | 345,3 | 10,6 | 1,165 | MMP19 |

| | | | | | | | | | | | | | | | | | | | | | | | | | | | | | | | |
|---|---|---|---|---|---|---|---|---|---|---|---|---|---|---|---|---|---|---|---|---|---|---|---|---|---|---|---|---|---|---|---|
| 10407 | B22 | B23 | 1462 | 933,7 | 15439 | 209 | 10,6 | 1,198 | H5A034 C | sample | Hm Drgbl | Hm Drgbl | Hm Drgbl | CPE002EB | CPE002EO | CPE002FR | XM 06189 | XM 0618 | XM 0618 | TRIM64 | TRIM64 | TRIM64 | tripartite motif-co | tripartite | tripartite | 120146 | 120146 | 120146 | s42355 | s42356 | s42357 |
| 10427 | C19 | C20 | 1589 | 893,5 | 16799 | 199 | 10,6 | 1,198 | H5A034 C | sample | Hm Drgbl | Hm Drgbl | Hm Drgbl | CPE002EB | CPE002EO | CPE002FR | NM 03287 | NM 0328 | NM 0328 | RELT | RELT | RELT | RELT tumor necro | RELT tumo | RELT tumo | 84957 | 84957 | 84957 | s39751 | s39752 | s224978 |
| 3051 | P11 | P12 | 666 | 716,1 | 7057 | 656,6 | 10,6 | 1,247 | H5A034 C | sample | Hm Drgbl | Hm Drgbl | Hm Drgbl | CPE002DP | CPE002EF | CPE002F5 | NM 02080 | NM 0208 | NM 0208 | PACSIN1 | PACSIN1 | PACSIN1 | protein kinase C a | protein ki | protein ki | 29993 | 29993 | 29993 | s26862 | s223837 | s26864 |
| 20012 | B5 | B6 | 911 | 1171 | 9645 | 235,3 | 10,6 | 1,293 | H5A034 C | sample | Hm Gen E | Hm Gen E | Hm Gen E | CPE002JQ | CPE002O5 | CPE002KL | NM 20330 | NM 2033 | NM 2033 | HEATR4 | HEATR4 | HEATR4 | HEAT repeat cont | HEAT repe | HEAT repe | 399671 | 399671 | 399671 | s53150 | s53151 | s53152 |
| 20022 | C14 | C15 | 936 | 1184 | 9891 | 142,3 | 10,6 | 1,293 | H5A034 C | sample | Hm Gen E | Hm Gen E | Hm Gen E | CPE002JQ | CPE002O5 | CPE002KL | XM 37926 | XM 3792 | XM 3792 | FLJ16686 | FLJ16686 | FLJ16686 | FLJ16686 protein | FLJ16686 p | FLJ16686 p | 401124 | 401124 | 401124 | s53478 | s53479 | s53480 |
| 20038 | C7 | C8 | 678 | 1112 | 7206 | 175,8 | 10,6 | 1,293 | H5A034 C | sample | Hm Gen E | Hm Gen E | Hm Gen E | CPE002JQ | CPE002O5 | CPE002KL | XM 37273 | XM 3727 | XM 3727 | LOC39093 | LOC39093 | LOC39093 | similar to hCG204 | similar to | similar to | 390937 | 390937 | 390937 | s52893 | s52894 | s52895 |
| 20195 | J19 | J20 | 762 | 1137 | 8108 | 179,2 | 10,6 | 1,293 | H5A034 C | sample | Hm Gen E | Hm Gen E | Hm Gen E | CPE002JQ | CPE002O5 | CPE002KL | XM 92819 | XM 9281 | XM 9281 | LOC40058 | LOC40058 | LOC40058 | GRB2-related ada | GRB2-rela | GRB2-rela | 400581 | 400581 | 400581 | s53309 | s53310 | s226535 |
| 20262 | M14 | M15 | 563 | 1272 | 5994 | 188,6 | 10,6 | 1,293 | H5A034 C | sample | Hm Gen E | Hm Gen E | Hm Gen E | CPE002JQ | CPE002O5 | CPE002KL | NM 20751 | NM 2075 | NM 2075 | FLJ45224 | FLJ45224 | FLJ45224 | FLJ45224 protein | FLJ45224 p | FLJ45224 p | 401562 | 401562 | 401562 | s53648 | s53649 | s53650 |
| 556 | H12 | H13 | 1606 | 1646 | 17034 | 294,9 | 10,6 | 1,547 | H5A034 C | sample | Hm Drgbl | Hm Drgbl | Hm Drgbl | CPE002E0 | CPE002EZ | CPE002FK | NM 00207 | NM 0020 | NM 0020 | GNAQ | GNAQ | GNAQ | guanine nucleotic | guanine n | guanine n | 2776 | 2776 | 2776 | s5888 | s5886 | s5887 |
| 7859 | H19 | H20 | 1510 | 604,4 | 16018 | 412,7 | 10,6 | 1,618 | H5A034 C | sample | Hm Drgbl | Hm Drgbl | Hm Drgbl | CPE002E4 | CPE002EV | CPE002FG | NM 00611 | NM 0061 | NM 0061 | MAP3K7IP | MAP3K7IP | MAP3K7IP | mitogen-activated | mitogen-a | mitogen-a | 10454 | 10454 | 10454 | s20451 | s20452 | s20453 |
| 7871 | H8 | H9 | 1690 | 558,7 | 17886 | 402,1 | 10,6 | 1,618 | H5A034 C | sample | Hm Drgbl | Hm Drgbl | Hm Drgbl | CPE002E4 | CPE002EV | CPE002FG | NM 00679 | NM 0067 | NM 0067 | EHD1 | EHD1 | EHD1 | EH-domain conta | EH-domai | EH-domai | 10938 | 10938 | 10938 | s21514 | s21515 | s21513 |
| 7877 | I13 | I14 | 589 | 537,5 | 6229 | 313,7 | 10,6 | 1,618 | H5A034 C | sample | Hm Drgbl | Hm Drgbl | Hm Drgbl | CPE002E4 | CPE002EV | CPE002FG | NM 00584 | NM 0058 | NM 0058 | SPRY3 | SPRY3 | SPRY3 | sprouty homolog | sprouty h | sprouty h | 10251 | 10251 | 10251 | s20023 | s20021 | s20022 |
| 3085 | A20 | A21 | 1553 | 1288 | 16646 | 348,1 | 10,7 | 0,663 | H5A034 C | sample | Hm Drgbl | Hm Drgbl | Hm Drgbl | CPE002DC | CPE002EG | CPE002F6 | NM 01636 | NM 0163 | NM 0163 | DUSP13 | DUSP13 | DUSP13 | dual specificity ph | dual speci | dual speci | 51207 | 51207 | 51207 | s27685 | s27686 | s27687 |
| 3277 | I20 | I21 | 1036 | 1179 | 11095 | 269,1 | 10,7 | 0,663 | H5A034 C | sample | Hm Drgbl | Hm Drgbl | Hm Drgbl | CPE002DC | CPE002EG | CPE002F6 | NM 00103 | NM 0010 | NM 0010 | ACPL2 | ACPL2 | ACPL2 | acid phosphatase | acid phos | acid phos | 92370 | 92370 | 92370 | s40941 | s40942 | s40943 |
| 6444 | M2 | M3 | 1039 | 525,6 | 11128 | 407,2 | 10,7 | 0,706 | H5A034 C | sample | Hm Drgbl | Hm Drgbl | Hm Drgbl | CPE002DZ | CPE002F0 | CPE002FL | NM 02108 | NM 0210 | NM 0210 | DAB1 | DAB1 | DAB1 | disabled homolog | disabled h | disabled h | 1600 | 1600 | 1600 | s3894 | s3893 | s3895 |
| 6493 | O20 | O21 | 2250 | 684,6 | 24158 | 377,3 | 10,7 | 0,706 | H5A034 C | sample | Hm Drgbl | Hm Drgbl | Hm Drgbl | CPE002DZ | CPE002F0 | CPE002FL | NM 20584 | NM 0013 | NM 0013 | AKR1C2 | AKR1C2 | AKR1C2 | aldo-keto reducta | aldo-keto | aldo-keto | 1646 | 1646 | 1646 | s3991 | s223393 | s194378 |
| 9505 | M1 | M2 | 1990 | 1223 | 21219 | 123,6 | 10,7 | 0,743 | H5A034 C | sample | Hm Drgbl | Hm Drgbl | Hm Drgbl | CPE002E8 | CPE002ER | CPE002FC | NM 01819 | NM 0181 | NM 0181 | RCBTB1 | RCBTB1 | RCBTB1 | regulator of chror | regulator | regulator | 55213 | 55213 | 55213 | s30455 | s30456 | s224351 |
| 9516 | M2 | M3 | 2051 | 1141 | 21863 | 109,6 | 10,7 | 0,743 | H5A034 C | sample | Hm Drgbl | Hm Drgbl | Hm Drgbl | CPE002E8 | CPE002ER | CPE002FC | NM 01911 | NM 0191 | NM 0191 | PCDHB9 | PCDHB9 | PCDHB9 | protocadherin be | protocadh | protocadh | 56127 | 56127 | 56127 | s31949 | s31950 | s31951 |
| 14473 | L1 | L2 | 1574 | 836,5 | 16901 | 132,3 | 10,7 | 0,762 | H5A034 C | sample | Hm Gen E | Hm Gen E | Hm Gen E | CPE002J7 | CPE002NC | CPE002K6 | NM 14499 | NM 1449 | NM 1449 | C21orf29 | C21orf29 | C21orf29 | chromosome 21 c | chromoso | chromoso | 54084 | 54084 | 54084 | s28854 | s28855 | s28856 |
| 11294 | G21 | G22 | 1247 | 802,8 | 13374 | 150,5 | 10,7 | 0,775 | H5A034 C | sample | Hm Drgbl | Hm Drgbl | Hm Drgbl | CPE002ED | CPE002F3 | CPE002FT | NM 17231 | NM 1723 | NM 1723 | SALF | SALF | SALF | stoned B/TFIIA-al | stoned B/ | stoned B/ | 286749 | 286749 | 286749 | s50197 | s50198 | s196005 |
| 7353 | C17 | C18 | 926 | 549,5 | 9923 | 390,9 | 10,7 | 0,817 | H5A034 C | sample | Hm Drgbl | Hm Drgbl | Hm Drgbl | CPE002E3 | CPE002EW | CPE002FH | NM 00103 | NM 0010 | NM 0010 | OAS2 | OAS2 | OAS2 | 2-5-oligoadenylat | 2-5-oligoa | 2-5-oligoa | 4939 | 4939 | 4939 | s9794 | s9796 | s9795 |
| 17209 | N1 | N2 | 1645 | 996,7 | 17672 | 123,8 | 10,7 | 0,826 | H5A034 C | sample | Hm Gen E | Hm Gen E | Hm Gen E | CPE002JI | CPE002NZ | CPE002KD | NM 13878 | NM 1815 | NM 1387 | C6orf72 | KRT25 | C6orf72 | chromosome 6 or | keratin 25 | chromoso | 116254 | 147183 | 116254 | s41978 | s44975 | s41980 |
| 7258 | O18 | O19 | 1190 | 577,9 | 12790 | 541,7 | 10,7 | 0,833 | H5A034 C | sample | Hm Drgbl | Hm Drgbl | Hm Drgbl | CPE002E2 | CPE002EX | CPE002FI | NM 00454 | NM 0045 | NM 0045 | NDUFA1 | NDUFA1 | NDUFA1 | NADH dehydroger | NADH deh | NADH deh | 4694 | 4694 | 4694 | s9323 | s9321 | s9322 |
| 5011 | A4 | A5 | 1613 | 684,4 | 17298 | 621,1 | 10,7 | 0,839 | H5A034 C | sample | Hm Drgbl | Hm Drgbl | Hm Drgbl | CPE002DV | CPE002EM | CPE002FO | NM 00542 | NM 0054 | NM 0054 | TNP2 | TNP2 | TNP2 | transition proteir | transition | transition | 7142 | 7142 | 7142 | s14293 | s14294 | s14295 |
| 5103 | E22 | E23 | 1752 | 644,8 | 18727 | 557,4 | 10,7 | 0,839 | H5A034 C | sample | Hm Drgbl | Hm Drgbl | Hm Drgbl | CPE002DV | CPE002EM | CPE002FO | NM 00036 | NM 0003 | NM 0003 | TSHR | TSHR | TSHR | thyroid stimulatir | thyroid st | thyroid st | 7253 | 7253 | 7253 | s14445 | s14446 | s14447 |
| 2355 | C11 | C12 | 1644 | 750,5 | 17605 | 440,7 | 10,7 | 0,843 | H5A034 C | sample | Hm Drgbl | Hm Drgbl | Hm Drgbl | CPE002DC | CPE002EE | CPE002F4 | NM 00131 | NM 0013 | NM 0013 | CSNK1G2 | CSNK1G2 | CSNK1G2 | casein kinase 1, g | casein kin | casein kin | 1455 | 1455 | 1455 | s3630 | s3631 | s3632 |
| 2389 | D20 | D21 | 1693 | 735,2 | 18178 | 387,3 | 10,7 | 0,843 | H5A034 C | sample | Hm Drgbl | Hm Drgbl | Hm Drgbl | CPE002DC | CPE002EE | CPE002F4 | NM 00274 | NM 0027 | NM 0027 | MAPK6 | MAPK6 | MAPK6 | mitogen-activated | mitogen-a | mitogen-a | 5597 | 5597 | 5597 | s11146 | s11147 | s11148 |
| 2424 | E9 | E10 | 671 | 703,4 | 7187 | 468,3 | 10,7 | 0,843 | H5A034 C | sample | Hm Drgbl | Hm Drgbl | Hm Drgbl | CPE002DC | CPE002EE | CPE002F4 | NM 00662 | NM 0066 | NM 0066 | PLK2 | PLK2 | PLK2 | polo-like kinase 2 | polo-like l | polo-like l | 10769 | 10769 | 10769 | s64 | s65 | s66 |
| 2484 | H2 | H3 | 1325 | 683,4 | 14237 | 374,6 | 10,7 | 0,843 | H5A034 C | sample | Hm Drgbl | Hm Drgbl | Hm Drgbl | CPE002DC | CPE002EE | CPE002F4 | NM 00275 | NM 0027 | NM 0027 | EIF2AK2 | EIF2AK2 | EIF2AK2 | eukaryotic transla | eukaryotic | eukaryotic | 5610 | 5610 | 5610 | s11185 | s11186 | s11187 |
| 2526 | J14 | J15 | 1086 | 680,2 | 11593 | 262,8 | 10,7 | 0,843 | H5A034 C | sample | Hm Drgbl | Hm Drgbl | Hm Drgbl | CPE002DC | CPE002EE | CPE002F4 | NM 02113 | NM 0211 | NM 0211 | RNASEL | RNASEL | RNASEL | ribonuclease L (2, | ribonuclea | ribonuclea | 6041 | 6041 | 6041 | s12064 | s12065 | s12066 |
| 2563 | K4 | K5 | 1040 | 670,3 | 11132 | 365,5 | 10,7 | 0,843 | H5A034 C | sample | Hm Drgbl | Hm Drgbl | Hm Drgbl | CPE002DC | CPE002EE | CPE002F4 | NM 00620 | NM 0062 | NM 0062 | PDGFRA | PDGFRA | PDGFRA | platelet-derived g | platelet-d | platelet-d | 5156 | 5156 | 5156 | s10234 | s10235 | s10236 |
| 2600 | M16 | M17 | 1889 | 762,4 | 20297 | 379,7 | 10,7 | 0,843 | H5A034 C | sample | Hm Drgbl | Hm Drgbl | Hm Drgbl | CPE002DC | CPE002EE | CPE002F4 | NM 00264 | NM 0026 | NM 0026 | PIK3C3 | PIK3C3 | PIK3C3 | phosphoinositide | phosphoir | phosphoir | 5289 | 5289 | 5289 | s10517 | s10518 | s10519 |
| 17995 | N4 | N5 | 1311 | 1041 | 14060 | 127,4 | 10,7 | 0,846 | H5A034 C | sample | Hm Gen E | Hm Gen E | Hm Gen E | CPE002JK | CPE002NZ | CPE002KF | NM 00100 | NM 0010 | NM 0010 | SH3D19 | SH3D19 | SH3D19 | SH3 domain conta | SH3 doma | SH3 doma | 152503 | 152503 | 152503 | s45697 | s45698 | s45699 |
| 8983 | G15 | G16 | 2251 | 1884 | 24067 | 141,4 | 10,7 | 0,859 | H5A034 C | sample | Hm Drgbl | Hm Drgbl | Hm Drgbl | CPE002E7 | CPE002ES | CPE002FD | NM 00107 | NM 0010 | NM 0010 | PDE11A | PDE11A | PDE11A | phosphodiesteras | phosphod | phosphod | 50940 | 50940 | 50940 | s27187 | s27188 | s27189 |
| 9121 | M1 | M2 | 2171 | 1856 | 23292 | 129,3 | 10,7 | 0,859 | H5A034 C | sample | Hm Drgbl | Hm Drgbl | Hm Drgbl | CPE002E7 | CPE002ES | CPE002FD | NM 01597 | NM 0159 | NM 0159 | CRYL1 | CRYL1 | CRYL1 | crystallin, lambda | crystallin, | crystallin, | 51084 | 51084 | 51084 | s27384 | s224184 | s27383 |
| 4243 | A4 | A5 | 1047 | 657,9 | 11237 | 308,5 | 10,7 | 0,903 | H5A034 C | sample | Hm Drgbl | Hm Drgbl | Hm Drgbl | CPE002DL | CPE002EK | CPE002FQ | NM 00651 | NM 0065 | NM 0065 | SCN10A | SCN10A | SCN10A | sodium channel, v | sodium ch | sodium ch | 6336 | 6336 | 6336 | s12540 | s12541 | s12542 |
| 4279 | C15 | C16 | 1746 | 728,6 | 18716 | 369 | 10,7 | 0,903 | H5A034 C | sample | Hm Drgbl | Hm Drgbl | Hm Drgbl | CPE002DL | CPE002EK | CPE002FQ | NM 17495 | NM 1749 | NM 1749 | ATP2A3 | ATP2A3 | ATP2A3 | ATPase, Ca++ trar | ATPase, C | ATPase, C | 489 | 489 | 489 | s1749 | s1750 | s1751 |
| 4311 | D22 | D23 | 1317 | 713,7 | 14065 | 364,9 | 10,7 | 0,903 | H5A034 C | sample | Hm Drgbl | Hm Drgbl | Hm Drgbl | CPE002DL | CPE002EK | CPE002FQ | NM 03146 | NM 0314 | NM 0314 | KCNK17 | KCNK17 | KCNK17 | potassium chann | potassium | potassium | 89822 | 89822 | 89822 | s40138 | s40139 | s40140 |
| 4446 | J14 | J15 | 1142 | 641,7 | 12245 | 315,2 | 10,7 | 0,903 | H5A034 C | sample | Hm Drgbl | Hm Drgbl | Hm Drgbl | CPE002DL | CPE002EK | CPE002FQ | NM 17801 | NM 1780 | NM 1780 | CATSPER3 | CATSPER3 | CATSPER3 | cation channel, s | cation cha | cation cha | 347732 | 347732 | 347732 | s51289 | s51290 | s51291 |
| 4455 | J22 | J23 | 1363 | 719,7 | 14549 | 388,9 | 10,7 | 0,903 | H5A034 C | sample | Hm Drgbl | Hm Drgbl | Hm Drgbl | CPE002DL | CPE002EK | CPE002FQ | NM 01228 | NM 0122 | NM 0122 | KCNG2 | KCNG2 | KCNG2 | potassium voltag | potassium | potassium | 26251 | 26251 | 26251 | s195125 | s195126 | s195127 |
| 4482 | K3 | K4 | 931 | 691,8 | 9945 | 370,6 | 10,7 | 0,903 | H5A034 C | sample | Hm Drgbl | Hm Drgbl | Hm Drgbl | CPE002DL | CPE002EK | CPE002FQ | NM 00008 | NM 0000 | NM 0000 | CLCN1 | CLCN1 | CLCN1 | chloride channel | chloride c | chloride c | 1180 | 1180 | 1180 | s3129 | s3130 | s3131 |
| 4539 | N11 | N12 | 934 | 630 | 9980 | 386 | 10,7 | 0,903 | H5A034 C | sample | Hm Drgbl | Hm Drgbl | Hm Drgbl | CPE002DL | CPE002EK | CPE002FQ | NM 00256 | NM 0025 | NM 0025 | P2RX4 | P2RX4 | P2RX4 | purinergic recept | purinergic | purinergic | 5025 | 5025 | 5025 | s9955 | s9956 | s9957 |
| 221 | J13 | J14 | 802 | 1639 | 8619 | 467,9 | 10,7 | 0,911 | H5A034 C | sample | Hm Drgbl | Hm Drgbl | Hm Drgbl | CPE002DT | CPE002EJ | CPE002F9 | NM 00417 | NM 0041 | NM 0041 | SLC1A2 | SLC1A2 | SLC1A2 | solute carrier fam | solute car | solute car | 6506 | 6506 | 6506 | s224637 | s12905 | s12906 |
| 247 | K15 | K16 | 515 | 1496 | 5510 | 436,7 | 10,7 | 0,911 | H5A034 C | sample | Hm Drgbl | Hm Drgbl | Hm Drgbl | CPE002DT | CPE002EJ | CPE002F9 | NM 19929 | NM 1992 | NM 1992 | TH | TH | TH | tyrosine hydroxyl | tyrosine h | tyrosine h | 7054 | 7054 | 7054 | s14094 | s14093 | s14092 |
| 271 | L15 | L16 | 559 | 1440 | 5979 | 468,7 | 10,7 | 0,911 | H5A034 C | sample | Hm Drgbl | Hm Drgbl | Hm Drgbl | CPE002DT | CPE002EJ | CPE002F9 | NM 00034 | NM 0003 | NM 0003 | SLC3A1 | SLC3A1 | SLC3A1 | solute carrier fam | solute car | solute car | 6519 | 6519 | 6519 | s12940 | s12941 | s12942 |
| 277 | L20 | L21 | 592 | 1496 | 6361 | 457,1 | 10,7 | 0,911 | H5A034 C | sample | Hm Drgbl | Hm Drgbl | Hm Drgbl | CPE002DT | CPE002EJ | CPE002F9 | NM 17753 | NM 1775 | NM 1775 | SULT1A1 | SULT1A1 | SULT1A1 | sulfotransferase f | sulfotrans | sulfotrans | 6817 | 6817 | 6817 | s13613 | s13614 | s13615 |
| 319 | N15 | N16 | 519 | 1301 | 5539 | 561,3 | 10,7 | 0,911 | H5A034 C | sample | Hm Drgbl | Hm Drgbl | Hm Drgbl | CPE002DT | CPE002EJ | CPE002F9 | NM 00104 | NM 0010 | NM 0010 | SLC6A3 | SLC6A3 | SLC6A3 | solute carrier fam | solute car | solute car | 6531 | 6531 | 6531 | s12977 | s12978 | s12976 |
| 1941 | A6 | A7 | 1292 | 752,5 | 13772 | 534,7 | 10,7 | 0,93 | H5A034 C | sample | Hm Drgbl | Hm Drgbl | Hm Drgbl | CPE002FX | CPE002G1 | CPE002G5 | NM 17353 | NM 1735 | NM 1735 | ZNF100 | ZNF100 | ZNF100 | zinc finger proteir | zinc finger | zinc finger | 163227 | 163227 | 163227 | s46441 | s46442 | s46443 |
| 2072 | G16 | G17 | 1901 | 645,2 | 20345 | 361,7 | 10,7 | 0,93 | H5A034 C | sample | Hm Drgbl | Hm Drgbl | Hm Drgbl | CPE002FX | CPE002G1 | CPE002G5 | NM 15275 | NM 1527 | NM 1527 | SCUBE3 | SCUBE3 | SCUBE3 | signal peptide, CU | signal pep | signal pep | 222663 | 222663 | 222663 | s48237 | s48238 | s48239 |
| 2086 | G7 | G8 | 515 | 786,2 | 5514 | 571,8 | 10,7 | 0,93 | H5A034 C | sample | Hm Drgbl | Hm Drgbl | Hm Drgbl | CPE002FX | CPE002G1 | CPE002G5 | NM 08073 | NM 0807 | NM 0807 | SYTL4 | SYTL4 | SYTL4 | synaptotagmin-lil | synaptota | synaptota | 94121 | 94121 | 94121 | s41272 | s41273 | s41274 |
| 3959 | E8 | E9 | 1115 | 754,3 | 11911 | 412,9 | 10,7 | 0,951 | H5A034 C | sample | Hm Drgbl | Hm Drgbl | Hm Drgbl | CPE002DS | CPE002EI | CPE002F8 | NM 18323 | NM 1832 | NM 1832 | RAB27A | RAB27A | RAB27A | RAB27A, member | RAB27A, r | RAB27A, r | 5873 | 5873 | 5873 | s11695 | s11693 | s11694 |
| 3972 | F2 | F3 | 1294 | 731,7 | 13813 | 277,7 | 10,7 | 0,951 | H5A034 C | sample | Hm Drgbl | Hm Drgbl | Hm Drgbl | CPE002DS | CPE002EI | CPE002F8 | NM 00032 | NM 0003 | NM 0003 | RHAG | RHAG | RHAG | Rh-associated gly | Rh-associa | Rh-associa | 6005 | 6005 | 6005 | s12010 | s12011 | s12012 |
| 4035 | I11 | I12 | 952 | 814 | 10145 | 445,3 | 10,7 | 0,951 | H5A034 C | sample | Hm Drgbl | Hm Drgbl | Hm Drgbl | CPE002DS | CPE002EI | CPE002F8 | NM 00268 | NM 0026 | NM 0026 | PNN | PNN | PNN | pinin, desmosom | pinin, des | pinin, des | 5411 | 5411 | 5411 | s10757 | s10758 | s10759 |
| 5990 | J21 | J22 | 1591 | 944,5 | 17051 | 358,8 | 10,7 | 0,977 | H5A034 C | sample | Hm Drgbl | Hm Drgbl | Hm Drgbl | CPE002DY | CPE002F1 | CPE002FM | NM 00116 | NM 0011 | NM 0011 | BIRC2 | BIRC2 | BIRC2 | baculoviral IAP re | baculovira | baculovira | 329 | 329 | 329 | s1450 | s1449 | s1448 |
| 6012 | K2 | K3 | 947 | 857 | 10121 | 495,8 | 10,7 | 0,977 | H5A034 C | sample | Hm Drgbl | Hm Drgbl | Hm Drgbl | CPE002DY | CPE002F1 | CPE002FM | NM 00168 | NM 0016 | NM 0016 | FXYD2 | FXYD2 | FXYD2 | FXYD domain con | FXYD dom | FXYD dom | 486 | 486 | 486 | s224129 | s194300 | s194301 |
| 1255 | E15 | E16 | 1605 | 789,8 | 17177 | 371,8 | 10,7 | 0,982 | H5A034 C | sample | Hm Drgbl | Hm Drgbl | Hm Drgbl | CPE002FV | CPE002FZ | CPE002G3 | NM 00697 | NM 0069 | NM 0069 | ZNF33A | ZNF33A | ZNF33A | zinc finger proteir | zinc finger | zinc finger | 7581 | 7581 | 7581 | s15072 | s15073 | s15074 |
| 1357 | I20 | I21 | 2024 | 748,5 | 21561 | 324,1 | 10,7 | 0,982 | H5A034 C | sample | Hm Drgbl | Hm Drgbl | Hm Drgbl | CPE002FV | CPE002FZ | CPE002G3 | NM 00582 | NM 0058 | NM 0058 | MSLN | MSLN | MSLN | mesothelin | mesotheli | mesotheli | 10232 | 10232 | 10232 | s19967 | s19968 | s223096 |
| 16310 | H21 | H22 | 1897 | 1027 | 20316 | 309,7 | 10,7 | 0,982 | H5A034 C | sample | Hm Gen E | Hm Gen E | Hm Gen E | CPE002JG | CPE002NV | CPE002KB | NM 03204 | NM 0320 | NM 0320 | REG4 | REG4 | REG4 | regenerating islet | regenerat | regenerat | 83998 | 83998 | 83998 | s38390 | s38391 | s38392 |
| 16417 | M1 | M2 | 1477 | 1063 | 15876 | 313,9 | 10,7 | 0,982 | H5A034 C | sample | Hm Gen E | Hm Gen E | Hm Gen E | CPE002JG | CPE002NV | CPE002KB | NM 03143 | NM 0314 | NM 0314 | NUDT12 | NUDT12 | NUDT12 | nudix (nucleoside | nudix (nuc | nudix (nuc | 83594 | 83594 | 83594 | s38024 | s38025 | s38026 |
| 16453 | N20 | N21 | 1698 | 975,9 | 18183 | 260,4 | 10,7 | 0,982 | H5A034 C | sample | Hm Gen E | Hm Gen E | Hm Gen E | CPE002JG | CPE002NV | CPE002KB | NM 00103 | NM 0010 | NM 0010 | MGC1296 | MGC1296 | MGC1296 | hypothetical prot | hypotheti | hypotheti | 84792 | 84792 | 84792 | s39436 | s39437 | s228389 |
| 13476 | B2 | B3 | 1356 | 1159 | 14467 | 144,6 | 10,7 | 1,005 | H5A034 C | sample | Hm Gen E | Hm Gen E | Hm Gen E | CPE002JC | CPE002NC | CPE002K4 | NM 01566 | NM 0156 | NM 0156 | GIMAP2 | GIMAP2 | GIMAP2 | GTPase, IMAP fan | GTPase, IM | GTPase, IM | 26157 | 26157 | 26157 | s25190 | s25191 | s25192 |
| 1062 | M14 | M15 | 1339 | 729,5 | 14273 | 411,6 | 10,7 | 1,014 | H5A034 C | sample | Hm Drgbl | Hm Drgbl | Hm Drgbl | CPE002FU | CPE002FY | CPE002G2 | NM 00269 | NM 0026 | NM 0026 | POU3F1 | POU3F1 | POU3F1 | POU domain, clas | POU doma | POU doma | 5453 | 5453 | 5453 | s10853 | s10854 | s10855 |
| 1100 | N5 | N6 | 1556 | 680,5 | 16706 | 365,3 | 10,7 | 1,014 | H5A034 C | sample | Hm Drgbl | Hm Drgbl | Hm Drgbl | CPE002FU | CPE002FY | CPE002G2 | NM 00216 | NM 0021 | NM 0021 | ID1 | ID1 | ID1 | inhibitor of DNA b | inhibitor c | inhibitor c | 3397 | 3397 | 3397 | s7104 | s7105 | s7106 |
| 1103 | N8 | N9 | 1537 | 721,4 | 16422 | 391,6 | 10,7 | 1,014 | H5A034 C | sample | Hm Drgbl | Hm Drgbl | Hm Drgbl | CPE002FU | CPE002FY | CPE002G2 | NM 00507 | NM 0050 | NM 0050 | TLE3 | TLE3 | TLE3 | transducin-like er | transducir | transducir | 7090 | 7090 | 7090 | s14173 | s14174 | s224733 |
| 5414 | B21 | B22 | 2402 | 792,5 | 25658 | 627 | 10,7 | 1,019 | H5A034 C | sample | Hm Drgbl | Hm Drgbl | Hm Drgbl | CPE002DX | CPE002F2 | CPE002FN | NM 00372 | NM 0037 | NM 0037 | JRK | JRK | JRK | jerky homolog (m | jerky hom | jerky hom | 8629 | 8629 | 8629 | s16414 | s16416 | s16415 |
| 5437 | C20 | C21 | 581 | 710,9 | 6208 | 431 | 10,7 | 1,019 | H5A034 C | sample | Hm Drgbl | Hm Drgbl | Hm Drgbl | CPE002DX | CPE002F2 | CPE002FN | NM 00388 | NM 0038 | NM 0038 | CDK5R1 | CDK5R1 | CDK5R1 | cyclin-dependent | cyclin-dep | cyclin-dep | 8851 | 8851 | 8851 | s225045 | s16898 | s16899 |
| 5581 | I20 | I21 | 1763 | 703,7 | 18890 | 419,6 | 10,7 | 1,019 | H5A034 C | sample | Hm Drgbl | Hm Drgbl | Hm Drgbl | CPE002DX | CPE002F2 | CPE002FN | NM 00392 | NM 0039 | NM 0039 | RAB7L1 | RAB7L1 | RAB7L1 | RAB7, member RA | RAB7, me | RAB7, me | 8934 | 8934 | 8934 | s17084 | s17082 | s17083 |
| 5582 | I21 | I22 | 2067 | 796,5 | 22146 | 535,3 | 10,7 | 1,019 | H5A034 C | sample | Hm Drgbl | Hm Drgbl | Hm Drgbl | CPE002DX | CPE002F2 | CPE002FN | NM 00100 | NM 0010 | NM 0010 | PKP4 | PKP4 | PKP4 | plakophilin 4 | plakophili | plakophili | 8502 | 8502 | 8502 | s16148 | s16149 | s16150 |
| 5636 | K5 | K6 | 1398 | 635,8 | 14949 | 554,7 | 10,7 | 1,019 | H5A034 C | sample | Hm Drgbl | Hm Drgbl | Hm Drgbl | CPE002DX | CPE002F2 | CPE002FN | NM 00363 | NM 0036 | NM 0036 | CNTNAP1 | CNTNAP1 | CNTNAP1 | contactin associat | contactin | contactin | 8506 | 8506 | 8506 | s16160 | s16161 | s16162 |
| 5711 | N8 | N9 | 1851 | 671,7 | 19855 | 562,9 | 10,7 | 1,019 | H5A034 C | sample | Hm Drgbl | Hm Drgbl | Hm Drgbl | CPE002DX | CPE002F2 | CPE002FN | NM 00424 | NM 0042 | NM 0042 | HMGN3 | HMGN3 | HMGN3 | high mobility grou | high mobi | high mobi | 9324 | 9324 | 9324 | s17818 | s17819 | s17817 |
| 5736 | O9 | O10 | 1894 | 730,3 | 20321 | 666,8 | 10,7 | 1,019 | H5A034 C | sample | Hm Drgbl | Hm Drgbl | Hm Drgbl | CPE002DX | CPE002F2 | CPE002FN | NM 00368 | NM 0036 | NM 0036 | YARS | YARS | YARS | tyrosyl-tRNA synt | tyrosyl-tR | tyrosyl-tR | 8565 | 8565 | 8565 | s443 | s444 | s442 |
| 5751 | P22 | P23 | 1671 | 696,8 | 17862 | 618,3 | 10,7 | 1,019 | H5A034 C | sample | Hm Drgbl | Hm Drgbl | Hm Drgbl | CPE002DX | CPE002F2 | CPE002FN | NM 00424 | NM 0042 | NM 0042 | RAB28 | RAB28 | RAB28 | RAB28, member R | RAB28, m | RAB28, m | 9364 | 9364 | 9364 | s17910 | s17911 | s17912 |
| 3504 | B9 | B10 | 987 | 1361 | 10542 | 343,5 | 10,7 | 1,029 | H5A034 C | sample | Hm Drgbl | Hm Drgbl | Hm Drgbl | CPE002DR | CPE002EH | CPE002F7 | NM 00528 | NM 0052 | NM 0052 | NPBWR1 | NPBWR1 | NPBWR1 | neuropeptides B/ | neuropep | neuropep | 2831 | 2831 | 2831 | s6010 | s223772 | s194452 |
| 3506 | C10 | C11 | 1328 | 1232 | 14170 | 333,8 | 10,7 | 1,029 | H5A034 C | sample | Hm Drgbl | Hm Drgbl | Hm Drgbl | CPE002DR | CPE002EH | CPE002F7 | NM 00106 | NM 0010 | NM 0010 | TBXA2R | TBXA2R | TBXA2R | thromboxane A2 | thrombox | thrombox | 6915 | 6915 | 6915 | s13841 | s13842 | s13843 |
| 3611 | G19 | G20 | 1607 | 1402 | 17272 | 317,7 | 10,7 | 1,029 | H5A034 C | sample | Hm Drgbl | Hm Drgbl | Hm Drgbl | CPE002DR | CPE002EH | CPE002F7 | NM 17832 | NM 1783 | NM 1783 | CCR3 | CCR3 | CCR3 | chemokine (C-C m | chemokin | chemokin | 1232 | 1232 | 1232 | s3203 | s3204 | s3205 |
| 3619 | G4 | G5 | 1235 | 1234 | 13224 | 354,1 | 10,7 | 1,029 | H5A034 C | sample | Hm Drgbl | Hm Drgbl | Hm Drgbl | CPE002DR | CPE002EH | CPE002F7 | NM 00396 | NM 0039 | NM 0039 | TAAR5 | TAAR5 | TAAR5 | trace amine assoc | trace amir | trace amir | 9038 | 9038 | 9038 | s17223 | s17224 | s17225 |
| 3632 | H16 | H17 | 1223 | 1336 | 13112 | 299,6 | 10,7 | 1,029 | H5A034 C | sample | Hm Drgbl | Hm Drgbl | Hm Drgbl | CPE002DR | CPE002EH | CPE002F7 | NM 03255 | NM 0325 | NM 0325 | KISS1R | KISS1R | KISS1R | KISS1 receptor | KISS1 rece | KISS1 rece | 84634 | 84634 | 84634 | s39221 | s39222 | s39223 |
| 3663 | I22 | I23 | 1259 | 1350 | 13482 | 403,8 | 10,7 | 1,029 | H5A034 C | sample | Hm Drgbl | Hm Drgbl | Hm Drgbl | CPE002DR | CPE002EH | CPE002F7 | NM 00656 | NM 0065 | NM 0065 | CXCR6 | CXCR6 | CXCR6 | chemokine (C-X-C | chemokin | chemokin | 10663 | 10663 | 10663 | s20963 | s20964 | s20965 |
| 3685 | J20 | J21 | 1172 | 1345 | 12596 | 290,2 | 10,7 | 1,029 | H5A034 C | sample | Hm Drgbl | Hm Drgbl | Hm Drgbl | CPE002DR | CPE002EH | CPE002F7 | NM 08086 | NM 0808 | NM 0808 | GPR62 | GPR62 | GPR62 | G protein-couplec | G protein- | G protein- | 118442 | 118442 | 118442 | s42204 | s42205 | s42206 |
| 3762 | M3 | M4 | 1177 | 1293 | 12552 | 313,9 | 10,7 | 1,029 | H5A034 C | sample | Hm Drgbl | Hm Drgbl | Hm Drgbl | CPE002DR | CPE002EH | CPE002F7 | NM 00140 | NM 0014 | NM 0014 | CELSR2 | CELSR2 | CELSR2 | cadherin, EGF LAG | cadherin, | cadherin, | 1952 | 1952 | 1952 | s4525 | s4526 | s4527 |
| 8074 | A18 | A19 | 1437 | 699,4 | 15444 | 414,1 | 10,7 | 1,029 | H5A034 C | sample | Hm Drgbl | Hm Drgbl | Hm Drgbl | CPE002E5 | CPE002EU | CPE002FF | NM 01505 | NM 0150 | NM 0150 | TXNDC4 | TXNDC4 | TXNDC4 | thioredoxin doma | thioredox | thioredox | 23071 | 23071 | 23071 | s22967 | s22966 | s22965 |
| 8085 | A6 | A7 | 1535 | 680 | 16365 | 462,7 | 10,7 | 1,029 | H5A034 C | sample | Hm Drgbl | Hm Drgbl | Hm Drgbl | CPE002E5 | CPE002EU | CPE002FF | XM 29054 | XM 2905 | XM 2905 | ENDOD1 | ENDOD1 | ENDOD1 | endonuclease dor | endonucle | endonucle | 23052 | 23052 | 23052 | s22923 | s22924 | s22925 |
| 8378 | N10 | N11 | 1478 | 664,4 | 15835 | 396 | 10,7 | 1,029 | H5A034 C | sample | Hm Drgbl | Hm Drgbl | Hm Drgbl | CPE002E5 | CPE002EU | CPE002FF | NM 01431 | NM 0143 | NM 0143 | RASD2 | RASD2 | RASD2 | RASD family, men | RASD fam | RASD fam | 23551 | 23551 | 23551 | s24072 | s24073 | s24071 |
| 8430 | P14 | P15 | 1961 | 699,7 | 20919 | 491,1 | 10,7 | 1,029 | H5A034 C | sample | Hm Drgbl | Hm Drgbl | Hm Drgbl | CPE002E5 | CPE002EU | CPE002FF | NM 01432 | NM 0143 | NM 0143 | ORC6L | ORC6L | ORC6L | origin recognition | origin reco | origin reco | 23594 | 23594 | 23594 | s24164 | s24165 | s24166 |
| 10249 | L1 | L2 | 1108 | 866,6 | 11843 | 148,5 | 10,7 | 1,029 | H5A034 C | sample | Hm Drgbl | Hm Drgbl | Hm Drgbl | CPE002EA | CPE002EP | CPE002FA | NM 14521 | NM 1452 | NM 1452 | TRIM11 | TRIM11 | TRIM11 | tripartite motif-co | tripartite | tripartite | 81559 | 81559 | 81559 | s37632 | s37633 | s37634 |
| 1573 | B20 | B21 | 1667 | 761,1 | 17920 | 381,2 | 10,7 | 1,12 | H5A034 C | sample | Hm Drgbl | Hm Drgbl | Hm Drgbl | CPE002FW | CPE002G0 | CPE002G4 | NM 02478 | NM 0247 | NM 0247 | ZBTB3 | ZBTB3 | ZBTB3 | zinc finger and BT | zinc finger | zinc finger | 79842 | 79842 | 79842 | s36462 | s36463 | s36464 |
| 1861 | N20 | N21 | 1026 | 724,8 | 10967 | 432,4 | 10,7 | 1,12 | H5A034 C | sample | Hm Drgbl | Hm Drgbl | Hm Drgbl | CPE002FW | CPE002G0 | CPE002G4 | NM 03267 | NM 0326 | NM 0326 | PCGF1 | PCGF1 | PCGF1 | polycomb group r | polycomb | polycomb | 84759 | 84759 | 84759 | s39408 | s39409 | s39410 |
| 21630 | F14 | F15 | 464 | 1007 | 4987 | 641,9 | 10,7 | 1,126 | H5A034 C | sample | Hm Gen E | Hm Gen E | Hm Gen E | CPE002JU | CPE002O9 | CPE002KP | XM 92703 | XM 9270 | XM 9270 | LOC65334 | LOC65334 | LOC65334 | similar to ADAMT | similar to | similar to | 653348 | 653348 | 653348 | s198048 | s198049 | s198050 |
| 21672 | G9 | G10 | 88 | 1154 | 945 | 958,6 | 10,7 | 1,126 | H5A034 C | sample | Hm Gen E | Hm Gen E | Hm Gen E | CPE002JU | CPE002O9 | CPE002KP | XM 93863 | XM 9386 | XM 9386 | LOC64956 | LOC64956 | LOC64956 | similar to gonado | similar to | similar to | 649563 | 649563 | 649563 | s197670 | s197671 | s197672 |
| 21816 | M9 | M10 | 264 | 977,8 | 2827 | 632,7 | 10,7 | 1,126 | H5A034 C | sample | Hm Gen E | Hm Gen E | Hm Gen E | CPE002JU | CPE002O9 | CPE002KP | XM 94408 | XM 9440 | XM 9440 | LOC65020 | LOC65020 | LOC65020 | hypothetical prot | hypotheti | hypotheti | 650203 | 650203 | 650203 | s57057 | s57058 | s57059 |
| 4659 | C11 | C12 | 1041 | 1057 | 11154 | 585,2 | 10,7 | 1,176 | H5A034 C | sample | Hm Drgbl | Hm Drgbl | Hm Drgbl | CPE002DV | CPE002EL | CPE002FP | NM 02125 | NM 0212 | NM 0212 | CAPN10 | CAPN10 | CAPN10 | calpain 10 | calpain 10 | calpain 10 | 11132 | 11132 | 11132 | s200487 | s200488 | s200489 |
| 4732 | F12 | F13 | 1705 | 791,2 | 18178 | 468 | 10,7 | 1,176 | H5A034 C | sample | Hm Drgbl | Hm Drgbl | Hm Drgbl | CPE002DV | CPE002EL | CPE002FP | NM 01211 | NM 0121 | NM 0121 | CASP14 | CASP14 | CASP14 | caspase 14, apopt | caspase 14 | caspase 14 | 23581 | 23581 | 23581 | s24128 | s24129 | s24130 |
| 4857 | K17 | K18 | 1584 | 890,3 | 16963 | 318 | 10,7 | 1,176 | H5A034 C | sample | Hm Drgbl | Hm Drgbl | Hm Drgbl | CPE002DV | CPE002EL | CPE002FP | NM 14778 | NM 1477 | NM 1477 | CTSB | CTSB | CTSB | cathepsin B | cathepsin | cathepsin | 1508 | 1508 | 1508 | s3738 | s3739 | s3740 |
| 4924 | N12 | N13 | 2020 | 777,3 | 21516 | 494,8 | 10,7 | 1,176 | H5A034 C | sample | Hm Drgbl | Hm Drgbl | Hm Drgbl | CPE002DV | CPE002EL | CPE002FP | NM 00108 | NM 0010 | NM 0010 | TMPRSS4 | TMPRSS4 | TMPRSS4 | transmembrane p | transmem | transmem | 56649 | 56649 | 56649 | s32202 | s32203 | s32204 |
| 4978 | P18 | P19 | 1955 | 886 | 20948 | 432,3 | 10,7 | 1,176 | H5A034 C | sample | Hm Drgbl | Hm Drgbl | Hm Drgbl | CPE002DV | CPE002EL | CPE002FP | NM 02162 | NM 0216 | NM 0216 | SCPEP1 | SCPEP1 | SCPEP1 | serine carboxypep | serine car | serine car | 59342 | 59342 | 59342 | s34004 | s34005 | s34006 |
| 2708 | A5 | A6 | 1363 | 664 | 14580 | 545 | 10,7 | 1,259 | H5A034 C | sample | Hm Drgbl | Hm Drgbl | Hm Drgbl | CPE002DP | CPE002EF | CPE002F5 | NM 00629 | NM 0062 | NM 0062 | VRK2 | VRK2 | VRK2 | vaccinia related k | vaccinia re | vaccinia re | 7444 | 7444 | 7444 | s14823 | s224777 | s14825 |
| 2747 | C19 | C20 | 1043 | 720,2 | 11110 | 541,4 | 10,7 | 1,259 | H5A034 C | sample | Hm Drgbl | Hm Drgbl | Hm Drgbl | CPE002DP | CPE002EF | CPE002F5 | NM 00368 | NM 0036 | NM 0036 | CASK | CASK | CASK | calcium/calmodul | calcium/ca | calcium/ca | 8573 | 8573 | 8573 | s16328 | s16329 | s16330 |
| 2834 | G10 | G11 | 905 | 638,3 | 9671 | 559,6 | 10,7 | 1,259 | H5A034 C | sample | Hm Drgbl | Hm Drgbl | Hm Drgbl | CPE002DP | CPE002EF | CPE002F5 | NM 02039 | NM 0203 | NM 0203 | CAMK1D | CAMK1D | CAMK1D | calcium/calmodul | calcium/ca | calcium/ca | 57118 | 57118 | 57118 | s32715 | s32716 | s32717 |
| 3025 | O1 | O2 | 481 | 573,5 | 5136 | 539,4 | 10,7 | 1,259 | H5A034 C | sample | Hm Drgbl | Hm Drgbl | Hm Drgbl | CPE002DP | CPE002EF | CPE002F5 | NM 18169 | NM 1816 | NM 1816 | AKT3 | AKT3 | AKT3 | v-akt murine thyr | v-akt muri | v-akt muri | 10000 | 10000 | 10000 | s19427 | s19428 | s19429 |
| 3030 | O14 | O15 | 1186 | 685,5 | 12710 | 519,8 | 10,7 | 1,259 | H5A034 C | sample | Hm Drgbl | Hm Drgbl | Hm Drgbl | CPE002DP | CPE002EF | CPE002F5 | NM 03128 | NM 0312 | NM 0312 | ADPGK | ADPGK | ADPGK | ADP-dependent g | ADP-depe | ADP-depe | 83440 | 83440 | 83440 | s37907 | s37908 | s37909 |
| 3063 | P22 | P23 | 1119 | 616,5 | 11945 | 551,7 | 10,7 | 1,259 | H5A034 C | sample | Hm Drgbl | Hm Drgbl | Hm Drgbl | CPE002DP | CPE002EF | CPE002F5 | NM 00429 | NM 0042 | NM 0042 | ABCB7 | ABCB7 | ABCB7 | ATP-binding casse | ATP-bindi | ATP-bindi | 22 | 22 | 22 | s855 | s856 | s857 |
| 19987 | A4 | A5 | 579 | 1124 | 6167 | 200,1 | 10,7 | 1,305 | H5A034 C | sample | Hm Gen E | Hm Gen E | Hm Gen E | CPE002JQ | CPE002O5 | CPE002KL | XM 94590 | XM 9459 | XM 9459 | PAIP2B | PAIP2B | PAIP2B | poly(A) binding p | poly(A) bi | poly(A) bi | 400961 | 400961 | 400961 | s53428 | s53429 | s53430 |
| 19994 | B10 | B11 | 328 | 1261 | 3509 | 198,1 | 10,7 | 1,305 | H5A034 C | sample | Hm Gen E | Hm Gen E | Hm Gen E | CPE002JQ | CPE002O5 | CPE002KL | NM 00108 | NM 0010 | NM 0010 | LOC40205 | LOC40205 | LOC40205 | similar to 40S ribo | similar to | similar to | 402057 | 402057 | 402057 | s53724 | s196460 | s226573 |
| 20030 | C21 | C22 | 798 | 1150 | 8516 | 197,4 | 10,7 | 1,305 | H5A034 C | sample | Hm Gen E | Hm Gen E | Hm Gen E | CPE002JQ | CPE002O5 | CPE002KL | XM 37277 | XM 0011 | XM 0011 | LOC39102 | LOC39102 | LOC39102 | similar to protein | similar to | similar to | 391025 | 391025 | 391025 | s52909 | s196370 | s196371 |
| 20037 | C6 | C7 | 653 | 1242 | 6972 | 155,2 | 10,7 | 1,305 | H5A034 C | sample | Hm Gen E | Hm Gen E | Hm Gen E | CPE002JQ | CPE002O5 | CPE002KL | XM 37626 | XM 3762 | XM 3762 | LOC40108 | LOC40108 | LOC40108 | FLJ43329 protein | FLJ43329 p | FLJ43329 p | 401089 | 401089 | 401089 | s53466 | s53467 | s53468 |
| 20043 | D11 | D12 | 1109 | 1105 | 11860 | 136,3 | 10,7 | 1,305 | H5A034 C | sample | Hm Gen E | Hm Gen E | Hm Gen E | CPE002JQ | CPE002O5 | CPE002KL | NM 00100 | NM 0010 | NM 0010 | FLJ41423 | FLJ41423 | FLJ41423 | FLJ41423 protein | FLJ41423 p | FLJ41423 p | 399886 | 399886 | 399886 | s53191 | s53192 | s53193 |
| 20207 | J8 | J9 | 673 | 1142 | 7197 | 145,8 | 10,7 | 1,305 | H5A034 C | sample | Hm Gen E | Hm Gen E | Hm Gen E | CPE002JQ | CPE002O5 | CPE002KL | NM 20696 | NM 2069 | NM 2069 | C16orf74 | C16orf74 | C16orf74 | chromosome 16 c | chromoso | chromoso | 404550 | 404550 | 404550 | s196485 | s196486 | s196487 |
| 20221 | K20 | K21 | 1106 | 1104 | 11844 | 134,5 | 10,7 | 1,305 | H5A034 C | sample | Hm Gen E | Hm Gen E | Hm Gen E | CPE002JQ | CPE002O5 | CPE002KL | XM 37682 | XM 3768 | XM 3768 | LOC40149 | LOC40149 | LOC40149 | similar to PRO273 | similar to | similar to | 401497 | 401497 | 401497 | s53621 | s53622 | s53623 |
| 20322 | O3 | O4 | 688 | 1090 | 7345 | 160,6 | 10,7 | 1,305 | H5A034 C | sample | Hm Gen E | Hm Gen E | Hm Gen E | CPE002JQ | CPE002O5 | CPE002KL | NM 00100 | NM 0010 | NM 0010 | TMEM195 | TMEM195 | TMEM195 | transmembrane p | transmem | transmem | 392636 | 392636 | 392636 | s53107 | s53108 | s53109 |
| 20330 | P10 | P11 | 990 | 1286 | 10617 | 176,2 | 10,7 | 1,305 | H5A034 C | sample | Hm Gen E | Hm Gen E | Hm Gen E | CPE002JQ | CPE002O5 | CPE002KL | NM 01501 | NM 0150 | NM 0150 | TMEM41B | TMEM41B | TMEM41B | transmembrane p | transmem | transmem | 440026 | 440026 | 440026 | s53991 | s53992 | s53993 |
| 768 | P9 | P10 | 2096 | 1842 | 22346 | 454,3 | 10,7 | 1,562 | H5A034 C | sample | Hm Drgbl | Hm Drgbl | Hm Drgbl | CPE002E0 | CPE002EZ | CPE002FK | NM 00411 | NM 0041 | NM 0041 | FKBP1B | FKBP1B | FKBP1B | FK506 binding pro | FK506 bin | FK506 bin | 2281 | 2281 | 2281 | s5208 | s5210 | s5209 |
| 7847 | G8 | G9 | 949 | 602,5 | 10127 | 374,1 | 10,7 | 1,634 | H5A034 C | sample | Hm Drgbl | Hm Drgbl | Hm Drgbl | CPE002E4 | CPE002EV | CPE002FG | NM 00647 | NM 0064 | NM 0064 | ATP5L | ATP5L | ATP5L | ATP synthase, H+ | ATP synth | ATP synth | 10632 | 10632 | 10632 | s20891 | s20892 | s20890 |
| 7882 | I18 | I19 | 790 | 545,3 | 8484 | 360 | 10,7 | 1,634 | H5A034 C | sample | Hm Drgbl | Hm Drgbl | Hm Drgbl | CPE002E4 | CPE002EV | CPE002FG | NM 00657 | NM 0065 | NM 0065 | RRAGA | RRAGA | RRAGA | Ras-related GTP b | Ras-relate | Ras-relate | 10670 | 10670 | 10670 | s20986 | s20985 | s20984 |
| 7992 | M9 | M10 | 251 | 787,2 | 2690 | 400,8 | 10,7 | 1,634 | H5A034 C | sample | Hm Drgbl | Hm Drgbl | Hm Drgbl | CPE002E4 | CPE002EV | CPE002FG | NM 00100 | NM 0010 | NM 0010 | SF3A1 | SF3A1 | SF3A1 | splicing factor 3a, | splicing fa | splicing fa | 10291 | 10291 | 10291 | s20116 | s20117 | s20118 |
| 3110 | B21 | B22 | 1562 | 1445 | 16921 | 422,5 | 10,8 | 0,669 | H5A034 C | sample | Hm Drgbl | Hm Drgbl | Hm Drgbl | CPE002DC | CPE002EG | CPE002F6 | NM 00282 | NM 0028 | NM 0028 | PTPN3 | PTPN3 | PTPN3 | protein tyrosine p | protein ty | protein ty | 5774 | 5774 | 5774 | s11512 | s11513 | s11514 |
| 6159 | A22 | A23 | 1583 | 542,3 | 17161 | 469,5 | 10,8 | 0,713 | H5A034 C | sample | Hm Drgbl | Hm Drgbl | Hm Drgbl | CPE002DZ | CPE002F0 | CPE002FL | NM 00009 | NM 0000 | NM 0000 | CST3 | CST3 | CST3 | cystatin C (amyloi | cystatin C | cystatin C | 1471 | 1471 | 1471 | s3667 | s3665 | s3666 |
| 20696 | O16 | O17 | 971 | 807,1 | 10504 | 176,6 | 10,8 | 0,72 | H5A034 C | sample | Hm Gen E | Hm Gen E | Hm Gen E | CPE002JR | CPE002O6 | CPE002KN | XM 93588 | XM 9358 | XM 9358 | LOC64182 | LOC64182 | LOC64182 | hypothetical prot | hypotheti | hypotheti | 641826 | 641826 | 641826 | s54858 | s196717 | s196718 |
| 19561 | P1 | P2 | 947 | 1356 | 10255 | 246,4 | 10,8 | 0,766 | H5A034 C | sample | Hm Gen E | Hm Gen E | Hm Gen E | CPE002JO | CPE002O3 | CPE002KJ | XM 94137 | XM 9413 | XM 9413 | LOC34165 | LOC34165 | LOC34165 | similar to Fumara | similar to | similar to | 341651 | 341651 | 341651 | s50861 | s50862 | s50863 |
| 11217 | D17 | D18 | 1082 | 869,7 | 11709 | 140,3 | 10,8 | 0,783 | H5A034 C | sample | Hm Drgbl | Hm Drgbl | Hm Drgbl | CPE002ED | CPE002F3 | CPE002FT | NM 19851 | NM 1985 | NM 1985 | DGAT2L6 | DGAT2L6 | DGAT2L6 | diacylglycerol O-a | diacylglyc | diacylglyc | 347516 | 347516 | 347516 | s51265 | s51266 | s51267 |
| 11520 | P9 | P10 | 1125 | 772,8 | 12175 | 112,3 | 10,8 | 0,783 | H5A034 C | sample | Hm Drgbl | Hm Drgbl | Hm Drgbl | CPE002ED | CPE002F3 | CPE002FT | XM 93793 | XM 9379 | XM 9379 | tAKR | tAKR | tAKR | aldo-keto reducta | aldo-keto | aldo-keto | 389932 | 389932 | 389932 | s52599 | s52598 | s52597 |
| 5187 | I11 | I12 | 999 | 577,3 | 10785 | 438,4 | 10,8 | 0,847 | H5A034 C | sample | Hm Drgbl | Hm Drgbl | Hm Drgbl | CPE002DV | CPE002EM | CPE002FO | NM 08038 | NM 0803 | NM 0803 | CPA5 | CPA5 | CPA5 | carboxypeptidase | carboxype | carboxype | 93979 | 93979 | 93979 | s41210 | s41211 | s41212 |
| 2415 | E22 | E23 | 1657 | 767,1 | 17870 | 406,2 | 10,8 | 0,85 | H5A034 C | sample | Hm Drgbl | Hm Drgbl | Hm Drgbl | CPE002DC | CPE002EE | CPE002F4 | NM 00193 | NM 0019 | NM 0019 | MPP3 | MPP3 | MPP3 | membrane protei | membran | membran | 4356 | 4356 | 4356 | s8946 | s8947 | s8948 |
| 2581 | L20 | L21 | 1037 | 772,6 | 11213 | 355,9 | 10,8 | 0,85 | H5A034 C | sample | Hm Drgbl | Hm Drgbl | Hm Drgbl | CPE002DC | CPE002EE | CPE002F4 | NM 08082 | NM 0808 | NM 0808 | SRMS | SRMS | SRMS | src-related kinase | src-relatec | src-relatec | 6725 | 6725 | 6725 | s13433 | s224679 | s194817 |
| 4372 | G12 | G13 | 910 | 697,4 | 9844 | 397,9 | 10,8 | 0,911 | H5A034 C | sample | Hm Drgbl | Hm Drgbl | Hm Drgbl | CPE002DL | CPE002EK | CPE002FQ | NM 01486 | NM 0148 | NM 0148 | ATP2C2 | ATP2C2 | ATP2C2 | ATPase, Ca++ trar | ATPase, C | ATPase, C | 9914 | 9914 | 9914 | s19224 | s19225 | s19226 |
| 4443 | J11 | J12 | 1044 | 658,5 | 11266 | 352,9 | 10,8 | 0,911 | H5A034 C | sample | Hm Drgbl | Hm Drgbl | Hm Drgbl | CPE002DL | CPE002EK | CPE002FQ | NM 00224 | NM 0022 | NM 0022 | KCNJ10 | KCNJ10 | KCNJ10 | potassium inwarc | potassium | potassium | 3766 | 3766 | 3766 | s7756 | s7757 | s223969 |
| 163 | G4 | G5 | 202 | 1136 | 2186 | 545,7 | 10,8 | 0,919 | H5A034 C | sample | Hm Drgbl | Hm Drgbl | Hm Drgbl | CPE002DT | CPE002EJ | CPE002F9 | NM 00306 | NM 0030 | NM 0030 | SLIT3 | SLIT3 | SLIT3 | slit homolog 3 (Dr | slit homol | slit homol | 6586 | 6586 | 6586 | s13122 | s13120 | s13121 |
| 174 | H14 | H15 | 378 | 1315 | 4082 | 425,9 | 10,8 | 0,919 | H5A034 C | sample | Hm Drgbl | Hm Drgbl | Hm Drgbl | CPE002DT | CPE002EJ | CPE002F9 | NM 00314 | NM 0031 | NM 0031 | TRIM21 | TRIM21 | TRIM21 | tripartite motif-co | tripartite | tripartite | 6737 | 6737 | 6737 | s13462 | s13463 | s13464 |
| 182 | H21 | H22 | 643 | 1439 | 6959 | 570,2 | 10,8 | 0,919 | H5A034 C | sample | Hm Drgbl | Hm Drgbl | Hm Drgbl | CPE002DT | CPE002EJ | CPE002F9 | NM 00506 | NM 0050 | NM 0050 | SIM1 | SIM1 | SIM1 | single-minded ho | single-mir | single-mir | 6492 | 6492 | 6492 | s12865 | s12866 | s12867 |
| 189 | H6 | H7 | 493 | 1286 | 5326 | 425,6 | 10,8 | 0,919 | H5A034 C | sample | Hm Drgbl | Hm Drgbl | Hm Drgbl | CPE002DT | CPE002EJ | CPE002F9 | NM 00313 | NM 0031 | NM 0031 | SRP54 | SRP54 | SRP54 | signal recognition | signal rec | signal rec | 6729 | 6729 | 6729 | s13441 | s13442 | s13443 |
| 262 | K7 | K8 | 624 | 1460 | 6748 | 412,6 | 10,8 | 0,919 | H5A034 C | sample | Hm Drgbl | Hm Drgbl | Hm Drgbl | CPE002DT | CPE002EJ | CPE002F9 | NM 00324 | NM 0032 | NM 0032 | TGIF1 | TGIF1 | TGIF1 | TGFB-induced fac | TGFB-indu | TGFB-indu | 7050 | 7050 | 7050 | s14082 | s14081 | s14080 |
| 300 | M2 | M3 | 336 | 1413 | 3637 | 562,3 | 10,8 | 0,919 | H5A034 C | sample | Hm Drgbl | Hm Drgbl | Hm Drgbl | CPE002DT | CPE002EJ | CPE002F9 | NM 02280 | NM 0228 | NM 0228 | SNRPN | SNRPN | SNRPN | small nuclear ribo | small nucl | small nucl | 6638 | 6638 | 6638 | s13246 | s13247 | s13248 |
| 321 | N17 | N18 | 349 | 1516 | 3778 | 470,4 | 10,8 | 0,919 | H5A034 C | sample | Hm Drgbl | Hm Drgbl | Hm Drgbl | CPE002DT | CPE002EJ | CPE002F9 | NM 00104 | NM 0010 | NM 0010 | SLC6A4 | SLC6A4 | SLC6A4 | solute carrier fam | solute car | solute car | 6532 | 6532 | 6532 | s12980 | s12981 | s12979 |
| 2037 | E6 | E7 | 1220 | 634,5 | 13127 | 426,6 | 10,8 | 0,939 | H5A034 C | sample | Hm Drgbl | Hm Drgbl | Hm Drgbl | CPE002FX | CPE002G1 | CPE002G5 | NM 14529 | NM 1452 | NM 1452 | ZNF627 | ZNF627 | ZNF627 | zinc finger proteir | zinc finger | zinc finger | 199692 | 199692 | 199692 | s47118 | s47119 | s47120 |
| 2223 | M22 | M23 | 1860 | 737,6 | 20000 | 390,1 | 10,8 | 0,939 | H5A034 C | sample | Hm Drgbl | Hm Drgbl | Hm Drgbl | CPE002FX | CPE002G1 | CPE002G5 | NM 18172 | NM 1817 | NM 1817 | EFHA2 | EFHA2 | EFHA2 | EF-hand domain f | EF-hand d | EF-hand d | 286097 | 286097 | 286097 | s50093 | s50094 | s50095 |
| 2239 | N15 | N16 | 1113 | 565,6 | 11987 | 414,1 | 10,8 | 0,939 | H5A034 C | sample | Hm Drgbl | Hm Drgbl | Hm Drgbl | CPE002FX | CPE002G1 | CPE002G5 | NM 14528 | NM 1452 | NM 1452 | ZNF519 | ZNF519 | ZNF519 | zinc finger proteir | zinc finger | zinc finger | 162655 | 162655 | 162655 | s46361 | s46362 | s46363 |
| 4103 | K8 | K9 | 1005 | 767,1 | 10813 | 383,2 | 10,8 | 0,96 | H5A034 C | sample | Hm Drgbl | Hm Drgbl | Hm Drgbl | CPE002DS | CPE002EI | CPE002F8 | NM 00565 | NM 0056 | NM 0056 | TERF2 | TERF2 | TERF2 | telomeric repeat b | telomeric | telomeric | 7014 | 7014 | 7014 | s13991 | s13993 | s13992 |
| 4137 | M17 | M18 | 1380 | 649 | 14921 | 363,7 | 10,8 | 0,96 | H5A034 C | sample | Hm Drgbl | Hm Drgbl | Hm Drgbl | CPE002DS | CPE002EI | CPE002F8 | NM 00101 | NM 0010 | NM 0010 | PON2 | PON2 | PON2 | paraoxonase 2 | paraoxona | paraoxona | 5445 | 5445 | 5445 | s10832 | s10833 | s10834 |
| 11771 | K19 | K20 | 845 | 867,2 | 9128 | 150,6 | 10,8 | 0,964 | H5A034 C | sample | Hm Gen E | Hm Gen E | Hm Gen E | CPE002JA | CPE002NJ | CPE002JZ | NM 00434 | NM 0043 | NM 0043 | CALD1 | CALD1 | CALD1 | caldesmon 1 | caldesmor | caldesmor | 800 | 800 | 800 | s2337 | s2338 | s2339 |
| 15505 | G1 | G2 | 1580 | 873,2 | 17060 | 144,2 | 10,8 | 0,982 | H5A034 C | sample | Hm Gen E | Hm Gen E | Hm Gen E | CPE002JE | CPE002NT | CPE002K9 | NM 02207 | NM 0220 | NM 0220 | SH2D4A | SH2D4A | SH2D4A | SH2 domain conta | SH2 doma | SH2 doma | 63898 | 63898 | 63898 | s34231 | s34232 | s34233 |
| 15639 | L22 | L23 | 1224 | 802,1 | 13218 | 138,5 | 10,8 | 0,982 | H5A034 C | sample | Hm Gen E | Hm Gen E | Hm Gen E | CPE002JE | CPE002NT | CPE002K9 | NM 02454 | NM 0245 | NM 0245 | CEP97 | CEP97 | CEP97 | centrosomal prot | centroson | centroson | 79598 | 79598 | 79598 | s35887 | s35888 | s35889 |
| 5931 | H11 | H12 | 898 | 812,3 | 9657 | 446,4 | 10,8 | 0,986 | H5A034 C | sample | Hm Drgbl | Hm Drgbl | Hm Drgbl | CPE002DY | CPE002F1 | CPE002FM | NM 00163 | NM 0016 | NM 0016 | SLC25A6 | SLC25A6 | SLC25A6 | solute carrier fam | solute car | solute car | 293 | 293 | 293 | s1377 | s1379 | s1378 |
| 5967 | I22 | I23 | 1726 | 822,9 | 18672 | 365 | 10,8 | 0,986 | H5A034 C | sample | Hm Drgbl | Hm Drgbl | Hm Drgbl | CPE002DY | CPE002F1 | CPE002FM | NM 00167 | NM 0016 | NM 0016 | ATP1B3 | ATP1B3 | ATP1B3 | ATPase, Na+/K+ tr | ATPase, N | ATPase, N | 483 | 483 | 483 | s1740 | s1739 | s1741 |
| 6014 | K21 | K22 | 2069 | 776,6 | 22297 | 537,7 | 10,8 | 0,986 | H5A034 C | sample | Hm Drgbl | Hm Drgbl | Hm Drgbl | CPE002DY | CPE002F1 | CPE002FM | NM 00002 | NM 0000 | NM 0000 | ADSL | ADSL | ADSL | adenylosuccinate | adenylosu | adenylosu | 158 | 158 | 158 | s1133 | s1134 | s1135 |
| 6023 | K8 | K9 | 1163 | 720,3 | 12510 | 486,4 | 10,8 | 0,986 | H5A034 C | sample | Hm Drgbl | Hm Drgbl | Hm Drgbl | CPE002DY | CPE002F1 | CPE002FM | XM 00112 | XM 0011 | XM 0011 | ALDH7A1 | ALDH7A1 | ALDH7A1 | aldehyde dehydro | aldehyde | aldehyde | 501 | 501 | 501 | s1770 | s1771 | s1772 |
| 6029 | L13 | L14 | 2126 | 842,8 | 22922 | 377,3 | 10,8 | 0,986 | H5A034 C | sample | Hm Drgbl | Hm Drgbl | Hm Drgbl | CPE002DY | CPE002F1 | CPE002FM | NM 00038 | NM 0003 | NM 0003 | APOB | APOB | APOB | apolipoprotein B | apolipopr | apolipopr | 338 | 338 | 338 | s1475 | s1476 | s1477 |
| 1215 | C22 | C23 | 1843 | 801,1 | 19828 | 459,3 | 10,8 | 0,991 | H5A034 C | sample | Hm Drgbl | Hm Drgbl | Hm Drgbl | CPE002FV | CPE002FZ | CPE002G3 | NM 01475 | NM 0147 | NM 0147 | SERTAD2 | SERTAD2 | SERTAD2 | SERTA domain co | SERTA dor | SERTA dor | 9792 | 9792 | 9792 | s18924 | s18925 | s18926 |
| 1231 | D15 | D16 | 1538 | 756,1 | 16647 | 342,9 | 10,8 | 0,991 | H5A034 C | sample | Hm Drgbl | Hm Drgbl | Hm Drgbl | CPE002FV | CPE002FZ | CPE002G3 | NM 00356 | NM 0035 | NM 0035 | STX7 | STX7 | STX7 | syntaxin 7 | syntaxin 7 | syntaxin 7 | 8417 | 8417 | 8417 | s15978 | s15979 | s15980 |
| 1341 | H6 | H7 | 519 | 846 | 5615 | 472 | 10,8 | 0,991 | H5A034 C | sample | Hm Drgbl | Hm Drgbl | Hm Drgbl | CPE002FV | CPE002FZ | CPE002G3 | NM 00708 | NM 0070 | NM 0070 | FSTL1 | FSTL1 | FSTL1 | follistatin-like 1 | follistatin | follistatin | 11167 | 11167 | 11167 | s22032 | s22033 | s22034 |
| 1381 | J20 | J21 | 1583 | 749,2 | 17051 | 344,7 | 10,8 | 0,991 | H5A034 C | sample | Hm Drgbl | Hm Drgbl | Hm Drgbl | CPE002FV | CPE002FZ | CPE002G3 | NM 01493 | NM 0149 | NM 0149 | ZNF510 | ZNF510 | ZNF510 | zinc finger proteir | zinc finger | zinc finger | 22869 | 22869 | 22869 | s22541 | s22542 | s22543 |
| 1520 | P16 | P17 | 1801 | 721,4 | 19525 | 434,6 | 10,8 | 0,991 | H5A034 C | sample | Hm Drgbl | Hm Drgbl | Hm Drgbl | CPE002FV | CPE002FZ | CPE002G3 | NM 03304 | NM 0330 | NM 0330 | MACF1 | MACF1 | MACF1 | microtubule-actin | microtubu | microtubu | 23499 | 23499 | 23499 | s23937 | s23938 | s23939 |
| 16158 | B14 | B15 | 1779 | 1015 | 19132 | 358,4 | 10,8 | 0,991 | H5A034 C | sample | Hm Gen E | Hm Gen E | Hm Gen E | CPE002JG | CPE002NV | CPE002KB | NM 03242 | NM 0324 | NM 0324 | MAML2 | MAML2 | MAML2 | mastermind-like 2 | mastermin | mastermin | 84441 | 84441 | 84441 | s39004 | s39005 | s39006 |
| 16441 | N1 | N2 | 1155 | 1104 | 12531 | 336,8 | 10,8 | 0,991 | H5A034 C | sample | Hm Gen E | Hm Gen E | Hm Gen E | CPE002JG | CPE002NV | CPE002KB | NM 03214 | NM 0321 | NM 0321 | FBXO30 | FBXO30 | FBXO30 | F-box protein 30 | F-box pro | F-box pro | 84085 | 84085 | 84085 | s38491 | s38492 | s38493 |
| 13565 | F13 | F14 | 1216 | 1038 | 13155 | 153,3 | 10,8 | 1,014 | H5A034 C | sample | Hm Gen E | Hm Gen E | Hm Gen E | CPE002JC | CPE002NC | CPE002K4 | NM 03088 | NM 0308 | NM 0308 | APOL2 | APOL2 | APOL2 | apolipoprotein L, | apolipopr | apolipopr | 23780 | 23780 | 23780 | s24394 | s24395 | s24396 |
| 13639 | I15 | I16 | 1397 | 908 | 15156 | 147,1 | 10,8 | 1,014 | H5A034 C | sample | Hm Gen E | Hm Gen E | Hm Gen E | CPE002JC | CPE002NC | CPE002K4 | NM 00108 | NM 0010 | NM 0010 | FBXO46 | FBXO46 | FBXO46 | F-box protein 46 | F-box pro | F-box pro | 23403 | 23403 | 23403 | s23748 | s23749 | s23750 |
| 988 | J12 | J13 | 1708 | 722,7 | 18410 | 370,6 | 10,8 | 1,024 | H5A034 C | sample | Hm Drgbl | Hm Drgbl | Hm Drgbl | CPE002FU | CPE002FY | CPE002G2 | NM 00695 | NM 0069 | NM 0069 | TAF5 | TAF5 | TAF5 | TAF5 RNA polyme | TAF5 RNA | TAF5 RNA | 6877 | 6877 | 6877 | s13742 | s13743 | s13744 |
| 1074 | M3 | M4 | 1298 | 724,9 | 13960 | 378 | 10,8 | 1,024 | H5A034 C | sample | Hm Drgbl | Hm Drgbl | Hm Drgbl | CPE002FU | CPE002FY | CPE002G2 | NM 00130 | NM 0013 | NM 0013 | KLF6 | KLF6 | KLF6 | Kruppel-like facto | Kruppel-li | Kruppel-li | 1316 | 1316 | 1316 | s3374 | s3375 | s3376 |
| 1102 | N7 | N8 | 1389 | 728,3 | 14961 | 393,4 | 10,8 | 1,024 | H5A034 C | sample | Hm Drgbl | Hm Drgbl | Hm Drgbl | CPE002FU | CPE002FY | CPE002G2 | NM 00216 | NM 0021 | NM 0021 | ID2 | ID2 | ID2 | inhibitor of DNA b | inhibitor c | inhibitor c | 3398 | 3398 | 3398 | s7107 | s7108 | s7109 |
| 1130 | P10 | P11 | 1191 | 805,3 | 12808 | 494,5 | 10,8 | 1,024 | H5A034 C | sample | Hm Drgbl | Hm Drgbl | Hm Drgbl | CPE002FU | CPE002FY | CPE002G2 | NM 02114 | NM 0211 | NM 0211 | UTX | UTX | UTX | ubiquitously tran | ubiquitou | ubiquitou | 7403 | 7403 | 7403 | s14735 | s14736 | s14737 |
| 5675 | M19 | M20 | 2298 | 788 | 24887 | 524,9 | 10,8 | 1,029 | H5A034 C | sample | Hm Drgbl | Hm Drgbl | Hm Drgbl | CPE002DX | CPE002F2 | CPE002FN | NM 00366 | NM 0036 | NM 0036 | PIR | PIR | PIR | pirin (iron-bindin | pirin (iron | pirin (iron | 8544 | 8544 | 8544 | s16260 | s16261 | s16262 |
| 5692 | N12 | N13 | 2450 | 766 | 26421 | 442,1 | 10,8 | 1,029 | H5A034 C | sample | Hm Drgbl | Hm Drgbl | Hm Drgbl | CPE002DX | CPE002F2 | CPE002FN | NM 01208 | NM 0120 | NM 0120 | GTF3C5 | GTF3C5 | GTF3C5 | general transcript | general tr | general tr | 9328 | 9328 | 9328 | s17826 | s17827 | s17828 |
| 5708 | N5 | N6 | 1621 | 813,3 | 17571 | 559,5 | 10,8 | 1,029 | H5A034 C | sample | Hm Drgbl | Hm Drgbl | Hm Drgbl | CPE002DX | CPE002F2 | CPE002FN | NM 00381 | NM 0038 | NM 0038 | PABPC4 | PABPC4 | PABPC4 | poly(A) binding p | poly(A) bi | poly(A) bi | 8761 | 8761 | 8761 | s16695 | s16693 | s16694 |
| 3607 | G15 | G16 | 1341 | 1289 | 14514 | 354,4 | 10,8 | 1,038 | H5A034 C | sample | Hm Drgbl | Hm Drgbl | Hm Drgbl | CPE002DR | CPE002EH | CPE002F7 | NM 00129 | NM 0012 | NM 0012 | CCR1 | CCR1 | CCR1 | chemokine (C-C m | chemokin | chemokin | 1230 | 1230 | 1230 | s3197 | s223268 | s3199 |
| 3692 | J5 | J6 | 1213 | 1284 | 13161 | 315,6 | 10,8 | 1,038 | H5A034 C | sample | Hm Drgbl | Hm Drgbl | Hm Drgbl | CPE002DR | CPE002EH | CPE002F7 | NM 00086 | NM 0008 | NM 0008 | HTR1D | HTR1D | HTR1D | 5-hydroxytryptam | 5-hydroxy | 5-hydroxy | 3352 | 3352 | 3352 | s7032 | s7033 | s7034 |
| 3704 | K16 | K17 | 1296 | 1325 | 13943 | 337,4 | 10,8 | 1,038 | H5A034 C | sample | Hm Drgbl | Hm Drgbl | Hm Drgbl | CPE002DR | CPE002EH | CPE002F7 | NM 00719 | NM 0071 | NM 0071 | FZD10 | FZD10 | FZD10 | frizzled homolog | frizzled ho | frizzled ho | 11211 | 11211 | 11211 | s22127 | s22128 | s22129 |
| 3791 | N8 | N9 | 1452 | 1174 | 15714 | 309,1 | 10,8 | 1,038 | H5A034 C | sample | Hm Drgbl | Hm Drgbl | Hm Drgbl | CPE002DR | CPE002EH | CPE002F7 | XM 00113 | XM 0011 | XM 0011 | GPR125 | GPR125 | GPR125 | G protein-couplec | G protein- | G protein- | 166647 | 166647 | 166647 | s46620 | s46621 | s46622 |
| 8071 | A15 | A16 | 1262 | 738 | 13646 | 417,5 | 10,8 | 1,038 | H5A034 C | sample | Hm Drgbl | Hm Drgbl | Hm Drgbl | CPE002E5 | CPE002EU | CPE002FF | NM 00701 | NM 0070 | NM 0070 | WWP1 | WWP1 | WWP1 | WW domain cont | WW doma | WW doma | 11059 | 11059 | 11059 | s21788 | s21790 | s21789 |
| 8282 | J10 | J11 | 1487 | 655,8 | 16112 | 423,9 | 10,8 | 1,038 | H5A034 C | sample | Hm Drgbl | Hm Drgbl | Hm Drgbl | CPE002E5 | CPE002EU | CPE002FF | NM 01220 | NM 0122 | NM 0122 | HAAO | HAAO | HAAO | 3-hydroxyanthrar | 3-hydroxy | 3-hydroxy | 23498 | 23498 | 23498 | s23936 | s23934 | s23935 |
| 8343 | L22 | L23 | 1481 | 638,5 | 16036 | 389,2 | 10,8 | 1,038 | H5A034 C | sample | Hm Drgbl | Hm Drgbl | Hm Drgbl | CPE002E5 | CPE002EU | CPE002FF | NM 01232 | NM 0123 | NM 0123 | MAPK8IP2 | MAPK8IP2 | MAPK8IP2 | mitogen-activated | mitogen-a | mitogen-a | 23542 | 23542 | 23542 | s24046 | s24044 | s24045 |
| 8448 | P9 | P10 | 1594 | 608,5 | 17254 | 493,8 | 10,8 | 1,038 | H5A034 C | sample | Hm Drgbl | Hm Drgbl | Hm Drgbl | CPE002E5 | CPE002EU | CPE002FF | NM 01501 | NM 0150 | NM 0150 | RBM34 | RBM34 | RBM34 | RNA binding moti | RNA bindi | RNA bindi | 23029 | 23029 | 23029 | s22863 | s22864 | s22865 |
| 10188 | I2 | I3 | 1599 | 926,9 | 17206 | 165,4 | 10,8 | 1,038 | H5A034 C | sample | Hm Drgbl | Hm Drgbl | Hm Drgbl | CPE002EA | CPE002EP | CPE002FA | NM 17586 | NM 1758 | NM 1758 | TMTC1 | TMTC1 | TMTC1 | transmembrane a | transmem | transmem | 83857 | 83857 | 83857 | s38231 | s38232 | s38230 |
| 10284 | M2 | M3 | 1030 | 933,7 | 11112 | 157,2 | 10,8 | 1,038 | H5A034 C | sample | Hm Drgbl | Hm Drgbl | Hm Drgbl | CPE002EA | CPE002EP | CPE002FA | NM 03213 | NM 0321 | NM 0321 | TKTL2 | TKTL2 | TKTL2 | transketolase-like | transketol | transketol | 84076 | 84076 | 84076 | s38468 | s38469 | s38470 |
| 10285 | M20 | M21 | 1213 | 837,9 | 13088 | 117,9 | 10,8 | 1,038 | H5A034 C | sample | Hm Drgbl | Hm Drgbl | Hm Drgbl | CPE002EA | CPE002EP | CPE002FA | NM 03218 | NM 0321 | NM 0321 | MYST1 | MYST1 | MYST1 | MYST histone ace | MYST hist | MYST hist | 84148 | 84148 | 84148 | s38571 | s38570 | s38569 |
| 1544 | A16 | A17 | 2178 | 893,6 | 23521 | 433,3 | 10,8 | 1,131 | H5A034 C | sample | Hm Drgbl | Hm Drgbl | Hm Drgbl | CPE002FW | CPE002G0 | CPE002G4 | NM 02071 | NM 0207 | NM 0207 | ZNF512B | ZNF512B | ZNF512B | zinc finger proteir | zinc finger | zinc finger | 57473 | 57473 | 57473 | s33103 | s33104 | s33105 |
| 1783 | K15 | K16 | 2047 | 763,9 | 22061 | 350,8 | 10,8 | 1,131 | H5A034 C | sample | Hm Drgbl | Hm Drgbl | Hm Drgbl | CPE002FW | CPE002G0 | CPE002G4 | NM 00100 | NM 0010 | NM 0010 | CLDN20 | CLDN20 | CLDN20 | claudin 20 | claudin 20 | claudin 20 | 49861 | 49861 | 49861 | s26989 | s26990 | s26991 |
| 1785 | K17 | K18 | 1872 | 741,8 | 20258 | 355,2 | 10,8 | 1,131 | H5A034 C | sample | Hm Drgbl | Hm Drgbl | Hm Drgbl | CPE002FW | CPE002G0 | CPE002G4 | NM 01591 | NM 0159 | NM 0159 | ZNF691 | ZNF691 | ZNF691 | zinc finger proteir | zinc finger | zinc finger | 51058 | 51058 | 51058 | s27316 | s27317 | s27318 |
| 1806 | L14 | L15 | 1832 | 674,4 | 19705 | 320,6 | 10,8 | 1,131 | H5A034 C | sample | Hm Drgbl | Hm Drgbl | Hm Drgbl | CPE002FW | CPE002G0 | CPE002G4 | NM 03237 | NM 0323 | NM 0323 | PCGF5 | PCGF5 | PCGF5 | polycomb group r | polycomb | polycomb | 84333 | 84333 | 84333 | s38939 | s38940 | s224942 |
| 1886 | O21 | O22 | 1654 | 723,1 | 17916 | 414 | 10,8 | 1,131 | H5A034 C | sample | Hm Drgbl | Hm Drgbl | Hm Drgbl | CPE002FW | CPE002G0 | CPE002G4 | NM 01625 | NM 0162 | NM 0162 | HPCAL4 | HPCAL4 | HPCAL4 | hippocalcin like 4 | hippocalc | hippocalc | 51440 | 51440 | 51440 | s28144 | s28145 | s224204 |
| 21580 | D12 | D13 | 354 | 896 | 3819 | 334,3 | 10,8 | 1,137 | H5A034 C | sample | Hm Gen E | Hm Gen E | Hm Gen E | CPE002JU | CPE002O9 | CPE002KP | XM 93235 | XM 9323 | XM 9323 | LOC65320 | LOC65320 | LOC65320 | similar to hypoth | similar to | similar to | 653203 | 653203 | 653203 | s57614 | s57615 | s57616 |
| 21648 | F9 | F10 | 228 | 936,4 | 2472 | 412,6 | 10,8 | 1,137 | H5A034 C | sample | Hm Gen E | Hm Gen E | Hm Gen E | CPE002JU | CPE002O9 | CPE002KP | XM 94031 | XM 9403 | XM 9403 | LOC65118 | LOC65118 | LOC65118 | similar to Zinc fin | similar to | similar to | 651186 | 651186 | 651186 | s57176 | s57177 | s227362 |
| 4637 | B13 | B14 | 1905 | 844,6 | 20647 | 504,4 | 10,8 | 1,187 | H5A034 C | sample | Hm Drgbl | Hm Drgbl | Hm Drgbl | CPE002DV | CPE002EL | CPE002FP | NM 03342 | NM 0334 | NM 0334 | GZMH | GZMH | GZMH | granzyme H (cath | granzyme | granzyme | 2999 | 2999 | 2999 | s6378 | s6379 | s6380 |
| 4735 | F15 | F16 | 1789 | 790,8 | 19396 | 396,9 | 10,8 | 1,187 | H5A034 C | sample | Hm Drgbl | Hm Drgbl | Hm Drgbl | CPE002DV | CPE002EL | CPE002FP | NM 00558 | NM 0055 | NM 0055 | MEP1A | MEP1A | MEP1A | meprin A, alpha ( | meprin A, | meprin A, | 4224 | 4224 | 4224 | s8691 | s8692 | s8693 |
| 4939 | N4 | N5 | 1341 | 814,9 | 14428 | 426,2 | 10,8 | 1,187 | H5A034 C | sample | Hm Drgbl | Hm Drgbl | Hm Drgbl | CPE002DV | CPE002EL | CPE002FP | NM 01780 | NM 0178 | NM 0178 | OSGEP | OSGEP | OSGEP | O-sialoglycoprote | O-sialogly | O-sialogly | 55644 | 55644 | 55644 | s31152 | s31153 | s31154 |
| 4955 | O19 | O20 | 2027 | 764,3 | 21798 | 463,1 | 10,8 | 1,187 | H5A034 C | sample | Hm Drgbl | Hm Drgbl | Hm Drgbl | CPE002DV | CPE002EL | CPE002FP | NM 00012 | NM 0001 | NM 0001 | F11 | F11 | F11 | coagulation facto | coagulatic | coagulatic | 2160 | 2160 | 2160 | s4949 | s4950 | s4951 |
| 10375 | A15 | A16 | 1763 | 899,9 | 19026 | 226,9 | 10,8 | 1,22 | H5A034 C | sample | Hm Drgbl | Hm Drgbl | Hm Drgbl | CPE002EB | CPE002EO | CPE002FR | NM 03280 | NM 0328 | NM 0328 | LINGO1 | LINGO1 | LINGO1 | leucine rich repea | leucine ric | leucine ric | 84894 | 84894 | 84894 | s39596 | s39597 | s39595 |
| 10549 | H20 | H21 | 1324 | 873,9 | 14315 | 183,9 | 10,8 | 1,22 | H5A034 C | sample | Hm Drgbl | Hm Drgbl | Hm Drgbl | CPE002EB | CPE002EO | CPE002FR | NM 15234 | NM 1523 | NM 1523 | CDYL2 | CDYL2 | CDYL2 | chromodomain p | chromodc | chromodc | 124359 | 124359 | 124359 | s42725 | s42724 | s42723 |
| 2691 | A11 | A12 | 722 | 628,6 | 7801 | 458,5 | 10,8 | 1,271 | H5A034 C | sample | Hm Drgbl | Hm Drgbl | Hm Drgbl | CPE002DP | CPE002EF | CPE002F5 | NM 00630 | NM 0063 | NM 0063 | MAP3K12 | MAP3K12 | MAP3K12 | mitogen-activated | mitogen-a | mitogen-a | 7786 | 7786 | 7786 | s15344 | s15345 | s15346 |
| 2700 | A2 | A3 | 1217 | 697,8 | 13103 | 648,9 | 10,8 | 1,271 | H5A034 C | sample | Hm Drgbl | Hm Drgbl | Hm Drgbl | CPE002DP | CPE002EF | CPE002F5 | NM 00102 | NM 0010 | NM 0010 | VRK3 | VRK3 | VRK3 | vaccinia related k | vaccinia re | vaccinia re | 51231 | 51231 | 51231 | s27724 | s27725 | s27726 |
| 2715 | B11 | B12 | 808 | 736,7 | 8757 | 607,8 | 10,8 | 1,271 | H5A034 C | sample | Hm Drgbl | Hm Drgbl | Hm Drgbl | CPE002DP | CPE002EF | CPE002F5 | NM 00638 | NM 0063 | NM 0063 | CIB1 | CIB1 | CIB1 | calcium and integ | calcium ar | calcium ar | 10519 | 10519 | 10519 | s20616 | s20617 | s20618 |
| 2856 | G9 | G10 | 542 | 702,2 | 5862 | 482 | 10,8 | 1,271 | H5A034 C | sample | Hm Drgbl | Hm Drgbl | Hm Drgbl | CPE002DP | CPE002EF | CPE002F5 | NM 00391 | NM 0039 | NM 0039 | PRPF4B | PRPF4B | PRPF4B | PRP4 pre-mRNA p | PRP4 pre- | PRP4 pre- | 8899 | 8899 | 8899 | s17017 | s17018 | s17019 |
| 3029 | O13 | O14 | 850 | 648,8 | 9163 | 580,4 | 10,8 | 1,271 | H5A034 C | sample | Hm Drgbl | Hm Drgbl | Hm Drgbl | CPE002DP | CPE002EF | CPE002F5 | NM 00101 | NM 0010 | NM 0010 | TNK2 | TNK2 | TNK2 | tyrosine kinase, n | tyrosine k | tyrosine k | 10188 | 10188 | 10188 | s19850 | s19851 | s223092 |
| 3054 | P14 | P15 | 1865 | 698,2 | 20155 | 543,5 | 10,8 | 1,271 | H5A034 C | sample | Hm Drgbl | Hm Drgbl | Hm Drgbl | CPE002DP | CPE002EF | CPE002F5 | NM 00100 | NM 0010 | NM 0010 | ATP5E | ATP5E | ATP5E | ATP synthase, H+ | ATP synth | ATP synth | 514 | 514 | 514 | s677 | s224217 | s679 |
| 20029 | C20 | C21 | 855 | 1250 | 9202 | 236,8 | 10,8 | 1,317 | H5A034 C | sample | Hm Gen E | Hm Gen E | Hm Gen E | CPE002JQ | CPE002O5 | CPE002KL | NM 20749 | NM 2074 | NM 2074 | MGC4862 | MGC4862 | MGC4862 | similar to KIAA16 | similar to | similar to | 401145 | 401145 | 401145 | s53490 | s53491 | s53492 |
| 20059 | D4 | D5 | 1353 | 1159 | 14644 | 201,3 | 10,8 | 1,317 | H5A034 C | sample | Hm Gen E | Hm Gen E | Hm Gen E | CPE002JQ | CPE002O5 | CPE002KL | XM 92634 | XM 9263 | XM 9263 | LOC40221 | LOC40221 | LOC40221 | similar to Tempo | similar to | similar to | 402217 | 402217 | 402217 | s53749 | s53750 | s53751 |
| 20066 | E10 | E11 | 413 | 1257 | 4460 | 183,8 | 10,8 | 1,317 | H5A034 C | sample | Hm Gen E | Hm Gen E | Hm Gen E | CPE002JQ | CPE002O5 | CPE002KL | XM 93738 | XM 9373 | XM 9373 | hCG 1640 | hCG 1640 | hCG 1640 | hCG1640785 | hCG16407 | hCG16407 | 401206 | 401206 | 401206 | s53506 | s53507 | s53508 |
| 20092 | F12 | F13 | 992 | 1026 | 10683 | 144,9 | 10,8 | 1,317 | H5A034 C | sample | Hm Gen E | Hm Gen E | Hm Gen E | CPE002JQ | CPE002O5 | CPE002KL | NM 00101 | NM 0010 | NM 0010 | UFSP1 | UFSP1 | UFSP1 | UFM1-specific pe | UFM1-spe | UFM1-spe | 402682 | 402682 | 402682 | s53789 | s53791 | s226577 |
| 20182 | I7 | I8 | 727 | 1163 | 7844 | 187,5 | 10,8 | 1,317 | H5A034 C | sample | Hm Gen E | Hm Gen E | Hm Gen E | CPE002JQ | CPE002O5 | CPE002KL | XM 49795 | XM 4979 | XM 4979 | LOC39160 | LOC39160 | LOC39160 | similar to CG3277 | similar to | similar to | 391609 | 391609 | 391609 | s196384 | s196385 | s196386 |
| 20307 | O11 | O12 | 1244 | 1049 | 13407 | 174,1 | 10,8 | 1,317 | H5A034 C | sample | Hm Gen E | Hm Gen E | Hm Gen E | CPE002JQ | CPE002O5 | CPE002KL | XM 37470 | XM 3747 | XM 3747 | LOC39307 | LOC39307 | LOC39307 | hypothetical LOC3 | hypotheti | hypotheti | 393078 | 393078 | 393078 | s53120 | s196416 | s196417 |
| 460 | D12 | D13 | 1780 | 1874 | 19176 | 315 | 10,8 | 1,577 | H5A034 C | sample | Hm Drgbl | Hm Drgbl | Hm Drgbl | CPE002E0 | CPE002EZ | CPE002FK | NM 00016 | NM 0001 | NM 0001 | GLA | GLA | GLA | galactosidase, alp | galactosid | galactosid | 2717 | 2717 | 2717 | s5791 | s5792 | s5790 |
| 7857 | H17 | H18 | 1301 | 599,2 | 14039 | 389,8 | 10,8 | 1,649 | H5A034 C | sample | Hm Drgbl | Hm Drgbl | Hm Drgbl | CPE002E4 | CPE002EV | CPE002FG | NM 00107 | NM 0010 | NM 0010 | VAV3 | VAV3 | VAV3 | vav 3 oncogene | vav 3 onco | vav 3 onco | 10451 | 10451 | 10451 | s347 | s348 | s346 |
| 7858 | H18 | H19 | 1243 | 547,6 | 13441 | 393 | 10,8 | 1,649 | H5A034 C | sample | Hm Drgbl | Hm Drgbl | Hm Drgbl | CPE002E4 | CPE002EV | CPE002FG | NM 00680 | NM 0068 | NM 0068 | STARD3 | STARD3 | STARD3 | START domain co | START dor | START dor | 10948 | 10948 | 10948 | s21540 | s21541 | s21542 |
| 7972 | M12 | M13 | 769 | 523,7 | 8286 | 401,7 | 10,8 | 1,649 | H5A034 C | sample | Hm Drgbl | Hm Drgbl | Hm Drgbl | CPE002E4 | CPE002EV | CPE002FG | NM 00661 | NM 0066 | NM 0066 | NES | NES | NES | nestin | nestin | nestin | 10763 | 10763 | 10763 | s21143 | s21141 | s21142 |
| 3274 | I18 | I19 | 925 | 1134 | 10092 | 257,9 | 10,9 | 0,675 | H5A034 C | sample | Hm Drgbl | Hm Drgbl | Hm Drgbl | CPE002DC | CPE002EG | CPE002F6 | NM 03321 | NM 0332 | NM 0332 | PPP1R3F | PPP1R3F | PPP1R3F | protein phosphat | protein pl | protein pl | 89801 | 89801 | 89801 | s40135 | s40136 | s40137 |
| 20484 | F2 | F3 | 1136 | 745,6 | 12423 | 132,4 | 10,9 | 0,727 | H5A034 C | sample | Hm Gen E | Hm Gen E | Hm Gen E | CPE002JR | CPE002O6 | CPE002KN | XM 93608 | XM 9360 | XM 9360 | LOC64201 | LOC64201 | LOC64201 | similar to cytochr | similar to | similar to | 642018 | 642018 | 642018 | s54915 | s196748 | s196749 |
| 20636 | L5 | L6 | 674 | 756,8 | 7332 | 166,8 | 10,9 | 0,727 | H5A034 C | sample | Hm Gen E | Hm Gen E | Hm Gen E | CPE002JR | CPE002O6 | CPE002KN | XM 00112 | XM 0011 | XM 0011 | hCG 1642 | hCG 1642 | hCG 1642 | hCG1642034 | hCG1642C | hCG1642C | 441898 | 441898 | 441898 | s54447 | s54448 | s54449 |
| 20697 | O17 | O18 | 1038 | 743,1 | 11340 | 160,4 | 10,9 | 0,727 | H5A034 C | sample | Hm Gen E | Hm Gen E | Hm Gen E | CPE002JR | CPE002O6 | CPE002KN | XM 49901 | XM 4990 | XM 4990 | LOC44109 | LOC44109 | LOC44109 | hypothetical LOC4 | hypotheti | hypotheti | 441097 | 441097 | 441097 | s54261 | s54262 | s54263 |
| 9463 | K15 | K16 | 1284 | 1186 | 14013 | 127,6 | 10,9 | 0,757 | H5A034 C | sample | Hm Drgbl | Hm Drgbl | Hm Drgbl | CPE002E8 | CPE002ER | CPE002FC | NM 01817 | NM 0181 | NM 0181 | MAP1S | MAP1S | MAP1S | microtubule-asso | microtubu | microtubu | 55201 | 55201 | 55201 | s30423 | s30424 | s30425 |
| 20990 | K21 | K22 | 1904 | 969,8 | 20848 | 211,2 | 10,9 | 0,773 | H5A034 C | sample | Hm Gen E | Hm Gen E | Hm Gen E | CPE002JS | CPE002O7 | CPE002KN | XM 93294 | XM 9329 | XM 9329 | LOC64327 | LOC64327 | LOC64327 | hypothetical LOC | hypotheti | hypotheti | 643275 | 643275 | 643275 | s55259 | s55260 | s55261 |
| 21176 | C16 | C17 | 1025 | 934 | 11138 | 325,7 | 10,9 | 0,773 | H5A034 C | sample | Hm Gen E | Hm Gen E | Hm Gen E | CPE002JT | CPE002O8 | CPE002KO | XM 92961 | XM 9296 | XM 9296 | LOC64667 | LOC64667 | LOC64667 | similar to COMM | similar to | similar to | 646670 | 646670 | 646670 | s56490 | s56491 | s56492 |
| 11442 | M3 | M4 | 1412 | 842,6 | 15361 | 134,6 | 10,9 | 0,79 | H5A034 C | sample | Hm Drgbl | Hm Drgbl | Hm Drgbl | CPE002ED | CPE002F3 | CPE002FT | NM 00100 | NM 0010 | NM 0010 | LINGO4 | LINGO4 | LINGO4 | leucine rich repea | leucine ric | leucine ric | 339398 | 339398 | 339398 | s50520 | s50519 | s50521 |
| 12444 | G2 | G3 | 1243 | 1076 | 13529 | 127,7 | 10,9 | 0,82 | H5A034 C | sample | Hm Gen E | Hm Gen E | Hm Gen E | CPE002J5 | CPE002NK | CPE002K1 | NM 00355 | NM 0035 | NM 0035 | OR1D4 | OR1D4 | OR1D4 | olfactory receptor | olfactory r | olfactory r | 8385 | 8385 | 8385 | s15913 | s15914 | s15915 |
| 7352 | C16 | C17 | 511 | 584,5 | 5553 | 394,9 | 10,9 | 0,832 | H5A034 C | sample | Hm Drgbl | Hm Drgbl | Hm Drgbl | CPE002E3 | CPE002EW | CPE002FH | NM 01464 | NM 0146 | NM 0146 | TTLL4 | TTLL4 | TTLL4 | tubulin tyrosine l | tubulin ty | tubulin ty | 9654 | 9654 | 9654 | s18574 | s18573 | s18572 |
| 16933 | B20 | B21 | 1844 | 1086 | 20048 | 140,4 | 10,9 | 0,842 | H5A034 C | sample | Hm Gen E | Hm Gen E | Hm Gen E | CPE002JI | CPE002NZ | CPE002KD | NM 00100 | NM 1525 | NM 0010 | OR4K14 | COMMD1 | OR4K14 | olfactory receptor | copper m | olfactory r | 122740 | 150684 | 122740 | s42569 | s45471 | s42571 |
| 16934 | B21 | B22 | 2156 | 1122 | 23573 | 203,8 | 10,9 | 0,842 | H5A034 C | sample | Hm Gen E | Hm Gen E | Hm Gen E | CPE002JI | CPE002NZ | CPE002KD | NM 05292 | NM 0010 | NM 0529 | ZNF452 | Gcom1 | ZNF452 | zinc finger proteir | GRINL1A c | zinc finger | 114821 | 145781 | 114821 | s41657 | s44775 | s41659 |
| 17202 | M3 | M4 | 1056 | 926,4 | 11469 | 103,5 | 10,9 | 0,842 | H5A034 C | sample | Hm Gen E | Hm Gen E | Hm Gen E | CPE002JI | CPE002NZ | CPE002KD | NM 14458 | NM 1446 | NM 1445 | TEX261 | GLT1D1 | TEX261 | testis expressed 2 | glycosyltra | testis expr | 113419 | 144423 | 113419 | s41470 | s44655 | s41472 |
| 7294 | P7 | P8 | 1032 | 573,7 | 11250 | 525,8 | 10,9 | 0,848 | H5A034 C | sample | Hm Drgbl | Hm Drgbl | Hm Drgbl | CPE002E2 | CPE002EX | CPE002FI | NM 02112 | NM 0010 | NM 0010 | MPST | MPST | MPST | mercaptopyruvate | mercapto | mercapto | 4357 | 4357 | 4357 | s8949 | s194617 | s194616 |
| 5012 | A5 | A6 | 1802 | 657,9 | 19561 | 588 | 10,9 | 0,855 | H5A034 C | sample | Hm Drgbl | Hm Drgbl | Hm Drgbl | CPE002DV | CPE002EM | CPE002FO | NM 02209 | NM 0220 | NM 0220 | XPNPEP3 | XPNPEP3 | XPNPEP3 | X-prolyl aminopep | X-prolyl ar | X-prolyl ar | 63929 | 63929 | 63929 | s34285 | s34286 | s34287 |
| 2435 | F19 | F20 | 1566 | 730,9 | 17059 | 408,6 | 10,9 | 0,858 | H5A034 C | sample | Hm Drgbl | Hm Drgbl | Hm Drgbl | CPE002DC | CPE002EE | CPE002F4 | NM 00021 | NM 0002 | NM 0002 | JAK3 | JAK3 | JAK3 | Janus kinase 3 (a | Janus kina | Janus kina | 3718 | 3718 | 3718 | s7652 | s7653 | s7654 |
| 16740 | J2 | J3 | 1325 | 932,1 | 14444 | 284,4 | 10,9 | 0,862 | H5A034 C | sample | Hm Gen E | Hm Gen E | Hm Gen E | CPE002JH | CPE002NV | CPE002KC | NM 13838 | NM 1383 | NM 1383 | NAF1 | NAF1 | NAF1 | nuclear assembly | nuclear as | nuclear as | 92345 | 92345 | 92345 | s40930 | s40931 | s40932 |
| 17977 | N1 | N2 | 1282 | 1014 | 13989 | 177,2 | 10,9 | 0,862 | H5A034 C | sample | Hm Gen E | Hm Gen E | Hm Gen E | CPE002JK | CPE002NZ | CPE002KF | NM 18153 | NM 1815 | NM 1815 | KRT25 | KRT25 | KRT25 | keratin 25 | keratin 25 | keratin 25 | 147183 | 147183 | 147183 | s44974 | s44975 | s44976 |
| 9114 | L3 | L4 | 1386 | 1688 | 15164 | 139,5 | 10,9 | 0,876 | H5A034 C | sample | Hm Drgbl | Hm Drgbl | Hm Drgbl | CPE002E7 | CPE002ES | CPE002FD | NM 01596 | NM 0159 | NM 0159 | MRPS17 | MRPS17 | MRPS17 | mitochondrial rib | mitochon | mitochon | 51373 | 51373 | 51373 | s28039 | s28040 | s28041 |
| 4459 | J4 | J5 | 1451 | 664,9 | 15774 | 325,2 | 10,9 | 0,92 | H5A034 C | sample | Hm Drgbl | Hm Drgbl | Hm Drgbl | CPE002DL | CPE002EK | CPE002FQ | NM 18258 | NM 1825 | NM 1825 | HTR3E | HTR3E | HTR3E | 5-hydroxytryptam | 5-hydroxy | 5-hydroxy | 285242 | 285242 | 285242 | s49862 | s49863 | s49864 |
| 4490 | L10 | L11 | 1127 | 631,9 | 12281 | 366,6 | 10,9 | 0,92 | H5A034 C | sample | Hm Drgbl | Hm Drgbl | Hm Drgbl | CPE002DL | CPE002EK | CPE002FQ | NM 00325 | NM 0032 | NM 0032 | TIMP1 | TIMP1 | TIMP1 | TIMP metallopept | TIMP met | TIMP met | 7076 | 7076 | 7076 | s14143 | s14144 | s14145 |
| 91 | D4 | D5 | 580 | 1618 | 6334 | 456,3 | 10,9 | 0,928 | H5A034 C | sample | Hm Drgbl | Hm Drgbl | Hm Drgbl | CPE002DT | CPE002EJ | CPE002F9 | NM 00312 | NM 0010 | NM 0031 | SPI1 | SPI1 | SPI1 | spleen focus form | spleen foc | spleen foc | 6688 | 6688 | 6688 | s13351 | s224672 | s13353 |
| 151 | G15 | G16 | 290 | 1363 | 3149 | 426,2 | 10,9 | 0,928 | H5A034 C | sample | Hm Drgbl | Hm Drgbl | Hm Drgbl | CPE002DT | CPE002EJ | CPE002F9 | NM 00107 | NM 0010 | NM 0010 | NR1I3 | NR1I3 | NR1I3 | nuclear receptor s | nuclear re | nuclear re | 9970 | 9970 | 9970 | s19368 | s19369 | s19370 |
| 211 | I4 | I5 | 634 | 1640 | 6930 | 568,9 | 10,9 | 0,928 | H5A034 C | sample | Hm Drgbl | Hm Drgbl | Hm Drgbl | CPE002DT | CPE002EJ | CPE002F9 | NM 00307 | NM 0030 | NM 0030 | SMARCD3 | SMARCD3 | SMARCD3 | SWI/SNF related, | SWI/SNF r | SWI/SNF r | 6604 | 6604 | 6604 | s13157 | s13158 | s13159 |
| 1934 | A21 | A22 | 1070 | 694,7 | 11663 | 436,8 | 10,9 | 0,948 | H5A034 C | sample | Hm Drgbl | Hm Drgbl | Hm Drgbl | CPE002FX | CPE002G1 | CPE002G5 | XM 93468 | XM 9346 | XM 9346 | ZNF799 | ZNF799 | ZNF799 | zinc finger proteir | zinc finger | zinc finger | 90576 | 90576 | 90576 | s40428 | s40429 | s40430 |

| | | | | | | | | | | | | | | | | | | | | | | |
|---|---|---|---|---|---|---|---|---|---|---|---|---|---|---|---|---|---|---|---|---|---|---|
| 1946 | B10 | B11 | 1257 | 636 | 13700 | 292,8 | 10,9 | 0,948 | H5A034 C | sample | Hm Drgbl | Hm Drgbl | Hm Drgbl | CPE002FX | CPE002G1 | CPE002G5 | NM 00103 | NM 0010 | NM 0010 | ZNF284 | ZNF284 | ZNF284 |
| 2035 | E4 | E5 | 834 | 621,1 | 9049 | 440,3 | 10,9 | 0,948 | H5A034 C | sample | Hm Drgbl | Hm Drgbl | Hm Drgbl | CPE002FX | CPE002G1 | CPE002G5 | NM 15265 | NM 1526 | NM 1526 | ZNF553 | ZNF553 | ZNF553 |
| 2055 | F22 | F23 | 1251 | 729,7 | 13658 | 469,2 | 10,9 | 0,948 | H5A034 C | sample | Hm Drgbl | Hm Drgbl | Hm Drgbl | CPE002FX | CPE002G1 | CPE002G5 | NM 0010 | NM 0010 | NM 0010 | SP5 | SP5 | SP5 |
| 2139 | J11 | J12 | 1374 | 717,7 | 14934 | 352,2 | 10,9 | 0,948 | H5A034 C | sample | Hm Drgbl | Hm Drgbl | Hm Drgbl | CPE002FX | CPE002G1 | CPE002G5 | NM 15279 | NM 1527 | NM 1527 | ZNF555 | ZNF555 | ZNF555 |
| 2199 | L22 | L23 | 1914 | 709 | 20911 | 463,3 | 10,9 | 0,948 | H5A034 C | sample | Hm Drgbl | Hm Drgbl | Hm Drgbl | CPE002FX | CPE002G1 | CPE002G5 | | | | | | |
| 4171 | N4 | N5 | 1674 | 690,9 | 18180 | 431,6 | 10,9 | 0,969 | H5A034 C | sample | Hm Drgbl | Hm Drgbl | Hm Drgbl | CPE002DS | CPE002EI | CPE002F8 | NM 00322 | NM 0032 | NM 0032 | TFR2 | TFR2 | TFR2 |
| 11641 | F1 | F2 | 1328 | 912,1 | 14411 | 148,8 | 10,9 | 0,973 | H5A034 C | sample | Hm Gen E | Hm Gen E | Hm Gen E | CPE002JA | CPE002NJ | CPE002JZ | NM 00436 | NM 0043 | NM 0043 | CNN2 | CNN2 | CNN2 |
| 11770 | K18 | K19 | 1168 | 790,1 | 12703 | 128,4 | 10,9 | 0,973 | H5A034 C | sample | Hm Gen E | Hm Gen E | Hm Gen E | CPE002JA | CPE002NJ | CPE002JZ | NM 0014 | NM 0014 | NM 0014 | FYB | FYB | FYB |
| 5842 | D18 | D19 | 1524 | 817,9 | 16559 | 567,5 | 10,9 | 0,995 | H5A034 C | sample | Hm Drgbl | Hm Drgbl | Hm Drgbl | CPE002DY | CPE002F1 | CPE002FM | NM 00071 | NM 0007 | NM 0007 | BLVRA | BLVRA | BLVRA |
| 6021 | K6 | K7 | 1759 | 791,3 | 19097 | 494,2 | 10,9 | 0,995 | H5A034 C | sample | Hm Drgbl | Hm Drgbl | Hm Drgbl | CPE002DY | CPE002F1 | CPE002FM | NM 00404 | NM 0040 | NM 0040 | ATP5A1 | ATP5A1 | ATP5A1 |
| 6047 | L8 | L9 | 1667 | 697,5 | 18162 | 501,4 | 10,9 | 0,995 | H5A034 C | sample | Hm Drgbl | Hm Drgbl | Hm Drgbl | CPE002DY | CPE002F1 | CPE002FM | NM 00121 | NM 0012 | NM 0012 | CA12 | CA12 | CA12 |
| 6077 | N13 | N14 | 2363 | 829,5 | 25752 | 475,5 | 10,9 | 0,995 | H5A034 C | sample | Hm Drgbl | Hm Drgbl | Hm Drgbl | CPE002DY | CPE002F1 | CPE002FM | NM 15287 | NM 0000 | NM 1528 | FAS | FAS | FAS |
| 6121 | P1 | P2 | 767 | 738,7 | 8379 | 480,9 | 10,9 | 0,995 | H5A034 C | sample | Hm Drgbl | Hm Drgbl | Hm Drgbl | CPE002DY | CPE002F1 | CPE002FM | NM 00165 | NM 0016 | NM 0016 | AQP5 | AQP5 | AQP5 |
| 1202 | C10 | C11 | 1222 | 688 | 13272 | 424,2 | 10,9 | 1 | H5A034 C | sample | Hm Drgbl | Hm Drgbl | Hm Drgbl | CPE002FV | CPE002FZ | CPE002G3 | NM 01467 | NM 0146 | NM 0146 | EDEM1 | EDEM1 | EDEM1 |
| 1443 | M11 | M12 | 1602 | 743,8 | 17450 | 423,8 | 10,9 | 1 | H5A034 C | sample | Hm Drgbl | Hm Drgbl | Hm Drgbl | CPE002FV | CPE002FZ | CPE002G3 | NM 00345 | NM 0034 | NM 0034 | ZNF177 | ZNF177 | ZNF177 |
| 1453 | M20 | M21 | 1678 | 721,6 | 18350 | 423,8 | 10,9 | 1 | H5A034 C | sample | Hm Drgbl | Hm Drgbl | Hm Drgbl | CPE002FV | CPE002FZ | CPE002G3 | NM 00638 | NM 0063 | NM 0063 | HYOU1 | HYOU1 | HYOU1 |
| 16159 | B15 | B16 | 1228 | 1157 | 13435 | 419,2 | 10,9 | 1 | H5A034 C | sample | Hm Gen E | Hm Gen E | Hm Gen E | CPE002JG | CPE002NV | CPE002KB | NM 0319 | NM 0319 | NM 0319 | FRMD8 | FRMD8 | FKSG44 |
| 16382 | K21 | K22 | 1328 | 1050 | 14472 | 313,1 | 10,9 | 1 | H5A034 C | sample | Hm Gen E | Hm Gen E | Hm Gen E | CPE002JG | CPE002NV | CPE002KB | NM 0314 | NM 0314 | NM 0314 | TMUB1 | TMUB1 | TMUB1 |
| 13692 | K2 | K3 | 1262 | 1063 | 13809 | 172,2 | 10,9 | 1,023 | H5A034 C | sample | Hm Gen E | Hm Gen E | Hm Gen E | CPE002JC | CPE002NC | CPE002K4 | NM 01547 | NM 0154 | NM 0154 | RAB11FIP5 | RAB11FIP5 | RAB11FIP5 |
| 1017 | K17 | K18 | 1736 | 749 | 18840 | 366,8 | 10,9 | 1,033 | H5A034 C | sample | Hm Drgbl | Hm Drgbl | Hm Drgbl | CPE002FU | CPE002FY | CPE002G2 | NM 0808 | NM 0808 | NM 0808 | COL13A1 | COL13A1 | COL13A1 |
| 1083 | N11 | N12 | 1461 | 667,9 | 15982 | 459 | 10,9 | 1,033 | H5A034 C | sample | Hm Drgbl | Hm Drgbl | Hm Drgbl | CPE002FU | CPE002FY | CPE002G2 | NM 00553 | NM 0055 | NM 0055 | IFI35 | IFI35 | IFI35 |
| 1136 | P16 | P17 | 1745 | 734 | 19062 | 384,6 | 10,9 | 1,033 | H5A034 C | sample | Hm Drgbl | Hm Drgbl | Hm Drgbl | CPE002FU | CPE002FY | CPE002G2 | NM 00338 | NM 0033 | NM 0033 | VSNL1 | VSNL1 | VSNL1 |
| 5420 | B5 | B6 | 1633 | 656,1 | 17808 | 645,8 | 10,9 | 1,038 | H5A034 C | sample | Hm Drgbl | Hm Drgbl | Hm Drgbl | CPE002DX | CPE002F2 | CPE002FN | NM 0037 | NM 0037 | NM 0037 | SLC25A12 | SLC25A12 | SLC25A12 |
| 5483 | E19 | E20 | 1739 | 626,1 | 18997 | 579,8 | 10,9 | 1,038 | H5A034 C | sample | Hm Drgbl | Hm Drgbl | Hm Drgbl | CPE002DX | CPE002F2 | CPE002FN | NM 01423 | NM 0142 | NM 0142 | GNPAT | GNPAT | GNPAT |
| 5606 | J21 | J22 | 1609 | 655,8 | 17513 | 409,9 | 10,9 | 1,038 | H5A034 C | sample | Hm Drgbl | Hm Drgbl | Hm Drgbl | CPE002DX | CPE002F2 | CPE002FN | NM 03316 | NM 0331 | NM 0331 | B3GALNT1 | B3GALNT1 | B3GALNT1 |
| 5628 | K2 | K3 | 689 | 641,6 | 7481 | 608,4 | 10,9 | 1,038 | H5A034 C | sample | Hm Drgbl | Hm Drgbl | Hm Drgbl | CPE002DX | CPE002F2 | CPE002FN | NM 00393 | NM 0039 | NM 0039 | TOP3B | TOP3B | TOP3B |
| 5655 | L22 | L23 | 1763 | 782,9 | 19206 | 527,7 | 10,9 | 1,038 | H5A034 C | sample | Hm Drgbl | Hm Drgbl | Hm Drgbl | CPE002DX | CPE002F2 | CPE002FN | NM 00423 | NM 0042 | NM 0042 | TRIP13 | TRIP13 | TRIP13 |
| 5734 | O7 | O8 | 1381 | 785,8 | 14987 | 663,2 | 10,9 | 1,038 | H5A034 C | sample | Hm Drgbl | Hm Drgbl | Hm Drgbl | CPE002DX | CPE002F2 | CPE002FN | NM 00367 | NM 0036 | NM 0036 | KMO | KMO | KMO |
| 5741 | P13 | P14 | 2199 | 743,5 | 24031 | 562,7 | 10,9 | 1,038 | H5A034 C | sample | Hm Drgbl | Hm Drgbl | Hm Drgbl | CPE002DX | CPE002F2 | CPE002FN | NM 00384 | NM 0038 | NM 0038 | SUCLG2 | SUCLG2 | SUCLG2 |
| 5742 | P14 | P15 | 1863 | 714,1 | 20385 | 643,9 | 10,9 | 1,038 | H5A034 C | sample | Hm Drgbl | Hm Drgbl | Hm Drgbl | CPE002DX | CPE002F2 | CPE002FN | NM 00479 | NM 1532 | NM 1532 | SLC22A6 | SLC22A6 | SLC22A6 |
| 3480 | A9 | A10 | 1320 | 1312 | 14339 | 358 | 10,9 | 1,048 | H5A034 C | sample | Hm Drgbl | Hm Drgbl | Hm Drgbl | CPE002DR | CPE002EH | CPE002F7 | NM 00067 | NM 0006 | NM 0006 | ADORA1 | ADORA1 | ADORA1 |
| 3509 | C13 | C14 | 1502 | 1229 | 16432 | 303,6 | 10,9 | 1,048 | H5A034 C | sample | Hm Drgbl | Hm Drgbl | Hm Drgbl | CPE002DR | CPE002EH | CPE002F7 | NM 00516 | NM 0051 | NM 0051 | AGTRL1 | AGTRL1 | AGTRL1 |
| 3620 | G5 | G6 | 1160 | 1221 | 12613 | 375,3 | 10,9 | 1,048 | H5A034 C | sample | Hm Drgbl | Hm Drgbl | Hm Drgbl | CPE002DR | CPE002EH | CPE002F7 | NM 07848 | NM 0784 | NM 0784 | CD97 | CD97 | CD97 |
| 3630 | H14 | H15 | 667 | 1298 | 7298 | 338,2 | 10,9 | 1,048 | H5A034 C | sample | Hm Drgbl | Hm Drgbl | Hm Drgbl | CPE002DR | CPE002EH | CPE002F7 | NM 0325 | NM 0325 | NM 0325 | MCHR2 | MCHR2 | MCHR2 |
| 3651 | I11 | I12 | 297 | 1495 | 3241 | 396,2 | 10,9 | 1,048 | H5A034 C | sample | Hm Drgbl | Hm Drgbl | Hm Drgbl | CPE002DR | CPE002EH | CPE002F7 | NM 00407 | NM 0040 | NM 0040 | CMKLR1 | CMKLR1 | CMKLR1 |
| 3708 | K2 | K3 | 972 | 1405 | 10558 | 405 | 10,9 | 1,048 | H5A034 C | sample | Hm Drgbl | Hm Drgbl | Hm Drgbl | CPE002DR | CPE002EH | CPE002F7 | NM 00658 | NM 0065 | NM 0065 | RRH | RRH | RRH |
| 3815 | O8 | O9 | 1498 | 1419 | 16388 | 416,3 | 10,9 | 1,048 | H5A034 C | sample | Hm Drgbl | Hm Drgbl | Hm Drgbl | CPE002DR | CPE002EH | CPE002F7 | NM 01848 | NM 0184 | NM 0184 | GPR77 | GPR77 | GPR77 |
| 8067 | A11 | A12 | 1611 | 676,8 | 17527 | 469,4 | 10,9 | 1,048 | H5A034 C | sample | Hm Drgbl | Hm Drgbl | Hm Drgbl | CPE002E5 | CPE002EU | CPE002FF | NM 00701 | NM 0070 | NM 0070 | DDX52 | DDX52 | DDX52 |
| 8112 | B9 | B10 | 983 | 672,1 | 10700 | 549,4 | 10,9 | 1,048 | H5A034 C | sample | Hm Drgbl | Hm Drgbl | Hm Drgbl | CPE002E5 | CPE002EU | CPE002FF | NM 00726 | NM 0072 | NM 0072 | XAB1 | XAB1 | XAB1 |
| 8223 | G22 | G23 | 1922 | 613,2 | 20928 | 386,5 | 10,9 | 1,048 | H5A034 C | sample | Hm Drgbl | Hm Drgbl | Hm Drgbl | CPE002E5 | CPE002EU | CPE002FF | NM 19833 | NM 1983 | NM 1983 | GANAB | GANAB | GANAB |
| 10162 | H18 | H19 | 1685 | 801,3 | 18402 | 118,9 | 10,9 | 1,048 | H5A034 C | sample | Hm Drgbl | Hm Drgbl | Hm Drgbl | CPE002EA | CPE002EP | CPE002FA | NM 03255 | NM 0325 | NM 0325 | HIATL1 | HIATL1 | HIATL1 |
| 1643 | E19 | E20 | 1608 | 732,7 | 17536 | 295,2 | 10,9 | 1,141 | H5A034 C | sample | Hm Drgbl | Hm Drgbl | Hm Drgbl | CPE002FW | CPE002G0 | CPE002G4 | NM 01442 | NM 0144 | NM 0144 | HSPB7 | HSPB7 | HSPB7 |
| 1724 | H5 | H6 | 1791 | 728,3 | 19600 | 442,7 | 10,9 | 1,141 | H5A034 C | sample | Hm Drgbl | Hm Drgbl | Hm Drgbl | CPE002FW | CPE002G0 | CPE002G4 | NM 01818 | NM 0181 | NM 0181 | ZNF532 | ZNF532 | ZNF532 |
| 1868 | N5 | N6 | 1591 | 779,6 | 17371 | 426,4 | 10,9 | 1,141 | H5A034 C | sample | Hm Drgbl | Hm Drgbl | Hm Drgbl | CPE002FW | CPE002G0 | CPE002G4 | NM 00101 | NM 0010 | NM 0010 | ZNF302 | ZNF302 | ZNF302 |
| 1870 | N7 | N8 | 1771 | 787,9 | 19238 | 446,7 | 10,9 | 1,141 | H5A034 C | sample | Hm Drgbl | Hm Drgbl | Hm Drgbl | CPE002FW | CPE002G0 | CPE002G4 | NM 01868 | NM 0186 | NM 0186 | ZNF313 | ZNF313 | ZNF313 |
| 21590 | D21 | D22 | 402 | 1039 | 4398 | 585,5 | 10,9 | 1,147 | H5A034 C | sample | Hm Gen E | Hm Gen E | Hm Gen E | CPE002JU | CPE002O9 | CPE002KP | XM 94449 | XM 9444 | XM 9444 | LOC65109 | LOC65109 | LOC65109 |
| 21602 | E10 | E11 | 120 | 948,4 | 1306 | 624,7 | 10,9 | 1,147 | H5A034 C | sample | Hm Gen E | Hm Gen E | Hm Gen E | CPE002JU | CPE002O9 | CPE002KP | XM 94214 | XM 9421 | XM 9421 | LOC65261 | LOC65261 | LOC65261 |
| 21674 | H10 | H11 | 290 | 851,1 | 3168 | 477,1 | 10,9 | 1,147 | H5A034 C | sample | Hm Gen E | Hm Gen E | Hm Gen E | CPE002JU | CPE002O9 | CPE002KP | XM 37055 | XM 3705 | XM 3705 | FAM21D | FAM21D | FAM21D |
| 21743 | J8 | J9 | 185 | 1170 | 2024 | 831,4 | 10,9 | 1,147 | H5A034 C | sample | Hm Gen E | Hm Gen E | Hm Gen E | CPE002JU | CPE002O9 | CPE002KP | XM 92822 | XM 9282 | XM 9282 | LOC65358 | LOC65358 | LOC65358 |
| 21872 | P16 | P17 | 129 | 1294 | 1410 | 457,8 | 10,9 | 1,147 | H5A034 C | sample | Hm Gen E | Hm Gen E | Hm Gen E | CPE002JU | CPE002O9 | CPE002KP | NM 00104 | NM 0010 | NM 0010 | IQCJ | IQCJ | IQCJ |
| 10524 | G2 | G3 | 962 | 832,9 | 10458 | 179,9 | 10,9 | 1,232 | H5A034 C | sample | Hm Drgbl | Hm Drgbl | Hm Drgbl | CPE002EB | CPE002EO | CPE002FR | NM 05283 | NM 0528 | NM 0528 | SLC26A7 | SLC26A7 | SLC26A7 |
| 10678 | M7 | M8 | 1134 | 948,7 | 12314 | 184,4 | 10,9 | 1,232 | H5A034 C | sample | Hm Drgbl | Hm Drgbl | Hm Drgbl | CPE002EB | CPE002EO | CPE002FR | NM 14717 | NM 1471 | NM 1471 | HS6ST2 | HS6ST2 | HS6ST2 |
| 2867 | H19 | H20 | 879 | 600,7 | 9617 | 463,9 | 10,9 | 1,282 | H5A034 C | sample | Hm Drgbl | Hm Drgbl | Hm Drgbl | CPE002DP | CPE002EF | CPE002F5 | NM 01509 | NM 0150 | NM 0150 | SMG1 | SMG1 | SMG1 |
| 20050 | D18 | D19 | 1133 | 1155 | 12325 | 175 | 10,9 | 1,329 | H5A034 C | sample | Hm Gen E | Hm Gen E | Hm Gen E | CPE002JQ | CPE002O5 | CPE002KL | NM 21255 | NM 2125 | NM 2125 | XKRX | XKRX | XKRX |
| 20142 | H14 | H15 | 708 | 1212 | 7727 | 137,8 | 10,9 | 1,329 | H5A034 C | sample | Hm Gen E | Hm Gen E | Hm Gen E | CPE002JQ | CPE002O5 | CPE002KL | XM 00113 | XM 0011 | XM 0011 | FAM92A3 | FAM92A3 | FAM92A3 |
| 20174 | I21 | I22 | 969 | 1129 | 10554 | 115,3 | 10,9 | 1,329 | H5A034 C | sample | Hm Gen E | Hm Gen E | Hm Gen E | CPE002JQ | CPE002O5 | CPE002KL | XM 92931 | XM 92931 | XM 92931 | LOC39177 | LOC39177 | LOC39177 |
| 20198 | J21 | J22 | 743 | 1158 | 8103 | 161,5 | 10,9 | 1,329 | H5A034 C | sample | Hm Gen E | Hm Gen E | Hm Gen E | CPE002JQ | CPE002O5 | CPE002KL | NM 20745 | NM 2074 | NM 2074 | FLJ44815 | FLJ44815 | FLJ44815 |
| 20206 | J7 | J8 | 780 | 1218 | 8501 | 127,2 | 10,9 | 1,329 | H5A034 C | sample | Hm Gen E | Hm Gen E | Hm Gen E | CPE002JQ | CPE002O5 | CPE002KL | XM 00112 | XM 0011 | XM 0011 | FLJ26245 | FLJ26245 | FLJ26245 |
| 391 | A15 | A16 | 2105 | 1899 | 23007 | 455,7 | 10,9 | 1,591 | H5A034 C | sample | Hm Drgbl | Hm Drgbl | Hm Drgbl | CPE002E0 | CPE002EZ | CPE002FK | NM 00194 | NM 0019 | NM 0019 | E2F3 | E2F3 | E2F3 |
| 417 | B17 | B18 | 1811 | 1779 | 19776 | 358 | 10,9 | 1,591 | H5A034 C | sample | Hm Drgbl | Hm Drgbl | Hm Drgbl | CPE002E0 | CPE002EZ | CPE002FK | NM 00012 | NM 0001 | NM 0001 | ETFA | ETFA | ETFA |
| 459 | D11 | D12 | 1775 | 1747 | 19276 | 327,8 | 10,9 | 1,591 | H5A034 C | sample | Hm Drgbl | Hm Drgbl | Hm Drgbl | CPE002E0 | CPE002EZ | CPE002FK | NM 00198 | NM 0019 | NM 0019 | EVPL | EVPL | EVPL |
| 746 | P10 | P11 | 1961 | 1774 | 21447 | 453,9 | 10,9 | 1,591 | H5A034 C | sample | Hm Drgbl | Hm Drgbl | Hm Drgbl | CPE002E0 | CPE002EZ | CPE002FK | NM 00531 | NM 0053 | NM 0053 | GSCL | GSCL | GSCL |
| 7853 | H13 | H14 | 1339 | 514,9 | 14620 | 338,3 | 10,9 | 1,664 | H5A034 C | sample | Hm Drgbl | Hm Drgbl | Hm Drgbl | CPE002E4 | CPE002EV | CPE002FG | NM 00611 | NM 0061 | NM 0061 | ACAA2 | ACAA2 | ACAA2 |
| 3273 | I17 | I18 | 1408 | 1168 | 15549 | 276,7 | 11 | 0,681 | H5A034 C | sample | Hm Drgbl | Hm Drgbl | Hm Drgbl | CPE002DC | CPE002EG | CPE002F6 | NM 00453 | NM 0045 | NM 0045 | NAP1L1 | NAP1L1 | NAP1L1 |
| 3333 | K6 | K7 | 858 | 1111 | 9432 | 353 | 11 | 0,681 | H5A034 C | sample | Hm Drgbl | Hm Drgbl | Hm Drgbl | CPE002DC | CPE002EG | CPE002F6 | NM 05411 | NM 0541 | NM 0541 | CIB3 | CIB3 | CIB3 |
| 3346 | L18 | L19 | 665 | 1310 | 7341 | 386,3 | 11 | 0,681 | H5A034 C | sample | Hm Drgbl | Hm Drgbl | Hm Drgbl | CPE002DC | CPE002EG | CPE002F6 | NM 00264 | NM 0026 | NM 0026 | PIGF | PIGF | PIGF |
| 20596 | K12 | K13 | 1318 | 745,7 | 14539 | 159 | 11 | 0,733 | H5A034 C | sample | Hm Gen E | Hm Gen E | Hm Gen E | CPE002JR | CPE002O6 | CPE002KM | NM 00103 | NM 0010 | NM 0010 | SERINC4 | SERINC4 | SERINC4 |
| 20605 | K20 | K21 | 1551 | 797 | 17099 | 165,9 | 11 | 0,733 | H5A034 C | sample | Hm Gen E | Hm Gen E | Hm Gen E | CPE002JR | CPE002O6 | CPE002KM | NM 00103 | NM 0010 | NM 0010 | C15orf45 | C15orf45 | C15orf45 |
| 9807 | I22 | I23 | 1209 | 1043 | 13303 | 126 | 11 | 0,769 | H5A034 C | sample | Hm Drgbl | Hm Drgbl | Hm Drgbl | CPE002E9 | CPE002EQ | CPE002FB | NM 00103 | NM 0010 | NM 0010 | SIL1 | SIL1 | SIL1 |
| 19489 | M1 | M2 | 1188 | 1133 | 13071 | 216,6 | 11 | 0,78 | H5A034 C | sample | Hm Gen E | Hm Gen E | Hm Gen E | CPE002JO | CPE002O3 | CPE002KJ | XM 29485 | XM 2948 | XM 2948 | LOC33920 | LOC33920 | LOC33920 |
| 21057 | N17 | N18 | 1585 | 1035 | 17398 | 268,2 | 11 | 0,78 | H5A034 C | sample | Hm Gen E | Hm Gen E | Hm Gen E | CPE002JS | CPE002O7 | CPE002KN | XM 93195 | XM 9319 | XM 9319 | LOC64397 | LOC64397 | LOC64397 |
| 14460 | K2 | K3 | 856 | 918,7 | 9421 | 157,7 | 11 | 0,783 | H5A034 C | sample | Hm Gen E | Hm Gen E | Hm Gen E | CPE002J7 | CPE002NC | CPE002K6 | NM 01756 | NM 0175 | NM 0175 | FAM20A | FAM20A | FAM20A |
| 11425 | M1 | M2 | 2022 | 882,1 | 22260 | 124,2 | 11 | 0,797 | H5A034 C | sample | Hm Drgbl | Hm Drgbl | Hm Drgbl | CPE002ED | CPE002F3 | CPE002FT | NM 00102 | NM 0010 | NM 0010 | NANOS2 | NANOS2 | NANOS2 |
| 12625 | O1 | O2 | 925 | 1034 | 10154 | 166,1 | 11 | 0,827 | H5A034 C | sample | Hm Gen E | Hm Gen E | Hm Gen E | CPE002J5 | CPE002NK | CPE002K1 | NM 00102 | NM 0010 | NM 0010 | TPD52 | TPD52 | TPD52 |
| 7377 | D17 | D18 | 1578 | 603 | 17322 | 398,8 | 11 | 0,84 | H5A034 C | sample | Hm Drgbl | Hm Drgbl | Hm Drgbl | CPE002E3 | CPE002EW | CPE002FH | NM 00481 | NM 0048 | NM 0048 | GRAP2 | GRAP2 | GRAP2 |
| 17232 | N9 | N10 | 2144 | 1024 | 23541 | 183,1 | 11 | 0,849 | H5A034 C | sample | Hm Gen E | Hm Gen E | Hm Gen E | CPE002JI | CPE002NZ | CPE002KD | XM 00112 | NM 1825 | XM 0011 | LOC11643 | CBLN2 | LOC11643 |
| 6975 | C22 | C23 | 1106 | 607,9 | 12140 | 453,4 | 11 | 0,856 | H5A034 C | sample | Hm Drgbl | Hm Drgbl | Hm Drgbl | CPE002E2 | CPE002EX | CPE002FI | NM 01529 | NM 0152 | NM 0152 | TRIM37 | TRIM37 | TRIM37 |
| 7210 | M18 | M19 | 1858 | 563,9 | 20478 | 431,6 | 11 | 0,856 | H5A034 C | sample | Hm Drgbl | Hm Drgbl | Hm Drgbl | CPE002E2 | CPE002EX | CPE002FI | NM 00453 | NM 0045 | NM 0045 | NAIP | NAIP | NAIP |
| 5353 | P1 | P2 | 1715 | 665,5 | 18815 | 510,1 | 11 | 0,863 | H5A034 C | sample | Hm Drgbl | Hm Drgbl | Hm Drgbl | CPE002DV | CPE002EM | CPE002FO | NM 13921 | NM 1392 | NM 1392 | TAF15 | TAF15 | TAF15 |
| 2413 | E20 | E21 | 1710 | 697,7 | 18760 | 337,3 | 11 | 0,866 | H5A034 C | sample | Hm Drgbl | Hm Drgbl | Hm Drgbl | CPE002DC | CPE002EE | CPE002F4 | NM 00537 | NM 0053 | NM 0053 | MPP2 | MPP2 | MPP2 |
| 2434 | F18 | F19 | 1421 | 620,1 | 15670 | 361,2 | 11 | 0,866 | H5A034 C | sample | Hm Drgbl | Hm Drgbl | Hm Drgbl | CPE002DC | CPE002EE | CPE002F4 | NM 14516 | NM 1451 | NM 1451 | MAP2K5 | MAP2K5 | MAP2K5 |
| 2491 | H4 | H5 | 761 | 672,4 | 8368 | 496,1 | 11 | 0,866 | H5A034 C | sample | Hm Drgbl | Hm Drgbl | Hm Drgbl | CPE002DC | CPE002EE | CPE002F4 | NM 00504 | NM 0050 | NM 0050 | PRKX | PRKX | PRKX |
| 2547 | K11 | K12 | 810 | 714,2 | 8912 | 454,5 | 11 | 0,866 | H5A034 C | sample | Hm Drgbl | Hm Drgbl | Hm Drgbl | CPE002DC | CPE002EE | CPE002F4 | NM 0010 | NM 0010 | NM 0010 | ERBB2 | ERBB2 | ERBB2 |
| 2555 | K19 | K20 | 1150 | 630,1 | 12621 | 267,4 | 11 | 0,866 | H5A034 C | sample | Hm Drgbl | Hm Drgbl | Hm Drgbl | CPE002DC | CPE002EE | CPE002F4 | NM 00562 | NM 0056 | NM 0056 | SGK | SGK | SGK |
| 2615 | M8 | M9 | 1583 | 708,9 | 17375 | 393,2 | 11 | 0,866 | H5A034 C | sample | Hm Drgbl | Hm Drgbl | Hm Drgbl | CPE002DC | CPE002EE | CPE002F4 | NM 00029 | NM 0002 | NM 0002 | PHKG2 | PHKG2 | PHKG2 |
| 2622 | N14 | N15 | 1449 | 723,1 | 16011 | 384 | 11 | 0,866 | H5A034 C | sample | Hm Drgbl | Hm Drgbl | Hm Drgbl | CPE002DC | CPE002EE | CPE002F4 | NM 00045 | NM 0004 | NM 0004 | STK11 | STK11 | STK11 |
| 2683 | P4 | P5 | 1118 | 703,6 | 12326 | 477,2 | 11 | 0,866 | H5A034 C | sample | Hm Drgbl | Hm Drgbl | Hm Drgbl | CPE002DC | CPE002EE | CPE002F4 | NM 00045 | NM 0004 | NM 0004 | TEK | TEK | TEK |
| 2688 | P9 | P10 | 1628 | 702,7 | 17902 | 431,9 | 11 | 0,866 | H5A034 C | sample | Hm Drgbl | Hm Drgbl | Hm Drgbl | CPE002DC | CPE002EE | CPE002F4 | NM 00015 | NM 0001 | NM 0001 | GALK1 | GALK1 | GALK1 |
| 16530 | A3 | A4 | 633 | 1095 | 6986 | 151,3 | 11 | 0,87 | H5A034 C | sample | Hm Gen E | Hm Gen E | Hm Gen E | CPE002JH | CPE002NV | CPE002KC | XM 00112 | XM 0011 | XM 0011 | MGC1612 | MGC1612 | MGC1612 |
| 16538 | B10 | B11 | 1421 | 981,9 | 15664 | 304,5 | 11 | 0,87 | H5A034 C | sample | Hm Gen E | Hm Gen E | Hm Gen E | CPE002JH | CPE002NV | CPE002KC | NM 00104 | NM 0010 | NM 0010 | C14orf43 | C14orf43 | C14orf43 |
| 16627 | E4 | E5 | 1521 | 1022 | 16777 | 247,2 | 11 | 0,87 | H5A034 C | sample | Hm Gen E | Hm Gen E | Hm Gen E | CPE002JH | CPE002NV | CPE002KC | NM 17812 | NM 1781 | NM 1781 | C1orf201 | C1orf201 | C1orf201 |
| 16825 | N1 | N2 | 1657 | 876,1 | 18201 | 208,4 | 11 | 0,87 | H5A034 C | sample | Hm Gen E | Hm Gen E | Hm Gen E | CPE002JH | CPE002NV | CPE002KC | NM 13834 | NM 1383 | NM 1383 | C14orf152 | C14orf152 | C14orf152 |
| 4310 | D21 | D22 | 1458 | 744 | 16026 | 333,5 | 11 | 0,928 | H5A034 C | sample | Hm Drgbl | Hm Drgbl | Hm Drgbl | CPE002DU | CPE002EK | CPE002FQ | NM 00021 | NM 0002 | NM 0002 | KCNA1 | KCNA1 | KCNA1 |
| 4502 | L21 | L22 | 1820 | 713,8 | 19952 | 352,1 | 11 | 0,928 | H5A034 C | sample | Hm Drgbl | Hm Drgbl | Hm Drgbl | CPE002DU | CPE002EK | CPE002FQ | NM 18179 | NM 1817 | NM 1817 | KCNQ1 | KCNQ1 | KCNQ1 |
| 198 | I14 | I15 | 619 | 1407 | 6783 | 487,1 | 11 | 0,936 | H5A034 C | sample | Hm Drgbl | Hm Drgbl | Hm Drgbl | CPE002DT | CPE002EJ | CPE002F9 | NM 02306 | NM 0230 | NM 0230 | SIGLEC1 | SIGLEC1 | SIGLEC1 |
| 215 | I8 | I9 | 313 | 1597 | 3428 | 487,1 | 11 | 0,936 | H5A034 C | sample | Hm Drgbl | Hm Drgbl | Hm Drgbl | CPE002DT | CPE002EJ | CPE002F9 | NM 00054 | NM 0005 | NM 0005 | SMPD1 | SMPD1 | SMPD1 |
| 274 | L18 | L19 | 517 | 1406 | 5683 | 514,2 | 11 | 0,936 | H5A034 C | sample | Hm Drgbl | Hm Drgbl | Hm Drgbl | CPE002DT | CPE002EJ | CPE002F9 | NM 14525 | NM 1452 | NM 1452 | STYX | STYX | STYX |
| 287 | L8 | L9 | 538 | 1405 | 5942 | 418,3 | 11 | 0,936 | H5A034 C | sample | Hm Drgbl | Hm Drgbl | Hm Drgbl | CPE002DT | CPE002EJ | CPE002F9 | NM 01745 | NM 0174 | NM 0174 | STAU1 | STAU1 | STAU1 |
| 291 | M11 | M12 | 710 | 1500 | 7786 | 490,4 | 11 | 0,936 | H5A034 C | sample | Hm Drgbl | Hm Drgbl | Hm Drgbl | CPE002DT | CPE002EJ | CPE002F9 | NM 00298 | NM 0029 | NM 0029 | CCL2 | CCL2 | CCL2 |
| 1952 | B16 | B17 | 1526 | 685,5 | 16744 | 446,6 | 11 | 0,957 | H5A034 C | sample | Hm Drgbl | Hm Drgbl | Hm Drgbl | CPE002FX | CPE002G1 | CPE002G5 | NM 00107 | NM 0010 | NM 0010 | ZNF391 | ZNF391 | ZNF391 |
| 2062 | F7 | F8 | 1534 | 751,6 | 16851 | 471,1 | 11 | 0,957 | H5A034 C | sample | Hm Drgbl | Hm Drgbl | Hm Drgbl | CPE002FX | CPE002G1 | CPE002G5 | NM 15245 | NM 1524 | NM 1524 | ZSCAN29 | ZSCAN29 | ZSCAN29 |
| 2111 | H8 | H9 | 1177 | 670,8 | 12932 | 381,5 | 11 | 0,957 | H5A034 C | sample | Hm Drgbl | Hm Drgbl | Hm Drgbl | CPE002FX | CPE002G1 | CPE002G5 | NM 00103 | NM 0010 | NM 0010 | LBXCOR1 | LBXCOR1 | LBXCOR1 |
| 2192 | L16 | L17 | 1559 | 604,1 | 17117 | 380,8 | 11 | 0,957 | H5A034 C | sample | Hm Drgbl | Hm Drgbl | Hm Drgbl | CPE002FX | CPE002G1 | CPE002G5 | NM 00104 | NM 0010 | NM 0010 | LOC73039 | LOC73039 | LOC73039 |
| 6591 | C22 | C23 | 1921 | 709,8 | 21124 | 413,3 | 11 | 0,965 | H5A034 C | sample | Hm Drgbl | Hm Drgbl | Hm Drgbl | CPE002E1 | CPE002EY | CPE002FJ | NM 00087 | NM 0008 | NM 0008 | IL1R1 | IL1R1 | IL1R1 |
| 4075 | J4 | J5 | 1285 | 655,9 | 14146 | 347 | 11 | 0,978 | H5A034 C | sample | Hm Drgbl | Hm Drgbl | Hm Drgbl | CPE002DS | CPE002EI | CPE002F8 | NM 00032 | NM 0003 | NM 0003 | RPGR | RPGR | RPGR |
| 4113 | L17 | L18 | 137 | 758 | 1507 | 316,4 | 11 | 0,978 | H5A034 C | sample | Hm Drgbl | Hm Drgbl | Hm Drgbl | CPE002DS | CPE002EI | CPE002F8 | NM 0028 | NM 0028 | NM 0028 | PSMC5 | PSMC5 | PSMC5 |
| 4175 | N8 | N9 | 880 | 668,2 | 9652 | 417,9 | 11 | 0,978 | H5A034 C | sample | Hm Drgbl | Hm Drgbl | Hm Drgbl | CPE002DS | CPE002EI | CPE002F8 | NM 00323 | NM 0032 | NM 0032 | TG | TG | TG |
| 11689 | H1 | H2 | 1392 | 904,6 | 15364 | 144,7 | 11 | 0,982 | H5A034 C | sample | Hm Gen E | Hm Gen E | Hm Gen E | CPE002JA | CPE002NJ | CPE002JZ | NM 00185 | NM 0018 | NM 0018 | COL9A1 | COL9A1 | COL9A1 |
| 15504 | F9 | F10 | 1365 | 904,3 | 14980 | 144,4 | 11 | 1 | H5A034 C | sample | Hm Gen E | Hm Gen E | Hm Gen E | CPE002JE | CPE002NT | CPE002K9 | NM 02275 | NM 0227 | NM 0227 | FAM59A | FAM59A | FAM59A |
| 5788 | B12 | B13 | 1720 | 804,1 | 18873 | 593,9 | 11 | 1,005 | H5A034 C | sample | Hm Drgbl | Hm Drgbl | Hm Drgbl | CPE002DY | CPE002F1 | CPE002FM | NM 00405 | NM 0040 | NM 0040 | BCL2L2 | BCL2L2 | BCL2L2 |
| 6111 | O22 | O23 | 1976 | 863,8 | 21786 | 566,9 | 11 | 1,005 | H5A034 C | sample | Hm Drgbl | Hm Drgbl | Hm Drgbl | CPE002DY | CPE002F1 | CPE002FM | NM 0007 | NM 0007 | NM 0007 | BCKDHA | BCKDHA | BCKDHA |
| 1399 | K15 | K16 | 1976 | 815,6 | 21687 | 331,9 | 11 | 1,009 | H5A034 C | sample | Hm Drgbl | Hm Drgbl | Hm Drgbl | CPE002FV | CPE002FZ | CPE002G3 | NM 00714 | NM 0071 | NM 0071 | PCGF2 | PCGF2 | PCGF2 |
| 1412 | K5 | K6 | 1376 | 782,2 | 15103 | 407,6 | 11 | 1,009 | H5A034 C | sample | Hm Drgbl | Hm Drgbl | Hm Drgbl | CPE002FV | CPE002FZ | CPE002G3 | NM 00343 | NM 0034 | NM 0034 | ZNF137 | ZNF137 | ZNF137 |
| 16427 | M19 | M20 | 1530 | 1014 | 16891 | 266 | 11 | 1,009 | H5A034 C | sample | Hm Gen E | Hm Gen E | Hm Gen E | CPE002JG | CPE002NV | CPE002KB | NM 03145 | NM 0314 | NM 0314 | TEX101 | TEX101 | TEX101 |
| 13610 | H10 | H11 | 1395 | 976,7 | 15343 | 140,3 | 11 | 1,033 | H5A034 C | sample | Hm Gen E | Hm Gen E | Hm Gen E | CPE002JC | CPE002NC | CPE002K4 | NM 01436 | NM 0143 | NM 0143 | SACS | SACS | SACS |
| 13631 | H8 | H9 | 996 | 1012 | 10980 | 155,9 | 11 | 1,033 | H5A034 C | sample | Hm Gen E | Hm Gen E | Hm Gen E | CPE002JC | CPE002NC | CPE002K4 | NM 01246 | NM 0124 | NM 0124 | TINF2 | TINF2 | TINF2 |
| 839 | C8 | C9 | 1770 | 751,6 | 19467 | 442,2 | 11 | 1,043 | H5A034 C | sample | Hm Drgbl | Hm Drgbl | Hm Drgbl | CPE002FU | CPE002FY | CPE002G2 | NM 00024 | NM 0002 | NM 0002 | MBL2 | MBL2 | MBL2 |
| 897 | F17 | F18 | 1234 | 754,5 | 13587 | 447,5 | 11 | 1,043 | H5A034 C | sample | Hm Drgbl | Hm Drgbl | Hm Drgbl | CPE002FU | CPE002FY | CPE002G2 | NM 00449 | NM 0044 | NM 0044 | GTF2A2 | GTF2A2 | GTF2A2 |
| 974 | I21 | I22 | 1091 | 784 | 11963 | 364,2 | 11 | 1,043 | H5A034 C | sample | Hm Drgbl | Hm Drgbl | Hm Drgbl | CPE002FU | CPE002FY | CPE002G2 | NM 00009 | NM 0000 | NM 0000 | COL5A1 | COL5A1 | COL5A1 |
| 1063 | M15 | M16 | 1268 | 832,5 | 13977 | 410,6 | 11 | 1,043 | H5A034 C | sample | Hm Drgbl | Hm Drgbl | Hm Drgbl | CPE002FU | CPE002FY | CPE002G2 | NM 00438 | NM 0043 | NM 0043 | NCAN | NCAN | NCAN |
| 5744 | P16 | P17 | 2176 | 704,2 | 24004 | 595,4 | 11 | 1,048 | H5A034 C | sample | Hm Drgbl | Hm Drgbl | Hm Drgbl | CPE002DX | CPE002F2 | CPE002FN | NM 00479 | NM 0047 | NM 0047 | PPIG | PPIG | PPIG |
| 5760 | P9 | P10 | 1912 | 658,4 | 20976 | 650,4 | 11 | 1,048 | H5A034 C | sample | Hm Drgbl | Hm Drgbl | Hm Drgbl | CPE002DX | CPE002F2 | CPE002FN | NM 00384 | NM 0038 | NM 0038 | TNFRSF10 | TNFRSF10 | TNFRSF10 |
| 3561 | E17 | E18 | 1444 | 1341 | 15850 | 324,3 | 11 | 1,058 | H5A034 C | sample | Hm Drgbl | Hm Drgbl | Hm Drgbl | CPE002DR | CPE002EH | CPE002F7 | NM 00173 | NM 0017 | NM 0017 | C5AR1 | C5AR1 | C5AR1 |
| 3705 | K17 | K18 | 1584 | 1314 | 17450 | 336,3 | 11 | 1,058 | H5A034 C | sample | Hm Drgbl | Hm Drgbl | Hm Drgbl | CPE002DR | CPE002EH | CPE002F7 | NM 00522 | NM 0052 | NM 0052 | EDG3 | EDG3 | EDG3 |
| 3729 | L17 | L18 | 1236 | 1495 | 13563 | 419,1 | 11 | 1,058 | H5A034 C | sample | Hm Drgbl | Hm Drgbl | Hm Drgbl | CPE002DR | CPE002EH | CPE002F7 | NM 00591 | NM 0059 | NM 0059 | MC5R | MC5R | MC5R |
| 8068 | A12 | A13 | 1348 | 601,5 | 14877 | 542,5 | 11 | 1,058 | H5A034 C | sample | Hm Drgbl | Hm Drgbl | Hm Drgbl | CPE002E5 | CPE002EU | CPE002FF | NM 01504 | NM 0150 | NM 0150 | TBC1D9B | TBC1D9B | TBC1D9B |
| 8366 | M21 | M22 | 1840 | 606,1 | 20194 | 457,4 | 11 | 1,058 | H5A034 C | sample | Hm Drgbl | Hm Drgbl | Hm Drgbl | CPE002E5 | CPE002EU | CPE002FF | NM 00723 | NM 0072 | NM 0072 | SLC6A14 | SLC6A14 | SLC6A14 |
| 8400 | N9 | N10 | 1669 | 597,6 | 18294 | 559,9 | 11 | 1,058 | H5A034 C | sample | Hm Drgbl | Hm Drgbl | Hm Drgbl | CPE002E5 | CPE002EU | CPE002FF | NM 01498 | NM 0149 | NM 0149 | RIMS1 | RIMS1 | RIMS1 |
| 1550 | A21 | A22 | 1425 | 847,4 | 15708 | 444,9 | 11 | 1,152 | H5A034 C | sample | Hm Drgbl | Hm Drgbl | Hm Drgbl | CPE002FW | CPE002G0 | CPE002G4 | NM 01542 | NM 0154 | NM 0154 | ZNF473 | ZNF473 | ZNF473 |
| 1597 | C20 | C21 | 1414 | 787,1 | 15581 | 416,7 | 11 | 1,152 | H5A034 C | sample | Hm Drgbl | Hm Drgbl | Hm Drgbl | CPE002FW | CPE002G0 | CPE002G4 | NM 02083 | NM 0208 | NM 0208 | ZNF687 | ZNF687 | ZNF687 |
| 1784 | K16 | K17 | 2265 | 788,1 | 24901 | 294,4 | 11 | 1,152 | H5A034 C | sample | Hm Drgbl | Hm Drgbl | Hm Drgbl | CPE002FW | CPE002G0 | CPE002G4 | NM 02237 | NM 0223 | NM 0223 | TOR3A | TOR3A | TOR3A |
| 1789 | K20 | K21 | 1402 | 691,9 | 15475 | 396,1 | 11 | 1,152 | H5A034 C | sample | Hm Drgbl | Hm Drgbl | Hm Drgbl | CPE002FW | CPE002G0 | CPE002G4 | NM 02246 | NM 0224 | NM 0224 | HS1BP3 | HS1BP3 | HS1BP3 |
| 21791 | L8 | L9 | 275 | 944,6 | 3026 | 701,6 | 11 | 1,158 | H5A034 C | sample | Hm Gen E | Hm Gen E | Hm Gen E | CPE002JU | CPE002O9 | CPE002KP | XM 92984 | XM 9298 | XM 9298 | LOC65378 | LOC65378 | LOC65378 |
| 21827 | N19 | N20 | 223 | 867,3 | 2460 | 634,5 | 11 | 1,158 | H5A034 C | sample | Hm Gen E | Hm Gen E | Hm Gen E | CPE002JU | CPE002O9 | CPE002KP | XM 94153 | XM 9415 | XM 9415 | LOC65218 | LOC65218 | LOC65218 |
| 21886 | P7 | P8 | 392 | 1082 | 4303 | 667,3 | 11 | 1,158 | H5A034 C | sample | Hm Gen E | Hm Gen E | Hm Gen E | CPE002JU | CPE002O9 | CPE002KP | XM 94165 | XM 9416 | XM 9416 | LOC65225 | LOC65225 | LOC65225 |
| 21887 | P8 | P9 | 472 | 1232 | 5195 | 568,8 | 11 | 1,158 | H5A034 C | sample | Hm Gen E | Hm Gen E | Hm Gen E | CPE002JU | CPE002O9 | CPE002KP | NM 00104 | NM 0010 | NM 0010 | TBC1D3G | TBC1D3G | TBC1D3G |
| 4622 | A21 | A22 | 1406 | 834,9 | 15500 | 481,7 | 11 | 1,209 | H5A034 C | sample | Hm Drgbl | Hm Drgbl | Hm Drgbl | CPE002DV | CPE002EL | CPE002FP | NM 00434 | NM 0043 | NM 0043 | CASP3 | CASP3 | CASP3 |
| 4799 | H8 | H9 | 1535 | 837,8 | 16899 | 413,6 | 11 | 1,209 | H5A034 C | sample | Hm Drgbl | Hm Drgbl | Hm Drgbl | CPE002DV | CPE002EL | CPE002FP | NM 01447 | NM 0144 | NM 0144 | ADAMDEC | ADAMDEC | ADAMDEC |
| 4873 | L1 | L2 | 1499 | 843,1 | 16504 | 535,1 | 11 | 1,209 | H5A034 C | sample | Hm Drgbl | Hm Drgbl | Hm Drgbl | CPE002DV | CPE002EL | CPE002FP | NM 2074 | NM 2074 | NM 2074 | TMPRSS11 | TMPRSS11 | TMPRSS11 |
| 10616 | K16 | K17 | 1624 | 852,5 | 17827 | 180,2 | 11 | 1,243 | H5A034 C | sample | Hm Drgbl | Hm Drgbl | Hm Drgbl | CPE002EB | CPE002EO | CPE002FR | NM 13871 | NM 1387 | NM 1387 | SLC26A8 | SLC26A8 | SLC26A8 |
| 2757 | C6 | C7 | 730 | 677,7 | 8049 | 604,1 | 11 | 1,294 | H5A034 C | sample | Hm Drgbl | Hm Drgbl | Hm Drgbl | CPE002DP | CPE002EF | CPE002F5 | NM 01842 | NM 0184 | NM 0184 | PI4KII | PI4KII | PI4KII |
| 2880 | H9 | H10 | 1221 | 677 | 13446 | 453,7 | 11 | 1,294 | H5A034 C | sample | Hm Drgbl | Hm Drgbl | Hm Drgbl | CPE002DP | CPE002EF | CPE002F5 | NM 01492 | NM 0149 | NM 0149 | ICK | ICK | ICK |
| 20018 | C10 | C11 | 660 | 1226 | 7274 | 176,6 | 11 | 1,341 | H5A034 C | sample | Hm Gen E | Hm Gen E | Hm Gen E | CPE002JQ | CPE002O5 | CPE002KL | XM 94135 | XM 9413 | XM 3762 | LOC40109 | LOC40109 | LOC40109 |
| 20035 | C4 | C5 | 983 | 1153 | 10820 | 201,6 | 11 | 1,341 | H5A034 C | sample | Hm Gen E | Hm Gen E | Hm Gen E | CPE002JQ | CPE002O5 | CPE002KL | XM 00112 | XM 0011 | XM 0011 | FLJ22763 | FLJ22763 | FLJ22763 |
| 20085 | E6 | E7 | 766 | 993 | 8397 | 146 | 11 | 1,341 | H5A034 C | sample | Hm Gen E | Hm Gen E | Hm Gen E | CPE002JQ | CPE002O5 | CPE002KL | XM 37932 | XM 3793 | XM 3793 | LOC40118 | LOC40118 | LOC40118 |
| 20099 | F19 | F20 | 938 | 1113 | 10335 | 145,2 | 11 | 1,341 | H5A034 C | sample | Hm Gen E | Hm Gen E | Hm Gen E | CPE002JQ | CPE002O5 | CPE002KL | XM 94423 | XM 9442 | XM 3783 | LOC40009 | LOC40009 | LOC40009 |
| 20223 | K22 | K23 | 552 | 1302 | 6088 | 237,7 | 11 | 1,341 | H5A034 C | sample | Hm Gen E | Hm Gen E | Hm Gen E | CPE002JQ | CPE002O5 | CPE002KL | NM 21255 | NM 2125 | NM 2125 | LOC40149 | LOC40149 | LOC40149 |
| 20331 | P11 | P12 | 617 | 1371 | 6761 | 147,1 | 11 | 1,341 | H5A034 C | sample | Hm Gen E | Hm Gen E | Hm Gen E | CPE002JQ | CPE002O5 | CPE002KL | NM 0010 | NM 0010 | NM 0010 | RP11-191L | RP11-191L | RP11-191L |
| 392 | A16 | A17 | 2370 | 1834 | 26071 | 372,4 | 11 | 1,606 | H5A034 C | sample | Hm Drgbl | Hm Drgbl | Hm Drgbl | CPE002E0 | CPE002EZ | CPE002FK | NM 00525 | NM 0052 | NM 0052 | FOXC2 | FOXC2 | FOXC2 |
| 7740 | C2 | C3 | 304 | 653 | 3345 | 388,1 | 11 | 1,679 | H5A034 C | sample | Hm Drgbl | Hm Drgbl | Hm Drgbl | CPE002E4 | CPE002EV | CPE002FG | NM 00642 | NM 0064 | NM 0064 | SLU7 | SLU7 | SLU7 |
| 7757 | D13 | D14 | 1105 | 552,4 | 12188 | 417,3 | 11 | 1,679 | H5A034 C | sample | Hm Drgbl | Hm Drgbl | Hm Drgbl | CPE002E4 | CPE002EV | CPE002FG | NM 14817 | NM 0071 | NM 0071 | PEMT | PEMT | PEMT |
| 8064 | P9 | P10 | 1113 | 547,6 | 12255 | 422,9 | 11 | 1,679 | H5A034 C | sample | Hm Drgbl | Hm Drgbl | Hm Drgbl | CPE002E4 | CPE002EV | CPE002FG | NM 00639 | NM 0063 | NM 0063 | ATG7 | ATG7 | ATG7 |
| 3203 | F19 | F20 | 1384 | 1326 | 15340 | 386 | 11,1 | 0,687 | H5A034 C | sample | Hm Drgbl | Hm Drgbl | Hm Drgbl | CPE002DC | CPE002EG | CPE002F6 | NM 00285 | NM 0028 | NM 0028 | PTPRZ1 | PTPRZ1 | PTPRZ1 |
| 3296 | J16 | J17 | 927 | 1071 | 10289 | 226,4 | 11,1 | 0,687 | H5A034 C | sample | Hm Drgbl | Hm Drgbl | Hm Drgbl | CPE002DC | CPE002EG | CPE002F6 | NM 00621 | NM 0062 | NM 0062 | SERPINA4 | SERPINA4 | SERPINA4 |
| 3347 | L19 | L20 | 887 | 1243 | 9879 | 378,7 | 11,1 | 0,687 | H5A034 C | sample | Hm Drgbl | Hm Drgbl | Hm Drgbl | CPE002DC | CPE002EG | CPE002F6 | NM 00580 | NM 0058 | NM 0010 | CTDSPL | CTDSPL | CTDSPL |
| 20583 | J22 | J23 | 988 | 765,6 | 10952 | 167,2 | 11,1 | 0,74 | H5A034 C | sample | Hm Gen E | Hm Gen E | Hm Gen E | CPE002JR | CPE002O6 | CPE002KM | XM 37696 | XM 3769 | XM 3769 | LOC64239 | LOC64239 | LOC64239 |
| 14545 | O1 | O2 | 1850 | 875,9 | 20520 | 154 | 11,1 | 0,79 | H5A034 C | sample | Hm Gen E | Hm Gen E | Hm Gen E | CPE002J7 | CPE002NC | CPE002K6 | NM 01650 | NM 0165 | NM 0165 | ZCCHC17 | ZCCHC17 | ZCCHC17 |
| 12469 | H20 | H21 | 1406 | 1036 | 15569 | 146,7 | 11,1 | 0,835 | H5A034 C | sample | Hm Gen E | Hm Gen E | Hm Gen E | CPE002J5 | CPE002NK | CPE002K1 | NM 00394 | NM 0039 | NM 0039 | NOL3 | NOL3 | NOL3 |
| 6969 | C17 | C18 | 914 | 549,2 | 10167 | 442,1 | 11,1 | 0,864 | H5A034 C | sample | Hm Drgbl | Hm Drgbl | Hm Drgbl | CPE002E2 | CPE002EX | CPE002FI | NM 01744 | NM 0174 | NM 0174 | LDHC | LDHC | LDHC |
| 6992 | D16 | D17 | 1417 | 543,8 | 15752 | 416,2 | 11,1 | 0,864 | H5A034 C | sample | Hm Drgbl | Hm Drgbl | Hm Drgbl | CPE002E2 | CPE002EX | CPE002FI | NM 00454 | NM 0045 | NM 0045 | NDUFB10 | NDUFB10 | NDUFB10 |
| 5039 | B8 | B9 | 1293 | 566,6 | 14294 | 512,7 | 11,1 | 0,871 | H5A034 C | sample | Hm Drgbl | Hm Drgbl | Hm Drgbl | CPE002DV | CPE002EM | CPE002FO | NM 18159 | NM 1815 | NM 1815 | UPP1 | UPP1 | UPP1 |
| 2346 | B3 | B4 | 2143 | 746,1 | 23733 | 491,6 | 11,1 | 0,874 | H5A034 C | sample | Hm Drgbl | Hm Drgbl | Hm Drgbl | CPE002DC | CPE002EE | CPE002F4 | NM 00433 | NM 0043 | NM 0043 | BRAF | BRAF | BRAF |
| 2402 | E10 | E11 | 777 | 738,6 | 8614 | 393 | 11,1 | 0,874 | H5A034 C | sample | Hm Drgbl | Hm Drgbl | Hm Drgbl | CPE002DC | CPE002EE | CPE002F4 | NM 03314 | NM 0331 | NM 0331 | MAP3K9 | MAP3K9 | MAP3K9 |
| 2463 | G22 | G23 | 1416 | 681,7 | 15660 | 362,5 | 11,1 | 0,874 | H5A034 C | sample | Hm Drgbl | Hm Drgbl | Hm Drgbl | CPE002DC | CPE002EE | CPE002F4 | NM 0010 | NM 0010 | NM 0010 | NTRK1 | NTRK1 | NTRK1 |
| 2538 | J3 | J4 | 1600 | 664,7 | 17685 | 367,1 | 11,1 | 0,874 | H5A034 C | sample | Hm Drgbl | Hm Drgbl | Hm Drgbl | CPE002DC | CPE002EE | CPE002F4 | NM 00134 | NM 0013 | NM 0010 | DGKG | DGKG | DGKG |
| 2574 | L14 | L15 | 1505 | 593,5 | 16687 | 301,4 | 11,1 | 0,874 | H5A034 C | sample | Hm Drgbl | Hm Drgbl | Hm Drgbl | CPE002DC | CPE002EE | CPE002F4 | NM 00301 | NM 0030 | NM 0030 | MAP2K4 | MAP2K4 | MAP2K4 |
| 2618 | N10 | N11 | 1593 | 666,8 | 17653 | 355,3 | 11,1 | 0,874 | H5A034 C | sample | Hm Drgbl | Hm Drgbl | Hm Drgbl | CPE002DC | CPE002EE | CPE002F4 | NM 00103 | NM 0010 | NM 0010 | CDKL5 | CDKL5 | CDKL5 |
| 16743 | J22 | J23 | 1512 | 1069 | 16773 | 201,8 | 11,1 | 0,877 | H5A034 C | sample | Hm Gen E | Hm Gen E | Hm Gen E | CPE002JH | CPE002NV | CPE002KC | NM 14523 | NM 1452 | NM 1452 | FANK1 | FANK1 | FANK1 |
| 17922 | K3 | K4 | 1165 | 1044 | 12963 | 197,1 | 11,1 | 0,877 | H5A034 C | sample | Hm Gen E | Hm Gen E | Hm Gen E | CPE002JK | CPE002NZ | CPE002KF | NM 00108 | NM 0010 | NM 0010 | OVOS2 | OVOS2 | OVOS2 |
| 9006 | H14 | H15 | 1380 | 1662 | 15264 | 137,9 | 11,1 | 0,892 | H5A034 C | sample | Hm Drgbl | Hm Drgbl | Hm Drgbl | CPE002E7 | CPE002ES | CPE002FD | NM 01906 | NM 0190 | NM 0190 | SDK2 | SDK2 | SDK2 |
| 4239 | A22 | A23 | 1888 | 789,2 | 21050 | 480,6 | 11,1 | 0,937 | H5A034 C | sample | Hm Drgbl | Hm Drgbl | Hm Drgbl | CPE002DU | CPE002EK | CPE002FQ | NM 00462 | NM 0046 | NM 0046 | TRPC6 | TRPC6 | TRPC6 |
| 72 | C9 | C10 | 785 | 1778 | 8718 | 562,6 | 11,1 | 0,945 | H5A034 C | sample | Hm Drgbl | Hm Drgbl | Hm Drgbl | CPE002DT | CPE002EJ | CPE002F9 | NM 00092 | NM 0009 | NM 0009 | PGR | PGR | PGR |
| 278 | L21 | L22 | 166 | 1471 | 1849 | 527 | 11,1 | 0,945 | H5A034 C | sample | Hm Drgbl | Hm Drgbl | Hm Drgbl | CPE002DT | CPE002EJ | CPE002F9 | NM 00304 | NM 0030 | NM 0030 | SLC4A2 | SLC4A2 | SLC4A2 |
| 350 | O21 | O22 | 603 | 1522 | 6714 | 748,8 | 11,1 | 0,945 | H5A034 C | sample | Hm Drgbl | Hm Drgbl | Hm Drgbl | CPE002DT | CPE002EJ | CPE002F9 | NM 00299 | NM 0029 | NM 0029 | CXCL6 | CXCL6 | CXCL6 |
| 4105 | L1 | L2 | 1769 | 708,8 | 19590 | 379,3 | 11,1 | 0,987 | H5A034 C | sample | Hm Drgbl | Hm Drgbl | Hm Drgbl | CPE002DS | CPE002EI | CPE002F8 | NM 00565 | NM 0056 | NM 0056 | TDO2 | TDO2 | TDO2 |
| 4136 | M16 | M17 | 1934 | 674,1 | 21452 | 317,9 | 11,1 | 0,987 | H5A034 C | sample | Hm Drgbl | Hm Drgbl | Hm Drgbl | CPE002DS | CPE002EI | CPE002F8 | NM 00289 | NM 0028 | NM 0028 | RBMS1 | RBMS1 | RBMS1 |
| 11806 | L7 | L8 | 1083 | 850,3 | 12007 | 158,5 | 11,1 | 0,991 | H5A034 C | sample | Hm Gen E | Hm Gen E | Hm Gen E | CPE002JA | CPE002NJ | CPE002JZ | NM 00189 | NM 0018 | NM 0018 | CSN2 | CSN2 | CSN2 |
| 15387 | B11 | B12 | 1178 | 905,1 | 13108 | 112,1 | 11,1 | 1,009 | H5A034 C | sample | Hm Gen E | Hm Gen E | Hm Gen E | CPE002JE | CPE002NT | CPE002K9 | NM 02249 | NM 0224 | NM 0224 | MRPS25 | MRPS25 | MRPS25 |
| 5780 | A5 | A6 | 1167 | 858,6 | 13003 | 448,7 | 11,1 | 1,014 | H5A034 C | sample | Hm Drgbl | Hm Drgbl | Hm Drgbl | CPE002DY | CPE002F1 | CPE002FM | NM 00001 | NM 0000 | NM 0000 | NAT2 | NAT2 | NAT2 |
| 5786 | B10 | B11 | 1829 | 761 | 20322 | 557,8 | 11,1 | 1,014 | H5A034 C | sample | Hm Drgbl | Hm Drgbl | Hm Drgbl | CPE002DY | CPE002F1 | CPE002FM | NM 13857 | NM 1385 | NM 0011 | BCL2L1 | BCL2L1 | BCL2L1 |
| 6036 | L2 | L3 | 1385 | 739,9 | 15328 | 378,5 | 11,1 | 1,014 | H5A034 C | sample | Hm Drgbl | Hm Drgbl | Hm Drgbl | CPE002DY | CPE002F1 | CPE002FM | NM 00405 | NM 0040 | NM 0040 | CA8 | CA8 | CA8 |
| 6039 | L22 | L23 | 1849 | 786,1 | 20573 | 536,1 | 11,1 | 1,014 | H5A034 C | sample | Hm Drgbl | Hm Drgbl | Hm Drgbl | CPE002DY | CPE002F1 | CPE002FM | NM 08059 | NM 0040 | NM 0040 | CAPS | CAPS | CAPS |
| 6043 | L4 | L5 | 934 | 853,7 | 10397 | 462,5 | 11,1 | 1,014 | H5A034 C | sample | Hm Drgbl | Hm Drgbl | Hm Drgbl | CPE002DY | CPE002F1 | CPE002FM | NM 00121 | NM 0012 | NM 0012 | CA9 | CA9 | CA9 |
| 6105 | O17 | O18 | 1681 | 713,9 | 18658 | 575,1 | 11,1 | 1,014 | H5A034 C | sample | Hm Drgbl | Hm Drgbl | Hm Drgbl | CPE002DY | CPE002F1 | CPE002FM | NM 00003 | NM 0000 | NM 0000 | ABCD1 | ABCD1 | ABCD1 |
| 6143 | P8 | P9 | 1017 | 816,3 | 11319 | 310,3 | 11,1 | 1,014 | H5A034 C | sample | Hm Drgbl | Hm Drgbl | Hm Drgbl | CPE002DY | CPE002F1 | CPE002FM | NM 00123 | NM 0012 | NM 0012 | CBR3 | CBR3 | CBR3 |
| 1272 | E9 | E10 | 1250 | 747,7 | 13821 | 383,8 | 11,1 | 1,018 | H5A034 C | sample | Hm Drgbl | Hm Drgbl | Hm Drgbl | CPE002FV | CPE002FZ | CPE002G3 | NM 00696 | NM 0069 | NM 0069 | ZNF28 | ZNF28 | ZNF28 |
| 1283 | F19 | F20 | 1776 | 800,1 | 19666 | 423,2 | 11,1 | 1,018 | H5A034 C | sample | Hm Drgbl | Hm Drgbl | Hm Drgbl | CPE002FV | CPE002FZ | CPE002G3 | NM 00372 | NM 0037 | NM 0037 | TP73L | TP73L | TP73L |
| 1287 | F22 | F23 | 1724 | 695,4 | 19054 | 390,5 | 11,1 | 1,018 | H5A034 C | sample | Hm Drgbl | Hm Drgbl | Hm Drgbl | CPE002FV | CPE002FZ | CPE002G3 | NM 01240 | NM 0124 | NM 0124 | PRDM4 | PRDM4 | PRDM4 |
| 1535 | P8 | P9 | 1965 | 733,6 | 21726 | 430,5 | 11,1 | 1,018 | H5A034 C | sample | Hm Drgbl | Hm Drgbl | Hm Drgbl | CPE002FV | CPE002FZ | CPE002G3 | NM 01531 | NM 0153 | NM 0153 | PPP1R13B | PPP1R13B | PPP1R13B |
| 16287 | G22 | G23 | 1050 | 980,7 | 11686 | 363,2 | 11,1 | 1,018 | H5A034 C | sample | Hm Gen E | Hm Gen E | Hm Gen E | CPE002JG | CPE002NV | CPE002KB | NM 00104 | NM 0010 | NM 0010 | MGC1305 | MGC1305 | MGC1305 |
| 16359 | J22 | J23 | 1239 | 997,8 | 13702 | 328,6 | 11,1 | 1,018 | H5A034 C | sample | Hm Gen E | Hm Gen E | Hm Gen E | CPE002JG | CPE002NV | CPE002KB | NM 0010 | NM 0010 | NM 0010 | FBXL10 | FBXL10 | FBXL10 |
| 831 | C22 | C23 | 1642 | 761,2 | 18307 | 490,5 | 11,1 | 1,052 | H5A034 C | sample | Hm Drgbl | Hm Drgbl | Hm Drgbl | CPE002FU | CPE002FY | CPE002G2 | NM 00593 | NM 0059 | NM 0059 | MICB | MICB | MICB |
| 865 | E1 | E2 | 775 | 879,5 | 8599 | 379,5 | 11,1 | 1,052 | H5A034 C | sample | Hm Drgbl | Hm Drgbl | Hm Drgbl | CPE002FU | CPE002FY | CPE002G2 | NM 0012 | NM 0012 | NM 0012 | KLF9 | KLF9 | KLF9 |
| 986 | J10 | J11 | 1694 | 751,3 | 18720 | 307,7 | 11,1 | 1,052 | H5A034 C | sample | Hm Drgbl | Hm Drgbl | Hm Drgbl | CPE002FU | CPE002FY | CPE002G2 | NM 00318 | NM 0031 | NM 0031 | SYT5 | SYT5 | SYT5 |
| 1006 | J7 | J8 | 1369 | 763,3 | 15256 | 336,7 | 11,1 | 1,052 | H5A034 C | sample | Hm Drgbl | Hm Drgbl | Hm Drgbl | CPE002FU | CPE002FY | CPE002G2 | NM 00215 | NM 0021 | NM 0021 | HRC | HRC | HRC |
| 1023 | K22 | K23 | 1220 | 771,5 | 13487 | 405,2 | 11,1 | 1,052 | H5A034 C | sample | Hm Drgbl | Hm Drgbl | Hm Drgbl | CPE002FU | CPE002FY | CPE002G2 | NM 00265 | NM 0026 | NM 0026 | PLAG1 | PLAG1 | PLAG1 |
| 1053 | L6 | L7 | 1720 | 717,8 | 19119 | 362,4 | 11,1 | 1,052 | H5A034 C | sample | Hm Drgbl | Hm Drgbl | Hm Drgbl | CPE002FU | CPE002FY | CPE002G2 | NM 18149 | NM 1814 | NM 1814 | TCF20 | TCF20 | TCF20 |
| 1060 | M12 | M13 | 1160 | 663,4 | 12909 | 397,1 | 11,1 | 1,052 | H5A034 C | sample | Hm Drgbl | Hm Drgbl | Hm Drgbl | CPE002FU | CPE002FY | CPE002G2 | NM 00269 | NM 0026 | NM 0026 | POU2F2 | POU2F2 | POU2F2 |
| 5595 | J11 | J12 | 1918 | 719,5 | 21288 | 558,5 | 11,1 | 1,057 | H5A034 C | sample | Hm Drgbl | Hm Drgbl | Hm Drgbl | CPE002DX | CPE002F2 | CPE002FN | NM 00377 | NM 0037 | NM 0037 | DNAH11 | DNAH11 | DNAH11 |
| 5726 | O21 | O22 | 2160 | 766,9 | 23988 | 515,2 | 11,1 | 1,057 | H5A034 C | sample | Hm Drgbl | Hm Drgbl | Hm Drgbl | CPE002DX | CPE002F2 | CPE002FN | NM 0037 | NM 0037 | NM 0037 | TNFSF11 | TNFSF11 | TNFSF11 |
| 5745 | P17 | P18 | 1975 | 690 | 21998 | 625,3 | 11,1 | 1,057 | H5A034 C | sample | Hm Drgbl | Hm Drgbl | Hm Drgbl | CPE002DX | CPE002F2 | CPE002FN | NM 00385 | NM 0038 | NM 0038 | SUCLA2 | SUCLA2 | SUCLA2 |
| 3587 | F19 | F20 | 1717 | 1432 | 19102 | 371,4 | 11,1 | 1,067 | H5A034 C | sample | Hm Drgbl | Hm Drgbl | Hm Drgbl | CPE002DR | CPE002EH | CPE002F7 | NM 00083 | NM 0008 | NM 0008 | GRM1 | GRM1 | GRM1 |
| 3751 | M15 | M16 | 1588 | 1224 | 17630 | 318 | 11,1 | 1,067 | H5A034 C | sample | Hm Drgbl | Hm Drgbl | Hm Drgbl | CPE002DR | CPE002EH | CPE002F7 | NM 0010 | NM 0010 | NM 0010 | FPRL1 | FPRL1 | FPRL1 |
| 3765 | M6 | M7 | 1011 | 1350 | 11217 | 348,8 | 11,1 | 1,067 | H5A034 C | sample | Hm Drgbl | Hm Drgbl | Hm Drgbl | CPE002DR | CPE002EH | CPE002F7 | NM 0010 | NM 0010 | NM 0010 | LPHN1 | LPHN1 | LPHN1 |
| 8090 | B10 | B11 | 1655 | 701,1 | 18305 | 451,5 | 11,1 | 1,067 | H5A034 C | sample | Hm Drgbl | Hm Drgbl | Hm Drgbl | CPE002E5 | CPE002EU | CPE002FF | NM 19831 | NM 1983 | NM 0153 | TENC1 | TENC1 | TENC1 |
| 8291 | J19 | J20 | 1659 | 603 | 18412 | 376,9 | 11,1 | 1,067 | H5A034 C | sample | Hm Drgbl | Hm Drgbl | Hm Drgbl | CPE002E5 | CPE002EU | CPE002FF | NM 02125 | NM 0212 | NM 0212 | RAB18 | RAB18 | RAB18 |
| 8339 | L19 | L20 | 1758 | 591,3 | 19548 | 437,7 | 11,1 | 1,067 | H5A034 C | sample | Hm Drgbl | Hm Drgbl | Hm Drgbl | CPE002E5 | CPE002EU | CPE002FF | NM 01497 | NM 0149 | NM 0149 | PDCD11 | PDCD11 | PDCD11 |
| 1561 | B1 | B2 | 1366 | 793,3 | 15206 | 492,8 | 11,1 | 1,162 | H5A034 C | sample | Hm Drgbl | Hm Drgbl | Hm Drgbl | CPE002FW | CPE002G0 | CPE002G4 | NM 01627 | NM 0162 | NM 0162 | RNF138 | RNF138 | RNF138 |
| 1630 | D7 | D8 | 1582 | 753 | 17502 | 444,9 | 11,1 | 1,162 | H5A034 C | sample | Hm Drgbl | Hm Drgbl | Hm Drgbl | CPE002FW | CPE002G0 | CPE002G4 | NM 02052 | NM 0205 | NM 0205 | PCBP3 | PCBP3 | PCBP3 |
| 1743 | I22 | I23 | 1912 | 773,6 | 21213 | 415,1 | 11,1 | 1,162 | H5A034 C | sample | Hm Drgbl | Hm Drgbl | Hm Drgbl | CPE002FW | CPE002G0 | CPE002G4 | NM 02211 | NM 0221 | NM 0221 | PRDM16 | PRDM16 | PRDM16 |
| 1852 | N12 | N13 | 1898 | 780,2 | 21145 | 393,6 | 11,1 | 1,162 | H5A034 C | sample | Hm Drgbl | Hm Drgbl | Hm Drgbl | CPE002FW | CPE002G0 | CPE002G4 | NM 03256 | NM 0325 | NM 0325 | SPZ1 | SPZ1 | SPZ1 |
| 21632 | F16 | F17 | 386 | 1028 | 4272 | 605,4 | 11,1 | 1,168 | H5A034 C | sample | Hm Gen E | Hm Gen E | Hm Gen E | CPE002JU | CPE002O9 | CPE002KP | XM 00112 | XM 0011 | XM 0011 | LOC65335 | LOC65335 | LOC65335 |
| 21706 | I18 | I19 | 261 | 1001 | 2892 | 679,3 | 11,1 | 1,168 | H5A034 C | sample | Hm Gen E | Hm Gen E | Hm Gen E | CPE002JU | CPE002O9 | CPE002KP | XM 94235 | XM 9423 | XM 9423 | LOC65272 | LOC65272 | LOC65272 |
| 21813 | M6 | M7 | 131 | 976,7 | 1449 | 734,3 | 11,1 | 1,168 | H5A034 C | sample | Hm Gen E | Hm Gen E | Hm Gen E | CPE002JU | CPE002O9 | CPE002KP | XM 94245 | XM 9424 | XM 9424 | LOC65279 | LOC65279 | LOC65279 |
| 21860 | O5 | O6 | 93 | 1090 | 1029 | 996,5 | 11,1 | 1,168 | H5A034 C | sample | Hm Gen E | Hm Gen E | Hm Gen E | CPE002JU | CPE002O9 | CPE002KP | XM 93960 | XM 9396 | XM 9396 | LOC65051 | LOC65051 | LOC65051 |
| 4655 | B8 | B9 | 1492 | 864,3 | 16621 | 298,5 | 11,1 | 1,22 | H5A034 C | sample | Hm Drgbl | Hm Drgbl | Hm Drgbl | CPE002DV | CPE002EL | CPE002FP | NM 02177 | NM 0217 | NM 0217 | ADAM28 | ADAM28 | ADAM28 |
| 4869 | K6 | K7 | 1204 | 793,4 | 13333 | 441,4 | 11,1 | 1,22 | H5A034 C | sample | Hm Drgbl | Hm Drgbl | Hm Drgbl | CPE002DV | CPE002EL | CPE002FP | NM 00471 | NM 0047 | NM 0047 | PCSK7 | PCSK7 | PCSK7 |
| 4898 | M10 | M11 | 1291 | 832,2 | 14380 | 490,8 | 11,1 | 1,22 | H5A034 C | sample | Hm Drgbl | Hm Drgbl | Hm Drgbl | CPE002DV | CPE002EL | CPE002FP | NM 01462 | NM 0146 | NM 0146 | ARHGEF10 | ARHGEF10 | ARHGEF10 |
| 4981 | P20 | P21 | 1614 | 787,8 | 17867 | 555,7 | 11,1 | 1,22 | H5A034 C | sample | Hm Drgbl | Hm Drgbl | Hm Drgbl | CPE002DV | CPE002EL | CPE002FP | NM 02162 | NM 0216 | NM 0216 | SENP2 | SENP2 | SENP2 |
| 4983 | P22 | P23 | 1319 | 842,9 | 14582 | 368 | 11,1 | 1,22 | H5A034 C | sample | Hm Drgbl | Hm Drgbl | Hm Drgbl | CPE002DV | CPE002EL | CPE002FP | NM 02192 | NM 0219 | NM 0219 | SPCS3 | SPCS3 | SPCS3 |
| 10500 | F2 | F3 | 783 | 992,5 | 8727 | 222,5 | 11,1 | 1,254 | H5A034 C | sample | Hm Drgbl | Hm Drgbl | Hm Drgbl | CPE002EB | CPE002EO | CPE002FR | NM 15304 | NM 1530 | NM 1530 | TDRD9 | TDRD9 | TDRD9 |
| 10591 | J15 | J16 | 964 | 796,8 | 10745 | 180,6 | 11,1 | 1,254 | H5A034 C | sample | Hm Drgbl | Hm Drgbl | Hm Drgbl | CPE002EB | CPE002EO | CPE002FR | NM 1489 | NM 1489 | NM 1489 | FOXP2 | FOXP2 | FOXP2 |
| 10614 | K14 | K15 | 1345 | 843,4 | 14866 | 170,3 | 11,1 | 1,254 | H5A034 C | sample | Hm Drgbl | Hm Drgbl | Hm Drgbl | CPE002EB | CPE002EO | CPE002FR | NM 05296 | NM 0529 | NM 0529 | RBP7 | RBP7 | RBP7 |
| 10659 | M11 | M12 | 1370 | 860,2 | 15179 | 186,1 | 11,1 | 1,254 | H5A034 C | sample | Hm Drgbl | Hm Drgbl | Hm Drgbl | CPE002EB | CPE002EO | CPE002FR | NM 13336 | NM 1333 | NM 1333 | UNC5A | UNC5A | UNC5A |
| 2699 | A19 | A20 | 1500 | 631 | 16708 | 480,7 | 11,1 | 1,306 | H5A034 C | sample | Hm Drgbl | Hm Drgbl | Hm Drgbl | CPE002DP | CPE002EF | CPE002F5 | NM 0036 | NM 0036 | NM 0036 | CDC42BPA | CDC42BPA | CDC42BPA |
| 2859 | H11 | H12 | 1653 | 659,1 | 18410 | 444,5 | 11,1 | 1,306 | H5A034 C | sample | Hm Drgbl | Hm Drgbl | Hm Drgbl | CPE002DP | CPE002EF | CPE002F5 | NM 01497 | NM 0149 | NM 0149 | MAST1 | MAST1 | MAST1 |

| | | | | | | | | | | | | | | | | | | | | | | | | | | | | | | | |
|---|---|---|---|---|---|---|---|---|---|---|---|---|---|---|---|---|---|---|---|---|---|---|---|---|---|---|---|---|---|---|---|
| 3026 | O10 | O11 | 1476 | 621,6 | 16388 | 548,2 | 11,1 | 1,306 | H5A034 C | sample | Hm Drgbl | Hm Drgbl | Hm Drgbl | CPE002DP | CPE002EF | CPE002F5 | NM 05284 | NM 0528 | NM 0528 | TSSK3 | TSSK3 | TSSK3 | testis-specific seri | testis-spe | testis-spe | 81629 | 81629 | 81629 | s37745 | s37746 | s37747 |
| 3038 | O21 | O22 | 1378 | 675,2 | 15282 | 501,8 | 11,1 | 1,306 | H5A034 C | sample | Hm Drgbl | Hm Drgbl | Hm Drgbl | CPE002DP | CPE002EF | CPE002F5 | NM 00585 | NM 0058 | NM 0058 | AKAP8 | AKAP8 | AKAP8 | A kinase (PRKA) a | A kinase ( | A kinase ( | 10270 | 10270 | 10270 | s20068 | s20069 | s20070 |
| 20040 | C9 | C10 | 1146 | 1136 | 12670 | 177 | 11,1 | 1,354 | H5A034 C | sample | Hm Gen E | Hm Gen E | Hm Gen E | CPE002JQ | CPE002O5 | CPE002KL | XM 00112 | XM 0011 | XM 0011 | LOC39094 | LOC39094 | LOC39094 | similar to R28379 | similar to | similar to | 390940 | 390940 | 390940 | s52896 | s52897 | s226484 |
| 20155 | H4 | H5 | 528 | 1255 | 5861 | 158,2 | 11,1 | 1,354 | H5A034 C | sample | Hm Gen E | Hm Gen E | Hm Gen E | CPE002JQ | CPE002O5 | CPE002KL | NM 00100 | NM 0010 | NM 0010 | OR5K4 | OR5K4 | OR5K4 | olfactory recepto | olfactory | olfactory | 403278 | 403278 | 403278 | s53819 | s53820 | s53821 |
| 20164 | I12 | I13 | 1071 | 1266 | 11858 | 197,7 | 11,1 | 1,354 | H5A034 C | sample | Hm Gen E | Hm Gen E | Hm Gen E | CPE002JQ | CPE002O5 | CPE002KL | NM 00100 | NM 0010 | NM 0010 | FLJ45974 | FLJ45974 | FLJ45974 | FLJ45974 protein | FLJ45974 | FLJ45974 | 401337 | 401337 | 401337 | s53573 | s53574 | s53575 |
| 20183 | I8 | I9 | 583 | 1124 | 6479 | 180,9 | 11,1 | 1,354 | H5A034 C | sample | Hm Gen E | Hm Gen E | Hm Gen E | CPE002JQ | CPE002O5 | CPE002KL | XM 37948 | XM 3794 | XM 3794 | LOC40132 | LOC40132 | LOC40132 | hypothetical LOC | hypotheti | hypotheti | 401321 | 401321 | 401321 | s53569 | s53570 | s53571 |
| 20323 | O4 | O5 | 294 | 1209 | 3263 | 312 | 11,1 | 1,354 | H5A034 C | sample | Hm Gen E | Hm Gen E | Hm Gen E | CPE002JQ | CPE002O5 | CPE002KL | NM 00100 | NM 0010 | NM 0010 | OR51T1 | OR51T1 | OR51T1 | olfactory recepto | olfactory | olfactory | 401665 | 401665 | 401665 | s53669 | s53670 | s53671 |
| 20341 | P20 | P21 | 713 | 1419 | 7898 | 129,2 | 11,1 | 1,354 | H5A034 C | sample | Hm Gen E | Hm Gen E | Hm Gen E | CPE002JQ | CPE002O5 | CPE002KL | XM 00112 | XM 0011 | XM 0011 | FAM66C | FAM66C | FAM66C | family with seque | family wit | family wit | 440078 | 440078 | 440078 | s54008 | s54009 | s54010 |
| 436 | C12 | C13 | 1503 | 1833 | 16649 | 315,1 | 11,1 | 1,62 | H5A034 C | sample | Hm Drgbl | Hm Drgbl | Hm Drgbl | CPE002E0 | CPE002EZ | CPE002FK | NM 00201 | NM 0020 | NM 0020 | FLII | FLII | FLII | flightless I homol | flightless I | flightless I | 2314 | 2314 | 2314 | s5269 | s5270 | s5271 |
| 3341 | L13 | L14 | 1123 | 1264 | 12626 | 377,8 | 11,2 | 0,693 | H5A034 C | sample | Hm Drgbl | Hm Drgbl | Hm Drgbl | CPE002DC | CPE002EG | CPE002F6 | NM 01487 | NM 0148 | NM 0148 | MFN2 | MFN2 | MFN2 | mitofusin 2 | mitofusin | mitofusin | 9927 | 9927 | 9927 | s19260 | s19261 | s19262 |
| 20454 | E14 | E15 | 999 | 791,5 | 11200 | 167,7 | 11,2 | 0,747 | H5A034 C | sample | Hm Gen E | Hm Gen E | Hm Gen E | CPE002JR | CPE002O6 | CPE002KN | NM 00100 | NM 0010 | NM 0010 | CHAC2 | CHAC2 | CHAC2 | ChaC, cation tran | ChaC, cati | ChaC, cati | 494143 | 494143 | 494143 | s54643 | s54644 | s54645 |
| 20607 | K22 | K23 | 1283 | 732,4 | 14379 | 179,9 | 11,2 | 0,747 | H5A034 C | sample | Hm Gen E | Hm Gen E | Hm Gen E | CPE002JR | CPE002O6 | CPE002KN | NM 00103 | NM 0010 | NM 0010 | ACOT1 | ACOT1 | ACOT1 | acyl-CoA thioeste | acyl-CoA t | acyl-CoA t | 641371 | 641371 | 641371 | s54800 | s54801 | s54802 |
| 17294 | A21 | A22 | 1693 | 1149 | 18998 | 299,9 | 11,2 | 0,765 | H5A034 C | sample | Hm Gen E | Hm Gen E | Hm Gen E | CPE002JJ | CPE002NY | CPE002KE | NM 14580 | NM 1458 | NM 1458 | LOC12614 | LOC12614 | LOC12614 | hypothetical prot | hypotheti | hypotheti | 126147 | 126147 | 126147 | s42966 | s42967 | s195667 |
| 9561 | O17 | O18 | 1671 | 1035 | 18796 | 105,1 | 11,2 | 0,778 | H5A034 C | sample | Hm Drgbl | Hm Drgbl | Hm Drgbl | CPE002E8 | CPE002ER | CPE002FC | NM 01829 | NM 0182 | NM 0182 | PGM2 | PGM2 | PGM2 | phosphoglucomu | phosphog | phosphog | 55276 | 55276 | 55276 | s30612 | s30613 | s30614 |
| 11402 | L10 | L11 | 1743 | 824,6 | 19513 | 125,7 | 11,2 | 0,812 | H5A034 C | sample | Hm Drgbl | Hm Drgbl | Hm Drgbl | CPE002ED | CPE002F3 | CPE002FT | | | | | | | | | | | | | | | |
| 11504 | P16 | P17 | 1789 | 858 | 20010 | 142,1 | 11,2 | 0,812 | H5A034 C | sample | Hm Drgbl | Hm Drgbl | Hm Drgbl | CPE002ED | CPE002F3 | CPE002FT | | | | | | | | | | | | | | | |
| 11120 | P16 | P17 | 1325 | 778,5 | 14846 | 442,7 | 11,2 | 0,848 | H5A034 C | sample | Hm Drgbl | Hm Drgbl | Hm Drgbl | CPE002EC | CPE002EN | CPE002FS | NM 03225 | NM 0322 | NM 0322 | CCDC88B | CCDC88B | CCDC88B | coiled-coil domai | coiled-coi | coiled-coi | 283234 | 283234 | 283234 | s49157 | s49158 | s49156 |
| 16976 | D16 | D17 | 1584 | 927,6 | 17809 | 175,7 | 11,2 | 0,865 | H5A034 C | sample | Hm Gen E | Hm Gen E | Hm Gen E | CPE002JI | CPE002NZ | CPE002KD | NM 14459 | XM 0011 | NM 1445 | C15orf40 | LOC15093 | C15orf40 | chromosome 15 c | hypotheti | chromoso | 123207 | 150935 | 123207 | s42626 | s45498 | s225546 |
| 7009 | E1 | E2 | 250 | 557,2 | 2793 | 420,6 | 11,2 | 0,872 | H5A034 C | sample | Hm Drgbl | Hm Drgbl | Hm Drgbl | CPE002E2 | CPE002EX | CPE002FI | NM 00023 | NM 0002 | NM 0002 | LEP | LEP | LEP | leptin (obesity ho | leptin (ob | leptin (ob | 3952 | 3952 | 3952 | s8133 | s8134 | s8135 |
| 5266 | L18 | L19 | 2175 | 656,9 | 24414 | 430,4 | 11,2 | 0,878 | H5A034 C | sample | Hm Drgbl | Hm Drgbl | Hm Drgbl | CPE002DV | CPE002EM | CPE002FO | NM 00344 | NM 0034 | NM 0034 | ZNF143 | ZNF143 | ZNF143 | zinc finger protei | zinc finge | zinc finge | 7702 | 7702 | 7702 | s15192 | s15193 | s15194 |
| 5371 | P4 | P5 | 1276 | 621,6 | 14312 | 263,4 | 11,2 | 0,878 | H5A034 C | sample | Hm Drgbl | Hm Drgbl | Hm Drgbl | CPE002DV | CPE002EM | CPE002FO | NM 00103 | NM 0010 | NM 0010 | ZNF226 | ZNF226 | ZNF226 | zinc finger protei | zinc finge | zinc finge | 7769 | 7769 | 7769 | s15309 | s224796 | s15308 |
| 2324 | A5 | A6 | 1487 | 676,9 | 16699 | 455,4 | 11,2 | 0,882 | H5A034 C | sample | Hm Drgbl | Hm Drgbl | Hm Drgbl | CPE002DC | CPE002EE | CPE002F4 | NM 00182 | NM 0018 | NM 0018 | CKB | CKB | CKB | creatine kinase, b | creatine k | creatine k | 1152 | 1152 | 1152 | s3082 | s223236 | s3084 |
| 2373 | C6 | C7 | 974 | 734,3 | 10893 | 292,5 | 11,2 | 0,882 | H5A034 C | sample | Hm Drgbl | Hm Drgbl | Hm Drgbl | CPE002DC | CPE002EE | CPE002F4 | NM 00231 | NM 0023 | NM 0023 | LIMK1 | LIMK1 | LIMK1 | LIM domain kinas | LIM doma | LIM doma | 3984 | 3984 | 3984 | s8188 | s8189 | s8190 |
| 2378 | D10 | D11 | 1698 | 730,8 | 19007 | 353 | 11,2 | 0,882 | H5A034 C | sample | Hm Drgbl | Hm Drgbl | Hm Drgbl | CPE002DC | CPE002EE | CPE002F4 | NM 00625 | NM 0062 | NM 0062 | PRKG1 | PRKG1 | PRKG1 | protein kinase, cG | protein ki | protein ki | 5592 | 5592 | 5592 | s11131 | s11132 | s11133 |
| 2540 | J5 | J6 | 1072 | 682 | 11988 | 307,6 | 11,2 | 0,882 | H5A034 C | sample | Hm Drgbl | Hm Drgbl | Hm Drgbl | CPE002DC | CPE002EE | CPE002F4 | NM 00134 | NM 0013 | NM 0013 | DGKQ | DGKQ | DGKQ | diacylglycerol kin | diacylglyc | diacylglyc | 1609 | 1609 | 1609 | s3920 | s3921 | s3922 |
| 2551 | K15 | K16 | 1471 | 691,4 | 16533 | 344,4 | 11,2 | 0,882 | H5A034 C | sample | Hm Drgbl | Hm Drgbl | Hm Drgbl | CPE002DC | CPE002EE | CPE002F4 | NM 00101 | NM 0010 | NM 0010 | AKT1 | AKT1 | AKT1 | v-akt murine thym | v-akt mur | v-akt mur | 207 | 207 | 207 | s659 | s660 | s661 |
| 2611 | M4 | M5 | 1206 | 631,4 | 13449 | 382,4 | 11,2 | 0,882 | H5A034 C | sample | Hm Drgbl | Hm Drgbl | Hm Drgbl | CPE002DC | CPE002EE | CPE002F4 | NM 13873 | NM 1387 | NM 1387 | PGK2 | PGK2 | PGK2 | phosphoglycerate | phosphog | phosphog | 5232 | 5232 | 5232 | s10403 | s10404 | s10405 |
| 16535 | A8 | A9 | 817 | 1075 | 9191 | 171 | 11,2 | 0,885 | H5A034 C | sample | Hm Gen E | Hm Gen E | Hm Gen E | CPE002JH | CPE002NV | CPE002KC | NM 14523 | NM 1452 | NM 1452 | ATPBD3 | ATPBD3 | ATPBD3 | ATP binding doma | ATP bindi | ATP bindi | 90353 | 90353 | 90353 | s40348 | s195548 | s228440 |
| 17898 | J3 | J4 | 1199 | 1048 | 13484 | 224,2 | 11,2 | 0,885 | H5A034 C | sample | Hm Gen E | Hm Gen E | Hm Gen E | CPE002JK | CPE002NZ | CPE002KF | NM 17350 | NM 1735 | NM 1735 | EFCAB3 | EFCAB3 | EFCAB3 | EF-hand calcium b | EF-hand c | EF-hand c | 146779 | 146779 | 146779 | s44906 | s44907 | s44908 |
| 17985 | N17 | N18 | 1510 | 1041 | 16949 | 221,3 | 11,2 | 0,885 | H5A034 C | sample | Hm Gen E | Hm Gen E | Hm Gen E | CPE002JK | CPE002NZ | CPE002KF | XM 08583 | XM 0858 | XM 0858 | LOC14764 | LOC14764 | LOC14764 | hypothetical prot | hypotheti | hypotheti | 147645 | 147645 | 147645 | s45009 | s45010 | s45011 |
| 17990 | N21 | N22 | 1761 | 1036 | 19666 | 225,4 | 11,2 | 0,885 | H5A034 C | sample | Hm Gen E | Hm Gen E | Hm Gen E | CPE002JK | CPE002NZ | CPE002KF | XM 49632 | XM 4963 | XM 4963 | ZNF534 | ZNF534 | ZNF534 | zinc finger protei | zinc finge | zinc finge | 147658 | 147658 | 147658 | s45018 | s45019 | s45020 |
| 4231 | A15 | A16 | 866 | 867,2 | 9683 | 422,6 | 11,2 | 0,945 | H5A034 C | sample | Hm Drgbl | Hm Drgbl | Hm Drgbl | CPE002DL | CPE002EK | CPE002FQ | NM 00072 | NM 0007 | NM 0007 | CACNA2D | CACNA2D | CACNA2D | calcium channel, | calcium ch | calcium ch | 781 | 781 | 781 | s809 | s810 | s811 |
| 4368 | F9 | F10 | 942 | 715,9 | 10590 | 409,5 | 11,2 | 0,945 | H5A034 C | sample | Hm Drgbl | Hm Drgbl | Hm Drgbl | CPE002DL | CPE002EK | CPE002FQ | NM 00223 | NM 0022 | NM 0022 | KCNA6 | KCNA6 | KCNA6 | potassium voltag | potassium | potassium | 3742 | 3742 | 3742 | s7691 | s7692 | s7693 |
| 4394 | H10 | H11 | 1264 | 672,4 | 14162 | 349,6 | 11,2 | 0,945 | H5A034 C | sample | Hm Drgbl | Hm Drgbl | Hm Drgbl | CPE002DL | CPE002EK | CPE002FQ | NM 13077 | NM 1307 | NM 1307 | HTR3C | HTR3C | HTR3C | 5-hydroxytryptam | 5-hydroxy | 5-hydroxy | 170572 | 170572 | 170572 | s46825 | s46826 | s46827 |
| 4404 | H2 | H3 | 1568 | 811,6 | 17627 | 414,3 | 11,2 | 0,945 | H5A034 C | sample | Hm Drgbl | Hm Drgbl | Hm Drgbl | CPE002DL | CPE002EK | CPE002FQ | NM 01766 | NM 0176 | NM 0176 | TRPM6 | TRPM6 | TRPM6 | transient recepto | transient | transient | 140803 | 140803 | 140803 | s44402 | s44403 | s44404 |
| 46 | B7 | B8 | 539 | 1435 | 6055 | 493,1 | 11,2 | 0,953 | H5A034 C | sample | Hm Drgbl | Hm Drgbl | Hm Drgbl | CPE002DT | CPE002EJ | CPE002F9 | NM 00103 | NM 0010 | NM 0010 | SDHC | SDHC | SDHC | succinate dehydr | succinate | succinate | 6391 | 6391 | 6391 | s12656 | s12657 | s12658 |
| 51 | C11 | C12 | 719 | 1575 | 8017 | 365,4 | 11,2 | 0,953 | H5A034 C | sample | Hm Drgbl | Hm Drgbl | Hm Drgbl | CPE002DT | CPE002EJ | CPE002F9 | NM 00100 | NM 0010 | NM 0010 | PPARA | PPARA | PPARA | peroxisome prolif | peroxison | peroxison | 5465 | 5465 | 5465 | s10880 | s10881 | s10882 |
| 143 | F8 | F9 | 153 | 1437 | 1714 | 534,4 | 11,2 | 0,953 | H5A034 C | sample | Hm Drgbl | Hm Drgbl | Hm Drgbl | CPE002DT | CPE002EJ | CPE002F9 | NM 00312 | NM 0031 | NM 0031 | SPTAN1 | SPTAN1 | SPTAN1 | spectrin, alpha, n | spectrin, a | spectrin, a | 6709 | 6709 | 6709 | s13404 | s13405 | s13403 |
| 148 | G12 | G13 | 819 | 1687 | 9158 | 406,4 | 11,2 | 0,953 | H5A034 C | sample | Hm Drgbl | Hm Drgbl | Hm Drgbl | CPE002DT | CPE002EJ | CPE002F9 | NM 00307 | NM 0030 | NM 0030 | SMARCA2 | SMARCA2 | SMARCA2 | SWI/SNF related, | SWI/SNF r | SWI/SNF r | 6595 | 6595 | 6595 | s13133 | s13134 | s13135 |
| 166 | G7 | G8 | 688 | 1521 | 7673 | 399 | 11,2 | 0,953 | H5A034 C | sample | Hm Drgbl | Hm Drgbl | Hm Drgbl | CPE002DT | CPE002EJ | CPE002F9 | NM 17319 | NM 1731 | NM 1731 | NR4A3 | NR4A3 | NR4A3 | nuclear receptor s | nuclear re | nuclear re | 8013 | 8013 | 8013 | s15541 | s15542 | s15543 |
| 178 | H18 | H19 | 641 | 1444 | 7165 | 471,8 | 11,2 | 0,953 | H5A034 C | sample | Hm Drgbl | Hm Drgbl | Hm Drgbl | CPE002DT | CPE002EJ | CPE002F9 | NM 00314 | NM 0031 | NM 0031 | SSB | SSB | SSB | Sjogren syndrome | Sjogren sy | Sjogren sy | 6741 | 6741 | 6741 | s13469 | s13470 | s13468 |
| 358 | O7 | O8 | 748 | 1652 | 8381 | 557 | 11,2 | 0,953 | H5A034 C | sample | Hm Drgbl | Hm Drgbl | Hm Drgbl | CPE002DT | CPE002EJ | CPE002F9 | NM 00627 | NM 0062 | NM 0062 | CCL19 | CCL19 | CCL19 | chemokine (C-C m | chemokin | chemokin | 6363 | 6363 | 6363 | s12600 | s12601 | s12599 |
| 1987 | C4 | C5 | 1069 | 679,3 | 12003 | 457,6 | 11,2 | 0,974 | H5A034 C | sample | Hm Drgbl | Hm Drgbl | Hm Drgbl | CPE002FX | CPE002G1 | CPE002G5 | NM 13346 | NM 1334 | NM 1334 | BMPER | BMPER | BMPER | BMP binding endo | BMP bind | BMP bind | 168667 | 168667 | 168667 | s46722 | s46723 | s46724 |
| 2124 | I2 | I3 | 1189 | 689 | 13311 | 481,9 | 11,2 | 0,974 | H5A034 C | sample | Hm Drgbl | Hm Drgbl | Hm Drgbl | CPE002FX | CPE002G1 | CPE002G5 | NM 17588 | NM 1758 | NM 1758 | ZNF620 | ZNF620 | ZNF620 | zinc finger protei | zinc finge | zinc finge | 253639 | 253639 | 253639 | s48432 | s48433 | s48434 |
| 2173 | K20 | K21 | 1486 | 597,1 | 16693 | 297,3 | 11,2 | 0,974 | H5A034 C | sample | Hm Drgbl | Hm Drgbl | Hm Drgbl | CPE002FX | CPE002G1 | CPE002G5 | NM 13346 | NM 1334 | NM 1334 | ZNF545 | ZNF545 | ZNF545 | zinc finger protei | zinc finge | zinc finge | 284406 | 284406 | 284406 | s49638 | s49639 | s49640 |
| 2183 | K8 | K9 | 1045 | 646,9 | 11688 | 399,4 | 11,2 | 0,974 | H5A034 C | sample | Hm Drgbl | Hm Drgbl | Hm Drgbl | CPE002FX | CPE002G1 | CPE002G5 | NM 17363 | NM 1736 | NM 1736 | ZNF547 | ZNF547 | ZNF547 | zinc finger protei | zinc finge | zinc finge | 284306 | 284306 | 284306 | s49565 | s49566 | s49567 |
| 2196 | L2 | L3 | 1426 | 680,6 | 16030 | 371,9 | 11,2 | 0,974 | H5A034 C | sample | Hm Drgbl | Hm Drgbl | Hm Drgbl | CPE002FX | CPE002G1 | CPE002G5 | NM 00623 | NM 0062 | NM 0062 | POU3F3 | POU3F3 | POU3F3 | POU domain, clas | POU dom | POU dom | 5455 | 5455 | 5455 | s194705 | s194706 | s194707 |
| 6557 | B13 | B14 | 642 | 747,8 | 7197 | 309,9 | 11,2 | 0,982 | H5A034 C | sample | Hm Drgbl | Hm Drgbl | Hm Drgbl | CPE002E1 | CPE002EY | CPE002FJ | NM 03126 | NM 0312 | NM 0312 | HNRPAB | HNRPAB | HNRPAB | heterogeneous nu | heteroger | heteroger | 3182 | 3182 | 3182 | s6717 | s6718 | s6716 |
| 6571 | B4 | B5 | 1193 | 637,3 | 13364 | 505,6 | 11,2 | 0,982 | H5A034 C | sample | Hm Drgbl | Hm Drgbl | Hm Drgbl | CPE002E1 | CPE002EY | CPE002FJ | NM 00220 | NM 0022 | NM 0022 | ITGA7 | ITGA7 | ITGA7 | integrin, alpha 7 | integrin, a | integrin, a | 3679 | 3679 | 3679 | s7550 | s7551 | s7552 |
| 6597 | C6 | C7 | 1297 | 667,9 | 14472 | 516,9 | 11,2 | 0,982 | H5A034 C | sample | Hm Drgbl | Hm Drgbl | Hm Drgbl | CPE002E1 | CPE002EY | CPE002FJ | NM 00061 | NM 0006 | NM 0006 | IGF2 | IGF2 | IGF2 | insulin-like growt | insulin-lik | insulin-lik | 3481 | 3481 | 3481 | s7214 | s7215 | s7216 |
| 6683 | G19 | G20 | 1058 | 592,4 | 11835 | 418,6 | 11,2 | 0,982 | H5A034 C | sample | Hm Drgbl | Hm Drgbl | Hm Drgbl | CPE002E1 | CPE002EY | CPE002FJ | NM 00051 | NM 0005 | NM 0005 | HBB | HBB | HBB | hemoglobin, beta | hemoglob | hemoglob | 3043 | 3043 | 3043 | s6464 | s6462 | s6463 |
| 6797 | L13 | L14 | 1162 | 659,3 | 13022 | 298,1 | 11,2 | 0,982 | H5A034 C | sample | Hm Drgbl | Hm Drgbl | Hm Drgbl | CPE002E1 | CPE002EY | CPE002FJ | NM 00552 | NM 0055 | NM 0055 | HES1 | HES1 | HES1 | hairy and enhanc | hairy and | hairy and | 3280 | 3280 | 3280 | s6922 | s6920 | s6921 |
| 3974 | F21 | F22 | 1263 | 624,8 | 14162 | 351,6 | 11,2 | 0,996 | H5A034 C | sample | Hm Drgbl | Hm Drgbl | Hm Drgbl | CPE002DS | CPE002EI | CPE002F8 | NM 00460 | NM 0046 | NM 0046 | TBX6 | TBX6 | TBX6 | T-box 6 | T-box 6 | T-box 6 | 6911 | 6911 | 6911 | s13837 | s13836 | s224707 |
| 4031 | H8 | H9 | 1619 | 720,6 | 18187 | 397,9 | 11,2 | 0,996 | H5A034 C | sample | Hm Drgbl | Hm Drgbl | Hm Drgbl | CPE002DS | CPE002EI | CPE002F8 | NM 00510 | NM 0051 | NM 0051 | BRD2 | BRD2 | BRD2 | bromodomain co | bromodo | bromodo | 6046 | 6046 | 6046 | s12071 | s12072 | s12070 |
| 4047 | I22 | I23 | 1576 | 709,1 | 17730 | 326,5 | 11,2 | 0,996 | H5A034 C | sample | Hm Drgbl | Hm Drgbl | Hm Drgbl | CPE002DS | CPE002EI | CPE002F8 | NM 00288 | NM 0028 | NM 0028 | RAP2B | RAP2B | RAP2B | RAP2B, member c | RAP2B, m | RAP2B, m | 5912 | 5912 | 5912 | s11794 | s11795 | s11796 |
| 5979 | J11 | J12 | 1176 | 750 | 13119 | 491,4 | 11,2 | 1,023 | H5A034 C | sample | Hm Drgbl | Hm Drgbl | Hm Drgbl | CPE002DY | CPE002F1 | CPE002FM | NM 00115 | NM 0011 | NM 0011 | AOC2 | AOC2 | AOC2 | amine oxidase, co | amine oxi | amine oxi | 314 | 314 | 314 | s1411 | s1412 | s1410 |
| 5991 | J22 | J23 | 1713 | 793,7 | 19173 | 568,8 | 11,2 | 1,023 | H5A034 C | sample | Hm Drgbl | Hm Drgbl | Hm Drgbl | CPE002DY | CPE002F1 | CPE002FM | NM 00518 | NM 0051 | NM 0051 | CA7 | CA7 | CA7 | carbonic anhydra | carbonic a | carbonic a | 766 | 766 | 766 | s2264 | s2263 | s2262 |
| 6128 | P16 | P17 | 1025 | 812,1 | 11452 | 628,5 | 11,2 | 1,023 | H5A034 C | sample | Hm Drgbl | Hm Drgbl | Hm Drgbl | CPE002DY | CPE002F1 | CPE002FM | NM 19445 | NM 1944 | NM 1944 | KRIT1 | KRIT1 | KRIT1 | KRIT1, ankyrin rep | KRIT1, ank | KRIT1, ank | 889 | 889 | 889 | s2511 | s2510 | s2509 |
| 1196 | B5 | B6 | 1263 | 773,5 | 14175 | 513,3 | 11,2 | 1,028 | H5A034 C | sample | Hm Drgbl | Hm Drgbl | Hm Drgbl | CPE002FV | CPE002FZ | CPE002G3 | NM 08070 | NM 0807 | NM 0807 | BAT3 | BAT3 | BAT3 | HLA-B associated | HLA-B ass | HLA-B ass | 7917 | 7917 | 7917 | s15466 | s15467 | s15468 |
| 1261 | E20 | E21 | 1965 | 738,7 | 21945 | 445,7 | 11,2 | 1,028 | H5A034 C | sample | Hm Drgbl | Hm Drgbl | Hm Drgbl | CPE002FV | CPE002FZ | CPE002G3 | NM 01483 | NM 0148 | NM 0148 | ZBTB39 | ZBTB39 | ZBTB39 | zinc finger and BT | zinc finge | zinc finge | 9880 | 9880 | 9880 | s19134 | s19135 | s19136 |
| 1426 | L18 | L19 | 1761 | 721,1 | 19700 | 397,9 | 11,2 | 1,028 | H5A034 C | sample | Hm Drgbl | Hm Drgbl | Hm Drgbl | CPE002FV | CPE002FZ | CPE002G3 | NM 01506 | NM 0150 | NM 0150 | ZNF423 | ZNF423 | ZNF423 | zinc finger protei | zinc finge | zinc finge | 23090 | 23090 | 23090 | s23007 | s23008 | s23009 |
| 1447 | M15 | M16 | 1181 | 785,5 | 13192 | 416,9 | 11,2 | 1,028 | H5A034 C | sample | Hm Drgbl | Hm Drgbl | Hm Drgbl | CPE002FV | CPE002FZ | CPE002G3 | NM 00715 | NM 0071 | NM 0071 | ZNF187 | ZNF187 | ZNF187 | zinc finger protei | zinc finge | zinc finge | 7741 | 7741 | 7741 | s15251 | s15252 | s15253 |
| 1451 | M19 | M20 | 1854 | 784,2 | 20768 | 408,7 | 11,2 | 1,028 | H5A034 C | sample | Hm Drgbl | Hm Drgbl | Hm Drgbl | CPE002FV | CPE002FZ | CPE002G3 | NM 00715 | NM 0071 | NM 0071 | ZNF195 | ZNF195 | ZNF195 | zinc finger protei | zinc finge | zinc finge | 7748 | 7748 | 7748 | s15263 | s15264 | s15265 |
| 1487 | N8 | N9 | 1629 | 730,4 | 18198 | 414,3 | 11,2 | 1,028 | H5A034 C | sample | Hm Drgbl | Hm Drgbl | Hm Drgbl | CPE002FV | CPE002FZ | CPE002G3 | NM 01511 | NM 0151 | NM 0151 | ZZEF1 | ZZEF1 | ZZEF1 | zinc finger, ZZ-typ | zinc finge | zinc finge | 23140 | 23140 | 23140 | s23121 | s23122 | s23123 |
| 16477 | O20 | O21 | 1379 | 1038 | 15411 | 379,6 | 11,2 | 1,028 | H5A034 C | sample | Hm Gen E | Hm Gen E | Hm Gen E | CPE002JG | CPE002NV | CPE002KB | NM 03239 | NM 0323 | NM 0323 | C17orf92 | C17orf92 | C17orf92 | chromosome 17 c | chromoso | chromoso | 84366 | 84366 | 84366 | s38968 | s38969 | s38970 |
| 13704 | K9 | K10 | 996 | 969,4 | 11164 | 151,9 | 11,2 | 1,052 | H5A034 C | sample | Hm Gen E | Hm Gen E | Hm Gen E | CPE002JC | CPE002NC | CPE002K4 | NM 01515 | NM 0151 | NM 0151 | PHF3 | PHF3 | PHF3 | PHD finger protei | PHD finge | PHD finge | 23469 | 23469 | 23469 | s23886 | s23887 | s23888 |
| 787 | A4 | A5 | 830 | 667,5 | 9309 | 483,6 | 11,2 | 1,062 | H5A034 C | sample | Hm Drgbl | Hm Drgbl | Hm Drgbl | CPE002FU | CPE002FY | CPE002G2 | NM 00557 | NM 0055 | NM 0055 | LMAN1 | LMAN1 | LMAN1 | lectin, mannose-b | lectin, ma | lectin, ma | 3998 | 3998 | 3998 | s8218 | s8219 | s8220 |
| 824 | C16 | C17 | 1689 | 729,4 | 18979 | 431,5 | 11,2 | 1,062 | H5A034 C | sample | Hm Drgbl | Hm Drgbl | Hm Drgbl | CPE002FU | CPE002FY | CPE002G2 | NM 00499 | NM 0049 | NM 0049 | MDS1 | MDS1 | MDS1 | myelodysplasia sy | myelodys | myelodys | 4197 | 4197 | 4197 | s8634 | s8635 | s8636 |
| 874 | E18 | E19 | 1595 | 683,1 | 17820 | 366,6 | 11,2 | 1,062 | H5A034 C | sample | Hm Drgbl | Hm Drgbl | Hm Drgbl | CPE002FU | CPE002FY | CPE002G2 | NM 17238 | NM 1723 | NM 1723 | NFATC1 | NFATC1 | NFATC1 | nuclear factor of a | nuclear fa | nuclear fa | 4772 | 4772 | 4772 | s9470 | s9471 | s9472 |
| 5603 | J19 | J20 | 2111 | 803,3 | 23645 | 378,7 | 11,2 | 1,067 | H5A034 C | sample | Hm Drgbl | Hm Drgbl | Hm Drgbl | CPE002DX | CPE002F2 | CPE002FN | NM 00378 | NM 0037 | NM 0037 | B3GALT4 | B3GALT4 | B3GALT4 | UDP-Gal:betaGlcN | UDP-Gal:b | UDP-Gal:b | 8705 | 8705 | 8705 | s16579 | s16580 | s16581 |
| 5650 | L18 | L19 | 2075 | 711 | 23235 | 519,3 | 11,2 | 1,067 | H5A034 C | sample | Hm Drgbl | Hm Drgbl | Hm Drgbl | CPE002DX | CPE002F2 | CPE002FN | NM 00100 | NM 0010 | NM 0010 | PTER | PTER | PTER | phosphotriestera | phosphot | phosphot | 9317 | 9317 | 9317 | s17799 | s17800 | s17801 |
| 5727 | O22 | O23 | 1751 | 679,6 | 19620 | 565,1 | 11,2 | 1,067 | H5A034 C | sample | Hm Drgbl | Hm Drgbl | Hm Drgbl | CPE002DX | CPE002F2 | CPE002FN | NM 00468 | NM 0046 | NM 0046 | EIF1AY | EIF1AY | EIF1AY | eukaryotic transla | eukaryoti | eukaryoti | 9086 | 9086 | 9086 | s17334 | s17335 | s17336 |
| 3476 | A5 | A6 | 1051 | 1258 | 11744 | 359 | 11,2 | 1,077 | H5A034 C | sample | Hm Drgbl | Hm Drgbl | Hm Drgbl | CPE002DR | CPE002EH | CPE002F7 | NM 00350 | NM 0035 | NM 0035 | FZD6 | FZD6 | FZD6 | frizzled homolog | frizzled hc | frizzled hc | 8323 | 8323 | 8323 | s728 | s729 | s730 |
| 3527 | C8 | C9 | 1032 | 1313 | 11535 | 361,1 | 11,2 | 1,077 | H5A034 C | sample | Hm Drgbl | Hm Drgbl | Hm Drgbl | CPE002DR | CPE002EH | CPE002F7 | NM 00105 | NM 0010 | NM 0010 | TACR3 | TACR3 | TACR3 | tachykinin recept | tachykinir | tachykinir | 6870 | 6870 | 6870 | s13724 | s13725 | s13726 |
| 3547 | D4 | D5 | 1430 | 1371 | 15950 | 385,3 | 11,2 | 1,077 | H5A034 C | sample | Hm Drgbl | Hm Drgbl | Hm Drgbl | CPE002DR | CPE002EH | CPE002F7 | NM 03305 | NM 0330 | NM 0330 | SUCNR1 | SUCNR1 | SUCNR1 | succinate recepto | succinate | succinate | 56670 | 56670 | 56670 | s32235 | s32236 | s32237 |
| 3554 | E10 | E11 | 962 | 1450 | 10792 | 404 | 11,2 | 1,077 | H5A034 C | sample | Hm Drgbl | Hm Drgbl | Hm Drgbl | CPE002DR | CPE002EH | CPE002F7 | NM 03186 | NM 0318 | NM 0318 | FZD8 | FZD8 | FZD8 | frizzled homolog | frizzled hc | frizzled hc | 8325 | 8325 | 8325 | s15844 | s15845 | s15846 |
| 3559 | E15 | E16 | 882 | 1257 | 9912 | 334 | 11,2 | 1,077 | H5A034 C | sample | Hm Drgbl | Hm Drgbl | Hm Drgbl | CPE002DR | CPE002EH | CPE002F7 | NM 00405 | NM 0040 | NM 0040 | C3AR1 | C3AR1 | C3AR1 | complement com | compleme | compleme | 719 | 719 | 719 | s2164 | s2165 | s2166 |
| 3646 | H7 | H8 | 1508 | 1667 | 16923 | 434,9 | 11,2 | 1,077 | H5A034 C | sample | Hm Drgbl | Hm Drgbl | Hm Drgbl | CPE002DR | CPE002EH | CPE002F7 | NM 00084 | NM 0008 | NM 0008 | GRM6 | GRM6 | GRM6 | glutamate recept | glutamate | glutamate | 2916 | 2916 | 2916 | s6206 | s6207 | s6208 |
| 3746 | M10 | M11 | 1289 | 1160 | 14381 | 339,8 | 11,2 | 1,077 | H5A034 C | sample | Hm Drgbl | Hm Drgbl | Hm Drgbl | CPE002DR | CPE002EH | CPE002F7 | NM 01523 | NM 0152 | NM 0152 | LPHN3 | LPHN3 | LPHN3 | latrophilin 3 | latrophilir | latrophilir | 23284 | 23284 | 23284 | s23473 | s23474 | s23475 |
| 3817 | P1 | P2 | 1259 | 1422 | 14099 | 421,7 | 11,2 | 1,077 | H5A034 C | sample | Hm Drgbl | Hm Drgbl | Hm Drgbl | CPE002DR | CPE002EH | CPE002F7 | NM 00091 | NM 0009 | NM 0009 | OXTR | OXTR | OXTR | oxytocin receptor | oxytocin r | oxytocin r | 5021 | 5021 | 5021 | s9946 | s9947 | s9948 |
| 3830 | P21 | P22 | 1977 | 1474 | 22166 | 562,3 | 11,2 | 1,077 | H5A034 C | sample | Hm Drgbl | Hm Drgbl | Hm Drgbl | CPE002DR | CPE002EH | CPE002F7 | NM 00095 | NM 0009 | NM 0009 | PTGER4 | PTGER4 | PTGER4 | prostaglandin E r | prostaglar | prostaglar | 5734 | 5734 | 5734 | s11454 | s11455 | s11456 |
| 8094 | B14 | B15 | 1492 | 572,2 | 16731 | 483,1 | 11,2 | 1,077 | H5A034 C | sample | Hm Drgbl | Hm Drgbl | Hm Drgbl | CPE002E5 | CPE002EU | CPE002FF | NM 01532 | NM 0153 | NM 0153 | KIAA0828 | KIAA0828 | KIAA0828 | adenosylhomocys | adenosylh | adenosylh | 23382 | 23382 | 23382 | s23697 | s23698 | s23699 |
| 8218 | G18 | G19 | 461 | 632,8 | 5184 | 515,5 | 11,2 | 1,077 | H5A034 C | sample | Hm Drgbl | Hm Drgbl | Hm Drgbl | CPE002E5 | CPE002EU | CPE002FF | NM 01460 | NM 0146 | NM 0146 | CYFIP1 | CYFIP1 | CYFIP1 | cytoplasmic FMR1 | cytoplasm | cytoplasm | 23191 | 23191 | 23191 | s23243 | s23242 | s23241 |
| 8394 | N3 | N4 | 1578 | 609,2 | 17622 | 464,7 | 11,2 | 1,077 | H5A034 C | sample | Hm Drgbl | Hm Drgbl | Hm Drgbl | CPE002E5 | CPE002EU | CPE002FF | NM 01497 | NM 0149 | NM 0149 | SV2C | SV2C | SV2C | synaptic vesicle gl | synaptic v | synaptic v | 22987 | 22987 | 22987 | s22781 | s22782 | s22783 |
| 8411 | O19 | O20 | 1659 | 554,9 | 18498 | 498,6 | 11,2 | 1,077 | H5A034 C | sample | Hm Drgbl | Hm Drgbl | Hm Drgbl | CPE002E5 | CPE002EU | CPE002FF | NM 00726 | NM 0072 | NM 0072 | LYPLA2 | LYPLA2 | LYPLA2 | lysophospholipas | lysophosc | lysophosc | 11313 | 11313 | 11313 | s22298 | s22299 | s22300 |
| 10159 | H15 | H16 | 1527 | 809 | 17109 | 129,5 | 11,2 | 1,077 | H5A034 C | sample | Hm Drgbl | Hm Drgbl | Hm Drgbl | CPE002EA | CPE002EP | CPE002FA | NM 03064 | NM 0306 | NM 0306 | SETD7 | SETD7 | SETD7 | SET domain conta | SET doma | SET doma | 80854 | 80854 | 80854 | s37452 | s37453 | s37451 |
| 1633 | E1 | E2 | 830 | 758,4 | 9326 | 431,4 | 11,2 | 1,173 | H5A034 C | sample | Hm Drgbl | Hm Drgbl | Hm Drgbl | CPE002FW | CPE002G0 | CPE002G4 | NM 15235 | NM 1523 | NM 1523 | ZNF285A | ZNF285A | ZNF285A | zinc finger protei | zinc finge | zinc finge | 26974 | 26974 | 26974 | s25655 | s25656 | s25657 |
| 21853 | O20 | O21 | 380 | 958,9 | 4256 | 543,2 | 11,2 | 1,179 | H5A034 C | sample | Hm Gen E | Hm Gen E | Hm Gen E | CPE002JU | CPE002O9 | CPE002KP | XM 92580 | XM 9258 | XM 9258 | LOC65305 | LOC65305 | LOC65305 | hypothetical LOC | hypotheti | hypotheti | 653052 | 653052 | 653052 | s57554 | s57555 | s57556 |
| 21867 | P11 | P12 | 174 | 1334 | 1956 | 721,9 | 11,2 | 1,179 | H5A034 C | sample | Hm Gen E | Hm Gen E | Hm Gen E | CPE002JU | CPE002O9 | CPE002KP | XM 94167 | XM 9416 | XM 9416 | LOC65226 | LOC65226 | LOC65226 | similar to opposit | similar to | similar to | 652260 | 652260 | 652260 | s57335 | s197867 | s197868 |
| 4844 | J5 | J6 | 1085 | 874,2 | 12161 | 429 | 11,2 | 1,231 | H5A034 C | sample | Hm Drgbl | Hm Drgbl | Hm Drgbl | CPE002DV | CPE002EL | CPE002FP | NM 02256 | NM 0225 | NM 0225 | MMP16 | MMP16 | MMP16 | matrix metallope | matrix me | matrix me | 4325 | 4325 | 4325 | s8883 | s8884 | s8885 |
| 4861 | K20 | K21 | 2138 | 813,3 | 23852 | 472 | 11,2 | 1,231 | H5A034 C | sample | Hm Drgbl | Hm Drgbl | Hm Drgbl | CPE002DV | CPE002EL | CPE002FP | NM 01424 | NM 0142 | NM 0142 | ADAMTS3 | ADAMTS3 | ADAMTS3 | ADAM metallopep | ADAM me | ADAM me | 9508 | 9508 | 9508 | s18230 | s18231 | s18232 |
| 10564 | I12 | I13 | 1316 | 810,1 | 14776 | 197,3 | 11,2 | 1,266 | H5A034 C | sample | Hm Drgbl | Hm Drgbl | Hm Drgbl | CPE002EB | CPE002EO | CPE002FR | NM 13845 | NM 1384 | NM 1384 | DHRS1 | DHRS1 | DHRS1 | dehydrogenase/re | dehydrog | dehydrog | 115817 | 115817 | 115817 | s41879 | s41878 | s41880 |
| 10613 | K13 | K14 | 1106 | 765,3 | 12359 | 173,3 | 11,2 | 1,266 | H5A034 C | sample | Hm Drgbl | Hm Drgbl | Hm Drgbl | CPE002EB | CPE002EO | CPE002FR | NM 08042 | NM 0804 | NM 0804 | AQP10 | AQP10 | AQP10 | aquaporin 10 | aquaporir | aquaporir | 89872 | 89872 | 89872 | s40172 | s40174 | s40173 |
| 20021 | C13 | C14 | 850 | 1091 | 9535 | 165,6 | 11,2 | 1,366 | H5A034 C | sample | Hm Gen E | Hm Gen E | Hm Gen E | CPE002JQ | CPE002O5 | CPE002KL | XM 37275 | XM 3727 | XM 3727 | LOC39098 | LOC39098 | LOC39098 | similar to hCG20 | similar to | similar to | 390988 | 390988 | 390988 | s196358 | s196359 | s196360 |
| 20034 | C3 | C4 | 1087 | 1152 | 12171 | 163,5 | 11,2 | 1,366 | H5A034 C | sample | Hm Gen E | Hm Gen E | Hm Gen E | CPE002JQ | CPE002O5 | CPE002KL | NM 00100 | NM 0010 | NM 0010 | OR7A10 | OR7A10 | OR7A10 | olfactory recepto | olfactory | olfactory | 390892 | 390892 | 390892 | s52881 | s52882 | s52883 |
| 20039 | C8 | C9 | 774 | 1199 | 8697 | 192,5 | 11,2 | 1,366 | H5A034 C | sample | Hm Gen E | Hm Gen E | Hm Gen E | CPE002JQ | CPE002O5 | CPE002KL | XM 37922 | XM 3792 | XM 3792 | hCG 1806 | hCG 1806 | hCG 1806 | hCG1806964 | hCG18069 | hCG18069 | 401093 | 401093 | 401093 | s53469 | s53470 | s53471 |
| 20060 | D5 | D6 | 945 | 1156 | 10551 | 199 | 11,2 | 1,366 | H5A034 C | sample | Hm Gen E | Hm Gen E | Hm Gen E | CPE002JQ | CPE002O5 | CPE002KL | NM 00100 | NM 0010 | NM 0010 | FLJ44653 | FLJ44653 | FLJ44653 | FLJ44653 protein | FLJ44653 | FLJ44653 | 399833 | 399833 | 399833 | s53183 | s53184 | s226520 |
| 20147 | H19 | H20 | 903 | 1153 | 10119 | 155,6 | 11,2 | 1,366 | H5A034 C | sample | Hm Gen E | Hm Gen E | Hm Gen E | CPE002JQ | CPE002O5 | CPE002KL | XM 00112 | XM 0011 | XM 0011 | LOC40045 | LOC40045 | LOC40045 | hypothetical gene | hypotheti | hypotheti | 400456 | 400456 | 400456 | s53279 | s53280 | s53281 |
| 20210 | K10 | K11 | 258 | 1268 | 2883 | 226,8 | 11,2 | 1,366 | H5A034 C | sample | Hm Gen E | Hm Gen E | Hm Gen E | CPE002JQ | CPE002O5 | CPE002KL | XM 37678 | XM 3767 | XM 3767 | RPS26P10 | RPS26P10 | RPS26P10 | ribosomal protein | ribosomal | ribosomal | 401470 | 401470 | 401470 | s53606 | s53607 | s226568 |
| 388 | A12 | A13 | 2312 | 1804 | 25884 | 465,3 | 11,2 | 1,635 | H5A034 C | sample | Hm Drgbl | Hm Drgbl | Hm Drgbl | CPE002E0 | CPE002EZ | CPE002FK | NM 01218 | NM 0121 | NM 0121 | FOXE3 | FOXE3 | FOXE3 | forkhead box E3 | forkhead | forkhead | 2301 | 2301 | 2301 | s5238 | s5239 | s223553 |
| 7751 | C8 | C9 | 652 | 519 | 7284 | 391,3 | 11,2 | 1,71 | H5A034 C | sample | Hm Drgbl | Hm Drgbl | Hm Drgbl | CPE002E4 | CPE002EV | CPE002FG | NM 00717 | NM 0071 | NM 0071 | POMT1 | POMT1 | POMT1 | protein-O-manno | protein-O | protein-O | 10585 | 10585 | 10585 | s20776 | s20777 | s20775 |
| 7971 | M11 | M12 | 714 | 542,8 | 7996 | 426,5 | 11,2 | 1,71 | H5A034 C | sample | Hm Drgbl | Hm Drgbl | Hm Drgbl | CPE002E4 | CPE002EV | CPE002FG | NM 00587 | NM 0058 | NM 0058 | TRAIP | TRAIP | TRAIP | TRAF interacting | TRAF inte | TRAF inte | 10293 | 10293 | 10293 | s20121 | s20120 | s223101 |
| 8010 | N3 | N4 | 889 | 579,3 | 9926 | 383 | 11,2 | 1,71 | H5A034 C | sample | Hm Drgbl | Hm Drgbl | Hm Drgbl | CPE002E4 | CPE002EV | CPE002FG | NM 00426 | NM 0042 | NM 0042 | SEMA4F | SEMA4F | SEMA4F | sema domain, im | sema dom | sema dom | 10505 | 10505 | 10505 | s20589 | s20591 | s20590 |
| 3177 | E17 | E18 | 1639 | 1316 | 18598 | 329,3 | 11,3 | 0,7 | H5A034 C | sample | Hm Drgbl | Hm Drgbl | Hm Drgbl | CPE002DC | CPE002EG | CPE002F6 | NM 00409 | NM 0040 | NM 0040 | DUSP3 | DUSP3 | DUSP3 | dual specificity pl | dual speci | dual speci | 1845 | 1845 | 1845 | s4369 | s4370 | s4371 |
| 3279 | I22 | I23 | 1266 | 1378 | 14300 | 253 | 11,3 | 0,7 | H5A034 C | sample | Hm Drgbl | Hm Drgbl | Hm Drgbl | CPE002DC | CPE002EG | CPE002F6 | NM 13078 | NM 1307 | NM 1307 | TPTE2 | TPTE2 | TPTE2 | transmembrane p | transmem | transmem | 93492 | 93492 | 93492 | s41150 | s41151 | s41152 |
| 3324 | K2 | K3 | 781 | 1141 | 8828 | 407 | 11,3 | 0,7 | H5A034 C | sample | Hm Drgbl | Hm Drgbl | Hm Drgbl | CPE002DC | CPE002EG | CPE002F6 | NM 03306 | NM 0330 | NM 0330 | ACPT | ACPT | ACPT | acid phosphatase | acid phos | acid phos | 93650 | 93650 | 93650 | s41186 | s41187 | s41188 |
| 3374 | M21 | M22 | 1395 | 1380 | 15719 | 385,9 | 11,3 | 0,7 | H5A034 C | sample | Hm Drgbl | Hm Drgbl | Hm Drgbl | CPE002DC | CPE002EG | CPE002F6 | NM 00100 | NM 0010 | NM 0010 | PPP2CB | PPP2CB | PPP2CB | protein phosphat | protein pl | protein pl | 5516 | 5516 | 5516 | s10960 | s10961 | s10962 |
| 20453 | E13 | E14 | 1025 | 689,5 | 11625 | 156,7 | 11,3 | 0,753 | H5A034 C | sample | Hm Gen E | Hm Gen E | Hm Gen E | CPE002JR | CPE002O6 | CPE002KN | NM 00102 | NM 0010 | NM 0010 | CTRB2 | CTRB2 | CTRB2 | chymotrypsinoge | chymotryp | chymotryp | 440387 | 440387 | 440387 | s54098 | s196535 | s196536 |
| 20688 | N9 | N10 | 1059 | 716,7 | 11915 | 146,1 | 11,3 | 0,753 | H5A034 C | sample | Hm Gen E | Hm Gen E | Hm Gen E | CPE002JR | CPE002O6 | CPE002KN | XM 94216 | XM 9421 | XM 9421 | LOC44213 | LOC44213 | LOC44213 | similar to hypoth | similar to | similar to | 442132 | 442132 | 442132 | s54492 | s196649 | s196650 |
| 12859 | H4 | H5 | 1266 | 1053 | 14348 | 130,3 | 11,3 | 0,766 | H5A034 C | sample | Hm Gen E | Hm Gen E | Hm Gen E | CPE002J6 | CPE002NN | CPE002K2 | NM 00606 | NM 0060 | NM 0060 | COX4NB | COX4NB | COX4NB | COX4 neighbor | COX4 neig | COX4 neig | 10328 | 10328 | 10328 | s20202 | s20200 | s225428 |
| 18769 | O1 | O2 | 1366 | 979,3 | 15504 | 226,3 | 11,3 | 0,766 | H5A034 C | sample | Hm Gen E | Hm Gen E | Hm Gen E | CPE002JM | CPE002O1 | CPE002KH | NM 00100 | NM 0010 | NM 0010 | OR10G8 | OR10G8 | OR10G8 | olfactory recepto | olfactory | olfactory | 219869 | 219869 | 219869 | s47745 | s47746 | s47747 |
| 17360 | D16 | D17 | 1137 | 1106 | 12820 | 230,1 | 11,3 | 0,771 | H5A034 C | sample | Hm Gen E | Hm Gen E | Hm Gen E | CPE002JJ | CPE002NY | CPE002KE | XM 05995 | XM 0599 | XM 0599 | C9orf57 | C9orf57 | C9orf57 | chromosome 9 op | chromoso | chromoso | 138240 | 138240 | 138240 | s44097 | s44098 | s44099 |
| 21061 | N20 | N21 | 1737 | 963,7 | 19687 | 170,3 | 11,3 | 0,801 | H5A034 C | sample | Hm Gen E | Hm Gen E | Hm Gen E | CPE002JS | CPE002O7 | CPE002KN | XM 92826 | XM 9282 | XM 9282 | LOC64522 | LOC64522 | LOC64522 | similar to ATP syn | similar to | similar to | 645225 | 645225 | 645225 | s55964 | s55966 | s227172 |
| 7320 | A9 | A10 | 1333 | 659,1 | 15020 | 459,6 | 11,3 | 0,863 | H5A034 C | sample | Hm Drgbl | Hm Drgbl | Hm Drgbl | CPE002E3 | CPE002EW | CPE002FH | NM 00090 | NM 0009 | NM 0009 | NPR3 | NPR3 | NPR3 | natriuretic peptid | natriureti | natriureti | 4883 | 4883 | 4883 | s9691 | s224132 | s9692 |
| 7579 | L4 | L5 | 572 | 648,9 | 6476 | 338,7 | 11,3 | 0,863 | H5A034 C | sample | Hm Drgbl | Hm Drgbl | Hm Drgbl | CPE002E3 | CPE002EW | CPE002FH | NM 02029 | NM 0202 | NM 0202 | ABCC9 | ABCC9 | ABCC9 | ATP-binding casse | ATP-bindi | ATP-bindi | 10060 | 10060 | 10060 | s19564 | s19563 | s19562 |
| 6926 | A21 | A22 | 1705 | 681,5 | 19251 | 535,7 | 11,3 | 0,879 | H5A034 C | sample | Hm Drgbl | Hm Drgbl | Hm Drgbl | CPE002E2 | CPE002EX | CPE002FI | NM 00229 | NM 1944 | NM 1944 | LBR | LBR | LBR | lamin B receptor | lamin B re | lamin B re | 3930 | 3930 | 3930 | s224003 | s8102 | s8100 |
| 7164 | K2 | K3 | 351 | 612,3 | 3974 | 441,5 | 11,3 | 0,879 | H5A034 C | sample | Hm Drgbl | Hm Drgbl | Hm Drgbl | CPE002E2 | CPE002EX | CPE002FI | NM 07942 | NM 0210 | NM 0794 | MYL6 | MYL6 | MYL6 | myosin, light chai | myosin, li | myosin, li | 4637 | 4637 | 4637 | s9192 | s224084 | s9190 |
| 5005 | A20 | A21 | 1728 | 759 | 19505 | 535,7 | 11,3 | 0,886 | H5A034 C | sample | Hm Drgbl | Hm Drgbl | Hm Drgbl | CPE002DV | CPE002EM | CPE002FO | NM 00667 | NM 0066 | NM 0066 | TPBG | TPBG | TPBG | trophoblast glyco | trophobla | trophobla | 7162 | 7162 | 7162 | s14322 | s14323 | s14324 |
| 5072 | D16 | D17 | 2241 | 599,5 | 25407 | 451,2 | 11,3 | 0,886 | H5A034 C | sample | Hm Drgbl | Hm Drgbl | Hm Drgbl | CPE002DV | CPE002EM | CPE002FO | NM 00542 | NM 0054 | NM 0054 | VEGFC | VEGFC | VEGFC | vascular endothe | vascular e | vascular e | 7424 | 7424 | 7424 | s14783 | s14784 | s14785 |
| 2360 | C16 | C17 | 1382 | 623,7 | 15580 | 331,6 | 11,3 | 0,89 | H5A034 C | sample | Hm Drgbl | Hm Drgbl | Hm Drgbl | CPE002DC | CPE002EE | CPE002F4 | NM 01865 | NM 0186 | NM 0186 | MARK1 | MARK1 | MARK1 | MAP/microtubule | MAP/micr | MAP/micr | 4139 | 4139 | 4139 | s8511 | s8512 | s8513 |
| 2399 | D8 | D9 | 614 | 749,9 | 6962 | 305,8 | 11,3 | 0,89 | H5A034 C | sample | Hm Drgbl | Hm Drgbl | Hm Drgbl | CPE002DC | CPE002EE | CPE002F4 | NM 00103 | NM 0010 | NM 0010 | PRKCZ | PRKCZ | PRKCZ | protein kinase C, | protein ki | protein ki | 5590 | 5590 | 5590 | s11128 | s11129 | s11130 |
| 2447 | F8 | F9 | 1087 | 746,9 | 12297 | 393,8 | 11,3 | 0,89 | H5A034 C | sample | Hm Drgbl | Hm Drgbl | Hm Drgbl | CPE002DC | CPE002EE | CPE002F4 | NM 13898 | NM 1389 | NM 1389 | MAPK10 | MAPK10 | MAPK10 | mitogen-activatec | mitogen-a | mitogen-a | 5602 | 5602 | 5602 | s11161 | s11162 | s11163 |
| 2456 | G16 | G17 | 1239 | 667,3 | 13966 | 272,1 | 11,3 | 0,89 | H5A034 C | sample | Hm Drgbl | Hm Drgbl | Hm Drgbl | CPE002DC | CPE002EE | CPE002F4 | NM 00101 | NM 0010 | NM 0010 | NME2 | NME2 | NME2 | non-metastatic ce | non-meta | non-meta | 4831 | 4831 | 4831 | s9591 | s224122 | s9593 |
| 2607 | M22 | M23 | 1949 | 763,5 | 21959 | 335,1 | 11,3 | 0,89 | H5A034 C | sample | Hm Drgbl | Hm Drgbl | Hm Drgbl | CPE002DC | CPE002EE | CPE002F4 | NM 00264 | NM 0026 | NM 0026 | PIM1 | PIM1 | PIM1 | pim-1 oncogene | pim-1 onc | pim-1 onc | 5292 | 5292 | 5292 | s10526 | s10527 | s10528 |
| 2628 | N2 | N3 | 1166 | 655,2 | 13222 | 438 | 11,3 | 0,89 | H5A034 C | sample | Hm Drgbl | Hm Drgbl | Hm Drgbl | CPE002DC | CPE002EE | CPE002F4 | NM 18269 | NM 1826 | NM 1826 | SRPK2 | SRPK2 | SRPK2 | SFRS protein kina | SFRS prot | SFRS prot | 6733 | 6733 | 6733 | s13453 | s13454 | s13455 |
| 2684 | P5 | P6 | 1459 | 692 | 16490 | 419,6 | 11,3 | 0,89 | H5A034 C | sample | Hm Drgbl | Hm Drgbl | Hm Drgbl | CPE002DC | CPE002EE | CPE002F4 | NM 15304 | NM 1530 | NM 1530 | FYN | FYN | FYN | FYN oncogene rel | FYN oncoj | FYN oncoj | 2534 | 2534 | 2534 | s5434 | s5435 | s5436 |
| 16713 | I17 | I18 | 1044 | 1005 | 11776 | 246,1 | 11,3 | 0,893 | H5A034 C | sample | Hm Gen E | Hm Gen E | Hm Gen E | CPE002JH | CPE002NV | CPE002KC | NM 03288 | NM 0328 | NM 0328 | PNMA6A | PNMA6A | PNMA6A | paraneoplastic an | paraneop | paraneop | 84968 | 84968 | 84968 | s39776 | s39777 | s39778 |
| 8977 | G1 | G2 | 1737 | 1862 | 19580 | 128,6 | 11,3 | 0,908 | H5A034 C | sample | Hm Drgbl | Hm Drgbl | Hm Drgbl | CPE002E7 | CPE002ES | CPE002FD | NM 01848 | NM 0184 | NM 0184 | DDEF1 | DDEF1 | DDEF1 | development and | developm | developm | 50807 | 50807 | 50807 | s27099 | s27100 | s27101 |
| 9156 | N2 | N3 | 1283 | 1581 | 14507 | 129,7 | 11,3 | 0,908 | H5A034 C | sample | Hm Drgbl | Hm Drgbl | Hm Drgbl | CPE002E7 | CPE002ES | CPE002FD | NM 01751 | NM 0175 | NM 0175 | RAB39 | RAB39 | RAB39 | RAB39, member R | RAB39, m | RAB39, m | 54734 | 54734 | 54734 | s29359 | s29360 | s29361 |
| 4382 | G21 | G22 | 1071 | 760,1 | 12126 | 506,7 | 11,3 | 0,954 | H5A034 C | sample | Hm Drgbl | Hm Drgbl | Hm Drgbl | CPE002DL | CPE002EK | CPE002FQ | NM 00074 | NM 0007 | NM 0007 | CHRNA2 | CHRNA2 | CHRNA2 | cholinergic recept | cholinergi | cholinergi | 1135 | 1135 | 1135 | s3040 | s3041 | s3042 |
| 4434 | I3 | I4 | 1417 | 678,8 | 15969 | 355,8 | 11,3 | 0,954 | H5A034 C | sample | Hm Drgbl | Hm Drgbl | Hm Drgbl | CPE002DL | CPE002EK | CPE002FQ | NM 00074 | NM 0007 | NM 0007 | CHRNA4 | CHRNA4 | CHRNA4 | cholinergic recept | cholinergi | cholinergi | 1137 | 1137 | 1137 | s3046 | s3047 | s3048 |
| 4474 | K18 | K19 | 1413 | 623,8 | 16012 | 286,9 | 11,3 | 0,954 | H5A034 C | sample | Hm Drgbl | Hm Drgbl | Hm Drgbl | CPE002DL | CPE002EK | CPE002FQ | NM 01455 | NM 0145 | NM 0145 | TRPM5 | TRPM5 | TRPM5 | transient recepto | transient | transient | 29850 | 29850 | 29850 | s26620 | s26621 | s26622 |
| 252 | K2 | K3 | 206 | 1459 | 2333 | 541 | 11,3 | 0,962 | H5A034 C | sample | Hm Drgbl | Hm Drgbl | Hm Drgbl | CPE002DT | CPE002EJ | CPE002F9 | NM 00308 | NM 0030 | NM 0030 | SNRP70 | SNRP70 | SNRP70 | small nuclear ribo | small nucl | small nucl | 6625 | 6625 | 6625 | s13210 | s13211 | s13212 |
| 296 | M16 | M17 | 588 | 1424 | 6652 | 472,4 | 11,3 | 0,962 | H5A034 C | sample | Hm Drgbl | Hm Drgbl | Hm Drgbl | CPE002DT | CPE002EJ | CPE002F9 | NM 00310 | NM 0031 | NM 0031 | SOD3 | SOD3 | SOD3 | superoxide dismu | superoxid | superoxid | 6649 | 6649 | 6649 | s13270 | s13271 | s13272 |
| 333 | N6 | N7 | 627 | 1460 | 7068 | 620,1 | 11,3 | 0,962 | H5A034 C | sample | Hm Drgbl | Hm Drgbl | Hm Drgbl | CPE002DT | CPE002EJ | CPE002F9 | NM 00316 | NM 0031 | NM 0031 | SULT2A1 | SULT2A1 | SULT2A1 | sulfotransferase f | sulfotrans | sulfotrans | 6822 | 6822 | 6822 | s13625 | s13626 | s13627 |
| 2005 | D20 | D21 | 1500 | 705,7 | 16963 | 425,3 | 11,3 | 0,983 | H5A034 C | sample | Hm Drgbl | Hm Drgbl | Hm Drgbl | CPE002FX | CPE002G1 | CPE002G5 | NM 19853 | NM 1985 | NM 1985 | ZNF568 | ZNF568 | ZNF568 | zinc finger protei | zinc finge | zinc finge | 374900 | 374900 | 374900 | s51615 | s51616 | s51617 |
| 2013 | D6 | D7 | 624 | 730 | 7024 | 450,7 | 11,3 | 0,983 | H5A034 C | sample | Hm Drgbl | Hm Drgbl | Hm Drgbl | CPE002FX | CPE002G1 | CPE002G5 | NM 00100 | NM 0010 | NM 0010 | ZNF429 | ZNF429 | ZNF429 | zinc finger protei | zinc finge | zinc finge | 353088 | 353088 | 353088 | s51414 | s51415 | s51416 |
| 2015 | D8 | D9 | 861 | 726,9 | 9708 | 421,3 | 11,3 | 0,983 | H5A034 C | sample | Hm Drgbl | Hm Drgbl | Hm Drgbl | CPE002FX | CPE002G1 | CPE002G5 | NM 18148 | NM 1814 | NM 1814 | ZNF445 | ZNF445 | ZNF445 | zinc finger protei | zinc finge | zinc finge | 353274 | 353274 | 353274 | s51456 | s51457 | s51458 |
| 2106 | H3 | H4 | 1166 | 727,6 | 13154 | 417,9 | 11,3 | 0,983 | H5A034 C | sample | Hm Drgbl | Hm Drgbl | Hm Drgbl | CPE002FX | CPE002G1 | CPE002G5 | NM 15247 | NM 1524 | NM 1524 | ZNF417 | ZNF417 | ZNF417 | zinc finger protei | zinc finge | zinc finge | 147687 | 147687 | 147687 | s45030 | s45031 | s45032 |
| 2146 | J18 | J19 | 1112 | 655,1 | 12535 | 351,7 | 11,3 | 0,983 | H5A034 C | sample | Hm Drgbl | Hm Drgbl | Hm Drgbl | CPE002FX | CPE002G1 | CPE002G5 | XM 00112 | XM 0011 | XM 0011 | MUC5B | MUC5B | MUC5B | mucin 5B, oligom | mucin 5B, | mucin 5B, | 727897 | 727897 | 727897 | s58030 | s58031 | s58032 |
| 2150 | J21 | J22 | 1486 | 690,6 | 16737 | 381,7 | 11,3 | 0,983 | H5A034 C | sample | Hm Drgbl | Hm Drgbl | Hm Drgbl | CPE002FX | CPE002G1 | CPE002G5 | NM 14896 | NM 1489 | NM 1489 | CLDN19 | CLDN19 | CLDN19 | claudin 19 | claudin 19 | claudin 19 | 149461 | 149461 | 149461 | s45292 | s45293 | s45294 |
| 2227 | M4 | M5 | 1619 | 682,1 | 18219 | 392,6 | 11,3 | 0,983 | H5A034 C | sample | Hm Drgbl | Hm Drgbl | Hm Drgbl | CPE002FX | CPE002G1 | CPE002G5 | NM 00104 | NM 0010 | NM 0010 | SYPL2 | SYPL2 | SYPL2 | synaptophysin-lik | synaptopl | synaptopl | 284612 | 284612 | 284612 | s49712 | s49713 | s49714 |
| 2271 | O22 | O23 | 2214 | 738,4 | 24955 | 489 | 11,3 | 0,983 | H5A034 C | sample | Hm Drgbl | Hm Drgbl | Hm Drgbl | CPE002FX | CPE002G1 | CPE002G5 | NM 21360 | NM 2136 | NM 2136 | ZNF517 | ZNF517 | ZNF517 | zinc finger protei | zinc finge | zinc finge | 340385 | 340385 | 340385 | s50710 | s50711 | s50712 |
| 6533 | A13 | A14 | 1138 | 719,2 | 12853 | 322 | 11,3 | 0,991 | H5A034 C | sample | Hm Drgbl | Hm Drgbl | Hm Drgbl | CPE002E1 | CPE002EY | CPE002FJ | NM 00085 | NM 0008 | NM 0008 | GSTP1 | GSTP1 | GSTP1 | glutathione S-trar | glutathion | glutathion | 2950 | 2950 | 2950 | s306 | s194476 | s223820 |
| 6562 | B18 | B19 | 615 | 759,9 | 6955 | 373,2 | 11,3 | 0,991 | H5A034 C | sample | Hm Drgbl | Hm Drgbl | Hm Drgbl | CPE002E1 | CPE002EY | CPE002FJ | NM 00088 | NM 0008 | NM 0008 | ITGAX | ITGAX | ITGAX | integrin, alpha X ( | integrin, a | integrin, a | 3687 | 3687 | 3687 | s7571 | s7572 | s7573 |
| 3949 | E20 | E21 | 1286 | 674,8 | 14503 | 394,2 | 11,3 | 1,004 | H5A034 C | sample | Hm Drgbl | Hm Drgbl | Hm Drgbl | CPE002DS | CPE002EI | CPE002F8 | NM 00690 | NM 0069 | NM 0069 | RAC1 | RAC1 | RAC1 | ras-related C3 bo | ras-relate | ras-relate | 5879 | 5879 | 5879 | s11711 | s11712 | s11713 |
| 4092 | K2 | K3 | 724 | 672,3 | 8185 | 267,9 | 11,3 | 1,004 | H5A034 C | sample | Hm Drgbl | Hm Drgbl | Hm Drgbl | CPE002DS | CPE002EI | CPE002F8 | NM 00505 | NM 0050 | NM 0050 | RAPSN | RAPSN | RAPSN | receptor-associat | receptor-a | receptor-a | 5913 | 5913 | 5913 | s11797 | s11798 | s11799 |
| 4095 | K22 | K23 | 718 | 735,5 | 8091 | 415,8 | 11,3 | 1,004 | H5A034 C | sample | Hm Drgbl | Hm Drgbl | Hm Drgbl | CPE002DS | CPE002EI | CPE002F8 | NM 00289 | NM 0028 | NM 0028 | RASGRF1 | RASGRF1 | RASGRF1 | Ras protein-speci | Ras prote | Ras prote | 5923 | 5923 | 5923 | s11825 | s11826 | s11827 |
| 4124 | L5 | L6 | 1053 | 683,3 | 11892 | 265,6 | 11,3 | 1,004 | H5A034 C | sample | Hm Drgbl | Hm Drgbl | Hm Drgbl | CPE002DS | CPE002EI | CPE002F8 | NM 18173 | NM 1817 | NM 0058 | PRDX2 | PRDX2 | PRDX2 | peroxiredoxin 2 | peroxired | peroxired | 7001 | 7001 | 7001 | s13958 | s13960 | s224716 |
| 15657 | M17 | M18 | 1225 | 778,7 | 13809 | 125,8 | 11,3 | 1,027 | H5A034 C | sample | Hm Gen E | Hm Gen E | Hm Gen E | CPE002JE | CPE002NT | CPE002K9 | NM 00108 | NM 0010 | NM 0010 | RGNEF | RGNEF | RGNEF | Rho-guanine nucl | Rho-guan | Rho-guan | 64283 | 64283 | 64283 | s34605 | s34606 | s34607 |
| 6018 | K3 | K4 | 475 | 777 | 5369 | 519,6 | 11,3 | 1,032 | H5A034 C | sample | Hm Drgbl | Hm Drgbl | Hm Drgbl | CPE002DY | CPE002F1 | CPE002FM | NM 00066 | NM 0006 | NM 0006 | ADH1B | ADH1B | ADH1B | alcohol dehydrog | alcohol de | alcohol de | 125 | 125 | 125 | s1063 | s1061 | s1062 |
| 6031 | L15 | L16 | 1527 | 754,1 | 17185 | 537,4 | 11,3 | 1,032 | H5A034 C | sample | Hm Drgbl | Hm Drgbl | Hm Drgbl | CPE002DY | CPE002F1 | CPE002FM | NM 00164 | NM 0016 | NM 0016 | APOBEC1 | APOBEC1 | APOBEC1 | apolipoprotein B | apolipopr | apolipopr | 339 | 339 | 339 | s1480 | s1479 | s1478 |
| 1242 | D3 | D4 | 1358 | 699,3 | 15383 | 431,2 | 11,3 | 1,037 | H5A034 C | sample | Hm Drgbl | Hm Drgbl | Hm Drgbl | CPE002FV | CPE002FZ | CPE002G3 | NM 00418 | NM 0041 | NM 0041 | JARID1C | JARID1C | JARID1C | jumonji, AT rich in | jumonji, A | jumonji, A | 8242 | 8242 | 8242 | s15748 | s15749 | s15750 |
| 1270 | E7 | E8 | 1278 | 686 | 14434 | 403,6 | 11,3 | 1,037 | H5A034 C | sample | Hm Drgbl | Hm Drgbl | Hm Drgbl | CPE002FV | CPE002FZ | CPE002G3 | NM 01959 | NM 0195 | NM 0195 | ZNF26 | ZNF26 | ZNF26 | zinc finger protei | zinc finge | zinc finge | 7574 | 7574 | 7574 | s15060 | s15061 | s15062 |
| 1465 | N1 | N2 | 1102 | 686,3 | 12444 | 445,3 | 11,3 | 1,037 | H5A034 C | sample | Hm Drgbl | Hm Drgbl | Hm Drgbl | CPE002FV | CPE002FZ | CPE002G3 | NM 00423 | NM 0042 | NM 0042 | ZNF235 | ZNF235 | ZNF235 | zinc finger protei | zinc finge | zinc finge | 9310 | 9310 | 9310 | s225111 | s17782 | s17783 |
| 1471 | N15 | N16 | 1687 | 685,6 | 19018 | 436,5 | 11,3 | 1,037 | H5A034 C | sample | Hm Drgbl | Hm Drgbl | Hm Drgbl | CPE002FV | CPE002FZ | CPE002G3 | NM 20162 | NM 2016 | NM 2016 | TJP2 | TJP2 | TJP2 | tight junction pro | tight junct | tight junct | 9414 | 9414 | 9414 | s18020 | s18021 | s18022 |
| 16397 | L13 | L14 | 1256 | 951,9 | 14251 | 265,6 | 11,3 | 1,037 | H5A034 C | sample | Hm Gen E | Hm Gen E | Hm Gen E | CPE002JG | CPE002NV | CPE002KB | NM 03213 | NM 0321 | NM 0321 | C3orf20 | C3orf20 | C3orf20 | chromosome 3 op | chromoso | chromoso | 84077 | 84077 | 84077 | s38471 | s38472 | s38473 |
| 16420 | M12 | M13 | 2079 | 1045 | 23413 | 280,6 | 11,3 | 1,037 | H5A034 C | sample | Hm Gen E | Hm Gen E | Hm Gen E | CPE002JG | CPE002NV | CPE002KB | NM 03235 | NM 0323 | NM 0323 | CCDC77 | CCDC77 | CCDC77 | coiled-coil domai | coiled-coi | coiled-coi | 84318 | 84318 | 84318 | s38908 | s38909 | s38910 |
| 13560 | E9 | E10 | 1278 | 959,4 | 14399 | 164,4 | 11,3 | 1,061 | H5A034 C | sample | Hm Gen E | Hm Gen E | Hm Gen E | CPE002JC | CPE002NC | CPE002K4 | XM 04859 | XM 0485 | XM 0485 | KIAA1045 | KIAA1045 | KIAA1045 | KIAA1045 | KIAA1045 | KIAA1045 | 23349 | 23349 | 23349 | s23617 | s23618 | s225949 |
| 1037 | L13 | L14 | 1060 | 652,8 | 11955 | 365 | 11,3 | 1,071 | H5A034 C | sample | Hm Drgbl | Hm Drgbl | Hm Drgbl | CPE002FU | CPE002FY | CPE002G2 | NM 00534 | NM 0053 | NM 0053 | HSP90AA1 | HSP90AA1 | HSP90AA1 | heat shock protei | heat shoc | heat shoc | 3320 | 3320 | 3320 | s6993 | s6994 | s6995 |
| 1044 | L2 | L3 | 1649 | 718,2 | 18607 | 364,8 | 11,3 | 1,071 | H5A034 C | sample | Hm Drgbl | Hm Drgbl | Hm Drgbl | CPE002FU | CPE002FY | CPE002G2 | NM 03075 | NM 0307 | NM 0307 | ZEB1 | ZEB1 | ZEB1 | zinc finger E-box b | zinc finge | zinc finge | 6935 | 6935 | 6935 | s13883 | s13884 | s13885 |
| 1075 | M4 | M5 | 866 | 701,7 | 9812 | 399,1 | 11,3 | 1,071 | H5A034 C | sample | Hm Drgbl | Hm Drgbl | Hm Drgbl | CPE002FU | CPE002FY | CPE002G2 | NM 00265 | NM 0026 | NM 0026 | PLAGL2 | PLAGL2 | PLAGL2 | pleiomorphic ade | pleiomorc | pleiomorc | 5326 | 5326 | 5326 | s10604 | s10605 | s10606 |
| 5455 | D15 | D16 | 1644 | 620,5 | 18539 | 531,6 | 11,3 | 1,076 | H5A034 C | sample | Hm Drgbl | Hm Drgbl | Hm Drgbl | CPE002DX | CPE002F2 | CPE002FN | NM 00373 | NM 0037 | NM 0037 | DCHS1 | DCHS1 | DCHS1 | dachsous 1 (Droso | dachsous | dachsous | 8642 | 8642 | 8642 | s16441 | s16442 | s16443 |
| 5627 | K19 | K20 | 1904 | 783,4 | 21449 | 518 | 11,3 | 1,076 | H5A034 C | sample | Hm Drgbl | Hm Drgbl | Hm Drgbl | CPE002DX | CPE002F2 | CPE002FN | NM 00364 | NM 0036 | NM 0036 | HAT1 | HAT1 | HAT1 | histone acetyltran | histone ac | histone ac | 8520 | 8520 | 8520 | s16194 | s16196 | s16195 |
| 5629 | K20 | K21 | 2411 | 755,2 | 27217 | 559,8 | 11,3 | 1,076 | H5A034 C | sample | Hm Drgbl | Hm Drgbl | Hm Drgbl | CPE002DX | CPE002F2 | CPE002FN | NM 00509 | NM 0050 | NM 0050 | PGLYRP1 | PGLYRP1 | PGLYRP1 | peptidoglycan rec | peptidogl | peptidogl | 8993 | 8993 | 8993 | s17156 | s17157 | s194971 |
| 5654 | L21 | L22 | 1522 | 585 | 17219 | 536,9 | 11,3 | 1,076 | H5A034 C | sample | Hm Drgbl | Hm Drgbl | Hm Drgbl | CPE002DX | CPE002F2 | CPE002FN | NM 00380 | NM 0038 | NM 0038 | HRK | HRK | HRK | harakiri, BCL2 inte | harakiri, B | harakiri, B | 8739 | 8739 | 8739 | s194953 | s194954 | s194952 |
| 5659 | L4 | L5 | 1652 | 740,3 | 18745 | 583,1 | 11,3 | 1,076 | H5A034 C | sample | Hm Drgbl | Hm Drgbl | Hm Drgbl | CPE002DX | CPE002F2 | CPE002FN | NM 00422 | NM 0174 | NM 0174 | PSCD2 | PSCD2 | PSCD2 | pleckstrin homolc | pleckstrin | pleckstrin | 9266 | 9266 | 9266 | s225107 | s17718 | s17719 |
| 3482 | B10 | B11 | 1292 | 1212 | 14637 | 302,8 | 11,3 | 1,087 | H5A034 C | sample | Hm Drgbl | Hm Drgbl | Hm Drgbl | CPE002DR | CPE002EH | CPE002F7 | NM 02391 | NM 0239 | NM 0239 | GPR87 | GPR87 | GPR87 | G protein-couplec | G protein | G protein | 53836 | 53836 | 53836 | s28783 | s28784 | s28785 |
| 3694 | J7 | J8 | 1491 | 1332 | 16818 | 271,5 | 11,3 | 1,087 | H5A034 C | sample | Hm Drgbl | Hm Drgbl | Hm Drgbl | CPE002DR | CPE002EH | CPE002F7 | NM 00086 | NM 0008 | NM 0008 | HTR1E | HTR1E | HTR1E | 5-hydroxytryptam | 5-hydroxy | 5-hydroxy | 3354 | 3354 | 3354 | s7035 | s7036 | s7037 |
| 3707 | K19 | K20 | 1559 | 1403 | 17576 | 330,3 | 11,3 | 1,087 | H5A034 C | sample | Hm Drgbl | Hm Drgbl | Hm Drgbl | CPE002DR | CPE002EH | CPE002F7 | NM 00195 | NM 0019 | NM 0019 | EDNRA | EDNRA | EDNRA | endothelin recept | endotheli | endotheli | 1909 | 1909 | 1909 | s4465 | s4466 | s4467 |
| 8087 | A8 | A9 | 1167 | 620,1 | 13183 | 518,5 | 11,3 | 1,087 | H5A034 C | sample | Hm Drgbl | Hm Drgbl | Hm Drgbl | CPE002E5 | CPE002EU | CPE002FF | NM 01407 | NM 0140 | NM 0140 | NCOA6 | NCOA6 | NCOA6 | nuclear receptor c | nuclear re | nuclear re | 23054 | 23054 | 23054 | s22931 | s22930 | s22929 |
| 8119 | C15 | C16 | 709 | 631,9 | 7997 | 347,5 | 11,3 | 1,087 | H5A034 C | sample | Hm Drgbl | Hm Drgbl | Hm Drgbl | CPE002E5 | CPE002EU | CPE002FF | NM 00703 | NM 0070 | NM 0070 | ESM1 | ESM1 | ESM1 | endothelial cell-sp | endotheli | endotheli | 11082 | 11082 | 11082 | s21850 | s21851 | s21852 |
| 8143 | D15 | D16 | 1792 | 695 | 20252 | 455,1 | 11,3 | 1,087 | H5A034 C | sample | Hm Drgbl | Hm Drgbl | Hm Drgbl | CPE002E5 | CPE002EU | CPE002FF | NM 01225 | NM 0122 | NM 0122 | XRN2 | XRN2 | XRN2 | 5-3 exoribonuclea | 5-3 exorib | 5-3 exorib | 22803 | 22803 | 22803 | s22414 | s22413 | s22412 |
| 8157 | D6 | D7 | 1771 | 646,4 | 19978 | 506,5 | 11,3 | 1,087 | H5A034 C | sample | Hm Drgbl | Hm Drgbl | Hm Drgbl | CPE002E5 | CPE002EU | CPE002FF | NM 01224 | NM 0122 | NM 0122 | SIRT4 | SIRT4 | SIRT4 | sirtuin (silent mat | sirtuin (sil | sirtuin (sil | 23409 | 23409 | 23409 | s23763 | s23764 | s23765 |
| 8185 | F1 | F2 | 1506 | 592,9 | 16983 | 505,9 | 11,3 | 1,087 | H5A034 C | sample | Hm Drgbl | Hm Drgbl | Hm Drgbl | CPE002E5 | CPE002EU | CPE002FF | NM 00736 | NM 0073 | NM 0073 | RASA3 | RASA3 | RASA3 | RAS p21 protein a | RAS p21 p | RAS p21 p | 22821 | 22821 | 22821 | s355 | s356 | s357 |
| 8424 | O9 | O10 | 2146 | 631,7 | 24183 | 558 | 11,3 | 1,087 | H5A034 C | sample | Hm Drgbl | Hm Drgbl | Hm Drgbl | CPE002E5 | CPE002EU | CPE002FF | NM 01638 | NM 0163 | NM 0163 | TREX1 | TREX1 | TREX1 | three prime repai | three prin | three prin | 11277 | 11277 | 11277 | s200494 | s200493 | s200495 |
| 10105 | F1 | F2 | 1362 | 759 | 15378 | 124,2 | 11,3 | 1,087 | H5A034 C | sample | Hm Drgbl | Hm Drgbl | Hm Drgbl | CPE002EA | CPE002EP | CPE002FA | NM 02523 | NM 0252 | NM 0252 | RNF39 | RNF39 | RNF39 | ring finger protei | ring finge | ring finge | 80352 | 80352 | 80352 | s37283 | s37282 | s37284 |
| 10161 | H17 | H18 | 1584 | 933,9 | 17899 | 121,3 | 11,3 | 1,087 | H5A034 C | sample | Hm Drgbl | Hm Drgbl | Hm Drgbl | CPE002EA | CPE002EP | CPE002FA | NM 03076 | NM 0307 | NM 0307 | NPL | NPL | NPL | N-acetylneuramin | N-acetylne | N-acetylne | 80896 | 80896 | 80896 | s37469 | s224867 | s37471 |
| 1551 | A22 | A23 | 1386 | 867,8 | 15706 | 412,2 | 11,3 | 1,183 | H5A034 C | sample | Hm Drgbl | Hm Drgbl | Hm Drgbl | CPE002FW | CPE002G0 | CPE002G4 | NM 01404 | NM 0140 | NM 0140 | MKL2 | MKL2 | MKL2 | MKL/myocardin-li | MKL/myo | MKL/myo | 57496 | 57496 | 57496 | s33157 | s33158 | s33159 |
| 1616 | D16 | D17 | 1336 | 729,2 | 15115 | 369,9 | 11,3 | 1,183 | H5A034 C | sample | Hm Drgbl | Hm Drgbl | Hm Drgbl | CPE002FW | CPE002G0 | CPE002G4 | NM 02492 | NM 0249 | NM 0249 | ZNF702 | ZNF702 | ZNF702 | zinc finger protei | zinc finge | zinc finge | 79986 | 79986 | 79986 | s36772 | s36773 | s36774 |
| 1623 | D22 | D23 | 1150 | 724,8 | 12952 | 418,9 | 11,3 | 1,183 | H5A034 C | sample | Hm Drgbl | Hm Drgbl | Hm Drgbl | CPE002FW | CPE002G0 | CPE002G4 | NM 03061 | NM 0306 | NM 0306 | ZFP2 | ZFP2 | ZFP2 | zinc finger protei | zinc finge | zinc finge | 80108 | 80108 | 80108 | s36902 | s36903 | s36904 |
| 21508 | A12 | A13 | 281 | 903,1 | 3175 | 755,7 | 11,3 | 1,189 | H5A034 C | sample | Hm Gen E | Hm Gen E | Hm Gen E | CPE002JU | CPE002O9 | CPE002KP | XM 94191 | XM 9419 | XM 9419 | LOC65246 | LOC65246 | LOC65246 | similar to Plectin | similar to | similar to | 652460 | 652460 | 652460 | s57370 | s197887 | s197888 |
| 21513 | A17 | A18 | 79 | 1273 | 891 | 657 | 11,3 | 1,189 | H5A034 C | sample | Hm Gen E | Hm Gen E | Hm Gen E | CPE002JU | CPE002O9 | CPE002KP | XM 93790 | XM 9379 | XM 9379 | LOC64883 | LOC64883 | LOC64883 | similar to Erythro | similar to | similar to | 648830 | 648830 | 648830 | s56885 | s56886 | s56887 |
| 21613 | E20 | E21 | 265 | 1004 | 3004 | 476,8 | 11,3 | 1,189 | H5A034 C | sample | Hm Gen E | Hm Gen E | Hm Gen E | CPE002JU | CPE002O9 | CPE002KP | XM 94217 | XM 9421 | XM 9421 | LOC65262 | LOC65262 | LOC65262 | hypothetical prot | hypotheti | hypotheti | 652629 | 652629 | 652629 | s57422 | s57423 | s57424 |
| 21618 | E3 | E4 | 58 | 1206 | 658 | 566,1 | 11,3 | 1,189 | H5A034 C | sample | Hm Gen E | Hm Gen E | Hm Gen E | CPE002JU | CPE002O9 | CPE002KP | XM 94362 | XM 9436 | XM 9436 | LOC64921 | LOC64921 | LOC64921 | hypothetical prot | hypotheti | hypotheti | 649217 | 649217 | 649217 | s197641 | s197642 | s197643 |
| 21633 | F17 | F18 | 380 | 985,7 | 4292 | 555,2 | 11,3 | 1,189 | H5A034 C | sample | Hm Gen E | Hm Gen E | Hm Gen E | CPE002JU | CPE002O9 | CPE002KP | XM 94051 | XM 9405 | XM 9405 | LOC65137 | LOC65137 | LOC65137 | similar to cystatin | similar to | similar to | 651373 | 651373 | 651373 | s57195 | s197782 | s197783 |
| 21639 | F22 | F23 | 396 | 1082 | 4470 | 697,2 | 11,3 | 1,189 | H5A034 C | sample | Hm Gen E | Hm Gen E | Hm Gen E | CPE002JU | CPE002O9 | CPE002KP | XM 93564 | XM 9356 | XM 9356 | LOC65337 | LOC65337 | LOC65337 | similar to RCC1-lil | similar to | similar to | 653375 | 653375 | 653375 | s57655 | s57656 | s57657 |
| 21663 | G22 | G23 | 283 | 1069 | 3188 | 696,7 | 11,3 | 1,189 | H5A034 C | sample | Hm Gen E | Hm Gen E | Hm Gen E | CPE002JU | CPE002O9 | CPE002KP | XM 94229 | XM 9422 | XM 9422 | LOC65268 | LOC65268 | LOC65268 | similar to Zinc fin | similar to | similar to | 652688 | 652688 | 652688 | s57437 | s57438 | s57439 |
| 4618 | A18 | A19 | 1450 | 896,1 | 16371 | 506,8 | 11,3 | 1,242 | H5A034 C | sample | Hm Drgbl | Hm Drgbl | Hm Drgbl | CPE002DV | CPE002EL | CPE002FP | NM 00563 | NM 0056 | NM 0056 | SOLH | SOLH | SOLH | small optic lobes | small opti | small opti | 6650 | 6650 | 6650 | s13273 | s13274 | s13275 |
| 4821 | I6 | I7 | 2123 | 824,8 | 23996 | 480,7 | 11,3 | 1,242 | H5A034 C | sample | Hm Drgbl | Hm Drgbl | Hm Drgbl | CPE002DV | CPE002EL | CPE002FP | NM 00387 | NM 0038 | NM 0038 | GGH | GGH | GGH | gamma-glutamyl | gamma-gl | gamma-gl | 8836 | 8836 | 8836 | s16861 | s16862 | s16863 |
| 4866 | K3 | K4 | 1204 | 822,5 | 13547 | 486,1 | 11,3 | 1,242 | H5A034 C | sample | Hm Drgbl | Hm Drgbl | Hm Drgbl | CPE002DV | CPE002EL | CPE002FP | NM 01641 | NM 0164 | NM 0164 | CPB2 | CPB2 | CPB2 | carboxypeptidase | carboxype | carboxype | 1361 | 1361 | 1361 | s3432 | s3433 | s3434 |
| 10369 | A1 | A2 | 745 | 948,7 | 8390 | 227 | 11,3 | 1,277 | H5A034 C | sample | Hm Drgbl | Hm Drgbl | Hm Drgbl | CPE002EB | CPE002EO | CPE002FR | NM 03279 | NM 0327 | NM 0327 | ZBTB45 | ZBTB45 | ZBTB45 | zinc finger and BT | zinc finge | zinc finge | 84878 | 84878 | 84878 | s39562 | s39561 | s39563 |
| 10454 | D21 | D22 | 1261 | 935,3 | 14235 | 210,5 | 11,3 | 1,277 | H5A034 C | sample | Hm Drgbl | Hm Drgbl | Hm Drgbl | CPE002EB | CPE002EO | CPE002FR | NM 13837 | NM 1383 | NM 1383 | TTC5 | TTC5 | TTC5 | tetratricopeptide | tetratrico | tetratrico | 91875 | 91875 | 91875 | s40808 | s40807 | s40806 |
| 10516 | G12 | G13 | 1199 | 896,7 | 13591 | 185,9 | 11,3 | 1,277 | H5A034 C | sample | Hm Drgbl | Hm Drgbl | Hm Drgbl | CPE002EB | CPE002EO | CPE002FR | NM 05293 | NM 0529 | NM 0529 | PCMTD1 | PCMTD1 | PCMTD1 | protein-L-isoaspa | protein-L- | protein-L- | 115294 | 115294 | 115294 | s41791 | s41792 | s41790 |
| 10623 | K22 | K23 | 1355 | 854,5 | 15356 | 199,1 | 11,3 | 1,277 | H5A034 C | sample | Hm Drgbl | Hm Drgbl | Hm Drgbl | CPE002EB | CPE002EO | CPE002FR | NM 17199 | NM 1719 | NM 1719 | RAB39B | RAB39B | RAB39B | RAB39B, member | RAB39B, r | RAB39B, r | 116442 | 116442 | 116442 | s42010 | s42009 | s42008 |
| 2784 | D9 | D10 | 788 | 662,6 | 8878 | 518,4 | 11,3 | 1,329 | H5A034 C | sample | Hm Drgbl | Hm Drgbl | Hm Drgbl | CPE002DP | CPE002EF | CPE002F5 | NM 14586 | NM 1458 | NM 1458 | CHEK2 | CHEK2 | CHEK2 | CHK2 checkpoint | CHK2 chec | CHK2 chec | 11200 | 11200 | 11200 | s22119 | s22120 | s22121 |
| 3044 | O5 | O6 | 1325 | 588,7 | 14940 | 517,6 | 11,3 | 1,329 | H5A034 C | sample | Hm Drgbl | Hm Drgbl | Hm Drgbl | CPE002DP | CPE002EF | CPE002F5 | NM 03136 | NM 0313 | NM 0313 | COL4A3BP | COL4A3BP | COL4A3BP | collagen, type IV, | collagen, | collagen, | 10087 | 10087 | 10087 | s19625 | s19626 | s19627 |
| 3058 | P18 | P19 | 1262 | 612,5 | 14267 | 503,2 | 11,3 | 1,329 | H5A034 C | sample | Hm Drgbl | Hm Drgbl | Hm Drgbl | CPE002DP | CPE002EF | CPE002F5 | NM 00318 | NM 0031 | NM 0031 | TAL1 | TAL1 | TAL1 | T-cell acute lymph | T-cell acut | T-cell acut | 6886 | 6886 | 6886 | s13770 | s224705 | s13769 |
| 20149 | H20 | H21 | 963 | 1262 | 10893 | 163,5 | 11,3 | 1,378 | H5A034 C | sample | Hm Gen E | Hm Gen E | Hm Gen E | CPE002JQ | CPE002O5 | CPE002KL | NM 02300 | NM 0230 | NM 0230 | HAPLN4 | HAPLN4 | HAPLN4 | hyaluronan and p | hyalurona | hyalurona | 404037 | 404037 | 404037 | s53846 | s53847 | s53848 |
| 20328 | O9 | O10 | 1077 | 1093 | 12159 | 174,2 | 11,3 | 1,378 | H5A034 C | sample | Hm Gen E | Hm Gen E | Hm Gen E | CPE002JQ | CPE002O5 | CPE002KL | NM 01236 | NM 0123 | NM 0123 | OR2A5 | OR2A5 | OR2A5 | olfactory recepto | olfactory | olfactory | 393046 | 393046 | 393046 | s53118 | s53119 | s196415 |
| 453 | C6 | C7 | 1627 | 1753 | 18336 | 274,6 | 11,3 | 1,65 | H5A034 C | sample | Hm Drgbl | Hm Drgbl | Hm Drgbl | CPE002E0 | CPE002EZ | CPE002FK | NM 00145 | NM 0014 | NM 0014 | FOXO3 | FOXO3 | FOXO3 | forkhead box O3 | forkhead | forkhead | 2309 | 2309 | 2309 | s5260 | s5261 | s5262 |
| 701 | N13 | N14 | 1514 | 1644 | 17051 | 401,9 | 11,3 | 1,65 | H5A034 C | sample | Hm Drgbl | Hm Drgbl | Hm Drgbl | CPE002E0 | CPE002EZ | CPE002FK | NM 00446 | NM 0044 | NM 0044 | FGD1 | FGD1 | FGD1 | FYVE, RhoGEF and | FYVE, Rho | FYVE, Rho | 2245 | 2245 | 2245 | s5121 | s5123 | s5122 |
| 7780 | E12 | E13 | 1081 | 566,2 | 12173 | 462,1 | 11,3 | 1,725 | H5A034 C | sample | Hm Drgbl | Hm Drgbl | Hm Drgbl | CPE002E4 | CPE002EV | CPE002FG | NM 03122 | NM 0312 | NM 0312 | RBCK1 | RBCK1 | RBCK1 | RanBP-type and C | RanBP-typ | RanBP-typ | 10616 | 10616 | 10616 | s20850 | s20848 | s20849 |
| 7844 | G5 | G6 | 941 | 555,9 | 10641 | 403,8 | 11,3 | 1,725 | H5A034 C | sample | Hm Drgbl | Hm Drgbl | Hm Drgbl | CPE002E4 | CPE002EV | CPE002FG | NM 00580 | NM 0058 | NM 0058 | OLIG2 | OLIG2 | OLIG2 | oligodendrocyte l | oligodend | oligodend | 10215 | 10215 | 10215 | s19922 | s19923 | s19924 |
| 23480 | C16 | C17 | 1005 | 1477 | 11485 | 283,9 | 11,4 | 0,605 | H5A034 C | sample | Hm Gen E | Hm Gen E | Hm Gen E | CPE002KV | CPE002OE | CPE002KU | XM 92602 | XM 9260 | XM 9260 | LOC64253 | LOC64253 | LOC64253 | similar to Sucrase | similar to | similar to | 642539 | 642539 | 642539 | s55050 | s227021 | s227022 |
| 6494 | O21 | O22 | 2273 | 729,5 | 25880 | 323,2 | 11,4 | 0,752 | H5A034 C | sample | Hm Drgbl | Hm Drgbl | Hm Drgbl | CPE002DZ | CPE002F0 | CPE002FL | NM 00181 | NM 0018 | NM 0018 | CENPB | CENPB | CENPB | centromere prote | centrome | centrome | 1059 | 1059 | 1059 | s2910 | s2911 | s2909 |
| 20433 | D17 | D18 | 1344 | 783,7 | 15327 | 153,9 | 11,4 | 0,76 | H5A034 C | sample | Hm Gen E | Hm Gen E | Hm Gen E | CPE002JR | CPE002O6 | CPE002KN | XM 94295 | XM 4991 | XM 4991 | FLJ46284 | FLJ46284 | FLJ46284 | hypothetical gene | hypotheti | hypotheti | 441369 | 441369 | 441369 | s54336 | s226639 | s226640 |
| 20449 | E1 | E2 | 696 | 793,9 | 7912 | 164,8 | 11,4 | 0,76 | H5A034 C | sample | Hm Gen E | Hm Gen E | Hm Gen E | CPE002JR | CPE002O6 | CPE002KN | XM 93539 | XM 9353 | XM 9353 | LOC44033 | LOC44033 | LOC44033 | hypothetical gene | hypotheti | hypotheti | 440335 | 440335 | 440335 | s196531 | s196532 | s196533 |
| 20553 | I17 | I18 | 1040 | 833,1 | 11845 | 174,9 | 11,4 | 0,76 | H5A034 C | sample | Hm Gen E | Hm Gen E | Hm Gen E | CPE002JR | CPE002O6 | CPE002KN | NM 00100 | NM 0010 | NM 0010 | C1orf186 | C1orf186 | C1orf186 | chromosome 1 op | chromoso | chromoso | 440712 | 440712 | 440712 | s54178 | s54179 | s54180 |
| 20578 | J18 | J19 | 1183 | 738,1 | 13507 | 193,1 | 11,4 | 0,76 | H5A034 C | sample | Hm Gen E | Hm Gen E | Hm Gen E | CPE002JR | CPE002O6 | CPE002KN | XM 93735 | XM 9373 | XM 9373 | LOC64238 | LOC64238 | LOC64238 | hypothetical LOC | hypotheti | hypotheti | 642384 | 642384 | 642384 | s55006 | s55007 | s55008 |
| 20606 | K21 | K22 | 1328 | 740,3 | 15157 | 176,5 | 11,4 | 0,76 | H5A034 C | sample | Hm Gen E | Hm Gen E | Hm Gen E | CPE002JR | CPE002O6 | CPE002KN | XM 49658 | XM 4965 | XM 4965 | LOC44089 | LOC44089 | LOC44089 | LIM and senescen | LIM and s | LIM and s | 440895 | 440895 | 440895 | s54211 | s54212 | s54213 |
| 12787 | E4 | E5 | 1364 | 965,9 | 15571 | 121,7 | 11,4 | 0,773 | H5A034 C | sample | Hm Gen E | Hm Gen E | Hm Gen E | CPE002J6 | CPE002NN | CPE002K2 | NM 01486 | NM 0148 | NM 0148 | SEC16A | SEC16A | SEC16A | SEC16 homolog A | SEC16 hor | SEC16 hor | 9919 | 9919 | 9919 | s19236 | s19237 | s19238 |
| 12858 | H3 | H4 | 1114 | 911,9 | 12722 | 138,3 | 11,4 | 0,773 | H5A034 C | sample | Hm Gen E | Hm Gen E | Hm Gen E | CPE002J6 | CPE002NN | CPE002K2 | NM 01472 | NM 0147 | NM 0147 | SNPH | SNPH | SNPH | syntaphilin | syntaphili | syntaphili | 9751 | 9751 | 9751 | s18821 | s18822 | s18823 |
| 12876 | I2 | I3 | 2243 | 1108 | 25651 | 174,4 | 11,4 | 0,773 | H5A034 C | sample | Hm Gen E | Hm Gen E | Hm Gen E | CPE002J6 | CPE002NN | CPE002K2 | NM 00101 | NM 0010 | NM 0010 | ABI1 | ABI1 | ABI1 | abl-interactor 1 | abl-intera | abl-intera | 10006 | 10006 | 10006 | s19444 | s19445 | s19446 |
| 17336 | C16 | C17 | 1290 | 944,5 | 14716 | 238,1 | 11,4 | 0,778 | H5A034 C | sample | Hm Gen E | Hm Gen E | Hm Gen E | CPE002JJ | CPE002NY | CPE002KE | NM 15383 | NM 1538 | NM 1538 | H1FOO | H1FOO | H1FOO | H1 histone family | H1 histon | H1 histon | 132243 | 132243 | 132243 | s43688 | s43689 | s43690 |
| 19507 | M4 | M5 | 1106 | 1171 | 12596 | 185,3 | 11,4 | 0,809 | H5A034 C | sample | Hm Gen E | Hm Gen E | Hm Gen E | CPE002JO | CPE002O3 | CPE002KJ | XM 00112 | XM 0011 | XM 0011 | GNAT3 | GNAT3 | GNAT3 | guanine nucleotic | guanine n | guanine n | 346562 | 346562 | 346562 | s51191 | s51192 | s51193 |
| 20794 | C18 | C19 | 1459 | 1002 | 16662 | 307 | 11,4 | 0,809 | H5A034 C | sample | Hm Gen E | Hm Gen E | Hm Gen E | CPE002JS | CPE002O7 | CPE002KN | XM 94299 | XM 9429 | XM 9429 | LOC64417 | LOC64417 | LOC64417 | hypothetical LOC | hypotheti | hypotheti | 644173 | 644173 | 644173 | s55590 | s55591 | s55592 |
| 14362 | G18 | G19 | 1388 | 888,4 | 15758 | 115,2 | 11,4 | 0,811 | H5A034 C | sample | Hm Gen E | Hm Gen E | Hm Gen E | CPE002J7 | CPE002NC | CPE002K6 | NM 01837 | NM 0183 | NM 0183 | TMEM106 | TMEM106 | TMEM106 | transmembrane p | transmem | transmem | 54664 | 54664 | 54664 | s29297 | s29298 | s29299 |
| 11065 | N1 | N2 | 1475 | 829,7 | 16875 | 414,2 | 11,4 | 0,864 | H5A034 C | sample | Hm Drgbl | Hm Drgbl | Hm Drgbl | CPE002EC | CPE002EN | CPE002FS | NM 00103 | NM 0010 | NM 0010 | ALDH1L2 | ALDH1L2 | ALDH1L2 | aldehyde dehydro | aldehyde | aldehyde | 160428 | 160428 | 160428 | s46203 | s46204 | s223376 |
| 7355 | C19 | C20 | 1052 | 582 | 11990 | 552,1 | 11,4 | 0,87 | H5A034 C | sample | Hm Drgbl | Hm Drgbl | Hm Drgbl | CPE002E3 | CPE002EW | CPE002FH | NM 00618 | NM 0061 | NM 0061 | OAS3 | OAS3 | OAS3 | 2-5-oligoadenylat | 2-5-oligoa | 2-5-oligoa | 4940 | 4940 | 4940 | s9799 | s9798 | s9797 |
| 7526 | J21 | J22 | 1159 | 588,7 | 13264 | 287,1 | 11,4 | 0,87 | H5A034 C | sample | Hm Drgbl | Hm Drgbl | Hm Drgbl | CPE002E3 | CPE002EW | CPE002FH | NM 00485 | NM 0048 | NM 0048 | PIGB | PIGB | PIGB | phosphatidylinos | phosphat | phosphat | 9488 | 9488 | 9488 | s18189 | s18188 | s18190 |
| 7617 | N17 | N18 | 988 | 656,8 | 11249 | 417,5 | 11,4 | 0,87 | H5A034 C | sample | Hm Drgbl | Hm Drgbl | Hm Drgbl | CPE002E3 | CPE002EW | CPE002FH | NM 00489 | NM 0048 | NM 0048 | SPAG7 | SPAG7 | SPAG7 | sperm associated | sperm ass | sperm ass | 9552 | 9552 | 9552 | s18343 | s18342 | s225159 |
| 17027 | F19 | F20 | 1629 | 1090 | 18546 | 83,25 | 11,4 | 0,88 | H5A034 C | sample | Hm Gen E | Hm Gen E | Hm Gen E | CPE002JI | CPE002NZ | CPE002KD | NM 02490 | NM 1533 | NM 0249 | FBXO17 | FBXL16 | FBXO17 | F-box protein 17 | F-box and | F-box pro | 115290 | 146330 | 115290 | s41787 | s44844 | s41789 |
| 7005 | D6 | D7 | 959 | 634,4 | 10970 | 457,9 | 11,4 | 0,887 | H5A034 C | sample | Hm Drgbl | Hm Drgbl | Hm Drgbl | CPE002E2 | CPE002EX | CPE002FI | NM 00249 | NM 0024 | NM 0024 | NDUFB5 | NDUFB5 | NDUFB5 | NADH dehydroge | NADH deh | NADH deh | 4711 | 4711 | 4711 | s9363 | s9362 | s9361 |
| 2364 | C2 | C3 | 1101 | 867,1 | 12601 | 487,7 | 11,4 | 0,898 | H5A034 C | sample | Hm Drgbl | Hm Drgbl | Hm Drgbl | CPE002DC | CPE002EE | CPE002F4 | NM 00155 | NM 0015 | NM 0015 | IKBKB | IKBKB | IKBKB | inhibitor of kappa | inhibitor o | inhibitor o | 3551 | 3551 | 3551 | s7263 | s223928 | s7265 |
| 2386 | D18 | D19 | 1309 | 763,8 | 14978 | 358,2 | 11,4 | 0,898 | H5A034 C | sample | Hm Drgbl | Hm Drgbl | Hm Drgbl | CPE002DC | CPE002EE | CPE002F4 | NM 00274 | NM 0027 | NM 0027 | MAPK4 | MAPK4 | MAPK4 | mitogen-activated | mitogen-a | mitogen-a | 5596 | 5596 | 5596 | s11143 | s224400 | s11145 |

| | | | | | | | | | |
|---|---|---|---|---|---|---|---|---|---|
| 2421 | E6 | E7 | 1360 | 703,5 | 15548 | 341,1 | 11,4 | 0,898 | H5A034 C |
| 2579 | L19 | L20 | 1459 | 639,6 | 16609 | 312,5 | 11,4 | 0,898 | H5A034 C |
| 4471 | K15 | K16 | 1380 | 705,2 | 15700 | 375,6 | 11,4 | 0,962 | H5A034 C |
| 224 | J16 | J17 | 515 | 1555 | 5847 | 359,6 | 11,4 | 0,97 | H5A034 C |
| 259 | K4 | K5 | 253 | 1223 | 2886 | 454,9 | 11,4 | 0,97 | H5A034 C |
| 285 | L6 | L7 | 604 | 1516 | 6896 | 500,9 | 11,4 | 0,97 | H5A034 C |
| 356 | O5 | O6 | 815 | 1797 | 9287 | 606,9 | 11,4 | 0,97 | H5A034 C |
| 383 | P8 | P9 | 757 | 1648 | 8610 | 591,5 | 11,4 | 0,97 | H5A034 C |
| 2102 | H21 | H22 | 1456 | 672,9 | 16669 | 324,5 | 11,4 | 0,991 | H5A034 C |
| 2159 | J8 | J9 | 916 | 626,8 | 10412 | 309,1 | 11,4 | 0,991 | H5A034 C |
| 2169 | K17 | K18 | 1524 | 624,3 | 17321 | 355,4 | 11,4 | 0,991 | H5A034 C |
| 2220 | M2 | M3 | 812 | 624,6 | 9265 | 284,7 | 11,4 | 0,991 | H5A034 C |
| 4119 | L22 | L23 | 1802 | 761,8 | 20457 | 384,6 | 11,4 | 1,013 | H5A034 C |
| 4194 | O3 | O4 | 873 | 706,5 | 9926 | 518,3 | 11,4 | 1,013 | H5A034 C |
| 11809 | M1 | M2 | 1603 | 946 | 18228 | 153 | 11,4 | 1,018 | H5A034 C |
| 15610 | K18 | K19 | 1187 | 841,6 | 13542 | 157,4 | 11,4 | 1,036 | H5A034 C |
| 6060 | M2 | M3 | 1126 | 747,3 | 12880 | 370,1 | 11,4 | 1,041 | H5A034 C |
| 6106 | O18 | O19 | 2486 | 883,6 | 28236 | 469,4 | 11,4 | 1,041 | H5A034 C |
| 6129 | P17 | P18 | 1719 | 785,4 | 19593 | 461,3 | 11,4 | 1,041 | H5A034 C |
| 1257 | E17 | E18 | 1218 | 730,4 | 13847 | 470,1 | 11,4 | 1,046 | H5A034 C |
| 1413 | K6 | K7 | 1022 | 773 | 11666 | 416,2 | 11,4 | 1,046 | H5A034 C |
| 16340 | I5 | I6 | 1103 | 1012 | 12587 | 328 | 11,4 | 1,046 | H5A034 C |
| 16413 | L6 | L7 | 1205 | 976,1 | 13706 | 308,7 | 11,4 | 1,046 | H5A034 C |
| 16495 | P15 | P16 | 940 | 1166 | 10676 | 440,4 | 11,4 | 1,046 | H5A034 C |
| 1068 | M2 | M3 | 1439 | 747,4 | 16444 | 380,5 | 11,4 | 1,081 | H5A034 C |
| 1070 | M21 | M22 | 1079 | 725,7 | 12330 | 475,2 | 11,4 | 1,081 | H5A034 C |
| 1110 | O14 | O15 | 2530 | 870,6 | 28927 | 476,6 | 11,4 | 1,081 | H5A034 C |
| 5401 | B1 | B2 | 1499 | 751,8 | 17071 | 660,4 | 11,4 | 1,086 | H5A034 C |
| 5707 | N4 | N5 | 1917 | 642,7 | 21851 | 569,1 | 11,4 | 1,086 | H5A034 C |
| 3648 | H9 | H10 | 1073 | 1442 | 12251 | 418,5 | 11,4 | 1,096 | H5A034 C |
| 3667 | I4 | I5 | 1210 | 1256 | 13767 | 336,3 | 11,4 | 1,096 | H5A034 C |
| 8259 | I11 | I12 | 1372 | 547,6 | 15653 | 386,9 | 11,4 | 1,096 | H5A034 C |
| 8439 | P22 | P23 | 1490 | 575,3 | 17032 | 480,4 | 11,4 | 1,096 | H5A034 C |
| 1622 | D21 | D22 | 1862 | 815,7 | 21173 | 416,1 | 11,4 | 1,194 | H5A034 C |
| 1753 | J1 | J2 | 1025 | 692,2 | 11663 | 403 | 11,4 | 1,194 | H5A034 C |
| 1757 | J13 | J14 | 1352 | 722,5 | 15372 | 290,7 | 11,4 | 1,194 | H5A034 C |
| 1762 | J18 | J19 | 1243 | 716 | 14216 | 320,7 | 11,4 | 1,194 | H5A034 C |
| 1770 | J3 | J4 | 1254 | 720,8 | 14288 | 358,2 | 11,4 | 1,194 | H5A034 C |
| 1775 | J8 | J9 | 1012 | 661,4 | 11571 | 265,1 | 11,4 | 1,194 | H5A034 C |
| 1894 | O7 | O8 | 1483 | 858,3 | 16976 | 498,9 | 11,4 | 1,194 | H5A034 C |
| 21534 | B14 | B15 | 376 | 904 | 4283 | 629,4 | 11,4 | 1,2 | H5A034 C |
| 21646 | F7 | F8 | 261 | 954,9 | 2964 | 644,5 | 11,4 | 1,2 | H5A034 C |
| 21824 | N16 | N17 | 391 | 980,7 | 4472 | 518,9 | 11,4 | 1,2 | H5A034 C |
| 21825 | N17 | N18 | 392 | 1040 | 4451 | 538,6 | 11,4 | 1,2 | H5A034 C |
| 4640 | B16 | B17 | 1542 | 833,6 | 17507 | 528,3 | 11,4 | 1,253 | H5A034 C |
| 4662 | C14 | C15 | 1351 | 829,3 | 15424 | 513,2 | 11,4 | 1,253 | H5A034 C |
| 4736 | F16 | F17 | 1649 | 789,8 | 18755 | 414,5 | 11,4 | 1,253 | H5A034 C |
| 4860 | K2 | K3 | 1194 | 741 | 13554 | 449,9 | 11,4 | 1,253 | H5A034 C |
| 4885 | L20 | L21 | 1815 | 784,9 | 20762 | 426,8 | 11,4 | 1,253 | H5A034 C |
| 4894 | L7 | L8 | 1671 | 814,1 | 19051 | 396,6 | 11,4 | 1,253 | H5A034 C |
| 4931 | N19 | N20 | 1589 | 796,3 | 18176 | 482 | 11,4 | 1,253 | H5A034 C |
| 4934 | N21 | N22 | 1165 | 800,1 | 13248 | 457,5 | 11,4 | 1,253 | H5A034 C |
| 10561 | I1 | I2 | 1740 | 866,2 | 19891 | 187,6 | 11,4 | 1,288 | H5A034 C |
| 10579 | I4 | I5 | 1635 | 888,7 | 18713 | 199,5 | 11,4 | 1,288 | H5A034 C |
| 10590 | J14 | J15 | 1192 | 871,4 | 13574 | 194,2 | 11,4 | 1,288 | H5A034 C |
| 10703 | N8 | N9 | 1519 | 840,7 | 17324 | 183,5 | 11,4 | 1,288 | H5A034 C |
| 2818 | F18 | F19 | 690 | 557,2 | 7838 | 455,4 | 11,4 | 1,341 | H5A034 C |
| 3052 | P12 | P13 | 1427 | 650,8 | 16227 | 632,5 | 11,4 | 1,341 | H5A034 C |
| 20016 | B9 | B10 | 1110 | 1165 | 12652 | 228,1 | 11,4 | 1,39 | H5A034 C |
| 20027 | C19 | C20 | 1286 | 1142 | 14669 | 227,4 | 11,4 | 1,39 | H5A034 C |
| 20062 | D7 | D8 | 1165 | 1218 | 13271 | 200,7 | 11,4 | 1,39 | H5A034 C |
| 20134 | G7 | G8 | 801 | 1031 | 9112 | 101,6 | 11,4 | 1,39 | H5A034 C |
| 20160 | H9 | H10 | 649 | 1226 | 7400 | 205,1 | 11,4 | 1,39 | H5A034 C |
| 20163 | I11 | I12 | 1179 | 1122 | 13396 | 172 | 11,4 | 1,39 | H5A034 C |
| 20194 | J18 | J19 | 1014 | 985 | 11597 | 122,4 | 11,4 | 1,39 | H5A034 C |
| 7916 | J5 | J6 | 448 | 614,1 | 5101 | 372,9 | 11,4 | 1,74 | H5A034 C |
| 8022 | O14 | O15 | 836 | 528,2 | 9551 | 405,5 | 11,4 | 1,74 | H5A034 C |
| 3423 | O22 | O23 | 1379 | 1320 | 15874 | 443,2 | 11,5 | 0,712 | H5A034 C |
| 20556 | I2 | I3 | 1256 | 779,6 | 14418 | 191,3 | 11,5 | 0,767 | H5A034 C |
| 20702 | O21 | O22 | 1116 | 808,9 | 12819 | 249,9 | 11,5 | 0,767 | H5A034 C |
| 12788 | E5 | E6 | 1482 | 1013 | 16998 | 116,7 | 11,5 | 0,78 | H5A034 C |
| 9577 | P1 | P2 | 1514 | 1002 | 17366 | 149,4 | 11,5 | 0,799 | H5A034 C |
| 19500 | M2 | M3 | 1013 | 1112 | 11609 | 195,7 | 11,5 | 0,816 | H5A034 C |
| 17196 | M2 | M3 | 1471 | 1126 | 16972 | 110,2 | 11,5 | 0,888 | H5A034 C |
| 17210 | N10 | N11 | 2715 | 1075 | 31253 | 227,9 | 11,5 | 0,888 | H5A034 C |
| 17223 | N22 | N23 | 2020 | 949,9 | 23137 | 199 | 11,5 | 0,888 | H5A034 C |
| 5020 | B12 | B13 | 2063 | 684,1 | 23765 | 598,5 | 11,5 | 0,902 | H5A034 C |
| 5073 | D17 | D18 | 1791 | 588,3 | 20647 | 474,1 | 11,5 | 0,902 | H5A034 C |
| 2366 | C21 | C22 | 839 | 760,7 | 9661 | 475 | 11,5 | 0,906 | H5A034 C |
| 2388 | D2 | D3 | 1853 | 687,1 | 21334 | 389,4 | 11,5 | 0,906 | H5A034 C |
| 2492 | H5 | H6 | 1631 | 671,5 | 18781 | 449 | 11,5 | 0,906 | H5A034 C |
| 2508 | I2 | I3 | 886 | 703,4 | 10162 | 448,9 | 11,5 | 0,906 | H5A034 C |
| 2517 | I6 | I7 | 1825 | 710,2 | 20910 | 397 | 11,5 | 0,906 | H5A034 C |
| 2532 | J2 | J3 | 1046 | 656,5 | 12051 | 372,7 | 11,5 | 0,906 | H5A034 C |
| 2567 | K8 | K9 | 1635 | 637,6 | 18739 | 305,6 | 11,5 | 0,906 | H5A034 C |
| 16688 | H16 | H17 | 1486 | 992,8 | 17024 | 232,6 | 11,5 | 0,909 | H5A034 C |
| 16831 | N15 | N16 | 1716 | 1103 | 19785 | 302 | 11,5 | 0,909 | H5A034 C |
| 17996 | N5 | N6 | 1604 | 996,7 | 18457 | 146,4 | 11,5 | 0,909 | H5A034 C |
| 9090 | K3 | K4 | 1809 | 1829 | 20841 | 143,1 | 11,5 | 0,924 | H5A034 C |
| 4230 | A14 | A15 | 1221 | 794,6 | 14061 | 441,5 | 11,5 | 0,97 | H5A034 C |
| 4235 | A19 | A20 | 1689 | 795,4 | 19374 | 319,3 | 11,5 | 0,97 | H5A034 C |
| 4301 | D13 | D14 | 1449 | 691,7 | 16607 | 420,9 | 11,5 | 0,97 | H5A034 C |
| 4535 | M8 | M9 | 1827 | 736,7 | 20955 | 397,5 | 11,5 | 0,97 | H5A034 C |
| 155 | G19 | G20 | 473 | 1272 | 5444 | 521,5 | 11,5 | 0,979 | H5A034 C |
| 195 | I11 | I12 | 487 | 1404 | 5590 | 542,2 | 11,5 | 0,979 | H5A034 C |
| 203 | I19 | I20 | 265 | 1367 | 3046 | 439 | 11,5 | 0,979 | H5A034 C |
| 314 | N10 | N11 | 499 | 1593 | 5714 | 655 | 11,5 | 0,979 | H5A034 C |
| 317 | N13 | N14 | 544 | 1468 | 6237 | 529 | 11,5 | 0,979 | H5A034 C |
| 373 | P20 | P21 | 692 | 1599 | 7926 | 832,5 | 11,5 | 0,979 | H5A034 C |
| 1998 | D14 | D15 | 1034 | 743,1 | 11936 | 339,5 | 11,5 | 1 | H5A034 C |
| 2026 | E18 | E19 | 1331 | 532,9 | 15315 | 303,6 | 11,5 | 1 | H5A034 C |
| 2039 | E8 | E9 | 1213 | 596,8 | 14005 | 457,9 | 11,5 | 1 | H5A034 C |
| 2268 | O2 | O3 | 1861 | 767,1 | 21339 | 489,1 | 11,5 | 1 | H5A034 C |
| 6615 | D22 | D23 | 1400 | 560 | 16144 | 421 | 11,5 | 1,009 | H5A034 C |
| 6649 | F1 | F2 | 1405 | 601,8 | 16153 | 433,2 | 11,5 | 1,009 | H5A034 C |
| 6827 | M19 | M20 | 1613 | 664 | 18497 | 472,2 | 11,5 | 1,009 | H5A034 C |
| 6877 | O20 | O21 | 1553 | 574,8 | 17836 | 487,3 | 11,5 | 1,009 | H5A034 C |
| 3922 | D18 | D19 | 1271 | 719,3 | 14675 | 363,3 | 11,5 | 1,022 | H5A034 C |
| 4032 | H9 | H10 | 652 | 744,1 | 7476 | 471,2 | 11,5 | 1,022 | H5A034 C |
| 4090 | K18 | K19 | 1470 | 718,4 | 16946 | 314,6 | 11,5 | 1,022 | H5A034 C |
| 4115 | L19 | L20 | 86 | 836,1 | 989 | 410,1 | 11,5 | 1,022 | H5A034 C |
| 4201 | P1 | P2 | 583 | 618,6 | 6691 | 432,4 | 11,5 | 1,022 | H5A034 C |
| 4218 | P3 | P4 | 977 | 740,4 | 11228 | 486 | 11,5 | 1,022 | H5A034 C |
| 11600 | D16 | D17 | 880 | 833,1 | 10084 | 115,2 | 11,5 | 1,027 | H5A034 C |
| 11707 | H4 | H5 | 1650 | 895,6 | 18916 | 155,8 | 11,5 | 1,027 | H5A034 C |
| 11713 | I1 | I2 | 1463 | 899,6 | 16890 | 131,7 | 11,5 | 1,027 | H5A034 C |
| 15417 | C17 | C18 | 816 | 784,3 | 9348 | 133,9 | 11,5 | 1,045 | H5A034 C |
| 15615 | K22 | K23 | 1167 | 839,9 | 13443 | 187,2 | 11,5 | 1,045 | H5A034 C |
| 6030 | L14 | L15 | 1530 | 774,9 | 17555 | 456,9 | 11,5 | 1,05 | H5A034 C |
| 6033 | L17 | L18 | 1256 | 850,5 | 14504 | 330,5 | 11,5 | 1,05 | H5A034 C |
| 6097 | O1 | O2 | 541 | 694 | 6229 | 264,6 | 11,5 | 1,05 | H5A034 C |
| 6124 | P12 | P13 | 1255 | 845,5 | 14376 | 547,2 | 11,5 | 1,05 | H5A034 C |
| 1273 | F1 | F2 | 1544 | 733,1 | 17827 | 440,8 | 11,5 | 1,055 | H5A034 C |
| 1280 | F16 | F17 | 1835 | 734,6 | 21149 | 360,1 | 11,5 | 1,055 | H5A034 C |
| 1351 | I15 | I16 | 1297 | 733,5 | 14854 | 418,1 | 11,5 | 1,055 | H5A034 C |
| 1374 | J14 | J15 | 1056 | 738 | 12104 | 335,8 | 11,5 | 1,055 | H5A034 C |
| 16166 | B21 | B22 | 1718 | 1000 | 19748 | 310,3 | 11,5 | 1,055 | H5A034 C |
| 1018 | K18 | K19 | 1690 | 806,5 | 19364 | 349,2 | 11,5 | 1,09 | H5A034 C |
| 1098 | N3 | N4 | 1391 | 663,7 | 15935 | 366,1 | 11,5 | 1,09 | H5A034 C |
| 1146 | P3 | P4 | 2156 | 802,9 | 24687 | 470,4 | 11,5 | 1,09 | H5A034 C |
| 5506 | F18 | F19 | 1869 | 672,3 | 21500 | 582,7 | 11,5 | 1,095 | H5A034 C |
| 5511 | F22 | F23 | 1782 | 717,1 | 20466 | 459,8 | 11,5 | 1,095 | H5A034 C |
| 5534 | G21 | G22 | 1775 | 668,1 | 20458 | 545,2 | 11,5 | 1,095 | H5A034 C |
| 3494 | B21 | B22 | 884 | 1423 | 10137 | 394,6 | 11,5 | 1,106 | H5A034 C |
| 3626 | H10 | H11 | 1429 | 1441 | 16377 | 310,9 | 11,5 | 1,106 | H5A034 C |
| 3677 | J13 | J14 | 1035 | 1147 | 11893 | 268,1 | 11,5 | 1,106 | H5A034 C |
| 3695 | J8 | J9 | 1069 | 1368 | 12271 | 304,9 | 11,5 | 1,106 | H5A034 C |
| 3835 | P4 | P5 | 1236 | 1507 | 14183 | 422,9 | 11,5 | 1,106 | H5A034 C |
| 8072 | A16 | A17 | 1094 | 685,1 | 12527 | 470,8 | 11,5 | 1,106 | H5A034 C |
| 8363 | M19 | M20 | 1806 | 680,1 | 20790 | 446,1 | 11,5 | 1,106 | H5A034 C |
| 8377 | N1 | N2 | 1601 | 620,9 | 18369 | 328,2 | 11,5 | 1,106 | H5A034 C |
| 8420 | O5 | O6 | 969 | 608,6 | 11127 | 467,5 | 11,5 | 1,106 | H5A034 C |
| 10006 | A7 | A8 | 1725 | 923,9 | 19857 | 200,3 | 11,5 | 1,106 | H5A034 C |
| 10191 | I22 | I23 | 1772 | 890,2 | 20403 | 177,7 | 11,5 | 1,106 | H5A034 C |
| 1593 | C17 | C18 | 913 | 706,7 | 10539 | 434 | 11,5 | 1,204 | H5A034 C |
| 1645 | E20 | E21 | 2021 | 808,8 | 23188 | 446,1 | 11,5 | 1,204 | H5A034 C |
| 1798 | K7 | K8 | 931 | 670,2 | 10667 | 400,7 | 11,5 | 1,204 | H5A034 C |
| 1884 | O2 | O3 | 1605 | 795,1 | 18507 | 419,3 | 11,5 | 1,204 | H5A034 C |
| 21614 | E21 | E22 | 456 | 1123 | 5254 | 538,7 | 11,5 | 1,211 | H5A034 C |
| 4635 | B11 | B12 | 1379 | 730,8 | 15790 | 521,6 | 11,5 | 1,264 | H5A034 C |
| 4668 | C2 | C3 | 1012 | 912,9 | 11639 | 340 | 11,5 | 1,264 | H5A034 C |
| 4678 | C7 | C8 | 869 | 858,9 | 9979 | 485,4 | 11,5 | 1,264 | H5A034 C |
| 4702 | D7 | D8 | 1233 | 734,2 | 14187 | 520 | 11,5 | 1,264 | H5A034 C |
| 4922 | N10 | N11 | 1497 | 720,9 | 17221 | 468,1 | 11,5 | 1,264 | H5A034 C |
| 10422 | C14 | C15 | 1202 | 803 | 13844 | 190 | 11,5 | 1,299 | H5A034 C |
| 10637 | L13 | L14 | 912 | 783,5 | 10447 | 185,3 | 11,5 | 1,299 | H5A034 C |
| 10647 | L22 | L23 | 1171 | 893,8 | 13451 | 207,8 | 11,5 | 1,299 | H5A034 C |
| 2722 | B18 | B19 | 1230 | 727 | 14185 | 499 | 11,5 | 1,353 | H5A034 C |
| 3027 | O11 | O12 | 1409 | 623,4 | 16166 | 522,4 | 11,5 | 1,353 | H5A034 C |
| 3048 | O9 | O10 | 1683 | 638,4 | 19336 | 555,5 | 11,5 | 1,353 | H5A034 C |
| 3066 | P3 | P4 | 1685 | 635,9 | 19391 | 571,8 | 11,5 | 1,353 | H5A034 C |
| 20031 | C22 | C23 | 899 | 1119 | 10328 | 180,6 | 11,5 | 1,402 | H5A034 C |
| 20158 | H7 | H8 | 924 | 1040 | 10653 | 140,1 | 11,5 | 1,402 | H5A034 C |
| 20346 | P3 | P4 | 545 | 1470 | 6265 | 135 | 11,5 | 1,402 | H5A034 C |
| 423 | B22 | B23 | 1888 | 1736 | 21705 | 385,8 | 11,5 | 1,679 | H5A034 C |
| 7805 | F13 | F14 | 1129 | 626,5 | 12936 | 438,1 | 11,5 | 1,756 | H5A034 C |
| 23754 | N3 | N4 | 1042 | 1445 | 12110 | 142,3 | 11,6 | 0,615 | H5A034 C |
| 3290 | J10 | J11 | 1403 | 1235 | 16260 | 333,6 | 11,6 | 0,718 | H5A034 C |
| 19670 | D21 | D22 | 1522 | 367,6 | 17687 | 206 | 11,6 | 0,756 | H5A034 C |
| 6422 | L21 | L22 | 1713 | 630,7 | 19928 | 331,7 | 11,6 | 0,766 | H5A034 C |
| 20591 | J8 | J9 | 1051 | 747,1 | 12233 | 167,2 | 11,6 | 0,773 | H5A034 C |
| 20676 | N2 | N3 | 752 | 779,5 | 8734 | 111,8 | 11,6 | 0,773 | H5A034 C |
| 12847 | H15 | H16 | 1167 | 1053 | 13523 | 131,2 | 11,6 | 0,786 | H5A034 C |
| 17484 | I2 | I3 | 1493 | 961,2 | 17309 | 215 | 11,6 | 0,792 | H5A034 C |
| 8749 | M20 | M21 | 1950 | 1054 | 22536 | 99,85 | 11,6 | 0,82 | H5A034 C |
| 11480 | O16 | O17 | 1773 | 879,2 | 20498 | 168 | 11,6 | 0,841 | H5A034 C |
| 7354 | C18 | C19 | 1282 | 624 | 14853 | 454,7 | 11,6 | 0,885 | H5A034 C |
| 7556 | K5 | K6 | 785 | 587,9 | 9132 | 415,2 | 11,6 | 0,885 | H5A034 C |
| 17220 | N2 | N3 | 1442 | 921,8 | 16784 | 115 | 11,6 | 0,896 | H5A034 C |
| 7236 | N2 | N3 | 1299 | 625 | 15013 | 378,8 | 11,6 | 0,903 | H5A034 C |
| 5007 | A22 | A23 | 1987 | 682,2 | 23040 | 622,9 | 11,6 | 0,91 | H5A034 C |
| 5164 | H12 | H13 | 1770 | 652,2 | 20586 | 391,8 | 11,6 | 0,91 | H5A034 C |
| 2426 | F10 | F11 | 1258 | 677,8 | 14542 | 456,3 | 11,6 | 0,913 | H5A034 C |
| 2511 | I22 | I23 | 1511 | 688,4 | 17513 | 406,3 | 11,6 | 0,913 | H5A034 C |
| 2529 | J17 | J18 | 1300 | 813,3 | 15059 | 336,5 | 11,6 | 0,913 | H5A034 C |
| 2549 | K13 | K14 | 1008 | 640,7 | 11709 | 346,9 | 11,6 | 0,913 | H5A034 C |
| 16719 | I22 | I23 | 1327 | 955 | 15362 | 282,3 | 11,6 | 0,917 | H5A034 C |
| 16842 | N3 | N4 | 1256 | 1077 | 14537 | 189,8 | 11,6 | 0,917 | H5A034 C |
| 17683 | A4 | A5 | 1029 | 1076 | 11952 | 346,4 | 11,6 | 0,917 | H5A034 C |
| 17833 | H1 | H2 | 999 | 984,4 | 11598 | 184,1 | 11,6 | 0,917 | H5A034 C |
| 8889 | C17 | C18 | 2135 | 2109 | 24714 | 200,8 | 11,6 | 0,932 | H5A034 C |
| 9018 | H3 | H4 | 1267 | 1538 | 14683 | 133,1 | 11,6 | 0,932 | H5A034 C |
| 4248 | A9 | A10 | 1578 | 708,8 | 18278 | 292,5 | 11,6 | 0,979 | H5A034 C |
| 4534 | M7 | M8 | 1404 | 758,7 | 16262 | 479,6 | 11,6 | 0,979 | H5A034 C |
| 4569 | O17 | O18 | 961 | 892,6 | 11143 | 469,7 | 11,6 | 0,979 | H5A034 C |
| 339 | O11 | O12 | 517 | 1449 | 5976 | 748,1 | 11,6 | 0,987 | H5A034 C |
| 1977 | C17 | C18 | 1101 | 733,5 | 12767 | 363,6 | 11,6 | 1,009 | H5A034 C |
| 2127 | I22 | I23 | 1076 | 678,4 | 12455 | 339,9 | 11,6 | 1,009 | H5A034 C |
| 2259 | O11 | O12 | 1777 | 729,6 | 20549 | 524,3 | 11,6 | 1,009 | H5A034 C |
| 2282 | P10 | P11 | 909 | 822,6 | 10586 | 575,5 | 11,6 | 1,009 | H5A034 C |
| 6671 | F8 | F9 | 910 | 595,4 | 10521 | 433,5 | 11,6 | 1,018 | H5A034 C |
| 3894 | C14 | C15 | 1430 | 726,4 | 16585 | 307,3 | 11,6 | 1,031 | H5A034 C |
| 3926 | D21 | D22 | 757 | 670,1 | 8769 | 333,3 | 11,6 | 1,031 | H5A034 C |
| 3932 | D5 | D6 | 1395 | 695,8 | 16240 | 305,4 | 11,6 | 1,031 | H5A034 C |
| 3937 | E1 | E2 | 983 | 673,6 | 11417 | 368,3 | 11,6 | 1,031 | H5A034 C |
| 4074 | J3 | J4 | 948 | 642,1 | 10993 | 345,5 | 11,6 | 1,031 | H5A034 C |
| 4116 | L2 | L3 | 1195 | 706,8 | 13881 | 391,2 | 11,6 | 1,031 | H5A034 C |
| 15565 | I20 | I21 | 1485 | 869,4 | 17237 | 137,2 | 11,6 | 1,055 | H5A034 C |
| 5779 | A4 | A5 | 1363 | 801,1 | 15805 | 640,6 | 11,6 | 1,059 | H5A034 C |
| 5846 | D21 | D22 | 1886 | 798,5 | 21836 | 409 | 11,6 | 1,059 | H5A034 C |
| 6071 | M8 | M9 | 1659 | 849,3 | 19183 | 401,1 | 11,6 | 1,059 | H5A034 C |
| 6080 | N16 | N17 | 1887 | 745,6 | 21844 | 536,4 | 11,6 | 1,059 | H5A034 C |
| 6118 | O7 | O8 | 1526 | 838,3 | 17748 | 584,3 | 11,6 | 1,059 | H5A034 C |
| 1349 | I13 | I14 | 1022 | 753,1 | 11884 | 348,3 | 11,6 | 1,064 | H5A034 C |
| 1375 | J15 | J16 | 978 | 747,5 | 11332 | 374,6 | 11,6 | 1,064 | H5A034 C |
| 1382 | J21 | J22 | 1541 | 823,8 | 17942 | 469,7 | 11,6 | 1,064 | H5A034 C |
| 1403 | K19 | K20 | 1718 | 779,9 | 19931 | 382,9 | 11,6 | 1,064 | H5A034 C |
| 1430 | L21 | L22 | 1855 | 756,3 | 21605 | 432,7 | 11,6 | 1,064 | H5A034 C |
| 1442 | M10 | M11 | 1467 | 755,2 | 16965 | 395,2 | 11,6 | 1,064 | H5A034 C |
| 1448 | M16 | M17 | 1862 | 744,3 | 21648 | 328,5 | 11,6 | 1,064 | H5A034 C |
| 1466 | N10 | N11 | 1489 | 676,5 | 17285 | 433,7 | 11,6 | 1,064 | H5A034 C |
| 16141 | A20 | A21 | 1107 | 1070 | 12793 | 329,4 | 11,6 | 1,064 | H5A034 C |
| 16387 | K4 | K5 | 916 | 937,4 | 10603 | 340,7 | 11,6 | 1,064 | H5A034 C |
| 16497 | P17 | P18 | 1008 | 1130 | 11659 | 293,1 | 11,6 | 1,064 | H5A034 C |
| 13629 | H6 | H7 | 1229 | 995,2 | 14285 | 151,9 | 11,6 | 1,089 | H5A034 C |
| 13633 | I1 | I2 | 1033 | 1007 | 11936 | 149,1 | 11,6 | 1,089 | H5A034 C |

| | | | | | | | | | | | | | | | | | | |
|---|---|---|---|---|---|---|---|---|---|---|---|---|---|---|---|---|---|---|
| 13655 | I8 | I9 | 953 | 1079 | 11082 | 149,6 | 11,6 | 1,089 | H5A034 C | sample | Hm Gen E | CPE002JC | CPE002NC | CPE002K4 | NM 01554 | RPAP1 | RNA polymerase I | 26015 |
| 994 | J18 | J19 | 1230 | 647,8 | 14216 | 340,2 | 11,6 | 1,1 | H5A034 C | sample | Hm Drgbl | CPE002FU | CPE002FY | CPE002G2 | NM 00628 | TAF10 | TAF10 RNA polym | 6881 |
| 1005 | J6 | J7 | 1176 | 752,8 | 13640 | 344,5 | 11,6 | 1,1 | H5A034 C | sample | Hm Drgbl | CPE002FU | CPE002FY | CPE002G2 | NM 00563 | SYT1 | synaptotagmin I | 6857 |
| 1013 | K13 | K14 | 1527 | 709,6 | 17650 | 358,8 | 11,6 | 1,1 | H5A034 C | sample | Hm Drgbl | CPE002FU | CPE002FY | CPE002G2 | NM 00049 | COL10A1 | collagen, type X, a | 1300 |
| 1080 | M9 | M10 | 1460 | 792,4 | 16904 | 416,6 | 11,6 | 1,1 | H5A034 C | sample | Hm Drgbl | CPE002FU | CPE002FY | CPE002G2 | NM 00130 | CLDN7 | claudin 7 | 1366 |
| 5413 | B20 | B21 | 1988 | 781,9 | 22975 | 469,8 | 11,6 | 1,105 | H5A034 C | sample | Hm Drgbl | CPE002DX | CPE002F2 | CPE002FN | NM 00469 | SLC16A4 | solute carrier fam | 9122 |
| 5421 | B6 | B7 | 1927 | 672,1 | 22348 | 629,4 | 11,6 | 1,105 | H5A034 C | sample | Hm Drgbl | CPE002DX | CPE002F2 | CPE002FN | NM 00514 | TBX19 | T-box 19 | 9095 |
| 5594 | J10 | J11 | 1729 | 720,4 | 20099 | 349,4 | 11,6 | 1,105 | H5A034 C | sample | Hm Drgbl | CPE002DX | CPE002F2 | CPE002FN | NM 00475 | GCNT3 | glucosaminyl (N-a | 9245 |
| 5634 | K3 | K4 | 510 | 659,6 | 5902 | 525,2 | 11,6 | 1,105 | H5A034 C | sample | Hm Drgbl | CPE002DX | CPE002F2 | CPE002FN | XM 93761 | PARG | poly (ADP-ribose) | 8505 |
| 5641 | L1 | L2 | 1516 | 674,7 | 17582 | 559,4 | 11,6 | 1,105 | H5A034 C | sample | Hm Drgbl | CPE002DX | CPE002F2 | CPE002FN | NM 00378 | B3GALT2 | UDP-Gal:betaGlcN | 8707 |
| 5691 | N11 | N12 | 1660 | 807,3 | 19270 | 595,3 | 11,6 | 1,105 | H5A034 C | sample | Hm Drgbl | CPE002DX | CPE002F2 | CPE002FN | NM 00382 | TNFRSF6B | tumor necrosis fa | 8771 |
| 5694 | N14 | N15 | 1882 | 673,7 | 21865 | 513,3 | 11,6 | 1,105 | H5A034 C | sample | Hm Drgbl | CPE002DX | CPE002F2 | CPE002FN | NM 00477 | B4GALT6 | UDP-Gal:betaGlcN | 9331 |
| 5701 | N20 | N21 | 1322 | 700,5 | 15379 | 606,3 | 11,6 | 1,105 | H5A034 C | sample | Hm Drgbl | CPE002DX | CPE002F2 | CPE002FN | NM 00477 | B4GALT5 | UDP-Gal:betaGlcN | 9334 |
| 5720 | O16 | O17 | 1225 | 666,7 | 14234 | 576,7 | 11,6 | 1,105 | H5A034 C | sample | Hm Drgbl | CPE002DX | CPE002F2 | CPE002FN | NM 17320 | PIAS2 | protein inhibitor | 9063 |
| 3524 | C5 | C6 | 1248 | 1260 | 14528 | 333,9 | 11,6 | 1,115 | H5A034 C | sample | Hm Drgbl | CPE002DR | CPE002EH | CPE002F7 | NM 00002 | ADRB2 | adrenergic, beta-2 | 154 |
| 3767 | M8 | M9 | 1359 | 1222 | 15790 | 319,5 | 11,6 | 1,115 | H5A034 C | sample | Hm Drgbl | CPE002DR | CPE002EH | CPE002F7 | NM 01230 | LPHN2 | latrophilin 2 | 23266 |
| 3782 | N21 | N22 | 1848 | 1505 | 21388 | 426 | 11,6 | 1,115 | H5A034 C | sample | Hm Drgbl | CPE002DR | CPE002EH | CPE002F7 | NM 00091 | OPRM1 | opioid receptor, r | 4988 |
| 3800 | O16 | O17 | 1229 | 1411 | 14208 | 455,4 | 11,6 | 1,115 | H5A034 C | sample | Hm Drgbl | CPE002DR | CPE002EH | CPE002F7 | NM 01334 | GPR132 | G protein-couple | 29933 |
| 8303 | J8 | J9 | 1750 | 631,3 | 20304 | 375,8 | 11,6 | 1,115 | H5A034 C | sample | Hm Drgbl | CPE002E5 | CPE002EU | CPE002FF | NM 01245 | TNFRSF13I | tumor necrosis fa | 23495 |
| 8341 | L20 | L21 | 1935 | 605,4 | 22364 | 478,2 | 11,6 | 1,115 | H5A034 C | sample | Hm Drgbl | CPE002E5 | CPE002EU | CPE002FF | NM 01242 | SEC14L2 | SEC14-like 2 (S. ce | 23541 |
| 8409 | O17 | O18 | 1513 | 646,9 | 17519 | 449,8 | 11,6 | 1,115 | H5A034 C | sample | Hm Drgbl | CPE002E5 | CPE002EU | CPE002FF | NM 00725 | SLCO2B1 | solute carrier org | 11309 |
| 10167 | H22 | H23 | 1422 | 782,8 | 16494 | 133,1 | 11,6 | 1,115 | H5A034 C | sample | Hm Drgbl | CPE002EA | CPE002EP | CPE002FA | NM 03256 | PLA2G12B | phospholipase A2 | 84647 |
| 1575 | B22 | B23 | 1435 | 774,1 | 16705 | 389,7 | 11,6 | 1,215 | H5A034 C | sample | Hm Drgbl | CPE002FW | CPE002G0 | CPE002G4 | NM 02480 | ZNF669 | zinc finger protein | 79862 |
| 1678 | F7 | F8 | 1107 | 755,3 | 12808 | 473,1 | 11,6 | 1,215 | H5A034 C | sample | Hm Drgbl | CPE002FW | CPE002G0 | CPE002G4 | NM 00107 | CASZ1 | castor zinc finger | 54897 |
| 1750 | I7 | I8 | 1388 | 744,4 | 16045 | 370,7 | 11,6 | 1,215 | H5A034 C | sample | Hm Drgbl | CPE002FW | CPE002G0 | CPE002G4 | NM 01332 | SNX8 | sorting nexin 8 | 29886 |
| 21516 | A2 | A3 | 154 | 1118 | 1781 | 696,1 | 11,6 | 1,221 | H5A034 C | sample | Hm Gen E | CPE002JU | CPE002O9 | CPE002KP | XM 94185 | LOC65241 | similar to CG9682 | 652417 |
| 21604 | E12 | E13 | 308 | 952,9 | 3586 | 650,4 | 11,6 | 1,221 | H5A034 C | sample | Hm Gen E | CPE002JU | CPE002O9 | CPE002KP | XM 94214 | LOC65261 | similar to HLA cla | 652614 |
| 21611 | E19 | E20 | 296 | 905,5 | 3435 | 508 | 11,6 | 1,221 | H5A034 C | sample | Hm Gen E | CPE002JU | CPE002O9 | CPE002KP | XM 00112 | LOC64930 | hypothetical prot | 649305 |
| 4619 | A19 | A20 | 1542 | 786 | 17822 | 504,1 | 11,6 | 1,275 | H5A034 C | sample | Hm Drgbl | CPE002DV | CPE002EL | CPE002FP | NM 00703 | ADAMTS5 | ADAM metallopep | 11096 |
| 4632 | A9 | A10 | 1448 | 892,7 | 16851 | 474,1 | 11,6 | 1,275 | H5A034 C | sample | Hm Drgbl | CPE002DV | CPE002EL | CPE002FP | NM 00133 | CTSZ | cathepsin Z | 1522 |
| 4666 | C18 | C19 | 1483 | 820,6 | 17180 | 509,8 | 11,6 | 1,275 | H5A034 C | sample | Hm Drgbl | CPE002DV | CPE002EL | CPE002FP | NM 00336 | USP4 | ubiquitin specific | 7375 |
| 4867 | K4 | K5 | 969 | 809,9 | 11230 | 502,7 | 11,6 | 1,275 | H5A034 C | sample | Hm Drgbl | CPE002DV | CPE002EL | CPE002FP | NM 00515 | USP8 | ubiquitin specific | 9101 |
| 4897 | M1 | M2 | 490 | 781,6 | 5662 | 525,2 | 11,6 | 1,275 | H5A034 C | sample | Hm Drgbl | CPE002DV | CPE002EL | CPE002FP | NM 00439 | CTSH | cathepsin H | 1512 |
| 4914 | M3 | M4 | 1087 | 737,9 | 12652 | 470,2 | 11,6 | 1,275 | H5A034 C | sample | Hm Drgbl | CPE002DV | CPE002EL | CPE002FP | NM 00039 | CTSK | cathepsin K | 1513 |
| 4943 | N8 | N9 | 1601 | 806,3 | 18520 | 465,3 | 11,6 | 1,275 | H5A034 C | sample | Hm Drgbl | CPE002DV | CPE002EL | CPE002FP | NM 01960 | CPXM1 | carboxypeptidase | 56265 |
| 10426 | C18 | C19 | 1460 | 901,9 | 16952 | 202,2 | 11,6 | 1,311 | H5A034 C | sample | Hm Drgbl | CPE002EB | CPE002EO | CPE002FR | NM 05292 | RHPN1 | rhophilin, Rho GT | 114822 |
| 10750 | P7 | P8 | 1773 | 823,4 | 20623 | 206,8 | 11,6 | 1,311 | H5A034 C | sample | Hm Drgbl | CPE002EB | CPE002EO | CPE002FR | NM 18307 | CYP2U1 | cytochrome P450, | 113612 |
| 2749 | C20 | C21 | 735 | 648,3 | 8559 | 531,6 | 11,6 | 1,365 | H5A034 C | sample | Hm Drgbl | CPE002DP | CPE002EF | CPE002F5 | NM 17220 | CAMKK1 | calcium/calmodul | 84254 |
| 2815 | F15 | F16 | 1076 | 676 | 12467 | 494,1 | 11,6 | 1,365 | H5A034 C | sample | Hm Drgbl | CPE002DP | CPE002EF | CPE002F5 | NM 01777 | PXK | PX domain contai | 54899 |
| 2924 | J5 | J6 | 989 | 570,8 | 11442 | 467,1 | 11,6 | 1,365 | H5A034 C | sample | Hm Drgbl | CPE002DP | CPE002EF | CPE002F5 | NM 01514 | PASK | PAS domain conta | 23178 |
| 20053 | D20 | D21 | 1195 | 1105 | 13904 | 159,5 | 11,6 | 1,415 | H5A034 C | sample | Hm Gen E | CPE002JQ | CPE002O5 | CPE002KL | XM 37978 | LOC40245 | similar to unc-93 | 402457 |
| 20136 | G9 | G10 | 731 | 1139 | 8503 | 204,3 | 11,6 | 1,415 | H5A034 C | sample | Hm Gen E | CPE002JQ | CPE002O5 | CPE002KL | XM 37287 | C21orf126 | chromosome 21 c | 391269 |
| 20203 | J4 | J5 | 698 | 1117 | 8118 | 171,8 | 11,6 | 1,415 | H5A034 C | sample | Hm Gen E | CPE002JQ | CPE002O5 | CPE002KL | NM 20683 | CTXN1 | cortexin 1 | 404217 |
| 393 | A17 | A18 | 2333 | 1829 | 26955 | 484,9 | 11,6 | 1,693 | H5A034 C | sample | Hm Drgbl | CPE002E0 | CPE002EZ | CPE002FK | NM 00195 | E2F4 | E2F transcription | 1874 |
| 415 | B15 | B16 | 1424 | 1639 | 16475 | 400,9 | 11,6 | 1,693 | H5A034 C | sample | Hm Drgbl | CPE002E0 | CPE002EZ | CPE002FK | NM 00473 | ETF1 | eukaryotic transla | 2107 |
| 552 | G9 | G10 | 1991 | 1674 | 23093 | 333,6 | 11,6 | 1,693 | H5A034 C | sample | Hm Drgbl | CPE002E0 | CPE002EZ | CPE002FK | NM 00196 | EHHADH | enoyl-Coenzyme A | 1962 |
| 7754 | D10 | D11 | 840 | 544,1 | 9709 | 418,4 | 11,6 | 1,771 | H5A034 C | sample | Hm Drgbl | CPE002E4 | CPE002EV | CPE002FG | NM 02100 | DHRS4 | dehydrogenase/r | 10901 |
| 3176 | E16 | E17 | 1595 | 1297 | 18607 | 380,2 | 11,7 | 0,724 | H5A034 C | sample | Hm Drgbl | CPE002DC | CPE002EG | CPE002F6 | NM 02078 | PDP2 | pyruvate dehydrog | 57546 |
| 3320 | K16 | K17 | 1343 | 1288 | 15658 | 306,3 | 11,7 | 0,724 | H5A034 C | sample | Hm Drgbl | CPE002DC | CPE002EG | CPE002F6 | NM 08087 | DUSP19 | dual specificity ph | 142679 |
| 3359 | L8 | L9 | 1074 | 1356 | 12600 | 401,1 | 11,7 | 0,724 | H5A034 C | sample | Hm Drgbl | CPE002DC | CPE002EG | CPE002F6 | NM 00502 | SERPINI1 | serpin peptidase | 5274 |
| 20577 | J17 | J18 | 1168 | 753,1 | 13649 | 166,3 | 11,7 | 0,78 | H5A034 C | sample | Hm Gen E | CPE002JR | CPE002O6 | CPE002KM | XM 49764 | LOC44186 | similar to cSH-PTP | 441868 |
| 20671 | N15 | N16 | 1216 | 759,7 | 14200 | 129 | 11,7 | 0,78 | H5A034 C | sample | Hm Gen E | CPE002JR | CPE002O6 | CPE002KM | NM 00113 | LOC44217 | similar to hCG181 | 442175 |
| 9272 | C16 | C17 | 1627 | 1174 | 19072 | 154,6 | 11,7 | 0,812 | H5A034 C | sample | Hm Drgbl | CPE002E8 | CPE002ER | CPE002FC | NM 01847 | ECHDC1 | enoyl Coenzyme A | 55862 |
| 9495 | L22 | L23 | 1746 | 1118 | 20390 | 172,9 | 11,7 | 0,812 | H5A034 C | sample | Hm Drgbl | CPE002E8 | CPE002ER | CPE002FC | NM 02041 | PCBP4 | poly(rC) binding p | 57060 |
| 19146 | N3 | N4 | 1774 | 1034 | 20816 | 117,1 | 11,7 | 0,815 | H5A034 C | sample | Hm Gen E | CPE002JN | CPE002O2 | CPE002KI | NM 00108 | C17orf60 | chromosome 17 c | 284021 |
| 20817 | D17 | D18 | 1238 | 1057 | 14516 | 261,4 | 11,7 | 0,83 | H5A034 C | sample | Hm Gen E | CPE002JS | CPE002O7 | CPE002KN | XM 93167 | LOC64359 | hypothetical LOC6 | 643599 |
| 5218 | J18 | J19 | 1783 | 629 | 20906 | 451,4 | 11,7 | 0,918 | H5A034 C | sample | Hm Drgbl | CPE002DV | CPE002EN | CPE002FO | NM 00340 | YWHAZ | tyrosine 3-monoo | 7534 |
| 5220 | J2 | J3 | 1860 | 628,9 | 21848 | 476 | 11,7 | 0,918 | H5A034 C | sample | Hm Drgbl | CPE002DV | CPE002EN | CPE002FO | NM 00629 | XRCC1 | X-ray repair comp | 7515 |
| 5360 | P16 | P17 | 1722 | 686,6 | 20207 | 528,9 | 11,7 | 0,918 | H5A034 C | sample | Hm Drgbl | CPE002DV | CPE002EN | CPE002FO | NM 00346 | ZP2 | zona pellucida gly | 7783 |
| 2446 | F7 | F8 | 1061 | 704,3 | 12437 | 428,2 | 11,7 | 0,921 | H5A034 C | sample | Hm Drgbl | CPE002DC | CPE002EE | CPE002F4 | NM 00554 | ITK | IL2-inducible T-ce | 3702 |
| 2631 | N22 | N23 | 2000 | 763,6 | 23317 | 425,7 | 11,7 | 0,921 | H5A034 C | sample | Hm Drgbl | CPE002DC | CPE002EE | CPE002F4 | NM 14533 | MAP3K7 | mitogen-activated | 6885 |
| 2673 | P17 | P18 | 1738 | 683,5 | 20252 | 415,5 | 11,7 | 0,921 | H5A034 C | sample | Hm Drgbl | CPE002DC | CPE002EE | CPE002F4 | NM 20339 | GK | glycerol kinase | 2710 |
| 16788 | L2 | L3 | 1032 | 920,4 | 12081 | 287,9 | 11,7 | 0,925 | H5A034 C | sample | Hm Gen E | CPE002JH | CPE002NV | CPE002KC | NM 08086 | ASB16 | ankyrin repeat an | 92591 |
| 17794 | F18 | F19 | 1827 | 1038 | 21464 | 220 | 11,7 | 0,925 | H5A034 C | sample | Hm Gen E | CPE002JK | CPE002NZ | CPE002KF | NM 17351 | SLC38A11 | solute carrier fam | 151258 |
| 9094 | K7 | K8 | 1190 | 1471 | 13899 | 124,1 | 11,7 | 0,94 | H5A034 C | sample | Hm Drgbl | CPE002E7 | CPE002ES | CPE002FD | NM 18159 | SPG3A | spastic paraplegia | 51062 |
| 4348 | F12 | F13 | 906 | 710,7 | 10609 | 394,1 | 11,7 | 0,987 | H5A034 C | sample | Hm Drgbl | CPE002DL | CPE002EK | CPE002FQ | NM 13344 | GRIN3A | glutamate recepto | 116443 |
| 4488 | K9 | K10 | 936 | 753,9 | 10939 | 389,9 | 11,7 | 0,987 | H5A034 C | sample | Hm Drgbl | CPE002DL | CPE002EK | CPE002FQ | NM 00183 | CLCN4 | chloride channel 4 | 1183 |
| 31 | B15 | B16 | 465 | 1523 | 5461 | 667,2 | 11,7 | 0,996 | H5A034 C | sample | Hm Drgbl | CPE002DT | CPE002EJ | CPE002F9 | NM 00065 | SELL | selectin L (lympho | 6402 |
| 37 | B20 | B21 | 607 | 1545 | 7092 | 594,7 | 11,7 | 0,996 | H5A034 C | sample | Hm Drgbl | CPE002DT | CPE002EJ | CPE002F9 | NM 15318 | SPAM1 | sperm adhesion m | 6677 |
| 90 | D3 | D4 | 141 | 1733 | 1646 | 598,2 | 11,7 | 0,996 | H5A034 C | sample | Hm Drgbl | CPE002DT | CPE002EJ | CPE002F9 | NM 00107 | SFRS1 | splicing factor, ar | 6426 |
| 105 | E17 | E18 | 733 | 1517 | 8557 | 389,8 | 11,7 | 0,996 | H5A034 C | sample | Hm Drgbl | CPE002DT | CPE002EJ | CPE002F9 | NM 00046 | THRB | thyroid hormone | 7068 |
| 152 | G16 | G17 | 553 | 1402 | 6467 | 400,2 | 11,7 | 0,996 | H5A034 C | sample | Hm Drgbl | CPE002DT | CPE002EJ | CPE002F9 | NM 00307 | SMARCA4 | SWI/SNF related, | 6597 |
| 293 | M13 | M14 | 375 | 1343 | 4401 | 723,1 | 11,7 | 0,996 | H5A034 C | sample | Hm Drgbl | CPE002DT | CPE002EJ | CPE002F9 | NM 00298 | CCL5 | chemokine (C-C m | 6352 |
| 299 | M19 | M20 | 504 | 1447 | 5878 | 519,3 | 11,7 | 0,996 | H5A034 C | sample | Hm Drgbl | CPE002DT | CPE002EJ | CPE002F9 | NM 00298 | CCL11 | chemokine (C-C m | 6356 |
| 310 | M7 | M8 | 647 | 1477 | 7564 | 471,8 | 11,7 | 0,996 | H5A034 C | sample | Hm Drgbl | CPE002DT | CPE002EJ | CPE002F9 | XM 94205 | SRL | sarcalumenin | 6345 |
| 1924 | A12 | A13 | 833 | 700 | 9708 | 460,8 | 11,7 | 1,017 | H5A034 C | sample | Hm Drgbl | CPE002FX | CPE002G1 | CPE002G5 | NM 18252 | CABP7 | calcium binding p | 164633 |
| 2096 | H16 | H17 | 1213 | 586,2 | 14148 | 306,2 | 11,7 | 1,017 | H5A034 C | sample | Hm Drgbl | CPE002FX | CPE002G1 | CPE002G5 | NM 00101 | SYT14L | synaptotagmin XI | 401135 |
| 2130 | I3 | I4 | 1502 | 690,6 | 17578 | 364,9 | 11,7 | 1,017 | H5A034 C | sample | Hm Drgbl | CPE002FX | CPE002G1 | CPE002G5 | NM 05292 | SMYD4 | SET and MYND dc | 114826 |
| 2148 | J2 | J3 | 822 | 704,4 | 9600 | 443,1 | 11,7 | 1,017 | H5A034 C | sample | Hm Drgbl | CPE002FX | CPE002G1 | CPE002G5 | NM 20692 | YY2 | YY2 transcription | 404281 |
| 2202 | L3 | L4 | 1772 | 681,8 | 20717 | 343,8 | 11,7 | 1,017 | H5A034 C | sample | Hm Drgbl | CPE002FX | CPE002G1 | CPE002G5 | NM 14517 | DNAJB7 | DnaJ (Hsp40) hom | 150353 |
| 3841 | A1 | A2 | 849 | 699,1 | 9907 | 447,3 | 11,7 | 1,04 | H5A034 C | sample | Hm Drgbl | CPE002DS | CPE002EI | CPE002F8 | NM 18174 | GPR120 | G protein-couple | 338557 |
| 3849 | A17 | A18 | 2019 | 807,6 | 23717 | 350,3 | 11,7 | 1,04 | H5A034 C | sample | Hm Drgbl | CPE002DS | CPE002EI | CPE002F8 | NM 20737 | GPR153 | G protein-couple | 387509 |
| 3890 | C10 | C11 | 1491 | 679,3 | 17471 | 328,6 | 11,7 | 1,04 | H5A034 C | sample | Hm Drgbl | CPE002DS | CPE002EI | CPE002F8 | NM 00416 | RAB1A | RAB1A, member R | 5861 |
| 3918 | D14 | D15 | 1072 | 609 | 12561 | 259,6 | 11,7 | 1,04 | H5A034 C | sample | Hm Drgbl | CPE002DS | CPE002EI | CPE002F8 | NM 00292 | RGS7 | regulator of G-pro | 6000 |
| 3964 | F12 | F13 | 1009 | 710 | 11836 | 418,8 | 11,7 | 1,04 | H5A034 C | sample | Hm Drgbl | CPE002DS | CPE002EI | CPE002F8 | NM 00293 | RIT2 | Ras-like without C | 6014 |
| 4015 | H15 | H16 | 1398 | 694,2 | 16397 | 331 | 11,7 | 1,04 | H5A034 C | sample | Hm Drgbl | CPE002DS | CPE002EI | CPE002F8 | NM 00319 | TCF4 | transcription facto | 6925 |
| 5854 | D7 | D8 | 1521 | 794,4 | 17805 | 614,7 | 11,7 | 1,068 | H5A034 C | sample | Hm Drgbl | CPE002DY | CPE002F1 | CPE002FM | NM 00113 | ALOX12B | arachidonate 12-l | 242 |
| 5875 | E4 | E5 | 1572 | 771,5 | 18411 | 612,9 | 11,7 | 1,068 | H5A034 C | sample | Hm Drgbl | CPE002DY | CPE002F1 | CPE002FM | NM 19900 | ARRB2 | arrestin, beta 2 | 409 |
| 6083 | N19 | N20 | 1825 | 787,5 | 21318 | 613,3 | 11,7 | 1,068 | H5A034 C | sample | Hm Drgbl | CPE002DY | CPE002F1 | CPE002FM | NM 00492 | AQP3 | aquaporin 3 (Gill | 360 |
| 1376 | J16 | J17 | 2001 | 760,3 | 23481 | 321,7 | 11,7 | 1,073 | H5A034 C | sample | Hm Drgbl | CPE002FV | CPE002FZ | CPE002G3 | NM 01491 | ZNF507 | zinc finger protein | 22847 |
| 1398 | K14 | K15 | 1950 | 699,9 | 22887 | 320,6 | 11,7 | 1,073 | H5A034 C | sample | Hm Drgbl | CPE002FV | CPE002FZ | CPE002G3 | NM 00699 | NPM3 | nucleophosmin/n | 10360 |
| 16332 | I2 | I3 | 1238 | 962,2 | 14452 | 222,6 | 11,7 | 1,073 | H5A034 C | sample | Hm Gen E | CPE002JG | CPE002NV | CPE002KB | NM 03232 | TMEM79 | transmembrane p | 84283 |
| 16335 | I22 | I23 | 1156 | 999 | 13514 | 305,8 | 11,7 | 1,073 | H5A034 C | sample | Hm Gen E | CPE002JG | CPE002NV | CPE002KB | NM 03233 | C12orf31 | chromosome 12 c | 84298 |
| 16370 | K10 | K11 | 1314 | 975,2 | 15372 | 313,4 | 11,7 | 1,073 | H5A034 C | sample | Hm Gen E | CPE002JG | CPE002NV | CPE002KB | NM 03234 | NUDT22 | nudix (nucleoside | 84304 |
| 16404 | L2 | L3 | 1194 | 1012 | 14006 | 276,2 | 11,7 | 1,073 | H5A034 C | sample | Hm Gen E | CPE002JG | CPE002NV | CPE002KB | NM 03259 | HINT2 | histidine triad nu | 84681 |
| 16502 | P21 | P22 | 1275 | 1134 | 14912 | 361,9 | 11,7 | 1,073 | H5A034 C | sample | Hm Gen E | CPE002JG | CPE002NV | CPE002KB | NM 03222 | CHD6 | chromodomain h | 84181 |
| 13683 | K11 | K12 | 929 | 948,4 | 10908 | 159 | 11,7 | 1,099 | H5A034 C | sample | Hm Gen E | CPE002JC | CPE002NC | CPE002K4 | NM 01429 | TRAM1 | translocation asso | 23471 |
| 870 | E14 | E15 | 1138 | 716,7 | 13267 | 439 | 11,7 | 1,109 | H5A034 C | sample | Hm Drgbl | CPE002FU | CPE002FY | CPE002G2 | NM 00596 | NAB1 | NGFI-A binding pr | 4664 |
| 971 | I19 | I20 | 1399 | 695,4 | 16344 | 301,4 | 11,7 | 1,109 | H5A034 C | sample | Hm Drgbl | CPE002FU | CPE002FY | CPE002G2 | NM 00184 | COL4A6 | collagen, type IV, | 1288 |
| 1085 | N13 | N14 | 1343 | 712,6 | 15686 | 443,4 | 11,7 | 1,109 | H5A034 C | sample | Hm Drgbl | CPE002FU | CPE002FY | CPE002G2 | NM 20583 | IGSF1 | immunoglobulin s | 3547 |
| 1101 | N6 | N7 | 1185 | 765,4 | 13886 | 397,1 | 11,7 | 1,109 | H5A034 C | sample | Hm Drgbl | CPE002FU | CPE002FY | CPE002G2 | NM 00326 | TLE2 | transducin-like er | 7089 |
| 5390 | A21 | A22 | 2231 | 807,5 | 26078 | 608,7 | 11,7 | 1,114 | H5A034 C | sample | Hm Drgbl | CPE002DX | CPE002F2 | CPE002FN | NM 01343 | KLRC4 | killer cell lectin-lik | 8302 |
| 5631 | K22 | K23 | 1559 | 719,5 | 18297 | 498 | 11,7 | 1,114 | H5A034 C | sample | Hm Drgbl | CPE002DX | CPE002F2 | CPE002FN | NM 00102 | KALRN | kalirin, RhoGEF ki | 8997 |
| 5676 | M2 | M3 | 922 | 613,3 | 10817 | 473,5 | 11,7 | 1,114 | H5A034 C | sample | Hm Drgbl | CPE002DX | CPE002F2 | CPE002FN | NM 02281 | SLC25A14 | solute carrier fam | 9016 |
| 5690 | N10 | N11 | 1970 | 702,7 | 23037 | 604,1 | 11,7 | 1,114 | H5A034 C | sample | Hm Drgbl | CPE002DX | CPE002F2 | CPE002FN | NM 01621 | TRIP4 | thyroid hormone | 9325 |
| 3483 | B11 | B12 | 1243 | 1463 | 14551 | 268,9 | 11,7 | 1,125 | H5A034 C | sample | Hm Drgbl | CPE002DR | CPE002EH | CPE002F7 | NM 00528 | NPBWR2 | neuropeptides B/ | 2832 |
| 3522 | C3 | C4 | 727 | 1372 | 8499 | 358 | 11,7 | 1,125 | H5A034 C | sample | Hm Drgbl | CPE002DR | CPE002EH | CPE002F7 | NM 00068 | ADRB1 | adrenergic, beta-1 | 153 |
| 3535 | D15 | D16 | 1060 | 1337 | 12383 | 330,2 | 11,7 | 1,125 | H5A034 C | sample | Hm Drgbl | CPE002DR | CPE002EH | CPE002F7 | NM 00529 | GPR25 | G protein-couple | 2848 |
| 3812 | O5 | O6 | 1436 | 1337 | 16734 | 487 | 11,7 | 1,125 | H5A034 C | sample | Hm Drgbl | CPE002DR | CPE002EH | CPE002F7 | NM 00016 | GCGR | glucagon recepto | 2642 |
| 8169 | E17 | E18 | 1711 | 625,7 | 20024 | 409,5 | 11,7 | 1,125 | H5A034 C | sample | Hm Drgbl | CPE002E5 | CPE002EU | CPE002FF | NM 01427 | SLC7A9 | solute carrier fam | 11136 |
| 8268 | I2 | I3 | 1001 | 637,4 | 11711 | 474,1 | 11,7 | 1,125 | H5A034 C | sample | Hm Drgbl | CPE002E5 | CPE002EU | CPE002FF | NM 01461 | ATP11B | ATPase, Class VI, t | 23200 |
| 10190 | I21 | I22 | 1213 | 878,1 | 14208 | 132,9 | 11,7 | 1,125 | H5A034 C | sample | Hm Drgbl | CPE002EA | CPE002EP | CPE002FA | NM 02483 | AYTL2 | acyltransferase lik | 79888 |
| 1620 | D2 | D3 | 937 | 721,2 | 10950 | 437,2 | 11,7 | 1,225 | H5A034 C | sample | Hm Drgbl | CPE002FW | CPE002G0 | CPE002G4 | NM 02481 | THSD4 | thrombospondin, | 79875 |
| 1760 | J16 | J17 | 1241 | 704,9 | 14534 | 279,3 | 11,7 | 1,225 | H5A034 C | sample | Hm Drgbl | CPE002FW | CPE002G0 | CPE002G4 | NM 03204 | EMILIN2 | elastin microfibril | 84034 |
| 1796 | K5 | K6 | 2158 | 779 | 25173 | 430,1 | 11,7 | 1,225 | H5A034 C | sample | Hm Drgbl | CPE002FW | CPE002G0 | CPE002G4 | NM 17319 | KCNIP2 | Kv channel intera | 30819 |
| 1832 | M16 | M17 | 1637 | 723,6 | 19172 | 332,1 | 11,7 | 1,225 | H5A034 C | sample | Hm Drgbl | CPE002FW | CPE002G0 | CPE002G4 | NM 02307 | ZNF643 | zinc finger protein | 65243 |
| 1833 | M17 | M18 | 1538 | 817,5 | 18020 | 468,9 | 11,7 | 1,225 | H5A034 C | sample | Hm Drgbl | CPE002FW | CPE002G0 | CPE002G4 | NM 01653 | ZNF571 | zinc finger protein | 51276 |
| 1920 | P9 | P10 | 1647 | 806,1 | 19293 | 445,4 | 11,7 | 1,225 | H5A034 C | sample | Hm Drgbl | CPE002FW | CPE002G0 | CPE002G4 | NM 02024 | CABC1 | chaperone, ABC1 | 56997 |
| 21531 | B11 | B12 | 256 | 1056 | 2992 | 707,2 | 11,7 | 1,232 | H5A034 C | sample | Hm Gen E | CPE002JU | CPE002O9 | CPE002KP | XM 93985 | LOC65077 | similar to pote pr | 650770 |
| 21603 | E11 | E12 | 225 | 873,6 | 2626 | 614,8 | 11,7 | 1,232 | H5A034 C | sample | Hm Gen E | CPE002JU | CPE002O9 | CPE002KP | XM 00113 | LOC64926 | similar to hCG403 | 649264 |
| 21684 | H2 | H3 | 393 | 981,9 | 4617 | 450 | 11,7 | 1,232 | H5A034 C | sample | Hm Gen E | CPE002JU | CPE002O9 | CPE002KP | XM 93913 | LOC65338 | similar to Sorbito | 653381 |
| 21728 | J16 | J17 | 372 | 953 | 4367 | 562,8 | 11,7 | 1,232 | H5A034 C | sample | Hm Gen E | CPE002JU | CPE002O9 | CPE002KP | XM 92891 | hCG 1731 | hCG1731871 | 653687 |
| 21826 | N18 | N19 | 528 | 1071 | 6185 | 504,8 | 11,7 | 1,232 | H5A034 C | sample | Hm Gen E | CPE002JU | CPE002O9 | CPE002KP | XM 93848 | LOC65405 | similar to Signal-r | 654056 |
| 4726 | E7 | E8 | 1412 | 741,1 | 16524 | 530,7 | 11,7 | 1,286 | H5A034 C | sample | Hm Drgbl | CPE002DV | CPE002EL | CPE002FP | NM 00112 | AEBP1 | AE binding protei | 165 |
| 4759 | G15 | G16 | 662 | 703,6 | 7742 | 398,4 | 11,7 | 1,286 | H5A034 C | sample | Hm Drgbl | CPE002DV | CPE002EL | CPE002FP | NM 03329 | CASP1 | caspase 1, apopto | 834 |
| 4762 | G18 | G19 | 951 | 773,2 | 11169 | 330,1 | 11,7 | 1,286 | H5A034 C | sample | Hm Drgbl | CPE002DV | CPE002EL | CPE002FP | NM 00381 | ADAM20 | ADAM metallopep | 8748 |
| 10653 | L6 | L7 | 1044 | 851,5 | 12170 | 191,6 | 11,7 | 1,322 | H5A034 C | sample | Hm Drgbl | CPE002EB | CPE002EO | CPE002FR | NM 13349 | ASAH3 | N-acylsphingosine | 125981 |
| 19974 | A14 | A15 | 725 | 1259 | 8455 | 219,3 | 11,7 | 1,427 | H5A034 C | sample | Hm Gen E | CPE002JQ | CPE002O5 | CPE002KL | NM 21360 | C2orf66 | chromosome 2 op | 401027 |
| 19975 | A15 | A16 | 1186 | 1271 | 13882 | 228,9 | 11,7 | 1,427 | H5A034 C | sample | Hm Gen E | CPE002JQ | CPE002O5 | CPE002KL | XM 93886 | LOC39085 | similar to 60S ribo | 390856 |
| 19982 | A21 | A22 | 823 | 1237 | 9633 | 189,2 | 11,7 | 1,427 | H5A034 C | sample | Hm Gen E | CPE002JQ | CPE002O5 | CPE002KL | NM 00100 | OR7G2 | olfactory recepto | 390882 |
| 20187 | J11 | J12 | 1075 | 1101 | 12545 | 164,5 | 11,7 | 1,427 | H5A034 C | sample | Hm Gen E | CPE002JQ | CPE002O5 | CPE002KL | NM 00100 | C16orf85 | chromosome 16 c | 400555 |
| 20205 | J6 | J7 | 899 | 1079 | 10535 | 189,5 | 11,7 | 1,427 | H5A034 C | sample | Hm Gen E | CPE002JQ | CPE002O5 | CPE002KL | NM 00108 | C6orf201 | chromosome 6 op | 404220 |
| 20326 | O7 | O8 | 405 | 1156 | 4755 | 175,8 | 11,7 | 1,427 | H5A034 C | sample | Hm Gen E | CPE002JQ | CPE002O5 | CPE002KL | NM 00108 | LOC39297 | similar to ribosom | 392979 |
| 387 | A11 | A12 | 2124 | 1845 | 24868 | 466 | 11,7 | 1,708 | H5A034 C | sample | Hm Drgbl | CPE002E0 | CPE002EZ | CPE002FK | NM 00522 | E2F1 | E2F transcription | 1869 |
| 399 | A22 | A23 | 2091 | 1834 | 24415 | 438,7 | 11,7 | 1,708 | H5A034 C | sample | Hm Drgbl | CPE002E0 | CPE002EZ | CPE002FK | NM 00447 | FOXD2 | forkhead box D2 | 2306 |
| 441 | C17 | C18 | 1246 | 1536 | 14617 | 357,7 | 11,7 | 1,708 | H5A034 C | sample | Hm Drgbl | CPE002E0 | CPE002EZ | CPE002FK | NM 19804 | PHC2 | polyhomeotic hor | 1912 |
| 7828 | G12 | G13 | 978 | 602,3 | 11479 | 414,1 | 11,7 | 1,786 | H5A034 C | sample | Hm Drgbl | CPE002E4 | CPE002EV | CPE002FG | NM 00648 | RGS14 | regulator of G-pro | 10636 |
| 7883 | I19 | I20 | 803 | 513,6 | 9418 | 364,4 | 11,7 | 1,786 | H5A034 C | sample | Hm Drgbl | CPE002E4 | CPE002EV | CPE002FG | NM 00584 | STAM2 | signal transducing | 10254 |
| 3363 | M11 | M12 | 1065 | 1270 | 12597 | 466,2 | 11,8 | 0,731 | H5A034 C | sample | Hm Drgbl | CPE002DC | CPE002EG | CPE002F6 | NM 00539 | PPP1R3C | protein phosphat | 5507 |
| 20604 | K2 | K3 | 767 | 782,7 | 9058 | 142,7 | 11,8 | 0,787 | H5A034 C | sample | Hm Gen E | CPE002JR | CPE002O6 | CPE002KM | NM 00103 | C8orf49 | chromosome 8 op | 606553 |
| 20683 | N4 | N5 | 813 | 771,6 | 9582 | 132,2 | 11,8 | 0,787 | H5A034 C | sample | Hm Gen E | CPE002JR | CPE002O6 | CPE002KM | XM 92602 | LOC64253 | similar to Matrin- | 642536 |
| 20703 | O22 | O23 | 1258 | 835,2 | 14887 | 191,2 | 11,8 | 0,787 | H5A034 C | sample | Hm Gen E | CPE002JR | CPE002O6 | CPE002KM | XM 93588 | LOC64183 | hypothetical prot | 641835 |
| 19519 | N15 | N16 | 1082 | 1202 | 12719 | 208,6 | 11,8 | 0,837 | H5A034 C | sample | Hm Gen E | CPE002JO | CPE002O3 | CPE002KJ | XM 94096 | PPIAP8 | peptidylprolyl iso | 341457 |
| 12458 | H10 | H11 | 1125 | 996 | 13300 | 148,8 | 11,8 | 0,887 | H5A034 C | sample | Hm Gen E | CPE002J5 | CPE002NK | CPE002K1 | NM 00602 | H1FX | H1 histone family | 8971 |
| 12466 | H18 | H19 | 1414 | 988 | 16752 | 135,9 | 11,8 | 0,887 | H5A034 C | sample | Hm Gen E | CPE002J5 | CPE002NK | CPE002K1 | NM 00509 | TNFSF18 | tumor necrosis fa | 8995 |
| 10809 | C17 | C18 | 1667 | 876,4 | 19599 | 456,8 | 11,8 | 0,894 | H5A034 C | sample | Hm Drgbl | CPE002EC | CPE002EN | CPE002FS | NM 00104 | CAPSL | calcyphosine-like | 133690 |
| 11058 | M3 | M4 | 1392 | 751,5 | 16380 | 409,3 | 11,8 | 0,894 | H5A034 C | sample | Hm Drgbl | CPE002EC | CPE002EN | CPE002FS | NM 00101 | LIPJ | lipase, family mem | 142910 |
| 6940 | B12 | B13 | 1153 | 602,8 | 13588 | 221,7 | 11,8 | 0,918 | H5A034 C | sample | Hm Drgbl | CPE002E2 | CPE002EX | CPE002FI | NM 00454 | NEB | nebulin | 4703 |
| 5143 | G15 | G16 | 500 | 642,7 | 5921 | 512,2 | 11,8 | 0,925 | H5A034 C | sample | Hm Drgbl | CPE002DV | CPE002EN | CPE002FO | NM 03280 | SPPL2A | signal peptide pe | 84888 |
| 5168 | H16 | H17 | 1441 | 564,9 | 16973 | 366,5 | 11,8 | 0,925 | H5A034 C | sample | Hm Drgbl | CPE002DV | CPE002EN | CPE002FO | NM 02108 | XK | X-linked Kx blood | 7504 |
| 5274 | L3 | L4 | 1543 | 597,5 | 18208 | 376,6 | 11,8 | 0,925 | H5A034 C | sample | Hm Drgbl | CPE002DV | CPE002EN | CPE002FO | NM 00464 | MLLT10 | myeloid/lymphoid | 8028 |
| 2336 | B16 | B17 | 1290 | 682,3 | 15176 | 414,7 | 11,8 | 0,929 | H5A034 C | sample | Hm Drgbl | CPE002DC | CPE002EE | CPE002F4 | NM 00625 | PRKCH | protein kinase C, | 5583 |
| 2358 | C14 | C15 | 1169 | 729 | 13767 | 499,6 | 11,8 | 0,929 | H5A034 C | sample | Hm Drgbl | CPE002DC | CPE002EE | CPE002F4 | NM 00590 | MAK | male germ cell-as | 4117 |
| 2438 | F21 | F22 | 1843 | 731,3 | 21768 | 400,3 | 11,8 | 0,929 | H5A034 C | sample | Hm Drgbl | CPE002DC | CPE002EE | CPE002F4 | NM 00225 | KDR | kinase insert dom | 3791 |
| 2442 | F3 | F4 | 588 | 725,1 | 6914 | 409,5 | 11,8 | 0,929 | H5A034 C | sample | Hm Drgbl | CPE002DC | CPE002EE | CPE002F4 | NM 01421 | INSRR | insulin receptor-r | 3645 |
| 2450 | G10 | G11 | 1242 | 661,2 | 14666 | 359 | 11,8 | 0,929 | H5A034 C | sample | Hm Drgbl | CPE002DC | CPE002EE | CPE002F4 | NM 01222 | NEK1 | NIMA (never in m | 4750 |
| 2562 | K3 | K4 | 536 | 668,5 | 6324 | 375,4 | 11,8 | 0,929 | H5A034 C | sample | Hm Drgbl | CPE002DC | CPE002EE | CPE002F4 | NM 00475 | MAPKAPK | mitogen-activated | 9261 |
| 2595 | M11 | M12 | 1525 | 674,3 | 18059 | 370 | 11,8 | 0,929 | H5A034 C | sample | Hm Drgbl | CPE002DC | CPE002EE | CPE002F4 | NM 02032 | ACVR1B | activin A receptor | 91 |
| 16692 | H2 | H3 | 1211 | 928 | 14245 | 180,5 | 11,8 | 0,933 | H5A034 C | sample | Hm Gen E | CPE002JH | CPE002NV | CPE002KC | NM 15227 | UBTD2 | ubiquitin domain | 92181 |
| 18013 | O20 | O21 | 1802 | 1070 | 21215 | 276,1 | 11,8 | 0,933 | H5A034 C | sample | Hm Gen E | CPE002JK | CPE002NZ | CPE002KF | NM 14591 | NFAM1 | NFAT activating p | 150372 |
| 8976 | F9 | F10 | 1624 | 1960 | 19152 | 157,8 | 11,8 | 0,948 | H5A034 C | sample | Hm Drgbl | CPE002E7 | CPE002ES | CPE002FD | NM 01648 | NT5C3 | 5-nucleotidase, cy | 51251 |
| 9145 | N1 | N2 | 1586 | 1546 | 18754 | 134 | 11,8 | 0,948 | H5A034 C | sample | Hm Drgbl | CPE002E7 | CPE002ES | CPE002FD | NM 01621 | POLK | polymerase (DNA | 51426 |
| 4266 | B3 | B4 | 1027 | 708,3 | 12095 | 429,8 | 11,8 | 0,996 | H5A034 C | sample | Hm Drgbl | CPE002DL | CPE002EK | CPE002FQ | NM 00082 | GLRB | glycine receptor, | 2743 |
| 4402 | H18 | H19 | 1169 | 718,9 | 13792 | 368,1 | 11,8 | 0,996 | H5A034 C | sample | Hm Drgbl | CPE002DL | CPE002EK | CPE002FQ | NM 13907 | TPCN2 | two pore segmen | 219931 |
| 4524 | M2 | M3 | 894 | 656,6 | 10547 | 367,2 | 11,8 | 0,996 | H5A034 C | sample | Hm Drgbl | CPE002DL | CPE002EK | CPE002FQ | NM 01660 | KCNK9 | potassium channe | 51305 |
| 42 | B3 | B4 | 717 | 1570 | 8484 | 618,7 | 11,8 | 1,004 | H5A034 C | sample | Hm Drgbl | CPE002DT | CPE002EJ | CPE002F9 | NM 00416 | SDHA | succinate dehydro | 6389 |
| 145 | G1 | G2 | 350 | 1334 | 4138 | 638,5 | 11,8 | 1,004 | H5A034 C | sample | Hm Drgbl | CPE002DT | CPE002EJ | CPE002F9 | NM 00329 | NR2C2 | nuclear receptor s | 7182 |
| 164 | G5 | G6 | 63 | 1229 | 744 | 596,1 | 11,8 | 1,004 | H5A034 C | sample | Hm Drgbl | CPE002DT | CPE002EJ | CPE002F9 | NM 00037 | VDR | vitamin D (1,25- d | 7421 |
| 1942 | A7 | A8 | 1654 | 763,6 | 19568 | 461,9 | 11,8 | 1,026 | H5A034 C | sample | Hm Drgbl | CPE002FX | CPE002G1 | CPE002G5 | NM 19432 | ZNF30 | zinc finger protein | 90075 |
| 2000 | D16 | D17 | 1345 | 665 | 15917 | 354 | 11,8 | 1,026 | H5A034 C | sample | Hm Drgbl | CPE002FX | CPE002G1 | CPE002G5 | NM 19852 | ZNF710 | zinc finger protein | 374655 |
| 2093 | H13 | H14 | 1402 | 661,2 | 16604 | 440,6 | 11,8 | 1,026 | H5A034 C | sample | Hm Drgbl | CPE002FX | CPE002G1 | CPE002G5 | NM 14527 | ZNF563 | zinc finger protein | 147837 |
| 2171 | K19 | K20 | 1336 | 633 | 15754 | 364,9 | 11,8 | 1,026 | H5A034 C | sample | Hm Drgbl | CPE002FX | CPE002G1 | CPE002G5 | NM 15232 | ZNF641 | zinc finger protein | 121274 |
| 3886 | B7 | B8 | 1336 | 684 | 15807 | 342,9 | 11,8 | 1,049 | H5A034 C | sample | Hm Drgbl | CPE002DS | CPE002EI | CPE002F8 | NM 00035 | TAT | tyrosine aminotra | 6898 |
| 3887 | B8 | B9 | 1148 | 628,7 | 13595 | 276,3 | 11,8 | 1,049 | H5A034 C | sample | Hm Drgbl | CPE002DS | CPE002EI | CPE002F8 | NM 00291 | REV3L | REV3-like, catalyti | 5980 |
| 4130 | M10 | M11 | 1499 | 665,6 | 17617 | 366,6 | 11,8 | 1,049 | H5A034 C | sample | Hm Drgbl | CPE002DS | CPE002EI | CPE002F8 | NM 00561 | RBL2 | retinoblastoma-li | 5934 |
| 4191 | O22 | O23 | 1623 | 730,1 | 19229 | 499,6 | 11,8 | 1,049 | H5A034 C | sample | Hm Drgbl | CPE002DS | CPE002EI | CPE002F8 | NM 19825 | TERT | telomerase revers | 7015 |
| 4198 | O7 | O8 | 1424 | 638,8 | 16810 | 449,3 | 11,8 | 1,049 | H5A034 C | sample | Hm Drgbl | CPE002DS | CPE002EI | CPE002F8 | NM 00477 | PPARBP | PPAR binding pro | 5469 |
| 11710 | H7 | H8 | 1238 | 756,5 | 14609 | 127,8 | 11,8 | 1,054 | H5A034 C | sample | Hm Gen E | CPE002JA | CPE002NJ | CPE002JZ | NM 00185 | COL19A1 | collagen, type XIX | 1310 |
| 5787 | B11 | B12 | 1510 | 853,1 | 17768 | 635,9 | 11,8 | 1,078 | H5A034 C | sample | Hm Drgbl | CPE002DY | CPE002F1 | CPE002FM | NM 00038 | ALDH3A2 | aldehyde dehydro | 224 |
| 6115 | O4 | O5 | 1175 | 762,7 | 13817 | 400,6 | 11,8 | 1,078 | H5A034 C | sample | Hm Drgbl | CPE002DY | CPE002F1 | CPE002FM | NM 00118 | BACH1 | BTB and CNC hon | 571 |
| 6119 | O8 | O9 | 1619 | 785,7 | 19054 | 570,8 | 11,8 | 1,078 | H5A034 C | sample | Hm Drgbl | CPE002DY | CPE002F1 | CPE002FM | NM 00118 | BAK1 | BCL2-antagonist/k | 578 |
| 1195 | B4 | B5 | 1277 | 698,5 | 15011 | 471,6 | 11,8 | 1,083 | H5A034 C | sample | Hm Drgbl | CPE002FV | CPE002FZ | CPE002G3 | NM 00725 | PNMA2 | paraneoplastic an | 10687 |
| 1327 | H15 | H16 | 27 | 884,5 | 319 | 519,6 | 11,8 | 1,083 | H5A034 C | sample | Hm Drgbl | CPE002FV | CPE002FZ | CPE002G3 | NM 00382 | NAPA | N-ethylmaleimide | 8775 |
| 1404 | K2 | K3 | 984 | 769,7 | 11608 | 384 | 11,8 | 1,083 | H5A034 C | sample | Hm Drgbl | CPE002FV | CPE002FZ | CPE002G3 | NM 00588 | DNAJA2 | DnaJ (Hsp40) hom | 10294 |
| 1435 | L4 | L5 | 1453 | 842,8 | 17084 | 498,6 | 11,8 | 1,083 | H5A034 C | sample | Hm Drgbl | CPE002FV | CPE002FZ | CPE002G3 | NM 01496 | TRAK1 | trafficking protein | 22906 |
| 1489 | O1 | O2 | 491 | 765,1 | 5809 | 454,1 | 11,8 | 1,083 | H5A034 C | sample | Hm Drgbl | CPE002FV | CPE002FZ | CPE002G3 | NM 00104 | ZNF205 | zinc finger protein | 7755 |
| 16207 | D15 | D16 | 1466 | 942,4 | 17363 | 289,1 | 11,8 | 1,083 | H5A034 C | sample | Hm Gen E | CPE002JG | CPE002NV | CPE002KB | NM 03194 | CDCA7 | cell division cycle | 83879 |
| 16209 | D17 | D18 | 1105 | 999,9 | 13029 | 307,4 | 11,8 | 1,083 | H5A034 C | sample | Hm Gen E | CPE002JG | CPE002NV | CPE002KB | NM 03194 | TSPAN10 | tetraspanin 10 | 83882 |
| 13740 | M2 | M3 | 928 | 1016 | 10905 | 165 | 11,8 | 1,108 | H5A034 C | sample | Hm Gen E | CPE002JC | CPE002NC | CPE002K4 | NM 01560 | TOR1AIP1 | torsin A interactin | 26092 |
| 13800 | O9 | O10 | 1249 | 1030 | 14693 | 131,1 | 11,8 | 1,108 | H5A034 C | sample | Hm Gen E | CPE002JC | CPE002NC | CPE002K4 | NM 02111 | SEZ6L | seizure related 6 | 23544 |
| 834 | C3 | C4 | 1413 | 801,5 | 16704 | 463,2 | 11,8 | 1,118 | H5A034 C | sample | Hm Drgbl | CPE002FU | CPE002FY | CPE002G2 | NM 00116 | APBA1 | amyloid beta (A4) | 320 |
| 973 | I20 | I21 | 1498 | 701,7 | 17608 | 295 | 11,8 | 1,118 | H5A034 C | sample | Hm Drgbl | CPE002FU | CPE002FY | CPE002G2 | NM 00258 | PBX1 | pre-B-cell leukem | 5087 |
| 1095 | N22 | N23 | 1351 | 809,3 | 15980 | 445,4 | 11,8 | 1,118 | H5A034 C | sample | Hm Drgbl | CPE002FU | CPE002FY | CPE002G2 | NM 01451 | UBP1 | upstream binding | 7342 |
| 3741 | L6 | L7 | 1164 | 1384 | 13744 | 380,3 | 11,8 | 1,135 | H5A034 C | sample | Hm Drgbl | CPE002DR | CPE002EH | CPE002F7 | NM 01637 | GPR175 | G protein-couple | 131601 |
| 3743 | L8 | L9 | 1462 | 1262 | 17269 | 340,4 | 11,8 | 1,135 | H5A034 C | sample | Hm Drgbl | CPE002DR | CPE002EH | CPE002F7 | NM 19425 | GPR151 | G protein-couple | 134391 |
| 3792 | N9 | N10 | 1631 | 1310 | 19185 | 361,3 | 11,8 | 1,135 | H5A034 C | sample | Hm Drgbl | CPE002DR | CPE002EH | CPE002F7 | NM 00617 | NPY5R | neuropeptide Y re | 4889 |
| 8195 | F19 | F20 | 1748 | 603,7 | 20568 | 466,9 | 11,8 | 1,135 | H5A034 C | sample | Hm Drgbl | CPE002E5 | CPE002EU | CPE002FF | NM 01493 | NLGN1 | neuroligin 1 | 22871 |
| 8281 | J1 | J2 | 1741 | 613,6 | 20464 | 461,8 | 11,8 | 1,135 | H5A034 C | sample | Hm Drgbl | CPE002E5 | CPE002EU | CPE002FF | NM 01673 | RALY | RNA binding prot | 22913 |
| 8313 | K17 | K18 | 1283 | 633,5 | 15135 | 545,5 | 11,8 | 1,135 | H5A034 C | sample | Hm Drgbl | CPE002E5 | CPE002EU | CPE002FF | NM 00720 | AKAP10 | A kinase (PRKA) a | 11216 |
| 8342 | L21 | L22 | 1983 | 687,2 | 23398 | 456,4 | 11,8 | 1,135 | H5A034 C | sample | Hm Drgbl | CPE002E5 | CPE002EU | CPE002FF | NM 01497 | ACIN1 | apoptotic chroma | 22985 |
| 8408 | O16 | O17 | 606 | 735,6 | 7175 | 425,9 | 11,8 | 1,135 | H5A034 C | sample | Hm Drgbl | CPE002E5 | CPE002EU | CPE002FF | NM 00108 | SR140 | U2-associated SR1 | 23350 |
| 10234 | K18 | K19 | 1915 | 853,9 | 22589 | 149,6 | 11,8 | 1,135 | H5A034 C | sample | Hm Drgbl | CPE002EA | CPE002EP | CPE002FA | NM 05284 | OBSCN | obscurin, cytoske | 84033 |
| 10335 | O22 | O23 | 1765 | 844,2 | 20877 | 162,2 | 11,8 | 1,135 | H5A034 C | sample | Hm Drgbl | CPE002EA | CPE002EP | CPE002FA | NM 03230 | HSDL2 | hydroxysteroid de | 84263 |
| 1639 | E15 | E16 | 1507 | 718,7 | 17840 | 409 | 11,8 | 1,236 | H5A034 C | sample | Hm Drgbl | CPE002FW | CPE002G0 | CPE002G4 | NM 01441 | ZBTB11 | zinc finger and BT | 27107 |
| 1722 | H3 | H4 | 1446 | 692,1 | 17049 | 400 | 11,8 | 1,236 | H5A034 C | sample | Hm Drgbl | CPE002FW | CPE002G0 | CPE002G4 | NM 01816 | DNAJC17 | DnaJ (Hsp40) hom | 55192 |
| 1828 | M12 | M13 | 1548 | 678,2 | 18281 | 368,9 | 11,8 | 1,236 | H5A034 C | sample | Hm Drgbl | CPE002FW | CPE002G0 | CPE002G4 | NM 20347 | PORCN | porcupine homol | 64840 |
| 1838 | M21 | M22 | 1533 | 712,9 | 18082 | 421,7 | 11,8 | 1,236 | H5A034 C | sample | Hm Drgbl | CPE002FW | CPE002G0 | CPE002G4 | NM 01664 | ZNF771 | zinc finger protein | 51333 |
| 1897 | P1 | P2 | 1364 | 689 | 16113 | 426,5 | 11,8 | 1,236 | H5A034 C | sample | Hm Drgbl | CPE002FW | CPE002G0 | CPE002G4 | NM 02011 | FSTL5 | follistatin-like 5 | 56884 |
| 21605 | E13 | E14 | 278 | 1049 | 3286 | 670,6 | 11,8 | 1,242 | H5A034 C | sample | Hm Gen E | CPE002JU | CPE002O9 | CPE002KP | XM 93832 | LOC64926 | similar to zinc fing | 649267 |
| 21658 | G18 | G19 | 282 | 968,6 | 3314 | 582 | 11,8 | 1,242 | H5A034 C | sample | Hm Gen E | CPE002JU | CPE002O9 | CPE002KP | XM 94225 | LOC65267 | similar to Recomb | 652675 |
| 21871 | P15 | P16 | 362 | 1122 | 4262 | 603,3 | 11,8 | 1,242 | H5A034 C | sample | Hm Gen E | CPE002JU | CPE002O9 | CPE002KP | XM 94171 | LOC65229 | similar to glutama | 652292 |
| 4630 | A7 | A8 | 1219 | 863,6 | 14331 | 426,5 | 11,8 | 1,297 | H5A034 C | sample | Hm Drgbl | CPE002DV | CPE002EL | CPE002FP | NM 00717 | PRSS23 | protease, serine, | 11098 |
| 4631 | A8 | A9 | 1389 | 819,6 | 16323 | 533 | 11,8 | 1,297 | H5A034 C | sample | Hm Drgbl | CPE002DV | CPE002EL | CPE002FP | NM 14891 | PSMB8 | proteasome (pros | 5696 |
| 4651 | B4 | B5 | 1601 | 796 | 18891 | 488,8 | 11,8 | 1,297 | H5A034 C | sample | Hm Drgbl | CPE002DV | CPE002EL | CPE002FP | NM 00661 | MASP2 | mannan-binding l | 10747 |
| 4676 | C5 | C6 | 998 | 794,7 | 11750 | 479,1 | 11,8 | 1,297 | H5A034 C | sample | Hm Drgbl | CPE002DV | CPE002EL | CPE002FP | NM 00518 | CAPN1 | calpain 1, (mu/I) l | 823 |
| 4725 | E6 | E7 | 1200 | 841 | 14143 | 611,2 | 11,8 | 1,297 | H5A034 C | sample | Hm Drgbl | CPE002DV | CPE002EL | CPE002FP | NM 00339 | XPNPEP2 | X-prolyl aminopep | 7512 |
| 4991 | P8 | P9 | 1707 | 776,6 | 20149 | 526,3 | 11,8 | 1,297 | H5A034 C | sample | Hm Drgbl | CPE002DV | CPE002EL | CPE002FP | NM 02509 | USP36 | ubiquitin specific | 57602 |
| 10423 | C15 | C16 | 1326 | 834,2 | 15625 | 186,4 | 11,8 | 1,333 | H5A034 C | sample | Hm Drgbl | CPE002EB | CPE002EO | CPE002FR | NM 03286 | SERAC1 | serine active site | 84947 |
| 10430 | C21 | C22 | 1031 | 879 | 12167 | 188,6 | 11,8 | 1,333 | H5A034 C | sample | Hm Drgbl | CPE002EB | CPE002EO | CPE002FR | NM 03287 | JUB | jub, ajuba homolo | 84962 |
| 10431 | C22 | C23 | 1141 | 948,7 | 13421 | 265,7 | 11,8 | 1,333 | H5A034 C | sample | Hm Drgbl | CPE002EB | CPE002EO | CPE002FR | XM 00113 | MST101 | MSTP101 | 114825 |
| 10683 | N11 | N12 | 1519 | 789,3 | 17992 | 171,4 | 11,8 | 1,333 | H5A034 C | sample | Hm Drgbl | CPE002EB | CPE002EO | CPE002FR | NM 05819 | RPUSD1 | RNA pseudouridy | 113000 |
| 2835 | G11 | G12 | 906 | 628,9 | 10682 | 462,3 | 11,8 | 1,388 | H5A034 C | sample | Hm Drgbl | CPE002DP | CPE002EF | CPE002F5 | NM 00394 | RPS6KA4 | ribosomal protein | 8986 |
| 2839 | G15 | G16 | 689 | 606,1 | 8112 | 483,4 | 11,8 | 1,388 | H5A034 C | sample | Hm Drgbl | CPE002DP | CPE002EF | CPE002F5 | NM 00395 | MAP3K14 | mitogen-activated | 9020 |
| 2866 | H18 | H19 | 834 | 582 | 9873 | 489,5 | 11,8 | 1,388 | H5A034 C | sample | Hm Drgbl | CPE002DP | CPE002EF | CPE002F5 | NM 14500 | STK32A | serine/threonine | 202374 |
| 2889 | I17 | I18 | 259 | 769,4 | 3060 | 564,6 | 11,8 | 1,388 | H5A034 C | sample | Hm Drgbl | CPE002DP | CPE002EF | CPE002F5 | NM 00474 | MAGI1 | membrane associ | 9223 |
| 20006 | B21 | B22 | 1119 | 1109 | 13187 | 227,3 | 11,8 | 1,439 | H5A034 C | sample | Hm Gen E | CPE002JQ | CPE002O5 | CPE002KL | NM 21255 | METTL10 | methyltransferase | 399818 |

| | | | | | | | | | | |
|---|---|---|---|---|---|---|---|---|---|---|
| 7771 | D4 | D5 | 859 | 517 | 10127 | 412,1 | 11,8 | 1,802 | H5A034 | RAB10 |
| 7923 | K11 | K12 | 895 | 552,5 | 10584 | 392,1 | 11,8 | 1,802 | H5A034 | STUB1 |
| 3183 | E22 | E23 | 1491 | 1421 | 17766 | 411,8 | 11,9 | 0,737 | H5A034 | DUSP21 |
| 3214 | F7 | F8 | 576 | 1251 | 6830 | 405,4 | 11,9 | 0,737 | H5A034 | PTPRM |
| 18258 | I3 | I4 | 1533 | 996,2 | 18187 | 338,7 | 11,9 | 0,765 | H5A034 | TMEM65 |
| 6158 | A21 | A22 | 1725 | 576,4 | 20468 | 440,5 | 11,9 | 0,785 | H5A034 | CD1D |
| 6182 | B21 | B22 | 1405 | 502,2 | 16739 | 449 | 11,9 | 0,785 | H5A034 | RCC1 |
| 6463 | N15 | N16 | 1806 | 600,4 | 21402 | 424 | 11,9 | 0,785 | H5A034 | CREBBP |
| 20675 | N19 | N20 | 1213 | 770,7 | 14477 | 145,4 | 11,9 | 0,793 | H5A034 | OR14J1 |
| 19321 | F1 | F2 | 926 | 1145 | 10975 | 268,9 | 11,9 | 0,844 | H5A034 | LOC34008 |
| 20775 | B22 | B23 | 1684 | 949,8 | 20043 | 233,7 | 11,9 | 0,844 | H5A034 | LOC64477 |
| 14264 | C16 | C17 | 1327 | 845,1 | 15745 | 136,5 | 11,9 | 0,847 | H5A034 | MRPL50 |
| 14370 | G3 | G4 | 1014 | 793,8 | 12073 | 145 | 11,9 | 0,847 | H5A034 | WNT16 |
| 12426 | F3 | F4 | 1260 | 1039 | 15026 | 143 | 11,9 | 0,895 | H5A034 | CLIP2 |
| 10925 | H13 | H14 | 835 | 814,1 | 9941 | 424,1 | 11,9 | 0,902 | H5A034 | MBOAT1 |
| 17175 | L22 | L23 | 1919 | 987,7 | 22857 | 238,6 | 11,9 | 0,919 | H5A034 | FLJ25006 |
| 7163 | K19 | K20 | 1669 | 579,8 | 19938 | 377 | 11,9 | 0,926 | H5A034 | LTB |
| 7244 | N5 | N6 | 1025 | 675,6 | 12164 | 506,6 | 11,9 | 0,926 | H5A034 | MLLT4 |
| 5097 | E17 | E18 | 1864 | 598,5 | 22219 | 399,3 | 11,9 | 0,933 | H5A034 | USP44 |
| 5150 | G21 | G22 | 2123 | 676,3 | 25265 | 476,7 | 11,9 | 0,933 | H5A034 | SEC11C |
| 2482 | H18 | H19 | 1042 | 721,9 | 12434 | 323,9 | 11,9 | 0,937 | H5A034 | PTK2 |
| 18001 | O1 | O2 | 829 | 970,1 | 9845 | 281,4 | 11,9 | 0,941 | H5A034 | C13orf30 |
| 8981 | G13 | G14 | 2075 | 1868 | 24666 | 131,9 | 11,9 | 0,956 | H5A034 | CDON |
| 4327 | E15 | E16 | 1399 | 660,7 | 16672 | 301,1 | 11,9 | 1,004 | H5A034 | HCN2 |
| 4374 | G14 | G15 | 788 | 677,1 | 9391 | 409,1 | 11,9 | 1,004 | H5A034 | KCNE2 |
| 4424 | I16 | I17 | 1333 | 798,9 | 15877 | 411,6 | 11,9 | 1,004 | H5A034 | KCNH4 |
| 4521 | M17 | M18 | 1812 | 675,9 | 21570 | 383,2 | 11,9 | 1,004 | H5A034 | GABRA5 |
| 75 | D11 | D12 | 382 | 1563 | 4529 | 451,7 | 11,9 | 1,013 | H5A034 | SFRS5 |
| 86 | D21 | D22 | 607 | 1346 | 7224 | 506,8 | 11,9 | 1,013 | H5A034 | SFTPA2B |
| 96 | D9 | D10 | 406 | 1521 | 4814 | 481,2 | 11,9 | 1,013 | H5A034 | SFRS4 |
| 97 | E1 | E2 | 273 | 1397 | 3258 | 559,3 | 11,9 | 1,013 | H5A034 | RORA |
| 320 | N16 | N17 | 675 | 1561 | 8021 | 457,1 | 11,9 | 1,013 | H5A034 | ABCC8 |
| 331 | N4 | N5 | 536 | 1496 | 6379 | 513 | 11,9 | 1,013 | H5A034 | SUOX |
| 351 | O22 | O23 | 489 | 1704 | 5809 | 645,2 | 11,9 | 1,013 | H5A034 | SOX15 |
| 1957 | B20 | B21 | 1540 | 724,7 | 18268 | 412,1 | 11,9 | 1,035 | H5A034 | ZNF81 |
| 1975 | C15 | C16 | 1675 | 639,4 | 19977 | 409,3 | 11,9 | 1,035 | H5A034 | ZNF502 |
| 2125 | I20 | I21 | 1754 | 757,1 | 20959 | 351,5 | 11,9 | 1,035 | H5A034 | ZNF311 |
| 2132 | I5 | I6 | 1201 | 677 | 14274 | 399,4 | 11,9 | 1,035 | H5A034 | C1QTNF2 |
| 2163 | K11 | K12 | 1448 | 688,7 | 17202 | 440,1 | 11,9 | 1,035 | H5A034 | ZMYND17 |
| 2229 | M6 | M7 | 2147 | 716,9 | 25509 | 392,7 | 11,9 | 1,035 | H5A034 | DNAJC5G |
| 2252 | N5 | N6 | 1117 | 686,8 | 13272 | 441,1 | 11,9 | 1,035 | H5A034 | FAM84B |
| 6906 | P3 | P4 | 1534 | 672,5 | 18210 | 416 | 11,9 | 1,044 | H5A034 | IBSP |
| 3927 | D22 | D23 | 1229 | 694,6 | 14635 | 433,1 | 11,9 | 1,058 | H5A034 | RGS16 |
| 3950 | E21 | E22 | 1276 | 758,5 | 15166 | 409,1 | 11,9 | 1,058 | H5A034 | PLS1 |
| 3980 | F5 | F6 | 1513 | 700,7 | 18038 | 421,8 | 11,9 | 1,058 | H5A034 | PROS1 |
| 4166 | N21 | N22 | 1161 | 732,4 | 13836 | 425,2 | 11,9 | 1,058 | H5A034 | PTGS2 |
| 11664 | F9 | F10 | 1261 | 847,1 | 14990 | 132 | 11,9 | 1,062 | H5A034 | COL4A1 |
| 11808 | L9 | L10 | 1716 | 861,3 | 20380 | 126,5 | 11,9 | 1,062 | H5A034 | CSN3 |
| 15373 | A20 | A21 | 1619 | 855 | 19253 | 164,5 | 11,9 | 1,082 | H5A034 | UPF3A |
| 15570 | I3 | I4 | 1719 | 893,5 | 20531 | 145,8 | 11,9 | 1,082 | H5A034 | C20orf177 |
| 15649 | M1 | M2 | 1281 | 811,2 | 15263 | 135,5 | 11,9 | 1,082 | H5A034 | NCAPG |
| 5774 | A21 | A22 | 1617 | 916,9 | 19256 | 537 | 11,9 | 1,087 | H5A034 | ABCF1 |
| 6110 | O21 | O22 | 1888 | 795,6 | 22522 | 675,1 | 11,9 | 1,087 | H5A034 | ALDH2 |
| 6141 | P6 | P7 | 1489 | 845,7 | 17670 | 464 | 11,9 | 1,087 | H5A034 | CBR1 |
| 1224 | C9 | C10 | 926 | 767,3 | 11023 | 462,2 | 11,9 | 1,092 | H5A034 | ZNF14 |
| 1338 | H3 | H4 | 1531 | 688 | 18199 | 347,4 | 11,9 | 1,092 | H5A034 | STX16 |
| 1378 | J18 | J19 | 1524 | 699,5 | 18183 | 357,3 | 11,9 | 1,092 | H5A034 | NTNG1 |
| 1379 | J19 | J20 | 1844 | 706,7 | 21892 | 353,1 | 11,9 | 1,092 | H5A034 | CLDN6 |
| 1380 | J2 | J3 | 1046 | 751,3 | 12445 | 381,5 | 11,9 | 1,092 | H5A034 | IKZF2 |
| 1472 | N16 | N17 | 1352 | 704,2 | 16080 | 453,6 | 11,9 | 1,092 | H5A034 | DTX4 |
| 1479 | N22 | N23 | 1449 | 775,6 | 17171 | 449,3 | 11,9 | 1,092 | H5A034 | EXOC7 |
| 16142 | A21 | A22 | 1259 | 1033 | 15029 | 355,1 | 11,9 | 1,092 | H5A034 | SNX27 |
| 16182 | C14 | C15 | 1369 | 1051 | 16358 | 409,2 | 11,9 | 1,092 | H5A034 | MAF1 |
| 16422 | M14 | M15 | 1851 | 966,3 | 21968 | 224,6 | 11,9 | 1,092 | H5A034 | C3orf26 |
| 16429 | M20 | M21 | 1472 | 1061 | 17513 | 260,9 | 11,9 | 1,092 | H5A034 | ZBED3 |
| 16460 | N5 | N6 | 1219 | 1066 | 14465 | 346,7 | 11,9 | 1,092 | H5A034 | C4orf17 |
| 887 | E8 | E9 | 1319 | 777,3 | 15653 | 465,9 | 11,9 | 1,128 | H5A034 | MNDA |
| 987 | J11 | J12 | 1123 | 696,4 | 13395 | 349,4 | 11,9 | 1,128 | H5A034 | DNAJA1 |
| 1056 | L9 | L10 | 745 | 759,9 | 8856 | 416,7 | 11,9 | 1,128 | H5A034 | HSPB1 |
| 1061 | M13 | M14 | 1019 | 793,6 | 12128 | 426,3 | 11,9 | 1,128 | H5A034 | VCAN |
| 1067 | M19 | M20 | 1125 | 717,8 | 13332 | 413,5 | 11,9 | 1,128 | H5A034 | DPP6 |
| 1113 | O17 | O18 | 1362 | 698 | 16210 | 430,6 | 11,9 | 1,128 | H5A034 | ETV1 |
| 1148 | P5 | P6 | 1644 | 688,2 | 19604 | 454 | 11,9 | 1,128 | H5A034 | LAMA3 |
| 5664 | L9 | L10 | 1597 | 645,6 | 18945 | 522,2 | 11,9 | 1,133 | H5A034 | TNFRSF25 |
| 5671 | M15 | M16 | 1137 | 573,1 | 13583 | 490,1 | 11,9 | 1,133 | H5A034 | AGPS |
| 5683 | M4 | M5 | 1260 | 783,9 | 15007 | 324,9 | 11,9 | 1,133 | H5A034 | MPZL1 |
| 5710 | N7 | N8 | 1643 | 608,2 | 19495 | 545,6 | 11,9 | 1,133 | H5A034 | TNFRSF14 |
| 5732 | O5 | O6 | 1588 | 762,3 | 18906 | 602,5 | 11,9 | 1,133 | H5A034 | DEGS1 |
| 5754 | P3 | P4 | 1650 | 738,2 | 19585 | 667,7 | 11,9 | 1,133 | H5A034 | TNFRSF11 |
| 3495 | B22 | B23 | 948 | 1522 | 11274 | 358 | 11,9 | 1,144 | H5A034 | GPRC5C |
| 3566 | E21 | E22 | 1381 | 1457 | 16422 | 445,1 | 11,9 | 1,144 | H5A034 | CASR |
| 3683 | J19 | J20 | 1554 | 1422 | 18511 | 321,2 | 11,9 | 1,144 | H5A034 | HTR5A |
| 8097 | B17 | B18 | 1480 | 747,7 | 17657 | 359,4 | 11,9 | 1,144 | H5A034 | CBX3 |
| 8103 | B22 | B23 | 1527 | 676,3 | 18134 | 444,3 | 11,9 | 1,144 | H5A034 | EXOSC2 |
| 8138 | D10 | D11 | 1481 | 603,7 | 17557 | 512,7 | 11,9 | 1,144 | H5A034 | SIRT1 |
| 8151 | D22 | D23 | 1553 | 631,1 | 18424 | 490,4 | 11,9 | 1,144 | H5A034 | GRIP1 |
| 8263 | I15 | I16 | 892 | 710,3 | 10631 | 180,3 | 11,9 | 1,144 | H5A034 | RASSF1 |
| 1578 | B3 | B4 | 996 | 675,1 | 11898 | 412,5 | 11,9 | 1,246 | H5A034 | CABP2 |
| 1605 | C6 | C7 | 1070 | 711,6 | 12683 | 437,7 | 11,9 | 1,246 | H5A034 | ZNF398 |
| 1640 | E16 | E17 | 2093 | 813,3 | 24898 | 420,3 | 11,9 | 1,246 | H5A034 | ZFP14 |
| 1692 | G2 | G3 | 1424 | 745,1 | 16980 | 487,8 | 11,9 | 1,246 | H5A034 | ZFYVE28 |
| 1764 | J2 | J3 | 766 | 760,1 | 9149 | 379,7 | 11,9 | 1,246 | H5A034 | CALN1 |
| 1766 | J21 | J22 | 1150 | 752,1 | 13636 | 356,5 | 11,9 | 1,246 | H5A034 | VPS35 |
| 1869 | N6 | N7 | 1085 | 627 | 12944 | 422,8 | 11,9 | 1,246 | H5A034 | ZBTB37 |
| 21542 | B21 | B22 | 313 | 1139 | 3724 | 400,4 | 11,9 | 1,253 | H5A034 | LOC65088 |
| 21647 | F8 | F9 | 209 | 909,9 | 2496 | 534,1 | 11,9 | 1,253 | H5A034 | FAM153C |
| 21795 | M11 | M12 | 270 | 815,8 | 3207 | 534 | 11,9 | 1,253 | H5A034 | LOC65021 |
| 4681 | D1 | D2 | 1278 | 777,2 | 15183 | 503,5 | 11,9 | 1,308 | H5A034 | HABP2 |
| 4785 | H17 | H18 | 1607 | 721,7 | 19067 | 356,4 | 11,9 | 1,308 | H5A034 | MMP11 |
| 4819 | I4 | I5 | 1455 | 852,3 | 17265 | 518 | 11,9 | 1,308 | H5A034 | ADAM7 |
| 4952 | O16 | O17 | 936 | 760,5 | 11148 | 483,8 | 11,9 | 1,308 | H5A034 | PITRM1 |
| 10575 | I22 | I23 | 1473 | 900 | 17478 | 198,8 | 11,9 | 1,345 | H5A034 | BATF2 |
| 10617 | K17 | K18 | 1498 | 808,9 | 17822 | 179,2 | 11,9 | 1,345 | H5A034 | LHX4 |
| 2822 | F21 | F22 | 1329 | 673,2 | 15766 | 571,4 | 11,9 | 1,4 | H5A034 | PNKP |
| 2844 | G2 | G3 | 650 | 698,2 | 7725 | 648,1 | 11,9 | 1,4 | H5A034 | TEX14 |
| 3055 | P15 | P16 | 1486 | 661,9 | 17693 | 587,1 | 11,9 | 1,4 | H5A034 | MINK1 |
| 20003 | B19 | B20 | 1419 | 1137 | 16896 | 183,4 | 11,9 | 1,451 | H5A034 | C10orf120 |
| 20264 | M16 | M17 | 751 | 1031 | 8924 | 167,3 | 11,9 | 1,451 | H5A034 | C9orf139 |
| 3278 | I21 | I22 | 1546 | 1343 | 18626 | 345,5 | 12 | 0,743 | H5A034 | NT5E |
| 3325 | K20 | K21 | 1387 | 1357 | 16665 | 299,3 | 12 | 0,743 | H5A034 | PPM1L |
| 18252 | I2 | I3 | 1090 | 1064 | 13066 | 361,7 | 12 | 0,772 | H5A034 | PSORS1C1 |
| 20496 | F9 | F10 | 1111 | 738,1 | 13319 | 154 | 12 | 0,8 | H5A034 | FAM22G |
| 20673 | N17 | N18 | 1001 | 789,3 | 12029 | 170,9 | 12 | 0,8 | H5A034 | OR2B3P |
| 9403 | H4 | H5 | 1613 | 1145 | 19323 | 141,6 | 12 | 0,833 | H5A034 | WRNIP1 |
| 19160 | O16 | O17 | 1497 | 1087 | 17951 | 277,1 | 12 | 0,836 | H5A034 | LOC28519 |
| 21022 | L7 | L8 | 856 | 978,5 | 10237 | 269,8 | 12 | 0,851 | H5A034 | CBLN3 |
| 16935 | B22 | B23 | 2214 | 1075 | 26493 | 209 | 12 | 0,927 | H5A034 | OR4L1 |
| 17161 | L1 | L2 | 1398 | 948,8 | 16768 | 176,9 | 12 | 0,927 | H5A034 | WDR92 |
| 5037 | B6 | B7 | 1677 | 662,1 | 20090 | 593,6 | 12 | 0,941 | H5A034 | UNG |
| 5054 | C21 | C22 | 2235 | 776,8 | 26750 | 437,5 | 12 | 0,941 | H5A034 | HM13 |
| 5098 | E18 | E19 | 1335 | 586,6 | 15969 | 531,9 | 12 | 0,941 | H5A034 | TSG101 |
| 5275 | L4 | L5 | 1636 | 635,9 | 19598 | 372,3 | 12 | 0,941 | H5A034 | ZNF24 |
| 5375 | P8 | P9 | 1309 | 616,3 | 15770 | 605,6 | 12 | 0,941 | H5A034 | SLC30A1 |
| 2423 | E8 | E9 | 855 | 641,5 | 10296 | 405 | 12 | 0,945 | H5A034 | SCGB2A1 |
| 2515 | I4 | I5 | 970 | 758,8 | 11648 | 447,6 | 12 | 0,945 | H5A034 | NTRK3 |
| 2556 | K2 | K3 | 1117 | 713,5 | 13384 | 411,8 | 12 | 0,945 | H5A034 | PCTK3 |
| 2565 | K6 | K7 | 988 | 730,9 | 11812 | 378,7 | 12 | 0,945 | H5A034 | PDGFRB |
| 2577 | L17 | L18 | 1378 | 713,4 | 16493 | 356,8 | 12 | 0,945 | H5A034 | EPHB3 |
| 2666 | P10 | P11 | 996 | 715,2 | 11955 | 438,4 | 12 | 0,945 | H5A034 | TGFBR2 |
| 17761 | E1 | E2 | 843 | 1026 | 10114 | 220,4 | 12 | 0,949 | H5A034 | ASB14 |
| 17980 | N12 | N13 | 1756 | 1076 | 21145 | 160,8 | 12 | 0,949 | H5A034 | FLJ30277 |
| 8929 | E1 | E2 | 1950 | 1706 | 23447 | 161,3 | 12 | 0,964 | H5A034 | CHST11 |
| 9003 | H11 | H12 | 1169 | 1358 | 14050 | 151,8 | 12 | 0,964 | H5A034 | PLUNC |
| 9134 | M21 | M22 | 2189 | 1902 | 26238 | 168,6 | 12 | 0,964 | H5A034 | ZDHHC9 |
| 4414 | H7 | H8 | 1181 | 745,8 | 14120 | 500,6 | 12 | 1,013 | H5A034 | KCNE1 |
| 70 | C7 | C8 | 184 | 1347 | 2199 | 300,1 | 12 | 1,021 | H5A034 | NR4A2 |
| 162 | G3 | G4 | 251 | 1201 | 3014 | 578,2 | 12 | 1,021 | H5A034 | NR1H2 |
| 229 | J20 | J21 | 709 | 1504 | 8516 | 486 | 12 | 1,021 | H5A034 | STAT3 |
| 269 | L13 | L14 | 223 | 1389 | 2673 | 775,1 | 12 | 1,021 | H5A034 | SLC2A5 |
| 303 | M22 | M23 | 341 | 1608 | 4080 | 579,6 | 12 | 1,021 | H5A034 | SORL1 |
| 361 | P1 | P2 | 619 | 1634 | 7438 | 649,7 | 12 | 1,021 | H5A034 | SLC6A8 |
| 1948 | B12 | B13 | 1960 | 770,1 | 23534 | 482,4 | 12 | 1,043 | H5A034 | ZNF677 |
| 2100 | H2 | H3 | 1494 | 712,5 | 17960 | 479,7 | 12 | 1,043 | H5A034 | ZNF662 |
| 2179 | K4 | K5 | 1827 | 697,5 | 21883 | 428,9 | 12 | 1,043 | H5A034 | LAMA1 |
| 2269 | O20 | O21 | 1520 | 705,3 | 18212 | 441,5 | 12 | 1,043 | H5A034 | ZNF680 |
| 2303 | P8 | P9 | 2014 | 790,4 | 24161 | 434,9 | 12 | 1,043 | H5A034 | |
| 6584 | C16 | C17 | 962 | 607,5 | 11565 | 338 | 12 | 1,053 | H5A034 | RBPJ |
| 6653 | F13 | F14 | 997 | 571,1 | 11981 | 180,2 | 12 | 1,053 | H5A034 | HOXB5 |
| 6667 | F4 | F5 | 1194 | 588,1 | 14342 | 468,5 | 12 | 1,053 | H5A034 | IVD |
| 3941 | E13 | E14 | 1125 | 693,5 | 13525 | 313,2 | 12 | 1,067 | H5A034 | TAP1 |
| 4018 | H18 | H19 | 986 | 665,2 | 11836 | 316,2 | 12 | 1,067 | H5A034 | ROM1 |
| 4026 | H3 | H4 | 1345 | 777,1 | 16183 | 409,1 | 12 | 1,067 | H5A034 | TBXAS1 |
| 4070 | J21 | J22 | 958 | 610,1 | 11488 | 358,4 | 12 | 1,067 | H5A034 | TDG |
| 4099 | K4 | K5 | 1270 | 634,7 | 15228 | 374,5 | 12 | 1,067 | H5A034 | TEP1 |
| 4102 | K7 | K8 | 1117 | 636,7 | 13364 | 359,7 | 12 | 1,067 | H5A034 | POLG |
| 4142 | M21 | M22 | 1557 | 646,4 | 18685 | 378,8 | 12 | 1,067 | H5A034 | POR |
| 11864 | O16 | O17 | 1331 | 844,8 | 16015 | 137,7 | 12 | 1,071 | H5A034 | GOLGA1 |
| 11889 | P17 | P18 | 876 | 822,5 | 10548 | 143,3 | 12 | 1,071 | H5A034 | DMWD |
| 15440 | D16 | D17 | 1034 | 809,7 | 12393 | 142,7 | 12 | 1,091 | H5A034 | C1orf35 |
| 5804 | B5 | B6 | 1409 | 791,4 | 16936 | 610,6 | 12 | 1,096 | H5A034 | ALDH3B1 |
| 5819 | C19 | C20 | 1638 | 806,7 | 19606 | 580,6 | 12 | 1,096 | H5A034 | ACADS |
| 5871 | E22 | E23 | 813 | 869 | 9770 | 653,8 | 12 | 1,096 | H5A034 | ART4 |
| 5889 | F17 | F18 | 373 | 887,3 | 4479 | 586,2 | 12 | 1,096 | H5A034 | ANG |
| 5958 | I14 | I15 | 1146 | 871,8 | 13781 | 527,2 | 12 | 1,096 | H5A034 | RERE |
| 1199 | B8 | B9 | 622 | 809,5 | 7448 | 582,4 | 12 | 1,101 | H5A034 | CCT8 |
| 1263 | E22 | E23 | 1606 | 719,1 | 19276 | 385,6 | 12 | 1,101 | H5A034 | ZBED4 |
| 1282 | F18 | F19 | 1011 | 796,3 | 12105 | 427,3 | 12 | 1,101 | H5A034 | DNAJB4 |
| 16172 | B5 | B6 | 1101 | 1012 | 13178 | 431 | 12 | 1,101 | H5A034 | FAM57B |
| 16213 | D20 | D21 | 1583 | 1008 | 19021 | 297,2 | 12 | 1,101 | H5A034 | ACRBP |
| 16333 | I20 | I21 | 1049 | 1107 | 12544 | 230,6 | 12 | 1,101 | H5A034 | GINS4 |
| 16465 | O1 | O2 | 1333 | 1060 | 15990 | 360,5 | 12 | 1,101 | H5A034 | CCDC3 |
| 16467 | O11 | O12 | 1650 | 1062 | 19724 | 487 | 12 | 1,101 | H5A034 | CD99L2 |
| 13589 | G13 | G14 | 1078 | 889,4 | 12903 | 148,2 | 12 | 1,127 | H5A034 | SMG5 |
| 807 | B22 | B23 | 1079 | 750,1 | 12914 | 464,8 | 12 | 1,137 | H5A034 | RING1 |
| 852 | D2 | D3 | 789 | 690,9 | 9468 | 220 | 12 | 1,137 | H5A034 | RNF2 |
| 1007 | J8 | J9 | 1232 | 730,1 | 14839 | 330,8 | 12 | 1,137 | H5A034 | SYT4 |
| 1055 | L8 | L9 | 948 | 884 | 11415 | 423 | 12 | 1,137 | H5A034 | TCP1 |
| 1116 | O2 | O3 | 2035 | 775,7 | 24491 | 433,4 | 12 | 1,137 | H5A034 | POU4F3 |
| 5389 | A20 | A21 | 1926 | 810,8 | 23026 | 547,5 | 12 | 1,143 | H5A034 | LIN7A |
| 5445 | C6 | C7 | 1928 | 721 | 23109 | 633,3 | 12 | 1,143 | H5A034 | CD84 |
| 5522 | G10 | G11 | 1353 | 695,6 | 16280 | 698,5 | 12 | 1,143 | H5A034 | EIF2B2 |
| 5688 | M9 | M10 | 1750 | 698,9 | 20949 | 503,4 | 12 | 1,143 | H5A034 | COPS3 |
| 5712 | N9 | N10 | 2091 | 685,5 | 25118 | 574,2 | 12 | 1,143 | H5A034 | RAB11A |
| 3599 | F8 | F9 | 1570 | 1422 | 18913 | 327,4 | 12 | 1,154 | H5A034 | NPVF |
| 3674 | J10 | J11 | 984 | 1299 | 11855 | 299,7 | 12 | 1,154 | H5A034 | MRGPRD |
| 8088 | A9 | A10 | 1331 | 746,9 | 16030 | 366,6 | 12 | 1,154 | H5A034 | OGFR |
| 8092 | B12 | B13 | 1742 | 584,5 | 20865 | 535,5 | 12 | 1,154 | H5A034 | SRGAP2 |
| 8266 | I18 | I19 | 1031 | 645,8 | 12399 | 493,7 | 12 | 1,154 | H5A034 | PLCL2 |
| 8382 | N14 | N15 | 1896 | 651,2 | 22829 | 494,6 | 12 | 1,154 | H5A034 | SNAPAP |
| 9999 | A22 | A23 | 1296 | 878,9 | 15521 | 152,7 | 12 | 1,154 | H5A034 | DDX59 |
| 10176 | H9 | H10 | 1402 | 815,7 | 16826 | 142,7 | 12 | 1,154 | H5A034 | APOL5 |
| 10177 | I1 | I2 | 1484 | 884 | 17795 | 135,6 | 12 | 1,154 | H5A034 | ZDHHC11 |
| 10179 | I11 | I12 | 1174 | 825,3 | 14104 | 117,8 | 12 | 1,154 | H5A034 | CBLL1 |
| 10316 | N5 | N6 | 1118 | 780,3 | 13377 | 139 | 12 | 1,154 | H5A034 | LOC81691 |
| 1641 | E17 | E18 | 1539 | 781,9 | 18478 | 450,2 | 12 | 1,257 | H5A034 | AFF4 |
| 1698 | G3 | G4 | 1149 | 728,4 | 13756 | 469,4 | 12 | 1,257 | H5A034 | ZNF777 |
| 1845 | M6 | M7 | 950 | 771,6 | 11356 | 444,1 | 12 | 1,257 | H5A034 | SMYD3 |
| 21546 | B3 | B4 | 353 | 982 | 4228 | 605,5 | 12 | 1,263 | H5A034 | LOC65071 |
| 21645 | F6 | F7 | 177 | 961,1 | 2130 | 793,8 | 12 | 1,263 | H5A034 | ASAH2B |
| 21682 | H18 | H19 | 434 | 1077 | 5214 | 730,2 | 12 | 1,263 | H5A034 | LOC65349 |
| 21842 | O10 | O11 | 470 | 1072 | 5634 | 912,6 | 12 | 1,263 | H5A034 | LOC65288 |
| 4645 | B20 | B21 | 1283 | 841,9 | 15418 | 491,4 | 12 | 1,319 | H5A034 | METAP2 |
| 4660 | C12 | C13 | 1499 | 854,3 | 17971 | 473,8 | 12 | 1,319 | H5A034 | TPSAB1 |
| 4730 | F10 | F11 | 1736 | 854,7 | 20860 | 450,2 | 12 | 1,319 | H5A034 | SEC11A |
| 10518 | G14 | G15 | 1433 | 892,1 | 17256 | 201,4 | 12 | 1,356 | H5A034 | FCRL1 |
| 2703 | A22 | A23 | 1502 | 712,7 | 17993 | 486,3 | 12 | 1,412 | H5A034 | RFK |
| 2721 | B17 | B18 | 848 | 724,7 | 10182 | 571,3 | 12 | 1,412 | H5A034 | PMVK |
| 2761 | D1 | D2 | 1632 | 645,5 | 19528 | 566,3 | 12 | 1,412 | H5A034 | NEK6 |
| 2764 | D12 | D13 | 1148 | 652,7 | 13790 | 562,6 | 12 | 1,412 | H5A034 | AK7 |
| 20093 | F13 | F14 | 421 | 1158 | 5051 | 164,7 | 12 | 1,463 | H5A034 | hCG 2652 |
| 20253 | L6 | L7 | 761 | 1029 | 9149 | 135 | 12 | 1,463 | H5A034 | C5orf40 |
| 394 | A18 | A19 | 1901 | 1739 | 22733 | 404,4 | 12 | 1,752 | H5A034 | FOXE1 |
| 3086 | A21 | A22 | 1531 | 1370 | 18505 | 411,7 | 12,1 | 0,749 | H5A034 | ACP2 |
| 3111 | B22 | B23 | 1533 | 1286 | 18500 | 365,8 | 12,1 | 0,749 | H5A034 | PDE9A |
| 3153 | D17 | D18 | 964 | 1160 | 11664 | 397,1 | 12,1 | 0,749 | H5A034 | PTPRE |
| 19603 | A4 | A5 | 618 | 360,1 | 7447 | 458,8 | 12,1 | 0,788 | H5A034 | LOC38881 |
| 20473 | F1 | F2 | 918 | 812,5 | 11121 | 168,9 | 12,1 | 0,807 | H5A034 | ANKRD20 |
| 20555 | I19 | I20 | 1025 | 749,7 | 12428 | 177,5 | 12,1 | 0,807 | H5A034 | LOC44073 |
| 20689 | O1 | O2 | 1027 | 762,4 | 12382 | 154,1 | 12,1 | 0,807 | H5A034 | LOC44101 |
| 13008 | N9 | N10 | 708 | 1159 | 8563 | 138,1 | 12,1 | 0,82 | H5A034 | SPATA2 |
| 17617 | O1 | O2 | 902 | 947,6 | 10880 | 212,8 | 12,1 | 0,826 | H5A034 | C1orf216 |
| 18873 | C17 | C18 | 1169 | 983,4 | 14093 | 226,4 | 12,1 | 0,843 | H5A034 | FAM44A |
| 20876 | F5 | F6 | 1116 | 974,1 | 13451 | 244,8 | 12,1 | 0,858 | H5A034 | LOC64364 |
| 21050 | N10 | N11 | 1303 | 879 | 15734 | 209 | 12,1 | 0,858 | H5A034 | LOC64519 |
| 21450 | N3 | N4 | 799 | 889,1 | 9688 | 159,3 | 12,1 | 0,858 | H5A034 | LOC64645 |
| 14329 | F1 | F2 | 1123 | 917,6 | 13615 | 141,1 | 12,1 | 0,861 | H5A034 | NUB1 |

| | | | | | | | | | | | | | | | | | | | | | | | | | | | | | | | |
|---|---|---|---|---|---|---|---|---|---|---|---|---|---|---|---|---|---|---|---|---|---|---|---|---|---|---|---|---|---|---|---|
| 11302 | G7 | G8 | 905 | 763,6 | 10988 | 155,3 | 12,1 | 0,877 | H5A034 C | sample | Hm Drgbl | Hm Drgbl | Hm Drgbl | CPE002ED | CPE002F3 | CPE002FT | NM 17367 | NM 1736 | NM 1736 | PNPLA1 | PNPLA1 | PNPLA1 | patatin-like phos | patatin-lik | patatin-lik | 285848 | 285848 | 285848 | s50041 | s50042 | s50043 |
| 11378 | K10 | K11 | 1411 | 809,9 | 17092 | 128,8 | 12,1 | 0,877 | H5A034 C | sample | Hm Drgbl | Hm Drgbl | Hm Drgbl | CPE002ED | CPE002F3 | CPE002FT | | | | | | | | | | | | | | | |
| 12385 | E1 | E2 | 1361 | 1017 | 16524 | 138,4 | 12,1 | 0,91 | H5A034 C | sample | Hm Gen E | Hm Gen E | Hm Gen E | CPE002J5 | CPE002NK | CPE002K1 | NM 00315 | NM 0031 | NM 0031 | STIM1 | STIM1 | STIM1 | stromal interactic | stromal in | stromal in | 6786 | 6786 | 6786 | s13561 | s13562 | s13563 |
| 7345 | C1 | C2 | 311 | 593,3 | 3749 | 566 | 12,1 | 0,924 | H5A034 C | sample | Hm Drgbl | Hm Drgbl | Hm Drgbl | CPE002E3 | CPE002EW | CPE002FH | NM 00617 | NM 0061 | NM 0061 | NSF | NSF | NSF | N-ethylmaleimide | N-ethylma | N-ethylma | 4905 | 4905 | 4905 | s182 | s180 | s181 |
| 17199 | M22 | M23 | 1796 | 1012 | 21706 | 231,1 | 12,1 | 0,934 | H5A034 C | sample | Hm Gen E | Hm Gen E | Hm Gen E | CPE002JI | CPE002NZ | CPE002KD | NM 20343 | NM 0010 | NM 2034 | ASCL4 | YDJC | ASCL4 | achaete-scute cor | YdjC hom | achaete-s | 121549 | 150223 | 121549 | s42482 | s195779 | s42484 |
| 7186 | L18 | L19 | 1327 | 571,9 | 16092 | 429,7 | 12,1 | 0,942 | H5A034 C | sample | Hm Drgbl | Hm Drgbl | Hm Drgbl | CPE002E2 | CPE002EX | CPE002FI | NM 01653 | NM 0165 | NM 0165 | NINJ2 | NINJ2 | NINJ2 | ninjurin 2 | ninjurin 2 | ninjurin 2 | 4815 | 4815 | 4815 | s9561 | s9560 | s224118 |
| 5021 | B13 | B14 | 1632 | 639,6 | 19697 | 572,9 | 12,1 | 0,949 | H5A034 C | sample | Hm Drgbl | Hm Drgbl | Hm Drgbl | CPE002DV | CPE002EM | CPE002FO | NM 18260 | NM 1826 | NM 1826 | TMPRSS11 | TMPRSS11 | TMPRSS11 | transmembrane p | transmem | transmem | 339967 | 339967 | 339967 | s50629 | s50630 | s50631 |
| 5163 | H11 | H12 | 1968 | 587,8 | 23822 | 373,7 | 12,1 | 0,949 | H5A034 C | sample | Hm Drgbl | Hm Drgbl | Hm Drgbl | CPE002DV | CPE002EM | CPE002FO | NM 17505 | NM 1750 | NM 1750 | ST8SIA4 | ST8SIA4 | ST8SIA4 | ST8 alpha-N-acety | ST8 alpha | ST8 alpha | 7903 | 7903 | 7903 | s15451 | s15452 | s15453 |
| 5179 | H4 | H5 | 1036 | 639,4 | 12581 | 549,6 | 12,1 | 0,949 | H5A034 C | sample | Hm Drgbl | Hm Drgbl | Hm Drgbl | CPE002DV | CPE002EM | CPE002FO | NM 00339 | NM 0033 | NM 0033 | WNT5A | WNT5A | WNT5A | wingless-type MM | wingless-t | wingless-t | 7474 | 7474 | 7474 | s14871 | s14872 | s14873 |
| 5186 | I10 | I11 | 1483 | 575,4 | 18011 | 341,4 | 12,1 | 0,949 | H5A034 C | sample | Hm Drgbl | Hm Drgbl | Hm Drgbl | CPE002DV | CPE002EM | CPE002FO | NM 00332 | NM 0033 | NM 0033 | TNFRSF4 | TNFRSF4 | TNFRSF4 | tumor necrosis fa | tumor nec | tumor nec | 7293 | 7293 | 7293 | s14531 | s14529 | s14530 |
| 5358 | P14 | P15 | 1226 | 734,6 | 14804 | 392,2 | 12,1 | 0,949 | H5A034 C | sample | Hm Drgbl | Hm Drgbl | Hm Drgbl | CPE002DV | CPE002EM | CPE002FO | NM 01330 | NM 0133 | NM 0133 | SLC30A4 | SLC30A4 | SLC30A4 | solute carrier fam | solute car | solute car | 7782 | 7782 | 7782 | s15337 | s15336 | s15335 |
| 2504 | I16 | I17 | 902 | 659,7 | 10932 | 280 | 12,1 | 0,953 | H5A034 C | sample | Hm Drgbl | Hm Drgbl | Hm Drgbl | CPE002DC | CPE002EE | CPE002F4 | NM 00257 | NM 0025 | NM 0025 | PAK3 | PAK3 | PAK3 | p21 (CDKN1A)-act | p21 (CDKN | p21 (CDKN | 5063 | 5063 | 5063 | s10025 | s10026 | s10027 |
| 2645 | O13 | O14 | 1649 | 696,3 | 19871 | 384,8 | 12,1 | 0,953 | H5A034 C | sample | Hm Drgbl | Hm Drgbl | Hm Drgbl | CPE002DC | CPE002EE | CPE002F4 | NM 13829 | NM 1382 | NM 1382 | ATM | ATM | ATM | ataxia telangiecta | ataxia tela | ataxia tela | 472 | 472 | 472 | s1708 | s1709 | s1710 |
| 2672 | P16 | P17 | 1613 | 646,2 | 19467 | 394,5 | 12,1 | 0,953 | H5A034 C | sample | Hm Drgbl | Hm Drgbl | Hm Drgbl | CPE002DC | CPE002EE | CPE002F4 | NM 00329 | NM 0032 | NM 0032 | TPR | TPR | TPR | translocated pror | translocat | translocat | 7175 | 7175 | 7175 | s14353 | s14354 | s14355 |
| 17941 | L20 | L21 | 1540 | 1018 | 18558 | 306,6 | 12,1 | 0,957 | H5A034 C | sample | Hm Gen E | Hm Gen E | Hm Gen E | CPE002JK | CPE002NZ | CPE002KF | NM 00101 | NM 0010 | NM 0010 | IGSF11 | IGSF11 | IGSF11 | immunoglobulin s | immunogl | immunogl | 152404 | 152404 | 152404 | s45688 | s45689 | s45690 |
| 9011 | H19 | H20 | 1809 | 1661 | 21858 | 123,9 | 12,1 | 0,972 | H5A034 C | sample | Hm Drgbl | Hm Drgbl | Hm Drgbl | CPE002E7 | CPE002ES | CPE002FD | NM 01659 | NM 0165 | NM 0165 | ZDHHC3 | ZDHHC3 | ZDHHC3 | zinc finger, DHHC- | zinc finger | zinc finger | 51304 | 51304 | 51304 | s27899 | s27900 | s27901 |
| 4238 | A21 | A22 | 1531 | 732,4 | 18485 | 518,1 | 12,1 | 1,021 | H5A034 C | sample | Hm Drgbl | Hm Drgbl | Hm Drgbl | CPE002DU | CPE002EK | CPE002FQ | NM 00164 | NM 0016 | NM 0016 | SHROOM2 | SHROOM2 | SHROOM2 | shroom family me | shroom fa | shroom fa | 357 | 357 | 357 | s1512 | s1513 | s1514 |
| 4270 | B7 | B8 | 1020 | 689,7 | 12375 | 346 | 12,1 | 1,021 | H5A034 C | sample | Hm Drgbl | Hm Drgbl | Hm Drgbl | CPE002DU | CPE002EK | CPE002FQ | NM 00108 | NM 0010 | NM 0010 | GRIA2 | GRIA2 | GRIA2 | glutamate recept | glutamate | glutamate | 2891 | 2891 | 2891 | s6134 | s6135 | s6136 |
| 4379 | G19 | G20 | 532 | 715,7 | 6416 | 355,1 | 12,1 | 1,021 | H5A034 C | sample | Hm Drgbl | Hm Drgbl | Hm Drgbl | CPE002DU | CPE002EK | CPE002FQ | NM 00103 | NM 0010 | NM 0010 | CHRNA1 | CHRNA1 | CHRNA1 | cholinergic recept | cholinergi | cholinergi | 1134 | 1134 | 1134 | s3037 | s3038 | s3039 |
| 4507 | L4 | L5 | 1231 | 665,1 | 14933 | 454,3 | 12,1 | 1,021 | H5A034 C | sample | Hm Drgbl | Hm Drgbl | Hm Drgbl | CPE002DU | CPE002EK | CPE002FQ | NM 13349 | NM 1334 | NM 1334 | KCNG4 | KCNG4 | KCNG4 | potassium voltage | potassium | potassium | 93107 | 93107 | 93107 | s195573 | s195574 | s195575 |
| 4575 | O22 | O23 | 1275 | 733,6 | 15398 | 449,9 | 12,1 | 1,021 | H5A034 C | sample | Hm Drgbl | Hm Drgbl | Hm Drgbl | CPE002DU | CPE002EK | CPE002FQ | NM 00104 | NM 0010 | NM 0010 | SCN3B | SCN3B | SCN3B | sodium channel, v | sodium ch | sodium ch | 55800 | 55800 | 55800 | s31541 | s31542 | s31543 |
| 4586 | P10 | P11 | 1223 | 772,9 | 14779 | 499,6 | 12,1 | 1,021 | H5A034 C | sample | Hm Drgbl | Hm Drgbl | Hm Drgbl | CPE002DU | CPE002EK | CPE002FQ | NM 00711 | NM 0071 | NM 0071 | TNFAIP6 | TNFAIP6 | TNFAIP6 | tumor necrosis fa | tumor nec | tumor nec | 7130 | 7130 | 7130 | s14263 | s14264 | s14262 |
| 168 | G9 | G10 | 620 | 1536 | 7496 | 360,8 | 12,1 | 1,03 | H5A034 C | sample | Hm Drgbl | Hm Drgbl | Hm Drgbl | CPE002DT | CPE002EJ | CPE002F9 | NM 02196 | NM 0219 | NM 0219 | NR0B2 | NR0B2 | NR0B2 | nuclear receptor s | nuclear re | nuclear re | 8431 | 8431 | 8431 | s15996 | s15997 | s15998 |
| 226 | J18 | J19 | 598 | 1492 | 7256 | 438,4 | 12,1 | 1,03 | H5A034 C | sample | Hm Drgbl | Hm Drgbl | Hm Drgbl | CPE002DT | CPE002EJ | CPE002F9 | NM 00541 | NM 0054 | NM 0054 | STAT2 | STAT2 | STAT2 | signal transducer | signal tran | signal tran | 6773 | 6773 | 6773 | s13530 | s13528 | s13529 |
| 234 | J3 | J4 | 447 | 1470 | 5423 | 493,9 | 12,1 | 1,03 | H5A034 C | sample | Hm Drgbl | Hm Drgbl | Hm Drgbl | CPE002DT | CPE002EJ | CPE002F9 | NM 15325 | NM 1532 | NM 0067 | SIPA1 | SIPA1 | SIPA1 | signal-induced pr | signal-ind | signal-ind | 6494 | 6494 | 6494 | s12873 | s12872 | s224633 |
| 327 | N22 | N23 | 650 | 1554 | 7883 | 679,2 | 12,1 | 1,03 | H5A034 C | sample | Hm Drgbl | Hm Drgbl | Hm Drgbl | CPE002DT | CPE002EJ | CPE002F9 | NM 02173 | NM 0217 | NM 0217 | SVIL | SVIL | SVIL | supervillin | supervillin | supervillin | 6840 | 6840 | 6840 | s13661 | s13662 | s13663 |
| 2099 | H19 | H20 | 1385 | 611,4 | 16792 | 338,5 | 12,1 | 1,052 | H5A034 C | sample | Hm Drgbl | Hm Drgbl | Hm Drgbl | CPE002FX | CPE002G1 | CPE002G5 | NM 15247 | NM 1524 | NM 1524 | ZNF565 | ZNF565 | ZNF565 | zinc finger protein | zinc finger | zinc finger | 147929 | 147929 | 147929 | s45083 | s45084 | s45085 |
| 2189 | L13 | L14 | 1688 | 675,9 | 20505 | 392,6 | 12,1 | 1,052 | H5A034 C | sample | Hm Drgbl | Hm Drgbl | Hm Drgbl | CPE002FX | CPE002G1 | CPE002G5 | NM 14471 | NM 1447 | NM 1447 | EFHB | EFHB | EFHB | EF-hand domain f | EF-hand d | EF-hand d | 151651 | 151651 | 151651 | s45605 | s45606 | s45607 |
| 2250 | N3 | N4 | 1233 | 694 | 14880 | 417,5 | 12,1 | 1,052 | H5A034 C | sample | Hm Drgbl | Hm Drgbl | Hm Drgbl | CPE002FX | CPE002G1 | CPE002G5 | NM 15255 | NM 1525 | NM 1525 | ZNF746 | ZNF746 | ZNF746 | zinc finger protein | zinc finger | zinc finger | 155061 | 155061 | 155061 | s45920 | s223367 | s45922 |
| 6849 | N17 | N18 | 1564 | 592,5 | 18864 | 511,5 | 12,1 | 1,061 | H5A034 C | sample | Hm Drgbl | Hm Drgbl | Hm Drgbl | CPE002E1 | CPE002EY | CPE002FJ | NM 00216 | NM 0021 | NM 0021 | TNC | TNC | TNC | tenascin C (hexab | tenascin C | tenascin C | 3371 | 3371 | 3371 | s7068 | s7069 | s7070 |
| 3845 | A13 | A14 | 657 | 712,7 | 7947 | 395,1 | 12,1 | 1,076 | H5A034 C | sample | Hm Drgbl | Hm Drgbl | Hm Drgbl | CPE002DS | CPE002EI | CPE002F8 | NM 18179 | NM 1817 | NM 1817 | GPR141 | GPR141 | GPR141 | G protein-couple | G protein- | G protein- | 353345 | 353345 | 353345 | s51470 | s51471 | s51472 |
| 3891 | C11 | C12 | 2172 | 763,5 | 26222 | 356,6 | 12,1 | 1,076 | H5A034 C | sample | Hm Drgbl | Hm Drgbl | Hm Drgbl | CPE002DS | CPE002EI | CPE002F8 | NM 00377 | NM 0037 | NM 0037 | EDG6 | EDG6 | EDG6 | endothelial differ | endotheli | endotheli | 8698 | 8698 | 8698 | s194948 | s225025 | s194950 |
| 3955 | E4 | E5 | 894 | 686 | 10787 | 356,3 | 12,1 | 1,076 | H5A034 C | sample | Hm Drgbl | Hm Drgbl | Hm Drgbl | CPE002DS | CPE002EI | CPE002F8 | NM 00286 | NM 0028 | NM 0028 | RAB6A | RAB6A | RAB6A | RAB6A, member R | RAB6A, m | RAB6A, m | 5870 | 5870 | 5870 | s11684 | s11685 | s11686 |
| 3994 | G18 | G19 | 761 | 694,7 | 9178 | 352,4 | 12,1 | 1,076 | H5A034 C | sample | Hm Drgbl | Hm Drgbl | Hm Drgbl | CPE002DS | CPE002EI | CPE002F8 | NM 13442 | NM 1344 | NM 1344 | RAD52 | RAD52 | RAD52 | RAD52 homolog ( | RAD52 ho | RAD52 ho | 5893 | 5893 | 5893 | s11746 | s11747 | s11748 |
| 4112 | L16 | L17 | 1267 | 646,4 | 15312 | 344,2 | 12,1 | 1,076 | H5A034 C | sample | Hm Drgbl | Hm Drgbl | Hm Drgbl | CPE002DS | CPE002EI | CPE002F8 | NM 00651 | NM 0065 | NM 0065 | RSC1A1 | RSC1A1 | RSC1A1 | regulatory solute | regulatory | regulatory | 6248 | 6248 | 6248 | s12370 | s12371 | s12369 |
| 4165 | N20 | N21 | 1551 | 654,2 | 18793 | 404,2 | 12,1 | 1,076 | H5A034 C | sample | Hm Drgbl | Hm Drgbl | Hm Drgbl | CPE002DS | CPE002EI | CPE002F8 | NM 00296 | NM 0029 | NM 0029 | SAFB | SAFB | SAFB | scaffold attachme | scaffold a | scaffold a | 6294 | 6294 | 6294 | s12452 | s224568 | s12454 |
| 15515 | G19 | G20 | 1502 | 844,9 | 18168 | 128,3 | 12,1 | 1,1 | H5A034 C | sample | Hm Gen E | Hm Gen E | Hm Gen E | CPE002JE | CPE002NT | CPE002K9 | NM 02210 | NM 0221 | NM 0221 | CXorf56 | CXorf56 | CXorf56 | chromosome X op | chromoso | chromoso | 63932 | 63932 | 63932 | s34291 | s34292 | s34293 |
| 15686 | N21 | N22 | 1074 | 786,1 | 13033 | 137,1 | 12,1 | 1,1 | H5A034 C | sample | Hm Gen E | Hm Gen E | Hm Gen E | CPE002JE | CPE002NT | CPE002K9 | NM 00377 | NM 0037 | NM 0037 | MRPL40 | MRPL40 | MRPL40 | mitochondrial rib | mitochon | mitochon | 64976 | 64976 | 64976 | s35133 | s35134 | s35135 |
| 6009 | K17 | K18 | 911 | 856,1 | 10997 | 607,8 | 12,1 | 1,105 | H5A034 C | sample | Hm Drgbl | Hm Drgbl | Hm Drgbl | CPE002DY | CPE002F1 | CPE002FM | NM 00161 | NM 0016 | NM 0016 | PARP1 | PARP1 | PARP1 | poly (ADP-ribose) | poly (ADP | poly (ADP | 142 | 142 | 142 | s1097 | s1098 | s1099 |
| 6015 | K22 | K23 | 1621 | 795,3 | 19563 | 552,8 | 12,1 | 1,105 | H5A034 C | sample | Hm Drgbl | Hm Drgbl | Hm Drgbl | CPE002DY | CPE002F1 | CPE002FM | NM 00710 | NM 0071 | NM 0071 | ATP5I | ATP5I | ATP5I | ATP synthase, H+ | ATP synth | ATP synth | 521 | 521 | 521 | s1785 | s1787 | s1786 |
| 6046 | L7 | L8 | 1670 | 754,8 | 20244 | 511,7 | 12,1 | 1,105 | H5A034 C | sample | Hm Drgbl | Hm Drgbl | Hm Drgbl | CPE002DY | CPE002F1 | CPE002FM | NM 00003 | NM 0000 | NM 0000 | APOA1 | APOA1 | APOA1 | apolipoprotein A- | apolipopr | apolipopr | 335 | 335 | 335 | s1466 | s223885 | s1468 |
| 6054 | M14 | M15 | 1168 | 773,2 | 14091 | 295,8 | 12,1 | 1,105 | H5A034 C | sample | Hm Drgbl | Hm Drgbl | Hm Drgbl | CPE002DY | CPE002F1 | CPE002FM | NM 00169 | NM 0016 | NM 0016 | ATP5O | ATP5O | ATP5O | ATP synthase, H+ | ATP synth | ATP synth | 539 | 539 | 539 | s224290 | s1819 | s1818 |
| 6114 | O3 | O4 | 941 | 736,4 | 11401 | 275,3 | 12,1 | 1,105 | H5A034 C | sample | Hm Drgbl | Hm Drgbl | Hm Drgbl | CPE002DY | CPE002F1 | CPE002FM | NM 00068 | NM 0006 | NM 0006 | AHCY | AHCY | AHCY | S-adenosylhomoc | S-adenosy | S-adenosy | 191 | 191 | 191 | s1197 | s1196 | s1195 |
| 6139 | P4 | P5 | 1724 | 759,2 | 20849 | 621,3 | 12,1 | 1,105 | H5A034 C | sample | Hm Drgbl | Hm Drgbl | Hm Drgbl | CPE002DY | CPE002F1 | CPE002FM | NM 00123 | NM 0012 | NM 0012 | SERPINH1 | SERPINH1 | SERPINH1 | serpin peptidase | serpin pep | serpin pep | 871 | 871 | 871 | s2485 | s2486 | s2487 |
| 1414 | K7 | K8 | 1384 | 697,2 | 16764 | 392,7 | 12,1 | 1,11 | H5A034 C | sample | Hm Drgbl | Hm Drgbl | Hm Drgbl | CPE002FV | CPE002FZ | CPE002G3 | NM 00652 | NM 0065 | NM 0065 | ZNF138 | ZNF138 | ZNF138 | zinc finger protein | zinc finger | zinc finger | 7697 | 7697 | 7697 | s15180 | s15181 | s15182 |
| 1416 | K9 | K10 | 905 | 696,9 | 10919 | 387,1 | 12,1 | 1,11 | H5A034 C | sample | Hm Drgbl | Hm Drgbl | Hm Drgbl | CPE002FV | CPE002FZ | CPE002G3 | NM 00344 | NM 0034 | NM 0034 | ZNF140 | ZNF140 | ZNF140 | zinc finger protein | zinc finger | zinc finger | 7699 | 7699 | 7699 | s224793 | s15184 | s15185 |
| 1463 | M8 | M9 | 984 | 763,3 | 11890 | 476,9 | 12,1 | 1,11 | H5A034 C | sample | Hm Drgbl | Hm Drgbl | Hm Drgbl | CPE002FV | CPE002FZ | CPE002G3 | NM 00634 | NM 0063 | NM 0063 | ZNHIT1 | ZNHIT1 | ZNHIT1 | zinc finger, HIT ty | zinc finger | zinc finger | 10467 | 10467 | 10467 | s20490 | s223121 | s20492 |
| 1515 | P11 | P12 | 1620 | 703,9 | 19604 | 534 | 12,1 | 1,11 | H5A034 C | sample | Hm Drgbl | Hm Drgbl | Hm Drgbl | CPE002FV | CPE002FZ | CPE002G3 | NM 00103 | NM 0010 | NM 0010 | CABP1 | CABP1 | CABP1 | calcium binding p | calcium bi | calcium bi | 9478 | 9478 | 9478 | s225147 | s18171 | s18172 |
| 16201 | D1 | D2 | 1162 | 1021 | 14050 | 397,5 | 12,1 | 1,11 | H5A034 C | sample | Hm Gen E | Hm Gen E | Hm Gen E | CPE002JG | CPE002NV | CPE002KB | NM 03191 | NM 0319 | NM 0319 | ROPN1L | ROPN1L | ROPN1L | ropporin 1-like | ropporin 1 | ropporin 1 | 83853 | 83853 | 83853 | s38218 | s38219 | s38220 |
| 16279 | G15 | G16 | 1661 | 1092 | 20117 | 337,1 | 12,1 | 1,11 | H5A034 C | sample | Hm Gen E | Hm Gen E | Hm Gen E | CPE002JG | CPE002NV | CPE002KB | XM 93709 | XM 92655 | XM 92655 | KRTAP2-1 | KRTAP2-1 | KRTAP2-1 | keratin associated | keratin as | keratin as | 81872 | 81872 | 81872 | s37853 | s195428 | s195429 |
| 16400 | L16 | L17 | 1543 | 985,6 | 18632 | 297,9 | 12,1 | 1,11 | H5A034 C | sample | Hm Gen E | Hm Gen E | Hm Gen E | CPE002JG | CPE002NV | CPE002KB | NM 03260 | NM 0326 | NM 0326 | MCEE | MCEE | MCEE | methylmalonyl Co | methylma | methylma | 84693 | 84693 | 84693 | s39339 | s39340 | s228384 |
| 16492 | P12 | P13 | 1441 | 1072 | 17382 | 403,1 | 12,1 | 1,11 | H5A034 C | sample | Hm Gen E | Hm Gen E | Hm Gen E | CPE002JG | CPE002NV | CPE002KB | NM 03273 | NM 0327 | NM 0327 | IL17RC | IL17RC | IL17RC | interleukin 17 rec | interleuki | interleuki | 84818 | 84818 | 84818 | s39470 | s39471 | s39472 |
| 13613 | H13 | H14 | 1275 | 968,3 | 15414 | 148,8 | 12,1 | 1,136 | H5A034 C | sample | Hm Gen E | Hm Gen E | Hm Gen E | CPE002JC | CPE002NC | CPE002K4 | NM 00100 | NM 0010 | NM 0010 | CBY1 | CBY1 | CBY1 | chibby homolog 1 | chibby ho | chibby ho | 25776 | 25776 | 25776 | s24462 | s24463 | s24464 |
| 813 | B6 | B7 | 1530 | 738,2 | 18565 | 457,7 | 12,1 | 1,147 | H5A034 C | sample | Hm Drgbl | Hm Drgbl | Hm Drgbl | CPE002FU | CPE002FY | CPE002G2 | NM 00290 | NM 0029 | NM 0029 | RCN1 | RCN1 | RCN1 | reticulocalbin 1, E | reticuloca | reticuloca | 5954 | 5954 | 5954 | s11882 | s11883 | s11884 |
| 879 | E22 | E23 | 1372 | 728,7 | 16604 | 424,6 | 12,1 | 1,147 | H5A034 C | sample | Hm Drgbl | Hm Drgbl | Hm Drgbl | CPE002FU | CPE002FY | CPE002G2 | NM 17316 | NM 1731 | NM 1731 | NFATC3 | NFATC3 | NFATC3 | nuclear factor of a | nuclear fa | nuclear fa | 4775 | 4775 | 4775 | s9479 | s9480 | s9481 |
| 1004 | J5 | J6 | 1866 | 740,8 | 22561 | 374,6 | 12,1 | 1,147 | H5A034 C | sample | Hm Drgbl | Hm Drgbl | Hm Drgbl | CPE002FU | CPE002FY | CPE002G2 | NM 00214 | NM 0021 | NM 0021 | HPCAL1 | HPCAL1 | HPCAL1 | hippocalcin-like 1 | hippocalc | hippocalc | 3241 | 3241 | 3241 | s6872 | s6873 | s6874 |
| 1065 | M17 | M18 | 1383 | 740,3 | 16758 | 390 | 12,1 | 1,147 | H5A034 C | sample | Hm Drgbl | Hm Drgbl | Hm Drgbl | CPE002FU | CPE002FY | CPE002G2 | NM 08076 | NM 0807 | NM 0807 | DACH1 | DACH1 | DACH1 | dachshund homo | dachshun | dachshun | 1602 | 1602 | 1602 | s3899 | s3900 | s3901 |
| 1114 | O18 | O19 | 1890 | 832,7 | 22935 | 494,3 | 12,1 | 1,147 | H5A034 C | sample | Hm Drgbl | Hm Drgbl | Hm Drgbl | CPE002FU | CPE002FY | CPE002G2 | NM 00285 | NM 0028 | NM 0028 | PVALB | PVALB | PVALB | parvalbumin | parvalbum | parvalbum | 5816 | 5816 | 5816 | s11597 | s11598 | s11599 |
| 5405 | B13 | B14 | 2146 | 729,8 | 25977 | 675,7 | 12,1 | 1,152 | H5A034 C | sample | Hm Drgbl | Hm Drgbl | Hm Drgbl | CPE002DX | CPE002F2 | CPE002FN | NM 00371 | NM 0037 | NM 0037 | STC2 | STC2 | STC2 | stanniocalcin 2 | stannioca | stannioca | 8614 | 8614 | 8614 | s225008 | s16387 | s16388 |
| 5481 | E17 | E18 | 1544 | 691,2 | 18704 | 428,1 | 12,1 | 1,152 | H5A034 C | sample | Hm Drgbl | Hm Drgbl | Hm Drgbl | CPE002DX | CPE002F2 | CPE002FN | NM 00100 | NM 0010 | NM 0010 | NCK2 | NCK2 | NCK2 | NCK adaptor prot | NCK adap | NCK adap | 8440 | 8440 | 8440 | s16020 | s16018 | s16019 |
| 5515 | F4 | F5 | 1799 | 620,7 | 21845 | 592,8 | 12,1 | 1,152 | H5A034 C | sample | Hm Drgbl | Hm Drgbl | Hm Drgbl | CPE002DX | CPE002F2 | CPE002FN | NM 00471 | NM 0047 | NM 0047 | COX7A2L | COX7A2L | COX7A2L | cytochrome c oxic | cytochron | cytochron | 9167 | 9167 | 9167 | s17517 | s17518 | s17519 |
| 5615 | J8 | J9 | 1876 | 617,4 | 22610 | 392,4 | 12,1 | 1,152 | H5A034 C | sample | Hm Drgbl | Hm Drgbl | Hm Drgbl | CPE002DX | CPE002F2 | CPE002FN | NM 00475 | NM 0047 | NM 0047 | CRLF1 | CRLF1 | CRLF1 | cytokine receptor | cytokine r | cytokine r | 9244 | 9244 | 9244 | s17675 | s17673 | s17674 |
| 5646 | L14 | L15 | 1639 | 688,3 | 19762 | 511,3 | 12,1 | 1,152 | H5A034 C | sample | Hm Drgbl | Hm Drgbl | Hm Drgbl | CPE002DX | CPE002F2 | CPE002FN | NM 00423 | NM 0042 | NM 0042 | ATP6V1F | ATP6V1F | ATP6V1F | ATPase, H+ transp | ATPase, H | ATPase, H | 9296 | 9296 | 9296 | s225109 | s17774 | s17772 |
| 5756 | P5 | P6 | 1807 | 725,1 | 21866 | 643,2 | 12,1 | 1,152 | H5A034 C | sample | Hm Drgbl | Hm Drgbl | Hm Drgbl | CPE002DX | CPE002F2 | CPE002FN | NM 00384 | NM 0038 | NM 0038 | TNFRSF10I | TNFRSF10 | TNFRSF10I | tumor necrosis fa | tumor nec | tumor nec | 8793 | 8793 | 8793 | s16751 | s16750 | s16752 |
| 3546 | D3 | D4 | 1155 | 1378 | 14032 | 376,2 | 12,1 | 1,163 | H5A034 C | sample | Hm Drgbl | Hm Drgbl | Hm Drgbl | CPE002DR | CPE002EH | CPE002F7 | NM 00614 | NM 0061 | NM 0061 | GPR19 | GPR19 | GPR19 | G protein-couple | G protein- | G protein- | 2842 | 2842 | 2842 | s6031 | s6032 | s6033 |
| 3552 | D9 | D10 | 1495 | 1403 | 18094 | 319,4 | 12,1 | 1,163 | H5A034 C | sample | Hm Drgbl | Hm Drgbl | Hm Drgbl | CPE002DR | CPE002EH | CPE002F7 | NM 00529 | NM 0052 | NM 0052 | GPR22 | GPR22 | GPR22 | G protein-couple | G protein- | G protein- | 2845 | 2845 | 2845 | s6039 | s223782 | s6041 |
| 3687 | J22 | J23 | 889 | 1300 | 10760 | 338,8 | 12,1 | 1,163 | H5A034 C | sample | Hm Drgbl | Hm Drgbl | Hm Drgbl | CPE002DR | CPE002EH | CPE002F7 | NM 13080 | NM 1308 | NM 1308 | RXFP2 | RXFP2 | RXFP2 | relaxin/insulin-lik | relaxin/in | relaxin/in | 122042 | 122042 | 122042 | s42516 | s42517 | s42518 |
| 3774 | N14 | N15 | 1502 | 1378 | 18188 | 352,1 | 12,1 | 1,163 | H5A034 C | sample | Hm Drgbl | Hm Drgbl | Hm Drgbl | CPE002DR | CPE002EH | CPE002F7 | NM 00103 | NM 0010 | NM 0010 | OPN5 | OPN5 | OPN5 | opsin 5 | opsin 5 | opsin 5 | 221391 | 221391 | 221391 | s48024 | s48025 | s48026 |
| 3783 | N22 | N23 | 1499 | 1312 | 18172 | 409,7 | 12,1 | 1,163 | H5A034 C | sample | Hm Drgbl | Hm Drgbl | Hm Drgbl | CPE002DR | CPE002EH | CPE002F7 | NM 14896 | NM 1489 | NM 1489 | GPRC6A | GPRC6A | GPRC6A | G protein-couple | G protein- | G protein- | 222545 | 222545 | 222545 | s48207 | s48208 | s48209 |
| 3789 | N6 | N7 | 1833 | 1495 | 22129 | 423,9 | 12,1 | 1,163 | H5A034 C | sample | Hm Drgbl | Hm Drgbl | Hm Drgbl | CPE002DR | CPE002EH | CPE002F7 | NM 15300 | NM 1530 | NM 1530 | GPR156 | GPR156 | GPR156 | G protein-couple | G protein- | G protein- | 165829 | 165829 | 165829 | s46593 | s46594 | s46595 |
| 3813 | O6 | O7 | 1706 | 1344 | 20667 | 398,2 | 12,1 | 1,163 | H5A034 C | sample | Hm Drgbl | Hm Drgbl | Hm Drgbl | CPE002DR | CPE002EH | CPE002F7 | NM 08081 | NM 0808 | NM 0808 | OXGR1 | OXGR1 | OXGR1 | oxoglutarate (alp | oxoglutar | oxoglutar | 27199 | 27199 | 27199 | s25983 | s25984 | s25985 |
| 3824 | P16 | P17 | 1212 | 1314 | 14698 | 438,2 | 12,1 | 1,163 | H5A034 C | sample | Hm Drgbl | Hm Drgbl | Hm Drgbl | CPE002DR | CPE002EH | CPE002F7 | NM 17385 | NM 1738 | NM 1738 | VN1R4 | VN1R4 | VN1R4 | vomeronasal 1 re | vomerona | vomerona | 317703 | 317703 | 317703 | s50226 | s50227 | s50228 |
| 8089 | B1 | B2 | 1394 | 621,6 | 16801 | 538,3 | 12,1 | 1,163 | H5A034 C | sample | Hm Drgbl | Hm Drgbl | Hm Drgbl | CPE002E5 | CPE002EU | CPE002FF | NM 00726 | NM 0072 | NM 0072 | PARK7 | PARK7 | PARK7 | Parkinson disease | Parkinson | Parkinson | 11315 | 11315 | 11315 | s22306 | s22304 | s22305 |
| 8258 | I10 | I11 | 1634 | 636,2 | 19796 | 383,1 | 12,1 | 1,163 | H5A034 C | sample | Hm Drgbl | Hm Drgbl | Hm Drgbl | CPE002E5 | CPE002EU | CPE002FF | NM 00108 | NM 0010 | NM 0010 | JMJD6 | JMJD6 | JMJD6 | jumonji domain c | jumonji d | jumonji d | 23210 | 23210 | 23210 | s23290 | s23291 | s23289 |
| 8338 | L18 | L19 | 1551 | 613,2 | 18721 | 486,7 | 12,1 | 1,163 | H5A034 C | sample | Hm Drgbl | Hm Drgbl | Hm Drgbl | CPE002E5 | CPE002EU | CPE002FF | NM 01335 | NM 0133 | NM 0133 | SLC16A8 | SLC16A8 | SLC16A8 | solute carrier fam | solute car | solute car | 23539 | 23539 | 23539 | s223611 | s24040 | s195105 |
| 8390 | N21 | N22 | 621 | 754,4 | 7539 | 202,3 | 12,1 | 1,163 | H5A034 C | sample | Hm Drgbl | Hm Drgbl | Hm Drgbl | CPE002E5 | CPE002EU | CPE002FF | NM 01401 | NM 0140 | NM 0140 | ASCC3L1 | ASCC3L1 | ASCC3L1 | activating signal c | activating | activating | 23020 | 23020 | 23020 | s22847 | s22845 | s22846 |
| 10135 | G15 | G16 | 1515 | 810,8 | 18320 | 125,6 | 12,1 | 1,163 | H5A034 C | sample | Hm Drgbl | Hm Drgbl | Hm Drgbl | CPE002EA | CPE002EP | CPE002FA | NM 02475 | NM 0247 | NM 0247 | AGMAT | AGMAT | AGMAT | agmatine ureohy | agmatine | agmatine | 79814 | 79814 | 79814 | s381 | s379 | s380 |
| 10332 | O2 | O3 | 1692 | 848,1 | 20395 | 205,7 | 12,1 | 1,163 | H5A034 C | sample | Hm Drgbl | Hm Drgbl | Hm Drgbl | CPE002EA | CPE002EP | CPE002FA | NM 01815 | NM 0181 | NM 0181 | ANTXR1 | ANTXR1 | ANTXR1 | anthrax toxin rec | anthrax to | anthrax to | 84168 | 84168 | 84168 | s38599 | s38597 | s38598 |
| 21540 | B2 | B3 | 74 | 1119 | 895 | 723 | 12,1 | 1,274 | H5A034 C | sample | Hm Gen E | Hm Gen E | Hm Gen E | CPE002JU | CPE002O9 | CPE002KP | XM 92594 | XM 92594 | XM 92594 | LOC65307 | LOC65307 | LOC65307 | similar to Golgin s | similar to | similar to | 653073 | 653073 | 653073 | s57557 | s57558 | s57559 |
| 21671 | G8 | G9 | 207 | 991,3 | 2509 | 577 | 12,1 | 1,274 | H5A034 C | sample | Hm Gen E | Hm Gen E | Hm Gen E | CPE002JU | CPE002O9 | CPE002KP | XM 94219 | XM 94219 | XM 94219 | LOC65264 | LOC65264 | LOC65264 | similar to double | similar to | similar to | 652641 | 652641 | 652641 | s197936 | s197937 | s197938 |
| 21747 | K11 | K12 | 163 | 880,5 | 1968 | 717,2 | 12,1 | 1,274 | H5A034 C | sample | Hm Gen E | Hm Gen E | Hm Gen E | CPE002JU | CPE002O9 | CPE002KP | XM 94400 | XM 94400 | XM 94400 | LOC65000 | LOC65000 | LOC65000 | hypothetical prot | hypotheti | hypotheti | 650004 | 650004 | 650004 | s197705 | s197706 | s197707 |
| 21789 | L6 | L7 | 259 | 991,5 | 3122 | 574 | 12,1 | 1,274 | H5A034 C | sample | Hm Gen E | Hm Gen E | Hm Gen E | CPE002JU | CPE002O9 | CPE002KP | XM 94134 | XM 94134 | XM 94134 | LOC65378 | LOC65378 | LOC65378 | similar to chromo | similar to | similar to | 653788 | 653788 | 653788 | s57764 | s57765 | s57766 |
| 4650 | B3 | B4 | 1009 | 809,7 | 12193 | 474,5 | 12,1 | 1,33 | H5A034 C | sample | Hm Drgbl | Hm Drgbl | Hm Drgbl | CPE002DV | CPE002EL | CPE002FP | NM 00447 | NM 0044 | NM 0044 | FOLH1 | FOLH1 | FOLH1 | folate hydrolase ( | folate hyd | folate hyd | 2346 | 2346 | 2346 | s5327 | s5328 | s5329 |
| 4684 | D12 | D13 | 1749 | 734,9 | 21192 | 472,6 | 12,1 | 1,33 | H5A034 C | sample | Hm Drgbl | Hm Drgbl | Hm Drgbl | CPE002DV | CPE002EL | CPE002FP | NM 01427 | NM 0142 | NM 0142 | ADAMTS7 | ADAMTS7 | ADAMTS7 | ADAM metallopep | ADAM me | ADAM me | 11173 | 11173 | 11173 | s22050 | s22051 | s22052 |
| 4804 | I12 | I13 | 1412 | 872,7 | 17097 | 377 | 12,1 | 1,33 | H5A034 C | sample | Hm Drgbl | Hm Drgbl | Hm Drgbl | CPE002DV | CPE002EL | CPE002FP | NM 00602 | NM 0060 | NM 0060 | P11 | P11 | P11 | 26 serine proteas | 26 serine | 26 serine | 8909 | 8909 | 8909 | s17038 | s17039 | s17040 |
| 4874 | L10 | L11 | 1654 | 816,3 | 20096 | 439,8 | 12,1 | 1,33 | H5A034 C | sample | Hm Drgbl | Hm Drgbl | Hm Drgbl | CPE002DV | CPE002EL | CPE002FP | NM 01771 | NM 0177 | NM 0177 | PGPEP1 | PGPEP1 | PGPEP1 | pyroglutamyl-pep | pyrogluta | pyrogluta | 54858 | 54858 | 54858 | s29605 | s29606 | s29607 |
| 4928 | N16 | N17 | 1710 | 818 | 20644 | 443,5 | 12,1 | 1,33 | H5A034 C | sample | Hm Drgbl | Hm Drgbl | Hm Drgbl | CPE002DV | CPE002EL | CPE002FP | NM 15298 | NM 1529 | NM 1529 | SPPL2B | SPPL2B | SPPL2B | signal peptide pe | signal pep | signal pep | 56928 | 56928 | 56928 | s32417 | s32418 | s32419 |
| 4933 | N20 | N21 | 1228 | 878 | 14879 | 420,6 | 12,1 | 1,33 | H5A034 C | sample | Hm Drgbl | Hm Drgbl | Hm Drgbl | CPE002DV | CPE002EL | CPE002FP | NM 02036 | NM 0203 | NM 0203 | CPA6 | CPA6 | CPA6 | carboxypeptidase | carboxype | carboxype | 57094 | 57094 | 57094 | s224448 | s32659 | s32660 |
| 10521 | G17 | G18 | 1000 | 934 | 12145 | 196,2 | 12,1 | 1,367 | H5A034 C | sample | Hm Drgbl | Hm Drgbl | Hm Drgbl | CPE002EB | CPE002EO | CPE002FR | NM 03340 | NM 0334 | NM 0334 | PRIC285 | PRIC285 | PRIC285 | peroxisomal proli | peroxison | peroxison | 85441 | 85441 | 85441 | s39997 | s39998 | s39999 |
| 10630 | K7 | K8 | 1458 | 823,1 | 17698 | 188,7 | 12,1 | 1,367 | H5A034 C | sample | Hm Drgbl | Hm Drgbl | Hm Drgbl | CPE002EB | CPE002EO | CPE002FR | NM 05300 | NM 0530 | NM 0530 | SIGLEC12 | SIGLEC12 | SIGLEC12 | sialic acid binding | sialic acid | sialic acid | 89858 | 89858 | 89858 | s40162 | s40163 | s40164 |
| 19989 | A6 | A7 | 904 | 1202 | 10917 | 258,5 | 12,1 | 1,476 | H5A034 C | sample | Hm Gen E | Hm Gen E | Hm Gen E | CPE002JQ | CPE002O5 | CPE002KL | XM 00113 | XM 00113 | XM 00113 | LOC40098 | LOC40098 | LOC40098 | protein immuno-r | protein im | protein im | 400986 | 400986 | 400986 | s53434 | s53435 | s53436 |
| 20000 | B16 | B17 | 1002 | 1209 | 12128 | 262,8 | 12,1 | 1,476 | H5A034 C | sample | Hm Gen E | Hm Gen E | Hm Gen E | CPE002JQ | CPE002O5 | CPE002KL | XM 94135 | XM 94135 | XM 49794 | LOC40214 | LOC40214 | LOC40214 | similar to A-kinas | similar to | similar to | 402145 | 402145 | 402145 | s53737 | s53738 | s196461 |
| 20015 | B8 | B9 | 771 | 1409 | 9327 | 225,8 | 12,1 | 1,476 | H5A034 C | sample | Hm Gen E | Hm Gen E | Hm Gen E | CPE002JQ | CPE002O5 | CPE002KL | NM 00101 | NM 0010 | NM 0010 | SRRD | SRRD | SRRD | SRR1 domain con | SRR1 dom | SRR1 dom | 402055 | 402055 | 402055 | s53721 | s53722 | s53723 |
| 20229 | K6 | K7 | 368 | 1150 | 4463 | 164,5 | 12,1 | 1,476 | H5A034 C | sample | Hm Gen E | Hm Gen E | Hm Gen E | CPE002JQ | CPE002O5 | CPE002KL | XM 37954 | XM 37954 | XM 37954 | LOC40144 | LOC40144 | LOC40144 | hypothetical gene | hypotheti | hypotheti | 401442 | 401442 | 401442 | s53600 | s53602 | s226567 |
| 20338 | P18 | P19 | 1171 | 1302 | 14162 | 155,3 | 12,1 | 1,476 | H5A034 C | sample | Hm Gen E | Hm Gen E | Hm Gen E | CPE002JQ | CPE002O5 | CPE002KL | NM 01523 | NM 0152 | NM 0152 | IQSEC3 | IQSEC3 | IQSEC3 | IQ motif and Sec7 | IQ motif a | IQ motif a | 440073 | 440073 | 440073 | s54002 | s54003 | s54004 |
| 7846 | G7 | G8 | 1251 | 604,3 | 15168 | 411 | 12,1 | 1,847 | H5A034 C | sample | Hm Drgbl | Hm Drgbl | Hm Drgbl | CPE002E4 | CPE002EV | CPE002FG | NM 02114 | NM 0211 | NM 0211 | ANGPTL7 | ANGPTL7 | ANGPTL7 | angiopoietin-like | angiopoie | angiopoie | 10218 | 10218 | 10218 | s19931 | s19932 | s19933 |
| 7850 | H10 | H11 | 928 | 572,2 | 11213 | 416,8 | 12,1 | 1,847 | H5A034 C | sample | Hm Drgbl | Hm Drgbl | Hm Drgbl | CPE002E4 | CPE002EV | CPE002FG | NM 00679 | NM 0067 | NM 0067 | UGT2A1 | UGT2A1 | UGT2A1 | UDP glucuronosyl | UDP glucu | UDP glucu | 10941 | 10941 | 10941 | s21522 | s21523 | s223166 |
| 3391 | N15 | N16 | 1043 | 1404 | 12688 | 463,2 | 12,2 | 0,755 | H5A034 C | sample | Hm Drgbl | Hm Drgbl | Hm Drgbl | CPE002DC | CPE002EG | CPE002F6 | NM 19904 | NM 1990 | NM 1990 | NUDT4 | NUDT4 | NUDT4 | nudix (nucleoside | nudix (nu | nudix (nu | 11163 | 11163 | 11163 | s22020 | s22021 | s22022 |
| 3435 | P11 | P12 | 1385 | 1260 | 16893 | 481,7 | 12,2 | 0,755 | H5A034 C | sample | Hm Drgbl | Hm Drgbl | Hm Drgbl | CPE002DC | CPE002EG | CPE002F6 | NM 01493 | NM 0149 | NM 0149 | INPP5F | INPP5F | INPP5F | inositol polyphos | inositol p | inositol p | 22876 | 22876 | 22876 | s22562 | s22563 | s22564 |
| 6274 | F18 | F19 | 623 | 651,1 | 7576 | 562,4 | 12,2 | 0,805 | H5A034 C | sample | Hm Drgbl | Hm Drgbl | Hm Drgbl | CPE002DZ | CPE002F0 | CPE002FL | NM 00010 | NM 0001 | NM 0001 | DLD | DLD | DLD | dihydrolipoamide | dihydrolip | dihydrolip | 1738 | 1738 | 1738 | s4116 | s4118 | s4117 |
| 6499 | O4 | O5 | 1465 | 607,2 | 17836 | 459,2 | 12,2 | 0,805 | H5A034 C | sample | Hm Drgbl | Hm Drgbl | Hm Drgbl | CPE002DZ | CPE002F0 | CPE002FL | NM 00521 | NM 0052 | NM 0052 | DCC | DCC | DCC | deleted in colore | deleted in | deleted in | 1630 | 1630 | 1630 | s3959 | s3960 | s3961 |
| 20618 | L10 | L11 | 776 | 813 | 9495 | 191,4 | 12,2 | 0,813 | H5A034 C | sample | Hm Gen E | Hm Gen E | Hm Gen E | CPE002JR | CPE002O6 | CPE002KM | XM 92622 | XM 92622 | XM 92622 | LOC64245 | LOC64245 | LOC64245 | hypothetical LOC | hypotheti | hypotheti | 642452 | 642452 | 642452 | s55027 | s55029 | s227019 |
| 13009 | O1 | O2 | 1860 | 1029 | 22761 | 188,9 | 12,2 | 0,827 | H5A034 C | sample | Hm Gen E | Hm Gen E | Hm Gen E | CPE002J6 | CPE002NN | CPE002K2 | NM 01463 | NM 0146 | NM 0146 | KIAA0372 | KIAA0372 | KIAA0372 | KIAA0372 | KIAA0372 | KIAA0372 | 9652 | 9652 | 9652 | s18566 | s18567 | s18568 |
| 17390 | E21 | E22 | 1611 | 925,9 | 19633 | 166 | 12,2 | 0,833 | H5A034 C | sample | Hm Gen E | Hm Gen E | Hm Gen E | CPE002JJ | CPE002NY | CPE002KE | NM 00100 | NM 0010 | NM 0010 | TCHHL1 | TCHHL1 | TCHHL1 | trichohyalin-like 1 | trichohyal | trichohyal | 126637 | 126637 | 126637 | s43057 | s43058 | s43059 |
| 19017 | I17 | I18 | 1255 | 968,9 | 15314 | 112,1 | 12,2 | 0,85 | H5A034 C | sample | Hm Gen E | Hm Gen E | Hm Gen E | CPE002JN | CPE002O2 | CPE002KI | NM 00100 | NM 0010 | NM 0010 | OR4C13 | OR4C13 | OR4C13 | olfactory recepto | olfactory r | olfactory r | 283092 | 283092 | 283092 | s49087 | s49088 | s49089 |
| 20895 | G22 | G23 | 1139 | 1032 | 13945 | 308,3 | 12,2 | 0,865 | H5A034 C | sample | Hm Gen E | Hm Gen E | Hm Gen E | CPE002JS | CPE002O7 | CPE002KN | XM 92752 | XM 92752 | XM 92752 | GCNT6 | GCNT6 | GCNT6 | glucosaminyl (N-a | glucosami | glucosami | 644378 | 644378 | 644378 | s55656 | s55657 | s55658 |
| 21015 | L22 | L23 | 1740 | 1034 | 21248 | 272,1 | 12,2 | 0,865 | H5A034 C | sample | Hm Gen E | Hm Gen E | Hm Gen E | CPE002JS | CPE002O7 | CPE002KN | XM 00112 | XM 00112 | XM 00112 | LOC64516 | LOC64516 | LOC64516 | similar to lympho | similar to | similar to | 645166 | 645166 | 645166 | s55937 | s55938 | s55939 |
| 11276 | F5 | F6 | 1411 | 852,8 | 17183 | 132,4 | 12,2 | 0,884 | H5A034 C | sample | Hm Drgbl | Hm Drgbl | Hm Drgbl | CPE002ED | CPE002F3 | CPE002FT | NM 17801 | NM 1780 | NM 1780 | TUBB2B | TUBB2B | TUBB2B | tubulin, beta 2B | tubulin, b | tubulin, b | 347733 | 347733 | 347733 | s223916 | s51294 | s51292 |
| 12522 | J3 | J4 | 1038 | 992,8 | 12691 | 145,8 | 12,2 | 0,917 | H5A034 C | sample | Hm Gen E | Hm Gen E | Hm Gen E | CPE002J5 | CPE002NK | CPE002K1 | NM 00566 | NM 0056 | NM 0056 | EVI5 | EVI5 | EVI5 | ecotropic viral int | ecotropic | ecotropic | 7813 | 7813 | 7813 | s15376 | s15377 | s15378 |
| 10825 | D1 | D2 | 1782 | 906,2 | 21804 | 444,7 | 12,2 | 0,924 | H5A034 C | sample | Hm Drgbl | Hm Drgbl | Hm Drgbl | CPE002EC | CPE002EN | CPE002FS | NM 18164 | NM 1816 | NM 1816 | MFSD4 | MFSD4 | MFSD4 | major facilitator s | major faci | major faci | 148808 | 148808 | 148808 | s45217 | s45216 | s45215 |
| 7340 | B5 | B6 | 640 | 672 | 7804 | 519,9 | 12,2 | 0,931 | H5A034 C | sample | Hm Drgbl | Hm Drgbl | Hm Drgbl | CPE002E3 | CPE002EW | CPE002FH | NM 00456 | NM 0045 | NM 0045 | PCK2 | PCK2 | PCK2 | phosphoenolpyru | phosphoe | phosphoe | 5106 | 5106 | 5106 | s10124 | s10125 | s10126 |
| 7611 | N11 | N12 | 1598 | 658,5 | 19524 | 555,3 | 12,2 | 0,931 | H5A034 C | sample | Hm Drgbl | Hm Drgbl | Hm Drgbl | CPE002E3 | CPE002EW | CPE002FH | NM 00428 | NM 0042 | NM 0042 | RAB3D | RAB3D | RAB3D | RAB3D, member R | RAB3D, m | RAB3D, m | 9545 | 9545 | 9545 | s18327 | s18328 | s18326 |
| 17012 | E5 | E6 | 1359 | 915,2 | 16609 | 172,3 | 12,2 | 0,942 | H5A034 C | sample | Hm Gen E | Hm Gen E | Hm Gen E | CPE002JI | CPE002NZ | CPE002KD | NM 01646 | NM 2073 | NM 0164 | ORMDL1 | C10orf129 | ORMDL1 | ORM1-like 1 (S. ce | chromoso | ORM1-like | 94101 | 142827 | 94101 | s41257 | s44493 | s41259 |
| 7290 | P3 | P4 | 1071 | 629,2 | 13045 | 364,7 | 12,2 | 0,949 | H5A034 C | sample | Hm Drgbl | Hm Drgbl | Hm Drgbl | CPE002E2 | CPE002EX | CPE002FI | NM 00537 | NM 0053 | NM 0053 | MPL | MPL | MPL | myeloproliferativ | myeloprol | myeloprol | 4352 | 4352 | 4352 | s8935 | s8936 | s8934 |
| 5049 | C17 | C18 | 1051 | 586,9 | 12838 | 467,8 | 12,2 | 0,957 | H5A034 C | sample | Hm Drgbl | Hm Drgbl | Hm Drgbl | CPE002DV | CPE002EM | CPE002FO | NM 14572 | NM 1457 | NM 1457 | LPAL2 | LPAL2 | LPAL2 | lipoprotein, Lp(a) | lipoprotei | lipoprotei | 80350 | 80350 | 80350 | s37276 | s37277 | s37278 |
| 5243 | K19 | K20 | 1850 | 705 | 22569 | 351,6 | 12,2 | 0,957 | H5A034 C | sample | Hm Drgbl | Hm Drgbl | Hm Drgbl | CPE002DV | CPE002EM | CPE002FO | NM 14469 | NM 1446 | NM 1446 | CAPN12 | CAPN12 | CAPN12 | calpain 12 | calpain 12 | calpain 12 | 147968 | 147968 | 147968 | s45098 | s45099 | s45100 |
| 5278 | L7 | L8 | 1833 | 718,3 | 22430 | 461,5 | 12,2 | 0,957 | H5A034 C | sample | Hm Drgbl | Hm Drgbl | Hm Drgbl | CPE002DV | CPE002EM | CPE002FO | NM 15270 | NM 1527 | NM 1527 | SLC25A16 | SLC25A16 | SLC25A16 | solute carrier fam | solute car | solute car | 8034 | 8034 | 8034 | s15569 | s15568 | s15570 |
| 5374 | P7 | P8 | 1608 | 647,8 | 19690 | 438,1 | 12,2 | 0,957 | H5A034 C | sample | Hm Drgbl | Hm Drgbl | Hm Drgbl | CPE002DV | CPE002EM | CPE002FO | NM 00348 | NM 0034 | NM 0034 | AKAP1 | AKAP1 | AKAP1 | A kinase (PRKA) a | A kinase ( | A kinase ( | 8165 | 8165 | 8165 | s15665 | s15666 | s15667 |
| 2311 | A15 | A16 | 1888 | 730,8 | 23057 | 403,2 | 12,2 | 0,961 | H5A034 C | sample | Hm Drgbl | Hm Drgbl | Hm Drgbl | CPE002DC | CPE002EE | CPE002F4 | NM 00399 | NM 0039 | NM 0039 | CLK2 | CLK2 | CLK2 | CDC-like kinase 2 | CDC-like k | CDC-like k | 1196 | 1196 | 1196 | s3165 | s3166 | s3167 |
| 2437 | F20 | F21 | 1253 | 707,2 | 15243 | 450,9 | 12,2 | 0,961 | H5A034 C | sample | Hm Drgbl | Hm Drgbl | Hm Drgbl | CPE002DC | CPE002EE | CPE002F4 | NM 00275 | NM 0027 | NM 0027 | MAP2K6 | MAP2K6 | MAP2K6 | mitogen-activated | mitogen-a | mitogen-a | 5608 | 5608 | 5608 | s11179 | s11180 | s11181 |
| 16595 | D19 | D20 | 1596 | 1072 | 19500 | 275,3 | 12,2 | 0,964 | H5A034 C | sample | Hm Gen E | Hm Gen E | Hm Gen E | CPE002JH | CPE002NV | CPE002KC | NM 03339 | NM 0333 | NM 0333 | KIAA1754 | KIAA1754 | KIAA1754 | KIAA1754 | KIAA1754 | KIAA1754 | 85450 | 85450 | 85450 | s40015 | s40016 | s40017 |
| 16622 | E21 | E22 | 1614 | 991,2 | 19740 | 271,8 | 12,2 | 0,964 | H5A034 C | sample | Hm Gen E | Hm Gen E | Hm Gen E | CPE002JH | CPE002NV | CPE002KC | NM 03284 | NM 0328 | NM 0328 | C13orf33 | C13orf33 | C13orf33 | chromosome 13 o | chromoso | chromoso | 84935 | 84935 | 84935 | s39697 | s39698 | s228399 |
| 16709 | I13 | I14 | 1959 | 965,5 | 23892 | 288,5 | 12,2 | 0,964 | H5A034 C | sample | Hm Gen E | Hm Gen E | Hm Gen E | CPE002JH | CPE002NV | CPE002KC | NM 03288 | NM 0328 | NM 0328 | IGSF21 | IGSF21 | IGSF21 | immunoglobin su | immunogl | immunogl | 84966 | 84966 | 84966 | s39770 | s39771 | s39772 |
| 16753 | K1 | K2 | 1139 | 968,2 | 13921 | 199,8 | 12,2 | 0,964 | H5A034 C | sample | Hm Gen E | Hm Gen E | Hm Gen E | CPE002JH | CPE002NV | CPE002KC | NM 03289 | NM 0328 | NM 0328 | DISP1 | DISP1 | DISP1 | dispatched homo | dispatche | dispatche | 84976 | 84976 | 84976 | s39791 | s39792 | s39793 |
| 16767 | K22 | K23 | 1357 | 999,2 | 16491 | 354,4 | 12,2 | 0,964 | H5A034 C | sample | Hm Gen E | Hm Gen E | Hm Gen E | CPE002JH | CPE002NV | CPE002KC | NM 05285 | NM 0528 | NM 0528 | ANKRD40 | ANKRD40 | ANKRD40 | ankyrin repeat do | ankyrin re | ankyrin re | 91369 | 91369 | 91369 | s40633 | s40634 | s40635 |
| 16789 | L20 | L21 | 1644 | 960,4 | 20025 | 277,6 | 12,2 | 0,964 | H5A034 C | sample | Hm Gen E | Hm Gen E | Hm Gen E | CPE002JH | CPE002NV | CPE002KC | NM 17816 | NM 1781 | NM 1781 | OTOP2 | OTOP2 | OTOP2 | otopetrin 2 | otopetrin | otopetrin | 92736 | 92736 | 92736 | s41007 | s41008 | s41009 |
| 17695 | B15 | B16 | 1465 | 1155 | 17828 | 339,7 | 12,2 | 0,964 | H5A034 C | sample | Hm Gen E | Hm Gen E | Hm Gen E | CPE002JK | CPE002NZ | CPE002KF | NM 20732 | NM 2073 | NM 2073 | FAM148A | FAM148A | NLF1 | family with seque | family wit | nuclear lo | 145741 | 145741 | 145741 | s44765 | s44766 | s225695 |
| 17850 | H3 | H4 | 1147 | 1002 | 13993 | 154,8 | 12,2 | 0,964 | H5A034 C | sample | Hm Gen E | Hm Gen E | Hm Gen E | CPE002JK | CPE002NZ | CPE002KF | XM 37099 | XM 37099 | XM 37099 | LOC14642 | LOC14642 | LOC14642 | hypothetical prot | hypotheti | hypotheti | 146429 | 146429 | 146429 | s195760 | s195761 | s195762 |
| 17999 | N8 | N9 | 1996 | 1059 | 24378 | 213,9 | 12,2 | 0,964 | H5A034 C | sample | Hm Gen E | Hm Gen E | Hm Gen E | CPE002JK | CPE002NZ | CPE002KF | NM 00108 | NM 0010 | NM 0010 | SHISA3 | SHISA3 | SHISA3 | shisa homolog 3 ( | shisa hom | shisa hom | 152573 | 152573 | 152573 | s45709 | s45710 | s45711 |
| 9164 | N5 | N6 | 1504 | 1581 | 18344 | 135,5 | 12,2 | 0,98 | H5A034 C | sample | Hm Drgbl | Hm Drgbl | Hm Drgbl | CPE002E7 | CPE002ES | CPE002FD | NM 01623 | NM 0162 | NM 0162 | ANAPC7 | ANAPC7 | ANAPC7 | anaphase promot | anaphase | anaphase | 51434 | 51434 | 51434 | s28134 | s28133 | s28132 |
| 4225 | A1 | A2 | 1829 | 741 | 22246 | 486,3 | 12,2 | 1,03 | H5A034 C | sample | Hm Drgbl | Hm Drgbl | Hm Drgbl | CPE002DU | CPE002EK | CPE002FQ | NM 00222 | NM 0022 | NM 0022 | ITPR3 | ITPR3 | ITPR3 | inositol 1,4,5-trip | inositol 1, | inositol 1, | 3710 | 3710 | 3710 | s265 | s266 | s267 |
| 4282 | C18 | C19 | 1093 | 669,3 | 13358 | 313,4 | 12,2 | 1,03 | H5A034 C | sample | Hm Drgbl | Hm Drgbl | Hm Drgbl | CPE002DU | CPE002EK | CPE002FQ | NM 00363 | NM 0036 | NM 0036 | KCNAB2 | KCNAB2 | KCNAB2 | potassium voltage | potassium | potassium | 8514 | 8514 | 8514 | s16177 | s16178 | s16179 |
| 4447 | J15 | J16 | 854 | 732,1 | 10413 | 389,7 | 12,2 | 1,03 | H5A034 C | sample | Hm Drgbl | Hm Drgbl | Hm Drgbl | CPE002DU | CPE002EK | CPE002FQ | NM 02101 | NM 0210 | NM 0210 | KCNJ12 | KCNJ12 | KCNJ12 | potassium inward | potassium | potassium | 3768 | 3768 | 3768 | s7762 | s7763 | s7764 |
| 4515 | M11 | M12 | 1146 | 718,1 | 13971 | 522 | 12,2 | 1,03 | H5A034 C | sample | Hm Drgbl | Hm Drgbl | Hm Drgbl | CPE002DU | CPE002EK | CPE002FQ | NM 00080 | NM 0008 | NM 0008 | GABRA2 | GABRA2 | GABRA2 | gamma-aminobut | gamma-ar | gamma-ar | 2555 | 2555 | 2555 | s5473 | s5474 | s5475 |
| 4527 | M22 | M23 | 1471 | 696,6 | 17949 | 380 | 12,2 | 1,03 | H5A034 C | sample | Hm Drgbl | Hm Drgbl | Hm Drgbl | CPE002DU | CPE002EK | CPE002FQ | NM 01899 | NM 0189 | NM 0189 | KCTD5 | KCTD5 | KCTD5 | potassium channe | potassium | potassium | 54442 | 54442 | 54442 | s28979 | s28980 | s28981 |
| 26 | B10 | B11 | 429 | 1529 | 5241 | 814,8 | 12,2 | 1,038 | H5A034 C | sample | Hm Drgbl | Hm Drgbl | Hm Drgbl | CPE002DT | CPE002EJ | CPE002F9 | NM 00311 | NM 0031 | NM 0031 | SP4 | SP4 | SP4 | Sp4 transcription | Sp4 transc | Sp4 transc | 6671 | 6671 | 6671 | s13329 | s13327 | s13328 |
| 87 | D22 | D23 | 520 | 1528 | 6334 | 380,2 | 12,2 | 1,038 | H5A034 C | sample | Hm Drgbl | Hm Drgbl | Hm Drgbl | CPE002DT | CPE002EJ | CPE002F9 | XM 00112 | XM 00112 | XM 00112 | SPRR1B | SPRR1B | SPRR1B | small proline-rich | small prol | small prol | 6699 | 6699 | 6699 | s13385 | s13383 | s13384 |
| 150 | G14 | G15 | 525 | 1375 | 6404 | 658,4 | 12,2 | 1,038 | H5A034 C | sample | Hm Drgbl | Hm Drgbl | Hm Drgbl | CPE002DT | CPE002EJ | CPE002F9 | NM 13904 | NM 1390 | NM 1390 | HLTF | HLTF | HLTF | helicase-like trans | helicase-li | helicase-li | 6596 | 6596 | 6596 | s13136 | s13138 | s13137 |
| 1931 | A19 | A20 | 1936 | 718 | 23565 | 465 | 12,2 | 1,061 | H5A034 C | sample | Hm Drgbl | Hm Drgbl | Hm Drgbl | CPE002FX | CPE002G1 | CPE002G5 | NM 18323 | NM 1832 | NM 1832 | ZNF605 | ZNF605 | ZNF605 | zinc finger protein | zinc finger | zinc finger | 90462 | 90462 | 90462 | s40394 | s40395 | s40396 |
| 2115 | I11 | I12 | 960 | 662,6 | 11678 | 361,9 | 12,2 | 1,061 | H5A034 C | sample | Hm Drgbl | Hm Drgbl | Hm Drgbl | CPE002FX | CPE002G1 | CPE002G5 | NM 13844 | NM 1384 | NM 1384 | KCTD12 | KCTD12 | KCTD12 | potassium channe | potassium | potassium | 115207 | 115207 | 115207 | s41772 | s41773 | s41774 |
| 2221 | M20 | M21 | 1447 | 707,2 | 17598 | 453 | 12,2 | 1,061 | H5A034 C | sample | Hm Drgbl | Hm Drgbl | Hm Drgbl | CPE002FX | CPE002G1 | CPE002G5 | NM 17383 | NM 1738 | NM 1738 | ZNF707 | ZNF707 | ZNF707 | zinc finger protein | zinc finger | zinc finger | 286075 | 286075 | 286075 | s50087 | s50088 | s50089 |
| 6582 | C14 | C15 | 648 | 559,2 | 7879 | 418 | 12,2 | 1,07 | H5A034 C | sample | Hm Drgbl | Hm Drgbl | Hm Drgbl | CPE002E1 | CPE002EY | CPE002FJ | NM 00218 | NM 0021 | NM 0021 | IGHMBP2 | IGHMBP2 | IGHMBP2 | immunoglobulin m | immunogl | immunogl | 3508 | 3508 | 3508 | s7245 | s7247 | s7246 |
| 6607 | D15 | D16 | 1652 | 623,6 | 20173 | 366,3 | 12,2 | 1,07 | H5A034 C | sample | Hm Drgbl | Hm Drgbl | Hm Drgbl | CPE002E1 | CPE002EY | CPE002FJ | NM 01910 | NM 0191 | NM 0191 | HOXA5 | HOXA5 | HOXA5 | homeobox A5 | homeobo | homeobo | 3202 | 3202 | 3202 | s6764 | s223862 | s6766 |
| 6643 | E4 | E5 | 1090 | 655,8 | 13335 | 343 | 12,2 | 1,07 | H5A034 C | sample | Hm Drgbl | Hm Drgbl | Hm Drgbl | CPE002E1 | CPE002EY | CPE002FJ | NM 17384 | NM 1738 | NM 1738 | IL1RN | IL1RN | IL1RN | interleukin 1 rece | interleuki | interleuki | 3557 | 3557 | 3557 | s7278 | s7279 | s7280 |
| 6846 | N14 | N15 | 1184 | 646,7 | 14424 | 508,6 | 12,2 | 1,07 | H5A034 C | sample | Hm Drgbl | Hm Drgbl | Hm Drgbl | CPE002E1 | CPE002EY | CPE002FJ | NM 15349 | NM 1534 | NM 1534 | KRT13 | KRT13 | KRT13 | keratin 13 | keratin 13 | keratin 13 | 3860 | 3860 | 3860 | s7980 | s7981 | s7982 |
| 4016 | H16 | H17 | 1083 | 696,9 | 13265 | 353,4 | 12,2 | 1,084 | H5A034 C | sample | Hm Drgbl | Hm Drgbl | Hm Drgbl | CPE002DS | CPE002EI | CPE002F8 | NM 00294 | NM 0029 | NM 0029 | ROBO1 | ROBO1 | ROBO1 | roundabout, axor | roundabo | roundabo | 6091 | 6091 | 6091 | s12093 | s12092 | s12091 |
| 4017 | H17 | H18 | 985 | 708,8 | 11986 | 322,4 | 12,2 | 1,084 | H5A034 C | sample | Hm Drgbl | Hm Drgbl | Hm Drgbl | CPE002DS | CPE002EI | CPE002F8 | NM 00599 | NM 0059 | NM 0059 | TBX3 | TBX3 | TBX3 | T-box 3 (ulnar ma | T-box 3 (u | T-box 3 (u | 6926 | 6926 | 6926 | s13865 | s13866 | s13867 |
| 4046 | I21 | I22 | 1240 | 611,7 | 15126 | 344,7 | 12,2 | 1,084 | H5A034 C | sample | Hm Drgbl | Hm Drgbl | Hm Drgbl | CPE002DS | CPE002EI | CPE002F8 | NM 00269 | NM 0026 | NM 0026 | POLD1 | POLD1 | POLD1 | polymerase (DNA | polymeras | polymeras | 5424 | 5424 | 5424 | s614 | s615 | s616 |
| 4179 | O11 | O12 | 1408 | 670,5 | 17227 | 542,8 | 12,2 | 1,084 | H5A034 C | sample | Hm Drgbl | Hm Drgbl | Hm Drgbl | CPE002DS | CPE002EI | CPE002F8 | NM 15229 | NM 1522 | NM 1522 | TARS | TARS | TARS | threonyl-tRNA syn | threonyl-t | threonyl-t | 6897 | 6897 | 6897 | s13795 | s13793 | s13794 |
| 4200 | O9 | O10 | 1145 | 648,4 | 14002 | 439,4 | 12,2 | 1,084 | H5A034 C | sample | Hm Drgbl | Hm Drgbl | Hm Drgbl | CPE002DS | CPE002EI | CPE002F8 | NM 00270 | NM 0027 | NM 0027 | PPAT | PPAT | PPAT | phosphoribosyl p | phosphor | phosphor | 5471 | 5471 | 5471 | s10895 | s10896 | s10897 |
| 15646 | L7 | L8 | 1385 | 784,8 | 16913 | 142,7 | 12,2 | 1,109 | H5A034 C | sample | Hm Gen E | Hm Gen E | Hm Gen E | CPE002JE | CPE002NT | CPE002K9 | NM 02284 | NM 0228 | NM 0228 | CDCP1 | CDCP1 | CDCP1 | CUB domain cont | CUB doma | CUB doma | 64866 | 64866 | 64866 | s35060 | s35061 | s35062 |
| 5781 | A6 | A7 | 1593 | 883,9 | 19477 | 449,7 | 12,2 | 1,114 | H5A034 C | sample | Hm Drgbl | Hm Drgbl | Hm Drgbl | CPE002DY | CPE002F1 | CPE002FM | NM 00166 | NM 0016 | NM 0016 | ARF6 | ARF6 | ARF6 | ADP-ribosylation | ADP-ribos | ADP-ribos | 382 | 382 | 382 | s223978 | s1566 | s1567 |
| 5799 | B22 | B23 | 1979 | 976,8 | 24127 | 490,2 | 12,2 | 1,114 | H5A034 C | sample | Hm Drgbl | Hm Drgbl | Hm Drgbl | CPE002DY | CPE002F1 | CPE002FM | NM 17073 | NM 1707 | NM 1707 | BDNF | BDNF | BDNF | brain-derived neu | brain-deri | brain-deri | 627 | 627 | 627 | s1962 | s1963 | s1964 |
| 5818 | C18 | C19 | 1017 | 840,8 | 12358 | 590,9 | 12,2 | 1,114 | H5A034 C | sample | Hm Drgbl | Hm Drgbl | Hm Drgbl | CPE002DY | CPE002F1 | CPE002FM | NM 00166 | NM 0016 | NM 0016 | ARL2 | ARL2 | ARL2 | ADP-ribosylation | ADP-ribos | ADP-ribos | 402 | 402 | 402 | s224019 | s1608 | s1609 |
| 5919 | G22 | G23 | 1824 | 767 | 22191 | 484,1 | 12,2 | 1,114 | H5A034 C | sample | Hm Drgbl | Hm Drgbl | Hm Drgbl | CPE002DY | CPE002F1 | CPE002FM | NM 00005 | NM 0000 | NM 0000 | ASS1 | ASS1 | ASS1 | argininosuccinate | argininosu | argininosu | 445 | 445 | 445 | s1684 | s1685 | s1686 |
| 5966 | I21 | I22 | 1186 | 899,6 | 14492 | 361,8 | 12,2 | 1,114 | H5A034 C | sample | Hm Drgbl | Hm Drgbl | Hm Drgbl | CPE002DY | CPE002F1 | CPE002FM | NM 00112 | NM 0011 | NM 0011 | ADD3 | ADD3 | ADD3 | adducin 3 (gamm | adducin 3 | adducin 3 | 120 | 120 | 120 | s1054 | s1052 | s1053 |
| 6079 | N15 | N16 | 1902 | 819,5 | 23237 | 515,9 | 12,2 | 1,114 | H5A034 C | sample | Hm Drgbl | Hm Drgbl | Hm Drgbl | CPE002DY | CPE002F1 | CPE002FM | NM 19809 | NM 1980 | NM 1980 | AQP1 | AQP1 | AQP1 | aquaporin 1 (Colt | aquaporin | aquaporin | 358 | 358 | 358 | s1517 | s1515 | s1516 |
| 6138 | P3 | P4 | 1541 | 838,4 | 18836 | 399,7 | 12,2 | 1,114 | H5A034 C | sample | Hm Drgbl | Hm Drgbl | Hm Drgbl | CPE002DY | CPE002F1 | CPE002FM | NM 00165 | NM 0016 | NM 0016 | AQP6 | AQP6 | AQP6 | aquaporin 6, kidn | aquaporin | aquaporin | 363 | 363 | 363 | s1529 | s1531 | s1530 |
| 1157 | A13 | A14 | 1353 | 732,7 | 16477 | 480,4 | 12,2 | 1,119 | H5A034 C | sample | Hm Drgbl | Hm Drgbl | Hm Drgbl | CPE002FV | CPE002FZ | CPE002G3 | NM 00712 | NM 0071 | NM 0071 | ZIC2 | ZIC2 | ZIC2 | Zic family membe | Zic family | Zic family | 7546 | 7546 | 7546 | s14998 | s14999 | s15000 |
| 1186 | B18 | B19 | 1631 | 763,3 | 19900 | 501,9 | 12,2 | 1,119 | H5A034 C | sample | Hm Drgbl | Hm Drgbl | Hm Drgbl | CPE002FV | CPE002FZ | CPE002G3 | NM 00661 | NM 0066 | NM 0066 | JARID1B | JARID1B | JARID1B | jumonji, AT rich in | jumonji, A | jumonji, A | 10765 | 10765 | 10765 | s21144 | s21145 | s21146 |
| 1249 | E1 | E2 | 979 | 772 | 11959 | 460,1 | 12,2 | 1,119 | H5A034 C | sample | Hm Drgbl | Hm Drgbl | Hm Drgbl | CPE002FV | CPE002FZ | CPE002G3 | NM 00696 | NM 0069 | NM 0069 | ZNF182 | ZNF182 | ZNF182 | zinc finger protein | zinc finger | zinc finger | 7569 | 7569 | 7569 | s15048 | s15049 | s15050 |
| 1291 | F4 | F5 | 1578 | 681,8 | 19257 | 405,5 | 12,2 | 1,119 | H5A034 C | sample | Hm Drgbl | Hm Drgbl | Hm Drgbl | CPE002FV | CPE002FZ | CPE002G3 | NM 00668 | NM 0066 | NM 0066 | CFHR4 | CFHR4 | CFHR4 | complement facto | compleme | compleme | 10877 | 10877 | 10877 | s21358 | s21359 | s21360 |
| 1340 | H5 | H6 | 1102 | 746,6 | 13395 | 454 | 12,2 | 1,119 | H5A034 C | sample | Hm Drgbl | Hm Drgbl | Hm Drgbl | CPE002FV | CPE002FZ | CPE002G3 | NM 00376 | NM 0037 | NM 0037 | STX11 | STX11 | STX11 | syntaxin 11 | syntaxin 1 | syntaxin 1 | 8676 | 8676 | 8676 | s16531 | s16532 | s16533 |
| 1502 | O21 | O22 | 1920 | 765,3 | 23431 | 505,2 | 12,2 | 1,119 | H5A034 C | sample | Hm Drgbl | Hm Drgbl | Hm Drgbl | CPE002FV | CPE002FZ | CPE002G3 | NM 00630 | NM 0063 | NM 0063 | ZNF230 | ZNF230 | ZNF230 | zinc finger protein | zinc finger | zinc finger | 7773 | 7773 | 7773 | s224797 | s15321 | s15322 |
| 16143 | A22 | A23 | 1152 | 1011 | 14017 | 362,8 | 12,2 | 1,119 | H5A034 C | sample | Hm Gen E | Hm Gen E | Hm Gen E | CPE002JG | CPE002NV | CPE002KB | NM 03226 | NM 0322 | NM 0322 | IQCG | IQCG | IQCG | IQ motif containin | IQ motif c | IQ motif c | 84223 | 84223 | 84223 | s38670 | s38671 | s38672 |
| 16170 | B3 | B4 | 1540 | 897,4 | 18816 | 310,5 | 12,2 | 1,119 | H5A034 C | sample | Hm Gen E | Hm Gen E | Hm Gen E | CPE002JG | CPE002NV | CPE002KB | NM 03147 | NM 0314 | NM 0314 | YPEL3 | YPEL3 | YPEL3 | yippee-like 3 (Dro | yippee-lik | yippee-lik | 83719 | 83719 | 83719 | s38145 | s38146 | s38147 |
| 16392 | K9 | K10 | 956 | 1032 | 11642 | 425 | 12,2 | 1,119 | H5A034 C | sample | Hm Gen E | Hm Gen E | Hm Gen E | CPE002JG | CPE002NV | CPE002KB | NM 03131 | NM 0313 | NM 0313 | PLVAP | PLVAP | PLVAP | plasmalemma ves | plasmaler | plasmaler | 83483 | 83483 | 83483 | s37972 | s37973 | s37974 |
| 16474 | O18 | O19 | 1589 | 965,5 | 19420 | 390,5 | 12,2 | 1,119 | H5A034 C | sample | Hm Gen E | Hm Gen E | Hm Gen E | CPE002JG | CPE002NV | CPE002KB | NM 03238 | NM 0323 | NM 0323 | HPS3 | HPS3 | HPS3 | Hermansky-Pudla | Hermansk | Hermansk | 84343 | 84343 | 84343 | s38959 | s38960 | s38961 |
| 13635 | I11 | I12 | 778 | 953,5 | 9467 | 164,2 | 12,2 | 1,146 | H5A034 C | sample | Hm Gen E | Hm Gen E | Hm Gen E | CPE002JC | CPE002NC | CPE002K4 | NM 01534 | NM 0153 | NM 0153 | DULLARD | DULLARD | DULLARD | dullard homolog | dullard ho | dullard ho | 23399 | 23399 | 23399 | s23739 | s23740 | s23741 |
| 13657 | J1 | J2 | 1237 | 1096 | 15103 | 178,5 | 12,2 | 1,146 | H5A034 C | sample | Hm Gen E | Hm Gen E | Hm Gen E | CPE002JC | CPE002NC | CPE002K4 | NM 01212 | NM 0121 | NM 0121 | CIZ1 | CIZ1 | CIZ1 | CDKN1A interacti | CDKN1A ir | CDKN1A ir | 25792 | 25792 | 25792 | s24488 | s24489 | s24490 |
| 13679 | J8 | J9 | 1050 | 999,6 | 12775 | 165,8 | 12,2 | 1,146 | H5A034 C | sample | Hm Gen E | Hm Gen E | Hm Gen E | CPE002JC | CPE002NC | CPE002K4 | NM 00104 | NM 0010 | NM 0010 | NUPR1 | NUPR1 | NUPR1 | nuclear protein 1 | nuclear pr | nuclear pr | 26471 | 26471 | 26471 | s25436 | s25437 | s25438 |
| 814 | B7 | B8 | 1330 | 752,2 | 16190 | 477,2 | 12,2 | 1,156 | H5A034 C | sample | Hm Drgbl | Hm Drgbl | Hm Drgbl | CPE002FU | CPE002FY | CPE002G2 | NM 00144 | NM 0014 | NM 0014 | FHL1 | FHL1 | FHL1 | four and a half LII | four and a | four and a | 2273 | 2273 | 2273 | s5194 | s5195 | s223534 |
| 1026 | K3 | K4 | 1376 | 720,5 | 16840 | 401,9 | 12,2 | 1,156 | H5A034 C | sample | Hm Drgbl | Hm Drgbl | Hm Drgbl | CPE002FU | CPE002FY | CPE002G2 | NM 05817 | NM 0581 | NM 0581 | COL6A2 | COL6A2 | COL6A2 | collagen, type VI, | collagen, t | collagen, t | 1292 | 1292 | 1292 | s3314 | s3315 | s3316 |
| 1051 | L4 | L5 | 1587 | 768 | 19322 | 413,4 | 12,2 | 1,156 | H5A034 C | sample | Hm Drgbl | Hm Drgbl | Hm Drgbl | CPE002FU | CPE002FY | CPE002G2 | NM 00564 | NM 0056 | NM 0056 | ZNF354A | ZNF354A | ZNF354A | zinc finger protein | zinc finger | zinc finger | 6940 | 6940 | 6940 | s13895 | s13896 | s13897 |
| 1115 | O19 | O20 | 1719 | 702,4 | 21002 | 471,6 | 12,2 | 1,156 | H5A034 C | sample | Hm Drgbl | Hm Drgbl | Hm Drgbl | CPE002FU | CPE002FY | CPE002G2 | NM 00198 | NM 0019 | NM 0019 | ETV4 | ETV4 | ETV4 | ets variant gene 4 | ets varian | ets varian | 2118 | 2118 | 2118 | s4860 | s4861 | s4862 |
| 5484 | E2 | E3 | 1133 | 731,6 | 13851 | 603,4 | 12,2 | 1,162 | H5A034 C | sample | Hm Drgbl | Hm Drgbl | Hm Drgbl | CPE002DX | CPE002F2 | CPE002FN | NM 00388 | NM 0038 | NM 0038 | DDEF2 | DDEF2 | DDEF2 | development and | developm | developm | 8853 | 8853 | 8853 | s16904 | s16903 | s16905 |
| 5497 | F1 | F2 | 1692 | 716 | 20610 | 643,1 | 12,2 | 1,162 | H5A034 C | sample | Hm Drgbl | Hm Drgbl | Hm Drgbl | CPE002DX | CPE002F2 | CPE002FN | NM 00374 | NM 0037 | NM 0037 | NUMB | NUMB | NUMB | numb homolog (D | numb hor | numb hor | 8650 | 8650 | 8650 | s16467 | s16465 | s16466 |
| 5611 | J4 | J5 | 2075 | 789,1 | 25266 | 387,9 | 12,2 | 1,162 | H5A034 C | sample | Hm Drgbl | Hm Drgbl | Hm Drgbl | CPE002DX | CPE002F2 | CPE002FN | NM 00545 | NM 0054 | NM 0054 | NOG | NOG | NOG | noggin | noggin | noggin | 9241 | 9241 | 9241 | s17668 | s17669 | s194994 |
| 5684 | M5 | M6 | 1331 | 826,2 | 16284 | 581,6 | 12,2 | 1,162 | H5A034 C | sample | Hm Drgbl | Hm Drgbl | Hm Drgbl | CPE002DX | CPE002F2 | CPE002FN | NM 00365 | NM 0036 | NM 0036 | CST7 | CST7 | CST7 | cystatin F (leukoc | cystatin F | cystatin F | 8530 | 8530 | 8530 | s16219 | s16220 | s16218 |
| 5698 | N18 | N19 | 1937 | 778 | 23575 | 425,7 | 12,2 | 1,162 | H5A034 C | sample | Hm Drgbl | Hm Drgbl | Hm Drgbl | CPE002DX | CPE002F2 | CPE002FN | NM 20163 | NM 2016 | NM 2016 | TGM5 | TGM5 | TGM5 | transglutaminase | transgluta | transgluta | 9333 | 9333 | 9333 | s17841 | s17842 | s17843 |
| 5739 | P11 | P12 | 1611 | 718,7 | 19614 | 548,4 | 12,2 | 1,162 | H5A034 C | sample | Hm Drgbl | Hm Drgbl | Hm Drgbl | CPE002DX | CPE002F2 | CPE002FN | NM 00384 | NM 0038 | NM 0038 | TNFRSF10A | TNFRSF10 | TNFRSF10 | tumor necrosis fa | tumor nec | tumor nec | 8797 | 8797 | 8797 | s16762 | s16763 | s16764 |
| 3594 | F3 | F4 | 1424 | 1485 | 17423 | 434,9 | 12,2 | 1,173 | H5A034 C | sample | Hm Drgbl | Hm Drgbl | Hm Drgbl | CPE002DR | CPE002EH | CPE002F7 | NM 00150 | NM 0015 | NM 0015 | GPR32 | GPR32 | GPR32 | G protein-couple | G protein- | G protein- | 2854 | 2854 | 2854 | s6057 | s6058 | s6059 |
| 3766 | M7 | M8 | 1162 | 1563 | 14174 | 382,2 | 12,2 | 1,173 | H5A034 C | sample | Hm Drgbl | Hm Drgbl | Hm Drgbl | CPE002DR | CPE002EH | CPE002F7 | NM 00199 | NM 0019 | NM 0019 | F2R | F2R | F2R | coagulation facto | coagulatic | coagulatic | 2149 | 2149 | 2149 | s4922 | s4923 | s4924 |
| 8076 | A2 | A3 | 1352 | 605,4 | 16452 | 503,4 | 12,2 | 1,173 | H5A034 C | sample | Hm Drgbl | Hm Drgbl | Hm Drgbl | CPE002E5 | CPE002EU | CPE002FF | NM 01503 | NM 0150 | NM 0150 | FNBP1 | FNBP1 | FNBP1 | formin binding pr | formin bir | formin bir | 23048 | 23048 | 23048 | s22914 | s22915 | s22916 |
| 8116 | C12 | C13 | 1226 | 659,1 | 15010 | 576,3 | 12,2 | 1,173 | H5A034 C | sample | Hm Drgbl | Hm Drgbl | Hm Drgbl | CPE002E5 | CPE002EU | CPE002FF | NM 01507 | NM 0150 | NM 0150 | SIPA1L3 | SIPA1L3 | SIPA1L3 | signal-induced pr | signal-ind | signal-ind | 23094 | 23094 | 23094 | s23021 | s23019 | s23020 |
| 8123 | C19 | C20 | 1346 | 622,2 | 16465 | 490,9 | 12,2 | 1,173 | H5A034 C | sample | Hm Drgbl | Hm Drgbl | Hm Drgbl | CPE002E5 | CPE002EU | CPE002FF | NM 00100 | NM 0010 | NM 0010 | ATE1 | ATE1 | ATE1 | arginyltransferase | arginyltra | arginyltra | 11101 | 11101 | 11101 | s21888 | s21886 | s21887 |
| 8222 | G21 | G22 | 1668 | 638,9 | 20422 | 487,7 | 12,2 | 1,173 | H5A034 C | sample | Hm Drgbl | Hm Drgbl | Hm Drgbl | CPE002E5 | CPE002EU | CPE002FF | NM 02114 | NM 0211 | NM 0211 | PSIP1 | PSIP1 | PSIP1 | PC4 and SFRS1 int | PC4 and S | PC4 and S | 11168 | 11168 | 11168 | s22036 | s22037 | s22035 |
| 8438 | P21 | P22 | 1504 | 588,9 | 18403 | 465,5 | 12,2 | 1,173 | H5A034 C | sample | Hm Drgbl | Hm Drgbl | Hm Drgbl | CPE002E5 | CPE002EU | CPE002FF | NM 01759 | NM 0175 | NM 0175 | KIF21B | KIF21B | KIF21B | kinesin family me | kinesin fa | kinesin fa | 23046 | 23046 | 23046 | s22909 | s22908 | s22910 |
| 10157 | H13 | H14 | 1185 | 761,3 | 14432 | 124,9 | 12,2 | 1,173 | H5A034 C | sample | Hm Drgbl | Hm Drgbl | Hm Drgbl | CPE002EA | CPE002EP | CPE002FA | NM 01434 | NM 0143 | NM 0143 | APOL3 | APOL3 | APOL3 | apolipoprotein L, | apolipopr | apolipopr | 80833 | 80833 | 80833 | s37435 | s37436 | s37434 |
| 10175 | H8 | H9 | 1413 | 824,3 | 17232 | 140,2 | 12,2 | 1,173 | H5A034 C | sample | Hm Drgbl | Hm Drgbl | Hm Drgbl | CPE002EA | CPE002EP | CPE002FA | XM 00113 | XM 00113 | XM 00113 | TUBB6 | TUBB6 | TUBB6 | tubulin, beta 6 | tubulin, b | tubulin, b | 84617 | 84617 | 84617 | s39181 | s39182 | s39180 |
| 1611 | D11 | D12 | 1830 | 740 | 22324 | 421,4 | 12,2 | 1,277 | H5A034 C | sample | Hm Drgbl | Hm Drgbl | Hm Drgbl | CPE002FW | CPE002G0 | CPE002G4 | NM 01907 | NM 0190 | NM 0190 | SGTB | SGTB | SGTB | small glutamine-r | small glut | small glut | 54557 | 54557 | 54557 | s29204 | s29205 | s29206 |
| 1725 | H6 | H7 | 1458 | 720,4 | 17856 | 390 | 12,2 | 1,277 | H5A034 C | sample | Hm Drgbl | Hm Drgbl | Hm Drgbl | CPE002FW | CPE002G0 | CPE002G4 | NM 03063 | NM 0306 | NM 0306 | ZNF436 | ZNF436 | ZNF436 | zinc finger protein | zinc finger | zinc finger | 80818 | 80818 | 80818 | s37407 | s37408 | s37409 |
| 1765 | J20 | J21 | 1209 | 806,7 | 14695 | 334,1 | 12,2 | 1,277 | H5A034 C | sample | Hm Drgbl | Hm Drgbl | Hm Drgbl | CPE002FW | CPE002G0 | CPE002G4 | NM 03215 | NM 0321 | NM 0321 | PCGF6 | PCGF6 | PCGF6 | polycomb group r | polycomb | polycomb | 84108 | 84108 | 84108 | s38515 | s38516 | s38517 |
| 1776 | J9 | J10 | 1140 | 676,1 | 13887 | 397,7 | 12,2 | 1,277 | H5A034 C | sample | Hm Drgbl | Hm Drgbl | Hm Drgbl | CPE002FW | CPE002G0 | CPE002G4 | NM 01787 | NM 0178 | NM 0178 | ZNF416 | ZNF416 | ZNF416 | zinc finger protein | zinc finger | zinc finger | 55659 | 55659 | 55659 | s31188 | s31189 | s31190 |
| 1815 | L22 | L23 | 1203 | 748,3 | 14654 | 418,8 | 12,2 | 1,277 | H5A034 C | sample | Hm Drgbl | Hm Drgbl | Hm Drgbl | CPE002FW | CPE002G0 | CPE002G4 | XM 94595 | XM 94595 | XM 94595 | ZNF527 | ZNF527 | ZNF527 | zinc finger protein | zinc finger | zinc finger | 84503 | 84503 | 84503 | s39073 | s39074 | s39075 |
| 21576 | C9 | C10 | 322 | 847,2 | 3915 | 630 | 12,2 | 1,284 | H5A034 C | sample | Hm Gen E | Hm Gen E | Hm Gen E | CPE002JU | CPE002O9 | CPE002KP | XM 93813 | XM 93813 | XM 93813 | LOC64905 | LOC64905 | LOC64905 | similar to ret fing | similar to | similar to | 649055 | 649055 | 649055 | s56911 | s56912 | s56913 |
| 21589 | D20 | D21 | 470 | 1073 | 5742 | 515,7 | 12,2 | 1,284 | H5A034 C | sample | Hm Gen E | Hm Gen E | Hm Gen E | CPE002JU | CPE002O9 | CPE002KP | XM 00113 | XM 00113 | XM 00113 | DKFZP686I | DKFZP686I | DKFZP686I | general transcript | general tr | general tr | 653238 | 653238 | 653238 | s200637 | s200638 | s200639 |
| 21591 | D22 | D23 | 318 | 1022 | 3883 | 507,4 | 12,2 | 1,284 | H5A034 C | sample | Hm Gen E | Hm Gen E | Hm Gen E | CPE002JU | CPE002O9 | CPE002KP | XM 92811 | XM 92811 | XM 92811 | TIMM23B | TIMM23B | TIMM23B | translocase of inn | translocas | translocas | 653252 | 653252 | 653252 | s57626 | s57627 | s57628 |
| 21634 | F18 | F19 | 262 | 1035 | 3199 | 551,3 | 12,2 | 1,284 | H5A034 C | sample | Hm Gen E | Hm Gen E | Hm Gen E | CPE002JU | CPE002O9 | CPE002KP | NM 00026 | NM 0002 | NM 0002 | NCF1 | NCF1 | NCF1 | neutrophil cytoso | neutrophi | neutrophi | 653361 | 653361 | 653361 | s57647 | s57648 | s57649 |
| 21668 | G5 | G6 | 293 | 1099 | 3581 | 771 | 12,2 | 1,284 | H5A034 C | sample | Hm Gen E | Hm Gen E | Hm Gen E | CPE002JU | CPE002O9 | CPE002KP | XM 00113 | XM 00113 | XM 00113 | LOC64945 | LOC64945 | LOC64945 | similar to hCG203 | similar to | similar to | 649458 | 649458 | 649458 | s56976 | s56977 | s56978 |
| 21675 | H11 | H12 | 336 | 935,2 | 4092 | 699,9 | 12,2 | 1,284 | H5A034 C | sample | Hm Gen E | Hm Gen E | Hm Gen E | CPE002JU | CPE002O9 | CPE002KP | XM 94077 | XM 94077 | XM 94077 | LOC65159 | LOC65159 | LOC65159 | similar to peptidy | similar to | similar to | 651593 | 651593 | 651593 | s57218 | s227370 | s227371 |
| 21862 | O7 | O8 | 149 | 956,5 | 1821 | 867 | 12,2 | 1,284 | H5A034 C | sample | Hm Gen E | Hm Gen E | Hm Gen E | CPE002JU | CPE002O9 | CPE002KP | XM 93964 | XM 93964 | XM 93964 | LOC65055 | LOC65055 | LOC65055 | similar to Phosph | similar to | similar to | 650556 | 650556 | 650556 | s57083 | s57084 | s227343 |
| 21864 | O9 | O10 | 254 | 829 | 3087 | 940 | 12,2 | 1,284 | H5A034 C | sample | Hm Gen E | Hm Gen E | Hm Gen E | CPE002JU | CPE002O9 | CPE002KP | XM 94224 | XM 94224 | XM 94224 | LOC65055 | LOC65055 | LOC65055 | similar to HLA cla | similar to | similar to | 650557 | 650557 | 650557 | s57085 | s57086 | s57087 |
| 4694 | D21 | D22 | 1260 | 818,5 | 15413 | 508,1 | 12,2 | 1,341 | H5A034 C | sample | Hm Drgbl | Hm Drgbl | Hm Drgbl | CPE002DV | CPE002EL | CPE002FP | NM 00225 | NM 0022 | NM 0022 | KLK1 | KLK1 | KLK1 | kallikrein 1 | kallikrein | kallikrein | 3816 | 3816 | 3816 | s7872 | s7873 | s7874 |
| 4728 | E9 | E10 | 1290 | 819,1 | 15788 | 439,9 | 12,2 | 1,341 | H5A034 C | sample | Hm Drgbl | Hm Drgbl | Hm Drgbl | CPE002DV | CPE002EL | CPE002FP | NM 00002 | NM 0000 | NM 0000 | AGA | AGA | AGA | aspartylglucosam | aspartylgl | aspartylgl | 175 | 175 | 175 | s1160 | s1161 | s1162 |
| 4760 | G16 | G17 | 1068 | 763,9 | 12979 | 432,6 | 12,2 | 1,341 | H5A034 C | sample | Hm Drgbl | Hm Drgbl | Hm Drgbl | CPE002DV | CPE002EL | CPE002FP | NM 00381 | NM 0038 | NM 0038 | ADAM21 | ADAM21 | ADAM21 | ADAM metallopep | ADAM me | ADAM me | 8747 | 8747 | 8747 | s16672 | s16673 | s16674 |
| 4775 | G8 | G9 | 1129 | 767,4 | 13829 | 490,6 | 12,2 | 1,341 | H5A034 C | sample | Hm Drgbl | Hm Drgbl | Hm Drgbl | CPE002DV | CPE002EL | CPE002FP | NM 00379 | NM 0037 | NM 0037 | CTSF | CTSF | CTSF | cathepsin F | cathepsin | cathepsin | 8722 | 8722 | 8722 | s16612 | s16613 | s16614 |
| 4778 | H10 | H11 | 1264 | 856,2 | 15416 | 465,9 | 12,2 | 1,341 | H5A034 C | sample | Hm Drgbl | Hm Drgbl | Hm Drgbl | CPE002DV | CPE002EL | CPE002FP | NM 01405 | NM 0140 | NM 0140 | TMPRSS11 | TMPRSS11 | TMPRSS11 | transmembrane p | transmem | transmem | 28983 | 28983 | 28983 | s26351 | s26352 | s26353 |
| 4797 | H6 | H7 | 1630 | 786,3 | 19900 | 465 | 12,2 | 1,341 | H5A034 C | sample | Hm Drgbl | Hm Drgbl | Hm Drgbl | CPE002DV | CPE002EL | CPE002FP | NM 00101 | NM 0010 | NM 0010 | USP21 | USP21 | USP21 | ubiquitin specific | ubiquitin | ubiquitin | 27005 | 27005 | 27005 | s223719 | s25690 | s25691 |
| 10725 | O6 | O7 | 1357 | 820,9 | 16582 | 192,6 | 12,2 | 1,379 | H5A034 C | sample | Hm Drgbl | Hm Drgbl | Hm Drgbl | CPE002EB | CPE002EO | CPE002FR | NM 01402 | NM 0140 | NM 0140 | SSX2IP | SSX2IP | SSX2IP | synovial sarcoma, | synovial s | synovial s | 117178 | 117178 | 117178 | s42131 | s42130 | s42132 |
| 2779 | D4 | D5 | 816 | 674,9 | 9944 | 526,5 | 12,2 | 1,435 | H5A034 C | sample | Hm Drgbl | Hm Drgbl | Hm Drgbl | CPE002DP | CPE002EF | CPE002F5 | NM 05411 | NM 0541 | NM 0541 | IHPK3 | IHPK3 | IHPK3 | inositol hexaphos | inositol h | inositol h | 117283 | 117283 | 117283 | s42154 | s42155 | s42156 |
| 2862 | H14 | H15 | 504 | 596,7 | 6141 | 499,4 | 12,2 | 1,435 | H5A034 C | sample | Hm Drgbl | Hm Drgbl | Hm Drgbl | CPE002DP | CPE002EF | CPE002F5 | NM 14505 | NM 1450 | NM 1450 | FUK | FUK | FUK | fucokinase | fucokinase | fucokinase | 197258 | 197258 | 197258 | s47084 | s47085 | s47086 |
| 3042 | O3 | O4 | 781 | 659,3 | 9535 | 551,3 | 12,2 | 1,435 | H5A034 C | sample | Hm Drgbl | Hm Drgbl | Hm Drgbl | CPE002DP | CPE002EF | CPE002F5 | NM 00547 | NM 0054 | NM 0054 | GNE | GNE | GNE | glucosamine (UDP | glucosami | glucosami | 10020 | 10020 | 10020 | s19478 | s19479 | s223068 |
| 19981 | A20 | A21 | 811 | 1171 | 9897 | 178,5 | 12,2 | 1,488 | H5A034 C | sample | Hm Gen E | Hm Gen E | Hm Gen E | CPE002JQ | CPE002O5 | CPE002KL | NM 00108 | NM 0010 | NM 0010 | IQCF3 | IQCF3 | IQCF3 | IQ motif containin | IQ motif c | IQ motif c | 401067 | 401067 | 401067 | s53455 | s53456 | s196437 |
| 20007 | B22 | B23 | 1076 | 1142 | 13162 | 185,8 | 12,2 | 1,488 | H5A034 C | sample | Hm Gen E | Hm Gen E | Hm Gen E | CPE002JQ | CPE002O5 | CPE002KL | XM 94113 | XM 49796 | XM 49796 | LOC40217 | LOC40217 | LOC40217 | hypothetical gene | hypotheti | hypotheti | 402175 | 402175 | 402175 | s53745 | s196462 | s196463 |
| 442 | C18 | C19 | 1468 | 1740 | 17897 | 283,1 | 12,2 | 1,781 | H5A034 C | sample | Hm Drgbl | Hm Drgbl | Hm Drgbl | CPE002E0 | CPE002EZ | CPE002FK | NM 00145 | NM 0014 | NM 0014 | FLT3LG | FLT3LG | FLT3LG | fms-related tyrosi | fms-relate | fms-relate | 2323 | 2323 | 2323 | s5293 | s194417 | s194418 |
| 7872 | H9 | H10 | 1191 | 536,6 | 14515 | 389,7 | 12,2 | 1,863 | H5A034 C | sample | Hm Drgbl | Hm Drgbl | Hm Drgbl | CPE002E4 | CPE002EV | CPE002FG | NM 00633 | NM 0063 | NM 0063 | OLFM1 | OLFM1 | OLFM1 | olfactomedin 1 | olfactome | olfactome | 10439 | 10439 | 10439 | s20423 | s195040 | s223117 |
| 3332 | K5 | K6 | 737 | 1272 | 9082 | 440,4 | 12,3 | 0,762 | H5A034 C | sample | Hm Drgbl | Hm Drgbl | Hm Drgbl | CPE002DC | CPE002EG | CPE002F6 | NM 00101 | NM 0010 | NM 0010 | PFKFB2 | PFKFB2 | PFKFB2 | 6-phosphofructo- | 6-phosph | 6-phosph | 5208 | 5208 | 5208 | s10354 | s10355 | s10356 |
| 3383 | M8 | M9 | 1325 | 1206 | 16326 | 374,1 | 12,3 | 0,762 | H5A034 C | sample | Hm Drgbl | Hm Drgbl | Hm Drgbl | CPE002DC | CPE002EG | CPE002F6 | NM 17753 | NM 1775 | NM 1775 | NUDT14 | NUDT14 | NUDT14 | nudix (nucleoside | nudix (nu | nudix (nu | 256281 | 256281 | 256281 | s48752 | s223649 | s223650 |
| 3387 | N11 | N12 | 845 | 1447 | 10415 | 513,7 | 12,3 | 0,762 | H5A034 C | sample | Hm Drgbl | Hm Drgbl | Hm Drgbl | CPE002DC | CPE002EG | CPE002F6 | NM 00705 | NM 0070 | NM 0070 | PTPRT | PTPRT | PTPRT | protein tyrosine p | protein ty | protein ty | 11122 | 11122 | 11122 | s21928 | s21929 | s21930 |
| 3438 | P14 | P15 | 1385 | 1355 | 17035 | 486,7 | 12,3 | 0,762 | H5A034 C | sample | Hm Drgbl | Hm Drgbl | Hm Drgbl | CPE002DC | CPE002EG | CPE002F6 | NM 00265 | NM 0026 | NM 0026 | PLAUR | PLAUR | PLAUR | plasminogen activ | plasminog | plasminog | 5329 | 5329 | 5329 | s10613 | s10614 | s10615 |
| 6166 | A7 | A8 | 662 | 686,9 | 8120 | 503,7 | 12,3 | 0,812 | H5A034 C | sample | Hm Drgbl | Hm Drgbl | Hm Drgbl | CPE002DZ | CPE002F0 | CPE002FL | NM 00176 | NM 0017 | NM 0017 | CCNF | CCNF | CCNF | cyclin F | cyclin F | cyclin F | 899 | 899 | 899 | s2529 | s2528 | s2527 |
| 20527 | H15 | H16 | 1156 | 778 | 14214 | 176 | 12,3 | 0,82 | H5A034 C | sample | Hm Gen E | Hm Gen E | Hm Gen E | CPE002JR | CPE002O6 | CPE002KM | NM 00100 | NM 0010 | NM 0010 | OR5B3 | OR5B3 | OR5B3 | olfactory recepto | olfactory r | olfactory r | 441608 | 441608 | 441608 | s54404 | s54405 | s54406 |
| 20551 | I15 | I16 | 637 | 832,2 | 7849 | 162,3 | 12,3 | 0,82 | H5A034 C | sample | Hm Gen E | Hm Gen E | Hm Gen E | CPE002JR | CPE002O6 | CPE002KM | XM 49642 | XM 49642 | XM 49642 | LOC44070 | LOC44070 | LOC44070 | similar to hCG204 | similar to | similar to | 440706 | 440706 | 440706 | s54175 | s54176 | s54177 |
| 12924 | K2 | K3 | 1350 | 1048 | 16548 | 130,5 | 12,3 | 0,834 | H5A034 C | sample | Hm Gen E | Hm Gen E | Hm Gen E | CPE002J6 | CPE002NN | CPE002K2 | NM 00569 | NM 0056 | NM 0056 | SCAMP2 | SCAMP2 | SCAMP2 | secretory carrier r | secretory | secretory | 10066 | 10066 | 10066 | s19574 | s19575 | s19576 |
| 9855 | K22 | K23 | 2519 | 1288 | 31015 | 164,4 | 12,3 | 0,86 | H5A034 C | sample | Hm Drgbl | Hm Drgbl | Hm Drgbl | CPE002E9 | CPE002EO | CPE002FB | NM 02248 | NM 0224 | NM 0224 | CENTD3 | CENTD3 | CENTD3 | centaurin, delta 3 | centaurin | centaurin | 64411 | 64411 | 64411 | s34710 | s34711 | s34712 |

| | | | | | | | | | | | |
|---|---|---|---|---|---|---|---|---|---|---|---|
| 15313 | O1 | O2 | 1608 | 813,6 | 19821 | 132,5 | 12,3 | 0,86 | TCEAL7 | 56849 | s32288 |
| 8741 | M13 | M14 | 1384 | 1004 | 16967 | 111,9 | 12,3 | 0,869 | PREI3 | 25843 | s24607 |
| 7309 | A20 | A21 | 1582 | 682,9 | 19474 | 528,7 | 12,3 | 0,939 | GUCA1C | 9626 | s18497 |
| 16990 | D7 | D8 | 1449 | 907,1 | 17766 | 242,5 | 12,3 | 0,95 | C1QTNF1 | 114897 | s225503 |
| 17094 | I14 | I15 | 1456 | 947,1 | 17878 | 224,9 | 12,3 | 0,95 | FAM55A | 120400 | s42376 |
| 17231 | N8 | N9 | 2199 | 1052 | 27048 | 176,4 | 12,3 | 0,95 | SHISA3 | 124944 | s225556 |
| 6951 | B22 | B23 | 1687 | 677 | 20735 | 342,7 | 12,3 | 0,957 | NDUFB2 | 4708 | s224101 |
| 6974 | C21 | C22 | 1812 | 598,2 | 22296 | 504,8 | 12,3 | 0,957 | LECT2 | 3950 | s8131 |
| 6999 | D22 | D23 | 1524 | 567,5 | 18739 | 487,2 | 12,3 | 0,957 | NDUFS1 | 4719 | s9382 |
| 5018 | B10 | B11 | 1421 | 663,3 | 17510 | 627,2 | 12,3 | 0,965 | UQCRB | 7381 | s14696 |
| 5079 | D22 | D23 | 1436 | 655,2 | 17646 | 643,6 | 12,3 | 0,965 | VIL2 | 7430 | s14795 |
| 5214 | J14 | J15 | 1906 | 607,1 | 23531 | 376,7 | 12,3 | 0,965 | YWHAG | 7532 | s14964 |
| 5352 | O9 | O10 | 1726 | 678,6 | 21210 | 398,6 | 12,3 | 0,965 | FLJ90650 | 206338 | s47576 |
| 2390 | D21 | D22 | 717 | 791,4 | 8817 | 467,5 | 12,3 | 0,969 | ILK | 3611 | s7406 |
| 2451 | G11 | G12 | 1599 | 716,6 | 19639 | 419,5 | 12,3 | 0,969 | RIPK2 | 8767 | s249 |
| 2494 | H7 | H8 | 1175 | 694,9 | 14484 | 369,4 | 12,3 | 0,969 | PIP5K1A | 8394 | s15932 |
| 2587 | L4 | L5 | 1354 | 708 | 16673 | 411,6 | 12,3 | 0,969 | RPS6KA3 | 6197 | s12281 |
| 2599 | M15 | M16 | 1055 | 641,1 | 12972 | 355,1 | 12,3 | 0,969 | ACVR2B | 93 | s985 |
| 16718 | I21 | I22 | 1099 | 1036 | 13542 | 238,9 | 12,3 | 0,972 | C1orf94 | 84970 | s39784 |
| 16801 | M1 | M2 | 1272 | 1058 | 15630 | 233,3 | 12,3 | 0,972 | TCEAL3 | 85012 | s195507 |
| 16803 | M11 | M12 | 1824 | 1030 | 22423 | 365,6 | 12,3 | 0,972 | TMEM60 | 85025 | s39851 |
| 17804 | F5 | F6 | 1240 | 965,3 | 15298 | 233,8 | 12,3 | 0,972 | OTOA | 146183 | s44823 |
| 4263 | B22 | B23 | 1533 | 734,6 | 18852 | 349,1 | 12,3 | 0,972 | HCN3 | 57657 | s33534 |
| 4281 | C17 | C18 | 947 | 666,5 | 11665 | 295,4 | 12,3 | 1,038 | ATP2B2 | 491 | s1754 |
| 4326 | E14 | E15 | 1293 | 715,4 | 15872 | 375,4 | 12,3 | 1,038 | P2RXL1 | 9127 | s17430 |
| 4342 | E7 | E8 | 493 | 823,7 | 6055 | 421,4 | 12,3 | 1,038 | ATP6V1B1 | 525 | s1796 |
| 4345 | F1 | F2 | 1102 | 716,5 | 13580 | 458,7 | 12,3 | 1,038 | KCNA2 | 3737 | s7681 |
| 4544 | N16 | N17 | 1213 | 694,2 | 14967 | 430,2 | 12,3 | 1,038 | GPR137B | 7107 | s14220 |
| 4559 | N8 | N9 | 1372 | 654,8 | 16896 | 412,1 | 12,3 | 1,038 | TLR3 | 7098 | s234 |
| 48 | B9 | B10 | 237 | 1451 | 2911 | 587,9 | 12,3 | 1,047 | SDHD | 6392 | s12661 |
| 205 | I20 | I21 | 615 | 1436 | 7539 | 581,2 | 12,3 | 1,047 | SNAPC4 | 6621 | s13203 |
| 382 | P7 | P8 | 570 | 1492 | 7038 | 672,8 | 12,3 | 1,047 | SLC6A12 | 6539 | s12999 |
| 1947 | B11 | B12 | 1170 | 610,6 | 14442 | 459,7 | 12,3 | 1,07 | ZNF513 | 130557 | s223284 |
| 2001 | D17 | D18 | 1319 | 676,5 | 16259 | 424 | 12,3 | 1,07 | GATA5 | 140628 | s44325 |
| 2017 | E1 | E2 | 623 | 697 | 7674 | 495,8 | 12,3 | 1,07 | ZNF300 | 91975 | s40828 |
| 2027 | E19 | E20 | 1621 | 558,3 | 19869 | 336,1 | 12,3 | 1,07 | MYOCD | 93649 | s41185 |
| 2036 | E5 | E6 | 1192 | 713,2 | 14695 | 413 | 12,3 | 1,07 | ZNF585B | 92285 | s40905 |
| 2135 | I8 | I9 | 1330 | 548,3 | 16418 | 301,1 | 12,3 | 1,07 | BCL6B | 255877 | s48714 |
| 2170 | K18 | K19 | 1663 | 588 | 20464 | 315 | 12,3 | 1,07 | ZNF763 | 284390 | s49628 |
| 4029 | H6 | H7 | 1659 | 687,4 | 20407 | 337,3 | 12,3 | 1,093 | RNASE6 | 6039 | s12062 |
| 4146 | M3 | M4 | 103 | 730 | 1265 | 360,4 | 12,3 | 1,093 | POLR2I | 5438 | s10818 |
| 4167 | N22 | N23 | 1455 | 708,7 | 17890 | 455,5 | 12,3 | 1,093 | SAG | 6295 | s12457 |
| 4187 | O19 | O20 | 1542 | 652,7 | 18913 | 441,3 | 12,3 | 1,093 | PPID | 5481 | s10914 |
| 4220 | P5 | P6 | 1655 | 676,5 | 20349 | 477,5 | 12,3 | 1,093 | QSOX1 | 5768 | s11503 |
| 11617 | E1 | E2 | 1483 | 823,9 | 18221 | 152 | 12,3 | 1,098 | A2GP1 | 563 | s1850 |
| 15648 | L9 | L10 | 1142 | 767,6 | 14027 | 132,2 | 12,3 | 1,118 | C12orf43 | 64897 | s35071 |
| 5797 | B20 | B21 | 1692 | 799 | 20821 | 586,3 | 12,3 | 1,123 | BDH1 | 622 | s224556 |
| 5815 | C15 | C16 | 1079 | 780,7 | 13257 | 520,7 | 12,3 | 1,123 | ACADL | 33 | s888 |
| 6127 | P15 | P16 | 1328 | 730,5 | 16286 | 527,8 | 12,3 | 1,123 | AREG | 374 | s1547 |
| 1173 | A6 | A7 | 1493 | 726,9 | 18315 | 462,9 | 12,3 | 1,128 | BAG2 | 9532 | s18295 |
| 1296 | F9 | F10 | 1130 | 777,6 | 13952 | 410,2 | 12,3 | 1,128 | FCN3 | 8547 | s16271 |
| 16163 | B19 | B20 | 1146 | 1007 | 14068 | 219,6 | 12,3 | 1,128 | FAM62C | 83850 | s38211 |
| 16318 | H7 | H8 | 1020 | 1066 | 12565 | 199,3 | 12,3 | 1,128 | TATDN1 | 83940 | s38349 |
| 16357 | J20 | J21 | 1440 | 964,6 | 17670 | 275,9 | 12,3 | 1,128 | FAM126A | 84668 | s39289 |
| 16383 | K22 | K23 | 1470 | 1011 | 18147 | 347,7 | 12,3 | 1,128 | BRMS1L | 84312 | s38893 |
| 16419 | M11 | M12 | 1772 | 1011 | 21725 | 382,6 | 12,3 | 1,128 | AMMECR1L | 83607 | s38045 |
| 13611 | H11 | H12 | 1084 | 1034 | 13321 | 166,8 | 12,3 | 1,155 | C22orf31 | 25770 | s226039 |
| 821 | C13 | C14 | 1964 | 780,8 | 24215 | 473,4 | 12,3 | 1,166 | ARNTL | 406 | s1618 |
| 999 | J22 | J23 | 1235 | 702,4 | 15166 | 408,6 | 12,3 | 1,166 | TCEB3 | 6924 | s13861 |
| 1033 | L1 | L2 | 351 | 732,6 | 4300 | 395,9 | 12,3 | 1,166 | HSPA5 | 3309 | s6981 |
| 1034 | L10 | L11 | 1774 | 763,4 | 21751 | 414 | 12,3 | 1,166 | TEAD1 | 7003 | s13963 |
| 1071 | M22 | M23 | 1216 | 741,6 | 14953 | 446,4 | 12,3 | 1,166 | POU4F2 | 5458 | s10867 |
| 1118 | O21 | O22 | 1331 | 732,6 | 16416 | 495,6 | 12,3 | 1,166 | HSPA5 | 2119 | s4865 |
| 5408 | B16 | B17 | 2263 | 743,5 | 27784 | 656 | 12,3 | 1,171 | SLC16A6 | 9120 | s17408 |
| 5423 | B8 | B9 | 1755 | 706,5 | 21584 | 654,8 | 12,3 | 1,171 | TBX18 | 9096 | s17356 |
| 5616 | J9 | J10 | 1296 | 617 | 15964 | 456,7 | 12,3 | 1,171 | CDC23 | 8697 | s16572 |
| 5653 | L20 | L21 | 1811 | 657,2 | 22323 | 529,4 | 12,3 | 1,171 | COPS2 | 9318 | s17804 |
| 3471 | A22 | A23 | 1158 | 1342 | 14199 | 351,9 | 12,3 | 1,183 | SSTR3 | 6753 | s224690 |
| 8091 | B11 | B12 | 1612 | 573,7 | 19819 | 484,5 | 12,3 | 1,183 | DDX42 | 11325 | s22330 |
| 8352 | L9 | L10 | 1360 | 562,6 | 16733 | 468,9 | 12,3 | 1,183 | TRIM32 | 22954 | s27741 |
| 8399 | N8 | N9 | 1399 | 593,1 | 17164 | 505,4 | 12,3 | 1,183 | DNPEP | 23549 | s24065 |
| 10133 | G13 | G14 | 1628 | 930,9 | 20085 | 138 | 12,3 | 1,183 | EHMT1 | 79813 | s36390 |
| 1564 | B12 | B13 | 1205 | 688,2 | 14877 | 435,1 | 12,3 | 1,288 | ZNF668 | 79759 | s36275 |
| 1684 | G12 | G13 | 1657 | 729 | 20353 | 434,2 | 12,3 | 1,288 | GRHL3 | 57822 | s49479 |
| 1772 | J5 | J6 | 1532 | 720,7 | 18778 | 379,1 | 12,3 | 1,288 | SUHW3 | 55609 | s31067 |
| 1823 | L8 | L9 | 1274 | 692 | 15678 | 372,4 | 12,3 | 1,288 | SYT3 | 84258 | s224939 |
| 1849 | N1 | N2 | 1234 | 756,1 | 15159 | 402,6 | 12,3 | 1,288 | MYNN | 55892 | s31732 |
| 21536 | B16 | B17 | 240 | 933,1 | 2946 | 589,8 | 12,3 | 1,288 | hCG1995786 | 653147 | s57590 |
| 21626 | F10 | F11 | 127 | 940,4 | 1567 | 494,6 | 12,3 | 1,288 | LOC653319 | 653319 | s57644 |
| 21673 | H1 | H2 | 254 | 967,9 | 3129 | 576,6 | 12,3 | 1,288 | FLJ38576 | 651430 | s227365 |
| 4609 | A1 | A2 | 472 | 847,8 | 5813 | 430,2 | 12,3 | 1,352 | TPP2 | 7174 | s69 |
| 4707 | E11 | E12 | 1758 | 872 | 21707 | 499 | 12,3 | 1,352 | APEH | 327 | s1443 |
| 4746 | F3 | F4 | 1335 | 753,4 | 16394 | 514,3 | 12,3 | 1,352 | KLKB1 | 3818 | s7880 |
| 4761 | G17 | G18 | 895 | 717,6 | 11039 | 482,2 | 12,3 | 1,352 | CASP2 | 835 | s2412 |
| 4782 | H14 | H15 | 1714 | 858,6 | 21119 | 388,4 | 12,3 | 1,352 | USP25 | 29761 | s26561 |
| 10391 | A8 | A9 | 1078 | 892,9 | 13281 | 227,2 | 12,3 | 1,39 | SLC2A9 | 114571 | s41562 |
| 10396 | B12 | B13 | 1539 | 920,6 | 18945 | 212,7 | 12,3 | 1,39 | CTGLF1 | 119016 | s42268 |
| 10482 | E3 | E4 | 1388 | 914,2 | 17112 | 190,9 | 12,3 | 1,39 | MFSD5 | 84975 | s39789 |
| 10643 | L19 | L20 | 1538 | 839,6 | 18974 | 200,1 | 12,3 | 1,39 | BIRC8 | 112401 | s41324 |
| 10728 | O9 | O10 | 1235 | 851,9 | 15154 | 183,5 | 12,3 | 1,39 | ZFAND2A | 90637 | s40453 |
| 2953 | L1 | L2 | 1517 | 692 | 18677 | 498,3 | 12,3 | 1,447 | PLXNB2 | 23654 | s24288 |
| 3056 | P16 | P17 | 847 | 667,7 | 10426 | 595,9 | 12,3 | 1,447 | ESD | 2098 | s748 |
| 20280 | M9 | M10 | 387 | 1194 | 4749 | 127,8 | 12,3 | 1,5 | LOC392444 | 392447 | s53086 |
| 429 | B6 | B7 | 1483 | 1646 | 18168 | 381,5 | 12,3 | 1,79 | GEM | 2669 | s5698 |
| 7825 | G1 | G2 | 107 | 871,6 | 1314 | 496,2 | 12,3 | 1,878 | PSMD14 | 10213 | s19919 |
| 7833 | G17 | G18 | 430 | 587,5 | 5271 | 278,9 | 12,3 | 1,878 | RCAN2 | 10231 | s19964 |
| 15800 | C16 | C17 | 1324 | 866,4 | 16390 | 133,1 | 12,4 | 0,691 | ATF7IP2 | 80063 | s36878 |
| 3158 | D21 | D22 | 1621 | 1469 | 20162 | 451,7 | 12,4 | 0,784 | PTPRG | 5793 | s11551 |
| 6176 | B16 | B17 | 1841 | 628,2 | 22866 | 395,8 | 12,4 | 0,818 | DDX11 | 1663 | s4026 |
| 6501 | O6 | O7 | 1320 | 563,6 | 16356 | 361,6 | 12,4 | 0,818 | DCI | 1632 | s194376 |
| 20456 | E16 | E17 | 976 | 814,2 | 12123 | 197,8 | 12,4 | 0,827 | FBXO47 | 494188 | s54648 |
| 20503 | G15 | G16 | 930 | 746,2 | 11533 | 168,5 | 12,4 | 0,827 | C10orf151 | 440574 | s54143 |
| 20646 | M14 | M15 | 1232 | 721,3 | 15256 | 166 | 12,4 | 0,827 | HEPN1 | 641654 | s58259 |
| 12862 | H7 | H8 | 1281 | 989,3 | 15908 | 128,5 | 12,4 | 0,841 | FRMPD4 | 9758 | s18836 |
| 17624 | O16 | O17 | 1525 | 928,9 | 18908 | 205,6 | 12,4 | 0,849 | LOC136288 | 136288 | s43994 |
| 15217 | K1 | K2 | 960 | 940,9 | 11883 | 127,9 | 12,4 | 0,867 | GKN1 | 56287 | s32125 |
| 8793 | O17 | O18 | 1757 | 1085 | 21797 | 101,2 | 12,4 | 0,869 | SIN3A | 25942 | s24800 |
| 20867 | F19 | F20 | 1202 | 1043 | 14877 | 231,7 | 12,4 | 0,879 | MS4A4E | 643680 | s55398 |
| 20936 | I16 | I17 | 1604 | 967,8 | 19893 | 165,1 | 12,4 | 0,879 | LOC644482 | 644482 | s227127 |
| 14250 | B3 | B4 | 919 | 823,2 | 11410 | 110,2 | 12,4 | 0,883 | NAG | 51594 | s28379 |
| 14394 | H3 | H4 | 1156 | 853,5 | 14356 | 152,1 | 12,4 | 0,883 | HIGD1B | 51751 | s226750 |
| 11179 | B4 | B5 | 1893 | 937 | 23405 | 175,5 | 12,4 | 0,899 | C20orf3 | | |
| 12476 | H5 | H6 | 1146 | 1017 | 14161 | 137,5 | 12,4 | 0,932 | WRB | 7485 | s14906 |
| 16993 | E1 | E2 | 1219 | 989,2 | 15135 | 144 | 12,4 | 0,958 | FTMT | 94033 | s44239 |
| 17017 | F1 | F2 | 1063 | 997,1 | 13209 | 146,2 | 12,4 | 0,958 | GPRASP2 | 114928 | s41727 |
| 7131 | J11 | J12 | 1286 | 577,5 | 16001 | 403,9 | 12,4 | 0,965 | MGAT2 | 4247 | s8734 |
| 5185 | I1 | I2 | 417 | 621,6 | 5164 | 259,1 | 12,4 | 0,973 | DPP9 | 91039 | s40557 |
| 2448 | F9 | F10 | 1214 | 661,6 | 15053 | 428,6 | 12,4 | 0,976 | ITPK1 | 3705 | s223955 |
| 2473 | H1 | H2 | 1406 | 720,1 | 17454 | 481,6 | 12,4 | 0,976 | KHK | 3795 | s7830 |
| 2619 | N11 | N12 | 1604 | 663,2 | 19835 | 385,5 | 12,4 | 0,976 | FGFR3 | 2261 | s5169 |
| 2643 | O11 | O12 | 1561 | 673,7 | 21827 | 427,2 | 12,4 | 0,976 | AMHR2 | 269 | s1327 |
| 2654 | O21 | O22 | 1754 | 716,8 | 21704 | 385,8 | 12,4 | 0,976 | BMPR2 | 659 | s2046 |
| 16856 | O16 | O17 | 917 | 1016 | 11412 | 352,8 | 12,4 | 0,976 | ECAT8 | 91646 | s40719 |
| 9050 | J10 | J11 | 1063 | 785,1 | 12667 | 135,2 | 12,4 | 0,976 | DDX56 | 54606 | s29253 |
| 4259 | B19 | B20 | 1563 | 729,3 | 19305 | 370,3 | 12,4 | 1,046 | GRIK2 | 2898 | s6156 |
| 4270 | C9 | C10 | 1427 | 701,5 | 17763 | 319,8 | 12,4 | 1,046 | ATP1A4 | 480 | s1732 |
| 4333 | E20 | E21 | 1063 | 701,7 | 13167 | 427,5 | 12,4 | 1,046 | KCNAB3 | 9196 | s225095 |
| 4383 | G22 | G23 | 1534 | 687 | 18974 | 433,5 | 12,4 | 1,046 | PKDREJ | 10343 | s20229 |
| 4388 | G5 | G6 | 1502 | 693,1 | 18658 | 419,2 | 12,4 | 1,046 | CACNA1F | 778 | s2294 |
| 4486 | K7 | K8 | 876 | 775,8 | 10858 | 412,5 | 12,4 | 1,046 | CLCN3 | 1182 | s3137 |
| 4545 | N17 | N18 | 1371 | 775,4 | 17029 | 505,4 | 12,4 | 1,046 | PKD2 | 5311 | s10566 |
| 4549 | N20 | N21 | 1356 | 651,8 | 16783 | 443,3 | 12,4 | 1,046 | TMEM1 | 7109 | s224738 |
| 4550 | O20 | O21 | 1150 | 737,3 | 14248 | 443,2 | 12,4 | 1,046 | CACNA2D3 | 55799 | s31540 |
| 4592 | P16 | P17 | 1279 | 720,5 | 15910 | 472,3 | 12,4 | 1,046 | TNNC1 | 7134 | s224762 |
| 225 | J17 | J18 | 616 | 1617 | 7653 | 545,5 | 12,4 | 1,047 | SLC4A3 | 6508 | s12910 |
| 2098 | H18 | H19 | 1422 | 577,5 | 17585 | 282,7 | 12,4 | 1,078 | SNX30 | 401548 | s53641 |
| 6867 | O11 | O12 | 1328 | 611,4 | 16408 | 394,4 | 12,4 | 1,083 | HMX1 | 3166 | s6683 |
| 6882 | O3 | O4 | 771 | 568,1 | 9539 | 446,4 | 12,4 | 1,083 | HMGA1 | 3159 | s6668 |
| 4117 | L20 | L21 | 1120 | 670,9 | 13937 | 329,3 | 12,4 | 1,093 | RSU1 | 6251 | s12375 |
| 4153 | N1 | N2 | 82 | 669,6 | 1017 | 479,8 | 12,4 | 1,093 | PSMD2 | 5708 | s11389 |
| 11736 | I9 | I10 | 827 | 856 | 10273 | 150,3 | 12,4 | 1,107 | C7 | 730 | s2193 |
| 11775 | K22 | K23 | 1395 | 877,9 | 17271 | 200,6 | 12,4 | 1,107 | GAGE1 | 2543 | s5454 |
| 5765 | A13 | A14 | 1272 | 844,6 | 15803 | 627,3 | 12,4 | 1,123 | AARS | 16 | s841 |
| 5876 | E5 | E6 | 1743 | 761,8 | 21534 | 592,5 | 12,4 | 1,123 | ACHE | 43 | s913 |
| 5894 | F21 | F22 | 1814 | 762,1 | 22441 | 571,1 | 12,4 | 1,123 | ANGPT2 | 285 | s1361 |
| 5954 | I10 | I11 | 907 | 743,7 | 11289 | 339,8 | 12,4 | 1,123 | ATF4 | 468 | s1703 |
| 5957 | I13 | I14 | 1099 | 740,6 | 13658 | 466,4 | 12,4 | 1,123 | ADCY9 | 115 | s1038 |
| 1235 | D19 | D20 | 1600 | 767,1 | 19694 | 401,5 | 12,4 | 1,128 | SDPR | 8436 | s16008 |
| 15641 | I5 | I6 | 1396 | 753,9 | 17248 | 443,8 | 12,4 | 1,128 | ZNF91 | 7644 | s15143 |
| 16323 | I11 | I12 | 1433 | 996 | 17751 | 402,1 | 12,4 | 1,128 | ZNHIT4 | 83444 | s37918 |
| 13700 | K5 | K6 | 974 | 968,8 | 12050 | 163,3 | 12,4 | 1,155 | SLC44A1 | 23446 | s225963 |
| 1003 | J4 | J5 | 1663 | 731,5 | 20650 | 379 | 12,4 | 1,166 | SYP | 6855 | s13690 |
| 1016 | K16 | K17 | 1307 | 757,4 | 16255 | 429,9 | 12,4 | 1,166 | PFDN5 | 5204 | s10346 |
| 1027 | K4 | K5 | 835 | 753,8 | 10345 | 406,6 | 12,4 | 1,166 | PCBP1 | 5093 | s224175 |
| 5598 | J14 | J15 | 2138 | 686,6 | 26582 | 488,9 | 12,4 | 1,171 | GCM2 | 9247 | s17682 |
| 5640 | K9 | K10 | 2199 | 785,4 | 27292 | 448,2 | 12,4 | 1,171 | NDST2 | 8509 | s16171 |
| 5643 | L11 | L12 | 1035 | 747,9 | 12858 | 458,8 | 12,4 | 1,171 | EDF1 | 8721 | s16609 |
| 5715 | O13 | O12 | 1260 | 660,1 | 15626 | 619,4 | 12,4 | 1,171 | KHSRP | 8570 | s16324 |
| 3533 | D13 | D14 | 636 | 1346 | 7882 | 293,9 | 12,4 | 1,183 | MCHR1 | 2847 | s4006 |
| 3557 | E13 | E14 | 827 | 1420 | 10295 | 407,3 | 12,4 | 1,183 | BRS3 | 680 | s2097 |
| 3579 | F11 | F12 | 844 | 1306 | 10474 | 362,3 | 12,4 | 1,183 | MLNR | 2862 | s6071 |
| 3610 | G18 | G19 | 1257 | 1350 | 15607 | 340,5 | 12,4 | 1,183 | GPR56 | 9289 | s17759 |
| 3731 | L19 | L20 | 855 | 1300 | 10633 | 332,8 | 12,4 | 1,183 | MTNR1A | 4543 | s9032 |
| 3764 | M5 | M6 | 1331 | 1268 | 16451 | 301,6 | 12,4 | 1,183 | EMR1 | 2015 | s4661 |
| 3768 | M9 | M10 | 958 | 1281 | 11868 | 334,4 | 12,4 | 1,183 | F2RL1 | 2150 | s223507 |
| 8099 | B19 | B20 | 1315 | 675,1 | 16367 | 380 | 12,4 | 1,183 | GABARAP | 11337 | s22361 |
| 8162 | E10 | E11 | 1533 | 620,7 | 19060 | 483,2 | 12,4 | 1,183 | SMC5 | 23137 | s23115 |
| 8192 | F16 | F17 | 1772 | 598,5 | 22023 | 449,2 | 12,4 | 1,183 | SLC35A3 | 23443 | s23841 |
| 8316 | K2 | K3 | 1185 | 602,6 | 14678 | 430,7 | 12,4 | 1,183 | ATP11A | 23250 | s23394 |
| 8332 | L12 | L13 | 1800 | 608,6 | 22283 | 440,6 | 12,4 | 1,183 | PIK3R5 | 23533 | s24029 |
| 1607 | C8 | C9 | 1653 | 689,6 | 20588 | 403,5 | 12,4 | 1,288 | ZNF624 | 57547 | s33284 |
| 1822 | L7 | L8 | 1453 | 705,7 | 17995 | 371,4 | 12,4 | 1,288 | ZNF415 | 55786 | s31507 |
| 1867 | N4 | N5 | 1452 | 736,2 | 17966 | 362,5 | 12,4 | 1,288 | PGBD1 | 84547 | s39142 |
| 1900 | P12 | P13 | 1299 | 748,2 | 16148 | 472,4 | 12,4 | 1,288 | ZNF382 | 84911 | s39639 |
| 21624 | E9 | E10 | 145 | 824,5 | 1794 | 365,3 | 12,4 | 1,288 | LOC649259 | 649259 | s57648 |
| 21724 | J12 | J13 | 77 | 1086 | 953 | 747,4 | 12,4 | 1,288 | LOC653658 | 653658 | s198083 |
| 4835 | J19 | J20 | 1726 | 858 | 21450 | 382,3 | 12,4 | 1,352 | PCSK6 | 5046 | s224155 |
| 4846 | J7 | J8 | 1614 | 747,5 | 19958 | 439,6 | 12,4 | 1,352 | MMP17 | 4326 | s8887 |
| 4890 | L3 | L4 | 918 | 698,8 | 11339 | 514,4 | 12,4 | 1,352 | USP27X | 389856 | s52575 |
| 4965 | O6 | O7 | 804 | 790,1 | 9984 | 526,4 | 12,4 | 1,352 | DPP3 | 10072 | s19589 |
| 10405 | B20 | B21 | 1297 | 922 | 16124 | 202,9 | 12,4 | 1,39 | FAT3 | 120114 | s42354 |
| 10543 | H15 | H16 | 1164 | 802,8 | 14463 | 202,7 | 12,4 | 1,401 | MARCH9 | 92979 | s40958 |
| 10563 | I11 | I12 | 1356 | 831,2 | 16477 | 192,6 | 12,4 | 1,401 | NUDCD1 | 84955 | s39750 |
| 2762 | D10 | D11 | 1218 | 675,8 | 15150 | 529,3 | 12,4 | 1,412 | CSNK1A1L | 122011 | s42514 |
| 20046 | D14 | D15 | 773 | 1148 | 9606 | 164,4 | 12,4 | 1,5 | SOHLH1 | 402381 | s53766 |
| 20135 | G8 | G9 | 741 | 1110 | 9165 | 158,8 | 12,4 | 1,5 | FLJ43093 | 401258 | s53590 |
| 20159 | H8 | H9 | 994 | 1037 | 12303 | 185,8 | 12,4 | 1,5 | OR6C68 | 403284 | s53826 |
| 20347 | P4 | P5 | 513 | 1368 | 6367 | 151,3 | 12,4 | 1,5 | LOC439992 | 439992 | s226596 |
| 7799 | E8 | E9 | 644 | 559,5 | 7915 | 339,1 | 12,4 | 1,878 | PDLIM5 | 10611 | s20834 |
| 7866 | H3 | H4 | 634 | 578,8 | 7866 | 368,2 | 12,4 | 1,878 | EMG1 | 10436 | s20415 |
| 7927 | K15 | K16 | 351 | 527,5 | 4360 | 380,3 | 12,4 | 1,878 | EFS | 10278 | s20080 |
| 3297 | J17 | J18 | 1077 | 1058 | 13135 | 231,7 | 12,5 | 0,774 | MTMR2 | 8898 | s17016 |
| 3334 | K7 | K8 | 269 | 1373 | 3366 | 423,1 | 12,5 | 0,774 | PFKFB3 | 5209 | s10359 |
| 6441 | M17 | M18 | 1986 | 628,5 | 24872 | 482,8 | 12,5 | 0,818 | CDKN2C | 1031 | s2848 |
| 20395 | B4 | B5 | 794 | 1117 | 14710 | 177,2 | 12,5 | 0,833 | LOC641857 | 641857 | s196727 |
| 20407 | C15 | C16 | 1087 | 757,7 | 13633 | 190 | 12,5 | 0,833 | LOC440292 | 440292 | s226609 |
| 20526 | H14 | H15 | 676 | 778,6 | 8458 | 198,4 | 12,5 | 0,833 | LOC642262 | 642262 | s54966 |
| 20642 | M10 | M11 | 1006 | 757 | 12679 | 180 | 12,5 | 0,833 | ARL17 | 641522 | s54818 |
| 20653 | M20 | M21 | 1253 | 771,6 | 15676 | 184,6 | 12,5 | 0,833 | FLJ44124 | 641737 | s54902 |
| 20698 | O18 | O19 | 1398 | 782,4 | 17387 | 189,6 | 12,5 | 0,833 | LOC642182 | 642182 | s54861 |
| 17498 | J10 | J11 | 1102 | 912,7 | 13805 | 242,8 | 12,5 | 0,847 | MAGEB10 | 139422 | s44210 |
| 19129 | N1 | N2 | 1593 | 943,8 | 19959 | 174,1 | 12,5 | 0,849 | C16orf58 | 284018 | s49447 |
| 9945 | O17 | O18 | 1801 | 1020 | 22501 | 178,5 | 12,5 | 0,874 | PCDHB16 | 57717 | s33467 |
| 21200 | D16 | D17 | 1311 | 860,6 | 16383 | 274,9 | 12,5 | 0,877 | LOC647278 | 647278 | s197524 |
| 10899 | G1 | G2 | 1198 | 754,4 | 15145 | 213,4 | 12,5 | 0,878 | NOBOX | 135935 | s43961 |
| 7006 | D7 | D8 | 1179 | 554 | 14699 | 509,5 | 12,5 | 0,957 | MB | 4151 | s8544 |
| 7116 | I2 | I3 | 1063 | 569,1 | 13211 | 425,8 | 12,5 | 0,957 | MYH6 | 4624 | s9161 |
| 7143 | J22 | J23 | 1265 | 536,9 | 15866 | 483,3 | 12,5 | 0,957 | NFKBIL1 | 4795 | s194653 |
| 7188 | L2 | L3 | 1500 | 607,2 | 18795 | 466,8 | 12,5 | 0,957 | NFKBIB | 4793 | s224114 |
| 7215 | M22 | M23 | 1440 | 618,2 | 17837 | 394,3 | 12,5 | 0,957 | NASP | 4678 | s9222 |
| 7226 | N10 | N11 | 1189 | 552,5 | 14893 | 499,3 | 12,5 | 0,957 | NMT1 | 4836 | s9601 |
| 5186 | F9 | F10 | 1540 | 703,3 | 19113 | 490,9 | 12,5 | 0,965 | LRPB | 7804 | s15366 |
| 5298 | M3 | M4 | 1147 | 571,2 | 14303 | 320 | 12,5 | 0,965 | RHBDL3 | 162494 | s46345 |
| 2384 | D16 | D17 | 215 | 757,7 | 2692 | 384,3 | 12,5 | 0,984 | MAPK3 | 5595 | s11142 |
| 2391 | D22 | D23 | 1538 | 730,1 | 19224 | 445,3 | 12,5 | 0,984 | MAPK7 | 5598 | s11151 |
| 2419 | E4 | E5 | 2023 | 679,2 | 25247 | 486,4 | 12,5 | 0,984 | MAP3K5 | 4217 | s8678 |
| 2521 | J1 | J2 | 1210 | 646,2 | 15106 | 390,7 | 12,5 | 0,984 | DGKB | 1607 | s3916 |
| 2539 | J4 | J5 | 1018 | 728,5 | 12756 | 387,5 | 12,5 | 0,984 | ALDH18A1 | 5832 | s13425 |
| 2578 | L18 | L19 | 834 | 818,4 | 10314 | 393,2 | 12,5 | 0,984 | SRC | 6714 | s13413 |
| 2655 | O22 | O23 | 1981 | 687,1 | 24810 | 403,9 | 12,5 | 0,984 | PRKACA | 5566 | s11067 |
| 2661 | O6 | O7 | 1922 | 761,8 | 24033 | 424,7 | 12,5 | 0,984 | PKACB | 5298 | s10543 |
| 16681 | H1 | H2 | 1244 | 983,7 | 15602 | 260,5 | 12,5 | 0,988 | TSLP | 85480 | s40074 |
| 16712 | I16 | I17 | 1364 | 956,9 | 17070 | 221,9 | 12,5 | 0,988 | TMEM67 | 91147 | s40584 |
| 16722 | I3 | I4 | 1069 | 970,7 | 13375 | 280,9 | 12,5 | 0,988 | NUDC | 84955 | s39750 |
| 16786 | L18 | L19 | 1252 | 987,7 | 15652 | 242,3 | 12,5 | 0,988 | WDR85 | 92715 | s41006 |
| 16826 | N10 | N11 | 1134 | 1016 | 14229 | 291,1 | 12,5 | 0,988 | CCDC102A | 92922 | s41047 |

| | | | | | | | | | | |
|---|---|---|---|---|---|---|---|---|---|---|
| 17940 | L2 | L3 | 854 | 911,6 | 10709 | 194,6 | 12,5 | 0,988 | H5A034 C | sample |
| 17943 | L22 | L23 | 1683 | 1084 | 20975 | 240,8 | 12,5 | 0,988 | H5A034 C | sample |
| 17966 | M21 | M22 | 1302 | 984,4 | 16315 | 201,2 | 12,5 | 0,988 | H5A034 C | sample |
| 8955 | F11 | F12 | 1761 | 1858 | 22068 | 136,2 | 12,5 | 1,004 | H5A034 C | sample |
| 4253 | B13 | B14 | 1195 | 711,8 | 14926 | 406,3 | 12,5 | 1,055 | H5A034 C | sample |
| 4302 | D14 | D15 | 365 | 809,2 | 4545 | 231,6 | 12,5 | 1,055 | H5A034 C | sample |
| 4303 | D15 | D16 | 1078 | 656,8 | 13442 | 301 | 12,5 | 1,055 | H5A034 C | sample |
| 4364 | F5 | F6 | 1316 | 769,1 | 16471 | 361,9 | 12,5 | 1,055 | H5A034 C | sample |
| 4550 | N21 | N22 | 1632 | 707,6 | 20475 | 443,5 | 12,5 | 1,055 | H5A034 C | sample |
| 196 | I12 | I13 | 610 | 1583 | 7608 | 617,9 | 12,5 | 1,064 | H5A034 C | sample |
| 308 | M5 | M6 | 606 | 1472 | 7551 | 589 | 12,5 | 1,064 | H5A034 C | sample |
| 368 | P16 | P17 | 338 | 1457 | 4223 | 663,9 | 12,5 | 1,064 | H5A034 C | sample |
| 1940 | A5 | A6 | 1197 | 684,2 | 14949 | 462,5 | 12,5 | 1,087 | H5A034 C | sample |
| 2016 | D9 | D10 | 1547 | 608,5 | 19387 | 409,8 | 12,5 | 1,087 | H5A034 C | sample |
| 2151 | J22 | J23 | 1094 | 620,9 | 13652 | 413,6 | 12,5 | 1,087 | H5A034 C | sample |
| 6609 | D17 | D18 | 1245 | 579,5 | 15577 | 355,6 | 12,5 | 1,096 | H5A034 C | sample |
| 6895 | P15 | P16 | 1534 | 628,5 | 19172 | 513,7 | 12,5 | 1,096 | H5A034 C | sample |
| 3968 | F16 | F17 | 1239 | 667,3 | 15470 | 333,1 | 12,5 | 1,111 | H5A034 C | sample |
| 3979 | F4 | F5 | 1094 | 701,7 | 13683 | 408,8 | 12,5 | 1,111 | H5A034 C | sample |
| 4023 | H22 | H23 | 1069 | 595,7 | 13383 | 384,7 | 12,5 | 1,111 | H5A034 C | sample |
| 4030 | H7 | H8 | 663 | 595,9 | 8285 | 330,6 | 12,5 | 1,111 | H5A034 C | sample |
| 4050 | I3 | I4 | 973 | 678,3 | 12124 | 378,5 | 12,5 | 1,111 | H5A034 C | sample |
| 11586 | C3 | C4 | 1088 | 810,3 | 13570 | 120,3 | 12,5 | 1,116 | H5A034 C | sample |
| 11754 | J3 | J4 | 1018 | 828,6 | 12732 | 158,9 | 12,5 | 1,116 | H5A034 C | sample |
| 5770 | A18 | A19 | 1165 | 871,2 | 14600 | 543,6 | 12,5 | 1,142 | H5A034 C | sample |
| 6034 | L18 | L19 | 1400 | 909,4 | 17569 | 434,9 | 12,5 | 1,142 | H5A034 C | sample |
| 6061 | M20 | M21 | 1113 | 793,8 | 13863 | 497,1 | 12,5 | 1,142 | H5A034 C | sample |
| 6144 | P9 | P10 | 1351 | 751,3 | 16825 | 477,9 | 12,5 | 1,142 | H5A034 C | sample |
| 1162 | A18 | A19 | 2129 | 846,6 | 26631 | 496,7 | 12,5 | 1,147 | H5A034 C | sample |
| 1214 | C21 | C22 | 1835 | 772,8 | 22916 | 450,9 | 12,5 | 1,147 | H5A034 C | sample |
| 1314 | G3 | G4 | 865 | 693,3 | 10835 | 421,9 | 12,5 | 1,147 | H5A034 C | sample |
| 1368 | I9 | I10 | 1540 | 742,4 | 19322 | 317,8 | 12,5 | 1,147 | H5A034 C | sample |
| 16171 | B4 | B5 | 854 | 1049 | 10707 | 417,3 | 12,5 | 1,147 | H5A034 C | sample |
| 16244 | E5 | E6 | 1196 | 1070 | 14979 | 334,5 | 12,5 | 1,147 | H5A034 C | sample |
| 16345 | J1 | J2 | 909 | 990,4 | 11384 | 382,7 | 12,5 | 1,147 | H5A034 C | sample |
| 16431 | M22 | M23 | 1492 | 970,8 | 18622 | 348,5 | 12,5 | 1,147 | H5A034 C | sample |
| 896 | F16 | F17 | 1059 | 800,4 | 13194 | 438,4 | 12,5 | 1,185 | H5A034 C | sample |
| 914 | G10 | G11 | 1363 | 772 | 17082 | 423,7 | 12,5 | 1,185 | H5A034 C | sample |
| 992 | J16 | J17 | 1065 | 825,3 | 13352 | 337,6 | 12,5 | 1,185 | H5A034 C | sample |
| 1019 | K19 | K20 | 1322 | 739,4 | 16460 | 327 | 12,5 | 1,185 | H5A034 C | sample |
| 1122 | O3 | O4 | 1132 | 731,4 | 14162 | 527,4 | 12,5 | 1,185 | H5A034 C | sample |
| 1140 | P2 | P3 | 1788 | 765,2 | 22267 | 571,8 | 12,5 | 1,185 | H5A034 C | sample |
| 5638 | K7 | K8 | 1751 | 654,9 | 21858 | 542 | 12,5 | 1,19 | H5A034 C | sample |
| 5702 | N21 | N22 | 1727 | 702,8 | 21526 | 520,3 | 12,5 | 1,19 | H5A034 C | sample |
| 5722 | O18 | O19 | 1722 | 700,6 | 21610 | 584,5 | 12,5 | 1,19 | H5A034 C | sample |
| 3477 | A6 | A7 | 997 | 1289 | 12480 | 309 | 12,5 | 1,202 | H5A034 C | sample |
| 3576 | E9 | E10 | 646 | 1399 | 8091 | 368,2 | 12,5 | 1,202 | H5A034 C | sample |
| 3642 | H3 | H4 | 1388 | 1406 | 17417 | 383,6 | 12,5 | 1,202 | H5A034 C | sample |
| 3655 | I15 | I16 | 998 | 1194 | 12441 | 312,2 | 12,5 | 1,202 | H5A034 C | sample |
| 8158 | D7 | D8 | 1383 | 631,8 | 17222 | 519,9 | 12,5 | 1,202 | H5A034 C | sample |
| 8353 | M1 | M2 | 572 | 611,4 | 7162 | 505,6 | 12,5 | 1,202 | H5A034 C | sample |
| 8361 | M17 | M18 | 1540 | 606,6 | 19290 | 486,7 | 12,5 | 1,202 | H5A034 C | sample |
| 8383 | N15 | N16 | 1637 | 610,5 | 20512 | 459,7 | 12,5 | 1,202 | H5A034 C | sample |
| 8395 | N4 | N5 | 1665 | 634,9 | 20757 | 496,6 | 12,5 | 1,202 | H5A034 C | sample |
| 8436 | P2 | P3 | 1565 | 557,1 | 19632 | 482,2 | 12,5 | 1,202 | H5A034 C | sample |
| 10022 | B21 | B22 | 1333 | 852,1 | 16663 | 174,7 | 12,5 | 1,202 | H5A034 C | sample |
| 10047 | C22 | C23 | 1461 | 823,5 | 18229 | 155,4 | 12,5 | 1,202 | H5A034 C | sample |
| 10232 | K16 | K17 | 1728 | 881,1 | 21548 | 162,5 | 12,5 | 1,202 | H5A034 C | sample |
| 10233 | K17 | K18 | 1934 | 906,6 | 24227 | 142,7 | 12,5 | 1,202 | H5A034 C | sample |
| 10238 | K21 | K22 | 1710 | 903,2 | 21429 | 168,5 | 12,5 | 1,202 | H5A034 C | sample |
| 1618 | D18 | D19 | 1528 | 781,2 | 19133 | 499,7 | 12,5 | 1,309 | H5A034 C | sample |
| 1652 | E5 | E6 | 1641 | 743,4 | 20558 | 475 | 12,5 | 1,309 | H5A034 C | sample |
| 1685 | G13 | G14 | 1308 | 756,3 | 16402 | 306,6 | 12,5 | 1,309 | H5A034 C | sample |
| 1699 | G4 | G5 | 952 | 743,2 | 11899 | 425,5 | 12,5 | 1,309 | H5A034 C | sample |
| 1758 | J14 | J15 | 938 | 717 | 11735 | 352,6 | 12,5 | 1,309 | H5A034 C | sample |
| 1911 | P22 | P23 | 1461 | 746,5 | 18271 | 468,7 | 12,5 | 1,309 | H5A034 C | sample |
| 21657 | G17 | G18 | 461 | 1100 | 5765 | 618,8 | 12,5 | 1,316 | H5A034 C | sample |
| 21851 | O19 | O20 | 178 | 1343 | 2229 | 1090 | 12,5 | 1,316 | H5A034 C | sample |
| 4642 | B18 | B19 | 1435 | 930,9 | 17937 | 503,9 | 12,5 | 1,374 | H5A034 C | sample |
| 4776 | G9 | G10 | 1646 | 821,4 | 20534 | 490,4 | 12,5 | 1,374 | H5A034 C | sample |
| 4910 | M21 | M22 | 1760 | 822,7 | 22079 | 484,5 | 12,5 | 1,374 | H5A034 C | sample |
| 10465 | E1 | E2 | 1001 | 832,9 | 12504 | 189,9 | 12,5 | 1,412 | H5A034 C | sample |
| 10726 | O7 | O8 | 1470 | 817,8 | 18409 | 194,5 | 12,5 | 1,412 | H5A034 C | sample |
| 19978 | A18 | A19 | 1051 | 1165 | 13163 | 321,5 | 12,5 | 1,524 | H5A034 C | sample |
| 20141 | H13 | H14 | 879 | 1139 | 10983 | 172,2 | 12,5 | 1,524 | H5A034 C | sample |
| 20144 | H16 | H17 | 910 | 1085 | 11354 | 140 | 12,5 | 1,524 | H5A034 C | sample |
| 20339 | P19 | P20 | 922 | 1415 | 11545 | 229,2 | 12,5 | 1,524 | H5A034 C | sample |
| 7772 | D5 | D6 | 860 | 519,2 | 10716 | 396,7 | 12,5 | 1,908 | H5A034 C | sample |
| 8021 | O13 | O14 | 524 | 510,1 | 6573 | 449,2 | 12,5 | 1,908 | H5A034 C | sample |
| 3295 | J15 | J16 | 1412 | 1098 | 17790 | 267 | 12,6 | 0,78 | H5A034 C | sample |
| 3318 | K14 | K15 | 1579 | 1284 | 19872 | 315,7 | 12,6 | 0,78 | H5A034 C | sample |
| 6354 | I3 | I4 | 781 | 594,5 | 9813 | 472,3 | 12,6 | 0,832 | H5A034 C | sample |
| 20409 | C17 | C18 | 622 | 814 | 7836 | 185,9 | 12,6 | 0,84 | H5A034 C | sample |
| 20662 | M7 | M8 | 804 | 760,1 | 10155 | 192,5 | 12,6 | 0,84 | H5A034 C | sample |
| 20666 | N10 | N11 | 961 | 751,8 | 12144 | 184,9 | 12,6 | 0,84 | H5A034 C | sample |
| 17466 | H3 | H4 | 1090 | 974,1 | 13758 | 117,9 | 12,6 | 0,86 | H5A034 C | sample |
| 17555 | L19 | L20 | 1559 | 961,8 | 19640 | 209,6 | 12,6 | 0,86 | H5A034 C | sample |
| 19354 | G18 | G19 | 1282 | 1209 | 16195 | 189,4 | 12,6 | 0,894 | H5A034 C | sample |
| 19531 | N4 | N5 | 1140 | 1190 | 14319 | 209,1 | 12,6 | 0,894 | H5A034 C | sample |
| 20774 | B21 | B22 | 1448 | 1039 | 18244 | 288,2 | 12,6 | 0,894 | H5A034 C | sample |
| 11287 | G15 | G16 | 1207 | 798,7 | 15265 | 141,6 | 12,6 | 0,913 | H5A034 C | sample |
| 11362 | J18 | J19 | 1793 | 952,2 | 22654 | 132,7 | 12,6 | 0,913 | H5A034 C | sample |
| 11413 | L20 | L21 | 1969 | 951,5 | 24822 | 142,9 | 12,6 | 0,913 | H5A034 C | sample |
| 11436 | M2 | M3 | 2028 | 941,9 | 25566 | 148,5 | 12,6 | 0,913 | H5A034 C | sample |
| 12417 | F17 | F18 | 1544 | 1075 | 19464 | 170,6 | 12,6 | 0,947 | H5A034 C | sample |
| 12477 | H6 | H7 | 1433 | 995,3 | 18091 | 143 | 12,6 | 0,947 | H5A034 C | sample |
| 12487 | I15 | I16 | 1660 | 979,1 | 20843 | 126,9 | 12,6 | 0,947 | H5A034 C | sample |
| 7606 | M7 | M8 | 1585 | 629 | 20023 | 528,1 | 12,6 | 0,962 | H5A034 C | sample |
| 7629 | N6 | N7 | 654 | 606,1 | 8233 | 525,6 | 12,6 | 0,962 | H5A034 C | sample |
| 17096 | I16 | I17 | 1245 | 1095 | 15721 | 212,9 | 12,6 | 0,973 | H5A034 C | sample |
| 17151 | K22 | K23 | 1595 | 976,4 | 20154 | 229,7 | 12,6 | 0,973 | H5A034 C | sample |
| 17240 | O16 | O17 | 1462 | 936,1 | 18367 | 207,3 | 12,6 | 0,973 | H5A034 C | sample |
| 7095 | H22 | H23 | 1583 | 615,7 | 19889 | 523,2 | 12,6 | 0,981 | H5A034 C | sample |
| 7140 | J2 | J3 | 894 | 564,7 | 11286 | 493,2 | 12,6 | 0,981 | H5A034 C | sample |
| 7142 | J21 | J22 | 742 | 680,1 | 9347 | 454,1 | 12,6 | 0,981 | H5A034 C | sample |
| 7200 | L9 | L10 | 769 | 570,8 | 9703 | 295 | 12,6 | 0,981 | H5A034 C | sample |
| 5028 | B2 | B3 | 1965 | 638,4 | 24681 | 627,6 | 12,6 | 0,988 | H5A034 C | sample |
| 5202 | I3 | I4 | 964 | 596,6 | 12178 | 422,2 | 12,6 | 0,988 | H5A034 C | sample |
| 5206 | I7 | I8 | 1843 | 571,4 | 23253 | 489,6 | 12,6 | 0,988 | H5A034 C | sample |
| 5255 | K8 | K9 | 1802 | 671,8 | 22773 | 459,9 | 12,6 | 0,988 | H5A034 C | sample |
| 5300 | M5 | M6 | 1393 | 632,7 | 17492 | 486,6 | 12,6 | 0,988 | H5A034 C | sample |
| 5361 | P17 | P18 | 2103 | 672,5 | 26591 | 666,7 | 12,6 | 0,988 | H5A034 C | sample |
| 2365 | C20 | C21 | 1555 | 662,4 | 19526 | 323,8 | 12,6 | 0,992 | H5A034 C | sample |
| 2443 | F4 | F5 | 1024 | 745,2 | 12866 | 433,8 | 12,6 | 0,992 | H5A034 C | sample |
| 2610 | M3 | M4 | 738 | 595,2 | 9319 | 348,4 | 12,6 | 0,992 | H5A034 C | sample |
| 2627 | N19 | N20 | 1620 | 738,9 | 20437 | 378,4 | 12,6 | 0,992 | H5A034 C | sample |
| 2650 | O18 | O19 | 1787 | 667,4 | 22483 | 395,6 | 12,6 | 0,992 | H5A034 C | sample |
| 2677 | P20 | P21 | 1510 | 688,7 | 19063 | 480,1 | 12,6 | 0,992 | H5A034 C | sample |
| 16527 | A22 | A23 | 1549 | 1068 | 19541 | 378,8 | 12,6 | 0,996 | H5A034 C | sample |
| 16570 | C18 | C19 | 1450 | 985,5 | 18249 | 349,3 | 12,6 | 0,996 | H5A034 C | sample |
| 16645 | F20 | F21 | 1323 | 921 | 16633 | 242,4 | 12,6 | 0,996 | H5A034 C | sample |
| 4370 | G10 | G11 | 1190 | 703,6 | 15040 | 363,3 | 12,6 | 1,063 | H5A034 C | sample |
| 4375 | G15 | G16 | 1424 | 701 | 17923 | 356 | 12,6 | 1,063 | H5A034 C | sample |
| 4392 | G9 | G10 | 614 | 681,2 | 7761 | 398 | 12,6 | 1,063 | H5A034 C | sample |
| 4485 | K6 | K7 | 1289 | 724,6 | 16261 | 434,6 | 12,6 | 1,063 | H5A034 C | sample |
| 22 | A7 | A8 | 568 | 1541 | 7153 | 738,1 | 12,6 | 1,072 | H5A034 C | sample |
| 80 | D16 | D17 | 336 | 1301 | 4237 | 426,8 | 12,6 | 1,072 | H5A034 C | sample |
| 322 | N18 | N19 | 648 | 1557 | 8170 | 565,3 | 12,6 | 1,072 | H5A034 C | sample |
| 1949 | B13 | B14 | 677 | 765,5 | 8497 | 548,9 | 12,6 | 1,096 | H5A034 C | sample |
| 1999 | D15 | D16 | 1589 | 663,6 | 20085 | 399,3 | 12,6 | 1,096 | H5A034 C | sample |
| 2144 | J16 | J17 | 1134 | 683,2 | 14308 | 318,6 | 12,6 | 1,096 | H5A034 C | sample |
| 2208 | L9 | L10 | 1013 | 623,1 | 12717 | 361,5 | 12,6 | 1,096 | H5A034 C | sample |
| 2264 | O16 | O17 | 1892 | 757,7 | 23768 | 486,6 | 12,6 | 1,096 | H5A034 C | sample |
| 6612 | D2 | D3 | 998 | 660,6 | 12548 | 486,9 | 12,6 | 1,105 | H5A034 C | sample |
| 6636 | E2 | E3 | 1297 | 641 | 16373 | 464,1 | 12,6 | 1,105 | H5A034 C | sample |
| 6659 | F19 | F20 | 1460 | 586,9 | 18377 | 429,1 | 12,6 | 1,105 | H5A034 C | sample |
| 6668 | F5 | F6 | 790 | 574,8 | 9975 | 471,9 | 12,6 | 1,105 | H5A034 C | sample |
| 11697 | H17 | H18 | 648 | 926,8 | 8197 | 128,3 | 12,6 | 1,125 | H5A034 C | sample |
| 11786 | L10 | L11 | 1261 | 796,3 | 15900 | 125,4 | 12,6 | 1,125 | H5A034 C | sample |
| 11826 | M3 | M4 | 1348 | 805,5 | 16923 | 134,4 | 12,6 | 1,125 | H5A034 C | sample |
| 5796 | B2 | B3 | 1318 | 822,8 | 16566 | 593,2 | 12,6 | 1,151 | H5A034 C | sample |
| 6126 | P14 | P15 | 1648 | 787,4 | 20725 | 369,8 | 12,6 | 1,151 | H5A034 C | sample |
| 1243 | D4 | D5 | 1596 | 693,9 | 20176 | 449 | 12,6 | 1,156 | H5A034 C | sample |
| 1253 | E13 | E14 | 1678 | 730,5 | 21176 | 502,7 | 12,6 | 1,156 | H5A034 C | sample |
| 1362 | I3 | I4 | 1513 | 770,7 | 19097 | 432,4 | 12,6 | 1,156 | H5A034 C | sample |
| 1377 | J17 | J18 | 1516 | 729,6 | 19028 | 414,6 | 12,6 | 1,156 | H5A034 C | sample |
| 16165 | B20 | B21 | 1174 | 982,5 | 14769 | 347,9 | 12,6 | 1,156 | H5A034 C | sample |
| 16259 | F19 | F20 | 1327 | 968,6 | 16704 | 313,1 | 12,6 | 1,156 | H5A034 C | sample |
| 16286 | G21 | G22 | 1596 | 990,1 | 20077 | 344,7 | 12,6 | 1,156 | H5A034 C | sample |
| 16358 | J21 | J22 | 1534 | 1013 | 19339 | 306,2 | 12,6 | 1,156 | H5A034 C | sample |
| 13563 | F11 | F12 | 1270 | 982,4 | 16047 | 165,6 | 12,6 | 1,183 | H5A034 C | sample |
| 13663 | J15 | J16 | 1484 | 1029 | 18724 | 151,6 | 12,6 | 1,183 | H5A034 C | sample |
| 5444 | C5 | C6 | 1795 | 683,8 | 22596 | 701,1 | 12,6 | 1,2 | H5A034 C | sample |
| 5602 | J18 | J19 | 1780 | 730,7 | 22371 | 527,2 | 12,6 | 1,2 | H5A034 C | sample |
| 5678 | M21 | M22 | 1796 | 663,2 | 22587 | 535,8 | 12,6 | 1,2 | H5A034 C | sample |
| 3623 | G8 | G9 | 809 | 1401 | 10217 | 356,6 | 12,6 | 1,212 | H5A034 C | sample |
| 3739 | L4 | L5 | 1187 | 1305 | 15000 | 382,3 | 12,6 | 1,212 | H5A034 C | sample |
| 3825 | P17 | P18 | 1318 | 1282 | 16578 | 410,1 | 12,6 | 1,212 | H5A034 C | sample |
| 3829 | P20 | P21 | 1494 | 1445 | 18872 | 458,2 | 12,6 | 1,212 | H5A034 C | sample |
| 8084 | A5 | A6 | 1061 | 633,3 | 13347 | 547,4 | 12,6 | 1,212 | H5A034 C | sample |
| 8181 | E6 | E7 | 1350 | 586,1 | 16990 | 506,7 | 12,6 | 1,212 | H5A034 C | sample |
| 8371 | M4 | M5 | 1281 | 583,3 | 16169 | 353,4 | 12,6 | 1,212 | H5A034 C | sample |
| 8388 | N2 | N3 | 1476 | 654,5 | 18588 | 444,7 | 12,6 | 1,212 | H5A034 C | sample |
| 8428 | P12 | P13 | 2012 | 642 | 25441 | 533,7 | 12,6 | 1,212 | H5A034 C | sample |
| 8429 | P13 | P14 | 1693 | 609,9 | 21260 | 596,4 | 12,6 | 1,212 | H5A034 C | sample |
| 8447 | P8 | P9 | 1453 | 584,2 | 18368 | 509,1 | 12,6 | 1,212 | H5A034 C | sample |
| 10201 | J1 | J2 | 1632 | 848,2 | 20628 | 151,9 | 12,6 | 1,212 | H5A034 C | sample |
| 10236 | K2 | K3 | 1749 | 931,5 | 21968 | 146,5 | 12,6 | 1,212 | H5A034 C | sample |
| 1559 | A8 | A9 | 1094 | 784,8 | 13814 | 456 | 12,6 | 1,319 | H5A034 C | sample |
| 1609 | D1 | D2 | 585 | 671,9 | 7366 | 350,4 | 12,6 | 1,319 | H5A034 C | sample |
| 1892 | O5 | O6 | 1333 | 761,6 | 16736 | 445,8 | 12,6 | 1,319 | H5A034 C | sample |
| 21523 | A4 | A5 | 78 | 1161 | 984 | 674,6 | 12,6 | 1,326 | H5A034 C | sample |
| 4652 | B5 | B6 | 1413 | 782,6 | 17783 | 551,4 | 12,6 | 1,385 | H5A034 C | sample |
| 4741 | F20 | F21 | 1457 | 707 | 18363 | 496,2 | 12,6 | 1,385 | H5A034 C | sample |
| 4852 | K12 | K13 | 949 | 699,7 | 12001 | 462,4 | 12,6 | 1,385 | H5A034 C | sample |
| 10377 | A17 | A18 | 851 | 932,5 | 10763 | 207,4 | 12,6 | 1,424 | H5A034 C | sample |
| 10418 | C10 | C11 | 1486 | 918,7 | 18691 | 196,4 | 12,6 | 1,424 | H5A034 C | sample |
| 10682 | N10 | N11 | 1222 | 785,9 | 15416 | 172,3 | 12,6 | 1,424 | H5A034 C | sample |
| 2850 | G3 | G4 | 785 | 719,1 | 9914 | 535 | 12,6 | 1,482 | H5A034 C | sample |
| 15972 | J2 | J3 | 1176 | 915,6 | 14978 | 123,6 | 12,7 | 0,708 | H5A034 C | sample |
| 14672 | D16 | D17 | 1217 | 819,7 | 15447 | 89,67 | 12,7 | 0,724 | H5A034 C | sample |
| 3246 | H14 | H15 | 1419 | 1242 | 18008 | 297,9 | 12,7 | 0,786 | H5A034 C | sample |
| 3350 | L21 | L22 | 1133 | 1329 | 14337 | 435,9 | 12,7 | 0,786 | H5A034 C | sample |
| 3375 | M22 | M23 | 1016 | 1186 | 12952 | 397,4 | 12,7 | 0,786 | H5A034 C | sample |
| 3406 | N7 | N8 | 1105 | 1235 | 14036 | 362,8 | 12,7 | 0,786 | H5A034 C | sample |
| 12025 | F1 | F2 | 1150 | 814,1 | 14552 | 96,37 | 12,7 | 0,814 | H5A034 C | sample |
| 12108 | I2 | I3 | 1804 | 785,5 | 22834 | 120,2 | 12,7 | 0,814 | H5A034 C | sample |
| 20564 | I5 | I6 | 727 | 733,7 | 9251 | 149 | 12,7 | 0,847 | H5A034 C | sample |
| 20611 | K4 | K5 | 662 | 740,1 | 8384 | 207,9 | 12,7 | 0,847 | H5A034 C | sample |
| 20664 | M9 | M10 | 1023 | 782,9 | 12982 | 198,8 | 12,7 | 0,847 | H5A034 C | sample |
| 20667 | N11 | N12 | 1207 | 788,3 | 15377 | 148,7 | 12,7 | 0,847 | H5A034 C | sample |
| 20672 | N16 | N17 | 987 | 772,2 | 12507 | 154,7 | 12,7 | 0,847 | H5A034 C | sample |
| 20699 | O19 | O20 | 978 | 804,5 | 12468 | 285,4 | 12,7 | 0,847 | H5A034 C | sample |
| 18745 | N1 | N2 | 1707 | 964,9 | 21631 | 195,8 | 12,7 | 0,861 | H5A034 C | sample |
| 17574 | M14 | M15 | 1541 | 935,5 | 19528 | 186,9 | 12,7 | 0,867 | H5A034 C | sample |
| 9546 | N3 | N4 | 1499 | 1045 | 19096 | 107,5 | 12,7 | 0,882 | H5A034 C | sample |
| 9773 | H13 | H14 | 1439 | 980,6 | 18329 | 112,1 | 12,7 | 0,888 | H5A034 C | sample |
| 9944 | O16 | O17 | 1799 | 1140 | 22890 | 165,5 | 12,7 | 0,888 | H5A034 C | sample |
| 8700 | K2 | K3 | 1664 | 1036 | 21152 | 114,7 | 12,7 | 0,898 | H5A034 C | sample |
| 20900 | G5 | G6 | 1297 | 1038 | 16503 | 252,8 | 12,7 | 0,901 | H5A034 C | sample |
| 14398 | H7 | H8 | 883 | 811,4 | 11212 | 102,3 | 12,7 | 0,904 | H5A034 C | sample |
| 11395 | K4 | K5 | 1821 | 860,1 | 23134 | 171,7 | 12,7 | 0,92 | H5A034 C | sample |
| 11447 | M8 | M9 | 1462 | 837,6 | 18620 | 125,1 | 12,7 | 0,92 | H5A034 C | sample |
| 12478 | H7 | H8 | 1227 | 1110 | 15592 | 169,8 | 12,7 | 0,955 | H5A034 C | sample |
| 12590 | M21 | M22 | 920 | 992,3 | 11660 | 154,8 | 12,7 | 0,955 | H5A034 C | sample |
| 17148 | K2 | K3 | 824 | 899,2 | 10471 | 167,7 | 12,7 | 0,981 | H5A034 C | sample |
| 17178 | L3 | L4 | 1266 | 948,2 | 16126 | 117,9 | 12,7 | 0,981 | H5A034 C | sample |
| 5058 | C3 | C4 | 1469 | 623,1 | 18657 | 498,9 | 12,7 | 0,996 | H5A034 C | sample |
| 5071 | D15 | D16 | 2072 | 601,4 | 26390 | 469,1 | 12,7 | 0,996 | H5A034 C | sample |
| 5096 | E16 | E17 | 649 | 553,4 | 8238 | 451,4 | 12,7 | 0,996 | H5A034 C | sample |
| 5113 | F1 | F2 | 1424 | 583,9 | 18082 | 353,8 | 12,7 | 0,996 | H5A034 C | sample |
| 2367 | C22 | C23 | 1756 | 696,5 | 22241 | 453,6 | 12,7 | 1 | H5A034 C | sample |
| 2554 | K18 | K19 | 1697 | 679,2 | 21597 | 331,2 | 12,7 | 1 | H5A034 C | sample |
| 2558 | K21 | K22 | 1516 | 643,3 | 19195 | 445,6 | 12,7 | 1 | H5A034 C | sample |
| 16555 | B4 | B5 | 1140 | 1019 | 14479 | 356,6 | 12,7 | 1,004 | H5A034 C | sample |
| 16640 | F16 | F17 | 1635 | 1020 | 20839 | 237,2 | 12,7 | 1,004 | H5A034 C | sample |
| 8886 | C14 | C15 | 2051 | 1970 | 26043 | 176,1 | 12,7 | 1,02 | H5A034 C | sample |
| 9004 | H12 | H13 | 1368 | 1531 | 17433 | 136 | 12,7 | 1,02 | H5A034 C | sample |
| 4226 | A10 | A11 | 1076 | 648,3 | 13621 | 332,4 | 12,7 | 1,072 | H5A034 C | sample |
| 4453 | J20 | J21 | 1234 | 690,9 | 15709 | 352,4 | 12,7 | 1,072 | H5A034 C | sample |
| 4512 | L9 | L10 | 1315 | 774,3 | 16748 | 385,8 | 12,7 | 1,072 | H5A034 C | sample |
| 4551 | N22 | N23 | 1406 | 699 | 17896 | 417,8 | 12,7 | 1,072 | H5A034 C | sample |
| 4556 | N5 | N6 | 1498 | 646,9 | 19022 | 393,2 | 12,7 | 1,072 | H5A034 C | sample |
| 4604 | P5 | P6 | 1638 | 696,8 | 20838 | 452,6 | 12,7 | 1,072 | H5A034 C | sample |
| 158 | G21 | G22 | 748 | 1623 | 9477 | 611,5 | 12,7 | 1,081 | H5A034 C | sample |
| 298 | M18 | M19 | 624 | 1457 | 7918 | 543,7 | 12,7 | 1,081 | H5A034 C | sample |

| | | | | | | | | | | | | | | | | |
|---|---|---|---|---|---|---|---|---|---|---|---|---|---|---|---|---|
| 1955 | B19 | B20 | 1781 | 774,8 | 22612 | 480,4 | 12,7 | 1,104 | H5A034 C | sample | Hm Drgbl | Hm Drgbl | Hm Drgbl | GRPEL2 | GRPEL2 | GRPEL2 |
| 1988 | C5 | C6 | 1279 | 652,2 | 16302 | 371,4 | 12,7 | 1,104 | H5A034 C | sample | Hm Drgbl | Hm Drgbl | Hm Drgbl | ZNF439 | ZNF439 | ZNF439 |
| 2023 | E15 | E16 | 1373 | 547 | 17467 | 341,2 | 12,7 | 1,104 | H5A034 C | sample | Hm Drgbl | Hm Drgbl | Hm Drgbl | ZNF561 | ZNF561 | ZNF561 |
| 2137 | J1 | J2 | 1755 | 676,5 | 22248 | 445,9 | 12,7 | 1,104 | H5A034 C | sample | Hm Drgbl | Hm Drgbl | Hm Drgbl | ZNF583 | ZNF583 | ZNF583 |
| 2161 | K1 | K2 | 1093 | 650,8 | 13913 | 399 | 12,7 | 1,104 | H5A034 C | sample | Hm Drgbl | Hm Drgbl | Hm Drgbl | ZMYND19 | ZMYND19 | ZMYND19 |
| 2218 | M18 | M19 | 1581 | 696,7 | 20039 | 467,9 | 12,7 | 1,104 | H5A034 C | sample | Hm Drgbl | Hm Drgbl | Hm Drgbl | ZNF789 | ZNF789 | ZNF789 |
| 6606 | D14 | D15 | 1065 | 678,3 | 13541 | 267,5 | 12,7 | 1,114 | H5A034 C | sample | Hm Drgbl | Hm Drgbl | Hm Drgbl | ITGB8 | ITGB8 | ITGB8 |
| 6626 | E10 | E11 | 1250 | 631,5 | 15910 | 481,2 | 12,7 | 1,114 | H5A034 C | sample | Hm Drgbl | Hm Drgbl | Hm Drgbl | IL2RB | IL2RB | IL2RB |
| 6635 | E19 | E20 | 1323 | 620,6 | 16821 | 406,1 | 12,7 | 1,114 | H5A034 C | sample | Hm Drgbl | Hm Drgbl | Hm Drgbl | HAGH | HAGH | HAGH |
| 6779 | K19 | K20 | 1718 | 648,2 | 21865 | 450,8 | 12,7 | 1,114 | H5A034 C | sample | Hm Drgbl | Hm Drgbl | Hm Drgbl | HIP2 | HIP2 | HIP2 |
| 6783 | K22 | K23 | 1754 | 752,3 | 22202 | 323,4 | 12,7 | 1,114 | H5A034 C | sample | Hm Drgbl | Hm Drgbl | Hm Drgbl | INS | INS | INS |
| 6806 | L21 | L22 | 1108 | 565,5 | 14105 | 498,5 | 12,7 | 1,114 | H5A034 C | sample | Hm Drgbl | Hm Drgbl | Hm Drgbl | HSD17B1 | HSD17B1 | HSD17B1 |
| 6858 | N3 | N4 | 1135 | 569,6 | 14459 | 427,9 | 12,7 | 1,114 | H5A034 C | sample | Hm Drgbl | Hm Drgbl | Hm Drgbl | HSD17B2 | HSD17B2 | HSD17B2 |
| 3868 | B12 | B13 | 1053 | 615,9 | 13419 | 361,1 | 12,7 | 1,129 | H5A034 C | sample | Hm Drgbl | Hm Drgbl | Hm Drgbl | RFC2 | RFC2 | RFC2 |
| 4154 | N10 | N11 | 1317 | 636,2 | 16761 | 389,5 | 12,7 | 1,129 | H5A034 C | sample | Hm Drgbl | Hm Drgbl | Hm Drgbl | TGFA | TGFA | TGFA |
| 4184 | O16 | O17 | 1365 | 716,1 | 17323 | 435,8 | 12,7 | 1,129 | H5A034 C | sample | Hm Drgbl | Hm Drgbl | Hm Drgbl | REL | REL | REL |
| 11658 | F3 | F4 | 1619 | 870,9 | 20589 | 151,7 | 12,7 | 1,134 | H5A034 C | sample | Hm Gen E | Hm Gen E | Hm Gen E | CNN3 | CNN3 | CNN3 |
| 11863 | O15 | O16 | 1481 | 914,6 | 18836 | 179,1 | 12,7 | 1,134 | H5A034 C | sample | Hm Gen E | Hm Gen E | Hm Gen E | CD72 | CD72 | CD72 |
| 15434 | D10 | D11 | 1198 | 875,1 | 15192 | 147,8 | 12,7 | 1,155 | H5A034 C | sample | Hm Gen E | Hm Gen E | Hm Gen E | C7orf23 | C7orf23 | C7orf23 |
| 15499 | F4 | F5 | 902 | 839,2 | 11430 | 156,5 | 12,7 | 1,155 | H5A034 C | sample | Hm Gen E | Hm Gen E | Hm Gen E | FBXL15 | FBXL15 | FBXL15 |
| 6001 | K1 | K2 | 655 | 753,1 | 8301 | 353,9 | 12,7 | 1,16 | H5A034 C | sample | Hm Drgbl | Hm Drgbl | Hm Drgbl | ADH1A | ADH1A | ADH1A |
| 6002 | K10 | K11 | 1563 | 835,7 | 19796 | 333,9 | 12,7 | 1,16 | H5A034 C | sample | Hm Drgbl | Hm Drgbl | Hm Drgbl | ATP5C1 | ATP5C1 | ATP5C1 |
| 6010 | K18 | K19 | 1516 | 835,7 | 19302 | 385,3 | 12,7 | 1,16 | H5A034 C | sample | Hm Drgbl | Hm Drgbl | Hm Drgbl | ATP5G2 | ATP5G2 | ATP5G2 |
| 6027 | L11 | L12 | 1183 | 783,2 | 14966 | 523,5 | 12,7 | 1,16 | H5A034 C | sample | Hm Drgbl | Hm Drgbl | Hm Drgbl | APOA4 | APOA4 | APOA4 |
| 6081 | N17 | N18 | 1926 | 822,4 | 24551 | 581,4 | 12,7 | 1,16 | H5A034 C | sample | Hm Drgbl | Hm Drgbl | Hm Drgbl | AQP2 | AQP2 | AQP2 |
| 1246 | D7 | D8 | 1120 | 780,8 | 14252 | 326,6 | 12,7 | 1,165 | H5A034 C | sample | Hm Drgbl | Hm Drgbl | Hm Drgbl | DYSF | DYSF | DYSF |
| 16442 | N10 | N11 | 1315 | 1086 | 16652 | 282,1 | 12,7 | 1,165 | H5A034 C | sample | Hm Gen E | Hm Gen E | Hm Gen E | MGC4655 | MGC4655 | MGC4655 |
| 13531 | D4 | D5 | 1106 | 1046 | 14009 | 171,9 | 12,7 | 1,192 | H5A034 C | sample | Hm Gen E | Hm Gen E | Hm Gen E | OR1J4 | OR1J4 | OR1J4 |
| 13592 | G16 | G17 | 1554 | 1012 | 19704 | 142,4 | 12,7 | 1,192 | H5A034 C | sample | Hm Gen E | Hm Gen E | Hm Gen E | RNF167 | RNF167 | RNF167 |
| 13615 | H15 | H16 | 913 | 892,3 | 11626 | 160,2 | 12,7 | 1,192 | H5A034 C | sample | Hm Gen E | Hm Gen E | Hm Gen E | UNC84B | UNC84B | UNC84B |
| 790 | A7 | A8 | 1782 | 702,6 | 22664 | 494,8 | 12,7 | 1,204 | H5A034 C | sample | Hm Drgbl | Hm Drgbl | Hm Drgbl | SREBF1 | SREBF1 | SREBF1 |
| 801 | B17 | B18 | 1365 | 781,8 | 17364 | 541,1 | 12,7 | 1,204 | H5A034 C | sample | Hm Drgbl | Hm Drgbl | Hm Drgbl | GABPB2 | GABPB2 | GABPB2 |
| 806 | B21 | B22 | 1442 | 766,1 | 18324 | 441,4 | 12,7 | 1,204 | H5A034 C | sample | Hm Drgbl | Hm Drgbl | Hm Drgbl | GATA2 | GATA2 | GATA2 |
| 991 | J15 | J16 | 1376 | 744 | 17493 | 296,2 | 12,7 | 1,204 | H5A034 C | sample | Hm Drgbl | Hm Drgbl | Hm Drgbl | HSPA1B | HSPA1B | HSPA1B |
| 5755 | P4 | P5 | 1867 | 723,7 | 23743 | 616 | 12,7 | 1,21 | H5A034 C | sample | Hm Drgbl | Hm Drgbl | Hm Drgbl | SLC9A3R2 | SLC9A3R2 | SLC9A3R2 |
| 3631 | H15 | H16 | 871 | 1474 | 11048 | 377,3 | 12,7 | 1,221 | H5A034 C | sample | Hm Drgbl | Hm Drgbl | Hm Drgbl | HCRTR1 | HCRTR1 | HCRTR1 |
| 3637 | H20 | H21 | 1383 | 1446 | 17562 | 353,3 | 12,7 | 1,221 | H5A034 C | sample | Hm Drgbl | Hm Drgbl | Hm Drgbl | EMR3 | EMR3 | EMR3 |
| 3735 | L22 | L23 | 806 | 1230 | 10207 | 365 | 12,7 | 1,221 | H5A034 C | sample | Hm Drgbl | Hm Drgbl | Hm Drgbl | PAQR7 | PAQR7 | PAQR7 |
| 3752 | M16 | M17 | 1201 | 1292 | 15288 | 313,2 | 12,7 | 1,221 | H5A034 C | sample | Hm Drgbl | Hm Drgbl | Hm Drgbl | OPN3 | OPN3 | OPN3 |
| 8274 | I3 | I4 | 610 | 560,8 | 7751 | 472,2 | 12,7 | 1,221 | H5A034 C | sample | Hm Drgbl | Hm Drgbl | Hm Drgbl | INSL6 | INSL6 | INSL6 |
| 10326 | O14 | O15 | 2148 | 884,8 | 27179 | 172,8 | 12,7 | 1,221 | H5A034 C | sample | Hm Drgbl | Hm Drgbl | Hm Drgbl | RKHD3 | RKHD3 | RKHD3 |
| 1727 | H8 | H9 | 1419 | 759,9 | 18019 | 411,2 | 12,7 | 1,33 | H5A034 C | sample | Hm Drgbl | Hm Drgbl | Hm Drgbl | EGFL8 | EGFL8 | EGFL8 |
| 21525 | A6 | A7 | 195 | 819 | 2473 | 689,7 | 12,7 | 1,337 | H5A034 C | sample | Hm Gen E | Hm Gen E | Hm Gen E | LOC65244 | LOC65244 | LOC65244 |
| 21631 | F15 | F16 | 282 | 906,3 | 3571 | 705,5 | 12,7 | 1,337 | H5A034 C | sample | Hm Gen E | Hm Gen E | Hm Gen E | LOC65133 | LOC65133 | LOC65133 |
| 4610 | A10 | A11 | 899 | 727,7 | 11420 | 507 | 12,7 | 1,396 | H5A034 C | sample | Hm Drgbl | Hm Drgbl | Hm Drgbl | PSMB9 | PSMB9 | PSMB9 |
| 4654 | B7 | B8 | 1601 | 903,7 | 20254 | 566,5 | 12,7 | 1,396 | H5A034 C | sample | Hm Drgbl | Hm Drgbl | Hm Drgbl | GGT1 | GGT1 | GGT1 |
| 4734 | F14 | F15 | 1758 | 803,3 | 22248 | 467,7 | 12,7 | 1,396 | H5A034 C | sample | Hm Drgbl | Hm Drgbl | Hm Drgbl | BACE1 | BACE1 | BACE1 |
| 4750 | F7 | F8 | 1037 | 737,2 | 13147 | 534,8 | 12,7 | 1,396 | H5A034 C | sample | Hm Drgbl | Hm Drgbl | Hm Drgbl | LPA | LPA | LPA |
| 4875 | L11 | L12 | 1976 | 833,8 | 25105 | 430,7 | 12,7 | 1,396 | H5A034 C | sample | Hm Drgbl | Hm Drgbl | Hm Drgbl | ASTL | ASTL | ASTL |
| 4926 | N14 | N15 | 1766 | 806,2 | 22484 | 534 | 12,7 | 1,396 | H5A034 C | sample | Hm Drgbl | Hm Drgbl | Hm Drgbl | NRIP3 | NRIP3 | NRIP3 |
| 4959 | O22 | O23 | 1188 | 766,9 | 15146 | 538,2 | 12,7 | 1,396 | H5A034 C | sample | Hm Drgbl | Hm Drgbl | Hm Drgbl | USP39 | USP39 | USP39 |
| 2792 | E16 | E17 | 955 | 684,3 | 12107 | 484,7 | 12,7 | 1,494 | H5A034 C | sample | Hm Drgbl | Hm Drgbl | Hm Drgbl | BMP2K | BMP2K | BMP2K |
| 2827 | F4 | F5 | 749 | 633,4 | 9519 | 498,7 | 12,7 | 1,494 | H5A034 C | sample | Hm Drgbl | Hm Drgbl | Hm Drgbl | PIP5KL1 | PIP5KL1 | PIP5KL1 |
| 20184 | I9 | I10 | 371 | 1218 | 4726 | 163,5 | 12,7 | 1,549 | H5A034 C | sample | Hm Gen E | Hm Gen E | Hm Gen E | LOC39165 | LOC39165 | LOC39165 |
| 408 | A9 | A10 | 2049 | 1978 | 25987 | 481 | 12,7 | 1,854 | H5A034 C | sample | Hm Drgbl | Hm Drgbl | Hm Drgbl | TOR1A | TOR1A | TOR1A |
| 7973 | M13 | M14 | 789 | 544,6 | 10010 | 394,8 | 12,7 | 1,939 | H5A034 C | sample | Hm Drgbl | Hm Drgbl | Hm Drgbl | APC2 | APC2 | APC2 |
| 3291 | J11 | J12 | 1546 | 1295 | 19792 | 366,7 | 12,8 | 0,793 | H5A034 C | sample | Hm Drgbl | Hm Drgbl | Hm Drgbl | SYNJ1 | SYNJ1 | SYNJ1 |
| 3415 | O15 | O16 | 1357 | 1400 | 17429 | 469,5 | 12,8 | 0,793 | H5A034 C | sample | Hm Drgbl | Hm Drgbl | Hm Drgbl | PPP2R5B | PPP2R5B | PPP2R5B |
| 6391 | K15 | K16 | 883 | 600,7 | 11342 | 213,3 | 12,8 | 0,845 | H5A034 C | sample | Hm Drgbl | Hm Drgbl | Hm Drgbl | CDH11 | CDH11 | CDH11 |
| 20548 | I12 | I13 | 851 | 741,8 | 10925 | 158,5 | 12,8 | 0,853 | H5A034 C | sample | Hm Gen E | Hm Gen E | Hm Gen E | CLLU1OS | CLLU1OS | CLLU1OS |
| 20597 | K13 | K14 | 1020 | 795,4 | 13033 | 180,6 | 12,8 | 0,853 | H5A034 C | sample | Hm Gen E | Hm Gen E | Hm Gen E | LOC44082 | LOC44082 | LOC44082 |
| 20709 | O6 | O7 | 848 | 782,7 | 10833 | 253,4 | 12,8 | 0,853 | H5A034 C | sample | Hm Gen E | Hm Gen E | Hm Gen E | LOC64176 | LOC64176 | LOC64176 |
| 20721 | P17 | P18 | 933 | 796,2 | 11919 | 157 | 12,8 | 0,853 | H5A034 C | sample | Hm Gen E | Hm Gen E | Hm Gen E | LOC44238 | LOC44238 | LOC44238 |
| 12972 | M2 | M3 | 1594 | 1014 | 20447 | 116,2 | 12,8 | 0,868 | H5A034 C | sample | Hm Gen E | Hm Gen E | Hm Gen E | TSPAN2 | TSPAN2 | TSPAN2 |
| 17386 | E18 | E19 | 1356 | 1047 | 17339 | 238,2 | 12,8 | 0,874 | H5A034 C | sample | Hm Gen E | Hm Gen E | Hm Gen E | PDCL2 | PDCL2 | PDCL2 |
| 17482 | I18 | I19 | 714 | 1013 | 9115 | 241,2 | 12,8 | 0,874 | H5A034 C | sample | Hm Gen E | Hm Gen E | Hm Gen E | LOC13412 | LOC13412 | LOC13412 |
| 17554 | L18 | L19 | 1405 | 940,4 | 17979 | 223,5 | 12,8 | 0,874 | H5A034 C | sample | Hm Gen E | Hm Gen E | Hm Gen E | ASB6 | ASB6 | ASB6 |
| 9450 | J3 | J4 | 1505 | 1173 | 19282 | 238,2 | 12,8 | 0,889 | H5A034 C | sample | Hm Drgbl | Hm Drgbl | Hm Drgbl | ITLN1 | ITLN1 | ITLN1 |
| 18896 | D16 | D17 | 1271 | 1022 | 16209 | 229,8 | 12,8 | 0,892 | H5A034 C | sample | Hm Gen E | Hm Gen E | Hm Gen E | RNF212 | RNF212 | RNF212 |
| 20805 | C6 | C7 | 1191 | 1036 | 15235 | 369,5 | 12,8 | 0,908 | H5A034 C | sample | Hm Gen E | Hm Gen E | Hm Gen E | WIPF3 | WIPF3 | WIPF3 |
| 20886 | G14 | G15 | 1387 | 1020 | 17729 | 296,1 | 12,8 | 0,908 | H5A034 C | sample | Hm Gen E | Hm Gen E | Hm Gen E | LOC64433 | LOC64433 | LOC64433 |
| 21255 | F22 | F23 | 1735 | 1014 | 22226 | 301,9 | 12,8 | 0,908 | H5A034 C | sample | Hm Gen E | Hm Gen E | Hm Gen E | hCG 1979 | hCG 1979 | hCG 1979 |
| 21452 | N5 | N6 | 1275 | 953,5 | 16301 | 194,7 | 12,8 | 0,908 | H5A034 C | sample | Hm Gen E | Hm Gen E | Hm Gen E | LOC64645 | LOC64645 | LOC64645 |
| 21502 | P7 | P8 | 1116 | 1029 | 14281 | 406,8 | 12,8 | 0,908 | H5A034 C | sample | Hm Gen E | Hm Gen E | Hm Gen E | LOC64654 | LOC64654 | LOC64654 |
| 11275 | F4 | F5 | 1928 | 923,4 | 24705 | 168,9 | 12,8 | 0,928 | H5A034 C | sample | Hm Drgbl | Hm Drgbl | Hm Drgbl | | | |
| 12603 | N11 | N12 | 1742 | 1038 | 22299 | 162,7 | 12,8 | 0,962 | H5A034 C | sample | Hm Gen E | Hm Gen E | Hm Gen E | TCL1A | TCL1A | TCL1A |
| 11082 | N3 | N4 | 1091 | 902,9 | 13994 | 502,2 | 12,8 | 0,97 | H5A034 C | sample | Hm Drgbl | Hm Drgbl | Hm Drgbl | SLC5A8 | SLC5A8 | SLC5A8 |
| 11097 | O17 | O18 | 1092 | 727,5 | 13960 | 413,4 | 12,8 | 0,97 | H5A034 C | sample | Hm Drgbl | Hm Drgbl | Hm Drgbl | CD300LF | CD300LF | CD300LF |
| 7298 | A10 | A11 | 1634 | 642,5 | 20881 | 551,9 | 12,8 | 0,977 | H5A034 C | sample | Hm Drgbl | Hm Drgbl | Hm Drgbl | NCOR2 | NCOR2 | NCOR2 |
| 7310 | A21 | A22 | 1416 | 623,5 | 18194 | 455,3 | 12,8 | 0,977 | H5A034 C | sample | Hm Drgbl | Hm Drgbl | Hm Drgbl | YBX1 | YBX1 | YBX1 |
| 7314 | A3 | A4 | 1698 | 651,7 | 21721 | 514,7 | 12,8 | 0,977 | H5A034 C | sample | Hm Drgbl | Hm Drgbl | Hm Drgbl | NPPB | NPPB | NPPB |
| 7503 | I22 | I23 | 1543 | 635,6 | 19686 | 366,2 | 12,8 | 0,977 | H5A034 C | sample | Hm Drgbl | Hm Drgbl | Hm Drgbl | RASSF2 | RASSF2 | RASSF2 |
| 7592 | M16 | M17 | 1050 | 633,7 | 13456 | 399,3 | 12,8 | 0,977 | H5A034 C | sample | Hm Drgbl | Hm Drgbl | Hm Drgbl | ELMO1 | ELMO1 | ELMO1 |
| 7654 | O7 | O8 | 1143 | 609,1 | 14629 | 538,5 | 12,8 | 0,977 | H5A034 C | sample | Hm Drgbl | Hm Drgbl | Hm Drgbl | PCBD1 | PCBD1 | PCBD1 |
| 16952 | C16 | C17 | 1221 | 988,3 | 15632 | 223,8 | 12,8 | 0,988 | H5A034 C | sample | Hm Gen E | Hm Gen E | Hm Gen E | PSTK | C1orf52 | PSTK |
| 17007 | E22 | E23 | 1931 | 1071 | 24637 | 267,3 | 12,8 | 0,988 | H5A034 C | sample | Hm Gen E | Hm Gen E | Hm Gen E | OR51A7 | MOBKL2C | OR51A7 |
| 6925 | A20 | A21 | 1822 | 607,9 | 23307 | 547,1 | 12,8 | 0,996 | H5A034 C | sample | Hm Drgbl | Hm Drgbl | Hm Drgbl | MTAP | MTAP | MTAP |
| 7239 | N22 | N23 | 1031 | 594,8 | 13203 | 231,3 | 12,8 | 0,996 | H5A034 C | sample | Hm Drgbl | Hm Drgbl | Hm Drgbl | NOS3 | NOS3 | NOS3 |
| 5027 | B19 | B20 | 1757 | 748,9 | 22500 | 351,2 | 12,8 | 1,004 | H5A034 C | sample | Hm Drgbl | Hm Drgbl | Hm Drgbl | TMPRSS7 | TMPRSS7 | TMPRSS7 |
| 5207 | I8 | I9 | 992 | 654,6 | 12692 | 291 | 12,8 | 1,004 | H5A034 C | sample | Hm Drgbl | Hm Drgbl | Hm Drgbl | TWIST1 | TWIST1 | TWIST1 |
| 5337 | O17 | O18 | 1416 | 616,4 | 18189 | 533,3 | 12,8 | 1,004 | H5A034 C | sample | Hm Drgbl | Hm Drgbl | Hm Drgbl | Klkbl4 | Klkbl4 | Klkbl4 |
| 2325 | A6 | A7 | 2045 | 715,6 | 26240 | 427,9 | 12,8 | 1,008 | H5A034 C | sample | Hm Drgbl | Hm Drgbl | Hm Drgbl | MKNK2 | MKNK2 | MKNK2 |
| 2469 | G6 | G7 | 1009 | 638,5 | 12905 | 416,7 | 12,8 | 1,008 | H5A034 C | sample | Hm Drgbl | Hm Drgbl | Hm Drgbl | MVK | MVK | MVK |
| 2548 | K12 | K13 | 1691 | 675,8 | 21573 | 386,6 | 12,8 | 1,008 | H5A034 C | sample | Hm Drgbl | Hm Drgbl | Hm Drgbl | PDK3 | PDK3 | PDK3 |
| 2589 | L6 | L7 | 1220 | 708,6 | 15644 | 348,2 | 12,8 | 1,008 | H5A034 C | sample | Hm Drgbl | Hm Drgbl | Hm Drgbl | RPS6KB1 | RPS6KB1 | RPS6KB1 |
| 2616 | M9 | M10 | 1249 | 656,8 | 15986 | 334,7 | 12,8 | 1,008 | H5A034 C | sample | Hm Drgbl | Hm Drgbl | Hm Drgbl | ACVR1 | ACVR1 | ACVR1 |
| 2660 | O5 | O6 | 1354 | 698,3 | 17376 | 414,9 | 12,8 | 1,008 | H5A034 C | sample | Hm Drgbl | Hm Drgbl | Hm Drgbl | AK3L1 | AK3L1 | AK3L1 |
| 2685 | P6 | P7 | 1391 | 774,2 | 17872 | 334,9 | 12,8 | 1,008 | H5A034 C | sample | Hm Drgbl | Hm Drgbl | Hm Drgbl | TESK1 | TESK1 | TESK1 |
| 16540 | B12 | B13 | 1192 | 884,9 | 15278 | 301,5 | 12,8 | 1,012 | H5A034 C | sample | Hm Gen E | Hm Gen E | Hm Gen E | KIAA1919 | KIAA1919 | KIAA1919 |
| 16598 | D21 | D22 | 1688 | 1058 | 21560 | 247,5 | 12,8 | 1,012 | H5A034 C | sample | Hm Gen E | Hm Gen E | Hm Gen E | UNK | UNK | UNK |
| 16646 | F21 | F22 | 1644 | 974,8 | 20981 | 262,1 | 12,8 | 1,012 | H5A034 C | sample | Hm Gen E | Hm Gen E | Hm Gen E | CCDC65 | CCDC65 | CCDC65 |
| 16811 | M19 | M20 | 1819 | 979,3 | 23273 | 269,4 | 12,8 | 1,012 | H5A034 C | sample | Hm Gen E | Hm Gen E | Hm Gen E | HIST1H2BI | HIST1H2BI | HIST1H2BI |
| 8982 | G14 | G15 | 1795 | 1947 | 22971 | 155,1 | 12,8 | 1,028 | H5A034 C | sample | Hm Drgbl | Hm Drgbl | Hm Drgbl | PADI3 | PADI3 | PADI3 |
| 9025 | I1 | I2 | 1377 | 1691 | 17612 | 130,1 | 12,8 | 1,028 | H5A034 C | sample | Hm Drgbl | Hm Drgbl | Hm Drgbl | COQ6 | COQ6 | COQ6 |
| 4323 | E11 | E12 | 1384 | 675,9 | 17781 | 397,3 | 12,8 | 1,08 | H5A034 C | sample | Hm Drgbl | Hm Drgbl | Hm Drgbl | ATP7A | ATP7A | ATP7A |
| 4378 | G18 | G19 | 1276 | 717,7 | 16389 | 351 | 12,8 | 1,08 | H5A034 C | sample | Hm Drgbl | Hm Drgbl | Hm Drgbl | HCN4 | HCN4 | HCN4 |
| 4470 | K14 | K15 | 1115 | 718,4 | 14227 | 355,6 | 12,8 | 1,08 | H5A034 C | sample | Hm Drgbl | Hm Drgbl | Hm Drgbl | KCNH5 | KCNH5 | KCNH5 |
| 4479 | K22 | K23 | 1468 | 729,8 | 18811 | 496,5 | 12,8 | 1,08 | H5A034 C | sample | Hm Drgbl | Hm Drgbl | Hm Drgbl | KCTD3 | KCTD3 | KCTD3 |
| 4496 | L16 | L17 | 1117 | 687 | 14260 | 360 | 12,8 | 1,08 | H5A034 C | sample | Hm Drgbl | Hm Drgbl | Hm Drgbl | TIMP4 | TIMP4 | TIMP4 |
| 4530 | M3 | M4 | 511 | 709,8 | 6524 | 340,8 | 12,8 | 1,08 | H5A034 C | sample | Hm Drgbl | Hm Drgbl | Hm Drgbl | CNGA1 | CNGA1 | CNGA1 |
| 21 | A6 | A7 | 659 | 1652 | 8436 | 614,5 | 12,8 | 1,089 | H5A034 C | sample | Hm Drgbl | Hm Drgbl | Hm Drgbl | SLC9A3 | SLC9A3 | SLC9A3 |
| 55 | C15 | C16 | 593 | 1439 | 7589 | 530,4 | 12,8 | 1,089 | H5A034 C | sample | Hm Drgbl | Hm Drgbl | Hm Drgbl | PPARG | PPARG | PPARG |
| 371 | P19 | P20 | 670 | 1550 | 8557 | 790,9 | 12,8 | 1,089 | H5A034 C | sample | Hm Drgbl | Hm Drgbl | Hm Drgbl | SLC8A1 | SLC8A1 | SLC8A1 |
| 374 | P21 | P22 | 756 | 1419 | 9661 | 799,5 | 12,8 | 1,089 | H5A034 C | sample | Hm Drgbl | Hm Drgbl | Hm Drgbl | SLC8A3 | SLC8A3 | SLC8A3 |
| 378 | P3 | P4 | 517 | 1432 | 6597 | 734 | 12,8 | 1,089 | H5A034 C | sample | Hm Drgbl | Hm Drgbl | Hm Drgbl | SLC6A9 | SLC6A9 | SLC6A9 |
| 1993 | D1 | D2 | 1606 | 681,5 | 20503 | 472 | 12,8 | 1,113 | H5A034 C | sample | Hm Drgbl | Hm Drgbl | Hm Drgbl | EMID2 | EMID2 | EMID2 |
| 2241 | N17 | N18 | 1101 | 620,2 | 14141 | 334,8 | 12,8 | 1,113 | H5A034 C | sample | Hm Drgbl | Hm Drgbl | Hm Drgbl | TCEB3C | TCEB3C | TCEB3C |
| 2295 | P22 | P23 | 1367 | 649,9 | 17480 | 547,9 | 12,8 | 1,113 | H5A034 C | sample | Hm Drgbl | Hm Drgbl | Hm Drgbl | | | |
| 6644 | E5 | E6 | 1547 | 651,5 | 19843 | 363,7 | 12,8 | 1,123 | H5A034 C | sample | Hm Drgbl | Hm Drgbl | Hm Drgbl | GUCY2F | GUCY2F | GUCY2F |
| 6799 | L15 | L16 | 1318 | 576,5 | 16899 | 473,1 | 12,8 | 1,123 | H5A034 C | sample | Hm Drgbl | Hm Drgbl | Hm Drgbl | HSD3B1 | HSD3B1 | HSD3B1 |
| 6804 | L2 | L3 | 755 | 515,4 | 9688 | 363,7 | 12,8 | 1,123 | H5A034 C | sample | Hm Drgbl | Hm Drgbl | Hm Drgbl | KPNA2 | KPNA2 | KPNA2 |
| 6847 | N15 | N16 | 1470 | 602,5 | 18858 | 469,6 | 12,8 | 1,123 | H5A034 C | sample | Hm Drgbl | Hm Drgbl | Hm Drgbl | FOXN2 | FOXN2 | FOXN2 |
| 6863 | N8 | N9 | 1382 | 593,7 | 17640 | 456,2 | 12,8 | 1,123 | H5A034 C | sample | Hm Drgbl | Hm Drgbl | Hm Drgbl | KRT9 | KRT9 | KRT9 |
| 3859 | A4 | A5 | 632 | 778,9 | 8070 | 437,7 | 12,8 | 1,138 | H5A034 C | sample | Hm Drgbl | Hm Drgbl | Hm Drgbl | PVRL2 | PVRL2 | PVRL2 |
| 3864 | A9 | A10 | 1471 | 648,3 | 18886 | 311,4 | 12,8 | 1,138 | H5A034 C | sample | Hm Drgbl | Hm Drgbl | Hm Drgbl | VN2R1P | VN2R1P | VN2R1P |
| 3883 | B4 | B5 | 607 | 775,6 | 7744 | 416,1 | 12,8 | 1,138 | H5A034 C | sample | Hm Drgbl | Hm Drgbl | Hm Drgbl | UPF1 | UPF1 | UPF1 |
| 3902 | C21 | C22 | 1119 | 636,1 | 14286 | 267,6 | 12,8 | 1,138 | H5A034 C | sample | Hm Drgbl | Hm Drgbl | Hm Drgbl | PLCG2 | PLCG2 | PLCG2 |
| 4063 | J15 | J16 | 997 | 574,4 | 12755 | 259,5 | 12,8 | 1,138 | H5A034 C | sample | Hm Drgbl | Hm Drgbl | Hm Drgbl | MLX | MLX | MLX |
| 4156 | N12 | N13 | 1414 | 697 | 18164 | 423,4 | 12,8 | 1,138 | H5A034 C | sample | Hm Drgbl | Hm Drgbl | Hm Drgbl | SORT1 | SORT1 | SORT1 |
| 11535 | A22 | A23 | 1497 | 942,6 | 19149 | 163 | 12,8 | 1,143 | H5A034 C | sample | Hm Gen E | Hm Gen E | Hm Gen E | EFNA4 | EFNA4 | EFNA4 |
| 11569 | C1 | C2 | 1317 | 936,7 | 16799 | 161,2 | 12,8 | 1,143 | H5A034 C | sample | Hm Gen E | Hm Gen E | Hm Gen E | AMELY | AMELY | AMELY |
| 5769 | A17 | A18 | 943 | 887,5 | 12068 | 466 | 12,8 | 1,169 | H5A034 C | sample | Hm Drgbl | Hm Drgbl | Hm Drgbl | ABCA2 | ABCA2 | ABCA2 |
| 5869 | E20 | E21 | 1714 | 830 | 21896 | 493,1 | 12,8 | 1,169 | H5A034 C | sample | Hm Drgbl | Hm Drgbl | Hm Drgbl | ART3 | ART3 | ART3 |
| 5943 | H22 | H23 | 1925 | 871,1 | 24640 | 453,4 | 12,8 | 1,169 | H5A034 C | sample | Hm Drgbl | Hm Drgbl | Hm Drgbl | TSPO | TSPO | TSPO |
| 6058 | M18 | M19 | 1569 | 786,6 | 20139 | 553,4 | 12,8 | 1,169 | H5A034 C | sample | Hm Drgbl | Hm Drgbl | Hm Drgbl | KIF1A | KIF1A | KIF1A |
| 6084 | N2 | N3 | 1326 | 796,5 | 16956 | 322,3 | 12,8 | 1,169 | H5A034 C | sample | Hm Drgbl | Hm Drgbl | Hm Drgbl | CAPZA1 | CAPZA1 | CAPZA1 |
| 6092 | N5 | N6 | 1486 | 777,6 | 18973 | 527,4 | 12,8 | 1,169 | H5A034 C | sample | Hm Drgbl | Hm Drgbl | Hm Drgbl | APOE | APOE | APOE |
| 6096 | N9 | N10 | 1542 | 706 | 19767 | 638,6 | 12,8 | 1,169 | H5A034 C | sample | Hm Drgbl | Hm Drgbl | Hm Drgbl | APP | APP | APP |
| 1352 | I16 | I17 | 1562 | 765 | 20054 | 343,8 | 12,8 | 1,174 | H5A034 C | sample | Hm Drgbl | Hm Drgbl | Hm Drgbl | CD96 | CD96 | CD96 |
| 1391 | J8 | J9 | 918 | 647,3 | 11788 | 311,7 | 12,8 | 1,174 | H5A034 C | sample | Hm Drgbl | Hm Drgbl | Hm Drgbl | HSPA4L | HSPA4L | HSPA4L |
| 16156 | B12 | B13 | 1401 | 974,8 | 17917 | 330,7 | 12,8 | 1,174 | H5A034 C | sample | Hm Gen E | Hm Gen E | Hm Gen E | HHIPL1 | HHIPL1 | HHIPL1 |
| 16215 | D22 | D23 | 1223 | 979,4 | 15709 | 351 | 12,8 | 1,174 | H5A034 C | sample | Hm Gen E | Hm Gen E | Hm Gen E | C14orf142 | C14orf142 | C14orf142 |
| 16362 | J3 | J4 | 810 | 994,5 | 10364 | 317,1 | 12,8 | 1,174 | H5A034 C | sample | Hm Gen E | Hm Gen E | Hm Gen E | WDR54 | WDR54 | WDR54 |
| 13638 | I14 | I15 | 965 | 955,2 | 12355 | 164,4 | 12,8 | 1,202 | H5A034 C | sample | Hm Gen E | Hm Gen E | Hm Gen E | PTCD1 | PTCD1 | PTCD1 |
| 13757 | N13 | N14 | 1624 | 1151 | 20799 | 160,4 | 12,8 | 1,202 | H5A034 C | sample | Hm Gen E | Hm Gen E | Hm Gen E | BRP44 | BRP44 | BRP44 |
| 876 | E2 | E3 | 655 | 805 | 8391 | 486 | 12,8 | 1,213 | H5A034 C | sample | Hm Drgbl | Hm Drgbl | Hm Drgbl | ATXN3 | ATXN3 | ATXN3 |
| 936 | G9 | G10 | 1165 | 788,1 | 14967 | 497,3 | 12,8 | 1,213 | H5A034 C | sample | Hm Drgbl | Hm Drgbl | Hm Drgbl | RUNX2 | RUNX2 | RUNX2 |
| 968 | I16 | I17 | 1693 | 717,3 | 21736 | 368,7 | 12,8 | 1,213 | H5A034 C | sample | Hm Drgbl | Hm Drgbl | Hm Drgbl | OVOL1 | OVOL1 | OVOL1 |
| 998 | J21 | J22 | 1632 | 807,9 | 20819 | 414,7 | 12,8 | 1,213 | H5A034 C | sample | Hm Drgbl | Hm Drgbl | Hm Drgbl | HSPA4 | HSPA4 | HSPA4 |
| 1009 | K1 | K2 | 843 | 755,8 | 10805 | 390,8 | 12,8 | 1,213 | H5A034 C | sample | Hm Drgbl | Hm Drgbl | Hm Drgbl | COL6A1 | COL6A1 | COL6A1 |
| 1047 | L22 | L23 | 1051 | 729,7 | 13456 | 456,9 | 12,8 | 1,213 | H5A034 C | sample | Hm Drgbl | Hm Drgbl | Hm Drgbl | TFCP2 | TFCP2 | TFCP2 |
| 1066 | M18 | M19 | 1264 | 704,9 | 16205 | 336,1 | 12,8 | 1,213 | H5A034 C | sample | Hm Drgbl | Hm Drgbl | Hm Drgbl | POU3F4 | POU3F4 | POU3F4 |
| 5395 | A4 | A5 | 1423 | 705,8 | 18146 | 701,7 | 12,8 | 1,219 | H5A034 C | sample | Hm Drgbl | Hm Drgbl | Hm Drgbl | IL18R1 | IL18R1 | IL18R1 |
| 5482 | E18 | E19 | 1571 | 703,7 | 20071 | 503 | 12,8 | 1,219 | H5A034 C | sample | Hm Drgbl | Hm Drgbl | Hm Drgbl | FUBP1 | FUBP1 | FUBP1 |
| 5527 | G15 | G16 | 1163 | 601,9 | 14928 | 420,8 | 12,8 | 1,219 | H5A034 C | sample | Hm Drgbl | Hm Drgbl | Hm Drgbl | SMARCA5 | SMARCA5 | SMARCA5 |
| 5570 | I10 | I11 | 2320 | 727 | 29624 | 579,4 | 12,8 | 1,219 | H5A034 C | sample | Hm Drgbl | Hm Drgbl | Hm Drgbl | HERC1 | HERC1 | HERC1 |
| 5645 | L13 | L14 | 1623 | 719,7 | 20707 | 557,8 | 12,8 | 1,219 | H5A034 C | sample | Hm Drgbl | Hm Drgbl | Hm Drgbl | CTNNAL1 | CTNNAL1 | CTNNAL1 |
| 5748 | P2 | P3 | 1645 | 665,1 | 21085 | 629,9 | 12,8 | 1,219 | H5A034 C | sample | Hm Drgbl | Hm Drgbl | Hm Drgbl | NDST3 | NDST3 | NDST3 |
| 3485 | B13 | B14 | 1030 | 1453 | 13187 | 382,8 | 12,8 | 1,231 | H5A034 C | sample | Hm Drgbl | Hm Drgbl | Hm Drgbl | CXCR3 | CXCR3 | CXCR3 |
| 3502 | B7 | B8 | 918 | 1382 | 11741 | 374,9 | 12,8 | 1,231 | H5A034 C | sample | Hm Drgbl | Hm Drgbl | Hm Drgbl | GPR6 | GPR6 | GPR6 |
| 3531 | D11 | D12 | 1355 | 1251 | 17367 | 377,6 | 12,8 | 1,231 | H5A034 C | sample | Hm Drgbl | Hm Drgbl | Hm Drgbl | GPR23 | GPR23 | GPR23 |
| 3583 | F15 | F16 | 1302 | 1436 | 16625 | 337,5 | 12,8 | 1,231 | H5A034 C | sample | Hm Drgbl | Hm Drgbl | Hm Drgbl | GPR42 | GPR42 | GPR42 |
| 3615 | G22 | G23 | 855 | 1465 | 10912 | 369,9 | 12,8 | 1,231 | H5A034 C | sample | Hm Drgbl | Hm Drgbl | Hm Drgbl | GPR52 | GPR52 | GPR52 |
| 3710 | K21 | K22 | 1235 | 1255 | 15793 | 377,8 | 12,8 | 1,231 | H5A034 C | sample | Hm Drgbl | Hm Drgbl | Hm Drgbl | EDNRB | EDNRB | EDNRB |
| 3715 | K4 | K5 | 1501 | 1353 | 19192 | 335,6 | 12,8 | 1,231 | H5A034 C | sample | Hm Drgbl | Hm Drgbl | Hm Drgbl | CYSLTR1 | CYSLTR1 | CYSLTR1 |
| 3726 | L14 | L15 | 898 | 1397 | 11452 | 384,1 | 12,8 | 1,231 | H5A034 C | sample | Hm Drgbl | Hm Drgbl | Hm Drgbl | GPR112 | GPR112 | GPR112 |
| 8070 | A14 | A15 | 1248 | 648,3 | 15971 | 468 | 12,8 | 1,231 | H5A034 C | sample | Hm Drgbl | Hm Drgbl | Hm Drgbl | GGA2 | GGA2 | GGA2 |
| 8203 | F4 | F5 | 1161 | 590,7 | 14839 | 530,8 | 12,8 | 1,231 | H5A034 C | sample | Hm Drgbl | Hm Drgbl | Hm Drgbl | AP4E1 | AP4E1 | AP4E1 |
| 8289 | J17 | J18 | 1349 | 697,4 | 17315 | 285 | 12,8 | 1,231 | H5A034 C | sample | Hm Drgbl | Hm Drgbl | Hm Drgbl | SEPHS1 | SEPHS1 | SEPHS1 |
| 8326 | K7 | K8 | 1866 | 636,4 | 23950 | 499,2 | 12,8 | 1,231 | H5A034 C | sample | Hm Drgbl | Hm Drgbl | Hm Drgbl | SUPT16H | SUPT16H | SUPT16H |
| 8437 | P20 | P21 | 1571 | 678,5 | 20127 | 365,8 | 12,8 | 1,231 | H5A034 C | sample | Hm Drgbl | Hm Drgbl | Hm Drgbl | PATZ1 | PATZ1 | PATZ1 |
| 10081 | E1 | E2 | 1192 | 803 | 15269 | 132,2 | 12,8 | 1,231 | H5A034 C | sample | Hm Drgbl | Hm Drgbl | Hm Drgbl | SUV39H2 | SUV39H2 | SUV39H2 |
| 10258 | L18 | L19 | 1896 | 864,4 | 24363 | 170 | 12,8 | 1,231 | H5A034 C | sample | Hm Drgbl | Hm Drgbl | Hm Drgbl | LNX1 | LNX1 | LNX1 |
| 1896 | O9 | O10 | 1617 | 761,1 | 20637 | 435,4 | 12,8 | 1,34 | H5A034 C | sample | Hm Drgbl | Hm Drgbl | Hm Drgbl | SNX7 | SNX7 | SNX7 |
| 1898 | P10 | P11 | 1377 | 708,4 | 17606 | 425,9 | 12,8 | 1,34 | H5A034 C | sample | Hm Drgbl | Hm Drgbl | Hm Drgbl | ZNF341 | ZNF341 | ZNF341 |
| 21571 | C4 | C5 | 207 | 913,8 | 2654 | 726 | 12,8 | 1,347 | H5A034 C | sample | Hm Gen E | Hm Gen E | Hm Gen E | LOC65253 | LOC65253 | LOC65253 |
| 21572 | C5 | C6 | 169 | 1025 | 2156 | 719,7 | 12,8 | 1,347 | H5A034 C | sample | Hm Gen E | Hm Gen E | Hm Gen E | LOC64899 | LOC64899 | LOC64899 |
| 21660 | G2 | G3 | 213 | 934,1 | 2725 | 498,3 | 12,8 | 1,347 | H5A034 C | sample | Hm Gen E | Hm Gen E | Hm Gen E | LOC65263 | LOC65263 | LOC65263 |
| 21778 | L18 | L19 | 309 | 913,4 | 3959 | 606,5 | 12,8 | 1,347 | H5A034 C | sample | Hm Gen E | Hm Gen E | Hm Gen E | LOC65387 | LOC65387 | LOC65387 |
| 21885 | P6 | P7 | 332 | 1247 | 4245 | 712,9 | 12,8 | 1,347 | H5A034 C | sample | Hm Gen E | Hm Gen E | Hm Gen E | LOC65426 | LOC65426 | LOC65426 |
| 4690 | D18 | D19 | 1165 | 767,4 | 14915 | 377,7 | 12,8 | 1,407 | H5A034 C | sample | Hm Drgbl | Hm Drgbl | Hm Drgbl | USP18 | USP18 | USP18 |
| 4699 | D4 | D5 | 1736 | 869,3 | 22188 | 493 | 12,8 | 1,407 | H5A034 C | sample | Hm Drgbl | Hm Drgbl | Hm Drgbl | ADAM29 | ADAM29 | ADAM29 |
| 4810 | I18 | I19 | 1162 | 717,5 | 14834 | 360,9 | 12,8 | 1,407 | H5A034 C | sample | Hm Drgbl | Hm Drgbl | Hm Drgbl | USP14 | USP14 | USP14 |
| 4929 | N17 | N18 | 1275 | 763,7 | 16303 | 438,7 | 12,8 | 1,407 | H5A034 C | sample | Hm Drgbl | Hm Drgbl | Hm Drgbl | PRSS8 | PRSS8 | PRSS8 |
| 10393 | B1 | B2 | 1403 | 864,1 | 17927 | 191,4 | 12,8 | 1,446 | H5A034 C | sample | Hm Drgbl | Hm Drgbl | Hm Drgbl | DHX57 | DHX57 | DHX57 |
| 10438 | C7 | C8 | 946 | 864 | 12135 | 240 | 12,8 | 1,446 | H5A034 C | sample | Hm Drgbl | Hm Drgbl | Hm Drgbl | FIBCD1 | FIBCD1 | FIBCD1 |
| 10660 | M12 | M13 | 1277 | 806,1 | 16289 | 163,5 | 12,8 | 1,446 | H5A034 C | sample | Hm Drgbl | Hm Drgbl | Hm Drgbl | ART5 | ART5 | ART5 |
| 443 | C19 | C20 | 1078 | 1689 | 13765 | 392,9 | 12,8 | 1,869 | H5A034 C | sample | Hm Drgbl | Hm Drgbl | Hm Drgbl | EEF1A1 | EEF1A1 | EEF1A1 |
| 549 | G6 | G7 | 1330 | 1590 | 17074 | 354,6 | 12,8 | 1,869 | H5A034 C | sample | Hm Drgbl | Hm Drgbl | Hm Drgbl | FOSL2 | FOSL2 | FOSL2 |
| 3178 | E18 | E19 | 1042 | 1144 | 13467 | 336,3 | 12,9 | 0,799 | H5A034 C | sample | Hm Drgbl | Hm Drgbl | Hm Drgbl | G6PC2 | G6PC2 | G6PC2 |
| 3429 | O6 | O7 | 1821 | 1401 | 23470 | 439,4 | 12,9 | 0,799 | H5A034 C | sample | Hm Drgbl | Hm Drgbl | Hm Drgbl | PCSK5 | PCSK5 | PCSK5 |
| 6157 | A20 | A21 | 1609 | 613 | 20679 | 460,3 | 12,9 | 0,851 | H5A034 C | sample | Hm Drgbl | Hm Drgbl | Hm Drgbl | CST2 | CST2 | CST2 |
| 20390 | B21 | B22 | 1185 | 854,6 | 15331 | 243,2 | 12,9 | 0,86 | H5A034 C | sample | Hm Gen E | Hm Gen E | Hm Gen E | LOC44125 | LOC44125 | LOC44125 |
| 20497 | G1 | G2 | 880 | 789,2 | 11366 | 137,5 | 12,9 | 0,86 | H5A034 C | sample | Hm Gen E | Hm Gen E | Hm Gen E | MGC2671 | MGC2671 | MGC2671 |
| 20550 | I14 | I15 | 1302 | 748,5 | 16850 | 160,6 | 12,9 | 0,86 | H5A034 C | sample | Hm Gen E | Hm Gen E | Hm Gen E | CLLU1 | CLLU1 | CLLU1 |
| 20623 | L15 | L16 | 934 | 759,8 | 12023 | 160,2 | 12,9 | 0,86 | H5A034 C | sample | Hm Gen E | Hm Gen E | Hm Gen E | OR13G1 | OR13G1 | OR13G1 |
| 20631 | L22 | L23 | 686 | 787,3 | 8861 | 232,1 | 12,9 | 0,86 | H5A034 C | sample | Hm Gen E | Hm Gen E | Hm Gen E | LOC64251 | LOC64251 | LOC64251 |
| 20668 | N12 | N13 | 1173 | 744,4 | 15097 | 150,7 | 12,9 | 0,86 | H5A034 C | sample | Hm Gen E | Hm Gen E | Hm Gen E | LOC64258 | LOC64258 | LOC64258 |
| 20701 | O20 | O21 | 1146 | 792,7 | 14799 | 239,1 | 12,9 | 0,86 | H5A034 C | sample | Hm Gen E | Hm Gen E | Hm Gen E | LOC64183 | LOC64183 | LOC64183 |
| 12691 | A4 | A5 | 1681 | 933,4 | 21734 | 141,9 | 12,9 | 0,875 | H5A034 C | sample | Hm Gen E | Hm Gen E | Hm Gen E | LBA1 | LBA1 | LBA1 |
| 17337 | C17 | C18 | 1120 | 1028 | 14493 | 251,7 | 12,9 | 0,881 | H5A034 C | sample | Hm Gen E | Hm Gen E | Hm Gen E | ZNF428 | ZNF428 | ZNF428 |
| 17343 | C22 | C23 | 1529 | 976,4 | 19734 | 284,2 | 12,9 | 0,881 | H5A034 C | sample | Hm Gen E | Hm Gen E | Hm Gen E | C4orf33 | C4orf33 | C4orf33 |
| 17467 | H4 | H5 | 1448 | 972,4 | 18738 | 148,8 | 12,9 | 0,881 | H5A034 C | sample | Hm Gen E | Hm Gen E | Hm Gen E | SPANXN3 | SPANXN3 | SPANXN3 |
| 17514 | J3 | J4 | 1257 | 897 | 16239 | 219,3 | 12,9 | 0,881 | H5A034 C | sample | Hm Gen E | Hm Gen E | Hm Gen E | CMPK2 | CMPK2 | CMPK2 |
| 19166 | O21 | O22 | 1567 | 999,5 | 20284 | 293,9 | 12,9 | 0,899 | H5A034 C | sample | Hm Gen E | Hm Gen E | Hm Gen E | CLEC9A | CLEC9A | CLEC9A |
| 19270 | C7 | C8 | 1424 | 1211 | 18321 | 399,7 | 12,9 | 0,915 | H5A034 C | sample | Hm Gen E | Hm Gen E | Hm Gen E | KRTAP6-2 | KRTAP6-2 | KRTAP6-2 |
| 19503 | M22 | M23 | 1337 | 1168 | 17252 | 334,4 | 12,9 | 0,915 | H5A034 C | sample | Hm Gen E | Hm Gen E | Hm Gen E | KIF24 | KIF24 | KIF24 |
| 20840 | E16 | E17 | 1698 | 921,8 | 21962 | 215,9 | 12,9 | 0,915 | H5A034 C | sample | Hm Gen E | Hm Gen E | Hm Gen E | LOC64424 | LOC64424 | LOC64424 |
| 20852 | E5 | E6 | 716 | 1004 | 9243 | 314,9 | 12,9 | 0,915 | H5A034 C | sample | Hm Gen E | Hm Gen E | Hm Gen E | C10orf141 | C10orf141 | C10orf141 |
| 20854 | E7 | E8 | 632 | 1068 | 8162 | 323,6 | 12,9 | 0,915 | H5A034 C | sample | Hm Gen E | Hm Gen E | Hm Gen E | LOC64294 | LOC64294 | LOC64294 |
| 20880 | F9 | F10 | 909 | 1011 | 11692 | 157,9 | 12,9 | 0,915 | H5A034 C | sample | Hm Gen E | Hm Gen E | Hm Gen E | hCG 2019 | hCG 2019 | hCG 2019 |
| 21205 | D20 | D21 | 1880 | 995 | 24195 | 246,7 | 12,9 | 0,915 | H5A034 C | sample | Hm Gen E | Hm Gen E | Hm Gen E | LOC64728 | LOC64728 | LOC64728 |
| 21464 | O16 | O17 | 778 | 863,6 | 10047 | 288,9 | 12,9 | 0,915 | H5A034 C | sample | Hm Gen E | Hm Gen E | Hm Gen E | LOC64710 | LOC64710 | LOC64710 |
| 11252 | E5 | E6 | 1365 | 865,9 | 17598 | 128 | 12,9 | 0,935 | H5A034 C | sample | Hm Drgbl | Hm Drgbl | Hm Drgbl | SEC14L4 | SEC14L4 | SEC14L4 |
| 11403 | L11 | L12 | 1288 | 773 | 16621 | 142,2 | 12,9 | 0,935 | H5A034 C | sample | Hm Drgbl | Hm Drgbl | Hm Drgbl | NHLRC1 | NHLRC1 | NHLRC1 |
| 11452 | N12 | N13 | 1774 | 924,3 | 22840 | 140,8 | 12,9 | 0,935 | H5A034 C | sample | Hm Drgbl | Hm Drgbl | Hm Drgbl | | | |
| 7532 | J5 | J6 | 999 | 621,6 | 12934 | 312,1 | 12,9 | 0,985 | H5A034 C | sample | Hm Drgbl | Hm Drgbl | Hm Drgbl | EIF4E2 | EIF4E2 | EIF4E2 |
| 16982 | D21 | D22 | 2138 | 1008 | 27485 | 220,7 | 12,9 | 0,996 | H5A034 C | sample | Hm Gen E | Hm Gen E | Hm Gen E | C8orf40 | NRG4 | C8orf40 |
| 16983 | D22 | D23 | 1470 | 1025 | 19034 | 227,3 | 12,9 | 0,996 | H5A034 C | sample | Hm Gen E | Hm Gen E | Hm Gen E | C15orf27 | C2orf67 | C15orf27 |
| 7223 | M8 | M9 | 1372 | 653,6 | 17718 | 434,2 | 12,9 | 1,004 | H5A034 C | sample | Hm Drgbl | Hm Drgbl | Hm Drgbl | MYOD1 | MYOD1 | MYOD1 |
| 5082 | D3 | D4 | 1835 | 650,1 | 23712 | 617,4 | 12,9 | 1,012 | H5A034 C | sample | Hm Drgbl | Hm Drgbl | Hm Drgbl | USP41 | USP41 | USP41 |
| 5102 | E21 | E22 | 2137 | 607,7 | 27581 | 421 | 12,9 | 1,012 | H5A034 C | sample | Hm Drgbl | Hm Drgbl | Hm Drgbl | USP48 | USP48 | USP48 |
| 5121 | F17 | F18 | 1769 | 653,3 | 22879 | 419 | 12,9 | 1,012 | H5A034 C | sample | Hm Drgbl | Hm Drgbl | Hm Drgbl | TUBA1A | TUBA1A | TUBA1A |
| 5246 | K21 | K22 | 2252 | 703,1 | 29041 | 554,8 | 12,9 | 1,012 | H5A034 C | sample | Hm Drgbl | Hm Drgbl | Hm Drgbl | SEC11B | SEC11B | SEC11B |
| 5256 | K9 | K10 | 1624 | 601,8 | 20962 | 497,7 | 12,9 | 1,012 | H5A034 C | sample | Hm Drgbl | Hm Drgbl | Hm Drgbl | TMPRSS11 | TMPRSS11 | TMPRSS11 |
| 17962 | M18 | M19 | 1362 | 979,9 | 17615 | 213,8 | 12,9 | 1,02 | H5A034 C | sample | Hm Gen E | Hm Gen E | Hm Gen E | NHEDC1 | NHEDC1 | NHEDC1 |

| | | | | | | | | | | | | | | | |
|---|---|---|---|---|---|---|---|---|---|---|---|---|---|---|---|
| 17979 | N11 | N12 | 2130 | 1086 | 27480 | 185,5 | 12,9 | 1,02 | H5A034 C | sample | Hm Gen E | Hm Gen E | Hm Gen E | CPE002JK | CPE002NZ |
| 4294 | C7 | C8 | 1447 | 677 | 18720 | 390,1 | 12,9 | 1,089 | H5A034 C | sample | Hm Drgbl | Hm Drgbl | Hm Drgbl | CPE002DU | CPE002EK |
| 4426 | I18 | I19 | 746 | 747,8 | 9617 | 381,9 | 12,9 | 1,089 | H5A034 C | sample | Hm Drgbl | Hm Drgbl | Hm Drgbl | CPE002DU | CPE002EK |
| 4431 | I22 | I23 | 1130 | 759,1 | 14600 | 417,4 | 12,9 | 1,089 | H5A034 C | sample | Hm Drgbl | Hm Drgbl | Hm Drgbl | CPE002DU | CPE002EK |
| 4451 | J19 | J20 | 834 | 591,8 | 10740 | 320,4 | 12,9 | 1,089 | H5A034 C | sample | Hm Drgbl | Hm Drgbl | Hm Drgbl | CPE002DU | CPE002EK |
| 4494 | L14 | L15 | 594 | 599,1 | 7636 | 375 | 12,9 | 1,089 | H5A034 C | sample | Hm Drgbl | Hm Drgbl | Hm Drgbl | CPE002DU | CPE002EK |
| 4605 | P6 | P7 | 1740 | 655,1 | 22371 | 471,4 | 12,9 | 1,089 | H5A034 C | sample | Hm Drgbl | Hm Drgbl | Hm Drgbl | CPE002DU | CPE002EK |
| 153 | G17 | G18 | 558 | 1387 | 7219 | 393,3 | 12,9 | 1,098 | H5A034 C | sample | Hm Drgbl | Hm Drgbl | Hm Drgbl | CPE002DT | CPE002EJ |
| 177 | H17 | H18 | 454 | 1321 | 5861 | 503,7 | 12,9 | 1,098 | H5A034 C | sample | Hm Drgbl | Hm Drgbl | Hm Drgbl | CPE002DT | CPE002EJ |
| 312 | M9 | M10 | 505 | 1456 | 6514 | 625,5 | 12,9 | 1,098 | H5A034 C | sample | Hm Drgbl | Hm Drgbl | Hm Drgbl | CPE002DT | CPE002EJ |
| 316 | N12 | N13 | 488 | 1497 | 6273 | 728,7 | 12,9 | 1,098 | H5A034 C | sample | Hm Drgbl | Hm Drgbl | Hm Drgbl | CPE002DT | CPE002EJ |
| 1928 | A16 | A17 | 1860 | 789,7 | 24014 | 472 | 12,9 | 1,122 | H5A034 C | sample | Hm Drgbl | Hm Drgbl | Hm Drgbl | CPE002FX | CPE002G1 |
| 1979 | C19 | C20 | 1311 | 692,5 | 16955 | 403,5 | 12,9 | 1,122 | H5A034 C | sample | Hm Drgbl | Hm Drgbl | Hm Drgbl | CPE002FX | CPE002G1 |
| 2182 | K7 | K8 | 1120 | 638 | 14408 | 361,5 | 12,9 | 1,122 | H5A034 C | sample | Hm Drgbl | Hm Drgbl | Hm Drgbl | CPE002FX | CPE002G1 |
| 6549 | A6 | A7 | 1386 | 624 | 17824 | 542,9 | 12,9 | 1,132 | H5A034 C | sample | Hm Drgbl | Hm Drgbl | Hm Drgbl | CPE002E1 | CPE002EY |
| 6590 | C21 | C22 | 1553 | 666,5 | 20017 | 370,5 | 12,9 | 1,132 | H5A034 C | sample | Hm Drgbl | Hm Drgbl | Hm Drgbl | CPE002E1 | CPE002EY |
| 6782 | K21 | K22 | 1703 | 681 | 22018 | 408,3 | 12,9 | 1,132 | H5A034 C | sample | Hm Drgbl | Hm Drgbl | Hm Drgbl | CPE002E1 | CPE002EY |
| 6816 | L9 | L10 | 1185 | 612,6 | 15247 | 334,6 | 12,9 | 1,132 | H5A034 C | sample | Hm Drgbl | Hm Drgbl | Hm Drgbl | CPE002E1 | CPE002EY |
| 3934 | D7 | D8 | 1290 | 661,6 | 16679 | 307,8 | 12,9 | 1,147 | H5A034 C | sample | Hm Drgbl | Hm Drgbl | Hm Drgbl | CPE002DS | CPE002EI |
| 4058 | J10 | J11 | 711 | 669,1 | 9185 | 360,2 | 12,9 | 1,147 | H5A034 C | sample | Hm Drgbl | Hm Drgbl | Hm Drgbl | CPE002DS | CPE002EI |
| 15704 | O16 | O17 | 1374 | 868,1 | 17785 | 185,3 | 12,9 | 1,173 | H5A034 C | sample | Hm Gen E | Hm Gen E | Hm Gen E | CPE002JE | CPE002NT |
| 5827 | C4 | C5 | 1530 | 790,3 | 19667 | 597,3 | 12,9 | 1,178 | H5A034 C | sample | Hm Drgbl | Hm Drgbl | Hm Drgbl | CPE002DY | CPE002F1 |
| 5847 | D22 | D23 | 1427 | 786,7 | 18391 | 602,2 | 12,9 | 1,178 | H5A034 C | sample | Hm Drgbl | Hm Drgbl | Hm Drgbl | CPE002DY | CPE002F1 |
| 5860 | E12 | E13 | 1759 | 801,9 | 22693 | 595,7 | 12,9 | 1,178 | H5A034 C | sample | Hm Drgbl | Hm Drgbl | Hm Drgbl | CPE002DY | CPE002F1 |
| 5955 | I11 | I12 | 1565 | 759,3 | 20202 | 429,4 | 12,9 | 1,178 | H5A034 C | sample | Hm Drgbl | Hm Drgbl | Hm Drgbl | CPE002DY | CPE002F1 |
| 5965 | I20 | I21 | 1156 | 798,1 | 14925 | 459,6 | 12,9 | 1,178 | H5A034 C | sample | Hm Drgbl | Hm Drgbl | Hm Drgbl | CPE002DY | CPE002F1 |
| 5981 | J13 | J14 | 1738 | 799,1 | 22390 | 431,4 | 12,9 | 1,178 | H5A034 C | sample | Hm Drgbl | Hm Drgbl | Hm Drgbl | CPE002DY | CPE002F1 |
| 6006 | K14 | K15 | 792 | 837,7 | 10195 | 509,5 | 12,9 | 1,178 | H5A034 C | sample | Hm Drgbl | Hm Drgbl | Hm Drgbl | CPE002DY | CPE002F1 |
| 6011 | K19 | K20 | 1935 | 829,1 | 25016 | 499,4 | 12,9 | 1,178 | H5A034 C | sample | Hm Drgbl | Hm Drgbl | Hm Drgbl | CPE002DY | CPE002F1 |
| 6056 | M16 | M17 | 1424 | 738,2 | 18370 | 541,4 | 12,9 | 1,178 | H5A034 C | sample | Hm Drgbl | Hm Drgbl | Hm Drgbl | CPE002DY | CPE002F1 |
| 1281 | F17 | F18 | 1889 | 760,6 | 24323 | 393,8 | 12,9 | 1,183 | H5A034 C | sample | Hm Drgbl | Hm Drgbl | Hm Drgbl | CPE002FV | CPE002FZ |
| 1473 | N17 | N18 | 2168 | 815,8 | 28038 | 529,1 | 12,9 | 1,183 | H5A034 C | sample | Hm Drgbl | Hm Drgbl | Hm Drgbl | CPE002FV | CPE002FZ |
| 16176 | B9 | B10 | 763 | 1014 | 9880 | 375 | 12,9 | 1,183 | H5A034 C | sample | Hm Gen E | Hm Gen E | Hm Gen E | CPE002JG | CPE002NV |
| 16180 | C12 | C13 | 1859 | 978,1 | 23959 | 358,8 | 12,9 | 1,183 | H5A034 C | sample | Hm Gen E | Hm Gen E | Hm Gen E | CPE002JG | CPE002NV |
| 16235 | E19 | E20 | 1430 | 1047 | 18479 | 238,1 | 12,9 | 1,183 | H5A034 C | sample | Hm Gen E | Hm Gen E | Hm Gen E | CPE002JG | CPE002NV |
| 16329 | I17 | I18 | 1534 | 946,2 | 19808 | 223,5 | 12,9 | 1,183 | H5A034 C | sample | Hm Gen E | Hm Gen E | Hm Gen E | CPE002JG | CPE002NV |
| 16440 | M9 | M10 | 1913 | 1035 | 24585 | 373,4 | 12,9 | 1,183 | H5A034 C | sample | Hm Gen E | Hm Gen E | Hm Gen E | CPE002JG | CPE002NV |
| 16461 | N6 | N7 | 1384 | 1024 | 17818 | 307,4 | 12,9 | 1,183 | H5A034 C | sample | Hm Gen E | Hm Gen E | Hm Gen E | CPE002JG | CPE002NV |
| 16493 | P13 | P14 | 472 | 1005 | 6105 | 155,7 | 12,9 | 1,183 | H5A034 C | sample | Hm Gen E | Hm Gen E | Hm Gen E | CPE002JG | CPE002NV |
| 16510 | P7 | P8 | 1353 | 1126 | 17488 | 443,9 | 12,9 | 1,183 | H5A034 C | sample | Hm Gen E | Hm Gen E | Hm Gen E | CPE002JG | CPE002NV |
| 13506 | C3 | C4 | 1219 | 1036 | 15685 | 161,2 | 12,9 | 1,211 | H5A034 C | sample | Hm Gen E | Hm Gen E | Hm Gen E | CPE002JC | CPE002NC |
| 13746 | M3 | M4 | 892 | 970,8 | 11529 | 186 | 12,9 | 1,211 | H5A034 C | sample | Hm Gen E | Hm Gen E | Hm Gen E | CPE002JC | CPE002NC |
| 888 | E9 | E10 | 1628 | 715,6 | 20947 | 485,9 | 12,9 | 1,223 | H5A034 C | sample | Hm Drgbl | Hm Drgbl | Hm Drgbl | CPE002FU | CPE002FY |
| 930 | G3 | G4 | 1109 | 773,8 | 14309 | 454,6 | 12,9 | 1,223 | H5A034 C | sample | Hm Drgbl | Hm Drgbl | Hm Drgbl | CPE002FU | CPE002FY |
| 964 | I12 | I13 | 1841 | 773,8 | 23758 | 419,5 | 12,9 | 1,223 | H5A034 C | sample | Hm Drgbl | Hm Drgbl | Hm Drgbl | CPE002FU | CPE002FY |
| 1150 | P7 | P8 | 1559 | 754 | 20071 | 434 | 12,9 | 1,223 | H5A034 C | sample | Hm Drgbl | Hm Drgbl | Hm Drgbl | CPE002FU | CPE002FY |
| 5380 | A12 | A13 | 2085 | 828,8 | 26931 | 679,2 | 12,9 | 1,229 | H5A034 C | sample | Hm Drgbl | Hm Drgbl | Hm Drgbl | CPE002DX | CPE002F2 |
| 5518 | F7 | F8 | 1321 | 619,6 | 17003 | 512,2 | 12,9 | 1,229 | H5A034 C | sample | Hm Drgbl | Hm Drgbl | Hm Drgbl | CPE002DX | CPE002F2 |
| 5580 | I2 | I3 | 1221 | 649,7 | 15764 | 546,4 | 12,9 | 1,229 | H5A034 C | sample | Hm Drgbl | Hm Drgbl | Hm Drgbl | CPE002DX | CPE002F2 |
| 5583 | I22 | I23 | 1238 | 696,2 | 15926 | 546,8 | 12,9 | 1,229 | H5A034 C | sample | Hm Drgbl | Hm Drgbl | Hm Drgbl | CPE002DX | CPE002F2 |
| 5587 | I4 | I5 | 1183 | 714,9 | 15243 | 600,7 | 12,9 | 1,229 | H5A034 C | sample | Hm Drgbl | Hm Drgbl | Hm Drgbl | CPE002DX | CPE002F2 |
| 5716 | O12 | O13 | 1511 | 773,6 | 19529 | 473,8 | 12,9 | 1,229 | H5A034 C | sample | Hm Drgbl | Hm Drgbl | Hm Drgbl | CPE002DX | CPE002F2 |
| 3601 | G1 | G2 | 734 | 1284 | 9447 | 403,1 | 12,9 | 1,24 | H5A034 C | sample | Hm Drgbl | Hm Drgbl | Hm Drgbl | CPE002DR | CPE002EH |
| 3621 | G6 | G7 | 1069 | 1407 | 13840 | 403,9 | 12,9 | 1,24 | H5A034 C | sample | Hm Drgbl | Hm Drgbl | Hm Drgbl | CPE002DR | CPE002EH |
| 3627 | H11 | H12 | 947 | 1506 | 12228 | 379,3 | 12,9 | 1,24 | H5A034 C | sample | Hm Drgbl | Hm Drgbl | Hm Drgbl | CPE002DR | CPE002EH |
| 3656 | I16 | I17 | 854 | 1422 | 11015 | 366,3 | 12,9 | 1,24 | H5A034 C | sample | Hm Drgbl | Hm Drgbl | Hm Drgbl | CPE002DR | CPE002EH |
| 3691 | J4 | J5 | 1027 | 1278 | 13257 | 357,6 | 12,9 | 1,24 | H5A034 C | sample | Hm Drgbl | Hm Drgbl | Hm Drgbl | CPE002DR | CPE002EH |
| 3734 | L21 | L22 | 1369 | 1334 | 17662 | 379 | 12,9 | 1,24 | H5A034 C | sample | Hm Drgbl | Hm Drgbl | Hm Drgbl | CPE002DR | CPE002EH |
| 8155 | D4 | D5 | 1595 | 607,3 | 20640 | 514,3 | 12,9 | 1,24 | H5A034 C | sample | Hm Drgbl | Hm Drgbl | Hm Drgbl | CPE002E5 | CPE002EU |
| 8304 | J9 | J10 | 477 | 649,6 | 6152 | 602,5 | 12,9 | 1,24 | H5A034 C | sample | Hm Drgbl | Hm Drgbl | Hm Drgbl | CPE002E5 | CPE002EU |
| 8359 | M15 | M16 | 1134 | 593,9 | 14658 | 429,6 | 12,9 | 1,24 | H5A034 C | sample | Hm Drgbl | Hm Drgbl | Hm Drgbl | CPE002E5 | CPE002EU |
| 8375 | M8 | M9 | 1608 | 586,2 | 20682 | 473,1 | 12,9 | 1,24 | H5A034 C | sample | Hm Drgbl | Hm Drgbl | Hm Drgbl | CPE002E5 | CPE002EU |
| 8425 | P1 | P2 | 1404 | 536,9 | 18048 | 513,9 | 12,9 | 1,24 | H5A034 C | sample | Hm Drgbl | Hm Drgbl | Hm Drgbl | CPE002E5 | CPE002EU |
| 10327 | O15 | O16 | 1672 | 880,7 | 21579 | 161,5 | 12,9 | 1,24 | H5A034 C | sample | Hm Drgbl | Hm Drgbl | Hm Drgbl | CPE002EA | CPE002EP |
| 1579 | B4 | B5 | 1256 | 712,1 | 16144 | 459,8 | 12,9 | 1,351 | H5A034 C | sample | Hm Drgbl | Hm Drgbl | Hm Drgbl | CPE002FW | CPE002G0 |
| 1687 | G15 | G16 | 1538 | 734,2 | 19847 | 372,6 | 12,9 | 1,351 | H5A034 C | sample | Hm Drgbl | Hm Drgbl | Hm Drgbl | CPE002FW | CPE002G0 |
| 1740 | I2 | I3 | 960 | 735,9 | 12357 | 453,6 | 12,9 | 1,351 | H5A034 C | sample | Hm Drgbl | Hm Drgbl | Hm Drgbl | CPE002FW | CPE002G0 |
| 1749 | I6 | I7 | 1355 | 673 | 17526 | 357,3 | 12,9 | 1,351 | H5A034 C | sample | Hm Drgbl | Hm Drgbl | Hm Drgbl | CPE002FW | CPE002G0 |
| 1754 | J10 | J11 | 1108 | 651,1 | 14300 | 331,3 | 12,9 | 1,351 | H5A034 C | sample | Hm Drgbl | Hm Drgbl | Hm Drgbl | CPE002FW | CPE002G0 |
| 1786 | K18 | K19 | 1403 | 705,9 | 18083 | 319,3 | 12,9 | 1,351 | H5A034 C | sample | Hm Drgbl | Hm Drgbl | Hm Drgbl | CPE002FW | CPE002G0 |
| 21506 | A10 | A11 | 222 | 834,3 | 2869 | 802,6 | 12,9 | 1,358 | H5A034 C | sample | Hm Gen E | Hm Gen E | Hm Gen E | CPE002JU | CPE002O9 |
| 21705 | I17 | I18 | 384 | 985,4 | 4944 | 509,5 | 12,9 | 1,358 | H5A034 C | sample | Hm Gen E | Hm Gen E | Hm Gen E | CPE002JU | CPE002O9 |
| 21773 | L13 | L14 | 251 | 913,4 | 3227 | 741,3 | 12,9 | 1,358 | H5A034 C | sample | Hm Gen E | Hm Gen E | Hm Gen E | CPE002JU | CPE002O9 |
| 4669 | C20 | C21 | 1239 | 895,7 | 16040 | 480,3 | 12,9 | 1,418 | H5A034 C | sample | Hm Drgbl | Hm Drgbl | Hm Drgbl | CPE002DV | CPE002EL |
| 4956 | O2 | O3 | 1292 | 750,9 | 16667 | 537,2 | 12,9 | 1,418 | H5A034 C | sample | Hm Drgbl | Hm Drgbl | Hm Drgbl | CPE002DV | CPE002EL |
| 10527 | G22 | G23 | 1076 | 857,1 | 13885 | 183,3 | 12,9 | 1,458 | H5A034 C | sample | Hm Drgbl | Hm Drgbl | Hm Drgbl | CPE002EB | CPE002EO |
| 10594 | J18 | J19 | 1174 | 918,3 | 15088 | 153,7 | 12,9 | 1,458 | H5A034 C | sample | Hm Drgbl | Hm Drgbl | Hm Drgbl | CPE002EB | CPE002EO |
| 10615 | K15 | K16 | 1246 | 814,6 | 16132 | 176,3 | 12,9 | 1,458 | H5A034 C | sample | Hm Drgbl | Hm Drgbl | Hm Drgbl | CPE002EB | CPE002EO |
| 10618 | K18 | K19 | 1005 | 821,6 | 13009 | 182,2 | 12,9 | 1,458 | H5A034 C | sample | Hm Drgbl | Hm Drgbl | Hm Drgbl | CPE002EB | CPE002EO |
| 10629 | K6 | K7 | 1734 | 854,5 | 22382 | 196,8 | 12,9 | 1,458 | H5A034 C | sample | Hm Drgbl | Hm Drgbl | Hm Drgbl | CPE002EB | CPE002EO |
| 10748 | P5 | P6 | 760 | 1023 | 9768 | 201,4 | 12,9 | 1,458 | H5A034 C | sample | Hm Drgbl | Hm Drgbl | Hm Drgbl | CPE002EB | CPE002EO |
| 10752 | P9 | P10 | 1290 | 774,2 | 16679 | 197,2 | 12,9 | 1,458 | H5A034 C | sample | Hm Drgbl | Hm Drgbl | Hm Drgbl | CPE002EB | CPE002EO |
| 3011 | N19 | N20 | 1219 | 644,7 | 15763 | 534,7 | 12,9 | 1,518 | H5A034 C | sample | Hm Drgbl | Hm Drgbl | Hm Drgbl | CPE002DP | CPE002EF |
| 20001 | B17 | B18 | 1084 | 1160 | 14027 | 238,8 | 12,9 | 1,573 | H5A034 C | sample | Hm Gen E | Hm Gen E | Hm Gen E | CPE002JQ | CPE002O5 |
| 20047 | D15 | D16 | 1050 | 1048 | 13543 | 155,3 | 12,9 | 1,573 | H5A034 C | sample | Hm Gen E | Hm Gen E | Hm Gen E | CPE002JQ | CPE002O5 |
| 20133 | G6 | G7 | 902 | 958,8 | 11601 | 133 | 12,9 | 1,573 | H5A034 C | sample | Hm Gen E | Hm Gen E | Hm Gen E | CPE002JQ | CPE002O5 |
| 13986 | G3 | G4 | 1289 | 1053 | 16811 | 127,5 | 13 | 0,745 | H5A034 C | sample | Hm Gen E | Hm Gen E | Hm Gen E | CPE002JF | CPE002NP |
| 3154 | D18 | D19 | 1000 | 1220 | 12991 | 283,7 | 13 | 0,805 | H5A034 C | sample | Hm Drgbl | Hm Drgbl | Hm Drgbl | CPE002DC | CPE002EG |
| 3201 | F17 | F18 | 1126 | 1370 | 14625 | 464,1 | 13 | 0,805 | H5A034 C | sample | Hm Drgbl | Hm Drgbl | Hm Drgbl | CPE002DC | CPE002EG |
| 18086 | B21 | B22 | 1768 | 1091 | 22948 | 370,4 | 13 | 0,836 | H5A034 C | sample | Hm Gen E | Hm Gen E | Hm Gen E | CPE002JL | CPE002O0 |
| 19897 | N1 | N2 | 1042 | 314,6 | 13528 | 256,9 | 13 | 0,847 | H5A034 C | sample | Hm Gen E | Hm Gen E | Hm Gen E | CPE002JP | CPE002O4 |
| 6351 | I22 | I23 | 1128 | 668,2 | 14632 | 284,8 | 13 | 0,858 | H5A034 C | sample | Hm Drgbl | Hm Drgbl | Hm Drgbl | CPE002DZ | CPE002F0 |
| 6365 | J13 | J14 | 1719 | 598,3 | 22366 | 278,2 | 13 | 0,858 | H5A034 C | sample | Hm Drgbl | Hm Drgbl | Hm Drgbl | CPE002DZ | CPE002F0 |
| 6397 | K20 | K21 | 1586 | 537,7 | 20669 | 481,1 | 13 | 0,858 | H5A034 C | sample | Hm Drgbl | Hm Drgbl | Hm Drgbl | CPE002DZ | CPE002F0 |
| 6398 | K21 | K22 | 1822 | 618,1 | 23699 | 457 | 13 | 0,858 | H5A034 C | sample | Hm Drgbl | Hm Drgbl | Hm Drgbl | CPE002DZ | CPE002F0 |
| 6405 | K6 | K7 | 1503 | 565,3 | 19529 | 383,4 | 13 | 0,858 | H5A034 C | sample | Hm Drgbl | Hm Drgbl | Hm Drgbl | CPE002DZ | CPE002F0 |
| 20506 | G18 | G19 | 1157 | 859,5 | 15083 | 159,9 | 13 | 0,867 | H5A034 C | sample | Hm Gen E | Hm Gen E | Hm Gen E | CPE002JR | CPE002O6 |
| 20627 | L19 | L20 | 684 | 790,7 | 8916 | 153,8 | 13 | 0,867 | H5A034 C | sample | Hm Gen E | Hm Gen E | Hm Gen E | CPE002JR | CPE002O6 |
| 20650 | M18 | M19 | 1026 | 789,7 | 13386 | 206,1 | 13 | 0,867 | H5A034 C | sample | Hm Gen E | Hm Gen E | Hm Gen E | CPE002JR | CPE002O6 |
| 20700 | O2 | O3 | 799 | 773,5 | 10416 | 228,5 | 13 | 0,867 | H5A034 C | sample | Hm Gen E | Hm Gen E | Hm Gen E | CPE002JR | CPE002O6 |
| 12828 | G2 | G3 | 1845 | 1033 | 24038 | 112,9 | 13 | 0,881 | H5A034 C | sample | Hm Gen E | Hm Gen E | Hm Gen E | CPE002J6 | CPE002NM |
| 17424 | F9 | F10 | 1448 | 963,5 | 18859 | 177,5 | 13 | 0,887 | H5A034 C | sample | Hm Gen E | Hm Gen E | Hm Gen E | CPE002JJ | CPE002NY |
| 9237 | A6 | A7 | 2060 | 1270 | 26822 | 212,9 | 13 | 0,903 | H5A034 C | sample | Hm Drgbl | Hm Drgbl | Hm Drgbl | CPE002E8 | CPE002ER |
| 15099 | F11 | F12 | 811 | 786,4 | 10566 | 118,6 | 13 | 0,909 | H5A034 C | sample | Hm Gen E | Hm Gen E | Hm Gen E | CPE002J8 | CPE002NS |
| 15282 | M3 | M4 | 1326 | 771,3 | 17205 | 121,7 | 13 | 0,909 | H5A034 C | sample | Hm Gen E | Hm Gen E | Hm Gen E | CPE002J8 | CPE002NS |
| 19434 | J3 | J4 | 744 | 1141 | 9666 | 218,1 | 13 | 0,922 | H5A034 C | sample | Hm Gen E | Hm Gen E | Hm Gen E | CPE002JO | CPE002O3 |
| 14412 | I2 | I3 | 1571 | 866,5 | 20351 | 131,2 | 13 | 0,925 | H5A034 C | sample | Hm Gen E | Hm Gen E | Hm Gen E | CPE002J7 | CPE002NC |
| 11453 | N13 | N14 | 927 | 813,7 | 12015 | 130 | 13 | 0,942 | H5A034 C | sample | Hm Drgbl | Hm Drgbl | Hm Drgbl | CPE002ED | CPE002F3 |
| 11473 | O1 | O2 | 1550 | 822,7 | 20177 | 163,8 | 13 | 0,942 | H5A034 C | sample | Hm Drgbl | Hm Drgbl | Hm Drgbl | CPE002ED | CPE002F3 |
| 11508 | P2 | P3 | 2083 | 907,8 | 27070 | 177,8 | 13 | 0,942 | H5A034 C | sample | Hm Drgbl | Hm Drgbl | Hm Drgbl | CPE002ED | CPE002F3 |
| 12435 | G11 | G12 | 691 | 1110 | 9013 | 154,5 | 13 | 0,977 | H5A034 C | sample | Hm Gen E | Hm Gen E | Hm Gen E | CPE002J5 | CPE002NK |
| 12601 | N1 | N2 | 918 | 960,5 | 11897 | 182 | 13 | 0,977 | H5A034 C | sample | Hm Gen E | Hm Gen E | Hm Gen E | CPE002J5 | CPE002NK |
| 17034 | F3 | F4 | 1371 | 934,2 | 17767 | 143,1 | 13 | 1,004 | H5A034 C | sample | Hm Gen E | Hm Gen E | Hm Gen E | CPE002JI | CPE002NZ |
| 17106 | I3 | I4 | 1412 | 925,9 | 18303 | 150,1 | 13 | 1,004 | H5A034 C | sample | Hm Gen E | Hm Gen E | Hm Gen E | CPE002JI | CPE002NZ |
| 17125 | J20 | J21 | 2154 | 1008 | 28102 | 211,3 | 13 | 1,004 | H5A034 C | sample | Hm Gen E | Hm Gen E | Hm Gen E | CPE002JI | CPE002NZ |
| 17170 | L18 | L19 | 2022 | 980,7 | 26276 | 208,6 | 13 | 1,004 | H5A034 C | sample | Hm Gen E | Hm Gen E | Hm Gen E | CPE002JI | CPE002NZ |
| 7154 | K10 | K11 | 526 | 622,6 | 6838 | 454,6 | 13 | 1,012 | H5A034 C | sample | Hm Drgbl | Hm Drgbl | Hm Drgbl | CPE002E2 | CPE002EX |
| 5036 | B5 | B6 | 1698 | 618,2 | 22091 | 610,8 | 13 | 1,02 | H5A034 C | sample | Hm Drgbl | Hm Drgbl | Hm Drgbl | CPE002DV | CPE002EM |
| 5232 | J9 | J10 | 1825 | 639 | 23728 | 342,2 | 13 | 1,02 | H5A034 C | sample | Hm Drgbl | Hm Drgbl | Hm Drgbl | CPE002DV | CPE002EM |
| 5295 | M22 | M23 | 1653 | 621 | 21422 | 581,7 | 13 | 1,02 | H5A034 C | sample | Hm Drgbl | Hm Drgbl | Hm Drgbl | CPE002DV | CPE002EM |
| 5303 | M8 | M9 | 1626 | 554,8 | 21179 | 525,9 | 13 | 1,02 | H5A034 C | sample | Hm Drgbl | Hm Drgbl | Hm Drgbl | CPE002DV | CPE002EM |
| 2337 | B17 | B18 | 1156 | 667,5 | 14981 | 458,6 | 13 | 1,024 | H5A034 C | sample | Hm Drgbl | Hm Drgbl | Hm Drgbl | CPE002DC | CPE002EE |
| 2497 | I1 | I2 | 628 | 652,8 | 8146 | 397 | 13 | 1,024 | H5A034 C | sample | Hm Drgbl | Hm Drgbl | Hm Drgbl | CPE002DC | CPE002EE |
| 2557 | K20 | K21 | 1465 | 708,8 | 18983 | 361,6 | 13 | 1,024 | H5A034 C | sample | Hm Drgbl | Hm Drgbl | Hm Drgbl | CPE002DC | CPE002EE |
| 2663 | O8 | O9 | 1579 | 689,3 | 20546 | 435,4 | 13 | 1,024 | H5A034 C | sample | Hm Drgbl | Hm Drgbl | Hm Drgbl | CPE002DC | CPE002EE |
| 16547 | B19 | B20 | 1548 | 1003 | 20143 | 289,7 | 13 | 1,028 | H5A034 C | sample | Hm Gen E | Hm Gen E | Hm Gen E | CPE002JH | CPE002NV |
| 16714 | I18 | I19 | 1323 | 912,6 | 17171 | 270,8 | 13 | 1,028 | H5A034 C | sample | Hm Gen E | Hm Gen E | Hm Gen E | CPE002JH | CPE002NV |
| 16746 | J3 | J4 | 1198 | 967,5 | 15565 | 288,4 | 13 | 1,028 | H5A034 C | sample | Hm Gen E | Hm Gen E | Hm Gen E | CPE002JH | CPE002NV |
| 16756 | K12 | K13 | 1895 | 1084 | 24696 | 291,4 | 13 | 1,028 | H5A034 C | sample | Hm Gen E | Hm Gen E | Hm Gen E | CPE002JH | CPE002NV |
| 16784 | L16 | L17 | 1459 | 907,9 | 18958 | 240,8 | 13 | 1,028 | H5A034 C | sample | Hm Gen E | Hm Gen E | Hm Gen E | CPE002JH | CPE002NV |
| 17838 | H14 | H15 | 1431 | 1100 | 18588 | 327,3 | 13 | 1,028 | H5A034 C | sample | Hm Gen E | Hm Gen E | Hm Gen E | CPE002JK | CPE002NZ |
| 17913 | K17 | K18 | 1117 | 1038 | 14564 | 232,4 | 13 | 1,028 | H5A034 C | sample | Hm Gen E | Hm Gen E | Hm Gen E | CPE002JK | CPE002NZ |
| 17946 | L3 | L4 | 948 | 1038 | 12341 | 183 | 13 | 1,028 | H5A034 C | sample | Hm Gen E | Hm Gen E | Hm Gen E | CPE002JK | CPE002NZ |
| 9135 | M22 | M23 | 2275 | 1909 | 29494 | 235,7 | 13 | 1,044 | H5A034 C | sample | Hm Drgbl | Hm Drgbl | Hm Drgbl | CPE002E7 | CPE002ES |
| 9159 | N22 | N23 | 2092 | 1848 | 27188 | 238 | 13 | 1,044 | H5A034 C | sample | Hm Drgbl | Hm Drgbl | Hm Drgbl | CPE002E7 | CPE002ES |
| 4369 | G1 | G2 | 1343 | 710,4 | 17399 | 481,3 | 13 | 1,097 | H5A034 C | sample | Hm Drgbl | Hm Drgbl | Hm Drgbl | CPE002DU | CPE002EK |
| 4593 | P17 | P18 | 377 | 816,3 | 4919 | 490,4 | 13 | 1,097 | H5A034 C | sample | Hm Drgbl | Hm Drgbl | Hm Drgbl | CPE002DU | CPE002EK |
| 117 | E6 | E7 | 721 | 1707 | 9349 | 366,2 | 13 | 1,106 | H5A034 C | sample | Hm Drgbl | Hm Drgbl | Hm Drgbl | CPE002DT | CPE002EJ |
| 2043 | F11 | F12 | 1125 | 699,9 | 14592 | 495,1 | 13 | 1,13 | H5A034 C | sample | Hm Drgbl | Hm Drgbl | Hm Drgbl | CPE002FX | CPE002G1 |
| 2051 | F19 | F20 | 1630 | 657,8 | 21227 | 450,1 | 13 | 1,13 | H5A034 C | sample | Hm Drgbl | Hm Drgbl | Hm Drgbl | CPE002FX | CPE002G1 |
| 2114 | I10 | I11 | 1010 | 610,9 | 13179 | 352 | 13 | 1,13 | H5A034 C | sample | Hm Drgbl | Hm Drgbl | Hm Drgbl | CPE002FX | CPE002G1 |
| 2122 | I18 | I19 | 1486 | 661,2 | 19383 | 332,3 | 13 | 1,13 | H5A034 C | sample | Hm Drgbl | Hm Drgbl | Hm Drgbl | CPE002FX | CPE002G1 |
| 2232 | M9 | M10 | 2012 | 750,9 | 26177 | 467,5 | 13 | 1,13 | H5A034 C | sample | Hm Drgbl | Hm Drgbl | Hm Drgbl | CPE002FX | CPE002G1 |
| 2294 | P21 | P22 | 2133 | 811 | 27678 | 511,4 | 13 | 1,13 | H5A034 C | sample | Hm Drgbl | Hm Drgbl | Hm Drgbl | CPE002FX | CPE002G1 |
| 6567 | B22 | B23 | 1272 | 610,9 | 16596 | 517,8 | 13 | 1,14 | H5A034 C | sample | Hm Drgbl | Hm Drgbl | Hm Drgbl | CPE002E1 | CPE002EY |
| 6622 | D7 | D8 | 1378 | 633,6 | 17949 | 470,5 | 13 | 1,14 | H5A034 C | sample | Hm Drgbl | Hm Drgbl | Hm Drgbl | CPE002E1 | CPE002EY |
| 6652 | F12 | F13 | 1073 | 588,4 | 13940 | 414,9 | 13 | 1,14 | H5A034 C | sample | Hm Drgbl | Hm Drgbl | Hm Drgbl | CPE002E1 | CPE002EY |
| 6710 | H21 | H22 | 1592 | 626,8 | 20690 | 457,6 | 13 | 1,14 | H5A034 C | sample | Hm Drgbl | Hm Drgbl | Hm Drgbl | CPE002E1 | CPE002EY |
| 3842 | A10 | A11 | 713 | 694 | 9304 | 369,5 | 13 | 1,156 | H5A034 C | sample | Hm Drgbl | Hm Drgbl | Hm Drgbl | CPE002DS | CPE002EI |
| 4101 | K6 | K7 | 614 | 711,4 | 7997 | 401,1 | 13 | 1,156 | H5A034 C | sample | Hm Drgbl | Hm Drgbl | Hm Drgbl | CPE002DS | CPE002EI |
| 4151 | M8 | M9 | 1183 | 687,5 | 15325 | 350,9 | 13 | 1,156 | H5A034 C | sample | Hm Drgbl | Hm Drgbl | Hm Drgbl | CPE002DS | CPE002EI |
| 4177 | O1 | O2 | 324 | 637,1 | 4197 | 374,7 | 13 | 1,156 | H5A034 C | sample | Hm Drgbl | Hm Drgbl | Hm Drgbl | CPE002DS | CPE002EI |
| 4180 | O12 | O13 | 1564 | 639,1 | 20311 | 488,1 | 13 | 1,156 | H5A034 C | sample | Hm Drgbl | Hm Drgbl | Hm Drgbl | CPE002DS | CPE002EI |
| 15606 | K14 | K15 | 876 | 818 | 11385 | 169,5 | 13 | 1,182 | H5A034 C | sample | Hm Gen E | Hm Gen E | Hm Gen E | CPE002JE | CPE002NT |
| 5838 | D14 | D15 | 1851 | 810,8 | 24150 | 578,1 | 13 | 1,187 | H5A034 C | sample | Hm Drgbl | Hm Drgbl | Hm Drgbl | CPE002DY | CPE002F1 |
| 6068 | M5 | M6 | 1494 | 810 | 19474 | 470,1 | 13 | 1,187 | H5A034 C | sample | Hm Drgbl | Hm Drgbl | Hm Drgbl | CPE002DY | CPE002F1 |
| 1236 | D2 | D3 | 1861 | 702,2 | 24269 | 420,3 | 13 | 1,193 | H5A034 C | sample | Hm Drgbl | Hm Drgbl | Hm Drgbl | CPE002FV | CPE002FZ |
| 1285 | F20 | F21 | 1325 | 757,2 | 17187 | 493,4 | 13 | 1,193 | H5A034 C | sample | Hm Drgbl | Hm Drgbl | Hm Drgbl | CPE002FV | CPE002FZ |
| 1420 | L12 | L13 | 1365 | 708,4 | 17770 | 404,6 | 13 | 1,193 | H5A034 C | sample | Hm Drgbl | Hm Drgbl | Hm Drgbl | CPE002FV | CPE002FZ |
| 1493 | O13 | O14 | 2074 | 741,5 | 26907 | 538,1 | 13 | 1,193 | H5A034 C | sample | Hm Drgbl | Hm Drgbl | Hm Drgbl | CPE002FV | CPE002FZ |
| 1536 | P9 | P10 | 1945 | 711 | 25305 | 446,5 | 13 | 1,193 | H5A034 C | sample | Hm Drgbl | Hm Drgbl | Hm Drgbl | CPE002FV | CPE002FZ |
| 16199 | C8 | C9 | 1000 | 918,9 | 12968 | 370,8 | 13 | 1,193 | H5A034 C | sample | Hm Gen E | Hm Gen E | Hm Gen E | CPE002JG | CPE002NV |
| 16403 | L19 | L20 | 1140 | 861,8 | 14805 | 247 | 13 | 1,193 | H5A034 C | sample | Hm Gen E | Hm Gen E | Hm Gen E | CPE002JG | CPE002NV |
| 16452 | N2 | N3 | 1203 | 1066 | 15585 | 239,3 | 13 | 1,193 | H5A034 C | sample | Hm Gen E | Hm Gen E | Hm Gen E | CPE002JG | CPE002NV |
| 16491 | P11 | P12 | 1331 | 1095 | 17257 | 414,4 | 13 | 1,193 | H5A034 C | sample | Hm Gen E | Hm Gen E | Hm Gen E | CPE002JG | CPE002NV |
| 769 | A1 | A2 | 1187 | 756,6 | 15440 | 583,1 | 13 | 1,232 | H5A034 C | sample | Hm Drgbl | Hm Drgbl | Hm Drgbl | CPE002FU | CPE002FY |
| 816 | B9 | B10 | 1718 | 760,2 | 22372 | 449,8 | 13 | 1,232 | H5A034 C | sample | Hm Drgbl | Hm Drgbl | Hm Drgbl | CPE002FU | CPE002FY |
| 848 | D16 | D17 | 1039 | 708,9 | 13490 | 388,5 | 13 | 1,232 | H5A034 C | sample | Hm Drgbl | Hm Drgbl | Hm Drgbl | CPE002FU | CPE002FY |
| 893 | F13 | F14 | 1651 | 749,1 | 21510 | 428,4 | 13 | 1,232 | H5A034 C | sample | Hm Drgbl | Hm Drgbl | Hm Drgbl | CPE002FU | CPE002FY |
| 925 | G20 | G21 | 2188 | 800,1 | 28352 | 416,1 | 13 | 1,232 | H5A034 C | sample | Hm Drgbl | Hm Drgbl | Hm Drgbl | CPE002FU | CPE002FY |
| 5435 | C19 | C20 | 1613 | 651,8 | 20897 | 462,2 | 13 | 1,238 | H5A034 C | sample | Hm Drgbl | Hm Drgbl | Hm Drgbl | CPE002DX | CPE002F2 |
| 5467 | D4 | D5 | 1566 | 592,7 | 20416 | 575 | 13 | 1,238 | H5A034 C | sample | Hm Drgbl | Hm Drgbl | Hm Drgbl | CPE002DX | CPE002F2 |
| 5571 | I11 | I12 | 1610 | 651,7 | 20963 | 536,1 | 13 | 1,238 | H5A034 C | sample | Hm Drgbl | Hm Drgbl | Hm Drgbl | CPE002DX | CPE002F2 |
| 5589 | I6 | I7 | 1340 | 771,4 | 17376 | 515,3 | 13 | 1,238 | H5A034 C | sample | Hm Drgbl | Hm Drgbl | Hm Drgbl | CPE002DX | CPE002F2 |
| 5626 | K18 | K19 | 658 | 773,4 | 8585 | 331,8 | 13 | 1,238 | H5A034 C | sample | Hm Drgbl | Hm Drgbl | Hm Drgbl | CPE002DX | CPE002F2 |
| 5759 | P8 | P9 | 2165 | 671,8 | 28120 | 640,1 | 13 | 1,238 | H5A034 C | sample | Hm Drgbl | Hm Drgbl | Hm Drgbl | CPE002DX | CPE002F2 |
| 3487 | B15 | B16 | 1133 | 1323 | 14683 | 339,1 | 13 | 1,25 | H5A034 C | sample | Hm Drgbl | Hm Drgbl | Hm Drgbl | CPE002DR | CPE002EH |
| 3500 | B5 | B6 | 1025 | 1260 | 13309 | 347 | 13 | 1,25 | H5A034 C | sample | Hm Drgbl | Hm Drgbl | Hm Drgbl | CPE002DR | CPE002EH |
| 3523 | C4 | C5 | 850 | 1268 | 11071 | 372,4 | 13 | 1,25 | H5A034 C | sample | Hm Drgbl | Hm Drgbl | Hm Drgbl | CPE002DR | CPE002EH |
| 3636 | H2 | H3 | 1120 | 1320 | 14506 | 376,3 | 13 | 1,25 | H5A034 C | sample | Hm Drgbl | Hm Drgbl | Hm Drgbl | CPE002DR | CPE002EH |
| 3786 | N3 | N4 | 1157 | 1294 | 15059 | 334,6 | 13 | 1,25 | H5A034 C | sample | Hm Drgbl | Hm Drgbl | Hm Drgbl | CPE002DR | CPE002EH |
| 3799 | O15 | O16 | 1194 | 1403 | 15519 | 471,3 | 13 | 1,25 | H5A034 C | sample | Hm Drgbl | Hm Drgbl | Hm Drgbl | CPE002DR | CPE002EH |
| 3802 | O18 | O19 | 1910 | 1368 | 24750 | 403 | 13 | 1,25 | H5A034 C | sample | Hm Drgbl | Hm Drgbl | Hm Drgbl | CPE002DR | CPE002EH |
| 8110 | B7 | B8 | 1628 | 615,3 | 21215 | 492,7 | 13 | 1,25 | H5A034 C | sample | Hm Drgbl | Hm Drgbl | Hm Drgbl | CPE002E5 | CPE002EU |
| 8206 | F7 | F8 | 1097 | 571,6 | 14237 | 476,5 | 13 | 1,25 | H5A034 C | sample | Hm Drgbl | Hm Drgbl | Hm Drgbl | CPE002E5 | CPE002EU |
| 8328 | K9 | K10 | 1720 | 570 | 22441 | 472,7 | 13 | 1,25 | H5A034 C | sample | Hm Drgbl | Hm Drgbl | Hm Drgbl | CPE002E5 | CPE002EU |
| 9994 | A18 | A19 | 1900 | 946,5 | 24758 | 190,2 | 13 | 1,25 | H5A034 C | sample | Hm Drgbl | Hm Drgbl | Hm Drgbl | CPE002EA | CPE002EP |
| 10065 | D17 | D18 | 1282 | 783,3 | 16643 | 148,9 | 13 | 1,25 | H5A034 C | sample | Hm Drgbl | Hm Drgbl | Hm Drgbl | CPE002EA | CPE002EP |
| 10146 | G3 | G4 | 1871 | 897 | 24240 | 128 | 13 | 1,25 | H5A034 C | sample | Hm Drgbl | Hm Drgbl | Hm Drgbl | CPE002EA | CPE002EP |
| 1893 | O6 | O7 | 1825 | 770,1 | 23698 | 452,2 | 13 | 1,361 | H5A034 C | sample | Hm Drgbl | Hm Drgbl | Hm Drgbl | CPE002FW | CPE002G0 |
| 21511 | A15 | A16 | 417 | 1065 | 5438 | 673,2 | 13 | 1,368 | H5A034 C | sample | Hm Gen E | Hm Gen E | Hm Gen E | CPE002JU | CPE002O9 |
| 21680 | H16 | H17 | 396 | 1142 | 5134 | 514,2 | 13 | 1,368 | H5A034 C | sample | Hm Gen E | Hm Gen E | Hm Gen E | CPE002JU | CPE002O9 |
| 21873 | P17 | P18 | 444 | 1216 | 5792 | 444 | 13 | 1,368 | H5A034 C | sample | Hm Gen E | Hm Gen E | Hm Gen E | CPE002JU | CPE002O9 |
| 4614 | A14 | A15 | 1017 | 873,4 | 13259 | 379,3 | 13 | 1,429 | H5A034 C | sample | Hm Drgbl | Hm Drgbl | Hm Drgbl | CPE002DV | CPE002EL |
| 4644 | B2 | B3 | 1282 | 831,9 | 16643 | 581,9 | 13 | 1,429 | H5A034 C | sample | Hm Drgbl | Hm Drgbl | Hm Drgbl | CPE002DV | CPE002EL |
| 4773 | G6 | G7 | 1210 | 939,5 | 15745 | 488,2 | 13 | 1,429 | H5A034 C | sample | Hm Drgbl | Hm Drgbl | Hm Drgbl | CPE002DV | CPE002EL |
| 4930 | N18 | N19 | 1589 | 840,4 | 20677 | 457,6 | 13 | 1,429 | H5A034 C | sample | Hm Drgbl | Hm Drgbl | Hm Drgbl | CPE002DV | CPE002EL |
| 10483 | E4 | E5 | 1261 | 844 | 16370 | 190,9 | 13 | 1,469 | H5A034 C | sample | Hm Drgbl | Hm Drgbl | Hm Drgbl | CPE002EB | CPE002EO |
| 10498 | F18 | F19 | 1203 | 862 | 15674 | 208,5 | 13 | 1,469 | H5A034 C | sample | Hm Drgbl | Hm Drgbl | Hm Drgbl | CPE002EB | CPE002EO |
| 10612 | K12 | K13 | 988 | 866,2 | 12837 | 184,7 | 13 | 1,469 | H5A034 C | sample | Hm Drgbl | Hm Drgbl | Hm Drgbl | CPE002EB | CPE002EO |
| 10751 | P8 | P9 | 1221 | 763,8 | 15832 | 204,4 | 13 | 1,469 | H5A034 C | sample | Hm Drgbl | Hm Drgbl | Hm Drgbl | CPE002EB | CPE002EO |
| 2735 | B8 | B9 | 1111 | 691,1 | 14438 | 642 | 13 | 1,529 | H5A034 C | sample | Hm Drgbl | Hm Drgbl | Hm Drgbl | CPE002DP | CPE002EF |
| 2739 | C11 | C12 | 1295 | 640,6 | 16787 | 581,4 | 13 | 1,529 | H5A034 C | sample | Hm Drgbl | Hm Drgbl | Hm Drgbl | CPE002DP | CPE002EF |
| 3068 | P5 | P6 | 1041 | 607,6 | 13576 | 552,1 | 13 | 1,529 | H5A034 C | sample | Hm Drgbl | Hm Drgbl | Hm Drgbl | CPE002DP | CPE002EF |
| 19979 | A19 | A20 | 1065 | 1116 | 13826 | 270,9 | 13 | 1,585 | H5A034 C | sample | Hm Gen E | Hm Gen E | Hm Gen E | CPE002JQ | CPE002O5 |
| 20315 | O19 | O20 | 942 | 1061 | 12218 | 187,1 | 13 | 1,585 | H5A034 C | sample | Hm Gen E | Hm Gen E | Hm Gen E | CPE002JQ | CPE002O5 |
| 7784 | E16 | E17 | 368 | 490,2 | 4769 | 348 | 13 | 1,985 | H5A034 C | sample | Hm Drgbl | Hm Drgbl | Hm Drgbl | CPE002E4 | CPE002EV |
| 3093 | A6 | A7 | 1587 | 1320 | 20833 | 367,2 | 13,1 | 0,811 | H5A034 C | sample | Hm Drgbl | Hm Drgbl | Hm Drgbl | CPE002DC | CPE002EG |
| 3349 | L20 | L21 | 1567 | 1417 | 20532 | 412,6 | 13,1 | 0,811 | H5A034 C | sample | Hm Drgbl | Hm Drgbl | Hm Drgbl | CPE002DC | CPE002EG |
| 12192 | L9 | L10 | 848 | 768,2 | 11075 | 73,98 | 13,1 | 0,84 | H5A034 C | sample | Hm Gen E | Hm Gen E | Hm Gen E | CPE002J4 | CPE002NL |
| 20461 | E20 | E21 | 1266 | 798,9 | 16610 | 161,2 | 13,1 | 0,873 | H5A034 C | sample | Hm Gen E | Hm Gen E | Hm Gen E | CPE002JR | CPE002O6 |
| 20552 | I16 | I17 | 1316 | 761,9 | 17222 | 143,2 | 13,1 | 0,873 | H5A034 C | sample | Hm Gen E | Hm Gen E | Hm Gen E | CPE002JR | CPE002O6 |
| 20620 | L12 | L13 | 862 | 769,2 | 11334 | 170,8 | 13,1 | 0,873 | H5A034 C | sample | Hm Gen E | Hm Gen E | Hm Gen E | CPE002JR | CPE002O6 |
| 20624 | L16 | L17 | 939 | 764,3 | 12274 | 125 | 13,1 | 0,873 | H5A034 C | sample | Hm Gen E | Hm Gen E | Hm Gen E | CPE002JR | CPE002O6 |
| 20644 | M12 | M13 | 1021 | 777 | 13354 | 182,8 | 13,1 | 0,873 | H5A034 C | sample | Hm Gen E | Hm Gen E | Hm Gen E | CPE002JR | CPE002O6 |
| 18519 | D22 | D23 | 1413 | 1011 | 18463 | 319 | 13,1 | 0,888 | H5A034 C | sample | Hm Gen E | Hm Gen E | Hm Gen E | CPE002JM | CPE002O1 |
| 17559 | L22 | L23 | 1831 | 984,2 | 24037 | 230,4 | 13,1 | 0,894 | H5A034 C | sample | Hm Gen E | Hm Gen E | Hm Gen E | CPE002JJ | CPE002NY |
| 17566 | L7 | L8 | 1283 | 890,8 | 16837 | 204,7 | 13,1 | 0,894 | H5A034 C | sample | Hm Gen E | Hm Gen E | Hm Gen E | CPE002JJ | CPE002NY |
| 17641 | P1 | P2 | 1256 | 1053 | 16479 | 200,9 | 13,1 | 0,894 | H5A034 C | sample | Hm Gen E | Hm Gen E | Hm Gen E | CPE002JJ | CPE002NY |
| 9681 | D17 | D18 | 2116 | 1128 | 27744 | 136,1 | 13,1 | 0,916 | H5A034 C | sample | Hm Drgbl | Hm Drgbl | Hm Drgbl | CPE002E9 | CPE002EQ |
| 15148 | H12 | H13 | 1589 | 862,6 | 20737 | 113,8 | 13,1 | 0,916 | H5A034 C | sample | Hm Gen E | Hm Gen E | Hm Gen E | CPE002J8 | CPE002NS |
| 19584 | P9 | P10 | 876 | 1296 | 11495 | 238,9 | 13,1 | 0,929 | H5A034 C | sample | Hm Gen E | Hm Gen E | Hm Gen E | CPE002JO | CPE002O3 |
| 20823 | D22 | D23 | 1388 | 1052 | 18115 | 241,2 | 13,1 | 0,929 | H5A034 C | sample | Hm Gen E | Hm Gen E | Hm Gen E | CPE002JS | CPE002O7 |
| 21043 | M4 | M5 | 1191 | 1041 | 15558 | 116,1 | 13,1 | 0,929 | H5A034 C | sample | Hm Gen E | Hm Gen E | Hm Gen E | CPE002JS | CPE002O7 |
| 7325 | B13 | B14 | 1469 | 643,3 | 19257 | 541,2 | 13,1 | 1 | H5A034 C | sample | Hm Drgbl | Hm Drgbl | Hm Drgbl | CPE002E3 | CPE002EW |
| 7374 | D14 | D15 | 1224 | 548,2 | 16075 | 531,7 | 13,1 | 1 | H5A034 C | sample | Hm Drgbl | Hm Drgbl | Hm Drgbl | CPE002E3 | CPE002EW |

| | | | | | | | | | | | | | | | | | | | | | | | | | | | | | | | |
|---|---|---|---|---|---|---|---|---|---|---|---|---|---|---|---|---|---|---|---|---|---|---|---|---|---|---|---|---|---|---|---|
| 16940 | B5 | B6 | 1546 | 957,6 | 20259 | 287,4 | 13,1 | 1,012 | H5A034 C | sample | Hm Gen E | Hm Gen E | Hm Gen E | CPE002JI | CPE002NZ | CPE002KD | NM 05290 | NM 1825 | NM 0529 | CSMD3 | THSD3 | CSMD3 | CUB and Sushi mu | thrombos | CUB and S | 114788 | 145501 | 114788 | s41603 | s44748 | s41605 |
| 17003 | E19 | E20 | 1547 | 1028 | 20302 | 262,4 | 13,1 | 1,012 | H5A034 C | sample | Hm Gen E | Hm Gen E | Hm Gen E | CPE002JI | CPE002NZ | CPE002KD | NM 03328 | NM 0010 | NM 0332 | TP53INP1 | OR52B4 | TP53INP1 | tumor protein p5 | olfactory r | tumor pro | 94241 | 143496 | 94241 | s41296 | s44541 | s41298 |
| 17029 | F20 | F21 | 1975 | 1001 | 25872 | 205,4 | 13,1 | 1,012 | H5A034 C | sample | Hm Gen E | Hm Gen E | Hm Gen E | CPE002JI | CPE002NZ | CPE002KD | NM 17347 | NM 1530 | NM 1734 | DCUN1D3 | CCDC140 | DCUN1D3 | DCN1, defective in | coiled-coil | DCN1, def | 123879 | 151278 | 123879 | s42682 | s45553 | s42684 |
| 7277 | P13 | P14 | 1586 | 582,7 | 20818 | 586,1 | 13,1 | 1,019 | H5A034 C | sample | Hm Drgbl | Hm Drgbl | Hm Drgbl | CPE002E2 | CPE002EX | CPE002FI | NM 00559 | NM 0055 | NM 0055 | MRE11A | MRE11A | MRE11A | MRE11 meiotic re | MRE11 me | MRE11 me | 4361 | 4361 | 4361 | s8961 | s8959 | s8960 |
| 2347 | B4 | B5 | 783 | 843,1 | 10274 | 525,8 | 13,1 | 1,031 | H5A034 C | sample | Hm Drgbl | Hm Drgbl | Hm Drgbl | CPE002DC | CPE002EE | CPE002F4 | NM 00273 | NM 0027 | NM 0027 | PRKACG | PRKACG | PRKACG | protein kinase, cA | protein ki | protein ki | 5568 | 5568 | 5568 | s11071 | s11072 | s224379 |
| 2418 | E3 | E4 | 1037 | 769,4 | 13627 | 381,2 | 13,1 | 1,031 | H5A034 C | sample | Hm Drgbl | Hm Drgbl | Hm Drgbl | CPE002DC | CPE002EE | CPE002F4 | NM 01511 | NM 0151 | NM 0151 | MAST2 | MAST2 | MAST2 | microtubule asso | microtubu | microtubu | 23139 | 23139 | 23139 | s42 | s43 | s44 |
| 2476 | H12 | H13 | 1441 | 797,9 | 18836 | 400,3 | 13,1 | 1,031 | H5A034 C | sample | Hm Drgbl | Hm Drgbl | Hm Drgbl | CPE002DC | CPE002EE | CPE002F4 | NM 00276 | NM 0027 | NM 0027 | PRPSAP1 | PRPSAP1 | PRPSAP1 | phosphoribosyl p | phosphor | phosphor | 5635 | 5635 | 5635 | s11239 | s11240 | s11241 |
| 2502 | I14 | I15 | 1065 | 637,3 | 13961 | 295,7 | 13,1 | 1,031 | H5A034 C | sample | Hm Drgbl | Hm Drgbl | Hm Drgbl | CPE002DC | CPE002EE | CPE002F4 | XM 00112 | XM 0011 | XM 0011 | PAK2 | PAK2 | PAK2 | p21 (CDKN1A)-act | p21 (CDKN | p21 (CDKN | 5062 | 5062 | 5062 | s10022 | s10023 | s10024 |
| 2572 | L12 | L13 | 1329 | 690,8 | 17416 | 398,3 | 13,1 | 1,031 | H5A034 C | sample | Hm Drgbl | Hm Drgbl | Hm Drgbl | CPE002DC | CPE002EE | CPE002F4 | NM 00296 | NM 0029 | NM 0029 | MAPK12 | MAPK12 | MAPK12 | mitogen-activated | mitogen-a | mitogen-a | 6300 | 6300 | 6300 | s12467 | s12468 | s12469 |
| 2606 | M21 | M22 | 1360 | 700,1 | 17837 | 354,8 | 13,1 | 1,031 | H5A034 C | sample | Hm Drgbl | Hm Drgbl | Hm Drgbl | CPE002DC | CPE002EE | CPE002F4 | NM 00161 | NM 0016 | NM 0016 | ADRBK1 | ADRBK1 | ADRBK1 | adrenergic, beta, | adrenergi | adrenergi | 156 | 156 | 156 | s1127 | s1128 | s1129 |
| 16711 | I15 | I16 | 1644 | 1020 | 21618 | 263 | 13,1 | 1,036 | H5A034 C | sample | Hm Gen E | Hm Gen E | Hm Gen E | CPE002JH | CPE002NV | CPE002KC | NM 03288 | NM 0328 | NM 0328 | LSM10 | LSM10 | LSM10 | LSM10, U7 small r | LSM10, U7 | LSM10, U7 | 84967 | 84967 | 84967 | s39773 | s39775 | s228404 |
| 16770 | K3 | K4 | 1183 | 964,6 | 15556 | 185,6 | 13,1 | 1,036 | H5A034 C | sample | Hm Gen E | Hm Gen E | Hm Gen E | CPE002JH | CPE002NV | CPE002KC | NM 03289 | NM 0328 | NM 0328 | FRMD5 | FRMD5 | FRMD5 | FERM domain cor | FERM don | FERM don | 84978 | 84978 | 84978 | s39794 | s39795 | s39796 |
| 16809 | M17 | M18 | 1388 | 1044 | 18163 | 369 | 13,1 | 1,036 | H5A034 C | sample | Hm Gen E | Hm Gen E | Hm Gen E | CPE002JH | CPE002NV | CPE002KC | NM 08059 | NM 0805 | NM 0805 | HIST1H2AI | HIST1H2AI | HIST1H2AI | histone cluster 1, | histone cl | histone cl | 85235 | 85235 | 85235 | s39856 | s195512 | s195513 |
| 16812 | M2 | M3 | 1113 | 951,1 | 14607 | 221,7 | 13,1 | 1,036 | H5A034 C | sample | Hm Gen E | Hm Gen E | Hm Gen E | CPE002JH | CPE002NV | CPE002KC | NM 20730 | NM 2073 | NM 2073 | UAP1L1 | UAP1L1 | UAP1L1 | UDP-N-acetylgluc | UDP-N-act | UDP-N-act | 91373 | 91373 | 91373 | s40636 | s40637 | s40638 |
| 17771 | E19 | E20 | 1414 | 1054 | 18482 | 317,8 | 13,1 | 1,036 | H5A034 C | sample | Hm Gen E | Hm Gen E | Hm Gen E | CPE002JK | CPE002NZ | CPE002KF | NM 00100 | NM 0010 | NM 0010 | OR52B4 | OR52B4 | OR52B4 | olfactory recepto | olfactory r | olfactory r | 143496 | 143496 | 143496 | s44540 | s44541 | s44542 |
| 17892 | J2 | J3 | 1087 | 1049 | 14293 | 339 | 13,1 | 1,036 | H5A034 C | sample | Hm Gen E | Hm Gen E | Hm Gen E | CPE002JK | CPE002NZ | CPE002KF | NM 15335 | NM 1533 | NM 1533 | LRRC34 | LRRC34 | LRRC34 | leucine rich repea | leucine ric | leucine ric | 151827 | 151827 | 151827 | s45614 | s45615 | s45616 |
| 17927 | K8 | K9 | 958 | 1057 | 12529 | 374,8 | 13,1 | 1,036 | H5A034 C | sample | Hm Gen E | Hm Gen E | Hm Gen E | CPE002JK | CPE002NZ | CPE002KF | NM 00102 | NM 0010 | NM 0010 | LOC14964 | LOC14964 | LOC14964 | hypothetical LOC1 | hypotheti | hypotheti | 149643 | 149643 | 149643 | s45325 | s45326 | s45327 |
| 17976 | M9 | M10 | 1329 | 1097 | 17473 | 193 | 13,1 | 1,036 | H5A034 C | sample | Hm Gen E | Hm Gen E | Hm Gen E | CPE002JK | CPE002NZ | CPE002KF | NM 00108 | NM 0010 | NM 0010 | KRT80 | KRT80 | KRT80 | keratin 80 | keratin 80 | keratin 80 | 144501 | 144501 | 144501 | s44669 | s44670 | s225684 |
| 8881 | C1 | C2 | 1553 | 1713 | 20383 | 142 | 13,1 | 1,052 | H5A034 C | sample | Hm Drgbl | Hm Drgbl | Hm Drgbl | CPE002E7 | CPE002ES | CPE002FD | NM 01459 | NM 0145 | NM 0145 | SOCS7 | SOCS7 | SOCS7 | suppressor of cyto | suppresso | suppresso | 30837 | 30837 | 30837 | s26952 | s26951 | s26950 |
| 8900 | C5 | C6 | 1698 | 1857 | 22160 | 155,8 | 13,1 | 1,052 | H5A034 C | sample | Hm Drgbl | Hm Drgbl | Hm Drgbl | CPE002E7 | CPE002ES | CPE002FD | NM 01460 | NM 0146 | NM 0146 | EHD3 | EHD3 | EHD3 | EH-domain conta | EH-domai | EH-domai | 30845 | 30845 | 30845 | s26958 | s26957 | s26956 |
| 9162 | N3 | N4 | 1153 | 1744 | 15055 | 140,4 | 13,1 | 1,052 | H5A034 C | sample | Hm Drgbl | Hm Drgbl | Hm Drgbl | CPE002E7 | CPE002ES | CPE002FD | NM 01622 | NM 0162 | NM 0162 | DDX41 | DDX41 | DDX41 | DEAD (Asp-Glu-Al | DEAD (Asp | DEAD (Asp | 51428 | 51428 | 51428 | s28121 | s224203 | s28120 |
| 4258 | B18 | B19 | 1016 | 822,4 | 13312 | 462,5 | 13,1 | 1,105 | H5A034 C | sample | Hm Drgbl | Hm Drgbl | Hm Drgbl | CPE002DU | CPE002EK | CPE002FQ | NM 02071 | NM 0207 | NM 0207 | SHROOM4 | SHROOM4 | SHROOM4 | shroom family me | shroom fa | shroom fa | 57477 | 57477 | 57477 | s33115 | s33116 | s33117 |
| 4386 | G3 | G4 | 1146 | 720,5 | 14958 | 415,5 | 13,1 | 1,105 | H5A034 C | sample | Hm Drgbl | Hm Drgbl | Hm Drgbl | CPE002DU | CPE002EK | CPE002FQ | NM 00072 | NM 0007 | NM 0007 | CACNA1E | CACNA1E | CACNA1E | calcium channel, v | calcium ch | calcium ch | 777 | 777 | 777 | s2289 | s2290 | s224799 |
| 4460 | J5 | J6 | 761 | 693,9 | 9946 | 337,4 | 13,1 | 1,105 | H5A034 C | sample | Hm Drgbl | Hm Drgbl | Hm Drgbl | CPE002DU | CPE002EK | CPE002FQ | NM 00224 | NM 0022 | NM 0022 | KCNJ6 | KCNJ6 | KCNJ6 | potassium inwarc | potassium | potassium | 3763 | 3763 | 3763 | s7747 | s7748 | s7749 |
| 4473 | K17 | K18 | 1349 | 710,6 | 17673 | 397,2 | 13,1 | 1,105 | H5A034 C | sample | Hm Drgbl | Hm Drgbl | Hm Drgbl | CPE002DU | CPE002EK | CPE002FQ | NM 00104 | NM 0010 | NM 0010 | CLCNKA | CLCNKA | CLCNKA | chloride channel | chloride c | chloride c | 1187 | 1187 | 1187 | s3150 | s3151 | s3152 |
| 4517 | M13 | M14 | 1352 | 726,8 | 17664 | 533,8 | 13,1 | 1,105 | H5A034 C | sample | Hm Drgbl | Hm Drgbl | Hm Drgbl | CPE002DU | CPE002EK | CPE002FQ | NM 00080 | NM 0008 | NM 0008 | GABRA3 | GABRA3 | GABRA3 | gamma-aminobut | gamma-ar | gamma-ar | 2556 | 2556 | 2556 | s5476 | s5477 | s5478 |
| 61 | C20 | C21 | 734 | 1567 | 9592 | 538,7 | 13,1 | 1,115 | H5A034 C | sample | Hm Drgbl | Hm Drgbl | Hm Drgbl | CPE002DT | CPE002EJ | CPE002F9 | NM 00305 | NM 0030 | NM 0030 | SLC18A2 | SLC18A2 | SLC18A2 | solute carrier fam | solute car | solute car | 6571 | 6571 | 6571 | s13078 | s13079 | s13080 |
| 100 | E12 | E13 | 478 | 1369 | 6246 | 497,9 | 13,1 | 1,115 | H5A034 C | sample | Hm Drgbl | Hm Drgbl | Hm Drgbl | CPE002DT | CPE002EJ | CPE002F9 | NM 00507 | NM 0050 | NM 0050 | SLCO1A2 | SLCO1A2 | SLCO1A2 | solute carrier org | solute car | solute car | 6579 | 6579 | 6579 | s13101 | s13099 | s13100 |
| 159 | G22 | G23 | 505 | 1553 | 6615 | 574,3 | 13,1 | 1,115 | H5A034 C | sample | Hm Drgbl | Hm Drgbl | Hm Drgbl | CPE002DT | CPE002EJ | CPE002F9 | NM 13906 | NM 1390 | NM 1390 | SMARCC2 | SMARCC2 | SMARCC2 | SWI/SNF related, | SWI/SNF r | SWI/SNF r | 6601 | 6601 | 6601 | s13149 | s13150 | s13148 |
| 326 | N21 | N22 | 543 | 1554 | 7095 | 537,7 | 13,1 | 1,115 | H5A034 C | sample | Hm Drgbl | Hm Drgbl | Hm Drgbl | CPE002DT | CPE002EJ | CPE002F9 | NM 01422 | NM 0142 | NM 0142 | SLC6A7 | SLC6A7 | SLC6A7 | solute carrier fam | solute car | solute car | 6534 | 6534 | 6534 | s12985 | s12986 | s12987 |
| 1966 | B7 | B8 | 1406 | 688,7 | 18386 | 495,2 | 13,1 | 1,139 | H5A034 C | sample | Hm Drgbl | Hm Drgbl | Hm Drgbl | CPE002FX | CPE002G1 | CPE002G5 | NM 13345 | NM 1334 | NM 1334 | EMID1 | EMID1 | EMID1 | EMI domain conta | EMI doma | EMI doma | 129080 | 129080 | 129080 | s43385 | s43386 | s43387 |
| 2006 | D21 | D22 | 1421 | 689,1 | 18589 | 374,3 | 13,1 | 1,139 | H5A034 C | sample | Hm Drgbl | Hm Drgbl | Hm Drgbl | CPE002FX | CPE002G1 | CPE002G5 | NM 08061 | NM 0806 | NM 0806 | CTCFL | CTCFL | CTCFL | CCCTC-binding fa | CCCTC-bir | CCCTC-bir | 140690 | 140690 | 140690 | s44347 | s44348 | s44349 |
| 2030 | E21 | E22 | 1740 | 631,7 | 22762 | 440 | 13,1 | 1,139 | H5A034 C | sample | Hm Drgbl | Hm Drgbl | Hm Drgbl | CPE002FX | CPE002G1 | CPE002G5 | NM 00100 | NM 0010 | NM 0010 | CADPS2 | CADPS2 | CADPS2 | Ca2+-dependent a | Ca2+-depe | Ca2+-depe | 93664 | 93664 | 93664 | s41195 | s41196 | s41197 |
| 2167 | K15 | K16 | 1178 | 730 | 15373 | 457,2 | 13,1 | 1,139 | H5A034 C | sample | Hm Drgbl | Hm Drgbl | Hm Drgbl | CPE002FX | CPE002G1 | CPE002G5 | NM 17379 | NM 1737 | NM 1737 | PDZD8 | PDZD8 | PDZD8 | PDZ domain conta | PDZ doma | PDZ doma | 118987 | 118987 | 118987 | s42265 | s42266 | s42267 |
| 2186 | L10 | L11 | 1327 | 622,9 | 17361 | 352,7 | 13,1 | 1,139 | H5A034 C | sample | Hm Drgbl | Hm Drgbl | Hm Drgbl | CPE002FX | CPE002G1 | CPE002G5 | NM 02393 | NM 0239 | NM 0239 | DLK2 | DLK2 | DLK2 | delta-like 2 homo | delta-like | delta-like | 65989 | 65989 | 65989 | s195362 | s195363 | s195364 |
| 2209 | M1 | M2 | 797 | 711,3 | 10433 | 458,1 | 13,1 | 1,139 | H5A034 C | sample | Hm Drgbl | Hm Drgbl | Hm Drgbl | CPE002FX | CPE002G1 | CPE002G5 | NM 15320 | NM 1532 | NM 1532 | AEBP2 | AEBP2 | AEBP2 | AE binding protei | AE bindin | AE bindin | 121536 | 121536 | 121536 | s42479 | s42480 | s42481 |
| 2215 | M15 | M16 | 1640 | 707,4 | 21462 | 448,1 | 13,1 | 1,139 | H5A034 C | sample | Hm Drgbl | Hm Drgbl | Hm Drgbl | CPE002FX | CPE002G1 | CPE002G5 | NM 00100 | NM 0010 | NM 0010 | ZNF813 | ZNF813 | ZNF813 | zinc finger protei | zinc finge | zinc finge | 126017 | 126017 | 126017 | s42936 | s42937 | s42938 |
| 2270 | O21 | O22 | 1609 | 652,2 | 21062 | 494,2 | 13,1 | 1,139 | H5A034 C | sample | Hm Drgbl | Hm Drgbl | Hm Drgbl | CPE002FX | CPE002G1 | CPE002G5 | NM 17348 | NM 1734 | NM 1734 | KLF17 | KLF17 | KLF17 | Kruppel-like facto | Kruppel-li | Kruppel-li | 128209 | 128209 | 128209 | s43260 | s43261 | s43262 |
| 6663 | F22 | F23 | 1203 | 601,4 | 15744 | 381,4 | 13,1 | 1,149 | H5A034 C | sample | Hm Drgbl | Hm Drgbl | Hm Drgbl | CPE002E1 | CPE002EY | CPE002FJ | NM 00225 | NM 0022 | NM 0022 | KCNS1 | KCNS1 | KCNS1 | potassium voltag | potassium | potassium | 3787 | 3787 | 3787 | s7814 | s7815 | s7813 |
| 6711 | H22 | H23 | 931 | 699,1 | 12159 | 286,7 | 13,1 | 1,149 | H5A034 C | sample | Hm Drgbl | Hm Drgbl | Hm Drgbl | CPE002E1 | CPE002EY | CPE002FJ | NM 21365 | NM 2136 | NM 2136 | KLRC1 | KLRC1 | KLRC1 | killer cell lectin-lik | killer cell l | killer cell l | 3821 | 3821 | 3821 | s7884 | s7885 | s7886 |
| 3861 | A6 | A7 | 1406 | 650,9 | 18450 | 318,9 | 13,1 | 1,164 | H5A034 C | sample | Hm Drgbl | Hm Drgbl | Hm Drgbl | CPE002DS | CPE002EI | CPE002F8 | NM 00285 | NM 0028 | NM 0028 | ABCD3 | ABCD3 | ABCD3 | ATP-binding casse | ATP-bindi | ATP-bindi | 5825 | 5825 | 5825 | s11617 | s11616 | s11615 |
| 3945 | E17 | E18 | 1426 | 694 | 18727 | 311,5 | 13,1 | 1,164 | H5A034 C | sample | Hm Drgbl | Hm Drgbl | Hm Drgbl | CPE002DS | CPE002EI | CPE002F8 | NM 00300 | NM 0030 | NM 0030 | PLOD1 | PLOD1 | PLOD1 | procollagen-lysine | procollage | procollage | 5351 | 5351 | 5351 | s413 | s414 | s412 |
| 4197 | O6 | O7 | 1194 | 670,4 | 15685 | 442,3 | 13,1 | 1,164 | H5A034 C | sample | Hm Drgbl | Hm Drgbl | Hm Drgbl | CPE002DS | CPE002EI | CPE002F8 | NM 00290 | NM 0029 | NM 0029 | RCVRN | RCVRN | RCVRN | recoverin | recoverin | recoverin | 5957 | 5957 | 5957 | s11890 | s11891 | s11892 |
| 11899 | P4 | P5 | 1521 | 845,8 | 19948 | 205,2 | 13,1 | 1,17 | H5A034 C | sample | Hm Gen E | Hm Gen E | Hm Gen E | CPE002JA | CPE002NJ | CPE002JZ | NM 00217 | NM 0021 | NM 0021 | IFNA10 | IFNA10 | IFNA10 | interferon, alpha | interferon | interferon | 3446 | 3446 | 3446 | s7172 | s7173 | s226339 |
| 5807 | B8 | B9 | 1427 | 808,6 | 18729 | 584,8 | 13,1 | 1,196 | H5A034 C | sample | Hm Drgbl | Hm Drgbl | Hm Drgbl | CPE002DY | CPE002F1 | CPE002FM | NM 00404 | NM 0040 | NM 0040 | BCL2A1 | BCL2A1 | BCL2A1 | BCL2-related prot | BCL2-relat | BCL2-relat | 597 | 597 | 597 | s1917 | s1919 | s1918 |
| 6042 | L3 | L4 | 1622 | 767,2 | 21312 | 372,2 | 13,1 | 1,196 | H5A034 C | sample | Hm Drgbl | Hm Drgbl | Hm Drgbl | CPE002DY | CPE002F1 | CPE002FM | NM 00116 | NM 0011 | NM 0011 | BIRC4 | BIRC4 | BIRC4 | baculoviral IAP re | baculovira | baculovira | 331 | 331 | 331 | s1456 | s1454 | s1455 |
| 6072 | M9 | M10 | 1830 | 758,7 | 23971 | 565,6 | 13,1 | 1,196 | H5A034 C | sample | Hm Drgbl | Hm Drgbl | Hm Drgbl | CPE002DY | CPE002F1 | CPE002FM | NM 00113 | NM 0011 | NM 0011 | AFM | AFM | AFM | afamin | afamin | afamin | 173 | 173 | 173 | s1156 | s1154 | s1155 |
| 6120 | O9 | O10 | 2213 | 824,6 | 29008 | 557,2 | 13,1 | 1,196 | H5A034 C | sample | Hm Drgbl | Hm Drgbl | Hm Drgbl | CPE002DY | CPE002F1 | CPE002FM | NM 00068 | NM 0006 | NM 0006 | ALAS1 | ALAS1 | ALAS1 | aminolevulinate, | aminolevu | aminolevu | 211 | 211 | 211 | s1221 | s1222 | s1223 |
| 1167 | A22 | A23 | 1303 | 895,1 | 17005 | 500 | 13,1 | 1,202 | H5A034 C | sample | Hm Drgbl | Hm Drgbl | Hm Drgbl | CPE002FV | CPE002FZ | CPE002G3 | NM 00489 | NM 0048 | NM 0048 | CLOCK | CLOCK | CLOCK | clock homolog (m | clock hom | clock hom | 9575 | 9575 | 9575 | s18392 | s18393 | s18394 |
| 1293 | F6 | F7 | 1402 | 712,1 | 18368 | 408,8 | 13,1 | 1,202 | H5A034 C | sample | Hm Drgbl | Hm Drgbl | Hm Drgbl | CPE002FV | CPE002FZ | CPE002G3 | NM 01326 | NM 0132 | NM 0132 | PPARGC1A | PPARGC1A | PPARGC1A | peroxisome prolif | peroxison | peroxison | 10891 | 10891 | 10891 | s21393 | s21394 | s21395 |
| 1370 | J10 | J11 | 1065 | 666,8 | 13966 | 384,5 | 13,1 | 1,202 | H5A034 C | sample | Hm Drgbl | Hm Drgbl | Hm Drgbl | CPE002FV | CPE002FZ | CPE002G3 | NM 01428 | NM 0142 | NM 0142 | DNAJC8 | DNAJC8 | DNAJC8 | DnaJ (Hsp40) hom | DnaJ (Hsp | DnaJ (Hsp | 22826 | 22826 | 22826 | s22442 | s22443 | s22444 |
| 1383 | J22 | J23 | 1429 | 793,9 | 18657 | 514,9 | 13,1 | 1,202 | H5A034 C | sample | Hm Drgbl | Hm Drgbl | Hm Drgbl | CPE002FV | CPE002FZ | CPE002G3 | NM 01495 | NM 0149 | NM 0149 | ZBTB1 | ZBTB1 | ZBTB1 | zinc finger and BT | zinc finge | zinc finge | 22890 | 22890 | 22890 | s22601 | s22602 | s22603 |
| 1415 | K8 | K9 | 1690 | 705,8 | 22121 | 355,2 | 13,1 | 1,202 | H5A034 C | sample | Hm Drgbl | Hm Drgbl | Hm Drgbl | CPE002FV | CPE002FZ | CPE002G3 | NM 00606 | NM 0060 | NM 0060 | IKZF1 | IKZF1 | IKZF1 | IKAROS family zin | IKAROS fa | IKAROS fa | 10320 | 10320 | 10320 | s20179 | s20180 | s20181 |
| 1518 | P14 | P15 | 1623 | 777,4 | 21231 | 499,2 | 13,1 | 1,202 | H5A034 C | sample | Hm Drgbl | Hm Drgbl | Hm Drgbl | CPE002FV | CPE002FZ | CPE002G3 | NM 01429 | NM 0142 | NM 0142 | NPTXR | NPTXR | NPTXR | neuronal pentrax | neuronal | neuronal | 23467 | 23467 | 23467 | s23880 | s23881 | s23882 |
| 16225 | E1 | E2 | 779 | 946,6 | 10241 | 271,9 | 13,1 | 1,202 | H5A034 C | sample | Hm Gen E | Hm Gen E | Hm Gen E | CPE002JG | CPE002NV | CPE002KB | NM 00103 | NM 0010 | NM 0010 | CCNL2 | CCNL2 | CCNL2 | cyclin L2 | cyclin L2 | cyclin L2 | 81669 | 81669 | 81669 | s37751 | s37752 | s37753 |
| 16355 | J19 | J20 | 1099 | 1050 | 14404 | 259,5 | 13,1 | 1,202 | H5A034 C | sample | Hm Gen E | Hm Gen E | Hm Gen E | CPE002JG | CPE002NV | CPE002KB | NM 00102 | NM 0010 | NM 0010 | SLC10A7 | SLC10A7 | SLC10A7 | solute carrier fam | solute car | solute car | 84068 | 84068 | 84068 | s38444 | s38445 | s38446 |
| 16394 | L10 | L11 | 1296 | 979,2 | 16938 | 306,8 | 13,1 | 1,202 | H5A034 C | sample | Hm Gen E | Hm Gen E | Hm Gen E | CPE002JG | CPE002NV | CPE002KB | NM 03259 | NM 0325 | NM 0325 | SPATA22 | SPATA22 | SPATA22 | spermatogenesis | spermato | spermato | 84690 | 84690 | 84690 | s39329 | s39330 | s39331 |
| 16428 | M2 | M3 | 1165 | 1072 | 15261 | 224,9 | 13,1 | 1,202 | H5A034 C | sample | Hm Gen E | Hm Gen E | Hm Gen E | CPE002JG | CPE002NV | CPE002KB | NM 03235 | NM 0323 | NM 0323 | VPS25 | VPS25 | VPS25 | vacuolar protein s | vacuolar p | vacuolar p | 84313 | 84313 | 84313 | s38894 | s38895 | s38896 |
| 13682 | K10 | K11 | 1486 | 1097 | 19536 | 145,9 | 13,1 | 1,23 | H5A034 C | sample | Hm Gen E | Hm Gen E | Hm Gen E | CPE002JC | CPE002NC | CPE002K4 | NM 01557 | NM 0155 | NM 0155 | LSM14A | LSM14A | LSM14A | LSM14A, SCD6 ho | LSM14A, S | LSM14A, S | 26065 | 26065 | 26065 | s25050 | s25051 | s25052 |
| 13776 | N9 | N10 | 1095 | 1074 | 14312 | 159,8 | 13,1 | 1,23 | H5A034 C | sample | Hm Gen E | Hm Gen E | Hm Gen E | CPE002JC | CPE002NC | CPE002K4 | NM 00102 | NM 0010 | NM 0010 | C3orf17 | C3orf17 | C3orf17 | chromosome 3 or | chromoso | chromoso | 25871 | 25871 | 25871 | s24650 | s24651 | s24652 |
| 777 | A17 | A18 | 1790 | 874,5 | 23464 | 578 | 13,1 | 1,242 | H5A034 C | sample | Hm Drgbl | Hm Drgbl | Hm Drgbl | CPE002FU | CPE002FY | CPE002G2 | NM 00113 | NM 0011 | NM 0011 | AES | AES | AES | amino-terminal e | amino-ter | amino-ter | 166 | 166 | 166 | s1148 | s1149 | s1150 |
| 796 | B12 | B13 | 1264 | 763,3 | 16594 | 526,7 | 13,1 | 1,242 | H5A034 C | sample | Hm Drgbl | Hm Drgbl | Hm Drgbl | CPE002FU | CPE002FY | CPE002G2 | NM 00561 | NM 0056 | NM 0056 | REST | REST | REST | RE1-silencing tran | RE1-silenc | RE1-silenc | 5978 | 5978 | 5978 | s11932 | s11933 | s11934 |
| 873 | E17 | E18 | 1703 | 753,3 | 22370 | 406,9 | 13,1 | 1,242 | H5A034 C | sample | Hm Drgbl | Hm Drgbl | Hm Drgbl | CPE002FU | CPE002FY | CPE002G2 | NM 00174 | NM 0017 | NM 0017 | CALB2 | CALB2 | CALB2 | calbindin 2, 29kDa | calbindin | calbindin | 794 | 794 | 794 | s2325 | s2326 | s224821 |
| 885 | E6 | E7 | 1224 | 724 | 16004 | 434,3 | 13,1 | 1,242 | H5A034 C | sample | Hm Drgbl | Hm Drgbl | Hm Drgbl | CPE002FU | CPE002FY | CPE002G2 | NM 00593 | NM 0059 | NM 0059 | AFF1 | AFF1 | AFF1 | AF4/FMR2 family, | AF4/FMR2 | AF4/FMR2 | 4299 | 4299 | 4299 | s8823 | s8824 | s8825 |
| 942 | H14 | H15 | 1670 | 772,8 | 21886 | 403 | 13,1 | 1,242 | H5A034 C | sample | Hm Drgbl | Hm Drgbl | Hm Drgbl | CPE002FU | CPE002FY | CPE002G2 | NM 00460 | NM 0046 | NM 0046 | STX1A | STX1A | STX1A | syntaxin 1A (brain | syntaxin 1 | syntaxin 1 | 6804 | 6804 | 6804 | s13589 | s13590 | s13591 |
| 951 | H22 | H23 | 1762 | 745,6 | 23143 | 456,2 | 13,1 | 1,242 | H5A034 C | sample | Hm Drgbl | Hm Drgbl | Hm Drgbl | CPE002FU | CPE002FY | CPE002G2 | NM 00316 | NM 0031 | NM 0031 | STXBP1 | STXBP1 | STXBP1 | syntaxin binding p | syntaxin b | syntaxin b | 6812 | 6812 | 6812 | s13601 | s13602 | s13603 |
| 995 | J19 | J20 | 1469 | 700,2 | 19219 | 380,3 | 13,1 | 1,242 | H5A034 C | sample | Hm Drgbl | Hm Drgbl | Hm Drgbl | CPE002FU | CPE002FY | CPE002G2 | NM 02197 | NM 0219 | NM 0219 | HSPA2 | HSPA2 | HSPA2 | heat shock 70kDa | heat shoc | heat shoc | 3306 | 3306 | 3306 | s6973 | s6974 | s6975 |
| 1030 | K7 | K8 | 1067 | 773,4 | 13955 | 399,5 | 13,1 | 1,242 | H5A034 C | sample | Hm Drgbl | Hm Drgbl | Hm Drgbl | CPE002FU | CPE002FY | CPE002G2 | NM 00009 | NM 0000 | NM 0000 | COL7A1 | COL7A1 | COL7A1 | collagen, type VII, | collagen, t | collagen, t | 1294 | 1294 | 1294 | s3320 | s3321 | s3322 |
| 1151 | P8 | P9 | 1707 | 735,8 | 22378 | 442,5 | 13,1 | 1,242 | H5A034 C | sample | Hm Drgbl | Hm Drgbl | Hm Drgbl | CPE002FU | CPE002FY | CPE002G2 | NM 00712 | NM 0071 | NM 0071 | USH2A | USH2A | USH2A | Usher syndrome 2 | Usher syn | Usher syn | 7399 | 7399 | 7399 | s14726 | s14727 | s14728 |
| 5610 | J3 | J4 | 1172 | 618,6 | 15342 | 576,4 | 13,1 | 1,248 | H5A034 C | sample | Hm Drgbl | Hm Drgbl | Hm Drgbl | CPE002DX | CPE002F2 | CPE002FN | NM 00377 | NM 0037 | NM 0037 | HYAL2 | HYAL2 | HYAL2 | hyaluronoglucosa | hyaluronc | hyaluronc | 8692 | 8692 | 8692 | s225023 | s427 | s428 |
| 3588 | F2 | F3 | 1160 | 1382 | 15140 | 349,9 | 13,1 | 1,26 | H5A034 C | sample | Hm Drgbl | Hm Drgbl | Hm Drgbl | CPE002DR | CPE002EH | CPE002F7 | NM 02163 | NM 0216 | NM 0216 | RXFP1 | RXFP1 | RXFP1 | relaxin/insulin-lik | relaxin/in | relaxin/in | 59350 | 59350 | 59350 | s34025 | s34026 | s34027 |
| 3603 | G11 | G12 | 884 | 1431 | 11588 | 340,5 | 13,1 | 1,26 | H5A034 C | sample | Hm Drgbl | Hm Drgbl | Hm Drgbl | CPE002DR | CPE002EH | CPE002F7 | NM 00074 | NM 0007 | NM 0007 | CHRM4 | CHRM4 | CHRM4 | cholinergic recept | cholinergi | cholinergi | 1132 | 1132 | 1132 | s3031 | s3032 | s3033 |
| 3604 | G12 | G13 | 1418 | 1404 | 18532 | 424,8 | 13,1 | 1,26 | H5A034 C | sample | Hm Drgbl | Hm Drgbl | Hm Drgbl | CPE002DR | CPE002EH | CPE002F7 | NM 00422 | NM 0042 | NM 0042 | GPR50 | GPR50 | GPR50 | G protein-couplec | G protein- | G protein- | 9248 | 9248 | 9248 | s17685 | s17686 | s17687 |
| 3672 | I9 | I10 | 1096 | 1308 | 14341 | 320,5 | 13,1 | 1,26 | H5A034 C | sample | Hm Drgbl | Hm Drgbl | Hm Drgbl | CPE002DR | CPE002EH | CPE002F7 | NM 00129 | NM 0012 | NM 0012 | CCBP2 | CCBP2 | CCBP2 | chemokine bindir | chemokin | chemokin | 1238 | 1238 | 1238 | s3221 | s3222 | s3223 |
| 3724 | L12 | L13 | 1137 | 1284 | 14868 | 343,3 | 13,1 | 1,26 | H5A034 C | sample | Hm Drgbl | Hm Drgbl | Hm Drgbl | CPE002DR | CPE002EH | CPE002F7 | NM 13832 | NM 1383 | NM 1383 | TAAR1 | TAAR1 | TAAR1 | trace amine assoc | trace amir | trace amir | 134864 | 134864 | 134864 | s43902 | s43903 | s223306 |
| 8069 | A13 | A14 | 883 | 612,1 | 11606 | 533,8 | 13,1 | 1,26 | H5A034 C | sample | Hm Drgbl | Hm Drgbl | Hm Drgbl | CPE002E5 | CPE002EU | CPE002FF | NM 00701 | NM 0070 | NM 0070 | ABHD2 | ABHD2 | ABHD2 | abhydrolase dom | abhydrola | abhydrola | 11057 | 11057 | 11057 | s21785 | s21786 | s21787 |
| 8142 | D14 | D15 | 1694 | 624,3 | 22264 | 506 | 13,1 | 1,26 | H5A034 C | sample | Hm Drgbl | Hm Drgbl | Hm Drgbl | CPE002E5 | CPE002EU | CPE002FF | NM 01208 | NM 0120 | NM 0120 | ZFPM2 | ZFPM2 | ZFPM2 | zinc finger protei | zinc finge | zinc finge | 23414 | 23414 | 23414 | s23779 | s23780 | s23778 |
| 8290 | J18 | J19 | 1711 | 620,9 | 22344 | 448,9 | 13,1 | 1,26 | H5A034 C | sample | Hm Drgbl | Hm Drgbl | Hm Drgbl | CPE002E5 | CPE002EU | CPE002FF | NM 01535 | NM 0153 | NM 0153 | MORC3 | MORC3 | MORC3 | MORC family CW- | MORC fan | MORC fan | 23515 | 23515 | 23515 | s23977 | s23978 | s23976 |
| 8298 | J3 | J4 | 832 | 650,9 | 10876 | 501,3 | 13,1 | 1,26 | H5A034 C | sample | Hm Drgbl | Hm Drgbl | Hm Drgbl | CPE002E5 | CPE002EU | CPE002FF | NM 00104 | NM 0010 | NM 0010 | NCBP2 | NCBP2 | NCBP2 | nuclear cap bindi | nuclear ca | nuclear ca | 22916 | 22916 | 22916 | s22664 | s22665 | s22666 |
| 10040 | C16 | C17 | 1254 | 832,3 | 16398 | 149,9 | 13,1 | 1,26 | H5A034 C | sample | Hm Drgbl | Hm Drgbl | Hm Drgbl | CPE002EA | CPE002EP | CPE002FA | NM 03143 | NM 0314 | NM 0314 | MFRP | MFRP | MFRP | membrane frizzle | membran | membran | 83552 | 83552 | 83552 | s38012 | s38010 | s38011 |
| 10260 | L2 | L3 | 1180 | 858,7 | 15473 | 150,9 | 13,1 | 1,26 | H5A034 C | sample | Hm Drgbl | Hm Drgbl | Hm Drgbl | CPE002EA | CPE002EP | CPE002FA | NM 03259 | NM 0325 | NM 0325 | PPP1R9B | PPP1R9B | PPP1R9B | protein phosphat | protein pl | protein pl | 84687 | 84687 | 84687 | s39320 | s39322 | s224958 |
| 10269 | L6 | L7 | 1533 | 844 | 20106 | 152,6 | 13,1 | 1,26 | H5A034 C | sample | Hm Drgbl | Hm Drgbl | Hm Drgbl | CPE002EA | CPE002EP | CPE002FA | NM 03260 | NM 0326 | NM 0326 | ABHD1 | ABHD1 | ABHD1 | abhydrolase dom | abhydrola | abhydrola | 84696 | 84696 | 84696 | s39349 | s39348 | s39347 |
| 10362 | P3 | P4 | 1435 | 831,1 | 18809 | 181,4 | 13,1 | 1,26 | H5A034 C | sample | Hm Drgbl | Hm Drgbl | Hm Drgbl | CPE002EA | CPE002EP | CPE002FA | NM 03096 | NM 0309 | NM 0309 | ST6GALNA | ST6GALNA | ST6GALNA | ST6 (alpha-N-acet | ST6 (alpha | ST6 (alpha | 81849 | 81849 | 81849 | s37829 | s224888 | s37827 |
| 1723 | H4 | H5 | 812 | 635,3 | 10600 | 348,5 | 13,1 | 1,372 | H5A034 C | sample | Hm Drgbl | Hm Drgbl | Hm Drgbl | CPE002FW | CPE002G0 | CPE002G4 | NM 03058 | NM 0305 | NM 0305 | COL18A1 | COL18A1 | COL18A1 | collagen, type XVI | collagen, t | collagen, t | 80781 | 80781 | 80781 | s37392 | s37393 | s37394 |
| 1737 | I17 | I18 | 924 | 630,6 | 12071 | 329,3 | 13,1 | 1,372 | H5A034 C | sample | Hm Drgbl | Hm Drgbl | Hm Drgbl | CPE002FW | CPE002G0 | CPE002G4 | NM 01336 | NM 0133 | NM 0133 | SERTAD3 | SERTAD3 | SERTAD3 | SERTA domain co | SERTA dor | SERTA dor | 29946 | 29946 | 29946 | s26766 | s26767 | s26768 |
| 1788 | K2 | K3 | 1104 | 716,2 | 14480 | 387,4 | 13,1 | 1,372 | H5A034 C | sample | Hm Drgbl | Hm Drgbl | Hm Drgbl | CPE002FW | CPE002G0 | CPE002G4 | NM 02211 | NM 0221 | NM 0221 | PRDM15 | PRDM15 | PRDM15 | PR domain contai | PR domai | PR domai | 63977 | 63977 | 63977 | s34349 | s34350 | s34351 |
| 1874 | O10 | O11 | 1206 | 780,9 | 15834 | 513,8 | 13,1 | 1,372 | H5A034 C | sample | Hm Drgbl | Hm Drgbl | Hm Drgbl | CPE002FW | CPE002G0 | CPE002G4 | NM 02431 | NM 0243 | NM 0243 | PLEKHF1 | PLEKHF1 | PLEKHF1 | pleckstrin homolo | pleckstrin | pleckstrin | 79156 | 79156 | 79156 | s35643 | s35644 | s35645 |
| 1904 | P16 | P17 | 1185 | 724,1 | 15532 | 390,2 | 13,1 | 1,372 | H5A034 C | sample | Hm Drgbl | Hm Drgbl | Hm Drgbl | CPE002FW | CPE002G0 | CPE002G4 | NM 03283 | NM 0328 | NM 0328 | FIZ1 | FIZ1 | FIZ1 | FLT3-interacting z | FLT3-inter | FLT3-inter | 84922 | 84922 | 84922 | s39664 | s39665 | s39666 |
| 21821 | N13 | N14 | 381 | 1087 | 4979 | 617,7 | 13,1 | 1,379 | H5A034 C | sample | Hm Gen E | Hm Gen E | Hm Gen E | CPE002JU | CPE002O9 | CPE002KP | XM 94522 | XM 9452 | XM 9452 | LOC65214 | LOC65214 | LOC65214 | hypothetical prot | hypotheti | hypotheti | 652148 | 652148 | 652148 | s57311 | s197850 | s197851 |
| 4653 | B6 | B7 | 1526 | 827,8 | 19932 | 513,2 | 13,1 | 1,44 | H5A034 C | sample | Hm Drgbl | Hm Drgbl | Hm Drgbl | CPE002DV | CPE002EL | CPE002FP | NM 00661 | NM 0066 | NM 0066 | CAPN9 | CAPN9 | CAPN9 | calpain 9 | calpain 9 | calpain 9 | 10753 | 10753 | 10753 | s21126 | s21127 | s21128 |
| 4972 | P12 | P13 | 1847 | 783,2 | 24138 | 500,6 | 13,1 | 1,44 | H5A034 C | sample | Hm Drgbl | Hm Drgbl | Hm Drgbl | CPE002DV | CPE002EL | CPE002FP | NM 02090 | NM 0209 | NM 0209 | USP29 | USP29 | USP29 | ubiquitin specific | ubiquitin | ubiquitin | 57663 | 57663 | 57663 | s33547 | s33548 | s33549 |
| 10395 | B11 | B12 | 888 | 1013 | 11668 | 233,6 | 13,1 | 1,48 | H5A034 C | sample | Hm Drgbl | Hm Drgbl | Hm Drgbl | CPE002EB | CPE002EO | CPE002FR | NM 03345 | NM 0334 | NM 0334 | TRIM47 | TRIM47 | TRIM47 | tripartite motif-cc | tripartite | tripartite | 91107 | 91107 | 91107 | s40570 | s40571 | s40572 |
| 10401 | B17 | B18 | 983 | 887,2 | 12864 | 198,9 | 13,1 | 1,48 | H5A034 C | sample | Hm Drgbl | Hm Drgbl | Hm Drgbl | CPE002EB | CPE002EO | CPE002FR | NM 15333 | NM 1533 | NM 1533 | SCARF2 | SCARF2 | SCARF2 | scavenger recepto | scavenger | scavenger | 91179 | 91179 | 91179 | s40592 | s40593 | s40591 |
| 10403 | B19 | B20 | 1288 | 905,3 | 16857 | 216,2 | 13,1 | 1,48 | H5A034 C | sample | Hm Drgbl | Hm Drgbl | Hm Drgbl | CPE002EB | CPE002EO | CPE002FR | NM 19844 | NM 1984 | NM 1984 | DERL3 | DERL3 | DERL3 | Der1-like domain | Der1-like | Der1-like | 91319 | 91319 | 91319 | s40621 | s40622 | s40623 |
| 10410 | B3 | B4 | 1111 | 858,4 | 14513 | 198 | 13,1 | 1,48 | H5A034 C | sample | Hm Drgbl | Hm Drgbl | Hm Drgbl | CPE002EB | CPE002EO | CPE002FR | NM 14575 | NM 1457 | NM 1457 | KIFC2 | KIFC2 | KIFC2 | kinesin family me | kinesin fa | kinesin fa | 90990 | 90990 | 90990 | s40544 | s40545 | s40543 |
| 10443 | D11 | D12 | 1223 | 934,6 | 15981 | 199,1 | 13,1 | 1,48 | H5A034 C | sample | Hm Drgbl | Hm Drgbl | Hm Drgbl | CPE002EB | CPE002EO | CPE002FR | NM 03325 | NM 0332 | NM 0332 | BOC | BOC | BOC | Boc homolog (mo | Boc homo | Boc homo | 91653 | 91653 | 91653 | s40723 | s40724 | s40725 |
| 10628 | K5 | K6 | 1319 | 898,2 | 17275 | 195,7 | 13,1 | 1,48 | H5A034 C | sample | Hm Drgbl | Hm Drgbl | Hm Drgbl | CPE002EB | CPE002EO | CPE002FR | NM 00108 | NM 0010 | NM 0010 | FGD3 | FGD3 | FGD3 | FYVE, RhoGEF anc | FYVE, Rho | FYVE, Rho | 89846 | 89846 | 89846 | s40147 | s40148 | s40149 |
| 2763 | D11 | D12 | 1217 | 672 | 15975 | 665,5 | 13,1 | 1,541 | H5A034 C | sample | Hm Drgbl | Hm Drgbl | Hm Drgbl | CPE002DP | CPE002EF | CPE002F5 | NM 00719 | NM 0071 | NM 0071 | IRAK3 | IRAK3 | IRAK3 | interleukin-1 rece | interleuki | interleuki | 11213 | 11213 | 11213 | s22133 | s22134 | s22135 |
| 20343 | P22 | P23 | 516 | 1355 | 6752 | 116,2 | 13,1 | 1,598 | H5A034 C | sample | Hm Gen E | Hm Gen E | Hm Gen E | CPE002JQ | CPE002O5 | CPE002KL | XM 49590 | XM 4959 | XM 4959 | DDX12 | DDX12 | DDX12 | DEAD/H (Asp-Glu- | DEAD/H (A | DEAD/H (A | 440081 | 440081 | 440081 | s54011 | s54012 | s54013 |
| 7996 | N12 | N13 | 755 | 603,8 | 9855 | 402,2 | 13,1 | 2 | H5A034 C | sample | Hm Drgbl | Hm Drgbl | Hm Drgbl | CPE002E4 | CPE002EV | CPE002FG | NM 00685 | NM 0068 | NM 0068 | ATF7 | ATF7 | ATF7 | activating transcr | activating | activating | 11016 | 11016 | 11016 | s21693 | s21694 | s21695 |
| 8020 | O12 | O13 | 761 | 548 | 9950 | 457,2 | 13,1 | 2 | H5A034 C | sample | Hm Drgbl | Hm Drgbl | Hm Drgbl | CPE002E4 | CPE002EV | CPE002FG | NM 00664 | NM 0066 | NM 0066 | UTP14A | UTP14A | UTP14A | UTP14, U3 small r | UTP14, U3 | UTP14, U3 | 10813 | 10813 | 10813 | s21250 | s21251 | s21252 |
| 22328 | C16 | C17 | 640 | 1497 | 8453 | 448,1 | 13,2 | 0,68 | H5A034 C | sample | Hm Gen E | Hm Gen E | Hm Gen E | CPE002JW | CPE002OB | CPE002KR | XM 00112 | XM 0011 | XM 0011 | LOC72902 | LOC72902 | LOC72902 | hypothetical prot | hypotheti | hypotheti | 729029 | 729029 | 729029 | s59284 | s59285 | s59286 |
| 3129 | C17 | C18 | 955 | 1306 | 12616 | 398,7 | 13,2 | 0,817 | H5A034 C | sample | Hm Drgbl | Hm Drgbl | Hm Drgbl | CPE002DC | CPE002EG | CPE002F6 | NM 00177 | NM 0017 | NM 0017 | ENTPD1 | ENTPD1 | ENTPD1 | ectonucleoside tr | ectonucle | ectonucle | 953 | 953 | 953 | s2660 | s2661 | s2662 |
| 3229 | G20 | G21 | 1237 | 1321 | 16270 | 347 | 13,2 | 0,817 | H5A034 C | sample | Hm Drgbl | Hm Drgbl | Hm Drgbl | CPE002DC | CPE002EG | CPE002F6 | NM 03064 | NM 0306 | NM 0306 | DUSP16 | DUSP16 | DUSP16 | dual specificity ph | dual speci | dual speci | 80824 | 80824 | 80824 | s37419 | s37420 | s37421 |
| 3276 | I2 | I3 | 722 | 1208 | 9529 | 384,2 | 13,2 | 0,817 | H5A034 C | sample | Hm Drgbl | Hm Drgbl | Hm Drgbl | CPE002DC | CPE002EG | CPE002F6 | NM 03079 | NM 0307 | NM 0307 | SGPP1 | SGPP1 | SGPP1 | sphingosine-1-ph | sphingosi | sphingosi | 81537 | 81537 | 81537 | s37587 | s37588 | s37589 |
| 6188 | B5 | B6 | 1451 | 527,6 | 19192 | 544,1 | 13,2 | 0,871 | H5A034 C | sample | Hm Drgbl | Hm Drgbl | Hm Drgbl | CPE002DZ | CPE002F0 | CPE002FL | NM 00126 | NM 0012 | NM 0012 | CES1 | CES1 | CES1 | carboxylesterase | carboxyle | carboxyle | 1066 | 1066 | 1066 | s2923 | s2921 | s2922 |
| 6453 | M6 | M7 | 1135 | 569,1 | 14993 | 498,9 | 13,2 | 0,871 | H5A034 C | sample | Hm Drgbl | Hm Drgbl | Hm Drgbl | CPE002DZ | CPE002F0 | CPE002FL | NM 00134 | NM 0013 | NM 0013 | DAD1 | DAD1 | DAD1 | defender against | defender | defender | 1603 | 1603 | 1603 | s3904 | s3903 | s3902 |
| 20652 | M2 | M3 | 845 | 791,6 | 11146 | 89,76 | 13,2 | 0,88 | H5A034 C | sample | Hm Gen E | Hm Gen E | Hm Gen E | CPE002JR | CPE002O6 | CPE002KM | NM 00103 | NM 0010 | NM 0010 | ACOT6 | ACOT6 | ACOT6 | acyl-CoA thioeste | acyl-CoA t | acyl-CoA t | 641372 | 641372 | 641372 | s54803 | s54804 | s54805 |
| 17485 | I20 | I21 | 1267 | 911,9 | 16663 | 178,4 | 13,2 | 0,901 | H5A034 C | sample | Hm Gen E | Hm Gen E | Hm Gen E | CPE002JJ | CPE002NY | CPE002KE | NM 19913 | NM 1991 | NM 1991 | LOC13414 | LOC13414 | LOC13414 | hypothetical prot | hypotheti | hypotheti | 134145 | 134145 | 134145 | s43830 | s43831 | s225636 |
| 17648 | P16 | P17 | 1287 | 1022 | 16990 | 202,2 | 13,2 | 0,901 | H5A034 C | sample | Hm Gen E | Hm Gen E | Hm Gen E | CPE002JJ | CPE002NY | CPE002KE | XM 00113 | XM 0011 | XM 0011 | ANKRD60 | ANKRD60 | ANKRD60 | ankyrin repeat dc | ankyrin re | ankyrin re | 140731 | 140731 | 140731 | s44374 | s44375 | s44376 |
| 9389 | H13 | H14 | 1065 | 1135 | 14095 | 162,4 | 13,2 | 0,917 | H5A034 C | sample | Hm Drgbl | Hm Drgbl | Hm Drgbl | CPE002E8 | CPE002ER | CPE002FC | NM 01754 | NM 1983 | NM 1983 | GALNT10 | GALNT10 | GALNT10 | UDP-N-acetyl-alph | UDP-N-ace | UDP-N-ace | 55568 | 55568 | 55568 | s224373 | s30993 | s30994 |
| 9529 | N1 | N2 | 1748 | 1093 | 22992 | 122,5 | 13,2 | 0,917 | H5A034 C | sample | Hm Drgbl | Hm Drgbl | Hm Drgbl | CPE002E8 | CPE002ER | CPE002FC | XM 00112 | XM 0011 | XM 0011 | VAC14 | VAC14 | VAC14 | Vac14 homolog (S | Vac14 hor | Vac14 hor | 55697 | 55697 | 55697 | s31277 | s31275 | s31276 |
| 9583 | P15 | P16 | 1662 | 1142 | 21945 | 167,4 | 13,2 | 0,917 | H5A034 C | sample | Hm Drgbl | Hm Drgbl | Hm Drgbl | CPE002E8 | CPE002ER | CPE002FC | NM 01830 | NM 0183 | NM 0183 | TBC1D23 | TBC1D23 | TBC1D23 | TBC1 domain fam | TBC1 dom | TBC1 dom | 55773 | 55773 | 55773 | s31476 | s31477 | s31478 |
| 19167 | O22 | O23 | 1465 | 1024 | 19397 | 282,6 | 13,2 | 0,92 | H5A034 C | sample | Hm Gen E | Hm Gen E | Hm Gen E | CPE002JN | CPE002O2 | CPE002KI | NM 17382 | NM 1738 | NM 1738 | C3orf38 | C3orf38 | C3orf38 | chromosome 3 op | chromoso | chromoso | 285237 | 285237 | 285237 | s49859 | s49860 | s226210 |
| 9969 | P17 | P18 | 1805 | 1071 | 23748 | 149,7 | 13,2 | 0,923 | H5A034 C | sample | Hm Drgbl | Hm Drgbl | Hm Drgbl | CPE002E9 | CPE002EQ | CPE002FB | NM 02216 | NM 0221 | NM 0221 | DMRTA1 | DMRTA1 | DMRTA1 | DMRT-like family | DMRT-like | DMRT-like | 63951 | 63951 | 63951 | s34327 | s34328 | s34329 |
| 15276 | M2 | M3 | 1310 | 830,1 | 17345 | 126,8 | 13,2 | 0,923 | H5A034 C | sample | Hm Gen E | Hm Gen E | Hm Gen E | CPE002J8 | CPE002NS | CPE002K8 | NM 02078 | NM 0207 | NM 0207 | TXNDC16 | TXNDC16 | TXNDC16 | thioredoxin doma | thioredox | thioredox | 57544 | 57544 | 57544 | s33274 | s33275 | s33276 |
| 19523 | N19 | N20 | 1455 | 1229 | 19184 | 261,8 | 13,2 | 0,936 | H5A034 C | sample | Hm Gen E | Hm Gen E | Hm Gen E | CPE002JO | CPE002O3 | CPE002KJ | NM 18178 | NM 1817 | NM 1817 | H1FNT | H1FNT | H1FNT | H1 histone family | H1 histon | H1 histon | 341567 | 341567 | 341567 | s50854 | s196071 | s196072 |
| 14514 | M3 | M4 | 1591 | 817,6 | 20931 | 116,5 | 13,2 | 0,94 | H5A034 C | sample | Hm Gen E | Hm Gen E | Hm Gen E | CPE002J7 | CPE002NC | CPE002K6 | NM 01646 | NM 0164 | NM 0164 | TMEM14C | TMEM14C | TMEM14C | transmembrane p | transmem | transmem | 51522 | 51522 | 51522 | s28255 | s28256 | s28257 |
| 14568 | O9 | O10 | 1306 | 850,8 | 17293 | 138,9 | 13,2 | 0,94 | H5A034 C | sample | Hm Gen E | Hm Gen E | Hm Gen E | CPE002J7 | CPE002NC | CPE002K6 | NM 01658 | NM 0165 | NM 0165 | MBIP | MBIP | MBIP | MAP3K12 binding | MAP3K12 | MAP3K12 | 51562 | 51562 | 51562 | s28332 | s28333 | s28334 |
| 7392 | D9 | D10 | 1013 | 639,4 | 13334 | 507 | 13,2 | 1,008 | H5A034 C | sample | Hm Drgbl | Hm Drgbl | Hm Drgbl | CPE002E3 | CPE002EW | CPE002FH | NM 00480 | NM 0048 | NM 0048 | HS6ST1 | HS6ST1 | HS6ST1 | heparan sulfate 6 | heparan s | heparan s | 9394 | 9394 | 9394 | s17976 | s225123 | s225124 |
| 7515 | J11 | J12 | 1371 | 588,1 | 18034 | 364,1 | 13,2 | 1,008 | H5A034 C | sample | Hm Drgbl | Hm Drgbl | Hm Drgbl | CPE002E3 | CPE002EW | CPE002FH | NM 00545 | NM 0054 | NM 0054 | MAPK8IP1 | MAPK8IP1 | MAPK8IP1 | mitogen-activated | mitogen-a | mitogen-a | 9479 | 9479 | 9479 | s18174 | s18175 | s18173 |
| 7535 | J8 | J9 | 1319 | 647,4 | 17447 | 353,8 | 13,2 | 1,008 | H5A034 C | sample | Hm Drgbl | Hm Drgbl | Hm Drgbl | CPE002E3 | CPE002EW | CPE002FH | NM 00549 | NM 0054 | NM 0054 | RANBP9 | RANBP9 | RANBP9 | RAN binding prot | RAN bindi | RAN bindi | 10048 | 10048 | 10048 | s19529 | s19531 | s19530 |
| 16921 | B1 | B2 | 1473 | 939,4 | 19508 | 245,8 | 13,2 | 1,019 | H5A034 C | sample | Hm Gen E | Hm Gen E | Hm Gen E | CPE002JI | CPE002NZ | CPE002KD | NM 05289 | NM 1524 | NM 0528 | XKR4 | C14orf44 | XKR4 | XK, Kell blood gro | chromoso | XK, Kell bl | 114786 | 145483 | 114786 | s41597 | s44742 | s41599 |
| 17174 | L21 | L22 | 2012 | 1092 | 26564 | 215 | 13,2 | 1,019 | H5A034 C | sample | Hm Gen E | Hm Gen E | Hm Gen E | CPE002JI | CPE002NZ | CPE002KD | NM 13846 | NM 1332 | NM 1384 | TLCD1 | WIPF2 | TLCD1 | TLC domain conta | WAS/WAS | TLC doma | 116238 | 147179 | 116238 | s41975 | s44973 | s41977 |
| 17276 | P5 | P6 | 730 | 1186 | 9649 | 246,6 | 13,2 | 1,019 | H5A034 C | sample | Hm Gen E | Hm Gen E | Hm Gen E | CPE002JI | CPE002NZ | CPE002KD | NM 00103 | XM 0972 | NM 0010 | CNTROB | LOC14771 | CNTROB | centrobin, centro | hypotheti | centrobin | 116840 | 147710 | 116840 | s42056 | s45043 | s42058 |
| 6927 | A22 | A23 | 1724 | 695 | 22789 | 454,5 | 13,2 | 1,027 | H5A034 C | sample | Hm Drgbl | Hm Drgbl | Hm Drgbl | CPE002E2 | CPE002EX | CPE002FI | NM 00595 | NM 0059 | NM 0059 | MTF1 | MTF1 | MTF1 | metal-regulatory | metal-reg | metal-reg | 4520 | 4520 | 4520 | s9029 | s9028 | s9027 |
| 6932 | A5 | A6 | 1039 | 608,8 | 13694 | 450,4 | 13,2 | 1,027 | H5A034 C | sample | Hm Drgbl | Hm Drgbl | Hm Drgbl | CPE002E2 | CPE002EX | CPE002FI | NM 00228 | NM 0022 | NM 0022 | KRT86 | KRT86 | KRT86 | keratin 86 | keratin 86 | keratin 86 | 3892 | 3892 | 3892 | s8032 | s8031 | s8030 |
| 7023 | E22 | E23 | 1088 | 548,3 | 14332 | 464,7 | 13,2 | 1,027 | H5A034 C | sample | Hm Drgbl | Hm Drgbl | Hm Drgbl | CPE002E2 | CPE002EX | CPE002FI | NM 00025 | NM 0002 | NM 0002 | MYBPC3 | MYBPC3 | MYBPC3 | myosin binding p | myosin bi | myosin bi | 4607 | 4607 | 4607 | s9124 | s9123 | s9125 |
| 7213 | M20 | M21 | 1474 | 563 | 19479 | 465,3 | 13,2 | 1,027 | H5A034 C | sample | Hm Drgbl | Hm Drgbl | Hm Drgbl | CPE002E2 | CPE002EX | CPE002FI | NM 00453 | NM 0045 | NM 0045 | NARS | NARS | NARS | asparaginyl-tRNA | asparagin | asparagin | 4677 | 4677 | 4677 | s9278 | s9279 | s9277 |
| 5170 | H18 | H19 | 1463 | 575,1 | 19267 | 454,7 | 13,2 | 1,035 | H5A034 C | sample | Hm Drgbl | Hm Drgbl | Hm Drgbl | CPE002DV | CPE002EM | CPE002FO | NM 00038 | NM 0003 | NM 0003 | XPA | XPA | XPA | xeroderma pigme | xeroderm | xeroderm | 7507 | 7507 | 7507 | s14927 | s14926 | s14925 |
| 5174 | H21 | H22 | 1949 | 652,4 | 25757 | 484,1 | 13,2 | 1,035 | H5A034 C | sample | Hm Drgbl | Hm Drgbl | Hm Drgbl | CPE002DV | CPE002EM | CPE002FO | NM 00697 | NM 0069 | NM 0069 | SLC39A7 | SLC39A7 | SLC39A7 | solute carrier fam | solute car | solute car | 7922 | 7922 | 7922 | s15478 | s15479 | s15480 |
| 5184 | H9 | H10 | 1300 | 577,9 | 17224 | 465,6 | 13,2 | 1,035 | H5A034 C | sample | Hm Drgbl | Hm Drgbl | Hm Drgbl | CPE002DV | CPE002EM | CPE002FO | NM 00463 | NM 0046 | NM 0046 | RAB7A | RAB7A | RAB7A | RAB7A, member R | RAB7A, m | RAB7A, m | 7879 | 7879 | 7879 | s15442 | s15443 | s15444 |
| 5199 | I22 | I23 | 1946 | 710,6 | 25592 | 540,4 | 13,2 | 1,035 | H5A034 C | sample | Hm Drgbl | Hm Drgbl | Hm Drgbl | CPE002DV | CPE002EM | CPE002FO | NM 00055 | NM 0005 | NM 0005 | TYRP1 | TYRP1 | TYRP1 | tyrosinase-relatec | tyrosinase | tyrosinase | 7306 | 7306 | 7306 | s14550 | s14551 | s14552 |
| 5310 | N14 | N15 | 2233 | 651 | 29374 | 599,6 | 13,2 | 1,035 | H5A034 C | sample | Hm Drgbl | Hm Drgbl | Hm Drgbl | CPE002DV | CPE002EM | CPE002FO | NM 00629 | NM 0062 | NM 0062 | ZNF193 | ZNF193 | ZNF193 | zinc finger protei | zinc finge | zinc finge | 7746 | 7746 | 7746 | s15260 | s15261 | s15262 |
| 5322 | N3 | N4 | 1163 | 622,9 | 15322 | 364,9 | 13,2 | 1,035 | H5A034 C | sample | Hm Drgbl | Hm Drgbl | Hm Drgbl | CPE002DV | CPE002EM | CPE002FO | NM 00653 | NM 0065 | NM 0065 | YEATS4 | YEATS4 | YEATS4 | YEATS domain co | YEATS dor | YEATS dor | 8089 | 8089 | 8089 | s15613 | s15614 | s15615 |
| 2370 | C3 | C4 | 744 | 692,6 | 9832 | 477,9 | 13,2 | 1,039 | H5A034 C | sample | Hm Drgbl | Hm Drgbl | Hm Drgbl | CPE002DC | CPE002EE | CPE002F4 | NM 00521 | NM 0052 | NM 0052 | CSF1R | CSF1R | CSF1R | colony stimulatin | colony sti | colony sti | 1436 | 1436 | 1436 | s3594 | s3595 | s3596 |
| 2455 | G15 | G16 | 1142 | 640 | 15092 | 357,3 | 13,2 | 1,039 | H5A034 C | sample | Hm Drgbl | Hm Drgbl | Hm Drgbl | CPE002DC | CPE002EE | CPE002F4 | NM 00367 | NM 0036 | NM 0036 | CDK10 | CDK10 | CDK10 | cyclin-dependent | cyclin-dep | cyclin-dep | 8558 | 8558 | 8558 | s262 | s263 | s264 |
| 2485 | H20 | H21 | 1166 | 659,3 | 15424 | 352,5 | 13,2 | 1,039 | H5A034 C | sample | Hm Drgbl | Hm Drgbl | Hm Drgbl | CPE002DC | CPE002EE | CPE002F4 | NM 00597 | NM 0059 | NM 0059 | PTK6 | PTK6 | PTK6 | PTK6 protein tyro | PTK6 prot | PTK6 prot | 5753 | 5753 | 5753 | s11487 | s11488 | s11489 |
| 16581 | C6 | C7 | 1327 | 994,9 | 17577 | 349,3 | 13,2 | 1,043 | H5A034 C | sample | Hm Gen E | Hm Gen E | Hm Gen E | CPE002JH | CPE002NV | CPE002KC | NM 00100 | NM 0010 | NM 0010 | BMF | BMF | BMF | Bcl2 modifying fac | Bcl2 modi | Bcl2 modi | 90427 | 90427 | 90427 | s40385 | s40386 | s40387 |
| 16616 | E16 | E17 | 1231 | 951,4 | 16266 | 229,4 | 13,2 | 1,043 | H5A034 C | sample | Hm Gen E | Hm Gen E | Hm Gen E | CPE002JH | CPE002NV | CPE002KC | NM 00103 | NM 0010 | NM 0010 | COX19 | COX19 | COX19 | COX19 cytochrom | COX19 cyt | COX19 cyt | 90639 | 90639 | 90639 | s40455 | s40456 | s40457 |
| 16650 | F3 | F4 | 1105 | 929,8 | 14590 | 227,2 | 13,2 | 1,043 | H5A034 C | sample | Hm Gen E | Hm Gen E | Hm Gen E | CPE002JH | CPE002NV | CPE002KC | NM 03351 | NM 0335 | NM 0335 | TSPYL5 | TSPYL5 | TSPYL5 | TSPY-like 5 | TSPY-like 5 | TSPY-like 5 | 85453 | 85453 | 85453 | s40024 | s40025 | s40026 |
| 16662 | G14 | G15 | 900 | 929,4 | 11854 | 323,3 | 13,2 | 1,043 | H5A034 C | sample | Hm Gen E | Hm Gen E | Hm Gen E | CPE002JH | CPE002NV | CPE002KC | NM 14456 | NM 1445 | NM 1445 | TMEM55B | TMEM55B | TMEM55B | transmembrane p | transmem | transmem | 90809 | 90809 | 90809 | s40497 | s40498 | s40499 |
| 16766 | K21 | K22 | 1493 | 938,5 | 19725 | 287,1 | 13,2 | 1,043 | H5A034 C | sample | Hm Gen E | Hm Gen E | Hm Gen E | CPE002JH | CPE002NV | CPE002KC | NM 00108 | NM 0010 | NM 0010 | FAM86B1 | FAM86B1 | FAM86B1 | family with seque | family wit | family wit | 85002 | 85002 | 85002 | s39824 | s39825 | s195505 |
| 16852 | O12 | O13 | 1633 | 947,9 | 21539 | 344,3 | 13,2 | 1,043 | H5A034 C | sample | Hm Gen E | Hm Gen E | Hm Gen E | CPE002JH | CPE002NV | CPE002KC | NM 00107 | NM 0010 | NM 0010 | DEPDC7 | DEPDC7 | DEPDC7 | DEP domain conta | DEP doma | DEP doma | 91614 | 91614 | 91614 | s40711 | s40712 | s40713 |
| 17781 | E6 | E7 | 1044 | 1052 | 13820 | 390,8 | 13,2 | 1,043 | H5A034 C | sample | Hm Gen E | Hm Gen E | Hm Gen E | CPE002JK | CPE002NZ | CPE002KF | NM 14469 | NM 1446 | NM 1446 | ANKRD35 | ANKRD35 | ANKRD35 | ankyrin repeat dc | ankyrin re | ankyrin re | 148741 | 148741 | 148741 | s45203 | s45204 | s45205 |
| 17799 | F22 | F23 | 1744 | 994 | 23012 | 241,7 | 13,2 | 1,043 | H5A034 C | sample | Hm Gen E | Hm Gen E | Hm Gen E | CPE002JK | CPE002NZ | CPE002KF | NM 14517 | NM 1451 | NM 1451 | FAM84A | FAM84A | FAM84A | family with seque | family wit | family wit | 151354 | 151354 | 151354 | s45560 | s45561 | s45562 |
| 4234 | A18 | A19 | 1898 | 785,5 | 25102 | 409,2 | 13,2 | 1,114 | H5A034 C | sample | Hm Drgbl | Hm Drgbl | Hm Drgbl | CPE002DU | CPE002EK | CPE002FQ | NM 01617 | NM 0161 | NM 0161 | TRPC4 | TRPC4 | TRPC4 | transient recepto | transient | transient | 7223 | 7223 | 7223 | s14415 | s14416 | s14417 |
| 4286 | C21 | C22 | 1461 | 700,1 | 19252 | 371,8 | 13,2 | 1,114 | H5A034 C | sample | Hm Drgbl | Hm Drgbl | Hm Drgbl | CPE002DU | CPE002EK | CPE002FQ | NM 00168 | NM 0016 | NM 0016 | ATP2B4 | ATP2B4 | ATP2B4 | ATPase, Ca++ trar | ATPase, C | ATPase, C | 493 | 493 | 493 | s1758 | s1759 | s1760 |
| 4377 | G17 | G18 | 1091 | 606,9 | 14379 | 337,7 | 13,2 | 1,114 | H5A034 C | sample | Hm Drgbl | Hm Drgbl | Hm Drgbl | CPE002DU | CPE002EK | CPE002FQ | NM 00049 | NM 0004 | NM 0004 | CFTR | CFTR | CFTR | cystic fibrosis trar | cystic fibr | cystic fibr | 1080 | 1080 | 1080 | s2945 | s2946 | s2947 |
| 4469 | K13 | K14 | 1263 | 749 | 16651 | 382 | 13,2 | 1,114 | H5A034 C | sample | Hm Drgbl | Hm Drgbl | Hm Drgbl | CPE002DU | CPE002EK | CPE002FQ | NM 02173 | NM 0012 | NM 0217 | CLCN6 | CLCN6 | CLCN6 | chloride channel | chloride c | chloride c | 1185 | 1185 | 1185 | s3144 | s223251 | s3146 |
| 4511 | L8 | L9 | 1226 | 713,4 | 16164 | 393,9 | 13,2 | 1,114 | H5A034 C | sample | Hm Drgbl | Hm Drgbl | Hm Drgbl | CPE002DU | CPE002EK | CPE002FQ | NM 00325 | NM 0032 | NM 0032 | TIAM1 | TIAM1 | TIAM1 | T-cell lymphoma i | T-cell lym | T-cell lym | 7074 | 7074 | 7074 | s14137 | s14138 | s14139 |
| 4558 | N7 | N8 | 1520 | 663,1 | 20135 | 440,5 | 13,2 | 1,114 | H5A034 C | sample | Hm Drgbl | Hm Drgbl | Hm Drgbl | CPE002DU | CPE002EK | CPE002FQ | NM 00255 | NM 0025 | NM 0025 | P2RX1 | P2RX1 | P2RX1 | purinergic recepto | purinergic | purinergic | 5023 | 5023 | 5023 | s9949 | s9950 | s9951 |
| 82 | D18 | D19 | 365 | 1548 | 4834 | 542,8 | 13,2 | 1,123 | H5A034 C | sample | Hm Drgbl | Hm Drgbl | Hm Drgbl | CPE002DT | CPE002EJ | CPE002F9 | NM 00312 | NM 0031 | NM 0031 | SPR | SPR | SPR | sepiapterin reduc | sepiapteri | sepiapteri | 6697 | 6697 | 6697 | s13378 | s13379 | s13380 |
| 99 | E11 | E12 | 644 | 1608 | 8487 | 457,6 | 13,2 | 1,123 | H5A034 C | sample | Hm Drgbl | Hm Drgbl | Hm Drgbl | CPE002DT | CPE002EJ | CPE002F9 | NM 00565 | NM 0056 | NM 0056 | NR2F1 | NR2F1 | NR2F1 | nuclear receptor s | nuclear re | nuclear re | 7025 | 7025 | 7025 | s14018 | s224723 | s14020 |
| 290 | M10 | M11 | 652 | 1442 | 8606 | 499,3 | 13,2 | 1,123 | H5A034 C | sample | Hm Drgbl | Hm Drgbl | Hm Drgbl | CPE002DT | CPE002EJ | CPE002F9 | NM 00310 | NM 0031 | NM 0031 | SOAT1 | SOAT1 | SOAT1 | sterol O-acyltrans | sterol O-a | sterol O-a | 6646 | 6646 | 6646 | s13264 | s13265 | s13266 |
| 380 | P5 | P6 | 540 | 1490 | 7113 | 718,4 | 13,2 | 1,123 | H5A034 C | sample | Hm Drgbl | Hm Drgbl | Hm Drgbl | CPE002DT | CPE002EJ | CPE002F9 | NM 01422 | NM 0142 | NM 0142 | SLC6A11 | SLC6A11 | SLC6A11 | solute carrier fam | solute car | solute car | 6538 | 6538 | 6538 | s12994 | s12995 | s12996 |
| 2014 | D7 | D8 | 1326 | 610,6 | 17448 | 438,5 | 13,2 | 1,148 | H5A034 C | sample | Hm Drgbl | Hm Drgbl | Hm Drgbl | CPE002FX | CPE002G1 | CPE002G5 | NM 15241 | NM 1524 | NM 1524 | ZNF572 | ZNF572 | ZNF572 | zinc finger protei | zinc finge | zinc finge | 137209 | 137209 | 137209 | s44027 | s44028 | s44029 |
| 2184 | K9 | K10 | 1517 | 778,4 | 19964 | 362,2 | 13,2 | 1,148 | H5A034 C | sample | Hm Drgbl | Hm Drgbl | Hm Drgbl | CPE002FX | CPE002G1 | CPE002G5 | NM 05823 | NM 0582 | NM 0582 | ZNF354B | ZNF354B | ZNF354B | zinc finger protei | zinc finge | zinc finge | 117608 | 117608 | 117608 | s42183 | s42184 | s42185 |
| 2197 | L20 | L21 | 1647 | 697 | 21702 | 417,1 | 13,2 | 1,148 | H5A034 C | sample | Hm Drgbl | Hm Drgbl | Hm Drgbl | CPE002FX | CPE002G1 | CPE002G5 | | | | | | | | | | | | | | | |
| 2257 | O1 | O2 | 987 | 598,4 | 12994 | 440,6 | 13,2 | 1,148 | H5A034 C | sample | Hm Drgbl | Hm Drgbl | Hm Drgbl | CPE002FX | CPE002G1 | CPE002G5 | NM 00100 | NM 0010 | NM 0010 | ZNF787 | ZNF787 | ZNF787 | zinc finger protei | zinc finge | zinc finge | 126208 | 126208 | 126208 | s42977 | s42978 | s42979 |
| 6807 | L22 | L23 | 1642 | 607,6 | 21622 | 456,3 | 13,2 | 1,158 | H5A034 C | sample | Hm Drgbl | Hm Drgbl | Hm Drgbl | CPE002E1 | CPE002EY | CPE002FJ | NM 00227 | NM 0022 | NM 0022 | KRT4 | KRT4 | KRT4 | keratin 4 | keratin 4 | keratin 4 | 3851 | 3851 | 3851 | s7953 | s7954 | s7955 |
| 6894 | P14 | P15 | 1401 | 595,9 | 18438 | 504,6 | 13,2 | 1,158 | H5A034 C | sample | Hm Drgbl | Hm Drgbl | Hm Drgbl | CPE002E1 | CPE002EY | CPE002FJ | NM 02101 | NM 0210 | NM 0210 | KRT34 | KRT34 | KRT34 | keratin 34 | keratin 34 | keratin 34 | 3885 | 3885 | 3885 | s8011 | s8012 | s8013 |
| 3899 | C19 | C20 | 415 | 806,9 | 5461 | 469,1 | 13,2 | 1,173 | H5A034 C | sample | Hm Drgbl | Hm Drgbl | Hm Drgbl | CPE002DS | CPE002EI | CPE002F8 | NM 18281 | NM 1828 | NM 1828 | PLCG1 | PLCG1 | PLCG1 | phospholipase C, | phospholi | phospholi | 5335 | 5335 | 5335 | s10631 | s10632 | s10633 |
| 3998 | G21 | G22 | 1405 | 724,2 | 18553 | 409 | 13,2 | 1,173 | H5A034 C | sample | Hm Drgbl | Hm Drgbl | Hm Drgbl | CPE002DS | CPE002EI | CPE002F8 | NM 00053 | NM 0005 | NM 0005 | PMS2 | PMS2 | PMS2 | PMS2 postmeiotic | PMS2 pos | PMS2 pos | 5395 | 5395 | 5395 | s10740 | s10742 | s10741 |
| 4062 | J14 | J15 | 187 | 720,7 | 2475 | 324,8 | 13,2 | 1,173 | H5A034 C | sample | Hm Drgbl | Hm Drgbl | Hm Drgbl | CPE002DS | CPE002EI | CPE002F8 | NM 00096 | NM 0009 | NM 0009 | RPL4 | RPL4 | RPL4 | ribosomal protein | ribosomal | ribosomal | 6124 | 6124 | 6124 | s12150 | s12149 | s12148 |
| 4138 | M18 | M19 | 1411 | 778,1 | 18682 | 459,2 | 13,2 | 1,173 | H5A034 C | sample | Hm Drgbl | Hm Drgbl | Hm Drgbl | CPE002DS | CPE002EI | CPE002F8 | NM 00289 | NM 0028 | NM 0028 | RBMS2 | RBMS2 | RBMS2 | RNA binding moti | RNA bindi | RNA bindi | 5939 | 5939 | 5939 | s11867 | s11868 | s11869 |
| 11767 | K15 | K16 | 1337 | 826 | 17623 | 128 | 13,2 | 1,179 | H5A034 C | sample | Hm Gen E | Hm Gen E | Hm Gen E | CPE002JA | CPE002NJ | CPE002JZ | NM 00613 | NM 0061 | NM 0061 | TMEM50B | TMEM50B | TMEM50B | transmembrane p | transmem | transmem | 757 | 757 | 757 | s2238 | s2239 | s2240 |
| 5795 | B19 | B20 | 1218 | 828,9 | 16049 | 423,9 | 13,2 | 1,205 | H5A034 C | sample | Hm Drgbl | Hm Drgbl | Hm Drgbl | CPE002DY | CPE002F1 | CPE002FM | NM 00516 | NM 0051 | NM 0051 | ALDOC | ALDOC | ALDOC | aldolase C, fructo | aldolase C | aldolase C | 230 | 230 | 230 | s1265 | s1263 | s1264 |
| 5934 | H14 | H15 | 2093 | 783,2 | 27598 | 472,3 | 13,2 | 1,205 | H5A034 C | sample | Hm Drgbl | Hm Drgbl | Hm Drgbl | CPE002DY | CPE002F1 | CPE002FM | NM 00433 | NM 0043 | NM 0043 | BST1 | BST1 | BST1 | bone marrow stro | bone mar | bone mar | 683 | 683 | 683 | s2102 | s2100 | s2101 |
| 6074 | N10 | N11 | 1869 | 793 | 24736 | 605,6 | 13,2 | 1,205 | H5A034 C | sample | Hm Drgbl | Hm Drgbl | Hm Drgbl | CPE002DY | CPE002F1 | CPE002FM | NM 00175 | NM 0017 | NM 0017 | CAT | CAT | CAT | catalase | catalase | catalase | 847 | 847 | 847 | s2443 | s2444 | s2445 |
| 1158 | A14 | A15 | 1389 | 741,8 | 18328 | 471,7 | 13,2 | 1,211 | H5A034 C | sample | Hm Drgbl | Hm Drgbl | Hm Drgbl | CPE002FV | CPE002FZ | CPE002G3 | NM 00489 | NM 0048 | NM 0048 | SEC22B | SEC22B | SEC22B | SEC22 vesicle traf | SEC22 ves | SEC22 ves | 9554 | 9554 | 9554 | s18347 | s225160 | s18349 |
| 1210 | C18 | C19 | 1691 | 707,8 | 22307 | 404,2 | 13,2 | 1,211 | H5A034 C | sample | Hm Drgbl | Hm Drgbl | Hm Drgbl | CPE002FV | CPE002FZ | CPE002G3 | NM 01473 | NM 0147 | NM 0147 | ZFYVE16 | ZFYVE16 | ZFYVE16 | zinc finger, FYVE c | zinc finge | zinc finge | 9765 | 9765 | 9765 | s18852 | s18853 | s18854 |
| 1219 | C4 | C5 | 1300 | 748,7 | 17139 | 513,1 | 13,2 | 1,211 | H5A034 C | sample | Hm Drgbl | Hm Drgbl | Hm Drgbl | CPE002FV | CPE002FZ | CPE002G3 | NM 01463 | NM 0146 | NM 0146 | ZNF592 | ZNF592 | ZNF592 | zinc finger protei | zinc finge | zinc finge | 9640 | 9640 | 9640 | s18533 | s18534 | s18535 |
| 1275 | F11 | F12 | 1358 | 707 | 17913 | 439,5 | 13,2 | 1,211 | H5A034 C | sample | Hm Drgbl | Hm Drgbl | Hm Drgbl | CPE002FV | CPE002FZ | CPE002G3 | NM 00370 | NM 0037 | NM 0037 | KLF7 | KLF7 | KLF7 | Kruppel-like facto | Kruppel-li | Kruppel-li | 8609 | 8609 | 8609 | s16375 | s16376 | s16377 |
| 1405 | K20 | K21 | 1919 | 772 | 25283 | 381,5 | 13,2 | 1,211 | H5A034 C | sample | Hm Drgbl | Hm Drgbl | Hm Drgbl | CPE002FV | CPE002FZ | CPE002G3 | NM 00607 | NM 0060 | NM 0060 | CBARA1 | CBARA1 | CBARA1 | calcium binding a | calcium bi | calcium bi | 10367 | 10367 | 10367 | s20272 | s20273 | s20274 |
| 16230 | E14 | E15 | 1481 | 948,6 | 19583 | 304,6 | 13,2 | 1,211 | H5A034 C | sample | Hm Gen E | Hm Gen E | Hm Gen E | CPE002JG | CPE002NV | CPE002KB | NM 02091 | NM 0209 | NM 0209 | FLYWCH1 | FLYWCH1 | FLYWCH1 | FLYWCH-type zinc | FLYWCH-t | FLYWCH-t | 84256 | 84256 | 84256 | s38739 | s38740 | s38741 |
| 16303 | H15 | H16 | 1377 | 972,5 | 18218 | 391,2 | 13,2 | 1,211 | H5A034 C | sample | Hm Gen E | Hm Gen E | Hm Gen E | CPE002JG | CPE002NV | CPE002KB | NM 03204 | NM 0320 | NM 0320 | CCDC8 | CCDC8 | CCDC8 | coiled-coil domai | coiled-coi | coiled-coi | 83987 | 83987 | 83987 | s38375 | s38376 | s228331 |
| 16421 | M13 | M14 | 1909 | 986,1 | 25146 | 273,2 | 13,2 | 1,211 | H5A034 C | sample | Hm Gen E | Hm Gen E | Hm Gen E | CPE002JG | CPE002NV | CPE002KB | NM 03144 | NM 0314 | NM 0314 | C18orf21 | C18orf21 | C18orf21 | chromosome 18 o | chromoso | chromoso | 83608 | 83608 | 83608 | s38046 | s38047 | s38048 |
| 13667 | J19 | J20 | 1924 | 1112 | 25366 | 191,8 | 13,2 | 1,239 | H5A034 C | sample | Hm Gen E | Hm Gen E | Hm Gen E | CPE002JC | CPE002NC | CPE002K4 | NM 00107 | NM 0010 | NM 0010 | TNFAIP8 | TNFAIP8 | TNFAIP8 | tumor necrosis fa | tumor nec | tumor nec | 25816 | 25816 | 25816 | s24539 | s24540 | s24541 |
| 13752 | M9 | M10 | 1186 | 945,2 | 15651 | 162,4 | 13,2 | 1,239 | H5A034 C | sample | Hm Gen E | Hm Gen E | Hm Gen E | CPE002JC | CPE002NC | CPE002K4 | NM 01535 | NM 0153 | NM 0153 | LRRC8B | LRRC8B | LRRC8B | leucine rich repea | leucine ric | leucine ric | 23507 | 23507 | 23507 | s23955 | s23956 | s23957 |
| 791 | A8 | A9 | 1764 | 750,6 | 23208 | 549,3 | 13,2 | 1,251 | H5A034 C | sample | Hm Drgbl | Hm Drgbl | Hm Drgbl | CPE002FU | CPE002FY | CPE002G2 | NM 00557 | NM 0055 | NM 0055 | LMO2 | LMO2 | LMO2 | LIM domain only | LIM doma | LIM doma | 4005 | 4005 | 4005 | s8230 | s224016 | s8232 |
| 884 | E5 | E6 | 1038 | 711,2 | 13654 | 419,3 | 13,2 | 1,251 | H5A034 C | sample | Hm Drgbl | Hm Drgbl | Hm Drgbl | CPE002FU | CPE002FY | CPE002G2 | NM 00121 | NM 0012 | NM 0012 | C1QBP | C1QBP | C1QBP | complement com | compleme | compleme | 708 | 708 | 708 | s2137 | s2138 | s2139 |
| 1035 | L11 | L12 | 1200 | 725,9 | 15783 | 395 | 13,2 | 1,251 | H5A034 C | sample | Hm Drgbl | Hm Drgbl | Hm Drgbl | CPE002FU | CPE002FY | CPE002G2 | NM 00154 | NM 0015 | NM 0015 | HSPB2 | HSPB2 | HSPB2 | heat shock 27kDa | heat shoc | heat shoc | 3316 | 3316 | 3316 | s6992 | s194539 | s194540 |
| 1076 | M5 | M6 | 1318 | 764 | 17349 | 436,2 | 13,2 | 1,251 | H5A034 C | sample | Hm Drgbl | Hm Drgbl | Hm Drgbl | CPE002FU | CPE002FY | CPE002G2 | NM 00130 | NM 0013 | NM 0013 | CLDN4 | CLDN4 | CLDN4 | claudin 4 | claudin 4 | claudin 4 | 1364 | 1364 | 1364 | s223316 | s3442 | s3443 |
| 5410 | B18 | B19 | 1013 | 681,9 | 13352 | 657 | 13,2 | 1,257 | H5A034 C | sample | Hm Drgbl | Hm Drgbl | Hm Drgbl | CPE002DX | CPE002F2 | CPE002FN | NM 00469 | NM 0046 | NM 0046 | SLC16A5 | SLC16A5 | SLC16A5 | solute carrier fam | solute car | solute car | 9121 | 9121 | 9121 | s17410 | s17411 | s17412 |
| 5468 | D5 | D6 | 1761 | 751,7 | 23312 | 517,3 | 13,2 | 1,257 | H5A034 C | sample | Hm Drgbl | Hm Drgbl | Hm Drgbl | CPE002DX | CPE002F2 | CPE002FN | NM 00372 | NM 0037 | NM 0037 | RTCD1 | RTCD1 | RTCD1 | RNA terminal pho | RNA term | RNA term | 8634 | 8634 | 8634 | s16426 | s16427 | s16428 |
| 5592 | I9 | I10 | 1732 | 689,4 | 22780 | 502 | 13,2 | 1,257 | H5A034 C | sample | Hm Drgbl | Hm Drgbl | Hm Drgbl | CPE002DX | CPE002F2 | CPE002FN | NM 00100 | NM 0010 | NM 0010 | SIP1 | SIP1 | SIP1 | survival of motor | survival o | survival o | 8487 | 8487 | 8487 | s16119 | s16120 | s16118 |
| 5605 | J20 | J21 | 1832 | 692,1 | 24103 | 496,9 | 13,2 | 1,257 | H5A034 C | sample | Hm Drgbl | Hm Drgbl | Hm Drgbl | CPE002DX | CPE002F2 | CPE002FN | NM 00475 | NM 0047 | NM 0047 | SCYE1 | SCYE1 | SCYE1 | small inducible cy | small indu | small indu | 9255 | 9255 | 9255 | s17700 | s17701 | s17702 |
| 5637 | K6 | K7 | 1490 | 674,3 | 19737 | 577,6 | 13,2 | 1,257 | H5A034 C | sample | Hm Drgbl | Hm Drgbl | Hm Drgbl | CPE002DX | CPE002F2 | CPE002FN | NM 00393 | NM 0039 | NM 0039 | KYNU | KYNU | KYNU | kynureninase (L-k | kynurenin | kynurenin | 8942 | 8942 | 8942 | s17103 | s17104 | s17105 |
| 5714 | O10 | O11 | 1866 | 660,1 | 24597 | 597,1 | 13,2 | 1,257 | H5A034 C | sample | Hm Drgbl | Hm Drgbl | Hm Drgbl | CPE002DX | CPE002F2 | CPE002FN | NM 00398 | NM 0039 | NM 0039 | SLC7A7 | SLC7A7 | SLC7A7 | solute carrier fam | solute car | solute car | 9056 | 9056 | 9056 | s17270 | s17271 | s17272 |
| 3589 | F20 | F21 | 979 | 1583 | 12936 | 385,5 | 13,2 | 1,269 | H5A034 C | sample | Hm Drgbl | Hm Drgbl | Hm Drgbl | CPE002DR | CPE002EH | CPE002F7 | NM 02498 | NM 0249 | NM 0249 | GPR157 | GPR157 | GPR157 | G protein-couplec | G protein | G protein | 80045 | 80045 | 80045 | s36865 | s36866 | s195393 |
| 3649 | I1 | I2 | 838 | 1301 | 11032 | 407,9 | 13,2 | 1,269 | H5A034 C | sample | Hm Drgbl | Hm Drgbl | Hm Drgbl | CPE002DR | CPE002EH | CPE002F7 | NM 00057 | NM 0005 | NM 0005 | CCR5 | CCR5 | CCR5 | chemokine (C-C m | chemokin | chemokin | 1234 | 1234 | 1234 | s3209 | s3210 | s3211 |
| 8170 | E18 | E19 | 1429 | 587,1 | 18884 | 425,1 | 13,2 | 1,269 | H5A034 C | sample | Hm Drgbl | Hm Drgbl | Hm Drgbl | CPE002E5 | CPE002EU | CPE002FF | NM 01512 | NM 0151 | NM 0151 | CIC | CIC | CIC | capicua homolog | capicua h | capicua h | 23152 | 23152 | 23152 | s23153 | s23152 | s23151 |
| 8239 | H15 | H16 | 1823 | 591,5 | 24023 | 418,2 | 13,2 | 1,269 | H5A034 C | sample | Hm Drgbl | Hm Drgbl | Hm Drgbl | CPE002E5 | CPE002EU | CPE002FF | NM 01495 | NM 0149 | NM 0149 | RPH3A | RPH3A | RPH3A | rabphilin 3A hom | rabphilin | rabphilin | 22895 | 22895 | 22895 | s223541 | s22612 | s22610 |
| 8284 | J12 | J13 | 1646 | 625,7 | 21761 | 395,4 | 13,2 | 1,269 | H5A034 C | sample | Hm Drgbl | Hm Drgbl | Hm Drgbl | CPE002E5 | CPE002EU | CPE002FF | XM 93819 | XM 9381 | XM 9381 | TTC9 | TTC9 | TTC9 | tetratricopeptide | tetratrico | tetratrico | 23508 | 23508 | 23508 | s23958 | s23960 | s23959 |
| 8312 | K16 | K17 | 727 | 670,4 | 9574 | 527,7 | 13,2 | 1,269 | H5A034 C | sample | Hm Drgbl | Hm Drgbl | Hm Drgbl | CPE002E5 | CPE002EU | CPE002FF | NM 01523 | NM 0152 | NM 0152 | NUP160 | NUP160 | NUP160 | nucleoporin 160k | nucleopor | nucleopor | 23279 | 23279 | 23279 | s23465 | s23466 | s23464 |
| 8419 | O4 | O5 | 1410 | 640,4 | 18646 | 560,2 | 13,2 | 1,269 | H5A034 C | sample | Hm Drgbl | Hm Drgbl | Hm Drgbl | CPE002E5 | CPE002EU | CPE002FF | NM 01527 | NM 0152 | NM 0152 | NEDD4L | NEDD4L | NEDD4L | neural precursor | neural pre | neural pre | 23327 | 23327 | 23327 | s23569 | s23570 | s23571 |
| 1592 | C16 | C17 | 950 | 776 | 12579 | 406,8 | 13,2 | 1,382 | H5A034 C | sample | Hm Drgbl | Hm Drgbl | Hm Drgbl | CPE002FW | CPE002G0 | CPE002G4 | NM 02083 | NM 0208 | NM 0208 | WDFY1 | WDFY1 | WDFY1 | WD repeat and FY | WD repea | WD repea | 57590 | 57590 | 57590 | s33388 | s33389 | s33390 |
| 1733 | I13 | I14 | 1334 | 700,3 | 17581 | 295,1 | 13,2 | 1,382 | H5A034 C | sample | Hm Drgbl | Hm Drgbl | Hm Drgbl | CPE002FW | CPE002G0 | CPE002G4 | NM 15224 | NM 1522 | NM 0133 | SNX11 | SNX11 | SNX11 | sorting nexin 11 | sorting ne | sorting ne | 29916 | 29916 | 29916 | s26697 | s26698 | s223830 |
| 1872 | N9 | N10 | 863 | 722,4 | 11382 | 422 | 13,2 | 1,382 | H5A034 C | sample | Hm Drgbl | Hm Drgbl | Hm Drgbl | CPE002FW | CPE002G0 | CPE002G4 | NM 01868 | NM 0186 | NM 0186 | BIN3 | BIN3 | BIN3 | bridging integrato | bridging in | bridging in | 55909 | 55909 | 55909 | s31762 | s31763 | s31764 |
| 21507 | A11 | A12 | 147 | 987 | 1947 | 854,6 | 13,2 | 1,389 | H5A034 C | sample | Hm Gen E | Hm Gen E | Hm Gen E | CPE002JU | CPE002O9 | CPE002KP | XM 94346 | XM 9434 | XM 9434 | LOC64879 | LOC64879 | LOC64879 | hypothetical prot | hypotheti | hypotheti | 648790 | 648790 | 648790 | s56880 | s56881 | s56882 |
| 21662 | G21 | G22 | 440 | 1024 | 5802 | 684,4 | 13,2 | 1,389 | H5A034 C | sample | Hm Gen E | Hm Gen E | Hm Gen E | CPE002JU | CPE002O9 | CPE002KP | XM 00113 | XM 0011 | XM 0011 | LOC64969 | LOC64969 | LOC64969 | similar to immuno | similar to | similar to | 649698 | 649698 | 649698 | s56992 | s56993 | s56994 |
| 21738 | J3 | J4 | 210 | 978,7 | 2771 | 642,2 | 13,2 | 1,389 | H5A034 C | sample | Hm Gen E | Hm Gen E | Hm Gen E | CPE002JU | CPE002O9 | CPE002KP | XM 94489 | XM 9448 | XM 9448 | LOC65173 | LOC65173 | LOC65173 | hypothetical prot | hypotheti | hypotheti | 651738 | 651738 | 651738 | s57233 | s57234 | s57235 |
| 21800 | M16 | M17 | 156 | 901 | 2052 | 533,8 | 13,2 | 1,389 | H5A034 C | sample | Hm Gen E | Hm Gen E | Hm Gen E | CPE002JU | CPE002O9 | CPE002KP | XM 94249 | XM 9424 | XM 9424 | LOC65282 | LOC65282 | LOC65282 | hypothetical prot | hypotheti | hypotheti | 652825 | 652825 | 652825 | s57507 | s57508 | s57509 |
| 4683 | D11 | D12 | 1190 | 816,5 | 15736 | 365,3 | 13,2 | 1,451 | H5A034 C | sample | Hm Drgbl | Hm Drgbl | Hm Drgbl | CPE002DV | CPE002EL | CPE002FP | NM 02099 | NM 0209 | NM 0209 | HPR | HPR | HPR | haptoglobin-relat | haptoglob | haptoglob | 3250 | 3250 | 3250 | s6884 | s6885 | s6886 |
| 4838 | J21 | J22 | 908 | 911,3 | 11956 | 470,2 | 13,2 | 1,451 | H5A034 C | sample | Hm Drgbl | Hm Drgbl | Hm Drgbl | CPE002DV | CPE002EL | CPE002FP | NM 00258 | NM 0025 | NM 0025 | PAPPA | PAPPA | PAPPA | pregnancy-associa | pregnancy | pregnancy | 5069 | 5069 | 5069 | s10040 | s10041 | s10042 |
| 10475 | E19 | E20 | 1561 | 830,8 | 20663 | 196,1 | 13,2 | 1,492 | H5A034 C | sample | Hm Drgbl | Hm Drgbl | Hm Drgbl | CPE002EB | CPE002EO | CPE002FR | NM 03309 | NM 0330 | NM 0330 | TRIM5 | TRIM5 | TRIM5 | tripartite motif-cc | tripartite | tripartite | 85363 | 85363 | 85363 | s39922 | s39923 | s39924 |
| 10490 | F10 | F11 | 995 | 848 | 13148 | 212,3 | 13,2 | 1,492 | H5A034 C | sample | Hm Drgbl | Hm Drgbl | Hm Drgbl | CPE002EB | CPE002EO | CPE002FR | NM 00101 | NM 0010 | NM 0010 | NAT12 | NAT12 | NAT12 | N-acetyltransfera | N-acetyltr | N-acetyltr | 122830 | 122830 | 122830 | s42590 | s42591 | s42592 |
| 10513 | G1 | G2 | 409 | 1025 | 5407 | 188,5 | 13,2 | 1,492 | H5A034 C | sample | Hm Drgbl | Hm Drgbl | Hm Drgbl | CPE002EB | CPE002EO | CPE002FR | NM 02046 | NM 0204 | NM 0204 | TUBGCP6 | TUBGCP6 | TUBGCP6 | tubulin, gamma c | tubulin, g | tubulin, g | 85378 | 85378 | 85378 | s39943 | s39944 | s224992 |
| 10607 | J8 | J9 | 946 | 795,6 | 12460 | 192 | 13,2 | 1,492 | H5A034 C | sample | Hm Drgbl | Hm Drgbl | Hm Drgbl | CPE002EB | CPE002EO | CPE002FR | NM 17489 | NM 1748 | NM 1748 | CD300LB | CD300LB | CD300LB | CD300 molecule-l | CD300 mo | CD300 mo | 124599 | 124599 | 124599 | s42769 | s42768 | s223276 |
| 10631 | K8 | K9 | 1219 | 847,7 | 16036 | 190,4 | 13,2 | 1,492 | H5A034 C | sample | Hm Drgbl | Hm Drgbl | Hm Drgbl | CPE002EB | CPE002EO | CPE002FR | NM 05295 | NM 0529 | NM 0529 | TGM7 | TGM7 | TGM7 | transglutaminase | transgluta | transgluta | 116179 | 116179 | 116179 | s41959 | s41958 | s41957 |
| 2853 | G6 | G7 | 211 | 642,2 | 2789 | 569,2 | 13,2 | 1,553 | H5A034 C | sample | Hm Drgbl | Hm Drgbl | Hm Drgbl | CPE002DP | CPE002EF | CPE002F5 | NM 02016 | NM 0201 | NM 0201 | PAK6 | PAK6 | PAK6 | p21(CDKN1A)-acti | p21(CDKN | p21(CDKN | 56924 | 56924 | 56924 | s32405 | s32406 | s32407 |
| 3032 | O16 | O17 | 758 | 718,2 | 10027 | 628,1 | 13,2 | 1,553 | H5A034 C | sample | Hm Drgbl | Hm Drgbl | Hm Drgbl | CPE002DP | CPE002EF | CPE002F5 | NM 03143 | NM 0314 | NM 0314 | UCK1 | UCK1 | UCK1 | uridine-cytidine k | uridine-cy | uridine-cy | 83549 | 83549 | 83549 | s38001 | s38002 | s38003 |
| 413 | B13 | B14 | 1545 | 1720 | 20322 | 364,2 | 13,2 | 1,927 | H5A034 C | sample | Hm Drgbl | Hm Drgbl | Hm Drgbl | CPE002E0 | CPE002EZ | CPE002FK | NM 00143 | NM 0014 | NM 0014 | FBL | FBL | FBL | fibrillarin | fibrillarin | fibrillarin | 2091 | 2091 | 2091 | s4820 | s4821 | s4822 |
| 7831 | G15 | G16 | 645 | 497,5 | 8505 | 342 | 13,2 | 2,015 | H5A034 C | sample | Hm Drgbl | Hm Drgbl | Hm Drgbl | CPE002E4 | CPE002EV | CPE002FG | NM 01613 | NM 0161 | NM 0161 | COQ7 | COQ7 | COQ7 | coenzyme Q7 hor | coenzyme | coenzyme | 10229 | 10229 | 10229 | s19963 | s19961 | s19962 |
| 8060 | P5 | P6 | 825 | 505,3 | 10894 | 428,5 | 13,2 | 2,015 | H5A034 C | sample | Hm Drgbl | Hm Drgbl | Hm Drgbl | CPE002E4 | CPE002EV | CPE002FG | NM 00639 | NM 0063 | NM 0063 | NOL5A | NOL5A | NOL5A | nucleolar protein | nucleolar | nucleolar | 10528 | 10528 | 10528 | s20643 | s20642 | s20641 |
| 13393 | O1 | O2 | 1518 | 990,7 | 20192 | 65,36 | 13,3 | 0,667 | H5A034 C | sample | Hm Gen E | Hm Gen E | Hm Gen E | CPE002JB | CPE002NN | CPE002K3 | NM 01664 | NM 0166 | NM 0166 | MRPS30 | MRPS30 | MRPS30 | mitochondrial rib | mitochon | mitochon | 10884 | 10884 | 10884 | s21375 | s21376 | s21377 |
| 14874 | L3 | L4 | 1129 | 872 | 14976 | 118,6 | 13,3 | 0,758 | H5A034 C | sample | Hm Gen E | Hm Gen E | Hm Gen E | CPE002JD | CPE002NR | CPE002K7 | NM 01827 | NM 0182 | NM 0182 | TMEM19 | TMEM19 | TMEM19 | transmembrane p | transmem | transmem | 55266 | 55266 | 55266 | s30587 | s30588 | s30589 |
| 3179 | E19 | E20 | 1252 | 1066 | 16710 | 440,3 | 13,3 | 0,824 | H5A034 C | sample | Hm Drgbl | Hm Drgbl | Hm Drgbl | CPE002DC | CPE002EG | CPE002F6 | NM 00139 | NM 0013 | NM 0013 | DUSP4 | DUSP4 | DUSP4 | dual specificity pl | dual speci | dual speci | 1846 | 1846 | 1846 | s223451 | s4373 | s4374 |
| 3182 | E21 | E22 | 1413 | 1362 | 18841 | 444,3 | 13,3 | 0,824 | H5A034 C | sample | Hm Drgbl | Hm Drgbl | Hm Drgbl | CPE002DC | CPE002EG | CPE002F6 | NM 00441 | NM 0044 | NM 0044 | DUSP5 | DUSP5 | DUSP5 | dual specificity pl | dual speci | dual speci | 1847 | 1847 | 1847 | s4375 | s4376 | s4377 |
| 3219 | G11 | G12 | 913 | 1204 | 12158 | 376,3 | 13,3 | 0,824 | H5A034 C | sample | Hm Drgbl | Hm Drgbl | Hm Drgbl | CPE002DC | CPE002EG | CPE002F6 | NM 00050 | NM 0005 | NM 0005 | FBP1 | FBP1 | FBP1 | fructose-1,6-bisph | fructose-1 | fructose-1 | 2203 | 2203 | 2203 | s5053 | s5054 | s5055 |
| 3294 | J14 | J15 | 1196 | 1092 | 15916 | 289,4 | 13,3 | 0,824 | H5A034 C | sample | Hm Drgbl | Hm Drgbl | Hm Drgbl | CPE002DC | CPE002EG | CPE002F6 | NM 00263 | NM 0026 | NM 0026 | PI3 | PI3 | PI3 | peptidase inhibito | peptidase | peptidase | 5266 | 5266 | 5266 | s10460 | s10461 | s224259 |
| 3342 | L14 | L15 | 1410 | 1201 | 18798 | 365,2 | 13,3 | 0,824 | H5A034 C | sample | Hm Drgbl | Hm Drgbl | Hm Drgbl | CPE002DC | CPE002EG | CPE002F6 | NM 00264 | NM 0026 | NM 0026 | PIGA | PIGA | PIGA | phosphatidylinos | phosphat | phosphat | 5277 | 5277 | 5277 | s10493 | s10494 | s224261 |
| 3368 | M16 | M17 | 1064 | 1226 | 14148 | 392,1 | 13,3 | 0,824 | H5A034 C | sample | Hm Drgbl | Hm Drgbl | Hm Drgbl | CPE002DC | CPE002EG | CPE002F6 | NM 19852 | NM 1985 | NM 1985 | HDDC3 | HDDC3 | HDDC3 | HD domain conta | HD domai | HD domai | 374659 | 374659 | 374659 | s51574 | s51575 | s51576 |
| 3427 | O4 | O5 | 1237 | 1121 | 16455 | 409,5 | 13,3 | 0,824 | H5A034 C | sample | Hm Drgbl | Hm Drgbl | Hm Drgbl | CPE002DC | CPE002EG | CPE002F6 | NM 00043 | NM 0004 | NM 0004 | PCSK1 | PCSK1 | PCSK1 | proprotein conve | proprotei | proprotei | 5122 | 5122 | 5122 | s10147 | s10148 | s10149 |
| 18105 | C17 | C18 | 1342 | 1026 | 17828 | 311,5 | 13,3 | 0,855 | H5A034 C | sample | Hm Gen E | Hm Gen E | Hm Gen E | CPE002JL | CPE002O0 | CPE002KG | NM 15255 | NM 1525 | NM 1525 | RNF217 | RNF217 | RNF217 | ring finger protei | ring finge | ring finge | 154214 | 154214 | 154214 | s45855 | s45856 | s45857 |

| | | | | | | | | | | | | | | | | | | | | | | | | | | | | | | | |
|---|---|---|---|---|---|---|---|---|---|---|---|---|---|---|---|---|---|---|---|---|---|---|---|---|---|---|---|---|---|---|---|
| 6505 | P1 | P2 | 1268 | 526,9 | 16860 | 503,8 | 13,3 | 0,878 | H5A034 C | sample | Hm Drgbl | Hm Drgbl | Hm Drgbl | CPE002DZ | CPE002F0 | CPE002FL | NM 00188 | NM 0018 | NM 0018 | CRHBP | CRHBP | CRHBP | corticotropin rele | corticotro | corticotro | 1393 | 1393 | 1393 | s3507 | s223333 | s223332 |
| 20405 | C13 | C14 | 886 | 745,3 | 11805 | 187,1 | 13,3 | 0,887 | H5A034 C | sample | Hm Gen E | Hm Gen E | Hm Gen E | CPE002JR | CPE002O6 | CPE002KN | NM 00102 | NM 0010 | NM 0010 | GOLGA8B | GOLGA8B | GOLGA8B | golgi autoantigen | golgi auto | golgi auto | 440270 | 440270 | 440270 | s54066 | s196520 | s196521 |
| 20523 | H11 | H12 | 878 | 759,5 | 11708 | 161,7 | 13,3 | 0,887 | H5A034 C | sample | Hm Gen E | Hm Gen E | Hm Gen E | CPE002JR | CPE002O6 | CPE002KN | NM 00102 | NM 0010 | NM 0010 | ARMETL1 | ARMETL1 | ARMETL1 | arginine-rich, mut | arginine-r | arginine-r | 441549 | 441549 | 441549 | s54395 | s54396 | s54397 |
| 20567 | I8 | I9 | 367 | 757,4 | 4884 | 138,7 | 13,3 | 0,887 | H5A034 C | sample | Hm Gen E | Hm Gen E | Hm Gen E | CPE002JR | CPE002O6 | CPE002KN | NM 00102 | NM 0010 | NM 0010 | LOC55423 | LOC55423 | LOC55423 | hypothetical prot | hypotheti | hypotheti | 554235 | 554235 | 554235 | s54741 | s54742 | s196698 |
| 20572 | J12 | J13 | 596 | 838,4 | 7952 | 207 | 13,3 | 0,887 | H5A034 C | sample | Hm Gen E | Hm Gen E | Hm Gen E | CPE002JR | CPE002O6 | CPE002KN | XM 92589 | XM 9258 | XM 9258 | LOC65243 | LOC65243 | LOC65243 | similar to triparti | similar to | similar to | 652436 | 652436 | 652436 | s54998 | s54999 | s196782 |
| 20629 | L20 | L21 | 795 | 821,9 | 10584 | 209 | 13,3 | 0,887 | H5A034 C | sample | Hm Gen E | Hm Gen E | Hm Gen E | CPE002JR | CPE002O6 | CPE002KN | XM 92659 | XM 9265 | XM 9265 | hCG 1776 | hCG 1776 | hCG 1776 | hCG1776980 | hCG17769 | hCG17769 | 642502 | 642502 | 642502 | s55037 | s55038 | s227020 |
| 12792 | E9 | E10 | 1439 | 1015 | 19195 | 126,8 | 13,3 | 0,902 | H5A034 C | sample | Hm Gen E | Hm Gen E | Hm Gen E | CPE002J6 | CPE002NN | CPE002K2 | NM 00425 | NM 0042 | NM 0042 | IGSF2 | IGSF2 | IGSF2 | immunoglobulin | immunogl | immunogl | 9398 | 9398 | 9398 | s17982 | s17983 | s17984 |
| 17651 | P19 | P20 | 1156 | 1033 | 15349 | 117,2 | 13,3 | 0,908 | H5A034 C | sample | Hm Gen E | Hm Gen E | Hm Gen E | CPE002JJ | CPE002NY | CPE002KE | NM 14463 | NM 1446 | NM 1446 | FAM131A | FAM131A | FAM131A | family with seque | family wit | family wit | 131408 | 131408 | 131408 | s43601 | s43602 | s43603 |
| 9387 | H11 | H12 | 1517 | 1116 | 20199 | 163,8 | 13,3 | 0,924 | H5A034 C | sample | Hm Drgbl | Hm Drgbl | Hm Drgbl | CPE002E8 | CPE002ER | CPE002FC | NM 01751 | NM 0175 | NM 0175 | ENOSF1 | ENOSF1 | ENOSF1 | enolase superfam | enolase su | enolase su | 55556 | 55556 | 55556 | s30974 | s30975 | s30976 |
| 9559 | O15 | O16 | 2249 | 1140 | 29866 | 152,1 | 13,3 | 0,924 | H5A034 C | sample | Hm Drgbl | Hm Drgbl | Hm Drgbl | CPE002E8 | CPE002ER | CPE002FC | NM 13332 | NM 1333 | NM 1333 | PHF10 | PHF10 | PHF10 | PHD finger protei | PHD finge | PHD finge | 55274 | 55274 | 55274 | s30606 | s30607 | s30608 |
| 9851 | K19 | K20 | 1876 | 1088 | 24960 | 169,9 | 13,3 | 0,93 | H5A034 C | sample | Hm Drgbl | Hm Drgbl | Hm Drgbl | CPE002E9 | CPE002EQ | CPE002FB | XM 00113 | XM 0011 | XM 0011 | BAHCC1 | BAHCC1 | BAHCC1 | BAH domain and | BAH doma | BAH doma | 57597 | 57597 | 57597 | s33409 | s33411 | s33410 |
| 20749 | A20 | A21 | 1570 | 1110 | 20805 | 372,7 | 13,3 | 0,943 | H5A034 C | sample | Hm Gen E | Hm Gen E | Hm Gen E | CPE002JS | CPE002O7 | CPE002KN | XM 00112 | XM 0011 | XM 0011 | LOC64411 | LOC64411 | LOC64411 | similar to ribosom | similar to | similar to | 644119 | 644119 | 644119 | s55563 | s55564 | s227108 |
| 21402 | L3 | L4 | 1038 | 975,8 | 13761 | 219,1 | 13,3 | 0,943 | H5A034 C | sample | Hm Gen E | Hm Gen E | Hm Gen E | CPE002JT | CPE002O8 | CPE002KO | XM 92933 | XM 9293 | XM 9293 | LOC64640 | LOC64640 | LOC64640 | hypothetical LOC | hypotheti | hypotheti | 646405 | 646405 | 646405 | s56360 | s56361 | s56362 |
| 14534 | N21 | N22 | 666 | 915,7 | 8826 | 96,53 | 13,3 | 0,947 | H5A034 C | sample | Hm Gen E | Hm Gen E | Hm Gen E | CPE002J7 | CPE002NC | CPE002K6 | NM 00100 | NM 0010 | NM 0010 | PRR13 | PRR13 | PRR13 | proline rich 13 | proline ric | proline ric | 54458 | 54458 | 54458 | s29000 | s29001 | s226771 |
| 11164 | B12 | B13 | 1679 | 909,1 | 22317 | 144,6 | 13,3 | 0,964 | H5A034 C | sample | Hm Drgbl | Hm Drgbl | Hm Drgbl | CPE002ED | CPE002F3 | CPE002FT | | | | | | | | | | | | | | | |
| 11257 | F1 | F2 | 1143 | 797,1 | 15231 | 141,2 | 13,3 | 0,964 | H5A034 C | sample | Hm Drgbl | Hm Drgbl | Hm Drgbl | CPE002ED | CPE002F3 | CPE002FT | NM 17883 | NM 1788 | NM 1788 | LRRTM1 | LRRTM1 | LRRTM1 | leucine rich repea | leucine ric | leucine ric | 347730 | 347730 | 347730 | s51283 | s51284 | s51285 |
| 11300 | G5 | G6 | 972 | 825,1 | 12900 | 132,4 | 13,3 | 0,964 | H5A034 C | sample | Hm Drgbl | Hm Drgbl | Hm Drgbl | CPE002ED | CPE002F3 | CPE002FT | NM 17367 | NM 1736 | NM 1736 | PPIL6 | PPIL6 | PPIL6 | peptidylprolyl iso | peptidylp | peptidylp | 285755 | 285755 | 285755 | s50029 | s50027 | s50028 |
| 11325 | H6 | H7 | 1568 | 863,7 | 20869 | 136,2 | 13,3 | 0,964 | H5A034 C | sample | Hm Drgbl | Hm Drgbl | Hm Drgbl | CPE002ED | CPE002F3 | CPE002FT | | | | | | | | | | | | | | | |
| 11354 | J10 | J11 | 1560 | 867,2 | 20766 | 130,6 | 13,3 | 0,964 | H5A034 C | sample | Hm Drgbl | Hm Drgbl | Hm Drgbl | CPE002ED | CPE002F3 | CPE002FT | | | | | | | | | | | | | | | |
| 11495 | O8 | O9 | 1737 | 859,4 | 23154 | 144,7 | 13,3 | 0,964 | H5A034 C | sample | Hm Drgbl | Hm Drgbl | Hm Drgbl | CPE002ED | CPE002F3 | CPE002FT | | | | | | | | | | | | | | | |
| 12660 | P2 | P3 | 1532 | 1064 | 20405 | 190,7 | 13,3 | 1 | H5A034 C | sample | Hm Gen E | Hm Gen E | Hm Gen E | CPE002J5 | CPE002NK | CPE002K1 | NM 00421 | NM 0042 | NM 0042 | EBAG9 | EBAG9 | EBAG9 | estrogen receptor | estrogen r | estrogen r | 9166 | 9166 | 9166 | s17514 | s17516 | s228466 |
| 7450 | G18 | G19 | 641 | 663,1 | 8538 | 510 | 13,3 | 1,015 | H5A034 C | sample | Hm Drgbl | Hm Drgbl | Hm Drgbl | CPE002E3 | CPE002EW | CPE002FH | NM 01470 | NM 0147 | NM 0147 | SART3 | SART3 | SART3 | squamous cell car | squamous | squamous | 9733 | 9733 | 9733 | s18771 | s18770 | s18772 |
| 17207 | M8 | M9 | 2015 | 1022 | 26806 | 251,5 | 13,3 | 1,027 | H5A034 C | sample | Hm Gen E | Hm Gen E | Hm Gen E | CPE002JI | CPE002NZ | CPE002KD | NM 14459 | NM 1825 | NM 1445 | GTSF1 | C20orf186 | GTSF1 | gametocyte speci | chromoso | gametocy | 121355 | 149954 | 121355 | s42462 | s45362 | s225535 |
| 17221 | N20 | N21 | 1917 | 972,3 | 25577 | 234 | 13,3 | 1,027 | H5A034 C | sample | Hm Gen E | Hm Gen E | Hm Gen E | CPE002JI | CPE002NZ | CPE002KD | NM 15234 | NM 1447 | NM 1523 | KRT222P | JAKMIP1 | KRT222P | keratin 222 pseud | janus kina | keratin 22 | 125113 | 152789 | 125113 | s42860 | s45731 | s225559 |
| 7273 | P1 | P2 | 1289 | 614,3 | 17170 | 557,7 | 13,3 | 1,035 | H5A034 C | sample | Hm Drgbl | Hm Drgbl | Hm Drgbl | CPE002E2 | CPE002EX | CPE002FI | NM 00243 | NM 0024 | NM 0024 | MPI | MPI | MPI | mannose phosph | mannose | mannose | 4351 | 4351 | 4351 | s8932 | s8933 | s8931 |
| 5002 | A18 | A19 | 1158 | 751,6 | 15431 | 329,7 | 13,3 | 1,043 | H5A034 C | sample | Hm Drgbl | Hm Drgbl | Hm Drgbl | CPE002DV | CPE002EM | CPE002FO | NM 00565 | NM 0056 | NM 0056 | TP53BP1 | TP53BP1 | TP53BP1 | tumor protein p5 | tumor pro | tumor pro | 7158 | 7158 | 7158 | s14313 | s14314 | s14315 |
| 5051 | C19 | C20 | 1756 | 643,1 | 23408 | 507,5 | 13,3 | 1,043 | H5A034 C | sample | Hm Drgbl | Hm Drgbl | Hm Drgbl | CPE002DV | CPE002EM | CPE002FO | NM 03077 | NM 0307 | NM 0307 | TMPRSS5 | TMPRSS5 | TMPRSS5 | transmembrane p | transmem | transmem | 80975 | 80975 | 80975 | s37472 | s37473 | s37474 |
| 5182 | H7 | H8 | 2177 | 696,3 | 28989 | 441,8 | 13,3 | 1,043 | H5A034 C | sample | Hm Drgbl | Hm Drgbl | Hm Drgbl | CPE002DV | CPE002EM | CPE002FO | NM 00463 | NM 0046 | NM 0046 | SEMA3B | SEMA3B | SEMA3B | sema domain, im | sema dom | sema dom | 7869 | 7869 | 7869 | s15431 | s15432 | s15430 |
| 5216 | J16 | J17 | 1695 | 675,5 | 22552 | 380,6 | 13,3 | 1,043 | H5A034 C | sample | Hm Drgbl | Hm Drgbl | Hm Drgbl | CPE002DV | CPE002EM | CPE002FO | NM 00340 | NM 0034 | NM 0034 | YWHAH | YWHAH | YWHAH | tyrosine 3-monoc | tyrosine 3 | tyrosine 3 | 7533 | 7533 | 7533 | s14967 | s14968 | s14969 |
| 5254 | K7 | K8 | 1772 | 602,2 | 23550 | 575,3 | 13,3 | 1,043 | H5A034 C | sample | Hm Drgbl | Hm Drgbl | Hm Drgbl | CPE002DV | CPE002EM | CPE002FO | NM 17307 | NM 1730 | NM 1730 | CPO | CPO | CPO | carboxypeptidase | carboxype | carboxype | 130749 | 130749 | 130749 | s223286 | s43538 | s43539 |
| 2524 | J12 | J13 | 1535 | 635,8 | 20383 | 338,1 | 13,3 | 1,047 | H5A034 C | sample | Hm Drgbl | Hm Drgbl | Hm Drgbl | CPE002DC | CPE002EE | CPE002F4 | NM 00292 | NM 0029 | NM 0029 | GRK1 | GRK1 | GRK1 | G protein-couplec | G protein | G protein | 6011 | 6011 | 6011 | s12025 | s12026 | s12027 |
| 2646 | O14 | O15 | 1636 | 709,1 | 21736 | 405,9 | 13,3 | 1,047 | H5A034 C | sample | Hm Drgbl | Hm Drgbl | Hm Drgbl | CPE002DC | CPE002EE | CPE002F4 | NM 03224 | NM 0322 | NM 0322 | PLXNA1 | PLXNA1 | PLXNA1 | plexin A1 | plexin A1 | plexin A1 | 5361 | 5361 | 5361 | s10696 | s10697 | s10698 |
| 2649 | O17 | O18 | 1221 | 724,7 | 16201 | 388,3 | 13,3 | 1,047 | H5A034 C | sample | Hm Drgbl | Hm Drgbl | Hm Drgbl | CPE002DC | CPE002EE | CPE002F4 | NM 00171 | NM 0017 | NM 0017 | BLK | BLK | BLK | B lymphoid tyrosi | B lymphoi | B lymphoi | 640 | 640 | 640 | s1994 | s1995 | s1996 |
| 16847 | N8 | N9 | 1448 | 965,1 | 19310 | 303,1 | 13,3 | 1,051 | H5A034 C | sample | Hm Gen E | Hm Gen E | Hm Gen E | CPE002JH | CPE002NV | CPE002KC | NM 14503 | NM 1450 | NM 1450 | MGC1638 | MGC1638 | MGC1638 | hypothetical prot | hypotheti | hypotheti | 92806 | 92806 | 92806 | s41028 | s41029 | s41030 |
| 17749 | D20 | D21 | 1670 | 1020 | 22184 | 213,7 | 13,3 | 1,051 | H5A034 C | sample | Hm Gen E | Hm Gen E | Hm Gen E | CPE002JK | CPE002NZ | CPE002KF | NM 14470 | NM 1447 | NM 1447 | PUS10 | PUS10 | PUS10 | pseudouridylate s | pseudour | pseudour | 150962 | 150962 | 150962 | s45503 | s45504 | s45505 |
| 17756 | D5 | D6 | 1116 | 1027 | 14897 | 358,6 | 13,3 | 1,051 | H5A034 C | sample | Hm Gen E | Hm Gen E | Hm Gen E | CPE002JK | CPE002NZ | CPE002KF | XM 37087 | XM 3708 | XM 3708 | LOC14583 | LOC14583 | LOC14583 | hypothetical prot | hypotheti | hypotheti | 145837 | 145837 | 145837 | s44783 | s44784 | s44785 |
| 17803 | F4 | F5 | 1082 | 957,3 | 14393 | 246,4 | 13,3 | 1,051 | H5A034 C | sample | Hm Gen E | Hm Gen E | Hm Gen E | CPE002JK | CPE002NZ | CPE002KF | XM 00113 | XM 0011 | XM 0011 | FAM132B | FAM132B | FAM132B | family with seque | family wit | family wit | 151176 | 151176 | 151176 | s45523 | s45524 | s225739 |
| 17874 | I3 | I4 | 1080 | 1006 | 14372 | 260,2 | 13,3 | 1,051 | H5A034 C | sample | Hm Gen E | Hm Gen E | Hm Gen E | CPE002JK | CPE002NZ | CPE002KF | NM 15370 | NM 1537 | NM 1537 | KDELC2 | KDELC2 | KDELC2 | KDEL (Lys-Asp-Glu | KDEL (Lys- | KDEL (Lys- | 143888 | 143888 | 143888 | s44583 | s44584 | s44585 |
| 17967 | M22 | M23 | 1632 | 1061 | 21744 | 286 | 13,3 | 1,051 | H5A034 C | sample | Hm Gen E | Hm Gen E | Hm Gen E | CPE002JK | CPE002NZ | CPE002KF | NM 00101 | NM 0010 | NM 0010 | YDJC | YDJC | YDJC | YdiC homolog (ba | YdiC hom | YdiC hom | 150223 | 150223 | 150223 | s45397 | s195779 | s195780 |
| 17998 | N7 | N8 | 1444 | 1122 | 19247 | 261,6 | 13,3 | 1,051 | H5A034 C | sample | Hm Gen E | Hm Gen E | Hm Gen E | CPE002JK | CPE002NZ | CPE002KF | NM 00100 | NM 0010 | NM 0010 | C18orf25 | C18orf25 | C18orf25 | chromosome 18 c | chromoso | chromoso | 147339 | 147339 | 147339 | s44985 | s44986 | s44987 |
| 9022 | H7 | H8 | 1476 | 1642 | 19633 | 137,3 | 13,3 | 1,068 | H5A034 C | sample | Hm Drgbl | Hm Drgbl | Hm Drgbl | CPE002E7 | CPE002ES | CPE002FD | NM 01658 | NM 0165 | NM 0165 | ECSIT | ECSIT | ECSIT | ECSIT homolog (D | ECSIT hon | ECSIT hon | 51295 | 51295 | 51295 | s27874 | s224197 | s27873 |
| 9084 | K2 | K3 | 1419 | 1561 | 18909 | 142,6 | 13,3 | 1,068 | H5A034 C | sample | Hm Drgbl | Hm Drgbl | Hm Drgbl | CPE002E7 | CPE002ES | CPE002FD | NM 01742 | NM 0174 | NM 0174 | COQ3 | COQ3 | COQ3 | coenzyme Q3 hor | coenzyme | coenzyme | 51805 | 51805 | 51805 | s28667 | s28668 | s28666 |
| 9168 | N9 | N10 | 1266 | 1620 | 16786 | 128,8 | 13,3 | 1,068 | H5A034 C | sample | Hm Drgbl | Hm Drgbl | Hm Drgbl | CPE002E7 | CPE002ES | CPE002FD | NM 01630 | NM 0163 | NM 0163 | PRRX2 | PRRX2 | PRRX2 | paired related ho | paired rel | paired rel | 51450 | 51450 | 51450 | s195224 | s224209 | s195223 |
| 4246 | A7 | A8 | 1069 | 801,6 | 14240 | 340,5 | 13,3 | 1,122 | H5A034 C | sample | Hm Drgbl | Hm Drgbl | Hm Drgbl | CPE002DL | CPE002EK | CPE002FQ | NM 00128 | NM 0012 | NM 0012 | CLIC1 | CLIC1 | CLIC1 | chloride intracellu | chloride in | chloride in | 1192 | 1192 | 1192 | s635 | s636 | s223253 |
| 4395 | H11 | H12 | 1160 | 729,2 | 15431 | 416,8 | 13,3 | 1,122 | H5A034 C | sample | Hm Drgbl | Hm Drgbl | Hm Drgbl | CPE002DL | CPE002EK | CPE002FQ | NM 00223 | NM 0022 | NM 0022 | KCNG1 | KCNG1 | KCNG1 | potassium voltag | potassium | potassium | 3755 | 3755 | 3755 | s7727 | s7728 | s194568 |
| 4519 | M15 | M16 | 1168 | 693,4 | 15528 | 384,9 | 13,3 | 1,122 | H5A034 C | sample | Hm Drgbl | Hm Drgbl | Hm Drgbl | CPE002DL | CPE002EK | CPE002FQ | NM 00080 | NM 0008 | NM 0008 | GABRA4 | GABRA4 | GABRA4 | gamma-aminobut | gamma-ar | gamma-ar | 2557 | 2557 | 2557 | s5479 | s5480 | s5481 |
| 4563 | O11 | O12 | 1498 | 719,9 | 19926 | 539,9 | 13,3 | 1,122 | H5A034 C | sample | Hm Drgbl | Hm Drgbl | Hm Drgbl | CPE002DL | CPE002EK | CPE002FQ | NM 00081 | NM 0008 | NM 0008 | GABRG2 | GABRG2 | GABRG2 | gamma-aminobut | gamma-ar | gamma-ar | 2566 | 2566 | 2566 | s5506 | s5507 | s5508 |
| 172 | H12 | H13 | 552 | 1565 | 7343 | 550,7 | 13,3 | 1,132 | H5A034 C | sample | Hm Drgbl | Hm Drgbl | Hm Drgbl | CPE002DT | CPE002EJ | CPE002F9 | NM 00314 | NM 0031 | NM 0031 | SRY | SRY | SRY | sex determining r | sex deterr | sex deterr | 6736 | 6736 | 6736 | s13459 | s13460 | s13461 |
| 1922 | A10 | A11 | 1320 | 655,3 | 17537 | 509,3 | 13,3 | 1,157 | H5A034 C | sample | Hm Drgbl | Hm Drgbl | Hm Drgbl | CPE002FX | CPE002G1 | CPE002G5 | NM 13870 | NM 1387 | NM 1387 | CALML6 | CALML6 | CALML6 | calmodulin-like 6 | calmoduli | calmoduli | 163688 | 163688 | 163688 | s46468 | s46469 | s46470 |
| 2233 | N1 | N2 | 1088 | 646,6 | 14502 | 437,1 | 13,3 | 1,157 | H5A034 C | sample | Hm Drgbl | Hm Drgbl | Hm Drgbl | CPE002FX | CPE002G1 | CPE002G5 | NM 00100 | NM 0010 | NM 0010 | ZNF783 | ZNF783 | ZNF783 | zinc finger protei | zinc finge | zinc finge | 155060 | 155060 | 155060 | s45917 | s45918 | s45919 |
| 2280 | O9 | O10 | 1255 | 688,8 | 16697 | 538 | 13,3 | 1,157 | H5A034 C | sample | Hm Drgbl | Hm Drgbl | Hm Drgbl | CPE002FX | CPE002G1 | CPE002G5 | NM 17587 | NM 1758 | NM 1758 | ZNF792 | ZNF792 | ZNF792 | zinc finger protei | zinc finge | zinc finge | 126375 | 126375 | 126375 | s43023 | s43024 | s43025 |
| 6879 | O22 | O23 | 1424 | 601,5 | 18945 | 497,6 | 13,3 | 1,167 | H5A034 C | sample | Hm Drgbl | Hm Drgbl | Hm Drgbl | CPE002E1 | CPE002EY | CPE002FJ | NM 00088 | NM 0008 | NM 0008 | ITGA4 | ITGA4 | ITGA4 | integrin, alpha 4 ( | integrin, a | integrin, a | 3676 | 3676 | 3676 | s7544 | s7546 | s7545 |
| 3855 | A22 | A23 | 1424 | 672,7 | 19006 | 401,9 | 13,3 | 1,182 | H5A034 C | sample | Hm Drgbl | Hm Drgbl | Hm Drgbl | CPE002DS | CPE002EI | CPE002F8 | NM 00286 | NM 0028 | NM 0028 | PYGL | PYGL | PYGL | phosphorylase, gl | phosphor | phosphor | 5836 | 5836 | 5836 | s11642 | s11643 | s11644 |
| 3958 | E7 | E8 | 1180 | 674,4 | 15667 | 390,8 | 13,3 | 1,182 | H5A034 C | sample | Hm Drgbl | Hm Drgbl | Hm Drgbl | CPE002DS | CPE002EI | CPE002F8 | NM 00266 | NM 0026 | NM 0026 | PLGLB2 | PLGLB2 | PLGLB2 | plasminogen-like | plasmino | plasmino | 5342 | 5342 | 5342 | s10652 | s10653 | s10654 |
| 4079 | J8 | J9 | 711 | 576,4 | 9477 | 267,6 | 13,3 | 1,182 | H5A034 C | sample | Hm Drgbl | Hm Drgbl | Hm Drgbl | CPE002DS | CPE002EI | CPE002F8 | NM 00294 | NM 0029 | NM 0029 | RPA2 | RPA2 | RPA2 | replication protei | replicatio | replicatio | 6118 | 6118 | 6118 | s12132 | s12131 | s12130 |
| 11769 | K17 | K18 | 1730 | 940 | 22929 | 153,8 | 13,3 | 1,188 | H5A034 C | sample | Hm Gen E | Hm Gen E | Hm Gen E | CPE002JA | CPE002NJ | CPE002JZ | NM 00104 | NM 0010 | NM 0010 | MPPED1 | MPPED1 | MPPED1 | metallophosphoe | metalloph | metalloph | 758 | 758 | 758 | s2241 | s2242 | s228158 |
| 11851 | N4 | N5 | 1052 | 796,8 | 13946 | 129 | 13,3 | 1,188 | H5A034 C | sample | Hm Gen E | Hm Gen E | Hm Gen E | CPE002JA | CPE002NJ | CPE002JZ | NM 00496 | NM 0049 | NM 0049 | ICA1 | ICA1 | ICA1 | islet cell autoanti | islet cell a | islet cell a | 3382 | 3382 | 3382 | s7084 | s7085 | s226292 |
| 5884 | F12 | F13 | 1338 | 849,4 | 17758 | 584 | 13,3 | 1,215 | H5A034 C | sample | Hm Drgbl | Hm Drgbl | Hm Drgbl | CPE002DY | CPE002F1 | CPE002FM | NM 00172 | NM 0017 | NM 0017 | POLR3D | POLR3D | POLR3D | polymerase (RNA | polymera | polymera | 661 | 661 | 661 | s2050 | s2051 | s2052 |
| 6082 | N18 | N19 | 1805 | 763,5 | 24048 | 607,3 | 13,3 | 1,215 | H5A034 C | sample | Hm Drgbl | Hm Drgbl | Hm Drgbl | CPE002DY | CPE002F1 | CPE002FM | NM 00435 | NM 0043 | NM 0043 | RUNX3 | RUNX3 | RUNX3 | runt-related trans | runt-relat | runt-relat | 864 | 864 | 864 | s2467 | s2468 | s2469 |
| 6103 | O15 | O16 | 1120 | 769,6 | 14893 | 543,6 | 13,3 | 1,215 | H5A034 C | sample | Hm Drgbl | Hm Drgbl | Hm Drgbl | CPE002DY | CPE002F1 | CPE002FM | NM 00162 | NM 0016 | NM 0016 | ALCAM | ALCAM | ALCAM | activated leukocy | activated | activated | 214 | 214 | 214 | s1230 | s1231 | s1232 |
| 1207 | C15 | C16 | 1100 | 667,9 | 14669 | 274,6 | 13,3 | 1,22 | H5A034 C | sample | Hm Drgbl | Hm Drgbl | Hm Drgbl | CPE002FV | CPE002FZ | CPE002G3 | NM 00695 | NM 0069 | NM 0069 | ZNF17 | ZNF17 | ZNF17 | zinc finger protei | zinc finge | zinc finge | 7565 | 7565 | 7565 | s15036 | s15037 | s15038 |
| 1258 | E18 | E19 | 1211 | 842,1 | 16049 | 483,6 | 13,3 | 1,22 | H5A034 C | sample | Hm Drgbl | Hm Drgbl | Hm Drgbl | CPE002FV | CPE002FZ | CPE002G3 | NM 01481 | NM 0148 | NM 0148 | PJA2 | PJA2 | PJA2 | praja 2, RING-H2 | praja 2, RI | praja 2, RI | 9867 | 9867 | 9867 | s19104 | s19105 | s19106 |
| 1425 | L17 | L18 | 1591 | 683,2 | 21169 | 382,8 | 13,3 | 1,22 | H5A034 C | sample | Hm Drgbl | Hm Drgbl | Hm Drgbl | CPE002FV | CPE002FZ | CPE002G3 | NM 00357 | NM 0035 | NM 0035 | VAPA | VAPA | VAPA | VAMP (vesicle-ass | VAMP (ve | VAMP (ve | 9218 | 9218 | 9218 | s17626 | s17627 | s17628 |
| 1438 | L7 | L8 | 1166 | 722,4 | 15489 | 373,4 | 13,3 | 1,22 | H5A034 C | sample | Hm Drgbl | Hm Drgbl | Hm Drgbl | CPE002FV | CPE002FZ | CPE002G3 | NM 00468 | NM 0046 | NM 0046 | RGN | RGN | RGN | regucalcin (senes | regucalcin | regucalcin | 9104 | 9104 | 9104 | s17374 | s17375 | s17376 |
| 1475 | N19 | N20 | 1539 | 683,6 | 20421 | 444,2 | 13,3 | 1,22 | H5A034 C | sample | Hm Drgbl | Hm Drgbl | Hm Drgbl | CPE002FV | CPE002FZ | CPE002G3 | NM 00482 | NM 0048 | NM 0048 | NTN1 | NTN1 | NTN1 | netrin 1 | netrin 1 | netrin 1 | 9423 | 9423 | 9423 | s18041 | s18042 | s18043 |
| 16326 | I14 | I15 | 1221 | 945 | 16201 | 287,7 | 13,3 | 1,22 | H5A034 C | sample | Hm Gen E | Hm Gen E | Hm Gen E | CPE002JG | CPE002NV | CPE002KB | NM 03233 | NM 0323 | NM 0323 | MORG1 | MORG1 | MORG1 | mitogen-activatec | mitogen-a | mitogen-a | 84292 | 84292 | 84292 | s38837 | s38838 | s228351 |
| 16374 | K14 | K15 | 1612 | 1016 | 21434 | 327,2 | 13,3 | 1,22 | H5A034 C | sample | Hm Gen E | Hm Gen E | Hm Gen E | CPE002JG | CPE002NV | CPE002KB | NM 03234 | NM 0323 | NM 0323 | PDCD2L | PDCD2L | PDCD2L | programmed cell | programm | programm | 84306 | 84306 | 84306 | s38876 | s38878 | s228356 |
| 16401 | L17 | L18 | 1442 | 987,7 | 19240 | 352,4 | 13,3 | 1,22 | H5A034 C | sample | Hm Gen E | Hm Gen E | Hm Gen E | CPE002JG | CPE002NV | CPE002KB | NM 03213 | NM 0321 | NM 0321 | ANKRD27 | ANKRD27 | ANKRD27 | ankyrin repeat dc | ankyrin re | ankyrin re | 84079 | 84079 | 84079 | s38477 | s38478 | s38479 |
| 770 | A10 | A11 | 1626 | 760,9 | 21670 | 439,2 | 13,3 | 1,261 | H5A034 C | sample | Hm Drgbl | Hm Drgbl | Hm Drgbl | CPE002FU | CPE002FY | CPE002G2 | NM 00235 | NM 0023 | NM 0023 | MXD1 | MXD1 | MXD1 | MAX dimerization | MAX dime | MAX dime | 4084 | 4084 | 4084 | s8388 | s8389 | s8390 |
| 835 | C4 | C5 | 1163 | 697 | 15453 | 507,9 | 13,3 | 1,261 | H5A034 C | sample | Hm Drgbl | Hm Drgbl | Hm Drgbl | CPE002FU | CPE002FY | CPE002G2 | NM 03058 | NM 0305 | NM 0305 | MATN2 | MATN2 | MATN2 | matrilin 2 | matrilin 2 | matrilin 2 | 4147 | 4147 | 4147 | s8532 | s8533 | s8534 |
| 890 | F10 | F11 | 1549 | 734 | 20669 | 399,2 | 13,3 | 1,261 | H5A034 C | sample | Hm Drgbl | Hm Drgbl | Hm Drgbl | CPE002FU | CPE002FY | CPE002G2 | NM 00598 | NM 0059 | NM 0059 | S100P | S100P | S100P | S100 calcium binc | S100 calci | S100 calci | 6286 | 6286 | 6286 | s12442 | s224565 | s224566 |
| 980 | I5 | I6 | 949 | 701,2 | 12606 | 347,9 | 13,3 | 1,261 | H5A034 C | sample | Hm Drgbl | Hm Drgbl | Hm Drgbl | CPE002FU | CPE002FY | CPE002G2 | NM 00436 | NM 0043 | NM 0043 | CLGN | CLGN | CLGN | calmegin | calmegin | calmegin | 1047 | 1047 | 1047 | s2882 | s2883 | s2884 |
| 1132 | P12 | P13 | 1620 | 771,8 | 21506 | 516,4 | 13,3 | 1,261 | H5A034 C | sample | Hm Drgbl | Hm Drgbl | Hm Drgbl | CPE002FU | CPE002FY | CPE002G2 | NM 18266 | NM 1826 | NM 1826 | UTY | UTY | UTY | ubiquitously tran | ubiquitou | ubiquitou | 7404 | 7404 | 7404 | s14738 | s14739 | s14740 |
| 1135 | P15 | P16 | 1679 | 771,6 | 22351 | 367,9 | 13,3 | 1,261 | H5A034 C | sample | Hm Drgbl | Hm Drgbl | Hm Drgbl | CPE002FU | CPE002FY | CPE002G2 | NM 00022 | NM 0002 | NM 0002 | LAMB3 | LAMB3 | LAMB3 | laminin, beta 3 | laminin, b | laminin, b | 3914 | 3914 | 3914 | s8074 | s223999 | s8076 |
| 5378 | A10 | A11 | 1588 | 760,4 | 21091 | 644,9 | 13,3 | 1,267 | H5A034 C | sample | Hm Drgbl | Hm Drgbl | Hm Drgbl | CPE002DX | CPE002F2 | CPE002FN | NM 00386 | NM 0038 | NM 0038 | DPM2 | DPM2 | DPM2 | dolichyl-phospha | dolichyl-p | dolichyl-p | 8818 | 8818 | 8818 | s16816 | s16817 | s16818 |
| 5454 | D14 | D15 | 1743 | 595,8 | 23097 | 486,8 | 13,3 | 1,267 | H5A034 C | sample | Hm Drgbl | Hm Drgbl | Hm Drgbl | CPE002DX | CPE002F2 | CPE002FN | NM 19900 | NM 1990 | NM 1990 | ARHGEF1 | ARHGEF1 | ARHGEF1 | Rho guanine nucl | Rho guani | Rho guani | 9138 | 9138 | 9138 | s17458 | s17459 | s17460 |
| 5591 | I8 | I9 | 1842 | 781,9 | 24570 | 432,2 | 13,3 | 1,267 | H5A034 C | sample | Hm Drgbl | Hm Drgbl | Hm Drgbl | CPE002DX | CPE002F2 | CPE002FN | NM 00466 | NM 0046 | NM 0046 | HERC2 | HERC2 | HERC2 | hect domain and | hect dom | hect dom | 8924 | 8924 | 8924 | s17064 | s17062 | s17063 |
| 5630 | K21 | K22 | 1583 | 624 | 21103 | 565,2 | 13,3 | 1,267 | H5A034 C | sample | Hm Drgbl | Hm Drgbl | Hm Drgbl | CPE002DX | CPE002F2 | CPE002FN | NM 00364 | NM 0036 | NM 0036 | GCM1 | GCM1 | GCM1 | glial cells missing | glial cells | glial cells | 8521 | 8521 | 8521 | s16199 | s16197 | s16198 |
| 5642 | L10 | L11 | 1456 | 638,4 | 19303 | 492,5 | 13,3 | 1,267 | H5A034 C | sample | Hm Drgbl | Hm Drgbl | Hm Drgbl | CPE002DX | CPE002F2 | CPE002FN | NM 00545 | NM 0054 | NM 0054 | WDR46 | WDR46 | WDR46 | WD repeat domai | WD repea | WD repea | 9277 | 9277 | 9277 | s17741 | s17739 | s17740 |
| 3597 | F6 | F7 | 1566 | 1343 | 20782 | 354,4 | 13,3 | 1,279 | H5A034 C | sample | Hm Drgbl | Hm Drgbl | Hm Drgbl | CPE002DR | CPE002EH | CPE002F7 | NM 02214 | NM 0221 | NM 0221 | NPFFR1 | NPFFR1 | NPFFR1 | neuropeptide FF r | neuropep | neuropep | 64106 | 64106 | 64106 | s34448 | s34449 | s195342 |
| 3638 | H21 | H22 | 1303 | 1345 | 17382 | 386,1 | 13,3 | 1,279 | H5A034 C | sample | Hm Drgbl | Hm Drgbl | Hm Drgbl | CPE002DR | CPE002EH | CPE002F7 | NM 02230 | NM 0223 | NM 0223 | HRH2 | HRH2 | HRH2 | histamine recepto | histamine | histamine | 3274 | 3274 | 3274 | s6911 | s6912 | s6913 |
| 8125 | C20 | C21 | 1328 | 614,2 | 17662 | 429,9 | 13,3 | 1,279 | H5A034 C | sample | Hm Drgbl | Hm Drgbl | Hm Drgbl | CPE002E5 | CPE002EU | CPE002FF | NM 01509 | NM 0150 | NM 0150 | NFASC | NFASC | NFASC | neurofascin homo | neurofasc | neurofasc | 23114 | 23114 | 23114 | s23065 | s23066 | s23064 |
| 8355 | M11 | M12 | 994 | 577,6 | 13267 | 525,9 | 13,3 | 1,279 | H5A034 C | sample | Hm Drgbl | Hm Drgbl | Hm Drgbl | CPE002E5 | CPE002EU | CPE002FF | NM 00722 | NM 0072 | NM 0072 | CA5B | CA5B | CA5B | carbonic anhydra | carbonic a | carbonic a | 11238 | 11238 | 11238 | s22186 | s22184 | s22185 |
| 10148 | G5 | G6 | 1471 | 847,2 | 19566 | 176,7 | 13,3 | 1,279 | H5A034 C | sample | Hm Drgbl | Hm Drgbl | Hm Drgbl | CPE002EA | CPE002EP | CPE002FA | NM 02472 | NM 0247 | NM 0247 | ACBD4 | ACBD4 | ACBD4 | acyl-Coenzyme A | acyl-Coen | acyl-Coen | 79777 | 79777 | 79777 | s224830 | s36303 | s36304 |
| 10164 | H2 | H3 | 1036 | 806,9 | 13791 | 118,7 | 13,3 | 1,279 | H5A034 C | sample | Hm Drgbl | Hm Drgbl | Hm Drgbl | CPE002EA | CPE002EP | CPE002FA | NM 02462 | NM 0246 | NM 0246 | SLC12A8 | SLC12A8 | SLC12A8 | solute carrier fam | solute car | solute car | 84561 | 84561 | 84561 | s39159 | s39160 | s39161 |
| 10363 | P4 | P5 | 1372 | 769,1 | 18241 | 206,7 | 13,3 | 1,279 | H5A034 C | sample | Hm Drgbl | Hm Drgbl | Hm Drgbl | CPE002EA | CPE002EP | CPE002FA | NM 03273 | NM 0327 | NM 0327 | FCRLA | FCRLA | FCRLA | Fc receptor-like A | Fc recepto | Fc recepto | 84824 | 84824 | 84824 | s39479 | s39481 | s39480 |
| 1580 | B5 | B6 | 1286 | 729,9 | 17138 | 467,7 | 13,3 | 1,393 | H5A034 C | sample | Hm Drgbl | Hm Drgbl | Hm Drgbl | CPE002FW | CPE002G0 | CPE002G4 | NM 01653 | NM 0165 | NM 0165 | ZNF581 | ZNF581 | ZNF581 | zinc finger protei | zinc finge | zinc finge | 51545 | 51545 | 51545 | s28300 | s28301 | s28302 |
| 1728 | H9 | H10 | 1095 | 697,9 | 14520 | 372,4 | 13,3 | 1,393 | H5A034 C | sample | Hm Drgbl | Hm Drgbl | Hm Drgbl | CPE002FW | CPE002G0 | CPE002G4 | NM 13911 | NM 1391 | NM 1391 | YY1AP1 | YY1AP1 | YY1AP1 | YY1 associated pr | YY1 assoc | YY1 assoc | 55249 | 55249 | 55249 | s30548 | s30549 | s30550 |
| 1908 | P2 | P3 | 1663 | 706 | 22187 | 461,2 | 13,3 | 1,393 | H5A034 C | sample | Hm Drgbl | Hm Drgbl | Hm Drgbl | CPE002FW | CPE002G0 | CPE002G4 | NM 03268 | NM 0326 | NM 0326 | ZNF607 | ZNF607 | ZNF607 | zinc finger protei | zinc finge | zinc finge | 84775 | 84775 | 84775 | s39423 | s39424 | s39425 |
| 21533 | B13 | B14 | 255 | 1168 | 3404 | 721,8 | 13,3 | 1,4 | H5A034 C | sample | Hm Gen E | Hm Gen E | Hm Gen E | CPE002JU | CPE002O9 | CPE002KP | XM 93985 | XM 9398 | XM 9398 | LOC65078 | LOC65078 | LOC65078 | similar to Immun | similar to | similar to | 650780 | 650780 | 650780 | s57123 | s57124 | s57125 |
| 21651 | G11 | G12 | 349 | 982,4 | 4634 | 580,2 | 13,3 | 1,4 | H5A034 C | sample | Hm Gen E | Hm Gen E | Hm Gen E | CPE002JU | CPE002O9 | CPE002KP | XM 93866 | XM 9386 | XM 9386 | LOC64959 | LOC64959 | LOC64959 | similar to CG3190 | similar to | similar to | 649597 | 649597 | 649597 | s56981 | s56982 | s227323 |
| 4951 | O15 | O16 | 1187 | 797,4 | 15728 | 549,7 | 13,3 | 1,462 | H5A034 C | sample | Hm Drgbl | Hm Drgbl | Hm Drgbl | CPE002DV | CPE002EL | CPE002FP | NM 00013 | NM 0001 | NM 0001 | F9 | F9 | F9 | coagulation facto | coagulatic | coagulatic | 2158 | 2158 | 2158 | s4943 | s4944 | s4945 |
| 10390 | A7 | A8 | 1361 | 908 | 18078 | 232 | 13,3 | 1,503 | H5A034 C | sample | Hm Drgbl | Hm Drgbl | Hm Drgbl | CPE002EB | CPE002EO | CPE002FR | NM 03279 | NM 0327 | NM 0327 | AIFM2 | AIFM2 | AIFM2 | apoptosis-inducir | apoptosis | apoptosis | 84883 | 84883 | 84883 | s39570 | s39571 | s39572 |
| 10477 | E20 | E21 | 1349 | 842,8 | 17877 | 200,7 | 13,3 | 1,503 | H5A034 C | sample | Hm Drgbl | Hm Drgbl | Hm Drgbl | CPE002EB | CPE002EO | CPE002FR | NM 13844 | NM 1384 | NM 1384 | VASN | VASN | VASN | vasorin | vasorin | vasorin | 114990 | 114990 | 114990 | s41742 | s41740 | s41741 |
| 10639 | L15 | L16 | 1059 | 800 | 14123 | 185 | 13,3 | 1,503 | H5A034 C | sample | Hm Drgbl | Hm Drgbl | Hm Drgbl | CPE002EB | CPE002EO | CPE002FR | NM 05304 | NM 0530 | NM 0530 | EGLN2 | EGLN2 | EGLN2 | egl nine homolog | egl nine h | egl nine h | 112398 | 112398 | 112398 | s223212 | s274 | s276 |
| 10674 | M3 | M4 | 879 | 813,2 | 11684 | 182,7 | 13,3 | 1,503 | H5A034 C | sample | Hm Drgbl | Hm Drgbl | Hm Drgbl | CPE002EB | CPE002EO | CPE002FR | NM 20152 | NM 2015 | NM 2015 | KLC4 | KLC4 | KLC4 | kinesin light chair | kinesin lig | kinesin lig | 89953 | 89953 | 89953 | s40215 | s40216 | s40217 |
| 19990 | A7 | A8 | 772 | 1172 | 10245 | 325,5 | 13,3 | 1,622 | H5A034 C | sample | Hm Gen E | Hm Gen E | Hm Gen E | CPE002JQ | CPE002O5 | CPE002KL | NM 00100 | NM 0010 | NM 0010 | OR3A4 | OR3A4 | OR3A4 | olfactory recepto | olfactory | olfactory | 390756 | 390756 | 390756 | s52858 | s196341 | s196342 |
| 20146 | H18 | H19 | 884 | 1049 | 11785 | 179,8 | 13,3 | 1,622 | H5A034 C | sample | Hm Gen E | Hm Gen E | Hm Gen E | CPE002JQ | CPE002O5 | CPE002KL | NM 20348 | NM 2034 | NM 2034 | MGC7087 | MGC7087 | MGC7087 | hypothetical LOC | hypotheti | hypotheti | 403340 | 403340 | 403340 | s53840 | s53841 | s53842 |
| 386 | A10 | A11 | 1737 | 1821 | 23097 | 493,6 | 13,3 | 1,942 | H5A034 C | sample | Hm Drgbl | Hm Drgbl | Hm Drgbl | CPE002E0 | CPE002EZ | CPE002FK | NM 00525 | NM 0052 | NM 0052 | FOXL1 | FOXL1 | FOXL1 | forkhead box L1 | forkhead | forkhead | 2300 | 2300 | 2300 | s5235 | s5236 | s5237 |
| 3205 | F20 | F21 | 1146 | 1259 | 15302 | 471,3 | 13,4 | 0,83 | H5A034 C | sample | Hm Drgbl | Hm Drgbl | Hm Drgbl | CPE002DC | CPE002EG | CPE002F6 | NM 01422 | NM 0142 | NM 0142 | PGA5 | PGA5 | PGA5 | pepsinogen 5, grc | pepsinog | pepsinog | 5222 | 5222 | 5222 | s10385 | s10386 | s10384 |
| 3236 | G5 | G6 | 1374 | 1341 | 18433 | 460,8 | 13,4 | 0,83 | H5A034 C | sample | Hm Drgbl | Hm Drgbl | Hm Drgbl | CPE002DC | CPE002EG | CPE002F6 | NM 00442 | NM 0044 | NM 0044 | DUSP8 | DUSP8 | DUSP8 | dual specificity pl | dual speci | dual speci | 1850 | 1850 | 1850 | s223452 | s4385 | s4386 |
| 3253 | H20 | H21 | 1340 | 1259 | 18005 | 352,3 | 13,4 | 0,83 | H5A034 C | sample | Hm Drgbl | Hm Drgbl | Hm Drgbl | CPE002DC | CPE002EG | CPE002F6 | NM 01885 | NM 0188 | NM 0188 | ABCB4 | ABCB4 | ABCB4 | ATP-binding casse | ATP-bindi | ATP-bindi | 5244 | 5244 | 5244 | s10421 | s10422 | s10423 |
| 3357 | L6 | L7 | 1432 | 1297 | 19164 | 393,9 | 13,4 | 0,83 | H5A034 C | sample | Hm Drgbl | Hm Drgbl | Hm Drgbl | CPE002DC | CPE002EG | CPE002F6 | NM 00502 | NM 0050 | NM 0050 | SERPINB1C | SERPINB1C | SERPINB1C | serpin peptidase | serpin pei | serpin pei | 5273 | 5273 | 5273 | s10481 | s10482 | s10483 |
| 6368 | J16 | J17 | 1905 | 543 | 25509 | 347,1 | 13,4 | 0,884 | H5A034 C | sample | Hm Drgbl | Hm Drgbl | Hm Drgbl | CPE002DZ | CPE002F0 | CPE002FL | NM 00100 | NM 0010 | NM 0010 | DNASE1L1 | DNASE1L1 | DNASE1L1 | deoxyribonucleas | deoxyribo | deoxyribo | 1774 | 1774 | 1774 | s4188 | s4189 | s223431 |
| 6436 | M12 | M13 | 566 | 688,5 | 7593 | 210,8 | 13,4 | 0,884 | H5A034 C | sample | Hm Drgbl | Hm Drgbl | Hm Drgbl | CPE002DZ | CPE002F0 | CPE002FL | NM 00134 | NM 0013 | NM 0013 | DARS | DARS | DARS | aspartyl-tRNA syr | aspartyl-t | aspartyl-t | 1615 | 1615 | 1615 | s3932 | s223380 | s3933 |
| 6452 | M5 | M6 | 806 | 617,2 | 10812 | 552,4 | 13,4 | 0,884 | H5A034 C | sample | Hm Drgbl | Hm Drgbl | Hm Drgbl | CPE002DZ | CPE002F0 | CPE002FL | NM 00493 | NM 0049 | NM 0049 | CDH18 | CDH18 | CDH18 | cadherin 18, type | cadherin | cadherin | 1016 | 1016 | 1016 | s2817 | s2818 | s2816 |
| 6474 | N3 | N4 | 1471 | 535,3 | 19706 | 441,2 | 13,4 | 0,884 | H5A034 C | sample | Hm Drgbl | Hm Drgbl | Hm Drgbl | CPE002DZ | CPE002F0 | CPE002FL | NM 00100 | NM 0010 | NM 0010 | CR2 | CR2 | CR2 | complement com | compleme | compleme | 1380 | 1380 | 1380 | s3477 | s3478 | s3479 |
| 6483 | O11 | O12 | 1756 | 552,5 | 23600 | 488,6 | 13,4 | 0,884 | H5A034 C | sample | Hm Drgbl | Hm Drgbl | Hm Drgbl | CPE002DZ | CPE002F0 | CPE002FL | NM 00519 | NM 0051 | NM 0051 | CEBPB | CEBPB | CEBPB | CCAAT/enhancer | CCAAT/en | CCAAT/en | 1051 | 1051 | 1051 | s2891 | s2892 | s2893 |
| 12882 | I3 | I4 | 1245 | 1069 | 16652 | 123,2 | 13,4 | 0,908 | H5A034 C | sample | Hm Gen E | Hm Gen E | Hm Gen E | CPE002J6 | CPE002NN | CPE002K2 | NM 13850 | NM 1385 | NM 1385 | GPSN2 | GPSN2 | GPSN2 | glycoprotein, syna | glycoprote | glycoprote | 9524 | 9524 | 9524 | s18269 | s18270 | s228511 |
| 12930 | K3 | K4 | 1204 | 1005 | 16116 | 144,5 | 13,4 | 0,908 | H5A034 C | sample | Hm Gen E | Hm Gen E | Hm Gen E | CPE002J6 | CPE002NN | CPE002K2 | NM 00485 | NM 0048 | NM 0048 | C14orf2 | C14orf2 | C14orf2 | chromosome 14 c | chromoso | chromoso | 9556 | 9556 | 9556 | s18353 | s18354 | s228514 |
| 17318 | B21 | B22 | 1473 | 1054 | 19796 | 238,9 | 13,4 | 0,915 | H5A034 C | sample | Hm Gen E | Hm Gen E | Hm Gen E | CPE002JJ | CPE002NY | CPE002KE | NM 00100 | NM 0010 | NM 0010 | OR6K6 | OR6K6 | OR6K6 | olfactory recepto | olfactory | olfactory | 128371 | 128371 | 128371 | s43299 | s43300 | s43301 |
| 9522 | M3 | M4 | 1685 | 1090 | 22556 | 124,3 | 13,4 | 0,931 | H5A034 C | sample | Hm Drgbl | Hm Drgbl | Hm Drgbl | CPE002E8 | CPE002ER | CPE002FC | NM 01819 | NM 0181 | NM 0181 | TMLHE | TMLHE | TMLHE | trimethyllysine hy | trimethyll | trimethyll | 55217 | 55217 | 55217 | s30467 | s30468 | s30469 |
| 9854 | K21 | K22 | 2038 | 1131 | 27240 | 203,4 | 13,4 | 0,937 | H5A034 C | sample | Hm Drgbl | Hm Drgbl | Hm Drgbl | CPE002E9 | CPE002EQ | CPE002FB | NM 02084 | NM 0208 | NM 0208 | PITPNM2 | PITPNM2 | PITPNM2 | phosphatidylinos | phosphat | phosphat | 57605 | 57605 | 57605 | s33423 | s224477 | s33421 |
| 8572 | F12 | F13 | 1904 | 1072 | 25575 | 105,3 | 13,4 | 0,947 | H5A034 C | sample | Hm Drgbl | Hm Drgbl | Hm Drgbl | CPE002E6 | CPE002ET | CPE002FE | NM 01416 | NM 0141 | NM 0141 | METTL5 | METTL5 | METTL5 | methyltransferase | methyltra | methyltra | 29081 | 29081 | 29081 | s26438 | s26439 | s26440 |
| 8751 | M22 | M23 | 1442 | 1063 | 19357 | 156,3 | 13,4 | 0,947 | H5A034 C | sample | Hm Drgbl | Hm Drgbl | Hm Drgbl | CPE002E6 | CPE002ET | CPE002FE | NM 03296 | NM 0329 | NM 0329 | PCDH11X | PCDH11X | PCDH11X | protocadherin 11 | protocadh | protocadh | 27328 | 27328 | 27328 | s26157 | s26158 | s26159 |
| 19544 | O16 | O17 | 1158 | 1206 | 15534 | 337 | 13,4 | 0,95 | H5A034 C | sample | Hm Gen E | Hm Gen E | Hm Gen E | CPE002JO | CPE002O3 | CPE002KJ | XM 29335 | XM 2933 | XM 2933 | WDR42C | WDR42C | WDR42C | WD repeat domai | WD repea | WD repea | 347442 | 347442 | 347442 | s51250 | s51251 | s51252 |
| 19569 | P17 | P18 | 1420 | 1229 | 19042 | 340,6 | 13,4 | 0,95 | H5A034 C | sample | Hm Gen E | Hm Gen E | Hm Gen E | CPE002JO | CPE002O3 | CPE002KJ | XM 29251 | XM 2925 | XM 2925 | hCG 1646 | hCG 1646 | hCG 1646 | brother of ataxin | brother o | brother o | 342371 | 342371 | 342371 | s50900 | s50901 | s50902 |
| 20791 | C15 | C16 | 1288 | 953,2 | 17195 | 332 | 13,4 | 0,95 | H5A034 C | sample | Hm Gen E | Hm Gen E | Hm Gen E | CPE002JS | CPE002O7 | CPE002KN | XM 93756 | XM 9375 | XM 9375 | FLJ16423 | FLJ16423 | FLJ16423 | hypothetical LOC | hypotheti | hypotheti | 642889 | 642889 | 642889 | s55146 | s55147 | s55148 |
| 21014 | L21 | L22 | 1854 | 969,1 | 24853 | 234,4 | 13,4 | 0,95 | H5A034 C | sample | Hm Gen E | Hm Gen E | Hm Gen E | CPE002JS | CPE002O7 | CPE002KN | XM 93191 | XM 9319 | XM 9319 | hCG 1815 | hCG 1815 | hCG 1815 | hCG1815491 | hCG18154 | hCG18154 | 643911 | 643911 | 643911 | s55495 | s55496 | s55497 |
| 21025 | M1 | M2 | 1434 | 993,3 | 19169 | 212,7 | 13,4 | 0,95 | H5A034 C | sample | Hm Gen E | Hm Gen E | Hm Gen E | CPE002JS | CPE002O7 | CPE002KN | XM 93146 | XM 9314 | XM 9314 | LOC64328 | LOC64328 | LOC64328 | similar to hCG204 | similar to | similar to | 643281 | 643281 | 643281 | s55262 | s55263 | s55264 |
| 11279 | F8 | F9 | 1618 | 822,2 | 21691 | 136,9 | 13,4 | 0,971 | H5A034 C | sample | Hm Drgbl | Hm Drgbl | Hm Drgbl | CPE002ED | CPE002F3 | CPE002FT | | | | | | | | | | | | | | | |
| 11281 | G1 | G2 | 1247 | 783,4 | 16660 | 112,1 | 13,4 | 0,971 | H5A034 C | sample | Hm Drgbl | Hm Drgbl | Hm Drgbl | CPE002ED | CPE002F3 | CPE002FT | NM 17366 | NM 1736 | NM 1736 | ARL10 | ARL10 | ARL10 | ADP-ribosylation | ADP-ribos | ADP-ribos | 285598 | 285598 | 285598 | s49975 | s49976 | s49977 |
| 11314 | H18 | H19 | 1674 | 864,3 | 22422 | 133,8 | 13,4 | 0,971 | H5A034 C | sample | Hm Drgbl | Hm Drgbl | Hm Drgbl | CPE002ED | CPE002F3 | CPE002FT | | | | | | | | | | | | | | | |
| 11353 | J1 | J2 | 1363 | 775,5 | 18238 | 134,5 | 13,4 | 0,971 | H5A034 C | sample | Hm Drgbl | Hm Drgbl | Hm Drgbl | CPE002ED | CPE002F3 | CPE002FT | NM 19851 | NM 1985 | NM 1985 | NHLRC2 | NHLRC2 | NHLRC2 | NHL repeat conta | NHL repea | NHL repea | 374354 | 374354 | 374354 | s51529 | s51530 | s51531 |
| 10930 | H18 | H19 | 1135 | 810,8 | 15211 | 396,7 | 13,4 | 1,015 | H5A034 C | sample | Hm Drgbl | Hm Drgbl | Hm Drgbl | CPE002EC | CPE002EN | CPE002FS | NM 00101 | NM 0010 | NM 0010 | SLC25A30 | SLC25A30 | SLC25A30 | solute carrier fam | solute car | solute car | 253512 | 253512 | 253512 | s48417 | s48418 | s48419 |
| 11007 | K22 | K23 | 1197 | 842,1 | 16004 | 631,6 | 13,4 | 1,015 | H5A034 C | sample | Hm Drgbl | Hm Drgbl | Hm Drgbl | CPE002EC | CPE002EN | CPE002FS | NM 00103 | NM 0010 | NM 0010 | ZBTB7C | ZBTB7C | ZBTB7C | zinc finger and BT | zinc finge | zinc finge | 201501 | 201501 | 201501 | s47371 | s47372 | s47373 |
| 11095 | O15 | O16 | 1808 | 952,6 | 24209 | 625,2 | 13,4 | 1,015 | H5A034 C | sample | Hm Drgbl | Hm Drgbl | Hm Drgbl | CPE002EC | CPE002EN | CPE002FS | NM 00100 | NM 0010 | NM 0010 | B3GNTL1 | B3GNTL1 | B3GNTL1 | UDP-GlcNAc:betal | UDP-GlcN | UDP-GlcN | 146712 | 146712 | 146712 | s44889 | s44890 | s223351 |
| 16927 | B15 | B16 | 1790 | 1115 | 23961 | 257,1 | 13,4 | 1,035 | H5A034 C | sample | Hm Gen E | Hm Gen E | Hm Gen E | CPE002JI | CPE002NZ | CPE002KD | NM 05291 | NM 2073 | NM 0529 | TMEM200 | FAM148A | TMEM200 | transmembrane p | family wit | transmem | 114801 | 145741 | 114801 | s41636 | s44766 | s41638 |
| 17011 | E4 | E5 | 1014 | 987,6 | 13581 | 172,7 | 13,4 | 1,035 | H5A034 C | sample | Hm Gen E | Hm Gen E | Hm Gen E | CPE002JI | CPE002NZ | CPE002KD | NM 14459 | NM 1452 | NM 1445 | C10orf32 | HFE2 | C10orf32 | chromosome 10 c | hemochro | chromoso | 119032 | 148738 | 119032 | s42271 | s45202 | s42273 |
| 17219 | N19 | N20 | 1576 | 926,9 | 21167 | 195,1 | 13,4 | 1,035 | H5A034 C | sample | Hm Gen E | Hm Gen E | Hm Gen E | CPE002JI | CPE002NZ | CPE002KD | NM 05305 | XM 0011 | NM 0530 | MRPL53 | LOC14764 | MRPL53 | mitochondrial rib | hypotheti | mitochon | 116540 | 147646 | 116540 | s42042 | s45013 | s42044 |
| 17243 | O19 | O20 | 1969 | 990,7 | 26307 | 253,6 | 13,4 | 1,035 | H5A034 C | sample | Hm Gen E | Hm Gen E | Hm Gen E | CPE002JI | CPE002NZ | CPE002KD | NM 05289 | XM 4985 | NM 0528 | CSMD2 | C14orf82 | CSMD2 | CUB and Sushi mu | chromoso | CUB and S | 114784 | 145438 | 114784 | s41591 | s44733 | s41593 |
| 17251 | O4 | O5 | 1167 | 969,8 | 15657 | 285,6 | 13,4 | 1,035 | H5A034 C | sample | Hm Gen E | Hm Gen E | Hm Gen E | CPE002JI | CPE002NZ | CPE002KD | NM 00100 | NM 1825 | NM 0010 | ALKBH2 | C22orf15 | ALKBH2 | alkB, alkylation re | chromoso | alkB, alkyl | 121642 | 150248 | 121642 | s42494 | s45402 | s42496 |
| 17265 | P17 | P18 | 1618 | 1175 | 21668 | 204,9 | 13,4 | 1,035 | H5A034 C | sample | Hm Gen E | Hm Gen E | Hm Gen E | CPE002JI | CPE002NZ | CPE002KD | NM 05305 | NM 1450 | NM 0530 | TADA1L | DACT3 | TADA1L | transcriptional ac | dapper, a | transcript | 117143 | 147906 | 117143 | s42098 | s45072 | s42100 |
| 6931 | A4 | A5 | 1243 | 641,2 | 16669 | 347,5 | 13,4 | 1,043 | H5A034 C | sample | Hm Drgbl | Hm Drgbl | Hm Drgbl | CPE002E2 | CPE002EX | CPE002FI | NM 00244 | NM 0024 | NM 0024 | MSH4 | MSH4 | MSH4 | mutS homolog 4 ( | mutS hom | mutS hom | 4438 | 4438 | 4438 | s8973 | s8972 | s8974 |
| 6958 | B7 | B8 | 909 | 715,9 | 12216 | 558,1 | 13,4 | 1,043 | H5A034 C | sample | Hm Drgbl | Hm Drgbl | Hm Drgbl | CPE002E2 | CPE002EX | CPE002FI | NM 00024 | NM 0002 | NM 0002 | MAOA | MAOA | MAOA | monoamine oxid | monoami | monoami | 4128 | 4128 | 4128 | s8490 | s8492 | s8491 |
| 7224 | M9 | M10 | 1151 | 621,7 | 15470 | 429,8 | 13,4 | 1,043 | H5A034 C | sample | Hm Drgbl | Hm Drgbl | Hm Drgbl | CPE002E2 | CPE002EX | CPE002FI | NM 14586 | NM 1458 | NM 1458 | LTC4S | LTC4S | LTC4S | leukotriene C4 sy | leukotrier | leukotrier | 4056 | 4056 | 4056 | s8328 | s8329 | s194598 |
| 5074 | D18 | D19 | 1294 | 632,5 | 17307 | 329,7 | 13,4 | 1,051 | H5A034 C | sample | Hm Drgbl | Hm Drgbl | Hm Drgbl | CPE002DV | CPE002EM | CPE002FO | NM 00055 | NM 0005 | NM 0005 | VHL | VHL | VHL | von Hippel-Linda | von Hippe | von Hippe | 7428 | 7428 | 7428 | s14789 | s14790 | s14791 |
| 5211 | J11 | J12 | 1779 | 604,2 | 23888 | 458,6 | 13,4 | 1,051 | H5A034 C | sample | Hm Drgbl | Hm Drgbl | Hm Drgbl | CPE002DV | CPE002EM | CPE002FO | NM 00652 | NM 0065 | NM 0065 | TFPI2 | TFPI2 | TFPI2 | tissue factor path | tissue fact | tissue fact | 7980 | 7980 | 7980 | s15514 | s224834 | s15516 |
| 5293 | M20 | M21 | 1365 | 708 | 18345 | 322,4 | 13,4 | 1,051 | H5A034 C | sample | Hm Drgbl | Hm Drgbl | Hm Drgbl | CPE002DV | CPE002EM | CPE002FO | NM 00335 | NM 0033 | NM 0033 | UCN | UCN | UCN | urocortin | urocortin | urocortin | 7349 | 7349 | 7349 | s14625 | s194844 | s194845 |
| 5315 | N19 | N20 | 1618 | 611,5 | 21695 | 541 | 13,4 | 1,051 | H5A034 C | sample | Hm Drgbl | Hm Drgbl | Hm Drgbl | CPE002DV | CPE002EM | CPE002FO | NM 02204 | NM 0220 | NM 0220 | GAN | GAN | GAN | giant axonal neur | giant axor | giant axor | 8139 | 8139 | 8139 | s15652 | s15650 | s15651 |
| 2483 | H19 | H20 | 1172 | 614,5 | 15752 | 366,8 | 13,4 | 1,055 | H5A034 C | sample | Hm Drgbl | Hm Drgbl | Hm Drgbl | CPE002DC | CPE002EE | CPE002F4 | NM 00358 | NM 0035 | NM 0035 | DYRK2 | DYRK2 | DYRK2 | dual-specificity ty | dual-spec | dual-spec | 8445 | 8445 | 8445 | s16027 | s16028 | s16029 |
| 2593 | M1 | M2 | 381 | 828,7 | 5108 | 446,8 | 13,4 | 1,055 | H5A034 C | sample | Hm Drgbl | Hm Drgbl | Hm Drgbl | CPE002DC | CPE002EE | CPE002F4 | NM 00108 | NM 0010 | NM 0010 | PRKDC | PRKDC | PRKDC | protein kinase, DI | protein ki | protein ki | 5591 | 5591 | 5591 | s773 | s774 | s775 |
| 2597 | M13 | M14 | 710 | 715,6 | 9521 | 447,2 | 13,4 | 1,055 | H5A034 C | sample | Hm Drgbl | Hm Drgbl | Hm Drgbl | CPE002DC | CPE002EE | CPE002F4 | NM 00161 | NM 0016 | NM 0016 | ACVR2A | ACVR2A | ACVR2A | activin A receptor | activin A r | activin A r | 92 | 92 | 92 | s980 | s981 | s982 |
| 2624 | N16 | N17 | 1442 | 657,9 | 19353 | 385,7 | 13,4 | 1,055 | H5A034 C | sample | Hm Drgbl | Hm Drgbl | Hm Drgbl | CPE002DC | CPE002EE | CPE002F4 | NM 00316 | NM 0031 | NM 0031 | AURKC | AURKC | AURKC | aurora kinase C | aurora kir | aurora kir | 6795 | 6795 | 6795 | s13582 | s13583 | s13584 |
| 16544 | B16 | B17 | 1261 | 1061 | 16845 | 276,9 | 13,4 | 1,059 | H5A034 C | sample | Hm Gen E | Hm Gen E | Hm Gen E | CPE002JH | CPE002NV | CPE002KC | NM 19425 | NM 1942 | NM 1942 | ZNF804A | ZNF804A | ZNF804A | zinc finger protei | zinc finge | zinc finge | 91752 | 91752 | 91752 | s40768 | s40769 | s40770 |
| 16551 | B22 | B23 | 1522 | 952 | 20393 | 280,7 | 13,4 | 1,059 | H5A034 C | sample | Hm Gen E | Hm Gen E | Hm Gen E | CPE002JH | CPE002NV | CPE002KC | NM 15227 | NM 1522 | NM 1522 | CHMP7 | CHMP7 | CHMP7 | CHMP family, mer | CHMP fam | CHMP fam | 91782 | 91782 | 91782 | s40780 | s40781 | s40782 |
| 16670 | G21 | G22 | 1350 | 935,3 | 18045 | 245,3 | 13,4 | 1,059 | H5A034 C | sample | Hm Gen E | Hm Gen E | Hm Gen E | CPE002JH | CPE002NV | CPE002KC | NM 03286 | NM 0328 | NM 0328 | MICALCL | MICALCL | MICALCL | MICAL C-terminal | MICAL C-t | MICAL C-t | 84953 | 84953 | 84953 | s39742 | s39743 | s39744 |
| 16764 | K2 | K3 | 1143 | 1042 | 15327 | 299,5 | 13,4 | 1,059 | H5A034 C | sample | Hm Gen E | Hm Gen E | Hm Gen E | CPE002JH | CPE002NV | CPE002KC | NM 13836 | NM 1383 | NM 1383 | FAM44B | FAM44B | FAM44B | family with seque | family wit | family wit | 91272 | 91272 | 91272 | s40603 | s40604 | s40605 |
| 16837 | N20 | N21 | 1882 | 1042 | 25190 | 285,1 | 13,4 | 1,059 | H5A034 C | sample | Hm Gen E | Hm Gen E | Hm Gen E | CPE002JH | CPE002NV | CPE002KC | NM 00108 | NM 0010 | NM 0010 | LINCR | LINCR | LINCR | likely ortholog of | likely orth | likely orth | 93082 | 93082 | 93082 | s41077 | s41078 | s41079 |
| 16861 | O20 | O21 | 1516 | 988,7 | 20283 | 283,3 | 13,4 | 1,059 | H5A034 C | sample | Hm Gen E | Hm Gen E | Hm Gen E | CPE002JH | CPE002NV | CPE002KC | NM 03329 | NM 0332 | NM 0332 | NLRP12 | NLRP12 | NLRP12 | NLR family, pyrin | NLR famil | NLR famil | 91662 | 91662 | 91662 | s40729 | s40730 | s40731 |
| 17798 | F21 | F22 | 1693 | 1078 | 22697 | 305,9 | 13,4 | 1,059 | H5A034 C | sample | Hm Gen E | Hm Gen E | Hm Gen E | CPE002JK | CPE002NZ | CPE002KF | XM 00113 | XM 0011 | XM 0011 | FLJ25404 | FLJ25404 | FLJ25404 | hypothetical prot | hypotheti | hypotheti | 146378 | 146378 | 146378 | s44846 | s44847 | s225700 |
| 17823 | G22 | G23 | 1637 | 1116 | 21944 | 322,7 | 13,4 | 1,059 | H5A034 C | sample | Hm Gen E | Hm Gen E | Hm Gen E | CPE002JK | CPE002NZ | CPE002KF | NM 00101 | NM 0010 | NM 0010 | FAM78B | FAM78B | FAM78B | family with seque | family wit | family wit | 149297 | 149297 | 149297 | s45274 | s45275 | s225731 |
| 8899 | C4 | C5 | 2020 | 2009 | 27076 | 191,5 | 13,4 | 1,076 | H5A034 C | sample | Hm Drgbl | Hm Drgbl | Hm Drgbl | CPE002E7 | CPE002ES | CPE002FD | NM 01590 | NM 0159 | NM 0159 | TRIM33 | TRIM33 | TRIM33 | tripartite motif-cc | tripartite | tripartite | 51592 | 51592 | 51592 | s28373 | s28371 | s28372 |
| 4262 | B21 | B22 | 1305 | 821,2 | 17520 | 442,3 | 13,4 | 1,131 | H5A034 C | sample | Hm Drgbl | Hm Drgbl | Hm Drgbl | CPE002DL | CPE002EK | CPE002FQ | NM 00083 | NM 0008 | NM 0008 | GRIK3 | GRIK3 | GRIK3 | glutamate recept | glutamate | glutamate | 2899 | 2899 | 2899 | s6158 | s6159 | s6160 |
| 4349 | F13 | F14 | 636 | 850,1 | 8500 | 438 | 13,4 | 1,131 | H5A034 C | sample | Hm Drgbl | Hm Drgbl | Hm Drgbl | CPE002DL | CPE002EK | CPE002FQ | NM 00554 | NM 0055 | NM 0055 | KCNA10 | KCNA10 | KCNA10 | potassium voltag | potassium | potassium | 3744 | 3744 | 3744 | s7697 | s7698 | s7699 |
| 4389 | G6 | G7 | 529 | 681,2 | 7102 | 416 | 13,4 | 1,131 | H5A034 C | sample | Hm Drgbl | Hm Drgbl | Hm Drgbl | CPE002DL | CPE002EK | CPE002FQ | NM 00482 | NM 0048 | NM 0048 | KCNK6 | KCNK6 | KCNK6 | potassium chann | potassium | potassium | 9424 | 9424 | 9424 | s18044 | s18045 | s18046 |
| 4417 | I1 | I2 | 1444 | 678,5 | 19337 | 348,3 | 13,4 | 1,131 | H5A034 C | sample | Hm Drgbl | Hm Drgbl | Hm Drgbl | CPE002DL | CPE002EK | CPE002FQ | NM 00074 | NM 0007 | NM 0007 | CHRNA3 | CHRNA3 | CHRNA3 | cholinergic recept | cholinergi | cholinergi | 1136 | 1136 | 1136 | s3043 | s3044 | s3045 |
| 4531 | M4 | M5 | 841 | 688,5 | 11286 | 407,2 | 13,4 | 1,131 | H5A034 C | sample | Hm Drgbl | Hm Drgbl | Hm Drgbl | CPE002DL | CPE002EK | CPE002FQ | NM 01611 | NM 0161 | NM 0161 | TRPV2 | TRPV2 | TRPV2 | transient recepto | transient | transient | 51393 | 51393 | 51393 | s28081 | s28082 | s28083 |
| 83 | D19 | D20 | 614 | 1455 | 8248 | 544,2 | 13,4 | 1,14 | H5A034 C | sample | Hm Drgbl | Hm Drgbl | Hm Drgbl | CPE002DT | CPE002EJ | CPE002F9 | NM 00455 | NM 0045 | NM 0045 | SFRS10 | SFRS10 | SFRS10 | splicing factor, arc | splicing fa | splicing fa | 6434 | 6434 | 6434 | s12749 | s12750 | s12751 |
| 98 | E10 | E11 | 708 | 1467 | 9476 | 432,7 | 13,4 | 1,14 | H5A034 C | sample | Hm Drgbl | Hm Drgbl | Hm Drgbl | CPE002DT | CPE002EJ | CPE002F9 | NM 00563 | NM 0056 | NM 0056 | SLCO2A1 | SLCO2A1 | SLCO2A1 | solute carrier org | solute car | solute car | 6578 | 6578 | 6578 | s13098 | s13096 | s13097 |
| 324 | N2 | N3 | 591 | 1486 | 7909 | 600,7 | 13,4 | 1,14 | H5A034 C | sample | Hm Drgbl | Hm Drgbl | Hm Drgbl | CPE002DT | CPE002EJ | CPE002F9 | NM 00460 | NM 0046 | NM 0046 | SULT2B1 | SULT2B1 | SULT2B1 | sulfotransferase f | sulfotrans | sulfotrans | 6820 | 6820 | 6820 | s13620 | s224701 | s13621 |
| 2031 | E22 | E23 | 1244 | 667,1 | 16615 | 478,3 | 13,4 | 1,165 | H5A034 C | sample | Hm Drgbl | Hm Drgbl | Hm Drgbl | CPE002FX | CPE002G1 | CPE002G5 | NM 14501 | NM 1450 | NM 1450 | ZNF25 | ZNF25 | ZNF25 | zinc finger protei | zinc finge | zinc finge | 219749 | 219749 | 219749 | s47712 | s47713 | s47714 |
| 2147 | J19 | J20 | 744 | 680,4 | 9956 | 408,9 | 13,4 | 1,165 | H5A034 C | sample | Hm Drgbl | Hm Drgbl | Hm Drgbl | CPE002FX | CPE002G1 | CPE002G5 | NM 14719 | NM 1471 | NM 1471 | GLIS1 | GLIS1 | GLIS1 | GLIS family zinc fi | GLIS famil | GLIS famil | 148979 | 148979 | 148979 | s45239 | s45240 | s45241 |
| 2230 | M7 | M8 | 1334 | 653,8 | 17852 | 462 | 13,4 | 1,165 | H5A034 C | sample | Hm Drgbl | Hm Drgbl | Hm Drgbl | CPE002FX | CPE002G1 | CPE002G5 | NM 15221 | NM 1522 | NM 1522 | GJC1 | GJC1 | GJC1 | gap junction prot | gap juncti | gap juncti | 125111 | 125111 | 125111 | s42857 | s42858 | s42859 |
| 2240 | N16 | N17 | 1195 | 710,9 | 16062 | 443,6 | 13,4 | 1,165 | H5A034 C | sample | Hm Drgbl | Hm Drgbl | Hm Drgbl | CPE002FX | CPE002G1 | CPE002G5 | | | | | | | | | | | | | | | |
| 2278 | O7 | O8 | 1678 | 737,7 | 22405 | 457 | 13,4 | 1,165 | H5A034 C | sample | Hm Drgbl | Hm Drgbl | Hm Drgbl | CPE002FX | CPE002G1 | CPE002G5 | NM 00108 | NM 0010 | NM 0010 | WTIP | WTIP | WTIP | Wilms tumor 1 in | Wilms tur | Wilms tur | 126374 | 126374 | 126374 | s43020 | s43021 | s43022 |
| 2304 | P9 | P10 | 1321 | 703 | 17669 | 417,8 | 13,4 | 1,165 | H5A034 C | sample | Hm Drgbl | Hm Drgbl | Hm Drgbl | CPE002FX | CPE002G1 | CPE002G5 | NM 14497 | NM 1449 | NM 1449 | ZNF564 | ZNF564 | ZNF564 | zinc finger protei | zinc finge | zinc finge | 163050 | 163050 | 163050 | s46404 | s46405 | s46406 |
| 6532 | A12 | A13 | 1188 | 615,8 | 15963 | 461,3 | 13,4 | 1,175 | H5A034 C | sample | Hm Drgbl | Hm Drgbl | Hm Drgbl | CPE002E1 | CPE002EY | CPE002FJ | NM 00103 | NM 0010 | NM 0010 | IFIT3 | IFIT3 | IFIT3 | interferon-induce | interferor | interferor | 3437 | 3437 | 3437 | s7153 | s7154 | s7155 |
| 6539 | A19 | A20 | 815 | 713,3 | 10923 | 240 | 13,4 | 1,175 | H5A034 C | sample | Hm Drgbl | Hm Drgbl | Hm Drgbl | CPE002E1 | CPE002EY | CPE002FJ | NM 14587 | NM 1458 | NM 1458 | GSTZ1 | GSTZ1 | GSTZ1 | glutathione trans | glutathior | glutathior | 2954 | 2954 | 2954 | s6283 | s6284 | s6285 |
| 6551 | A8 | A9 | 1075 | 596,4 | 14355 | 494,9 | 13,4 | 1,175 | H5A034 C | sample | Hm Drgbl | Hm Drgbl | Hm Drgbl | CPE002E1 | CPE002EY | CPE002FJ | NM 00154 | NM 0015 | NM 0015 | IFIT2 | IFIT2 | IFIT2 | interferon-induce | interferor | interferor | 3433 | 3433 | 3433 | s7147 | s7148 | s7149 |
| 6781 | K20 | K21 | 1119 | 650 | 14999 | 342,9 | 13,4 | 1,175 | H5A034 C | sample | Hm Drgbl | Hm Drgbl | Hm Drgbl | CPE002E1 | CPE002EY | CPE002FJ | NM 00553 | NM 0055 | NM 0055 | INHBC | INHBC | INHBC | inhibin, beta C | inhibin, b | inhibin, b | 3626 | 3626 | 3626 | s7440 | s7441 | s223942 |
| 3993 | G17 | G18 | 1409 | 681,3 | 18855 | 331,7 | 13,4 | 1,191 | H5A034 C | sample | Hm Drgbl | Hm Drgbl | Hm Drgbl | CPE002DS | CPE002EI | CPE002F8 | NM 00100 | NM 0010 | NM 0010 | PMS2L3 | PMS2L3 | PMS2L3 | postmeiotic segre | postmeio | postmeio | 5387 | 5387 | 5387 | s10733 | s194697 | s194698 |
| 4157 | N13 | N14 | 1278 | 652,3 | 17132 | 382,8 | 13,4 | 1,191 | H5A034 C | sample | Hm Drgbl | Hm Drgbl | Hm Drgbl | CPE002DS | CPE002EI | CPE002F8 | NM 00095 | NM 0009 | NM 0009 | PTGDS | PTGDS | PTGDS | prostaglandin D2 | prostagla | prostagla | 5730 | 5730 | 5730 | s11446 | s11447 | s194724 |
| 4199 | O8 | O9 | 928 | 588 | 12438 | 414,5 | 13,4 | 1,191 | H5A034 C | sample | Hm Drgbl | Hm Drgbl | Hm Drgbl | CPE002DS | CPE002EI | CPE002F8 | NM 00290 | NM 0029 | NM 0029 | RDH5 | RDH5 | RDH5 | retinol dehydroge | retinol de | retinol de | 5959 | 5959 | 5959 | s224525 | s11895 | s11893 |
| 11802 | L3 | L4 | 1112 | 774,6 | 14855 | 141 | 13,4 | 1,196 | H5A034 C | sample | Hm Gen E | Hm Gen E | Hm Gen E | CPE002JA | CPE002NJ | CPE002JZ | NM 00075 | NM 0007 | NM 0007 | CSF3 | CSF3 | CSF3 | colony stimulatin | colony sti | colony sti | 1440 | 1440 | 1440 | s3606 | s3607 | s3608 |
| 15423 | C22 | C23 | 806 | 966,2 | 10824 | 198,5 | 13,4 | 1,218 | H5A034 C | sample | Hm Gen E | Hm Gen E | Hm Gen E | CPE002JE | CPE002NT | CPE002K9 | NM 00104 | NM 0010 | NM 0010 | PHACTR4 | PHACTR4 | PHACTR4 | phosphatase and | phosphat | phosphat | 65979 | 65979 | 65979 | s35290 | s35291 | s35292 |
| 15534 | H14 | H15 | 1045 | 846,2 | 14005 | 141,2 | 13,4 | 1,218 | H5A034 C | sample | Hm Gen E | Hm Gen E | Hm Gen E | CPE002JE | CPE002NT | CPE002K9 | NM 03076 | NM 0307 | NM 0307 | NSBP1 | NSBP1 | NSBP1 | nucleosomal binc | nucleosor | nucleosor | 79366 | 79366 | 79366 | s35758 | s35759 | s35760 |
| 15559 | I15 | I16 | 1440 | 847,3 | 19226 | 132,3 | 13,4 | 1,218 | H5A034 C | sample | Hm Gen E | Hm Gen E | Hm Gen E | CPE002JE | CPE002NT | CPE002K9 | NM 03141 | NM 0314 | NM 0314 | TMEM16C | TMEM16C | TMEM16C | transmembrane p | transmem | transmem | 63982 | 63982 | 63982 | s34358 | s34359 | s34360 |
| 15684 | N2 | N3 | 1175 | 791,4 | 15779 | 134,6 | 13,4 | 1,218 | H5A034 C | sample | Hm Gen E | Hm Gen E | Hm Gen E | CPE002JE | CPE002NT | CPE002K9 | NM 00108 | NM 0010 | NM 0010 | TCTN1 | TCTN1 | TCTN1 | tectonic family m | tectonic fa | tectonic fa | 79600 | 79600 | 79600 | s35890 | s35891 | s35892 |
| 5803 | B4 | B5 | 1574 | 753,8 | 21159 | 571,6 | 13,4 | 1,224 | H5A034 C | sample | Hm Drgbl | Hm Drgbl | Hm Drgbl | CPE002DY | CPE002F1 | CPE002FM | NM 05305 | NM 0530 | NM 0530 | CCND1 | CCND1 | CCND1 | cyclin D1 | cyclin D1 | cyclin D1 | 595 | 595 | 595 | s230 | s228 | s229 |
| 5880 | E9 | E10 | 1422 | 757,1 | 19087 | 607,5 | 13,4 | 1,224 | H5A034 C | sample | Hm Drgbl | Hm Drgbl | Hm Drgbl | CPE002DY | CPE002F1 | CPE002FM | NM 00219 | NM 0021 | NM 0021 | ACO1 | ACO1 | ACO1 | aconitase 1, solut | aconitase | aconitase | 48 | 48 | 48 | s673 | s672 | s224137 |
| 6069 | M6 | M7 | 1342 | 735,3 | 18003 | 565,2 | 13,4 | 1,224 | H5A034 C | sample | Hm Drgbl | Hm Drgbl | Hm Drgbl | CPE002DY | CPE002F1 | CPE002FM | NM 00103 | NM 0010 | NM 0010 | ATP6V1E1 | ATP6V1E1 | ATP6V1E1 | ATPase, H+ transp | ATPase, H | ATPase, H | 529 | 529 | 529 | s1804 | s1803 | s1805 |
| 1174 | A7 | A8 | 1552 | 713,3 | 20750 | 515,4 | 13,4 | 1,229 | H5A034 C | sample | Hm Drgbl | Hm Drgbl | Hm Drgbl | CPE002FV | CPE002FZ | CPE002G3 | NM 00341 | NM 0034 | NM 0034 | ZFX | ZFX | ZFX | zinc finger protei | zinc finge | zinc finge | 7543 | 7543 | 7543 | s14989 | s14990 | s14991 |
| 1188 | B2 | B3 | 1266 | 711,3 | 16964 | 516,3 | 13,4 | 1,229 | H5A034 C | sample | Hm Drgbl | Hm Drgbl | Hm Drgbl | CPE002FV | CPE002FZ | CPE002G3 | NM 00658 | NM 0065 | NM 0065 | CLDN16 | CLDN16 | CLDN16 | claudin 16 | claudin 16 | claudin 16 | 10686 | 10686 | 10686 | s21011 | s21012 | s21013 |
| 1229 | D13 | D14 | 1409 | 736,1 | 18875 | 441,3 | 13,4 | 1,229 | H5A034 C | sample | Hm Drgbl | Hm Drgbl | Hm Drgbl | CPE002FV | CPE002FZ | CPE002G3 | NM 00356 | NM 0035 | NM 0035 | EEA1 | EEA1 | EEA1 | early endosome a | early endo | early endo | 8411 | 8411 | 8411 | s15969 | s15970 | s15971 |
| 1239 | D22 | D23 | 1488 | 734,8 | 19884 | 455,4 | 13,4 | 1,229 | H5A034 C | sample | Hm Drgbl | Hm Drgbl | Hm Drgbl | CPE002FV | CPE002FZ | CPE002G3 | NM 00666 | NM 0066 | NM 0066 | CLPX | CLPX | CLPX | ClpX caseinolytic | ClpX casei | ClpX casei | 10845 | 10845 | 10845 | s21287 | s21288 | s21289 |
| 1260 | E2 | E3 | 1267 | 748,6 | 17011 | 527,9 | 13,4 | 1,229 | H5A034 C | sample | Hm Drgbl | Hm Drgbl | Hm Drgbl | CPE002FV | CPE002FZ | CPE002G3 | NM 20350 | NM 2035 | NM 2035 | KEAP1 | KEAP1 | KEAP1 | kelch-like ECH-ass | kelch-like | kelch-like | 9817 | 9817 | 9817 | s18981 | s18982 | s18983 |
| 1302 | G14 | G15 | 967 | 727,2 | 13004 | 467,4 | 13,4 | 1,229 | H5A034 C | sample | Hm Drgbl | Hm Drgbl | Hm Drgbl | CPE002FV | CPE002FZ | CPE002G3 | NM 01323 | NM 0132 | NM 0132 | PDCD6 | PDCD6 | PDCD6 | programmed cell | programm | programm | 10016 | 10016 | 10016 | s19468 | s19469 | s19470 |
| 1353 | I17 | I18 | 1407 | 698,9 | 18847 | 375,1 | 13,4 | 1,229 | H5A034 C | sample | Hm Drgbl | Hm Drgbl | Hm Drgbl | CPE002FV | CPE002FZ | CPE002G3 | NM 00343 | NM 0034 | NM 0034 | ZNF132 | ZNF132 | ZNF132 | zinc finger protei | zinc finge | zinc finge | 7691 | 7691 | 7691 | s15162 | s15163 | s15164 |
| 1460 | M5 | M6 | 1180 | 686,3 | 15759 | 377,2 | 13,4 | 1,229 | H5A034 C | sample | Hm Drgbl | Hm Drgbl | Hm Drgbl | CPE002FV | CPE002FZ | CPE002G3 | NM 00344 | NM 0034 | NM 0034 | ZNF157 | ZNF157 | ZNF157 | zinc finger protei | zinc finge | zinc finge | 7712 | 7712 | 7712 | s15219 | s15220 | s15221 |
| 1496 | O16 | O17 | 1787 | 756 | 24016 | 492,8 | 13,4 | 1,229 | H5A034 C | sample | Hm Drgbl | Hm Drgbl | Hm Drgbl | CPE002FV | CPE002FZ | CPE002G3 | NM 00655 | NM 0065 | NM 0065 | YKT6 | YKT6 | YKT6 | YKT6 v-SNARE hor | YKT6 v-SN | YKT6 v-SN | 10652 | 10652 | 10652 | s20936 | s20937 | s20938 |
| 1500 | O2 | O3 | 1407 | 692,6 | 18791 | 483,9 | 13,4 | 1,229 | H5A034 C | sample | Hm Drgbl | Hm Drgbl | Hm Drgbl | CPE002FV | CPE002FZ | CPE002G3 | NM 00639 | NM 0063 | NM 0063 | BATF | BATF | BATF | basic leucine zipp | basic leuc | basic leuc | 10538 | 10538 | 10538 | s20665 | s20666 | s223128 |
| 16328 | I16 | I17 | 1252 | 956 | 16719 | 253,4 | 13,4 | 1,229 | H5A034 C | sample | Hm Gen E | Hm Gen E | Hm Gen E | CPE002JG | CPE002NV | CPE002KB | NM 03233 | NM 0323 | NM 0323 | C10orf58 | C10orf58 | C10orf58 | chromosome 10 c | chromoso | chromoso | 84293 | 84293 | 84293 | s38840 | s38841 | s38842 |
| 16338 | I3 | I4 | 879 | 998,1 | 11739 | 323,3 | 13,4 | 1,229 | H5A034 C | sample | Hm Gen E | Hm Gen E | Hm Gen E | CPE002JG | CPE002NV | CPE002KB | NM 00101 | NM 0010 | NM 0010 | SEH1L | SEH1L | SEH1L | SEH1-like (S. cerev | SEH1-like | SEH1-like | 81929 | 81929 | 81929 | s37878 | s37879 | s37880 |
| 16373 | K13 | K14 | 1575 | 1126 | 21088 | 331,6 | 13,4 | 1,229 | H5A034 C | sample | Hm Gen E | Hm Gen E | Hm Gen E | CPE002JG | CPE002NV | CPE002KB | NM 00104 | NM 0010 | NM 0010 | FAM110A | FAM110A | FAM110A | family with seque | family wit | family wit | 83541 | 83541 | 83541 | s37984 | s37985 | s228292 |
| 16418 | M10 | M11 | 1635 | 994 | 21971 | 371,4 | 13,4 | 1,229 | H5A034 C | sample | Hm Gen E | Hm Gen E | Hm Gen E | CPE002JG | CPE002NV | CPE002KB | NM 03235 | NM 0323 | NM 0323 | CCDC115 | CCDC115 | CCDC115 | coiled-coil domai | coiled-coi | coiled-coi | 84317 | 84317 | 84317 | s38905 | s38906 | s38907 |
| 13774 | N7 | N8 | 1483 | 1024 | 19835 | 182,5 | 13,4 | 1,258 | H5A034 C | sample | Hm Gen E | Hm Gen E | Hm Gen E | CPE002JC | CPE002NC | CPE002K4 | NM 00104 | NM 0010 | NM 0010 | SUMF2 | SUMF2 | SUMF2 | sulfatase modifyi | sulfatase | sulfatase | 25870 | 25870 | 25870 | s24647 | s24648 | s24649 |
| 843 | D11 | D12 | 778 | 707,1 | 10407 | 517 | 13,4 | 1,27 | H5A034 C | sample | Hm Drgbl | Hm Drgbl | Hm Drgbl | CPE002FU | CPE002FY | CPE002G2 | NM 00016 | NM 0001 | NM 0001 | GJA1 | GJA1 | GJA1 | gap junction prot | gap juncti | gap juncti | 2697 | 2697 | 2697 | s5757 | s5758 | s5759 |
| 5379 | A11 | A12 | 1506 | 753,9 | 20184 | 685 | 13,4 | 1,276 | H5A034 C | sample | Hm Drgbl | Hm Drgbl | Hm Drgbl | CPE002DX | CPE002F2 | CPE002FN | NM 00349 | NM 0034 | NM 0034 | ARD1A | ARD1A | ARD1A | ARD1 homolog A, | ARD1 hon | ARD1 hon | 8260 | 8260 | 8260 | s15755 | s15756 | s15754 |
| 5391 | A22 | A23 | 1774 | 751,7 | 23838 | 632,4 | 13,4 | 1,276 | H5A034 C | sample | Hm Drgbl | Hm Drgbl | Hm Drgbl | CPE002DX | CPE002F2 | CPE002FN | NM 00387 | NM 0038 | NM 0038 | IQGAP1 | IQGAP1 | IQGAP1 | IQ motif containi | IQ motif c | IQ motif c | 8826 | 8826 | 8826 | s16839 | s16838 | s16837 |
| 5590 | I7 | I8 | 2008 | 728,9 | 26926 | 519,9 | 13,4 | 1,276 | H5A034 C | sample | Hm Drgbl | Hm Drgbl | Hm Drgbl | CPE002DX | CPE002F2 | CPE002FN | NM 00361 | NM 0036 | NM 0036 | CILP | CILP | CILP | cartilage interme | cartilage i | cartilage i | 8483 | 8483 | 8483 | s16115 | s16116 | s16114 |
| 5697 | N17 | N18 | 1706 | 673 | 22860 | 597,7 | 13,4 | 1,276 | H5A034 C | sample | Hm Drgbl | Hm Drgbl | Hm Drgbl | CPE002DX | CPE002F2 | CPE002FN | NM 00419 | NM 0041 | NM 0041 | TNFRSF18 | TNFRSF18 | TNFRSF18 | tumor necrosis fa | tumor nec | tumor nec | 8784 | 8784 | 8784 | s200468 | s194959 | s194960 |
| 3558 | E14 | E15 | 1211 | 1301 | 16191 | 313,4 | 13,4 | 1,288 | H5A034 C | sample | Hm Drgbl | Hm Drgbl | Hm Drgbl | CPE002DR | CPE002EH | CPE002F7 | NM 00360 | NM 0036 | NM 0036 | GPR65 | GPR65 | GPR65 | G protein-couplec | G protein | G protein | 8477 | 8477 | 8477 | s16099 | s16100 | s16101 |
| 3602 | G10 | G11 | 603 | 1307 | 8076 | 392,3 | 13,4 | 1,288 | H5A034 C | sample | Hm Drgbl | Hm Drgbl | Hm Drgbl | CPE002DR | CPE002EH | CPE002F7 | NM 00473 | NM 0047 | NM 0047 | XPR1 | XPR1 | XPR1 | xenotropic and po | xenotropi | xenotropi | 9213 | 9213 | 9213 | s17614 | s17615 | s17616 |
| 3719 | K8 | K9 | 1392 | 1368 | 18650 | 413 | 13,4 | 1,288 | H5A034 C | sample | Hm Drgbl | Hm Drgbl | Hm Drgbl | CPE002DR | CPE002EH | CPE002F7 | NM 00488 | NM 0048 | NM 0048 | NPFFR2 | NPFFR2 | NPFFR2 | neuropeptide FF r | neuropep | neuropep | 10886 | 10886 | 10886 | s21381 | s21382 | s223161 |
| 3781 | N20 | N21 | 1079 | 1346 | 14427 | 355,4 | 13,4 | 1,288 | H5A034 C | sample | Hm Drgbl | Hm Drgbl | Hm Drgbl | CPE002DR | CPE002EH | CPE002F7 | XM 00112 | XM 0011 | XM 0011 | GPR97 | GPR97 | GPR97 | G protein-couplec | G protein | G protein | 222487 | 222487 | 222487 | s48198 | s48199 | s48200 |
| 3787 | N4 | N5 | 690 | 1164 | 9237 | 337,4 | 13,4 | 1,288 | H5A034 C | sample | Hm Drgbl | Hm Drgbl | Hm Drgbl | CPE002DR | CPE002EH | CPE002F7 | NM 14896 | NM 1489 | NM 1489 | OXER1 | OXER1 | OXER1 | oxoeicosanoid (O | oxoeicosa | oxoeicosa | 165140 | 165140 | 165140 | s46563 | s46564 | s46565 |
| 3831 | P22 | P23 | 1643 | 1325 | 21939 | 473,5 | 13,4 | 1,288 | H5A034 C | sample | Hm Drgbl | Hm Drgbl | Hm Drgbl | CPE002DR | CPE002EH | CPE002F7 | NM 00108 | NM 0010 | NM 0010 | EMR4 | EMR4 | EMR4 | egf-like module c | egf-like m | egf-like m | 326342 | 326342 | 326342 | s50259 | s50260 | s50261 |
| 8204 | F5 | F6 | 1136 | 576,3 | 15254 | 561,9 | 13,4 | 1,288 | H5A034 C | sample | Hm Drgbl | Hm Drgbl | Hm Drgbl | CPE002E5 | CPE002EU | CPE002FF | NM 01489 | NM 0148 | NM 0148 | RBM16 | RBM16 | RBM16 | RNA binding moti | RNA bindi | RNA bindi | 22828 | 22828 | 22828 | s22448 | s22449 | s22450 |
| 8214 | G14 | G15 | 697 | 547,8 | 9352 | 490,4 | 13,4 | 1,288 | H5A034 C | sample | Hm Drgbl | Hm Drgbl | Hm Drgbl | CPE002E5 | CPE002EU | CPE002FF | NM 01515 | NM 0151 | NM 0151 | LARP5 | LARP5 | LARP5 | La ribonucleoprot | La ribonu | La ribonu | 23185 | 23185 | 23185 | s23228 | s23227 | s23226 |
| 8283 | J11 | J12 | 1474 | 600,9 | 19820 | 376,4 | 13,4 | 1,288 | H5A034 C | sample | Hm Drgbl | Hm Drgbl | Hm Drgbl | CPE002E5 | CPE002EU | CPE002FF | NM 00100 | NM 0010 | NM 0010 | PLA2R1 | PLA2R1 | PLA2R1 | phospholipase A2 | phospholi | phospholi | 22925 | 22925 | 22925 | s22685 | s22686 | s22687 |
| 9997 | A20 | A21 | 1616 | 912,9 | 21652 | 172,5 | 13,4 | 1,288 | H5A034 C | sample | Hm Drgbl | Hm Drgbl | Hm Drgbl | CPE002EA | CPE002EP | CPE002FA | XM 00112 | XM 0011 | XM 0011 | KATNAL2 | KATNAL2 | KATNAL2 | katanin p60 subu | katanin p | katanin p | 83473 | 83473 | 83473 | s37954 | s37955 | s37956 |
| 10070 | D21 | D22 | 1481 | 880,5 | 19838 | 146 | 13,4 | 1,288 | H5A034 C | sample | Hm Drgbl | Hm Drgbl | Hm Drgbl | CPE002EA | CPE002EP | CPE002FA | NM 02523 | NM 0252 | NM 0252 | TNKS2 | TNKS2 | TNKS2 | tankyrase, TRF1-i | tankyrase | tankyrase | 80351 | 80351 | 80351 | s37280 | s37281 | s37279 |
| 10256 | L16 | L17 | 1660 | 850,4 | 22277 | 145,7 | 13,4 | 1,288 | H5A034 C | sample | Hm Drgbl | Hm Drgbl | Hm Drgbl | CPE002EA | CPE002EP | CPE002FA | NM 13344 | NM 1334 | NM 1334 | GPT2 | GPT2 | GPT2 | glutamic pyruvate | glutamic p | glutamic p | 84706 | 84706 | 84706 | s39365 | s39364 | s39366 |
| 1626 | D3 | D4 | 1515 | 710 | 20305 | 412,9 | 13,4 | 1,403 | H5A034 C | sample | Hm Drgbl | Hm Drgbl | Hm Drgbl | CPE002FW | CPE002G0 | CPE002G4 | NM 01743 | NM 0174 | NM 0174 | PTOV1 | PTOV1 | PTOV1 | prostate tumor o | prostate t | prostate t | 53635 | 53635 | 53635 | s28748 | s28749 | s28750 |
| 1701 | G6 | G7 | 970 | 674,5 | 12976 | 407,6 | 13,4 | 1,403 | H5A034 C | sample | Hm Drgbl | Hm Drgbl | Hm Drgbl | CPE002FW | CPE002G0 | CPE002G4 | NM 02303 | NM 0230 | NM 0230 | ANKRA2 | ANKRA2 | ANKRA2 | ankyrin repeat, fa | ankyrin re | ankyrin re | 57763 | 57763 | 57763 | s33711 | s33712 | s33713 |
| 21801 | M17 | M18 | 345 | 888,1 | 4627 | 605,3 | 13,4 | 1,411 | H5A034 C | sample | Hm Gen E | Hm Gen E | Hm Gen E | CPE002JU | CPE002O9 | CPE002KP | XM 93949 | XM 9394 | XM 9394 | LOC65041 | LOC65041 | LOC65041 | similar to dynein, | similar to | similar to | 650412 | 650412 | 650412 | s57066 | s57067 | s57068 |
| 4686 | D14 | D15 | 1395 | 839,5 | 18683 | 463,9 | 13,4 | 1,473 | H5A034 C | sample | Hm Drgbl | Hm Drgbl | Hm Drgbl | CPE002DV | CPE002EL | CPE002FP | NM 19794 | NM 1979 | NM 1979 | ADAMTS6 | ADAMTS6 | ADAMTS6 | ADAM metallopep | ADAM me | ADAM me | 11174 | 11174 | 11174 | s22053 | s22054 | s22055 |
| 4809 | I17 | I18 | 1117 | 762,7 | 14974 | 404,6 | 13,4 | 1,473 | H5A034 C | sample | Hm Drgbl | Hm Drgbl | Hm Drgbl | CPE002DV | CPE002EL | CPE002FP | NM 00186 | NM 0018 | NM 0018 | CPA1 | CPA1 | CPA1 | carboxypeptidase | carboxype | carboxype | 1357 | 1357 | 1357 | s3420 | s3421 | s3422 |
| 10495 | F15 | F16 | 1395 | 913,7 | 18743 | 211,1 | 13,4 | 1,514 | H5A034 C | sample | Hm Drgbl | Hm Drgbl | Hm Drgbl | CPE002EB | CPE002EO | CPE002FR | XM 04416 | XM 0441 | XM 0441 | RKHD4 | RKHD4 | RKHD4 | ring finger and KH | ring finge | ring finge | 92312 | 92312 | 92312 | s40918 | s40919 | s225103 |
| 3004 | N12 | N13 | 1137 | 661,1 | 15234 | 676,1 | 13,4 | 1,576 | H5A034 C | sample | Hm Drgbl | Hm Drgbl | Hm Drgbl | CPE002DP | CPE002EF | CPE002F5 | NM 00100 | NM 0010 | NM 0010 | DNM1 | DNM1 | DNM1 | dynamin 1 | dynamin | dynamin | 1759 | 1759 | 1759 | s144 | s145 | s146 |
| 15801 | C17 | C18 | 684 | 893,4 | 9217 | 131,2 | 13,5 | 0,752 | H5A034 C | sample | Hm Gen E | Hm Gen E | Hm Gen E | CPE002J9 | CPE002NL | CPE002KA | NM 02462 | NM 0246 | NM 0246 | VTCN1 | VTCN1 | VTCN1 | V-set domain con | V-set dom | V-set dom | 79679 | 79679 | 79679 | s36082 | s36083 | s36084 |
| 14713 | F1 | F2 | 1825 | 912,9 | 24688 | 106,3 | 13,5 | 0,769 | H5A034 C | sample | Hm Gen E | Hm Gen E | Hm Gen E | CPE002JD | CPE002NR | CPE002K7 | NM 01816 | NM 0181 | NM 0181 | C12orf35 | C12orf35 | C12orf35 | chromosome 12 c | chromoso | chromoso | 55196 | 55196 | 55196 | s30408 | s30409 | s30410 |
| 3082 | A18 | A19 | 1635 | 1321 | 22133 | 358,6 | 13,5 | 0,836 | H5A034 C | sample | Hm Drgbl | Hm Drgbl | Hm Drgbl | CPE002DC | CPE002EG | CPE002F6 | NM 01636 | NM 0163 | NM 0163 | ACP6 | ACP6 | ACP6 | acid phosphatase | acid phos | acid phos | 51205 | 51205 | 51205 | s27679 | s27680 | s27681 |
| 3286 | I7 | I8 | 1206 | 1285 | 16290 | 423,1 | 13,5 | 0,836 | H5A034 C | sample | Hm Drgbl | Hm Drgbl | Hm Drgbl | CPE002DC | CPE002EG | CPE002F6 | NM 00156 | NM 0015 | NM 0015 | INPPL1 | INPPL1 | INPPL1 | inositol polyphos | inositol p | inositol p | 3636 | 3636 | 3636 | s7462 | s7463 | s7464 |
| 3331 | K4 | K5 | 757 | 1241 | 10212 | 437 | 13,5 | 0,836 | H5A034 C | sample | Hm Drgbl | Hm Drgbl | Hm Drgbl | CPE002DC | CPE002EG | CPE002F6 | NM 03325 | NM 0332 | NM 0332 | PPP1R14A | PPP1R14A | PPP1R14A | protein phosphat | protein pl | protein pl | 94274 | 94274 | 94274 | s41299 | s41300 | s195581 |

| | | | | | | | | | | | |
|---|---|---|---|---|---|---|---|---|---|---|---|
| 3371 | M19 | M20 | 1628 | 1216 | 22028 | 386,1 | 13,5 | 0,836 | HSA034 C | sample | Hm Drgbl |
| 18374 | N21 | N22 | 1720 | 1075 | 23265 | 269 | 13,5 | 0,868 | HSA034 C | sample | Hm Gen E |
| 20485 | F20 | F21 | 1591 | 815,9 | 21450 | 165 | 13,5 | 0,9 | HSA034 C | sample | Hm Gen E |
| 20538 | H3 | H4 | 736 | 776,3 | 9948 | 148,8 | 13,5 | 0,9 | HSA034 C | sample | Hm Gen E |
| 20543 | H8 | H9 | 734 | 751 | 9940 | 226,3 | 13,5 | 0,9 | HSA034 C | sample | Hm Gen E |
| 12867 | I11 | I12 | 1334 | 1044 | 18074 | 125,5 | 13,5 | 0,915 | HSA034 C | sample | Hm Gen E |
| 12956 | L5 | L6 | 1043 | 1026 | 14049 | 148,4 | 13,5 | 0,915 | HSA034 C | sample | Hm Gen E |
| 18512 | D16 | D17 | 1128 | 1046 | 15226 | 280,2 | 13,5 | 0,915 | HSA034 C | sample | Hm Gen E |
| 17434 | G18 | G19 | 1506 | 959,1 | 20368 | 180,3 | 13,5 | 0,922 | HSA034 C | sample | Hm Gen E |
| 17631 | O22 | O23 | 1732 | 1020 | 23334 | 296,8 | 13,5 | 0,922 | HSA034 C | sample | Hm Gen E |
| 17662 | P7 | P8 | 1004 | 1063 | 13522 | 242,7 | 13,5 | 0,922 | HSA034 C | sample | Hm Gen E |
| 19138 | N18 | N19 | 1084 | 929,6 | 14588 | 133,1 | 13,5 | 0,941 | HSA034 C | sample | Hm Gen E |
| 9919 | N15 | N16 | 2572 | 1216 | 34786 | 149,1 | 13,5 | 0,944 | HSA034 C | sample | Hm Drgbl |
| 19256 | C16 | C17 | 1213 | 1189 | 16389 | 364,5 | 13,5 | 0,957 | HSA034 C | sample | Hm Gen E |
| 19386 | H3 | H4 | 969 | 1111 | 13085 | 194,8 | 13,5 | 0,957 | HSA034 C | sample | Hm Gen E |
| 19428 | J2 | J3 | 925 | 1111 | 12450 | 284,6 | 13,5 | 0,957 | HSA034 C | sample | Hm Gen E |
| 19482 | L3 | L4 | 773 | 1191 | 10400 | 268,6 | 13,5 | 0,957 | HSA034 C | sample | Hm Gen E |
| 20793 | C17 | C18 | 737 | 1015 | 9953 | 246,1 | 13,5 | 0,957 | HSA034 C | sample | Hm Gen E |
| 20858 | F10 | F11 | 1032 | 970,8 | 13917 | 198,1 | 13,5 | 0,957 | HSA034 C | sample | Hm Gen E |
| 20868 | F2 | F3 | 1106 | 1009 | 14897 | 223,6 | 13,5 | 0,957 | HSA034 C | sample | Hm Gen E |
| 20941 | I20 | I21 | 1092 | 944,3 | 14734 | 261,5 | 13,5 | 0,957 | HSA034 C | sample | Hm Gen E |
| 20970 | J3 | J4 | 983 | 927,9 | 13289 | 281,1 | 13,5 | 0,957 | HSA034 C | sample | Hm Gen E |
| 21085 | O20 | O21 | 1300 | 1031 | 17576 | 260 | 13,5 | 0,957 | HSA034 C | sample | Hm Gen E |
| 21139 | A4 | A5 | 830 | 1066 | 11205 | 435,1 | 13,5 | 0,957 | HSA034 C | sample | Hm Gen E |
| 11430 | M14 | M15 | 1775 | 870,7 | 24025 | 132,1 | 13,5 | 0,978 | HSA034 C | sample | Hm Drgbl |
| 12594 | M3 | M4 | 1557 | 936,6 | 20949 | 138,9 | 13,5 | 1,015 | HSA034 C | sample | Hm Gen E |
| 7316 | A5 | A6 | 669 | 646,6 | 9012 | 334,9 | 13,5 | 1,031 | HSA034 C | sample | Hm Drgbl |
| 17000 | E16 | E17 | 1449 | 1117 | 19619 | 154,7 | 13,5 | 1,042 | HSA034 C | sample | Hm Gen E |
| 17130 | J3 | J4 | 1032 | 899,9 | 13905 | 176,5 | 13,5 | 1,042 | HSA034 C | sample | Hm Gen E |
| 17198 | M21 | M22 | 1557 | 933,6 | 21037 | 244,7 | 13,5 | 1,042 | HSA034 C | sample | Hm Gen E |
| 17206 | M7 | M8 | 1375 | 979,9 | 18536 | 139,5 | 13,5 | 1,042 | HSA034 C | sample | Hm Gen E |
| 6956 | B5 | B6 | 1396 | 554,3 | 18801 | 495,1 | 13,5 | 1,051 | HSA034 C | sample | Hm Drgbl |
| 5019 | B11 | B12 | 2022 | 654,7 | 27364 | 618,8 | 13,5 | 1,059 | HSA034 C | sample | Hm Drgbl |
| 5070 | D14 | D15 | 1504 | 562,4 | 20332 | 530,8 | 13,5 | 1,059 | HSA034 C | sample | Hm Drgbl |
| 5312 | N16 | N17 | 1556 | 676,4 | 20968 | 579,9 | 13,5 | 1,059 | HSA034 C | sample | Hm Drgbl |
| 5316 | N2 | N3 | 1333 | 649,3 | 18059 | 584,2 | 13,5 | 1,059 | HSA034 C | sample | Hm Drgbl |
| 2397 | D6 | D7 | 905 | 734,7 | 12207 | 458,2 | 13,5 | 1,063 | HSA034 C | sample | Hm Drgbl |
| 16562 | C10 | C11 | 1237 | 997 | 16758 | 210,2 | 13,5 | 1,067 | HSA034 C | sample | Hm Gen E |
| 16657 | G1 | G2 | 1060 | 992,7 | 14258 | 226,8 | 13,5 | 1,067 | HSA034 C | sample | Hm Gen E |
| 16765 | K20 | K21 | 1590 | 1003 | 21427 | 247,3 | 13,5 | 1,067 | HSA034 C | sample | Hm Gen E |
| 17783 | E8 | E9 | 972 | 1068 | 13147 | 348,7 | 13,5 | 1,067 | HSA034 C | sample | Hm Gen E |
| 17950 | L7 | L8 | 909 | 925,3 | 12283 | 176 | 13,5 | 1,067 | HSA034 C | sample | Hm Gen E |
| 9008 | H16 | H17 | 1224 | 1658 | 16536 | 144,7 | 13,5 | 1,084 | HSA034 C | sample | Hm Drgbl |
| 4257 | B17 | B18 | 1590 | 712,7 | 21537 | 369,9 | 13,5 | 1,139 | HSA034 C | sample | Hm Drgbl |
| 4280 | C16 | C17 | 1022 | 660 | 13820 | 337,7 | 13,5 | 1,139 | HSA034 C | sample | Hm Drgbl |
| 4425 | I17 | I18 | 1147 | 732,9 | 15452 | 394,7 | 13,5 | 1,139 | HSA034 C | sample | Hm Drgbl |
| 4464 | J9 | J10 | 1126 | 684,7 | 15188 | 334,4 | 13,5 | 1,139 | HSA034 C | sample | Hm Drgbl |
| 63 | C22 | C23 | 721 | 1759 | 9711 | 494,8 | 13,5 | 1,149 | HSA034 C | sample | Hm Drgbl |
| 357 | O6 | O7 | 777 | 1746 | 10486 | 609,3 | 13,5 | 1,149 | HSA034 C | sample | Hm Drgbl |
| 2108 | H5 | H6 | 1114 | 667,1 | 15082 | 462,5 | 13,5 | 1,174 | HSA034 C | sample | Hm Drgbl |
| 2160 | J9 | J10 | 1586 | 671,3 | 21373 | 369,8 | 13,5 | 1,174 | HSA034 C | sample | Hm Drgbl |
| 6543 | A22 | A23 | 1841 | 619,9 | 24929 | 532,5 | 13,5 | 1,184 | HSA034 C | sample | Hm Drgbl |
| 6552 | A9 | A10 | 1261 | 623,5 | 17019 | 226 | 13,5 | 1,184 | HSA034 C | sample | Hm Drgbl |
| 3875 | B19 | B20 | 1699 | 765,3 | 22919 | 429,7 | 13,5 | 1,2 | HSA034 C | sample | Hm Drgbl |
| 3930 | D3 | D4 | 820 | 634 | 11097 | 407 | 13,5 | 1,2 | HSA034 C | sample | Hm Drgbl |
| 4010 | H10 | H11 | 1170 | 776,3 | 15847 | 393,8 | 13,5 | 1,2 | HSA034 C | sample | Hm Drgbl |
| 4051 | I4 | I5 | 1210 | 619,8 | 16379 | 359,2 | 13,5 | 1,2 | HSA034 C | sample | Hm Drgbl |
| 4110 | L14 | L15 | 975 | 605,1 | 13120 | 282,5 | 13,5 | 1,2 | HSA034 C | sample | Hm Drgbl |
| 4131 | M11 | M12 | 1421 | 746,2 | 19226 | 434 | 13,5 | 1,2 | HSA034 C | sample | Hm Drgbl |
| 4204 | P12 | P13 | 1412 | 634,8 | 19067 | 445,5 | 13,5 | 1,2 | HSA034 C | sample | Hm Drgbl |
| 11575 | C15 | C16 | 1413 | 891,8 | 19088 | 212,9 | 13,5 | 1,205 | HSA034 C | sample | Hm Gen E |
| 11587 | C4 | C5 | 1397 | 901,5 | 18840 | 198,5 | 13,5 | 1,205 | HSA034 C | sample | Hm Gen E |
| 11724 | I2 | I3 | 1463 | 851,5 | 19743 | 196,7 | 13,5 | 1,205 | HSA034 C | sample | Hm Gen E |
| 11847 | N22 | N23 | 1324 | 960,9 | 17935 | 221,2 | 13,5 | 1,205 | HSA034 C | sample | Hm Gen E |
| 15705 | O17 | O18 | 1110 | 736,6 | 15035 | 142 | 13,5 | 1,227 | HSA034 C | sample | Hm Gen E |
| 5899 | F4 | F5 | 1747 | 735,4 | 23500 | 602,2 | 13,5 | 1,233 | HSA034 C | sample | Hm Drgbl |
| 5911 | G15 | G16 | 929 | 733,3 | 12506 | 356,1 | 13,5 | 1,233 | HSA034 C | sample | Hm Drgbl |
| 5988 | J2 | J3 | 1295 | 789 | 17503 | 498 | 13,5 | 1,233 | HSA034 C | sample | Hm Drgbl |
| 6019 | K4 | K5 | 799 | 783,7 | 10784 | 545,4 | 13,5 | 1,233 | HSA034 C | sample | Hm Drgbl |
| 6057 | M17 | M18 | 1472 | 735,5 | 19876 | 582,4 | 13,5 | 1,233 | HSA034 C | sample | Hm Drgbl |
| 6078 | N14 | N15 | 1702 | 745,3 | 22929 | 506,7 | 13,5 | 1,233 | HSA034 C | sample | Hm Drgbl |
| 6107 | O19 | O20 | 1968 | 912,9 | 26625 | 577,1 | 13,5 | 1,233 | HSA034 C | sample | Hm Drgbl |
| 6131 | P19 | P20 | 1674 | 746,1 | 22618 | 659,3 | 13,5 | 1,233 | HSA034 C | sample | Hm Drgbl |
| 1248 | D9 | D10 | 585 | 696,4 | 7900 | 250,6 | 13,5 | 1,239 | HSA034 C | sample | Hm Drgbl |
| 1499 | O19 | O20 | 2013 | 751,9 | 27081 | 478,8 | 13,5 | 1,239 | HSA034 C | sample | Hm Drgbl |
| 1532 | P5 | P6 | 1839 | 741,7 | 24790 | 544,2 | 13,5 | 1,239 | HSA034 C | sample | Hm Drgbl |
| 16147 | A4 | A5 | 824 | 1060 | 11126 | 350 | 13,5 | 1,239 | HSA034 C | sample | Hm Gen E |
| 16148 | A5 | A6 | 717 | 1098 | 9650 | 392 | 13,5 | 1,239 | HSA034 C | sample | Hm Gen E |
| 16219 | D4 | D5 | 758 | 1107 | 10237 | 424,5 | 13,5 | 1,239 | HSA034 C | sample | Hm Gen E |
| 16222 | D7 | D8 | 1319 | 990,1 | 17847 | 359,3 | 13,5 | 1,239 | HSA034 C | sample | Hm Gen E |
| 16399 | L15 | L16 | 1304 | 1035 | 17545 | 308,8 | 13,5 | 1,239 | HSA034 C | sample | Hm Gen E |
| 16449 | N17 | N18 | 1392 | 980,1 | 18828 | 357,2 | 13,5 | 1,239 | HSA034 C | sample | Hm Gen E |
| 16468 | O12 | O13 | 1368 | 996,8 | 18434 | 439,7 | 13,5 | 1,239 | HSA034 C | sample | Hm Gen E |
| 13579 | F4 | F5 | 1305 | 1011 | 17565 | 151,2 | 13,5 | 1,268 | HSA034 C | sample | Hm Gen E |
| 13614 | H14 | H15 | 855 | 883,6 | 11567 | 157,9 | 13,5 | 1,268 | HSA034 C | sample | Hm Gen E |
| 13656 | I9 | I10 | 890 | 1099 | 12010 | 173,9 | 13,5 | 1,268 | HSA034 C | sample | Hm Gen E |
| 935 | G8 | G9 | 1275 | 784,8 | 17153 | 482,6 | 13,5 | 1,28 | HSA034 C | sample | Hm Drgbl |
| 1078 | M7 | M8 | 1361 | 713,6 | 18435 | 361,3 | 13,5 | 1,28 | HSA034 C | sample | Hm Drgbl |
| 5397 | A6 | A7 | 1290 | 745,9 | 17462 | 786,7 | 13,5 | 1,286 | HSA034 C | sample | Hm Drgbl |
| 5434 | C18 | C19 | 1631 | 592,9 | 22004 | 540,1 | 13,5 | 1,286 | HSA034 C | sample | Hm Drgbl |
| 5540 | G5 | G6 | 1794 | 669,3 | 24244 | 620,3 | 13,5 | 1,286 | HSA034 C | sample | Hm Drgbl |
| 5555 | H19 | H20 | 2180 | 737,1 | 29480 | 474 | 13,5 | 1,286 | HSA034 C | sample | Hm Drgbl |
| 5623 | K15 | K16 | 1152 | 727 | 15543 | 340,8 | 13,5 | 1,286 | HSA034 C | sample | Hm Drgbl |
| 5740 | P12 | P13 | 1893 | 745,2 | 25616 | 495 | 13,5 | 1,286 | HSA034 C | sample | Hm Drgbl |
| 3475 | A4 | A5 | 802 | 1263 | 10806 | 362,2 | 13,5 | 1,298 | HSA034 C | sample | Hm Drgbl |
| 3629 | H13 | H14 | 1720 | 1527 | 23293 | 399,7 | 13,5 | 1,298 | HSA034 C | sample | Hm Drgbl |
| 3773 | N13 | N14 | 1038 | 1192 | 14028 | 366,5 | 13,5 | 1,298 | HSA034 C | sample | Hm Drgbl |
| 3818 | P10 | P11 | 846 | 1550 | 11444 | 389,8 | 13,5 | 1,298 | HSA034 C | sample | Hm Drgbl |
| 8122 | C18 | C19 | 1304 | 630,3 | 17547 | 497,1 | 13,5 | 1,298 | HSA034 C | sample | Hm Drgbl |
| 8164 | E12 | E13 | 1563 | 573 | 21125 | 548,9 | 13,5 | 1,298 | HSA034 C | sample | Hm Drgbl |
| 8198 | F21 | F22 | 1640 | 626,3 | 22152 | 466,7 | 13,5 | 1,298 | HSA034 C | sample | Hm Drgbl |
| 8267 | I19 | I20 | 1236 | 635,6 | 16696 | 481,5 | 13,5 | 1,298 | HSA034 C | sample | Hm Drgbl |
| 8286 | J14 | J15 | 1625 | 620,2 | 21950 | 413,8 | 13,5 | 1,298 | HSA034 C | sample | Hm Drgbl |
| 8315 | K19 | K20 | 1726 | 623,7 | 23325 | 426,2 | 13,5 | 1,298 | HSA034 C | sample | Hm Drgbl |
| 8323 | K4 | K5 | 1303 | 631,4 | 17530 | 425,7 | 13,5 | 1,298 | HSA034 C | sample | Hm Drgbl |
| 8340 | L2 | L3 | 1571 | 573,9 | 21275 | 511,4 | 13,5 | 1,298 | HSA034 C | sample | Hm Drgbl |
| 8380 | N12 | N13 | 1729 | 647,2 | 23384 | 516 | 13,5 | 1,298 | HSA034 C | sample | Hm Drgbl |
| 10074 | D3 | D4 | 1414 | 822,9 | 19107 | 142,6 | 13,5 | 1,298 | HSA034 C | sample | Hm Drgbl |
| 10115 | F19 | F20 | 1206 | 843,4 | 16262 | 142,2 | 13,5 | 1,298 | HSA034 C | sample | Hm Drgbl |
| 10230 | K14 | K15 | 1313 | 867,5 | 17776 | 143,9 | 13,5 | 1,298 | HSA034 C | sample | Hm Drgbl |
| 10309 | N20 | N21 | 1836 | 843,9 | 24869 | 159,2 | 13,5 | 1,298 | HSA034 C | sample | Hm Drgbl |
| 1563 | B11 | B12 | 1040 | 722,9 | 14069 | 473 | 13,5 | 1,414 | HSA034 C | sample | Hm Drgbl |
| 1670 | F21 | F22 | 1563 | 783,4 | 21077 | 445,4 | 13,5 | 1,414 | HSA034 C | sample | Hm Drgbl |
| 1875 | O11 | O12 | 1547 | 702,9 | 20845 | 474,5 | 13,5 | 1,414 | HSA034 C | sample | Hm Drgbl |
| 1899 | P11 | P12 | 1361 | 680,5 | 18356 | 435,7 | 13,5 | 1,414 | HSA034 C | sample | Hm Drgbl |
| 21692 | H5 | H6 | 127 | 873,5 | 1709 | 500,1 | 13,5 | 1,421 | HSA034 C | sample | Hm Gen E |
| 21843 | O11 | O12 | 367 | 854 | 4969 | 722,1 | 13,5 | 1,421 | HSA034 C | sample | Hm Gen E |
| 4992 | P9 | P10 | 515 | 939 | 6954 | 626 | 13,5 | 1,484 | HSA034 C | sample | Hm Drgbl |
| 10420 | C12 | C13 | 1311 | 881,2 | 17641 | 211,7 | 13,5 | 1,525 | HSA034 C | sample | Hm Drgbl |
| 10651 | L4 | L5 | 1215 | 804,1 | 16439 | 191,5 | 13,5 | 1,525 | HSA034 C | sample | Hm Drgbl |
| 10740 | P2 | P3 | 1182 | 808,5 | 15948 | 204,7 | 13,5 | 1,525 | HSA034 C | sample | Hm Drgbl |
| 10746 | P3 | P4 | 1098 | 838,4 | 14792 | 229,1 | 13,5 | 1,525 | HSA034 C | sample | Hm Drgbl |
| 3060 | P2 | P3 | 1003 | 621,9 | 13585 | 563,7 | 13,5 | 1,588 | HSA034 C | sample | Hm Drgbl |
| 20317 | O20 | O21 | 632 | 1039 | 8555 | 172,4 | 13,5 | 1,646 | HSA034 C | sample | Hm Gen E |
| 23634 | I3 | I4 | 793 | 1724 | 10746 | 183,1 | 13,6 | 0,721 | HSA034 C | sample | Hm Gen E |
| 14127 | M22 | M23 | 1650 | 1060 | 22480 | 176 | 13,6 | 0,779 | HSA034 C | sample | Hm Gen E |
| 3079 | A15 | A16 | 1249 | 1203 | 17020 | 387,1 | 13,6 | 0,842 | HSA034 C | sample | Hm Drgbl |
| 3187 | E4 | E5 | 883 | 1211 | 12046 | 377,1 | 13,6 | 0,842 | HSA034 C | sample | Hm Drgbl |
| 3221 | G13 | G14 | 985 | 1113 | 13364 | 442 | 13,6 | 0,842 | HSA034 C | sample | Hm Drgbl |
| 3384 | M9 | M10 | 1331 | 1282 | 18079 | 391,2 | 13,6 | 0,842 | HSA034 C | sample | Hm Drgbl |
| 12284 | P5 | P6 | 1119 | 771,3 | 15176 | 70,29 | 13,6 | 0,872 | HSA034 C | sample | Hm Gen E |
| 6468 | N2 | N3 | 1475 | 504 | 20129 | 446,4 | 13,6 | 0,898 | HSA034 C | sample | Hm Drgbl |
| 20396 | B5 | B6 | 887 | 798,3 | 12040 | 258,9 | 13,6 | 0,907 | HSA034 C | sample | Hm Gen E |
| 20466 | E3 | E4 | 847 | 761,3 | 11477 | 160,3 | 13,6 | 0,907 | HSA034 C | sample | Hm Gen E |
| 20474 | F10 | F11 | 701 | 793,4 | 9518 | 188,3 | 13,6 | 0,907 | HSA034 C | sample | Hm Gen E |
| 20549 | I13 | I14 | 908 | 762,6 | 12383 | 164,3 | 13,6 | 0,907 | HSA034 C | sample | Hm Gen E |
| 20610 | K3 | K4 | 686 | 750,1 | 9334 | 155,6 | 13,6 | 0,907 | HSA034 C | sample | Hm Gen E |
| 18759 | N22 | N23 | 1874 | 1026 | 25432 | 365,4 | 13,6 | 0,922 | HSA034 C | sample | Hm Gen E |
| 9537 | N17 | N18 | 1990 | 1210 | 27150 | 179,5 | 13,6 | 0,944 | HSA034 C | sample | Hm Drgbl |
| 19045 | J20 | J21 | 1229 | 1084 | 16748 | 268,1 | 13,6 | 0,948 | HSA034 C | sample | Hm Gen E |
| 19136 | N16 | N17 | 1540 | 975,6 | 20884 | 206 | 13,6 | 0,948 | HSA034 C | sample | Hm Gen E |
| 9730 | F18 | F19 | 1753 | 1118 | 23922 | 98,31 | 13,6 | 0,951 | HSA034 C | sample | Hm Drgbl |
| 15169 | I1 | I2 | 1412 | 798,1 | 19138 | 116,3 | 13,6 | 0,951 | HSA034 C | sample | Hm Gen E |
| 8473 | B1 | B2 | 1734 | 1079 | 23640 | 178,7 | 13,6 | 0,961 | HSA034 C | sample | Hm Drgbl |
| 21018 | L3 | L4 | 1010 | 1014 | 13769 | 237,9 | 13,6 | 0,965 | HSA034 C | sample | Hm Gen E |
| 21083 | O19 | O20 | 1298 | 1025 | 17670 | 340,9 | 13,6 | 0,965 | HSA034 C | sample | Hm Gen E |
| 21453 | N6 | N7 | 1046 | 961,9 | 14203 | 337,4 | 13,6 | 0,965 | HSA034 C | sample | Hm Gen E |
| 11293 | G20 | G21 | 979 | 884,2 | 13317 | 141,1 | 13,6 | 0,986 | HSA034 C | sample | Hm Drgbl |
| 11330 | I10 | I11 | 1090 | 791,4 | 14795 | 147,8 | 13,6 | 0,986 | HSA034 C | sample | Hm Drgbl |
| 11389 | K20 | K21 | 1993 | 921,7 | 27007 | 170,6 | 13,6 | 0,986 | HSA034 C | sample | Hm Drgbl |
| 12529 | K1 | K2 | 1559 | 1099 | 21209 | 148,3 | 13,6 | 1,023 | HSA034 C | sample | Hm Gen E |
| 7376 | D16 | D17 | 1328 | 644,2 | 18009 | 457,3 | 13,6 | 1,038 | HSA034 C | sample | Hm Drgbl |
| 7557 | K6 | K7 | 1121 | 590,3 | 15190 | 459,6 | 13,6 | 1,038 | HSA034 C | sample | Hm Drgbl |
| 16951 | C15 | C16 | 1715 | 1017 | 23399 | 252,1 | 13,6 | 1,05 | HSA034 C | sample | Hm Gen E |
| 17123 | J19 | J20 | 2217 | 1031 | 30226 | 216,9 | 13,6 | 1,05 | HSA034 C | sample | Hm Gen E |
| 17218 | N18 | N19 | 1538 | 995,8 | 20881 | 213,5 | 13,6 | 1,05 | HSA034 C | sample | Hm Gen E |
| 17234 | O10 | O11 | 1606 | 963,4 | 21787 | 271,8 | 13,6 | 1,05 | HSA034 C | sample | Hm Gen E |
| 5060 | C5 | C6 | 1357 | 659,1 | 18483 | 644,8 | 13,6 | 1,067 | HSA034 C | sample | Hm Drgbl |
| 5107 | E4 | E5 | 1456 | 611,1 | 19862 | 617,2 | 13,6 | 1,067 | HSA034 C | sample | Hm Drgbl |
| 5205 | I6 | I7 | 1999 | 611 | 27220 | 517,2 | 13,6 | 1,067 | HSA034 C | sample | Hm Drgbl |
| 5259 | L11 | L12 | 2135 | 678 | 29130 | 529,7 | 13,6 | 1,067 | HSA034 C | sample | Hm Drgbl |
| 5267 | L19 | L20 | 1822 | 597 | 24865 | 576,6 | 13,6 | 1,067 | HSA034 C | sample | Hm Drgbl |
| 5359 | P15 | P16 | 1882 | 662,7 | 25619 | 547,7 | 13,6 | 1,067 | HSA034 C | sample | Hm Drgbl |
| 2351 | B8 | B9 | 1322 | 652,3 | 17936 | 472,5 | 13,6 | 1,071 | HSA034 C | sample | Hm Drgbl |
| 2383 | D15 | D16 | 839 | 815,4 | 11418 | 560,6 | 13,6 | 1,071 | HSA034 C | sample | Hm Drgbl |
| 2472 | G9 | G10 | 1207 | 676,3 | 16363 | 374,6 | 13,6 | 1,071 | HSA034 C | sample | Hm Drgbl |
| 2569 | L1 | L2 | 1491 | 680,5 | 20208 | 384 | 13,6 | 1,071 | HSA034 C | sample | Hm Drgbl |
| 2582 | L21 | L22 | 1306 | 649,7 | 17806 | 349,7 | 13,6 | 1,071 | HSA034 C | sample | Hm Drgbl |
| 2648 | O16 | O17 | 1333 | 725,2 | 18169 | 395,8 | 13,6 | 1,071 | HSA034 C | sample | Hm Drgbl |
| 16644 | F2 | F3 | 1328 | 1022 | 18093 | 292,9 | 13,6 | 1,075 | HSA034 C | sample | Hm Gen E |
| 16652 | F5 | F6 | 1234 | 989,3 | 16826 | 239,5 | 13,6 | 1,075 | HSA034 C | sample | Hm Gen E |
| 16850 | O10 | O11 | 1357 | 941 | 18488 | 401,7 | 13,6 | 1,075 | HSA034 C | sample | Hm Gen E |
| 17747 | D19 | D20 | 1809 | 1108 | 24636 | 255,2 | 13,6 | 1,075 | HSA034 C | sample | Hm Gen E |
| 17844 | H2 | H3 | 1057 | 946,1 | 14367 | 157,5 | 13,6 | 1,075 | HSA034 C | sample | Hm Gen E |
| 17909 | K13 | K14 | 1439 | 1002 | 19638 | 225,6 | 13,6 | 1,075 | HSA034 C | sample | Hm Gen E |
| 4256 | B16 | B17 | 912 | 751,9 | 12375 | 452,2 | 13,6 | 1,148 | HSA034 C | sample | Hm Drgbl |
| 4276 | C12 | C13 | 1315 | 659,5 | 17867 | 362,6 | 13,6 | 1,148 | HSA034 C | sample | Hm Drgbl |
| 4285 | C20 | C21 | 1283 | 714 | 17454 | 438,2 | 13,6 | 1,148 | HSA034 C | sample | Hm Drgbl |
| 4318 | D7 | D8 | 961 | 696,3 | 13096 | 406,6 | 13,6 | 1,148 | HSA034 C | sample | Hm Drgbl |
| 4423 | I15 | I16 | 1025 | 758,9 | 13955 | 388,7 | 13,6 | 1,148 | HSA034 C | sample | Hm Drgbl |
| 4516 | M12 | M13 | 1424 | 659,1 | 19376 | 478,5 | 13,6 | 1,148 | HSA034 C | sample | Hm Drgbl |
| 32 | B16 | B17 | 522 | 1429 | 7115 | 547,6 | 13,6 | 1,157 | HSA034 C | sample | Hm Drgbl |
| 59 | C19 | C20 | 628 | 1511 | 8547 | 501,3 | 13,6 | 1,157 | HSA034 C | sample | Hm Drgbl |
| 301 | M20 | M21 | 685 | 1607 | 9307 | 648,8 | 13,6 | 1,157 | HSA034 C | sample | Hm Drgbl |
| 2002 | D18 | D19 | 673 | 658,1 | 9175 | 482,1 | 13,6 | 1,183 | HSA034 C | sample | Hm Drgbl |
| 2073 | G17 | G18 | 900 | 569,6 | 12230 | 402,4 | 13,6 | 1,183 | HSA034 C | sample | Hm Drgbl |
| 2074 | G18 | G19 | 1137 | 667,1 | 15438 | 383,7 | 13,6 | 1,183 | HSA034 C | sample | Hm Drgbl |
| 2236 | N12 | N13 | 2076 | 676,1 | 28257 | 431,1 | 13,6 | 1,183 | HSA034 C | sample | Hm Drgbl |
| 2237 | N13 | N14 | 1599 | 664,7 | 21790 | 445 | 13,6 | 1,183 | HSA034 C | sample | Hm Drgbl |
| 2258 | O10 | O11 | 1898 | 690,5 | 25810 | 467,6 | 13,6 | 1,183 | HSA034 C | sample | Hm Drgbl |
| 6634 | E18 | E19 | 1025 | 548,1 | 13920 | 384,3 | 13,6 | 1,193 | HSA034 C | sample | Hm Drgbl |
| 6831 | M22 | M23 | 1323 | 590,2 | 17946 | 369,8 | 13,6 | 1,193 | HSA034 C | sample | Hm Drgbl |
| 6843 | N11 | N12 | 1321 | 636,5 | 17970 | 445,6 | 13,6 | 1,193 | HSA034 C | sample | Hm Drgbl |
| 4203 | P11 | P12 | 1180 | 666,2 | 16037 | 472,9 | 13,6 | 1,209 | HSA034 C | sample | Hm Drgbl |
| 11634 | E3 | E4 | 1282 | 846,4 | 17443 | 140,9 | 13,6 | 1,214 | HSA034 C | sample | Hm Gen E |
| 11895 | P22 | P23 | 1467 | 898,8 | 19972 | 199,8 | 13,6 | 1,214 | HSA034 C | sample | Hm Gen E |
| 15456 | D9 | D10 | 1272 | 826,4 | 17244 | 147,2 | 13,6 | 1,236 | HSA034 C | sample | Hm Gen E |
| 15625 | L1 | L2 | 1033 | 889,7 | 14050 | 143,2 | 13,6 | 1,236 | HSA034 C | sample | Hm Gen E |
| 15667 | M4 | M5 | 1158 | 858,5 | 15789 | 191,5 | 13,6 | 1,236 | HSA034 C | sample | Hm Gen E |
| 5839 | D15 | D16 | 1230 | 843,9 | 16674 | 594 | 13,6 | 1,242 | HSA034 C | sample | Hm Drgbl |
| 5870 | E21 | E22 | 1243 | 754,9 | 16886 | 528,2 | 13,6 | 1,242 | HSA034 C | sample | Hm Drgbl |
| 1175 | A8 | A9 | 1539 | 747,2 | 20873 | 541 | 13,6 | 1,248 | HSA034 C | sample | Hm Drgbl |
| 1205 | C13 | C14 | 1565 | 685,6 | 21269 | 439,7 | 13,6 | 1,248 | HSA034 C | sample | Hm Drgbl |
| 1303 | G15 | G16 | 1344 | 697,8 | 18234 | 388,4 | 13,6 | 1,248 | HSA034 C | sample | Hm Drgbl |
| 1309 | G20 | G21 | 2153 | 802,3 | 29205 | 438,9 | 13,6 | 1,248 | HSA034 C | sample | Hm Drgbl |
| 1355 | I19 | I20 | 1487 | 692 | 20201 | 353,1 | 13,6 | 1,248 | HSA034 C | sample | Hm Drgbl |
| 1372 | J12 | J13 | 1897 | 730,8 | 25725 | 343,5 | 13,6 | 1,248 | HSA034 C | sample | Hm Drgbl |
| 1407 | K22 | K23 | 1701 | 743,5 | 23120 | 435,9 | 13,6 | 1,248 | HSA034 C | sample | Hm Drgbl |
| 16162 | B18 | B19 | 1460 | 980,4 | 19873 | 389,1 | 13,6 | 1,248 | HSA034 C | sample | Hm Gen E |
| 16183 | C15 | C16 | 1140 | 1069 | 15483 | 368,2 | 13,6 | 1,248 | HSA034 C | sample | Hm Gen E |
| 16260 | F2 | F3 | 1241 | 1083 | 16826 | 359 | 13,6 | 1,248 | HSA034 C | sample | Hm Gen E |
| 16262 | F21 | F22 | 736 | 1014 | 10002 | 286,1 | 13,6 | 1,248 | HSA034 C | sample | Hm Gen E |
| 16271 | F8 | F9 | 949 | 1050 | 12923 | 328,8 | 13,6 | 1,248 | HSA034 C | sample | Hm Gen E |
| 16396 | L12 | L13 | 1224 | 918,5 | 16670 | 309,1 | 13,6 | 1,248 | HSA034 C | sample | Hm Gen E |
| 13520 | D16 | D17 | 888 | 1074 | 12070 | 180,5 | 13,6 | 1,277 | HSA034 C | sample | Hm Gen E |
| 13532 | D5 | D6 | 976 | 1039 | 13249 | 165,4 | 13,6 | 1,277 | HSA034 C | sample | Hm Gen E |
| 849 | D17 | D18 | 1533 | 751 | 20800 | 432,4 | 13,6 | 1,289 | HSA034 C | sample | Hm Drgbl |
| 5412 | B2 | B3 | 1741 | 734,1 | 23707 | 560,1 | 13,6 | 1,295 | HSA034 C | sample | Hm Drgbl |
| 5492 | E5 | E6 | 1341 | 570,6 | 18251 | 515,8 | 13,6 | 1,295 | HSA034 C | sample | Hm Drgbl |
| 5717 | O13 | O14 | 742 | 621,5 | 10088 | 678,8 | 13,6 | 1,295 | HSA034 C | sample | Hm Drgbl |
| 3670 | I7 | I8 | 1306 | 1460 | 17751 | 302,7 | 13,6 | 1,308 | HSA034 C | sample | Hm Drgbl |
| 3693 | J6 | J7 | 979 | 1253 | 13309 | 323,3 | 13,6 | 1,308 | HSA034 C | sample | Hm Drgbl |
| 8161 | E1 | E2 | 660 | 616,7 | 8991 | 518,9 | 13,6 | 1,308 | HSA034 C | sample | Hm Drgbl |
| 8410 | O18 | O19 | 1595 | 609,5 | 21767 | 495,5 | 13,6 | 1,308 | HSA034 C | sample | Hm Drgbl |
| 10039 | C15 | C16 | 1388 | 863,6 | 18891 | 167,2 | 13,6 | 1,308 | HSA034 C | sample | Hm Drgbl |
| 10139 | G19 | G20 | 1667 | 899,3 | 22631 | 140,3 | 13,6 | 1,308 | HSA034 C | sample | Hm Drgbl |

| | | | | | | | | | | | | |
|---|---|---|---|---|---|---|---|---|---|---|---|---|
| 10158 | H14 | H15 | 1483 | 807,5 | 20122 | 128,2 | 13,6 | 1,308 | H5A034 C | sample | Hm Drgbl | [illegible] |
| 10351 | P15 | P16 | 1271 | 805,9 | 17343 | 218,8 | 13,6 | 1,308 | H5A034 C | sample | Hm Drgbl | [illegible] |
| 1571 | B19 | B20 | 1648 | 777,8 | 22438 | 404,8 | 13,6 | 1,424 | H5A034 C | sample | Hm Drgbl | [illegible] |
| 1587 | C11 | C12 | 1618 | 689,4 | 21930 | 371,4 | 13,6 | 1,424 | H5A034 C | sample | Hm Drgbl | [illegible] |
| 1708 | H12 | H13 | 1169 | 766,3 | 15926 | 414,7 | 13,6 | 1,424 | H5A034 C | sample | Hm Drgbl | [illegible] |
| 1741 | I20 | I21 | 1467 | 750,5 | 19924 | 288,4 | 13,6 | 1,424 | H5A034 C | sample | Hm Drgbl | [illegible] |
| 1774 | J7 | J8 | 1137 | 672,1 | 15503 | 336,2 | 13,6 | 1,424 | H5A034 C | sample | Hm Drgbl | [illegible] |
| 1803 | L11 | L12 | 1743 | 757,9 | 23686 | 365,5 | 13,6 | 1,424 | H5A034 C | sample | Hm Drgbl | [illegible] |
| 1831 | M15 | M16 | 934 | 702,8 | 12732 | 343,5 | 13,6 | 1,424 | H5A034 C | sample | Hm Drgbl | [illegible] |
| 21741 | J6 | J7 | 125 | 1049 | 1705 | 665,7 | 13,6 | 1,432 | H5A034 C | sample | Hm Gen E | [illegible] |
| 21855 | O22 | O23 | 323 | 911,5 | 4405 | 679,2 | 13,6 | 1,432 | H5A034 C | sample | Hm Gen E | [illegible] |
| 4612 | A12 | A13 | 1407 | 972,9 | 19179 | 510,7 | 13,6 | 1,495 | H5A034 C | sample | Hm Drgbl | [illegible] |
| 4807 | I15 | I16 | 736 | 739,9 | 10007 | 432,6 | 13,6 | 1,495 | H5A034 C | sample | Hm Drgbl | [illegible] |
| 4989 | P6 | P7 | 1066 | 710,5 | 14508 | 458,4 | 13,6 | 1,495 | H5A034 C | sample | Hm Drgbl | [illegible] |
| 10411 | B4 | B5 | 1102 | 871,4 | 14974 | 225,5 | 13,6 | 1,537 | H5A034 C | sample | Hm Drgbl | [illegible] |
| 10417 | C1 | C2 | 1173 | 890,9 | 15948 | 218,5 | 13,6 | 1,537 | H5A034 C | sample | Hm Drgbl | [illegible] |
| 2980 | M12 | M13 | 911 | 584,6 | 12406 | 489,8 | 13,6 | 1,6 | H5A034 C | sample | Hm Drgbl | [illegible] |
| 20094 | F14 | F15 | 451 | 1169 | 6117 | 200,2 | 13,6 | 1,659 | H5A034 C | sample | Hm Gen E | [illegible] |
| 23768 | O16 | O17 | 1150 | 1524 | 15777 | 271,6 | 13,7 | 0,727 | H5A034 C | sample | Hm Gen E | [illegible] |
| 14953 | P1 | P2 | 951 | 761,4 | 13036 | 111,8 | 13,7 | 0,781 | H5A034 C | sample | Hm Gen E | [illegible] |
| 3131 | C19 | C20 | 500 | 1283 | 6829 | 461,5 | 13,7 | 0,848 | H5A034 C | sample | Hm Drgbl | [illegible] |
| 3143 | C8 | C9 | 1054 | 1275 | 14419 | 413,5 | 13,7 | 0,848 | H5A034 C | sample | Hm Drgbl | [illegible] |
| 3338 | L10 | L11 | 1170 | 1163 | 15974 | 373,3 | 13,7 | 0,848 | H5A034 C | sample | Hm Drgbl | [illegible] |
| 3447 | P22 | P23 | 1355 | 1331 | 18502 | 505,4 | 13,7 | 0,848 | H5A034 C | sample | Hm Drgbl | [illegible] |
| 18104 | C16 | C17 | 1403 | 998,5 | 19287 | 263 | 13,7 | 0,881 | H5A034 C | sample | Hm Gen E | [illegible] |
| 18348 | M2 | M3 | 1627 | 986,4 | 22238 | 158,6 | 13,7 | 0,881 | H5A034 C | sample | Hm Gen E | [illegible] |
| 6349 | I20 | I21 | 1024 | 659,3 | 14058 | 360,8 | 13,7 | 0,904 | H5A034 C | sample | Hm Drgbl | [illegible] |
| 6457 | N1 | N2 | 1382 | 469,1 | 18909 | 465,9 | 13,7 | 0,904 | H5A034 C | sample | Hm Drgbl | [illegible] |
| 20477 | F13 | F14 | 836 | 755,5 | 11440 | 153,4 | 13,7 | 0,913 | H5A034 C | sample | Hm Gen E | [illegible] |
| 20492 | F5 | F6 | 1148 | 789,7 | 15700 | 152,3 | 13,7 | 0,913 | H5A034 C | sample | Hm Gen E | [illegible] |
| 20574 | J14 | J15 | 1027 | 779,1 | 14113 | 142,4 | 13,7 | 0,913 | H5A034 C | sample | Hm Gen E | [illegible] |
| 20600 | K16 | K17 | 596 | 777,2 | 8189 | 167,9 | 13,7 | 0,913 | H5A034 C | sample | Hm Gen E | [illegible] |
| 20619 | L11 | L12 | 855 | 781,2 | 11672 | 181,4 | 13,7 | 0,913 | H5A034 C | sample | Hm Gen E | [illegible] |
| 20651 | M19 | M20 | 1061 | 746,8 | 14532 | 174,5 | 13,7 | 0,913 | H5A034 C | sample | Hm Gen E | [illegible] |
| 12793 | F1 | F2 | 1305 | 961,3 | 17912 | 127,6 | 13,7 | 0,929 | H5A034 C | sample | Hm Gen E | [illegible] |
| 13007 | N8 | N9 | 1352 | 947,9 | 18477 | 107,1 | 13,7 | 0,929 | H5A034 C | sample | Hm Gen E | [illegible] |
| 17281 | A1 | A2 | 813 | 1027 | 11116 | 222,1 | 13,7 | 0,935 | H5A034 C | sample | Hm Gen E | [illegible] |
| 17490 | I3 | I4 | 1099 | 911,5 | 15009 | 202,8 | 13,7 | 0,935 | H5A034 C | sample | Hm Gen E | [illegible] |
| 17511 | J22 | J23 | 1660 | 987,5 | 22682 | 231,6 | 13,7 | 0,935 | H5A034 C | sample | Hm Gen E | [illegible] |
| 9243 | B11 | B12 | 1173 | 1155 | 16037 | 188,7 | 13,7 | 0,951 | H5A034 C | sample | Hm Drgbl | [illegible] |
| 9297 | D17 | D18 | 1255 | 1100 | 17216 | 157,3 | 13,7 | 0,951 | H5A034 C | sample | Hm Drgbl | [illegible] |
| 9447 | J22 | J23 | 1837 | 1129 | 25103 | 144,6 | 13,7 | 0,951 | H5A034 C | sample | Hm Drgbl | [illegible] |
| 19107 | M11 | M12 | 1499 | 942,3 | 20529 | 238,6 | 13,7 | 0,955 | H5A034 C | sample | Hm Gen E | [illegible] |
| 15171 | I11 | I12 | 1231 | 790,7 | 16925 | 128,9 | 13,7 | 0,958 | H5A034 C | sample | Hm Gen E | [illegible] |
| 15204 | J2 | J3 | 1401 | 774,2 | 19245 | 120,1 | 13,7 | 0,958 | H5A034 C | sample | Hm Gen E | [illegible] |
| 8622 | H14 | H15 | 1502 | 1020 | 20511 | 109 | 13,7 | 0,968 | H5A034 C | sample | Hm Drgbl | [illegible] |
| 8809 | P1 | P2 | 1381 | 970 | 18908 | 128,7 | 13,7 | 0,968 | H5A034 C | sample | Hm Drgbl | [illegible] |
| 20750 | A21 | A22 | 1170 | 1067 | 16062 | 387,9 | 13,7 | 0,972 | H5A034 C | sample | Hm Gen E | [illegible] |
| 20807 | C8 | C9 | 1272 | 959 | 17380 | 290,3 | 13,7 | 0,972 | H5A034 C | sample | Hm Gen E | [illegible] |
| 20942 | I21 | I22 | 1294 | 949 | 17717 | 193,6 | 13,7 | 0,972 | H5A034 C | sample | Hm Gen E | [illegible] |
| 20988 | K2 | K3 | 1075 | 953 | 14702 | 259,4 | 13,7 | 0,972 | H5A034 C | sample | Hm Gen E | [illegible] |
| 21081 | O17 | O18 | 1129 | 958,9 | 15452 | 228,5 | 13,7 | 0,972 | H5A034 C | sample | Hm Gen E | [illegible] |
| 21087 | O22 | O23 | 1460 | 991,4 | 19994 | 358,3 | 13,7 | 0,972 | H5A034 C | sample | Hm Gen E | [illegible] |
| 21433 | N1 | N2 | 1561 | 992,3 | 21342 | 248,5 | 13,7 | 0,972 | H5A034 C | sample | Hm Gen E | [illegible] |
| 14552 | O16 | O17 | 1587 | 799 | 21804 | 168,8 | 13,7 | 0,975 | H5A034 C | sample | Hm Gen E | [illegible] |
| 11216 | D16 | D17 | 1562 | 902,3 | 21447 | 157 | 13,7 | 0,993 | H5A034 C | sample | Hm Drgbl | [illegible] |
| 11311 | H15 | H16 | 579 | 782,6 | 7905 | 143,3 | 13,7 | 0,993 | H5A034 C | sample | Hm Drgbl | [illegible] |
| 11379 | K11 | K12 | 1479 | 829,8 | 20196 | 142,8 | 13,7 | 0,993 | H5A034 C | sample | Hm Drgbl | [illegible] |
| 12596 | M5 | M6 | 688 | 1153 | 9401 | 166,6 | 13,7 | 1,03 | H5A034 C | sample | Hm Gen E | [illegible] |
| 7327 | B15 | B16 | 1495 | 563,6 | 20419 | 507,9 | 13,7 | 1,046 | H5A034 C | sample | Hm Drgbl | [illegible] |
| 7497 | I17 | I18 | 1264 | 591,2 | 17263 | 385,3 | 13,7 | 1,046 | H5A034 C | sample | Hm Drgbl | [illegible] |
| 7502 | I21 | I22 | 1306 | 603,9 | 17870 | 409,3 | 13,7 | 1,046 | H5A034 C | sample | Hm Drgbl | [illegible] |
| 7536 | J9 | J10 | 1097 | 556,9 | 15001 | 402,6 | 13,7 | 1,046 | H5A034 C | sample | Hm Drgbl | [illegible] |
| 16923 | B11 | B12 | 1316 | 1003 | 18049 | 228,1 | 13,7 | 1,058 | H5A034 C | sample | Hm Gen E | [illegible] |
| 16932 | B2 | B3 | 1102 | 942,7 | 15140 | 220 | 13,7 | 1,058 | H5A034 C | sample | Hm Gen E | [illegible] |
| 17139 | K11 | K12 | 1815 | 960,2 | 24779 | 202,6 | 13,7 | 1,058 | H5A034 C | sample | Hm Gen E | [illegible] |
| 17172 | L2 | L3 | 1174 | 950,2 | 16050 | 146,7 | 13,7 | 1,058 | H5A034 C | sample | Hm Gen E | [illegible] |
| 17246 | O21 | O22 | 1734 | 1027 | 23758 | 275,3 | 13,7 | 1,058 | H5A034 C | sample | Hm Gen E | [illegible] |
| 7029 | E6 | E7 | 1348 | 575,8 | 18417 | 478,8 | 13,7 | 1,066 | H5A034 C | sample | Hm Drgbl | [illegible] |
| 7182 | L14 | L15 | 1032 | 621,8 | 14107 | 209,4 | 13,7 | 1,066 | H5A034 C | sample | Hm Drgbl | [illegible] |
| 7279 | P15 | P16 | 971 | 598,5 | 13339 | 565,6 | 13,7 | 1,066 | H5A034 C | sample | Hm Drgbl | [illegible] |
| 5061 | C6 | C7 | 1405 | 647,6 | 19311 | 549,6 | 13,7 | 1,075 | H5A034 C | sample | Hm Drgbl | [illegible] |
| 5188 | I12 | I13 | 1215 | 581,1 | 16702 | 436,9 | 13,7 | 1,075 | H5A034 C | sample | Hm Drgbl | [illegible] |
| 5198 | I21 | I22 | 2029 | 661,8 | 27749 | 436,1 | 13,7 | 1,075 | H5A034 C | sample | Hm Drgbl | [illegible] |
| 5208 | I9 | I10 | 1891 | 644,1 | 25978 | 467,7 | 13,7 | 1,075 | H5A034 C | sample | Hm Drgbl | [illegible] |
| 5365 | P20 | P21 | 1319 | 659,3 | 18127 | 580,2 | 13,7 | 1,075 | H5A034 C | sample | Hm Drgbl | [illegible] |
| 2436 | F2 | F3 | 1173 | 716,9 | 16017 | 465,9 | 13,7 | 1,079 | H5A034 C | sample | Hm Drgbl | [illegible] |
| 2571 | L11 | L12 | 1418 | 696,1 | 19404 | 296,2 | 13,7 | 1,079 | H5A034 C | sample | Hm Drgbl | [illegible] |
| 2634 | N3 | N4 | 1062 | 698,7 | 14558 | 425,7 | 13,7 | 1,079 | H5A034 C | sample | Hm Drgbl | [illegible] |
| 2662 | O7 | O8 | 1872 | 746,5 | 25623 | 411,1 | 13,7 | 1,079 | H5A034 C | sample | Hm Drgbl | [illegible] |
| 2669 | P13 | P14 | 1464 | 703,7 | 20035 | 427,9 | 13,7 | 1,079 | H5A034 C | sample | Hm Drgbl | [illegible] |
| 16567 | C15 | C16 | 1400 | 918 | 19213 | 297,9 | 13,7 | 1,083 | H5A034 C | sample | Hm Gen E | [illegible] |
| 16580 | C5 | C6 | 983 | 1006 | 13501 | 366,2 | 13,7 | 1,083 | H5A034 C | sample | Hm Gen E | [illegible] |
| 16686 | H14 | H15 | 1372 | 978,2 | 18770 | 294,2 | 13,7 | 1,083 | H5A034 C | sample | Hm Gen E | [illegible] |
| 16716 | I2 | I3 | 1126 | 939,1 | 15422 | 237,4 | 13,7 | 1,083 | H5A034 C | sample | Hm Gen E | [illegible] |
| 16741 | J20 | J21 | 1632 | 936 | 22368 | 269,4 | 13,7 | 1,083 | H5A034 C | sample | Hm Gen E | [illegible] |
| 16790 | L21 | L22 | 1499 | 965,4 | 20516 | 297,5 | 13,7 | 1,083 | H5A034 C | sample | Hm Gen E | [illegible] |
| 16791 | L22 | L23 | 1360 | 960 | 18646 | 295,9 | 13,7 | 1,083 | H5A034 C | sample | Hm Gen E | [illegible] |
| 17678 | A21 | A22 | 1162 | 1183 | 15973 | 404,9 | 13,7 | 1,083 | H5A034 C | sample | Hm Gen E | [illegible] |
| 17701 | B20 | B21 | 1538 | 1041 | 21060 | 314 | 13,7 | 1,083 | H5A034 C | sample | Hm Gen E | [illegible] |
| 17965 | M20 | M21 | 1417 | 909,1 | 19422 | 225,4 | 13,7 | 1,083 | H5A034 C | sample | Hm Gen E | [illegible] |
| 8853 | A6 | A7 | 1999 | 1944 | 27470 | 191,2 | 13,7 | 1,1 | H5A034 C | sample | Hm Drgbl | [illegible] |
| 8854 | A7 | A8 | 2019 | 1972 | 27715 | 234,4 | 13,7 | 1,1 | H5A034 C | sample | Hm Drgbl | [illegible] |
| 8908 | D12 | D13 | 1466 | 2024 | 20067 | 146,1 | 13,7 | 1,1 | H5A034 C | sample | Hm Drgbl | [illegible] |
| 4269 | B6 | B7 | 1657 | 754,7 | 22644 | 463,6 | 13,7 | 1,156 | H5A034 C | sample | Hm Drgbl | [illegible] |
| 4293 | C6 | C7 | 924 | 690,7 | 12617 | 481,8 | 13,7 | 1,156 | H5A034 C | sample | Hm Drgbl | [illegible] |
| 4321 | E1 | E2 | 911 | 734,1 | 12436 | 467,2 | 13,7 | 1,156 | H5A034 C | sample | Hm Drgbl | [illegible] |
| 4338 | E3 | E4 | 613 | 687,8 | 8417 | 473,2 | 13,7 | 1,156 | H5A034 C | sample | Hm Drgbl | [illegible] |
| 4418 | I10 | I11 | 1181 | 781,8 | 16142 | 397,4 | 13,7 | 1,156 | H5A034 C | sample | Hm Drgbl | [illegible] |
| 4427 | I19 | I20 | 699 | 660,5 | 9569 | 364,2 | 13,7 | 1,156 | H5A034 C | sample | Hm Drgbl | [illegible] |
| 4560 | N9 | N10 | 1544 | 810 | 21203 | 468,8 | 13,7 | 1,156 | H5A034 C | sample | Hm Drgbl | [illegible] |
| 4582 | O7 | O8 | 1315 | 687,7 | 17992 | 485,2 | 13,7 | 1,156 | H5A034 C | sample | Hm Drgbl | [illegible] |
| 4591 | P15 | P16 | 1063 | 802,2 | 14591 | 479,8 | 13,7 | 1,156 | H5A034 C | sample | Hm Drgbl | [illegible] |
| 36 | B2 | B3 | 569 | 1590 | 7780 | 662,5 | 13,7 | 1,166 | H5A034 C | sample | Hm Drgbl | [illegible] |
| 294 | M14 | M15 | 409 | 1502 | 5621 | 498,3 | 13,7 | 1,166 | H5A034 C | sample | Hm Drgbl | [illegible] |
| 2024 | E16 | E17 | 1053 | 556,6 | 14454 | 330,1 | 13,7 | 1,191 | H5A034 C | sample | Hm Drgbl | [illegible] |
| 2126 | I21 | I22 | 1403 | 757,8 | 19189 | 318 | 13,7 | 1,191 | H5A034 C | sample | Hm Drgbl | [illegible] |
| 6619 | D4 | D5 | 1154 | 546,6 | 15844 | 483,8 | 13,7 | 1,202 | H5A034 C | sample | Hm Drgbl | [illegible] |
| 6680 | G16 | G17 | 625 | 766,6 | 8548 | 518,8 | 13,7 | 1,202 | H5A034 C | sample | Hm Drgbl | [illegible] |
| 6900 | P2 | P3 | 1485 | 619,1 | 20334 | 443,1 | 13,7 | 1,202 | H5A034 C | sample | Hm Drgbl | [illegible] |
| 3901 | C20 | C21 | 1009 | 667,1 | 13840 | 426,4 | 13,7 | 1,218 | H5A034 C | sample | Hm Drgbl | [illegible] |
| 3919 | D15 | D16 | 1096 | 640,1 | 15068 | 367,1 | 13,7 | 1,218 | H5A034 C | sample | Hm Drgbl | [illegible] |
| 3962 | F10 | F11 | 1508 | 645,7 | 20619 | 311,3 | 13,7 | 1,218 | H5A034 C | sample | Hm Drgbl | [illegible] |
| 4027 | H4 | H5 | 1323 | 714,1 | 18142 | 418,6 | 13,7 | 1,218 | H5A034 C | sample | Hm Drgbl | [illegible] |
| 4028 | H5 | H6 | 1445 | 688 | 19790 | 513,6 | 13,7 | 1,218 | H5A034 C | sample | Hm Drgbl | [illegible] |
| 4182 | O14 | O15 | 1009 | 636,5 | 13861 | 403,1 | 13,7 | 1,218 | H5A034 C | sample | Hm Drgbl | [illegible] |
| 4212 | P2 | P3 | 1226 | 628,4 | 16784 | 432,4 | 13,7 | 1,218 | H5A034 C | sample | Hm Drgbl | [illegible] |
| 15638 | L21 | L22 | 1232 | 803,7 | 16873 | 131,8 | 13,7 | 1,245 | H5A034 C | sample | Hm Gen E | [illegible] |
| 5761 | A1 | A2 | 889 | 777,6 | 12188 | 594,4 | 13,7 | 1,251 | H5A034 C | sample | Hm Drgbl | [illegible] |
| 5866 | E18 | E19 | 1371 | 786,4 | 18724 | 573,8 | 13,7 | 1,251 | H5A034 C | sample | Hm Drgbl | [illegible] |
| 5960 | I16 | I17 | 662 | 835,3 | 9050 | 446,1 | 13,7 | 1,251 | H5A034 C | sample | Hm Drgbl | [illegible] |
| 6094 | N7 | N8 | 1253 | 700,7 | 17179 | 520,4 | 13,7 | 1,251 | H5A034 C | sample | Hm Drgbl | [illegible] |
| 1166 | A21 | A22 | 1529 | 819,6 | 20992 | 537,8 | 13,7 | 1,257 | H5A034 C | sample | Hm Drgbl | [illegible] |
| 1307 | G19 | G20 | 1711 | 740,5 | 23445 | 528,6 | 13,7 | 1,257 | H5A034 C | sample | Hm Drgbl | [illegible] |
| 1329 | H17 | H18 | 1081 | 715,2 | 14794 | 391,2 | 13,7 | 1,257 | H5A034 C | sample | Hm Drgbl | [illegible] |
| 1525 | P20 | P21 | 1963 | 793,3 | 26848 | 525,6 | 13,7 | 1,257 | H5A034 C | sample | Hm Drgbl | [illegible] |
| 1530 | P3 | P4 | 1327 | 730,9 | 18236 | 436 | 13,7 | 1,257 | H5A034 C | sample | Hm Drgbl | [illegible] |
| 16187 | C19 | C20 | 1079 | 954,6 | 14794 | 282 | 13,7 | 1,257 | H5A034 C | sample | Hm Gen E | [illegible] |
| 16261 | F20 | F21 | 1722 | 984,6 | 23651 | 259,2 | 13,7 | 1,257 | H5A034 C | sample | Hm Gen E | [illegible] |
| 16266 | F3 | F4 | 1230 | 977,2 | 16877 | 325,5 | 13,7 | 1,257 | H5A034 C | sample | Hm Gen E | [illegible] |
| 16293 | G6 | G7 | 1510 | 988,4 | 20759 | 274,9 | 13,7 | 1,257 | H5A034 C | sample | Hm Gen E | [illegible] |
| 16294 | G7 | G8 | 1013 | 914,9 | 13861 | 264,1 | 13,7 | 1,257 | H5A034 C | sample | Hm Gen E | [illegible] |
| 16314 | H3 | H4 | 884 | 980,7 | 12117 | 194,1 | 13,7 | 1,257 | H5A034 C | sample | Hm Gen E | [illegible] |
| 16339 | I4 | I5 | 1228 | 974,4 | 16879 | 248,7 | 13,7 | 1,257 | H5A034 C | sample | Hm Gen E | [illegible] |
| 16405 | L20 | L21 | 1312 | 963,1 | 17931 | 260,8 | 13,7 | 1,257 | H5A034 C | sample | Hm Gen E | [illegible] |
| 16450 | N18 | N19 | 1108 | 972,2 | 15157 | 380,8 | 13,7 | 1,257 | H5A034 C | sample | Hm Gen E | [illegible] |
| 16458 | N3 | N4 | 1111 | 1023 | 15262 | 247,9 | 13,7 | 1,257 | H5A034 C | sample | Hm Gen E | [illegible] |
| 16462 | N7 | N8 | 1263 | 896,4 | 17352 | 369 | 13,7 | 1,257 | H5A034 C | sample | Hm Gen E | [illegible] |
| 16482 | O3 | O4 | 586 | 1087 | 8040 | 511,8 | 13,7 | 1,257 | H5A034 C | sample | Hm Gen E | [illegible] |
| 13459 | A4 | A5 | 1337 | 1134 | 18264 | 197,5 | 13,7 | 1,286 | H5A034 C | sample | Hm Gen E | [illegible] |
| 13478 | B21 | B22 | 1720 | 1113 | 23629 | 191 | 13,7 | 1,286 | H5A034 C | sample | Hm Gen E | [illegible] |
| 13484 | B5 | B6 | 1088 | 1109 | 14865 | 154,9 | 13,7 | 1,286 | H5A034 C | sample | Hm Gen E | [illegible] |
| 13728 | L9 | L10 | 1007 | 1050 | 13808 | 169,1 | 13,7 | 1,286 | H5A034 C | sample | Hm Gen E | [illegible] |
| 13807 | P15 | P16 | 1572 | 995,3 | 21507 | 185,4 | 13,7 | 1,286 | H5A034 C | sample | Hm Gen E | [illegible] |
| 878 | E21 | E22 | 1428 | 892,8 | 19506 | 303,8 | 13,7 | 1,299 | H5A034 C | sample | Hm Drgbl | [illegible] |
| 985 | J1 | J2 | 967 | 695,3 | 13262 | 449,1 | 13,7 | 1,299 | H5A034 C | sample | Hm Drgbl | [illegible] |
| 5422 | B7 | B8 | 1802 | 651,5 | 24672 | 627 | 13,7 | 1,305 | H5A034 C | sample | Hm Drgbl | [illegible] |
| 5550 | H14 | H15 | 1730 | 657,9 | 23661 | 563,2 | 13,7 | 1,305 | H5A034 C | sample | Hm Drgbl | [illegible] |
| 5554 | H18 | H19 | 1480 | 689,5 | 20211 | 485,4 | 13,7 | 1,305 | H5A034 C | sample | Hm Drgbl | [illegible] |
| 5607 | J22 | J23 | 1672 | 725,8 | 22948 | 472,4 | 13,7 | 1,305 | H5A034 C | sample | Hm Drgbl | [illegible] |
| 5647 | L15 | L16 | 1550 | 608,5 | 21176 | 457,8 | 13,7 | 1,305 | H5A034 C | sample | Hm Drgbl | [illegible] |
| 5758 | P7 | P8 | 1289 | 683,3 | 17605 | 559 | 13,7 | 1,305 | H5A034 C | sample | Hm Drgbl | [illegible] |
| 3488 | B16 | B17 | 1175 | 1295 | 16150 | 329,5 | 13,7 | 1,317 | H5A034 C | sample | Hm Drgbl | [illegible] |
| 3498 | B3 | B4 | 1254 | 1308 | 17212 | 449,2 | 13,7 | 1,317 | H5A034 C | sample | Hm Drgbl | [illegible] |
| 3514 | C18 | C19 | 1099 | 1362 | 15040 | 366,2 | 13,7 | 1,317 | H5A034 C | sample | Hm Drgbl | [illegible] |
| 3540 | D2 | D3 | 1311 | 1415 | 17946 | 335,8 | 13,7 | 1,317 | H5A034 C | sample | Hm Drgbl | [illegible] |
| 3653 | I13 | I14 | 996 | 1482 | 13626 | 362,4 | 13,7 | 1,317 | H5A034 C | sample | Hm Drgbl | [illegible] |
| 3747 | M11 | M12 | 638 | 1283 | 8769 | 292,5 | 13,7 | 1,317 | H5A034 C | sample | Hm Drgbl | [illegible] |
| 3806 | O21 | O22 | 1493 | 1434 | 20483 | 405 | 13,7 | 1,317 | H5A034 C | sample | Hm Drgbl | [illegible] |
| 3822 | P14 | P15 | 1379 | 1417 | 18854 | 452,5 | 13,7 | 1,317 | H5A034 C | sample | Hm Drgbl | [illegible] |
| 8065 | A1 | A2 | 365 | 630,5 | 4993 | 628,7 | 13,7 | 1,317 | H5A034 C | sample | Hm Drgbl | [illegible] |
| 8130 | C3 | C4 | 701 | 712,7 | 9627 | 501,4 | 13,7 | 1,317 | H5A034 C | sample | Hm Drgbl | [illegible] |
| 8207 | F8 | F9 | 1212 | 631 | 16614 | 560,2 | 13,7 | 1,317 | H5A034 C | sample | Hm Drgbl | [illegible] |
| 8362 | M18 | M19 | 1730 | 585,6 | 23666 | 476,7 | 13,7 | 1,317 | H5A034 C | sample | Hm Drgbl | [illegible] |
| 10002 | A3 | A4 | 1452 | 904,5 | 19934 | 123,8 | 13,7 | 1,317 | H5A034 C | sample | Hm Drgbl | [illegible] |
| 10239 | K22 | K23 | 1842 | 950,1 | 25216 | 169,7 | 13,7 | 1,317 | H5A034 C | sample | Hm Drgbl | [illegible] |
| 1656 | E9 | E10 | 1248 | 719,6 | 17131 | 454,4 | 13,7 | 1,435 | H5A034 C | sample | Hm Drgbl | [illegible] |
| 1704 | G9 | G10 | 792 | 674,4 | 10874 | 482,2 | 13,7 | 1,435 | H5A034 C | sample | Hm Drgbl | [illegible] |
| 1801 | L1 | L2 | 1141 | 805,4 | 15594 | 415 | 13,7 | 1,435 | H5A034 C | sample | Hm Drgbl | [illegible] |
| 1827 | M11 | M12 | 1188 | 684,5 | 16329 | 373,1 | 13,7 | 1,435 | H5A034 C | sample | Hm Drgbl | [illegible] |
| 1916 | P5 | P6 | 1447 | 699,5 | 19814 | 436,9 | 13,7 | 1,435 | H5A034 C | sample | Hm Drgbl | [illegible] |
| 21510 | A14 | A15 | 281 | 859,7 | 3856 | 697,3 | 13,7 | 1,442 | H5A034 C | sample | Hm Gen E | [illegible] |
| 21581 | D13 | D14 | 367 | 940,5 | 5040 | 534,1 | 13,7 | 1,442 | H5A034 C | sample | Hm Gen E | [illegible] |
| 21852 | O2 | O3 | 359 | 851,2 | 4918 | 783,4 | 13,7 | 1,442 | H5A034 C | sample | Hm Gen E | [illegible] |
| 4693 | D20 | D21 | 1129 | 773,7 | 15470 | 466,4 | 13,7 | 1,505 | H5A034 C | sample | Hm Drgbl | [illegible] |
| 4964 | O5 | O6 | 836 | 721,7 | 11483 | 608,3 | 13,7 | 1,505 | H5A034 C | sample | Hm Drgbl | [illegible] |
| 10439 | C8 | C9 | 901 | 858,9 | 12376 | 215,8 | 13,7 | 1,548 | H5A034 C | sample | Hm Drgbl | [illegible] |
| 10450 | D18 | D19 | 1192 | 901,3 | 16271 | 224,1 | 13,7 | 1,548 | H5A034 C | sample | Hm Drgbl | [illegible] |
| 10532 | G5 | G6 | 935 | 823,1 | 12846 | 196,7 | 13,7 | 1,548 | H5A034 C | sample | Hm Drgbl | [illegible] |
| 10573 | I20 | I21 | 920 | 881 | 12639 | 201,2 | 13,7 | 1,548 | H5A034 C | sample | Hm Drgbl | [illegible] |
| 10709 | O13 | O14 | 1215 | 795,9 | 16586 | 170,1 | 13,7 | 1,548 | H5A034 C | sample | Hm Drgbl | [illegible] |
| 20082 | E3 | E4 | 229 | 1208 | 3134 | 159,3 | 13,7 | 1,671 | H5A034 C | sample | Hm Gen E | [illegible] |
| 3231 | G22 | G23 | 926 | 1277 | 12756 | 463,8 | 13,8 | 0,854 | H5A034 C | sample | Hm Drgbl | [illegible] |
| 3285 | I6 | I7 | 1425 | 1199 | 19688 | 363,8 | 13,8 | 0,854 | H5A034 C | sample | Hm Drgbl | [illegible] |
| 3367 | M15 | M16 | 692 | 1290 | 9544 | 402,3 | 13,8 | 0,854 | H5A034 C | sample | Hm Drgbl | [illegible] |
| 3402 | N3 | N4 | 1572 | 1312 | 21709 | 389,8 | 13,8 | 0,854 | H5A034 C | sample | Hm Drgbl | [illegible] |
| 3416 | O16 | O17 | 1125 | 1291 | 15490 | 428,9 | 13,8 | 0,854 | H5A034 C | sample | Hm Drgbl | [illegible] |
| 12241 | O1 | O2 | 1667 | 844,5 | 22925 | 132,1 | 13,8 | 0,885 | H5A034 C | sample | Hm Gen E | [illegible] |
| 12272 | P16 | P17 | 1521 | 810,2 | 20968 | 81,24 | 13,8 | 0,885 | H5A034 C | sample | Hm Gen E | [illegible] |
| 19717 | F20 | F21 | 1199 | 372,9 | 16574 | 269,8 | 13,8 | 0,899 | H5A034 C | sample | Hm Gen E | [illegible] |
| 19861 | L20 | L21 | 1240 | 345,2 | 17067 | 251,2 | 13,8 | 0,899 | H5A034 C | sample | Hm Gen E | [illegible] |
| 19914 | N3 | N4 | 920 | 360 | 12739 | 232,1 | 13,8 | 0,899 | H5A034 C | sample | Hm Gen E | [illegible] |
| 6238 | D7 | D8 | 1134 | 543,3 | 15599 | 476,2 | 13,8 | 0,911 | H5A034 C | sample | Hm Drgbl | [illegible] |
| 6260 | E5 | E6 | 1317 | 494,5 | 18111 | 515,8 | 13,8 | 0,911 | H5A034 C | sample | Hm Drgbl | [illegible] |
| 6489 | O17 | O18 | 1119 | 528,6 | 15407 | 486,4 | 13,8 | 0,911 | H5A034 C | sample | Hm Drgbl | [illegible] |
| 20476 | F12 | F13 | 888 | 794,6 | 12276 | 207,2 | 13,8 | 0,92 | H5A034 C | sample | Hm Gen E | [illegible] |
| 20614 | K7 | K8 | 448 | 736,1 | 6188 | 179,6 | 13,8 | 0,92 | H5A034 C | sample | Hm Gen E | [illegible] |
| 20637 | L6 | L7 | 520 | 833,6 | 7160 | 143,6 | 13,8 | 0,92 | H5A034 C | sample | Hm Gen E | [illegible] |
| 20686 | N7 | N8 | 700 | 746,8 | 9674 | 193,2 | 13,8 | 0,92 | H5A034 C | sample | Hm Gen E | [illegible] |
| 12834 | G3 | G4 | 1009 | 1070 | 13960 | 123,6 | 13,8 | 0,936 | H5A034 C | sample | Hm Gen E | [illegible] |
| 18708 | L2 | L3 | 1265 | 927,7 | 17492 | 148,4 | 13,8 | 0,936 | H5A034 C | sample | Hm Gen E | [illegible] |
| 17317 | B20 | B21 | 1391 | 983,9 | 19205 | 223,2 | 13,8 | 0,942 | H5A034 C | sample | Hm Gen E | [illegible] |
| 17415 | F22 | F23 | 1396 | 995,7 | 19210 | 176 | 13,8 | 0,942 | H5A034 C | sample | Hm Gen E | [illegible] |
| 9408 | H9 | H10 | 1466 | 1103 | 20175 | 124,4 | 13,8 | 0,958 | H5A034 C | sample | Hm Drgbl | [illegible] |
| 9480 | K9 | K10 | 1366 | 1100 | 18841 | 137,3 | 13,8 | 0,958 | H5A034 C | sample | Hm Drgbl | [illegible] |
| 9882 | L3 | L4 | 1028 | 1096 | 14176 | 151 | 13,8 | 0,965 | H5A034 C | sample | Hm Drgbl | [illegible] |
| 20838 | E14 | E15 | 882 | 1010 | 12202 | 337,8 | 13,8 | 0,979 | H5A034 C | sample | Hm Gen E | [illegible] |
| 20847 | E22 | E23 | 1197 | 955,1 | 16552 | 251,7 | 13,8 | 0,979 | H5A034 C | sample | Hm Gen E | [illegible] |
| 20965 | J20 | J21 | 1326 | 1005 | 18273 | 319 | 13,8 | 0,979 | H5A034 C | sample | Hm Gen E | [illegible] |
| 21054 | N14 | N15 | 1383 | 919,4 | 19135 | 256,3 | 13,8 | 0,979 | H5A034 C | sample | Hm Gen E | [illegible] |
| 14305 | E1 | E2 | 823 | 798,8 | 11352 | 135,3 | 13,8 | 0,982 | H5A034 C | sample | Hm Gen E | [illegible] |
| 11259 | F11 | F12 | 1030 | 792,7 | 14212 | 132 | 13,8 | 1 | H5A034 C | sample | Hm Drgbl | [illegible] |
| 12570 | L3 | L4 | 1202 | 1000 | 16617 | 151,7 | 13,8 | 1,038 | H5A034 C | sample | Hm Gen E | [illegible] |
| 10941 | H6 | H7 | 805 | 714,9 | 11088 | 410,6 | 13,8 | 1,045 | H5A034 C | sample | Hm Drgbl | [illegible] |
| 7307 | A19 | A20 | 1321 | 662,4 | 18194 | 555,2 | 13,8 | 1,053 | H5A034 C | sample | Hm Drgbl | [illegible] |
| 7439 | F8 | F9 | 756 | 573,8 | 10396 | 479,8 | 13,8 | 1,053 | H5A034 C | sample | Hm Drgbl | [illegible] |
| 17036 | F5 | F6 | 985 | 914,7 | 13610 | 174,3 | 13,8 | 1,066 | H5A034 C | sample | Hm Gen E | [illegible] |
| 17156 | K5 | K6 | 943 | 1036 | 12971 | 229,5 | 13,8 | 1,066 | H5A034 C | sample | Hm Gen E | [illegible] |
| 17182 | L7 | L8 | 1326 | 999,7 | 18237 | 181,8 | 13,8 | 1,066 | H5A034 C | sample | Hm Gen E | [illegible] |
| 17189 | M13 | M14 | 2016 | 1098 | 27839 | 174,3 | 13,8 | 1,066 | H5A034 C | sample | Hm Gen E | [illegible] |
| 17192 | M16 | M17 | 1705 | 969,5 | 23580 | 165,8 | 13,8 | 1,066 | H5A034 C | sample | Hm Gen E | [illegible] |
| 7191 | L22 | L23 | 1322 | 569,4 | 18275 | 513,9 | 13,8 | 1,074 | H5A034 C | sample | Hm Drgbl | [illegible] |
| 7272 | O9 | O10 | 1192 | 508,4 | 16427 | 514,7 | 13,8 | 1,074 | H5A034 C | sample | Hm Drgbl | [illegible] |
| 5078 | D21 | D22 | 1609 | 621,7 | 22127 | 540,6 | 13,8 | 1,082 | H5A034 C | sample | Hm Drgbl | [illegible] |
| 5127 | F22 | F23 | 1320 | 611,8 | 18214 | 560,2 | 13,8 | 1,082 | H5A034 C | sample | Hm Drgbl | [illegible] |
| 5183 | H8 | H9 | 1316 | 666,5 | 18174 | 464,9 | 13,8 | 1,082 | H5A034 C | sample | Hm Drgbl | [illegible] |

| | | | | | | | | | | | | | | | | | | | | | | | | | | | | | | | |
|---|---|---|---|---|---|---|---|---|---|---|---|---|---|---|---|---|---|---|---|---|---|---|---|---|---|---|---|---|---|---|---|
| 2326 | A7 | A8 | 1098 | 665,7 | 15184 | 417,7 | 13,8 | 1,087 | H5A034 C | sample | Hm Drgbl | Hm Drgbl | Hm Drgbl | CPE002DC | CPE002EE | CPE002F4 | NM 00182 | NM 0018 | NM 0018 | CKM | CKM | CKM | creatine kinase, n | creatine k | creatine k | 1158 | 1158 | 1158 | s3094 | s3095 | s3096 |
| 2387 | D19 | D20 | 1281 | 830,6 | 17644 | 451,5 | 13,8 | 1,087 | H5A034 C | sample | Hm Drgbl | Hm Drgbl | Hm Drgbl | CPE002DC | CPE002EE | CPE002F4 | NM 00126 | NM 0012 | NM 0012 | CDK9 | CDK9 | CDK9 | cyclin-dependent | cyclin-dep | cyclin-dep | 1025 | 1025 | 1025 | s2834 | s2835 | s2836 |
| 2535 | J22 | J23 | 1039 | 687,6 | 14379 | 438,5 | 13,8 | 1,087 | H5A034 C | sample | Hm Drgbl | Hm Drgbl | Hm Drgbl | CPE002DC | CPE002EE | CPE002F4 | NM 00100 | NM 0010 | NM 0010 | RPS6KA1 | RPS6KA1 | RPS6KA1 | ribosomal protei | ribosomal | ribosomal | 6195 | 6195 | 6195 | s12273 | s12274 | s12275 |
| 2614 | M7 | M8 | 895 | 640,6 | 12342 | 371,9 | 13,8 | 1,087 | H5A034 C | sample | Hm Drgbl | Hm Drgbl | Hm Drgbl | CPE002DC | CPE002EE | CPE002F4 | NM 00731 | NM 0073 | NM 0073 | ABL2 | ABL2 | ABL2 | v-abl Abelson mu | v-abl Abel | v-abl Abel | 27 | 27 | 27 | s870 | s871 | s872 |
| 2647 | O15 | O16 | 1469 | 735,2 | 20258 | 523,5 | 13,8 | 1,087 | H5A034 C | sample | Hm Drgbl | Hm Drgbl | Hm Drgbl | CPE002DC | CPE002EE | CPE002F4 | NM 02191 | NM 0219 | NM 0219 | AXL | AXL | AXL | AXL receptor tyro | AXL recep | AXL recep | 558 | 558 | 558 | s1845 | s1846 | s1847 |
| 16573 | C20 | C21 | 1398 | 980,8 | 19351 | 318,2 | 13,8 | 1,091 | H5A034 C | sample | Hm Gen E | Hm Gen E | Hm Gen E | CPE002JH | CPE002NV | CPE002KC | NM 13856 | NM 1385 | NM 1385 | C6orf142 | C6orf142 | C6orf142 | chromosome 6 op | chromoso | chromoso | 90523 | 90523 | 90523 | s40410 | s40411 | s40412 |
| 16683 | H11 | H12 | 1248 | 926,6 | 17240 | 234 | 13,8 | 1,091 | H5A034 C | sample | Hm Gen E | Hm Gen E | Hm Gen E | CPE002JH | CPE002NV | CPE002KC | NM 03311 | NM 0331 | NM 0331 | C6orf153 | C6orf153 | C6orf153 | chromosome 6 or | chromoso | chromoso | 88745 | 88745 | 88745 | s40096 | s40097 | s40098 |
| 16777 | L1 | L2 | 1118 | 978,5 | 15389 | 291,3 | 13,8 | 1,091 | H5A034 C | sample | Hm Gen E | Hm Gen E | Hm Gen E | CPE002JH | CPE002NV | CPE002KC | NM 05284 | NM 0528 | NM 0528 | WDR34 | WDR34 | WDR34 | WD repeat domai | WD repea | WD repea | 89891 | 89891 | 89891 | s40194 | s40195 | s40196 |
| 16787 | L19 | L20 | 1308 | 976,5 | 18020 | 265,5 | 13,8 | 1,091 | H5A034 C | sample | Hm Gen E | Hm Gen E | Hm Gen E | CPE002JH | CPE002NV | CPE002KC | XM 02821 | XM 02821 | XM 02821 | FLJ20021 | FLJ20021 | FLJ20021 | hypothetical LOC | hypotheti | hypotheti | 90024 | 90024 | 90024 | s40232 | s40233 | s40234 |
| 16808 | M16 | M17 | 1071 | 946,9 | 14775 | 371,6 | 13,8 | 1,091 | H5A034 C | sample | Hm Gen E | Hm Gen E | Hm Gen E | CPE002JH | CPE002NV | CPE002KC | NM 15369 | NM 1536 | NM 1536 | ANKRD44 | ANKRD44 | ANKRD44 | ankyrin repeat dc | ankyrin re | ankyrin re | 91526 | 91526 | 91526 | s40681 | s40682 | s40683 |
| 16829 | N13 | N14 | 1304 | 1005 | 18053 | 309 | 13,8 | 1,091 | H5A034 C | sample | Hm Gen E | Hm Gen E | Hm Gen E | CPE002JH | CPE002NV | CPE002KC | NM 05816 | NM 0581 | NM 0581 | TSR2 | TSR2 | TSR2 | TSR2, 20S rRNA a | TSR2, 20S | TSR2, 20S | 90121 | 90121 | 90121 | s40258 | s40259 | s40260 |
| 16835 | N19 | N20 | 1662 | 984,8 | 22942 | 273,1 | 13,8 | 1,091 | H5A034 C | sample | Hm Gen E | Hm Gen E | Hm Gen E | CPE002JH | CPE002NV | CPE002KC | NM 14523 | NM 1452 | NM 1452 | C14orf143 | C14orf143 | C14orf143 | chromosome 14 c | chromoso | chromoso | 90141 | 90141 | 90141 | s40270 | s40271 | s40272 |
| 17835 | H11 | H12 | 1226 | 937,1 | 16916 | 200,1 | 13,8 | 1,091 | H5A034 C | sample | Hm Gen E | Hm Gen E | Hm Gen E | CPE002JK | CPE002NZ | CPE002KF | NM 15245 | NM 1524 | NM 1524 | ZNF785 | ZNF785 | ZNF785 | zinc finger protei | zinc finge | zinc finge | 146540 | 146540 | 146540 | s44864 | s44865 | s44866 |
| 18037 | P20 | P21 | 1472 | 1178 | 20366 | 382,2 | 13,8 | 1,091 | H5A034 C | sample | Hm Gen E | Hm Gen E | Hm Gen E | CPE002JK | CPE002NZ | CPE002KF | NM 14528 | NM 1452 | NM 1452 | LOC15332 | LOC15332 | LOC15332 | similar to CG4995 | similar to | similar to | 153328 | 153328 | 153328 | s45769 | s45770 | s45771 |
| 8877 | B6 | B7 | 1373 | 1746 | 19006 | 206 | 13,8 | 1,108 | H5A034 C | sample | Hm Drgbl | Hm Drgbl | Hm Drgbl | CPE002E7 | CPE002ES | CPE002FD | NM 01894 | NM 0189 | NM 0189 | NANS | NANS | NANS | N-acetylneuramir | N-acetyln | N-acetyln | 54187 | 54187 | 54187 | s28894 | s28895 | s28893 |
| 8890 | C18 | C19 | 2267 | 2053 | 31268 | 156 | 13,8 | 1,108 | H5A034 C | sample | Hm Drgbl | Hm Drgbl | Hm Drgbl | CPE002E7 | CPE002ES | CPE002FD | NM 01597 | NM 0159 | NM 0159 | TAF9B | TAF9B | TAF9B | TAF9B RNA polym | TAF9B RN | TAF9B RN | 51616 | 51616 | 51616 | s28414 | s28415 | s224223 |
| 9002 | H10 | H11 | 1641 | 1891 | 22623 | 154,5 | 13,8 | 1,108 | H5A034 C | sample | Hm Drgbl | Hm Drgbl | Hm Drgbl | CPE002E7 | CPE002ES | CPE002FD | NM 01905 | NM 0190 | NM 0190 | NDUFB11 | NDUFB11 | NDUFB11 | NADH dehydroger | NADH deh | NADH deh | 54539 | 54539 | 54539 | s29165 | s29163 | s224300 |
| 9036 | I2 | I3 | 1766 | 1844 | 24365 | 193,8 | 13,8 | 1,108 | H5A034 C | sample | Hm Drgbl | Hm Drgbl | Hm Drgbl | CPE002E7 | CPE002ES | CPE002FD | NM 01632 | NM 0163 | NM 0163 | UPB1 | UPB1 | UPB1 | ureidopropionase | ureidopro | ureidopro | 51733 | 51733 | 51733 | s28591 | s28592 | s28593 |
| 9098 | L10 | L11 | 1679 | 1694 | 23233 | 142,3 | 13,8 | 1,108 | H5A034 C | sample | Hm Drgbl | Hm Drgbl | Hm Drgbl | CPE002E7 | CPE002ES | CPE002FD | NM 02115 | NM 0211 | NM 0211 | CROT | CROT | CROT | carnitine O-octan | carnitine | carnitine | 54677 | 54677 | 54677 | s29314 | s29312 | s29313 |
| 9186 | O3 | O4 | 1536 | 1648 | 21250 | 129,3 | 13,8 | 1,108 | H5A034 C | sample | Hm Drgbl | Hm Drgbl | Hm Drgbl | CPE002E7 | CPE002ES | CPE002FD | NM 18152 | NM 1815 | NM 1815 | NAT5 | NAT5 | NAT5 | N-acetyltransfera | N-acetyltr | N-acetyltr | 51126 | 51126 | 51126 | s27500 | s27501 | s27502 |
| 4255 | B15 | B16 | 1328 | 727,4 | 18306 | 416,4 | 13,8 | 1,165 | H5A034 C | sample | Hm Drgbl | Hm Drgbl | Hm Drgbl | CPE002DU | CPE002EK | CPE002FQ | NM 00151 | NM 0015 | NM 0015 | GRID2 | GRID2 | GRID2 | glutamate recept | glutamate | glutamate | 2895 | 2895 | 2895 | s6146 | s6147 | s6148 |
| 4261 | B20 | B21 | 1477 | 774,8 | 20402 | 432,2 | 13,8 | 1,165 | H5A034 C | sample | Hm Drgbl | Hm Drgbl | Hm Drgbl | CPE002DU | CPE002EK | CPE002FQ | NM 02082 | NM 0208 | NM 0208 | KCNT1 | KCNT1 | KCNT1 | potassium chann | potassium | potassium | 57582 | 57582 | 57582 | s33367 | s33368 | s224474 |
| 4268 | B5 | B6 | 1556 | 786 | 21418 | 460,2 | 13,8 | 1,165 | H5A034 C | sample | Hm Drgbl | Hm Drgbl | Hm Drgbl | CPE002DU | CPE002EK | CPE002FQ | NM 00082 | NM 0008 | NM 0008 | GRIA1 | GRIA1 | GRIA1 | glutamate recept | glutamate | glutamate | 2890 | 2890 | 2890 | s6131 | s6132 | s6133 |
| 4271 | B8 | B9 | 995 | 649,1 | 13763 | 373,4 | 13,8 | 1,165 | H5A034 C | sample | Hm Drgbl | Hm Drgbl | Hm Drgbl | CPE002DU | CPE002EK | CPE002FQ | NM 02205 | NM 0220 | NM 0220 | KCNK12 | KCNK12 | KCNK12 | potassium chann | potassium | potassium | 56660 | 56660 | 56660 | s32226 | s32227 | s32228 |
| 4291 | C4 | C5 | 926 | 650,3 | 12814 | 492,7 | 13,8 | 1,165 | H5A034 C | sample | Hm Drgbl | Hm Drgbl | Hm Drgbl | CPE002DU | CPE002EK | CPE002FQ | NM 00337 | NM 0033 | NM 0033 | VDAC1 | VDAC1 | VDAC1 | voltage-depender | voltage-de | voltage-de | 7416 | 7416 | 7416 | s14768 | s14769 | s14770 |
| 4297 | D1 | D2 | 1630 | 728,9 | 22479 | 443,5 | 13,8 | 1,165 | H5A034 C | sample | Hm Drgbl | Hm Drgbl | Hm Drgbl | CPE002DU | CPE002EK | CPE002FQ | NM 01461 | NM 0146 | NM 0146 | GRIK4 | GRIK4 | GRIK4 | glutamate recept | glutamate | glutamate | 2900 | 2900 | 2900 | s6161 | s6162 | s223812 |
| 4407 | H22 | H23 | 1144 | 714,2 | 15769 | 524,5 | 13,8 | 1,165 | H5A034 C | sample | Hm Drgbl | Hm Drgbl | Hm Drgbl | CPE002DU | CPE002EK | CPE002FQ | NM 05286 | NM 0528 | NM 0528 | NALCN | NALCN | NALCN | sodium leak chan | sodium le | sodium le | 259232 | 259232 | 259232 | s48917 | s48918 | s48919 |
| 4449 | J17 | J18 | 1180 | 638,4 | 16264 | 301,4 | 13,8 | 1,165 | H5A034 C | sample | Hm Drgbl | Hm Drgbl | Hm Drgbl | CPE002DU | CPE002EK | CPE002FQ | NM 00224 | NM 0022 | NM 0022 | KCNJ13 | KCNJ13 | KCNJ13 | potassium inward | potassium | potassium | 3769 | 3769 | 3769 | s7765 | s7766 | s7767 |
| 4491 | L11 | L12 | 817 | 687,5 | 11264 | 444,8 | 13,8 | 1,165 | H5A034 C | sample | Hm Drgbl | Hm Drgbl | Hm Drgbl | CPE002DU | CPE002EK | CPE002FQ | NM 00413 | NM 0041 | NM 0041 | KCNMB1 | KCNMB1 | KCNMB1 | potassium large c | potassium | potassium | 3779 | 3779 | 3779 | s7789 | s7790 | s7791 |
| 4514 | M10 | M11 | 925 | 803 | 12770 | 628,8 | 13,8 | 1,165 | H5A034 C | sample | Hm Drgbl | Hm Drgbl | Hm Drgbl | CPE002DU | CPE002EK | CPE002FQ | NM 01692 | NM 0169 | NM 0169 | CLIC5 | CLIC5 | CLIC5 | chloride intracellu | chloride ir | chloride ir | 53405 | 53405 | 53405 | s28730 | s28731 | s28732 |
| 33 | B17 | B18 | 585 | 1382 | 8096 | 614,7 | 13,8 | 1,174 | H5A034 C | sample | Hm Drgbl | Hm Drgbl | Hm Drgbl | CPE002DT | CPE002EJ | CPE002F9 | NM 00300 | NM 0030 | NM 0030 | SELP | SELP | SELP | selectin P (granul | selectin P | selectin P | 6403 | 6403 | 6403 | s12683 | s12684 | s12685 |
| 2040 | E9 | E10 | 1174 | 611,1 | 16222 | 443,8 | 13,8 | 1,2 | H5A034 C | sample | Hm Drgbl | Hm Drgbl | Hm Drgbl | CPE002FX | CPE002G1 | CPE002G5 | NM 03341 | NM 0334 | NM 0334 | ZNF764 | ZNF764 | ZNF764 | zinc finger protei | zinc finge | zinc finge | 92595 | 92595 | 92595 | s40974 | s40975 | s40976 |
| 2090 | H10 | H11 | 1213 | 681 | 16703 | 409,9 | 13,8 | 1,2 | H5A034 C | sample | Hm Drgbl | Hm Drgbl | Hm Drgbl | CPE002FX | CPE002G1 | CPE002G5 | NM 00101 | NM 0010 | NM 0010 | ZNF793 | ZNF793 | ZNF793 | zinc finger protei | zinc finge | zinc finge | 390927 | 390927 | 390927 | s52887 | s52888 | s52889 |
| 2109 | H6 | H7 | 1840 | 726 | 25316 | 428 | 13,8 | 1,2 | H5A034 C | sample | Hm Drgbl | Hm Drgbl | Hm Drgbl | CPE002FX | CPE002G1 | CPE002G5 | NM 00101 | NM 0010 | NM 0010 | C1QL3 | C1QL3 | C1QL3 | complement com | compleme | compleme | 389941 | 389941 | 389941 | s52603 | s52604 | s52605 |
| 2110 | H7 | H8 | 1150 | 665,3 | 15834 | 398,2 | 13,8 | 1,2 | H5A034 C | sample | Hm Drgbl | Hm Drgbl | Hm Drgbl | CPE002FX | CPE002G1 | CPE002G5 | NM 15247 | NM 1524 | NM 1524 | ZNF560 | ZNF560 | ZNF560 | zinc finger protei | zinc finge | zinc finge | 147741 | 147741 | 147741 | s45048 | s45049 | s45050 |
| 6614 | D21 | D22 | 1182 | 548,2 | 16270 | 481,3 | 13,8 | 1,211 | H5A034 C | sample | Hm Drgbl | Hm Drgbl | Hm Drgbl | CPE002E1 | CPE002EY | CPE002FJ | NM 15273 | NM 1527 | NM 1527 | HOXA9 | HOXA9 | HOXA9 | homeobox A9 | homeobo | homeobo | 3205 | 3205 | 3205 | s6773 | s223865 | s6775 |
| 6655 | F15 | F16 | 1334 | 561,5 | 18405 | 429,9 | 13,8 | 1,211 | H5A034 C | sample | Hm Drgbl | Hm Drgbl | Hm Drgbl | CPE002E1 | CPE002EY | CPE002FJ | NM 01895 | NM 0189 | NM 0189 | HOXB6 | HOXB6 | HOXB6 | homeobox B6 | homeobo | homeobo | 3216 | 3216 | 3216 | s6803 | s6805 | s6804 |
| 6684 | G2 | G3 | 607 | 635 | 8374 | 408,6 | 13,8 | 1,211 | H5A034 C | sample | Hm Drgbl | Hm Drgbl | Hm Drgbl | CPE002E1 | CPE002EY | CPE002FJ | NM 00060 | NM 0006 | NM 0006 | IL6 | IL6 | IL6 | interleukin 6 (inte | interleuki | interleuki | 3569 | 3569 | 3569 | s7313 | s7311 | s7312 |
| 6899 | P19 | P20 | 1402 | 572 | 19408 | 488,5 | 13,8 | 1,211 | H5A034 C | sample | Hm Drgbl | Hm Drgbl | Hm Drgbl | CPE002E1 | CPE002EY | CPE002FJ | NM 00413 | NM 0041 | NM 0041 | IDH3G | IDH3G | IDH3G | isocitrate dehydrc | isocitrate | isocitrate | 3421 | 3421 | 3421 | s153 | s155 | s154 |
| 6908 | P5 | P6 | 978 | 577,9 | 13452 | 541 | 13,8 | 1,211 | H5A034 C | sample | Hm Drgbl | Hm Drgbl | Hm Drgbl | CPE002E1 | CPE002EY | CPE002FJ | NM 00020 | NM 0002 | NM 0002 | ICAM1 | ICAM1 | ICAM1 | intercellular adhe | intercellul | intercellul | 3383 | 3383 | 3383 | s7087 | s7088 | s7086 |
| 3850 | A18 | A19 | 1508 | 650,1 | 20761 | 337,6 | 13,8 | 1,227 | H5A034 C | sample | Hm Drgbl | Hm Drgbl | Hm Drgbl | CPE002DS | CPE002EI | CPE002F8 | NM 00286 | NM 0028 | NM 0028 | PCYT2 | PCYT2 | PCYT2 | phosphate cytidy | phosphat | phosphat | 5833 | 5833 | 5833 | s11636 | s11637 | s11638 |
| 3889 | C1 | C2 | 707 | 703,7 | 9763 | 465,8 | 13,8 | 1,227 | H5A034 C | sample | Hm Drgbl | Hm Drgbl | Hm Drgbl | CPE002DS | CPE002EI | CPE002F8 | NM 00068 | NM 0006 | NM 0006 | ADRA2C | ADRA2C | ADRA2C | adrenergic, alpha | adrenergi | adrenergi | 152 | 152 | 152 | s194275 | s194276 | s194277 |
| 3944 | E16 | E17 | 953 | 638,2 | 13167 | 321 | 13,8 | 1,227 | H5A034 C | sample | Hm Drgbl | Hm Drgbl | Hm Drgbl | CPE002DS | CPE002EI | CPE002F8 | NM 00287 | NM 0028 | NM 0028 | RABIF | RABIF | RABIF | RAB interacting fa | RAB intera | RAB intera | 5877 | 5877 | 5877 | s11707 | s11705 | s11706 |
| 4036 | I12 | I13 | 1075 | 722,8 | 14812 | 380,2 | 13,8 | 1,227 | H5A034 C | sample | Hm Drgbl | Hm Drgbl | Hm Drgbl | CPE002DS | CPE002EI | CPE002F8 | NM 00626 | NM 0062 | NM 0062 | RANBP2 | RANBP2 | RANBP2 | RAN binding prot | RAN bindi | RAN bindi | 5903 | 5903 | 5903 | s11773 | s11774 | s11775 |
| 4122 | L3 | L4 | 1240 | 685,6 | 17134 | 385 | 13,8 | 1,227 | H5A034 C | sample | Hm Drgbl | Hm Drgbl | Hm Drgbl | CPE002DS | CPE002EI | CPE002F8 | NM 00416 | NM 0041 | NM 0041 | PYY | PYY | PYY | peptide YY | peptide Y | peptide Y | 5697 | 5697 | 5697 | s11364 | s11363 | s224443 |
| 4186 | O18 | O19 | 1379 | 601 | 19090 | 390,8 | 13,8 | 1,227 | H5A034 C | sample | Hm Drgbl | Hm Drgbl | Hm Drgbl | CPE002DS | CPE002EI | CPE002F8 | NM 00290 | NM 0029 | NM 0029 | REG1A | REG1A | REG1A | regenerating islet | regenerat | regenerat | 5967 | 5967 | 5967 | s11910 | s11908 | s11909 |
| 15399 | B22 | B23 | 1564 | 866,4 | 21559 | 169,6 | 13,8 | 1,255 | H5A034 C | sample | Hm Gen E | Hm Gen E | Hm Gen E | CPE002JE | CPE002NT | CPE002K9 | NM 00101 | NM 0010 | NM 0010 | CHCHD7 | CHCHD7 | CHCHD7 | coiled-coil-helix-c | coiled-coi | coiled-coi | 79145 | 79145 | 79145 | s35620 | s35621 | s35622 |
| 6055 | M15 | M16 | 1220 | 774,7 | 16851 | 410,1 | 13,8 | 1,26 | H5A034 C | sample | Hm Drgbl | Hm Drgbl | Hm Drgbl | CPE002DY | CPE002F1 | CPE002FM | NM 00002 | NM 0000 | NM 0000 | AGL | AGL | AGL | amylo-1, 6-glucos | amylo-1, 6 | amylo-1, 6 | 178 | 178 | 178 | s1169 | s1171 | s1170 |
| 6095 | N8 | N9 | 1215 | 703,4 | 16776 | 601 | 13,8 | 1,26 | H5A034 C | sample | Hm Drgbl | Hm Drgbl | Hm Drgbl | CPE002DY | CPE002F1 | CPE002FM | NM 00101 | NM 0010 | NM 0010 | CARS | CARS | CARS | cysteinyl-tRNA sy | cysteinyl-t | cysteinyl-t | 833 | 833 | 833 | s2405 | s2406 | s2404 |
| 1194 | B3 | B4 | 1793 | 705,9 | 24810 | 464,8 | 13,8 | 1,266 | H5A034 C | sample | Hm Drgbl | Hm Drgbl | Hm Drgbl | CPE002FV | CPE002FZ | CPE002G3 | NM 00100 | NM 0010 | NM 0010 | PRDM2 | PRDM2 | PRDM2 | PR domain contai | PR domai | PR domai | 7799 | 7799 | 7799 | s15356 | s15357 | s15358 |
| 1204 | C12 | C13 | 1488 | 729,6 | 20546 | 461,8 | 13,8 | 1,266 | H5A034 C | sample | Hm Drgbl | Hm Drgbl | Hm Drgbl | CPE002FV | CPE002FZ | CPE002G3 | NM 01468 | NM 0146 | NM 0146 | ST18 | ST18 | ST18 | suppression of tu | suppressi | suppressi | 9705 | 9705 | 9705 | s18701 | s18702 | s18703 |
| 1308 | G2 | G3 | 890 | 746,5 | 12276 | 444,4 | 13,8 | 1,266 | H5A034 C | sample | Hm Drgbl | Hm Drgbl | Hm Drgbl | CPE002FV | CPE002FZ | CPE002G3 | NM 01486 | NM 0148 | NM 0148 | ARNT2 | ARNT2 | ARNT2 | aryl-hydrocarbon | aryl-hydrc | aryl-hydrc | 9915 | 9915 | 9915 | s19227 | s19228 | s19229 |
| 1419 | L11 | L12 | 1355 | 738,5 | 18700 | 415,9 | 13,8 | 1,266 | H5A034 C | sample | Hm Drgbl | Hm Drgbl | Hm Drgbl | CPE002FV | CPE002FZ | CPE002G3 | NM 00103 | NM 0010 | NM 0010 | CBFA2T2 | CBFA2T2 | CBFA2T2 | core-binding facto | core-bind | core-bind | 9139 | 9139 | 9139 | s17461 | s17462 | s17463 |
| 1424 | L16 | L17 | 1187 | 694,7 | 16373 | 447,6 | 13,8 | 1,266 | H5A034 C | sample | Hm Drgbl | Hm Drgbl | Hm Drgbl | CPE002FV | CPE002FZ | CPE002G3 | NM 01506 | NM 0150 | NM 0150 | PPRC1 | PPRC1 | PPRC1 | peroxisome prolif | peroxison | peroxison | 23082 | 23082 | 23082 | s223567 | s22993 | s22994 |
| 16255 | F15 | F16 | 1387 | 918,4 | 19155 | 312,2 | 13,8 | 1,266 | H5A034 C | sample | Hm Gen E | Hm Gen E | Hm Gen E | CPE002JG | CPE002NV | CPE002KB | NM 03196 | NM 0319 | NM 0319 | KRTAP9-2 | KRTAP9-2 | KRTAP9-2 | keratin associated | keratin as | keratin as | 83899 | 83899 | 83899 | s38311 | s38312 | s38313 |
| 16369 | K1 | K2 | 789 | 1069 | 10852 | 327,7 | 13,8 | 1,266 | H5A034 C | sample | Hm Gen E | Hm Gen E | Hm Gen E | CPE002JG | CPE002NV | CPE002KB | NM 00101 | NM 0010 | NM 0010 | TMEM93 | TMEM93 | TMEM93 | transmembrane p | transmem | transmem | 83460 | 83460 | 83460 | s37940 | s37942 | s228288 |
| 16434 | M3 | M4 | 1102 | 1012 | 15226 | 212,6 | 13,8 | 1,266 | H5A034 C | sample | Hm Gen E | Hm Gen E | Hm Gen E | CPE002JG | CPE002NV | CPE002KB | NM 03144 | NM 0314 | NM 0314 | RTP3 | RTP3 | RTP3 | receptor (chemos | receptor ( | receptor ( | 83597 | 83597 | 83597 | s38031 | s38032 | s38033 |
| 16506 | P3 | P4 | 1206 | 1099 | 16690 | 322,2 | 13,8 | 1,266 | H5A034 C | sample | Hm Gen E | Hm Gen E | Hm Gen E | CPE002JG | CPE002NV | CPE002KB | XM 08618 | XM 08618 | XM 08618 | SYDE2 | SYDE2 | SYDE2 | synapse defective | synapse d | synapse d | 84144 | 84144 | 84144 | s38563 | s38564 | s38565 |
| 13618 | H18 | H19 | 1318 | 972,3 | 18144 | 153,6 | 13,8 | 1,296 | H5A034 C | sample | Hm Gen E | Hm Gen E | Hm Gen E | CPE002JC | CPE002NC | CPE002K4 | NM 01911 | NM 0191 | NM 0191 | FGF21 | FGF21 | FGF21 | fibroblast growth | fibroblast | fibroblast | 26291 | 26291 | 26291 | s25388 | s25390 | s226076 |
| 820 | C12 | C13 | 1621 | 704,6 | 22426 | 471,6 | 13,8 | 1,308 | H5A034 C | sample | Hm Drgbl | Hm Drgbl | Hm Drgbl | CPE002FU | CPE002FY | CPE002G2 | NM 00558 | NM 0055 | NM 0055 | MDFI | MDFI | MDFI | MyoD family inhil | MyoD fam | MyoD fam | 4188 | 4188 | 4188 | s8613 | s8614 | s8615 |
| 931 | G4 | G5 | 1322 | 717,8 | 18202 | 481,6 | 13,8 | 1,308 | H5A034 C | sample | Hm Drgbl | Hm Drgbl | Hm Drgbl | CPE002FU | CPE002FY | CPE002G2 | NM 00320 | NM 0032 | NM 0032 | NFE2L1 | NFE2L1 | NFE2L1 | nuclear factor (er | nuclear fa | nuclear fa | 4779 | 4779 | 4779 | s9488 | s9489 | s9490 |
| 1152 | P9 | P10 | 1607 | 765,6 | 22128 | 446,7 | 13,8 | 1,308 | H5A034 C | sample | Hm Drgbl | Hm Drgbl | Hm Drgbl | CPE002FU | CPE002FY | CPE002G2 | NM 00556 | NM 0055 | NM 0055 | LAMA5 | LAMA5 | LAMA5 | laminin, alpha 5 | laminin, a | laminin, a | 3911 | 3911 | 3911 | s8065 | s8066 | s8067 |
| 5520 | F9 | F10 | 1527 | 666,8 | 21144 | 577,3 | 13,8 | 1,314 | H5A034 C | sample | Hm Drgbl | Hm Drgbl | Hm Drgbl | CPE002DX | CPE002F2 | CPE002FN | NM 00374 | NM 0037 | NM 0037 | TNKS | TNKS | TNKS | tankyrase, TRF1-ir | tankyrase | tankyrase | 8658 | 8658 | 8658 | s16480 | s16482 | s16481 |
| 5737 | P1 | P2 | 659 | 829,7 | 9076 | 602,3 | 13,8 | 1,314 | H5A034 C | sample | Hm Drgbl | Hm Drgbl | Hm Drgbl | CPE002DX | CPE002F2 | CPE002FN | NM 00383 | NM 0038 | NM 0038 | FPGT | FPGT | FPGT | fucose-1-phospha | fucose-1-p | fucose-1-p | 8790 | 8790 | 8790 | s16744 | s16745 | s16746 |
| 3516 | C2 | C3 | 638 | 1396 | 8822 | 431,4 | 13,8 | 1,327 | H5A034 C | sample | Hm Drgbl | Hm Drgbl | Hm Drgbl | CPE002DR | CPE002EH | CPE002F7 | NM 00105 | NM 0010 | NM 0010 | SSTR4 | SSTR4 | SSTR4 | somatostatin rece | somatosta | somatosta | 6754 | 6754 | 6754 | s13504 | s224691 | s194819 |
| 3699 | K11 | K12 | 829 | 1274 | 11451 | 339,6 | 13,8 | 1,327 | H5A034 C | sample | Hm Drgbl | Hm Drgbl | Hm Drgbl | CPE002DR | CPE002EH | CPE002F7 | NM 00495 | NM 0049 | NM 0049 | EBI2 | EBI2 | EBI2 | Epstein-Barr virus | Epstein-Ba | Epstein-Ba | 1880 | 1880 | 1880 | s4429 | s4430 | s4431 |
| 8095 | B15 | B16 | 1321 | 633,6 | 18186 | 488,1 | 13,8 | 1,327 | H5A034 C | sample | Hm Drgbl | Hm Drgbl | Hm Drgbl | CPE002E5 | CPE002EU | CPE002FF | NM 18186 | NM 1818 | NM 1818 | ACOT7 | ACOT7 | ACOT7 | acyl-CoA thioeste | acyl-CoA t | acyl-CoA t | 11332 | 11332 | 11332 | s22346 | s22347 | s22348 |
| 8136 | C9 | C10 | 1653 | 691,9 | 22737 | 527,5 | 13,8 | 1,327 | H5A034 C | sample | Hm Drgbl | Hm Drgbl | Hm Drgbl | CPE002E5 | CPE002EU | CPE002FF | NM 00702 | NM 0070 | NM 0070 | TRIM31 | TRIM31 | TRIM31 | tripartite motif-co | tripartite | tripartite | 11074 | 11074 | 11074 | s21826 | s223187 | s21828 |
| 8141 | D13 | D14 | 1657 | 612,6 | 22895 | 514,3 | 13,8 | 1,327 | H5A034 C | sample | Hm Drgbl | Hm Drgbl | Hm Drgbl | CPE002E5 | CPE002EU | CPE002FF | NM 00100 | NM 0010 | NM 0010 | ITGA11 | ITGA11 | ITGA11 | integrin, alpha 11 | integrin, a | integrin, a | 22801 | 22801 | 22801 | s240 | s241 | s242 |
| 8160 | D9 | D10 | 1505 | 647,8 | 20759 | 527,5 | 13,8 | 1,327 | H5A034 C | sample | Hm Drgbl | Hm Drgbl | Hm Drgbl | CPE002E5 | CPE002EU | CPE002FF | NM 01225 | NM 0122 | NM 0122 | TFEC | TFEC | TFEC | transcription fact | transcript | transcript | 22797 | 22797 | 22797 | s22400 | s22401 | s22402 |
| 8205 | F6 | F7 | 1504 | 613,3 | 20762 | 567,7 | 13,8 | 1,327 | H5A034 C | sample | Hm Drgbl | Hm Drgbl | Hm Drgbl | CPE002E5 | CPE002EU | CPE002FF | NM 01224 | NM 0122 | NM 0122 | RHOQ | RHOQ | RHOQ | ras homolog gene | ras homol | ras homol | 23433 | 23433 | 23433 | s223595 | s23825 | s23823 |
| 8381 | N13 | N14 | 1571 | 686,2 | 21756 | 399 | 13,8 | 1,327 | H5A034 C | sample | Hm Drgbl | Hm Drgbl | Hm Drgbl | CPE002E5 | CPE002EU | CPE002FF | NM 01499 | NM 0149 | NM 0149 | PLCH1 | PLCH1 | PLCH1 | phospholipase C, | phospholi | phospholi | 23007 | 23007 | 23007 | s22817 | s22818 | s22819 |
| 10089 | E17 | E18 | 1764 | 869,5 | 24382 | 137,6 | 13,8 | 1,327 | H5A034 C | sample | Hm Drgbl | Hm Drgbl | Hm Drgbl | CPE002EA | CPE002EP | CPE002FA | NM 02469 | NM 0246 | NM 0246 | ZFAND1 | ZFAND1 | ZFAND1 | zinc finger, AN1-ty | zinc finge | zinc finge | 79752 | 79752 | 79752 | s36259 | s36258 | s36260 |
| 10194 | I3 | I4 | 1882 | 858,7 | 25880 | 161,9 | 13,8 | 1,327 | H5A034 C | sample | Hm Drgbl | Hm Drgbl | Hm Drgbl | CPE002EA | CPE002EP | CPE002FA | NM 02479 | NM 0247 | NM 0247 | FAM57A | FAM57A | FAM57A | family with seque | family wit | family wit | 79850 | 79850 | 79850 | s36488 | s36487 | s36486 |
| 1556 | A5 | A6 | 1384 | 785,3 | 19040 | 500 | 13,8 | 1,445 | H5A034 C | sample | Hm Drgbl | Hm Drgbl | Hm Drgbl | CPE002FW | CPE002G0 | CPE002G4 | NM 01456 | NM 0145 | NM 0145 | ZKSCAN5 | ZKSCAN5 | ZKSCAN5 | zinc finger with KI | zinc finge | zinc finge | 23660 | 23660 | 23660 | s24298 | s24299 | s24300 |
| 1583 | B8 | B9 | 902 | 769,2 | 12473 | 497,6 | 13,8 | 1,445 | H5A034 C | sample | Hm Drgbl | Hm Drgbl | Hm Drgbl | CPE002FW | CPE002G0 | CPE002G4 | NM 02468 | NM 0246 | NM 0246 | BBS10 | BBS10 | BBS10 | Bardet-Biedl synd | Bardet-Bie | Bardet-Bie | 79738 | 79738 | 79738 | s36222 | s36223 | s36224 |
| 1584 | B9 | B10 | 1255 | 795,8 | 17293 | 506,5 | 13,8 | 1,445 | H5A034 C | sample | Hm Drgbl | Hm Drgbl | Hm Drgbl | CPE002FW | CPE002G0 | CPE002G4 | NM 01626 | NM 0162 | NM 0162 | ZNF44 | ZNF44 | ZNF44 | zinc finger protei | zinc finge | zinc finge | 51710 | 51710 | 51710 | s28561 | s28562 | s28563 |
| 1603 | C4 | C5 | 1077 | 710,9 | 14827 | 509,1 | 13,8 | 1,445 | H5A034 C | sample | Hm Drgbl | Hm Drgbl | Hm Drgbl | CPE002FW | CPE002G0 | CPE002G4 | NM 02077 | NM 0207 | NM 0207 | CGN | CGN | CGN | cingulin | cingulin | cingulin | 57530 | 57530 | 57530 | s33235 | s33236 | s33237 |
| 1706 | H10 | H11 | 1467 | 704,2 | 20282 | 375,6 | 13,8 | 1,445 | H5A034 C | sample | Hm Drgbl | Hm Drgbl | Hm Drgbl | CPE002FW | CPE002G0 | CPE002G4 | NM 03077 | NM 0307 | NM 0307 | GJA10 | GJA10 | GJA10 | gap junction prot | gap juncti | gap juncti | 81025 | 81025 | 81025 | s37475 | s37476 | s37477 |
| 4731 | F11 | F12 | 1503 | 829,3 | 20672 | 366,3 | 13,8 | 1,516 | H5A034 C | sample | Hm Drgbl | Hm Drgbl | Hm Drgbl | CPE002DV | CPE002EL | CPE002FP | NM 00234 | NM 0023 | NM 0023 | LTF | LTF | LTF | lactotransferrin | lactotrans | lactotrans | 4057 | 4057 | 4057 | s8330 | s8331 | s8332 |
| 4808 | I16 | I17 | 569 | 694,1 | 7845 | 321,4 | 13,8 | 1,516 | H5A034 C | sample | Hm Drgbl | Hm Drgbl | Hm Drgbl | CPE002DV | CPE002EL | CPE002FP | NM 00396 | NM 0039 | NM 0039 | RHBDL1 | RHBDL1 | RHBDL1 | rhomboid, veinlet | rhomboid | rhomboid | 9028 | 9028 | 9028 | s17206 | s17207 | s194977 |
| 10381 | A20 | A21 | 878 | 916,9 | 12130 | 238,1 | 13,8 | 1,559 | H5A034 C | sample | Hm Drgbl | Hm Drgbl | Hm Drgbl | CPE002EB | CPE002EO | CPE002FR | NM 00107 | NM 0010 | NM 0010 | PKD1L2 | PKD1L2 | PKD1L2 | polycystic kidney | polycystic | polycystic | 114780 | 114780 | 114780 | s41582 | s41583 | s41584 |
| 10444 | D12 | D13 | 1221 | 882,8 | 16873 | 190,3 | 13,8 | 1,559 | H5A034 C | sample | Hm Drgbl | Hm Drgbl | Hm Drgbl | CPE002EB | CPE002EO | CPE002FR | NM 14459 | NM 1445 | NM 1445 | RHEBL1 | RHEBL1 | RHEBL1 | Ras homolog enri | Ras homo | Ras homo | 121268 | 121268 | 121268 | s42445 | s42444 | s42443 |
| 10466 | E10 | E11 | 1406 | 877,2 | 19370 | 205,9 | 13,8 | 1,559 | H5A034 C | sample | Hm Drgbl | Hm Drgbl | Hm Drgbl | CPE002EB | CPE002EO | CPE002FR | NM 14890 | NM 1489 | NM 1489 | OSBPL9 | OSBPL9 | OSBPL9 | oxysterol binding | oxysterol | oxysterol | 114883 | 114883 | 114883 | s41695 | s41694 | s41693 |
| 10509 | F6 | F7 | 1163 | 879,5 | 16032 | 242,5 | 13,8 | 1,559 | H5A034 C | sample | Hm Drgbl | Hm Drgbl | Hm Drgbl | CPE002EB | CPE002EO | CPE002FR | NM 13833 | NM 1383 | NM 1383 | RNASE8 | RNASE8 | RNASE8 | ribonuclease, RNa | ribonucle | ribonucle | 122665 | 122665 | 122665 | s42562 | s42561 | s223266 |
| 10662 | M14 | M15 | 1383 | 812,1 | 19046 | 171 | 13,8 | 1,559 | H5A034 C | sample | Hm Drgbl | Hm Drgbl | Hm Drgbl | CPE002EB | CPE002EO | CPE002FR | NM 03064 | NM 0306 | NM 0306 | CENTB5 | CENTB5 | CENTB5 | centaurin, beta 5 | centaurin | centaurin | 116983 | 116983 | 116983 | s42080 | s42082 | s42081 |
| 20002 | B18 | B19 | 1114 | 1188 | 15392 | 205,6 | 13,8 | 1,683 | H5A034 C | sample | Hm Gen E | Hm Gen E | Hm Gen E | CPE002JQ | CPE002O5 | CPE002KL | XM 93804 | XM 93804 | XM 93804 | LOC40216 | LOC40216 | LOC40216 | hypothetical LOC | hypotheti | hypotheti | 402160 | 402160 | 402160 | s53739 | s53740 | s53741 |
| 23785 | P1 | P2 | 843 | 1619 | 11710 | 269,5 | 13,9 | 0,737 | H5A034 C | sample | Hm Gen E | Hm Gen E | Hm Gen E | CPE002KV | CPE002OE | CPE002KU | NM 00101 | NM 0010 | NM 0010 | LOC13035 | LOC13035 | LOC13035 | hypothetical prot | hypotheti | hypotheti | 130355 | 130355 | 130355 | s43493 | s225619 | s225620 |
| 15763 | A4 | A5 | 1485 | 902,8 | 20601 | 189,5 | 13,9 | 0,774 | H5A034 C | sample | Hm Gen E | Hm Gen E | Hm Gen E | CPE002J9 | CPE002NU | CPE002KA | NM 02493 | NM 0249 | NM 0249 | RBM35B | RBM35B | RBM35B | RNA binding moti | RNA bindi | RNA bindi | 80004 | 80004 | 80004 | s36796 | s36797 | s228220 |
| 14012 | H5 | H6 | 1676 | 1082 | 23255 | 116,1 | 13,9 | 0,797 | H5A034 C | sample | Hm Gen E | Hm Gen E | Hm Gen E | CPE002JF | CPE002NP | CPE002K5 | NM 01417 | NM 0141 | NM 0141 | STXBP6 | STXBP6 | STXBP6 | syntaxin binding | syntaxin b | syntaxin b | 29091 | 29091 | 29091 | s26465 | s26466 | s26467 |
| 3227 | G19 | G20 | 1519 | 1300 | 21083 | 343,6 | 13,9 | 0,861 | H5A034 C | sample | Hm Drgbl | Hm Drgbl | Hm Drgbl | CPE002DC | CPE002EG | CPE002F6 | NM 01421 | NM 0142 | NM 0142 | IMPA2 | IMPA2 | IMPA2 | inositol(myo)-1(o | inositol(m | inositol(m | 3613 | 3613 | 3613 | s7410 | s7411 | s7412 |
| 19699 | E4 | E5 | 1595 | 350,1 | 22164 | 330,2 | 13,9 | 0,906 | H5A034 C | sample | Hm Gen E | Hm Gen E | Hm Gen E | CPE002JP | CPE002O4 | CPE002KK | XM 94419 | XM 94419 | XM 94419 | LOC38902 | LOC38902 | LOC38902 | hypothetical LOC | hypotheti | hypotheti | 389024 | 389024 | 389024 | s52341 | s52342 | s52343 |
| 19945 | P1 | P2 | 905 | 370,1 | 12596 | 400,7 | 13,9 | 0,906 | H5A034 C | sample | Hm Gen E | Hm Gen E | Hm Gen E | CPE002JP | CPE002O4 | CPE002KK | NM 00101 | NM 0010 | NM 0010 | C1orf189 | C1orf189 | C1orf189 | chromosome 1 op | chromoso | chromoso | 388701 | 388701 | 388701 | s52241 | s52242 | s196249 |
| 6212 | C5 | C6 | 888 | 607,2 | 12301 | 575 | 13,9 | 0,917 | H5A034 C | sample | Hm Drgbl | Hm Drgbl | Hm Drgbl | CPE002DZ | CPE002F0 | CPE002FL | NM 00007 | NM 0000 | NM 0000 | CD3G | CD3G | CD3G | CD3g molecule, ga | CD3g mol | CD3g mol | 917 | 917 | 917 | s2572 | s2573 | s2574 |
| 6407 | K8 | K9 | 1143 | 499,9 | 15896 | 439,3 | 13,9 | 0,917 | H5A034 C | sample | Hm Drgbl | Hm Drgbl | Hm Drgbl | CPE002DZ | CPE002F0 | CPE002FL | NM 00049 | NM 0004 | NM 0004 | CYP11B2 | CYP11B2 | CYP11B2 | cytochrome P450, | cytochron | cytochron | 1585 | 1585 | 1585 | s3870 | s3869 | s3871 |
| 6432 | L9 | L10 | 818 | 569,4 | 11397 | 527,5 | 13,9 | 0,917 | H5A034 C | sample | Hm Drgbl | Hm Drgbl | Hm Drgbl | CPE002DZ | CPE002F0 | CPE002FL | NM 00009 | NM 0000 | NM 0000 | CP | CP | CP | ceruloplasmin (fe | ceruloplas | ceruloplas | 1356 | 1356 | 1356 | s3417 | s3418 | s3419 |
| 6491 | O19 | O20 | 2183 | 647,8 | 30428 | 459,7 | 13,9 | 0,917 | H5A034 C | sample | Hm Drgbl | Hm Drgbl | Hm Drgbl | CPE002DZ | CPE002F0 | CPE002FL | NM 00180 | NM 0018 | NM 0018 | CEL | CEL | CEL | carboxyl ester lip | carboxyl e | carboxyl e | 1056 | 1056 | 1056 | s2905 | s2904 | s2903 |
| 6517 | P20 | P21 | 1742 | 576,7 | 24146 | 478,8 | 13,9 | 0,917 | H5A034 C | sample | Hm Drgbl | Hm Drgbl | Hm Drgbl | CPE002DZ | CPE002F0 | CPE002FL | NM 00495 | NM 0049 | NM 0049 | EPYC | EPYC | EPYC | epiphycan | epiphycar | epiphycar | 1833 | 1833 | 1833 | s4336 | s4337 | s4338 |
| 20487 | F22 | F23 | 912 | 815,6 | 12690 | 267,3 | 13,9 | 0,927 | H5A034 C | sample | Hm Gen E | Hm Gen E | Hm Gen E | CPE002JR | CPE002O6 | CPE002KN | XM 93628 | XM 93628 | XM 93628 | LOC64214 | LOC64214 | LOC64214 | similar to Polypep | similar to | similar to | 642141 | 642141 | 642141 | s54946 | s54947 | s227012 |
| 20507 | G19 | G20 | 865 | 747,8 | 12032 | 199,9 | 13,9 | 0,927 | H5A034 C | sample | Hm Gen E | Hm Gen E | Hm Gen E | CPE002JR | CPE002O6 | CPE002KN | XM 49636 | XM 49636 | XM 49636 | hCG 2317 | hCG 2317 | hCG 2317 | hCG23177 | hCG23177 | hCG23177 | 440585 | 440585 | 440585 | s54148 | s54149 | s196544 |
| 20522 | H10 | H11 | 569 | 809,2 | 7918 | 195,4 | 13,9 | 0,927 | H5A034 C | sample | Hm Gen E | Hm Gen E | Hm Gen E | CPE002JR | CPE002O6 | CPE002KN | XM 93638 | XM 93638 | XM 93638 | LOC64224 | LOC64224 | LOC64224 | similar to CXXC fi | similar to | similar to | 642242 | 642242 | 642242 | s54957 | s54958 | s54959 |
| 20691 | O11 | O12 | 813 | 727,1 | 11308 | 250,7 | 13,9 | 0,927 | H5A034 C | sample | Hm Gen E | Hm Gen E | Hm Gen E | CPE002JR | CPE002O6 | CPE002KN | XM 93952 | XM 93952 | XM 93952 | hCG 2026 | hCG 2026 | hCG 2026 | hCG2026038 | hCG20260 | hCG20260 | 441050 | 441050 | 441050 | s54252 | s54253 | s54254 |
| 12991 | N15 | N16 | 1443 | 1019 | 20071 | 135,1 | 13,9 | 0,942 | H5A034 C | sample | Hm Gen E | Hm Gen E | Hm Gen E | CPE002J6 | CPE002NM | CPE002K2 | NM 01479 | NM 0147 | NM 0147 | JAKMIP2 | JAKMIP2 | JAKMIP2 | janus kinase and | janus kina | janus kina | 9832 | 9832 | 9832 | s19023 | s19024 | s19025 |
| 9407 | H8 | H9 | 1034 | 1130 | 14332 | 119,3 | 13,9 | 0,965 | H5A034 C | sample | Hm Drgbl | Hm Drgbl | Hm Drgbl | CPE002E8 | CPE002ER | CPE002FC | NM 02014 | NM 0201 | NM 0201 | PAPOLB | PAPOLB | PAPOLB | poly(A) polymera | poly(A) pc | poly(A) pc | 56903 | 56903 | 56903 | s32355 | s32356 | s32354 |
| 9471 | K22 | K23 | 1934 | 1190 | 26898 | 190,5 | 13,9 | 0,965 | H5A034 C | sample | Hm Drgbl | Hm Drgbl | Hm Drgbl | CPE002E8 | CPE002ER | CPE002FC | NM 01893 | NM 0189 | NM 0189 | PCDHB10 | PCDHB10 | PCDHB10 | protocadherin be | protocadh | protocadh | 56126 | 56126 | 56126 | s31948 | s31947 | s31946 |
| 9498 | L3 | L4 | 961 | 1022 | 13380 | 136 | 13,9 | 0,965 | H5A034 C | sample | Hm Drgbl | Hm Drgbl | Hm Drgbl | CPE002E8 | CPE002ER | CPE002FC | NM 01779 | NM 0177 | NM 0177 | FLVCR2 | FLVCR2 | FLVCR2 | feline leukemia vi | feline leul | feline leul | 55640 | 55640 | 55640 | s31146 | s31148 | s31147 |
| 9499 | L4 | L5 | 1083 | 1017 | 15013 | 134,7 | 13,9 | 0,965 | H5A034 C | sample | Hm Drgbl | Hm Drgbl | Hm Drgbl | CPE002E8 | CPE002ER | CPE002FC | NM 02029 | NM 0202 | NM 0202 | AKR1B10 | AKR1B10 | AKR1B10 | aldo-keto reducta | aldo-keto | aldo-keto | 57016 | 57016 | 57016 | s224446 | s32584 | s32583 |
| 18949 | F20 | F21 | 1369 | 949,3 | 19086 | 143,6 | 13,9 | 0,969 | H5A034 C | sample | Hm Gen E | Hm Gen E | Hm Gen E | CPE002JN | CPE002O2 | CPE002KI | XM 20833 | XM 20833 | XM 20833 | RP5-964H1 | RP5-964H1 | RP5-964H1 | hypothetical prot | hypotheti | hypotheti | 285622 | 285622 | 285622 | s49985 | s195994 | s195995 |
| 19149 | N6 | N7 | 1368 | 1064 | 18972 | 248,1 | 13,9 | 0,969 | H5A034 C | sample | Hm Gen E | Hm Gen E | Hm Gen E | CPE002JN | CPE002O2 | CPE002KI | NM 00100 | NM 0010 | NM 0010 | C9orf47 | C9orf47 | C9orf47 | chromosome 9 op | chromoso | chromoso | 286223 | 286223 | 286223 | s50126 | s50127 | s50128 |
| 9786 | H3 | H4 | 1358 | 1010 | 18902 | 121,1 | 13,9 | 0,972 | H5A034 C | sample | Hm Drgbl | Hm Drgbl | Hm Drgbl | CPE002E9 | CPE002EQ | CPE002FB | NM 02124 | NM 0212 | NM 0212 | RRAGD | RRAGD | RRAGD | Ras-related GTP b | Ras-relate | Ras-relate | 58528 | 58528 | 58528 | s33929 | s33927 | s33928 |
| 9978 | P3 | P4 | 1641 | 1051 | 22731 | 127,2 | 13,9 | 0,972 | H5A034 C | sample | Hm Drgbl | Hm Drgbl | Hm Drgbl | CPE002E9 | CPE002EQ | CPE002FB | NM 02209 | NM 0220 | NM 0220 | TNN | TNN | TNN | tenascin N | tenascin N | tenascin N | 63923 | 63923 | 63923 | s34272 | s34271 | s34270 |
| 8716 | L12 | L13 | 1562 | 1041 | 21680 | 112 | 13,9 | 0,982 | H5A034 C | sample | Hm Drgbl | Hm Drgbl | Hm Drgbl | CPE002E6 | CPE002ET | CPE002FE | NM 01332 | NM 0133 | NM 0133 | PYCR2 | PYCR2 | PYCR2 | pyrroline-5-carbo | pyrroline- | pyrroline- | 29920 | 29920 | 29920 | s26703 | s26705 | s26704 |
| 20966 | J21 | J22 | 936 | 980 | 13018 | 235,3 | 13,9 | 0,986 | H5A034 C | sample | Hm Gen E | Hm Gen E | Hm Gen E | CPE002JS | CPE002O7 | CPE002KN | NM 00103 | NM 0010 | NM 0010 | FLJ45032 | FLJ45032 | FLJ45032 | similar to F40B5.2 | similar to | similar to | 643853 | 643853 | 643853 | s55468 | s55469 | s55470 |
| 21005 | L13 | L14 | 1294 | 956,7 | 17967 | 298,7 | 13,9 | 0,986 | H5A034 C | sample | Hm Gen E | Hm Gen E | Hm Gen E | CPE002JS | CPE002O7 | CPE002KN | XM 92715 | XM 92715 | XM 92715 | LOC64388 | LOC64388 | LOC64388 | similar to Cytokin | similar to | similar to | 643884 | 643884 | 643884 | s55487 | s55488 | s55489 |
| 21013 | L20 | L21 | 1391 | 945,8 | 19298 | 256,8 | 13,9 | 0,986 | H5A034 C | sample | Hm Gen E | Hm Gen E | Hm Gen E | CPE002JS | CPE002O7 | CPE002KN | XM 92819 | XM 92819 | XM 92819 | RPL12P8 | RPL12P8 | RPL12P8 | ribosomal protei | ribosomal | ribosomal | 645161 | 645161 | 645161 | s55934 | s55935 | s55936 |
| 21036 | M2 | M3 | 1135 | 972,7 | 15744 | 204 | 13,9 | 0,986 | H5A034 C | sample | Hm Gen E | Hm Gen E | Hm Gen E | CPE002JS | CPE002O7 | CPE002KN | XM 92768 | XM 92768 | XM 92768 | LOC64456 | LOC64456 | LOC64456 | similar to Glutam | similar to | similar to | 644567 | 644567 | 644567 | s55714 | s55715 | s227132 |
| 21039 | M22 | M23 | 1021 | 1072 | 14144 | 251,7 | 13,9 | 0,986 | H5A034 C | sample | Hm Gen E | Hm Gen E | Hm Gen E | CPE002JS | CPE002O7 | CPE002KN | XM 93227 | XM 93227 | XM 93227 | LOC64463 | LOC64463 | LOC64463 | hypothetical LOC | hypotheti | hypotheti | 644638 | 644638 | 644638 | s55736 | s55737 | s55738 |
| 21060 | N2 | N3 | 1032 | 928 | 14313 | 158,5 | 13,9 | 0,986 | H5A034 C | sample | Hm Gen E | Hm Gen E | Hm Gen E | CPE002JS | CPE002O7 | CPE002KN | XM 92821 | XM 92821 | XM 92821 | LOC64517 | LOC64517 | LOC64517 | hypothetical LOC | hypotheti | hypotheti | 645177 | 645177 | 645177 | s55940 | s55941 | s55942 |
| 21236 | E5 | E6 | 745 | 932,5 | 10375 | 281,5 | 13,9 | 0,986 | H5A034 C | sample | Hm Gen E | Hm Gen E | Hm Gen E | CPE002JT | CPE002O8 | CPE002KO | XM 00112 | XM 00112 | XM 00112 | LOC64551 | LOC64551 | LOC64551 | similar to septin 7 | similar to | similar to | 645513 | 645513 | 645513 | s56077 | s56078 | s56079 |
| 21444 | N2 | N3 | 1177 | 918,9 | 16322 | 188,1 | 13,9 | 0,986 | H5A034 C | sample | Hm Gen E | Hm Gen E | Hm Gen E | CPE002JT | CPE002O8 | CPE002KO | XM 93833 | XM 93833 | XM 93833 | LOC64808 | LOC64808 | LOC64808 | similar to membra | similar to | similar to | 648089 | 648089 | 648089 | s56818 | s56820 | s227304 |
| 14323 | E4 | E5 | 1293 | 840,9 | 17983 | 129,3 | 13,9 | 0,989 | H5A034 C | sample | Hm Gen E | Hm Gen E | Hm Gen E | CPE002J7 | CPE002NO | CPE002K6 | NM 01883 | NM 0188 | NM 0188 | RC3H2 | RC3H2 | RC3H2 | ring finger and CC | ring finge | ring finge | 54542 | 54542 | 54542 | s29169 | s29170 | s29171 |
| 11364 | J2 | J3 | 1773 | 944,8 | 24562 | 161,5 | 13,9 | 1,007 | H5A034 C | sample | Hm Drgbl | Hm Drgbl | Hm Drgbl | CPE002ED | CPE002F3 | CPE002FT | | | | | | | | | | | | | | | |
| 11422 | L7 | L8 | 1363 | 806,3 | 18962 | 140,7 | 13,9 | 1,007 | H5A034 C | sample | Hm Drgbl | Hm Drgbl | Hm Drgbl | CPE002ED | CPE002F3 | CPE002FT | NM 19858 | NM 1985 | NM 1985 | CA13 | CA13 | CA13 | carbonic anhydra | carbonic a | carbonic a | 377677 | 377677 | 377677 | s51780 | s51781 | s51782 |
| 12368 | D16 | D17 | 1211 | 962,9 | 16855 | 158,7 | 13,9 | 1,045 | H5A034 C | sample | Hm Gen E | Hm Gen E | Hm Gen E | CPE002J5 | CPE002NK | CPE002K1 | NM 00601 | NM 0060 | NM 0060 | PROM1 | PROM1 | PROM1 | prominin 1 | prominin | prominin | 8842 | 8842 | 8842 | s16879 | s16880 | s16881 |
| 12481 | I1 | I2 | 1998 | 1142 | 27721 | 155,2 | 13,9 | 1,045 | H5A034 C | sample | Hm Gen E | Hm Gen E | Hm Gen E | CPE002J5 | CPE002NK | CPE002K1 | NM 00106 | NM 0010 | NM 0010 | TCN1 | TCN1 | TCN1 | transcobalamin I | transcoba | transcoba | 6947 | 6947 | 6947 | s13913 | s13914 | s13915 |
| 10836 | D2 | D3 | 1389 | 867,4 | 19314 | 427,7 | 13,9 | 1,053 | H5A034 C | sample | Hm Drgbl | Hm Drgbl | Hm Drgbl | CPE002EC | CPE002EN | CPE002FS | NM 15369 | NM 1536 | NM 1536 | GSTA5 | GSTA5 | GSTA5 | glutathione S-tran | glutathior | glutathior | 221357 | 221357 | 221357 | s48021 | s48022 | s48023 |
| 10894 | F7 | F8 | 1430 | 937,3 | 19830 | 530,1 | 13,9 | 1,053 | H5A034 C | sample | Hm Drgbl | Hm Drgbl | Hm Drgbl | CPE002EC | CPE002EN | CPE002FS | NM 17335 | NM 1733 | NM 1733 | UPP2 | UPP2 | UPP2 | uridine phosphor | uridine ph | uridine ph | 151531 | 151531 | 151531 | s45584 | s45585 | s45586 |
| 10942 | H7 | H8 | 362 | 741,1 | 5017 | 417,2 | 13,9 | 1,053 | H5A034 C | sample | Hm Drgbl | Hm Drgbl | Hm Drgbl | CPE002EC | CPE002EN | CPE002FS | NM 19815 | NM 1981 | NM 1981 | ARSK | ARSK | ARSK | arylsulfatase fami | arylsulfat | arylsulfat | 153642 | 153642 | 153642 | s45806 | s45807 | s45805 |
| 7301 | A13 | A14 | 1084 | 600,9 | 15036 | 515,1 | 13,9 | 1,061 | H5A034 C | sample | Hm Drgbl | Hm Drgbl | Hm Drgbl | CPE002E3 | CPE002EW | CPE002FH | NM 00617 | NM 0061 | NM 0061 | NRAP | NRAP | NRAP | nebulin-related a | nebulin-re | nebulin-re | 4892 | 4892 | 4892 | s9712 | s9713 | s9711 |
| 7454 | G21 | G22 | 958 | 657,3 | 13316 | 452,5 | 13,9 | 1,061 | H5A034 C | sample | Hm Drgbl | Hm Drgbl | Hm Drgbl | CPE002E3 | CPE002EW | CPE002FH | NM 00710 | NM 0071 | NM 0071 | SLC22A18 | SLC22A18 | SLC22A18 | solute carrier fam | solute car | solute car | 5003 | 5003 | 5003 | s9907 | s9908 | s9909 |
| 7491 | I11 | I12 | 922 | 637,4 | 12844 | 395,3 | 13,9 | 1,061 | H5A034 C | sample | Hm Drgbl | Hm Drgbl | Hm Drgbl | CPE002E3 | CPE002EW | CPE002FH | NM 00255 | NM 0025 | NM 0025 | OVGP1 | OVGP1 | OVGP1 | oviductal glycopr | oviductal | oviductal | 5016 | 5016 | 5016 | s9935 | s9936 | s9934 |
| 7511 | I8 | I9 | 1058 | 561,6 | 14739 | 310,8 | 13,9 | 1,061 | H5A034 C | sample | Hm Drgbl | Hm Drgbl | Hm Drgbl | CPE002E3 | CPE002EW | CPE002FH | NM 01471 | NM 0147 | NM 0147 | ZNF536 | ZNF536 | ZNF536 | zinc finger protei | zinc finge | zinc finge | 9745 | 9745 | 9745 | s18804 | s18803 | s18805 |
| 7614 | N14 | N15 | 1756 | 666,7 | 24399 | 574,7 | 13,9 | 1,061 | H5A034 C | sample | Hm Drgbl | Hm Drgbl | Hm Drgbl | CPE002E3 | CPE002EW | CPE002FH | NM 00677 | NM 0067 | NM 0067 | TRIM10 | TRIM10 | TRIM10 | tripartite motif-co | tripartite | tripartite | 10107 | 10107 | 10107 | s19667 | s19668 | s19669 |
| 7667 | P19 | P20 | 1714 | 603,5 | 23758 | 525,1 | 13,9 | 1,061 | H5A034 C | sample | Hm Drgbl | Hm Drgbl | Hm Drgbl | CPE002E3 | CPE002EW | CPE002FH | NM 00491 | NM 0049 | NM 0049 | PITPNM1 | PITPNM1 | PITPNM1 | phosphatidylinos | phosphat | phosphat | 9600 | 9600 | 9600 | s18445 | s18443 | s18444 |
| 16915 | A4 | A5 | 1381 | 1135 | 19128 | 346 | 13,9 | 1,073 | H5A034 C | sample | Hm Gen E | Hm Gen E | Hm Gen E | CPE002JI | CPE002NZ | CPE002KD | NM 05328 | NM 1532 | NM 0532 | SH2D1B | MGC3544 | SH2D1B | SH2 domain conta | hypotheti | SH2 doma | 117157 | 147990 | 117157 | s42119 | s45103 | s225518 |
| 16946 | C10 | C11 | 1559 | 981,5 | 21685 | 224,7 | 13,9 | 1,073 | H5A034 C | sample | Hm Gen E | Hm Gen E | Hm Gen E | CPE002JI | CPE002NZ | CPE002KD | NM 00100 | NM 1446 | NM 0010 | C10orf90 | C1orf58 | C10orf90 | chromosome 10 c | chromoso | chromoso | 118611 | 148362 | 118611 | s42227 | s45175 | s42229 |
| 16980 | D2 | D3 | 1342 | 1024 | 18668 | 160 | 13,9 | 1,073 | H5A034 C | sample | Hm Gen E | Hm Gen E | Hm Gen E | CPE002JI | CPE002NZ | CPE002KD | NM 00100 | NM 0010 | NM 0010 | OR11H6 | FBXO41 | OR11H6 | olfactory recepto | F-box pro | olfactory r | 122748 | 150726 | 122748 | s42575 | s45480 | s42577 |
| 16986 | D3 | D4 | 1457 | 934,9 | 20278 | 159,6 | 13,9 | 1,073 | H5A034 C | sample | Hm Gen E | Hm Gen E | Hm Gen E | CPE002JI | CPE002NZ | CPE002KD | XM 93488 | XM 92797 | XM 93488 | FHAD1 | LOC14581 | FHAD1 | forkhead-associat | hypotheti | forkhead- | 114827 | 145814 | 114827 | s41675 | s44782 | s41677 |
| 17076 | H2 | H3 | 1596 | 940,5 | 22142 | 126,2 | 13,9 | 1,073 | H5A034 C | sample | Hm Gen E | Hm Gen E | Hm Gen E | CPE002JI | CPE002NZ | CPE002KD | NM 19844 | NM 1447 | NM 1984 | NRN1L | FAM82A | NRN1L | neuritin 1-like | family wit | neuritin 1 | 123904 | 151393 | 123904 | s42688 | s45564 | s225549 |
| 17089 | I1 | I2 | 1431 | 934,9 | 19960 | 174,3 | 13,9 | 1,073 | H5A034 C | sample | Hm Gen E | Hm Gen E | Hm Gen E | CPE002JI | CPE002NZ | CPE002KD | NM 05287 | NM 1524 | NM 0528 | C14orf179 | CWF19L2 | C14orf179 | chromosome 14 c | CWF19-lik | chromoso | 112752 | 143884 | 112752 | s41364 | s44581 | s41366 |
| 17092 | I12 | I13 | 1631 | 1024 | 22749 | 183 | 13,9 | 1,073 | H5A034 C | sample | Hm Gen E | Hm Gen E | Hm Gen E | CPE002JI | CPE002NZ | CPE002KD | NM 00108 | NM 1825 | NM 0010 | PIH1D2 | C1orf210 | PIH1D2 | PIH1 domain cont | chromoso | PIH1 doma | 120379 | 149466 | 120379 | s42371 | s45299 | s42373 |
| 17185 | M1 | M2 | 1282 | 889,5 | 17849 | 142,9 | 13,9 | 1,073 | H5A034 C | sample | Hm Gen E | Hm Gen E | Hm Gen E | CPE002JI | CPE002NZ | CPE002KD | NM 14516 | NM 1446 | NM 1451 | SFT2D1 | WDR66 | SFT2D1 | SFT2 domain cont | WD repea | SFT2 dom | 113402 | 144406 | 113402 | s41467 | s44653 | s41469 |
| 17239 | O15 | O16 | 1595 | 954,2 | 22248 | 282,7 | 13,9 | 1,073 | H5A034 C | sample | Hm Gen E | Hm Gen E | Hm Gen E | CPE002JI | CPE002NZ | CPE002KD | NM 05288 | NM 1538 | NM 0528 | LRRC37B | SLC38A6 | LRRC37B | leucine rich repea | solute car | leucine ric | 114659 | 145389 | 114659 | s41567 | s44727 | s41569 |
| 17264 | P16 | P17 | 1405 | 1167 | 19595 | 204,4 | 13,9 | 1,073 | H5A034 C | sample | Hm Gen E | Hm Gen E | Hm Gen E | CPE002JI | CPE002NZ | CPE002KD | NM 00104 | NM 1536 | NM 0010 | C18orf51 | C5orf41 | C18orf51 | chromosome 18 c | chromoso | chromoso | 125704 | 153222 | 125704 | s42892 | s45764 | s42894 |
| 6943 | B15 | B16 | 1404 | 622,5 | 19451 | 284,2 | 13,9 | 1,082 | H5A034 C | sample | Hm Drgbl | Hm Drgbl | Hm Drgbl | CPE002E2 | CPE002EX | CPE002FI | NM 03184 | NM 0318 | NM 0318 | MAP2 | MAP2 | MAP2 | microtubule-asso | microtubu | microtubu | 4133 | 4133 | 4133 | s8504 | s8503 | s8502 |
| 6949 | B20 | B21 | 1059 | 630,7 | 14691 | 540,9 | 13,9 | 1,082 | H5A034 C | sample | Hm Drgbl | Hm Drgbl | Hm Drgbl | CPE002E2 | CPE002EX | CPE002FI | NM 00454 | NM 0045 | NM 0045 | NDUFB1 | NDUFB1 | NDUFB1 | NADH dehydroger | NADH deh | NADH deh | 4707 | 4707 | 4707 | s9352 | s9354 | s224098 |
| 6950 | B21 | B22 | 1323 | 616,5 | 18367 | 408,2 | 13,9 | 1,082 | H5A034 C | sample | Hm Drgbl | Hm Drgbl | Hm Drgbl | CPE002E2 | CPE002EX | CPE002FI | NM 00042 | NM 0004 | NM 0004 | MAT1A | MAT1A | MAT1A | methionine aden | methionir | methionir | 4143 | 4143 | 4143 | s8525 | s8524 | s8523 |
| 7130 | J10 | J11 | 919 | 561,1 | 12811 | 214,2 | 13,9 | 1,082 | H5A034 C | sample | Hm Drgbl | Hm Drgbl | Hm Drgbl | CPE002E2 | CPE002EX | CPE002FI | NM 00616 | NM 0061 | NM 0061 | NFE2L2 | NFE2L2 | NFE2L2 | nuclear factor (er | nuclear fa | nuclear fa | 4780 | 4780 | 4780 | s9493 | s9492 | s9491 |
| 7141 | J20 | J21 | 1245 | 528,5 | 17282 | 373,8 | 13,9 | 1,082 | H5A034 C | sample | Hm Drgbl | Hm Drgbl | Hm Drgbl | CPE002E2 | CPE002EX | CPE002FI | NM 00455 | NM 0045 | NM 0045 | NFKBIE | NFKBIE | NFKBIE | nuclear factor of k | nuclear fa | nuclear fa | 4794 | 4794 | 4794 | s9514 | s9516 | s9515 |
| 7151 | J8 | J9 | 1194 | 673 | 16557 | 474 | 13,9 | 1,082 | H5A034 C | sample | Hm Drgbl | Hm Drgbl | Hm Drgbl | CPE002E2 | CPE002EX | CPE002FI | NM 00616 | NM 0061 | NM 0061 | NFE2 | NFE2 | NFE2 | nuclear factor (er | nuclear fa | nuclear fa | 4778 | 4778 | 4778 | s9487 | s9486 | s9485 |
| 7238 | N21 | N22 | 723 | 570,4 | 10021 | 211,7 | 13,9 | 1,082 | H5A034 C | sample | Hm Drgbl | Hm Drgbl | Hm Drgbl | CPE002E2 | CPE002EX | CPE002FI | NM 00243 | NM 0024 | NM 0024 | MPG | MPG | MPG | N-methylpurine-D | N-methylp | N-methylp | 4350 | 4350 | 4350 | s8928 | s8929 | s8930 |
| 5132 | F5 | F6 | 1703 | 600,7 | 23714 | 593 | 13,9 | 1,09 | H5A034 C | sample | Hm Drgbl | Hm Drgbl | Hm Drgbl | CPE002DV | CPE002EM | CPE002FO | NM 00101 | NM 0010 | NM 0010 | ZYX | ZYX | ZYX | zyxin | zyxin | zyxin | 7791 | 7791 | 7791 | s15351 | s224804 | s15350 |
| 5226 | J3 | J4 | 1811 | 642,3 | 25205 | 547,4 | 13,9 | 1,09 | H5A034 C | sample | Hm Drgbl | Hm Drgbl | Hm Drgbl | CPE002DV | CPE002EM | CPE002FO | NM 00290 | NM 0029 | NM 0029 | RDBP | RDBP | RDBP | RD RNA binding p | RD RNA bi | RD RNA bi | 7936 | 7936 | 7936 | s15488 | s15489 | s15487 |
| 5247 | K22 | K23 | 2095 | 686,4 | 29178 | 583,5 | 13,9 | 1,09 | H5A034 C | sample | Hm Drgbl | Hm Drgbl | Hm Drgbl | CPE002DV | CPE002EM | CPE002FO | NM 00334 | NM 0033 | NM 0033 | UBE2G1 | UBE2G1 | UBE2G1 | ubiquitin-conjuga | ubiquitin- | ubiquitin- | 7326 | 7326 | 7326 | s638 | s640 | s639 |
| 5280 | L9 | L10 | 1651 | 672,3 | 22896 | 419,6 | 13,9 | 1,09 | H5A034 C | sample | Hm Drgbl | Hm Drgbl | Hm Drgbl | CPE002DV | CPE002EM | CPE002FO | NM 00737 | NM 0073 | NM 0073 | SHOC2 | SHOC2 | SHOC2 | soc-2 suppressor | soc-2 supp | soc-2 supp | 8036 | 8036 | 8036 | s15572 | s15573 | s15571 |
| 2315 | A19 | A20 | 1837 | 769,1 | 25527 | 412,4 | 13,9 | 1,094 | H5A034 C | sample | Hm Drgbl | Hm Drgbl | Hm Drgbl | CPE002DC | CPE002EE | CPE002F4 | NM 00407 | NM 0040 | NM 0040 | PLK3 | PLK3 | PLK3 | polo-like kinase 3 | polo-like k | polo-like k | 1263 | 1263 | 1263 | s3245 | s223278 | s3247 |
| 2377 | D1 | D2 | 1230 | 734,1 | 17048 | 494,6 | 13,9 | 1,094 | H5A034 C | sample | Hm Drgbl | Hm Drgbl | Hm Drgbl | CPE002DC | CPE002EE | CPE002F4 | NM 17211 | NM 1721 | NM 1721 | CAMK2D | CAMK2D | CAMK2D | calcium/calmodul | calcium/ca | calcium/ca | 817 | 817 | 817 | s2364 | s2365 | s2366 |
| 2404 | E12 | E13 | 1151 | 759,3 | 15982 | 345 | 13,9 | 1,094 | H5A034 C | sample | Hm Drgbl | Hm Drgbl | Hm Drgbl | CPE002DC | CPE002EE | CPE002F4 | NM 00244 | NM 0024 | NM 0024 | MAP3K10 | MAP3K10 | MAP3K10 | mitogen-activated | mitogen-a | mitogen-a | 4294 | 4294 | 4294 | s8808 | s8809 | s8810 |
| 2474 | H10 | H11 | 1564 | 711 | 21803 | 388,7 | 13,9 | 1,094 | H5A034 C | sample | Hm Drgbl | Hm Drgbl | Hm Drgbl | CPE002DC | CPE002EE | CPE002F4 | NM 00103 | NM 0010 | NM 0010 | PRPS2 | PRPS2 | PRPS2 | phosphoribosyl p | phosphor | phosphor | 5634 | 5634 | 5634 | s11236 | s11237 | s11238 |
| 2588 | L5 | L6 | 1197 | 639,3 | 16629 | 351,2 | 13,9 | 1,094 | H5A034 C | sample | Hm Drgbl | Hm Drgbl | Hm Drgbl | CPE002DC | CPE002EE | CPE002F4 | NM 00443 | NM 0044 | NM 0044 | EPHA4 | EPHA4 | EPHA4 | EPH receptor A4 | EPH recep | EPH recep | 2043 | 2043 | 2043 | s4725 | s4726 | s223493 |
| 2617 | N1 | N2 | 1618 | 699,2 | 22562 | 410,2 | 13,9 | 1,094 | H5A034 C | sample | Hm Drgbl | Hm Drgbl | Hm Drgbl | CPE002DC | CPE002EE | CPE002F4 | NM 00523 | NM 0052 | NM 0052 | ERBB4 | ERBB4 | ERBB4 | v-erb-a erythrobla | v-erb-a er | v-erb-a er | 2066 | 2066 | 2066 | s4781 | s4782 | s4783 |
| 2640 | N9 | N10 | 1436 | 676,5 | 19890 | 357 | 13,9 | 1,094 | H5A034 C | sample | Hm Drgbl | Hm Drgbl | Hm Drgbl | CPE002DC | CPE002EE | CPE002F4 | NM 02310 | NM 0231 | NM 0231 | FGFR1 | FGFR1 | FGFR1 | fibroblast growth | fibroblast | fibroblast | 2260 | 2260 | 2260 | s5164 | s5165 | s5166 |
| 16735 | J15 | J16 | 903 | 1015 | 12518 | 337,6 | 13,9 | 1,099 | H5A034 C | sample | Hm Gen E | Hm Gen E | Hm Gen E | CPE002JH | CPE002NV | CPE002KC | NM 03336 | NM 0333 | NM 0333 | C3orf15 | C3orf15 | C3orf15 | chromosome 3 op | chromoso | chromoso | 89876 | 89876 | 89876 | s40178 | s40179 | s228433 |
| 17691 | B11 | B12 | 1115 | 1082 | 15484 | 447,5 | 13,9 | 1,099 | H5A034 C | sample | Hm Gen E | Hm Gen E | Hm Gen E | CPE002JK | CPE002NZ | CPE002KF | NM 15244 | NM 1524 | NM 1524 | LRFN5 | LRFN5 | LRFN5 | leucine rich repea | leucine ric | leucine ric | 145581 | 145581 | 145581 | s44759 | s44760 | s44761 |
| 8840 | A16 | A17 | 1710 | 1938 | 23832 | 188,5 | 13,9 | 1,116 | H5A034 C | sample | Hm Drgbl | Hm Drgbl | Hm Drgbl | CPE002E7 | CPE002ES | CPE002FD | NM 01661 | NM 0166 | NM 0166 | TTRAP | TTRAP | TTRAP | TRAF and TNF rec | TRAF and | TRAF and | 51567 | 51567 | 51567 | s28341 | s28342 | s28343 |
| 9201 | P17 | P18 | 1686 | 1627 | 23445 | 161,3 | 13,9 | 1,116 | H5A034 C | sample | Hm Drgbl | Hm Drgbl | Hm Drgbl | CPE002E7 | CPE002ES | CPE002FD | NM 17334 | NM 1733 | NM 1733 | PHF7 | PHF7 | PHF7 | PHD finger protei | PHD finge | PHD finge | 51533 | 51533 | 51533 | s28282 | s28284 | s28283 |
| 4359 | F22 | F23 | 974 | 703,5 | 13529 | 459,6 | 13,9 | 1,173 | H5A034 C | sample | Hm Drgbl | Hm Drgbl | Hm Drgbl | CPE002DU | CPE002EK | CPE002FQ | NM 13345 | NM 1334 | NM 1334 | SHROOM1 | SHROOM1 | SHROOM1 | shroom family me | shroom fa | shroom fa | 134549 | 134549 | 134549 | s43881 | s43882 | s43883 |
| 4376 | G16 | G17 | 950 | 664,1 | 13208 | 390,2 | 13,9 | 1,173 | H5A034 C | sample | Hm Drgbl | Hm Drgbl | Hm Drgbl | CPE002DU | CPE002EK | CPE002FQ | NM 00547 | NM 0054 | NM 0054 | KCNE3 | KCNE3 | KCNE3 | potassium voltag | potassium | potassium | 10008 | 10008 | 10008 | s19447 | s19448 | s19449 |
| 4484 | K5 | K6 | 1302 | 734,3 | 18159 | 433,5 | 13,9 | 1,173 | H5A034 C | sample | Hm Drgbl | Hm Drgbl | Hm Drgbl | CPE002DU | CPE002EK | CPE002FQ | NM 00436 | NM 0043 | NM 0043 | CLCN2 | CLCN2 | CLCN2 | chloride channel | chloride c | chloride c | 1181 | 1181 | 1181 | s3132 | s3133 | s3134 |
| 4493 | L13 | L14 | 1415 | 711,1 | 19667 | 410,9 | 13,9 | 1,173 | H5A034 C | sample | Hm Drgbl | Hm Drgbl | Hm Drgbl | CPE002DU | CPE002EK | CPE002FQ | NM 00224 | NM 0022 | NM 0022 | KCNN1 | KCNN1 | KCNN1 | potassium interm | potassium | potassium | 3780 | 3780 | 3780 | s7792 | s7793 | s7794 |
| 4509 | L6 | L7 | 1054 | 681,2 | 14640 | 408,5 | 13,9 | 1,173 | H5A034 C | sample | Hm Drgbl | Hm Drgbl | Hm Drgbl | CPE002DU | CPE002EK | CPE002FQ | NM 00103 | NM 0010 | NM 0010 | TIAL1 | TIAL1 | TIAL1 | TIA1 cytotoxic gra | TIA1 cytot | TIA1 cytot | 7073 | 7073 | 7073 | s14136 | s14134 | s14135 |
| 103 | E15 | E16 | 535 | 1373 | 7463 | 342 | 13,9 | 1,183 | H5A034 C | sample | Hm Drgbl | Hm Drgbl | Hm Drgbl | CPE002DT | CPE002EJ | CPE002F9 | NM 19933 | NM 1993 | NM 1993 | THRA | THRA | THRA | thyroid hormone | thyroid hc | thyroid hc | 7067 | 7067 | 7067 | s14116 | s14117 | s14118 |
| 110 | E21 | E22 | 720 | 1590 | 9982 | 435 | 13,9 | 1,183 | H5A034 C | sample | Hm Drgbl | Hm Drgbl | Hm Drgbl | CPE002DT | CPE002EJ | CPE002F9 | NM 00103 | NM 0010 | NM 0010 | NR2C1 | NR2C1 | NR2C1 | nuclear receptor s | nuclear re | nuclear re | 7181 | 7181 | 7181 | s14367 | s14368 | s14369 |
| 201 | I17 | I18 | 673 | 1376 | 9374 | 540,1 | 13,9 | 1,183 | H5A034 C | sample | Hm Drgbl | Hm Drgbl | Hm Drgbl | CPE002DT | CPE002EJ | CPE002F9 | NM 00066 | NM 0006 | NM 0006 | TGFB1 | TGFB1 | TGFB1 | transforming grov | transform | transform | 7040 | 7040 | 7040 | s14055 | s14054 | s14056 |
| 2178 | K3 | K4 | 759 | 626,4 | 10530 | 408,7 | 13,9 | 1,209 | H5A034 C | sample | Hm Drgbl | Hm Drgbl | Hm Drgbl | CPE002FX | CPE002G1 | CPE002G5 | NM 05297 | NM 0529 | NM 0529 | HSPA12B | HSPA12B | HSPA12B | heat shock 70kD p | heat shoc | heat shoc | 116835 | 116835 | 116835 | s42053 | s42054 | s42055 |
| 2265 | O17 | O18 | 1190 | 685,5 | 16566 | 426,6 | 13,9 | 1,209 | H5A034 C | sample | Hm Drgbl | Hm Drgbl | Hm Drgbl | CPE002FX | CPE002G1 | CPE002G5 | NM 00100 | NM 0010 | NM 0010 | ZNF648 | ZNF648 | ZNF648 | zinc finger protei | zinc finge | zinc finge | 127665 | 127665 | 127665 | s43205 | s43206 | s43207 |
| 2288 | P16 | P17 | 1320 | 678,4 | 18360 | 470,7 | 13,9 | 1,209 | H5A034 C | sample | Hm Drgbl | Hm Drgbl | Hm Drgbl | CPE002FX | CPE002G1 | CPE002G5 | | | | | | | | | | | | | | | |
| 6576 | B9 | B10 | 1288 | 713,3 | 17934 | 408,4 | 13,9 | 1,219 | H5A034 C | sample | Hm Drgbl | Hm Drgbl | Hm Drgbl | CPE002E1 | CPE002EY | CPE002FJ | NM 00213 | NM 0021 | NM 0021 | HNRPA1 | HNRPA1 | HNRPA1 | heterogeneous nu | heteroger | heteroger | 3178 | 3178 | 3178 | s6712 | s223860 | s6711 |
| 6639 | E22 | E23 | 1227 | 553,4 | 17077 | 468,7 | 13,9 | 1,219 | H5A034 C | sample | Hm Drgbl | Hm Drgbl | Hm Drgbl | CPE002E1 | CPE002EY | CPE002FJ | NM 00056 | NM 0005 | NM 0005 | IL5RA | IL5RA | IL5RA | interleukin 5 rece | interleuki | interleuki | 3568 | 3568 | 3568 | s7309 | s7308 | s7310 |
| 6889 | P1 | P2 | 673 | 583,5 | 9344 | 383,9 | 13,9 | 1,219 | H5A034 C | sample | Hm Drgbl | Hm Drgbl | Hm Drgbl | CPE002E1 | CPE002EY | CPE002FJ | NM 01341 | NM 0134 | NM 0134 | IARS | IARS | IARS | isoleucyl-tRNA sy | isoleucyl-t | isoleucyl-t | 3376 | 3376 | 3376 | s7079 | s7077 | s7078 |
| 3844 | A12 | A13 | 807 | 564,1 | 11197 | 329,7 | 13,9 | 1,236 | H5A034 C | sample | Hm Drgbl | Hm Drgbl | Hm Drgbl | CPE002DS | CPE002EI | CPE002F8 | NM 00285 | NM 0028 | NM 0028 | PXN | PXN | PXN | paxillin | paxillin | paxillin | 5829 | 5829 | 5829 | s11629 | s11627 | s11628 |
| 3908 | C5 | C6 | 1044 | 707,8 | 14527 | 413,4 | 13,9 | 1,236 | H5A034 C | sample | Hm Drgbl | Hm Drgbl | Hm Drgbl | CPE002DS | CPE002EI | CPE002F8 | NM 01894 | NM 0189 | NM 0189 | UTS2R | UTS2R | UTS2R | urotensin 2 recep | urotensin | urotensin | 2837 | 2837 | 2837 | s194454 | s194455 | s194456 |
| 4019 | H19 | H20 | 1010 | 695,8 | 14050 | 363,3 | 13,9 | 1,236 | H5A034 C | sample | Hm Drgbl | Hm Drgbl | Hm Drgbl | CPE002DS | CPE002EI | CPE002F8 | NM 00054 | NM 0005 | NM 0005 | TCF1 | TCF1 | TCF1 | transcription fact | transcript | transcript | 6927 | 6927 | 6927 | s13868 | s13869 | s13870 |
| 4037 | I13 | I14 | 897 | 647,4 | 12483 | 335,7 | 13,9 | 1,236 | H5A034 C | sample | Hm Drgbl | Hm Drgbl | Hm Drgbl | CPE002DS | CPE002EI | CPE002F8 | NM 00268 | NM 0026 | NM 0026 | SEPT5 | SEPT5 | SEPT5 | septin 5 | septin 5 | septin 5 | 5413 | 5413 | 5413 | s10763 | s10764 | s224294 |
| 4125 | L6 | L7 | 986 | 701,5 | 13660 | 393,7 | 13,9 | 1,236 | H5A034 C | sample | Hm Drgbl | Hm Drgbl | Hm Drgbl | CPE002DS | CPE002EI | CPE002F8 | NM 00458 | NM 0045 | NM 0045 | RRBP1 | RRBP1 | RRBP1 | ribosome binding | ribosome | ribosome | 6238 | 6238 | 6238 | s12351 | s12352 | s12353 |
| 4149 | M6 | M7 | 517 | 807,6 | 7174 | 459 | 13,9 | 1,236 | H5A034 C | sample | Hm Drgbl | Hm Drgbl | Hm Drgbl | CPE002DS | CPE002EI | CPE002F8 | NM 00691 | NM 0069 | NM 0069 | RBBP6 | RBBP6 | RBBP6 | retinoblastoma b | retinoblas | retinoblas | 5930 | 5930 | 5930 | s11844 | s11845 | s11843 |
| 4208 | P16 | P17 | 1462 | 805,1 | 20260 | 483,1 | 13,9 | 1,236 | H5A034 C | sample | Hm Drgbl | Hm Drgbl | Hm Drgbl | CPE002DS | CPE002EI | CPE002F8 | NM 00674 | NM 0010 | NM 0067 | SC4MOL | SC4MOL | SC4MOL | sterol-C4-methyl | sterol-C4- | sterol-C4- | 6307 | 6307 | 6307 | s12486 | s224569 | s12485 |
| 5852 | D5 | D6 | 1310 | 773 | 18230 | 552,3 | 13,9 | 1,269 | H5A034 C | sample | Hm Drgbl | Hm Drgbl | Hm Drgbl | CPE002DY | CPE002F1 | CPE002FM | NM 00162 | NM 0016 | NM 0016 | ALOX5AP | ALOX5AP | ALOX5AP | arachidonate 5-lip | arachidon | arachidon | 241 | 241 | 241 | s1279 | s223633 | s1278 |
| 6122 | P10 | P11 | 1592 | 728,9 | 22091 | 590,4 | 13,9 | 1,269 | H5A034 C | sample | Hm Drgbl | Hm Drgbl | Hm Drgbl | CPE002DY | CPE002F1 | CPE002FM | NM 00007 | NM 0000 | NM 0000 | CBS | CBS | CBS | cystathionine-bet | cystathior | cystathior | 875 | 875 | 875 | s290 | s289 | s291 |
| 1458 | M3 | M4 | 922 | 712,2 | 12809 | 400,2 | 13,9 | 1,275 | H5A034 C | sample | Hm Drgbl | Hm Drgbl | Hm Drgbl | CPE002FV | CPE002FZ | CPE002G3 | NM 00344 | NM 1980 | NM 1980 | ZNF155 | ZNF155 | ZNF155 | zinc finger protei | zinc finge | zinc finge | 7711 | 7711 | 7711 | s224794 | s15217 | s15218 |
| 1462 | M7 | M8 | 1306 | 764,4 | 18182 | 377,4 | 13,9 | 1,275 | H5A034 C | sample | Hm Drgbl | Hm Drgbl | Hm Drgbl | CPE002FV | CPE002FZ | CPE002G3 | NM 00103 | NM 0010 | NM 0010 | ZNF174 | ZNF174 | ZNF174 | zinc finger protei | zinc finge | zinc finge | 7727 | 7727 | 7727 | s15231 | s15232 | s194852 |
| 1478 | N21 | N22 | 1302 | 655,2 | 18159 | 407,5 | 13,9 | 1,275 | H5A034 C | sample | Hm Drgbl | Hm Drgbl | Hm Drgbl | CPE002FV | CPE002FZ | CPE002G3 | NM 00426 | NM 0042 | NM 0042 | CRSP6 | CRSP6 | CRSP6 | cofactor required | cofactor r | cofactor r | 9440 | 9440 | 9440 | s18071 | s18072 | s18073 |
| 16161 | B17 | B18 | 1255 | 1032 | 17496 | 391,3 | 13,9 | 1,275 | H5A034 C | sample | Hm Gen E | Hm Gen E | Hm Gen E | CPE002JG | CPE002NV | CPE002KB | NM 03190 | NM 0319 | NM 0319 | ARMC10 | ARMC10 | ARMC10 | armadillo repeat | armadillo | armadillo | 83787 | 83787 | 83787 | s38197 | s38198 | s38199 |
| 16311 | H22 | H23 | 1446 | 892,8 | 20112 | 301,9 | 13,9 | 1,275 | H5A034 C | sample | Hm Gen E | Hm Gen E | Hm Gen E | CPE002JG | CPE002NV | CPE002KB | NM 00108 | NM 0010 | NM 0010 | TNRC18 | TNRC18 | KIAA1856 | trinucleotide repe | trinucleot | KIAA1856 | 84629 | 84629 | 84629 | s39210 | s39211 | s228375 |
| 16348 | J12 | J13 | 1153 | 968,2 | 16082 | 396,4 | 13,9 | 1,275 | H5A034 C | sample | Hm Gen E | Hm Gen E | Hm Gen E | CPE002JG | CPE002NV | CPE002KB | NM 03257 | NM 0325 | NM 0325 | DPY30 | DPY30 | DPY30 | dpy-30 homolog ( | dpy-30 ho | dpy-30 ho | 84661 | 84661 | 84661 | s39269 | s39270 | s39271 |
| 16366 | J7 | J8 | 643 | 1039 | 8944 | 421,1 | 13,9 | 1,275 | H5A034 C | sample | Hm Gen E | Hm Gen E | Hm Gen E | CPE002JG | CPE002NV | CPE002KB | NM 03212 | NM 0321 | NM 0321 | DTNBP1 | DTNBP1 | DTNBP1 | dystrobrevin binc | dystrobre | dystrobre | 84062 | 84062 | 84062 | s38426 | s38427 | s38428 |
| 13464 | A9 | A10 | 1494 | 1116 | 20836 | 197,6 | 13,9 | 1,305 | H5A034 C | sample | Hm Gen E | Hm Gen E | Hm Gen E | CPE002JC | CPE002NC | CPE002K4 | NM 15255 | NM 1525 | NM 1525 | IQCE | IQCE | IQCE | IQ motif containir | IQ motif c | IQ motif c | 23288 | 23288 | 23288 | s23482 | s23484 | s225942 |
| 778 | A18 | A19 | 1802 | 922,8 | 25133 | 498,2 | 13,9 | 1,318 | H5A034 C | sample | Hm Drgbl | Hm Drgbl | Hm Drgbl | CPE002FU | CPE002FY | CPE002G2 | NM 00558 | NM 0055 | NM 0055 | SMAD6 | SMAD6 | SMAD6 | SMAD family men | SMAD fan | SMAD fan | 4091 | 4091 | 4091 | s8409 | s8410 | s8411 |
| 921 | G17 | G18 | 1916 | 724,4 | 26619 | 371,4 | 13,9 | 1,318 | H5A034 C | sample | Hm Drgbl | Hm Drgbl | Hm Drgbl | CPE002FU | CPE002FY | CPE002G2 | NM 00175 | NM 0017 | NM 0017 | CBFB | CBFB | CBFB | core-binding facto | core-bind | core-bind | 865 | 865 | 865 | s2470 | s2471 | s2472 |
| 939 | H11 | H12 | 1871 | 794,1 | 26056 | 453,7 | 13,9 | 1,318 | H5A034 C | sample | Hm Drgbl | Hm Drgbl | Hm Drgbl | CPE002FU | CPE002FY | CPE002G2 | NM 00533 | NM 0053 | NM 0053 | HCLS1 | HCLS1 | HCLS1 | hematopoietic ce | hematopc | hematopc | 3059 | 3059 | 3059 | s6482 | s6483 | s6484 |
| 1091 | N19 | N20 | 1457 | 710,5 | 20257 | 562,4 | 13,9 | 1,318 | H5A034 C | sample | Hm Drgbl | Hm Drgbl | Hm Drgbl | CPE002FU | CPE002FY | CPE002G2 | NM 00020 | NM 0002 | NM 0002 | PDX1 | PDX1 | PDX1 | pancreatic and du | pancreati | pancreati | 3651 | 3651 | 3651 | s7486 | s7487 | s223945 |
| 1119 | O22 | O23 | 1698 | 775,5 | 23579 | 511,9 | 13,9 | 1,318 | H5A034 C | sample | Hm Drgbl | Hm Drgbl | Hm Drgbl | CPE002FU | CPE002FY | CPE002G2 | NM 00104 | NM 0010 | NM 0010 | JARID1A | JARID1A | JARID1A | jumonji, AT rich ir | jumonji, A | jumonji, A | 5927 | 5927 | 5927 | s11834 | s11835 | s11836 |
| 1126 | O7 | O8 | 706 | 816,3 | 9811 | 487,3 | 13,9 | 1,318 | H5A034 C | sample | Hm Drgbl | Hm Drgbl | Hm Drgbl | CPE002FU | CPE002FY | CPE002G2 | NM 00442 | NM 0044 | NM 0044 | E4F1 | E4F1 | E4F1 | E4F transcription | E4F transc | E4F transc | 1877 | 1877 | 1877 | s223457 | s4424 | s4425 |
| 1138 | P18 | P19 | 1833 | 727,7 | 25405 | 447,4 | 13,9 | 1,318 | H5A034 C | sample | Hm Drgbl | Hm Drgbl | Hm Drgbl | CPE002FU | CPE002FY | CPE002G2 | NM 00055 | NM 0005 | NM 0005 | VWF | VWF | VWF | von Willebrand fa | von Willel | von Willel | 7450 | 7450 | 7450 | s14832 | s14833 | s14834 |
| 1147 | P4 | P5 | 1855 | 790,3 | 25813 | 510,4 | 13,9 | 1,318 | H5A034 C | sample | Hm Drgbl | Hm Drgbl | Hm Drgbl | CPE002FU | CPE002FY | CPE002G2 | NM 20700 | NM 2070 | NM 2070 | USF1 | USF1 | USF1 | upstream transcri | upstream | upstream | 7391 | 7391 | 7391 | s14717 | s14718 | s14719 |
| 5428 | C12 | C13 | 1932 | 698,9 | 26811 | 732,6 | 13,9 | 1,324 | H5A034 C | sample | Hm Drgbl | Hm Drgbl | Hm Drgbl | CPE002DX | CPE002F2 | CPE002FN | NM 08083 | NM 0808 | NM 0808 | WISP1 | WISP1 | WISP1 | WNT1 inducible s | WNT1 ind | WNT1 ind | 8840 | 8840 | 8840 | s16874 | s16875 | s16873 |
| 5526 | G14 | G15 | 643 | 539,9 | 8922 | 499,1 | 13,9 | 1,324 | H5A034 C | sample | Hm Drgbl | Hm Drgbl | Hm Drgbl | CPE002DX | CPE002F2 | CPE002FN | NM 00390 | NM 0039 | NM 0039 | EIF2S2 | EIF2S2 | EIF2S2 | eukaryotic transla | eukaryoti | eukaryoti | 8894 | 8894 | 8894 | s17004 | s17002 | s17003 |
| 5635 | K4 | K5 | 1440 | 663,6 | 20038 | 549,9 | 13,9 | 1,324 | H5A034 C | sample | Hm Drgbl | Hm Drgbl | Hm Drgbl | CPE002DX | CPE002F2 | CPE002FN | NM 00393 | NM 0039 | NM 0039 | CDK5R2 | CDK5R2 | CDK5R2 | cyclin-dependent | cyclin-dep | cyclin-dep | 8941 | 8941 | 8941 | s17102 | s17101 | s17100 |
| 5672 | M16 | M17 | 1023 | 685,4 | 14232 | 470,6 | 13,9 | 1,324 | H5A034 C | sample | Hm Drgbl | Hm Drgbl | Hm Drgbl | CPE002DX | CPE002F2 | CPE002FN | NM 00396 | NM 0039 | NM 0039 | UBE2M | UBE2M | UBE2M | ubiquitin-conjuga | ubiquitin- | ubiquitin- | 9040 | 9040 | 9040 | s17231 | s17229 | s225073 |
| 5693 | N13 | N14 | 1895 | 765,1 | 26290 | 420,8 | 13,9 | 1,324 | H5A034 C | sample | Hm Drgbl | Hm Drgbl | Hm Drgbl | CPE002DX | CPE002F2 | CPE002FN | NM 00382 | NM 0038 | NM 0038 | MPDZ | MPDZ | MPDZ | multiple PDZ dom | multiple F | multiple F | 8777 | 8777 | 8777 | s16720 | s16721 | s16722 |
| 5735 | O8 | O9 | 1090 | 695,8 | 15103 | 566,2 | 13,9 | 1,324 | H5A034 C | sample | Hm Drgbl | Hm Drgbl | Hm Drgbl | CPE002DX | CPE002F2 | CPE002FN | NM 19941 | NM 1994 | NM 1994 | PRC1 | PRC1 | PRC1 | protein regulator | protein re | protein re | 9055 | 9055 | 9055 | s17269 | s17268 | s17267 |
| 3492 | B2 | B3 | 1091 | 1276 | 15202 | 420,5 | 13,9 | 1,337 | H5A034 C | sample | Hm Drgbl | Hm Drgbl | Hm Drgbl | CPE002DR | CPE002EH | CPE002F7 | NM 01623 | NM 0162 | NM 0162 | GPRC5B | GPRC5B | GPRC5B | G protein-couplec | G protein- | G protein- | 51704 | 51704 | 51704 | s28552 | s28553 | s28554 |
| 3618 | G3 | G4 | 1207 | 1257 | 16772 | 417,6 | 13,9 | 1,337 | H5A034 C | sample | Hm Drgbl | Hm Drgbl | Hm Drgbl | CPE002DR | CPE002EH | CPE002F7 | NM 17687 | NM 1768 | NM 1768 | CCKBR | CCKBR | CCKBR | cholecystokinin B | cholecyst | cholecyst | 887 | 887 | 887 | s2506 | s2507 | s2508 |
| 3633 | H17 | H18 | 1043 | 1428 | 14502 | 386,9 | 13,9 | 1,337 | H5A034 C | sample | Hm Drgbl | Hm Drgbl | Hm Drgbl | CPE002DR | CPE002EH | CPE002F7 | NM 00152 | NM 0015 | NM 0015 | HCRTR2 | HCRTR2 | HCRTR2 | hypocretin (orexir | hypocreti | hypocreti | 3062 | 3062 | 3062 | s6487 | s6488 | s6489 |
| 8148 | D2 | D3 | 1208 | 591,9 | 16811 | 362,9 | 13,9 | 1,337 | H5A034 C | sample | Hm Drgbl | Hm Drgbl | Hm Drgbl | CPE002E5 | CPE002EU | CPE002FF | NM 17743 | NM 1774 | NM 1774 | DICER1 | DICER1 | DICER1 | Dicer1, Dcr-1 hom | Dicer1, Dc | Dicer1, Dc | 23405 | 23405 | 23405 | s23756 | s23754 | s23755 |
| 8190 | F14 | F15 | 1611 | 618,9 | 22433 | 474 | 13,9 | 1,337 | H5A034 C | sample | Hm Drgbl | Hm Drgbl | Hm Drgbl | CPE002E5 | CPE002EU | CPE002FF | NM 03210 | NM 0321 | NM 0321 | OTP | OTP | OTP | orthopedia home | orthopedi | orthopedi | 23440 | 23440 | 23440 | s223597 | s195104 | s195102 |
| 8350 | L7 | L8 | 1670 | 638 | 23136 | 488 | 13,9 | 1,337 | H5A034 C | sample | Hm Drgbl | Hm Drgbl | Hm Drgbl | CPE002E5 | CPE002EU | CPE002FF | NM 01815 | NM 0181 | NM 0181 | SLC4A1AP | SLC4A1AP | SLC4A1AP | solute carrier fam | solute car | solute car | 22950 | 22950 | 22950 | s22735 | s22734 | s22733 |
| 8384 | N16 | N17 | 1662 | 569,9 | 23122 | 485,5 | 13,9 | 1,337 | H5A034 C | sample | Hm Drgbl | Hm Drgbl | Hm Drgbl | CPE002E5 | CPE002EU | CPE002FF | NM 01234 | NM 0123 | NM 0123 | GTPBP4 | GTPBP4 | GTPBP4 | GTP binding prote | GTP bindi | GTP bindi | 23560 | 23560 | 23560 | s24098 | s24099 | s24100 |
| 10033 | C1 | C2 | 854 | 900,2 | 11873 | 177 | 13,9 | 1,337 | H5A034 C | sample | Hm Drgbl | Hm Drgbl | Hm Drgbl | CPE002EA | CPE002EP | CPE002FA | NM 02462 | NM 0246 | NM 0246 | OGFOD2 | OGFOD2 | OGFOD2 | 2-oxoglutarate ar | 2-oxoglut | 2-oxoglut | 79676 | 79676 | 79676 | s36076 | s36077 | s36078 |
| 10091 | E19 | E20 | 1855 | 902,3 | 25707 | 170,9 | 13,9 | 1,337 | H5A034 C | sample | Hm Drgbl | Hm Drgbl | Hm Drgbl | CPE002EA | CPE002EP | CPE002FA | NM 00103 | NM 0247 | NM 0247 | DHRS12 | DHRS12 | DHRS12 | dehydrogenase/r | dehydrog | dehydrog | 79758 | 79758 | 79758 | s224829 | s36271 | s36272 |
| 10330 | O18 | O19 | 1372 | 800 | 19018 | 159,3 | 13,9 | 1,337 | H5A034 C | sample | Hm Drgbl | Hm Drgbl | Hm Drgbl | CPE002EA | CPE002EP | CPE002FA | NM 03228 | NM 0322 | NM 0322 | ZDHHC18 | ZDHHC18 | ZDHHC18 | zinc finger, DHHC- | zinc finge | zinc finge | 84243 | 84243 | 84243 | s38708 | s38706 | s38707 |
| 1562 | B10 | B11 | 1407 | 818 | 19586 | 504,9 | 13,9 | 1,455 | H5A034 C | sample | Hm Drgbl | Hm Drgbl | Hm Drgbl | CPE002FW | CPE002G0 | CPE002G4 | NM 02469 | NM 0246 | NM 0246 | ZNF419 | ZNF419 | ZNF419 | zinc finger protei | zinc finge | zinc finge | 79744 | 79744 | 79744 | s36237 | s36238 | s36239 |
| 1581 | B6 | B7 | 1361 | 741,4 | 18959 | 441,3 | 13,9 | 1,455 | H5A034 C | sample | Hm Drgbl | Hm Drgbl | Hm Drgbl | CPE002FW | CPE002G0 | CPE002G4 | NM 02467 | NM 0246 | NM 0246 | ZNF768 | ZNF768 | ZNF768 | zinc finger protei | zinc finge | zinc finge | 79724 | 79724 | 79724 | s36186 | s36187 | s36188 |
| 1655 | E8 | E9 | 1334 | 654,6 | 18591 | 455,4 | 13,9 | 1,455 | H5A034 C | sample | Hm Drgbl | Hm Drgbl | Hm Drgbl | CPE002FW | CPE002G0 | CPE002G4 | NM 00102 | NM 0010 | NM 0010 | ZFAT1 | ZFAT1 | ZFAT1 | ZFAT zinc finger 1 | ZFAT zinc | ZFAT zinc | 57623 | 57623 | 57623 | s33466 | s33467 | s224479 |
| 1667 | F19 | F20 | 1549 | 707 | 21569 | 422,4 | 13,9 | 1,455 | H5A034 C | sample | Hm Drgbl | Hm Drgbl | Hm Drgbl | CPE002FW | CPE002G0 | CPE002G4 | NM 01795 | NM 0179 | NM 0179 | EPN3 | EPN3 | EPN3 | epsin 3 | epsin 3 | epsin 3 | 55040 | 55040 | 55040 | s30052 | s30053 | s30054 |
| 1714 | H18 | H19 | 736 | 758 | 10214 | 333,6 | 13,9 | 1,455 | H5A034 C | sample | Hm Drgbl | Hm Drgbl | Hm Drgbl | CPE002FW | CPE002G0 | CPE002G4 | NM 03097 | NM 0309 | NM 0309 | MED25 | MED25 | MED25 | mediator of RNA | mediator | mediator | 81857 | 81857 | 81857 | s37845 | s224889 | s37847 |

| | | | | | | | | | | | | | | | | | | | | | | | |
|---|---|---|---|---|---|---|---|---|---|---|---|---|---|---|---|---|---|---|---|---|---|---|---|
| 1738 | I18 | I19 | 1260 | 684 | 17474 | 316,9 | 13,9 | 1,455 | H5A034 C | sample | NM | ZNF335 | ZNF335 | ZNF335 | zinc finger protei | zinc finge | zinc finge | 63925 | 63925 | 63925 | s34276 | s34277 | s34278 |
| 1895 | O8 | O9 | 1307 | 730,4 | 18151 | 475,8 | 13,9 | 1,455 | H5A034 C | sample | NM | ZSCAN5 | ZSCAN5 | ZSCAN5 | zinc finger and SC | zinc finge | zinc finge | 79149 | 79149 | 79149 | s35628 | s224813 | s35630 |
| 21681 | H17 | H18 | 385 | 890,6 | 5369 | 569,4 | 13,9 | 1,463 | H5A034 C | sample | XM | LOC65167 | LOC65167 | LOC65167 | similar to double | similar to | similar to | 651672 | 651672 | 651672 | s197803 | s197804 | s197805 |
| 4698 | D3 | D4 | 1230 | 791 | 17073 | 437,7 | 13,9 | 1,527 | H5A034 C | sample | NM | HGF | HGF | HGF | hepatocyte growt | hepatocyt | hepatocyt | 3082 | 3082 | 3082 | s6528 | s6529 | s6530 |
| 4915 | M4 | M5 | 929 | 772,2 | 12948 | 514,5 | 13,9 | 1,527 | H5A034 C | sample | NM | PMPCB | PMPCB | PMPCB | peptidase (mitoc | peptidase | peptidase | 9512 | 9512 | 9512 | s18239 | s18240 | s18241 |
| 10453 | D20 | D21 | 1341 | 797,9 | 18587 | 194 | 13,9 | 1,571 | H5A034 C | sample | NM | SPIC | SPIC | SPIC | Spi-C transcriptio | Spi-C tran | Spi-C tran | 121599 | 121599 | 121599 | s42490 | s42489 | s42488 |
| 10460 | D5 | D6 | 1018 | 890,3 | 14200 | 196,4 | 13,9 | 1,571 | H5A034 C | sample | NM | ACBD5 | ACBD5 | ACBD5 | acyl-Coenzyme A | acyl-Coen | acyl-Coen | 91452 | 91452 | 91452 | s40667 | s40666 | s40668 |
| 10664 | M16 | M17 | 1593 | 808,3 | 22153 | 202,6 | 13,9 | 1,571 | H5A034 C | sample | NM | CENTD1 | CENTD1 | CENTD1 | centaurin, delta 1 | centaurin | centaurin | 116984 | 116984 | 116984 | s42085 | s42083 | s42084 |
| 3053 | P13 | P14 | 1544 | 652,2 | 21470 | 659,7 | 13,9 | 1,635 | H5A034 C | sample | NM | HUNK | HUNK | HUNK | hormonally upreg | hormonal | hormonal | 30811 | 30811 | 30811 | s26908 | s26909 | s26910 |
| 3059 | P19 | P20 | 1323 | 630,7 | 18403 | 596,4 | 13,9 | 1,635 | H5A034 C | sample | NM | TNNI3K | TNNI3K | TNNI3K | TNNI3 interacting | TNNI3 int | TNNI3 int | 51086 | 51086 | 51086 | s27389 | s27390 | s27391 |
| 7829 | G13 | G14 | 651 | 547,4 | 9017 | 367,6 | 13,9 | 2,122 | H5A034 C | sample | NM | TETRAN | TETRAN | TETRAN | tetracycline trans | tetracyclir | tetracyclir | 10227 | 10227 | 10227 | s19955 | s19957 | s19956 |
| 22753 | E1 | E2 | 1058 | 1676 | 14787 | 189,2 | 14 | 0,657 | H5A034 C | sample | XM | LOC72945 | LOC72945 | LOC72945 | hypothetical prot | hypotheti | hypotheti | 729451 | 729451 | 729451 | s59741 | s59742 | s59743 |
| 22291 | A4 | A5 | 1096 | 1352 | 15358 | 424,6 | 14 | 0,722 | H5A034 C | sample | XM | LOC72900 | LOC72900 | LOC72900 | hypothetical prot | hypotheti | hypotheti | 729002 | 729002 | 729002 | s59244 | s59245 | s227714 |
| 3321 | K17 | K18 | 854 | 1251 | 11947 | 414 | 14 | 0,867 | H5A034 C | sample | NM | PPM1A | PPM1A | PPM1A | protein phosphat | protein pl | protein pl | 5494 | 5494 | 5494 | s10919 | s10920 | s10921 |
| 3323 | K19 | K20 | 1493 | 1304 | 20852 | 342,2 | 14 | 0,867 | H5A034 C | sample | NM | PPM1B | PPM1B | PPM1B | protein phosphat | protein pl | protein pl | 5495 | 5495 | 5495 | s224329 | s10923 | s224330 |
| 6378 | J3 | J4 | 1253 | 610 | 17602 | 524,6 | 14 | 0,924 | H5A034 C | sample | NM | SLC31A1 | SLC31A1 | SLC31A1 | solute carrier fam | solute car | solute car | 1317 | 1317 | 1317 | s3377 | s3378 | s3379 |
| 20425 | D1 | D2 | 920 | 764,6 | 12901 | 183,8 | 14 | 0,933 | H5A034 C | sample | NM | LOC44126 | LOC44126 | LOC44126 | hypothetical gene | hypotheti | hypotheti | 441268 | 441268 | 441268 | s54301 | s54302 | s54303 |
| 20677 | N20 | N21 | 616 | 776,1 | 8607 | 211,7 | 14 | 0,933 | H5A034 C | sample | XM | LOC64263 | LOC64263 | LOC64263 | hypothetical LOC | hypotheti | hypotheti | 642633 | 642633 | 642633 | s55072 | s55073 | s227026 |
| 20695 | O15 | O16 | 1469 | 808,7 | 20604 | 228 | 14 | 0,933 | H5A034 C | sample | XM | LOC44106 | LOC44106 | LOC44106 | similar to membr | similar to | similar to | 441061 | 441061 | 441061 | s54258 | s54259 | s54260 |
| 12819 | G11 | G12 | 939 | 1013 | 13186 | 126,3 | 14 | 0,949 | H5A034 C | sample | NM | PSMF1 | PSMF1 | PSMF1 | proteasome (pros | proteasor | proteasor | 9491 | 9491 | 9491 | s18194 | s18195 | s18196 |
| 13002 | N3 | N4 | 1104 | 994,5 | 15408 | 120 | 14 | 0,949 | H5A034 C | sample | NM | CUL7 | CUL7 | CUL7 | cullin 7 | cullin 7 | cullin 7 | 9820 | 9820 | 9820 | s18990 | s18991 | s18992 |
| 18738 | M3 | M4 | 1097 | 892,9 | 15325 | 141,8 | 14 | 0,949 | H5A034 C | sample | NM | C10orf35 | C10orf35 | C10orf35 | chromosome 10 c | chromoso | chromoso | 219738 | 219738 | 219738 | s47706 | s47707 | s225872 |
| 17367 | D22 | D23 | 1438 | 971,6 | 20147 | 220,6 | 14 | 0,956 | H5A034 C | sample | NM | FAM69B | FAM69B | FAM69B | family with seque | family wit | family wit | 138311 | 138311 | 138311 | s44109 | s44110 | s195724 |
| 17401 | F1 | F2 | 1201 | 1074 | 16871 | 302,1 | 14 | 0,956 | H5A034 C | sample | NM | C20orf94 | C20orf94 | C20orf94 | chromosome 20 c | chromoso | chromoso | 128710 | 128710 | 128710 | s43341 | s43342 | s43343 |
| 17486 | I21 | I22 | 1368 | 959,8 | 19130 | 167,8 | 14 | 0,956 | H5A034 C | sample | NM | OR2T4 | OR2T4 | OR2T4 | olfactory recepto | olfactory | olfactory | 127074 | 127074 | 127074 | s43130 | s43131 | s195683 |
| 17563 | L4 | L5 | 1289 | 957,9 | 18057 | 231,8 | 14 | 0,956 | H5A034 C | sample | NM | PAGE3 | PAGE3 | PAGE3 | P antigen family, | P antigen | P antigen | 139793 | 139793 | 139793 | s44246 | s44247 | s44248 |
| 17643 | P11 | P12 | 803 | 1045 | 11280 | 331,3 | 14 | 0,956 | H5A034 C | sample | NM | ZPLD1 | ZPLD1 | ZPLD1 | zona pellucida-lik | zona pellu | zona pellu | 131368 | 131368 | 131368 | s43589 | s43590 | s43591 |
| 9369 | G17 | G18 | 1788 | 1145 | 25089 | 129,7 | 14 | 0,972 | H5A034 C | sample | NM | FANCL | FANCL | FANCL | Fanconi anemia, | Fanconi a | Fanconi a | 55120 | 55120 | 55120 | s30218 | s30219 | s30220 |
| 9485 | L13 | L14 | 1179 | 1122 | 16465 | 134,8 | 14 | 0,972 | H5A034 C | sample | NM | HIF1AN | HIF1AN | HIF1AN | hypoxia-inducible | hypoxia-ir | hypoxia-ir | 55662 | 55662 | 55662 | s31199 | s31197 | s31198 |
| 18930 | E3 | E4 | 1317 | 966,8 | 18455 | 202,4 | 14 | 0,976 | H5A034 C | sample | NM | TAS2R43 | TAS2R43 | TAS2R43 | taste receptor, ty | taste rece | taste rece | 259289 | 259289 | 259289 | s48949 | s48951 | s226055 |
| 19153 | O1 | O2 | 1126 | 942,3 | 15775 | 264,9 | 14 | 0,976 | H5A034 C | sample | NM | C11orf36 | C11orf36 | C11orf36 | chromosome 11 c | chromoso | chromoso | 283303 | 283303 | 283303 | s49183 | s49184 | s49185 |
| 9775 | H15 | H16 | 1566 | 1092 | 21992 | 137,1 | 14 | 0,979 | H5A034 C | sample | NM | HIVEP3 | HIVEP3 | HIVEP3 | human immunod | human im | human im | 59269 | 59269 | 59269 | s33959 | s33958 | s33960 |
| 9967 | P15 | P16 | 1769 | 1034 | 24806 | 156,8 | 14 | 0,979 | H5A034 C | sample | XM | DMRTA2 | DMRTA2 | DMRTA2 | DMRT-like family | DMRT-like | DMRT-like | 63950 | 63950 | 63950 | s34324 | s34325 | s224591 |
| 15234 | K3 | K4 | 767 | 771,5 | 10770 | 135,1 | 14 | 0,979 | H5A034 C | sample | NM | IL1F9 | IL1F9 | IL1F9 | interleukin 1 fami | interleuki | interleuki | 56300 | 56300 | 56300 | s32129 | s32130 | s32131 |
| 8730 | L3 | L4 | 1703 | 1063 | 23766 | 134,4 | 14 | 0,989 | H5A034 C | sample | NM | KLHL3 | KLHL3 | KLHL3 | kelch-like 3 (Dros | kelch-like | kelch-like | 26249 | 26249 | 26249 | s25292 | s25293 | s25294 |
| 19458 | K3 | K4 | 1100 | 1162 | 15353 | 245 | 14 | 0,993 | H5A034 C | sample | NM | TMEM202 | TMEM202 | TMEM202 | transmembrane p | transmem | transmem | 338949 | 338949 | 338949 | s50440 | s50441 | s50442 |
| 20770 | B18 | B19 | 1489 | 1031 | 20910 | 316,4 | 14 | 0,993 | H5A034 C | sample | XM | LOC64475 | LOC64475 | LOC64475 | similar to serine p | similar to | similar to | 644759 | 644759 | 644759 | s55797 | s55798 | s55799 |
| 20819 | D19 | D20 | 1518 | 971,8 | 21214 | 238,2 | 14 | 0,993 | H5A034 C | sample | XM | LOC64362 | LOC64362 | LOC64362 | hypothetical LOC | hypotheti | hypotheti | 643623 | 643623 | 643623 | s55368 | s55369 | s55370 |
| 21029 | M13 | M14 | 1536 | 940 | 21480 | 252,6 | 14 | 0,993 | H5A034 C | sample | XM | LOC64333 | LOC64333 | LOC64333 | similar to Nonsec | similar to | similar to | 643332 | 643332 | 643332 | s55277 | s55278 | s55279 |
| 21447 | N22 | N23 | 1440 | 902,2 | 20113 | 342,9 | 14 | 0,993 | H5A034 C | sample | XM | LOC64849 | LOC64849 | LOC64849 | similar to IQ moti | similar to | similar to | 648494 | 648494 | 648494 | s56840 | s56841 | s56842 |
| 11156 | A5 | A6 | 950 | 943,6 | 13303 | 142,6 | 14 | 1,014 | H5A034 C | sample | NM | B4GALNT3 | B4GALNT3 | B4GALNT3 | beta-1,4-N-acetyl- | beta-1,4-N | beta-1,4-N | 283358 | 283358 | 283358 | s49201 | s49202 | s49203 |
| 11233 | E1 | E2 | 1582 | 827,5 | 22176 | 153,8 | 14 | 1,014 | H5A034 C | sample | NM | SLC25A34 | SLC25A34 | SLC25A34 | solute carrier fam | solute car | solute car | 284723 | 284723 | 284723 | s49746 | s49744 | s49745 |
| 11375 | J8 | J9 | 1535 | 816,6 | 21417 | 133,3 | 14 | 1,014 | H5A034 C | sample | | | | | | | | | | | | | |
| 11399 | K8 | K9 | 1468 | 807,9 | 20528 | 140,3 | 14 | 1,014 | H5A034 C | sample | | | | | | | | | | | | | |
| 11478 | O14 | O15 | 1930 | 898,4 | 26988 | 189,3 | 14 | 1,014 | H5A034 C | sample | | | | | | | | | | | | | |
| 7530 | J3 | J4 | 1024 | 595,1 | 14316 | 328,4 | 14 | 1,069 | H5A034 C | sample | NM | CHST3 | CHST3 | CHST3 | carbohydrate (ch | carbohydr | carbohydr | 9469 | 9469 | 9469 | s18147 | s18148 | s225142 |
| 16925 | B13 | B14 | 1440 | 985,2 | 20224 | 251,3 | 14 | 1,081 | H5A034 C | sample | NM | CCDC85A | C15orf43 | CCDC85A | coiled-coil domai | chromoso | coiled-coi | 114800 | 145645 | 114800 | s41633 | s44763 | s41635 |
| 17030 | F21 | F22 | 1720 | 1069 | 24036 | 198,3 | 14 | 1,081 | H5A034 C | sample | NM | LRRC42 | FLJ25404 | LRRC42 | leucine rich repea | hypotheti | leucine ric | 115353 | 146378 | 115353 | s41802 | s44847 | s41804 |
| 17047 | G15 | G16 | 923 | 823,2 | 12906 | 213 | 14 | 1,081 | H5A034 C | sample | NM | RWDD2A | SESN3 | RWDD2A | RWD domain con | sestrin 3 | RWD dom | 112611 | 143686 | 112611 | s41347 | s44569 | s41349 |
| 17100 | I2 | I3 | 1381 | 975,2 | 19395 | 231,7 | 14 | 1,081 | H5A034 C | sample | NM | GYLTL1B | SHISA4 | GYLTL1B | glycosyltransferas | shisa hom | glycosyltra | 120071 | 149345 | 120071 | s42346 | s45278 | s42348 |
| 6971 | C19 | C20 | 1531 | 608 | 21421 | 531,8 | 14 | 1,089 | H5A034 C | sample | NM | LDLR | LDLR | LDLR | low density lipop | low densi | low densi | 3949 | 3949 | 3949 | s224008 | s6 | s4 |
| 5046 | C14 | C15 | 1518 | 591,2 | 21257 | 614,5 | 14 | 1,098 | H5A034 C | sample | NM | TPO | TPO | TPO | thyroid peroxidas | thyroid pe | thyroid pe | 7173 | 7173 | 7173 | s14350 | s14351 | s224750 |
| 5089 | E1 | E2 | 419 | 558,3 | 5883 | 539,9 | 14 | 1,098 | H5A034 C | sample | NM | ADAMTS1 | ADAMTS1 | ADAMTS1 | ADAM metallope | ADAM me | ADAM me | 81792 | 81792 | 81792 | s37788 | s37789 | s37790 |
| 5099 | E19 | E20 | 1910 | 605,3 | 26813 | 600,1 | 14 | 1,098 | H5A034 C | sample | NM | USP42 | USP42 | USP42 | ubiquitin specific | ubiquitin | ubiquitin | 84132 | 84132 | 84132 | s38539 | s38540 | s38541 |
| 5302 | M7 | M8 | 1331 | 564 | 18700 | 573,3 | 14 | 1,098 | H5A034 C | sample | NM | TMPRSS6 | TMPRSS6 | TMPRSS6 | transmembrane p | transmem | transmem | 164656 | 164656 | 164656 | s46533 | s46534 | s46535 |
| 2394 | D3 | D4 | 1162 | 651,1 | 16229 | 307,2 | 14 | 1,102 | H5A034 C | sample | NM | CAMK2G | CAMK2G | CAMK2G | calcium/calmodul | calcium/c | calcium/c | 818 | 818 | 818 | s2367 | s2368 | s224890 |
| 2480 | H16 | H17 | 1333 | 633,1 | 18617 | 281,1 | 14 | 1,102 | H5A034 C | sample | NM | PSKH1 | PSKH1 | PSKH1 | protein serine kin | protein se | protein se | 5681 | 5681 | 5681 | s11324 | s11325 | s11326 |
| 2552 | K16 | K17 | 1199 | 865,8 | 16751 | 384,8 | 14 | 1,102 | H5A034 C | sample | NM | PDPK1 | PDPK1 | PDPK1 | 3-phosphoinositi | 3-phosph | 3-phosph | 5170 | 5170 | 5170 | s10273 | s10274 | s10275 |
| 2594 | M10 | M11 | 1459 | 633,8 | 20481 | 370 | 14 | 1,102 | H5A034 C | sample | NM | PIK3C2A | PIK3C2A | PIK3C2A | phosphoinositide | phosphoi | phosphoi | 5286 | 5286 | 5286 | s10508 | s10509 | s10510 |
| 2686 | P7 | P8 | 1204 | 778,5 | 16863 | 458,3 | 14 | 1,102 | H5A034 C | sample | XM | GAK | GAK | GAK | cyclin G associate | cyclin G a | cyclin G a | 2580 | 2580 | 2580 | s5527 | s5528 | s5529 |
| 16546 | B18 | B19 | 1300 | 1004 | 18155 | 286,9 | 14 | 1,107 | H5A034 C | sample | NM | CABLES1 | CABLES1 | CABLES1 | Cdk5 and Abl enz | Cdk5 and | Cdk5 and | 91768 | 91768 | 91768 | s40774 | s40775 | s40776 |
| 16671 | G22 | G23 | 1254 | 960,2 | 17520 | 281,7 | 14 | 1,107 | H5A034 C | sample | NM | HN1L | HN1L | HN1L | hematological an | hematolo | hematolo | 90861 | 90861 | 90861 | s40515 | s40516 | s40517 |
| 17787 | F11 | F12 | 1153 | 1093 | 16168 | 259,4 | 14 | 1,107 | H5A034 C | sample | NM | CMTM2 | CMTM2 | CMTM2 | CKLF-like MARVEL | CKLF-like | CKLF-like | 146225 | 146225 | 146225 | s44833 | s44834 | s44835 |
| 17802 | F3 | F4 | 1092 | 1004 | 15319 | 241,4 | 14 | 1,107 | H5A034 C | sample | NM | SLC38A8 | SLC38A8 | SLC38A8 | solute carrier fam | solute car | solute car | 146167 | 146167 | 146167 | s44818 | s44819 | s44820 |
| 17826 | G3 | G4 | 799 | 1048 | 11216 | 174,1 | 14 | 1,107 | H5A034 C | sample | NM | OR51E1 | OR51E1 | OR51E1 | olfactory recepto | olfactory | olfactory | 143503 | 143503 | 143503 | s44549 | s44550 | s44551 |
| 17937 | L17 | L18 | 1268 | 1073 | 17805 | 238,3 | 14 | 1,107 | H5A034 C | sample | NM | TMC8 | TMC8 | TMC8 | transmembrane c | transmem | transmem | 147138 | 147138 | 147138 | s44965 | s44966 | s225707 |
| 18045 | P6 | P7 | 1074 | 1156 | 15005 | 335,1 | 14 | 1,107 | H5A034 C | sample | NM | C4orf23 | C4orf23 | C4orf23 | chromosome 4 op | chromoso | chromoso | 152992 | 152992 | 152992 | s45751 | s45752 | s45753 |
| 9073 | K1 | K2 | 1987 | 1766 | 27764 | 141,2 | 14 | 1,124 | H5A034 C | sample | NM | ST8SIA3 | ST8SIA3 | ST8SIA3 | ST8 alpha-N-acety | ST8 alpha | ST8 alpha | 51046 | 51046 | 51046 | s27299 | s27300 | s27301 |
| 4249 | B1 | B2 | 1207 | 665,3 | 16938 | 446,6 | 14 | 1,181 | H5A034 C | sample | NM | GLRA2 | GLRA2 | GLRA2 | glycine receptor, | glycine re | glycine re | 2742 | 2742 | 2742 | s5832 | s5833 | s5834 |
| 4254 | B14 | B15 | 1442 | 695 | 20204 | 419,2 | 14 | 1,181 | H5A034 C | sample | NM | ATP13A1 | ATP13A1 | ATP13A1 | ATPase type 13A1 | ATPase ty | ATPase ty | 57130 | 57130 | 57130 | s32748 | s32749 | s32750 |
| 4274 | C10 | C11 | 969 | 683,2 | 13522 | 376,1 | 14 | 1,181 | H5A034 C | sample | NM | BEST1 | BEST1 | BEST1 | bestrophin 1 | bestrophi | bestrophi | 7439 | 7439 | 7439 | s14813 | s14814 | s14815 |
| 4275 | C11 | C12 | 1325 | 724,4 | 18543 | 372,9 | 14 | 1,181 | H5A034 C | sample | NM | ATP2A1 | ATP2A1 | ATP2A1 | ATPase, Ca++ trar | ATPase, C | ATPase, C | 487 | 487 | 487 | s1743 | s1744 | s1745 |
| 4354 | F18 | F19 | 1192 | 685,1 | 16666 | 372,3 | 14 | 1,181 | H5A034 C | sample | NM | CATSPER2 | CATSPER2 | CATSPER2 | cation channel, sp | cation cha | cation cha | 117155 | 117155 | 117155 | s42113 | s42114 | s42115 |
| 4420 | I12 | I13 | 1347 | 722,3 | 18839 | 475,4 | 14 | 1,181 | H5A034 C | sample | NM | P2RX2 | P2RX2 | P2RX2 | purinergic recept | purinergic | purinergic | 22953 | 22953 | 22953 | s22736 | s22737 | s223546 |
| 81 | D17 | D18 | 358 | 1655 | 5012 | 411,2 | 14 | 1,191 | H5A034 C | sample | NM | SFRS8 | SFRS8 | SFRS8 | splicing factor, ar | splicing fa | splicing fa | 6433 | 6433 | 6433 | s12748 | s12746 | s12747 |
| 191 | H8 | H9 | 556 | 1515 | 7787 | 590,1 | 14 | 1,191 | H5A034 C | sample | NM | SRP68 | SRP68 | SRP68 | signal recognition | signal rec | signal rec | 6730 | 6730 | 6730 | s13446 | s13444 | s13445 |
| 200 | I16 | I17 | 320 | 1588 | 4471 | 435,8 | 14 | 1,191 | H5A034 C | sample | NM | SNAI1 | SNAI1 | SNAI1 | snail homolog 1 ( | snail hom | snail hom | 6615 | 6615 | 6615 | s13186 | s13187 | s13185 |
| 1943 | A8 | A9 | 1245 | 733,9 | 17436 | 464 | 14 | 1,217 | H5A034 C | sample | NM | ZNF540 | ZNF540 | ZNF540 | zinc finger protei | zinc finge | zinc finge | 163255 | 163255 | 163255 | s223388 | s46445 | s46446 |
| 2107 | H4 | H5 | 1014 | 722,9 | 14159 | 477,5 | 14 | 1,217 | H5A034 C | sample | NM | FEZF1 | FEZF1 | FEZF1 | FEZ family zinc fin | FEZ family | FEZ family | 389549 | 389549 | 389549 | s52493 | s52494 | s52495 |
| 6541 | A20 | A21 | 1333 | 667,2 | 18663 | 423,4 | 14 | 1,228 | H5A034 C | sample | NM | IFNB1 | IFNB1 | IFNB1 | interferon, beta 1 | interferon | interferon | 3456 | 3456 | 3456 | s7187 | s7188 | s7189 |
| 6604 | D12 | D13 | 1394 | 601,8 | 19478 | 448,5 | 14 | 1,228 | H5A034 C | sample | NM | ITGB7 | ITGB7 | ITGB7 | integrin, beta 7 | integrin, b | integrin, b | 3695 | 3695 | 3695 | s7597 | s7596 | s7595 |
| 6669 | F6 | F7 | 1132 | 582,5 | 15886 | 487,3 | 14 | 1,228 | H5A034 C | sample | NM | JAG2 | JAG2 | JAG2 | jagged 2 | jagged 2 | jagged 2 | 3714 | 3714 | 3714 | s7643 | s7644 | s7645 |
| 6687 | G22 | G23 | 1705 | 604,4 | 23933 | 414,8 | 14 | 1,228 | H5A034 C | sample | NM | IL11RA | IL11RA | IL11RA | interleukin 11 rec | interleuki | interleuki | 3590 | 3590 | 3590 | s7354 | s7355 | s7353 |
| 6828 | M2 | M3 | 894 | 553,7 | 12477 | 239,5 | 14 | 1,228 | H5A034 C | sample | NM | INPP4A | INPP4A | INPP4A | inositol polyphos | inositol p | inositol p | 3631 | 3631 | 3631 | s7450 | s7452 | s7451 |
| 6851 | N19 | N20 | 1111 | 646,7 | 15526 | 325,9 | 14 | 1,228 | H5A034 C | sample | NM | HYAL1 | HYAL1 | HYAL1 | hyaluronoglucosa | hyaluronc | hyaluronc | 3373 | 3373 | 3373 | s7071 | s7072 | s7073 |
| 6897 | P17 | P18 | 1016 | 632,2 | 14206 | 391,2 | 14 | 1,228 | H5A034 C | sample | NM | IDH3B | IDH3B | IDH3B | isocitrate dehydr | isocitrate | isocitrate | 3420 | 3420 | 3420 | s797 | s798 | s799 |
| 3951 | E22 | E23 | 875 | 692,6 | 12272 | 404,1 | 14 | 1,244 | H5A034 C | sample | NM | RAC2 | RAC2 | RAC2 | ras-related C3 bo | ras-relate | ras-relate | 5880 | 5880 | 5880 | s11714 | s11715 | s11716 |
| 4064 | J16 | J17 | 232 | 749,3 | 3253 | 321,4 | 14 | 1,244 | H5A034 C | sample | NM | RPL17 | RPL17 | RPL17 | ribosomal protein | ribosomal | ribosomal | 6139 | 6139 | 6139 | s12177 | s12178 | s12179 |
| 4065 | J17 | J18 | 878 | 656,9 | 12335 | 281,5 | 14 | 1,244 | H5A034 C | sample | NM | TCOF1 | TCOF1 | TCOF1 | Treacher Collins-F | Treacher ( | Treacher ( | 6949 | 6949 | 6949 | s13920 | s13921 | s13919 |
| 11533 | A20 | A21 | 1028 | 935,9 | 14418 | 213 | 14 | 1,25 | H5A034 C | sample | NM | EFNA3 | EFNA3 | EFNA3 | ephrin-A3 | ephrin-A3 | ephrin-A3 | 1944 | 1944 | 1944 | s4501 | s4502 | s4503 |
| 11702 | H21 | H22 | 1097 | 891,7 | 15369 | 141,4 | 14 | 1,25 | H5A034 C | sample | NM | CRYBA1 | CRYBA1 | CRYBA1 | crystallin, beta A1 | crystallin, | crystallin, | 1411 | 1411 | 1411 | s3546 | s3547 | s3548 |
| 11732 | I5 | I6 | 1002 | 875,1 | 14042 | 137,8 | 14 | 1,25 | H5A034 C | sample | NM | C4BPB | C4BPB | C4BPB | complement com | compleme | compleme | 725 | 725 | 725 | s2176 | s2177 | s2178 |
| 11807 | L8 | L9 | 1261 | 792 | 17608 | 171,8 | 14 | 1,25 | H5A034 C | sample | NM | HNRNPH2 | HNRNPH2 | HNRNPH2 | heterogeneous nu | heteroger | heteroger | 3188 | 3188 | 3188 | s6731 | s6732 | s6733 |
| 11844 | N2 | N3 | 761 | 901,6 | 10624 | 142,8 | 14 | 1,25 | H5A034 C | sample | NM | HUS1 | HUS1 | HUS1 | HUS1 checkpoint | HUS1 che | HUS1 che | 3364 | 3364 | 3364 | s7065 | s7066 | s7067 |
| 15560 | I16 | I17 | 1361 | 840,9 | 19011 | 131,1 | 14 | 1,273 | H5A034 C | sample | NM | DBNDD1 | DBNDD1 | DBNDD1 | dysbindin (dystro | dysbindin | dysbindin | 79007 | 79007 | 79007 | s35414 | s195370 | s195371 |
| 15729 | P17 | P18 | 1146 | 752,9 | 16066 | 140,8 | 14 | 1,273 | H5A034 C | sample | NM | GPBP1 | GPBP1 | GPBP1 | GC-rich promoter | GC-rich pr | GC-rich pr | 65056 | 65056 | 65056 | s35172 | s35173 | s35174 |
| 5820 | C2 | C3 | 940 | 932,6 | 13192 | 546,4 | 14 | 1,279 | H5A034 C | sample | NM | ARHGAP4 | ARHGAP4 | ARHGAP4 | Rho GTPase activ | Rho GTPa | Rho GTPa | 393 | 393 | 393 | s1586 | s1587 | s1588 |
| 5837 | D13 | D14 | 1564 | 896,9 | 21910 | 476,3 | 14 | 1,279 | H5A034 C | sample | NM | ALOX15B | ALOX15B | ALOX15B | arachidonate 15-l | arachidon | arachidon | 247 | 247 | 247 | s1290 | s1291 | s1292 |
| 6086 | N21 | N22 | 1613 | 865,7 | 22633 | 380,7 | 14 | 1,279 | H5A034 C | sample | NM | AQP4 | AQP4 | AQP4 | aquaporin 4 | aquaporir | aquaporir | 361 | 361 | 361 | s1524 | s1525 | s1526 |
| 1161 | A17 | A18 | 1193 | 821,8 | 16721 | 525,9 | 14 | 1,284 | H5A034 C | sample | NM | ZNF2 | ZNF2 | ZNF2 | zinc finger protei | zinc finge | zinc finge | 7549 | 7549 | 7549 | s15004 | s15005 | s15006 |
| 1178 | B10 | B11 | 1376 | 729,6 | 19217 | 480,4 | 14 | 1,284 | H5A034 C | sample | NM | AP4B1 | AP4B1 | AP4B1 | adaptor-related p | adaptor-r | adaptor-r | 10717 | 10717 | 10717 | s21050 | s21051 | s21052 |
| 1284 | F2 | F3 | 1158 | 712,3 | 16236 | 472,5 | 14 | 1,284 | H5A034 C | sample | NM | ARID5A | ARID5A | ARID5A | AT rich interactive | AT rich int | AT rich int | 10865 | 10865 | 10865 | s21327 | s21328 | s21329 |
| 1328 | H16 | H17 | 1162 | 665,2 | 16260 | 374,5 | 14 | 1,284 | H5A034 C | sample | NM | KLF8 | KLF8 | KLF8 | Kruppel-like facto | Kruppel-li | Kruppel-li | 11279 | 11279 | 11279 | s22271 | s22272 | s22273 |
| 1373 | J13 | J14 | 1021 | 720,3 | 14278 | 462,9 | 14 | 1,284 | H5A034 C | sample | NM | ASH2L | ASH2L | ASH2L | ash2 (absent, sma | ash2 (abs | ash2 (abs | 9070 | 9070 | 9070 | s17300 | s17301 | s17302 |
| 1397 | K13 | K14 | 1187 | 683,3 | 16595 | 340,7 | 14 | 1,284 | H5A034 C | sample | NM | ZNF142 | ZNF142 | ZNF142 | zinc finger protei | zinc finge | zinc finge | 7701 | 7701 | 7701 | s15189 | s15190 | s15191 |
| 1427 | L19 | L20 | 1362 | 688 | 19024 | 394,6 | 14 | 1,284 | H5A034 C | sample | NM | DLG5 | DLG5 | DLG5 | discs, large homo | discs, larg | discs, larg | 9231 | 9231 | 9231 | s17650 | s17651 | s17652 |
| 16134 | A14 | A15 | 884 | 1114 | 12354 | 396,3 | 14 | 1,284 | H5A034 C | sample | NM | FLJ23356 | FLJ23356 | FLJ23356 | hypothetical prot | hypotheti | hypotheti | 84197 | 84197 | 84197 | s38645 | s38646 | s38647 |
| 16164 | B2 | B3 | 968 | 999,7 | 13595 | 354,5 | 14 | 1,284 | H5A034 C | sample | NM | C2orf40 | C2orf40 | C2orf40 | chromosome 2 op | chromoso | chromoso | 84417 | 84417 | 84417 | s38974 | s38975 | s38976 |
| 16274 | G10 | G11 | 783 | 1096 | 10977 | 218,8 | 14 | 1,284 | H5A034 C | sample | NM | CHCHD5 | CHCHD5 | CHCHD5 | coiled-coil-helix-c | coiled-coi | coiled-coi | 84269 | 84269 | 84269 | s38772 | s38773 | s195463 |
| 16306 | H18 | H19 | 1288 | 934 | 18077 | 263,5 | 14 | 1,284 | H5A034 C | sample | NM | KRBA1 | KRBA1 | KRBA1 | KRAB-A domain c | KRAB-A d | KRAB-A d | 84626 | 84626 | 84626 | s39200 | s39201 | s39202 |
| 13572 | F2 | F3 | 1085 | 1077 | 15154 | 166,6 | 14 | 1,315 | H5A034 C | sample | NM | OR2L2 | OR2L2 | OR2L2 | olfactory recepto | olfactory | olfactory | 26246 | 26246 | 26246 | s25286 | s25287 | s25288 |
| 932 | G5 | G6 | 1400 | 845,2 | 19571 | 503,2 | 14 | 1,327 | H5A034 C | sample | NM | CASQ1 | CASQ1 | CASQ1 | calsequestrin 1 (f | calseques | calseques | 844 | 844 | 844 | s2434 | s2435 | s2436 |
| 1045 | L20 | L21 | 1198 | 734,9 | 16724 | 371,9 | 14 | 1,327 | H5A034 C | sample | NM | TFAP2C | TFAP2C | TFAP2C | transcription fact | transcript | transcript | 7022 | 7022 | 7022 | s14009 | s14010 | s14011 |
| 1046 | L21 | L22 | 1356 | 699 | 19044 | 383,6 | 14 | 1,327 | H5A034 C | sample | NM | DNAJB1 | DNAJB1 | DNAJB1 | DnaJ (Hsp40) hom | DnaJ (Hsp | DnaJ (Hsp | 3337 | 3337 | 3337 | s7008 | s7009 | s223882 |
| 1105 | O1 | O2 | 881 | 765 | 12368 | 447,6 | 14 | 1,327 | H5A034 C | sample | NM | TSC22D3 | TSC22D3 | TSC22D3 | TSC22 domain far | TSC22 dor | TSC22 dor | 1831 | 1831 | 1831 | s4330 | s4331 | s4332 |
| 1134 | P14 | P15 | 1511 | 802,1 | 21117 | 329,8 | 14 | 1,327 | H5A034 C | sample | NM | VBP1 | VBP1 | VBP1 | von Hippel-Lindau | von Hippe | von Hippe | 7411 | 7411 | 7411 | s14756 | s14757 | s14758 |
| 5461 | D20 | D21 | 1186 | 695,1 | 16617 | 607,3 | 14 | 1,333 | H5A034 C | sample | NM | SLC28A2 | SLC28A2 | SLC28A2 | solute carrier fam | solute car | solute car | 9153 | 9153 | 9153 | s17498 | s17499 | s17500 |
| 5476 | E12 | E13 | 1597 | 715,9 | 22371 | 668,8 | 14 | 1,333 | H5A034 C | sample | NM | VNN2 | VNN2 | VNN2 | vanin 2 | vanin 2 | vanin 2 | 8875 | 8875 | 8875 | s16952 | s16951 | s16953 |
| 5613 | J6 | J7 | 1752 | 650,9 | 24542 | 496,4 | 14 | 1,333 | H5A034 C | sample | NM | MSC | MSC | MSC | musculin (activat | musculin | musculin | 9242 | 9242 | 9242 | s17672 | s17670 | s17671 |
| 5733 | O6 | O7 | 1399 | 695,7 | 19537 | 622,1 | 14 | 1,333 | H5A034 C | sample | NM | NFS1 | NFS1 | NFS1 | NFS1 nitrogen fix | NFS1 nitro | NFS1 nitro | 9054 | 9054 | 9054 | s17266 | s17265 | s17264 |
| 3780 | N2 | N3 | 1291 | 1289 | 18125 | 387,2 | 14 | 1,346 | H5A034 C | sample | NM | GPR113 | GPR113 | GPR113 | G protein-couple | G protein | G protein | 165082 | 165082 | 165082 | s46557 | s46558 | s223394 |
| 8287 | J15 | J16 | 1756 | 622,4 | 24632 | 377,6 | 14 | 1,346 | H5A034 C | sample | NM | SEPHS2 | SEPHS2 | SEPHS2 | selenophosphate | selenopho | selenopho | 22928 | 22928 | 22928 | s22695 | s22696 | s22694 |
| 8322 | K3 | K4 | 852 | 635,6 | 11918 | 284,2 | 14 | 1,346 | H5A034 C | sample | NM | SEC23IP | SEC23IP | SEC23IP | SEC23 interacting | SEC23 inte | SEC23 inte | 11196 | 11196 | 11196 | s22108 | s22107 | s22109 |
| 8354 | M10 | M11 | 1491 | 602,4 | 20881 | 504,5 | 14 | 1,346 | H5A034 C | sample | NM | UBR2 | UBR2 | UBR2 | ubiquitin protein | ubiquitin | ubiquitin | 23304 | 23304 | 23304 | s23512 | s23513 | s23514 |
| 8387 | N19 | N20 | 1363 | 603,2 | 19143 | 535,5 | 14 | 1,346 | H5A034 C | sample | NM | EXOSC7 | EXOSC7 | EXOSC7 | exosome compon | exosome | exosome | 23016 | 23016 | 23016 | s223551 | s22838 | s22837 |
| 10277 | M13 | M14 | 1363 | 792,8 | 19127 | 141 | 14 | 1,346 | H5A034 C | sample | NM | SLC24A6 | SLC24A6 | SLC24A6 | solute carrier fam | solute car | solute car | 80024 | 80024 | 80024 | s224842 | s36845 | s36846 |
| 1538 | A10 | A11 | 1341 | 777,8 | 18768 | 454,5 | 14 | 1,466 | H5A034 C | sample | NM | ZNF304 | ZNF304 | ZNF304 | zinc finger protei | zinc finge | zinc finge | 57343 | 57343 | 57343 | s32965 | s32966 | s32967 |
| 1628 | D5 | D6 | 1077 | 732,5 | 15067 | 462,1 | 14 | 1,466 | H5A034 C | sample | XM | CLDN22 | CLDN22 | CLDN22 | claudin 22 | claudin 22 | claudin 22 | 53842 | 53842 | 53842 | s28792 | s28793 | s195239 |
| 1777 | K1 | K2 | 903 | 681,1 | 12673 | 416 | 14 | 1,466 | H5A034 C | sample | NM | PILRA | PILRA | PILRA | paired immunogl | paired im | paired im | 29992 | 29992 | 29992 | s26860 | s26861 | s223835 |
| 1907 | P19 | P20 | 1041 | 638,3 | 14541 | 399,4 | 14 | 1,466 | H5A034 C | sample | NM | ADAMTSL3 | ADAMTSL3 | ADAMTSL3 | ADAMTS-like 3 | ADAMTS-l | ADAMTS-l | 57188 | 57188 | 57188 | s224461 | s32864 | s32865 |
| 21588 | D2 | D3 | 130 | 1078 | 1825 | 564,6 | 14 | 1,474 | H5A034 C | sample | XM | LOC65316 | LOC65316 | LOC65316 | similar to 40S ribo | similar to | similar to | 653162 | 653162 | 653162 | s57599 | s57600 | s57601 |
| 4705 | E1 | E2 | 673 | 753,3 | 9451 | 471,9 | 14 | 1,538 | H5A034 C | sample | NM | ACY1 | ACY1 | ACY1 | aminoacylase 1 | aminoacyl | aminoacyl | 95 | 95 | 95 | s225163 | s990 | s991 |
| 4747 | F4 | F5 | 1229 | 792,3 | 17185 | 489,3 | 14 | 1,538 | H5A034 C | sample | XM | USP22 | USP22 | USP22 | ubiquitin specific | ubiquitin | ubiquitin | 23326 | 23326 | 23326 | s23566 | s23567 | s23568 |
| 4850 | K10 | K11 | 1224 | 765 | 17189 | 432,1 | 14 | 1,538 | H5A034 C | sample | NM | LONP1 | LONP1 | LONP1 | lon peptidase 1, r | lon peptic | lon peptic | 9361 | 9361 | 9361 | s17901 | s17902 | s17903 |
| 4948 | O12 | O13 | 1346 | 771,4 | 18867 | 534,4 | 14 | 1,538 | H5A034 C | sample | NM | PRSS16 | PRSS16 | PRSS16 | protease, serine, | protease, | protease, | 10279 | 10279 | 10279 | s20083 | s20084 | s20085 |
| 4950 | O14 | O15 | 1175 | 748,7 | 16411 | 545,8 | 14 | 1,538 | H5A034 C | sample | NM | PGCP | PGCP | PGCP | plasma glutamate | plasma gl | plasma gl | 10404 | 10404 | 10404 | s20353 | s20354 | s20355 |
| 2870 | H21 | H22 | 402 | 694,7 | 5610 | 546 | 14 | 1,647 | H5A034 C | sample | NM | CDC2L6 | CDC2L6 | CDC2L6 | cell division cycle | cell divisic | cell divisic | 23097 | 23097 | 23097 | s23028 | s23029 | s23030 |
| 3043 | O4 | O5 | 771 | 566,5 | 10759 | 441,5 | 14 | 1,647 | H5A034 C | sample | NM | ITPKC | ITPKC | ITPKC | inositol 1,4,5-tris | inositol 1, | inositol 1, | 80271 | 80271 | 80271 | s37150 | s37151 | s37152 |
| 19991 | A8 | A9 | 697 | 1094 | 9751 | 234,7 | 14 | 1,707 | H5A034 C | sample | XM | LOC40098 | LOC40098 | LOC40098 | similar to ankyrin | similar to | similar to | 400987 | 400987 | 400987 | s53437 | s53438 | s53439 |
| 14028 | I2 | I3 | 2028 | 1116 | 28612 | 187,1 | 14,1 | 0,808 | H5A034 C | sample | NM | TAS2R9 | TAS2R9 | TAS2R9 | taste receptor, ty | taste rece | taste rece | 50835 | 50835 | 50835 | s27129 | s27130 | s27131 |
| 3198 | F14 | F15 | 1162 | 1306 | 16389 | 403,1 | 14,1 | 0,873 | H5A034 C | sample | NM | PEX14 | PEX14 | PEX14 | peroxisomal biog | peroxison | peroxison | 5195 | 5195 | 5195 | s10324 | s10325 | s224254 |
| 3225 | G17 | G18 | 1336 | 1226 | 18880 | 346,5 | 14,1 | 0,873 | H5A034 C | sample | NM | IMPA1 | IMPA1 | IMPA1 | inositol(myo)-1(o | inositol(m | inositol(m | 3612 | 3612 | 3612 | s7407 | s7408 | s7409 |
| 3230 | G21 | G22 | 1318 | 1323 | 18546 | 351,5 | 14,1 | 0,873 | H5A034 C | sample | NM | INPP1 | INPP1 | INPP1 | inositol polyphos | inositol p | inositol p | 3628 | 3628 | 3628 | s7446 | s7447 | s7448 |
| 3298 | J18 | J19 | 889 | 1139 | 12576 | 304,8 | 14,1 | 0,873 | H5A034 C | sample | NM | SERPINB5 | SERPINB5 | SERPINB5 | serpin peptidase | serpin pe | serpin pe | 5268 | 5268 | 5268 | s10467 | s10468 | s10466 |
| 3386 | N10 | N11 | 1490 | 1259 | 21004 | 437,3 | 14,1 | 0,873 | H5A034 C | sample | NM | PITX1 | PITX1 | PITX1 | paired-like home | paired-lik | paired-lik | 5307 | 5307 | 5307 | s10556 | s194685 | s194686 |
| 3399 | N22 | N23 | 1267 | 1454 | 17894 | 504,7 | 14,1 | 0,873 | H5A034 C | sample | NM | PKP2 | PKP2 | PKP2 | plakophilin 2 | plakophili | plakophili | 5318 | 5318 | 5318 | s10583 | s10584 | s10585 |
| 3418 | O18 | O19 | 1271 | 1282 | 17961 | 449,6 | 14,1 | 0,873 | H5A034 C | sample | NM | PDE2A | PDE2A | PDE2A | phosphodiesteras | phosphod | phosphod | 5138 | 5138 | 5138 | s779 | s781 | s780 |
| 6181 | B20 | B21 | 1687 | 576,8 | 23836 | 423,2 | 14,1 | 0,931 | H5A034 C | sample | NM | DECR1 | DECR1 | DECR1 | 2,4-dienoyl CoA r | 2,4-dieno | 2,4-dieno | 1666 | 1666 | 1666 | s4031 | s4032 | s223400 |
| 6443 | M19 | M20 | 1993 | 645,9 | 28061 | 392,2 | 14,1 | 0,931 | H5A034 C | sample | NM | CDKN2D | CDKN2D | CDKN2D | cyclin-dependent | cyclin-dep | cyclin-dep | 1032 | 1032 | 1032 | s2849 | s2850 | s2851 |
| 20621 | L13 | L14 | 1124 | 769,6 | 15891 | 148 | 14,1 | 0,94 | H5A034 C | sample | XM | LOC44192 | LOC44192 | LOC44192 | similar to hCG164 | similar to | similar to | 441925 | 441925 | 441925 | s54459 | s54460 | s54461 |
| 20622 | L14 | L15 | 715 | 727,1 | 10068 | 170,6 | 14,1 | 0,94 | H5A034 C | sample | XM | C8orf73 | C8orf73 | C8orf73 | chromosome 8 op | chromoso | chromoso | 642475 | 642475 | 642475 | s55033 | s196792 | s196793 |
| 18781 | O20 | O21 | 1542 | 922,3 | 21710 | 309,5 | 14,1 | 0,956 | H5A034 C | sample | NM | TBC1D28 | TBC1D28 | TBC1D28 | TBC1 domain fam | TBC1 dom | TBC1 dom | 254272 | 254272 | 254272 | s48528 | s48529 | s195877 |
| 17565 | L6 | L7 | 1366 | 883,9 | 19251 | 185,3 | 14,1 | 0,962 | H5A034 C | sample | NM | SPIN4 | SPIN4 | SPIN4 | spindlin family, m | spindlin fa | spindlin fa | 139886 | 139886 | 139886 | s44252 | s44253 | s44254 |
| 9523 | M4 | M5 | 1575 | 1147 | 22268 | 169,5 | 14,1 | 0,979 | H5A034 C | sample | NM | PCDHB8 | PCDHB8 | PCDHB8 | protocadherin be | protocadh | protocadh | 56128 | 56128 | 56128 | s31952 | s31953 | s31954 |
| 9542 | N21 | N22 | 1897 | 1172 | 26833 | 128,4 | 14,1 | 0,979 | H5A034 C | sample | NM | PARVA | PARVA | PARVA | parvin, alpha | parvin, al | parvin, al | 55742 | 55742 | 55742 | s224386 | s31390 | s31391 |
| 18923 | E19 | E20 | 1460 | 961,9 | 20591 | 174,8 | 14,1 | 0,983 | H5A034 C | sample | NM | NPHP4 | NPHP4 | NPHP4 | nephronophthisis | nephrono | nephrono | 261734 | 261734 | 261734 | s48994 | s48995 | s48996 |
| 19151 | N8 | N9 | 1526 | 991,1 | 21451 | 234,6 | 14,1 | 0,983 | H5A034 C | sample | NM | C9orf79 | C9orf79 | C9orf79 | chromosome 9 op | chromoso | chromoso | 286234 | 286234 | 286234 | s50129 | s50130 | s50131 |
| 15140 | G5 | G6 | 1084 | 860,1 | 15263 | 125,1 | 14,1 | 0,986 | H5A034 C | sample | NM | MLZE | MLZE | MLZE | melanoma-derive | melanom | melanom | 56169 | 56169 | 56169 | s32042 | s32043 | s32044 |
| 8625 | H17 | H18 | 1668 | 1038 | 23521 | 95,37 | 14,1 | 0,996 | H5A034 C | sample | NM | TES | TES | TES | testis derived tra | testis deri | testis deri | 26136 | 26136 | 26136 | s25146 | s25145 | s25147 |
| 8692 | K12 | K13 | 1921 | 1099 | 27049 | 142,1 | 14,1 | 0,996 | H5A034 C | sample | NM | SRPX2 | SRPX2 | SRPX2 | sushi-repeat-cont | sushi-repe | sushi-repe | 27286 | 27286 | 27286 | s26078 | s26079 | s26080 |
| 8698 | K18 | K19 | 1539 | 947,5 | 21697 | 125,4 | 14,1 | 0,996 | H5A034 C | sample | NM | RND1 | RND1 | RND1 | Rho family GTPas | Rho famil | Rho famil | 27289 | 27289 | 27289 | s26088 | s26089 | s26087 |
| 8822 | P21 | P22 | 1830 | 1175 | 25880 | 146,6 | 14,1 | 0,996 | H5A034 C | sample | NM | RGS17 | RGS17 | RGS17 | regulator of G-pro | regulator | regulator | 26575 | 26575 | 26575 | s25542 | s25543 | s25544 |
| 19263 | C22 | C23 | 1097 | 1281 | 15451 | 390,3 | 14,1 | 1 | H5A034 C | sample | NM | OR14K1 | OR14K1 | OR14K1 | olfactory recepto | olfactory | olfactory | 343170 | 343170 | 343170 | s50989 | s50990 | s50991 |
| 19280 | D16 | D17 | 1228 | 1161 | 17366 | 307,4 | 14,1 | 1 | H5A034 C | sample | NM | FAM131C | FAM131C | FAM131C | family with seque | family wit | family wit | 348487 | 348487 | 348487 | s51349 | s51350 | s196120 |
| 19281 | D17 | D18 | 1131 | 1267 | 15904 | 364,4 | 14,1 | 1 | H5A034 C | sample | NM | LOC33997 | LOC33997 | LOC33997 | similar to hypoth | similar to | similar to | 339977 | 339977 | 339977 | s50638 | s50639 | s50640 |
| 20815 | D15 | D16 | 1499 | 972,1 | 21190 | 252,5 | 14,1 | 1 | H5A034 C | sample | XM | LOC64359 | LOC64359 | LOC64359 | similar to C06A5.8 | similar to | similar to | 643596 | 643596 | 643596 | s55359 | s55361 | s227073 |
| 21062 | N21 | N22 | 1107 | 1060 | 15564 | 215,3 | 14,1 | 1 | H5A034 C | sample | XM | LOC64398 | LOC64398 | LOC64398 | hypothetical LOC | hypotheti | hypotheti | 643988 | 643988 | 643988 | s55514 | s55515 | s197002 |
| 21396 | L2 | L3 | 783 | 958,4 | 11027 | 228,3 | 14,1 | 1 | H5A034 C | sample | XM | LOC64786 | LOC64786 | LOC64786 | similar to PR-dom | similar to | similar to | 647868 | 647868 | 647868 | s197574 | s197575 | s197576 |
| 11305 | H1 | H2 | 1460 | 791,2 | 20596 | 135,2 | 14,1 | 1,022 | H5A034 C | sample | NM | RTN4RL2 | RTN4RL2 | RTN4RL2 | reticulon 4 recept | reticulon | reticulon | 349667 | 349667 | 349667 | s51401 | s196126 | s51400 |
| 11439 | M22 | M23 | 1987 | 917 | 28056 | 192,2 | 14,1 | 1,022 | H5A034 C | sample | | | | | | | | | | | | | |
| 12302 | A21 | A22 | 1281 | 1028 | 18052 | 175,4 | 14,1 | 1,06 | H5A034 C | sample | NM | SPTBN1 | SPTBN1 | SPTBN1 | spectrin, beta, no | spectrin, b | spectrin, b | 6711 | 6711 | 6711 | s629 | s630 | s631 |
| 12308 | A5 | A6 | 1501 | 1159 | 21131 | 224,2 | 14,1 | 1,06 | H5A034 C | sample | NM | SNAPC2 | SNAPC2 | SNAPC2 | small nuclear RNA | small nucl | small nucl | 6618 | 6618 | 6618 | s13194 | s194803 | s194804 |
| 12505 | J1 | J2 | 1014 | 1017 | 14339 | 155,9 | 14,1 | 1,06 | H5A034 C | sample | NM | CSDE1 | CSDE1 | CSDE1 | cold shock domai | cold shocl | cold shocl | 7812 | 7812 | 7812 | s15373 | s15374 | s15375 |
| 7334 | B21 | B22 | 1436 | 618,5 | 20190 | 470,1 | 14,1 | 1,076 | H5A034 C | sample | NM | NRXN2 | NRXN2 | NRXN2 | neurexin 2 | neurexin | neurexin | 9379 | 9379 | 9379 | s17949 | s17950 | s17951 |
| 7445 | G13 | G14 | 872 | 587,3 | 12282 | 411,2 | 14,1 | 1,076 | H5A034 C | sample | NM | ORC2L | ORC2L | ORC2L | origin recognition | origin rec | origin rec | 4999 | 4999 | 4999 | s9897 | s9895 | s9896 |
| 17141 | K13 | K14 | 1634 | 953,4 | 23115 | 202,5 | 14,1 | 1,089 | H5A034 C | sample | XM | LOC11323 | FLJ32894 | LOC11323 | hypothetical prot | hypotheti | hypotheti | 113230 | 144360 | 113230 | s41447 | s44639 | s225492 |
| 17171 | L19 | L20 | 1715 | 893,6 | 24158 | 209,1 | 14,1 | 1,089 | H5A034 C | sample | NM | LOC11623 | TRIM16L | LOC11623 | hypothetical prot | tripartite | hypotheti | 116236 | 147166 | 116236 | s41972 | s44969 | s41974 |
| 17247 | O22 | O23 | 1421 | 901,1 | 20066 | 268,3 | 14,1 | 1,089 | H5A034 C | sample | NM | ANKRD9 | PNPLA5 | ANKRD9 | ankyrin repeat dc | patatin-lik | ankyrin re | 122416 | 150379 | 122416 | s195641 | s45435 | s195643 |
| 7146 | J3 | J4 | 811 | 526,3 | 11433 | 466,4 | 14,1 | 1,097 | H5A034 C | sample | NM | MFGE8 | MFGE8 | MFGE8 | milk fat globule-E | milk fat gl | milk fat gl | 4240 | 4240 | 4240 | s8718 | s8719 | s224038 |
| 7167 | K22 | K23 | 1402 | 582,3 | 19772 | 418,8 | 14,1 | 1,097 | H5A034 C | sample | NM | MYO9A | MYO9A | MYO9A | myosin IXA | myosin IX | myosin IX | 4649 | 4649 | 4649 | s9220 | s9222 | s9221 |
| 7183 | L15 | L16 | 1259 | 572,6 | 17738 | 449,6 | 14,1 | 1,097 | H5A034 C | sample | NM | MIP | MIP | MIP | major intrinsic pr | major intr | major intr | 4284 | 4284 | 4284 | s8784 | s8786 | s8785 |
| 5016 | A9 | A10 | 1533 | 682,8 | 21631 | 581,6 | 14,1 | 1,106 | H5A034 C | sample | NM | MMP27 | MMP27 | MMP27 | matrix metallope | matrix me | matrix me | 64066 | 64066 | 64066 | s34379 | s34380 | s34381 |
| 5029 | B20 | B21 | 1469 | 663,8 | 20737 | 569,5 | 14,1 | 1,106 | H5A034 C | sample | NM | UTRN | UTRN | UTRN | utrophin | utrophin | utrophin | 7402 | 7402 | 7402 | s14732 | s14733 | s14734 |
| 5030 | B21 | B22 | 1794 | 656 | 25264 | 637 | 14,1 | 1,106 | H5A034 C | sample | NM | ESSPL | ESSPL | ESSPL | epidermis-specific | epidermis | epidermis | 345062 | 345062 | 345062 | s51122 | s51123 | s51124 |
| 5178 | H3 | H4 | 1753 | 612,3 | 24664 | 553,1 | 14,1 | 1,106 | H5A034 C | sample | NM | SCG2 | SCG2 | SCG2 | secretogranin II ( | secretogra | secretogra | 7857 | 7857 | 7857 | s15418 | s15419 | s15420 |
| 5197 | I20 | I21 | 1188 | 680,5 | 16727 | 361,3 | 14,1 | 1,106 | H5A034 C | sample | NM | TYROBP | TYROBP | TYROBP | TYRO protein tyrc | TYRO prot | TYRO prot | 7305 | 7305 | 7305 | s14547 | s14548 | s14549 |
| 5245 | K20 | K21 | 1605 | 698,2 | 22595 | 480,5 | 14,1 | 1,106 | H5A034 C | sample | NM | UBE2E2 | UBE2E2 | UBE2E2 | ubiquitin-conjuga | ubiquitin- | ubiquitin- | 7325 | 7325 | 7325 | s14582 | s14580 | s14581 |
| 5257 | L1 | L2 | 1597 | 623,2 | 22595 | 345,3 | 14,1 | 1,106 | H5A034 C | sample | NM | STAM | STAM | STAM | signal transducin | signal tra | signal tra | 8027 | 8027 | 8027 | s15554 | s224853 | s15553 |
| 5269 | L20 | L21 | 1786 | 617,5 | 25217 | 611,6 | 14,1 | 1,106 | H5A034 C | sample | NM | TRIM25 | TRIM25 | TRIM25 | tripartite motif-co | tripartite | tripartite | 7706 | 7706 | 7706 | s15206 | s15205 | s15204 |
| 5339 | O19 | O20 | 1328 | 619,8 | 18708 | 647,6 | 14,1 | 1,106 | H5A034 C | sample | NM | MAP1D | MAP1D | MAP1D | methionine amin | methionir | methionir | 254042 | 254042 | 254042 | s48480 | s48481 | s48482 |
| 5376 | P9 | P10 | 1313 | 661,5 | 18504 | 529,2 | 14,1 | 1,106 | H5A034 C | sample | NM | SLC14A2 | SLC14A2 | SLC14A2 | solute carrier fam | solute car | solute car | 8170 | 8170 | 8170 | s15668 | s15669 | s15670 |
| 2330 | B10 | B11 | 1265 | 671,6 | 17811 | 487,7 | 14,1 | 1,11 | H5A034 C | sample | NM | PRKCD | PRKCD | PRKCD | protein kinase C, | protein ki | protein ki | 5580 | 5580 | 5580 | s11098 | s11099 | s11100 |
| 2363 | C19 | C20 | 1448 | 716,9 | 20401 | 331,8 | 14,1 | 1,11 | H5A034 C | sample | NM | PDGFRL | PDGFRL | PDGFRL | platelet-derived g | platelet-d | platelet-d | 5157 | 5157 | 5157 | s10 | s11 | s224220 |
| 2642 | O10 | O11 | 871 | 596,6 | 12261 | 414,5 | 14,1 | 1,11 | H5A034 C | sample | NM | PKLR | PKLR | PKLR | pyruvate kinase, l | pyruvate l | pyruvate l | 5313 | 5313 | 5313 | s10568 | s10569 | s10570 |
| 16541 | B13 | B14 | 1397 | 1003 | 19664 | 321,7 | 14,1 | 1,115 | H5A034 C | sample | XM | KIAA1666 | KIAA1666 | KIAA1666 | KIAA1666 protein | KIAA1666 | KIAA1666 | 85376 | 85376 | 85376 | s39937 | s39938 | s39939 |
| 16549 | B20 | B21 | 897 | 1029 | 12682 | 295,5 | 14,1 | 1,115 | H5A034 C | sample | NM | FAM55C | FAM55C | FAM55C | family with seque | family wit | family wit | 91775 | 91775 | 91775 | s40777 | s40778 | s40779 |
| 16592 | D16 | D17 | 1120 | 920,3 | 15821 | 296,7 | 14,1 | 1,115 | H5A034 C | sample | NM | NDUFAF2 | NDUFAF2 | NDUFAF2 | NADH dehydroge | NADH deh | NADH deh | 91942 | 91942 | 91942 | s40815 | s40816 | s40817 |
| 16618 | E18 | E19 | 1487 | 937,5 | 20922 | 283,2 | 14,1 | 1,115 | H5A034 C | sample | NM | TBL1Y | TBL1Y | TBL1Y | transducin (beta)- | transduci | transduci | 90665 | 90665 | 90665 | s40464 | s40465 | s40466 |
| 16658 | G10 | G11 | 783 | 1010 | 11012 | 275 | 14,1 | 1,115 | H5A034 C | sample | NM | PYGO2 | PYGO2 | PYGO2 | pygopus homolog | pygopus h | pygopus h | 90780 | 90780 | 90780 | s40488 | s40489 | s40490 |
| 16821 | M6 | M7 | 1080 | 987,3 | 15258 | 356 | 14,1 | 1,115 | H5A034 C | sample | NM | RCCD1 | RCCD1 | RCCD1 | RCC1 domain con | RCC1 dom | RCC1 dom | 91433 | 91433 | 91433 | s40657 | s40658 | s228458 |
| 17734 | C7 | C8 | 844 | 1029 | 11927 | 409,1 | 14,1 | 1,115 | H5A034 C | sample | NM | SFRS12 | SFRS12 | SFRS12 | splicing factor, ar | splicing fa | splicing fa | 140890 | 140890 | 140890 | s44452 | s44453 | s225669 |
| 17769 | E17 | E18 | 1099 | 1100 | 15484 | 309,8 | 14,1 | 1,115 | H5A034 C | sample | NM | C10orf46 | C10orf46 | C10orf46 | chromosome 10 c | chromoso | chromoso | 143384 | 143384 | 143384 | s44528 | s44529 | s44530 |
| 17774 | E21 | E22 | 1238 | 1020 | 17467 | 286,6 | 14,1 | 1,115 | H5A034 C | sample | NM | C11orf40 | C11orf40 | C11orf40 | chromosome 11 c | chromoso | chromoso | 143501 | 143501 | 143501 | s44543 | s44544 | s44545 |
| 9001 | H1 | H2 | 1496 | 1640 | 21116 | 155,4 | 14,1 | 1,133 | H5A034 C | sample | NM | GMIP | GMIP | GMIP | GEM interacting p | GEM inter | GEM inter | 51291 | 51291 | 51291 | s27860 | s27861 | s27862 |
| 9049 | J1 | J2 | 1684 | 1769 | 23702 | 150,5 | 14,1 | 1,133 | H5A034 C | sample | NM | TLR8 | TLR8 | TLR8 | toll-like receptor | toll-like re | toll-like re | 51311 | 51311 | 51311 | s27920 | s27921 | s27922 |
| 4468 | K12 | K13 | 1407 | 716,4 | 19773 | 391,2 | 14,1 | 1,19 | H5A034 C | sample | NM | KCNMB3 | KCNMB3 | KCNMB3 | potassium large c | potassium | potassium | 27094 | 27094 | 27094 | s25804 | s25805 | s25806 |
| 4538 | N10 | N11 | 1395 | 712,2 | 19666 | 463,6 | 14,1 | 1,19 | H5A034 C | sample | NM | TLR4 | TLR4 | TLR4 | toll-like receptor | toll-like re | toll-like re | 7099 | 7099 | 7099 | s14195 | s14196 | s14194 |
| 4567 | O15 | O16 | 1498 | 765,5 | 21056 | 479,9 | 14,1 | 1,19 | H5A034 C | sample | NM | GABRP | GABRP | GABRP | gamma-aminobut | gamma-ar | gamma-ar | 2568 | 2568 | 2568 | s5512 | s5513 | s5514 |
| 4607 | P8 | P9 | 1370 | 715,1 | 19329 | 523,9 | 14,1 | 1,19 | H5A034 C | sample | NM | TNFAIP3 | TNFAIP3 | TNFAIP3 | tumor necrosis fa | tumor nec | tumor nec | 7128 | 7128 | 7128 | s14260 | s14261 | s14259 |
| 9 | A17 | A18 | 657 | 1624 | 9246 | 798,6 | 14,1 | 1,2 | H5A034 C | sample | NM | NR5A2 | NR5A2 | NR5A2 | nuclear receptor | nuclear re | nuclear re | 2494 | 2494 | 2494 | s5380 | s5381 | s5382 |
| 1964 | B5 | B6 | 1408 | 748,9 | 19841 | 480,3 | 14,1 | 1,226 | H5A034 C | sample | NM | SUHW1 | SUHW1 | SUHW1 | suppressor of hai | suppresso | suppresso | 129025 | 129025 | 129025 | s43379 | s43380 | s43381 |
| 2071 | G15 | G16 | 845 | 575,4 | 11879 | 395,1 | 14,1 | 1,226 | H5A034 C | sample | NM | VPS26B | VPS26B | VPS26B | vacuolar protein | vacuolar p | vacuolar p | 112936 | 112936 | 112936 | s41397 | s41398 | s41399 |
| 2255 | N8 | N9 | 1337 | 689,1 | 18867 | 391,2 | 14,1 | 1,226 | H5A034 C | sample | | | | | | | | | | | | | |
| 6547 | A4 | A5 | 1412 | 712 | 19958 | 442,2 | 14,1 | 1,237 | H5A034 C | sample | NM | IDUA | IDUA | IDUA | iduronidase, alph | iduronida | iduronida | 3425 | 3425 | 3425 | s7132 | s7131 | s223903 |
| 6732 | I2 | I3 | 1065 | 667,3 | 14999 | 302 | 14,1 | 1,237 | H5A034 C | sample | NM | IL12B | IL12B | IL12B | interleukin 12B (n | interleuki | interleuki | 3593 | 3593 | 3593 | s7360 | s7361 | s7359 |
| 6788 | K5 | K6 | 904 | 627,7 | 12732 | 309,4 | 14,1 | 1,237 | H5A034 C | sample | NM | HEXA | HEXA | HEXA | hexosaminidase A | hexosami | hexosami | 3073 | 3073 | 3073 | s6511 | s6512 | s6513 |
| 6850 | N18 | N19 | 1437 | 548,6 | 20194 | 516,4 | 14,1 | 1,237 | H5A034 C | sample | NM | KRT15 | KRT15 | KRT15 | keratin 15 | keratin 15 | keratin 15 | 3866 | 3866 | 3866 | s7986 | s7987 | s7988 |
| 6885 | O6 | O7 | 818 | 561 | 11518 | 497,7 | 14,1 | 1,237 | H5A034 C | sample | NM | IRF7 | IRF7 | IRF7 | interferon regulat | interferon | interferon | 3665 | 3665 | 3665 | s7519 | s194563 | s223948 |
| 6886 | O7 | O8 | 1241 | 625,6 | 17517 | 455,9 | 14,1 | 1,237 | H5A034 C | sample | NM | HMOX1 | HMOX1 | HMOX1 | heme oxygenase | heme oxy | heme oxy | 3162 | 3162 | 3162 | s6673 | s223854 | s194530 |
| 6903 | P22 | P23 | 1023 | 574,8 | 14397 | 344,8 | 14,1 | 1,237 | H5A034 C | sample | NM | KRT83 | KRT83 | KRT83 | keratin 83 | keratin 83 | keratin 83 | 3889 | 3889 | 3889 | s8022 | s8023 | s194589 |
| 3851 | A19 | A20 | 1373 | 806,9 | 19336 | 416,3 | 14,1 | 1,253 | H5A034 C | sample | NM | GPR152 | GPR152 | GPR152 | G protein-couple | G protein | G protein | 390212 | 390212 | 390212 | s52741 | s52742 | s52743 |
| 3900 | C2 | C3 | 1085 | 717,7 | 15299 | 505,3 | 14,1 | 1,253 | H5A034 C | sample | NM | PYGM | PYGM | PYGM | phosphorylase, gl | phosphor | phosphor | 5837 | 5837 | 5837 | s11645 | s11647 | s11646 |
| 3969 | F17 | F18 | 1352 | 697,5 | 19063 | 426,4 | 14,1 | 1,253 | H5A034 C | sample | NM | TBX2 | TBX2 | TBX2 | T-box 2 | T-box 2 | T-box 2 | 6909 | 6909 | 6909 | s13831 | s13829 | s13830 |
| 4053 | I6 | I7 | 1252 | 651 | 17701 | 364,5 | 14,1 | 1,253 | H5A034 C | sample | NM | RALGDS | RALGDS | RALGDS | ral guanine nucle | ral guanin | ral guanin | 5900 | 5900 | 5900 | s11764 | s11765 | s11766 |
| 4134 | M14 | M15 | 842 | 585,9 | 11836 | 327,9 | 14,1 | 1,253 | H5A034 C | sample | NM | RBM4 | RBM4 | RBM4 | RNA binding moti | RNA bindi | RNA bindi | 5936 | 5936 | 5936 | s11861 | s11862 | s11863 |
| 4181 | O13 | O14 | 630 | 693,7 | 8888 | 496,7 | 14,1 | 1,253 | H5A034 C | sample | NM | PPIA | PPIA | PPIA | peptidylprolyl iso | peptidylp | peptidylp | 5478 | 5478 | 5478 | s312 | s311 | s310 |

| | | | | | | | | | | | | |
|---|---|---|---|---|---|---|---|---|---|---|---|---|
| 11781 | K6 | K7 | 1114 | 795 | 15709 | 165,8 | 14,1 | 1,259 | H5A034 C | sample | Hm Gen E | [illegible] |
| 11794 | L18 | L19 | 1085 | 782,3 | 15326 | 159,8 | 14,1 | 1,259 | H5A034 C | sample | Hm Gen E | [illegible] |
| 11898 | P3 | P4 | 1403 | 831,4 | 19825 | 164 | 14,1 | 1,259 | H5A034 C | sample | Hm Gen E | [illegible] |
| 15500 | F5 | F6 | 1027 | 870,5 | 14524 | 153,5 | 14,1 | 1,282 | H5A034 C | sample | Hm Gen E | [illegible] |
| 5782 | A7 | A8 | 1226 | 787 | 17278 | 525,2 | 14,1 | 1,288 | H5A034 C | sample | Hm Drgbl | [illegible] |
| 5887 | F15 | F16 | 1974 | 775,1 | 27781 | 547 | 14,1 | 1,288 | H5A034 C | sample | Hm Drgbl | [illegible] |
| 5900 | F5 | F6 | 792 | 785,4 | 11196 | 622 | 14,1 | 1,288 | H5A034 C | sample | Hm Drgbl | [illegible] |
| 6024 | K9 | K10 | 1562 | 786,8 | 22082 | 496,4 | 14,1 | 1,288 | H5A034 C | sample | Hm Drgbl | [illegible] |
| 6025 | L1 | L2 | 1526 | 793 | 21578 | 407,1 | 14,1 | 1,288 | H5A034 C | sample | Hm Drgbl | [illegible] |
| 6134 | P21 | P22 | 1783 | 740,2 | 25201 | 577,3 | 14,1 | 1,288 | H5A034 C | sample | Hm Drgbl | [illegible] |
| 1159 | A15 | A16 | 1638 | 764,4 | 23121 | 546,3 | 14,1 | 1,294 | H5A034 C | sample | Hm Drgbl | [illegible] |
| 1512 | O9 | O10 | 1834 | 690,1 | 25781 | 462,1 | 14,1 | 1,294 | H5A034 C | sample | Hm Drgbl | [illegible] |
| 16197 | C6 | C7 | 1105 | 996 | 15527 | 417,8 | 14,1 | 1,294 | H5A034 C | sample | Hm Gen E | [illegible] |
| 16232 | E16 | E17 | 1271 | 968 | 17860 | 282,3 | 14,1 | 1,294 | H5A034 C | sample | Hm Gen E | [illegible] |
| 16291 | G4 | G5 | 967 | 973,5 | 13676 | 270,3 | 14,1 | 1,294 | H5A034 C | sample | Hm Gen E | [illegible] |
| 13580 | F5 | F6 | 1022 | 972,7 | 14361 | 157,9 | 14,1 | 1,324 | H5A034 C | sample | Hm Gen E | [illegible] |
| 927 | G22 | G23 | 1521 | 788,9 | 21392 | 463,4 | 14,1 | 1,336 | H5A034 C | sample | Hm Drgbl | [illegible] |
| 962 | I10 | I11 | 1608 | 726,7 | 22681 | 350,5 | 14,1 | 1,336 | H5A034 C | sample | Hm Drgbl | [illegible] |
| 966 | I14 | I15 | 1137 | 800,6 | 16037 | 410,9 | 14,1 | 1,336 | H5A034 C | sample | Hm Drgbl | [illegible] |
| 1059 | M11 | M12 | 1298 | 653 | 18270 | 388,6 | 14,1 | 1,336 | H5A034 C | sample | Hm Drgbl | [illegible] |
| 1109 | O13 | O14 | 1639 | 767,7 | 23032 | 437,9 | 14,1 | 1,336 | H5A034 C | sample | Hm Drgbl | [illegible] |
| 5396 | A5 | A6 | 1366 | 667 | 19323 | 680,6 | 14,1 | 1,343 | H5A034 C | sample | Hm Drgbl | [illegible] |
| 5400 | A9 | A10 | 1060 | 688,1 | 14895 | 410,8 | 14,1 | 1,343 | H5A034 C | sample | Hm Drgbl | [illegible] |
| 5453 | D13 | D14 | 2087 | 727,9 | 29395 | 606,4 | 14,1 | 1,343 | H5A034 C | sample | Hm Drgbl | [illegible] |
| 5480 | E16 | E17 | 1458 | 638,1 | 20486 | 542,8 | 14,1 | 1,343 | H5A034 C | sample | Hm Drgbl | [illegible] |
| 5713 | O1 | O2 | 535 | 637,5 | 7548 | 624,8 | 14,1 | 1,343 | H5A034 C | sample | Hm Drgbl | [illegible] |
| 5750 | P21 | P22 | 1487 | 682,4 | 20985 | 457,1 | 14,1 | 1,343 | H5A034 C | sample | Hm Drgbl | [illegible] |
| 3458 | A10 | A11 | 589 | 1534 | 8286 | 406,8 | 14,1 | 1,356 | H5A034 C | sample | Hm Drgbl | [illegible] |
| 3529 | D1 | D2 | 1045 | 1393 | 14696 | 450,5 | 14,1 | 1,356 | H5A034 C | sample | Hm Drgbl | [illegible] |
| 3541 | D20 | D21 | 1175 | 1234 | 16550 | 343,5 | 14,1 | 1,356 | H5A034 C | sample | Hm Drgbl | [illegible] |
| 3647 | H8 | H9 | 1423 | 1532 | 20110 | 410,3 | 14,1 | 1,356 | H5A034 C | sample | Hm Drgbl | [illegible] |
| 3657 | I17 | I18 | 1095 | 1343 | 15402 | 359,8 | 14,1 | 1,356 | H5A034 C | sample | Hm Drgbl | [illegible] |
| 3723 | L11 | L12 | 975 | 1427 | 13743 | 348,6 | 14,1 | 1,356 | H5A034 C | sample | Hm Drgbl | [illegible] |
| 3749 | M13 | M14 | 1001 | 1101 | 14104 | 336 | 14,1 | 1,356 | H5A034 C | sample | Hm Drgbl | [illegible] |
| 3820 | P12 | P13 | 1602 | 1457 | 22521 | 498,8 | 14,1 | 1,356 | H5A034 C | sample | Hm Drgbl | [illegible] |
| 8096 | B16 | B17 | 1511 | 650,3 | 21342 | 420,8 | 14,1 | 1,356 | H5A034 C | sample | Hm Drgbl | [illegible] |
| 8165 | E13 | E14 | 1323 | 571,7 | 18684 | 522,2 | 14,1 | 1,356 | H5A034 C | sample | Hm Drgbl | [illegible] |
| 8293 | J20 | J21 | 1211 | 634,7 | 17090 | 508,2 | 14,1 | 1,356 | H5A034 C | sample | Hm Drgbl | [illegible] |
| 8348 | L5 | L6 | 1553 | 632,9 | 21899 | 561,8 | 14,1 | 1,356 | H5A034 C | sample | Hm Drgbl | [illegible] |
| 8398 | N7 | N8 | 1636 | 586,4 | 23110 | 512,1 | 14,1 | 1,356 | H5A034 C | sample | Hm Drgbl | [illegible] |
| 10045 | C20 | C21 | 1485 | 888,7 | 20896 | 167,6 | 14,1 | 1,356 | H5A034 C | sample | Hm Drgbl | [illegible] |
| 10053 | C6 | C7 | 1174 | 817 | 16503 | 178,1 | 14,1 | 1,356 | H5A034 C | sample | Hm Drgbl | [illegible] |
| 10182 | I14 | I15 | 2045 | 861,7 | 28934 | 158,6 | 14,1 | 1,356 | H5A034 C | sample | Hm Drgbl | [illegible] |
| 10184 | I16 | I17 | 1744 | 823,9 | 24602 | 146,6 | 14,1 | 1,356 | H5A034 C | sample | Hm Drgbl | [illegible] |
| 1661 | F13 | F14 | 1856 | 718 | 26181 | 437,2 | 14,1 | 1,476 | H5A034 C | sample | Hm Drgbl | [illegible] |
| 1778 | K10 | K11 | 1438 | 705,2 | 20225 | 326,4 | 14,1 | 1,476 | H5A034 C | sample | Hm Drgbl | [illegible] |
| 1790 | K21 | K22 | 1377 | 731,5 | 19370 | 405,4 | 14,1 | 1,476 | H5A034 C | sample | Hm Drgbl | [illegible] |
| 21722 | J10 | J11 | 210 | 823,6 | 2971 | 725,2 | 14,1 | 1,484 | H5A034 C | sample | Hm Gen E | [illegible] |
| 21802 | M18 | M19 | 265 | 1053 | 3737 | 440,6 | 14,1 | 1,484 | H5A034 C | sample | Hm Gen E | [illegible] |
| 21820 | N12 | N13 | 171 | 866,5 | 2415 | 564,2 | 14,1 | 1,484 | H5A034 C | sample | Hm Gen E | [illegible] |
| 4719 | E22 | E23 | 1109 | 779,5 | 15685 | 476,6 | 14,1 | 1,549 | H5A034 C | sample | Hm Drgbl | [illegible] |
| 4918 | M7 | M8 | 944 | 810,6 | 13303 | 442,8 | 14,1 | 1,549 | H5A034 C | sample | Hm Drgbl | [illegible] |
| 4973 | P13 | P14 | 518 | 897,1 | 7287 | 607,9 | 14,1 | 1,549 | H5A034 C | sample | Hm Drgbl | [illegible] |
| 10501 | F20 | F21 | 1352 | 890,3 | 19086 | 227,4 | 14,1 | 1,593 | H5A034 C | sample | Hm Drgbl | [illegible] |
| 10506 | F3 | F4 | 1051 | 862,3 | 14779 | 214,8 | 14,1 | 1,593 | H5A034 C | sample | Hm Drgbl | [illegible] |
| 2716 | B12 | B13 | 782 | 633,9 | 11057 | 553,4 | 14,1 | 1,659 | H5A034 C | sample | Hm Drgbl | [illegible] |
| 20058 | D3 | D4 | 522 | 1053 | 7362 | 143,7 | 14,1 | 1,72 | H5A034 C | sample | Hm Gen E | [illegible] |
| 20308 | O12 | O13 | 935 | 1030 | 13157 | 149,6 | 14,1 | 1,72 | H5A034 C | sample | Hm Gen E | [illegible] |
| 20332 | P12 | P13 | 602 | 1370 | 8490 | 166,5 | 14,1 | 1,72 | H5A034 C | sample | Hm Gen E | [illegible] |
| 7856 | H16 | H17 | 461 | 685,7 | 6510 | 432 | 14,1 | 2,153 | H5A034 C | sample | Hm Drgbl | [illegible] |
| 22482 | I3 | I4 | 1028 | 1411 | 14619 | 335 | 14,2 | 0,732 | H5A034 C | sample | Hm Gen E | [illegible] |
| 3362 | M10 | M11 | 1146 | 1270 | 16302 | 376,7 | 14,2 | 0,879 | H5A034 C | sample | Hm Drgbl | [illegible] |
| 3393 | N17 | N18 | 993 | 1289 | 14123 | 414,6 | 14,2 | 0,879 | H5A034 C | sample | Hm Drgbl | [illegible] |
| 11971 | C4 | C5 | 1093 | 831,7 | 15539 | 77,71 | 14,2 | 0,91 | H5A034 C | sample | Hm Gen E | [illegible] |
| 12231 | N22 | N23 | 1623 | 803 | 22997 | 166,6 | 14,2 | 0,91 | H5A034 C | sample | Hm Gen E | [illegible] |
| 19873 | M1 | M2 | 1321 | 362 | 18792 | 295,8 | 14,2 | 0,925 | H5A034 C | sample | Hm Gen E | [illegible] |
| 6255 | E22 | E23 | 1585 | 530 | 22558 | 519 | 14,2 | 0,937 | H5A034 C | sample | Hm Drgbl | [illegible] |
| 20603 | K19 | K20 | 1162 | 764,5 | 16547 | 176,3 | 14,2 | 0,947 | H5A034 C | sample | Hm Gen E | [illegible] |
| 20710 | O7 | O8 | 860 | 814,4 | 12176 | 195,1 | 14,2 | 0,947 | H5A034 C | sample | Hm Gen E | [illegible] |
| 17443 | G4 | G5 | 1132 | 955,1 | 16041 | 213,8 | 14,2 | 0,969 | H5A034 C | sample | Hm Gen E | [illegible] |
| 9420 | I2 | I3 | 1538 | 1145 | 21899 | 192,6 | 14,2 | 0,986 | H5A034 C | sample | Hm Drgbl | [illegible] |
| 18897 | D17 | D18 | 1265 | 877,2 | 17914 | 194,9 | 14,2 | 0,99 | H5A034 C | sample | Hm Gen E | [illegible] |
| 19095 | L22 | L23 | 1213 | 981,3 | 17202 | 222,8 | 14,2 | 0,99 | H5A034 C | sample | Hm Gen E | [illegible] |
| 19139 | N19 | N20 | 1832 | 990,3 | 26017 | 244,2 | 14,2 | 0,99 | H5A034 C | sample | Hm Gen E | [illegible] |
| 9804 | I2 | I3 | 1523 | 1064 | 21610 | 185,6 | 14,2 | 0,993 | H5A034 C | sample | Hm Drgbl | [illegible] |
| 19551 | O22 | O23 | 1627 | 1229 | 23031 | 446,5 | 14,2 | 1,007 | H5A034 C | sample | Hm Gen E | [illegible] |
| 20783 | B8 | B9 | 1216 | 994,2 | 17242 | 304,6 | 14,2 | 1,007 | H5A034 C | sample | Hm Gen E | [illegible] |
| 21370 | K18 | K19 | 1096 | 938,4 | 15527 | 327,6 | 14,2 | 1,007 | H5A034 C | sample | Hm Gen E | [illegible] |
| 14391 | H22 | H23 | 1228 | 910,2 | 17444 | 157,5 | 14,2 | 1,011 | H5A034 C | sample | Hm Gen E | [illegible] |
| 11355 | J11 | J12 | 713 | 896,2 | 10139 | 148,4 | 14,2 | 1,029 | H5A034 C | sample | Hm Drgbl | [illegible] |
| 11377 | K1 | K2 | 986 | 887,3 | 14030 | 147,8 | 14,2 | 1,029 | H5A034 C | sample | Hm Drgbl | [illegible] |
| 11382 | K14 | K15 | 1653 | 864,9 | 23463 | 142,3 | 14,2 | 1,029 | H5A034 C | sample | Hm Drgbl | [illegible] |
| 12620 | N5 | N6 | 1630 | 1018 | 23121 | 187,4 | 14,2 | 1,068 | H5A034 C | sample | Hm Gen E | [illegible] |
| 11010 | K3 | K4 | 1193 | 772,1 | 16964 | 544,2 | 14,2 | 1,076 | H5A034 C | sample | Hm Drgbl | [illegible] |
| 7311 | A22 | A23 | 1517 | 648,7 | 21548 | 531,4 | 14,2 | 1,084 | H5A034 C | sample | Hm Drgbl | [illegible] |
| 7514 | J10 | J11 | 1182 | 551,7 | 16758 | 453,2 | 14,2 | 1,084 | H5A034 C | sample | Hm Drgbl | [illegible] |
| 7546 | K18 | K19 | 1236 | 583,1 | 17536 | 396,2 | 14,2 | 1,084 | H5A034 C | sample | Hm Drgbl | [illegible] |
| 7582 | L7 | L8 | 919 | 512 | 13081 | 415,5 | 14,2 | 1,084 | H5A034 C | sample | Hm Drgbl | [illegible] |
| 7646 | O21 | O22 | 1539 | 703,5 | 21845 | 508,3 | 14,2 | 1,084 | H5A034 C | sample | Hm Drgbl | [illegible] |
| 16924 | B12 | B13 | 1249 | 978,1 | 17783 | 195,7 | 14,2 | 1,097 | H5A034 C | sample | Hm Gen E | [illegible] |
| 17082 | H3 | H4 | 1334 | 960,5 | 18966 | 127,5 | 14,2 | 1,097 | H5A034 C | sample | Hm Gen E | [illegible] |
| 17194 | M18 | M19 | 1804 | 992,5 | 25539 | 221,7 | 14,2 | 1,097 | H5A034 C | sample | Hm Gen E | [illegible] |
| 7040 | F16 | F17 | 1401 | 558,2 | 19955 | 463,9 | 14,2 | 1,105 | H5A034 C | sample | Hm Drgbl | [illegible] |
| 4998 | A14 | A15 | 1441 | 693,2 | 20521 | 544,1 | 14,2 | 1,114 | H5A034 C | sample | Hm Drgbl | [illegible] |
| 5038 | B7 | B8 | 1629 | 595 | 23119 | 542,2 | 14,2 | 1,114 | H5A034 C | sample | Hm Drgbl | [illegible] |
| 5047 | C15 | C16 | 828 | 672,3 | 11790 | 646,9 | 14,2 | 1,114 | H5A034 C | sample | Hm Drgbl | [illegible] |
| 5122 | F18 | F19 | 1553 | 626,2 | 22025 | 529,3 | 14,2 | 1,114 | H5A034 C | sample | Hm Drgbl | [illegible] |
| 5222 | J21 | J22 | 1226 | 579,6 | 17420 | 537,2 | 14,2 | 1,114 | H5A034 C | sample | Hm Drgbl | [illegible] |
| 5319 | N22 | N23 | 1406 | 650,4 | 19964 | 555,9 | 14,2 | 1,114 | H5A034 C | sample | Hm Drgbl | [illegible] |
| 2328 | A9 | A10 | 1456 | 699,1 | 20615 | 446,8 | 14,2 | 1,118 | H5A034 C | sample | Hm Drgbl | [illegible] |
| 2352 | B9 | B10 | 487 | 851,1 | 6932 | 535,3 | 14,2 | 1,118 | H5A034 C | sample | Hm Drgbl | [illegible] |
| 2420 | E5 | E6 | 1218 | 615,8 | 17355 | 379,7 | 14,2 | 1,118 | H5A034 C | sample | Hm Drgbl | [illegible] |
| 2573 | L13 | L14 | 1435 | 612,1 | 20405 | 325,6 | 14,2 | 1,118 | H5A034 C | sample | Hm Drgbl | [illegible] |
| 2576 | L16 | L17 | 1124 | 611 | 15980 | 317,1 | 14,2 | 1,118 | H5A034 C | sample | Hm Drgbl | [illegible] |
| 16564 | C12 | C13 | 1501 | 1027 | 21350 | 331,4 | 14,2 | 1,123 | H5A034 C | sample | Hm Gen E | [illegible] |
| 16632 | E9 | E10 | 1035 | 939,3 | 14742 | 302,5 | 14,2 | 1,123 | H5A034 C | sample | Hm Gen E | [illegible] |
| 16668 | G2 | G3 | 750 | 972,6 | 10687 | 230 | 14,2 | 1,123 | H5A034 C | sample | Hm Gen E | [illegible] |
| 16704 | H9 | H10 | 769 | 990,4 | 10947 | 177 | 14,2 | 1,123 | H5A034 C | sample | Hm Gen E | [illegible] |
| 17711 | B8 | B9 | 1032 | 1113 | 14603 | 348,8 | 14,2 | 1,123 | H5A034 C | sample | Hm Gen E | [illegible] |
| 17797 | F20 | F21 | 1544 | 1022 | 21869 | 266,7 | 14,2 | 1,123 | H5A034 C | sample | Hm Gen E | [illegible] |
| 17806 | F7 | F8 | 1355 | 999,9 | 19187 | 342,1 | 14,2 | 1,123 | H5A034 C | sample | Hm Gen E | [illegible] |
| 17947 | L4 | L5 | 1045 | 955,3 | 14824 | 251,5 | 14,2 | 1,123 | H5A034 C | sample | Hm Gen E | [illegible] |
| 9021 | H6 | H7 | 1624 | 1592 | 23011 | 126,6 | 14,2 | 1,141 | H5A034 C | sample | Hm Drgbl | [illegible] |
| 4396 | H12 | H13 | 1275 | 650,8 | 18166 | 439,8 | 14,2 | 1,198 | H5A034 C | sample | Hm Drgbl | [illegible] |
| 44 | B5 | B6 | 451 | 1595 | 6393 | 663,1 | 14,2 | 1,209 | H5A034 C | sample | Hm Drgbl | [illegible] |
| 66 | C3 | C4 | 93 | 1209 | 1320 | 716,3 | 14,2 | 1,209 | H5A034 C | sample | Hm Drgbl | [illegible] |
| 71 | C8 | C9 | 547 | 1753 | 7789 | 453,3 | 14,2 | 1,209 | H5A034 C | sample | Hm Drgbl | [illegible] |
| 1972 | C12 | C13 | 1254 | 652,1 | 17754 | 477,2 | 14,2 | 1,235 | H5A034 C | sample | Hm Drgbl | [illegible] |
| 1986 | C3 | C4 | 1002 | 644,4 | 14189 | 330,5 | 14,2 | 1,235 | H5A034 C | sample | Hm Drgbl | [illegible] |
| 2076 | G2 | G3 | 897 | 654,5 | 12728 | 415,8 | 14,2 | 1,235 | H5A034 C | sample | Hm Drgbl | [illegible] |
| 2077 | G20 | G21 | 1277 | 752,7 | 18131 | 421,7 | 14,2 | 1,235 | H5A034 C | sample | Hm Drgbl | [illegible] |
| 2140 | J12 | J13 | 916 | 660,9 | 13049 | 378,2 | 14,2 | 1,235 | H5A034 C | sample | Hm Drgbl | [illegible] |
| 2234 | N10 | N11 | 1313 | 650 | 18632 | 403,3 | 14,2 | 1,235 | H5A034 C | sample | Hm Drgbl | [illegible] |
| 2251 | N4 | N5 | 1444 | 683,7 | 20499 | 432,7 | 14,2 | 1,235 | H5A034 C | sample | Hm Drgbl | [illegible] |
| 3854 | A21 | A22 | 885 | 780,4 | 12607 | 418,5 | 14,2 | 1,262 | H5A034 C | sample | Hm Drgbl | [illegible] |
| 3987 | G11 | G12 | 1446 | 650,4 | 20597 | 393,3 | 14,2 | 1,262 | H5A034 C | sample | Hm Drgbl | [illegible] |
| 11701 | H20 | H21 | 1209 | 784,6 | 17202 | 126,9 | 14,2 | 1,268 | H5A034 C | sample | Hm Gen E | [illegible] |
| 11722 | I18 | I19 | 1967 | 903,2 | 27841 | 163,2 | 14,2 | 1,268 | H5A034 C | sample | Hm Gen E | [illegible] |
| 11787 | L11 | L12 | 738 | 806 | 10475 | 157,1 | 14,2 | 1,268 | H5A034 C | sample | Hm Gen E | [illegible] |
| 15637 | L20 | L21 | 1397 | 821,1 | 19831 | 139,3 | 14,2 | 1,291 | H5A034 C | sample | Hm Gen E | [illegible] |
| 5885 | F13 | F14 | 1552 | 746,2 | 22023 | 598 | 14,2 | 1,297 | H5A034 C | sample | Hm Drgbl | [illegible] |
| 5893 | F20 | F21 | 1711 | 826,3 | 24226 | 600,5 | 14,2 | 1,297 | H5A034 C | sample | Hm Drgbl | [illegible] |
| 5933 | H13 | H14 | 1508 | 765,9 | 21442 | 434,3 | 14,2 | 1,297 | H5A034 C | sample | Hm Drgbl | [illegible] |
| 5986 | J18 | J19 | 1684 | 903,5 | 23893 | 548 | 14,2 | 1,297 | H5A034 C | sample | Hm Drgbl | [illegible] |
| 1187 | B19 | B20 | 1248 | 701,4 | 17666 | 478,1 | 14,2 | 1,303 | H5A034 C | sample | Hm Drgbl | [illegible] |
| 1292 | F5 | F6 | 1240 | 681 | 17630 | 415,8 | 14,2 | 1,303 | H5A034 C | sample | Hm Drgbl | [illegible] |
| 1513 | P1 | P2 | 734 | 650,7 | 10405 | 410,3 | 14,2 | 1,303 | H5A034 C | sample | Hm Drgbl | [illegible] |
| 1527 | P22 | P23 | 1265 | 634,5 | 18015 | 532 | 14,2 | 1,303 | H5A034 C | sample | Hm Drgbl | [illegible] |
| 16228 | E12 | E13 | 1192 | 1024 | 16901 | 412,9 | 14,2 | 1,303 | H5A034 C | sample | Hm Gen E | [illegible] |
| 16309 | H20 | H21 | 1028 | 956,4 | 14561 | 288,1 | 14,2 | 1,303 | H5A034 C | sample | Hm Gen E | [illegible] |
| 16488 | O9 | O10 | 1321 | 1005 | 18747 | 477,4 | 14,2 | 1,303 | H5A034 C | sample | Hm Gen E | [illegible] |
| 16509 | P6 | P7 | 1023 | 1033 | 14512 | 344,3 | 14,2 | 1,303 | H5A034 C | sample | Hm Gen E | [illegible] |
| 13496 | C16 | C17 | 1154 | 985,4 | 16332 | 156,9 | 14,2 | 1,333 | H5A034 C | sample | Hm Gen E | [illegible] |
| 13724 | L5 | L6 | 1029 | 1011 | 14579 | 160,1 | 14,2 | 1,333 | H5A034 C | sample | Hm Gen E | [illegible] |
| 877 | E20 | E21 | 1249 | 732,7 | 17756 | 444,8 | 14,2 | 1,346 | H5A034 C | sample | Hm Drgbl | [illegible] |
| 979 | I4 | I5 | 1397 | 696,3 | 19854 | 423,2 | 14,2 | 1,346 | H5A034 C | sample | Hm Drgbl | [illegible] |
| 982 | I7 | I8 | 1341 | 714,1 | 19039 | 450,2 | 14,2 | 1,346 | H5A034 C | sample | Hm Drgbl | [illegible] |
| 1011 | K11 | K12 | 720 | 695,8 | 10199 | 427,3 | 14,2 | 1,346 | H5A034 C | sample | Hm Drgbl | [illegible] |
| 1081 | N1 | N2 | 1183 | 681,5 | 16761 | 406,3 | 14,2 | 1,346 | H5A034 C | sample | Hm Drgbl | [illegible] |
| 5409 | B17 | B18 | 1559 | 732,1 | 22169 | 633,4 | 14,2 | 1,352 | H5A034 C | sample | Hm Drgbl | [illegible] |
| 5438 | C21 | C22 | 1579 | 646,4 | 22463 | 618,1 | 14,2 | 1,352 | H5A034 C | sample | Hm Drgbl | [illegible] |
| 5443 | C4 | C5 | 1584 | 711,7 | 22569 | 638,3 | 14,2 | 1,352 | H5A034 C | sample | Hm Drgbl | [illegible] |
| 5458 | D18 | D19 | 1475 | 570 | 20922 | 478,6 | 14,2 | 1,352 | H5A034 C | sample | Hm Drgbl | [illegible] |
| 5471 | D8 | D9 | 1555 | 716,5 | 22032 | 625,2 | 14,2 | 1,352 | H5A034 C | sample | Hm Drgbl | [illegible] |
| 5485 | E20 | E21 | 1630 | 768,3 | 23142 | 591,7 | 14,2 | 1,352 | H5A034 C | sample | Hm Drgbl | [illegible] |
| 5508 | F2 | F3 | 2046 | 666,5 | 29127 | 610,3 | 14,2 | 1,352 | H5A034 C | sample | Hm Drgbl | [illegible] |
| 5523 | G11 | G12 | 1324 | 706,3 | 18791 | 671,1 | 14,2 | 1,352 | H5A034 C | sample | Hm Drgbl | [illegible] |
| 3469 | A20 | A21 | 937 | 1404 | 13335 | 334,1 | 14,2 | 1,365 | H5A034 C | sample | Hm Drgbl | [illegible] |
| 3555 | E11 | E12 | 703 | 1326 | 10015 | 367,5 | 14,2 | 1,365 | H5A034 C | sample | Hm Drgbl | [illegible] |
| 3556 | E12 | E13 | 962 | 1376 | 13622 | 326,6 | 14,2 | 1,365 | H5A034 C | sample | Hm Drgbl | [illegible] |
| 8124 | C2 | C3 | 1026 | 660 | 14563 | 536,8 | 14,2 | 1,365 | H5A034 C | sample | Hm Drgbl | [illegible] |
| 8277 | I6 | I7 | 1469 | 671 | 20899 | 484,5 | 14,2 | 1,365 | H5A034 C | sample | Hm Drgbl | [illegible] |
| 8370 | M3 | M4 | 1016 | 578,4 | 14436 | 552,2 | 14,2 | 1,365 | H5A034 C | sample | Hm Drgbl | [illegible] |
| 10011 | B11 | B12 | 1737 | 834,7 | 24689 | 164 | 14,2 | 1,365 | H5A034 C | sample | Hm Drgbl | [illegible] |
| 10023 | B22 | B23 | 1049 | 950,3 | 14929 | 217,2 | 14,2 | 1,365 | H5A034 C | sample | Hm Drgbl | [illegible] |
| 1653 | E6 | E7 | 1344 | 818 | 19068 | 478,4 | 14,2 | 1,487 | H5A034 C | sample | Hm Drgbl | [illegible] |
| 1662 | F14 | F15 | 1590 | 710,8 | 22554 | 364,7 | 14,2 | 1,487 | H5A034 C | sample | Hm Drgbl | [illegible] |
| 1876 | O12 | O13 | 1649 | 753,3 | 23472 | 425,9 | 14,2 | 1,487 | H5A034 C | sample | Hm Drgbl | [illegible] |
| 21566 | C21 | C22 | 350 | 973,1 | 4973 | 639,1 | 14,2 | 1,495 | H5A034 C | sample | Hm Gen E | [illegible] |
| 21567 | C22 | C23 | 223 | 1271 | 3176 | 805,6 | 14,2 | 1,495 | H5A034 C | sample | Hm Gen E | [illegible] |
| 21627 | F11 | F12 | 292 | 944,6 | 4145 | 575,1 | 14,2 | 1,495 | H5A034 C | sample | Hm Gen E | [illegible] |
| 21690 | H3 | H4 | 198 | 932,1 | 2815 | 377,1 | 14,2 | 1,495 | H5A034 C | sample | Hm Gen E | [illegible] |
| 4641 | B17 | B18 | 1514 | 816,4 | 21522 | 562 | 14,2 | 1,56 | H5A034 C | sample | Hm Drgbl | [illegible] |
| 4677 | C6 | C7 | 997 | 792,9 | 14126 | 475,1 | 14,2 | 1,56 | H5A034 C | sample | Hm Drgbl | [illegible] |
| 4794 | H3 | H4 | 540 | 961,4 | 7643 | 447,4 | 14,2 | 1,56 | H5A034 C | sample | Hm Drgbl | [illegible] |
| 4824 | I9 | I10 | 1500 | 822,8 | 21268 | 419,4 | 14,2 | 1,56 | H5A034 C | sample | Hm Drgbl | [illegible] |
| 3037 | O20 | O21 | 1195 | 646,8 | 16991 | 593,1 | 14,2 | 1,671 | H5A034 C | sample | Hm Drgbl | [illegible] |
| 3072 | P9 | P10 | 945 | 589 | 13423 | 462,8 | 14,2 | 1,671 | H5A034 C | sample | Hm Drgbl | [illegible] |
| 3226 | G18 | G19 | 1262 | 1254 | 18002 | 380,2 | 14,3 | 0,885 | H5A034 C | sample | Hm Drgbl | [illegible] |
| 3247 | H15 | H16 | 1125 | 1318 | 16072 | 365,5 | 14,3 | 0,885 | H5A034 C | sample | Hm Drgbl | [illegible] |
| 3360 | L9 | L10 | 1043 | 1187 | 14891 | 347,4 | 14,3 | 0,885 | H5A034 C | sample | Hm Drgbl | [illegible] |
| 6348 | I2 | I3 | 830 | 569,8 | 11862 | 516,9 | 14,3 | 0,944 | H5A034 C | sample | Hm Drgbl | [illegible] |
| 6438 | M14 | M15 | 1379 | 576,7 | 19670 | 481,6 | 14,3 | 0,944 | H5A034 C | sample | Hm Drgbl | [illegible] |
| 6446 | M21 | M22 | 1421 | 570,6 | 20358 | 390,7 | 14,3 | 0,944 | H5A034 C | sample | Hm Drgbl | [illegible] |
| 20458 | E18 | E19 | 898 | 812,5 | 12852 | 208,5 | 14,3 | 0,953 | H5A034 C | sample | Hm Gen E | [illegible] |
| 20472 | E9 | E10 | 304 | 735,2 | 4349 | 230,3 | 14,3 | 0,953 | H5A034 C | sample | Hm Gen E | [illegible] |
| 20480 | F16 | F17 | 774 | 743,7 | 11056 | 165,3 | 14,3 | 0,953 | H5A034 C | sample | Hm Gen E | [illegible] |
| 20502 | G14 | G15 | 964 | 828,2 | 13742 | 211,2 | 14,3 | 0,953 | H5A034 C | sample | Hm Gen E | [illegible] |
| 20557 | I20 | I21 | 878 | 747,5 | 12569 | 128 | 14,3 | 0,953 | H5A034 C | sample | Hm Gen E | [illegible] |
| 20562 | I3 | I4 | 450 | 838,6 | 6419 | 196,5 | 14,3 | 0,953 | H5A034 C | sample | Hm Gen E | [illegible] |
| 20594 | K10 | K11 | 980 | 807,1 | 14035 | 176,8 | 14,3 | 0,953 | H5A034 C | sample | Hm Gen E | [illegible] |
| 20616 | K9 | K10 | 630 | 726 | 9019 | 188,3 | 14,3 | 0,953 | H5A034 C | sample | Hm Gen E | [illegible] |
| 17361 | D17 | D18 | 1263 | 961,8 | 18061 | 188,1 | 14,3 | 0,976 | H5A034 C | sample | Hm Gen E | [illegible] |
| 17630 | O21 | O22 | 1444 | 1072 | 20655 | 211,8 | 14,3 | 0,976 | H5A034 C | sample | Hm Gen E | [illegible] |
| 15261 | L6 | L7 | 716 | 834,7 | 10237 | 151,6 | 14,3 | 1 | H5A034 C | sample | Hm Gen E | [illegible] |
| 8826 | P3 | P4 | 1221 | 991,9 | 17421 | 184,6 | 14,3 | 1,011 | H5A034 C | sample | Hm Drgbl | [illegible] |
| 20986 | K18 | K19 | 1317 | 956,1 | 18776 | 252,1 | 14,3 | 1,014 | H5A034 C | sample | Hm Gen E | [illegible] |
| 20991 | K22 | K23 | 1577 | 1006 | 22487 | 302 | 14,3 | 1,014 | H5A034 C | sample | Hm Gen E | [illegible] |
| 21058 | N18 | N19 | 1411 | 977,6 | 20151 | 287 | 14,3 | 1,014 | H5A034 C | sample | Hm Gen E | [illegible] |
| 21178 | C18 | C19 | 1075 | 967,6 | 15406 | 301,9 | 14,3 | 1,014 | H5A034 C | sample | Hm Gen E | [illegible] |
| 21395 | L19 | L20 | 1446 | 911,9 | 20643 | 322,6 | 14,3 | 1,014 | H5A034 C | sample | Hm Gen E | [illegible] |
| 21409 | M1 | M2 | 1410 | 980,3 | 20115 | 193,1 | 14,3 | 1,014 | H5A034 C | sample | Hm Gen E | [illegible] |
| 11155 | A4 | A5 | 1954 | 971,9 | 27986 | 223,2 | 14,3 | 1,036 | H5A034 C | sample | Hm Drgbl | [illegible] |
| 11414 | L21 | L22 | 1593 | 881,9 | 22771 | 148,9 | 14,3 | 1,036 | H5A034 C | sample | Hm Drgbl | [illegible] |
| 11487 | O22 | O23 | 1870 | 906,8 | 26740 | 170,6 | 14,3 | 1,036 | H5A034 C | sample | Hm Drgbl | [illegible] |
| 11131 | P4 | P5 | 1526 | 829,9 | 21774 | 1010 | 14,3 | 1,083 | H5A034 C | sample | Hm Drgbl | [illegible] |
| 7324 | B12 | B13 | 1514 | 559,7 | 21610 | 517,5 | 14,3 | 1,092 | H5A034 C | sample | Hm Drgbl | [illegible] |
| 7460 | G5 | G6 | 852 | 550,7 | 12208 | 409,6 | 14,3 | 1,092 | H5A034 C | sample | Hm Drgbl | [illegible] |
| 7593 | M17 | M18 | 1503 | 603,8 | 21433 | 331,9 | 14,3 | 1,092 | H5A034 C | sample | Hm Drgbl | [illegible] |
| 7633 | O1 | O2 | 495 | 530,8 | 7069 | 496 | 14,3 | 1,092 | H5A034 C | sample | Hm Drgbl | [illegible] |
| 17002 | E18 | E19 | 1755 | 1005 | 25025 | 200,9 | 14,3 | 1,104 | H5A034 C | sample | Hm Gen E | [illegible] |
| 17132 | J5 | J6 | 1478 | 928 | 21166 | 151 | 14,3 | 1,104 | H5A034 C | sample | Hm Gen E | [illegible] |
| 17222 | N21 | N22 | 1629 | 951,2 | 23350 | 220,2 | 14,3 | 1,104 | H5A034 C | sample | Hm Gen E | [illegible] |
| 7166 | K21 | K22 | 1243 | 560,2 | 17733 | 459 | 14,3 | 1,113 | H5A034 C | sample | Hm Drgbl | [illegible] |
| 5292 | M2 | M3 | 893 | 614,7 | 12781 | 621,4 | 14,3 | 1,122 | H5A034 C | sample | Hm Drgbl | [illegible] |
| 5294 | M21 | M22 | 1928 | 641,5 | 27602 | 501 | 14,3 | 1,122 | H5A034 C | sample | Hm Drgbl | [illegible] |
| 5307 | N11 | N12 | 2043 | 621,5 | 29155 | 592 | 14,3 | 1,122 | H5A034 C | sample | Hm Drgbl | [illegible] |
| 5372 | P5 | P6 | 1718 | 650,3 | 24624 | 409,9 | 14,3 | 1,122 | H5A034 C | sample | Hm Drgbl | [illegible] |
| 2348 | B5 | B6 | 1341 | 703,9 | 19223 | 498,1 | 14,3 | 1,126 | H5A034 C | sample | Hm Drgbl | [illegible] |
| 2408 | E16 | E17 | 1558 | 717,9 | 22352 | 428,7 | 14,3 | 1,126 | H5A034 C | sample | Hm Drgbl | [illegible] |
| 2530 | J18 | J19 | 1344 | 675,1 | 19156 | 365,7 | 14,3 | 1,126 | H5A034 C | sample | Hm Drgbl | [illegible] |
| 16833 | N17 | N18 | 1269 | 892,4 | 18154 | 278,4 | 14,3 | 1,13 | H5A034 C | sample | Hm Gen E | [illegible] |
| 16859 | O19 | O20 | 1627 | 943,2 | 23291 | 406,7 | 14,3 | 1,13 | H5A034 C | sample | Hm Gen E | [illegible] |
| 17916 | K2 | K3 | 917 | 976,8 | 13115 | 212,4 | 14,3 | 1,13 | H5A034 C | sample | Hm Gen E | [illegible] |
| 17963 | M19 | M20 | 1106 | 973,6 | 15830 | 225,4 | 14,3 | 1,13 | H5A034 C | sample | Hm Gen E | [illegible] |
| 17972 | M5 | M6 | 943 | 1023 | 13511 | 179,1 | 14,3 | 1,13 | H5A034 C | sample | Hm Gen E | [illegible] |

| | | | | | | | | | |
|---|---|---|---|---|---|---|---|---|---|
| 8913 | D17 | D18 | 1187 | 1675 | 16981 | 127,5 | 14,3 | 1,149 | DDX47 |
| 8938 | E18 | E19 | 1717 | 1799 | 24470 | 215,2 | 14,3 | 1,149 | ZFR |
| 4272 | B9 | B10 | 1003 | 662,3 | 14294 | 352,7 | 14,3 | 1,207 | GRIA3 |
| 4339 | E4 | E5 | 796 | 614 | 11393 | 403,3 | 14,3 | 1,207 | CACNA1G |
| 4344 | E9 | E10 | 1145 | 849,2 | 16394 | 514,7 | 14,3 | 1,207 | ATP6V1B2 |
| 4410 | H3 | H4 | 1298 | 716,3 | 18574 | 408,1 | 14,3 | 1,207 | KCND2 |
| 4463 | J8 | J9 | 1236 | 733,2 | 17718 | 349,8 | 14,3 | 1,207 | KCNK18 |
| 4581 | O6 | O7 | 998 | 667,4 | 14301 | 476,8 | 14,3 | 1,207 | TRPM4 |
| 74 | D10 | D11 | 505 | 1482 | 7241 | 466,5 | 14,3 | 1,217 | SPINK2 |
| 95 | D8 | D9 | 539 | 1634 | 7691 | 508,2 | 14,3 | 1,217 | SPINK1 |
| 323 | N19 | N20 | 606 | 1415 | 8684 | 720 | 14,3 | 1,217 | SLC6A6 |
| 384 | P9 | P10 | 498 | 1325 | 7122 | 756 | 14,3 | 1,217 | SLC6A13 |
| 1956 | B2 | B3 | 1450 | 781,6 | 20754 | 593,7 | 14,3 | 1,243 | SYT10 |
| 2053 | F20 | F21 | 1452 | 645,2 | 20771 | 456,5 | 14,3 | 1,243 | ZNF324B |
| 2174 | K21 | K22 | 1201 | 596,4 | 17151 | 380,2 | 14,3 | 1,243 | SP7 |
| 2289 | P17 | P18 | 1434 | 695,9 | 20494 | 511,6 | 14,3 | 1,243 | ZNF567 |
| 6574 | B7 | B8 | 1284 | 625 | 18402 | 486,4 | 14,3 | 1,254 | SLC29A2 |
| 3858 | A3 | A4 | 553 | 819,4 | 7932 | 507,7 | 14,3 | 1,271 | RXFP4 |
| 4060 | J12 | J13 | 539 | 723,4 | 7702 | 388,3 | 14,3 | 1,271 | RPE65 |
| 4118 | L21 | L22 | 19 | 784,3 | 272 | 482 | 14,3 | 1,271 | PSMD1 |
| 4123 | L4 | L5 | 621 | 697,3 | 8907 | 403 | 14,3 | 1,271 | RRAS |
| 4147 | M4 | M5 | 1184 | 659,7 | 16890 | 382,6 | 14,3 | 1,271 | RB1 |
| 11534 | A21 | A22 | 1409 | 918,8 | 20157 | 187 | 14,3 | 1,277 | AMELX |
| 11756 | J5 | J6 | 1284 | 856,1 | 18330 | 158,2 | 14,3 | 1,277 | CRYBB1 |
| 11772 | K2 | K3 | 1190 | 824,6 | 16962 | 149,8 | 14,3 | 1,277 | AFF2 |
| 11774 | K21 | K22 | 966 | 811,6 | 13816 | 160,1 | 14,3 | 1,277 | CAMLG |
| 11820 | M2 | M3 | 1358 | 822,4 | 19385 | 127,1 | 14,3 | 1,277 | GAP43 |
| 15441 | D17 | D18 | 884 | 738,7 | 12622 | 136,3 | 14,3 | 1,3 | WDR13 |
| 15467 | E2 | E3 | 1267 | 794,6 | 18072 | 132,6 | 14,3 | 1,3 | C4orf41 |
| 5813 | C13 | C14 | 1011 | 721 | 14446 | 582,6 | 14,3 | 1,306 | ACACB |
| 5814 | C14 | C15 | 1234 | 780,3 | 17683 | 565,8 | 14,3 | 1,306 | ARL1 |
| 5918 | G21 | G22 | 1659 | 865,8 | 23685 | 567,3 | 14,3 | 1,306 | ADCY1 |
| 6067 | M4 | M5 | 1357 | 754,4 | 19434 | 513,1 | 14,3 | 1,306 | ATP6V1C1 |
| 1300 | G12 | G13 | 1764 | 739 | 25153 | 473,8 | 14,3 | 1,312 | ZBTB33 |
| 1350 | I14 | I15 | 1229 | 749,4 | 17628 | 381,1 | 14,3 | 1,312 | ZNF443 |
| 1436 | L5 | L6 | 1182 | 675 | 16916 | 441,6 | 14,3 | 1,312 | DNAJA3 |
| 1467 | N11 | N12 | 1258 | 719,8 | 17952 | 414,8 | 14,3 | 1,312 | TCEAL1 |
| 16414 | L7 | L8 | 800 | 1100 | 11461 | 402,7 | 14,3 | 1,312 | MYCBPAP |
| 13479 | B22 | B23 | 1746 | 1094 | 25045 | 194,8 | 14,3 | 1,343 | OR2F1 |
| 13499 | C19 | C20 | 1447 | 1076 | 20659 | 174,1 | 14,3 | 1,343 | DPY19L1 |
| 13670 | J21 | J22 | 1646 | 1125 | 23514 | 190,8 | 14,3 | 1,343 | FAM19A5 |
| 13706 | L10 | L11 | 441 | 1015 | 6288 | 175,8 | 14,3 | 1,343 | IL1F5 |
| 13808 | P16 | P17 | 970 | 976,4 | 13836 | 155,5 | 14,3 | 1,343 | OR2W1 |
| 815 | B8 | B9 | 1556 | 725,4 | 22244 | 452,4 | 14,3 | 1,355 | RCN2 |
| 911 | F8 | F9 | 1117 | 796 | 16012 | 476 | 14,3 | 1,355 | S100B |
| 961 | I1 | I2 | 1282 | 733,4 | 18373 | 444,3 | 14,3 | 1,355 | LRBA |
| 1089 | N17 | N18 | 1220 | 700,7 | 17466 | 439,4 | 14,3 | 1,355 | ING2 |
| 1133 | P13 | P14 | 1019 | 799,8 | 14548 | 428,8 | 14,3 | 1,355 | LAMB2 |
| 5618 | K10 | K11 | 1741 | 701,1 | 24900 | 507,9 | 14,3 | 1,362 | BTRC |
| 3658 | I18 | I19 | 1151 | 1393 | 16498 | 413,6 | 14,3 | 1,375 | LANCL1 |
| 3728 | L16 | L17 | 966 | 1377 | 13857 | 404,3 | 14,3 | 1,375 | GPR119 |
| 8210 | G10 | G11 | 2251 | 662,6 | 32163 | 457,5 | 14,3 | 1,375 | GPD1L |
| 8251 | H4 | H5 | 1527 | 602,9 | 21897 | 521,5 | 14,3 | 1,375 | ABCB9 |
| 8252 | H5 | H6 | 1410 | 611,3 | 20188 | 519,1 | 14,3 | 1,375 | CLSTN1 |
| 10237 | K20 | K21 | 1580 | 897,5 | 22516 | 154,9 | 14,3 | 1,375 | KATNAL1 |
| 1615 | D15 | D16 | 1055 | 735 | 15061 | 499,1 | 14,3 | 1,497 | DNAJB12 |
| 1734 | I14 | I15 | 1501 | 721 | 21439 | 339,4 | 14,3 | 1,497 | HEATR6 |
| 4724 | E5 | E6 | 1134 | 795,1 | 16178 | 539,9 | 14,3 | 1,571 | ADAM10 |
| 4845 | J6 | J7 | 1594 | 793,4 | 22867 | 496,4 | 14,3 | 1,571 | ELA2B |
| 4856 | K16 | K17 | 1170 | 750,8 | 16756 | 477,4 | 14,3 | 1,571 | NAPSA |
| 4963 | O4 | O5 | 1009 | 822,5 | 14454 | 548,9 | 14,3 | 1,571 | NAALADL1 |
| 10399 | B15 | B16 | 1339 | 990,1 | 19083 | 231,7 | 14,3 | 1,616 | TIGD7 |
| 10595 | J19 | J20 | 1166 | 889,1 | 16659 | 225,6 | 14,3 | 1,616 | SFXN1 |
| 10596 | J2 | J3 | 363 | 917,9 | 5199 | 215,2 | 14,3 | 1,616 | EARS2 |
| 10700 | N5 | N6 | 1252 | 804,1 | 17892 | 191,6 | 14,3 | 1,616 | PHF21B |
| 7842 | G3 | G4 | 570 | 495,4 | 8163 | 356,5 | 14,3 | 2,183 | SSX3 |
| 22177 | M1 | M2 | 1092 | 1440 | 15726 | 249,8 | 14,4 | 0,684 | LOC727912 |
| 22359 | D22 | D23 | 1002 | 1394 | 14472 | 306,6 | 14,4 | 0,742 | LOC729245 |
| 13867 | B4 | B5 | 1329 | 1109 | 19162 | 156,6 | 14,4 | 0,825 | MRPL2 |
| 3124 | C12 | C13 | 1216 | 1210 | 17560 | 498,4 | 14,4 | 0,892 | PPP1R14D |
| 3192 | E9 | E10 | 594 | 1008 | 8568 | 419 | 14,4 | 0,892 | CDC25C |
| 12116 | K2 | K3 | 1586 | 826,8 | 22888 | 118,2 | 14,4 | 0,923 | ROBO2 |
| 6372 | J2 | J3 | 1384 | 611 | 19989 | 331,7 | 14,4 | 0,95 | SARDH |
| 6423 | L22 | L23 | 2048 | 648,7 | 29439 | 465,7 | 14,4 | 0,95 | DOCK1 |
| 20439 | D22 | D23 | 1067 | 829,4 | 15364 | 246,2 | 14,4 | 0,96 | LOC642014 |
| 20509 | G20 | G21 | 913 | 761,8 | 13154 | 206,1 | 14,4 | 0,96 | LOC541473 |
| 20519 | G8 | G9 | 602 | 788,4 | 8649 | 229,2 | 14,4 | 0,96 | MS4A13 |
| 20530 | H18 | H19 | 919 | 797,7 | 13219 | 189,6 | 14,4 | 0,96 | LOC642277 |
| 12891 | J11 | J12 | 1449 | 946,5 | 20880 | 128,1 | 14,4 | 0,976 | KIAA0586 |
| 12996 | N2 | N3 | 1193 | 1037 | 17165 | 142,6 | 14,4 | 0,976 | PFDN6 |
| 18763 | N4 | N5 | 1511 | 959,9 | 21797 | 196,5 | 14,4 | 0,976 | LOC256483 |
| 9337 | F1 | F2 | 1257 | 1153 | 18060 | 162,3 | 14,4 | 1 | SIRPG |
| 18947 | F19 | F20 | 1426 | 1019 | 20533 | 249,7 | 14,4 | 1,003 | OR4N4 |
| 19012 | I12 | I13 | 1471 | 1041 | 21138 | 221,3 | 14,4 | 1,003 | RSPO1 |
| 19046 | J21 | J22 | 1506 | 991,3 | 21680 | 213,1 | 14,4 | 1,003 | LOC283953 |
| 19150 | N7 | N8 | 1450 | 1057 | 20860 | 314,4 | 14,4 | 1,003 | CDRT4 |
| 9750 | G14 | G15 | 1604 | 1041 | 23049 | 148,4 | 14,4 | 1,007 | MS4A5 |
| 9774 | H14 | H15 | 1559 | 999,7 | 22373 | 125,6 | 14,4 | 1,007 | DDX50 |
| 9924 | N2 | N3 | 1643 | 1081 | 23574 | 126,3 | 14,4 | 1,007 | IRX3 |
| 15092 | E5 | E6 | 1166 | 814,3 | 16772 | 137,1 | 14,4 | 1,007 | C19orf10 |
| 15306 | N3 | N4 | 1149 | 797,1 | 16526 | 126,7 | 14,4 | 1,007 | MRPL47 |
| 8818 | P18 | P19 | 1653 | 1153 | 23767 | 212 | 14,4 | 1,018 | TNPO2 |
| 19387 | H4 | H5 | 1069 | 1181 | 15356 | 252,7 | 14,4 | 1,021 | RILPL1 |
| 19525 | N20 | N21 | 611 | 1417 | 8789 | 284 | 14,4 | 1,021 | FLJ44968 |

| | | | | | | | | | | | | |
|---|---|---|---|---|---|---|---|---|---|---|---|---|
| 19532 | N5 | N6 | 920 | 1211 | 13300 | 304,5 | 14,5 | 1,028 | H5A034 | C | sample | [illegible] |
| 20756 | A5 | A6 | 948 | 1093 | 13769 | 368,9 | 14,5 | 1,028 | H5A034 | C | sample | [illegible] |
| 21042 | M3 | M4 | 913 | 1032 | 13250 | 164,2 | 14,5 | 1,028 | H5A034 | C | sample | [illegible] |
| 21059 | N19 | N20 | 1388 | 950 | 20081 | 210,1 | 14,5 | 1,028 | H5A034 | C | sample | [illegible] |
| 21241 | F1 | F2 | 742 | 962,3 | 10757 | 251,3 | 14,5 | 1,028 | H5A034 | C | sample | [illegible] |
| 21244 | F12 | F13 | 1333 | 933,8 | 19334 | 306,9 | 14,5 | 1,028 | H5A034 | C | sample | [illegible] |
| 21249 | F17 | F18 | 1246 | 944,8 | 18125 | 282,5 | 14,5 | 1,028 | H5A034 | C | sample | [illegible] |
| 14353 | G1 | G2 | 1521 | 848,1 | 22068 | 123 | 14,5 | 1,032 | H5A034 | C | sample | [illegible] |
| 14532 | N2 | N3 | 669 | 890,7 | 9696 | 139,7 | 14,5 | 1,032 | H5A034 | C | sample | [illegible] |
| 14565 | O6 | O7 | 1589 | 850,4 | 23099 | 161,6 | 14,5 | 1,032 | H5A034 | C | sample | [illegible] |
| 11162 | B10 | B11 | 1648 | 859,6 | 23970 | 228,5 | 14,5 | 1,051 | H5A034 | C | sample | [illegible] |
| 11190 | C14 | C15 | 1211 | 859,9 | 17600 | 169,3 | 14,5 | 1,051 | H5A034 | C | sample | [illegible] |
| 11329 | I1 | I2 | 1294 | 804,1 | 18704 | 132,8 | 14,5 | 1,051 | H5A034 | C | sample | [illegible] |
| 11358 | J14 | J15 | 1610 | 843,4 | 23354 | 132 | 14,5 | 1,051 | H5A034 | C | sample | [illegible] |
| 11396 | K5 | K6 | 1396 | 812,6 | 20274 | 151 | 14,5 | 1,051 | H5A034 | C | sample | [illegible] |
| 12467 | H19 | H20 | 1108 | 999,8 | 16085 | 141,4 | 14,5 | 1,09 | H5A034 | C | sample | [illegible] |
| 12498 | I3 | I4 | 1378 | 1039 | 20049 | 157,6 | 14,5 | 1,09 | H5A034 | C | sample | [illegible] |
| 10951 | I15 | I16 | 1521 | 730 | 22118 | 436,5 | 14,5 | 1,098 | H5A034 | C | sample | [illegible] |
| 7300 | A12 | A13 | 1282 | 591,4 | 18551 | 509,3 | 14,5 | 1,107 | H5A034 | C | sample | [illegible] |
| 7348 | C12 | C13 | 1219 | 599,8 | 17640 | 524,1 | 14,5 | 1,107 | H5A034 | C | sample | [illegible] |
| 16973 | D13 | D14 | 1199 | 997,7 | 17353 | 230,9 | 14,5 | 1,12 | H5A034 | C | sample | [illegible] |
| 17065 | H1 | H2 | 1092 | 991,4 | 15887 | 120,5 | 14,5 | 1,12 | H5A034 | C | sample | [illegible] |
| 17204 | M5 | M6 | 1262 | 959,9 | 18259 | 163,2 | 14,5 | 1,12 | H5A034 | C | sample | [illegible] |
| 17259 | P11 | P12 | 1480 | 1080 | 21512 | 310,8 | 14,5 | 1,12 | H5A034 | C | sample | [illegible] |
| 6972 | C2 | C3 | 903 | 542,1 | 13114 | 535,9 | 14,5 | 1,128 | H5A034 | C | sample | [illegible] |
| 7165 | K20 | K21 | 1076 | 605,3 | 15629 | 388,8 | 14,5 | 1,128 | H5A034 | C | sample | [illegible] |
| 7278 | P14 | P15 | 1274 | 598,8 | 18441 | 545,9 | 14,5 | 1,128 | H5A034 | C | sample | [illegible] |
| 5087 | D8 | D9 | 1591 | 687,2 | 23041 | 486,5 | 14,5 | 1,137 | H5A034 | C | sample | [illegible] |
| 5271 | L22 | L23 | 1718 | 600,5 | 24938 | 594,3 | 14,5 | 1,137 | H5A034 | C | sample | [illegible] |
| 5324 | N5 | N6 | 1671 | 607,8 | 24268 | 431,3 | 14,5 | 1,137 | H5A034 | C | sample | [illegible] |
| 5343 | O22 | O23 | 1895 | 670,7 | 27402 | 509,7 | 14,5 | 1,137 | H5A034 | C | sample | [illegible] |
| 5347 | O4 | O5 | 1055 | 552,9 | 15282 | 396,6 | 14,5 | 1,137 | H5A034 | C | sample | [illegible] |
| 2452 | G12 | G13 | 1002 | 674,5 | 14543 | 583,1 | 14,5 | 1,142 | H5A034 | C | sample | [illegible] |
| 2626 | N18 | N19 | 1470 | 704,4 | 21254 | 506,6 | 14,5 | 1,142 | H5A034 | C | sample | [illegible] |
| 2635 | N4 | N5 | 1022 | 729,5 | 14773 | 436,3 | 14,5 | 1,142 | H5A034 | C | sample | [illegible] |
| 16682 | H10 | H11 | 946 | 1012 | 13674 | 234,6 | 14,5 | 1,146 | H5A034 | C | sample | [illegible] |
| 16805 | M13 | M14 | 1717 | 954,1 | 24963 | 308,3 | 14,5 | 1,146 | H5A034 | C | sample | [illegible] |
| 16844 | N5 | N6 | 1243 | 1035 | 17983 | 357,5 | 14,5 | 1,146 | H5A034 | C | sample | [illegible] |
| 16849 | O1 | O2 | 847 | 1098 | 12323 | 340,6 | 14,5 | 1,146 | H5A034 | C | sample | [illegible] |
| 16883 | P19 | P20 | 1539 | 1102 | 22328 | 395,7 | 14,5 | 1,146 | H5A034 | C | sample | [illegible] |
| 16886 | P21 | P22 | 1341 | 1091 | 19456 | 374,1 | 14,5 | 1,146 | H5A034 | C | sample | [illegible] |
| 17718 | C14 | C15 | 977 | 1010 | 14187 | 303,3 | 14,5 | 1,146 | H5A034 | C | sample | [illegible] |
| 17739 | D11 | D12 | 1090 | 946,2 | 15788 | 182,8 | 14,5 | 1,146 | H5A034 | C | sample | [illegible] |
| 17857 | I1 | I2 | 1161 | 1035 | 16837 | 227,6 | 14,5 | 1,146 | H5A034 | C | sample | [illegible] |
| 18000 | N9 | N10 | 1542 | 1031 | 22407 | 204 | 14,5 | 1,146 | H5A034 | C | sample | [illegible] |
| 8869 | B20 | B21 | 1775 | 1894 | 25750 | 179,9 | 14,5 | 1,165 | H5A034 | C | sample | [illegible] |
| 9111 | L22 | L23 | 2049 | 2047 | 29638 | 224,5 | 14,5 | 1,165 | H5A034 | C | sample | [illegible] |
| 9154 | N18 | N19 | 2122 | 1945 | 30671 | 198,2 | 14,5 | 1,165 | H5A034 | C | sample | [illegible] |
| 9157 | N20 | N21 | 2170 | 1871 | 31379 | 196 | 14,5 | 1,165 | H5A034 | C | sample | [illegible] |
| 4331 | E19 | E20 | 1205 | 689,8 | 17490 | 394,8 | 14,5 | 1,224 | H5A034 | C | sample | [illegible] |
| 4454 | J21 | J22 | 888 | 627,6 | 12891 | 373,5 | 14,5 | 1,224 | H5A034 | C | sample | [illegible] |
| 45 | B6 | B7 | 322 | 1379 | 4684 | 570,5 | 14,5 | 1,234 | H5A034 | C | sample | [illegible] |
| 342 | O14 | O15 | 522 | 1473 | 7566 | 744,3 | 14,5 | 1,234 | H5A034 | C | sample | [illegible] |
| 1981 | C20 | C21 | 818 | 690,9 | 11864 | 475,7 | 14,5 | 1,261 | H5A034 | C | sample | [illegible] |
| 2117 | I13 | I14 | 902 | 792 | 13108 | 294,6 | 14,5 | 1,261 | H5A034 | C | sample | [illegible] |
| 2235 | N11 | N12 | 1197 | 617,8 | 17390 | 404,2 | 14,5 | 1,261 | H5A034 | C | sample | [illegible] |
| 2277 | O6 | O7 | 1465 | 768 | 21174 | 470,8 | 14,5 | 1,261 | H5A034 | C | sample | [illegible] |
| 2283 | P11 | P12 | 1481 | 664,5 | 21420 | 501,2 | 14,5 | 1,261 | H5A034 | C | sample | [illegible] |
| 6628 | E12 | E13 | 856 | 646,4 | 12424 | 479,9 | 14,5 | 1,272 | H5A034 | C | sample | [illegible] |
| 6859 | N4 | N5 | 1110 | 551,8 | 16148 | 493,7 | 14,5 | 1,272 | H5A034 | C | sample | [illegible] |
| 3865 | B1 | B2 | 878 | 704,3 | 12762 | 360,8 | 14,5 | 1,289 | H5A034 | C | sample | [illegible] |
| 3885 | B6 | B7 | 859 | 703,9 | 12492 | 337,2 | 14,5 | 1,289 | H5A034 | C | sample | [illegible] |
| 3895 | C15 | C16 | 1029 | 588,2 | 14882 | 327,9 | 14,5 | 1,289 | H5A034 | C | sample | [illegible] |
| 3915 | D11 | D12 | 455 | 741,9 | 6578 | 389,8 | 14,5 | 1,289 | H5A034 | C | sample | [illegible] |
| 3982 | F7 | F8 | 1142 | 724,6 | 16549 | 430,8 | 14,5 | 1,289 | H5A034 | C | sample | [illegible] |
| 4002 | G3 | G4 | 1191 | 709,3 | 17292 | 421 | 14,5 | 1,289 | H5A034 | C | sample | [illegible] |
| 4126 | L7 | L8 | 724 | 684,6 | 10483 | 384,6 | 14,5 | 1,289 | H5A034 | C | sample | [illegible] |
| 11748 | J2 | J3 | 1031 | 864 | 14969 | 164,9 | 14,5 | 1,295 | H5A034 | C | sample | [illegible] |
| 11846 | N21 | N22 | 1277 | 789,3 | 18548 | 185 | 14,5 | 1,295 | H5A034 | C | sample | [illegible] |
| 15439 | D15 | D16 | 1218 | 865,4 | 17654 | 181,3 | 14,5 | 1,318 | H5A034 | C | sample | [illegible] |
| 15679 | N15 | N16 | 1300 | 816,7 | 18846 | 168,8 | 14,5 | 1,318 | H5A034 | C | sample | [illegible] |
| 15687 | N22 | N23 | 1354 | 822,6 | 19581 | 133,5 | 14,5 | 1,318 | H5A034 | C | sample | [illegible] |
| 15721 | P1 | P2 | 917 | 721,6 | 13292 | 155,7 | 14,5 | 1,318 | H5A034 | C | sample | [illegible] |
| 5794 | B18 | B19 | 1767 | 845,4 | 25681 | 602,3 | 14,5 | 1,324 | H5A034 | C | sample | [illegible] |
| 5802 | B3 | B4 | 1768 | 847,8 | 25552 | 518,8 | 14,5 | 1,324 | H5A034 | C | sample | [illegible] |
| 5821 | C20 | C21 | 1600 | 779,6 | 23227 | 617,8 | 14,5 | 1,324 | H5A034 | C | sample | [illegible] |
| 5888 | F16 | F17 | 2020 | 821,5 | 29218 | 588,9 | 14,5 | 1,324 | H5A034 | C | sample | [illegible] |
| 5901 | F6 | F7 | 1589 | 769,7 | 22993 | 563,5 | 14,5 | 1,324 | H5A034 | C | sample | [illegible] |
| 6075 | N11 | N12 | 1273 | 707,6 | 18417 | 605 | 14,5 | 1,324 | H5A034 | C | sample | [illegible] |
| 6116 | O5 | O6 | 1279 | 711,4 | 18561 | 504,5 | 14,5 | 1,324 | H5A034 | C | sample | [illegible] |
| 1172 | A5 | A6 | 1250 | 702,6 | 18161 | 469,9 | 14,5 | 1,33 | H5A034 | C | sample | [illegible] |
| 1304 | G16 | G17 | 1019 | 659,6 | 14774 | 357,8 | 14,5 | 1,33 | H5A034 | C | sample | [illegible] |
| 1347 | I11 | I12 | 1021 | 781,4 | 14782 | 451,1 | 14,5 | 1,33 | H5A034 | C | sample | [illegible] |
| 1470 | N14 | N15 | 1403 | 762,2 | 20390 | 426,2 | 14,5 | 1,33 | H5A034 | C | sample | [illegible] |
| 16157 | B13 | B14 | 1325 | 971 | 19271 | 354,1 | 14,5 | 1,33 | H5A034 | C | sample | [illegible] |
| 16195 | C4 | C5 | 855 | 980,8 | 12387 | 404,2 | 14,5 | 1,33 | H5A034 | C | sample | [illegible] |
| 16381 | K20 | K21 | 1190 | 960,4 | 17295 | 318,2 | 14,5 | 1,33 | H5A034 | C | sample | [illegible] |
| 16445 | N13 | N14 | 1630 | 976,7 | 23678 | 352,1 | 14,5 | 1,33 | H5A034 | C | sample | [illegible] |
| 16459 | N4 | N5 | 1290 | 966,7 | 18714 | 341,2 | 14,5 | 1,33 | H5A034 | C | sample | [illegible] |
| 16486 | O7 | O8 | 676 | 1069 | 9778 | 497,4 | 14,5 | 1,33 | H5A034 | C | sample | [illegible] |
| 13551 | E22 | E23 | 1656 | 1062 | 24059 | 187 | 14,5 | 1,362 | H5A034 | C | sample | [illegible] |
| 13607 | G8 | G9 | 1442 | 1044 | 20900 | 180,7 | 14,5 | 1,362 | H5A034 | C | sample | [illegible] |
| 1008 | J9 | J10 | 1331 | 727,6 | 19359 | 310,7 | 14,5 | 1,374 | H5A034 | C | sample | [illegible] |
| 1041 | L17 | L18 | 862 | 668 | 12487 | 417,1 | 14,5 | 1,374 | H5A034 | C | sample | [illegible] |
| 1104 | N9 | N10 | 1095 | 668,5 | 15923 | 428 | 14,5 | 1,374 | H5A034 | C | sample | [illegible] |
| 1108 | O12 | O13 | 1267 | 866,7 | 18403 | 491,6 | 14,5 | 1,374 | H5A034 | C | sample | [illegible] |
| 5407 | B15 | B16 | 1296 | 600,5 | 18778 | 683,7 | 14,5 | 1,381 | H5A034 | C | sample | [illegible] |
| 5450 | D10 | D11 | 1647 | 659,3 | 23928 | 576,3 | 14,5 | 1,381 | H5A034 | C | sample | [illegible] |
| 5459 | D19 | D20 | 1722 | 723,9 | 25031 | 612,4 | 14,5 | 1,381 | H5A034 | C | sample | [illegible] |
| 5460 | D2 | D3 | 1147 | 678,6 | 16594 | 617,7 | 14,5 | 1,381 | H5A034 | C | sample | [illegible] |
| 5473 | E1 | E2 | 796 | 715,6 | 11579 | 362 | 14,5 | 1,381 | H5A034 | C | sample | [illegible] |
| 5549 | H13 | H14 | 1471 | 609,6 | 21291 | 546 | 14,5 | 1,381 | H5A034 | C | sample | [illegible] |
| 5669 | M13 | M14 | 998 | 667,8 | 14502 | 340,9 | 14,5 | 1,381 | H5A034 | C | sample | [illegible] |
| 3551 | D8 | D9 | 1163 | 1281 | 16844 | 385 | 14,5 | 1,394 | H5A034 | C | sample | [illegible] |
| 3581 | F13 | F14 | 532 | 1400 | 7731 | 388,6 | 14,5 | 1,394 | H5A034 | C | sample | [illegible] |
| 3810 | O3 | O4 | 1017 | 1281 | 14740 | 434,5 | 14,5 | 1,394 | H5A034 | C | sample | [illegible] |
| 8101 | B20 | B21 | 1013 | 648 | 14664 | 378 | 14,5 | 1,394 | H5A034 | C | sample | [illegible] |
| 8131 | C4 | C5 | 906 | 586,7 | 13169 | 578,3 | 14,5 | 1,394 | H5A034 | C | sample | [illegible] |
| 8172 | E2 | E3 | 900 | 599,7 | 13076 | 584 | 14,5 | 1,394 | H5A034 | C | sample | [illegible] |
| 8182 | E7 | E8 | 1063 | 600,9 | 15414 | 504,4 | 14,5 | 1,394 | H5A034 | C | sample | [illegible] |
| 8247 | H22 | H23 | 1030 | 591,3 | 14984 | 420,9 | 14,5 | 1,394 | H5A034 | C | sample | [illegible] |
| 8278 | I7 | I8 | 1530 | 608,5 | 22146 | 413,3 | 14,5 | 1,394 | H5A034 | C | sample | [illegible] |
| 8295 | J22 | J23 | 1227 | 592,5 | 17736 | 477,3 | 14,5 | 1,394 | H5A034 | C | sample | [illegible] |
| 8319 | K22 | K23 | 1020 | 658,9 | 14819 | 476,8 | 14,5 | 1,394 | H5A034 | C | sample | [illegible] |
| 10124 | F5 | F6 | 1307 | 784,5 | 18922 | 137,3 | 14,5 | 1,394 | H5A034 | C | sample | [illegible] |
| 1574 | B21 | B22 | 1145 | 818 | 16601 | 432,5 | 14,5 | 1,518 | H5A034 | C | sample | [illegible] |
| 1602 | C3 | C4 | 1167 | 795,6 | 16884 | 501,8 | 14,5 | 1,518 | H5A034 | C | sample | [illegible] |
| 1761 | J17 | J18 | 1167 | 680,1 | 16879 | 256,1 | 14,5 | 1,518 | H5A034 | C | sample | [illegible] |
| 1781 | K13 | K14 | 1460 | 843 | 21204 | 376,4 | 14,5 | 1,518 | H5A034 | C | sample | [illegible] |
| 1810 | L18 | L19 | 1301 | 802,3 | 18928 | 332,8 | 14,5 | 1,518 | H5A034 | C | sample | [illegible] |
| 21558 | C14 | C15 | 302 | 994,6 | 4367 | 705,7 | 14,5 | 1,526 | H5A034 | C | sample | [illegible] |
| 21575 | C8 | C9 | 212 | 864,7 | 3083 | 679,6 | 14,5 | 1,526 | H5A034 | C | sample | [illegible] |
| 21587 | D19 | D20 | 462 | 1009 | 6693 | 515,1 | 14,5 | 1,526 | H5A034 | C | sample | [illegible] |
| 21650 | G10 | G11 | 231 | 1003 | 3361 | 772,7 | 14,5 | 1,526 | H5A034 | C | sample | [illegible] |
| 4661 | C13 | C14 | 1338 | 848,5 | 19414 | 502,7 | 14,5 | 1,593 | H5A034 | C | sample | [illegible] |
| 4685 | D13 | D14 | 1373 | 817,3 | 19904 | 494,2 | 14,5 | 1,593 | H5A034 | C | sample | [illegible] |
| 10584 | I9 | I10 | 1093 | 922,7 | 15874 | 204,7 | 14,5 | 1,638 | H5A034 | C | sample | [illegible] |
| 10592 | J16 | J17 | 1131 | 778,5 | 16445 | 173,7 | 14,5 | 1,638 | H5A034 | C | sample | [illegible] |
| 10602 | J3 | J4 | 901 | 814,1 | 13102 | 224,4 | 14,5 | 1,638 | H5A034 | C | sample | [illegible] |
| 10622 | K21 | K22 | 975 | 848,9 | 14135 | 201,5 | 14,5 | 1,638 | H5A034 | C | sample | [illegible] |
| 10688 | N16 | N17 | 1466 | 817,1 | 21301 | 206 | 14,5 | 1,638 | H5A034 | C | sample | [illegible] |
| 7873 | I1 | I2 | 394 | 487,4 | 5708 | 457,1 | 14,5 | 2,214 | H5A034 | C | sample | [illegible] |
| 23498 | D10 | D11 | 851 | 1541 | 12383 | 128,1 | 14,6 | 0,775 | H5A034 | C | sample | [illegible] |
| 3202 | F18 | F19 | 1014 | 1264 | 14840 | 402,2 | 14,6 | 0,904 | H5A034 | C | sample | [illegible] |
| 3254 | H21 | H22 | 1501 | 1425 | 21962 | 429,3 | 14,6 | 0,904 | H5A034 | C | sample | [illegible] |
| 3373 | M20 | M21 | 1496 | 1252 | 21774 | 374,3 | 14,6 | 0,904 | H5A034 | C | sample | [illegible] |
| 18385 | O1 | O2 | 1274 | 994,8 | 18630 | 319,5 | 14,6 | 0,939 | H5A034 | C | sample | [illegible] |
| 6214 | C7 | C8 | 937 | 553,3 | 13702 | 472,9 | 14,6 | 0,964 | H5A034 | C | sample | [illegible] |
| 6369 | J17 | J18 | 1107 | 597,7 | 16170 | 440,1 | 14,6 | 0,964 | H5A034 | C | sample | [illegible] |
| 6509 | P13 | P14 | 1528 | 629,9 | 22344 | 338,6 | 14,6 | 0,964 | H5A034 | C | sample | [illegible] |
| 20382 | B14 | B15 | 995 | 813,5 | 14478 | 258,3 | 14,6 | 0,973 | H5A034 | C | sample | [illegible] |
| 20483 | F19 | F20 | 827 | 789,6 | 12099 | 177,8 | 14,6 | 0,973 | H5A034 | C | sample | [illegible] |
| 20535 | H22 | H23 | 1199 | 809 | 17542 | 189 | 14,6 | 0,973 | H5A034 | C | sample | [illegible] |
| 9279 | C22 | C23 | 1491 | 1111 | 21721 | 180,8 | 14,6 | 1,014 | H5A034 | C | sample | [illegible] |
| 9330 | E3 | E4 | 1476 | 1155 | 21528 | 146,9 | 14,6 | 1,014 | H5A034 | C | sample | [illegible] |
| 9354 | F3 | F4 | 1603 | 1065 | 23483 | 116,4 | 14,6 | 1,014 | H5A034 | C | sample | [illegible] |
| 9404 | H5 | H6 | 1132 | 1122 | 16544 | 150,4 | 14,6 | 1,014 | H5A034 | C | sample | [illegible] |
| 18922 | E18 | E19 | 1032 | 1085 | 15091 | 291,1 | 14,6 | 1,017 | H5A034 | C | sample | [illegible] |
| 19109 | M13 | M14 | 1683 | 959,6 | 24639 | 189,9 | 14,6 | 1,017 | H5A034 | C | sample | [illegible] |
| 19158 | O14 | O15 | 1297 | 988,4 | 18978 | 270,4 | 14,6 | 1,017 | H5A034 | C | sample | [illegible] |
| 9830 | J21 | J22 | 1983 | 1140 | 29008 | 152,1 | 14,6 | 1,021 | H5A034 | C | sample | [illegible] |
| 9867 | L11 | L12 | 1125 | 983,6 | 16465 | 129,8 | 14,6 | 1,021 | H5A034 | C | sample | [illegible] |
| 15265 | M1 | M2 | 938 | 764,5 | 13711 | 142 | 14,6 | 1,021 | H5A034 | C | sample | [illegible] |
| 8724 | L2 | L3 | 1660 | 1058 | 24158 | 149,2 | 14,6 | 1,032 | H5A034 | C | sample | [illegible] |
| 19471 | L15 | L16 | 1082 | 1309 | 15800 | 382,2 | 14,6 | 1,035 | H5A034 | C | sample | [illegible] |
| 20857 | F1 | F2 | 796 | 987,8 | 11642 | 217,6 | 14,6 | 1,035 | H5A034 | C | sample | [illegible] |
| 20977 | K1 | K2 | 1100 | 989,1 | 16020 | 247,3 | 14,6 | 1,035 | H5A034 | C | sample | [illegible] |
| 21393 | L17 | L18 | 1636 | 927,8 | 23955 | 247,7 | 14,6 | 1,035 | H5A034 | C | sample | [illegible] |
| 21438 | N14 | N15 | 948 | 934,5 | 13818 | 265,6 | 14,6 | 1,035 | H5A034 | C | sample | [illegible] |
| 14270 | C21 | C22 | 1405 | 837 | 20443 | 126,8 | 14,6 | 1,039 | H5A034 | C | sample | [illegible] |
| 14271 | C22 | C23 | 1446 | 947,9 | 21098 | 238,5 | 14,6 | 1,039 | H5A034 | C | sample | [illegible] |
| 14396 | H5 | H6 | 1203 | 883,1 | 17619 | 130,6 | 14,6 | 1,039 | H5A034 | C | sample | [illegible] |
| 11163 | B11 | B12 | 850 | 817,6 | 12396 | 150,1 | 14,6 | 1,058 | H5A034 | C | sample | [illegible] |
| 11356 | J12 | J13 | 1750 | 855,1 | 25499 | 135,8 | 14,6 | 1,058 | H5A034 | C | sample | [illegible] |
| 11391 | K22 | K23 | 1879 | 866,2 | 27348 | 193,5 | 14,6 | 1,058 | H5A034 | C | sample | [illegible] |
| 11408 | L16 | L17 | 1734 | 834,3 | 25321 | 147,4 | 14,6 | 1,058 | H5A034 | C | sample | [illegible] |
| 11463 | N22 | N23 | 1902 | 889,4 | 27806 | 189,6 | 14,6 | 1,058 | H5A034 | C | sample | [illegible] |
| 12450 | G3 | G4 | 1048 | 1113 | 15327 | 160,3 | 14,6 | 1,098 | H5A034 | C | sample | [illegible] |
| 10833 | D17 | D18 | 1359 | 857,9 | 19819 | 663,4 | 14,6 | 1,106 | H5A034 | C | sample | [illegible] |
| 10849 | E1 | E2 | 1525 | 858,4 | 22274 | 597,8 | 14,6 | 1,106 | H5A034 | C | sample | [illegible] |
| 7575 | L22 | L23 | 1192 | 635,1 | 17427 | 573,1 | 14,6 | 1,115 | H5A034 | C | sample | [illegible] |
| 16949 | C13 | C14 | 1699 | 911,6 | 24831 | 244,5 | 14,6 | 1,127 | H5A034 | C | sample | [illegible] |
| 17005 | E20 | E21 | 1713 | 1007 | 25018 | 200,8 | 14,6 | 1,127 | H5A034 | C | sample | [illegible] |
| 17124 | J2 | J3 | 1369 | 994,5 | 20026 | 160,9 | 14,6 | 1,127 | H5A034 | C | sample | [illegible] |
| 17227 | N4 | N5 | 1612 | 930,4 | 23494 | 169,6 | 14,6 | 1,127 | H5A034 | C | sample | [illegible] |
| 17245 | O20 | O21 | 1578 | 993,1 | 22987 | 250,1 | 14,6 | 1,127 | H5A034 | C | sample | [illegible] |
| 7212 | M2 | M3 | 996 | 547 | 14525 | 461,8 | 14,6 | 1,136 | H5A034 | C | sample | [illegible] |
| 7257 | O17 | O18 | 959 | 538,2 | 13966 | 509,3 | 14,6 | 1,136 | H5A034 | C | sample | [illegible] |
| 5117 | F13 | F14 | 1459 | 689,4 | 21355 | 300,6 | 14,6 | 1,145 | H5A034 | C | sample | [illegible] |
| 5146 | G18 | G19 | 1510 | 596,4 | 22060 | 400,8 | 14,6 | 1,145 | H5A034 | C | sample | [illegible] |
| 5329 | O1 | O2 | 395 | 550,3 | 5779 | 482,4 | 14,6 | 1,145 | H5A034 | C | sample | [illegible] |
| 2329 | B1 | B2 | 659 | 698,5 | 9642 | 535,7 | 14,6 | 1,15 | H5A034 | C | sample | [illegible] |
| 2350 | B7 | B8 | 1069 | 712,8 | 15595 | 442,6 | 14,6 | 1,15 | H5A034 | C | sample | [illegible] |
| 16802 | M10 | M11 | 1334 | 902,8 | 19529 | 329,1 | 14,6 | 1,154 | H5A034 | C | sample | [illegible] |
| 16845 | N6 | N7 | 1360 | 934,8 | 19870 | 361,1 | 14,6 | 1,154 | H5A034 | C | sample | [illegible] |
| 17702 | B21 | B22 | 1540 | 1077 | 22500 | 348,8 | 14,6 | 1,154 | H5A034 | C | sample | [illegible] |
| 17716 | C12 | C13 | 1075 | 971 | 15748 | 302,9 | 14,6 | 1,154 | H5A034 | C | sample | [illegible] |
| 17807 | F8 | F9 | 1178 | 1074 | 17237 | 258,2 | 14,6 | 1,154 | H5A034 | C | sample | [illegible] |
| 8895 | C22 | C23 | 1809 | 1691 | 26428 | 205,7 | 14,6 | 1,173 | H5A034 | C | sample | [illegible] |
| 8931 | E11 | E12 | 1534 | 1933 | 22337 | 165,4 | 14,6 | 1,173 | H5A034 | C | sample | [illegible] |
| 9043 | I4 | I5 | 1809 | 1819 | 26401 | 172,3 | 14,6 | 1,173 | H5A034 | C | sample | [illegible] |
| 9071 | J8 | J9 | 1588 | 1744 | 23171 | 149,3 | 14,6 | 1,173 | H5A034 | C | sample | [illegible] |
| 9076 | K12 | K13 | 1199 | 1787 | 17452 | 129,4 | 14,6 | 1,173 | H5A034 | C | sample | [illegible] |
| 4244 | A5 | A6 | 826 | 690 | 12072 | 306,2 | 14,6 | 1,232 | H5A034 | C | sample | [illegible] |
| 4251 | B11 | B12 | 1165 | 702,9 | 17041 | 374,6 | 14,6 | 1,232 | H5A034 | C | sample | [illegible] |
| 4290 | C3 | C4 | 892 | 637,9 | 13055 | 430 | 14,6 | 1,232 | H5A034 | C | sample | [illegible] |
| 4439 | I8 | I9 | 1559 | 728,9 | 22695 | 427,6 | 14,6 | 1,232 | H5A034 | C | sample | [illegible] |
| 4501 | L20 | L21 | 983 | 618,5 | 14378 | 380,6 | 14,6 | 1,232 | H5A034 | C | sample | [illegible] |
| 4603 | P4 | P5 | 1468 | 641 | 21396 | 459 | 14,6 | 1,232 | H5A034 | C | sample | [illegible] |
| 134 | F21 | F22 | 634 | 1503 | 9253 | 431,7 | 14,6 | 1,243 | H5A034 | C | sample | [illegible] |
| 138 | F3 | F4 | 316 | 1243 | 4617 | 480,5 | 14,6 | 1,243 | H5A034 | C | sample | [illegible] |
| 365 | P13 | P14 | 571 | 1555 | 8340 | 789,8 | 14,6 | 1,243 | H5A034 | C | sample | [illegible] |
| 2003 | D19 | D20 | 1046 | 592,4 | 15323 | 337,2 | 14,6 | 1,27 | H5A034 | C | sample | [illegible] |
| 2042 | F10 | F11 | 1323 | 655 | 19283 | 471,5 | 14,6 | 1,27 | H5A034 | C | sample | [illegible] |
| 2113 | I1 | I2 | 940 | 676,5 | 13718 | 493,3 | 14,6 | 1,27 | H5A034 | C | sample | [illegible] |
| 2145 | J17 | J18 | 1325 | 625,8 | 19389 | 355 | 14,6 | 1,27 | H5A034 | C | sample | [illegible] |
| 2206 | L7 | L8 | 1215 | 639,7 | 17721 | 405,5 | 14,6 | 1,27 | H5A034 | C | sample | [illegible] |
| 6534 | A14 | A15 | 982 | 631,2 | 14324 | 523,7 | 14,6 | 1,281 | H5A034 | C | sample | [illegible] |
| 6579 | C11 | C12 | 1389 | 625,9 | 20334 | 486,5 | 14,6 | 1,281 | H5A034 | C | sample | [illegible] |
| 6605 | D13 | D14 | 1258 | 572,7 | 18411 | 320,4 | 14,6 | 1,281 | H5A034 | C | sample | [illegible] |
| 3893 | C13 | C14 | 614 | 707,2 | 8941 | 395,7 | 14,6 | 1,298 | H5A034 | C | sample | [illegible] |
| 3965 | F13 | F14 | 1415 | 670,6 | 20616 | 322,3 | 14,6 | 1,298 | H5A034 | C | sample | [illegible] |
| 4196 | O5 | O6 | 1295 | 649,6 | 18894 | 401,7 | 14,6 | 1,298 | H5A034 | C | sample | [illegible] |
| 4205 | P13 | P14 | 640 | 774,6 | 9352 | 495,2 | 14,6 | 1,298 | H5A034 | C | sample | [illegible] |
| 11582 | C21 | C22 | 1460 | 896,6 | 21292 | 151,1 | 14,6 | 1,304 | H5A034 | C | sample | [illegible] |
| 11610 | D3 | D4 | 1353 | 905,5 | 19765 | 141,2 | 14,6 | 1,304 | H5A034 | C | sample | [illegible] |
| 11792 | L16 | L17 | 848 | 871,3 | 12366 | 171,1 | 14,6 | 1,304 | H5A034 | C | sample | [illegible] |
| 11857 | O1 | O2 | 1264 | 835,6 | 18516 | 163,5 | 14,6 | 1,304 | H5A034 | C | sample | [illegible] |
| 5778 | A3 | A4 | 1517 | 828,5 | 22145 | 461,2 | 14,6 | 1,333 | H5A034 | C | sample | [illegible] |
| 1198 | B7 | B8 | 1328 | 781,6 | 19335 | 522,2 | 14,6 | 1,339 | H5A034 | C | sample | [illegible] |
| 1316 | G5 | G6 | 1242 | 747,4 | 18149 | 435 | 14,6 | 1,339 | H5A034 | C | sample | [illegible] |
| 1318 | G7 | G8 | 951 | 740,5 | 13896 | 520,4 | 14,6 | 1,339 | H5A034 | C | sample | [illegible] |
| 1346 | I10 | I11 | 1219 | 733 | 17796 | 393,7 | 14,6 | 1,339 | H5A034 | C | sample | [illegible] |
| 1402 | K18 | K19 | 1119 | 694,4 | 16282 | 370,3 | 14,6 | 1,339 | H5A034 | C | sample | [illegible] |
| 1490 | O10 | O11 | 1579 | 760,9 | 23023 | 529,5 | 14,6 | 1,339 | H5A034 | C | sample | [illegible] |
| 1498 | O18 | O19 | 1647 | 756,1 | 24059 | 454,2 | 14,6 | 1,339 | H5A034 | C | sample | [illegible] |

| | | | | | | | | | | | | | | |
|---|---|---|---|---|---|---|---|---|---|---|---|---|---|---|
| 1510 | O7 | O8 | 1430 | 673,6 | 20882 | 477,3 | 14,6 | 1,339 | H5A034 C | sample | Hm Drgbl | Hm Drgbl | Hm Drgbl | CPE002FV |
| 1519 | P15 | P16 | 1616 | 704,5 | 23550 | 475,5 | 14,6 | 1,339 | H5A034 C | sample | Hm Drgbl | Hm Drgbl | Hm Drgbl | CPE002FV |
| 16253 | F13 | F14 | 1140 | 953,2 | 16673 | 342,4 | 14,6 | 1,339 | H5A034 C | sample | Hm Gen E | Hm Gen E | Hm Gen E | CPE002JG |
| 16346 | J10 | J11 | 1284 | 975,4 | 18810 | 376,7 | 14,6 | 1,339 | H5A034 C | sample | Hm Gen E | Hm Gen E | Hm Gen E | CPE002JG |
| 16356 | J2 | J3 | 785 | 885,7 | 11500 | 325,9 | 14,6 | 1,339 | H5A034 C | sample | Hm Gen E | Hm Gen E | Hm Gen E | CPE002JG |
| 16390 | K7 | K8 | 756 | 981,4 | 11004 | 362,2 | 14,6 | 1,339 | H5A034 C | sample | Hm Gen E | Hm Gen E | Hm Gen E | CPE002JG |
| 16415 | L8 | L9 | 999 | 972,7 | 14566 | 362,4 | 14,6 | 1,339 | H5A034 C | sample | Hm Gen E | Hm Gen E | Hm Gen E | CPE002JG |
| 16425 | M17 | M18 | 1167 | 912,2 | 17029 | 261 | 14,6 | 1,339 | H5A034 C | sample | Hm Gen E | Hm Gen E | Hm Gen E | CPE002JG |
| 13691 | K19 | K20 | 1602 | 1015 | 23423 | 174,6 | 14,6 | 1,371 | H5A034 C | sample | Hm Gen E | Hm Gen E | Hm Gen E | CPE002JC |
| 771 | A11 | A12 | 1372 | 755,4 | 20082 | 547,8 | 14,6 | 1,384 | H5A034 C | sample | Hm Drgbl | Hm Drgbl | Hm Drgbl | CPE002FU |
| 773 | A13 | A14 | 1402 | 753,7 | 20490 | 493,4 | 14,6 | 1,384 | H5A034 C | sample | Hm Drgbl | Hm Drgbl | Hm Drgbl | CPE002FU |
| 826 | C18 | C19 | 1504 | 754,1 | 21894 | 411,9 | 14,6 | 1,384 | H5A034 C | sample | Hm Drgbl | Hm Drgbl | Hm Drgbl | CPE002FU |
| 875 | E19 | E20 | 1329 | 726,7 | 19366 | 440,5 | 14,6 | 1,384 | H5A034 C | sample | Hm Drgbl | Hm Drgbl | Hm Drgbl | CPE002FU |
| 965 | I13 | I14 | 1579 | 753,5 | 23018 | 345,6 | 14,6 | 1,384 | H5A034 C | sample | Hm Drgbl | Hm Drgbl | Hm Drgbl | CPE002FU |
| 5386 | A18 | A19 | 1577 | 768,1 | 23036 | 632,5 | 14,6 | 1,39 | H5A034 C | sample | Hm Drgbl | Hm Drgbl | Hm Drgbl | CPE002DX |
| 5531 | G19 | G20 | 1349 | 684,1 | 19674 | 511,6 | 14,6 | 1,39 | H5A034 C | sample | Hm Drgbl | Hm Drgbl | Hm Drgbl | CPE002DX |
| 3491 | B19 | B20 | 1192 | 1339 | 17437 | 381,6 | 14,6 | 1,404 | H5A034 C | sample | Hm Drgbl | Hm Drgbl | Hm Drgbl | CPE002DR |
| 3518 | C21 | C22 | 652 | 1344 | 9508 | 439,6 | 14,6 | 1,404 | H5A034 C | sample | Hm Drgbl | Hm Drgbl | Hm Drgbl | CPE002DR |
| 3582 | F14 | F15 | 866 | 1164 | 12684 | 355,9 | 14,6 | 1,404 | H5A034 C | sample | Hm Drgbl | Hm Drgbl | Hm Drgbl | CPE002DR |
| 3714 | K3 | K4 | 780 | 1297 | 11374 | 329,4 | 14,6 | 1,404 | H5A034 C | sample | Hm Drgbl | Hm Drgbl | Hm Drgbl | CPE002DR |
| 3778 | N18 | N19 | 1282 | 1262 | 18749 | 367,1 | 14,6 | 1,404 | H5A034 C | sample | Hm Drgbl | Hm Drgbl | Hm Drgbl | CPE002DR |
| 8230 | G7 | G8 | 1318 | 612,6 | 19270 | 506,8 | 14,6 | 1,404 | H5A034 C | sample | Hm Drgbl | Hm Drgbl | Hm Drgbl | CPE002E5 |
| 8255 | H8 | H9 | 1791 | 655,2 | 26227 | 520,3 | 14,6 | 1,404 | H5A034 C | sample | Hm Drgbl | Hm Drgbl | Hm Drgbl | CPE002E5 |
| 8376 | M9 | M10 | 1205 | 580,4 | 17553 | 416,3 | 14,6 | 1,404 | H5A034 C | sample | Hm Drgbl | Hm Drgbl | Hm Drgbl | CPE002E5 |
| 8389 | N20 | N21 | 1323 | 666,3 | 19337 | 498,3 | 14,6 | 1,404 | H5A034 C | sample | Hm Drgbl | Hm Drgbl | Hm Drgbl | CPE002E5 |
| 10037 | C13 | C14 | 1731 | 901,9 | 25256 | 177,8 | 14,6 | 1,404 | H5A034 C | sample | Hm Drgbl | Hm Drgbl | Hm Drgbl | CPE002EA |
| 10143 | G22 | G23 | 1481 | 789,1 | 21602 | 144,9 | 14,6 | 1,404 | H5A034 C | sample | Hm Drgbl | Hm Drgbl | Hm Drgbl | CPE002EA |
| 10215 | J22 | J23 | 1312 | 838,2 | 19163 | 160,6 | 14,6 | 1,404 | H5A034 C | sample | Hm Drgbl | Hm Drgbl | Hm Drgbl | CPE002EA |
| 1669 | F20 | F21 | 1266 | 784,2 | 18506 | 443,9 | 14,6 | 1,529 | H5A034 C | sample | Hm Drgbl | Hm Drgbl | Hm Drgbl | CPE002FW |
| 1829 | M13 | M14 | 982 | 674 | 14312 | 372,5 | 14,6 | 1,529 | H5A034 C | sample | Hm Drgbl | Hm Drgbl | Hm Drgbl | CPE002FW |
| 1843 | M4 | M5 | 636 | 815,2 | 9307 | 433,1 | 14,6 | 1,529 | H5A034 C | sample | Hm Drgbl | Hm Drgbl | Hm Drgbl | CPE002FW |
| 1882 | O18 | O19 | 1508 | 720,4 | 22015 | 506,6 | 14,6 | 1,529 | H5A034 C | sample | Hm Drgbl | Hm Drgbl | Hm Drgbl | CPE002FW |
| 21695 | H8 | H9 | 258 | 931,6 | 3762 | 678 | 14,6 | 1,537 | H5A034 C | sample | Hm Gen E | Hm Gen E | Hm Gen E | CPE002JU |
| 4692 | D2 | D3 | 1515 | 751,4 | 22174 | 553,2 | 14,6 | 1,604 | H5A034 C | sample | Hm Drgbl | Hm Drgbl | Hm Drgbl | CPE002DV |
| 10412 | B5 | B6 | 942 | 877 | 13733 | 207,7 | 14,6 | 1,65 | H5A034 C | sample | Hm Drgbl | Hm Drgbl | Hm Drgbl | CPE002EB |
| 10557 | H6 | H7 | 803 | 766,8 | 11711 | 199,7 | 14,6 | 1,65 | H5A034 C | sample | Hm Drgbl | Hm Drgbl | Hm Drgbl | CPE002EB |
| 3028 | O12 | O13 | 841 | 567,1 | 12304 | 540,7 | 14,6 | 1,718 | H5A034 C | sample | Hm Drgbl | Hm Drgbl | Hm Drgbl | CPE002DP |
| 7736 | C16 | C17 | 176 | 637,8 | 2566 | 369,1 | 14,6 | 2,229 | H5A034 C | sample | Hm Drgbl | Hm Drgbl | Hm Drgbl | CPE002E4 |
| 22407 | F22 | F23 | 978 | 1353 | 14351 | 241,2 | 14,7 | 0,758 | H5A034 C | sample | Hm Gen E | Hm Gen E | Hm Gen E | CPE002JW |
| 23805 | P6 | P7 | 1376 | 1835 | 20177 | 364,8 | 14,7 | 0,78 | H5A034 C | sample | Hm Gen E | Hm Gen E | Hm Gen E | CPE002KV |
| 14868 | L2 | L3 | 768 | 849,6 | 11310 | 152,2 | 14,7 | 0,838 | H5A034 C | sample | Hm Gen E | Hm Gen E | Hm Gen E | CPE002JD |
| 13969 | G1 | G2 | 1056 | 1051 | 15536 | 128,9 | 14,7 | 0,842 | H5A034 C | sample | Hm Gen E | Hm Gen E | Hm Gen E | CPE002JF |
| 13975 | G15 | G16 | 1687 | 1096 | 24758 | 144,5 | 14,7 | 0,842 | H5A034 C | sample | Hm Gen E | Hm Gen E | Hm Gen E | CPE002JF |
| 3151 | D15 | D16 | 973 | 1152 | 14260 | 376,9 | 14,7 | 0,91 | H5A034 C | sample | Hm Drgbl | Hm Drgbl | Hm Drgbl | CPE002DC |
| 3169 | E1 | E2 | 846 | 1501 | 12442 | 455,9 | 14,7 | 0,91 | H5A034 C | sample | Hm Drgbl | Hm Drgbl | Hm Drgbl | CPE002DC |
| 3299 | J19 | J20 | 1120 | 1112 | 16449 | 335,8 | 14,7 | 0,91 | H5A034 C | sample | Hm Drgbl | Hm Drgbl | Hm Drgbl | CPE002DC |
| 3300 | J2 | J3 | 714 | 1074 | 10490 | 462,7 | 14,7 | 0,91 | H5A034 C | sample | Hm Drgbl | Hm Drgbl | Hm Drgbl | CPE002DC |
| 12068 | G5 | G6 | 1441 | 862,8 | 21224 | 89,07 | 14,7 | 0,942 | H5A034 C | sample | Hm Gen E | Hm Gen E | Hm Gen E | CPE002J4 |
| 12114 | I3 | I4 | 1022 | 856,2 | 15061 | 93,24 | 14,7 | 0,942 | H5A034 C | sample | Hm Gen E | Hm Gen E | Hm Gen E | CPE002J4 |
| 18360 | M9 | M10 | 1551 | 974,8 | 22801 | 281,9 | 14,7 | 0,945 | H5A034 C | sample | Hm Gen E | Hm Gen E | Hm Gen E | CPE002JL |
| 6454 | M7 | M8 | 822 | 581,3 | 12116 | 421,3 | 14,7 | 0,97 | H5A034 C | sample | Hm Drgbl | Hm Drgbl | Hm Drgbl | CPE002DZ |
| 6488 | O16 | O17 | 1185 | 531,4 | 17383 | 534,2 | 14,7 | 0,97 | H5A034 C | sample | Hm Drgbl | Hm Drgbl | Hm Drgbl | CPE002DZ |
| 20384 | B16 | B17 | 1107 | 829,6 | 16260 | 292,5 | 14,7 | 0,98 | H5A034 C | sample | Hm Gen E | Hm Gen E | Hm Gen E | CPE002JR |
| 20429 | D13 | D14 | 1134 | 778 | 16657 | 252,4 | 14,7 | 0,98 | H5A034 C | sample | Hm Gen E | Hm Gen E | Hm Gen E | CPE002JR |
| 20706 | O3 | O4 | 663 | 758 | 9752 | 245,6 | 14,7 | 0,98 | H5A034 C | sample | Hm Gen E | Hm Gen E | Hm Gen E | CPE002JR |
| 12714 | B3 | B4 | 1394 | 991,5 | 20499 | 116,7 | 14,7 | 0,997 | H5A034 C | sample | Hm Gen E | Hm Gen E | Hm Gen E | CPE002J6 |
| 17444 | G5 | G6 | 1141 | 1024 | 16720 | 203,5 | 14,7 | 1,003 | H5A034 C | sample | Hm Gen E | Hm Gen E | Hm Gen E | CPE002JJ |
| 17525 | K13 | K14 | 1376 | 987,1 | 20234 | 261,5 | 14,7 | 1,003 | H5A034 C | sample | Hm Gen E | Hm Gen E | Hm Gen E | CPE002JJ |
| 17557 | L20 | L21 | 1609 | 947,4 | 23605 | 226,9 | 14,7 | 1,003 | H5A034 C | sample | Hm Gen E | Hm Gen E | Hm Gen E | CPE002JJ |
| 17583 | M22 | M23 | 1589 | 986,2 | 23342 | 252,4 | 14,7 | 1,003 | H5A034 C | sample | Hm Gen E | Hm Gen E | Hm Gen E | CPE002JJ |
| 17649 | P17 | P18 | 1181 | 1031 | 17355 | 182,5 | 14,7 | 1,003 | H5A034 C | sample | Hm Gen E | Hm Gen E | Hm Gen E | CPE002JJ |
| 18998 | H21 | H22 | 1665 | 956,3 | 24558 | 167,5 | 14,7 | 1,024 | H5A034 C | sample | Hm Gen E | Hm Gen E | Hm Gen E | CPE002JN |
| 19066 | K18 | K19 | 1202 | 920 | 17728 | 271,2 | 14,7 | 1,024 | H5A034 C | sample | Hm Gen E | Hm Gen E | Hm Gen E | CPE002JN |
| 19177 | P1 | P2 | 997 | 1076 | 14702 | 254,2 | 14,7 | 1,024 | H5A034 C | sample | Hm Gen E | Hm Gen E | Hm Gen E | CPE002JN |
| 15147 | H11 | H12 | 1267 | 787,6 | 18595 | 116,7 | 14,7 | 1,028 | H5A034 C | sample | Hm Gen E | Hm Gen E | Hm Gen E | CPE002J8 |
| 8544 | D9 | D10 | 938 | 1125 | 13782 | 129,5 | 14,7 | 1,039 | H5A034 C | sample | Hm Drgbl | Hm Drgbl | Hm Drgbl | CPE002E6 |
| 8634 | H3 | H4 | 1314 | 1090 | 19363 | 96,5 | 14,7 | 1,039 | H5A034 C | sample | Hm Drgbl | Hm Drgbl | Hm Drgbl | CPE002E6 |
| 8727 | L22 | L23 | 1852 | 1168 | 27189 | 161,9 | 14,7 | 1,039 | H5A034 C | sample | Hm Drgbl | Hm Drgbl | Hm Drgbl | CPE002E6 |
| 19524 | N2 | N3 | 732 | 1061 | 10796 | 186,7 | 14,7 | 1,043 | H5A034 C | sample | Hm Gen E | Hm Gen E | Hm Gen E | CPE002JO |
| 19550 | O21 | O22 | 1206 | 1149 | 17786 | 359,3 | 14,7 | 1,043 | H5A034 C | sample | Hm Gen E | Hm Gen E | Hm Gen E | CPE002JO |
| 20766 | B14 | B15 | 1300 | 1053 | 19066 | 262,6 | 14,7 | 1,043 | H5A034 C | sample | Hm Gen E | Hm Gen E | Hm Gen E | CPE002JS |
| 20768 | B16 | B17 | 1156 | 1117 | 16936 | 308 | 14,7 | 1,043 | H5A034 C | sample | Hm Gen E | Hm Gen E | Hm Gen E | CPE002JS |
| 20771 | B19 | B20 | 1318 | 1030 | 19427 | 215,8 | 14,7 | 1,043 | H5A034 C | sample | Hm Gen E | Hm Gen E | Hm Gen E | CPE002JS |
| 20822 | D21 | D22 | 1432 | 999,3 | 21114 | 229,9 | 14,7 | 1,043 | H5A034 C | sample | Hm Gen E | Hm Gen E | Hm Gen E | CPE002JS |
| 20915 | H19 | H20 | 814 | 1223 | 11960 | 312,2 | 14,7 | 1,043 | H5A034 C | sample | Hm Gen E | Hm Gen E | Hm Gen E | CPE002JS |
| 21035 | M19 | M20 | 1357 | 931,2 | 19938 | 188,4 | 14,7 | 1,043 | H5A034 C | sample | Hm Gen E | Hm Gen E | Hm Gen E | CPE002JS |
| 21247 | F15 | F16 | 1297 | 931,6 | 19064 | 321,3 | 14,7 | 1,043 | H5A034 C | sample | Hm Gen E | Hm Gen E | Hm Gen E | CPE002JT |
| 21445 | N20 | N21 | 1444 | 956,6 | 21187 | 259,5 | 14,7 | 1,043 | H5A034 C | sample | Hm Gen E | Hm Gen E | Hm Gen E | CPE002JT |
| 21446 | N21 | N22 | 1015 | 924 | 14899 | 307,4 | 14,7 | 1,043 | H5A034 C | sample | Hm Gen E | Hm Gen E | Hm Gen E | CPE002JT |
| 21471 | O22 | O23 | 1381 | 875,3 | 20274 | 406,6 | 14,7 | 1,043 | H5A034 C | sample | Hm Gen E | Hm Gen E | Hm Gen E | CPE002JT |
| 11168 | B16 | B17 | 1688 | 917,4 | 24867 | 179,3 | 14,7 | 1,065 | H5A034 C | sample | Hm Drgbl | Hm Drgbl | Hm Drgbl | CPE002ED |
| 11172 | B2 | B3 | 1666 | 895,3 | 24427 | 152,4 | 14,7 | 1,065 | H5A034 C | sample | Hm Drgbl | Hm Drgbl | Hm Drgbl | CPE002ED |
| 11271 | F22 | F23 | 2008 | 951 | 29418 | 203 | 14,7 | 1,065 | H5A034 C | sample | Hm Drgbl | Hm Drgbl | Hm Drgbl | CPE002ED |
| 11336 | I16 | I17 | 1011 | 705,9 | 14826 | 126,5 | 14,7 | 1,065 | H5A034 C | sample | Hm Drgbl | Hm Drgbl | Hm Drgbl | CPE002ED |
| 11380 | K12 | K13 | 1782 | 873,9 | 26150 | 162,4 | 14,7 | 1,065 | H5A034 C | sample | Hm Drgbl | Hm Drgbl | Hm Drgbl | CPE002ED |
| 11419 | L4 | L5 | 1900 | 885 | 27926 | 169,2 | 14,7 | 1,065 | H5A034 C | sample | Hm Drgbl | Hm Drgbl | Hm Drgbl | CPE002ED |
| 11498 | P10 | P11 | 1618 | 836,8 | 23815 | 152,4 | 14,7 | 1,065 | H5A034 C | sample | Hm Drgbl | Hm Drgbl | Hm Drgbl | CPE002ED |
| 11517 | P6 | P7 | 1676 | 827,7 | 24623 | 212,2 | 14,7 | 1,065 | H5A034 C | sample | Hm Drgbl | Hm Drgbl | Hm Drgbl | CPE002ED |
| 12427 | F4 | F5 | 842 | 1143 | 12417 | 163,6 | 14,7 | 1,105 | H5A034 C | sample | Hm Gen E | Hm Gen E | Hm Gen E | CPE002J5 |
| 12526 | J7 | J8 | 1288 | 1055 | 18898 | 163,4 | 14,7 | 1,105 | H5A034 C | sample | Hm Gen E | Hm Gen E | Hm Gen E | CPE002J5 |
| 12668 | P5 | P6 | 1238 | 947,1 | 18220 | 211,2 | 14,7 | 1,105 | H5A034 C | sample | Hm Gen E | Hm Gen E | Hm Gen E | CPE002J5 |
| 10923 | H11 | H12 | 1325 | 869,3 | 19469 | 481,1 | 14,7 | 1,114 | H5A034 C | sample | Hm Drgbl | Hm Drgbl | Hm Drgbl | CPE002EC |
| 7357 | C20 | C21 | 1633 | 638,5 | 23991 | 535,6 | 14,7 | 1,122 | H5A034 C | sample | Hm Drgbl | Hm Drgbl | Hm Drgbl | CPE002E3 |
| 7620 | N2 | N3 | 1332 | 612,1 | 19522 | 571,1 | 14,7 | 1,122 | H5A034 C | sample | Hm Drgbl | Hm Drgbl | Hm Drgbl | CPE002E3 |
| 16963 | C4 | C5 | 1072 | 1057 | 15726 | 137,9 | 14,7 | 1,135 | H5A034 C | sample | Hm Gen E | Hm Gen E | Hm Gen E | CPE002JI |
| 17121 | J17 | J18 | 1068 | 1082 | 15711 | 219,7 | 14,7 | 1,135 | H5A034 C | sample | Hm Gen E | Hm Gen E | Hm Gen E | CPE002JI |
| 17126 | J21 | J22 | 1605 | 979,4 | 23667 | 239,6 | 14,7 | 1,135 | H5A034 C | sample | Hm Gen E | Hm Gen E | Hm Gen E | CPE002JI |
| 7195 | L4 | L5 | 659 | 568,2 | 9693 | 393,2 | 14,7 | 1,144 | H5A034 C | sample | Hm Drgbl | Hm Drgbl | Hm Drgbl | CPE002E2 |
| 5135 | F8 | F9 | 1488 | 623,5 | 21934 | 457,5 | 14,7 | 1,153 | H5A034 C | sample | Hm Drgbl | Hm Drgbl | Hm Drgbl | CPE002DV |
| 5180 | H5 | H6 | 1582 | 601,2 | 23228 | 459,9 | 14,7 | 1,153 | H5A034 C | sample | Hm Drgbl | Hm Drgbl | Hm Drgbl | CPE002DV |
| 2323 | A4 | A5 | 809 | 676,8 | 11917 | 444,1 | 14,7 | 1,157 | H5A034 C | sample | Hm Drgbl | Hm Drgbl | Hm Drgbl | CPE002DC |
| 2331 | B11 | B12 | 940 | 661,8 | 13779 | 443,7 | 14,7 | 1,157 | H5A034 C | sample | Hm Drgbl | Hm Drgbl | Hm Drgbl | CPE002DC |
| 2432 | F16 | F17 | 949 | 695,1 | 13991 | 354,1 | 14,7 | 1,157 | H5A034 C | sample | Hm Drgbl | Hm Drgbl | Hm Drgbl | CPE002DC |
| 2525 | J13 | J14 | 963 | 673,2 | 14168 | 455,6 | 14,7 | 1,157 | H5A034 C | sample | Hm Drgbl | Hm Drgbl | Hm Drgbl | CPE002DC |
| 2630 | N21 | N22 | 1146 | 665,1 | 16818 | 382,3 | 14,7 | 1,157 | H5A034 C | sample | Hm Drgbl | Hm Drgbl | Hm Drgbl | CPE002DC |
| 2653 | O20 | O21 | 1357 | 696,4 | 19953 | 492,2 | 14,7 | 1,157 | H5A034 C | sample | Hm Drgbl | Hm Drgbl | Hm Drgbl | CPE002DC |
| 16532 | A5 | A6 | 997 | 1081 | 14611 | 407,1 | 14,7 | 1,162 | H5A034 C | sample | Hm Gen E | Hm Gen E | Hm Gen E | CPE002JH |
| 16635 | F11 | F12 | 1044 | 968,4 | 15344 | 265,6 | 14,7 | 1,162 | H5A034 C | sample | Hm Gen E | Hm Gen E | Hm Gen E | CPE002JH |
| 16736 | J16 | J17 | 1251 | 918,3 | 18338 | 234 | 14,7 | 1,162 | H5A034 C | sample | Hm Gen E | Hm Gen E | Hm Gen E | CPE002JH |
| 16759 | K15 | K16 | 1142 | 986,4 | 16800 | 332,9 | 14,7 | 1,162 | H5A034 C | sample | Hm Gen E | Hm Gen E | Hm Gen E | CPE002JH |
| 16771 | K4 | K5 | 1049 | 985,1 | 15394 | 373,9 | 14,7 | 1,162 | H5A034 C | sample | Hm Gen E | Hm Gen E | Hm Gen E | CPE002JH |
| 16873 | P1 | P2 | 609 | 1106 | 8977 | 278 | 14,7 | 1,162 | H5A034 C | sample | Hm Gen E | Hm Gen E | Hm Gen E | CPE002JH |
| 17727 | C22 | C23 | 1328 | 1028 | 19456 | 344,4 | 14,7 | 1,162 | H5A034 C | sample | Hm Gen E | Hm Gen E | Hm Gen E | CPE002JK |
| 17864 | I16 | I17 | 1763 | 1027 | 25927 | 174 | 14,7 | 1,162 | H5A034 C | sample | Hm Gen E | Hm Gen E | Hm Gen E | CPE002JK |
| 17942 | L21 | L22 | 1423 | 1059 | 20912 | 353,8 | 14,7 | 1,162 | H5A034 C | sample | Hm Gen E | Hm Gen E | Hm Gen E | CPE002JK |
| 18024 | O9 | O10 | 1172 | 1018 | 17269 | 348,2 | 14,7 | 1,162 | H5A034 C | sample | Hm Gen E | Hm Gen E | Hm Gen E | CPE002JK |
| 8833 | A1 | A2 | 1370 | 1778 | 20134 | 147,9 | 14,7 | 1,181 | H5A034 C | sample | Hm Drgbl | Hm Drgbl | Hm Drgbl | CPE002E7 |
| 8868 | B2 | B3 | 1402 | 1823 | 20551 | 158,1 | 14,7 | 1,181 | H5A034 C | sample | Hm Drgbl | Hm Drgbl | Hm Drgbl | CPE002E7 |
| 8887 | C15 | C16 | 1976 | 1782 | 29082 | 211,5 | 14,7 | 1,181 | H5A034 C | sample | Hm Drgbl | Hm Drgbl | Hm Drgbl | CPE002E7 |
| 8984 | G16 | G17 | 2258 | 2056 | 33158 | 158,5 | 14,7 | 1,181 | H5A034 C | sample | Hm Drgbl | Hm Drgbl | Hm Drgbl | CPE002E7 |
| 9149 | N13 | N14 | 2141 | 1915 | 31531 | 160,7 | 14,7 | 1,181 | H5A034 C | sample | Hm Drgbl | Hm Drgbl | Hm Drgbl | CPE002E7 |
| 4508 | L5 | L6 | 808 | 685,3 | 11866 | 418 | 14,7 | 1,241 | H5A034 C | sample | Hm Drgbl | Hm Drgbl | Hm Drgbl | CPE002DU |
| 4568 | O16 | O17 | 1293 | 730,3 | 19013 | 502,6 | 14,7 | 1,241 | H5A034 C | sample | Hm Drgbl | Hm Drgbl | Hm Drgbl | CPE002DU |
| 4595 | P19 | P20 | 1482 | 663,9 | 21836 | 582,7 | 14,7 | 1,241 | H5A034 C | sample | Hm Drgbl | Hm Drgbl | Hm Drgbl | CPE002DU |
| 73 | D1 | D2 | 246 | 1286 | 3627 | 848,4 | 14,7 | 1,251 | H5A034 C | sample | Hm Drgbl | Hm Drgbl | Hm Drgbl | CPE002DT |
| 279 | L22 | L23 | 436 | 1480 | 6429 | 559,6 | 14,7 | 1,251 | H5A034 C | sample | Hm Drgbl | Hm Drgbl | Hm Drgbl | CPE002DT |
| 338 | O10 | O11 | 739 | 1644 | 10855 | 727,7 | 14,7 | 1,251 | H5A034 C | sample | Hm Drgbl | Hm Drgbl | Hm Drgbl | CPE002DT |
| 362 | P10 | P11 | 768 | 1494 | 11296 | 715,9 | 14,7 | 1,251 | H5A034 C | sample | Hm Drgbl | Hm Drgbl | Hm Drgbl | CPE002DT |
| 1980 | C2 | C3 | 1335 | 620,4 | 19653 | 533,1 | 14,7 | 1,278 | H5A034 C | sample | Hm Drgbl | Hm Drgbl | Hm Drgbl | CPE002FX |
| 6662 | F21 | F22 | 1004 | 659,4 | 14778 | 427,4 | 14,7 | 1,289 | H5A034 C | sample | Hm Drgbl | Hm Drgbl | Hm Drgbl | CPE002E1 |
| 6697 | H1 | H2 | 733 | 542,9 | 10773 | 427,6 | 14,7 | 1,289 | H5A034 C | sample | Hm Drgbl | Hm Drgbl | Hm Drgbl | CPE002E1 |
| 6802 | L18 | L19 | 1207 | 555,7 | 17769 | 461,4 | 14,7 | 1,289 | H5A034 C | sample | Hm Drgbl | Hm Drgbl | Hm Drgbl | CPE002E1 |
| 6860 | N5 | N6 | 1013 | 602,9 | 14885 | 493,6 | 14,7 | 1,289 | H5A034 C | sample | Hm Drgbl | Hm Drgbl | Hm Drgbl | CPE002E1 |
| 4052 | I5 | I6 | 618 | 742,7 | 9060 | 432,8 | 14,7 | 1,307 | H5A034 C | sample | Hm Drgbl | Hm Drgbl | Hm Drgbl | CPE002DS |
| 4213 | P20 | P21 | 1306 | 656,3 | 19165 | 458,8 | 14,7 | 1,307 | H5A034 C | sample | Hm Drgbl | Hm Drgbl | Hm Drgbl | CPE002DS |
| 11615 | D8 | D9 | 919 | 824,4 | 13541 | 140 | 14,7 | 1,312 | H5A034 C | sample | Hm Gen E | Hm Gen E | Hm Gen E | CPE002JA |
| 11660 | F5 | F6 | 964 | 801,3 | 14217 | 143,4 | 14,7 | 1,312 | H5A034 C | sample | Hm Gen E | Hm Gen E | Hm Gen E | CPE002JA |
| 15690 | N3 | N4 | 1079 | 798,1 | 15834 | 138,4 | 14,7 | 1,336 | H5A034 C | sample | Hm Gen E | Hm Gen E | Hm Gen E | CPE002JE |
| 5864 | E16 | E17 | 1200 | 806,5 | 17626 | 350,5 | 14,7 | 1,342 | H5A034 C | sample | Hm Drgbl | Hm Drgbl | Hm Drgbl | CPE002DY |
| 5881 | F1 | F2 | 669 | 741,3 | 9813 | 393,5 | 14,7 | 1,342 | H5A034 C | sample | Hm Drgbl | Hm Drgbl | Hm Drgbl | CPE002DY |
| 5942 | H21 | H22 | 1425 | 816,6 | 20975 | 577 | 14,7 | 1,342 | H5A034 C | sample | Hm Drgbl | Hm Drgbl | Hm Drgbl | CPE002DY |
| 5978 | J10 | J11 | 638 | 849 | 9358 | 439,5 | 14,7 | 1,342 | H5A034 C | sample | Hm Drgbl | Hm Drgbl | Hm Drgbl | CPE002DY |
| 1223 | C8 | C9 | 853 | 687,4 | 12569 | 455,6 | 14,7 | 1,349 | H5A034 C | sample | Hm Drgbl | Hm Drgbl | Hm Drgbl | CPE002FV |
| 1256 | E16 | E17 | 1396 | 749,8 | 20567 | 420,7 | 14,7 | 1,349 | H5A034 C | sample | Hm Drgbl | Hm Drgbl | Hm Drgbl | CPE002FV |
| 1310 | G21 | G22 | 1276 | 772,9 | 18717 | 516 | 14,7 | 1,349 | H5A034 C | sample | Hm Drgbl | Hm Drgbl | Hm Drgbl | CPE002FV |
| 1421 | L13 | L14 | 1439 | 690,6 | 21109 | 361,1 | 14,7 | 1,349 | H5A034 C | sample | Hm Drgbl | Hm Drgbl | Hm Drgbl | CPE002FV |
| 1533 | P6 | P7 | 1763 | 750,1 | 25926 | 483,2 | 14,7 | 1,349 | H5A034 C | sample | Hm Drgbl | Hm Drgbl | Hm Drgbl | CPE002FV |
| 16191 | C22 | C23 | 1161 | 1017 | 17015 | 414,1 | 14,7 | 1,349 | H5A034 C | sample | Hm Gen E | Hm Gen E | Hm Gen E | CPE002JG |
| 16331 | I19 | I20 | 1054 | 963,6 | 15498 | 231,6 | 14,7 | 1,349 | H5A034 C | sample | Hm Gen E | Hm Gen E | Hm Gen E | CPE002JG |
| 13465 | B1 | B2 | 1146 | 1064 | 16851 | 169,3 | 14,7 | 1,38 | H5A034 C | sample | Hm Gen E | Hm Gen E | Hm Gen E | CPE002JC |
| 13502 | C21 | C22 | 1446 | 1083 | 21282 | 187,9 | 14,7 | 1,38 | H5A034 C | sample | Hm Gen E | Hm Gen E | Hm Gen E | CPE002JC |
| 864 | D9 | D10 | 1489 | 677 | 21871 | 419,3 | 14,7 | 1,393 | H5A034 C | sample | Hm Drgbl | Hm Drgbl | Hm Drgbl | CPE002FU |
| 912 | F9 | F10 | 936 | 718,5 | 13727 | 473,9 | 14,7 | 1,393 | H5A034 C | sample | Hm Drgbl | Hm Drgbl | Hm Drgbl | CPE002FU |
| 944 | H16 | H17 | 1255 | 748,6 | 18490 | 356,2 | 14,7 | 1,393 | H5A034 C | sample | Hm Drgbl | Hm Drgbl | Hm Drgbl | CPE002FU |
| 1015 | K15 | K16 | 1313 | 709,7 | 19281 | 368,2 | 14,7 | 1,393 | H5A034 C | sample | Hm Drgbl | Hm Drgbl | Hm Drgbl | CPE002FU |
| 1031 | K8 | K9 | 946 | 650,8 | 13890 | 353,7 | 14,7 | 1,393 | H5A034 C | sample | Hm Drgbl | Hm Drgbl | Hm Drgbl | CPE002FU |
| 1032 | K9 | K10 | 1006 | 807,9 | 14813 | 392,8 | 14,7 | 1,393 | H5A034 C | sample | Hm Drgbl | Hm Drgbl | Hm Drgbl | CPE002FU |
| 1124 | O5 | O6 | 1021 | 827,7 | 14960 | 603,6 | 14,7 | 1,393 | H5A034 C | sample | Hm Drgbl | Hm Drgbl | Hm Drgbl | CPE002FU |
| 5516 | F5 | F6 | 1693 | 620,3 | 24966 | 581,2 | 14,7 | 1,4 | H5A034 C | sample | Hm Drgbl | Hm Drgbl | Hm Drgbl | CPE002DX |
| 5663 | L8 | L9 | 427 | 631,1 | 6278 | 573,7 | 14,7 | 1,4 | H5A034 C | sample | Hm Drgbl | Hm Drgbl | Hm Drgbl | CPE002DX |
| 5666 | M10 | M11 | 1017 | 658,8 | 14973 | 518,2 | 14,7 | 1,4 | H5A034 C | sample | Hm Drgbl | Hm Drgbl | Hm Drgbl | CPE002DX |
| 3803 | O19 | O20 | 1510 | 1258 | 22249 | 464,7 | 14,7 | 1,413 | H5A034 C | sample | Hm Drgbl | Hm Drgbl | Hm Drgbl | CPE002DR |
| 3837 | P6 | P7 | 656 | 1496 | 9657 | 389,2 | 14,7 | 1,413 | H5A034 C | sample | Hm Drgbl | Hm Drgbl | Hm Drgbl | CPE002DR |
| 8139 | D11 | D12 | 1146 | 566,8 | 16848 | 571,2 | 14,7 | 1,413 | H5A034 C | sample | Hm Drgbl | Hm Drgbl | Hm Drgbl | CPE002E5 |
| 8236 | H12 | H13 | 1634 | 632,3 | 24023 | 405,2 | 14,7 | 1,413 | H5A034 C | sample | Hm Drgbl | Hm Drgbl | Hm Drgbl | CPE002E5 |
| 8238 | H14 | H15 | 1546 | 622,6 | 22662 | 423 | 14,7 | 1,413 | H5A034 C | sample | Hm Drgbl | Hm Drgbl | Hm Drgbl | CPE002E5 |
| 8325 | K6 | K7 | 1174 | 613 | 17281 | 517,4 | 14,7 | 1,413 | H5A034 C | sample | Hm Drgbl | Hm Drgbl | Hm Drgbl | CPE002E5 |
| 8422 | O7 | O8 | 1205 | 566,9 | 17726 | 585,5 | 14,7 | 1,413 | H5A034 C | sample | Hm Drgbl | Hm Drgbl | Hm Drgbl | CPE002E5 |
| 8435 | P19 | P20 | 1727 | 656,3 | 25314 | 514,3 | 14,7 | 1,413 | H5A034 C | sample | Hm Drgbl | Hm Drgbl | Hm Drgbl | CPE002E5 |
| 8445 | P6 | P7 | 1242 | 526,6 | 18212 | 492,4 | 14,7 | 1,413 | H5A034 C | sample | Hm Drgbl | Hm Drgbl | Hm Drgbl | CPE002E5 |
| 10271 | L8 | L9 | 977 | 789,7 | 14343 | 145 | 14,7 | 1,413 | H5A034 C | sample | Hm Drgbl | Hm Drgbl | Hm Drgbl | CPE002EA |
| 10352 | P16 | P17 | 1027 | 735,2 | 15131 | 137,4 | 14,7 | 1,413 | H5A034 C | sample | Hm Drgbl | Hm Drgbl | Hm Drgbl | CPE002EA |
| 1569 | B17 | B18 | 1402 | 715,5 | 20563 | 462 | 14,7 | 1,539 | H5A034 C | sample | Hm Drgbl | Hm Drgbl | Hm Drgbl | CPE002FW |
| 1654 | E7 | E8 | 1696 | 774,5 | 24975 | 442 | 14,7 | 1,539 | H5A034 C | sample | Hm Drgbl | Hm Drgbl | Hm Drgbl | CPE002FW |
| 1715 | H19 | H20 | 967 | 742,5 | 14212 | 384 | 14,7 | 1,539 | H5A034 C | sample | Hm Drgbl | Hm Drgbl | Hm Drgbl | CPE002FW |
| 1804 | L12 | L13 | 1254 | 739,1 | 18428 | 370,5 | 14,7 | 1,539 | H5A034 C | sample | Hm Drgbl | Hm Drgbl | Hm Drgbl | CPE002FW |
| 21532 | B12 | B13 | 267 | 950,2 | 3924 | 683,3 | 14,7 | 1,547 | H5A034 C | sample | Hm Gen E | Hm Gen E | Hm Gen E | CPE002JU |
| 21876 | P2 | P3 | 235 | 1335 | 3457 | 578,3 | 14,7 | 1,547 | H5A034 C | sample | Hm Gen E | Hm Gen E | Hm Gen E | CPE002JU |
| 4656 | B9 | B10 | 1476 | 751,9 | 21722 | 508,9 | 14,7 | 1,615 | H5A034 C | sample | Hm Drgbl | Hm Drgbl | Hm Drgbl | CPE002DV |
| 10486 | E7 | E8 | 1129 | 897,1 | 16583 | 254,1 | 14,7 | 1,661 | H5A034 C | sample | Hm Drgbl | Hm Drgbl | Hm Drgbl | CPE002EB |
| 10488 | E9 | E10 | 885 | 880,2 | 13013 | 209,6 | 14,7 | 1,661 | H5A034 C | sample | Hm Drgbl | Hm Drgbl | Hm Drgbl | CPE002EB |
| 10510 | F7 | F8 | 892 | 836,3 | 13068 | 236,4 | 14,7 | 1,661 | H5A034 C | sample | Hm Drgbl | Hm Drgbl | Hm Drgbl | CPE002EB |
| 10519 | G15 | G16 | 1082 | 794,8 | 15904 | 168,4 | 14,7 | 1,661 | H5A034 C | sample | Hm Drgbl | Hm Drgbl | Hm Drgbl | CPE002EB |
| 10711 | O15 | O16 | 1261 | 839,9 | 18501 | 202,9 | 14,7 | 1,661 | H5A034 C | sample | Hm Drgbl | Hm Drgbl | Hm Drgbl | CPE002EB |
| 20014 | B7 | B8 | 854 | 1308 | 12530 | 314,3 | 14,7 | 1,793 | H5A034 C | sample | Hm Gen E | Hm Gen E | Hm Gen E | CPE002JQ |
| 20042 | D10 | D11 | 546 | 1113 | 8017 | 182,3 | 14,7 | 1,793 | H5A034 C | sample | Hm Gen E | Hm Gen E | Hm Gen E | CPE002JQ |
| 7852 | H12 | H13 | 850 | 526,9 | 12471 | 345,3 | 14,7 | 2,244 | H5A034 C | sample | Hm Drgbl | Hm Drgbl | Hm Drgbl | CPE002E4 |
| 23748 | N2 | N3 | 1197 | 1516 | 17699 | 164,1 | 14,8 | 0,785 | H5A034 C | sample | Hm Gen E | Hm Gen E | Hm Gen E | CPE002KV |
| 14861 | L13 | L14 | 948 | 906 | 14018 | 108,3 | 14,8 | 0,843 | H5A034 C | sample | Hm Gen E | Hm Gen E | Hm Gen E | CPE002JD |
| 3100 | B12 | B13 | 738 | 1176 | 10920 | 468,9 | 14,8 | 0,916 | H5A034 C | sample | Hm Drgbl | Hm Drgbl | Hm Drgbl | CPE002DC |
| 3272 | I16 | I17 | 575 | 1222 | 8490 | 267,1 | 14,8 | 0,916 | H5A034 C | sample | Hm Drgbl | Hm Drgbl | Hm Drgbl | CPE002DC |
| 6200 | C16 | C17 | 708 | 597,6 | 10495 | 478,5 | 14,8 | 0,977 | H5A034 C | sample | Hm Drgbl | Hm Drgbl | Hm Drgbl | CPE002DZ |
| 6513 | P17 | P18 | 1553 | 612,2 | 22915 | 480,6 | 14,8 | 0,977 | H5A034 C | sample | Hm Drgbl | Hm Drgbl | Hm Drgbl | CPE002DZ |
| 20528 | H16 | H17 | 921 | 727,9 | 13600 | 177 | 14,8 | 0,987 | H5A034 C | sample | Hm Gen E | Hm Gen E | Hm Gen E | CPE002JR |
| 20529 | H17 | H18 | 1012 | 751,9 | 14952 | 180,2 | 14,8 | 0,987 | H5A034 C | sample | Hm Gen E | Hm Gen E | Hm Gen E | CPE002JR |
| 20612 | K5 | K6 | 472 | 724,2 | 6973 | 199,3 | 14,8 | 0,987 | H5A034 C | sample | Hm Gen E | Hm Gen E | Hm Gen E | CPE002JR |
| 18582 | G14 | G15 | 1260 | 1027 | 18620 | 236,3 | 14,8 | 1,003 | H5A034 C | sample | Hm Gen E | Hm Gen E | Hm Gen E | CPE002JM |
| 17314 | B18 | B19 | 1579 | 1043 | 23336 | 264,7 | 14,8 | 1,01 | H5A034 C | sample | Hm Gen E | Hm Gen E | Hm Gen E | CPE002JJ |
| 17457 | H17 | H18 | 1341 | 994,2 | 19907 | 204,7 | 14,8 | 1,01 | H5A034 C | sample | Hm Gen E | Hm Gen E | Hm Gen E | CPE002JJ |
| 17476 | I12 | I13 | 1773 | 981,2 | 26271 | 232 | 14,8 | 1,01 | H5A034 C | sample | Hm Gen E | Hm Gen E | Hm Gen E | CPE002JJ |
| 17505 | J17 | J18 | 1483 | 1021 | 21938 | 181,4 | 14,8 | 1,01 | H5A034 C | sample | Hm Gen E | Hm Gen E | Hm Gen E | CPE002JJ |
| 9637 | B20 | B21 | 2111 | 1219 | 31259 | 187,2 | 14,8 | 1,035 | H5A034 C | sample | Hm Drgbl | Hm Drgbl | Hm Drgbl | CPE002E9 |
| 9888 | L9 | L10 | 1197 | 1067 | 17741 | 140,1 | 14,8 | 1,035 | H5A034 C | sample | Hm Drgbl | Hm Drgbl | Hm Drgbl | CPE002E9 |
| 9913 | N1 | N2 | 1405 | 1004 | 20738 | 129,2 | 14,8 | 1,035 | H5A034 C | sample | Hm Drgbl | Hm Drgbl | Hm Drgbl | CPE002E9 |
| 8792 | O16 | O17 | 1665 | 1059 | 24662 | 107,7 | 14,8 | 1,046 | H5A034 C | sample | Hm Drgbl | Hm Drgbl | Hm Drgbl | CPE002E6 |
| 20918 | H21 | H22 | 1319 | 1032 | 19524 | 297,5 | 14,8 | 1,05 | H5A034 C | sample | Hm Gen E | Hm Gen E | Hm Gen E | CPE002JS |
| 20924 | H5 | H6 | 864 | 1066 | 12799 | 258,9 | 14,8 | 1,05 | H5A034 C | sample | Hm Gen E | Hm Gen E | Hm Gen E | CPE002JS |
| 20960 | J16 | J17 | 1101 | 934,9 | 16316 | 278,9 | 14,8 | 1,05 | H5A034 C | sample | Hm Gen E | Hm Gen E | Hm Gen E | CPE002JS |
| 21024 | L9 | L10 | 695 | 1023 | 10260 | 319,4 | 14,8 | 1,05 | H5A034 C | sample | Hm Gen E | Hm Gen E | Hm Gen E | CPE002JS |
| 21206 | D21 | D22 | 1261 | 964,7 | 18655 | 283,2 | 14,8 | 1,05 | H5A034 C | sample | Hm Gen E | Hm Gen E | Hm Gen E | CPE002JT |
| 21469 | O20 | O21 | 1306 | 889,5 | 19347 | 409,6 | 14,8 | 1,05 | H5A034 C | sample | Hm Gen E | Hm Gen E | Hm Gen E | CPE002JT |
| 14343 | F22 | F23 | 1585 | 838,1 | 23477 | 192,9 | 14,8 | 1,053 | H5A034 C | sample | Hm Gen E | Hm Gen E | Hm Gen E | CPE002J7 |
| 11165 | B13 | B14 | 1131 | 821,5 | 16734 | 170,7 | 14,8 | 1,072 | H5A034 C | sample | Hm Drgbl | Hm Drgbl | Hm Drgbl | CPE002ED |
| 11174 | B21 | B22 | 1483 | 880,3 | 21951 | 196,9 | 14,8 | 1,072 | H5A034 C | sample | Hm Drgbl | Hm Drgbl | Hm Drgbl | CPE002ED |
| 11238 | E14 | E15 | 1017 | 825,8 | 15041 | 177,3 | 14,8 | 1,072 | H5A034 C | sample | Hm Drgbl | Hm Drgbl | Hm Drgbl | CPE002ED |
| 11264 | F16 | F17 | 1864 | 851,2 | 27579 | 147,4 | 14,8 | 1,072 | H5A034 C | sample | Hm Drgbl | Hm Drgbl | Hm Drgbl | CPE002ED |
| 11323 | H4 | H5 | 1630 | 842,4 | 24170 | 131,8 | 14,8 | 1,072 | H5A034 C | sample | Hm Drgbl | Hm Drgbl | Hm Drgbl | CPE002ED |
| 7299 | A11 | A12 | 1361 | 610,3 | 20179 | 554,7 | 14,8 | 1,13 | H5A034 C | sample | Hm Drgbl | Hm Drgbl | Hm Drgbl | CPE002E3 |
| 7407 | E22 | E23 | 1427 | 588,6 | 21053 | 504,4 | 14,8 | 1,13 | H5A034 C | sample | Hm Drgbl | Hm Drgbl | Hm Drgbl | CPE002E3 |
| 7426 | F18 | F19 | 1502 | 604,6 | 22177 | 517 | 14,8 | 1,13 | H5A034 C | sample | Hm Drgbl | Hm Drgbl | Hm Drgbl | CPE002E3 |
| 17026 | F18 | F19 | 1710 | 999 | 25277 | 167,8 | 14,8 | 1,143 | H5A034 C | sample | Hm Gen E | Hm Gen E | Hm Gen E | CPE002JI |
| 17150 | K21 | K22 | 1539 | 976,1 | 22834 | 270,2 | 14,8 | 1,143 | H5A034 C | sample | Hm Gen E | Hm Gen E | Hm Gen E | CPE002JI |
| 17197 | M20 | M21 | 1511 | 991,6 | 22355 | 237,6 | 14,8 | 1,143 | H5A034 C | sample | Hm Gen E | Hm Gen E | Hm Gen E | CPE002JI |
| 17203 | M4 | M5 | 1424 | 986,7 | 21073 | 253,3 | 14,8 | 1,143 | H5A034 C | sample | Hm Gen E | Hm Gen E | Hm Gen E | CPE002JI |
| 5067 | D11 | D12 | 1580 | 589,8 | 23317 | 618 | 14,8 | 1,161 | H5A034 C | sample | Hm Drgbl | Hm Drgbl | Hm Drgbl | CPE002DV |
| 5258 | L10 | L11 | 1340 | 653,8 | 19862 | 615,9 | 14,8 | 1,161 | H5A034 C | sample | Hm Drgbl | Hm Drgbl | Hm Drgbl | CPE002DV |

| | | | | | | | | | |
|---|---|---|---|---|---|---|---|---|---|
| 5350 | O7 | O8 | 1836 | 610,2 | 27237 | 582,2 | 14,8 | 1,161 | SENP5 |
| 2403 | E11 | E12 | 1020 | 689,9 | 15120 | 399,8 | 14,8 | 1,165 | NUAK1 |
| 2625 | N17 | N18 | 1092 | 694,7 | 16141 | 349,8 | 14,8 | 1,165 | FGR |
| 2636 | N5 | N6 | 1389 | 738,1 | 20594 | 396,3 | 14,8 | 1,165 | FER |
| 16526 | A21 | A22 | 1214 | 1103 | 18021 | 328,7 | 14,8 | 1,17 | C1orf198 |
| 16596 | D2 | D3 | 1121 | 972,3 | 16643 | 350,8 | 14,8 | 1,17 | C14orf73 |
| 16633 | F1 | F2 | 1109 | 969,3 | 16367 | 231,2 | 14,8 | 1,17 | KIAA1751 |
| 16754 | K10 | K11 | 1482 | 891,6 | 21952 | 350,6 | 14,8 | 1,17 | C19orf22 |
| 16780 | L12 | L13 | 931 | 954,8 | 13759 | 388,3 | 14,8 | 1,17 | TMEM169 |
| 16807 | M15 | M16 | 1374 | 961 | 20331 | 257,4 | 14,8 | 1,17 | MST150 |
| 17715 | C11 | C12 | 1283 | 978,7 | 19000 | 346,7 | 14,8 | 1,17 | C20orf152 |
| 17775 | E22 | E23 | 1379 | 1041 | 20454 | 338,6 | 14,8 | 1,17 | MOBKL2C |
| 17827 | G4 | G5 | 1181 | 959,9 | 17500 | 323,2 | 14,8 | 1,17 | RC3H1 |
| 8851 | A4 | A5 | 1563 | 1670 | 23151 | 176,3 | 14,8 | 1,189 | SIRT6 |
| 8940 | E2 | E3 | 1717 | 1959 | 25380 | 191,3 | 14,8 | 1,189 | RBMX2 |
| 9110 | L21 | L22 | 2118 | 1906 | 31370 | 184,1 | 14,8 | 1,189 | PRKAG2 |
| 4351 | F15 | F16 | 1329 | 701,7 | 19658 | 384,5 | 14,8 | 1,249 | KCNB1 |
| 4436 | I5 | I6 | 1295 | 649,7 | 19104 | 440,5 | 14,8 | 1,249 | CHRNA5 |
| 4499 | L19 | L20 | 1189 | 712,1 | 17589 | 394,5 | 14,8 | 1,249 | KCNN4 |
| 4555 | N4 | N5 | 1085 | 722,6 | 16053 | 440,6 | 14,8 | 1,249 | TLR1 |
| 4580 | O5 | O6 | 428 | 774,5 | 6343 | 536,5 | 14,8 | 1,249 | GABRD |
| 1950 | B14 | B15 | 870 | 638,3 | 12870 | 463,1 | 14,8 | 1,287 | ZSCAN22 |
| 2142 | J14 | J15 | 1180 | 703,3 | 17434 | 398,3 | 14,8 | 1,287 | ZNF674 |
| 2211 | M11 | M12 | 1144 | 646,3 | 16877 | 444,7 | 14,8 | 1,287 | ZNF543 |
| 6599 | C8 | C9 | 537 | 560,7 | 7924 | 490,3 | 14,8 | 1,298 | IGF2R |
| 6624 | D9 | D10 | 1090 | 712,7 | 16089 | 490,3 | 14,8 | 1,298 | HOXA2 |
| 6660 | F2 | F3 | 1287 | 550,7 | 18988 | 487,2 | 14,8 | 1,298 | STT3A |
| 3873 | B17 | B18 | 1309 | 656,1 | 19339 | 356,2 | 14,8 | 1,316 | PRKAB1 |
| 3981 | F6 | F7 | 700 | 683,4 | 10395 | 435,7 | 14,8 | 1,316 | RHD |
| 4004 | G5 | G6 | 1103 | 635,4 | 16331 | 395,1 | 14,8 | 1,316 | PLTP |
| 4109 | L13 | L14 | 82 | 819,4 | 1210 | 384,5 | 14,8 | 1,316 | PSMC3 |
| 11703 | H22 | H23 | 1036 | 902,5 | 15372 | 176,7 | 14,8 | 1,321 | HLA-DPA1 |
| 11833 | N1 | N2 | 977 | 714,1 | 14432 | 145,6 | 14,8 | 1,321 | CYBA |
| 15415 | C15 | C16 | 1066 | 777 | 15737 | 157,2 | 14,8 | 1,345 | RINT1 |
| 15467 | E19 | E20 | 1114 | 790,7 | 16525 | 156,5 | 14,8 | 1,345 | C14orf133 |
| 5832 | C9 | C10 | 1006 | 810,1 | 14862 | 667,8 | 14,8 | 1,352 | ACAA1 |
| 5844 | D2 | D3 | 1770 | 817,1 | 26200 | 648,6 | 14,8 | 1,352 | BFSP1 |
| 5891 | F19 | F20 | 2025 | 821,4 | 29960 | 596,7 | 14,8 | 1,352 | ANGPT1 |
| 5902 | F7 | F8 | 1004 | 796,7 | 14826 | 472,3 | 14,8 | 1,352 | AMPD3 |
| 5923 | G4 | G5 | 839 | 733,7 | 12379 | 615,4 | 14,8 | 1,352 | ASCL1 |
| 5997 | J6 | J7 | 1336 | 696 | 19727 | 511,1 | 14,8 | 1,352 | MPPED2 |
| 6053 | M13 | M14 | 906 | 767,1 | 13367 | 281,9 | 14,8 | 1,352 | AGER |
| 6085 | N20 | N21 | 1363 | 790,4 | 20184 | 518,6 | 14,8 | 1,352 | SERPINA6 |
| 1417 | L1 | L2 | 1548 | 705,9 | 22915 | 401,7 | 14,8 | 1,358 | CLDN1 |
| 16257 | F17 | F18 | 1157 | 1031 | 17167 | 327,3 | 14,8 | 1,358 | KRTAP17-1 |
| 16319 | H8 | H9 | 946 | 1062 | 13984 | 269,9 | 14,8 | 1,358 | LYZL1 |
| 16371 | K11 | K12 | 1247 | 953,1 | 18478 | 357,7 | 14,8 | 1,358 | TTC25 |
| 16430 | M21 | M22 | 920 | 1094 | 13578 | 230,8 | 14,8 | 1,358 | FAM103A1 |
| 16487 | O8 | O9 | 1146 | 1051 | 16906 | 450,5 | 14,8 | 1,358 | DYDC2 |
| 13694 | K21 | K22 | 1273 | 1026 | 18780 | 196 | 14,8 | 1,39 | CBX7 |
| 772 | A12 | A13 | 1251 | 683,6 | 18488 | 522,8 | 14,8 | 1,403 | SMAD1 |
| 913 | G1 | G2 | 1284 | 695,9 | 19009 | 424,6 | 14,8 | 1,403 | CALU |
| 943 | H15 | H16 | 1665 | 733,3 | 24586 | 379,6 | 14,8 | 1,403 | HLA-A |
| 1139 | P19 | P20 | 1501 | 745,2 | 22279 | 403,4 | 14,8 | 1,403 | LAMC2 |
| 5451 | D11 | D12 | 1492 | 742,3 | 22058 | 542,2 | 14,8 | 1,41 | AOC3 |
| 5533 | G20 | G21 | 1657 | 727,1 | 24533 | 458,7 | 14,8 | 1,41 | CPNE1 |
| 3548 | D5 | D6 | 1279 | 1388 | 18868 | 414 | 14,8 | 1,423 | GPR20 |
| 8280 | I9 | I10 | 1269 | 570,7 | 18720 | 465 | 14,8 | 1,423 | TREH |
| 8374 | M7 | M8 | 1294 | 582 | 19139 | 527 | 14,8 | 1,423 | POLG2 |
| 1547 | A19 | A20 | 1431 | 753,6 | 21172 | 478,9 | 14,8 | 1,55 | ZNF345 |
| 1572 | B2 | B3 | 1319 | 756,8 | 19568 | 517,7 | 14,8 | 1,55 | ZNF329 |
| 1610 | D10 | D11 | 1568 | 735,5 | 23141 | 337,8 | 14,8 | 1,55 | ZNF696 |
| 1705 | H1 | H2 | 1243 | 731,3 | 18383 | 451,3 | 14,8 | 1,55 | CRTAC1 |
| 1780 | K12 | K13 | 962 | 709,2 | 14209 | 384,3 | 14,8 | 1,55 | LEPRE1 |
| 1883 | O19 | O20 | 1585 | 794,9 | 23536 | 396,9 | 14,8 | 1,55 | SNX9 |
| 21642 | F3 | F4 | 222 | 1069 | 3283 | 595,4 | 14,8 | 1,558 | LOC65111 |
| 21849 | O17 | O18 | 229 | 1066 | 3384 | 604,9 | 14,8 | 1,558 | PP8961 |
| 4667 | C19 | C20 | 1433 | 826,7 | 21195 | 524,3 | 14,8 | 1,626 | PRCP |
| 10670 | M21 | M22 | 1043 | 818,2 | 15449 | 186,8 | 14,8 | 1,672 | MCFD2 |
| 10708 | O12 | O13 | 1574 | 899,7 | 23226 | 246,9 | 14,8 | 1,672 | GALNTL2 |
| 2737 | C1 | C2 | 503 | 625,6 | 7425 | 475,2 | 14,8 | 1,741 | IKBKG |
| 3067 | P4 | P5 | 532 | 818,5 | 7884 | 744,9 | 14,8 | 1,741 | CLTC |
| 20126 | G21 | G22 | 322 | 1184 | 4781 | 150,5 | 14,8 | 1,805 | LOC391370 |
| 20279 | M8 | M9 | 452 | 1046 | 6705 | 169,9 | 14,8 | 1,805 | C9orf152 |
| 22604 | N5 | N6 | 938 | 1476 | 13965 | 246,7 | 14,9 | 0,768 | LOC72896 |
| 14879 | L8 | L9 | 1216 | 860,6 | 18110 | 134,3 | 14,9 | 0,849 | IL26 |
| 14148 | N2 | N3 | 1532 | 1016 | 22842 | 111,5 | 14,9 | 0,854 | SHISA5 |
| 3081 | A17 | A18 | 1549 | 1394 | 23113 | 387,8 | 14,9 | 0,923 | G6PC3 |
| 3146 | D10 | D11 | 1219 | 1154 | 18195 | 369,3 | 14,9 | 0,923 | PDHB |
| 3223 | G15 | G16 | 1045 | 1189 | 15602 | 353,5 | 14,9 | 0,923 | HINT1 |
| 3266 | I10 | I11 | 1146 | 1284 | 17040 | 321,9 | 14,9 | 0,923 | PPP1R1B |
| 3310 | J7 | J8 | 1277 | 1216 | 19091 | 358,7 | 14,9 | 0,923 | MTMR1 |
| 3326 | K21 | K22 | 1160 | 1272 | 17336 | 410,4 | 14,9 | 0,923 | PPM1G |
| 3395 | N19 | N20 | 1259 | 1275 | 18791 | 377,3 | 14,9 | 0,923 | NUDT3 |
| 3410 | O10 | O11 | 833 | 1354 | 12394 | 490,8 | 14,9 | 0,923 | PCYT1A |
| 3411 | O11 | O12 | 1361 | 1192 | 20281 | 464,5 | 14,9 | 0,923 | PPP2R4 |
| 12078 | H14 | H15 | 1006 | 794,3 | 14944 | 97,05 | 14,9 | 0,955 | VPS52 |
| 19871 | L8 | L9 | 905 | 365,1 | 13478 | 400,1 | 14,9 | 0,971 | OR6M1 |
| 6221 | D13 | D14 | 1793 | 588,2 | 26791 | 450,4 | 14,9 | 0,983 | CHGA |
| 6343 | I15 | I16 | 649 | 718,3 | 9657 | 392,3 | 14,9 | 0,983 | CDC42 |
| 6367 | J15 | J16 | 1730 | 562 | 25797 | 376,1 | 14,9 | 0,983 | COX6B1 |
| 20427 | D11 | D12 | 849 | 724,2 | 12643 | 191 | 14,9 | 0,993 | LOC441344 |
| 20435 | D19 | D20 | 1005 | 810,7 | 15022 | 217,8 | 14,9 | 0,993 | LOC441370 |
| 20505 | G17 | G18 | 1193 | 744,5 | 17786 | 171,4 | 14,9 | 0,993 | LOC440057 |
| 20514 | G3 | G4 | 774 | 758,6 | 11525 | 145,6 | 14,9 | 0,993 | LOC440490 |
| 20545 | I1 | I2 | 768 | 795,9 | 11427 | 173,6 | 14,9 | 0,993 | LOC440589 |
| 20639 | L8 | L9 | 931 | 753,4 | 13836 | 221,2 | 14,9 | 0,993 | LOC642451 |
| 20659 | M4 | M5 | 869 | 765,2 | 12973 | 170,7 | 14,9 | 0,993 | TMEM75 |
| 20678 | N21 | N22 | 965 | 804,9 | 14386 | 175,6 | 14,9 | 0,993 | OR10C1 |
| 20694 | O14 | O15 | 894 | 846,3 | 13350 | 297 | 14,9 | 0,993 | LOC641808 |
| 18636 | I2 | I3 | 1128 | 936,1 | 16782 | 327,1 | 14,9 | 1,01 | GAS2L2 |
| 17503 | J15 | J16 | 1242 | 976,3 | 18496 | 235,8 | 14,9 | 1,017 | BBS5 |
| 17601 | N17 | N18 | 1431 | 934,2 | 21302 | 205 | 14,9 | 1,017 | MTERFD2 |
| 9351 | F22 | F23 | 1452 | 1066 | 21593 | 150 | 14,9 | 1,035 | AGPAT4 |
| 9359 | F8 | F9 | 1660 | 1098 | 24708 | 184,5 | 14,9 | 1,035 | ZC3HAV1 |
| 9570 | O3 | O4 | 1672 | 1066 | 24953 | 180,4 | 14,9 | 1,035 | TYW1 |
| 18841 | B1 | B2 | 1278 | 1073 | 18995 | 350,3 | 14,9 | 1,038 | C12orf36 |
| 18853 | B20 | B21 | 1572 | 1064 | 23422 | 247,5 | 14,9 | 1,038 | PRRT3 |
| 18860 | B5 | B6 | 763 | 991,6 | 11397 | 344,4 | 14,9 | 1,038 | LOC283440 |
| 9755 | G19 | G20 | 2150 | 1178 | 32034 | 173,5 | 14,9 | 1,042 | MTA3 |
| 15156 | H2 | H3 | 1148 | 749,3 | 17143 | 117,4 | 14,9 | 1,042 | TNRC6C |
| 8468 | A5 | A6 | 1643 | 1084 | 24557 | 196,1 | 14,9 | 1,053 | MKRN1 |
| 19431 | J22 | J23 | 1120 | 1153 | 16647 | 278 | 14,9 | 1,057 | USP50 |
| 19517 | N13 | N14 | 962 | 1128 | 14360 | 226,7 | 14,9 | 1,057 | OR6C4 |
| 20790 | C14 | C15 | 1111 | 1159 | 16524 | 326,6 | 14,9 | 1,057 | DRGX |
| 20830 | D7 | D8 | 828 | 973,8 | 12325 | 342,3 | 14,9 | 1,057 | LOC643554 |
| 20870 | F21 | F22 | 1020 | 969,7 | 15183 | 227,8 | 14,9 | 1,057 | LOC643684 |
| 20875 | F4 | F5 | 1018 | 970,2 | 15191 | 196,6 | 14,9 | 1,057 | LOC644885 |
| 20891 | G19 | G20 | 1617 | 936 | 24154 | 224,9 | 14,9 | 1,057 | LOC643081 |
| 20940 | I2 | I3 | 1059 | 1020 | 15779 | 292,8 | 14,9 | 1,057 | LOC644397 |
| 21199 | D15 | D16 | 1425 | 1049 | 21274 | 326,5 | 14,9 | 1,057 | LOC646051 |
| 11268 | F2 | F3 | 1612 | 866,5 | 23988 | 136,2 | 14,9 | 1,08 | |
| 11313 | H17 | H18 | 1168 | 738,7 | 17396 | 129,6 | 14,9 | 1,08 | GSTK1 |
| 11372 | J5 | J6 | 969 | 862,5 | 14470 | 133,6 | 14,9 | 1,08 | KRT77 |
| 12589 | M20 | M21 | 1304 | 1081 | 19404 | 169,4 | 14,9 | 1,12 | SOCS1 |
| 10866 | E3 | E4 | 432 | 927,8 | 6429 | 677,4 | 14,9 | 1,129 | STARD4 |
| 7597 | M20 | M21 | 1590 | 596,7 | 23644 | 454,9 | 14,9 | 1,137 | LRIG2 |
| 7631 | N8 | N9 | 753 | 613,6 | 11245 | 632,8 | 14,9 | 1,137 | ACTR2 |
| 16959 | C22 | C23 | 1349 | 977,9 | 20037 | 267,8 | 14,9 | 1,151 | C10orf4 |
| 17006 | E21 | E22 | 1575 | 1002 | 23502 | 227,1 | 14,9 | 1,151 | FNIP1 |
| 17028 | F2 | F3 | 1461 | 864,6 | 21762 | 149,2 | 14,9 | 1,151 | NIPA1 |
| 17208 | M9 | M10 | 1361 | 920,9 | 20309 | 176 | 14,9 | 1,151 | CMTM1 |
| 6936 | A9 | A10 | 1154 | 614,3 | 17150 | 550,3 | 14,9 | 1,16 | L1CAM |
| 7113 | I17 | I18 | 722 | 576,2 | 10726 | 457,8 | 14,9 | 1,16 | LPO |
| 7189 | L20 | L21 | 965 | 584,4 | 14378 | 411,3 | 14,9 | 1,16 | NIT1 |
| 7231 | N15 | N16 | 1325 | 630,5 | 19757 | 235,5 | 14,9 | 1,16 | MOCS2 |
| 5040 | B9 | B10 | 1609 | 633,8 | 23977 | 512,8 | 14,9 | 1,169 | MPN2 |
| 5090 | E10 | E11 | 1621 | 590,9 | 24076 | 579,7 | 14,9 | 1,169 | TRO |
| 5144 | G16 | G17 | 803 | 591,2 | 12000 | 462,5 | 14,9 | 1,169 | TTPA |
| 5231 | J8 | J9 | 1212 | 593,9 | 18085 | 477,1 | 14,9 | 1,169 | XRCC5 |
| 5306 | N10 | N11 | 1781 | 600,1 | 26576 | 539,2 | 14,9 | 1,169 | RNF113A |
| 5348 | O5 | O6 | 493 | 686,6 | 7357 | 418,1 | 14,9 | 1,169 | ADAM32 |
| 2487 | H22 | H23 | 1074 | 667,9 | 16022 | 346,8 | 14,9 | 1,173 | PTK7 |
| 2671 | P15 | P16 | 1518 | 682,1 | 22692 | 413,2 | 14,9 | 1,173 | GCKR |
| 16543 | B15 | B16 | 1388 | 1008 | 20640 | 319,5 | 14,9 | 1,178 | MICALL1 |
| 16669 | G20 | G21 | 1273 | 898,8 | 18919 | 242,1 | 14,9 | 1,178 | SPOCD1 |
| 16690 | H18 | H19 | 1341 | 920,6 | 19956 | 287,7 | 14,9 | 1,178 | TMEM129 |
| 16862 | O21 | O22 | 1081 | 940,9 | 16115 | 373,4 | 14,9 | 1,178 | FBF1 |
| 17760 | D9 | D10 | 1291 | 1078 | 19193 | 167,3 | 14,9 | 1,178 | LOC14585 |
| 17773 | E20 | E21 | 1808 | 1064 | 26944 | 276,6 | 14,9 | 1,178 | KNCN |
| 17813 | G13 | G14 | 1324 | 1075 | 19668 | 279,4 | 14,9 | 1,178 | FAM76B |
| 17816 | G16 | G17 | 1252 | 1013 | 18667 | 207,9 | 14,9 | 1,178 | MANEAL |
| 17820 | G2 | G3 | 854 | 1052 | 12685 | 197,3 | 14,9 | 1,178 | LELP1 |
| 17846 | H21 | H22 | 1300 | 1057 | 19384 | 392,2 | 14,9 | 1,178 | HRNBP3 |
| 17958 | M14 | M15 | 1201 | 1036 | 17871 | 224,6 | 14,9 | 1,178 | IGSF5 |
| 18008 | O16 | O17 | 867 | 982,8 | 12913 | 320,1 | 14,9 | 1,178 | MEI1 |
| 8855 | A8 | A9 | 1934 | 1890 | 28913 | 209,6 | 14,9 | 1,197 | PEX5L |
| 8888 | C16 | C17 | 1133 | 1739 | 16859 | 174,4 | 14,9 | 1,197 | DPH5 |
| 4300 | D12 | D13 | 1263 | 766,2 | 18866 | 469,8 | 14,9 | 1,257 | KCTD17 |
| 4397 | H13 | H14 | 989 | 671,5 | 14755 | 369,5 | 14,9 | 1,257 | KCNH1 |
| 4398 | H14 | H15 | 969 | 681 | 14446 | 400,8 | 14,9 | 1,257 | HTR3D |
| 4440 | I9 | I10 | 997 | 755,9 | 14831 | 440 | 14,9 | 1,257 | CHRNB1 |
| 4461 | J6 | J7 | 1172 | 642,9 | 17512 | 395,4 | 14,9 | 1,257 | SLC9A10 |
| 109 | E20 | E21 | 409 | 1495 | 6100 | 459,4 | 14,9 | 1,268 | SLC22A4 |
| 2028 | E2 | E3 | 772 | 624,5 | 11541 | 471,6 | 14,9 | 1,296 | DTX3 |
| 2067 | G11 | G12 | 1034 | 760,4 | 15439 | 445,6 | 14,9 | 1,296 | SNAG1 |
| 2118 | I14 | I15 | 1516 | 729,3 | 22552 | 400,3 | 14,9 | 1,296 | SH3PX3 |
| 2212 | M12 | M13 | 1544 | 648,4 | 22982 | 442,9 | 14,9 | 1,296 | ZNF660 |
| 6530 | A10 | A11 | 1086 | 682,6 | 16230 | 401 | 14,9 | 1,307 | IFIT1 |
| 6645 | E6 | E7 | 1131 | 592,9 | 16878 | 487,5 | 14,9 | 1,307 | IL2 |
| 6681 | G17 | G18 | 695 | 634,3 | 10327 | 456,8 | 14,9 | 1,307 | HBM |
| 6793 | L1 | L2 | 1127 | 583,9 | 16739 | 297,7 | 14,9 | 1,307 | HRAS |
| 6805 | L20 | L21 | 1124 | 567,2 | 16697 | 464,1 | 14,9 | 1,307 | KRT3 |
| 6901 | P20 | P21 | 1165 | 593 | 17385 | 465,1 | 14,9 | 1,307 | KRT82 |
| 3985 | G1 | G2 | 696 | 733,9 | 10349 | 478,9 | 14,9 | 1,324 | PLS3 |
| 4006 | G7 | G8 | 1038 | 729,7 | 15513 | 437,7 | 14,9 | 1,324 | PML |
| 11694 | H14 | H15 | 1287 | 780,9 | 19145 | 127,1 | 14,9 | 1,33 | HLA-C |
| 11700 | H2 | H3 | 1203 | 848,4 | 17898 | 130,3 | 14,9 | 1,33 | NCKAP1L |
| 11745 | J17 | J18 | 1317 | 819,1 | 19565 | 140,2 | 14,9 | 1,33 | CRYGD |
| 15390 | B14 | B15 | 1132 | 790,9 | 16881 | 141 | 14,9 | 1,355 | FAM134A |
| 15547 | H4 | H5 | 1083 | 769,3 | 16109 | 132,9 | 14,9 | 1,355 | OR4K5 |
| 15556 | I12 | I13 | 1169 | 872,3 | 17361 | 135,2 | 14,9 | 1,355 | SCNM1 |
| 15661 | M20 | M21 | 1124 | 834,3 | 16784 | 162,7 | 14,9 | 1,355 | FASTKD3 |
| 5771 | A19 | A20 | 1629 | 817,3 | 24213 | 620,1 | 14,9 | 1,361 | ABCA3 |
| 5836 | D12 | D13 | 1383 | 843,4 | 20603 | 514 | 14,9 | 1,361 | BIK |
| 5924 | G5 | G6 | 1142 | 750,8 | 17038 | 501,1 | 14,9 | 1,361 | ACTL6A |
| 5937 | H17 | H18 | 1057 | 710,7 | 15778 | 459 | 14,9 | 1,361 | ANXA3 |
| 5971 | I4 | I5 | 1430 | 751 | 21305 | 573,1 | 14,9 | 1,361 | ATBF1 |
| 6028 | L12 | L13 | 1553 | 813,8 | 23144 | 535 | 14,9 | 1,361 | SLC25A20 |
| 1155 | A11 | A12 | 799 | 670,6 | 11896 | 511,7 | 14,9 | 1,367 | ZIC1 |
| 1184 | B16 | B17 | 1030 | 734,1 | 15299 | 537 | 14,9 | 1,367 | RAI1 |
| 1197 | B6 | B7 | 1463 | 754,9 | 21773 | 542,9 | 14,9 | 1,367 | CCT6B |
| 1203 | C11 | C12 | 2017 | 807,5 | 30138 | 461,3 | 14,9 | 1,367 | ZNF708 |
| 1333 | H20 | H21 | 762 | 765,5 | 11388 | 485,8 | 14,9 | 1,367 | NID2 |
| 1394 | K10 | K11 | 915 | 721,5 | 13619 | 389,4 | 14,9 | 1,367 | SMYD5 |
| 1506 | O3 | O4 | 880 | 790,4 | 13069 | 529,5 | 14,9 | 1,367 | ZNF208 |
| 1526 | P21 | P22 | 1530 | 727,3 | 22866 | 488,1 | 14,9 | 1,367 | BAG5 |
| 16268 | F5 | F6 | 1218 | 1059 | 18184 | 300,8 | 14,9 | 1,367 | SPATA16 |
| 16475 | O19 | O20 | 1368 | 870,6 | 20364 | 398,2 | 14,9 | 1,367 | JAM3 |
| 13644 | I2 | I3 | 1129 | 1010 | 16853 | 177,9 | 14,9 | 1,399 | LOC26010 |
| 13775 | N8 | N9 | 794 | 1066 | 11792 | 176,2 | 14,9 | 1,399 | MYEOV |
| 776 | A16 | A17 | 1637 | 796,1 | 24409 | 541,2 | 14,9 | 1,412 | SMAD5 |
| 891 | F11 | F12 | 1466 | 744,5 | 21849 | 438,2 | 14,9 | 1,412 | GLI3 |
| 978 | I3 | I4 | 245 | 882,4 | 3641 | 492,5 | 14,9 | 1,412 | CDC20 |
| 1010 | K10 | K11 | 1269 | 744,4 | 18904 | 345,9 | 14,9 | 1,412 | PEG3 |
| 1069 | M20 | M21 | 1027 | 767 | 15351 | 399,2 | 14,9 | 1,412 | POU4F1 |
| 5665 | M1 | M2 | 947 | 659,7 | 14076 | 591,5 | 14,9 | 1,419 | DDO |
| 5670 | M14 | M15 | 1046 | 646,5 | 15605 | 488 | 14,9 | 1,419 | UBE1C |
| 5730 | O3 | O4 | 1230 | 668,9 | 18380 | 594,8 | 14,9 | 1,419 | TCAP |
| 3671 | I8 | I9 | 888 | 1284 | 13223 | 295,7 | 14,9 | 1,433 | CELSR1 |
| 3745 | M1 | M2 | 488 | 1349 | 7257 | 419,1 | 14,9 | 1,433 | CELSR3 |
| 8100 | B2 | B3 | 1149 | 623,5 | 17173 | 515,5 | 14,9 | 1,433 | ARHGEF12 |
| 10311 | N22 | N23 | 1369 | 822,2 | 20438 | 197,7 | 14,9 | 1,433 | TXNL5 |
| 1590 | C14 | C15 | 1424 | 675,6 | 21211 | 451 | 14,9 | 1,56 | SYT13 |
| 1683 | G11 | G12 | 1318 | 715,3 | 19588 | 390,5 | 14,9 | 1,56 | KLF15 |
| 1858 | N18 | N19 | 1291 | 741 | 19285 | 534 | 14,9 | 1,56 | CAPS2 |
| 1885 | O20 | O21 | 1359 | 769,5 | 20307 | 421,9 | 14,9 | 1,56 | ZXDC |
| 21562 | C18 | C19 | 224 | 1103 | 3343 | 716,5 | 14,9 | 1,568 | LOC652580 |
| 21729 | J17 | J18 | 297 | 902,8 | 4429 | 630,4 | 14,9 | 1,568 | LOC651789 |
| 21863 | O8 | O9 | 233 | 895,3 | 3474 | 860,5 | 14,9 | 1,568 | LOC652879 |
| 21866 | P10 | P11 | 238 | 1177 | 3543 | 611,5 | 14,9 | 1,568 | LOC654342 |
| 4616 | A16 | A17 | 477 | 845,3 | 7101 | 579,9 | 14,9 | 1,637 | SHH |
| 4663 | C15 | C16 | 316 | 948 | 4722 | 623,3 | 14,9 | 1,637 | PSMB7 |
| 4801 | I1 | I2 | 470 | 760,6 | 7012 | 533,2 | 14,9 | 1,637 | CASP6 |
| 10416 | B9 | B10 | 606 | 964,2 | 9019 | 231,2 | 14,9 | 1,684 | ANKRD30A |
| 10484 | E5 | E6 | 649 | 917,1 | 9676 | 229,6 | 14,9 | 1,684 | RBM17 |

| | | | | | | | | | | |
|---|---|---|---|---|---|---|---|---|---|---|
| 2781 | D6 | D7 | 732 | 673,2 | 10895 | 559,8 | 14,9 | 1,753 | H5A034 C | sample |
| 2887 | I15 | I16 | 289 | 641,1 | 4295 | 406,4 | 14,9 | 1,753 | H5A034 C | sample |
| 22669 | A20 | A21 | 741 | 1911 | 11129 | 101,2 | 15 | 0,704 | H5A034 C | sample |
| 15864 | E9 | E10 | 1266 | 852,3 | 19048 | 137,7 | 15 | 0,836 | H5A034 C | sample |
| 3149 | D13 | D14 | 990 | 1220 | 14864 | 442,2 | 15 | 0,929 | H5A034 C | sample |
| 3189 | E6 | E7 | 903 | 1156 | 13513 | 384,2 | 15 | 0,929 | H5A034 C | sample |
| 3242 | H10 | H11 | 658 | 1228 | 9843 | 381,8 | 15 | 0,929 | H5A034 C | sample |
| 3265 | I1 | I2 | 569 | 1170 | 8516 | 406,4 | 15 | 0,929 | H5A034 C | sample |
| 3311 | J8 | J9 | 1255 | 1296 | 18768 | 377,5 | 15 | 0,929 | H5A034 C | sample |
| 3312 | J9 | J10 | 627 | 1142 | 9435 | 337,8 | 15 | 0,929 | H5A034 C | sample |
| 3398 | N21 | N22 | 1253 | 1313 | 18787 | 447,3 | 15 | 0,929 | H5A034 C | sample |
| 3441 | P17 | P18 | 1155 | 1254 | 17349 | 405,9 | 15 | 0,929 | H5A034 C | sample |
| 11961 | C17 | C18 | 1383 | 804,5 | 20746 | 99,71 | 15 | 0,962 | H5A034 C | sample |
| 12186 | L3 | L4 | 1102 | 709 | 16518 | 91,09 | 15 | 0,962 | H5A034 C | sample |
| 6373 | J20 | J21 | 1441 | 510,2 | 21674 | 453,7 | 15 | 0,99 | H5A034 C | sample |
| 6382 | J7 | J8 | 1311 | 618 | 19607 | 305,3 | 15 | 0,99 | H5A034 C | sample |
| 20500 | G12 | G13 | 1074 | 778,8 | 16086 | 199,3 | 15 | 1 | H5A034 C | sample |
| 20521 | H1 | H2 | 774 | 823,1 | 11606 | 164,4 | 15 | 1 | H5A034 C | sample |
| 20581 | J20 | J21 | 784 | 783,7 | 11755 | 187,6 | 15 | 1 | H5A034 C | sample |
| 20660 | M5 | M6 | 443 | 720 | 6635 | 153,4 | 15 | 1 | H5A034 C | sample |
| 20661 | M6 | M7 | 1029 | 722,5 | 15385 | 203,2 | 15 | 1 | H5A034 C | sample |
| 20707 | O4 | O5 | 690 | 781,5 | 10346 | 267,2 | 15 | 1 | H5A034 C | sample |
| 12934 | K7 | K8 | 960 | 981,1 | 14364 | 135,9 | 15 | 1,017 | H5A034 C | sample |
| 12961 | M1 | M2 | 1985 | 1089 | 29765 | 138 | 15 | 1,017 | H5A034 C | sample |
| 17384 | E16 | E17 | 1313 | 930,6 | 19745 | 188,6 | 15 | 1,024 | H5A034 C | sample |
| 17395 | E4 | E5 | 1023 | 903 | 15297 | 229,8 | 15 | 1,024 | H5A034 C | sample |
| 17410 | F18 | F19 | 1336 | 951,3 | 20060 | 190,8 | 15 | 1,024 | H5A034 C | sample |
| 17425 | G1 | G2 | 1110 | 976 | 16688 | 194,5 | 15 | 1,024 | H5A034 C | sample |
| 17521 | K1 | K2 | 974 | 943,8 | 14591 | 168,1 | 15 | 1,024 | H5A034 C | sample |
| 9290 | D10 | D11 | 1176 | 1039 | 17657 | 110,4 | 15 | 1,042 | H5A034 C | sample |
| 9455 | J8 | J9 | 1497 | 1053 | 22424 | 136,4 | 15 | 1,042 | H5A034 C | sample |
| 18871 | C15 | C16 | 1445 | 961,7 | 21615 | 269,9 | 15 | 1,045 | H5A034 C | sample |
| 18886 | C7 | C8 | 1023 | 1003 | 15297 | 331,7 | 15 | 1,045 | H5A034 C | sample |
| 19113 | M17 | M18 | 910 | 1017 | 13658 | 230,2 | 15 | 1,045 | H5A034 C | sample |
| 19127 | M8 | M9 | 1512 | 969,9 | 22637 | 306,6 | 15 | 1,045 | H5A034 C | sample |
| 9821 | J13 | J14 | 1603 | 1075 | 24073 | 119,2 | 15 | 1,049 | H5A034 C | sample |
| 15033 | C17 | C18 | 1384 | 788,7 | 20712 | 124,4 | 15 | 1,049 | H5A034 C | sample |
| 15130 | G18 | G19 | 1074 | 840,9 | 16106 | 126,3 | 15 | 1,049 | H5A034 C | sample |
| 8593 | G1 | G2 | 1586 | 1076 | 23774 | 121,9 | 15 | 1,06 | H5A034 C | sample |
| 19369 | H1 | H2 | 709 | 1166 | 10605 | 244,5 | 15 | 1,064 | H5A034 C | sample |
| 20826 | D3 | D4 | 1074 | 993,3 | 16136 | 217,5 | 15 | 1,064 | H5A034 C | sample |
| 20905 | H1 | H2 | 849 | 948,3 | 12763 | 191,7 | 15 | 1,064 | H5A034 C | sample |
| 20943 | I22 | I23 | 1116 | 958,5 | 16792 | 193,3 | 15 | 1,064 | H5A034 C | sample |
| 21028 | M12 | M13 | 1504 | 1023 | 22540 | 305,7 | 15 | 1,064 | H5A034 C | sample |
| 11448 | M9 | M10 | 1136 | 799,4 | 17028 | 137,2 | 15 | 1,087 | H5A034 C | sample |
| 12343 | C15 | C16 | 1372 | 1051 | 20541 | 147,9 | 15 | 1,128 | H5A034 C | sample |
| 12420 | F2 | F3 | 1443 | 1005 | 21714 | 149,1 | 15 | 1,128 | H5A034 C | sample |
| 12608 | N16 | N17 | 1319 | 1026 | 19729 | 177,4 | 15 | 1,128 | H5A034 C | sample |
| 10957 | I20 | I21 | 1755 | 847,4 | 26248 | 508,8 | 15 | 1,136 | H5A034 C | sample |
| 11121 | P17 | P18 | 900 | 781,6 | 13463 | 512,9 | 15 | 1,136 | H5A034 C | sample |
| 11126 | P21 | P22 | 1388 | 825,1 | 20805 | 624,6 | 15 | 1,136 | H5A034 C | sample |
| 7342 | B7 | B8 | 1430 | 599,3 | 21519 | 551,5 | 15 | 1,145 | H5A034 C | sample |
| 7616 | N16 | N17 | 1363 | 645,9 | 20487 | 277,4 | 15 | 1,145 | H5A034 C | sample |
| 7628 | N5 | N6 | 1025 | 624,2 | 15324 | 406 | 15 | 1,145 | H5A034 C | sample |
| 7651 | O4 | O5 | 946 | 584,3 | 14164 | 384,7 | 15 | 1,145 | H5A034 C | sample |
| 16979 | D19 | D20 | 1569 | 995,4 | 23463 | 184,6 | 15 | 1,158 | H5A034 C | sample |
| 17101 | I20 | I21 | 1557 | 990,9 | 23368 | 211,5 | 15 | 1,158 | H5A034 C | sample |
| 6973 | C20 | C21 | 1407 | 600 | 21042 | 462,4 | 15 | 1,167 | H5A034 C | sample |
| 7137 | J17 | J18 | 1511 | 583,1 | 22667 | 427,9 | 15 | 1,167 | H5A034 C | sample |
| 5000 | A16 | A17 | 955 | 606 | 14370 | 641,7 | 15 | 1,176 | H5A034 C | sample |
| 5253 | K6 | K7 | 1374 | 614,7 | 20646 | 541,2 | 15 | 1,176 | H5A034 C | sample |
| 2429 | F13 | F14 | 1241 | 730,6 | 18652 | 378,4 | 15 | 1,181 | H5A034 C | sample |
| 2430 | F14 | F15 | 1194 | 703,2 | 17922 | 455 | 15 | 1,181 | H5A034 C | sample |
| 2667 | P11 | P12 | 1528 | 714,2 | 22981 | 483 | 15 | 1,181 | H5A034 C | sample |
| 16620 | E2 | E3 | 1086 | 971,3 | 16261 | 315,5 | 15 | 1,186 | H5A034 C | sample |
| 16738 | J18 | J19 | 1462 | 1048 | 21964 | 240,1 | 15 | 1,186 | H5A034 C | sample |
| 17714 | C10 | C11 | 1081 | 1048 | 16195 | 368,6 | 15 | 1,186 | H5A034 C | sample |
| 17754 | D3 | D4 | 1342 | 1025 | 20112 | 264,1 | 15 | 1,186 | H5A034 C | sample |
| 17789 | F13 | F14 | 1696 | 1076 | 25490 | 235,2 | 15 | 1,186 | H5A034 C | sample |
| 17796 | F2 | F3 | 1146 | 993,6 | 17242 | 236,3 | 15 | 1,186 | H5A034 C | sample |
| 17949 | L6 | L7 | 990 | 1001 | 14806 | 264 | 15 | 1,186 | H5A034 C | sample |
| 8843 | A19 | A20 | 2132 | 1966 | 31962 | 226,5 | 15 | 1,205 | H5A034 C | sample |
| 8857 | B1 | B2 | 1531 | 1972 | 23022 | 153,7 | 15 | 1,205 | H5A034 C | sample |
| 8998 | G7 | G8 | 1700 | 1803 | 25464 | 141,8 | 15 | 1,205 | H5A034 C | sample |
| 9023 | H8 | H9 | 1517 | 1576 | 22773 | 138,7 | 15 | 1,205 | H5A034 C | sample |
| 9026 | I10 | I11 | 1076 | 1525 | 16167 | 159,1 | 15 | 1,205 | H5A034 C | sample |
| 9158 | N21 | N22 | 2211 | 1905 | 33102 | 207,5 | 15 | 1,205 | H5A034 C | sample |
| 4441 | J1 | J2 | 1119 | 639,3 | 16752 | 437,3 | 15 | 1,266 | H5A034 C | sample |
| 4546 | N18 | N19 | 1252 | 720,5 | 18762 | 442,5 | 15 | 1,266 | H5A034 C | sample |
| 4562 | O10 | O11 | 1436 | 711,2 | 21582 | 588,5 | 15 | 1,266 | H5A034 C | sample |
| 4598 | P21 | P22 | 1396 | 699,3 | 20875 | 498,9 | 15 | 1,266 | H5A034 C | sample |
| 58 | C18 | C19 | 592 | 1606 | 8855 | 439,2 | 15 | 1,277 | H5A034 C | sample |
| 341 | O13 | O14 | 638 | 1589 | 9599 | 580,4 | 15 | 1,277 | H5A034 C | sample |
| 2048 | F16 | F17 | 722 | 563,8 | 10800 | 385,2 | 15 | 1,304 | H5A034 C | sample |
| 2121 | I17 | I18 | 1333 | 653 | 19976 | 304,7 | 15 | 1,304 | H5A034 C | sample |
| 2133 | I6 | I7 | 1713 | 701,1 | 25724 | 383,2 | 15 | 1,304 | H5A034 C | sample |
| 2134 | I7 | I8 | 1103 | 608,5 | 16507 | 428,9 | 15 | 1,304 | H5A034 C | sample |
| 2210 | M10 | M11 | 1273 | 653,3 | 19105 | 398,2 | 15 | 1,304 | H5A034 C | sample |
| 6618 | D3 | D4 | 880 | 574,5 | 13177 | 493,1 | 15 | 1,316 | H5A034 C | sample |
| 4163 | N19 | N20 | 1449 | 696,7 | 21668 | 395,1 | 15 | 1,333 | H5A034 C | sample |
| 4190 | O21 | O22 | 1151 | 750,4 | 17289 | 499,5 | 15 | 1,333 | H5A034 C | sample |
| 11591 | C8 | C9 | 1227 | 855,1 | 18380 | 140,7 | 15 | 1,339 | H5A034 C | sample |
| 11699 | H19 | H20 | 1269 | 843,5 | 19045 | 178,3 | 15 | 1,339 | H5A034 C | sample |
| 11746 | J18 | J19 | 1207 | 865,4 | 18139 | 158,7 | 15 | 1,339 | H5A034 C | sample |
| 15578 | J10 | J11 | 1121 | 809,1 | 16796 | 134,6 | 15 | 1,364 | H5A034 C | sample |
| 15605 | K13 | K14 | 1008 | 799,4 | 15128 | 156,1 | 15 | 1,364 | H5A034 C | sample |
| 5828 | C5 | C6 | 1155 | 759,1 | 17353 | 589,5 | 15 | 1,37 | H5A034 C | sample |
| 1315 | G4 | G5 | 799 | 754,1 | 11946 | 459,2 | 15 | 1,376 | H5A034 C | sample |
| 1389 | J6 | J7 | 1414 | 726,9 | 21256 | 391,6 | 15 | 1,376 | H5A034 C | sample |
| 1464 | M9 | M10 | 1100 | 743,1 | 16480 | 394,8 | 15 | 1,376 | H5A034 C | sample |
| 1488 | N9 | N10 | 1515 | 712,4 | 22712 | 443,1 | 15 | 1,376 | H5A034 C | sample |
| 16133 | A13 | A14 | 1135 | 950,7 | 17057 | 436,7 | 15 | 1,376 | H5A034 C | sample |
| 16173 | B6 | B7 | 904 | 1059 | 13556 | 370,7 | 15 | 1,376 | H5A034 C | sample |
| 16378 | K18 | K19 | 1181 | 976,5 | 17712 | 326 | 15 | 1,376 | H5A034 C | sample |
| 16379 | K19 | K20 | 915 | 1015 | 13734 | 333,4 | 15 | 1,376 | H5A034 C | sample |
| 16438 | M7 | M8 | 1216 | 1077 | 18181 | 386,3 | 15 | 1,376 | H5A034 C | sample |
| 16446 | N14 | N15 | 964 | 969,3 | 14416 | 420,5 | 15 | 1,376 | H5A034 C | sample |
| 16476 | O2 | O3 | 1089 | 1000 | 16312 | 471,5 | 15 | 1,376 | H5A034 C | sample |
| 13545 | E17 | E18 | 1275 | 993 | 19072 | 171,9 | 15 | 1,408 | H5A034 C | sample |
| 774 | A14 | A15 | 1588 | 806,9 | 23870 | 510,4 | 15 | 1,422 | H5A034 C | sample |
| 871 | E15 | E16 | 1369 | 719,4 | 20469 | 421,8 | 15 | 1,422 | H5A034 C | sample |
| 1054 | L7 | L8 | 986 | 622,1 | 14832 | 370,8 | 15 | 1,422 | H5A034 C | sample |
| 1079 | M8 | M9 | 945 | 831,5 | 14157 | 458,1 | 15 | 1,422 | H5A034 C | sample |
| 5429 | C13 | C14 | 1086 | 672,3 | 16247 | 741,6 | 15 | 1,429 | H5A034 C | sample |
| 5472 | D9 | D10 | 1403 | 673,8 | 21012 | 573 | 15 | 1,429 | H5A034 C | sample |
| 5546 | H10 | H11 | 1553 | 696,7 | 23318 | 365,2 | 15 | 1,429 | H5A034 C | sample |
| 5619 | K11 | K12 | 1094 | 654,5 | 16410 | 602,4 | 15 | 1,429 | H5A034 C | sample |
| 3673 | J1 | J2 | 1202 | 1354 | 18065 | 427,4 | 15 | 1,442 | H5A034 C | sample |
| 3769 | N1 | N2 | 1248 | 1428 | 18696 | 454 | 15 | 1,442 | H5A034 C | sample |
| 8306 | K10 | K11 | 1040 | 597,1 | 15603 | 551,4 | 15 | 1,442 | H5A034 C | sample |
| 8349 | L6 | L7 | 1354 | 564,3 | 20339 | 471,4 | 15 | 1,442 | H5A034 C | sample |
| 9991 | A15 | A16 | 1757 | 883,7 | 26366 | 222,4 | 15 | 1,442 | H5A034 C | sample |
| 10018 | B18 | B19 | 1909 | 930,2 | 28557 | 193,1 | 15 | 1,442 | H5A034 C | sample |
| 21514 | A18 | A19 | 168 | 1083 | 2526 | 743,4 | 15 | 1,579 | H5A034 C | sample |
| 21515 | A19 | A20 | 133 | 1166 | 1997 | 736,3 | 15 | 1,579 | H5A034 C | sample |
| 21661 | G20 | G21 | 408 | 976,1 | 6139 | 551,8 | 15 | 1,579 | H5A034 C | sample |
| 21691 | H4 | H5 | 290 | 948 | 4354 | 602,9 | 15 | 1,579 | H5A034 C | sample |
| 4613 | A13 | A14 | 1264 | 876,6 | 18903 | 577,1 | 15 | 1,648 | H5A034 C | sample |
| 4708 | E12 | E13 | 1158 | 767,7 | 17370 | 427,8 | 15 | 1,648 | H5A034 C | sample |
| 10511 | F8 | F9 | 949 | 819,4 | 14241 | 196,3 | 15 | 1,695 | H5A034 C | sample |
| 10544 | H16 | H17 | 1043 | 872,4 | 15667 | 179,4 | 15 | 1,695 | H5A034 C | sample |
| 10741 | P20 | P21 | 1174 | 767,9 | 17608 | 206,3 | 15 | 1,695 | H5A034 C | sample |
| 454 | C7 | C8 | 997 | 1929 | 14995 | 300,4 | 15 | 2,19 | H5A034 C | sample |
| 7711 | B15 | B16 | 645 | 692,3 | 9703 | 410,5 | 15 | 2,29 | H5A034 C | sample |
| 22329 | C17 | C18 | 904 | 1373 | 13621 | 384,6 | 15,1 | 0,778 | H5A034 C | sample |
| 23604 | H2 | H3 | 1362 | 1529 | 20613 | 175,1 | 15,1 | 0,801 | H5A034 C | sample |
| 14937 | O17 | O18 | 1605 | 810,7 | 24190 | 110,8 | 15,1 | 0,86 | H5A034 C | sample |
| 12101 | I13 | I14 | 1288 | 784,3 | 19507 | 92,88 | 15,1 | 0,968 | H5A034 C | sample |
| 18090 | B3 | B4 | 1097 | 1004 | 16529 | 350,8 | 15,1 | 0,971 | H5A034 C | sample |
| 18207 | G22 | G23 | 1587 | 1002 | 24017 | 317 | 15,1 | 0,971 | H5A034 C | sample |
| 6213 | C6 | C7 | 971 | 507,2 | 14663 | 523,6 | 15,1 | 0,997 | H5A034 C | sample |
| 6225 | D17 | D18 | 1284 | 593,8 | 19364 | 419 | 15,1 | 0,997 | H5A034 C | sample |
| 6299 | G19 | G20 | 1175 | 620,8 | 17766 | 443,3 | 15,1 | 0,997 | H5A034 C | sample |
| 6426 | L3 | L4 | 1758 | 619,2 | 26493 | 360,8 | 15,1 | 0,997 | H5A034 C | sample |
| 20378 | B10 | B11 | 718 | 811,4 | 10874 | 275,2 | 15,1 | 1,007 | H5A034 C | sample |
| 20462 | E21 | E22 | 1082 | 789,6 | 16342 | 223,4 | 15,1 | 1,007 | H5A034 C | sample |
| 20475 | F11 | F12 | 722 | 758,7 | 10922 | 185,9 | 15,1 | 1,007 | H5A034 C | sample |
| 20495 | F8 | F9 | 670 | 775,3 | 10121 | 211,7 | 15,1 | 1,007 | H5A034 C | sample |
| 18513 | D17 | D18 | 1445 | 957,1 | 21874 | 250,9 | 15,1 | 1,024 | H5A034 C | sample |
| 18732 | M2 | M3 | 1077 | 876,5 | 16290 | 126 | 15,1 | 1,024 | H5A034 C | sample |
| 18734 | M21 | M22 | 1168 | 965,9 | 17619 | 195,2 | 15,1 | 1,024 | H5A034 C | sample |
| 17334 | C14 | C15 | 1521 | 971,6 | 22923 | 247,5 | 15,1 | 1,031 | H5A034 C | sample |
| 17427 | G11 | G12 | 1195 | 1013 | 17999 | 128,3 | 15,1 | 1,031 | H5A034 C | sample |
| 17455 | H15 | H16 | 785 | 986,6 | 11876 | 286,9 | 15,1 | 1,031 | H5A034 C | sample |
| 17579 | M19 | M20 | 1501 | 1065 | 22737 | 174 | 15,1 | 1,031 | H5A034 C | sample |
| 17655 | P22 | P23 | 1260 | 1018 | 18969 | 248,5 | 15,1 | 1,031 | H5A034 C | sample |
| 9372 | G2 | G3 | 1643 | 1162 | 24875 | 146 | 15,1 | 1,049 | H5A034 C | sample |
| 9380 | G5 | G6 | 1404 | 1084 | 21167 | 187,7 | 15,1 | 1,049 | H5A034 C | sample |
| 9468 | K2 | K3 | 1110 | 1092 | 16746 | 146,7 | 15,1 | 1,049 | H5A034 C | sample |
| 19137 | N17 | N18 | 1439 | 909,6 | 21742 | 173,5 | 15,1 | 1,052 | H5A034 C | sample |
| 9777 | H17 | H18 | 1906 | 1114 | 28826 | 126,9 | 15,1 | 1,056 | H5A034 C | sample |
| 14998 | A7 | A8 | 1986 | 899,5 | 30084 | 250,3 | 15,1 | 1,056 | H5A034 C | sample |
| 15240 | K9 | K10 | 828 | 745 | 12533 | 146,7 | 15,1 | 1,056 | H5A034 C | sample |
| 19239 | B22 | B23 | 1210 | 1120 | 18291 | 352,1 | 15,1 | 1,071 | H5A034 C | sample |
| 19242 | B3 | B4 | 1061 | 1188 | 16059 | 270,2 | 15,1 | 1,071 | H5A034 C | sample |
| 19455 | K22 | K23 | 1309 | 1250 | 19776 | 288,7 | 15,1 | 1,071 | H5A034 C | sample |
| 20782 | B7 | B8 | 1122 | 1002 | 16929 | 403,4 | 15,1 | 1,071 | H5A034 C | sample |
| 20869 | F20 | F21 | 1040 | 1024 | 15670 | 260,5 | 15,1 | 1,071 | H5A034 C | sample |
| 20946 | I3 | I4 | 1341 | 946,3 | 20246 | 236,1 | 15,1 | 1,071 | H5A034 C | sample |
| 21055 | N15 | N16 | 1231 | 1013 | 18595 | 262,5 | 15,1 | 1,071 | H5A034 C | sample |
| 21066 | N3 | N4 | 1184 | 973,9 | 17858 | 167,8 | 15,1 | 1,071 | H5A034 C | sample |
| 21119 | P8 | P9 | 1086 | 1119 | 16368 | 357,6 | 15,1 | 1,071 | H5A034 C | sample |
| 21423 | M22 | M23 | 1408 | 1053 | 21313 | 313,6 | 15,1 | 1,071 | H5A034 C | sample |
| 21437 | N13 | N14 | 1281 | 940 | 19366 | 271 | 15,1 | 1,071 | H5A034 C | sample |
| 21442 | N18 | N19 | 1296 | 898,1 | 19605 | 266,1 | 15,1 | 1,071 | H5A034 C | sample |
| 14364 | G2 | G3 | 694 | 778,2 | 10500 | 135,6 | 15,1 | 1,075 | H5A034 C | sample |
| 11175 | B22 | B23 | 1731 | 916,2 | 26205 | 188,4 | 15,1 | 1,094 | H5A034 C | sample |
| 12335 | B8 | B9 | 1438 | 1068 | 21745 | 197,4 | 15,1 | 1,135 | H5A034 C | sample |
| 12375 | D22 | D23 | 1124 | 1025 | 16968 | 172,1 | 15,1 | 1,135 | H5A034 C | sample |
| 12486 | I14 | I15 | 1785 | 950,8 | 26946 | 151,4 | 15,1 | 1,135 | H5A034 C | sample |
| 7647 | O22 | O23 | 1647 | 611,7 | 24884 | 478,5 | 15,1 | 1,153 | H5A034 C | sample |
| 16928 | B16 | B17 | 1794 | 1155 | 27016 | 213 | 15,1 | 1,166 | H5A034 C | sample |
| 17022 | F14 | F15 | 1592 | 915,6 | 24003 | 199,4 | 15,1 | 1,166 | H5A034 C | sample |
| 17102 | I21 | I22 | 1209 | 864,8 | 18217 | 166,2 | 15,1 | 1,166 | H5A034 C | sample |
| 17142 | K14 | K15 | 1543 | 958,8 | 23235 | 222 | 15,1 | 1,166 | H5A034 C | sample |
| 17263 | P15 | P16 | 1032 | 1120 | 15613 | 268,3 | 15,1 | 1,166 | H5A034 C | sample |
| 7173 | K6 | K7 | 1377 | 548,6 | 20754 | 467,4 | 15,1 | 1,175 | H5A034 C | sample |
| 7266 | O3 | O4 | 974 | 577,1 | 14717 | 418 | 15,1 | 1,175 | H5A034 C | sample |
| 7292 | P5 | P6 | 1433 | 548 | 21603 | 555,7 | 15,1 | 1,175 | H5A034 C | sample |
| 5085 | D6 | D7 | 1296 | 660 | 19551 | 608,6 | 15,1 | 1,184 | H5A034 C | sample |
| 5213 | J13 | J14 | 1740 | 597,8 | 26240 | 469,1 | 15,1 | 1,184 | H5A034 C | sample |
| 5217 | J17 | J18 | 1898 | 606,6 | 28593 | 452,4 | 15,1 | 1,184 | H5A034 C | sample |
| 2519 | I8 | I9 | 1230 | 757,1 | 18539 | 336,8 | 15,1 | 1,189 | H5A034 C | sample |
| 2542 | J7 | J8 | 978 | 647,3 | 14771 | 274,8 | 15,1 | 1,189 | H5A034 C | sample |
| 2659 | O4 | O5 | 1133 | 655,7 | 17129 | 444 | 15,1 | 1,189 | H5A034 C | sample |
| 16525 | A20 | A21 | 1234 | 1125 | 18694 | 391,7 | 15,1 | 1,194 | H5A034 C | sample |
| 16578 | C3 | C4 | 669 | 957,9 | 10112 | 226,4 | 15,1 | 1,194 | H5A034 C | sample |
| 16651 | F4 | F5 | 1183 | 900,9 | 17918 | 280,6 | 15,1 | 1,194 | H5A034 C | sample |
| 16656 | F9 | F10 | 842 | 932,7 | 12739 | 231,9 | 15,1 | 1,194 | H5A034 C | sample |
| 16674 | G3 | G4 | 1185 | 974,1 | 17943 | 241,8 | 15,1 | 1,194 | H5A034 C | sample |
| 16832 | N16 | N17 | 1471 | 945,4 | 22146 | 286,5 | 15,1 | 1,194 | H5A034 C | sample |
| 17706 | B3 | B4 | 1182 | 1001 | 17845 | 280,8 | 15,1 | 1,194 | H5A034 C | sample |
| 17733 | C6 | C7 | 1002 | 1033 | 15093 | 401,8 | 15,1 | 1,194 | H5A034 C | sample |
| 17904 | J9 | J10 | 1121 | 983,7 | 16941 | 261,7 | 15,1 | 1,194 | H5A034 C | sample |
| 17918 | K21 | K22 | 1360 | 970,9 | 20597 | 314,6 | 15,1 | 1,194 | H5A034 C | sample |
| 8933 | E13 | E14 | 1924 | 1867 | 29011 | 159,5 | 15,1 | 1,213 | H5A034 C | sample |
| 9078 | K14 | K15 | 2059 | 1969 | 31113 | 146,8 | 15,1 | 1,213 | H5A034 C | sample |
| 4305 | D17 | D18 | 379 | 805,5 | 5721 | 374,5 | 15,1 | 1,274 | H5A034 C | sample |
| 4306 | D18 | D19 | 1368 | 715,9 | 20660 | 424 | 15,1 | 1,274 | H5A034 C | sample |
| 4308 | D2 | D3 | 1196 | 714,1 | 18044 | 453,8 | 15,1 | 1,274 | H5A034 C | sample |
| 4357 | F20 | F21 | 907 | 779,3 | 13709 | 473 | 15,1 | 1,274 | H5A034 C | sample |
| 4371 | G11 | G12 | 1124 | 652,5 | 16927 | 382,9 | 15,1 | 1,274 | H5A034 C | sample |
| 4498 | L18 | L19 | 1280 | 666,2 | 19334 | 358,7 | 15,1 | 1,274 | H5A034 C | sample |
| 92 | D5 | D6 | 315 | 1508 | 4772 | 387,9 | 15,1 | 1,285 | H5A034 C | sample |
| 157 | G20 | G21 | 650 | 1657 | 9783 | 487,3 | 15,1 | 1,285 | H5A034 C | sample |
| 242 | K10 | K11 | 234 | 1509 | 3541 | 609,2 | 15,1 | 1,285 | H5A034 C | sample |
| 2181 | K6 | K7 | 959 | 631,7 | 14500 | 382,7 | 15,1 | 1,313 | H5A034 C | sample |
| 2301 | P6 | P7 | 1426 | 704,3 | 21599 | 485,5 | 15,1 | 1,313 | H5A034 C | sample |
| 6602 | D10 | D11 | 1412 | 596,4 | 21270 | 450 | 15,1 | 1,325 | H5A034 C | sample |
| 3986 | G10 | G11 | 279 | 706,7 | 4216 | 427,6 | 15,1 | 1,342 | H5A034 C | sample |
| 4071 | J22 | J23 | 262 | 866,3 | 3964 | 399,3 | 15,1 | 1,342 | H5A034 C | sample |
| 4174 | N7 | N8 | 24 | 832,5 | 362 | 373,7 | 15,1 | 1,342 | H5A034 C | sample |
| 15369 | A17 | A18 | 1472 | 846,4 | 22273 | 171,5 | 15,1 | 1,373 | H5A034 C | sample |
| 15388 | B12 | B13 | 961 | 859,5 | 14483 | 148,5 | 15,1 | 1,373 | H5A034 C | sample |
| 1343 | H8 | H9 | 1013 | 647,5 | 15319 | 383,7 | 15,1 | 1,385 | H5A034 C | sample |
| 1423 | L15 | L16 | 1027 | 737,2 | 15511 | 417,5 | 15,1 | 1,385 | H5A034 C | sample |

| | | | | | | | | | | | |
|---|---|---|---|---|---|---|---|---|---|---|---|
| 1455 | M22 | M23 | 1349 | 733,1 | 20343 | 404,4 | 15,1 | 1,385 | H5A034 ( | sample | [illegible] |
| 1492 | O12 | O13 | 1098 | 749,4 | 16582 | 606,5 | 15,1 | 1,385 | H5A034 ( | sample | [illegible] |
| 16237 | E20 | E21 | 1575 | 1004 | 23808 | 293,4 | 15,1 | 1,385 | H5A034 ( | sample | [illegible] |
| 13809 | P17 | P18 | 1320 | 1028 | 19919 | 212,7 | 15,1 | 1,418 | H5A034 ( | sample | [illegible] |
| 836 | C5 | C6 | 1093 | 720 | 16460 | 529 | 15,1 | 1,431 | H5A034 ( | sample | [illegible] |
| 918 | G14 | G15 | 1104 | 785,9 | 16687 | 482 | 15,1 | 1,431 | H5A034 ( | sample | [illegible] |
| 919 | G15 | G16 | 1405 | 706,3 | 21208 | 396 | 15,1 | 1,431 | H5A034 ( | sample | [illegible] |
| 1090 | N18 | N19 | 792 | 840,6 | 11938 | 529,5 | 15,1 | 1,431 | H5A034 ( | sample | [illegible] |
| 5544 | G9 | G10 | 1531 | 578,8 | 23157 | 571,3 | 15,1 | 1,438 | H5A034 ( | sample | [illegible] |
| 5577 | I17 | I18 | 1198 | 705,7 | 18041 | 555,3 | 15,1 | 1,438 | H5A034 ( | sample | [illegible] |
| 5617 | K1 | K2 | 369 | 719,1 | 5555 | 277,2 | 15,1 | 1,438 | H5A034 ( | sample | [illegible] |
| 3696 | J9 | J10 | 936 | 1286 | 14133 | 284,6 | 15,1 | 1,452 | H5A034 ( | sample | [illegible] |
| 8183 | E8 | E9 | 873 | 646,5 | 13201 | 626 | 15,1 | 1,452 | H5A034 ( | sample | [illegible] |
| 8193 | F17 | F18 | 1639 | 594,6 | 24823 | 442,7 | 15,1 | 1,452 | H5A034 ( | sample | [illegible] |
| 10174 | H7 | H8 | 1118 | 719,8 | 16841 | 126 | 15,1 | 1,452 | H5A034 ( | sample | [illegible] |
| 10214 | J21 | J22 | 986 | 755,9 | 14904 | 148,9 | 15,1 | 1,452 | H5A034 ( | sample | [illegible] |
| 10219 | J4 | J5 | 1587 | 936,4 | 24002 | 145,3 | 15,1 | 1,452 | H5A034 ( | sample | [illegible] |
| 1566 | B14 | B15 | 1564 | 774,8 | 23645 | 459,1 | 15,1 | 1,581 | H5A034 ( | sample | [illegible] |
| 21509 | A13 | A14 | 155 | 1010 | 2340 | 755,6 | 15,1 | 1,589 | H5A034 ( | sample | [illegible] |
| 21573 | C6 | C7 | 213 | 893,3 | 3220 | 788,6 | 15,1 | 1,589 | H5A034 ( | sample | [illegible] |
| 21607 | E15 | E16 | 332 | 943,9 | 5025 | 539,7 | 15,1 | 1,589 | H5A034 ( | sample | [illegible] |
| 4706 | E10 | E11 | 1297 | 766,1 | 19570 | 553,4 | 15,1 | 1,659 | H5A034 ( | sample | [illegible] |
| 4900 | M12 | M13 | 1046 | 782,2 | 15766 | 378,9 | 15,1 | 1,659 | H5A034 ( | sample | [illegible] |
| 4902 | M14 | M15 | 1067 | 813,4 | 16108 | 395,7 | 15,1 | 1,659 | H5A034 ( | sample | [illegible] |
| 4917 | M6 | M7 | 1259 | 789,6 | 18951 | 491,5 | 15,1 | 1,659 | H5A034 ( | sample | [illegible] |
| 10473 | E17 | E18 | 1489 | 858,7 | 22507 | 211,5 | 15,1 | 1,706 | H5A034 ( | sample | [illegible] |
| 10522 | G18 | G19 | 777 | 827 | 11706 | 171,4 | 15,1 | 1,706 | H5A034 ( | sample | [illegible] |
| 10606 | J7 | J8 | 870 | 844,6 | 13102 | 157,8 | 15,1 | 1,706 | H5A034 ( | sample | [illegible] |
| 2765 | D13 | D14 | 769 | 639 | 11621 | 515,3 | 15,1 | 1,776 | H5A034 ( | sample | [illegible] |
| 22609 | O1 | O2 | 892 | 1290 | 13525 | 416,3 | 15,2 | 0,784 | H5A034 ( | sample | [illegible] |
| 23682 | K3 | K4 | 963 | 1552 | 14623 | 184,2 | 15,2 | 0,806 | H5A034 ( | sample | [illegible] |
| 23724 | M2 | M3 | 915 | 1535 | 13886 | 168,1 | 15,2 | 0,806 | H5A034 ( | sample | [illegible] |
| 3106 | B18 | B19 | 1469 | 1299 | 22377 | 375,2 | 15,2 | 0,941 | H5A034 ( | sample | [illegible] |
| 3322 | K18 | K19 | 861 | 1338 | 13113 | 427 | 15,2 | 0,941 | H5A034 ( | sample | [illegible] |
| 3389 | N13 | N14 | 859 | 1243 | 13059 | 481,8 | 15,2 | 0,941 | H5A034 ( | sample | [illegible] |
| 18301 | K20 | K21 | 833 | 1098 | 12639 | 290,4 | 15,2 | 0,977 | H5A034 ( | sample | [illegible] |
| 19647 | C22 | C23 | 1027 | 370 | 15643 | 413,6 | 15,2 | 0,99 | H5A034 ( | sample | [illegible] |
| 19921 | O1 | O2 | 1137 | 306,9 | 17251 | 365,6 | 15,2 | 0,99 | H5A034 ( | sample | [illegible] |
| 6286 | F7 | F8 | 1156 | 504,3 | 17533 | 498,2 | 15,2 | 1,003 | H5A034 ( | sample | [illegible] |
| 6363 | J11 | J12 | 1493 | 517,6 | 22737 | 345,6 | 15,2 | 1,003 | H5A034 ( | sample | [illegible] |
| 6383 | J8 | J9 | 912 | 656,1 | 13886 | 365,6 | 15,2 | 1,003 | H5A034 ( | sample | [illegible] |
| 6428 | L5 | L6 | 629 | 621 | 9539 | 442,9 | 15,2 | 1,003 | H5A034 ( | sample | [illegible] |
| 20460 | E2 | E3 | 901 | 752,7 | 13662 | 182,6 | 15,2 | 1,013 | H5A034 ( | sample | [illegible] |
| 20531 | H19 | H20 | 1150 | 756,3 | 17466 | 162,9 | 15,2 | 1,013 | H5A034 ( | sample | [illegible] |
| 20570 | J10 | J11 | 708 | 775,7 | 10765 | 191,7 | 15,2 | 1,013 | H5A034 ( | sample | [illegible] |
| 20588 | J5 | J6 | 1143 | 764,9 | 17395 | 165 | 15,2 | 1,013 | H5A034 ( | sample | [illegible] |
| 20634 | L3 | L4 | 319 | 791,3 | 4835 | 146,1 | 15,2 | 1,013 | H5A034 ( | sample | [illegible] |
| 20649 | M17 | M18 | 770 | 791,9 | 11692 | 174,4 | 15,2 | 1,013 | H5A034 ( | sample | [illegible] |
| 20692 | O12 | O13 | 1078 | 731,1 | 16395 | 239,5 | 15,2 | 1,013 | H5A034 ( | sample | [illegible] |
| 13031 | O8 | O9 | 1094 | 1044 | 16680 | 213,8 | 15,2 | 1,031 | H5A034 ( | sample | [illegible] |
| 18642 | I3 | I4 | 975 | 978,7 | 14821 | 254 | 15,2 | 1,031 | H5A034 ( | sample | [illegible] |
| 17305 | B1 | B2 | 1414 | 1011 | 21515 | 311,2 | 15,2 | 1,038 | H5A034 ( | sample | [illegible] |
| 17363 | D19 | D20 | 1485 | 981,1 | 22580 | 165,4 | 15,2 | 1,038 | H5A034 ( | sample | [illegible] |
| 17628 | O2 | O3 | 1382 | 971,7 | 20985 | 312,3 | 15,2 | 1,038 | H5A034 ( | sample | [illegible] |
| 9326 | E21 | E22 | 1672 | 1109 | 25487 | 167,9 | 15,2 | 1,056 | H5A034 ( | sample | [illegible] |
| 9423 | I22 | I23 | 1239 | 1153 | 18865 | 190,7 | 15,2 | 1,056 | H5A034 ( | sample | [illegible] |
| 9590 | P21 | P22 | 1578 | 1073 | 24047 | 214,4 | 15,2 | 1,056 | H5A034 ( | sample | [illegible] |
| 18950 | F21 | F22 | 1506 | 1072 | 22832 | 125,4 | 15,2 | 1,059 | H5A034 ( | sample | [illegible] |
| 19141 | N20 | N21 | 1520 | 1001 | 23033 | 208,5 | 15,2 | 1,059 | H5A034 ( | sample | [illegible] |
| 9817 | J1 | J2 | 1657 | 1043 | 25167 | 134,5 | 15,2 | 1,063 | H5A034 ( | sample | [illegible] |
| 9925 | N20 | N21 | 2371 | 1235 | 36150 | 195,2 | 15,2 | 1,063 | H5A034 ( | sample | [illegible] |
| 15107 | F19 | F20 | 1093 | 898,5 | 16635 | 195 | 15,2 | 1,063 | H5A034 ( | sample | [illegible] |
| 15172 | I12 | I13 | 1463 | 873,7 | 22205 | 124,3 | 15,2 | 1,063 | H5A034 ( | sample | [illegible] |
| 8528 | D16 | D17 | 1577 | 1004 | 23902 | 104,1 | 15,2 | 1,074 | H5A034 ( | sample | [illegible] |
| 8714 | L10 | L11 | 756 | 1033 | 11503 | 114,1 | 15,2 | 1,074 | H5A034 ( | sample | [illegible] |
| 8772 | N2 | N3 | 1559 | 1003 | 23699 | 121,4 | 15,2 | 1,074 | H5A034 ( | sample | [illegible] |
| 8780 | N5 | N6 | 1278 | 1023 | 19461 | 108,8 | 15,2 | 1,074 | H5A034 ( | sample | [illegible] |
| 19311 | E22 | E23 | 1162 | 1290 | 17693 | 321,9 | 15,2 | 1,078 | H5A034 ( | sample | [illegible] |
| 19335 | F22 | F23 | 1552 | 1190 | 23622 | 326,8 | 15,2 | 1,078 | H5A034 ( | sample | [illegible] |
| 19410 | I3 | I4 | 799 | 1141 | 12154 | 251,9 | 15,2 | 1,078 | H5A034 ( | sample | [illegible] |
| 19572 | P2 | P3 | 1286 | 1397 | 19568 | 397 | 15,2 | 1,078 | H5A034 ( | sample | [illegible] |
| 21202 | D18 | D19 | 976 | 1007 | 14826 | 346,6 | 15,2 | 1,078 | H5A034 ( | sample | [illegible] |
| 21326 | I21 | I22 | 1336 | 936,8 | 20312 | 342,8 | 15,2 | 1,078 | H5A034 ( | sample | [illegible] |
| 11146 | A18 | A19 | 1894 | 923,5 | 28771 | 205,2 | 15,2 | 1,101 | H5A034 ( | sample | [illegible] |
| 11333 | I13 | I14 | 1285 | 861,7 | 19498 | 159,3 | 15,2 | 1,101 | H5A034 ( | sample | [illegible] |
| 11384 | K16 | K17 | 1825 | 916,7 | 27789 | 163,7 | 15,2 | 1,101 | H5A034 ( | sample | [illegible] |
| 11409 | L17 | L18 | 1190 | 830 | 18113 | 144,2 | 15,2 | 1,101 | H5A034 ( | sample | [illegible] |
| 11461 | N20 | N21 | 1922 | 937,2 | 29250 | 192,9 | 15,2 | 1,101 | H5A034 ( | sample | [illegible] |
| 11101 | O20 | O21 | 1282 | 812,5 | 19527 | 828,8 | 15,2 | 1,152 | H5A034 ( | sample | [illegible] |
| 7412 | E5 | E6 | 1289 | 564,7 | 19657 | 482,5 | 15,2 | 1,16 | H5A034 ( | sample | [illegible] |
| 7662 | P14 | P15 | 1125 | 558,3 | 17061 | 515,3 | 15,2 | 1,16 | H5A034 ( | sample | [illegible] |
| 16947 | C11 | C12 | 1647 | 990,5 | 24959 | 142,1 | 15,2 | 1,174 | H5A034 ( | sample | [illegible] |
| 16987 | D4 | D5 | 1157 | 949,6 | 17639 | 258,1 | 15,2 | 1,174 | H5A034 ( | sample | [illegible] |
| 16995 | E11 | E12 | 1092 | 991,5 | 16602 | 218,4 | 15,2 | 1,174 | H5A034 ( | sample | [illegible] |
| 17165 | L13 | L14 | 1387 | 952,3 | 21078 | 181,8 | 15,2 | 1,174 | H5A034 ( | sample | [illegible] |
| 17168 | L16 | L17 | 678 | 1045 | 10293 | 200,2 | 15,2 | 1,174 | H5A034 ( | sample | [illegible] |
| 17195 | M19 | M20 | 1429 | 942,9 | 21667 | 254,3 | 15,2 | 1,174 | H5A034 ( | sample | [illegible] |
| 17212 | N12 | N13 | 1454 | 936,4 | 22101 | 184,4 | 15,2 | 1,174 | H5A034 ( | sample | [illegible] |
| 7203 | M11 | M12 | 600 | 591,1 | 9120 | 408,6 | 15,2 | 1,183 | H5A034 ( | sample | [illegible] |
| 5004 | A2 | A3 | 1301 | 633,8 | 19797 | 590,4 | 15,2 | 1,192 | H5A034 ( | sample | [illegible] |
| 5015 | A8 | A9 | 1390 | 663,8 | 21168 | 590,7 | 15,2 | 1,192 | H5A034 ( | sample | [illegible] |
| 5068 | D12 | D13 | 1609 | 613,1 | 24472 | 553,1 | 15,2 | 1,192 | H5A034 ( | sample | [illegible] |
| 5069 | D13 | D14 | 1391 | 597,3 | 21088 | 572,4 | 15,2 | 1,192 | H5A034 ( | sample | [illegible] |
| 5265 | L17 | L18 | 1628 | 658,7 | 24725 | 496,3 | 15,2 | 1,192 | H5A034 ( | sample | [illegible] |
| 5363 | P19 | P20 | 1967 | 598,9 | 29883 | 671,8 | 15,2 | 1,192 | H5A034 ( | sample | [illegible] |
| 5364 | P2 | P3 | 1458 | 622,4 | 22168 | 386,8 | 15,2 | 1,192 | H5A034 ( | sample | [illegible] |
| 2333 | B13 | B14 | 789 | 714,3 | 12008 | 497,6 | 15,2 | 1,197 | H5A034 ( | sample | [illegible] |
| 16554 | B3 | B4 | 1106 | 938,5 | 16817 | 349,3 | 15,2 | 1,202 | H5A034 ( | sample | [illegible] |
| 16563 | C11 | C12 | 1273 | 960 | 19397 | 264,3 | 15,2 | 1,202 | H5A034 ( | sample | [illegible] |
| 16591 | D15 | D16 | 1189 | 951,3 | 18025 | 355,2 | 15,2 | 1,202 | H5A034 ( | sample | [illegible] |
| 16626 | E3 | E4 | 1127 | 962,9 | 17184 | 257,3 | 15,2 | 1,202 | H5A034 ( | sample | [illegible] |
| 16705 | I1 | I2 | 982 | 872,6 | 14917 | 188,5 | 15,2 | 1,202 | H5A034 ( | sample | [illegible] |
| 16707 | I11 | I12 | 994 | 932,6 | 15114 | 268,5 | 15,2 | 1,202 | H5A034 ( | sample | [illegible] |
| 16860 | O2 | O3 | 1186 | 937,5 | 18059 | 449,7 | 15,2 | 1,202 | H5A034 ( | sample | [illegible] |
| 17732 | C5 | C6 | 783 | 1045 | 11935 | 356,2 | 15,2 | 1,202 | H5A034 ( | sample | [illegible] |
| 17745 | D17 | D18 | 1330 | 1062 | 20192 | 244,1 | 15,2 | 1,202 | H5A034 ( | sample | [illegible] |
| 17746 | D18 | D19 | 1050 | 1030 | 15952 | 293,3 | 15,2 | 1,202 | H5A034 ( | sample | [illegible] |
| 17779 | E4 | E5 | 935 | 939,6 | 14234 | 316,8 | 15,2 | 1,202 | H5A034 ( | sample | [illegible] |
| 17847 | H22 | H23 | 1759 | 1033 | 26734 | 298,6 | 15,2 | 1,202 | H5A034 ( | sample | [illegible] |
| 17895 | J22 | J23 | 1025 | 1076 | 15557 | 360 | 15,2 | 1,202 | H5A034 ( | sample | [illegible] |
| 17923 | K4 | K5 | 686 | 1040 | 10404 | 387,3 | 15,2 | 1,202 | H5A034 ( | sample | [illegible] |
| 17984 | N16 | N17 | 1176 | 888,5 | 17916 | 138,6 | 15,2 | 1,202 | H5A034 ( | sample | [illegible] |
| 8918 | D21 | D22 | 1609 | 1781 | 24392 | 175,5 | 15,2 | 1,221 | H5A034 ( | sample | [illegible] |
| 8932 | E12 | E13 | 1995 | 1920 | 30373 | 163,6 | 15,2 | 1,221 | H5A034 ( | sample | [illegible] |
| 9120 | L9 | L10 | 1631 | 1755 | 24713 | 158,2 | 15,2 | 1,221 | H5A034 ( | sample | [illegible] |
| 4228 | A12 | A13 | 1012 | 678 | 15414 | 311 | 15,2 | 1,283 | H5A034 ( | sample | [illegible] |
| 4232 | A16 | A17 | 1610 | 738,7 | 24546 | 419,9 | 15,2 | 1,283 | H5A034 ( | sample | [illegible] |
| 4332 | E2 | E3 | 1140 | 682,9 | 17349 | 465 | 15,2 | 1,283 | H5A034 ( | sample | [illegible] |
| 4421 | I13 | I14 | 920 | 693 | 14025 | 445,9 | 15,2 | 1,283 | H5A034 ( | sample | [illegible] |
| 4489 | L1 | L2 | 672 | 684,2 | 10208 | 416,1 | 15,2 | 1,283 | H5A034 ( | sample | [illegible] |
| 4513 | M1 | M2 | 1081 | 680,3 | 16463 | 406,2 | 15,2 | 1,283 | H5A034 ( | sample | [illegible] |
| 4599 | P22 | P23 | 1244 | 691,7 | 18872 | 518 | 15,2 | 1,283 | H5A034 ( | sample | [illegible] |
| 28 | B12 | B13 | 604 | 1508 | 9168 | 694,9 | 15,2 | 1,294 | H5A034 ( | sample | [illegible] |
| 54 | C14 | C15 | 579 | 1497 | 8827 | 487,2 | 15,2 | 1,294 | H5A034 ( | sample | [illegible] |
| 128 | F16 | F17 | 447 | 1374 | 6816 | 350,7 | 15,2 | 1,294 | H5A034 ( | sample | [illegible] |
| 227 | J19 | J20 | 477 | 1327 | 7259 | 525 | 15,2 | 1,294 | H5A034 ( | sample | [illegible] |
| 372 | P2 | P3 | 607 | 1575 | 9220 | 777,8 | 15,2 | 1,294 | H5A034 ( | sample | [illegible] |
| 1967 | B8 | B9 | 1040 | 705,9 | 15803 | 534,1 | 15,2 | 1,322 | H5A034 ( | sample | [illegible] |
| 2061 | F6 | F7 | 1328 | 621,1 | 20224 | 417,8 | 15,2 | 1,322 | H5A034 ( | sample | [illegible] |
| 2190 | L14 | L15 | 1200 | 666,4 | 18189 | 443,4 | 15,2 | 1,322 | H5A034 ( | sample | [illegible] |
| 2198 | L21 | L22 | 849 | 731,9 | 12942 | 472 | 15,2 | 1,322 | H5A034 ( | sample | [illegible] |
| 2217 | M17 | M18 | 1089 | 675,4 | 16583 | 393,9 | 15,2 | 1,322 | H5A034 ( | sample | [illegible] |
| 6715 | H4 | H5 | 974 | 628,9 | 14783 | 456,7 | 15,2 | 1,333 | H5A034 ( | sample | [illegible] |
| 6815 | L8 | L9 | 1064 | 563,5 | 16155 | 477,1 | 15,2 | 1,333 | H5A034 ( | sample | [illegible] |
| 6845 | N13 | N14 | 1398 | 615,8 | 21319 | 487,7 | 15,2 | 1,333 | H5A034 ( | sample | [illegible] |
| 11541 | A6 | A7 | 1599 | 969,9 | 24280 | 223,2 | 15,2 | 1,357 | H5A034 ( | sample | [illegible] |
| 11874 | O3 | O4 | 1182 | 792,5 | 18025 | 172,5 | 15,2 | 1,357 | H5A034 ( | sample | [illegible] |
| 15402 | B3 | B4 | 893 | 912,7 | 13559 | 150,2 | 15,2 | 1,382 | H5A034 ( | sample | [illegible] |
| 5810 | C10 | C11 | 899 | 725,7 | 13667 | 549,6 | 15,2 | 1,388 | H5A034 ( | sample | [illegible] |
| 5874 | E3 | E4 | 1628 | 775,2 | 24691 | 624,2 | 15,2 | 1,388 | H5A034 ( | sample | [illegible] |
| 5980 | J12 | J13 | 1259 | 730,3 | 19131 | 482,3 | 15,2 | 1,388 | H5A034 ( | sample | [illegible] |
| 6093 | N6 | N7 | 1052 | 776,7 | 15959 | 600,4 | 15,2 | 1,388 | H5A034 ( | sample | [illegible] |
| 1277 | F13 | F14 | 1051 | 679,5 | 15959 | 445,6 | 15,2 | 1,394 | H5A034 ( | sample | [illegible] |
| 1295 | F8 | F9 | 509 | 740,8 | 7728 | 457,7 | 15,2 | 1,394 | H5A034 ( | sample | [illegible] |
| 1494 | O14 | O15 | 1325 | 688,9 | 20084 | 503,7 | 15,2 | 1,394 | H5A034 ( | sample | [illegible] |
| 16251 | F11 | F12 | 1531 | 982,3 | 23208 | 326,4 | 15,2 | 1,394 | H5A034 ( | sample | [illegible] |
| 16278 | G14 | G15 | 1357 | 957,3 | 20660 | 300,9 | 15,2 | 1,394 | H5A034 ( | sample | [illegible] |
| 16301 | H13 | H14 | 883 | 898,8 | 13451 | 355,5 | 15,2 | 1,394 | H5A034 ( | sample | [illegible] |
| 16327 | I15 | I16 | 1165 | 952,6 | 17684 | 344,3 | 15,2 | 1,394 | H5A034 ( | sample | [illegible] |
| 16363 | J4 | J5 | 991 | 1048 | 15090 | 403 | 15,2 | 1,394 | H5A034 ( | sample | [illegible] |
| 13503 | C22 | C23 | 1348 | 1038 | 20554 | 206,6 | 15,2 | 1,427 | H5A034 ( | sample | [illegible] |
| 13636 | I12 | I13 | 1186 | 974,8 | 18083 | 155,9 | 15,2 | 1,427 | H5A034 ( | sample | [illegible] |
| 13658 | J10 | J11 | 1163 | 1040 | 17628 | 158 | 15,2 | 1,427 | H5A034 ( | sample | [illegible] |
| 13686 | K14 | K15 | 1440 | 1021 | 21824 | 174,8 | 15,2 | 1,427 | H5A034 ( | sample | [illegible] |
| 13690 | K18 | K19 | 969 | 1145 | 14696 | 179,4 | 15,2 | 1,427 | H5A034 ( | sample | [illegible] |
| 779 | A19 | A20 | 1657 | 779,9 | 25185 | 547,5 | 15,2 | 1,441 | H5A034 ( | sample | [illegible] |
| 1143 | P22 | P23 | 988 | 770,6 | 15051 | 613,9 | 15,2 | 1,441 | H5A034 ( | sample | [illegible] |
| 5388 | A2 | A3 | 1549 | 718,7 | 23498 | 650,9 | 15,2 | 1,448 | H5A034 ( | sample | [illegible] |
| 5452 | D12 | D13 | 1934 | 725,2 | 29355 | 672,6 | 15,2 | 1,448 | H5A034 ( | sample | [illegible] |
| 5491 | E4 | E5 | 482 | 794,8 | 7335 | 569,6 | 15,2 | 1,448 | H5A034 ( | sample | [illegible] |
| 5519 | F8 | F9 | 1058 | 623,4 | 16033 | 568,9 | 15,2 | 1,448 | H5A034 ( | sample | [illegible] |
| 5601 | J17 | J18 | 1190 | 680,9 | 18029 | 429 | 15,2 | 1,448 | H5A034 ( | sample | [illegible] |
| 5696 | N16 | N17 | 974 | 809,6 | 14795 | 387,3 | 15,2 | 1,448 | H5A034 ( | sample | [illegible] |
| 3650 | I10 | I11 | 900 | 1281 | 13675 | 328,5 | 15,2 | 1,462 | H5A034 ( | sample | [illegible] |
| 3771 | N11 | N12 | 1093 | 1203 | 16587 | 345,8 | 15,2 | 1,462 | H5A034 ( | sample | [illegible] |
| 8211 | G11 | G12 | 1287 | 602,3 | 19581 | 513,7 | 15,2 | 1,462 | H5A034 ( | sample | [illegible] |
| 8279 | I8 | I9 | 972 | 654,9 | 14775 | 512,1 | 15,2 | 1,462 | H5A034 ( | sample | [illegible] |
| 8302 | J7 | J8 | 1094 | 584,3 | 16620 | 536,8 | 15,2 | 1,462 | H5A034 ( | sample | [illegible] |
| 8358 | M14 | M15 | 936 | 681,7 | 14255 | 374,1 | 15,2 | 1,462 | H5A034 ( | sample | [illegible] |
| 8396 | N5 | N6 | 1440 | 557,6 | 21934 | 493,3 | 15,2 | 1,462 | H5A034 ( | sample | [illegible] |
| 10209 | J17 | J18 | 1271 | 869,2 | 19358 | 139,7 | 15,2 | 1,462 | H5A034 ( | sample | [illegible] |
| 10218 | J3 | J4 | 1075 | 843,6 | 16366 | 132,7 | 15,2 | 1,462 | H5A034 ( | sample | [illegible] |
| 1637 | E13 | E14 | 1275 | 714,8 | 19400 | 516,1 | 15,2 | 1,592 | H5A034 ( | sample | [illegible] |
| 21557 | C13 | C14 | 540 | 970 | 8215 | 555,3 | 15,2 | 1,6 | H5A034 ( | sample | [illegible] |
| 21654 | G14 | G15 | 399 | 984,8 | 6068 | 688,8 | 15,2 | 1,6 | H5A034 ( | sample | [illegible] |
| 4947 | O11 | O12 | 1282 | 833,4 | 19503 | 587,1 | 15,2 | 1,67 | H5A034 ( | sample | [illegible] |
| 10610 | K10 | K11 | 1006 | 790,3 | 15294 | 178,4 | 15,2 | 1,718 | H5A034 ( | sample | [illegible] |
| 10636 | L12 | L13 | 368 | 1010 | 5596 | 243,7 | 15,2 | 1,718 | H5A034 ( | sample | [illegible] |
| 10645 | L20 | L21 | 1210 | 826,7 | 18388 | 224,6 | 15,2 | 1,718 | H5A034 ( | sample | [illegible] |
| 10665 | M17 | M18 | 1345 | 813 | 20470 | 178,6 | 15,2 | 1,718 | H5A034 ( | sample | [illegible] |
| 20013 | B6 | B7 | 871 | 1107 | 13255 | 252,7 | 15,2 | 1,854 | H5A034 ( | sample | [illegible] |
| 14910 | N14 | N15 | 1251 | 794,3 | 19174 | 104,8 | 15,3 | 0,872 | H5A034 ( | sample | [illegible] |
| 14113 | M1 | M2 | 1336 | 1009 | 20502 | 116,3 | 15,3 | 0,877 | H5A034 ( | sample | [illegible] |
| 3078 | A14 | A15 | 898 | 1240 | 13711 | 393,1 | 15,3 | 0,947 | H5A034 ( | sample | [illegible] |
| 3248 | H16 | H17 | 1253 | 1324 | 19133 | 404,6 | 15,3 | 0,947 | H5A034 ( | sample | [illegible] |
| 3419 | O19 | O20 | 1467 | 1300 | 22417 | 455,1 | 15,3 | 0,947 | H5A034 ( | sample | [illegible] |
| 18276 | J2 | J3 | 1221 | 1008 | 18721 | 328,9 | 15,3 | 0,984 | H5A034 ( | sample | [illegible] |
| 6231 | D22 | D23 | 1311 | 540,9 | 20121 | 562,1 | 15,3 | 1,01 | H5A034 ( | sample | [illegible] |
| 6284 | F5 | F6 | 727 | 572,7 | 11151 | 532,6 | 15,3 | 1,01 | H5A034 ( | sample | [illegible] |
| 20403 | C11 | C12 | 779 | 776,8 | 11910 | 215,9 | 15,3 | 1,02 | H5A034 ( | sample | [illegible] |
| 20423 | C8 | C9 | 584 | 797,7 | 8951 | 226,8 | 15,3 | 1,02 | H5A034 ( | sample | [illegible] |
| 20426 | D10 | D11 | 808 | 790,4 | 12396 | 212,1 | 15,3 | 1,02 | H5A034 ( | sample | [illegible] |
| 20443 | D4 | D5 | 1283 | 802,2 | 19625 | 264,7 | 15,3 | 1,02 | H5A034 ( | sample | [illegible] |
| 20469 | E6 | E7 | 765 | 799,6 | 11678 | 245,3 | 15,3 | 1,02 | H5A034 ( | sample | [illegible] |
| 20482 | F18 | F19 | 561 | 789,4 | 8591 | 172,8 | 15,3 | 1,02 | H5A034 ( | sample | [illegible] |
| 20533 | H20 | H21 | 935 | 779,4 | 14299 | 149,4 | 15,3 | 1,02 | H5A034 ( | sample | [illegible] |
| 20568 | I9 | I10 | 491 | 765,9 | 7523 | 189,1 | 15,3 | 1,02 | H5A034 ( | sample | [illegible] |
| 20595 | K11 | K12 | 558 | 767,9 | 8545 | 194 | 15,3 | 1,02 | H5A034 ( | sample | [illegible] |
| 20626 | L18 | L19 | 886 | 824,2 | 13561 | 186,8 | 15,3 | 1,02 | H5A034 ( | sample | [illegible] |
| 20655 | M22 | M23 | 1021 | 822,1 | 15606 | 177,6 | 15,3 | 1,02 | H5A034 ( | sample | [illegible] |
| 20674 | N18 | N19 | 827 | 760,6 | 12639 | 169,5 | 15,3 | 1,02 | H5A034 ( | sample | [illegible] |
| 20711 | O8 | O9 | 649 | 781,1 | 9904 | 182 | 15,3 | 1,02 | H5A034 ( | sample | [illegible] |
| 20712 | O9 | O10 | 874 | 768,1 | 13381 | 250,8 | 15,3 | 1,02 | H5A034 ( | sample | [illegible] |
| 12810 | F3 | F4 | 1385 | 1047 | 21142 | 120,4 | 15,3 | 1,037 | H5A034 ( | sample | [illegible] |
| 18488 | C16 | C17 | 1123 | 1004 | 17191 | 371,2 | 15,3 | 1,037 | H5A034 ( | sample | [illegible] |
| 18714 | L3 | L4 | 1066 | 1018 | 16323 | 171,6 | 15,3 | 1,037 | H5A034 ( | sample | [illegible] |
| 18777 | O17 | O18 | 1420 | 1010 | 21684 | 286,1 | 15,3 | 1,037 | H5A034 ( | sample | [illegible] |
| 17409 | F17 | F18 | 1507 | 970,2 | 22995 | 192,3 | 15,3 | 1,044 | H5A034 ( | sample | [illegible] |
| 17453 | H13 | H14 | 1553 | 1006 | 23782 | 213 | 15,3 | 1,044 | H5A034 ( | sample | [illegible] |
| 17586 | M3 | M4 | 1049 | 996,7 | 16024 | 147 | 15,3 | 1,044 | H5A034 ( | sample | [illegible] |
| 17591 | M8 | M9 | 1304 | 881,8 | 19996 | 229,4 | 15,3 | 1,044 | H5A034 ( | sample | [illegible] |
| 17640 | O9 | O10 | 1049 | 945,3 | 16058 | 335,6 | 15,3 | 1,044 | H5A034 ( | sample | [illegible] |
| 9229 | A20 | A21 | 1849 | 1151 | 28203 | 207,1 | 15,3 | 1,063 | H5A034 ( | sample | [illegible] |
| 9509 | M13 | M14 | 1587 | 1080 | 24336 | 120,4 | 15,3 | 1,063 | H5A034 ( | sample | [illegible] |
| 9551 | N8 | N9 | 1444 | 1254 | 22044 | 192,8 | 15,3 | 1,063 | H5A034 ( | sample | [illegible] |
| 9661 | C20 | C21 | 2232 | 1198 | 34055 | 172,1 | 15,3 | 1,07 | H5A034 ( | sample | [illegible] |
| 15357 | P6 | P7 | 1387 | 768,6 | 21166 | 227,1 | 15,3 | 1,07 | H5A034 ( | sample | [illegible] |
| 8553 | E17 | E18 | 1396 | 1087 | 21401 | 145 | 15,3 | 1,081 | H5A034 ( | sample | [illegible] |
| 19284 | D2 | D3 | 1073 | 1256 | 16402 | 263,4 | 15,3 | 1,085 | H5A034 ( | sample | [illegible] |
| 19297 | E1 | E2 | 873 | 1130 | 13342 | 252,3 | 15,3 | 1,085 | H5A034 ( | sample | [illegible] |
| 19393 | I1 | I2 | 771 | 1131 | 11804 | 233 | 15,3 | 1,085 | H5A034 ( | sample | [illegible] |
| 19574 | P21 | P22 | 1317 | 1306 | 20201 | 419,6 | 15,3 | 1,085 | H5A034 ( | sample | [illegible] |
| 20814 | D14 | D15 | 1061 | 992,6 | 16194 | 271 | 15,3 | 1,085 | H5A034 ( | sample | [illegible] |
| 20938 | I18 | I19 | 1308 | 975,2 | 20017 | 299,3 | 15,3 | 1,085 | H5A034 ( | sample | [illegible] |
| 20985 | K17 | K18 | 842 | 983,8 | 12859 | 199,8 | 15,3 | 1,085 | H5A034 ( | sample | [illegible] |
| 21078 | O14 | O15 | 1182 | 969,8 | 18081 | 310,1 | 15,3 | 1,085 | H5A034 ( | sample | [illegible] |

| | | | | | | | | | | | | | | | | | | | | | | | | | | | | | | | |
|---|---|---|---|---|---|---|---|---|---|---|---|---|---|---|---|---|---|---|---|---|---|---|---|---|---|---|---|---|---|---|---|
| 21207 | D22 | D23 | 1082 | 1026 | 16523 | 319,2 | 15,3 | 1,085 | H5A034 C | sample | Hm Gen E | Hm Gen E | Hm Gen E | CPE002JT | CPE002O8 | CPE002KO | XM 93468 | XM 93468 | XM 93468 | LOC64728 | LOC64728 | LOC64728 | hCG1820717 | hCG18207 | hCG18207 | 647283 | 647283 | 647283 | s56715 | s56716 | s197526 |
| 14454 | K14 | K15 | 1632 | 824,9 | 24951 | 131,5 | 15,3 | 1,089 | H5A034 C | sample | Hm Gen E | Hm Gen E | Hm Gen E | CPE002J7 | CPE002NC | CPE002K6 | NM 01755 | NM 0175 | NM 0175 | HYDIN | HYDIN | HYDIN | hydrocephalus in | hydrocepl | hydrocepl | 54768 | 54768 | 54768 | s29414 | s29415 | s29416 |
| 11367 | J22 | J23 | 1821 | 914 | 27879 | 201,1 | 15,3 | 1,109 | H5A034 C | sample | Hm Drgbl | Hm Drgbl | Hm Drgbl | CPE002ED | CPE002F3 | CPE002FT | | | | | | | | | | | | | | | |
| 11376 | J9 | J10 | 1220 | 789,1 | 18606 | 136,3 | 15,3 | 1,109 | H5A034 C | sample | Hm Drgbl | Hm Drgbl | Hm Drgbl | CPE002ED | CPE002F3 | CPE002FT | NM 19853 | NM 1985 | NM 1985 | ATP9B | ATP9B | ATP9B | ATPase, Class II, t | ATPase, C | ATPase, C | 374868 | 374868 | 374868 | s51592 | s51593 | s51594 |
| 11415 | L22 | L23 | 1716 | 875,4 | 26308 | 184,1 | 15,3 | 1,109 | H5A034 C | sample | Hm Drgbl | Hm Drgbl | Hm Drgbl | CPE002ED | CPE002F3 | CPE002FT | | | | | | | | | | | | | | | |
| 12351 | C22 | C23 | 1264 | 1028 | 19326 | 197,3 | 15,3 | 1,15 | H5A034 C | sample | Hm Gen E | Hm Gen E | Hm Gen E | CPE002J5 | CPE002NK | CPE002K1 | NM 00353 | NM 0035 | NM 0035 | HIST1H3B | HIST1H3B | HIST1H3B | histone cluster 1, | histone cl | histone cl | 8358 | 8358 | 8358 | s15881 | s15882 | s194915 |
| 12364 | D12 | D13 | 835 | 991,6 | 12735 | 164,2 | 15,3 | 1,15 | H5A034 C | sample | Hm Gen E | Hm Gen E | Hm Gen E | CPE002J5 | CPE002NK | CPE002K1 | NM 00387 | NM 0038 | NM 0038 | SOCS2 | SOCS2 | SOCS2 | suppressor of cyto | suppressc | suppressc | 8835 | 8835 | 8835 | s16858 | s16859 | s16860 |
| 12402 | E3 | E4 | 780 | 1086 | 11964 | 161,1 | 15,3 | 1,15 | H5A034 C | sample | Hm Gen E | Hm Gen E | Hm Gen E | CPE002J5 | CPE002NK | CPE002K1 | NM 00316 | NM 0031 | NM 0031 | STRN | STRN | STRN | striatin, calmodul | striatin, c | striatin, c | 6801 | 6801 | 6801 | s13586 | s13587 | s13588 |
| 12419 | F19 | F20 | 972 | 1067 | 14861 | 177,1 | 15,3 | 1,15 | H5A034 C | sample | Hm Gen E | Hm Gen E | Hm Gen E | CPE002J5 | CPE002NK | CPE002K1 | NM 00462 | NM 0046 | NM 0046 | WNT11 | WNT11 | WNT11 | wingless-type MM | wingless-t | wingless-t | 7481 | 7481 | 7481 | s14892 | s14893 | s14894 |
| 10962 | I3 | I4 | 1312 | 840,1 | 20081 | 595,1 | 15,3 | 1,159 | H5A034 C | sample | Hm Drgbl | Hm Drgbl | Hm Drgbl | CPE002EC | CPE002EN | CPE002FS | NM 17307 | NM 1730 | NM 1730 | SLITRK4 | SLITRK4 | SLITRK4 | SLIT and NTRK-lik | SLIT and N | SLIT and N | 139065 | 139065 | 139065 | s44157 | s44158 | s44159 |
| 11017 | L1 | L2 | 1365 | 875,4 | 20925 | 562,2 | 15,3 | 1,159 | H5A034 C | sample | Hm Drgbl | Hm Drgbl | Hm Drgbl | CPE002EC | CPE002EN | CPE002FS | NM 13928 | NM 1392 | NM 1392 | OTEX | OTEX | OTEX | paired-like home | paired-lik | paired-lik | 158800 | 158800 | 158800 | s46119 | s46118 | s223373 |
| 11034 | L3 | L4 | 826 | 774,6 | 12618 | 510,5 | 15,3 | 1,159 | H5A034 C | sample | Hm Drgbl | Hm Drgbl | Hm Drgbl | CPE002EC | CPE002EN | CPE002FS | NM 00101 | NM 0010 | NM 0010 | DGAT2L3 | DGAT2L3 | DGAT2L3 | diacylglycerol O-a | diacylglyc | diacylglyc | 158833 | 158833 | 158833 | s46126 | s46127 | s46128 |
| 7323 | B11 | B12 | 1308 | 624,8 | 19998 | 544,7 | 15,3 | 1,168 | H5A034 C | sample | Hm Drgbl | Hm Drgbl | Hm Drgbl | CPE002E3 | CPE002EW | CPE002FH | NM 00276 | NM 0027 | NM 0010 | CHMP1A | CHMP1A | CHMP1A | chromatin modify | chromatin | chromatin | 5119 | 5119 | 5119 | s10142 | s10143 | s194678 |
| 16938 | B3 | B4 | 1446 | 1117 | 22176 | 211 | 15,3 | 1,181 | H5A034 C | sample | Hm Gen E | Hm Gen E | Hm Gen E | CPE002JI | CPE002NZ | CPE002KD | NM 05285 | NM 0010 | NM 0528 | GPRIN1 | C14orf166 | GPRIN1 | G protein regulat | chromoso | G protein | 114787 | 145497 | 114787 | s41600 | s44745 | s41602 |
| 17183 | L8 | L9 | 1198 | 978,4 | 18378 | 267,7 | 15,3 | 1,181 | H5A034 C | sample | Hm Gen E | Hm Gen E | Hm Gen E | CPE002JI | CPE002NZ | CPE002KD | NM 15234 | XM 0981 | NM 1523 | C17orf46 | LOC15211 | C17orf46 | chromosome 17 c | hypotheti | chromoso | 124783 | 152118 | 124783 | s42791 | s45665 | s42793 |
| 17235 | O11 | O12 | 1540 | 864,1 | 23488 | 290,7 | 15,3 | 1,181 | H5A034 C | sample | Hm Gen E | Hm Gen E | Hm Gen E | CPE002JI | CPE002NZ | CPE002KD | NM 01810 | NM 1387 | NM 0181 | EFHC1 | MIPOL1 | EFHC1 | EF-hand domain ( | mirror-im | EF-hand d | 114327 | 145282 | 114327 | s41551 | s44721 | s41553 |
| 6990 | D14 | D15 | 1301 | 529,3 | 19898 | 525,9 | 15,3 | 1,191 | H5A034 C | sample | Hm Drgbl | Hm Drgbl | Hm Drgbl | CPE002E2 | CPE002EX | CPE002FI | NM 0050 | NM 0050 | NM 0050 | NDUFB9 | NDUFB9 | NDUFB9 | NADH dehydroge | NADH deh | NADH deh | 4715 | 4715 | 4715 | s9372 | s9371 | s9373 |
| 7047 | F22 | F23 | 1373 | 550,9 | 21008 | 483 | 15,3 | 1,191 | H5A034 C | sample | Hm Drgbl | Hm Drgbl | Hm Drgbl | CPE002E2 | CPE002EX | CPE002FI | NM 19840 | NM 1984 | NM 1984 | NEDD4 | NEDD4 | NEDD4 | neural precursor | neural pre | neural pre | 4734 | 4734 | 4734 | s9415 | s9416 | s9417 |
| 7171 | K4 | K5 | 586 | 518,4 | 8937 | 409,4 | 15,3 | 1,191 | H5A034 C | sample | Hm Drgbl | Hm Drgbl | Hm Drgbl | CPE002E2 | CPE002EX | CPE002FI | NM 00537 | NM 0053 | NM 0053 | MYO1A | MYO1A | MYO1A | myosin IA | myosin IA | myosin IA | 4640 | 4640 | 4640 | s9198 | s9197 | s9196 |
| 7287 | P22 | P23 | 1219 | 585,6 | 18646 | 549 | 15,3 | 1,191 | H5A034 C | sample | Hm Drgbl | Hm Drgbl | Hm Drgbl | CPE002E2 | CPE002EX | CPE002FI | NM 00464 | NM 0046 | NM 0046 | NPHS1 | NPHS1 | NPHS1 | nephrosis 1, cong | nephrosis | nephrosis | 4868 | 4868 | 4868 | s9675 | s9673 | s9674 |
| 5022 | B14 | B15 | 1386 | 626,8 | 21185 | 619,2 | 15,3 | 1,2 | H5A034 C | sample | Hm Drgbl | Hm Drgbl | Hm Drgbl | CPE002DV | CPE002EM | CPE002FO | NM 00600 | NM 0060 | NM 0060 | UQCRH | UQCRH | UQCRH | ubiquinol-cytochr | ubiquinol | ubiquinol | 7388 | 7388 | 7388 | s14708 | s14709 | s14710 |
| 5023 | B15 | B16 | 1652 | 709,1 | 25310 | 603,4 | 15,3 | 1,2 | H5A034 C | sample | Hm Drgbl | Hm Drgbl | Hm Drgbl | CPE002DV | CPE002EM | CPE002FO | NM 19818 | NM 1981 | NM 1981 | OVCH2 | OVCH2 | OVCH2 | ovochymase 2 | ovochyma | ovochyma | 341277 | 341277 | 341277 | s50823 | s50824 | s50825 |
| 5138 | G10 | G11 | 1615 | 663 | 24732 | 497,3 | 15,3 | 1,2 | H5A034 C | sample | Hm Drgbl | Hm Drgbl | Hm Drgbl | CPE002DV | CPE002EM | CPE002FO | NM 00331 | NM 0033 | NM 0033 | TTC3 | TTC3 | TTC3 | tetratricopeptide | tetratrico | tetratrico | 7267 | 7267 | 7267 | s14475 | s14476 | s14477 |
| 5165 | H13 | H14 | 1422 | 568,2 | 21802 | 459,1 | 15,3 | 1,2 | H5A034 C | sample | Hm Drgbl | Hm Drgbl | Hm Drgbl | CPE002DV | CPE002EM | CPE002FO | NM 00347 | NM 0034 | NM 0034 | DEK | DEK | DEK | DEK oncogene (DI | DEK onco | DEK onco | 7913 | 7913 | 7913 | s15459 | s15458 | s15457 |
| 2306 | A10 | A11 | 917 | 731,3 | 14008 | 522,1 | 15,3 | 1,205 | H5A034 C | sample | Hm Drgbl | Hm Drgbl | Hm Drgbl | CPE002DC | CPE002EE | CPE002F4 | NM 00205 | NM 0020 | NM 0020 | GSK3B | GSK3B | GSK3B | glycogen synthase | glycogen s | glycogen s | 2932 | 2932 | 2932 | s6239 | s6240 | s6241 |
| 2409 | E17 | E18 | 773 | 696,2 | 11796 | 338,2 | 15,3 | 1,205 | H5A034 C | sample | Hm Drgbl | Hm Drgbl | Hm Drgbl | CPE002DC | CPE002EE | CPE002F4 | NM 00331 | NM 0033 | NM 0033 | TTK | TTK | TTK | TTK protein kinas | TTK prote | TTK prote | 7272 | 7272 | 7272 | s120 | s224757 | s122 |
| 2481 | H17 | H18 | 777 | 650,4 | 11853 | 326,6 | 15,3 | 1,205 | H5A034 C | sample | Hm Drgbl | Hm Drgbl | Hm Drgbl | CPE002DC | CPE002EE | CPE002F4 | NM 00100 | NM 0010 | NM 0010 | DYRK3 | DYRK3 | DYRK3 | dual-specificity ty | dual-spec | dual-spec | 8444 | 8444 | 8444 | s16024 | s16025 | s16026 |
| 16569 | C17 | C18 | 871 | 944,9 | 13318 | 261,4 | 15,3 | 1,209 | H5A034 C | sample | Hm Gen E | Hm Gen E | Hm Gen E | CPE002JH | CPE002NV | CPE002KC | NM 03282 | NM 0328 | NM 0328 | FAM136A | FAM136A | FAM136A | family with seque | family wit | family wit | 84908 | 84908 | 84908 | s39628 | s39629 | s39630 |
| 16617 | E17 | E18 | 1283 | 948,3 | 19681 | 277,3 | 15,3 | 1,209 | H5A034 C | sample | Hm Gen E | Hm Gen E | Hm Gen E | CPE002JH | CPE002NV | CPE002KC | NM 03284 | NM 0328 | NM 0328 | C8orf76 | C8orf76 | C8orf76 | chromosome 8 o | chromoso | chromoso | 84933 | 84933 | 84933 | s39691 | s39692 | s39693 |
| 16715 | I19 | I20 | 1071 | 1042 | 16349 | 333,7 | 15,3 | 1,209 | H5A034 C | sample | Hm Gen E | Hm Gen E | Hm Gen E | CPE002JH | CPE002NV | CPE002KC | NM 03288 | NM 0328 | NM 0328 | TOX2 | TOX2 | TOX2 | TOX high mobility | TOX high | TOX high | 84969 | 84969 | 84969 | s39779 | s39780 | s39781 |
| 16815 | M22 | M23 | 1191 | 1037 | 18169 | 384,2 | 15,3 | 1,209 | H5A034 C | sample | Hm Gen E | Hm Gen E | Hm Gen E | CPE002JH | CPE002NV | CPE002KC | NM 15226 | NM 1522 | NM 1522 | C12orf65 | C12orf65 | C12orf65 | chromosome 12 c | chromoso | chromoso | 91574 | 91574 | 91574 | s40690 | s40691 | s40692 |
| 16867 | O4 | O5 | 628 | 1059 | 9581 | 455,8 | 15,3 | 1,209 | H5A034 C | sample | Hm Gen E | Hm Gen E | Hm Gen E | CPE002JH | CPE002NV | CPE002KC | NM 18177 | NM 1817 | NM 1817 | PLXNA4 | PLXNA4 | PLXNA4 | plexin A4 | plexin A4 | plexin A4 | 91584 | 91584 | 91584 | s40696 | s40697 | s40698 |
| 17808 | F9 | F10 | 742 | 1016 | 11383 | 284,5 | 15,3 | 1,209 | H5A034 C | sample | Hm Gen E | Hm Gen E | Hm Gen E | CPE002JK | CPE002NZ | CPE002KF | NM 17881 | NM 1788 | NM 1788 | CMTM4 | CMTM4 | CMTM4 | CKLF-like MARVEL | CKLF-like | CKLF-like | 146223 | 146223 | 146223 | s44830 | s44831 | s44832 |
| 8839 | A15 | A16 | 1525 | 1750 | 23372 | 215,7 | 15,3 | 1,229 | H5A034 C | sample | Hm Drgbl | Hm Drgbl | Hm Drgbl | CPE002E7 | CPE002ES | CPE002FD | NM 01458 | NM 0145 | NM 0145 | PLA2G2E | PLA2G2E | PLA2G2E | phospholipase A2 | phospholi | phospholi | 30814 | 30814 | 30814 | s26916 | s26914 | s26915 |
| 8852 | A5 | A6 | 1699 | 1784 | 25941 | 172,3 | 15,3 | 1,229 | H5A034 C | sample | Hm Drgbl | Hm Drgbl | Hm Drgbl | CPE002E7 | CPE002ES | CPE002FD | NM 02102 | NM 0210 | NM 0210 | TLX3 | TLX3 | TLX3 | T-cell leukemia ho | T-cell leuk | T-cell leuk | 30012 | 30012 | 30012 | s26898 | s26899 | s26900 |
| 8856 | A9 | A10 | 1465 | 1618 | 22428 | 174,7 | 15,3 | 1,229 | H5A034 C | sample | Hm Drgbl | Hm Drgbl | Hm Drgbl | CPE002E7 | CPE002ES | CPE002FD | NM 01343 | NM 0134 | NM 0134 | RAX | RAX | RAX | retina and anteri | retina and | retina and | 30062 | 30062 | 30062 | s26906 | s223840 | s26905 |
| 8880 | B9 | B10 | 1802 | 1857 | 27556 | 213,5 | 15,3 | 1,229 | H5A034 C | sample | Hm Drgbl | Hm Drgbl | Hm Drgbl | CPE002E7 | CPE002ES | CPE002FD | NM 01623 | NM 0162 | NM 0162 | MYO15A | MYO15A | MYO15A | myosin XVA | myosin XV | myosin XV | 51168 | 51168 | 51168 | s27598 | s27599 | s27600 |
| 9042 | I3 | I4 | 1652 | 1660 | 25278 | 173,6 | 15,3 | 1,229 | H5A034 C | sample | Hm Drgbl | Hm Drgbl | Hm Drgbl | CPE002E7 | CPE002ES | CPE002FD | NM 01594 | NM 0159 | NM 0159 | AMDHD2 | AMDHD2 | AMDHD2 | amidohydrolase c | amidohyd | amidohyd | 51005 | 51005 | 51005 | s27219 | s27217 | s224177 |
| 9086 | K21 | K22 | 1706 | 1704 | 26100 | 199,3 | 15,3 | 1,229 | H5A034 C | sample | Hm Drgbl | Hm Drgbl | Hm Drgbl | CPE002E7 | CPE002ES | CPE002FD | NM 01597 | NM 0159 | NM 0159 | POLR1D | POLR1D | POLR1D | polymerase (RNA | polymeras | polymeras | 51082 | 51082 | 51082 | s200516 | s200514 | s200515 |
| 9212 | P5 | P6 | 1777 | 1614 | 27209 | 172 | 15,3 | 1,229 | H5A034 C | sample | Hm Drgbl | Hm Drgbl | Hm Drgbl | CPE002E7 | CPE002ES | CPE002FD | NM 01637 | NM 0163 | NM 0163 | HSD17B7 | HSD17B7 | HSD17B7 | hydroxysteroid (1 | hydroxyst | hydroxyst | 51478 | 51478 | 51478 | s718 | s200519 | s200518 |
| 4341 | E6 | E7 | 1104 | 671,3 | 16916 | 359,5 | 15,3 | 1,291 | H5A034 C | sample | Hm Drgbl | Hm Drgbl | Hm Drgbl | CPE002DL | CPE002EK | CPE002FQ | NM 00415 | NM 0041 | NM 0041 | CHRNA6 | CHRNA6 | CHRNA6 | cholinergic recept | cholinergi | cholinergi | 8973 | 8973 | 8973 | s17123 | s17124 | s17125 |
| 4435 | I4 | I5 | 853 | 702,2 | 13051 | 494,4 | 15,3 | 1,291 | H5A034 C | sample | Hm Drgbl | Hm Drgbl | Hm Drgbl | CPE002DL | CPE002EK | CPE002FQ | NM 00607 | NM 0060 | NM 0060 | CACNG2 | CACNG2 | CACNG2 | calcium channel, | calcium ch | calcium ch | 10369 | 10369 | 10369 | s20278 | s20279 | s20280 |
| 4583 | O8 | O9 | 1034 | 701,4 | 15869 | 502,8 | 15,3 | 1,291 | H5A034 C | sample | Hm Drgbl | Hm Drgbl | Hm Drgbl | CPE002DL | CPE002EK | CPE002FQ | NM 01767 | NM 0176 | NM 0176 | TRPM7 | TRPM7 | TRPM7 | transient recepto | transient | transient | 54822 | 54822 | 54822 | s29516 | s29517 | s29518 |
| 4587 | P11 | P12 | 1331 | 709,2 | 20423 | 505,8 | 15,3 | 1,291 | H5A034 C | sample | Hm Drgbl | Hm Drgbl | Hm Drgbl | CPE002DL | CPE002EK | CPE002FQ | NM 00458 | NM 0045 | NM 0045 | SCN2B | SCN2B | SCN2B | sodium channel, | sodium ch | sodium ch | 6327 | 6327 | 6327 | s12516 | s12517 | s12518 |
| 10 | A18 | A19 | 579 | 1539 | 8860 | 781,2 | 15,3 | 1,302 | H5A034 C | sample | Hm Drgbl | Hm Drgbl | Hm Drgbl | CPE002DT | CPE002EJ | CPE002F9 | NM 00104 | NM 0010 | NM 0010 | SLC12A2 | SLC12A2 | SLC12A2 | solute carrier fam | solute car | solute car | 6558 | 6558 | 6558 | s13046 | s13047 | s13045 |
| 122 | F10 | F11 | 410 | 1393 | 6262 | 434,8 | 15,3 | 1,302 | H5A034 C | sample | Hm Drgbl | Hm Drgbl | Hm Drgbl | CPE002DT | CPE002EJ | CPE002F9 | NM 00102 | NM 0010 | NM 0010 | SPTB | SPTB | SPTB | spectrin, beta, er | spectrin, b | spectrin, b | 6710 | 6710 | 6710 | s13408 | s13406 | s13407 |
| 130 | F18 | F19 | 654 | 1600 | 10037 | 388,7 | 15,3 | 1,302 | H5A034 C | sample | Hm Drgbl | Hm Drgbl | Hm Drgbl | CPE002DT | CPE002EJ | CPE002F9 | NM 19890 | NM 1989 | NM 1989 | SRI | SRI | SRI | sorcin | sorcin | sorcin | 6717 | 6717 | 6717 | s13421 | s13422 | s13423 |
| 325 | N20 | N21 | 439 | 1426 | 6726 | 670,8 | 15,3 | 1,302 | H5A034 C | sample | Hm Drgbl | Hm Drgbl | Hm Drgbl | CPE002DT | CPE002EJ | CPE002F9 | NM 00317 | NM 0031 | NM 0031 | SUV39H1 | SUV39H1 | SUV39H1 | suppressor of var | suppressc | suppressc | 6839 | 6839 | 6839 | s13660 | s13658 | s13659 |
| 1963 | B4 | B5 | 783 | 668,5 | 11960 | 490,8 | 15,3 | 1,33 | H5A034 C | sample | Hm Drgbl | Hm Drgbl | Hm Drgbl | CPE002FX | CPE002G1 | CPE002G5 | NM 00100 | NM 0010 | NM 0010 | ZNF774 | ZNF774 | ZNF774 | zinc finger protein | zinc finger | zinc finger | 342132 | 342132 | 342132 | s50885 | s50886 | s50887 |
| 6735 | I22 | I23 | 1131 | 603,2 | 17264 | 545,8 | 15,3 | 1,342 | H5A034 C | sample | Hm Drgbl | Hm Drgbl | Hm Drgbl | CPE002E1 | CPE002EY | CPE002FJ | XM 00113 | XM 0011 | XM 0011 | FOXK2 | FOXK2 | FOXK2 | forkhead box K2 | forkhead | forkhead | 3607 | 3607 | 3607 | s7395 | s7396 | s7397 |
| 6873 | O17 | O18 | 1306 | 556,6 | 19960 | 532,6 | 15,3 | 1,342 | H5A034 C | sample | Hm Drgbl | Hm Drgbl | Hm Drgbl | CPE002E1 | CPE002EY | CPE002FJ | NM 15367 | NM 1536 | NM 1536 | FOXA2 | FOXA2 | FOXA2 | forkhead box A2 | forkhead | forkhead | 3170 | 3170 | 3170 | s6690 | s6691 | s6692 |
| 3948 | E2 | E3 | 894 | 688,9 | 13720 | 436,1 | 15,3 | 1,36 | H5A034 C | sample | Hm Drgbl | Hm Drgbl | Hm Drgbl | CPE002DS | CPE002EI | CPE002F8 | NM 00286 | NM 0028 | NM 0028 | RAB5B | RAB5B | RAB5B | RAB5B, member R | RAB5B, m | RAB5B, m | 5869 | 5869 | 5869 | s11683 | s11681 | s11682 |
| 4020 | H2 | H3 | 1250 | 666 | 19155 | 416 | 15,3 | 1,36 | H5A034 C | sample | Hm Drgbl | Hm Drgbl | Hm Drgbl | CPE002DS | CPE002EI | CPE002F8 | NM 00293 | NM 0029 | NM 0029 | RNASE3 | RNASE3 | RNASE3 | ribonuclease, RNa | ribonucle | ribonucle | 6037 | 6037 | 6037 | s12055 | s12056 | s12057 |
| 4084 | K12 | K13 | 1024 | 660,2 | 15706 | 367,9 | 15,3 | 1,36 | H5A034 C | sample | Hm Drgbl | Hm Drgbl | Hm Drgbl | CPE002DS | CPE002EI | CPE002F8 | NM 00288 | NM 0028 | NM 0028 | RARRES1 | RARRES1 | RARRES1 | retinoic acid rece | retinoic a | retinoic a | 5918 | 5918 | 5918 | s11812 | s224518 | s11814 |
| 4183 | O15 | O16 | 1466 | 706,4 | 22415 | 501,1 | 15,3 | 1,36 | H5A034 C | sample | Hm Drgbl | Hm Drgbl | Hm Drgbl | CPE002DS | CPE002EI | CPE002F8 | NM 00094 | NM 0009 | NM 0009 | PPIB | PPIB | PPIB | peptidylprolyl iso | peptidylp | peptidylp | 5479 | 5479 | 5479 | s10907 | s10908 | s224317 |
| 4209 | P17 | P18 | 977 | 612,2 | 14960 | 383,6 | 15,3 | 1,36 | H5A034 C | sample | Hm Drgbl | Hm Drgbl | Hm Drgbl | CPE002DS | CPE002EI | CPE002F8 | NM 00585 | NM 0058 | NM 0058 | PURA | PURA | PURA | purine-rich eleme | purine-ric | purine-ric | 5813 | 5813 | 5813 | s11592 | s11593 | s11591 |
| 4219 | P4 | P5 | 1238 | 733,5 | 18973 | 434,8 | 15,3 | 1,36 | H5A034 C | sample | Hm Drgbl | Hm Drgbl | Hm Drgbl | CPE002DS | CPE002EI | CPE002F8 | NM 00540 | NM 0054 | NM 0054 | SALL2 | SALL2 | SALL2 | sal-like 2 (Drosop | sal-like 2 ( | sal-like 2 ( | 6297 | 6297 | 6297 | s12461 | s12462 | s12463 |
| 11650 | F18 | F19 | 1326 | 827,3 | 20322 | 170,1 | 15,3 | 1,366 | H5A034 C | sample | Hm Gen E | Hm Gen E | Hm Gen E | CPE002JA | CPE002NJ | CPE002JZ | NM 00532 | NM 0053 | NM 0053 | HIST1H1A | HIST1H1A | HIST1H1A | histone cluster 1, | histone cl | histone cl | 3024 | 3024 | 3024 | s6427 | s6428 | s226274 |
| 11678 | G21 | G22 | 1381 | 853,5 | 21070 | 169,1 | 15,3 | 1,366 | H5A034 C | sample | Hm Gen E | Hm Gen E | Hm Gen E | CPE002JA | CPE002NJ | CPE002JZ | NM 00049 | NM 0004 | NM 0004 | C1QB | C1QB | C1QB | complement com | compleme | compleme | 713 | 713 | 713 | s2146 | s2147 | s2148 |
| 15384 | A9 | A10 | 1333 | 859,3 | 20461 | 201,2 | 15,3 | 1,391 | H5A034 C | sample | Hm Gen E | Hm Gen E | Hm Gen E | CPE002JE | CPE002NT | CPE002K9 | NM 02163 | NM 0216 | NM 0216 | GPBP1L1 | GPBP1L1 | GPBP1L1 | GC-rich promoter | GC-rich pr | GC-rich pr | 60313 | 60313 | 60313 | s34040 | s34041 | s34042 |
| 15491 | F19 | F20 | 1403 | 830,6 | 21406 | 158,6 | 15,3 | 1,391 | H5A034 C | sample | Hm Gen E | Hm Gen E | Hm Gen E | CPE002JE | CPE002NT | CPE002K9 | NM 00104 | NM 0010 | NM 0010 | FLJ21865 | FLJ21865 | FLJ21865 | endo-beta-N-acet | endo-beta | endo-beta | 64772 | 64772 | 64772 | s34908 | s34909 | s34910 |
| 15673 | N1 | N2 | 984 | 802,5 | 15022 | 148,6 | 15,3 | 1,391 | H5A034 C | sample | Hm Gen E | Hm Gen E | Hm Gen E | CPE002JE | CPE002NT | CPE002K9 | NM 02290 | NM 0229 | NM 0229 | NT5DC2 | NT5DC2 | NT5DC2 | 5'-nucleotidase d | 5'-nucleot | 5'-nucleot | 64943 | 64943 | 64943 | s35105 | s35106 | s35107 |
| 15674 | N10 | N11 | 1535 | 832,7 | 23415 | 146 | 15,3 | 1,391 | H5A034 C | sample | Hm Gen E | Hm Gen E | Hm Gen E | CPE002JE | CPE002NT | CPE002K9 | NM 02455 | NM 0245 | NM 0245 | RIC3 | RIC3 | RIC3 | resistance to inhi | resistance | resistance | 79608 | 79608 | 79608 | s35905 | s35906 | s35907 |
| 5806 | B7 | B8 | 1069 | 852,3 | 16407 | 585,2 | 15,3 | 1,397 | H5A034 C | sample | Hm Drgbl | Hm Drgbl | Hm Drgbl | CPE002DY | CPE002F1 | CPE002FM | NM 00103 | NM 0010 | NM 0010 | ALDH3B2 | ALDH3B2 | ALDH3B2 | aldehyde dehydro | aldehyde | aldehyde | 222 | 222 | 222 | s1252 | s1253 | s1251 |
| 5865 | E17 | E18 | 1243 | 783,5 | 19000 | 388,5 | 15,3 | 1,397 | H5A034 C | sample | Hm Drgbl | Hm Drgbl | Hm Drgbl | CPE002DY | CPE002F1 | CPE002FM | NM 00161 | NM 0016 | NM 0016 | ACTA2 | ACTA2 | ACTA2 | actin, alpha 2, sm | actin, alph | actin, alph | 59 | 59 | 59 | s945 | s944 | s946 |
| 5925 | G6 | G7 | 1842 | 849,8 | 28254 | 471,6 | 15,3 | 1,397 | H5A034 C | sample | Hm Drgbl | Hm Drgbl | Hm Drgbl | CPE002DY | CPE002F1 | CPE002FM | NM 00517 | NM 0051 | NM 0051 | ASCL2 | ASCL2 | ASCL2 | achaete-scute cor | achaete-s | achaete-s | 430 | 430 | 430 | s224049 | s1660 | s1661 |
| 5974 | I7 | I8 | 1038 | 734,6 | 15916 | 407,5 | 15,3 | 1,397 | H5A034 C | sample | Hm Drgbl | Hm Drgbl | Hm Drgbl | CPE002DY | CPE002F1 | CPE002FM | NM 02098 | NM 0209 | NM 0209 | ADCY6 | ADCY6 | ADCY6 | adenylate cyclase | adenylate | adenylate | 112 | 112 | 112 | s1030 | s1028 | s1029 |
| 1227 | D11 | D12 | 1385 | 758,7 | 21193 | 456 | 15,3 | 1,404 | H5A034 C | sample | Hm Drgbl | Hm Drgbl | Hm Drgbl | CPE002FV | CPE002FZ | CPE002G3 | NM 00100 | NM 0010 | NM 0010 | SPOP | SPOP | SPOP | speckle-type POZ | speckle-ty | speckle-ty | 8405 | 8405 | 8405 | s15954 | s15955 | s15956 |
| 1238 | D21 | D22 | 1053 | 711,1 | 16074 | 427,8 | 15,3 | 1,404 | H5A034 C | sample | Hm Drgbl | Hm Drgbl | Hm Drgbl | CPE002FV | CPE002FZ | CPE002G3 | XM 00112 | XM 0011 | XM 0011 | KLF11 | KLF11 | KLF11 | Kruppel-like facto | Kruppel-li | Kruppel-li | 8462 | 8462 | 8462 | s16072 | s16073 | s16074 |
| 1276 | F12 | F13 | 1472 | 731,2 | 22543 | 441,3 | 15,3 | 1,404 | H5A034 C | sample | Hm Drgbl | Hm Drgbl | Hm Drgbl | CPE002FV | CPE002FZ | CPE002G3 | NM 00681 | NM 0068 | NM 0068 | LMAN2 | LMAN2 | LMAN2 | lectin, mannose-b | lectin, ma | lectin, ma | 10960 | 10960 | 10960 | s21573 | s21574 | s21575 |
| 1393 | K1 | K2 | 823 | 660,5 | 12589 | 422,4 | 15,3 | 1,404 | H5A034 C | sample | Hm Drgbl | Hm Drgbl | Hm Drgbl | CPE002FV | CPE002FZ | CPE002G3 | NM 00343 | NM 0034 | NM 0034 | ZNF135 | ZNF135 | ZNF135 | zinc finger protein | zinc finger | zinc finger | 7694 | 7694 | 7694 | s15171 | s15172 | s15173 |
| 1511 | O8 | O9 | 978 | 825,6 | 14952 | 486 | 15,3 | 1,404 | H5A034 C | sample | Hm Drgbl | Hm Drgbl | Hm Drgbl | CPE002FV | CPE002FZ | CPE002G3 | NM 00643 | NM 0064 | NM 0064 | CCT2 | CCT2 | CCT2 | chaperonin conta | chaperoni | chaperoni | 10576 | 10576 | 10576 | s20756 | s20757 | s20758 |
| 16139 | A19 | A20 | 977 | 1110 | 14980 | 374,1 | 15,3 | 1,404 | H5A034 C | sample | Hm Gen E | Hm Gen E | Hm Gen E | CPE002JG | CPE002NV | CPE002KB | NM 03091 | NM 0309 | NM 0309 | FIP1L1 | FIP1L1 | FIP1L1 | FIP1 like 1 (S. cere | FIP1 like 1 | FIP1 like 1 | 81608 | 81608 | 81608 | s37692 | s37693 | s37694 |
| 16364 | J5 | J6 | 1049 | 1007 | 16071 | 367,2 | 15,3 | 1,404 | H5A034 C | sample | Hm Gen E | Hm Gen E | Hm Gen E | CPE002JG | CPE002NV | CPE002KB | NM 03212 | NM 0321 | NM 0321 | DKFZP564 | DKFZP564 | DKFZP564 | hypothetical prot | hypotheti | hypotheti | 84060 | 84060 | 84060 | s38421 | s38422 | s228332 |
| 16391 | K8 | K9 | 703 | 989,8 | 10763 | 388,5 | 15,3 | 1,404 | H5A034 C | sample | Hm Gen E | Hm Gen E | Hm Gen E | CPE002JG | CPE002NV | CPE002KB | NM 03234 | NM 0323 | NM 0323 | CHCHD6 | CHCHD6 | CHCHD6 | coiled-coil-helix-c | coiled-coi | coiled-coi | 84303 | 84303 | 84303 | s38867 | s38868 | s38869 |
| 16466 | O10 | O11 | 919 | 1076 | 14029 | 422,1 | 15,3 | 1,404 | H5A034 C | sample | Hm Gen E | Hm Gen E | Hm Gen E | CPE002JG | CPE002NV | CPE002KB | NM 03237 | NM 0323 | NM 0323 | C14orf153 | C14orf153 | C14orf153 | chromosome 14 c | chromoso | chromoso | 84334 | 84334 | 84334 | s38942 | s38943 | s38944 |
| 13643 | I19 | I20 | 1142 | 1018 | 17437 | 162,1 | 15,3 | 1,437 | H5A034 C | sample | Hm Gen E | Hm Gen E | Hm Gen E | CPE002JC | CPE002NC | CPE002K4 | NM 01207 | NM 0120 | NM 0120 | COMMD3 | COMMD3 | COMMD3 | COMM domain co | COMM dc | COMM dc | 23412 | 23412 | 23412 | s23772 | s23773 | s23774 |
| 906 | F3 | F4 | 983 | 725,8 | 15043 | 479,6 | 15,3 | 1,45 | H5A034 C | sample | Hm Drgbl | Hm Drgbl | Hm Drgbl | CPE002FU | CPE002FY | CPE002G2 | NM 02400 | NM 0240 | NM 0240 | GJB3 | GJB3 | GJB3 | gap junction prot | gap juncti | gap juncti | 2707 | 2707 | 2707 | s5778 | s5779 | s5780 |
| 908 | F5 | F6 | 1362 | 737,7 | 20827 | 490,6 | 15,3 | 1,45 | H5A034 C | sample | Hm Drgbl | Hm Drgbl | Hm Drgbl | CPE002FU | CPE002FY | CPE002G2 | NM 00526 | NM 0052 | NM 0052 | GJB5 | GJB5 | GJB5 | gap junction prot | gap juncti | gap juncti | 2709 | 2709 | 2709 | s5781 | s5782 | s223727 |
| 1117 | O20 | O21 | 1456 | 728,9 | 22344 | 522,3 | 15,3 | 1,45 | H5A034 C | sample | Hm Drgbl | Hm Drgbl | Hm Drgbl | CPE002FU | CPE002FY | CPE002G2 | NM 00285 | NM 0028 | NM 0028 | ARID4A | ARID4A | ARID4A | AT rich interactive | AT rich int | AT rich int | 5926 | 5926 | 5926 | s11831 | s11832 | s11833 |
| 1141 | P20 | P21 | 1089 | 723,4 | 16702 | 438,6 | 15,3 | 1,45 | H5A034 C | sample | Hm Drgbl | Hm Drgbl | Hm Drgbl | CPE002FU | CPE002FY | CPE002G2 | NM 00508 | NM 0050 | NM 0050 | XBP1 | XBP1 | XBP1 | X-box binding pro | X-box bin | X-box bin | 7494 | 7494 | 7494 | s14913 | s14914 | s14915 |
| 3681 | J17 | J18 | 1201 | 1294 | 18382 | 345,8 | 15,3 | 1,471 | H5A034 C | sample | Hm Drgbl | Hm Drgbl | Hm Drgbl | CPE002DR | CPE002EH | CPE002F7 | NM 00087 | NM 0008 | NM 0008 | HTR4 | HTR4 | HTR4 | 5-hydroxytryptam | 5-hydroxy | 5-hydroxy | 3360 | 3360 | 3360 | s7053 | s7054 | s7055 |
| 3811 | O4 | O5 | 1203 | 1311 | 18414 | 435,2 | 15,3 | 1,471 | H5A034 C | sample | Hm Drgbl | Hm Drgbl | Hm Drgbl | CPE002DR | CPE002EH | CPE002F7 | NM 03255 | NM 0325 | NM 0325 | GPR81 | GPR81 | GPR81 | G protein-couplec | G protein | G protein | 27198 | 27198 | 27198 | s25980 | s25981 | s25982 |
| 3814 | O7 | O8 | 1576 | 1512 | 24167 | 438,7 | 15,3 | 1,471 | H5A034 C | sample | Hm Drgbl | Hm Drgbl | Hm Drgbl | CPE002DR | CPE002EH | CPE002F7 | NM 00051 | NM 0005 | NM 0005 | OPN1MW | OPN1MW | OPN1MW | opsin 1 (cone pig | opsin 1 (c | opsin 1 (c | 2652 | 2652 | 2652 | s5669 | s5670 | s5671 |
| 3840 | P9 | P10 | 685 | 1671 | 10451 | 414,9 | 15,3 | 1,471 | H5A034 C | sample | Hm Drgbl | Hm Drgbl | Hm Drgbl | CPE002DR | CPE002EH | CPE002F7 | NM 17679 | NM 1767 | NM 0041 | P2RY6 | P2RY6 | P2RY6 | pyrimidinergic rec | pyrimidin | pyrimidin | 5031 | 5031 | 5031 | s9971 | s9972 | s224151 |
| 10128 | F9 | F10 | 1188 | 791,7 | 18151 | 146,4 | 15,3 | 1,471 | H5A034 C | sample | Hm Drgbl | Hm Drgbl | Hm Drgbl | CPE002EA | CPE002EP | CPE002FA | NM 02526 | NM 0252 | NM 0252 | THUMPD2 | THUMPD2 | THUMPD2 | THUMP domain c | THUMP dc | THUMP dc | 80745 | 80745 | 80745 | s37344 | s37345 | s37346 |
| 10181 | I13 | I14 | 1865 | 833,9 | 28596 | 155,8 | 15,3 | 1,471 | H5A034 C | sample | Hm Drgbl | Hm Drgbl | Hm Drgbl | CPE002EA | CPE002EP | CPE002FA | NM 02481 | XM 0011 | XM 0011 | RABEP2 | RABEP2 | RABEP2 | rabaptin, RAB GT | rabaptin, | rabaptin, | 79874 | 79874 | 79874 | s195385 | s36543 | s36544 |
| 10262 | L21 | L22 | 964 | 755,9 | 14717 | 155,8 | 15,3 | 1,471 | H5A034 C | sample | Hm Drgbl | Hm Drgbl | Hm Drgbl | CPE002EA | CPE002EP | CPE002FA | NM 00100 | NM 0010 | NM 0010 | NIPA2 | NIPA2 | NIPA2 | non imprinted in | non impri | non impri | 81614 | 81614 | 81614 | s37704 | s37705 | s37706 |
| 1598 | C21 | C22 | 897 | 699,8 | 13754 | 425,3 | 15,3 | 1,602 | H5A034 C | sample | Hm Drgbl | Hm Drgbl | Hm Drgbl | CPE002FW | CPE002G0 | CPE002G4 | NM 01225 | NM 0122 | NM 0122 | HBP1 | HBP1 | HBP1 | HMG-box transcri | HMG-box | HMG-box | 26959 | 26959 | 26959 | s25646 | s25647 | s25648 |
| 1853 | N13 | N14 | 1303 | 724,1 | 19922 | 412,7 | 15,3 | 1,602 | H5A034 C | sample | Hm Drgbl | Hm Drgbl | Hm Drgbl | CPE002FW | CPE002G0 | CPE002G4 | NM 02104 | NM 0210 | NM 0210 | ZNF253 | ZNF253 | ZNF253 | zinc finger protein | zinc finger | zinc finger | 56242 | 56242 | 56242 | s32066 | s32067 | s32068 |
| 21565 | C20 | C21 | 313 | 1002 | 4792 | 750,4 | 15,3 | 1,611 | H5A034 C | sample | Hm Gen E | Hm Gen E | Hm Gen E | CPE002JU | CPE002O9 | CPE002KP | XM 94211 | XM 9421 | XM 9421 | LOC65259 | LOC65259 | LOC65259 | similar to tripartit | similar to | similar to | 652591 | 652591 | 652591 | s57399 | s57401 | s227402 |
| 21583 | D15 | D16 | 410 | 1011 | 6263 | 680,6 | 15,3 | 1,611 | H5A034 C | sample | Hm Gen E | Hm Gen E | Hm Gen E | CPE002JU | CPE002O9 | CPE002KP | XM 94012 | XM 9401 | XM 9401 | LOC65100 | LOC65100 | LOC65100 | similar to mitoger | similar to | similar to | 651008 | 651008 | 651008 | s57159 | s197766 | s227356 |
| 21594 | D3 | D4 | 312 | 876,4 | 4761 | 455 | 15,3 | 1,611 | H5A034 C | sample | Hm Gen E | Hm Gen E | Hm Gen E | CPE002JU | CPE002O9 | CPE002KP | XM 94601 | XM 9460 | XM 9460 | LOC65090 | LOC65090 | LOC65090 | similar to ribosom | similar to | similar to | 650901 | 650901 | 650901 | s197759 | s197760 | s197761 |
| 4837 | J20 | J21 | 1355 | 732,3 | 20712 | 439,8 | 15,3 | 1,681 | H5A034 C | sample | Hm Drgbl | Hm Drgbl | Hm Drgbl | CPE002DV | CPE002EL | CPE002FP | NM 00104 | NM 0010 | NM 0010 | ARTS-1 | ARTS-1 | ARTS-1 | type 1 tumor nec | type 1 tur | type 1 tur | 51752 | 51752 | 51752 | s28616 | s28617 | s28618 |
| 4958 | O21 | O22 | 1237 | 865,4 | 18926 | 339,2 | 15,3 | 1,681 | H5A034 C | sample | Hm Drgbl | Hm Drgbl | Hm Drgbl | CPE002DV | CPE002EL | CPE002FP | NM 00050 | NM 0005 | NM 0005 | F12 | F12 | F12 | coagulation facto | coagulatic | coagulatic | 2161 | 2161 | 2161 | s4952 | s4953 | s4954 |
| 10581 | I6 | I7 | 1048 | 818,4 | 16030 | 203,7 | 15,3 | 1,729 | H5A034 C | sample | Hm Drgbl | Hm Drgbl | Hm Drgbl | CPE002EB | CPE002EO | CPE002FR | NM 17060 | NM 1706 | NM 1706 | RASGRP4 | RASGRP4 | RASGRP4 | RAS guanyl releas | RAS guany | RAS guany | 115727 | 115727 | 115727 | s41862 | s41861 | s41860 |
| 13398 | O14 | O15 | 1723 | 1153 | 26468 | 179,4 | 15,4 | 0,772 | H5A034 C | sample | Hm Gen E | Hm Gen E | Hm Gen E | CPE002JB | CPE002NN | CPE002K3 | NM 01504 | NM 0150 | NM 0150 | WAPAL | WAPAL | WAPAL | wings apart-like h | wings apa | wings apa | 23063 | 23063 | 23063 | s22947 | s22948 | s22949 |
| 22626 | O3 | O4 | 1292 | 1306 | 19873 | 449,8 | 15,4 | 0,794 | H5A034 C | sample | Hm Gen E | Hm Gen E | Hm Gen E | CPE002JW | CPE002OB | CPE002KR | NM 00108 | NM 0010 | NM 0010 | hCG 1789 | hCG 1789 | hCG 1789 | protein (peptidylp | protein (p | protein (p | 728758 | 728758 | 728758 | s58975 | s58976 | s58977 |
| 23803 | P4 | P5 | 936 | 1816 | 14419 | 289,2 | 15,4 | 0,817 | H5A034 C | sample | Hm Gen E | Hm Gen E | Hm Gen E | CPE002KV | CPE002OE | CPE002KU | | | | | | | | | | | | | | | |
| 14743 | G15 | G16 | 1342 | 870,7 | 20718 | 81,59 | 15,4 | 0,877 | H5A034 C | sample | Hm Gen E | Hm Gen E | Hm Gen E | CPE002JD | CPE002NR | CPE002K7 | NM 18135 | NM 1813 | NM 1813 | OXR1 | OXR1 | OXR1 | oxidation resistar | oxidation | oxidation | 55074 | 55074 | 55074 | s30111 | s30112 | s30113 |
| 13859 | B19 | B20 | 1249 | 1121 | 19241 | 103,4 | 15,4 | 0,883 | H5A034 C | sample | Hm Gen E | Hm Gen E | Hm Gen E | CPE002JF | CPE002NP | CPE002K5 | NM 02468 | NM 0246 | NM 0246 | C11orf67 | C11orf67 | C11orf67 | chromosome 11 c | chromoso | chromoso | 28971 | 28971 | 28971 | s26325 | s226236 | s226237 |
| 13904 | D16 | D17 | 1149 | 938,7 | 17742 | 142,5 | 15,4 | 0,883 | H5A034 C | sample | Hm Gen E | Hm Gen E | Hm Gen E | CPE002JF | CPE002NP | CPE002K5 | NM 01601 | NM 0160 | NM 0160 | NDUFAF1 | NDUFAF1 | NDUFAF1 | NADH dehydroge | NADH deh | NADH deh | 51103 | 51103 | 51103 | s27437 | s27438 | s27439 |
| 3104 | B16 | B17 | 973 | 1186 | 15012 | 356,6 | 15,4 | 0,954 | H5A034 C | sample | Hm Drgbl | Hm Drgbl | Hm Drgbl | CPE002DC | CPE002EG | CPE002F6 | NM 00620 | NM 0062 | NM 0062 | PDE6H | PDE6H | PDE6H | phosphodiesteras | phosphod | phosphod | 5149 | 5149 | 5149 | s10213 | s10214 | s10215 |
| 3381 | M6 | M7 | 496 | 1211 | 7626 | 380,4 | 15,4 | 0,954 | H5A034 C | sample | Hm Drgbl | Hm Drgbl | Hm Drgbl | CPE002DC | CPE002EG | CPE002F6 | NM 00103 | NM 0010 | NM 0010 | PPAPDC1A | PPAPDC1A | PPAPDC1A | phosphatidic acid | phosphat | phosphat | 196051 | 196051 | 196051 | s46967 | s223470 | s46969 |
| 3445 | P20 | P21 | 1377 | 1337 | 21150 | 558,9 | 15,4 | 0,954 | H5A034 C | sample | Hm Drgbl | Hm Drgbl | Hm Drgbl | CPE002DC | CPE002EG | CPE002F6 | NM 00093 | NM 0009 | NM 0009 | PLCB4 | PLCB4 | PLCB4 | phospholipase C, | phospholi | phospholi | 5332 | 5332 | 5332 | s10622 | s10623 | s10624 |
| 18087 | B22 | B23 | 1560 | 1070 | 24090 | 308,8 | 15,4 | 0,99 | H5A034 C | sample | Hm Gen E | Hm Gen E | Hm Gen E | CPE002JL | CPE002OC | CPE002KG | NM 14575 | NM 1457 | NM 1457 | TTC21A | TTC21A | TTC21A | tetratricopeptide | tetratrico | tetratrico | 199223 | 199223 | 199223 | s47112 | s47113 | s47114 |
| 19791 | I22 | I23 | 938 | 387,7 | 14443 | 364,8 | 15,4 | 1,003 | H5A034 C | sample | Hm Gen E | Hm Gen E | Hm Gen E | CPE002JP | CPE002O4 | CPE002KK | NM 20740 | NM 2074 | NM 2074 | FLJ27505 | FLJ27505 | FLJ27505 | FLJ27505 protein | FLJ27505 | FLJ27505 | 389320 | 389320 | 389320 | s52436 | s52437 | s52438 |
| 6162 | A3 | A4 | 1302 | 556,5 | 20035 | 557 | 15,4 | 1,017 | H5A034 C | sample | Hm Drgbl | Hm Drgbl | Hm Drgbl | CPE002DZ | CPE002F0 | CPE002FL | NM 00176 | NM 0017 | NM 0017 | CCND3 | CCND3 | CCND3 | cyclin D3 | cyclin D3 | cyclin D3 | 896 | 896 | 896 | s2523 | s2522 | s2521 |
| 6216 | C9 | C10 | 804 | 501,3 | 12384 | 578 | 15,4 | 1,017 | H5A034 C | sample | Hm Drgbl | Hm Drgbl | Hm Drgbl | CPE002DZ | CPE002F0 | CPE002FL | NM 00061 | NM 0006 | NM 0006 | CD4 | CD4 | CD4 | CD4 molecule | CD4 mole | CD4 mole | 920 | 920 | 920 | s2580 | s225098 | s2578 |
| 6222 | D14 | D15 | 1455 | 548,1 | 22413 | 464,1 | 15,4 | 1,017 | H5A034 C | sample | Hm Drgbl | Hm Drgbl | Hm Drgbl | CPE002DZ | CPE002F0 | CPE002FL | NM 00079 | NM 0007 | NM 0007 | DHFR | DHFR | DHFR | dihydrofolate red | dihydrofo | dihydrofo | 1719 | 1719 | 1719 | s4080 | s4082 | s4081 |
| 6277 | F20 | F21 | 1711 | 608,7 | 26390 | 534,6 | 15,4 | 1,017 | H5A034 C | sample | Hm Drgbl | Hm Drgbl | Hm Drgbl | CPE002DZ | CPE002F0 | CPE002FL | NM 00408 | NM 0040 | NM 0040 | DLG1 | DLG1 | DLG1 | discs, large homo | discs, larg | discs, larg | 1739 | 1739 | 1739 | s4119 | s4120 | s4121 |
| 6350 | I21 | I22 | 1675 | 581,4 | 25805 | 415,9 | 15,4 | 1,017 | H5A034 C | sample | Hm Drgbl | Hm Drgbl | Hm Drgbl | CPE002DZ | CPE002F0 | CPE002FL | NM 00179 | NM 0017 | NM 0017 | CDH3 | CDH3 | CDH3 | cadherin 3, type 1 | cadherin | cadherin | 1001 | 1001 | 1001 | s2775 | s2776 | s2774 |
| 6404 | K5 | K6 | 1162 | 537,1 | 17847 | 502,5 | 15,4 | 1,017 | H5A034 C | sample | Hm Drgbl | Hm Drgbl | Hm Drgbl | CPE002DZ | CPE002F0 | CPE002FL | NM 00493 | NM 0049 | NM 0049 | CDH6 | CDH6 | CDH6 | cadherin 6, type 2 | cadherin | cadherin | 1004 | 1004 | 1004 | s2785 | s2784 | s2783 |
| 6450 | M3 | M4 | 1121 | 589,3 | 17251 | 492,6 | 15,4 | 1,017 | H5A034 C | sample | Hm Drgbl | Hm Drgbl | Hm Drgbl | CPE002DZ | CPE002F0 | CPE002FL | NM 00406 | NM 0040 | NM 0040 | CDH17 | CDH17 | CDH17 | cadherin 17, LI ca | cadherin | cadherin | 1015 | 1015 | 1015 | s2813 | s2814 | s2815 |
| 6507 | P11 | P12 | 1262 | 566,3 | 19486 | 541,1 | 15,4 | 1,017 | H5A034 C | sample | Hm Drgbl | Hm Drgbl | Hm Drgbl | CPE002DZ | CPE002F0 | CPE002FL | NM 02111 | NM 0211 | NM 0211 | CRY2 | CRY2 | CRY2 | cryptochrome 2 (i | cryptochr | cryptochr | 1408 | 1408 | 1408 | s3537 | s3538 | s3539 |
| 20436 | D2 | D3 | 714 | 737,5 | 10967 | 181,9 | 15,4 | 1,027 | H5A034 C | sample | Hm Gen E | Hm Gen E | Hm Gen E | CPE002JR | CPE002O6 | CPE002KM | XM 00113 | XM 0011 | XM 0011 | LOC64193 | LOC64193 | LOC64193 | similar to zinc fing | similar to | similar to | 641930 | 641930 | 641930 | s54890 | s196736 | s196737 |
| 20471 | E8 | E9 | 865 | 745,2 | 13300 | 207,9 | 15,4 | 1,027 | H5A034 C | sample | Hm Gen E | Hm Gen E | Hm Gen E | CPE002JR | CPE002O6 | CPE002KM | NM 00100 | NM 0010 | NM 0010 | LOC49386 | LOC49386 | LOC49386 | similar to RIKEN c | similar to | similar to | 493869 | 493869 | 493869 | s54628 | s54629 | s54630 |
| 20516 | G5 | G6 | 860 | 759,7 | 13203 | 161,7 | 15,4 | 1,027 | H5A034 C | sample | Hm Gen E | Hm Gen E | Hm Gen E | CPE002JR | CPE002O6 | CPE002KM | XM 49870 | XM 4987 | XM 4987 | LOC44049 | LOC44049 | LOC44049 | hypothetical gene | hypotheti | hypotheti | 440498 | 440498 | 440498 | s54121 | s54123 | s226619 |
| 20524 | H12 | H13 | 785 | 757,9 | 12074 | 190,4 | 15,4 | 1,027 | H5A034 C | sample | Hm Gen E | Hm Gen E | Hm Gen E | CPE002JR | CPE002O6 | CPE002KM | XM 00112 | XM 0011 | XM 0011 | LOC64224 | LOC64224 | LOC64224 | hypothetical prot | hypotheti | hypotheti | 642249 | 642249 | 642249 | s54960 | s54961 | s196774 |
| 20540 | H5 | H6 | 926 | 811,2 | 14287 | 158,4 | 15,4 | 1,027 | H5A034 C | sample | Hm Gen E | Hm Gen E | Hm Gen E | CPE002JR | CPE002O6 | CPE002KM | NM 00100 | NM 0010 | NM 0010 | CT45-5 | CT45-5 | CT45-5 | cancer/testis anti | cancer/tes | cancer/tes | 441521 | 441521 | 441521 | s54385 | s54386 | s54387 |
| 20544 | H9 | H10 | 958 | 777,2 | 14723 | 204 | 15,4 | 1,027 | H5A034 C | sample | Hm Gen E | Hm Gen E | Hm Gen E | CPE002JR | CPE002O6 | CPE002KM | XM 49919 | XM 4991 | XM 4991 | LOC44154 | LOC44154 | LOC44154 | hypothetical LOC | hypotheti | hypotheti | 441546 | 441546 | 441546 | s54394 | s196623 | s196624 |
| 20640 | L9 | L10 | 586 | 745,4 | 9009 | 193,9 | 15,4 | 1,027 | H5A034 C | sample | Hm Gen E | Hm Gen E | Hm Gen E | CPE002JR | CPE002O6 | CPE002KM | NM 00100 | NM 0010 | NM 0010 | OR10J3 | OR10J3 | OR10J3 | olfactory receptor | olfactory | olfactory | 441911 | 441911 | 441911 | s54453 | s54454 | s54455 |
| 18457 | B1 | B2 | 1380 | 1044 | 21252 | 379,3 | 15,4 | 1,044 | H5A034 C | sample | Hm Gen E | Hm Gen E | Hm Gen E | CPE002JM | CPE002O1 | CPE002KH | NM 00100 | NM 0010 | NM 0010 | OR1S2 | OR1S2 | OR1S2 | olfactory receptor | olfactory | olfactory | 219958 | 219958 | 219958 | s47790 | s47791 | s47792 |
| 18754 | N18 | N19 | 1403 | 904,2 | 21539 | 268,5 | 15,4 | 1,044 | H5A034 C | sample | Hm Gen E | Hm Gen E | Hm Gen E | CPE002JM | CPE002O1 | CPE002KH | NM 15278 | NM 1527 | NM 1527 | MAP7D2 | MAP7D2 | MAP7D2 | MAP7 domain co | MAP7 dor | MAP7 dor | 256714 | 256714 | 256714 | s48811 | s48812 | s48813 |
| 18757 | N20 | N21 | 1501 | 955,4 | 23149 | 262,2 | 15,4 | 1,044 | H5A034 C | sample | Hm Gen E | Hm Gen E | Hm Gen E | CPE002JM | CPE002O1 | CPE002KH | NM 18275 | NM 1827 | NM 1827 | WDR72 | WDR72 | WDR72 | WD repeat domai | WD repea | WD repea | 256764 | 256764 | 256764 | s48814 | s48815 | s48816 |
| 17351 | C8 | C9 | 931 | 1021 | 14337 | 351,4 | 15,4 | 1,051 | H5A034 C | sample | Hm Gen E | Hm Gen E | Hm Gen E | CPE002JJ | CPE002NY | CPE002KE | NM 15239 | NM 1523 | NM 1523 | IQCF1 | IQCF1 | IQCF1 | IQ motif containi | IQ motif c | IQ motif c | 132141 | 132141 | 132141 | s43667 | s43668 | s43669 |
| 9340 | F12 | F13 | 1532 | 1045 | 23595 | 142,4 | 15,4 | 1,069 | H5A034 C | sample | Hm Drgbl | Hm Drgbl | Hm Drgbl | CPE002E8 | CPE002ER | CPE002FC | NM 02016 | NM 0201 | NM 0201 | RAD18 | RAD18 | RAD18 | RAD18 homolog ( | RAD18 ho | RAD18 ho | 56852 | 56852 | 56852 | s32295 | s32296 | s32297 |
| 9419 | I19 | I20 | 1648 | 1088 | 25394 | 158,1 | 15,4 | 1,069 | H5A034 C | sample | Hm Drgbl | Hm Drgbl | Hm Drgbl | CPE002E8 | CPE002ER | CPE002FC | NM 01812 | NM 0181 | NM 0181 | DARS2 | DARS2 | DARS2 | aspartyl-tRNA syr | aspartyl-t | aspartyl-t | 55157 | 55157 | 55157 | s30308 | s30307 | s30309 |
| 9433 | J1 | J2 | 1259 | 1191 | 19424 | 154,9 | 15,4 | 1,069 | H5A034 C | sample | Hm Drgbl | Hm Drgbl | Hm Drgbl | CPE002E8 | CPE002ER | CPE002FC | NM 01761 | NM 0176 | NM 0176 | ZCCHC8 | ZCCHC8 | ZCCHC8 | zinc finger, CCHC | zinc finger | zinc finger | 55596 | 55596 | 55596 | s31040 | s31039 | s31038 |
| 9457 | K1 | K2 | 1544 | 1092 | 23837 | 138,6 | 15,4 | 1,069 | H5A034 C | sample | Hm Drgbl | Hm Drgbl | Hm Drgbl | CPE002E8 | CPE002ER | CPE002FC | NM 01812 | NM 0181 | NM 0181 | PNPO | PNPO | PNPO | pyridoxamine 5-p | pyridoxan | pyridoxan | 55163 | 55163 | 55163 | s30319 | s30320 | s30321 |
| 9502 | L7 | L8 | 1555 | 1102 | 23888 | 133 | 15,4 | 1,069 | H5A034 C | sample | Hm Drgbl | Hm Drgbl | Hm Drgbl | CPE002E8 | CPE002ER | CPE002FC | NM 01781 | NM 0178 | NM 0178 | RAB20 | RAB20 | RAB20 | RAB20, member F | RAB20, m | RAB20, m | 55647 | 55647 | 55647 | s31159 | s31160 | s31158 |
| 19061 | K13 | K14 | 1447 | 962,4 | 22324 | 205,8 | 15,4 | 1,073 | H5A034 C | sample | Hm Gen E | Hm Gen E | Hm Gen E | CPE002JN | CPE002O2 | CPE002KI | NM 00100 | NM 0010 | NM 0010 | OR8D1 | OR8D1 | OR8D1 | olfactory receptor | olfactory | olfactory | 283159 | 283159 | 283159 | s49121 | s49122 | s49123 |
| 19069 | K20 | K21 | 1160 | 1055 | 17881 | 308,5 | 15,4 | 1,073 | H5A034 C | sample | Hm Gen E | Hm Gen E | Hm Gen E | CPE002JN | CPE002O2 | CPE002KI | NM 17364 | NM 1736 | NM 1736 | DNHL1 | DNHL1 | DNHL1 | dynein heavy cha | dynein he | dynein he | 284944 | 284944 | 284944 | s49773 | s49774 | s49775 |
| 19074 | K3 | K4 | 830 | 1041 | 12759 | 218,5 | 15,4 | 1,073 | H5A034 C | sample | Hm Gen E | Hm Gen E | Hm Gen E | CPE002JN | CPE002O2 | CPE002KI | NM 00103 | NM 0010 | NM 0010 | LOC28312 | LOC28312 | LOC28312 | hypothetical prot | hypotheti | hypotheti | 283129 | 283129 | 283129 | s49102 | s49103 | s49104 |
| 9657 | C17 | C18 | 1670 | 1051 | 25644 | 157,5 | 15,4 | 1,077 | H5A034 C | sample | Hm Drgbl | Hm Drgbl | Hm Drgbl | CPE002E9 | CPE002EQ | CPE002FB | NM 02067 | NM 0206 | NM 0206 | RAB22A | RAB22A | RAB22A | RAB22A, member | RAB22A, r | RAB22A, r | 57403 | 57403 | 57403 | s32992 | s32993 | s32994 |
| 9790 | H7 | H8 | 1864 | 1053 | 28640 | 125,5 | 15,4 | 1,077 | H5A034 C | sample | Hm Drgbl | Hm Drgbl | Hm Drgbl | CPE002E9 | CPE002EQ | CPE002FB | NM 02125 | NM 0212 | NM 0212 | IL22RA1 | IL22RA1 | IL22RA1 | interleukin 22 rec | interleuki | interleuki | 58985 | 58985 | 58985 | s33943 | s33944 | s33945 |
| 9968 | P16 | P17 | 1195 | 1044 | 18390 | 161,7 | 15,4 | 1,077 | H5A034 C | sample | Hm Drgbl | Hm Drgbl | Hm Drgbl | CPE002E9 | CPE002EQ | CPE002FB | NM 02455 | NM 0245 | NM 0245 | ADIPOR2 | ADIPOR2 | ADIPOR2 | adiponectin recep | adiponect | adiponect | 79602 | 79602 | 79602 | s35893 | s35895 | s35894 |
| 8479 | B15 | B16 | 1637 | 1086 | 25208 | 186,8 | 15,4 | 1,088 | H5A034 C | sample | Hm Drgbl | Hm Drgbl | Hm Drgbl | CPE002E6 | CPE002ET | CPE002FE | NM 01552 | NM 0155 | NM 0155 | MOXD1 | MOXD1 | MOXD1 | monooxygenase, | monooxy | monooxy | 26002 | 26002 | 26002 | s24910 | s24911 | s24912 |
| 8555 | E19 | E20 | 1611 | 1048 | 24877 | 128,2 | 15,4 | 1,088 | H5A034 C | sample | Hm Drgbl | Hm Drgbl | Hm Drgbl | CPE002E6 | CPE002ET | CPE002FE | NM 01232 | NM 0123 | NM 0123 | MAFF | MAFF | MAFF | v-maf musculoap | v-maf mu | v-maf mu | 23764 | 23764 | 23764 | s24371 | s24370 | s24372 |
| 8620 | H12 | H13 | 1572 | 1092 | 24236 | 112,2 | 15,4 | 1,088 | H5A034 C | sample | Hm Drgbl | Hm Drgbl | Hm Drgbl | CPE002E6 | CPE002ET | CPE002FE | NM 01326 | NM 0132 | NM 0132 | DDX25 | DDX25 | DDX25 | DEAD (Asp-Glu-Al | DEAD (Asp | DEAD (Asp | 29118 | 29118 | 29118 | s26527 | s26528 | s26529 |
| 8816 | P16 | P17 | 1718 | 1126 | 26519 | 129,4 | 15,4 | 1,088 | H5A034 C | sample | Hm Drgbl | Hm Drgbl | Hm Drgbl | CPE002E6 | CPE002ET | CPE002FE | NM 01345 | NM 0134 | NM 0134 | BAZ2B | BAZ2B | BAZ2B | bromodomain ad | bromodo | bromodo | 29994 | 29994 | 29994 | s26866 | s26867 | s26865 |
| 8823 | P22 | P23 | 1619 | 1089 | 24901 | 224,5 | 15,4 | 1,088 | H5A034 C | sample | Hm Drgbl | Hm Drgbl | Hm Drgbl | CPE002E6 | CPE002ET | CPE002FE | NM 01693 | NM 0169 | NM 0169 | EFEMP2 | EFEMP2 | EFEMP2 | EGF-containing fil | EGF-conta | EGF-conta | 30008 | 30008 | 30008 | s26888 | s26886 | s26887 |
| 19214 | A21 | A22 | 1250 | 1189 | 19272 | 361,5 | 15,4 | 1,092 | H5A034 C | sample | Hm Gen E | Hm Gen E | Hm Gen E | CPE002JO | CPE002O3 | CPE002KJ | NM 17585 | NM 1758 | NM 1758 | KRTAP8-1 | KRTAP8-1 | KRTAP8-1 | keratin associatec | keratin as | keratin as | 337879 | 337879 | 337879 | s50286 | s226288 | s226289 |
| 19324 | F12 | F13 | 611 | 1459 | 9408 | 353,3 | 15,4 | 1,092 | H5A034 C | sample | Hm Gen E | Hm Gen E | Hm Gen E | CPE002JO | CPE002O3 | CPE002KJ | NM 19828 | NM 1982 | NM 1982 | WDR86 | WDR86 | WDR86 | WD repeat domai | WD repea | WD repea | 349136 | 349136 | 349136 | s51381 | s196124 | s226367 |
| 20757 | A6 | A7 | 1213 | 1041 | 18730 | 370,3 | 15,4 | 1,092 | H5A034 C | sample | Hm Gen E | Hm Gen E | Hm Gen E | CPE002JS | CPE002O7 | CPE002KN | XM 93010 | XM 9301 | XM 9301 | LOC64407 | LOC64407 | LOC64407 | hypothetical LOC | hypotheti | hypotheti | 644075 | 644075 | 644075 | s55548 | s55549 | s55550 |
| 20874 | F3 | F4 | 1018 | 961,6 | 15707 | 221,2 | 15,4 | 1,092 | H5A034 C | sample | Hm Gen E | Hm Gen E | Hm Gen E | CPE002JS | CPE002O7 | CPE002KN | XM 92694 | XM 9269 | XM 9269 | LOC64363 | LOC64363 | LOC64363 | similar to hCG172 | similar to | similar to | 643637 | 643637 | 643637 | s55374 | s55375 | s55376 |
| 21109 | P20 | P21 | 1264 | 1063 | 19428 | 330,4 | 15,4 | 1,092 | H5A034 C | sample | Hm Gen E | Hm Gen E | Hm Gen E | CPE002JS | CPE002O7 | CPE002KN | XM 93492 | XM 9349 | XM 9349 | LOC64531 | LOC64531 | LOC64531 | similar to 16.7Kd | similar to | similar to | 645317 | 645317 | 645317 | s55997 | s55998 | s55999 |
| 21111 | P22 | P23 | 948 | 1111 | 14624 | 310,9 | 15,4 | 1,092 | H5A034 C | sample | Hm Gen E | Hm Gen E | Hm Gen E | CPE002JS | CPE002O7 | CPE002KN | XM 92835 | XM 9283 | XM 9283 | LOC64532 | LOC64532 | LOC64532 | hypothetical LOC | hypotheti | hypotheti | 645320 | 645320 | 645320 | s56000 | s56001 | s56002 |
| 21404 | L5 | L6 | 1043 | 879,5 | 16095 | 255,6 | 15,4 | 1,092 | H5A034 C | sample | Hm Gen E | Hm Gen E | Hm Gen E | CPE002JT | CPE002O8 | CPE002KO | XM 92933 | XM 9293 | XM 9293 | DGAT2L7 | DGAT2L7 | DGAT2L7 | diacylglycerol O-a | diacylglyc | diacylglyc | 646409 | 646409 | 646409 | s56363 | s56364 | s227233 |
| 21440 | N16 | N17 | 1023 | 884,6 | 15752 | 292,5 | 15,4 | 1,092 | H5A034 C | sample | Hm Gen E | Hm Gen E | Hm Gen E | CPE002JT | CPE002O8 | CPE002KO | XM 93731 | XM 9373 | XM 9373 | LOC64826 | LOC64826 | LOC64826 | similar to capicua | similar to | similar to | 648262 | 648262 | 648262 | s56835 | s56836 | s197603 |
| 21487 | P15 | P16 | 1165 | 1060 | 17988 | 387,6 | 15,4 | 1,092 | H5A034 C | sample | Hm Gen E | Hm Gen E | Hm Gen E | CPE002JT | CPE002O8 | CPE002KO | XM 92950 | XM 9295 | XM 9295 | LOC64656 | LOC64656 | LOC64656 | similar to melano | similar to | similar to | 646569 | 646569 | 646569 | s56440 | s56441 | s56442 |
| 11394 | K3 | K4 | 669 | 918 | 10313 | 146,5 | 15,4 | 1,116 | H5A034 C | sample | Hm Drgbl | Hm Drgbl | Hm Drgbl | CPE002ED | CPE002F3 | CPE002FT | NM 18162 | NM 1816 | NM 1816 | KRTAP23-1 | KRTAP23- | KRTAP23- | keratin associatec | keratin as | keratin as | 337963 | 337963 | 337963 | s50298 | s196016 | s196015 |
| 11494 | O7 | O8 | 1390 | 902,1 | 21426 | 192,2 | 15,4 | 1,116 | H5A034 C | sample | Hm Drgbl | Hm Drgbl | Hm Drgbl | CPE002ED | CPE002F3 | CPE002FT | NM 00100 | NM 0010 | NM 0010 | NANOS1 | NANOS1 | NANOS1 | nanos homolog 1 | nanos hor | nanos hor | 340719 | 340719 | 340719 | s50774 | s50773 | s50772 |
| 11509 | P20 | P21 | 1856 | 911,2 | 28494 | 208,9 | 15,4 | 1,116 | H5A034 C | sample | Hm Drgbl | Hm Drgbl | Hm Drgbl | CPE002ED | CPE002F3 | CPE002FT | | | | | | | | | | | | | | | |
| 12366 | D14 | D15 | 1283 | 1025 | 19725 | 183,3 | 15,4 | 1,158 | H5A034 C | sample | Hm Gen E | Hm Gen E | Hm Gen E | CPE002J5 | CPE002NK | CPE002K1 | NM 00388 | NM 0038 | NM 0038 | WISP3 | WISP3 | WISP3 | WNT1 inducible s | WNT1 ind | WNT1 ind | 8838 | 8838 | 8838 | s16867 | s16868 | s16869 |
| 12382 | D7 | D8 | 1096 | 1047 | 16895 | 155,7 | 15,4 | 1,158 | H5A034 C | sample | Hm Gen E | Hm Gen E | Hm Gen E | CPE002J5 | CPE002NK | CPE002K1 | NM 00676 | NM 0067 | NM 0067 | UPK2 | UPK2 | UPK2 | uroplakin 2 | uroplakin | uroplakin | 7379 | 7379 | 7379 | s14690 | s14691 | s14692 |
| 12418 | F18 | F19 | 1119 | 1008 | 17236 | 176,4 | 15,4 | 1,158 | H5A034 C | sample | Hm Gen E | Hm Gen E | Hm Gen E | CPE002J5 | CPE002NK | CPE002K1 | NM 00392 | NM 0039 | NM 0039 | PHOX2B | PHOX2B | PHOX2B | paired-like home | paired-lik | paired-lik | 8929 | 8929 | 8929 | s17073 | s17074 | s17075 |
| 12432 | F9 | F10 | 953 | 1024 | 14693 | 176 | 15,4 | 1,158 | H5A034 C | sample | Hm Gen E | Hm Gen E | Hm Gen E | CPE002J5 | CPE002NK | CPE002K1 | NM 00339 | NM 0033 | NM 0033 | WNT2 | WNT2 | WNT2 | wingless-type MM | wingless-t | wingless-t | 7472 | 7472 | 7472 | s14865 | s14866 | s14867 |
| 10992 | J9 | J10 | 1174 | 878,6 | 18123 | 458,9 | 15,4 | 1,167 | H5A034 C | sample | Hm Drgbl | Hm Drgbl | Hm Drgbl | CPE002EC | CPE002EN | CPE002FS | NM 19894 | NM 1989 | NM 1989 | LCN6 | LCN6 | LCN6 | lipocalin 6 | lipocalin 6 | lipocalin 6 | 158062 | 158062 | 158062 | s46025 | s46026 | s223369 |
| 11041 | M1 | M2 | 1338 | 870,4 | 20663 | 470 | 15,4 | 1,167 | H5A034 C | sample | Hm Drgbl | Hm Drgbl | Hm Drgbl | CPE002EC | CPE002EN | CPE002FS | NM 08087 | NM 0808 | NM 0807 | RAB40A | RAB40A | RAB40A | RAB40A, member | RAB40A, r | RAB40A, r | 142684 | 142684 | 142684 | s44481 | s223342 | s223341 |
| 11112 | O9 | O10 | 1537 | 833,2 | 23673 | 795,5 | 15,4 | 1,167 | H5A034 C | sample | Hm Drgbl | Hm Drgbl | Hm Drgbl | CPE002EC | CPE002EN | CPE002FS | NM 15244 | NM 1524 | NM 1524 | ZADH1 | ZADH1 | ZADH1 | zinc binding alcoh | zinc bindi | zinc bindi | 145482 | 145482 | 145482 | s44739 | s44738 | s44740 |
| 7383 | D22 | D23 | 1467 | 581,5 | 22628 | 509,3 | 15,4 | 1,176 | H5A034 C | sample | Hm Drgbl | Hm Drgbl | Hm Drgbl | CPE002E3 | CPE002EW | CPE002FH | NM 00511 | NM 0051 | NM 0051 | THRAP3 | THRAP3 | THRAP3 | thyroid hormone | thyroid hc | thyroid hc | 9967 | 9967 | 9967 | s19360 | s19361 | s19359 |
| 16899 | A11 | A12 | 917 | 994,1 | 14152 | 295,3 | 15,4 | 1,189 | H5A034 C | sample | Hm Gen E | Hm Gen E | Hm Gen E | CPE002JI | CPE002NZ | CPE002KD | XM 00113 | NM 0807 | XM 0011 | LOC93432 | C20orf52 | LOC93432 | maltase-glucoam | chromoso | maltase-g | 93432 | 140823 | 93432 | s41138 | s44412 | s41140 |
| 16948 | C12 | C13 | 1340 | 944,2 | 20697 | 222,6 | 15,4 | 1,189 | H5A034 C | sample | Hm Gen E | Hm Gen E | Hm Gen E | CPE002JI | CPE002NZ | CPE002KD | NM 14458 | NM 1524 | NM 1445 | BTBD16 | SAMD11 | BTBD16 | BTB (POZ) domair | sterile alp | BTB (POZ) | 118663 | 148398 | 118663 | s42230 | s45178 | s225522 |
| 17043 | G11 | G12 | 837 | 954,1 | 12917 | 206,6 | 15,4 | 1,189 | H5A034 C | sample | Hm Gen E | Hm Gen E | Hm Gen E | CPE002JI | CPE002NZ | CPE002KD | NM 05287 | NM 0010 | NM 0528 | C2orf59 | LOC14367 | C2orf59 | chromosome 2 op | hypotheti | chromoso | 112597 | 143678 | 112597 | s195584 | s195748 | s225478 |
| 17083 | H4 | H5 | 1048 | 1048 | 16111 | 195,8 | 15,4 | 1,189 | H5A034 C | sample | Hm Gen E | Hm Gen E | Hm Gen E | CPE002JI | CPE002NZ | CPE002KD | NM 00104 | NM 1828 | NM 0010 | CMTM3 | GDF7 | CMTM3 | CKLF-like MARVEL | growth di | CKLF-like | 123920 | 151449 | 123920 | s42690 | s45567 | s42692 |
| 17146 | K18 | K19 | 1120 | 924,4 | 17276 | 215,9 | 15,4 | 1,189 | H5A034 C | sample | Hm Gen E | Hm Gen E | Hm Gen E | CPE002JI | CPE002NZ | CPE002KD | XM 92681 | XM 0011 | XM 9268 | LOC12100 | LOC14977 | LOC12100 | hypothetical prot | hypotheti | hypotheti | 121006 | 149773 | 121006 | s42422 | s45344 | s42424 |
| 17258 | P10 | P11 | 1433 | 1128 | 22114 | 253,6 | 15,4 | 1,189 | H5A034 C | sample | Hm Gen E | Hm Gen E | Hm Gen E | CPE002JI | CPE002NZ | CPE002KD | NM 14461 | NM 1735 | NM 1446 | LOXHD1 | SLC38A9 | LOXHD1 | lipoxygenase hon | solute car | lipoxygen | 125336 | 153129 | 125336 | s42883 | s45755 | s225562 |
| 6991 | D15 | D16 | 1416 | 572,3 | 21767 | 511,2 | 15,4 | 1,198 | H5A034 C | sample | Hm Drgbl | Hm Drgbl | Hm Drgbl | CPE002E2 | CPE002EX | CPE002FI | NM 02161 | NM 0216 | NM 0216 | CHST6 | CHST6 | CHST6 | carbohydrate (N-a | carbohydr | carbohydr | 4166 | 4166 | 4166 | s8578 | s8579 | s8577 |
| 7069 | G20 | G21 | 1780 | 607,6 | 27494 | 451,6 | 15,4 | 1,198 | H5A034 C | sample | Hm Drgbl | Hm Drgbl | Hm Drgbl | CPE002E2 | CPE002EX | CPE002FI | NM 00247 | NM 0024 | NM 0024 | MYH3 | MYH3 | MYH3 | myosin, heavy ch | myosin, h | myosin, h | 4621 | 4621 | 4621 | s9156 | s9154 | s9155 |
| 7228 | N12 | N13 | 1898 | 627,2 | 29250 | 525,2 | 15,4 | 1,198 | H5A034 C | sample | Hm Drgbl | Hm Drgbl | Hm Drgbl | CPE002E2 | CPE002EX | CPE002FI | NM 00616 | NM 0061 | NM 0061 | NNMT | NNMT | NNMT | nicotinamide N-m | nicotinam | nicotinam | 4837 | 4837 | 4837 | s9603 | s9604 | s9605 |
| 7267 | O4 | O5 | 833 | 614,8 | 12859 | 377,9 | 15,4 | 1,198 | H5A034 C | sample | Hm Drgbl | Hm Drgbl | Hm Drgbl | CPE002E2 | CPE002EX | CPE002FI | NM 18135 | NM 1813 | NM 1813 | NCAM1 | NCAM1 | NCAM1 | neural cell adhesi | neural cel | neural cel | 4684 | 4684 | 4684 | s9296 | s9295 | s9294 |
| 7274 | P10 | P11 | 1347 | 574,4 | 20683 | 561,3 | 15,4 | 1,198 | H5A034 C | sample | Hm Drgbl | Hm Drgbl | Hm Drgbl | CPE002E2 | CPE002EX | CPE002FI | NM 00455 | NM 0045 | NM 0045 | NOTCH4 | NOTCH4 | NOTCH4 | Notch homolog 4 | Notch hor | Notch hor | 4855 | 4855 | 4855 | s9644 | s9645 | s224126 |
| 5045 | C13 | C14 | 1232 | 594,2 | 18921 | 528,5 | 15,4 | 1,208 | H5A034 C | sample | Hm Drgbl | Hm Drgbl | Hm Drgbl | CPE002DV | CPE002EM | CPE002FO | NM 02508 | NM 0250 | NM 0250 | ASRGL1 | ASRGL1 | ASRGL1 | asparaginase like | asparagin | asparagin | 80150 | 80150 | 80150 | s36972 | s36973 | s36974 |
| 5077 | D20 | D21 | 1642 | 564,2 | 25311 | 569,9 | 15,4 | 1,208 | H5A034 C | sample | Hm Drgbl | Hm Drgbl | Hm Drgbl | CPE002DV | CPE002EM | CPE002FO | NM 00712 | NM 0071 | NM 0071 | VIL1 | VIL1 | VIL1 | villin 1 | villin 1 | villin 1 | 7429 | 7429 | 7429 | s14792 | s224774 | s14794 |
| 5114 | F10 | F11 | 651 | 577,9 | 10008 | 589,4 | 15,4 | 1,208 | H5A034 C | sample | Hm Drgbl | Hm Drgbl | Hm Drgbl | CPE002DV | CPE002EM | CPE002FO | NM 00418 | NM 0041 | NM 0041 | WARS | WARS | WARS | tryptophanyl-tRN | tryptopha | tryptopha | 7453 | 7453 | 7453 | s686 | s688 | s687 |
| 5123 | F19 | F20 | 1314 | 610,4 | 20211 | 532 | 15,4 | 1,208 | H5A034 C | sample | Hm Drgbl | Hm Drgbl | Hm Drgbl | CPE002DV | CPE002EM | CPE002FO | NM 01395 | NM 0139 | NM 0139 | PAX8 | PAX8 | PAX8 | paired box gene 8 | paired bo | paired bo | 7849 | 7849 | 7849 | s15404 | s15405 | s15403 |
| 5126 | F21 | F22 | 1912 | 648,3 | 29361 | 589,8 | 15,4 | 1,208 | H5A034 C | sample | Hm Drgbl | Hm Drgbl | Hm Drgbl | CPE002DV | CPE002EM | CPE002FO | NM 00463 | NM 0046 | NM 0046 | IL1R2 | IL1R2 | IL1R2 | interleukin 1 rece | interleuki | interleuki | 7850 | 7850 | 7850 | s15407 | s15408 | s15406 |
| 5172 | H2 | H3 | 1334 | 560,4 | 20519 | 489,3 | 15,4 | 1,208 | H5A034 C | sample | Hm Drgbl | Hm Drgbl | Hm Drgbl | CPE002DV | CPE002EM | CPE002FO | NM 03075 | NM 0307 | NM 0307 | WNT3 | WNT3 | WNT3 | wingless-type MM | wingless-t | wingless-t | 7473 | 7473 | 7473 | s14868 | s14869 | s14870 |
| 5233 | K1 | K2 | 515 | 578,8 | 7939 | 368,7 | 15,4 | 1,208 | H5A034 C | sample | Hm Drgbl | Hm Drgbl | Hm Drgbl | CPE002DV | CPE002EM | CPE002FO | NM 19814 | NM 1981 | NM 1981 | CPXM2 | CPXM2 | CPXM2 | carboxypeptidase | carboxype | carboxype | 119587 | 119587 | 119587 | s42299 | s42300 | s42301 |
| 5289 | M17 | M18 | 1889 | 620,5 | 29169 | 584,9 | 15,4 | 1,208 | H5A034 C | sample | Hm Drgbl | Hm Drgbl | Hm Drgbl | CPE002DV | CPE002EM | CPE002FO | NM 13905 | NM 1390 | NM 1390 | ADAMTS1 | ADAMTS1 | ADAMTS1 | ADAM metallopep | ADAM me | ADAM me | 170692 | 170692 | 170692 | s46860 | s46861 | s46862 |
| 5351 | O8 | O9 | 1836 | 624,6 | 28245 | 618,7 | 15,4 | 1,208 | H5A034 C | sample | Hm Drgbl | Hm Drgbl | Hm Drgbl | CPE002DV | CPE002EM | CPE002FO | NM 00335 | NM 0033 | NM 0033 | UGCG | UGCG | UGCG | UDP-glucose cera | UDP-gluc | UDP-gluc | 7357 | 7357 | 7357 | s14642 | s14643 | s14644 |
| 2332 | B12 | B13 | 1669 | 686,5 | 25686 | 424,4 | 15,4 | 1,213 | H5A034 C | sample | Hm Drgbl | Hm Drgbl | Hm Drgbl | CPE002DC | CPE002EE | CPE002F4 | NM 00540 | NM 0054 | NM 0054 | PRKCE | PRKCE | PRKCE | protein kinase C, | protein ki | protein ki | 5581 | 5581 | 5581 | s11101 | s11102 | s11103 |
| 2372 | C5 | C6 | 1293 | 601,1 | 19894 | 305,2 | 15,4 | 1,213 | H5A034 C | sample | Hm Drgbl | Hm Drgbl | Hm Drgbl | CPE002DC | CPE002EE | CPE002F4 | NM 00438 | NM 0043 | NM 0043 | CSK | CSK | CSK | c-src tyrosine kina | c-src tyros | c-src tyros | 1445 | 1445 | 1445 | s3612 | s223346 | s3614 |
| 2375 | C8 | C9 | 1077 | 670 | 16578 | 412,8 | 15,4 | 1,213 | H5A034 C | sample | Hm Drgbl | Hm Drgbl | Hm Drgbl | CPE002DC | CPE002EE | CPE002F4 | NM 00103 | NM 0010 | NM 0010 | LIMK2 | LIMK2 | LIMK2 | LIM domain kinas | LIM doma | LIM doma | 3985 | 3985 | 3985 | s8191 | s8192 | s8193 |
| 2400 | D9 | D10 | 737 | 662,3 | 11382 | 405 | 15,4 | 1,213 | H5A034 C | sample | Hm Drgbl | Hm Drgbl | Hm Drgbl | CPE002DC | CPE002EE | CPE002F4 | NM 00125 | NM 0012 | NM 0012 | CDK3 | CDK3 | CDK3 | cyclin-dependent | cyclin-dep | cyclin-dep | 1018 | 1018 | 1018 | s2819 | s2820 | s2821 |
| 2534 | J21 | J22 | 818 | 662,1 | 12602 | 380,6 | 15,4 | 1,213 | H5A034 C | sample | Hm Drgbl | Hm Drgbl | Hm Drgbl | CPE002DC | CPE002EE | CPE002F4 | NM 00103 | NM 0010 | NM 0010 | MARK2 | MARK2 | MARK2 | MAP/microtubule | MAP/micr | MAP/micr | 2011 | 2011 | 2011 | s4648 | s4649 | s4650 |
| 16575 | C22 | C23 | 919 | 960,7 | 14133 | 412,2 | 15,4 | 1,217 | H5A034 C | sample | Hm Gen E | Hm Gen E | Hm Gen E | CPE002JH | CPE002NV | CPE002KC | NM 13835 | NM 1383 | NM 1383 | SHF | SHF | SHF | Src homology 2 d | Src homol | Src homol | 90525 | 90525 | 90525 | s40413 | s40414 | s40415 |
| 16599 | D22 | D23 | 1484 | 1008 | 22912 | 337,6 | 15,4 | 1,217 | H5A034 C | sample | Hm Gen E | Hm Gen E | Hm Gen E | CPE002JH | CPE002NV | CPE002KC | NM 17812 | NM 1781 | NM 1781 | CXorf40A | CXorf40A | CXorf40A | chromosome X op | chromoso | chromoso | 91966 | 91966 | 91966 | s40824 | s40825 | s195560 |
| 16653 | F6 | F7 | 1076 | 908,5 | 16591 | 295,1 | 15,4 | 1,217 | H5A034 C | sample | Hm Gen E | Hm Gen E | Hm Gen E | CPE002JH | CPE002NV | CPE002KC | NM 05286 | NM 0528 | NM 0528 | C4orf42 | C4orf42 | C4orf42 | chromosome 4 o | chromoso | chromoso | 92070 | 92070 | 92070 | s40841 | s40842 | s40843 |
| 16702 | H7 | H8 | 1140 | 929,7 | 17507 | 213,6 | 15,4 | 1,217 | H5A034 C | sample | Hm Gen E | Hm Gen E | Hm Gen E | CPE002JH | CPE002NV | CPE002KC | NM 15258 | NM 1525 | NM 1525 | HSFY1 | HSFY1 | HSFY1 | heat shock transc | heat shoc | heat shoc | 86614 | 86614 | 86614 | s200552 | s200553 | s200554 |
| 16823 | M8 | M9 | 1496 | 1033 | 22971 | 328,4 | 15,4 | 1,217 | H5A034 C | sample | Hm Gen E | Hm Gen E | Hm Gen E | CPE002JH | CPE002NV | CPE002KC | NM 15226 | NM 1522 | NM 1522 | C19orf40 | C19orf40 | C19orf40 | chromosome 19 c | chromoso | chromoso | 91442 | 91442 | 91442 | s40660 | s40661 | s40662 |
| 16828 | N12 | N13 | 1159 | 1032 | 17802 | 292,1 | 15,4 | 1,217 | H5A034 C | sample | Hm Gen E | Hm Gen E | Hm Gen E | CPE002JH | CPE002NV | CPE002KC | NM 08066 | NM 0806 | NM 0806 | PEX11G | PEX11G | PEX11G | peroxisomal biog | peroxison | peroxison | 92960 | 92960 | 92960 | s41055 | s41056 | s41057 |
| 16853 | O13 | O14 | 1545 | 1038 | 23856 | 457,2 | 15,4 | 1,217 | H5A034 C | sample | Hm Gen E | Hm Gen E | Hm Gen E | CPE002JH | CPE002NV | CPE002KC | NM 03318 | NM 0331 | NM 0331 | KRTAP3-3 | KRTAP3-3 | KRTAP3-3 | keratin associatec | keratin as | keratin as | 85293 | 85293 | 85293 | s39881 | s39882 | s228416 |
| 17703 | B22 | B23 | 1213 | 1070 | 18692 | 373,2 | 15,4 | 1,217 | H5A034 C | sample | Hm Gen E | Hm Gen E | Hm Gen E | CPE002JK | CPE002NZ | CPE002KF | NM 14470 | NM 1447 | NM 1447 | ANKAR | ANKAR | ANKAR | ankyrin and arma | ankyrin ar | ankyrin ar | 150709 | 150709 | 150709 | s45476 | s45477 | s45478 |
| 17751 | D22 | D23 | 1438 | 1030 | 22103 | 304,3 | 15,4 | 1,217 | H5A034 C | sample | Hm Gen E | Hm Gen E | Hm Gen E | CPE002JK | CPE002NZ | CPE002KF | NM 15251 | NM 1525 | NM 1525 | C2orf67 | C2orf67 | C2orf67 | chromosome 2 op | chromoso | chromoso | 151050 | 151050 | 151050 | s45509 | s45510 | s45511 |
| 17770 | E18 | E19 | 1075 | 1097 | 16586 | 265,9 | 15,4 | 1,217 | H5A034 C | sample | Hm Gen E | Hm Gen E | Hm Gen E | CPE002JK | CPE002NZ | CPE002KF | NM 00100 | NM 0010 | NM 0010 | C1orf213 | C1orf213 | C1orf213 | chromosome 1 o | chromoso | chromoso | 148898 | 148898 | 148898 | s45230 | s45231 | s45232 |
| 18009 | O17 | O18 | 1156 | 929 | 17823 | 344 | 15,4 | 1,217 | H5A034 C | sample | Hm Gen E | Hm Gen E | Hm Gen E | CPE002JK | CPE002NZ | CPE002KF | NM 00100 | NM 0010 | NM 0010 | C14orf37 | C14orf37 | C14orf37 | chromosome 14 c | chromoso | chromoso | 145407 | 145407 | 145407 | s44729 | s44730 | s44731 |
| 8953 | F1 | F2 | 1144 | 1751 | 17660 | 162 | 15,4 | 1,237 | H5A034 C | sample | Hm Drgbl | Hm Drgbl | Hm Drgbl | CPE002E7 | CPE002ES | CPE002FD | NM 01642 | NM 0164 | NM 0164 | TCEB3B | TCEB3B | TCEB3B | transcription elor | transcript | transcript | 51224 | 51224 | 51224 | s27707 | s27708 | s27706 |
| 9173 | O13 | O14 | 2044 | 1629 | 31474 | 215,9 | 15,4 | 1,237 | H5A034 C | sample | Hm Drgbl | Hm Drgbl | Hm Drgbl | CPE002E7 | CPE002ES | CPE002FD | NM 01613 | NM 0161 | NM 0161 | CHCHD2 | CHCHD2 | CHCHD2 | coiled-coil-helix-c | coiled-coi | coiled-coi | 51142 | 51142 | 51142 | s27541 | s27540 | s224192 |
| 9180 | O2 | O3 | 1956 | 1876 | 30046 | 207,8 | 15,4 | 1,237 | H5A034 C | sample | Hm Drgbl | Hm Drgbl | Hm Drgbl | CPE002E7 | CPE002ES | CPE002FD | NM 01594 | NM 0159 | NM 0159 | PELO | PELO | PELO | pelota homolog ( | pelota ho | pelota ho | 53918 | 53918 | 53918 | s28806 | s28807 | s28808 |
| 4401 | H17 | H18 | 796 | 818,6 | 12278 | 443,3 | 15,4 | 1,3 | H5A034 C | sample | Hm Drgbl | Hm Drgbl | Hm Drgbl | CPE002DL | CPE002EK | CPE002FQ | NM 15376 | NM 1537 | NM 0002 | KCNJ1 | KCNJ1 | KCNJ1 | potassium inward | potassium | potassium | 3758 | 3758 | 3758 | s7733 | s7734 | s223966 |
| 52 | C12 | C13 | 677 | 1573 | 10451 | 475,5 | 15,4 | 1,311 | H5A034 C | sample | Hm Drgbl | Hm Drgbl | Hm Drgbl | CPE002DT | CPE002EJ | CPE002F9 | NM 00651 | NM 0065 | NM 0065 | SLC16A2 | SLC16A2 | SLC16A2 | solute carrier fam | solute car | solute car | 6567 | 6567 | 6567 | s13066 | s13067 | s13068 |
| 102 | E14 | E15 | 484 | 1304 | 7464 | 379 | 15,4 | 1,311 | H5A034 C | sample | Hm Drgbl | Hm Drgbl | Hm Drgbl | CPE002DT | CPE002EJ | CPE002F9 | NM 15318 | NM 1531 | NM 1531 | SLC22A1 | SLC22A1 | SLC22A1 | solute carrier fam | solute car | solute car | 6580 | 6580 | 6580 | s13102 | s13103 | s13104 |
| 1953 | B17 | B18 | 1047 | 628,3 | 16074 | 469,6 | 15,4 | 1,339 | H5A034 C | sample | Hm Drgbl | Hm Drgbl | Hm Drgbl | CPE002FX | CPE002G1 | CPE002G5 | NM 13326 | NM 1332 | NM 1332 | PPARGC1E | PPARGC1E | PPARGC1E | peroxisome prolif | peroxison | peroxison | 133522 | 133522 | 133522 | s43784 | s43785 | s43786 |
| 2025 | E17 | E18 | 1205 | 499,2 | 18521 | 292,1 | 15,4 | 1,339 | H5A034 C | sample | Hm Drgbl | Hm Drgbl | Hm Drgbl | CPE002FX | CPE002G1 | CPE002G5 | NM 03321 | NM 0332 | NM 0332 | ZNF670 | ZNF670 | ZNF670 | zinc finger protein | zinc finger | zinc finger | 93474 | 93474 | 93474 | s41144 | s41145 | s41146 |
| 2286 | P14 | P15 | 1595 | 701 | 24598 | 516 | 15,4 | 1,339 | H5A034 C | sample | Hm Drgbl | Hm Drgbl | Hm Drgbl | CPE002FX | CPE002G1 | CPE002G5 | | | | | | | | | | | | | | | |
| 6556 | B12 | B13 | 1292 | 624 | 19851 | 455,2 | 15,4 | 1,351 | H5A034 C | sample | Hm Drgbl | Hm Drgbl | Hm Drgbl | CPE002E1 | CPE002EY | CPE002FJ | NM 00220 | NM 0022 | NM 0022 | ITGAL | ITGAL | ITGAL | integrin, alpha L ( | integrin, a | integrin, a | 3683 | 3683 | 3683 | s7563 | s7562 | s7564 |
| 6587 | C19 | C20 | 1062 | 565 | 16305 | 506,2 | 15,4 | 1,351 | H5A034 C | sample | Hm Drgbl | Hm Drgbl | Hm Drgbl | CPE002E1 | CPE002EY | CPE002FJ | NM 00710 | NM 0071 | NM 0071 | GUCA2B | GUCA2B | GUCA2B | guanylate cyclase | guanylate | guanylate | 2981 | 2981 | 2981 | s6343 | s6344 | s194480 |
| 6666 | F3 | F4 | 1214 | 563,7 | 18665 | 476,4 | 15,4 | 1,351 | H5A034 C | sample | Hm Drgbl | Hm Drgbl | Hm Drgbl | CPE002E1 | CPE002EY | CPE002FJ | NM 00552 | NM 0055 | NM 0055 | HOXA11 | HOXA11 | HOXA11 | homeobox A11 | homeobo | homeobo | 3207 | 3207 | 3207 | s6781 | s6779 | s6780 |
| 3924 | D2 | D3 | 1054 | 663,9 | 16227 | 304,1 | 15,4 | 1,369 | H5A034 C | sample | Hm Drgbl | Hm Drgbl | Hm Drgbl | CPE002DS | CPE002EI | CPE002F8 | NM 02102 | NM 0210 | NM 0210 | RFPL1 | RFPL1 | RFPL1 | ret finger protein | ret finger | ret finger | 5988 | 5988 | 5988 | s11964 | s11962 | s11963 |
| 3957 | E6 | E7 | 866 | 656,5 | 13315 | 364,2 | 15,4 | 1,369 | H5A034 C | sample | Hm Drgbl | Hm Drgbl | Hm Drgbl | CPE002DS | CPE002EI | CPE002F8 | NM 00287 | NM 0028 | NM 0028 | RAB13 | RAB13 | RAB13 | RAB13, member F | RAB13, m | RAB13, m | 5872 | 5872 | 5872 | s11690 | s11691 | s11692 |
| 4176 | N9 | N10 | 672 | 712,9 | 10363 | 396,8 | 15,4 | 1,369 | H5A034 C | sample | Hm Drgbl | Hm Drgbl | Hm Drgbl | CPE002DS | CPE002EI | CPE002F8 | NM 00281 | NM 0028 | NM 0028 | PSMD13 | PSMD13 | PSMD13 | proteasome (pros | proteasor | proteasor | 5719 | 5719 | 5719 | s11419 | s11421 | s11420 |
| 11579 | C19 | C20 | 978 | 872,4 | 15022 | 187,3 | 15,4 | 1,375 | H5A034 C | sample | Hm Gen E | Hm Gen E | Hm Gen E | CPE002JA | CPE002NJ | CPE002JZ | NM 00431 | NM 0043 | NM 0043 | ASTN1 | ASTN1 | ASTN1 | astrotactin 1 | astrotacti | astrotacti | 460 | 460 | 460 | s1687 | s1688 | s1689 |
| 11613 | D6 | D7 | 958 | 884,5 | 14710 | 150,6 | 15,4 | 1,375 | H5A034 C | sample | Hm Gen E | Hm Gen E | Hm Gen E | CPE002JA | CPE002NJ | CPE002JZ | NM 00209 | NM 0020 | NM 0020 | GYPA | GYPA | GYPA | glycophorin A (MI | glycophor | glycophor | 2993 | 2993 | 2993 | s6361 | s6362 | s6363 |
| 11653 | F20 | F21 | 1414 | 845,7 | 21811 | 188 | 15,4 | 1,375 | H5A034 C | sample | Hm Gen E | Hm Gen E | Hm Gen E | CPE002JA | CPE002NJ | CPE002JZ | NM 00152 | NM 0015 | NM 0015 | HCRT | HCRT | HCRT | hypocretin (orexi | hypocreti | hypocreti | 3060 | 3060 | 3060 | s194516 | s194517 | s194518 |
| 11733 | I6 | I7 | 854 | 971,9 | 13173 | 197 | 15,4 | 1,375 | H5A034 C | sample | Hm Gen E | Hm Gen E | Hm Gen E | CPE002JA | CPE002NJ | CPE002JZ | NM 00201 | NM 0020 | NM 0020 | FGF9 | FGF9 | FGF9 | fibroblast growth | fibroblast | fibroblast | 2254 | 2254 | 2254 | s5146 | s5147 | s225906 |
| 15601 | K1 | K2 | 1169 | 858,7 | 18059 | 135,7 | 15,4 | 1,4 | H5A034 C | sample | Hm Gen E | Hm Gen E | Hm Gen E | CPE002JE | CPE002NT | CPE002K9 | NM 02213 | NM 0221 | NM 0221 | SAMSN1 | SAMSN1 | SAMSN1 | SAM domain, SH3 | SAM dom | SAM dom | 64092 | 64092 | 64092 | s34418 | s34419 | s34420 |
| 5811 | C11 | C12 | 1635 | 768,8 | 25151 | 598,7 | 15,4 | 1,406 | H5A034 C | sample | Hm Drgbl | Hm Drgbl | Hm Drgbl | CPE002DY | CPE002F1 | CPE002FM | NM 19883 | NM 1988 | NM 1988 | ACACA | ACACA | ACACA | acetyl-Coenzyme | acetyl-Coe | acetyl-Coe | 31 | 31 | 31 | s882 | s883 | s884 |
| 5867 | E19 | E20 | 1726 | 932 | 26656 | 443,6 | 15,4 | 1,406 | H5A034 C | sample | Hm Drgbl | Hm Drgbl | Hm Drgbl | CPE002DY | CPE002F1 | CPE002FM | NM 00515 | NM 0051 | NM 0051 | ACTC1 | ACTC1 | ACTC1 | actin, alpha, card | actin, alph | actin, alph | 70 | 70 | 70 | s950 | s951 | s952 |
| 5906 | G10 | G11 | 984 | 700,3 | 15112 | 463 | 15,4 | 1,406 | H5A034 C | sample | Hm Drgbl | Hm Drgbl | Hm Drgbl | CPE002DY | CPE002F1 | CPE002FM | NM 08091 | NM 0011 | NM 0011 | ASGR2 | ASGR2 | ASGR2 | asialoglycoprotei | asialoglyc | asialoglyc | 433 | 433 | 433 | s1665 | s194297 | s194296 |
| 6087 | N22 | N23 | 864 | 816,6 | 13320 | 637,5 | 15,4 | 1,406 | H5A034 C | sample | Hm Drgbl | Hm Drgbl | Hm Drgbl | CPE002DY | CPE002F1 | CPE002FM | NM 00518 | NM 0051 | NM 0051 | CBL | CBL | CBL | Cas-Br-M (murine | Cas-Br-M | Cas-Br-M | 867 | 867 | 867 | s2476 | s2477 | s2478 |
| 1163 | A19 | A20 | 1339 | 765,4 | 20613 | 513,5 | 15,4 | 1,413 | H5A034 C | sample | Hm Drgbl | Hm Drgbl | Hm Drgbl | CPE002FV | CPE002FZ | CPE002G3 | NM 01771 | NM 0329 | NM 0177 | ZNF3 | ZNF3 | ZNF3 | zinc finger protein | zinc finger | zinc finger | 7551 | 7551 | 7551 | s224785 | s15008 | s194851 |
| 1324 | H12 | H13 | 743 | 694,1 | 11437 | 474,9 | 15,4 | 1,413 | H5A034 C | sample | Hm Drgbl | Hm Drgbl | Hm Drgbl | CPE002FV | CPE002FZ | CPE002G3 | NM 00721 | NM 0072 | NM 0072 | SEC63 | SEC63 | SEC63 | SEC63 homolog (S | SEC63 hor | SEC63 hor | 11231 | 11231 | 11231 | s22166 | s22167 | s22168 |
| 1482 | N3 | N4 | 1076 | 915,4 | 16596 | 383,2 | 15,4 | 1,413 | H5A034 C | sample | Hm Drgbl | Hm Drgbl | Hm Drgbl | CPE002FV | CPE002FZ | CPE002G3 | NM 00423 | NM 0042 | NM 0042 | KLF4 | KLF4 | KLF4 | Kruppel-like facto | Kruppel-li | Kruppel-li | 9314 | 9314 | 9314 | s17793 | s17794 | s17795 |
| 1524 | P2 | P3 | 1318 | 657,4 | 20328 | 488,9 | 15,4 | 1,413 | H5A034 C | sample | Hm Drgbl | Hm Drgbl | Hm Drgbl | CPE002FV | CPE002FZ | CPE002G3 | NM 01526 | NM 0152 | NM 0152 | DNAJC13 | DNAJC13 | DNAJC13 | DnaJ (Hsp40) hon | DnaJ (Hsp | DnaJ (Hsp | 23317 | 23317 | 23317 | s23548 | s23549 | s23550 |

| | | | | | | | | | | | | | | | | | | | | | | | | | | | | | | | | |
|---|---|---|---|---|---|---|---|---|---|---|---|---|---|---|---|---|---|---|---|---|---|---|---|---|---|---|---|---|---|---|---|---|
| 16234 | E18 | E19 | 1099 | 981,5 | 16911 | 256 | 15,4 | 1,413 | H5A034 C | sample | Hm Gen E | Hm Gen E | Hm Gen E | Hm Gen E | CPE002JG | CPE002NV | CPE002KB | NM 03230 | NM 0323 | NM 0323 | TCHP | TCHP | TCHP | trichoplein, kerat | trichoplei | trichoplei | 84260 | 84260 | 84260 | s38748 | s38749 | s38750 |
| 16256 | F16 | F17 | 1217 | 914,4 | 18801 | 314,8 | 15,4 | 1,413 | H5A034 C | sample | Hm Gen E | Hm Gen E | Hm Gen E | Hm Gen E | CPE002JG | CPE002NV | CPE002KB | NM 03250 | NM 0325 | NM 0325 | KBTBD8 | KBTBD8 | KBTBD8 | kelch repeat and | kelch repe | kelch repe | 84541 | 84541 | 84541 | s39132 | s39133 | s39134 |
| 16298 | H10 | H11 | 789 | 1055 | 12159 | 277,4 | 15,4 | 1,413 | H5A034 C | sample | Hm Gen E | Hm Gen E | Hm Gen E | Hm Gen E | CPE002JG | CPE002NV | CPE002KB | NM 03251 | NM 0325 | NM 0325 | COL25A1 | COL25A1 | COL25A1 | collagen, type XX | collagen, t | collagen, t | 84570 | 84570 | 84570 | s39165 | s39166 | s39167 |
| 16304 | H16 | H17 | 838 | 989,7 | 12925 | 286,8 | 15,4 | 1,413 | H5A034 C | sample | Hm Gen E | Hm Gen E | Hm Gen E | Hm Gen E | CPE002JG | CPE002NV | CPE002KB | NM 03253 | NM 0325 | NM 0325 | FNDC1 | FNDC1 | FNDC1 | fibronectin type I | fibronecti | fibronecti | 84624 | 84624 | 84624 | s39197 | s39198 | s39199 |
| 16354 | J18 | J19 | 1313 | 938,7 | 20282 | 246,2 | 15,4 | 1,413 | H5A034 C | sample | Hm Gen E | Hm Gen E | Hm Gen E | Hm Gen E | CPE002JG | CPE002NV | CPE002KB | NM 03257 | NM 0325 | NM 0325 | RETNLB | RETNLB | RETNLB | resistin like beta | resistin lik | resistin lik | 84666 | 84666 | 84666 | s39281 | s39282 | s39283 |
| 13468 | B12 | B13 | 1400 | 1095 | 21535 | 174,1 | 15,4 | 1,446 | H5A034 C | sample | Hm Gen E | Hm Gen E | Hm Gen E | Hm Gen E | CPE002JC | CPE002NC | CPE002K4 | NM 01235 | NM 0123 | NM 0123 | OR1C1 | OR1C1 | OR1C1 | olfactory receptor | olfactory r | olfactory r | 26188 | 26188 | 26188 | s25214 | s25215 | s25216 |
| 13665 | J17 | J18 | 1483 | 984,8 | 22869 | 152,4 | 15,4 | 1,446 | H5A034 C | sample | Hm Gen E | Hm Gen E | Hm Gen E | Hm Gen E | CPE002JC | CPE002NC | CPE002K4 | NM 01323 | NM 0132 | NM 0132 | ATXN10 | ATXN10 | ATXN10 | ataxin 10 | ataxin 10 | ataxin 10 | 25814 | 25814 | 25814 | s24536 | s24537 | s24538 |
| 13726 | L7 | L8 | 942 | 853,1 | 14507 | 162 | 15,4 | 1,446 | H5A034 C | sample | Hm Gen E | Hm Gen E | Hm Gen E | Hm Gen E | CPE002JC | CPE002NC | CPE002K4 | NM 01538 | NM 0153 | NM 0153 | NIPBL | NIPBL | NIPBL | Nipped-B homolo | Nipped-B | Nipped-B | 25836 | 25836 | 25836 | s24587 | s24588 | s24589 |
| 13803 | P11 | P12 | 1124 | 1028 | 17273 | 194,1 | 15,4 | 1,446 | H5A034 C | sample | Hm Gen E | Hm Gen E | Hm Gen E | Hm Gen E | CPE002JC | CPE002NC | CPE002K4 | NM 01543 | NM 0154 | NM 0154 | INTS7 | INTS7 | INTS7 | integrator comple | integrator | integrator | 25896 | 25896 | 25896 | s24697 | s24699 | s226049 |
| 1029 | K6 | K7 | 853 | 676,8 | 13127 | 312,4 | 15,4 | 1,46 | H5A034 C | sample | Hm Drgbl | Hm Drgbl | Hm Drgbl | Hm Drgbl | CPE002FU | CPE002FY | CPE002G2 | NM 00501 | NM 0050 | NM 0050 | PCBP2 | PCBP2 | PCBP2 | poly(rC) binding p | poly(rC) b | poly(rC) b | 5094 | 5094 | 5094 | s10097 | s10098 | s10099 |
| 1087 | N15 | N16 | 707 | 869 | 10862 | 459,8 | 15,4 | 1,46 | H5A034 C | sample | Hm Drgbl | Hm Drgbl | Hm Drgbl | Hm Drgbl | CPE002FU | CPE002FY | CPE002G2 | NM 19821 | NM 0055 | NM 1982 | ING1 | ING1 | ING1 | inhibitor of growt | inhibitor c | inhibitor c | 3621 | 3621 | 3621 | s7428 | s223939 | s7430 |
| 5494 | E7 | E8 | 1918 | 682,8 | 29567 | 600,2 | 15,4 | 1,467 | H5A034 C | sample | Hm Drgbl | Hm Drgbl | Hm Drgbl | Hm Drgbl | CPE002DX | CPE002F2 | CPE002FN | NM 00357 | NM 0035 | NM 0035 | LTBP4 | LTBP4 | LTBP4 | latent transformi | latent trai | latent trai | 8425 | 8425 | 8425 | s15987 | s15988 | s15989 |
| 5524 | G12 | G13 | 1226 | 600,7 | 18847 | 670,1 | 15,4 | 1,467 | H5A034 C | sample | Hm Drgbl | Hm Drgbl | Hm Drgbl | Hm Drgbl | CPE002DX | CPE002F2 | CPE002FN | NM 00390 | NM 0039 | NM 0039 | EIF2B5 | EIF2B5 | EIF2B5 | eukaryotic transla | eukaryotic | eukaryotic | 8893 | 8893 | 8893 | s225051 | s16999 | s17000 |
| 5724 | O2 | O3 | 1305 | 715,3 | 20126 | 689,4 | 15,4 | 1,467 | H5A034 C | sample | Hm Drgbl | Hm Drgbl | Hm Drgbl | Hm Drgbl | CPE002DX | CPE002F2 | CPE002FN | NM 00397 | NM 0039 | NM 0039 | PSTPIP1 | PSTPIP1 | PSTPIP1 | proline-serine-thr | proline-se | proline-se | 9051 | 9051 | 9051 | s17255 | s17256 | s17257 |
| 5747 | P19 | P20 | 1697 | 730,6 | 26071 | 532,9 | 15,4 | 1,467 | H5A034 C | sample | Hm Drgbl | Hm Drgbl | Hm Drgbl | Hm Drgbl | CPE002DX | CPE002F2 | CPE002FN | NM 01590 | NM 0159 | NM 0159 | TRIM24 | TRIM24 | TRIM24 | tripartite motif-co | tripartite | tripartite | 8805 | 8805 | 8805 | s16788 | s16786 | s16787 |
| 3534 | D14 | D15 | 730 | 1561 | 11239 | 350,1 | 15,4 | 1,481 | H5A034 C | sample | Hm Drgbl | Hm Drgbl | Hm Drgbl | Hm Drgbl | CPE002DR | CPE002EH | CPE002F7 | NM 02063 | NM 0206 | NM 0206 | VN1R1 | VN1R1 | VN1R1 | vomeronasal 1 re | vomerona | vomerona | 57191 | 57191 | 57191 | s32872 | s32873 | s32874 |
| 3605 | G13 | G14 | 748 | 1221 | 11532 | 370,1 | 15,4 | 1,481 | H5A034 C | sample | Hm Drgbl | Hm Drgbl | Hm Drgbl | Hm Drgbl | CPE002DR | CPE002EH | CPE002F7 | NM 01212 | NM 0121 | NM 0121 | CHRM5 | CHRM5 | CHRM5 | cholinergic recept | cholinergi | cholinergi | 1133 | 1133 | 1133 | s3034 | s3035 | s3036 |
| 8117 | C13 | C14 | 1235 | 647,4 | 19080 | 500,6 | 15,4 | 1,481 | H5A034 C | sample | Hm Drgbl | Hm Drgbl | Hm Drgbl | Hm Drgbl | CPE002E5 | CPE002EU | CPE002FF | NM 00703 | NM 0070 | NM 0070 | KERA | KERA | KERA | keratocan | keratocan | keratocan | 11081 | 11081 | 11081 | s21848 | s21849 | s223190 |
| 8118 | C14 | C15 | 762 | 592,9 | 11718 | 523,9 | 15,4 | 1,481 | H5A034 C | sample | Hm Drgbl | Hm Drgbl | Hm Drgbl | Hm Drgbl | CPE002E5 | CPE002EU | CPE002FF | NM 18341 | NM 1834 | NM 1834 | KIF1B | KIF1B | KIF1B | kinesin family me | kinesin fa | kinesin fa | 23095 | 23095 | 23095 | s23022 | s23023 | s23024 |
| 8180 | E5 | E6 | 1203 | 588,3 | 18505 | 503,5 | 15,4 | 1,481 | H5A034 C | sample | Hm Drgbl | Hm Drgbl | Hm Drgbl | Hm Drgbl | CPE002E5 | CPE002EU | CPE002FF | NM 00704 | NM 0070 | NM 0070 | BTN3A2 | BTN3A2 | BTN3A2 | butyrophilin, sub | butyrophi | butyrophi | 11118 | 11118 | 11118 | s21921 | s223196 | s21919 |
| 8270 | I21 | I22 | 1108 | 626,8 | 17115 | 414,7 | 15,4 | 1,481 | H5A034 C | sample | Hm Drgbl | Hm Drgbl | Hm Drgbl | Hm Drgbl | CPE002E5 | CPE002EU | CPE002FF | NM 00718 | NM 0071 | NM 0071 | TNRC4 | TNRC4 | TNRC4 | trinucleotide repe | trinucleot | trinucleot | 11189 | 11189 | 11189 | s22095 | s223209 | s22097 |
| 8356 | M12 | M13 | 918 | 641,3 | 14147 | 384,4 | 15,4 | 1,481 | H5A034 C | sample | Hm Drgbl | Hm Drgbl | Hm Drgbl | Hm Drgbl | CPE002E5 | CPE002EU | CPE002FF | NM 00100 | NM 0010 | NM 0010 | ACSL6 | ACSL6 | ACSL6 | acyl-CoA syntheta | acyl-CoA s | acyl-CoA s | 23305 | 23305 | 23305 | s23516 | s23515 | s23517 |
| 8443 | P4 | P5 | 1070 | 510,6 | 16463 | 323,2 | 15,4 | 1,481 | H5A034 C | sample | Hm Drgbl | Hm Drgbl | Hm Drgbl | Hm Drgbl | CPE002E5 | CPE002EU | CPE002FF | NM 01213 | NM 0121 | NM 0121 | DDAH1 | DDAH1 | DDAH1 | dimethylarginine | dimethyla | dimethyla | 23576 | 23576 | 23576 | s223613 | s24123 | s24124 |
| 10038 | C14 | C15 | 1389 | 878,8 | 21419 | 224,3 | 15,4 | 1,481 | H5A034 C | sample | Hm Drgbl | Hm Drgbl | Hm Drgbl | Hm Drgbl | CPE002EA | CPE002EP | CPE002FA | NM 03143 | NM 0314 | NM 0314 | RILP | RILP | RILP | Rab interacting ly | Rab intera | Rab intera | 83547 | 83547 | 83547 | s37995 | s37996 | s37997 |
| 10171 | H4 | H5 | 1379 | 873,1 | 21230 | 140,1 | 15,4 | 1,481 | H5A034 C | sample | Hm Drgbl | Hm Drgbl | Hm Drgbl | Hm Drgbl | CPE002EA | CPE002EP | CPE002FA | NM 03252 | NM 0325 | NM 0325 | GNPTG | GNPTG | GNPTG | N-acetylglucosam | N-acetylgl | N-acetylgl | 84572 | 84572 | 84572 | s39168 | s39169 | s39170 |
| 1835 | M19 | M20 | 1209 | 728,2 | 18567 | 402 | 15,4 | 1,613 | H5A034 C | sample | Hm Drgbl | Hm Drgbl | Hm Drgbl | Hm Drgbl | CPE002FW | CPE002G0 | CPE002G4 | NM 01663 | NM 0166 | NM 0166 | ERAF | ERAF | ERAF | erythroid associat | erythroid | erythroid | 51327 | 51327 | 51327 | s27959 | s27960 | s27961 |
| 21744 | J9 | J10 | 127 | 1017 | 1961 | 728,8 | 15,4 | 1,621 | H5A034 C | sample | Hm Gen E | Hm Gen E | Hm Gen E | Hm Gen E | CPE002JU | CPE002O9 | CPE002KP | XM 94097 | XM 94097 | XM 94097 | LOC651758 | LOC65175 | LOC65175 | similar to ciliary r | similar to | similar to | 651758 | 651758 | 651758 | s57240 | s197816 | s197817 |
| 4615 | A15 | A16 | 996 | 806,5 | 15346 | 574,7 | 15,4 | 1,692 | H5A034 C | sample | Hm Drgbl | Hm Drgbl | Hm Drgbl | Hm Drgbl | CPE002DV | CPE002EL | CPE002FP | NM 01588 | NM 0158 | NM 0158 | MBTPS2 | MBTPS2 | MBTPS2 | membrane-bound | membran | membran | 51360 | 51360 | 51360 | s343 | s344 | s345 |
| 4966 | O7 | O8 | 1263 | 784 | 19392 | 529,5 | 15,4 | 1,692 | H5A034 C | sample | Hm Drgbl | Hm Drgbl | Hm Drgbl | Hm Drgbl | CPE002DV | CPE002EL | CPE002FP | NM 00050 | NM 0005 | NM 0005 | F2 | F2 | F2 | coagulation facto | coagulatic | coagulatic | 2147 | 2147 | 2147 | s4919 | s4920 | s4921 |
| 4980 | P2 | P3 | 892 | 787,1 | 13746 | 596,1 | 15,4 | 1,692 | H5A034 C | sample | Hm Drgbl | Hm Drgbl | Hm Drgbl | Hm Drgbl | CPE002DV | CPE002EL | CPE002FP | NM 02071 | NM 0207 | NM 0207 | USP31 | USP31 | USP31 | ubiquitin specific | ubiquitin | ubiquitin | 57478 | 57478 | 57478 | s33118 | s33119 | s33120 |
| 10428 | C2 | C3 | 913 | 927,2 | 14089 | 221,7 | 15,4 | 1,74 | H5A034 C | sample | Hm Drgbl | Hm Drgbl | Hm Drgbl | Hm Drgbl | CPE002EB | CPE002EO | CPE002FR | NM 05290 | NM 0529 | NM 0529 | FMNL2 | FMNL2 | FMNL2 | formin-like 2 | formin-lik | formin-lik | 114793 | 114793 | 114793 | s41620 | s41619 | s41618 |
| 10550 | H21 | H22 | 978 | 879,3 | 15103 | 205,8 | 15,4 | 1,74 | H5A034 C | sample | Hm Drgbl | Hm Drgbl | Hm Drgbl | Hm Drgbl | CPE002EB | CPE002EO | CPE002FR | NM 14516 | NM 1451 | NM 1451 | PIGM | PIGM | PIGM | phosphatidylinos | phosphat | phosphat | 93183 | 93183 | 93183 | s41100 | s41101 | s41102 |
| 10690 | N18 | N19 | 1559 | 842,9 | 23978 | 239,9 | 15,4 | 1,74 | H5A034 C | sample | Hm Drgbl | Hm Drgbl | Hm Drgbl | Hm Drgbl | CPE002EB | CPE002EO | CPE002FR | NM 14462 | NM 1446 | NM 1446 | WDR64 | WDR64 | WDR64 | WD repeat domai | WD repea | WD repea | 128025 | 128025 | 128025 | s43244 | s43245 | s43246 |
| 22369 | E1 | E2 | 823 | 1384 | 12773 | 335,3 | 15,5 | 0,799 | H5A034 C | sample | Hm Gen E | Hm Gen E | Hm Gen E | Hm Gen E | CPE002JW | CPE002OB | CPE002KR | XM 00113 | XM 00113 | XM 00113 | LOC728626 | LOC72862 | LOC72862 | hypothetical prot | hypotheti | hypotheti | 728626 | 728626 | 728626 | s58833 | s58834 | s227645 |
| 14169 | O17 | O18 | 1578 | 1037 | 24526 | 130,9 | 15,5 | 0,888 | H5A034 C | sample | Hm Gen E | Hm Gen E | Hm Gen E | Hm Gen E | CPE002JF | CPE002NP | CPE002K5 | XM 00112 | XM 00112 | XM 00112 | CECR8 | CECR8 | CECR8 | cat eye syndrome | cat eye sy | cat eye sy | 27437 | 27437 | 27437 | s26237 | s26238 | s26239 |
| 3094 | A7 | A8 | 1166 | 1250 | 18113 | 414,8 | 15,5 | 0,96 | H5A034 C | sample | Hm Drgbl | Hm Drgbl | Hm Drgbl | Hm Drgbl | CPE002DC | CPE002EG | CPE002F6 | NM 00031 | NM 0003 | NM 0003 | PTEN | PTEN | PTEN | phosphatase and | phosphat | phosphat | 5728 | 5728 | 5728 | s325 | s326 | s327 |
| 3098 | B10 | B11 | 840 | 1376 | 12998 | 431,8 | 15,5 | 0,96 | H5A034 C | sample | Hm Drgbl | Hm Drgbl | Hm Drgbl | Hm Drgbl | CPE002DC | CPE002EG | CPE002F6 | NM 00620 | NM 0062 | NM 0062 | PDE6C | PDE6C | PDE6C | phosphodiesteras | phosphod | phosphod | 5146 | 5146 | 5146 | s10204 | s10205 | s224213 |
| 3335 | K8 | K9 | 1115 | 1235 | 17230 | 347,5 | 15,5 | 0,96 | H5A034 C | sample | Hm Drgbl | Hm Drgbl | Hm Drgbl | Hm Drgbl | CPE002DC | CPE002EG | CPE002F6 | NM 13879 | NM 1387 | NM 1387 | CANT1 | CANT1 | CANT1 | calcium activated | calcium ac | calcium ac | 124583 | 124583 | 124583 | s42761 | s42762 | s42763 |
| 3443 | P19 | P20 | 1573 | 1384 | 24340 | 536,1 | 15,5 | 0,96 | H5A034 C | sample | Hm Drgbl | Hm Drgbl | Hm Drgbl | Hm Drgbl | CPE002DC | CPE002EG | CPE002F6 | NM 01229 | NM 0122 | NM 0122 | CABIN1 | CABIN1 | CABIN1 | calcineurin bindir | calcineuri | calcineuri | 23523 | 23523 | 23523 | s24000 | s24001 | s24002 |
| 18337 | M1 | M2 | 1566 | 973,2 | 24234 | 177,1 | 15,5 | 0,997 | H5A034 C | sample | Hm Gen E | Hm Gen E | Hm Gen E | Hm Gen E | CPE002JL | CPE002OO | CPE002KG | NM 15370 | NM 1537 | NM 1537 | FAM154A | FAM154A | FAM154A | family with seque | family wit | family wit | 158297 | 158297 | 158297 | s46055 | s46056 | s46057 |
| 18355 | M4 | M5 | 672 | 1051 | 10397 | 219,8 | 15,5 | 0,997 | H5A034 C | sample | Hm Gen E | Hm Gen E | Hm Gen E | Hm Gen E | CPE002JL | CPE002OO | CPE002KG | NM 15263 | NM 1526 | NM 1526 | METT5D1 | METT5D1 | METT5D1 | methyltransferase | methyltra | methyltra | 196074 | 196074 | 196074 | s46970 | s46971 | s46972 |
| 18356 | M5 | M6 | 934 | 1113 | 14514 | 270,2 | 15,5 | 0,997 | H5A034 C | sample | Hm Gen E | Hm Gen E | Hm Gen E | Hm Gen E | CPE002JL | CPE002OO | CPE002KG | NM 00101 | NM 0010 | NM 0010 | KIAA2026 | KIAA2026 | KIAA2026 | KIAA2026 | KIAA2026 | KIAA2026 | 158358 | 158358 | 158358 | s46061 | s46062 | s225766 |
| 19640 | C16 | C17 | 997 | 327,3 | 15449 | 413,2 | 15,5 | 1,01 | H5A034 C | sample | Hm Gen E | Hm Gen E | Hm Gen E | Hm Gen E | CPE002JP | CPE002O4 | CPE002KK | NM 00108 | NM 0010 | NM 0010 | FLJ43987 | FLJ43987 | FLJ43987 | similar to RIKEN c | similar to | similar to | 388960 | 388960 | 388960 | s52319 | s52320 | s52321 |
| 6324 | H2 | H3 | 1301 | 618,6 | 20143 | 383,8 | 15,5 | 1,023 | H5A034 C | sample | Hm Drgbl | Hm Drgbl | Hm Drgbl | Hm Drgbl | CPE002DZ | CPE002F0 | CPE002FL | NM 02073 | NM 0207 | NM 0207 | DLG3 | DLG3 | DLG3 | discs, large homo | discs, larg | discs, larg | 1741 | 1741 | 1741 | s4127 | s4125 | s4126 |
| 6374 | J21 | J22 | 1542 | 504,1 | 23837 | 409,4 | 15,5 | 1,023 | H5A034 C | sample | Hm Drgbl | Hm Drgbl | Hm Drgbl | Hm Drgbl | CPE002DZ | CPE002F0 | CPE002FL | NM 00186 | NM 0018 | NM 0018 | COX7B | COX7B | COX7B | cytochrome c oxic | cytochron | cytochron | 1349 | 1349 | 1349 | s3402 | s223307 | s3401 |
| 6384 | J9 | J10 | 1591 | 557,8 | 24625 | 405,8 | 15,5 | 1,023 | H5A034 C | sample | Hm Drgbl | Hm Drgbl | Hm Drgbl | Hm Drgbl | CPE002DZ | CPE002F0 | CPE002FL | NM 00186 | NM 0018 | NM 0018 | COX5B | COX5B | COX5B | cytochrome c oxic | cytochron | cytochron | 1329 | 1329 | 1329 | s3389 | s194345 | s194344 |
| 20589 | J6 | J7 | 637 | 793,6 | 9891 | 195,6 | 15,5 | 1,033 | H5A034 C | sample | Hm Gen E | Hm Gen E | Hm Gen E | Hm Gen E | CPE002JR | CPE002O6 | CPE002KM | XM 93071 | XM 93071 | XM 93071 | LOC642340 | LOC64234 | LOC64234 | hypothetical LOC | hypotheti | hypotheti | 642340 | 642340 | 642340 | s54986 | s54987 | s54988 |
| 20690 | O10 | O11 | 1015 | 774,9 | 15741 | 264,2 | 15,5 | 1,033 | H5A034 C | sample | Hm Gen E | Hm Gen E | Hm Gen E | Hm Gen E | CPE002JR | CPE002O6 | CPE002KM | XM 00112 | XM 00112 | XM 00112 | LOC641796 | LOC64179 | LOC64179 | similar to Protein | similar to | similar to | 641796 | 641796 | 641796 | s54847 | s54848 | s54849 |
| 18764 | N5 | N6 | 1301 | 916,8 | 20157 | 213,8 | 15,5 | 1,051 | H5A034 C | sample | Hm Gen E | Hm Gen E | Hm Gen E | Hm Gen E | CPE002JM | CPE002O1 | CPE002KH | NM 15273 | NM 1527 | NM 1527 | ZBTB9 | ZBTB9 | ZBTB9 | zinc finger and BT | zinc finger | zinc finger | 221504 | 221504 | 221504 | s48073 | s48074 | s48075 |
| 18770 | O10 | O11 | 1252 | 991 | 19388 | 464,3 | 15,5 | 1,051 | H5A034 C | sample | Hm Gen E | Hm Gen E | Hm Gen E | Hm Gen E | CPE002JM | CPE002O1 | CPE002KH | NM 15276 | NM 1527 | NM 1527 | TSGA10IP | TSGA10IP | TSGA10IP | testis specific, 10 | testis spec | testis spec | 254187 | 254187 | 254187 | s48507 | s48508 | s48509 |
| 17483 | I19 | I20 | 1315 | 971,6 | 20406 | 238,6 | 15,5 | 1,058 | H5A034 C | sample | Hm Gen E | Hm Gen E | Hm Gen E | Hm Gen E | CPE002JJ | CPE002NY | CPE002KE | NM 00100 | NM 0010 | NM 0010 | OR2T10 | OR2T10 | OR2T10 | olfactory receptor | olfactory r | olfactory r | 127069 | 127069 | 127069 | s43127 | s43128 | s43129 |
| 17556 | L2 | L3 | 1155 | 899 | 17906 | 197,5 | 15,5 | 1,058 | H5A034 C | sample | Hm Gen E | Hm Gen E | Hm Gen E | Hm Gen E | CPE002JJ | CPE002NY | CPE002KE | XM 06685 | XM 06685 | XM 06685 | ZFP92 | ZFP92 | ZFP92 | zinc finger protein | zinc finger | zinc finger | 139735 | 139735 | 139735 | s44238 | s44239 | s195727 |
| 17558 | L21 | L22 | 1745 | 1034 | 26980 | 209,5 | 15,5 | 1,058 | H5A034 C | sample | Hm Gen E | Hm Gen E | Hm Gen E | Hm Gen E | CPE002JJ | CPE002NY | CPE002KE | NM 17796 | NM 1779 | NM 1779 | LOC130576 | LOC13057 | LOC13057 | hypothetical prot | hypotheti | hypotheti | 130576 | 130576 | 130576 | s43521 | s43522 | s43523 |
| 17576 | M16 | M17 | 1051 | 954,6 | 16268 | 259,5 | 15,5 | 1,058 | H5A034 C | sample | Hm Gen E | Hm Gen E | Hm Gen E | Hm Gen E | CPE002JJ | CPE002NY | CPE002KE | NM 08087 | NM 0808 | NM 0808 | DPCR1 | DPCR1 | DPCR1 | diffuse panbroncl | diffuse pa | diffuse pa | 135656 | 135656 | 135656 | s43941 | s43942 | s43943 |
| 9267 | C11 | C12 | 1532 | 1166 | 23751 | 196,8 | 15,5 | 1,076 | H5A034 C | sample | Hm Drgbl | Hm Drgbl | Hm Drgbl | Hm Drgbl | CPE002E8 | CPE002ER | CPE002FC | NM 01783 | NM 0178 | NM 0178 | AYTL1 | AYTL1 | AYTL1 | acyltransferase lik | acyltransf | acyltransf | 54947 | 54947 | 54947 | s29823 | s29824 | s29825 |
| 9395 | H19 | H20 | 1541 | 1070 | 23849 | 130 | 15,5 | 1,076 | H5A034 C | sample | Hm Drgbl | Hm Drgbl | Hm Drgbl | Hm Drgbl | CPE002E8 | CPE002ER | CPE002FC | NM 01758 | NM 0175 | NM 0175 | UBE2Q1 | UBE2Q1 | UBE2Q1 | ubiquitin-conjuga | ubiquitin- | ubiquitin- | 55585 | 55585 | 55585 | s31021 | s31020 | s31019 |
| 9396 | H2 | H3 | 888 | 1190 | 13762 | 119,3 | 15,5 | 1,076 | H5A034 C | sample | Hm Drgbl | Hm Drgbl | Hm Drgbl | Hm Drgbl | CPE002E8 | CPE002ER | CPE002FC | NM 02013 | NM 0201 | NM 0201 | DPYSL5 | DPYSL5 | DPYSL5 | dihydropyrimidin | dihydropy | dihydropy | 56896 | 56896 | 56896 | s32336 | s32335 | s32334 |
| 19143 | N22 | N23 | 952 | 1011 | 14739 | 228,4 | 15,5 | 1,08 | H5A034 C | sample | Hm Gen E | Hm Gen E | Hm Gen E | Hm Gen E | CPE002JN | CPE002O2 | CPE002KI | NM 00100 | NM 0010 | NM 0010 | OR13D1 | OR13D1 | OR13D1 | olfactory receptor | olfactory r | olfactory r | 286365 | 286365 | 286365 | s50153 | s50154 | s50155 |
| 19189 | P20 | P21 | 1594 | 1092 | 24680 | 295,6 | 15,5 | 1,08 | H5A034 C | sample | Hm Gen E | Hm Gen E | Hm Gen E | Hm Gen E | CPE002JN | CPE002O2 | CPE002KI | XM 21050 | XM 21050 | XM 21050 | LOC286528 | LOC28652 | LOC28652 | similar to Ssu72 R | similar to | similar to | 286528 | 286528 | 286528 | s50188 | s50189 | s50190 |
| 9961 | P1 | P2 | 1156 | 1034 | 17891 | 115,4 | 15,5 | 1,084 | H5A034 C | sample | Hm Drgbl | Hm Drgbl | Hm Drgbl | Hm Drgbl | CPE002E9 | CPE002EQ | CPE002FB | NM 02208 | NM 0220 | NM 0220 | GALNT11 | GALNT11 | GALNT11 | UDP-N-acetyl-alph | UDP-N-ac | UDP-N-ac | 63917 | 63917 | 63917 | s34263 | s34262 | s34261 |
| 8470 | A7 | A8 | 1711 | 1134 | 26449 | 223,5 | 15,5 | 1,095 | H5A034 C | sample | Hm Drgbl | Hm Drgbl | Hm Drgbl | Hm Drgbl | CPE002E6 | CPE002ET | CPE002FE | NM 01416 | NM 0141 | NM 0141 | MKRN2 | MKRN2 | MKRN2 | makorin, ring fing | makorin, r | makorin, r | 23609 | 23609 | 23609 | s24197 | s24198 | s24199 |
| 8573 | F13 | F14 | 1250 | 1044 | 19335 | 108,6 | 15,5 | 1,095 | H5A034 C | sample | Hm Drgbl | Hm Drgbl | Hm Drgbl | Hm Drgbl | CPE002E6 | CPE002ET | CPE002FE | NM 01556 | NM 0155 | NM 0155 | DNM3 | DNM3 | DNM3 | dynamin 3 | dynamin 3 | dynamin 3 | 26052 | 26052 | 26052 | s25017 | s25018 | s25019 |
| 8602 | G18 | G19 | 1585 | 1013 | 24528 | 97,34 | 15,5 | 1,095 | H5A034 C | sample | Hm Drgbl | Hm Drgbl | Hm Drgbl | Hm Drgbl | CPE002E6 | CPE002ET | CPE002FE | NM 01444 | NM 0144 | NM 0144 | SIGLEC9 | SIGLEC9 | SIGLEC9 | sialic acid binding | sialic acid | sialic acid | 27180 | 27180 | 27180 | s25961 | s25962 | s25960 |
| 8754 | M3 | M4 | 1725 | 1039 | 26792 | 119,3 | 15,5 | 1,095 | H5A034 C | sample | Hm Drgbl | Hm Drgbl | Hm Drgbl | Hm Drgbl | CPE002E6 | CPE002ET | CPE002FE | NM 01324 | NM 0132 | NM 0132 | MGAT4C | MGAT4C | MGAT4C | mannosyl (alpha- | mannosyl | mannosyl | 25834 | 25834 | 25834 | s24584 | s24585 | s24586 |
| 19207 | A15 | A16 | 1386 | 1311 | 21538 | 430,7 | 15,5 | 1,099 | H5A034 C | sample | Hm Gen E | Hm Gen E | Hm Gen E | Hm Gen E | CPE002JO | CPE002O3 | CPE002KJ | NM 17561 | NM 1756 | NM 1756 | ZAR1 | ZAR1 | ZAR1 | zygote arrest 1 | zygote arr | zygote arr | 326340 | 326340 | 326340 | s50256 | s50257 | s50258 |
| 19215 | A22 | A23 | 1433 | 1238 | 22256 | 330,5 | 15,5 | 1,099 | H5A034 C | sample | Hm Gen E | Hm Gen E | Hm Gen E | Hm Gen E | CPE002JO | CPE002O3 | CPE002KJ | XM 00112 | XM 00112 | XM 00112 | hCG 1657 | hCG 1657 | hCG 1657 | hCG1657112 | hCG16571 | hCG16571 | 342900 | 342900 | 342900 | s50941 | s50943 | s226330 |
| 19243 | B4 | B5 | 730 | 1196 | 11284 | 453 | 15,5 | 1,099 | H5A034 C | sample | Hm Gen E | Hm Gen E | Hm Gen E | Hm Gen E | CPE002JO | CPE002O3 | CPE002KJ | XM 29340 | XM 29340 | XM 29340 | MAGEB5 | MAGEB5 | MAGEB5 | melanoma antige | melanoma | melanoma | 347541 | 347541 | 347541 | s51274 | s51275 | s51276 |
| 19454 | K21 | K22 | 1550 | 1270 | 24063 | 353,5 | 15,5 | 1,099 | H5A034 C | sample | Hm Gen E | Hm Gen E | Hm Gen E | Hm Gen E | CPE002JO | CPE002O3 | CPE002KJ | NM 17854 | NM 1785 | NM 1785 | C17orf65 | C17orf65 | C17orf65 | chromosome 17 c | chromoso | chromoso | 339201 | 339201 | 339201 | s50471 | s50472 | s50473 |
| 19543 | O15 | O16 | 780 | 1339 | 12125 | 402,6 | 15,5 | 1,099 | H5A034 C | sample | Hm Gen E | Hm Gen E | Hm Gen E | Hm Gen E | CPE002JO | CPE002O3 | CPE002KJ | NM 17855 | NM 1785 | NM 1785 | C1orf110 | C1orf110 | C1orf110 | chromosome 1 op | chromoso | chromoso | 339512 | 339512 | 339512 | s50556 | s50557 | s50558 |
| 19575 | P22 | P23 | 1203 | 1368 | 18652 | 337,4 | 15,5 | 1,099 | H5A034 C | sample | Hm Gen E | Hm Gen E | Hm Gen E | Hm Gen E | CPE002JO | CPE002O3 | CPE002KJ | NM 19855 | NM 1985 | NM 1985 | MIA3 | MIA3 | MIA3 | melanoma inhibit | melanoma | melanoma | 375056 | 375056 | 375056 | s51657 | s51658 | s51659 |
| 20806 | C7 | C8 | 793 | 1029 | 12289 | 300,6 | 15,5 | 1,099 | H5A034 C | sample | Hm Gen E | Hm Gen E | Hm Gen E | Hm Gen E | CPE002JS | CPE002O7 | CPE002KN | XM 00112 | XM 00112 | XM 00112 | LOC642861 | LOC64286 | LOC64286 | hypothetical LOC | hypotheti | hypotheti | 642861 | 642861 | 642861 | s196840 | s196841 | s196842 |
| 20932 | I12 | I13 | 1478 | 1014 | 22856 | 300,6 | 15,5 | 1,099 | H5A034 C | sample | Hm Gen E | Hm Gen E | Hm Gen E | Hm Gen E | CPE002JS | CPE002O7 | CPE002KN | XM 92758 | XM 92758 | XM 92758 | TMEM30C | TMEM30C | TMEM30C | transmembrane p | transmem | transmem | 644444 | 644444 | 644444 | s55671 | s55672 | s227126 |
| 21063 | N22 | N23 | 1247 | 999,6 | 19346 | 250,3 | 15,5 | 1,099 | H5A034 C | sample | Hm Gen E | Hm Gen E | Hm Gen E | Hm Gen E | CPE002JS | CPE002O7 | CPE002KN | XM 93480 | XM 93480 | XM 93480 | LOC645227 | LOC64522 | LOC64522 | hypothetical LOC | hypotheti | hypotheti | 645227 | 645227 | 645227 | s55967 | s55968 | s227173 |
| 21084 | O2 | O3 | 1113 | 1030 | 17208 | 365,7 | 15,5 | 1,099 | H5A034 C | sample | Hm Gen E | Hm Gen E | Hm Gen E | Hm Gen E | CPE002JS | CPE002O7 | CPE002KN | XM 00112 | XM 00112 | XM 00112 | LOC644659 | LOC64465 | LOC64465 | similar to High m | similar to | similar to | 644659 | 644659 | 644659 | s55742 | s55743 | s55744 |
| 21235 | E4 | E5 | 919 | 904,5 | 14206 | 305,3 | 15,5 | 1,099 | H5A034 C | sample | Hm Gen E | Hm Gen E | Hm Gen E | Hm Gen E | CPE002JT | CPE002O8 | CPE002KO | XM 93385 | XM 93385 | XM 93385 | LOC646730 | LOC64673 | LOC64673 | similar to hCG203 | similar to | similar to | 646730 | 646730 | 646730 | s56504 | s56505 | s56506 |
| 14348 | F5 | F6 | 699 | 838,7 | 10841 | 140,5 | 15,5 | 1,103 | H5A034 C | sample | Hm Gen E | Hm Gen E | Hm Gen E | Hm Gen E | CPE002J7 | CPE002NC | CPE002K6 | NM 01612 | NM 0161 | NM 0161 | TMEM66 | TMEM66 | TMEM66 | transmembrane p | transmem | transmem | 51669 | 51669 | 51669 | s28509 | s28510 | s28511 |
| 11432 | M16 | M17 | 1819 | 876,1 | 28225 | 155,7 | 15,5 | 1,123 | H5A034 C | sample | Hm Drgbl | Hm Drgbl | Hm Drgbl | Hm Drgbl | CPE002ED | CPE002F3 | CPE002FT | | | | | | | | | | | | | | | |
| 11467 | N4 | N5 | 1755 | 859 | 27163 | 145,7 | 15,5 | 1,123 | H5A034 C | sample | Hm Drgbl | Hm Drgbl | Hm Drgbl | Hm Drgbl | CPE002ED | CPE002F3 | CPE002FT | | | | | | | | | | | | | | | |
| 12330 | B3 | B4 | 1404 | 1067 | 21770 | 161,7 | 15,5 | 1,165 | H5A034 C | sample | Hm Gen E | Hm Gen E | Hm Gen E | Hm Gen E | CPE002J5 | CPE002NK | CPE002K1 | NM 00037 | NM 0003 | NM 0003 | TTR | TTR | TTR | transthyretin (pre | transthyre | transthyre | 7276 | 7276 | 7276 | s14493 | s14494 | s14495 |
| 7317 | A6 | A7 | 1470 | 687,8 | 22774 | 557 | 15,5 | 1,183 | H5A034 C | sample | Hm Drgbl | Hm Drgbl | Hm Drgbl | Hm Drgbl | CPE002E3 | CPE002EW | CPE002FH | NM 00429 | NM 0042 | NM 0042 | RIN1 | RIN1 | RIN1 | Ras and Rab inter | Ras and R | Ras and R | 9610 | 9610 | 9610 | s18464 | s18466 | s225168 |
| 7558 | K7 | K8 | 1053 | 589,7 | 16372 | 499,9 | 15,5 | 1,183 | H5A034 C | sample | Hm Drgbl | Hm Drgbl | Hm Drgbl | Hm Drgbl | CPE002E3 | CPE002EW | CPE002FH | NM 00257 | NM 0025 | NM 0025 | PAEP | PAEP | PAEP | progestagen-asso | progestag | progestag | 5047 | 5047 | 5047 | s9993 | s9994 | s9995 |
| 7612 | N12 | N13 | 1628 | 607,9 | 25265 | 434,9 | 15,5 | 1,183 | H5A034 C | sample | Hm Drgbl | Hm Drgbl | Hm Drgbl | Hm Drgbl | CPE002E3 | CPE002EW | CPE002FH | NM 00572 | NM 0057 | NM 0057 | PPIF | PPIF | PPIF | peptidylprolyl iso | peptidylp | peptidylp | 10105 | 10105 | 10105 | s19662 | s19663 | s19661 |
| 17024 | F16 | F17 | 1300 | 1014 | 20202 | 196,3 | 15,5 | 1,197 | H5A034 C | sample | Hm Gen E | Hm Gen E | Hm Gen E | Hm Gen E | CPE002JI | CPE002NZ | CPE002KD | XM 06387 | NM 1525 | XM 06387 | LOC12385 | ALS2CR11 | LOC12385 | similar to hCG204 | amyotrop | similar to | 123855 | 151254 | 123855 | s42674 | s45547 | s42676 |
| 17035 | F4 | F5 | 1021 | 967,7 | 15875 | 180,5 | 15,5 | 1,197 | H5A034 C | sample | Hm Gen E | Hm Gen E | Hm Gen E | Hm Gen E | CPE002JI | CPE002NZ | CPE002KD | NM 15233 | XM 00113 | NM 15233 | AGBL1 | FAM132B | AGBL1 | ATP/GTP binding | family wit | ATP/GTP I | 123624 | 151176 | 123624 | s42650 | s45524 | s42652 |
| 17093 | I13 | I14 | 1962 | 977,7 | 30472 | 194,2 | 15,5 | 1,197 | H5A034 C | sample | Hm Gen E | Hm Gen E | Hm Gen E | Hm Gen E | CPE002JI | CPE002NZ | CPE002KD | NM 14458 | NM 1942 | NM 1445 | C14orf149 | SPTY2D1 | C14orf149 | chromosome 14 c | SPT2, Sup | chromoso | 112849 | 144108 | 112849 | s41385 | s44597 | s41387 |
| 17099 | I19 | I20 | 1277 | 940,4 | 19765 | 222 | 15,5 | 1,197 | H5A034 C | sample | Hm Gen E | Hm Gen E | Hm Gen E | Hm Gen E | CPE002JI | CPE002NZ | CPE002KD | NM 08066 | NM 0010 | NM 0806 | CCDC104 | OR2AG1 | CCDC104 | coiled-coil domai | olfactory r | coiled-coi | 112942 | 144125 | 112942 | s41406 | s44606 | s41408 |
| 17114 | J10 | J11 | 1279 | 1023 | 19786 | 208,3 | 15,5 | 1,197 | H5A034 C | sample | Hm Gen E | Hm Gen E | Hm Gen E | Hm Gen E | CPE002JI | CPE002NZ | CPE002KD | NM 14525 | NM 0010 | NM 1452 | TMEM170 | BTLA | TMEM170 | transmembrane p | B and T ly | transmem | 124491 | 151888 | 124491 | s42743 | s45627 | s42745 |
| 17154 | K3 | K4 | 498 | 971,8 | 7712 | 125,9 | 15,5 | 1,197 | H5A034 C | sample | Hm Gen E | Hm Gen E | Hm Gen E | Hm Gen E | CPE002JI | CPE002NZ | CPE002KD | XM 00113 | NM 0010 | XM 00113 | AHNAK2 | OVOS2 | AHNAK2 | AHNAK nucleoprc | ovostatin | AHNAK nu | 113146 | 144203 | 113146 | s41427 | s44621 | s41429 |
| 17160 | K9 | K10 | 1502 | 949,8 | 23342 | 238,6 | 15,5 | 1,197 | H5A034 C | sample | Hm Gen E | Hm Gen E | Hm Gen E | Hm Gen E | CPE002JI | CPE002NZ | CPE002KD | NM 13842 | NM 1524 | NM 1384 | ADAT3 | GLIPR1L2 | ADAT3 | adenosine deami | GLI patho | adenosine | 113179 | 144321 | 113179 | s195590 | s44630 | s225488 |
| 17167 | L15 | L16 | 1601 | 980,9 | 24770 | 191 | 15,5 | 1,197 | H5A034 C | sample | Hm Gen E | Hm Gen E | Hm Gen E | Hm Gen E | CPE002JI | CPE002NZ | CPE002KD | NM 13833 | NM 1784 | NM 1383 | FAM122A | NOTUM | FAM122A | family with seque | notum pe | family wit | 116224 | 147111 | 116224 | s41963 | s44964 | s41965 |
| 17179 | L4 | L5 | 1217 | 905,5 | 18863 | 171,9 | 15,5 | 1,197 | H5A034 C | sample | Hm Gen E | Hm Gen E | Hm Gen E | Hm Gen E | CPE002JI | CPE002NZ | CPE002KD | NM 21359 | XM 9421 | NM 2135 | KRBA2 | C3orf55 | KRBA2 | KRAB-A domain c | chromoso | KRAB-A d | 124751 | 152078 | 124751 | s42785 | s45653 | s42787 |
| 17261 | P13 | P14 | 1383 | 1081 | 21479 | 251 | 15,5 | 1,197 | H5A034 C | sample | Hm Gen E | Hm Gen E | Hm Gen E | Hm Gen E | CPE002JI | CPE002NZ | CPE002KD | NM 17071 | NM 1825 | NM 1707 | WDR17 | SPC24 | WDR17 | WD repeat domai | SPC24, NE | WD repea | 116966 | 147841 | 116966 | s42074 | s45066 | s42076 |
| 6959 | B8 | B9 | 1435 | 595,7 | 22259 | 535,2 | 15,5 | 1,206 | H5A034 C | sample | Hm Drgbl | Hm Drgbl | Hm Drgbl | Hm Drgbl | CPE002E2 | CPE002EX | CPE002FI | NM 00500 | NM 0050 | NM 0050 | NDUFA7 | NDUFA7 | NDUFA7 | NADH dehydrogen | NADH deh | NADH deh | 4701 | 4701 | 4701 | s9337 | s224097 | s9339 |
| 5031 | B22 | B23 | 1510 | 703,7 | 23471 | 503,6 | 15,5 | 1,216 | H5A034 C | sample | Hm Drgbl | Hm Drgbl | Hm Drgbl | Hm Drgbl | CPE002DV | CPE002EN | CPE002FO | XM 00113 | XM 00113 | XM 00113 | VARS | VARS | VARS | valyl-tRNA synthe | valyl-tRNA | valyl-tRNA | 7407 | 7407 | 7407 | s14744 | s14745 | s14746 |
| 5063 | C8 | C9 | 745 | 610,6 | 11524 | 599 | 15,5 | 1,216 | H5A034 C | sample | Hm Drgbl | Hm Drgbl | Hm Drgbl | Hm Drgbl | CPE002DV | CPE002EN | CPE002FO | NM 15226 | NM 0010 | NM 0010 | TPM3 | TPM3 | TPM3 | tropomyosin 3 | tropomyo | tropomyo | 7170 | 7170 | 7170 | s14343 | s224749 | s194839 |
| 2457 | G17 | G18 | 702 | 676 | 10878 | 468,5 | 15,5 | 1,22 | H5A034 C | sample | Hm Drgbl | Hm Drgbl | Hm Drgbl | Hm Drgbl | CPE002DC | CPE002EE | CPE002F4 | NM 00432 | NM 0043 | NM 0043 | BMPR1A | BMPR1A | BMPR1A | bone morphogen | bone mor | bone mor | 657 | 657 | 657 | s280 | s281 | s282 |
| 2541 | J6 | J7 | 917 | 642 | 14243 | 318 | 15,5 | 1,22 | H5A034 C | sample | Hm Drgbl | Hm Drgbl | Hm Drgbl | Hm Drgbl | CPE002DC | CPE002EE | CPE002F4 | NM 00457 | NM 0045 | NM 0045 | MAP4K2 | MAP4K2 | MAP4K2 | mitogen-activatec | mitogen-a | mitogen-a | 5871 | 5871 | 5871 | s11687 | s11688 | s11689 |
| 2596 | M12 | M13 | 925 | 707,3 | 14368 | 456,8 | 15,5 | 1,22 | H5A034 C | sample | Hm Drgbl | Hm Drgbl | Hm Drgbl | Hm Drgbl | CPE002DC | CPE002EE | CPE002F4 | NM 00264 | NM 0026 | NM 0026 | PIK3C2B | PIK3C2B | PIK3C2B | phosphoinositide | phosphoi | phosphoi | 5287 | 5287 | 5287 | s10511 | s10512 | s10513 |
| 16531 | A4 | A5 | 896 | 1027 | 13916 | 403,1 | 15,5 | 1,225 | H5A034 C | sample | Hm Gen E | Hm Gen E | Hm Gen E | Hm Gen E | CPE002JH | CPE002NV | CPE002KC | NM 13856 | NM 1385 | NM 1385 | EXOC3L2 | EXOC3L2 | EXOC3L2 | exocyst complex c | exocyst cc | exocyst cc | 90332 | 90332 | 90332 | s40336 | s40337 | s40338 |
| 16615 | E15 | E16 | 1489 | 1001 | 23113 | 307,8 | 15,5 | 1,225 | H5A034 C | sample | Hm Gen E | Hm Gen E | Hm Gen E | Hm Gen E | CPE002JH | CPE002NV | CPE002KC | NM 03284 | NM 0328 | NM 0328 | TMEM209 | TMEM209 | TMEM209 | transmembrane p | transmem | transmem | 84928 | 84928 | 84928 | s39679 | s39680 | s39681 |
| 16678 | G7 | G8 | 1247 | 905,4 | 19340 | 223 | 15,5 | 1,225 | H5A034 C | sample | Hm Gen E | Hm Gen E | Hm Gen E | Hm Gen E | CPE002JH | CPE002NV | CPE002KC | NM 03285 | NM 0328 | NM 0328 | WDR73 | WDR73 | WDR73 | WD repeat domai | WD repea | WD repea | 84942 | 84942 | 84942 | s39718 | s39719 | s39720 |
| 16685 | H13 | H14 | 1421 | 923,6 | 22051 | 243,6 | 15,5 | 1,225 | H5A034 C | sample | Hm Gen E | Hm Gen E | Hm Gen E | Hm Gen E | CPE002JH | CPE002NV | CPE002KC | NM 08086 | NM 0808 | NM 0808 | RSPH1 | RSPH1 | RSPH1 | radial spoke head | radial spo | radial spo | 89765 | 89765 | 89765 | s40102 | s40103 | s40104 |
| 16689 | H17 | H18 | 1033 | 976,6 | 15974 | 236,2 | 15,5 | 1,225 | H5A034 C | sample | Hm Gen E | Hm Gen E | Hm Gen E | Hm Gen E | CPE002JH | CPE002NV | CPE002KC | NM 02208 | NM 0220 | NM 0220 | HPS4 | HPS4 | HPS4 | Hermansky-Pudla | Hermansk | Hermansk | 89781 | 89781 | 89781 | s40114 | s40115 | s40116 |
| 16695 | H22 | H23 | 1457 | 1083 | 22546 | 282,7 | 15,5 | 1,225 | H5A034 C | sample | Hm Gen E | Hm Gen E | Hm Gen E | Hm Gen E | CPE002JH | CPE002NV | CPE002KC | NM 15228 | NM 1522 | NM 1522 | SCYL1BP1 | SCYL1BP1 | SCYL1BP1 | SCY1-like 1 bindir | SCY1-like | SCY1-like | 92344 | 92344 | 92344 | s40927 | s40928 | s40929 |
| 16728 | I9 | I10 | 720 | 1008 | 11178 | 294,6 | 15,5 | 1,225 | H5A034 C | sample | Hm Gen E | Hm Gen E | Hm Gen E | Hm Gen E | CPE002JH | CPE002NV | CPE002KC | NM 03287 | NM 0328 | NM 0328 | FBXL20 | FBXL20 | FBXL20 | F-box and leucine | F-box and | F-box and | 84961 | 84961 | 84961 | s39763 | s39764 | s39765 |
| 16781 | L13 | L14 | 903 | 978,9 | 14027 | 342,2 | 15,5 | 1,225 | H5A034 C | sample | Hm Gen E | Hm Gen E | Hm Gen E | Hm Gen E | CPE002JH | CPE002NV | CPE002KC | NM 17740 | NM 1774 | NM 1774 | MIDN | MIDN | MIDN | midnolin | midnolin | midnolin | 90007 | 90007 | 90007 | s40225 | s40226 | s195541 |
| 17845 | H20 | H21 | 1143 | 1114 | 17773 | 306,4 | 15,5 | 1,225 | H5A034 C | sample | Hm Gen E | Hm Gen E | Hm Gen E | Hm Gen E | CPE002JK | CPE002NZ | CPE002KF | NM 14471 | NM 1447 | NM 1447 | C3orf48 | C3orf48 | C3orf48 | chromosome 3 op | chromoso | chromoso | 151649 | 151649 | 151649 | s45602 | s45603 | s45604 |
| 17871 | I22 | I23 | 1005 | 1067 | 15580 | 354,9 | 15,5 | 1,225 | H5A034 C | sample | Hm Gen E | Hm Gen E | Hm Gen E | Hm Gen E | CPE002JK | CPE002NZ | CPE002KF | NM 14470 | NM 1447 | NM 1447 | C1orf92 | C1orf92 | C1orf92 | chromosome 1 op | chromoso | chromoso | 149499 | 149499 | 149499 | s45313 | s45314 | s45315 |
| 17903 | J8 | J9 | 1166 | 1023 | 18028 | 249,8 | 15,5 | 1,225 | H5A034 C | sample | Hm Gen E | Hm Gen E | Hm Gen E | Hm Gen E | CPE002JK | CPE002NZ | CPE002KF | NM 19951 | NM 1995 | NM 1995 | CCDC80 | CCDC80 | CCDC80 | coiled-coil domai | coiled-coi | coiled-coi | 151887 | 151887 | 151887 | s45623 | s45624 | s45625 |
| 17907 | K11 | K12 | 1425 | 953,2 | 22029 | 221,5 | 15,5 | 1,225 | H5A034 C | sample | Hm Gen E | Hm Gen E | Hm Gen E | Hm Gen E | CPE002JK | CPE002NZ | CPE002KF | NM 18170 | NM 1817 | NM 1817 | FAM101A | FAM101A | FAM101A | family with seque | family wit | family wit | 144347 | 144347 | 144347 | s44632 | s44633 | s225679 |
| 17935 | L15 | L16 | 874 | 1000 | 13544 | 300,6 | 15,5 | 1,225 | H5A034 C | sample | Hm Gen E | Hm Gen E | Hm Gen E | Hm Gen E | CPE002JK | CPE002NZ | CPE002KF | NM 17849 | NM 1784 | NM 1784 | NOTUM | NOTUM | NOTUM | notum pectinacet | notum pe | notum pe | 147111 | 147111 | 147111 | s44963 | s44964 | s225706 |
| 18025 | P1 | P2 | 680 | 1073 | 10568 | 193,5 | 15,5 | 1,225 | H5A034 C | sample | Hm Gen E | Hm Gen E | Hm Gen E | Hm Gen E | CPE002JK | CPE002NZ | CPE002KF | NM 15247 | NM 1524 | NM 1524 | C19orf18 | C19orf18 | C19orf18 | chromosome 19 c | chromoso | chromoso | 147685 | 147685 | 147685 | s45024 | s45025 | s45026 |
| 18042 | P3 | P4 | 1031 | 1139 | 15970 | 335,7 | 15,5 | 1,225 | H5A034 C | sample | Hm Gen E | Hm Gen E | Hm Gen E | Hm Gen E | CPE002JK | CPE002NZ | CPE002KF | NM 00108 | NM 0010 | NM 0010 | FLJ40125 | FLJ40125 | FLJ40125 | hypothetical prot | hypotheti | hypotheti | 147699 | 147699 | 147699 | s45036 | s45038 | s225712 |
| 9066 | J3 | J4 | 1551 | 1657 | 24027 | 167,4 | 15,5 | 1,245 | H5A034 C | sample | Hm Drgbl | Hm Drgbl | Hm Drgbl | Hm Drgbl | CPE002E7 | CPE002ES | CPE002FD | NM 01661 | NM 0166 | NM 0166 | SLC25A37 | SLC25A37 | SLC25A37 | solute carrier fam | solute car | solute car | 51312 | 51312 | 51312 | s27925 | s27923 | s27924 |
| 9077 | K13 | K14 | 1892 | 1806 | 29265 | 148,1 | 15,5 | 1,245 | H5A034 C | sample | Hm Drgbl | Hm Drgbl | Hm Drgbl | Hm Drgbl | CPE002E7 | CPE002ES | CPE002FD | NM 01595 | NM 0159 | NM 0159 | APIP | APIP | APIP | APAF1 interacting | APAF1 int | APAF1 int | 51074 | 51074 | 51074 | s27360 | s27361 | s27359 |
| 9082 | K18 | K19 | 1959 | 1845 | 30395 | 182,7 | 15,5 | 1,245 | H5A034 C | sample | Hm Drgbl | Hm Drgbl | Hm Drgbl | Hm Drgbl | CPE002E7 | CPE002ES | CPE002FD | NM 01896 | NM 0189 | NM 0189 | UBASH3A | UBASH3A | UBASH3A | ubiquitin associat | ubiquitin | ubiquitin | 53347 | 53347 | 53347 | s28711 | s28709 | s28710 |
| 9087 | K22 | K23 | 1891 | 1687 | 29394 | 190 | 15,5 | 1,245 | H5A034 C | sample | Hm Drgbl | Hm Drgbl | Hm Drgbl | Hm Drgbl | CPE002E7 | CPE002ES | CPE002FD | NM 01693 | NM 0169 | NM 0169 | STX18 | STX18 | STX18 | syntaxin 18 | syntaxin 1 | syntaxin 1 | 53407 | 53407 | 53407 | s28733 | s28734 | s28735 |
| 4570 | O18 | O19 | 1316 | 704,7 | 20344 | 498,8 | 15,5 | 1,308 | H5A034 C | sample | Hm Drgbl | Hm Drgbl | Hm Drgbl | Hm Drgbl | CPE002DU | CPE002EK | CPE002FQ | NM 01758 | NM 0175 | NM 0175 | CHRNA9 | CHRNA9 | CHRNA9 | cholinergic recept | cholinergi | cholinergi | 55584 | 55584 | 55584 | s31016 | s31017 | s31018 |
| 4584 | O9 | O10 | 1331 | 680,3 | 20638 | 526,8 | 15,5 | 1,308 | H5A034 C | sample | Hm Drgbl | Hm Drgbl | Hm Drgbl | Hm Drgbl | CPE002DU | CPE002EK | CPE002FQ | NM 17353 | NM 1735 | NM 1735 | GABRG1 | GABRG1 | GABRG1 | gamma-aminobut | gamma-ar | gamma-ar | 2565 | 2565 | 2565 | s5503 | s5504 | s5505 |
| 3 | A11 | A12 | 591 | 1560 | 9153 | 812,9 | 15,5 | 1,319 | H5A034 C | sample | Hm Drgbl | Hm Drgbl | Hm Drgbl | Hm Drgbl | CPE002DT | CPE002EJ | CPE002F9 | NM 00445 | NM 0044 | NM 0044 | ESRRA | ESRRA | ESRRA | estrogen-related | estrogen-r | estrogen-r | 2101 | 2101 | 2101 | s4829 | s4830 | s4831 |
| 101 | E13 | E14 | 568 | 1427 | 8803 | 471,3 | 15,5 | 1,319 | H5A034 C | sample | Hm Drgbl | Hm Drgbl | Hm Drgbl | Hm Drgbl | CPE002DT | CPE002EJ | CPE002F9 | NM 02100 | NM 0210 | NM 0210 | NR2F2 | NR2F2 | NR2F2 | nuclear receptor s | nuclear re | nuclear re | 7026 | 7026 | 7026 | s14021 | s14022 | s14023 |
| 2010 | D3 | D4 | 943 | 625 | 14656 | 267,7 | 15,5 | 1,348 | H5A034 C | sample | Hm Drgbl | Hm Drgbl | Hm Drgbl | Hm Drgbl | CPE002FX | CPE002G1 | CPE002G5 | NM 13869 | NM 1386 | NM 1386 | KLF14 | KLF14 | KLF14 | Kruppel-like facto | Kruppel-li | Kruppel-li | 136259 | 136259 | 136259 | s43985 | s43986 | s43987 |
| 2060 | F5 | F6 | 820 | 614,5 | 12739 | 375,9 | 15,5 | 1,348 | H5A034 C | sample | Hm Drgbl | Hm Drgbl | Hm Drgbl | Hm Drgbl | CPE002FX | CPE002G1 | CPE002G5 | NM 15243 | NM 1524 | NM 1524 | ZNF664 | ZNF664 | ZNF664 | zinc finger protein | zinc finger | zinc finger | 144348 | 144348 | 144348 | s44635 | s223345 | s44637 |
| 2085 | G6 | G7 | 1109 | 657,5 | 17142 | 528,5 | 15,5 | 1,348 | H5A034 C | sample | Hm Drgbl | Hm Drgbl | Hm Drgbl | Hm Drgbl | CPE002FX | CPE002G1 | CPE002G5 | NM 00424 | NM 0327 | NM 0042 | JMJD1C | JMJD1C | JMJD1C | jumonji domain c | jumonji d | jumonji d | 221037 | 221037 | 221037 | s47941 | s47942 | s47943 |
| 2154 | J3 | J4 | 1263 | 683,8 | 19518 | 458,3 | 15,5 | 1,348 | H5A034 C | sample | Hm Drgbl | Hm Drgbl | Hm Drgbl | Hm Drgbl | CPE002FX | CPE002G1 | CPE002G5 | NM 00100 | NM 0010 | NM 0010 | ZNF599 | ZNF599 | ZNF599 | zinc finger protein | zinc finger | zinc finger | 148103 | 148103 | 148103 | s45120 | s45121 | s45122 |
| 2266 | O18 | O19 | 1774 | 721,1 | 27570 | 493,4 | 15,5 | 1,348 | H5A034 C | sample | Hm Drgbl | Hm Drgbl | Hm Drgbl | Hm Drgbl | CPE002FX | CPE002G1 | CPE002G5 | NM 19849 | NM 1984 | NM 1984 | ZNF642 | ZNF642 | ZNF642 | zinc finger protein | zinc finger | zinc finger | 339559 | 339559 | 339559 | s50566 | s50567 | s50568 |
| 6565 | B20 | B21 | 915 | 638,7 | 14194 | 516,6 | 15,5 | 1,36 | H5A034 C | sample | Hm Drgbl | Hm Drgbl | Hm Drgbl | Hm Drgbl | CPE002E1 | CPE002EY | CPE002FJ | NM 03366 | NM 0336 | NM 0336 | ITGB1 | ITGB1 | ITGB1 | integrin, beta 1 (f | integrin, b | integrin, b | 3688 | 3688 | 3688 | s7575 | s7576 | s7574 |
| 6589 | C20 | C21 | 1423 | 602,3 | 22090 | 537,5 | 15,5 | 1,36 | H5A034 C | sample | Hm Drgbl | Hm Drgbl | Hm Drgbl | Hm Drgbl | CPE002E1 | CPE002EY | CPE002FJ | NM 00057 | NM 0005 | NM 0005 | IL1B | IL1B | IL1B | interleukin 1, bet | interleuki | interleuki | 3553 | 3553 | 3553 | s7271 | s223930 | s7270 |
| 6719 | H8 | H9 | 1582 | 590,9 | 24445 | 353 | 15,5 | 1,36 | H5A034 C | sample | Hm Drgbl | Hm Drgbl | Hm Drgbl | Hm Drgbl | CPE002E1 | CPE002EY | CPE002FJ | NM 00225 | NM 0022 | NM 0022 | KIF3C | KIF3C | KIF3C | kinesin family me | kinesin fa | kinesin fa | 3797 | 3797 | 3797 | s7836 | s223973 | s7834 |
| 6830 | M21 | M22 | 1080 | 523 | 16789 | 457,6 | 15,5 | 1,36 | H5A034 C | sample | Hm Drgbl | Hm Drgbl | Hm Drgbl | Hm Drgbl | CPE002E1 | CPE002EY | CPE002FJ | NM 00213 | NM 0021 | NM 0021 | HMGCS1 | HMGCS1 | HMGCS1 | 3-hydroxy-3-meth | 3-hydroxy | 3-hydroxy | 3157 | 3157 | 3157 | s6663 | s6661 | s6662 |
| 6864 | N9 | N10 | 1134 | 602,7 | 17535 | 429,7 | 15,5 | 1,36 | H5A034 C | sample | Hm Drgbl | Hm Drgbl | Hm Drgbl | Hm Drgbl | CPE002E1 | CPE002EY | CPE002FJ | NM 00450 | NM 0045 | NM 0045 | HSF2 | HSF2 | HSF2 | heat shock transc | heat shoc | heat shoc | 3298 | 3298 | 3298 | s6953 | s6954 | s6955 |
| 6872 | O16 | O17 | 989 | 561,3 | 15365 | 631,3 | 15,5 | 1,36 | H5A034 C | sample | Hm Drgbl | Hm Drgbl | Hm Drgbl | Hm Drgbl | CPE002E1 | CPE002EY | CPE002FJ | NM 00220 | NM 0022 | NM 0022 | ITGA2 | ITGA2 | ITGA2 | integrin, alpha 2 ( | integrin, a | integrin, a | 3673 | 3673 | 3673 | s7537 | s7536 | s7535 |
| 6893 | P13 | P14 | 1524 | 681,1 | 23598 | 530,1 | 15,5 | 1,36 | H5A034 C | sample | Hm Drgbl | Hm Drgbl | Hm Drgbl | Hm Drgbl | CPE002E1 | CPE002EY | CPE002FJ | NM 00216 | NM 0021 | NM 0021 | IDH2 | IDH2 | IDH2 | isocitrate dehydro | isocitrate | isocitrate | 3418 | 3418 | 3418 | s227 | s225 | s226 |
| 11739 | J11 | J12 | 935 | 824,6 | 14447 | 161,2 | 15,5 | 1,384 | H5A034 C | sample | Hm Gen E | Hm Gen E | Hm Gen E | Hm Gen E | CPE002JA | CPE002NJ | CPE002JZ | NM 01461 | NM 0146 | NM 0146 | CRYGA | CRYGA | CRYGA | crystallin, gamma | crystallin, | crystallin, | 1418 | 1418 | 1418 | s3564 | s3565 | s3566 |
| 15392 | B16 | B17 | 1291 | 861,1 | 20014 | 141,6 | 15,5 | 1,409 | H5A034 C | sample | Hm Gen E | Hm Gen E | Hm Gen E | Hm Gen E | CPE002JE | CPE002NT | CPE002K9 | NM 02429 | NM 0242 | NM 0242 | CCDC28B | CCDC28B | CCDC28B | coiled-coil domai | coiled-coi | coiled-coi | 79140 | 79140 | 79140 | s35608 | s35609 | s228177 |
| 15714 | O3 | O4 | 1524 | 794,1 | 23613 | 180,1 | 15,5 | 1,409 | H5A034 C | sample | Hm Gen E | Hm Gen E | Hm Gen E | Hm Gen E | CPE002JE | CPE002NT | CPE002K9 | NM 00100 | NM 0010 | NM 0010 | NFKBIZ | NFKBIZ | NFKBIZ | nuclear factor of l | nuclear fa | nuclear fa | 64332 | 64332 | 64332 | s34641 | s34642 | s34643 |
| 5908 | G12 | G13 | 788 | 744,2 | 12197 | 592,1 | 15,5 | 1,416 | H5A034 C | sample | Hm Drgbl | Hm Drgbl | Hm Drgbl | Hm Drgbl | CPE002DY | CPE002F1 | CPE002FM | NM 00167 | NM 0016 | NM 0016 | ASIP | ASIP | ASIP | agouti signaling p | agouti sig | agouti sig | 434 | 434 | 434 | s1668 | s1666 | s1667 |
| 6050 | M10 | M11 | 1254 | 740,1 | 19379 | 582,9 | 15,5 | 1,416 | H5A034 C | sample | Hm Drgbl | Hm Drgbl | Hm Drgbl | Hm Drgbl | CPE002DY | CPE002F1 | CPE002FM | NM 00517 | NM 0051 | NM 0051 | ATP6V0A1 | ATP6V0A1 | ATP6V0A1 | ATPase, H+ transp | ATPase, H | ATPase, H | 535 | 535 | 535 | s1810 | s1811 | s1809 |
| 6076 | N12 | N13 | 1790 | 762,2 | 27684 | 603 | 15,5 | 1,416 | H5A034 C | sample | Hm Drgbl | Hm Drgbl | Hm Drgbl | Hm Drgbl | CPE002DY | CPE002F1 | CPE002FM | NM 00175 | NM 0017 | NM 0017 | CAV1 | CAV1 | CAV1 | caveolin 1, caveol | caveolin 1 | caveolin 1 | 857 | 857 | 857 | s2446 | s2447 | s2448 |
| 6125 | P13 | P14 | 1280 | 763 | 19901 | 534,5 | 15,5 | 1,416 | H5A034 C | sample | Hm Drgbl | Hm Drgbl | Hm Drgbl | Hm Drgbl | CPE002DY | CPE002F1 | CPE002FM | NM 03322 | NM 0332 | NM 0332 | TRIM23 | TRIM23 | TRIM23 | tripartite motif-co | tripartite | tripartite | 373 | 373 | 373 | s1545 | s1546 | s1544 |
| 1334 | H21 | H22 | 929 | 706,2 | 14417 | 491,5 | 15,5 | 1,422 | H5A034 C | sample | Hm Drgbl | Hm Drgbl | Hm Drgbl | Hm Drgbl | CPE002FV | CPE002FZ | CPE002G3 | NM 02281 | NM 0228 | NM 0228 | PER2 | PER2 | PER2 | period homolog 2 | period ho | period ho | 8864 | 8864 | 8864 | s16930 | s16931 | s16932 |
| 1400 | K16 | K17 | 1430 | 797,7 | 22181 | 369,4 | 15,5 | 1,422 | H5A034 C | sample | Hm Drgbl | Hm Drgbl | Hm Drgbl | Hm Drgbl | CPE002FV | CPE002FZ | CPE002G3 | NM 18279 | NM 1827 | NM 1827 | NPM2 | NPM2 | NPM2 | nucleophosmin/n | nucleopho | nucleopho | 10361 | 10361 | 10361 | s20260 | s20261 | s20262 |
| 16250 | F10 | F11 | 1284 | 952,2 | 19879 | 280,6 | 15,5 | 1,422 | H5A034 C | sample | Hm Gen E | Hm Gen E | Hm Gen E | Hm Gen E | CPE002JG | CPE002NV | CPE002KB | NM 19428 | NM 1942 | NM 1942 | KIAA1853 | KIAA1853 | KIAA1853 | KIAA1853 | KIAA1853 | KIAA1853 | 84530 | 84530 | 84530 | s39118 | s39119 | s39120 |
| 16324 | I12 | I13 | 870 | 1002 | 13447 | 303,3 | 15,5 | 1,422 | H5A034 C | sample | Hm Gen E | Hm Gen E | Hm Gen E | Hm Gen E | CPE002JG | CPE002NV | CPE002KB | NM 03233 | NM 0323 | NM 0323 | ECE2 | ECE2 | ECE2 | endothelin conve | endotheli | endotheli | 9718 | 9718 | 9718 | s38834 | s38835 | s38836 |
| 16386 | K3 | K4 | 533 | 1055 | 8253 | 339,4 | 15,5 | 1,422 | H5A034 C | sample | Hm Gen E | Hm Gen E | Hm Gen E | Hm Gen E | CPE002JG | CPE002NV | CPE002KB | NM 03129 | NM 0312 | NM 0312 | CDCA3 | CDCA3 | CDCA3 | cell division cycle | cell divisio | cell divisio | 83461 | 83461 | 83461 | s37943 | s37944 | s37945 |
| 16398 | L14 | L15 | 1180 | 1046 | 18320 | 365,3 | 15,5 | 1,422 | H5A034 C | sample | Hm Gen E | Hm Gen E | Hm Gen E | Hm Gen E | CPE002JG | CPE002NV | CPE002KB | NM 03260 | NM 0326 | NM 0326 | CCDC54 | CCDC54 | CCDC54 | coiled-coil domai | coiled-coi | coiled-coi | 84692 | 84692 | 84692 | s39335 | s39336 | s39337 |
| 16471 | O15 | O16 | 952 | 1014 | 14801 | 442,1 | 15,5 | 1,422 | H5A034 C | sample | Hm Gen E | Hm Gen E | Hm Gen E | Hm Gen E | CPE002JG | CPE002NV | CPE002KB | NM 03146 | NM 0314 | NM 0314 | NIBP | NIBP | NIBP | NIK and IKK{beta} | NIK and IK | NIK and IK | 83696 | 83696 | 83696 | s38115 | s38116 | s38117 |
| 13483 | B4 | B5 | 1228 | 1139 | 18996 | 166,9 | 15,5 | 1,455 | H5A034 C | sample | Hm Gen E | Hm Gen E | Hm Gen E | Hm Gen E | CPE002JC | CPE002NC | CPE002K4 | NM 01566 | NM 0156 | NM 0156 | IFT172 | IFT172 | IFT172 | intraflagellar tran | intraflage | intraflage | 26160 | 26160 | 26160 | s25193 | s25194 | s25195 |
| 13688 | K16 | K17 | 1225 | 967 | 18929 | 164,9 | 15,5 | 1,455 | H5A034 C | sample | Hm Gen E | Hm Gen E | Hm Gen E | Hm Gen E | CPE002JC | CPE002NC | CPE002K4 | NM 01558 | NM 0155 | NM 0155 | C20orf26 | C20orf26 | C20orf26 | chromosome 20 c | chromoso | chromoso | 26074 | 26074 | 26074 | s25058 | s25059 | s25060 |
| 902 | F21 | F22 | 772 | 677,3 | 11971 | 471 | 15,5 | 1,469 | H5A034 C | sample | Hm Drgbl | Hm Drgbl | Hm Drgbl | Hm Drgbl | CPE002FU | CPE002FY | CPE002G2 | NM 00551 | NM 0055 | NM 0055 | GTF2E1 | GTF2E1 | GTF2E1 | general transcript | general tr | general tr | 2960 | 2960 | 2960 | s6298 | s6299 | s6300 |
| 922 | G18 | G19 | 1842 | 701,3 | 28603 | 388,6 | 15,5 | 1,469 | H5A034 C | sample | Hm Drgbl | Hm Drgbl | Hm Drgbl | Hm Drgbl | CPE002FU | CPE002FY | CPE002G2 | NM 00251 | NM 0025 | NM 0025 | NPAS2 | NPAS2 | NPAS2 | neuronal PAS dor | neuronal | neuronal | 4862 | 4862 | 4862 | s9661 | s9662 | s9663 |
| 1038 | L14 | L15 | 697 | 739,6 | 10829 | 399,1 | 15,5 | 1,469 | H5A034 C | sample | Hm Drgbl | Hm Drgbl | Hm Drgbl | Hm Drgbl | CPE002FU | CPE002FY | CPE002G2 | NM 00321 | NM 0032 | NM 0032 | TEAD3 | TEAD3 | TEAD3 | TEA domain famil | TEA doma | TEA doma | 7005 | 7005 | 7005 | s13967 | s13968 | s13969 |
| 5470 | D7 | D8 | 909 | 608,6 | 14093 | 616 | 15,5 | 1,476 | H5A034 C | sample | Hm Drgbl | Hm Drgbl | Hm Drgbl | Hm Drgbl | CPE002DX | CPE002F2 | CPE002FN | NM 00373 | NM 0037 | NM 0037 | RNASET2 | RNASET2 | RNASET2 | ribonuclease T2 | ribonuclea | ribonuclea | 8635 | 8635 | 8635 | s16429 | s16430 | s16431 |
| 5530 | G18 | G19 | 1514 | 669,6 | 23426 | 524,5 | 15,5 | 1,476 | H5A034 C | sample | Hm Drgbl | Hm Drgbl | Hm Drgbl | Hm Drgbl | CPE002DX | CPE002F2 | CPE002FN | NM 00391 | NM 0039 | NM 0039 | CCNA1 | CCNA1 | CCNA1 | cyclin A1 | cyclin A1 | cyclin A1 | 8900 | 8900 | 8900 | s17020 | s17021 | s17022 |
| 5651 | L19 | L20 | 1348 | 696,6 | 20870 | 573,5 | 15,5 | 1,476 | H5A034 C | sample | Hm Drgbl | Hm Drgbl | Hm Drgbl | Hm Drgbl | CPE002DX | CPE002F2 | CPE002FN | NM 00380 | NM 0038 | NM 0038 | MYH13 | MYH13 | MYH13 | myosin, heavy cha | myosin, h | myosin, h | 8735 | 8735 | 8735 | s16646 | s16647 | s16645 |
| 5685 | M6 | M7 | 1179 | 766 | 18305 | 440,2 | 15,5 | 1,476 | H5A034 C | sample | Hm Drgbl | Hm Drgbl | Hm Drgbl | Hm Drgbl | CPE002DX | CPE002F2 | CPE002FN | NM 00395 | NM 0039 | NM 0039 | CH25H | CH25H | CH25H | cholesterol 25-hy | cholester | cholester | 9023 | 9023 | 9023 | s225065 | s17196 | s17194 |
| 3532 | D12 | D13 | 862 | 1324 | 13357 | 295,5 | 15,5 | 1,49 | H5A034 C | sample | Hm Drgbl | Hm Drgbl | Hm Drgbl | Hm Drgbl | CPE002DR | CPE002EH | CPE002F7 | NM 02040 | NM 0204 | NM 0204 | GPR92 | GPR92 | GPR92 | G protein-couplec | G protein- | G protein- | 57121 | 57121 | 57121 | s32724 | s32725 | s32726 |
| 8137 | D1 | D2 | 1188 | 586,1 | 18434 | 550,2 | 15,5 | 1,49 | H5A034 C | sample | Hm Drgbl | Hm Drgbl | Hm Drgbl | Hm Drgbl | CPE002E5 | CPE002EU | CPE002FF | NM 18150 | NM 1815 | NM 1815 | EXOSC8 | EXOSC8 | EXOSC8 | exosome compon | exosome c | exosome c | 11340 | 11340 | 11340 | s22372 | s22371 | s22370 |
| 8442 | P3 | P4 | 1341 | 624,3 | 20739 | 389,9 | 15,5 | 1,49 | H5A034 C | sample | Hm Drgbl | Hm Drgbl | Hm Drgbl | Hm Drgbl | CPE002E5 | CPE002EU | CPE002FF | NM 00108 | NM 0010 | NM 0010 | UNC13A | UNC13A | UNC13A | unc-13 homolog A | unc-13 ho | unc-13 ho | 23025 | 23025 | 23025 | s22858 | s22859 | s22857 |
| 10251 | L11 | L12 | 1554 | 816,2 | 24139 | 138,3 | 15,5 | 1,49 | H5A034 C | sample | Hm Drgbl | Hm Drgbl | Hm Drgbl | Hm Drgbl | CPE002EA | CPE002EP | CPE002FA | NM 03082 | NM 0308 | NM 0308 | COL21A1 | COL21A1 | COL21A1 | collagen, type XXI | collagen, t | collagen, t | 81578 | 81578 | 81578 | s37671 | s37672 | s37673 |
| 1541 | A13 | A14 | 1171 | 758 | 18203 | 491,7 | 15,5 | 1,623 | H5A034 C | sample | Hm Drgbl | Hm Drgbl | Hm Drgbl | Hm Drgbl | CPE002FW | CPE002G0 | CPE002G4 | NM 01219 | NM 0121 | NM 0121 | GCA | GCA | GCA | grancalcin, EF-har | grancalcin | grancalcin | 25801 | 25801 | 25801 | s24512 | s24513 | s24514 |
| 1763 | J19 | J20 | 807 | 615 | 12525 | 347,3 | 15,5 | 1,623 | H5A034 C | sample | Hm Drgbl | Hm Drgbl | Hm Drgbl | Hm Drgbl | CPE002FW | CPE002G0 | CPE002G4 | NM 01819 | NM 0181 | NM 0181 | DNAJC11 | DNAJC11 | DNAJC11 | DnaJ (Hsp40) hom | DnaJ (Hsp | DnaJ (Hsp | 55735 | 55735 | 55735 | s31371 | s31372 | s31373 |
| 1919 | P8 | P9 | 1465 | 762,5 | 22732 | 518,1 | 15,5 | 1,623 | H5A034 C | sample | Hm Drgbl | Hm Drgbl | Hm Drgbl | Hm Drgbl | CPE002FW | CPE002G0 | CPE002G4 | NM 03280 | NM 0328 | NM 0328 | ZSCAN10 | ZSCAN10 | ZSCAN10 | zinc finger and SC | zinc finger | zinc finger | 84891 | 84891 | 84891 | s39586 | s39587 | s39588 |
| 21519 | A22 | A23 | 153 | 912,8 | 2370 | 565,4 | 15,5 | 1,632 | H5A034 C | sample | Hm Gen E | Hm Gen E | Hm Gen E | Hm Gen E | CPE002JU | CPE002O9 | CPE002KP | XM 94199 | XM 94199 | XM 94199 | LOC652517 | LOC65251 | LOC65251 | similar to stereoc | similar to | similar to | 652517 | 652517 | 652517 | s57381 | s57382 | s197898 |
| 21731 | J19 | J20 | 226 | 949,2 | 3510 | 599,6 | 15,5 | 1,632 | H5A034 C | sample | Hm Gen E | Hm Gen E | Hm Gen E | Hm Gen E | CPE002JU | CPE002O9 | CPE002KP | XM 94107 | XM 94107 | XM 94107 | LOC651825 | LOC65182 | LOC65182 | similar to ribosom | similar to | similar to | 651825 | 651825 | 651825 | s57257 | s197826 | s227376 |
| 21884 | P5 | P6 | 291 | 1111 | 4503 | 675,5 | 15,5 | 1,632 | H5A034 C | sample | Hm Gen E | Hm Gen E | Hm Gen E | Hm Gen E | CPE002JU | CPE002O9 | CPE002KP | XM 94162 | XM 94162 | XM 94162 | LOC652231 | LOC65223 | LOC65223 | L1 protein | L1 protein | L1 protein | 652231 | 652231 | 652231 | s57329 | s57330 | s57331 |
| 4774 | G7 | G8 | 650 | 784,2 | 10047 | 409,3 | 15,5 | 1,703 | H5A034 C | sample | Hm Drgbl | Hm Drgbl | Hm Drgbl | Hm Drgbl | CPE002DV | CPE002EL | CPE002FP | NM 00405 | NM 0040 | NM 0040 | CAPN5 | CAPN5 | CAPN5 | calpain 5 | calpain 5 | calpain 5 | 726 | 726 | 726 | s2179 | s2180 | s2181 |
| 10449 | D17 | D18 | 920 | 829,2 | 14300 | 197,7 | 15,5 | 1,751 | H5A034 C | sample | Hm Drgbl | Hm Drgbl | Hm Drgbl | Hm Drgbl | CPE002EB | CPE002EO | CPE002FR | NM 13877 | NM 1387 | NM 1387 | ALKBH8 | ALKBH8 | ALKBH8 | alkB, alkylation re | alkB, alkyl | alkB, alkyl | 91801 | 91801 | 91801 | s40784 | s40783 | s40785 |
| 10558 | H7 | H8 | 618 | 761,5 | 9575 | 176,7 | 15,5 | 1,751 | H5A034 C | sample | Hm Drgbl | Hm Drgbl | Hm Drgbl | Hm Drgbl | CPE002EB | CPE002EO | CPE002FR | NM 03341 | NM 0334 | NM 0334 | IMP4 | IMP4 | IMP4 | IMP4, U3 small nu | IMP4, U3 s | IMP4, U3 s | 92856 | 92856 | 92856 | s41039 | s41037 | s41038 |
| 10562 | I10 | I11 | 1003 | 871,8 | 15541 | 200,2 | 15,5 | 1,751 | H5A034 C | sample | Hm Drgbl | Hm Drgbl | Hm Drgbl | Hm Drgbl | CPE002EB | CPE002EO | CPE002FR | NM 13845 | NM 1384 | NM 1384 | ARL11 | ARL11 | ARL11 | ADP-ribosylation | ADP-ribos | ADP-ribos | 115761 | 115761 | 115761 | s41870 | s41871 | s41869 |
| 10719 | O22 | O23 | 1188 | 816,7 | 18424 | 225,4 | 15,5 | 1,751 | H5A034 C | sample | Hm Drgbl | Hm Drgbl | Hm Drgbl | Hm Drgbl | CPE002EB | CPE002EO | CPE002FR | NM 19431 | NM 1943 | NM 1943 | UBE2J2 | UBE2J2 | UBE2J2 | ubiquitin-conjuga | ubiquitin- | ubiquitin- | 118424 | 118424 | 118424 | s42191 | s42189 | s42190 |
| 22354 | D18 | D19 | 1067 | 1425 | 16691 | 320,9 | 15,6 | 0,804 | H5A034 C | sample | Hm Gen E | Hm Gen E | Hm Gen E | Hm Gen E | CPE002JW | CPE002OB | CPE002KR | XM 00112 | XM 00112 | XM 00112 | LOC729243 | LOC72924 | LOC72924 | similar to RNA me | similar to | similar to | 729243 | 729243 | 729243 | s59523 | s227762 | s227763 |
| 23761 | O1 | O2 | 765 | 1502 | 11963 | 231,1 | 15,6 | 0,828 | H5A034 C | sample | Hm Gen E | Hm Gen E | Hm Gen E | Hm Gen E | CPE002KV | CPE002OE | CPE002KU | XM 00113 | XM 00113 | XM 00113 | LOC732307 | LOC73230 | LOC73230 | similar to zinc fin | similar to | similar to | 732307 | 732307 | 732307 | s61622 | s61623 | s200338 |
| 14922 | N3 | N4 | 1301 | 771,3 | 20352 | 86,52 | 15,6 | 0,889 | H5A034 C | sample | Hm Gen E | Hm Gen E | Hm Gen E | Hm Gen E | CPE002JD | CPE002NR | CPE002K7 | NM 01830 | NM 0183 | NM 0183 | TMEM40 | TMEM40 | TMEM40 | transmembrane p | transmem | transmem | 55287 | 55287 | 55287 | s30645 | s30646 | s30647 |
| 3084 | A2 | A3 | 503 | 1296 | 7851 | 529,4 | 15,6 | 0,966 | H5A034 C | sample | Hm Drgbl | Hm Drgbl | Hm Drgbl | Hm Drgbl | CPE002DC | CPE002EG | CPE002F6 | NM 01556 | NM 0155 | NM 0155 | PPP1R16B | PPP1R16B | PPP1R16B | protein phosphat | protein pl | protein pl | 26051 | 26051 | 26051 | s25014 | s25015 | s25016 |
| 3292 | J12 | J13 | 1107 | 1235 | 17284 | 344,7 | 15,6 | 0,966 | H5A034 C | sample | Hm Drgbl | Hm Drgbl | Hm Drgbl | Hm Drgbl | CPE002DC | CPE002EG | CPE002F6 | NM 00029 | NM 0002 | NM 0002 | SERPINA1 | SERPINA1 | SERPINA1 | serpin peptidase | serpin pei | serpin pei | 5265 | 5265 | 5265 | s10457 | s10458 | s10459 |
| 3396 | N2 | N3 | 1217 | 1216 | 18999 | 389,9 | 15,6 | 0,966 | H5A034 C | sample | Hm Drgbl | Hm Drgbl | Hm Drgbl | Hm Drgbl | CPE002DC | CPE002EG | CPE002F6 | NM 18152 | NM 1815 | NM 1815 | PIK3R1 | PIK3R1 | PIK3R1 | phosphoinositide | phosphoi | phosphoi | 5295 | 5295 | 5295 | s10535 | s10537 | s10536 |
| 12018 | E3 | E4 | 1474 | 828,1 | 23064 | 91,24 | 15,6 | 1 | H5A034 C | sample | Hm Gen E | Hm Gen E | Hm Gen E | Hm Gen E | CPE002J4 | CPE002NL | CPE002K0 | NM 00228 | NM 0022 | NM 0022 | LAG3 | LAG3 | LAG3 | lymphocyte-activa | lymphocy | lymphocy | 3902 | 3902 | 3902 | s8045 | s8046 | s8047 |
| 12165 | K6 | K7 | 1043 | 813,9 | 16316 | 140,5 | 15,6 | 1 | H5A034 C | sample | Hm Gen E | Hm Gen E | Hm Gen E | Hm Gen E | CPE002J4 | CPE002NL | CPE002K0 | NM 00626 | NM 0062 | NM 0062 | RP1 | RP1 | RP1 | retinitis pigmento | retinitis p | retinitis p | 6101 | 6101 | 6101 | s12118 | s12119 | s12120 |
| 12267 | P11 | P12 | 1716 | 786,3 | 26703 | 149,7 | 15,6 | 1 | H5A034 C | sample | Hm Gen E | Hm Gen E | Hm Gen E | Hm Gen E | CPE002J4 | CPE002NL | CPE002K0 | NM 00266 | NM 0026 | NM 0026 | PLEK | PLEK | PLEK | pleckstrin | pleckstrin | pleckstrin | 5341 | 5341 | 5341 | s10649 | s10650 | s10651 |
| 18344 | M16 | M17 | 1183 | 1033 | 18510 | 209,9 | 15,6 | 1,003 | H5A034 C | sample | Hm Gen E | Hm Gen E | Hm Gen E | Hm Gen E | CPE002JL | CPE002OO | CPE002KG | NM 00108 | NM 0010 | NM 0010 | DNAH10 | DNAH10 | DNAH10 | dynein, axonemal | dynein, ax | dynein, ax | 196385 | 196385 | 196385 | s46997 | s46998 | s225822 |
| 18382 | N7 | N8 | 1659 | 983,8 | 25954 | 393,8 | 15,6 | 1,003 | H5A034 C | sample | Hm Gen E | Hm Gen E | Hm Gen E | Hm Gen E | CPE002JL | CPE002OO | CPE002KG | NM 20341 | NM 2034 | NM 2034 | UBL4B | UBL4B | UBL4B | ubiquitin-like 4B | ubiquitin- | ubiquitin- | 164153 | 164153 | 164153 | s46512 | s46513 | s46514 |
| 6218 | D10 | D11 | 1173 | 591,3 | 18329 | 516 | 15,6 | 1,03 | H5A034 C | sample | Hm Drgbl | Hm Drgbl | Hm Drgbl | Hm Drgbl | CPE002DZ | CPE002F0 | CPE002FL | NM 00136 | NM 0013 | NM 0013 | DHCR7 | DHCR7 | DHCR7 | 7-dehydrocholest | 7-dehydro | 7-dehydro | 1717 | 1717 | 1717 | s4074 | s4075 | s4076 |
| 6272 | F16 | F17 | 1499 | 585,8 | 23361 | 384,6 | 15,6 | 1,03 | H5A034 C | sample | Hm Drgbl | Hm Drgbl | Hm Drgbl | Hm Drgbl | CPE002DZ | CPE002F0 | CPE002FL | NM 00193 | NM 0019 | NM 0019 | DLAT | DLAT | DLAT | dihydrolipoamide | dihydrolic | dihydrolic | 1737 | 1737 | 1737 | s4113 | s4115 | s4114 |
| 6461 | N13 | N14 | 1926 | 651,4 | 30009 | 432,2 | 15,6 | 1,03 | H5A034 C | sample | Hm Drgbl | Hm Drgbl | Hm Drgbl | Hm Drgbl | CPE002DZ | CPE002F0 | CPE002FL | NM 00188 | NM 0018 | NM 0018 | ATF2 | ATF2 | ATF2 | activating transcri | activating | activating | 1386 | 1386 | 1386 | s3492 | s3494 | s3493 |
| 6481 | O1 | O2 | 290 | 636,6 | 4527 | 534,5 | 15,6 | 1,03 | H5A034 C | sample | Hm Drgbl | Hm Drgbl | Hm Drgbl | Hm Drgbl | CPE002DZ | CPE002F0 | CPE002FL | NM 00180 | NM 0018 | NM 0018 | CDR2 | CDR2 | CDR2 | cerebellar degene | cerebellar | cerebellar | 1039 | 1039 | 1039 | s2862 | s2863 | s2861 |
| 12841 | H1 | H2 | 1146 | 1036 | 17882 | 154,4 | 15,6 | 1,058 | H5A034 C | sample | Hm Gen E | Hm Gen E | Hm Gen E | Hm Gen E | CPE002J6 | CPE002NN | CPE002K2 | NM 01586 | NM 0158 | NM 0158 | C6orf32 | C6orf32 | C6orf32 | chromosome 6 op | chromoso | chromoso | 9750 | 9750 | 9750 | s18818 | s18819 | s18820 |
| 12906 | J3 | J4 | 1069 | 1016 | 16650 | 129,1 | 15,6 | 1,058 | H5A034 C | sample | Hm Gen E | Hm Gen E | Hm Gen E | Hm Gen E | CPE002J6 | CPE002NN | CPE002K2 | NM 01474 | NM 0147 | NM 0147 | KIAA0232 | KIAA0232 | KIAA0232 | KIAA0232 | KIAA0232 | KIAA0232 | 9778 | 9778 | 9778 | s18885 | s18886 | s18887 |
| 17346 | C3 | C4 | 1042 | 1012 | 16218 | 189,1 | 15,6 | 1,065 | H5A034 C | sample | Hm Gen E | Hm Gen E | Hm Gen E | Hm Gen E | CPE002JJ | CPE002NY | CPE002KE | NM 17681 | NM 1768 | NM 1768 | NLRP8 | NLRP8 | NLRP8 | NLR family, pyrin | NLR family | NLR family | 126205 | 126205 | 126205 | s42971 | s42972 | s42973 |
| 17460 | H2 | H3 | 998 | 955,8 | 15531 | 158,4 | 15,6 | 1,065 | H5A034 C | sample | Hm Gen E | Hm Gen E | Hm Gen E | Hm Gen E | CPE002JJ | CPE002NY | CPE002KE | NM 01236 | NM 0123 | NM 0123 | OR1N1 | OR1N1 | OR1N1 | olfactory receptor | olfactory r | olfactory r | 138883 | 138883 | 138883 | s44154 | s44155 | s44156 |
| 17600 | N16 | N17 | 1191 | 892,4 | 18597 | 226,3 | 15,6 | 1,065 | H5A034 C | sample | Hm Gen E | Hm Gen E | Hm Gen E | Hm Gen E | CPE002JJ | CPE002NY | CPE002KE | NM 08057 | NM 0805 | NM 0805 | C20orf70 | C20orf70 | C20orf70 | chromosome 20 c | chromoso | chromoso | 140683 | 140683 | 140683 | s44332 | s44333 | s44334 |
| 9409 | I1 | I2 | 1279 | 1097 | 19954 | 129,1 | 15,6 | 1,083 | H5A034 C | sample | Hm Drgbl | Hm Drgbl | Hm Drgbl | Hm Drgbl | CPE002E8 | CPE002ER | CPE002FC | NM 01807 | NM 0180 | NM 0180 | RBM28 | RBM28 | RBM28 | RNA binding moti | RNA bindi | RNA bindi | 55131 | 55131 | 55131 | s30243 | s30242 | s30244 |
| 9478 | K7 | K8 | 409 | 1028 | 6363 | 146,1 | 15,6 | 1,083 | H5A034 C | sample | Hm Drgbl | Hm Drgbl | Hm Drgbl | Hm Drgbl | CPE002E8 | CPE002ER | CPE002FC | NM 01815 | NM 0181 | NM 0181 | SLC25A36 | SLC25A36 | SLC25A36 | solute carrier fam | solute car | solute car | 55186 | 55186 | 55186 | s30382 | s30383 | s30384 |
| 18927 | E22 | E23 | 1786 | 1034 | 27833 | 324,9 | 15,6 | 1,087 | H5A034 C | sample | Hm Gen E | Hm Gen E | Hm Gen E | Hm Gen E | CPE002JN | CPE002O2 | CPE002KI | NM 17494 | NM 1749 | NM 1749 | C19orf30 | C19orf30 | C19orf30 | chromosome 19 c | chromoso | chromoso | 284424 | 284424 | 284424 | s49653 | s49654 | s49655 |
| 18944 | F16 | F17 | 1404 | 1002 | 21887 | 221,7 | 15,6 | 1,087 | H5A034 C | sample | Hm Gen E | Hm Gen E | Hm Gen E | Hm Gen E | CPE002JN | CPE002O2 | CPE002KI | NM 17366 | NM 1736 | NM 1736 | DTWD2 | DTWD2 | DTWD2 | DTW domain con | DTW dom | DTW dom | 285605 | 285605 | 285605 | s49982 | s49983 | s49984 |
| 18974 | G21 | G22 | 766 | 948,3 | 11936 | 234,1 | 15,6 | 1,087 | H5A034 C | sample | Hm Gen E | Hm Gen E | Hm Gen E | Hm Gen E | CPE002JN | CPE002O2 | CPE002KI | NM 00100 | NM 0010 | NM 0010 | OR51B5 | OR51B5 | OR51B5 | olfactory receptor | olfactory r | olfactory r | 282763 | 282763 | 282763 | s49054 | s49055 | s49056 |
| 19154 | O10 | O11 | 1363 | 956,3 | 21214 | 373,9 | 15,6 | 1,087 | H5A034 C | sample | Hm Gen E | Hm Gen E | Hm Gen E | Hm Gen E | CPE002JN | CPE002O2 | CPE002KI | NM 17382 | NM 1738 | NM 1738 | FAM126B | FAM126B | FAM126B | family with seque | family wit | family wit | 285172 | 285172 | 285172 | s49831 | s49832 | s49833 |
| 8598 | G14 | G15 | 1678 | 941,9 | 26244 | 111,1 | 15,6 | 1,102 | H5A034 C | sample | Hm Drgbl | Hm Drgbl | Hm Drgbl | Hm Drgbl | CPE002E6 | CPE002ET | CPE002FE | NM 01443 | NM 0144 | NM 0144 | SLC39A1 | SLC39A1 | SLC39A1 | solute carrier fam | solute car | solute car | 27173 | 27173 | 27173 | s25946 | s25947 | s223736 |
| 8748 | M2 | M3 | 2106 | 1187 | 32764 | 115,9 | 15,6 | 1,102 | H5A034 C | sample | Hm Drgbl | Hm Drgbl | Hm Drgbl | Hm Drgbl | CPE002E6 | CPE002ET | CPE002FE | NM 01447 | NM 0144 | NM 0144 | SMPDL3B | SMPDL3B | SMPDL3B | sphingomyelin ph | sphingom | sphingom | 27293 | 27293 | 27293 | s26099 | s26100 | s26101 |
| 19536 | N9 | N10 | 1099 | 1119 | 17105 | 285,7 | 15,6 | 1,106 | H5A034 C | sample | Hm Gen E | Hm Gen E | Hm Gen E | Hm Gen E | CPE002JO | CPE002O3 | CPE002KJ | NM 18260 | NM 1826 | NM 1826 | ANKRD33 | ANKRD33 | ANKRD33 | ankyrin repeat dc | ankyrin re | ankyrin re | 341405 | 341405 | 341405 | s50842 | s50843 | s226321 |
| 20845 | E20 | E21 | 1042 | 961,8 | 16282 | 202,5 | 15,6 | 1,106 | H5A034 C | sample | Hm Gen E | Hm Gen E | Hm Gen E | Hm Gen E | CPE002JS | CPE002O7 | CPE002KN | XM 92744 | XM 92744 | XM 92744 | LOC644265 | LOC64426 | LOC64426 | similar to hCG179 | similar to | similar to | 644265 | 644265 | 644265 | s55622 | s55624 | s227114 |
| 20850 | E3 | E4 | 1106 | 963,8 | 17221 | 214 | 15,6 | 1,106 | H5A034 C | sample | Hm Gen E | Hm Gen E | Hm Gen E | Hm Gen E | CPE002JS | CPE002O7 | CPE002KN | XM 92631 | XM 92631 | XM 92631 | LOC642936 | LOC64293 | LOC64293 | similar to zinc fin | similar to | similar to | 642936 | 642936 | 642936 | s55158 | s55159 | s55160 |
| 20947 | I4 | I5 | 951 | 906 | 14817 | 264,1 | 15,6 | 1,106 | H5A034 C | sample | Hm Gen E | Hm Gen E | Hm Gen E | Hm Gen E | CPE002JS | CPE002O7 | CPE002KN | XM 92754 | XM 92754 | XM 92754 | LOC644402 | LOC64440 | LOC64440 | hypothetical LOC | hypotheti | hypotheti | 644402 | 644402 | 644402 | s55662 | s55663 | s55664 |
| 20984 | K16 | K17 | 850 | 945,6 | 13296 | 288,7 | 15,6 | 1,106 | H5A034 C | sample | Hm Gen E | Hm Gen E | Hm Gen E | Hm Gen E | CPE002JS | CPE002O7 | CPE002KN | XM 92767 | XM 92767 | XM 92767 | LOC644548 | LOC64454 | LOC64454 | similar to LOC531 | similar to | similar to | 644548 | 644548 | 644548 | s55705 | s55706 | s55707 |
| 21387 | L11 | L12 | 588 | 941,1 | 9169 | 331,7 | 15,6 | 1,106 | H5A034 C | sample | Hm Gen E | Hm Gen E | Hm Gen E | Hm Gen E | CPE002JT | CPE002O8 | CPE002KO | XM 92936 | XM 92936 | XM 92936 | hCG 1794 | hCG 1794 | hCG 1794 | hCG1794003 | hCG17940 | hCG17940 | 646433 | 646433 | 646433 | s56371 | s56372 | s56373 |
| 11280 | F9 | F10 | 1187 | 831,5 | 18570 | 143,7 | 15,6 | 1,13 | H5A034 C | sample | Hm Drgbl | Hm Drgbl | Hm Drgbl | Hm Drgbl | CPE002ED | CPE002F3 | CPE002FT | NM 00599 | NM 0059 | NM 0059 | TBX10 | TBX10 | TBX10 | T-box 10 | T-box 10 | T-box 10 | 347853 | 347853 | 347853 | s51312 | s51311 | s51310 |
| 11404 | L12 | L13 | 1769 | 874,1 | 27647 | 165,8 | 15,6 | 1,13 | H5A034 C | sample | Hm Drgbl | Hm Drgbl | Hm Drgbl | Hm Drgbl | CPE002ED | CPE002F3 | CPE002FT | | | | | | | | | | | | | | | |
| 11451 | N11 | N12 | 1646 | 841,3 | 25740 | 146,9 | 15,6 | 1,13 | H5A034 C | sample | Hm Drgbl | Hm Drgbl | Hm Drgbl | Hm Drgbl | CPE002ED | CPE002F3 | CPE002FT | NM 00101 | NM 0010 | NM 0010 | HES5 | HES5 | HES5 | hairy and enhanc | hairy and | hairy and | 388585 | 388585 | 388585 | s223986 | s52196 | s52197 |
| 11511 | P22 | P23 | 1748 | 849,6 | 27279 | 251,1 | 15,6 | 1,13 | H5A034 C | sample | Hm Drgbl | Hm Drgbl | Hm Drgbl | Hm Drgbl | CPE002ED | CPE002F3 | CPE002FT | | | | | | | | | | | | | | | |
| 12511 | J15 | J16 | 1421 | 991,3 | 22115 | 139,9 | 15,6 | 1,173 | H5A034 C | sample | Hm Gen E | Hm Gen E | Hm Gen E | Hm Gen E | CPE002J5 | CPE002NK | CPE002K1 | NM 00715 | NM 0071 | NM 0071 | SLMAP | SLMAP | SLMAP | sarcolemma asso | sarcolemr | sarcolemr | 7871 | 7871 | 7871 | s15433 | s15434 | s15435 |
| 12662 | P21 | P22 | 1359 | 1186 | 21223 | 243,7 | 15,6 | 1,173 | H5A034 C | sample | Hm Gen E | Hm Gen E | Hm Gen E | Hm Gen E | CPE002J5 | CPE002NK | CPE002K1 | NM 00349 | NM 0034 | NM 0034 | SNN | SNN | SNN | stannin | stannin | stannin | 8303 | 8303 | 8303 | s15805 | s15806 | s15807 |
| 10934 | H21 | H22 | 1589 | 890,9 | 24843 | 605,7 | 15,6 | 1,182 | H5A034 C | sample | Hm Drgbl | Hm Drgbl | Hm Drgbl | Hm Drgbl | CPE002EC | CPE002EN | CPE002FS | NM 17203 | NM 1720 | NM 1720 | RDH10 | RDH10 | RDH10 | retinol dehydroge | retinol de | retinol de | 157506 | 157506 | 157506 | s45959 | s45960 | s45961 |
| 10944 | H9 | H10 | 1091 | 754 | 17034 | 483,7 | 15,6 | 1,182 | H5A034 C | sample | Hm Drgbl | Hm Drgbl | Hm Drgbl | Hm Drgbl | CPE002EC | CPE002EN | CPE002FS | NM 14472 | NM 1447 | NM 1447 | RNF145 | RNF145 | RNF145 | ring finger protein | ring finger | ring finger | 153830 | 153830 | 153830 | s45831 | s45830 | s45829 |
| 7331 | B19 | B20 | 1315 | 598,3 | 20563 | 555,9 | 15,6 | 1,191 | H5A034 C | sample | Hm Drgbl | Hm Drgbl | Hm Drgbl | Hm Drgbl | CPE002E3 | CPE002EW | CPE002FH | NM 00480 | NM 0048 | NM 0048 | NRXN1 | NRXN1 | NRXN1 | neurexin 1 | neurexin 1 | neurexin 1 | 9378 | 9378 | 9378 | s17946 | s225122 | s17948 |
| 7435 | F4 | F5 | 937 | 534,9 | 14597 | 516,7 | 15,6 | 1,191 | H5A034 C | sample | Hm Drgbl | Hm Drgbl | Hm Drgbl | Hm Drgbl | CPE002E3 | CPE002EW | CPE002FH | NM 00512 | NM 0051 | NM 0051 | THRAP1 | THRAP1 | THRAP1 | thyroid hormone | thyroid hc | thyroid hc | 9969 | 9969 | 9969 | s19366 | s19367 | s19365 |
| 7455 | G22 | G23 | 1300 | 649,7 | 20293 | 514 | 15,6 | 1,191 | H5A034 C | sample | Hm Drgbl | Hm Drgbl | Hm Drgbl | Hm Drgbl | CPE002E3 | CPE002EW | CPE002FH | NM 01471 | NM 0147 | NM 0147 | SETD1A | SETD1A | SETD1A | SET domain conta | SET doma | SET doma | 9739 | 9739 | 9739 | s18788 | s18789 | s18790 |
| 7469 | H13 | H14 | 1371 | 625,9 | 21351 | 504,5 | 15,6 | 1,191 | H5A034 C | sample | Hm Drgbl | Hm Drgbl | Hm Drgbl | Hm Drgbl | CPE002E3 | CPE002EW | CPE002FH | NM 00427 | NM 0042 | NM 0042 | HOMER1 | HOMER1 | HOMER1 | homer homolog 1 | homer ho | homer ho | 9456 | 9456 | 9456 | s18117 | s18118 | s18116 |
| 7495 | I15 | I16 | 971 | 576,1 | 15171 | 401,8 | 15,6 | 1,191 | H5A034 C | sample | Hm Drgbl | Hm Drgbl | Hm Drgbl | Hm Drgbl | CPE002E3 | CPE002EW | CPE002FH | NM 00091 | NM 0009 | NM 0009 | OXT | OXT | OXT | oxytocin, prepro- | oxytocin, | oxytocin, | 5020 | 5020 | 5020 | s194671 | s194672 | s194670 |
| 7635 | O11 | O12 | 1163 | 568,5 | 18162 | 577,6 | 15,6 | 1,191 | H5A034 C | sample | Hm Drgbl | Hm Drgbl | Hm Drgbl | Hm Drgbl | CPE002E3 | CPE002EW | CPE002FH | NM 00053 | NM 0005 | NM 0005 | PCCB | PCCB | PCCB | propionyl Coenzy | propionyl | propionyl | 5096 | 5096 | 5096 | s10103 | s10104 | s10105 |
| 7643 | O19 | O20 | 1610 | 591,6 | 25174 | 531,3 | 15,6 | 1,191 | H5A034 C | sample | Hm Drgbl | Hm Drgbl | Hm Drgbl | Hm Drgbl | CPE002E3 | CPE002EW | CPE002FH | NM 00259 | NM 0025 | NM 0025 | PCDH8 | PCDH8 | PCDH8 | protocadherin 8 | protocadh | protocadh | 5100 | 5100 | 5100 | s10113 | s10114 | s10112 |
| 16900 | A12 | A13 | 1033 | 991,8 | 16106 | 277,8 | 15,6 | 1,205 | H5A034 C | sample | Hm Gen E | Hm Gen E | Hm Gen E | Hm Gen E | CPE002JI | CPE002NZ | CPE002KD | NM 13869 | NM 1524 | NM 1386 | TMC1 | C19orf23 | TMC1 | transmembrane c | chromoso | transmem | 117531 | 148046 | 117531 | s42166 | s45117 | s42168 |
| 16917 | A6 | A7 | 1409 | 1069 | 22002 | 282,5 | 15,6 | 1,205 | H5A034 C | sample | Hm Gen E | Hm Gen E | Hm Gen E | Hm Gen E | CPE002JI | CPE002NZ | CPE002KD | NM 05328 | NM 2073 | NM 0532 | DCD | DPY19L3 | DCD | dermcidin | dpy-19-lik | dermcidin | 117159 | 147991 | 117159 | s42122 | s45105 | s42124 |
| 16918 | A7 | A8 | 1297 | 1083 | 20291 | 342,9 | 15,6 | 1,205 | H5A034 C | sample | Hm Gen E | Hm Gen E | Hm Gen E | Hm Gen E | CPE002JI | CPE002NZ | CPE002KD | NM 17347 | NM 0807 | NM 1734 | TMEM32 | KRT72 | TMEM32 | transmembrane p | keratin 72 | transmem | 93380 | 140807 | 93380 | s41129 | s44406 | s41131 |
| 17063 | G8 | G9 | 1143 | 958 | 17849 | 198 | 15,6 | 1,205 | H5A034 C | sample | Hm Gen E | Hm Gen E | Hm Gen E | Hm Gen E | CPE002JI | CPE002NZ | CPE002KD | NM 13878 | NM 1524 | NM 1387 | C11orf74 | ZNF362 | C11orf74 | chromosome 11 c | zinc finger | chromoso | 119710 | 149076 | 119710 | s42323 | s45251 | s42325 |
| 17109 | I6 | I7 | 487 | 1173 | 7586 | 339 | 15,6 | 1,205 | H5A034 C | sample | Hm Gen E | Hm Gen E | Hm Gen E | Hm Gen E | CPE002JI | CPE002NZ | CPE002KD | NM 13878 | NM 1758 | NM 1387 | TMEM45B | EXOC8 | TMEM45B | transmembrane p | exocyst cc | transmem | 120224 | 149371 | 120224 | s42358 | s45284 | s42360 |
| 17113 | J1 | J2 | 1052 | 835,9 | 16372 | 163,5 | 15,6 | 1,205 | H5A034 C | sample | Hm Gen E | Hm Gen E | Hm Gen E | Hm Gen E | CPE002JI | CPE002NZ | CPE002KD | NM 17347 | NM 1524 | NM 1734 | C3orf24 | C17orf77 | C3orf24 | chromosome 3 or | chromoso | chromoso | 115795 | 146723 | 115795 | s41872 | s44898 | s41874 |

| | | | | | | | | | | | | | | | | | | | | | | | | | | | | | | | |
|---|---|---|---|---|---|---|---|---|---|---|---|---|---|---|---|---|---|---|---|---|---|---|---|---|---|---|---|---|---|---|---|
| 17147 | K19 | K20 | 968 | 929,7 | 15087 | 248 | 15,6 | 1,205 | H5A034 C | sample | Hm Gen E | Hm Gen E | Hm Gen E | CPE002JI | CPE002NZ | CPE002KI | NM 14504 | NM 1536 | NM 1450 | TMEM106 | CPNE8 | TMEM106 | transmembrane | copine VIII | transmem | 113277 | 144402 | 113277 | s41461 | s44646 | s225493 |
| 17159 | K8 | K9 | 1130 | 1020 | 17669 | 261,4 | 15,6 | 1,205 | H5A034 C | sample | Hm Gen E | Hm Gen E | Hm Gen E | CPE002JI | CPE002NZ | CPE002KI | NM 00100 | NM 0010 | NM 0010 | OR56A1 | LOC14964 | OR56A1 | olfactory receptor | hypotheti | olfactory | 120796 | 149643 | 120796 | s42404 | s45326 | s42406 |
| 17256 | O9 | O10 | 1390 | 947,1 | 21700 | 278,1 | 15,6 | 1,205 | H5A034 C | sample | Hm Gen E | Hm Gen E | Hm Gen E | CPE002JI | CPE002NZ | CPE002KI | NM 00100 | NM 1780 | NM 0010 | PALM2 | PRIMA1 | PALM2 | paralemmin 2 | proline ric | paralemm | 114299 | 145270 | 114299 | s41548 | s44737 | s41550 |
| 17268 | P2 | P3 | 967 | 1119 | 15080 | 204,6 | 15,6 | 1,205 | H5A034 C | sample | Hm Gen E | Hm Gen E | Hm Gen E | CPE002JI | CPE002NZ | CPE002KI | NM 00100 | NM 0010 | NM 0010 | ZSWIM7 | FAM53A | ZSWIM7 | zinc finger, SWIM | family wit | zinc finge | 125150 | 152877 | 125150 | s42869 | s45743 | s42871 |
| 6941 | B13 | B14 | 1810 | 652,2 | 28252 | 476,4 | 15,6 | 1,214 | H5A034 C | sample | Hm Drgbl | Hm Drgbl | Hm Drgbl | CPE002E2 | CPE002EX | CPE002FI | NM 03203 | NM 0320 | NM 0320 | MAP1B | MAP1B | MAP1B | microtubule-assoc | microtubu | microtubu | 4131 | 4131 | 4131 | s8501 | s8500 | s8499 |
| 7211 | M19 | M20 | 1710 | 560,8 | 26745 | 448,2 | 15,6 | 1,214 | H5A034 C | sample | Hm Drgbl | Hm Drgbl | Hm Drgbl | CPE002E2 | CPE002EX | CPE002FI | NM 00027 | NM 0002 | NM 0002 | LYZ | LYZ | LYZ | lysozyme (renal a | lysozyme | lysozyme | 4069 | 4069 | 4069 | s8361 | s8362 | s8363 |
| 5286 | M14 | M15 | 865 | 723 | 13484 | 438,2 | 15,6 | 1,224 | H5A034 C | sample | Hm Drgbl | Hm Drgbl | Hm Drgbl | CPE002DV | CPE002EM | CPE002FI | NM 00333 | NM 0033 | NM 0033 | UBE2V2 | UBE2V2 | UBE2V2 | ubiquitin-conjugat | ubiquitin- | ubiquitin- | 7336 | 7336 | 7336 | s14602 | s224769 | s14601 |
| 5328 | N9 | N10 | 1970 | 637,6 | 30703 | 594,4 | 15,6 | 1,224 | H5A034 C | sample | Hm Drgbl | Hm Drgbl | Hm Drgbl | CPE002DV | CPE002EM | CPE002FI | NM 00464 | NM 0046 | NM 0046 | CDK2AP1 | CDK2AP1 | CDK2AP1 | CDK2-associated | CDK2-assc | CDK2-assc | 8099 | 8099 | 8099 | s15622 | s15621 | s15620 |
| 5349 | O6 | O7 | 1431 | 633,4 | 22318 | 541 | 15,6 | 1,224 | H5A034 C | sample | Hm Drgbl | Hm Drgbl | Hm Drgbl | CPE002DV | CPE002EM | CPE002FI | NM 00566 | NM 0010 | NM 0010 | SLC35A2 | SLC35A2 | SLC35A2 | solute carrier fam | solute car | solute car | 7355 | 7355 | 7355 | s14639 | s224772 | s194846 |
| 2445 | F6 | F7 | 1012 | 659,8 | 15817 | 427,4 | 15,6 | 1,228 | H5A034 C | sample | Hm Drgbl | Hm Drgbl | Hm Drgbl | CPE002DC | CPE002EE | CPE002FA | NM 00275 | NM 0027 | NM 0027 | MAPK9 | MAPK9 | MAPK9 | mitogen-activated | mitogen-a | mitogen-a | 5601 | 5601 | 5601 | s11158 | s11159 | s11160 |
| 2470 | G7 | G8 | 683 | 662,8 | 10679 | 436 | 15,6 | 1,228 | H5A034 C | sample | Hm Drgbl | Hm Drgbl | Hm Drgbl | CPE002DC | CPE002EE | CPE002FA | NM 00583 | NM 0058 | NM 0058 | PRKD3 | PRKD3 | PRKD3 | protein kinase D3 | protein ki | protein ki | 23683 | 23683 | 23683 | s31 | s232 | s233 |
| 2500 | I12 | I13 | 1152 | 653,7 | 18011 | 359,6 | 15,6 | 1,228 | H5A034 C | sample | Hm Drgbl | Hm Drgbl | Hm Drgbl | CPE002DC | CPE002EE | CPE002FA | NM 00257 | NM 0025 | NM 0025 | PAK1 | PAK1 | PAK1 | p21/Cdc42/Rac1-a | p21/Cdc4 | p21/Cdc4 | 5058 | 5058 | 5058 | s10019 | s10020 | s10021 |
| 16558 | B7 | B8 | 942 | 914,4 | 14724 | 386,6 | 15,6 | 1,233 | H5A034 C | sample | Hm Gen E | Hm Gen E | Hm Gen E | CPE002JH | CPE002NV | CPE002KC | NM 00108 | NM 0010 | NM 0010 | SHANK3 | SHANK3 | SHANK3 | SH3 and multiple | SH3 and n | SH3 and n | 85358 | 85358 | 85358 | s39915 | s39916 | s39917 |
| 16602 | D3 | D4 | 1274 | 952,6 | 19921 | 280,3 | 15,6 | 1,233 | H5A034 C | sample | Hm Gen E | Hm Gen E | Hm Gen E | CPE002JH | CPE002NV | CPE002KC | NM 03312 | NM 0331 | NM 0331 | NKD2 | NKD2 | NKD2 | naked cuticle hom | naked cut | naked cut | 85409 | 85409 | 85409 | s39964 | s39965 | s39966 |
| 16607 | D8 | D9 | 1354 | 930,8 | 21150 | 356,6 | 15,6 | 1,233 | H5A034 C | sample | Hm Gen E | Hm Gen E | Hm Gen E | CPE002JH | CPE002NV | CPE002KC | NM 00103 | NM 0010 | NM 0010 | CALML4 | CALML4 | CALML4 | calmodulin-like 4 | calmoduli | calmoduli | 91860 | 91860 | 91860 | s40798 | s40799 | s40800 |
| 16806 | M14 | M15 | 1369 | 970,6 | 21359 | 344,5 | 15,6 | 1,233 | H5A034 C | sample | Hm Gen E | Hm Gen E | Hm Gen E | CPE002JH | CPE002NV | CPE002KC | NM 13837 | NM 1383 | NM 1383 | FAM113B | FAM113B | FAM113B | family with seque | family wit | family wit | 91523 | 91523 | 91523 | s40678 | s40679 | s40680 |
| 16843 | N4 | N5 | 1266 | 1095 | 19773 | 356,7 | 15,6 | 1,233 | H5A034 C | sample | Hm Gen E | Hm Gen E | Hm Gen E | CPE002JH | CPE002NV | CPE002KC | NM 14507 | NM 1450 | NM 1450 | C2orf39 | C2orf39 | C2orf39 | chromosome 2 op | chromoso | chromoso | 92749 | 92749 | 92749 | s41019 | s41020 | s41021 |
| 16846 | N7 | N8 | 1616 | 988,2 | 25203 | 331,5 | 15,6 | 1,233 | H5A034 C | sample | Hm Gen E | Hm Gen E | Hm Gen E | CPE002JH | CPE002NV | CPE002KC | NM 14537 | NM 1453 | NM 1453 | PHLDB2 | PHLDB2 | PHLDB2 | pleckstrin homolo | pleckstrin | pleckstrin | 90102 | 90102 | 90102 | s40249 | s40250 | s40251 |
| 17685 | A6 | A7 | 955 | 1103 | 14905 | 322,9 | 15,6 | 1,233 | H5A034 C | sample | Hm Gen E | Hm Gen E | Hm Gen E | CPE002JK | CPE002NZ | CPE002KI | NM 20732 | NM 2073 | NM 2073 | DPY19L3 | DPY19L3 | DPY19L3 | dpy-19-like 3 (C. e | dpy-19-lik | dpy-19-lik | 147991 | 147991 | 147991 | s45104 | s45105 | s225718 |
| 17744 | D16 | D17 | 1038 | 977,3 | 16225 | 245,8 | 15,6 | 1,233 | H5A034 C | sample | Hm Gen E | Hm Gen E | Hm Gen E | CPE002JK | CPE002NZ | CPE002KI | XM 00113 | XM 0011 | XM 0011 | LOC15093 | LOC15093 | LOC15093 | hypothetical prot | hypotheti | hypotheti | 150935 | 150935 | 150935 | s45497 | s45498 | s45499 |
| 17989 | N20 | N21 | 1128 | 996,5 | 17550 | 222,2 | 15,6 | 1,233 | H5A034 C | sample | Hm Gen E | Hm Gen E | Hm Gen E | CPE002JK | CPE002NZ | CPE002KI | NM 14472 | NM 1447 | NM 1447 | JAKMIP1 | JAKMIP1 | JAKMIP1 | janus kinase and | janus kina | janus kina | 152789 | 152789 | 152789 | s45730 | s45731 | s45732 |
| 8876 | B5 | B6 | 1036 | 1740 | 16150 | 151,4 | 15,6 | 1,253 | H5A034 C | sample | Hm Drgbl | Hm Drgbl | Hm Drgbl | CPE002E7 | CPE002ES | CPE002FD | NM 18266 | NM 1826 | NM 1826 | AADAT | AADAT | AADAT | aminoadipate am | aminoadi | aminoadi | 51166 | 51166 | 51166 | s27592 | s27593 | s27594 |
| 8939 | E19 | E20 | 1607 | 1610 | 25074 | 187,4 | 15,6 | 1,253 | H5A034 C | sample | Hm Drgbl | Hm Drgbl | Hm Drgbl | CPE002E7 | CPE002ES | CPE002FD | NM 01557 | NM 0157 | NM 0157 | RDH8 | RDH8 | RDH8 | retinol dehydroge | retinol de | retinol de | 50700 | 50700 | 50700 | s27087 | s27088 | s27086 |
| 9061 | J20 | J21 | 1771 | 1727 | 27673 | 155,8 | 15,6 | 1,253 | H5A034 C | sample | Hm Drgbl | Hm Drgbl | Hm Drgbl | CPE002E7 | CPE002ES | CPE002FD | NM 00104 | NM 0010 | NM 0010 | FAM63B | FAM63B | FAM63B | family with seque | family wit | family wit | 54629 | 54629 | 54629 | s29281 | s29280 | s29280 |
| 9127 | M15 | M16 | 1577 | 1663 | 24545 | 145,5 | 15,6 | 1,253 | H5A034 C | sample | Hm Drgbl | Hm Drgbl | Hm Drgbl | CPE002E7 | CPE002ES | CPE002FD | NM 01602 | NM 0160 | NM 0160 | TFB1M | TFB1M | TFB1M | transcription fact | transcript | transcript | 51106 | 51106 | 51106 | s27446 | s27447 | s27448 |
| 4363 | F4 | F5 | 1121 | 716,2 | 17540 | 421 | 15,6 | 1,316 | H5A034 C | sample | Hm Drgbl | Hm Drgbl | Hm Drgbl | CPE002DL | CPE002EK | CPE002FI | NM 17316 | NM 1731 | NM 1731 | KCNH7 | KCNH7 | KCNH7 | potassium voltag | potassium | potassium | 90134 | 90134 | 90134 | s40261 | s40262 | s40263 |
| 4533 | M6 | M7 | 1344 | 659,2 | 21024 | 518,8 | 15,6 | 1,316 | H5A034 C | sample | Hm Drgbl | Hm Drgbl | Hm Drgbl | CPE002DL | CPE002EK | CPE002FI | NM 01743 | NM 0174 | NM 0174 | ACCN5 | ACCN5 | ACCN5 | amiloride-sensitiv | amiloride | amiloride | 51802 | 51802 | 51802 | s28660 | s28661 | s28662 |
| 4564 | O12 | O13 | 879 | 726,6 | 13681 | 575,4 | 15,6 | 1,316 | H5A034 C | sample | Hm Drgbl | Hm Drgbl | Hm Drgbl | CPE002DL | CPE002EK | CPE002FI | NM 01828 | NM 0182 | NM 0182 | MCOLN3 | MCOLN3 | MCOLN3 | mucolipin 3 | mucolipin | mucolipin | 55283 | 55283 | 55283 | s30633 | s30634 | s30635 |
| 132 | F2 | F3 | 416 | 1396 | 6470 | 370 | 15,6 | 1,328 | H5A034 C | sample | Hm Drgbl | Hm Drgbl | Hm Drgbl | CPE002DT | CPE002EJ | CPE002F9 | NM 00649 | NM 0010 | NM 0010 | SPRR2B | SPRR2B | SPRR2B | small proline-rich | small prol | small prol | 6701 | 6701 | 6701 | s13390 | s13389 | s13388 |
| 363 | P11 | P12 | 543 | 1395 | 8486 | 670,3 | 15,6 | 1,328 | H5A034 C | sample | Hm Drgbl | Hm Drgbl | Hm Drgbl | CPE002DT | CPE002EJ | CPE002F9 | NM 00304 | NM 0030 | NM 0030 | SLC7A1 | SLC7A1 | SLC7A1 | solute carrier fam | solute car | solute car | 6541 | 6541 | 6541 | s13005 | s13003 | s13004 |
| 1935 | A22 | A23 | 1403 | 787,5 | 21864 | 500,6 | 15,6 | 1,357 | H5A034 C | sample | Hm Drgbl | Hm Drgbl | Hm Drgbl | CPE002FX | CPE002G1 | CPE002GS | NM 00107 | NM 0071 | NM 0071 | ZNF92 | ZNF92 | ZNF92 | zinc finger protei | zinc finge | zinc finge | 168374 | 168374 | 168374 | s46692 | s46693 | s46694 |
| 1965 | B6 | B7 | 1088 | 626 | 16978 | 493,8 | 15,6 | 1,357 | H5A034 C | sample | Hm Drgbl | Hm Drgbl | Hm Drgbl | CPE002FX | CPE002G1 | CPE002GS | NM 00107 | NM 0010 | NM 0010 | ZKSCAN2 | ZKSCAN2 | ZKSCAN2 | zinc finger with K | zinc finge | zinc finge | 342357 | 342357 | 342357 | s50897 | s50898 | s50899 |
| 2004 | D2 | D3 | 1184 | 645,9 | 18434 | 430,6 | 15,6 | 1,357 | H5A034 C | sample | Hm Drgbl | Hm Drgbl | Hm Drgbl | CPE002FX | CPE002G1 | CPE002GS | NM 18263 | NM 1826 | NM 1826 | ZNF713 | ZNF713 | ZNF713 | zinc finger protei | zinc finge | zinc finge | 349075 | 349075 | 349075 | s51378 | s51379 | s51380 |
| 2066 | G10 | G11 | 296 | 685,6 | 4603 | 468,2 | 15,6 | 1,357 | H5A034 C | sample | Hm Drgbl | Hm Drgbl | Hm Drgbl | CPE002FX | CPE002G1 | CPE002GS | NM 18184 | NM 1818 | NM 1818 | ZBTB12 | ZBTB12 | ZBTB12 | zinc finger and BT | zinc finge | zinc finge | 221527 | 221527 | 221527 | s48076 | s48077 | s48078 |
| 6514 | E22 | E23 | 1175 | 626,2 | 18324 | 497,1 | 15,6 | 1,368 | H5A034 C | sample | Hm Drgbl | Hm Drgbl | Hm Drgbl | CPE002E1 | CPE002EU | CPE002FE | NM 0022 | NM 0022 | NM 0022 | ITGA5 | ITGA5 | ITGA5 | integrin, alpha 5 ( | integrin, a | integrin, a | 3678 | 3678 | 3678 | s7547 | s7548 | s7549 |
| 6585 | C17 | C18 | 902 | 553,6 | 14097 | 406,9 | 15,6 | 1,368 | H5A034 C | sample | Hm Drgbl | Hm Drgbl | Hm Drgbl | CPE002E1 | CPE002EU | CPE002FE | NM 00335 | NM 0033 | NM 0033 | GUCA2A | GUCA2A | GUCA2A | guanylate cyclase | guanylate | guanylate | 2980 | 2980 | 2980 | s6341 | s6342 | s6340 |
| 6620 | D5 | D6 | 1227 | 572,6 | 19081 | 462,5 | 15,6 | 1,368 | H5A034 C | sample | Hm Drgbl | Hm Drgbl | Hm Drgbl | CPE002E1 | CPE002EU | CPE002FE | NM 01635 | NM 0163 | NM 0163 | TLX2 | TLX2 | TLX2 | T-cell leukemia ho | T-cell leuk | T-cell leuk | 3196 | 3196 | 3196 | s6749 | s6750 | s6751 |
| 6633 | E17 | E18 | 1261 | 550,9 | 19678 | 348,7 | 15,6 | 1,368 | H5A034 C | sample | Hm Drgbl | Hm Drgbl | Hm Drgbl | CPE002E1 | CPE002EU | CPE002FE | NM 00101 | NM 0010 | NM 0010 | HSD17B10 | HSD17B10 | HSD17B10 | hydroxysteroid (1 | hydroxyst | hydroxyst | 3028 | 3028 | 3028 | s6434 | s6435 | s6433 |
| 6657 | F17 | F18 | 1420 | 558,9 | 22114 | 436,9 | 15,6 | 1,368 | H5A034 C | sample | Hm Drgbl | Hm Drgbl | Hm Drgbl | CPE002E1 | CPE002EU | CPE002FE | NM 00450 | NM 0045 | NM 0045 | HOXB7 | HOXB7 | HOXB7 | homeobox B7 | homeobo | homeobo | 3217 | 3217 | 3217 | s6807 | s6806 | s6808 |
| 6890 | P10 | P11 | 1223 | 562,1 | 19134 | 506,5 | 15,6 | 1,368 | H5A034 C | sample | Hm Drgbl | Hm Drgbl | Hm Drgbl | CPE002E1 | CPE002EU | CPE002FE | NM 00413 | NM 0041 | NM 0041 | KRT33A | KRT33A | KRT33A | keratin 33A | keratin 33 | keratin 33 | 3883 | 3883 | 3883 | s8007 | s194587 | s194586 |
| 3913 | D1 | D2 | 1536 | 752,5 | 23999 | 394,8 | 15,6 | 1,387 | H5A034 C | sample | Hm Drgbl | Hm Drgbl | Hm Drgbl | CPE002DS | CPE002EI | CPE002F8 | NM 00324 | NM 0032 | NM 0032 | PKIB | PKIB | PKIB | protein kinase (cA | protein ki | protein ki | 5570 | 5570 | 5570 | s11077 | s11078 | s224383 |
| 3939 | E11 | E12 | 776 | 587,1 | 12105 | 339,2 | 15,6 | 1,387 | H5A034 C | sample | Hm Drgbl | Hm Drgbl | Hm Drgbl | CPE002DS | CPE002EI | CPE002F8 | NM 00268 | NM 0026 | NM 0026 | PLIN | PLIN | PLIN | perilipin | perilipin | perilipin | 5346 | 5346 | 5346 | s10663 | s10661 | s10662 |
| 3989 | G13 | G14 | 772 | 610,1 | 12035 | 410,3 | 15,6 | 1,387 | H5A034 C | sample | Hm Drgbl | Hm Drgbl | Hm Drgbl | CPE002DS | CPE002EI | CPE002F8 | NM 00267 | NM 0026 | NM 0026 | PMP2 | PMP2 | PMP2 | peripheral myelin | peripheral | peripheral | 5375 | 5375 | 5375 | s10723 | s10724 | s10722 |
| 4061 | J13 | J14 | 1121 | 659,2 | 17444 | 364,4 | 15,6 | 1,387 | H5A034 C | sample | Hm Drgbl | Hm Drgbl | Hm Drgbl | CPE002DS | CPE002EI | CPE002F8 | NM 00595 | NM 0059 | NM 0059 | VPS72 | VPS72 | VPS72 | vacuolar protein | vacuolar p | vacuolar p | 6944 | 6944 | 6944 | s13909 | s13907 | s13908 |
| 4100 | K5 | K6 | 1026 | 567,2 | 16017 | 372,4 | 15,6 | 1,387 | H5A034 C | sample | Hm Drgbl | Hm Drgbl | Hm Drgbl | CPE002DS | CPE002EI | CPE002F8 | NM 00269 | NM 0026 | NM 0026 | POLE2 | POLE2 | POLE2 | polymerase (DNA | polymeras | polymeras | 5427 | 5427 | 5427 | s10785 | s10786 | s10784 |
| 11525 | A13 | A14 | 1323 | 901,1 | 20598 | 206,7 | 15,6 | 1,393 | H5A034 C | sample | Hm Drgbl | Hm Drgbl | Hm Drgbl | CPE002JA | CPE002NI | CPE002JZ | NM 00113 | NM 0011 | NM 0011 | ACAN | ACAN | ACAN | aggrecan | aggrecan | aggrecan | 176 | 176 | 176 | s1163 | s1164 | s1165 |
| 15414 | C14 | C15 | 1083 | 791,2 | 16874 | 132,5 | 15,6 | 1,418 | H5A034 C | sample | Hm Gen E | Hm Gen E | Hm Gen E | CPE002JG | CPE002NS | CPE002K9 | NM 02303 | NM 0230 | NM 0230 | UNKL | C16orf28 | UNKL | unkempt homolo | chromoso | unkempt | 64718 | 65259 | 64718 | s35260 | s35261 | s35262 |
| 5763 | A11 | A12 | 1299 | 910,1 | 20207 | 558,9 | 15,6 | 1,425 | H5A034 C | sample | Hm Drgbl | Hm Drgbl | Hm Drgbl | CPE002DY | CPE002EO | CPE002FN | NM 00106 | NM 0010 | NM 0010 | AANAT | AANAT | AANAT | arylalkylamine N- | arylalkyla | arylalkyla | 15 | 15 | 15 | s837 | s838 | s839 |
| 5785 | B1 | B2 | 1647 | 802,9 | 25770 | 664 | 15,6 | 1,425 | H5A034 C | sample | Hm Drgbl | Hm Drgbl | Hm Drgbl | CPE002DY | CPE002EO | CPE002FN | NM 00068 | NM 0006 | NM 0006 | ALDH3A1 | ALDH3A1 | ALDH3A1 | aldehyde dehydro | aldehyde | aldehyde | 218 | 218 | 218 | s1243 | s1242 | s1244 |
| 5845 | D20 | D21 | 1479 | 756,8 | 23085 | 595,7 | 15,6 | 1,425 | H5A034 C | sample | Hm Drgbl | Hm Drgbl | Hm Drgbl | CPE002DY | CPE002EO | CPE002FN | NM 00071 | NM 0007 | NM 0007 | BLVRB | BLVRB | BLVRB | biliverdin reducta | biliverdin | biliverdin | 645 | 645 | 645 | s2009 | s2010 | s194312 |
| 5859 | D6 | D7 | 1138 | 773,7 | 17747 | 610,7 | 15,6 | 1,425 | H5A034 C | sample | Hm Drgbl | Hm Drgbl | Hm Drgbl | CPE002DY | CPE002EO | CPE002FN | NM 00173 | NM 0017 | NM 0017 | CEACAM1 | CEACAM1 | CEACAM1 | carcinoembryoni | carcinoen | carcinoen | 634 | 634 | 634 | s1978 | s1976 | s1977 |
| 5862 | E14 | E15 | 894 | 799 | 13947 | 533,2 | 15,6 | 1,425 | H5A034 C | sample | Hm Drgbl | Hm Drgbl | Hm Drgbl | CPE002DY | CPE002EO | CPE002FN | NM 00004 | NM 0000 | NM 0000 | ARSE | ARSE | ARSE | arylsulfatase E (c | arylsulfat | arylsulfat | 415 | 415 | 415 | s1638 | s1639 | s1640 |
| 1191 | B22 | B23 | 1163 | 743,1 | 18181 | 452,9 | 15,6 | 1,431 | H5A034 C | sample | Hm Drgbl | Hm Drgbl | Hm Drgbl | CPE002FV | CPE002FZ | CPE002G3 | NM 00066 | NM 0066 | NM 0066 | ZBTB6 | ZBTB6 | ZBTB6 | zinc finger and BT | zinc finge | zinc finge | 10773 | 10773 | 10773 | s21159 | s21160 | s21161 |
| 1321 | H1 | H2 | 963 | 772,5 | 15026 | 482 | 15,6 | 1,431 | H5A034 C | sample | Hm Drgbl | Hm Drgbl | Hm Drgbl | CPE002FV | CPE002FZ | CPE002G3 | NM 00378 | NM 0037 | NM 0037 | VAMP8 | VAMP8 | VAMP8 | vesicle-associated | vesicle-as | vesicle-as | 8673 | 8673 | 8673 | s16522 | s16523 | s16524 |
| 1367 | I8 | I9 | 1342 | 745,9 | 20895 | 462,3 | 15,6 | 1,431 | H5A034 C | sample | Hm Drgbl | Hm Drgbl | Hm Drgbl | CPE002FV | CPE002FZ | CPE002G3 | NM 00577 | NM 0057 | NM 0057 | ZNF256 | ZNF256 | ZNF256 | zinc finger protei | zinc finge | zinc finge | 10172 | 10172 | 10172 | s19823 | s19824 | s19825 |
| 1371 | J11 | J12 | 986 | 810,6 | 15406 | 430,8 | 15,6 | 1,431 | H5A034 C | sample | Hm Drgbl | Hm Drgbl | Hm Drgbl | CPE002FV | CPE002FZ | CPE002G3 | NM 00426 | NM 0042 | NM 0042 | SYT7 | SYT7 | SYT7 | synaptotagmin VII | synaptota | synaptota | 9066 | 9066 | 9066 | s17291 | s17292 | s17293 |
| 1491 | O11 | O12 | 1855 | 796,9 | 29016 | 486,9 | 15,6 | 1,431 | H5A034 C | sample | Hm Drgbl | Hm Drgbl | Hm Drgbl | CPE002FV | CPE002FZ | CPE002G3 | NM 01338 | NM 0133 | NM 0133 | ZNF224 | ZNF224 | ZNF224 | zinc finger protei | zinc finge | zinc finge | 7767 | 7767 | 7767 | s15302 | s15303 | s15304 |
| 16130 | A10 | A11 | 601 | 1053 | 9362 | 383,3 | 15,6 | 1,431 | H5A034 C | sample | Hm Gen E | Hm Gen E | Hm Gen E | CPE002JG | CPE002NU | CPE002KB | NM 00103 | NM 0010 | NM 0010 | FAM96A | FAM96A | FAM96A | family with seque | family wit | family wit | 84191 | 84191 | 84191 | s38636 | s38637 | s38638 |
| 16202 | D10 | D11 | 813 | 1076 | 12651 | 410,2 | 15,6 | 1,431 | H5A034 C | sample | Hm Gen E | Hm Gen E | Hm Gen E | CPE002JG | CPE002NU | CPE002KB | NM 03244 | NM 0324 | NM 0324 | FAM120B | FAM120B | FAM120B | family with seque | family wit | family wit | 84498 | 84498 | 84498 | s39064 | s39065 | s39066 |
| 16352 | J16 | J17 | 910 | 927,8 | 14218 | 288,2 | 15,6 | 1,431 | H5A034 C | sample | Hm Gen E | Hm Gen E | Hm Gen E | CPE002JG | CPE002NU | CPE002KB | NM 03253 | NM 0325 | NM 0325 | MYPN | MYPN | MYPN | myopalladin | myopallac | myopallac | 84665 | 84665 | 84665 | s39278 | s39279 | s39280 |
| 16353 | J17 | J18 | 975 | 963,1 | 15162 | 248,9 | 15,6 | 1,431 | H5A034 C | sample | Hm Gen E | Hm Gen E | Hm Gen E | CPE002JG | CPE002NU | CPE002KB | NM 03321 | NM 0321 | NM 0321 | C11orf56 | C11orf56 | C11orf56 | chromosome 11 o | chromoso | chromoso | 84067 | 84067 | 84067 | s38441 | s38442 | s38443 |
| 16365 | J6 | J7 | 493 | 1081 | 7694 | 267,3 | 15,6 | 1,431 | H5A034 C | sample | Hm Gen E | Hm Gen E | Hm Gen E | CPE002JG | CPE002NU | CPE002KB | NM 03256 | NM 0325 | NM 0325 | C22orf23 | C22orf23 | C22orf23 | chromosome 22 o | chromoso | chromoso | 84645 | 84645 | 84645 | s39239 | s39240 | s39241 |
| 16498 | P18 | P19 | 1143 | 1141 | 17872 | 414,3 | 15,6 | 1,431 | H5A034 C | sample | Hm Gen E | Hm Gen E | Hm Gen E | CPE002JG | CPE002NU | CPE002KB | NM 03272 | NM 0327 | NM 0327 | C6orf105 | C6orf105 | C6orf105 | chromosome 6 op | chromoso | chromoso | 84830 | 84830 | 84830 | s39485 | s39486 | s39487 |
| 13660 | J12 | J13 | 1331 | 936,8 | 20825 | 172,7 | 15,6 | 1,465 | H5A034 C | sample | Hm Gen E | Hm Gen E | Hm Gen E | CPE002JC | CPE002NJ | CPE002K4 | NM 00101 | NM 0010 | NM 0010 | ORBG2 | ORBG2 | ORBG2 | olfactory recepto | olfactory | olfactory | 26492 | 26492 | 26492 | s25443 | s25444 | s25445 |
| 13796 | O5 | O6 | 1031 | 1069 | 16104 | 160 | 15,6 | 1,465 | H5A034 C | sample | Hm Gen E | Hm Gen E | Hm Gen E | CPE002JC | CPE002NJ | CPE002K4 | NM 00613 | NM 0061 | NM 0061 | PRAME | PRAME | PRAME | preferentially exp | preferenti | preferenti | 23532 | 23532 | 23532 | s24024 | s24025 | s24026 |
| 827 | C19 | C20 | 881 | 732 | 13726 | 514,1 | 15,6 | 1,479 | H5A034 C | sample | Hm Drgbl | Hm Drgbl | Hm Drgbl | CPE002FU | CPE002FY | CPE002G2 | NM 19913 | NM 1991 | NM 1991 | BGLAP | BGLAP | BGLAP | bone gamma-carb | bone gam | bone gam | 632 | 632 | 632 | s1971 | s1972 | s224571 |
| 898 | F18 | F19 | 926 | 629,7 | 14491 | 333,6 | 15,6 | 1,479 | H5A034 C | sample | Hm Drgbl | Hm Drgbl | Hm Drgbl | CPE002FU | CPE002FY | CPE002G2 | NM 00303 | NM 0030 | NM 0030 | SKI | SKI | SKI | v-ski sarcoma vira | v-ski sarc | v-ski sarc | 6497 | 6497 | 6497 | s12880 | s12881 | s12882 |
| 901 | F20 | F21 | 869 | 718,5 | 13523 | 412,3 | 15,6 | 1,479 | H5A034 C | sample | Hm Drgbl | Hm Drgbl | Hm Drgbl | CPE002FU | CPE002FY | CPE002G2 | NM 00543 | NM 0054 | NM 0054 | SKIL | SKIL | SKIL | SKI-like oncogene | SKI-like on | SKI-like on | 6498 | 6498 | 6498 | s12883 | s12884 | s12885 |
| 909 | F6 | F7 | 1324 | 769,2 | 20661 | 531,3 | 15,6 | 1,479 | H5A034 C | sample | Hm Drgbl | Hm Drgbl | Hm Drgbl | CPE002FU | CPE002FY | CPE002G2 | NM 00101 | NM 0010 | NM 0010 | S100A13 | S100A13 | S100A13 | S100 calcium bind | S100 calci | S100 calci | 6284 | 6284 | 6284 | s12436 | s12437 | s12438 |
| 945 | H17 | H18 | 1555 | 732,5 | 24281 | 328,1 | 15,6 | 1,479 | H5A034 C | sample | Hm Drgbl | Hm Drgbl | Hm Drgbl | CPE002FU | CPE002FY | CPE002G2 | NM 00211 | NM 0021 | NM 0021 | HLA-B | HLA-B | HLA-B | major histocomp | major hist | major hist | 3106 | 3106 | 3106 | s6571 | s6572 | s6573 |
| 5404 | B12 | B13 | 1825 | 757,7 | 28530 | 634,2 | 15,6 | 1,486 | H5A034 C | sample | Hm Drgbl | Hm Drgbl | Hm Drgbl | CPE002DX | CPE002EN | CPE002FJ | NM 00466 | NM 0046 | NM 0046 | ATP6V0D1 | ATP6V0D1 | ATP6V0D1 | ATPase, H+ transp | ATPase, H | ATPase, H | 9114 | 9114 | 9114 | s17396 | s17397 | s17395 |
| 5448 | C9 | C10 | 1105 | 638,1 | 17256 | 576,7 | 15,6 | 1,486 | H5A034 C | sample | Hm Drgbl | Hm Drgbl | Hm Drgbl | CPE002DX | CPE002EN | CPE002FJ | NM 00544 | NM 0054 | NM 0054 | EOMES | EOMES | EOMES | eomesodermin h | eomesode | eomesode | 8320 | 8320 | 8320 | s15832 | s15833 | s15834 |
| 5588 | I5 | I6 | 956 | 655,9 | 14898 | 597,1 | 15,6 | 1,486 | H5A034 C | sample | Hm Drgbl | Hm Drgbl | Hm Drgbl | CPE002DX | CPE002EN | CPE002FJ | NM 00636 | NM 0063 | NM 0063 | SEMA7A | SEMA7A | SEMA7A | semaphorin 7A, G | semaphor | semaphor | 8482 | 8482 | 8482 | s16111 | s16112 | s16113 |
| 8147 | D19 | D20 | 766 | 733,4 | 11944 | 612,6 | 15,6 | 1,5 | H5A034 C | sample | Hm Drgbl | Hm Drgbl | Hm Drgbl | CPE002E5 | CPE002EQ | CPE002FB | NM 01605 | NM 0160 | NM 0160 | COPZ1 | COPZ1 | COPZ1 | coatomer protein | coatomer | coatomer | 22818 | 22818 | 22818 | s22427 | s22428 | s22429 |
| 8300 | J5 | J6 | 1252 | 554,3 | 19508 | 506 | 15,6 | 1,5 | H5A034 C | sample | Hm Drgbl | Hm Drgbl | Hm Drgbl | CPE002E5 | CPE002EQ | CPE002FB | NM 01209 | NM 0120 | NM 0120 | CD93 | CD93 | CD93 | CD93 molecule | CD93 mol | CD93 mol | 22918 | 22918 | 22918 | s22670 | s22671 | s22672 |
| 8305 | L8 | L9 | 1179 | 569 | 18367 | 477,3 | 15,6 | 1,5 | H5A034 C | sample | Hm Drgbl | Hm Drgbl | Hm Drgbl | CPE002E5 | CPE002EQ | CPE002FB | NM 18292 | NM 1829 | NM 1829 | NNT | NNT | NNT | nicotinamide nuc | nicotinam | nicotinam | 23530 | 23530 | 23530 | s24020 | s24018 | s24019 |
| 8360 | M16 | M17 | 1378 | 626,8 | 21494 | 463,8 | 15,6 | 1,5 | H5A034 C | sample | Hm Drgbl | Hm Drgbl | Hm Drgbl | CPE002E5 | CPE002EQ | CPE002FB | NM 01523 | NM 0152 | NM 0152 | SATB2 | SATB2 | SATB2 | SATB homeobox 2 | SATB hom | SATB hom | 23314 | 23314 | 23314 | s23539 | s23540 | s223583 |
| 1666 | F18 | F19 | 832 | 751,3 | 13009 | 498,7 | 15,6 | 1,634 | H5A034 C | sample | Hm Drgbl | Hm Drgbl | Hm Drgbl | CPE002FW | CPE002G0 | CPE002G4 | NM 02522 | NM 0252 | NM 0252 | KCNIP4 | KCNIP4 | KCNIP4 | Kv channel intera | Kv channe | Kv channe | 80333 | 80333 | 80333 | s37243 | s37244 | s37245 |
| 21861 | O6 | O7 | 85 | 1042 | 1327 | 926,3 | 15,6 | 1,642 | H5A034 C | sample | Hm Gen E | Hm Gen E | Hm Gen E | CPE002JU | CPE002OI | CPE002KX | NM 94258 | NM 9425 | NM 9425 | LOC65286 | LOC65286 | LOC65286 | similar to E2F tra | similar to | similar to | 652869 | 652869 | 652869 | s57531 | s57532 | s57533 |
| 4710 | E14 | E15 | 1158 | 832,6 | 17558 | 329,8 | 15,6 | 1,673 | H5A034 C | sample | Hm Drgbl | Hm Drgbl | Hm Drgbl | CPE002DM | CPE002EJ | CPE002FI | NM 00660 | NM 0060 | NM 0060 | CLPP | CLPP | CLPP | ClpP caseinolytic | ClpP case | ClpP case | 8192 | 8192 | 8192 | s15686 | s15687 | s15688 |
| 4788 | H2 | H3 | 1411 | 770,9 | 21988 | 483,8 | 15,6 | 1,673 | H5A034 C | sample | Hm Drgbl | Hm Drgbl | Hm Drgbl | CPE002DM | CPE002EJ | CPE002FI | NM 01559 | NM 0155 | NM 0155 | KLK13 | KLK13 | KLK13 | kallikrein-related | kallikrein- | kallikrein- | 26085 | 26085 | 26085 | s223677 | s25068 | s25069 |
| 4849 | K1 | K2 | 374 | 757,7 | 5835 | 520,8 | 15,6 | 1,673 | H5A034 C | sample | Hm Drgbl | Hm Drgbl | Hm Drgbl | CPE002DM | CPE002EJ | CPE002FI | NM 00187 | NM 0018 | NM 0018 | CPB1 | CPB1 | CPB1 | carboxypeptidase | carboxype | carboxype | 1360 | 1360 | 1360 | s223313 | s3430 | s3431 |
| 4908 | M2 | M3 | 794 | 845,8 | 12420 | 452,1 | 15,6 | 1,673 | H5A034 C | sample | Hm Drgbl | Hm Drgbl | Hm Drgbl | CPE002DM | CPE002EJ | CPE002FI | NM 00694 | NM 0069 | NM 0069 | ADAMTS1 | ADAMTS1 | ADAMTS1 | ADAM metallopep | ADAM me | ADAM me | 9510 | 9510 | 9510 | s18236 | s18237 | s18238 |
| 10551 | H22 | H23 | 824 | 920,2 | 12864 | 211,6 | 15,6 | 1,763 | H5A034 C | sample | Hm Drgbl | Hm Drgbl | Hm Drgbl | CPE002EB | CPE002EW | CPE002FH | NM 14466 | NM 1446 | NM 1446 | SEPT12 | SEPT12 | SEPT12 | septin 12 | septin 12 | septin 12 | 124404 | 124404 | 124404 | s42734 | s42732 | s42733 |
| 10646 | L21 | L22 | 1263 | 836,6 | 19745 | 245,5 | 15,6 | 1,763 | H5A034 C | sample | Hm Drgbl | Hm Drgbl | Hm Drgbl | CPE002EB | CPE002EW | CPE002FH | NM 13334 | NM 1334 | NM 1334 | SAT2 | SAT2 | SAT2 | spermidine/sperm | spermidin | spermidin | 112483 | 112483 | 112483 | s41332 | s41333 | s41334 |
| 10717 | O20 | O21 | 1553 | 826,2 | 24182 | 229,8 | 15,6 | 1,763 | H5A034 C | sample | Hm Drgbl | Hm Drgbl | Hm Drgbl | CPE002EB | CPE002EW | CPE002FH | NM 00100 | NM 0010 | NM 0010 | TRIM6 | TRIM6 | TRIM6 | tripartite motif-co | tripartite | tripartite | 117854 | 117854 | 117854 | s42187 | s42188 | s42186 |
| 2778 | D3 | D4 | 596 | 564,5 | 9273 | 422,5 | 15,6 | 1,835 | H5A034 C | sample | Hm Drgbl | Hm Drgbl | Hm Drgbl | CPE002DH | CPE002ER | CPE002FC | NM 03300 | NM 0067 | NM 0330 | FASTK | FASTK | FASTK | Fas-activated seri | Fas-activa | Fas-activa | 10922 | 10922 | 10922 | s21477 | s223164 | s21479 |
| 19976 | A16 | A17 | 593 | 1193 | 9224 | 244,8 | 15,6 | 1,902 | H5A034 C | sample | Hm Gen E | Hm Gen E | Hm Gen E | CPE002Q | CPE002OS | CPE002KS | NM 21255 | NM 2125 | NM 2125 | ASB18 | ASB18 | ASB18 | ankyrin repeat an | ankyrin re | ankyrin re | 401036 | 401036 | 401036 | s53449 | s53450 | s226553 |
| 22236 | O2 | O3 | 1163 | 1396 | 18253 | 331,6 | 15,6 | 0,746 | H5A034 C | sample | Hm Gen E | Hm Gen E | Hm Gen E | CPE002JV | CPE002ON | CPE002KY | XM 00112 | XM 0011 | XM 0011 | LOC72833 | LOC72833 | LOC72833 | hypothetical prot | hypotheti | hypotheti | 728335 | 728335 | 728335 | s58528 | s58529 | s198494 |
| 13284 | J2 | J3 | 1164 | 1092 | 18283 | 92,6 | 15,7 | 0,787 | H5A034 C | sample | Hm Gen E | Hm Gen E | Hm Gen E | CPE002JB | CPE002NH | CPE002K3 | NM 01236 | NM 0123 | NM 0123 | FBXL7 | FBXL7 | FBXL7 | F-box and leucine | F-box and | F-box and | 23194 | 23194 | 23194 | s23250 | s23251 | s23252 |
| 22506 | J3 | J4 | 972 | 1317 | 15236 | 339,8 | 15,7 | 0,809 | H5A034 C | sample | Hm Gen E | Hm Gen E | Hm Gen E | CPE002JV | CPE002ON | CPE002KY | XM 00112 | XM 0011 | XM 0011 | LOC72887 | LOC72887 | LOC72887 | hypothetical prot | hypotheti | hypotheti | 728870 | 728870 | 728870 | s198828 | s198829 | s198830 |
| 15807 | C22 | C23 | 1208 | 871,2 | 18956 | 159,5 | 15,7 | 0,85 | H5A034 C | sample | Hm Gen E | Hm Gen E | Hm Gen E | CPE002J9 | CPE002NT | CPE002KA | NM 02506 | NM 0250 | NM 0250 | CCDC15 | CCDC15 | CCDC15 | coiled-coil domai | coiled-coi | coiled-coi | 80071 | 80071 | 80071 | s36888 | s36889 | s36890 |
| 13829 | A13 | A14 | 1718 | 871,8 | 27013 | 175,4 | 15,7 | 0,9 | H5A034 C | sample | Hm Gen E | Hm Gen E | Hm Gen E | CPE002JD | CPE002NP | CPE002K5 | NM 01560 | NM 0156 | NM 0156 | NBEA | NBEA | NBEA | neurobeachin | neurobea | neurobea | 26960 | 26960 | 26960 | s25649 | s25650 | s25651 |
| 3309 | J6 | J7 | 943 | 1238 | 14763 | 368,3 | 15,7 | 0,972 | H5A034 C | sample | Hm Drgbl | Hm Drgbl | Hm Drgbl | CPE002DP | CPE002EH | CPE002FB | NM 00025 | NM 0002 | NM 0002 | PHKA2 | PHKA2 | PHKA2 | phosphorylase kin | phosphor | phosphor | 5256 | 5256 | 5256 | s10442 | s10443 | s10444 |
| 18075 | B11 | B12 | 1023 | 1138 | 16079 | 457,6 | 15,7 | 1,01 | H5A034 C | sample | Hm Gen E | Hm Gen E | Hm Gen E | CPE002JL | CPE002O1 | CPE002KK | NM 15258 | NM 1525 | NM 1525 | C11orf65 | C11orf65 | C11orf65 | chromosome 11 o | chromoso | chromoso | 160140 | 160140 | 160140 | s46179 | s46180 | s46181 |
| 6464 | N16 | N17 | 1500 | 643,2 | 23524 | 514 | 15,7 | 1,036 | H5A034 C | sample | Hm Drgbl | Hm Drgbl | Hm Drgbl | CPE002E0 | CPE002ET | CPE002FD | NM 00318 | NM 0031 | NM 0031 | SLC26A3 | SLC26A3 | SLC26A3 | solute carrier fam | solute car | solute car | 1811 | 1811 | 1811 | s4280 | s4279 | s4278 |
| 6476 | N5 | N6 | 1437 | 528,9 | 22631 | 486,4 | 15,7 | 1,036 | H5A034 C | sample | Hm Drgbl | Hm Drgbl | Hm Drgbl | CPE002E0 | CPE002ET | CPE002FD | NM 00437 | NM 0043 | NM 0043 | CRABP1 | CRABP1 | CRABP1 | cellular retinoic a | cellular re | cellular re | 1381 | 1381 | 1381 | s3482 | s3480 | s3481 |
| 6506 | P10 | P11 | 1583 | 561,2 | 24827 | 518,7 | 15,7 | 1,036 | H5A034 C | sample | Hm Drgbl | Hm Drgbl | Hm Drgbl | CPE002E0 | CPE002ET | CPE002FD | NM 00138 | NM 0013 | NM 0013 | DSCAM | DSCAM | DSCAM | Down syndrome c | Down syn | Down syn | 1826 | 1826 | 1826 | s4316 | s4315 | s223447 |
| 20371 | A4 | A5 | 407 | 758,6 | 6373 | 274,7 | 15,7 | 1,047 | H5A034 C | sample | Hm Gen E | Hm Gen E | Hm Gen E | CPE002JR | CPE002OC | CPE002KT | NM 93566 | XM 9356 | XM 9356 | LOC44250 | LOC44250 | LOC44250 | similar to zinc fin | similar to | similar to | 442501 | 442501 | 442501 | s196666 | s196667 | s196668 |
| 20470 | E7 | E8 | 1035 | 835,8 | 16236 | 261,6 | 15,7 | 1,047 | H5A034 C | sample | Hm Gen E | Hm Gen E | Hm Gen E | CPE002JR | CPE002OC | CPE002KT | XM 00110 | XM 0011 | XM 0011 | LOC44035 | LOC44035 | LOC44035 | similar to nuclear | similar to | similar to | 440350 | 440350 | 440350 | s54090 | s54091 | s54092 |
| 20532 | H2 | H3 | 914 | 763,3 | 14326 | 123 | 15,7 | 1,047 | H5A034 C | sample | Hm Gen E | Hm Gen E | Hm Gen E | CPE002JR | CPE002OC | CPE002KT | XM 93631 | XM 9363 | XM 9363 | LOC64216 | LOC64216 | LOC64216 | similar to Glutath | similar to | similar to | 642160 | 642160 | 642160 | s196769 | s196770 | s196771 |
| 18663 | J22 | J23 | 1476 | 993,7 | 23152 | 294,6 | 15,7 | 1,064 | H5A034 C | sample | Hm Gen E | Hm Gen E | Hm Gen E | CPE002JM | CPE002O3 | CPE002KM | NM 17860 | NM 1786 | NM 1786 | TMEM196 | TMEM196 | TMEM196 | transmembrane p | transmem | transmem | 256130 | 256130 | 256130 | s48737 | s48738 | s48739 |
| 18753 | N17 | N18 | 938 | 939,7 | 14721 | 265,8 | 15,7 | 1,064 | H5A034 C | sample | Hm Gen E | Hm Gen E | Hm Gen E | CPE002JM | CPE002O3 | CPE002KM | NM 15277 | NM 1527 | NM 1527 | C6orf218 | C6orf218 | C6orf218 | chromosome 6 op | chromoso | chromoso | 221718 | 221718 | 221718 | s48101 | s48101 | s48102 |
| 17430 | G14 | G15 | 1188 | 878,2 | 18602 | 190,4 | 15,7 | 1,072 | H5A034 C | sample | Hm Gen E | Hm Gen E | Hm Gen E | CPE002JJ | CPE002NY | CPE002KH | NM 17644 | XM 1764 | XM 1764 | LOC13349 | LOC13349 | LOC13349 | hypothetical prot | hypotheti | hypotheti | 133491 | 133491 | 133491 | s43780 | s43781 | s43782 |
| 17446 | G7 | G8 | 1042 | 967,1 | 16391 | 153,7 | 15,7 | 1,072 | H5A034 C | sample | Hm Gen E | Hm Gen E | Hm Gen E | CPE002JJ | CPE002NY | CPE002KH | NM 00101 | NM 0010 | NM 0010 | SHE | SHE | SHE | Src homology 2 d | Src homol | Src homol | 126669 | 126669 | 126669 | s43069 | s43070 | s225578 |
| 17487 | I22 | I23 | 1435 | 988,6 | 22497 | 257,5 | 15,7 | 1,072 | H5A034 C | sample | Hm Gen E | Hm Gen E | Hm Gen E | CPE002JJ | CPE002NY | CPE002KH | NM 15323 | NM 1532 | NM 1532 | POU5F2 | POU5F2 | POU5F2 | POU domain class | POU dom | POU dom | 134187 | 134187 | 134187 | s43836 | s43838 | s225637 |
| 17535 | K22 | K23 | 1559 | 976,3 | 24529 | 272,2 | 15,7 | 1,072 | H5A034 C | sample | Hm Gen E | Hm Gen E | Hm Gen E | CPE002JJ | CPE002NY | CPE002KH | NM 00102 | NM 0010 | NM 0010 | RLBP1L2 | RLBP1L2 | RLBP1L2 | retinaldehyde bin | retinalde | retinalde | 134829 | 134829 | 134829 | s43897 | s43897 | s43898 |
| 17594 | N10 | N11 | 1059 | 872,8 | 16590 | 222,1 | 15,7 | 1,072 | H5A034 C | sample | Hm Gen E | Hm Gen E | Hm Gen E | CPE002JJ | CPE002NY | CPE002KH | NM 08035 | NM 0803 | NM 0803 | TCEAL2 | TCEAL2 | TCEAL2 | transcription elo | transcript | transcript | 140597 | 140597 | 140597 | s44311 | s225661 | s225662 |
| 17598 | N14 | N15 | 1115 | 910,1 | 17381 | 282,6 | 15,7 | 1,072 | H5A034 C | sample | Hm Gen E | Hm Gen E | Hm Gen E | CPE002JJ | CPE002NY | CPE002KH | NM 15326 | NM 1532 | NM 1532 | C20orf96 | C20orf96 | C20orf96 | chromosome 20 o | chromoso | chromoso | 140680 | 140680 | 140680 | s44329 | s44330 | s44331 |
| 9313 | E1 | E2 | 1367 | 1125 | 21496 | 160,2 | 15,7 | 1,09 | H5A034 C | sample | Hm Drgbl | Hm Drgbl | Hm Drgbl | CPE002E8 | CPE002EV | CPE002FE | NM 03010 | NM 0301 | NM 0301 | ZSCAN2 | ZSCAN2 | ZSCAN2 | zinc finger and SC | zinc finge | zinc finge | 54993 | 54993 | 54993 | s29939 | s29940 | s224832 |
| 9475 | K4 | K5 | 1067 | 1148 | 16730 | 193,6 | 15,7 | 1,09 | H5A034 C | sample | Hm Drgbl | Hm Drgbl | Hm Drgbl | CPE002E8 | CPE002EV | CPE002FE | NM 01889 | NM 0189 | NM 0189 | PCDHGA4 | PCDHGA4 | PCDHGA4 | protocadherin ga | protocadl | protocadl | 56111 | 56111 | 56111 | s31919 | s31920 | s31921 |
| 18937 | F1 | F2 | 1182 | 950,9 | 18539 | 233,1 | 15,7 | 1,094 | H5A034 C | sample | Hm Gen E | Hm Gen E | Hm Gen E | CPE002JN | CPE002O6 | CPE002KN | NM 01530 | NM 0150 | NM 0150 | KIAA0284 | KIAA0284 | KIAA0284 | KIAA0284 | KIAA0284 | KIAA0284 | 283638 | 283638 | 283638 | s49322 | s49323 | s49324 |
| 19020 | I2 | I3 | 1126 | 935,3 | 17639 | 244,1 | 15,7 | 1,094 | H5A034 C | sample | Hm Gen E | Hm Gen E | Hm Gen E | CPE002JN | CPE002O6 | CPE002KN | NM 00103 | NM 0010 | NM 0010 | FAM102B | FAM102B | FAM102B | family with seque | family wit | family wit | 284611 | 284611 | 284611 | s49709 | s49710 | s49711 |
| 9847 | K15 | K16 | 1658 | 1117 | 26006 | 130,6 | 15,7 | 1,098 | H5A034 C | sample | Hm Drgbl | Hm Drgbl | Hm Drgbl | CPE002E9 | CPE002EU | CPE002FF | NM 02094 | NM 0209 | NM 0209 | ARHGAP2 | ARHGAP2 | ARHGAP2 | Rho GTPase activa | Rho GTPa | Rho GTPa | 57584 | 57584 | 57584 | s33373 | s33374 | s33375 |
| 9900 | M2 | M3 | 1842 | 1025 | 28857 | 121,8 | 15,7 | 1,098 | H5A034 C | sample | Hm Drgbl | Hm Drgbl | Hm Drgbl | CPE002E9 | CPE002EU | CPE002FF | NM 02248 | NM 0224 | NM 0224 | ZDHHC6 | ZDHHC6 | ZDHHC6 | zinc finger, DHHC | zinc finge | zinc finge | 64429 | 64429 | 64429 | s34749 | s34750 | s34751 |
| 9974 | P21 | P22 | 1649 | 1029 | 25942 | 236,2 | 15,7 | 1,098 | H5A034 C | sample | Hm Drgbl | Hm Drgbl | Hm Drgbl | CPE002E9 | CPE002EU | CPE002FF | NM 02403 | NM 0240 | NM 0240 | NEUROG2 | NEUROG2 | NEUROG2 | neurogenin 2 | neurogen | neurogen | 63973 | 63973 | 63973 | s34340 | s34342 | s224592 |
| 15125 | G13 | G14 | 1353 | 805,4 | 21188 | 113,6 | 15,7 | 1,098 | H5A034 C | sample | Hm Gen E | Hm Gen E | Hm Gen E | CPE002JF | CPE002NR | CPE002K8 | NM 19827 | NM 1982 | NM 1982 | LMOD3 | LMOD3 | LMOD3 | leiomodin 3 (feta | leiomodir | leiomodir | 56203 | 56203 | 56203 | s32058 | s32059 | s32059 |
| 8490 | B3 | B4 | 1502 | 1098 | 23592 | 135,5 | 15,7 | 1,11 | H5A034 C | sample | Hm Drgbl | Hm Drgbl | Hm Drgbl | CPE002E6 | CPE002ER | CPE002FC | NM 01555 | NM 0155 | NM 0155 | TIPARP | TIPARP | TIPARP | TCDD-inducible p | TCDD-indi | TCDD-indi | 25976 | 25976 | 25976 | s24856 | s24857 | s24858 |
| 8587 | F4 | F5 | 1552 | 976,1 | 24302 | 130,9 | 15,7 | 1,11 | H5A034 C | sample | Hm Drgbl | Hm Drgbl | Hm Drgbl | CPE002E6 | CPE002ER | CPE002FC | NM 01415 | NM 0141 | NM 0141 | ZC3H7A | ZC3H7A | ZC3H7A | zinc finger CCCH-t | zinc finge | zinc finge | 29066 | 29066 | 29066 | s26411 | s26412 | s26413 |
| 8715 | L11 | L12 | 1317 | 1079 | 20668 | 113,4 | 15,7 | 1,11 | H5A034 C | sample | Hm Drgbl | Hm Drgbl | Hm Drgbl | CPE002E6 | CPE002ER | CPE002FC | NM 01221 | NM 0121 | NM 0121 | TSPAN17 | TSPAN17 | TSPAN17 | tetraspanin 17 | tetraspan | tetraspan | 26262 | 26262 | 26262 | s25310 | s25309 | s223689 |
| 8820 | P2 | P3 | 1680 | 1028 | 26425 | 192,4 | 15,7 | 1,11 | H5A034 C | sample | Hm Drgbl | Hm Drgbl | Hm Drgbl | CPE002E6 | CPE002ER | CPE002FC | NM 02117 | NM 0211 | NM 0211 | PSAT1 | PSAT1 | PSAT1 | phosphoserine am | phosphos | phosphos | 29968 | 29968 | 29968 | s26817 | s26816 | s26815 |
| 20892 | G2 | G3 | 1347 | 981,4 | 21147 | 232,6 | 15,7 | 1,113 | H5A034 C | sample | Hm Gen E | Hm Gen E | Hm Gen E | CPE002JS | CPE002OE | CPE002KV | NM 49776 | XM 4977 | XM 4977 | LOC64428 | LOC64428 | LOC64428 | hypothetical LOC | hypotheti | hypotheti | 644280 | 644280 | 644280 | s55628 | s55629 | s197050 |
| 21026 | M10 | M11 | 1068 | 922,8 | 16804 | 332,2 | 15,7 | 1,113 | H5A034 C | sample | Hm Gen E | Hm Gen E | Hm Gen E | CPE002JS | CPE002OE | CPE002KV | NM 92779 | XM 9277 | XM 9277 | LOC64459 | LOC64459 | LOC64459 | similar to SYN1 | similar to | similar to | 644590 | 644590 | 644590 | s55773 | s197086 | s197087 |
| 21027 | M11 | M12 | 1432 | 1069 | 22440 | 202,6 | 15,7 | 1,113 | H5A034 C | sample | Hm Gen E | Hm Gen E | Hm Gen E | CPE002JS | CPE002OE | CPE002KV | NM 92666 | XM 0011 | XM 0011 | LOC64333 | LOC64333 | LOC64333 | similar to mCG10 | similar to | similar to | 643330 | 643330 | 643330 | s55774 | s55276 | s227063 |
| 21086 | O21 | O22 | 1091 | 908,4 | 17098 | 341,7 | 15,7 | 1,113 | H5A034 C | sample | Hm Gen E | Hm Gen E | Hm Gen E | CPE002JS | CPE002OE | CPE002KV | NM 93155 | XM 9315 | XM 9315 | LOC64344 | LOC64344 | LOC64344 | similar to hCG20 | similar to | similar to | 643449 | 643449 | 643449 | s55323 | s227065 | s227066 |
| 21373 | K20 | K21 | 1134 | 967,8 | 17828 | 332 | 15,7 | 1,113 | H5A034 C | sample | Hm Gen E | Hm Gen E | Hm Gen E | CPE002JT | CPE002OF | CPE002KW | XM 94116 | XM 9411 | XM 9411 | LOC64699 | LOC64699 | LOC64699 | hypothetical LOC | hypotheti | hypotheti | 646992 | 646992 | 646992 | s56609 | s56610 | s56611 |
| 14265 | C17 | C18 | 958 | 947,2 | 14681 | 149,6 | 15,7 | 1,117 | H5A034 C | sample | Hm Gen E | Hm Gen E | Hm Gen E | CPE002JD | CPE002NL | CPE002K6 | NM 01666 | NM 0166 | NM 0166 | TNFRSF12 | TNFRSF12 | TNFRSF12 | tumor necrosis fa | tumor nec | tumor nec | 51330 | 51330 | 51330 | s195218 | s195219 | s195220 |
| 11292 | G2 | G3 | 550 | 985,4 | 8616 | 151,2 | 15,7 | 1,138 | H5A034 C | sample | Hm Drgbl | Hm Drgbl | Hm Drgbl | CPE002JA | CPE002NF | CPE002JY | NM 00100 | NM 0010 | NM 0010 | FKSG30 | FKSG30 | FKSG30 | kappa-actin | kappa-act | kappa-act | 440915 | 440915 | 440915 | s54217 | s54218 | s54218 |
| 11332 | I12 | I13 | 1503 | 779,6 | 23664 | 146,1 | 15,7 | 1,138 | H5A034 C | sample | Hm Drgbl | Hm Drgbl | Hm Drgbl | CPE002JA | CPE002NF | CPE002JY | NM 93986 | XM 9398 | XM 9398 | PERF15 | PERF15 | PERF15 | lipid-binding prot | lipid-bind | lipid-bind | 646480 | 646480 | 646480 | s56399 | s56400 | s56401 |
| 12354 | C3 | C4 | 1180 | 1099 | 18520 | 152,5 | 15,7 | 1,145 | H5A034 C | sample | Hm Gen E | Hm Gen E | Hm Gen E | CPE002JA | CPE002NF | CPE002K1 | NM 00321 | NM 0032 | NM 0032 | SRP9 | SRP9 | SRP9 | signal recognition | signal rec | signal rec | 6726 | 6726 | 6726 | s13435 | s13436 | s13437 |
| 10846 | D7 | D8 | 1204 | 850 | 18888 | 418,1 | 15,7 | 1,177 | H5A034 C | sample | Hm Drgbl | Hm Drgbl | Hm Drgbl | CPE002EC | CPE002F0 | CPE002FK | NM 14466 | NM 1446 | NM 1446 | WFDC5 | WFDC5 | WFDC5 | WAP four-disulfid | WAP four- | WAP four- | 149708 | 149708 | 149708 | s45340 | s45341 | s45340 |
| 10956 | I2 | I3 | 1460 | 865,1 | 22912 | 349,3 | 15,7 | 1,189 | H5A034 C | sample | Hm Drgbl | Hm Drgbl | Hm Drgbl | CPE002EC | CPE002F0 | CPE002FK | NM 14489 | NM 1448 | NM 1448 | U2AF1L4 | U2AF1L4 | U2AF1L4 | U2 small nuclear | U2 small r | U2 small r | 199746 | 199746 | 199746 | s47136 | s47135 | s195829 |
| 7521 | J17 | J18 | 1010 | 556,4 | 15844 | 511,9 | 15,7 | 1,198 | H5A034 C | sample | Hm Drgbl | Hm Drgbl | Hm Drgbl | CPE002E3 | CPE002EY | CPE002FH | NM 00488 | NM 0048 | NM 0048 | CHST10 | CHST10 | CHST10 | carbohydrate sulf | carbohydr | carbohydr | 9486 | 9486 | 9486 | s431 | s432 | s432 |
| 7570 | L18 | L19 | 592 | 638,3 | 9308 | 511,9 | 15,7 | 1,198 | H5A034 C | sample | Hm Drgbl | Hm Drgbl | Hm Drgbl | CPE002E3 | CPE002EY | CPE002FH | NM 00100 | NM 0010 | NM 0010 | UST | UST | UST | uronyl-2-sulfotran | uronyl-2-s | uronyl-2-s | 10090 | 10090 | 10090 | s19629 | s19630 | s19628 |
| 7678 | P7 | P8 | 1310 | 551,1 | 20540 | 479,3 | 15,7 | 1,198 | H5A034 C | sample | Hm Drgbl | Hm Drgbl | Hm Drgbl | CPE002E3 | CPE002EY | CPE002FH | NM 00568 | NM 0056 | NM 0056 | SOX13 | SOX13 | SOX13 | SRY (sex determin | SRY (sex d | SRY (sex d | 9580 | 9580 | 9580 | s18405 | s18406 | s18404 |
| 17091 | I11 | I12 | 1176 | 918,5 | 18486 | 197,8 | 15,7 | 1,212 | H5A034 C | sample | Hm Gen E | Hm Gen E | Hm Gen E | CPE002JI | CPE002NX | CPE002KF | NM 00104 | NM 0010 | NM 0010 | WDR89 | WDR89 | WDR89 | WD repeat doma | WD repea | WD repea | 112840 | 112840 | 112840 | s41382 | s41383 | s41384 |
| 17103 | I22 | I23 | 1040 | 981,9 | 16306 | 237,2 | 15,7 | 1,212 | H5A034 C | sample | Hm Gen E | Hm Gen E | Hm Gen E | CPE002JI | CPE002NX | CPE002KF | NM 00101 | NM 0010 | NM 0010 | OR2T2 | C1orf92 | OR2T2 | olfactory recepto | chromoso | olfactory | 120065 | 149499 | 120065 | s42389 | s45314 | s42391 |
| 7177 | L1 | L2 | 1211 | 584,2 | 18992 | 310,2 | 15,7 | 1,217 | H5A034 C | sample | Hm Drgbl | Hm Drgbl | Hm Drgbl | CPE002E2 | CPE002EX | CPE002FI | NM 14578 | NM 1457 | NM 1457 | MGST1 | MGST1 | MGST1 | microsomal gluta | microsom | microsom | 4257 | 4257 | 4257 | s8757 | s8758 | s8756 |
| 7248 | N9 | N10 | 719 | 605,3 | 11270 | 522 | 15,7 | 1,217 | H5A034 C | sample | Hm Drgbl | Hm Drgbl | Hm Drgbl | CPE002E2 | CPE002EX | CPE002FI | NM 00519 | NM 0051 | NM 0051 | FOXO4 | FOXO4 | FOXO4 | forkhead box O4 | forkhead | forkhead | 4303 | 4303 | 4303 | s8835 | s8836 | s8837 |
| 5050 | C18 | C19 | 1017 | 632,1 | 15991 | 516 | 15,7 | 1,217 | H5A034 C | sample | Hm Drgbl | Hm Drgbl | Hm Drgbl | CPE002DU | CPE002EL | CPE002FH | NM 00518 | NM 0051 | NM 0051 | TRAF1 | TRAF1 | TRAF1 | TNF receptor-asso | TNF recep | TNF recep | 7185 | 7185 | 7185 | s14376 | s224753 | s14377 |
| 5141 | G13 | G14 | 1273 | 627,9 | 19948 | 462,8 | 15,7 | 1,217 | H5A034 C | sample | Hm Drgbl | Hm Drgbl | Hm Drgbl | CPE002DU | CPE002EL | CPE002FH | NM 03266 | NM 0326 | NM 0326 | USP30 | USP30 | USP30 | ubiquitin specific | ubiquitin | ubiquitin | 84749 | 84749 | 84749 | s39399 | s39400 | s39401 |
| 5219 | J19 | J20 | 1429 | 576,1 | 22485 | 563,8 | 15,7 | 1,217 | H5A034 C | sample | Hm Drgbl | Hm Drgbl | Hm Drgbl | CPE002DU | CPE002EL | CPE002FH | NM 02475 | NM 0247 | NM 0247 | NUP214 | NUP214 | NUP214 | nucleoporin 214k | nucleopor | nucleopor | 8021 | 8021 | 8021 | s15547 | s15548 | s224849 |
| 5279 | L8 | L9 | 1064 | 561,1 | 16597 | 560,6 | 15,7 | 1,217 | H5A034 C | sample | Hm Drgbl | Hm Drgbl | Hm Drgbl | CPE002DU | CPE002EL | CPE002FH | NM 14459 | NM 1445 | NM 1445 | ZSCAN21 | ZSCAN21 | ZSCAN21 | zinc finger and SC | zinc finge | zinc finge | 7589 | 7589 | 7589 | s15087 | s15088 | s15089 |
| 5313 | N17 | N18 | 1848 | 646,1 | 29000 | 552 | 15,7 | 1,217 | H5A034 C | sample | Hm Drgbl | Hm Drgbl | Hm Drgbl | CPE002DU | CPE002EL | CPE002FH | NM 00603 | NM 0060 | NM 0060 | ST8SIA2 | ST8SIA2 | ST8SIA2 | ST8 alpha-N-acet | ST8 alpha | ST8 alpha | 8128 | 8128 | 8128 | s15646 | s15644 | s224874 |
| 2362 | C18 | C19 | 1484 | 726,2 | 23266 | 452,9 | 15,7 | 1,223 | H5A034 C | sample | Hm Drgbl | Hm Drgbl | Hm Drgbl | CPE002DC | CPE002ED | CPE002FA | NM 00582 | NM 0058 | NM 0058 | MARK3 | MARK3 | MARK3 | MAP/microtubule | MAP/micr | MAP/micr | 4140 | 4140 | 4140 | s8514 | s8515 | s8516 |
| 2412 | E2 | E3 | 691 | 762,3 | 10832 | 407,5 | 15,7 | 1,223 | H5A034 C | sample | Hm Drgbl | Hm Drgbl | Hm Drgbl | CPE002DC | CPE002ED | CPE002FA | NM 00591 | NM 0059 | NM 0059 | MAP3K4 | MAP3K4 | MAP3K4 | mitogen-activated | mitogen-a | mitogen-a | 4216 | 4216 | 4216 | s8673 | s8674 | s8675 |
| 16623 | E22 | E23 | 1131 | 1030 | 17788 | 383,4 | 15,7 | 1,241 | H5A034 C | sample | Hm Gen E | Hm Gen E | Hm Gen E | CPE002JH | CPE002NW | CPE002KD | NM 92702 | XM 9270 | XM 9270 | PPP1R3E | PPP1R3E | PPP1R3E | protein phosphat | protein pl | protein pl | 90673 | 90673 | 90673 | s40470 | s40471 | s228448 |
| 16660 | G12 | G13 | 843 | 946,4 | 13228 | 346,2 | 15,7 | 1,241 | H5A034 C | sample | Hm Gen E | Hm Gen E | Hm Gen E | CPE002JH | CPE002NW | CPE002KD | NM 13838 | NM 1383 | NM 1383 | CCDC45 | CCDC45 | CCDC45 | coiled-coil domai | coiled-coi | coiled-coi | 90799 | 90799 | 90799 | s40491 | s40492 | s40493 |
| 16723 | I4 | I5 | 820 | 938,5 | 12880 | 344,8 | 15,7 | 1,241 | H5A034 C | sample | Hm Gen E | Hm Gen E | Hm Gen E | CPE002JH | CPE002NW | CPE002KD | NM 03344 | NM 0334 | NM 0334 | C9orf123 | C9orf123 | C9orf123 | chromosome 9 op | chromoso | chromoso | 90871 | 90871 | 90871 | s40524 | s40525 | s40526 |
| 16747 | J4 | J5 | 834 | 960,1 | 13060 | 344,3 | 15,7 | 1,241 | H5A034 C | sample | Hm Gen E | Hm Gen E | Hm Gen E | CPE002JH | CPE002NW | CPE002KD | NM 13834 | NM 1383 | NM 1383 | C1orf165 | C1orf165 | C1orf165 | chromosome 1 op | chromoso | chromoso | 92346 | 92346 | 92346 | s40934 | s40935 | s40935 |
| 16794 | L3 | L4 | 861 | 947,7 | 13544 | 257,6 | 15,7 | 1,241 | H5A034 C | sample | Hm Gen E | Hm Gen E | Hm Gen E | CPE002JH | CPE002NW | CPE002KD | NM 13834 | NM 1383 | NM 1383 | TMEM116 | TMEM116 | TMEM116 | transmembrane p | transmem | transmem | 89894 | 89894 | 89894 | s40197 | s40198 | s40199 |
| 17815 | G15 | G16 | 1374 | 1143 | 21514 | 263 | 15,7 | 1,241 | H5A034 C | sample | Hm Gen E | Hm Gen E | Hm Gen E | CPE002JK | CPE002O0 | CPE002KJ | NM 14462 | NM 1446 | NM 1446 | SESN3 | SESN3 | SESN3 | sestrin 3 | sestrin 3 | sestrin 3 | 143686 | 143686 | 143686 | s44569 | s44570 | s44570 |
| 17856 | H9 | H10 | 1279 | 1069 | 20089 | 285,4 | 15,7 | 1,241 | H5A034 C | sample | Hm Gen E | Hm Gen E | Hm Gen E | CPE002JK | CPE002O0 | CPE002KJ | NM 14461 | NM 1446 | NM 1446 | TMED6 | TMED6 | TMED6 | transmembrane e | transmem | transmem | 146456 | 146456 | 146456 | s44862 | s44863 | s44864 |
| 17859 | I11 | I12 | 1011 | 1165 | 15864 | 382 | 15,7 | 1,241 | H5A034 C | sample | Hm Gen E | Hm Gen E | Hm Gen E | CPE002JK | CPE002O0 | CPE002KJ | NM 17502 | NM 1750 | NM 1750 | PLEKHA7 | PLEKHA7 | PLEKHA7 | pleckstrin homol | pleckstrin | pleckstrin | 144100 | 144100 | 144100 | s44593 | s44595 | s44595 |
| 17861 | I13 | I14 | 1415 | 1077 | 22145 | 254,6 | 15,7 | 1,241 | H5A034 C | sample | Hm Gen E | Hm Gen E | Hm Gen E | CPE002JK | CPE002O0 | CPE002KJ | NM 19425 | NM 1942 | NM 1942 | SPTY2D1 | SPTY2D1 | SPTY2D1 | SPT2, Suppressor | SPT2, Sup | SPT2, Sup | 144108 | 144108 | 144108 | s44596 | s44597 | s44598 |
| 9014 | H21 | H22 | 1145 | 1765 | 17837 | 151,7 | 15,7 | 1,249 | H5A034 C | sample | Hm Drgbl | Hm Drgbl | Hm Drgbl | CPE002E7 | CPE002ES | CPE002FD | NM 01660 | NM 0166 | NM 0166 | SLC22A17 | SLC22A17 | SLC22A17 | solute carrier fam | solute car | solute car | 51310 | 51310 | 51310 | s27917 | s27918 | s27919 |
| 9133 | M20 | M21 | 1525 | 1776 | 23978 | 151,7 | 15,7 | 1,249 | H5A034 C | sample | Hm Drgbl | Hm Drgbl | Hm Drgbl | CPE002E7 | CPE002ES | CPE002FD | NM 01616 | NM 0161 | NM 0161 | RAB4B | RAB4B | RAB4B | RAB4B, member R | RAB4B, m | RAB4B, m | 53916 | 53916 | 53916 | s28802 | s28800 | s28801 |
| 4287 | C22 | C23 | 942 | 624,2 | 14787 | 350,6 | 15,7 | 1,259 | H5A034 C | sample | Hm Drgbl | Hm Drgbl | Hm Drgbl | CPE002DL | CPE002EK | CPE002FI | NM 00107 | NM 0010 | NM 0010 | CACNA1I | CACNA1I | CACNA1I | calcium channel, | calcium c | calcium c | 8911 | 8911 | 8911 | s17044 | s17045 | s17046 |
| 4358 | F21 | F22 | 1272 | 710,8 | 19911 | 431,9 | 15,7 | 1,259 | H5A034 C | sample | Hm Drgbl | Hm Drgbl | Hm Drgbl | CPE002DL | CPE002EK | CPE002FI | NM 00049 | NM 0004 | NM 0004 | KCNC3 | KCNC3 | KCNC3 | potassium voltag | potassium | potassium | 3748 | 3748 | 3748 | s7709 | s7710 | s7711 |
| 4422 | I14 | I15 | 1418 | 768,7 | 22298 | 434 | 15,7 | 1,259 | H5A034 C | sample | Hm Drgbl | Hm Drgbl | Hm Drgbl | CPE002DL | CPE002EK | CPE002FI | NM 00220 | NM 0022 | NM 0022 | ATP13A2 | ATP13A2 | ATP13A2 | ATPase type 13A2 | ATPase ty | ATPase ty | 23400 | 23400 | 23400 | s23742 | s23743 | s23744 |
| 12 | A2 | A3 | 503 | 1614 | 7914 | 803,2 | 15,7 | 1,264 | H5A034 C | sample | Hm Drgbl | Hm Drgbl | Hm Drgbl | CPE002DT | CPE002EJ | CPE002F9 | NM 00309 | NM 0030 | NM 0030 | SLC9A1 | SLC9A1 | SLC9A1 | solute carrier fam | solute car | solute car | 6548 | 6548 | 6548 | s13022 | s13023 | s224645 |
| 106 | E18 | E19 | 542 | 1580 | 8576 | 465,4 | 15,7 | 1,264 | H5A034 C | sample | Hm Drgbl | Hm Drgbl | Hm Drgbl | CPE002DT | CPE002EJ | CPE002F9 | NM 00300 | NM 0030 | NM 0030 | SLC22A2 | SLC22A2 | SLC22A2 | solute carrier fam | solute car | solute car | 6582 | 6582 | 6582 | s13109 | s13110 | s13108 |
| 2059 | F4 | F5 | 783 | 552,8 | 12298 | 429,5 | 15,7 | 1,264 | H5A034 C | sample | Hm Drgbl | Hm Drgbl | Hm Drgbl | CPE002FX | CPE002G1 | CPE002GS | NM 19835 | NM 1983 | NM 1983 | KCTD8 | KCTD8 | KCTD8 | potassium channe | potassium | potassium | 386617 | 386617 | 386617 | s51813 | s51814 | s51815 |
| 2245 | N20 | N21 | 1087 | 704,3 | 17102 | 475,5 | 15,7 | 1,264 | H5A034 C | sample | Hm Drgbl | Hm Drgbl | Hm Drgbl | CPE002FX | CPE002G1 | CPE002GS | | | | | | | | | | | | | | | |
| 2279 | O8 | O9 | 708 | 652,2 | 11058 | 465,6 | 15,7 | 1,264 | H5A034 C | sample | Hm Drgbl | Hm Drgbl | Hm Drgbl | CPE002FX | CPE002G1 | CPE002GS | NM 00101 | NM 0010 | NM 0010 | ZNF181 | ZNF181 | ZNF181 | zinc finger protei | zinc finge | zinc finge | 339318 | 339318 | 339318 | s50499 | s50500 | s50501 |
| 2292 | P2 | P3 | 1275 | 686,1 | 20008 | 514,1 | 15,7 | 1,264 | H5A034 C | sample | Hm Drgbl | Hm Drgbl | Hm Drgbl | CPE002FX | CPE002G1 | CPE002GS | | | | | | | | | | | | | | | |
| 6610 | D18 | D19 | 1580 | 624,2 | 17638 | 496,5 | 15,7 | 1,377 | H5A034 C | sample | Hm Drgbl | Hm Drgbl | Hm Drgbl | CPE002E1 | CPE002EU | CPE002FE | NM 00222 | NM 0022 | NM 0022 | ITIH2 | ITIH2 | ITIH2 | inter-alpha (globu | inter-alph | inter-alph | 3698 | 3698 | 3698 | s7604 | s7605 | s7606 |
| 6648 | E9 | E10 | 1139 | 561,7 | 17895 | 395,1 | 15,7 | 1,377 | H5A034 C | sample | Hm Drgbl | Hm Drgbl | Hm Drgbl | CPE002E1 | CPE002EU | CPE002FE | NM 00441 | NM 0041 | NM 0041 | GYG1 | GYG1 | GYG1 | glycogenin 1 | glycogeni | glycogeni | 2992 | 2992 | 2992 | s6358 | s6359 | s6360 |
| 6892 | P12 | P13 | 1617 | 598,2 | 25338 | 497,7 | 15,7 | 1,377 | H5A034 C | sample | Hm Drgbl | Hm Drgbl | Hm Drgbl | CPE002E1 | CPE002EU | CPE002FE | NM 00413 | NM 0041 | NM 0041 | KRT33B | KRT33B | KRT33B | keratin 33B | keratin 33 | keratin 33 | 3884 | 3884 | 3884 | s8008 | s8009 | s8010 |
| 4104 | K9 | K10 | 993 | 658,3 | 15565 | 380,8 | 15,7 | 1,396 | H5A034 C | sample | Hm Drgbl | Hm Drgbl | Hm Drgbl | CPE002DS | CPE002EI | CPE002F8 | NM 00656 | NM 0065 | NM 0065 | POLH | POLH | POLH | polymerase (DNA | polymeras | polymeras | 5429 | 5429 | 5429 | s10790 | s10791 | s10792 |
| 4158 | N14 | N15 | 1100 | 677,3 | 17284 | 431,8 | 15,7 | 1,396 | H5A034 C | sample | Hm Drgbl | Hm Drgbl | Hm Drgbl | CPE002DS | CPE002EI | CPE002F8 | NM 00621 | NM 0062 | NM 0062 | PTGDS | PTGDS | PTGDS | prostaglandin D2 | prostagla | prostagla | 5730 | 5730 | 5730 | s11359 | s11360 | s11361 |
| 11576 | C16 | C17 | 1374 | 849,6 | 21558 | 239,3 | 15,7 | 1,401 | H5A034 C | sample | Hm Drgbl | Hm Drgbl | Hm Drgbl | CPE002JA | CPE002NI | CPE002JZ | NM 00104 | NM 0010 | NM 0010 | S100A6 | S100A6 | S100A6 | S100 calcium bind | S100 calci | S100 calci | 6277 | 6277 | 6277 | s12418 | s194777 | s194778 |
| 15420 | C2 | C3 | 1121 | 806,5 | 17614 | 149 | 15,7 | 1,411 | H5A034 C | sample | Hm Gen E | Hm Gen E | Hm Gen E | CPE002JG | CPE002NS | CPE002K9 | NM 02303 | NM 0230 | NM 0230 | EMP3 | EMP3 | EMP3 | epithelial membr | epithelial | epithelial | 2014 | 2014 | 2014 | s4657 | s4658 | s194394 |
| 15446 | D21 | D22 | 1088 | 806,8 | 17168 | 150,9 | 15,7 | 1,411 | H5A034 C | sample | Hm Gen E | Hm Gen E | Hm Gen E | CPE002JG | CPE002NS | CPE002K9 | NM 02303 | NM 0230 | NM 0230 | PRAMEF2 | PRAMEF2 | PRAMEF2 | PRAME family me | PRAME fa | PRAME fa | 65122 | 65122 | 65122 | s35224 | s35225 | s35226 |
| 15621 | K6 | K7 | 737 | 894,8 | 11604 | 171,4 | 15,7 | 1,411 | H5A034 C | sample | Hm Gen E | Hm Gen E | Hm Gen E | CPE002JG | CPE002NS | CPE002K9 | NM 02247 | NM 0224 | NM 0224 | ACBD3 | ACBD3 | ACBD3 | acyl-Coenzyme A | acyl-Coen | acyl-Coen | 64746 | 64746 | 64746 | s34848 | s34849 | s34850 |
| 15719 | O8 | O9 | 1227 | 887,7 | 19223 | 234,4 | 15,7 | 1,411 | H5A034 C | sample | Hm Gen E | Hm Gen E | Hm Gen E | CPE002JG | CPE002NS | CPE002K9 | NM 02401 | NM 0240 | NM 0240 | C1orf50 | C1orf50 | C1orf50 | chromosome 1 op | chromoso | chromoso | 79078 | 79078 | 79078 | s35534 | s35535 | s35536 |
| 5772 | A2 | A3 | 1363 | 824,7 | 21365 | 541,1 | 15,7 | 1,433 | H5A034 C | sample | Hm Drgbl | Hm Drgbl | Hm Drgbl | CPE002DY | CPE002EO | CPE002FN | NM 00112 | NM 0011 | NM 0011 | ARL4D | ARL4D | ARL4D | ADP-ribosylation | ADP-ribos | ADP-ribos | 379 | 379 | 379 | s1560 | s1559 | s1561 |
| 5792 | B16 | B17 | 1522 | 857,2 | 23866 | 624,9 | 15,7 | 1,433 | H5A034 C | sample | Hm Drgbl | Hm Drgbl | Hm Drgbl | CPE002DY | CPE002EO | CPE002FN | NM 00446 | NM 0044 | NM 0044 | BCR | BCR | BCR | breakpoint cluste | breakpoin | breakpoin | 613 | 613 | 613 | s1930 | s1931 | s1932 |
| 5863 | E15 | E16 | 748 | 786,9 | 11777 | 445,1 | 15,7 | 1,433 | H5A034 C | sample | Hm Drgbl | Hm Drgbl | Hm Drgbl | CPE002DY | CPE002EO | CPE002FN | NM 00110 | NM 0011 | NM 0011 | ACTA1 | ACTA1 | ACTA1 | actin, alpha 1, ske | actin, alpl | actin, alpl | 58 | 58 | 58 | s942 | s941 | s943 |
| 5929 | H1 | H2 | 1501 | 745,9 | 23608 | 487,1 | 15,7 | 1,433 | H5A034 C | sample | Hm Drgbl | Hm Drgbl | Hm Drgbl | CPE002DY | CPE002EO | CPE002FN | NM 00114 | NM 0011 | NM 0011 | ANK1 | ANK1 | ANK1 | ankyrin 1, erythro | ankyrin 1, | ankyrin 1, | 286 | 286 | 286 | s1362 | s223810 | s1364 |
| 5939 | H19 | H20 | 1398 | 774 | 20565 | 473,5 | 15,7 | 1,433 | H5A034 C | sample | Hm Drgbl | Hm Drgbl | Hm Drgbl | CPE002DY | CPE002EO | CPE002FN | NM 00115 | NM 0011 | NM 0011 | ANXA4 | ANXA4 | ANXA4 | annexin A4 | annexin A | annexin A | 307 | 307 | 307 | s1390 | s1391 | s1389 |
| 5985 | J7 | J8 | 1469 | 771,5 | 23128 | 514,6 | 15,7 | 1,433 | H5A034 C | sample | Hm Drgbl | Hm Drgbl | Hm Drgbl | CPE002DY | CPE002EO | CPE002FN | NM 00003 | NM 0000 | NM 0000 | APC | APC | APC | adenomatosis pol | adenoma | adenoma | 324 | 324 | 324 | s1433 | s1434 | s1435 |
| 1179 | B11 | B12 | 812 | 798,9 | 12696 | 448,5 | 15,7 | 1,434 | H5A034 C | sample | Hm Drgbl | Hm Drgbl | Hm Drgbl | CPE002FV | CPE002FZ | CPE002G3 | NM 00652 | NM 0065 | NM 0065 | NCOA4 | NCOA4 | NCOA4 | nuclear receptor | nuclear re | nuclear re | 8031 | 8031 | 8031 | s15565 | s15566 | s15567 |
| 1484 | N5 | N6 | 657 | 776,5 | 10336 | 424,9 | 15,7 | 1,434 | H5A034 C | sample | Hm Drgbl | Hm Drgbl | Hm Drgbl | CPE002FV | CPE002FZ | CPE002G3 | NM 00447 | NM 0044 | NM 0044 | ZNHIT3 | ZNHIT3 | ZNHIT3 | zinc finger, HIT ty | zinc finge | zinc finge | 9326 | 9326 | 9326 | s17823 | s17824 | s17825 |
| 16131 | A11 | A12 | 725 | 953,3 | 11399 | 466,9 | 15,7 | 1,44 | H5A034 C | sample | Hm Gen E | Hm Gen E | Hm Gen E | CPE002JG | CPE002NU | CPE002KB | NM 03308 | NM 0330 | NM 0330 | APOLD1 | APOLD1 | APOLD1 | apolipoprotein L d | apolipopr | apolipopr | 81575 | 81575 | 81575 | s37662 | s37663 | s37664 |
| 16175 | B8 | B9 | 1236 | 1018 | 19433 | 447,7 | 15,7 | 1,44 | H5A034 C | sample | Hm Gen E | Hm Gen E | Hm Gen E | CPE002JG | CPE002NU | CPE002KB | NM 00114 | NM 0011 | NM 0011 | PROK1 | PROK1 | PROK1 | prokineticin 1 | prokinetic | prokinetic | 84432 | 84432 | 84432 | s38982 | s38983 | s38984 |
| 16424 | M16 | M17 | 1310 | 938,4 | 20624 | 370,3 | 15,7 | 1,44 | H5A034 C | sample | Hm Gen E | Hm Gen E | Hm Gen E | CPE002JG | CPE002NU | CPE002KB | NM 03234 | NM 0323 | NM 0323 | ACBD6 | ACBD6 | ACBD6 | acyl-Coenzyme A | acyl-Coen | acyl-Coen | 84320 | 84320 | 84320 | s38914 | s38915 | s38916 |
| 16507 | P4 | P5 | 848 | 1085 | 13351 | 367,9 | 15,7 | 1,44 | H5A034 C | sample | Hm Gen E | Hm Gen E | Hm Gen E | CPE002JG | CPE002NU | CPE002KB | NM 03254 | NM 0325 | NM 0325 | NFKBID | NFKBID | NFKBID | nuclear factor of | nuclear fa | nuclear fa | 84807 | 84807 | 84807 | s39472 | s39473 | s39474 |
| 16508 | P5 | P6 | 1170 | 1083 | 18379 | 355,4 | 15,7 | 1,44 | H5A034 C | sample | Hm Gen E | Hm Gen E | Hm Gen E | CPE002JG | CPE002NU | CPE002KB | NM 03215 | NM 0321 | NM 0321 | RNASEH2C | RNASEH2C | RNASEH2C | ribonuclease H2, | ribonucle | ribonucle | 84153 | 84153 | 84153 | s195454 | s195455 | s195455 |
| 13454 | A21 | A22 | 996 | 1037 | 15137 | 172,6 | 15,7 | 1,474 | H5A034 C | sample | Hm Gen E | Hm Gen E | Hm Gen E | CPE002JC | CPE002NJ | CPE002K4 | NM 01581 | NM 0158 | NM 0158 | ICOSLG | ICOSLG | ICOSLG | inducible T-cell co | inducible | inducible | 23308 | 23308 | 23308 | s23525 | s23526 | s225947 |
| 13664 | J16 | J17 | 951 | 954,1 | 14942 | 160,1 | 15,7 | 1,474 | H5A034 C | sample | Hm Gen E | Hm Gen E | Hm Gen E | CPE002JC | CPE002NJ | CPE002K4 | NM 00100 | NM 0010 | NM 0010 | ORBG1 | ORBG1 | ORBG1 | olfactory recepto | olfactory | olfactory | 26494 | 26494 | 26494 | s25449 | s25450 | s226078 |
| 850 | D18 | D19 | 1320 | 739 | 20749 | 339 | 15,7 | 1,482 | H5A034 C | sample | Hm Drgbl | Hm Drgbl | Hm Drgbl | CPE002FU | CPE002FY | CPE002G2 | NM 00296 | NM 0029 | NM 0029 | S100A7 | S100A7 | S100A7 | S100 calcium bind | S100 calci | S100 calci | 6278 | 6278 | 6278 | s12419 | s12420 | s12421 |
| 948 | H2 | H3 | 1643 | 728,3 | 25632 | 467,1 | 15,7 | 1,482 | H5A034 C | sample | Hm Drgbl | Hm Drgbl | Hm Drgbl | CPE002FU | CPE002FY | CPE002G2 | NM 00303 | NM 0030 | NM 0030 | SNCA | SNCA | SNCA | synuclein, alpha ( | synuclein, | synuclein, | 6622 | 6622 | 6622 | s13204 | s226658 | s13206 |
| 5466 | D3 | D4 | 1434 | 652 | 22559 | 647,9 | 15,7 | 1,493 | H5A034 C | sample | Hm Drgbl | Hm Drgbl | Hm Drgbl | CPE002DX | CPE002EN | CPE002FJ | NM 00338 | NM 0033 | NM 0033 | UNC5C | UNC5C | UNC5C | unc-5 homolog C | unc-5 hon | unc-5 hon | 8633 | 8633 | 8633 | s16031 | s16032 | s16033 |
| 5499 | F11 | F12 | 1652 | 616,9 | 25889 | 687,9 | 15,7 | 1,493 | H5A034 C | sample | Hm Drgbl | Hm Drgbl | Hm Drgbl | CPE002DX | CPE002EN | CPE002FJ | NM 00374 | NM 0037 | NM 0037 | ALDH4A1 | ALDH4A1 | ALDH4A1 | aldehyde dehydro | aldehyde | aldehyde | 8659 | 8659 | 8659 | s16485 | s16484 | s16484 |
| 3590 | F21 | F22 | 434 | 1736 | 6804 | 225 | 15,7 | 1,51 | H5A034 C | sample | Hm Drgbl | Hm Drgbl | Hm Drgbl | CPE002DP | CPE002EH | CPE002F7 | NM 00083 | NM 0008 | NM 0008 | GRM2 | GRM2 | GRM2 | glutamate recept | glutamate | glutamate | 2912 | 2912 | 2912 | s6194 | s6195 | s6196 |
| 3686 | J21 | J22 | 843 | 1385 | 12953 | 323 | 15,7 | 1,51 | H5A034 C | sample | Hm Drgbl | Hm Drgbl | Hm Drgbl | CPE002DP | CPE002EH | CPE002F7 | NM 00086 | NM 0008 | NM 0008 | HTR6 | HTR6 | HTR6 | 5-hydroxytryptam | 5-hydroxy | 5-hydroxy | 3362 | 3362 | 3362 | s7059 | s7060 | s7061 |
| 3717 | K6 | K7 | 695 | 1314 | 10939 | 353,7 | 15,7 | 1,51 | H5A034 C | sample | Hm Drgbl | Hm Drgbl | Hm Drgbl | CPE002DP | CPE002EH | CPE002F7 | NM 00126 | NM 0012 | NM 0012 | CCR9 | CCR9 | CCR9 | chemokine (C-C m | chemokin | chemokin | 10803 | 10803 | 10803 | s21228 | s21229 | s21230 |
| 3838 | P7 | P8 | 1470 | 1484 | 23012 | 337,1 | 15,7 | 1,51 | H5A034 C | sample | Hm Drgbl | Hm Drgbl | Hm Drgbl | CPE002DP | CPE002EH | CPE002F7 | NM 00269 | NM 0026 | NM 0026 | P2RY4 | P2RY4 | P2RY4 | pyrimidinergic re | pyrimidine | pyrimidine | 5030 | 5030 | 5030 | s9968 | s9969 | s9970 |
| 8134 | C7 | C8 | 1107 | 589,9 | 17346 | 576,1 | 15,7 | 1,512 | H5A034 C | sample | Hm Drgbl | Hm Drgbl | Hm Drgbl | CPE002E5 | CPE002EQ | CPE002FB | NM 00007 | NM 0007 | NM 0007 | TOPBP1 | TOPBP1 | TOPBP1 | topoisomerase (D | topoisom | topoisom | 11073 | 11073 | 11073 | s21825 | s21826 | s21824 |
| 8168 | E16 | E17 | 948 | 618,9 | 14919 | 512,8 | 15,7 | 1,512 | H5A034 C | sample | Hm Drgbl | Hm Drgbl | Hm Drgbl | CPE002E5 | CPE002EQ | CPE002FB | NM 00113 | NM 0011 | NM 0011 | FRMD4B | FRMD4B | FRMD4B | FERM domain con | FERM dor | FERM dor | 23150 | 23150 | 23150 | s23147 | s23145 | s23146 |
| 8308 | K12 | K13 | 1551 | 672,6 | 24266 | 551,5 | 15,7 | 1,512 | H5A034 C | sample | Hm Drgbl | Hm Drgbl | Hm Drgbl | CPE002E5 | CPE002EQ | CPE002FB | NM 01531 | NM 0153 | NM 0153 | POFUT2 | POFUT2 | POFUT2 | protein O-fucosyl | protein O- | protein O- | 23275 | 23275 | 23275 | s23430 | s23431 | s23432 |
| 8334 | L14 | L15 | 1478 | 591 | 23243 | 466 | 15,7 | 1,512 | H5A034 C | sample | Hm Drgbl | Hm Drgbl | Hm Drgbl | CPE002E5 | CPE002EQ | CPE002FB | NM 01212 | NM 0121 | NM 0121 | TNPO3 | TNPO3 | TNPO3 | transportin 3 | transport | transport | 23534 | 23534 | 23534 | s24030 | s24031 | s24032 |
| 10064 | D16 | D17 | 1037 | 777,4 | 16255 | 156,5 | 15,7 | 1,51 | H5A034 C | sample | Hm Drgbl | Hm Drgbl | Hm Drgbl | CPE002EA | CPE002EV | CPE002FG | NM 03245 | NM 0324 | NM 0324 | SYVN1 | SYVN1 | SYVN1 | synovial apoptosi | synovial a | synovial a | 84447 | 84447 | 84447 | s39019 | s39020 | s39021 |
| 10069 | D20 | D21 | 1193 | 850,5 | 18716 | 156,9 | 15,7 | 1,51 | H5A034 C | sample | Hm Drgbl | Hm Drgbl | Hm Drgbl | CPE002EA | CPE002EV | CPE002FG | NM 01610 | NM 0161 | NM 0161 | LDOC1 | LDOC1 | LDOC1 | leucine zipper, do | leucine zi | leucine zi | 23641 | 23641 | 23641 | s24042 | s24043 | s24044 |
| 10140 | G2 | G3 | 1693 | 820,3 | 26635 | 145,2 | 15,7 | 1,51 | H5A034 C | sample | Hm Drgbl | Hm Drgbl | Hm Drgbl | CPE002EA | CPE002EV | CPE002FG | NM 03314 | NM 0331 | NM 0331 | SLC4A9 | SLC4A9 | SLC4A9 | solute carrier fam | solute car | solute car | 83697 | 83697 | 83697 | s38118 | s38119 | s38120 |
| 10225 | K1 | K2 | 1232 | 791,5 | 19315 | 141,8 | 15,7 | 1,51 | H5A034 C | sample | Hm Drgbl | Hm Drgbl | Hm Drgbl | CPE002EA | CPE002EV | CPE002FG | NM 02247 | NM 0224 | NM 0224 | ATP8B4 | ATP8B4 | ATP8B4 | ATPase, Class I, ty | ATPase, C | ATPase, C | 79895 | 79895 | 79895 | s36593 | s36594 | s36595 |
| 1702 | G7 | G8 | 935 | 701,2 | 14637 | 427,2 | 15,7 | 1,64 | H5A034 C | sample | Hm Drgbl | Hm Drgbl | Hm Drgbl | CPE002FW | CPE002G0 | CPE002G4 | NM 01448 | NM 0144 | NM 0144 | FRAG1 | FRAG1 | FRAG1 | FGF receptor activ | FGF recep | FGF recep | 27315 | 27315 | 27315 | s26139 | s26140 | s26141 |
| 21527 | A8 | A9 | 196 | 953,3 | 3084 | 580,9 | 15,7 | 1,643 | H5A034 C | sample | Hm Gen E | Hm Gen E | Hm Gen E | CPE002JU | CPE002OH | CPE002KX | NM 94196 | XM 9419 | XM 9419 | LOC65245 | LOC65245 | LOC65245 | similar to Prograr | similar to | similar to | 652455 | 652455 | 652455 | s57395 | s197986 | s227394 |
| 21858 | O3 | O4 | 54 | 897,8 | 839 | 729,9 | 15,7 | 1,643 | H5A034 C | sample | Hm Gen E | Hm Gen E | Hm Gen E | CPE002JU | CPE002OH | CPE002KX | NM 94258 | XM 9425 | XM 9425 | LOC65286 | LOC65286 | LOC65286 | similar to PDZ an | similar to | similar to | 652865 | 652865 | 652865 | s57526 | s57527 | s227441 |
| 21883 | P4 | P5 | 200 | 1162 | 3136 | 755,7 | 15,7 | 1,643 | H5A034 C | sample | Hm Gen E | Hm Gen E | Hm Gen E | CPE002JU | CPE002OH | CPE002KX | NM 29066 | XM 2906 | XM 2906 | LOC65425 | LOC65425 | LOC65425 | similar to transm | similar to | similar to | 654259 | 654259 | 654259 | s198129 | s198130 | s198130 |
| 4658 | C10 | C11 | 777 | 790,4 | 12221 | 685 | 15,7 | 1,676 | H5A034 C | sample | Hm Drgbl | Hm Drgbl | Hm Drgbl | CPE002DM | CPE002EJ | CPE002FI | NM 00102 | NM 0010 | NM 0010 | TMPRSS2 | TMPRSS2 | TMPRSS2 | transmembrane p | transmem | transmem | 7113 | 7113 | 7113 | s14236 | s14237 | s14238 |
| 10372 | A12 | A13 | 1317 | 857,2 | 20626 | 219,2 | 15,7 | 1,774 | H5A034 C | sample | Hm Drgbl | Hm Drgbl | Hm Drgbl | CPE002EB | CPE002EW | CPE002FH | NM 00101 | NM 0010 | NM 0010 | ERMAP | ERMAP | ERMAP | erythroblast mem | erythrobl | erythrobl | 114625 | 114625 | 114625 | s41564 | s41565 | s41566 |
| 10413 | B6 | B7 | 1049 | 881,6 | 16452 | 231,6 | 15,7 | 1,774 | H5A034 C | sample | Hm Drgbl | Hm Drgbl | Hm Drgbl | CPE002EB | CPE002EW | CPE002FH | NM 14451 | NM 1451 | NM 1451 | TTC18 | TTC18 | TTC18 | tetratricopeptide | tetratrico | tetratrico | 118491 | 118491 | 118491 | s42225 | s42224 | s42224 |
| 10634 | L10 | L11 | 607 | 924,8 | 9553 | 210 | 15,7 | 1,774 | H5A034 C | sample | Hm Drgbl | Hm Drgbl | Hm Drgbl | CPE002EB | CPE002EW | CPE002FH | NM 15323 | NM 1523 | NM 1523 | CPT1C | CPT1C | CPT1C | carnitine palmitoy | carnitine | carnitine | 126129 | 126129 | 126129 | s42960 | s42961 | s42962 |
| 10642 | L18 | L19 | 714 | 901,9 | 11178 | 283,2 | 15,7 | 1,774 | H5A034 C | sample | Hm Drgbl | Hm Drgbl | Hm Drgbl | CPE002EB | CPE002EW | CPE002FH | NM 17566 | NM 1756 | NM 1756 | NDUFA11 | NDUFA11 | NDUFA11 | NADH dehydroge | NADH del | NADH del | 126328 | 126328 | 126328 | s195674 | s195675 | s195673 |
| 10742 | P21 | P22 | 1321 | 871,2 | 20694 | 247,1 | 15,7 | 1,774 | H5A034 C | sample | Hm Drgbl | Hm Drgbl | Hm Drgbl | CPE002EB | CPE002EW | CPE002FH | NM 00111 | NM 0011 | NM 0011 | TXNRD3 | TXNRD3 | TXNRD3 | thioredoxin redu | thioredox | thioredox | 114112 | 114112 | 114112 | s41537 | s41536 | s41535 |
| 10749 | P6 | P7 | 1363 | 898,5 | 21446 | 260,1 | 15,7 | 1,774 | H5A034 C | sample | Hm Drgbl | Hm Drgbl | Hm Drgbl | CPE002EB | CPE002EW | CPE002FH | NM 14491 | NM 1449 | NM 1449 | KIRREL3 | KIRREL3 | KIRREL3 | kin actin-like | kin of IRR | kin of IRR | 84623 | 84623 | 84623 | s43403 | s43404 | s43403 |
| 22534 | K7 | K8 | 680 | 1163 | 10767 | 419,8 | 15,7 | 0,826 | H5A034 C | sample | Hm Gen E | Hm Gen E | Hm Gen E | CPE002JV | CPE002ON | CPE002KY | XM 00112 | XM 0011 | XM 0011 | LOC72871 | LOC72871 | LOC72871 | hypothetical prot | hypotheti | hypotheti | 728710 | 728710 | 728710 | s58917 | s58918 | s58918 |
| 23481 | C17 | C18 | 983 | 1594 | 15543 | 265,1 | 15,8 | 0,838 | H5A034 C | sample | Hm Gen E | Hm Gen E | Hm Gen E | CPE002KV | CPE002OP | CPE002L0 | XM 00112 | XM 0011 | XM 0011 | LOC73192 | LOC73192 | LOC73192 | hypothetical prot | hypotheti | hypotheti | 731929 | 731929 | 731929 | s61466 | s61467 | s61468 |
| 3243 | H11 | H12 | 1092 | 1095 | 17201 | 359,2 | 15,8 | 0,972 | H5A034 C | sample | Hm Drgbl | Hm Drgbl | Hm Drgbl | CPE002DP | CPE002EH | CPE002F7 | NM 00054 | NM 0005 | NM 0005 | HDHD1A | HDHD1A | HDHD1A | haloacid dehalog | haloacid c | haloacid c | 8226 | 8226 | 8226 | s15727 | s223957 | s15729 |
| 3260 | H5 | H6 | 1208 | 1165 | 19045 | 238,3 | 15,8 | 0,972 | H5A034 C | sample | Hm Drgbl | Hm Drgbl | Hm Drgbl | CPE002DP | CPE002EH | CPE002F7 | NM 00346 | NM 0034 | NM 0034 | PTP4A1 | PTP4A1 | PTP4A1 | protein tyrosine p | protein ty | protein ty | 7803 | 7803 | 7803 | s15362 | s15363 | s15364 |
| 3336 | K9 | K10 | 963 | 1231 | 15193 | 400,1 | 15,8 | 0,978 | H5A034 C | sample | Hm Drgbl | Hm Drgbl | Hm Drgbl | CPE002DP | CPE002EH | CPE002F7 | NM 00045 | NM 0004 | NM 0004 | PFKFB4 | PFKFB4 | PFKFB4 | 6-phosphofructo- | 6-phosph | 6-phosph | 5210 | 5210 | 5210 | s10360 | s10361 | s10362 |
| 3363 | M1 | M2 | 812 | 1212 | 12846 | 359,8 | 15,8 | 0,978 | H5A034 C | sample | Hm Drgbl | Hm Drgbl | Hm Drgbl | CPE002DP | CPE002EH | CPE002F7 | NM 00018 | NM 0001 | NM 0001 | PPP1CA | PPP1CA | PPP1CA | protein phosphat | protein pl | protein pl | 5499 | 5499 | 5499 | s10930 | s10931 | s10932 |
| 3243 | P2 | P3 | 1415 | 1148 | 22397 | 430,8 | 15,8 | 0,978 | H5A034 C | sample | Hm Drgbl | Hm Drgbl | Hm Drgbl | CPE002DP | CPE002EH | CPE002F7 | NM 00100 | NM 0010 | NM 0010 | PIP4K2A | PIP4K2A | PIP4K2A | phosphatidylinos | phosphati | phosphati | 5305 | 5305 | 5305 | s10558 | s10559 | s10560 |
| 19770 | H3 | H4 | 769 | 1096 | 12136 | 235,9 | 15,8 | 1,029 | H5A034 C | sample | Hm Gen E | Hm Gen E | Hm Gen E | CPE002JP | CPE002O4 | CPE002KR | NM 20710 | NM 2071 | NM 2071 | C17orf87 | C17orf87 | C17orf87 | chromosome 17 o | chromoso | chromoso | 388325 | 388325 | 388325 | s52096 | s52096 | s226425 |

| | | | | | | | | | | | |
|---|---|---|---|---|---|---|---|---|---|---|---|
| 19818 | J3 | J4 | 702 | 322,7 | 11061 | 389 | 15,8 | 1,029 | H5A034 C | sample | [illegible] |
| 6171 | B11 | B12 | 1547 | 584,8 | 24435 | 578,4 | 15,8 | 1,043 | H5A034 C | sample | [illegible] |
| 6510 | P14 | P15 | 1841 | 613,1 | 29036 | 537,3 | 15,8 | 1,043 | H5A034 C | sample | [illegible] |
| 20479 | F15 | F16 | 941 | 847,3 | 14847 | 206,7 | 15,8 | 1,053 | H5A034 C | sample | [illegible] |
| 20547 | I11 | I12 | 570 | 786,3 | 9014 | 217,2 | 15,8 | 1,053 | H5A034 C | sample | [illegible] |
| 20559 | I22 | I23 | 840 | 760,5 | 13242 | 195,5 | 15,8 | 1,053 | H5A034 C | sample | [illegible] |
| 20573 | J13 | J14 | 983 | 811,8 | 15486 | 160,3 | 15,8 | 1,053 | H5A034 C | sample | [illegible] |
| 20685 | N6 | N7 | 645 | 782,8 | 10181 | 198,6 | 15,8 | 1,053 | H5A034 C | sample | [illegible] |
| 13033 | P1 | P2 | 1198 | 1001 | 18985 | 153,1 | 15,8 | 1,071 | H5A034 C | sample | [illegible] |
| 13040 | P16 | P17 | 1375 | 952,8 | 21759 | 169,3 | 15,8 | 1,071 | H5A034 C | sample | [illegible] |
| 18527 | D8 | D9 | 1200 | 1026 | 18990 | 392,2 | 15,8 | 1,071 | H5A034 C | sample | [illegible] |
| 18538 | E18 | E19 | 1517 | 962,2 | 23895 | 215,9 | 15,8 | 1,071 | H5A034 C | sample | [illegible] |
| 17293 | A20 | A21 | 1599 | 1118 | 25297 | 298,4 | 15,8 | 1,078 | H5A034 C | sample | [illegible] |
| 17404 | F12 | F13 | 1598 | 1026 | 25224 | 212,1 | 15,8 | 1,078 | H5A034 C | sample | [illegible] |
| 17516 | J5 | J6 | 1279 | 913 | 20245 | 234,7 | 15,8 | 1,078 | H5A034 C | sample | [illegible] |
| 17551 | L15 | L16 | 1498 | 973,4 | 23641 | 202,9 | 15,8 | 1,078 | H5A034 C | sample | [illegible] |
| 9249 | B17 | B18 | 1565 | 1117 | 24760 | 145,4 | 15,8 | 1,097 | H5A034 C | sample | [illegible] |
| 9461 | K13 | K14 | 1384 | 1022 | 21875 | 158,7 | 15,8 | 1,097 | H5A034 C | sample | [illegible] |
| 18845 | B13 | B14 | 1277 | 1058 | 20120 | 355,6 | 15,8 | 1,101 | H5A034 C | sample | [illegible] |
| 19142 | N21 | N22 | 1083 | 930,4 | 17064 | 187,5 | 15,8 | 1,101 | H5A034 C | sample | [illegible] |
| 19147 | N4 | N5 | 1168 | 1064 | 18433 | 195,3 | 15,8 | 1,101 | H5A034 C | sample | [illegible] |
| 19165 | O20 | O21 | 1351 | 990,6 | 21409 | 220,7 | 15,8 | 1,101 | H5A034 C | sample | [illegible] |
| 19197 | P6 | P7 | 797 | 1152 | 12586 | 403,1 | 15,8 | 1,101 | H5A034 C | sample | [illegible] |
| 15186 | I3 | I4 | 1249 | 758,5 | 19785 | 123,5 | 15,8 | 1,105 | H5A034 C | sample | [illegible] |
| 15195 | J11 | J12 | 1066 | 753 | 16800 | 129 | 15,8 | 1,105 | H5A034 C | sample | [illegible] |
| 19232 | B16 | B17 | 1254 | 1208 | 19800 | 328,6 | 15,8 | 1,121 | H5A034 C | sample | [illegible] |
| 21056 | N16 | N17 | 1155 | 984,5 | 18270 | 305,5 | 15,8 | 1,121 | H5A034 C | sample | [illegible] |
| 21075 | O11 | O12 | 1537 | 937,8 | 24224 | 383,6 | 15,8 | 1,121 | H5A034 C | sample | [illegible] |
| 21177 | C17 | C18 | 950 | 900,2 | 15033 | 351,1 | 15,8 | 1,121 | H5A034 C | sample | [illegible] |
| 21451 | N4 | N5 | 1086 | 905,3 | 17130 | 195,9 | 15,8 | 1,121 | H5A034 C | sample | [illegible] |
| 14490 | L3 | L4 | 1212 | 785,7 | 19177 | 116,2 | 15,8 | 1,125 | H5A034 C | sample | [illegible] |
| 11206 | C7 | C8 | 1266 | 871,1 | 19975 | 221,8 | 15,8 | 1,145 | H5A034 C | sample | [illegible] |
| 11250 | E3 | E4 | 1258 | 812,6 | 19836 | 151,1 | 15,8 | 1,145 | H5A034 C | sample | [illegible] |
| 11443 | M4 | M5 | 1943 | 925 | 30658 | 195,2 | 15,8 | 1,145 | H5A034 C | sample | [illegible] |
| 11468 | N5 | N6 | 1291 | 772,9 | 20433 | 134,8 | 15,8 | 1,145 | H5A034 C | sample | [illegible] |
| 12362 | D10 | D11 | 823 | 1019 | 12985 | 149,1 | 15,8 | 1,188 | H5A034 C | sample | [illegible] |
| 12661 | P20 | P21 | 1802 | 1005 | 28387 | 227,4 | 15,8 | 1,188 | H5A034 C | sample | [illegible] |
| 10873 | F1 | F2 | 880 | 773,9 | 13927 | 520,5 | 15,8 | 1,197 | H5A034 C | sample | [illegible] |
| 10952 | I16 | I17 | 1153 | 724 | 18183 | 419 | 15,8 | 1,197 | H5A034 C | sample | [illegible] |
| 10986 | J3 | J4 | 1024 | 772,2 | 16209 | 499,4 | 15,8 | 1,197 | H5A034 C | sample | [illegible] |
| 11134 | P7 | P8 | 1376 | 798,7 | 21767 | 683,1 | 15,8 | 1,197 | H5A034 C | sample | [illegible] |
| 7501 | I20 | I21 | 1079 | 530,9 | 17063 | 359,5 | 15,8 | 1,206 | H5A034 C | sample | [illegible] |
| 7656 | O9 | O10 | 1853 | 588,5 | 29264 | 530,1 | 15,8 | 1,206 | H5A034 C | sample | [illegible] |
| 7680 | P9 | P10 | 1200 | 556,5 | 18900 | 541,3 | 15,8 | 1,206 | H5A034 C | sample | [illegible] |
| 16978 | D18 | D19 | 1386 | 953,2 | 21855 | 193,3 | 15,8 | 1,22 | H5A034 C | sample | [illegible] |
| 17004 | E2 | E3 | 1481 | 982,9 | 23374 | 194,6 | 15,8 | 1,22 | H5A034 C | sample | [illegible] |
| 17053 | G20 | G21 | 1584 | 968,2 | 25101 | 201,1 | 15,8 | 1,22 | H5A034 C | sample | [illegible] |
| 17087 | H8 | H9 | 1341 | 941 | 21240 | 165,3 | 15,8 | 1,22 | H5A034 C | sample | [illegible] |
| 17097 | I17 | I18 | 1479 | 877,8 | 23412 | 168 | 15,8 | 1,22 | H5A034 C | sample | [illegible] |
| 17275 | P4 | P5 | 791 | 1127 | 12532 | 253,3 | 15,8 | 1,22 | H5A034 C | sample | [illegible] |
| 6942 | B14 | B15 | 1589 | 643,2 | 25052 | 459 | 15,8 | 1,23 | H5A034 C | sample | [illegible] |
| 6984 | C9 | C10 | 1185 | 650,7 | 18708 | 546,6 | 15,8 | 1,23 | H5A034 C | sample | [illegible] |
| 7205 | M13 | M14 | 759 | 562 | 11994 | 472,9 | 15,8 | 1,23 | H5A034 C | sample | [illegible] |
| 7276 | P12 | P13 | 1450 | 573,3 | 22848 | 507,9 | 15,8 | 1,23 | H5A034 C | sample | [illegible] |
| 5106 | E3 | E4 | 1369 | 640 | 21587 | 567,5 | 15,8 | 1,239 | H5A034 C | sample | [illegible] |
| 5119 | F15 | F16 | 1458 | 588,3 | 23088 | 547,4 | 15,8 | 1,239 | H5A034 C | sample | [illegible] |
| 5148 | G2 | G3 | 1136 | 588,3 | 17953 | 468,3 | 15,8 | 1,239 | H5A034 C | sample | [illegible] |
| 5191 | I15 | I16 | 1074 | 591,5 | 16957 | 462,4 | 15,8 | 1,239 | H5A034 C | sample | [illegible] |
| 2395 | D4 | D5 | 1364 | 672,3 | 21601 | 450 | 15,8 | 1,244 | H5A034 C | sample | [illegible] |
| 2495 | H8 | H9 | 1463 | 687,4 | 23096 | 335,8 | 15,8 | 1,244 | H5A034 C | sample | [illegible] |
| 16548 | B2 | B3 | 987 | 1031 | 15623 | 332,6 | 15,8 | 1,249 | H5A034 C | sample | [illegible] |
| 16621 | E20 | E21 | 1353 | 1007 | 21385 | 304 | 15,8 | 1,249 | H5A034 C | sample | [illegible] |
| 16700 | H5 | H6 | 1042 | 1033 | 16468 | 303,1 | 15,8 | 1,249 | H5A034 C | sample | [illegible] |
| 16799 | L8 | L9 | 1305 | 941,8 | 20564 | 328,4 | 15,8 | 1,249 | H5A034 C | sample | [illegible] |
| 16830 | N14 | N15 | 1579 | 1003 | 24935 | 335,2 | 15,8 | 1,249 | H5A034 C | sample | [illegible] |
| 17828 | G5 | G6 | 1109 | 1055 | 17531 | 282,6 | 15,8 | 1,249 | H5A034 C | sample | [illegible] |
| 17925 | K6 | K7 | 829 | 1030 | 13102 | 405,8 | 15,8 | 1,249 | H5A034 C | sample | [illegible] |
| 8874 | B3 | B4 | 1721 | 1693 | 27155 | 154,7 | 15,8 | 1,269 | H5A034 C | sample | [illegible] |
| 8884 | C12 | C13 | 1570 | 1788 | 24836 | 160,5 | 15,8 | 1,269 | H5A034 C | sample | [illegible] |
| 9193 | P1 | P2 | 1134 | 1490 | 17871 | 147,5 | 15,8 | 1,269 | H5A034 C | sample | [illegible] |
| 4543 | N15 | N16 | 1024 | 714 | 16180 | 442,5 | 15,8 | 1,333 | H5A034 C | sample | [illegible] |
| 4602 | P3 | P4 | 1144 | 681,3 | 18096 | 466,8 | 15,8 | 1,333 | H5A034 C | sample | [illegible] |
| 53 | C13 | C14 | 545 | 1418 | 8616 | 735,1 | 15,8 | 1,345 | H5A034 C | sample | [illegible] |
| 104 | E16 | E17 | 574 | 1520 | 9047 | 422,9 | 15,8 | 1,345 | H5A034 C | sample | [illegible] |
| 126 | F14 | F15 | 605 | 1461 | 9560 | 394,1 | 15,8 | 1,345 | H5A034 C | sample | [illegible] |
| 135 | F22 | F23 | 624 | 1507 | 9839 | 519,9 | 15,8 | 1,345 | H5A034 C | sample | [illegible] |
| 244 | K12 | K13 | 94 | 1409 | 1484 | 634,9 | 15,8 | 1,345 | H5A034 C | sample | [illegible] |
| 347 | O19 | O20 | 486 | 1541 | 7673 | 657,8 | 15,8 | 1,345 | H5A034 C | sample | [illegible] |
| 1989 | C6 | C7 | 562 | 634,2 | 8879 | 431,8 | 15,8 | 1,374 | H5A034 C | sample | [illegible] |
| 2058 | F3 | F4 | 1269 | 623,8 | 20094 | 467 | 15,8 | 1,374 | H5A034 C | sample | [illegible] |
| 2095 | H15 | H16 | 974 | 604,3 | 15405 | 312,8 | 15,8 | 1,374 | H5A034 C | sample | [illegible] |
| 2207 | L8 | L9 | 1490 | 682 | 23613 | 444,5 | 15,8 | 1,374 | H5A034 C | sample | [illegible] |
| 6553 | B1 | B2 | 959 | 611 | 15136 | 519,3 | 15,8 | 1,386 | H5A034 C | sample | [illegible] |
| 4206 | P14 | P15 | 1279 | 687 | 20176 | 438,4 | 15,8 | 1,404 | H5A034 C | sample | [illegible] |
| 11574 | C14 | C15 | 1011 | 923,2 | 15948 | 163,7 | 15,8 | 1,411 | H5A034 C | sample | [illegible] |
| 11580 | C2 | C3 | 1272 | 876,5 | 20119 | 207 | 15,8 | 1,411 | H5A034 C | sample | [illegible] |
| 11601 | D17 | D18 | 1162 | 793,4 | 18415 | 163 | 15,8 | 1,411 | H5A034 C | sample | [illegible] |
| 11683 | G4 | G5 | 1320 | 848 | 20841 | 201,8 | 15,8 | 1,411 | H5A034 C | sample | [illegible] |
| 15370 | A18 | A19 | 1628 | 868,7 | 25690 | 186,7 | 15,8 | 1,436 | H5A034 C | sample | [illegible] |
| 15529 | H1 | H2 | 495 | 884 | 7840 | 174,4 | 15,8 | 1,436 | H5A034 C | sample | [illegible] |
| 6098 | O10 | O11 | 1833 | 762 | 28886 | 612,3 | 15,8 | 1,443 | H5A034 C | sample | [illegible] |
| 16203 | D11 | D12 | 1304 | 1032 | 20629 | 427,2 | 15,8 | 1,45 | H5A034 C | sample | [illegible] |
| 16221 | D6 | D7 | 1013 | 925,1 | 15999 | 369,4 | 15,8 | 1,45 | H5A034 C | sample | [illegible] |
| 16229 | E13 | E14 | 1372 | 963,3 | 21685 | 296,9 | 15,8 | 1,45 | H5A034 C | sample | [illegible] |
| 16258 | F18 | F19 | 1582 | 1068 | 25010 | 273,2 | 15,8 | 1,45 | H5A034 C | sample | [illegible] |
| 16269 | F6 | F7 | 938 | 1107 | 14851 | 308,4 | 15,8 | 1,45 | H5A034 C | sample | [illegible] |
| 16305 | H17 | H18 | 1294 | 951,6 | 20466 | 279,4 | 15,8 | 1,45 | H5A034 C | sample | [illegible] |
| 16375 | K15 | K16 | 864 | 993 | 13650 | 334,1 | 15,8 | 1,45 | H5A034 C | sample | [illegible] |
| 13820 | P5 | P6 | 1571 | 1023 | 24874 | 197,4 | 15,8 | 1,484 | H5A034 C | sample | [illegible] |
| 946 | H18 | H19 | 1433 | 746,5 | 22571 | 355,9 | 15,8 | 1,498 | H5A034 C | sample | [illegible] |
| 1052 | L5 | L6 | 1073 | 708,6 | 16924 | 387,3 | 15,8 | 1,498 | H5A034 C | sample | [illegible] |
| 5496 | E9 | E10 | 1354 | 678,3 | 21427 | 621,5 | 15,8 | 1,505 | H5A034 C | sample | [illegible] |
| 5545 | H1 | H2 | 571 | 693,3 | 9025 | 545,6 | 15,8 | 1,505 | H5A034 C | sample | [illegible] |
| 5556 | H2 | H3 | 1670 | 680,7 | 26333 | 558,6 | 15,8 | 1,505 | H5A034 C | sample | [illegible] |
| 5578 | I18 | I19 | 1243 | 659,5 | 19667 | 510,8 | 15,8 | 1,505 | H5A034 C | sample | [illegible] |
| 3567 | E22 | E23 | 684 | 1652 | 10824 | 438,7 | 15,8 | 1,519 | H5A034 C | sample | [illegible] |
| 3775 | N15 | N16 | 881 | 1309 | 13900 | 345,5 | 15,8 | 1,519 | H5A034 C | sample | [illegible] |
| 8106 | B3 | B4 | 1179 | 584,9 | 18614 | 519,5 | 15,8 | 1,519 | H5A034 C | sample | [illegible] |
| 8331 | L11 | L12 | 1602 | 587,1 | 25358 | 532,4 | 15,8 | 1,519 | H5A034 C | sample | [illegible] |
| 8406 | O14 | O15 | 884 | 550,6 | 13948 | 617,2 | 15,8 | 1,519 | H5A034 C | sample | [illegible] |
| 10004 | A5 | A6 | 1381 | 896,2 | 21788 | 195,1 | 15,8 | 1,519 | H5A034 C | sample | [illegible] |
| 10019 | B19 | B20 | 1716 | 987,1 | 27156 | 181,4 | 15,8 | 1,519 | H5A034 C | sample | [illegible] |
| 10046 | C21 | C22 | 1473 | 818,6 | 23252 | 152,7 | 15,8 | 1,519 | H5A034 C | sample | [illegible] |
| 10052 | C5 | C6 | 1406 | 854,3 | 22229 | 155,1 | 15,8 | 1,519 | H5A034 C | sample | [illegible] |
| 10102 | E7 | E8 | 1297 | 810,7 | 20502 | 197,4 | 15,8 | 1,519 | H5A034 C | sample | [illegible] |
| 10155 | H11 | H12 | 1098 | 744,6 | 17379 | 131,1 | 15,8 | 1,519 | H5A034 C | sample | [illegible] |
| 10183 | I15 | I16 | 1575 | 931,1 | 24849 | 144 | 15,8 | 1,519 | H5A034 C | sample | [illegible] |
| 10273 | M1 | M2 | 1429 | 833,1 | 22512 | 146,9 | 15,8 | 1,519 | H5A034 C | sample | [illegible] |
| 1736 | I16 | I17 | 605 | 719 | 9537 | 363,7 | 15,8 | 1,654 | H5A034 C | sample | [illegible] |
| 1830 | M14 | M15 | 1097 | 682,5 | 17367 | 352,8 | 15,8 | 1,654 | H5A034 C | sample | [illegible] |
| 1906 | P18 | P19 | 1476 | 683,9 | 23339 | 451,1 | 15,8 | 1,654 | H5A034 C | sample | [illegible] |
| 21549 | B6 | B7 | 142 | 871,4 | 2250 | 837,1 | 15,8 | 1,663 | H5A034 C | sample | [illegible] |
| 21552 | B9 | B10 | 153 | 955,2 | 2411 | 727,9 | 15,8 | 1,663 | H5A034 C | sample | [illegible] |
| 21629 | F13 | F14 | 357 | 1010 | 5658 | 612,3 | 15,8 | 1,663 | H5A034 C | sample | [illegible] |
| 21685 | H20 | H21 | 557 | 1114 | 8806 | 867,6 | 15,8 | 1,663 | H5A034 C | sample | [illegible] |
| 4977 | P17 | P18 | 433 | 846,3 | 6836 | 640,4 | 15,8 | 1,736 | H5A034 C | sample | [illegible] |
| 10730 | P10 | P11 | 1445 | 799,6 | 22896 | 214 | 15,8 | 1,785 | H5A034 C | sample | [illegible] |
| 10735 | P15 | P16 | 1275 | 798,1 | 20201 | 216,3 | 15,8 | 1,785 | H5A034 C | sample | [illegible] |
| 21944 | C16 | C17 | 653 | 1419 | 10383 | 369,2 | 15,9 | 0,755 | H5A034 C | sample | [illegible] |
| 22215 | N22 | N23 | 1140 | 1390 | 18144 | 295,1 | 15,9 | 0,755 | H5A034 C | sample | [illegible] |
| 22378 | E18 | E19 | 999 | 1341 | 15861 | 305,3 | 15,9 | 0,82 | H5A034 C | sample | [illegible] |
| 22591 | N15 | N16 | 829 | 1355 | 13192 | 366,7 | 15,9 | 0,82 | H5A034 C | sample | [illegible] |
| 23706 | L3 | L4 | 541 | 1586 | 8575 | 184,2 | 15,9 | 0,844 | H5A034 C | sample | [illegible] |
| 23759 | N8 | N9 | 1098 | 1540 | 17442 | 184 | 15,9 | 0,844 | H5A034 C | sample | [illegible] |
| 15876 | F2 | F3 | 1594 | 843 | 25367 | 112,5 | 15,9 | 0,886 | H5A034 C | sample | [illegible] |
| 3095 | A8 | A9 | 806 | 1189 | 12825 | 401,4 | 15,9 | 0,985 | H5A034 C | sample | [illegible] |
| 3101 | B13 | B14 | 1081 | 1272 | 17216 | 408,8 | 15,9 | 0,985 | H5A034 C | sample | [illegible] |
| 3244 | H12 | H13 | 1048 | 1283 | 16700 | 439,5 | 15,9 | 0,985 | H5A034 C | sample | [illegible] |
| 3340 | L12 | L13 | 1166 | 1304 | 18541 | 448,6 | 15,9 | 0,985 | H5A034 C | sample | [illegible] |
| 11931 | B11 | B12 | 1183 | 839,8 | 18837 | 125,6 | 15,9 | 1,019 | H5A034 C | sample | [illegible] |
| 12145 | K1 | K2 | 1551 | 794,6 | 24663 | 114,6 | 15,9 | 1,019 | H5A034 C | sample | [illegible] |
| 18298 | K18 | K19 | 607 | 1050 | 9671 | 340,9 | 15,9 | 1,023 | H5A034 C | sample | [illegible] |
| 18373 | N20 | N21 | 1603 | 1004 | 25412 | 260 | 15,9 | 1,023 | H5A034 C | sample | [illegible] |
| 6199 | C15 | C16 | 755 | 516,8 | 11990 | 545,9 | 15,9 | 1,05 | H5A034 C | sample | [illegible] |
| 6357 | I6 | I7 | 1318 | 588,7 | 20983 | 425,9 | 15,9 | 1,05 | H5A034 C | sample | [illegible] |
| 6511 | P15 | P16 | 1706 | 539,6 | 27071 | 547,5 | 15,9 | 1,05 | H5A034 C | sample | [illegible] |
| 20410 | C18 | C19 | 1028 | 799 | 16341 | 189,4 | 15,9 | 1,06 | H5A034 C | sample | [illegible] |
| 20428 | D12 | D13 | 739 | 739 | 11761 | 168,7 | 15,9 | 1,06 | H5A034 C | sample | [illegible] |
| 20437 | D20 | D21 | 758 | 782,2 | 12059 | 159,7 | 15,9 | 1,06 | H5A034 C | sample | [illegible] |
| 20444 | D5 | D6 | 1242 | 787,8 | 19709 | 248 | 15,9 | 1,06 | H5A034 C | sample | [illegible] |
| 20455 | E15 | E16 | 798 | 805,7 | 12689 | 230,1 | 15,9 | 1,06 | H5A034 C | sample | [illegible] |
| 20511 | G22 | G23 | 1074 | 800,6 | 17089 | 210,7 | 15,9 | 1,06 | H5A034 C | sample | [illegible] |
| 20601 | K17 | K18 | 1052 | 740,7 | 16778 | 146,6 | 15,9 | 1,06 | H5A034 C | sample | [illegible] |
| 12865 | I1 | I2 | 2140 | 1140 | 33963 | 177,7 | 15,9 | 1,078 | H5A034 C | sample | [illegible] |
| 17292 | A2 | A3 | 742 | 1069 | 11804 | 262,1 | 15,9 | 1,085 | H5A034 C | sample | [illegible] |
| 17308 | B12 | B13 | 1365 | 932,3 | 21749 | 312,6 | 15,9 | 1,085 | H5A034 C | sample | [illegible] |
| 17335 | C15 | C16 | 964 | 1014 | 15351 | 292,9 | 15,9 | 1,085 | H5A034 C | sample | [illegible] |
| 17357 | D13 | D14 | 1319 | 1062 | 20932 | 183,7 | 15,9 | 1,085 | H5A034 C | sample | [illegible] |
| 17373 | D6 | D7 | 1356 | 971,3 | 21610 | 248,5 | 15,9 | 1,085 | H5A034 C | sample | [illegible] |
| 17403 | F11 | F12 | 1401 | 953,5 | 22342 | 193,5 | 15,9 | 1,085 | H5A034 C | sample | [illegible] |
| 17414 | F21 | F22 | 1569 | 894,5 | 24939 | 204 | 15,9 | 1,085 | H5A034 C | sample | [illegible] |
| 17621 | O13 | O14 | 1376 | 906,9 | 21919 | 318,2 | 15,9 | 1,085 | H5A034 C | sample | [illegible] |
| 17646 | P14 | P15 | 1153 | 1007 | 18390 | 219 | 15,9 | 1,085 | H5A034 C | sample | [illegible] |
| 9223 | A15 | A16 | 1599 | 1184 | 25446 | 202,8 | 15,9 | 1,104 | H5A034 C | sample | [illegible] |
| 9411 | I11 | I12 | 1415 | 1101 | 22538 | 129,9 | 15,9 | 1,104 | H5A034 C | sample | [illegible] |
| 19070 | K21 | K22 | 1277 | 976,8 | 20254 | 239,9 | 15,9 | 1,108 | H5A034 C | sample | [illegible] |
| 19105 | M1 | M2 | 1584 | 1015 | 25153 | 191,8 | 15,9 | 1,108 | H5A034 C | sample | [illegible] |
| 9638 | B21 | B22 | 1740 | 1171 | 27718 | 185,7 | 15,9 | 1,112 | H5A034 C | sample | [illegible] |
| 9678 | D14 | D15 | 1564 | 1127 | 24865 | 166,7 | 15,9 | 1,112 | H5A034 C | sample | [illegible] |
| 9810 | I3 | I4 | 1518 | 1007 | 24123 | 180,7 | 15,9 | 1,112 | H5A034 C | sample | [illegible] |
| 8647 | I15 | I16 | 1705 | 997,4 | 27075 | 105,5 | 15,9 | 1,124 | H5A034 C | sample | [illegible] |
| 8702 | K21 | K22 | 1817 | 1121 | 28898 | 187,3 | 15,9 | 1,124 | H5A034 C | sample | [illegible] |
| 19328 | F16 | F17 | 1342 | 1218 | 21275 | 246,3 | 15,9 | 1,128 | H5A034 C | sample | [illegible] |
| 19440 | J9 | J10 | 1044 | 1202 | 16586 | 267,2 | 15,9 | 1,128 | H5A034 C | sample | [illegible] |
| 19520 | N16 | N17 | 1056 | 1188 | 16843 | 302,9 | 15,9 | 1,128 | H5A034 C | sample | [illegible] |
| 19557 | O6 | O7 | 924 | 1162 | 14691 | 350,8 | 15,9 | 1,128 | H5A034 C | sample | [illegible] |
| 19583 | P8 | P9 | 1289 | 1296 | 20542 | 399,6 | 15,9 | 1,128 | H5A034 C | sample | [illegible] |
| 20934 | I14 | I15 | 1239 | 959,1 | 19677 | 268,8 | 15,9 | 1,128 | H5A034 C | sample | [illegible] |
| 21048 | M9 | M10 | 695 | 1126 | 11025 | 352,9 | 15,9 | 1,128 | H5A034 C | sample | [illegible] |
| 21165 | B6 | B7 | 779 | 988,7 | 12421 | 358,3 | 15,9 | 1,128 | H5A034 C | sample | [illegible] |
| 21213 | D6 | D7 | 1147 | 954,4 | 18288 | 355 | 15,9 | 1,128 | H5A034 C | sample | [illegible] |
| 21260 | F5 | F6 | 753 | 858,7 | 11959 | 248,4 | 15,9 | 1,128 | H5A034 C | sample | [illegible] |
| 14425 | J1 | J2 | 1075 | 803,1 | 17061 | 126,1 | 15,9 | 1,132 | H5A034 C | sample | [illegible] |
| 11220 | D2 | D3 | 1341 | 872,9 | 21334 | 159,3 | 15,9 | 1,152 | H5A034 C | sample | [illegible] |
| 11319 | H22 | H23 | 1675 | 850,8 | 26674 | 187,1 | 15,9 | 1,152 | H5A034 C | sample | [illegible] |
| 11351 | I8 | I9 | 1126 | 814,1 | 17863 | 149,3 | 15,9 | 1,152 | H5A034 C | sample | [illegible] |
| 11373 | J6 | J7 | 1659 | 836,3 | 26377 | 148,8 | 15,9 | 1,152 | H5A034 C | sample | [illegible] |
| 11481 | O17 | O18 | 970 | 791,8 | 15393 | 151 | 15,9 | 1,152 | H5A034 C | sample | [illegible] |
| 11085 | N6 | N7 | 1173 | 776,6 | 18702 | 452,2 | 15,9 | 1,205 | H5A034 C | sample | [illegible] |
| 11125 | P20 | P21 | 1185 | 900,8 | 18860 | 1045 | 15,9 | 1,205 | H5A034 C | sample | [illegible] |
| 7315 | A4 | A5 | 723 | 580,4 | 11462 | 600,8 | 15,9 | 1,214 | H5A034 C | sample | [illegible] |
| 7326 | B14 | B15 | 1823 | 593,7 | 28899 | 482,7 | 15,9 | 1,214 | H5A034 C | sample | [illegible] |
| 7350 | C14 | C15 | 719 | 532,4 | 11455 | 454,7 | 15,9 | 1,214 | H5A034 C | sample | [illegible] |
| 7506 | I3 | I4 | 650 | 587,4 | 10306 | 506,6 | 15,9 | 1,214 | H5A034 C | sample | [illegible] |
| 16904 | A16 | A17 | 1515 | 1188 | 24098 | 340,4 | 15,9 | 1,228 | H5A034 C | sample | [illegible] |
| 16950 | C14 | C15 | 1356 | 1010 | 21556 | 241,8 | 15,9 | 1,228 | H5A034 C | sample | [illegible] |
| 17018 | F10 | F11 | 1149 | 946,4 | 18253 | 169,1 | 15,9 | 1,228 | H5A034 C | sample | [illegible] |
| 17140 | K12 | K13 | 1517 | 1044 | 24155 | 258,2 | 15,9 | 1,228 | H5A034 C | sample | [illegible] |
| 17279 | P8 | P9 | 1152 | 1079 | 18271 | 220,5 | 15,9 | 1,228 | H5A034 C | sample | [illegible] |
| 6994 | D18 | D19 | 1229 | 559,8 | 19512 | 529,5 | 15,9 | 1,237 | H5A034 C | sample | [illegible] |
| 7007 | D8 | D9 | 1106 | 552,2 | 17561 | 516 | 15,9 | 1,237 | H5A034 C | sample | [illegible] |
| 7027 | E4 | E5 | 1262 | 541,9 | 20072 | 518 | 15,9 | 1,237 | H5A034 C | sample | [illegible] |
| 7280 | P16 | P17 | 928 | 520 | 14795 | 545,2 | 15,9 | 1,237 | H5A034 C | sample | [illegible] |
| 5062 | C7 | C8 | 813 | 618,5 | 12903 | 590,3 | 15,9 | 1,247 | H5A034 C | sample | [illegible] |
| 5142 | G14 | G15 | 1080 | 619,9 | 17223 | 438,9 | 15,9 | 1,247 | H5A034 C | sample | [illegible] |
| 5212 | J12 | J13 | 1312 | 592,6 | 20871 | 531,9 | 15,9 | 1,247 | H5A034 C | sample | [illegible] |
| 5221 | J20 | J21 | 1220 | 660,3 | 19409 | 549,5 | 15,9 | 1,247 | H5A034 C | sample | [illegible] |
| 5261 | L13 | L14 | 1557 | 594,9 | 24765 | 523,3 | 15,9 | 1,247 | H5A034 C | sample | [illegible] |
| 5287 | M15 | M16 | 1393 | 670,1 | 22187 | 498,6 | 15,9 | 1,247 | H5A034 C | sample | [illegible] |
| 5336 | O16 | O17 | 1367 | 564,7 | 21742 | 605,7 | 15,9 | 1,247 | H5A034 C | sample | [illegible] |
| 16755 | K11 | K12 | 1350 | 990,6 | 21433 | 345,2 | 15,9 | 1,257 | H5A034 C | sample | [illegible] |
| 16884 | P2 | P3 | 368 | 1059 | 5862 | 171,1 | 15,9 | 1,257 | H5A034 C | sample | [illegible] |
| 17700 | B2 | B3 | 828 | 1038 | 13150 | 278,2 | 15,9 | 1,257 | H5A034 C | sample | [illegible] |
| 17722 | C18 | C19 | 749 | 983,5 | 11940 | 234,3 | 15,9 | 1,257 | H5A034 C | sample | [illegible] |
| 17755 | D4 | D5 | 756 | 1038 | 12026 | 376,9 | 15,9 | 1,257 | H5A034 C | sample | [illegible] |
| 17831 | G8 | G9 | 1144 | 1108 | 18138 | 317,3 | 15,9 | 1,257 | H5A034 C | sample | [illegible] |
| 17891 | J19 | J20 | 1681 | 967,5 | 26771 | 244,1 | 15,9 | 1,257 | H5A034 C | sample | [illegible] |
| 17906 | K10 | K11 | 1033 | 955,6 | 16409 | 240,4 | 15,9 | 1,257 | H5A034 C | sample | [illegible] |
| 18010 | O18 | O19 | 1355 | 1062 | 21496 | 428,6 | 15,9 | 1,257 | H5A034 C | sample | [illegible] |
| 8846 | A21 | A22 | 1387 | 1742 | 22120 | 180,5 | 15,9 | 1,277 | H5A034 C | sample | [illegible] |
| 8943 | E22 | E23 | 1785 | 1654 | 28374 | 236,4 | 15,9 | 1,277 | H5A034 C | sample | [illegible] |
| 9130 | M18 | M19 | 2062 | 1730 | 32836 | 231 | 15,9 | 1,277 | H5A034 C | sample | [illegible] |
| 4252 | B12 | B13 | 1058 | 659,1 | 16836 | 376,8 | 15,9 | 1,342 | H5A034 C | sample | [illegible] |
| 4462 | J7 | J8 | 606 | 673,5 | 9615 | 417,4 | 15,9 | 1,342 | H5A034 C | sample | [illegible] |
| 116 | E5 | E6 | 128 | 1178 | 2040 | 639 | 15,9 | 1,353 | H5A034 C | sample | [illegible] |
| 379 | P4 | P5 | 613 | 1503 | 9760 | 834,2 | 15,9 | 1,353 | H5A034 C | sample | [illegible] |

| | | | | | | | | | | | | | | | | | | | | | | | | | | | | | | | |
|---|---|---|---|---|---|---|---|---|---|---|---|---|---|---|---|---|---|---|---|---|---|---|---|---|---|---|---|---|---|---|---|
| 381 | P6 | P7 | 424 | 1341 | 6721 | 746,5 | 15,9 | 1,353 | H5A034 C | sample | Hm Drgbl | Hm Drgbl | Hm Drgbl | CPE002DT | CPE002EJ | CPE002F9 | NM_13362 | NM_1336 | NM_1336 | SYN2 | SYN2 | SYN2 | synapsin II | synapsin I | synapsin I | 6854 | 6854 | 6854 | s13685 | s13686 | s13687 |
| 1939 | A4 | A5 | 1023 | 689 | 16280 | 511,9 | 15,9 | 1,383 | H5A034 C | sample | Hm Drgbl | Hm Drgbl | Hm Drgbl | CPE002FX | CPE002G1 | CPE002G5 | NM_00100 | NM_0010 | NM_0010 | ZNF676 | ZNF676 | ZNF676 | zinc finger protei | zinc finge | zinc finge | 163223 | 163223 | 163223 | s223385 | s46439 | s46440 |
| 6685 | G20 | G21 | 1084 | 643,3 | 17192 | 457,5 | 15,9 | 1,395 | H5A034 C | sample | Hm Drgbl | Hm Drgbl | Hm Drgbl | CPE002E1 | CPE002EY | CPE002FJ | NM_00062 | NM_0006 | NM_0006 | IL10RB | IL10RB | IL10RB | interleukin 10 rec | interleuki | interleuki | 3588 | 3588 | 3588 | s7347 | s7348 | s7349 |
| 6803 | L19 | L20 | 791 | 520,7 | 12587 | 483,5 | 15,9 | 1,395 | H5A034 C | sample | Hm Drgbl | Hm Drgbl | Hm Drgbl | CPE002E1 | CPE002EY | CPE002FJ | NM_00019 | NM_0001 | NM_0001 | HSD11B2 | HSD11B2 | HSD11B2 | hydroxysteroid (1 | hydroxyst | hydroxyst | 3291 | 3291 | 3291 | s6935 | s6936 | s6937 |
| 4162 | N18 | N19 | 822 | 743,5 | 13083 | 400,3 | 15,9 | 1,413 | H5A034 C | sample | Hm Drgbl | Hm Drgbl | Hm Drgbl | CPE002DS | CPE002EI | CPE002F8 | NM_00651 | NM_0065 | NM_0065 | SAA4 | SAA4 | SAA4 | serum amyloid A4 | serum am | serum am | 6291 | 6291 | 6291 | s12448 | s224567 | s12447 |
| 4207 | P15 | P16 | 1138 | 616,9 | 18147 | 452,6 | 15,9 | 1,413 | H5A034 C | sample | Hm Drgbl | Hm Drgbl | Hm Drgbl | CPE002DS | CPE002EI | CPE002F8 | NM_00285 | NM_0028 | NM_0028 | RAD1 | RAD1 | RAD1 | RAD1 homolog (S | RAD1 hon | RAD1 hon | 5810 | 5810 | 5810 | s11590 | s11588 | s11589 |
| 11598 | D14 | D15 | 739 | 853,6 | 11760 | 160,1 | 15,9 | 1,42 | H5A034 C | sample | Hm Gen E | Hm Gen E | Hm Gen E | CPE002JA | CPE002NJ | CPE002JZ | NM_00531 | NM_0053 | NM_0053 | H1F0 | H1F0 | H1F0 | H1 histone family | H1 histon | H1 histon | 3005 | 3005 | 3005 | s6394 | s6395 | s6396 |
| 11696 | H16 | H17 | 1188 | 790,1 | 18926 | 145,9 | 15,9 | 1,42 | H5A034 C | sample | Hm Gen E | Hm Gen E | Hm Gen E | CPE002JA | CPE002NJ | CPE002JZ | NM_00211 | NM_0021 | NM_0021 | HLA-DMB | HLA-DMB | HLA-DMB | major histocompa | major hist | major hist | 3109 | 3109 | 3109 | s6579 | s6580 | s6581 |
| 11779 | K4 | K5 | 908 | 844,8 | 14477 | 180,3 | 15,9 | 1,42 | H5A034 C | sample | Hm Gen E | Hm Gen E | Hm Gen E | CPE002JA | CPE002NJ | CPE002JZ | NM_00014 | NM_0001 | NM_0001 | FXN | FXN | FXN | frataxin | frataxin | frataxin | 2395 | 2395 | 2395 | s5360 | s5361 | s5362 |
| 15368 | A16 | A17 | 972 | 966,5 | 15478 | 232,5 | 15,9 | 1,445 | H5A034 C | sample | Hm Gen E | Hm Gen E | Hm Gen E | CPE002JE | CPE002NT | CPE002K9 | NM_02300 | NM_0230 | NM_0230 | MARCKSL1 | MARCKSL1 | MARCKSL1 | MARCKS-like 1 | MARCKS-l | MARCKS-l | 65108 | 65108 | 65108 | s35209 | s35210 | s35211 |
| 15530 | H10 | H11 | 984 | 762,7 | 15611 | 141,6 | 15,9 | 1,445 | H5A034 C | sample | Hm Gen E | Hm Gen E | Hm Gen E | CPE002JE | CPE002NT | CPE002K9 | NM_03318 | NM_0331 | NM_0331 | OR51B2 | OR51B2 | OR51B2 | olfactory receptor | olfactory r | olfactory r | 79345 | 79345 | 79345 | s35746 | s35747 | s35748 |
| 15564 | I2 | I3 | 1279 | 890,4 | 20274 | 169,6 | 15,9 | 1,445 | H5A034 C | sample | Hm Gen E | Hm Gen E | Hm Gen E | CPE002JE | CPE002NT | CPE002K9 | NM_02403 | NM_0240 | NM_0240 | GDAP1L1 | GDAP1L1 | GDAP1L1 | ganglioside-induc | gangliosid | gangliosid | 78997 | 78997 | 78997 | s35390 | s35391 | s228169 |
| 15604 | K12 | K13 | 1449 | 859,6 | 22968 | 185,3 | 15,9 | 1,445 | H5A034 C | sample | Hm Gen E | Hm Gen E | Hm Gen E | CPE002JE | CPE002NT | CPE002K9 | NM_00162 | NM_0016 | NM_0016 | AHNAK | AHNAK | AHNAK | AHNAK nucleopro | AHNAK nu | AHNAK nu | 79026 | 79026 | 79026 | s195372 | s200547 | s200548 |
| 15666 | M3 | M4 | 815 | 880,7 | 12978 | 152,3 | 15,9 | 1,445 | H5A034 C | sample | Hm Gen E | Hm Gen E | Hm Gen E | CPE002JE | CPE002NT | CPE002K9 | NM_02234 | NM_0223 | NM_0223 | IFRG15 | IFRG15 | IFRG15 | interferon respon | interferon | interferon | 64163 | 64163 | 64163 | s34516 | s34517 | s34518 |
| 5878 | E7 | E8 | 793 | 736,7 | 12629 | 613,7 | 15,9 | 1,452 | H5A034 C | sample | Hm Drgbl | Hm Drgbl | Hm Drgbl | CPE002DY | CPE002F1 | CPE002FM | NM_00109 | NM_0010 | NM_0010 | ACLY | ACLY | ACLY | ATP citrate lyase | ATP citrat | ATP citrat | 47 | 47 | 47 | s915 | s916 | s917 |
| 5975 | I8 | I9 | 729 | 667,2 | 11579 | 309,9 | 15,9 | 1,452 | H5A034 C | sample | Hm Drgbl | Hm Drgbl | Hm Drgbl | CPE002DY | CPE002F1 | CPE002FM | NM_00103 | NM_0016 | NM_0016 | ATF3 | ATF3 | ATF3 | activating transcr | activating | activating | 467 | 467 | 467 | s224089 | s1699 | s1701 |
| 1279 | F15 | F16 | 1098 | 694,9 | 17408 | 415,5 | 15,9 | 1,459 | H5A034 C | sample | Hm Drgbl | Hm Drgbl | Hm Drgbl | CPE002FV | CPE002FZ | CPE002G3 | NM_18339 | NM_1833 | NM_1833 | CADPS | CADPS | CADPS | Ca2+-dependent | Ca2+-depe | Ca2+-depe | 8618 | 8618 | 8618 | s16393 | s16394 | s16395 |
| 16198 | C7 | C8 | 1030 | 970,8 | 16382 | 354,4 | 15,9 | 1,459 | H5A034 C | sample | Hm Gen E | Hm Gen E | Hm Gen E | CPE002JG | CPE002NV | CPE002KB | NM_03092 | NM_0309 | NM_0309 | TSPAN14 | TSPAN14 | TSPAN14 | tetraspanin 14 | tetraspan | tetraspan | 81619 | 81619 | 81619 | s37719 | s37720 | s37721 |
| 16243 | E4 | E5 | 946 | 942,3 | 15004 | 364,1 | 15,9 | 1,459 | H5A034 C | sample | Hm Gen E | Hm Gen E | Hm Gen E | CPE002JG | CPE002NV | CPE002KB | NM_00101 | NM_0010 | NM_0010 | FYTTD1 | FYTTD1 | FYTTD1 | forty-two-three d | forty-two- | forty-two- | 84248 | 84248 | 84248 | s38718 | s38719 | s38720 |
| 16317 | H6 | H7 | 416 | 1067 | 6634 | 339,2 | 15,9 | 1,459 | H5A034 C | sample | Hm Gen E | Hm Gen E | Hm Gen E | CPE002JG | CPE002NV | CPE002KB | NM_03293 | NM_0329 | NM_0329 | MT4 | MT4 | MT4 | metallothionein 4 | metalloth | metalloth | 84560 | 84560 | 84560 | s39158 | s195476 | s195477 |
| 16416 | L9 | L10 | 956 | 862,6 | 15210 | 350,7 | 15,9 | 1,459 | H5A034 C | sample | Hm Gen E | Hm Gen E | Hm Gen E | CPE002JG | CPE002NV | CPE002KB | NM_03213 | NM_0321 | NM_0321 | QRICH2 | QRICH2 | QRICH2 | glutamine rich 2 | glutamine | glutamine | 84074 | 84074 | 84074 | s38462 | s38463 | s38464 |
| 16483 | O4 | O5 | 863 | 943,5 | 13709 | 434,8 | 15,9 | 1,459 | H5A034 C | sample | Hm Gen E | Hm Gen E | Hm Gen E | CPE002JG | CPE002NV | CPE002KB | NM_03237 | NM_0323 | NM_0323 | ZNF414 | ZNF414 | ZNF414 | zinc finger protei | zinc finge | zinc finge | 84330 | 84330 | 84330 | s38932 | s195468 | s228361 |
| 13555 | E4 | E5 | 824 | 960,9 | 13065 | 178,6 | 15,9 | 1,493 | H5A034 C | sample | Hm Gen E | Hm Gen E | Hm Gen E | CPE002JC | CPE002NC | CPE002K4 | NM_01590 | NM_0159 | NM_0159 | NUDT13 | NUDT13 | NUDT13 | nudix (nucleoside | nudix (nu | nudix (nu | 25961 | 25961 | 25961 | s24829 | s24830 | s24831 |
| 13573 | F20 | F21 | 1194 | 1032 | 19014 | 170,4 | 15,9 | 1,493 | H5A034 C | sample | Hm Gen E | Hm Gen E | Hm Gen E | CPE002JC | CPE002NC | CPE002K4 | NM_01234 | NM_0123 | NM_0123 | FBXO9 | FBXO9 | FBXO9 | F-box protein 9 | F-box pro | F-box pro | 26268 | 26268 | 26268 | s25331 | s25332 | s25333 |
| 13647 | I22 | I23 | 1235 | 995,9 | 19681 | 167 | 15,9 | 1,493 | H5A034 C | sample | Hm Gen E | Hm Gen E | Hm Gen E | CPE002JC | CPE002NC | CPE002K4 | NM_01557 | NM_0155 | NM_0155 | AUTS2 | AUTS2 | AUTS2 | autism susceptibi | autism su | autism su | 26053 | 26053 | 26053 | s25020 | s25022 | s226064 |
| 13718 | L21 | L22 | 1254 | 1022 | 19896 | 200,5 | 15,9 | 1,493 | H5A034 C | sample | Hm Gen E | Hm Gen E | Hm Gen E | CPE002JC | CPE002NC | CPE002K4 | NM_01539 | NM_0153 | NM_0153 | WDR40A | WDR40A | WDR40A | WD repeat domai | WD repea | WD repea | 25853 | 25853 | 25853 | s24626 | s24627 | s24628 |
| 5436 | C2 | C3 | 742 | 589,8 | 11834 | 377,1 | 15,9 | 1,514 | H5A034 C | sample | Hm Drgbl | Hm Drgbl | Hm Drgbl | CPE002DX | CPE002F2 | CPE002FN | NM_20127 | NM_2012 | NM_2012 | NRP2 | NRP2 | NRP2 | neuropilin 2 | neuropilir | neuropilir | 8828 | 8828 | 8828 | s16840 | s16841 | s16842 |
| 5621 | K13 | K14 | 798 | 667,4 | 12676 | 481 | 15,9 | 1,514 | H5A034 C | sample | Hm Drgbl | Hm Drgbl | Hm Drgbl | CPE002DX | CPE002F2 | CPE002FN | NM_00363 | NM_0036 | NM_0036 | ITGA10 | ITGA10 | ITGA10 | integrin, alpha 10 | integrin, a | integrin, a | 8515 | 8515 | 8515 | s16182 | s16180 | s16181 |
| 5743 | P15 | P16 | 1573 | 624,4 | 24986 | 612,1 | 15,9 | 1,514 | H5A034 C | sample | Hm Drgbl | Hm Drgbl | Hm Drgbl | CPE002DX | CPE002F2 | CPE002FN | NM_00384 | NM_0038 | NM_0038 | SUCLG1 | SUCLG1 | SUCLG1 | succinate-CoA liga | succinate- | succinate- | 8802 | 8802 | 8802 | s16779 | s16778 | s16777 |
| 3634 | H18 | H19 | 1060 | 1324 | 16834 | 346,6 | 15,9 | 1,529 | H5A034 C | sample | Hm Drgbl | Hm Drgbl | Hm Drgbl | CPE002DR | CPE002EH | CPE002F7 | NM_03255 | NM_0325 | NM_0325 | GPR174 | GPR174 | GPR174 | G protein-coupled | G protein | G protein | 84636 | 84636 | 84636 | s39224 | s39225 | s39226 |
| 8132 | C5 | C6 | 813 | 616,7 | 12966 | 566,8 | 15,9 | 1,529 | H5A034 C | sample | Hm Drgbl | Hm Drgbl | Hm Drgbl | CPE002E5 | CPE002EU | CPE002FF | NM_00702 | NM_0070 | NM_0070 | RAPGEF4 | RAPGEF4 | RAPGEF4 | Rap guanine nucl | Rap guani | Rap guani | 11069 | 11069 | 11069 | s21816 | s21814 | s21815 |
| 8188 | F12 | F13 | 1068 | 571,7 | 16935 | 535,8 | 15,9 | 1,529 | H5A034 C | sample | Hm Drgbl | Hm Drgbl | Hm Drgbl | CPE002E5 | CPE002EU | CPE002FF | NM_01206 | NM_0120 | NM_0120 | ATP1B4 | ATP1B4 | ATP1B4 | ATPase, (Na+)/K+ | ATPase, (N | ATPase, (N | 23439 | 23439 | 23439 | s23838 | s23839 | s23840 |
| 8305 | K1 | K2 | 487 | 560,9 | 7732 | 494,2 | 15,9 | 1,529 | H5A034 C | sample | Hm Drgbl | Hm Drgbl | Hm Drgbl | CPE002E5 | CPE002EU | CPE002FF | NM_00718 | NM_0071 | NM_0071 | ABCB8 | ABCB8 | ABCB8 | ATP-binding casse | ATP-bindi | ATP-bindi | 11194 | 11194 | 11194 | s22105 | s22106 | s22104 |
| 8330 | L10 | L11 | 1135 | 562,9 | 18082 | 472,7 | 15,9 | 1,529 | H5A034 C | sample | Hm Drgbl | Hm Drgbl | Hm Drgbl | CPE002E5 | CPE002EU | CPE002FF | NM_01232 | NM_0123 | NM_0123 | MMD | MMD | MMD | monocyte to mac | monocyte | monocyte | 23531 | 23531 | 23531 | s24021 | s24022 | s24023 |
| 8426 | P10 | P11 | 979 | 552,3 | 15556 | 517,1 | 15,9 | 1,529 | H5A034 C | sample | Hm Drgbl | Hm Drgbl | Hm Drgbl | CPE002E5 | CPE002EU | CPE002FF | NM_01431 | NM_0143 | NM_0143 | CARHSP1 | CARHSP1 | CARHSP1 | calcium regulated | calcium re | calcium re | 23589 | 23589 | 23589 | s24153 | s24152 | s24154 |
| 10187 | I19 | I20 | 1692 | 920,8 | 26892 | 187,5 | 15,9 | 1,529 | H5A034 C | sample | Hm Drgbl | Hm Drgbl | Hm Drgbl | CPE002EA | CPE002EP | CPE002FA | NM_02482 | NM_0248 | NM_0248 | HDAC11 | HDAC11 | HDAC11 | histone deacetyla | histone de | histone de | 79885 | 79885 | 79885 | s36567 | s36568 | s36566 |
| 10270 | L7 | L8 | 1226 | 886,4 | 19518 | 164,2 | 15,9 | 1,529 | H5A034 C | sample | Hm Drgbl | Hm Drgbl | Hm Drgbl | CPE002EA | CPE002EP | CPE002FA | NM_03081 | NM_0308 | NM_0308 | CLPB | CLPB | CLPB | ClpB caseinolytic | ClpB casei | ClpB casei | 81570 | 81570 | 81570 | s37653 | s224882 | s37655 |
| 10305 | N17 | N18 | 1485 | 849,9 | 23645 | 183,8 | 15,9 | 1,529 | H5A034 C | sample | Hm Drgbl | Hm Drgbl | Hm Drgbl | CPE002EA | CPE002EP | CPE002FA | NM_01809 | NM_0180 | NM_0180 | NETO2 | NETO2 | NETO2 | neuropilin (NRP) a | neuropilir | neuropilir | 81831 | 81831 | 81831 | s37803 | s37804 | s37805 |
| 10338 | O3 | O4 | 1206 | 844,6 | 19117 | 186 | 15,9 | 1,529 | H5A034 C | sample | Hm Drgbl | Hm Drgbl | Hm Drgbl | CPE002EA | CPE002EP | CPE002FA | NM_02504 | NM_0250 | NM_0250 | PIF1 | PIF1 | PIF1 | PIF1 5-to-3 DNA h | PIF1 5-to-: | PIF1 5-to-: | 80119 | 80119 | 80119 | s36919 | s36920 | s36921 |
| 1596 | C2 | C3 | 921 | 710,2 | 14688 | 487,9 | 15,9 | 1,665 | H5A034 C | sample | Hm Drgbl | Hm Drgbl | Hm Drgbl | CPE002FW | CPE002GG | CPE002G4 | NM_02076 | NM_0207 | NM_0207 | KCTD16 | KCTD16 | KCTD16 | potassium channe | potassium | potassium | 57528 | 57528 | 57528 | s33229 | s33230 | s33231 |
| 1807 | L15 | L16 | 823 | 653,5 | 13124 | 325,7 | 15,9 | 1,665 | H5A034 C | sample | Hm Drgbl | Hm Drgbl | Hm Drgbl | CPE002FW | CPE002GG | CPE002G4 | NM_01844 | NM_0184 | NM_0184 | CAND1 | CAND1 | CAND1 | cullin-associated | cullin-ass | cullin-ass | 55832 | 55832 | 55832 | s31616 | s31617 | s31618 |
| 21538 | B18 | B19 | 213 | 990,8 | 3377 | 789,5 | 15,9 | 1,674 | H5A034 C | sample | Hm Gen E | Hm Gen E | Hm Gen E | CPE002JU | CPE002O9 | CPE002KP | XM_92621 | XM_9262 | XM_9262 | NBPF6 | NBPF6 | NBPF6 | neuroblastoma b | neuroblas | neuroblas | 653149 | 653149 | 653149 | s57591 | s57592 | s57593 |
| 21874 | P18 | P19 | 391 | 1153 | 6233 | 851,5 | 15,9 | 1,674 | H5A034 C | sample | Hm Gen E | Hm Gen E | Hm Gen E | CPE002JU | CPE002O9 | CPE002KP | XM_92946 | XM_9294 | XM_9294 | PCP4L1 | PCP4L1 | PCP4L1 | Purkinje cell prot | Purkinje c | Purkinje c | 654790 | 654790 | 654790 | s57854 | s57855 | s57856 |
| 21879 | P22 | P23 | 327 | 1125 | 5189 | 689,9 | 15,9 | 1,674 | H5A034 C | sample | Hm Gen E | Hm Gen E | Hm Gen E | CPE002JU | CPE002O9 | CPE002KP | NM_00104 | NM_0010 | NM_0010 | PC-3 | PC-3 | PC-3 | secreted micropro | secreted r | secreted r | 692094 | 692094 | 692094 | s57860 | s57861 | s198138 |
| 4709 | E13 | E14 | 1135 | 814 | 18081 | 439,8 | 15,9 | 1,747 | H5A034 C | sample | Hm Drgbl | Hm Drgbl | Hm Drgbl | CPE002DV | CPE002EL | CPE002FP | NM_00004 | NM_0000 | NM_0000 | ASPA | ASPA | ASPA | aspartoacylase (C | aspartoac | aspartoac | 443 | 443 | 443 | s1681 | s1682 | s1683 |
| 4757 | G13 | G14 | 1181 | 806,9 | 18770 | 439,5 | 15,9 | 1,747 | H5A034 C | sample | Hm Drgbl | Hm Drgbl | Hm Drgbl | CPE002DV | CPE002EL | CPE002FP | NM_01428 | NM_0142 | NM_0142 | CAPN6 | CAPN6 | CAPN6 | calpain 6 | calpain 6 | calpain 6 | 827 | 827 | 827 | s2388 | s2389 | s2390 |
| 4806 | I14 | I15 | 709 | 703,5 | 11281 | 385,2 | 15,9 | 1,747 | H5A034 C | sample | Hm Drgbl | Hm Drgbl | Hm Drgbl | CPE002DV | CPE002EL | CPE002FP | NM_00394 | NM_0039 | NM_0039 | USP13 | USP13 | USP13 | ubiquitin specific | ubiquitin | ubiquitin | 8975 | 8975 | 8975 | s17129 | s17130 | s17131 |
| 4949 | O13 | O14 | 938 | 803,7 | 14909 | 580,4 | 15,9 | 1,747 | H5A034 C | sample | Hm Drgbl | Hm Drgbl | Hm Drgbl | CPE002DV | CPE002EL | CPE002FP | NM_00013 | NM_0001 | NM_0001 | F8 | F8 | F8 | coagulation facto | coagulatic | coagulatic | 2157 | 2157 | 2157 | s4940 | s4941 | s4942 |
| 10371 | A11 | A12 | 748 | 998,7 | 11870 | 245,8 | 15,9 | 1,797 | H5A034 C | sample | Hm Drgbl | Hm Drgbl | Hm Drgbl | CPE002EB | CPE002EO | CPE002FR | NM_00104 | NM_0010 | NM_0010 | SLC7A3 | SLC7A3 | SLC7A3 | solute carrier fam | solute car | solute car | 84889 | 84889 | 84889 | s39580 | s39581 | s39582 |
| 10502 | F21 | F22 | 1051 | 802,8 | 16690 | 206 | 15,9 | 1,797 | H5A034 C | sample | Hm Drgbl | Hm Drgbl | Hm Drgbl | CPE002EB | CPE002EO | CPE002FR | NM_08082 | NM_0808 | NM_0808 | DTD1 | DTD1 | DTD1 | D-tyrosyl-tRNA de | D-tyrosyl- | D-tyrosyl- | 92675 | 92675 | 92675 | s40990 | s40989 | s40991 |
| 10520 | G16 | G17 | 1038 | 826,6 | 16463 | 197,9 | 15,9 | 1,797 | H5A034 C | sample | Hm Drgbl | Hm Drgbl | Hm Drgbl | CPE002EB | CPE002EO | CPE002FR | NM_05293 | NM_0529 | NM_0529 | FCRL3 | FCRL3 | FCRL3 | Fc receptor-like 3 | Fc recepto | Fc recepto | 115352 | 115352 | 115352 | s41801 | s41800 | s41799 |
| 10605 | J6 | J7 | 776 | 954,3 | 12355 | 221,8 | 15,9 | 1,797 | H5A034 C | sample | Hm Drgbl | Hm Drgbl | Hm Drgbl | CPE002EB | CPE002EO | CPE002FR | NM_13896 | NM_1389 | NM_1389 | MSI2 | MSI2 | MSI2 | musashi homolog | musashi h | musashi h | 124540 | 124540 | 124540 | s42757 | s42756 | s42755 |
| 10621 | K20 | K21 | 815 | 873,6 | 12962 | 176,2 | 15,9 | 1,797 | H5A034 C | sample | Hm Drgbl | Hm Drgbl | Hm Drgbl | CPE002EB | CPE002EO | CPE002FR | NM_18131 | NM_1813 | NM_1813 | IL22RA2 | IL22RA2 | IL22RA2 | interleukin 22 rec | interleuki | interleuki | 116379 | 116379 | 116379 | s41999 | s42001 | s42000 |
| 13260 | I2 | I3 | 1680 | 1047 | 26806 | 125,5 | 16 | 0,802 | H5A034 C | sample | Hm Gen E | Hm Gen E | Hm Gen E | CPE002JB | CPE002NN | CPE002K3 | NM_01496 | NM_0149 | NM_0149 | WDR47 | WDR47 | WDR47 | WD repeat domai | WD repea | WD repea | 22911 | 22911 | 22911 | s22652 | s22653 | s22654 |
| 22285 | A20 | A21 | 904 | 1433 | 14464 | 411,2 | 16 | 0,825 | H5A034 C | sample | Hm Gen E | Hm Gen E | Hm Gen E | CPE002JW | CPE002OB | CPE002KR | XM_00112 | XM_0011 | XM_0011 | LOC72901 | LOC72901 | LOC72901 | hypothetical prot | hypotheti | hypotheti | 729014 | 729014 | 729014 | s59267 | s59268 | s227719 |
| 14866 | L18 | L19 | 1218 | 805,7 | 19435 | 128,7 | 16 | 0,912 | H5A034 C | sample | Hm Gen E | Hm Gen E | Hm Gen E | CPE002JD | CPE002NR | CPE002K7 | NM_01844 | NM_0184 | NM_0184 | PAG1 | PAG1 | PAG1 | phosphoprotein a | phosphoc | phosphoc | 55824 | 55824 | 55824 | s31598 | s31599 | s31600 |
| 3255 | H22 | H23 | 1130 | 1479 | 18075 | 457,2 | 16 | 0,991 | H5A034 C | sample | Hm Drgbl | Hm Drgbl | Hm Drgbl | CPE002DC | CPE002EG | CPE002F6 | NM_00588 | NM_0058 | NM_0058 | SLC25A3 | SLC25A3 | SLC25A3 | solute carrier fam | solute car | solute car | 5250 | 5250 | 5250 | s10429 | s10427 | s10428 |
| 3397 | N20 | N21 | 548 | 1413 | 8747 | 505 | 16 | 0,991 | H5A034 C | sample | Hm Drgbl | Hm Drgbl | Hm Drgbl | CPE002DC | CPE002EG | CPE002F6 | NM_00100 | NM_0010 | NM_0010 | PKP1 | PKP1 | PKP1 | plakophilin 1 (ect | plakophili | plakophili | 5317 | 5317 | 5317 | s10580 | s10581 | s10582 |
| 12097 | I1 | I2 | 1807 | 835,2 | 28831 | 91,56 | 16 | 1,026 | H5A034 C | sample | Hm Gen E | Hm Gen E | Hm Gen E | CPE002J4 | CPE002NL | CPE002K0 | NM_01422 | NM_0142 | NM_0142 | TM4SF1 | TM4SF1 | TM4SF1 | transmembrane 4 | transmem | transmem | 4071 | 4071 | 4071 | s8367 | s8368 | s8369 |
| 18398 | O21 | O22 | 1432 | 1013 | 22954 | 481,3 | 16 | 1,029 | H5A034 C | sample | Hm Gen E | Hm Gen E | Hm Gen E | CPE002JL | CPE002OC | CPE002KG | NM_13881 | NM_1388 | NM_1388 | FAM122C | FAM122C | FAM122C | family with seque | family wit | family wit | 159091 | 159091 | 159091 | s46150 | s46151 | s46152 |
| 19718 | F21 | F22 | 1452 | 371,9 | 23283 | 299,9 | 16 | 1,042 | H5A034 C | sample | Hm Gen E | Hm Gen E | Hm Gen E | CPE002JP | CPE002O4 | CPE002KK | NM_00101 | NM_0010 | NM_0010 | GLTPD2 | GLTPD2 | GLTPD2 | glycolipid transfe | glycolipid | glycolipid | 388323 | 388323 | 388323 | s52090 | s52091 | s196222 |
| 19719 | F22 | F23 | 1292 | 353,9 | 20659 | 359 | 16 | 1,042 | H5A034 C | sample | Hm Gen E | Hm Gen E | Hm Gen E | CPE002JP | CPE002O4 | CPE002KK | NM_00100 | NM_0010 | NM_0010 | OR56A3 | OR56A3 | OR56A3 | olfactory receptor | olfactory r | olfactory r | 390083 | 390083 | 390083 | s52669 | s52670 | s52671 |
| 19902 | N14 | N15 | 1111 | 325,3 | 17796 | 236,5 | 16 | 1,042 | H5A034 C | sample | Hm Gen E | Hm Gen E | Hm Gen E | CPE002JP | CPE002O4 | CPE002KK | NM_00100 | NM_0010 | NM_0010 | OR4N5 | OR4N5 | OR4N5 | olfactory receptor | olfactory r | olfactory r | 390437 | 390437 | 390437 | s52803 | s52804 | s226477 |
| 6366 | J14 | J15 | 1324 | 632,4 | 21142 | 399,4 | 16 | 1,056 | H5A034 C | sample | Hm Drgbl | Hm Drgbl | Hm Drgbl | CPE002DZ | CPE002F0 | CPE002FL | NM_00522 | NM_0052 | NM_0052 | DNASE1 | DNASE1 | DNASE1 | deoxyribonucleas | deoxyribo | deoxyribo | 1773 | 1773 | 1773 | s4187 | s4185 | s4186 |
| 6385 | K1 | K2 | 435 | 515,7 | 6977 | 491,3 | 16 | 1,056 | H5A034 C | sample | Hm Drgbl | Hm Drgbl | Hm Drgbl | CPE002DZ | CPE002F0 | CPE002FL | NM_00179 | NM_0017 | NM_0017 | CDH4 | CDH4 | CDH4 | cadherin 4, type 1 | cadherin 4 | cadherin 4 | 1002 | 1002 | 1002 | s2777 | s2778 | s2779 |
| 6462 | N14 | N15 | 1709 | 588,8 | 27296 | 447,7 | 16 | 1,056 | H5A034 C | sample | Hm Drgbl | Hm Drgbl | Hm Drgbl | CPE002DZ | CPE002F0 | CPE002FL | NM_00138 | NM_0013 | NM_0013 | DPYSL3 | DPYSL3 | DPYSL3 | dihydropyrimidin | dihydropy | dihydropy | 1809 | 1809 | 1809 | s4272 | s4274 | s4273 |
| 20546 | I10 | I11 | 659 | 784,8 | 10574 | 229,1 | 16 | 1,067 | H5A034 C | sample | Hm Gen E | Hm Gen E | Hm Gen E | CPE002JR | CPE002O6 | CPE002KN | NM_00102 | NM_0010 | NM_0010 | LOC55425 | LOC55425 | LOC55425 | hypothetical prot | hypotheti | hypotheti | 554251 | 554251 | 554251 | s54743 | s54744 | s54745 |
| 20593 | K1 | K2 | 401 | 837,9 | 6399 | 181,1 | 16 | 1,067 | H5A034 C | sample | Hm Gen E | Hm Gen E | Hm Gen E | CPE002JR | CPE002O6 | CPE002KN | NM_00100 | NM_0010 | NM_0010 | MAP1LC3C | MAP1LC3C | MAP1LC3C | microtubule-asso | microtubu | microtubu | 440738 | 440738 | 440738 | s54188 | s54189 | s54190 |
| 20598 | K14 | K15 | 1006 | 816,7 | 16061 | 211,6 | 16 | 1,067 | H5A034 C | sample | Hm Gen E | Hm Gen E | Hm Gen E | CPE002JR | CPE002O6 | CPE002KN | NM_00103 | NM_0010 | NM_0010 | LOC61920 | LOC61920 | LOC61920 | hypothetical prot | hypotheti | hypotheti | 619208 | 619208 | 619208 | s54782 | s54784 | s226968 |
| 12801 | F17 | F18 | 1727 | 1147 | 27552 | 117,1 | 16 | 1,085 | H5A034 C | sample | Hm Gen E | Hm Gen E | Hm Gen E | CPE002J6 | CPE002NN | CPE002K2 | NM_01471 | NM_0147 | NM_0147 | LAPTM4A | LAPTM4A | LAPTM4A | lysosomal-associa | lysosomal | lysosomal | 9741 | 9741 | 9741 | s18791 | s18792 | s18793 |
| 12861 | H6 | H7 | 958 | 971 | 15327 | 128,3 | 16 | 1,085 | H5A034 C | sample | Hm Gen E | Hm Gen E | Hm Gen E | CPE002J6 | CPE002NN | CPE002K2 | NM_01425 | NM_0142 | NM_0142 | TMEM5 | TMEM5 | TMEM5 | transmembrane p | transmem | transmem | 10329 | 10329 | 10329 | s20203 | s20204 | s20205 |
| 18514 | D18 | D19 | 1554 | 1095 | 24930 | 277,4 | 16 | 1,085 | H5A034 C | sample | Hm Gen E | Hm Gen E | Hm Gen E | CPE002JM | CPE002O1 | CPE002KH | NM_15276 | NM_1527 | NM_1527 | C19orf26 | C19orf26 | C19orf26 | chromosome 19 o | chromoso | chromoso | 255057 | 255057 | 255057 | s48604 | s48605 | s195882 |
| 18618 | H3 | H4 | 1290 | 1045 | 20619 | 185,6 | 16 | 1,085 | H5A034 C | sample | Hm Gen E | Hm Gen E | Hm Gen E | CPE002JM | CPE002O1 | CPE002KH | NM_00103 | NM_0010 | NM_0010 | C10orf25 | C10orf25 | C10orf25 | chromosome 10 o | chromoso | chromoso | 220979 | 220979 | 220979 | s47923 | s47924 | s47925 |
| 17366 | D21 | D22 | 1490 | 968,2 | 23780 | 202,4 | 16 | 1,092 | H5A034 C | sample | Hm Gen E | Hm Gen E | Hm Gen E | CPE002JJ | CPE002NY | CPE002KE | NM_08073 | NM_0807 | NM_0807 | C20orf141 | C20orf141 | C20orf141 | chromosome 20 o | chromoso | chromoso | 128653 | 128653 | 128653 | s195700 | s195702 | s225602 |
| 17479 | I15 | I16 | 1344 | 868,2 | 21478 | 171,6 | 16 | 1,092 | H5A034 C | sample | Hm Gen E | Hm Gen E | Hm Gen E | CPE002JJ | CPE002NY | CPE002KE | NM_00100 | NM_0010 | NM_0010 | OR14C36 | OR14C36 | OR14C36 | olfactory receptor | olfactory r | olfactory r | 127066 | 127066 | 127066 | s43121 | s43122 | s225581 |
| 17577 | M17 | M18 | 1313 | 1042 | 20967 | 239,5 | 16 | 1,092 | H5A034 C | sample | Hm Gen E | Hm Gen E | Hm Gen E | CPE002JJ | CPE002NY | CPE002KE | NM_17200 | NM_1720 | NM_1720 | TEDDM1 | TEDDM1 | TEDDM1 | transmembrane e | transmem | transmem | 127670 | 127670 | 127670 | s43208 | s43210 | s225589 |
| 17582 | M21 | M22 | 1320 | 872,7 | 21152 | 188,1 | 16 | 1,092 | H5A034 C | sample | Hm Gen E | Hm Gen E | Hm Gen E | CPE002JJ | CPE002NY | CPE002KE | NM_14504 | NM_1450 | NM_1450 | C1orf102 | C1orf102 | C1orf102 | chromosome 1 op | chromoso | chromoso | 127700 | 127700 | 127700 | s43214 | s43215 | s225591 |
| 9230 | A21 | A22 | 1259 | 1143 | 20167 | 180,9 | 16 | 1,111 | H5A034 C | sample | Hm Drgbl | Hm Drgbl | Hm Drgbl | CPE002E8 | CPE002ER | CPE002FC | NM_01778 | NM_0177 | NM_0177 | SEMA4C | SEMA4C | SEMA4C | sema domain, imi | sema dom | sema dom | 54910 | 54910 | 54910 | s29736 | s29734 | s29735 |
| 9388 | H12 | H13 | 1096 | 1215 | 17553 | 133,4 | 16 | 1,111 | H5A034 C | sample | Hm Drgbl | Hm Drgbl | Hm Drgbl | CPE002E8 | CPE002ER | CPE002FC | NM_02015 | NM_0201 | NM_0201 | C1GALT1 | C1GALT1 | C1GALT1 | core 1 synthase, g | core 1 syn | core 1 syn | 56913 | 56913 | 56913 | s525 | s526 | s527 |
| 9414 | I14 | I15 | 1642 | 1048 | 26241 | 132,1 | 16 | 1,111 | H5A034 C | sample | Hm Drgbl | Hm Drgbl | Hm Drgbl | CPE002E8 | CPE002ER | CPE002FC | NM_01891 | NM_0189 | NM_0189 | PCDHGA1 | PCDHGA1 | PCDHGA1 | protocadherin ga | protocadh | protocadh | 56105 | 56105 | 56105 | s31903 | s31902 | s31901 |
| 9543 | N22 | N23 | 1593 | 1054 | 25500 | 142,4 | 16 | 1,111 | H5A034 C | sample | Hm Drgbl | Hm Drgbl | Hm Drgbl | CPE002E8 | CPE002ER | CPE002FC | NM_02039 | NM_0203 | NM_0203 | PGLYRP4 | PGLYRP4 | PGLYRP4 | peptidoglycan rec | peptidogl | peptidogl | 57115 | 57115 | 57115 | s32708 | s32706 | s32707 |
| 9609 | A17 | A18 | 1891 | 1134 | 30235 | 174,6 | 16 | 1,119 | H5A034 C | sample | Hm Drgbl | Hm Drgbl | Hm Drgbl | CPE002E9 | CPE002EO | CPE002FB | NM_02045 | NM_0204 | NM_0204 | ATP10D | ATP10D | ATP10D | ATPase, Class V, ty | ATPase, C | ATPase, C | 57205 | 57205 | 57205 | s32884 | s32885 | s32886 |
| 9654 | C14 | C15 | 1496 | 1154 | 23988 | 141,7 | 16 | 1,119 | H5A034 C | sample | Hm Drgbl | Hm Drgbl | Hm Drgbl | CPE002E9 | CPE002EO | CPE002FB | NM_02215 | NM_0221 | NM_0221 | SLC39A8 | SLC39A8 | SLC39A8 | solute carrier fam | solute car | solute car | 64116 | 64116 | 64116 | s34469 | s34470 | s34468 |
| 9787 | H4 | H5 | 1226 | 1051 | 19613 | 125,4 | 16 | 1,119 | H5A034 C | sample | Hm Drgbl | Hm Drgbl | Hm Drgbl | CPE002E9 | CPE002EO | CPE002FB | NM_00102 | NM_0010 | NM_0010 | SLC2A11 | SLC2A11 | SLC2A11 | solute carrier fam | solute car | solute car | 66035 | 66035 | 66035 | s35351 | s35352 | s35353 |
| 9801 | I17 | I18 | 1855 | 1060 | 29608 | 144 | 16 | 1,119 | H5A034 C | sample | Hm Drgbl | Hm Drgbl | Hm Drgbl | CPE002E9 | CPE002EO | CPE002FB | NM_02079 | NM_0207 | NM_0207 | NLGN2 | NLGN2 | NLGN2 | neuroligin 2 | neuroligin | neuroligin | 57555 | 57555 | 57555 | s33298 | s33299 | s33300 |
| 9902 | M21 | M22 | 2277 | 1186 | 36422 | 207,6 | 16 | 1,119 | H5A034 C | sample | Hm Drgbl | Hm Drgbl | Hm Drgbl | CPE002E9 | CPE002EO | CPE002FB | NM_02091 | NM_0209 | NM_0209 | GPAM | GPAM | GPAM | glycerol-3-phosph | glycerol-3 | glycerol-3 | 57678 | 57678 | 57678 | s33574 | s33575 | s224484 |
| 9926 | N21 | N22 | 2239 | 1072 | 35869 | 227,8 | 16 | 1,119 | H5A034 C | sample | Hm Drgbl | Hm Drgbl | Hm Drgbl | CPE002E9 | CPE002EO | CPE002FB | NM_20128 | NM_2012 | NM_2012 | MUTED | MUTED | MUTED | muted homolog ( | muted ho | muted ho | 63915 | 63915 | 63915 | s34257 | s34256 | s34255 |
| 15252 | L2 | L3 | 862 | 823,5 | 13805 | 140,7 | 16 | 1,119 | H5A034 C | sample | Hm Gen E | Hm Gen E | Hm Gen E | CPE002J8 | CPE002NS | CPE002K8 | NM_02117 | NM_0211 | NM_0211 | KIAA1967 | KIAA1967 | KIAA1967 | KIAA1967 | KIAA1967 | KIAA1967 | 57805 | 57805 | 57805 | s33736 | s33737 | s33738 |
| 15279 | M22 | M23 | 1692 | 849,2 | 27022 | 151,4 | 16 | 1,119 | H5A034 C | sample | Hm Gen E | Hm Gen E | Hm Gen E | CPE002J8 | CPE002NS | CPE002K8 | XM_94196 | XM_9419 | XM_9419 | KIAA1394 | KIAA1394 | KIAA1394 | KIAA1394 protein | KIAA1394 | KIAA1394 | 57571 | 57571 | 57571 | s33337 | s33338 | s33339 |
| 8457 | A17 | A18 | 990 | 1048 | 15855 | 160,4 | 16 | 1,131 | H5A034 C | sample | Hm Drgbl | Hm Drgbl | Hm Drgbl | CPE002E6 | CPE002ET | CPE002FE | NM_01231 | NM_0123 | NM_0123 | KPNA6 | KPNA6 | KPNA6 | karyopherin alph | karyopher | karyopher | 23633 | 23633 | 23633 | s24241 | s24242 | s24243 |
| 8534 | D21 | D22 | 1563 | 1040 | 25047 | 139,2 | 16 | 1,131 | H5A034 C | sample | Hm Drgbl | Hm Drgbl | Hm Drgbl | CPE002E6 | CPE002ET | CPE002FE | NM_19893 | NM_1989 | NM_1989 | SS18L1 | SS18L1 | SS18L1 | synovial sarcoma | synovial s | synovial s | 26039 | 26039 | 26039 | s24990 | s24991 | s24992 |
| 8828 | P5 | P6 | 1802 | 1121 | 28847 | 169,1 | 16 | 1,131 | H5A034 C | sample | Hm Drgbl | Hm Drgbl | Hm Drgbl | CPE002E6 | CPE002ET | CPE002FE | NM_00103 | NM_0010 | NM_0010 | INTS6 | INTS6 | INTS6 | integrator comple | integrator | integrator | 26512 | 26512 | 26512 | s25482 | s25483 | s25484 |
| 19364 | G5 | G6 | 956 | 1119 | 15317 | 242,7 | 16 | 1,135 | H5A034 C | sample | Hm Gen E | Hm Gen E | Hm Gen E | CPE002JO | CPE002O3 | CPE002KJ | NM_00100 | NM_0010 | NM_0010 | LUZP2 | LUZP2 | LUZP2 | leucine zipper pro | leucine zi | leucine zi | 338645 | 338645 | 338645 | s50365 | s50366 | s50367 |
| 19383 | H22 | H23 | 1204 | 1119 | 19237 | 398,2 | 16 | 1,135 | H5A034 C | sample | Hm Gen E | Hm Gen E | Hm Gen E | CPE002JO | CPE002O3 | CPE002KJ | NM_18172 | NM_1817 | NM_1817 | ANKRD37 | ANKRD37 | ANKRD37 | ankyrin repeat do | ankyrin re | ankyrin re | 353322 | 353322 | 353322 | s51462 | s51463 | s51464 |
| 19548 | O2 | O3 | 1116 | 1176 | 17810 | 398,3 | 16 | 1,135 | H5A034 C | sample | Hm Gen E | Hm Gen E | Hm Gen E | CPE002JO | CPE002O3 | CPE002KJ | NM_00100 | NM_0010 | NM_0010 | IGFBPL1 | IGFBPL1 | IGFBPL1 | insulin-like growt | insulin-lik | insulin-lik | 347252 | 347252 | 347252 | s51223 | s51224 | s226350 |
| 19563 | P11 | P12 | 1309 | 1326 | 20957 | 438,8 | 16 | 1,135 | H5A034 C | sample | Hm Gen E | Hm Gen E | Hm Gen E | CPE002JO | CPE002O3 | CPE002KJ | XM_94333 | XM_9433 | XM_9433 | FMN1 | FMN1 | FMN1 | formin 1 | formin 1 | formin 1 | 342184 | 342184 | 342184 | s50888 | s50889 | s50890 |
| 19581 | P6 | P7 | 1267 | 1282 | 20223 | 406,8 | 16 | 1,135 | H5A034 C | sample | Hm Gen E | Hm Gen E | Hm Gen E | CPE002JO | CPE002O3 | CPE002KJ | NM_19934 | NM_1993 | NM_1993 | LOC37492 | LOC37492 | LOC37492 | hypothetical prot | hypotheti | hypotheti | 374920 | 374920 | 374920 | s51624 | s51625 | s51626 |
| 20930 | I10 | I11 | 859 | 942,8 | 13715 | 306,9 | 16 | 1,135 | H5A034 C | sample | Hm Gen E | Hm Gen E | Hm Gen E | CPE002JS | CPE002O7 | CPE002KN | XM_93217 | XM_9321 | XM_9321 | LOC64441 | LOC64441 | LOC64441 | hypothetical LOC | hypotheti | hypotheti | 644419 | 644419 | 644419 | s197060 | s197061 | s197062 |
| 20961 | J17 | J18 | 1040 | 974,9 | 16661 | 247,7 | 16 | 1,135 | H5A034 C | sample | Hm Gen E | Hm Gen E | Hm Gen E | CPE002JS | CPE002O7 | CPE002KN | NM_00107 | NM_0010 | NM_0010 | KRTAP27-1 | KRTAP27-1 | KRTAP27-1 | keratin associated | keratin as | keratin as | 643812 | 643812 | 643812 | s55456 | s55457 | s55458 |
| 14367 | G22 | G23 | 1408 | 800,5 | 22558 | 138,7 | 16 | 1,139 | H5A034 C | sample | Hm Gen E | Hm Gen E | Hm Gen E | CPE002J7 | CPE002NC | CPE002K6 | NM_01795 | NM_0179 | NM_0179 | C1orf181 | C1orf181 | C1orf181 | chromosome 1 op | chromoso | chromoso | 54680 | 54680 | 54680 | s29315 | s29316 | s29317 |
| 11260 | F12 | F13 | 1849 | 936,1 | 29588 | 179 | 16 | 1,159 | H5A034 C | sample | Hm Drgbl | Hm Drgbl | Hm Drgbl | CPE002ED | CPE002F3 | CPE002FT | | | | | | | | | | | | | | | |
| 11352 | I9 | I10 | 979 | 812,2 | 15618 | 135,3 | 16 | 1,159 | H5A034 C | sample | Hm Drgbl | Hm Drgbl | Hm Drgbl | CPE002ED | CPE002F3 | CPE002FT | NM_17336 | NM_1733 | NM_1733 | RFESD | RFESD | RFESD | Rieske (Fe-S) dom | Rieske (Fe | Rieske (Fe | 317671 | 317671 | 317671 | s50222 | s50220 | s50221 |
| 11407 | L15 | L16 | 1152 | 821,2 | 18422 | 130,7 | 16 | 1,159 | H5A034 C | sample | Hm Drgbl | Hm Drgbl | Hm Drgbl | CPE002ED | CPE002F3 | CPE002FT | NM_19872 | NM_1987 | NM_1987 | AMIGO3 | AMIGO3 | AMIGO3 | adhesion molecul | adhesion | adhesion | 386724 | 386724 | 386724 | s51824 | s51825 | s51826 |
| 11493 | O6 | O7 | 1572 | 852,3 | 25177 | 197,6 | 16 | 1,159 | H5A034 C | sample | Hm Drgbl | Hm Drgbl | Hm Drgbl | CPE002ED | CPE002F3 | CPE002FT | | | | | | | | | | | | | | | |
| 11497 | P1 | P2 | 750 | 811,3 | 12035 | 145,7 | 16 | 1,159 | H5A034 C | sample | Hm Drgbl | Hm Drgbl | Hm Drgbl | CPE002ED | CPE002F3 | CPE002FT | NM_20339 | NM_2033 | NM_2033 | IYD | IYD | IYD | iodotyrosine deic | iodotyros | iodotyros | 389434 | 389434 | 389434 | s223989 | s52484 | s52485 |
| 12395 | E19 | E20 | 1120 | 1009 | 17925 | 174,9 | 16 | 1,203 | H5A034 C | sample | Hm Gen E | Hm Gen E | Hm Gen E | CPE002J5 | CPE002NK | CPE002K1 | NM_01423 | NM_0142 | NM_0142 | VAMP2 | VAMP2 | VAMP2 | vesicle-associated | vesicle-as | vesicle-as | 6844 | 6844 | 6844 | s13667 | s13668 | s13669 |
| 12538 | K18 | K19 | 1208 | 1017 | 19316 | 167,5 | 16 | 1,203 | H5A034 C | sample | Hm Gen E | Hm Gen E | Hm Gen E | CPE002J5 | CPE002NK | CPE002K1 | NM_00366 | NM_0036 | NM_0036 | BLZF1 | BLZF1 | BLZF1 | basic leucine zipp | basic leuc | basic leuc | 8548 | 8548 | 8548 | s16272 | s16273 | s16274 |
| 7308 | A2 | A3 | 1206 | 648,7 | 19325 | 450,7 | 16 | 1,221 | H5A034 C | sample | Hm Drgbl | Hm Drgbl | Hm Drgbl | CPE002E3 | CPE002EW | CPE002FH | NM_00429 | NM_0042 | NM_0042 | RNF14 | RNF14 | RNF14 | ring finger protei | ring finger | ring finger | 9604 | 9604 | 9604 | s18453 | s18454 | s18452 |
| 7321 | B1 | B2 | 1208 | 564,3 | 19332 | 575,8 | 16 | 1,221 | H5A034 C | sample | Hm Drgbl | Hm Drgbl | Hm Drgbl | CPE002E3 | CPE002EW | CPE002FH | NM_00062 | NM_0006 | NM_0006 | SERPINA5 | SERPINA5 | SERPINA5 | serpin peptidase | serpin pe | serpin pe | 5104 | 5104 | 5104 | s10118 | s10119 | s10120 |
| 7341 | B6 | B7 | 1309 | 594,9 | 20915 | 528,2 | 16 | 1,221 | H5A034 C | sample | Hm Drgbl | Hm Drgbl | Hm Drgbl | CPE002E3 | CPE002EW | CPE002FH | NM_01229 | NM_0122 | NM_0122 | G3BP2 | G3BP2 | G3BP2 | GTPase activating | GTPase ac | GTPase ac | 9908 | 9908 | 9908 | s19206 | s19207 | s19208 |
| 7572 | L2 | L3 | 810 | 613,8 | 12925 | 572,5 | 16 | 1,221 | H5A034 C | sample | Hm Drgbl | Hm Drgbl | Hm Drgbl | CPE002E3 | CPE002EW | CPE002FH | NM_01206 | NM_0120 | NM_0120 | DNM1L | DNM1L | DNM1L | dynamin 1-like | dynamin 1 | dynamin 1 | 10059 | 10059 | 10059 | s19559 | s19560 | s19561 |
| 7595 | M19 | M20 | 1622 | 664,2 | 25962 | 472,5 | 16 | 1,221 | H5A034 C | sample | Hm Drgbl | Hm Drgbl | Hm Drgbl | CPE002E3 | CPE002EW | CPE002FH | NM_00160 | NM_0016 | NM_0016 | PAX6 | PAX6 | PAX6 | paired box gene 6 | paired bo | paired bo | 5080 | 5080 | 5080 | s10067 | s10068 | s10069 |
| 16897 | A1 | A2 | 793 | 995 | 12657 | 244,2 | 16 | 1,236 | H5A034 C | sample | Hm Gen E | Hm Gen E | Hm Gen E | CPE002JI | CPE002NZ | CPE002KD | NM_13840 | NM_0807 | NM_1384 | FAM125A | NRSN1 | FAM125A | family with seque | neurensin | family wit | 93343 | 140767 | 93343 | s41121 | s44394 | s228489 |
| 16944 | B9 | B10 | 1034 | 1027 | 16569 | 217,9 | 16 | 1,236 | H5A034 C | sample | Hm Gen E | Hm Gen E | Hm Gen E | CPE002JI | CPE002NZ | CPE002KD | NM_05290 | NM_1384 | NM_0529 | KLHL32 | MDP-1 | KLHL32 | kelch-like 32 (Dro | magnesiu | kelch-like | 114792 | 145553 | 114792 | s41615 | s44754 | s41617 |
| 17186 | M10 | M11 | 1323 | 1090 | 21179 | 291,3 | 16 | 1,236 | H5A034 C | sample | Hm Gen E | Hm Gen E | Hm Gen E | CPE002JI | CPE002NZ | CPE002KD | NM_00100 | NM_1447 | NM_0010 | OR10A7 | LSM14B | OR10A7 | olfactory receptor | LSM14B, S | olfactory r | 121364 | 149986 | 121364 | s42464 | s45365 | s42466 |
| 17215 | N15 | N16 | 1235 | 973,4 | 19772 | 212,4 | 16 | 1,236 | H5A034 C | sample | Hm Gen E | Hm Gen E | Hm Gen E | CPE002JI | CPE002NZ | CPE002KD | NM_05296 | NM_1530 | NM_0529 | C1orf19 | APCDD1 | C1orf19 | chromosome 1 op | adenomat | chromoso | 116461 | 147495 | 116461 | s42026 | s45007 | s42028 |
| 17226 | N3 | N4 | 1390 | 938,2 | 22269 | 120 | 16 | 1,236 | H5A034 C | sample | Hm Gen E | Hm Gen E | Hm Gen E | CPE002JI | CPE002NZ | CPE002KD | NM_05295 | NM_1452 | NM_0529 | C8orf34 | TMEM99 | C8orf34 | chromosome 8 op | transmem | chromoso | 116328 | 147184 | 116328 | s41987 | s44978 | s41989 |
| 7106 | I10 | I11 | 1443 | 584,4 | 23084 | 439,3 | 16 | 1,245 | H5A034 C | sample | Hm Drgbl | Hm Drgbl | Hm Drgbl | CPE002E2 | CPE002EX | CPE002FI | NM_00596 | NM_0059 | NM_0059 | MYH10 | MYH10 | MYH10 | myosin, heavy cha | myosin, h | myosin, h | 4628 | 4628 | 4628 | s9169 | s9170 | s9171 |
| 7124 | I5 | I6 | 1000 | 548,7 | 16032 | 444,1 | 16 | 1,245 | H5A034 C | sample | Hm Drgbl | Hm Drgbl | Hm Drgbl | CPE002E2 | CPE002EX | CPE002FI | NM_00231 | NM_0023 | NM_0023 | LMX1B | LMX1B | LMX1B | LIM homeobox tr | LIM home | LIM home | 4010 | 4010 | 4010 | s8244 | s8242 | s8243 |
| 5109 | E6 | E7 | 1200 | 665,5 | 19227 | 565,3 | 16 | 1,255 | H5A034 C | sample | Hm Drgbl | Hm Drgbl | Hm Drgbl | CPE002DV | CPE002EM | CPE002FO | NM_00711 | NM_0071 | NM_0071 | TRIO | TRIO | TRIO | triple functional d | triple func | triple func | 7204 | 7204 | 7204 | s14400 | s14401 | s14402 |
| 5288 | M16 | M17 | 1513 | 630,7 | 24190 | 535,2 | 16 | 1,255 | H5A034 C | sample | Hm Drgbl | Hm Drgbl | Hm Drgbl | CPE002DV | CPE002EM | CPE002FO | NM_13083 | NM_1308 | NM_1308 | UBE3A | UBE3A | UBE3A | ubiquitin protein | ubiquitin | ubiquitin | 7337 | 7337 | 7337 | s14604 | s14605 | s14606 |
| 5305 | N1 | N2 | 1029 | 566,6 | 16470 | 377,5 | 16 | 1,255 | H5A034 C | sample | Hm Drgbl | Hm Drgbl | Hm Drgbl | CPE002DV | CPE002EM | CPE002FO | NM_00101 | NM_0010 | NM_0010 | FXR1 | FXR1 | FXR1 | fragile X mental r | fragile X n | fragile X n | 8087 | 8087 | 8087 | s15610 | s15611 | s15612 |
| 5325 | N6 | N7 | 1604 | 639,3 | 25658 | 561,2 | 16 | 1,255 | H5A034 C | sample | Hm Drgbl | Hm Drgbl | Hm Drgbl | CPE002DV | CPE002EM | CPE002FO | NM_00344 | NM_0034 | NM_0034 | TRIM26 | TRIM26 | TRIM26 | tripartite motif-co | tripartite | tripartite | 7726 | 7726 | 7726 | s15228 | s15229 | s15230 |
| 2527 | J15 | J16 | 932 | 607,6 | 14877 | 361,4 | 16 | 1,26 | H5A034 C | sample | Hm Drgbl | Hm Drgbl | Hm Drgbl | CPE002DC | CPE002EE | CPE002F4 | NM_01214 | NM_0121 | NM_0121 | DTYMK | DTYMK | DTYMK | deoxythymidylate | deoxythyr | deoxythyr | 1841 | 1841 | 1841 | s4357 | s4358 | s4359 |
| 2620 | N12 | N13 | 1189 | 663,6 | 19019 | 425 | 16 | 1,26 | H5A034 C | sample | Hm Drgbl | Hm Drgbl | Hm Drgbl | CPE002DC | CPE002EE | CPE002F4 | NM_00599 | NM_0059 | NM_0059 | STK10 | STK10 | STK10 | serine/threonine | serine/thr | serine/thr | 6793 | 6793 | 6793 | s13576 | s13577 | s13578 |
| 2621 | N13 | N14 | 955 | 637,1 | 15275 | 385 | 16 | 1,26 | H5A034 C | sample | Hm Drgbl | Hm Drgbl | Hm Drgbl | CPE002DC | CPE002EE | CPE002F4 | NM_02297 | NM_0229 | NM_0229 | FGFR2 | FGFR2 | FGFR2 | fibroblast growth | fibroblast | fibroblast | 2263 | 2263 | 2263 | s5173 | s5174 | s5175 |
| 2638 | N7 | N8 | 1175 | 671,4 | 18853 | 412,5 | 16 | 1,26 | H5A034 C | sample | Hm Drgbl | Hm Drgbl | Hm Drgbl | CPE002DC | CPE002EE | CPE002F4 | NM_00200 | NM_0020 | NM_0020 | FES | FES | FES | feline sarcoma or | feline sarc | feline sarc | 2242 | 2242 | 2242 | s5112 | s5113 | s5114 |
| 2641 | O1 | O2 | 722 | 664,5 | 11581 | 467,8 | 16 | 1,26 | H5A034 C | sample | Hm Drgbl | Hm Drgbl | Hm Drgbl | CPE002DC | CPE002EE | CPE002F4 | NM_00516 | NM_0051 | NM_0051 | ADRBK2 | ADRBK2 | ADRBK2 | adrenergic, beta, | adrenergi | adrenergi | 157 | 157 | 157 | s1130 | s1131 | s1132 |
| 2668 | P12 | P13 | 1307 | 703,7 | 20869 | 457,4 | 16 | 1,26 | H5A034 C | sample | Hm Drgbl | Hm Drgbl | Hm Drgbl | CPE002DC | CPE002EE | CPE002F4 | NM_00542 | NM_0054 | NM_0054 | TIE1 | TIE1 | TIE1 | tyrosine kinase w | tyrosine k | tyrosine k | 7075 | 7075 | 7075 | s14140 | s224730 | s14142 |
| 16524 | A2 | A3 | 941 | 1019 | 15024 | 412,1 | 16 | 1,265 | H5A034 C | sample | Hm Gen E | Hm Gen E | Hm Gen E | CPE002JH | CPE002NV | CPE002KC | NM_05284 | NM_0528 | NM_0528 | CCDC97 | CCDC97 | CCDC97 | coiled-coil domai | coiled-coi | coiled-coi | 90324 | 90324 | 90324 | s40330 | s40331 | s40332 |
| 16730 | J10 | J11 | 901 | 958,8 | 14454 | 324,6 | 16 | 1,265 | H5A034 C | sample | Hm Gen E | Hm Gen E | Hm Gen E | CPE002JH | CPE002NV | CPE002KC | NM_13877 | NM_1387 | NM_1387 | MRRF | MRRF | MRRF | mitochondrial rib | mitochon | mitochon | 92399 | 92399 | 92399 | s40947 | s40948 | s40949 |
| 16732 | J12 | J13 | 1237 | 922,4 | 19744 | 310,8 | 16 | 1,265 | H5A034 C | sample | Hm Gen E | Hm Gen E | Hm Gen E | CPE002JH | CPE002NV | CPE002KC | NM_03311 | NM_0331 | NM_0331 | RBM18 | RBM18 | RBM18 | RNA binding moti | RNA bindi | RNA bindi | 92400 | 92400 | 92400 | s40950 | s40951 | s40952 |
| 17692 | B12 | B13 | 1189 | 1002 | 19020 | 351,9 | 16 | 1,265 | H5A034 C | sample | Hm Gen E | Hm Gen E | Hm Gen E | CPE002JK | CPE002NZ | CPE002KF | NM_14470 | NM_1447 | NM_1447 | C2orf15 | C2orf15 | C2orf15 | chromosome 2 op | chromoso | chromoso | 150590 | 150590 | 150590 | s45461 | s45462 | s45463 |
| 17960 | M16 | M17 | 837 | 961,9 | 13432 | 203,9 | 16 | 1,265 | H5A034 C | sample | Hm Gen E | Hm Gen E | Hm Gen E | CPE002JK | CPE002NZ | CPE002KF | NM_15250 | NM_1525 | NM_1525 | C21orf129 | C21orf129 | C21orf129 | chromosome 21 o | chromoso | chromoso | 150135 | 150135 | 150135 | s45379 | s45380 | s45381 |
| 18014 | O21 | O22 | 1193 | 995,1 | 19108 | 363,1 | 16 | 1,265 | H5A034 C | sample | Hm Gen E | Hm Gen E | Hm Gen E | CPE002JK | CPE002NZ | CPE002KF | NM_18153 | NM_1815 | NM_1815 | ABHD12B | ABHD12B | ABHD12B | abhydrolase dom | abhydrola | abhydrola | 145447 | 145447 | 145447 | s44735 | s44736 | s44737 |
| 18048 | P9 | P10 | 933 | 1110 | 14972 | 279,8 | 16 | 1,265 | H5A034 C | sample | Hm Gen E | Hm Gen E | Hm Gen E | CPE002JK | CPE002NZ | CPE002KF | NM_13917 | NM_1391 | NM_1391 | TMEM190 | TMEM190 | TMEM190 | transmembrane p | transmem | transmem | 147744 | 147744 | 147744 | s45051 | s195768 | s195769 |
| 8901 | C6 | C7 | 1251 | 1639 | 20037 | 187,4 | 16 | 1,285 | H5A034 C | sample | Hm Drgbl | Hm Drgbl | Hm Drgbl | CPE002E7 | CPE002ES | CPE002FD | NM_20583 | NM_2058 | NM_2058 | LSR | LSR | LSR | lipolysis stimulate | lipolysis s | lipolysis s | 51599 | 51599 | 51599 | s28383 | s28384 | s28385 |
| 9144 | M9 | M10 | 784 | 1318 | 12548 | 142,2 | 16 | 1,285 | H5A034 C | sample | Hm Drgbl | Hm Drgbl | Hm Drgbl | CPE002E7 | CPE002ES | CPE002FD | NM_01599 | NM_0159 | NM_0159 | ADIPOR1 | ADIPOR1 | ADIPOR1 | adiponectin recep | adiponect | adiponect | 51094 | 51094 | 51094 | s27412 | s27410 | s27411 |
| 9179 | O19 | O20 | 2096 | 1872 | 33543 | 219,6 | 16 | 1,285 | H5A034 C | sample | Hm Drgbl | Hm Drgbl | Hm Drgbl | CPE002E7 | CPE002ES | CPE002FD | NM_01616 | NM_0161 | NM_0161 | A4GNT | A4GNT | A4GNT | alpha-1,4-N-acety | alpha-1,4- | alpha-1,4- | 51146 | 51146 | 51146 | s27549 | s27548 | s27550 |
| 4328 | E16 | E17 | 503 | 806,9 | 8063 | 404,4 | 16 | 1,35 | H5A034 C | sample | Hm Drgbl | Hm Drgbl | Hm Drgbl | CPE002DL | CPE002EK | CPE002FQ | NM_00470 | NM_0047 | NM_0047 | KCNQ4 | KCNQ4 | KCNQ4 | potassium voltage | potassium | potassium | 9132 | 9132 | 9132 | s17443 | s17444 | s17445 |
| 4355 | F19 | F20 | 600 | 729,6 | 9607 | 377,3 | 16 | 1,35 | H5A034 C | sample | Hm Drgbl | Hm Drgbl | Hm Drgbl | CPE002DL | CPE002EK | CPE002FQ | NM_13913 | NM_1391 | NM_1391 | KCNC2 | KCNC2 | KCNC2 | potassium voltage | potassium | potassium | 3747 | 3747 | 3747 | s7706 | s7707 | s7708 |
| 4492 | L12 | L13 | 1227 | 698,5 | 19642 | 403,2 | 16 | 1,35 | H5A034 C | sample | Hm Drgbl | Hm Drgbl | Hm Drgbl | CPE002DL | CPE002EK | CPE002FQ | NM_00325 | NM_0032 | NM_0032 | TIMP2 | TIMP2 | TIMP2 | TIMP metallopept | TIMP met | TIMP met | 7077 | 7077 | 7077 | s656 | s658 | s657 |
| 4525 | M20 | M21 | 1156 | 705,1 | 18541 | 454,6 | 16 | 1,35 | H5A034 C | sample | Hm Drgbl | Hm Drgbl | Hm Drgbl | CPE002DL | CPE002EK | CPE002FQ | NM_02116 | NM_0211 | NM_0211 | KCNK10 | KCNK10 | KCNK10 | potassium channe | potassium | potassium | 54207 | 54207 | 54207 | s28902 | s28903 | s28904 |
| 47 | B8 | B9 | 361 | 1420 | 5769 | 628,4 | 16 | 1,362 | H5A034 C | sample | Hm Drgbl | Hm Drgbl | Hm Drgbl | CPE002DT | CPE002EJ | CPE002F9 | NM_00101 | NM_0010 | NM_0010 | SP3 | SP3 | SP3 | Sp3 transcription | Sp3 transc | Sp3 transc | 6670 | 6670 | 6670 | s13324 | s13325 | s13326 |
| 249 | K17 | K18 | 410 | 1287 | 6578 | 461,4 | 16 | 1,362 | H5A034 C | sample | Hm Drgbl | Hm Drgbl | Hm Drgbl | CPE002DT | CPE002EJ | CPE002F9 | NM_00036 | NM_0003 | NM_0003 | THBD | THBD | THBD | thrombomodulin | thrombon | thrombon | 7056 | 7056 | 7056 | s14097 | s14095 | s14096 |
| 1974 | C14 | C15 | 1523 | 695,8 | 24370 | 460,1 | 16 | 1,391 | H5A034 C | sample | Hm Drgbl | Hm Drgbl | Hm Drgbl | CPE002FX | CPE002G1 | CPE002G5 | XM_37564 | XM_3756 | XM_3756 | ZNF525 | ZNF525 | ZNF525 | zinc finger protei | zinc finge | zinc finge | 170958 | 170958 | 170958 | s46875 | s46876 | s46877 |
| 2038 | E7 | E8 | 1175 | 718 | 18763 | 419,2 | 16 | 1,391 | H5A034 C | sample | Hm Drgbl | Hm Drgbl | Hm Drgbl | CPE002FX | CPE002G1 | CPE002G5 | XM_92685 | XM_9268 | XM_9268 | ZFP62 | ZFP62 | ZFP62 | zinc finger protei | zinc finge | zinc finge | 92379 | 92379 | 92379 | s40944 | s40945 | s40946 |
| 2097 | H17 | H18 | 594 | 502 | 9494 | 327,7 | 16 | 1,391 | H5A034 C | sample | Hm Drgbl | Hm Drgbl | Hm Drgbl | CPE002FX | CPE002G1 | CPE002G5 | NM_14468 | NM_1446 | NM_1446 | ZNF420 | ZNF420 | ZNF420 | zinc finger protei | zinc finge | zinc finge | 147923 | 147923 | 147923 | s45080 | s45081 | s45082 |
| 2254 | N7 | N8 | 1026 | 601,1 | 16381 | 438,7 | 16 | 1,391 | H5A034 C | sample | Hm Drgbl | Hm Drgbl | Hm Drgbl | CPE002FX | CPE002G1 | CPE002G5 | NM_00100 | NM_0010 | NM_0010 | ZNF483 | ZNF483 | ZNF483 | zinc finger protei | zinc finge | zinc finge | 158399 | 158399 | 158399 | s46070 | s46071 | s46072 |
| 3847 | A15 | A16 | 1028 | 785,3 | 16478 | 346,4 | 16 | 1,422 | H5A034 C | sample | Hm Drgbl | Hm Drgbl | Hm Drgbl | CPE002DS | CPE002EI | CPE002F8 | NM_20717 | NM_2071 | NM_2071 | NPSR1 | NPSR1 | NPSR1 | neuropeptide S re | neuropep | neuropep | 387129 | 387129 | 387129 | s51845 | s51846 | s51847 |
| 3990 | G14 | G15 | 929 | 705 | 14886 | 441,2 | 16 | 1,422 | H5A034 C | sample | Hm Drgbl | Hm Drgbl | Hm Drgbl | CPE002DS | CPE002EI | CPE002F8 | NM_13351 | NM_1335 | NM_1335 | RAD51L1 | RAD51L1 | RAD51L1 | RAD51-like 1 (S. c | RAD51-lik | RAD51-lik | 5890 | 5890 | 5890 | s11742 | s11741 | s11740 |
| 4013 | H13 | H14 | 764 | 712,6 | 12225 | 374,4 | 16 | 1,422 | H5A034 C | sample | Hm Drgbl | Hm Drgbl | Hm Drgbl | CPE002DS | CPE002EI | CPE002F8 | NM_00564 | NM_0056 | NM_0056 | TCEB1 | TCEB1 | TCEB1 | transcription elor | transcript | transcript | 6921 | 6921 | 6921 | s13858 | s13856 | s224708 |
| 4087 | K15 | K16 | 632 | 680,2 | 10120 | 337,5 | 16 | 1,422 | H5A034 C | sample | Hm Drgbl | Hm Drgbl | Hm Drgbl | CPE002DS | CPE002EI | CPE002F8 | NM_03294 | NM_0329 | NM_0329 | POLR2C | POLR2C | POLR2C | polymerase (RNA | polymeras | polymeras | 5432 | 5432 | 5432 | s10799 | s10800 | s10801 |
| 4152 | M9 | M10 | 462 | 667,8 | 7396 | 354,9 | 16 | 1,422 | H5A034 C | sample | Hm Drgbl | Hm Drgbl | Hm Drgbl | CPE002DS | CPE002EI | CPE002F8 | NM_02112 | NM_0211 | NM_0211 | POLR2L | POLR2L | POLR2L | polymerase (RNA | polymeras | polymeras | 5441 | 5441 | 5441 | s246 | s194701 | s194702 |
| 4155 | N11 | N12 | 1140 | 713,3 | 18264 | 451,9 | 16 | 1,422 | H5A034 C | sample | Hm Drgbl | Hm Drgbl | Hm Drgbl | CPE002DS | CPE002EI | CPE002F8 | NM_17584 | NM_1758 | NM_1758 | PTBP1 | PTBP1 | PTBP1 | polypyrimidine tr | polypyrim | polypyrim | 5725 | 5725 | 5725 | s11436 | s11435 | s11434 |
| 11621 | E13 | E14 | 1569 | 967,8 | 25051 | 198,3 | 16 | 1,429 | H5A034 C | sample | Hm Gen E | Hm Gen E | Hm Gen E | CPE002JA | CPE002NJ | CPE002JZ | NM_00517 | NM_0051 | NM_0051 | BCL3 | BCL3 | BCL3 | B-cell CLL/lymphc | B-cell CLL/ | B-cell CLL/ | 602 | 602 | 602 | s1926 | s1927 | s1928 |
| 11652 | F2 | F3 | 1236 | 778,4 | 19796 | 151,3 | 16 | 1,429 | H5A034 C | sample | Hm Gen E | Hm Gen E | Hm Gen E | CPE002JA | CPE002NJ | CPE002JZ | NM_00532 | NM_0053 | NM_0053 | HIST1H1T | HIST1H1T | HIST1H1T | histone cluster 1, | histone cl | histone cl | 3010 | 3010 | 3010 | s6406 | s6407 | s6408 |
| 11695 | H15 | H16 | 727 | 813,5 | 11663 | 175,8 | 16 | 1,429 | H5A034 C | sample | Hm Gen E | Hm Gen E | Hm Gen E | CPE002JA | CPE002NJ | CPE002JZ | NM_00520 | NM_0052 | NM_0052 | CRK | CRK | CRK | v-crk sarcoma viru | v-crk sarco | v-crk sarco | 1398 | 1398 | 1398 | s3519 | s3520 | s3521 |
| 11712 | H9 | H10 | 643 | 828,5 | 10270 | 152,4 | 16 | 1,429 | H5A034 C | sample | Hm Gen E | Hm Gen E | Hm Gen E | CPE002JA | CPE002NJ | CPE002JZ | NM_00130 | NM_0013 | NM_0013 | CORT | CORT | CORT | cortistatin | cortistatir | cortistatir | 1325 | 1325 | 1325 | s194341 | s194342 | s194343 |
| 15611 | K19 | K20 | 1025 | 835,8 | 16428 | 145,5 | 16 | 1,455 | H5A034 C | sample | Hm Gen E | Hm Gen E | Hm Gen E | CPE002JE | CPE002NT | CPE002K9 | NM_02234 | NM_0223 | NM_0223 | ZFYVE20 | ZFYVE20 | ZFYVE20 | zinc finger, FYVE c | zinc finge | zinc finge | 64145 | 64145 | 64145 | s34504 | s34505 | s34506 |
| 15728 | P16 | P17 | 1063 | 786 | 17049 | 157,5 | 16 | 1,455 | H5A034 C | sample | Hm Gen E | Hm Gen E | Hm Gen E | CPE002JE | CPE002NT | CPE002K9 | NM_02458 | NM_0245 | NM_0245 | C6orf60 | C6orf60 | C6orf60 | chromosome 6 op | chromoso | chromoso | 79632 | 79632 | 79632 | s35962 | s35963 | s35964 |
| 5941 | H20 | H21 | 2187 | 859,5 | 34988 | 543,1 | 16 | 1,461 | H5A034 C | sample | Hm Drgbl | Hm Drgbl | Hm Drgbl | CPE002DY | CPE002F1 | CPE002FM | NM_00173 | NM_0017 | NM_0017 | BTN1A1 | BTN1A1 | BTN1A1 | butyrophilin, sub | butyrophi | butyrophi | 696 | 696 | 696 | s2128 | s2129 | s2127 |
| 1437 | L6 | L7 | 1310 | 718,9 | 20974 | 366,5 | 16 | 1,468 | H5A034 C | sample | Hm Drgbl | Hm Drgbl | Hm Drgbl | CPE002FV | CPE002FZ | CPE002G3 | NM_01208 | NM_0120 | NM_0120 | ELL2 | ELL2 | ELL2 | elongation factor | elongatio | elongatio | 22936 | 22936 | 22936 | s22712 | s22713 | s22714 |
| 1522 | P18 | P19 | 1321 | 693,2 | 21144 | 530,2 | 16 | 1,468 | H5A034 C | sample | Hm Drgbl | Hm Drgbl | Hm Drgbl | CPE002FV | CPE002FZ | CPE002G3 | NM_01534 | NM_0153 | NM_0153 | ZFYVE26 | ZFYVE26 | ZFYVE26 | zinc finger, FYVE c | zinc finge | zinc finge | 23503 | 23503 | 23503 | s23943 | s23944 | s23945 |
| 16238 | E21 | E22 | 1390 | 1026 | 22253 | 255,4 | 16 | 1,468 | H5A034 C | sample | Hm Gen E | Hm Gen E | Hm Gen E | CPE002JG | CPE002NV | CPE002KB | NM_03096 | NM_0309 | NM_0309 | SPACA1 | SPACA1 | SPACA1 | sperm acrosome a | sperm acr | sperm acr | 81833 | 81833 | 81833 | s37809 | s37810 | s37811 |
| 16252 | F12 | F13 | 1538 | 944,3 | 24581 | 295,5 | 16 | 1,468 | H5A034 C | sample | Hm Gen E | Hm Gen E | Hm Gen E | CPE002JG | CPE002NV | CPE002KB | NM_05818 | NM_0581 | NM_0581 | C21orf67 | C21orf67 | C21orf67 | chromosome 21 o | chromoso | chromoso | 84536 | 84536 | 84536 | s39124 | s39125 | s195474 |
| 16267 | F4 | F5 | 1082 | 1032 | 17295 | 338,6 | 16 | 1,468 | H5A034 C | sample | Hm Gen E | Hm Gen E | Hm Gen E | CPE002JG | CPE002NV | CPE002KB | NM_03249 | NM_0324 | NM_0324 | ZC3H8 | ZC3H8 | ZC3H8 | zinc finger CCCH-t | zinc finge | zinc finge | 84524 | 84524 | 84524 | s39103 | s39104 | s39105 |
| 16283 | G19 | G20 | 1088 | 1063 | 17401 | 305,3 | 16 | 1,468 | H5A034 C | sample | Hm Gen E | Hm Gen E | Hm Gen E | CPE002JG | CPE002NV | CPE002KB | NM_03120 | NM_0312 | NM_0312 | LAS1L | LAS1L | LAS1L | LAS1-like (S. cerev | LAS1-like ( | LAS1-like ( | 81887 | 81887 | 81887 | s37860 | s37861 | s37862 |
| 16285 | G20 | G21 | 1172 | 1077 | 18697 | 426,3 | 16 | 1,468 | H5A034 C | sample | Hm Gen E | Hm Gen E | Hm Gen E | CPE002JG | CPE002NV | CPE002KB | NM_03231 | NM_0323 | NM_0323 | C2orf7 | C2orf7 | C2orf7 | chromosome 2 op | chromoso | chromoso | 84279 | 84279 | 84279 | s38798 | s38799 | s38800 |
| 16292 | G5 | G6 | 1431 | 982,3 | 22897 | 276,5 | 16 | 1,468 | H5A034 C | sample | Hm Gen E | Hm Gen E | Hm Gen E | CPE002JG | CPE002NV | CPE002KB | NM_03096 | NM_0309 | NM_0309 | KRTAP1-3 | KRTAP1-3 | KRTAP1-3 | keratin associated | keratin as | keratin as | 81850 | 81850 | 81850 | s37830 | s37831 | s37832 |
| 16334 | I21 | I22 | 742 | 1062 | 11890 | 293,2 | 16 | 1,468 | H5A034 C | sample | Hm Gen E | Hm Gen E | Hm Gen E | CPE002JG | CPE002NV | CPE002KB | NM_03129 | NM_0312 | NM_0312 | LRRC48 | LRRC48 | LRRC48 | leucine rich repea | leucine ric | leucine ric | 83450 | 83450 | 83450 | s37934 | s37935 | s37936 |
| 16349 | J13 | J14 | 1293 | 956,8 | 20708 | 319,7 | 16 | 1,468 | H5A034 C | sample | Hm Gen E | Hm Gen E | Hm Gen E | CPE002JG | CPE002NV | CPE002KB | NM_03212 | NM_0321 | NM_0321 | C1orf160 | C1orf160 | C1orf160 | chromosome 1 op | chromoso | chromoso | 84065 | 84065 | 84065 | s38435 | s38436 | s38437 |
| 13574 | F21 | F22 | 1480 | 1067 | 23741 | 186,5 | 16 | 1,502 | H5A034 C | sample | Hm Gen E | Hm Gen E | Hm Gen E | CPE002JC | CPE002NC | CPE002K4 | NM_01434 | NM_0143 | NM_0143 | FJX1 | FJX1 | FJX1 | four jointed box 1 | four jointe | four jointe | 24147 | 24147 | 24147 | s24429 | s24430 | s225983 |
| 13586 | G10 | G11 | 882 | 935,1 | 14135 | 157,9 | 16 | 1,502 | H5A034 C | sample | Hm Gen E | Hm Gen E | Hm Gen E | CPE002JC | CPE002NC | CPE002K4 | NM_01552 | NM_0155 | NM_0155 | IBTK | IBTK | IBTK | inhibitor of Bruto | inhibitor c | inhibitor c | 25998 | 25998 | 25998 | s24898 | s24899 | s24900 |
| 13702 | K7 | K8 | 1203 | 955,7 | 19295 | 175,3 | 16 | 1,502 | H5A034 C | sample | Hm Gen E | Hm Gen E | Hm Gen E | CPE002JC | CPE002NC | CPE002K4 | NM_01429 | NM_0142 | NM_0142 | CBX6 | CBX6 | CBX6 | chromobox homo | chromobc | chromobc | 23466 | 23466 | 23466 | s23877 | s23878 | s23879 |
| 13703 | K8 | K9 | 977 | 960 | 15614 | 171,7 | 16 | 1,502 | H5A034 C | sample | Hm Gen E | Hm Gen E | Hm Gen E | CPE002JC | CPE002NC | CPE002K4 | NM_01557 | NM_0155 | NM_0155 | RAI14 | RAI14 | RAI14 | retinoic acid indu | retinoic ac | retinoic ac | 26064 | 26064 | 26064 | s25047 | s25048 | s25049 |
| 13772 | N5 | N6 | 1048 | 958,8 | 16801 | 170,1 | 16 | 1,502 | H5A034 C | sample | Hm Gen E | Hm Gen E | Hm Gen E | CPE002JC | CPE002NC | CPE002K4 | NM_01540 | NM_0154 | NM_0154 | ABHD14A | ABHD14A | ABHD14A | abhydrolase dom | abhydrola | abhydrola | 25864 | 25864 | 25864 | s24641 | s24642 | s24643 |
| 792 | A9 | A10 | 1221 | 719,4 | 19476 | 484,6 | 16 | 1,517 | H5A034 C | sample | Hm Drgbl | Hm Drgbl | Hm Drgbl | CPE002FU | CPE002FY | CPE002G2 | NM_01629 | NM_0162 | NM_0162 | TRAP1 | TRAP1 | TRAP1 | TNF receptor-asso | TNF recep | TNF recep | 10131 | 10131 | 10131 | s177 | s178 | s179 |
| 1039 | L15 | L16 | 1334 | 734,5 | 21353 | 370 | 16 | 1,517 | H5A034 C | sample | Hm Drgbl | Hm Drgbl | Hm Drgbl | CPE002FU | CPE002FY | CPE002G2 | NM_00735 | NM_0073 | NM_0073 | HSP90AB1 | HSP90AB1 | HSP90AB1 | heat shock protei | heat shoc | heat shoc | 3326 | 3326 | 3326 | s6999 | s7000 | s7001 |
| 5553 | H17 | H18 | 1465 | 670,1 | 23446 | 529,3 | 16 | 1,524 | H5A034 C | sample | Hm Drgbl | Hm Drgbl | Hm Drgbl | CPE002DX | CPE002F2 | CPE002FN | NM_00677 | NM_0067 | NM_0067 | KRT38 | KRT38 | KRT38 | keratin 38 | keratin 38 | keratin 38 | 8687 | 8687 | 8687 | s16554 | s16552 | s16553 |
| 5757 | P6 | P7 | 1123 | 640,6 | 17932 | 656 | 16 | 1,524 | H5A034 C | sample | Hm Drgbl | Hm Drgbl | Hm Drgbl | CPE002DX | CPE002F2 | CPE002FN | NM_00478 | NM_0047 | NM_0047 | TXNL1 | TXNL1 | TXNL1 | thioredoxin-like 1 | thioredox | thioredox | 9352 | 9352 | 9352 | s17880 | s17881 | s17882 |
| 3804 | O2 | O3 | 1034 | 1395 | 16593 | 398,3 | 16 | 1,538 | H5A034 C | sample | Hm Drgbl | Hm Drgbl | Hm Drgbl | CPE002DR | CPE002EH | CPE002F7 | NM_08081 | NM_0808 | NM_0808 | GPR82 | GPR82 | GPR82 | G protein-coupled | G protein | G protein | 27197 | 27197 | 27197 | s25977 | s25978 | s25979 |
| 8385 | N17 | N18 | 1081 | 597 | 17279 | 607,6 | 16 | 1,538 | H5A034 C | sample | Hm Drgbl | Hm Drgbl | Hm Drgbl | CPE002E5 | CPE002EU | CPE002FF | NM_01500 | NM_0150 | NM_0150 | SPEN | SPEN | SPEN | spen homolog, tra | spen hom | spen hom | 23013 | 23013 | 23013 | s22831 | s22830 | s22829 |
| 8423 | O8 | O9 | 1316 | 617,5 | 21103 | 535,7 | 16 | 1,538 | H5A034 C | sample | Hm Drgbl | Hm Drgbl | Hm Drgbl | CPE002E5 | CPE002EU | CPE002FF | NM_14572 | NM_1457 | NM_1457 | DMN | DMN | DMN | desmuslin | desmuslin | desmuslin | 23336 | 23336 | 23336 | s23593 | s23594 | s23595 |
| 10100 | E5 | E6 | 1016 | 800,9 | 16289 | 156,3 | 16 | 1,538 | H5A034 C | sample | Hm Drgbl | Hm Drgbl | Hm Drgbl | CPE002EA | CPE002EP | CPE002FA | NM_02467 | NM_0246 | NM_0246 | NARS2 | NARS2 | NARS2 | asparaginyl-tRNA | asparagin | asparagin | 79731 | 79731 | 79731 | s36209 | s36207 | s36208 |
| 10116 | F2 | F3 | 1471 | 790,2 | 23604 | 152,5 | 16 | 1,538 | H5A034 C | sample | Hm Drgbl | Hm Drgbl | Hm Drgbl | CPE002EA | CPE002EP | CPE002FA | NM_03244 | NM_0324 | NM_0324 | MEGF10 | MEGF10 | MEGF10 | multiple EGF-like- | multiple E | multiple E | 84466 | 84466 | 84466 | s39059 | s39060 | s224948 |
| 10185 | I17 | I18 | 1295 | 753,7 | 20681 | 131,2 | 16 | 1,538 | H5A034 C | sample | Hm Drgbl | Hm Drgbl | Hm Drgbl | CPE002EA | CPE002EP | CPE002FA | NM_02482 | NM_0248 | NM_0248 | PODNL1 | PODNL1 | PODNL1 | podocan-like 1 | podocan-l | podocan-l | 79883 | 79883 | 79883 | s224838 | s36562 | s36560 |
| 1629 | D6 | D7 | 1293 | 723,7 | 20751 | 421,5 | 16 | 1,675 | H5A034 C | sample | Hm Drgbl | Hm Drgbl | Hm Drgbl | CPE002FW | CPE002GG | CPE002G4 | NM_02483 | NM_0248 | NM_0248 | ZNF672 | ZNF672 | ZNF672 | zinc finger protei | zinc finge | zinc finge | 79894 | 79894 | 79894 | s36590 | s36591 | s36592 |
| 1847 | M8 | M9 | 613 | 809,5 | 9827 | 451,6 | 16 | 1,675 | H5A034 C | sample | Hm Drgbl | Hm Drgbl | Hm Drgbl | CPE002FW | CPE002GG | CPE002G4 | NM_00104 | NM_0010 | NM_0010 | ATPAF1 | ATPAF1 | ATPAF1 | ATP synthase mit | ATP synth | ATP synth | 64756 | 64756 | 64756 | s34869 | s34870 | s34871 |
| 1918 | P7 | P8 | 907 | 738,2 | 14498 | 532,4 | 16 | 1,675 | H5A034 C | sample | Hm Drgbl | Hm Drgbl | Hm Drgbl | CPE002FW | CPE002GG | CPE002G4 | NM_02022 | NM_0202 | NM_0202 | PRDM9 | PRDM9 | PRDM9 | PR domain contai | PR domai | PR domai | 56979 | 56979 | 56979 | s32519 | s32520 | s224442 |
| 21655 | G15 | G16 | 131 | 1146 | 2100 | 879,1 | 16 | 1,684 | H5A034 C | sample | Hm Gen E | Hm Gen E | Hm Gen E | CPE002JU | CPE002O9 | CPE002KP | XM_93869 | XM_9386 | XM_9386 | LOC64962 | LOC64962 | LOC64962 | similar to Cell div | similar to | similar to | 649620 | 649620 | 649620 | s56987 | s56988 | s56989 |
| 4723 | E4 | E5 | 730 | 809,7 | 11681 | 507,3 | 16 | 1,758 | H5A034 C | sample | Hm Drgbl | Hm Drgbl | Hm Drgbl | CPE002DV | CPE002EL | CPE002FP | NM_02038 | NM_0203 | NM_0203 | XPNPEP1 | XPNPEP1 | XPNPEP1 | X-prolyl aminopep | X-prolyl ai | X-prolyl ai | 7511 | 7511 | 7511 | s14931 | s14932 | s14933 |
| 10499 | F19 | F20 | 1043 | 853,5 | 16654 | 205,9 | 16 | 1,808 | H5A034 C | sample | Hm Drgbl | Hm Drgbl | Hm Drgbl | CPE002EB | CPE002EO | CPE002FR | NM_17346 | NM_1734 | NM_1734 | MOBKL1A | MOBKL1A | MOBKL1A | MOB1, Mps One B | MOB1, Mp | MOB1, Mp | 92597 | 92597 | 92597 | s40977 | s40978 | s40979 |
| 10701 | N6 | N7 | 1323 | 827,7 | 21171 | 215,7 | 16 | 1,808 | H5A034 C | sample | Hm Drgbl | Hm Drgbl | Hm Drgbl | CPE002EB | CPE002EO | CPE002FR | NM_13332 | NM_1333 | NM_1332 | ATP6V1G3 | ATP6V1G3 | ATP6V1G3 | ATPase, H+ transp | ATPase, H | ATPase, H | 127124 | 127124 | 127124 | s43140 | s43138 | s223279 |
| 10710 | O14 | O15 | 1124 | 963,4 | 18025 | 239,6 | 16 | 1,808 | H5A034 C | sample | Hm Drgbl | Hm Drgbl | Hm Drgbl | CPE002EB | CPE002EO | CPE002FR | NM_05411 | NM_0541 | NM_0541 | TAGAP | TAGAP | TAGAP | T-cell activation G | T-cell acti | T-cell acti | 117289 | 117289 | 117289 | s42165 | s42164 | s42163 |
| 10743 | P22 | P23 | 1059 | 840,4 | 16955 | 313,2 | 16 | 1,808 | H5A034 C | sample | Hm Drgbl | Hm Drgbl | Hm Drgbl | CPE002EB | CPE002EO | CPE002FR | NM_14526 | NM_1452 | NM_1452 | OSR1 | OSR1 | OSR1 | odd-skipped relat | odd-skipp | odd-skipp | 130497 | 130497 | 130497 | s43500 | s43501 | s43502 |
| 22129 | K1 | K2 | 676 | 1504 | 10892 | 178,2 | 16,1 | 0,765 | H5A034 C | sample | Hm Gen E | Hm Gen E | Hm Gen E | CPE002JV | CPE002OA | CPE002KQ | XM_00112 | XM_0011 | XM_0011 | LOC72787 | LOC72787 | LOC72787 | similar to hCG203 | similar to | similar to | 727878 | 727878 | 727878 | s58016 | s58017 | s58018 |
| 22162 | L18 | L19 | 1182 | 1384 | 19049 | 339,1 | 16,1 | 0,765 | H5A034 C | sample | Hm Gen E | Hm Gen E | Hm Gen E | CPE002JV | CPE002OA | CPE002KQ | XM_00112 | XM_0011 | XM_0011 | LOC72851 | LOC72851 | LOC72851 | hypothetical LOC | hypotheti | hypotheti | 728510 | 728510 | 728510 | s58701 | s58702 | s58703 |
| 13297 | K1 | K2 | 1421 | 1106 | 22838 | 124,1 | 16,1 | 0,807 | H5A034 C | sample | Hm Gen E | Hm Gen E | Hm Gen E | CPE002JB | CPE002NN | CPE002K3 | NM_00664 | NM_0066 | NM_0066 | SDCCAG8 | SDCCAG8 | SDCCAG8 | serologically defir | serologica | serologica | 10806 | 10806 | 10806 | s21234 | s21235 | s21236 |
| 22500 | J2 | J3 | 792 | 1340 | 12744 | 367,7 | 16,1 | 0,83 | H5A034 C | sample | Hm Gen E | Hm Gen E | Hm Gen E | CPE002JW | CPE002OB | CPE002KR | XM_00112 | XM_0011 | XM_0011 | LOC72929 | LOC72929 | LOC72929 | similar to hCG201 | similar to | similar to | 729296 | 729296 | 729296 | s59594 | s59595 | s59596 |
| 22573 | M20 | M21 | 921 | 1416 | 14863 | 319,9 | 16,1 | 0,83 | H5A034 C | sample | Hm Gen E | Hm Gen E | Hm Gen E | CPE002JW | CPE002OB | CPE002KR | XM_00112 | XM_0011 | XM_0011 | LOC72916 | LOC72916 | LOC72916 | hypothetical prot | hypotheti | hypotheti | 729167 | 729167 | 729167 | s59439 | s59440 | s227747 |
| 23593 | H1 | H2 | 1268 | 1641 | 20421 | 196,3 | 16,1 | 0,854 | H5A034 C | sample | Hm Gen E | Hm Gen E | Hm Gen E | CPE002KV | CPE002OE | CPE002KU | XM_00113 | XM_0011 | XM_0011 | LOC73247 | LOC73247 | LOC73247 | hypothetical prot | hypotheti | hypotheti | 732472 | 732472 | 732472 | s200403 | s200404 | s200405 |
| 23652 | J2 | J3 | 947 | 1546 | 15240 | 268,4 | 16,1 | 0,854 | H5A034 C | sample | Hm Gen E | Hm Gen E | Hm Gen E | CPE002KV | CPE002OE | CPE002KU | | | | | | | | | | | | | | | |
| 14876 | L5 | L6 | 679 | 840,4 | 10924 | 128,7 | 16,1 | 0,917 | H5A034 C | sample | Hm Gen E | Hm Gen E | Hm Gen E | CPE002JD | CPE002NR | CPE002K7 | NM_01828 | NM_0182 | NM_0182 | C22orf26 | C22orf26 | C22orf26 | chromosome 22 o | chromoso | chromoso | 55267 | 55267 | 55267 | s30590 | s30591 | s30592 |
| 13997 | H13 | H14 | 1360 | 1063 | 21907 | 115,6 | 16,1 | 0,923 | H5A034 C | sample | Hm Gen E | Hm Gen E | Hm Gen E | CPE002JF | CPE002NP | CPE002K5 | NM_01649 | NM_0164 | NM_0164 | RANGRF | RANGRF | RANGRF | RAN guanine nucl | RAN guan | RAN guan | 29098 | 29098 | 29098 | s26480 | s195166 | s195167 |
| 14031 | I22 | I23 | 1400 | 1084 | 22560 | 185,3 | 16,1 | 0,923 | H5A034 C | sample | Hm Gen E | Hm Gen E | Hm Gen E | CPE002JF | CPE002NP | CPE002K5 | NM_01589 | NM_0158 | NM_0158 | STMN3 | STMN3 | STMN3 | stathmin-like 3 | stathmin- | stathmin- | 50861 | 50861 | 50861 | s27170 | s27171 | s195201 |
| 3080 | A16 | A17 | 1213 | 1177 | 19472 | 394,3 | 16,1 | 0,997 | H5A034 C | sample | Hm Drgbl | Hm Drgbl | Hm Drgbl | CPE002DC | CPE002EG | CPE002F6 | NM_01323 | NM_0132 | NM_0132 | PPP2R3B | PPP2R3B | PPP2R3B | protein phosphat | protein pl | protein pl | 28227 | 28227 | 28227 | s26252 | s26253 | s26254 |
| 3096 | A9 | A10 | 1151 | 1115 | 18570 | 374,7 | 16,1 | 0,997 | H5A034 C | sample | Hm Drgbl | Hm Drgbl | Hm Drgbl | CPE002DC | CPE002EG | CPE002F6 | NM_08039 | NM_0803 | NM_0803 | PTP4A2 | PTP4A2 | PTP4A2 | protein tyrosine p | protein ty | protein ty | 8073 | 8073 | 8073 | s224860 | s582 | s583 |
| 3157 | D20 | D21 | 1157 | 1296 | 18652 | 446,1 | 16,1 | 0,997 | H5A034 C | sample | Hm Drgbl | Hm Drgbl | Hm Drgbl | CPE002DC | CPE002EG | CPE002F6 | NM_00044 | NM_0004 | NM_0004 | PECAM1 | PECAM1 | PECAM1 | platelet/endothe | platelet/e | platelet/e | 5175 | 5175 | 5175 | s10285 | s10286 | s10287 |
| 3339 | L11 | L12 | 1007 | 1307 | 16254 | 400,8 | 16,1 | 0,997 | H5A034 C | sample | Hm Drgbl | Hm Drgbl | Hm Drgbl | CPE002DC | CPE002EG | CPE002F6 | NM_01463 | NM_0146 | NM_0146 | PPM1F | PPM1F | PPM1F | protein phosphat | protein pl | protein pl | 9647 | 9647 | 9647 | s18551 | s18552 | s18553 |
| 3409 | O1 | O2 | 116 | 1310 | 1864 | 568,8 | 16,1 | 0,997 | H5A034 C | sample | Hm Drgbl | Hm Drgbl | Hm Drgbl | CPE002DC | CPE002EG | CPE002F6 | NM_01422 | NM_0142 | NM_0142 | PPP2R1A | PPP2R1A | PPP2R1A | protein phosphat | protein pl | protein pl | 5518 | 5518 | 5518 | s10963 | s10964 | s10965 |
| 12001 | E1 | E2 | 1401 | 803,1 | 22489 | 107,5 | 16,1 | 1,032 | H5A034 C | sample | Hm Gen E | Hm Gen E | Hm Gen E | CPE002J4 | CPE002NL | CPE002K0 | NM_00555 | NM_0055 | NM_0055 | LAD1 | LAD1 | LAD1 | ladinin 1 | ladinin 1 | ladinin 1 | 3898 | 3898 | 3898 | s8039 | s8040 | s8041 |
| 12092 | H5 | H6 | 1078 | 735,5 | 17308 | 71,5 | 16,1 | 1,032 | H5A034 C | sample | Hm Gen E | Hm Gen E | Hm Gen E | CPE002J4 | CPE002NL | CPE002K0 | NM_00538 | NM_0053 | NM_0053 | NNAT | NNAT | NNAT | neuronatin | neuronati | neuronati | 4826 | 4826 | 4826 | s9579 | s9580 | s226688 |
| 12223 | N15 | N16 | 1033 | 776,6 | 16671 | 72,17 | 16,1 | 1,032 | H5A034 C | sample | Hm Gen E | Hm Gen E | Hm Gen E | CPE002J4 | CPE002NL | CPE002K0 | NM_05327 | NM_0532 | NM_0532 | VIT | VIT | VIT | vitrin | vitrin | vitrin | 5212 | 5212 | 5212 | s10366 | s10367 | s10368 |
| 18183 | F22 | F23 | 1449 | 962,7 | 23317 | 222,5 | 16,1 | 1,035 | H5A034 C | sample | Hm Gen E | Hm Gen E | Hm Gen E | CPE002JL | CPE002OC | CPE002KG | NM_15266 | NM_1526 | NM_1526 | TCTEX1D1 | TCTEX1D1 | TCTEX1D1 | Tctex1 domain co | Tctex1 do | Tctex1 do | 200132 | 200132 | 200132 | s47191 | s47192 | s47193 |
| 18302 | K21 | K22 | 1426 | 977,6 | 22932 | 400,9 | 16,1 | 1,035 | H5A034 C | sample | Hm Gen E | Hm Gen E | Hm Gen E | CPE002JL | CPE002OC | CPE002KG | NM_19884 | NM_1988 | NM_1988 | FAM120A | FAM120A | FAM120A | family with seque | family wit | family wit | 158293 | 158293 | 158293 | s46052 | s46053 | s46054 |
| 18335 | L8 | L9 | 1106 | 1128 | 17804 | 400,1 | 16,1 | 1,035 | H5A034 C | sample | Hm Gen E | Hm Gen E | Hm Gen E | CPE002JL | CPE002OC | CPE002KG | NM_15267 | NM_1526 | NM_1526 | MUC20 | MUC20 | MUC20 | mucin 20, cell sur | mucin 20, | mucin 20, | 200958 | 200958 | 200958 | s47302 | s47303 | s47304 |
| 18357 | M6 | M7 | 421 | 1067 | 6772 | 262,2 | 16,1 | 1,035 | H5A034 C | sample | Hm Gen E | Hm Gen E | Hm Gen E | CPE002JL | CPE002OC | CPE002KG | NM_19827 | NM_1982 | NM_1982 | MPZL3 | MPZL3 | MPZL3 | myelin protein ze | myelin pro | myelin pro | 196264 | 196264 | 196264 | s46976 | s46977 | s46978 |
| 18408 | O9 | O10 | 1442 | 1007 | 23234 | 524,6 | 16,1 | 1,035 | H5A034 C | sample | Hm Gen E | Hm Gen E | Hm Gen E | CPE002JL | CPE002OC | CPE002KG | NM_17352 | NM_1735 | NM_1735 | MAGEB6 | MAGEB6 | MAGEB6 | melanoma antige | melanoma | melanoma | 158809 | 158809 | 158809 | s46120 | s46121 | s46122 |
| 6263 | E8 | E9 | 660 | 587,8 | 10615 | 575,6 | 16,1 | 1,063 | H5A034 C | sample | Hm Drgbl | Hm Drgbl | Hm Drgbl | CPE002DZ | CPE002F0 | CPE002FL | XM_00112 | XM_0011 | XM_0011 | CTNND1 | CTNND1 | CTNND1 | catenin (cadherin | catenin (c | catenin (c | 1500 | 1500 | 1500 | s3725 | s3726 | s3727 |
| 6518 | P21 | P22 | 1491 | 601,7 | 24002 | 451,6 | 16,1 | 1,063 | H5A034 C | sample | Hm Drgbl | Hm Drgbl | Hm Drgbl | CPE002DZ | CPE002F0 | CPE002FL | NM_00407 | NM_0040 | NM_0040 | CS | CS | CS | citrate synthase | citrate syr | citrate syr | 1431 | 1431 | 1431 | s3583 | s3584 | s3582 |
| 20391 | B22 | B23 | 921 | 810,6 | 14873 | 233,6 | 16,1 | 1,073 | H5A034 C | sample | Hm Gen E | Hm Gen E | Hm Gen E | CPE002JR | CPE002O6 | CPE002KM | NM_00103 | NM_0010 | NM_0010 | FLJ16734 | FLJ16734 | FLJ16734 | hypothetical LOC | hypotheti | hypotheti | 641928 | 641928 | 641928 | s54887 | s54888 | s54889 |
| 20422 | C7 | C8 | 611 | 793,7 | 9838 | 250,8 | 16,1 | 1,073 | H5A034 C | sample | Hm Gen E | Hm Gen E | Hm Gen E | CPE002JR | CPE002O6 | CPE002KM | NM_00100 | NM_0010 | NM_0010 | FLJ41170 | FLJ41170 | FLJ41170 | hypothetical LOC | hypotheti | hypotheti | 440200 | 440200 | 440200 | s54061 | s54062 | s226608 |
| 20459 | E19 | E20 | 812 | 759,5 | 13057 | 229,2 | 16,1 | 1,073 | H5A034 C | sample | Hm Gen E | Hm Gen E | Hm Gen E | CPE002JR | CPE002O6 | CPE002KM | XM_49619 | XM_4961 | XM_4961 | TRE17 | TRE17 | TRE17 | TRE17 protein | TRE17 pro | TRE17 pro | 440414 | 440414 | 440414 | s54105 | s54106 | s54107 |
| 20587 | J4 | J5 | 414 | 728,3 | 6649 | 183,9 | 16,1 | 1,073 | H5A034 C | sample | Hm Gen E | Hm Gen E | Hm Gen E | CPE002JR | CPE002O6 | CPE002KM | XM_94337 | XM_9433 | XM_9433 | LOC64233 | LOC64233 | LOC64233 | hypothetical LOC | hypotheti | hypotheti | 642335 | 642335 | 642335 | s54983 | s54984 | s54985 |
| 12811 | F4 | F5 | 1099 | 1059 | 17669 | 133,9 | 16,1 | 1,092 | H5A034 C | sample | Hm Gen E | Hm Gen E | Hm Gen E | CPE002J6 | CPE002NN | CPE002K2 | NM_00586 | NM_0058 | NM_0058 | DSCR4 | DSCR4 | DSCR4 | Down syndrome c | Down syn | Down syn | 10281 | 10281 | 10281 | s20089 | s20090 | s20091 |

| | | | | | | | | | | | | | |
|---|---|---|---|---|---|---|---|---|---|---|---|---|---|
| 12900 | J2 | J3 | 1428 | 945,8 | 22995 | 153,4 | 16,1 | 1,092 | H5A034 C | sample | Hm Gen E | Hm Gen E | Hm Gen E |
| 18486 | C14 | C15 | 1462 | 991,1 | 23466 | 336,5 | 16,1 | 1,092 | H5A034 C | sample | Hm Gen E | Hm Gen E | Hm Gen E |
| 18756 | N2 | N3 | 1290 | 918,4 | 20813 | 159,2 | 16,1 | 1,092 | H5A034 C | sample | Hm Gen E | Hm Gen E | Hm Gen E |
| 17319 | B22 | B23 | 1478 | 1029 | 23863 | 288,9 | 16,1 | 1,099 | H5A034 C | sample | Hm Gen E | Hm Gen E | Hm Gen E |
| 17439 | G22 | G23 | 1340 | 960,2 | 21577 | 232,7 | 16,1 | 1,099 | H5A034 C | sample | Hm Gen E | Hm Gen E | Hm Gen E |
| 17481 | I17 | I18 | 1015 | 991 | 16299 | 183,2 | 16,1 | 1,099 | H5A034 C | sample | Hm Gen E | Hm Gen E | Hm Gen E |
| 17517 | J6 | J7 | 1037 | 975,6 | 16725 | 131,4 | 16,1 | 1,099 | H5A034 C | sample | Hm Gen E | Hm Gen E | Hm Gen E |
| 17542 | K7 | K8 | 568 | 943,1 | 9154 | 252,8 | 16,1 | 1,099 | H5A034 C | sample | Hm Gen E | Hm Gen E | Hm Gen E |
| 17642 | P10 | P11 | 1013 | 1025 | 16344 | 290,8 | 16,1 | 1,099 | H5A034 C | sample | Hm Gen E | Hm Gen E | Hm Gen E |
| 9373 | G20 | G21 | 1360 | 1091 | 21839 | 137,6 | 16,1 | 1,118 | H5A034 C | sample | Hm Drgbl | Hm Drgbl | Hm Drgbl |
| 9466 | K18 | K19 | 1600 | 1020 | 25717 | 147,7 | 16,1 | 1,118 | H5A034 C | sample | Hm Drgbl | Hm Drgbl | Hm Drgbl |
| 9486 | L14 | L15 | 1301 | 1048 | 21005 | 126,7 | 16,1 | 1,118 | H5A034 C | sample | Hm Drgbl | Hm Drgbl | Hm Drgbl |
| 18878 | C21 | C22 | 1255 | 965,3 | 20250 | 245,2 | 16,1 | 1,122 | H5A034 C | sample | Hm Gen E | Hm Gen E | Hm Gen E |
| 19040 | J16 | J17 | 1465 | 939,1 | 23609 | 170,5 | 16,1 | 1,122 | H5A034 C | sample | Hm Gen E | Hm Gen E | Hm Gen E |
| 9622 | A7 | A8 | 1792 | 1240 | 28817 | 225,6 | 16,1 | 1,126 | H5A034 C | sample | Hm Drgbl | Hm Drgbl | Hm Drgbl |
| 9680 | D16 | D17 | 1407 | 1127 | 22590 | 175,1 | 16,1 | 1,126 | H5A034 C | sample | Hm Drgbl | Hm Drgbl | Hm Drgbl |
| 9838 | J7 | J8 | 1126 | 1067 | 18112 | 175,6 | 16,1 | 1,126 | H5A034 C | sample | Hm Drgbl | Hm Drgbl | Hm Drgbl |
| 9879 | L22 | L23 | 1199 | 1175 | 19255 | 206,4 | 16,1 | 1,126 | H5A034 C | sample | Hm Drgbl | Hm Drgbl | Hm Drgbl |
| 9921 | N17 | N18 | 1705 | 1169 | 27449 | 145,8 | 16,1 | 1,126 | H5A034 C | sample | Hm Drgbl | Hm Drgbl | Hm Drgbl |
| 9975 | P22 | P23 | 1713 | 1028 | 27512 | 237,2 | 16,1 | 1,126 | H5A034 C | sample | Hm Drgbl | Hm Drgbl | Hm Drgbl |
| 15227 | K19 | K20 | 1343 | 837,2 | 21592 | 142,6 | 16,1 | 1,126 | H5A034 C | sample | Hm Gen E | Hm Gen E | Hm Gen E |
| 19262 | C21 | C22 | 1064 | 1271 | 17171 | 302,7 | 16,1 | 1,142 | H5A034 C | sample | Hm Gen E | Hm Gen E | Hm Gen E |
| 21074 | O10 | O11 | 891 | 984,3 | 14347 | 360,9 | 16,1 | 1,142 | H5A034 C | sample | Hm Gen E | Hm Gen E | Hm Gen E |
| 21102 | P14 | P15 | 642 | 1136 | 10327 | 415,6 | 16,1 | 1,142 | H5A034 C | sample | Hm Gen E | Hm Gen E | Hm Gen E |
| 21155 | B19 | B20 | 1148 | 1033 | 18506 | 377,3 | 16,1 | 1,142 | H5A034 C | sample | Hm Gen E | Hm Gen E | Hm Gen E |
| 21191 | C8 | C9 | 1037 | 996,8 | 16653 | 287 | 16,1 | 1,142 | H5A034 C | sample | Hm Gen E | Hm Gen E | Hm Gen E |
| 14216 | A16 | A17 | 1416 | 906,8 | 22770 | 168,4 | 16,1 | 1,146 | H5A034 C | sample | Hm Gen E | Hm Gen E | Hm Gen E |
| 14352 | F9 | F10 | 1075 | 833,3 | 17361 | 130,8 | 16,1 | 1,146 | H5A034 C | sample | Hm Gen E | Hm Gen E | Hm Gen E |
| 14525 | N13 | N14 | 1618 | 817,4 | 26070 | 132,3 | 16,1 | 1,146 | H5A034 C | sample | Hm Gen E | Hm Gen E | Hm Gen E |
| 11410 | L18 | L19 | 1836 | 864,1 | 29589 | 194,7 | 16,1 | 1,167 | H5A034 C | sample | Hm Drgbl | Hm Drgbl | Hm Drgbl |
| 11505 | P17 | P18 | 1454 | 793,8 | 23412 | 182,1 | 16,1 | 1,167 | H5A034 C | sample | Hm Drgbl | Hm Drgbl | Hm Drgbl |
| 12372 | D2 | D3 | 1329 | 983,4 | 21366 | 148,5 | 16,1 | 1,211 | H5A034 C | sample | Hm Gen E | Hm Gen E | Hm Gen E |
| 12399 | E22 | E23 | 1681 | 1017 | 27144 | 209,7 | 16,1 | 1,211 | H5A034 C | sample | Hm Gen E | Hm Gen E | Hm Gen E |
| 12632 | O16 | O17 | 1188 | 976,4 | 19102 | 156,9 | 16,1 | 1,211 | H5A034 C | sample | Hm Gen E | Hm Gen E | Hm Gen E |
| 10932 | H2 | H3 | 1089 | 812,6 | 17493 | 438,7 | 16,1 | 1,22 | H5A034 C | sample | Hm Drgbl | Hm Drgbl | Hm Drgbl |
| 7559 | K8 | K9 | 1481 | 630 | 23909 | 466,7 | 16,1 | 1,229 | H5A034 C | sample | Hm Drgbl | Hm Drgbl | Hm Drgbl |
| 17046 | G14 | G15 | 1152 | 892,8 | 18544 | 187 | 16,1 | 1,243 | H5A034 C | sample | Hm Gen E | Hm Gen E | Hm Gen E |
| 17127 | J22 | J23 | 1318 | 926,6 | 21229 | 216,4 | 16,1 | 1,243 | H5A034 C | sample | Hm Gen E | Hm Gen E | Hm Gen E |
| 7194 | L3 | L4 | 1177 | 551,7 | 18942 | 419,9 | 16,1 | 1,253 | H5A034 C | sample | Hm Drgbl | Hm Drgbl | Hm Drgbl |
| 7275 | P11 | P12 | 1063 | 562,3 | 17089 | 510,9 | 16,1 | 1,253 | H5A034 C | sample | Hm Drgbl | Hm Drgbl | Hm Drgbl |
| 4994 | A10 | A11 | 1346 | 642,7 | 21608 | 661,6 | 16,1 | 1,263 | H5A034 C | sample | Hm Drgbl | Hm Drgbl | Hm Drgbl |
| 5124 | F2 | F3 | 1634 | 596,9 | 26245 | 600,3 | 16,1 | 1,263 | H5A034 C | sample | Hm Drgbl | Hm Drgbl | Hm Drgbl |
| 5158 | G7 | G8 | 954 | 589,8 | 15379 | 436,9 | 16,1 | 1,263 | H5A034 C | sample | Hm Drgbl | Hm Drgbl | Hm Drgbl |
| 5234 | K10 | K11 | 1234 | 601 | 19879 | 524,9 | 16,1 | 1,263 | H5A034 C | sample | Hm Drgbl | Hm Drgbl | Hm Drgbl |
| 5240 | K16 | K17 | 835 | 600,7 | 13443 | 363,6 | 16,1 | 1,263 | H5A034 C | sample | Hm Drgbl | Hm Drgbl | Hm Drgbl |
| 2546 | K10 | K11 | 1152 | 655,2 | 18571 | 321,6 | 16,1 | 1,268 | H5A034 C | sample | Hm Drgbl | Hm Drgbl | Hm Drgbl |
| 2678 | P21 | P22 | 1666 | 710,6 | 26745 | 521,9 | 16,1 | 1,268 | H5A034 C | sample | Hm Drgbl | Hm Drgbl | Hm Drgbl |
| 16539 | B11 | B12 | 851 | 950,2 | 13739 | 397 | 16,1 | 1,273 | H5A034 C | sample | Hm Gen E | Hm Gen E | Hm Gen E |
| 16609 | E1 | E2 | 901 | 991,3 | 14474 | 289,8 | 16,1 | 1,273 | H5A034 C | sample | Hm Gen E | Hm Gen E | Hm Gen E |
| 16775 | K8 | K9 | 782 | 942,2 | 12618 | 336,4 | 16,1 | 1,273 | H5A034 C | sample | Hm Gen E | Hm Gen E | Hm Gen E |
| 16851 | O11 | O12 | 1028 | 906,8 | 16583 | 475,9 | 16,1 | 1,273 | H5A034 C | sample | Hm Gen E | Hm Gen E | Hm Gen E |
| 17738 | D10 | D11 | 1194 | 983,1 | 19266 | 300,4 | 16,1 | 1,273 | H5A034 C | sample | Hm Gen E | Hm Gen E | Hm Gen E |
| 17750 | D21 | D22 | 1340 | 1081 | 21555 | 293,1 | 16,1 | 1,273 | H5A034 C | sample | Hm Gen E | Hm Gen E | Hm Gen E |
| 17784 | E9 | E10 | 777 | 991,1 | 12482 | 272 | 16,1 | 1,273 | H5A034 C | sample | Hm Gen E | Hm Gen E | Hm Gen E |
| 17792 | F16 | F17 | 1196 | 1013 | 19207 | 290,2 | 16,1 | 1,273 | H5A034 C | sample | Hm Gen E | Hm Gen E | Hm Gen E |
| 8996 | G5 | G6 | 1154 | 1794 | 18556 | 135,7 | 16,1 | 1,293 | H5A034 C | sample | Hm Drgbl | Hm Drgbl | Hm Drgbl |
| 9039 | I22 | I23 | 1788 | 1702 | 28713 | 218 | 16,1 | 1,293 | H5A034 C | sample | Hm Drgbl | Hm Drgbl | Hm Drgbl |
| 4352 | F16 | F17 | 948 | 660,9 | 15236 | 374,9 | 16,1 | 1,359 | H5A034 C | sample | Hm Drgbl | Hm Drgbl | Hm Drgbl |
| 4574 | O21 | O22 | 788 | 718,7 | 12675 | 485,5 | 16,1 | 1,359 | H5A034 C | sample | Hm Drgbl | Hm Drgbl | Hm Drgbl |
| 181 | H20 | H21 | 510 | 1417 | 8210 | 602,5 | 16,1 | 1,37 | H5A034 C | sample | Hm Drgbl | Hm Drgbl | Hm Drgbl |
| 1930 | A18 | A19 | 1470 | 720,8 | 23601 | 469,2 | 16,1 | 1,4 | H5A034 C | sample | Hm Drgbl | Hm Drgbl | Hm Drgbl |
| 2156 | J5 | J6 | 890 | 638,5 | 14297 | 363,6 | 16,1 | 1,4 | H5A034 C | sample | Hm Drgbl | Hm Drgbl | Hm Drgbl |
| 6546 | A3 | A4 | 1114 | 614,8 | 17891 | 533,6 | 16,1 | 1,412 | H5A034 C | sample | Hm Drgbl | Hm Drgbl | Hm Drgbl |
| 6658 | F18 | F19 | 1209 | 591 | 19421 | 394,5 | 16,1 | 1,412 | H5A034 C | sample | Hm Drgbl | Hm Drgbl | Hm Drgbl |
| 6738 | I3 | I4 | 784 | 562,6 | 12599 | 468,3 | 16,1 | 1,412 | H5A034 C | sample | Hm Drgbl | Hm Drgbl | Hm Drgbl |
| 6874 | O18 | O19 | 1411 | 556,5 | 22686 | 538,4 | 16,1 | 1,412 | H5A034 C | sample | Hm Drgbl | Hm Drgbl | Hm Drgbl |
| 3874 | B18 | B19 | 1203 | 663,8 | 19364 | 403,9 | 16,1 | 1,431 | H5A034 C | sample | Hm Drgbl | Hm Drgbl | Hm Drgbl |
| 3984 | F9 | F10 | 852 | 633,7 | 13729 | 365,2 | 16,1 | 1,431 | H5A034 C | sample | Hm Drgbl | Hm Drgbl | Hm Drgbl |
| 4076 | J5 | J6 | 1031 | 695,2 | 16634 | 320,1 | 16,1 | 1,431 | H5A034 C | sample | Hm Drgbl | Hm Drgbl | Hm Drgbl |
| 4127 | L8 | L9 | 793 | 625,7 | 12746 | 409,5 | 16,1 | 1,431 | H5A034 C | sample | Hm Drgbl | Hm Drgbl | Hm Drgbl |
| 4172 | N5 | N6 | 62 | 850,9 | 998 | 449,9 | 16,1 | 1,431 | H5A034 C | sample | Hm Drgbl | Hm Drgbl | Hm Drgbl |
| 11551 | B15 | B16 | 1264 | 897,4 | 20331 | 222,9 | 16,1 | 1,438 | H5A034 C | sample | Hm Gen E | Hm Gen E | Hm Gen E |
| 11698 | H18 | H19 | 1549 | 822,1 | 24883 | 141,9 | 16,1 | 1,438 | H5A034 C | sample | Hm Gen E | Hm Gen E | Hm Gen E |
| 11858 | O10 | O11 | 924 | 974,2 | 14852 | 323 | 16,1 | 1,438 | H5A034 C | sample | Hm Gen E | Hm Gen E | Hm Gen E |
| 15516 | G2 | G3 | 1030 | 846 | 16565 | 158,4 | 16,1 | 1,464 | H5A034 C | sample | Hm Gen E | Hm Gen E | Hm Gen E |
| 15577 | J1 | J2 | 1039 | 802,8 | 16705 | 146,8 | 16,1 | 1,464 | H5A034 C | sample | Hm Gen E | Hm Gen E | Hm Gen E |
| 15583 | J15 | J16 | 1261 | 850,9 | 20280 | 141,7 | 16,1 | 1,464 | H5A034 C | sample | Hm Gen E | Hm Gen E | Hm Gen E |
| 15589 | J20 | J21 | 1070 | 775,4 | 17203 | 135,5 | 16,1 | 1,464 | H5A034 C | sample | Hm Gen E | Hm Gen E | Hm Gen E |
| 15711 | O22 | O23 | 1489 | 791 | 24042 | 178,7 | 16,1 | 1,464 | H5A034 C | sample | Hm Gen E | Hm Gen E | Hm Gen E |
| 15734 | P21 | P22 | 948 | 848,5 | 15273 | 218 | 16,1 | 1,464 | H5A034 C | sample | Hm Gen E | Hm Gen E | Hm Gen E |
| 1156 | A12 | A13 | 774 | 825,6 | 12473 | 529,7 | 16,1 | 1,477 | H5A034 C | sample | Hm Drgbl | Hm Drgbl | Hm Drgbl |
| 1268 | E5 | E6 | 757 | 726,1 | 12217 | 470,2 | 16,1 | 1,477 | H5A034 C | sample | Hm Drgbl | Hm Drgbl | Hm Drgbl |
| 1339 | H4 | H5 | 869 | 686,9 | 14018 | 438,7 | 16,1 | 1,477 | H5A034 C | sample | Hm Drgbl | Hm Drgbl | Hm Drgbl |
| 1345 | I1 | I2 | 832 | 766,6 | 13436 | 531,9 | 16,1 | 1,477 | H5A034 C | sample | Hm Drgbl | Hm Drgbl | Hm Drgbl |
| 1422 | L14 | L15 | 1071 | 703,7 | 17276 | 370,3 | 16,1 | 1,477 | H5A034 C | sample | Hm Drgbl | Hm Drgbl | Hm Drgbl |
| 16330 | I18 | I19 | 1078 | 942,6 | 17307 | 360,7 | 16,1 | 1,477 | H5A034 C | sample | Hm Gen E | Hm Gen E | Hm Gen E |
| 16470 | O14 | O15 | 1252 | 1066 | 20217 | 496,7 | 16,1 | 1,477 | H5A034 C | sample | Hm Gen E | Hm Gen E | Hm Gen E |
| 13564 | F12 | F13 | 1023 | 969,7 | 16504 | 155,5 | 16,1 | 1,512 | H5A034 C | sample | Hm Gen E | Hm Gen E | Hm Gen E |
| 13604 | G5 | G6 | 1216 | 1009 | 19599 | 175,4 | 16,1 | 1,512 | H5A034 C | sample | Hm Gen E | Hm Gen E | Hm Gen E |
| 956 | H5 | H6 | 1605 | 759,3 | 25773 | 465,7 | 16,1 | 1,526 | H5A034 C | sample | Hm Drgbl | Hm Drgbl | Hm Drgbl |
| 967 | I15 | I16 | 575 | 642,8 | 9255 | 445,3 | 16,1 | 1,526 | H5A034 C | sample | Hm Drgbl | Hm Drgbl | Hm Drgbl |
| 5387 | A19 | A20 | 1074 | 708,4 | 17286 | 685,2 | 16,1 | 1,533 | H5A034 C | sample | Hm Drgbl | Hm Drgbl | Hm Drgbl |
| 5430 | C14 | C15 | 958 | 575,6 | 15396 | 686,5 | 16,1 | 1,533 | H5A034 C | sample | Hm Drgbl | Hm Drgbl | Hm Drgbl |
| 5498 | F10 | F11 | 1596 | 662,5 | 25641 | 612,3 | 16,1 | 1,533 | H5A034 C | sample | Hm Drgbl | Hm Drgbl | Hm Drgbl |
| 5502 | F14 | F15 | 1385 | 657,2 | 22330 | 541,3 | 16,1 | 1,533 | H5A034 C | sample | Hm Drgbl | Hm Drgbl | Hm Drgbl |
| 5667 | M11 | M12 | 936 | 740,5 | 15071 | 402,7 | 16,1 | 1,533 | H5A034 C | sample | Hm Drgbl | Hm Drgbl | Hm Drgbl |
| 5719 | O15 | O16 | 1236 | 698,9 | 19910 | 638,7 | 16,1 | 1,533 | H5A034 C | sample | Hm Drgbl | Hm Drgbl | Hm Drgbl |
| 8365 | M20 | M21 | 1363 | 706,4 | 21983 | 488,1 | 16,1 | 1,548 | H5A034 C | sample | Hm Drgbl | Hm Drgbl | Hm Drgbl |
| 8403 | O11 | O12 | 1223 | 626,7 | 19654 | 519,4 | 16,1 | 1,548 | H5A034 C | sample | Hm Drgbl | Hm Drgbl | Hm Drgbl |
| 10010 | B10 | B11 | 1466 | 828,7 | 23602 | 184,6 | 16,1 | 1,548 | H5A034 C | sample | Hm Drgbl | Hm Drgbl | Hm Drgbl |
| 10017 | B17 | B18 | 1570 | 821,2 | 25339 | 194,4 | 16,1 | 1,548 | H5A034 C | sample | Hm Drgbl | Hm Drgbl | Hm Drgbl |
| 10229 | K13 | K14 | 939 | 828,7 | 15114 | 172,2 | 16,1 | 1,548 | H5A034 C | sample | Hm Drgbl | Hm Drgbl | Hm Drgbl |
| 10357 | P20 | P21 | 1564 | 851,3 | 25165 | 174 | 16,1 | 1,548 | H5A034 C | sample | Hm Drgbl | Hm Drgbl | Hm Drgbl |
| 1585 | C1 | C2 | 464 | 744,8 | 7475 | 464,3 | 16,1 | 1,686 | H5A034 C | sample | Hm Drgbl | Hm Drgbl | Hm Drgbl |
| 1690 | G18 | G19 | 802 | 697,9 | 12937 | 387,8 | 16,1 | 1,686 | H5A034 C | sample | Hm Drgbl | Hm Drgbl | Hm Drgbl |
| 1877 | O13 | O14 | 1253 | 695,3 | 20189 | 438,3 | 16,1 | 1,686 | H5A034 C | sample | Hm Drgbl | Hm Drgbl | Hm Drgbl |
| 21564 | C2 | C3 | 187 | 1106 | 3012 | 855,8 | 16,1 | 1,695 | H5A034 C | sample | Hm Gen E | Hm Gen E | Hm Gen E |
| 21628 | F12 | F13 | 113 | 867,4 | 1814 | 635,7 | 16,1 | 1,695 | H5A034 C | sample | Hm Gen E | Hm Gen E | Hm Gen E |
| 21698 | I10 | I11 | 140 | 980,9 | 2256 | 635,8 | 16,1 | 1,695 | H5A034 C | sample | Hm Gen E | Hm Gen E | Hm Gen E |
| 21845 | O13 | O14 | 424 | 966,9 | 6842 | 743,4 | 16,1 | 1,695 | H5A034 C | sample | Hm Gen E | Hm Gen E | Hm Gen E |
| 21850 | O18 | O19 | 289 | 1009 | 4652 | 744,5 | 16,1 | 1,695 | H5A034 C | sample | Hm Gen E | Hm Gen E | Hm Gen E |
| 4749 | F6 | F7 | 1108 | 749,4 | 17838 | 512,2 | 16,1 | 1,769 | H5A034 C | sample | Hm Drgbl | Hm Drgbl | Hm Drgbl |
| 23713 | M1 | M2 | 914 | 1568 | 14823 | 170,8 | 16,2 | 0,859 | H5A034 C | sample | Hm Gen E | Hm Gen E | Hm Gen E |
| 23756 | N5 | N6 | 837 | 1526 | 13598 | 191,3 | 16,2 | 0,859 | H5A034 C | sample | Hm Gen E | Hm Gen E | Hm Gen E |
| 14673 | D17 | D18 | 1165 | 827,6 | 18830 | 86,07 | 16,2 | 0,923 | H5A034 C | sample | Hm Gen E | Hm Gen E | Hm Gen E |
| 14761 | H1 | H2 | 974 | 855,8 | 15819 | 112,6 | 16,2 | 0,923 | H5A034 C | sample | Hm Gen E | Hm Gen E | Hm Gen E |
| 14765 | H13 | H14 | 797 | 826,3 | 12886 | 101 | 16,2 | 0,923 | H5A034 C | sample | Hm Gen E | Hm Gen E | Hm Gen E |
| 14844 | K2 | K3 | 1535 | 903,4 | 24871 | 115,1 | 16,2 | 0,923 | H5A034 C | sample | Hm Gen E | Hm Gen E | Hm Gen E |
| 3076 | A12 | A13 | 750 | 1257 | 12176 | 483,4 | 16,2 | 1,003 | H5A034 C | sample | Hm Drgbl | Hm Drgbl | Hm Drgbl |
| 3120 | B9 | B10 | 732 | 1273 | 11886 | 405,5 | 16,2 | 1,003 | H5A034 C | sample | Hm Drgbl | Hm Drgbl | Hm Drgbl |
| 3130 | C18 | C19 | 634 | 1129 | 10247 | 454,9 | 16,2 | 1,003 | H5A034 C | sample | Hm Drgbl | Hm Drgbl | Hm Drgbl |
| 3134 | C21 | C22 | 1173 | 1319 | 19010 | 480,1 | 16,2 | 1,003 | H5A034 C | sample | Hm Drgbl | Hm Drgbl | Hm Drgbl |
| 3172 | E12 | E13 | 1112 | 1196 | 18031 | 352,1 | 16,2 | 1,003 | H5A034 C | sample | Hm Drgbl | Hm Drgbl | Hm Drgbl |
| 3262 | H7 | H8 | 909 | 1263 | 14711 | 426 | 16,2 | 1,003 | H5A034 C | sample | Hm Drgbl | Hm Drgbl | Hm Drgbl |
| 3271 | I15 | I16 | 914 | 1141 | 14764 | 355,6 | 16,2 | 1,003 | H5A034 C | sample | Hm Drgbl | Hm Drgbl | Hm Drgbl |
| 3455 | P8 | P9 | 1388 | 1246 | 22487 | 486,3 | 16,2 | 1,003 | H5A034 C | sample | Hm Drgbl | Hm Drgbl | Hm Drgbl |
| 11913 | A17 | A18 | 2004 | 861,4 | 32492 | 139,8 | 16,2 | 1,038 | H5A034 C | sample | Hm Gen E | Hm Gen E | Hm Gen E |
| 18091 | B4 | B5 | 1108 | 1042 | 17921 | 440,4 | 16,2 | 1,042 | H5A034 C | sample | Hm Gen E | Hm Gen E | Hm Gen E |
| 19906 | N18 | N19 | 1137 | 322,8 | 18403 | 211,8 | 16,2 | 1,055 | H5A034 C | sample | Hm Gen E | Hm Gen E | Hm Gen E |
| 6315 | H11 | H12 | 1613 | 641,7 | 26125 | 443,8 | 16,2 | 1,069 | H5A034 C | sample | Hm Drgbl | Hm Drgbl | Hm Drgbl |
| 6364 | J12 | J13 | 1692 | 572,2 | 27482 | 346,4 | 16,2 | 1,069 | H5A034 C | sample | Hm Drgbl | Hm Drgbl | Hm Drgbl |
| 6498 | O3 | O4 | 1252 | 557,9 | 20261 | 554,9 | 16,2 | 1,069 | H5A034 C | sample | Hm Drgbl | Hm Drgbl | Hm Drgbl |
| 20388 | B2 | B3 | 802 | 821,5 | 12961 | 248,4 | 16,2 | 1,08 | H5A034 C | sample | Hm Gen E | Hm Gen E | Hm Gen E |
| 20448 | D9 | D10 | 895 | 790,6 | 14461 | 266,9 | 16,2 | 1,08 | H5A034 C | sample | Hm Gen E | Hm Gen E | Hm Gen E |
| 20451 | E11 | E12 | 556 | 743,1 | 9008 | 191,7 | 16,2 | 1,08 | H5A034 C | sample | Hm Gen E | Hm Gen E | Hm Gen E |
| 20582 | J21 | J22 | 600 | 830,7 | 9741 | 235,8 | 16,2 | 1,08 | H5A034 C | sample | Hm Gen E | Hm Gen E | Hm Gen E |
| 12823 | G15 | G16 | 1072 | 893,7 | 17331 | 129,6 | 16,2 | 1,098 | H5A034 C | sample | Hm Gen E | Hm Gen E | Hm Gen E |
| 12963 | M11 | M12 | 1203 | 946,2 | 19438 | 137 | 16,2 | 1,098 | H5A034 C | sample | Hm Gen E | Hm Gen E | Hm Gen E |
| 18572 | F5 | F6 | 1199 | 1000 | 19441 | 214,8 | 16,2 | 1,098 | H5A034 C | sample | Hm Gen E | Hm Gen E | Hm Gen E |
| 18711 | L22 | L23 | 1597 | 991,5 | 25850 | 263,4 | 16,2 | 1,098 | H5A034 C | sample | Hm Gen E | Hm Gen E | Hm Gen E |
| 17499 | J11 | J12 | 910 | 975,6 | 14742 | 251,8 | 16,2 | 1,106 | H5A034 C | sample | Hm Gen E | Hm Gen E | Hm Gen E |
| 17538 | K3 | K4 | 988 | 896,8 | 16025 | 164,2 | 16,2 | 1,106 | H5A034 C | sample | Hm Gen E | Hm Gen E | Hm Gen E |
| 17612 | N5 | N6 | 1569 | 1014 | 25377 | 249 | 16,2 | 1,106 | H5A034 C | sample | Hm Gen E | Hm Gen E | Hm Gen E |
| 17623 | O15 | O16 | 868 | 1033 | 14038 | 305,6 | 16,2 | 1,106 | H5A034 C | sample | Hm Gen E | Hm Gen E | Hm Gen E |
| 9286 | C7 | C8 | 1141 | 1151 | 18507 | 204,8 | 16,2 | 1,125 | H5A034 C | sample | Hm Drgbl | Hm Drgbl | Hm Drgbl |
| 9306 | D3 | D4 | 1423 | 1157 | 23040 | 171,9 | 16,2 | 1,125 | H5A034 C | sample | Hm Drgbl | Hm Drgbl | Hm Drgbl |
| 9370 | G18 | G19 | 1325 | 1044 | 21462 | 155,3 | 16,2 | 1,125 | H5A034 C | sample | Hm Drgbl | Hm Drgbl | Hm Drgbl |
| 9406 | H7 | H8 | 860 | 1052 | 13895 | 123,3 | 16,2 | 1,125 | H5A034 C | sample | Hm Drgbl | Hm Drgbl | Hm Drgbl |
| 9500 | L5 | L6 | 1152 | 1214 | 18710 | 230,9 | 16,2 | 1,125 | H5A034 C | sample | Hm Drgbl | Hm Drgbl | Hm Drgbl |
| 9585 | P17 | P18 | 1388 | 1064 | 22541 | 151,4 | 16,2 | 1,125 | H5A034 C | sample | Hm Drgbl | Hm Drgbl | Hm Drgbl |
| 18885 | C6 | C7 | 901 | 967,5 | 14580 | 309,8 | 16,2 | 1,129 | H5A034 C | sample | Hm Gen E | Hm Gen E | Hm Gen E |
| 19110 | M14 | M15 | 1291 | 1011 | 20907 | 155,9 | 16,2 | 1,129 | H5A034 C | sample | Hm Gen E | Hm Gen E | Hm Gen E |
| 9937 | O1 | O2 | 1209 | 1005 | 19549 | 163,9 | 16,2 | 1,133 | H5A034 C | sample | Hm Drgbl | Hm Drgbl | Hm Drgbl |
| 15162 | H3 | H4 | 1271 | 775,9 | 20628 | 121,5 | 16,2 | 1,133 | H5A034 C | sample | Hm Gen E | Hm Gen E | Hm Gen E |
| 19380 | H2 | H3 | 921 | 1147 | 14944 | 219,6 | 16,2 | 1,149 | H5A034 C | sample | Hm Gen E | Hm Gen E | Hm Gen E |
| 20764 | B12 | B13 | 1422 | 983,9 | 23055 | 320 | 16,2 | 1,149 | H5A034 C | sample | Hm Gen E | Hm Gen E | Hm Gen E |
| 20780 | B5 | B6 | 870 | 1046 | 14083 | 309,4 | 16,2 | 1,149 | H5A034 C | sample | Hm Gen E | Hm Gen E | Hm Gen E |
| 20871 | F22 | F23 | 1130 | 983,3 | 18349 | 268,4 | 16,2 | 1,149 | H5A034 C | sample | Hm Gen E | Hm Gen E | Hm Gen E |
| 20963 | J19 | J20 | 1242 | 958,5 | 20150 | 248,8 | 16,2 | 1,149 | H5A034 C | sample | Hm Gen E | Hm Gen E | Hm Gen E |
| 21364 | K12 | K13 | 1084 | 955,1 | 17613 | 391,8 | 16,2 | 1,149 | H5A034 C | sample | Hm Gen E | Hm Gen E | Hm Gen E |
| 14239 | B15 | B16 | 1042 | 891,6 | 16829 | 123,5 | 16,2 | 1,153 | H5A034 C | sample | Hm Gen E | Hm Gen E | Hm Gen E |
| 14586 | P3 | P4 | 1227 | 843,9 | 19934 | 139,5 | 16,2 | 1,153 | H5A034 C | sample | Hm Gen E | Hm Gen E | Hm Gen E |
| 14591 | P8 | P9 | 1411 | 870,2 | 22884 | 211,9 | 16,2 | 1,153 | H5A034 C | sample | Hm Gen E | Hm Gen E | Hm Gen E |
| 11166 | B14 | B15 | 1608 | 873,1 | 26129 | 166,3 | 16,2 | 1,174 | H5A034 C | sample | Hm Drgbl | Hm Drgbl | Hm Drgbl |
| 11227 | D4 | D5 | 1655 | 878,4 | 26746 | 176,7 | 16,2 | 1,174 | H5A034 C | sample | Hm Drgbl | Hm Drgbl | Hm Drgbl |
| 11241 | E17 | E18 | 1330 | 770 | 21568 | 188,9 | 16,2 | 1,174 | H5A034 C | sample | Hm Drgbl | Hm Drgbl | Hm Drgbl |
| 11283 | G11 | G12 | 1061 | 801,7 | 17141 | 152,3 | 16,2 | 1,174 | H5A034 C | sample | Hm Drgbl | Hm Drgbl | Hm Drgbl |
| 11444 | M5 | M6 | 1189 | 862 | 19270 | 172,7 | 16,2 | 1,174 | H5A034 C | sample | Hm Drgbl | Hm Drgbl | Hm Drgbl |
| 11514 | P3 | P4 | 1323 | 784,9 | 21491 | 146,5 | 16,2 | 1,174 | H5A034 C | sample | Hm Drgbl | Hm Drgbl | Hm Drgbl |
| 11515 | P4 | P5 | 1948 | 900,7 | 31604 | 231,2 | 16,2 | 1,174 | H5A034 C | sample | Hm Drgbl | Hm Drgbl | Hm Drgbl |
| 10778 | B10 | B11 | 1688 | 903,8 | 27379 | 695 | 16,2 | 1,227 | H5A034 C | sample | Hm Drgbl | Hm Drgbl | Hm Drgbl |
| 7390 | D7 | D8 | 577 | 585,3 | 9361 | 544,1 | 16,2 | 1,237 | H5A034 C | sample | Hm Drgbl | Hm Drgbl | Hm Drgbl |
| 7594 | M18 | M19 | 1639 | 577,9 | 26568 | 486,3 | 16,2 | 1,237 | H5A034 C | sample | Hm Drgbl | Hm Drgbl | Hm Drgbl |
| 7607 | M8 | M9 | 1002 | 571 | 16238 | 485,9 | 16,2 | 1,237 | H5A034 C | sample | Hm Drgbl | Hm Drgbl | Hm Drgbl |
| 7670 | P21 | P22 | 1829 | 621,9 | 29548 | 575 | 16,2 | 1,237 | H5A034 C | sample | Hm Drgbl | Hm Drgbl | Hm Drgbl |
| 17015 | E8 | E9 | 1161 | 881,2 | 18776 | 225,1 | 16,2 | 1,251 | H5A034 C | sample | Hm Gen E | Hm Gen E | Hm Gen E |
| 17050 | G18 | G19 | 1608 | 961,3 | 26050 | 174,8 | 16,2 | 1,251 | H5A034 C | sample | Hm Gen E | Hm Gen E | Hm Gen E |
| 17072 | H16 | H17 | 1522 | 969,6 | 24723 | 164,9 | 16,2 | 1,251 | H5A034 C | sample | Hm Gen E | Hm Gen E | Hm Gen E |
| 17079 | H22 | H23 | 1417 | 977,6 | 22903 | 220,8 | 16,2 | 1,251 | H5A034 C | sample | Hm Gen E | Hm Gen E | Hm Gen E |
| 17180 | L5 | L6 | 1022 | 979,7 | 16556 | 201,5 | 16,2 | 1,251 | H5A034 C | sample | Hm Gen E | Hm Gen E | Hm Gen E |
| 17216 | N16 | N17 | 1481 | 896,8 | 23990 | 182 | 16,2 | 1,251 | H5A034 C | sample | Hm Gen E | Hm Gen E | Hm Gen E |
| 6938 | B10 | B11 | 1617 | 580,7 | 26179 | 541,8 | 16,2 | 1,261 | H5A034 C | sample | Hm Drgbl | Hm Drgbl | Hm Drgbl |
| 6983 | C8 | C9 | 808 | 571,1 | 13126 | 434,7 | 16,2 | 1,261 | H5A034 C | sample | Hm Drgbl | Hm Drgbl | Hm Drgbl |
| 6995 | D19 | D20 | 1104 | 573,2 | 17883 | 470,9 | 16,2 | 1,261 | H5A034 C | sample | Hm Drgbl | Hm Drgbl | Hm Drgbl |
| 7030 | E7 | E8 | 897 | 642 | 14525 | 242,2 | 16,2 | 1,261 | H5A034 C | sample | Hm Drgbl | Hm Drgbl | Hm Drgbl |
| 7246 | N7 | N8 | 918 | 535,6 | 14883 | 512,4 | 16,2 | 1,261 | H5A034 C | sample | Hm Drgbl | Hm Drgbl | Hm Drgbl |
| 7259 | O19 | O20 | 1650 | 534 | 26765 | 513 | 16,2 | 1,261 | H5A034 C | sample | Hm Drgbl | Hm Drgbl | Hm Drgbl |
| 5034 | B3 | B4 | 1676 | 645,9 | 27126 | 556,2 | 16,2 | 1,271 | H5A034 C | sample | Hm Drgbl | Hm Drgbl | Hm Drgbl |
| 5118 | F14 | F15 | 1290 | 653,4 | 20882 | 550,6 | 16,2 | 1,271 | H5A034 C | sample | Hm Drgbl | Hm Drgbl | Hm Drgbl |
| 5291 | M19 | M20 | 1467 | 765,4 | 23729 | 415,8 | 16,2 | 1,271 | H5A034 C | sample | Hm Drgbl | Hm Drgbl | Hm Drgbl |
| 2353 | C1 | C2 | 874 | 653,4 | 14154 | 418,5 | 16,2 | 1,276 | H5A034 C | sample | Hm Drgbl | Hm Drgbl | Hm Drgbl |
| 2382 | D14 | D15 | 820 | 693,1 | 13256 | 395,2 | 16,2 | 1,276 | H5A034 C | sample | Hm Drgbl | Hm Drgbl | Hm Drgbl |
| 16603 | D4 | D5 | 1037 | 899,6 | 16770 | 342,1 | 16,2 | 1,281 | H5A034 C | sample | Hm Gen E | Hm Gen E | Hm Gen E |
| 16785 | L17 | L18 | 1531 | 966,7 | 24857 | 283,3 | 16,2 | 1,281 | H5A034 C | sample | Hm Gen E | Hm Gen E | Hm Gen E |
| 16848 | N9 | N10 | 1707 | 947,2 | 27586 | 311,7 | 16,2 | 1,281 | H5A034 C | sample | Hm Gen E | Hm Gen E | Hm Gen E |
| 17782 | E7 | E8 | 1169 | 1096 | 18889 | 418,9 | 16,2 | 1,281 | H5A034 C | sample | Hm Gen E | Hm Gen E | Hm Gen E |
| 17853 | H6 | H7 | 1032 | 964 | 16720 | 286,2 | 16,2 | 1,281 | H5A034 C | sample | Hm Gen E | Hm Gen E | Hm Gen E |
| 17902 | J7 | J8 | 1265 | 1018 | 20441 | 318,5 | 16,2 | 1,281 | H5A034 C | sample | Hm Gen E | Hm Gen E | Hm Gen E |
| 18003 | O11 | O12 | 833 | 1058 | 13513 | 510,9 | 16,2 | 1,281 | H5A034 C | sample | Hm Gen E | Hm Gen E | Hm Gen E |
| 8966 | F21 | F22 | 1679 | 1813 | 27185 | 226 | 16,2 | 1,301 | H5A034 C | sample | Hm Drgbl | Hm Drgbl | Hm Drgbl |
| 8972 | F5 | F6 | 1111 | 1638 | 17955 | 148,9 | 16,2 | 1,301 | H5A034 C | sample | Hm Drgbl | Hm Drgbl | Hm Drgbl |
| 9093 | K6 | K7 | 1185 | 1460 | 19196 | 156,4 | 16,2 | 1,301 | H5A034 C | sample | Hm Drgbl | Hm Drgbl | Hm Drgbl |
| 1951 | B15 | B16 | 690 | 704,4 | 11210 | 497,6 | 16,2 | 1,409 | H5A034 C | sample | Hm Drgbl | Hm Drgbl | Hm Drgbl |
| 2158 | J7 | J8 | 1382 | 657 | 22334 | 342,6 | 16,2 | 1,409 | H5A034 C | sample | Hm Drgbl | Hm Drgbl | Hm Drgbl |
| 2246 | N21 | N22 | 877 | 627,4 | 14223 | 523,6 | 16,2 | 1,409 | H5A034 C | sample | Hm Drgbl | Hm Drgbl | Hm Drgbl |
| 2263 | O15 | O16 | 1360 | 599,2 | 22026 | 522,5 | 16,2 | 1,409 | H5A034 C | sample | Hm Drgbl | Hm Drgbl | Hm Drgbl |
| 6572 | B5 | B6 | 912 | 655,2 | 14809 | 498,2 | 16,2 | 1,421 | H5A034 C | sample | Hm Drgbl | Hm Drgbl | Hm Drgbl |
| 6583 | C15 | C16 | 706 | 527,2 | 11460 | 425,3 | 16,2 | 1,421 | H5A034 C | sample | Hm Drgbl | Hm Drgbl | Hm Drgbl |
| 6596 | C5 | C6 | 877 | 571,5 | 14171 | 481,8 | 16,2 | 1,421 | H5A034 C | sample | Hm Drgbl | Hm Drgbl | Hm Drgbl |
| 6656 | F16 | F17 | 1534 | 587,2 | 24791 | 456,1 | 16,2 | 1,421 | H5A034 C | sample | Hm Drgbl | Hm Drgbl | Hm Drgbl |
| 3898 | C18 | C19 | 1121 | 701,8 | 18178 | 471,2 | 16,2 | 1,44 | H5A034 C | sample | Hm Drgbl | Hm Drgbl | Hm Drgbl |
| 3942 | E14 | E15 | 811 | 689,6 | 13129 | 429,4 | 16,2 | 1,44 | H5A034 C | sample | Hm Drgbl | Hm Drgbl | Hm Drgbl |
| 11521 | A1 | A2 | 1128 | 812,5 | 18296 | 167,8 | 16,2 | 1,446 | H5A034 C | sample | Hm Gen E | Hm Gen E | Hm Gen E |
| 11602 | D18 | D19 | 1022 | 822,9 | 16543 | 161,6 | 16,2 | 1,446 | H5A034 C | sample | Hm Gen E | Hm Gen E | Hm Gen E |
| 11642 | F10 | F11 | 997 | 949,7 | 16199 | 182,6 | 16,2 | 1,446 | H5A034 C | sample | Hm Gen E | Hm Gen E | Hm Gen E |
| 11735 | I8 | I9 | 1070 | 803,3 | 17359 | 155,1 | 16,2 | 1,446 | H5A034 C | sample | Hm Gen E | Hm Gen E | Hm Gen E |
| 11827 | M4 | M5 | 1286 | 932,9 | 20892 | 223,1 | 16,2 | 1,446 | H5A034 C | sample | Hm Gen E | Hm Gen E | Hm Gen E |
| 15407 | B8 | B9 | 1453 | 855,4 | 23495 | 164,5 | 16,2 | 1,473 | H5A034 C | sample | Hm Gen E | Hm Gen E | Hm Gen E |
| 15416 | C16 | C17 | 846 | 832,7 | 13735 | 166,3 | 16,2 | 1,473 | H5A034 C | sample | Hm Gen E | Hm Gen E | Hm Gen E |
| 15540 | H2 | H3 | 764 | 793,8 | 12404 | 155,7 | 16,2 | 1,473 | H5A034 C | sample | Hm Gen E | Hm Gen E | Hm Gen E |
| 15594 | J3 | J4 | 917 | 825,9 | 14811 | 147,9 | 16,2 | 1,473 | H5A034 C | sample | Hm Gen E | Hm Gen E | Hm Gen E |
| 15598 | J7 | J8 | 1125 | 835,3 | 18182 | 138,6 | 16,2 | 1,473 | H5A034 C | sample | Hm Gen E | Hm Gen E | Hm Gen E |
| 5850 | D3 | D4 | 1461 | 745,6 | 23723 | 628,8 | 16,2 | 1,479 | H5A034 C | sample | Hm Drgbl | Hm Drgbl | Hm Drgbl |
| 5956 | I12 | I13 | 1234 | 739,2 | 19977 | 502,3 | 16,2 | 1,479 | H5A034 C | sample | Hm Drgbl | Hm Drgbl | Hm Drgbl |
| 1154 | A10 | A11 | 1138 | 744,9 | 18469 | 539,3 | 16,2 | 1,486 | H5A034 C | sample | Hm Drgbl | Hm Drgbl | Hm Drgbl |
| 16444 | N12 | N13 | 1444 | 963,5 | 23460 | 328,3 | 16,2 | 1,486 | H5A034 C | sample | Hm Gen E | Hm Gen E | Hm Gen E |

| | | | | | | | | | | |
|---|---|---|---|---|---|---|---|---|---|---|
| 16464 | N9 | N10 | 1141 | 965,6 | 18474 | 325,4 | 16,2 | 1,486 | H5A034 C | sample |
| 13509 | C6 | C7 | 782 | 1115 | 12679 | 166,1 | 16,2 | 1,521 | H5A034 C | sample |
| 13676 | J5 | J6 | 1314 | 1108 | 21326 | 166,4 | 16,2 | 1,521 | H5A034 C | sample |
| 13710 | L14 | L15 | 1334 | 1028 | 21662 | 175,6 | 16,2 | 1,521 | H5A034 C | sample |
| 13743 | M22 | M23 | 1448 | 1054 | 23471 | 220,1 | 16,2 | 1,521 | H5A034 C | sample |
| 3669 | I6 | I7 | 1149 | 1318 | 18600 | 385,9 | 16,2 | 1,558 | H5A034 C | sample |
| 3744 | L9 | L10 | 1386 | 1336 | 22436 | 379 | 16,2 | 1,558 | H5A034 C | sample |
| 3839 | P8 | P9 | 1173 | 1509 | 19037 | 495,9 | 16,2 | 1,558 | H5A034 C | sample |
| 8215 | G15 | G16 | 602 | 589,3 | 9770 | 526,7 | 16,2 | 1,558 | H5A034 C | sample |
| 8246 | H21 | H22 | 1151 | 678,6 | 18604 | 486,4 | 16,2 | 1,558 | H5A034 C | sample |
| 8276 | I5 | I6 | 1054 | 691,1 | 17069 | 334,3 | 16,2 | 1,558 | H5A034 C | sample |
| 8427 | P11 | P12 | 1471 | 601,7 | 23824 | 588,4 | 16,2 | 1,558 | H5A034 C | sample |
| 8444 | P5 | P6 | 1158 | 549,3 | 18771 | 502,9 | 16,2 | 1,558 | H5A034 C | sample |
| 10304 | N16 | N17 | 1451 | 838,6 | 23525 | 178,4 | 16,2 | 1,558 | H5A034 C | sample |
| 1621 | D20 | D21 | 1174 | 703 | 18987 | 403,6 | 16,2 | 1,696 | H5A034 C | sample |
| 1663 | F15 | F16 | 854 | 903,1 | 13875 | 445 | 16,2 | 1,696 | H5A034 C | sample |
| 1703 | G8 | G9 | 1076 | 743,6 | 17389 | 504,4 | 16,2 | 1,696 | H5A034 C | sample |
| 1905 | P17 | P18 | 1158 | 692,5 | 18750 | 423,3 | 16,2 | 1,696 | H5A034 C | sample |
| 21719 | I8 | I9 | 65 | 932,1 | 1053 | 752,2 | 16,2 | 1,705 | H5A034 C | sample |
| 21819 | N11 | N12 | 122 | 1019 | 1973 | 627,6 | 16,2 | 1,705 | H5A034 C | sample |
| 21882 | P3 | P4 | 271 | 1137 | 4396 | 627,1 | 16,2 | 1,705 | H5A034 C | sample |
| 4842 | J3 | J4 | 1047 | 863,6 | 16979 | 383,5 | 16,2 | 1,78 | H5A034 C | sample |
| 2724 | B2 | B3 | 832 | 643,3 | 13444 | 638 | 16,2 | 1,906 | H5A034 C | sample |
| 20234 | L10 | L11 | 180 | 1153 | 2920 | 181,5 | 16,2 | 1,976 | H5A034 C | sample |
| 7785 | E17 | E18 | 339 | 471,4 | 5486 | 382,3 | 16,2 | 2,473 | H5A034 C | sample |
| 21985 | E1 | E2 | 703 | 1360 | 11474 | 191 | 16,3 | 0,774 | H5A034 C | sample |
| 22225 | O1 | O2 | 1155 | 1344 | 18770 | 268,1 | 16,3 | 0,774 | H5A034 C | sample |
| 22368 | D9 | D10 | 827 | 1411 | 13441 | 396,1 | 16,3 | 0,84 | H5A034 C | sample |
| 22393 | F1 | F2 | 935 | 1397 | 15239 | 354,2 | 16,3 | 0,84 | H5A034 C | sample |
| 23457 | B17 | B18 | 1133 | 1847 | 18512 | 243,5 | 16,3 | 0,865 | H5A034 C | sample |
| 3358 | L7 | L8 | 446 | 1272 | 7278 | 440 | 16,3 | 1,009 | H5A034 C | sample |
| 3408 | N9 | N10 | 805 | 1166 | 13100 | 454 | 16,3 | 1,009 | H5A034 C | sample |
| 3414 | O14 | O15 | 960 | 1208 | 15622 | 452,4 | 16,3 | 1,009 | H5A034 C | sample |
| 11924 | A5 | A6 | 962 | 749,8 | 15718 | 125,3 | 16,3 | 1,045 | H5A034 C | sample |
| 11962 | C18 | C19 | 1417 | 886,7 | 23112 | 147,4 | 16,3 | 1,045 | H5A034 C | sample |
| 18130 | D18 | D19 | 1469 | 1036 | 23913 | 313,5 | 16,3 | 1,048 | H5A034 C | sample |
| 6164 | A5 | A6 | 962 | 469,3 | 15667 | 559,4 | 16,3 | 1,076 | H5A034 C | sample |
| 6394 | K18 | K19 | 1450 | 548,3 | 23677 | 459,6 | 16,3 | 1,076 | H5A034 C | sample |
| 6396 | K2 | K3 | 870 | 580,7 | 14215 | 492,2 | 16,3 | 1,076 | H5A034 C | sample |
| 6399 | K22 | K23 | 1423 | 608,7 | 23212 | 474,8 | 16,3 | 1,076 | H5A034 C | sample |
| 6413 | L13 | L14 | 1710 | 534,3 | 27892 | 443,3 | 16,3 | 1,076 | H5A034 C | sample |
| 6442 | M18 | M19 | 1552 | 587,1 | 25322 | 536 | 16,3 | 1,076 | H5A034 C | sample |
| 20415 | C22 | C23 | 721 | 903,5 | 11761 | 296 | 16,3 | 1,087 | H5A034 C | sample |
| 20554 | I18 | I19 | 859 | 718,7 | 14041 | 182,4 | 16,3 | 1,087 | H5A034 C | sample |
| 12728 | C16 | C17 | 1031 | 1003 | 16802 | 153,9 | 16,3 | 1,105 | H5A034 C | sample |
| 18687 | K22 | K23 | 1212 | 982,7 | 19804 | 209,6 | 16,3 | 1,105 | H5A034 C | sample |
| 17324 | B5 | B6 | 1101 | 987,4 | 17949 | 288,7 | 16,3 | 1,113 | H5A034 C | sample |
| 17391 | E22 | E23 | 1310 | 1017 | 21348 | 294,8 | 16,3 | 1,113 | H5A034 C | sample |
| 17451 | H11 | H12 | 1149 | 932,4 | 18767 | 156,9 | 16,3 | 1,113 | H5A034 C | sample |
| 17552 | L16 | L17 | 1015 | 974,7 | 16585 | 221 | 16,3 | 1,113 | H5A034 C | sample |
| 9346 | F18 | F19 | 1296 | 1005 | 21160 | 145,9 | 16,3 | 1,132 | H5A034 C | sample |
| 9374 | G21 | G22 | 1446 | 1072 | 23528 | 161,9 | 16,3 | 1,132 | H5A034 C | sample |
| 9391 | H15 | H16 | 1016 | 1010 | 16571 | 115,7 | 16,3 | 1,132 | H5A034 C | sample |
| 9519 | M22 | M23 | 1589 | 1094 | 25920 | 194 | 16,3 | 1,132 | H5A034 C | sample |
| 19159 | O15 | O16 | 1020 | 991,8 | 16673 | 364,2 | 16,3 | 1,136 | H5A034 C | sample |
| 19161 | O17 | O18 | 965 | 840,7 | 15727 | 169,4 | 16,3 | 1,136 | H5A034 C | sample |
| 9633 | B17 | B18 | 2032 | 1172 | 33216 | 150 | 16,3 | 1,14 | H5A034 C | sample |
| 9754 | G18 | G19 | 1437 | 1143 | 23424 | 158,7 | 16,3 | 1,14 | H5A034 C | sample |
| 15116 | F5 | F6 | 1098 | 732,4 | 17901 | 115,1 | 16,3 | 1,14 | H5A034 C | sample |
| 15288 | M9 | M10 | 585 | 818,5 | 9544 | 125,6 | 16,3 | 1,14 | H5A034 C | sample |
| 19213 | A20 | A21 | 794 | 1313 | 12979 | 318,2 | 16,3 | 1,156 | H5A034 C | sample |
| 19254 | C14 | C15 | 937 | 1163 | 15280 | 328,1 | 16,3 | 1,156 | H5A034 C | sample |
| 19292 | D5 | D6 | 1094 | 1225 | 17836 | 334 | 16,3 | 1,156 | H5A034 C | sample |
| 19538 | O10 | O11 | 1156 | 1170 | 18855 | 295,6 | 16,3 | 1,156 | H5A034 C | sample |
| 19558 | O7 | O8 | 743 | 1195 | 12124 | 363 | 16,3 | 1,156 | H5A034 C | sample |
| 21163 | B4 | B5 | 993 | 993,2 | 16167 | 429 | 16,3 | 1,156 | H5A034 C | sample |
| 21204 | D2 | D3 | 956 | 987 | 15537 | 307,8 | 16,3 | 1,156 | H5A034 C | sample |
| 21323 | I19 | I20 | 1164 | 932 | 18962 | 303,9 | 16,3 | 1,156 | H5A034 C | sample |
| 14263 | C15 | C16 | 1515 | 874,6 | 24724 | 156 | 16,3 | 1,16 | H5A034 C | sample |
| 14324 | E5 | E6 | 767 | 909,9 | 12530 | 147,6 | 16,3 | 1,16 | H5A034 C | sample |
| 11147 | A19 | A20 | 1493 | 908,4 | 24361 | 201,7 | 16,3 | 1,181 | H5A034 C | sample |
| 11338 | I18 | I19 | 1415 | 838,2 | 23022 | 146,8 | 16,3 | 1,181 | H5A034 C | sample |
| 11426 | M10 | M11 | 1659 | 875,9 | 27083 | 164,4 | 16,3 | 1,181 | H5A034 C | sample |
| 12299 | A19 | A20 | 1795 | 1102 | 29197 | 193,5 | 16,3 | 1,226 | H5A034 C | sample |
| 12579 | M11 | M12 | 1495 | 1039 | 24434 | 193,3 | 16,3 | 1,226 | H5A034 C | sample |
| 12622 | N7 | N8 | 989 | 950,5 | 16130 | 231,1 | 16,3 | 1,226 | H5A034 C | sample |
| 12631 | O15 | O16 | 1741 | 994,1 | 28296 | 197,6 | 16,3 | 1,226 | H5A034 C | sample |
| 10753 | A1 | A2 | 1321 | 942,5 | 21517 | 642,5 | 16,3 | 1,235 | H5A034 C | sample |
| 10902 | G14 | G15 | 1489 | 807,3 | 24279 | 592,4 | 16,3 | 1,235 | H5A034 C | sample |
| 7318 | A7 | A8 | 960 | 607,3 | 15621 | 596,1 | 16,3 | 1,244 | H5A034 C | sample |
| 7328 | B16 | B17 | 1251 | 581,2 | 20369 | 493 | 16,3 | 1,244 | H5A034 C | sample |
| 7413 | E6 | E7 | 662 | 502,7 | 10812 | 413,1 | 16,3 | 1,244 | H5A034 C | sample |
| 7453 | G20 | G21 | 1348 | 605,4 | 21921 | 516,8 | 16,3 | 1,244 | H5A034 C | sample |
| 7499 | I19 | I20 | 1229 | 590,7 | 20006 | 448,1 | 16,3 | 1,244 | H5A034 C | sample |
| 17134 | J7 | J8 | 1570 | 1008 | 25557 | 233 | 16,3 | 1,259 | H5A034 C | sample |
| 17173 | L20 | L21 | 1404 | 1029 | 22856 | 215,7 | 16,3 | 1,259 | H5A034 C | sample |
| 17236 | O12 | O13 | 1440 | 866,1 | 23409 | 270,3 | 16,3 | 1,259 | H5A034 C | sample |
| 6915 | A11 | A12 | 1270 | 577 | 20676 | 535,2 | 16,3 | 1,268 | H5A034 C | sample |
| 6923 | A19 | A20 | 1223 | 576,9 | 19905 | 561,3 | 16,3 | 1,268 | H5A034 C | sample |
| 5120 | F16 | F17 | 1677 | 621 | 27331 | 525,3 | 16,3 | 1,278 | H5A034 C | sample |
| 5314 | N18 | N19 | 1371 | 675,2 | 22391 | 636 | 16,3 | 1,278 | H5A034 C | sample |
| 16542 | B14 | B15 | 910 | 976 | 14852 | 339,7 | 16,3 | 1,289 | H5A034 C | sample |
| 16629 | E6 | E7 | 1006 | 915,8 | 16380 | 389,3 | 16,3 | 1,289 | H5A034 C | sample |
| 16701 | H6 | H7 | 713 | 906,4 | 11588 | 240,2 | 16,3 | 1,289 | H5A034 C | sample |
| 16739 | J19 | J20 | 970 | 1022 | 15847 | 245,1 | 16,3 | 1,289 | H5A034 C | sample |
| 16797 | L6 | L7 | 1257 | 902,5 | 20434 | 275,2 | 16,3 | 1,289 | H5A034 C | sample |
| 16824 | M9 | M10 | 1387 | 970,3 | 22665 | 355,3 | 16,3 | 1,289 | H5A034 C | sample |
| 16866 | O3 | O4 | 859 | 1098 | 13989 | 448,4 | 16,3 | 1,289 | H5A034 C | sample |
| 17735 | C8 | C9 | 989 | 1039 | 16151 | 347,3 | 16,3 | 1,289 | H5A034 C | sample |
| 17881 | J1 | J2 | 882 | 982,1 | 14357 | 237,6 | 16,3 | 1,289 | H5A034 C | sample |
| 17900 | J5 | J6 | 752 | 1075 | 12225 | 238,8 | 16,3 | 1,289 | H5A034 C | sample |
| 17912 | K16 | K17 | 904 | 989,8 | 14728 | 296,5 | 16,3 | 1,289 | H5A034 C | sample |
| 17954 | M10 | M11 | 1478 | 1014 | 24038 | 204,9 | 16,3 | 1,289 | H5A034 C | sample |
| 18006 | O14 | O15 | 1399 | 1114 | 22753 | 415 | 16,3 | 1,289 | H5A034 C | sample |
| 18012 | O2 | O3 | 716 | 1115 | 11676 | 275,8 | 16,3 | 1,289 | H5A034 C | sample |
| 18023 | O8 | O9 | 907 | 1013 | 14762 | 461,9 | 16,3 | 1,289 | H5A034 C | sample |
| 4448 | J16 | J17 | 1013 | 777 | 16552 | 332,4 | 16,3 | 1,376 | H5A034 C | sample |
| 4523 | M19 | M20 | 901 | 744 | 14730 | 496,3 | 16,3 | 1,376 | H5A034 C | sample |
| 4590 | P14 | P15 | 1440 | 733,5 | 23482 | 451,4 | 16,3 | 1,376 | H5A034 C | sample |
| 332 | N5 | N6 | 247 | 1432 | 4014 | 769,8 | 16,3 | 1,387 | H5A034 C | sample |
| 1983 | C22 | C23 | 1142 | 734,1 | 18643 | 545,8 | 16,3 | 1,417 | H5A034 C | sample |
| 2011 | D4 | D5 | 1036 | 636 | 16853 | 414,8 | 16,3 | 1,417 | H5A034 C | sample |
| 2157 | J6 | J7 | 1150 | 608,6 | 18801 | 439,2 | 16,3 | 1,417 | H5A034 C | sample |
| 2203 | L4 | L5 | 580 | 635,7 | 9434 | 398,2 | 16,3 | 1,417 | H5A034 C | sample |
| 6600 | C9 | C10 | 888 | 573,7 | 14480 | 421 | 16,3 | 1,43 | H5A034 C | sample |
| 6692 | G5 | G6 | 800 | 596 | 13049 | 489,7 | 16,3 | 1,43 | H5A034 C | sample |
| 6796 | L12 | L13 | 1200 | 623 | 19522 | 357,4 | 16,3 | 1,43 | H5A034 C | sample |
| 4223 | P8 | P9 | 743 | 727,5 | 12143 | 407,2 | 16,3 | 1,449 | H5A034 C | sample |
| 15450 | D3 | D4 | 708 | 1122 | 11531 | 171 | 16,3 | 1,482 | H5A034 C | sample |
| 15480 | E9 | E10 | 998 | 837,7 | 16259 | 202 | 16,3 | 1,482 | H5A034 C | sample |
| 15498 | F3 | F4 | 910 | 795,5 | 14840 | 140,7 | 16,3 | 1,482 | H5A034 C | sample |
| 15588 | J2 | J3 | 874 | 809,1 | 14286 | 165,1 | 16,3 | 1,482 | H5A034 C | sample |
| 15663 | M22 | M23 | 1069 | 810,4 | 17384 | 156,1 | 16,3 | 1,482 | H5A034 C | sample |
| 5935 | H15 | H16 | 959 | 697,9 | 15673 | 348,7 | 16,3 | 1,489 | H5A034 C | sample |
| 5940 | H2 | H3 | 1487 | 775,6 | 24220 | 416,1 | 16,3 | 1,489 | H5A034 C | sample |
| 5950 | H7 | H8 | 1510 | 760 | 24580 | 531,5 | 16,3 | 1,489 | H5A034 C | sample |
| 5962 | I18 | I19 | 682 | 707,6 | 11091 | 509,7 | 16,3 | 1,489 | H5A034 C | sample |
| 1410 | K3 | K4 | 660 | 629,3 | 10758 | 365,4 | 16,3 | 1,495 | H5A034 C | sample |
| 1446 | M14 | M15 | 1323 | 741,2 | 21503 | 416,1 | 16,3 | 1,495 | H5A034 C | sample |
| 16160 | B16 | B17 | 1014 | 1195 | 16558 | 331,5 | 16,3 | 1,495 | H5A034 C | sample |
| 16282 | G18 | G19 | 976 | 1090 | 15948 | 292,8 | 16,3 | 1,495 | H5A034 C | sample |
| 13544 | E16 | E17 | 1398 | 943,5 | 22817 | 187,8 | 16,3 | 1,531 | H5A034 C | sample |
| 13603 | G4 | G5 | 1316 | 983,2 | 21470 | 214,6 | 16,3 | 1,531 | H5A034 C | sample |
| 975 | I22 | I23 | 1087 | 689,4 | 17710 | 429,2 | 16,3 | 1,545 | H5A034 C | sample |
| 5402 | B10 | B11 | 1572 | 625,6 | 25584 | 632,9 | 16,3 | 1,552 | H5A034 C | sample |
| 3645 | H6 | H7 | 1359 | 1333 | 22140 | 403,7 | 16,3 | 1,567 | H5A034 C | sample |
| 10015 | B15 | B16 | 1449 | 851,6 | 23656 | 168,1 | 16,3 | 1,567 | H5A034 C | sample |
| 10016 | B16 | B17 | 1522 | 878 | 24851 | 173,6 | 16,3 | 1,567 | H5A034 C | sample |
| 10041 | C17 | C18 | 1242 | 742,9 | 20282 | 149,4 | 16,3 | 1,567 | H5A034 C | sample |
| 10134 | G14 | G15 | 1454 | 801,5 | 23699 | 144,7 | 16,3 | 1,567 | H5A034 C | sample |
| 10160 | H16 | H17 | 1324 | 806,4 | 21622 | 128,9 | 16,3 | 1,567 | H5A034 C | sample |
| 10243 | K4 | K5 | 1705 | 810 | 27771 | 209,2 | 16,3 | 1,567 | H5A034 C | sample |
| 10300 | N12 | N13 | 1929 | 966,2 | 31359 | 187,4 | 16,3 | 1,567 | H5A034 C | sample |
| 1677 | F6 | F7 | 980 | 694,1 | 15985 | 448,9 | 16,3 | 1,707 | H5A034 C | sample |
| 1771 | J4 | J5 | 729 | 634,9 | 11908 | 337,9 | 16,3 | 1,707 | H5A034 C | sample |
| 21550 | B7 | B8 | 181 | 937,4 | 2955 | 829,1 | 16,3 | 1,716 | H5A034 C | sample |
| 21597 | D6 | D7 | 208 | 1002 | 3391 | 674,4 | 16,3 | 1,716 | H5A034 C | sample |
| 21653 | G13 | G14 | 243 | 940,5 | 3958 | 778,6 | 16,3 | 1,716 | H5A034 C | sample |
| 4920 | M9 | M10 | 1403 | 794,4 | 22937 | 527,2 | 16,3 | 1,791 | H5A034 C | sample |
| 4968 | O9 | O10 | 535 | 724,7 | 8707 | 569,3 | 16,3 | 1,791 | H5A034 C | sample |
| 10445 | D13 | D14 | 1337 | 871,4 | 21785 | 212,3 | 16,3 | 1,842 | H5A034 C | sample |
| 10497 | F17 | F18 | 1352 | 799,5 | 22082 | 179,3 | 16,3 | 1,842 | H5A034 C | sample |
| 10598 | J21 | J22 | 1011 | 881,4 | 16499 | 200,4 | 16,3 | 1,842 | H5A034 C | sample |
| 19998 | B14 | B15 | 402 | 1248 | 6563 | 242,3 | 16,3 | 1,988 | H5A034 C | sample |
| 20017 | C1 | C2 | 372 | 1291 | 6072 | 223,7 | 16,3 | 1,988 | H5A034 C | sample |
| 22043 | G19 | G20 | 1137 | 1493 | 18623 | 330 | 16,4 | 0,779 | H5A034 C | sample |
| 22174 | L7 | L8 | 614 | 1466 | 10067 | 290,5 | 16,4 | 0,779 | H5A034 C | sample |
| 22431 | G22 | G23 | 1091 | 1357 | 17867 | 365,4 | 16,4 | 0,845 | H5A034 C | sample |
| 14771 | H19 | H20 | 1050 | 812,7 | 17220 | 85,64 | 16,4 | 0,934 | H5A034 C | sample |
| 14102 | L21 | L22 | 1358 | 990,4 | 22306 | 128,8 | 16,4 | 0,94 | H5A034 C | sample |
| 3125 | C13 | C14 | 1514 | 1250 | 24897 | 496,8 | 16,4 | 1,015 | H5A034 C | sample |
| 3174 | E14 | E15 | 715 | 1053 | 11747 | 383,9 | 16,4 | 1,015 | H5A034 C | sample |
| 3200 | F16 | F17 | 1028 | 1273 | 16834 | 372,7 | 16,4 | 1,015 | H5A034 C | sample |
| 3287 | I8 | I9 | 1057 | 1175 | 17361 | 321,5 | 16,4 | 1,015 | H5A034 C | sample |
| 3306 | J3 | J4 | 583 | 1407 | 9545 | 435,2 | 16,4 | 1,015 | H5A034 C | sample |
| 3404 | N5 | N6 | 1339 | 1444 | 21950 | 415 | 16,4 | 1,015 | H5A034 C | sample |
| 12158 | K21 | K22 | 1240 | 788,9 | 20363 | 141,4 | 16,4 | 1,051 | H5A034 C | sample |
| 18393 | O17 | O18 | 1467 | 990,3 | 24029 | 256,6 | 16,4 | 1,055 | H5A034 C | sample |
| 19860 | L2 | L3 | 1164 | 327,4 | 19049 | 310,3 | 16,4 | 1,068 | H5A034 C | sample |
| 19862 | L21 | L22 | 1353 | 354,7 | 22216 | 263 | 16,4 | 1,068 | H5A034 C | sample |
| 19908 | N2 | N3 | 859 | 325,4 | 14087 | 200,1 | 16,4 | 1,068 | H5A034 C | sample |
| 19929 | O17 | O18 | 1018 | 324,3 | 16738 | 286,1 | 16,4 | 1,068 | H5A034 C | sample |
| 6275 | F19 | F20 | 1590 | 534,6 | 25998 | 498,9 | 16,4 | 1,083 | H5A034 C | sample |
| 6345 | I17 | I18 | 1260 | 652,8 | 20652 | 425,5 | 16,4 | 1,083 | H5A034 C | sample |
| 6362 | J10 | J11 | 1326 | 547,9 | 21768 | 377,8 | 16,4 | 1,083 | H5A034 C | sample |
| 6371 | J19 | J20 | 1245 | 606,9 | 20442 | 363,6 | 16,4 | 1,083 | H5A034 C | sample |
| 6519 | P22 | P23 | 1400 | 563,1 | 22962 | 595,1 | 16,4 | 1,083 | H5A034 C | sample |
| 20397 | B6 | B7 | 745 | 812,9 | 12226 | 224,9 | 16,4 | 1,093 | H5A034 C | sample |
| 20515 | G4 | G5 | 718 | 773,7 | 11798 | 214,9 | 16,4 | 1,093 | H5A034 C | sample |
| 20576 | J16 | J17 | 810 | 769,6 | 13293 | 178,3 | 16,4 | 1,093 | H5A034 C | sample |
| 12816 | F9 | F10 | 1482 | 1028 | 24283 | 133 | 16,4 | 1,112 | H5A034 C | sample |
| 12871 | I15 | I16 | 1528 | 971,2 | 25009 | 132,6 | 16,4 | 1,112 | H5A034 C | sample |
| 12957 | L6 | L7 | 1075 | 1027 | 17592 | 144,2 | 16,4 | 1,112 | H5A034 C | sample |
| 18721 | M1 | M2 | 1106 | 905,3 | 18130 | 169,2 | 16,4 | 1,112 | H5A034 C | sample |
| 17313 | B17 | B18 | 1556 | 1062 | 25588 | 222,3 | 16,4 | 1,119 | H5A034 C | sample |
| 17413 | F20 | F21 | 1663 | 974,1 | 27326 | 200,7 | 16,4 | 1,119 | H5A034 C | sample |
| 17461 | H20 | H21 | 1542 | 986,3 | 25348 | 230,1 | 16,4 | 1,119 | H5A034 C | sample |
| 17543 | K8 | K9 | 1016 | 912,3 | 16652 | 266,3 | 16,4 | 1,119 | H5A034 C | sample |
| 9263 | B8 | B9 | 1433 | 1187 | 23462 | 186,8 | 16,4 | 1,139 | H5A034 C | sample |
| 18946 | F18 | F19 | 1453 | 949,7 | 23761 | 223,9 | 16,4 | 1,143 | H5A034 C | sample |
| 18996 | H2 | H3 | 966 | 910 | 15824 | 165,8 | 16,4 | 1,143 | H5A034 C | sample |
| 19062 | K14 | K15 | 1190 | 918,8 | 19565 | 164,9 | 16,4 | 1,143 | H5A034 C | sample |
| 19090 | L18 | L19 | 1250 | 1076 | 20501 | 276,2 | 16,4 | 1,143 | H5A034 C | sample |
| 19133 | N13 | N14 | 1379 | 957 | 22615 | 257,6 | 16,4 | 1,143 | H5A034 C | sample |
| 9875 | L19 | L20 | 1891 | 1072 | 30937 | 175,1 | 16,4 | 1,147 | H5A034 C | sample |
| 15180 | I2 | I3 | 1507 | 783,7 | 24788 | 149,6 | 16,4 | 1,147 | H5A034 C | sample |
| 8541 | D6 | D7 | 1187 | 1056 | 19470 | 122 | 16,4 | 1,159 | H5A034 C | sample |
| 8713 | L1 | L2 | 1606 | 1064 | 26375 | 133,3 | 16,4 | 1,159 | H5A034 C | sample |
| 19338 | F3 | F4 | 648 | 1120 | 10658 | 239,2 | 16,4 | 1,163 | H5A034 C | sample |
| 20762 | B10 | B11 | 1349 | 1002 | 22066 | 373,9 | 16,4 | 1,163 | H5A034 C | sample |
| 20763 | B11 | B12 | 1161 | 1010 | 19097 | 393,1 | 16,4 | 1,163 | H5A034 C | sample |
| 20816 | D16 | D17 | 590 | 1107 | 9703 | 207 | 16,4 | 1,163 | H5A034 C | sample |
| 20971 | J4 | J5 | 1392 | 974,8 | 22897 | 256,2 | 16,4 | 1,163 | H5A034 C | sample |
| 21324 | I2 | I3 | 876 | 930,7 | 14367 | 283,8 | 16,4 | 1,163 | H5A034 C | sample |
| 21374 | K21 | K22 | 1225 | 877,3 | 20036 | 331,3 | 16,4 | 1,163 | H5A034 C | sample |
| 21493 | P20 | P21 | 1438 | 1007 | 23581 | 454 | 16,4 | 1,163 | H5A034 C | sample |
| 14217 | A17 | A18 | 1475 | 906,2 | 24250 | 179,6 | 16,4 | 1,167 | H5A034 C | sample |
| 14328 | E9 | E10 | 1454 | 857,3 | 23881 | 139 | 16,4 | 1,167 | H5A034 C | sample |
| 14501 | M13 | M14 | 1594 | 816,9 | 26154 | 136 | 16,4 | 1,167 | H5A034 C | sample |
| 14509 | M20 | M21 | 1829 | 802,2 | 29917 | 145 | 16,4 | 1,167 | H5A034 C | sample |
| 11161 | B1 | B2 | 1332 | 833,8 | 21843 | 167,1 | 16,4 | 1,188 | H5A034 C | sample |
| 11277 | F6 | F7 | 1842 | 884,4 | 30119 | 205,4 | 16,4 | 1,188 | H5A034 C | sample |
| 11386 | K18 | K19 | 1892 | 890,5 | 30969 | 166,9 | 16,4 | 1,188 | H5A034 C | sample |
| 11454 | N14 | N15 | 1827 | 889,6 | 30043 | 177,7 | 16,4 | 1,188 | H5A034 C | sample |
| 12482 | I10 | I11 | 1047 | 1052 | 17195 | 153,6 | 16,4 | 1,233 | H5A034 C | sample |
| 12492 | I2 | I3 | 1370 | 1042 | 22444 | 213,6 | 16,4 | 1,233 | H5A034 C | sample |
| 12615 | N22 | N23 | 1146 | 981,7 | 18754 | 169,8 | 16,4 | 1,233 | H5A034 C | sample |
| 7565 | L13 | L14 | 1097 | 551 | 18011 | 456,6 | 16,4 | 1,252 | H5A034 C | sample |
| 16971 | D11 | D12 | 1329 | 875,6 | 21743 | 206,4 | 16,4 | 1,266 | H5A034 C | sample |
| 16972 | D12 | D13 | 1228 | 1008 | 20107 | 257,3 | 16,4 | 1,266 | H5A034 C | sample |
| 17039 | F8 | F9 | 1332 | 935,6 | 21871 | 213,9 | 16,4 | 1,266 | H5A034 C | sample |
| 17041 | G1 | G2 | 894 | 947 | 14659 | 107,3 | 16,4 | 1,266 | H5A034 C | sample |
| 17067 | H11 | H12 | 1027 | 1012 | 16858 | 167,7 | 16,4 | 1,266 | H5A034 C | sample |
| 17169 | L17 | L18 | 1734 | 984,3 | 28410 | 187,8 | 16,4 | 1,266 | H5A034 C | sample |
| 17242 | O18 | O19 | 1477 | 886,9 | 24166 | 309,7 | 16,4 | 1,266 | H5A034 C | sample |
| 17244 | O2 | O3 | 1376 | 1001 | 22501 | 273 | 16,4 | 1,266 | H5A034 C | sample |

| | | | | | | | | | | | |
|---|---|---|---|---|---|---|---|---|---|---|---|
| 6917 | A13 | A14 | 993 | 572,5 | 16328 | 561,8 | 16,4 | 1,276 | H5A034 C | sample | LAIR2 |
| 6993 | D17 | D18 | 1092 | 506,9 | 17926 | 452,6 | 16,4 | 1,276 | H5A034 C | sample | MCF2 |
| 7207 | M15 | M16 | 1114 | 511,5 | 18323 | 419,4 | 16,4 | 1,276 | H5A034 C | sample | LY75 |
| 7243 | N4 | N5 | 1153 | 559,2 | 18865 | 420,7 | 16,4 | 1,276 | H5A034 C | sample | NKX6-1 |
| 7245 | N6 | N7 | 1480 | 565,4 | 24256 | 518,5 | 16,4 | 1,276 | H5A034 C | sample | NMB |
| 7281 | P17 | P18 | 1410 | 519,2 | 23063 | 532,6 | 16,4 | 1,276 | H5A034 C | sample | MYO1B |
| 4993 | A1 | A2 | 504 | 624,5 | 8245 | 623,7 | 16,4 | 1,286 | H5A034 C | sample | PAPPA2 |
| 4997 | A13 | A14 | 1315 | 657,5 | 21605 | 522,9 | 16,4 | 1,286 | H5A034 C | sample | OSGEPL1 |
| 5116 | F12 | F13 | 1704 | 666,4 | 27984 | 598,5 | 16,4 | 1,286 | H5A034 C | sample | ZAN |
| 5149 | G20 | G21 | 2008 | 635,3 | 32966 | 523,1 | 16,4 | 1,286 | H5A034 C | sample | TUBA3C |
| 5203 | I4 | I5 | 1358 | 607,7 | 22336 | 578,4 | 16,4 | 1,286 | H5A034 C | sample | TUFM |
| 5370 | P3 | P4 | 1326 | 690,8 | 21756 | 517,1 | 16,4 | 1,286 | H5A034 C | sample | RND2 |
| 2623 | N15 | N16 | 1133 | 613,4 | 18538 | 387,9 | 16,4 | 1,291 | H5A034 C | sample | FGFR4 |
| 16612 | E12 | E13 | 864 | 1056 | 14188 | 366,9 | 16,4 | 1,296 | H5A034 C | sample | LYRM7 |
| 16647 | F22 | F23 | 932 | 1030 | 15283 | 257 | 16,4 | 1,296 | H5A034 C | sample | TMEM88 |
| 16664 | G16 | G17 | 852 | 1036 | 13970 | 248,3 | 16,4 | 1,296 | H5A034 C | sample | LOC90826 |
| 16694 | H21 | H22 | 1400 | 962,7 | 23022 | 285,2 | 16,4 | 1,296 | H5A034 C | sample | NAV1 |
| 16819 | M4 | M5 | 1302 | 1022 | 21320 | 280,4 | 16,4 | 1,296 | H5A034 C | sample | SESTD1 |
| 16868 | O5 | O6 | 488 | 1018 | 7998 | 485,3 | 16,4 | 1,296 | H5A034 C | sample | KRTAP4-1 |
| 16896 | P9 | P10 | 1255 | 1059 | 20562 | 396,5 | 16,4 | 1,296 | H5A034 C | sample | UCN2 |
| 17743 | D15 | D16 | 1392 | 997,1 | 22835 | 336,4 | 16,4 | 1,296 | H5A034 C | sample | MESP2 |
| 17936 | L16 | L17 | 449 | 954,9 | 7351 | 244,5 | 16,4 | 1,296 | H5A034 C | sample | CMTM8 |
| 8917 | D20 | D21 | 2089 | 1969 | 34231 | 210 | 16,4 | 1,317 | H5A034 C | sample | ANKIB1 |
| 8937 | E17 | E18 | 2039 | 1988 | 33540 | 187,3 | 16,4 | 1,317 | H5A034 C | sample | NEUROG3 |
| 8987 | G19 | G20 | 1757 | 1718 | 28853 | 150,4 | 16,4 | 1,317 | H5A034 C | sample | TBX22 |
| 9024 | H9 | H10 | 1373 | 1709 | 22462 | 130,3 | 16,4 | 1,317 | H5A034 C | sample | SLC15A3 |
| 9052 | J12 | J13 | 1347 | 1648 | 22055 | 161,8 | 16,4 | 1,317 | H5A034 C | sample | INOC1 |
| 9097 | L1 | L2 | 1728 | 1851 | 28372 | 159,2 | 16,4 | 1,317 | H5A034 C | sample | UBR5 |
| 4406 | H21 | H22 | 959 | 612,8 | 15774 | 414,9 | 16,4 | 1,384 | H5A034 C | sample | KCNJ3 |
| 4429 | I20 | I21 | 840 | 663,2 | 13809 | 373,5 | 16,4 | 1,384 | H5A034 C | sample | KCTD2 |
| 4478 | K21 | K22 | 784 | 675,4 | 12830 | 471,7 | 16,4 | 1,384 | H5A034 C | sample | CLIC2 |
| 4495 | L15 | L16 | 1058 | 665,1 | 17371 | 384,3 | 16,4 | 1,384 | H5A034 C | sample | KCNN2 |
| 4542 | N14 | N15 | 953 | 623,7 | 15626 | 380,6 | 16,4 | 1,384 | H5A034 C | sample | TSPAN4 |
| 4554 | N3 | N4 | 881 | 639,7 | 14475 | 371,1 | 16,4 | 1,384 | H5A034 C | sample | KCNQ3 |
| 14 | A21 | A22 | 449 | 1534 | 7378 | 877,7 | 16,4 | 1,396 | H5A034 C | sample | NR6A1 |
| 62 | C21 | C22 | 325 | 1367 | 5329 | 683,7 | 16,4 | 1,396 | H5A034 C | sample | RARG |
| 2116 | I12 | I13 | 1093 | 613,6 | 17931 | 376,2 | 16,4 | 1,426 | H5A034 C | sample | ZNF549 |
| 6887 | O8 | O9 | 1225 | 562 | 20087 | 481,4 | 16,4 | 1,439 | H5A034 C | sample | ISG20 |
| 6912 | P9 | P10 | 1352 | 610 | 22213 | 481 | 16,4 | 1,439 | H5A034 C | sample | ICAM4 |
| 4106 | L10 | L11 | 43 | 553,2 | 707 | 442,4 | 16,4 | 1,458 | H5A034 C | sample | RRM1 |
| 4210 | P18 | P19 | 1235 | 718,3 | 20219 | 536,5 | 16,4 | 1,458 | H5A034 C | sample | SC5DL |
| 11721 | I17 | I18 | 1158 | 793,8 | 18975 | 131,6 | 16,4 | 1,464 | H5A034 C | sample | C9 |
| 11810 | M10 | M11 | 1060 | 743,9 | 17427 | 150,8 | 16,4 | 1,464 | H5A034 C | sample | GBAS |
| 1177 | B1 | B2 | 1341 | 743,9 | 21984 | 531,5 | 16,4 | 1,505 | H5A034 C | sample | ZNF236 |
| 1271 | E8 | E9 | 1320 | 711,2 | 21662 | 438,7 | 16,4 | 1,505 | H5A034 C | sample | ZNF623 |
| 1395 | K11 | K12 | 729 | 678,4 | 11950 | 415,3 | 16,4 | 1,505 | H5A034 C | sample | ZNF141 |
| 1531 | P4 | P5 | 1279 | 694,9 | 20986 | 472,9 | 16,4 | 1,505 | H5A034 C | sample | DNAJC16 |
| 16138 | A18 | A19 | 837 | 1057 | 13760 | 456,3 | 16,4 | 1,505 | H5A034 C | sample | WDR24 |
| 16485 | O6 | O7 | 832 | 970,4 | 13647 | 478,7 | 16,4 | 1,505 | H5A034 C | sample | C16orf14 |
| 13725 | L6 | L7 | 1423 | 996,5 | 23288 | 169,9 | 16,4 | 1,54 | H5A034 C | sample | FER1L3 |
| 844 | D12 | D13 | 940 | 702,2 | 15385 | 486,3 | 16,4 | 1,555 | H5A034 C | sample | S100A3 |
| 916 | G12 | G13 | 1278 | 886,7 | 20970 | 598,7 | 16,4 | 1,555 | H5A034 C | sample | NFX1 |
| 926 | G21 | G22 | 1193 | 727,3 | 19563 | 437 | 16,4 | 1,555 | H5A034 C | sample | CD74 |
| 937 | H1 | H2 | 1077 | 741,1 | 17617 | 443,1 | 16,4 | 1,555 | H5A034 C | sample | GTF2E2 |
| 1002 | J3 | J4 | 816 | 704,2 | 13376 | 409,8 | 16,4 | 1,555 | H5A034 C | sample | HPCA |
| 5411 | B19 | B20 | 1270 | 741,7 | 20882 | 640 | 16,4 | 1,562 | H5A034 C | sample | ASMTL |
| 5541 | G6 | G7 | 1121 | 696,2 | 18434 | 590,3 | 16,4 | 1,562 | H5A034 C | sample | EIF2B4 |
| 3474 | A3 | A4 | 771 | 1252 | 12628 | 425 | 16,4 | 1,577 | H5A034 C | sample | GPR173 |
| 3542 | D21 | D22 | 1190 | 1407 | 19517 | 460,3 | 16,4 | 1,577 | H5A034 C | sample | GPER |
| 3776 | N16 | N17 | 1124 | 1147 | 18425 | 348,3 | 16,4 | 1,577 | H5A034 C | sample | GPR115 |
| 8245 | H20 | H21 | 974 | 641,3 | 15967 | 534,7 | 16,4 | 1,577 | H5A034 C | sample | QPRT |
| 8260 | I12 | I13 | 1045 | 553,4 | 17190 | 430,4 | 16,4 | 1,577 | H5A034 C | sample | SULF1 |
| 8337 | L17 | L18 | 1373 | 611,9 | 22475 | 459,4 | 16,4 | 1,577 | H5A034 C | sample | RP4-691N |
| 8418 | O3 | O4 | 817 | 643,4 | 13431 | 415,1 | 16,4 | 1,577 | H5A034 C | sample | SP140 |
| 10055 | C8 | C9 | 1472 | 813,5 | 24159 | 136 | 16,4 | 1,577 | H5A034 C | sample | CHST9 |
| 10071 | D22 | D23 | 1416 | 855,9 | 23159 | 152,5 | 16,4 | 1,577 | H5A034 C | sample | ZMAT1 |
| 10235 | K19 | K20 | 1192 | 783,7 | 19541 | 142,7 | 16,4 | 1,577 | H5A034 C | sample | CNTNAP3 |
| 10334 | O21 | O22 | 1410 | 857 | 23170 | 156,9 | 16,4 | 1,577 | H5A034 C | sample | RNF34 |
| 10346 | P10 | P11 | 1790 | 841,2 | 29283 | 302,7 | 16,4 | 1,577 | H5A034 C | sample | HPDL |
| 1557 | A6 | A7 | 1347 | 795,5 | 22150 | 502 | 16,4 | 1,717 | H5A034 C | sample | ZNF286A |
| 1588 | C12 | C13 | 1375 | 682,2 | 22515 | 466,7 | 16,4 | 1,717 | H5A034 C | sample | ZNF471 |
| 1688 | G16 | G17 | 960 | 696,8 | 15727 | 359,6 | 16,4 | 1,717 | H5A034 C | sample | ZNF77 |
| 1739 | I19 | I20 | 913 | 687,8 | 14976 | 380,2 | 16,4 | 1,717 | H5A034 C | sample | SERTAD1 |
| 21599 | D8 | D9 | 297 | 880,8 | 4871 | 592,1 | 16,4 | 1,726 | H5A034 C | sample | ABCC6P1 |
| 21670 | G7 | G8 | 254 | 979,4 | 4164 | 657,1 | 16,4 | 1,726 | H5A034 C | sample | LOC64948 |
| 10640 | L16 | L17 | 1218 | 877,2 | 19924 | 219,3 | 16,4 | 1,853 | H5A034 C | sample | GIPC3 |
| 19969 | A1 | A2 | 181 | 1311 | 2966 | 231,2 | 16,4 | 2 | H5A034 C | sample | LOC39073 |
| 19992 | A9 | A10 | 520 | 1311 | 8502 | 247 | 16,4 | 2 | H5A034 C | sample | LOC39080 |
| 22027 | F4 | F5 | 884 | 1385 | 14625 | 317,3 | 16,5 | 0,784 | H5A034 C | sample | LOC72842 |
| 22230 | O14 | O15 | 1458 | 1404 | 23992 | 424,4 | 16,5 | 0,784 | H5A034 C | sample | LOC72834 |
| 23517 | D6 | D7 | 942 | 1596 | 15575 | 310,1 | 16,5 | 0,875 | H5A034 C | sample | |
| 14826 | J3 | J4 | 1256 | 867,2 | 20768 | 100,2 | 16,5 | 0,94 | H5A034 C | sample | UQCC |
| 3250 | H18 | H19 | 1055 | 1325 | 17414 | 351,2 | 16,5 | 1,022 | H5A034 C | sample | ABCB1 |
| 3355 | L4 | L5 | 1303 | 1288 | 21447 | 478,2 | 16,5 | 1,022 | H5A034 C | sample | SERPINB9 |
| 12069 | G6 | G7 | 850 | 782,8 | 14008 | 95,14 | 16,5 | 1,058 | H5A034 C | sample | PSME2 |
| 12217 | N1 | N2 | 1500 | 753,9 | 24755 | 89,34 | 16,5 | 1,058 | H5A034 C | sample | PEX7 |
| 18164 | E5 | E6 | 1117 | 969,2 | 18469 | 379,1 | 16,5 | 1,061 | H5A034 C | sample | C6orf129 |
| 18392 | O16 | O17 | 1291 | 997,1 | 21250 | 340,8 | 16,5 | 1,061 | H5A034 C | sample | FLJ30092 |
| 19887 | M22 | M23 | 1245 | 312,3 | 20545 | 326,8 | 16,5 | 1,075 | H5A034 C | sample | LOC38964 |
| 6229 | D20 | D21 | 1379 | 538,1 | 22782 | 512,5 | 16,5 | 1,089 | H5A034 C | sample | CYB5R3 |
| 6235 | D4 | D5 | 1125 | 520,6 | 18592 | 523,1 | 16,5 | 1,089 | H5A034 C | sample | DFFB |
| 6386 | K10 | K11 | 996 | 542,9 | 16409 | 502,3 | 16,5 | 1,089 | H5A034 C | sample | CYP17A1 |
| 6508 | P12 | P13 | 1621 | 586,6 | 26783 | 500,2 | 16,5 | 1,089 | H5A034 C | sample | RCAN1 |
| 20418 | C3 | C4 | 703 | 817,3 | 11588 | 192,2 | 16,5 | 1,1 | H5A034 C | sample | MGC8837 |
| 20563 | I4 | I5 | 728 | 722,4 | 11988 | 178,7 | 16,5 | 1,1 | H5A034 C | sample | LOC55063 |
| 12696 | A9 | A10 | 1369 | 1064 | 22617 | 197,5 | 16,5 | 1,119 | H5A034 C | sample | CCPG1 |
| 18487 | C15 | C16 | 1324 | 1039 | 21877 | 353,7 | 16,5 | 1,119 | H5A034 C | sample | SUSD3 |
| 18495 | C22 | C23 | 1476 | 998,7 | 24417 | 332,1 | 16,5 | 1,119 | H5A034 C | sample | FERD3L |
| 18567 | F22 | F23 | 1405 | 1054 | 23142 | 293,2 | 16,5 | 1,119 | H5A034 C | sample | C3orf46 |
| 18773 | O13 | O14 | 1380 | 957,6 | 22773 | 362 | 16,5 | 1,119 | H5A034 C | sample | SPATA19 |
| 17572 | M12 | M13 | 1446 | 913,7 | 23913 | 193,3 | 16,5 | 1,126 | H5A034 C | sample | PM20D2 |
| 9335 | E8 | E9 | 1104 | 1062 | 18259 | 167,9 | 16,5 | 1,146 | H5A034 C | sample | NKRF |
| 9454 | J7 | J8 | 1014 | 1129 | 16727 | 176,6 | 16,5 | 1,146 | H5A034 C | sample | OTUB1 |
| 9469 | K20 | K21 | 1462 | 1015 | 24097 | 153,1 | 16,5 | 1,146 | H5A034 C | sample | PCDHB11 |
| 9673 | D1 | D2 | 1236 | 1171 | 20454 | 196,9 | 16,5 | 1,154 | H5A034 C | sample | ZNF410 |
| 9710 | E21 | E22 | 2051 | 1124 | 33766 | 185,7 | 16,5 | 1,154 | H5A034 C | sample | AMIGO1 |
| 15031 | C15 | C16 | 1284 | 810,2 | 21124 | 169,8 | 16,5 | 1,154 | H5A034 C | sample | KLHL9 |
| 15173 | I13 | I14 | 1370 | 819,5 | 22577 | 133,5 | 16,5 | 1,154 | H5A034 C | sample | KIAA1434 |
| 8467 | A4 | A5 | 1471 | 1123 | 24276 | 229,3 | 16,5 | 1,166 | H5A034 C | sample | DUX5 |
| 8481 | B17 | B18 | 1852 | 1145 | 30620 | 194,6 | 16,5 | 1,166 | H5A034 C | sample | L3MBTL |
| 8667 | J11 | J12 | 1458 | 1110 | 24016 | 137,2 | 16,5 | 1,166 | H5A034 C | sample | PCDHB5 |
| 8723 | L19 | L20 | 1208 | 968,4 | 19880 | 155,9 | 16,5 | 1,166 | H5A034 C | sample | HIBCH |
| 8819 | P19 | P20 | 1877 | 1055 | 30878 | 186,2 | 16,5 | 1,166 | H5A034 C | sample | DAZAP1 |
| 19208 | A16 | A17 | 1259 | 1287 | 20776 | 432,3 | 16,5 | 1,17 | H5A034 C | sample | VSTM2B |
| 19230 | B14 | B15 | 1049 | 1275 | 17275 | 402,2 | 16,5 | 1,17 | H5A034 C | sample | C6orf52 |
| 19537 | O1 | O2 | 1052 | 1111 | 17375 | 276,2 | 16,5 | 1,17 | H5A034 C | sample | ANKRD45 |
| 20761 | B1 | B2 | 648 | 1029 | 10682 | 418,9 | 16,5 | 1,17 | H5A034 C | sample | LOC64346 |
| 20841 | E17 | E18 | 1205 | 1000 | 19940 | 275,1 | 16,5 | 1,17 | H5A034 C | sample | LOC64299 |
| 20952 | I9 | I10 | 810 | 964,1 | 13329 | 284 | 16,5 | 1,17 | H5A034 C | sample | LOC64313 |
| 20955 | J11 | J12 | 1238 | 1019 | 20404 | 319,7 | 16,5 | 1,17 | H5A034 C | sample | FLJ37512 |
| 21076 | O12 | O13 | 1289 | 887,6 | 21280 | 345,1 | 16,5 | 1,17 | H5A034 C | sample | LOC64468 |
| 21201 | D17 | D18 | 888 | 915,5 | 14688 | 262,6 | 16,5 | 1,17 | H5A034 C | sample | LOC64605 |
| 21337 | J1 | J2 | 761 | 937,2 | 12529 | 229,9 | 16,5 | 1,17 | H5A034 C | sample | LOC64626 |
| 21403 | L4 | L5 | 793 | 938,8 | 13065 | 284,9 | 16,5 | 1,17 | H5A034 C | sample | LOC64786 |
| 21488 | P16 | P17 | 969 | 998,3 | 15988 | 322,4 | 16,5 | 1,17 | H5A034 C | sample | LOC64862 |
| 11214 | D14 | D15 | 1690 | 859,2 | 27927 | 167,5 | 16,5 | 1,196 | H5A034 C | sample | |
| 11256 | E9 | E10 | 1088 | 843,4 | 17900 | 172,1 | 16,5 | 1,196 | H5A034 C | sample | SLC9A9 |
| 11406 | L14 | L15 | 1702 | 854,1 | 28072 | 175,4 | 16,5 | 1,196 | H5A034 C | sample | |
| 12409 | F1 | F2 | 1189 | 1054 | 19563 | 185,1 | 16,5 | 1,241 | H5A034 C | sample | WIPF1 |
| 12528 | J9 | J10 | 644 | 1022 | 10624 | 150,2 | 16,5 | 1,241 | H5A034 C | sample | ALMS1 |
| 12633 | O17 | O18 | 1017 | 1054 | 16802 | 170,1 | 16,5 | 1,241 | H5A034 C | sample | TSSC1 |
| 10791 | B22 | B23 | 1529 | 864,5 | 25288 | 721,8 | 16,5 | 1,25 | H5A034 C | sample | CES7 |
| 11015 | K8 | K9 | 623 | 915,7 | 10296 | 539,1 | 16,5 | 1,25 | H5A034 C | sample | FBXO45 |
| 11036 | L5 | L6 | 1180 | 902,4 | 19504 | 664,6 | 16,5 | 1,25 | H5A034 C | sample | DGAT2L4 |
| 11052 | M2 | M3 | 1510 | 874,5 | 24846 | 547,2 | 16,5 | 1,25 | H5A034 C | sample | PTPLB |
| 11122 | P18 | P19 | 1617 | 842,4 | 26686 | 839,1 | 16,5 | 1,25 | H5A034 C | sample | TTC9C |
| 7641 | O17 | O18 | 1062 | 565,6 | 17475 | 406,7 | 16,5 | 1,26 | H5A034 C | sample | PCDH7 |
| 7653 | O6 | O7 | 1112 | 510,8 | 18399 | 547 | 16,5 | 1,26 | H5A034 C | sample | SETDB1 |
| 7664 | P16 | P17 | 1246 | 547,9 | 20538 | 491 | 16,5 | 1,26 | H5A034 C | sample | BCAP31 |
| 16920 | A9 | A10 | 1189 | 1150 | 19609 | 182,8 | 16,5 | 1,274 | H5A034 C | sample | SYCE1 |
| 16926 | B14 | B15 | 1467 | 1027 | 24192 | 223,6 | 16,5 | 1,274 | H5A034 C | sample | TPPP2 |
| 16931 | B19 | B20 | 1549 | 1081 | 25588 | 218 | 16,5 | 1,274 | H5A034 C | sample | KLHL29 |
| 16966 | C7 | C8 | 898 | 1041 | 14824 | 283,9 | 16,5 | 1,274 | H5A034 C | sample | ACRC |
| 17060 | G5 | G6 | 1124 | 989,1 | 18495 | 170,3 | 16,5 | 1,274 | H5A034 C | sample | EXOD1 |
| 17250 | O3 | O4 | 549 | 997,9 | 9033 | 196,8 | 16,5 | 1,274 | H5A034 C | sample | C21orf88 |
| 7043 | F19 | F20 | 1210 | 605,4 | 19969 | 527,7 | 16,5 | 1,284 | H5A034 C | sample | ME1 |
| 7230 | N14 | N15 | 1227 | 584,7 | 20186 | 513,7 | 16,5 | 1,284 | H5A034 C | sample | NOL1 |
| 7237 | N20 | N21 | 867 | 516,8 | 14338 | 416,3 | 16,5 | 1,284 | H5A034 C | sample | NOS2A |
| 7249 | O1 | O2 | 474 | 570,9 | 7820 | 376,1 | 16,5 | 1,284 | H5A034 C | sample | M6PR |
| 7296 | P9 | P10 | 1140 | 586,2 | 18848 | 437 | 16,5 | 1,284 | H5A034 C | sample | MPZ |
| 5064 | C9 | C10 | 1367 | 610,3 | 22620 | 589,6 | 16,5 | 1,294 | H5A034 C | sample | ERMP1 |
| 5193 | I17 | I18 | 1203 | 655,8 | 19830 | 452,6 | 16,5 | 1,294 | H5A034 C | sample | COP1 |
| 5229 | J6 | J7 | 1412 | 709,5 | 23357 | 302 | 16,5 | 1,294 | H5A034 C | sample | XRCC3 |
| 5330 | O10 | O11 | 1710 | 590,1 | 28279 | 635,4 | 16,5 | 1,294 | H5A034 C | sample | UGDH |
| 2433 | F17 | F18 | 690 | 737,5 | 11372 | 404,1 | 16,5 | 1,299 | H5A034 C | sample | JAK2 |
| 2466 | G3 | G4 | 889 | 668,7 | 14631 | 408,5 | 16,5 | 1,299 | H5A034 C | sample | CDK2 |
| 2651 | O19 | O20 | 1562 | 817,6 | 25796 | 487,1 | 16,5 | 1,299 | H5A034 C | sample | BMPR1B |
| 2652 | O2 | O3 | 1326 | 745,5 | 21877 | 561,6 | 16,5 | 1,299 | H5A034 C | sample | PIK3CD |
| 16667 | G19 | G20 | 1075 | 879,4 | 17785 | 257,7 | 16,5 | 1,304 | H5A034 C | sample | CGNL1 |
| 17817 | G17 | G18 | 969 | 1087 | 15972 | 242,6 | 16,5 | 1,304 | H5A034 C | sample | PIWIL4 |
| 17821 | G20 | G21 | 1056 | 974 | 17420 | 302,5 | 16,5 | 1,304 | H5A034 C | sample | C1orf184 |
| 17852 | H5 | H6 | 904 | 952,9 | 14920 | 230,6 | 16,5 | 1,304 | H5A034 C | sample | C16orf77 |
| 17915 | K19 | K20 | 1129 | 1033 | 18680 | 345,4 | 16,5 | 1,304 | H5A034 C | sample | CPNE8 |
| 18022 | O7 | O8 | 491 | 981,1 | 8098 | 452,2 | 16,5 | 1,304 | H5A034 C | sample | C14orf72 |
| 8860 | B12 | B13 | 1575 | 1713 | 26027 | 191,6 | 16,5 | 1,325 | H5A034 C | sample | TREM1 |
| 8875 | B4 | B5 | 1359 | 1560 | 22366 | 198,1 | 16,5 | 1,325 | H5A034 C | sample | MRPL39 |
| 8914 | D18 | D19 | 2020 | 1927 | 33283 | 194,4 | 16,5 | 1,325 | H5A034 C | sample | XRN1 |
| 8964 | F2 | F3 | 1296 | 1857 | 21381 | 169,8 | 16,5 | 1,325 | H5A034 C | sample | TRIAD3 |
| 8991 | G22 | G23 | 1920 | 1911 | 31726 | 213,2 | 16,5 | 1,325 | H5A034 C | sample | POLR3K |
| 9035 | I19 | I20 | 1543 | 1658 | 25433 | 156,4 | 16,5 | 1,325 | H5A034 C | sample | ZNF593 |
| 4317 | D6 | D7 | 1166 | 693,2 | 19204 | 334,1 | 16,5 | 1,392 | H5A034 C | sample | CACNG7 |
| 4380 | G2 | G3 | 1223 | 745 | 20203 | 418,6 | 16,5 | 1,392 | H5A034 C | sample | ACCN3 |
| 125 | F13 | F14 | 479 | 1410 | 7904 | 604,8 | 16,5 | 1,404 | H5A034 C | sample | SHC1 |
| 6853 | N20 | N21 | 1311 | 569,9 | 21579 | 501,4 | 16,5 | 1,447 | H5A034 C | sample | KRT16 |
| 3912 | C9 | C10 | 1180 | 656,7 | 19426 | 353,2 | 16,5 | 1,467 | H5A034 C | sample | PTGER1 |
| 3923 | D19 | D20 | 969 | 696,6 | 16017 | 355,1 | 16,5 | 1,467 | H5A034 C | sample | PRLR |
| 3973 | F20 | F21 | 935 | 610 | 15385 | 335,8 | 16,5 | 1,467 | H5A034 C | sample | RNASE1 |
| 4215 | P22 | P23 | 1257 | 658 | 20748 | 484,5 | 16,5 | 1,467 | H5A034 C | sample | ATXN2 |
| 11623 | E15 | E16 | 1263 | 878,9 | 20835 | 122,6 | 16,5 | 1,473 | H5A034 C | sample | BCL7A |
| 11655 | F22 | F23 | 918 | 836,3 | 15123 | 187,3 | 16,5 | 1,473 | H5A034 C | sample | HDGF |
| 11674 | G18 | G19 | 1321 | 761,3 | 21812 | 132,3 | 16,5 | 1,473 | H5A034 C | sample | FGF4 |
| 11720 | I16 | I17 | 1495 | 833,4 | 24724 | 149,2 | 16,5 | 1,473 | H5A034 C | sample | FHIT |
| 11738 | J10 | J11 | 1257 | 785,5 | 20793 | 148,7 | 16,5 | 1,473 | H5A034 C | sample | HLA-DRA |
| 11768 | K16 | K17 | 641 | 987,7 | 10591 | 139,3 | 16,5 | 1,473 | H5A034 C | sample | FTL |
| 15366 | A14 | A15 | 1217 | 875,5 | 20044 | 189,3 | 16,5 | 1,5 | H5A034 C | sample | KRI1 |
| 15431 | C8 | C9 | 860 | 809,1 | 14190 | 138,1 | 16,5 | 1,5 | H5A034 C | sample | SPATS2 |
| 15451 | D4 | D5 | 1292 | 849,8 | 21305 | 170 | 16,5 | 1,5 | H5A034 C | sample | TNIP2 |
| 15596 | J5 | J6 | 884 | 907,7 | 14581 | 176,5 | 16,5 | 1,5 | H5A034 C | sample | ARV1 |
| 15623 | K8 | K9 | 855 | 822,3 | 14124 | 193,9 | 16,5 | 1,5 | H5A034 C | sample | NUP37 |
| 5961 | I17 | I18 | 1107 | 723,9 | 18264 | 525,9 | 16,5 | 1,507 | H5A034 C | sample | ADD1 |
| 5976 | I9 | I10 | 1315 | 688,8 | 21697 | 417,3 | 16,5 | 1,507 | H5A034 C | sample | ADCY7 |
| 6051 | M11 | M12 | 1400 | 782,3 | 23047 | 540,8 | 16,5 | 1,507 | H5A034 C | sample | AFP |
| 1218 | C3 | C4 | 1079 | 699,2 | 17773 | 508,9 | 16,5 | 1,514 | H5A034 C | sample | ZNF8 |
| 1226 | D10 | D11 | 1198 | 796,6 | 19800 | 480,1 | 16,5 | 1,514 | H5A034 C | sample | ZNF273 |
| 1228 | D12 | D13 | 1354 | 729,3 | 22395 | 496,5 | 16,5 | 1,514 | H5A034 C | sample | ZNF460 |
| 1344 | H9 | H10 | 1501 | 703,3 | 24731 | 415 | 16,5 | 1,514 | H5A034 C | sample | SNX4 |
| 1508 | O5 | O6 | 1352 | 700,4 | 22260 | 515,5 | 16,5 | 1,514 | H5A034 C | sample | ZNF214 |
| 16200 | C9 | C10 | 1069 | 1024 | 17623 | 355,9 | 16,5 | 1,514 | H5A034 C | sample | CDT1 |
| 16206 | D14 | D15 | 914 | 1018 | 15080 | 408,7 | 16,5 | 1,514 | H5A034 C | sample | PPAPDC1B |
| 16372 | K12 | K13 | 1110 | 999,8 | 18347 | 353,7 | 16,5 | 1,514 | H5A034 C | sample | WIBG |
| 16451 | N19 | N20 | 1259 | 945,9 | 20741 | 298,3 | 16,5 | 1,514 | H5A034 C | sample | FLJ13305 |
| 13516 | D12 | D13 | 1401 | 1033 | 23149 | 169,5 | 16,5 | 1,549 | H5A034 C | sample | FBXO2 |
| 13525 | D20 | D21 | 1310 | 1037 | 21676 | 189,3 | 16,5 | 1,549 | H5A034 C | sample | FAM50B |
| 804 | B2 | B3 | 935 | 658,9 | 15424 | 494,1 | 16,5 | 1,564 | H5A034 C | sample | RBBP5 |
| 5398 | A7 | A8 | 932 | 767,6 | 15342 | 613 | 16,5 | 1,571 | H5A034 C | sample | RBM10 |
| 5624 | K16 | K17 | 864 | 643,5 | 14253 | 517,3 | 16,5 | 1,571 | H5A034 C | sample | WASL |
| 3797 | O13 | O14 | 1282 | 1376 | 21181 | 489 | 16,5 | 1,587 | H5A034 C | sample | GIPR |
| 8086 | A7 | A8 | 819 | 651,3 | 13479 | 518,1 | 16,5 | 1,587 | H5A034 C | sample | CPSF6 |
| 8093 | B13 | B14 | 872 | 596,6 | 14416 | 513,6 | 16,5 | 1,587 | H5A034 C | sample | FKBP9 |
| 8335 | L15 | L16 | 1325 | 637,7 | 21898 | 473,1 | 16,5 | 1,587 | H5A034 C | sample | AKR7A3 |
| 10008 | A9 | A10 | 1317 | 885,5 | 21716 | 191,3 | 16,5 | 1,587 | H5A034 C | sample | SRD5A2L |
| 10318 | N7 | N8 | 1303 | 769,8 | 21543 | 168,9 | 16,5 | 1,587 | H5A034 C | sample | DOCK8 |
| 1632 | D9 | D10 | 1117 | 739,6 | 18479 | 422,2 | 16,5 | 1,728 | H5A034 C | sample | TAF7L |
| 1675 | F4 | F5 | 1174 | 784,7 | 19385 | 523 | 16,5 | 1,728 | H5A034 C | sample | RUFY1 |
| 1731 | I11 | I12 | 747 | 673,6 | 12292 | 363,9 | 16,5 | 1,728 | H5A034 C | sample | HOOK2 |
| 21554 | C10 | C11 | 341 | 992,9 | 5637 | 745 | 16,5 | 1,737 | H5A034 C | sample | LOC65255 |
| 21693 | H6 | H7 | 204 | 968,6 | 3362 | 569,7 | 16,5 | 1,737 | H5A034 C | sample | LOC65344 |
| 21710 | I21 | I22 | 163 | 1128 | 2685 | 721,3 | 16,5 | 1,737 | H5A034 C | sample | LOC64991 |
| 4711 | E15 | E16 | 1059 | 726,6 | 17505 | 479,3 | 16,5 | 1,813 | H5A034 C | sample | AZU1 |
| 4876 | L12 | L13 | 1166 | 787,5 | 19232 | 484,8 | 16,5 | 1,813 | H5A034 C | sample | DPP8 |
| 10421 | C13 | C14 | 1220 | 880,4 | 20123 | 214,3 | 16,5 | 1,864 | H5A034 C | sample | ATG4C |
| 389 | A13 | A14 | 1078 | 1773 | 17742 | 411,3 | 16,5 | 2,409 | H5A034 C | sample | E2F2 |
| 22355 | D19 | D20 | 877 | 1445 | 14538 | 318,8 | 16,6 | 0,856 | H5A034 C | sample | LOC28643 |
| 22460 | H5 | H6 | 926 | 1402 | 15374 | 408,8 | 16,6 | 0,856 | H5A034 C | sample | LOC72885 |
| 23564 | F5 | F6 | 580 | 1625 | 9642 | 240 | 16,6 | 0,881 | H5A034 C | sample | LOC73244 |
| 15788 | B5 | B6 | 890 | 838,5 | 14754 | 118,2 | 16,6 | 0,925 | H5A034 C | sample | ARHGAP2 |

| | | | | | | | | | | | | | | | | | | | | | | | | | | | | | | | |
|---|---|---|---|---|---|---|---|---|---|---|---|---|---|---|---|---|---|---|---|---|---|---|---|---|---|---|---|---|---|---|---|
| 15975 | J22 | J23 | 1319 | 768,5 | 21926 | 168,2 | 16,6 | 0,925 | H5A034 C | sample | Hm Gen E | Hm Gen E | Hm Gen E | CPE002J9 | CPE002NU | CPE002KA | NM 03078 | NM 0307 | NM 0307 | CLPTM1L | CLPTM1L | CLPTM1L | CLPTM1-like | CLPTM1-li | CLPTM1-li | 81037 | 81037 | 81037 | s37499 | s37500 | s37501 |
| 16020 | L2 | L3 | 1595 | 836,7 | 26511 | 114,5 | 16,6 | 0,925 | H5A034 C | sample | Hm Gen E | Hm Gen E | Hm Gen E | CPE002J9 | CPE002NU | CPE002KA | NM 05410 | NM 0541 | NM 0541 | OR5AC2 | OR5AC2 | OR5AC2 | olfactory receptor | olfactory | olfactory | 81050 | 81050 | 81050 | s37502 | s37503 | s37504 |
| 16089 | O17 | O18 | 1118 | 758,7 | 18611 | 111,6 | 16,6 | 0,925 | H5A034 C | sample | Hm Gen E | Hm Gen E | Hm Gen E | CPE002J9 | CPE002NU | CPE002KA | NM 02475 | NM 0247 | NM 0247 | MMRN2 | MMRN2 | MMRN2 | multimerin 2 | multimeri | multimeri | 79812 | 79812 | 79812 | s36387 | s36388 | s36389 |
| 14954 | P10 | P11 | 1200 | 807,8 | 19962 | 204,9 | 16,6 | 0,946 | H5A034 C | sample | Hm Gen E | Hm Gen E | Hm Gen E | CPE002JD | CPE002NR | CPE002K7 | NM 01847 | NM 0184 | NM 0184 | FAM45B | FAM45B | FAM45B | family with seque | family wit | family wit | 55855 | 55855 | 55855 | s31668 | s31669 | s195291 |
| 13881 | C17 | C18 | 1165 | 1025 | 19317 | 145,9 | 16,6 | 0,951 | H5A034 C | sample | Hm Gen E | Hm Gen E | Hm Gen E | CPE002JF | CPE002NP | CPE002K5 | NM 00104 | NM 0010 | NM 0010 | D4S234E | D4S234E | D4S234E | DNA segment on | DNA segm | DNA segm | 27065 | 27065 | 27065 | s25753 | s25754 | s25755 |
| 13921 | E1 | E2 | 1404 | 1012 | 23310 | 135,1 | 16,6 | 0,951 | H5A034 C | sample | Hm Gen E | Hm Gen E | Hm Gen E | CPE002JF | CPE002NP | CPE002K5 | NM 01439 | NM 0143 | NM 0143 | DAPP1 | DAPP1 | DAPP1 | dual adaptor of p | dual adap | dual adap | 27071 | 27071 | 27071 | s25765 | s25767 | s226100 |
| 14082 | K3 | K4 | 1762 | 1154 | 29227 | 136,1 | 16,6 | 0,951 | H5A034 C | sample | Hm Gen E | Hm Gen E | Hm Gen E | CPE002JF | CPE002NP | CPE002K5 | NM 01444 | NM 0144 | NM 0144 | SERP1 | SERP1 | SERP1 | stress-associated | stress-ass | stress-ass | 27230 | 27230 | 27230 | s25992 | s25993 | s25994 |
| 14185 | P1 | P2 | 1336 | 1046 | 22191 | 127,5 | 16,6 | 0,951 | H5A034 C | sample | Hm Gen E | Hm Gen E | Hm Gen E | CPE002JF | CPE002NP | CPE002K5 | NM 00108 | NM 0010 | NM 0010 | DSE | DSE | DSE | dermatan sulfate | dermatan | dermatan | 29940 | 29940 | 29940 | s26748 | s26749 | s26750 |
| 3119 | B8 | B9 | 882 | 1358 | 14612 | 410,3 | 16,6 | 1,028 | H5A034 C | sample | Hm Drgbl | Hm Drgbl | Hm Drgbl | CPE002DC | CPE002EG | CPE002F6 | NM 00044 | NM 0004 | NM 0004 | PDE6A | PDE6A | PDE6A | phosphodiesteras | phosphod | phosphod | 5145 | 5145 | 5145 | s10201 | s10202 | s10203 |
| 3126 | C14 | C15 | 1166 | 1231 | 19413 | 448,6 | 16,6 | 1,028 | H5A034 C | sample | Hm Drgbl | Hm Drgbl | Hm Drgbl | CPE002DC | CPE002EG | CPE002F6 | NM 01776 | NM 0177 | NM 0177 | MTMR10 | MTMR10 | MTMR10 | myotubularin rela | myotubul | myotubul | 54893 | 54893 | 54893 | s29695 | s29696 | s29697 |
| 3141 | C6 | C7 | 948 | 1236 | 15716 | 412,7 | 16,6 | 1,028 | H5A034 C | sample | Hm Drgbl | Hm Drgbl | Hm Drgbl | CPE002DC | CPE002EG | CPE002F6 | NM 00104 | NM 0010 | NM 0010 | MTMR12 | MTMR12 | MTMR12 | myotubularin rela | myotubul | myotubul | 54545 | 54545 | 54545 | s29177 | s29178 | s29179 |
| 3440 | P16 | P17 | 947 | 1201 | 15760 | 448,4 | 16,6 | 1,028 | H5A034 C | sample | Hm Drgbl | Hm Drgbl | Hm Drgbl | CPE002DC | CPE002EG | CPE002F6 | NM 00457 | NM 0045 | NM 0045 | PLCB2 | PLCB2 | PLCB2 | phospholipase C, | phospholi | phospholi | 5330 | 5330 | 5330 | s10616 | s10617 | s10618 |
| 12228 | N2 | N3 | 1120 | 803,9 | 18551 | 78,86 | 16,6 | 1,064 | H5A034 C | sample | Hm Gen E | Hm Gen E | Hm Gen E | CPE002J4 | CPE002NL | CPE002K0 | NM 00301 | NM 0030 | NM 0030 | SFRP5 | SFRP5 | SFRP5 | secreted frizzled-r | secreted f | secreted f | 6425 | 6425 | 6425 | s12722 | s12723 | s12724 |
| 12288 | P9 | P10 | 1351 | 748,3 | 22404 | 111,7 | 16,6 | 1,064 | H5A034 C | sample | Hm Gen E | Hm Gen E | Hm Gen E | CPE002J4 | CPE002NL | CPE002K0 | NM 00044 | NM 0004 | NM 0004 | PLEC1 | PLEC1 | PLEC1 | plectin 1, interme | plectin 1, | plectin 1, | 5339 | 5339 | 5339 | s10643 | s10644 | s10645 |
| 18062 | A21 | A22 | 1169 | 1077 | 19459 | 367,9 | 16,6 | 1,068 | H5A034 C | sample | Hm Gen E | Hm Gen E | Hm Gen E | CPE002JL | CPE002O0 | CPE002KG | NM 13089 | NM 1308 | NM 1308 | FAM71B | FAM71B | FAM71B | family with seque | family wit | family wit | 153745 | 153745 | 153745 | s45817 | s45818 | s225757 |
| 18068 | A5 | A6 | 1222 | 1053 | 20305 | 432 | 16,6 | 1,068 | H5A034 C | sample | Hm Gen E | Hm Gen E | Hm Gen E | CPE002JL | CPE002O0 | CPE002KG | NM 15254 | NM 1525 | NM 1525 | SRFBP1 | SRFBP1 | SRFBP1 | serum response f | serum res | serum res | 153443 | 153443 | 153443 | s45781 | s45782 | s45783 |
| 18399 | O22 | O23 | 1477 | 991,1 | 24482 | 384,8 | 16,6 | 1,068 | H5A034 C | sample | Hm Gen E | Hm Gen E | Hm Gen E | CPE002JL | CPE002O0 | CPE002KG | NM 00103 | NM 0010 | NM 0010 | C10orf72 | C10orf72 | C10orf72 | chromosome 10 c | chromoso | chromoso | 196740 | 196740 | 196740 | s47048 | s47049 | s47050 |
| 6198 | C14 | C15 | 863 | 538,1 | 14309 | 469,4 | 16,6 | 1,096 | H5A034 C | sample | Hm Drgbl | Hm Drgbl | Hm Drgbl | CPE002DZ | CPE002F0 | CPE002FL | NM 00438 | NM 0043 | NM 0043 | NKX2-5 | NKX2-5 | NKX2-5 | NK2 transcription | NK2 trans | NK2 trans | 1482 | 1482 | 1482 | s3691 | s3692 | s3690 |
| 6408 | K9 | K10 | 169 | 738,1 | 2798 | 493,9 | 16,6 | 1,096 | H5A034 C | sample | Hm Drgbl | Hm Drgbl | Hm Drgbl | CPE002DZ | CPE002F0 | CPE002FL | NM 00179 | NM 0017 | NM 0017 | CDH8 | CDH8 | CDH8 | cadherin 8, type 2 | cadherin 8 | cadherin 8 | 1006 | 1006 | 1006 | s2789 | s2790 | s2791 |
| 6411 | L11 | L12 | 1848 | 581,7 | 30709 | 435,7 | 16,6 | 1,096 | H5A034 C | sample | Hm Drgbl | Hm Drgbl | Hm Drgbl | CPE002DZ | CPE002F0 | CPE002FL | XM 94207 | XM 94207 | XM 94207 | CPN2 | CPN2 | CPN2 | carboxypeptidase | carboxype | carboxype | 1370 | 1370 | 1370 | s3456 | s3457 | s3458 |
| 20504 | G16 | G17 | 835 | 752,5 | 13825 | 162,7 | 16,6 | 1,107 | H5A034 C | sample | Hm Gen E | Hm Gen E | Hm Gen E | CPE002JR | CPE002O6 | CPE002KM | NM 00101 | NM 0010 | NM 0010 | C1orf190 | C1orf190 | C1orf190 | chromosome 1 or | chromoso | chromoso | 541468 | 541468 | 541468 | s54709 | s54710 | s54711 |
| 12791 | E8 | E9 | 800 | 953,3 | 13315 | 136,2 | 16,6 | 1,125 | H5A034 C | sample | Hm Gen E | Hm Gen E | Hm Gen E | CPE002J6 | CPE002NM | CPE002K2 | NM 01486 | NM 0148 | NM 0148 | RNF10 | RNF10 | RNF10 | ring finger protei | ring finger | ring finger | 9921 | 9921 | 9921 | s19242 | s19243 | s19244 |
| 12814 | F7 | F8 | 1533 | 1028 | 25406 | 131,3 | 16,6 | 1,125 | H5A034 C | sample | Hm Gen E | Hm Gen E | Hm Gen E | CPE002J6 | CPE002NM | CPE002K2 | NM 01470 | NM 0147 | NM 0147 | KIAA0562 | KIAA0562 | KIAA0562 | KIAA0562 | KIAA0562 | KIAA0562 | 9731 | 9731 | 9731 | s18764 | s18765 | s18766 |
| 12875 | I19 | I20 | 1552 | 1058 | 25824 | 148,4 | 16,6 | 1,125 | H5A034 C | sample | Hm Gen E | Hm Gen E | Hm Gen E | CPE002J6 | CPE002NM | CPE002K2 | NM 00488 | NM 0048 | NM 0048 | CXCL14 | CXCL14 | CXCL14 | chemokine (C-X-C | chemokin | chemokin | 9547 | 9547 | 9547 | s18332 | s18333 | s18334 |
| 12893 | J13 | J14 | 1114 | 997,6 | 18437 | 130,7 | 16,6 | 1,125 | H5A034 C | sample | Hm Gen E | Hm Gen E | Hm Gen E | CPE002J6 | CPE002NM | CPE002K2 | NM 01475 | NM 0147 | NM 0147 | DLG7 | DLG7 | DLG7 | discs, large homo | discs, larg | discs, larg | 9787 | 9787 | 9787 | s18912 | s18913 | s18914 |
| 13017 | O17 | O18 | 1327 | 962,9 | 21962 | 173,5 | 16,6 | 1,125 | H5A034 C | sample | Hm Gen E | Hm Gen E | Hm Gen E | CPE002J6 | CPE002NM | CPE002K2 | NM 01465 | NM 0146 | NM 0146 | KIAA0406 | KIAA0406 | KIAA0406 | KIAA0406 | KIAA0406 | KIAA0406 | 9675 | 9675 | 9675 | s18620 | s18621 | s18622 |
| 18434 | A10 | A11 | 463 | 969 | 7704 | 382,4 | 16,6 | 1,125 | H5A034 C | sample | Hm Gen E | Hm Gen E | Hm Gen E | CPE002JM | CPE002O1 | CPE002KH | NM 15289 | NM 1528 | NM 1528 | LRWD1 | LRWD1 | LRWD1 | leucine-rich repea | leucine-ric | leucine-ric | 222229 | 222229 | 222229 | s48174 | s48175 | s48176 |
| 18526 | D7 | D8 | 1191 | 923,7 | 19732 | 141,9 | 16,6 | 1,125 | H5A034 C | sample | Hm Gen E | Hm Gen E | Hm Gen E | CPE002JM | CPE002O1 | CPE002KH | NM 14501 | NM 1450 | NM 1450 | C11orf66 | C11orf66 | C11orf66 | chromosome 11 c | chromoso | chromoso | 220004 | 220004 | 220004 | s47838 | s47839 | s225877 |
| 18710 | L21 | L22 | 1487 | 929,6 | 24755 | 235,6 | 16,6 | 1,125 | H5A034 C | sample | Hm Gen E | Hm Gen E | Hm Gen E | CPE002JM | CPE002O1 | CPE002KH | NM 14502 | NM 1450 | NM 1450 | C6orf81 | C6orf81 | C6orf81 | chromosome 6 or | chromoso | chromoso | 221481 | 221481 | 221481 | s48066 | s48067 | s48068 |
| 17408 | F16 | F17 | 1115 | 851,7 | 18456 | 177,6 | 16,6 | 1,133 | H5A034 C | sample | Hm Gen E | Hm Gen E | Hm Gen E | CPE002JJ | CPE002NY | CPE002KE | NM 00100 | NM 0010 | NM 0010 | OR13C4 | OR13C4 | OR13C4 | olfactory recepto | olfactory | olfactory | 138804 | 138804 | 138804 | s44142 | s44143 | s44144 |
| 17470 | H7 | H8 | 1366 | 934,4 | 22608 | 177,4 | 16,6 | 1,133 | H5A034 C | sample | Hm Gen E | Hm Gen E | Hm Gen E | CPE002JJ | CPE002NY | CPE002KE | NM 13879 | NM 1387 | NM 1387 | ANKRD54 | ANKRD54 | ANKRD54 | ankyrin repeat dc | ankyrin re | ankyrin re | 129138 | 129138 | 129138 | s43388 | s43389 | s43390 |
| 17473 | I1 | I2 | 1171 | 968,9 | 19493 | 169 | 16,6 | 1,133 | H5A034 C | sample | Hm Gen E | Hm Gen E | Hm Gen E | CPE002JJ | CPE002NY | CPE002KE | NM 14469 | NM 1446 | NM 1446 | C1orf125 | C1orf125 | C1orf125 | chromosome 1 or | chromoso | chromoso | 126859 | 126859 | 126859 | s43096 | s43097 | s43098 |
| 17507 | J19 | J20 | 1067 | 937 | 17717 | 255,1 | 16,6 | 1,133 | H5A034 C | sample | Hm Gen E | Hm Gen E | Hm Gen E | CPE002JJ | CPE002NY | CPE002KE | NM 13846 | NM 1384 | NM 1384 | ICA1L | ICA1L | ICA1L | islet cell autoanti | islet cell a | islet cell a | 130026 | 130026 | 130026 | s43461 | s43462 | s43463 |
| 17508 | J2 | J3 | 852 | 899,3 | 14115 | 246,6 | 16,6 | 1,133 | H5A034 C | sample | Hm Gen E | Hm Gen E | Hm Gen E | CPE002JJ | CPE002NY | CPE002KE | NM 19845 | NM 1984 | NM 1984 | APOOL | APOOL | APOOL | apolipoprotein O | apolipopr | apolipopr | 139322 | 139322 | 139322 | s44193 | s44194 | s225655 |
| 17564 | L5 | L6 | 1040 | 896,9 | 17229 | 236,1 | 16,6 | 1,133 | H5A034 C | sample | Hm Gen E | Hm Gen E | Hm Gen E | CPE002JJ | CPE002NY | CPE002KE | NM 00100 | NM 0010 | NM 0010 | REG3G | REG3G | REG3G | regenerating islet | regenerat | regenerat | 130120 | 130120 | 130120 | s43476 | s43477 | s225618 |
| 17581 | M20 | M21 | 1252 | 955,5 | 20823 | 222,7 | 16,6 | 1,133 | H5A034 C | sample | Hm Gen E | Hm Gen E | Hm Gen E | CPE002JJ | CPE002NY | CPE002KE | NM 17882 | NM 1788 | NM 1788 | C7orf34 | C7orf34 | C7orf34 | chromosome 7 or | chromoso | chromoso | 135927 | 135927 | 135927 | s43953 | s43954 | s43955 |
| 9253 | B20 | B21 | 1662 | 1164 | 27607 | 173,7 | 16,6 | 1,153 | H5A034 C | sample | Hm Drgbl | Hm Drgbl | Hm Drgbl | CPE002E8 | CPE002ER | CPE002FC | NM 01985 | NM 0198 | NM 0198 | METTL3 | METTL3 | METTL3 | methyltransferase | methyltra | methyltra | 56339 | 56339 | 56339 | s32143 | s32141 | s32142 |
| 18934 | E7 | E8 | 1332 | 968 | 22052 | 273,9 | 16,6 | 1,157 | H5A034 C | sample | Hm Gen E | Hm Gen E | Hm Gen E | CPE002JN | CPE002O2 | CPE002KI | NM 17688 | NM 1768 | NM 1768 | TAS2R48 | TAS2R48 | TAS2R48 | taste receptor, ty | taste rece | taste rece | 259294 | 259294 | 259294 | s48961 | s48962 | s48963 |
| 9845 | K13 | K14 | 909 | 1057 | 15070 | 186,3 | 16,6 | 1,161 | H5A034 C | sample | Hm Drgbl | Hm Drgbl | Hm Drgbl | CPE002E9 | CPE002EQ | CPE002FB | NM 02081 | NM 0208 | NM 0208 | KIF17 | KIF17 | KIF17 | kinesin family me | kinesin fa | kinesin fa | 57576 | 57576 | 57576 | s33352 | s33353 | s33354 |
| 9939 | O11 | O12 | 1927 | 1093 | 32081 | 223 | 16,6 | 1,161 | H5A034 C | sample | Hm Drgbl | Hm Drgbl | Hm Drgbl | CPE002E9 | CPE002EQ | CPE002FB | NM 00107 | NM 0010 | NM 0010 | MIER1 | MIER1 | MIER1 | mesoderm induct | mesoderm | mesoderm | 57708 | 57708 | 57708 | s33645 | s33643 | s33644 |
| 9943 | O15 | O16 | 1888 | 1072 | 31264 | 158,2 | 16,6 | 1,161 | H5A034 C | sample | Hm Drgbl | Hm Drgbl | Hm Drgbl | CPE002E9 | CPE002EQ | CPE002FB | NM 01789 | NM 0178 | NM 0178 | SEMA4G | SEMA4G | SEMA4G | sema domain, im | sema dom | sema dom | 57715 | 57715 | 57715 | s33661 | s33662 | s33663 |
| 15275 | M19 | M20 | 1487 | 850,6 | 24743 | 156,5 | 16,6 | 1,161 | H5A034 C | sample | Hm Gen E | Hm Gen E | Hm Gen E | CPE002J8 | CPE002NS | CPE002K8 | NM 02012 | NM 0201 | NM 0201 | IFNK | IFNK | IFNK | interferon, kappa | interferon | interferon | 56832 | 56832 | 56832 | s32275 | s32276 | s32277 |
| 8461 | A20 | A21 | 1403 | 1076 | 23235 | 172,5 | 16,6 | 1,173 | H5A034 C | sample | Hm Drgbl | Hm Drgbl | Hm Drgbl | CPE002E6 | CPE002ET | CPE002FE | NM 01437 | NM 0143 | NM 0143 | CYFIP2 | CYFIP2 | CYFIP2 | cytoplasmic FMR1 | cytoplasm | cytoplasm | 26999 | 26999 | 26999 | s25682 | s25683 | s25684 |
| 19285 | D20 | D21 | 1099 | 1193 | 18250 | 253,4 | 16,6 | 1,177 | H5A034 C | sample | Hm Gen E | Hm Gen E | Hm Gen E | CPE002JO | CPE002O3 | CPE002KJ | NM 18262 | NM 1826 | NM 1826 | C2orf48 | C2orf48 | C2orf48 | chromosome 2 or | chromoso | chromoso | 348738 | 348738 | 348738 | s51354 | s51355 | s51356 |
| 19315 | E4 | E5 | 1065 | 1107 | 17653 | 390,4 | 16,6 | 1,177 | H5A034 C | sample | Hm Gen E | Hm Gen E | Hm Gen E | CPE002JO | CPE002O3 | CPE002KJ | NM 00101 | NM 0010 | NM 0010 | MYBPHL | MYBPHL | MYBPHL | myosin binding p | myosin bi | myosin bi | 343263 | 343263 | 343263 | s50998 | s50999 | s51000 |
| 19359 | G22 | G23 | 1181 | 1217 | 19564 | 355 | 16,6 | 1,177 | H5A034 C | sample | Hm Gen E | Hm Gen E | Hm Gen E | CPE002JO | CPE002O3 | CPE002KJ | NM 00108 | NM 0010 | NM 0010 | LOC34465 | LOC34465 | LOC34465 | similar to LETHal | similar to | similar to | 344657 | 344657 | 344657 | s51074 | s51075 | s51076 |
| 19540 | O12 | O13 | 1410 | 1190 | 23428 | 442,9 | 16,6 | 1,177 | H5A034 C | sample | Hm Gen E | Hm Gen E | Hm Gen E | CPE002JO | CPE002O3 | CPE002KJ | NM 19851 | NM 1985 | NM 1985 | LANCL3 | LANCL3 | LANCL3 | LanC lantibiotic sy | LanC lanti | LanC lanti | 347404 | 347404 | 347404 | s51244 | s51245 | s51246 |
| 19568 | P16 | P17 | 961 | 1301 | 15910 | 307,1 | 16,6 | 1,177 | H5A034 C | sample | Hm Gen E | Hm Gen E | Hm Gen E | CPE002JO | CPE002O3 | CPE002KJ | NM 19854 | NM 1985 | NM 1985 | FAM73A | FAM73A | FAM73A | family with seque | family wit | family wit | 374986 | 374986 | 374986 | s51645 | s51646 | s51647 |
| 20742 | A14 | A15 | 1155 | 1050 | 19154 | 380,3 | 16,6 | 1,177 | H5A034 C | sample | Hm Gen E | Hm Gen E | Hm Gen E | CPE002JS | CPE002O7 | CPE002KN | NM 00104 | NM 0010 | NM 0010 | LOC64409 | LOC64409 | LOC64409 | hypothetical prot | hypotheti | hypotheti | 644096 | 644096 | 644096 | s55556 | s55557 | s227106 |
| 20785 | C1 | C2 | 995 | 975,4 | 16510 | 337,6 | 16,6 | 1,177 | H5A034 C | sample | Hm Gen E | Hm Gen E | Hm Gen E | CPE002JS | CPE002O7 | CPE002KN | XM 92623 | XM 92623 | XM 92623 | LOC64283 | LOC64283 | LOC64283 | similar to SR prot | similar to | similar to | 642837 | 642837 | 642837 | s55133 | s55134 | s196836 |
| 20809 | D1 | D2 | 1034 | 953,5 | 17159 | 288,9 | 16,6 | 1,177 | H5A034 C | sample | Hm Gen E | Hm Gen E | Hm Gen E | CPE002JS | CPE002O7 | CPE002KN | XM 93160 | XM 93160 | XM 93160 | LOC64352 | LOC64352 | LOC64352 | hypothetical LOC | hypotheti | hypotheti | 643524 | 643524 | 643524 | s55351 | s196917 | s196918 |
| 20885 | G13 | G14 | 1630 | 1034 | 27047 | 251,6 | 16,6 | 1,177 | H5A034 C | sample | Hm Gen E | Hm Gen E | Hm Gen E | CPE002JS | CPE002O7 | CPE002KN | XM 92642 | XM 92642 | XM 92642 | LOC64305 | LOC64305 | LOC64305 | hypothetical LOC | hypotheti | hypotheti | 643052 | 643052 | 643052 | s55198 | s55199 | s196866 |
| 20929 | I1 | I2 | 954 | 980,3 | 15846 | 240,8 | 16,6 | 1,177 | H5A034 C | sample | Hm Gen E | Hm Gen E | Hm Gen E | CPE002JS | CPE002O7 | CPE002KN | XM 92646 | XM 92646 | XM 92646 | LOC64308 | LOC64308 | LOC64308 | similar to scaveng | similar to | similar to | 643086 | 643086 | 643086 | s55211 | s227051 | s227052 |
| 21183 | C22 | C23 | 921 | 1007 | 15285 | 389,5 | 16,6 | 1,177 | H5A034 C | sample | Hm Gen E | Hm Gen E | Hm Gen E | CPE002JT | CPE002O8 | CPE002KO | XM 92964 | XM 92964 | XM 92964 | LOC64669 | LOC64669 | LOC64669 | similar to zinc fin | similar to | similar to | 646698 | 646698 | 646698 | s56497 | s56498 | s56499 |
| 21283 | G4 | G5 | 1145 | 999,1 | 19049 | 254,4 | 16,6 | 1,177 | H5A034 C | sample | Hm Gen E | Hm Gen E | Hm Gen E | CPE002JT | CPE002O8 | CPE002KO | XM 92977 | XM 92977 | XM 92977 | LOC64681 | LOC64681 | LOC64681 | hypothetical LOC | hypotheti | hypotheti | 646817 | 646817 | 646817 | s56542 | s56543 | s56544 |
| 21372 | K2 | K3 | 951 | 943,6 | 15756 | 278,7 | 16,6 | 1,177 | H5A034 C | sample | Hm Gen E | Hm Gen E | Hm Gen E | CPE002JT | CPE002O8 | CPE002KO | XM 92990 | XM 92990 | XM 92990 | LOC64693 | LOC64693 | LOC64693 | similar to thyroid | similar to | similar to | 646939 | 646939 | 646939 | s56586 | s56587 | s56588 |
| 21391 | L15 | L16 | 1195 | 898,8 | 19855 | 323,4 | 16,6 | 1,177 | H5A034 C | sample | Hm Gen E | Hm Gen E | Hm Gen E | CPE002JT | CPE002O8 | CPE002KO | XM 93361 | XM 93361 | XM 93361 | LOC64643 | LOC64643 | LOC64643 | hypothetical LOC | hypotheti | hypotheti | 646437 | 646437 | 646437 | s56377 | s197379 | s227234 |
| 14245 | B20 | B21 | 1571 | 914,4 | 26044 | 164,8 | 16,6 | 1,181 | H5A034 C | sample | Hm Gen E | Hm Gen E | Hm Gen E | CPE002J7 | CPE002NC | CPE002K6 | NM 01772 | NM 0177 | NM 0177 | CC2D1A | CC2D1A | CC2D1A | coiled-coil and C2 | coiled-coi | coiled-coi | 54862 | 54862 | 54862 | s29617 | s29619 | s226792 |
| 11149 | A20 | A21 | 1758 | 882,3 | 29164 | 228,1 | 16,6 | 1,203 | H5A034 C | sample | Hm Drgbl | Hm Drgbl | Hm Drgbl | CPE002ED | CPE002F3 | CPE002FT | NM 00108 | NM 0010 | NM 0010 | RKHD1 | RKHD1 | RKHD1 | ring finger and KH | ring finger | ring finger | 399664 | 399664 | 399664 | s53136 | s53135 | s53137 |
| 11170 | B18 | B19 | 1698 | 890,9 | 28223 | 212,2 | 16,6 | 1,203 | H5A034 C | sample | Hm Drgbl | Hm Drgbl | Hm Drgbl | CPE002ED | CPE002F3 | CPE002FT | | | | | | | | | | | | | | | |
| 11193 | C17 | C18 | 1055 | 826,4 | 17472 | 165,7 | 16,6 | 1,203 | H5A034 C | sample | Hm Drgbl | Hm Drgbl | Hm Drgbl | CPE002ED | CPE002F3 | CPE002FT | NM 17852 | NM 1785 | NM 1785 | SLC25A42 | SLC25A42 | SLC25A42 | solute carrier fam | solute car | solute car | 284439 | 284439 | 284439 | s49667 | s49666 | s49665 |
| 11202 | C3 | C4 | 1168 | 832,7 | 19332 | 148,4 | 16,6 | 1,203 | H5A034 C | sample | Hm Drgbl | Hm Drgbl | Hm Drgbl | CPE002ED | CPE002F3 | CPE002FT | NM 17851 | NM 1785 | NM 1785 | PIGW | PIGW | PIGW | phosphatidylinos | phosphat | phosphat | 284098 | 284098 | 284098 | s49488 | s49489 | s49490 |
| 11456 | N16 | N17 | 1666 | 876,5 | 27626 | 161,6 | 16,6 | 1,203 | H5A034 C | sample | Hm Drgbl | Hm Drgbl | Hm Drgbl | CPE002ED | CPE002F3 | CPE002FT | | | | | | | | | | | | | | | |
| 11506 | P18 | P19 | 1917 | 896,7 | 31913 | 234,9 | 16,6 | 1,203 | H5A034 C | sample | Hm Drgbl | Hm Drgbl | Hm Drgbl | CPE002ED | CPE002F3 | CPE002FT | | | | | | | | | | | | | | | |
| 12345 | C17 | C18 | 867 | 983 | 14388 | 161,7 | 16,6 | 1,248 | H5A034 C | sample | Hm Gen E | Hm Gen E | Hm Gen E | CPE002J5 | CPE002NK | CPE002K1 | NM 00541 | NM 0054 | NM 0054 | ST5 | ST5 | ST5 | suppression of tu | suppressi | suppressi | 6764 | 6764 | 6764 | s13513 | s13515 | s227458 |
| 12428 | F5 | F6 | 1297 | 967,6 | 21550 | 156,6 | 16,6 | 1,248 | H5A034 C | sample | Hm Gen E | Hm Gen E | Hm Gen E | CPE002J5 | CPE002NK | CPE002K1 | NM 01414 | NM 0141 | NM 0141 | LAT2 | LAT2 | LAT2 | linker for activati | linker for | linker for | 7462 | 7462 | 7462 | s14850 | s14852 | s228152 |
| 12546 | K3 | K4 | 1457 | 1098 | 24209 | 159,9 | 16,6 | 1,248 | H5A034 C | sample | Hm Gen E | Hm Gen E | Hm Gen E | CPE002J5 | CPE002NK | CPE002K1 | NM 00542 | NM 0054 | NM 0054 | TFF2 | TFF2 | TFF2 | trefoil factor 2 (sc | trefoil fac | trefoil fac | 7032 | 7032 | 7032 | s14036 | s14038 | s227469 |
| 12636 | O2 | O3 | 1335 | 1131 | 22211 | 167,4 | 16,6 | 1,248 | H5A034 C | sample | Hm Gen E | Hm Gen E | Hm Gen E | CPE002J5 | CPE002NK | CPE002K1 | NM 00376 | NM 0037 | NM 0037 | VAMP4 | VAMP4 | VAMP4 | vesicle-associated | vesicle-as | vesicle-as | 8674 | 8674 | 8674 | s16525 | s16526 | s16527 |
| 10819 | C4 | C5 | 1407 | 823,7 | 23340 | 636 | 16,6 | 1,258 | H5A034 C | sample | Hm Drgbl | Hm Drgbl | Hm Drgbl | CPE002EC | CPE002EN | CPE002FS | NM 13917 | NM 1391 | NM 1391 | RNF133 | RNF133 | RNF133 | ring finger protei | ring finger | ring finger | 168433 | 168433 | 168433 | s46702 | s46703 | s46701 |
| 7346 | C10 | C11 | 912 | 618,4 | 15157 | 540,5 | 16,6 | 1,267 | H5A034 C | sample | Hm Drgbl | Hm Drgbl | Hm Drgbl | CPE002E3 | CPE002EW | CPE002FH | NM 01463 | NM 0146 | NM 0146 | MICAL2 | MICAL2 | MICAL2 | microtubule asso | microtubu | microtubu | 9645 | 9645 | 9645 | s18547 | s18546 | s18545 |
| 7359 | C22 | C23 | 1344 | 609,1 | 22288 | 501,9 | 16,6 | 1,267 | H5A034 C | sample | Hm Drgbl | Hm Drgbl | Hm Drgbl | CPE002E3 | CPE002EW | CPE002FH | NM 02500 | NM 0250 | NM 0250 | CEP135 | CEP135 | CEP135 | centrosomal prot | centroson | centroson | 9662 | 9662 | 9662 | s18587 | s18588 | s18589 |
| 7427 | F19 | F20 | 1563 | 602,2 | 25998 | 505,6 | 16,6 | 1,267 | H5A034 C | sample | Hm Drgbl | Hm Drgbl | Hm Drgbl | CPE002E3 | CPE002EW | CPE002FH | NM 00426 | NM 0042 | NM 0042 | CHST2 | CHST2 | CHST2 | carbohydrate (N-a | carbohydr | carbohydr | 9435 | 9435 | 9435 | s18059 | s18060 | s225134 |
| 7551 | K22 | K23 | 711 | 741,2 | 11787 | 535,5 | 16,6 | 1,267 | H5A034 C | sample | Hm Drgbl | Hm Drgbl | Hm Drgbl | CPE002E3 | CPE002EW | CPE002FH | NM 01475 | NM 0147 | NM 0147 | PHYHIP | PHYHIP | PHYHIP | phytanoyl-CoA 2- | phytanoyl | phytanoyl | 9796 | 9796 | 9796 | s18933 | s18935 | s18934 |
| 7554 | K3 | K4 | 783 | 583,6 | 12980 | 535,3 | 16,6 | 1,267 | H5A034 C | sample | Hm Drgbl | Hm Drgbl | Hm Drgbl | CPE002E3 | CPE002EW | CPE002FH | NM 00256 | NM 0025 | NM 0025 | PEBP1 | PEBP1 | PEBP1 | phosphatidyletha | phosphat | phosphat | 5037 | 5037 | 5037 | s9982 | s9983 | s9981 |
| 7555 | K4 | K5 | 106 | 667,3 | 1759 | 478,3 | 16,6 | 1,267 | H5A034 C | sample | Hm Drgbl | Hm Drgbl | Hm Drgbl | CPE002E3 | CPE002EW | CPE002FH | NM 01474 | NM 0147 | NM 0147 | EIF4A3 | EIF4A3 | EIF4A3 | eukaryotic transla | eukaryoti | eukaryoti | 9775 | 9775 | 9775 | s18877 | s18876 | s18878 |
| 16974 | D14 | D15 | 1432 | 994,5 | 23842 | 192,6 | 16,6 | 1,282 | H5A034 C | sample | Hm Gen E | Hm Gen E | Hm Gen E | CPE002JI | CPE002NZ | CPE002KD | XM 06348 | NM 1757 | XM 0634 | LOC12310 | TCF23 | LOC12310 | similar to kelch-lil | transcript | similar to | 123103 | 150921 | 123103 | s42620 | s45496 | s42622 |
| 17149 | K20 | K21 | 1605 | 974,7 | 26650 | 220,6 | 16,6 | 1,282 | H5A034 C | sample | Hm Gen E | Hm Gen E | Hm Gen E | CPE002JI | CPE002NZ | CPE002KD | NM 15231 | NM 1775 | NM 1523 | C12orf45 | PRNT | C12orf45 | chromosome 12 c | prion prot | chromoso | 121053 | 149830 | 121053 | s42426 | s45348 | s225534 |
| 17162 | L10 | L11 | 970 | 888,6 | 16096 | 168 | 16,6 | 1,282 | H5A034 C | sample | Hm Gen E | Hm Gen E | Hm Gen E | CPE002JI | CPE002NZ | CPE002KD | NM 14460 | NM 1749 | NM 1446 | HEXIM2 | CCDC50 | HEXIM2 | hexamethylene bis | coiled-coi | hexamthy | 124790 | 152137 | 124790 | s42794 | s45668 | s42796 |
| 17181 | L6 | L7 | 1387 | 858,9 | 22975 | 227,4 | 16,6 | 1,282 | H5A034 C | sample | Hm Gen E | Hm Gen E | Hm Gen E | CPE002JI | CPE002NZ | CPE002KD | NM 18170 | NM 0010 | NM 1817 | C17orf64 | ZCWPW2 | C17orf64 | chromosome 17 c | zinc finger | chromoso | 124773 | 152098 | 124773 | s42788 | s45656 | s225554 |
| 17193 | M17 | M18 | 1282 | 996,4 | 21226 | 248,7 | 16,6 | 1,282 | H5A034 C | sample | Hm Gen E | Hm Gen E | Hm Gen E | CPE002JI | CPE002NZ | CPE002KD | NM 05288 | NM 1758 | NM 0528 | PIK3IP1 | C12orf60 | PIK3IP1 | phosphoinositide | chromoso | phosphoi | 113791 | 144608 | 113791 | s41504 | s44682 | s41506 |
| 17230 | N7 | N8 | 1530 | 1016 | 25325 | 223,6 | 16,6 | 1,282 | H5A034 C | sample | Hm Gen E | Hm Gen E | Hm Gen E | CPE002JI | CPE002NZ | CPE002KD | XM 37566 | NM 0010 | XM 3756 | LOC11641 | C18orf25 | LOC11641 | hypothetical prot | chromoso | hypotheti | 116412 | 147339 | 116412 | s42002 | s44986 | s225510 |
| 17271 | P22 | P23 | 1086 | 1116 | 18033 | 342 | 16,6 | 1,282 | H5A034 C | sample | Hm Gen E | Hm Gen E | Hm Gen E | CPE002JI | CPE002NZ | CPE002KD | NM 00100 | NM 1749 | NM 0010 | OR7D4 | TMEM167 | OR7D4 | olfactory recepto | transmem | olfactory | 125958 | 153339 | 125958 | s42909 | s45774 | s225564 |
| 7284 | P2 | P3 | 975 | 596,4 | 16176 | 366,3 | 16,6 | 1,292 | H5A034 C | sample | Hm Drgbl | Hm Drgbl | Hm Drgbl | CPE002E2 | CPE002EX | CPE002FI | NM 01331 | NM 0133 | NM 0133 | CNOT4 | CNOT4 | CNOT4 | CCR4-NOT transcr | CCR4-NOT | CCR4-NOT | 4850 | 4850 | 4850 | s9631 | s9630 | s9632 |
| 5088 | D9 | D10 | 1525 | 651,4 | 25382 | 549 | 16,6 | 1,302 | H5A034 C | sample | Hm Drgbl | Hm Drgbl | Hm Drgbl | CPE002DV | CPE002EM | CPE002FO | NM 00425 | NM 0042 | NM 0042 | RAB9A | RAB9A | RAB9A | RAB9A, member R | RAB9A, m | RAB9A, m | 9367 | 9367 | 9367 | s17916 | s17918 | s17917 |
| 5285 | M13 | M14 | 931 | 567 | 15434 | 569,3 | 16,6 | 1,302 | H5A034 C | sample | Hm Drgbl | Hm Drgbl | Hm Drgbl | CPE002DV | CPE002EM | CPE002FO | NM 13905 | NM 1390 | NM 1390 | ADAMTS1 | ADAMTS1 | ADAMTS1 | ADAM metallopep | ADAM me | ADAM me | 170690 | 170690 | 170690 | s223419 | s46855 | s46856 |
| 5323 | N4 | N5 | 1382 | 629,4 | 22945 | 644 | 16,6 | 1,302 | H5A034 C | sample | Hm Drgbl | Hm Drgbl | Hm Drgbl | CPE002DV | CPE002EM | CPE002FO | NM 00344 | NM 0034 | NM 0034 | ZNF165 | ZNF165 | ZNF165 | zinc finger protei | zinc finger | zinc finger | 7718 | 7718 | 7718 | s15225 | s15226 | s15227 |
| 2334 | B14 | B15 | 1536 | 720,3 | 25491 | 532,2 | 16,6 | 1,307 | H5A034 C | sample | Hm Drgbl | Hm Drgbl | Hm Drgbl | CPE002DC | CPE002EE | CPE002F4 | NM 00273 | NM 0027 | NM 0027 | PRKCG | PRKCG | PRKCG | protein kinase C, | protein ki | protein ki | 5582 | 5582 | 5582 | s11104 | s11105 | s11106 |
| 2339 | B19 | B20 | 788 | 680 | 13056 | 483,1 | 16,6 | 1,307 | H5A034 C | sample | Hm Drgbl | Hm Drgbl | Hm Drgbl | CPE002DC | CPE002EE | CPE002F4 | NM 01598 | NM 0159 | NM 0159 | CAMK2A | CAMK2A | CAMK2A | calcium/calmodul | calcium/ca | calcium/ca | 815 | 815 | 815 | s2358 | s2359 | s2360 |
| 2471 | G8 | G9 | 781 | 688,2 | 12966 | 464,4 | 16,6 | 1,307 | H5A034 C | sample | Hm Drgbl | Hm Drgbl | Hm Drgbl | CPE002DC | CPE002EE | CPE002F4 | NM 05302 | NM 0530 | NM 0530 | MYLK | MYLK | MYLK | myosin, light chai | myosin, li | myosin, li | 4638 | 4638 | 4638 | s9193 | s9194 | s9195 |
| 2612 | M5 | M6 | 668 | 714,1 | 11081 | 329,7 | 16,6 | 1,307 | H5A034 C | sample | Hm Drgbl | Hm Drgbl | Hm Drgbl | CPE002DC | CPE002EE | CPE002F4 | NM 00731 | NM 0073 | NM 0073 | ABL1 | ABL1 | ABL1 | v-abl Abelson mu | v-abl Abel | v-abl Abel | 25 | 25 | 25 | s864 | s865 | s866 |
| 16708 | I12 | I13 | 923 | 921 | 15355 | 283,4 | 16,6 | 1,312 | H5A034 C | sample | Hm Gen E | Hm Gen E | Hm Gen E | CPE002JH | CPE002NV | CPE002KC | NM 03077 | NM 0307 | NM 0307 | CCDC34 | CCDC34 | CCDC34 | coiled-coil domai | coiled-coi | coiled-coi | 91057 | 91057 | 91057 | s40564 | s40565 | s228454 |
| 16733 | J13 | J14 | 1178 | 911,1 | 19541 | 336,9 | 16,6 | 1,312 | H5A034 C | sample | Hm Gen E | Hm Gen E | Hm Gen E | CPE002JH | CPE002NV | CPE002KC | NM 03312 | NM 0331 | NM 0331 | SEC16B | SEC16B | SEC16B | SEC16 homolog B | SEC16 hor | SEC16 hor | 89866 | 89866 | 89866 | s40165 | s40166 | s40167 |
| 16834 | N18 | N19 | 866 | 1127 | 14353 | 430,2 | 16,6 | 1,312 | H5A034 C | sample | Hm Gen E | Hm Gen E | Hm Gen E | CPE002JH | CPE002NV | CPE002KC | NM 13877 | NM 1387 | NM 1387 | C13orf27 | C13orf27 | C13orf27 | chromosome 13 c | chromoso | chromoso | 93081 | 93081 | 93081 | s41074 | s41075 | s41076 |
| 16881 | P17 | P18 | 662 | 1015 | 10988 | 247,8 | 16,6 | 1,312 | H5A034 C | sample | Hm Gen E | Hm Gen E | Hm Gen E | CPE002JH | CPE002NV | CPE002KC | NM 03354 | NM 0335 | NM 0335 | CEACAM2 | CEACAM2 | CEACAM2 | carcinoembryonic | carcinoem | carcinoem | 90273 | 90273 | 90273 | s40310 | s40311 | s40312 |
| 17814 | G14 | G15 | 1008 | 1090 | 16739 | 193,4 | 16,6 | 1,312 | H5A034 C | sample | Hm Gen E | Hm Gen E | Hm Gen E | CPE002JK | CPE002NZ | CPE002KF | XM 00113 | XM 0011 | XM 0011 | LOC14915 | LOC14915 | LOC14915 | hypothetical prot | hypotheti | hypotheti | 149157 | 149157 | 149157 | s45259 | s45260 | s45261 |
| 17888 | J16 | J17 | 1301 | 1037 | 21594 | 279,9 | 16,6 | 1,312 | H5A034 C | sample | Hm Gen E | Hm Gen E | Hm Gen E | CPE002JK | CPE002NZ | CPE002KF | NM 15253 | NM 1525 | NM 1525 | C3orf21 | C3orf21 | C3orf21 | chromosome 3 or | chromoso | chromoso | 152002 | 152002 | 152002 | s45638 | s45639 | s45640 |
| 17901 | J6 | J7 | 1124 | 1022 | 18713 | 291,4 | 16,6 | 1,312 | H5A034 C | sample | Hm Gen E | Hm Gen E | Hm Gen E | CPE002JK | CPE002NZ | CPE002KF | NM 13881 | NM 1388 | NM 1388 | DPPA2 | DPPA2 | DPPA2 | developmental pl | developm | developm | 151871 | 151871 | 151871 | s45620 | s45621 | s45622 |
| 17929 | L1 | L2 | 832 | 959 | 13802 | 244,9 | 16,6 | 1,312 | H5A034 C | sample | Hm Gen E | Hm Gen E | Hm Gen E | CPE002JK | CPE002NZ | CPE002KF | NM 00103 | NM 0010 | NM 0010 | UNC45B | UNC45B | UNC45B | unc-45 homolog B | unc-45 ho | unc-45 ho | 146862 | 146862 | 146862 | s44933 | s44934 | s44935 |
| 17938 | L18 | L19 | 1169 | 891,7 | 19361 | 247 | 16,6 | 1,312 | H5A034 C | sample | Hm Gen E | Hm Gen E | Hm Gen E | CPE002JK | CPE002NZ | CPE002KF | NM 14471 | NM 1447 | NM 1447 | CCDC13 | CCDC13 | CCDC13 | coiled-coil domai | coiled-coi | coiled-coi | 152206 | 152206 | 152206 | s45679 | s45680 | s45681 |
| 17956 | M12 | M13 | 1273 | 972,3 | 21089 | 193,8 | 16,6 | 1,312 | H5A034 C | sample | Hm Gen E | Hm Gen E | Hm Gen E | CPE002JK | CPE002NZ | CPE002KF | NM 15250 | NM 1525 | NM 1525 | LCA5L | LCA5L | LCA5L | Leber congenital a | Leber con | Leber con | 150082 | 150082 | 150082 | s45370 | s45371 | s45372 |
| 18004 | O12 | O13 | 1391 | 937,1 | 23039 | 391,2 | 16,6 | 1,312 | H5A034 C | sample | Hm Gen E | Hm Gen E | Hm Gen E | CPE002JK | CPE002NZ | CPE002KF | NM 00101 | NM 0010 | NM 0010 | LOC15029 | LOC15029 | LOC15029 | hypothetical prot | hypotheti | hypotheti | 150297 | 150297 | 150297 | s45416 | s45417 | s45418 |
| 8845 | A20 | A21 | 1325 | 1746 | 21968 | 173,9 | 16,6 | 1,333 | H5A034 C | sample | Hm Drgbl | Hm Drgbl | Hm Drgbl | CPE002E7 | CPE002ES | CPE002FD | NM 01545 | NM 0154 | NM 0154 | LARP7 | LARP7 | LARP7 | La ribonucleoprot | La ribonu | La ribonu | 51574 | 51574 | 51574 | s28353 | s28354 | s28355 |
| 8864 | B16 | B17 | 1806 | 1783 | 29917 | 170,2 | 16,6 | 1,333 | H5A034 C | sample | Hm Drgbl | Hm Drgbl | Hm Drgbl | CPE002E7 | CPE002ES | CPE002FD | NM 01896 | NM 0189 | NM 0189 | SNTG2 | SNTG2 | SNTG2 | syntrophin, gamm | syntrophi | syntrophi | 54221 | 54221 | 54221 | s28914 | s28915 | s28916 |
| 8894 | C21 | C22 | 1719 | 1810 | 28541 | 196,5 | 16,6 | 1,333 | H5A034 C | sample | Hm Drgbl | Hm Drgbl | Hm Drgbl | CPE002E7 | CPE002ES | CPE002FD | NM 01571 | NM 0157 | NM 0157 | NOX3 | NOX3 | NOX3 | NADPH oxidase 3 | NADPH ox | NADPH ox | 50508 | 50508 | 50508 | s27018 | s27016 | s27017 |
| 8915 | D19 | D20 | 1763 | 1788 | 29342 | 161,7 | 16,6 | 1,333 | H5A034 C | sample | Hm Drgbl | Hm Drgbl | Hm Drgbl | CPE002E7 | CPE002ES | CPE002FD | NM 01637 | NM 0163 | NM 0163 | RAB9B | RAB9B | RAB9B | RAB9B, member R | RAB9B, m | RAB9B, m | 51209 | 51209 | 51209 | s27691 | s27692 | s27693 |
| 8942 | E21 | E22 | 1843 | 1784 | 30553 | 192,2 | 16,6 | 1,333 | H5A034 C | sample | Hm Drgbl | Hm Drgbl | Hm Drgbl | CPE002E7 | CPE002ES | CPE002FD | NM 01635 | NM 0163 | NM 0163 | IRX4 | IRX4 | IRX4 | iroquois homeob | iroquois h | iroquois h | 50805 | 50805 | 50805 | s27097 | s27098 | s224173 |
| 8956 | F12 | F13 | 1377 | 1641 | 22876 | 153,2 | 16,6 | 1,333 | H5A034 C | sample | Hm Drgbl | Hm Drgbl | Hm Drgbl | CPE002E7 | CPE002ES | CPE002FD | NM 17583 | NM 1758 | NM 1758 | SMOX | SMOX | SMOX | spermine oxidase | spermine | spermine | 54498 | 54498 | 54498 | s29082 | s29083 | s29084 |
| 4299 | D11 | D12 | 1453 | 711,6 | 24146 | 496,4 | 16,6 | 1,401 | H5A034 C | sample | Hm Drgbl | Hm Drgbl | Hm Drgbl | CPE002DU | CPE002EK | CPE002FQ | NM 00083 | NM 0008 | NM 0008 | GRIN2C | GRIN2C | GRIN2C | glutamate recepto | glutamate | glutamate | 2905 | 2905 | 2905 | s6176 | s6177 | s6178 |
| 4510 | L7 | L8 | 877 | 777 | 14553 | 399,6 | 16,6 | 1,401 | H5A034 C | sample | Hm Drgbl | Hm Drgbl | Hm Drgbl | CPE002DU | CPE002EK | CPE002FQ | NM 00224 | NM 0022 | NM 0022 | KCNK3 | KCNK3 | KCNK3 | potassium chann | potassium | potassium | 3777 | 3777 | 3777 | s7783 | s7784 | s7785 |
| 302 | M21 | M22 | 465 | 1363 | 7715 | 561,5 | 16,6 | 1,413 | H5A034 C | sample | Hm Drgbl | Hm Drgbl | Hm Drgbl | CPE002DT | CPE002EJ | CPE002F9 | NM 00540 | NM 0054 | NM 0054 | CCL13 | CCL13 | CCL13 | chemokine (C-C m | chemokin | chemokin | 6357 | 6357 | 6357 | s12588 | s12589 | s12587 |
| 1932 | A2 | A3 | 436 | 743,5 | 7231 | 599,2 | 16,6 | 1,443 | H5A034 C | sample | Hm Drgbl | Hm Drgbl | Hm Drgbl | CPE002FX | CPE002G1 | CPE002G5 | NM 00100 | NM 0010 | NM 0010 | ZNF780B | ZNF780B | ZNF780B | zinc finger protei | zinc finger | zinc finger | 163131 | 163131 | 163131 | s46428 | s46429 | s46430 |
| 6588 | C2 | C3 | 699 | 634,1 | 11575 | 476,5 | 16,6 | 1,456 | H5A034 C | sample | Hm Drgbl | Hm Drgbl | Hm Drgbl | CPE002E1 | CPE002EY | CPE002FJ | NM 00553 | NM 0055 | NM 0055 | IFNGR2 | IFNGR2 | IFNGR2 | interferon gamma | interferon | interferon | 3460 | 3460 | 3460 | s7198 | s7196 | s7197 |
| 6810 | L3 | L4 | 1103 | 574,7 | 18267 | 484,3 | 16,6 | 1,456 | H5A034 C | sample | Hm Drgbl | Hm Drgbl | Hm Drgbl | CPE002E1 | CPE002EY | CPE002FJ | NM 18153 | NM 1815 | NM 1815 | ERAS | ERAS | ERAS | ES cell expressed | ES cell exp | ES cell exp | 3266 | 3266 | 3266 | s6895 | s6894 | s6893 |
| 6862 | N7 | N8 | 1378 | 553,8 | 22879 | 511,7 | 16,6 | 1,456 | H5A034 C | sample | Hm Drgbl | Hm Drgbl | Hm Drgbl | CPE002E1 | CPE002EY | CPE002FJ | NM 00552 | NM 0055 | NM 0055 | HSF1 | HSF1 | HSF1 | heat shock transc | heat shoc | heat shoc | 3297 | 3297 | 3297 | s6950 | s6951 | s6952 |
| 6911 | P8 | P9 | 1235 | 591,8 | 20526 | 509,3 | 16,6 | 1,456 | H5A034 C | sample | Hm Drgbl | Hm Drgbl | Hm Drgbl | CPE002E1 | CPE002EY | CPE002FJ | NM 00227 | NM 0022 | NM 0022 | KRT32 | KRT32 | KRT32 | keratin 32 | keratin 32 | keratin 32 | 3882 | 3882 | 3882 | s8005 | s8006 | s8004 |
| 3983 | F8 | F9 | 1063 | 677,3 | 17680 | 404,1 | 16,6 | 1,476 | H5A034 C | sample | Hm Drgbl | Hm Drgbl | Hm Drgbl | CPE002DS | CPE002EI | CPE002F8 | NM 00561 | NM 0056 | NM 0056 | RHEB | RHEB | RHEB | Ras homolog enri | Ras homo | Ras homo | 6009 | 6009 | 6009 | s12019 | s12020 | s12021 |
| 4007 | G8 | G9 | 875 | 670,6 | 14482 | 362,8 | 16,6 | 1,476 | H5A034 C | sample | Hm Drgbl | Hm Drgbl | Hm Drgbl | CPE002DS | CPE002EI | CPE002F8 | NM 00287 | NM 0028 | NM 0028 | RAD23B | RAD23B | RAD23B | RAD23 homolog B | RAD23 ho | RAD23 ho | 5887 | 5887 | 5887 | s11731 | s11732 | s11733 |
| 4056 | I9 | I10 | 877 | 607,8 | 14532 | 327,4 | 16,6 | 1,476 | H5A034 C | sample | Hm Drgbl | Hm Drgbl | Hm Drgbl | CPE002DS | CPE002EI | CPE002F8 | NM 00268 | NM 0026 | NM 0026 | PNMT | PNMT | PNMT | phenylethanolam | phenyleth | phenyleth | 5409 | 5409 | 5409 | s10756 | s10755 | s224292 |
| 11659 | F4 | F5 | 857 | 811,7 | 14192 | 164,8 | 16,6 | 1,482 | H5A034 C | sample | Hm Gen E | Hm Gen E | Hm Gen E | CPE002JA | CPE002NJ | CPE002JZ | NM 02105 | NM 0210 | NM 0210 | HIST1H2AI | HIST1H2AI | HIST1H2AI | histone cluster 1, | histone cl | histone cl | 3012 | 3012 | 3012 | s6409 | s6410 | s194489 |
| 11759 | J8 | J9 | 717 | 860,7 | 11882 | 201,9 | 16,6 | 1,482 | H5A034 C | sample | Hm Gen E | Hm Gen E | Hm Gen E | CPE002JA | CPE002NJ | CPE002JZ | NM 18254 | NM 1825 | NM 1825 | HLA-DOB2 | HLA-DOB2 | HLA-DOB2 | major histocompa | major hist | major hist | 3120 | 3120 | 3120 | s6604 | s6605 | s226279 |
| 11886 | P14 | P15 | 1203 | 848,4 | 19979 | 221,3 | 16,6 | 1,482 | H5A034 C | sample | Hm Gen E | Hm Gen E | Hm Gen E | CPE002JA | CPE002NJ | CPE002JZ | NM 00155 | NM 0015 | NM 0015 | IGBP1 | IGBP1 | IGBP1 | immunoglobulin ( | immunogl | immunogl | 3476 | 3476 | 3476 | s7205 | s7206 | s7207 |
| 15421 | C20 | C21 | 1215 | 831 | 20179 | 150,6 | 16,6 | 1,509 | H5A034 C | sample | Hm Gen E | Hm Gen E | Hm Gen E | CPE002JE | CPE002NT | CPE002K9 | NM 01909 | NM 0190 | NM 0190 | PLEKHA3 | PLEKHA3 | PLEKHA3 | pleckstrin homolo | pleckstrin | pleckstrin | 65977 | 65977 | 65977 | s35287 | s35288 | s35289 |
| 15432 | C9 | C10 | 670 | 879,7 | 11102 | 157,2 | 16,6 | 1,509 | H5A034 C | sample | Hm Gen E | Hm Gen E | Hm Gen E | CPE002JE | CPE002NT | CPE002K9 | NM 02182 | NM 0218 | NM 0218 | CCDC81 | CCDC81 | CCDC81 | coiled-coil domai | coiled-coi | coiled-coi | 60494 | 60494 | 60494 | s34105 | s34106 | s34107 |
| 15619 | K4 | K5 | 865 | 860,1 | 14357 | 170,7 | 16,6 | 1,509 | H5A034 C | sample | Hm Gen E | Hm Gen E | Hm Gen E | CPE002JE | CPE002NT | CPE002K9 | NM 02405 | NM 0240 | NM 0240 | C7orf25 | C7orf25 | C7orf25 | chromosome 7 or | chromoso | chromoso | 79020 | 79020 | 79020 | s35433 | s35434 | s35435 |
| 5791 | B15 | B16 | 902 | 710,4 | 14998 | 480,8 | 16,6 | 1,516 | H5A034 C | sample | Hm Drgbl | Hm Drgbl | Hm Drgbl | CPE002DY | CPE002F1 | CPE002FM | NM 00003 | NM 0000 | NM 0000 | ALDOA | ALDOA | ALDOA | aldolase A, fructo | aldolase A | aldolase A | 226 | 226 | 226 | s71 | s70 | s72 |
| 5809 | C1 | C2 | 252 | 812,9 | 4193 | 530,9 | 16,6 | 1,516 | H5A034 C | sample | Hm Drgbl | Hm Drgbl | Hm Drgbl | CPE002DY | CPE002F1 | CPE002FM | NM 00035 | NM 0003 | NM 0003 | ABCA4 | ABCA4 | ABCA4 | ATP-binding casse | ATP-bindi | ATP-bindi | 24 | 24 | 24 | s863 | s861 | s862 |
| 5912 | G16 | G17 | 726 | 757,9 | 12084 | 481,7 | 16,6 | 1,516 | H5A034 C | sample | Hm Drgbl | Hm Drgbl | Hm Drgbl | CPE002DY | CPE002F1 | CPE002FM | NM 00404 | NM 0040 | NM 0040 | ASMT | ASMT | ASMT | acetylserotonin O | acetylserc | acetylserc | 438 | 438 | 438 | s224055 | s1673 | s1674 |
| 5915 | G19 | G20 | 1440 | 806,5 | 23973 | 536,6 | 16,6 | 1,516 | H5A034 C | sample | Hm Drgbl | Hm Drgbl | Hm Drgbl | CPE002DY | CPE002F1 | CPE002FM | NM 01870 | NM 0187 | NM 0187 | ADARB2 | ADARB2 | ADARB2 | adenosine deami | adenosine | adenosine | 105 | 105 | 105 | s1015 | s1014 | s1013 |
| 6037 | L20 | L21 | 1153 | 831,3 | 19095 | 505,5 | 16,6 | 1,516 | H5A034 C | sample | Hm Drgbl | Hm Drgbl | Hm Drgbl | CPE002DY | CPE002F1 | CPE002FM | NM 00434 | NM 0043 | NM 0043 | CAMP | CAMP | CAMP | cathelicidin antim | cathelicid | cathelicid | 820 | 820 | 820 | s2375 | s2373 | s2374 |
| 1247 | D8 | D9 | 699 | 772,9 | 11624 | 464,1 | 16,6 | 1,523 | H5A034 C | sample | Hm Drgbl | Hm Drgbl | Hm Drgbl | CPE002FV | CPE002FZ | CPE002G3 | NM 01632 | NM 0163 | NM 0163 | ZNF274 | ZNF274 | ZNF274 | zinc finger protei | zinc finger | zinc finger | 10782 | 10782 | 10782 | s223157 | s21181 | s21182 |
| 1486 | N7 | N8 | 1020 | 699,3 | 16892 | 438,1 | 16,6 | 1,523 | H5A034 C | sample | Hm Drgbl | Hm Drgbl | Hm Drgbl | CPE002FV | CPE002FZ | CPE002G3 | NM 01208 | NM 0120 | NM 0120 | GTF3C3 | GTF3C3 | GTF3C3 | general transcript | general tr | general tr | 9330 | 9330 | 9330 | s17832 | s17833 | s17834 |
| 16135 | A15 | A16 | 1108 | 1066 | 18371 | 432,8 | 16,6 | 1,523 | H5A034 C | sample | Hm Gen E | Hm Gen E | Hm Gen E | CPE002JG | CPE002NV | CPE002KB | NM 03091 | NM 0309 | NM 0309 | URM1 | URM1 | URM1 | ubiquitin related | ubiquitin | ubiquitin | 81605 | 81605 | 81605 | s37684 | s37685 | s228263 |
| 16300 | H12 | H13 | 770 | 1034 | 12765 | 324,1 | 16,6 | 1,523 | H5A034 C | sample | Hm Gen E | Hm Gen E | Hm Gen E | CPE002JG | CPE002NV | CPE002KB | NM 03252 | NM 0325 | NM 0325 | KRTAP4-4 | KRTAP4-4 | KRTAP4-4 | keratin associated | keratin as | keratin as | 84616 | 84616 | 84616 | s39177 | s39178 | s39179 |
| 16302 | H14 | H15 | 1098 | 909,6 | 18202 | 349,2 | 16,6 | 1,523 | H5A034 C | sample | Hm Gen E | Hm Gen E | Hm Gen E | CPE002JG | CPE002NV | CPE002KB | NM 00108 | NM 0010 | NM 0010 | ZGPAT | ZGPAT | ZGPAT | zinc finger, CCCH- | zinc finger | zinc finger | 84619 | 84619 | 84619 | s39186 | s39187 | s195478 |
| 16463 | N8 | N9 | 1266 | 946,2 | 21034 | 432,8 | 16,6 | 1,523 | H5A034 C | sample | Hm Gen E | Hm Gen E | Hm Gen E | CPE002JG | CPE002NV | CPE002KB | NM 00108 | NM 0010 | NM 0010 | UNC119B | UNC119B | UNC119B | unc-119 homolog | unc-119 h | unc-119 h | 84747 | 84747 | 84747 | s39396 | s39397 | s228388 |
| 13541 | E13 | E14 | 1328 | 937,9 | 22007 | 162,2 | 16,6 | 1,559 | H5A034 C | sample | Hm Gen E | Hm Gen E | Hm Gen E | CPE002JC | CPE002NC | CPE002K4 | NM 02515 | NM 0251 | NM 0251 | UNC84A | UNC84A | UNC84A | unc-84 homolog A | unc-84 ho | unc-84 ho | 23353 | 23353 | 23353 | s23629 | s23630 | s23631 |
| 13722 | L3 | L4 | 640 | 956,8 | 10651 | 170,3 | 16,6 | 1,559 | H5A034 C | sample | Hm Gen E | Hm Gen E | Hm Gen E | CPE002JC | CPE002NC | CPE002K4 | NM 01226 | NM 0122 | NM 0122 | TMEM184 | TMEM184 | TMEM184 | transmembrane p | transmem | transmem | 25829 | 25829 | 25829 | s24569 | s24570 | s24571 |
| 817 | C1 | C2 | 737 | 818,2 | 12238 | 525,9 | 16,6 | 1,573 | H5A034 C | sample | Hm Drgbl | Hm Drgbl | Hm Drgbl | CPE002FU | CPE002FY | CPE002G2 | NM 00430 | NM 0043 | NM 0043 | BIN1 | BIN1 | BIN1 | bridging integrato | bridging ir | bridging ir | 274 | 274 | 274 | s1340 | s1341 | s1342 |
| 1123 | O4 | O5 | 1201 | 781,8 | 19909 | 619,6 | 16,6 | 1,573 | H5A034 C | sample | Hm Drgbl | Hm Drgbl | Hm Drgbl | CPE002FU | CPE002FY | CPE002G2 | NM 20328 | NM 2032 | NM 2032 | POU5F1 | POU5F1 | POU5F1 | POU domain, clas | POU doma | POU doma | 5460 | 5460 | 5460 | s10871 | s10872 | s10873 |
| 5566 | H7 | H8 | 1294 | 727 | 21440 | 541,7 | 16,6 | 1,581 | H5A034 C | sample | Hm Drgbl | Hm Drgbl | Hm Drgbl | CPE002DX | CPE002F2 | CPE002FN | NM 00375 | NM 0037 | NM 0037 | SLC4A4 | SLC4A4 | SLC4A4 | solute carrier fam | solute car | solute car | 8671 | 8671 | 8671 | s16516 | s16518 | s16517 |
| 5749 | P20 | P21 | 1481 | 642 | 24639 | 600,8 | 16,6 | 1,581 | H5A034 C | sample | Hm Drgbl | Hm Drgbl | Hm Drgbl | CPE002DX | CPE002F2 | CPE002FN | NM 00479 | NM 0047 | NM 0047 | RAB33A | RAB33A | RAB33A | RAB33A, member | RAB33A, r | RAB33A, r | 9363 | 9363 | 9363 | s225118 | s17907 | s17908 |
| 3507 | C11 | C12 | 1209 | 1277 | 20097 | 350,9 | 16,6 | 1,596 | H5A034 C | sample | Hm Drgbl | Hm Drgbl | Hm Drgbl | CPE002DR | CPE002EH | CPE002F7 | NM 00068 | NM 0006 | NM 0006 | AGTR2 | AGTR2 | AGTR2 | angiotensin II rec | angiotens | angiotens | 186 | 186 | 186 | s1183 | s1184 | s1185 |
| 3790 | N7 | N8 | 996 | 1217 | 16564 | 392,1 | 16,6 | 1,596 | H5A034 C | sample | Hm Drgbl | Hm Drgbl | Hm Drgbl | CPE002DR | CPE002EH | CPE002F7 | NM 00091 | NM 0009 | NM 0009 | NPY2R | NPY2R | NPY2R | neuropeptide Y re | neuropep | neuropep | 4887 | 4887 | 4887 | s9702 | s9703 | s9704 |
| 8307 | K11 | K12 | 865 | 637,4 | 14372 | 514,2 | 16,6 | 1,596 | H5A034 C | sample | Hm Drgbl | Hm Drgbl | Hm Drgbl | CPE002E5 | CPE002EU | CPE002FF | NM 00719 | NM 0071 | NM 0071 | POLI | POLI | POLI | polymerase (DNA | polymeras | polymeras | 11201 | 11201 | 11201 | s22122 | s22123 | s22124 |
| 9993 | A17 | A18 | 1377 | 898,9 | 22892 | 190,7 | 16,6 | 1,596 | H5A034 C | sample | Hm Drgbl | Hm Drgbl | Hm Drgbl | CPE002EA | CPE002EP | CPE002FA | XM 93108 | XM 93108 | XM 93108 | DYNC2H1 | DYNC2H1 | DYNC2H1 | dynein, cytoplasn | dynein, cy | dynein, cy | 79659 | 79659 | 79659 | s36031 | s36032 | s36033 |
| 10028 | B5 | B6 | 549 | 869,6 | 9110 | 193,2 | 16,6 | 1,596 | H5A034 C | sample | Hm Drgbl | Hm Drgbl | Hm Drgbl | CPE002EA | CPE002EP | CPE002FA | NM 02513 | NM 0251 | NM 0251 | CHD9 | CHD9 | CHD9 | chromodomain h | chromodo | chromodo | 80205 | 80205 | 80205 | s37051 | s37052 | s37050 |
| 10044 | C2 | C3 | 1282 | 857,7 | 21246 | 150,6 | 16,6 | 1,596 | H5A034 C | sample | Hm Drgbl | Hm Drgbl | Hm Drgbl | CPE002EA | CPE002EP | CPE002FA | NM 03130 | NM 0313 | NM 0313 | PUS3 | PUS3 | PUS3 | pseudouridylate s | pseudour | pseudour | 83480 | 83480 | 83480 | s37965 | s37963 | s37964 |
| 1549 | A20 | A21 | 911 | 741,2 | 15168 | 502,1 | 16,6 | 1,738 | H5A034 C | sample | Hm Drgbl | Hm Drgbl | Hm Drgbl | CPE002FW | CPE002G0 | CPE002G4 | NM 02073 | NM 0207 | NM 0207 | AHRR | AHRR | AHRR | aryl-hydrocarbon | aryl-hydrc | aryl-hydrc | 57491 | 57491 | 57491 | s33142 | s33143 | s33144 |
| 21574 | C7 | C8 | 195 | 1024 | 3236 | 787,4 | 16,6 | 1,747 | H5A034 C | sample | Hm Gen E | Hm Gen E | Hm Gen E | CPE002JU | CPE002O9 | CPE002KP | XM 94170 | XM 9417 | XM 9417 | LOC64900 | LOC64900 | LOC64900 | similar to parasp | similar to | similar to | 649009 | 649009 | 649009 | s56908 | s56909 | s56910 |
| 21888 | P9 | P10 | 216 | 1147 | 3587 | 772,6 | 16,6 | 1,747 | H5A034 C | sample | Hm Gen E | Hm Gen E | Hm Gen E | CPE002JU | CPE002O9 | CPE002KP | XM 94166 | XM 94166 | XM 94166 | LOC65225 | LOC65225 | LOC65225 | hypothetical prot | hypotheti | hypotheti | 652259 | 652259 | 652259 | s57332 | s57333 | s57334 |
| 4987 | P4 | P5 | 1394 | 746,6 | 23171 | 563 | 16,6 | 1,824 | H5A034 C | sample | Hm Drgbl | Hm Drgbl | Hm Drgbl | CPE002DV | CPE002EL | CPE002FP | NM 02072 | NM 0207 | NM 0207 | NLN | NLN | NLN | neurolysin (metal | neurolysir | neurolysir | 57486 | 57486 | 57486 | s33133 | s33134 | s33135 |
| 10468 | E12 | E13 | 1176 | 931,3 | 19480 | 231,4 | 16,6 | 1,876 | H5A034 C | sample | Hm Drgbl | Hm Drgbl | Hm Drgbl | CPE002EB | CPE002EO | CPE002FR | NM 01778 | NM 0177 | NM 0177 | OSBPL10 | OSBPL10 | OSBPL10 | oxysterol binding | oxysterol | oxysterol | 114884 | 114884 | 114884 | s360 | s359 | s223235 |
| 10514 | G10 | G11 | 1191 | 830,1 | 19777 | 182,6 | 16,6 | 1,876 | H5A034 C | sample | Hm Drgbl | Hm Drgbl | Hm Drgbl | CPE002EB | CPE002EO | CPE002FR | NM 17347 | NM 1734 | NM 1734 | SLC25A26 | SLC25A26 | SLC25A26 | solute carrier fam | solute car | solute car | 115286 | 115286 | 115286 | s41786 | s41784 | s41785 |
| 10635 | L11 | L12 | 921 | 818,5 | 15292 | 175,4 | 16,6 | 1,876 | H5A034 C | sample | Hm Drgbl | Hm Drgbl | Hm Drgbl | CPE002EB | CPE002EO | CPE002FR | NM 02483 | NM 0248 | NM 0248 | TGS1 | TGS1 | TGS1 | trimethylguanosir | trimethyl | trimethyl | 96764 | 96764 | 96764 | s41314 | s41315 | s41313 |
| 10689 | N17 | N18 | 1186 | 889 | 19708 | 207,5 | 16,6 | 1,876 | H5A034 C | sample | Hm Drgbl | Hm Drgbl | Hm Drgbl | CPE002EB | CPE002EO | CPE002FR | NM 13046 | NM 1304 | NM 1304 | CHST14 | CHST14 | CHST14 | carbohydrate (N-a | carbohydr | carbohydr | 113189 | 113189 | 113189 | s41438 | s41439 | s41440 |
| 10734 | P14 | P15 | 1081 | 872,7 | 17956 | 201,9 | 16,6 | 1,876 | H5A034 C | sample | Hm Drgbl | Hm Drgbl | Hm Drgbl | CPE002EB | CPE002EO | CPE002FR | NM 15294 | NM 1529 | NM 1529 | DRB1 | DRB1 | DRB1 | developmentally | developm | developm | 129831 | 129831 | 129831 | s43442 | s43441 | s43440 |
| 22245 | O6 | O7 | 898 | 1419 | 14984 | 408,4 | 16,7 | 0,793 | H5A034 C | sample | Hm Gen E | Hm Gen E | Hm Gen E | CPE002JV | CPE002OA | CPE002KQ | XM 00112 | XM 0011 | XM 0011 | LOC72833 | LOC72833 | LOC72833 | hypothetical prot | hypotheti | hypotheti | 728339 | 728339 | 728339 | s58533 | s58534 | s58535 |
| 13311 | K22 | K23 | 1772 | 1129 | 29677 | 145,2 | 16,7 | 0,837 | H5A034 C | sample | Hm Gen E | Hm Gen E | Hm Gen E | CPE002JB | CPE002NN | CPE002K3 | XM 94066 | XM 94066 | XM 94066 | IGSF9B | IGSF9B | IGSF9B | immunoglobulin s | immunogl | immunogl | 22997 | 22997 | 22997 | s22802 | s22803 | s22804 |
| 15954 | I3 | I4 | 970 | 880,8 | 16151 | 130,5 | 16,7 | 0,93 | H5A034 C | sample | Hm Gen E | Hm Gen E | Hm Gen E | CPE002J9 | CPE002NU | CPE002KA | NM 02467 | NM 0246 | NM 0246 | NSUN7 | NSUN7 | NSUN7 | NOL1/NOP2/Sun ( | NOL1/NOI | NOL1/NOI | 79730 | 79730 | 79730 | s36205 | s36206 | s228196 |
| 14671 | D15 | D16 | 1372 | 857,1 | 22919 | 120,2 | 16,7 | 0,952 | H5A034 C | sample | Hm Gen E | Hm Gen E | Hm Gen E | CPE002JD | CPE002NR | CPE002K7 | NM 01815 | NM 0181 | NM 0181 | RIC8B | RIC8B | RIC8B | resistance to inhi | resistance | resistance | 55188 | 55188 | 55188 | s30388 | s30389 | s30390 |
| 14774 | H21 | H22 | 1425 | 813,9 | 23789 | 128 | 16,7 | 0,952 | H5A034 C | sample | Hm Gen E | Hm Gen E | Hm Gen E | CPE002JD | CPE002NR | CPE002K7 | NM 00100 | NM 0010 | NM 0010 | STEAP3 | STEAP3 | STEAP3 | STEAP family mer | STEAP fan | STEAP fan | 55240 | 55240 | 55240 | s30527 | s30528 | s30529 |
| 14857 | L1 | L2 | 1418 | 887,4 | 23731 | 106,2 | 16,7 | 0,952 | H5A034 C | sample | Hm Gen E | Hm Gen E | Hm Gen E | CPE002JD | CPE002NR | CPE002K7 | NM 01827 | NM 0182 | NM 0182 | C7orf43 | C7orf43 | C7orf43 | chromosome 7 or | chromoso | chromoso | 55262 | 55262 | 55262 | s30584 | s30585 | s30586 |
| 13857 | B17 | B18 | 1737 | 1124 | 28941 | 143,7 | 16,7 | 0,957 | H5A034 C | sample | Hm Gen E | Hm Gen E | Hm Gen E | CPE002JF | CPE002NP | CPE002K5 | NM 01403 | NM 0140 | NM 0140 | C11orf54 | C11orf54 | C11orf54 | chromosome 11 c | chromoso | chromoso | 28970 | 28970 | 28970 | s26321 | s26322 | s26323 |
| 14034 | I3 | I4 | 1316 | 1010 | 22007 | 170 | 16,7 | 0,957 | H5A034 C | sample | Hm Gen E | Hm Gen E | Hm Gen E | CPE002JF | CPE002NP | CPE002K5 | NM 01568 | NM 0156 | NM 0156 | DENND2A | DENND2A | DENND2A | DENN/MADD don | DENN/MA | DENN/MA | 27147 | 27147 | 27147 | s25903 | s25905 | s226110 |
| 14145 | N17 | N18 | 1376 | 1064 | 22986 | 212 | 16,7 | 0,957 | H5A034 C | sample | Hm Gen E | Hm Gen E | Hm Gen E | CPE002JF | CPE002NP | CPE002K5 | NM 01333 | NM 0133 | NM 0133 | TIMM22 | TIMM22 | TIMM22 | translocase of inr | translocas | translocas | 29928 | 29928 | 29928 | s26724 | s26725 | s26726 |
| 3348 | L2 | L3 | 1212 | 1222 | 20260 | 387,1 | 16,7 | 1,034 | H5A034 C | sample | Hm Drgbl | Hm Drgbl | Hm Drgbl | CPE002DC | CPE002EG | CPE002F6 | NM 00264 | NM 0026 | NM 0026 | SERPINB8 | SERPINB8 | SERPINB8 | serpin peptidase | serpin pe | serpin pe | 5271 | 5271 | 5271 | s10475 | s10476 | s10477 |
| 3365 | M13 | M14 | 903 | 1174 | 15085 | 435,4 | 16,7 | 1,034 | H5A034 C | sample | Hm Drgbl | Hm Drgbl | Hm Drgbl | CPE002DC | CPE002EG | CPE002F6 | NM 00624 | NM 0062 | NM 0062 | PPP1R3D | PPP1R3D | PPP1R3D | protein phosphat | protein pl | protein pl | 5509 | 5509 | 5509 | s10948 | s10949 | s10950 |
| 12079 | H15 | H16 | 1458 | 776,4 | 24331 | 95,66 | 16,7 | 1,071 | H5A034 C | sample | Hm Gen E | Hm Gen E | Hm Gen E | CPE002J4 | CPE002NL | CPE002K0 | NM 00455 | NM 0045 | NM 0045 | NRTN | NRTN | NRTN | neurturin | neurturin | neurturin | 4902 | 4902 | 4902 | s9729 | s9730 | s194665 |
| 18129 | D17 | D18 | 1343 | 951,3 | 22419 | 335,9 | 16,7 | 1,074 | H5A034 C | sample | Hm Gen E | Hm Gen E | Hm Gen E | CPE002JL | CPE002O0 | CPE002KG | NM 15259 | NM 1525 | NM 1525 | C14orf49 | C14orf49 | C14orf49 | chromosome 14 c | chromoso | chromoso | 161176 | 161176 | 161176 | s46245 | s46246 | s46247 |
| 18343 | M15 | M16 | 1396 | 993,6 | 23341 | 288 | 16,7 | 1,074 | H5A034 C | sample | Hm Gen E | Hm Gen E | Hm Gen E | CPE002JL | CPE002O0 | CPE002KG | NM 13881 | NM 1388 | NM 1388 | PRUNE2 | PRUNE2 | PRUNE2 | prune homolog 2 | prune hor | prune hor | 158471 | 158471 | 158471 | s46085 | s46086 | s46087 |
| 19953 | P17 | P18 | 1167 | 381,8 | 19450 | 287,6 | 16,7 | 1,088 | H5A034 C | sample | Hm Gen E | Hm Gen E | Hm Gen E | CPE002JP | CPE002O4 | CPE002KK | XM 37390 | XM 37390 | XM 37390 | LOC38878 | LOC38878 | LOC38878 | hypothetical LOC | hypotheti | hypotheti | 388789 | 388789 | 388789 | s52262 | s52263 | s52264 |
| 6318 | H14 | H15 | 1297 | 614,6 | 21623 | 435,5 | 16,7 | 1,102 | H5A034 C | sample | Hm Drgbl | Hm Drgbl | Hm Drgbl | CPE002DZ | CPE002F0 | CPE002FL | NM 00193 | NM 0019 | NM 0019 | DLX4 | DLX4 | DLX4 | distal-less homeo | distal-less | distal-less | 1748 | 1748 | 1748 | s4144 | s4145 | s223426 |
| 6359 | I8 | I9 | 949 | 512,6 | 15856 | 411,2 | 16,7 | 1,102 | H5A034 C | sample | Hm Drgbl | Hm Drgbl | Hm Drgbl | CPE002DZ | CPE002F0 | CPE002FL | NM 00077 | NM 0007 | NM 0007 | CYP2E1 | CYP2E1 | CYP2E1 | cytochrome P450, | cytochron | cytochron | 1571 | 1571 | 1571 | s3838 | s3839 | s3837 |
| 6395 | K19 | K20 | 1594 | 543,1 | 26660 | 458,5 | 16,7 | 1,102 | H5A034 C | sample | Hm Drgbl | Hm Drgbl | Hm Drgbl | CPE002DZ | CPE002F0 | CPE002FL | NM 00125 | NM 0012 | NM 0012 | CDH13 | CDH13 | CDH13 | cadherin 13, H-ca | cadherin 1 | cadherin 1 | 1012 | 1012 | 1012 | s2804 | s2806 | s2805 |
| 20366 | A21 | A22 | 782 | 813,4 | 13027 | 258,2 | 16,7 | 1,113 | H5A034 C | sample | Hm Gen E | Hm Gen E | Hm Gen E | CPE002JR | CPE002O6 | CPE002KM | NM 00101 | NM 0010 | NM 0010 | LOC44015 | LOC44015 | LOC44015 | hypothetical gene | hypotheti | hypotheti | 440157 | 440157 | 440157 | s54048 | s54049 | s226605 |
| 20387 | B19 | B20 | 1136 | 827 | 18949 | 216 | 16,7 | 1,113 | H5A034 C | sample | Hm Gen E | Hm Gen E | Hm Gen E | CPE002JR | CPE002O6 | CPE002KM | XM 49930 | XM 49930 | XM 49930 | LOC44123 | LOC44123 | LOC44123 | hypothetical gene | hypotheti | hypotheti | 441239 | 441239 | 441239 | s54294 | s54295 | s226635 |
| 20406 | C14 | C15 | 1088 | 787,2 | 18147 | 221,9 | 16,7 | 1,113 | H5A034 C | sample | Hm Gen E | Hm Gen E | Hm Gen E | CPE002JR | CPE002O6 | CPE002KM | NM 00101 | NM 0010 | NM 0010 | H2AFB2 | H2AFB2 | H2AFB2 | H2A histone fami | H2A histo | H2A histo | 474381 | 474381 | 474381 | s54605 | s54606 | s54607 |
| 20478 | F14 | F15 | 742 | 775,5 | 12372 | 204,2 | 16,7 | 1,113 | H5A034 C | sample | Hm Gen E | Hm Gen E | Hm Gen E | CPE002JR | CPE002O6 | CPE002KM | XM 93623 | XM 93623 | XM 93623 | LOC64210 | LOC64210 | LOC64210 | similar to Maltase | similar to | similar to | 642103 | 642103 | 642103 | s54931 | s54932 | s54933 |
| 20715 | P11 | P12 | 673 | 745,9 | 11206 | 204 | 16,7 | 1,113 | H5A034 C | sample | Hm Gen E | Hm Gen E | Hm Gen E | CPE002JR | CPE002O6 | CPE002KM | XM 00113 | XM 0011 | XM 0011 | LOC44228 | LOC44228 | LOC44228 | similar to Zgc:731 | similar to | similar to | 442287 | 442287 | 442287 | s54527 | s54528 | s196659 |
| 12704 | B16 | B17 | 1663 | 1178 | 27781 | 138,7 | 16,7 | 1,132 | H5A034 C | sample | Hm Gen E | Hm Gen E | Hm Gen E | CPE002J6 | CPE002NM | CPE002K2 | NM 00699 | NM 0069 | NM 0069 | LRRC23 | LRRC23 | LRRC23 | leucine rich repea | leucine ric | leucine ric | 10233 | 10233 | 10233 | s19970 | s19971 | s19972 |
| 12887 | I8 | I9 | 986 | 985,7 | 16451 | 160,4 | 16,7 | 1,132 | H5A034 C | sample | Hm Gen E | Hm Gen E | Hm Gen E | CPE002J6 | CPE002NM | CPE002K2 | NM 00547 | NM 0054 | NM 0054 | INSL5 | INSL5 | INSL5 | insulin-like 5 | insulin-lik | insulin-lik | 10022 | 10022 | 10022 | s19484 | s19485 | s19486 |
| 12984 | M9 | M10 | 1077 | 867 | 17988 | 130,6 | 16,7 | 1,132 | H5A034 C | sample | Hm Gen E | Hm Gen E | Hm Gen E | CPE002J6 | CPE002NM | CPE002K2 | NM 00104 | NM 0010 | NM 0010 | FEZ2 | FEZ2 | FEZ2 | fasciculation and | fasciculati | fasciculati | 9637 | 9637 | 9637 | s18525 | s18526 | s18527 |
| 18511 | D15 | D16 | 1374 | 1034 | 22943 | 375,7 | 16,7 | 1,132 | H5A034 C | sample | Hm Gen E | Hm Gen E | Hm Gen E | CPE002JM | CPE002O1 | CPE002KH | NM 14530 | NM 1453 | NM 1453 | LRRC51 | LRRC51 | LRRC51 | leucine rich repea | leucine ric | leucine ric | 220074 | 220074 | 220074 | s47853 | s47854 | s225878 |
| 18543 | E22 | E23 | 1324 | 995,4 | 22126 | 339,5 | 16,7 | 1,132 | H5A034 C | sample | Hm Gen E | Hm Gen E | Hm Gen E | CPE002JM | CPE002O1 | CPE002KH | NM 00103 | NM 0010 | NM 0010 | DEFB113 | DEFB113 | DEFB113 | defensin, beta 11 | defensin, | defensin, | 245927 | 245927 | 245927 | s48302 | s48303 | s48304 |
| 18731 | M19 | M20 | 1273 | 958,9 | 21300 | 240,6 | 16,7 | 1,132 | H5A034 C | sample | Hm Gen E | Hm Gen E | Hm Gen E | CPE002JM | CPE002O1 | CPE002KH | NM 00100 | NM 0010 | NM 0010 | OR8B12 | OR8B12 | OR8B12 | olfactory recepto | olfactory | olfactory | 219858 | 219858 | 219858 | s47739 | s47740 | s225875 |
| 18735 | M22 | M23 | 1008 | 1047 | 16812 | 244,8 | 16,7 | 1,132 | H5A034 C | sample | Hm Gen E | Hm Gen E | Hm Gen E | CPE002JM | CPE002O1 | CPE002KH | NM 15325 | NM 1532 | NM 1532 | BRWD3 | BRWD3 | BRWD3 | bromodomain an | bromodoi | bromodoi | 254065 | 254065 | 254065 | s48489 | s48490 | s48491 |
| 17322 | B3 | B4 | 886 | 1003 | 14817 | 236,5 | 16,7 | 1,14 | H5A034 C | sample | Hm Gen E | Hm Gen E | Hm Gen E | CPE002JJ | CPE002NY | CPE002KE | NM 14477 | NM 1447 | NM 1447 | APOA1BP | APOA1BP | APOA1BP | apolipoprotein A- | apolipopr | apolipopr | 128240 | 128240 | 128240 | s43271 | s43272 | s43273 |
| 17435 | G19 | G20 | 1230 | 948,9 | 20485 | 179,8 | 16,7 | 1,14 | H5A034 C | sample | Hm Gen E | Hm Gen E | Hm Gen E | CPE002JJ | CPE002NY | CPE002KE | NM 14517 | NM 1451 | NM 1451 | WDR63 | WDR63 | WDR63 | WD repeat domai | WD repea | WD repea | 126820 | 126820 | 126820 | s43090 | s43091 | s43092 |
| 17463 | H22 | H23 | 1438 | 936,8 | 23986 | 259,9 | 16,7 | 1,14 | H5A034 C | sample | Hm Gen E | Hm Gen E | Hm Gen E | CPE002JJ | CPE002NY | CPE002KE | NM 15242 | NM 1524 | NM 1524 | FAM123B | FAM123B | FAM123B | family with seque | family wit | family wit | 139285 | 139285 | 139285 | s44190 | s44191 | s44192 |
| 17478 | I14 | I15 | 1328 | 955,7 | 22147 | 206,8 | 16,7 | 1,14 | H5A034 C | sample | Hm Gen E | Hm Gen E | Hm Gen E | CPE002JJ | CPE002NY | CPE002KE | NM 00100 | NM 0010 | NM 0010 | OR2Y1 | OR2Y1 | OR2Y1 | olfactory recepto | olfactory | olfactory | 134083 | 134083 | 134083 | s43821 | s43822 | s43823 |
| 17637 | O6 | O7 | 920 | 912,1 | 15393 | 314,3 | 16,7 | 1,14 | H5A034 C | sample | Hm Gen E | Hm Gen E | Hm Gen E | CPE002JJ | CPE002NY | CPE002KE | NM 00100 | NM 0010 | NM 0010 | OR2F2 | OR2F2 | OR2F2 | olfactory recepto | olfactory | olfactory | 135948 | 135948 | 135948 | s43968 | s43969 | s43970 |
| 9227 | A19 | A20 | 1636 | 1133 | 27254 | 177,2 | 16,7 | 1,16 | H5A034 C | sample | Hm Drgbl | Hm Drgbl | Hm Drgbl | CPE002E8 | CPE002ER | CPE002FC | NM 01778 | NM 0177 | NM 0177 | CYP2W1 | CYP2W1 | CYP2W1 | cytochrome P450, | cytochron | cytochron | 54905 | 54905 | 54905 | s195255 | s195256 | s195254 |
| 9434 | J10 | J11 | 1095 | 1086 | 18253 | 141,9 | 16,7 | 1,16 | H5A034 C | sample | Hm Drgbl | Hm Drgbl | Hm Drgbl | CPE002E8 | CPE002ER | CPE002FC | NM 02020 | NM 0202 | NM 0202 | LHX9 | LHX9 | LHX9 | LIM homeobox 9 | LIM home | LIM home | 56956 | 56956 | 56956 | s32486 | s32487 | s32488 |
| 9479 | K8 | K9 | 956 | 1081 | 15959 | 200,9 | 16,7 | 1,16 | H5A034 C | sample | Hm Drgbl | Hm Drgbl | Hm Drgbl | CPE002E8 | CPE002ER | CPE002FC | NM 03200 | NM 0320 | NM 0320 | PCDHGA2 | PCDHGA2 | PCDHGA2 | protocadherin ga | protocadh | protocadh | 56113 | 56113 | 56113 | s31927 | s31926 | s31925 |
| 9552 | N9 | N10 | 1172 | 1069 | 19607 | 187,8 | 16,7 | 1,16 | H5A034 C | sample | Hm Drgbl | Hm Drgbl | Hm Drgbl | CPE002E8 | CPE002ER | CPE002FC | NM 01809 | NM 0180 | NM 0180 | MLSTD1 | MLSTD1 | MLSTD1 | male sterility don | male steri | male steri | 55711 | 55711 | 55711 | s31311 | s31313 | s31312 |
| 19081 | L1 | L2 | 787 | 1013 | 13159 | 284 | 16,7 | 1,164 | H5A034 C | sample | Hm Gen E | Hm Gen E | Hm Gen E | CPE002JN | CPE002O2 | CPE002KI | XM 20893 | XM 20893 | XM 20893 | LOC28395 | LOC28395 | LOC28395 | similar to hCG154 | similar to | hypotheti | 283953 | 283953 | 283953 | s49416 | s195946 | s226165 |
| 19173 | O6 | O7 | 637 | 929,1 | 10650 | 399,5 | 16,7 | 1,164 | H5A034 C | sample | Hm Gen E | Hm Gen E | Hm Gen E | CPE002JN | CPE002O2 | CPE002KI | NM 00103 | NM 0010 | NM 0010 | IAH1 | IAH1 | IAH1 | isoamyl acetate-h | isoamyl ac | isoamyl ac | 285148 | 285148 | 285148 | s49825 | s49826 | s49827 |
| 19182 | P14 | P15 | 1191 | 1178 | 19889 | 211,1 | 16,7 | 1,164 | H5A034 C | sample | Hm Gen E | Hm Gen E | Hm Gen E | CPE002JN | CPE002O2 | CPE002KI | NM 17369 | NM 1736 | NM 1736 | MAGEB18 | MAGEB18 | MAGEB18 | melanoma antige | melanom | melanom | 286514 | 286514 | 286514 | s50179 | s50180 | s50181 |
| 9662 | C21 | C22 | 1874 | 1157 | 31244 | 167,3 | 16,7 | 1,168 | H5A034 C | sample | Hm Drgbl | Hm Drgbl | Hm Drgbl | CPE002E9 | CPE002EQ | CPE002FB | NM 02067 | NM 0206 | NM 0206 | ABHD6 | ABHD6 | ABHD6 | abhydrolase dom | abhydrola | abhydrola | 57406 | 57406 | 57406 | s33002 | s33003 | s33001 |
| 9778 | H18 | H19 | 1732 | 1047 | 28919 | 128,7 | 16,7 | 1,168 | H5A034 C | sample | Hm Drgbl | Hm Drgbl | Hm Drgbl | CPE002E9 | CPE002EQ | CPE002FB | NM 02406 | NM 0240 | NM 0240 | SPATA5L1 | SPATA5L1 | SPATA5L1 | spermatogenesis | spermato | spermato | 79029 | 79029 | 79029 | s35445 | s35447 | s35446 |
| 9860 | K5 | K6 | 1486 | 994,8 | 24833 | 150,2 | 16,7 | 1,168 | H5A034 C | sample | Hm Drgbl | Hm Drgbl | Hm Drgbl | CPE002E9 | CPE002EQ | CPE002FB | NM 02081 | NM 0208 | NM 0208 | TRMT5 | TRMT5 | TRMT5 | TRM5 tRNA meth | TRM5 tRN | TRM5 tRN | 57570 | 57570 | 57570 | s33334 | s33335 | s33336 |
| 15164 | H5 | H6 | 1021 | 834,5 | 17065 | 120,6 | 16,7 | 1,168 | H5A034 C | sample | Hm Gen E | Hm Gen E | Hm Gen E | CPE002J8 | CPE002NS | CPE002K8 | NM 02022 | NM 0202 | NM 0202 | STOX2 | STOX2 | STOX2 | storkhead box 2 | storkhead | storkhead | 56977 | 56977 | 56977 | s32513 | s32514 | s32515 |
| 8511 | C22 | C23 | 1769 | 1122 | 29577 | 181 | 16,7 | 1,18 | H5A034 C | sample | Hm Drgbl | Hm Drgbl | Hm Drgbl | CPE002E6 | CPE002ET | CPE002FE | NM 01439 | NM 0143 | NM 0143 | SND1 | SND1 | SND1 | staphylococcal nu | staphyloc | staphyloc | 27044 | 27044 | 27044 | s25747 | s25748 | s25749 |
| 8617 | H1 | H2 | 1275 | 1012 | 21265 | 134,9 | 16,7 | 1,18 | H5A034 C | sample | Hm Drgbl | Hm Drgbl | Hm Drgbl | CPE002E6 | CPE002ET | CPE002FE | NM 02066 | NM 0206 | NM 0206 | DECR2 | DECR2 | DECR2 | 2,4-dienoyl CoA r | 2,4-dienoy | 2,4-dienoy | 26063 | 26063 | 26063 | s25044 | s25046 | s25045 |
| 8630 | H21 | H22 | 1823 | 1065 | 30416 | 151,6 | 16,7 | 1,18 | H5A034 C | sample | Hm Drgbl | Hm Drgbl | Hm Drgbl | CPE002E6 | CPE002ET | CPE002FE | NM 01564 | NM 0156 | NM 0156 | TTLL3 | TTLL3 | TTLL3 | tubulin tyrosine l | tubulin ty | tubulin ty | 26140 | 26140 | 26140 | s25151 | s25152 | s25153 |
| 8673 | J17 | J18 | 1900 | 955 | 31787 | 132,2 | 16,7 | 1,18 | H5A034 C | sample | Hm Drgbl | Hm Drgbl | Hm Drgbl | CPE002E6 | CPE002ET | CPE002FE | NM 00662 | NM 0066 | NM 0066 | PHGDH | PHGDH | PHGDH | phosphoglycerate | phosphog | phosphog | 26227 | 26227 | 26227 | s516 | s515 | s223687 |
| 8782 | N7 | N8 | 1589 | 1006 | 26586 | 155,4 | 16,7 | 1,18 | H5A034 C | sample | Hm Drgbl | Hm Drgbl | Hm Drgbl | CPE002E6 | CPE002ET | CPE002FE | NM 01213 | NM 0121 | NM 0121 | SERGEF | SERGEF | SERGEF | secretion regulati | secretion | secretion | 26297 | 26297 | 26297 | s25394 | s25395 | s25396 |
| 20833 | E1 | E2 | 842 | 987,8 | 14047 | 239,8 | 16,7 | 1,184 | H5A034 C | sample | Hm Gen E | Hm Gen E | Hm Gen E | CPE002JS | CPE002O7 | CPE002KN | XM 94299 | XM 94299 | XM 94299 | LOC64293 | LOC64293 | LOC64293 | hypothetical prot | hypotheti | hypotheti | 642934 | 642934 | 642934 | s55155 | s55156 | s55157 |
| 21099 | P11 | P12 | 1171 | 1155 | 19544 | 377,9 | 16,7 | 1,184 | H5A034 C | sample | Hm Gen E | Hm Gen E | Hm Gen E | CPE002JS | CPE002O7 | CPE002KN | XM 93687 | XM 93687 | XM 93687 | DKFZP781 | DKFZP781 | DKFZP781 | hypothetical prot | hypotheti | hypotheti | 644041 | 644041 | 644041 | s55533 | s55534 | s55535 |
| 21499 | P4 | P5 | 1132 | 1028 | 18955 | 400 | 16,7 | 1,184 | H5A034 C | sample | Hm Gen E | Hm Gen E | Hm Gen E | CPE002JT | CPE002O8 | CPE002KO | NM 00104 | NM 0010 | NM 0010 | LOC64857 | LOC64857 | LOC64857 | hypothetical LOC | hypotheti | hypotheti | 648570 | 648570 | 648570 | s56846 | s56847 | s227309 |
| 11192 | C16 | C17 | 1174 | 793,3 | 19560 | 171,6 | 16,7 | 1,21 | H5A034 C | sample | Hm Drgbl | Hm Drgbl | Hm Drgbl | CPE002ED | CPE002F3 | CPE002FT | NM 00103 | NM 0010 | NM 0010 | ACBD7 | ACBD7 | ACBD7 | acyl-Coenzyme A | acyl-Coen | acyl-Coen | 414149 | 414149 | 414149 | s53904 | s53902 | s224029 |
| 11245 | E20 | E21 | 1511 | 849,2 | 25256 | 160,9 | 16,7 | 1,21 | H5A034 C | sample | Hm Drgbl | Hm Drgbl | Hm Drgbl | CPE002ED | CPE002F3 | CPE002FT | NM 00100 | NM 0010 | NM 0010 | LRRC52 | LRRC52 | LRRC52 | leucine rich repea | leucine ric | leucine ric | 440699 | 440699 | 440699 | s224060 | s54173 | s54174 |
| 11401 | L1 | L2 | 1109 | 808,7 | 18542 | 154,5 | 16,7 | 1,21 | H5A034 C | sample | Hm Drgbl | Hm Drgbl | Hm Drgbl | CPE002ED | CPE002F3 | CPE002FT | XM 37211 | XM 37211 | XM 37211 | PTAR1 | PTAR1 | PTAR1 | protein prenyltra | protein pr | protein pr | 375743 | 375743 | 375743 | s51741 | s51740 | s51742 |
| 12365 | D13 | D14 | 814 | 1128 | 13580 | 177,2 | 16,7 | 1,256 | H5A034 C | sample | Hm Gen E | Hm Gen E | Hm Gen E | CPE002J5 | CPE002NK | CPE002K1 | NM 00336 | NM 0033 | NM 0033 | UVRAG | UVRAG | UVRAG | UV radiation resis | UV radiati | UV radiati | 7405 | 7405 | 7405 | s14741 | s14742 | s14743 |
| 11027 | L19 | L20 | 1237 | 782 | 20679 | 581,9 | 16,7 | 1,265 | H5A034 C | sample | Hm Drgbl | Hm Drgbl | Hm Drgbl | CPE002EC | CPE002EN | CPE002FS | NM 15258 | NM 1525 | NM 1525 | TMTC2 | TMTC2 | TMTC2 | transmembrane a | transmem | transmem | 160335 | 160335 | 160335 | s46188 | s46189 | s46190 |
| 16919 | A8 | A9 | 888 | 1159 | 14813 | 337,1 | 16,7 | 1,29 | H5A034 C | sample | Hm Gen E | Hm Gen E | Hm Gen E | CPE002JI | CPE002NZ | CPE002KD | NM 05410 | XM 0860 | NM 0541 | HRASLS5 | LOC14800 | HRASLS5 | HRAS-like suppres | similar to | HRAS-like | 117245 | 148003 | 117245 | s42142 | s45108 | s42144 |
| 16969 | D1 | D2 | 817 | 1138 | 13641 | 295,7 | 16,7 | 1,29 | H5A034 C | sample | Hm Gen E | Hm Gen E | Hm Gen E | CPE002JI | CPE002NZ | CPE002KD | NM 05292 | XM 9343 | NM 0529 | PNMA5 | LOC14578 | PNMA5 | paraneoplastic ar | hypotheti | paraneop | 114824 | 145783 | 114824 | s41666 | s44778 | s41668 |
| 6930 | A3 | A4 | 1303 | 627,1 | 21759 | 574,4 | 16,7 | 1,3 | H5A034 C | sample | Hm Drgbl | Hm Drgbl | Hm Drgbl | CPE002E2 | CPE002EX | CPE002FI | NM 00228 | NM 0022 | NM 0022 | KRT85 | KRT85 | KRT85 | keratin 85 | keratin 85 | keratin 85 | 3891 | 3891 | 3891 | s8027 | s8029 | s8028 |
| 6935 | A8 | A9 | 893 | 682,9 | 14926 | 594,4 | 16,7 | 1,3 | H5A034 C | sample | Hm Drgbl | Hm Drgbl | Hm Drgbl | CPE002E2 | CPE002EX | CPE002FI | NM 00244 | NM 0024 | NM 0024 | MSI1 | MSI1 | MSI1 | musashi homolog | musashi h | musashi h | 4440 | 4440 | 4440 | s8980 | s8978 | s8979 |
| 7122 | I3 | I4 | 376 | 509,1 | 6267 | 433,5 | 16,7 | 1,3 | H5A034 C | sample | Hm Drgbl | Hm Drgbl | Hm Drgbl | CPE002E2 | CPE002EX | CPE002FI | NM 00103 | NM 0010 | NM 0010 | LMX1A | LMX1A | LMX1A | LIM homeobox tr | LIM home | LIM home | 4009 | 4009 | 4009 | s8239 | s8240 | s8241 |
| 7178 | L10 | L11 | 773 | 475,8 | 12929 | 455 | 16,7 | 1,3 | H5A034 C | sample | Hm Drgbl | Hm Drgbl | Hm Drgbl | CPE002E2 | CPE002EX | CPE002FI | NM 00250 | NM 0025 | NM 0025 | NGFR | NGFR | NGFR | nerve growth fact | nerve grov | nerve grov | 4804 | 4804 | 4804 | s9540 | s194655 | s194654 |
| 7232 | N16 | N17 | 831 | 534,2 | 13891 | 264,2 | 16,7 | 1,3 | H5A034 C | sample | Hm Drgbl | Hm Drgbl | Hm Drgbl | CPE002E2 | CPE002EX | CPE002FI | NM 00736 | NM 0073 | NM 0073 | NONO | NONO | NONO | non-POU domain | non-POU | non-POU | 4841 | 4841 | 4841 | s9612 | s9613 | s9614 |
| 7269 | O6 | O7 | 1283 | 556,3 | 21374 | 547,7 | 16,7 | 1,3 | H5A034 C | sample | Hm Drgbl | Hm Drgbl | Hm Drgbl | CPE002E2 | CPE002EX | CPE002FI | NM 00454 | NM 0045 | NM 0045 | NCAM2 | NCAM2 | NCAM2 | neural cell adhesi | neural cel | neural cel | 4685 | 4685 | 4685 | s9299 | s9298 | s9297 |
| 7270 | O7 | O8 | 1024 | 522,5 | 17054 | 506,6 | 16,7 | 1,3 | H5A034 C | sample | Hm Drgbl | Hm Drgbl | Hm Drgbl | CPE002E2 | CPE002EX | CPE002FI | NM 00535 | NM 0053 | NM 0053 | SMAD4 | SMAD4 | SMAD4 | SMAD family men | SMAD fam | SMAD fam | 4089 | 4089 | 4089 | s8405 | s8403 | s8404 |
| 5160 | G9 | G10 | 1136 | 632,5 | 19008 | 498,5 | 16,7 | 1,31 | H5A034 C | sample | Hm Drgbl | Hm Drgbl | Hm Drgbl | CPE002DV | CPE002EM | CPE002FO | NM 03258 | NM 0325 | NM 0325 | USP32 | USP32 | USP32 | ubiquitin specific | ubiquitin | ubiquitin | 84669 | 84669 | 84669 | s39290 | s39291 | s39292 |
| 5171 | H19 | H20 | 830 | 599 | 13871 | 534,6 | 16,7 | 1,31 | H5A034 C | sample | Hm Drgbl | Hm Drgbl | Hm Drgbl | CPE002DV | CPE002EM | CPE002FO | NM 00464 | NM 0046 | NM 0046 | BAT1 | BAT1 | BAT1 | HLA-B associated | HLA-B ass | HLA-B ass | 7919 | 7919 | 7919 | s15472 | s15473 | s15474 |

| | | | | | | | | | | | | | | | | | | | | | | | | | | | | | | | |
|---|---|---|---|---|---|---|---|---|---|---|---|---|---|---|---|---|---|---|---|---|---|---|---|---|---|---|---|---|---|---|---|
| 2545 | K1 | K2 | 437 | 684,8 | 7293 | 390,7 | 16,7 | 1,315 | H5A034 C | sample | Hm Drgbl | Hm Drgbl | Hm Drgbl | CPE002DC | CPE002EE | CPE002F4 | NM 00522 | NM 0052 | NM 0052 | EGFR | EGFR | EGFR | epidermal growth | epidermal | epidermal | 1956 | 1956 | 1956 | s563 | s564 | s565 |
| 2590 | L7 | L8 | 1223 | 664,6 | 20422 | 346,1 | 16,7 | 1,315 | H5A034 C | sample | Hm Drgbl | Hm Drgbl | Hm Drgbl | CPE002DC | CPE002EE | CPE002F4 | NM 00443 | NM 0044 | NM 0044 | EPHA5 | EPHA5 | EPHA5 | EPH receptor A5 | EPH recep | EPH recep | 2044 | 2044 | 2044 | s4728 | s4729 | s4730 |
| 16590 | D14 | D15 | 1552 | 1004 | 25861 | 377,3 | 16,7 | 1,32 | H5A034 C | sample | Hm Gen E | Hm Gen E | Hm Gen E | CPE002JH | CPE002NV | CPE002KC | NM 13837 | NM 1383 | NM 1383 | TIMD4 | TIMD4 | TIMD4 | T-cell immunoglo | T-cell imm | T-cell imm | 91937 | 91937 | 91937 | s40812 | s40813 | s40814 |
| 16608 | D9 | D10 | 1146 | 934,7 | 19101 | 291,4 | 16,7 | 1,32 | H5A034 C | sample | Hm Gen E | Hm Gen E | Hm Gen E | CPE002JH | CPE002NV | CPE002KC | NM 03312 | NM 0331 | NM 0331 | C4orf35 | C4orf35 | C4orf35 | chromosome 4 or | chromoso | chromoso | 85438 | 85438 | 85438 | s39988 | s39989 | s39990 |
| 16659 | G11 | G12 | 1231 | 941,1 | 20598 | 258,4 | 16,7 | 1,32 | H5A034 C | sample | Hm Gen E | Hm Gen E | Hm Gen E | CPE002JH | CPE002NV | CPE002KC | NM 03285 | NM 0328 | NM 0328 | ABHD13 | ABHD13 | ABHD13 | abhydrolase dom | abhydrola | abhydrola | 84945 | 84945 | 84945 | s39724 | s39725 | s228401 |
| 16724 | I5 | I6 | 663 | 946,3 | 11092 | 312,3 | 16,7 | 1,32 | H5A034 C | sample | Hm Gen E | Hm Gen E | Hm Gen E | CPE002JH | CPE002NV | CPE002KC | NM 03287 | NM 0328 | NM 0328 | UBASH3B | UBASH3B | UBASH3B | ubiquitin associat | ubiquitin | ubiquitin | 84959 | 84959 | 84959 | s39757 | s39758 | s39759 |
| 16748 | J5 | J6 | 1378 | 989,4 | 23010 | 323,3 | 16,7 | 1,32 | H5A034 C | sample | Hm Gen E | Hm Gen E | Hm Gen E | CPE002JH | CPE002NV | CPE002KC | NM 03344 | NM 0334 | NM 0334 | FCHSD1 | FCHSD1 | FCHSD1 | FCH and double S | FCH and d | FCH and d | 89848 | 89848 | 89848 | s40150 | s40151 | s228431 |
| 17709 | B6 | B7 | 1061 | 1138 | 17733 | 307,4 | 16,7 | 1,32 | H5A034 C | sample | Hm Gen E | Hm Gen E | Hm Gen E | CPE002JK | CPE002NZ | CPE002KF | NM 14470 | NM 1447 | NM 1447 | TEKT4 | TEKT4 | TEKT4 | tektin 4 | tektin 4 | tektin 4 | 150483 | 150483 | 150483 | s45449 | s45450 | s45451 |
| 17710 | B7 | B8 | 930 | 1114 | 15558 | 443,8 | 16,7 | 1,32 | H5A034 C | sample | Hm Gen E | Hm Gen E | Hm Gen E | CPE002JK | CPE002NZ | CPE002KF | NM 15244 | NM 1524 | NM 1524 | C14orf145 | C14orf145 | C14orf145 | chromosome 14 c | chromoso | chromoso | 145508 | 145508 | 145508 | s44750 | s44751 | s225694 |
| 17851 | H4 | H5 | 499 | 935 | 8321 | 289,8 | 16,7 | 1,32 | H5A034 C | sample | Hm Gen E | Hm Gen E | Hm Gen E | CPE002JK | CPE002NZ | CPE002KF | NM 18282 | NM 1828 | NM 1828 | GDF7 | GDF7 | GDF7 | growth differentia | growth di | growth di | 151449 | 151449 | 151449 | s45566 | s45567 | s45568 |
| 17953 | M1 | M2 | 630 | 1127 | 10552 | 143,5 | 16,7 | 1,32 | H5A034 C | sample | Hm Gen E | Hm Gen E | Hm Gen E | CPE002JK | CPE002NZ | CPE002KF | NM 14466 | NM 1446 | NM 1446 | WDR66 | WDR66 | WDR66 | WD repeat domai | WD repea | WD repea | 144406 | 144406 | 144406 | s44651 | s44653 | s225680 |
| 18046 | P7 | P8 | 1109 | 1132 | 18494 | 371,4 | 16,7 | 1,32 | H5A034 C | sample | Hm Gen E | Hm Gen E | Hm Gen E | CPE002JK | CPE002NZ | CPE002KF | NM 17350 | NM 1735 | NM 1735 | LYPD4 | LYPD4 | LYPD4 | LY6/PLAUR domai | LY6/PLAUI | LY6/PLAUI | 147719 | 147719 | 147719 | s45045 | s45046 | s45047 |
| 9038 | I21 | I22 | 1765 | 1956 | 29412 | 190,8 | 16,7 | 1,341 | H5A034 C | sample | Hm Drgbl | Hm Drgbl | Hm Drgbl | CPE002E7 | CPE002ES | CPE002FD | NM 01587 | NM 0158 | NM 0158 | ZBTB7B | ZBTB7B | ZBTB7B | zinc finger and BT | zinc finger | zinc finger | 51043 | 51043 | 51043 | s27296 | s27297 | s27298 |
| 4260 | B2 | B3 | 1097 | 661,1 | 18323 | 427,6 | 16,7 | 1,409 | H5A034 C | sample | Hm Drgbl | Hm Drgbl | Hm Drgbl | CPE002DU | CPE002EK | CPE002FQ | NM 01984 | NM 0198 | NM 0198 | TRPV5 | TRPV5 | TRPV5 | transient recepto | transient | transient | 56302 | 56302 | 56302 | s32135 | s32136 | s32137 |
| 4585 | P1 | P2 | 655 | 852,6 | 10938 | 512,7 | 16,7 | 1,409 | H5A034 C | sample | Hm Drgbl | Hm Drgbl | Hm Drgbl | CPE002DU | CPE002EK | CPE002FQ | NM 00054 | NM 0005 | NM 0005 | RYR1 | RYR1 | RYR1 | ryanodine recepto | ryanodine | ryanodine | 6261 | 6261 | 6261 | s12393 | s12394 | s12395 |
| 11 | A19 | A20 | 422 | 1559 | 7041 | 784,8 | 16,7 | 1,421 | H5A034 C | sample | Hm Drgbl | Hm Drgbl | Hm Drgbl | CPE002DT | CPE002EJ | CPE002F9 | NM 00495 | NM 0049 | NM 0049 | NR5A1 | NR5A1 | NR5A1 | nuclear receptor s | nuclear re | nuclear re | 2516 | 2516 | 2516 | s5392 | s5393 | s5394 |
| 1971 | C11 | C12 | 1322 | 646,1 | 22119 | 440,8 | 16,7 | 1,452 | H5A034 C | sample | Hm Drgbl | Hm Drgbl | Hm Drgbl | CPE002FX | CPE002G1 | CPE002G5 | XM 93781 | XM 00112 | XM 00112 | ZNF251 | ZNF251 | ZNF251 | zinc finger protei | zinc finger | zinc finger | 90987 | 90987 | 90987 | s40542 | s225079 | s195555 |
| 2204 | L5 | L6 | 1122 | 655,3 | 18791 | 444 | 16,7 | 1,452 | H5A034 C | sample | Hm Drgbl | Hm Drgbl | Hm Drgbl | CPE002FX | CPE002G1 | CPE002G5 | NM 19827 | NM 1982 | NM 1982 | SMYD1 | SMYD1 | SMYD1 | SET and MYND dc | SET and M | SET and M | 150572 | 150572 | 150572 | s45455 | s223362 | s45457 |
| 6734 | I21 | I22 | 1301 | 563,8 | 21713 | 532,9 | 16,7 | 1,465 | H5A034 C | sample | Hm Drgbl | Hm Drgbl | Hm Drgbl | CPE002E1 | CPE002EY | CPE002FJ | NM 00211 | NM 0021 | NM 0021 | HDC | HDC | HDC | histidine decarbo | histidine c | histidine c | 3067 | 3067 | 3067 | s6496 | s6497 | s6498 |
| 6813 | L6 | L7 | 931 | 631,5 | 15563 | 525,9 | 16,7 | 1,465 | H5A034 C | sample | Hm Drgbl | Hm Drgbl | Hm Drgbl | CPE002E1 | CPE002EY | CPE002FJ | NM 00226 | NM 0022 | NM 0022 | KPNA4 | KPNA4 | KPNA4 | karyopherin alpha | karyopher | karyopher | 3840 | 3840 | 3840 | s7926 | s7927 | s7928 |
| 11571 | C11 | C12 | 850 | 857,5 | 14165 | 167,3 | 16,7 | 1,491 | H5A034 C | sample | Hm Gen E | Hm Gen E | Hm Gen E | CPE002JA | CPE002NJ | CPE002JZ | NM 00164 | NM 0016 | NM 0016 | APLP2 | APLP2 | APLP2 | amyloid beta (A4 | amyloid b | amyloid b | 334 | 334 | 334 | s1463 | s1464 | s226287 |
| 11578 | C18 | C19 | 1474 | 870,2 | 24679 | 176,9 | 16,7 | 1,491 | H5A034 C | sample | Hm Gen E | Hm Gen E | Hm Gen E | CPE002JA | CPE002NJ | CPE002JZ | NM 00443 | NM 0044 | NM 0044 | EPB41 | EPB41 | EPB41 | erythrocyte meml | erythrocyt | erythrocyt | 2035 | 2035 | 2035 | s4701 | s4702 | s4703 |
| 11644 | F12 | F13 | 861 | 848,8 | 14356 | 179,2 | 16,7 | 1,491 | H5A034 C | sample | Hm Gen E | Hm Gen E | Hm Gen E | CPE002JA | CPE002NJ | CPE002JZ | NM 02106 | NM 0210 | NM 0210 | HIST1H2BE | HIST1H2BI | HIST1H2BI | histone cluster 1, | histone cl | histone cl | 3018 | 3018 | 3018 | s6419 | s6420 | s194493 |
| 11762 | K10 | K11 | 901 | 783 | 15050 | 138,9 | 16,7 | 1,491 | H5A034 C | sample | Hm Gen E | Hm Gen E | Hm Gen E | CPE002JA | CPE002NJ | CPE002JZ | NM 00051 | NM 0005 | NM 0005 | FSHB | FSHB | FSHB | follicle stimulatin | follicle sti | follicle sti | 2488 | 2488 | 2488 | s5371 | s5372 | s5373 |
| 11783 | K8 | K9 | 717 | 750,2 | 11991 | 162,6 | 16,7 | 1,491 | H5A034 C | sample | Hm Gen E | Hm Gen E | Hm Gen E | CPE002JA | CPE002NJ | CPE002JZ | NM 00146 | NM 0014 | NM 0014 | FRZB | FRZB | FRZB | frizzled-related pr | frizzled-re | frizzled-re | 2487 | 2487 | 2487 | s5368 | s5369 | s5370 |
| 15380 | A5 | A6 | 1260 | 845,8 | 21046 | 182,9 | 16,7 | 1,518 | H5A034 C | sample | Hm Gen E | Hm Gen E | Hm Gen E | CPE002JE | CPE002NT | CPE002K9 | NM 02163 | NM 0216 | NM 0216 | TMEM35 | TMEM35 | TMEM35 | transmembrane p | transmem | transmem | 59353 | 59353 | 59353 | s34034 | s34035 | s34036 |
[truncated: 2,097,861 more chars]
